# Effective Local and Secondary Protein Structure Prediction by Combining a Neural Network-Based Approach with Extensive Feature Design and Selection without Reliance on Evolutionary Information

**DOI:** 10.3390/ijms242115656

**Published:** 2023-10-27

**Authors:** Yury V. Milchevskiy, Vladislava Y. Milchevskaya, Alexei M. Nikitin, Yury V. Kravatsky

**Affiliations:** 1 Engelhardt Institute of Molecular Biology, Russian Academy of Sciences, Vavilov Str., 32, 119991 Moscow, Russiajiri@eimb.ru (Y.V.K.); 2 Institute of Medical Statistics and Bioinformatics, University of Cologne, 50931 Cologne, Germany; 3 Center for Precision Genome Editing and Genetic Technologies for Biomedicine, Engelhardt Institute of Molecular Biology, Russian Academy of Sciences, 119991 Moscow, Russia

**Keywords:** protein blocks (PBs), protein local structure, protein local structure prediction, DSSP (Dictionary of Secondary Structure in Proteins), protein secondary structure prediction, machine learning (ML)

## Abstract

Protein structure prediction continues to pose multiple challenges despite outstanding progress that is largely attributable to the use of novel machine learning techniques. One of the widely used representations of local 3D structure—protein blocks (PBs)—can be treated in a similar way to secondary structure classes. Here, we present a new approach for predicting local conformation in terms of PB classes solely from amino acid sequences. We apply the RMSD metric to ensure unambiguous future 3D protein structure recovery. The selection of statistically assessed features is a key component of the proposed method. We suggest that ML input features should be created from the statistically significant predictors that are derived from the amino acids’ physicochemical properties and the resolved structures’ statistics. The statistical significance of the suggested features was assessed using a stepwise regression analysis that permitted the evaluation of the contribution and statistical significance of each predictor. We used the set of 380 statistically significant predictors as a learning model for the regression neural network that was trained using the PISCES30 dataset. When using the same dataset and metrics for benchmarking, our method outperformed all other methods reported in the literature for the CB513 nonredundant dataset (for the PBs, Q16 = 81.01%, and for the DSSP, Q3 = 85.99% and Q8 = 79.35%).

## 1. Introduction

Recent advances in machine learning approaches have allowed for substantial improvement in the prediction of protein structures and functions from their sequences [1,2,3,4]. When describing and analyzing the secondary structures of proteins, for a long time, researchers used the DSSP (Dictionary of Secondary Structure in Proteins) assignment, which includes structures such as α-helixes, β-strands, coils, π-helixes, turns, bridges, etc. [5]. However, the DSSP does not reflect subtle changes in the local geometry of a protein’s structure. In particular, a coil represents a large and heterogeneous class prominently present among the structures in the Protein Data Bank (PDB), which requires a more fine-grained description [6]. One of the strategies to address these limitations involved analyzing backbone angles that define the protein structure. A number of methods addressed the prediction of backbone torsion angles (ϕ and ψ) [7,8,9,10,11] as well as the backbone Cα angle θ (the angle between the Cα_i−1_ − Cα − Cα_i+1_ atoms) and the rotation angle τ (dihedral angle of rotation around the Cα_i_ − Cα_i+1_ bond) [12].

Another strategy to grasp the diversity of local protein backbone conformations involved generalizing based on the DSSP assignment: multiple protein structure alphabets have been proposed. One of the most widely used alphabets is the 16-class protein blocks (PBs), proposed by de Brevern et al. [13]. This alphabet offers refined subgroups for classical structural elements (e.g., α-helix, β-sheet, and coil) as well as their adjacent structures (e.g., N-caps–β/α and C-caps–β/α) [13,14]. In particular, there are four classes describing different coil structures, and they capture the abovementioned diversity in more detail. 

In this context, a protein block (PB) is a five-residue long structural fragment defined by its dihedral angles. Further, the authors defined a structural class by its center—a protein block (PB)—and the five-residue long structures closest to it in terms of the RMSDA (Root Mean Square Deviation of Angular values) [15] between the corresponding backbone atoms. De Brevern et al. define the RMSDA between two protein blocks as follows:

(1) $$RMSDA(PB_{1},PB_{2})=\sqrt{\frac{\sum_{i=1}^{M-1}\left[{\left(\psi_{1,i}-\psi_{2,i}\right)}^{2}+{\left(\varphi_{1,i+1}-\varphi_{2,i+1}\right)}^{2}\right]}{2(M-1)}}$$

where $\left\{\varphi_{k,i},\psi_{k,i}\right\}$ denotes the series of the (2*M* − 1) dihedral angles for *k*-th protein block **PB***_k_* and *M* = 5 for the PBs (as they are pentapeptides) [13]. Each class is then labeled with a letter, and if two classes are structurally similar, their labels are close in the alphabetical order. For instance, the PBs ‘d’, ‘e’, and ‘f’ correspond to classes with structures similar in three dimensions (Supplemental Table S1). The complete list of protein blocks and the dihedral angles through which they are defined can be found in Supplemental Table S1, along with their mapping to the ‘classic’ secondary protein structures.

In our recent work [16], we proposed using the same protein blocks as cluster centers, but we assigned cluster labels based on the root mean square deviation (RMSD) [17] distance instead of the RMSDA. The RMSD for protein blocks can be written as follows:

(2) $$RMSD(PB_{1},PB_{2})=\min\sqrt{\frac{\sum_{i=1}^{3M}\left[{\left(x_{1,i}-x_{2,i}\right)}^{2}+{\left(y_{1,i}-y_{2,i}\right)}^{2}+{\left(z_{1,i}-z_{2,i}\right)}^{2}\right]}{3M}}$$

where *x_k_*_,*i*_ = *x_i_* (**PB***_k_*); *y_k_*_,*i*_ = *y_i_* (**PB***_k_*); *z_k_*_,*i*_ = *z_i_* (**PB***_k_*); and *i* = 1, …, 3*M* denote the Cartesian coordinates of the backbone atoms N, Cα, and C, respectively, of the *M*–residue protein block **PB***_k_* (*M* = 5 for the PBs as in (1)), whereas the minimum is taken over by all of the spatial colocalization of the two protein blocks **PB**_1_ and **PB**_2_. By definition, an RMSD is close to zero only when two structures are identical in three dimensions, and using the RMSD may therefore be preferable to using the RMSDA, which does not always satisfy this criterion [16]. However, RMSD calculations require assessing all possible alignments between two query fragments, and thus, they have higher computational costs than RMSDA calculations.

Further investigations revealed that for regular structures and compact clusters (such as ‘m’ and ‘d’), label assignments based on RMSDA values largely agree with those based on RMSD values. However, the discrepancies between these two assignment systems become higher as the structural irregularities increase, and for clusters covering irregular structures, the two distances have qualitatively different meanings. Since we further aimed at reconstructing the 3D coordinates rather than the fragment classifications, we needed to approximate the distances between the atoms. For this purpose, we decided in favor of using the RMSD despite its computational cost. The optimization procedure proposed by Kabsch [17] was shown to reach the minimum RMSD [18], and its implementation in Qmol [19] was previously integrated into our pipeline.

In the context of 3D coordinate reconstruction, cluster centers are treated as basic structures, and distances from the query fragment to these basic structures are referred to as “structural coordinates”. We previously demonstrated that, given a large enough set of basic structures, one can reconstruct the coordinates of the backbone atoms of a protein fragment [16]. The number of basic structures required for such a reconstruction depends on the number of degrees of freedom (i.e., bond angles, bond lengths, and dihedral angles). For instance, for a five-residue fragment with all bond lengths and bond angles considered to be fixed, one would need at least twelve basic structures to reconstruct the conformation of the backbone. We previously developed and implemented an algorithm [16] that could unambiguously reconstruct a protein’s backbone coordinates from its “structural coordinates” representation.

Applying structural alphabets to problems of protein structure predictions requires efficient tools for local conformation predictions directly from an amino acid sequence. Early methods for predicting PBs relied on statistical approaches that did not incorporate evolutionary information [13,20]. In subsequent research, the incorporation of evolutionary information boosted prediction performance [21,22].

A variety of machine learning (ML) methods have been applied to tackle sequence-based structure predictions. SVM-PB-Pred [23] slightly improved the prediction quality using sequence profiles (PSSM) compared to the Bayesian approach [20], despite its more complex computational model. LOCUSTRA [24] employs a 15-amino-acid sliding-window encoding method, with each residue represented by a PSI-BLAST [25] PSSM profile and a 21-length, one-hot encoding vector (20 amino acids plus 1 extremity flag) per position. PYTHIA [26] utilizes a hybrid encoding approach, incorporating a 20 one-hot encoding vector, physicochemical properties from the AAindex [27] database, and a PSSM profile [28] derived from a multiple-sequence alignment. PYTHIA has achieved an impressive performance in predicting the PBs (Q16 = 59.9%) of challenging proteins from the CASP14 (Critical Assessment of methods of protein Structure Prediction) [29] free modeling category [30].

In several studies, authors integrated evolutionary information and the physicochemical properties of amino acids, decreasing the reliance on homologous sequences and achieving a Q3 accuracy above 80% [31,32,33]. In another study [34], incorporated into the SPIDER2 package [35], a dimension-reduced set of physicochemical properties [36] was employed for artificial neural networks. This approach aimed to predict four distinct structural properties: secondary structure, torsion angles; Cα-atom-based angles; dihedral angles; and solvent-accessible surface area. Notably, in predicting secondary structure, this method yielded an accuracy of Q3 = 82% [35].

Revolutionary ML approaches such as AlphaFold [2] focus solely on 3D structure predictions and do not engage in local or secondary protein structure predictions. Although AlphaFold performs exceptionally well overall, it shows a drop in performance on specific CASP14 targets, such as flexible protein regions and components of multiprotein complexes. Authors reported a high prediction quality (the original performance measure pLDDT > 90) in only 35.7% of cases and a confident prediction (pLDDT > 70) in 58.7% of cases [37], indicating the need for further investigation into certain protein families.

In our previous work [16], we did not utilize evolutionary information. Instead, our feature encoding relied solely on amino acids’ physicochemical properties and resolved structures’ statistics. We employed the stepwise linear regression with bidirectional elimination of predictors to build the prediction model, achieving a classification accuracy of Q16 at 67.9%. During the model’s development process, we found that a well-curated set of predictors played a fundamental role in achieving high prediction accuracy. Each predictor was designed to formalize a hypothesis about the factors influencing the local structure, and the model-building procedure assessed their statistical significance. To facilitate the mass assignment of such predictors, we implemented an ad hoc scripting language [38].

In the current study, we present a novel approach that combines our previous predictor generation scheme with the use of deep learning methods, and this improved the prediction accuracy of the method. Similar to our previous work, the method does not rely on evolutionary information, but it managed to surpass the known accuracy of the local structure prediction methods for both the CB513 dataset [39] and the CASP14 free modeling targets [30]. Furthermore, it provides sufficient information to reconstruct the Cartesian coordinates of the protein backbone. We also applied the same set of statistically significant predictors to perform classic secondary structure predictions using an ML method.

## 2. Results

### 2.1. Improved Q16 Classification with RMSD-Based Protein Blocks

One of the most common tasks in protein local structure prediction is the classification of structural fragments to a given set of classes. Here, we used the 16 protein blocks from the studies by de Brevern et al. [13,20] with the RMSD distance and the three most commonly used datasets to assess our method’s performance: PISCES30 [40], CB513 [39], and a small set of proteins without known homologues from CASP14 (the so-called free modeling category [30]). Table 1 shows the performance of our method on several datasets. Because it is the largest, the PISCES30 dataset was used to train, validate, and test the model. On the CB513 dataset, which is widely used for benchmarking secondary structure prediction methods, our algorithm achieved a classification accuracy of Q16 = 81.01%. This is 13.5% higher than the classification accuracy reported by the latest leading method, PYTHIA [26].

One of the most challenging scenarios in secondary structure prediction is when a query protein does not have homologues with resolved structures. In this case, we benchmarked our model against the leading prediction methods reported in the literature (LOCUSTRA [24] and PYTHIA [26]) by examining 10 targets from the CASP14 contest in the free modeling category. These targets were particularly challenging for the 3D structure prediction as they featured distinct folds and lacked close homologues with resolved structures that could serve as templates. Table 2 presents the results for each of the CASP14 free modeling targets, and for 8 of the 10 targets, our model showed higher overall accuracy.

To benchmark our predictions against those of AlphaFold [2], we used the targets from the CASP14 [29] free modeling category [30]. For each five-residue fragment in a given target, we calculated its Q16 classification accuracy based on the RMSD distance from the structure predicted by AlphaFold and compared it to the classification accuracy predicted by our method. For T1027 7D2OA, our method outperformed AlphaFold by 20% (Q16 = 68.3% for our method and Q16 = 48.2% for AlphaFold); for T1029 6UF2A, our method outperformed AlphaFold by 0.9% (Q16 = 71.9% for our method and Q16 = 71.0% for AlphaFold); and for T1049 6Y4FA, AlphaFold outperformed our method by 3.1% (Q16 = 72.3% for our method and Q16 = 75.4% for AlphaFold). The detailed RMSD profiles and correlation values between the predicted and native structures can be found in Supplemental Figure S1.

### 2.2. Accurate Prediction of the Distances to the Cluster Centers as the First Step in 3D Coordinate Reconstruction

**Figure 1:**
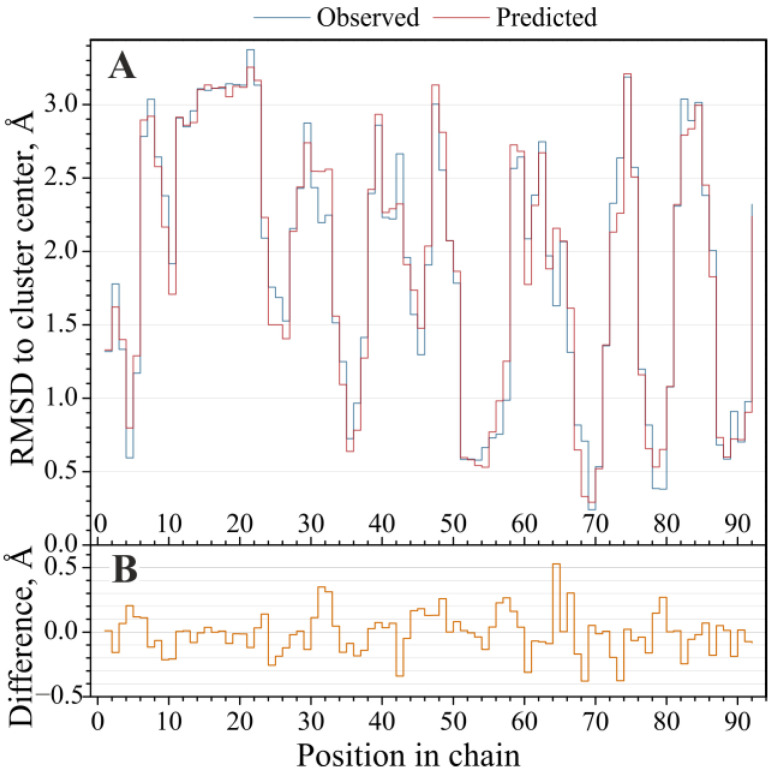
The observed and predicted RMSDs to the cluster center PB (‘d’). (**A**) The observed and predicted RMSD profiles from the five-residue fragments of the 1 gmp chain ‘B’ to the protein block (‘d’) cluster center. The observed and predicted distances are displayed for the central residue of a five-residue fragment. The Pearson correlation between the predicted and observed profiles is 0.95. (**B**) The difference between the observed and predicted RMSDs.

In order to reconstruct the Cartesian coordinates of a query for the protein backbone, it is crucial to predict the distances from the query peptides to the PB cluster centers as accurately as possible [16]. We examined, observed, and predicted the RMSD profiles for each PB class individually in all three datasets. For all the datasets and all the PB classes, our method achieved a Pearson’s correlation coefficient of ≳ 0.9 (*r* = 0.91 ± 0.02). The complete correlation table can be found in Supplemental Table S2. Figure 1 shows a representative example of the predicted and observed RMSD distances to the protein block ‘d’ and for each five-residue fragment (always shifted by one amino acid) in the displayed chain, illustrating substantial agreement between the observed data and our model’s output.

The correlation coefficients for the validation and test datasets are nearly identical (Supplemental Table S2) for all the PBs. The training dataset demonstrated higher correlation values, as it was used for neural network training. These consistent results indicate that the neural network yielded robust outcomes.

### 2.3. Improved Performance of the DSSP-Based Secondary Structure Prediction

Using the same feature set and network architecture, we retrained the network for the Q3 and Q8 classification tasks based on the DSSP classes. Table 1 shows the percentages of correct classifications for each task. The normalized Q3 and Q8 confusion matrices can be found in Supplemental Table S3.

For the Q3 and Q8 classification tasks on the CB513 dataset, our method outperformed the state-of-the-art secondary structure prediction methods reported in the literature (the values shown in Table 3 and Table 4 are as reported in the original articles [41,42,43]).

## 3. Discussion

In this study, we introduced a model for predicting protein local conformations in terms of protein blocks (PBs). Modifying the approach originally presented by de Brevern et al. [13,14], we used the same PBs as cluster centers but represented them using 3D coordinates rather than dihedral angles. When classifying query protein fragments, we also determined the closest PBs in terms of the RMSD (root mean square deviation) distance.

It is worth noting that within a single PB class, there can be significant structural variations. Therefore, when a fragment is assigned a single label (the closest PB), some information is inevitably lost. One way to circumvent this is to view the task as a regression rather than a classification problem, i.e., to retain the distances to all the cluster centers. When combined into a vector, these distances can be regarded as ‘structural coordinates’ that encapsulate more information about the 3D structure of the query protein fragment’s backbone than a single class label. Moreover, for any five-residue protein fragment, its Cartesian coordinates can be recovered from its structural coordinates with any accuracy if certain conditions are met (i.e., if there are enough basic structures that are sufficiently different from one another).

Further, our prediction method does not rely on evolutionary information and is independent of the availability of structural data for the homologous proteins. Particularly for the proteins in the CASP14 free modeling category (or artificial proteins), this is an advantage because none of the target proteins had a homologue with a resolved structure.

On the other hand, for protein chains involved in interactions with other protein chains, such as disulfide bridges or multiple interchain hydrogen bonding, predictions may be significantly distorted. For instance, for a class of proteins known as ‘small proteins’, their tertiary structure is typically maintained by disulfide bridges [52], metal ligands [53], and/or cofactors such as heme [54]. In such cases, a prediction model based solely on an amino acid sequence lacks the necessary information about the interactions between the protein chains or ligands, but an alignment-based method may greatly benefit from the existence of homologous proteins with resolved structures.

Overall, the application of neural network-based ML methods substantially improved the prediction accuracy compared to the traditional methods that we previously employed. For local conformation predictions in terms of protein blocks Q16, the accuracy increased from 67.9% [16] to 76.8%, and for the secondary structure prediction Q3, it rose from 81.3% [38] to 84.9%. To inspect our method’s performance in greater detail, we analyzed the confusion matrices for the following three classification tasks: Q16 (based on protein blocks), Q8, and Q3 (based on the DSSP classes).

### 3.1. Q16 Confusion Matrix

**Figure 2:**
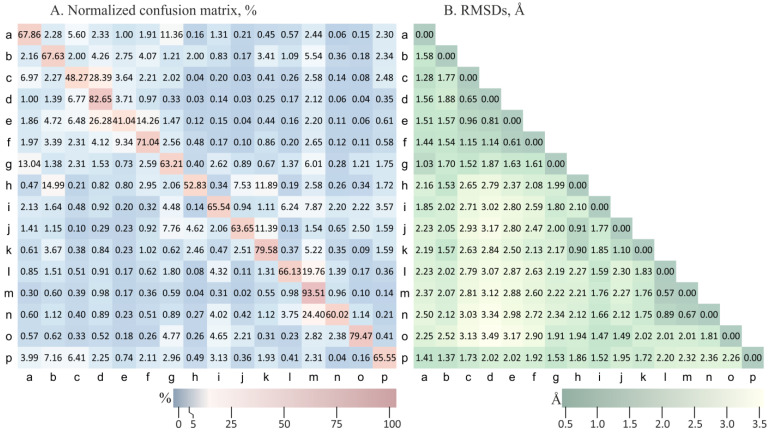
Local structure prediction performance analysis. (**A**) The normalized confusion matrix (in percentages) for the local structure by the PB prediction. The rows and columns represent the true and the predicted class assignments of a fragment corresponding to our model. The values reflect the percentages of the fragments of a given class that were predicted to have each of the possible 16 classes. For example, the value 2.28 in row ‘a’ and column ‘b’ is the percentage of true ‘a’ fragments that were classified as ‘b’ by our model. (**B**) The RMSDs (Å) between the protein block cluster centers.

Figure 2A reveals that many errors in the PB16 classification were associated with structurally similar classes. For example, for the PB ‘c’ (one of the variations of the N-caps of the β-sheet), with 48.3% correct predictions, 28.4% were predicted as structurally similar to PB ‘d’ (β-sheet). Indeed, these PBs had very close conformations based on the RMSD metric (RMSD (‘c’, ‘d’) = 0.647 Å, see Figure 2B). For such small RMSDs, the 3D structure recovery step would yield approximately the same Cartesian coordinates. This shows that when a misclassification falls within structurally similar classes, it does not prevent the accurate recovery of the Cartesian coordinates.

Misclassifications that occurred between relatively distant classes in the alphabet (e.g., for ‘a’ and ‘g’, 11.4% of ‘a’ predicted as ‘g’ and 13% of ‘g’ predicted as ‘a’) could also be explained by the proximity of these classes in terms of the RMSDs (RMSD (‘a’, ‘g’) = 1.035 Å). PB ‘a’ was the closest neighbor to PB ‘g’ and vice versa, although this is not the case for all the PB pairs (Figure 2B). For example, for the PB pair with the maximum RMSD (‘d’, ‘o’) = 3.494 Å, these PBs (corresponding to secondary structure classes C-caps of the α-helix and β-sheet) have few misclassifications between them.

### 3.2. Q8 Confusion Matrix

In the Q8 classification problem, the class that suffered from the most misclassifications was ‘B’ (β-bridge).

This class represents a single amino acid’s residue (a single pair of β-sheet hydrogen bond formations), which is determined by hydrogen bonds and allows for significant conformational variations in the protein chains. Unsurprisingly, for such a heterogeneous class, 50.8% of its misclassifications fell into the ‘coil’ class, and 27.62% were labeled as class ‘E’ (β-sheet). The rarely occurring class ‘I’ is structurally closer to the well-represented class ‘H’ than it is to ‘G’ or ‘T’, and class ‘G’ fell between ‘H’ and ‘T’, which aligned with the prediction data for these classes (Supplemental Table S3B).

### 3.3. Q3 Confusion Matrix

The table in Supplemental Table S3A is essentially a condensed version of the more comprehensive confusion matrix for Q8 (Supplemental Table S3B). As with the eight DSSP classes, for the three classes, predictions for class ‘H’ were more accurate than they were for the others. This could be explained by the α-helix’s more distinct (than other classes) structure, which is solely based on the main chain conformation, and so it could be effectively recognized. Errors in the predictions for class ‘E’ (β-sheet) and vice versa were minimal at 1.32% and 2.57%, respectively.

A slightly higher level of “leakage” occurred from class ‘H’ to ‘coil’, which was due to the presence of the structurally close class ‘T.’ Among the conformations assigned to the ‘coil’ class, a significant proportion closely resembles the classical β-sheet, but they lack specific β-sheet hydrogen bonds (e.g., the polyproline II helix). As a result, there were significant misclassifications between these classes, with 18.82% of ‘E’ predicted as ‘coil’ and 8.80% being the opposite.

### 3.4. Structure Alphabets and Their Applicability

A classification task can be substantially influenced by the choice of categories to which objects are classified. There are a number of structure classification schemes in the literature, with varying numbers and compositions of classes. The two class assignments we addressed here—PB-based and DSSP-based classes—are very similar for regular structures such as α-helix and β-sheet. The classical α-helix corresponds to the ‘H’ class in DSSP and the ‘m’ class in PB, and similarly for a β-sheet, it is ‘E’ in DSSP and ‘d’ in PB, respectively. For other classes, there are no clear one-to-one correspondences between PBs and the DSSP. This is because not all structures can be as precisely defined, and they may not always fit neatly into compact, homogeneous classes.

The rule of thumb for good partitioning is that intraclass similarity (between objects from different clusters) should be high and the interclass similarity (objects within the same class) should be low. In the case of short fragment structures, object similarity is reflected by the RMSD, and the lower the RMSD, the more similar two structures are. Since the PBs and the DSSP assignments were not designed to satisfy this criterion, introducing other structural alphabets could potentially improve the descriptive potential as well as the prediction accuracy of RMSD-based methods. It is important that the distance measure used when designing new alphabets is the same as the one used for labeling or building predictive algorithms afterwards. We suppose that the structural alphabets suitable for the 3D coordinate reconstruction of a protein backbone would contain more categories than the PBs and the DSSP assignments, and they would possibly be based on protein fragments larger than five residues.

### 3.5. Feature Selection Prior to Model Training

Our method involves the following two main steps: feature design and selection, followed by model training. As an established approach in classical machine learning, in training a neural network, it is less common to separate feature selection and model training. Here, doing so allowed us to train a shallow network and avoid fitting too many parameters, making our model faster to train and less prone to overfitting. Second, it increased the interpretability of the model and allowed us to evaluate each feature’s importance, which is a task that is usually less straightforward when dealing with deep learning models.

While leaving feature selection to the model or performing it separately can be debated, we considered that a more crucial step in our approach was feature design. We constructed features based on the physicochemical properties of the amino acids, the occurrence statistics of the structural elements corresponding to the same fixed sequence, and the various hypotheses about the factors that influence protein structure (for instance, the periodicity of hydrophobic amino acids). Attempting to access as many factors as possible that influence the local protein structure, we developed a method that streamlined feature generation on a large scale. For instance, it designed features with the different periodicities of hydrophobic acids, and for features based on occurrence statistics, it attempted different regular expressions to group sequences of proteins with resolved structures.

### 3.6. Recovering Tertiary Structure

Recovering the tertiary structure of a protein directly from alternative structural coordinates can be challenging, especially for long heterogeneous protein fragments where small deviations in each part may accumulate and lead to substantial errors in the final tertiary structure. To restore the tertiary structure effectively, an algorithm that involves scanning a database of resolved protein structures to identify conformations that closely resemble the predicted extended fragment would be needed. Particularly, various advanced sequence alignment methods could be applied to protein block sequences rather than to amino acid sequences. However, it is important to note that the accuracy of such a recovery can depend on the availability of data and the quality of similar structures in a database. For complex and unique protein structures, specialized methods and algorithms may yet be required.

## 4. Methods and Materials

### 4.1. Dataset Preparation

The PISCES30 dataset [40] is a curated subset of the Protein Data Bank (PDB) [6], stratified by the resolution of resolved structures and sequence similarity. Here, we applied a resolution cutoff of 2.5 Å and an R-factor cutoff of 1.0, and we allowed a sequence identity of up to 30%. This led to the selection of 17,148 protein chains, all of which had their structures resolved through X-ray methods (i.e., no NMR-resolved structures).

The CB513 dataset [39] was originally designed by Cuff and Barton to evaluate secondary structure prediction methods, and it remains a widely used benchmark. The dataset consists of 513 nonhomologous protein domains accounting for 438 protein chains in total (some chains contain two or more domains).

The CASP14 dataset consists of 10 proteins from the free modeling category of the CASP14 contest [30]. Importantly, the proteins in this dataset do not have homologues with resolved structures, making this dataset an important benchmarking tool.

### 4.2. Feature Design and Selection

Dataset composition and feature design is a crucial step not only for classic ML model development but also for many deep learning methods. A curated feature set has the following two main characteristics: it is comprehensive, which enables learning, and it does not contain too many redundant predictors, which makes the learning more effective.

In this work, we used 5-residue long protein fragments, but the design principles described below can be applied to a protein fragment of any size. The feature design involved two conceptually different strategies. The first type of feature was based on the physicochemical properties of the amino acids acquired from the AAindex database [27]. The initial feature set was composed by applying a number of functional transformations of AAindex parameters, such as scales of hydrophobicity, flexibilities of amino acid residues, solvation free energy, etc. The following example illustrates feature generation: assume there is a sequence fragment of *n* residues {a_1_, …, a*_n_*}. Each amino acid {a*_i_*, *i* = 1, …, *n*} has a certain value of hydrophobicity {*H_i_*, *i* = 1, …, *n*}. Further, a functional transformation *F*(*H*_1_, …, *H_n_*) is applied to the array of property values {*H*_1_, …,*H_n_*}. In this particular example, *F*(*H*_1_, …, *H_n_*) reflects a periodic change in hydrophobicity with period *T* in a given fragment of *n* residues:

(3) $$F\left(H_{1},\ldots ,H_{n}\right)=\sqrt{{\left(\sum_{k=1}^{n}H_{k}\cos\left(k\frac{2\pi }{T}\right)\right)}^{2}+{\left(\sum_{k=1}^{n}H_{k}\sin\left(k\frac{2\pi }{T}\right)\right)}^{2}}$$

which reaches its maximum when the hydrophobicity of the amino acids along the backbone chain varies harmoniously with the *T* period and thus captures the similarity between the query structure and the standard α-helix periodicity of 3.6 with high precision. The complete features generation procedure involves various transformations *F*(). Initially we created more than 10,000 initial predictors, which were substantially decreased by using the stepwise regression analysis. Details can be found in Supplemental Methods S5 as well as in our previous works [16,38]. The C++ implementation of the method is available at Github (https://github.com/Milchevskiy/ML.Proteins.2023, https://github.com/Milchevskiy/protein-encoding-projects (accessed on 26 October 2023)).

To generate predictors based on the resolved structures’ statistics, we split all the resolved structures into classes of 5-residue fragments with the same amino acid sequence. For each structural element in a given class, we calculated the RMSD to each of the 16 PBs, and we recorded the means and standard deviations of such distances within a class. Then, different characteristics of the distributions could be calculated, for instance, the first and second momentums. A detailed description of the algorithm can also be found in our previous work [38].

Having generated an extensive pool of putatively predictive features, we applied a stepwise regression analysis to select statistically significant features for each of the 16 regression tasks individually. Features with F-statistics below a specified threshold were excluded. We then selected predictors with F-statistics greater than 100, ranked them by descending F-statistic values, and removed duplicates. Subsequently, we examined the Pearson correlations between the included predictors. For pairs with Pearson correlation coefficients exceeding 0.9, we kept the feature with the higher F-statistic. This process resulted in a set of 76 features associated with a single 5-residue fragment (see Supplemental Table S4 for the full list and the Supplemental Methods S5 for the full description of all possible features and their parameters).

To account for a possible interaction effect from flanking amino acids, we extended our view field to 9 residues, which included the 5 original residues and the 2 before and the 2 after the pentapeptide. This resulted in four additional fragments: a fragment shifted by one residue to the left, two residues to the left, one residue to the right, and two residues to the right from the target. For each of the additional fragments, we included the same 76 features. This led to the final set of 380 (76 × 5) features, which were then used as inputs to the neural network.

### 4.3. Neural Network Architecture and Training

We trained, tested, and validated the model using the PISCES30 dataset. First, the protein chains from the dataset were split into training, test, and validation sets in the following ratio: 6:3:1. By sampling complete chains instead of 5-residue fragments, we ensured that overlapping fragments could only appear in the same dataset, but no two fragments from different datasets would have an overlap. Then, for each 5-residue fragment, we calculated the 380 features described above, and we used them as inputs to the neural network.

For the local structure prediction of the 16 PBs, we utilized a multioutput regression neural network with four hidden layers, and we used the standard mean squared error loss between the predicted and observed RMSDs, the sigmoid (nn.Sigmoid()) as the activation function, and kaiming_uniform_() for the initialization of the weights. The input and output layers’ sizes were determined by the predictor set (380 nodes) and the number of PB classes (16), respectively. The model was implemented in PyTorch 1.12.1+cu116 [55] using Python 3.8 as well as the pandas 1.5.1 and numpy 1.22.3 libraries.

For the 3- and 8-group classification tasks based on the DSSP assignments, we utilized the same network architecture as above but adjusted the output layer dimensions. By using one-hot encoding, we converted a multioutput regression into a classification task, e.g., (1, 0, 0, 0, 0, 0, 0, 0) corresponded to the first class ‘H’ in the DSSP-8 classification list. In other words, the resulting class label corresponded to the DSSP class that was predicted to have the smallest RMSD to the query protein fragment.

In both cases, we used the torch.optim.Adam() optimizer with an initial learning rate of 10^−2^. We gradually reduced the learning rate by a factor of five whenever the optimizer’s improvement stopped, ultimately reaching a final learning rate of 10^−5^. Even despite the differences in the sizes of the test and validation sets, we observed a high similarity between the performance metrics of our model—for both the classification error and confusion rates for the individual clusters—which indicated the reproducibility of our results. The training and prediction scripts and the prediction model are available on GitHub (https://github.com/Milchevskiy/ML.Proteins.2023 (accessed on 26 October 2023).

### 4.4. Measures for the Evaluation of the Model’s Performance

In this work, we applied the most widely used measure to evaluate the local and secondary protein structure prediction performance, namely, the Q measure, which was defined as the percentage of residues predicted correctly. This measure was formulated in [56], and for three DSSP classes, it can be written as follows:

(4) $$Q3=\sum_{S\in \{C,H,E\}}\frac{O_{+}(S)}{N}$$

where *O*_+_(*S*) is the observed number of correctly predicted residues in the class *S*, and *N* is the total number of residues in the query protein. This measure does not depend on the number of prediction classes, and so it can be used for 3-classes, 8-classes, and 16-classes (protein blocks).

Confusion matrices [57] can supply extended information about the interrelationships of predictions for different classes of secondary structures or PBs. Each row of the confusion matrix represents the instances in an actual class, while each column represents the instances in a predicted class. Thus, we can analyze the mispredictions, i.e., how the prediction model confuse two classes (mislabeled one as another).

## 5. Conclusions

The method we presented here predicts local and secondary protein structures from sequences. We observed that combining an extensive feature design and selection procedure with a shallow neural network architecture yielded results that outperformed the other local or secondary structure prediction methods reported in the literature. An analysis of the CASP14 results revealed that at some targets from the free modeling classification, our set of statistically significant features with a nonintricate neural network can outperform other local structure prediction methods, even such powerful methods as AlphaFold.

Another contribution to the method came from the choice of distance measure between the structural fragments. The RMSD-based distance appeared to allow for the 3D coordinate reconstruction of a query fragment. Lastly, since our method does not rely on structural alignment, it is especially beneficial for synthetic proteins or proteins that do not have homologues with resolved structures. There are several paths that could further improve the model’s accuracy. A different set of basic structures could be beneficial for the classification tasks, as it is important that the central structures are sufficiently different from one another in terms of their RMSDs. For the efficient and accurate reconstruction of 3D coordinates, a larger set of basic structures or even the consideration of fragments of larger sizes appears to be promising. We are currently addressing these challenges.

## Figures and Tables

**Table 1:** Q16, Q8, and Q3 classification accuracies for the datasets with known Cartesian coordinates.

Dataset	Q16, %	Q8, %	Q3, %	Dataset Size, Residues
Validation	76.75	78.15	84.93	357,338
Test	76.53	77.91	84.81	1,155,341
Train	79.37	80.27	86.42	2,334,579
CB513	81.01	79.35	85.99	102,719
CASP14	76.35	76.11	83.51	2110

The table shows the percentages of correct classifications for the Q16, Q8, and Q3 tasks. The CASP14 subset included free modeling targets only. The training, test, and validation subsets of the PISCES30 dataset were designed by randomly sampling protein chains with the 6:3:1 ratio, correspondingly. The test set was used for hyperparameter optimization, and the validation set was only used to evaluate the performance of the final model. The last column reflects the total number of five-residue fragments with known Cartesian coordinates in each dataset. As each PB is a pentapeptide with known Cartesian coordinates, we excluded two amino acids’ residues at both the N-end and the C-end. The same approach was applied to flank all the amino acids’ residues without known Cartesian coordinates, and so two residues to the left and two to the right were excluded for each residue/chain with the unknown Cartesian coordinates.

**Table 2:** Benchmarking the Q16 classification on the CASP14 dataset.

PDB ID and Chain ID (Last Letter)	Query Length	Our Model	PYTHIA (Balanced)	LOCUSTRA
6UF2A	125	71.9	69.4	47.7
6XC0C	105	84.7	61.1	59.5
6Y4FA	141	72.3	57.7	48.2
6YA2A	199	66.9	47.8	45.5
6ZYCA	148	63.9	68.1	57.0
7D2OA	174	68.9	42.1	30.4
7JTLA	107	73.4	43.6	37.6
7K7WA	197	65.2	69.2	56.4
7M7AA	590	83.1	69.6	64.5
7M7AB	590	84.3	70.5	66.5
Mean	–	73.5	59.9	51.3

The table shows the Q16 classification accuracy (% of correctly classified fragments) on the CASP14 free modeling targets for our method, the PYTHIA model (balanced accuracy), and the LOCUSTRA method. The last row (‘Mean’) presents the prediction accuracies averaged across all 10 targets, where each target was weighted equally (independent of the length of the protein chain). This was performed to ensure our results were comparable to those reported for PYTHIA and LOCUSTRA.

**Table 3:** CB513 Q3 secondary structure prediction method benchmarks.

Method	FSVM [44]	GApred [41]	Jpred4 [45]	MUFold-SS [46]	SPIDER3 [47]	SSpro5 [48]	Our Method
Q3, %	83.0	85.3	65.6	85.1	84.5	82.0	86.0

**Table 4:** CB513 Q8 secondary structure prediction method benchmarks.

Method	BLSTM [47]	DCRNN2 ^a^ [49]	DeepCNF [50]	eCRRNN ^a^ [42]	MUFold-SS [46]	NCCNN ^a^ [51]	Our Method
Q8, %	67.4	70.4	68.3	74.0	72.4	71.4	79.4

‘^a^’ index indicates employment of the so called ‘ensemble model’ described in the original article [42]

## Data Availability

The source codes of neural networks applied in this work and final learning models are available at https://github.com/Milchevskiy/ML.Proteins.2023 (accessed on 26 October 2023).

## Supplementary Materials

The supporting information can be downloaded at: https://www.mdpi.com/article/10.3390/ijms242115656/s1.
