# Supplementary material for: Effective Local and Secondary Protein Structure Prediction by Combining a Neural Network-Based Approach with Extensive Feature Design and Selection without Reliance on Evolutionary Information
Source: Int J Mol Sci. 2023 Oct 27;24(21):15656. doi: 10.3390/ijms242115656 (PMC10648199; doi:10.3390/ijms242115656)
Supplement: Supplementary file 1 [file ijms-24-15656-s001.zip › Figure S1 caption.pdf]

Figure S1. Observed and predicted RMSDs to protein blocks cluster centers for CASP14 targets T1027 7D2OA, T1029 6UF2A and T1049 6Y4FA for AlphaFold and our method.
