# Supplementary material for: Effective Local and Secondary Protein Structure Prediction by Combining a Neural Network-Based Approach with Extensive Feature Design and Selection without Reliance on Evolutionary Information
Source: Int J Mol Sci. 2023 Oct 27;24(21):15656. doi: 10.3390/ijms242115656 (PMC10648199; doi:10.3390/ijms242115656)
Supplement: Supplementary file 1 [file ijms-24-15656-s001.zip › Figure S1.T1027.7D2OA.pdf]

# T 1027   7D2OA  PB 'a':  N-cap β

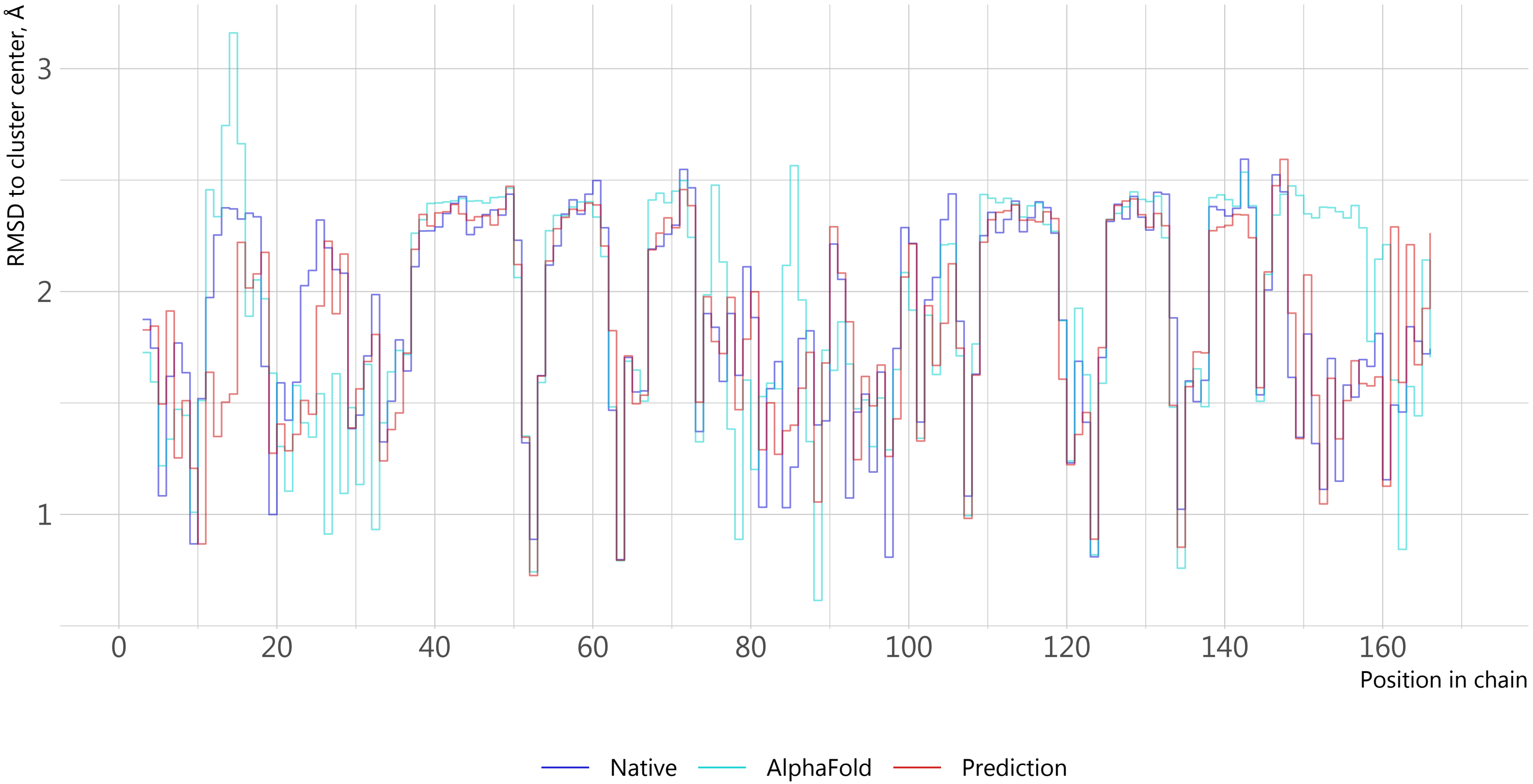


Corr(Native,AlphaFold) = 0.6549
Corr(Native, Prediction) = 0.8535

# T 1027   7D2OA  PB 'b':  N-cap β

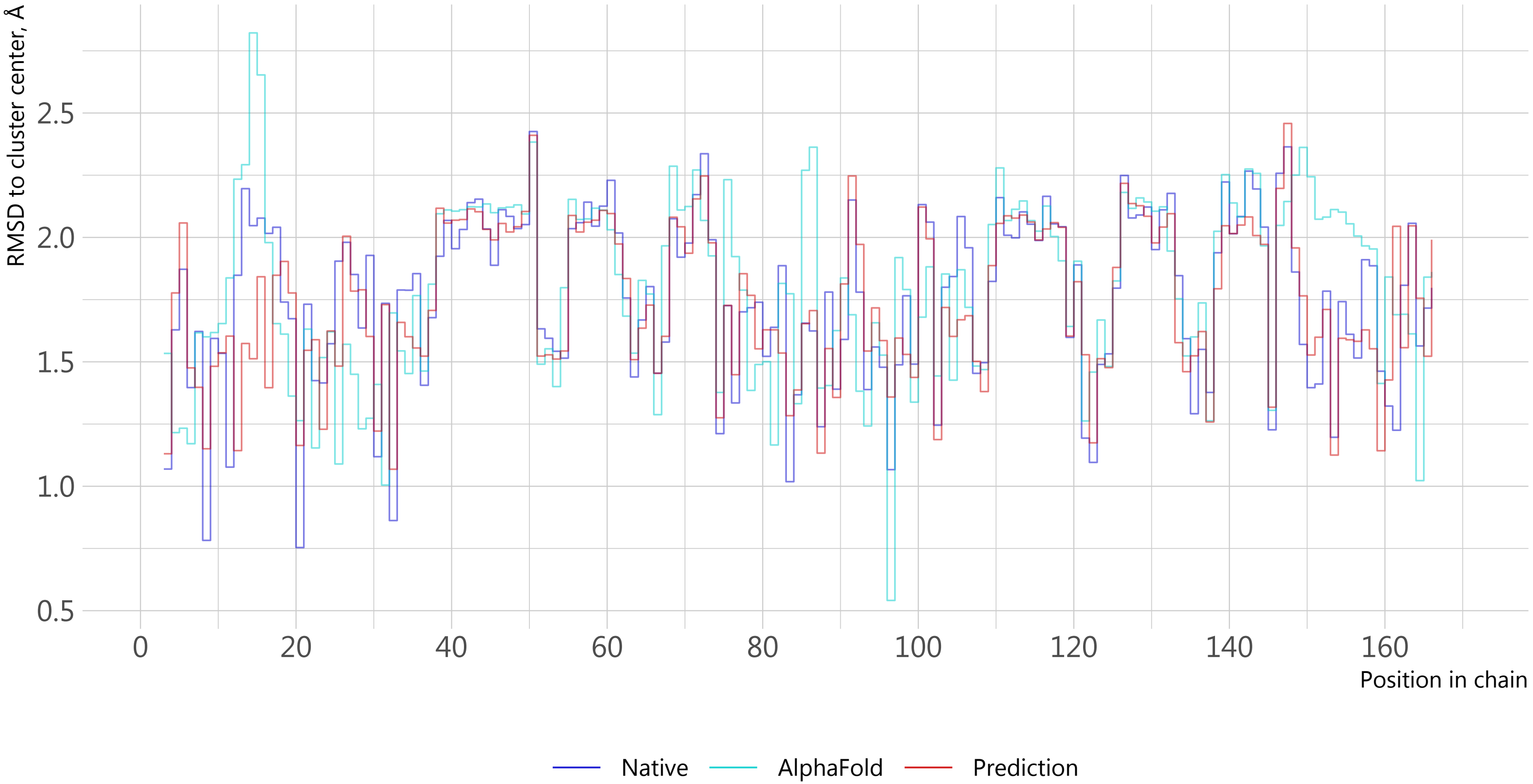


Corr(Native,AlphaFold) = 0.5877
Corr(Native, Prediction) = 0.8308

# T 1027   7D2OA  PB 'c':  N-cap β

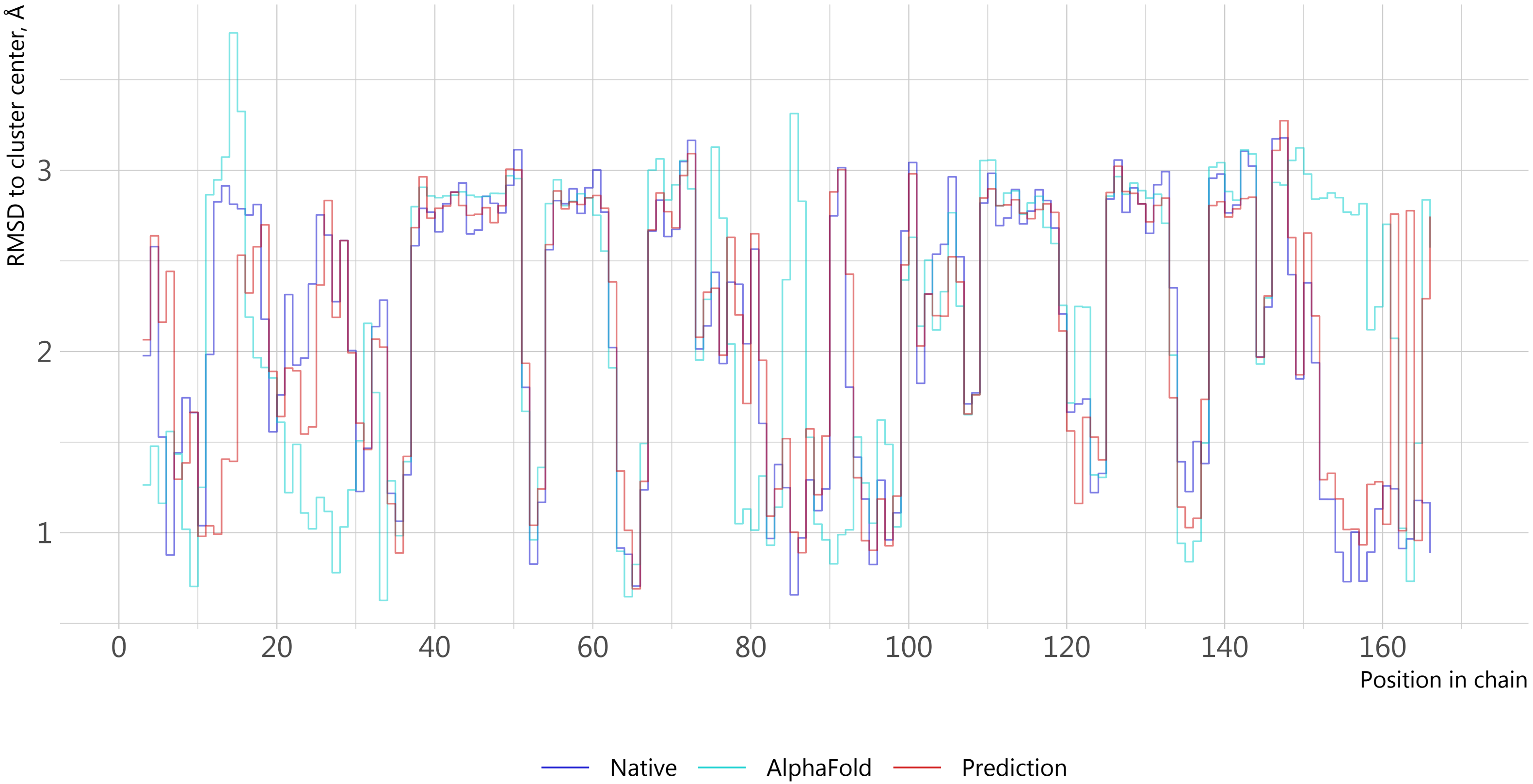


Corr(Native,AlphaFold) = 0.5493
Corr(Native, Prediction) = 0.8396

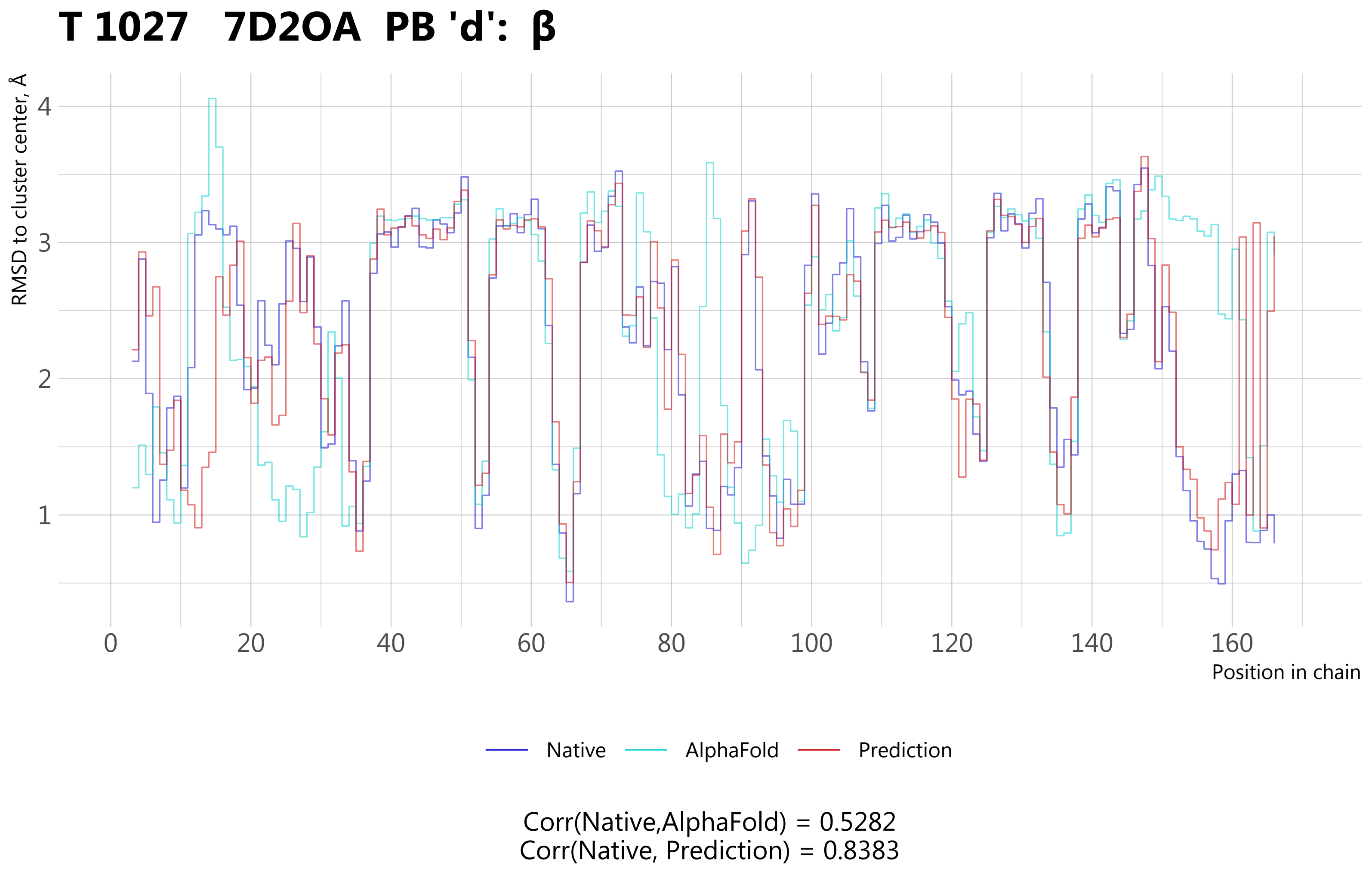

T 1027 7D2OA PB 'd': β
RMSD to cluster center, Å
4
3
2
1
0
20
40
60
80
100
120
140
160
Position in chain
Native
AlphaFold
Prediction
Corr(Native,AlphaFold) = 0.5282
Corr(Native, Prediction) = 0.8383

# T 1027   7D2OA  PB 'e':  C-cap β

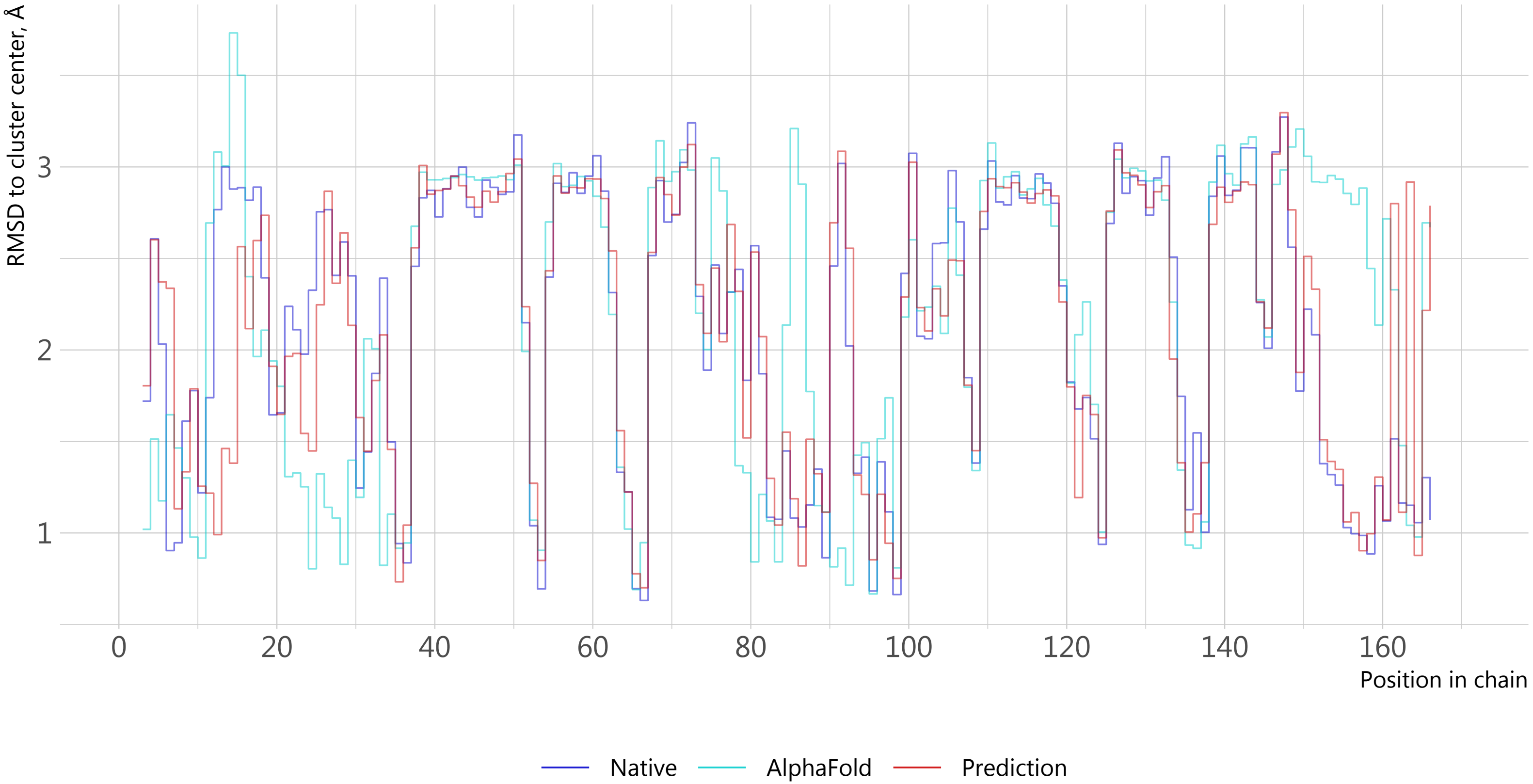


Corr(Native,AlphaFold) = 0.5937
Corr(Native, Prediction) = 0.8651

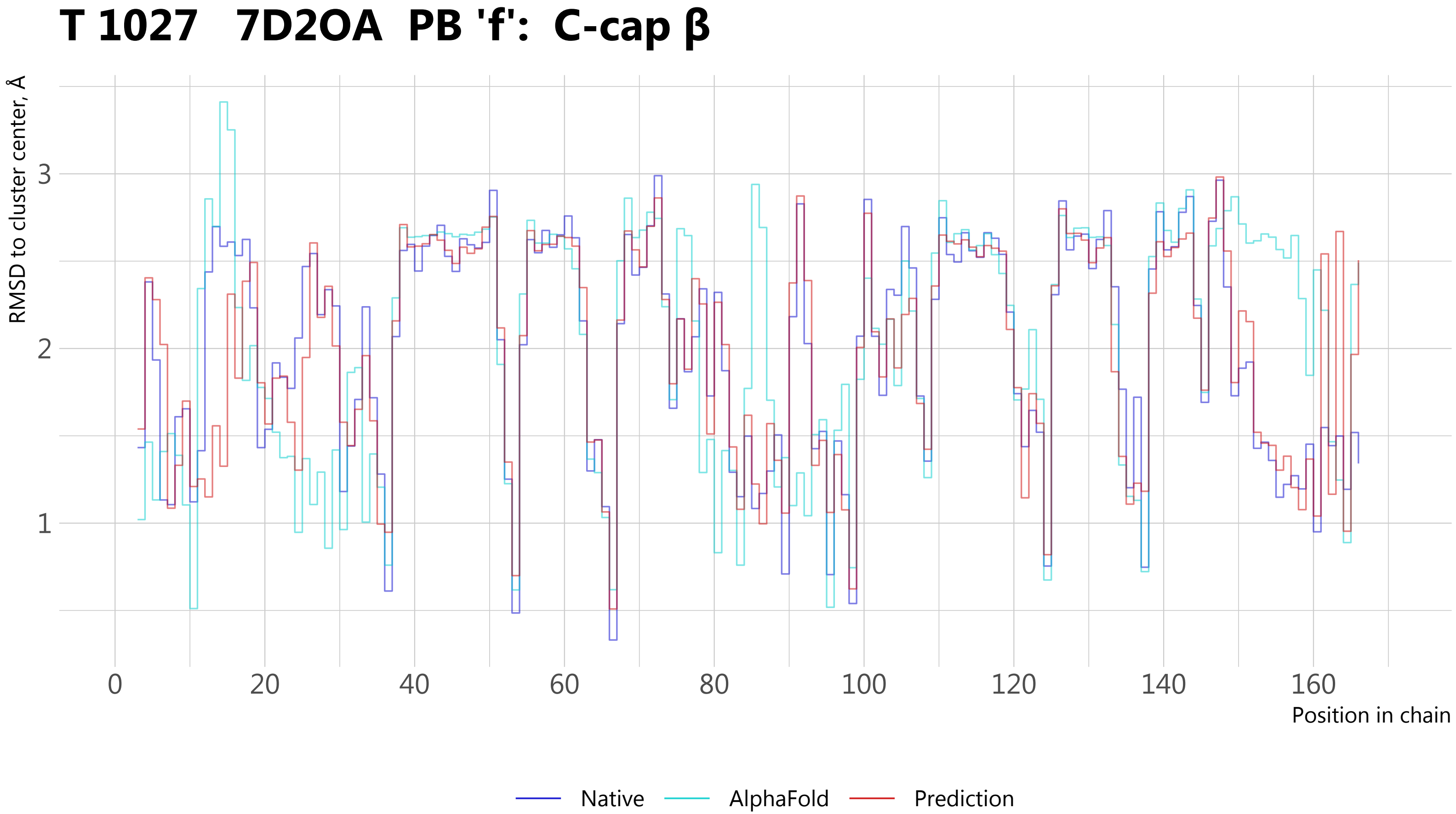


Corr(Native,AlphaFold) = 0.6274
Corr(Native, Prediction) = 0.8804

# T 1027   7D2OA  PB 'g':  mainly coil

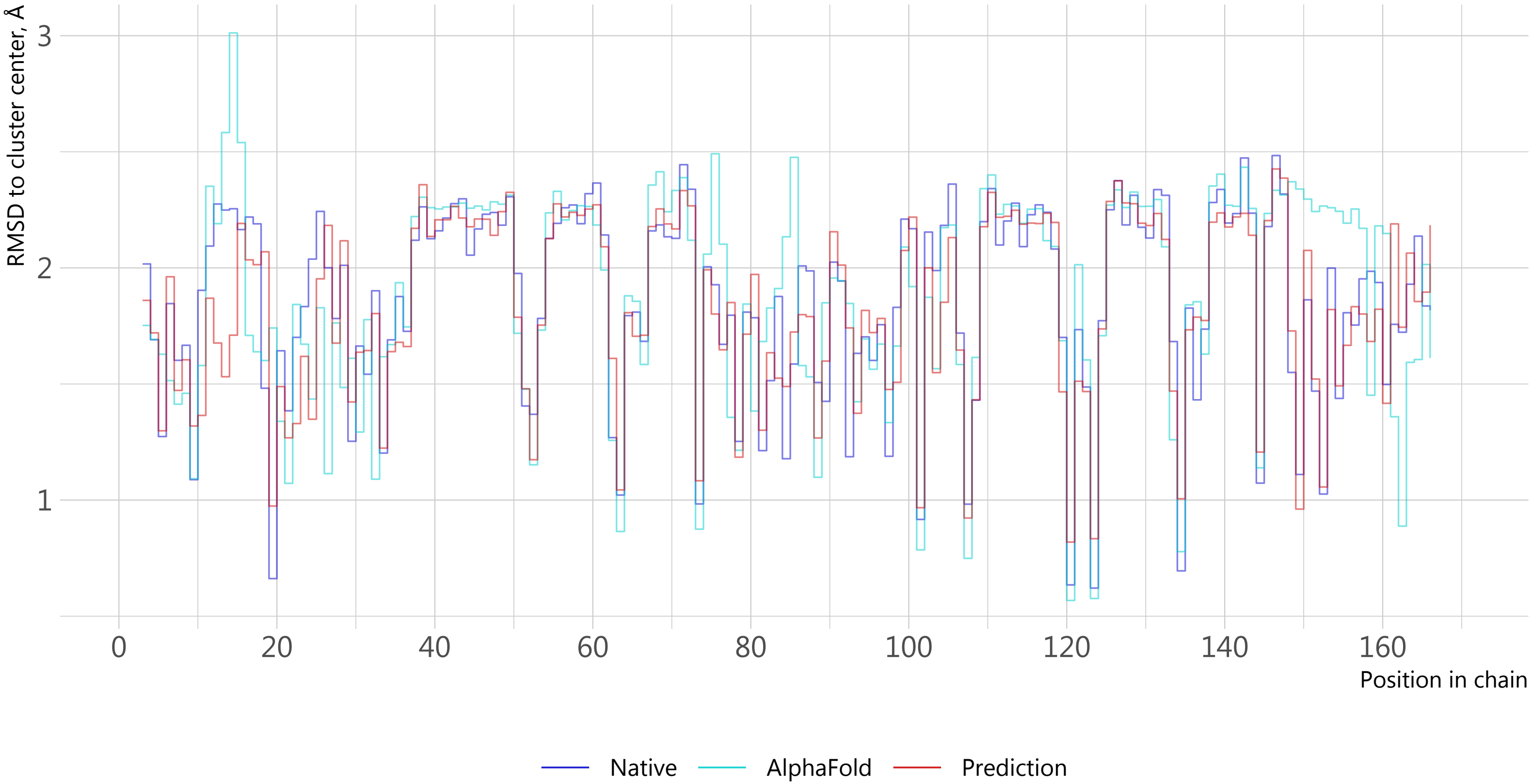


Corr(Native,AlphaFold) = 0.6904
Corr(Native, Prediction) = 0.8854

# T 1027 7D2OA PB 'h': mainly coil

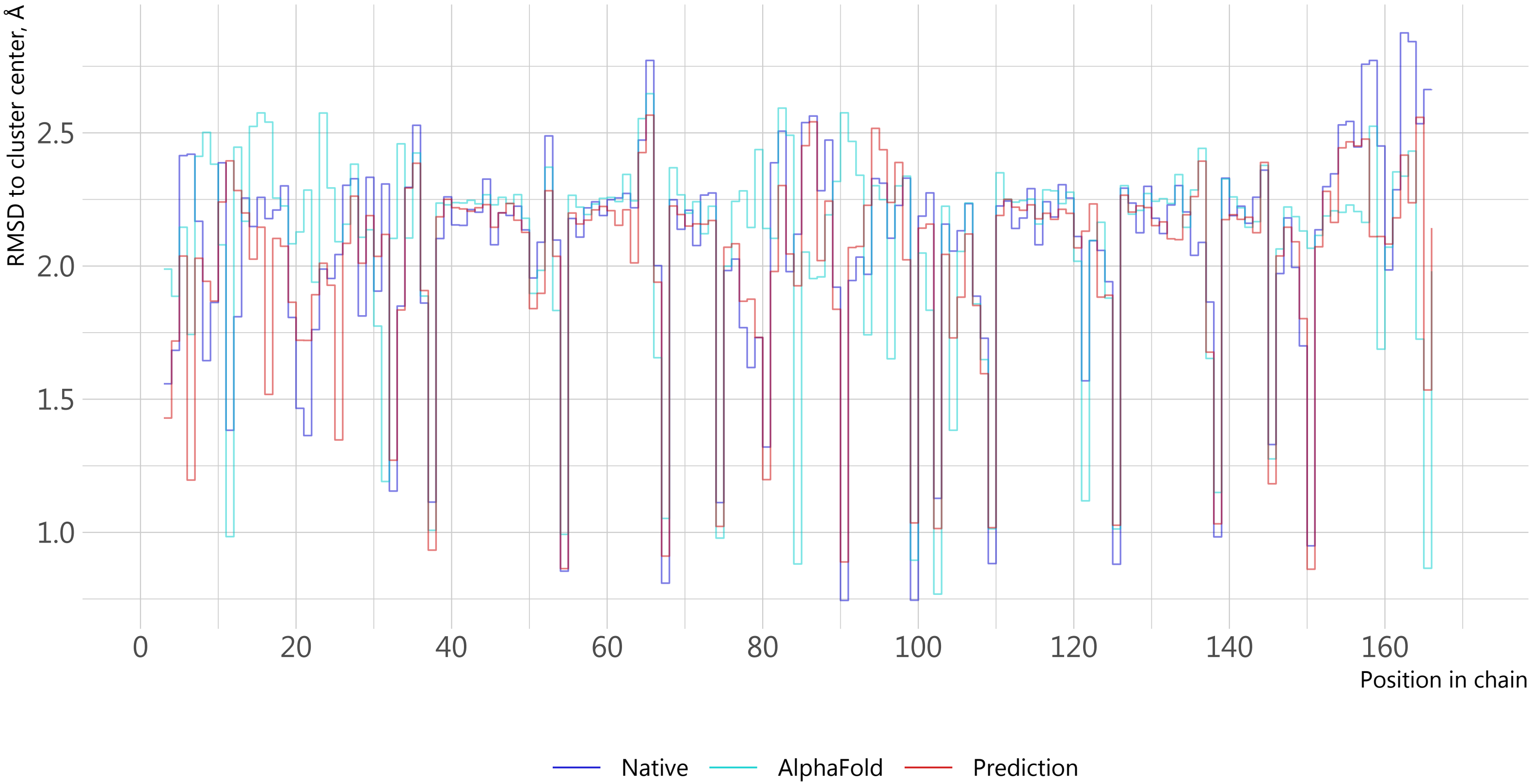


Corr(Native,AlphaFold) = 0.5301
Corr(Native, Prediction) = 0.8280

# T 1027 7D2OA PB 'i': mainly coil

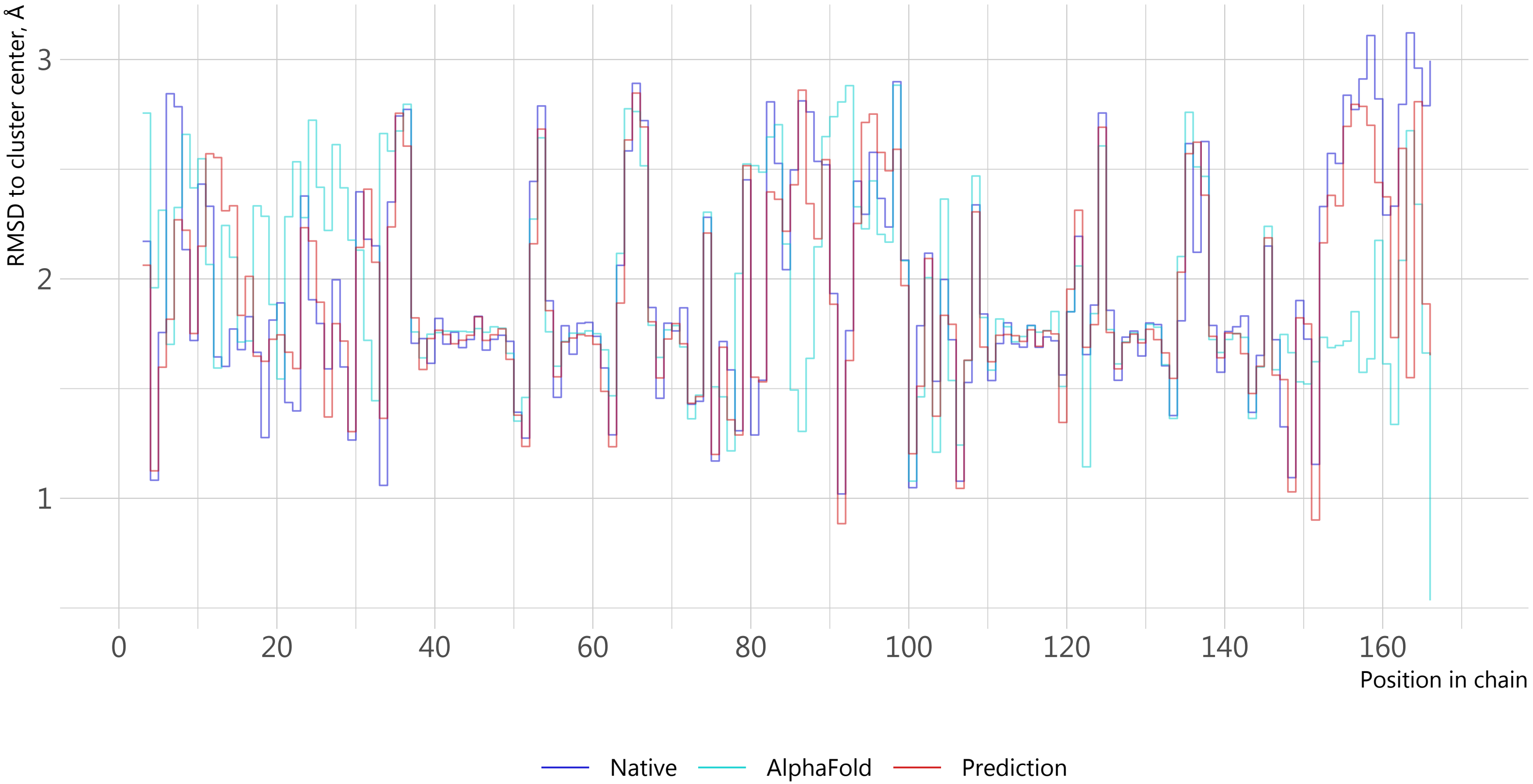


Corr(Native,AlphaFold) = 0.3415
Corr(Native, Prediction) = 0.8432

# T 1027   7D2OA  PB 'j':  mainly coil

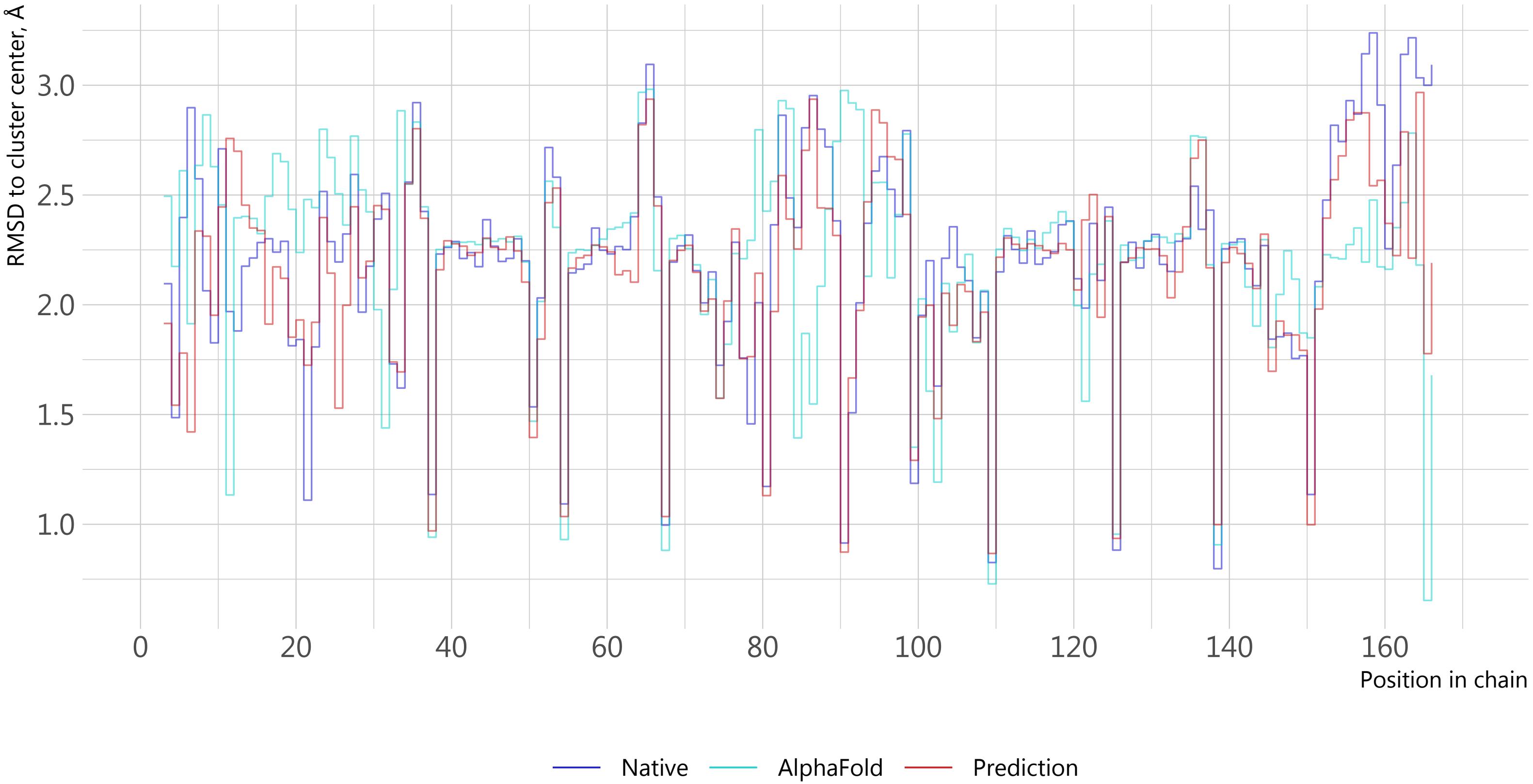


Corr(Native,AlphaFold) = 0.3997
Corr(Native, Prediction) = 0.8307

# T 1027 7D2OA PB 'k': N-cap α

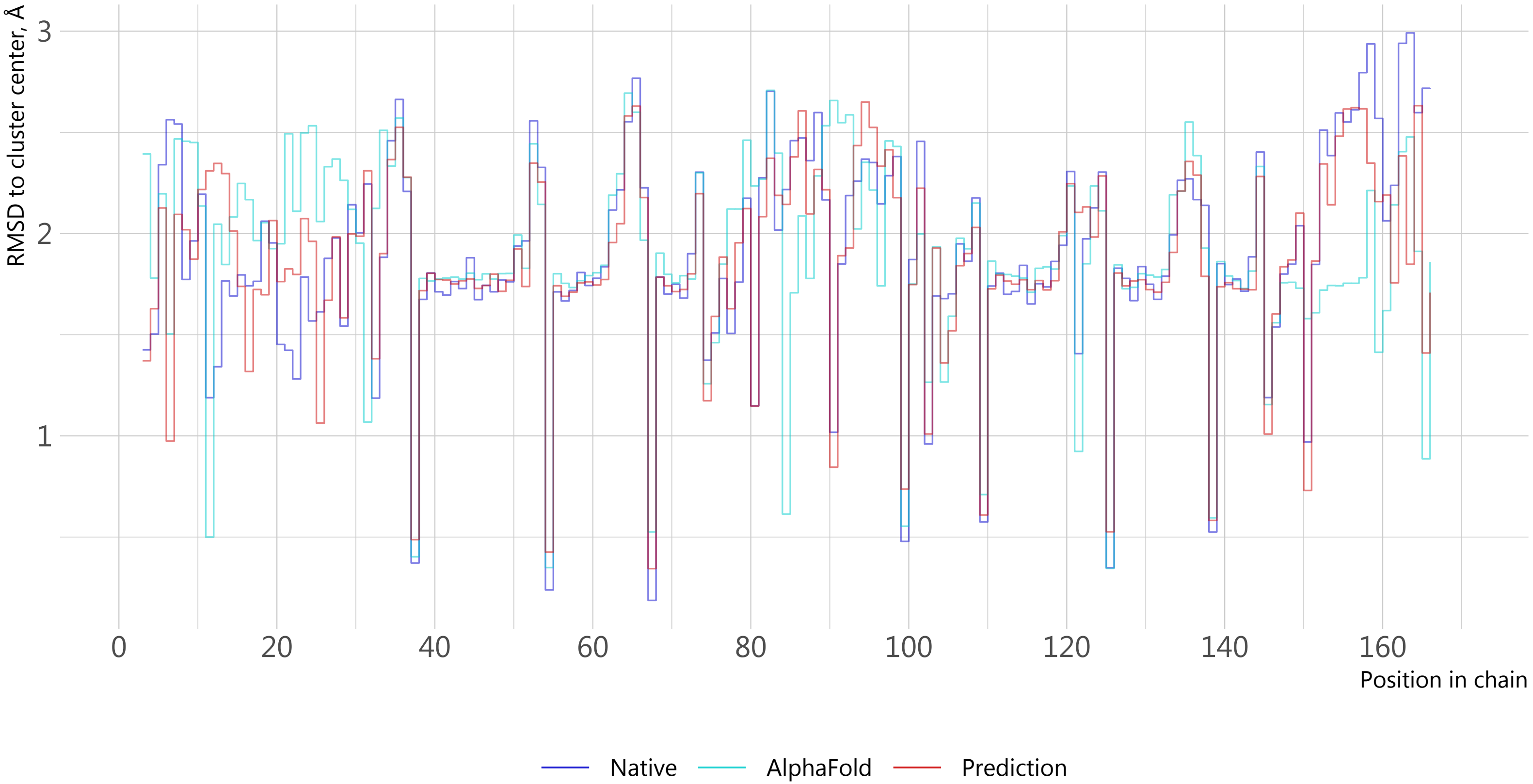


Corr(Native,AlphaFold) = 0.5688
Corr(Native, Prediction) = 0.8173

# T 1027 7D2OA PB 'l': N-cap α

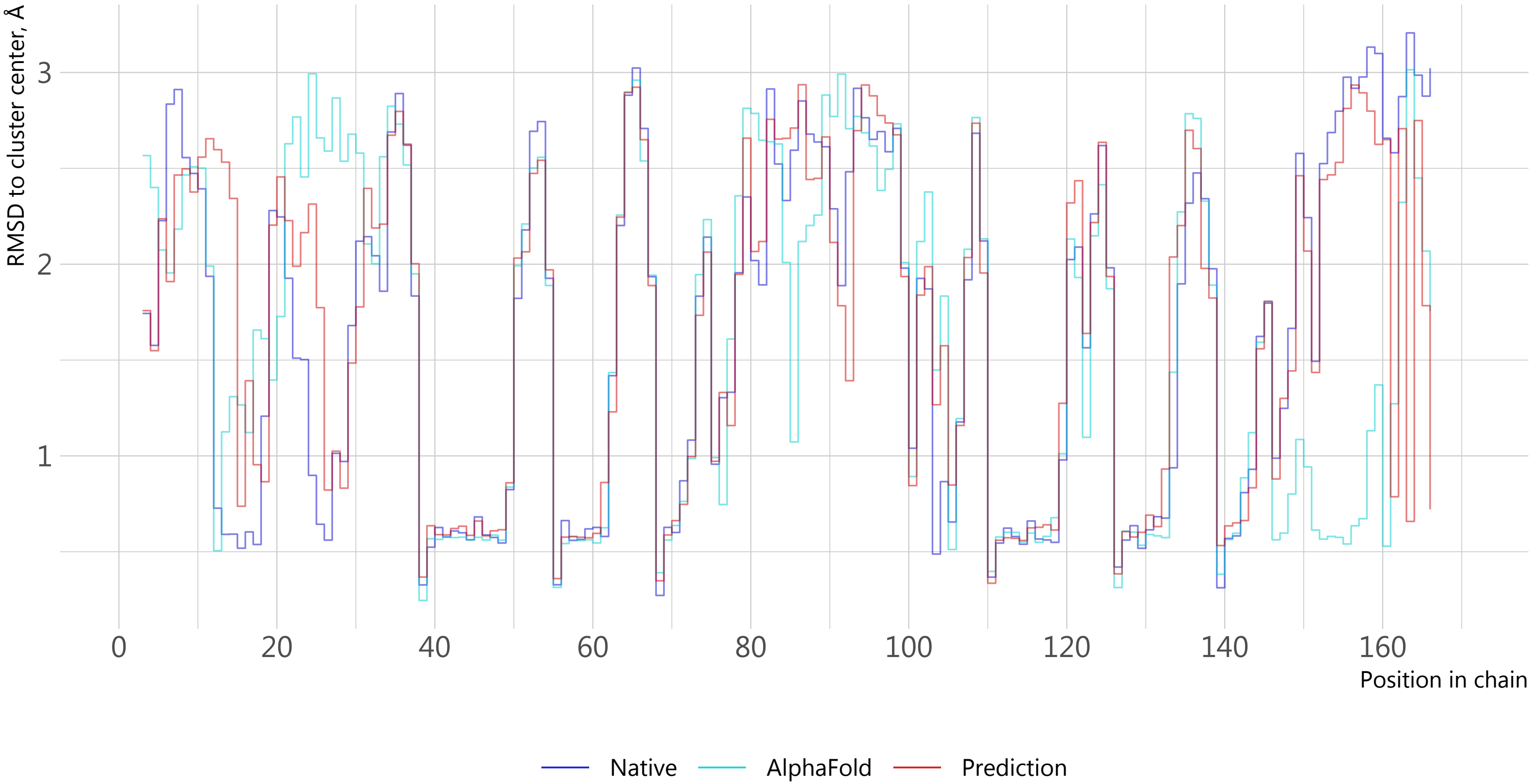


Corr(Native,AlphaFold) = 0.6461
Corr(Native, Prediction) = 0.8420

# T 1027   7D2OA  PB 'm':  α

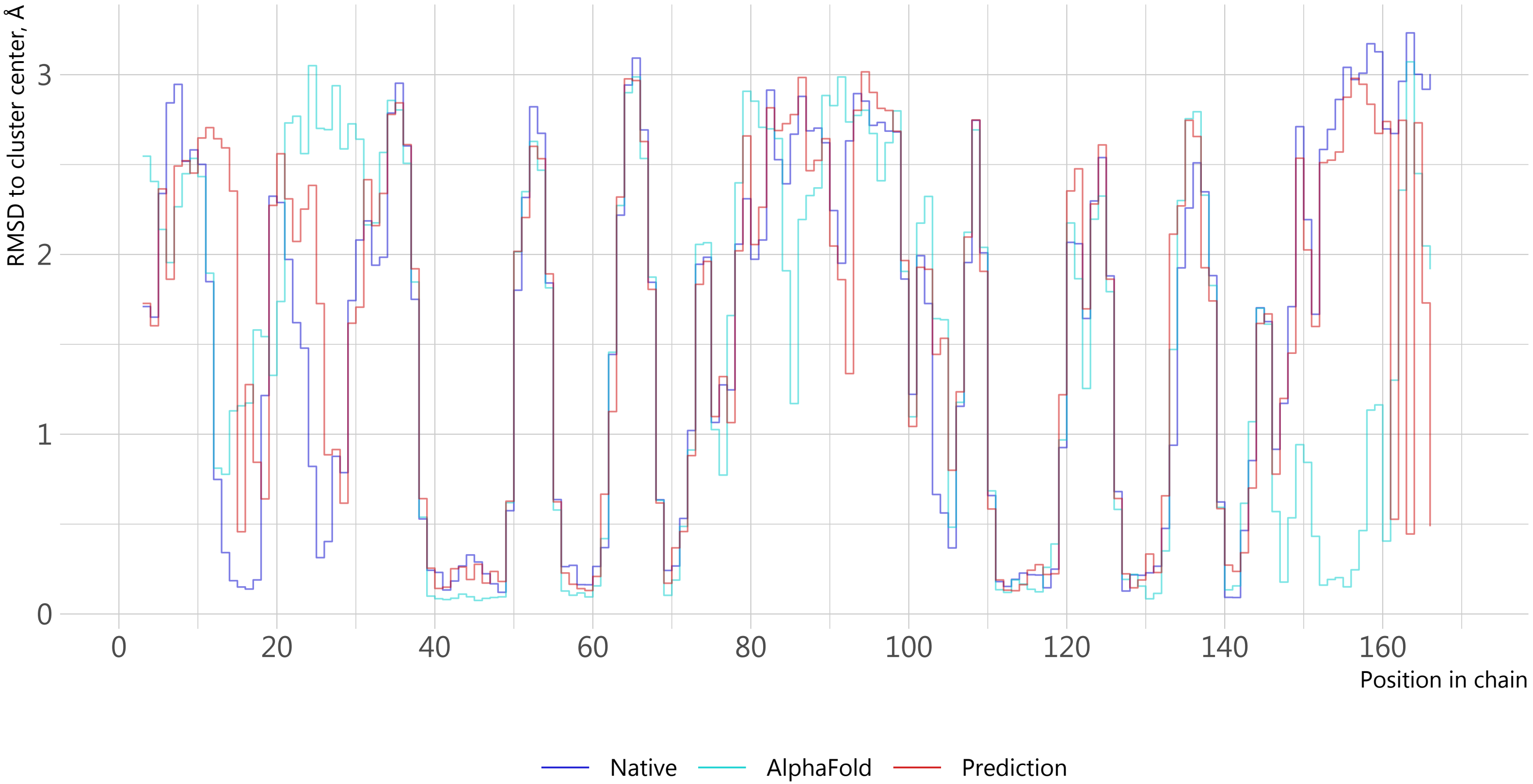


Corr(Native,AlphaFold) = 0.6627
Corr(Native, Prediction) = 0.8451

# T 1027   7D2OA  PB 'n':  C-cap α

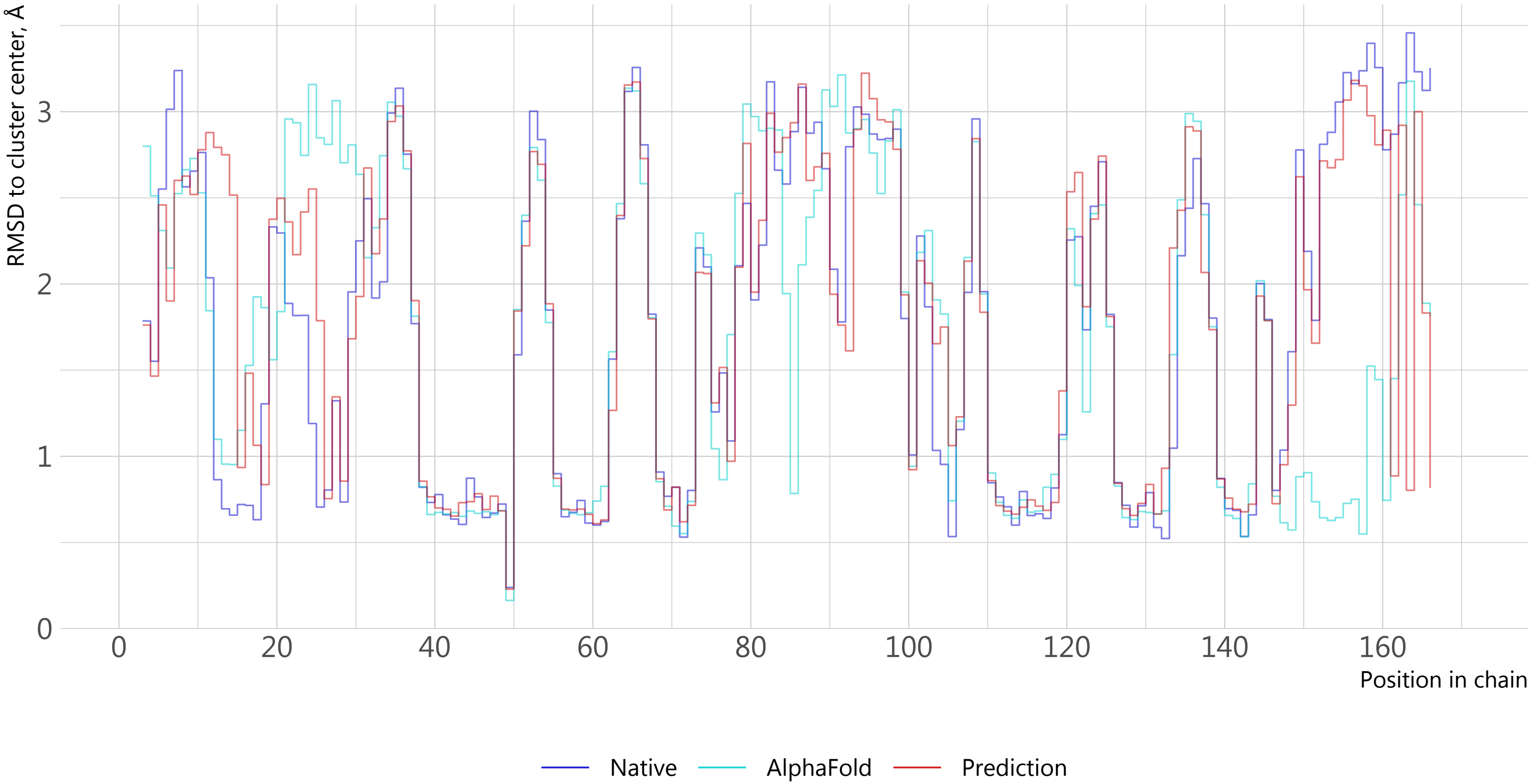


Corr(Native,AlphaFold) = 0.6210
Corr(Native, Prediction) = 0.8385

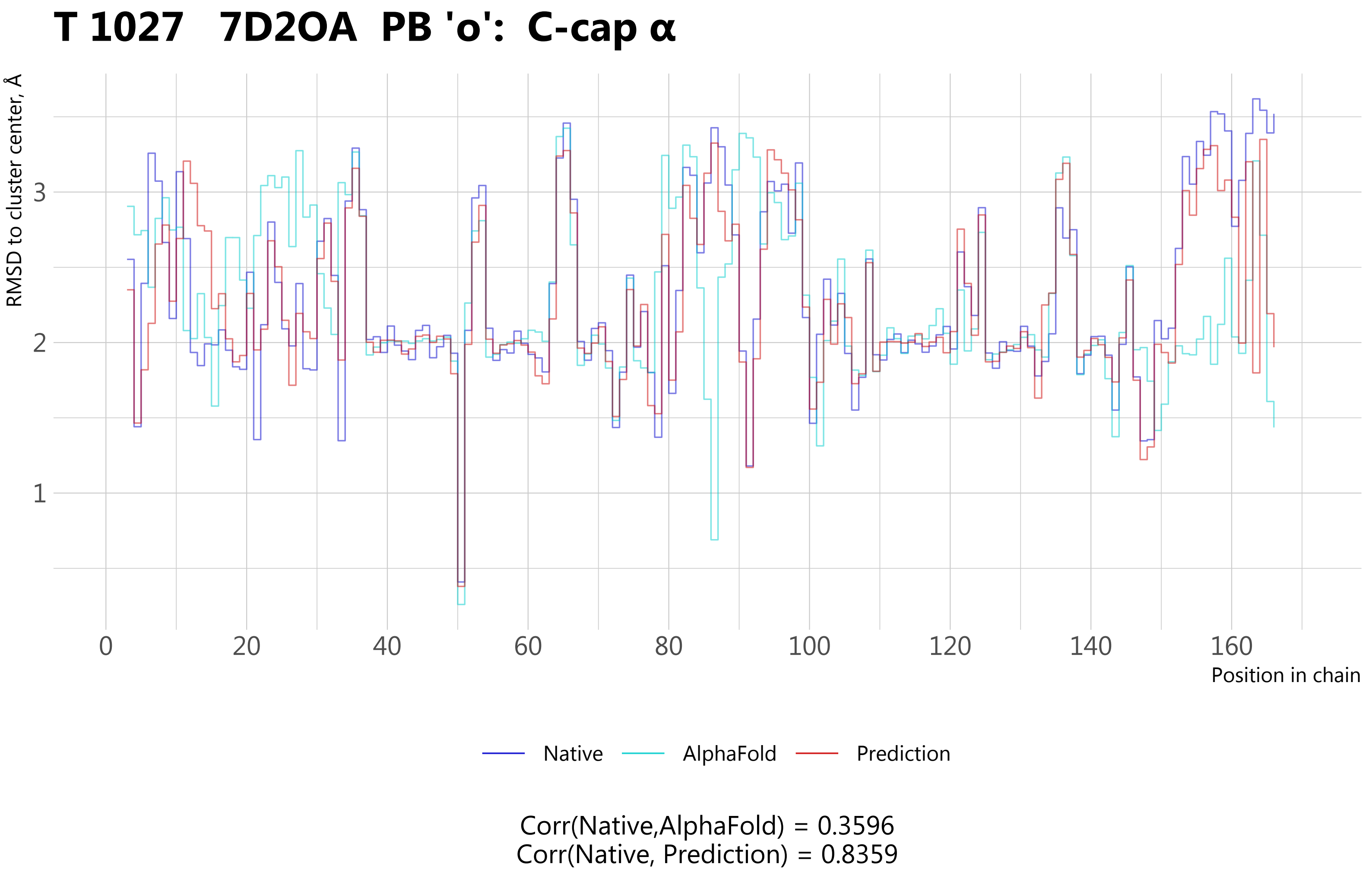
T 1027 7D2OA PB 'o': C-cap α
RMSD to cluster center, Å
Position in chain
Native
AlphaFold
Prediction
Corr(Native,AlphaFold) = 0.3596
Corr(Native, Prediction) = 0.8359

# T 1027   7D2OA  PB 'p':  C-cap α to N-cap β

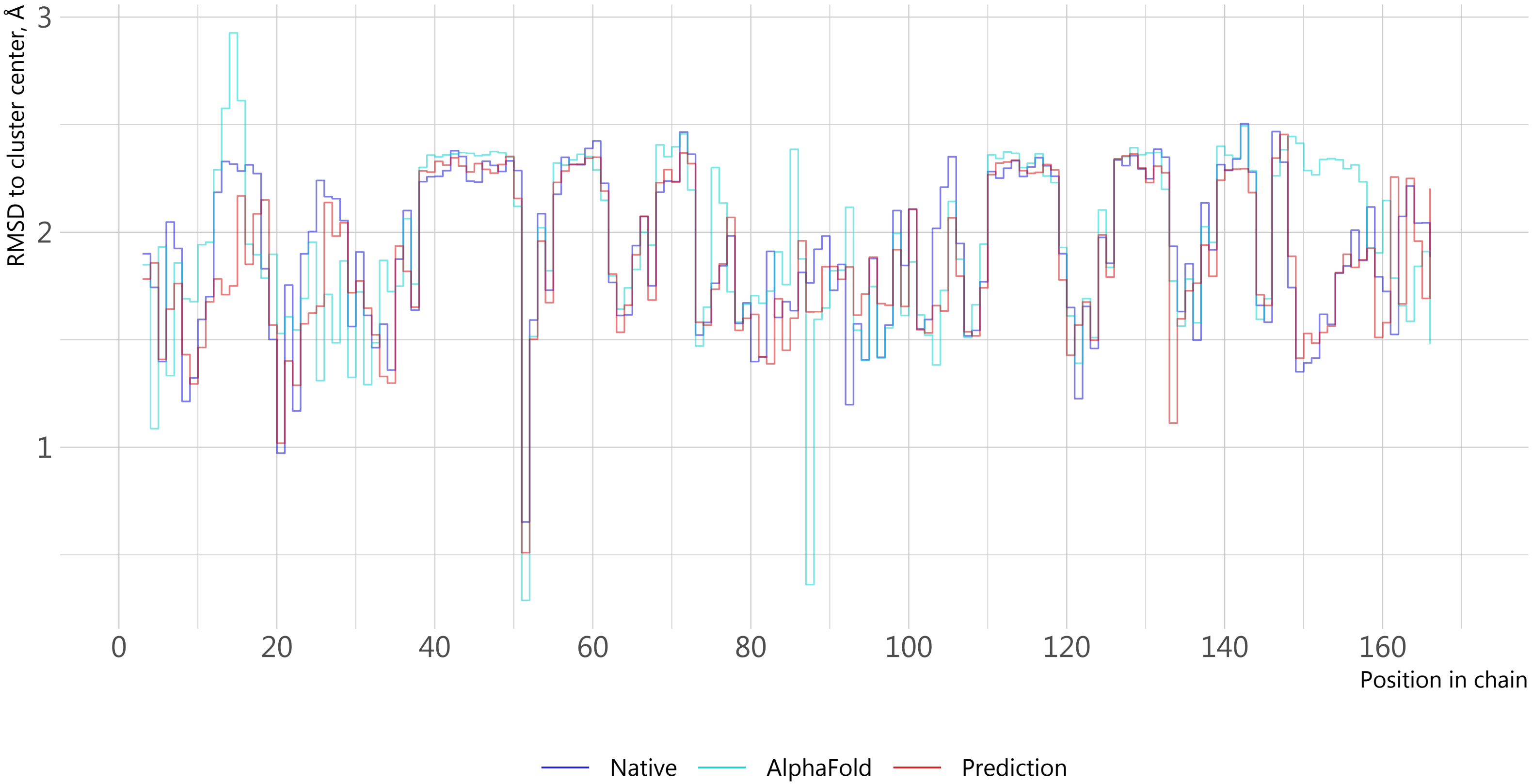


Corr(Native,AlphaFold) = 0.6347
Corr(Native, Prediction) = 0.8467
