# Supplementary material for: Effective Local and Secondary Protein Structure Prediction by Combining a Neural Network-Based Approach with Extensive Feature Design and Selection without Reliance on Evolutionary Information
Source: Int J Mol Sci. 2023 Oct 27;24(21):15656. doi: 10.3390/ijms242115656 (PMC10648199; doi:10.3390/ijms242115656)
Supplement: Supplementary file 1 [file ijms-24-15656-s001.zip › Figure S1.T1029.6UF2A.pdf]

# T 1029 6UF2A PB 'a': N-cap β

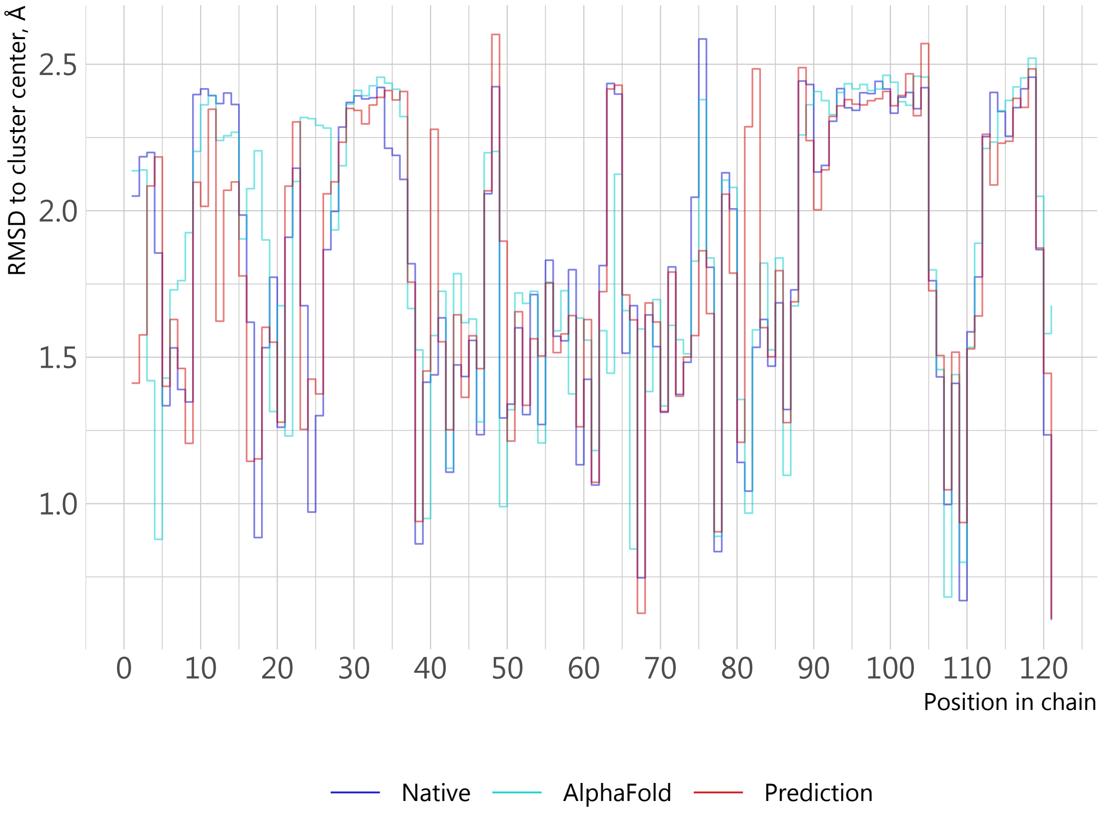


Corr(Native,AlphaFold) = 0.7460
Corr(Native, Prediction) = 0.8588

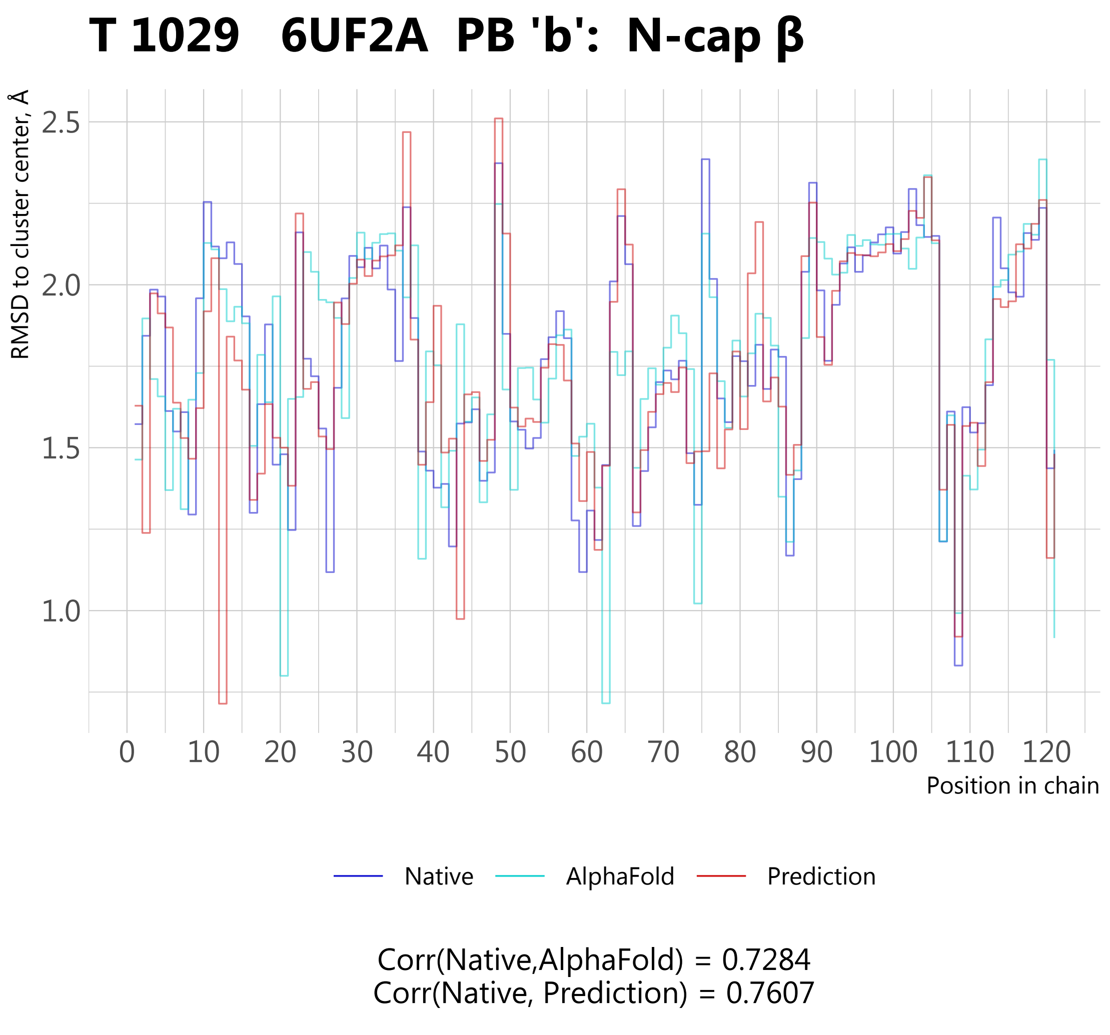

T 1029 6UF2A PB 'b': N-cap β
RMSD to cluster center, Å
2.5
2.0
1.5
1.0
0
10
20
30
40
50
60
70
80
90
100
110
120
Position in chain
Native
AlphaFold
Prediction
Corr(Native,AlphaFold) = 0.7284
Corr(Native, Prediction) = 0.7607

# T 1029   6UF2A  PB 'c':  N-cap β

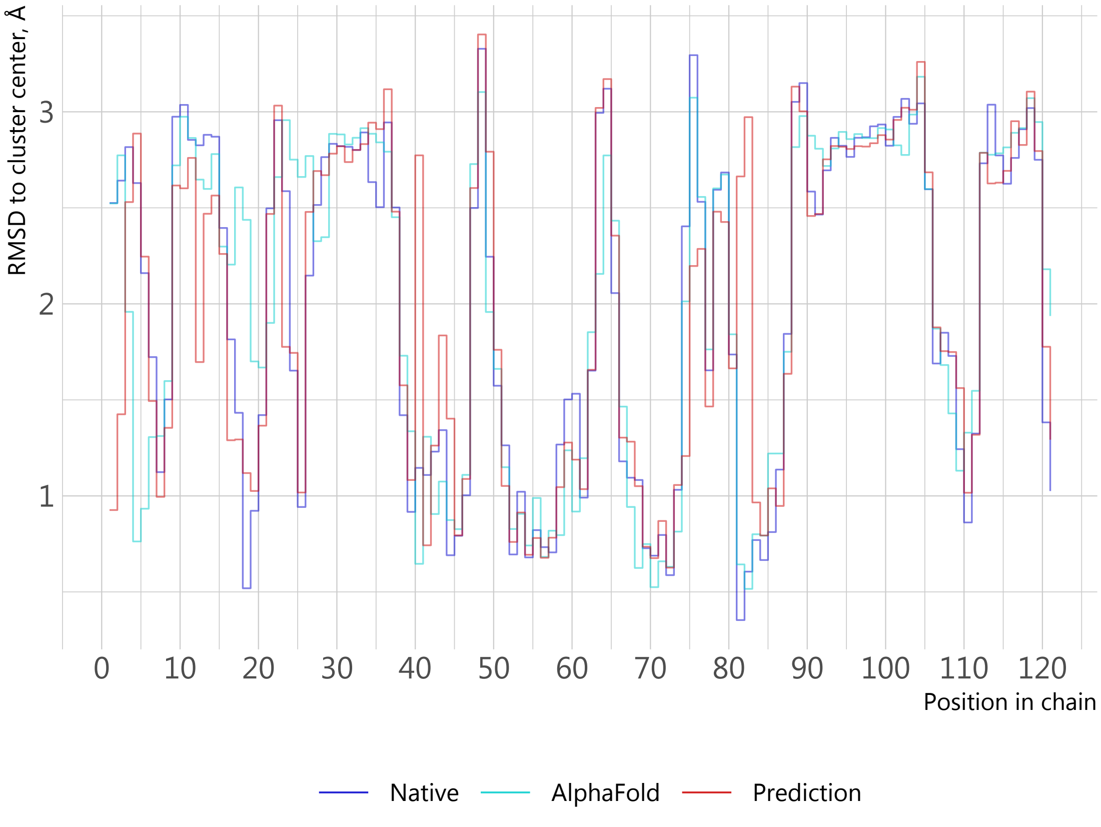


Corr(Native,AlphaFold) = 0.8676
Corr(Native, Prediction) = 0.8464

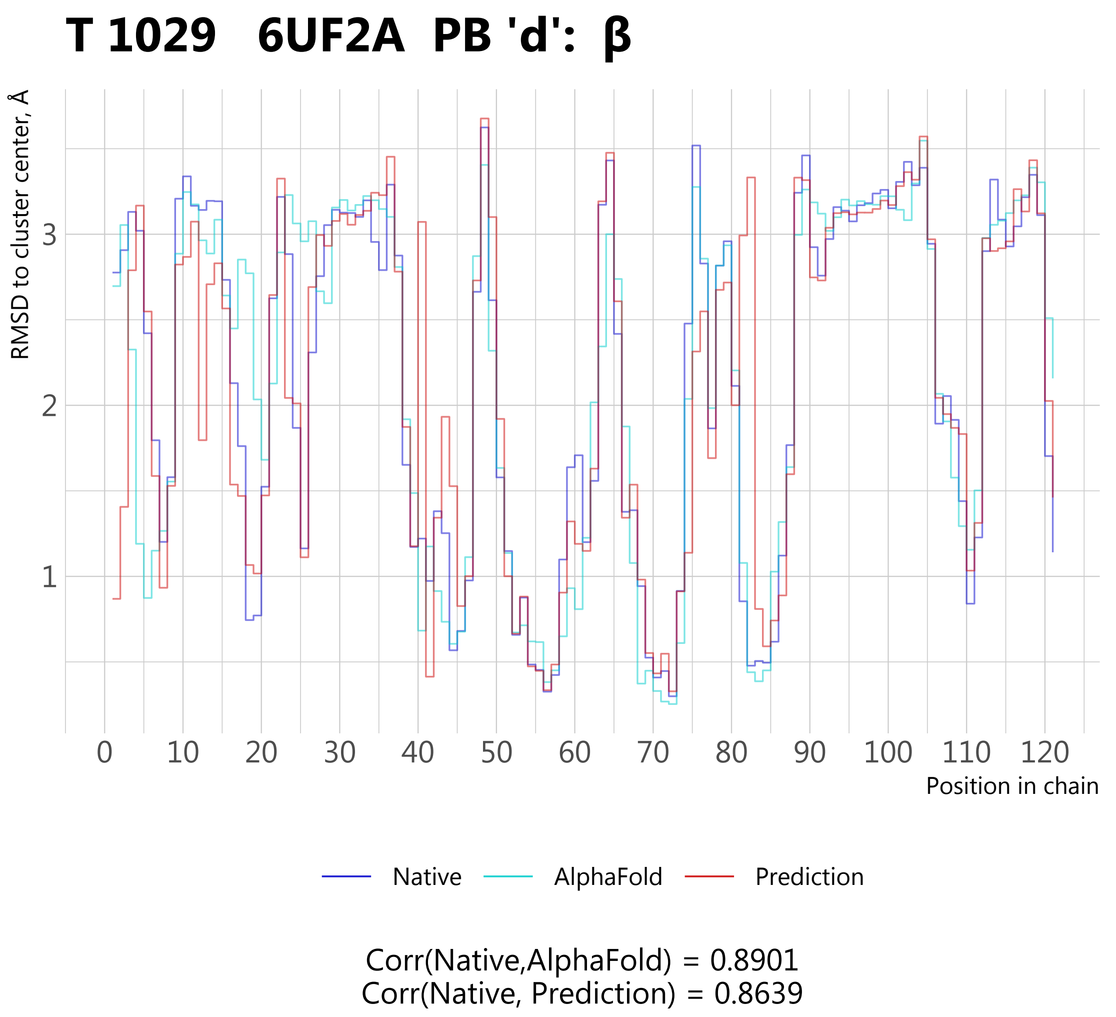
T 1029 6UF2A PB 'd': β
RMSD to cluster center, Å
3
2
1
0
10
20
30
40
50
60
70
80
90
100
110
120
Position in chain
Native
AlphaFold
Prediction
Corr(Native,AlphaFold) = 0.8901
Corr(Native, Prediction) = 0.8639

# T 1029   6UF2A  PB 'e':  C-cap β

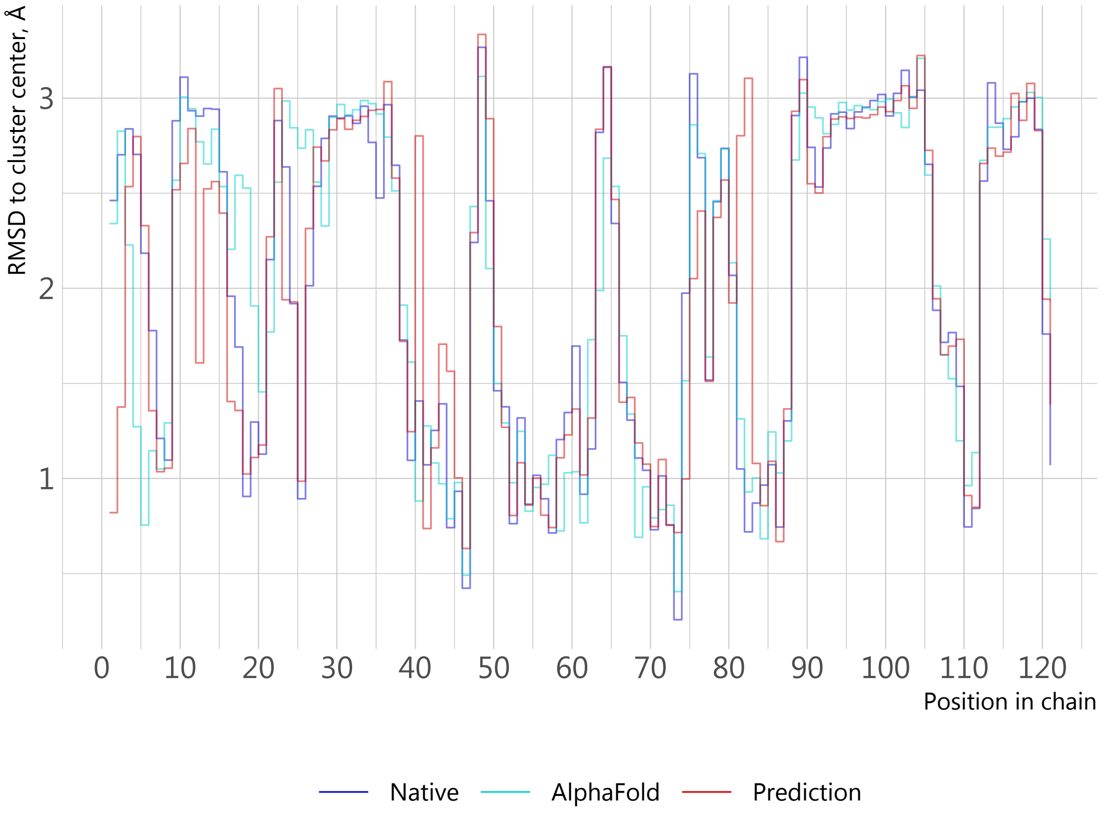


Corr(Native,AlphaFold) = 0.8814
Corr(Native, Prediction) = 0.8633

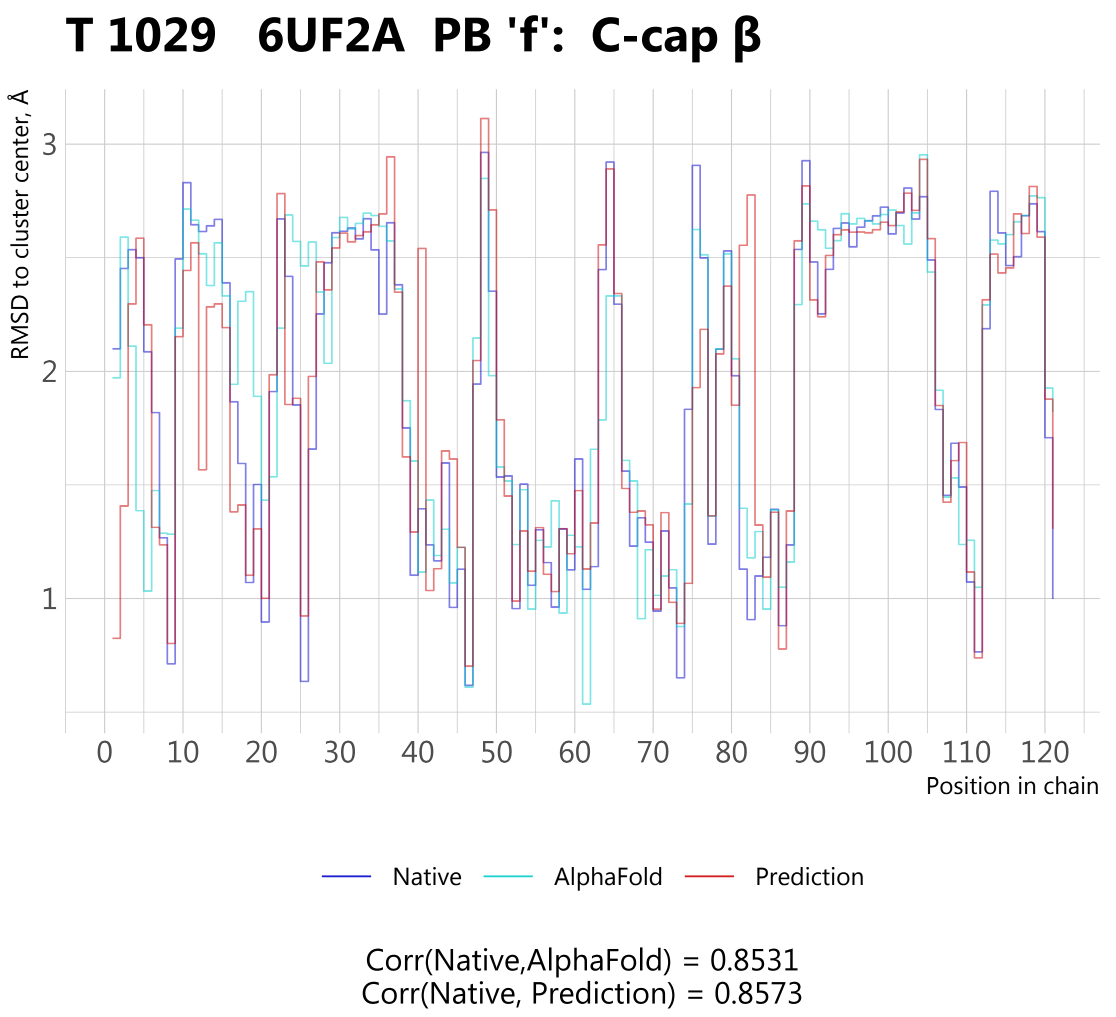
T 1029 6UF2A PB 'f': C-cap β
RMSD to cluster center, Å
3
2
1
0
10
20
30
40
50
60
70
80
90
100
110
120
Position in chain
Native
AlphaFold
Prediction
Corr(Native,AlphaFold) = 0.8531
Corr(Native, Prediction) = 0.8573

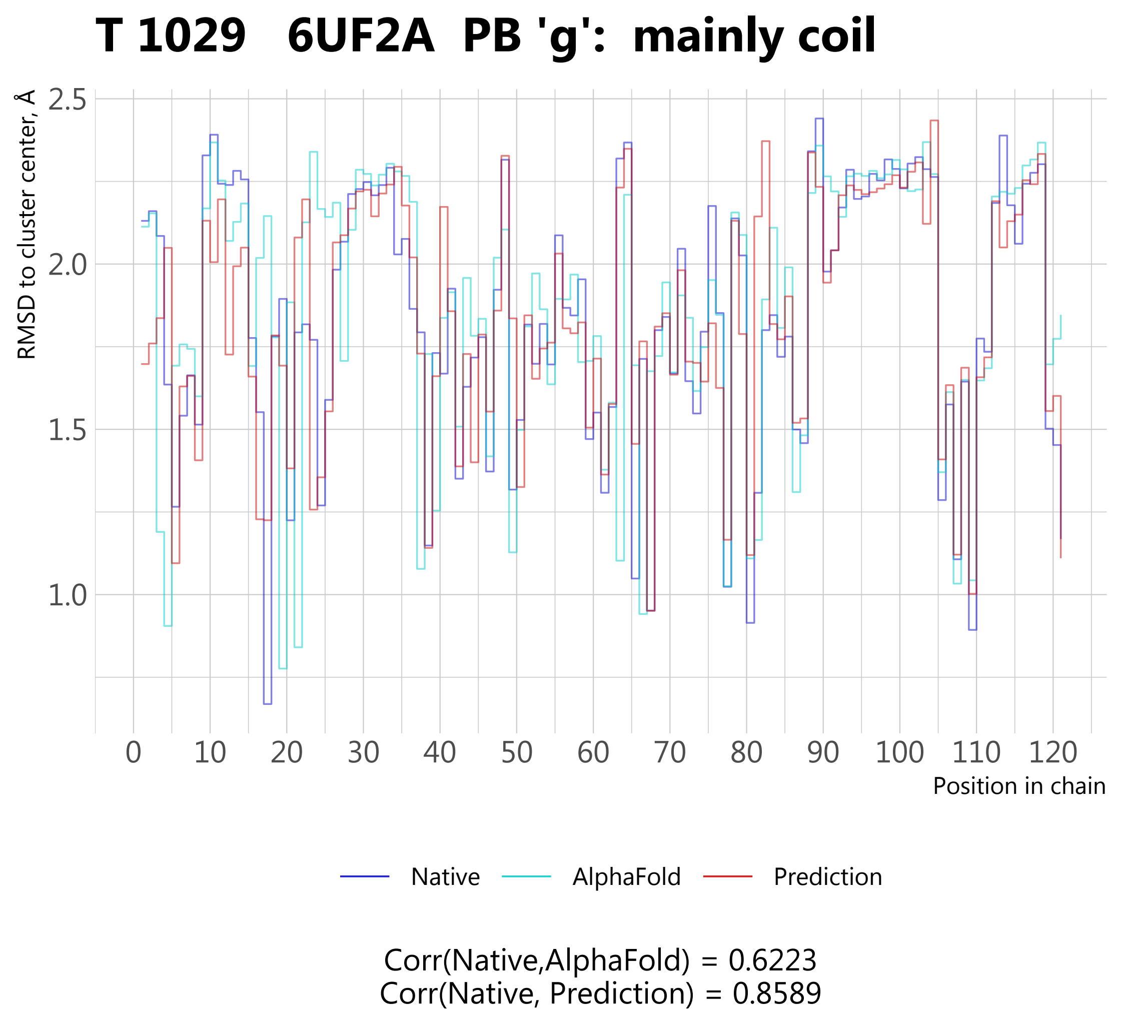

T 1029 6UF2A PB 'g': mainly coil
RMSD to cluster center, Å
2.5
2.0
1.5
1.0
0
10
20
30
40
50
60
70
80
90
100
110
120
Position in chain
Native
AlphaFold
Prediction
Corr(Native,AlphaFold) = 0.6223
Corr(Native, Prediction) = 0.8589

# T 1029   6UF2A  PB 'h':  mainly coil

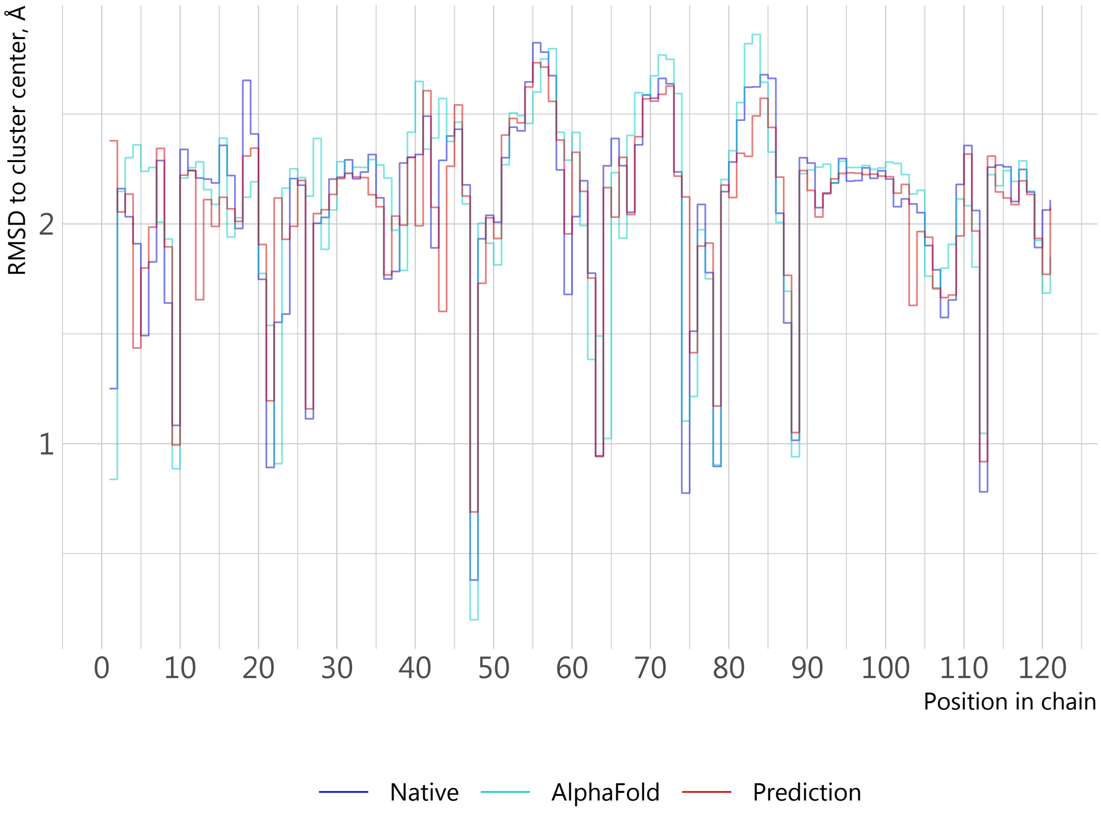


Corr(Native,AlphaFold) = 0.8072
Corr(Native, Prediction) = 0.8430

# T 1029   6UF2A  PB 'i':  mainly coil

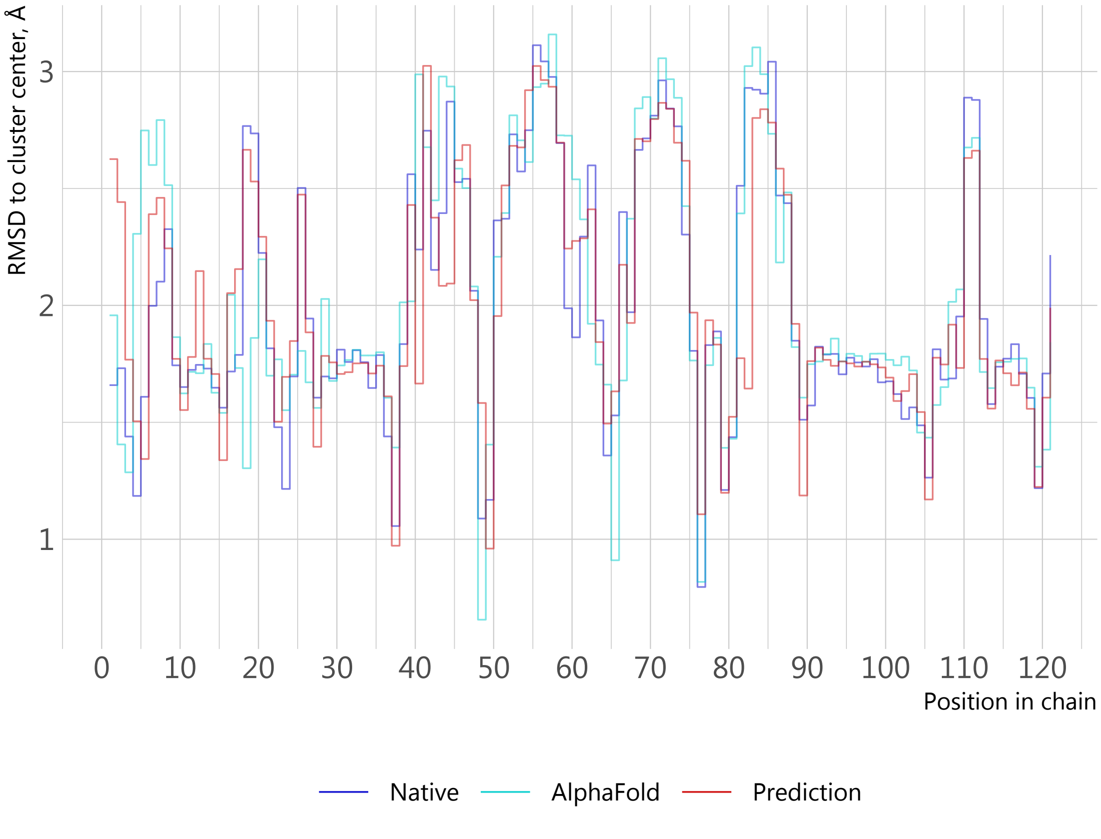


Corr(Native,AlphaFold) = 0.8101
Corr(Native, Prediction) = 0.8729

# T 1029   6UF2A  PB 'j':  mainly coil

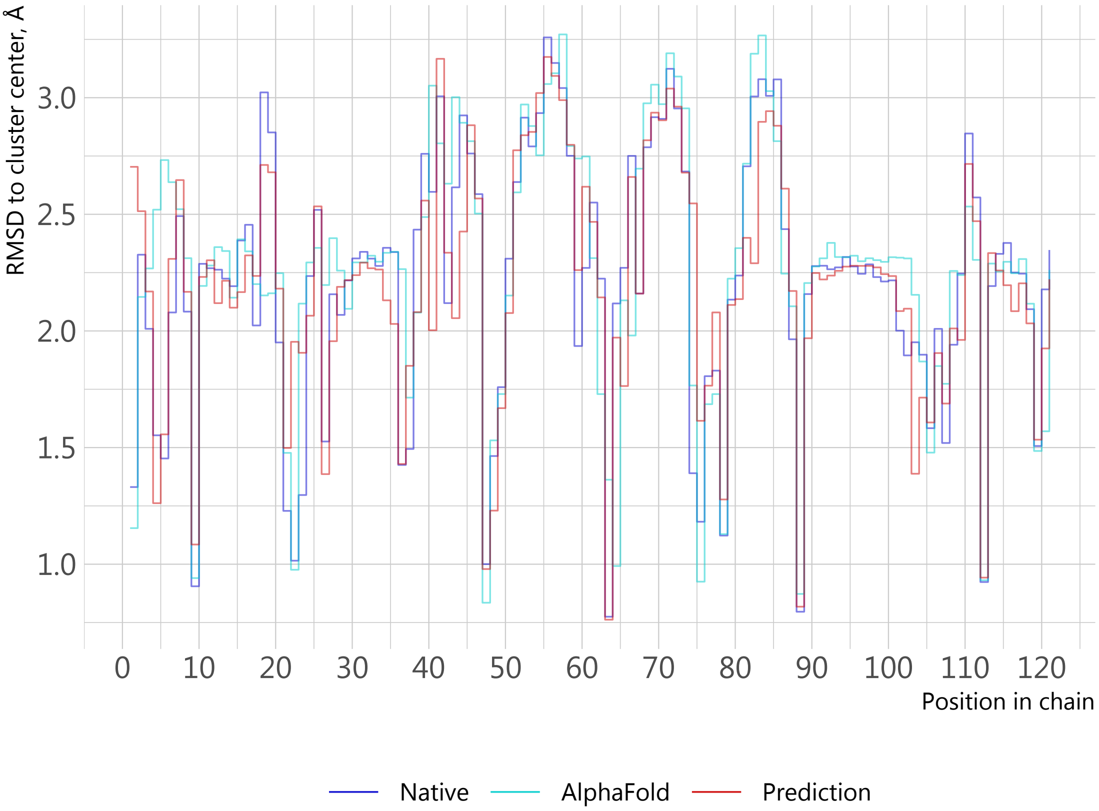


Corr(Native,AlphaFold) = 0.8246
Corr(Native, Prediction) = 0.8671

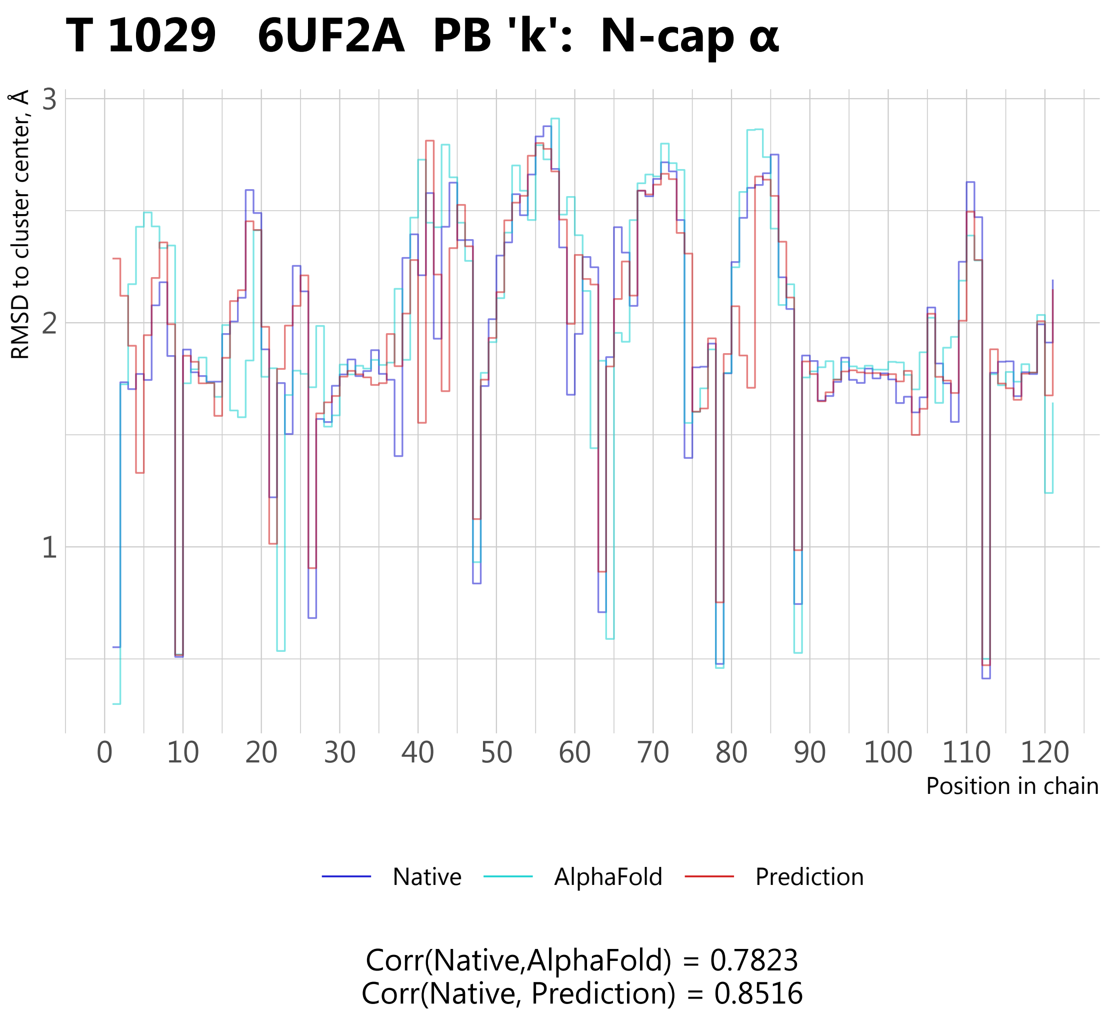
T 1029 6UF2A PB 'k': N-cap α
RMSD to cluster center, Å
3
2
1
0
10
20
30
40
50
60
70
80
90
100
110
120
Position in chain
Native
AlphaFold
Prediction
Corr(Native,AlphaFold) = 0.7823
Corr(Native, Prediction) = 0.8516

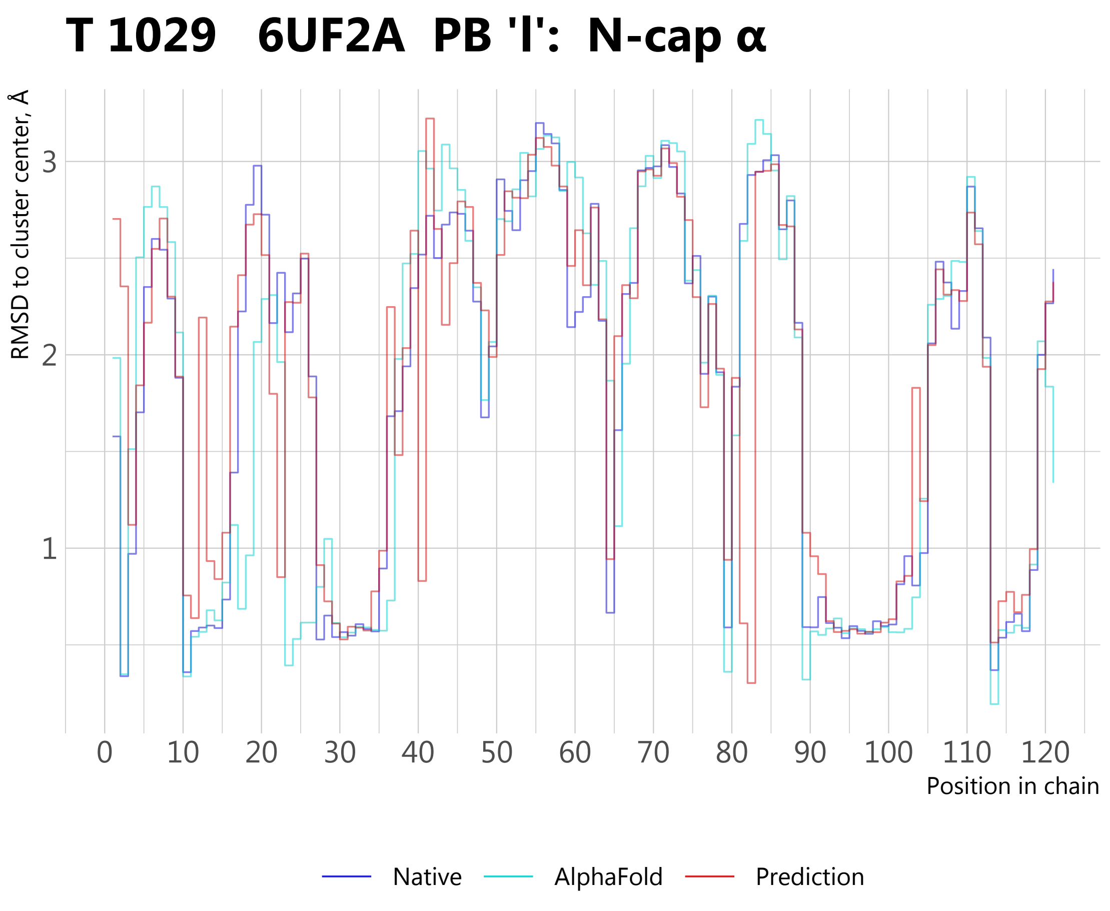


Corr(Native,AlphaFold) = 0.8792
Corr(Native, Prediction) = 0.8484

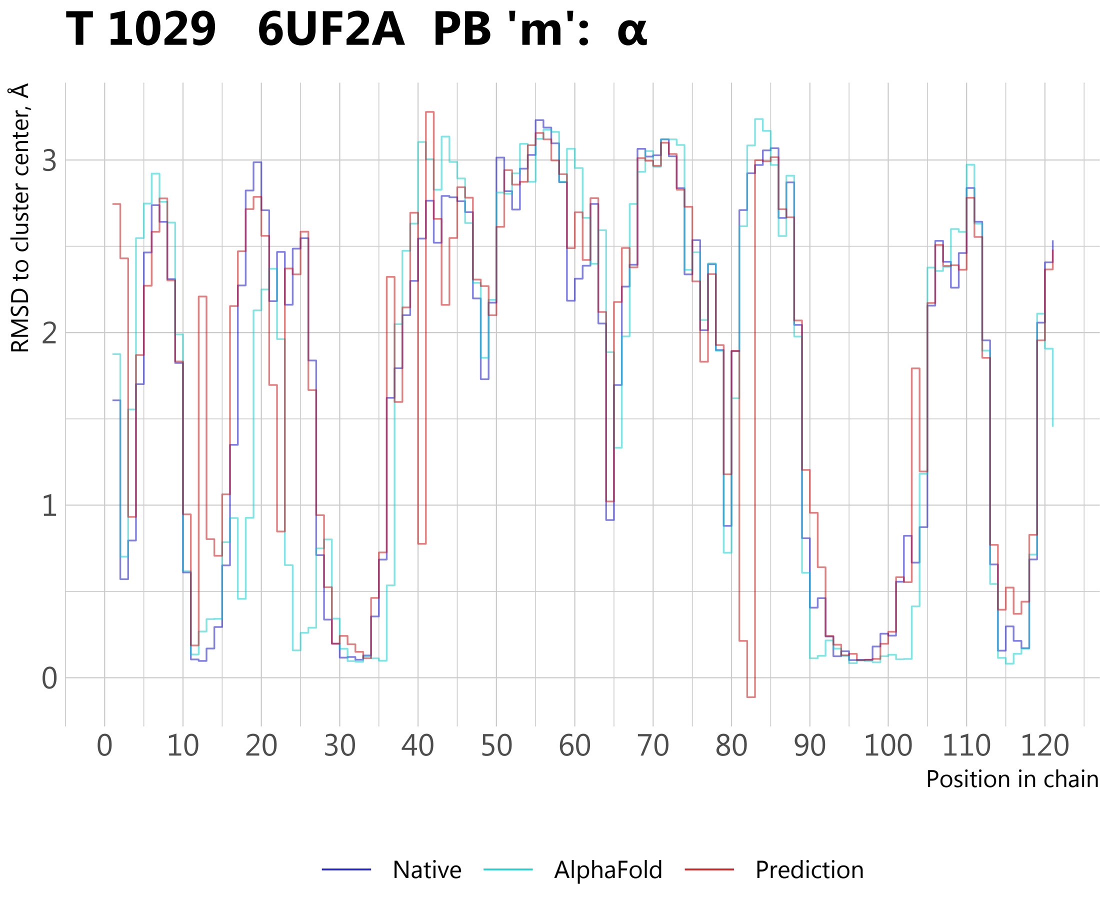


Corr(Native,AlphaFold) = 0.8896
Corr(Native, Prediction) = 0.8571

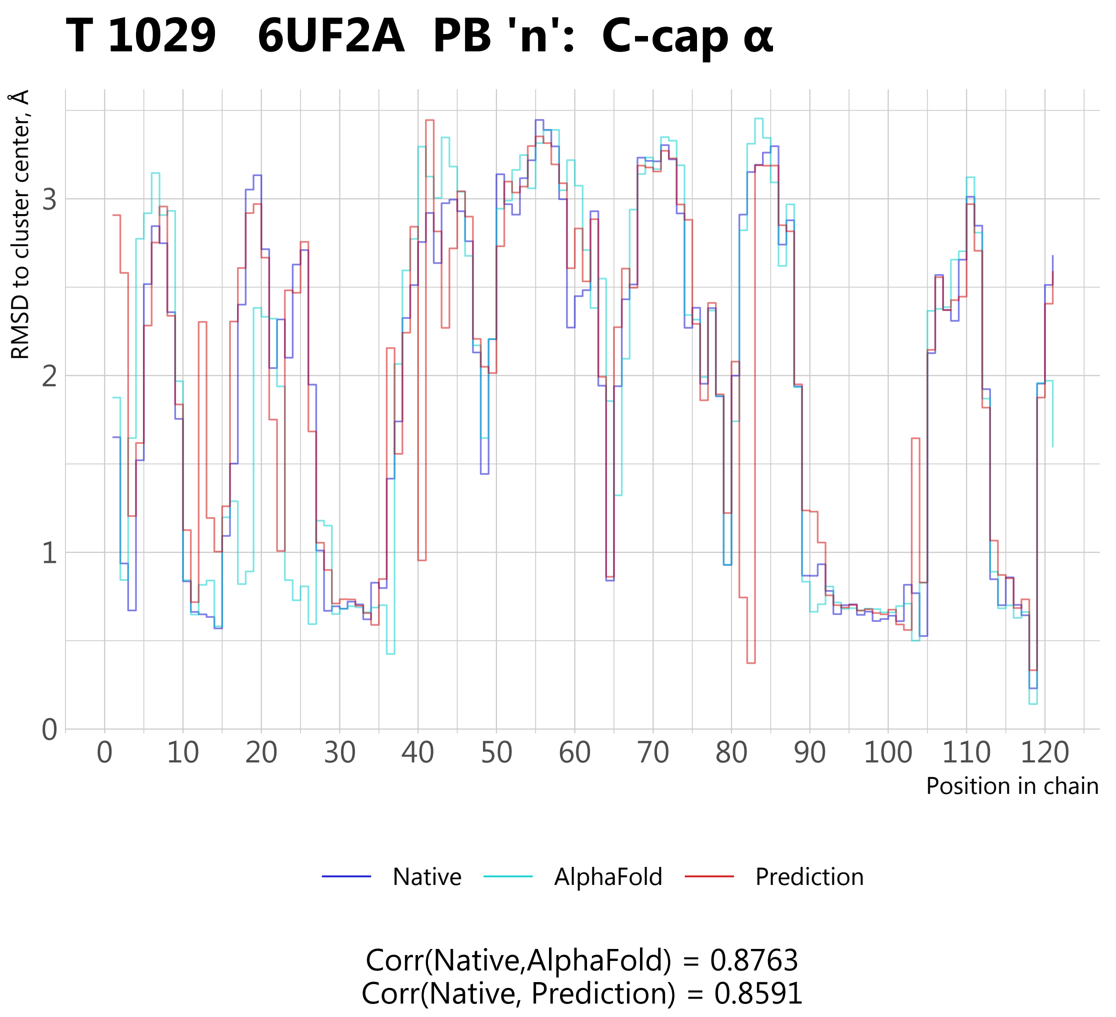

T 1029 6UF2A PB 'n': C-cap α
RMSD to cluster center, Å
0
1
2
3
0
10
20
30
40
50
60
70
80
90
100
110
120
Position in chain
Native
AlphaFold
Prediction
Corr(Native,AlphaFold) = 0.8763
Corr(Native, Prediction) = 0.8591

# T 1029   6UF2A  PB 'o':  C-cap α

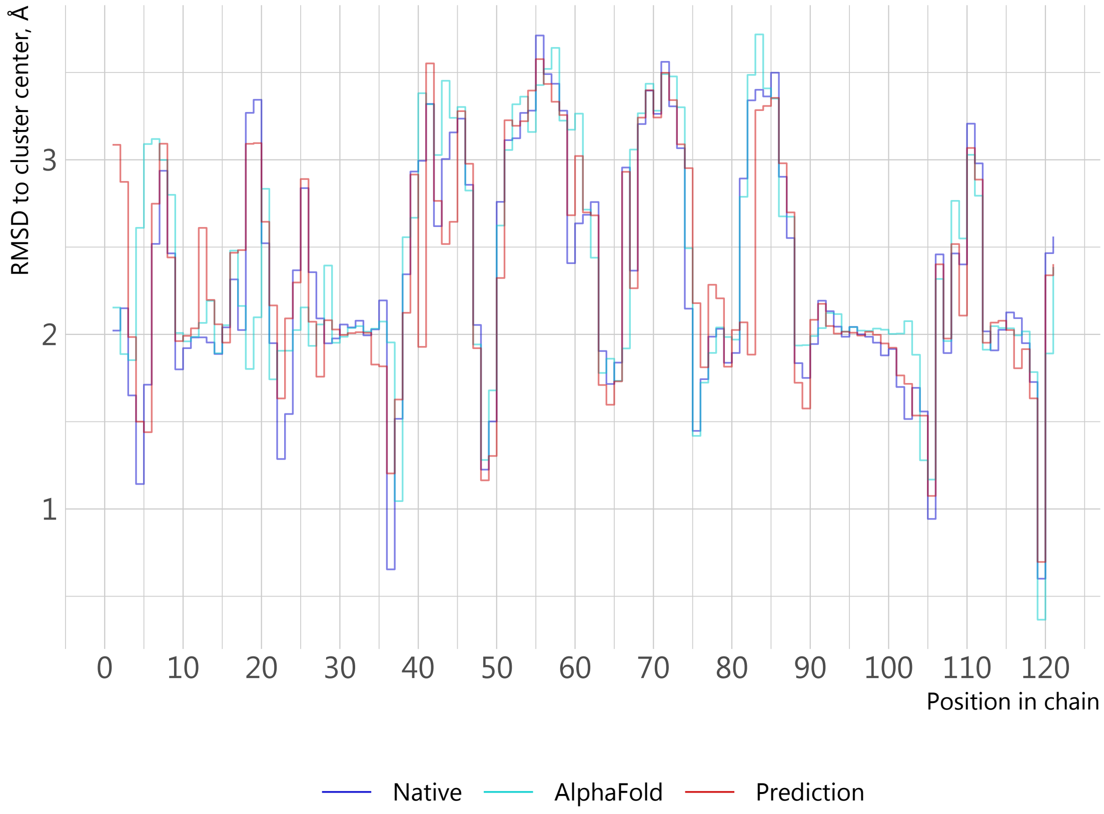


Corr(Native,AlphaFold) = 0.8346
Corr(Native, Prediction) = 0.8897

# T 1029   6UF2A  PB 'p':  C-cap α to N-cap β

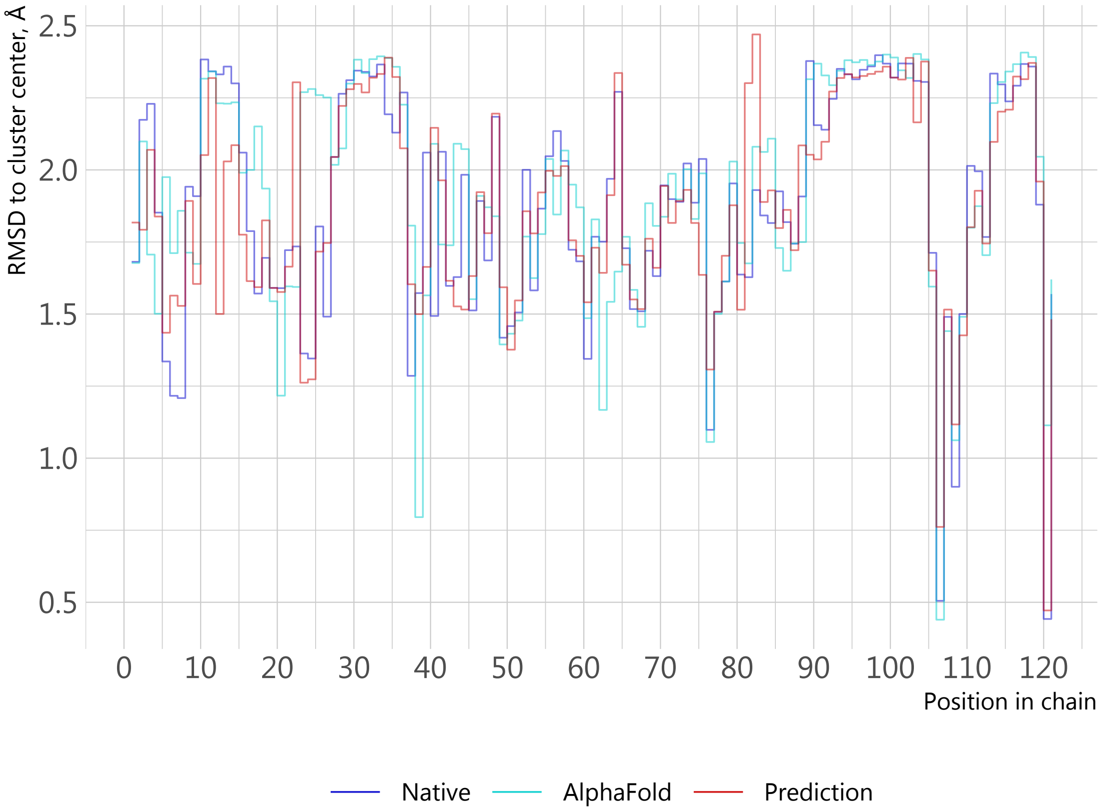


Corr(Native,AlphaFold) = 0.7532
Corr(Native, Prediction) = 0.8637
