# Supplementary material for: Effective Local and Secondary Protein Structure Prediction by Combining a Neural Network-Based Approach with Extensive Feature Design and Selection without Reliance on Evolutionary Information
Source: Int J Mol Sci. 2023 Oct 27;24(21):15656. doi: 10.3390/ijms242115656 (PMC10648199; doi:10.3390/ijms242115656)
Supplement: Supplementary file 1 [file ijms-24-15656-s001.zip › Figure S1.T1049.6Y4FA.pdf]

T 1049   6Y4FA PB 'a': N-cap β

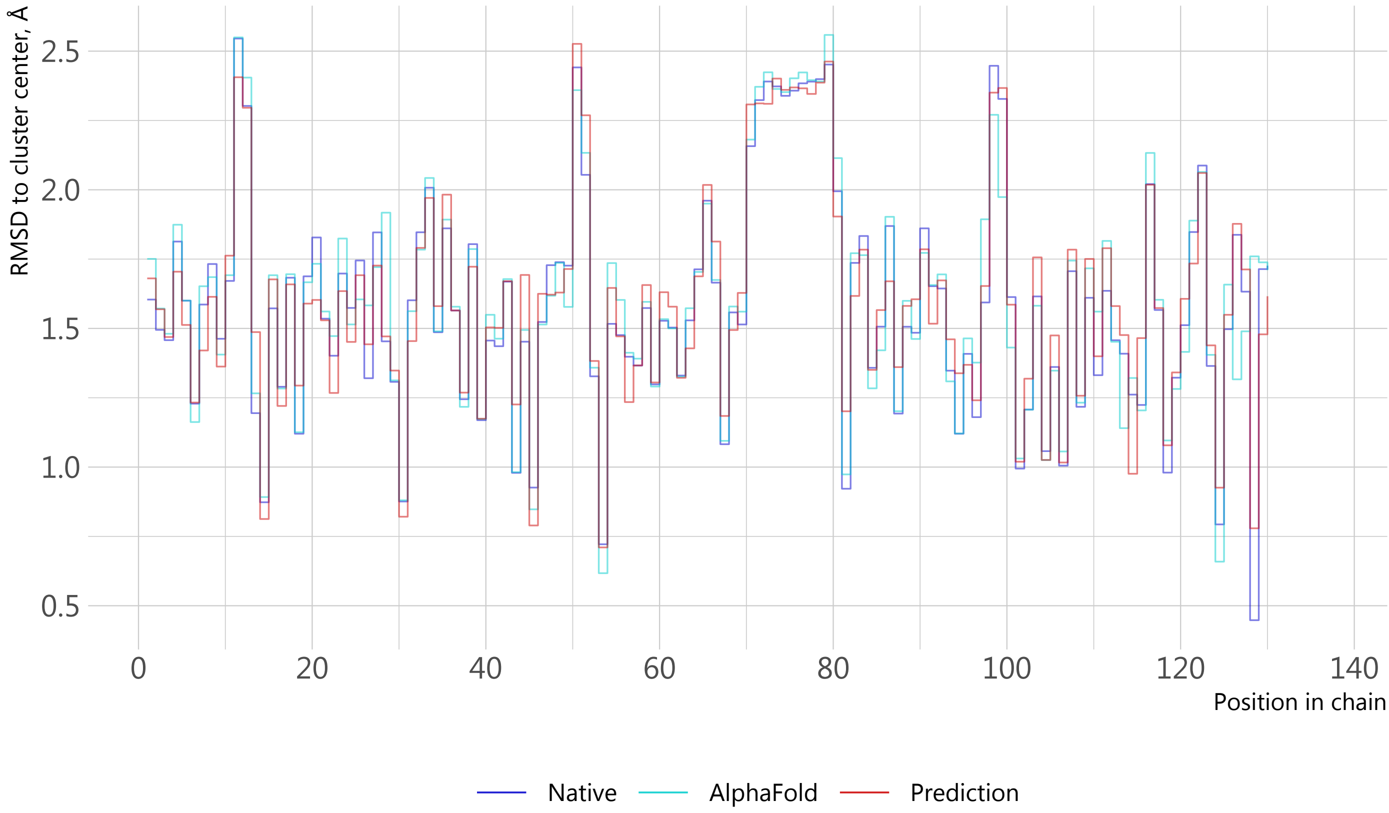

Corr(Native,AlphaFold) = 0.9227
Corr(Native, Prediction) = 0.9613

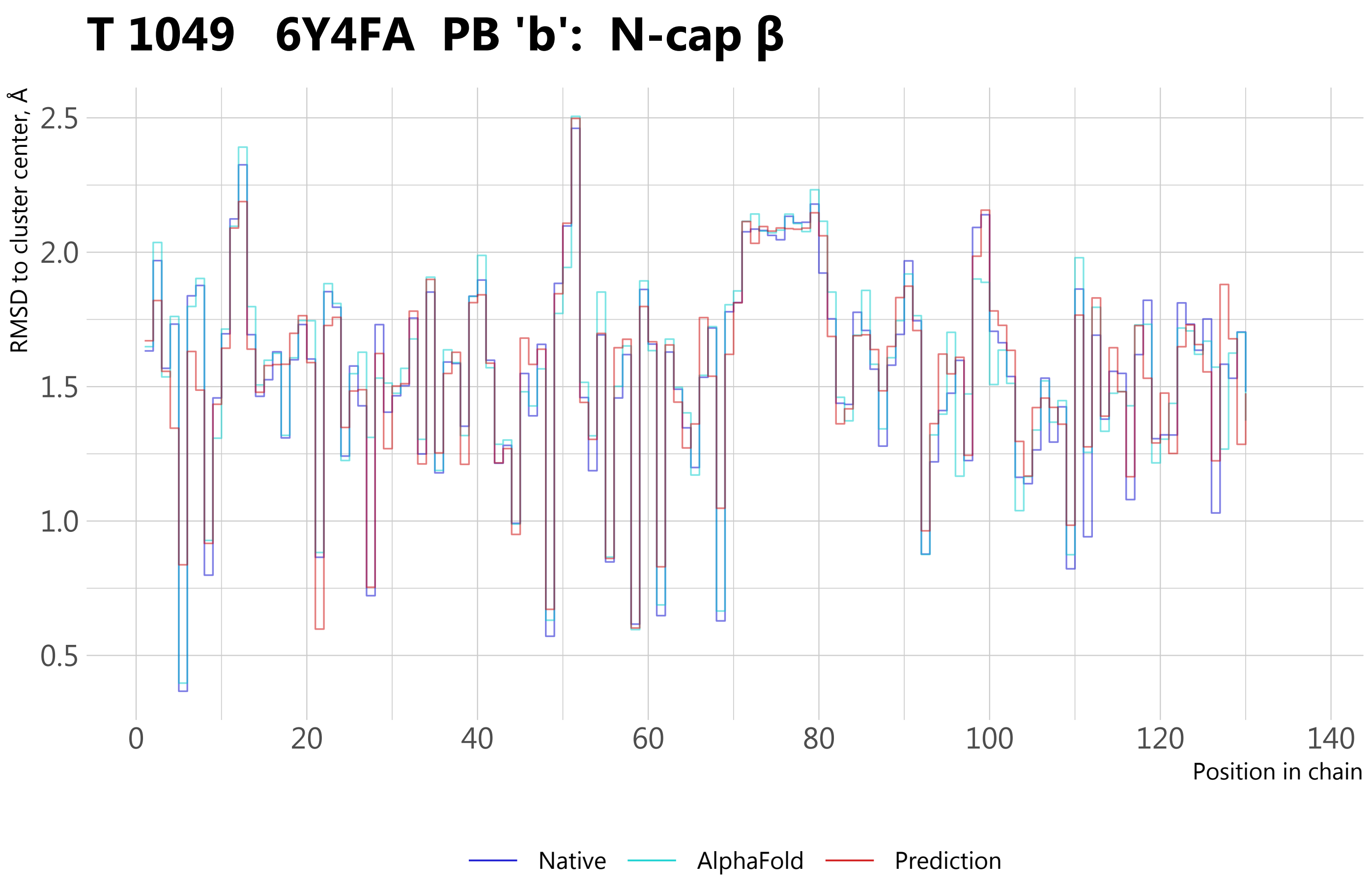

Corr(Native,AlphaFold) = 0.9477
Corr(Native, Prediction) = 0.9366

T 1049   6Y4FA PB 'c': N-cap β

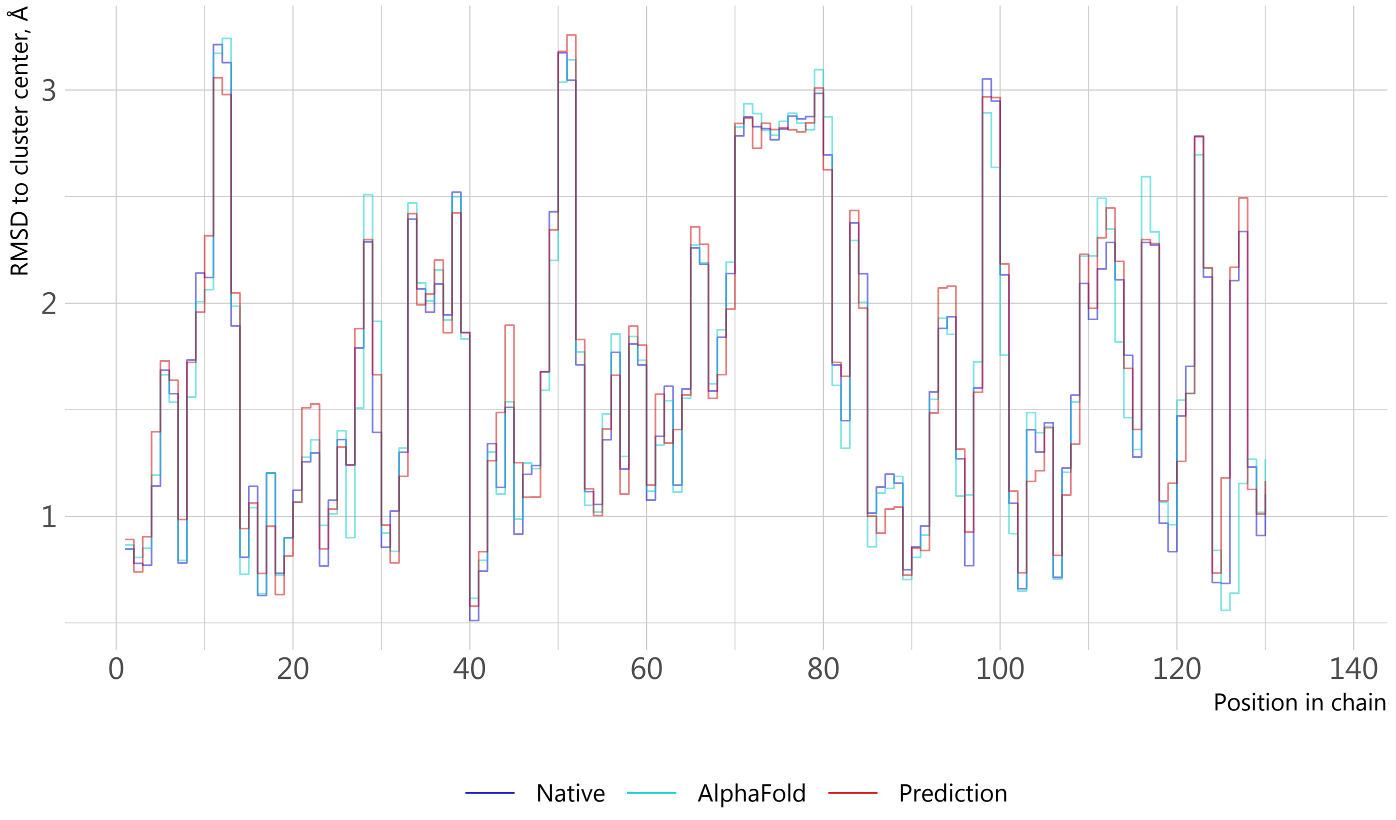

Corr(Native,AlphaFold) = 0.9561
Corr(Native, Prediction) = 0.9800

T 1049   6Y4FA PB 'd': β

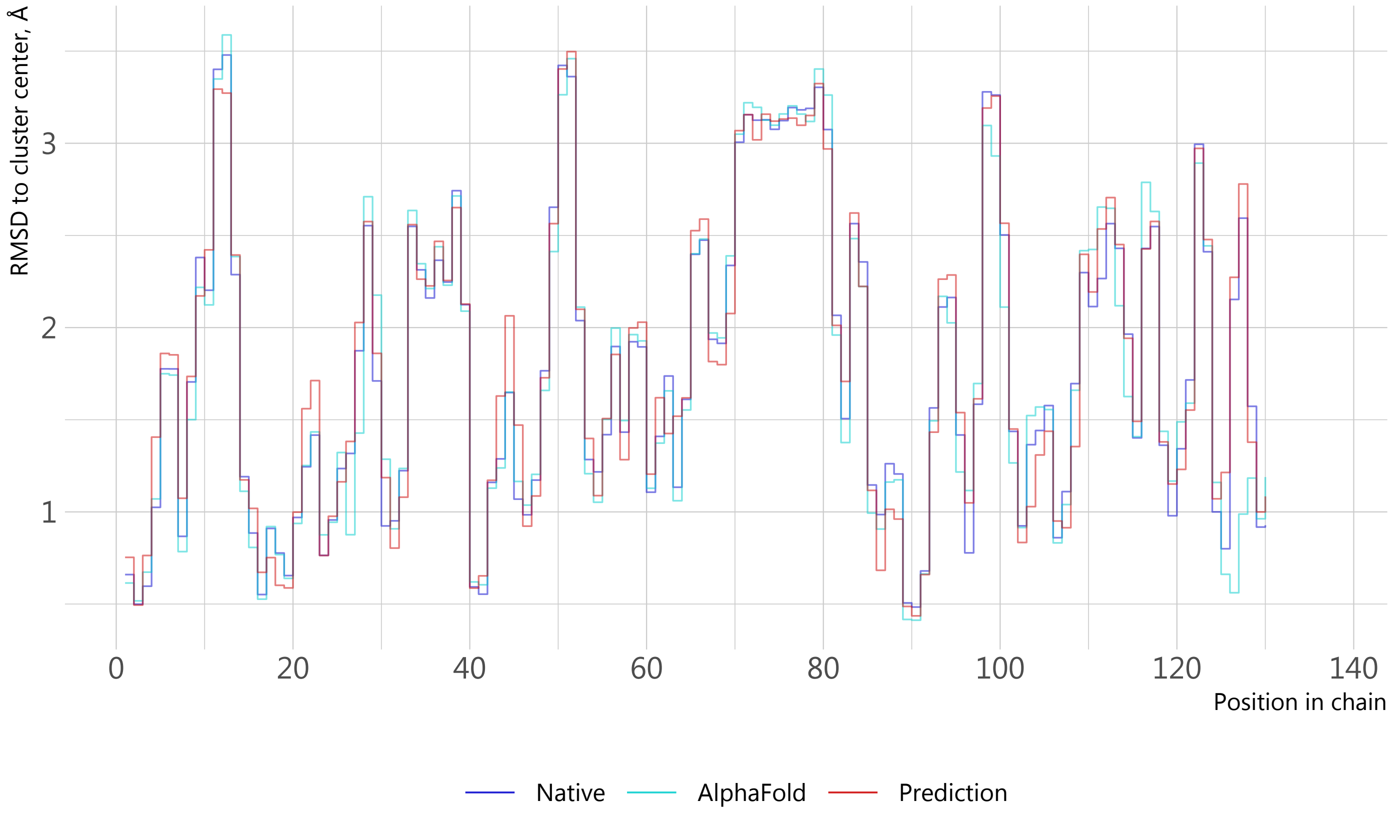

Corr(Native,AlphaFold) = 0.9555
Corr(Native, Prediction) = 0.9819

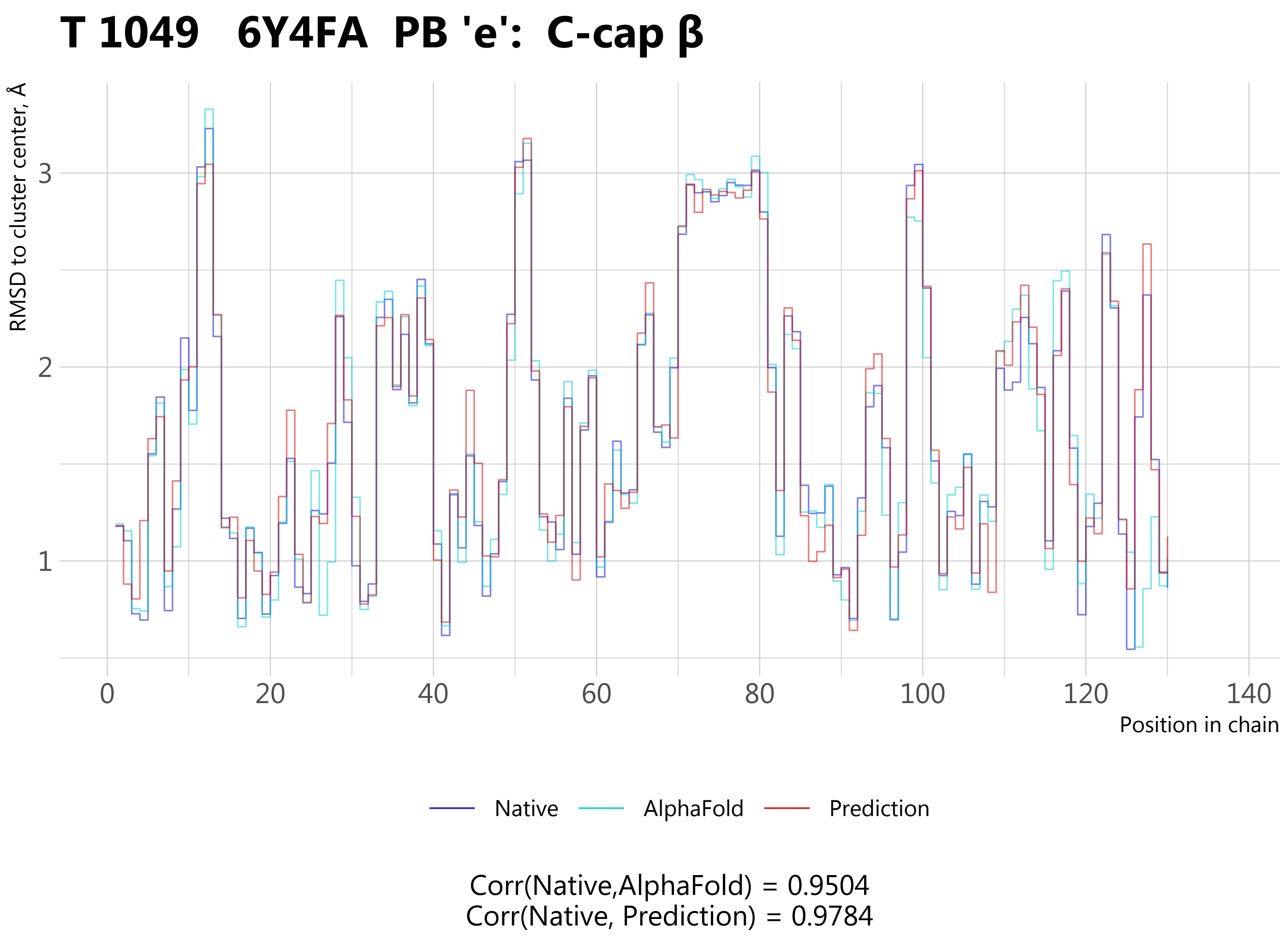
T 1049 6Y4FA PB 'e': C-cap β
RMSD to cluster center, Å
1
2
3
0
20
40
60
80
100
120
140
Position in chain
Native
AlphaFold
Prediction
Corr(Native,AlphaFold) = 0.9504
Corr(Native, Prediction) = 0.9784

T 1049   6Y4FA PB 'f': C-cap β

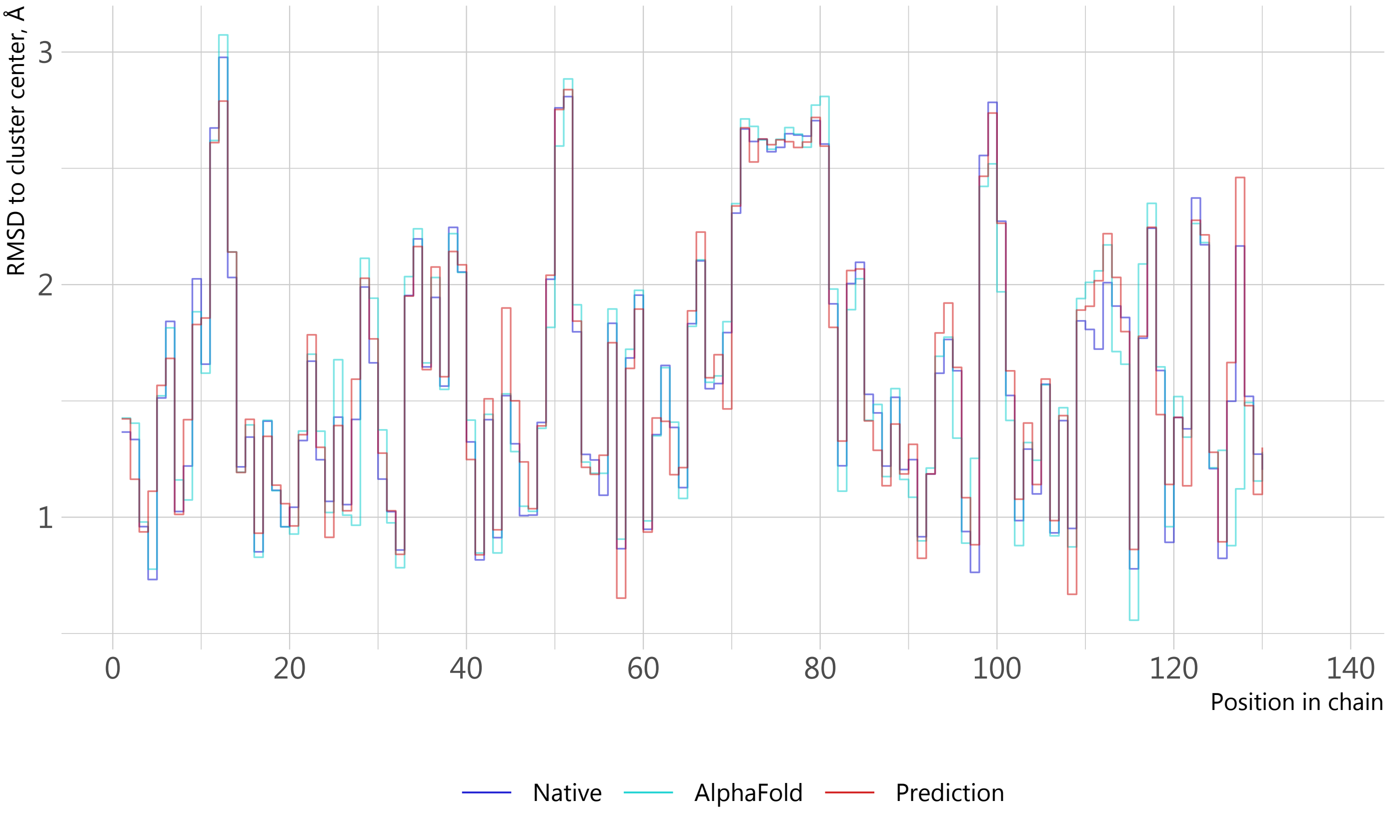

Corr(Native,AlphaFold) = 0.9567
Corr(Native, Prediction) = 0.9760

T 1049   6Y4FA PB 'g': mainly coil

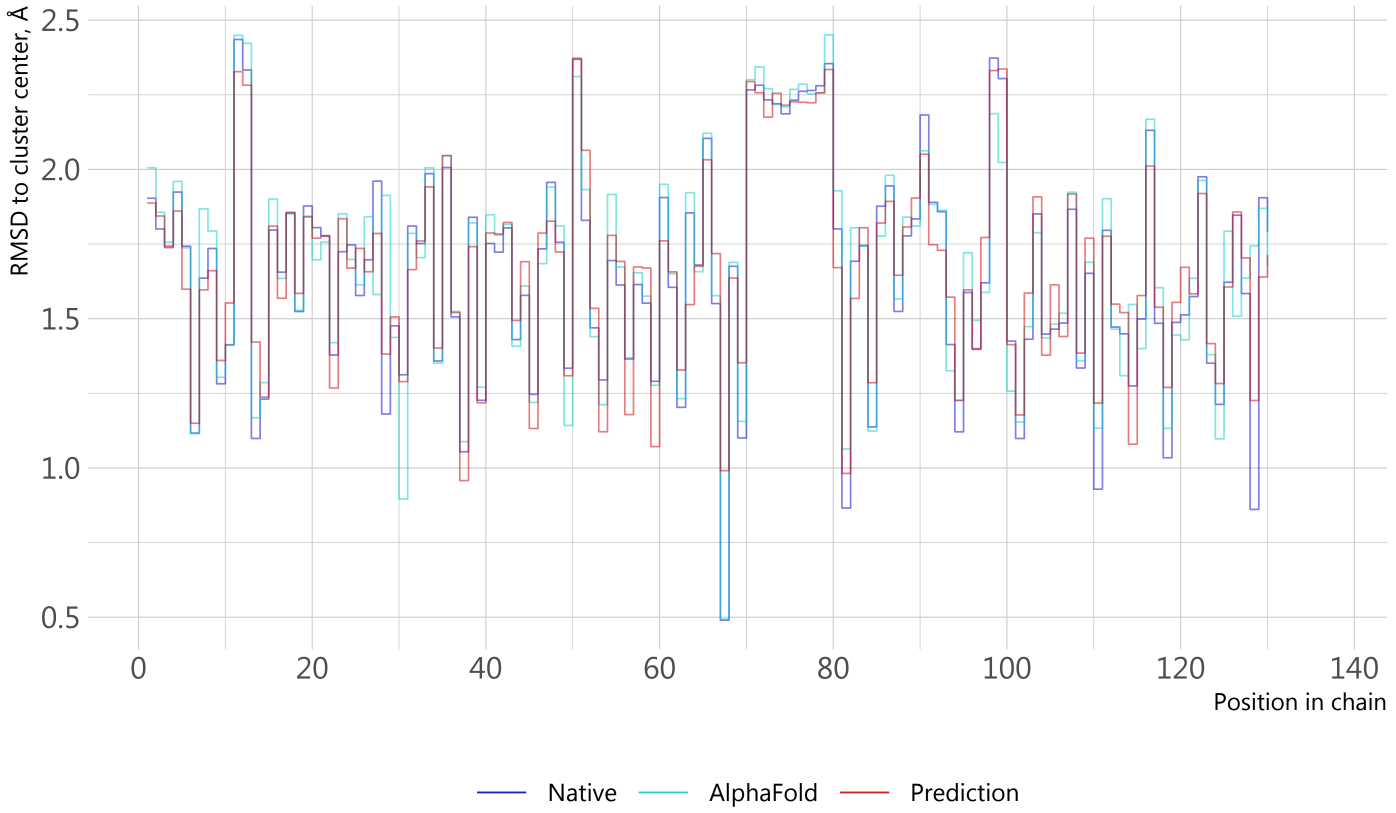

Corr(Native,AlphaFold) = 0.9243
Corr(Native, Prediction) = 0.9464

T 1049   6Y4FA PB 'h': mainly coil

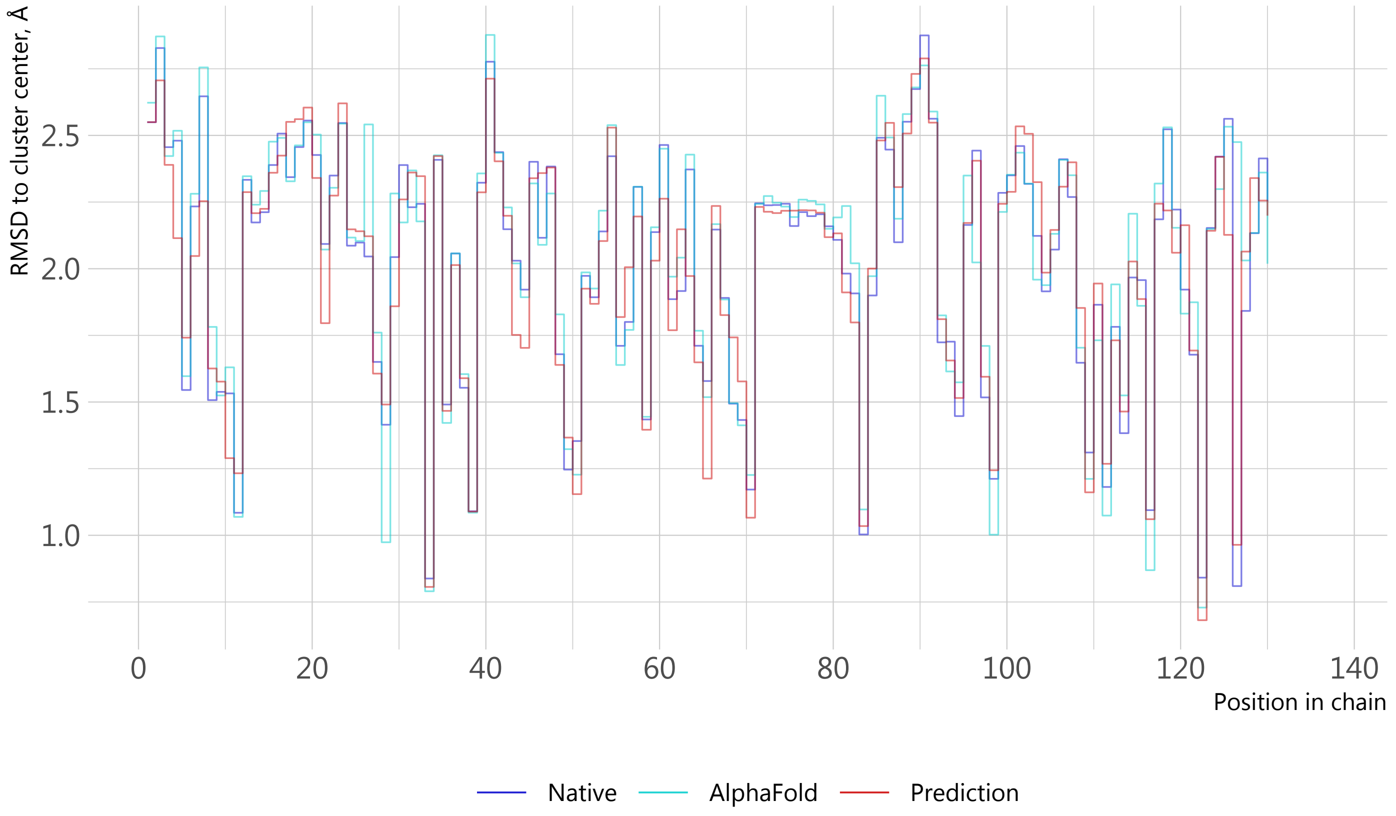

Corr(Native,AlphaFold) = 0.9179
Corr(Native, Prediction) = 0.9526

T 1049 6Y4FA PB 'i': mainly coil

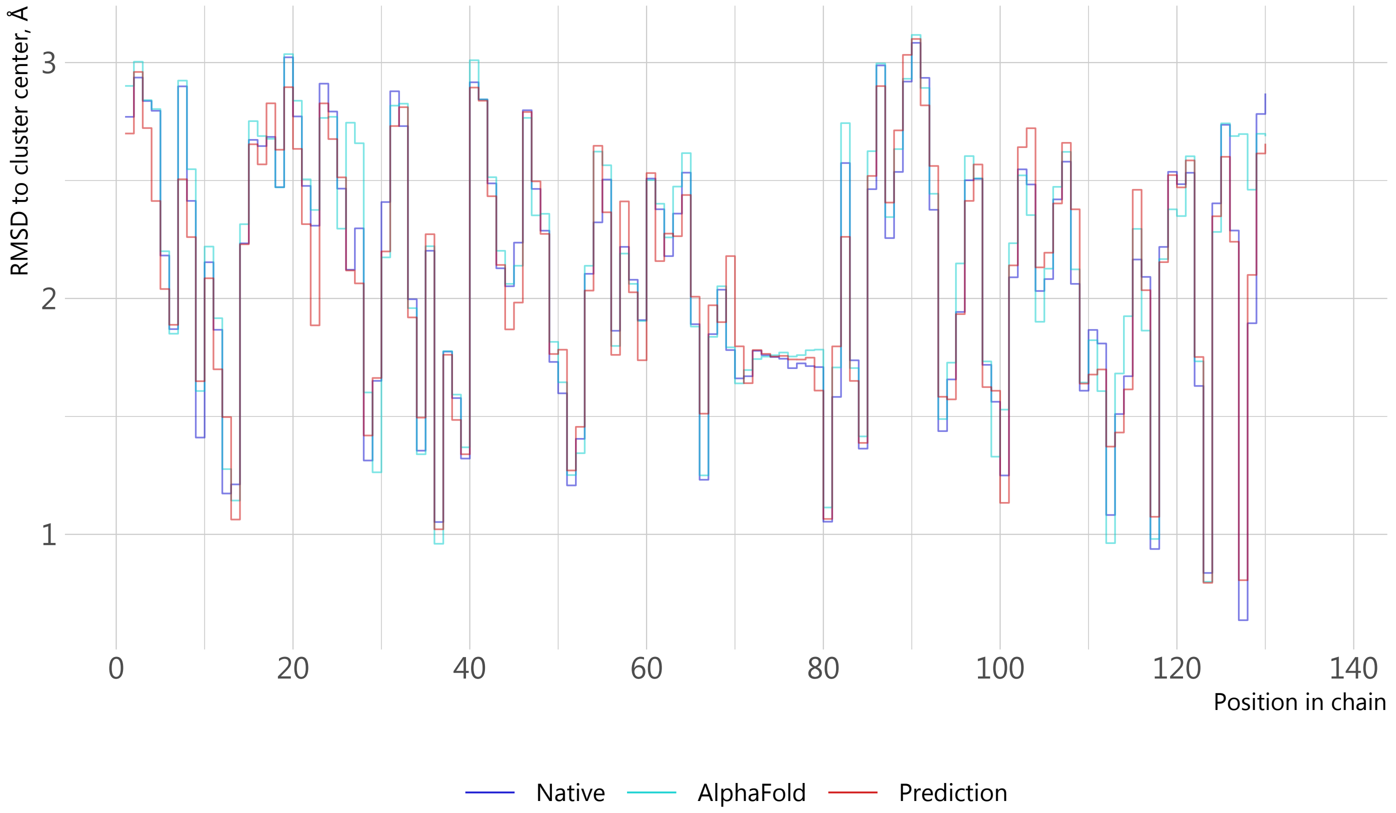

Corr(Native,AlphaFold) = 0.9173
Corr(Native, Prediction) = 0.9633

T 1049   6Y4FA PB 'j': mainly coil

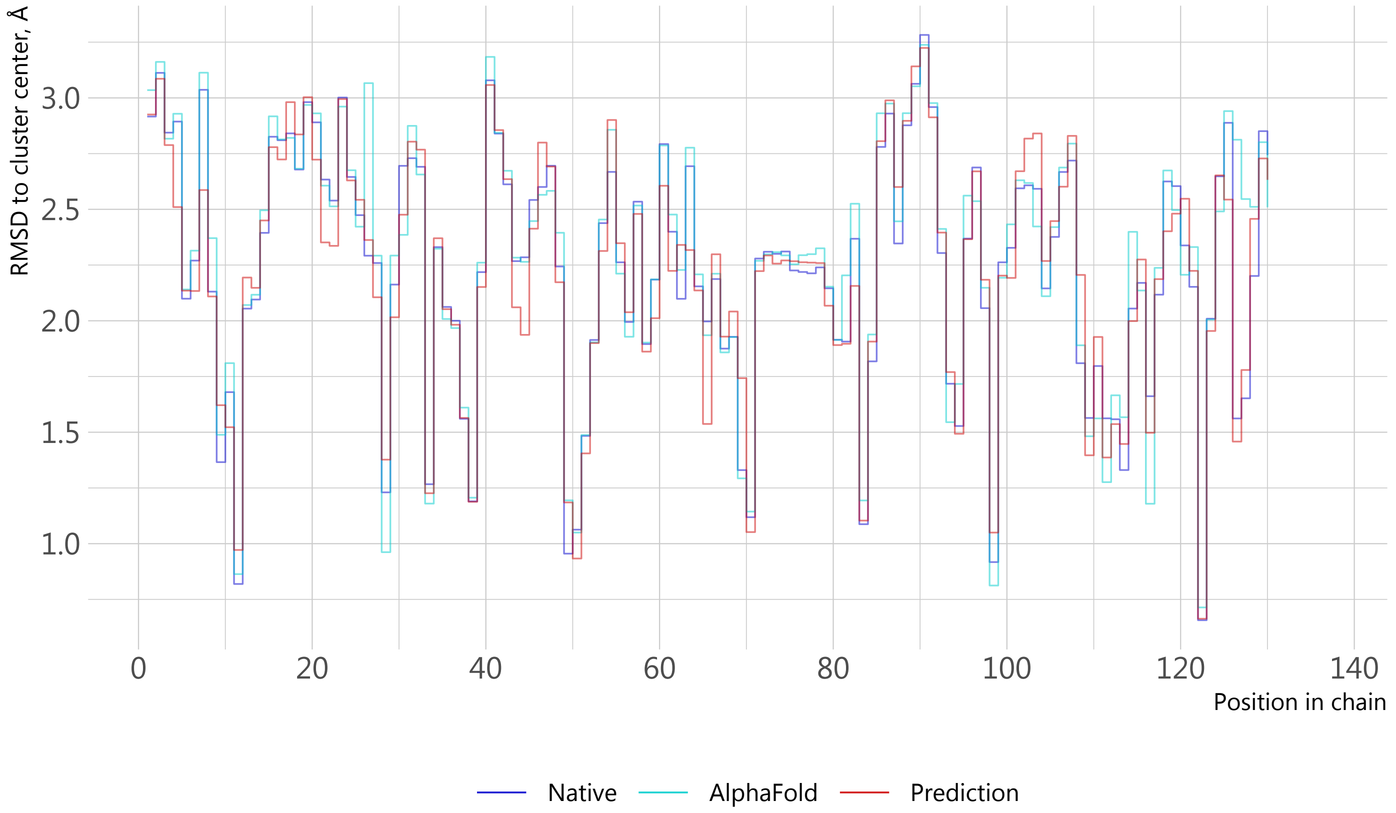

Corr(Native,AlphaFold) = 0.9428
Corr(Native, Prediction) = 0.9612

T 1049   6Y4FA PB 'k': N-cap α

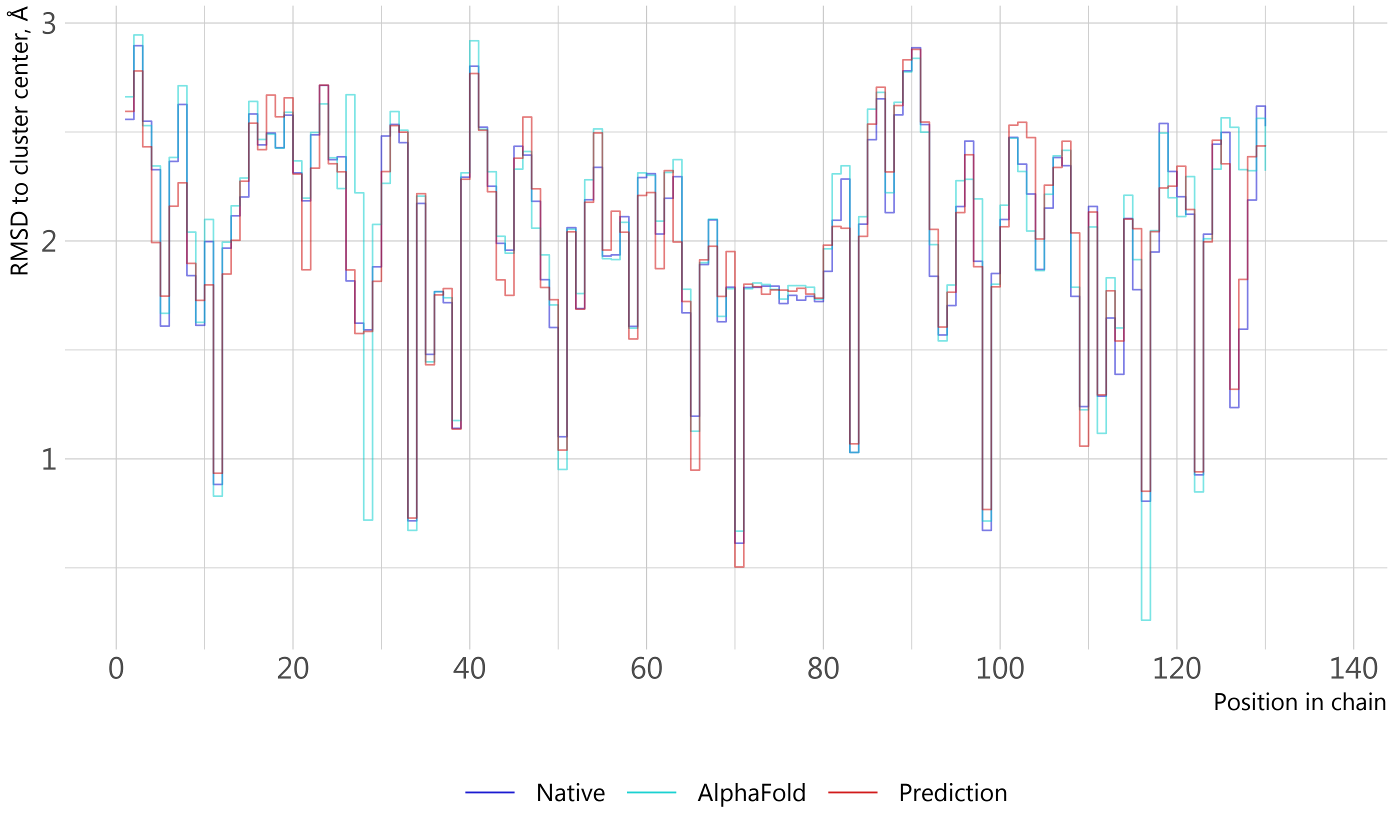

Corr(Native,AlphaFold) = 0.9202
Corr(Native, Prediction) = 0.9662

T 1049   6Y4FA PB 'l': N-cap α

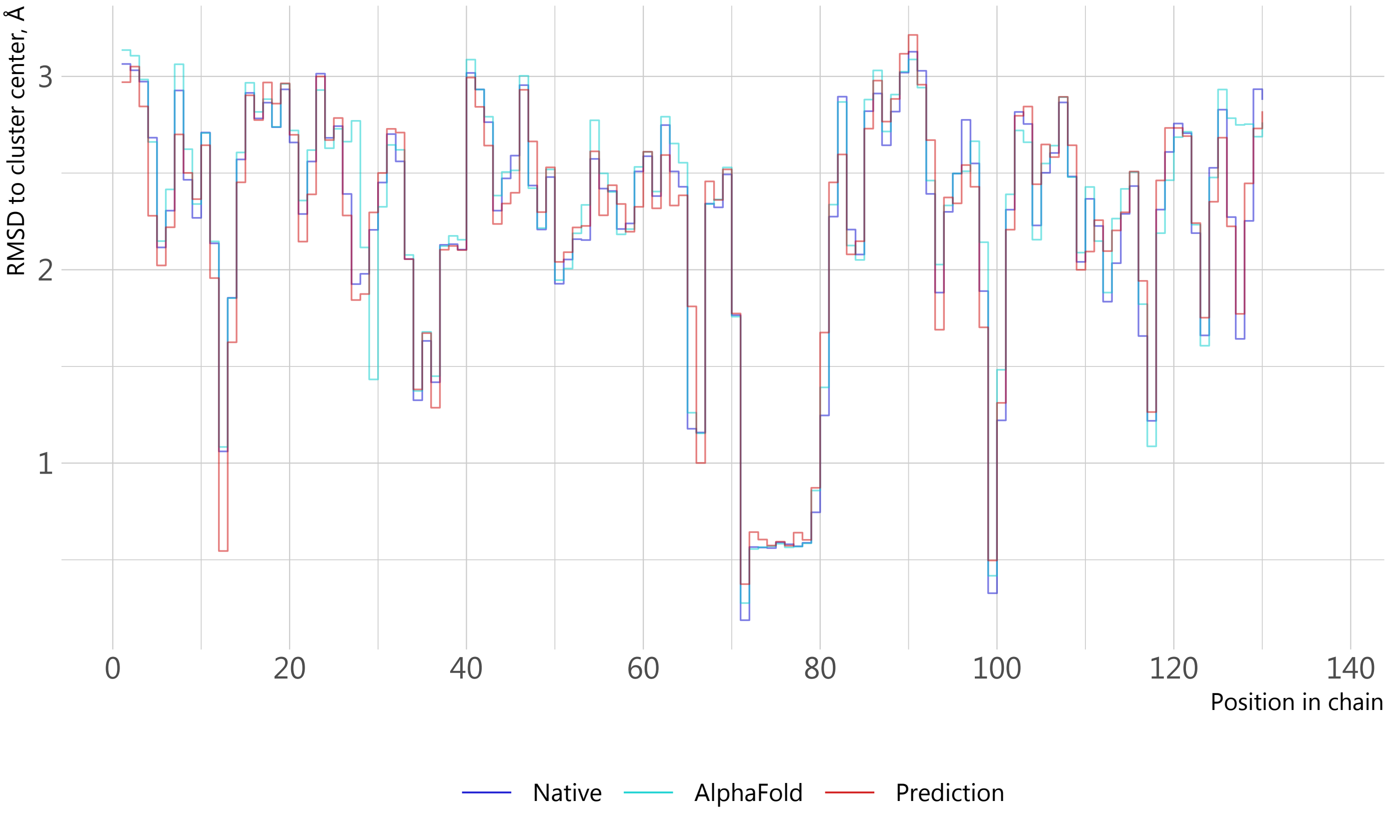

Corr(Native,AlphaFold) = 0.9667
Corr(Native, Prediction) = 0.9748

T 1049   6Y4FA PB 'm': α

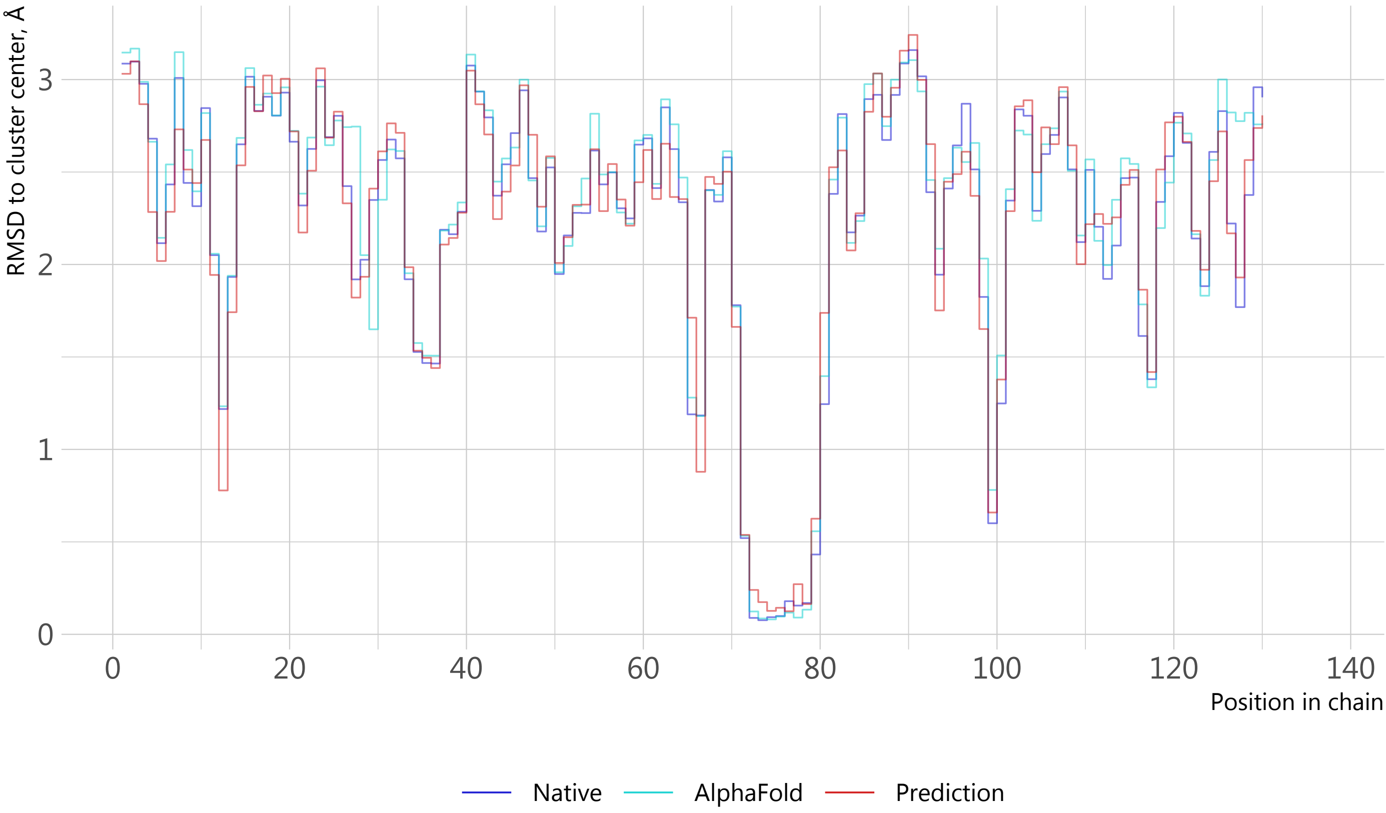

Corr(Native,AlphaFold) = 0.9742
Corr(Native, Prediction) = 0.9795

T 1049   6Y4FA PB 'n': C-cap α

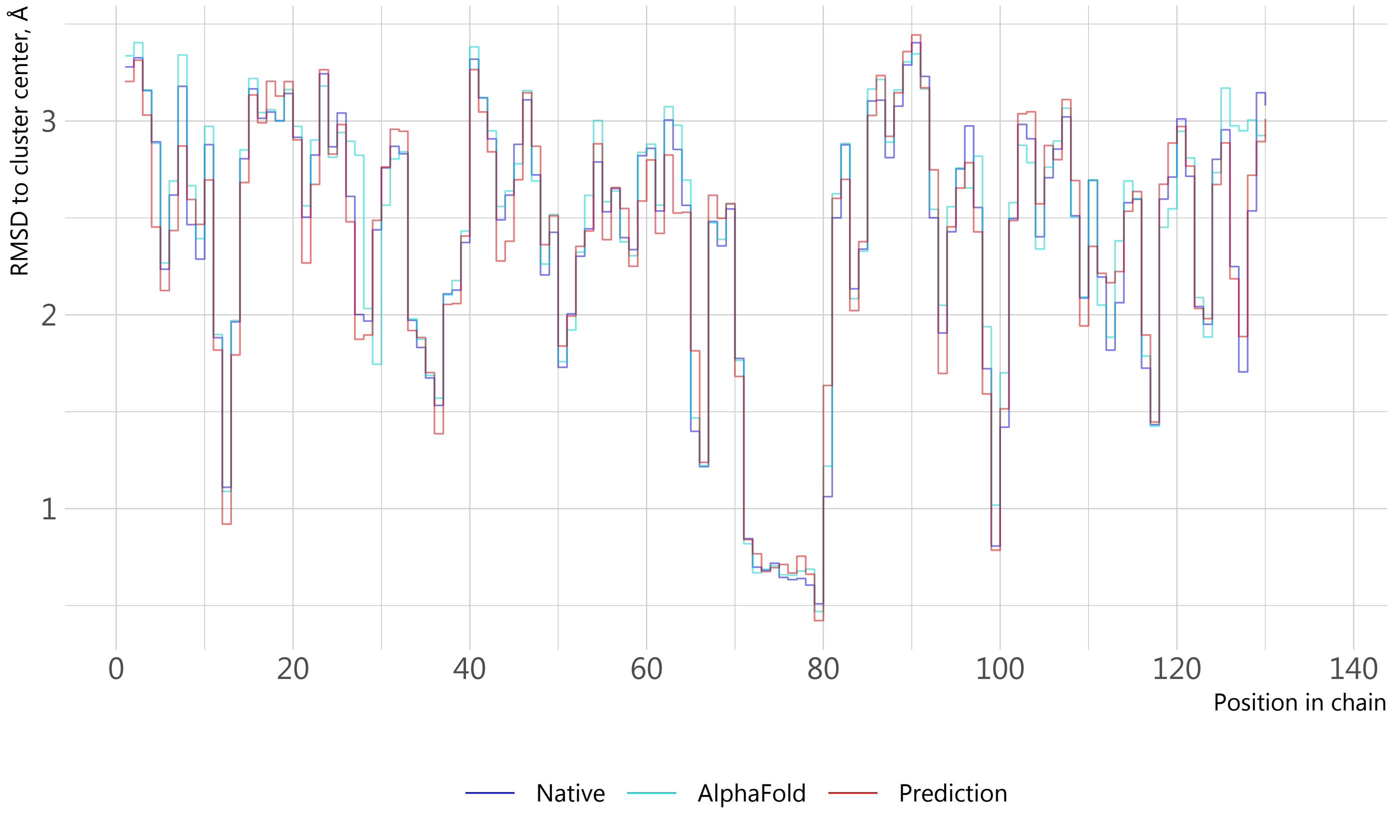

Corr(Native,AlphaFold) = 0.9653
Corr(Native, Prediction) = 0.9783

T 1049   6Y4FA PB 'o': C-cap α

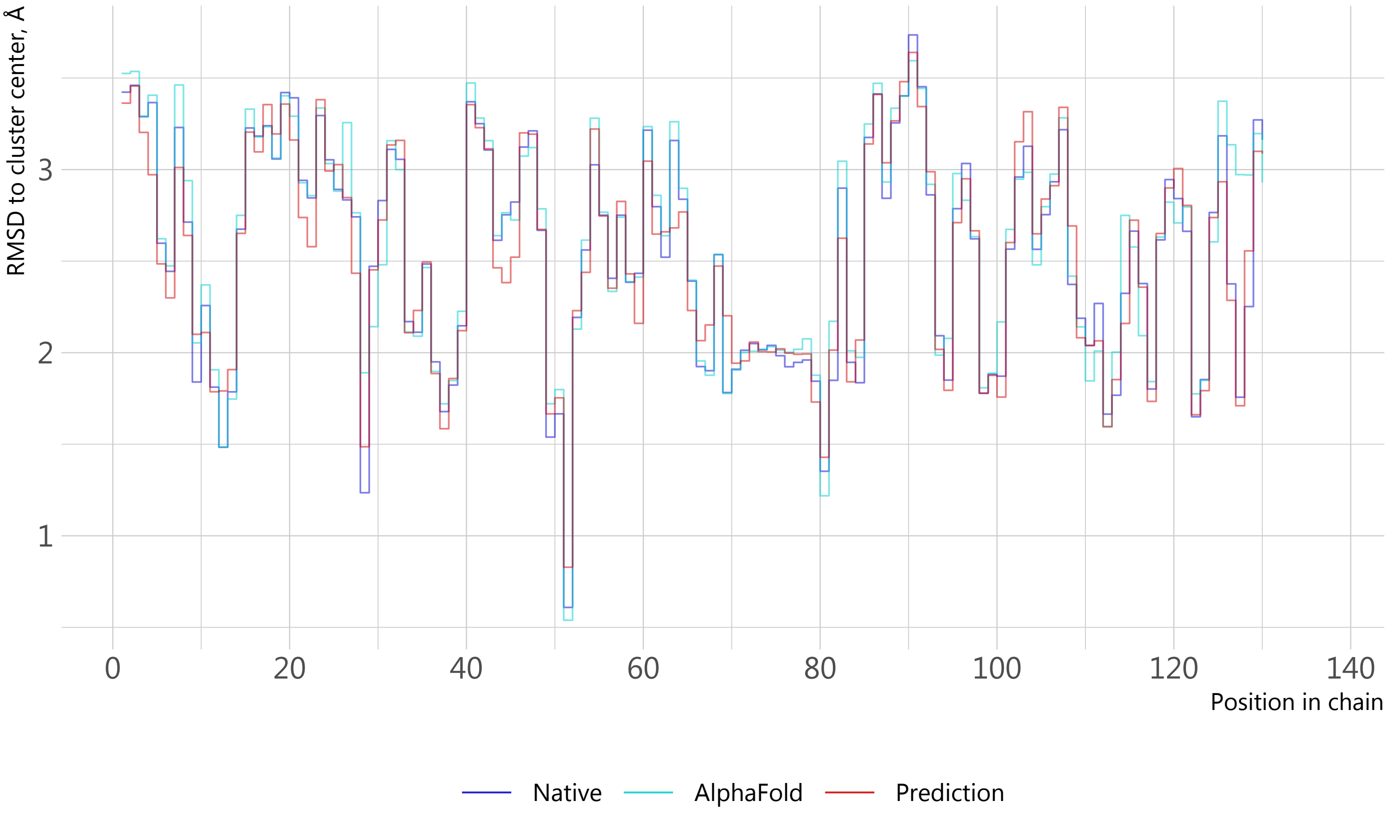

Corr(Native,AlphaFold) = 0.9491
Corr(Native, Prediction) = 0.9694

T 1049 6Y4FA PB 'p': C-cap α to N-cap β

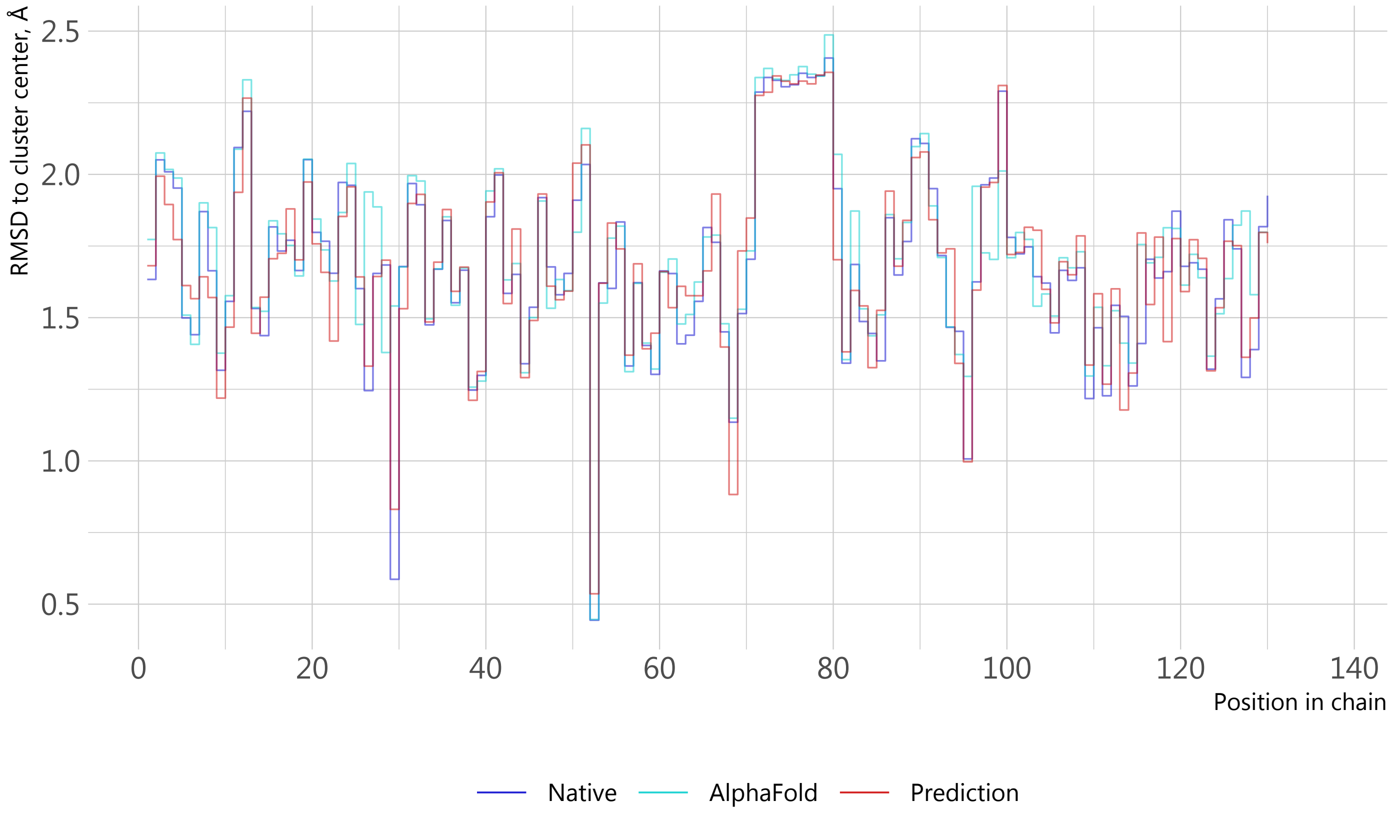

Corr(Native,AlphaFold) = 0.8913
Corr(Native, Prediction) = 0.9391
