# Supplementary material for: Effective Local and Secondary Protein Structure Prediction by Combining a Neural Network-Based Approach with Extensive Feature Design and Selection without Reliance on Evolutionary Information
Source: Int J Mol Sci. 2023 Oct 27;24(21):15656. doi: 10.3390/ijms242115656 (PMC10648199; doi:10.3390/ijms242115656)
Supplement: Supplementary file 1 [file ijms-24-15656-s001.zip › S5.Methods.pdf]

Supplemental Methods S5. Databases and predictors generation. Statistical significant features assessment.

After unpacking the files, all databases are now prepared for the assessment of statistically significant features. We utilized the PDB databank, specifically the PISCES30 [1] PDB subset, with the following selection criteria: a resolution cutoff of 2.5 Å and an R-factor cutoff of 1.0. Additionally, we allowed a sequence identity of up to 30%. These criteria resulted in the selection of 17,148 protein chains, all of which had their structures resolved through X-ray methods (*i.e.*, no NMR-resolved structures). If you wish to utilize a different PDB subset, you will need to regenerate all databases, following the process described below.

Databases generation

1. Get PDB databank:
```
rsync -rlpt -v -z --delete --port=33444 \
rsync.rcsb.org::ftp_data/structures/divided/pdb/ /home/username/PDB
```
2. Unpack PDB databank.
3. Edit file _config that contains paths to directories for the databases:

```
# Path to directory containing PDB (Protein Data Bank) files
Path_to_PDB_store                  /home/username/PDB/

# Path do directory containing binary chain representation
Path_to_Chain_store                Store/Chain_store/

# directory for RMSD based database
Path_to_Frequency_extrapolation    Store/Frequency_extrapolation/

# definition of 16 Protein Blocks by dihedral angles
Path_to_Cluster_set                Store/Cluster_set/

# path to 20Aminoacids templates
Path_to_20Aminoacids_template      Store/20Aminoacids/
```

There are vector models for 20 amino acids for calculation Cartesian coordinates by dihedral angles. *I.e.* it contains information about bond angles and bond lengths for 20 amino acids.

```
# path to predictive models
Path_to_Model_store                Store/Model_store/
```

This directory contains predictive models.

4. Generation a presentations of protein main chain of datasets.

`all_for_dssp`	– all chains
`CaspDeBrew`	– 10 targets from the CASP 14 free modeling classification, as in [2]
`CB513`	– CB513 benchmarking dataset [3]
`test_for_dssp`	– test sample
`train_for_dssp`	– train sample
`validation_for_dssp`	– validation sample<br>e.g.: first line in `all_for_dssp` - `1A0TQ`<br>`1A0T` – PDB ID<br>`Q` – chain ID

These chain files contain the lists (PDB subsets usually) that will be used for databases generation and features statistical assessment. *E.g.* `all_for_dssp` file contains the list of protein chains from PISCES30 PDB subset. The `train_for_dssp`, `test_for_dssp`, and `validation_for_dssp` files are subsets of the `all_for_dssp` generated by randomly sampling with the 6:3:1 ratio correspondingly. The CB513 dataset [3] was designed to evaluate secondary structure prediction methods, and it remains a widely used benchmark. The dataset consists of 513 non-homologous protein domains accounting for 438 protein chains in total (some chains contain two or more domains). The `CaspDeBrew` dataset [2] consists of 10 proteins from the free modeling category [4] of the CASP14 contest.

Before compilation you should uncomment two lines in the file `foundation/main.cpp`:

```
//    Chain_store_test chain_store_test_;
//    chain_store_test_.run();      /// uncomment for 1_MakeChainBinary
```

and then compile by `make -j8 makefile`
Alternatively, you can run precompiled Linux file
`./1_MakeChainBinary`
When process will finish you will get:
`*.bin` files will appear in `Store/Chain_store/binary/` directory. They are the binary representations of the proteins main chain coordinates for the database entry.
`*.protocol` files will appear in `Store/Chain_store/protocol/` directory. They are the protocols of processing the main chain coordinates.

Example: at the beginning of the Store/Chain_store/protocol/4OGEA.protocol

`MET`	`MET`	`***`	`--`	'*' means missing Cartesian coordinates
`TRP`	`TRP`	`***`	`--`	'*' means missing Cartesian coordinates
……………				
`HIS`	`HIS`	`10`	`CG`	'HIS' by SEQRES record in PDB file, 'HIS' by ATOM record in PDB file, 10 – residue number according to PDB entry, C – means that Cartesian coordinates present, G – bond angles, while bond length satisfies the constraints from the control file `Store/Chain_store/sheduler`

5. Generation of a database reflecting statistics of distances from various sequence elements to Protein Blocks centers

In each of the subdirectories in `Store/Frequency_Extrapolation/` there is a file `'sheduler'` that defines the database parameters:

#This file defines the rules by which some amino acids are considered indistinguishable
`DEGENERATION_ASSIGNMENT_FILE current.degeneration`

Cluster set name PB
`CLUSTER_SET_NAME              PB`

In addition to regular RMSD, the database stores inverse distances 'inv_D':
#inv_D = 1/(DENOMINATOR_CONSTANT + RMSD)
`DENOMINATOR_CONSTANT          0.1`

#List of PDB chains used for database generation
```
CHAIN_LIST                      all_for_dssp
```

Below you can see the example of `current.degeneration` file for `Store/Frequency_extrapolation/PB_W7_tail_GP/databases`:

```
//
-3   A V L I P M C F Y W K R H D E N Q S T G O X
-2   A V L I P M C F Y W K R H D E N Q S T G O X
-1   ALMC VIFYWKRHDENQST GP O X
 0   ALMC VIFYWKRHDENQST GP O X
 1   ALMC VIFYWKRHDENQST GP O X
 2   A V L I P M C F Y W K R H D E N Q S T G O X
 3   A V L I P M C F Y W K R H D E N Q S T G O X
```

The first column is the relative chain position. At position -3, all amino acids are different. At position -1, `ALMC` amino acids are indistinguishable, as are `VIFYWKRHDENQST`, etc.

With the above degeneration, the following fragments of the sequence:
```
AAAAAAA
AALLLMC
AALMCAA
```
are indistinguishable.

The database contains the number of such indistinguishable fragments, the average RMSD of each PB and the corresponding standard deviations. For each database, the type of degeneration and the size of the current window were specified in file `current.degeneration` file. `PB_W7_tail_GP` database has window size = 7.

Before compilation you should uncomment two lines in the file foundation/main.cpp:
```
//Frequency_extrapolation_test frequency_extrapolation_test_;
//frequency_extrapolation_test_.run();
```
and then compile by `make –j8 makefile`
Alternatively, you can run precompiled Linux file
`./2_MakeFrequencyDatabases`
When process will finish a database file `base/together.freq_data` should appear in each subdirectory of `Store/Frequency Extrapolation/` for all 11 databases that are used to create RMSD-based predictors.

Generation a predictive model and identifying a set of statistically significant predictors for each of Protein Blocks.

Configuration files & directories are located in the subdirectory `PredictorSelectModel` of the directory that configured by variable `Path_to_Model_store` in `_config` file (by default it's `Store/Model_store/`).

`sheduler`
file contains model main configuration, *i.e.* filenames that describe various model parameters.

`cluster_function.task`
file determines the order of Protein Blocks for a multiple regression.

`CowardVariables.task`
file contains the full list of predictors. This list is common for all Protein Blocks. The current set of predictors was generated in the process of predictive model iterative enhancements. The parameters of these predictors were adjusted during these iterations.

`cross_sum\together.cross_sum`
file contains the fitted model.

`plain_results`
subdirectory contains lists of significant predictors both for regular RMSD and inverted distances for each of Protein Blocks/distance. E.g. for PB ‘b’ it’s files `together_1.plain_result` and `together_17.plain_result`

`regression_options`
file contains parameters for stepwise regression analysis

```
#F-statistics lower threshold to include predictor to the model. I.e. if during stepwise regression
#analysis F statistics increases above 100 for some predictor then it is added to the model.
FISHER_INCLUDE    100
#F-statistics lower threshold to exclude predictor from the model. I.e. if during stepwise regression
#analysis F statistics decreases below 100 for some predictor then it is excluded from the model.
FISHER_EXCLUDE    100
#Provides calculations stability for the highly correlated predictors cases
TOLERANCE         0.1
```

The higher the `FISHER_INCLUDE` and `FISHER_EXCLUDE` values, the fewer predictors will be included to the model.

Before compilation you should uncomment two lines in the file `foundation/main.cpp`:

```
//    Abu_Maimonides_Rambam_test abu_maimonides_rambam_test_;
//    abu_maimonides_rambam_test_.run();
```

and then compile by `make –j8 makefile`
Alternatively, you can run precompiled Linux file
`./3_MakeRegressStepwiseModel`

When process will finish, a set of `*.plain_result` files will appear in the directory `Model_store/PredictorSelectModel/` Files with indices from 0 to 15 are direct RMSDs, while files with indices from 16 to 31 are inverse RMSDs (1/(const +RMSD)).
In this work we do not use inverse RMSDs to obtain a set of predictors for the neural network.

E.g., first predictor in the file
`Store/Model_store/PredictorSelectModel/plain_results/together_0.plain_result`

```
0 7 1322.416 7 -0.13177601 0.00362370 7 # Log_occurence_differrence 0 DUMB PB_w11_tail 0 2
```

`0`	serial number of included predictor
`7`	serial number in original predictor’s set
`1322.416`	F-statistics value
`7`	serial number in original predictor’s set
`-0.13177601`	regression coefficient
`0.00362370`	regression coefficient standard deviation

The predictors from all 16 corresponding PBs were combined into a single unified set, eliminating any duplicates. In the subsequent step, for any pairs of predictors displaying a Pearson correlation exceeding 0.9, the one with the lower F-statistics was eliminated.

Features generation

1. Physicochemical amino acids properties based predictors.

There are two types of functional transformations that are implemented right now: Dull_Sum (the sum of current property values in the window), c_Fourier_Smoothed (periodicity search for the current property with the smoothing by bell-shape function). Amino acid sequence is mapping to an array, corresponding to the selected property from the AAindex [5] database. *E.g.*, for the amino acid sequence AGP and the property PTIO830101 (helix-coil equilibrium constant, https://www.genome.jp/dbget-bin/www_bget?aaindex:PTIO830101) [5], the corresponding array would be {1.10, 0.60, 0.10}, according to the values from the following table:

A/L	R/K	N/M	D/F	C/P	Q/S	E/T	G/W	H/Y	I/V
1.10	0.95	0.80	0.65	0.95	1.00	1.00	0.60	0.85	1.10
1.25	1.00	1.15	1.10	0.10	0.75	0.75	1.10	1.10	0.95

Further, a functional transformation of this predictor is performing according to the rules that are determined by a configuration line for this predictor in the control file. *E.g.*

```
Dull_Sum    0      DUMB  CHOP780208  0      15     1
```

`Dull_Sum` Type of transformation function (mandatory item)
`0 DUMB` Service fields for generating combined predictors (mandatory item)
`CHOP780208` Physicochemical property from AAINDEX database
`0 15` window start and end relatively to the current position in the protein backbone chain
`1` the power of predictor (linear, quadratic, square root, etc).

This predictor calculates the sum of the properties corresponding to CHOP780208 (normalized frequency of N-terminal beta-sheet, https://www.genome.jp/dbget-bin/www_bget?aaindex:CHOP780208 [5]) within the amino acid sequence, ranging from position 0 (the current position in the protein backbone chain) to 15. The final value is determined by raising the sum to a power, which can be any decimal value.

The initial construction of preliminary prediction models relies on an extensive set of input predictors. This set is derived using various transformation functions with different parameters. The stepwise regression analysis method, employed in constructing these preliminary models, is capable of selecting statistically significant predictors. This helps in identifying the most appropriate transformation functions and their corresponding parameters.

Predictors serve to formalize our assumptions about the factors influencing the protein's structure. For instance, if we assume that the property PTIO830101 is significant within the range of -5 to 5, the predictor will take the following form:

```
Dull_Sum    0      DUMB  PTIO830101  -5     5      1
```

If we presume that the relationship may be nonlinear, it makes sense to include the following predictors in the model:

```
Dull_Sum    0      DUMB  PTIO830101  -5     5      2
Dull_Sum    0      DUMB  PTIO830101  -5     5      3
Dull_Sum    0      DUMB  PTIO830101  -5     5      0.5
```

The example of more complex predictor:

```
c_FourierSmoothed      0      DUMB   WERD780101  3.6   3     1
```

The functional transformation of this predictor reflects a periodic change in hydrophobicity with a period of T=3.6. The parameter "3" characterizes the diminishing influence as you move away from the current position. It's a smoothing parameter, where a larger value implies stronger smoothing. You can find detailed implementation information in the file `CowardVariables/c_FourierSmoothed.cpp`. The value of this predictor reach maximum when the variation of the WERD780101 property (propensity to be buried inside, https://www.genome.jp/dbget-bin/www_bget?aaindex:WERD780101, [5]) along the chain corresponds to a period of 3.6. The mass generation of predictors for this transformation function involves testing a range of suitable properties from AAindex, along with all other relevant parameters:

```
c_FourierSmoothed      0     DUMB  WERD780101  1.2   3     1
...
c_FourierSmoothed      0     DUMB  WERD780101  3.6   3     1
...
c_FourierSmoothed      0     DUMB  PTIO830101  3.6   3     1
c_FourierSmoothed      0     DUMB  PTIO830101  1.2   3     1
c_FourierSmoothed      0     DUMB  PTIO830101  1.2   4     1
c_FourierSmoothed      0     DUMB  PTIO830101  1.2   4     2
```

In this work in the initial predictors set were included all energy-associated properties from AAindex database [5]:

`EISD860101`	solvation free energy
`GUYH850101`	partition energy
`JANJ790102`	transfer free energy
`MIYS850101`	effective partition energy
`NOZY710101`	transfer energy, organic solvent/water
`RADA880107`	energy transfer from out to in (95% buried)
`ROBB790101`	hydration free energy
`YUTK870101`	unfolding Gibbs energy in water at pH 7.0
`MUNV940105`	free energy in beta-strand region
`WOLR810101`	hydration potential
`PTIO830101`	helix-coil equilibrium constant
`PTIO830102`	beta-coil equilibrium constant

with the following parameters ranges:

T was tested in the range [1, 10] with the step 0.1
D was tested in the range [4, 9] with the step 0.1
P was tested in the range [1, 2] with the step 0.1

The example of initial combinations of physicochemical-associated predictors for the further processing is located in the file `Store/Model_store/DebuggingPredictorParameters/CowardVariables.task`

2. RMSD structure-based predictors.

There are 4 types of structure-based predictors. First three types of predictors are based upon t-test comparing the means of two independent samples. Let us consider one of the sixteen protein blocks PB_j, $j \in \{1, 2, 3, . . . , 16\}$, and a 5-residue sequence *seq*. Also, let $N_{occ}(seq)$ be the number of times

sequence *seq* occurs among the sequences with known structures (the training sample), $\bar{\mu}_j = \bar{\mu}_j(seq)$ be the mean distance between the structures with that sequence and the PB_j. Further, let $\bar{\mu}_j$ be the average distance between PB_j and all 5-residue fragments in the training sample, and s_j^2 be its sampling variance and N to be the size of the training sample. Then, according to Student t-statistics, one example of the predictors is

$$t_j(seq) = \frac{\mu_j - \bar{\mu}_j(seq)}{s_j(seq)}, \text{ where } s(seq) = \frac{\sigma_j^2(seq)}{N_{occ}(seq)} + \frac{s_j^2}{N}$$

Note that if $N \gg N_{occ}(seq) > 1$, $s \cong \frac{\sigma_j^2(seq)}{N_{occ}(seq)}$, the following holds:

$$t_j(seq) \cong \frac{\mu_j - \bar{\mu}_j(seq)}{s_j(seq)} \sqrt{N_{occ}(seq)} \quad (1)$$

Thus, the number of occurrences of as certain sequence in the sample, $N_{occ}(seq)$ is crucial for correct estimation of $t_j(seq)$. Namely, small values of $N_{occ}(seq)$ may yield unreliable estimates of $\sigma_j(seq)$ as well as $t_j(seq)$. To alleviate this, we tried various reduced alphabets [6].

a. `Student_emasculate` T-statistics value, calculated by formula (1)
b. `T_statistics_single` Probability by t-test statistics, calculated by formula (1)
c. `T_statistics_window` Sum the t-test probabilities in the window
d. `Log_occurence_differrence` Occurrence difference, calculated by formula $value = \log(1+\log(1+N_{occ}))*(\mu_j - \bar{\mu}_j(seq))$

Each predictor can be both for direct and inverse RMSDs. In the case of the inverse RMSDs predictor name concatenates with the _inv suffix. E.g. coupled to `T_statistics_window` predictor for the inverse RMSDs has the name `T_statistics_window_inv` with the same parameters. There are 11 ready-to-use RMSD databases, any database can be applied to any predictor. Parameters of each database can be found in the '`current.degeneration`' and '`sheduler`' files of the appropriate subdirectory of `Store/Frequency_extrapolation/` directory.

a. `PB_w11_tail` Window size = 11, parameters to find β-turn
b. `PB_w11_tail_GP` Window size = 11, parameters to find β-turn, P and G are identical in the range [-2, 2]
c. `PB_W4C` Window size = 5, parameters to find α-helix ends
d. `PB_W4N` Window size = 5, parameters to find α-helix
e. `PB_W5_noDEG_ZIP` Window size = 5, no degeneration at all
f. `PB_W5_noDEG_ZIP_denom` Window size = 5, no degeneration at all, optimized for reverse RMSD
g. `PB_W6_3_trivial_PG` Window size = 6, coordinates [-2, 3], classification by amino acids classes (aliphatic, polar, acidic, basic, unique). Origin in the window is in '2' position.
h. `PB_W6_4_trivial_PG` The same as above, but origin in the window is in '3' position.
i. `PB_W7_tail_GP` Window size = 7, parameters to find turn with the possible interaction at window's ends.
j. `PB_W7_trivial_PG` Window size = 7, classification by amino acids classes (as in 'g').
k. `PB_w9_tail` Window size = 9, parameters to find any turn at longer window size.

Examples of complete records for structure-based predictors are below:

```
Student_emasculate      0     DUMB  PB_W5_noDEG_ZIP         10    0     3     1
```

`Student_emasculate`	functional transformation name
`0     DUMB`	Service fields for generating combined predictors (mandatory item)
`PB_W5_noDEG_ZIP`	RMSD database (mean values, standard deviation and occurrence number for each 'word' in learning sample (e.g. '`AGGPL`')
`10`	Protein Block serial number (PB 'i' in this case)
`0     3`	considered interval in the chain (positions from the current to +3)
`1`	the power of predictor (linear, quadratic, square root, etc)

```
Log_occurence_differrence     0     DUMB  PB_W5_noDEG_ZIP_denom   7     0.5
```

`Log_occurence_differrence`	functional transformation name
`0     DUMB`	Service fields for generating combined predictors (mandatory item)
`PB_W5_noDEG_ZIP_denom`	RMSD database (mean values, standard deviation and occurrence number for each 'word' in learning sample (e.g. '`AGGPL`')
`7`	Protein Block serial number (PB 'g' in this case)
`0.5`	the power of predictor (linear, quadratic, square root, etc)

```
T_statistics_window     0     DUMB  PB_W7_trivial_PG  3     -2    2     2.7
```

`T_statistics_window`	functional transformation name
`0     DUMB`	Service fields for generating combined predictors (mandatory item)
`PB_W7_trivial_PG`	RMSD database (mean values, standard deviation and occurrence number for each 'word' in learning sample (e.g. '`AGGPL`')
`3`	Protein Block serial number (PB 'd' in this case)
`-2    2`	considered interval in the chain (positions from –2 to +2)
`2.7`	the power of predictor (linear, quadratic, square root, etc)

```
T_statistics_single     0     DUMB  PB_w9_tail  11    1.3
```

`T_statistics_single`	functional transformation name
`0     DUMB`	Service fields for generating combined predictors (mandatory item)
`PB_w9_tail`	RMSD database (mean values, standard deviation and occurrence number for each 'word' in learning sample (e.g. '`AGGPL`')
`11`	Protein Block serial number (PB 'h' in this case)
`1.3`	the power of predictor (linear, quadratic, square root, etc)

3. Assessing of statistically significant predictors

The source list of predictors for statistical assessment was obtained from our previous publication [6]. This list can be found in the `Store/Model_store/PredictorSelectModel/CowardVariables.task` file. The primary determinant of the number of significant predictors is the F-statistics. For each of the 16 Protein Blocks (PB) clusters, a distinct set of statistically significant predictors was obtained. To create a unified set for analysis, all these predictors were merged and duplicates were eliminated (duplicates invariably exist because the original set of predictors is common to all 16 PB clusters). Subsequently, the remaining predictors were arranged in order of their F-statistics magnitude, signifying their level of significance. Next, predictors exhibiting a Pearson correlation coefficient exceeding 0.9 were excluded from the set. In cases of correlated predictors, the one with the higher F-statistics value was retained. At last, a specific number of the most significant predictors were chosen as inputs for neural network models. Through the application of neural networks, predictive models were fitted and tested, their final quality was assessed, and adjustments to the F-statistics threshold value were made. After numerous iterations, we settled on an F-statistics threshold of 100. As a result of this rigorous process, a working set comprising 76 (predictors) x 5 (PB clusters) = 380 predictors was selected for final processing.

If you wish to create your own machine learning model, you'll need to craft your version of the 'Store/Model_store/PredictorSelectModel/CowardVariables.task' file (this can be achieved through an automated program, MassivePredictorGeneration). Afterward, run the './3_MakeRegressStepwiseModel' program. Upon completion of the processing, you will receive a list of statistically significant predictors for each protein block. These results will be stored in the directory 'Store/Model_store/PredictorSelectModel/plain_results'. The first 16 files pertain to direct RMSDs, while the subsequent 16 files concern reverse RMSDs. The first (or last) 16 files must be merged into a single file and processed as described earlier, which involves removing one of the correlated predictors in each pair, sorting the remaining predictors by their F-statistics values, and eliminating any features with F-statistics below a specified threshold.
The processed file should then be saved within the Store/Model_store/PredictorSelectModel directory as 'NN_CowardVariables.task' file.
Configuration of the dataset that will be used for generating datasets is determined by the 'CHAIN_LIST' parameter within the 'sheduler' file (e.g., 'train_dssp'). An appropriate list of PDB chains should be in the 'Store/Chain_store' directory under a filename corresponding to the 'CHAIN_LIST' variable (e.g., 'train_dssp' file).
Following this, execute the './4_PredictorListToNN' program, resulting in the appearance of two files in the 'Store/Model_store/NN_data_creation/NN_data' directory. These files should be converted into numpy arrays using Python scripts and employed as input for neural networks, serving as the dataset (X suffix) and feature set (Y suffix). To create next pair of files you should change 'CHAIN_LIST' variable (e.g. to CB513), corresponding PDB chain ID list should be in the file 'Store/Chain_store/CB513', and re-run './4_PredictorListToNN' program.

References

1. Wang, G.; Dunbrack, R. L., Jr., PISCES: recent improvements to a PDB sequence culling server. *Nucleic Acids Res* **2005,** 33, (Web Server issue), W94-8.
2. Cretin, G.; Galochkina, T.; de Brevern, A. G.; Gelly, J. C., PYTHIA: Deep Learning Approach for Local Protein Conformation Prediction. *Int J Mol Sci* **2021,** 22, (16).
3. Cuff, J. A.; Barton, G. J., Evaluation and improvement of multiple sequence methods for protein secondary structure prediction. *Proteins* **1999,** 34, (4), 508-19.
4. Kryshtafovych, A.; Schwede, T.; Topf, M.; Fidelis, K.; Moult, J., Critical assessment of methods of protein structure prediction (CASP)-Round XIV. *Proteins* **2021,** 89, (12), 1607-1617.
5. Kawashima, S.; Pokarowski, P.; Pokarowska, M.; Kolinski, A.; Katayama, T.; Kanehisa, M., AAindex: amino acid index database, progress report 2008. *Nucleic Acids Res* **2008,** 36, (Database issue), D202-5.
6. Milchevskaya, V.; Nikitin, A. M.; Lukshin, S. A.; Filatov, I. V.; Kravatsky, Y. V.; Tumanyan, V. G.; Esipova, N. G.; Milchevskiy, Y. V., Structural coordinates: A novel approach to predict protein backbone conformation. *PLoS One* **2021,** 16, (5), e0239793.
