# Supplementary material for: Effective Local and Secondary Protein Structure Prediction by Combining a Neural Network-Based Approach with Extensive Feature Design and Selection without Reliance on Evolutionary Information
Source: Int J Mol Sci. 2023 Oct 27;24(21):15656. doi: 10.3390/ijms242115656 (PMC10648199; doi:10.3390/ijms242115656)
Supplement: Supplementary file 1 [file ijms-24-15656-s001.zip › Generation.Databases.And.Predictors.tar.gz!/foundation/Minimizer_NR/Manual_numberical_recipies/Numerical.Recipes.3ed.pdf]

NUMERICAL RECIPES

The Art of Scientific Computing

THIRD EDITION

William H. Press
Saul A. Teukolsky
William T. Vetterling
Brian P. Flannery

CAMBRIDGE www.cambridge.org/9780521880688

This page intentionally left blank

NUMERICAL RECIPES

The Art of Scientific Computing

Third Edition

NUMERICAL RECIPES

The Art of Scientific Computing

Third Edition

William H. Press

Raymer Chair in Computer Sciences and Integrative Biology
The University of Texas at Austin

Saul A. Teukolsky

Hans A. Bethe Professor of Physics and Astrophysics
Cornell University

William T. Vetterling

Research Fellow and Director of Image Science
ZINK Imaging, LLC

Brian P. Flannery

Science, Strategy and Programs Manager
Exxon Mobil Corporation

CAMBRIDGE
UNIVERSITY PRESS

CAMBRIDGE UNIVERSITY PRESS
Cambridge, New York, Melbourne, Madrid, Cape Town, Singapore, São Paulo

Cambridge University Press
The Edinburgh Building, Cambridge CB2 8RU, UK
Published in the United States of America by Cambridge University Press, New York

www.cambridge.org
Information on this title: www.cambridge.org/9780521880688

© Cambridge University Press 1988, 1992, 2002, 2007 except for 13.10, which is placed into the public domain, and except for all other computer programs and procedures, which are

This publication is in copyright. Subject to statutory exception and to the provision of relevant collective licensing agreements, no reproduction of any part may take place without the written permission of Cambridge University Press.

First published in print format 2007

ISBN-13 978-0-511-33555-6 eBook (NetLibrary)
ISBN-10 0-511-33555-5 eBook (NetLibrary)

ISBN-13 978-0-521-88068-8 hardback
ISBN-10 0-521-88068-8 hardback

Cambridge University Press has no responsibility for the persistence or accuracy of urls for external or third-party internet websites referred to in this publication, and does not guarantee that any content on such websites is, or will remain, accurate or appropriate.

Without an additional license to use the contained software, this book is intended as a text and reference book, for reading and study purposes only. However, a restricted, limited free license for use of the software by the individual owner of a copy of this book who personally keyboards one or more routines into a single computer is granted under terms described on p. xix. See the section "License and Legal Information" (pp. xix–xxi) for information on obtaining more general licenses. Machine-readable media containing the software in this book, with included license for use by a single individual, are available from Cambridge University Press. The software may also be downloaded, with immediate purchase of a license also possible, from the Numerical Recipes Software Web site (http://www.nr.com). Unlicensed transfer of Numerical Recipes programs to any other format, or to any computer except one that is specifically licensed, is strictly prohibited. Technical questions, corrections, and requests for information should be addressed to Numerical Recipes Software, P.O. Box 380243, Cambridge, MA 02238-0243 (USA), email info@nr.com, or fax 781-863-1739.

Contents

Preface to the Third Edition (2007) **xi**

Preface to the Second Edition (1992) **xiv**

Preface to the First Edition (1985) **xvii**

License and Legal Information **xix**

1 Preliminaries **1**

1.0 Introduction 1
1.1 Error, Accuracy, and Stability 8
1.2 C Family Syntax 12
1.3 Objects, Classes, and Inheritance 17
1.4 Vector and Matrix Objects 24
1.5 Some Further Conventions and Capabilities 30

2 Solution of Linear Algebraic Equations **37**

2.0 Introduction 37
2.1 Gauss-Jordan Elimination 41
2.2 Gaussian Elimination with Backsubstitution 46
2.3 LU Decomposition and Its Applications 48
2.4 Tridiagonal and Band-Diagonal Systems of Equations 56
2.5 Iterative Improvement of a Solution to Linear Equations 61
2.6 Singular Value Decomposition 65
2.7 Sparse Linear Systems 75
2.8 Vandermonde Matrices and Toeplitz Matrices 93
2.9 Cholesky Decomposition 100
2.10 QR Decomposition 102
2.11 Is Matrix Inversion an N^3 Process? 106

3 Interpolation and Extrapolation **110**

3.0 Introduction 110
3.1 Preliminaries: Searching an Ordered Table 114
3.2 Polynomial Interpolation and Extrapolation 118
3.3 Cubic Spline Interpolation 120
3.4 Rational Function Interpolation and Extrapolation 124

3.5 Coefficients of the Interpolating Polynomial 129
3.6 Interpolation on a Grid in Multidimensions 132
3.7 Interpolation on Scattered Data in Multidimensions 139
3.8 Laplace Interpolation 150

4 Integration of Functions **155**
4.0 Introduction 155
4.1 Classical Formulas for Equally Spaced Abscissas 156
4.2 Elementary Algorithms 162
4.3 Romberg Integration 166
4.4 Improper Integrals 167
4.5 Quadrature by Variable Transformation 172
4.6 Gaussian Quadratures and Orthogonal Polynomials 179
4.7 Adaptive Quadrature 194
4.8 Multidimensional Integrals 196

5 Evaluation of Functions **201**
5.0 Introduction 201
5.1 Polynomials and Rational Functions 201
5.2 Evaluation of Continued Fractions 206
5.3 Series and Their Convergence 209
5.4 Recurrence Relations and Clenshaw's Recurrence Formula 219
5.5 Complex Arithmetic 225
5.6 Quadratic and Cubic Equations 227
5.7 Numerical Derivatives 229
5.8 Chebyshev Approximation 233
5.9 Derivatives or Integrals of a Chebyshev-Approximated Function . . 240
5.10 Polynomial Approximation from Chebyshev Coefficients 241
5.11 Economization of Power Series 243
5.12 Padé Approximants 245
5.13 Rational Chebyshev Approximation 247
5.14 Evaluation of Functions by Path Integration 251

6 Special Functions **255**
6.0 Introduction 255
6.1 Gamma Function, Beta Function, Factorials, Binomial Coefficients 256
6.2 Incomplete Gamma Function and Error Function 259
6.3 Exponential Integrals 266
6.4 Incomplete Beta Function 270
6.5 Bessel Functions of Integer Order 274
6.6 Bessel Functions of Fractional Order, Airy Functions, Spherical Bessel Functions 283
6.7 Spherical Harmonics 292
6.8 Fresnel Integrals, Cosine and Sine Integrals 297
6.9 Dawson's Integral 302
6.10 Generalized Fermi-Dirac Integrals 304
6.11 Inverse of the Function $x \log(x)$ 307
6.12 Elliptic Integrals and Jacobian Elliptic Functions 309

6.13 Hypergeometric Functions . . . 318
6.14 Statistical Functions . . . 320

7 Random Numbers **340**
7.0 Introduction . . . 340
7.1 Uniform Deviates . . . 341
7.2 Completely Hashing a Large Array . . . 358
7.3 Deviates from Other Distributions . . . 361
7.4 Multivariate Normal Deviates . . . 378
7.5 Linear Feedback Shift Registers . . . 380
7.6 Hash Tables and Hash Memories . . . 386
7.7 Simple Monte Carlo Integration . . . 397
7.8 Quasi- (that is, Sub-) Random Sequences . . . 403
7.9 Adaptive and Recursive Monte Carlo Methods . . . 410

8 Sorting and Selection **419**
8.0 Introduction . . . 419
8.1 Straight Insertion and Shell's Method . . . 420
8.2 Quicksort . . . 423
8.3 Heapsort . . . 426
8.4 Indexing and Ranking . . . 428
8.5 Selecting the Mth Largest . . . 431
8.6 Determination of Equivalence Classes . . . 439

9 Root Finding and Nonlinear Sets of Equations **442**
9.0 Introduction . . . 442
9.1 Bracketing and Bisection . . . 445
9.2 Secant Method, False Position Method, and Ridders' Method . . . 449
9.3 Van Wijngaarden-Dekker-Brent Method . . . 454
9.4 Newton-Raphson Method Using Derivative . . . 456
9.5 Roots of Polynomials . . . 463
9.6 Newton-Raphson Method for Nonlinear Systems of Equations . . . 473
9.7 Globally Convergent Methods for Nonlinear Systems of Equations 477

10 Minimization or Maximization of Functions **487**
10.0 Introduction . . . 487
10.1 Initially Bracketing a Minimum . . . 490
10.2 Golden Section Search in One Dimension . . . 492
10.3 Parabolic Interpolation and Brent's Method in One Dimension . . . 496
10.4 One-Dimensional Search with First Derivatives . . . 499
10.5 Downhill Simplex Method in Multidimensions . . . 502
10.6 Line Methods in Multidimensions . . . 507
10.7 Direction Set (Powell's) Methods in Multidimensions . . . 509
10.8 Conjugate Gradient Methods in Multidimensions . . . 515
10.9 Quasi-Newton or Variable Metric Methods in Multidimensions . . 521
10.10 Linear Programming: The Simplex Method . . . 526
10.11 Linear Programming: Interior-Point Methods . . . 537
10.12 Simulated Annealing Methods . . . 549
10.13 Dynamic Programming . . . 555

11 Eigensystems **563**
11.0 Introduction . . . 563
11.1 Jacobi Transformations of a Symmetric Matrix . . . 570
11.2 Real Symmetric Matrices . . . 576
11.3 Reduction of a Symmetric Matrix to Tridiagonal Form: Givens and Householder Reductions . . . 578
11.4 Eigenvalues and Eigenvectors of a Tridiagonal Matrix . . . 583
11.5 Hermitian Matrices . . . 590
11.6 Real Nonsymmetric Matrices . . . 590
11.7 The QR Algorithm for Real Hessenberg Matrices . . . 596
11.8 Improving Eigenvalues and/or Finding Eigenvectors by Inverse Iteration . . . 597

12 Fast Fourier Transform **600**
12.0 Introduction . . . 600
12.1 Fourier Transform of Discretely Sampled Data . . . 605
12.2 Fast Fourier Transform (FFT) . . . 608
12.3 FFT of Real Functions . . . 617
12.4 Fast Sine and Cosine Transforms . . . 620
12.5 FFT in Two or More Dimensions . . . 627
12.6 Fourier Transforms of Real Data in Two and Three Dimensions . . . 631
12.7 External Storage or Memory-Local FFTs . . . 637

13 Fourier and Spectral Applications **640**
13.0 Introduction . . . 640
13.1 Convolution and Deconvolution Using the FFT . . . 641
13.2 Correlation and Autocorrelation Using the FFT . . . 648
13.3 Optimal (Wiener) Filtering with the FFT . . . 649
13.4 Power Spectrum Estimation Using the FFT . . . 652
13.5 Digital Filtering in the Time Domain . . . 667
13.6 Linear Prediction and Linear Predictive Coding . . . 673
13.7 Power Spectrum Estimation by the Maximum Entropy (All-Poles) Method . . . 681
13.8 Spectral Analysis of Unevenly Sampled Data . . . 685
13.9 Computing Fourier Integrals Using the FFT . . . 692
13.10 Wavelet Transforms . . . 699
13.11 Numerical Use of the Sampling Theorem . . . 717

14 Statistical Description of Data **720**
14.0 Introduction . . . 720
14.1 Moments of a Distribution: Mean, Variance, Skewness, and So Forth 721
14.2 Do Two Distributions Have the Same Means or Variances? . . . 726
14.3 Are Two Distributions Different? . . . 730
14.4 Contingency Table Analysis of Two Distributions . . . 741
14.5 Linear Correlation . . . 745
14.6 Nonparametric or Rank Correlation . . . 748
14.7 Information-Theoretic Properties of Distributions . . . 754
14.8 Do Two-Dimensional Distributions Differ? . . . 762

14.9 Savitzky-Golay Smoothing Filters . . . 766

15 Modeling of Data **773**
15.0 Introduction . . . 773
15.1 Least Squares as a Maximum Likelihood Estimator . . . 776
15.2 Fitting Data to a Straight Line . . . 780
15.3 Straight-Line Data with Errors in Both Coordinates . . . 785
15.4 General Linear Least Squares . . . 788
15.5 Nonlinear Models . . . 799
15.6 Confidence Limits on Estimated Model Parameters . . . 807
15.7 Robust Estimation . . . 818
15.8 Markov Chain Monte Carlo . . . 824
15.9 Gaussian Process Regression . . . 836

16 Classification and Inference **840**
16.0 Introduction . . . 840
16.1 Gaussian Mixture Models and k-Means Clustering . . . 842
16.2 Viterbi Decoding . . . 850
16.3 Markov Models and Hidden Markov Modeling . . . 856
16.4 Hierarchical Clustering by Phylogenetic Trees . . . 868
16.5 Support Vector Machines . . . 883

17 Integration of Ordinary Differential Equations **899**
17.0 Introduction . . . 899
17.1 Runge-Kutta Method . . . 907
17.2 Adaptive Stepsize Control for Runge-Kutta . . . 910
17.3 Richardson Extrapolation and the Bulirsch-Stoer Method . . . 921
17.4 Second-Order Conservative Equations . . . 928
17.5 Stiff Sets of Equations . . . 931
17.6 Multistep, Multivalue, and Predictor-Corrector Methods . . . 942
17.7 Stochastic Simulation of Chemical Reaction Networks . . . 946

18 Two-Point Boundary Value Problems **955**
18.0 Introduction . . . 955
18.1 The Shooting Method . . . 959
18.2 Shooting to a Fitting Point . . . 962
18.3 Relaxation Methods . . . 964
18.4 A Worked Example: Spheroidal Harmonics . . . 971
18.5 Automated Allocation of Mesh Points . . . 981
18.6 Handling Internal Boundary Conditions or Singular Points . . . 983

19 Integral Equations and Inverse Theory **986**
19.0 Introduction . . . 986
19.1 Fredholm Equations of the Second Kind . . . 989
19.2 Volterra Equations . . . 992
19.3 Integral Equations with Singular Kernels . . . 995
19.4 Inverse Problems and the Use of A Priori Information . . . 1001
19.5 Linear Regularization Methods . . . 1006
19.6 Backus-Gilbert Method . . . 1014

19.7 Maximum Entropy Image Restoration 1016

20 Partial Differential Equations **1024**
20.0 Introduction 1024
20.1 Flux-Conservative Initial Value Problems 1031
20.2 Diffusive Initial Value Problems 1043
20.3 Initial Value Problems in Multidimensions 1049
20.4 Fourier and Cyclic Reduction Methods for Boundary Value Problems 1053
20.5 Relaxation Methods for Boundary Value Problems 1059
20.6 Multigrid Methods for Boundary Value Problems 1066
20.7 Spectral Methods 1083

21 Computational Geometry **1097**
21.0 Introduction 1097
21.1 Points and Boxes 1099
21.2 KD Trees and Nearest-Neighbor Finding 1101
21.3 Triangles in Two and Three Dimensions 1111
21.4 Lines, Line Segments, and Polygons 1117
21.5 Spheres and Rotations 1128
21.6 Triangulation and Delaunay Triangulation 1131
21.7 Applications of Delaunay Triangulation 1141
21.8 Quadtrees and Octrees: Storing Geometrical Objects 1149

22 Less-Numerical Algorithms **1160**
22.0 Introduction 1160
22.1 Plotting Simple Graphs 1160
22.2 Diagnosing Machine Parameters 1163
22.3 Gray Codes 1166
22.4 Cyclic Redundancy and Other Checksums 1168
22.5 Huffman Coding and Compression of Data 1175
22.6 Arithmetic Coding 1181
22.7 Arithmetic at Arbitrary Precision 1185

Index **1195**

Preface to the Third Edition (2007)

"I was just going to say, when I was interrupted..." begins Oliver Wendell Holmes in the second series of his famous essays, *The Autocrat of the Breakfast Table*. The interruption referred to was a gap of 25 years. In our case, as the autocrats of *Numerical Recipes*, the gap between our second and third editions has been "only" 15 years. Scientific computing has changed enormously in that time.

The first edition of *Numerical Recipes* was roughly coincident with the first commercial success of the personal computer. The second edition came at about the time that the Internet, as we know it today, was created. Now, as we launch the third edition, the practice of science and engineering, and thus scientific computing, has been profoundly altered by the mature Internet and Web. It is no longer difficult to find *somebody's* algorithm, and usually free code, for almost any conceivable scientific application. The critical questions have instead become, "How does it work?" and "Is it any good?" Correspondingly, the second edition of *Numerical Recipes* has come to be valued more and more for its text explanations, concise mathematical derivations, critical judgments, and advice, and less for its code implementations per se.

Recognizing the change, we have expanded and improved the text in many places in this edition and added many completely new sections. We seriously considered leaving the code out entirely, or making it available only on the Web. However, in the end, we decided that without code, it wouldn't be *Numerical Recipes*. That is, without code you, the reader, could never know whether our advice was in fact honest, implementable, and practical. Many discussions of algorithms in the literature and on the Web omit crucial details that can only be uncovered by actually coding (our job) or reading compilable code (your job). Also, we needed actual code to teach and illustrate the large number of lessons about object-oriented programming that are implicit and explicit in this edition.

Our wholehearted embrace of a style of object-oriented computing for scientific applications should be evident throughout this book. We say "*a* style," because, contrary to the claims of various self-appointed experts, there can be no one rigid style of programming that serves all purposes, not even all scientific purposes. Our style is ecumenical. If a simple, global, C-style function will fill the need, then we use it. On the other hand, you will find us building some fairly complicated structures for something as complicated as, e.g., integrating ordinary differential equations. For more on the approach taken in this book, see §1.3 – §1.5.

In bringing the text up to date, we have luckily not had to bridge a full 15-year gap. Significant modernizations were incorporated into the second edition versions in Fortran 90 (1996)* and C++ (2002), in which, notably, the last vestiges of unit-based arrays were expunged in favor of C-style zero-based indexing. Only with this third edition, however, have we incorporated a substantial amount (several hundred pages!) of completely new material. Highlights include:

- a new chapter on classification and inference, including such topics as Gaussian mixture models, hidden Markov modeling, hierarchical clustering (phylogenetic trees), and support vector machines

*"Alas, poor Fortran 90! We knew him, Horatio: a programming language of infinite jest, of most excellent fancy: he hath borne us on his back a thousand times."

- a new chapter on computational geometry, including topics like KD trees, quad- and octrees, Delaunay triangulation and applications, and many useful algorithms for lines, polygons, triangles, spheres, etc.
- many new statistical distributions, with pdfs, cdfs, and inverse cdfs
- an expanded treatment of ODEs, emphasizing recent advances, and with completely new routines
- much expanded sections on uniform random deviates and on deviates from many other statistical distributions
- an introduction to spectral and pseudospectral methods for PDEs
- interior point methods for linear programming
- more on sparse matrices
- interpolation on scattered data in multidimensions
- curve interpolation in multidimensions
- quadrature by variable transformation and adaptive quadrature
- more on Gaussian quadratures and orthogonal polynomials
- more on accelerating the convergence of series
- improved incomplete gamma and beta functions and new inverse functions
- improved spherical harmonics and fast spherical harmonic transforms
- generalized Fermi-Dirac integrals
- multivariate Gaussian deviates
- algorithms and implementations for hash memory functions
- incremental quantile estimation
- chi-square with small numbers of counts
- dynamic programming
- hard and soft error correction and Viterbi decoding
- eigensystem routines for real, nonsymmetric matrices
- multitaper methods for power spectral estimation
- wavelets on the interval
- information-theoretic properties of distributions
- Markov chain Monte Carlo
- Gaussian process regression and kriging
- stochastic simulation of chemical reaction networks
- code for plotting simple graphs from within programs

The *Numerical Recipes* Web site, www.nr.com, is one of the oldest active sites on the Internet, as evidenced by its two-letter domain name. We will continue to make the Web site useful to readers of this edition. Go there to find the latest bug reports, to purchase the machine-readable source code, or to participate in our readers' forum. With this third edition, we also plan to offer, by subscription, a completely electronic version of *Numerical Recipes* — accessible via the Web, downloadable, printable, and, unlike any paper version, always up to date with the latest corrections. Since the electronic version does not share the page limits of the print version, it will grow over time by the addition of completely new sections, available only electronically. This, we think, is the future of *Numerical Recipes* and perhaps of technical reference books generally. If it sounds interesting to you, look at http://www.nr.com/electronic.

This edition also incorporates some "user-friendly" typographical and stylistic improvements: Color is used for headings and to highlight executable code. For code, a label in the margin gives the name of the source file in the machine-readable distribution. Instead of printing repetitive `#include` statements, we provide a con-

venient Web tool at http://www.nr.com/dependencies that will generate exactly the statements you need for any combination of routines. Subsections are now numbered and referred to by number. References to journal articles now include, in most cases, the article title, as an aid to easy Web searching. Many references have been updated; but we have kept references to the grand old literature of classical numerical analysis when we think that books and articles deserve to be remembered.

Acknowledgments

Regrettably, over 15 years, we were not able to maintain a systematic record of the many dozens of colleagues and readers who have made important suggestions, pointed us to new material, corrected errors, and otherwise improved the *Numerical Recipes* enterprise. It is a tired cliché to say that "you know who you are." Actually, in most cases, *we* know who you are, and we are grateful. But a list of names would be incomplete, and therefore offensive to those whose contributions are no less important than those listed. We apologize to both groups, those we might have listed and those we might have missed.

We prepared this book for publication on Windows and Linux machines, generally with Intel Pentium processors, using LaTeX in the TeTeX and MiKTeX implementations. Packages used include amsmath, amsfonts, txfonts, and graphicx, among others. Our principal development environments were Microsoft Visual Studio / Microsoft Visual C++ and GNU C++. We used the SourceJammer cross-platform source control system. Many tasks were automated with Perl scripts. We could not live without GNU Emacs. To all the developers: "You know who you are," and we thank you.

Research by the authors on computational methods was supported in part by the U.S. National Science Foundation and the U.S. Department of Energy.

Preface to the Second Edition (1992)

Our aim in writing the original edition of *Numerical Recipes* was to provide a book that combined general discussion, analytical mathematics, algorithmics, and actual working programs. The success of the first edition puts us now in a difficult, though hardly unenviable, position. We wanted, then and now, to write a book that is informal, fearlessly editorial, unesoteric, and above all useful. There is a danger that, if we are not careful, we might produce a second edition that is weighty, balanced, scholarly, and boring.

It is a mixed blessing that we know more now than we did six years ago. Then, we were making educated guesses, based on existing literature and our own research, about which numerical techniques were the most important and robust. Now, we have the benefit of direct feedback from a large reader community. Letters to our alter-ego enterprise, Numerical Recipes Software, are in the thousands per year. (Please, *don't telephone* us.) Our post office box has become a magnet for letters pointing out that we have omitted some particular technique, well known to be important in a particular field of science or engineering. We value such letters and digest them carefully, especially when they point us to specific references in the literature.

The inevitable result of this input is that this second edition of *Numerical Recipes* is substantially larger than its predecessor, in fact about 50% larger in both words and number of included programs (the latter now numbering well over 300). "Don't let the book grow in size," is the advice that we received from several wise colleagues. We have tried to follow the intended spirit of that advice, even as we violate the letter of it. We have not lengthened, or increased in difficulty, the book's principal discussions of mainstream topics. Many new topics are presented at this same accessible level. Some topics, both from the earlier edition and new to this one, are now set in smaller type that labels them as being "advanced." The reader who ignores such advanced sections completely will not, we think, find any lack of continuity in the shorter volume that results.

Here are some highlights of the new material in this second edition:

- a new chapter on integral equations and inverse methods
- a detailed treatment of multigrid methods for solving elliptic partial differential equations
- routines for band-diagonal linear systems
- improved routines for linear algebra on sparse matrices
- Cholesky and QR decomposition
- orthogonal polynomials and Gaussian quadratures for arbitrary weight functions
- methods for calculating numerical derivatives
- Padé approximants and rational Chebyshev approximation
- Bessel functions, and modified Bessel functions, of fractional order and several other new special functions
- improved random number routines
- quasi-random sequences
- routines for adaptive and recursive Monte Carlo integration in high-dimensional spaces
- globally convergent methods for sets of nonlinear equations
- simulated annealing minimization for continuous control spaces

- fast Fourier transform (FFT) for real data in two and three dimensions
- fast Fourier transform using external storage
- improved fast cosine transform routines
- wavelet transforms
- Fourier integrals with upper and lower limits
- spectral analysis on unevenly sampled data
- Savitzky-Golay smoothing filters
- fitting straight line data with errors in both coordinates
- a two-dimensional Kolmogorov-Smirnoff test
- the statistical bootstrap method
- embedded Runge-Kutta-Fehlberg methods for differential equations
- high-order methods for stiff differential equations
- a new chapter on "less-numerical" algorithms, including Huffman and arithmetic coding, arbitrary precision arithmetic, and several other topics

Consult the Preface to the first edition, following, or the Contents, for a list of the more "basic" subjects treated.

Acknowledgments

It is not possible for us to list by name here all the readers who have made useful suggestions; we are grateful for these. In the text, we attempt to give specific attribution for ideas that appear to be original and are not known in the literature. We apologize in advance for any omissions.

Some readers and colleagues have been particularly generous in providing us with ideas, comments, suggestions, and programs for this second edition. We especially want to thank George Rybicki, Philip Pinto, Peter Lepage, Robert Lupton, Douglas Eardley, Ramesh Narayan, David Spergel, Alan Oppenheim, Sallie Baliunas, Scott Tremaine, Glennys Farrar, Steven Block, John Peacock, Thomas Loredo, Matthew Choptuik, Gregory Cook, L. Samuel Finn, P. Deuflhard, Harold Lewis, Peter Weinberger, David Syer, Richard Ferch, Steven Ebstein, Bradley Keister, and William Gould. We have been helped by Nancy Lee Snyder's mastery of a complicated TeX manuscript. We express appreciation to our editors Lauren Cowles and Alan Harvey at Cambridge University Press, and to our production editor Russell Hahn. We remain, of course, grateful to the individuals acknowledged in the Preface to the first edition.

Special acknowledgment is due to programming consultant Seth Finkelstein, who wrote, rewrote, or influenced many of the routines in this book, as well as in its Fortran-language twin and the companion Example books. Our project has benefited enormously from Seth's talent for detecting, and following the trail of, even very slight anomalies (often compiler bugs, but occasionally our errors), and from his good programming sense. To the extent that this edition of *Numerical Recipes in C* has a more graceful and "C-like" programming style than its predecessor, most of the credit goes to Seth. (Of course, we accept the blame for the Fortranish lapses that still remain.)

We prepared this book for publication on DEC and Sun workstations running the UNIX operating system and on a 486/33 PC compatible running MS-DOS 5.0 / Windows 3.0. We enthusiastically recommend the principal software used: GNU Emacs, TeX, Perl, Adobe Illustrator, and PostScript. Also used were a variety of C

compilers — too numerous (and sometimes too buggy) for individual acknowledgment. It is a sobering fact that our standard test suite (exercising all the routines in this book) has uncovered compiler bugs in many of the compilers tried. When possible, we work with developers to see that such bugs get fixed; we encourage interested compiler developers to contact us about such arrangements.

WHP and SAT acknowledge the continued support of the U.S. National Science Foundation for their research on computational methods. DARPA support is acknowledged for §13.10 on wavelets.

Preface to the First Edition (1985)

We call this book *Numerical Recipes* for several reasons. In one sense, this book is indeed a "cookbook" on numerical computation. However, there is an important distinction between a cookbook and a restaurant menu. The latter presents choices among complete dishes in each of which the individual flavors are blended and disguised. The former — and this book — reveals the individual ingredients and explains how they are prepared and combined.

Another purpose of the title is to connote an eclectic mixture of presentational techniques. This book is unique, we think, in offering, for each topic considered, a certain amount of general discussion, a certain amount of analytical mathematics, a certain amount of discussion of algorithmics, and (most important) actual implementations of these ideas in the form of working computer routines. Our task has been to find the right balance among these ingredients for each topic. You will find that for some topics we have tilted quite far to the analytic side; this where we have felt there to be gaps in the "standard" mathematical training. For other topics, where the mathematical prerequisites are universally held, we have tilted toward more in-depth discussion of the nature of the computational algorithms, or toward practical questions of implementation.

We admit, therefore, to some unevenness in the "level" of this book. About half of it is suitable for an advanced undergraduate course on numerical computation for science or engineering majors. The other half ranges from the level of a graduate course to that of a professional reference. Most cookbooks have, after all, recipes at varying levels of complexity. An attractive feature of this approach, we think, is that the reader can use the book at increasing levels of sophistication as his/her experience grows. Even inexperienced readers should be able to use our most advanced routines as black boxes. Having done so, we hope that these readers will subsequently go back and learn what secrets are inside.

If there is a single dominant theme in this book, it is that practical methods of numerical computation can be simultaneously efficient, clever, and — important — clear. The alternative viewpoint, that efficient computational methods must necessarily be so arcane and complex as to be useful only in "black box" form, we firmly reject.

Our purpose in this book is thus to open up a large number of computational black boxes to your scrutiny. We want to teach you to take apart these black boxes and to put them back together again, modifying them to suit your specific needs. We assume that you are mathematically literate, i.e., that you have the normal mathematical preparation associated with an undergraduate degree in a physical science, or engineering, or economics, or a quantitative social science. We assume that you know how to program a computer. We do not assume that you have any prior formal knowledge of numerical analysis or numerical methods.

The scope of *Numerical Recipes* is supposed to be "everything up to, but not including, partial differential equations." We honor this in the breach: First, we *do* have one introductory chapter on methods for partial differential equations. Second, we obviously cannot include *everything* else. All the so-called "standard" topics of a numerical analysis course have been included in this book: linear equations, interpolation and extrapolation, integration, nonlinear root finding, eigensystems, and ordinary differential equations. Most of these topics have been taken beyond their

standard treatments into some advanced material that we have felt to be particularly important or useful.

Some other subjects that we cover in detail are not usually found in the standard numerical analysis texts. These include the evaluation of functions and of particular special functions of higher mathematics; random numbers and Monte Carlo methods; sorting; optimization, including multidimensional methods; Fourier transform methods, including FFT methods and other spectral methods; two chapters on the statistical description and modeling of data; and two-point boundary value problems, both shooting and relaxation methods.

Acknowledgments

Many colleagues have been generous in giving us the benefit of their numerical and computational experience, in providing us with programs, in commenting on the manuscript, or with general encouragement. We particularly wish to thank George Rybicki, Douglas Eardley, Philip Marcus, Stuart Shapiro, Paul Horowitz, Bruce Musicus, Irwin Shapiro, Stephen Wolfram, Henry Abarbanel, Larry Smarr, Richard Muller, John Bahcall, and A.G.W. Cameron.

We also wish to acknowledge two individuals whom we have never met: Forman Acton, whose 1970 textbook *Numerical Methods That Work* (New York: Harper and Row) has surely left its stylistic mark on us; and Donald Knuth, both for his series of books on *The Art of Computer Programming* (Reading, MA: Addison-Wesley), and for TeX, the computer typesetting language that immensely aided production of this book.

Research by the authors on computational methods was supported in part by the U.S. National Science Foundation.

License and Legal Information

You must read this section if you intend to use the code in this book on a computer. You'll need to read the following Disclaimer of Warranty, acquire a Numerical Recipes software license, and get the code onto your computer. Without the license, which can be the limited, free "immediate license" under terms described below, this book is intended as a text and reference book, for reading and study purposes only.

For purposes of licensing, the electronic version of the *Numerical Recipes* book is equivalent to the paper version. It is not equivalent to a Numerical Recipes software license, which must still be acquired separately or as part of a combined electronic product. For information on Numerical Recipes electronic products, go to http://www.nr.com/electronic.

Disclaimer of Warranty

We make no warranties, express or implied, that the programs contained in this volume are free of error, or are consistent with any particular standard of merchantability, or that they will meet your requirements for any particular application. They should not be relied on for solving a problem whose incorrect solution could result in injury to a person or loss of property. If you do use the programs in such a manner, it is at your own risk. The authors and publisher disclaim all liability for direct or consequential damages resulting from your use of the programs.

The Restricted, Limited Free License

We recognize that readers may have an immediate, urgent wish to copy a small amount of code from this book for use in their own applications. If you personally keyboard no more than 10 routines from this book into your computer, then we authorize you (and only you) to use those routines (and only those routines) on that single computer. You are not authorized to transfer or distribute the routines to any other person or computer, nor to have any other person keyboard the programs into a computer on your behalf. We do not want to hear bug reports from you, because experience has shown that *virtually all* reported bugs in such cases are typing errors! This free license is not a GNU General Public License.

Regular Licenses

When you purchase a code subscription or one-time code download from the Numerical Recipes Web site (http://www.nr.com), or when you buy physical Numerical Recipes media published by Cambridge University Press, you automatically get a *Numerical Recipes Personal Single-User License*. This license lets you personally use Numerical Recipes code on any one computer at a time, but not to allow anyone else access to the code. You may also, under this license, transfer precompiled, executable programs incorporating the code to other, unlicensed, users or computers, providing that (i) your application is noncommercial (i.e., does not involve the selling of your program for a fee); (ii) the programs were first developed, compiled, and successfully run by you; and (iii) our routines are bound into the programs in such a manner that they cannot be accessed as individual routines and cannot practicably be

unbound and used in other programs. That is, under this license, your program user must not be able to use our programs as part of a program library or "mix-and-match" workbench. See the Numerical Recipes Web site for further details.

Businesses and organizations that purchase code subscriptions, downloads, or media, and that thus acquire one or more Numerical Recipes Personal Single-User Licenses, may permanently assign those licenses, in the number acquired, to individual employees. In most cases, however, businesses and organizations will instead want to purchase Numerical Recipes licenses "by the seat," allowing them to be used by a pool of individuals rather than being individually permanently assigned. See http://www.nr.com/licenses for information on such licenses.

Instructors at accredited educational institutions who have adopted this book for a course may purchase on behalf of their students one-semester subscriptions to both the electronic version of the *Numerical Recipes* book and to the Numerical Recipes code. During the subscription term, students may download, view, save, and print all of the book and code. See http://www.nr.com/licenses for further information.

Other types of corporate licenses are also available. Please see the Numerical Recipes Web site.

About Copyrights on Computer Programs

Like artistic or literary compositions, computer programs are protected by copyright. Generally it is an infringement for you to copy into your computer a program from a copyrighted source. (It is also not a friendly thing to do, since it deprives the program's author of compensation for his or her creative effort.) Under copyright law, all "derivative works" (modified versions, or translations into another computer language) also come under the same copyright as the original work.

Copyright does not protect ideas, but only the expression of those ideas in a particular form. In the case of a computer program, the ideas consist of the program's methodology and algorithm, including the necessary sequence of steps adopted by the programmer. The expression of those ideas is the program source code (particularly any arbitrary or stylistic choices embodied in it), its derived object code, and any other derivative works.

If you analyze the ideas contained in a program, and then express those ideas in your own completely different implementation, then that new program implementation belongs to you. That is what we have done for those programs in this book that are not entirely of our own devising. When programs in this book are said to be "based" on programs published in copyright sources, we mean that the ideas are the same. The expression of these ideas as source code is our own. We believe that no material in this book infringes on an existing copyright.

Trademarks

Several registered trademarks appear within the text of this book. Words that are known to be trademarks are shown with an initial capital letter. However, the capitalization of any word is not an expression of the authors' or publisher's opinion as to whether or not it is subject to proprietary rights, nor is it to be regarded as affecting the validity of any trademark.

Numerical Recipes, NR, and nr.com (when identifying our products) are trademarks of Numerical Recipes Software.

Attributions

The fact that ideas are legally "free as air" in no way supersedes the ethical requirement that ideas be credited to their known originators. When programs in this book are based on known sources, whether copyrighted or in the public domain, published or "handed-down," we have attempted to give proper attribution. Unfortunately, the lineage of many programs in common circulation is often unclear. We would be grateful to readers for new or corrected information regarding attributions, which we will attempt to incorporate in subsequent printings.

Routines by Chapter and Section

Previous editions included a table of all the routines in the book, along with a short description, arranged by chapter and section. This information is now available as an interactive Web page at http://www.nr.com/routines. The following illustration gives the idea.

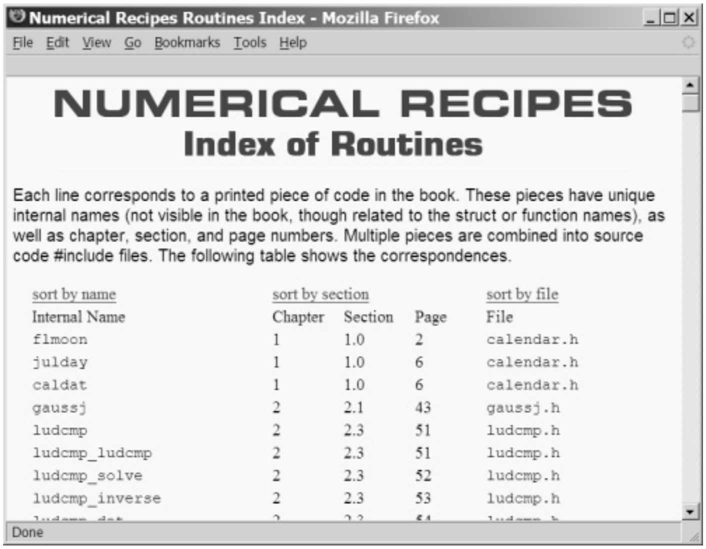

Numerical Recipes Routines Index - Mozilla Firefox

File Edit View Go Bookmarks Tools Help

NUMERICAL RECIPES

Index of Routines

Each line corresponds to a printed piece of code in the book. These pieces have unique internal names (not visible in the book, though related to the struct or function names), as well as chapter, section, and page numbers. Multiple pieces are combined into source code #include files. The following table shows the correspondences.

sort by name	sort by section			sort by file
Internal Name	Chapter	Section	Page	File
flmoon	1	1.0	2	calendar.h
julday	1	1.0	6	calendar.h
caldat	1	1.0	6	calendar.h
gaussj	2	2.1	43	gaussj.h
ludcmp	2	2.3	51	ludcmp.h
ludcmp_ludcmp	2	2.3	51	ludcmp.h
ludcmp_solve	2	2.3	52	ludcmp.h
ludcmp_inverse	2	2.3	53	ludcmp.h
[illegible]	[illegible]	[illegible]	[illegible]	[illegible]

Done

Preliminaries

CHAPTER 1

1.0 Introduction

This book is supposed to teach you methods of numerical computing that are practical, efficient, and (insofar as possible) elegant. We presume throughout this book that you, the reader, have particular tasks that you want to get done. We view our job as educating you on how to proceed. Occasionally we may try to reroute you briefly onto a particularly beautiful side road; but by and large, we will guide you along main highways that lead to practical destinations.

Throughout this book, you will find us fearlessly editorializing, telling you what you should and shouldn't do. This prescriptive tone results from a conscious decision on our part, and we hope that you will not find it irritating. We do not claim that our advice is infallible! Rather, we are reacting against a tendency, in the textbook literature of computation, to discuss every possible method that has ever been invented, without ever offering a practical judgment on relative merit. We do, therefore, offer you our practical judgments whenever we can. As you gain experience, you will form your own opinion of how reliable our advice is. Be assured that it is not perfect!

We presume that you are able to read computer programs in C++. The question, "Why C++?", is a complicated one. For now, suffice it to say that we wanted a language with a C-like syntax in the small (because that is most universally readable by our audience), which had a rich set of facilities for object-oriented programming (because that is an emphasis of this third edition), and which was highly backward-compatible with some old, but established and well-tested, tricks in numerical programming. That pretty much led us to C++, although Java (and the closely related C#) were close contenders.

Honesty compels us to point out that in the 20-year history of *Numerical Recipes*, we have never been correct in our predictions about the future of programming languages for scientific programming, *not once*! At various times we convinced ourselves that the wave of the scientific future would be ... Fortran ... Pascal ... C ... Fortran 90 (or 95 or 2000) ... Mathematica ... Matlab ... C++ or Java .... Indeed, several of these enjoy continuing success and have significant followings (not including Pascal!). None, however, currently command a majority, or even a large plurality, of scientific users.

With this edition, we are no longer trying to predict the future of programming languages. Rather, we want a serviceable way of communicating ideas about scientific programming. We hope that these ideas transcend the language, C++, in which we are expressing them.

When we include programs in the text, they look like this:

calendar.h

```
void flmoon(const Int n, const Int nph, Int &jd, Doub &frac) {
```

Our routines begin with an introductory comment summarizing their purpose and explaining their calling sequence. This routine calculates the phases of the moon. Given an integer n and a code nph for the phase desired (nph $= 0$ for new moon, 1 for first quarter, 2 for full, 3 for last quarter), the routine returns the Julian Day Number jd, and the fractional part of a day frac to be added to it, of the nth such phase since January, 1900. Greenwich Mean Time is assumed.

```
    const Doub RAD=3.141592653589793238/180.0;
    Int i;
    Doub am,as,c,t,t2,xtra;
    c=n+nph/4.0;                              This is how we comment an individual line.
    t=c/1236.85;
    t2=t*t;
    as=359.2242+29.105356*c;                  You aren't really intended to understand
    am=306.0253+385.816918*c+0.010730*t2;         this algorithm, but it does work!
    jd=2415020+28*n+7*nph;
    xtra=0.75933+1.53058868*c+((1.178e-4)-(1.55e-7)*t)*t2;
    if (nph == 0 || nph == 2)
        xtra += (0.1734-3.93e-4*t)*sin(RAD*as)-0.4068*sin(RAD*am);
    else if (nph == 1 || nph == 3)
        xtra += (0.1721-4.0e-4*t)*sin(RAD*as)-0.6280*sin(RAD*am);
    else throw("nph is unknown in flmoon");   This indicates an error condition.
    i=Int(xtra >= 0.0 ? floor(xtra) : ceil(xtra-1.0));
    jd += i;
    frac=xtra-i;
}
```

Note our convention of handling all errors and exceptional cases with a statement like `throw("some error message");`. Since C++ has no built-in exception class for type `char*`, executing this statement results in a fairly rude program abort. However we will explain in §1.5.1 how to get a more elegant result without having to modify the source code.

1.0.1 *What* Numerical Recipes *Is Not*

We want to use the platform of this introductory section to emphasize what *Numerical Recipes* is *not*:

1. *Numerical Recipes* is not a textbook on programming, or on best programming practices, or on C++, or on software engineering. We are not opposed to good programming. We try to communicate good programming practices whenever we can — but only incidentally to our main purpose, which is to teach how practical numerical methods actually work. The unity of style and subordination of function to standardization that is necessary in a good programming (or software engineering) textbook is just not what we have in mind for this book. Each section in this book has as its focus a particular computational method. Our goal is to explain and illustrate *that* method as clearly as possible. No single programming style is best for all such methods, and, accordingly, our style varies from section to section.

2. *Numerical Recipes* is not a program library. That may surprise you if you are one of the many scientists and engineers who use our source code regularly. What

makes our code *not* a program library is that it demands a greater intellectual commitment from the user than a program library ought to do. If you haven't read a routine's accompanying section and gone through the routine line by line to understand how it works, then you use it at great peril! We consider this a feature, not a bug, because our primary purpose is to teach methods, not provide packaged solutions. This book does not include formal exercises, in part because we consider each section's code to be the exercise: If you can understand each line of the code, then you have probably mastered the section.

There are some fine commercial program libraries [1,2] and integrated numerical environments [3-5] available. Comparable free resources are available, both program libraries [6,7] and integrated environments [8-10]. When you want a packaged solution, we recommend that you use one of these. *Numerical Recipes* is intended as a cookbook for cooks, not a restaurant menu for diners.

1.0.2 Frequently Asked Questions

This section is for people who want to jump right in.

1. *How do I use NR routines with my own program?*

The easiest way is to put a bunch of `#include`'s at the top of your program. Always start with `nr3.h`, since that defines some necessary utility classes and functions (see §1.4 for a lot more about this). For example, here's how you compute the mean and variance of the Julian Day numbers of the first 20 full moons after January 1900. (Now *there's* a useful pair of quantities!)

```
#include "nr3.h"
#include "calendar.h"
#include "moment.h"

Int main(void) {
    const Int NTOT=20;
    Int i,jd,nph=2;
    Doub frac,ave,vrnce;
    VecDoub data(NTOT);
    for (i=0;i<NTOT;i++) {
        flmoon(i,nph,jd,frac);
        data[i]=jd;
    }
    avevar(data,ave,vrnce);
    cout << "Average = " << setw(12) << ave;
    cout << " Variance = " << setw(13) << vrnce << endl;
    return 0;
}
```

Be sure that the NR source code files are in a place that your compiler can find them to `#include`. Compile and run the above file. (We can't tell you how to do this part.) Output should be something like this:

```
    Average =  2.41532e+06 Variance =       30480.7
```

2. *Yes, but where do I actually get the NR source code as computer files?*

You can buy a code subscription, or a one-time code download, at the Web site http://www.nr.com, or you can get the code on media published by Cambridge

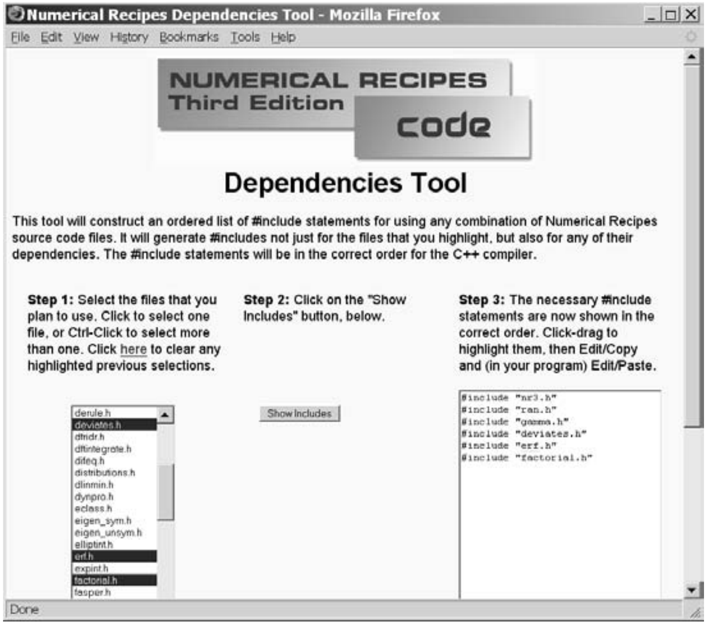

Figure 1.0.1. The interactive page located at http://www.nr.com/dependencies sorts out the dependencies for any combination of *Numerical Recipes* routines, giving an ordered list of the necessary #include files.

University Press (e.g., from Amazon.com or your favorite online or physical bookstore). The code comes with a personal, single-user license (see License and Legal Information on p. xix). The reason that the book (or its electronic version) and the code license are sold separately is to help keep down the price of each. Also, making these products separate meets the needs of more users: Your company or educational institution may have a site license — ask them.

3. *How do I know which files to* `#include`*? It's hard to sort out the dependencies among all the routines.*

In the margin next to each code listing is the name of the source code file that it is in. Make a list of the source code files that you are using. Then go to http://www.nr.com/dependencies and click on the name of each source code file. The interactive Web page will return a list of the necessary `#include`s, in the correct order, to satisfy all dependencies. Figure 1.0.1 will give you an idea of how this works.

4. *What is all this* `Doub`, `Int`, `VecDoub`, *etc., stuff?*

We always use defined types, not built-in types, so that they can be redefined if necessary. The definitions are in `nr3.h`. Generally, as you can guess, `Doub` means `double`, `Int` means `int`, and so forth. Our convention is to begin all defined types with an uppercase letter. `VecDoub` is a vector class type. Details on our types are in §1.4.

5. *What are* Numerical Recipes *Webnotes?*

Numerical Recipes Webnotes are documents, accessible on the Web, that include some code implementation listings, or other highly specialized topics, that are not included in the paper version of the book. A list of all Webnotes is at

Tested Operating Systems and Compilers	
O/S	Compiler
Microsoft Windows XP SP2	Visual C++ ver. 14.00 (Visual Studio 2005)
Microsoft Windows XP SP2	Visual C++ ver. 13.10 (Visual Studio 2003)
Microsoft Windows XP SP2	Intel C++ Compiler ver. 9.1
Novell SUSE Linux 10.1	GNU GCC (g++) ver. 4.1.0
Red Hat Enterprise Linux 4 (64-bit)	GNU GCC (g++) ver. 3.4.6 and ver. 4.1.0
Red Hat Linux 7.3	Intel C++ Compiler ver. 9.1
Apple Mac OS X 10.4 (Tiger) Intel Core	GNU GCC (g++) ver. 4.0.1

http://www.nr.com/webnotes. By moving some specialized material into Webnotes, we are able to keep down the size and price of the paper book. Webnotes are automatically included in the electronic version of the book; see next question.

6. *I am a post-paper person. I want* Numerical Recipes *on my laptop. Where do I get the complete, fully electronic version?*

A fully electronic version of *Numerical Recipes* is available by annual subscription. You can subscribe instead of, or in addition to, owning a paper copy of the book. A subscription is accessible via the Web, downloadable, printable, and, unlike any paper version, always up to date with the latest corrections. Since the electronic version does not share the page limits of the printed version, it will grow over time by the addition of completely new sections, available only electronically. This, we think, is the future of *Numerical Recipes* and perhaps of technical reference books generally. We anticipate various electronic formats, changing with time as technologies for display and rights management continuously improve: We place a big emphasis on user convenience and usability. See http://www.nr.com/electronic for further information.

7. *Are there bugs in NR?*

Of course! By now, most NR code has the benefit of long-time use by a large user community, but new bugs are sure to creep in. Look at http://www.nr.com for information about known bugs, or to report apparent new ones.

1.0.3 Computational Environment and Program Validation

The code in this book should run without modification on any compiler that implements the ANSI/ISO C++ standard, as described, for example, in Stroustrup's book [11].

As surrogates for the large number of hardware and software configurations, we have tested all the code in this book on the combinations of operating systems and compilers shown in the table above.

In validating the code, we have taken it directly from the machine-readable form of the book's manuscript, so that we have tested exactly what is printed. (This does not, of course, mean that the code is bug-free!)

1.0.4 About References

You will find references, and suggestions for further reading, listed at the end of most sections of this book. References are cited in the text by bracketed numbers like this [12].

We do not pretend to any degree of bibliographical completeness in this book. For topics where a substantial secondary literature exists (discussion in textbooks, reviews, etc.) we often limit our references to a few of the more useful secondary sources, especially those with good references to the primary literature. Where the existing secondary literature is insufficient, we give references to a few primary sources that are intended to serve as starting points for further reading, not as complete bibliographies for the field.

Since progress is ongoing, it is inevitable that our references for many topics are already, or will soon become, out of date. We have tried to include older references that are good for "forward" Web searching: A search for more recent papers that cite the references given should lead you to the most current work.

Web references and URLs present a problem, because there is no way for us to guarantee that they will still be there when you look for them. A date like 2007+ means "it was there in 2007." We try to give citations that are complete enough for you to find the document by Web search, even if it has moved from the location listed.

The order in which references are listed is not necessarily significant. It reflects a compromise between listing cited references in the order cited, and listing suggestions for further reading in a roughly prioritized order, with the most useful ones first.

1.0.5 About "Advanced Topics"

Material set in smaller type, like this, signals an "advanced topic," either one outside of the main argument of the chapter, or else one requiring of you more than the usual assumed mathematical background, or else (in a few cases) a discussion that is more speculative or an algorithm that is less well tested. Nothing important will be lost if you skip the advanced topics on a first reading of the book.

Here is a function for getting the Julian Day Number from a calendar date.

calendar.h

```
Int julday(const Int mm, const Int id, const Int iyyy) {
```

In this routine julday returns the Julian Day Number that begins at noon of the calendar date specified by month mm, day id, and year iyyy, all integer variables. Positive year signifies A.D.; negative, B.C. Remember that the year after 1 B.C. was 1 A.D.

```
    const Int IGREG=15+31*(10+12*1582);        Gregorian Calendar adopted Oct. 15, 1582.
    Int ja,jul,jy=iyyy,jm;
    if (jy == 0) throw("julday: there is no year zero.");
    if (jy < 0) ++jy;
    if (mm > 2) {
        jm=mm+1;
    } else {
        --jy;
        jm=mm+13;
    }
    jul = Int(floor(365.25*jy)+floor(30.6001*jm)+id+1720995);
    if (id+31*(mm+12*iyyy) >= IGREG) {        Test whether to change to Gregorian Calendar.
        ja=Int(0.01*jy);
        jul += 2-ja+Int(0.25*ja);
    }
    return jul;
}
```

And here is its inverse.

```
void caldat(const Int julian, Int &mm, Int &id, Int &iyyy) {
```
calendar.h

Inverse of the function julday given above. Here julian is input as a Julian Day Number, and the routine outputs mm,id, and iyyy as the month, day, and year on which the specified Julian Day started at noon.

```
    const Int IGREG=2299161;
    Int ja,jalpha,jb,jc,jd,je;

    if (julian >= IGREG) {          Cross-over to Gregorian Calendar produces this correc-
        jalpha=Int((Doub(julian-1867216)-0.25)/36524.25);              tion.
        ja=julian+1+jalpha-Int(0.25*jalpha);
    } else if (julian < 0) {        Make day number positive by adding integer number of
        ja=julian+36525*(1-julian/36525);      Julian centuries, then subtract them off
    } else                                     at the end.
        ja=julian;
    jb=ja+1524;
    jc=Int(6680.0+(Doub(jb-2439870)-122.1)/365.25);
    jd=Int(365*jc+(0.25*jc));
    je=Int((jb-jd)/30.6001);
    id=jb-jd-Int(30.6001*je);
    mm=je-1;
    if (mm > 12) mm -= 12;
    iyyy=jc-4715;
    if (mm > 2) --iyyy;
    if (iyyy <= 0) --iyyy;
    if (julian < 0) iyyy -= 100*(1-julian/36525);
}
```

As an exercise, you might try using these functions, along with `flmoon` in §1.0, to search for future occurrences of a full moon on Friday the 13th. (Answers, in time zone GMT minus 5: 9/13/2019 and 8/13/2049.) For additional calendrical algorithms, applicable to various historical calendars, see [13].

CITED REFERENCES AND FURTHER READING:

Visual Numerics, 2007+, *IMSL Numerical Libraries*, at `http://www.vni.com`.[1]

Numerical Algorithms Group, 2007+, *NAG Numerical Library*, at `http://www.nag.co.uk`.[2]

Wolfram Research, Inc., 2007+, *Mathematica*, at `http://www.wolfram.com`.[3]

The MathWorks, Inc., 2007+, *MATLAB*, at `http://www.mathworks.com`.[4]

Maplesoft, Inc., 2007+, *Maple*, at `http://www.maplesoft.com`.[5]

GNU Scientific Library, 2007+, at `http://www.gnu.org/software/gsl`.[6]

Netlib Repository, 2007+, at `http://www.netlib.org`.[7]

Scilab Scientific Software Package, 2007+, at `http://www.scilab.org`.[8]

GNU Octave, 2007+, at `http://www.gnu.org/software/octave`.[9]

R Software Environment for Statistical Computing and Graphics, 2007+, at `http://www.r-project.org`.[10]

Stroustrup, B. 1997, *The C++ Programming Language*, 3rd ed. (Reading, MA: Addison-Wesley).[11]

Meeus, J. 1982, *Astronomical Formulae for Calculators*, 2nd ed., revised and enlarged (Richmond, VA: Willmann-Bell).[12]

Hatcher, D.A. 1984, "Simple Formulae for Julian Day Numbers and Calendar Dates," *Quarterly Journal of the Royal Astronomical Society*, vol. 25, pp. 53–55; see also *op. cit.* 1985, vol. 26, pp. 151–155, and 1986, vol. 27, pp. 506–507.[13]

1.1 Error, Accuracy, and Stability

Computers store numbers not with infinite precision but rather in some approximation that can be packed into a fixed number of *bits* (binary digits) or *bytes* (groups of 8 bits). Almost all computers allow the programmer a choice among several different such *representations* or *data types*. Data types can differ in the number of bits utilized (the *wordlength*), but also in the more fundamental respect of whether the stored number is represented in *fixed-point* (like `int`) or *floating-point* (like `float` or `double`) format.

A number in integer representation is exact. Arithmetic between numbers in integer representation is also exact, with the provisos that (i) the answer is not outside the range of (usually, signed) integers that can be represented, and (ii) that division is interpreted as producing an integer result, throwing away any integer remainder.

1.1.1 Floating-Point Representation

In a floating-point representation, a number is represented internally by a sign bit S (interpreted as plus or minus), an exact integer exponent E, and an exactly represented binary mantissa M. Taken together these represent the number

$$S \times M \times b^{E-e} \tag{1.1.1}$$

where b is the base of the representation ($b = 2$ almost always), and e is the *bias* of the exponent, a fixed integer constant for any given machine and representation.

	S	E	F	Value
`float`	any	1–254	any	$(-1)^S \times 2^{E-127} \times 1.F$
	any	0	nonzero	$(-1)^S \times 2^{-126} \times 0.F*$
	0	0	0	$+0.0$
	1	0	0	-0.0
	0	255	0	$+\infty$
	1	255	0	$-\infty$
	any	255	nonzero	`NaN`
`double`	any	1–2046	any	$(-1)^S \times 2^{E-1023} \times 1.F$
	any	0	nonzero	$(-1)^S \times 2^{-1022} \times 0.F*$
	0	0	0	$+0.0$
	1	0	0	-0.0
	0	2047	0	$+\infty$
	1	2047	0	$-\infty$
	any	2047	nonzero	`NaN`
*unnormalized values				

Several floating-point bit patterns can in principle represent the same number. If $b = 2$, for example, a mantissa with leading (high-order) zero bits can be left-shifted, i.e., multiplied by a power of 2, if the exponent is decreased by a compensating amount. Bit patterns that are "as left-shifted as they can be" are termed *normalized*.

Virtually all modern processors share the same floating-point data representations, namely those specified in IEEE Standard 754-1985 [1]. (For some discussion of nonstandard processors, see §22.2.) For 32-bit `float` values, the exponent is represented in 8 bits (with $e = 127$), the mantissa in 23; for 64-bit `double` values, the exponent is 11 bits (with $e = 1023$), the mantissa, 52. An additional trick is used for the mantissa for most nonzero floating values: Since the high-order bit of a properly normalized mantissa is *always* one, the stored mantissa bits are viewed as being preceded by a "phantom" bit with the value 1. In other words, the mantissa M has the numerical value $1.F$, where F (called the *fraction*) consists of the bits (23 or 52 in number) that are actually stored. This trick gains a little "bit" of precision.

Here are some examples of IEEE 754 representations of `double` values:

$$
\begin{aligned}
&\texttt{0 01111111111 0000} \text{ (+ 48 more zeros)} = +1 \times 2^{1023-1023} \times 1.0_2 = 1. \\
&\texttt{1 01111111111 0000} \text{ (+ 48 more zeros)} = -1 \times 2^{1023-1023} \times 1.0_2 = -1. \\
&\texttt{0 01111111111 1000} \text{ (+ 48 more zeros)} = +1 \times 2^{1023-1023} \times 1.1_2 = 1.5 \\
&\texttt{0 10000000000 0000} \text{ (+ 48 more zeros)} = +1 \times 2^{1024-1023} \times 1.0_2 = 2. \\
&\texttt{0 10000000001 1010} \text{ (+ 48 more zeros)} = +1 \times 2^{1025-1023} \times 1.1010_2 = 6.5
\end{aligned}
\tag{1.1.2}
$$

You can examine the representation of any value by code like this:

```
union Udoub {
    double d;
    unsigned char c[8];
};

void main() {
    Udoub u;
    u.d = 6.5;
    for (int i=7;i>=0;i--) printf("%02x",u.c[i]);
    printf("\n");
}
```

This is C, and deprecated style, but it will work. On most processors, including Intel Pentium and successors, you'll get the printed result `401a000000000000`, which (writing out each hex digit as four binary digits) is the last line in equation (1.1.2). If you get the bytes (groups of two hex digits) in reverse order, then your processor is *big-endian* instead of *little-endian*: The IEEE 754 standard does not specify (or care) in which order the bytes in a floating-point value are stored.

The IEEE 754 standard includes representations of positive and negative infinity, positive and negative zero (treated as computationally equivalent, of course), and also `NaN` ("not a number"). The table on the previous page gives details of how these are represented.

The reason for representing some *unnormalized* values, as shown in the table, is to make "underflow to zero" more graceful. For a sequence of smaller and smaller values, after you pass the smallest normalizable value (with magnitude 2^{-127} or 2^{-1023}; see table), you start right-shifting the leading bit of the mantissa. Although

you gradually lose precision, you don't actually underflow to zero until 23 or 52 bits later.

When a routine needs to know properties of the floating-point representation, it can reference the `numeric_limits` class, which is part of the C++ Standard Library. For example, `numeric_limits<double>::min()` returns the smallest normalized `double` value, usually $2^{-1022} \approx 2.23 \times 10^{-308}$. For more on this, see §22.2.

1.1.2 Roundoff Error

Arithmetic among numbers in floating-point representation is not exact, even if the operands happen to be exactly represented (i.e., have exact values in the form of equation 1.1.1). For example, two floating numbers are added by first right-shifting (dividing by two) the mantissa of the smaller (in magnitude) one and simultaneously increasing its exponent until the two operands have the same exponent. Low-order (least significant) bits of the smaller operand are lost by this shifting. If the two operands differ too greatly in magnitude, then the smaller operand is effectively replaced by zero, since it is right-shifted to oblivion.

The smallest (in magnitude) floating-point number that, when added to the floating-point number 1.0, produces a floating-point result different from 1.0 is termed the *machine accuracy* ϵ_m. IEEE 754 standard `float` has ϵ_m about 1.19×10^{-7}, while `double` has about 2.22×10^{-16}. Values like this are accessible as, e.g., `numeric _limits <double>::epsilon()`. (A more detailed discussion of machine characteristics is in §22.2.) Roughly speaking, the machine accuracy ϵ_m is the fractional accuracy to which floating-point numbers are represented, corresponding to a change of one in the least significant bit of the mantissa. Pretty much any arithmetic operation among floating numbers should be thought of as introducing an additional fractional error of at least ϵ_m. This type of error is called *roundoff error*.

It is important to understand that ϵ_m is not the smallest floating-point number that can be represented on a machine. *That* number depends on how many bits there are in the exponent, while ϵ_m depends on how many bits there are in the mantissa.

Roundoff errors accumulate with increasing amounts of calculation. If, in the course of obtaining a calculated value, you perform N such arithmetic operations, you *might* be so lucky as to have a total roundoff error on the order of $\sqrt{N}\epsilon_m$, if the roundoff errors come in randomly up or down. (The square root comes from a random-walk.) However, this estimate can be very badly off the mark for two reasons:

(1) It very frequently happens that the regularities of your calculation, or the peculiarities of your computer, cause the roundoff errors to accumulate preferentially in one direction. In this case the total will be of order $N\epsilon_m$.

(2) Some especially unfavorable occurrences can vastly increase the roundoff error of single operations. Generally these can be traced to the subtraction of two very nearly equal numbers, giving a result whose only significant bits are those (few) low-order ones in which the operands differed. You might think that such a "coincidental" subtraction is unlikely to occur. Not always so. Some mathematical expressions magnify its probability of occurrence tremendously. For example, in the familiar formula for the solution of a quadratic equation,

$$x = \frac{-b + \sqrt{b^2 - 4ac}}{2a} \tag{1.1.3}$$

the addition becomes delicate and roundoff-prone whenever $b > 0$ and $|ac| \ll b^2$. (In §5.6 we will learn how to avoid the problem in this particular case.)

1.1.3 Truncation Error

Roundoff error is a characteristic of computer hardware. There is another, different, kind of error that is a characteristic of the program or algorithm used, independent of the hardware on which the program is executed. Many numerical algorithms compute "discrete" approximations to some desired "continuous" quantity. For example, an integral is evaluated numerically by computing a function at a discrete set of points, rather than at "every" point. Or, a function may be evaluated by summing a finite number of leading terms in its infinite series, rather than all infinity terms. In cases like this, there is an adjustable parameter, e.g., the number of points or of terms, such that the "true" answer is obtained only when that parameter goes to infinity. Any practical calculation is done with a finite, but sufficiently large, choice of that parameter.

The discrepancy between the true answer and the answer obtained in a practical calculation is called the *truncation error*. Truncation error would persist even on a hypothetical, "perfect" computer that had an infinitely accurate representation and no roundoff error. As a general rule there is not much that a programmer can do about roundoff error, other than to choose algorithms that do not magnify it unnecessarily (see discussion of "stability" below). Truncation error, on the other hand, is entirely under the programmer's control. In fact, it is only a slight exaggeration to say that clever minimization of truncation error is practically the entire content of the field of numerical analysis!

Most of the time, truncation error and roundoff error do not strongly interact with one another. A calculation can be imagined as having, first, the truncation error that it would have if run on an infinite-precision computer, "plus" the roundoff error associated with the number of operations performed.

1.1.4 Stability

Sometimes an otherwise attractive method can be *unstable*. This means that any roundoff error that becomes "mixed into" the calculation at an early stage is successively magnified until it comes to swamp the true answer. An unstable method would be useful on a hypothetical, perfect computer; but in this imperfect world it is necessary for us to require that algorithms be stable — or if unstable that we use them with great caution.

Here is a simple, if somewhat artificial, example of an unstable algorithm: Suppose that it is desired to calculate all integer powers of the so-called "Golden Mean," the number given by

$$\phi \equiv \frac{\sqrt{5}-1}{2} \approx 0.61803398 \tag{1.1.4}$$

It turns out (you can easily verify) that the powers ϕ^n satisfy a simple recursion relation,

$$\phi^{n+1} = \phi^{n-1} - \phi^n \tag{1.1.5}$$

Thus, knowing the first two values $\phi^0 = 1$ and $\phi^1 = 0.61803398$, we can successively apply (1.1.5) performing only a single subtraction, rather than a slower

multiplication by ϕ, at each stage.

Unfortunately, the recurrence (1.1.5) also has *another* solution, namely the value $-\frac{1}{2}(\sqrt{5}+1)$. Since the recurrence is linear, and since this undesired solution has magnitude greater than unity, any small admixture of it introduced by roundoff errors will grow exponentially. On a typical machine, using a 32-bit `float`, (1.1.5) starts to give completely wrong answers by about $n = 16$, at which point ϕ^n is down to only 10^{-4}. The recurrence (1.1.5) is *unstable* and cannot be used for the purpose stated.

We will encounter the question of stability in many more sophisticated guises later in this book.

CITED REFERENCES AND FURTHER READING:

IEEE, 1985, *ANSI/IEEE Std 754–1985: IEEE Standard for Binary Floating-Point Numbers* (New York: IEEE).[1]

Stoer, J., and Bulirsch, R. 2002, *Introduction to Numerical Analysis*, 3rd ed. (New York: Springer), Chapter 1.

Kahaner, D., Moler, C., and Nash, S. 1989, *Numerical Methods and Software* (Englewood Cliffs, NJ: Prentice-Hall), Chapter 2.

Johnson, L.W., and Riess, R.D. 1982, *Numerical Analysis*, 2nd ed. (Reading, MA: Addison-Wesley), §1.3.

Wilkinson, J.H. 1964, *Rounding Errors in Algebraic Processes* (Englewood Cliffs, NJ: Prentice-Hall).

1.2 C Family Syntax

Not only C++, but also Java, C#, and (to varying degrees) other computer languages, share a lot of small-scale syntax with the older C language [1]. By small scale, we mean operations on built-in types, simple expressions, control structures, and the like. In this section, we review some of the basics, give some hints on good programming, and mention some of our conventions and habits.

1.2.1 Operators

A first piece of advice might seem superfluous if it were not so often ignored: You should learn all the C operators and their precedence and associativity rules. You might not yourself want to write

```
n << 1 | 1
```

as a synonym for `2*n+1` (for positive integer `n`), but you definitely do need to be able to see at a glance that

```
n << 1 + 1
```

is not at all the same thing! Please study the table on the next page while you brush your teeth every night. While the occasional set of unnecessary parentheses, for clarity, is hardly a sin, code that is habitually overparenthesized is annoying and hard to read.

Operator Precedence and Associativity Rules in C and C++		
`::`	scope resolution	left-to-right
`()`	function call	left-to-right
`[]`	array element (subscripting)	
`.`	member selection	
`->`	member selection (by pointer)	
`++`	post increment	**right-to-left**
`--`	post decrement	
`!`	logical not	**right-to-left**
`~`	bitwise complement	
`-`	unary minus	
`++`	pre increment	
`--`	pre decrement	
`&`	address of	
`*`	contents of (dereference)	
`new`	create	
`delete`	destroy	
`(type)`	cast to `type`	
`sizeof`	size in bytes	
`*`	multiply	left-to-right
`/`	divide	
`%`	remainder	
`+`	add	left-to-right
`-`	subtract	
`<<`	bitwise left shift	left-to-right
`>>`	bitwise right shift	
`<`	arithmetic less than	left-to-right
`>`	arithmetic greater than	
`<=`	arithmetic less than or equal to	
`>=`	arithmetic greater than or equal to	
`==`	arithmetic equal	left-to-right
`!=`	arithmetic not equal	
`&`	bitwise and	left-to-right
`^`	bitwise exclusive or	left-to-right
`|`	bitwise or	left-to-right
`&&`	logical and	left-to-right
`||`	logical or	left-to-right
`? :`	conditional expression	**right-to-left**
`=`	assignment operator	**right-to-left**
also `+= -= *= /= %=` `<<= >>= &= ^= |=`		
`,`	sequential expression	left-to-right

1.2.2 Control Structures

These should all be familiar to you.

Iteration. In C family languages simple iteration is performed with a `for` loop, for example

```
for (j=2;j<=1000;j++) {
    b[j]=a[j-1];
    a[j-1]=j;
}
```

It is conventional to indent the block of code that is acted upon by the control structure, leaving the structure itself unindented. We like to put the initial curly brace on the same line as the `for` statement, instead of on the next line. This saves a full line of white space, and our publisher loves us for it.

Conditional. The conditional or `if` structure looks, in full generality, like this:

```
if (...) {
    ...
}
else if (...) {
    ...
}
else {
    ...
}
```

However, since compound-statement curly braces are required only when there is more than one statement in a block, the `if` construction can be somewhat less explicit than that shown above. Some care must be exercised in constructing nested `if` clauses. For example, consider the following:

```
if (b > 3)
    if (a > 3) b += 1;
else b -= 1;                    /* questionable! */
```

As judged by the indentation used on successive lines, the intent of the writer of this code is the following: 'If b is greater than 3 and a is greater than 3, then increment b. If b is not greater than 3, then decrement b.' According to the rules, however, the actual meaning is 'If b is greater than 3, then evaluate a. If a is greater than 3, then increment b, and if a is less than or equal to 3, decrement b.' The point is that an `else` clause is associated with the most recent open `if` statement, no matter how you lay it out on the page. Such confusions in meaning are easily resolved by the inclusion of braces that clarify your intent and improve the program. The above fragment should be written as

```
if (b > 3) {
    if (a > 3) b += 1;
} else {
    b -= 1;
}
```

While iteration. Alternative to the `for` iteration is the `while` structure, for example,

```
while (n < 1000) {
    n *= 2;
    j += 1;
}
```

The control clause (in this case `n < 1000`) is evaluated before each iteration. If the clause is not true, the enclosed statements will not be executed. In particular, if this code is encountered at a time when `n` is greater than or equal to 1000, the statements will not even be executed once.

Do-While iteration. Companion to the `while` iteration is a related control structure that tests its control clause at the *end* of each iteration:

```
do {
    n *= 2;
    j += 1;
} while (n < 1000);
```

In this case, the enclosed statements will be executed at least once, independent of the initial value of `n`.

Break and Continue. You use the `break` statement when you have a loop that is to be repeated indefinitely until some condition *tested somewhere in the middle of the loop* (and possibly tested in more than one place) becomes true. At that point you wish to exit the loop and proceed with what comes after it. In C family languages the simple `break` statement terminates execution of the innermost `for`, `while`, `do`, or `switch` construction and proceeds to the next sequential instruction. A typical usage might be

```
for(;;) {
    ...                         (statements before the test)
    if (...) break;
    ...                         (statements after the test)
}
...                             (next sequential instruction)
```

Companion to `break` is `continue`, which transfers program control to the end of the body of the smallest enclosing `for`, `while`, or `do` statement, but *just inside* that body's terminating curly brace. In general, this results in the execution of the next loop test associated with that body.

1.2.3 How Tricky Is Too Tricky?

Every programmer is occasionally tempted to write a line or two of code that is so elegantly tricky that all who read it will stand in awe of its author's intelligence. Poetic justice is that it is usually that same programmer who gets stumped, later on, trying to understand his or her own creation. You might momentarily be proud of yourself at writing the single line

```
k=(2-j)*(1+3*j)/2;
```

if you want to permute cyclically one of the values $j = (0, 1, 2)$ into respectively $k = (1, 2, 0)$. You will regret it later, however. Better, and likely also faster, is

```
k=j+1;
if (k == 3) k=0;
```

On the other hand, it can also be a mistake, or at least suboptimal, to be too ploddingly literal, as in

```
switch (j) {
    case 0: k=1; break;
    case 1: k=2; break;
    case 2: k=0; break;
    default: {
        cerr << "unexpected value for j";
        exit(1);
    }
}
```

This (or similar) might be the house style if you are one of 10^5 programmers working for a megacorporation, but if you are programming for your own research, or within a small group of collaborators, this kind of style will soon cause you to lose the forest for the trees. You need to find the right personal balance between obscure trickery and boring prolixity. A good rule is that you should always write code that is *slightly less tricky than you are willing to read, but only slightly.*

There is a fine line between being tricky (bad) and being idiomatic (good). *Idioms* are short expressions that are sufficiently common, or sufficiently self-explanatory, that you can use them freely. For example, testing an integer n's even- or odd-ness by

```
if (n & 1) ...
```

is, we think, much preferable to

```
if (n % 2 == 1) ...
```

We similarly like to double a positive integer by writing

```
n <<= 1;
```

or construct a mask of n bits by writing

```
(1 << n) - 1
```

and so forth.

Some idioms are worthy of consideration even when they are not so immediately obvious. S.E. Anderson [2] has collected a number of "bit-twiddling hacks," of which we show three here:

The test

```
if ((v&(v-1))==0) {}            Is a power of 2 or zero.
```

tests whether v is a power of 2. If you care about the case $v = 0$, you have to write

```
if (v&&((v&(v-1))==0)) {}       Is a power of 2.
```

The idiom

```
for (c=0;v;c++) v &= v - 1;
```

gives as c the number of set ($= 1$) bits in a positive or unsigned integer v (destroying v in the process). The number of iterations is only as many as the number of bits set.

The idiom

```
v--;
v |= v >> 1; v |= v >> 2; v |= v >> 4; v |= v >> 8; v |= v >> 16;
v++;
```

rounds a positive (or unsigned) 32-bit integer v up to the next power of 2 that is $\geq$ v.

When we use the bit-twiddling hacks, we'll include an explanatory comment in the code.

1.2.4 Utility Macros or Templated Functions

The file nr3.h includes, among other things, definitions for the functions

```
MAX(a,b)
MIN(a,b)
SWAP(a,b)
SIGN(a,b)
```

These are all self-explanatory, except possibly the last. SIGN(a,b) returns a value with the same magnitude as a and the same sign as b. These functions are all implemented as templated inline functions, so that they can be used for all argument types that make sense semantically. Implementation as macros is also possible.

CITED REFERENCES AND FURTHER READING:

Harbison, S.P., and Steele, G.L., Jr. 2002, *C: A Reference Manual*, 5th ed. (Englewood Cliffs, NJ: Prentice-Hall).[1]

Anderson, S.E. 2006, "Bit Twiddling Hacks," at http://graphics.stanford.edu/~seander/bithacks.html.[2]

1.3 Objects, Classes, and Inheritance

An *object* or *class* (the terms are interchangeable) is a program structure that groups together some variables, or functions, or both, in such a way that all the included variables or functions "see" each other and can interact intimately, while most of this internal structure is hidden from other program structures and units. Objects make possible *object-oriented programming* (OOP), which has become recognized as the almost unique successful paradigm for creating complex software. The key insight in OOP is that objects have *state* and *behavior*. The state of the object is described by the values stored in its member variables, while the possible behavior is determined by the member functions. We will use objects in other ways as well.

The terminology surrounding OOP can be confusing. Objects, classes, and structures pretty much refer to the same thing. Member functions in a class are often referred to as *methods* belonging to that class. In C++, objects are defined with either the keyword class or the keyword struct. These differ, however, in the details of how rigorously they hide the object's internals from public view. Specifically,

```
struct SomeName { ...
```

is defined as being the same as

```
class SomeName {
public: ...
```

In this book we *always* use struct. This is not because we deprecate the use of public and private access specifiers in OOP, but only because such access control would add little to understanding the underlying numerical methods that are the focus of this book. In fact, access specifiers could impede your understanding, because

you would be constantly moving things from private to public (and back again) as you program different test cases and want to examine different internal, normally private, variables.

Because our classes are declared by `struct`, not `class`, use of the word "class" is potentially confusing, and we will usually try to avoid it. So "object" means `struct`, which is really a class!

If you are an OOP beginner, it is important to understand the distinction between defining an object and instantiating it. You define an object by writing code like this:

```
struct Twovar {
    Doub a,b;
    Twovar(const Doub aa, const Doub bb) : a(aa), b(bb) {}
    Doub sum() {return a+b;}
    Doub diff() {return a-b;}
};
```

This code does not create a `Twovar` object. It only tells the compiler how to create one when, later in your program, you tell it to do so, for example by a declaration like,

```
Twovar mytwovar(3.,5.);
```

which invokes the `Twovar` constructor and creates an instance of (or *instantiates*) a `Twovar`. In this example, the constructor also sets the internal variables `a` and `b` to 3 and 5, respectively. You can have any number of simultaneously existing, noninteracting, instances:

```
Twovar anothertwovar(4.,6.);
Twovar athirdtwovar(7.,8.);
```

We have already promised you that this book is not a textbook in OOP, or the C++ language; so we will go no farther here. If you need more, good references are [1-4].

1.3.1 Simple Uses of Objects

We use objects in various ways, ranging from trivial to quite complex, depending on the needs of the specific numerical method that is being discussed. As mentioned in §1.0, this lack of consistency means that *Numerical Recipes* is not a useful examplar of a program library (or, in an OOP context, a *class library*). It also means that, somewhere in this book, you can probably find an example of every possible way to think about objects in numerical computing! (We hope that you will find this a plus.)

Object for Grouping Functions. Sometimes an object just collects together a group of closely related functions, not too differently from the way that you might use a `namespace`. For example, a simplification of Chapter 6's object `Erf` looks like:

```
struct Erf {                              No constructor needed.
    Doub erf(Doub x);
    Doub erfc(Doub x);
    Doub inverf(Doub p);
    Doub inverfc(Doub p);
    Doub erfccheb(Doub z);
};
```

As will be explained in §6.2, the first four methods are the ones intended to be called by the user, giving the error function, complementary error function, and the two

corresponding inverse functions. But these methods share some code and also use common code in the last method, `erfccheb`, which the user will normally ignore completely. It therefore makes sense to group the whole collection as an `Erf` object. About the only disadvantage of this is that you must instantiate an `Erf` object before you can use (say) the `erf` function:

```
Erf myerf;                          The name myerf is arbitrary.
...
Doub y = myerf.erf(3.);
```

Instantiating the object doesn't actually *do* anything here, because `Erf` contains no variables (i.e., has no stored state). It just tells the compiler what local name you are going to use in referring to its member functions. (We would normally use the name `erf` for the instance of `Erf`, but we thought that `erf.erf(3.)` would be confusing in the above example.)

Object for Standardizing an Interface. In §6.14 we'll discuss a number of useful standard probability distributions, for example, normal, Cauchy, binomial, Poisson, etc. Each gets its own object definition, for example,

```
struct Cauchydist {
    Doub mu, sig;
    Cauchydist(Doub mmu = 0., Doub ssig = 1.) : mu(mmu), sig(ssig) {}
    Doub p(Doub x);
    Doub cdf(Doub x);
    Doub invcdf(Doub p);
};
```

where the function `p` returns the probability density, the function `cdf` returns the cumulative distribution function (cdf), and the function `invcdf` returns the inverse of the cdf. Because the interface is consistent across all the different probability distributions, you can change which distribution a program is using by changing a single program line, for example from

```
Cauchydist mydist();
```

to

```
Normaldist mydist();
```

All subsequent references to functions like `mydist.p`, `mydist.cdf`, and so on, are thus changed automatically. This is hardly OOP at all, but it can be very convenient.

Object for Returning Multiple Values. It often happens that a function computes more than one useful quantity, but you don't know which one or ones the user is actually interested in on that particular function call. A convenient use of objects is to save all the potentially useful results and then let the user grab those that are of interest. For example, a simplified version of the `Fitab` structure in Chapter 15, which fits a straight line $y = a + bx$ to a set of data points `xx` and `yy`, looks like this:

```
struct Fitab {
    Doub a, b;
    Fitab(const VecDoub &xx, const VecDoub &yy);        Constructor.
};
```

(We'll discuss `VecDoub` and related matters below, in §1.4.) The user calculates the fit by calling the constructor with the data points as arguments,

```
Fitab myfit(xx,yy);
```

Then the two "answers" a and b are separately available as `myfit.a` and `myfit.b`. We will see more elaborate examples throughout the book.

Objects That Save Internal State for Multiple Uses. This is classic OOP, worthy of the name. A good example is Chapter 2's `LUdcmp` object, which (in abbreviated form) looks like this:

```
struct LUdcmp {
    Int n;
    MatDoub lu;
    LUdcmp(const MatDoub &a);  Constructor.
    void solve(const VecDoub &b, VecDoub &x);
    void inverse(MatDoub &ainv);
    Doub det();
};
```

This object is used to solve linear equations and/or invert a matrix. You use it by creating an instance with your matrix `a` as the argument in the constructor. The constructor then computes and stores, in the internal matrix `lu`, a so-called LU decomposition of your matrix (see §2.3). Normally you won't use the matrix `lu` directly (though you could if you wanted to). Rather, you now have available the methods `solve()`, which returns a solution vector `x` for any right-hand side `b`, `inverse()`, which returns the inverse matrix, and `det()`, which returns the determinant of your matrix.

You can call any or all of `LUdcmp`'s methods in any order; you might well want to call `solve` multiple times, with different right-hand sides. If you have more than one matrix in your problem, you create a separate instance of `LUdcmp` for each one, for example,

```
LUdcmp alu(a), aalu(aa);
```

after which `alu.solve()` and `aalu.solve()` are the methods for solving linear equations for each respective matrix, `a` and `aa`; `alu.det()` and `aalu.det()` return the two determinants; and so forth.

We are not finished listing ways to use objects: Several more are discussed in the next few sections.

1.3.2 Scope Rules and Object Destruction

This last example, `LUdcmp`, raises the important issue of how to manage an object's time and memory usage within your program.

For a large matrix, the `LUdcmp` constructor does a lot of computation. You choose exactly where in your program you want this to occur in the obvious way, by putting the declaration

```
LUdcmp alu(a);
```

in just that place. The important distinction between a non-OOP language (like C) and an OOP language (like C++) is that, in the latter, declarations are not passive instructions to the compiler, but executable statments at run-time.

The `LUdcmp` constructor also, for a large matrix, grabs a lot of memory, to store the matrix `lu`. How do you take charge of this? That is, how do you communicate that it should save this state for as long as you might need it for calls to methods like `alu.solve()`, but not indefinitely?

The answer lies in C++'s strict and predictable rules about *scope*. You can start a temporary scope at any point by writing an open bracket, "{". You end that scope by a matching close bracket, "}". You can nest scopes in the obvious way. Any objects that are declared within a scope are destroyed (and their memory resources returned) when the end of the scope is reached. An example might look like this:

```
MatDoub a(1000,1000);               Create a big matrix,
VecDoub b(1000),x(1000);            and a couple of vectors.
...
{                                   Begin temporary scope.
    LUdcmp alu(a);                  Create object alu.
    ...
    alu.solve(b,x);                 Use alu.
    ...
}                                   End temporary scope. Resources in alu are freed.
...
Doub d = alu.det();                 ERROR! alu is out of scope.
```

This example presumes that you have some other use for the matrix a later on. If not, then the the declaration of a should itself probably be inside the temporary scope.

Be aware that *all* program blocks delineated by braces are scope units. This includes the main block associated with a function definition and also blocks associated with control structures. In code like this,

```
for (;;) {
    ...
    LUdcmp alu(a);
    ...
}
```

a new instance of alu is created at each iteration and then destroyed at the end of that iteration. This might sometimes be what you intend (if the matrix a changes on each iteration, for example); but you should be careful not to let it happen unintentionally.

1.3.3 Functions and Functors

Many routines in this book take functions as input. For example, the quadrature (integration) routines in Chapter 4 take as input the function $f(x)$ to be integrated. For a simple case like $f(x) = x^2$, you code such a function simply as

```
Doub f(const Doub x) {
    return x*x;
}
```

and pass f as an argument to the routine. However, it is often useful to use a more general object to communicate the function to the routine. For example, $f(x)$ may depend on other variables or parameters that need to be communicated from the calling program. Or the computation of $f(x)$ may be associated with other sub-calculations or information from other parts of the program. In non-OOP programing, this communication is usually accomplished with global variables that pass the information "over the head" of the routine that receives the function argument f.

C++ provides a better and more elegant solution: *function objects* or *functors*. A functor is simply an object in which the operator () has been overloaded to play the role of returning a function value. (There is no relation between this use of the word functor and its different meaning in pure mathematics.) The case $f(x) = x^2$ would now be coded as

```
struct Square {
    Doub operator()(const Doub x) {
        return x*x;
    }
};
```

To use this with a quadrature or other routine, you declare an instance of `Square`

```
Square g;
```

and pass `g` to the routine. Inside the quadrature routine, an invocation of `g(x)` returns the function value in the usual way.

In the above example, there's no point in using a functor instead of a plain function. But suppose you have a parameter in the problem, for example, $f(x) = cx^p$, where c and p are to be communicated from somewhere else in your program. You can set the parameters via a constructor:

```
struct Contimespow {
    Doub c,p;
    Contimespow(const Doub cc, const Doub pp) : c(cc), p(pp) {}
    Doub operator()(const Doub x) {
        return c*pow(x,p);
    }
};
```

In the calling program, you might declare the instance of `Contimespow` by

```
Contimespow h(4.,0.5);          Communicate c and p to the functor.
```

and later pass `h` to the routine. Clearly you can make the functor much more complicated. For example, it can contain other helper functions to aid in the calculation of the function value.

So should we implement all our routines to accept only functors and not functions? Luckily, we don't have to decide. We can write the routines so they can accept *either* a function or a functor. A routine accepting only a function to be integrated from a to b might be declared as

```
Doub someQuadrature(Doub func(const Doub), const Doub a, const Doub b);
```

To allow it to accept either functions or functors, we instead make it a *templated* function:

```
template <class T>
Doub someQuadrature(T &func, const Doub a, const Doub b);
```

Now the compiler figures out whether you are calling `someQuadrature` with a function or a functor and generates the appropriate code. If you call the routine in one place in your program with a function and in another with a functor, the compiler will handle that too.

We will use this capability to pass functors as arguments in many different places in the book where function arguments are required. There is a tremendous gain in flexibility and ease of use.

As a convention, when we write `Ftor`, we mean a functor like `Square` or `Contimespow` above; when we write `fbare`, we mean a "bare" function like `f` above; and when we write `ftor` (all in lower case), we mean an instantiation of a functor, that is, something declared like

```
Ftor ftor(...);          Replace the dots by your parameters, if any.
```

Of course your names for functors and their instantiations will be different.

Slightly more complicated syntax is involved in passing a function to an *object*

that is templated to accept either a function or functor. So if the object is

```
template <class T>
struct SomeStruct {
    SomeStruct(T &func, ...);  constructor
    ...
```

we would instantiate it with a functor like this:

```
Ftor ftor;
SomeStruct<Ftor> s(ftor, ...
```

but with a function like this:

```
SomeStruct<Doub (const Doub)> s(fbare, ...
```

In this example, `fbare` takes a single `const Doub` argument and returns a `Doub`. You must use the arguments and return type for your specific case, of course.

1.3.4 Inheritance

Objects can be defined as deriving from other, already defined, objects. In such *inheritance*, the "parent" class is called a *base class*, while the "child" class is called a *derived class*. A derived class has all the methods and stored state of its base class, plus it can add any new ones.

"Is-a" Relationships. The most straightforward use of inheritance is to describe so-called *is-a* relationships. OOP texts are full of examples where the base class is `ZooAnimal` and a derived class is `Lion`. In other words, `Lion` "is-a" `ZooAnimal`. The base class has methods common to all `ZooAnimals`, for example `eat()` and `sleep()`, while the derived class extends the base class with additional methods specific to `Lion`, for example `roar()` and `eat_visitor()`.

In this book we use is-a inheritance less often than you might expect. Except in some highly stylized situations, like optimized matrix classes ("triangular matrix is-a matrix"), we find that the diversity of tasks in scientific computing does not lend itself to strict is-a hierarchies. There are exceptions, however. For example, in Chapter 7, we define an object `Ran` with methods for returning uniform random deviates of various types (e.g., `Int` or `Doub`). Later in the chapter, we define objects for returning other kinds of random deviates, for example normal or binomial. These are defined as derived classes of `Ran`, for example,

```
struct Binomialdev : Ran {};
```

so that they can share the machinery already in `Ran`. This is a true is-a relationship, because "binomial deviate is-a random deviate."

Another example occurs in Chapter 13, where objects `Daub4`, `Daub4i`, and `Daubs` are all derived from the `Wavelet` base class. Here `Wavelet` is an *abstract base class* or *ABC* [1,4] that has no content of its own. Rather, it merely specifies interfaces for all the methods that any `Wavelet` is required to have. The relationship is nevertheless is-a: "`Daub4` is-a `Wavelet`".

"Prerequisite" Relationships. Not for any dogmatic reason, but simply because it is convenient, we frequently use inheritance to pass on to an object a set of methods that it needs as prerequisites. This is especially true when the same set of prerequisites is used by more than one object. In this use of inheritance, the base class has no particular `ZooAnimal` unity; it may be a grab-bag. There is not a logical is-a relationship between the base and derived classes.

An example in Chapter 10 is `Bracketmethod`, which is a base class for several

minimization routines, but which simply provides a common method for the initial bracketing of a minimum. In Chapter 7, the `Hashtable` object provides prerequisite methods to its derived classes `Hash` and `Mhash`, but one cannot say, "`Mhash` is-a `Hashtable`" in any meaningful way. An extreme example, in Chapter 6, is the base class `Gauleg18`, which does nothing except provide a bunch of constants for Gauss-Legendre integration to derived classes `Beta` and `Gamma`, both of which need them. Similarly, long lists of constants are provided to the routines `StepperDopr853` and `StepperRoss` in Chapter 17 by base classes to avoid cluttering the coding of the algorithms.

Partial Abstraction. Inheritance can be used in more complicated or situation-specific ways. For example, consider Chapter 4, where elementary quadrature rules such as `Trapzd` and `Midpnt` are used as building blocks to construct more elaborate quadrature algorithms. The key feature these simple rules share is a mechanism for adding more points to an existing approximation to an integral to get the "next" stage of refinement. This suggests deriving these objects from an abstract base clase called `Quadrature`, which specifies that all objects derived from it must have a `next()` method. This is not a complete specification of a common is-a interface; it abstracts only one feature that turns out to be useful.

For example, in §4.6, the `Stiel` object invokes, in different situations, two different quadrature objects, `Trapzd` and `DErule`. These are not interchangeable. They have different constructor arguments and could not easily both be made `ZooAnimals` (as it were). `Stiel` of course knows about their differences. However, one of `Stiel`'s methods, `quad()`, doesn't (and shouldn't) know about these differences. It uses only the method `next()`, which exists, with different definitions, in both `Trapzd` and `DErule`.

While there are several different ways to deal with situations like this, an easy one is available once `Trapzd` and `DErule` have been given a common abstract base class `Quadrature` that contains nothing except a virtual interface to `next`. In a case like this, the base class is a minor design feature as far as the implementation of `Stiel` is concerned, almost an afterthought, rather than being the apex of a top-down design. As long as the usage is clear, there is nothing wrong with this.

Chapter 17, which discusses ordinary differential equations, has some even more complicated examples that combine inheritance and templating. We defer further discussion to there.

CITED REFERENCES AND FURTHER READING:

Stroustrup, B. 1997, *The C++ Programming Language*, 3rd ed. (Reading, MA: Addison-Wesley).[1]

Lippman, S.B., Lajoie, J., and Moo, B.E. 2005, *C++ Primer*, 4th ed. (Boston: Addison-Wesley).[2]

Keogh, J., and Giannini, M. 2004, *OOP Demystified* (Emeryville, CA: McGraw-Hill/Osborne).[3]

Cline, M., Lomow, G., and Girou, M. 1999, *C++ FAQs*, 2nd ed. (Boston: Addison-Wesley).[4]

1.4 Vector and Matrix Objects

The C++ Standard Library [1] includes a perfectly good `vector<>` template class. About the only criticism that one can make of it is that it is so feature-rich

that some compiler vendors neglect to squeeze the last little bit of performance out of its most elementary operations, for example returning an element by its subscript. That performance is extremely important in scientific applications; its occasional absence in C++ compilers is a main reason that many scientists still (as we write) program in C, or even in Fortran!

Also included in the C++ Standard Library is the class `valarray<>`. At one time, this was supposed to be a vector-like class that was optimized for numerical computation, including some features associated with matrices and multidimensional arrays. However, as reported by one participant,

> The valarray classes were not designed very well. In fact, nobody tried to determine whether the final specification worked. This happened because nobody felt "responsible" for these classes. The people who introduced valarrays to the C++ standard library left the committee a long time before the standard was finished. [1]

The result of this history is that C++, at least now, has a good (but not always reliably optimized) class for vectors and no dependable class at all for matrices or higher-dimensional arrays. What to do? We will adopt a strategy that emphasizes flexibility and assumes only a minimal set of properties for vectors and matrices. We will then provide our own, basic, classes for vectors and matrices. For most compilers, these are at least as efficient as `vector<>` and other vector and matrix classes in common use. But if, for you, they're not, then it is easy to change to a different set of classes, as we will explain.

1.4.1 Typedefs

Flexibility is achieved by having several layers of `typedef` type-indirection, resolved at compile time so that there is no run-time performance penalty. The first level of type-indirection, not just for vectors and matrices but for virtually all variables, is that we use user-defined type names instead of C++ fundamental types. These are defined in `nr3.h`. If you ever encounter a compiler with peculiar built-in types, these definitions are the "hook" for making any necessary changes. The complete list of such definitions is

NR Type	*Usual Definition*	*Intent*
`Char`	`char`	8-bit signed integer
`Uchar`	`unsigned char`	8-bit unsigned integer
`Int`	`int`	32-bit signed integer
`Uint`	`unsigned int`	32-bit unsigned integer
`Llong`	`long long int`	64-bit signed integer
`Ullong`	`unsigned long long int`	64-bit unsigned integer
`Doub`	`double`	64-bit floating point
`Ldoub`	`long double`	[reserved for future use]
`Complex`	`complex<double>`	2×64-bit floating complex
`Bool`	`bool`	`true` or `false`

An example of when you might need to change the `typedefs` in `nr3.h` is if your compiler's `int` is not 32 bits, or if it doesn't recognize the type `long long int`.

You might need to substitute vendor-specific types like (in the case of Microsoft) `__int32` and `__int64`.

The second level of type-indirection returns us to the discussion of vectors and matrices. The vector and matrix types that appear in *Numerical Recipes* source code are as follows. Vectors: `VecInt`, `VecUint`, `VecChar`, `VecUchar`, `VecCharp`, `VecLlong`, `VecUllong`, `VecDoub`, `VecDoubp`, `VecComplex`, and `VecBool`. Matrices: `MatInt`, `MatUint`, `MatChar`, `MatUchar`, `MatLlong`, `MatUllong`, `MatDoub`, `MatComplex`, and `MatBool`. These should all be understandable, semantically, as vectors and matrices whose elements are the corresponding user-defined types, above. Those ending in a "p" have elements that are pointers, e.g., `VecCharp` is a vector of pointers to `char`, that is, `char*`. If you are wondering why the above list is not combinatorially complete, it is because we don't happen to use all possible combinations of `Vec`, `Mat`, fundamental type, and pointer in this book. You can add further analogous types as you need them.

Wait, there's more! For every vector and matrix type above, we also define types with the same names plus one of the suffixes "`_I`", "`_O`", and "`_IO`", for example `VecDoub_IO`. We use these suffixed types for specifying argument types in function definitions. The meaning, respectively, is that the argument is "input", "output", or "both input and output".* The `_I` types are automatically defined to be `const`. We discuss this further in §1.5.2 under the topic of `const` correctness.

It may seem capricious for us to define such a long list of types when a much smaller number of templated types would do. The rationale is flexibility: You have a hook into redefining each and every one of the types individually, according to your needs for program efficiency, local coding standards, const-correctness, or whatever. In fact, in `nr3.h`, all these types *are* `typedef`'d to one vector and one matrix class, along the following lines:

```
typedef NRvector<Int> VecInt, VecInt_O, VecInt_IO;
typedef const NRvector<Int> VecInt_I;
...
typedef NRvector<Doub> VecDoub, VecDoub_O, VecDoub_IO;
typedef const NRvector<Doub> VecDoub_I;
...
typedef NRmatrix<Int> MatInt, MatInt_O, MatInt_IO;
typedef const NRmatrix<Int> MatInt_I;
...
typedef NRmatrix<Doub> MatDoub, MatDoub_O, MatDoub_IO;
typedef const NRmatrix<Doub> MatDoub_I;
...
```

So (flexibility, again) you can change the definition of one particular type, like `VecDoub`, or else you can change the implementation of all vectors by changing the definition of `NRvector<>`. Or, you can just leave things the way we have them in `nr3.h`. That ought to work fine in 99.9% of all applications.

1.4.2 Required Methods for Vector and Matrix Classes

The important thing about the vector and matrix classes is not what names they are `typedef`'d to, but what methods are assumed for them (and are provided in the `NRvector` and `NRmatrix` template classes). For vectors, the assumed methods are a

*This is a bit of history, and derives from Fortran 90's very useful `INTENT` attributes.

subset of those in the C++ Standard Library vector<> class. If v is a vector of type NRvector<T>, then we assume the methods:

v()	Constructor, zero-length vector.
v(Int n)	Constructor, vector of length n.
v(Int n, const T &a)	Constructor, initialize all elements to the value a.
v(Int n, const T *a)	Constructor, initialize elements to values in a C-style array, a[0], a[1], ...
v(const NRvector &rhs)	Copy constructor.
v.size()	Returns number of elements in v.
v.resize(Int newn)	Resizes v to size newn. We do not assume that contents are preserved.
v.assign(Int newn, const T &a)	Resize v to size newn, and set all elements to the value a.
v[Int i]	Element of v by subscript, either an l-value and an r-value.
v = rhs	Assignment operator. Resizes v if necessary and makes it a copy of the vector rhs.
typedef T value_type;	Makes T available externally (useful in templated functions or classes).

As we will discuss later in more detail, you can use any vector class you like with *Numerical Recipes*, as long as it provides the above basic functionality. For example, a brute force way to use the C++ Standard Library vector<> class instead of NRvector is by the preprocessor directive

```
#define NRvector vector
```

(In fact, there is a compiler switch, _USESTDVECTOR_, in the file nr3.h that will do just this.)

The methods for matrices are closely analogous. If vv is a matrix of type NRmatrix<T>, then we assume the methods:

vv()	Constructor, zero-length vector.
vv(Int n, Int m)	Constructor, n × m matrix.
vv(Int n, Int m, const T &a)	Constructor, initialize all elements to the value a.
vv(Int n, Int m, const T *a)	Constructor, initialize elements by rows to the values in a C-style array.
vv(const NRmatrix &rhs)	Copy constructor.
vv.nrows()	Returns number of rows n.
vv.ncols()	Returns number of columns m.
vv.resize(Int newn, Int newm)	Resizes vv to newn×newm. We do not assume that contents are preserved.
vv.assign(Int newn, Int newm, const t &a)	Resizes vv to newn × newm, and sets all elements to the value a.
vv[Int i]	Return a pointer to the first element in row i (not often used by itself).
v[Int i][Int j]	Element of vv by subscript, either an l-value and an r-value.
vv = rhs	Assignment operator. Resizes vv if necessary and makes it a copy of the matrix rhs.
typedef T value_type;	Makes T available externally.

For more precise specifications, see §1.4.3.

There is one additional property that we assume of the vector and matrix classes, namely that all of an object's elements are stored in sequential order. For a vector, this means that its elements can be addressed by pointer arithmetic relative to the first element. For example, if we have

```
VecDoub a(100);
Doub *b = &a[0];
```

then a[i] and b[i] reference the same element, both as an l-value and as an r-value. This capability is sometimes important for inner-loop efficiency, and it is also useful for interfacing with legacy code that can handle Doub* arrays, but not VecDoub vectors. Although the original C++ Standard Library did not guarantee this behavior, all known implementations of it do so, and the behavior is now required by an amendment to the standard [2].

For matrices, we analogously assume that storage is by rows within a single sequential block so that, for example,

```
Int n=97, m=103;
MatDoub a(n,m);
Doub *b = &a[0][0];
```

implies that a[i][j] and b[m*i+j] are equivalent.

A few of our routines need the capability of taking as an argument either a vector or else one row of a matrix. For simplicity, we usually code this using overloading, as, e.g.,

```
void someroutine(Doub *v, Int m) {          Version for a matrix row.
    ...
}
inline void someroutine(VecDoub &v) {       Version for a vector.
    someroutine(&v[0],v.size());
}
```

For a vector v, a call looks like someroutine(v), while for row i of a matrix vv it is someroutine(&vv[i][0],vv.ncols()). While the simpler argument vv[i] would in fact work in our implementation of NRmatrix, it might not work in some other matrix class that guarantees sequential storage but has the return type for a single subscript different from T*.

1.4.3 Implementations in nr3.h

For reference, here is a complete declaration of NRvector.

```
template <class T>
class NRvector {
private:
    int nn;                                     Size of array, indices 0..nn-1.
    T *v;                                       Pointer to data array.
public:
    NRvector();                                 Default constructor.
    explicit NRvector(int n);                   Construct vector of size n.
    NRvector(int n, const T &a);                Initialize to constant value a.
    NRvector(int n, const T *a);                Initialize to values in C-style array a.
    NRvector(const NRvector &rhs);              Copy constructor.
    NRvector & operator=(const NRvector &rhs);  Assignment operator.
    typedef T value_type;                       Make T available.
    inline T & operator[](const int i);         Return element number i.
    inline const T & operator[](const int i) const;     const version.
    inline int size() const;                    Return size of vector.
    void resize(int newn);                      Resize, losing contents.
    void assign(int newn, const T &a);          Resize and assign a to every element.
    ~NRvector();                                Destructor.
};
```

The implementations are straightforward and can be found in the file `nr3.h`. The only issues requiring finesse are the consistent treatment of zero-length vectors and the avoidance of unnecessary resize operations.

A complete declaration of `NRmatrix` is

```
template <class T>
class NRmatrix {
private:
    int nn;                                          Number of rows and columns. Index
    int mm;                                             range is 0..nn-1, 0..mm-1.
    T **v;                                           Storage for data.
public:
    NRmatrix();                                      Default constructor.
    NRmatrix(int n, int m);                          Construct n × m matrix.
    NRmatrix(int n, int m, const T &a);              Initialize to constant value a.
    NRmatrix(int n, int m, const T *a);              Initialize to values in C-style array a.
    NRmatrix(const NRmatrix &rhs);                   Copy constructor.
    NRmatrix & operator=(const NRmatrix &rhs);       Assignment operator.
    typedef T value_type;                            Make T available.
    inline T* operator[](const int i);               Subscripting: pointer to row i.
    inline const T* operator[](const int i) const;           const version.
    inline int nrows() const;                        Return number of rows.
    inline int ncols() const;                        Return number of columns.
    void resize(int newn, int newm);                 Resize, losing contents.
    void assign(int newn, int newm, const T &a);     Resize and assign a to every element.
    ~NRmatrix();                                     Destructor.
};
```

A couple of implementation details in `NRmatrix` are worth commenting on. The private variable `**v` points not to the data but rather to an array of pointers to the data rows. Memory allocation of this array is separate from the allocation of space for the actual data. The data space is allocated as a single block, not separately for each row. For matrices of zero size, we have to account for the separate possibilities that there are zero rows, or that there are a finite number of rows, but each with zero columns. So, for example, one of the constructors looks like this:

```
template <class T>
NRmatrix<T>::NRmatrix(int n, int m) : nn(n), mm(m), v(n>0 ? new T*[n] : NULL)
{
    int i,nel=m*n;
    if (v) v[0] = nel>0 ? new T[nel] : NULL;
    for (i=1;i<n;i++) v[i] = v[i-1] + m;
}
```

Finally, it matters *a lot* whether your compiler honors the `inline` directives in `NRvector` and `NRmatrix` above. If it doesn't, then you may be doing full function calls, saving and restoring context within the processor, every time you address a vector or matrix element. This is tantamount to making C++ useless for most numerical computing! Luckily, as we write, the most commonly used compilers are all "honorable" in this respect.

CITED REFERENCES AND FURTHER READING:

Josuttis, N.M. 1999, *The C++ Standard Library: A Tutorial and Reference* (Boston: Addison-Wesley).[1]

International Standardization Organization 2003, *Technical Corrigendum ISO 14882:2003*.[2]

1.5 Some Further Conventions and Capabilities

We collect in this section some further explanation of C++ language capabilities and how we use them in this book.

1.5.1 Error and Exception Handling

We already mentioned that we code error conditions with simple `throw` statements, like this

```
throw("error foo in routine bah");
```

If you are programming in an environment that has a defined set of error classes, and you want to use them, then you'll need to change these lines in the routines that you use. Alternatively, without any additional machinery, you can choose between a couple of different, useful behaviors just by making small changes in `nr3.h`.

By default, `nr3.h` redefines `throw()` by a preprocessor macro,

```
#define throw(message) \
    {printf("ERROR: %s\n in file %s at line %d\n", \
     message,__FILE__,__LINE__); \
     exit(1);}
```

This uses standard ANSI C features, also present in C++, to print the source code file name and line number at which the error occurs. It is inelegant, but perfectly functional.

Somewhat more functional, and definitely more elegant, is to set `nr3.h`'s compiler switch `_USENRERRORCLASS_`, which instead substitutes the following code:

```
struct NRerror {
    char *message;
    char *file;
    int line;
    NRerror(char *m, char *f, int l) : message(m), file(f), line(l) {}
};

void NRcatch(NRerror err) {
    printf("ERROR: %s\n     in file %s at line %d\n",
        err.message, err.file, err.line);
    exit(1);
}

#define throw(message) throw(NRerror(message,__FILE__,__LINE__));
```

Now you have a (rudimentary) error class, `NRerror`, available. You use it by putting a `try...catch` control structure at any desired point (or points) in your code, for example (§2.9),

```
...
try {
    Cholesky achol(a);
}
catch (NRerror err) {
    NRcatch(err);                 Executed if Cholesky throws an exception.
}
```

As shown, the use of the `NRcatch` function above simply mimics the behavior of the previous macro in the global context. But you don't have to use `NRcatch` at all: You

can substitute any code that you want for the body of the catch statement. If you want to distinguish between different kinds of exceptions that may be thrown, you can use the information returned in err. We'll let you figure this out yourself. And of course you are welcome to add more complicated error classes to your own copy of nr3.h.

1.5.2 Const Correctness

Few topics in discussions about C++ evoke more heat than questions about the keyword const. We are firm believers in using const wherever possible, to achieve what is called "const correctness." Many coding errors are automatically trapped by the compiler if you have qualified identifiers that should not change with const when they are declared. Also, using const makes your code much more readable: When you see const in front of an argument to a function, you know immediately that the function will not modify the object. Conversely, if const is absent, you should be able to count on the object being changed somewhere.

We are such strong const believers that we insert const even where it is theoretically redundant: If an argument is passed *by value* to a function, then the function makes a copy of it. Even if this copy is modified by the function, the original value is unchanged after the function exits. While this allows you to change, with impunity, the values of arguments that have been passed by value, this usage is error-prone and hard to read. If your intention in passing something by value is that it is an input variable only, then make it clear. So we declare a function $f(x)$ as, for example,

```
Doub f(const Doub x);
```

If in the function you want to use a local variable that is initialized to x but then gets changed, define a new quantity — don't use x. If you put const in the declaration, the compiler will not let you get this wrong.

Using const in your function arguments makes your function more general: Calling a function that expects a const argument with a non-const variable involves a "trivial" conversion. But trying to pass a const quantity to a non-const argument is an error.

A final reason for using const is that it allows certain user-defined conversions to be made. As discussed in [1], this can be useful if you want to use *Numerical Recipes* routines with another matrix/vector class library.

We now need to elaborate on what exactly const does for a nonsimple type such as a class that is an argument of a function. Basically, it guarantees that the object is not modified by the function. In other words, the object's data members are unchanged. But if a data member is a *pointer* to some data, and the data itself is not a member variable, then *the data can be changed* even though the pointer cannot be.

Let's look at the implications of this for a function f that takes an NRvector<Doub> argument a. To avoid unnecessary copying, we always pass vectors and matrices by reference. Consider the difference between declaring the argument of a function with and without const:

```
void f(NRvector<Doub> &a)      versus      void f(const NRvector<Doub> &a)
```

The const version promises that f does not modify the data members of a. But a statement like

```
a[i] = 4.;
```

inside the function definition is in principle perfectly OK — you are modifying the data pointed to, not the pointer itself.

"Isn't there some way to protect the data?" you may ask. Yes, there is: You can declare the *return type* of the subscript operator, operator[], to be const. This is why there are two versions of operator[] in the NRvector class,

```
T & operator[](const int i);
const T & operator[](const int i) const;
```

The first form returns a reference to a modifiable vector element, while the second returns a nonmodifiable vector element (because the return type has a const in front).

But how does the compiler know which version to invoke when you just write a[i]? That is specified by the *trailing* word const in the second version. It refers not to the returned element, nor to the argument i, but to the object whose operator[] is being invoked, in our example the vector a. Taken together, the two versions say this to the compiler: "If the vector a is const, then transfer that const'ness to the returned element a[i]. If it isn't, then don't."

The remaining question is thus how the compiler determines whether a is const. In our example, where a is a function argument, it is trivial: The argument is either declared as const or else it isn't. In other contexts, a might be const because you originally declared it as such (and initialized it via constructor arguments), or because it is a const reference data member in some other object, or for some other, more arcane, reason.

As you can see, getting const to protect the data is a little complicated. Judging from the large number of matrix/vector libraries that follow this scheme, many people feel that the payoff is worthwhile. We urge you *always* to declare as const those objects and variables that are not intended to be modified. You do this both at the time an object is actually created and in the arguments of function declarations and definitions. You won't regret making a habit of const correctness.

In §1.4 we defined vector and matrix type names with trailing _I labels, for example, VecDoub_I and MatInt_I. The _I, which stands for "input to a function," means that the type is declared as const. (This is already done in the typedef statement; you don't have to repeat it.) The corresponding labels _O and _IO are to remind you when arguments are not just non-const, but will actually be modified by the function in whose argument list they appear.

Having rightly put all this emphasis on const correctness, duty compels us also to recognize the existence of an alternative philosophy, which is to stick with the more rudimentary view "const protects the container, not the contents." In this case you would want only *one* form of operator[], namely

```
T & operator[](const int i) const;
```

It would be invoked whether your vector was passed by const reference or not. In both cases element i is returned as potentially modifiable. While we are opposed to this philosophically, it turns out that it does make possible a tricky kind of automatic type conversion that allows you to use your favorite vector and matrix classes instead of NRvector and NRmatrix, even if your classes use a syntax completely different from what we have assumed. For information on this very specialized application, see [1].

1.5.3 Abstract Base Class (ABC), or Template?

There is sometimes more than one good way to achieve some end in C++. Heck, let's be honest: There is *always* more than one way. Sometimes the differences amount to small tweaks, but at other times they embody very different views about the language. When we make one such choice, and you prefer another, you are going to be quite annoyed with us. Our defense against this is to avoid foolish consistencies,* and to illustrate as many viewpoints as possible.

A good example is the question of when to use an abstract base class (ABC) versus a template, when their capabilities overlap. Suppose we have a function `func` that can do its (useful) thing on, or using, several different types of objects, call them `ObjA`, `ObjB`, and `ObjC`. Moreover, `func` doesn't need to know much about the object it interacts with, only that it has some method `tellme`.

We could implement this setup as an abstract base class:

```
struct ObjABC {                        Abstract Base Class for objects with tellme.
    virtual void tellme() = 0;
};

struct ObjA : ObjABC {                 Derived class.
    ...
    void tellme() {...}
};
struct ObjB : ObjABC {                 Derived class.
    ...
    void tellme() {...}
};
struct ObjC : ObjABC {                 Derived class.
    ...
    void tellme() {...}
};

void func(ObjABC &x) {
    ...
    x.tellme();                        References the appropriate tellme.
}
```

On the other hand, using a template, we can write code for `func` without ever seeing (or even knowing the names of) the objects for which it is intended:

```
template<class T>
void func(T &x) {
    ...
    x.tellme();
}
```

That certainly seems easier! Is it better?

Maybe. A disadvantage of templates is that the template must be available to the compiler every time it encounters a call to `func`. This is because it actually compiles a different version of `func` for every different type of argument `T` that it encounters. Unless your code is so large that it cannot easily be compiled as a single unit, however, this is not much of a disadvantage. On the other side, favoring templates, is the fact that virtual functions incur a small run-time penalty when they are called. But this is rarely significant.

The deciding factors in this example relate to software engineering, not performance, and are hidden in the lines with ellipses (`...`). We haven't really told

*"A foolish consistency is the hobgoblin of little minds." —Emerson

you how closely related `ObjA`, `ObjB`, and `ObjC` are. If they are close, then the ABC approach offers possibilities for putting more than just `tellme` into the base class. Putting things into the base class, whether data or pure virtual methods, lets the compiler enforce consistency across the derived classes. If you later write another derived object `ObjD`, its consistency will also be enforced. For example, the compiler will require you to implement a method in every derived class corresponding to every pure virtual method in the base class.

By contrast, in the template approach, the only enforced consistency will be that the method `tellme` exists, and this will only be enforced at the point in the code where `func` is actually called with an `ObjD` argument (if such a point exists), not at the point where `ObjD` is defined. Consistency checking in the template approach is thus somewhat more haphazard.

Laid-back programmers will opt for templates. Up-tight programmers will opt for ABCs. We opt for... both, on different occasions. There can also be other reasons, having to do with subtle features of class derivation or of templates, for choosing one approach over the other. We will point these out as we encounter them in later chapters. For example, in Chapter 17 we define an abstract base class called `StepperBase` for the various "stepper" routines for solving ODEs. The derived classes implement particular stepping algorithms, and they are templated so they can accept function or functor arguments (see §1.3.3).

1.5.4 NaN and Floating Point Exceptions

We mentioned in §1.1.1 that the IEEE floating-point standard includes a representation for NaN, meaning "not a number." NaN is distinct from positive and negative infinity, as well as from every representable number. It can be both a blessing and a curse.

The blessing is that it can be useful to have a value that can be used with meanings like "don't process me" or "missing data" or "not yet initialized." To use NaN in this fashion, you need to be able to *set* variables to it, and you need to be able to *test* for its having been set.

Setting is easy. The "approved" method is to use `numeric_limits`. In `nr3.h` the line

```
static const Doub NaN = numeric_limits<Doub>::quiet_NaN();
```

defines a global value `NaN`, so that you can write things like

```
x = NaN;
```

at will. If you ever encounter a compiler that doesn't do this right (it's a pretty obscure corner of the standard library!), then try either

```
Uint proto_nan[2]=0xffffffff, 0x7fffffff;
double NaN = *( double* )proto_nan;
```

(which assumes little-endian behavior; cf. §1.1.1) or the self-explanatory

```
Doub NaN = sqrt(-1.);
```

which may, however, throw an immediate exception (see below) and thus not work for this purpose. But, one way or another, you can generally figure out how to get a `NaN` constant into your environment.

Testing also requires a bit of (one-time) experimentation: According to the IEEE standard, NaN is guaranteed to be the only value that is not equal to itself!

So, the "approved" method of testing whether Doub value x has been set to NaN is

```
if (x != x) {...}                    It's a NaN!
```

(or test for equality to determine that it's not a NaN). Unfortunately, at time of writing, some otherwise perfectly good compilers don't do this right. Instead, they provide a macro `isnan()` that returns `true` if the argument is NaN, otherwise `false`. (Check carefully whether the required `#include` is `math.h` or `float.h` — it varies.)

What, then, is the *curse* of NaN? It is that some compilers, notably Microsoft, have poorly thought-out default behaviors in distinguishing *quiet NaNs* from *signalling NaNs*. Both kinds of NaNs are defined in the IEEE floating-point standard. Quiet NaNs are supposed to be for uses like those above: You can set them, test them, and propagate them by assignment, or even through other floating operations. In such uses they are not supposed to signal an exception that causes your program to abort. Signalling NaNs, on the other hand, are, as the name implies, supposed to signal exceptions. Signalling NaNs should be generated by invalid operations, such as the square root or logarithm of a negative number, or `pow(0.,0.)`.

If all NaNs are treated as signalling exceptions, then you can't make use of them as we have suggested above. That's annoying, but OK. On the other hand, if all NaNs are treated as quiet (the Microsoft default at time of writing), then you will run long calculations only to find that all the results are NaN — and you have no way of locating the invalid operation that triggered the propagating cascade of (quiet) NaNs. That's *not* OK. It makes debugging a nightmare. (You can get the same disease if other floating-point exceptions propagate, for example overflow or division-by-zero.)

Tricks for specific compilers are not within our normal scope. But this one is so essential that we make it an "exception": If you are living on planet Microsoft, then the lines of code,

```
int cw = _controlfp(0,0);
cw &=~(EM_INVALID | EM_OVERFLOW | EM_ZERODIVIDE );
_controlfp(cw,MCW_EM);
```

at the beginning of your program will turn NaNs from invalid operations, overflows, and divides-by-zero into signalling NaNs, and leave all the other NaNs quiet. There is a compiler switch, `_TURNONFPES_` in `nr3.h` that will do this for you automatically. (Further options are `EM_UNDERFLOW`, `EM_INEXACT`, and `EM_DENORMAL`, but we think these are best left quiet.)

1.5.5 Miscellany

- Bounds checking in vectors and matrices, that is, verifying that subscripts are in range, is expensive. It can easily double or triple the access time to subscripted elements. In their default configuration, the `NRvector` and `NRmatrix` classes never do bounds checking. However, `nr3.h` has a compiler switch, `_CHECKBOUNDS_`, that turns bounds checking on. This is implemented by preprocessor directives for conditional compilation so there is no performance penalty when you leave it turned off. This is ugly, but effective.

  The `vector<>` class in the C++ Standard Library takes a different tack. If you access a vector element by the syntax `v[i]`, there is no bounds checking. If you instead use the `at()` method, as `v.at(i)`, then bounds checking is performed. The obvious weakness of this approach is that you can't easily change a lengthy program from one method to the other, as you might want to

do when debugging.

- The importance to performance of avoiding unnecessary copying of large objects, such as vectors and matrices, cannot be overemphasized. As already mentioned, they should always be passed by reference in function arguments. But you also need to be careful about, or avoid completely, the use of functions whose return type is a large object. This is true even if the return type is a reference (which is a tricky business anyway). Our experience is that compilers often create temporary objects, using the copy constructor, when the need to do so is obscure or nonexistent. That is why we so frequently write `void` functions that have an argument of type (e.g.) `MatDoub_O` for returning the "answer." (When we do use vector or matrix return types, our excuse is either that the code is pedagogical, or that the overhead is negligible compared to some big calculation that has just been done.)

  You can check up on your compiler by instrumenting the vector and matrix classes: Add a static integer variable to the class definition, increment it within the copy constructor and assignment operator, and look at its value before and after operations that (you think) should not require any copies. You might be surprised.

- There are only two routines in *Numerical Recipes* that use three-dimensional arrays, `rlft3` in §12.6, and `solvde` in §18.3. The file `nr3.h` includes a rudimentary class for three-dimensional arrays, mainly to service these two routines. In many applications, a better way to proceed is to declare a vector of matrices, for example,

```
vector<MatDoub> threedee(17);
for (Int i=0;i<17;i++) threedee[i].resize(19,21);
```

  which creates, in effect, a three-dimensional array of size $17 \times 19 \times 21$. You can address individual components as `threedee[i][j][k]`.

- "Why no `namespace`?" Industrial-strength programmers will notice that, unlike the second edition, this third edition of *Numerical Recipes* does not shield its function and class names by a `NR::` namespace. Therefore, if you are so bold as to `#include` every single file in the book, you are dumping on the order of 500 names into the global namespace, definitely a bad idea!

  The explanation, quite simply, is that the vast majority of our users are not industrial-strength programmers, and most found the `NR::` namespace annoying and confusing. As we emphasized, strongly, in §1.0.1, NR is not a program library. If you want to create your own personal namespace for NR, please go ahead.

- In the distant past, *Numerical Recipes* included provisions for unit- or one-based, instead of zero-based, array indices. The last such version was published in 1992. Zero-based arrays have become so universally accepted that we no longer support any other option.

CITED REFERENCES AND FURTHER READING:

Numerical Recipes Software 2007, "Using Other Vector and Matrix Libraries," *Numerical Recipes Webnote No. 1*, at `http://www.nr.com/webnotes?1` [1]

CHAPTER 2

Solution of Linear Algebraic Equations

2.0 Introduction

The most basic task in linear algebra, and perhaps in all of scientific computing, is to solve for the unknowns in a set of linear algebraic equations. In general, a set of linear algebraic equations looks like this:

$$\begin{aligned} a_{00}x_0 + a_{01}x_1 + a_{02}x_2 + \cdots + a_{0,N-1}x_{N-1} &= b_0 \\ a_{10}x_0 + a_{11}x_1 + a_{12}x_2 + \cdots + a_{1,N-1}x_{N-1} &= b_1 \\ a_{20}x_0 + a_{21}x_1 + a_{22}x_2 + \cdots + a_{2,N-1}x_{N-1} &= b_2 \\ \cdots \qquad\qquad & \quad \cdots \\ a_{M-1,0}x_0 + a_{M-1,1}x_1 + \cdots + a_{M-1,N-1}x_{N-1} &= b_{M-1} \end{aligned} \tag{2.0.1}$$

Here the N unknowns x_j, $j = 0, 1, \ldots, N-1$ are related by M equations. The coefficients a_{ij} with $i = 0, 1, \ldots, M-1$ and $j = 0, 1, \ldots, N-1$ are known numbers, as are the *right-hand side* quantities b_i, $i = 0, 1, \ldots, M-1$.

If $N = M$, then there are as many equations as unknowns, and there is a good chance of solving for a unique solution set of x_j's. Otherwise, if $N \neq M$, things are even more interesting; we'll have more to say about this below.

If we write the coefficients a_{ij} as a matrix, and the right-hand sides b_i as a column vector,

$$\mathbf{A} = \begin{bmatrix} a_{00} & a_{01} & \ldots & a_{0,N-1} \\ a_{10} & a_{11} & \ldots & a_{1,N-1} \\ & \cdots & & \\ a_{M-1,0} & a_{M-1,1} & \ldots & a_{M-1,N-1} \end{bmatrix} \qquad \mathbf{b} = \begin{bmatrix} b_0 \\ b_1 \\ \cdots \\ b_{M-1} \end{bmatrix} \tag{2.0.2}$$

then equation (2.0.1) can be written in matrix form as

$$\mathbf{A} \cdot \mathbf{x} = \mathbf{b} \tag{2.0.3}$$

Here, and throughout the book, we use a raised dot to denote matrix multiplication, *or* the multiplication of a matrix and a vector, *or* the dot product of two vectors.

This usage is nonstandard, but we think it adds clarity: the dot is, in all of these cases, a *contraction* operator that represents the sum over a pair of indices, for example

$$\begin{aligned}
\mathbf{C} = \mathbf{A} \cdot \mathbf{B} \quad &\Longleftrightarrow \quad c_{ik} = \sum_j a_{ij} b_{jk} \\
\mathbf{b} = \mathbf{A} \cdot \mathbf{x} \quad &\Longleftrightarrow \quad b_i = \sum_j a_{ij} x_j \\
\mathbf{d} = \mathbf{x} \cdot \mathbf{A} \quad &\Longleftrightarrow \quad d_j = \sum_i x_i a_{ij} \\
q = \mathbf{x} \cdot \mathbf{y} \quad &\Longleftrightarrow \quad q = \sum_i x_i y_i
\end{aligned} \tag{2.0.4}$$

In matrices, by convention, the first index on an element a_{ij} denotes its row and the second index its column. For most purposes you don't need to know how a matrix is stored in a computer's physical memory; you just reference matrix elements by their two-dimensional addresses, e.g., a_{34} = `a[3][4]`. This C++ notation can in fact hide a variety of subtle and versatile physical storage schemes, see §1.4 and §1.5.

2.0.1 Nonsingular versus Singular Sets of Equations

You might be wondering why, above, and for the case $M = N$, we credited only a "good" chance of solving for the unknowns. Analytically, there can fail to be a solution (or a unique solution) if one or more of the M equations is a linear combination of the others, a condition called *row degeneracy*, or if all equations contain certain variables only in exactly the same linear combination, called *column degeneracy*. It turns out that, for square matrices, row degeneracy implies column degeneracy, and vice versa. A set of equations that is degenerate is called *singular*. We will consider singular matrices in some detail in §2.6.

Numerically, at least two additional things prevent us from getting a good solution:

- While not exact linear combinations of each other, some of the equations may be so close to linearly dependent that roundoff errors in the machine render them linearly dependent at some stage in the solution process. In this case your numerical procedure will fail, and it can tell you that it has failed.
- Accumulated roundoff errors in the solution process can swamp the true solution. This problem particularly emerges if N is too large. The numerical procedure does not fail algorithmically. However, it returns a set of x's that are wrong, as can be discovered by direct substitution back into the original equations. The closer a set of equations is to being singular, the more likely this is to happen, since increasingly close cancellations will occur during the solution. In fact, the preceding item can be viewed as the special case in which the loss of significance is unfortunately total.

Much of the sophistication of well-written "linear equation-solving packages" is devoted to the detection and/or correction of these two pathologies. It is difficult to give any firm guidelines for when such sophistication is needed, since there is no such thing as a "typical" linear problem. But here is a rough idea: Linear sets with N no larger than 20 or 50 are routine if they are not close to singular; they rarely

require more than the most straightforward methods, even in only single (that is, `float`) precision. With `double` precision, this number can readily be extended to N as large as perhaps 1000, after which point the limiting factor anyway soon becomes machine time, not accuracy.

Even larger linear sets, N in the thousands or millions, can be solved when the coefficients are sparse (that is, mostly zero), by methods that take advantage of the sparseness. We discuss this further in §2.7.

Unfortunately, one seems just as often to encounter linear problems that, by their underlying nature, are close to singular. In this case, you *might* need to resort to sophisticated methods even for the case of $N = 10$ (though rarely for $N = 5$). Singular value decomposition (§2.6) is a technique that can sometimes turn singular problems into nonsingular ones, in which case additional sophistication becomes unnecessary.

2.0.2 Tasks of Computational Linear Algebra

There is much more to linear algebra than just solving a single set of equations with a single right-hand side. Here, we list the major topics treated in this chapter. (Chapter 11 continues the subject with discussion of eigenvalue/eigenvector problems.)

When $M = N$:

- Solution of the matrix equation $\mathbf{A} \cdot \mathbf{x} = \mathbf{b}$ for an unknown vector $\mathbf{x}$ (§2.1 – §2.10).
- Solution of more than one matrix equation $\mathbf{A} \cdot \mathbf{x}_j = \mathbf{b}_j$, for a set of vectors $\mathbf{x}_j$, $j = 0, 1, \ldots$, each corresponding to a different, known right-hand side vector $\mathbf{b}_j$. In this task the key simplification is that the matrix $\mathbf{A}$ is held constant, while the right-hand sides, the $\mathbf{b}$'s, are changed (§2.1 – §2.10).
- Calculation of the matrix $\mathbf{A}^{-1}$ that is the matrix inverse of a square matrix $\mathbf{A}$, i.e., $\mathbf{A} \cdot \mathbf{A}^{-1} = \mathbf{A}^{-1} \cdot \mathbf{A} = \mathbf{1}$, where $\mathbf{1}$ is the identity matrix (all zeros except for ones on the diagonal). This task is equivalent, for an $N \times N$ matrix $\mathbf{A}$, to the previous task with N different $\mathbf{b}_j$'s ($j = 0, 1, \ldots, N-1$), namely the unit vectors ($\mathbf{b}_j$ = all zero elements except for 1 in the jth component). The corresponding $\mathbf{x}$'s are then the columns of the matrix inverse of $\mathbf{A}$ (§2.1 and §2.3).
- Calculation of the determinant of a square matrix $\mathbf{A}$ (§2.3).

If $M < N$, or if $M = N$ but the equations are degenerate, then there are effectively fewer equations than unknowns. In this case there can be either no solution, or else more than one solution vector $\mathbf{x}$. In the latter event, the solution space consists of a particular solution $\mathbf{x}_p$ added to any linear combination of (typically) $N - M$ vectors (which are said to be in the nullspace of the matrix $\mathbf{A}$). The task of finding the solution space of $\mathbf{A}$ involves

- Singular value decomposition of a matrix $\mathbf{A}$ (§2.6).

If there are more equations than unknowns, $M > N$, there is in general no solution vector $\mathbf{x}$ to equation (2.0.1), and the set of equations is said to be *overdetermined*. It happens frequently, however, that the best "compromise" solution is sought, the one that comes closest to satisfying all equations simultaneously. If closeness is defined in the least-squares sense, i.e., that the sum of the squares of the differences between the left- and right-hand sides of equation (2.0.1) be mini-

mized, then the overdetermined linear problem reduces to a (usually) solvable linear problem, called the

- Linear least-squares problem.

The reduced set of equations to be solved can be written as the $N \times N$ set of equations

$$(\mathbf{A}^T \cdot \mathbf{A}) \cdot \mathbf{x} = (\mathbf{A}^T \cdot \mathbf{b}) \tag{2.0.5}$$

where $\mathbf{A}^T$ denotes the transpose of the matrix $\mathbf{A}$. Equations (2.0.5) are called the *normal equations* of the linear least-squares problem. There is a close connection between singular value decomposition and the linear least-squares problem, and the latter is also discussed in §2.6. You should be warned that direct solution of the normal equations (2.0.5) is not generally the best way to find least-squares solutions.

Some other topics in this chapter include

- Iterative improvement of a solution (§2.5)
- Various special forms: symmetric positive-definite (§2.9), tridiagonal (§2.4), band-diagonal (§2.4), Toeplitz (§2.8), Vandermonde (§2.8), sparse (§2.7)
- Strassen's "fast matrix inversion" (§2.11).

2.0.3 Software for Linear Algebra

Going beyond what we can include in this book, several good software packages for linear algebra are available, though not always in C++. LAPACK, a successor to the venerable LINPACK, was developed at Argonne National Laboratories and deserves particular mention because it is published, documented, and available for free use. ScaLAPACK is a version available for parallel architectures. Packages available commercially include those in the IMSL and NAG libraries.

Sophisticated packages are designed with very large linear systems in mind. They therefore go to great effort to minimize not only the number of operations, but also the required storage. Routines for the various tasks are usually provided in several versions, corresponding to several possible simplifications in the form of the input coefficient matrix: symmetric, triangular, banded, positive-definite, etc. If you have a large matrix in one of these forms, you should certainly take advantage of the increased efficiency provided by these different routines, and not just use the form provided for general matrices.

There is also a great watershed dividing routines that are *direct* (i.e., execute in a predictable number of operations) from routines that are *iterative* (i.e., attempt to converge to the desired answer in however many steps are necessary). Iterative methods become preferable when the battle against loss of significance is in danger of being lost, either due to large N or because the problem is close to singular. We will treat iterative methods only incompletely in this book, in §2.7 and in Chapters 19 and 20. These methods are important but mostly beyond our scope. We will, however, discuss in detail a technique that is on the borderline between direct and iterative methods, namely the iterative improvement of a solution that has been obtained by direct methods (§2.5).

CITED REFERENCES AND FURTHER READING:

Golub, G.H., and Van Loan, C.F. 1996, *Matrix Computations*, 3rd ed. (Baltimore: Johns Hopkins University Press).

Gill, P.E., Murray, W., and Wright, M.H. 1991, *Numerical Linear Algebra and Optimization*, vol. 1 (Redwood City, CA: Addison-Wesley).

Stoer, J., and Bulirsch, R. 2002, *Introduction to Numerical Analysis*, 3rd ed. (New York: Springer), Chapter 4.

Ueberhuber, C.W. 1997, *Numerical Computation: Methods, Software, and Analysis*, 2 vols. (Berlin: Springer), Chapter 13.

Coleman, T.F., and Van Loan, C. 1988, *Handbook for Matrix Computations* (Philadelphia: S.I.A.M.).

Forsythe, G.E., and Moler, C.B. 1967, *Computer Solution of Linear Algebraic Systems* (Englewood Cliffs, NJ: Prentice-Hall).

Wilkinson, J.H., and Reinsch, C. 1971, *Linear Algebra*, vol. II of *Handbook for Automatic Computation* (New York: Springer).

Westlake, J.R. 1968, *A Handbook of Numerical Matrix Inversion and Solution of Linear Equations* (New York: Wiley).

Johnson, L.W., and Riess, R.D. 1982, *Numerical Analysis*, 2nd ed. (Reading, MA: Addison-Wesley), Chapter 2.

Ralston, A., and Rabinowitz, P. 1978, *A First Course in Numerical Analysis*, 2nd ed.; reprinted 2001 (New York: Dover), Chapter 9.

2.1 Gauss-Jordan Elimination

Gauss-Jordan elimination is probably the way you learned to solve linear equations in high school. (You may have learned it as "Gaussian elimination," but, strictly speaking, that term refers to the somewhat different technique discussed in §2.2.) The basic idea is to add or subtract linear combinations of the given equations until each equation contains only one of the unknowns, thus giving an immediate solution. You might also have learned to use the same technique for calculating the inverse of a matrix.

For solving sets of linear equations, Gauss-Jordan elimination produces *both* the solution of the equations for one or more right-hand side vectors $\mathbf{b}$, and also the matrix inverse $\mathbf{A}^{-1}$. However, its principal deficiencies are (i) that it requires all the right-hand sides to be stored and manipulated at the same time, and (ii) that when the inverse matrix is *not* desired, Gauss-Jordan is three times slower than the best alternative technique for solving a single linear set (§2.3). The method's principal strength is that it is as stable as any other direct method, perhaps even a bit more stable when full pivoting is used (see §2.1.2).

For inverting a matrix, Gauss-Jordan elimination is about as efficient as any other direct method. We know of no reason not to use it in this application if you are sure that the matrix inverse is what you really want.

You might wonder about deficiency (i) above: If we are getting the matrix inverse anyway, can't we later let it multiply a new right-hand side to get an additional solution? This does work, but it gives an answer that is very susceptible to roundoff error and not nearly as good as if the new vector had been included with the set of right-hand side vectors in the first instance.

Thus, Gauss-Jordan elimination should not be your method of first choice for solving linear equations. The decomposition methods in §2.3 are better. Why do we discuss Gauss-Jordan at all? Because it is straightforward, solid as a rock, and a good place for us to introduce the important concept of *pivoting* which will also

be important for the methods described later. The actual sequence of operations performed in Gauss-Jordan elimination is very closely related to that performed by the routines in the next two sections.

2.1.1 Elimination on Column-Augmented Matrices

For clarity, and to avoid writing endless ellipses ($\cdots$) we will write out equations only for the case of four equations and four unknowns, and with three different right-hand side vectors that are known in advance. You can write bigger matrices and extend the equations to the case of $N \times N$ matrices, with M sets of right-hand side vectors, in completely analogous fashion. The routine implemented below in §2.1.2 is, of course, general.

Consider the linear matrix equation

$$\begin{bmatrix} a_{00} & a_{01} & a_{02} & a_{03} \\ a_{10} & a_{11} & a_{12} & a_{13} \\ a_{20} & a_{21} & a_{22} & a_{23} \\ a_{30} & a_{31} & a_{32} & a_{33} \end{bmatrix} \cdot \left[\begin{pmatrix} x_{00} \\ x_{10} \\ x_{20} \\ x_{30} \end{pmatrix} \sqcup \begin{pmatrix} x_{01} \\ x_{11} \\ x_{21} \\ x_{31} \end{pmatrix} \sqcup \begin{pmatrix} x_{02} \\ x_{12} \\ x_{22} \\ x_{32} \end{pmatrix} \sqcup \begin{pmatrix} y_{00} & y_{01} & y_{02} & y_{03} \\ y_{10} & y_{11} & y_{12} & y_{13} \\ y_{20} & y_{21} & y_{22} & y_{23} \\ y_{30} & y_{31} & y_{32} & y_{33} \end{pmatrix} \right]$$
$$= \left[\begin{pmatrix} b_{00} \\ b_{10} \\ b_{20} \\ b_{30} \end{pmatrix} \sqcup \begin{pmatrix} b_{01} \\ b_{11} \\ b_{21} \\ b_{31} \end{pmatrix} \sqcup \begin{pmatrix} b_{02} \\ b_{12} \\ b_{22} \\ b_{32} \end{pmatrix} \sqcup \begin{pmatrix} 1 & 0 & 0 & 0 \\ 0 & 1 & 0 & 0 \\ 0 & 0 & 1 & 0 \\ 0 & 0 & 0 & 1 \end{pmatrix} \right] \tag{2.1.1}$$

Here the raised dot ($\cdot$) signifies matrix multiplication, while the operator $\sqcup$ just signifies column augmentation, that is, removing the abutting parentheses and making a wider matrix out of the operands of the $\sqcup$ operator.

It should not take you long to write out equation (2.1.1) and to see that it simply states that x_{ij} is the ith component ($i = 0, 1, 2, 3$) of the vector solution of the jth right-hand side ($j = 0, 1, 2$), the one whose coefficients are $b_{ij}, i = 0, 1, 2, 3$; and that the matrix of unknown coefficients y_{ij} is the inverse matrix of a_{ij}. In other words, the matrix solution of

$$[\mathbf{A}] \cdot [\mathbf{x}_0 \sqcup \mathbf{x}_1 \sqcup \mathbf{x}_2 \sqcup \mathbf{Y}] = [\mathbf{b}_0 \sqcup \mathbf{b}_1 \sqcup \mathbf{b}_2 \sqcup \mathbf{1}] \tag{2.1.2}$$

where $\mathbf{A}$ and $\mathbf{Y}$ are square matrices, the $\mathbf{b}_i$'s and $\mathbf{x}_i$'s are column vectors, and $\mathbf{1}$ is the identity matrix, simultaneously solves the linear sets

$$\mathbf{A} \cdot \mathbf{x}_0 = \mathbf{b}_0 \qquad \mathbf{A} \cdot \mathbf{x}_1 = \mathbf{b}_1 \qquad \mathbf{A} \cdot \mathbf{x}_2 = \mathbf{b}_2 \tag{2.1.3}$$

and

$$\mathbf{A} \cdot \mathbf{Y} = \mathbf{1} \tag{2.1.4}$$

Now it is also elementary to verify the following facts about (2.1.1):

- Interchanging any two *rows* of $\mathbf{A}$ and the corresponding *rows* of the $\mathbf{b}$'s and of $\mathbf{1}$ does not change (or scramble in any way) the solution $\mathbf{x}$'s and $\mathbf{Y}$. Rather, it just corresponds to writing the same set of linear equations in a different order.
- Likewise, the solution set is unchanged and in no way scrambled if we replace any row in $\mathbf{A}$ by a linear combination of itself and any other row, as long as we do the same linear combination of the rows of the $\mathbf{b}$'s and $\mathbf{1}$ (which then is no longer the identity matrix, of course).

- Interchanging any two *columns* of $\mathbf{A}$ gives the same solution set only if we simultaneously interchange corresponding *rows* of the $\mathbf{x}$'s and of $\mathbf{Y}$. In other words, this interchange scrambles the order of the rows in the solution. If we do this, we will need to unscramble the solution by restoring the rows to their original order.

Gauss-Jordan elimination uses one or more of the above operations to reduce the matrix $\mathbf{A}$ to the identity matrix. When this is accomplished, the right-hand side becomes the solution set, as one sees instantly from (2.1.2).

2.1.2 Pivoting

In "Gauss-Jordan elimination with no pivoting," only the second operation in the above list is used. The zeroth row is divided by the element a_{00} (this being a trivial linear combination of the zeroth row with any other row — zero coefficient for the other row). Then the right amount of the zeroth row is subtracted from each other row to make all the remaining a_{i0}'s zero. The zeroth column of $\mathbf{A}$ now agrees with the identity matrix. We move to column 1 and divide row 1 by a_{11}, then subtract the right amount of row 1 from rows 0, 2, and 3, so as to make their entries in column 1 zero. Column 1 is now reduced to the identity form. And so on for columns 2 and 3. As we do these operations to $\mathbf{A}$, we of course also do the corresponding operations to the $\mathbf{b}$'s and to $\mathbf{1}$ (which by now no longer resembles the identity matrix in any way!).

Obviously we will run into trouble if we ever encounter a zero element on the (then current) diagonal when we are going to divide by the diagonal element. (The element that we divide by, incidentally, is called the *pivot element* or *pivot*.) Not so obvious, but true, is the fact that Gauss-Jordan elimination with no pivoting (no use of the first or third procedures in the above list) is numerically unstable in the presence of any roundoff error, even when a zero pivot is not encountered. You must *never* do Gauss-Jordan elimination (or Gaussian elimination; see below) without pivoting!

So what *is* this magic pivoting? Nothing more than interchanging rows (*partial pivoting*) or rows and columns (*full pivoting*), so as to put a particularly desirable element in the diagonal position from which the pivot is about to be selected. Since we don't want to mess up the part of the identity matrix that we have already built up, we can choose among elements that are both (i) on rows below (or on) the one that is about to be normalized, and also (ii) on columns to the right (or on) the column we are about to eliminate. Partial pivoting is easier than full pivoting, because we don't have to keep track of the permutation of the solution vector. Partial pivoting makes available as pivots only the elements already in the correct column. It turns out that partial pivoting is "almost" as good as full pivoting, in a sense that can be made mathematically precise, but which need not concern us here (for discussion and references, see [1]). To show you both variants, we do full pivoting in the routine in this section and partial pivoting in §2.3.

We have to state how to recognize a particularly desirable pivot when we see one. The answer to this is not completely known theoretically. It is known, both theoretically and in practice, that simply picking the largest (in magnitude) available element as the pivot is a very good choice. A curiosity of this procedure, however, is that the choice of pivot will depend on the original scaling of the equations. If we take the third linear equation in our original set and multiply it by a factor of a million, it is almost guaranteed that it will contribute the first pivot; yet the underlying solution

of the equations is not changed by this multiplication! One therefore sometimes sees routines which choose as pivot that element which *would* have been largest if the original equations had all been scaled to have their largest coefficient normalized to unity. This is called *implicit pivoting*. There is some extra bookkeeping to keep track of the scale factors by which the rows would have been multiplied. (The routines in §2.3 include implicit pivoting, but the routine in this section does not.)

Finally, let us consider the storage requirements of the method. With a little reflection you will see that at every stage of the algorithm, *either* an element of **A** is predictably a one or zero (if it is already in a part of the matrix that has been reduced to identity form) *or else* the exactly corresponding element of the matrix that started as **1** is predictably a one or zero (if its mate in **A** has not been reduced to the identity form). Therefore the matrix **1** does not have to exist as separate storage: The matrix inverse of **A** is gradually built up in **A** as the original **A** is destroyed. Likewise, the solution vectors **x** can gradually replace the right-hand side vectors **b** and share the same storage, since after each column in **A** is reduced, the corresponding row entry in the **b**'s is never again used.

Here is a routine that does Gauss-Jordan elimination with full pivoting, replacing its input matrices by the desired answers. Immediately following is an overloaded version for use when there are no right-hand sides, i.e., when you want only the matrix inverse.

gaussj.h

```
void gaussj(MatDoub_IO &a, MatDoub_IO &b)
```

Linear equation solution by Gauss-Jordan elimination, equation (2.1.1) above. The input matrix is `a[0..n-1][0..n-1]`. `b[0..n-1][0..m-1]` is input containing the `m` right-hand side vectors. On output, `a` is replaced by its matrix inverse, and `b` is replaced by the corresponding set of solution vectors.

```
{
    Int i,icol,irow,j,k,l,ll,n=a.nrows(),m=b.ncols();
    Doub big,dum,pivinv;
    VecInt indxc(n),indxr(n),ipiv(n);   These integer arrays are used for bookkeeping on
                                            the pivoting.
    for (j=0;j<n;j++) ipiv[j]=0;
    for (i=0;i<n;i++) {                 This is the main loop over the columns to be
                                            reduced.
        big=0.0;
        for (j=0;j<n;j++)               This is the outer loop of the search for a pivot
                                            element.
            if (ipiv[j] != 1)
                for (k=0;k<n;k++) {
                    if (ipiv[k] == 0) {
                        if (abs(a[j][k]) >= big) {
                            big=abs(a[j][k]);
                            irow=j;
                            icol=k;
                        }
                    }
                }
        ++(ipiv[icol]);
```

We now have the pivot element, so we interchange rows, if needed, to put the pivot element on the diagonal. The columns are not physically interchanged, only relabeled: `indxc[i]`, the column of the $(i+1)$th pivot element, is the $(i+1)$th column that is reduced, while `indxr[i]` is the row in which that pivot element was originally located. If `indxr[i]` $\neq$ `indxc[i]`, there is an implied column interchange. With this form of bookkeeping, the solution `b`'s will end up in the correct order, and the inverse matrix will be scrambled by columns.

```
        if (irow != icol) {
            for (l=0;l<n;l++) SWAP(a[irow][l],a[icol][l]);
            for (l=0;l<m;l++) SWAP(b[irow][l],b[icol][l]);
        }
        indxr[i]=irow;                  We are now ready to divide the pivot row by the
                                            pivot element, located at irow and icol.
```

```
        indxc[i]=icol;
        if (a[icol][icol] == 0.0) throw("gaussj: Singular Matrix");
        pivinv=1.0/a[icol][icol];
        a[icol][icol]=1.0;
        for (l=0;l<n;l++) a[icol][l] *= pivinv;
        for (l=0;l<m;l++) b[icol][l] *= pivinv;
        for (ll=0;ll<n;ll++)              Next, we reduce the rows...
            if (ll != icol) {             ...except for the pivot one, of course.
                dum=a[ll][icol];
                a[ll][icol]=0.0;
                for (l=0;l<n;l++) a[ll][l] -= a[icol][l]*dum;
                for (l=0;l<m;l++) b[ll][l] -= b[icol][l]*dum;
            }
    }
    This is the end of the main loop over columns of the reduction. It only remains to unscram-
    ble the solution in view of the column interchanges. We do this by interchanging pairs of
    columns in the reverse order that the permutation was built up.
    for (l=n-1;l>=0;l--) {
        if (indxr[l] != indxc[l])
            for (k=0;k<n;k++)
                SWAP(a[k][indxr[l]],a[k][indxc[l]]);
    }                                     And we are done.
}

void gaussj(MatDoub_IO &a)
Overloaded version with no right-hand sides. Replaces a by its inverse.
{
    MatDoub b(a.nrows(),0);            Dummy vector with zero columns.
    gaussj(a,b);
}
```

2.1.3 Row versus Column Elimination Strategies

The above discussion can be amplified by a modest amount of formalism. Row operations on a matrix $\mathbf{A}$ correspond to pre- (that is, left-) multiplication by some simple matrix $\mathbf{R}$. For example, the matrix $\mathbf{R}$ with components

$$R_{ij} = \begin{cases} 1 & \text{if } i = j \text{ and } i \neq 2, 4 \\ 1 & \text{if } i = 2,\ j = 4 \\ 1 & \text{if } i = 4,\ j = 2 \\ 0 & \text{otherwise} \end{cases} \tag{2.1.5}$$

effects the interchange of rows 2 and 4. Gauss-Jordan elimination by row operations alone (including the possibility of *partial* pivoting) consists of a series of such left-multiplications, yielding successively

$$\begin{aligned} \mathbf{A} \cdot \mathbf{x} &= \mathbf{b} \\ (\cdots \mathbf{R}_2 \cdot \mathbf{R}_1 \cdot \mathbf{R}_0 \cdot \mathbf{A}) \cdot \mathbf{x} &= \cdots \mathbf{R}_2 \cdot \mathbf{R}_1 \cdot \mathbf{R}_0 \cdot \mathbf{b} \\ (\mathbf{1}) \cdot \mathbf{x} &= \cdots \mathbf{R}_2 \cdot \mathbf{R}_1 \cdot \mathbf{R}_0 \cdot \mathbf{b} \\ \mathbf{x} &= \cdots \mathbf{R}_2 \cdot \mathbf{R}_1 \cdot \mathbf{R}_0 \cdot \mathbf{b} \end{aligned} \tag{2.1.6}$$

The key point is that since the $\mathbf{R}$'s build from right to left, the right-hand side is simply transformed at each stage from one vector to another.

Column operations, on the other hand, correspond to post-, or right-, multiplications by simple matrices, call them $\mathbf{C}$. The matrix in equation (2.1.5), if right-multiplied onto a matrix $\mathbf{A}$, will interchange *columns* 2 and 4 of $\mathbf{A}$. Elimination by column operations involves (conceptually) inserting a column operator, *and also its inverse,* between the matrix $\mathbf{A}$ and the

unknown vector $\mathbf{x}$:

$$\begin{aligned}
\mathbf{A} \cdot \mathbf{x} &= \mathbf{b} \\
\mathbf{A} \cdot \mathbf{C}_0 \cdot \mathbf{C}_0^{-1} \cdot \mathbf{x} &= \mathbf{b} \\
\mathbf{A} \cdot \mathbf{C}_0 \cdot \mathbf{C}_1 \cdot \mathbf{C}_1^{-1} \cdot \mathbf{C}_0^{-1} \cdot \mathbf{x} &= \mathbf{b} \\
(\mathbf{A} \cdot \mathbf{C}_0 \cdot \mathbf{C}_1 \cdot \mathbf{C}_2 \cdots) \cdots \mathbf{C}_2^{-1} \cdot \mathbf{C}_1^{-1} \cdot \mathbf{C}_0^{-1} \cdot \mathbf{x} &= \mathbf{b} \\
(\mathbf{1}) \cdots \mathbf{C}_2^{-1} \cdot \mathbf{C}_1^{-1} \cdot \mathbf{C}_0^{-1} \cdot \mathbf{x} &= \mathbf{b}
\end{aligned} \tag{2.1.7}$$

which (peeling off the $\mathbf{C}^{-1}$'s one at a time) implies a solution

$$\mathbf{x} = \mathbf{C}_0 \cdot \mathbf{C}_1 \cdot \mathbf{C}_2 \cdots \mathbf{b} \tag{2.1.8}$$

Notice the essential difference between equation (2.1.8) and equation (2.1.6). In the latter case, the $\mathbf{C}$'s must be applied to $\mathbf{b}$ in the *reverse order* from that in which they become known. That is, they must all be stored along the way. This requirement greatly reduces the usefulness of column operations, generally restricting them to simple permutations, for example in support of full pivoting.

CITED REFERENCES AND FURTHER READING:

Wilkinson, J.H. 1965, *The Algebraic Eigenvalue Problem* (New York: Oxford University Press).[1]

Carnahan, B., Luther, H.A., and Wilkes, J.O. 1969, *Applied Numerical Methods* (New York: Wiley), Example 5.2, p. 282.

Bevington, P.R., and Robinson, D.K. 2002, *Data Reduction and Error Analysis for the Physical Sciences*, 3rd ed. (New York: McGraw-Hill), p. 247.

Westlake, J.R. 1968, *A Handbook of Numerical Matrix Inversion and Solution of Linear Equations* (New York: Wiley).

Ralston, A., and Rabinowitz, P. 1978, *A First Course in Numerical Analysis*, 2nd ed.; reprinted 2001 (New York: Dover), §9.3–1.

2.2 Gaussian Elimination with Backsubstitution

Any discussion of *Gaussian elimination with backsubstitution* is primarily pedagogical. The method stands between full elimination schemes such as Gauss-Jordan, and triangular decomposition schemes such as will be discussed in the next section. Gaussian elimination reduces a matrix not all the way to the identity matrix, but only halfway, to a matrix whose components on the diagonal and above (say) remain nontrivial. Let us now see what advantages accrue.

Suppose that in doing Gauss-Jordan elimination, as described in §2.1, we at each stage subtract away rows only *below* the then-current pivot element. When a_{11} is the pivot element, for example, we divide the row 1 by its value (as before), but now use the pivot row to zero only a_{21} and a_{31}, not a_{01} (see equation 2.1.1). Suppose, also, that we do only partial pivoting, never interchanging columns, so that the order of the unknowns never needs to be modified.

Then, when we have done this for all the pivots, we will be left with a reduced equation that looks like this (in the case of a single right-hand side vector):

$$\begin{bmatrix} a'_{00} & a'_{01} & a'_{02} & a'_{03} \\ 0 & a'_{11} & a'_{12} & a'_{13} \\ 0 & 0 & a'_{22} & a'_{23} \\ 0 & 0 & 0 & a'_{33} \end{bmatrix} \cdot \begin{bmatrix} x_0 \\ x_1 \\ x_2 \\ x_3 \end{bmatrix} = \begin{bmatrix} b'_0 \\ b'_1 \\ b'_2 \\ b'_3 \end{bmatrix} \tag{2.2.1}$$

Here the primes signify that the a's and b's do not have their original numerical values, but have been modified by all the row operations in the elimination to this point. The procedure up to this point is termed *Gaussian elimination*.

2.2.1 Backsubstitution

But how do we solve for the x's? The last x (x_3 in this example) is already isolated, namely

$$x_3 = b'_3/a'_{33} \tag{2.2.2}$$

With the last x known we can move to the penultimate x,

$$x_2 = \frac{1}{a'_{22}}[b'_2 - x_3 a'_{23}] \tag{2.2.3}$$

and then proceed with the x before that one. The typical step is

$$x_i = \frac{1}{a'_{ii}}\left[b'_i - \sum_{j=i+1}^{N-1} a'_{ij} x_j\right] \tag{2.2.4}$$

The procedure defined by equation (2.2.4) is called *backsubstitution*. The combination of Gaussian elimination and backsubstitution yields a solution to the set of equations.

The advantage of Gaussian elimination and backsubstitution over Gauss-Jordan elimination is simply that the former is faster in raw operations count: The innermost loops of Gauss-Jordan elimination, each containing one subtraction and one multiplication, are executed N^3 and N^2m times (where there are N equations and unknowns, and m different right-hand sides). The corresponding loops in Gaussian elimination are executed only $\frac{1}{3}N^3$ times (only half the matrix is reduced, and the increasing numbers of predictable zeros reduce the count to one-third), and $\frac{1}{2}N^2m$ times, respectively. Each backsubstitution of a right-hand side is $\frac{1}{2}N^2$ executions of a similar loop (one multiplication plus one subtraction). For $m \ll N$ (only a few right-hand sides) Gaussian elimination thus has about a factor three advantage over Gauss-Jordan. (We could reduce this advantage to a factor 1.5 by *not* computing the inverse matrix as part of the Gauss-Jordan scheme.)

For computing the inverse matrix (which we can view as the case of $m = N$ right-hand sides, namely the N unit vectors that are the columns of the identity matrix), Gaussian elimination and backsubstitution at first glance require $\frac{1}{3}N^3$ (matrix reduction) $+\frac{1}{2}N^3$ (right-hand side manipulations) $+\frac{1}{2}N^3$ (N backsubstitutions) $= \frac{4}{3}N^3$ loop executions, which is more than the N^3 for Gauss-Jordan. However, the unit vectors are quite special in containing all zeros except for one element. If this

is taken into account, the right-side manipulations can be reduced to only $\frac{1}{6}N^3$ loop executions, and, for matrix inversion, the two methods have identical efficiencies.

Both Gaussian elimination and Gauss-Jordan elimination share the disadvantage that all right-hand sides must be known in advance. The LU decomposition method in the next section does not share that deficiency, and also has an equally small operations count, both for solution with any number of right-hand sides and for matrix inversion.

CITED REFERENCES AND FURTHER READING:

Ralston, A., and Rabinowitz, P. 1978, *A First Course in Numerical Analysis*, 2nd ed.; reprinted 2001 (New York: Dover), §9.3–1.

Isaacson, E., and Keller, H.B. 1966, *Analysis of Numerical Methods*; reprinted 1994 (New York: Dover), §2.1.

Johnson, L.W., and Riess, R.D. 1982, *Numerical Analysis*, 2nd ed. (Reading, MA: Addison-Wesley), §2.2.1.

Westlake, J.R. 1968, *A Handbook of Numerical Matrix Inversion and Solution of Linear Equations* (New York: Wiley).

2.3 LU Decomposition and Its Applications

Suppose we are able to write the matrix $\mathbf{A}$ as a product of two matrices,

$$\mathbf{L} \cdot \mathbf{U} = \mathbf{A} \tag{2.3.1}$$

where $\mathbf{L}$ is *lower triangular* (has elements only on the diagonal and below) and $\mathbf{U}$ is *upper triangular* (has elements only on the diagonal and above). For the case of a 4×4 matrix $\mathbf{A}$, for example, equation (2.3.1) would look like this:

$$\begin{bmatrix} \alpha_{00} & 0 & 0 & 0 \\ \alpha_{10} & \alpha_{11} & 0 & 0 \\ \alpha_{20} & \alpha_{21} & \alpha_{22} & 0 \\ \alpha_{30} & \alpha_{31} & \alpha_{32} & \alpha_{33} \end{bmatrix} \cdot \begin{bmatrix} \beta_{00} & \beta_{01} & \beta_{02} & \beta_{03} \\ 0 & \beta_{11} & \beta_{12} & \beta_{13} \\ 0 & 0 & \beta_{22} & \beta_{23} \\ 0 & 0 & 0 & \beta_{33} \end{bmatrix} = \begin{bmatrix} a_{00} & a_{01} & a_{02} & a_{03} \\ a_{10} & a_{11} & a_{12} & a_{13} \\ a_{20} & a_{21} & a_{22} & a_{23} \\ a_{30} & a_{31} & a_{32} & a_{33} \end{bmatrix} \tag{2.3.2}$$

We can use a decomposition such as (2.3.1) to solve the linear set

$$\mathbf{A} \cdot \mathbf{x} = (\mathbf{L} \cdot \mathbf{U}) \cdot \mathbf{x} = \mathbf{L} \cdot (\mathbf{U} \cdot \mathbf{x}) = \mathbf{b} \tag{2.3.3}$$

by first solving for the vector $\mathbf{y}$ such that

$$\mathbf{L} \cdot \mathbf{y} = \mathbf{b} \tag{2.3.4}$$

and then solving

$$\mathbf{U} \cdot \mathbf{x} = \mathbf{y} \tag{2.3.5}$$

What is the advantage of breaking up one linear set into two successive ones? The advantage is that the solution of a triangular set of equations is quite trivial, as

we have already seen in §2.2 (equation 2.2.4). Thus, equation (2.3.4) can be solved by *forward substitution* as follows:

$$y_0 = \frac{b_0}{\alpha_{00}}$$
$$y_i = \frac{1}{\alpha_{ii}}\left[b_i - \sum_{j=0}^{i-1} \alpha_{ij} y_j\right] \qquad i = 1, 2, \ldots, N-1 \tag{2.3.6}$$

while (2.3.5) can then be solved by *backsubstitution* exactly as in equations (2.2.2) – (2.2.4),

$$x_{N-1} = \frac{y_{N-1}}{\beta_{N-1,N-1}}$$
$$x_i = \frac{1}{\beta_{ii}}\left[y_i - \sum_{j=i+1}^{N-1} \beta_{ij} x_j\right] \qquad i = N-2, N-3, \ldots, 0 \tag{2.3.7}$$

Equations (2.3.6) and (2.3.7) total (for each right-hand side **b**) N^2 executions of an inner loop containing one multiply and one add. If we have N right-hand sides that are the unit column vectors (which is the case when we are inverting a matrix), then taking into account the leading zeros reduces the total execution count of (2.3.6) from $\frac{1}{2}N^3$ to $\frac{1}{6}N^3$, while (2.3.7) is unchanged at $\frac{1}{2}N^3$.

Notice that, once we have the LU decomposition of **A**, we can solve with as many right-hand sides as we then care to, one at a time. This is a distinct advantage over the methods of §2.1 and §2.2.

2.3.1 Performing the LU Decomposition

How then can we solve for **L** and **U**, given **A**? First, we write out the i,jth component of equation (2.3.1) or (2.3.2). That component always is a sum beginning with

$$\alpha_{i0}\beta_{0j} + \cdots = a_{ij}$$

The number of terms in the sum depends, however, on whether i or j is the smaller number. We have, in fact, the three cases,

$$i < j: \qquad \alpha_{i0}\beta_{0j} + \alpha_{i1}\beta_{1j} + \cdots + \alpha_{ii}\beta_{ij} = a_{ij} \tag{2.3.8}$$
$$i = j: \qquad \alpha_{i0}\beta_{0j} + \alpha_{i1}\beta_{1j} + \cdots + \alpha_{ii}\beta_{jj} = a_{ij} \tag{2.3.9}$$
$$i > j: \qquad \alpha_{i0}\beta_{0j} + \alpha_{i1}\beta_{1j} + \cdots + \alpha_{ij}\beta_{jj} = a_{ij} \tag{2.3.10}$$

Equations (2.3.8) – (2.3.10) total N^2 equations for the $N^2 + N$ unknown α's and β's (the diagonal being represented twice). Since the number of unknowns is greater than the number of equations, we are invited to specify N of the unknowns arbitrarily and then try to solve for the others. In fact, as we shall see, it is always possible to take

$$\alpha_{ii} \equiv 1 \qquad i = 0, \ldots, N-1 \tag{2.3.11}$$

A surprising procedure, now, is *Crout's algorithm*, which quite trivially solves the set of N^2+N equations (2.3.8) – (2.3.11) for all the α's and β's by just arranging the equations in a certain order! That order is as follows:

- Set $\alpha_{ii} = 1, i = 0, \ldots, N-1$ (equation 2.3.11).
- For each $j = 0, 1, 2, \ldots, N-1$ do these two procedures: First, for $i = 0, 1, \ldots, j$, use (2.3.8), (2.3.9), and (2.3.11) to solve for β_{ij}, namely

$$\beta_{ij} = a_{ij} - \sum_{k=0}^{i-1} \alpha_{ik}\beta_{kj} \tag{2.3.12}$$

(When $i = 0$ in 2.3.12 the summation term is taken to mean zero.) Second, for $i = j+1, j+2, \ldots, N-1$ use (2.3.10) to solve for α_{ij}, namely

$$\alpha_{ij} = \frac{1}{\beta_{jj}}\left(a_{ij} - \sum_{k=0}^{j-1} \alpha_{ik}\beta_{kj}\right) \tag{2.3.13}$$

Be sure to do both procedures before going on to the next j.

If you work through a few iterations of the above procedure, you will see that the α's and β's that occur on the right-hand side of equations (2.3.12) and (2.3.13) are already determined by the time they are needed. You will also see that every a_{ij} is used only once and never again. This means that the corresponding α_{ij} or β_{ij} can be stored in the location that the a used to occupy: the decomposition is "in place." [The diagonal unity elements α_{ii} (equation 2.3.11) are not stored at all.] In brief, Crout's method fills in the combined matrix of α's and β's,

$$\begin{bmatrix} \beta_{00} & \beta_{01} & \beta_{02} & \beta_{03} \\ \alpha_{10} & \beta_{11} & \beta_{12} & \beta_{13} \\ \alpha_{20} & \alpha_{21} & \beta_{22} & \beta_{23} \\ \alpha_{30} & \alpha_{31} & \alpha_{32} & \beta_{33} \end{bmatrix} \tag{2.3.14}$$

by columns from left to right, and within each column from top to bottom (see Figure 2.3.1).

What about pivoting? Pivoting (i.e., selection of a salubrious pivot element for the division in equation 2.3.13) is absolutely essential for the stability of Crout's method. Only partial pivoting (interchange of rows) can be implemented efficiently. However this is enough to make the method stable. This means, incidentally, that we don't actually decompose the matrix $\mathbf{A}$ into LU form, but rather we decompose a rowwise permutation of $\mathbf{A}$. (If we keep track of what that permutation is, this decomposition is just as useful as the original one would have been.)

Pivoting is slightly subtle in Crout's algorithm. The key point to notice is that equation (2.3.12) in the case of $i = j$ (its final application) is *exactly the same* as equation (2.3.13) except for the division in the latter equation; in both cases the upper limit of the sum is $k = j - 1$ $(= i - 1)$. This means that we don't have to commit ourselves as to whether the diagonal element β_{jj} is the one that happens to fall on the diagonal in the first instance, or whether one of the (undivided) α_{ij}'s below it in the column, $i = j+1, \ldots, N-1$, is to be "promoted" to become the diagonal β. This can be decided after all the candidates in the column are in hand. As you should be able to guess by now, we will choose the largest one as the diagonal β (pivot

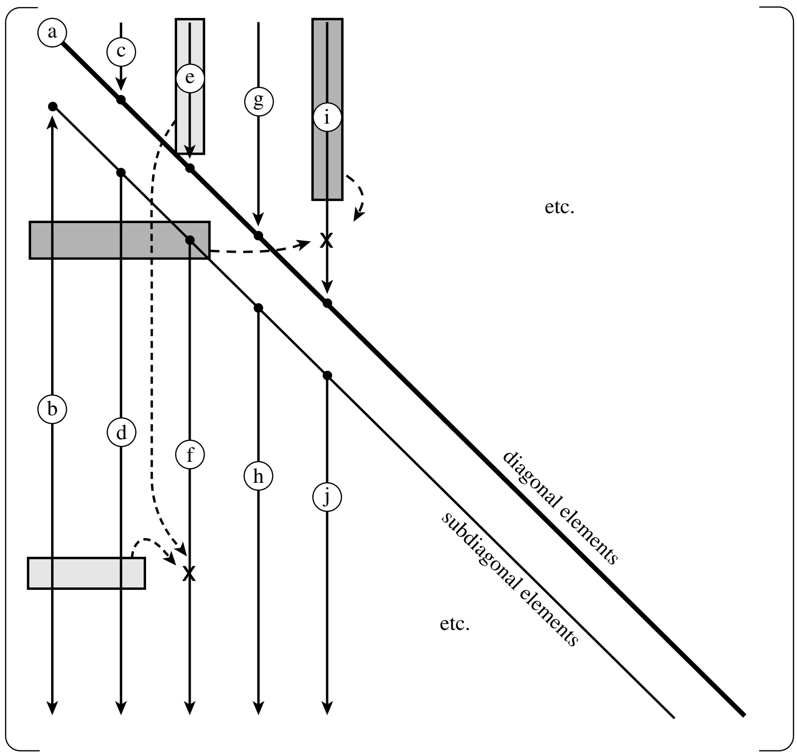

Figure 2.3.1. Crout's algorithm for LU decomposition of a matrix. Elements of the original matrix are modified in the order indicated by lowercase letters: a, b, c, etc. Shaded boxes show the previously modified elements that are used in modifying two typical elements, each indicated by an "x".

element), and then do all the divisions by that element *en masse*. This is *Crout's method with partial pivoting*. Our implementation has one additional wrinkle: It initially finds the largest element in each row, and subsequently (when it is looking for the maximal pivot element) scales the comparison *as if* we had initially scaled all the equations to make their maximum coefficient equal to unity; this is the *implicit pivoting* mentioned in §2.1.

The inner loop of the LU decomposition, equations (2.3.12) and (2.3.13), resembles the inner loop of matrix multiplication. There is a triple loop over the indices i, j, and k. There are six permutations of the order in which these loops can be done. The straightforward implementation of Crout's algorithm corresponds to the jik permutation, where the order of the indices is the order of the loops from outermost to innermost. On modern processors with a hierarchy of cache memory, and when matrices are stored by rows, the fastest execution time is usually the kij or ikj ordering. Pivoting is easier with kij ordering, so that is the implementation we use. This is called "outer product Gaussian elimination" by Golub and Van Loan [1].

LU decomposition is well suited for implementation as an object (a `class` or `struct`). The constructor performs the decomposition, and the object itself stores the result. Then, a method for forward- and backsubstitution can be called once, or many times, to solve for one or more right-hand sides. Methods for additional functionality are also easy to include. The object's declaration looks like this:

ludcmp.h

```
struct LUdcmp
```
Object for solving linear equations $\mathbf{A} \cdot \mathbf{x} = \mathbf{b}$ using LU decomposition, and related functions.
```
{
    Int n;
    MatDoub lu;                                 Stores the decomposition.
    VecInt indx;                                Stores the permutation.
    Doub d;                                     Used by det.
    LUdcmp(MatDoub_I &a);                       Constructor. Argument is the matrix A.
    void solve(VecDoub_I &b, VecDoub_O &x);     Solve for a single right-hand side.
    void solve(MatDoub_I &b, MatDoub_O &x);     Solve for multiple right-hand sides.
    void inverse(MatDoub_O &ainv);              Calculate matrix inverse A^-1.
    Doub det();                                 Return determinant of A.
    void mprove(VecDoub_I &b, VecDoub_IO &x);   Discussed in §2.5.
    MatDoub_I &aref;                            Used only by mprove.
};
```

Here is the implementation of the constructor, whose argument is the input matrix that is to be LU decomposed. The input matrix is not altered; a copy is made, on which outer product Gaussian elimination is then done in-place.

ludcmp.h

```
LUdcmp::LUdcmp(MatDoub_I &a) : n(a.nrows()), lu(a), aref(a), indx(n) {
```
Given a matrix `a[0..n-1][0..n-1]`, this routine replaces it by the LU decomposition of a rowwise permutation of itself. `a` is input. On output, it is arranged as in equation (2.3.14) above; `indx[0..n-1]` is an output vector that records the row permutation effected by the partial pivoting; `d` is output as ± 1 depending on whether the number of row interchanges was even or odd, respectively. This routine is used in combination with `solve` to solve linear equations or invert a matrix.
```
    const Doub TINY=1.0e-40;                A small number.
    Int i,imax,j,k;
    Doub big,temp;
    VecDoub vv(n);                          vv stores the implicit scaling of each row.
    d=1.0;                                  No row interchanges yet.
    for (i=0;i<n;i++) {                     Loop over rows to get the implicit scaling infor-
        big=0.0;                                mation.
        for (j=0;j<n;j++)
            if ((temp=abs(lu[i][j])) > big) big=temp;
        if (big == 0.0) throw("Singular matrix in LUdcmp");
        No nonzero largest element.
        vv[i]=1.0/big;                      Save the scaling.
    }
    for (k=0;k<n;k++) {                     This is the outermost kij loop.
        big=0.0;                            Initialize for the search for largest pivot element.
        for (i=k;i<n;i++) {
            temp=vv[i]*abs(lu[i][k]);
            if (temp > big) {               Is the figure of merit for the pivot better than
                big=temp;                       the best so far?
                imax=i;
            }
        }
        if (k != imax) {                    Do we need to interchange rows?
            for (j=0;j<n;j++) {             Yes, do so...
                temp=lu[imax][j];
                lu[imax][j]=lu[k][j];
                lu[k][j]=temp;
            }
            d = -d;                         ...and change the parity of d.
            vv[imax]=vv[k];                 Also interchange the scale factor.
        }
        indx[k]=imax;
        if (lu[k][k] == 0.0) lu[k][k]=TINY;
```
If the pivot element is zero, the matrix is singular (at least to the precision of the algorithm). For some applications on singular matrices, it is desirable to substitute

```
        TINY for zero.
        for (i=k+1;i<n;i++) {
            temp=lu[i][k] /= lu[k][k];   Divide by the pivot element.
            for (j=k+1;j<n;j++)          Innermost loop: reduce remaining submatrix.
                lu[i][j] -= temp*lu[k][j];
        }
    }
}
```

Once the LUdcmp object is constructed, two functions implementing equations (2.3.6) and (2.3.7) are available for solving linear equations. The first solves a single right-hand side vector $\mathbf{b}$ for a solution vector $\mathbf{x}$. The second simultaneously solves multiple right-hand vectors, arranged as the columns of a matrix $\mathbf{B}$. In other words, it calculates the matrix $\mathbf{A}^{-1} \cdot \mathbf{B}$.

ludcmp.h

```
void LUdcmp::solve(VecDoub_I &b, VecDoub_O &x)
```

Solves the set of n linear equations $\mathbf{A} \cdot \mathbf{x} = \mathbf{b}$ using the stored LU decomposition of $\mathbf{A}$. b[0..n-1] is input as the right-hand side vector $\mathbf{b}$, while x returns the solution vector $\mathbf{x}$; b and x may reference the same vector, in which case the solution overwrites the input. This routine takes into account the possibility that b will begin with many zero elements, so it is efficient for use in matrix inversion.

```
{
    Int i,ii=0,ip,j;
    Doub sum;
    if (b.size() != n || x.size() != n)
        throw("LUdcmp::solve bad sizes");
    for (i=0;i<n;i++) x[i] = b[i];
    for (i=0;i<n;i++) {          When ii is set to a positive value, it will become the
        ip=indx[i];                  index of the first nonvanishing element of b. We now
        sum=x[ip];                   do the forward substitution, equation (2.3.6). The
        x[ip]=x[i];                  only new wrinkle is to unscramble the permutation
        if (ii != 0)                 as we go.
            for (j=ii-1;j<i;j++) sum -= lu[i][j]*x[j];
        else if (sum != 0.0)     A nonzero element was encountered, so from now on we
            ii=i+1;                  will have to do the sums in the loop above.
        x[i]=sum;
    }
    for (i=n-1;i>=0;i--) {       Now we do the backsubstitution, equation (2.3.7).
        sum=x[i];
        for (j=i+1;j<n;j++) sum -= lu[i][j]*x[j];
        x[i]=sum/lu[i][i];       Store a component of the solution vector X.
    }                            All done!
}
```

```
void LUdcmp::solve(MatDoub_I &b, MatDoub_O &x)
```

Solves m sets of n linear equations $\mathbf{A} \cdot \mathbf{X} = \mathbf{B}$ using the stored LU decomposition of $\mathbf{A}$. The matrix b[0..n-1][0..m-1] inputs the right-hand sides, while x[0..n-1][0..m-1] returns the solution $\mathbf{A}^{-1} \cdot \mathbf{B}$. b and x may reference the same matrix, in which case the solution overwrites the input.

```
{
    int i,j,m=b.ncols();
    if (b.nrows() != n || x.nrows() != n || b.ncols() != x.ncols())
        throw("LUdcmp::solve bad sizes");
    VecDoub xx(n);
    for (j=0;j<m;j++) {              Copy and solve each column in turn.
        for (i=0;i<n;i++) xx[i] = b[i][j];
        solve(xx,xx);
        for (i=0;i<n;i++) x[i][j] = xx[i];
    }
}
```

The LU decomposition in `LUdcmp` requires about $\frac{1}{3}N^3$ executions of the inner loops (each with one multiply and one add). This is thus the operation count for solving one (or a few) right-hand sides, and is a factor of 3 better than the Gauss-Jordan routine `gaussj` that was given in §2.1, and a factor of 1.5 better than a Gauss-Jordan routine (not given) that does not compute the inverse matrix. For inverting a matrix, the total count (including the forward- and backsubstitution as discussed following equation 2.3.7 above) is $(\frac{1}{3}+\frac{1}{6}+\frac{1}{2})N^3 = N^3$, the same as `gaussj`.

To summarize, this is the preferred way to solve the linear set of equations $\mathbf{A}\cdot\mathbf{x}=\mathbf{b}$:

```
const Int n = ...
MatDoub a(n,n);
VecDoub b(n),x(n);
...
LUdcmp alu(a);
alu.solve(b,x);
```

The answer will be given back in `x`. Your original matrix `a` and vector `b` are not altered. If you need to recover the storage in the object `alu`, then start a temporary scope with "{" before `alu` is declared, and end that scope with "}" when you want `alu` to be destroyed.

If you subsequently want to solve a set of equations with the same $\mathbf{A}$ but a different right-hand side $\mathbf{b}$, you repeat *only*

```
alu.solve(b,x);
```

2.3.2 Inverse of a Matrix

`LUdcmp` has a member function that gives the inverse of the matrix $\mathbf{A}$. Simply, it creates an identity matrix and then invokes the appropriate `solve` method.

ludcmp.h

```
void LUdcmp::inverse(MatDoub_O &ainv)
Using the stored LU decomposition, return in ainv the matrix inverse A^-1.
{
    Int i,j;
    ainv.resize(n,n);
    for (i=0;i<n;i++) {
        for (j=0;j<n;j++) ainv[i][j] = 0.;
        ainv[i][i] = 1.;
    }
    solve(ainv,ainv);
}
```

The matrix `ainv` will now contain the inverse of the original matrix `a`. Alternatively, there is nothing wrong with using a Gauss-Jordan routine like `gaussj` (§2.1) to invert a matrix in place, destroying the original. Both methods have practically the same operations count.

2.3.3 Determinant of a Matrix

The determinant of an LU decomposed matrix is just the product of the diagonal elements,

$$\det = \prod_{j=0}^{N-1} \beta_{jj} \tag{2.3.15}$$

We don't, recall, compute the decomposition of the original matrix, but rather a decomposition of a rowwise permutation of it. Luckily, we have kept track of whether the number of row interchanges was even or odd, so we just preface the product by the corresponding sign. (You now finally know the purpose of `d` in the `LUdcmp` structure.)

ludcmp.h

```
Doub LUdcmp::det()
Using the stored LU decomposition, return the determinant of the matrix A.
{
    Doub dd = d;
    for (Int i=0;i<n;i++) dd *= lu[i][i];
    return dd;
}
```

For a matrix of any substantial size, it is quite likely that the determinant will overflow or underflow your computer's floating-point dynamic range. In such a case you can easily add another member function that, e.g., divides by powers of ten, to keep track of the scale separately, or, e.g., accumulates the sum of logarithms of the absolute values of the factors and the sign separately.

2.3.4 Complex Systems of Equations

If your matrix $\mathbf{A}$ is real, but the right-hand side vector is complex, say $\mathbf{b} + i\,\mathbf{d}$, then (i) LU decompose $\mathbf{A}$ in the usual way, (ii) backsubstitute $\mathbf{b}$ to get the real part of the solution vector, and (iii) backsubstitute $\mathbf{d}$ to get the imaginary part of the solution vector.

If the matrix itself is complex, so that you want to solve the system

$$(\mathbf{A} + i\,\mathbf{C}) \cdot (\mathbf{x} + i\,\mathbf{y}) = (\mathbf{b} + i\,\mathbf{d}) \tag{2.3.16}$$

then there are two possible ways to proceed. The best way is to rewrite `LUdcmp` with complex routines. Complex modulus substitutes for absolute value in the construction of the scaling vector `vv` and in the search for the largest pivot elements. Everything else goes through in the obvious way, with complex arithmetic used as needed.

A quick-and-dirty way to solve complex systems is to take the real and imaginary parts of (2.3.16), giving

$$\begin{aligned} \mathbf{A} \cdot \mathbf{x} - \mathbf{C} \cdot \mathbf{y} &= \mathbf{b} \\ \mathbf{C} \cdot \mathbf{x} + \mathbf{A} \cdot \mathbf{y} &= \mathbf{d} \end{aligned} \tag{2.3.17}$$

which can be written as a $2N \times 2N$ set of *real* equations,

$$\begin{pmatrix} \mathbf{A} & -\mathbf{C} \\ \mathbf{C} & \mathbf{A} \end{pmatrix} \cdot \begin{pmatrix} \mathbf{x} \\ \mathbf{y} \end{pmatrix} = \begin{pmatrix} \mathbf{b} \\ \mathbf{d} \end{pmatrix} \tag{2.3.18}$$

and then solved with `LUdcmp`'s routines in their present forms. This scheme is a factor of 2 inefficient in storage, since $\mathbf{A}$ and $\mathbf{C}$ are stored twice. It is also a factor of 2 inefficient in time, since the complex multiplies in a complexified version of the routines would each use 4 real multiplies, while the solution of a $2N \times 2N$ problem involves 8 times the work of an $N \times N$ one. If you can tolerate these factor-of-two inefficiencies, then equation (2.3.18) is an easy way to proceed.

CITED REFERENCES AND FURTHER READING:

Golub, G.H., and Van Loan, C.F. 1996, *Matrix Computations*, 3rd ed. (Baltimore: Johns Hopkins University Press), Chapter 4.[1]

Anderson, E., et al. 1999, LAPACK User's Guide, 3rd ed. (Philadelphia: S.I.A.M.). Online with software at 2007+, `http://www.netlib.org/lapack`.

Forsythe, G.E., Malcolm, M.A., and Moler, C.B. 1977, *Computer Methods for Mathematical Computations* (Englewood Cliffs, NJ: Prentice-Hall), §3.3, and p. 50.

Forsythe, G.E., and Moler, C.B. 1967, *Computer Solution of Linear Algebraic Systems* (Englewood Cliffs, NJ: Prentice-Hall), Chapters 9, 16, and 18.

Westlake, J.R. 1968, *A Handbook of Numerical Matrix Inversion and Solution of Linear Equations* (New York: Wiley).

Stoer, J., and Bulirsch, R. 2002, *Introduction to Numerical Analysis*, 3rd ed. (New York: Springer), §4.1.

Ralston, A., and Rabinowitz, P. 1978, *A First Course in Numerical Analysis*, 2nd ed.; reprinted 2001 (New York: Dover), §9.11.

Horn, R.A., and Johnson, C.R. 1985, *Matrix Analysis* (Cambridge: Cambridge University Press).

2.4 Tridiagonal and Band-Diagonal Systems of Equations

The special case of a system of linear equations that is *tridiagonal*, that is, has nonzero elements only on the diagonal plus or minus one column, is one that occurs frequently. Also common are systems that are *band-diagonal*, with nonzero elements only along a few diagonal lines adjacent to the main diagonal (above and below).

For tridiagonal sets, the procedures of LU decomposition, forward- and back-substitution each take only $O(N)$ operations, and the whole solution can be encoded very concisely. The resulting routine `tridag` is one that we will use in later chapters.

Naturally, one does not reserve storage for the full $N \times N$ matrix, but only for the nonzero components, stored as three vectors. The set of equations to be solved is

$$\begin{bmatrix} b_0 & c_0 & 0 & \cdots & & & \\ a_1 & b_1 & c_1 & \cdots & & & \\ & & & \cdots & & & \\ & & & \cdots & a_{N-2} & b_{N-2} & c_{N-2} \\ & & & \cdots & 0 & a_{N-1} & b_{N-1} \end{bmatrix} \cdot \begin{bmatrix} u_0 \\ u_1 \\ \cdots \\ u_{N-2} \\ u_{N-1} \end{bmatrix} = \begin{bmatrix} r_0 \\ r_1 \\ \cdots \\ r_{N-2} \\ r_{N-1} \end{bmatrix} \tag{2.4.1}$$

Notice that a_0 and c_{N-1} are undefined and are not referenced by the routine that follows.

tridag.h

```
void tridag(VecDoub_I &a, VecDoub_I &b, VecDoub_I &c, VecDoub_I &r, VecDoub_O &u)
Solves for a vector u[0..n-1] the tridiagonal linear set given by equation (2.4.1). a[0..n-1],
b[0..n-1], c[0..n-1], and r[0..n-1] are input vectors and are not modified.
{
    Int j,n=a.size();
    Doub bet;
    VecDoub gam(n);                     One vector of workspace, gam, is needed.
    if (b[0] == 0.0) throw("Error 1 in tridag");
    If this happens, then you should rewrite your equations as a set of order N − 1, with u1
    trivially eliminated.
    u[0]=r[0]/(bet=b[0]);
    for (j=1;j<n;j++) {                 Decomposition and forward substitution.
        gam[j]=c[j-1]/bet;
        bet=b[j]-a[j]*gam[j];
```

```
        if (bet == 0.0) throw("Error 2 in tridag");       Algorithm fails; see below.
        u[j]=(r[j]-a[j]*u[j-1])/bet;
    }
    for (j=(n-2);j>=0;j--)
        u[j] -= gam[j+1]*u[j+1];          Backsubstitution.
}
```

There is no pivoting in `tridag`. It is for this reason that `tridag` can fail even when the underlying matrix is nonsingular: A zero pivot can be encountered even for a nonsingular matrix. In practice, this is not something to lose sleep about. The kinds of problems that lead to tridiagonal linear sets usually have additional properties which guarantee that the algorithm in `tridag` will succeed. For example, if

$$|b_j| > |a_j| + |c_j| \qquad j = 0, \ldots, N-1 \tag{2.4.2}$$

(called *diagonal dominance*), then it can be shown that the algorithm cannot encounter a zero pivot.

It is possible to construct special examples in which the lack of pivoting in the algorithm causes numerical instability. In practice, however, such instability is almost never encountered — unlike the general matrix problem where pivoting is essential.

The tridiagonal algorithm is the rare case of an algorithm that, in practice, is more robust than theory says it should be. Of course, should you ever encounter a problem for which `tridag` fails, you can instead use the more general method for band-diagonal systems, described below (the `Bandec` object).

Some other matrix forms consisting of tridiagonal with a small number of additional elements (e.g., upper right and lower left corners) also allow rapid solution; see §2.7.

2.4.1 Parallel Solution of Tridiagonal Systems

It is possible to solve tridiagonal systems doing many of the operations in parallel. We illustrate by the special case with $N = 7$:

$$\begin{bmatrix} b_0 & c_0 & & & & & \\ a_1 & b_1 & c_1 & & & & \\ & a_2 & b_2 & c_2 & & & \\ & & a_3 & b_3 & c_3 & & \\ & & & a_4 & b_4 & c_4 & \\ & & & & a_5 & b_5 & c_5 \\ & & & & & a_6 & b_6 \end{bmatrix} \cdot \begin{bmatrix} u_0 \\ u_1 \\ u_2 \\ u_3 \\ u_4 \\ u_5 \\ u_6 \end{bmatrix} = \begin{bmatrix} r_0 \\ r_1 \\ r_2 \\ r_3 \\ r_4 \\ r_5 \\ r_6 \end{bmatrix} \tag{2.4.3}$$

The basic idea is to partition the problem into even and odd elements, recurse to solve the latter, and then solve the former in parallel. Specifically, we first rewrite equation (2.4.3) by permuting its rows and columns,

$$\begin{bmatrix} b_0 & & & & c_0 & & \\ & b_2 & & & a_2 & c_2 & \\ & & b_4 & & & a_4 & c_4 \\ & & & b_6 & & & a_6 \\ a_1 & c_1 & & & b_1 & & \\ & a_3 & c_3 & & & b_3 & \\ & & a_5 & c_5 & & & b_5 \end{bmatrix} \cdot \begin{bmatrix} u_0 \\ u_2 \\ u_4 \\ u_6 \\ u_1 \\ u_3 \\ u_5 \end{bmatrix} = \begin{bmatrix} r_0 \\ r_2 \\ r_4 \\ r_6 \\ r_1 \\ r_3 \\ r_5 \end{bmatrix} \tag{2.4.4}$$

Now observe that, by row operations that subtract multiples of the first four rows from each of the last three rows, we can eliminate all nonzero elements in the lower-left quadrant. The price we pay is bringing some new elements into the lower-right quadrant, whose

nonzero elements we now call x's, y's, and z's. We call the modified right-hand sides q. The transformed problem is now

$$\begin{bmatrix} b_0 & & & & c_0 & & \\ & b_2 & & & a_2 & c_2 & \\ & & b_4 & & & a_4 & c_4 \\ & & & b_6 & & & a_6 \\ & & & & y_0 & z_0 & \\ & & & & x_1 & y_1 & z_1 \\ & & & & & x_2 & y_2 \end{bmatrix} \cdot \begin{bmatrix} u_0 \\ u_2 \\ u_4 \\ u_6 \\ u_1 \\ u_3 \\ u_5 \end{bmatrix} = \begin{bmatrix} r_0 \\ r_2 \\ r_4 \\ r_6 \\ q_0 \\ q_1 \\ q_2 \end{bmatrix} \tag{2.4.5}$$

Notice that the last three rows form a new, smaller, tridiagonal problem, which we can solve simply by recursing. Once its solution is known, the first four rows can be solved by a simple, parallelizable, substitution. For discussion of this and related methods for parallelizing tridiagonal systems, and references to the literature, see [2].

2.4.2 Band-Diagonal Systems

Where tridiagonal systems have nonzero elements only on the diagonal plus or minus one, band-diagonal systems are slightly more general and have (say) $m_1 \geq 0$ nonzero elements immediately to the left of (below) the diagonal and $m_2 \geq 0$ nonzero elements immediately to its right (above it). Of course, this is only a useful classification if m_1 and m_2 are both $\ll N$. In that case, the solution of the linear system by LU decomposition can be accomplished much faster, and in much less storage, than for the general $N \times N$ case.

The precise definition of a band-diagonal matrix with elements a_{ij} is that

$$a_{ij} = 0 \quad \text{when} \quad j > i + m_2 \quad \text{or} \quad i > j + m_1 \tag{2.4.6}$$

Band-diagonal matrices are stored and manipulated in a so-called compact form, which results if the matrix is tilted 45° clockwise, so that its nonzero elements lie in a long, narrow matrix with $m_1 + 1 + m_2$ columns and N rows. This is best illustrated by an example: The band-diagonal matrix

$$\begin{pmatrix} 3 & 1 & 0 & 0 & 0 & 0 & 0 \\ 4 & 1 & 5 & 0 & 0 & 0 & 0 \\ 9 & 2 & 6 & 5 & 0 & 0 & 0 \\ 0 & 3 & 5 & 8 & 9 & 0 & 0 \\ 0 & 0 & 7 & 9 & 3 & 2 & 0 \\ 0 & 0 & 0 & 3 & 8 & 4 & 6 \\ 0 & 0 & 0 & 0 & 2 & 4 & 4 \end{pmatrix} \tag{2.4.7}$$

which has $N = 7$, $m_1 = 2$, and $m_2 = 1$, is stored compactly as the 7×4 matrix,

$$\begin{pmatrix} x & x & 3 & 1 \\ x & 4 & 1 & 5 \\ 9 & 2 & 6 & 5 \\ 3 & 5 & 8 & 9 \\ 7 & 9 & 3 & 2 \\ 3 & 8 & 4 & 6 \\ 2 & 4 & 4 & x \end{pmatrix} \tag{2.4.8}$$

Here x denotes elements that are wasted space in the compact format; these will not be referenced by any manipulations and can have arbitrary values. Notice that the diagonal of the original matrix appears in column m_1, with subdiagonal elements to its left and superdiagonal elements to its right.

The simplest manipulation of a band-diagonal matrix, stored compactly, is to multiply it by a vector to its right. Although this is algorithmically trivial, you might want to study the following routine as an example of how to pull nonzero elements a_{ij} out of the compact storage format in an orderly fashion.

```
void banmul(MatDoub_I &a, const Int m1, const Int m2, VecDoub_I &x,
    VecDoub_O &b)
```
banded.h

Matrix multiply $\mathbf{b} = \mathbf{A} \cdot \mathbf{x}$, where $\mathbf{A}$ is band-diagonal with `m1` rows below the diagonal and `m2` rows above. The input vector is `x[0..n-1]` and the output vector is `b[0..n-1]`. The array `a[0..n-1][0..m1+m2]` stores $\mathbf{A}$ as follows: The diagonal elements are in `a[0..n-1][m1]`. Subdiagonal elements are in `a[`j`..n-1][0..m1-1]` with $j > 0$ appropriate to the number of elements on each subdiagonal. Superdiagonal elements are in `a[0..`j`][m1+1..m1+m2]` with $j <$ `n-1` appropriate to the number of elements on each superdiagonal.

```
{
    Int i,j,k,tmploop,n=a.nrows();
    for (i=0;i<n;i++) {
        k=i-m1;
        tmploop=MIN(m1+m2+1,Int(n-k));
        b[i]=0.0;
        for (j=MAX(0,-k);j<tmploop;j++) b[i] += a[i][j]*x[j+k];
    }
}
```

It is not possible to store the LU decomposition of a band-diagonal matrix $\mathbf{A}$ quite as compactly as the compact form of $\mathbf{A}$ itself. The decomposition (essentially by Crout's method; see §2.3) produces additional nonzero "fill-ins." One straightforward storage scheme is to store the upper triangular factor (U) in a space with the same shape as $\mathbf{A}$, and to store the lower triangular factor (L) in a separate compact matrix of size $N \times m_1$. The diagonal elements of U (whose product, times `d` $= \pm 1$, gives the determinant) are in the first column of U.

Here is an object, analogous to `LUdcmp` in §2.3, for solving band-diagonal linear equations:

```
struct Bandec {
```
banded.h

Object for solving linear equations $\mathbf{A} \cdot \mathbf{x} = \mathbf{b}$ for a band-diagonal matrix $\mathbf{A}$, using LU decomposition.

```
    Int n,m1,m2;
    MatDoub au,al;                Upper and lower triangular matrices, stored compactly.
    VecInt indx;
    Doub d;
    Bandec(MatDoub_I &a, const int mm1, const int mm2);   Constructor.
    void solve(VecDoub_I &b, VecDoub_O &x);      Solve a right-hand side vector.
    Doub det();                                  Return determinant of A.
};
```

The constructor takes as arguments the compactly stored matrix $\mathbf{A}$, and the integers m_1 and m_2. (One could of course define a "band-diagonal matrix object" to encapsulate these quantities, but in this brief treatment we want to keep things simple.)

```
Bandec::Bandec(MatDoub_I &a, const Int mm1, const Int mm2)
    : n(a.nrows()), au(a), m1(mm1), m2(mm2), al(n,m1), indx(n)
```
banded.h

Constructor. Given an `n`×`n` band-diagonal matrix $\mathbf{A}$ with `m1` subdiagonal rows and `m2` superdiagonal rows, compactly stored in the array `a[0..n-1][0..m1+m2]` as described in the comment for routine `banmul`, an LU decomposition of a rowwise permutation of $\mathbf{A}$ is constructed. The upper and lower triangular matrices are stored in `au` and `al`, respectively. The stored vector `indx[0..n-1]` records the row permutation effected by the partial pivoting; `d` is ± 1 depending on whether the number of row interchanges was even or odd, respectively.

```
{
    const Doub TINY=1.0e-40;
    Int i,j,k,l,mm;
    Doub dum;
    mm=m1+m2+1;
    l=m1;
    for (i=0;i<m1;i++) {              Rearrange the storage a bit.
        for (j=m1-i;j<mm;j++) au[i][j-l]=au[i][j];
        l--;
        for (j=mm-l-1;j<mm;j++) au[i][j]=0.0;
    }
```

```
    d=1.0;
    l=m1;
    for (k=0;k<n;k++) {                     For each row...
        dum=au[k][0];
        i=k;
        if (l<n) l++;
        for (j=k+1;j<l;j++) {               Find the pivot element.
            if (abs(au[j][0]) > abs(dum)) {
                dum=au[j][0];
                i=j;
            }
        }
        indx[k]=i+1;
        if (dum == 0.0) au[k][0]=TINY;
        Matrix is algorithmically singular, but proceed anyway with TINY pivot (desirable in
        some applications).
        if (i != k) {                       Interchange rows.
            d = -d;
            for (j=0;j<mm;j++) SWAP(au[k][j],au[i][j]);
        }
        for (i=k+1;i<l;i++) {               Do the elimination.
            dum=au[i][0]/au[k][0];
            al[k][i-k-1]=dum;
            for (j=1;j<mm;j++) au[i][j-1]=au[i][j]-dum*au[k][j];
            au[i][mm-1]=0.0;
        }
    }
}
```

Some pivoting is possible within the storage limitations of bandec, and the above routine does take advantage of the opportunity. In general, when TINY is returned as a diagonal element of U, then the original matrix (perhaps as modified by roundoff error) is in fact singular. In this regard, bandec is somewhat more robust than tridag above, which can fail algorithmically even for nonsingular matrices; bandec is thus also useful (with $m_1 = m_2 = 1$) for some ill-behaved tridiagonal systems.

Once the matrix $\mathbf{A}$ has been decomposed, any number of right-hand sides can be solved in turn by repeated calls to the solve method, the forward- and backsubstitution routine analogous to its same-named cousin in §2.3.

banded.h

```
void Bandec::solve(VecDoub_I &b, VecDoub_O &x)
```

Given a right-hand side vector b[0..n-1], solves the band-diagonal linear equations $\mathbf{A} \cdot \mathbf{x} = \mathbf{b}$. The solution vector $\mathbf{x}$ is returned as x[0..n-1].

```
{
    Int i,j,k,l,mm;
    Doub dum;
    mm=m1+m2+1;
    l=m1;
    for (k=0;k<n;k++) x[k] = b[k];
    for (k=0;k<n;k++) {                  Forward substitution, unscrambling the permuted rows
        j=indx[k]-1;                        as we go.
        if (j!=k) SWAP(x[k],x[j]);
        if (l<n) l++;
        for (j=k+1;j<l;j++) x[j] -= al[k][j-k-1]*x[k];
    }
    l=1;
    for (i=n-1;i>=0;i--) {               Backsubstitution.
        dum=x[i];
        for (k=1;k<l;k++) dum -= au[i][k]*x[k+i];
        x[i]=dum/au[i][0];
        if (l<mm) l++;
    }
}
```

And, finally, a method for getting the determinant:

banded.h

```
Doub Bandec::det() {
Using the stored decomposition, return the determinant of the matrix A.
    Doub dd = d;
    for (int i=0;i<n;i++) dd *= au[i][0];
    return dd;
}
```

The routines in `Bandec` are based on the Handbook routines *bandet1* and *bansol1* in [1].

CITED REFERENCES AND FURTHER READING:

Keller, H.B. 1968, *Numerical Methods for Two-Point Boundary-Value Problems*; reprinted 1991 (New York: Dover), p. 74.

Dahlquist, G., and Bjorck, A. 1974, *Numerical Methods* (Englewood Cliffs, NJ: Prentice-Hall); reprinted 2003 (New York: Dover), Example 5.4.3, p. 166.

Ralston, A., and Rabinowitz, P. 1978, *A First Course in Numerical Analysis*, 2nd ed.; reprinted 2001 (New York: Dover), §9.11.

Wilkinson, J.H., and Reinsch, C. 1971, *Linear Algebra*, vol. II of *Handbook for Automatic Computation* (New York: Springer), Chapter I/6.[1]

Golub, G.H., and Van Loan, C.F. 1996, *Matrix Computations*, 3rd ed. (Baltimore: Johns Hopkins University Press), §4.3.

Hockney, R.W., and Jesshope, C.R. 1988, *Parallel Computers 2: Architecture, Programming, and Algorithms* (Bristol and Philadelphia: Adam Hilger), §5.4.[2]

2.5 Iterative Improvement of a Solution to Linear Equations

Obviously it is not easy to obtain greater precision for the solution of a linear set than the precision of your computer's floating-point word. Unfortunately, for large sets of linear equations, it is not always easy to obtain precision equal to, or even comparable to, the computer's limit. In direct methods of solution, roundoff errors accumulate, and they are magnified to the extent that your matrix is close to singular. You can easily lose two or three significant figures for matrices that (you thought) were *far* from singular.

If this happens to you, there is a neat trick to restore the full machine precision, called *iterative improvement* of the solution. The theory is straightforward (see Figure 2.5.1): Suppose that a vector $\mathbf{x}$ is the exact solution of the linear set

$$\mathbf{A} \cdot \mathbf{x} = \mathbf{b} \tag{2.5.1}$$

You don't, however, know $\mathbf{x}$. You only know some slightly wrong solution $\mathbf{x} + \delta\mathbf{x}$, where $\delta\mathbf{x}$ is the unknown error. When multiplied by the matrix $\mathbf{A}$, your slightly wrong solution gives a product slightly discrepant from the desired right-hand side $\mathbf{b}$, namely

$$\mathbf{A} \cdot (\mathbf{x} + \delta\mathbf{x}) = \mathbf{b} + \delta\mathbf{b} \tag{2.5.2}$$

Subtracting (2.5.1) from (2.5.2) gives

$$\mathbf{A} \cdot \delta\mathbf{x} = \delta\mathbf{b} \tag{2.5.3}$$

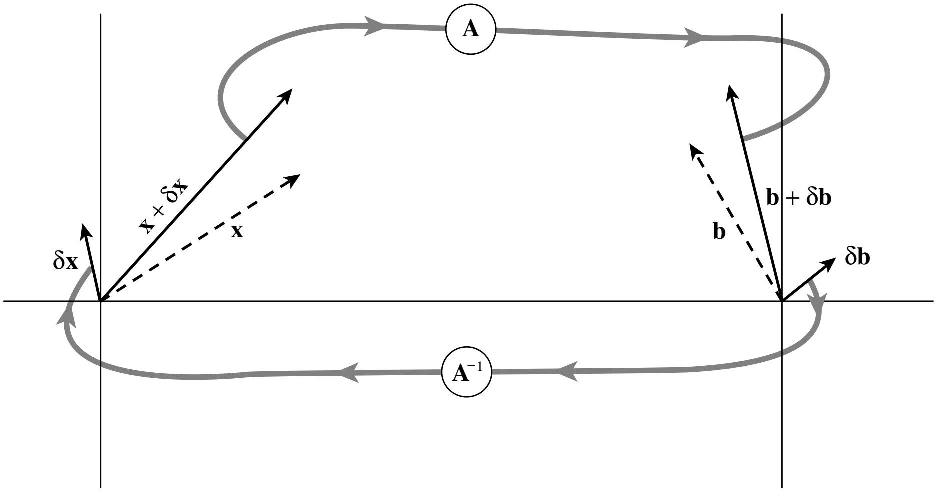

Figure 2.5.1. Iterative improvement of the solution to $\mathbf{A}\cdot\mathbf{x} = \mathbf{b}$. The first guess $\mathbf{x} + \delta\mathbf{x}$ is multiplied by $\mathbf{A}$ to produce $\mathbf{b} + \delta\mathbf{b}$. The known vector $\mathbf{b}$ is subtracted, giving $\delta\mathbf{b}$. The linear set with this right-hand side is inverted, giving $\delta\mathbf{x}$. This is subtracted from the first guess giving an improved solution $\mathbf{x}$.

But (2.5.2) can also be solved, trivially, for $\delta\mathbf{b}$. Substituting this into (2.5.3) gives

$$\mathbf{A}\cdot\delta\mathbf{x} = \mathbf{A}\cdot(\mathbf{x} + \delta\mathbf{x}) - \mathbf{b} \tag{2.5.4}$$

In this equation, the whole right-hand side is known, since $\mathbf{x} + \delta\mathbf{x}$ is the wrong solution that you want to improve. It is good to calculate the right-hand side in higher precision than the original solution, if you can, since there will be a lot of cancellation in the subtraction of $\mathbf{b}$. Then, we need only solve (2.5.4) for the error $\delta\mathbf{x}$, and then subtract this from the wrong solution to get an improved solution.

An important extra benefit occurs if we obtained the original solution by LU decomposition. In this case we already have the LU decomposed form of $\mathbf{A}$, and all we need do to solve (2.5.4) is compute the right-hand side, forward- and backsubstitute.

Because so much of the necessary machinery is already in `LUdcmp`, we implement iterative improvement as a member function of that class. Since iterative improvement requires the matrix $\mathbf{A}$ (as well as its LU decomposition), we have, with foresight, caused `LUdcmp` to save a reference to the matrix `a` from which it was constructed. If you plan to use iterative improvement, you must not modify `a` or let it go out of scope. (No other method in `LUdcmp` makes use of this reference to `a`.)

ludcmp.h

```
void LUdcmp::mprove(VecDoub_I &b, VecDoub_IO &x)
```

Improves a solution vector `x[0..n-1]` of the linear set of equations $\mathbf{A}\cdot\mathbf{x} = \mathbf{b}$. The vectors `b[0..n-1]` and `x[0..n-1]` are input. On output, `x[0..n-1]` is modified, to an improved set of values.

```
{
    Int i,j;
    VecDoub r(n);
    for (i=0;i<n;i++) {                         Calculate the right-hand side, accumulating
        Ldoub sdp = -b[i];                        the residual in higher precision.
        for (j=0;j<n;j++)
            sdp += (Ldoub)aref[i][j] * (Ldoub)x[j];
```

```
        r[i]=sdp;
    }
    solve(r,r);                               Solve for the error term,
    for (i=0;i<n;i++) x[i] -= r[i];           and subtract it from the old solution.
}
```

Iterative improvement is *highly* recommended: It is a process of order only N^2 operations (multiply vector by matrix, forward- and backsubstitute — see discussion following equation 2.3.7); it never hurts; and it can really give you your money's worth if it saves an otherwise ruined solution on which you have already spent of order N^3 operations.

You can call `mprove` several times in succession if you want. Unless you are starting quite far from the true solution, one call is generally enough; but a second call to verify convergence can be reassuring.

If you cannot compute the right-hand side in equation (2.5.4) in higher precision, iterative refinement will still often improve the quality of a solution, although not in all cases as much as if higher precision is available. Many textbooks assert the contrary, but you will find the proof in [1].

2.5.1 More on Iterative Improvement

It is illuminating (and will be useful later in the book) to give a somewhat more solid analytical foundation for equation (2.5.4), and also to give some additional results. Implicit in the previous discussion was the notion that the solution vector $\mathbf{x} + \delta\mathbf{x}$ has an error term; but we neglected the fact that the LU decomposition of $\mathbf{A}$ is itself not exact.

A different analytical approach starts with some matrix $\mathbf{B}_0$ that is assumed to be an *approximate* inverse of the matrix $\mathbf{A}$, so that $\mathbf{B}_0 \cdot \mathbf{A}$ is approximately the identity matrix $\mathbf{1}$. Define the *residual matrix* $\mathbf{R}$ of $\mathbf{B}_0$ as

$$\mathbf{R} \equiv \mathbf{1} - \mathbf{B}_0 \cdot \mathbf{A} \tag{2.5.5}$$

which is supposed to be "small" (we will be more precise below). Note that therefore

$$\mathbf{B}_0 \cdot \mathbf{A} = \mathbf{1} - \mathbf{R} \tag{2.5.6}$$

Next consider the following formal manipulation:

$$\begin{aligned}\mathbf{A}^{-1} &= \mathbf{A}^{-1} \cdot (\mathbf{B}_0^{-1} \cdot \mathbf{B}_0) = (\mathbf{A}^{-1} \cdot \mathbf{B}_0^{-1}) \cdot \mathbf{B}_0 = (\mathbf{B}_0 \cdot \mathbf{A})^{-1} \cdot \mathbf{B}_0 \\ &= (\mathbf{1} - \mathbf{R})^{-1} \cdot \mathbf{B}_0 = (\mathbf{1} + \mathbf{R} + \mathbf{R}^2 + \mathbf{R}^3 + \cdots) \cdot \mathbf{B}_0\end{aligned} \tag{2.5.7}$$

We can define the nth partial sum of the last expression by

$$\mathbf{B}_n \equiv (\mathbf{1} + \mathbf{R} + \cdots + \mathbf{R}^n) \cdot \mathbf{B}_0 \tag{2.5.8}$$

so that $\mathbf{B}_\infty \to \mathbf{A}^{-1}$, if the limit exists.

It now is straightforward to verify that equation (2.5.8) satisfies some interesting recurrence relations. As regards solving $\mathbf{A} \cdot \mathbf{x} = \mathbf{b}$, where $\mathbf{x}$ and $\mathbf{b}$ are vectors, define

$$\mathbf{x}_n \equiv \mathbf{B}_n \cdot \mathbf{b} \tag{2.5.9}$$

Then it is easy to show that

$$\mathbf{x}_{n+1} = \mathbf{x}_n + \mathbf{B}_0 \cdot (\mathbf{b} - \mathbf{A} \cdot \mathbf{x}_n) \tag{2.5.10}$$

This is immediately recognizable as equation (2.5.4), with $-\delta\mathbf{x} = \mathbf{x}_{n+1} - \mathbf{x}_n$, and with $\mathbf{B}_0$ taking the role of $\mathbf{A}^{-1}$. We see, therefore, that equation (2.5.4) does not require that the LU decomposition of $\mathbf{A}$ be exact, but only that the implied residual $\mathbf{R}$ be small. In rough terms,

if the residual is smaller than the square root of your computer's roundoff error, then after one application of equation (2.5.10) (that is, going from $\mathbf{x}_0 \equiv \mathbf{B}_0 \cdot \mathbf{b}$ to $\mathbf{x}_1$) the first neglected term, of order $\mathbf{R}^2$, will be smaller than the roundoff error. Equation (2.5.10), like equation (2.5.4), moreover, can be applied more than once, since it uses only $\mathbf{B}_0$, and not any of the higher $\mathbf{B}$'s.

A much more surprising recurrence that follows from equation (2.5.8) is one that more than *doubles* the order n at each stage:

$$\mathbf{B}_{2n+1} = 2\mathbf{B}_n - \mathbf{B}_n \cdot \mathbf{A} \cdot \mathbf{B}_n \qquad n = 0, 1, 3, 7, \ldots \tag{2.5.11}$$

Repeated application of equation (2.5.11), from a suitable starting matrix $\mathbf{B}_0$, converges *quadratically* to the unknown inverse matrix $\mathbf{A}^{-1}$ (see §9.4 for the definition of "quadratically"). Equation (2.5.11) goes by various names, including *Schultz's Method* and *Hotelling's Method*; see Pan and Reif [2] for references. In fact, equation (2.5.11) is simply the iterative Newton-Raphson method of root finding (§9.4) applied to matrix inversion.

Before you get too excited about equation (2.5.11), however, you should notice that it involves two full matrix multiplications at each iteration. Each matrix multiplication involves N^3 adds and multiplies. But we already saw in §2.1 – §2.3 that direct inversion of $\mathbf{A}$ requires only N^3 adds and N^3 multiplies *in toto*. Equation (2.5.11) is therefore practical only when special circumstances allow it to be evaluated much more rapidly than is the case for general matrices. We will meet such circumstances later, in §13.10.

In the spirit of delayed gratification, let us nevertheless pursue the two related issues: When does the series in equation (2.5.7) converge; and what is a suitable initial guess $\mathbf{B}_0$ (if, for example, an initial LU decomposition is not feasible)?

We can define the norm of a matrix as the largest amplification of length that it is able to induce on a vector,

$$\|\mathbf{R}\| \equiv \max_{\mathbf{v}\neq 0} \frac{|\mathbf{R}\cdot\mathbf{v}|}{|\mathbf{v}|} \tag{2.5.12}$$

If we let equation (2.5.7) act on some arbitrary right-hand side $\mathbf{b}$, as one wants a matrix inverse to do, it is obvious that a sufficient condition for convergence is

$$\|\mathbf{R}\| < 1 \tag{2.5.13}$$

Pan and Reif [2] point out that a suitable initial guess for $\mathbf{B}_0$ is any sufficiently small constant ϵ times the matrix transpose of $\mathbf{A}$, that is,

$$\mathbf{B}_0 = \epsilon\mathbf{A}^T \qquad \text{or} \qquad \mathbf{R} = \mathbf{1} - \epsilon\mathbf{A}^T\cdot\mathbf{A} \tag{2.5.14}$$

To see why this is so involves concepts from Chapter 11; we give here only the briefest sketch: $\mathbf{A}^T \cdot \mathbf{A}$ is a symmetric, positive-definite matrix, so it has real, positive eigenvalues. In its diagonal representation, $\mathbf{R}$ takes the form

$$\mathbf{R} = \text{diag}(1-\epsilon\lambda_0, 1-\epsilon\lambda_1, \ldots, 1-\epsilon\lambda_{N-1}) \tag{2.5.15}$$

where all the λ_i's are positive. Evidently any ϵ satisfying $0 < \epsilon < 2/(\max_i \lambda_i)$ will give $\|\mathbf{R}\| < 1$. It is not difficult to show that the optimal choice for ϵ, giving the most rapid convergence for equation (2.5.11), is

$$\epsilon = 2/(\max_i \lambda_i + \min_i \lambda_i) \tag{2.5.16}$$

Rarely does one know the eigenvalues of $\mathbf{A}^T\cdot\mathbf{A}$ in equation (2.5.16). Pan and Reif derive several interesting bounds, which are computable directly from $\mathbf{A}$. The following choices guarantee the convergence of $\mathbf{B}_n$ as $n \to \infty$:

$$\epsilon \le 1\Big/\sum_{j,k} a_{jk}^2 \qquad \text{or} \qquad \epsilon \le 1\Big/\Big(\max_i \sum_j |a_{ij}| \times \max_j \sum_i |a_{ij}|\Big) \tag{2.5.17}$$

The latter expression is truly a remarkable formula, which Pan and Reif derive by noting that the vector norm in equation (2.5.12) need not be the usual L_2 norm, but can instead be either the L_∞ (max) norm, or the L_1 (absolute value) norm. See their work for details.

Another approach, with which we have had some success, is to estimate the largest eigenvalue statistically, by calculating $s_i \equiv |\mathbf{A}\cdot\mathbf{v}_i|^2$ for several unit vector $\mathbf{v}_i$'s with randomly chosen directions in N-space. The largest eigenvalue λ can then be bounded by the maximum of $2\max s_i$ and $2N\,\mathrm{Var}(s_i)/\mu(s_i)$, where Var and μ denote the sample variance and mean, respectively.

CITED REFERENCES AND FURTHER READING:

Johnson, L.W., and Riess, R.D. 1982, *Numerical Analysis*, 2nd ed. (Reading, MA: Addison-Wesley), §2.3.4, p. 55.

Golub, G.H., and Van Loan, C.F. 1996, *Matrix Computations*, 3rd ed. (Baltimore: Johns Hopkins University Press), §3.5.3.

Dahlquist, G., and Bjorck, A. 1974, *Numerical Methods* (Englewood Cliffs, NJ: Prentice-Hall); reprinted 2003 (New York: Dover), §5.5.6, p. 183.

Forsythe, G.E., and Moler, C.B. 1967, *Computer Solution of Linear Algebraic Systems* (Englewood Cliffs, NJ: Prentice-Hall), Chapter 13.

Ralston, A., and Rabinowitz, P. 1978, *A First Course in Numerical Analysis*, 2nd ed.; reprinted 2001 (New York: Dover), §9.5, p. 437.

Higham, N.J. 1997, "Iterative Refinement for Linear Systems and LAPACK," *IMA Journal of Numerical Analysis*, vol. 17, pp. 495–509.[1]

Pan, V., and Reif, J. 1985, "Efficient Parallel Solution of Linear Systems," in *Proceedings of the Seventeenth Annual ACM Symposium on Theory of Computing* (New York: Association for Computing Machinery).[2]

2.6 Singular Value Decomposition

There exists a very powerful set of techniques for dealing with sets of equations or matrices that are either singular or else numerically very close to singular. In many cases where Gaussian elimination and LU decomposition fail to give satisfactory results, this set of techniques, known as *singular value decomposition*, or *SVD*, will diagnose for you precisely what the problem is. In some cases, SVD will not only diagnose the problem, it will also solve it, in the sense of giving you a useful numerical answer, although, as we shall see, not necessarily "the" answer that you thought you should get.

SVD is also the method of choice for solving most *linear least-squares* problems. We will outline the relevant theory in this section, but defer detailed discussion of the use of SVD in this application to Chapter 15, whose subject is the parametric modeling of data.

SVD methods are based on the following theorem of linear algebra, whose proof is beyond our scope: Any $M\times N$ matrix $\mathbf{A}$ can be written as the product of an $M\times N$ column-orthogonal matrix $\mathbf{U}$, an $N\times N$ diagonal matrix $\mathbf{W}$ with positive or zero elements (the *singular values*), and the transpose of an $N\times N$ orthogonal matrix $\mathbf{V}$. The various shapes of these matrices are clearer when shown as tableaus. If $M > N$ (which corresponds to the *overdetermined* situation of more equations than

unknowns), the decomposition looks like this:

$$\begin{pmatrix} \\ \\ \mathbf{A} \\ \\ \\ \end{pmatrix} = \begin{pmatrix} \\ \\ \mathbf{U} \\ \\ \\ \end{pmatrix} \cdot \begin{pmatrix} w_0 & & & \\ & w_1 & & \\ & & \cdots & \\ & & \cdots & \\ & & & w_{N-1} \end{pmatrix} \cdot \begin{pmatrix} & \\ & \mathbf{V}^T & \\ & \end{pmatrix} \tag{2.6.1}$$

If $M < N$ (the *undetermined* situation of fewer equations than unknowns), it looks like this:

$$\begin{pmatrix} & \mathbf{A} & \end{pmatrix} = \begin{pmatrix} & \mathbf{U} & \end{pmatrix} \cdot \begin{pmatrix} w_0 & & & \\ & w_1 & & \\ & & \cdots & \\ & & \cdots & \\ & & & w_{N-1} \end{pmatrix} \cdot \begin{pmatrix} & \\ & \mathbf{V}^T & \\ & \end{pmatrix} \tag{2.6.2}$$

The matrix $\mathbf{V}$ is orthogonal in the sense that its columns are orthonormal,

$$\sum_{j=0}^{N-1} V_{jk} V_{jn} = \delta_{kn} \qquad \begin{array}{l} 0 \le k \le N-1 \\ 0 \le n \le N-1 \end{array} \tag{2.6.3}$$

that is, $\mathbf{V}^T \cdot \mathbf{V} = \mathbf{1}$. Since $\mathbf{V}$ is square, it is also row-orthonormal, $\mathbf{V} \cdot \mathbf{V}^T = 1$. When $M \ge N$, the matrix $\mathbf{U}$ is also column-orthogonal,

$$\sum_{i=0}^{M-1} U_{ik} U_{in} = \delta_{kn} \qquad \begin{array}{l} 0 \le k \le N-1 \\ 0 \le n \le N-1 \end{array} \tag{2.6.4}$$

that is, $\mathbf{U}^T \cdot \mathbf{U} = \mathbf{1}$. In the case $M < N$, however, two things happen: (i) The singular values w_j for $j = M, \ldots, N-1$ are all zero, and (ii) the corresponding columns of $\mathbf{U}$ are also zero. Equation (2.6.4) then holds only for $k, n \le M-1$.

The decomposition (2.6.1) or (2.6.2) can always be done, no matter how singular the matrix is, and it is "almost" unique. That is to say, it is unique up to (i) making the same permutation of the columns of $\mathbf{U}$, elements of $\mathbf{W}$, and columns of $\mathbf{V}$ (or rows of $\mathbf{V}^T$); or (ii) performing an orthogonal rotation on any set of columns of $\mathbf{U}$ and $\mathbf{V}$ whose corresponding elements of $\mathbf{W}$ happen to be exactly equal. (A special case is multiplying any column of $\mathbf{U}$, and the corresponding column of $\mathbf{V}$ by -1.) A consequence of the permutation freedom is that for the case $M < N$, a numerical algorithm for the decomposition need not return zero w_j's in the canonical positions $j = M, \ldots, N-1$; the $N - M$ zero singular values can be scattered among all positions $j = 0, 1, \ldots, N-1$, and one needs to perform a sort to get the canonical order. In any case, it is conventional to sort *all* the singular values into descending order.

A Webnote [1] gives the details of the routine that actually performs SVD on an arbitrary matrix $\mathbf{A}$, yielding $\mathbf{U}$, $\mathbf{W}$, and $\mathbf{V}$. The routine is based on a routine

by Forsythe et al. [2], which is in turn based on the original routine of Golub and Reinsch, found, in various forms, in [4-6] and elsewhere. These references include extensive discussion of the algorithm used. As much as we dislike the use of black-box routines, we need to ask you to accept this one, since it would take us too far afield to cover its necessary background material here. The algorithm is very stable, and it is very unusual for it ever to misbehave. Most of the concepts that enter the algorithm (Householder reduction to bidiagonal form, diagonalization by QR procedure with shifts) will be discussed further in Chapter 11.

As we did for LU decomposition, we encapsulate the singular value decomposition and also the methods that depend on it into an object, SVD. We give its declaration here. The rest of this section will give the details on how to use it.

svd.h

```
struct SVD {
```
Object for singular value decomposition of a matrix $\mathbf{A}$, and related functions.
```
    Int m,n;
    MatDoub u,v;                          The matrices U and V.
    VecDoub w;                            The diagonal matrix W.
    Doub eps, tsh;
    SVD(MatDoub_I &a) : m(a.nrows()), n(a.ncols()), u(a), v(n,n), w(n) {
```
Constructor. The single argument is $\mathbf{A}$. The SVD computation is done by decompose, and the results are sorted by reorder.
```
        eps = numeric_limits<Doub>::epsilon();
        decompose();
        reorder();
        tsh = 0.5*sqrt(m+n+1.)*w[0]*eps;      Default threshold for nonzero singular
    }                                             values.

    void solve(VecDoub_I &b, VecDoub_O &x, Doub thresh);
    void solve(MatDoub_I &b, MatDoub_O &x, Doub thresh);
```
Solve with (apply the pseudoinverse to) one or more right-hand sides.
```
    Int rank(Doub thresh);                Quantities associated with the range and
    Int nullity(Doub thresh);                 nullspace of A.
    MatDoub range(Doub thresh);
    MatDoub nullspace(Doub thresh);

    Doub inv_condition() {                Return reciprocal of the condition num-
        return (w[0] <= 0. || w[n-1] <= 0.) ? 0. : w[n-1]/w[0];     ber of A.
    }

    void decompose();                     Functions used by the constructor.
    void reorder();
    Doub pythag(const Doub a, const Doub b);
};
```

2.6.1 Range, Nullspace, and All That

Consider the familiar set of simultaneous equations

$$\mathbf{A} \cdot \mathbf{x} = \mathbf{b} \tag{2.6.5}$$

where $\mathbf{A}$ is an $M \times N$ matrix, and $\mathbf{x}$ and $\mathbf{b}$ are vectors of dimension N and M respectively. Equation (2.6.5) defines $\mathbf{A}$ as a linear mapping from an N-dimensional vector space to (generally) an M-dimensional one. But the map *might* be able to reach only a lesser-dimensional subspace of the full M-dimensional one. That subspace is called the *range* of $\mathbf{A}$. The dimension of the range is called the *rank* of $\mathbf{A}$. The rank

of **A** is equal to its number of linearly independent columns, and also (perhaps less obviously) to its number of linearly independent rows. If **A** is not identically zero, its rank is at least 1, and at most $\min(M, N)$.

Sometimes there are nonzero vectors **x** that are mapped to zero by **A**, that is, $\mathbf{A} \cdot \mathbf{x} = 0$. The space of such vectors (a subspace of the N-dimensional space that **x** lives in) is called the *nullspace* of **A**, and its dimension is called **A**'s *nullity*. The nullity can have any value from zero to N. The *rank-nullity theorem* states that, for any **A**, the rank plus the nullity is N, the number of columns.

An important special case is $M = N$, so the **A** is square, $N \times N$. If the rank of **A** is N, its maximum possible value, then **A** is nonsingular and invertible: $\mathbf{A} \cdot \mathbf{x} = \mathbf{b}$ has a unique solution for any **b**, and only the zero vector is mapped to zero. This is a case where LU decomposition (§2.3) is the preferred solution method for **x**. However, if **A** has rank less than N (i.e., has nullity greater than zero), then two things happen: (i) most right-hand side vectors **b** yield no solution, but (ii) some have multiple solutions (in fact a whole subspace of them). We consider this situation further, below.

What has all this to do with singular value decomposition? SVD explicitly constructs orthonormal bases for the nullspace and range of a matrix! Specifically, the columns of **U** whose same-numbered elements w_j are *nonzero* are an orthonormal set of basis vectors that span the range; the columns of **V** whose same-numbered elements w_j are *zero* are an orthonormal basis for the nullspace. Our `SVD` object has methods that return the rank or nullity (integers), and also the range and nullspace, each of these packaged as a matrix whose columns form an orthonormal basis for the respective subspace.

svd.h

```
Int SVD::rank(Doub thresh = -1.) {
```
Return the rank of **A**, after zeroing any singular values smaller than `thresh`. If `thresh` is negative, a default value based on estimated roundoff is used.
```
    Int j,nr=0;
    tsh = (thresh >= 0. ? thresh : 0.5*sqrt(m+n+1.)*w[0]*eps);
    for (j=0;j<n;j++) if (w[j] > tsh) nr++;
    return nr;
}

Int SVD::nullity(Doub thresh = -1.) {
```
Return the nullity of **A**, after zeroing any singular values smaller than `thresh`. Default value as above.
```
    Int j,nn=0;
    tsh = (thresh >= 0. ? thresh : 0.5*sqrt(m+n+1.)*w[0]*eps);
    for (j=0;j<n;j++) if (w[j] <= tsh) nn++;
    return nn;
}

MatDoub SVD::range(Doub thresh = -1.){
```
Give an orthonormal basis for the range of **A** as the columns of a returned matrix. `thresh` as above.
```
    Int i,j,nr=0;
    MatDoub rnge(m,rank(thresh));
    for (j=0;j<n;j++) {
        if (w[j] > tsh) {
            for (i=0;i<m;i++) rnge[i][nr] = u[i][j];
            nr++;
        }
    }
    return rnge;
}
```

```
MatDoub SVD::nullspace(Doub thresh = -1.){
```
Give an orthonormal basis for the nullspace of **A** as the columns of a returned matrix. `thresh` as above.
```
    Int j,jj,nn=0;
    MatDoub nullsp(n,nullity(thresh));
    for (j=0;j<n;j++) {
        if (w[j] <= tsh) {
            for (jj=0;jj<n;jj++) nullsp[jj][nn] = v[jj][j];
            nn++;
        }
    }
    return nullsp;
}
```

The meaning of the optional parameter `thresh` is discussed below.

2.6.2 SVD of a Square Matrix

We return to the case of a square $N \times N$ matrix $\mathbf{A}$. $\mathbf{U}$, $\mathbf{V}$, and $\mathbf{W}$ are also square matrices of the same size. Their inverses are also trivial to compute: $\mathbf{U}$ and $\mathbf{V}$ are orthogonal, so their inverses are equal to their transposes; $\mathbf{W}$ is diagonal, so its inverse is the diagonal matrix whose elements are the reciprocals of the elements w_j. From (2.6.1) it now follows immediately that the inverse of $\mathbf{A}$ is

$$\mathbf{A}^{-1} = \mathbf{V} \cdot \left[\operatorname{diag}(1/w_j)\right] \cdot \mathbf{U}^T \tag{2.6.6}$$

The only thing that can go wrong with this construction is for one of the w_j's to be zero, or (numerically) for it to be so small that its value is dominated by roundoff error and therefore unknowable. If more than one of the w_j's has this problem, then the matrix is even more singular. So, first of all, SVD gives you a clear diagnosis of the situation.

Formally, the *condition number* of a matrix is defined as the ratio of the largest (in magnitude) of the w_j's to the smallest of the w_j's. A matrix is singular if its condition number is infinite, and it is *ill-conditioned* if its condition number is too large, that is, if its reciprocal approaches the machine's floating-point precision (for example, less than about 10^{-15} for values of type `double`). A function returning the condition number (or, rather, its reciprocal, to avoid overflow) is implemented in `SVD`.

Now let's have another look at solving the set of simultaneous linear equations (2.6.5) in the case that $\mathbf{A}$ is singular. We already saw that the set of *homogeneous* equations, where $\mathbf{b} = 0$, is solved immediately by SVD. The solution is any linear combination of the columns returned by the `nullspace` method above.

When the vector $\mathbf{b}$ on the right-hand side is not zero, the important question is whether it lies in the range of $\mathbf{A}$ or not. If it does, then the singular set of equations *does* have a solution $\mathbf{x}$; in fact it has more than one solution, since any vector in the nullspace (any column of $\mathbf{V}$ with a corresponding zero w_j) can be added to $\mathbf{x}$ in any linear combination.

If we want to single out one particular member of this solution set of vectors as a representative, we might want to pick the one with the smallest length $|\mathbf{x}|^2$. Here is how to find that vector using SVD: Simply *replace* $1/w_j$ *by zero if* $w_j = 0$. (It is not very often that one gets to set $\infty = 0$!) Then compute, working from right to

left,

$$\mathbf{x} = \mathbf{V} \cdot \left[\text{diag}\,(1/w_j)\right] \cdot (\mathbf{U}^T \cdot \mathbf{b}) \tag{2.6.7}$$

This will be the solution vector of smallest length; the columns of $\mathbf{V}$ that are in the nullspace complete the specification of the solution set.

Proof: Consider $|\mathbf{x} + \mathbf{x}'|$, where $\mathbf{x}'$ lies in the nullspace. Then, if $\mathbf{W}^{-1}$ denotes the modified inverse of $\mathbf{W}$ with some elements zeroed,

$$\begin{aligned} \left|\mathbf{x} + \mathbf{x}'\right| &= \left|\mathbf{V} \cdot \mathbf{W}^{-1} \cdot \mathbf{U}^T \cdot \mathbf{b} + \mathbf{x}'\right| \\ &= \left|\mathbf{V} \cdot (\mathbf{W}^{-1} \cdot \mathbf{U}^T \cdot \mathbf{b} + \mathbf{V}^T \cdot \mathbf{x}')\right| \\ &= \left|\mathbf{W}^{-1} \cdot \mathbf{U}^T \cdot \mathbf{b} + \mathbf{V}^T \cdot \mathbf{x}'\right| \end{aligned} \tag{2.6.8}$$

Here the first equality follows from (2.6.7), and the second and third from the orthonormality of $\mathbf{V}$. If you now examine the two terms that make up the sum on the right-hand side, you will see that the first one has nonzero j components only where $w_j \neq 0$, while the second one, since $\mathbf{x}'$ is in the nullspace, has nonzero j components only where $w_j = 0$. Therefore the minimum length obtains for $\mathbf{x}' = 0$, q.e.d.

If $\mathbf{b}$ is not in the range of the singular matrix $\mathbf{A}$, then the set of equations (2.6.5) has no solution. But here is some good news: If $\mathbf{b}$ is not in the range of $\mathbf{A}$, then equation (2.6.7) can still be used to construct a "solution" vector $\mathbf{x}$. This vector $\mathbf{x}$ will not exactly solve $\mathbf{A} \cdot \mathbf{x} = \mathbf{b}$. But, among all possible vectors $\mathbf{x}$, it will do the closest possible job in the least-squares sense. In other words, (2.6.7) finds

$$\mathbf{x} \quad \text{which minimizes} \quad r \equiv |\mathbf{A} \cdot \mathbf{x} - \mathbf{b}| \tag{2.6.9}$$

The number r is called the *residual* of the solution.

The proof is similar to (2.6.8): Suppose we modify $\mathbf{x}$ by adding some arbitrary $\mathbf{x}'$. Then $\mathbf{A} \cdot \mathbf{x} - \mathbf{b}$ is modified by adding some $\mathbf{b}' \equiv \mathbf{A} \cdot \mathbf{x}'$. Obviously $\mathbf{b}'$ is in the range of $\mathbf{A}$. We then have

$$\begin{aligned} \left|\mathbf{A} \cdot \mathbf{x} - \mathbf{b} + \mathbf{b}'\right| &= \left|(\mathbf{U} \cdot \mathbf{W} \cdot \mathbf{V}^T) \cdot (\mathbf{V} \cdot \mathbf{W}^{-1} \cdot \mathbf{U}^T \cdot \mathbf{b}) - \mathbf{b} + \mathbf{b}'\right| \\ &= \left|(\mathbf{U} \cdot \mathbf{W} \cdot \mathbf{W}^{-1} \cdot \mathbf{U}^T - 1) \cdot \mathbf{b} + \mathbf{b}'\right| \\ &= \left|\mathbf{U} \cdot \left[(\mathbf{W} \cdot \mathbf{W}^{-1} - 1) \cdot \mathbf{U}^T \cdot \mathbf{b} + \mathbf{U}^T \cdot \mathbf{b}'\right]\right| \\ &= \left|(\mathbf{W} \cdot \mathbf{W}^{-1} - 1) \cdot \mathbf{U}^T \cdot \mathbf{b} + \mathbf{U}^T \cdot \mathbf{b}'\right| \end{aligned} \tag{2.6.10}$$

Now, $(\mathbf{W} \cdot \mathbf{W}^{-1} - 1)$ is a diagonal matrix that has nonzero j components only for $w_j = 0$, while $\mathbf{U}^T\mathbf{b}'$ has nonzero j components only for $w_j \neq 0$, since $\mathbf{b}'$ lies in the range of $\mathbf{A}$. Therefore the minimum obtains for $\mathbf{b}' = 0$, q.e.d.

Equation (2.6.7), which is also equation (2.6.6) applied associatively to $\mathbf{b}$, is thus very general. If no w_j's are zero, it solves a nonsingular system of linear equations. If some w_j's are zero, and their reciprocals are made zero, then it gives a "best" solution, either the one of shortest length among many, or the one of minimum residual when no exact solution exists. Equation (2.6.6), with the singular $1/w_j$'s zeroized, is called the *Moore-Penrose inverse* or *pseudoinverse* of $\mathbf{A}$.

Equation (2.6.7) is implemented in the SVD object as the method solve. (As in LUdcmp, we also include an overloaded form that solves for multiple right-hand sides simultaneously.) The argument thresh inputs a value below which w_j's are to be considered as being zero; if you omit this argument, or set it to a negative value, then the program uses a default value based on expected roundoff error.

svd.h

```
void SVD::solve(VecDoub_I &b, VecDoub_O &x, Doub thresh = -1.) {
```

Solve $\mathbf{A} \cdot \mathbf{x} = \mathbf{b}$ for a vector $\mathbf{x}$ using the pseudoinverse of $\mathbf{A}$ as obtained by SVD. If positive, thresh is the threshold value below which singular values are considered as zero. If thresh is negative, a default based on expected roundoff error is used.

```
    Int i,j,jj;
    Doub s;
    if (b.size() != m || x.size() != n) throw("SVD::solve bad sizes");
    VecDoub tmp(n);
    tsh = (thresh >= 0. ? thresh : 0.5*sqrt(m+n+1.)*w[0]*eps);
    for (j=0;j<n;j++) {                 Calculate U^T B.
        s=0.0;
        if (w[j] > tsh) {               Nonzero result only if w_j is nonzero.
            for (i=0;i<m;i++) s += u[i][j]*b[i];
            s /= w[j];                  This is the divide by w_j.
        }
        tmp[j]=s;
    }
    for (j=0;j<n;j++) {                 Matrix multiply by V to get answer.
        s=0.0;
        for (jj=0;jj<n;jj++) s += v[j][jj]*tmp[jj];
        x[j]=s;
    }
}
```

```
void SVD::solve(MatDoub_I &b, MatDoub_O &x, Doub thresh = -1.)
```

Solves m sets of n equations $\mathbf{A} \cdot \mathbf{X} = \mathbf{B}$ using the pseudoinverse of $\mathbf{A}$. The right-hand sides are input as b[0..n-1][0..m-1], while x[0..n-1][0..m-1] returns the solutions. thresh as above.

```
{
    int i,j,m=b.ncols();
    if (b.nrows() != n || x.nrows() != n || b.ncols() != x.ncols())
        throw("SVD::solve bad sizes");
    VecDoub xx(n);
    for (j=0;j<m;j++) {                 Copy and solve each column in turn.
        for (i=0;i<n;i++) xx[i] = b[i][j];
        solve(xx,xx,thresh);
        for (i=0;i<n;i++) x[i][j] = xx[i];
    }
}
```

Figure 2.6.1 summarizes the situation for the SVD of square matrices.

There are cases in which you may want to set the value of thresh to larger than its default. (You can retrieve the default as the member value tsh.) In the discussion since equation (2.6.5), we have been pretending that a matrix either is singular or else isn't. Numerically, however, the more common situation is that some of the w_j's are very small but nonzero, so that the matrix is ill-conditioned. In that case, the direct solution methods of LU decomposition or Gaussian elimination may actually give a formal solution to the set of equations (that is, a zero pivot may not be encountered); but the solution vector may have wildly large components whose algebraic cancellation, when multiplying by the matrix $\mathbf{A}$, may give a very poor approximation to the right-hand vector $\mathbf{b}$. In such cases, the solution vector $\mathbf{x}$ obtained by *zeroing* the small w_j's and then using equation (2.6.7) is very often better (in the sense of the residual $|\mathbf{A} \cdot \mathbf{x} - \mathbf{b}|$ being smaller) than *both* the direct-method

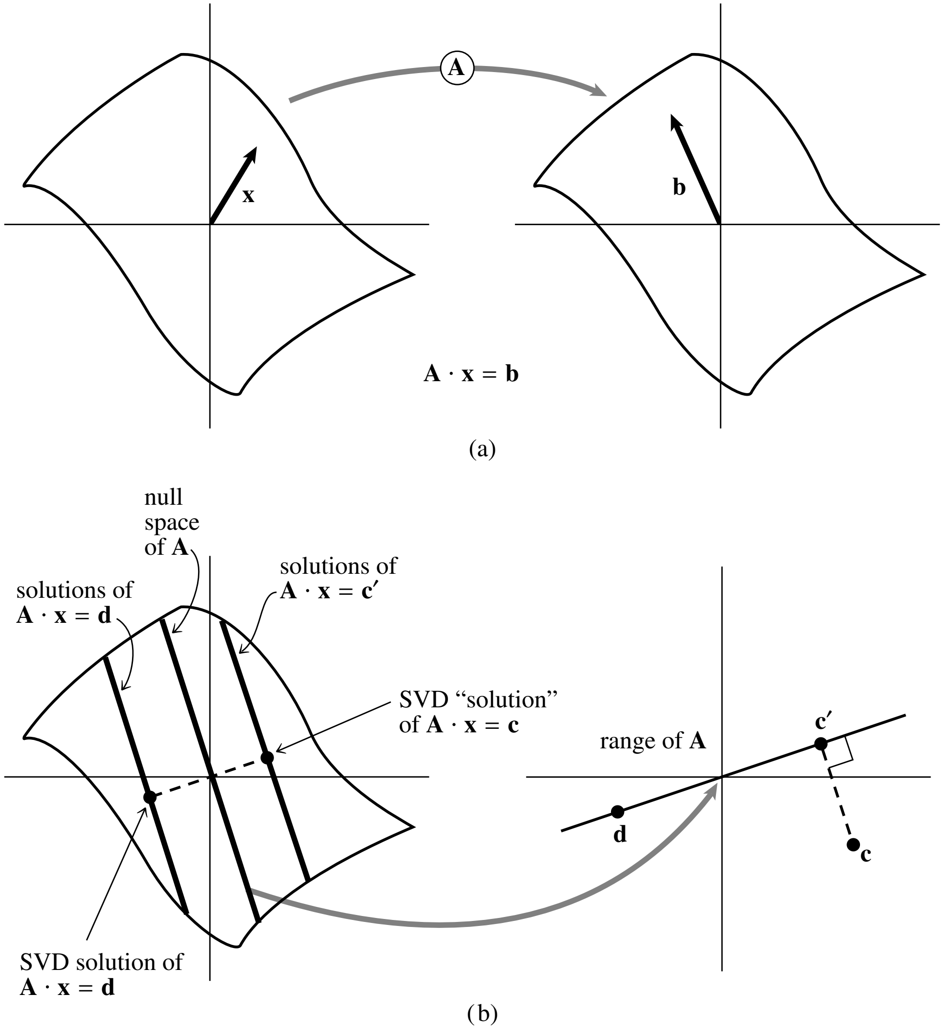

Figure 2.6.1. (a) A nonsingular matrix $\mathbf{A}$ maps a vector space into one of the same dimension. The vector $\mathbf{x}$ is mapped into $\mathbf{b}$, so that $\mathbf{x}$ satisfies the equation $\mathbf{A}\cdot\mathbf{x} = \mathbf{b}$. (b) A singular matrix $\mathbf{A}$ maps a vector space into one of lower dimensionality, here a plane into a line, called the "range" of $\mathbf{A}$. The "nullspace" of $\mathbf{A}$ is mapped to zero. The solutions of $\mathbf{A}\cdot\mathbf{x} = \mathbf{d}$ consist of any one particular solution plus any vector in the nullspace, here forming a line parallel to the nullspace. Singular value decomposition (SVD) selects the particular solution closest to zero, as shown. The point $\mathbf{c}$ lies outside of the range of $\mathbf{A}$, so $\mathbf{A}\cdot\mathbf{x} = \mathbf{c}$ has no solution. SVD finds the least-squares best compromise solution, namely a solution of $\mathbf{A}\cdot\mathbf{x} = \mathbf{c}'$, as shown.

solution *and* the SVD solution where the small w_j's are left nonzero.

It may seem paradoxical that this can be so, since zeroing a singular value corresponds to throwing away one linear combination of the set of equations that we are trying to solve. The resolution of the paradox is that we are throwing away precisely a combination of equations that is so corrupted by roundoff error as to be at best useless; usually it is worse than useless since it "pulls" the solution vector way off toward infinity along some direction that is almost a nullspace vector. In doing this,

it compounds the roundoff problem and makes the residual $|\mathbf{A}\cdot\mathbf{x}-\mathbf{b}|$ larger.

You therefore have the opportunity of deciding at what threshold `thresh` to zero the small w_j's, based on some idea of what size of computed residual $|\mathbf{A}\cdot\mathbf{x}-\mathbf{b}|$ is acceptable.

For discussion of how the singular value decomposition of a matrix is related to its eigenvalues and eigenvectors, see §11.0.6.

2.6.3 SVD for Fewer Equations than Unknowns

If you have fewer linear equations M than unknowns N, then you are not expecting a unique solution. Usually there will be an $N-M$ dimensional family of solutions (which is the nullity, absent any other degeneracies), but the number could be larger. If you want to find this whole solution space, then `SVD` can readily do the job: Use `solve` to get one (the shortest) solution, then use `nullspace` to get a set of basis vectors for the nullspace. Your solutions are the former plus any linear combination of the latter.

2.6.4 SVD for More Equations than Unknowns

This situation will occur in Chapter 15, when we wish to find the least-squares solution to an overdetermined set of linear equations. In tableau, the equations to be solved are

$$\left(\begin{array}{c} \\ \\ \\ \quad\quad\mathbf{A}\quad\quad \\ \\ \\ \\ \end{array}\right)\cdot\left(\begin{array}{c} \\ \mathbf{x} \\ \\ \end{array}\right)=\left(\begin{array}{c} \\ \\ \\ \mathbf{b} \\ \\ \\ \\ \end{array}\right) \tag{2.6.11}$$

The proofs that we gave above for the square case apply without modification to the case of more equations than unknowns. The least-squares solution vector $\mathbf{x}$ is given by applying the pseudoinverse (2.6.7), which, with nonsquare matrices, looks like this,

$$\left(\begin{array}{c} \\ \mathbf{x} \\ \\ \end{array}\right)=\left(\begin{array}{c} \\ \quad\mathbf{V}\quad \\ \\ \end{array}\right)\cdot\left(\begin{array}{c} \\ \mathrm{diag}(1/w_j) \\ \\ \end{array}\right)\cdot\left(\begin{array}{c} \\ \quad\quad\mathbf{U}^T\quad\quad \\ \\ \end{array}\right)\cdot\left(\begin{array}{c} \\ \\ \\ \mathbf{b} \\ \\ \\ \\ \end{array}\right) \tag{2.6.12}$$

In general, the matrix $\mathbf{W}$ will not be singular, and no w_j's will need to be set to zero. Occasionally, however, there might be column degeneracies in $\mathbf{A}$. In this case you will need to zero some small w_j values after all. The corresponding column in $\mathbf{V}$ gives the linear combination of $\mathbf{x}$'s that is then ill-determined even by the supposedly overdetermined set.

Sometimes, although you do not need to zero any w_j's for *computational* reasons, you may nevertheless want to take note of any that are unusually small: Their corresponding columns in $\mathbf{V}$ are linear combinations of $\mathbf{x}$'s that are insensitive to your data. In fact, you may then wish to zero these w_j's, by increasing the value of `thresh`, to reduce the number of free parameters in the fit. These matters are discussed more fully in Chapter 15.

2.6.5 Constructing an Orthonormal Basis

Suppose that you have N vectors in an M-dimensional vector space, with $N \leq M$. Then the N vectors span some subspace of the full vector space. Often you want to construct an orthonormal set of N vectors that span the same subspace. The elementary textbook way to do this is by Gram-Schmidt orthogonalization, starting with one vector and then expanding the subspace one dimension at a time. Numerically, however, because of the build-up of roundoff errors, naive Gram-Schmidt orthogonalization is *terrible*.

The right way to construct an orthonormal basis for a subspace is by SVD: Form an $M \times N$ matrix $\mathbf{A}$ whose N columns are your vectors. Construct an `SVD` object from the matrix. The columns of the matrix $\mathbf{U}$ are your desired orthonormal basis vectors.

You might also want to check the w_j's for zero values. If any occur, then the spanned subspace was not, in fact, N-dimensional; the columns of $\mathbf{U}$ corresponding to zero w_j's should be discarded from the orthonormal basis set. The method `range` does this.

QR factorization, discussed in §2.10, also constructs an orthonormal basis; see [3].

2.6.6 Approximation of Matrices

Note that equation (2.6.1) can be rewritten to express any matrix A_{ij} as a sum of outer products of columns of $\mathbf{U}$ and rows of $\mathbf{V}^T$, with the "weighting factors" being the singular values w_j,

$$A_{ij} = \sum_{k=0}^{N-1} w_k \, U_{ik} V_{jk} \tag{2.6.13}$$

If you ever encounter a situation where *most* of the singular values w_j of a matrix $\mathbf{A}$ are very small, then $\mathbf{A}$ will be well-approximated by only a few terms in the sum (2.6.13). This means that you have to store only a few columns of $\mathbf{U}$ and $\mathbf{V}$ (the same k ones) and you will be able to recover, with good accuracy, the whole matrix.

Note also that it is very efficient to multiply such an approximated matrix by a vector $\mathbf{x}$: You just dot $\mathbf{x}$ with each of the stored columns of $\mathbf{V}$, multiply the resulting scalar by the corresponding w_k, and accumulate that multiple of the corresponding column of $\mathbf{U}$. If your matrix is approximated by a small number K of singular values, then this computation of $\mathbf{A} \cdot \mathbf{x}$ takes only about $K(M + N)$ multiplications, instead of MN for the full matrix.

2.6.7 Newer Algorithms

Analogous to the newer methods for eigenvalues of symmetric tridiagonal matrices mentioned in §11.4.4, there are newer methods for SVD. There is a divide-and-conquer algorithm, implemented in LAPACK as `dgesdd`, which is typically faster by a factor of about 5 for large matrices than the algorithm we give (which is similar to the LAPACK routine `dgesvd`). Another routine based on the MRRR algorithm (see §11.4.4) promises to be even better, but it is not available in LAPACK as of 2006. It will appear as routine `dbdscr`.

CITED REFERENCES AND FURTHER READING:

Numerical Recipes Software 2007, "SVD Implementation Code," *Numerical Recipes Webnote No. 2*, at `http://www.nr.com/webnotes?2` [1]

Forsythe, G.E., Malcolm, M.A., and Moler, C.B. 1977, *Computer Methods for Mathematical Computations* (Englewood Cliffs, NJ: Prentice-Hall), Chapter 9.[2]

Golub, G.H., and Van Loan, C.F. 1996, *Matrix Computations*, 3rd ed. (Baltimore: Johns Hopkins University Press), §8.6 and Chapter 12 (SVD). QR decomposition is discussed in §5.2.6.[3]

Lawson, C.L., and Hanson, R. 1974, *Solving Least Squares Problems* (Englewood Cliffs, NJ: Prentice-Hall); reprinted 1995 (Philadelphia: S.I.A.M.), Chapter 18.

Wilkinson, J.H., and Reinsch, C. 1971, *Linear Algebra*, vol. II of *Handbook for Automatic Computation* (New York: Springer), Chapter I.10 by G.H. Golub and C. Reinsch.[4]

Anderson, E., et al. 1999, LAPACK User's Guide, 3rd ed. (Philadelphia: S.I.A.M.). Online with software at 2007+, `http://www.netlib.org/lapack`.[5]

Smith, B.T., et al. 1976, *Matrix Eigensystem Routines — EISPACK Guide*, 2nd ed., vol. 6 of Lecture Notes in Computer Science (New York: Springer).

Stoer, J., and Bulirsch, R. 2002, *Introduction to Numerical Analysis*, 3rd ed. (New York: Springer), §6.7.[6]

2.7 Sparse Linear Systems

A system of linear equations is called *sparse* if only a relatively small number of its matrix elements a_{ij} are nonzero. It is wasteful to use general methods of linear algebra on such problems, because most of the $O(N^3)$ arithmetic operations devoted to solving the set of equations or inverting the matrix involve zero operands. Furthermore, you might wish to work problems so large as to tax your available memory space, and it is wasteful to reserve storage for unfruitful zero elements. Note that there are two distinct (and not always compatible) goals for any sparse matrix method: saving time and/or saving space.

We considered one archetypal sparse form in §2.4, the band-diagonal matrix. In the tridiagonal case, e.g., we saw that it was possible to save both time (order N instead of N^3) and space (order N instead of N^2). The method of solution was not different in principle from the general method of LU decomposition; it was just applied cleverly, and with due attention to the bookkeeping of zero elements. Many practical schemes for dealing with sparse problems have this same character. They are fundamentally decomposition schemes, or else elimination schemes akin to Gauss-Jordan, but carefully optimized so as to minimize the number of so-called

fill-ins, initially zero elements that must become nonzero during the solution process, and for which storage must be reserved.

Direct methods for solving sparse equations, then, depend crucially on the precise pattern of sparsity of the matrix. Patterns that occur frequently, or that are useful as way stations in the reduction of more general forms, already have special names and special methods of solution. We do not have space here for any detailed review of these. References listed at the end of this section will furnish you with an "in" to the specialized literature, and the following list of buzz words (and Figure 2.7.1) will at least let you hold your own at cocktail parties:

- tridiagonal
- band-diagonal (or banded) with bandwidth M
- band triangular
- block diagonal
- block tridiagonal
- block triangular
- cyclic banded
- singly (or doubly) bordered block diagonal
- singly (or doubly) bordered block triangular
- singly (or doubly) bordered band-diagonal
- singly (or doubly) bordered band triangular
- other (!)

You should also be aware of some of the special sparse forms that occur in the solution of partial differential equations in two or more dimensions. See Chapter 20.

If your particular pattern of sparsity is not a simple one, then you may wish to try an *analyze/factorize/operate* package, which automates the procedure of figuring out how fill-ins are to be minimized. The *analyze* stage is done once only for each pattern of sparsity. The *factorize* stage is done once for each particular matrix that fits the pattern. The *operate* stage is performed once for each right-hand side to be used with the particular matrix. Consult [2,3] for references on this. The NAG library [4] has an analyze/factorize/operate capability. A substantial collection of routines for sparse matrix calculation is also available from IMSL [5] as the *Yale Sparse Matrix Package* [6].

You should be aware that the special order of interchanges and eliminations, prescribed by a sparse matrix method so as to minimize fill-ins and arithmetic operations, generally acts to decrease the method's numerical stability as compared to, e.g., regular LU decomposition with pivoting. Scaling your problem so as to make its nonzero matrix elements have comparable magnitudes (if you can do it) will sometimes ameliorate this problem.

In the remainder of this section, we present some concepts that are applicable to some general classes of sparse matrices, and which do not necessarily depend on details of the pattern of sparsity.

2.7.1 Sherman-Morrison Formula

Suppose that you have already obtained, by herculean effort, the inverse matrix $\mathbf{A}^{-1}$ of a square matrix $\mathbf{A}$. Now you want to make a "small" change in $\mathbf{A}$, for example change one element a_{ij}, or a few elements, or one row, or one column. Is there any

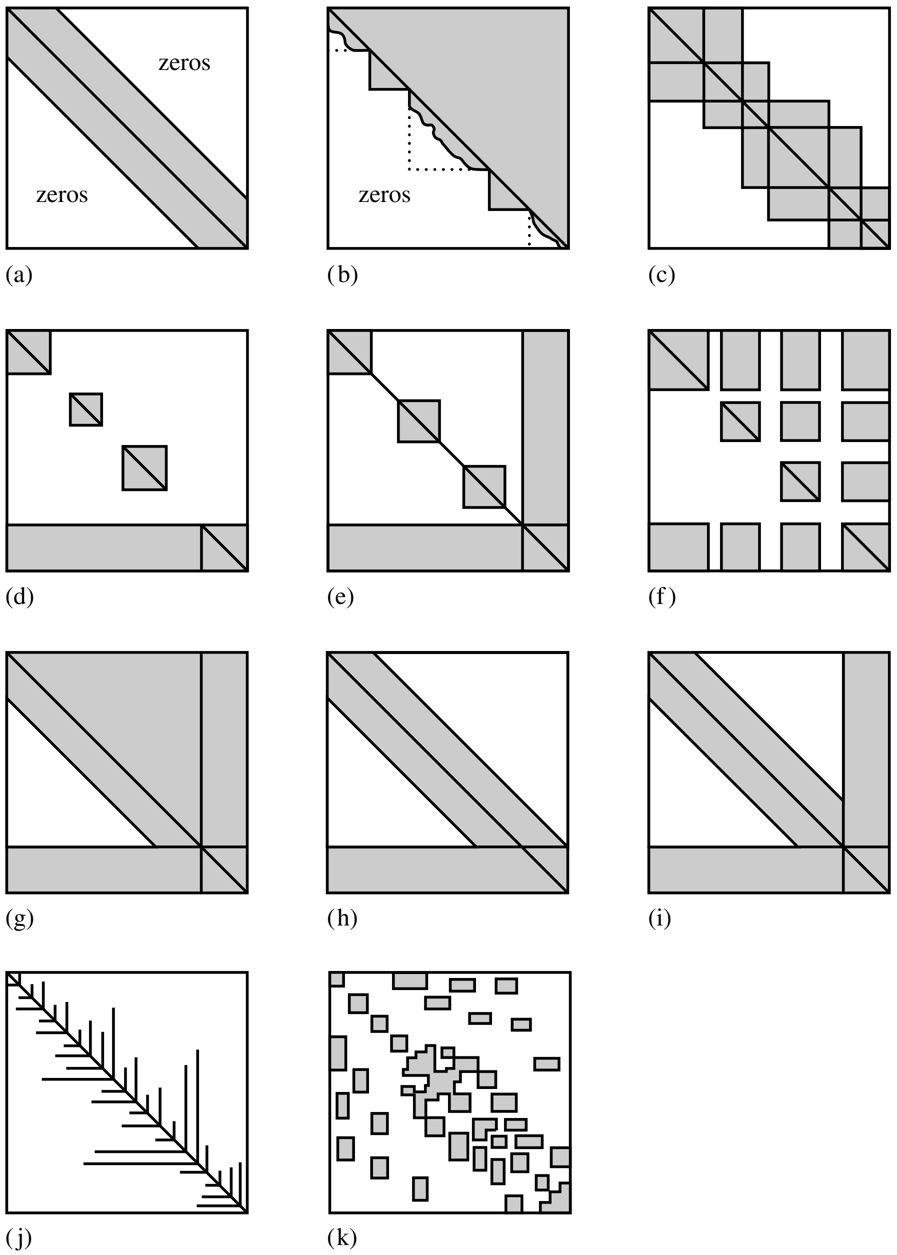

Figure 2.7.1. Some standard forms for sparse matrices. (a) Band-diagonal; (b) block triangular; (c) block tridiagonal; (d) singly bordered block diagonal; (e) doubly bordered block diagonal; (f) singly bordered block triangular; (g) bordered band-triangular; (h) and (i) singly and doubly bordered band-diagonal; (j) and (k) other! (after Tewarson) [1].

way of calculating the corresponding change in $\mathbf{A}^{-1}$ without repeating your difficult labors? Yes, if your change is of the form

$$\mathbf{A} \;\rightarrow\; (\mathbf{A} + \mathbf{u} \otimes \mathbf{v}) \tag{2.7.1}$$

for some vectors $\mathbf{u}$ and $\mathbf{v}$. If $\mathbf{u}$ is a unit vector $\mathbf{e}_i$, then (2.7.1) adds the components of $\mathbf{v}$ to the ith row. (Recall that $\mathbf{u} \otimes \mathbf{v}$ is a matrix whose i, jth element is the product of the ith component of $\mathbf{u}$ and the jth component of $\mathbf{v}$.) If $\mathbf{v}$ is a unit vector $\mathbf{e}_j$, then (2.7.1) adds the components of $\mathbf{u}$ to the jth column. If both $\mathbf{u}$ and $\mathbf{v}$ are proportional to unit vectors $\mathbf{e}_i$ and $\mathbf{e}_j$, respectively, then a term is added only to the element a_{ij}.

The *Sherman-Morrison* formula gives the inverse $(\mathbf{A} + \mathbf{u} \otimes \mathbf{v})^{-1}$ and is derived briefly as follows:

$$\begin{aligned}
(\mathbf{A} + \mathbf{u} \otimes \mathbf{v})^{-1} &= (\mathbf{1} + \mathbf{A}^{-1} \cdot \mathbf{u} \otimes \mathbf{v})^{-1} \cdot \mathbf{A}^{-1} \\
&= (\mathbf{1} - \mathbf{A}^{-1} \cdot \mathbf{u} \otimes \mathbf{v} + \mathbf{A}^{-1} \cdot \mathbf{u} \otimes \mathbf{v} \cdot \mathbf{A}^{-1} \cdot \mathbf{u} \otimes \mathbf{v} - \ldots) \cdot \mathbf{A}^{-1} \\
&= \mathbf{A}^{-1} - \mathbf{A}^{-1} \cdot \mathbf{u} \otimes \mathbf{v} \cdot \mathbf{A}^{-1} (1 - \lambda + \lambda^2 - \ldots) \\
&= \mathbf{A}^{-1} - \frac{(\mathbf{A}^{-1} \cdot \mathbf{u}) \otimes (\mathbf{v} \cdot \mathbf{A}^{-1})}{1 + \lambda}
\end{aligned} \tag{2.7.2}$$

where

$$\lambda \equiv \mathbf{v} \cdot \mathbf{A}^{-1} \cdot \mathbf{u} \tag{2.7.3}$$

The second line of (2.7.2) is a formal power series expansion. In the third line, the associativity of outer and inner products is used to factor out the scalars λ.

The use of (2.7.2) is this: Given $\mathbf{A}^{-1}$ and the vectors $\mathbf{u}$ and $\mathbf{v}$, we need only perform two matrix multiplications and a vector dot product,

$$\mathbf{z} \equiv \mathbf{A}^{-1} \cdot \mathbf{u} \qquad \mathbf{w} \equiv (\mathbf{A}^{-1})^T \cdot \mathbf{v} \qquad \lambda = \mathbf{v} \cdot \mathbf{z} \tag{2.7.4}$$

to get the desired change in the inverse

$$\mathbf{A}^{-1} \quad \rightarrow \quad \mathbf{A}^{-1} - \frac{\mathbf{z} \otimes \mathbf{w}}{1 + \lambda} \tag{2.7.5}$$

The whole procedure requires only $3N^2$ multiplies and a like number of adds (an even smaller number if $\mathbf{u}$ or $\mathbf{v}$ is a unit vector).

The Sherman-Morrison formula can be directly applied to a class of sparse problems. If you already have a fast way of calculating the inverse of $\mathbf{A}$ (e.g., a tridiagonal matrix or some other standard sparse form), then (2.7.4) – (2.7.5) allow you to build up to your related but more complicated form, adding for example a row or column at a time. Notice that you can apply the Sherman-Morrison formula more than once successively, using at each stage the most recent update of $\mathbf{A}^{-1}$ (equation 2.7.5). Of course, if you have to modify *every* row, then you are back to an N^3 method. The constant in front of the N^3 is only a few times worse than the better direct methods, but you have deprived yourself of the stabilizing advantages of pivoting — so be careful.

For some other sparse problems, the Sherman-Morrison formula cannot be directly applied for the simple reason that storage of the whole inverse matrix $\mathbf{A}^{-1}$ is not feasible. If you want to add only a single correction of the form $\mathbf{u} \otimes \mathbf{v}$ and solve the linear system

$$(\mathbf{A} + \mathbf{u} \otimes \mathbf{v}) \cdot \mathbf{x} = \mathbf{b} \tag{2.7.6}$$

then you proceed as follows. Using the fast method that is presumed available for the matrix $\mathbf{A}$, solve the two auxiliary problems

$$\mathbf{A} \cdot \mathbf{y} = \mathbf{b} \qquad \mathbf{A} \cdot \mathbf{z} = \mathbf{u} \tag{2.7.7}$$

for the vectors $\mathbf{y}$ and $\mathbf{z}$. In terms of these,

$$\mathbf{x} = \mathbf{y} - \left[\frac{\mathbf{v} \cdot \mathbf{y}}{1 + (\mathbf{v} \cdot \mathbf{z})} \right] \mathbf{z} \tag{2.7.8}$$

as we see by multiplying (2.7.2) on the right by $\mathbf{b}$.

2.7.2 Cyclic Tridiagonal Systems

So-called *cyclic tridiagonal systems* occur quite frequently and are a good example of how to use the Sherman-Morrison formula in the manner just described. The equations have the form

$$\begin{bmatrix} b_0 & c_0 & 0 & \cdots & & & \beta \\ a_1 & b_1 & c_1 & \cdots & & & \\ & & & \cdots & & & \\ & & & \cdots & a_{N-2} & b_{N-2} & c_{N-2} \\ \alpha & & & \cdots & 0 & a_{N-1} & b_{N-1} \end{bmatrix} \cdot \begin{bmatrix} x_0 \\ x_1 \\ \cdots \\ x_{N-2} \\ x_{N-1} \end{bmatrix} = \begin{bmatrix} r_0 \\ r_1 \\ \cdots \\ r_{N-2} \\ r_{N-1} \end{bmatrix} \tag{2.7.9}$$

This is a tridiagonal system, except for the matrix elements α and β in the corners. Forms like this are typically generated by finite differencing differential equations with periodic boundary conditions (§20.4).

We use the Sherman-Morrison formula, treating the system as tridiagonal plus a correction. In the notation of equation (2.7.6), define vectors $\mathbf{u}$ and $\mathbf{v}$ to be

$$\mathbf{u} = \begin{bmatrix} \gamma \\ 0 \\ \vdots \\ 0 \\ \alpha \end{bmatrix} \qquad \mathbf{v} = \begin{bmatrix} 1 \\ 0 \\ \vdots \\ 0 \\ \beta/\gamma \end{bmatrix} \tag{2.7.10}$$

Here γ is arbitrary for the moment. Then the matrix $\mathbf{A}$ is the tridiagonal part of the matrix in (2.7.9), with two terms modified:

$$b'_0 = b_0 - \gamma, \qquad b'_{N-1} = b_{N-1} - \alpha\beta/\gamma \tag{2.7.11}$$

We now solve equations (2.7.7) with the standard tridiagonal algorithm and then get the solution from equation (2.7.8).

The routine `cyclic` below implements this algorithm. We choose the arbitrary parameter $\gamma = -b_0$ to avoid loss of precision by subtraction in the first of equations (2.7.11). In the unlikely event that this causes loss of precision in the second of these equations, you can make a different choice.

```
void cyclic(VecDoub_I &a, VecDoub_I &b, VecDoub_I &c, const Doub alpha,
    const Doub beta, VecDoub_I &r, VecDoub_O &x)
```
tridag.h

Solves for a vector `x[0..n-1]` the "cyclic" set of linear equations given by equation (2.7.9). `a`, `b`, `c`, and `r` are input vectors, all dimensioned as `[0..n-1]`, while `alpha` and `beta` are the corner entries in the matrix. The input is not modified.

```
{
    Int i,n=a.size();
    Doub fact,gamma;
    if (n <= 2) throw("n too small in cyclic");
```

```
    VecDoub bb(n),u(n),z(n);
    gamma = -b[0];                          Avoid subtraction error in forming bb[0].
    bb[0]=b[0]-gamma;                       Set up the diagonal of the modified tridi-
    bb[n-1]=b[n-1]-alpha*beta/gamma;            agonal system.
    for (i=1;i<n-1;i++) bb[i]=b[i];
    tridag(a,bb,c,r,x);                     Solve A · x = r.
    u[0]=gamma;                             Set up the vector u.
    u[n-1]=alpha;
    for (i=1;i<n-1;i++) u[i]=0.0;
    tridag(a,bb,c,u,z);                     Solve A · z = u.
    fact=(x[0]+beta*x[n-1]/gamma)/          Form v · x/(1 + v · z).
        (1.0+z[0]+beta*z[n-1]/gamma);
    for (i=0;i<n;i++) x[i] -= fact*z[i];    Now get the solution vector x.
}
```

2.7.3 Woodbury Formula

If you want to add more than a single correction term, then you cannot use (2.7.8) repeatedly, since without storing a new $\mathbf{A}^{-1}$ you will not be able to solve the auxiliary problems (2.7.7) efficiently after the first step. Instead, you need the *Woodbury formula*, which is the block-matrix version of the Sherman-Morrison formula,

$$\begin{aligned}(\mathbf{A} + \mathbf{U}\cdot\mathbf{V}^T)^{-1} \\ = \mathbf{A}^{-1} - \left[\mathbf{A}^{-1}\cdot\mathbf{U}\cdot(\mathbf{1}+\mathbf{V}^T\cdot\mathbf{A}^{-1}\cdot\mathbf{U})^{-1}\cdot\mathbf{V}^T\cdot\mathbf{A}^{-1}\right]\end{aligned} \tag{2.7.12}$$

Here A is, as usual, an $N \times N$ matrix, while $\mathbf{U}$ and $\mathbf{V}$ are $N \times P$ matrices with $P < N$ and usually $P \ll N$. The inner piece of the correction term may become clearer if written as the tableau,

$$\left[\begin{array}{c} \\ \\ \mathbf{U} \\ \\ \\ \end{array}\right] \cdot \left[\mathbf{1} + \mathbf{V}^T\cdot\mathbf{A}^{-1}\cdot\mathbf{U}\right]^{-1} \cdot \left[\qquad \mathbf{V}^T \qquad\right] \tag{2.7.13}$$

where you can see that the matrix whose inverse is needed is only $P \times P$ rather than $N \times N$.

The relation between the Woodbury formula and successive applications of the Sherman-Morrison formula is now clarified by noting that, if $\mathbf{U}$ is the matrix formed by columns out of the P vectors $\mathbf{u}_0, \ldots, \mathbf{u}_{P-1}$, and $\mathbf{V}$ is the matrix formed by columns out of the P vectors $\mathbf{v}_0, \ldots, \mathbf{v}_{P-1}$,

$$\mathbf{U} \equiv \left[\left[\mathbf{u}_0\right] \cdots \left[\mathbf{u}_{P-1}\right]\right] \qquad \mathbf{V} \equiv \left[\left[\mathbf{v}_0\right] \cdots \left[\mathbf{v}_{P-1}\right]\right] \tag{2.7.14}$$

then two ways of expressing the same correction to $\mathbf{A}$ are

$$\left(\mathbf{A} + \sum_{k=0}^{P-1} \mathbf{u}_k \otimes \mathbf{v}_k\right) = (\mathbf{A} + \mathbf{U}\cdot\mathbf{V}^T) \tag{2.7.15}$$

(Note that the subscripts on $\mathbf{u}$ and $\mathbf{v}$ do *not* denote components, but rather distinguish the different column vectors.)

Equation (2.7.15) reveals that, if you have $\mathbf{A}^{-1}$ in storage, then you can either make the P corrections in one fell swoop by using (2.7.12) and inverting a $P \times P$ matrix, or else make them by applying (2.7.5) P successive times.

If you don't have storage for $\mathbf{A}^{-1}$, then you *must* use (2.7.12) in the following way: To solve the linear equation

$$\left(\mathbf{A} + \sum_{k=0}^{P-1} \mathbf{u}_k \otimes \mathbf{v}_k\right) \cdot \mathbf{x} = \mathbf{b} \tag{2.7.16}$$

first solve the P auxiliary problems

$$\begin{aligned} \mathbf{A} \cdot \mathbf{z}_0 &= \mathbf{u}_0 \\ \mathbf{A} \cdot \mathbf{z}_1 &= \mathbf{u}_1 \\ &\cdots \\ \mathbf{A} \cdot \mathbf{z}_{P-1} &= \mathbf{u}_{P-1} \end{aligned} \tag{2.7.17}$$

and construct the matrix $\mathbf{Z}$ by columns from the $\mathbf{z}$'s obtained,

$$\mathbf{Z} \equiv \left[\begin{array}{c} \\ \mathbf{z}_0 \\ \\ \end{array}\right] \cdots \left[\begin{array}{c} \\ \mathbf{z}_{P-1} \\ \\ \end{array}\right] \tag{2.7.18}$$

Next, do the $P \times P$ matrix inversion

$$\mathbf{H} \equiv (\mathbf{1} + \mathbf{V}^T \cdot \mathbf{Z})^{-1} \tag{2.7.19}$$

Finally, solve the one further auxiliary problem

$$\mathbf{A} \cdot \mathbf{y} = \mathbf{b} \tag{2.7.20}$$

In terms of these quantities, the solution is given by

$$\mathbf{x} = \mathbf{y} - \mathbf{Z} \cdot \left[\mathbf{H} \cdot (\mathbf{V}^T \cdot \mathbf{y})\right] \tag{2.7.21}$$

2.7.4 Inversion by Partitioning

Once in a while, you will encounter a matrix (not even necessarily sparse) that can be inverted efficiently by partitioning. Suppose that the $N \times N$ matrix $\mathbf{A}$ is partitioned into

$$\mathbf{A} = \begin{bmatrix} \mathbf{P} & \mathbf{Q} \\ \mathbf{R} & \mathbf{S} \end{bmatrix} \tag{2.7.22}$$

where $\mathbf{P}$ and $\mathbf{S}$ are square matrices of size $p \times p$ and $s \times s$, respectively ($p + s = N$). The matrices $\mathbf{Q}$ and $\mathbf{R}$ are not necessarily square and have sizes $p \times s$ and $s \times p$, respectively.

If the inverse of $\mathbf{A}$ is partitioned in the same manner,

$$\mathbf{A}^{-1} = \begin{bmatrix} \widetilde{\mathbf{P}} & \widetilde{\mathbf{Q}} \\ \widetilde{\mathbf{R}} & \widetilde{\mathbf{S}} \end{bmatrix} \tag{2.7.23}$$

then $\widetilde{\mathbf{P}}$, $\widetilde{\mathbf{Q}}$, $\widetilde{\mathbf{R}}$, $\widetilde{\mathbf{S}}$, which have the same sizes as $\mathbf{P}$, $\mathbf{Q}$, $\mathbf{R}$, $\mathbf{S}$, respectively, can be found by either the formulas

$$\begin{aligned}
\widetilde{\mathbf{P}} &= (\mathbf{P} - \mathbf{Q}\cdot\mathbf{S}^{-1}\cdot\mathbf{R})^{-1} \\
\widetilde{\mathbf{Q}} &= -(\mathbf{P} - \mathbf{Q}\cdot\mathbf{S}^{-1}\cdot\mathbf{R})^{-1}\cdot(\mathbf{Q}\cdot\mathbf{S}^{-1}) \\
\widetilde{\mathbf{R}} &= -(\mathbf{S}^{-1}\cdot\mathbf{R})\cdot(\mathbf{P} - \mathbf{Q}\cdot\mathbf{S}^{-1}\cdot\mathbf{R})^{-1} \\
\widetilde{\mathbf{S}} &= \mathbf{S}^{-1} + (\mathbf{S}^{-1}\cdot\mathbf{R})\cdot(\mathbf{P} - \mathbf{Q}\cdot\mathbf{S}^{-1}\cdot\mathbf{R})^{-1}\cdot(\mathbf{Q}\cdot\mathbf{S}^{-1})
\end{aligned} \tag{2.7.24}$$

or else by the equivalent formulas

$$\begin{aligned}
\widetilde{\mathbf{P}} &= \mathbf{P}^{-1} + (\mathbf{P}^{-1}\cdot\mathbf{Q})\cdot(\mathbf{S} - \mathbf{R}\cdot\mathbf{P}^{-1}\cdot\mathbf{Q})^{-1}\cdot(\mathbf{R}\cdot\mathbf{P}^{-1}) \\
\widetilde{\mathbf{Q}} &= -(\mathbf{P}^{-1}\cdot\mathbf{Q})\cdot(\mathbf{S} - \mathbf{R}\cdot\mathbf{P}^{-1}\cdot\mathbf{Q})^{-1} \\
\widetilde{\mathbf{R}} &= -(\mathbf{S} - \mathbf{R}\cdot\mathbf{P}^{-1}\cdot\mathbf{Q})^{-1}\cdot(\mathbf{R}\cdot\mathbf{P}^{-1}) \\
\widetilde{\mathbf{S}} &= (\mathbf{S} - \mathbf{R}\cdot\mathbf{P}^{-1}\cdot\mathbf{Q})^{-1}
\end{aligned} \tag{2.7.25}$$

The parentheses in equations (2.7.24) and (2.7.25) highlight repeated factors that you may wish to compute only once. (Of course, by associativity, you can instead do the matrix multiplications in any order you like.) The choice between using equations (2.7.24) and (2.7.25) depends on whether you want $\widetilde{\mathbf{P}}$ or $\widetilde{\mathbf{S}}$ to have the simpler formula; or on whether the repeated expression $(\mathbf{S}-\mathbf{R}\cdot\mathbf{P}^{-1}\cdot\mathbf{Q})^{-1}$ is easier to calculate than the expression $(\mathbf{P}-\mathbf{Q}\cdot\mathbf{S}^{-1}\cdot\mathbf{R})^{-1}$; or on the relative sizes of $\mathbf{P}$ and $\mathbf{S}$; or on whether $\mathbf{P}^{-1}$ or $\mathbf{S}^{-1}$ is already known.

Another sometimes useful formula is for the determinant of the partitioned matrix,

$$\det\mathbf{A} = \det\mathbf{P}\,\det(\mathbf{S} - \mathbf{R}\cdot\mathbf{P}^{-1}\cdot\mathbf{Q}) = \det\mathbf{S}\,\det(\mathbf{P} - \mathbf{Q}\cdot\mathbf{S}^{-1}\cdot\mathbf{R}) \tag{2.7.26}$$

2.7.5 Indexed Storage of Sparse Matrices

We have already seen (§2.4) that tri- or band-diagonal matrices can be stored in a compact format that allocates storage only to elements that can be nonzero, plus perhaps a few wasted locations to make the bookkeeping easier. What about more general sparse matrices? When a sparse matrix of dimension $M \times N$ contains only a few times M or N nonzero elements (a typical case), it is surely inefficient — and often physically impossible — to allocate storage for all MN elements. Even if one did allocate such storage, it would be inefficient or prohibitive in machine time to loop over all of it in search of nonzero elements.

Obviously some kind of indexed storage scheme is required, one that stores only nonzero matrix elements, along with sufficient auxiliary information to determine where an element logically belongs and how the various elements can be looped over in common matrix operations. Unfortunately, there is no one standard scheme in general use. Each scheme has its own pluses and minuses, depending on the application.

Before we look at sparse matrices, let's consider the simpler problem of a *sparse vector*. The obvious data structure is a list of the nonzero values and another list of the corresponding locations:

sparse.h

```
struct NRsparseCol
Sparse vector data structure.
{
    Int nrows;                              Number of rows.
    Int nvals;                              Maximum number of nonzeros.
```

```
    VecInt row_ind;                              Row indices of nonzeros.
    VecDoub val;                                 Array of nonzero values.

    NRsparseCol(Int m,Int nnvals) : nrows(m), nvals(nnvals),
    row_ind(nnvals,0),val(nnvals,0.0) {}         Constructor. Initializes vector to zero.

    NRsparseCol() : nrows(0),nvals(0),row_ind(),val() {}     Default constructor.

    void resize(Int m, Int nnvals) {
        nrows = m;
        nvals = nnvals;
        row_ind.assign(nnvals,0);
        val.assign(nnvals,0.0);
    }

};
```

While we think of this as defining a column vector, you can use exactly the same data structure for a row vector — just mentally interchange the meaning of row and column for the variables. For matrices, however, we have to decide ahead of time whether to use row-oriented or column-oriented storage.

One simple scheme is to use a vector of sparse columns:

```
    NRvector<NRsparseCol *> a;
    for (i=0;i<n;i++) {
        nvals=...
        a[i]=new NRsparseCol(m,nvals);
    }
```

Each column is filled with statements like

```
    count=0;
    for (j=...) {
        a[i]->row_ind[count]=...
        a[i]->val[count]=...
        count++;
    }
```

This data structure is good for an algorithm that primarily works with columns of the matrix, but it is not very efficient when one needs to loop over all elements of the matrix.

A good general storage scheme is the *compressed column storage* format. It is sometimes called the Harwell-Boeing format, after the two large organizations that first systematically provided a standard collection of sparse matrices for research purposes. In this scheme, three vectors are used: `val` for the nonzero values as they are traversed column by column, `row_ind` for the corresponding row indices of each value, and `col_ptr` for the locations in the other two arrays that start a column. In other words, if `val[k]=a[i][j]`, then `row_ind[k]=i`. The first nonzero in column `j` is at `col_ptr[j]`. The last is at `col_ptr[j+1]-1`. Note that `col_ptr[0]` is always 0, and by convention we define `col_ptr[n]` equal to the number of nonzeros. Note also that the dimension of the `col_ptr` array is $N+1$, not N. The advantage of this scheme is that it requires storage of only about two times the number of nonzero matrix elements. (Other methods can require as much as three or five times.)

As an example, consider the matrix

$$\begin{bmatrix} 3.0 & 0.0 & 1.0 & 2.0 & 0.0 \\ 0.0 & 4.0 & 0.0 & 0.0 & 0.0 \\ 0.0 & 7.0 & 5.0 & 9.0 & 0.0 \\ 0.0 & 0.0 & 0.0 & 0.0 & 0.0 \\ 0.0 & 0.0 & 0.0 & 6.0 & 5.0 \end{bmatrix} \tag{2.7.27}$$

In compressed column storage mode, matrix (2.7.27) is represented by two arrays of length 9 and an array of length 6, as follows

index k	0	1	2	3	4	5	6	7	8
val[k]	3.0	4.0	7.0	1.0	5.0	2.0	9.0	6.0	5.0
row_ind[k]	0	1	2	0	2	0	2	4	4

index i	0	1	2	3	4	5
col_ptr[i]	0	1	3	5	8	9

(2.7.28)

Notice that, according to the storage rules, the value of N (namely 5) is the maximum valid index in col_ptr. The value of col_ptr[5] is 9, the length of the other two arrays. The elements 1.0 and 5.0 in column number 2, for example, are located in positions col_ptr[2] $\leq k <$ col_ptr[3].

Here is a data structure to handle this storage scheme:

sparse.h
```
struct NRsparseMat
Sparse matrix data structure for compressed column storage.
{
    Int nrows;                          Number of rows.
    Int ncols;                          Number of columns.
    Int nvals;                          Maximum number of nonzeros.
    VecInt col_ptr;                     Pointers to start of columns. Length is ncols+1.
    VecInt row_ind;                     Row indices of nonzeros.
    VecDoub val;                        Array of nonzero values.

    NRsparseMat();                             Default constructor.
    NRsparseMat(Int m,Int n,Int nnvals);       Constructor. Initializes vector to zero.
    VecDoub ax(const VecDoub &x) const;        Multiply A by a vector x[0..ncols-1].
    VecDoub atx(const VecDoub &x) const;       Multiply A^T by a vector x[0..nrows-1].
    NRsparseMat transpose() const;             Form A^T.
};
```

The code for the constructors is standard:

sparse.h
```
NRsparseMat::NRsparseMat() : nrows(0),ncols(0),nvals(0),col_ptr(),
    row_ind(),val() {}
NRsparseMat::NRsparseMat(Int m,Int n,Int nnvals) : nrows(m),ncols(n),
    nvals(nnvals),col_ptr(n+1,0),row_ind(nnvals,0),val(nnvals,0.0) {}
```

The single most important use of a matrix in compressed column storage mode is to multiply a vector to its right. Don't implement this by traversing the rows of $\mathbf{A}$, which is extremely inefficient in this storage mode. Here's the right way to do it:

sparse.h
```
VecDoub NRsparseMat::ax(const VecDoub &x) const {
    VecDoub y(nrows,0.0);
    for (Int j=0;j<ncols;j++) {
        for (Int i=col_ptr[j];i<col_ptr[j+1];i++)
            y[row_ind[i]] += val[i]*x[j];
    }
    return y;
}
```

Some inefficiency occurs because of the indirect addressing. While there are other storage modes that minimize this, they have their own drawbacks.

It is also simple to multiply the *transpose* of a matrix by a vector to its right, since we just traverse the columns directly. (Indirect addressing is still required.) Note that the transpose matrix is not actually constructed.

sparse.h
```
VecDoub NRsparseMat::atx(const VecDoub &x) const {
    VecDoub y(ncols);
    for (Int i=0;i<ncols;i++) {
        y[i]=0.0;
        for (Int j=col_ptr[i];j<col_ptr[i+1];j++)
            y[i] += val[j]*x[row_ind[j]];
    }
    return y;
}
```

Because the choice of compressed column storage treats rows and columns quite differently, it is rather an involved operation to construct the transpose of a matrix, given the matrix itself in compressed column storage mode. When the operation cannot be avoided, it is

sparse.h
```
NRsparseMat NRsparseMat::transpose() const {
    Int i,j,k,index,m=nrows,n=ncols;
    NRsparseMat at(n,m,nvals);                  Initialized to zero.
    First find the column lengths for A^T, i.e. the row lengths of A.
    VecInt count(m,0);                          Temporary counters for each row of A.
    for (i=0;i<n;i++)
        for (j=col_ptr[i];j<col_ptr[i+1];j++) {
            k=row_ind[j];
            count[k]++;
        }
    for (j=0;j<m;j++)                           Now set at.col_ptr. 0th entry stays 0.
        at.col_ptr[j+1]=at.col_ptr[j]+count[j];
    for(j=0;j<m;j++)                            Reset counters to zero.
        count[j]=0;
    for (i=0;i<n;i++)                           Main loop.
        for (j=col_ptr[i];j<col_ptr[i+1];j++) {
            k=row_ind[j];
            index=at.col_ptr[k]+count[k];       Element's position in column of A^T.
            at.row_ind[index]=i;
            at.val[index]=val[j];
            count[k]++;                         Increment counter for next element in that
        }                                           column.
    return at;
}
```

The only sparse matrix-matrix multiply routine we give is to form the product $\mathbf{ADA}^T$, where $\mathbf{D}$ is a diagonal matrix. This particular product is used to form the so-called normal equations in the interior-point method for linear programming (§10.11). We encapsulate the algorithm in its own structure, ADAT:

sparse.h
```
struct ADAT {
    const NRsparseMat &a,&at;                   Store references to A and A^T.
    NRsparseMat *adat;                          This will hold ADA^T.

    ADAT(const NRsparseMat &A,const NRsparseMat &AT);
    Allocates compressed column storage for AA^T, where A and A^T are input in compressed
    column format, and fills in values of col_ptr and row_ind. Each column must be in sorted
    order in input matrices. Matrix is output with each column sorted.
    void updateD(const VecDoub &D);
    Computes ADA^T, where D is a diagonal matrix. This function can be called repeatedly
    to update ADA^T for fixed A.
    NRsparseMat &ref();
    Returns reference to adat, which holds ADA^T.
    ~ADAT();
};
```

The algorithm proceeds in two steps. First, the nonzero pattern of $\mathbf{AA}^T$ is found by a call to the constructor. Since $\mathbf{D}$ is diagonal, $\mathbf{AA}^T$ and $\mathbf{ADA}^T$ have the same nonzero structure. Algorithms using `ADAT` will typically have both $\mathbf{A}$ and $\mathbf{A}^T$ available, so we pass them both to the constructor rather than recompute $\mathbf{A}^T$ from $\mathbf{A}$. The constructor allocates storage and assigns values to `col_ptr` and `row_ind`. The structure of `ADAT` is returned with columns in sorted order because routines like the AMD ordering algorithm used in §10.11 require it.

sparse.h

```
ADAT::ADAT(const NRsparseMat &A,const NRsparseMat &AT) : a(A), at(AT) {
    Int h,i,j,k,l,nvals,m=AT.ncols;
    VecInt done(m);
    for (i=0;i<m;i++)                       Initialize to not done.
        done[i]=-1;
    nvals=0;                                First pass determines number of nonzeros.
    for (j=0;j<m;j++) {                     Outer loop over columns of A^T in AA^T.
        for (i=AT.col_ptr[j];i<AT.col_ptr[j+1];i++) {
            k=AT.row_ind[i];                A^T_kj ≠ 0. Find column k in first matrix, A.
            for (l=A.col_ptr[k];l<A.col_ptr[k+1];l++) {
                h=A.row_ind[l];             A_hl ≠ 0.
                if (done[h] != j) {         Test if contribution already included.
                    done[h]=j;
                    nvals++;
                }
            }
        }
    }
    adat = new NRsparseMat(m,m,nvals);      Allocate storage for ADAT.
    for (i=0;i<m;i++)                       Re-initialize.
        done[i]=-1;
    nvals=0;
```

Second pass: Determine columns of `adat`. Code is identical to first pass except `adat->col_ptr` and `adat->row_ind` get assigned at appropriate places.

```
    for (j=0;j<m;j++) {
        adat->col_ptr[j]=nvals;
        for (i=AT.col_ptr[j];i<AT.col_ptr[j+1];i++) {
            k=AT.row_ind[i];
            for (l=A.col_ptr[k];l<A.col_ptr[k+1];l++) {
                h=A.row_ind[l];
                if (done[h] != j) {
                    done[h]=j;
                    adat->row_ind[nvals]=h;
                    nvals++;
                }
            }
        }
    }
    adat->col_ptr[m]=nvals;                 Set last value.
    for (j=0;j<m;j++) {                     Sort columns
        i=adat->col_ptr[j];
        Int size=adat->col_ptr[j+1]-i;
        if (size > 1) {
            VecInt col(size,&adat->row_ind[i]);
            sort(col);
            for (k=0;k<size;k++)
                adat->row_ind[i+k]=col[k];
        }
    }
}
```

The next routine, `updateD`, actually fills in the values in the `val` array. It can be called repeatedly to update $\mathbf{ADA}^T$ for fixed $\mathbf{A}$.

sparse.h

```
void ADAT::updateD(const VecDoub &D) {
    Int h,i,j,k,l,m=a.nrows,n=a.ncols;
    VecDoub temp(n),temp2(m,0.0);
    for (i=0;i<m;i++) {                          Outer loop over columns of A^T.
        for (j=at.col_ptr[i];j< at.col_ptr[i+1];j++) {
            k=at.row_ind[j];                     Scale elements of each column with D and
            temp[k]=at.val[j]*D[k];                  store in temp.
        }
        for (j=at.col_ptr[i];j<at.col_ptr[i+1];j++) {       Go down column again.
            k=at.row_ind[j];
            for (l=a.col_ptr[k];l<a.col_ptr[k+1];l++) {     Go down column k in
                h=a.row_ind[l];                                 A.
                temp2[h] += temp[k]*a.val[l];     All terms from temp[k] used here.
            }
        }
        for (j=adat->col_ptr[i];j<adat->col_ptr[i+1];j++) {
        Store temp2 in column of answer.
            k=adat->row_ind[j];
            adat->val[j]=temp2[k];
            temp2[k]=0.0;                        Restore temp2.
        }
    }
}
```

The final two functions are simple. The `ref` routine returns a reference to the matrix $\mathbf{ADA}^T$ stored in the structure for other routines that may need to work with it. And the destructor releases the storage.

sparse.h

```
NRsparseMat & ADAT::ref() {
    return *adat;
}

ADAT::~ADAT() {
    delete adat;
}
```

By the way, if you invoke `ADAT` with *different* matrices $\mathbf{A}$ and $\mathbf{B}^T$, everything will work fine as long as $\mathbf{A}$ and $\mathbf{B}$ have the same nonzero pattern.

In *Numerical Recipes* second edition, we gave a related sparse matrix storage mode in which the diagonal of the matrix is stored first, followed by the off-diagonal elements. We now feel that the added complexity of that scheme is not worthwhile for any of the uses in this book. For a discussion of this and other storage schemes, see [7,8]. To see how to work with the diagonal in the compressed column mode, look at the code for `asolve` at the end of this section.

2.7.6 Conjugate Gradient Method for a Sparse System

So-called *conjugate gradient methods* provide a quite general means for solving the $N \times N$ linear system

$$\mathbf{A} \cdot \mathbf{x} = \mathbf{b} \tag{2.7.29}$$

The attractiveness of these methods for large sparse systems is that they reference $\mathbf{A}$ only through its multiplication of a vector, or the multiplication of its transpose and a vector. As we have seen, these operations can be very efficient for a properly stored sparse matrix. You, the "owner" of the matrix $\mathbf{A}$, can be asked to provide functions that perform these sparse matrix multiplications as efficiently as possible. We, the "grand strategists," supply an abstract base class, `Linbcg` below, that contains the method for solving the set of linear equations, (2.7.29), using your functions.

The simplest, "ordinary" conjugate gradient algorithm [9-11] solves (2.7.29) only in the case that $\mathbf{A}$ is symmetric and positive-definite. It is based on the idea of minimizing the function

$$f(\mathbf{x}) = \frac{1}{2}\, \mathbf{x} \cdot \mathbf{A} \cdot \mathbf{x} - \mathbf{b} \cdot \mathbf{x} \tag{2.7.30}$$

This function is minimized when its gradient

$$\nabla f = \mathbf{A} \cdot \mathbf{x} - \mathbf{b} \tag{2.7.31}$$

is zero, which is equivalent to (2.7.29). The minimization is carried out by generating a succession of search directions $\mathbf{p}_k$ and improved minimizers $\mathbf{x}_k$. At each stage a quantity α_k is found that minimizes $f(\mathbf{x}_k + \alpha_k \mathbf{p}_k)$, and $\mathbf{x}_{k+1}$ is set equal to the new point $\mathbf{x}_k + \alpha_k \mathbf{p}_k$. The $\mathbf{p}_k$ and $\mathbf{x}_k$ are built up in such a way that $\mathbf{x}_{k+1}$ is also the minimizer of f over the whole vector space of directions already taken, $\{\mathbf{p}_0, \mathbf{p}_1, \ldots, \mathbf{p}_{k-1}\}$. After N iterations you arrive at the minimizer over the entire vector space, i.e., the solution to (2.7.29).

Later, in §10.8, we will generalize this "ordinary" conjugate gradient algorithm to the minimization of arbitrary nonlinear functions. Here, where our interest is in solving linear, but not necessarily positive-definite or symmetric, equations, a different generalization is important, the *biconjugate gradient method*. This method does not, in general, have a simple connection with function minimization. It constructs four sequences of vectors, $\mathbf{r}_k$, $\overline{\mathbf{r}}_k$, $\mathbf{p}_k$, $\overline{\mathbf{p}}_k$, $k = 0, 1, \ldots$. You supply the initial vectors $\mathbf{r}_0$ and $\overline{\mathbf{r}}_0$, and set $\mathbf{p}_0 = \mathbf{r}_0$, $\overline{\mathbf{p}}_0 = \overline{\mathbf{r}}_0$. Then you carry out the following recurrence:

$$\begin{aligned}
\alpha_k &= \frac{\overline{\mathbf{r}}_k \cdot \mathbf{r}_k}{\overline{\mathbf{p}}_k \cdot \mathbf{A} \cdot \mathbf{p}_k} \\
\mathbf{r}_{k+1} &= \mathbf{r}_k - \alpha_k \mathbf{A} \cdot \mathbf{p}_k \\
\overline{\mathbf{r}}_{k+1} &= \overline{\mathbf{r}}_k - \alpha_k \mathbf{A}^T \cdot \overline{\mathbf{p}}_k \\
\beta_k &= \frac{\overline{\mathbf{r}}_{k+1} \cdot \mathbf{r}_{k+1}}{\overline{\mathbf{r}}_k \cdot \mathbf{r}_k} \\
\mathbf{p}_{k+1} &= \mathbf{r}_{k+1} + \beta_k \mathbf{p}_k \\
\overline{\mathbf{p}}_{k+1} &= \overline{\mathbf{r}}_{k+1} + \beta_k \overline{\mathbf{p}}_k
\end{aligned} \tag{2.7.32}$$

This sequence of vectors satisfies the *biorthogonality* condition

$$\overline{\mathbf{r}}_i \cdot \mathbf{r}_j = \mathbf{r}_i \cdot \overline{\mathbf{r}}_j = 0, \qquad j < i \tag{2.7.33}$$

and the *biconjugacy* condition

$$\overline{\mathbf{p}}_i \cdot \mathbf{A} \cdot \mathbf{p}_j = \mathbf{p}_i \cdot \mathbf{A}^T \cdot \overline{\mathbf{p}}_j = 0, \qquad j < i \tag{2.7.34}$$

There is also a mutual orthogonality,

$$\overline{\mathbf{r}}_i \cdot \mathbf{p}_j = \mathbf{r}_i \cdot \overline{\mathbf{p}}_j = 0, \qquad j < i \tag{2.7.35}$$

The proof of these properties proceeds by straightforward induction [12]. As long as the recurrence does not break down earlier because one of the denominators is zero, it must terminate after $m \leq N$ steps with $\mathbf{r}_m = \overline{\mathbf{r}}_m = 0$. This is basically because after at most N steps you run out of new orthogonal directions to the vectors you've already constructed.

To use the algorithm to solve the system (2.7.29), make an initial guess $\mathbf{x}_0$ for the solution. Choose $\mathbf{r}_0$ to be the *residual*

$$\mathbf{r}_0 = \mathbf{b} - \mathbf{A} \cdot \mathbf{x}_0 \tag{2.7.36}$$

and choose $\overline{\mathbf{r}}_0 = \mathbf{r}_0$. Then form the sequence of improved estimates

$$\mathbf{x}_{k+1} = \mathbf{x}_k + \alpha_k \mathbf{p}_k \tag{2.7.37}$$

while carrying out the recurrence (2.7.32). Equation (2.7.37) guarantees that $\mathbf{r}_{k+1}$ from the recurrence is in fact the residual $\mathbf{b} - \mathbf{A} \cdot \mathbf{x}_{k+1}$ corresponding to $\mathbf{x}_{k+1}$. Since $\mathbf{r}_m = 0$, $\mathbf{x}_m$ is the solution to equation (2.7.29).

While there is no guarantee that this whole procedure will not break down or become unstable for general $\mathbf{A}$, in practice this is rare. More importantly, the exact termination in at most N iterations occurs only with exact arithmetic. Roundoff error means that you should

regard the process as a genuinely iterative procedure, to be halted when some appropriate error criterion is met.

The ordinary conjugate gradient algorithm is the special case of the biconjugate gradient algorithm when $\mathbf{A}$ is symmetric, and we choose $\overline{\mathbf{r}}_0 = \mathbf{r}_0$. Then $\overline{\mathbf{r}}_k = \mathbf{r}_k$ and $\overline{\mathbf{p}}_k = \mathbf{p}_k$ for all k; you can omit computing them and halve the work of the algorithm. This conjugate gradient version has the interpretation of minimizing equation (2.7.30). If $\mathbf{A}$ is positive-definite as well as symmetric, the algorithm cannot break down (in theory!). The `solve` routine `Linbcg` below indeed reduces to the ordinary conjugate gradient method if you input a symmetric $\mathbf{A}$, but it does all the redundant computations.

Another variant of the general algorithm corresponds to a symmetric but non-positive-definite $\mathbf{A}$, with the choice $\overline{\mathbf{r}}_0 = \mathbf{A} \cdot \mathbf{r}_0$ instead of $\overline{\mathbf{r}}_0 = \mathbf{r}_0$. In this case $\overline{\mathbf{r}}_k = \mathbf{A} \cdot \mathbf{r}_k$ and $\overline{\mathbf{p}}_k = \mathbf{A} \cdot \mathbf{p}_k$ for all k. This algorithm is thus equivalent to the ordinary conjugate gradient algorithm, but with all dot products $\mathbf{a} \cdot \mathbf{b}$ replaced by $\mathbf{a} \cdot \mathbf{A} \cdot \mathbf{b}$. It is called the *minimum residual* algorithm, because it corresponds to successive minimizations of the function

$$\Phi(\mathbf{x}) = \frac{1}{2}\, \mathbf{r} \cdot \mathbf{r} = \frac{1}{2}\, |\mathbf{A} \cdot \mathbf{x} - \mathbf{b}|^2 \tag{2.7.38}$$

where the successive iterates $\mathbf{x}_k$ minimize Φ over the same set of search directions $\mathbf{p}_k$ generated in the conjugate gradient method. This algorithm has been generalized in various ways for unsymmetric matrices. The *generalized minimum residual* method (GMRES; see [13,14]) is probably the most robust of these methods.

Note that equation (2.7.38) gives

$$\nabla\Phi(\mathbf{x}) = \mathbf{A}^T \cdot (\mathbf{A} \cdot \mathbf{x} - \mathbf{b}) \tag{2.7.39}$$

For any nonsingular matrix $\mathbf{A}$, $\mathbf{A}^T \cdot \mathbf{A}$ is symmetric and positive-definite. You might therefore be tempted to solve equation (2.7.29) by applying the ordinary conjugate gradient algorithm to the problem

$$(\mathbf{A}^T \cdot \mathbf{A}) \cdot \mathbf{x} = \mathbf{A}^T \cdot \mathbf{b} \tag{2.7.40}$$

Don't! The condition number of the matrix $\mathbf{A}^T \cdot \mathbf{A}$ is the square of the condition number of $\mathbf{A}$ (see §2.6 for definition of condition number). A large condition number both increases the number of iterations required and limits the accuracy to which a solution can be obtained. It is almost always better to apply the biconjugate gradient method to the original matrix $\mathbf{A}$.

So far we have said nothing about the *rate* of convergence of these methods. The ordinary conjugate gradient method works well for matrices that are well-conditioned, i.e., "close" to the identity matrix. This suggests applying these methods to the *preconditioned* form of equation (2.7.29),

$$(\widetilde{\mathbf{A}}^{-1} \cdot \mathbf{A}) \cdot \mathbf{x} = \widetilde{\mathbf{A}}^{-1} \cdot \mathbf{b} \tag{2.7.41}$$

The idea is that you might already be able to solve your linear system easily for some $\widetilde{\mathbf{A}}$ close to $\mathbf{A}$, in which case $\widetilde{\mathbf{A}}^{-1} \cdot \mathbf{A} \approx \mathbf{1}$, allowing the algorithm to converge in fewer steps. The matrix $\widetilde{\mathbf{A}}$ is called a *preconditioner* [9], and the overall scheme given here is known as the *preconditioned biconjugate gradient method* or *PBCG*. In the code below, the user-supplied routine `atimes` does sparse matrix multiplication by $\mathbf{A}$, while the user-supplied routine `asolve` effects matrix multiplication by the inverse of the preconditioner $\widetilde{\mathbf{A}}^{-1}$.

For efficient implementation, the PBCG algorithm introduces an additional set of vectors $\mathbf{z}_k$ and $\overline{\mathbf{z}}_k$ defined by

$$\widetilde{\mathbf{A}} \cdot \mathbf{z}_k = \mathbf{r}_k \qquad \text{and} \qquad \widetilde{\mathbf{A}}^T \cdot \overline{\mathbf{z}}_k = \overline{\mathbf{r}}_k \tag{2.7.42}$$

and modifies the definitions of α_k, β_k, $\mathbf{p}_k$, and $\overline{\mathbf{p}}_k$ in equation (2.7.32):

$$\begin{aligned}
\alpha_k &= \frac{\overline{\mathbf{r}}_k \cdot \mathbf{z}_k}{\overline{\mathbf{p}}_k \cdot \mathbf{A} \cdot \mathbf{p}_k} \\
\beta_k &= \frac{\overline{\mathbf{r}}_{k+1} \cdot \mathbf{z}_{k+1}}{\overline{\mathbf{r}}_k \cdot \mathbf{z}_k} \\
\mathbf{p}_{k+1} &= \mathbf{z}_{k+1} + \beta_k \mathbf{p}_k \\
\overline{\mathbf{p}}_{k+1} &= \overline{\mathbf{z}}_{k+1} + \beta_k \overline{\mathbf{p}}_k
\end{aligned} \tag{2.7.43}$$

To use `Linbcg`, below, you will need to supply routines that solve the auxiliary linear systems (2.7.42). If you have no idea what to use for the preconditioner $\widetilde{\mathbf{A}}$, then use the diagonal part of $\mathbf{A}$, or even the identity matrix, in which case the burden of convergence will be entirely on the biconjugate gradient method itself.

`Linbcg`'s routine `solve`, below, is based on a program originally written by Anne Greenbaum. (See [11] for a different, less sophisticated, implementation.) There are a few wrinkles you should know about.

What constitutes "good" convergence is rather application-dependent. The routine `solve` therefore provides for four possibilities, selected by setting the flag `itol` on input. If `itol=1`, iteration stops when the quantity $|\mathbf{A}\cdot\mathbf{x}-\mathbf{b}|/|\mathbf{b}|$ is less than the input quantity `tol`. If `itol=2`, the required criterion is

$$|\widetilde{\mathbf{A}}^{-1}\cdot(\mathbf{A}\cdot\mathbf{x}-\mathbf{b})|/|\widetilde{\mathbf{A}}^{-1}\cdot\mathbf{b}| < \mathtt{tol} \tag{2.7.44}$$

If `itol=3`, the routine uses its own estimate of the error in $\mathbf{x}$ and requires its magnitude, divided by the magnitude of $\mathbf{x}$, to be less than `tol`. The setting `itol=4` is the same as `itol=3`, except that the largest (in absolute value) component of the error and largest component of $\mathbf{x}$ are used instead of the vector magnitude (that is, the L_∞ norm instead of the L_2 norm). You may need to experiment to find which of these convergence criteria is best for your problem.

On output, `err` is the tolerance actually achieved. If the returned count `iter` does not indicate that the maximum number of allowed iterations `itmax` was exceeded, then `err` should be less than `tol`. If you want to do further iterations, leave all returned quantities as they are and call the routine again. The routine loses its memory of the spanned conjugate gradient subspace between calls, however, so you should not force it to return more often than about every N iterations.

linbcg.h

```
struct Linbcg {
```
Abstract base class for solving sparse linear equations by the preconditioned biconjugate gradient method. To use, declare a derived class in which the methods `atimes` and `asolve` are defined for your problem, along with any data that they need. Then call the `solve` method.
```
    virtual void asolve(VecDoub_I &b, VecDoub_O &x, const Int itrnsp) = 0;
    virtual void atimes(VecDoub_I &x, VecDoub_O &r, const Int itrnsp) = 0;
    void solve(VecDoub_I &b, VecDoub_IO &x, const Int itol, const Doub tol,
        const Int itmax, Int &iter, Doub &err);
    Doub snrm(VecDoub_I &sx, const Int itol);        Utility used by solve.
};

void Linbcg::solve(VecDoub_I &b, VecDoub_IO &x, const Int itol, const Doub tol,
    const Int itmax, Int &iter, Doub &err)
```
Solves $\mathbf{A}\cdot\mathbf{x}=\mathbf{b}$ for `x[0..n-1]`, given `b[0..n-1]`, by the iterative biconjugate gradient method. On input `x[0..n-1]` should be set to an initial guess of the solution (or all zeros); `itol` is 1,2,3, or 4, specifying which convergence test is applied (see text); `itmax` is the maximum number of allowed iterations; and `tol` is the desired convergence tolerance. On output, `x[0..n-1]` is reset to the improved solution, `iter` is the number of iterations actually taken, and `err` is the estimated error. The matrix $\mathbf{A}$ is referenced only through the user-supplied routines `atimes`, which computes the product of either $\mathbf{A}$ or its transpose on a vector, and `asolve`, which solves $\widetilde{\mathbf{A}}\cdot\mathbf{x}=\mathbf{b}$ or $\widetilde{\mathbf{A}}^T\cdot\mathbf{x}=\mathbf{b}$ for some preconditioner matrix $\widetilde{\mathbf{A}}$ (possibly the trivial diagonal part of $\mathbf{A}$). This routine can be called repeatedly, with `itmax`$\lesssim$`n`, to monitor how `err` decreases; or it can be called once with a sufficiently large value of `itmax` so that convergence to `tol` is achieved.
```
{
    Doub ak,akden,bk,bkden=1.0,bknum,bnrm,dxnrm,xnrm,zm1nrm,znrm;
    const Doub EPS=1.0e-14;
    Int j,n=b.size();
    VecDoub p(n),pp(n),r(n),rr(n),z(n),zz(n);
    iter=0;                              Calculate initial residual.
    atimes(x,r,0);                       Input to atimes is x[0..n-1], output is r[0..n-1];
    for (j=0;j<n;j++) {                      the final 0 indicates that the matrix (not its
        r[j]=b[j]-r[j];                      transpose) is to be used.
        rr[j]=r[j];
    }
```

```
//atimes(r,rr,0);                     Uncomment this line to get the "minimum resid-
if (itol == 1) {                          ual" variant of the algorithm.
    bnrm=snrm(b,itol);
    asolve(r,z,0);                    Input to asolve is r[0..n-1], output is z[0..n-1];
}                                         the final 0 indicates that the matrix Ã (not
else if (itol == 2) {                     its transpose) is to be used.
    asolve(b,z,0);
    bnrm=snrm(z,itol);
    asolve(r,z,0);
}
else if (itol == 3 || itol == 4) {
    asolve(b,z,0);
    bnrm=snrm(z,itol);
    asolve(r,z,0);
    znrm=snrm(z,itol);
} else throw("illegal itol in linbcg");
while (iter < itmax) {                Main loop.
    ++iter;
    asolve(rr,zz,1);                  Final 1 indicates use of transpose matrix Ã^T.
    for (bknum=0.0,j=0;j<n;j++) bknum += z[j]*rr[j];
    Calculate coefficient bk and direction vectors p and pp.
    if (iter == 1) {
        for (j=0;j<n;j++) {
            p[j]=z[j];
            pp[j]=zz[j];
        }
    } else {
        bk=bknum/bkden;
        for (j=0;j<n;j++) {
            p[j]=bk*p[j]+z[j];
            pp[j]=bk*pp[j]+zz[j];
        }
    }
    bkden=bknum;                      Calculate coefficient ak, new iterate x, and new
    atimes(p,z,0);                        residuals r and rr.
    for (akden=0.0,j=0;j<n;j++) akden += z[j]*pp[j];
    ak=bknum/akden;
    atimes(pp,zz,1);
    for (j=0;j<n;j++) {
        x[j] += ak*p[j];
        r[j] -= ak*z[j];
        rr[j] -= ak*zz[j];
    }
    asolve(r,z,0);                    Solve Ã · z = r and check stopping criterion.
    if (itol == 1)
        err=snrm(r,itol)/bnrm;
    else if (itol == 2)
        err=snrm(z,itol)/bnrm;
    else if (itol == 3 || itol == 4) {
        zm1nrm=znrm;
        znrm=snrm(z,itol);
        if (abs(zm1nrm-znrm) > EPS*znrm) {
            dxnrm=abs(ak)*snrm(p,itol);
            err=znrm/abs(zm1nrm-znrm)*dxnrm;
        } else {
            err=znrm/bnrm;            Error may not be accurate, so loop again.
            continue;
        }
        xnrm=snrm(x,itol);
        if (err <= 0.5*xnrm) err /= xnrm;
        else {
            err=znrm/bnrm;            Error may not be accurate, so loop again.
            continue;
        }
```

```
        }
        if (err <= tol) break;
    }
}
```

The solve routine uses this short utility for computing vector norms:

linbcg.h

```
Doub Linbcg::snrm(VecDoub_I &sx, const Int itol)
Compute one of two norms for a vector sx[0..n-1], as signaled by itol. Used by solve.
{
    Int i,isamax,n=sx.size();
    Doub ans;
    if (itol <= 3) {
        ans = 0.0;
        for (i=0;i<n;i++) ans += SQR(sx[i]);       Vector magnitude norm.
        return sqrt(ans);
    } else {
        isamax=0;
        for (i=0;i<n;i++) {                          Largest component norm.
            if (abs(sx[i]) > abs(sx[isamax])) isamax=i;
        }
        return abs(sx[isamax]);
    }
}
```

Here is an example of a derived class that solves $\mathbf{A} \cdot \mathbf{x} = \mathbf{b}$ for a matrix $\mathbf{A}$ in NRsparseMat's compressed column sparse format. A naive diagonal preconditioner is used.

asolve.h

```
struct NRsparseLinbcg : Linbcg {
    NRsparseMat &mat;
    Int n;
    NRsparseLinbcg(NRsparseMat &matrix) : mat(matrix), n(mat.nrows) {}
    The constructor just binds a reference to your sparse matrix, making it available to asolve
    and atimes. To solve for a right-hand side, you call this object's solve method, as defined
    in the base class.
    void atimes(VecDoub_I &x, VecDoub_O &r, const Int itrnsp) {
        if (itrnsp) r=mat.atx(x);
        else r=mat.ax(x);
    }
    void asolve(VecDoub_I &b, VecDoub_O &x, const Int itrnsp) {
        Int i,j;
        Doub diag;
        for (i=0;i<n;i++) {
            diag=0.0;
            for (j=mat.col_ptr[i];j<mat.col_ptr[i+1];j++)
                if (mat.row_ind[j] == i) {
                    diag=mat.val[j];
                    break;
                }
            x[i]=(diag != 0.0 ? b[i]/diag : b[i]);
            The matrix Ã is the diagonal part of A. Since the transpose matrix has the same
            diagonal, the flag itrnsp is not used in this example.
        }
    }
};
```

For another example of using a class derived from Linbcg to solve a sparse matrix problem, see §3.8.

CITED REFERENCES AND FURTHER READING:

Tewarson, R.P. 1973, *Sparse Matrices* (New York: Academic Press).[1]

Jacobs, D.A.H. (ed.) 1977, *The State of the Art in Numerical Analysis* (London: Academic Press), Chapter I.3 (by J.K. Reid).[2]

George, A., and Liu, J.W.H. 1981, *Computer Solution of Large Sparse Positive Definite Systems* (Englewood Cliffs, NJ: Prentice-Hall).[3]

NAG Fortran Library (Oxford, UK: Numerical Algorithms Group), see 2007+, `http://www.nag.co.uk`.[4]

IMSL Math/Library Users Manual (Houston: IMSL Inc.), see 2007+, `http://www.vni.com/products/imsl`.[5]

Eisenstat, S.C., Gursky, M.C., Schultz, M.H., and Sherman, A.H. 1977, *Yale Sparse Matrix Package*, Technical Reports 112 and 114 (Yale University Department of Computer Science).[6]

Bai, Z., Demmel, J., Dongarra, J. Ruhe, A., and van der Vorst, H. (eds.) 2000, *Templates for the Solution of Algebraic Eigenvalue Problems: A Practical Guide* Ch. 10 (Philadelphia: S.I.A.M.). Online at URL in `http://www.cs.ucdavis.edu/~bai/ET/contents.html`.[7]

SPARSKIT, 2007+, at `http://www-users.cs.umn.edu/~saad/software/SPARSKIT/sparskit.html`.[8]

Golub, G.H., and Van Loan, C.F. 1996, *Matrix Computations*, 3rd ed. (Baltimore: Johns Hopkins University Press), Chapters 4 and 10, particularly §10.2–§10.3.[9]

Stoer, J., and Bulirsch, R. 2002, *Introduction to Numerical Analysis*, 3rd ed. (New York: Springer), Chapter 8.[10]

Baker, L. 1991, *More C Tools for Scientists and Engineers* (New York: McGraw-Hill).[11]

Fletcher, R. 1976, in *Numerical Analysis Dundee 1975*, Lecture Notes in Mathematics, vol. 506, A. Dold and B Eckmann, eds. (Berlin: Springer), pp. 73–89.[12]

PCGPAK User's Guide (New Haven: Scientific Computing Associates, Inc.).[13]

Saad, Y., and Schulz, M. 1986, *SIAM Journal on Scientific and Statistical Computing*, vol. 7, pp. 856–869.[14]

Ueberhuber, C.W. 1997, *Numerical Computation: Methods, Software, and Analysis*, 2 vols. (Berlin: Springer), Chapter 13.

Bunch, J.R., and Rose, D.J. (eds.) 1976, *Sparse Matrix Computations* (New York: Academic Press).

Duff, I.S., and Stewart, G.W. (eds.) 1979, *Sparse Matrix Proceedings 1978* (Philadelphia: S.I.A.M.).

2.8 Vandermonde Matrices and Toeplitz Matrices

In §2.4 the case of a tridiagonal matrix was treated specially, because that particular type of linear system admits a solution in only of order N operations, rather than of order N^3 for the general linear problem. When such particular types exist, it is important to know about them. Your computational savings, should you ever happen to be working on a problem that involves the right kind of particular type, can be enormous.

This section treats two special types of matrices that can be solved in of order N^2 operations, not as good as tridiagonal, but a lot better than the general case. (Other than the operations count, these two types having nothing in common.) Matrices of the first type, termed *Vandermonde matrices*, occur in some problems having to do with the fitting of polynomials, the reconstruction of distributions from their moments, and also other contexts. In this book, for example, a Vandermonde problem crops up in §3.5. Matrices of the second type, termed *Toeplitz matrices*, tend

to occur in problems involving deconvolution and signal processing. In this book, a Toeplitz problem is encountered in §13.7.

These are not the only special types of matrices worth knowing about. The *Hilbert matrices*, whose components are of the form $a_{ij} = 1/(i + j + 1)$, $i, j = 0, \ldots, N - 1$, can be inverted by an exact integer algorithm and are very difficult to invert in any other way, since they are notoriously ill-conditioned (see [1] for details). The Sherman-Morrison and Woodbury formulas, discussed in §2.7, can sometimes be used to convert new special forms into old ones. Reference [2] gives some other special forms. We have not found these additional forms to arise as frequently as the two that we now discuss.

2.8.1 Vandermonde Matrices

A Vandermonde matrix of size $N \times N$ is completely determined by N arbitrary numbers $x_0, x_1, \ldots, x_{N-1}$, in terms of which its N^2 components are the integer powers x_i^j, $i, j = 0, \ldots, N - 1$. Evidently there are two possible such forms, depending on whether we view the i's as rows and j's as columns, or vice versa. In the former case, we get a linear system of equations that looks like this,

$$\begin{bmatrix} 1 & x_0 & x_0^2 & \cdots & x_0^{N-1} \\ 1 & x_1 & x_1^2 & \cdots & x_1^{N-1} \\ \vdots & \vdots & \vdots & & \vdots \\ 1 & x_{N-1} & x_{N-1}^2 & \cdots & x_{N-1}^{N-1} \end{bmatrix} \cdot \begin{bmatrix} c_0 \\ c_1 \\ \vdots \\ c_{N-1} \end{bmatrix} = \begin{bmatrix} y_0 \\ y_1 \\ \vdots \\ y_{N-1} \end{bmatrix} \tag{2.8.1}$$

Performing the matrix multiplication, you will see that this equation solves for the unknown coefficients c_i that fit a polynomial to the N pairs of abscissas and ordinates (x_j, y_j). Precisely this problem will arise in §3.5, and the routine given there will solve (2.8.1) by the method that we are about to describe.

The alternative identification of rows and columns leads to the set of equations

$$\begin{bmatrix} 1 & 1 & \cdots & 1 \\ x_0 & x_1 & \cdots & x_{N-1} \\ x_0^2 & x_1^2 & \cdots & x_{N-1}^2 \\ & & \cdots & \\ x_0^{N-1} & x_1^{N-1} & \cdots & x_{N-1}^{N-1} \end{bmatrix} \cdot \begin{bmatrix} w_0 \\ w_1 \\ w_2 \\ \cdots \\ w_{N-1} \end{bmatrix} = \begin{bmatrix} q_0 \\ q_1 \\ q_2 \\ \cdots \\ q_{N-1} \end{bmatrix} \tag{2.8.2}$$

Write this out and you will see that it relates to the *problem of moments*: Given the values of N points x_i, find the unknown weights w_i, assigned so as to match the given values q_j of the first N moments. (For more on this problem, consult [3].) The routine given in this section solves (2.8.2).

The method of solution of both (2.8.1) and (2.8.2) is closely related to Lagrange's polynomial interpolation formula, which we will not formally meet until §3.2. Notwithstanding, the following derivation should be comprehensible:

Let $P_j(x)$ be the polynomial of degree $N - 1$ defined by

$$P_j(x) = \prod_{\substack{n=0 \\ n \neq j}}^{N-1} \frac{x - x_n}{x_j - x_n} = \sum_{k=0}^{N-1} A_{jk} x^k \tag{2.8.3}$$

Here the meaning of the last equality is to define the components of the matrix A_{ij} as the coefficients that arise when the product is multiplied out and like terms collected.

The polynomial $P_j(x)$ is a function of x generally. But you will notice that it is specifically designed so that it takes on a value of zero at all x_i with $i \neq j$ and has a value of unity at $x = x_j$. In other words,

$$P_j(x_i) = \delta_{ij} = \sum_{k=0}^{N-1} A_{jk} x_i^k \tag{2.8.4}$$

But (2.8.4) says that A_{jk} is exactly the inverse of the matrix of components x_i^k, which appears in (2.8.2), with the subscript as the column index. Therefore the solution of (2.8.2) is just that matrix inverse times the right-hand side,

$$w_j = \sum_{k=0}^{N-1} A_{jk} q_k \tag{2.8.5}$$

As for the transpose problem (2.8.1), we can use the fact that the inverse of the transpose is the transpose of the inverse, so

$$c_j = \sum_{k=0}^{N-1} A_{kj} y_k \tag{2.8.6}$$

The routine in §3.5 implements this.

It remains to find a good way of multiplying out the monomial terms in (2.8.3), in order to get the components of A_{jk}. This is essentially a bookkeeping problem, and we will let you read the routine itself to see how it can be solved. One trick is to define a master $P(x)$ by

$$P(x) \equiv \prod_{n=0}^{N-1} (x - x_n) \tag{2.8.7}$$

work out its coefficients, and then obtain the numerators and denominators of the specific P_j's via synthetic division by the one supernumerary term. (See §5.1 for more on synthetic division.) Since each such division is only a process of order N, the total procedure is of order N^2.

You should be warned that Vandermonde systems are notoriously ill-conditioned, by their very nature. (As an aside anticipating §5.8, the reason is the same as that which makes Chebyshev fitting so impressively accurate: There exist high-order polynomials that are very good uniform fits to zero. Hence roundoff error can introduce rather substantial coefficients of the leading terms of these polynomials.) It is a good idea always to compute Vandermonde problems in double precision or higher.

The routine for (2.8.2) that follows is due to G.B. Rybicki.

vander.h

```
void vander(VecDoub_I &x, VecDoub_O &w, VecDoub_I &q)
```
Solves the Vandermonde linear system $\sum_{i=0}^{N-1} x_i^k w_i = q_k$ $(k = 0, \ldots, N-1)$. Input consists of the vectors `x[0..n-1]` and `q[0..n-1]`; the vector `w[0..n-1]` is output.
```
{
    Int i,j,k,n=q.size();
    Doub b,s,t,xx;
    VecDoub c(n);
    if (n == 1) w[0]=q[0];
    else {
        for (i=0;i<n;i++) c[i]=0.0;          Initialize array.
        c[n-1] = -x[0];                      Coefficients of the master polynomial are found
        for (i=1;i<n;i++) {                      by recursion.
            xx = -x[i];
            for (j=(n-1-i);j<(n-1);j++) c[j] += xx*c[j+1];
            c[n-1] += xx;
        }
        for (i=0;i<n;i++) {                  Each subfactor in turn
```

```
                xx=x[i];
                t=b=1.0;
                s=q[n-1];
                for (k=n-1;k>0;k--) {           is synthetically divided,
                    b=c[k]+xx*b;
                    s += q[k-1]*b;              matrix-multiplied by the right-hand side,
                    t=xx*t+b;
                }
                w[i]=s/t;                       and supplied with a denominator.
            }
        }
}
```

2.8.2 Toeplitz Matrices

An $N \times N$ Toeplitz matrix is specified by giving $2N-1$ numbers R_k, where the index k ranges over $k = -N+1, \ldots, -1, 0, 1, \ldots, N-1$. Those numbers are then emplaced as matrix elements constant along the (upper-left to lower-right) diagonals of the matrix:

$$\begin{bmatrix} R_0 & R_{-1} & R_{-2} & \cdots & R_{-(N-2)} & R_{-(N-1)} \\ R_1 & R_0 & R_{-1} & \cdots & R_{-(N-3)} & R_{-(N-2)} \\ R_2 & R_1 & R_0 & \cdots & R_{-(N-4)} & R_{-(N-3)} \\ \cdots & & & \cdots & & \\ R_{N-2} & R_{N-3} & R_{N-4} & \cdots & R_0 & R_{-1} \\ R_{N-1} & R_{N-2} & R_{N-3} & \cdots & R_1 & R_0 \end{bmatrix} \tag{2.8.8}$$

The linear Toeplitz problem can thus be written as

$$\sum_{j=0}^{N-1} R_{i-j} x_j = y_i \qquad (i = 0, \ldots, N-1) \tag{2.8.9}$$

where the x_j's, $j = 0, \ldots, N-1$, are the unknowns to be solved for.

The Toeplitz matrix is symmetric if $R_k = R_{-k}$ for all k. Levinson [4] developed an algorithm for fast solution of the symmetric Toeplitz problem, by a *bordering method*, that is, a recursive procedure that solves the $(M+1)$-dimensional Toeplitz problem

$$\sum_{j=0}^{M} R_{i-j} x_j^{(M)} = y_i \qquad (i = 0, \ldots, M) \tag{2.8.10}$$

in turn for $M = 0, 1, \ldots$ until $M = N-1$, the desired result, is finally reached. The vector $x_j^{(M)}$ is the result at the Mth stage and becomes the desired answer only when $N-1$ is reached.

Levinson's method is well documented in standard texts (e.g., [5]). The useful fact that the method generalizes to the *nonsymmetric* case seems to be less well known. At some risk of excessive detail, we therefore give a derivation here, due to G.B. Rybicki.

In following a recursion from step M to step $M+1$ we find that our developing solution $x^{(M)}$ changes in this way:

$$\sum_{j=0}^{M} R_{i-j} x_j^{(M)} = y_i \qquad i = 0, \ldots, M \tag{2.8.11}$$

becomes

$$\sum_{j=0}^{M} R_{i-j} x_j^{(M+1)} + R_{i-(M+1)} x_{M+1}^{(M+1)} = y_i \qquad i = 0, \ldots, M+1 \tag{2.8.12}$$

By eliminating y_i we find

$$\sum_{j=0}^{M} R_{i-j}\left(\frac{x_j^{(M)} - x_j^{(M+1)}}{x_{M+1}^{(M+1)}}\right) = R_{i-(M+1)} \qquad i = 0, \ldots, M \tag{2.8.13}$$

or by letting $i \to M - i$ and $j \to M - j$,

$$\sum_{j=0}^{M} R_{j-i} G_j^{(M)} = R_{-(i+1)} \tag{2.8.14}$$

where

$$G_j^{(M)} \equiv \frac{x_{M-j}^{(M)} - x_{M-j}^{(M+1)}}{x_{M+1}^{(M+1)}} \tag{2.8.15}$$

To put this another way,

$$x_{M-j}^{(M+1)} = x_{M-j}^{(M)} - x_{M+1}^{(M+1)} G_j^{(M)} \qquad j = 0, \ldots, M \tag{2.8.16}$$

Thus, if we can use recursion to find the order M quantities $x^{(M)}$ and $G^{(M)}$ *and* the single order $M + 1$ quantity $x_{M+1}^{(M+1)}$, then all of the other $x_j^{(M+1)}$'s will follow. Fortunately, the quantity $x_{M+1}^{(M+1)}$ follows from equation (2.8.12) with $i = M + 1$,

$$\sum_{j=0}^{M} R_{M+1-j} x_j^{(M+1)} + R_0 x_{M+1}^{(M+1)} = y_{M+1} \tag{2.8.17}$$

For the unknown order $M + 1$ quantities $x_j^{(M+1)}$ we can substitute the previous order quantities in G since

$$G_{M-j}^{(M)} = \frac{x_j^{(M)} - x_j^{(M+1)}}{x_{M+1}^{(M+1)}} \tag{2.8.18}$$

The result of this operation is

$$x_{M+1}^{(M+1)} = \frac{\sum_{j=0}^{M} R_{M+1-j} x_j^{(M)} - y_{M+1}}{\sum_{j=0}^{M} R_{M+1-j} G_{M-j}^{(M)} - R_0} \tag{2.8.19}$$

The only remaining problem is to develop a recursion relation for G. Before we do that, however, we should point out that there are actually two distinct sets of solutions to the original linear problem for a nonsymmetric matrix, namely right-hand solutions (which we have been discussing) and left-hand solutions z_i. The formalism for the left-hand solutions differs only in that we deal with the equations

$$\sum_{j=0}^{M} R_{j-i} z_j^{(M)} = y_i \qquad i = 0, \ldots, M \tag{2.8.20}$$

Then, the same sequence of operations on this set leads to

$$\sum_{j=0}^{M} R_{i-j} H_j^{(M)} = R_{i+1} \tag{2.8.21}$$

where

$$H_j^{(M)} \equiv \frac{z_{M-j}^{(M)} - z_{M-j}^{(M+1)}}{z_{M+1}^{(M+1)}} \tag{2.8.22}$$

(compare with 2.8.14 – 2.8.15). The reason for mentioning the left-hand solutions now is that, by equation (2.8.21), the H_j's satisfy exactly the same equation as the x_j's except for the substitution $y_i \to R_{i+1}$ on the right-hand side. Therefore we can quickly deduce from equation (2.8.19) that

$$H_{M+1}^{(M+1)} = \frac{\sum_{j=0}^{M} R_{M+1-j} H_j^{(M)} - R_{M+2}}{\sum_{j=0}^{M} R_{M+1-j} G_{M-j}^{(M)} - R_0} \tag{2.8.23}$$

By the same token, G satisfies the same equation as z, except for the substitution $y_i \to R_{-(i+1)}$. This gives

$$G_{M+1}^{(M+1)} = \frac{\sum_{j=0}^{M} R_{j-M-1} G_j^{(M)} - R_{-M-2}}{\sum_{j=0}^{M} R_{j-M-1} H_{M-j}^{(M)} - R_0} \tag{2.8.24}$$

The same "morphism" also turns equation (2.8.16), and its partner for z, into the final equations

$$\begin{aligned} G_j^{(M+1)} &= G_j^{(M)} - G_{M+1}^{(M+1)} H_{M-j}^{(M)} \\ H_j^{(M+1)} &= H_j^{(M)} - H_{M+1}^{(M+1)} G_{M-j}^{(M)} \end{aligned} \tag{2.8.25}$$

Now, starting with the initial values

$$x_0^{(0)} = y_0/R_0 \qquad G_0^{(0)} = R_{-1}/R_0 \qquad H_0^{(0)} = R_1/R_0 \tag{2.8.26}$$

we can recurse away. At each stage M we use equations (2.8.23) and (2.8.24) to find $H_{M+1}^{(M+1)}$, $G_{M+1}^{(M+1)}$, and then equation (2.8.25) to find the other components of $H^{(M+1)}, G^{(M+1)}$. From there the vectors $x^{(M+1)}$ and/or $z^{(M+1)}$ are easily calculated.

The program below does this. It incorporates the second equation in (2.8.25) in the form

$$H_{M-j}^{(M+1)} = H_{M-j}^{(M)} - H_{M+1}^{(M+1)} G_j^{(M)} \tag{2.8.27}$$

so that the computation can be done "in place."

Notice that the above algorithm fails if $R_0 = 0$. In fact, because the bordering method does not allow pivoting, the algorithm will fail if any of the diagonal principal minors of the original Toeplitz matrix vanish. (Compare with discussion of the tridiagonal algorithm in §2.4.) If the algorithm fails, your matrix is not necessarily singular — you might just have to solve your problem by a slower and more general algorithm such as LU decomposition with pivoting.

The routine that implements equations (2.8.23) – (2.8.27) is also due to Rybicki. Note that the routine's `r[n-1+j]` is equal to R_j above, so that subscripts on the `r` array vary from 0 to $2N - 2$.

toeplz.h

```
void toeplz(VecDoub_I &r, VecDoub_O &x, VecDoub_I &y)
```

Solves the Toeplitz system $\sum_{j=0}^{N-1} R_{(N-1+i-j)} x_j = y_i \; (i = 0, \ldots, N-1)$. The Toeplitz matrix need not be symmetric. `y[0..n-1]` and `r[0..2*n-2]` are input arrays; `x[0..n-1]` is the output array.

```
{
    Int j,k,m,m1,m2,n1,n=y.size();
    Doub pp,pt1,pt2,qq,qt1,qt2,sd,sgd,sgn,shn,sxn;
    n1=n-1;
```

```
    if (r[n1] == 0.0) throw("toeplz-1 singular principal minor");
    x[0]=y[0]/r[n1];                    Initialize for the recursion.
    if (n1 == 0) return;
    VecDoub g(n1),h(n1);
    g[0]=r[n1-1]/r[n1];
    h[0]=r[n1+1]/r[n1];
    for (m=0;m<n;m++) {                 Main loop over the recursion.
        m1=m+1;
        sxn = -y[m1];                   Compute numerator and denominator for x from eq.
        sd = -r[n1];                        (2.8.19),
        for (j=0;j<m+1;j++) {
            sxn += r[n1+m1-j]*x[j];
            sd += r[n1+m1-j]*g[m-j];
        }
        if (sd == 0.0) throw("toeplz-2 singular principal minor");
        x[m1]=sxn/sd;                   whence x.
        for (j=0;j<m+1;j++)             Eq. (2.8.16).
            x[j] -= x[m1]*g[m-j];
        if (m1 == n1) return;
        sgn = -r[n1-m1-1];              Compute numerator and denominator for G and H,
        shn = -r[n1+m1+1];                  eqs. (2.8.24) and (2.8.23),
        sgd = -r[n1];
        for (j=0;j<m+1;j++) {
            sgn += r[n1+j-m1]*g[j];
            shn += r[n1+m1-j]*h[j];
            sgd += r[n1+j-m1]*h[m-j];
        }
        if (sgd == 0.0) throw("toeplz-3 singular principal minor");
        g[m1]=sgn/sgd;                  whence G and H.
        h[m1]=shn/sd;
        k=m;
        m2=(m+2) >> 1;
        pp=g[m1];
        qq=h[m1];
        for (j=0;j<m2;j++) {
            pt1=g[j];
            pt2=g[k];
            qt1=h[j];
            qt2=h[k];
            g[j]=pt1-pp*qt2;
            g[k]=pt2-pp*qt1;
            h[j]=qt1-qq*pt2;
            h[k--]=qt2-qq*pt1;
        }
    }                                   Back for another recurrence.
    throw("toeplz - should not arrive here!");
}
```

If you are in the business of solving *very* large Toeplitz systems, you should find out about so-called "new, fast" algorithms, which require only on the order of $N(\log N)^2$ operations, compared to N^2 for Levinson's method. These methods are too complicated to include here. Papers by Bunch [6] and de Hoog [7] will give entry to the literature.

CITED REFERENCES AND FURTHER READING:

Golub, G.H., and Van Loan, C.F. 1996, *Matrix Computations*, 3rd ed. (Baltimore: Johns Hopkins University Press), Chapter 5 [also treats some other special forms].

Forsythe, G.E., and Moler, C.B. 1967, *Computer Solution of Linear Algebraic Systems* (Englewood Cliffs, NJ: Prentice-Hall), §19.[1]

Westlake, J.R. 1968, *A Handbook of Numerical Matrix Inversion and Solution of Linear Equations* (New York: Wiley).[2]

von Mises, R. 1964, *Mathematical Theory of Probability and Statistics* (New York: Academic Press), pp. 394ff.[3]

Levinson, N., Appendix B of N. Wiener, 1949, *Extrapolation, Interpolation and Smoothing of Stationary Time Series* (New York: Wiley).[4]

Robinson, E.A., and Treitel, S. 1980, *Geophysical Signal Analysis* (Englewood Cliffs, NJ: Prentice-Hall), pp. 163ff.[5]

Bunch, J.R. 1985, "Stability of Methods for Solving Toeplitz Systems of Equations," *SIAM Journal on Scientific and Statistical Computing*, vol. 6, pp. 349–364.[6]

de Hoog, F. 1987, "A New Algorithm for Solving Toeplitz Systems of Equations," *Linear Algebra and Its Applications*, vol. 88/89, pp. 123–138.[7]

2.9 Cholesky Decomposition

If a square matrix $\mathbf{A}$ happens to be symmetric and positive-definite, then it has a special, more efficient, triangular decomposition. *Symmetric* means that $a_{ij} = a_{ji}$ for $i, j = 0, \ldots, N-1$, while *positive-definite* means that

$$\mathbf{v} \cdot \mathbf{A} \cdot \mathbf{v} > 0 \quad \text{for all vectors } \mathbf{v} \tag{2.9.1}$$

(In Chapter 11 we will see that positive-definite has the equivalent interpretation that $\mathbf{A}$ has all positive eigenvalues.) While symmetric, positive-definite matrices are rather special, they occur quite frequently in some applications, so their special factorization, called *Cholesky decomposition*, is good to know about. When you can use it, Cholesky decomposition is about a factor of two faster than alternative methods for solving linear equations.

Instead of seeking arbitrary lower and upper triangular factors $\mathbf{L}$ and $\mathbf{U}$, Cholesky decomposition constructs a lower triangular matrix $\mathbf{L}$ whose transpose $\mathbf{L}^T$ can itself serve as the upper triangular part. In other words we replace equation (2.3.1) by

$$\mathbf{L} \cdot \mathbf{L}^T = \mathbf{A} \tag{2.9.2}$$

This factorization is sometimes referred to as "taking the square root" of the matrix $\mathbf{A}$, though, because of the transpose, it is not literally that. The components of $\mathbf{L}^T$ are of course related to those of $\mathbf{L}$ by

$$L^T_{ij} = L_{ji} \tag{2.9.3}$$

Writing out equation (2.9.2) in components, one readily obtains the analogs of equations (2.3.12) – (2.3.13),

$$L_{ii} = \left(a_{ii} - \sum_{k=0}^{i-1} L_{ik}^2 \right)^{1/2} \tag{2.9.4}$$

and

$$L_{ji} = \frac{1}{L_{ii}} \left(a_{ij} - \sum_{k=0}^{i-1} L_{ik} L_{jk} \right) \qquad j = i+1, i+2, \ldots, N-1 \tag{2.9.5}$$

If you apply equations (2.9.4) and (2.9.5) in the order $i = 0, 1, \ldots, N-1$, you will see that the L's that occur on the right-hand side are already determined by the time they are needed. Also, only components a_{ij} with $j \geq i$ are referenced. (Since $\mathbf{A}$ is symmetric, these have complete information.) If storage is at a premium, it is possible to have the factor $\mathbf{L}$ overwrite the subdiagonal (lower triangular but not including the diagonal) part of $\mathbf{A}$, preserving the input upper triangular values of $\mathbf{A}$; one extra vector of length N is then needed to store the diagonal part of $\mathbf{L}$. The operations count is $N^3/6$ executions of the inner loop (consisting of one multiply and one subtract), with also N square roots. As already mentioned, this is about a factor 2 better than LU decomposition of $\mathbf{A}$ (where its symmetry would be ignored).

You might wonder about pivoting. The pleasant answer is that Cholesky decomposition is extremely stable numerically, without any pivoting at all. Failure of the decomposition simply indicates that the matrix $\mathbf{A}$ (or, with roundoff error, another very nearby matrix) is not positive-definite. In fact, this is an efficient way to test *whether* a symmetric matrix is positive-definite. (In this application, you may want to replace the `throw` in the code below with some less drastic signaling method.)

By now you should be familiar with, if not bored by, our conventions for objects implementing decomposition methods, so we list the object `Cholesky` as a single big mouthful. The methods `elmult` and `elsolve` perform manipulations using the matrix $\mathbf{L}$. The first multiplies $\mathbf{L} \cdot \mathbf{y} = \mathbf{c}$ for a given $\mathbf{y}$, returning $\mathbf{c}$. The second solves this same equation, given $\mathbf{c}$ and returning $\mathbf{y}$. These manipulations are useful in contexts such as multivariate Gaussians (§7.4 and §16.5) and in the analysis of covariance matrices (§15.6).

cholesky.h

```
struct Cholesky{
Object for Cholesky decomposition of a matrix A, and related functions.
    Int n;
    MatDoub el;                         Stores the decomposition.
    Cholesky(MatDoub_I &a) : n(a.nrows()), el(a) {
    Constructor. Given a positive-definite symmetric matrix a[0..n-1][0..n-1], construct
    and store its Cholesky decomposition, A = L · L^T.
        Int i,j,k;
        VecDoub tmp;
        Doub sum;
        if (el.ncols() != n) throw("need square matrix");
        for (i=0;i<n;i++) {
            for (j=i;j<n;j++) {
                for (sum=el[i][j],k=i-1;k>=0;k--) sum -= el[i][k]*el[j][k];
                if (i == j) {
                    if (sum <= 0.0)          A, with rounding errors, is not positive-definite.
                        throw("Cholesky failed");
                    el[i][i]=sqrt(sum);
                } else el[j][i]=sum/el[i][i];
            }
        }
        for (i=0;i<n;i++) for (j=0;j<i;j++) el[j][i] = 0.;
    }
    void solve(VecDoub_I &b, VecDoub_O &x) {
    Solve the set of n linear equations A·x = b, where a is a positive-definite symmetric matrix
    whose Cholesky decomposition has been stored. b[0..n-1] is input as the right-hand side
    vector. The solution vector is returned in x[0..n-1].
        Int i,k;
        Doub sum;
        if (b.size() != n || x.size() != n) throw("bad lengths in Cholesky");
        for (i=0;i<n;i++) {             Solve L · y = b, storing y in x.
            for (sum=b[i],k=i-1;k>=0;k--) sum -= el[i][k]*x[k];
            x[i]=sum/el[i][i];
        }
        for (i=n-1;i>=0;i--) {          Solve L^T · x = y.
            for (sum=x[i],k=i+1;k<n;k++) sum -= el[k][i]*x[k];
            x[i]=sum/el[i][i];
        }
    }
    void elmult(VecDoub_I &y, VecDoub_O &b) {
    Multiply L · y = b, where L is the lower triangular matrix in the stored Cholesky decom-
    position. y[0..n-1] is input. The result is returned in b[0..n-1].
        Int i,j;
        if (b.size() != n || y.size() != n) throw("bad lengths");
        for (i=0;i<n;i++) {
            b[i] = 0.;
            for (j=0;j<=i;j++) b[i] += el[i][j]*y[j];
        }
    }
```

```
    void elsolve(VecDoub_I &b, VecDoub_O &y) {
    Solve L · y = b, where L is the lower triangular matrix in the stored Cholesky decomposi-
    tion. b[0..n-1] is input as the right-hand side vector. The solution vector is returned in
    y[0..n-1].
        Int i,j;
        Doub sum;
        if (b.size() != n || y.size() != n) throw("bad lengths");
        for (i=0;i<n;i++) {
            for (sum=b[i],j=0; j<i; j++) sum -= el[i][j]*y[j];
            y[i] = sum/el[i][i];
        }
    }
    void inverse(MatDoub_O &ainv) {
    Set ainv[0..n-1][0..n-1] to the matrix inverse of A, the matrix whose Cholesky decom-
    position has been stored.
        Int i,j,k;
        Doub sum;
        ainv.resize(n,n);
        for (i=0;i<n;i++) for (j=0;j<=i;j++){
            sum = (i==j? 1. : 0.);
            for (k=i-1;k>=j;k--) sum -= el[i][k]*ainv[j][k];
            ainv[j][i]= sum/el[i][i];
        }
        for (i=n-1;i>=0;i--) for (j=0;j<=i;j++){
            sum = (i<j? 0. : ainv[j][i]);
            for (k=i+1;k<n;k++) sum -= el[k][i]*ainv[j][k];
            ainv[i][j] = ainv[j][i] = sum/el[i][i];
        }
    }
    Doub logdet() {
    Return the logarithm of the determinant of A, the matrix whose Cholesky decomposition
    has been stored.
        Doub sum = 0.;
        for (Int i=0; i<n; i++) sum += log(el[i][i]);
        return 2.*sum;
    }
};
```

CITED REFERENCES AND FURTHER READING:

Wilkinson, J.H., and Reinsch, C. 1971, *Linear Algebra*, vol. II of *Handbook for Automatic Computation* (New York: Springer), Chapter I/1.

Gill, P.E., Murray, W., and Wright, M.H. 1991, *Numerical Linear Algebra and Optimization*, vol. 1 (Redwood City, CA: Addison-Wesley), §4.9.2.

Dahlquist, G., and Bjorck, A. 1974, *Numerical Methods* (Englewood Cliffs, NJ: Prentice-Hall); reprinted 2003 (New York: Dover), §5.3.5.

Golub, G.H., and Van Loan, C.F. 1996, *Matrix Computations*, 3rd ed. (Baltimore: Johns Hopkins University Press), §4.2.

2.10 QR Decomposition

There is another matrix factorization that is sometimes very useful, the so-called *QR decomposition*,

$$\mathbf{A} = \mathbf{Q} \cdot \mathbf{R} \tag{2.10.1}$$

Here $\mathbf{R}$ is upper triangular, while $\mathbf{Q}$ is orthogonal, that is,

$$\mathbf{Q}^T \cdot \mathbf{Q} = \mathbf{1} \tag{2.10.2}$$

where $\mathbf{Q}^T$ is the transpose matrix of $\mathbf{Q}$. Although the decomposition exists for a general rectangular matrix, we shall restrict our treatment to the case when all the matrices are square, with dimensions $N \times N$.

Like the other matrix factorizations we have met (LU, SVD, Cholesky), QR decomposition can be used to solve systems of linear equations. To solve

$$\mathbf{A} \cdot \mathbf{x} = \mathbf{b} \tag{2.10.3}$$

first form $\mathbf{Q}^T \cdot \mathbf{b}$ and then solve

$$\mathbf{R} \cdot \mathbf{x} = \mathbf{Q}^T \cdot \mathbf{b} \tag{2.10.4}$$

by backsubstitution. Since QR decomposition involves about twice as many operations as LU decomposition, it is not used for typical systems of linear equations. However, we will meet special cases where QR is the method of choice.

The standard algorithm for the QR decomposition involves successive Householder transformations (to be discussed later in §11.3). We write a Householder matrix in the form $\mathbf{1} - \mathbf{u} \otimes \mathbf{u}/c$, where $c = \frac{1}{2}\mathbf{u} \cdot \mathbf{u}$. An appropriate Householder matrix applied to a given matrix can zero all elements in a column of the matrix situated below a chosen element. Thus we arrange for the first Householder matrix $\mathbf{Q}_0$ to zero all elements in column 0 of $\mathbf{A}$ below the zeroth element. Similarly, $\mathbf{Q}_1$ zeroes all elements in column 1 below element 1, and so on up to $\mathbf{Q}_{n-2}$. Thus

$$\mathbf{R} = \mathbf{Q}_{n-2} \cdots \mathbf{Q}_0 \cdot \mathbf{A} \tag{2.10.5}$$

Since the Householder matrices are orthogonal,

$$\mathbf{Q} = (\mathbf{Q}_{n-2} \cdots \mathbf{Q}_0)^{-1} = \mathbf{Q}_0 \cdots \mathbf{Q}_{n-2} \tag{2.10.6}$$

In many applications $\mathbf{Q}$ is not needed explicitly, and it is sufficient to store only the factored form (2.10.6). (We do, however, store $\mathbf{Q}$, or rather its transpose, in the code below.) Pivoting is not usually necessary unless the matrix $\mathbf{A}$ is very close to singular. A general QR algorithm for rectangular matrices including pivoting is given in [1]. For square matrices and without pivoting, an implementation is as follows:

qrdcmp.h

```
struct QRdcmp {
```

Object for QR decomposition of a matrix $\mathbf{A}$, and related functions.

```
    Int n;
    MatDoub qt, r;                                  Stored Q^T and R.
    Bool sing;                                      Indicates whether A is singular.
    QRdcmp(MatDoub_I &a);                           Constructor from A.
    void solve(VecDoub_I &b, VecDoub_O &x);         Solve A·x = b for x.
    void qtmult(VecDoub_I &b, VecDoub_O &x);        Multiply Q^T·b = x.
    void rsolve(VecDoub_I &b, VecDoub_O &x);        Solve R·x = b for x.
    void update(VecDoub_I &u, VecDoub_I &v);        See next subsection.
    void rotate(const Int i, const Doub a, const Doub b);     Used by update.
};
```

As usual, the constructor performs the actual decomposition:

qrdcmp.h

```
QRdcmp::QRdcmp(MatDoub_I &a)
    : n(a.nrows()), qt(n,n), r(a), sing(false) {
```

Construct the QR decomposition of `a[0..n-1][0..n-1]`. The upper triangular matrix $\mathbf{R}$ and the transpose of the orthogonal matrix $\mathbf{Q}$ are stored. `sing` is set to `true` if a singularity is encountered during the decomposition, but the decomposition is still completed in this case; otherwise it is set to `false`.

```
    Int i,j,k;
    VecDoub c(n), d(n);
    Doub scale,sigma,sum,tau;
```

```
    for (k=0;k<n-1;k++) {
        scale=0.0;
        for (i=k;i<n;i++) scale=MAX(scale,abs(r[i][k]));
        if (scale == 0.0) {                          Singular case.
            sing=true;
            c[k]=d[k]=0.0;
        } else {                                     Form Q_k and Q_k · A.
            for (i=k;i<n;i++) r[i][k] /= scale;
            for (sum=0.0,i=k;i<n;i++) sum += SQR(r[i][k]);
            sigma=SIGN(sqrt(sum),r[k][k]);
            r[k][k] += sigma;
            c[k]=sigma*r[k][k];
            d[k] = -scale*sigma;
            for (j=k+1;j<n;j++) {
                for (sum=0.0,i=k;i<n;i++) sum += r[i][k]*r[i][j];
                tau=sum/c[k];
                for (i=k;i<n;i++) r[i][j] -= tau*r[i][k];
            }
        }
    }
    d[n-1]=r[n-1][n-1];
    if (d[n-1] == 0.0) sing=true;
    for (i=0;i<n;i++) {                              Form Q^T explicitly.
        for (j=0;j<n;j++) qt[i][j]=0.0;
        qt[i][i]=1.0;
    }
    for (k=0;k<n-1;k++) {
        if (c[k] != 0.0) {
            for (j=0;j<n;j++) {
                sum=0.0;
                for (i=k;i<n;i++)
                    sum += r[i][k]*qt[i][j];
                sum /= c[k];
                for (i=k;i<n;i++)
                    qt[i][j] -= sum*r[i][k];
            }
        }
    }
    for (i=0;i<n;i++) {                              Form R explicitly.
        r[i][i]=d[i];
        for (j=0;j<i;j++) r[i][j]=0.0;
    }
}
```

The next set of member functions is used to solve linear systems. In many applications only the part (2.10.4) of the algorithm is needed, so we put in separate routines the multiplication $\mathbf{Q}^T \cdot \mathbf{b}$ and the backsubstitution on $\mathbf{R}$.

qrdcmp.h

```
void QRdcmp::solve(VecDoub_I &b, VecDoub_O &x) {
```

Solve the set of n linear equations $\mathbf{A} \cdot \mathbf{x} = \mathbf{b}$. b[0..n-1] is input as the right-hand side vector, and x[0..n-1] is returned as the solution vector.

```
    qtmult(b,x);                  Form Q^T · b.
    rsolve(x,x);                  Solve R · x = Q^T · b.
}
```

```
void QRdcmp::qtmult(VecDoub_I &b, VecDoub_O &x) {
```

Multiply $\mathbf{Q}^T \cdot \mathbf{b}$ and put the result in $\mathbf{x}$. Since $\mathbf{Q}$ is orthogonal, this is equivalent to solving $\mathbf{Q} \cdot \mathbf{x} = \mathbf{b}$ for $\mathbf{x}$.

```
    Int i,j;
    Doub sum;
    for (i=0;i<n;i++) {
        sum = 0.;
```

```
        for (j=0;j<n;j++) sum += qt[i][j]*b[j];
        x[i] = sum;
    }
}

void QRdcmp::rsolve(VecDoub_I &b, VecDoub_O &x) {
```
Solve the triangular set of n linear equations $\mathbf{R} \cdot \mathbf{x} = \mathbf{b}$. b[0..n-1] is input as the right-hand side vector, and x[0..n-1] is returned as the solution vector.
```
    Int i,j;
    Doub sum;
    if (sing) throw("attempting solve in a singular QR");
    for (i=n-1;i>=0;i--) {
        sum=b[i];
        for (j=i+1;j<n;j++) sum -= r[i][j]*x[j];
        x[i]=sum/r[i][i];
    }
}
```

See [2] for details on how to use QR decomposition for constructing orthogonal bases, and for solving least-squares problems. (We prefer to use SVD, §2.6, for these purposes, because of its greater diagnostic capability in pathological cases.)

2.10.1 Updating a QR decomposition

Some numerical algorithms involve solving a succession of linear systems each of which differs only slightly from its predecessor. Instead of doing $O(N^3)$ operations each time to solve the equations from scratch, one can often update a matrix factorization in $O(N^2)$ operations and use the new factorization to solve the next set of linear equations. The LU decomposition is complicated to update because of pivoting. However, QR turns out to be quite simple for a very common kind of update,

$$\mathbf{A} \rightarrow \mathbf{A} + \mathbf{s} \otimes \mathbf{t} \tag{2.10.7}$$

(compare equation 2.7.1). In practice it is more convenient to work with the equivalent form

$$\mathbf{A} = \mathbf{Q} \cdot \mathbf{R} \quad \rightarrow \quad \mathbf{A}' = \mathbf{Q}' \cdot \mathbf{R}' = \mathbf{Q} \cdot (\mathbf{R} + \mathbf{u} \otimes \mathbf{v}) \tag{2.10.8}$$

One can go back and forth between equations (2.10.7) and (2.10.8) using the fact that $\mathbf{Q}$ is orthogonal, giving

$$\mathbf{t} = \mathbf{v} \quad \text{and either} \quad \mathbf{s} = \mathbf{Q} \cdot \mathbf{u} \quad \text{or} \quad \mathbf{u} = \mathbf{Q}^T \cdot \mathbf{s} \tag{2.10.9}$$

The algorithm [2] has two phases. In the first we apply $N-1$ Jacobi rotations (§11.1) to reduce $\mathbf{R} + \mathbf{u} \otimes \mathbf{v}$ to upper Hessenberg form. Another $N-1$ Jacobi rotations transform this upper Hessenberg matrix to the new upper triangular matrix $\mathbf{R}'$. The matrix $\mathbf{Q}'$ is simply the product of $\mathbf{Q}$ with the $2(N-1)$ Jacobi rotations. In applications we usually want $\mathbf{Q}^T$, so the algorithm is arranged to work with this matrix (which is stored in the QRdcmp object) instead of with $\mathbf{Q}$.

qrdcmp.h
```
void QRdcmp::update(VecDoub_I &u, VecDoub_I &v) {
```
Starting from the stored QR decomposition $\mathbf{A} = \mathbf{Q} \cdot \mathbf{R}$, update it to be the QR decomposition of the matrix $\mathbf{Q} \cdot (\mathbf{R} + \mathbf{u} \otimes \mathbf{v})$. Input quantities are u[0..n-1], and v[0..n-1].
```
    Int i,k;
    VecDoub w(u);
    for (k=n-1;k>=0;k--)                 Find largest k such that u[k] ≠ 0.
        if (w[k] != 0.0) break;
    if (k < 0) k=0;
    for (i=k-1;i>=0;i--) {               Transform R + u ⊗ v to upper Hessenberg.
        rotate(i,w[i],-w[i+1]);
        if (w[i] == 0.0)
            w[i]=abs(w[i+1]);
```

```
        else if (abs(w[i]) > abs(w[i+1]))
            w[i]=abs(w[i])*sqrt(1.0+SQR(w[i+1]/w[i]));
        else w[i]=abs(w[i+1])*sqrt(1.0+SQR(w[i]/w[i+1]));
    }
    for (i=0;i<n;i++) r[0][i] += w[0]*v[i];
    for (i=0;i<k;i++)                       Transform upper Hessenberg matrix to upper tri-
        rotate(i,r[i][i],-r[i+1][i]);           angular.
    for (i=0;i<n;i++)
        if (r[i][i] == 0.0) sing=true;
}
```

`void QRdcmp::rotate(const Int i, const Doub a, const Doub b)`

Utility used by `update`. Given matrices `r[0..n-1][0..n-1]` and `qt[0..n-1][0..n-1]`, carry out a Jacobi rotation on rows `i` and $i+1$ of each matrix. `a` and `b` are the parameters of the rotation: $\cos\theta = a/\sqrt{a^2+b^2}$, $\sin\theta = b/\sqrt{a^2+b^2}$.

```
{
    Int j;
    Doub c,fact,s,w,y;
    if (a == 0.0) {                 Avoid unnecessary overflow or underflow.
        c=0.0;
        s=(b >= 0.0 ? 1.0 : -1.0);
    } else if (abs(a) > abs(b)) {
        fact=b/a;
        c=SIGN(1.0/sqrt(1.0+(fact*fact)),a);
        s=fact*c;
    } else {
        fact=a/b;
        s=SIGN(1.0/sqrt(1.0+(fact*fact)),b);
        c=fact*s;
    }
    for (j=i;j<n;j++) {             Premultiply r by Jacobi rotation.
        y=r[i][j];
        w=r[i+1][j];
        r[i][j]=c*y-s*w;
        r[i+1][j]=s*y+c*w;
    }
    for (j=0;j<n;j++) {             Premultiply qt by Jacobi rotation.
        y=qt[i][j];
        w=qt[i+1][j];
        qt[i][j]=c*y-s*w;
        qt[i+1][j]=s*y+c*w;
    }
}
```

We will make use of QR decomposition, and its updating, in §9.7.

CITED REFERENCES AND FURTHER READING:

Wilkinson, J.H., and Reinsch, C. 1971, *Linear Algebra*, vol. II of *Handbook for Automatic Computation* (New York: Springer), Chapter I/8.[1]

Golub, G.H., and Van Loan, C.F. 1996, *Matrix Computations*, 3rd ed. (Baltimore: Johns Hopkins University Press), §5.2, §5.3, §12.5.[2]

2.11 Is Matrix Inversion an N^3 Process?

We close this chapter with a little entertainment, a bit of algorithmic prestidigitation that probes more deeply into the subject of matrix inversion. We start with a seemingly simple question:

How many individual multiplications does it take to perform the matrix multiplication of two 2×2 matrices,

$$\begin{pmatrix} a_{00} & a_{01} \\ a_{10} & a_{11} \end{pmatrix} \cdot \begin{pmatrix} b_{00} & b_{01} \\ b_{10} & b_{11} \end{pmatrix} = \begin{pmatrix} c_{00} & c_{01} \\ c_{10} & c_{11} \end{pmatrix} \tag{2.11.1}$$

Eight, right? Here they are written explicitly:

$$\begin{aligned} c_{00} &= a_{00} \times b_{00} + a_{01} \times b_{10} \\ c_{01} &= a_{00} \times b_{01} + a_{01} \times b_{11} \\ c_{10} &= a_{10} \times b_{00} + a_{11} \times b_{10} \\ c_{11} &= a_{10} \times b_{01} + a_{11} \times b_{11} \end{aligned} \tag{2.11.2}$$

Do you think that one can write formulas for the c's that involve only *seven* multiplications? (Try it yourself, before reading on.)

Such a set of formulas was, in fact, discovered by Strassen [1]. The formulas are

$$\begin{aligned} Q_0 &\equiv (a_{00} + a_{11}) \times (b_{00} + b_{11}) \\ Q_1 &\equiv (a_{10} + a_{11}) \times b_{00} \\ Q_2 &\equiv a_{00} \times (b_{01} - b_{11}) \\ Q_3 &\equiv a_{11} \times (-b_{00} + b_{10}) \\ Q_4 &\equiv (a_{00} + a_{01}) \times b_{11} \\ Q_5 &\equiv (-a_{00} + a_{10}) \times (b_{00} + b_{01}) \\ Q_6 &\equiv (a_{01} - a_{11}) \times (b_{10} + b_{11}) \end{aligned} \tag{2.11.3}$$

in terms of which

$$\begin{aligned} c_{00} &= Q_0 + Q_3 - Q_4 + Q_6 \\ c_{10} &= Q_1 + Q_3 \\ c_{01} &= Q_2 + Q_4 \\ c_{11} &= Q_0 + Q_2 - Q_1 + Q_5 \end{aligned} \tag{2.11.4}$$

What's the use of this? There is one fewer multiplication than in equation (2.11.2), but *many more* additions and subtractions. It is not clear that anything has been gained. But notice that in (2.11.3) the a's and b's are never commuted. Therefore (2.11.3) and (2.11.4) are valid when the a's and b's are themselves matrices. The problem of multiplying two very large matrices (of order $N = 2^m$ for some integer m) can now be broken down recursively by partitioning the matrices into quarters, sixteenths, etc. And note the key point: The savings is not just a factor "7/8"; it is that factor at *each* hierarchical level of the recursion. In total it reduces the process of matrix multiplication to order $N^{\log_2 7}$ instead of N^3.

What about all the extra additions in (2.11.3) – (2.11.4)? Don't they outweigh the advantage of the fewer multiplications? For large N, it turns out that there are six times as many additions as multiplications implied by (2.11.3) – (2.11.4). But, if N is very large, this constant factor is no match for the change in the *exponent* from N^3 to $N^{\log_2 7}$.

With this "fast" matrix multiplication, Strassen also obtained a surprising result for matrix inversion [1]. Suppose that the matrices

$$\begin{pmatrix} a_{00} & a_{01} \\ a_{10} & a_{11} \end{pmatrix} \quad \text{and} \quad \begin{pmatrix} c_{00} & c_{01} \\ c_{10} & c_{11} \end{pmatrix} \tag{2.11.5}$$

are inverses of each other. Then the c's can be obtained from the a's by the following operations (compare equations 2.7.11 and 2.7.25):

$$\begin{aligned}
R_0 &= \text{Inverse}(a_{00}) \\
R_1 &= a_{10} \times R_0 \\
R_2 &= R_0 \times a_{01} \\
R_3 &= a_{10} \times R_2 \\
R_4 &= R_3 - a_{11} \\
R_5 &= \text{Inverse}(R_4) \\
c_{01} &= R_2 \times R_5 \\
c_{10} &= R_5 \times R_1 \\
R_6 &= R_2 \times c_{10} \\
c_{00} &= R_0 - R_6 \\
c_{11} &= -R_5
\end{aligned} \tag{2.11.6}$$

In (2.11.6) the "inverse" operator occurs just twice. It is to be interpreted as the reciprocal if the a's and c's are scalars, but as matrix inversion if the a's and c's are themselves submatrices. Imagine doing the inversion of a very large matrix, of order $N = 2^m$, recursively by partitions in half. At each step, halving the order *doubles* the number of inverse operations. But this means that there are only N divisions in all! So divisions don't dominate in the recursive use of (2.11.6). Equation (2.11.6) is dominated, in fact, by its 6 multiplications. Since these can be done by an $N^{\log_2 7}$ algorithm, so can the matrix inversion!

This is fun, but let's look at practicalities: If you estimate how large N has to be before the difference between exponent 3 and exponent $\log_2 7 = 2.807$ is substantial enough to outweigh the bookkeeping overhead, arising from the complicated nature of the recursive Strassen algorithm, you will find that LU decomposition is in no immediate danger of becoming obsolete. However, the fast matrix multiplication routine itself is beginning to appear in libraries like BLAS, where it is typically used for $N \gtrsim 100$.

Strassen's original result for matrix multiplication has been steadily improved. The fastest currently known algorithm [2] has an asymptotic order of $N^{2.376}$, but it is not likely to be practical to implement it.

If you like this kind of fun, then try these: (1) Can you multiply the complex numbers $(a+ib)$ and $(c+id)$ in only *three* real multiplications? [Answer: See §5.5.] (2) Can you evaluate a general fourth-degree polynomial in x for many different values of x with only *three* multiplications per evaluation? [Answer: See §5.1.]

CITED REFERENCES AND FURTHER READING:

Strassen, V. 1969, "Gaussian Elimination Is Not Optimal," *Numerische Mathematik*, vol. 13, pp. 354–356.[1]

Coppersmith, D., and Winograd, S. 1990, "Matrix Multiplications via Arithmetic Progressions," *Journal of Symbolic Computation*, vol. 9, pp. 251–280.[2]

Kronsjö, L. 1987, *Algorithms: Their Complexity and Efficiency*, 2nd ed. (New York: Wiley).

Winograd, S. 1971, "On the Multiplication of 2 by 2 Matrices," *Linear Algebra and Its Applications*, vol. 4, pp. 381–388.

Pan, V. Ya. 1980, "New Fast Algorithms for Matrix Operations," *SIAM Journal on Computing*, vol. 9, pp. 321–342.

Pan, V. 1984, *How to Multiply Matrices Faster*, Lecture Notes in Computer Science, vol. 179 (New York: Springer)

Pan, V. 1984, "How Can We Speed Up Matrix Multiplication?", *SIAM Review*, vol. 26, pp. 393–415.

Interpolation and Extrapolation

CHAPTER 3

3.0 Introduction

We sometimes know the value of a function $f(x)$ at a set of points $x_0, x_1, \ldots, x_{N-1}$ (say, with $x_0 < \ldots < x_{N-1}$), but we don't have an analytic expression for $f(x)$ that lets us calculate its value at an arbitrary point. For example, the $f(x_i)$'s might result from some physical measurement or from long numerical calculation that cannot be cast into a simple functional form. Often the x_i's are equally spaced, but not necessarily.

The task now is to estimate $f(x)$ for arbitrary x by, in some sense, drawing a smooth curve through (and perhaps beyond) the x_i. If the desired x is in between the largest and smallest of the x_i's, the problem is called *interpolation*; if x is outside that range, it is called *extrapolation*, which is considerably more hazardous (as many former investment analysts can attest).

Interpolation and extrapolation schemes must model the function, between or beyond the known points, by some plausible functional form. The form should be sufficiently general so as to be able to approximate large classes of functions that might arise in practice. By far most common among the functional forms used are polynomials (§3.2). Rational functions (quotients of polynomials) also turn out to be extremely useful (§3.4). Trigonometric functions, sines and cosines, give rise to *trigonometric interpolation* and related Fourier methods, which we defer to Chapters 12 and 13.

There is an extensive mathematical literature devoted to theorems about what sort of functions can be well approximated by which interpolating functions. These theorems are, alas, almost completely useless in day-to-day work: If we know enough about our function to apply a theorem of any power, we are usually not in the pitiful state of having to interpolate on a table of its values!

Interpolation is related to, but distinct from, *function approximation*. That task consists of finding an approximate (but easily computable) function to use in place of a more complicated one. In the case of interpolation, you are given the function f at points *not of your own choosing*. For the case of function approximation, you are allowed to compute the function f at *any* desired points for the purpose of developing

your approximation. We deal with function approximation in Chapter 5.

One can easily find pathological functions that make a mockery of any interpolation scheme. Consider, for example, the function

$$f(x) = 3x^2 + \frac{1}{\pi^4} \ln\left[(\pi - x)^2\right] + 1 \tag{3.0.1}$$

which is well-behaved everywhere except at $x = \pi$, very mildly singular at $x = \pi$, and otherwise takes on all positive and negative values. Any interpolation based on the values $x = 3.13, 3.14, 3.15, 3.16$, will assuredly get a very wrong answer for the value $x = 3.1416$, even though a graph plotting those five points looks really quite smooth! (Try it.)

Because pathologies can lurk anywhere, it is highly desirable that an interpolation and extrapolation routine should provide an estimate of its own error. Such an error estimate can never be foolproof, of course. We could have a function that, for reasons known only to its maker, takes off wildly and unexpectedly between two tabulated points. Interpolation always presumes some degree of smoothness for the function interpolated, but within this framework of presumption, deviations from smoothness can be detected.

Conceptually, the interpolation process has two stages: (1) Fit (once) an interpolating function to the data points provided. (2) Evaluate (as many times as you wish) that interpolating function at a target point x.

However, this two-stage method is usually not the best way to proceed in practice. Typically it is computationally less efficient, and more susceptible to roundoff error, than methods that construct a functional estimate $f(x)$ directly from the N tabulated values every time one is desired. Many practical schemes start at a nearby point $f(x_i)$, and then add a sequence of (hopefully) decreasing corrections, as information from other nearby $f(x_i)$'s is incorporated. The procedure typically takes $O(M^2)$ operations, where $M \ll N$ is the number of local points used. If everything is well behaved, the last correction will be the smallest, and it can be used as an informal (though not rigorous) bound on the error. In schemes like this, we might also say that there are two stages, but now they are: (1) Find the right starting position in the table (x_i or i). (2) Perform the interpolation using M nearby values (for example, centered on x_i).

In the case of polynomial interpolation, it sometimes does happen that the coefficients of the interpolating polynomial are of interest, even though their use in *evaluating* the interpolating function should be frowned on. We deal with this possibility in §3.5.

Local interpolation, using M nearest-neighbor points, gives interpolated values $f(x)$ that do not, in general, have continuous first or higher derivatives. That happens because, as x crosses the tabulated values x_i, the interpolation scheme switches which tabulated points are the "local" ones. (If such a switch is allowed to occur anywhere *else*, then there will be a discontinuity in the interpolated function itself at that point. Bad idea!)

In situations where continuity of derivatives is a concern, one must use the "stiffer" interpolation provided by a so-called *spline* function. A spline is a polynomial between each pair of table points, but one whose coefficients are determined "slightly" nonlocally. The nonlocality is designed to guarantee global smoothness in the interpolated function up to some order of derivative. Cubic splines (§3.3) are the

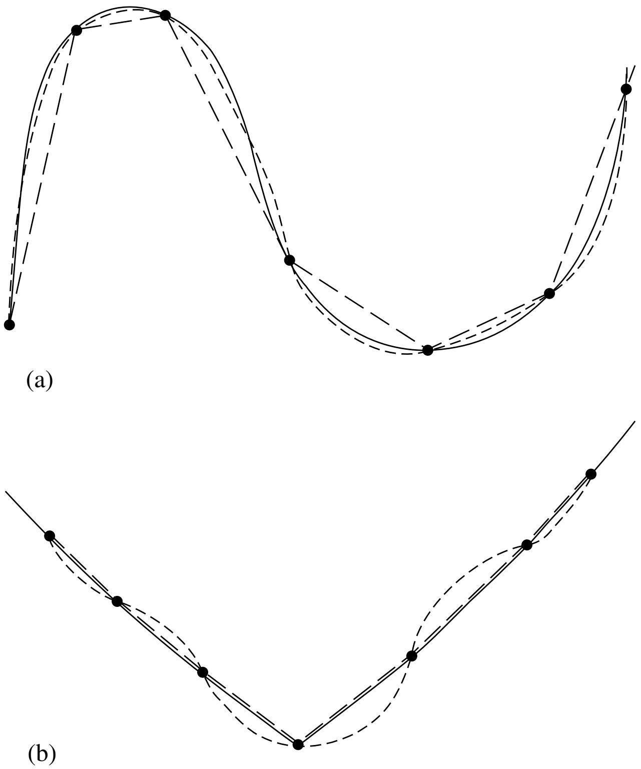

Figure 3.0.1. (a) A smooth function (solid line) is more accurately interpolated by a high-order polynomial (shown schematically as dotted line) than by a low-order polynomial (shown as a piecewise linear dashed line). (b) A function with sharp corners or rapidly changing higher derivatives is *less* accurately approximated by a high-order polynomial (dotted line), which is too "stiff," than by a low-order polynomial (dashed lines). Even some smooth functions, such as exponentials or rational functions, can be badly approximated by high-order polynomials.

most popular. They produce an interpolated function that is continuous through the second derivative. Splines tend to be stabler than polynomials, with less possibility of wild oscillation between the tabulated points.

The number M of points used in an interpolation scheme, minus 1, is called the *order* of the interpolation. Increasing the order does not necessarily increase the accuracy, especially in polynomial interpolation. If the added points are distant from the point of interest x, the resulting higher-order polynomial, with its additional constrained points, tends to oscillate wildly between the tabulated values. This oscillation may have no relation at all to the behavior of the "true" function (see Figure 3.0.1). Of course, adding points *close* to the desired point usually does help, but a finer mesh implies a larger table of values, which is not always available.

For polynomial interpolation, it turns out that the *worst* possible arrangement of the x_i's is for them to be equally spaced. Unfortunately, this is by far the most common way that tabulated data are gathered or presented. High-order polynomial interpolation on equally spaced data is *ill-conditioned*: small changes in the data can give large differences in the oscillations between the points. The disease is particularly bad if you are interpolating on values of an analytic function that has poles in

the complex plane lying inside a certain oval region whose major axis is the M-point interval. But even if you have a function with no nearby poles, roundoff error can, in effect, create nearby poles and cause big interpolation errors. In §5.8 we will see that these issues go away if you are allowed to choose an optimal set of x_i's. But when you are handed a table of function values, that option is not available.

As the order is increased, it is typical for interpolation error to decrease at first, but only up to a certain point. Larger orders result in the error exploding.

For the reasons mentioned, it is a good idea to be cautious about high-order interpolation. We can enthusiastically endorse polynomial interpolation with 3 or 4 points; we are perhaps tolerant of 5 or 6; but we rarely go higher than that unless there is quite rigorous monitoring of estimated errors. Most of the interpolation methods in this chapter are applied *piecewise* using only M points at a time, so that the order is a fixed value $M - 1$, no matter how large N is. As mentioned, *splines* (§3.3) are a special case where the function and various derivatives are required to be continuous from one interval to the next, but the order is nevertheless held fixed a a small value (usually 3).

In §3.4 we discuss *rational function interpolation*. In many, but not all, cases, rational function interpolation is more robust, allowing higher orders to give higher accuracy. The standard algorithm, however, allows poles on the real axis or nearby in the complex plane. (This is not necessarily bad: You may be trying to approximate a function with such poles.) A newer method, *barycentric rational interpolation* (§3.4.1) suppresses all nearby poles. This is the only method in this chapter for which we might actually encourage experimentation with high order (say, > 6). Barycentric rational interpolation competes very favorably with splines: its error is often smaller, and the resulting approximation is infinitely smooth (unlike splines).

The interpolation methods below are also methods for extrapolation. An important application, in Chapter 17, is their use in the integration of ordinary differential equations. There, considerable care *is* taken with the monitoring of errors. Otherwise, the dangers of extrapolation cannot be overemphasized: An interpolating function, which is perforce an extrapolating function, will typically go berserk when the argument x is outside the range of tabulated values by more (and often significantly less) than the typical spacing of tabulated points.

Interpolation can be done in more than one dimension, e.g., for a function $f(x, y, z)$. Multidimensional interpolation is often accomplished by a sequence of one-dimensional interpolations, but there are also other techniques applicable to scattered data. We discuss multidimensional methods in §3.6 – §3.8.

CITED REFERENCES AND FURTHER READING:

Abramowitz, M., and Stegun, I.A. 1964, *Handbook of Mathematical Functions* (Washington: National Bureau of Standards); reprinted 1968 (New York: Dover); online at `http://www.nr.com/aands`, §25.2.

Ueberhuber, C.W. 1997, *Numerical Computation: Methods, Software, and Analysis*, vol. 1 (Berlin: Springer), Chapter 9.

Stoer, J., and Bulirsch, R. 2002, *Introduction to Numerical Analysis*, 3rd ed. (New York: Springer), Chapter 2.

Acton, F.S. 1970, *Numerical Methods That Work*; 1990, corrected edition (Washington, DC: Mathematical Association of America), Chapter 3.

Johnson, L.W., and Riess, R.D. 1982, *Numerical Analysis*, 2nd ed. (Reading, MA: Addison-Wesley), Chapter 5.

Ralston, A., and Rabinowitz, P. 1978, *A First Course in Numerical Analysis*, 2nd ed.; reprinted 2001 (New York: Dover), Chapter 3.

Isaacson, E., and Keller, H.B. 1966, *Analysis of Numerical Methods*; reprinted 1994 (New York: Dover), Chapter 6.

3.1 Preliminaries: Searching an Ordered Table

We want to define an interpolation object that knows everything about interpolation except one thing — how to actually interpolate! Then we can plug mathematically different interpolation methods into the object to get different objects sharing a common user interface. A key task common to all objects in this framework is finding your place in the table of x_i's, given some particular value x at which the function evaluation is desired. It is worth some effort to do this efficiently; otherwise you can easily spend more time searching the table than doing the actual interpolation.

Our highest-level object for one-dimensional interpolation is an abstract base class containing just one function intended to be called by the user: `interp(x)` returns the interpolated function value at x. The base class "promises," by declaring a virtual function `rawinterp(jlo,x)`, that every derived interpolation class will provide a method for local interpolation when given an appropriate local starting point in the table, an offset `jlo`. Interfacing between `interp` and `rawinterp` must thus be a method for calculating `jlo` from x, that is, for searching the table. In fact, we will use two such methods.

interp_1d.h

```
struct Base_interp
Abstract base class used by all interpolation routines in this chapter. Only the routine interp
is called directly by the user.
{
    Int n, mm, jsav, cor, dj;
    const Doub *xx, *yy;
    Base_interp(VecDoub_I &x, const Doub *y, Int m)
    Constructor: Set up for interpolating on a table of x's and y's of length m. Normally called
    by a derived class, not by the user.
        : n(x.size()), mm(m), jsav(0), cor(0), xx(&x[0]), yy(y) {
        dj = MIN(1,(int)pow((Doub)n,0.25));
    }

    Doub interp(Doub x) {
    Given a value x, return an interpolated value, using data pointed to by xx and yy.
        Int jlo = cor ? hunt(x) : locate(x);
        return rawinterp(jlo,x);
    }

    Int locate(const Doub x);            See definitions below.
    Int hunt(const Doub x);

    Doub virtual rawinterp(Int jlo, Doub x) = 0;
    Derived classes provide this as the actual interpolation method.

};
```

Formally, the problem is this: Given an array of abscissas x_j, $j = 0, \ldots, N-1$, with the abscissas either monotonically increasing or monotonically decreasing, and given an integer $M \leq N$, and a number x, find an integer $j_{\rm lo}$ such that x is centered

among the M abscissas $x_{j_{lo}}, \ldots, x_{j_{lo}+M-1}$. By centered we mean that x lies between x_m and x_{m+1} insofar as possible, where

$$m = j_{lo} + \left\lfloor \frac{M-2}{2} \right\rfloor \tag{3.1.1}$$

By "insofar as possible" we mean that j_{lo} should never be less than zero, nor should $j_{lo} + M - 1$ be greater than $N - 1$.

In most cases, when all is said and done, it is hard to do better than *bisection*, which will find the right place in the table in about $\log_2 N$ tries.

```
Int Base_interp::locate(const Doub x)
```
interp_1d.h

Given a value `x`, return a value `j` such that `x` is (insofar as possible) centered in the subrange `xx[j..j+mm-1]`, where `xx` is the stored pointer. The values in `xx` must be monotonic, either increasing or decreasing. The returned value is not less than 0, nor greater than `n-1`.

```
{
    Int ju,jm,jl;
    if (n < 2 || mm < 2 || mm > n) throw("locate size error");
    Bool ascnd=(xx[n-1] >= xx[0]);      True if ascending order of table, false otherwise.
    jl=0;                               Initialize lower
    ju=n-1;                             and upper limits.
    while (ju-jl > 1) {                 If we are not yet done,
        jm = (ju+jl) >> 1;              compute a midpoint,
        if (x >= xx[jm] == ascnd)
            jl=jm;                      and replace either the lower limit
        else
            ju=jm;                      or the upper limit, as appropriate.
    }                                   Repeat until the test condition is satisfied.
    cor = abs(jl-jsav) > dj ? 0 : 1;    Decide whether to use hunt or locate next time.
    jsav = jl;
    return MAX(0,MIN(n-mm,jl-((mm-2)>>1)));
}
```

The above `locate` routine accesses the array of values `xx[]` via a pointer stored by the base class. This rather primitive method of access, avoiding the use of a higher-level vector class like `VecDoub`, is here preferable for two reasons: (1) It's usually faster; and (2) for two-dimensional interpolation, we will later need to point directly into a row of a matrix. The peril of this design choice is that it assumes that consecutive values of a vector are stored consecutively, and similarly for consecutive values of a single row of a matrix. See discussion in §1.4.2.

3.1.1 Search with Correlated Values

Experience shows that in many, perhaps even most, applications, interpolation routines are called with nearly identical abscissas on consecutive searches. For example, you may be generating a function that is used on the right-hand side of a differential equation: Most differential equation integrators, as we shall see in Chapter 17, call for right-hand side evaluations at points that hop back and forth a bit, but whose trend moves slowly in the direction of the integration.

In such cases it is wasteful to do a full bisection, *ab initio*, on each call. Much more desirable is to give our base class a tiny bit of intelligence: If it sees two calls that are "close," it anticipates that the next call will also be. Of course, there must not be too big a penalty if it anticipates wrongly.

The `hunt` method starts with a guessed position in the table. It first "hunts," either up or down, in increments of 1, then 2, then 4, etc., until the desired value is bracketed. It then bisects in the bracketed interval. At worst, this routine is about a

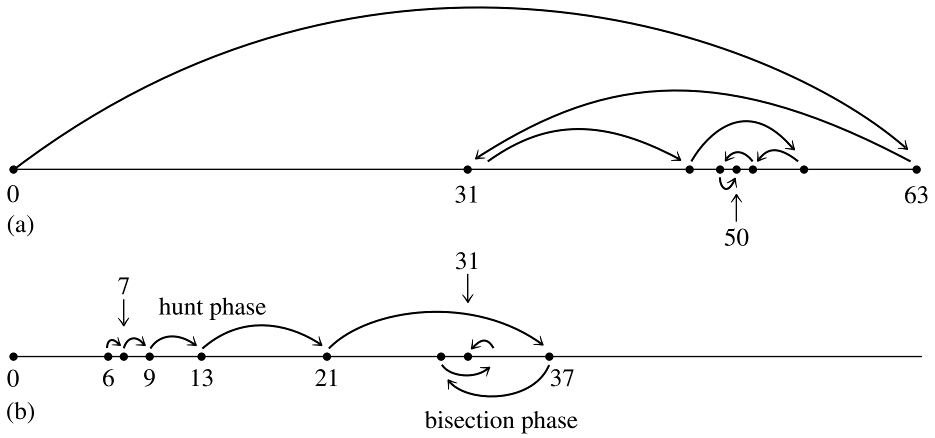

Figure 3.1.1. Finding a table entry by bisection. Shown here is the sequence of steps that converge to element 50 in a table of length 64. (b) The routine `hunt` searches from a previous known position in the table by increasing steps and then converges by bisection. Shown here is a particularly unfavorable example, converging to element 31 from element 6. A favorable example would be convergence to an element near 6, such as 8, which would require just three "hops."

factor of 2 slower than `locate` above (if the hunt phase expands to include the whole table). At best, it can be a factor of $\log_2 n$ faster than `locate`, if the desired point is usually quite close to the input guess. Figure 3.1.1 compares the two routines.

interp_1d.h

```
Int Base_interp::hunt(const Doub x)
```
Given a value `x`, return a value `j` such that `x` is (insofar as possible) centered in the subrange `xx[j..j+mm-1]`, where `xx` is the stored pointer. The values in `xx` must be monotonic, either increasing or decreasing. The returned value is not less than 0, nor greater than `n-1`.
```
{
    Int jl=jsav, jm, ju, inc=1;
    if (n < 2 || mm < 2 || mm > n) throw("hunt size error");
    Bool ascnd=(xx[n-1] >= xx[0]);        True if ascending order of table, false otherwise.
    if (jl < 0 || jl > n-1) {             Input guess not useful. Go immediately to bisec-
        jl=0;                                 tion.
        ju=n-1;
    } else {
        if (x >= xx[jl] == ascnd) {       Hunt up:
            for (;;) {
                ju = jl + inc;
                if (ju >= n-1) { ju = n-1; break;}          Off end of table.
                else if (x < xx[ju] == ascnd) break;        Found bracket.
                else {                    Not done, so double the increment and try again.
                    jl = ju;
                    inc += inc;
                }
            }
        } else {                          Hunt down:
            ju = jl;
            for (;;) {
                jl = jl - inc;
                if (jl <= 0) { jl = 0; break;}              Off end of table.
                else if (x >= xx[jl] == ascnd) break;       Found bracket.
                else {                    Not done, so double the increment and try again.
                    ju = jl;
                    inc += inc;
```

```
                }
            }
        }
    }
    while (ju-jl > 1) {                    Hunt is done, so begin the final bisection phase:
        jm = (ju+jl) >> 1;
        if (x >= xx[jm] == ascnd)
            jl=jm;
        else
            ju=jm;
    }
    cor = abs(jl-jsav) > dj ? 0 : 1;       Decide whether to use hunt or locate next
    jsav = jl;                                 time.
    return MAX(0,MIN(n-mm,jl-((mm-2)>>1)));
}
```

The methods `locate` and `hunt` each update the boolean variable `cor` in the base class, indicating whether consecutive calls seem correlated. That variable is then used by `interp` to decide whether to use `locate` or `hunt` on the next call. This is all invisible to the user, of course.

3.1.2 Example: Linear Interpolation

You may think that, at this point, we have wandered far from the subject of interpolation methods. To show that we are actually on track, here is a class that efficiently implements piecewise linear interpolation.

interp_linear.h

```
struct Linear_interp : Base_interp
Piecewise linear interpolation object. Construct with x and y vectors, then call interp for
interpolated values.
{
    Linear_interp(VecDoub_I &xv, VecDoub_I &yv)
        : Base_interp(xv,&yv[0],2)  {}
    Doub rawinterp(Int j, Doub x) {
        if (xx[j]==xx[j+1]) return yy[j];      Table is defective, but we can recover.
        else return yy[j] + ((x-xx[j])/(xx[j+1]-xx[j]))*(yy[j+1]-yy[j]);
    }
};
```

You construct a linear interpolation object by declaring an instance with your filled vectors of abscissas x_i and function values $y_i = f(x_i)$,

```
Int n=...;
VecDoub xx(n), yy(n);
...
Linear_interp myfunc(xx,yy);
```

Behind the scenes, the base class constructor is called with $M = 2$ because linear interpolation uses just the two points bracketing a value. Also, pointers to the data are saved. (You must ensure that the vectors `xx` and `yy` don't go out of scope while `myfunc` is in use.)

When you want an interpolated value, it's as simple as

```
Doub x,y;
...
y = myfunc.interp(x);
```

If you have several functions that you want to interpolate, you declare a separate instance of `Linear_interp` for each one.

We will now use the same interface for more advanced interpolation methods.

CITED REFERENCES AND FURTHER READING:

Knuth, D.E. 1997, *Sorting and Searching*, 3rd ed., vol. 3 of *The Art of Computer Programming* (Reading, MA: Addison-Wesley), §6.2.1.

3.2 Polynomial Interpolation and Extrapolation

Through any two points there is a unique line. Through any three points there is a unique quadratic. *Et cetera.* The interpolating polynomial of degree $M-1$ through the M points $y_0 = f(x_0), y_1 = f(x_1), \ldots, y_{M-1} = f(x_{M-1})$ is given explicitly by Lagrange's classical formula,

$$\begin{aligned} P(x) = &\frac{(x-x_1)(x-x_2)...(x-x_{M-1})}{(x_0-x_1)(x_0-x_2)...(x_0-x_{M-1})}y_0 \\ &+ \frac{(x-x_0)(x-x_2)...(x-x_{M-1})}{(x_1-x_0)(x_1-x_2)...(x_1-x_{M-1})}y_1 + \cdots \\ &+ \frac{(x-x_0)(x-x_1)...(x-x_{M-2})}{(x_{M-1}-x_0)(x_{M-1}-x_1)...(x_{M-1}-x_{M-2})}y_{M-1} \end{aligned} \tag{3.2.1}$$

There are M terms, each a polynomial of degree $M-1$ and each constructed to be zero at all of the x_i's except one, at which it is constructed to be y_i.

It is not terribly wrong to implement the Lagrange formula straightforwardly, but it is not terribly right either. The resulting algorithm gives no error estimate, and it is also somewhat awkward to program. A much better algorithm (for constructing the same, unique, interpolating polynomial) is *Neville's algorithm*, closely related to and sometimes confused with *Aitken's algorithm*, the latter now considered obsolete.

Let P_0 be the value at x of the unique polynomial of degree zero (i.e., a constant) passing through the point (x_0, y_0); so $P_0 = y_0$. Likewise define $P_1, P_2, \ldots, P_{M-1}$. Now let P_{01} be the value at x of the unique polynomial of degree one passing through both (x_0, y_0) and (x_1, y_1). Likewise $P_{12}, P_{23}, \ldots, P_{(M-2)(M-1)}$. Similarly, for higher-order polynomials, up to $P_{012...(M-1)}$, which is the value of the unique interpolating polynomial through all M points, i.e., the desired answer. The various P's form a "tableau" with "ancestors" on the left leading to a single "descendant" at the extreme right. For example, with $M = 4$,

$$\begin{array}{lllll} x_0: & y_0 = P_0 & & & \\ & & P_{01} & & \\ x_1: & y_1 = P_1 & & P_{012} & \\ & & P_{12} & & P_{0123} \\ x_2: & y_2 = P_2 & & P_{123} & \\ & & P_{23} & & \\ x_3: & y_3 = P_3 & & & \end{array} \tag{3.2.2}$$

Neville's algorithm is a recursive way of filling in the numbers in the tableau a column at a time, from left to right. It is based on the relationship between a

"daughter" P and its two "parents,"

$$P_{i(i+1)\ldots(i+m)} = \frac{(x - x_{i+m})P_{i(i+1)\ldots(i+m-1)} + (x_i - x)P_{(i+1)(i+2)\ldots(i+m)}}{x_i - x_{i+m}} \tag{3.2.3}$$

This recurrence works because the two parents already agree at points $x_{i+1} \ldots x_{i+m-1}$.

An improvement on the recurrence (3.2.3) is to keep track of the small *differences* between parents and daughters, namely to define (for $m = 1, 2, \ldots, M - 1$),

$$\begin{aligned} C_{m,i} &\equiv P_{i\ldots(i+m)} - P_{i\ldots(i+m-1)} \\ D_{m,i} &\equiv P_{i\ldots(i+m)} - P_{(i+1)\ldots(i+m)}. \end{aligned} \tag{3.2.4}$$

Then one can easily derive from (3.2.3) the relations

$$\begin{aligned} D_{m+1,i} &= \frac{(x_{i+m+1} - x)(C_{m,i+1} - D_{m,i})}{x_i - x_{i+m+1}} \\ C_{m+1,i} &= \frac{(x_i - x)(C_{m,i+1} - D_{m,i})}{x_i - x_{i+m+1}} \end{aligned} \tag{3.2.5}$$

At each level m, the C's and D's are the corrections that make the interpolation one order higher. The final answer $P_{0\ldots(M-1)}$ is equal to the sum of *any* y_i plus a set of C's and/or D's that form a path through the family tree to the rightmost daughter.

Here is the class implementing polynomial interpolation or extrapolation. All of its "support infrastructure" is in the base class `Base_interp`. It needs only to provide a `rawinterp` method that contains Neville's algorithm.

interp_1d.h

```
struct Poly_interp : Base_interp
```
Polynomial interpolation object. Construct with **x** and **y** vectors, and the number M of points to be used locally (polynomial order plus one), then call `interp` for interpolated values.
```
{
    Doub dy;
    Poly_interp(VecDoub_I &xv, VecDoub_I &yv, Int m)
        : Base_interp(xv,&yv[0],m), dy(0.) {}
    Doub rawinterp(Int jl, Doub x);
};
```

```
Doub Poly_interp::rawinterp(Int jl, Doub x)
```
Given a value `x`, and using pointers to data `xx` and `yy`, this routine returns an interpolated value `y`, and stores an error estimate `dy`. The returned value is obtained by `mm`-point polynomial interpolation on the subrange `xx[jl..jl+mm-1]`.
```
{
    Int i,m,ns=0;
    Doub y,den,dif,dift,ho,hp,w;
    const Doub *xa = &xx[jl], *ya = &yy[jl];
    VecDoub c(mm),d(mm);
    dif=abs(x-xa[0]);
    for (i=0;i<mm;i++) {            Here we find the index ns of the closest table entry,
        if ((dift=abs(x-xa[i])) < dif) {
            ns=i;
            dif=dift;
        }
        c[i]=ya[i];                 and initialize the tableau of c's and d's.
        d[i]=ya[i];
    }
    y=ya[ns--];                     This is the initial approximation to y.
    for (m=1;m<mm;m++) {            For each column of the tableau,
```

```
        for (i=0;i<mm-m;i++) {          we loop over the current c's and d's and update
            ho=xa[i]-x;                     them.
            hp=xa[i+m]-x;
            w=c[i+1]-d[i];
            if ((den=ho-hp) == 0.0) throw("Poly_interp error");
            This error can occur only if two input xa's are (to within roundoff) identical.
            den=w/den;
            d[i]=hp*den;                Here the c's and d's are updated.
            c[i]=ho*den;
        }
        y += (dy=(2*(ns+1) < (mm-m) ? c[ns+1] : d[ns--]));
        After each column in the tableau is completed, we decide which correction, c or d, we
        want to add to our accumulating value of y, i.e., which path to take through the tableau
        — forking up or down. We do this in such a way as to take the most "straight line"
        route through the tableau to its apex, updating ns accordingly to keep track of where
        we are. This route keeps the partial approximations centered (insofar as possible) on
        the target x. The last dy added is thus the error indication.
    }
    return y;
}
```

The user interface to `Poly_interp` is virtually the same as for `Linear_interp` (end of §3.1), except that an additional argument in the constructor sets M, the number of points used (the order plus one). A cubic interpolator looks like this:

```
Int n=...;
VecDoub xx(n), yy(n);
...
Poly_interp myfunc(xx,yy,4);
```

`Poly_interp` stores an error estimate `dy` for the most recent call to its `interp` function:

```
Doub x,y,err;
...
y = myfunc.interp(x);
err = myfunc.dy;
```

CITED REFERENCES AND FURTHER READING:

Abramowitz, M., and Stegun, I.A. 1964, *Handbook of Mathematical Functions* (Washington: National Bureau of Standards); reprinted 1968 (New York: Dover); online at `http://www.nr.com/aands`, §25.2.

Stoer, J., and Bulirsch, R. 2002, *Introduction to Numerical Analysis*, 3rd ed. (New York: Springer), §2.1.

Gear, C.W. 1971, *Numerical Initial Value Problems in Ordinary Differential Equations* (Englewood Cliffs, NJ: Prentice-Hall), §6.1.

3.3 Cubic Spline Interpolation

Given a tabulated function $y_i = y(x_i)$, $i = 0...N-1$, focus attention on one particular interval, between x_j and x_{j+1}. Linear interpolation in that interval gives the interpolation formula

$$y = Ay_j + By_{j+1} \tag{3.3.1}$$

where
$$A \equiv \frac{x_{j+1} - x}{x_{j+1} - x_j} \qquad B \equiv 1 - A = \frac{x - x_j}{x_{j+1} - x_j} \tag{3.3.2}$$

Equations (3.3.1) and (3.3.2) are a special case of the general Lagrange interpolation formula (3.2.1).

Since it is (piecewise) linear, equation (3.3.1) has zero second derivative in the interior of each interval and an undefined, or infinite, second derivative at the abscissas x_j. The goal of cubic spline interpolation is to get an interpolation formula that is smooth in the first derivative and continuous in the second derivative, both within an interval and at its boundaries.

Suppose, contrary to fact, that in addition to the tabulated values of y_i, we also have tabulated values for the function's second derivatives, y'', that is, a set of numbers y_i''. Then, within each interval, we can add to the right-hand side of equation (3.3.1) a cubic polynomial whose second derivative varies linearly from a value y_j'' on the left to a value y_{j+1}'' on the right. Doing so, we will have the desired continuous second derivative. If we also construct the cubic polynomial to have zero *values* at x_j and x_{j+1}, then adding it in will not spoil the agreement with the tabulated functional values y_j and y_{j+1} at the endpoints x_j and x_{j+1}.

A little side calculation shows that there is only one way to arrange this construction, namely replacing (3.3.1) by

$$y = Ay_j + By_{j+1} + Cy_j'' + Dy_{j+1}'' \tag{3.3.3}$$

where A and B are defined in (3.3.2) and

$$C \equiv \tfrac{1}{6}(A^3 - A)(x_{j+1} - x_j)^2 \qquad D \equiv \tfrac{1}{6}(B^3 - B)(x_{j+1} - x_j)^2 \tag{3.3.4}$$

Notice that the dependence on the independent variable x in equations (3.3.3) and (3.3.4) is entirely through the linear x-dependence of A and B, and (through A and B) the cubic x-dependence of C and D.

We can readily check that y'' is in fact the second derivative of the new interpolating polynomial. We take derivatives of equation (3.3.3) with respect to x, using the definitions of A, B, C, and D to compute dA/dx, dB/dx, dC/dx, and dD/dx. The result is

$$\frac{dy}{dx} = \frac{y_{j+1} - y_j}{x_{j+1} - x_j} - \frac{3A^2 - 1}{6}(x_{j+1} - x_j)y_j'' + \frac{3B^2 - 1}{6}(x_{j+1} - x_j)y_{j+1}'' \tag{3.3.5}$$

for the first derivative, and

$$\frac{d^2y}{dx^2} = Ay_j'' + By_{j+1}'' \tag{3.3.6}$$

for the second derivative. Since $A = 1$ at x_j, $A = 0$ at x_{j+1}, while B is just the other way around, (3.3.6) shows that y'' is just the tabulated second derivative, and also that the second derivative will be continuous across, e.g., the boundary between the two intervals (x_{j-1}, x_j) and (x_j, x_{j+1}).

The only problem now is that we supposed the y_i'''s to be known, when, actually, they are not. However, we have not yet required that the *first* derivative, computed from equation (3.3.5), be continuous across the boundary between two intervals. The

key idea of a cubic spline is to require this continuity and to use it to get equations for the second derivatives y_i''.

The required equations are obtained by setting equation (3.3.5) evaluated for $x = x_j$ in the interval (x_{j-1}, x_j) equal to the same equation evaluated for $x = x_j$ but in the interval (x_j, x_{j+1}). With some rearrangement, this gives (for $j = 1, \ldots, N-2$)

$$\frac{x_j - x_{j-1}}{6} y_{j-1}'' + \frac{x_{j+1} - x_{j-1}}{3} y_j'' + \frac{x_{j+1} - x_j}{6} y_{j+1}'' = \frac{y_{j+1} - y_j}{x_{j+1} - x_j} - \frac{y_j - y_{j-1}}{x_j - x_{j-1}} \tag{3.3.7}$$

These are $N-2$ linear equations in the N unknowns $y_i'', i = 0, \ldots, N-1$. Therefore there is a two-parameter family of possible solutions.

For a unique solution, we need to specify two further conditions, typically taken as boundary conditions at x_0 and x_{N-1}. The most common ways of doing this are either

- set one or both of y_0'' and y_{N-1}'' equal to zero, giving the so-called *natural cubic spline*, which has zero second derivative on one or both of its boundaries, or
- set either of y_0'' and y_{N-1}'' to values calculated from equation (3.3.5) so as to make the first derivative of the interpolating function have a specified value on either or both boundaries.

Although the boundary condition for natural splines is commonly used, another possibility is to estimate the first derivatives at the endpoints from the first and last few tabulated points. For details of how to do this, see the end of §3.7. Best, of course, is if you can compute the endpoint first derivatives analytically.

One reason that cubic splines are especially practical is that the set of equations (3.3.7), along with the two additional boundary conditions, are not only linear, but also *tridiagonal*. Each y_j'' is coupled only to its nearest neighbors at $j \pm 1$. Therefore, the equations can be solved in $O(N)$ operations by the tridiagonal algorithm (§2.4). That algorithm is concise enough to build right into the function called by the constructor.

The object for cubic spline interpolation looks like this:

interp_1d.h

```
struct Spline_interp : Base_interp
```
Cubic spline interpolation object. Construct with **x** and **y** vectors, and (optionally) values of the first derivative at the endpoints, then call `interp` for interpolated values.
```
{
    VecDoub y2;

    Spline_interp(VecDoub_I &xv, VecDoub_I &yv, Doub yp1=1.e99, Doub ypn=1.e99)
    : Base_interp(xv,&yv[0],2), y2(xv.size())
    {sety2(&xv[0],&yv[0],yp1,ypn);}

    Spline_interp(VecDoub_I &xv, const Doub *yv, Doub yp1=1.e99, Doub ypn=1.e99)
    : Base_interp(xv,yv,2), y2(xv.size())
    {sety2(&xv[0],yv,yp1,ypn);}

    void sety2(const Doub *xv, const Doub *yv, Doub yp1, Doub ypn);
    Doub rawinterp(Int jl, Doub xv);
};
```

For now, you can ignore the second constructor; it will be used later for two-dimensional spline interpolation.

The user interface differs from previous ones only in the addition of two constructor arguments, used to set the values of the first derivatives at the endpoints, y'_0 and y'_{N-1}. These are coded with default values that signal that you want a natural spline, so they can be omitted in most situations. Both constructors invoke sety2 to do the actual work of computing, and storing, the second derivatives.

```
void Spline_interp::sety2(const Doub *xv, const Doub *yv, Doub yp1, Doub ypn)
```
interp_1d.h

This routine stores an array y2[0..n-1] with second derivatives of the interpolating function at the tabulated points pointed to by xv, using function values pointed to by yv. If yp1 and/or ypn are equal to 1×10^{99} or larger, the routine is signaled to set the corresponding boundary condition for a natural spline, with zero second derivative on that boundary; otherwise, they are the values of the first derivatives at the endpoints.

```
{
    Int i,k;
    Doub p,qn,sig,un;
    Int n=y2.size();
    VecDoub u(n-1);
    if (yp1 > 0.99e99)              The lower boundary condition is set either to be "nat-
        y2[0]=u[0]=0.0;                 ural"
    else {                          or else to have a specified first derivative.
        y2[0] = -0.5;
        u[0]=(3.0/(xv[1]-xv[0]))*((yv[1]-yv[0])/(xv[1]-xv[0])-yp1);
    }
    for (i=1;i<n-1;i++) {           This is the decomposition loop of the tridiagonal al-
        sig=(xv[i]-xv[i-1])/(xv[i+1]-xv[i-1]);    gorithm. y2 and u are used for tem-
        p=sig*y2[i-1]+2.0;                        porary storage of the decomposed
        y2[i]=(sig-1.0)/p;                        factors.
        u[i]=(yv[i+1]-yv[i])/(xv[i+1]-xv[i]) - (yv[i]-yv[i-1])/(xv[i]-xv[i-1]);
        u[i]=(6.0*u[i]/(xv[i+1]-xv[i-1])-sig*u[i-1])/p;
    }
    if (ypn > 0.99e99)              The upper boundary condition is set either to be
        qn=un=0.0;                      "natural"
    else {                          or else to have a specified first derivative.
        qn=0.5;
        un=(3.0/(xv[n-1]-xv[n-2]))*(ypn-(yv[n-1]-yv[n-2])/(xv[n-1]-xv[n-2]));
    }
    y2[n-1]=(un-qn*u[n-2])/(qn*y2[n-2]+1.0);
    for (k=n-2;k>=0;k--)            This is the backsubstitution loop of the tridiagonal
        y2[k]=y2[k]*y2[k+1]+u[k];       algorithm.
}
```

Note that, unlike the previous object Poly_interp, Spline_interp stores data that depend on the contents of your array of y_i's at its time of creation — a whole vector y2. Although we didn't point it out, the previous interpolation object actually allowed the misuse of altering the contents of their x and y arrays on the fly (as long as the lengths didn't change). If you do that with Spline_interp, you'll get definitely wrong answers!

The required rawinterp method, never called directly by the users, uses the stored y2 and implements equation (3.3.3):

```
Doub Spline_interp::rawinterp(Int jl, Doub x)
```
interp_1d.h

Given a value x, and using pointers to data xx and yy, and the stored vector of second derivatives y2, this routine returns the cubic spline interpolated value y.

```
{
    Int klo=jl,khi=jl+1;
    Doub y,h,b,a;
    h=xx[khi]-xx[klo];
    if (h == 0.0) throw("Bad input to routine splint");     The xa's must be dis-
    a=(xx[khi]-x)/h;                                             tinct.
```

```
    b=(x-xx[klo])/h;                         Cubic spline polynomial is now evaluated.
    y=a*yy[klo]+b*yy[khi]+((a*a*a-a)*y2[klo]
        +(b*b*b-b)*y2[khi])*(h*h)/6.0;
    return y;
}
```

Typical use looks like this:

```
Int n=...;
VecDoub xx(n), yy(n);
...
Spline_interp myfunc(xx,yy);
```

and then, as often as you like,

```
Doub x,y;
...
y = myfunc.interp(x);
```

Note that no error estimate is available.

CITED REFERENCES AND FURTHER READING:

De Boor, C. 1978, *A Practical Guide to Splines* (New York: Springer).

Ueberhuber, C.W. 1997, *Numerical Computation: Methods, Software, and Analysis*, vol. 1 (Berlin: Springer), Chapter 9.

Forsythe, G.E., Malcolm, M.A., and Moler, C.B. 1977, *Computer Methods for Mathematical Computations* (Englewood Cliffs, NJ: Prentice-Hall), §4.4 – §4.5.

Stoer, J., and Bulirsch, R. 2002, *Introduction to Numerical Analysis*, 3rd ed. (New York: Springer), §2.4.

Ralston, A., and Rabinowitz, P. 1978, *A First Course in Numerical Analysis*, 2nd ed.; reprinted 2001 (New York: Dover), §3.8.

3.4 Rational Function Interpolation and Extrapolation

Some functions are not well approximated by polynomials but *are* well approximated by rational functions, that is quotients of polynomials. We denote by $R_{i(i+1)\dots(i+m)}$ a rational function passing through the $m+1$ points $(x_i, y_i), \dots, (x_{i+m}, y_{i+m})$. More explicitly, suppose

$$R_{i(i+1)\dots(i+m)} = \frac{P_\mu(x)}{Q_\nu(x)} = \frac{p_0 + p_1 x + \dots + p_\mu x^\mu}{q_0 + q_1 x + \dots + q_\nu x^\nu} \tag{3.4.1}$$

Since there are $\mu + \nu + 1$ unknown p's and q's (q_0 being arbitrary), we must have

$$m + 1 = \mu + \nu + 1 \tag{3.4.2}$$

In specifying a rational function interpolating function, you must give the desired order of both the numerator and the denominator.

Rational functions are sometimes superior to polynomials, roughly speaking, because of their ability to model functions with poles, that is, zeros of the denominator of equation (3.4.1). These poles might occur for real values of x, if the function

to be interpolated itself has poles. More often, the function $f(x)$ is finite for all finite *real* x but has an analytic continuation with poles in the complex x-plane. Such poles can themselves ruin a polynomial approximation, even one restricted to real values of x, just as they can ruin the convergence of an infinite power series in x. If you draw a circle in the complex plane around your m tabulated points, then you should not expect polynomial interpolation to be good unless the nearest pole is rather far outside the circle. A rational function approximation, by contrast, will stay "good" as long as it has enough powers of x in its denominator to account for (cancel) any nearby poles.

For the interpolation problem, a rational function is constructed so as to go through a chosen set of tabulated functional values. However, we should also mention in passing that rational function approximations can be used in analytic work. One sometimes constructs a rational function approximation by the criterion that the rational function of equation (3.4.1) itself have a power series expansion that agrees with the first $m+1$ terms of the power series expansion of the desired function $f(x)$. This is called *Padé approximation* and is discussed in §5.12.

Bulirsch and Stoer found an algorithm of the Neville type that performs rational function extrapolation on tabulated data. A tableau like that of equation (3.2.2) is constructed column by column, leading to a result and an error estimate. The Bulirsch-Stoer algorithm produces the so-called *diagonal* rational function, with the degrees of the numerator and denominator equal (if m is even) or with the degree of the denominator larger by one (if m is odd; cf. equation 3.4.2 above). For the derivation of the algorithm, refer to [1]. The algorithm is summarized by a recurrence relation exactly analogous to equation (3.2.3) for polynomial approximation:

$$\begin{aligned} R_{i(i+1)\dots(i+m)} &= R_{(i+1)\dots(i+m)} \\ &+ \frac{R_{(i+1)\dots(i+m)} - R_{i\dots(i+m-1)}}{\left(\frac{x-x_i}{x-x_{i+m}}\right)\left(1 - \frac{R_{(i+1)\dots(i+m)}-R_{i\dots(i+m-1)}}{R_{(i+1)\dots(i+m)}-R_{(i+1)\dots(i+m-1)}}\right) - 1} \end{aligned} \tag{3.4.3}$$

This recurrence generates the rational functions through $m+1$ points from the ones through m and (the term $R_{(i+1)\dots(i+m-1)}$ in equation 3.4.3) $m-1$ points. It is started with

$$R_i = y_i \tag{3.4.4}$$

and with

$$R \equiv [R_{i(i+1)\dots(i+m)} \quad \text{with} \quad m = -1] = 0 \tag{3.4.5}$$

Now, exactly as in equations (3.2.4) and (3.2.5) above, we can convert the recurrence (3.4.3) to one involving only the small differences

$$\begin{aligned} C_{m,i} &\equiv R_{i\dots(i+m)} - R_{i\dots(i+m-1)} \\ D_{m,i} &\equiv R_{i\dots(i+m)} - R_{(i+1)\dots(i+m)} \end{aligned} \tag{3.4.6}$$

Note that these satisfy the relation

$$C_{m+1,i} - D_{m+1,i} = C_{m,i+1} - D_{m,i} \tag{3.4.7}$$

which is useful in proving the recurrences

$$D_{m+1,i} = \frac{C_{m,i+1}(C_{m,i+1} - D_{m,i})}{\left(\frac{x-x_i}{x-x_{i+m+1}}\right) D_{m,i} - C_{m,i+1}}$$
$$C_{m+1,i} = \frac{\left(\frac{x-x_i}{x-x_{i+m+1}}\right) D_{m,i}(C_{m,i+1} - D_{m,i})}{\left(\frac{x-x_i}{x-x_{i+m+1}}\right) D_{m,i} - C_{m,i+1}} \tag{3.4.8}$$

The class for rational function interpolation is identical to that for polynomial interpolation in every way, except, of course, for the different method implemented in `rawinterp`. See the end of §3.2 for usage. Plausible values for M are in the range 4 to 7.

interp_1d.h

```
struct Rat_interp : Base_interp
```
Diagonal rational function interpolation object. Construct with **x** and **y** vectors, and the number `m` of points to be used locally, then call `interp` for interpolated values.
```
{
    Doub dy;
    Rat_interp(VecDoub_I &xv, VecDoub_I &yv, Int m)
        : Base_interp(xv,&yv[0],m), dy(0.) {}
    Doub rawinterp(Int jl, Doub x);
};

Doub Rat_interp::rawinterp(Int jl, Doub x)
```
Given a value `x`, and using pointers to data `xx` and `yy`, this routine returns an interpolated value `y`, and stores an error estimate `dy`. The returned value is obtained by `mm`-point diagonal rational function interpolation on the subrange `xx[jl..jl+mm-1]`.
```
{
    const Doub TINY=1.0e-99;          A small number.
    Int m,i,ns=0;
    Doub y,w,t,hh,h,dd;
    const Doub *xa = &xx[jl], *ya = &yy[jl];
    VecDoub c(mm),d(mm);
    hh=abs(x-xa[0]);
    for (i=0;i<mm;i++) {
        h=abs(x-xa[i]);
        if (h == 0.0) {
            dy=0.0;
            return ya[i];
        } else if (h < hh) {
            ns=i;
            hh=h;
        }
        c[i]=ya[i];
        d[i]=ya[i]+TINY;             The TINY part is needed to prevent a rare zero-over-zero
    }                                    condition.
    y=ya[ns--];
    for (m=1;m<mm;m++) {
        for (i=0;i<mm-m;i++) {
            w=c[i+1]-d[i];
            h=xa[i+m]-x;             h will never be zero, since this was tested in the initial-
            t=(xa[i]-x)*d[i]/h;          izing loop.
            dd=t-c[i+1];
            if (dd == 0.0) throw("Error in routine ratint");
```
This error condition indicates that the interpolating function has a pole at the requested value of `x`.
```
            dd=w/dd;
            d[i]=c[i+1]*dd;
            c[i]=t*dd;
```

```
        }
        y += (dy=(2*(ns+1) < (mm-m) ? c[ns+1] : d[ns--]));
    }
    return y;
}
```

3.4.1 Barycentric Rational Interpolation

Suppose one tries to use the above algorithm to construct a global approximation on the entire table of values using all the given nodes $x_0, x_1, \ldots, x_{N-1}$. One potential drawback is that the approximation can have poles inside the interpolation interval where the denominator in (3.4.1) vanishes, even if the original function has no poles there. An even greater (related) hazard is that we have allowed the order of the approximation to grow to $N-1$, probably much too large.

An alternative algorithm can be derived [2] that has no poles anywhere on the real axis, and that allows the actual order of the approximation to be specified to be any integer $d < N$. The trick is to make the degree of both the numerator and the denominator in equation (3.4.1) be $N-1$. This requires that the p's and the q's not be independent, so that equation (3.4.2) no longer holds.

The algorithm utilizes the *barycentric form* of the rational interpolant

$$R(x) = \frac{\displaystyle\sum_{i=0}^{N-1} \frac{w_i}{x - x_i} y_i}{\displaystyle\sum_{i=0}^{N-1} \frac{w_i}{x - x_i}} \tag{3.4.9}$$

One can show that by a suitable choice of the weights w_i, *every* rational interpolant can be written in this form, and that, as a special case, so can polynomial interpolants [3]. It turns out that this form has many nice numerical properties. Barycentric rational interpolation competes very favorably with splines: its error is often smaller, and the resulting approximation is infinitely smooth (unlike splines).

Suppose we want our rational interpolant to have approximation order d, i.e., if the spacing of the points is $O(h)$, the error is $O(h^{d+1})$ as $h \to 0$. Then the formula for the weights is

$$w_k = \sum_{\substack{i=k-d \\ 0 \le i < N-d}}^{k} (-1)^k \prod_{\substack{j=i \\ j \ne k}}^{i+d} \frac{1}{x_k - x_j} \tag{3.4.10}$$

For example,

$$\begin{aligned} w_k &= (-1)^k, & d &= 0 \\ w_k &= (-1)^{k-1} \left[\frac{1}{x_k - x_{k-1}} + \frac{1}{x_{k+1} - x_k} \right], & d &= 1 \end{aligned} \tag{3.4.11}$$

In the last equation, you omit the terms in w_0 and w_{N-1} that refer to out-of-range values of x_k.

Here is a routine that implements barycentric rational interpolation. Given a set of N nodes and a desired order d, with $d < N$, it first computes the weights w_k. Then subsequent calls to `interp` evaluate the interpolant using equation (3.4.9). Note that the parameter `jl` of `rawinterp` is not used, since the algorithm is designed to construct an approximation on the entire interval at once.

The workload to construct the weights is of order $O(Nd)$ operations. For small d, this is not too different from splines. Note, however, that the workload for each subsequent interpolated value is $O(N)$, not $O(d)$ as for splines.

interp_1d.h

```
struct BaryRat_interp : Base_interp
```
Barycentric rational interpolation object. After constructing the object, call `interp` for interpolated values. Note that no error estimate `dy` is calculated.
```
{
    VecDoub w;
    Int d;
    BaryRat_interp(VecDoub_I &xv, VecDoub_I &yv, Int dd);
    Doub rawinterp(Int jl, Doub x);
    Doub interp(Doub x);
};

BaryRat_interp::BaryRat_interp(VecDoub_I &xv, VecDoub_I &yv, Int dd)
        : Base_interp(xv,&yv[0],xv.size()), w(n), d(dd)
```
Constructor arguments are $\mathbf{x}$ and $\mathbf{y}$ vectors of length `n`, and order d of desired approximation.
```
{
    if (n<=d) throw("d too large for number of points in BaryRat_interp");
    for (Int k=0;k<n;k++) {              Compute weights from equation (3.4.10).
        Int imin=MAX(k-d,0);
        Int imax = k >= n-d ? n-d-1 : k;
        Doub temp = imin & 1 ? -1.0 : 1.0;
        Doub sum=0.0;
        for (Int i=imin;i<=imax;i++) {
            Int jmax=MIN(i+d,n-1);
            Doub term=1.0;
            for (Int j=i;j<=jmax;j++) {
                if (j==k) continue;
                term *= (xx[k]-xx[j]);
            }
            term=temp/term;
            temp=-temp;
            sum += term;
        }
        w[k]=sum;
    }
}
Doub BaryRat_interp::rawinterp(Int jl, Doub x)
```
Use equation (3.4.9) to compute the barycentric rational interpolant. Note that `jl` is not used since the approximation is global; it is included only for compatibility with `Base_interp`.
```
{
    Doub num=0,den=0;
    for (Int i=0;i<n;i++) {
        Doub h=x-xx[i];
        if (h == 0.0) {
            return yy[i];
        } else {
            Doub temp=w[i]/h;
            num += temp*yy[i];
            den += temp;
        }
    }
    return num/den;
}
Doub BaryRat_interp::interp(Doub x) {
```
No need to invoke `hunt` or `locate` since the interpolation is global, so override `interp` to simply call `rawinterp` directly with a dummy value of `jl`.
```
    return rawinterp(1,x);
}
```

It is wise to start with small values of d before trying larger values.

CITED REFERENCES AND FURTHER READING:

Stoer, J., and Bulirsch, R. 2002, *Introduction to Numerical Analysis*, 3rd ed. (New York: Springer), §2.2.[1]

Floater, M.S., and Hormann, K. 2006+, "Barycentric Rational Interpolation with No Poles and High Rates of Approximation," at `http://www.in.tu-clausthal.de/fileadmin/homes/techreports/ifi0606hormann.pdf`.[2]

Berrut, J.-P., and Trefethen, L.N. 2004, "Barycentric Lagrange Interpolation," *SIAM Review*, vol. 46, pp. 501–517.[3]

Gear, C.W. 1971, *Numerical Initial Value Problems in Ordinary Differential Equations* (Englewood Cliffs, NJ: Prentice-Hall), §6.2.

Cuyt, A., and Wuytack, L. 1987, *Nonlinear Methods in Numerical Analysis* (Amsterdam: North-Holland), Chapter 3.

3.5 Coefficients of the Interpolating Polynomial

Occasionally you may wish to know not the value of the interpolating polynomial that passes through a (small!) number of points, but the coefficients of that polynomial. A valid use of the coefficients might be, for example, to compute simultaneous interpolated values of the function and of several of its derivatives (see §5.1), or to convolve a segment of the tabulated function with some other function, where the moments of that other function (i.e., its convolution with powers of x) are known analytically.

Please be certain, however, that the coefficients are what you need. Generally the coefficients of the interpolating polynomial can be determined much less accurately than its value at a desired abscissa. Therefore, it is not a good idea to determine the coefficients only for use in calculating interpolating values. Values thus calculated will not pass exactly through the tabulated points, for example, while values computed by the routines in §3.1 – §3.3 will pass exactly through such points.

Also, you should not mistake the interpolating polynomial (and its coefficients) for its cousin, the *best-fit* polynomial through a data set. Fitting is a *smoothing* process, since the number of fitted coefficients is typically much less than the number of data points. Therefore, fitted coefficients can be accurately and stably determined even in the presence of statistical errors in the tabulated values. (See §14.9.) Interpolation, where the number of coefficients and number of tabulated points are equal, takes the tabulated values as perfect. If they in fact contain statistical errors, these can be magnified into oscillations of the interpolating polynomial in between the tabulated points.

As before, we take the tabulated points to be $y_i \equiv y(x_i)$. If the interpolating polynomial is written as

$$y = c_0 + c_1 x + c_2 x^2 + \cdots + c_{N-1} x^{N-1} \tag{3.5.1}$$

then the c_i's are required to satisfy the linear equation

$$\begin{bmatrix} 1 & x_0 & x_0^2 & \cdots & x_0^{N-1} \\ 1 & x_1 & x_1^2 & \cdots & x_1^{N-1} \\ \vdots & \vdots & \vdots & & \vdots \\ 1 & x_{N-1} & x_{N-1}^2 & \cdots & x_{N-1}^{N-1} \end{bmatrix} \cdot \begin{bmatrix} c_0 \\ c_1 \\ \vdots \\ c_{N-1} \end{bmatrix} = \begin{bmatrix} y_0 \\ y_1 \\ \vdots \\ y_{N-1} \end{bmatrix} \tag{3.5.2}$$

This is a *Vandermonde matrix*, as described in §2.8. One could in principle solve equation (3.5.2) by standard techniques for linear equations generally (§2.3); however, the special method that was derived in §2.8 is more efficient by a large factor, of order N, so it is much better.

Remember that Vandermonde systems can be quite ill-conditioned. In such a case, *no* numerical method is going to give a very accurate answer. Such cases do not, please note, imply any difficulty in finding interpolated *values* by the methods of §3.2, but only difficulty in finding *coefficients*.

Like the routine in §2.8, the following is due to G.B. Rybicki.

polcoef.h

```
void polcoe(VecDoub_I &x, VecDoub_I &y, VecDoub_O &cof)
```

Given arrays x[0..n-1] and y[0..n-1] containing a tabulated function $y_i = f(x_i)$, this routine returns an array of coefficients cof[0..n-1], such that $y_i = \sum_{j=0}^{n-1} \mathrm{cof}_j x_i^j$.

```
{
    Int k,j,i,n=x.size();
    Doub phi,ff,b;
    VecDoub s(n);
    for (i=0;i<n;i++) s[i]=cof[i]=0.0;
    s[n-1]= -x[0];
    for (i=1;i<n;i++) {                    Coefficients s_i of the master polynomial P(x) are
        for (j=n-1-i;j<n-1;j++)                found by recurrence.
            s[j] -= x[i]*s[j+1];
        s[n-1] -= x[i];
    }
    for (j=0;j<n;j++) {
        phi=n;
        for (k=n-1;k>0;k--)                The quantity phi = ∏_{j≠k}(x_j − x_k) is found as a
            phi=k*s[k]+x[j]*phi;               derivative of P(x_j).
        ff=y[j]/phi;
        b=1.0;                             Coefficients of polynomials in each term of the La-
        for (k=n-1;k>=0;k--) {                 grange formula are found by synthetic division of
            cof[k] += b*ff;                    P(x) by (x − x_j). The solution c_k is accumu-
            b=s[k]+x[j]*b;                     lated.
        }
    }
}
```

3.5.1 Another Method

Another technique is to make use of the function value interpolation routine already given (polint; §3.2). If we interpolate (or extrapolate) to find the value of the interpolating polynomial at $x = 0$, then this value will evidently be c_0. Now we can subtract c_0 from the y_i's and divide each by its corresponding x_i. Throwing out one point (the one with smallest x_i is a good candidate), we can repeat the procedure to find c_1, and so on.

It is not instantly obvious that this procedure is stable, but we have generally found it to be somewhat *more* stable than the routine immediately preceding. This method is of order N^3, while the preceding one was of order N^2. You will find, however, that neither works very well for large N, because of the intrinsic ill-condition of the Vandermonde problem. In single precision, N up to 8 or 10 is satisfactory; about double this in double precision.

polcoef.h

```
void polcof(VecDoub_I &xa, VecDoub_I &ya, VecDoub_O &cof)
```
Given arrays xa[0..n-1] and ya[0..n-1] containing a tabulated function $ya_i = f(xa_i)$, this routine returns an array of coefficients cof[0..n-1], such that $ya_i = \sum_{j=0}^{n-1} cof_j \, xa_i^j$.
```
{
    Int k,j,i,n=xa.size();
    Doub xmin;
    VecDoub x(n),y(n);
    for (j=0;j<n;j++) {
        x[j]=xa[j];
        y[j]=ya[j];
    }
    for (j=0;j<n;j++) {                       Fill a temporary vector whose size
        VecDoub x_t(n-j),y_t(n-j);              decreases as each coefficient is
        for (k=0;k<n-j;k++) {                   found.
            x_t[k]=x[k];
            y_t[k]=y[k];
        }
        Poly_interp interp(x,y,n-j);
        cof[j] = interp.rawinterp(0,0.);       Extrapolate to x = 0.
        xmin=1.0e99;
        k = -1;
        for (i=0;i<n-j;i++) {                  Find the remaining x_i of smallest
            if (abs(x[i]) < xmin) {             absolute value
                xmin=abs(x[i]);
                k=i;
            }
            if (x[i] != 0.0)                   (meanwhile reducing all the terms)
                y[i]=(y[i]-cof[j])/x[i];
        }
        for (i=k+1;i<n-j;i++) {                and eliminate it.
            y[i-1]=y[i];
            x[i-1]=x[i];
        }
    }
}
```

If the point $x = 0$ is not in (or at least close to) the range of the tabulated x_i's, then the coefficients of the interpolating polynomial will in general become very large. However, the real "information content" of the coefficients is in small differences from the "translation-induced" large values. This is one cause of ill-conditioning, resulting in loss of significance and poorly determined coefficients. In this case, you should consider redefining the origin of the problem, to put $x = 0$ in a sensible place.

Another pathology is that, if too high a degree of interpolation is attempted on a smooth function, the interpolating polynomial will attempt to use its high-degree coefficients, in combinations with large and almost precisely canceling combinations, to match the tabulated values down to the last possible epsilon of accuracy. This effect is the same as the intrinsic tendency of the interpolating polynomial values to oscillate (wildly) between its constrained points and would be present even if the machine's floating precision were infinitely good. The above routines `polcoe` and `polcof` have slightly different sensitivities to the pathologies that can occur.

Are you still quite certain that using the *coefficients* is a good idea?

CITED REFERENCES AND FURTHER READING:

Isaacson, E., and Keller, H.B. 1966, *Analysis of Numerical Methods*; reprinted 1994 (New York: Dover), §5.2.

3.6 Interpolation on a Grid in Multidimensions

In multidimensional interpolation, we seek an estimate of a function of more than one independent variable, $y(x_1, x_2, \ldots, x_n)$. The Great Divide is, Are we given a complete set of tabulated values on an n-dimensional grid? Or, do we know function values only on some scattered set of points in the n-dimensional space? In one dimension, the question never arose, because any set of x_i's, once sorted into ascending order, could be viewed as a valid one-dimensional grid (regular spacing not being a requirement).

As the number of dimensions n gets large, maintaining a full grid becomes rapidly impractical, because of the explosion in the number of gridpoints. Methods that work with scattered data, to be considered in §3.7, then become the methods of choice. Don't, however, make the mistake of thinking that such methods are intrinsically more accurate than grid methods. In general they are less accurate. Like the proverbial three-legged dog, they are remarkable because they work at all, not because they work, necessarily, well!

Both kinds of methods are practical in two dimensions, and some other kinds as well. For example, *finite element methods*, of which *triangulation* is the most common, find ways to impose some kind of geometrically regular structure on scattered points, and then use that structure for interpolation. We will treat two-dimensional interpolation by triangulation in detail in §21.6; that section should be considered as a continuation of the discussion here.

In the remainder of this section, we consider only the case of interpolating on a grid, and we implement in code only the (most common) case of two dimensions. All of the methods given generalize to three dimensions in an obvious way. When we implement methods for scattered data, in §3.7, the treatment will be for general n.

In two dimensions, we imagine that we are given a matrix of functional values y_{ij}, with $i = 0, \ldots, M-1$ and $j = 0, \ldots, N-1$. We are also given an array of x_1 values x_{1i}, and an array of x_2 values x_{2j}, with i and j as just stated. The relation of these input quantities to an underlying function $y(x_1, x_2)$ is just

$$y_{ij} = y(x_{1i}, x_{2j}) \tag{3.6.1}$$

We want to estimate, by interpolation, the function y at some untabulated point (x_1, x_2).

An important concept is that of the *grid square* in which the point (x_1, x_2) falls, that is, the four tabulated points that surround the desired interior point. For convenience, we will number these points from 0 to 3, counterclockwise starting from the lower left. More precisely, if

$$\begin{aligned} x_{1i} &\le x_1 \le x_{1(i+1)} \\ x_{2j} &\le x_2 \le x_{2(j+1)} \end{aligned} \tag{3.6.2}$$

defines values of i and j, then

$$\begin{aligned} y_0 &\equiv y_{ij} \\ y_1 &\equiv y_{(i+1)j} \\ y_2 &\equiv y_{(i+1)(j+1)} \\ y_3 &\equiv y_{i(j+1)} \end{aligned} \tag{3.6.3}$$

The simplest interpolation in two dimensions is *bilinear interpolation* on the grid square. Its formulas are

$$t \equiv (x_1 - x_{1i})/(x_{1(i+1)} - x_{1i})$$
$$u \equiv (x_2 - x_{2j})/(x_{2(j+1)} - x_{2j}) \qquad (3.6.4)$$

(so that t and u each lie between 0 and 1) and

$$y(x_1, x_2) = (1-t)(1-u)y_0 + t(1-u)y_1 + tuy_2 + (1-t)uy_3 \qquad (3.6.5)$$

Bilinear interpolation is frequently "close enough for government work." As the interpolating point wanders from grid square to grid square, the interpolated function value changes continuously. However, the gradient of the interpolated function changes discontinuously at the boundaries of each grid square.

We can easily implement an object for bilinear interpolation by grabbing pieces of "machinery" from our one-dimensional interpolation classes:

interp_2d.h

```
struct Bilin_interp {
```
Object for bilinear interpolation on a matrix. Construct with a vector of x_1 values, a vector of x_2 values, and a matrix of tabulated function values y_{ij}. Then call interp for interpolated values.
```
    Int m,n;
    const MatDoub &y;
    Linear_interp x1terp, x2terp;

    Bilin_interp(VecDoub_I &x1v, VecDoub_I &x2v, MatDoub_I &ym)
        : m(x1v.size()), n(x2v.size()), y(ym),
        x1terp(x1v,x1v), x2terp(x2v,x2v) {}
```
Construct dummy 1-dim interpolations for their locate and hunt functions.
```
    Doub interp(Doub x1p, Doub x2p) {
        Int i,j;
        Doub yy, t, u;
        i = x1terp.cor ? x1terp.hunt(x1p) : x1terp.locate(x1p);
        j = x2terp.cor ? x2terp.hunt(x2p) : x2terp.locate(x2p);
```
Find the grid square.
```
        t = (x1p-x1terp.xx[i])/(x1terp.xx[i+1]-x1terp.xx[i]);
```
Interpolate.
```
        u = (x2p-x2terp.xx[j])/(x2terp.xx[j+1]-x2terp.xx[j]);
        yy = (1.-t)*(1.-u)*y[i][j] + t*(1.-u)*y[i+1][j]
            + (1.-t)*u*y[i][j+1] + t*u*y[i+1][j+1];
        return yy;
    }
};
```

Here we declare two instances of `Linear_interp`, one for each direction, and use them merely to do the bookkeeping on the arrays x_{1i} and x_{2j} — in particular, to provide the "intelligent" table-searching mechanisms that we have come to rely on. (The second occurrence of `x1v` and `x2v` in the constructors is just a placeholder; there are not really any one-dimensional "y" arrays.)

Usage of `Bilin_interp` is just what you'd expect:

```
    Int m=..., n=...;
    MatDoub yy(m,n);
    VecDoub x1(m), x2(n);
    ...
    Bilin_interp myfunc(x1,x2,yy);
```

followed (any number of times) by

```
    Doub x1,x2,y;
    ...
    y = myfunc.interp(x1,x2);
```

Bilinear interpolation is a good place to start, in two dimensions, unless you positively know that you need something fancier.

There are two distinctly different directions that one can take in going beyond bilinear interpolation to higher-order methods: One can use higher order to obtain increased accuracy for the interpolated function (for sufficiently smooth functions!), without necessarily trying to fix up the continuity of the gradient and higher derivatives. Or, one can make use of higher order to enforce smoothness of some of these derivatives as the interpolating point crosses grid-square boundaries. We will now consider each of these two directions in turn.

3.6.1 Higher Order for Accuracy

The basic idea is to break up the problem into a succession of one-dimensional interpolations. If we want to do `m-1` order interpolation in the x_1 direction, and `n-1` order in the x_2 direction, we first locate an $m \times n$ sub-block of the tabulated function matrix that contains our desired point (x_1, x_2). We then do `m` one-dimensional interpolations in the x_2 direction, i.e., on the rows of the sub-block, to get function values at the points (x_{1i}, x_2), with m values of i. Finally, we do a last interpolation in the x_1 direction to get the answer.

Again using the previous one-dimensional machinery, this can all be coded very concisely as

interp_2d.h

```
struct Poly2D_interp {
Object for two-dimensional polynomial interpolation on a matrix. Construct with a vector of x1
values, a vector of x2 values, a matrix of tabulated function values yij, and integers to specify
the number of points to use locally in each direction. Then call interp for interpolated values.
    Int m,n,mm,nn;
    const MatDoub &y;
    VecDoub yv;
    Poly_interp x1terp, x2terp;

    Poly2D_interp(VecDoub_I &x1v, VecDoub_I &x2v, MatDoub_I &ym,
        Int mp, Int np) : m(x1v.size()), n(x2v.size()),
        mm(mp), nn(np), y(ym), yv(m),
        x1terp(x1v,yv,mm), x2terp(x2v,x2v,nn) {}    Dummy 1-dim interpolations for their
                                                        locate and hunt functions.
    Doub interp(Doub x1p, Doub x2p) {
        Int i,j,k;
        i = x1terp.cor ? x1terp.hunt(x1p) : x1terp.locate(x1p);
        j = x2terp.cor ? x2terp.hunt(x2p) : x2terp.locate(x2p);
        Find grid block.
        for (k=i;k<i+mm;k++) {                      mm interpolations in the x2 direction.
            x2terp.yy = &y[k][0];
            yv[k] = x2terp.rawinterp(j,x2p);
        }
        return x1terp.rawinterp(i,x1p);             A final interpolation in the x1 direc-
    }                                                   tion.
};
```

The user interface is the same as for `Bilin_interp`, except that the constructor has two additional arguments that specify the number of points (order plus one) to be used locally in, respectively, the x_1 and x_2 interpolations. Typical values will be in the range 3 to 7.

Code stylists won't like some of the details in `Poly2D_interp` (see discussion in §3.1 immediately following `Base_interp`). As we loop over the rows of the sub-block, we reach into the guts of `x2terp` and repoint its `yy` array to a row of our y matrix. Further, we alter the contents of the array `yv`, for which `x1terp` has stored a pointer, on the fly. None of this is particularly dangerous as long as we control the implementations in both `Base_interp` and `Poly2D_interp`; and it makes for a very efficient implementation. You should view these two classes as not just (implicitly) `friend` classes, but as *really intimate* friends.

3.6.2 Higher Order for Smoothness: Bicubic Spline

A favorite technique for obtaining smoothness in two-dimensional interpolation is the *bicubic spline*. To set up a bicubic spline, you (one time) construct M one-dimensional splines across the rows of the two-dimensional matrix of function values. Then, for each desired interpolated value you proceed as follows: (1) Perform M spline interpolations to get a vector of values $y(x_{1i}, x_2)$, $i = 0, \ldots, M-1$. (2) Construct a one-dimensional spline through those values. (3) Finally, spline-interpolate to the desired value $y(x_1, x_2)$.

If this sounds like a lot of work, well, yes, it is. The one-time setup work scales as the table size $M \times N$, while the work per interpolated value scales roughly as $M \log M + N$, both with pretty hefty constants in front. This is the price that you pay for the desirable characteristics of splines that derive from their nonlocality. For tables with modest M and N, less than a few hundred, say, the cost is usually tolerable. If it's not, then fall back to the previous local methods.

Again a very concise implementation is possible:

interp_2d.h

```
struct Spline2D_interp {
```
Object for two-dimensional cubic spline interpolation on a matrix. Construct with a vector of x_1 values, a vector of x_2 values, and a matrix of tabulated function values y_{ij}. Then call `interp` for interpolated values.
```
    Int m,n;
    const MatDoub &y;
    const VecDoub &x1;
    VecDoub yv;
    NRvector<Spline_interp*> srp;

    Spline2D_interp(VecDoub_I &x1v, VecDoub_I &x2v, MatDoub_I &ym)
        : m(x1v.size()), n(x2v.size()), y(ym), yv(m), x1(x1v), srp(m) {
        for (Int i=0;i<m;i++) srp[i] = new Spline_interp(x2v,&y[i][0]);
        Save an array of pointers to 1-dim row splines.
    }

    ~Spline2D_interp(){
        for (Int i=0;i<m;i++) delete srp[i];        We need a destructor to clean up.
    }

    Doub interp(Doub x1p, Doub x2p) {
        for (Int i=0;i<m;i++) yv[i] = (*srp[i]).interp(x2p);
        Interpolate on each row.
        Spline_interp scol(x1,yv);                  Construct the column spline,
        return scol.interp(x1p);                    and evaluate it.
    }
};
```

The reason for that ugly vector of pointers to `Spline_interp` objects is that we need to initialize each row spline separately, with data from the appropriate row. The user interface is the same as `Bilin_interp`, above.

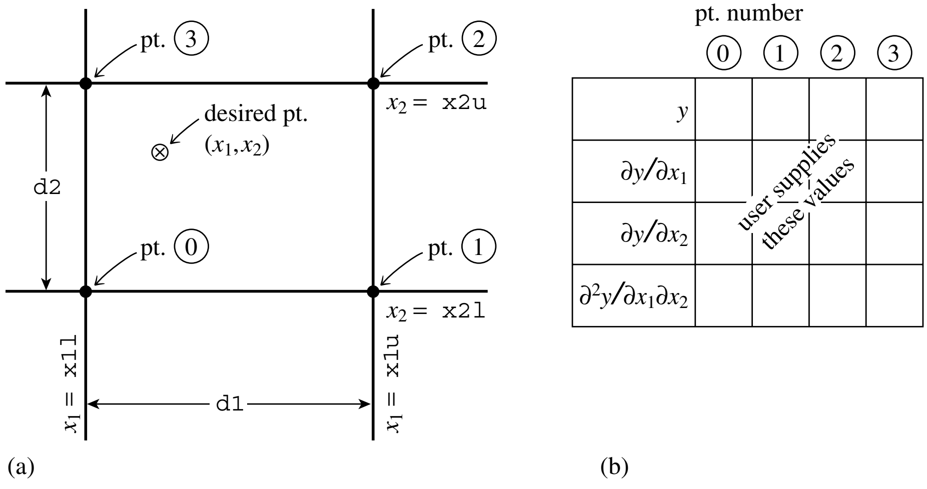

Figure 3.6.1. (a) Labeling of points used in the two-dimensional interpolation routines `bcuint` and `bcucof`. (b) For each of the four points in (a), the user supplies one function value, two first derivatives, and one cross-derivative, a total of 16 numbers.

3.6.3 Higher Order for Smoothness: Bicubic Interpolation

Bicubic interpolation gives the same degree of smoothness as bicubic spline interpolation, but it has the advantage of being a local method. Thus, after you set it up, a function interpolation costs only a constant, plus $\log M + \log N$, to find your place in the table. Unfortunately, this advantage comes with a lot of complexity in coding. Here, we will give only some building blocks for the method, not a complete user interface.

Bicubic splines are in fact a special case of bicubic interpolation. In the general case, however, we leave the values of all derivatives at the grid points as freely specifiable. You, the user, can specify them *any way you want*. In other words, you specify at each grid point not just the function $y(x_1, x_2)$, but also the gradients $\partial y/\partial x_1 \equiv y_{,1}$, $\partial y/\partial x_2 \equiv y_{,2}$ and the cross derivative $\partial^2 y/\partial x_1 \partial x_2 \equiv y_{,12}$ (see Figure 3.6.1). Then an interpolating function that is *cubic* in the scaled coordinates t and u (equation 3.6.4) can be found, with the following properties: (i) The values of the function and the specified derivatives are reproduced exactly on the grid points, and (ii) the values of the function and the specified derivatives change continuously as the interpolating point crosses from one grid square to another.

It is important to understand that nothing in the equations of bicubic interpolation requires you to specify the extra derivatives *correctly*! The smoothness properties are tautologically "forced," and have nothing to do with the "accuracy" of the specified derivatives. It is a separate problem for you to decide how to obtain the values that are specified. The better you do, the more *accurate* the interpolation will be. But it will be *smooth* no matter what you do.

Best of all is to know the derivatives analytically, or to be able to compute them accurately by numerical means, at the grid points. Next best is to determine them by numerical differencing from the functional values already tabulated on the grid. The relevant code would be something like this (using centered differencing):

```
    y1a[j][k]=(ya[j+1][k]-ya[j-1][k])/(x1a[j+1]-x1a[j-1]);
    y2a[j][k]=(ya[j][k+1]-ya[j][k-1])/(x2a[k+1]-x2a[k-1]);
    y12a[j][k]=(ya[j+1][k+1]-ya[j+1][k-1]-ya[j-1][k+1]+ya[j-1][k-1])
          /((x1a[j+1]-x1a[j-1])*(x2a[k+1]-x2a[k-1]));
```

To do a bicubic interpolation within a grid square, given the function y and the derivatives y1, y2, y12 at each of the four corners of the square, there are two steps: First obtain the 16 quantities c_{ij}, $i, j = 0, \ldots, 3$ using the routine bcucof below. (The formulas that obtain the c's from the function and derivative values are just a complicated linear transformation, with coefficients that, having been determined once in the mists of numerical history, can be tabulated and forgotten.) Next, substitute the c's into any or all of the following bicubic formulas for function and derivatives, as desired:

$$\begin{aligned}
y(x_1, x_2) &= \sum_{i=0}^{3}\sum_{j=0}^{3} c_{ij} t^i u^j \\
y_{,1}(x_1, x_2) &= \sum_{i=0}^{3}\sum_{j=0}^{3} i c_{ij} t^{i-1} u^j (dt/dx_1) \\
y_{,2}(x_1, x_2) &= \sum_{i=0}^{3}\sum_{j=0}^{3} j c_{ij} t^i u^{j-1} (du/dx_2) \\
y_{,12}(x_1, x_2) &= \sum_{i=0}^{3}\sum_{j=0}^{3} i j c_{ij} t^{i-1} u^{j-1} (dt/dx_1)(du/dx_2)
\end{aligned} \tag{3.6.6}$$

where t and u are again given by equation (3.6.4).

interp_2d.h

```
void bcucof(VecDoub_I &y, VecDoub_I &y1, VecDoub_I &y2, VecDoub_I &y12,
    const Doub d1, const Doub d2, MatDoub_O &c) {
```

Given arrays y[0..3], y1[0..3], y2[0..3], and y12[0..3], containing the function, gradients, and cross-derivative at the four grid points of a rectangular grid cell (numbered counterclockwise from the lower left), and given d1 and d2, the length of the grid cell in the 1 and 2 directions, this routine returns the table c[0..3][0..3] that is used by routine bcuint for bicubic interpolation.

```
    static Int wt_d[16*16]=
        {1, 0, 0, 0, 0, 0, 0, 0, 0, 0, 0, 0, 0, 0, 0, 0,
        0, 0, 0, 0, 0, 0, 0, 0, 1, 0, 0, 0, 0, 0, 0, 0,
        -3, 0, 0, 3, 0, 0, 0, 0,-2, 0, 0,-1, 0, 0, 0, 0,
        2, 0, 0,-2, 0, 0, 0, 0, 1, 0, 0, 1, 0, 0, 0, 0,
        0, 0, 0, 0, 1, 0, 0, 0, 0, 0, 0, 0, 0, 0, 0, 0,
        0, 0, 0, 0, 0, 0, 0, 0, 0, 0, 0, 0, 1, 0, 0, 0,
        0, 0, 0, 0,-3, 0, 0, 3, 0, 0, 0, 0,-2, 0, 0,-1,
        0, 0, 0, 0, 2, 0, 0,-2, 0, 0, 0, 0, 1, 0, 0, 1,
        -3, 3, 0, 0,-2,-1, 0, 0, 0, 0, 0, 0, 0, 0, 0, 0,
        0, 0, 0, 0, 0, 0, 0, 0,-3, 3, 0, 0,-2,-1, 0, 0,
        9,-9, 9,-9, 6, 3,-3,-6, 6,-6,-3, 3, 4, 2, 1, 2,
        -6, 6,-6, 6,-4,-2, 2, 4,-3, 3, 3,-3,-2,-1,-1,-2,
        2,-2, 0, 0, 1, 1, 0, 0, 0, 0, 0, 0, 0, 0, 0, 0,
        0, 0, 0, 0, 0, 0, 0, 0, 2,-2, 0, 0, 1, 1, 0, 0,
        -6, 6,-6, 6,-3,-3, 3, 3,-4, 4, 2,-2,-2,-2,-1,-1,
        4,-4, 4,-4, 2, 2,-2,-2, 2,-2,-2, 2, 1, 1, 1, 1};
    Int l,k,j,i;
    Doub xx,d1d2=d1*d2;
    VecDoub cl(16),x(16);
    static MatInt wt(16,16,wt_d);
    for (i=0;i<4;i++) {            Pack a temporary vector x.
```

```
        x[i]=y[i];
        x[i+4]=y1[i]*d1;
        x[i+8]=y2[i]*d2;
        x[i+12]=y12[i]*d1d2;
    }
    for (i=0;i<16;i++) {          Matrix-multiply by the stored table.
        xx=0.0;
        for (k=0;k<16;k++) xx += wt[i][k]*x[k];
        cl[i]=xx;
    }
    l=0;
    for (i=0;i<4;i++)             Unpack the result into the output table.
        for (j=0;j<4;j++) c[i][j]=cl[l++];
}
```

The implementation of equation (3.6.6), which performs a bicubic interpolation, gives back the interpolated function value and the two gradient values, and uses the above routine bcucof, is simply:

interp_2d.h

```
void bcuint(VecDoub_I &y, VecDoub_I &y1, VecDoub_I &y2, VecDoub_I &y12,
    const Doub x1l, const Doub x1u, const Doub x2l, const Doub x2u,
    const Doub x1, const Doub x2, Doub &ansy, Doub &ansy1, Doub &ansy2) {
Bicubic interpolation within a grid square. Input quantities are y,y1,y2,y12 (as described in
bcucof); x1l and x1u, the lower and upper coordinates of the grid square in the 1 direction;
x2l and x2u likewise for the 2 direction; and x1,x2, the coordinates of the desired point for
the interpolation. The interpolated function value is returned as ansy, and the interpolated
gradient values as ansy1 and ansy2. This routine calls bcucof.
    Int i;
    Doub t,u,d1=x1u-x1l,d2=x2u-x2l;
    MatDoub c(4,4);
    bcucof(y,y1,y2,y12,d1,d2,c);          Get the c's.
    if (x1u == x1l || x2u == x2l)
        throw("Bad input in routine bcuint");
    t=(x1-x1l)/d1;                        Equation (3.6.4).
    u=(x2-x2l)/d2;
    ansy=ansy2=ansy1=0.0;
    for (i=3;i>=0;i--) {                  Equation (3.6.6).
        ansy=t*ansy+((c[i][3]*u+c[i][2])*u+c[i][1])*u+c[i][0];
        ansy2=t*ansy2+(3.0*c[i][3]*u+2.0*c[i][2])*u+c[i][1];
        ansy1=u*ansy1+(3.0*c[3][i]*t+2.0*c[2][i])*t+c[1][i];
    }
    ansy1 /= d1;
    ansy2 /= d2;
}
```

You can combine the best features of bicubic interpolation and bicubic splines by using splines to compute values for the necessary derivatives at the grid points, storing these values, and then using bicubic interpolation, with an efficient table-searching method, for the actual function interpolations. Unfortunately this is beyond our scope here.

CITED REFERENCES AND FURTHER READING:

Abramowitz, M., and Stegun, I.A. 1964, *Handbook of Mathematical Functions* (Washington: National Bureau of Standards); reprinted 1968 (New York: Dover); online at http://www.nr.com/aands, §25.2.

Kinahan, B.F., and Harm, R. 1975, "Chemical Composition and the Hertzsprung Gap," *Astrophysical Journal*, vol. 200, pp. 330–335.

Johnson, L.W., and Riess, R.D. 1982, *Numerical Analysis*, 2nd ed. (Reading, MA: Addison-Wesley), §5.2.7.

Dahlquist, G., and Bjorck, A. 1974, *Numerical Methods* (Englewood Cliffs, NJ: Prentice-Hall); reprinted 2003 (New York: Dover), §7.7.

3.7 Interpolation on Scattered Data in Multidimensions

We now leave behind, if with some trepidation, the orderly world of regular grids. Courage is required. We are given an arbitrarily scattered set of N data points $(\mathbf{x}_i, y_i)$, $i = 0, \ldots, N-1$ in n-dimensional space. Here $\mathbf{x}_i$ denotes an n-dimensional vector of independent variables, $(x_{1i}, x_{2i}, \ldots, x_{ni})$, and y_i is the value of the function at that point.

In this section we discuss two of the most widely used *general* methods for this problem, *radial basis function (RBF)* interpolation, and *kriging*. Both of these methods are expensive. By that we mean that they require $O(N^3)$ operations to initially digest a set of data points, followed by $O(N)$ operations for each interpolated value. Kriging is also able to supply an error estimate — but at the rather high cost of $O(N^2)$ per value. Shepard interpolation, discussed below, is a variant of RBF that at least avoids the $O(N^3)$ initial work; otherwise these workloads effectively limit the usefulness of these general methods to values of $N \lesssim 10^4$. It is therefore worthwhile for you to consider whether you have any other options. Two of these are

- If n is not too large (meaning, usually, $n = 2$), and if the data points are fairly dense, then consider triangulation, discussed in §21.6. Triangulation is an example of a finite element method. Such methods construct some semblance of geometric regularity and then exploit that construction to advantage. Mesh generation is a closely related subject.

- If your accuracy goals will tolerate it, consider moving each data point to the nearest point on a regular Cartesian grid and then using Laplace interpolation (§3.8) to fill in the rest of the grid points. After that, you can interpolate on the grid by the methods of §3.6. You will need to compromise between making the grid very fine (to minimize the error introduced when you move the points) and the compute time workload of the Laplace method.

If neither of these options seem attractive, and you can't think of another one that is, then try one or both of the two methods that we now discuss. RBF interpolation is probably the more widely used of the two, but kriging is our personal favorite. Which works better will depend on the details of your problem.

The related, but easier, problem of *curve interpolation* in multidimensions is discussed at the end of this section.

3.7.1 Radial Basis Function Interpolation

The idea behind RBF interpolation is very simple: Imagine that every known point j "influences" its surroundings the same way in all directions, according to

some assumed functional form $\phi(r)$ — the radial basis function — that is a function only of radial distance $r = |\mathbf{x} - \mathbf{x}_j|$ from the point. Let us try to approximate the interpolating function everywhere by a linear combination of the ϕ's, centered on all the known points,

$$y(\mathbf{x}) = \sum_{i=0}^{N-1} w_i \phi(|\mathbf{x} - \mathbf{x}_i|) \tag{3.7.1}$$

where the w_i's are some unknown set of weights. How do we find these weights? Well, we haven't used the function values y_i yet. The weights are determined by requiring that the interpolation be exact at all the known data points. That is equivalent to solving a set of N linear equations in N unknowns for the w_i's:

$$y_j = \sum_{i=0}^{N-1} w_i \phi(|\mathbf{x}_j - \mathbf{x}_i|) \tag{3.7.2}$$

For many functional forms ϕ, it can be proved, under various general assumptions, that this set of equations is nondegenerate and can be readily solved by, e.g., LU decomposition (§2.3). References [1,2] provide entry to the literature.

A variant on RBF interpolation is *normalized radial basis function (NRBF)* interpolation, in which we require the sum of the basis functions to be unity or, equivalently, replace equations (3.7.1) and (3.7.2) by

$$y(\mathbf{x}) = \frac{\sum_{i=0}^{N-1} w_i \phi(|\mathbf{x} - \mathbf{x}_i|)}{\sum_{i=0}^{N-1} \phi(|\mathbf{x} - \mathbf{x}_i|)} \tag{3.7.3}$$

and

$$y_j \sum_{i=0}^{N-1} \phi(|\mathbf{x}_j - \mathbf{x}_i|) = \sum_{i=0}^{N-1} w_i \phi(|\mathbf{x}_j - \mathbf{x}_i|) \tag{3.7.4}$$

Equations (3.7.3) and (3.7.4) arise more naturally from a Bayesian statistical perspective [3]. However, there is no evidence that either the NRBF method is consistently superior to the RBF method, or vice versa. It is easy to implement both methods in the same code, leaving the choice to the user.

As we already mentioned, for N data points the one-time work to solve for the weights by LU decomposition is $O(N^3)$. After that, the cost is $O(N)$ for each interpolation. Thus $N \sim 10^3$ is a rough dividing line (at 2007 desktop speeds) between "easy" and "difficult." If your N is larger, however, don't despair: There are *fast multipole methods*, beyond our scope here, with much more favorable scaling [1,4,5]. Another, much lower-tech, option is to use *Shepard interpolation* discussed later in this section.

Here are a couple of objects that implement everything discussed thus far. `RBF_fn` is a virtual base class whose derived classes will embody different functional forms for `rbf`$(r) \equiv \phi(r)$. `RBF_interp`, via its constructor, digests your data and solves the equations for the weights. The data points $\mathbf{x}_i$ are input as an $N \times n$ matrix, and the code works for any dimension n. A boolean argument `nrbf` inputs whether NRBF is to be used instead of RBF. You call `interp` to get an interpolated function value at a new point $\mathbf{x}$.

interp_rbf.h

```
struct RBF_fn {
```
Abstract base class template for any particular radial basis function. See specific examples below.
```
    virtual Doub rbf(Doub r) = 0;
};

struct RBF_interp {
```
Object for radial basis function interpolation using n points in dim dimensions. Call constructor once, then interp as many times as desired.
```
    Int dim, n;
    const MatDoub &pts;
    const VecDoub &vals;
    VecDoub w;
    RBF_fn &fn;
    Bool norm;

    RBF_interp(MatDoub_I &ptss, VecDoub_I &valss, RBF_fn &func, Bool nrbf=false)
    : dim(ptss.ncols()), n(ptss.nrows()) , pts(ptss), vals(valss),
    w(n), fn(func), norm(nrbf) {
```
Constructor. The $n \times dim$ matrix ptss inputs the data points, the vector valss the function values. func contains the chosen radial basis function, derived from the class RBF_fn. The default value of nrbf gives RBF interpolation; set it to 1 for NRBF.
```
        Int i,j;
        Doub sum;
        MatDoub rbf(n,n);
        VecDoub rhs(n);
        for (i=0;i<n;i++) {                Fill the matrix φ(|r_i − r_j|) and the r.h.s. vector.
            sum = 0.;
            for (j=0;j<n;j++) {
                sum += (rbf[i][j] = fn.rbf(rad(&pts[i][0],&pts[j][0])));
            }
            if (norm) rhs[i] = sum*vals[i];
            else rhs[i] = vals[i];
        }
        LUdcmp lu(rbf);                    Solve the set of linear equations.
        lu.solve(rhs,w);
    }

    Doub interp(VecDoub_I &pt) {
```
Return the interpolated function value at a dim-dimensional point pt.
```
        Doub fval, sum=0., sumw=0.;
        if (pt.size() != dim) throw("RBF_interp bad pt size");
        for (Int i=0;i<n;i++) {            Sum over all tabulated points.
            fval = fn.rbf(rad(&pt[0],&pts[i][0]));
            sumw += w[i]*fval;
            sum += fval;
        }
        return norm ? sumw/sum : sumw;
    }

    Doub rad(const Doub *p1, const Doub *p2) {
```
Euclidean distance.
```
        Doub sum = 0.;
        for (Int i=0;i<dim;i++) sum += SQR(p1[i]-p2[i]);
        return sqrt(sum);
    }
};
```

3.7.2 Radial Basis Functions in General Use

The most often used radial basis function is the *multiquadric* first used by Hardy, circa 1970. The functional form is

$$\phi(r) = (r^2 + r_0^2)^{1/2} \tag{3.7.5}$$

where r_0 is a scale factor that you get to choose. Multiquadrics are said to be less sensitive to the choice of r_0 than some other functional forms.

In general, both for multiquadrics and for other functions, below, r_0 should be larger than the typical separation of points but smaller than the "outer scale" or feature size of the function that you are interpolating. There can be several orders of magnitude difference between the interpolation accuracy with a good choice for r_0, versus a poor choice, so it is definitely worth some experimentation. One way to experiment is to construct an RBF interpolator omitting one data point at a time and measuring the interpolation error at the omitted point.

The *inverse multiquadric*

$$\phi(r) = (r^2 + r_0^2)^{-1/2} \tag{3.7.6}$$

gives results that are comparable to the multiquadric, sometimes better.

It might seem odd that a function and its inverse (actually, reciprocal) work about equally well. The explanation is that what really matters is smoothness, and certain properties of the function's Fourier transform that are not very different between the multiquadric and its reciprocal. The fact that one increases monotonically and the other decreases turns out to be almost irrelevant. However, if you want the extrapolated function to go to zero far from all the data (where an accurate value is impossible anyway), then the inverse multiquadric is a good choice.

The *thin-plate spline* radial basis function is

$$\phi(r) = r^2 \log(r/r_0) \tag{3.7.7}$$

with the limiting value $\phi(0) = 0$ assumed. This function has some physical justification in the energy minimization problem associated with warping a thin elastic plate. There is no indication that it is generally better than either of the above forms, however.

The *Gaussian* radial basis function is just what you'd expect,

$$\phi(r) = \exp\left(-\tfrac{1}{2}r^2/r_0^2\right) \tag{3.7.8}$$

The interpolation accuracy using Gaussian basis functions can be very sensitive to r_0, and they are often avoided for this reason. However, for smooth functions and with an optimal r_0, very high accuracy can be achieved. The Gaussian also will extrapolate any function to zero far from the data, and it gets to zero quickly.

Other functions are also in use, for example those of Wendland [6]. There is a large literature in which the above choices for basis functions are tested against specific functional forms or experimental data sets [1,2,7]. Few, if any, general recommendations emerge. We suggest that you try the alternatives in the order listed above, starting with multiquadrics, and that you not omit experimenting with different choices of the scale parameters r_0.

The functions discussed are implemented in code as:

interp_rbf.h

```
struct RBF_multiquadric : RBF_fn {
Instantiate this and send to RBF_interp to get multiquadric interpolation.
    Doub r02;
    RBF_multiquadric(Doub scale=1.) : r02(SQR(scale)) {}
    Constructor argument is the scale factor. See text.
    Doub rbf(Doub r) { return sqrt(SQR(r)+r02); }
};

struct RBF_thinplate : RBF_fn {
Same as above, but for thin-plate spline.
    Doub r0;
    RBF_thinplate(Doub scale=1.) : r0(scale) {}
    Doub rbf(Doub r) { return r <= 0. ? 0. : SQR(r)*log(r/r0); }
};

struct RBF_gauss : RBF_fn {
Same as above, but for Gaussian.
    Doub r0;
    RBF_gauss(Doub scale=1.) : r0(scale) {}
    Doub rbf(Doub r) { return exp(-0.5*SQR(r/r0)); }
};

struct RBF_inversemultiquadric : RBF_fn {
Same as above, but for inverse multiquadric.
    Doub r02;
    RBF_inversemultiquadric(Doub scale=1.) : r02(SQR(scale)) {}
    Doub rbf(Doub r) { return 1./sqrt(SQR(r)+r02); }
};
```

Typical use of the objects in this section should look something like this:

```
Int npts=...,ndim=...;
Doub r0=...;
MatDoub pts(npts,ndim);
VecDoub y(npts);
...
RBF_multiquadric multiquadric(r0);
RBF_interp myfunc(pts,y,multiquadric,0);
```

followed by any number of interpolation calls,

```
VecDoub pt(ndim);
Doub val;
...
val = myfunc.interp(pt);
```

3.7.3 Shepard Interpolation

An interesting special case of normalized radial basis function interpolation (equations 3.7.3 and 3.7.4) occurs if the function $\phi(r)$ goes to infinity as $r \to 0$, and is finite (e.g., decreasing) for $r > 0$. In that case it is easy to see that the weights w_i are just equal to the respective function values y_i, and the interpolation formula is simply

$$y(\mathbf{x}) = \frac{\sum_{i=0}^{N-1} y_i \phi(|\mathbf{x} - \mathbf{x}_i|)}{\sum_{i=0}^{N-1} \phi(|\mathbf{x} - \mathbf{x}_i|)} \tag{3.7.9}$$

(with appropriate provision for the limiting case where $\mathbf{x}$ is equal to one of the $\mathbf{x}_i$'s). Note that no solution of linear equations is required. The one-time work is negligible, while the work for each interpolation is $O(N)$, tolerable even for very large N.

Shepard proposed the simple power-law function

$$\phi(r) = r^{-p} \tag{3.7.10}$$

with (typically) $1 < p \lesssim 3$, as well as some more complicated functions with different exponents in an inner and outer region (see [8]). You can see that what is going on is basically interpolation by a nearness-weighted average, with nearby points contributing more strongly than distant ones.

Shepard interpolation is rarely as accurate as the well-tuned application of one of the other radial basis functions, above. On the other hand, it is simple, fast, and often just the thing for quick and dirty applications. It, and variants, are thus widely used.

An implementing object is

interp_rbf.h

```
struct Shep_interp {
```
Object for Shepard interpolation using `n` points in `dim` dimensions. Call constructor once, then `interp` as many times as desired.
```
    Int dim, n;
    const MatDoub &pts;
    const VecDoub &vals;
    Doub pneg;

    Shep_interp(MatDoub_I &ptss, VecDoub_I &valss, Doub p=2.)
    : dim(ptss.ncols()), n(ptss.nrows()) , pts(ptss),
    vals(valss), pneg(-p) {}
```
Constructor. The $n \times dim$ matrix `ptss` inputs the data points, the vector `valss` the function values. Set `p` to the desired exponent. The default value is typical.
```
    Doub interp(VecDoub_I &pt) {
```
Return the interpolated function value at a `dim`-dimensional point `pt`.
```
        Doub r, w, sum=0., sumw=0.;
        if (pt.size() != dim) throw("RBF_interp bad pt size");
        for (Int i=0;i<n;i++) {
            if ((r=rad(&pt[0],&pts[i][0])) == 0.) return vals[i];
            sum += (w = pow(r,pneg));
            sumw += w*vals[i];
        }
        return sumw/sum;
    }

    Doub rad(const Doub *p1, const Doub *p2) {
```
Euclidean distance.
```
        Doub sum = 0.;
        for (Int i=0;i<dim;i++) sum += SQR(p1[i]-p2[i]);
        return sqrt(sum);
    }
};
```

3.7.4 Interpolation by Kriging

Kriging is a technique named for South African mining engineer D.G. Krige. It is basically a form of linear prediction (§13.6), also known in different communities as *Gauss-Markov estimation* or *Gaussian process regression*.

Kriging can be either an interpolation method or a fitting method. The distinction between the two is whether the fitted/interpolated function goes exactly through all the input data points (interpolation), or whether it allows measurement errors to be specified and then "smooths" to get a statistically better predictor that does not

generally go through the data points (does not "honor the data"). In this section we consider only the former case, that is, interpolation. We will return to the latter case in §15.9.

At this point in the book, it is beyond our scope to derive the equations for kriging. You can turn to §13.6 to get a flavor, and look to references [9,10,11] for details. To use kriging, you must be able to estimate the mean square variation of your function $y(\mathbf{x})$ as a function of offset distance $\mathbf{r}$, a so-called *variogram*,

$$v(\mathbf{r}) \sim \frac{1}{2}\left\langle [y(\mathbf{x}+\mathbf{r}) - y(\mathbf{x})]^2 \right\rangle \tag{3.7.11}$$

where the average is over all $\mathbf{x}$ with fixed $\mathbf{r}$. If this seems daunting, don't worry. For interpolation, even very crude variogram estimates work fine, and we will give below a routine to estimate $v(\mathbf{r})$ from your input data points $\mathbf{x}_i$ and $y_i = y(\mathbf{x}_i)$, $i = 0, \ldots, N-1$, automatically. One usually takes $v(\mathbf{r})$ to be a function only of the magnitude $r = |\mathbf{r}|$ and writes it as $v(r)$.

Let v_{ij} denote $v(|\mathbf{x}_i - \mathbf{x}_j|)$, where i and j are input points, and let v_{*j} denote $v(|\mathbf{x}_* - \mathbf{x}_j|)$, $\mathbf{x}_*$ being a point at which we want an interpolated value $y(\mathbf{x}_*)$. Now define two vectors of length $N+1$,

$$\begin{aligned} \mathbf{Y} &= (y_0, y_1, \ldots, y_{N-1}, 0) \\ \mathbf{V}_* &= (v_{*1}, v_{*2}, \ldots, v_{*,N-1}, 1) \end{aligned} \tag{3.7.12}$$

and an $(N+1) \times (N+1)$ symmetric matrix,

$$\mathbf{V} = \begin{pmatrix} v_{00} & v_{01} & \ldots & v_{0,N-1} & 1 \\ v_{10} & v_{11} & \ldots & v_{1,N-1} & 1 \\ & & \ldots & & \ldots \\ v_{N-1,0} & v_{N-1,1} & \ldots & v_{N-1,N-1} & 1 \\ 1 & 1 & \ldots & 1 & 0 \end{pmatrix} \tag{3.7.13}$$

Then the kriging interpolation estimate $\widehat{y}_* \approx y(\mathbf{x}_*)$ is given by

$$\widehat{y}_* = \mathbf{V}_* \cdot \mathbf{V}^{-1} \cdot \mathbf{Y} \tag{3.7.14}$$

and its variance is given by

$$\text{Var}(\widehat{y}_*) = \mathbf{V}_* \cdot \mathbf{V}^{-1} \cdot \mathbf{V}_* \tag{3.7.15}$$

Notice that if we compute, once, the LU decomposition of $\mathbf{V}$, and then backsubstitute, once, to get the vector $\mathbf{V}^{-1} \cdot \mathbf{Y}$, then the individual interpolations cost only $O(N)$: Compute the vector $\mathbf{V}_*$ and take a vector dot product. On the other hand, every computation of a variance, equation (3.7.15), requires an $O(N^2)$ backsubstitution.

As an aside (if you have looked ahead to §13.6) the purpose of the extra row and column in $\mathbf{V}$, and extra last components in $\mathbf{V}_*$ and $\mathbf{Y}$, is to automatically calculate, and correct for, an appropriately weighted average of the data, and thus to make equation (3.7.14) an unbiased estimator.

Here is an implementation of equations (3.7.12) – (3.7.15). The constructor does the one-time work, while the two overloaded `interp` methods calculate either an interpolated value or else a value and a standard deviation (square root of the variance). You should leave the optional argument `err` set to the default value of `NULL` until you read §15.9.

krig.h

```
template<class T>
struct Krig {
```
Object for interpolation by kriging, using npt points in ndim dimensions. Call constructor once, then interp as many times as desired.
```
    const MatDoub &x;
    const T &vgram;
    Int ndim, npt;
    Doub lastval, lasterr;               Most recently computed value and (if computed) error.
    VecDoub y,dstar,vstar,yvi;
    MatDoub v;
    LUdcmp *vi;

    Krig(MatDoub_I &xx, VecDoub_I &yy, T &vargram, const Doub *err=NULL)
    : x(xx),vgram(vargram),npt(xx.nrows()),ndim(xx.ncols()),dstar(npt+1),
    vstar(npt+1),v(npt+1,npt+1),y(npt+1),yvi(npt+1) {
```
Constructor. The npt × ndim matrix xx inputs the data points, the vector yy the function values. vargram is the variogram function or functor. The argument err is not used for interpolation; see §15.9.
```
        Int i,j;
        for (i=0;i<npt;i++) {            Fill Y and V.
            y[i] = yy[i];
            for (j=i;j<npt;j++) {
                v[i][j] = v[j][i] = vgram(rdist(&x[i][0],&x[j][0]));
            }
            v[i][npt] = v[npt][i] = 1.;
        }
        v[npt][npt] = y[npt] = 0.;
        if (err) for (i=0;i<npt;i++) v[i][i] -= SQR(err[i]);    §15.9.
        vi = new LUdcmp(v);
        vi->solve(y,yvi);
    }
    ~Krig() { delete vi; }

    Doub interp(VecDoub_I &xstar) {
```
Return an interpolated value at the point xstar.
```
        Int i;
        for (i=0;i<npt;i++) vstar[i] = vgram(rdist(&xstar[0],&x[i][0]));
        vstar[npt] = 1.;
        lastval = 0.;
        for (i=0;i<=npt;i++) lastval += yvi[i]*vstar[i];
        return lastval;
    }

    Doub interp(VecDoub_I &xstar, Doub &esterr) {
```
Return an interpolated value at the point xstar, and return its estimated error as esterr.
```
        lastval = interp(xstar);
        vi->solve(vstar,dstar);
        lasterr = 0;
        for (Int i=0;i<=npt;i++) lasterr += dstar[i]*vstar[i];
        esterr = lasterr = sqrt(MAX(0.,lasterr));
        return lastval;
    }

    Doub rdist(const Doub *x1, const Doub *x2) {
```
Utility used internally. Cartesian distance between two points.
```
        Doub d=0.;
        for (Int i=0;i<ndim;i++) d += SQR(x1[i]-x2[i]);
        return sqrt(d);
    }
};
```

The constructor argument vgram, the variogram function, can be either a func-

tion or functor (§1.3.3). For interpolation, you can use a Powvargram object that fits a simple model

$$v(r) = \alpha r^{\beta} \tag{3.7.16}$$

where β is considered fixed and α is fitted by unweighted least squares over all pairs of data points i and j. We'll get more sophisticated about variograms in §15.9; but for interpolation, excellent results can be obtained with this simple choice. The value of β should be in the range $1 \leq \beta < 2$. A good general choice is 1.5, but for functions with a strong linear trend, you may want to experiment with values as large as 1.99. (The value 2 gives a degenerate matrix and meaningless results.) The optional argument nug will be explained in §15.9.

krig.h

```
struct Powvargram {
```
Functor for variogram $v(r) = \alpha r^{\beta}$, where β is specified, α is fitted from the data.
```
    Doub alph, bet, nugsq;

    Powvargram(MatDoub_I &x, VecDoub_I &y, const Doub beta=1.5, const Doub nug=0.)
    : bet(beta), nugsq(nug*nug) {
```
Constructor. The npt $\times$ ndim matrix x inputs the data points, the vector y the function values, beta the value of β. For interpolation, the default value of beta is usually adequate. For the (rare) use of nug see §15.9.
```
        Int i,j,k,npt=x.nrows(),ndim=x.ncols();
        Doub rb,num=0.,denom=0.;
        for (i=0;i<npt;i++) for (j=i+1;j<npt;j++) {
            rb = 0.;
            for (k=0;k<ndim;k++) rb += SQR(x[i][k]-x[j][k]);
            rb = pow(rb,0.5*beta);
            num += rb*(0.5*SQR(y[i]-y[j]) - nugsq);
            denom += SQR(rb);
        }
        alph = num/denom;
    }

    Doub operator() (const Doub r) const {return nugsq+alph*pow(r,bet);}
};
```

Sample code for interpolating on a set of data points is

```
    MatDoub x(npts,ndim);
    VecDoub y(npts), xstar(ndim);
    ...
    Powvargram vgram(x,y);
    Krig<Powvargram> krig(x,y,vgram);
```

followed by any number of interpolations of the form

```
    ystar = krig.interp(xstar);
```

Be aware that while the interpolated values are quite insensitive to the variogram model, the estimated errors are rather sensitive to it. You should thus consider the error estimates as being order of magnitude only. Since they are also relatively expensive to compute, their value in this application is not great. They will be much more useful in §15.9, when our model includes measurement errors.

3.7.5 Curve Interpolation in Multidimensions

A different kind of interpolation, worth a brief mention here, is when you have an ordered set of N tabulated points in n dimensions that lie on a one-dimensional curve, $\mathbf{x}_0, \ldots \mathbf{x}_{N-1}$, and you want to interpolate other values along the curve. Two

cases worth distinguishing are: (i) The curve is an open curve, so that $\mathbf{x}_0$ and $\mathbf{x}_{N-1}$ represent endpoints. (ii) The curve is a closed curve, so that there is an implied curve segment connecting $\mathbf{x}_{N-1}$ back to $\mathbf{x}_0$.

A straightforward solution, using methods already at hand, is first to approximate distance along the curve by the sum of chord lengths between the tabulated points, and then to construct spline interpolations for each of the coordinates, $0, \ldots, n-1$, as a function of that parameter. Since the derivative of any single coordinate with respect to arc length can be no greater than 1, it is guaranteed that the spline interpolations will be well-behaved.

Probably 90% of applications require nothing more complicated than the above. If you are in the unhappy 10%, then you will need to learn about *Bézier curves*, *B-splines*, and *interpolating splines* more generally [12,13,14]. For the happy majority, an implementation is

interp_curve.h

```
struct Curve_interp {
Object for interpolating a curve specified by n points in dim dimensions.
    Int dim, n, in;
    Bool cls;                              Set if a closed curve.
    MatDoub pts;
    VecDoub s;
    VecDoub ans;
    NRvector<Spline_interp*> srp;

    Curve_interp(MatDoub &ptsin, Bool close=0)
    : n(ptsin.nrows()), dim(ptsin.ncols()), in(close ? 2*n : n),
    cls(close), pts(dim,in), s(in), ans(dim), srp(dim) {
    Constructor. The n × dim matrix ptsin inputs the data points. Input close as 0 for
    an open curve, 1 for a closed curve. (For a closed curve, the last data point should not
    duplicate the first — the algorithm will connect them.)
        Int i,ii,im,j,ofs;
        Doub ss,soff,db,de;
        ofs = close ? n/2 : 0;             The trick for closed curves is to duplicate half a
        s[0] = 0.;                             period at the beginning and end, and then
        for (i=0;i<in;i++) {                   use the middle half of the resulting spline.
            ii = (i-ofs+n) % n;
            im = (ii-1+n) % n;
            for (j=0;j<dim;j++) pts[j][i] = ptsin[ii][j];        Store transpose.
            if (i>0) {                     Accumulate arc length.
                s[i] = s[i-1] + rad(&ptsin[ii][0],&ptsin[im][0]);
                if (s[i] == s[i-1]) throw("error in Curve_interp");
                Consecutive points may not be identical. For a closed curve, the last data
                point should not duplicate the first.
            }
        }
        ss = close ? s[ofs+n]-s[ofs] : s[n-1]-s[0];  Rescale parameter so that the
        soff = s[ofs];                     interval [0,1] is the whole curve (or one period).
        for (i=0;i<in;i++) s[i] = (s[i]-soff)/ss;
        for (j=0;j<dim;j++) {              Construct the splines using endpoint derivatives.
            db = in < 4 ? 1.e99 : fprime(&s[0],&pts[j][0],1);
            de = in < 4 ? 1.e99 : fprime(&s[in-1],&pts[j][in-1],-1);
            srp[j] = new Spline_interp(s,&pts[j][0],db,de);
        }
    }
    ~Curve_interp() {for (Int j=0;j<dim;j++) delete srp[j];}

    VecDoub &interp(Doub t) {
    Interpolate a point on the stored curve. The point is parameterized by t, in the range [0,1].
    For open curves, values of t outside this range will return extrapolations (dangerous!). For
    closed curves, t is periodic with period 1.
```

```
        if (cls) t = t - floor(t);
        for (Int j=0;j<dim;j++) ans[j] = (*srp[j]).interp(t);
        return ans;
    }

    Doub fprime(Doub *x, Doub *y, Int pm) {
```
Utility for estimating the derivatives at the endpoints. x and y point to the abscissa and ordinate of the endpoint. If pm is $+1$, points to the right will be used (left endpoint); if it is -1, points to the left will be used (right endpoint). See text, below.
```
        Doub s1 = x[0]-x[pm*1], s2 = x[0]-x[pm*2], s3 = x[0]-x[pm*3],
            s12 = s1-s2, s13 = s1-s3, s23 = s2-s3;
        return -(s1*s2/(s13*s23*s3))*y[pm*3]+(s1*s3/(s12*s2*s23))*y[pm*2]
            -(s2*s3/(s1*s12*s13))*y[pm*1]+(1./s1+1./s2+1./s3)*y[0];
    }

    Doub rad(const Doub *p1, const Doub *p2) {
```
Euclidean distance.
```
        Doub sum = 0.;
        for (Int i=0;i<dim;i++) sum += SQR(p1[i]-p2[i]);
        return sqrt(sum);
    }

};
```

The utility routine fprime estimates the derivative of a function at a tabulated abscissa x_0 using four consecutive tabulated abscissa-ordinate pairs, $(x_0, y_0), \dots, (x_3, y_3)$. The formula for this, readily derived by power-series expansion, is

$$y_0' = -C_0 y_0 + C_1 y_1 - C_2 y_2 + C_3 y_3 \tag{3.7.17}$$

where

$$\begin{aligned} C_0 &= \frac{1}{s_1} + \frac{1}{s_2} + \frac{1}{s_3} \\ C_1 &= \frac{s_2 s_3}{s_1(s_2 - s_1)(s_3 - s_1)} \\ C_2 &= \frac{s_1 s_3}{(s_2 - s_1)s_2(s_3 - s_2)} \\ C_3 &= \frac{s_1 s_2}{(s_3 - s_1)(s_3 - s_2)s_3} \end{aligned} \tag{3.7.18}$$

with

$$\begin{aligned} s_1 &\equiv x_1 - x_0 \\ s_2 &\equiv x_2 - x_0 \\ s_3 &\equiv x_3 - x_0 \end{aligned} \tag{3.7.19}$$

CITED REFERENCES AND FURTHER READING:

Buhmann, M.D. 2003, *Radial Basis Functions: Theory and Implementations* (Cambridge, UK: Cambridge University Press).[1]

Powell, M.J.D. 1992, "The Theory of Radial Basis Function Approximation" in *Advances in Numerical Analysis II: Wavelets, Subdivision Algorithms and Radial Functions*, ed. W. A. Light (Oxford: Oxford University Press), pp. 105–210.[2]

Wikipedia. 2007+, "Radial Basis Functions," at http://en.wikipedia.org/.[3]

Beatson, R.K. and Greengard, L. 1997, "A Short Course on Fast Multipole Methods", in *Wavelets, Multilevel Methods and Elliptic PDEs*, eds. M. Ainsworth, J. Levesley, W. Light, and M. Marletta (Oxford: Oxford University Press), pp. 1–37.[4]

Beatson, R.K. and Newsam, G.N. 1998, "Fast Evaluation of Radial Basis Functions: Moment-Based Methods" in *SIAM Journal on Scientific Computing*, vol. 19, pp. 1428-1449.[5]

Wendland, H. 2005, *Scattered Data Approximation* (Cambridge, UK: Cambridge University Press).[6]

Franke, R. 1982, "Scattered Data Interpolation: Tests of Some Methods," *Mathematics of Computation*, vol. 38, pp. 181–200.[7]

Shepard, D. 1968, "A Two-dimensional Interpolation Function for Irregularly-spaced Data," in *Proceedings of the 1968 23rd ACM National Conference* (New York: ACM Press), pp. 517–524.[8]

Cressie, N. 1991, *Statistics for Spatial Data* (New York: Wiley).[9]

Wackernagel, H. 1998, *Multivariate Geostatistics*, 2nd ed. (Berlin: Springer).[10]

Rybicki, G.B., and Press, W.H. 1992, "Interpolation, Realization, and Reconstruction of Noisy, Irregularly Sampled Data," *Astrophysical Journal*, vol. 398, pp. 169–176.[11]

Isaaks, E.H., and Srivastava, R.M. 1989, *Applied Geostatistics* (New York: Oxford University Press).

Deutsch, C.V., and Journel, A.G. 1992, *GSLIB: Geostatistical Software Library and User's Guide* (New York: Oxford University Press).

Knott, G.D. 1999, *Interpolating Cubic Splines* (Boston: Birkhäuser).[12]

De Boor, C. 2001, *A Practical Guide to Splines* (Berlin: Springer).[13]

Prautzsch, H., Boehm, W., and Paluszny, M. 2002, *Bézier and B-Spline Techniques* (Berlin: Springer).[14]

3.8 Laplace Interpolation

In this section we look at a *missing data* or *gridding* problem, namely, how to restore missing or unmeasured values on a regular grid. Evidently some kind of interpolation from the not-missing values is required, but how shall we do this in a principled way?

One good method, already in use at the dawn of the computer age [1,2], is *Laplace interpolation*, sometimes called *Laplace/Poisson interpolation*. The general idea is to find an interpolating function y that satisfies Laplace's equation in n dimensions,

$$\nabla^2 y = 0 \tag{3.8.1}$$

wherever there is no data, and which satisfies

$$y(\mathbf{x}_i) = y_i \tag{3.8.2}$$

at all measured data points. Generically, such a function does exist. The reason for choosing Laplace's equation (among all possible partial differential equations, say) is that the solution to Laplace's equation selects, in some sense, the smoothest possible interpolant. In particular, its solution minimizes the integrated square of the gradient,

$$\int_\Omega |\nabla y|^2 \, d\Omega \tag{3.8.3}$$

where Ω denotes the n-dimensional domain of interest. This is a very general idea, and it can be applied to irregular meshes as well as to regular grids. Here, however, we consider only the latter.

For purposes of illustration (and because it is the most useful example) we further specialize to the case of two dimensions, and to the case of a Cartesian grid whose x_1 and x_2 values are evenly spaced — like a checkerboard.

In this geometry, the finite difference approximation to Laplace's equation has a particularly simple form, one that echos the *mean value theorem* for continuous solutions of the Laplace equation: The value of the solution at any free gridpoint (i.e., not a point with a measured value) equals the average of its four Cartesian neighbors. (See §20.0.) Indeed, this already sounds a lot like interpolation.

If y_0 denotes the value at a free point, while y_u, y_d, y_l, and y_r denote the values at its up, down, left, and right neighbors, respectively, then the equation satisfied is

$$y_0 - \tfrac{1}{4}y_u - \tfrac{1}{4}y_d - \tfrac{1}{4}y_l - \tfrac{1}{4}y_r = 0 \tag{3.8.4}$$

For gridpoints with measured values, on the other hand, a different (simple) equation is satisfied,

$$y_0 = y_{0(\text{measured})} \tag{3.8.5}$$

Note that these nonzero right-hand sides are what make an inhomogeneous, and therefore generally solvable, set of linear equations.

We are not quite done, since we must provide special forms for the top, bottom, left, and right boundaries, and for the four corners. Homogeneous choices that embody "natural" boundary conditions (with no preferred function values) are

$$\begin{aligned}
y_0 - \tfrac{1}{2}y_u - \tfrac{1}{2}y_d &= 0 && \text{(left and right boundaries)}\\
y_0 - \tfrac{1}{2}y_l - \tfrac{1}{2}y_r &= 0 && \text{(top and bottom boundaries)}\\
y_0 - \tfrac{1}{2}y_r - \tfrac{1}{2}y_d &= 0 && \text{(top-left corner)}\\
y_0 - \tfrac{1}{2}y_l - \tfrac{1}{2}y_d &= 0 && \text{(top-right corner)}\\
y_0 - \tfrac{1}{2}y_r - \tfrac{1}{2}y_u &= 0 && \text{(bottom-left corner)}\\
y_0 - \tfrac{1}{2}y_l - \tfrac{1}{2}y_u &= 0 && \text{(bottom-right corner)}
\end{aligned} \tag{3.8.6}$$

Since every gridpoint corresponds to exactly one of the equations in (3.8.4), (3.8.5), or (3.8.4), we have exactly as many equations as there are unknowns. If the grid is M by N, then there are MN of each. This can be quite a large number; but the equations are evidently very sparse. We solve them by defining a derived class from §2.7's `Linbcg` base class. You can readily identify all the cases of equations (3.8.4) – (3.8.6) in the code for `atimes`, below.

interp_laplace.h

```
struct Laplace_interp : Linbcg {
```
Object for interpolating missing data in a matrix by solving Laplace's equation. Call constructor once, then `solve` one or more times (see text).
```
    MatDoub &mat;
    Int ii,jj;
    Int nn,iter;
    VecDoub b,y,mask;

    Laplace_interp(MatDoub_IO &matrix) : mat(matrix), ii(mat.nrows()),
    jj(mat.ncols()), nn(ii*jj), iter(0), b(nn), y(nn), mask(nn) {
```
Constructor. Values greater than `1.e99` in the input matrix `mat` are deemed to be missing data. The matrix is not altered until `solve` is called.
```
        Int i,j,k;
        Doub vl = 0.;
```

```
        for (k=0;k<nn;k++) {                    Fill the r.h.s. vector, the initial guess,
            i = k/jj;                              and a mask of the missing data.
            j = k - i*jj;
            if (mat[i][j] < 1.e99) {
                b[k] =  y[k] = vl = mat[i][j];
                mask[k] = 1;
            } else {
                b[k] = 0.;
                y[k] = vl;
                mask[k] = 0;
            }
        }
    }

    void asolve(VecDoub_I &b, VecDoub_O &x, const Int itrnsp);
    void atimes(VecDoub_I &x, VecDoub_O &r, const Int itrnsp);
```

See definitions below. These are the real algorithmic content.

```
    Doub solve(Doub tol=1.e-6, Int itmax=-1) {
```

Invoke Linbcg::solve with appropriate arguments. The default argument values will usually work, in which case this routine need be called only once. The original matrix mat is refilled with the interpolated solution.

```
        Int i,j,k;
        Doub err;
        if (itmax <= 0) itmax = 2*MAX(ii,jj);
        Linbcg::solve(b,y,1,tol,itmax,iter,err);
        for (k=0,i=0;i<ii;i++) for (j=0;j<jj;j++) mat[i][j] = y[k++];
        return err;
    }
};

void Laplace_interp::asolve(VecDoub_I &b, VecDoub_O &x, const Int itrnsp) {
```

Diagonal preconditioner. (Diagonal elements all unity.)

```
    Int i,n=b.size();
    for (i=0;i<n;i++) x[i] = b[i];
}

void Laplace_interp::atimes(VecDoub_I &x, VecDoub_O &r, const Int itrnsp) {
```

Sparse matrix, and matrix transpose, multiply. This routine embodies eqs. (3.8.4), (3.8.5), and (3.8.6).

```
    Int i,j,k,n=r.size(),jjt,it;
    Doub del;
    for (k=0;k<n;k++) r[k] = 0.;
    for (k=0;k<n;k++) {
        i = k/jj;
        j = k - i*jj;
        if (mask[k]) {                                  Measured point, eq. (3.8.5).
            r[k] += x[k];
        } else if (i>0 && i<ii-1 && j>0 && j<jj-1) {    Interior point, eq. (3.8.4).
            if (itrnsp) {
                r[k] += x[k];
                del = -0.25*x[k];
                r[k-1] += del;
                r[k+1] += del;
                r[k-jj] += del;
                r[k+jj] += del;
            } else {
                r[k] = x[k] - 0.25*(x[k-1]+x[k+1]+x[k+jj]+x[k-jj]);
            }
        } else if (i>0 && i<ii-1) {                     Left or right edge, eq. (3.8.6).
            if (itrnsp) {
                r[k] += x[k];
                del = -0.5*x[k];
                r[k-jj] += del;
```

```
                r[k+jj] += del;
            } else {
                r[k] = x[k] - 0.5*(x[k+jj]+x[k-jj]);
            }
        } else if (j>0 && j<jj-1) {                          Top or bottom edge, eq. (3.8.6).
            if (itrnsp) {
                r[k] += x[k];
                del = -0.5*x[k];
                r[k-1] += del;
                r[k+1] += del;
            } else {
                r[k] = x[k] - 0.5*(x[k+1]+x[k-1]);
            }
        } else {                                             Corners, eq. (3.8.6).
            jjt = i==0 ? jj : -jj;
            it = j==0 ? 1 : -1;
            if (itrnsp) {
                r[k] += x[k];
                del = -0.5*x[k];
                r[k+jjt] += del;
                r[k+it] += del;
            } else {
                r[k] = x[k] - 0.5*(x[k+jjt]+x[k+it]);
            }
        }
    }
}
```

Usage is quite simple. Just fill a matrix with function values where they are known, and with `1.e99` where they are not; send the matrix to the constructor; and call the `solve` routine. The missing values will be interpolated. The default arguments should serve for most cases.

```
Int m=...,n=...;
MatDoub mat(m,n);
...
Laplace_interp mylaplace(mat);
mylaplace.solve();
```

Quite decent results are obtained for smooth functions on 300×300 matrices in which a random 10% of gridpoints have known function values, with 90% interpolated. However, since compute time scales as $MN \max(M, N)$ (that is, as the cube), this is not a method to use for much larger matrices, unless you break them up into overlapping tiles. If you experience convergence difficulties, then you should call `solve`, with appropriate nondefault arguments, several times in succession, and look at the returned error estimate after each call returns.

3.8.1 Minimum Curvature Methods

Laplace interpolation has a tendency to yield cone-like cusps around any small islands of known data points that are surrounded by a sea of unknowns. The reason is that, in two dimensions, the solution of Laplace's equation near a point source is logarithmically singular. When the known data is spread fairly evenly (if randomly) across the grid, this is not generally a problem. *Minimum curvature methods* deal with the problem at a more fundamental level by being based on the biharmonic equation

$$\nabla(\nabla y) = 0 \tag{3.8.7}$$

instead of Laplace's equation. Solutions of the biharmonic equation minimize the integrated square of the curvature,

$$\int_\Omega |\nabla^2 y|^2 \, d\Omega \tag{3.8.8}$$

Minimum curvature methods are widely used in the earth-science community [3,4].

The references give a variety of other methods that can be used for missing data interpolation and gridding.

CITED REFERENCES AND FURTHER READING:

Noma, A.A. and Misulia, M.G. 1959, "Programming Topographic Maps for Automatic Terrain Model Construction," *Surveying and Mapping*, vol. 19, pp. 355–366.[1]

Crain, I.K. 1970, "Computer Interpolation and Contouring of Two-dimensional Data: a Review," *Geoexploration*, vol. 8, pp. 71–86.[2]

Burrough, P.A. 1998, *Principles of Geographical Information Systems*, 2nd ed. (Oxford, UK: Clarendon Press)

Watson, D.F. 1982, *Contouring: A Guide to the Analysis and Display of Spatial Data* (Oxford, UK: Pergamon).

Briggs, I.C. 1974, "Machine Contouring Using Minimum Curvature," *Geophysics*, vol. 39, pp. 39–48.[3]

Smith, W.H.F. and Wessel, P. 1990, "Gridding With Continuous Curvature Splines in Tension," *Geophysics*, vol. 55, pp. 293–305.[4]

Integration of Functions

CHAPTER 4

4.0 Introduction

Numerical integration, which is also called *quadrature*, has a history extending back to the invention of calculus and before. The fact that integrals of elementary functions could not, in general, be computed analytically, while derivatives *could* be, served to give the field a certain panache, and to set it a cut above the arithmetic drudgery of numerical analysis during the whole of the 18th and 19th centuries.

With the invention of automatic computing, quadrature became just one numerical task among many, and not a very interesting one at that. Automatic computing, even the most primitive sort involving desk calculators and rooms full of "computers" (that were, until the 1950s, people rather than machines), opened to feasibility the much richer field of numerical integration of differential equations. Quadrature is merely the simplest special case: The evaluation of the integral

$$I = \int_a^b f(x)dx \tag{4.0.1}$$

is precisely equivalent to solving for the value $I \equiv y(b)$ the differential equation

$$\frac{dy}{dx} = f(x) \tag{4.0.2}$$

with the boundary condition

$$y(a) = 0 \tag{4.0.3}$$

Chapter 17 of this book deals with the numerical integration of differential equations. In that chapter, much emphasis is given to the concept of "variable" or "adaptive" choices of stepsize. We will not, therefore, develop that material here. If the function that you propose to integrate is sharply concentrated in one or more peaks, or if its shape is not readily characterized by a single length scale, then it is likely that you should cast the problem in the form of (4.0.2) – (4.0.3) and use the methods of Chapter 17. (But take a look at §4.7 first.)

The quadrature methods in this chapter are based, in one way or another, on the obvious device of adding up the value of the integrand at a sequence of abscissas

within the range of integration. The game is to obtain the integral as accurately as possible with the smallest number of function evaluations of the integrand. Just as in the case of interpolation (Chapter 3), one has the freedom to choose methods of various *orders*, with higher order sometimes, but not always, giving higher accuracy. *Romberg integration*, which is discussed in §4.3, is a general formalism for making use of integration methods of a variety of different orders, and we recommend it highly.

Apart from the methods of this chapter and of Chapter 17, there are yet other methods for obtaining integrals. One important class is based on function approximation. We discuss explicitly the integration of functions by Chebyshev approximation (*Clenshaw-Curtis* quadrature) in §5.9. Although not explicitly discussed here, you ought to be able to figure out how to do *cubic spline quadrature* using the output of the routine `spline` in §3.3. (Hint: Integrate equation 3.3.3 over x analytically. See [1].)

Some integrals related to Fourier transforms can be calculated using the fast Fourier transform (FFT) algorithm. This is discussed in §13.9. A related problem is the evaluation of integrals with long oscillatory tails. This is discussed at the end of §5.3.

Multidimensional integrals are a whole 'nother multidimensional bag of worms. Section 4.8 is an introductory discussion in this chapter; the important technique of *Monte Carlo integration* is treated in Chapter 7.

CITED REFERENCES AND FURTHER READING:

Carnahan, B., Luther, H.A., and Wilkes, J.O. 1969, *Applied Numerical Methods* (New York: Wiley), Chapter 2.

Isaacson, E., and Keller, H.B. 1966, *Analysis of Numerical Methods*; reprinted 1994 (New York: Dover), Chapter 7.

Acton, F.S. 1970, *Numerical Methods That Work*; 1990, corrected edition (Washington, DC: Mathematical Association of America), Chapter 4.

Stoer, J., and Bulirsch, R. 2002, *Introduction to Numerical Analysis*, 3rd ed. (New York: Springer), Chapter 3.

Ralston, A., and Rabinowitz, P. 1978, *A First Course in Numerical Analysis*, 2nd ed.; reprinted 2001 (New York: Dover), Chapter 4.

Dahlquist, G., and Bjorck, A. 1974, *Numerical Methods* (Englewood Cliffs, NJ: Prentice-Hall); reprinted 2003 (New York: Dover), §7.4.

Kahaner, D., Moler, C., and Nash, S. 1989, *Numerical Methods and Software* (Englewood Cliffs, NJ: Prentice-Hall), Chapter 5.

Forsythe, G.E., Malcolm, M.A., and Moler, C.B. 1977, *Computer Methods for Mathematical Computations* (Englewood Cliffs, NJ: Prentice-Hall), §5.2, p. 89.[1]

Davis, P., and Rabinowitz, P. 1984, *Methods of Numerical Integration*, 2nd ed. (Orlando, FL: Academic Press).

4.1 Classical Formulas for Equally Spaced Abscissas

Where would any book on numerical analysis be without Mr. Simpson and his "rule"? The classical formulas for integrating a function whose value is known at

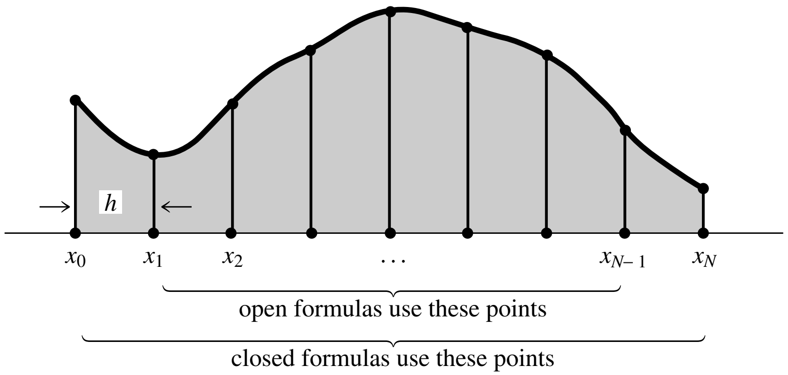

Figure 4.1.1. Quadrature formulas with equally spaced abscissas compute the integral of a function between x_0 and x_N. Closed formulas evaluate the function on the boundary points, while open formulas refrain from doing so (useful if the evaluation algorithm breaks down on the boundary points).

equally spaced steps have a certain elegance about them, and they are redolent with historical association. Through them, the modern numerical analyst communes with the spirits of his or her predecessors back across the centuries, as far as the time of Newton, if not farther. Alas, times *do* change; with the exception of two of the most modest formulas ("extended trapezoidal rule," equation 4.1.11, and "extended midpoint rule," equation 4.1.19; see §4.2), the classical formulas are almost entirely useless. They are museum pieces, but beautiful ones; we now enter the museum. (You can skip to §4.2 if you are not touristically inclined.)

Some notation: We have a sequence of abscissas, denoted $x_0, x_1, \ldots, x_{N-1}, x_N$, that are spaced apart by a constant step h,

$$x_i = x_0 + ih \qquad i = 0, 1, \ldots, N \tag{4.1.1}$$

A function $f(x)$ has known values at the x_i's,

$$f(x_i) \equiv f_i \tag{4.1.2}$$

We want to integrate the function $f(x)$ between a lower limit a and an upper limit b, where a and b are each equal to one or the other of the x_i's. An integration formula that uses the value of the function at the endpoints, $f(a)$ or $f(b)$, is called a *closed* formula. Occasionally, we want to integrate a function whose value at one or both endpoints is difficult to compute (e.g., the computation of f goes to a limit of zero over zero there, or worse yet has an integrable singularity there). In this case we want an *open* formula, which estimates the integral using only x_i's strictly *between* a and b (see Figure 4.1.1).

The basic building blocks of the classical formulas are rules for integrating a function over a small number of intervals. As that number increases, we can find rules that are exact for polynomials of increasingly high order. (Keep in mind that higher order does not always imply higher accuracy in real cases.) A sequence of such closed formulas is now given.

4.1.1 Closed Newton-Cotes Formulas

Trapezoidal rule:

$$\int_{x_0}^{x_1} f(x)dx = h\left[\frac{1}{2}f_0 + \frac{1}{2}f_1\right] \quad + O(h^3 f'') \tag{4.1.3}$$

Here the error term $O(\ )$ signifies that the true answer differs from the estimate by an amount that is the product of some numerical coefficient times h^3 times the value of the function's second derivative somewhere in the interval of integration. The coefficient is knowable, and it can be found in all the standard references on this subject. The point at which the second derivative is to be evaluated is, however, unknowable. If we knew it, we could evaluate the function there and have a higher-order method! Since the product of a knowable and an unknowable is unknowable, we will streamline our formulas and write only $O(\ )$, instead of the coefficient.

Equation (4.1.3) is a two-point formula (x_0 and x_1). It is exact for polynomials up to and including degree 1, i.e., $f(x) = x$. One anticipates that there is a three-point formula exact up to polynomials of degree 2. This is true; moreover, by a cancellation of coefficients due to left-right symmetry of the formula, the three-point formula is exact for polynomials up to and including degree 3, i.e., $f(x) = x^3$.

Simpson's rule:

$$\int_{x_0}^{x_2} f(x)dx = h\left[\frac{1}{3}f_0 + \frac{4}{3}f_1 + \frac{1}{3}f_2\right] \quad + O(h^5 f^{(4)}) \tag{4.1.4}$$

Here $f^{(4)}$ means the fourth derivative of the function f evaluated at an unknown place in the interval. Note also that the formula gives the integral over an interval of size $2h$, so the coefficients add up to 2.

There is no lucky cancellation in the four-point formula, so it is also exact for polynomials up to and including degree 3.

Simpson's $\frac{3}{8}$ rule:

$$\int_{x_0}^{x_3} f(x)dx = h\left[\frac{3}{8}f_0 + \frac{9}{8}f_1 + \frac{9}{8}f_2 + \frac{3}{8}f_3\right] \quad + O(h^5 f^{(4)}) \tag{4.1.5}$$

The five-point formula again benefits from a cancellation:

Bode's rule:

$$\int_{x_0}^{x_4} f(x)dx = h\left[\frac{14}{45}f_0 + \frac{64}{45}f_1 + \frac{24}{45}f_2 + \frac{64}{45}f_3 + \frac{14}{45}f_4\right] \quad + O(h^7 f^{(6)}) \tag{4.1.6}$$

This is exact for polynomials up to and including degree 5.

At this point the formulas stop being named after famous personages, so we will not go any further. Consult [1] for additional formulas in the sequence.

4.1.2 Extrapolative Formulas for a Single Interval

We are going to depart from historical practice for a moment. Many texts would give, at this point, a sequence of "Newton-Cotes Formulas of Open Type." Here is

an example:

$$\int_{x_0}^{x_5} f(x)dx = h\left[\frac{55}{24}f_1 + \frac{5}{24}f_2 + \frac{5}{24}f_3 + \frac{55}{24}f_4\right] \quad + O(h^5 f^{(4)})$$

Notice that the integral from $a = x_0$ to $b = x_5$ is estimated, using only the interior points x_1, x_2, x_3, x_4. In our opinion, formulas of this type are not useful for the reasons that (i) they cannot usefully be strung together to get "extended" rules, as we are about to do with the closed formulas, and (ii) for all other possible uses they are dominated by the Gaussian integration formulas, which we will introduce in §4.6.

Instead of the Newton-Cotes open formulas, let us set out the formulas for estimating the integral in the single interval from x_0 to x_1, using values of the function f at $x_1, x_2, \ldots$. These will be useful building blocks later for the "extended" open formulas.

$$\int_{x_0}^{x_1} f(x)dx = h[f_1] \quad + O(h^2 f') \tag{4.1.7}$$

$$\int_{x_0}^{x_1} f(x)dx = h\left[\frac{3}{2}f_1 - \frac{1}{2}f_2\right] \quad + O(h^3 f'') \tag{4.1.8}$$

$$\int_{x_0}^{x_1} f(x)dx = h\left[\frac{23}{12}f_1 - \frac{16}{12}f_2 + \frac{5}{12}f_3\right] \quad + O(h^4 f^{(3)}) \tag{4.1.9}$$

$$\int_{x_0}^{x_1} f(x)dx = h\left[\frac{55}{24}f_1 - \frac{59}{24}f_2 + \frac{37}{24}f_3 - \frac{9}{24}f_4\right] \quad + O(h^5 f^{(4)}) \tag{4.1.10}$$

Perhaps a word here would be in order about how formulas like the above can be derived. There are elegant ways, but the most straightforward is to write down the basic form of the formula, replacing the numerical coefficients with unknowns, say p, q, r, s. Without loss of generality take $x_0 = 0$ and $x_1 = 1$, so $h = 1$. Substitute in turn for $f(x)$ (and for f_1, f_2, f_3, f_4) the functions $f(x) = 1$, $f(x) = x$, $f(x) = x^2$, and $f(x) = x^3$. Doing the integral in each case reduces the left-hand side to a number and the right-hand side to a linear equation for the unknowns p, q, r, s. Solving the four equations produced in this way gives the coefficients.

4.1.3 Extended Formulas (Closed)

If we use equation (4.1.3) $N - 1$ times to do the integration in the intervals $(x_0, x_1), (x_1, x_2), \ldots, (x_{N-2}, x_{N-1})$ and then add the results, we obtain an "extended" or "composite" formula for the integral from x_0 to x_{N-1}.

Extended trapezoidal rule:

$$\begin{aligned}\int_{x_0}^{x_{N-1}} f(x)dx = h\Bigg[&\frac{1}{2}f_0 + f_1 + f_2 + \\ &\cdots + f_{N-2} + \frac{1}{2}f_{N-1}\Bigg] \quad + O\left(\frac{(b-a)^3 f''}{N^2}\right)\end{aligned} \tag{4.1.11}$$

Here we have written the error estimate in terms of the interval $b - a$ and the number of points N instead of in terms of h. This is clearer, since one is usually holding a and

b fixed and wanting to know, e.g., how much the error will be decreased by taking twice as many steps (in this case, it is by a factor of 4). In subsequent equations we will show *only* the scaling of the error term with the number of steps.

For reasons that will not become clear until §4.2, equation (4.1.11) is in fact the most important equation in this section; it is the basis for most practical quadrature schemes.

The *extended formula of order* $1/N^3$ is

$$\int_{x_0}^{x_{N-1}} f(x)dx = h\left[\frac{5}{12}f_0 + \frac{13}{12}f_1 + f_2 + f_3 + \cdots + f_{N-3} + \frac{13}{12}f_{N-2} + \frac{5}{12}f_{N-1}\right] + O\left(\frac{1}{N^3}\right) \tag{4.1.12}$$

(We will see in a moment where this comes from.)

If we apply equation (4.1.4) to successive, nonoverlapping *pairs* of intervals, we get the *extended Simpson's rule:*

$$\int_{x_0}^{x_{N-1}} f(x)dx = h\left[\frac{1}{3}f_0 + \frac{4}{3}f_1 + \frac{2}{3}f_2 + \frac{4}{3}f_3 + \cdots + \frac{2}{3}f_{N-3} + \frac{4}{3}f_{N-2} + \frac{1}{3}f_{N-1}\right] + O\left(\frac{1}{N^4}\right) \tag{4.1.13}$$

Notice that the 2/3, 4/3 alternation continues throughout the interior of the evaluation. Many people believe that the wobbling alternation somehow contains deep information about the integral of their function that is not apparent to mortal eyes. In fact, the alternation is an artifact of using the building block (4.1.4). Another extended formula with the same order as Simpson's rule is

$$\int_{x_0}^{x_{N-1}} f(x)dx = h\left[\frac{3}{8}f_0 + \frac{7}{6}f_1 + \frac{23}{24}f_2 + f_3 + f_4 + \cdots + f_{N-5} + f_{N-4} + \frac{23}{24}f_{N-3} + \frac{7}{6}f_{N-2} + \frac{3}{8}f_{N-1}\right] + O\left(\frac{1}{N^4}\right) \tag{4.1.14}$$

This equation is constructed by fitting cubic polynomials through successive groups of four points; we defer details to §19.3, where a similar technique is used in the solution of integral equations. We can, however, tell you where equation (4.1.12) came from. It is Simpson's extended rule, averaged with a modified version of itself in which the first and last steps are done with the trapezoidal rule (4.1.3). The trapezoidal step is *two* orders lower than Simpson's rule; however, its contribution to the integral goes down as an additional power of N (since it is used only twice, not N times). This makes the resulting formula of degree *one* less than Simpson.

4.1.4 Extended Formulas (Open and Semi-Open)

We can construct open and semi-open extended formulas by adding the closed formulas (4.1.11) – (4.1.14), evaluated for the second and subsequent steps, to the

extrapolative open formulas for the first step, (4.1.7) – (4.1.10). As discussed immediately above, it is consistent to use an end step that is of one order lower than the (repeated) interior step. The resulting formulas for an interval open at both ends are as follows.

Equations (4.1.7) and (4.1.11) give

$$\int_{x_0}^{x_{N-1}} f(x)dx = h\left[\frac{3}{2}f_1 + f_2 + f_3 + \cdots + f_{N-3} + \frac{3}{2}f_{N-2}\right] \quad + O\left(\frac{1}{N^2}\right) \tag{4.1.15}$$

Equations (4.1.8) and (4.1.12) give

$$\int_{x_0}^{x_{N-1}} f(x)dx = h\left[\frac{23}{12}f_1 + \frac{7}{12}f_2 + f_3 + f_4 + \cdots + f_{N-4} + \frac{7}{12}f_{N-3} + \frac{23}{12}f_{N-2}\right] \quad + O\left(\frac{1}{N^3}\right) \tag{4.1.16}$$

Equations (4.1.9) and (4.1.13) give

$$\int_{x_0}^{x_{N-1}} f(x)dx = h\left[\frac{27}{12}f_1 + 0 + \frac{13}{12}f_3 + \frac{4}{3}f_4 + \cdots + \frac{4}{3}f_{N-5} + \frac{13}{12}f_{N-4} + 0 + \frac{27}{12}f_{N-2}\right] \quad + O\left(\frac{1}{N^4}\right) \tag{4.1.17}$$

The interior points alternate 4/3 and 2/3. If we want to avoid this alternation, we can combine equations (4.1.9) and (4.1.14), giving

$$\int_{x_0}^{x_{N-1}} f(x)dx = h\left[\frac{55}{24}f_1 - \frac{1}{6}f_2 + \frac{11}{8}f_3 + f_4 + f_5 + f_6 + \cdots + f_{N-6} + f_{N-5} + \frac{11}{8}f_{N-4} - \frac{1}{6}f_{N-3} + \frac{55}{24}f_{N-2}\right] + O\left(\frac{1}{N^4}\right) \tag{4.1.18}$$

We should mention in passing another extended open formula, for use where the limits of integration are located halfway between tabulated abscissas. This one is known as the *extended midpoint rule* and is accurate to the same order as (4.1.15):

$$\int_{x_0}^{x_{N-1}} f(x)dx = h[f_{1/2} + f_{3/2} + f_{5/2} + \cdots + f_{N-5/2} + f_{N-3/2}] \quad + O\left(\frac{1}{N^2}\right) \tag{4.1.19}$$

There are also formulas of higher order for this situation, but we will refrain from giving them.

The *semi-open formulas* are just the obvious combinations of equations (4.1.11) – (4.1.14) with (4.1.15) – (4.1.18), respectively. At the closed end of the integration, use the weights from the former equations; at the open end, use the weights from

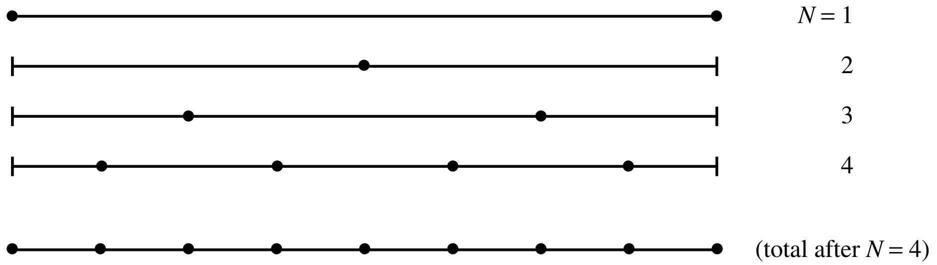

Figure 4.2.1. Sequential calls to the routine `Trapzd` incorporate the information from previous calls and evaluate the integrand only at those new points necessary to refine the grid. The bottom line shows the totality of function evaluations after the fourth call. The routine `qsimp`, by weighting the intermediate results, transforms the trapezoid rule into Simpson's rule with essentially no additional overhead.

the latter equations. One example should give the idea, the formula with error term decreasing as $1/N^3$, which is closed on the right and open on the left:

$$\int_{x_0}^{x_{N-1}} f(x)dx = h\left[\frac{23}{12}f_1 + \frac{7}{12}f_2 + f_3 + f_4 + \cdots + f_{N-3} + \frac{13}{12}f_{N-2} + \frac{5}{12}f_{N-1}\right] + O\left(\frac{1}{N^3}\right) \tag{4.1.20}$$

CITED REFERENCES AND FURTHER READING:

Abramowitz, M., and Stegun, I.A. 1964, *Handbook of Mathematical Functions* (Washington: National Bureau of Standards); reprinted 1968 (New York: Dover); online at `http://www.nr.com/aands`, §25.4.[1]

Isaacson, E., and Keller, H.B. 1966, *Analysis of Numerical Methods*; reprinted 1994 (New York: Dover), §7.1.

4.2 Elementary Algorithms

Our starting point is equation (4.1.11), the extended trapezoidal rule. There are two facts about the trapezoidal rule that make it the starting point for a variety of algorithms. One fact is rather obvious, while the second is rather "deep."

The obvious fact is that, for a fixed function $f(x)$ to be integrated between fixed limits a and b, one can double the number of intervals in the extended trapezoidal rule without losing the benefit of previous work. The coarsest implementation of the trapezoidal rule is to average the function at its endpoints a and b. The first stage of refinement is to add to this average the value of the function at the halfway point. The second stage of refinement is to add the values at the 1/4 and 3/4 points. And so on (see Figure 4.2.1).

As we will see, a number of elementary quadrature algorithms involve adding successive stages of refinement. It is convenient to encapsulate this feature in a `Quadrature` structure:

quadrature.h

```
struct Quadrature{
Abstract base class for elementary quadrature algorithms.
    Int n;                                   Current level of refinement.
    virtual Doub next() = 0;
    Returns the value of the integral at the nth stage of refinement. The function next() must
    be defined in the derived class.
};
```

Then the Trapzd structure is derived from this as follows:

quadrature.h

```
template<class T>
struct Trapzd : Quadrature {
Routine implementing the extended trapezoidal rule.
    Doub a,b,s;                              Limits of integration and current value of integral.
    T &func;
    Trapzd() {};
    Trapzd(T &funcc, const Doub aa, const Doub bb) :
        func(funcc), a(aa), b(bb) {n=0;}
        The constructor takes as inputs func, the function or functor to be integrated between
        limits a and b, also input.
    Doub next() {
    Returns the nth stage of refinement of the extended trapezoidal rule. On the first call (n=1),
    the routine returns the crudest estimate of ∫_a^b f(x)dx. Subsequent calls set n=2,3,... and
    improve the accuracy by adding 2^(n-2) additional interior points.
        Doub x,tnm,sum,del;
        Int it,j;
        n++;
        if (n == 1) {
            return (s=0.5*(b-a)*(func(a)+func(b)));
        } else {
            for (it=1,j=1;j<n-1;j++) it <<= 1;
            tnm=it;
            del=(b-a)/tnm;                   This is the spacing of the points to be added.
            x=a+0.5*del;
            for (sum=0.0,j=0;j<it;j++,x+=del) sum += func(x);
            s=0.5*(s+(b-a)*sum/tnm);         This replaces s by its refined value.
            return s;
        }
    }
};
```

Note that Trapzd is templated on the whole struct and does not just contain a templated function. This is necessary because it retains a reference to the supplied function or functor as a member variable.

The Trapzd structure is a workhorse that can be harnessed in several ways. The simplest and crudest is to integrate a function by the extended trapezoidal rule where you know in advance (we can't imagine how!) the number of steps you want. If you want $2^M + 1$, you can accomplish this by the fragment

```
Ftor func;                               Functor func here has no parameters.
Trapzd<Ftor> s(func,a,b);
for(j=1;j<=m+1;j++) val=s.next();
```

with the answer returned as val. Here Ftor is a functor containing the function to be integrated.

Much better, of course, is to refine the trapezoidal rule until some specified degree of accuracy has been achieved. A function for this is

quadrature.h

```
template<class T>
Doub qtrap(T &func, const Doub a, const Doub b, const Doub eps=1.0e-10) {
Returns the integral of the function or functor func from a to b. The constants EPS can be
set to the desired fractional accuracy and JMAX so that 2 to the power JMAX-1 is the maximum
allowed number of steps. Integration is performed by the trapezoidal rule.
    const Int JMAX=20;
    Doub s,olds=0.0;              Initial value of olds is arbitrary.
    Trapzd<T> t(func,a,b);
    for (Int j=0;j<JMAX;j++) {
        s=t.next();
        if (j > 5)                Avoid spurious early convergence.
            if (abs(s-olds) < eps*abs(olds) ||
                (s == 0.0 && olds == 0.0)) return s;
        olds=s;
    }
    throw("Too many steps in routine qtrap");
}
```

The optional argument eps sets the desired fractional accuracy. Unsophisticated as it is, routine qtrap is in fact a fairly robust way of doing integrals of functions that are not very smooth. Increased sophistication will usually translate into a higher-order method whose efficiency will be greater only for sufficiently smooth integrands. qtrap is the method of choice, e.g., for an integrand that is a function of a variable that is linearly interpolated between measured data points. Be sure that you do not require too stringent an eps, however: If qtrap takes too many steps in trying to achieve your required accuracy, accumulated roundoff errors may start increasing, and the routine may never converge. A value of 10^{-10} or even smaller is usually no problem in double precision when the convergence is moderately rapid, but not otherwise. (Of course, very few problems really require such precision.)

We come now to the "deep" fact about the extended trapezoidal rule, equation (4.1.11). It is this: The error of the approximation, which begins with a term of order $1/N^2$, is in fact *entirely even* when expressed in powers of $1/N$. This follows directly from the *Euler-Maclaurin summation formula*,

$$\int_{x_0}^{x_{N-1}} f(x)dx = h\left[\frac{1}{2}f_0 + f_1 + f_2 + \cdots + f_{N-2} + \frac{1}{2}f_{N-1}\right] - \frac{B_2 h^2}{2!}(f'_{N-1} - f'_0) - \cdots - \frac{B_{2k} h^{2k}}{(2k)!}(f_{N-1}^{(2k-1)} - f_0^{(2k-1)}) - \cdots \tag{4.2.1}$$

Here B_{2k} is a *Bernoulli number*, defined by the generating function

$$\frac{t}{e^t - 1} = \sum_{n=0}^{\infty} B_n \frac{t^n}{n!} \tag{4.2.2}$$

with the first few even values (odd values vanish except for $B_1 = -1/2$)

$$\begin{aligned} B_0 = 1 \quad B_2 = \frac{1}{6} \quad B_4 = -\frac{1}{30} \quad B_6 = \frac{1}{42} \\ B_8 = -\frac{1}{30} \quad B_{10} = \frac{5}{66} \quad B_{12} = -\frac{691}{2730} \end{aligned} \tag{4.2.3}$$

Equation (4.2.1) is not a convergent expansion, but rather only an asymptotic expansion whose error when truncated at any point is always less than twice the magnitude

of the first neglected term. The reason that it is not convergent is that the Bernoulli numbers become very large, e.g.,

$$B_{50} = \frac{495057205241079648212477525}{66}$$

The key point is that only even powers of h occur in the error series of (4.2.1). This fact is not, in general, shared by the higher-order quadrature rules in §4.1. For example, equation (4.1.12) has an error series beginning with $O(1/N^3)$, but continuing with all subsequent powers of N: $1/N^4$, $1/N^5$, etc.

Suppose we evaluate (4.1.11) with N steps, getting a result S_N, and then again with $2N$ steps, getting a result S_{2N}. (This is done by any two consecutive calls of `Trapzd`.) The leading error term in the second evaluation will be 1/4 the size of the error in the first evaluation. Therefore the combination

$$S = \tfrac{4}{3}S_{2N} - \tfrac{1}{3}S_N \tag{4.2.4}$$

will cancel out the leading order error term. But there *is* no error term of order $1/N^3$, by (4.2.1). The surviving error is of order $1/N^4$, the same as Simpson's rule. In fact, it should not take long for you to see that (4.2.4) is *exactly* Simpson's rule (4.1.13), alternating 2/3's, 4/3's, and all. This is the preferred method for evaluating that rule, and we can write it as a routine exactly analogous to `qtrap` above:

quadrature.h

```
template<class T>
Doub qsimp(T &func, const Doub a, const Doub b, const Doub eps=1.0e-10) {
```
Returns the integral of the function or functor `func` from `a` to `b`. The constants `EPS` can be set to the desired fractional accuracy and `JMAX` so that 2 to the power `JMAX-1` is the maximum allowed number of steps. Integration is performed by Simpson's rule.
```
    const Int JMAX=20;
    Doub s,st,ost=0.0,os=0.0;
    Trapzd<T> t(func,a,b);
    for (Int j=0;j<JMAX;j++) {
        st=t.next();
        s=(4.0*st-ost)/3.0;            Compare equation (4.2.4), above.
        if (j > 5)                     Avoid spurious early convergence.
            if (abs(s-os) < eps*abs(os) ||
                (s == 0.0 && os == 0.0)) return s;
        os=s;
        ost=st;
    }
    throw("Too many steps in routine qsimp");
}
```

The routine `qsimp` will in general be more efficient than `qtrap` (i.e., require fewer function evaluations) when the function to be integrated has a finite fourth derivative (i.e., a continuous third derivative). The combination of `qsimp` and its necessary workhorse `Trapzd` is a good one for light-duty work.

CITED REFERENCES AND FURTHER READING:

Stoer, J., and Bulirsch, R. 2002, *Introduction to Numerical Analysis*, 3rd ed. (New York: Springer), §3.1.

Dahlquist, G., and Bjorck, A. 1974, *Numerical Methods* (Englewood Cliffs, NJ: Prentice-Hall); reprinted 2003 (New York: Dover), §7.4.1 – §7.4.2.

Forsythe, G.E., Malcolm, M.A., and Moler, C.B. 1977, *Computer Methods for Mathematical Computations* (Englewood Cliffs, NJ: Prentice-Hall), §5.3.

4.3 Romberg Integration

We can view Romberg's method as the natural generalization of the routine qsimp in the last section to integration schemes that are of higher order than Simpson's rule. The basic idea is to use the results from k successive refinements of the extended trapezoidal rule (implemented in trapzd) to remove all terms in the error series up to but not including $O(1/N^{2k})$. The routine qsimp is the case of $k = 2$. This is one example of a very general idea that goes by the name of *Richardson's deferred approach to the limit*: Perform some numerical algorithm for various values of a parameter h, and then extrapolate the result to the continuum limit $h = 0$.

Equation (4.2.4), which subtracts off the leading error term, is a special case of polynomial extrapolation. In the more general Romberg case, we can use Neville's algorithm (see §3.2) to extrapolate the successive refinements to zero stepsize. Neville's algorithm can in fact be coded very concisely within a Romberg integration routine. For clarity of the program, however, it seems better to do the extrapolation by a function call to Poly_interp::rawinterp, as given in §3.2.

romberg.h

```
template <class T>
Doub qromb(T &func, Doub a, Doub b, const Doub eps=1.0e-10) {
Returns the integral of the function or functor func from a to b. Integration is performed by
Romberg's method of order 2K, where, e.g., K=2 is Simpson's rule.
    const Int JMAX=20, JMAXP=JMAX+1, K=5;
    Here EPS is the fractional accuracy desired, as determined by the extrapolation error es-
    timate; JMAX limits the total number of steps; K is the number of points used in the
    extrapolation.
    VecDoub s(JMAX),h(JMAXP);          These store the successive trapezoidal approxi-
    Poly_interp polint(h,s,K);            mations and their relative stepsizes.
    h[0]=1.0;
    Trapzd<T> t(func,a,b);
    for (Int j=1;j<=JMAX;j++) {
        s[j-1]=t.next();
        if (j >= K) {
            Doub ss=polint.rawinterp(j-K,0.0);
            if (abs(polint.dy) <= eps*abs(ss)) return ss;
        }
        h[j]=0.25*h[j-1];
        This is a key step: The factor is 0.25 even though the stepsize is decreased by only
        0.5. This makes the extrapolation a polynomial in h^2 as allowed by equation (4.2.1),
        not just a polynomial in h.
    }
    throw("Too many steps in routine qromb");
}
```

The routine qromb is quite powerful for sufficiently smooth (e.g., analytic) integrands, integrated over intervals that contain no singularities, and where the endpoints are also nonsingular. qromb, in such circumstances, takes many, *many* fewer function evaluations than either of the routines in §4.2. For example, the integral

$$\int_0^2 x^4 \log(x + \sqrt{x^2 + 1})dx$$

converges (with parameters as shown above) on the second extrapolation, after just 6 calls to trapzd, while qsimp requires 11 calls (32 times as many evaluations of the integrand) and qtrap requires 19 calls (8192 times as many evaluations of the integrand).

CITED REFERENCES AND FURTHER READING:

Stoer, J., and Bulirsch, R. 2002, *Introduction to Numerical Analysis*, 3rd ed. (New York: Springer), §3.4 – §3.5.

Dahlquist, G., and Bjorck, A. 1974, *Numerical Methods* (Englewood Cliffs, NJ: Prentice-Hall); reprinted 2003 (New York: Dover), §7.4.1 – §7.4.2.

Ralston, A., and Rabinowitz, P. 1978, *A First Course in Numerical Analysis*, 2nd ed.; reprinted 2001 (New York: Dover), §4.10–2.

4.4 Improper Integrals

For our present purposes, an integral will be "improper" if it has any of the following problems:

- its integrand goes to a finite limiting value at finite upper and lower limits, but cannot be evaluated *right on* one of those limits (e.g., $\sin x/x$ at $x = 0$)
- its upper limit is ∞ , or its lower limit is $-\infty$
- it has an integrable singularity at either limit (e.g., $x^{-1/2}$ at $x = 0$)
- it has an integrable singularity at a known place between its upper and lower limits
- it has an integrable singularity at an unknown place between its upper and lower limits

If an integral is infinite (e.g., $\int_1^\infty x^{-1}dx$), or does not exist in a limiting sense (e.g., $\int_{-\infty}^\infty \cos x dx$), we do not call it improper; we call it impossible. No amount of clever algorithmics will return a meaningful answer to an ill-posed problem.

In this section we will generalize the techniques of the preceding two sections to cover the first four problems on the above list. A more advanced discussion of quadrature with integrable singularities occurs in Chapter 19, notably §19.3. The fifth problem, singularity at an unknown location, can really only be handled by the use of a variable stepsize differential equation integration routine, as will be given in Chapter 17, or an adaptive quadrature routine such as in §4.7.

We need a workhorse like the extended trapezoidal rule (equation 4.1.11), but one that is an *open* formula in the sense of §4.1, i.e., does not require the integrand to be evaluated at the endpoints. Equation (4.1.19), the extended midpoint rule, is the best choice. The reason is that (4.1.19) shares with (4.1.11) the "deep" property of having an error series that is entirely even in h. Indeed there is a formula, not as well known as it ought to be, called the *Second Euler-Maclaurin summation formula*,

$$\begin{aligned}\int_{x_0}^{x_{N-1}} f(x)dx = h[f_{1/2} + f_{3/2} + f_{5/2} + \cdots + f_{N-5/2} + f_{N-3/2}]\\ + \frac{B_2h^2}{4}(f'_{N-1} - f'_0) + \cdots\\ + \frac{B_{2k}h^{2k}}{(2k)!}(1 - 2^{-2k+1})(f^{(2k-1)}_{N-1} - f^{(2k-1)}_0) + \cdots\end{aligned} \tag{4.4.1}$$

This equation can be derived by writing out (4.2.1) with stepsize h, then writing it out again with stepsize $h/2$, and then subtracting the first from twice the second.

It is not possible to double the number of steps in the extended midpoint rule and still have the benefit of previous function evaluations (try it!). However, it is possible to *triple* the number of steps and do so. Shall we do this, or double and accept the loss? On the average, tripling does a factor $\sqrt{3}$ of unnecessary work, since the "right" number of steps for a desired accuracy criterion may in fact fall anywhere in the logarithmic interval implied by tripling. For doubling, the factor is only $\sqrt{2}$, but we lose an extra factor of 2 in being unable to use all the previous evaluations. Since $1.732 < 2 \times 1.414$, it is better to triple.

Here is the resulting structure, which is directly comparable to `Trapzd`.

quadrature.h

```
template <class T>
struct Midpnt : Quadrature {
Routine implementing the extended midpoint rule.
    Doub a,b,s;                         Limits of integration and current value of inte-
    T &funk;                                gral.
    Midpnt(T &funcc, const Doub aa, const Doub bb) :
        funk(funcc), a(aa), b(bb) {n=0;}
        The constructor takes as inputs func, the function or functor to be integrated between
        limits a and b, also input.
    Doub next(){
    Returns the nth stage of refinement of the extended midpoint rule. On the first call (n=1),
    the routine returns the crudest estimate of ∫_a^b f(x)dx. Subsequent calls set n=2,3,... and
    improve the accuracy by adding (2/3) × 3^(n-1) additional interior points.
        Int it,j;
        Doub x,tnm,sum,del,ddel;
        n++;
        if (n == 1) {
            return (s=(b-a)*func(0.5*(a+b)));
        } else {
            for(it=1,j=1;j<n-1;j++) it *= 3;
            tnm=it;
            del=(b-a)/(3.0*tnm);
            ddel=del+del;                   The added points alternate in spacing be-
            x=a+0.5*del;                        tween del and ddel.
            sum=0.0;
            for (j=0;j<it;j++) {
                sum += func(x);
                x += ddel;
                sum += func(x);
                x += del;
            }
            s=(s+(b-a)*sum/tnm)/3.0;        The new sum is combined with the old inte-
            return s;                           gral to give a refined integral.
        }
    }
    virtual Doub func(const Doub x) {return funk(x);}     Identity mapping.
};
```

You may have spotted a seemingly unnecessary extra level of indirection in `Midpnt`, namely its calling the user-supplied function `funk` through an identity function `func`. The reason for this is that we are going to use mappings other than the identity mapping between `funk` and `func` to solve the problems of improper integrals listed above. The new quadratures will simply be derived from `Midpnt` with `func` overridden.

The structure `Midpnt` could be used to exactly replace `Trapzd` in a driver routine like `qtrap` (§4.2); one could simply change `Trapzd<T> t(func,a,b)` to `Midpnt<T> t(func,a,b)`, and perhaps also decrease the parameter `JMAX` since

$3^{\text{JMAX}-1}$ (from step tripling) is a much larger number than $2^{\text{JMAX}-1}$ (step doubling). The open formula implementation analogous to Simpson's rule (qsimp in §4.2) could also substitute Midpnt for Trapzd, decreasing JMAX as above, but now also changing the extrapolation step to be

```
s=(9.0*st-ost)/8.0;
```

since, when the number of steps is tripled, the error decreases to 1/9th its size, not 1/4th as with step doubling.

Either the thus modified qtrap or qsimp will fix the first problem on the list at the beginning of this section. More sophisticated, and allowing us to fix more problems, is to generalize Romberg integration in like manner:

romberg.h

```
template<class T>
Doub qromo(Midpnt<T> &q, const Doub eps=3.0e-9) {
```

Romberg integration on an open interval. Returns the integral of a function using any specified elementary quadrature algorithm q and Romberg's method. Normally q will be an open formula, not evaluating the function at the endpoints. It is assumed that q triples the number of steps on each call, and that its error series contains only even powers of the number of steps. The routines midpnt, midinf, midsql, midsqu, midexp are possible choices for q. The constants below have the same meanings as in qromb.

```
    const Int JMAX=14, JMAXP=JMAX+1, K=5;
    VecDoub h(JMAXP),s(JMAX);
    Poly_interp polint(h,s,K);
    h[0]=1.0;
    for (Int j=1;j<=JMAX;j++) {
        s[j-1]=q.next();
        if (j >= K) {
            Doub ss=polint.rawinterp(j-K,0.0);
            if (abs(polint.dy) <= eps*abs(ss)) return ss;
        }
        h[j]=h[j-1]/9.0;          This is where the assumption of step tripling and an even
    }                                 error series is used.
    throw("Too many steps in routine qromo");
}
```

Notice that we now pass a Midpnt object instead of the user function and limits of integration. There is a good reason for this, as we will see below. It does, however, mean that you have to bind things together before calling qromo, something like this, where we integrate from a to b:

```
Midpnt<Ftor> q(ftor,a,b);
Doub integral=qromo(q);
```

or, for a bare function,

```
Midpnt<Doub(Doub)> q(fbare,a,b);
Doub integral=qromo(q);
```

Laid back C++ compilers will let you condense these to

```
Doub integral = qromo(Midpnt<Ftor>(Ftor(),a,b));
```

or

```
Doub integral = qromo(Midpnt<Doub(Doub)>(fbare,a,b));
```

but uptight compilers may object to the way that a temporary is passed by reference, in which case use the two-line forms above.

As we shall now see, the function qromo, with its peculiar interface, is an excellent driver routine for solving all the other problems of improper integrals in our first list (except the intractable fifth).

The basic trick for improper integrals is to make a change of variables to eliminate the singularity or to map an infinite range of integration to a finite one. For example, the identity

$$\int_a^b f(x)dx = \int_{1/b}^{1/a} \frac{1}{t^2} f\left(\frac{1}{t}\right) dt \qquad ab > 0 \tag{4.4.2}$$

can be used with *either* $b \to \infty$ and a positive, *or* with $a \to -\infty$ and b negative, and works for any function that decreases toward infinity faster than $1/x^2$.

You can make the change of variable implied by (4.4.2) either analytically and then use, e.g., `qromo` and `Midpnt` to do the numerical evaluation, *or* you can let the numerical algorithm make the change of variable for you. We prefer the latter method as being more transparent to the user. To implement equation (4.4.2) we simply write a modified version of `Midpnt`, called `Midinf`, which allows b to be infinite (or, more precisely, a very large number on your particular machine, such as 1×10^{99}), or a to be negative and infinite. Since all the machinery is already in place in `Midpnt`, we write `Midinf` as a derived class and simply override the mapping function.

quadrature.h

```
template <class T>
struct Midinf : Midpnt<T>{
```

This routine is an exact replacement for `midpnt`, i.e., returns the `n`th stage of refinement of the integral of `funcc` from `aa` to `bb`, except that the function is evaluated at evenly spaced points in $1/x$ rather than in x. This allows the upper limit `bb` to be as large and positive as the computer allows, or the lower limit `aa` to be as large and negative, but not both. `aa` and `bb` must have the same sign.

```
    Doub func(const Doub x) {
        return Midpnt<T>::funk(1.0/x)/(x*x);        Effect the change of variable.
    }
    Midinf(T &funcc, const Doub aa, const Doub bb) :
        Midpnt<T>(funcc, aa, bb) {
        Midpnt<T>::a=1.0/bb;                        Set the limits of integration.
        Midpnt<T>::b=1.0/aa;
    }
};
```

An integral from 2 to ∞, for example, might be calculated by

```
Midinf<Ftor> q(ftor,2.,1.e99);
Doub integral=qromo(q);
```

If you need to integrate from a negative lower limit to positive infinity, you do this by breaking the integral into two pieces at some positive value, for example,

```
Midpnt<Ftor> q1(ftor,-5.,2.);
Midinf<Ftor> q2(ftor,2.,1.e99);
integral=qromo(q1)+qromo(q2);
```

Where should you choose the breakpoint? At a sufficiently large positive value so that the function `funk` is at least beginning to approach its asymptotic decrease to zero value at infinity. The polynomial extrapolation implicit in the second call to `qromo` deals with a polynomial in $1/x$, not in x.

To deal with an integral that has an integrable power-law singularity at its lower limit, one also makes a change of variable. If the integrand diverges as $(x-a)^{-\gamma}$, $0 \le \gamma < 1$, near $x = a$, use the identity

$$\int_a^b f(x)dx = \frac{1}{1-\gamma} \int_0^{(b-a)^{1-\gamma}} t^{\frac{\gamma}{1-\gamma}} f(t^{\frac{1}{1-\gamma}} + a)dt \qquad (b > a) \tag{4.4.3}$$

If the singularity is at the upper limit, use the identity

$$\int_a^b f(x)dx = \frac{1}{1-\gamma}\int_0^{(b-a)^{1-\gamma}} t^{\frac{\gamma}{1-\gamma}} f(b - t^{\frac{1}{1-\gamma}})dt \qquad (b > a) \qquad (4.4.4)$$

If there is a singularity at both limits, divide the integral at an interior breakpoint as in the example above.

Equations (4.4.3) and (4.4.4) are particularly simple in the case of inverse square-root singularities, a case that occurs frequently in practice:

$$\int_a^b f(x)dx = \int_0^{\sqrt{b-a}} 2tf(a+t^2)dt \qquad (b > a) \qquad (4.4.5)$$

for a singularity at a, and

$$\int_a^b f(x)dx = \int_0^{\sqrt{b-a}} 2tf(b-t^2)dt \qquad (b > a) \qquad (4.4.6)$$

for a singularity at b. Once again, we can implement these changes of variable transparently to the user by defining substitute routines for `Midpnt` that make the change of variable automatically:

quadrature.h

```
template <class T>
struct Midsql : Midpnt<T>{
```

This routine is an exact replacement for `midpnt`, except that it allows for an inverse square-root singularity in the integrand at the lower limit `aa`.

```
    Doub aorig;
    Doub func(const Doub x) {
        return 2.0*x*Midpnt<T>::funk(aorig+x*x);      Effect the change of variable.
    }
    Midsql(T &funcc, const Doub aa, const Doub bb) :
        Midpnt<T>(funcc, aa, bb), aorig(aa) {
        Midpnt<T>::a=0;
        Midpnt<T>::b=sqrt(bb-aa);
    }
};
```

Similarly,

quadrature.h

```
template <class T>
struct Midsqu : Midpnt<T>{
```

This routine is an exact replacement for `midpnt`, except that it allows for an inverse square-root singularity in the integrand at the upper limit `bb`.

```
    Doub borig;
    Doub func(const Doub x) {
        return 2.0*x*Midpnt<T>::funk(borig-x*x);      Effect the change of variable.
    }
    Midsqu(T &funcc, const Doub aa, const Doub bb) :
        Midpnt<T>(funcc, aa, bb), borig(bb) {
        Midpnt<T>::a=0;
        Midpnt<T>::b=sqrt(bb-aa);
    }
};
```

One last example should suffice to show how these formulas are derived in general. Suppose the upper limit of integration is infinite and the integrand falls off exponentially. Then we want a change of variable that maps $e^{-x}dx$ into $(\pm)dt$ (with the sign chosen to keep the upper limit of the new variable larger than the lower limit). Doing the integration gives by inspection

$$t = e^{-x} \qquad \text{or} \qquad x = -\log t \tag{4.4.7}$$

so that

$$\int_{x=a}^{x=\infty} f(x)dx = \int_{t=0}^{t=e^{-a}} f(-\log t)\frac{dt}{t} \tag{4.4.8}$$

The user-transparent implementation would be

quadrature.h

```
template <class T>
struct Midexp : Midpnt<T>{
```

This routine is an exact replacement for `midpnt`, except that `bb` is assumed to be infinite (value passed not actually used). It is assumed that the function `funk` decreases exponentially rapidly at infinity.

```
    Doub func(const Doub x) {
        return Midpnt<T>::funk(-log(x))/x;            Effect the change of variable.
    }
    Midexp(T &funcc, const Doub aa, const Doub bb) :
        Midpnt<T>(funcc, aa, bb) {
        Midpnt<T>::a=0.0;
        Midpnt<T>::b=exp(-aa);
    }
};
```

CITED REFERENCES AND FURTHER READING:

Acton, F.S. 1970, *Numerical Methods That Work*; 1990, corrected edition (Washington, DC: Mathematical Association of America), Chapter 4.

Dahlquist, G., and Bjorck, A. 1974, *Numerical Methods* (Englewood Cliffs, NJ: Prentice-Hall); reprinted 2003 (New York: Dover), §7.4.3, p. 294.

Stoer, J., and Bulirsch, R. 2002, *Introduction to Numerical Analysis*, 3rd ed. (New York: Springer), §3.7.

4.5 Quadrature by Variable Transformation

Imagine a simple general quadrature algorithm that is very rapidly convergent and allows you to ignore endpoint singularities completely. Sound too good to be true? In this section we'll describe an algorithm that in fact handles large classes of integrals in exactly this way.

Consider evaluating the integral

$$I = \int_a^b f(x)dx \tag{4.5.1}$$

As we saw in the construction of equations (4.1.11) – (4.1.20), quadrature formulas of arbitrarily high order can be constructed with interior weights unity, just by tuning the weights near the endpoints. But if a function dies off rapidly enough near

the endpoints, then those weights don't matter at all. In such a case, an N-point quadrature with uniform weights converges converges exponentially with N. (For a more rigorous motivation of this idea, see §4.5.1. For the connection to Gaussian quadrature, see the discussion at the end of §20.7.4.)

What about a function that doesn't vanish at the endpoints? Consider a change of variables $x = x(t)$, such that $x \in [a,b] \to t \in [c,d]$:

$$I = \int_c^d f[x(t)] \frac{dx}{dt}\, dt \tag{4.5.2}$$

Choose the transformation such that the factor dx/dt goes rapidly to zero at the endpoints of the interval. Then the simple trapezoidal rule applied to (4.5.2) will give extremely accurate results. (In this section, we'll call quadrature with uniform weights trapezoidal quadrature, with the understanding that it's a matter of taste whether you weight the endpoints with weight 1/2 or 1, since they don't count anyway.)

Even when $f(x)$ has integrable singularities at the endpoints of the interval, their effect can be overwhelmed by a suitable transformation $x = x(t)$. One need not tailor the transformation to the specific nature of the singularity: We will discuss several transformations that are effective at obliterating just about any kind of endpoint singularity.

The first transformation of this kind was introduced by Schwartz [1] and has become known as the TANH rule:

$$\begin{aligned} x &= \frac{1}{2}(b+a) + \frac{1}{2}(b-a)\tanh t, \qquad x \in [a,b] \to t \in [-\infty, \infty] \\ \frac{dx}{dt} &= \frac{1}{2}(b-a)\,\text{sech}^2\, t = \frac{2}{b-a}(b-x)(x-a) \end{aligned} \tag{4.5.3}$$

The sharp decrease of $\text{sech}^2\, t$ as $t \to \pm\infty$ explains the efficiency of the algorithm and its ability to deal with singularities. Another similar algorithm is the IMT rule [2]. However, $x(t)$ for the IMT rule is not given by a simple analytic expression, and its performance is not too different from the TANH rule.

There are two kinds of errors to consider when using something like the TANH rule. The *discretization error* is just the truncation error because you are using the trapezoidal rule to approximate I. The *trimming error* is the result of truncating the infinite sum in the trapezoidal rule at a finite value of N. (Recall that the limits are now $\pm\infty$.) You might think that the sharper the decrease of dx/dt as $t \to \pm\infty$, the more efficient the algorithm. But if the decrease is too sharp, then the density of quadrature points near the center of the original interval $[a,b]$ is low and the discretization error is large. The optimal strategy is to try to arrange that the discretization and trimming errors are approximately equal.

For the TANH rule, Schwartz [1] showed that the discretization error is of order

$$\epsilon_d \sim e^{-2\pi w/h} \tag{4.5.4}$$

where w is the distance from the real axis to the nearest singularity of the integrand. There is a pole when $\text{sech}^2\, t \to \infty$, i.e., when $t = \pm i\pi/2$. If there are no poles closer to the real axis in $f(x)$, then $w = \pi/2$. The trimming error, on the other hand, is

$$\epsilon_t \sim \text{sech}^2\, t_N \sim e^{-2Nh} \tag{4.5.5}$$

Setting $\epsilon_d \sim \epsilon_t$, we find

$$h \sim \frac{\pi}{(2N)^{1/2}}, \qquad \epsilon \sim e^{-\pi(2N)^{1/2}} \tag{4.5.6}$$

as the optimum h and the corresponding error. Note that ϵ decreases with N faster than any power of N. If f is singular at the endpoints, this can modify equation (4.5.5) for ϵ_t. This usually results in the constant π in (4.5.6) being reduced. Rather than developing an algorithm where we try to estimate the optimal h for each integrand a priori, we recommend simple step doubling and testing for convergence. We expect convergence to set in for h around the value given by equation (4.5.6).

The TANH rule essentially uses an exponential mapping to achieve the desired rapid fall-off at infinity. On the theory that more is better, one can try repeating the procedure. This leads to the DE (double exponential) rule:

$$\begin{aligned} x &= \frac{1}{2}(b+a) + \frac{1}{2}(b-a)\tanh(c\sinh t), \qquad x \in [a,b] \to t \in [-\infty,\infty] \\ \frac{dx}{dt} &= \frac{1}{2}(b-a)\,\text{sech}^2(c\sinh t)c\cosh t \sim \exp(-c\exp|t|) \quad \text{as} \quad |t| \to \infty \end{aligned} \tag{4.5.7}$$

Here the constant c is usually taken to be 1 or $\pi/2$. (Values larger than $\pi/2$ are not useful since $w = \pi/2$ for $0 < c \le \pi/2$, but w decreases rapidly for larger c.) By an analysis similar to equations (4.5.4) – (4.5.6), one can show that the optimal h and corresponding error for the DE rule are of order

$$h \sim \frac{\log(2\pi N w/c)}{N}, \qquad \epsilon \sim e^{-kN/\log N} \tag{4.5.8}$$

where k is a constant. The improved performance of the DE rule over the TANH rule indicated by comparing equations (4.5.6) and (4.5.8) is borne out in practice.

4.5.1 Exponential Convergence of the Trapezoidal Rule

The error in evaluating the integral (4.5.1) by the trapezoidal rule is given by the Euler-Maclaurin summation formula,

$$I \approx \frac{h}{2}[f(a)+f(b)]+h\sum_{j=1}^{N-1} f(a+jh)-\sum_{k=1}^{\infty}\frac{B_{2k}h^{2k}}{(2k)!}[f^{(2k-1)}(b)-f^{(2k-1)}(a)] \tag{4.5.9}$$

Note that this is in general an asymptotic expansion, not a convergent series. If all the derivatives of the function f vanish at the endpoints, then all the "correction terms" in equation (4.5.9) are zero. The error in this case is very small — it goes to zero with h faster than any power of h. We say that the method converges *exponentially*. The straight trapezoidal rule is thus an excellent method for integrating functions such as $\exp(-x^2)$ on $(-\infty,\infty)$, whose derivatives all vanish at the endpoints.

The class of transformations that will produce exponential convergence for a function whose derivatives do not all vanish at the endpoints is those for which dx/dt and all its derivatives go to zero at the endpoints of the interval. For functions with singularities at the endpoints, we require that $f(x)\,dx/dt$ and all its derivatives vanish at the endpoints. This is a more precise statement of "dx/dt goes rapidly to zero" given above.

4.5.2 Implementation

Implementing the DE rule is a little tricky. It's not a good idea to simply use `Trapzd` on the function $f(x)\,dx/dt$. First, the factor $\text{sech}^2(c \sinh t)$ in equation (4.5.7) can overflow if sech is computed as $1/\cosh$. We follow [3] and avoid this by using the variable q defined by

$$q = e^{-2\sinh t} \tag{4.5.10}$$

(we take $c = 1$ for simplicity) so that

$$\frac{dx}{dt} = 2(b-a)\frac{q}{(1+q)^2}\cosh t \tag{4.5.11}$$

For large positive t, q just underflows harmlessly to zero. Negative t is handled by using the symmetry of the trapezoidal rule about the midpoint of the interval. We write

$$\begin{aligned} I &\simeq h \sum_{j=-N}^{N} f(x_j) \left.\frac{dx}{dt}\right|_j \\ &= h\left\{ f[(a+b)/2] \left.\frac{dx}{dt}\right|_0 + \sum_{j=1}^{N}[f(a+\delta_j) + f(b-\delta_j)] \left.\frac{dx}{dt}\right|_j \right\} \end{aligned} \tag{4.5.12}$$

where

$$\delta = b - x = (b-a)\frac{q}{1+q} \tag{4.5.13}$$

A second possible problem is that cancellation errors in computing $a+\delta$ or $b-\delta$ can cause the computed value of $f(x)$ to blow up near the endpoint singularities. To handle this, you should code the function $f(x)$ as a function of two arguments, $f(x,\delta)$. Then compute the singular part using δ directly. For example, code the function $x^{-\alpha}(1-x)^{-\beta}$ as $\delta^{-\alpha}(1-x)^{-\beta}$ near $x = 0$ and $x^{-\alpha}\delta^{-\beta}$ near $x = 1$. (See §6.10 for another example of a $f(x,\delta)$.) Accordingly, the routine `DErule` below expects the function f to have two arguments. If your function has no singularities, or the singularities are "mild" (e.g., no worse than logarithmic), you can ignore δ when coding $f(x,\delta)$ and code it as if it were just $f(x)$.

The routine `DErule` implements equation (4.5.12). It contains an argument $h_{\max}$ that corresponds to the upper limit for t. The first approximation to I is given by the first term on the right-hand side of (4.5.12) with $h = h_{\max}$. Subsequent refinements correspond to halving h as usual. We typically take $h_{\max} = 3.7$ in double precision, corresponding to $q = 3 \times 10^{-18}$. This is generally adequate for "mild" singularities, like logarithms. If you want high accuracy for stronger singularities, you may have to increase $h_{\max}$. For example, for $1/\sqrt{x}$ you need $h_{\max} = 4.3$ to get full double precision. This corresponds to $q = 10^{-32} = (10^{-16})^2$, as you might expect.

derule.h

```
template<class T>
struct DErule : Quadrature {
```
Structure for implementing the DE rule.
```
    Doub a,b,hmax,s;
    T &func;
```

```
    DErule(T &funcc, const Doub aa, const Doub bb, const Doub hmaxx=3.7)
        : func(funcc), a(aa), b(bb), hmax(hmaxx) {n=0;}
```

Constructor. `funcc` is the function or functor that provides the function to be integrated between limits `aa` and `bb`, also input. The function operator in `funcc` takes two arguments, x and δ, as described in the text. The range of integration in the transformed variable t is $(-\texttt{hmaxx}, \texttt{hmaxx})$. Typical values of `hmaxx` are 3.7 for logarithmic or milder singularities, and 4.3 for square-root singularities, as discussed in the text.

```
    Doub next() {
```

On the first call to the function `next` $(n = 1)$, the routine returns the crudest estimate of $\int_a^b f(x)dx$. Subsequent calls to `next` $(n = 2, 3, \ldots)$ will improve the accuracy by adding 2^{n-1} additional interior points.

```
        Doub del,fact,q,sum,t,twoh;
        Int it,j;
        n++;
        if (n == 1) {
            fact=0.25;
            return s=hmax*2.0*(b-a)*fact*func(0.5*(b+a),0.5*(b-a));
        } else {
            for (it=1,j=1;j<n-1;j++) it <<= 1;
            twoh=hmax/it;                       Twice the spacing of the points to be added.
            t=0.5*twoh;
            for (sum=0.0,j=0;j<it;j++) {
                q=exp(-2.0*sinh(t));
                del=(b-a)*q/(1.0+q);
                fact=q/SQR(1.0+q)*cosh(t);
                sum += fact*(func(a+del,del)+func(b-del,del));
                t += twoh;
            }
            return s=0.5*s+(b-a)*twoh*sum;  Replace s by its refined value and return.
        }
    }
};
```

If the double exponential rule (DE rule) is generally better than the single exponential rule (TANH rule), why don't we keep going and use a triple exponential rule, quadruple exponential rule, ...? As we mentioned earlier, the discretization error is dominated by the pole nearest to the real axis. It turns out that beyond the double exponential the poles come nearer and nearer to the real axis, so the methods tend to get worse, not better.

If the function to be integrated itself has a pole near the real axis (much nearer than the $\pi/2$ that comes from the DE or TANH rules), the convergence of the method slows down. In analytically tractable cases, one can find a "pole correction term" to add to the trapezoidal rule to restore rapid convergence [4].

4.5.3 Infinite Ranges

Simple variations of the TANH or DE rules can be used if either or both of the limits of integration is infinite:

Range	TANH Rule	DE Rule	Mixed Rule
$(0,\infty)$	$x = e^t$	$x = e^{2c\sinh t}$	$x = e^{t-e^{-t}}$
$(-\infty,\infty)$	$x = \sinh t$	$x = \sinh(c\sinh t)$	—

(4.5.14)

The last column gives a mixed rule for functions that fall off rapidly (e^{-x} or e^{-x^2}) at infinity. It is a DE rule at $x = 0$ but only a single exponential at infinity. The expo-

nential fall-off of the integrand makes it behave like a DE rule there too. The mixed rule for $(-\infty, \infty)$ is constructed by splitting the range into $(-\infty, 0)$ and $(0, \infty)$ and making the substitution $x \to -x$ in the first range. This gives two integrals on $(0, \infty)$.

To implement the DE rule for infinite ranges we don't need the precautions we used in coding the finite range DE rule. It's fine to simply use the routine Trapzd directly as a function of t, with the function func that it calls returning $f(x)\,dx/dt$. So if funk is your function returning $f(x)$, then you define the function func as a function of t by code of the following form (for the mixed rule)

```
x=exp(t-exp(-t));
dxdt=x*(1.0+exp(-t));
return funk(x)*dxdt;
```

and pass func to Trapzd. The only care required is in deciding the range of integration. You want the contribution to the integral from the endpoints of the integration to be negligible. For example, $(-4, 4)$ is typically adequate for $x = \exp(\pi \sinh t)$.

4.5.4 Examples

As examples of the power of these methods, consider the following integrals:

$$\int_0^1 \log x \log(1-x)\,dx = 2 - \frac{\pi^2}{6} \tag{4.5.15}$$

$$\int_0^\infty \frac{1}{x^{1/2}(1+x)} dx = \pi \tag{4.5.16}$$

$$\int_0^\infty x^{-3/2} \sin\frac{x}{2} e^{-x}\,dx = [\pi(\sqrt{5}-2)]^{1/2} \tag{4.5.17}$$

$$\int_0^\infty x^{-2/7} e^{-x^2}\,dx = \tfrac{1}{2}\Gamma(\tfrac{5}{14}) \tag{4.5.18}$$

The integral (4.5.15) is easily handled by DErule. The routine converges to machine precision (10^{-16}) with about 30 function evaluations, completely unfazed by the singularities at the endpoints. The integral (4.5.16) is an example of an integrand that is singular at the origin and falls off slowly at infinity. The routine Midinf fails miserably because of the slow fall-off. Yet the transformation $x = \exp(\pi \sinh t)$ again gives machine precision in about 30 function evaluations, integrating t over the range $(-4, 4)$. By comparison, the transformation $x = e^t$ for t in the range $(-90, 90)$ requires about 500 function evaluations for the same accuracy.

The integral (4.5.17) combines a singularity at the origin with exponential fall-off at infinity. Here the "mixed" transformation $x = \exp(t - e^{-t})$ is best, requiring about 60 function evaluations for t in the range $(-4.5, 4)$. Note that the exponential fall-off is crucial here; these transformations fail completely for slowly decaying oscillatory functions like $x^{-3/2} \sin x$. Fortunately the series acceleration algorithms of §5.3 work well in such cases.

The final integral (4.5.18) is similar to (4.5.17), and using the same transformation requires about the same number of function evaluations to achieve machine precision. The range of t can be smaller, say $(-4, 3)$, because of the more rapid fall-off of the integrand. Note that for all these integrals the number of function evaluations would be double the number we quote if we are using step doubling to

decide when the integrals have converged, since we need one extra set of trapezoidal evaluations to confirm convergence. In many cases, however, you don't need this extra set of function evaluations: Once the method starts converging, the number of significant digits approximately doubles with each iteration. Accordingly, you can set the convergence criterion to stop the procedure when two successive iterations agree to the *square root* of the desired precision. The last iteration will then have approximately the required precision. Even without this trick, the method is quite remarkable for the range of difficult integrals that it can tame efficiently.

An extended example of the use of the DE rule for finite and infinite ranges is given in §6.10. There we give a routine for computing the generalized Fermi-Dirac integrals

$$F_k(\eta,\theta) = \int_0^\infty \frac{x^k(1+\frac{1}{2}\theta x)^{1/2}}{e^{x-\eta}+1}\,dx \tag{4.5.19}$$

Another example is given in the routine `Stiel` in §4.6.

4.5.5 Relation to the Sampling Theorem

The *sinc expansion* of a function is

$$f(x) \simeq \sum_{k=-\infty}^{\infty} f(kh)\,\mathrm{sinc}\left[\frac{\pi}{h}(x-kh)\right] \tag{4.5.20}$$

where $\mathrm{sinc}(x) \equiv \sin x/x$. The expansion is exact for a limited class of analytic functions. However, it can be a good approximation for other functions too, and the sampling theorem characterizes these functions, as will be discussed in §13.11. There we will use the sinc expansion of e^{-x^2} to get an approximation for the complex error function. Functions well-approximated by the sinc expansion typically fall off rapidly as $x \to \pm\infty$, so truncating the expansion at $k = \pm N$ still gives a good approximation to $f(x)$.

If we integrate both sides of equation (4.5.20), we find

$$\int_{-\infty}^{\infty} f(x)\,dx \simeq h \sum_{k=-\infty}^{\infty} f(kh) \tag{4.5.21}$$

which is just the trapezoidal formula! Thus, rapid convergence of the trapezoidal formula for the integral of f corresponds to f being well-approximated by its sinc expansion. The various transformations described earlier can be used to map $x \to x(t)$ and produce good sinc approximations with uniform samples in t. These approximations can be used not only for the trapezoidal quadrature of f, but also for good approximations to derivatives, integral transforms, Cauchy principal value integrals, and solving differential and integral equations [5].

CITED REFERENCES AND FURTHER READING:

Schwartz, C. 1969, "Numerical Integration of Analytic Functions," *Journal of Computational Physics*, vol. 4, pp. 19–29.[1]

Iri, M., Moriguti, S., and Takasawa, Y. 1987, "On a Certain Quadrature Formula," *Journal of Computational and Applied Mathematics*, vol. 17, pp. 3–20. (English version of Japanese article originally published in 1970.)[2]

Evans, G.A., Forbes, R.C., and Hyslop, J. 1984, "The Tanh Transformation for Singular Integrals," *International Journal of Computer Mathematics*, vol. 15, pp. 339–358.[3]

Bialecki, B. 1989, *BIT*, "A Modified Sinc Quadrature Rule for Functions with Poles near the Arc of Integration," vol. 29, pp. 464–476.[4]

Stenger, F. 1981, "Numerical Methods Based on Whittaker Cardinal or Sinc Functions," *SIAM Review*, vol. 23, pp. 165–224.[5]

Takahasi, H., and Mori, H. 1973, "Quadrature Formulas Obtained by Variable Transformation," *Numerische Mathematik*, vol. 21, pp. 206–219.

Mori, M. 1985, "Quadrature Formulas Obtained by Variable Transformation and DE Rule," *Journal of Computational and Applied Mathematics*, vol. 12&13, pp. 119–130.

Sikorski, K., and Stenger, F. 1984, "Optimal Quadratures in H_p Spaces," *ACM Transactions on Mathematical Software*, vol. 10, pp. 140–151; *op. cit.*, pp. 152–160.

4.6 Gaussian Quadratures and Orthogonal Polynomials

In the formulas of §4.1, the integral of a function was approximated by the sum of its functional values at a set of equally spaced points, multiplied by certain aptly chosen weighting coefficients. We saw that as we allowed ourselves more freedom in choosing the coefficients, we could achieve integration formulas of higher and higher order. The idea of *Gaussian quadratures* is to give ourselves the freedom to choose not only the weighting coefficients, but also the location of the abscissas at which the function is to be evaluated. They will no longer be equally spaced. Thus, we will have *twice* the number of degrees of freedom at our disposal; it will turn out that we can achieve Gaussian quadrature formulas whose order is, essentially, twice that of the Newton-Cotes formula with the same number of function evaluations.

Does this sound too good to be true? Well, in a sense it is. The catch is a familiar one, which cannot be overemphasized: High order is not the same as high accuracy. High order translates to high accuracy only when the integrand is very smooth, in the sense of being "well-approximated by a polynomial."

There is, however, one additional feature of Gaussian quadrature formulas that adds to their usefulness: We can arrange the choice of weights and abscissas to make the integral exact for a class of integrands "polynomials times some known function $W(x)$" rather than for the usual class of integrands "polynomials." The function $W(x)$ can then be chosen to remove integrable singularities from the desired integral. Given $W(x)$, in other words, and given an integer N, we can find a set of weights w_j and abscissas x_j such that the approximation

$$\int_a^b W(x) f(x) dx \approx \sum_{j=0}^{N-1} w_j f(x_j) \tag{4.6.1}$$

is exact if $f(x)$ is a polynomial. For example, to do the integral

$$\int_{-1}^{1} \frac{\exp(-\cos^2 x)}{\sqrt{1-x^2}} dx \tag{4.6.2}$$

(not a very natural looking integral, it must be admitted), we might well be interested in a Gaussian quadrature formula based on the choice

$$W(x) = \frac{1}{\sqrt{1-x^2}} \tag{4.6.3}$$

in the interval $(-1, 1)$. (This particular choice is called *Gauss-Chebyshev integration*, for reasons that will become clear shortly.)

Notice that the integration formula (4.6.1) can also be written with the weight function $W(x)$ not overtly visible: Define $g(x) \equiv W(x) f(x)$ and $v_j \equiv w_j / W(x_j)$. Then (4.6.1) becomes

$$\int_a^b g(x)dx \approx \sum_{j=0}^{N-1} v_j g(x_j) \tag{4.6.4}$$

Where did the function $W(x)$ go? It is lurking there, ready to give high-order accuracy to integrands of the form polynomials times $W(x)$, and ready to *deny* high-order accuracy to integrands that are otherwise perfectly smooth and well-behaved. When you find tabulations of the weights and abscissas for a given $W(x)$, you have to determine carefully whether they are to be used with a formula in the form of (4.6.1), or like (4.6.4).

So far our introduction to Gaussian quadrature is pretty standard. However, there is an aspect of the method that is not as widely appreciated as it should be: For smooth integrands (after factoring out the appropriate weight function), Gaussian quadrature converges *exponentially* fast as N increases, because the order of the method, not just the density of points, increases with N. This behavior should be contrasted with the power-law behavior (e.g., $1/N^2$ or $1/N^4$) of the Newton-Cotes based methods in which the order remains fixed (e.g., 2 or 4) even as the density of points increases. For a more rigorous discussion, see §20.7.4.

Here is an example of a quadrature routine that contains the tabulated abscissas and weights for the case $W(x) = 1$ and $N = 10$. Since the weights and abscissas are, in this case, symmetric around the midpoint of the range of integration, there are actually only five distinct values of each:

qgaus.h

```
template <class T>
Doub qgaus(T &func, const Doub a, const Doub b)
```

Returns the integral of the function or functor `func` between `a` and `b`, by ten-point Gauss-Legendre integration: the function is evaluated exactly ten times at interior points in the range of integration.

```
{
```

Here are the abscissas and weights:

```
    static const Doub x[]={0.1488743389816312,0.4333953941292472,
        0.6794095682990244,0.8650633666889845,0.9739065285171717};
    static const Doub w[]={0.2955242247147529,0.2692667193099963,
        0.2190863625159821,0.1494513491505806,0.0666713443086881};
    Doub xm=0.5*(b+a);
    Doub xr=0.5*(b-a);
    Doub s=0;                       Will be twice the average value of the function, since the
    for (Int j=0;j<5;j++) {             ten weights (five numbers above each used twice)
        Doub dx=xr*x[j];                sum to 2.
        s += w[j]*(func(xm+dx)+func(xm-dx));
    }
    return s *= xr;                 Scale the answer to the range of integration.
}
```

The above routine illustrates that one can use Gaussian quadratures without necessarily understanding the theory behind them: One just locates tabulated weights and abscissas in a book (e.g., [1] or [2]). However, the theory is very pretty, and it will come in handy if you ever need to construct your own tabulation of weights and abscissas for an unusual choice of $W(x)$. We will therefore give, without any proofs, some useful results that will enable you to do this. Several of the results assume that $W(x)$ does not change sign inside (a, b), which is usually the case in practice.

The theory behind Gaussian quadratures goes back to Gauss in 1814, who used continued fractions to develop the subject. In 1826, Jacobi rederived Gauss's results by means of orthogonal polynomials. The systematic treatment of arbitrary weight functions $W(x)$ using orthogonal polynomials is largely due to Christoffel in 1877. To introduce these orthogonal polynomials, let us fix the interval of interest to be (a, b). We can define the "scalar product of two functions f and g over a weight function W" as

$$\langle f|g\rangle \equiv \int_a^b W(x)f(x)g(x)dx \tag{4.6.5}$$

The scalar product is a number, not a function of x. Two functions are said to be *orthogonal* if their scalar product is zero. A function is said to be *normalized* if its scalar product with itself is unity. A set of functions that are all mutually orthogonal and also all individually normalized is called an *orthonormal* set.

We can find a set of polynomials (i) that includes exactly one polynomial of order j, called $p_j(x)$, for each $j = 0, 1, 2, \ldots$, and (ii) all of which are mutually orthogonal over the specified weight function $W(x)$. A constructive procedure for finding such a set is the recurrence relation

$$\begin{aligned} p_{-1}(x) &\equiv 0 \\ p_0(x) &\equiv 1 \\ p_{j+1}(x) &= (x - a_j)p_j(x) - b_j p_{j-1}(x) \qquad j = 0, 1, 2, \ldots \end{aligned} \tag{4.6.6}$$

where

$$\begin{aligned} a_j &= \frac{\langle xp_j|p_j\rangle}{\langle p_j|p_j\rangle} \qquad j = 0, 1, \ldots \\ b_j &= \frac{\langle p_j|p_j\rangle}{\langle p_{j-1}|p_{j-1}\rangle} \qquad j = 1, 2, \ldots \end{aligned} \tag{4.6.7}$$

The coefficient b_0 is arbitrary; we can take it to be zero.

The polynomials defined by (4.6.6) are *monic*, that is, the coefficient of their leading term [x^j for $p_j(x)$] is unity. If we divide each $p_j(x)$ by the constant $[\langle p_j|p_j\rangle]^{1/2}$, we can render the set of polynomials orthonormal. One also encounters orthogonal polynomials with various other normalizations. You can convert from a given normalization to monic polynomials if you know that the coefficient of x^j in p_j is λ_j, say; then the monic polynomials are obtained by dividing each p_j by λ_j. Note that the coefficients in the recurrence relation (4.6.6) depend on the adopted normalization.

The polynomial $p_j(x)$ can be shown to have exactly j distinct roots in the interval (a, b). Moreover, it can be shown that the roots of $p_j(x)$ "interleave" the $j - 1$ roots of $p_{j-1}(x)$, i.e., there is exactly one root of the former in between each two adjacent roots of the latter. This fact comes in handy if you need to find all the

roots. You can start with the one root of $p_1(x)$ and then, in turn, bracket the roots of each higher j, pinning them down at each stage more precisely by Newton's rule or some other root-finding scheme (see Chapter 9).

Why would you ever want to find all the roots of an orthogonal polynomial $p_j(x)$? Because the abscissas of the N-point Gaussian quadrature formulas (4.6.1) and (4.6.4) with weighting function $W(x)$ in the interval (a, b) are precisely the roots of the orthogonal polynomial $p_N(x)$ for the same interval and weighting function. This is the fundamental theorem of Gaussian quadratures, and it lets you find the abscissas for any particular case.

Once you know the abscissas $x_0, \ldots, x_{N-1}$, you need to find the weights w_j, $j = 0, \ldots, N-1$. One way to do this (not the most efficient) is to solve the set of linear equations

$$\begin{bmatrix} p_0(x_0) & \ldots & p_0(x_{N-1}) \\ p_1(x_0) & \ldots & p_1(x_{N-1}) \\ \vdots & & \vdots \\ p_{N-1}(x_0) & \ldots & p_{N-1}(x_{N-1}) \end{bmatrix} \begin{bmatrix} w_0 \\ w_1 \\ \vdots \\ w_{N-1} \end{bmatrix} = \begin{bmatrix} \int_a^b W(x) p_0(x) dx \\ 0 \\ \vdots \\ 0 \end{bmatrix} \tag{4.6.8}$$

Equation (4.6.8) simply solves for those weights such that the quadrature (4.6.1) gives the correct answer for the integral of the first N orthogonal polynomials. Note that the zeros on the right-hand side of (4.6.8) appear because $p_1(x), \ldots, p_{N-1}(x)$ are all orthogonal to $p_0(x)$, which is a constant. It can be shown that, with those weights, the integral of the *next* $N - 1$ polynomials is also exact, so that the quadrature is exact for all polynomials of degree $2N - 1$ or less. Another way to evaluate the weights (though one whose proof is beyond our scope) is by the formula

$$w_j = \frac{\langle p_{N-1} | p_{N-1} \rangle}{p_{N-1}(x_j) p'_N(x_j)} \tag{4.6.9}$$

where $p'_N(x_j)$ is the derivative of the orthogonal polynomial at its zero x_j.

The computation of Gaussian quadrature rules thus involves two distinct phases: (i) the generation of the orthogonal polynomials $p_0, \ldots, p_N$, i.e., the computation of the coefficients a_j, b_j in (4.6.6), and (ii) the determination of the zeros of $p_N(x)$, and the computation of the associated weights. For the case of the "classical" orthogonal polynomials, the coefficients a_j and b_j are explicitly known (equations 4.6.10 – 4.6.14 below) and phase (i) can be omitted. However, if you are confronted with a "nonclassical" weight function $W(x)$, and you don't know the coefficients a_j and b_j, the construction of the associated set of orthogonal polynomials is not trivial. We discuss it at the end of this section.

4.6.1 Computation of the Abscissas and Weights

This task can range from easy to difficult, depending on how much you already know about your weight function and its associated polynomials. In the case of classical, well-studied, orthogonal polynomials, practically everything is known, including good approximations for their zeros. These can be used as starting guesses, enabling Newton's method (to be discussed in §9.4) to converge very rapidly. Newton's method requires the derivative $p'_N(x)$, which is evaluated by standard relations in terms of p_N and p_{N-1}. The weights are then conveniently evaluated by equation

(4.6.9). For the following named cases, this direct root finding is faster, by a factor of 3 to 5, than any other method.

Here are the weight functions, intervals, and recurrence relations that generate the most commonly used orthogonal polynomials and their corresponding Gaussian quadrature formulas.

Gauss-Legendre:

$$\begin{aligned} W(x) &= 1 \qquad -1 < x < 1 \\ (j+1)P_{j+1} &= (2j+1)xP_j - jP_{j-1} \end{aligned} \tag{4.6.10}$$

Gauss-Chebyshev:

$$\begin{aligned} W(x) &= (1-x^2)^{-1/2} \qquad -1 < x < 1 \\ T_{j+1} &= 2xT_j - T_{j-1} \end{aligned} \tag{4.6.11}$$

Gauss-Laguerre:

$$\begin{aligned} W(x) &= x^{\alpha}e^{-x} \qquad 0 < x < \infty \\ (j+1)L^{\alpha}_{j+1} &= (-x+2j+\alpha+1)L^{\alpha}_j - (j+\alpha)L^{\alpha}_{j-1} \end{aligned} \tag{4.6.12}$$

Gauss-Hermite:

$$\begin{aligned} W(x) &= e^{-x^2} \qquad -\infty < x < \infty \\ H_{j+1} &= 2xH_j - 2jH_{j-1} \end{aligned} \tag{4.6.13}$$

Gauss-Jacobi:

$$\begin{aligned} W(x) &= (1-x)^{\alpha}(1+x)^{\beta} \qquad -1 < x < 1 \\ c_j P^{(\alpha,\beta)}_{j+1} &= (d_j + e_j x)P^{(\alpha,\beta)}_j - f_j P^{(\alpha,\beta)}_{j-1} \end{aligned} \tag{4.6.14}$$

where the coefficients c_j, d_j, e_j, and f_j are given by

$$\begin{aligned} c_j &= 2(j+1)(j+\alpha+\beta+1)(2j+\alpha+\beta) \\ d_j &= (2j+\alpha+\beta+1)(\alpha^2-\beta^2) \\ e_j &= (2j+\alpha+\beta)(2j+\alpha+\beta+1)(2j+\alpha+\beta+2) \\ f_j &= 2(j+\alpha)(j+\beta)(2j+\alpha+\beta+2) \end{aligned} \tag{4.6.15}$$

We now give individual routines that calculate the abscissas and weights for these cases. First comes the most common set of abscissas and weights, those of Gauss-Legendre. The routine, due to G.B. Rybicki, uses equation (4.6.9) in the special form for the Gauss-Legendre case,

$$w_j = \frac{2}{(1-x_j^2)[P'_N(x_j)]^2} \tag{4.6.16}$$

The routine also scales the range of integration from (x_1, x_2) to $(-1, 1)$, and provides abscissas x_j and weights w_j for the Gaussian formula

$$\int_{x_1}^{x_2} f(x)dx = \sum_{j=0}^{N-1} w_j f(x_j) \tag{4.6.17}$$

gauss_wgts.h

```
void gauleg(const Doub x1, const Doub x2, VecDoub_O &x, VecDoub_O &w)
```

Given the lower and upper limits of integration x1 and x2, this routine returns arrays x[0..n-1] and w[0..n-1] of length n, containing the abscissas and weights of the Gauss-Legendre n-point quadrature formula.

```
{
    const Doub EPS=1.0e-14;                EPS is the relative precision.
    Doub z1,z,xm,xl,pp,p3,p2,p1;
    Int n=x.size();
    Int m=(n+1)/2;                         The roots are symmetric in the interval, so
    xm=0.5*(x2+x1);                            we only have to find half of them.
    xl=0.5*(x2-x1);
    for (Int i=0;i<m;i++) {                Loop over the desired roots.
        z=cos(3.141592654*(i+0.75)/(n+0.5));
        Starting with this approximation to the ith root, we enter the main loop of refinement
        by Newton's method.
        do {
            p1=1.0;
            p2=0.0;
            for (Int j=0;j<n;j++) {        Loop up the recurrence relation to get the
                p3=p2;                         Legendre polynomial evaluated at z.
                p2=p1;
                p1=((2.0*j+1.0)*z*p2-j*p3)/(j+1);
            }
            p1 is now the desired Legendre polynomial. We next compute pp, its derivative,
            by a standard relation involving also p2, the polynomial of one lower order.
            pp=n*(z*p1-p2)/(z*z-1.0);
            z1=z;
            z=z1-p1/pp;                    Newton's method.
        } while (abs(z-z1) > EPS);
        x[i]=xm-xl*z;                      Scale the root to the desired interval,
        x[n-1-i]=xm+xl*z;                  and put in its symmetric counterpart.
        w[i]=2.0*xl/((1.0-z*z)*pp*pp);     Compute the weight
        w[n-1-i]=w[i];                     and its symmetric counterpart.
    }
}
```

Next we give three routines that use initial approximations for the roots given by Stroud and Secrest [2]. The first is for Gauss-Laguerre abscissas and weights, to be used with the integration formula

$$\int_0^\infty x^\alpha e^{-x} f(x)dx = \sum_{j=0}^{N-1} w_j f(x_j) \qquad (4.6.18)$$

gauss_wgts.h

```
void gaulag(VecDoub_O &x, VecDoub_O &w, const Doub alf)
```

Given alf, the parameter α of the Laguerre polynomials, this routine returns arrays x[0..n-1] and w[0..n-1] containing the abscissas and weights of the n-point Gauss-Laguerre quadrature formula. The smallest abscissa is returned in x[0], the largest in x[n-1].

```
{
    const Int MAXIT=10;
    const Doub EPS=1.0e-14;                EPS is the relative precision.
    Int i,its,j;
    Doub ai,p1,p2,p3,pp,z,z1;
    Int n=x.size();
    for (i=0;i<n;i++) {                    Loop over the desired roots.
        if (i == 0) {                      Initial guess for the smallest root.
            z=(1.0+alf)*(3.0+0.92*alf)/(1.0+2.4*n+1.8*alf);
        } else if (i == 1) {               Initial guess for the second root.
            z += (15.0+6.25*alf)/(1.0+0.9*alf+2.5*n);
        } else {                           Initial guess for the other roots.
            ai=i-1;
```

```
            z += ((1.0+2.55*ai)/(1.9*ai)+1.26*ai*alf/
                (1.0+3.5*ai))*(z-x[i-2])/(1.0+0.3*alf);
        }
        for (its=0;its<MAXIT;its++) {          Refinement by Newton's method.
            p1=1.0;
            p2=0.0;
            for (j=0;j<n;j++) {                Loop up the recurrence relation to get the
                p3=p2;                             Laguerre polynomial evaluated at z.
                p2=p1;
                p1=((2*j+1+alf-z)*p2-(j+alf)*p3)/(j+1);
            }
            p1 is now the desired Laguerre polynomial. We next compute pp, its derivative,
            by a standard relation involving also p2, the polynomial of one lower order.
            pp=(n*p1-(n+alf)*p2)/z;
            z1=z;
            z=z1-p1/pp;                        Newton's formula.
            if (abs(z-z1) <= EPS) break;
        }
        if (its >= MAXIT) throw("too many iterations in gaulag");
        x[i]=z;                                Store the root and the weight.
        w[i] = -exp(gammln(alf+n)-gammln(Doub(n)))/(pp*n*p2);
    }
}
```

Next is a routine for Gauss-Hermite abscissas and weights. If we use the "standard" normalization of these functions, as given in equation (4.6.13), we find that the computations overflow for large N because of various factorials that occur. We can avoid this by using instead the orthonormal set of polynomials $\widetilde{H}_j$. They are generated by the recurrence

$$\widetilde{H}_{-1} = 0, \quad \widetilde{H}_0 = \frac{1}{\pi^{1/4}}, \quad \widetilde{H}_{j+1} = x\sqrt{\frac{2}{j+1}}\,\widetilde{H}_j - \sqrt{\frac{j}{j+1}}\,\widetilde{H}_{j-1} \tag{4.6.19}$$

The formula for the weights becomes

$$w_j = \frac{2}{[\widetilde{H}'_N(x_j)]^2} \tag{4.6.20}$$

while the formula for the derivative with this normalization is

$$\widetilde{H}'_j = \sqrt{2j}\,\widetilde{H}_{j-1} \tag{4.6.21}$$

The abscissas and weights returned by `gauher` are used with the integration formula

$$\int_{-\infty}^{\infty} e^{-x^2} f(x)dx = \sum_{j=0}^{N-1} w_j f(x_j) \tag{4.6.22}$$

gauss_wgts.h

```
void gauher(VecDoub_O &x, VecDoub_O &w)
```
This routine returns arrays `x[0..n-1]` and `w[0..n-1]` containing the abscissas and weights of the `n`-point Gauss-Hermite quadrature formula. The largest abscissa is returned in `x[0]`, the most negative in `x[n-1]`.

```
{
    const Doub EPS=1.0e-14,PIM4=0.7511255444649425;
    Relative precision and 1/π^(1/4).
    const Int MAXIT=10;                        Maximum iterations.
    Int i,its,j,m;
```

```
    Doub p1,p2,p3,pp,z,z1;
    Int n=x.size();
    m=(n+1)/2;
    The roots are symmetric about the origin, so we have to find only half of them.
    for (i=0;i<m;i++) {                          Loop over the desired roots.
        if (i == 0) {                            Initial guess for the largest root.
            z=sqrt(Doub(2*n+1))-1.85575*pow(Doub(2*n+1),-0.16667);
        } else if (i == 1) {                     Initial guess for the second largest root.
            z -= 1.14*pow(Doub(n),0.426)/z;
        } else if (i == 2) {                     Initial guess for the third largest root.
            z=1.86*z-0.86*x[0];
        } else if (i == 3) {                     Initial guess for the fourth largest root.
            z=1.91*z-0.91*x[1];
        } else {                                 Initial guess for the other roots.
            z=2.0*z-x[i-2];
        }
        for (its=0;its<MAXIT;its++) {            Refinement by Newton's method.
            p1=PIM4;
            p2=0.0;
            for (j=0;j<n;j++) {                  Loop up the recurrence relation to get
                p3=p2;                               the Hermite polynomial evaluated at
                p2=p1;                               z.
                p1=z*sqrt(2.0/(j+1))*p2-sqrt(Doub(j)/(j+1))*p3;
            }
            p1 is now the desired Hermite polynomial. We next compute pp, its derivative, by
            the relation (4.6.21) using p2, the polynomial of one lower order.
            pp=sqrt(Doub(2*n))*p2;
            z1=z;
            z=z1-p1/pp;                          Newton's formula.
            if (abs(z-z1) <= EPS) break;
        }
        if (its >= MAXIT) throw("too many iterations in gauher");
        x[i]=z;                                  Store the root
        x[n-1-i] = -z;                           and its symmetric counterpart.
        w[i]=2.0/(pp*pp);                        Compute the weight
        w[n-1-i]=w[i];                           and its symmetric counterpart.
    }
}
```

Finally, here is a routine for Gauss-Jacobi abscissas and weights, which implement the integration formula

$$\int_{-1}^{1} (1-x)^{\alpha}(1+x)^{\beta} f(x)dx = \sum_{j=0}^{N-1} w_j f(x_j) \tag{4.6.23}$$

gauss_wgts.h

```
void gaujac(VecDoub_O &x, VecDoub_O &w, const Doub alf, const Doub bet)
```

Given alf and bet, the parameters α and β of the Jacobi polynomials, this routine returns arrays x[0..n-1] and w[0..n-1] containing the abscissas and weights of the n-point Gauss-Jacobi quadrature formula. The largest abscissa is returned in x[0], the smallest in x[n-1].

```
{
    const Int MAXIT=10;
    const Doub EPS=1.0e-14;                  EPS is the relative precision.
    Int i,its,j;
    Doub alfbet,an,bn,r1,r2,r3;
    Doub a,b,c,p1,p2,p3,pp,temp,z,z1;
    Int n=x.size();
    for (i=0;i<n;i++) {                      Loop over the desired roots.
        if (i == 0) {                        Initial guess for the largest root.
            an=alf/n;
```

```
            bn=bet/n;
            r1=(1.0+alf)*(2.78/(4.0+n*n)+0.768*an/n);
            r2=1.0+1.48*an+0.96*bn+0.452*an*an+0.83*an*bn;
            z=1.0-r1/r2;
        } else if (i == 1) {                Initial guess for the second largest root.
            r1=(4.1+alf)/((1.0+alf)*(1.0+0.156*alf));
            r2=1.0+0.06*(n-8.0)*(1.0+0.12*alf)/n;
            r3=1.0+0.012*bet*(1.0+0.25*abs(alf))/n;
            z -= (1.0-z)*r1*r2*r3;
        } else if (i == 2) {                Initial guess for the third largest root.
            r1=(1.67+0.28*alf)/(1.0+0.37*alf);
            r2=1.0+0.22*(n-8.0)/n;
            r3=1.0+8.0*bet/((6.28+bet)*n*n);
            z -= (x[0]-z)*r1*r2*r3;
        } else if (i == n-2) {              Initial guess for the second smallest root.
            r1=(1.0+0.235*bet)/(0.766+0.119*bet);
            r2=1.0/(1.0+0.639*(n-4.0)/(1.0+0.71*(n-4.0)));
            r3=1.0/(1.0+20.0*alf/((7.5+alf)*n*n));
            z += (z-x[n-4])*r1*r2*r3;
        } else if (i == n-1) {              Initial guess for the smallest root.
            r1=(1.0+0.37*bet)/(1.67+0.28*bet);
            r2=1.0/(1.0+0.22*(n-8.0)/n);
            r3=1.0/(1.0+8.0*alf/((6.28+alf)*n*n));
            z += (z-x[n-3])*r1*r2*r3;
        } else {                            Initial guess for the other roots.
            z=3.0*x[i-1]-3.0*x[i-2]+x[i-3];
        }
        alfbet=alf+bet;
        for (its=1;its<=MAXIT;its++) {      Refinement by Newton's method.
            temp=2.0+alfbet;                Start the recurrence with P0 and P1 to avoid
            p1=(alf-bet+temp*z)/2.0;            a division by zero when α + β = 0 or
            p2=1.0;                             −1.
            for (j=2;j<=n;j++) {            Loop up the recurrence relation to get the
                p3=p2;                          Jacobi polynomial evaluated at z.
                p2=p1;
                temp=2*j+alfbet;
                a=2*j*(j+alfbet)*(temp-2.0);
                b=(temp-1.0)*(alf*alf-bet*bet+temp*(temp-2.0)*z);
                c=2.0*(j-1+alf)*(j-1+bet)*temp;
                p1=(b*p2-c*p3)/a;
            }
            pp=(n*(alf-bet-temp*z)*p1+2.0*(n+alf)*(n+bet)*p2)/(temp*(1.0-z*z));
            p1 is now the desired Jacobi polynomial. We next compute pp, its derivative, by
            a standard relation involving also p2, the polynomial of one lower order.
            z1=z;
            z=z1-p1/pp;                     Newton's formula.
            if (abs(z-z1) <= EPS) break;
        }
        if (its > MAXIT) throw("too many iterations in gaujac");
        x[i]=z;                             Store the root and the weight.
        w[i]=exp(gammln(alf+n)+gammln(bet+n)-gammln(n+1.0)-
            gammln(n+alfbet+1.0))*temp*pow(2.0,alfbet)/(pp*p2);
    }
}
```

Legendre polynomials are special cases of Jacobi polynomials with $\alpha = \beta = 0$, but it is worth having the separate routine for them, gauleg, given above. Chebyshev polynomials correspond to $\alpha = \beta = -1/2$ (see §5.8). They have analytic abscissas and weights:

$$
\begin{aligned}
x_j &= \cos\left(\frac{\pi(j+\frac{1}{2})}{N}\right) \\
w_j &= \frac{\pi}{N}
\end{aligned}
\tag{4.6.24}
$$

4.6.2 Case of Known Recurrences

Turn now to the case where you do not know good initial guesses for the zeros of your orthogonal polynomials, but you do have available the coefficients a_j and b_j that generate them. As we have seen, the zeros of $p_N(x)$ are the abscissas for the N-point Gaussian quadrature formula. The most useful computational formula for the weights is equation (4.6.9) above, since the derivative p'_N can be efficiently computed by the derivative of (4.6.6) in the general case, or by special relations for the classical polynomials. Note that (4.6.9) is valid as written only for monic polynomials; for other normalizations, there is an extra factor of λ_N/λ_{N-1}, where λ_N is the coefficient of x^N in p_N.

Except in those special cases already discussed, the best way to find the abscissas is *not* to use a root-finding method like Newton's method on $p_N(x)$. Rather, it is generally faster to use the Golub-Welsch [3] algorithm, which is based on a result of Wilf [4]. This algorithm notes that if you bring the term xp_j to the left-hand side of (4.6.6) and the term p_{j+1} to the right-hand side, the recurrence relation can be written in matrix form as

$$
x\begin{bmatrix} p_0 \\ p_1 \\ \vdots \\ p_{N-2} \\ p_{N-1} \end{bmatrix} = \begin{bmatrix} a_0 & 1 & & & \\ b_1 & a_1 & 1 & & \\ & \vdots & \vdots & & \\ & & b_{N-2} & a_{N-2} & 1 \\ & & & b_{N-1} & a_{N-1} \end{bmatrix} \cdot \begin{bmatrix} p_0 \\ p_1 \\ \vdots \\ p_{N-2} \\ p_{N-1} \end{bmatrix} + \begin{bmatrix} 0 \\ 0 \\ \vdots \\ 0 \\ p_N \end{bmatrix}
\tag{4.6.25}
$$

or

$$
x\mathbf{p} = \mathbf{T}\cdot\mathbf{p} + p_N\mathbf{e}_{N-1}
\tag{4.6.26}
$$

Here $\mathbf{T}$ is a tridiagonal matrix; $\mathbf{p}$ is a column vector of $p_0, p_1, \ldots, p_{N-1}$; and $\mathbf{e}_{N-1}$ is a unit vector with a 1 in the $(N-1)$st (last) position and zeros elsewhere. The matrix $\mathbf{T}$ can be symmetrized by a diagonal similarity transformation $\mathbf{D}$ to give

$$
\mathbf{J} = \mathbf{DTD}^{-1} = \begin{bmatrix} a_0 & \sqrt{b_1} & & & \\ \sqrt{b_1} & a_1 & \sqrt{b_2} & & \\ & \vdots & \vdots & & \\ & & \sqrt{b_{N-2}} & a_{N-2} & \sqrt{b_{N-1}} \\ & & & \sqrt{b_{N-1}} & a_{N-1} \end{bmatrix}
\tag{4.6.27}
$$

The matrix $\mathbf{J}$ is called the *Jacobi matrix* (not to be confused with other matrices named after Jacobi that arise in completely different problems!). Now we see from (4.6.26) that $p_N(x_j) = 0$ is equivalent to x_j being an eigenvalue of $\mathbf{T}$. Since eigenvalues are preserved by a similarity transformation, x_j is an eigenvalue of the symmetric tridiagonal matrix $\mathbf{J}$. Moreover, Wilf [4] shows that if $\mathbf{v}_j$ is the eigenvector corresponding to the eigenvalue x_j, normalized so that $\mathbf{v}\cdot\mathbf{v} = 1$, then

$$
w_j = \mu_0 v_{j,0}^2
\tag{4.6.28}
$$

where

$$
\mu_0 = \int_a^b W(x)\,dx
\tag{4.6.29}
$$

and where $v_{j,0}$ is the zeroth component of $\mathbf{v}$. As we shall see in Chapter 11, finding all eigenvalues and eigenvectors of a symmetric tridiagonal matrix is a relatively efficient and well-conditioned procedure. We accordingly give a routine, `gaucof`, for finding the abscissas and weights, given the coefficients a_j and b_j. Remember that if you know the recurrence relation for orthogonal polynomials that are not normalized to be monic, you can easily convert it to monic form by means of the quantities λ_j.

```
void gaucof(VecDoub_IO &a, VecDoub_IO &b, const Doub amu0, VecDoub_O &x,
    VecDoub_O &w)
```
gauss_wgts2.h

Computes the abscissas and weights for a Gaussian quadrature formula from the Jacobi matrix. On input, `a[0..n-1]` and `b[0..n-1]` are the coefficients of the recurrence relation for the set of monic orthogonal polynomials. The quantity $\mu_0 \equiv \int_a^b W(x)\,dx$ is input as `amu0`. The abscissas `x[0..n-1]` are returned in descending order, with the corresponding weights in `w[0..n-1]`. The arrays `a` and `b` are modified. Execution can be speeded up by modifying `tqli` and `eigsrt` to compute only the zeroth component of each eigenvector.

```
{
    Int n=a.size();
    for (Int i=0;i<n;i++)
        if (i != 0) b[i]=sqrt(b[i]);            Set up superdiagonal of Jacobi matrix.
    Symmeig sym(a,b);
    for (Int i=0;i<n;i++) {
        x[i]=sym.d[i];
        w[i]=amu0*sym.z[0][i]*sym.z[0][i];        Equation (4.6.28).
    }
}
```

4.6.3 Orthogonal Polynomials with Nonclassical Weights

What do you do if your weight function is not one of the classical ones dealt with above and you do not know the a_j's and b_j's of the recurrence relation (4.6.6) to use in `gaucof`? Obviously, you need a method of finding the a_j's and b_j's.

The best general method is the *Stieltjes procedure*: First compute a_0 from (4.6.7), and then $p_1(x)$ from (4.6.6). Knowing p_0 and p_1, compute a_1 and b_1 from (4.6.7), and so on. But how are we to compute the inner products in (4.6.7)?

The textbook approach is to represent each $p_j(x)$ explicitly as a polynomial in x and to compute the inner products by multiplying out term by term. This will be feasible if we know the first $2N$ moments of the weight function,

$$\mu_j = \int_a^b x^j W(x)dx \qquad j = 0, 1, \dots, 2N-1 \tag{4.6.30}$$

However, the solution of the resulting set of algebraic equations for the coefficients a_j and b_j in terms of the moments μ_j is in general *extremely* ill-conditioned. Even in double precision, it is not unusual to lose all accuracy by the time $N = 12$. We thus reject any procedure based on the moments (4.6.30).

Gautschi [5] showed that the Stieltjes procedure is feasible if the inner products in (4.6.7) are computed directly by numerical quadrature. This is only practicable if you can find a quadrature scheme that can compute the integrals to high accuracy despite the singularities in the weight function $W(x)$. Gautschi advocates the Fejér quadrature scheme [5] as a general-purpose scheme for handling singularities when no better method is available. We have personally had much better experience with the transformation methods of §4.5, particularly the DE rule and its variants.

We use a structure `Stiel` that implements the Stieltjes procedure. Its member function `get_weights` generates the coefficients a_j and b_j of the recurrence relation, and then calls `gaucof` to find the abscissas and weights. You can easily modify it to return the a_j's and b_j's if you want them as well. Internally, the routine calls the function `quad` to do the integrals in (4.6.7). For a finite range of integration, the routine uses the straight DE rule. This is effected by invoking the constructor with five parameters: the number of quadrature abscissas (and weights) desired, the lower and upper limits of integration, the parameter $h_{\max}$ to be passed to the DE rule (see §4.5), and the weight function $W(x)$. For an infinite range of integration, the routine invokes the trapezoidal rule with one of the coordinate transformations discussed in §4.5. For this case you invoke the constructor that has no $h_{\max}$, but takes the mapping function $x = x(t)$ and its derivative dx/dt in addition to $W(x)$. Now the range of integration you input is the finite range of the trapezoidal rule.

This will all be clearer with some examples. Consider first the weight function

$$W(x) = -\log x \tag{4.6.31}$$

on the finite interval (0, 1). Normally, for the finite range case (DE rule), the weight function must be coded as a function of two variables, $W(x, \delta)$, where δ is the distance from the endpoint singularity. Since the logarithmic singularity at the endpoint $x = 0$ is "mild," there is no need to use the argument δ in coding the function:

```
Doub wt(const Doub x, const Doub del)
{
    return -log(x);
}
```

A value of $h_{\max} = 3.7$ will give full double precision, as discussed in §4.5, so the calling code looks like this:

```
n= ...
VecDoub x(n),w(n);
Stiel s(n,0.0,1.0,3.7,wt);
s.get_weights(x,w);
```

For the infinite range case, in addition to the weight function $W(x)$, you have to supply two functions for the coordinate transformation you want to use (see equation 4.5.14). We'll denote the mapping $x = x(t)$ by `fx` and dx/dt by `fdxdt`, but you can use any names you like. All these functions are coded as functions of one variable.

Here is an example of the user-supplied functions for the weight function

$$W(x) = \frac{x^{1/2}}{e^x + 1} \tag{4.6.32}$$

on the interval $(0, \infty)$. Gaussian quadrature based on $W(x)$ has been proposed for evaluating generalized Fermi-Dirac integrals [6] (cf. §4.5). We use the "mixed" DE rule of equation (4.5.14), $x = e^{t-e^{-t}}$. As is typical with the Stieltjes procedure, you get abscissas and weights within about one or two significant digits of machine accuracy for N of a few dozen.

```
Doub wt(const Doub x)
{
    Doub s=exp(-x);
    return sqrt(x)*s/(1.0+s);
}

Doub fx(const Doub t)
{
    return exp(t-exp(-t));
}

Doub fdxdt(const Doub t)
{
    Doub s=exp(-t);
    return exp(t-s)*(1.0+s);
}
    ...
Stiel ss(n,-5.5,6.5,wt,fx,fdxdt);
ss.get_weights(x,w);
```

The listing of the `Stiel` object, and discussion of some of the C++ intricacies of its coding, are in a Webnote [9].

Two other algorithms exist [7,8] for finding abscissas and weights for Gaussian quadratures. The first starts similarly to the Stieltjes procedure by representing the inner product integrals in equation (4.6.7) as discrete quadratures using some quadrature rule. This defines a matrix whose elements are formed from the abscissas and weights in your chosen quadrature rule, together with the given weight function. Then an algorithm due to Lanczos is used to transform this to a matrix that is essentially the Jacobi matrix (4.6.27).

The second algorithm is based on the idea of *modified moments*. Instead of using powers of x as a set of basis functions to represent the p_j's, one uses some other known set of orthogonal polynomials $\pi_j(x)$, say. Then the inner products in equation (4.6.7) will be expressible

in terms of the modified moments

$$\nu_j = \int_a^b \pi_j(x)W(x)dx \qquad j = 0, 1, \ldots, 2N-1 \tag{4.6.33}$$

The *modified Chebyshev algorithm* (due to Sack and Donovan [10] and later improved by Wheeler [11]) is an efficient algorithm that generates the desired a_j's and b_j's from the modified moments. Roughly speaking, the improved stability occurs because the polynomial basis "samples" the interval (a, b) better than the power basis when the inner product integrals are evaluated, especially if its weight function resembles $W(x)$. The algorithm requires that the modified moments (4.6.33) be accurately computed. Sometimes there is a closed form, for example, for the important case of the $\log x$ weight function [12,8]. Otherwise you have to use a suitable discretization procedure to compute the modified moments [7,8], just as we did for the inner products in the Stieltjes procedure. There is some art in choosing the auxiliary polynomials π_j, and in practice it is not always possible to find a set that removes the ill-conditioning.

Gautschi [8] has given an extensive suite of routines that handle all three of the algorithms we have described, together with many other aspects of orthogonal polynomials and Gaussian quadrature. However, for most straightforward applications, you should find `Stiel` together with a suitable DE rule quadrature more than adequate.

4.6.4 Extensions of Gaussian Quadrature

There are many different ways in which the ideas of Gaussian quadrature have been extended. One important extension is the case of *preassigned nodes*: Some points are required to be included in the set of abscissas, and the problem is to choose the weights and the remaining abscissas to maximize the degree of exactness of the the quadrature rule. The most common cases are *Gauss-Radau* quadrature, where one of the nodes is an endpoint of the interval, either a or b, and *Gauss-Lobatto* quadrature, where both a and b are nodes. Golub [13,8] has given an algorithm similar to `gaucof` for these cases.

An N-point Gauss-Radau rule has the form of equation (4.6.1), where x_1 is chosen to be either a or b (x_1 must be finite). You can construct the rule from the coefficients for the corresponding ordinary N-point Gaussian quadrature. Simply set up the Jacobi matrix equation (4.6.27), but modify the entry a_{N-1}:

$$a'_{N-1} = x_1 - b_{N-1}\frac{p_{N-2}(x_1)}{p_{N-1}(x_1)} \tag{4.6.34}$$

Here is the routine:

gauss_wgts2.h

```
void radau(VecDoub_IO &a, VecDoub_IO &b, const Doub amu0, const Doub x1,
    VecDoub_O &x, VecDoub_O &w)
```

Computes the abscissas and weights for a Gauss-Radau quadrature formula. On input, `a[0..n-1]` and `b[0..n-1]` are the coefficients of the recurrence relation for the set of monic orthogonal polynomials corresponding to the weight function. (`b[0]` is not referenced.) The quantity $\mu_0 \equiv \int_a^b W(x)\,dx$ is input as `amu0`. `x1` is input as either endpoint of the interval. The abscissas `x[0..n-1]` are returned in descending order, with the corresponding weights in `w[0..n-1]`. The arrays `a` and `b` are modified.

```
{
    Int n=a.size();
    if (n == 1) {
        x[0]=x1;
        w[0]=amu0;
    } else {                                Compute p_{N-1} and p_{N-2} by recurrence.
        Doub p=x1-a[0];
```

```
        Doub pm1=1.0;
        Doub p1=p;
        for (Int i=1;i<n-1;i++) {
            p=(x1-a[i])*p1-b[i]*pm1;
            pm1=p1;
            p1=p;
        }
        a[n-1]=x1-b[n-1]*pm1/p;           Equation (4.6.34).
        gaucof(a,b,amu0,x,w);
    }
}
```

An N-point Gauss-Lobatto rule has the form of equation (4.6.1) where $x_1 = a$, $x_N = b$ (both finite). This time you modify the entries a_{N-1} and b_{N-1} in equation (4.6.27) by solving two linear equations:

$$\begin{bmatrix} p_{N-1}(x_1) & p_{N-2}(x_1) \\ p_{N-1}(x_N) & p_{N-2}(x_N) \end{bmatrix} \begin{bmatrix} a'_{N-1} \\ b'_{N-1} \end{bmatrix} = \begin{bmatrix} x_1 p_{N-1}(x_1) \\ x_N p_{N-1}(x_N) \end{bmatrix} \tag{4.6.35}$$

gauss_wgts2.h

```
void lobatto(VecDoub_IO &a, VecDoub_IO &b, const Doub amu0, const Doub x1,
    const Doub xn, VecDoub_O &x, VecDoub_O &w)
```

Computes the abscissas and weights for a Gauss-Lobatto quadrature formula. On input, the vectors `a[0..n-1]` and `b[0..n-1]` are the coefficients of the recurrence relation for the set of monic orthogonal polynomials corresponding to the weight function. (`b[0]` is not referenced.) The quantity $\mu_0 \equiv \int_a^b W(x)\,dx$ is input as `amu0`. `x1` amd `xn` are input as the endpoints of the interval. The abscissas `x[0..n-1]` are returned in descending order, with the corresponding weights in `w[0..n-1]`. The arrays `a` and `b` are modified.

```
{
    Doub det,pl,pr,p1l,p1r,pm1l,pm1r;
    Int n=a.size();
    if (n <= 1)
        throw("n must be bigger than 1 in lobatto");
    pl=x1-a[0];                        Compute p_{N-1} and p_{N-2} at x_1 and x_N by recur-
    pr=xn-a[0];                            rence.
    pm1l=1.0;
    pm1r=1.0;
    p1l=pl;
    p1r=pr;
    for (Int i=1;i<n-1;i++) {
        pl=(x1-a[i])*p1l-b[i]*pm1l;
        pr=(xn-a[i])*p1r-b[i]*pm1r;
        pm1l=p1l;
        pm1r=p1r;
        p1l=pl;
        p1r=pr;
    }
    det=pl*pm1r-pr*pm1l;               Solve equation (4.6.35).
    a[n-1]=(x1*pl*pm1r-xn*pr*pm1l)/det;
    b[n-1]=(xn-x1)*pl*pr/det;
    gaucof(a,b,amu0,x,w);
}
```

The second important extension of Gaussian quadrature is the *Gauss-Kronrod* formulas. For ordinary Gaussian quadrature formulas, as N increases, the sets of abscissas have no points in common. This means that if you compare results with increasing N as a way of estimating the quadrature error, you cannot reuse the previous function evaluations. Kronrod [14] posed the problem of searching for optimal sequences of rules, each of which reuses all abscissas of its predecessor. If one starts with $N = m$, say, and then adds n new points, one has $2n + m$ free parameters: the

n new abscissas and weights, and m new weights for the fixed previous abscissas. The maximum degree of exactness one would expect to achieve would therefore be $2n + m - 1$. The question is whether this maximum degree of exactness can actually be achieved in practice, when the abscissas are required to all lie inside (a, b). The answer to this question is not known in general.

Kronrod showed that if you choose $n = m + 1$, an optimal extension can be found for Gauss-Legendre quadrature. Patterson [15] showed how to compute continued extensions of this kind. Sequences such as $N = 10, 21, 43, 87, \ldots$ are popular in automatic quadrature routines [16] that attempt to integrate a function until some specified accuracy has been achieved.

CITED REFERENCES AND FURTHER READING:

Abramowitz, M., and Stegun, I.A. 1964, *Handbook of Mathematical Functions* (Washington: National Bureau of Standards); reprinted 1968 (New York: Dover); online at `http://www.nr.com/aands`, §25.4.[1]

Stroud, A.H., and Secrest, D. 1966, *Gaussian Quadrature Formulas* (Englewood Cliffs, NJ: Prentice-Hall).[2]

Golub, G.H., and Welsch, J.H. 1969, "Calculation of Gauss Quadrature Rules," *Mathematics of Computation*, vol. 23, pp. 221–230 and A1–A10.[3]

Wilf, H.S. 1962, *Mathematics for the Physical Sciences* (New York: Wiley), Problem 9, p. 80.[4]

Gautschi, W. 1968, "Construction of Gauss-Christoffel Quadrature Formulas," *Mathematics of Computation*, vol. 22, pp. 251–270.[5]

Sagar, R.P. 1991, "A Gaussian Quadrature for the Calculation of Generalized Fermi-Dirac Integrals," *Computer Physics Communications*, vol. 66, pp. 271–275.[6]

Gautschi, W. 1982, "On Generating Orthogonal Polynomials," *SIAM Journal on Scientific and Statistical Computing*, vol. 3, pp. 289–317.[7]

Gautschi, W. 1994, "ORTHPOL: A Package of Routines for Generating Orthogonal Polynomials and Gauss-type Quadrature Rules," *ACM Transactions on Mathematical Software*, vol. 20, pp. 21–62 (Algorithm 726 available from netlib).[8]

Numerical Recipes Software 2007, "Implementation of Stiel," *Numerical Recipes Webnote No. 3*, at `http://www.nr.com/webnotes?3` [9]

Sack, R.A., and Donovan, A.F. 1971/72, "An Algorithm for Gaussian Quadrature Given Modified Moments," *Numerische Mathematik*, vol. 18, pp. 465–478.[10]

Wheeler, J.C. 1974, "Modified Moments and Gaussian Quadratures," *Rocky Mountain Journal of Mathematics*, vol. 4, pp. 287–296.[11]

Gautschi, W. 1978, in *Recent Advances in Numerical Analysis*, C. de Boor and G.H. Golub, eds. (New York: Academic Press), pp. 45–72.[12]

Golub, G.H. 1973, "Some Modified Matrix Eigenvalue Problems," *SIAM Review*, vol. 15, pp. 318–334.[13]

Kronrod, A.S. 1964, *Doklady Akademii Nauk SSSR*, vol. 154, pp. 283–286 (in Russian); translated as *Soviet Physics "Doklady"*.[14]

Patterson, T.N.L. 1968, "The Optimum Addition of Points to Quadrature Formulae," *Mathematics of Computation*, vol. 22, pp. 847–856 and C1–C11; 1969, *op. cit.*, vol. 23, p. 892.[15]

Piessens, R., de Doncker-Kapenga, E., Überhuber, C., and Kahaner, D. 1983 *QUADPACK, A Subroutine Package for Automatic Integration* (New York: Springer). Software at `http://www.netlib.org/quadpack`.[16]

Gautschi, W. 1981, in *E.B. Christoffel*, P.L. Butzer and F. Fehér, eds. (Basel: Birkhäuser), pp. 72–147.

Gautschi, W. 1990, in *Orthogonal Polynomials*, P. Nevai, ed. (Dordrecht: Kluwer Academic Publishers), pp. 181–216.

Stoer, J., and Bulirsch, R. 2002, *Introduction to Numerical Analysis*, 3rd ed. (New York: Springer), §3.6.

4.7 Adaptive Quadrature

The idea behind adaptive quadrature is very simple. Suppose you have two different numerical estimates I_1 and I_2 of the integral

$$I = \int_a^b f(x)\,dx \tag{4.7.1}$$

Suppose I_1 is more accurate. Use the relative difference between I_1 and I_2 as an error estimate. If it is less than ϵ, accept I_1 as the answer. Otherwise divide the interval $[a,b]$ into two subintervals,

$$I = \int_a^m f(x)\,dx + \int_m^b f(x)\,dx \qquad m = (a+b)/2 \tag{4.7.2}$$

and compute the two integrals independently. For each one, compute an I_1 and I_2, estimate the error, and continue subdividing if necessary. Dividing any given subinterval stops when its contribution to ϵ is sufficiently small. (Obviously recursion will be a good way to implement this algorithm.)

The most important criterion for an adaptive quadrature routine is reliability: If you request an accuracy of 10^{-6}, you would like to be sure that the answer is at least that good. From a theoretical point of view, however, it is impossible to design an adaptive quadrature routine that will work for all possible functions. The reason is simple: A quadrature is based on the value of the integrand $f(x)$ at a *finite* set of points. You can alter the function at all the other points in an arbitrary way without affecting the estimate your algorithm returns, while the true value of the integral changes unpredictably. Despite this point of principle, however, in practice good routines are reliable for a high fraction of functions they encounter. Our favorite routine is one proposed by Gander and Gautschi [1], which we now describe. It is relatively simple, yet scores well on reliability and efficiency.

A key component of a good adaptive algorithm is the termination criterion. The usual criterion

$$|I_1 - I_2| < \epsilon |I_1| \tag{4.7.3}$$

is problematic. In the neighborhood of a singularity, I_1 and I_2 might never agree to the requested tolerance, even if it's not particularly small. Instead, you need to somehow come up with an estimate of the *whole* integral I of equation (4.7.1). Then you can terminate when the error in I_1 is negligible compared to the whole integral:

$$|I_1 - I_2| < \epsilon |I_s| \tag{4.7.4}$$

where I_s is the estimate of I. Gander and Gautschi implement this test by writing

```
if (is + (i1-i2) == is)
```

which is equivalent to setting ϵ to the machine precision. However, modern optimizing compilers have become too good at recognizing that this is algebraically equivalent to

```
if (i1-i2 == 0.0)
```

which might never be satisfied in floating point arithmetic. Accordingly, we implement the test with an explicit ϵ.

The other problem you need to take care of is when an interval gets subdivided so small that it contains no interior machine-representable point. You then need to terminate the recursion and alert the user that the full accuracy might not have been attained. In the case where the points in an interval are supposed to be $\{a, m = (a+b)/2, b\}$, you can test for $m \le a$ or $b \le m$.

The lowest order integration method in the Gander-Gautschi method is the four-point Gauss-Lobatto quadrature (cf. §4.6)

$$\int_{-1}^{1} f(x)\,dx = \tfrac{1}{6}\Big[f(-1) + f(1)\Big] + \tfrac{5}{6}\Big[f\Big(-\tfrac{1}{\sqrt{5}}\Big) + f\Big(\tfrac{1}{\sqrt{5}}\Big)\Big] \tag{4.7.5}$$

This formula, which is exact for polynomials of degree 5, is used to compute I_2. To reuse these function evaluations in computing I_1, they find the seven-point Kronrod extension,

$$\begin{aligned}\int_{-1}^{1} f(x)\,dx = \tfrac{11}{210}\Big[f(-1) + f(1)\Big] + \tfrac{72}{245}\Big[f\Big(-\sqrt{\tfrac{2}{3}}\Big) + f\Big(\sqrt{\tfrac{2}{3}}\Big)\Big] \\ + \tfrac{125}{294}\Big[f\Big(-\tfrac{1}{\sqrt{5}}\Big) + f\Big(\tfrac{1}{\sqrt{5}}\Big)\Big] + \tfrac{16}{35} f(0)\end{aligned} \tag{4.7.6}$$

whose degree of exactness is nine. The formulas (4.7.5) and (4.7.6) get scaled from $[-1, 1]$ to an arbitrary subinterval $[a, b]$.

For I_s, Gander and Gautschi find a 13-point Kronrod extension of equation (4.7.6), which lets them reuse the previous function evaluations. The formula is coded into the routine below. You can think of this initial 13-point evaluation as a kind of Monte Carlo sampling to get an idea of the order of magnitude of the integral. But if the integrand is smooth, this initial evaluation will itself be quite accurate already. The routine below takes advantage of this.

Note that to reuse the four function evaluations in (4.7.5) in the seven-point formula (4.7.6), you can't simply bisect intervals. But dividing into six subintervals works (there are six intervals between seven points).

To use the routine, you need to initialize an `Adapt` object with your required tolerance,

```
Adapt s(1.0e-6);
```

and then call the `integrate` function:

```
ans=s.integrate(func,a,b);
```

You should check that the desired tolerance could be met:

```
if (s.out_of_tolerance)
    cout << "Required tolerance may not be met" << endl;
```

The smallest allowed tolerance is 10 times the machine precision. If you enter a smaller tolerance, it gets reset internally. (The routine will work using the machine precision itself, but then it usually just takes lots of function evaluations for little additional benefit.)

The implementation of the `Adapt` object is given in a Webnote [2].

Adaptive quadrature is no panacea. The above routine has no special machinery to deal with singularities other than to refine the neighboring intervals. By using

suitable schemes for I_1 and I_2, one can customize an adaptive routine to deal with a particular kind of singularity (cf. [3]).

CITED REFERENCES AND FURTHER READING:

Gander, W., and Gautschi, W. 2000, "Adaptive Quadrature — Revisited," *BIT* vol. 40, pp. 84–101.[1]

Numerical Recipes Software 2007, "Implementation of Adapt," *Numerical Recipes Webnote No. 4*, at `http://www.nr.com/webnotes?4` [2]

Piessens, R., de Doncker-Kapenga, E., Überhuber, C., and Kahaner, D. 1983 *QUADPACK, A Subroutine Package for Automatic Integration* (New York: Springer). Software at `http://www.netlib.org/quadpack`.[3]

Davis, P.J., and Rabinowitz, P. 1984, *Methods of Numerical Integration*, 2nd ed., (Orlando, FL: Academic Press), Chapter 6.

4.8 Multidimensional Integrals

Integrals of functions of several variables, over regions with dimension greater than one, are *not easy*. There are two reasons for this. First, the number of function evaluations needed to sample an N-dimensional space increases as the Nth power of the number needed to do a one-dimensional integral. If you need 30 function evaluations to do a one-dimensional integral crudely, then you will likely need on the order of 30000 evaluations to reach the same crude level for a three-dimensional integral. Second, the region of integration in N-dimensional space is defined by an $N-1$ dimensional boundary that can itself be terribly complicated: It need not be convex or simply connected, for example. By contrast, the boundary of a one-dimensional integral consists of two numbers, its upper and lower limits.

The first question to be asked, when faced with a multidimensional integral, is, can it be reduced analytically to a lower dimensionality? For example, so-called *iterated integrals* of a function of one variable $f(t)$ can be reduced to one-dimensional integrals by the formula

$$\int_0^x dt_n \int_0^{t_n} dt_{n-1} \cdots \int_0^{t_3} dt_2 \int_0^{t_2} f(t_1)\, dt_1 = \frac{1}{(n-1)!} \int_0^x (x-t)^{n-1} f(t)\, dt \tag{4.8.1}$$

Alternatively, the function may have some special symmetry in the way it depends on its independent variables. If the boundary also has this symmetry, then the dimension can be reduced. In three dimensions, for example, the integration of a spherically symmetric function over a spherical region reduces, in polar coordinates, to a one-dimensional integral.

The next questions to be asked will guide your choice between two entirely different approaches to doing the problem. The questions are: Is the shape of the boundary of the region of integration simple or complicated? Inside the region, is the integrand smooth and simple, or complicated, or locally strongly peaked? Does the problem require high accuracy, or does it require an answer accurate only to a percent, or a few percent?

If your answers are that the boundary is complicated, the integrand is *not* strongly peaked in very small regions, and relatively low accuracy is tolerable, then your problem is a good candidate for *Monte Carlo integration*. This method is very straightforward to program, in its cruder forms. One needs only to know a region with simple boundaries that *includes* the complicated region of integration, plus a method of determining whether a random point is inside or outside the region of integration. Monte Carlo integration evaluates the function at a random sample of points and estimates its integral based on that random sample. We will discuss it in more detail, and with more sophistication, in Chapter 7.

If the boundary is simple, and the function is very smooth, then the remaining approaches, breaking up the problem into repeated one-dimensional integrals, or multidimensional Gaussian quadratures, will be effective and relatively fast [1]. If you require high accuracy, these approaches are in any case the *only* ones available to you, since Monte Carlo methods are by nature asymptotically slow to converge.

For low accuracy, use repeated one-dimensional integration or multidimensional Gaussian quadratures when the integrand is slowly varying and smooth in the region of integration, Monte Carlo when the integrand is oscillatory or discontinuous but not strongly peaked in small regions.

If the integrand *is* strongly peaked in small regions, and you know where those regions are, break the integral up into several regions so that the integrand is smooth in each, and do each separately. If you don't know where the strongly peaked regions are, you might as well (at the level of sophistication of this book) quit: It is hopeless to expect an integration routine to search out unknown pockets of large contribution in a huge N-dimensional space. (But see §7.9.)

If, on the basis of the above guidelines, you decide to pursue the repeated one-dimensional integration approach, here is how it works. For definiteness, we will consider the case of a three-dimensional integral in x, y, z-space. Two dimensions, or more than three dimensions, are entirely analogous.

The first step is to specify the region of integration by (i) its lower and upper limits in x, which we will denote x_1 and x_2; (ii) its lower and upper limits in y at a specified value of x, denoted $y_1(x)$ and $y_2(x)$; and (iii) its lower and upper limits in z at specified x and y, denoted $z_1(x, y)$ and $z_2(x, y)$. In other words, find the numbers x_1 and x_2, and the functions $y_1(x)$, $y_2(x)$, $z_1(x, y)$, and $z_2(x, y)$ such that

$$\begin{aligned} I &\equiv \iiint dx\, dy\, dz\, f(x, y, z) \\ &= \int_{x_1}^{x_2} dx \int_{y_1(x)}^{y_2(x)} dy \int_{z_1(x,y)}^{z_2(x,y)} dz\, f(x, y, z) \end{aligned} \tag{4.8.2}$$

For example, a two-dimensional integral over a circle of radius one centered on the origin becomes

$$\int_{-1}^{1} dx \int_{-\sqrt{1-x^2}}^{\sqrt{1-x^2}} dy\, f(x, y) \tag{4.8.3}$$

Now we can define a function $G(x, y)$ that does the innermost integral,

$$G(x, y) \equiv \int_{z_1(x,y)}^{z_2(x,y)} f(x, y, z)\, dz \tag{4.8.4}$$

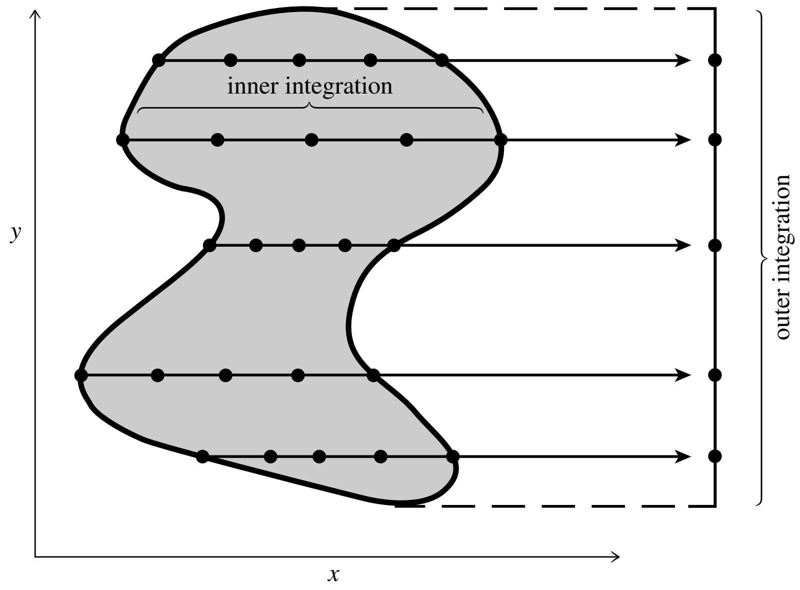

Figure 4.8.1. Function evaluations for a two-dimensional integral over an irregular region, shown schematically. The outer integration routine, in y, requests values of the inner, x, integral at locations along the y-axis of its own choosing. The inner integration routine then evaluates the function at x locations suitable to *it*. This is more accurate in general than, e.g., evaluating the function on a Cartesian mesh of points.

and a function $H(x)$ that does the integral of $G(x, y)$,

$$H(x) \equiv \int_{y_1(x)}^{y_2(x)} G(x, y)\, dy \tag{4.8.5}$$

and finally our answer as an integral over $H(x)$

$$I = \int_{x_1}^{x_2} H(x)\, dx \tag{4.8.6}$$

In an implementation of equations (4.8.4) – (4.8.6), some basic one-dimensional integration routine (e.g., qgaus in the program following) gets called recursively: once to evaluate the outer integral I, then many times to evaluate the middle integral H, then even more times to evaluate the inner integral G (see Figure 4.8.1). Current values of x and y, and the pointers to the user-supplied functions for the integrand and the boundaries, are passed "over the head" of the intermediate calls through member variables in the three functors defining the integrands for G, H and I.

quad3d.h

```
struct NRf3 {
    Doub xsav,ysav;
    Doub (*func3d)(const Doub, const Doub, const Doub);
    Doub operator()(const Doub z)     The integrand f(x,y,z) evaluated at fixed x and
    {                                 y.
        return func3d(xsav,ysav,z);
    }
};
```

```
struct NRf2 {
    NRf3 f3;
    Doub (*z1)(Doub, Doub);
    Doub (*z2)(Doub, Doub);
    NRf2(Doub zz1(Doub, Doub), Doub zz2(Doub, Doub)) : z1(zz1), z2(zz2) {}
    Doub operator()(const Doub y)    This is G of eq. (4.8.4).
    {
        f3.ysav=y;
        return qgaus(f3,z1(f3.xsav,y),z2(f3.xsav,y));
    }
};
struct NRf1 {
    Doub (*y1)(Doub);
    Doub (*y2)(Doub);
    NRf2 f2;
    NRf1(Doub yy1(Doub), Doub yy2(Doub), Doub z1(Doub, Doub),
        Doub z2(Doub, Doub)) : y1(yy1),y2(yy2), f2(z1,z2) {}
    Doub operator()(const Doub x)    This is H of eq. (4.8.5).
    {
        f2.f3.xsav=x;
        return qgaus(f2,y1(x),y2(x));
    }
};

template <class T>
Doub quad3d(T &func, const Doub x1, const Doub x2, Doub y1(Doub), Doub y2(Doub),
    Doub z1(Doub, Doub), Doub z2(Doub, Doub))
```

Returns the integral of a user-supplied function `func` over a three-dimensional region specified by the limits `x1`, `x2`, and by the user-supplied functions `y1`, `y2`, `z1`, and `z2`, as defined in (4.8.2). Integration is performed by calling `qgaus` recursively.

```
{
    NRf1 f1(y1,y2,z1,z2);
    f1.f2.f3.func3d=func;
    return qgaus(f1,x1,x2);
}
```

Note that while the function to be integrated can be supplied either as a simple function

```
    Doub func(const Doub x, const Doub y, const Doub z);
```

or as the equivalent functor, the functions defining the boundary can only be functions:

```
    Doub y1(const Doub x);
    Doub y2(const Doub x);
    Doub z1(const Doub x, const Doub y);
    Doub z2(const Doub x, const Doub y);
```

This is for simplicity; you can easily modify the code to take functors if you need to.

The Gaussian quadrature routine used in `quad3d` is simple, but its accuracy is not controllable. An alternative is to use a one-dimensional integration routine like `qtrap`, `qsimp` or `qromb`, which have a user-definable tolerance `eps`. Simply replace all occurrences of `qgaus` in `quad3d` by `qromb`, say.

Note that multidimensional integration is likely to be very slow if you try for too much accuracy. You should almost certainly increase the default `eps` in `qromb` from 10^{-10} to 10^{-6} or bigger. You should also decrease `JMAX` to avoid a lot of waiting around for an answer. Some people advocate using a smaller `eps` for the inner quadrature (over z in our routine) than for the outer quadratures (over x or y).

CITED REFERENCES AND FURTHER READING:

Stroud, A.H. 1971, *Approximate Calculation of Multiple Integrals* (Englewood Cliffs, NJ: Prentice-Hall).[1]

Dahlquist, G., and Bjorck, A. 1974, *Numerical Methods* (Englewood Cliffs, NJ: Prentice-Hall); reprinted 2003 (New York: Dover), §7.7, p. 318.

Johnson, L.W., and Riess, R.D. 1982, *Numerical Analysis*, 2nd ed. (Reading, MA: Addison-Wesley), §6.2.5, p. 307.

Abramowitz, M., and Stegun, I.A. 1964, *Handbook of Mathematical Functions* (Washington: National Bureau of Standards); reprinted 1968 (New York: Dover); online at `http://www.nr.com/aands`, equations 25.4.58ff.

Evaluation of Functions

CHAPTER 5

5.0 Introduction

The purpose of this chapter is to acquaint you with a selection of the techniques that are frequently used in evaluating functions. In Chapter 6, we will apply and illustrate these techniques by giving routines for a variety of specific functions. The purposes of this chapter and the next are thus mostly congruent. Occasionally, however, the method of choice for a particular special function in Chapter 6 is peculiar to that function. By comparing this chapter to the next one, you should get some idea of the balance between "general" and "special" methods that occurs in practice.

Insofar as that balance favors general methods, this chapter should give you ideas about how to write your own routine for the evaluation of a function that, while "special" to you, is not so special as to be included in Chapter 6 or the standard function libraries.

CITED REFERENCES AND FURTHER READING:

Fike, C.T. 1968, *Computer Evaluation of Mathematical Functions* (Englewood Cliffs, NJ: Prentice-Hall).

Lanczos, C. 1956, *Applied Analysis*; reprinted 1988 (New York: Dover), Chapter 7.

5.1 Polynomials and Rational Functions

A polynomial of degree N is represented numerically as a stored array of coefficients, `c[j]` with `j`$= 0, \ldots, N$. We will always take `c[0]` to be the constant term in the polynomial and `c[`N`]` the coefficient of x^N; but of course other conventions are possible. There are two kinds of manipulations that you can do with a polynomial: *numerical* manipulations (such as evaluation), where you are given the numerical value of its argument, or *algebraic* manipulations, where you want to transform the coefficient array in some way without choosing any particular argument. Let's start with the numerical.

We assume that you know enough *never* to evaluate a polynomial this way:

```
p=c[0]+c[1]*x+c[2]*x*x+c[3]*x*x*x+c[4]*x*x*x*x;
```

or (even worse!),

```
p=c[0]+c[1]*x+c[2]*pow(x,2.0)+c[3]*pow(x,3.0)+c[4]*pow(x,4.0);
```

Come the (computer) revolution, all persons found guilty of such criminal behavior will be summarily executed, and their programs won't be! It is a matter of taste, however, whether to write

```
p=c[0]+x*(c[1]+x*(c[2]+x*(c[3]+x*c[4])));
```

or

```
p=(((c[4]*x+c[3])*x+c[2])*x+c[1])*x+c[0];
```

If the number of coefficients `c[0..n-1]` is large, one writes

```
p=c[n-1];
for(j=n-2;j>=0;j--) p=p*x+c[j];
```

or

```
p=c[j=n-1];
while (j>0) p=p*x+c[--j];
```

We can formalize this by defining a function object (or functor) that binds a reference to an array of coefficients and endows them with a polynomial evaluation function,

poly.h

```
struct Poly {
Polynomial function object that binds a reference to a vector of coefficients.
    VecDoub &c;
    Poly(VecDoub &cc) : c(cc) {}
    Doub operator() (Doub x) {
    Returns the value of the polynomial at x.
        Int j;
        Doub p = c[j=c.size()-1];
        while (j>0) p = p*x + c[--j];
        return p;
    }
};
```

which allows you to write things like

```
y = Poly(c)(x);
```

where `c` is a coefficient vector.

Another useful trick is for evaluating a polynomial $P(x)$ and its derivative $dP(x)/dx$ simultaneously:

```
p=c[n-1];
dp=0.;
for(j=n-2;j>=0;j--) {dp=dp*x+p; p=p*x+c[j];}
```

or

```
p=c[j=n-1];
dp=0.;
while (j>0) {dp=dp*x+p; p=p*x+c[--j];}
```

which yields the polynomial as `p` and its derivative as `dp` using coefficients `c[0..n-1]`.

The above trick, which is basically *synthetic division* [1,2], generalizes to the evaluation of the polynomial and `nd` of its derivatives simultaneously:

```
void ddpoly(VecDoub_I &c, const Doub x, VecDoub_O &pd)
```
poly.h

Given the coefficients of a polynomial of degree nc as an array c[0..nc] of size nc+1 (with c[0] being the constant term), and given a value x, this routine fills an output array pd of size nd+1 with the value of the polynomial evaluated at x in pd[0], and the first nd derivatives at x in pd[1..nd].

```
{
    Int nnd,j,i,nc=c.size()-1,nd=pd.size()-1;
    Doub cnst=1.0;
    pd[0]=c[nc];
    for (j=1;j<nd+1;j++) pd[j]=0.0;
    for (i=nc-1;i>=0;i--) {
        nnd=(nd < (nc-i) ? nd : nc-i);
        for (j=nnd;j>0;j--) pd[j]=pd[j]*x+pd[j-1];
        pd[0]=pd[0]*x+c[i];
    }
    for (i=2;i<nd+1;i++) {        After the first derivative, factorial constants come in.
        cnst *= i;
        pd[i] *= cnst;
    }
}
```

As a curiosity, you might be interested to know that polynomials of degree $n > 3$ can be evaluated in *fewer* than n multiplications, at least if you are willing to precompute some auxiliary coefficients and, in some cases, do some extra addition. For example, the polynomial

$$P(x) = a_0 + a_1 x + a_2 x^2 + a_3 x^3 + a_4 x^4 \tag{5.1.1}$$

where $a_4 > 0$, can be evaluated with three multiplications and five additions as follows:

$$P(x) = [(Ax + B)^2 + Ax + C][(Ax + B)^2 + D] + E \tag{5.1.2}$$

where A, B, C, D, and E are to be precomputed by

$$\begin{aligned}
A &= (a_4)^{1/4} \\
B &= \frac{a_3 - A^3}{4A^3} \\
D &= 3B^2 + 8B^3 + \frac{a_1 A - 2a_2 B}{A^2} \\
C &= \frac{a_2}{A^2} - 2B - 6B^2 - D \\
E &= a_0 - B^4 - B^2(C + D) - CD
\end{aligned} \tag{5.1.3}$$

Fifth-degree polynomials can be evaluated in four multiplies and five adds; sixth-degree polynomials can be evaluated in four multiplies and seven adds; if any of this strikes you as interesting, consult references [3-5]. The subject has something of the same flavor as that of fast matrix multiplication, discussed in §2.11.

Turn now to algebraic manipulations. You multiply a polynomial of degree $n - 1$ (array of range [0..n-1]) by a monomial factor $x - a$ by a bit of code like the following,

```
c[n]=c[n-1];
for (j=n-1;j>=1;j--) c[j]=c[j-1]-c[j]*a;
c[0] *= (-a);
```

Likewise, you divide a polynomial of degree n by a monomial factor $x - a$ (synthetic division again) using

```
rem=c[n];
c[n]=0.;
for(i=n-1;i>=0;i--) {
    swap=c[i];
    c[i]=rem;
    rem=swap+rem*a;
}
```

which leaves you with a new polynomial array and a numerical remainder rem.

Multiplication of two general polynomials involves straightforward summing of the products, each involving one coefficient from each polynomial. Division of two general polynomials, while it can be done awkwardly in the fashion taught using pencil and paper, is susceptible to a good deal of streamlining. Witness the following routine based on the algorithm in [3].

poly.h

```
void poldiv(VecDoub_I &u, VecDoub_I &v, VecDoub_O &q, VecDoub_O &r)
```

Divide a polynomial u by a polynomial v, and return the quotient and remainder polynomials in q and r, respectively. The four polynomials are represented as vectors of coefficients, each starting with the constant term. There is no restriction on the relative lengths of u and v, and either may have trailing zeros (represent a lower degree polynomial than its length allows). q and r are returned with the size of u, but will usually have trailing zeros.

```
{
    Int k,j,n=u.size()-1,nv=v.size()-1;
    while (nv >= 0 && v[nv] == 0.) nv--;
    if (nv < 0) throw("poldiv divide by zero polynomial");
    r = u;                                        May do a resize.
    q.assign(u.size(),0.);                        May do a resize.
    for (k=n-nv;k>=0;k--) {
        q[k]=r[nv+k]/v[nv];
        for (j=nv+k-1;j>=k;j--) r[j] -= q[k]*v[j-k];
    }
    for (j=nv;j<=n;j++) r[j]=0.0;
}
```

5.1.1 Rational Functions

You evaluate a rational function like

$$R(x) = \frac{P_\mu(x)}{Q_\nu(x)} = \frac{p_0 + p_1 x + \cdots + p_\mu x^\mu}{q_0 + q_1 x + \cdots + q_\nu x^\nu} \tag{5.1.4}$$

in the obvious way, namely as two separate polynomials followed by a divide. As a matter of convention one usually chooses $q_0 = 1$, obtained by dividing the numerator and denominator by any other q_0. In that case, it is often convenient to have both sets of coefficients, omitting q_0, stored in a single array, in the order

$$(p_0, p_1, \ldots, p_\mu, q_1, \ldots, q_\nu) \tag{5.1.5}$$

The following object encapsulates a rational function. It provides constructors from either separate numerator and denominator polynomials, or a single array like (5.1.5) with explicit values for $\texttt{n} = \mu + 1$ and $\texttt{d} = \nu + 1$. The evaluation function makes Ratfn a functor, like Poly. We'll make use of this object in §5.12 and §5.13.

poly.h

```
struct Ratfn {
Function object for a rational function.
    VecDoub cofs;
    Int nn,dd;                      Number of numerator, denominator coefficients.

    Ratfn(VecDoub_I &num, VecDoub_I &den) : cofs(num.size()+den.size()-1),
    nn(num.size()), dd(den.size()) {
    Constructor from numerator, denominator polyomials (as coefficient vectors).
        Int j;
        for (j=0;j<nn;j++) cofs[j] = num[j]/den[0];
        for (j=1;j<dd;j++) cofs[j+nn-1] = den[j]/den[0];
    }

    Ratfn(VecDoub_I &coffs, const Int n, const Int d) : cofs(coffs), nn(n),
    dd(d) {}
    Constructor from coefficients already normalized and in a single array.

    Doub operator() (Doub x) const {
    Evaluate the rational function at x and return result.
        Int j;
        Doub sumn = 0., sumd = 0.;
        for (j=nn-1;j>=0;j--) sumn = sumn*x + cofs[j];
        for (j=nn+dd-2;j>=nn;j--) sumd = sumd*x + cofs[j];
        return sumn/(1.0+x*sumd);
    }

};
```

5.1.2 Parallel Evaluation of a Polynomial

A polynomial of degree N can be evaluated in about $\log_2 N$ parallel steps [6]. This is best illustrated by an example, for example with $N = 5$. Start with the vector of coefficients, imagining appended zeros:

$$c_0, \quad c_1, \quad c_2, \quad c_3, \quad c_4, \quad c_5, \quad 0, \quad \dots \tag{5.1.6}$$

Now add the elements by pairs, multiplying the second of each pair by x:

$$c_0 + c_1 x, \quad c_2 + c_3 x, \quad c_4 + c_5 x, \quad 0, \quad \dots \tag{5.1.7}$$

Now the same operation, but with the multiplier x^2:

$$(c_0 + c_1 x) + (c_2 + c_3 x)x^2, \quad (c_4 + c_5 x) + (0)x^2, \quad 0 \quad \dots \tag{5.1.8}$$

And a final time with multiplier x^4:

$$[(c_0 + c_1 x) + (c_2 + c_3 x)x^2] + [(c_4 + c_5 x) + (0)x^2]x^4, \quad 0 \quad \dots \tag{5.1.9}$$

We are left with a vector of (active) length 1, whose value is the desired polynomial evaluation. You can see that the zeros are just a bookkeeping device for taking care of the case where the active subvector has an odd length; in an actual implementation you can avoid most operations on the zeros. This parallel method generally has better roundoff properties than the standard sequential coding.

CITED REFERENCES AND FURTHER READING:

Acton, F.S. 1970, *Numerical Methods That Work*; 1990, corrected edition (Washington, DC: Mathematical Association of America), pp. 183, 190.[1]

Mathews, J., and Walker, R.L. 1970, *Mathematical Methods of Physics*, 2nd ed. (Reading, MA: W.A. Benjamin/Addison-Wesley), pp. 361–363.[2]

Knuth, D.E. 1997, *Seminumerical Algorithms*, 3rd ed., vol. 2 of *The Art of Computer Programming* (Reading, MA: Addison-Wesley), §4.6.[3]

Fike, C.T. 1968, *Computer Evaluation of Mathematical Functions* (Englewood Cliffs, NJ: Prentice-Hall), Chapter 4.

Winograd, S. 1970, "On the number of multiplications necessary to compute certain functions," *Communications on Pure and Applied Mathematics*, vol. 23, pp. 165–179.[4]

Kronsjö, L. 1987, *Algorithms: Their Complexity and Efficiency*, 2nd ed. (New York: Wiley).[5]

Estrin, G. 1960, quoted in Knuth, D.E. 1997, *Seminumerical Algorithms*, 3rd ed., vol. 2 of *The Art of Computer Programming* (Reading, MA: Addison-Wesley), §4.6.4.[6]

5.2 Evaluation of Continued Fractions

Continued fractions are often powerful ways of evaluating functions that occur in scientific applications. A continued fraction looks like this:

$$f(x) = b_0 + \cfrac{a_1}{b_1 + \cfrac{a_2}{b_2 + \cfrac{a_3}{b_3 + \cfrac{a_4}{b_4 + \cfrac{a_5}{b_5 + \cdots}}}}} \tag{5.2.1}$$

Printers prefer to write this as

$$f(x) = b_0 + \frac{a_1}{b_1 +}\ \frac{a_2}{b_2 +}\ \frac{a_3}{b_3 +}\ \frac{a_4}{b_4 +}\ \frac{a_5}{b_5 +} \cdots \tag{5.2.2}$$

In either (5.2.1) or (5.2.2), the a's and b's can themselves be functions of x, usually linear or quadratic monomials at worst (i.e., constants times x or times x^2). For example, the continued fraction representation of the tangent function is

$$\tan x = \frac{x}{1 -}\ \frac{x^2}{3 -}\ \frac{x^2}{5 -}\ \frac{x^2}{7 -} \cdots \tag{5.2.3}$$

Continued fractions frequently converge much more rapidly than power series expansions, and in a much larger domain in the complex plane (not necessarily including the domain of convergence of the series, however). Sometimes the continued fraction converges best where the series does worst, although this is not a general rule. Blanch [1] gives a good review of the most useful convergence tests for continued fractions.

There are standard techniques, including the important *quotient-difference algorithm*, for going back and forth between continued fraction approximations, power series approximations, and rational function approximations. Consult Acton [2] for an introduction to this subject, and Fike [3] for further details and references.

How do you tell how far to go when evaluating a continued fraction? Unlike a series, you can't just evaluate equation (5.2.1) from left to right, stopping when the change is small. Written in the form of (5.2.1), the only way to evaluate the continued fraction is from right to left, first (blindly!) guessing how far out to start. This is not the right way.

The right way is to use a result that relates continued fractions to rational approximations, and that gives a means of evaluating (5.2.1) or (5.2.2) from left to right. Let f_n denote the result of evaluating (5.2.2) with coefficients through a_n and b_n. Then

$$f_n = \frac{A_n}{B_n} \tag{5.2.4}$$

where A_n and B_n are given by the following recurrence:

$$\begin{aligned}
A_{-1} &\equiv 1 \qquad B_{-1} \equiv 0 \\
A_0 &\equiv b_0 \qquad B_0 \equiv 1 \\
A_j = b_j A_{j-1} + a_j A_{j-2} \qquad & B_j = b_j B_{j-1} + a_j B_{j-2} \qquad j = 1, 2, \ldots, n
\end{aligned} \tag{5.2.5}$$

This method was invented by J. Wallis in 1655 (!) and is discussed in his *Arithmetica Infinitorum* [4]. You can easily prove it by induction.

In practice, this algorithm has some unattractive features: The recurrence (5.2.5) frequently generates very large or very small values for the partial numerators and denominators A_j and B_j. There is thus the danger of overflow or underflow of the floating-point representation. However, the recurrence (5.2.5) is linear in the A's and B's. At any point you can rescale the currently saved two levels of the recurrence, e.g., divide A_j, B_j, A_{j-1}, and B_{j-1} all by B_j. This incidentally makes $A_j = f_j$ and is convenient for testing whether you have gone far enough: See if f_j and f_{j-1} from the last iteration are as close as you would like them to be. If B_j happens to be zero, which can happen, just skip the renormalization for this cycle. A fancier level of optimization is to renormalize only when an overflow is imminent, saving the unnecessary divides. In fact, the C library function `ldexp` can be used to avoid division entirely. (See the end of §6.5 for an example.)

Two newer algorithms have been proposed for evaluating continued fractions. *Steed's method* does not use A_j and B_j explicitly, but only the *ratio* $D_j = B_{j-1}/B_j$. One calculates D_j and $\Delta f_j = f_j - f_{j-1}$ recursively using

$$D_j = 1/(b_j + a_j D_{j-1}) \tag{5.2.6}$$

$$\Delta f_j = (b_j D_j - 1)\Delta f_{j-1} \tag{5.2.7}$$

Steed's method (see, e.g., [5]) avoids the need for rescaling of intermediate results. However, for certain continued fractions you can occasionally run into a situation where the denominator in (5.2.6) approaches zero, so that D_j and Δf_j are very large. The next Δf_{j+1} will typically cancel this large change, but with loss of accuracy in the numerical running sum of the f_j's. It is awkward to program around this, so Steed's method can be recommended only for cases where you know in advance that no denominator can vanish. We will use it for a special purpose in the routine `besselik` (§6.6).

The best general method for evaluating continued fractions seems to be the *modified Lentz's method* [6]. The need for rescaling intermediate results is avoided by using *both* the ratios

$$C_j = A_j/A_{j-1}, \qquad D_j = B_{j-1}/B_j \tag{5.2.8}$$

and calculating f_j by

$$f_j = f_{j-1} C_j D_j \tag{5.2.9}$$

From equation (5.2.5), one easily shows that the ratios satisfy the recurrence relations

$$D_j = 1/(b_j + a_j D_{j-1}), \qquad C_j = b_j + a_j/C_{j-1} \tag{5.2.10}$$

In this algorithm there is the danger that the denominator in the expression for D_j, or the quantity C_j itself, might approach zero. Either of these conditions invalidates (5.2.10). However, Thompson and Barnett [5] show how to modify Lentz's algorithm to fix this: Just shift the offending term by a small amount, e.g., 10^{-30}. If you work through a cycle of the algorithm with this prescription, you will see that f_{j+1} is accurately calculated.

In detail, the modified Lentz's algorithm is this:

- Set $f_0 = b_0$; if $b_0 = 0$, set $f_0 = \texttt{tiny}$.
- Set $C_0 = f_0$.
- Set $D_0 = 0$.
- For $j = 1, 2, \ldots$

  Set $D_j = b_j + a_j D_{j-1}$.
  If $D_j = 0$, set $D_j = \texttt{tiny}$.
  Set $C_j = b_j + a_j/C_{j-1}$.
  If $C_j = 0$, set $C_j = \texttt{tiny}$.
  Set $D_j = 1/D_j$.
  Set $\Delta_j = C_j D_j$.
  Set $f_j = f_{j-1}\Delta_j$.
  If $|\Delta_j - 1| < \texttt{eps}$, then exit.

Here eps is your floating-point precision, say 10^{-7} or 10^{-15}. The parameter tiny should be less than typical values of eps $|b_j|$, say 10^{-30}.

The above algorithm assumes that you can terminate the evaluation of the continued fraction when $|f_j - f_{j-1}|$ is sufficiently small. This is usually the case, but by no means guaranteed. Jones [7] gives a list of theorems that can be used to justify this termination criterion for various kinds of continued fractions.

There is at present no rigorous analysis of error propagation in Lentz's algorithm. However, empirical tests suggest that it is at least as good as other methods.

5.2.1 Manipulating Continued Fractions

Several important properties of continued fractions can be used to rewrite them in forms that can speed up numerical computation. An *equivalence transformation*

$$a_n \to \lambda a_n, \quad b_n \to \lambda b_n, \quad a_{n+1} \to \lambda a_{n+1} \tag{5.2.11}$$

leaves the value of a continued fraction unchanged. By a suitable choice of the scale factor λ you can often simplify the form of the a's and the b's. Of course, you can carry out successive equivalence transformations, possibly with different λ's, on successive terms of the continued fraction.

The *even* and *odd* parts of a continued fraction are continued fractions whose successive convergents are f_{2n} and f_{2n+1}, respectively. Their main use is that they converge twice as fast as the original continued fraction, and so if their terms are not much more complicated than the terms in the original, there can be a big savings in computation. The formula for the even part of (5.2.2) is

$$f_{\text{even}} = d_0 + \frac{c_1}{d_1 +}\,\frac{c_2}{d_2 +}\cdots \tag{5.2.12}$$

where in terms of intermediate variables

$$\begin{aligned}\alpha_1 &= \frac{a_1}{b_1} \\ \alpha_n &= \frac{a_n}{b_n b_{n-1}}, \qquad n \ge 2\end{aligned} \tag{5.2.13}$$

we have

$$\begin{aligned}d_0 = b_0, \quad c_1 = \alpha_1, \quad d_1 = 1 + \alpha_2 \\ c_n = -\alpha_{2n-1}\alpha_{2n-2}, \quad d_n = 1 + \alpha_{2n-1} + \alpha_{2n}, \qquad n \ge 2\end{aligned} \tag{5.2.14}$$

You can find the similar formula for the odd part in the review by Blanch [1]. Often a combination of the transformations (5.2.14) and (5.2.11) is used to get the best form for numerical work.

We will make frequent use of continued fractions in the next chapter.

CITED REFERENCES AND FURTHER READING:

Abramowitz, M., and Stegun, I.A. 1964, *Handbook of Mathematical Functions* (Washington: National Bureau of Standards); reprinted 1968 (New York: Dover); online at `http://www.nr.com/aands`, §3.10.

Blanch, G. 1964, "Numerical Evaluation of Continued Fractions," *SIAM Review*, vol. 6, pp. 383–421.[1]

Acton, F.S. 1970, *Numerical Methods That Work*; 1990, corrected edition (Washington, DC: Mathematical Association of America), Chapter 11.[2]

Cuyt, A., and Wuytack, L. 1987, *Nonlinear Methods in Numerical Analysis* (Amsterdam: North-Holland), Chapter 1.

Fike, C.T. 1968, *Computer Evaluation of Mathematical Functions* (Englewood Cliffs, NJ: Prentice-Hall), §8.2, §10.4, and §10.5.[3]

Wallis, J. 1695, in *Opera Mathematica*, vol. 1, p. 355, Oxoniae e Theatro Shedoniano. Reprinted by Georg Olms Verlag, Hildeshein, New York (1972).[4]

Thompson, I.J., and Barnett, A.R. 1986, "Coulomb and Bessel Functions of Complex Arguments and Order," *Journal of Computational Physics*, vol. 64, pp. 490–509.[5]

Lentz, W.J. 1976, "Generating Bessel Functions in Mie Scattering Calculations Using Continued Fractions," *Applied Optics*, vol. 15, pp. 668–671.[6]

Jones, W.B. 1973, in *Padé Approximants and Their Applications*, P.R. Graves-Morris, ed. (London: Academic Press), p. 125.[7]

5.3 Series and Their Convergence

Everybody knows that an analytic function can be expanded in the neighborhood of a point x_0 in a power series,

$$f(x) = \sum_{k=0}^{\infty} a_k (x - x_0)^k \tag{5.3.1}$$

Such series are straightforward to evaluate. You don't, of course, evaluate the kth power of $x - x_0$ *ab initio* for each term; rather, you keep the $k-1$st power and update

it with a multiply. Similarly, the form of the coefficients a_k is often such as to make use of previous work: Terms like $k!$ or $(2k)!$ can be updated in a multiply or two.

How do you know when you have summed enough terms? In practice, the terms had better be getting small fast, otherwise the series is not a good technique to use in the first place. While not mathematically rigorous in all cases, standard practice is to quit when the term you have just added is smaller in magnitude than some small ϵ times the magnitude of the sum thus far accumulated. (But watch out if isolated instances of $a_k = 0$ are possible!)

Sometimes you will want to compute a function from a series representation even when the computation is *not* efficient. For example, you may be using the values obtained to fit the function to an approximating form that you will use subsequently (cf. §5.8). If you are summing very large numbers of slowly convergent terms, pay attention to roundoff errors! In floating-point representation it is more accurate to sum a list of numbers in the order starting with the smallest one, rather than starting with the largest one. It is even better to group terms pairwise, then in pairs of pairs, etc., so that all additions involve operands of comparable magnitude.

A weakness of a power series representation is that it is guaranteed *not* to converge farther than that distance from x_0 at which a singularity is encountered *in the complex plane*. This catastrophe is not usually unexpected: When you find a power series in a book (or when you work one out yourself), you will generally also know the radius of convergence. An insidious problem occurs with series that converge everywhere (in the mathematical sense), but almost nowhere fast enough to be useful in a numerical method. Two familiar examples are the sine function and the Bessel function of the first kind,

$$\sin x = \sum_{k=0}^{\infty} \frac{(-1)^k}{(2k+1)!} x^{2k+1} \tag{5.3.2}$$

$$J_n(x) = \left(\frac{x}{2}\right)^n \sum_{k=0}^{\infty} \frac{(-\frac{1}{4}x^2)^k}{k!(k+n)!} \tag{5.3.3}$$

Both of these series converge for all x. But both don't even start to converge until $k \gg |x|$; before this, their terms are increasing. Even worse, the terms alternate in sign, leading to large cancellation errors with finite precision arithmetic. This makes these series useless for large x.

5.3.1 Divergent Series

Divergent series are often very useful. One class consists of power series outside their radius of convergence, which can often be summed by the acceleration techniques we will describe below. Another class is asymptotic series, such as the Euler series that comes from Euler's integral (related to the exponential integral E_1):

$$E(x) = \int_0^\infty \frac{e^{-t}}{1+xt}dt \simeq \sum_{k=0}^{\infty} (-1)^k k!\, x^k \tag{5.3.4}$$

Here the series is derived by expanding $(1+xt)^{-1}$ in powers of x and integrating term by term. The series diverges for all $x \neq 0$. For $x = 0.1$, the series gives only three significant digits before diverging. Nevertheless, convergence acceleration

techniques allow effortless evaluation of the function $E(x)$, even for $x \sim 2$, when the series is wildly divergent!

5.3.2 Accelerating the Convergence of Series

There are several tricks for accelerating the rate of convergence of a series or, equivalently, of a sequence of partial sums

$$s_n = \sum_{k=0}^{n} a_k \tag{5.3.5}$$

(We'll use the terms sequence and series interchangeably in this section.) An excellent review has been given by Weniger [1]. Before we can describe the tricks and when to use them, we need to classify some of the ways in which a sequence can converge. Suppose s_n converges to s, say, and that

$$\lim_{n\to\infty} \frac{a_{n+1}}{a_n} = \rho \tag{5.3.6}$$

If $0 < |\rho| < 1$, we say the convergence is *linear*; if $\rho = 1$, it is *logarithmic*; and if $\rho = 0$, it is *hyperlinear*. Of course, if $|\rho| > 1$, the sequence diverges. (More rigorously, this classification should be given in terms of the so-called remainders $s_n - s$ [1]. However, our definition is more practical and is equivalent if we restrict the logarithmic case to terms of the same sign.)

The prototype of linear convergence is a geometric series,

$$s_n = \sum_{k=0}^{n} x^k = \frac{1 - x^{n+1}}{1 - x} \tag{5.3.7}$$

It is easy to see that $\rho = x$, and so we have linear convergence for $0 < |x| < 1$. The prototype of logarithmic convergence is the series for the Riemann zeta function,

$$\zeta(x) = \sum_{k=1}^{\infty} \frac{1}{k^x}, \qquad x > 1 \tag{5.3.8}$$

which is notoriously slowly convergent, especially as $x \to 1$. The series (5.3.2) and (5.3.3), or the series for e^x, exemplify hyperlinear convergence. We see that hyperlinear convergence doesn't necessarily imply that the series is easy to evaluate for all values of x. Sometimes convergence acceleration is helpful only once the terms start decreasing.

Probably the most famous series transformation for accelerating convergence is the Euler transformation (see, e.g., [2,3]), which dates from 1755. Euler's transformation works on *alternating series* (where the terms in the sum alternate in sign). Generally it is advisable to do a small number of terms directly, through term $n - 1$, say, and then apply the transformation to the rest of the series beginning with term n. The formula (for n even) is

$$\sum_{s=0}^{\infty} (-1)^s a_s = a_0 - a_1 + a_2 \ldots - a_{n-1} + \sum_{s=0}^{\infty} \frac{(-1)^s}{2^{s+1}} [\Delta^s a_n] \tag{5.3.9}$$

Here Δ is the *forward difference operator*, i.e.,

$$\begin{aligned} \Delta a_n &\equiv a_{n+1} - a_n \\ \Delta^2 a_n &\equiv a_{n+2} - 2a_{n+1} + a_n \\ \Delta^3 a_n &\equiv a_{n+3} - 3a_{n+2} + 3a_{n+1} - a_n \qquad \text{etc.} \end{aligned} \tag{5.3.10}$$

Of course you don't actually do the infinite sum on the right-hand side of (5.3.9), but only the first, say, p terms, thus requiring the first p differences (5.3.10) obtained from the terms starting at a_n. There is an elegant and subtle implementation of Euler's transformation due to van Wijngaarden [6], discussed in full in a Webnote [7].

Euler's transformation is an example of a *linear* transformation: The partial sums of the transformed series are linear combinations of the partial sums of the original series. Euler's transformation and other linear transformations, while still important theoretically, have generally been superseded by newer *nonlinear* transformations that are considerably more powerful. As usual in numerical work, there is no free lunch: While the nonlinear transformations are more powerful, they are somewhat riskier than linear transformations in that they can occasionally fail spectacularly. But if you follow the guidance below, we think that you will never again resort to puny linear transformations.

The oldest example of a nonlinear sequence transformation is *Aitken's Δ^2-process*. If s_n, s_{n+1}, s_{n+2} are three successive partial sums, then an improved estimate is

$$s'_n \equiv s_n - \frac{(s_{n+1} - s_n)^2}{s_{n+2} - 2s_{n+1} + s_n} = s_n - \frac{(\Delta s_n)^2}{\Delta^2 s_n} \tag{5.3.11}$$

The formula (5.3.11) is exact for a geometric series, which is one way of deriving it. If you form the sequence of s'_i's, you can apply (5.3.11) a second time to *that* sequence, and so on. (In practice, this iteration will only rarely do much for you after the first stage.) Note that equation (5.3.11) should be computed as written; there exist algebraically equivalent forms that are much more susceptible to roundoff error.

Aitken's Δ^2-process works only on linearly convergent sequences. Like Euler's transformation, it has also been superseded by algorithms such as the two we will now describe. After giving routines for these algorithms, we will supply some rules of thumb on when to use them.

The first "modern" nonlinear transformation was proposed by Shanks. An efficient recursive implementation was given by Wynn, called the ϵ algorithm. Aitken's Δ^2-process is a special case of the ϵ algorithm, corresponding to using just three terms at a time. Although we will not give a derivation here, it is easy to state exactly what the ϵ algorithm does: If you input the partial sums of a power series, the ϵ algorithm returns the "diagonal" Padé approximants (§5.12) evaluated at the value of x used in the power series. (The coefficients in the approximant itself are not calculated.) That is, if $[M/N]$ denotes the Padé approximant with a polynomial of degree M in the numerator and degree N in the denominator, the algorithm returns the numerical values of the approximants

$$[0,0], \quad [1/0], \quad [1/1], \quad [2/1], \quad [2/2], \quad [3,2], \quad [3,3] \quad \dots \tag{5.3.12}$$

(The object `Epsalg` below is roughly equivalent to `pade` in §5.12 followed by an evaluation of the resulting rational function.)

In the object `Epsalg`, which is based on a routine in [1], you supply the sequence term by term and monitor the output for convergence in the calling program. Internally, the routine contains a check for division by zero and substitutes a large number

for the result. There are three conditions under which this check can be triggered: (i) Most likely, the algorithm has already converged, and should have been stopped earlier; (ii) there is an "accidental" zero term, and the program will recover; (iii) hardly ever in practice, the algorithm can actually fail because of a perverse combination of terms. Because (i) and (ii) are vastly more common than (iii), `Epsalg` hides the check condition and instead returns the last-known good estimate.

series.h

```
struct Epsalg {
```

Convergence acceleration of a sequence by the ϵ algorithm. Initialize by calling the constructor with arguments `nmax`, an upper bound on the number of terms to be summed, and `epss`, the desired accuracy. Then make successive calls to the function `next`, with argument the next partial sum of the sequence. The current estimate of the limit of the sequence is returned by `next`. The flag `cnvgd` is set when convergence is detected.

```
    VecDoub e;                                   Workspace.
    Int n,ncv;
    Bool cnvgd;
    Doub eps,small,big,lastval,lasteps;          Numbers near machine underflow and
                                                     overflow limits.
    Epsalg(Int nmax, Doub epss) : e(nmax), n(0), ncv(0),
    cnvgd(0), eps(epss), lastval(0.) {
        small = numeric_limits<Doub>::min()*10.0;
        big = numeric_limits<Doub>::max();
    }

    Doub next(Doub sum) {
        Doub diff,temp1,temp2,val;
        e[n]=sum;
        temp2=0.0;
        for (Int j=n; j>0; j--) {
            temp1=temp2;
            temp2=e[j-1];
            diff=e[j]-temp2;
            if (abs(diff) <= small)
                e[j-1]=big;
            else
                e[j-1]=temp1+1.0/diff;
        }
        n++;
        val = (n & 1) ? e[0] : e[1];             Cases of n even or odd.
        if (abs(val) > 0.01*big) val = lastval;
        lasteps = abs(val-lastval);
        if (lasteps > eps) ncv = 0;
        else ncv++;
        if (ncv >= 3) cnvgd = 1;
        return (lastval = val);
    }

};
```

The last few lines above implement a simple criterion for deciding whether the sequence has converged. For problems whose convergence is robust, you can simply put your calls to `next` inside a `while` loop like this:

```
    Doub val, partialsum, eps=...;
    Epsalg mysum(1000,eps);
    while (! mysum.cnvgd) {
        partialsum = ...
        val = mysum.next(partialsum);
    }
```

For more delicate cases, you can ignore the `cnvgd` flag and just keep calling `next` until you are satisfied with the convergence.

A large class of modern nonlinear transformations can be derived by using the concept of a *model sequence*. The idea is to choose a "simple" sequence that approximates the asymptotic form of the given sequence and construct a transformation that sums the model sequence exactly. Presumably the transformation will work well for other sequences with similar asymptotic properties. For example, a geometric series provides the model sequence for Aitken's Δ^2-process.

The *Levin transformation* is probably the best single sequence acceleration method currently known. It is based on approximating a sequence asymptotically by an expression of the form

$$s_n = s + \omega_n \sum_{j=0}^{k-1} \frac{c_j}{(n+\beta)^j} \tag{5.3.13}$$

Here ω_n is the dominant term in the remainder of the sequence:

$$s_n - s = \omega_n[c + O(n^{-1})], \qquad n \to \infty \tag{5.3.14}$$

The constants c_j are arbitrary, and β is a parameter that is restricted to be positive. Levin showed that for a model sequence of the form (5.3.13), the following transformation gives the exact value of the series:

$$s = \frac{\displaystyle\sum_{j=0}^{k} (-1)^j \binom{k}{j} \frac{(\beta+n+j)^{k-1}}{(\beta+n+k)^{k-1}} \frac{s_{n+j}}{\omega_{n+j}}}{\displaystyle\sum_{j=0}^{k} (-1)^j \binom{k}{j} \frac{(\beta+n+j)^{k-1}}{(\beta+n+k)^{k-1}} \frac{1}{\omega_{n+j}}} \tag{5.3.15}$$

(The common factor $(\beta + n + k)^{k-1}$ in the numerator and denominator reduces the chances of overflow for large k.) A derivation of equation (5.3.15) is given in a Webnote [4].

The numerator and denominator in (5.3.15) are not computed as written. Instead, they can be computed efficiently from a single recurrence relation with different starting values (see [1] for a derivation):

$$D_{k+1}^n(\beta) = D_k^{n+1}(\beta) - \frac{(\beta+n)(\beta+n+k)^{k-1}}{(\beta+n+k+1)^k} D_k^n(\beta) \tag{5.3.16}$$

The starting values are

$$D_0^n(\beta) = \begin{cases} s_n/\omega_n, & \text{numerator} \\ 1/\omega_n, & \text{denominator} \end{cases} \tag{5.3.17}$$

Although D_k^n is a two-dimensional object, the recurrence can be coded in a one-dimensional array proceeding up the counterdiagonal $n + k =$ constant.

The choice (5.3.14) doesn't determine ω_n uniquely, but if you have analytic information about your series, this is where you can make use of it. Usually you won't

be so lucky, in which case you can make a choice based on heuristics. For example, the remainder in an alternating series is approximately half the first neglected term, which suggests setting ω_n equal to a_n or a_{n+1}. These are called the Levin t and d transformations, respectively. Similarly, the remainder for a geometric series is the difference between the partial sum (5.3.7) and its limit $1/(1-x)$. This can be written as $a_n a_{n+1}/(a_n - a_{n+1})$, which defines the Levin v transformation. The most popular choice comes from approximating the remainder in the ζ function (5.3.8) by an integral:

$$\sum_{k=n+1}^{\infty} \frac{1}{k^x} \approx \int_{n+1}^{\infty} \frac{dk}{k^x} = \frac{(n+1)^{1-x}}{x-1} = \frac{(n+1)a_{n+1}}{x-1} \tag{5.3.18}$$

This motivates the choice $(n+\beta)a_n$ (Levin u transformation), where β is usually chosen to be 1. To summarize:

$$\omega_n = \begin{cases} (\beta+n)a_n, & u \text{ transformation} \\ a_n, & t \text{ transformation} \\ a_{n+1}, & d \text{ transformation (modified } t \text{ transformation)} \\ \dfrac{a_n a_{n+1}}{a_n - a_{n+1}}, & v \text{ transformation} \end{cases} \tag{5.3.19}$$

For sequences that are not partial sums, so that the individual a_n's are not defined, replace a_n by Δs_{n-1} in (5.3.19).

Here is the routine for Levin's transformation, also based on the routine in [1]:

series.h

```
struct Levin {
```
Convergence acceleration of a sequence by the Levin transformation. Initialize by calling the constructor with arguments `nmax`, an upper bound on the number of terms to be summed, and `epss`, the desired accuracy. Then make successive calls to the function `next`, which returns the current estimate of the limit of the sequence. The flag `cnvgd` is set when convergence is detected.
```
    VecDoub numer,denom;          Numerator and denominator computed via (5.3.16).
    Int n,ncv;
    Bool cnvgd;
    Doub small,big;               Numbers near machine underflow and overflow limits.
    Doub eps,lastval,lasteps;

    Levin(Int nmax, Doub epss) : numer(nmax), denom(nmax), n(0), ncv(0),
    cnvgd(0), eps(epss), lastval(0.) {
        small=numeric_limits<Doub>::min()*10.0;
        big=numeric_limits<Doub>::max();
    }

    Doub next(Doub sum, Doub omega, Doub beta=1.) {
```
Arguments: `sum`, the nth partial sum of the sequence; `omega`, the nth remainder estimate ω_n, usually from (5.3.19); and the parameter `beta`, which should usually be set to 1, but sometimes 0.5 works better. The current estimate of the limit of the sequence is returned.
```
        Int j;
        Doub fact,ratio,term,val;
        term=1.0/(beta+n);
        denom[n]=term/omega;
        numer[n]=sum*denom[n];
        if (n > 0) {
            ratio=(beta+n-1)*term;
            for (j=1;j<=n;j++) {
```

```
                fact=(n-j+beta)*term;
                numer[n-j]=numer[n-j+1]-fact*numer[n-j];
                denom[n-j]=denom[n-j+1]-fact*denom[n-j];
                term=term*ratio;
            }
        }
        n++;
        val = abs(denom[0]) < small ? lastval : numer[0]/denom[0];
        lasteps = abs(val-lastval);
        if (lasteps <= eps) ncv++;
        if (ncv >= 2) cnvgd = 1;
        return (lastval = val);
    }
};
```

You can use, or not use, the `cnvgd` flag exactly as previously discussed for `Epsalg`.

An alternative to the model sequence method of deriving sequence transformations is to use extrapolation of a polynomial or rational function approximation to a series, e.g., as in Wynn's ρ algorithm [1]. Since none of these methods generally beats the two we have given, we won't say any more about them.

5.3.3 Practical Hints and an Example

There is no general theoretical understanding of nonlinear sequence transformations. Accordingly, most of the practical advice is based on numerical experiments [5]. You might have thought that summing a wildly divergent series is the hardest problem for a sequence transformation. However, the difficulty of a problem depends more on whether the terms are all of the same sign or whether the signs alternate, rather than whether the sequence actually converges or not. In particular, logarithmically convergent series with terms all of the same sign are generally the most difficult to sum. Even the best acceleration methods are corrupted by rounding errors when accelerating logarithmic convergence. You should always use double precision and be prepared for some loss of significant digits. Typically one observes convergence up to some optimum number of terms, and then a loss of significant digits if one tries to go further. Moreover, there is no single algorithm that can accelerate every logarithmically convergent sequence. Nevertheless, there are some good rules of thumb.

First, note that among divergent series it is useful to separate out asymptotic series, where the terms first decrease before increasing, as a separate class from other divergent series, e.g., power series outside their radius of convergence. For alternating series, whether convergent, asymptotic, or divergent power series, Levin's u transformation is almost always the best choice. For monotonic linearly convergent or monotonic divergent power series, the ϵ algorithm typically is the first choice, but the u transformation often does a reasonable job. For logarithmic convergence, the u transformation is clearly the best. (The ϵ algorithm fails completely.) For series with irregular signs or other nonstandard features, typically the ϵ algorithm is relatively robust, often succeeding where other algorithms fail. Finally, for monotonic asymptotic series, such as (6.3.11) for $\text{Ei}(x)$, there is nothing better than direct summation without acceleration.

The v and t transformations are almost as good as the u transformation, except that the t transformation typically fails for logarithmic convergence.

If you have only a few numerical terms of some sequence and no theoretical insight, blindly applying a convergence accelerator can be dangerous. The algorithm

can sometimes display "convergence" that is only apparent, not real. The remedy is to try two different transformations as a check.

Since convergence acceleration is so much more difficult for a series of positive terms than for an alternating series, occasionally it is useful to convert a series of positive terms into an alternating series. Van Wijngaarden has given a transformation for accomplishing this [6]:

$$\sum_{r=1}^{\infty} v_r = \sum_{r=1}^{\infty} (-1)^{r-1} w_r \tag{5.3.20}$$

where

$$w_r \equiv v_r + 2v_{2r} + 4v_{4r} + 8v_{8r} + \cdots \tag{5.3.21}$$

Equations (5.3.20) and (5.3.21) replace a simple sum by a two-dimensional sum, each term in (5.3.20) being itself an infinite sum (5.3.21). This may seem a strange way to save on work! Since, however, the indices in (5.3.21) increase tremendously rapidly, as powers of 2, it often requires only a few terms to converge (5.3.21) to extraordinary accuracy. You do, however, need to be able to compute the v_r's efficiently for "random" values r. The standard "updating" tricks for sequential r's, mentioned above following equation (5.3.1), can't be used.

Once you've generated the alternating series by Van Wijngaarden's transformation, the Levin d transformation is particularly effective at summing the series [8]. This strategy is most useful for linearly convergent series with ρ close to 1. For logarithmically convergent series, even the transformed series (5.3.21) is often too slowly convergent to be useful numerically.

As an example of how to call the routines `Epsalg` or `Levin`, consider the problem of evaluating the integral

$$I = \int_0^{\infty} \frac{x}{1+x^2} J_0(x)\, dx = K_0(1) = 0.4210244382\ldots \tag{5.3.22}$$

Standard quadrature methods such as `qromo` fail because the integrand has a long oscillatory tail, giving alternating positive and negative contributions that tend to cancel. A good way of evaluating such an integral is to split it into a sum of integrals between successive zeros of $J_0(x)$:

$$I = \int_0^{\infty} f(x)\, dx = \sum_{j=0}^{\infty} I_j \tag{5.3.23}$$

where

$$I_j = \int_{x_{j-1}}^{x_j} f(x)\, dx, \qquad f(x_j) = 0, \quad j = 0, 1, \ldots \tag{5.3.24}$$

We take x_{-1} equal to the lower limit of the integral, zero in this example. The idea is to evaluate the relatively simple integrals I_j by `qromb` or Gaussian quadrature, and then accelerate the convergence of the series (5.3.23), since we expect the contributions to alternate in sign. For the example (5.3.22), we don't even need accurate values of the zeros of $J_0(x)$. It is good enough to take $x_j = (j+1)\pi$, which is asymptotically correct. Here is the code:

levex.h

```
Doub func(const Doub x)
```
Integrand for (5.3.22).
```
{
    if (x == 0.0)
        return 0.0;
    else {
        Bessel bess;
        return x*bess.jnu(0.0,x)/(1.0+x*x);
    }
}
```

```
Int main_levex(void)
```
This sample program shows how to use the Levin u transformation to evaluate an oscillatory integral, equation (5.3.22).
```
{
    const Doub PI=3.141592653589793;
    Int nterm=12;
    Doub beta=1.0,a=0.0,b=0.0,sum=0.0;
    Levin series(100,0.0);
    cout << setw(5) << "N" << setw(19) << "Sum (direct)" << setw(21)
        << "Sum (Levin)" << endl;
    for (Int n=0; n<=nterm; n++) {
        b+=PI;
        Doub s=qromb(func,a,b,1.e-8);
        a=b;
        sum+=s;
        Doub omega=(beta+n)*s;          Use u transformation.
        Doub ans=series.next(sum,omega,beta);
        cout << setw(5) << n << fixed << setprecision(14) << setw(21)
            << sum << setw(21) << ans << endl;
    }
    return 0;
}
```

Setting eps to 1×10^{-8} in qromb, we get 9 significant digits with about 200 function evaluations by $n = 8$. Replacing qromb with a Gaussian quadrature routine cuts the number of function evaluations in half. Note that $n = 8$ corresponds to an upper limit in the integral of 9π, where the amplitude of the integrand is still of order 10^{-2}. This shows the remarkable power of convergence acceleration. (For more on oscillatory integrals, see §13.9.)

CITED REFERENCES AND FURTHER READING:

Weniger, E.J. 1989, "Nonlinear Sequence Transformations for the Acceleration of Convergence and the Summation of Divergent Series," *Computer Physics Reports*, vol. 10, pp. 189–371.[1]

Abramowitz, M., and Stegun, I.A. 1964, *Handbook of Mathematical Functions* (Washington: National Bureau of Standards); reprinted 1968 (New York: Dover); online at http://www.nr.com/aands, §3.6.[2]

Mathews, J., and Walker, R.L. 1970, *Mathematical Methods of Physics*, 2nd ed. (Reading, MA: W.A. Benjamin/Addison-Wesley), §2.3.[3]

Numerical Recipes Software 2007, "Derivation of the Levin Transformation," *Numerical Recipes Webnote No. 6*, at http://www.nr.com/webnotes?6 [4]

Smith, D.A., and Ford, W.F. 1982, "Numerical Comparisons of Nonlinear Convergence Accelerators," *Mathematics of Computation*, vol. 38, pp. 481–499.[5]

Goodwin, E.T. (ed.) 1961, *Modern Computing Methods*, 2nd ed. (New York: Philosophical Library), Chapter 13 [van Wijngaarden's transformations].[6]

Numerical Recipes Software 2007, "Implementation of the Euler Transformation," *Numerical Recipes Webnote No. 5*, at `http://www.nr.com/webnotes?5` [7]

Jentschura, U.D., Mohr, P.J., Soff, G., and Weniger, E.J. 1999, "Convergence Acceleration via Combined Nonlinear-Condensation Transformations," *Computer Physics Communications*, vol. 116, pp. 28–54.[8]

Dahlquist, G., and Bjorck, A. 1974, *Numerical Methods* (Englewood Cliffs, NJ: Prentice-Hall); reprinted 2003 (New York: Dover), Chapter 3.

5.4 Recurrence Relations and Clenshaw's Recurrence Formula

Many useful functions satisfy recurrence relations, e.g.,

$$(n+1)P_{n+1}(x) = (2n+1)xP_n(x) - nP_{n-1}(x) \tag{5.4.1}$$

$$J_{n+1}(x) = \frac{2n}{x}J_n(x) - J_{n-1}(x) \tag{5.4.2}$$

$$nE_{n+1}(x) = e^{-x} - xE_n(x) \tag{5.4.3}$$

$$\cos n\theta = 2\cos\theta\cos(n-1)\theta - \cos(n-2)\theta \tag{5.4.4}$$

$$\sin n\theta = 2\cos\theta\sin(n-1)\theta - \sin(n-2)\theta \tag{5.4.5}$$

where the first three functions are Legendre polynomials, Bessel functions of the first kind, and exponential integrals, respectively. (For notation see [1].) These relations are useful for extending computational methods from two successive values of n to other values, either larger or smaller.

Equations (5.4.4) and (5.4.5) motivate us to say a few words about trigonometric functions. If your program's running time is dominated by evaluating trigonometric functions, you are probably doing something wrong. Trig functions whose arguments form a linear sequence $\theta = \theta_0 + n\delta$, $n = 0, 1, 2, \ldots$, are efficiently calculated by the recurrence

$$\begin{aligned}\cos(\theta+\delta) &= \cos\theta - [\alpha\cos\theta + \beta\sin\theta]\\ \sin(\theta+\delta) &= \sin\theta - [\alpha\sin\theta - \beta\cos\theta]\end{aligned} \tag{5.4.6}$$

where α and β are the precomputed coefficients

$$\alpha \equiv 2\sin^2\left(\frac{\delta}{2}\right) \qquad \beta \equiv \sin\delta \tag{5.4.7}$$

The reason for doing things this way, rather than with the standard (and equivalent) identities for sums of angles, is that here α and β do not lose significance if the incremental δ is small. Likewise, the adds in equation (5.4.6) should be done in the order indicated by the square brackets. We will use (5.4.6) repeatedly in Chapter 12, when we deal with Fourier transforms.

Another trick, occasionally useful, is to note that both $\sin\theta$ and $\cos\theta$ can be calculated via a single call to tan:

$$t \equiv \tan\left(\frac{\theta}{2}\right) \qquad \cos\theta = \frac{1-t^2}{1+t^2} \qquad \sin\theta = \frac{2t}{1+t^2} \tag{5.4.8}$$

The cost of getting both sin and cos, if you need them, is thus the cost of tan plus 2 multiplies, 2 divides, and 2 adds. On machines with slow trig functions, this can be a savings. *However*, note that special treatment is required if $\theta \to \pm\pi$. And also note that many modern machines have *very fast* trig functions; so you should not assume that equation (5.4.8) is faster without testing.

5.4.1 Stability of Recurrences

You need to be aware that recurrence relations are not necessarily *stable* against roundoff error in the direction that you propose to go (either increasing n or decreasing n). A three-term linear recurrence relation

$$y_{n+1} + a_n y_n + b_n y_{n-1} = 0, \qquad n = 1, 2, \ldots \tag{5.4.9}$$

has two linearly independent solutions, f_n and g_n, say. Only one of these corresponds to the sequence of functions f_n that you are trying to generate. The other one, g_n, *may* be exponentially growing in the direction that you want to go, or exponentially damped, or exponentially neutral (growing or dying as some power law, for example). If it is exponentially growing, then the recurrence relation is of little or no practical use in that direction. This is the case, e.g., for (5.4.2) in the direction of increasing n, when $x < n$. You cannot generate Bessel functions of high n by forward recurrence on (5.4.2).

To state things a bit more formally, if

$$f_n/g_n \to 0 \quad \text{as} \quad n \to \infty \tag{5.4.10}$$

then f_n is called the *minimal* solution of the recurrence relation (5.4.9). Nonminimal solutions like g_n are called *dominant* solutions. The minimal solution is unique, if it exists, but dominant solutions are not — you can add an arbitrary multiple of f_n to a given g_n. You can evaluate any dominant solution by forward recurrence, *but not the minimal solution*. (Unfortunately it is sometimes the one you want.)

Abramowitz and Stegun (in their Introduction!) [1] give a list of recurrences that are stable in the increasing or decreasing direction. That list does not contain all possible formulas, of course. Given a recurrence relation for some function $f_n(x)$, you can test it yourself with about five minutes of (human) labor: For a fixed x in your range of interest, start the recurrence not with true values of $f_j(x)$ and $f_{j+1}(x)$, but (first) with the values 1 and 0, respectively, and then (second) with 0 and 1, respectively. Generate 10 or 20 terms of the recursive sequences in the direction that you want to go (increasing or decreasing from j), for each of the two starting conditions. Look at the differences between the corresponding members of the two sequences. If the differences stay of order unity (absolute value less than 10, say), then the recurrence is stable. If they increase slowly, then the recurrence may be mildly unstable but quite tolerably so. If they increase catastrophically, then there is an exponentially growing solution of the recurrence. If you know that the function that you want actually corresponds to the growing solution, then you can keep the recurrence formula anyway (e.g., the case of the Bessel function $Y_n(x)$ for increasing n; see §6.5). If you don't know which solution your function corresponds to, you must at this point reject the recurrence formula. Notice that you can do this test *before* you go to the trouble of finding a numerical method for computing the two

starting functions $f_j(x)$ and $f_{j+1}(x)$: Stability is a property of the recurrence, not of the starting values.

An alternative heuristic procedure for testing stability is to replace the recurrence relation by a similar one that is linear with constant coefficients. For example, the relation (5.4.2) becomes

$$y_{n+1} - 2\gamma y_n + y_{n-1} = 0 \qquad (5.4.11)$$

where $\gamma \equiv n/x$ is treated as a constant. You solve such recurrence relations by trying solutions of the form $y_n = a^n$. Substituting into the above recurrence gives

$$a^2 - 2\gamma a + 1 = 0 \qquad \text{or} \qquad a = \gamma \pm \sqrt{\gamma^2 - 1} \qquad (5.4.12)$$

The recurrence is stable if $|a| \le 1$ for all solutions a. This holds (as you can verify) if $|\gamma| \le 1$ or $n \le x$. The recurrence (5.4.2) thus cannot be used, starting with $J_0(x)$ and $J_1(x)$, to compute $J_n(x)$ for large n.

Possibly you would at this point like the security of some real theorems on this subject (although we ourselves always follow one of the heuristic procedures). Here are two theorems, due to Perron [2]:

Theorem A. If in (5.4.9) $a_n \sim an^\alpha$, $b_n \sim bn^\beta$ as $n \to \infty$, and $\beta < 2\alpha$, then

$$g_{n+1}/g_n \sim -an^\alpha, \qquad f_{n+1}/f_n \sim -(b/a)n^{\beta-\alpha} \qquad (5.4.13)$$

and f_n is the minimal solution to (5.4.9).

Theorem B. Under the same conditions as Theorem A, but with $\beta = 2\alpha$, consider the *characteristic polynomial*

$$t^2 + at + b = 0 \qquad (5.4.14)$$

If the roots t_1 and t_2 of (5.4.14) have distinct moduli, $|t_1| > |t_2|$ say, then

$$g_{n+1}/g_n \sim t_1 n^\alpha, \qquad f_{n+1}/f_n \sim t_2 n^\alpha \qquad (5.4.15)$$

and f_n is again the minimal solution to (5.4.9). Cases other than those in these two theorems are inconclusive for the existence of minimal solutions. (For more on the stability of recurrences, see [3].)

How do you proceed if the solution that you desire *is* the minimal solution? The answer lies in that old aphorism, that every cloud has a silver lining: If a recurrence relation is catastrophically unstable in one direction, then that (undesired) solution will decrease very rapidly in the reverse direction. This means that you can start with *any* seed values for the consecutive f_j and f_{j+1} and (when you have gone enough steps in the stable direction) you will converge to the sequence of functions that you want, times an unknown normalization factor. If there is some other way to normalize the sequence (e.g., by a formula for the sum of the f_n's), then this can be a practical means of function evaluation. The method is called *Miller's algorithm.* An example often given [1,4] uses equation (5.4.2) in just this way, along with the normalization formula

$$1 = J_0(x) + 2J_2(x) + 2J_4(x) + 2J_6(x) + \cdots \qquad (5.4.16)$$

Incidentally, there is an important relation between three-term recurrence relations and *continued fractions*. Rewrite the recurrence relation (5.4.9) as

$$\frac{y_n}{y_{n-1}} = -\frac{b_n}{a_n + y_{n+1}/y_n} \tag{5.4.17}$$

Iterating this equation, starting with n, gives

$$\frac{y_n}{y_{n-1}} = -\frac{b_n}{a_n -}\,\frac{b_{n+1}}{a_{n+1} -}\cdots \tag{5.4.18}$$

Pincherle's theorem [2] tells us that (5.4.18) converges if and only if (5.4.9) has a minimal solution f_n, in which case it converges to f_n/f_{n-1}. This result, usually for the case $n = 1$ and combined with some way to determine f_0, underlies many of the practical methods for computing special functions that we give in the next chapter.

5.4.2 Clenshaw's Recurrence Formula

Clenshaw's recurrence formula [5] is an elegant and efficient way to evaluate a sum of coefficients times functions that obey a recurrence formula, e.g.,

$$f(\theta) = \sum_{k=0}^{N} c_k \cos k\theta \qquad \text{or} \qquad f(x) = \sum_{k=0}^{N} c_k P_k(x)$$

Here is how it works: Suppose that the desired sum is

$$f(x) = \sum_{k=0}^{N} c_k F_k(x) \tag{5.4.19}$$

and that F_k obeys the recurrence relation

$$F_{n+1}(x) = \alpha(n,x)F_n(x) + \beta(n,x)F_{n-1}(x) \tag{5.4.20}$$

for some functions $\alpha(n,x)$ and $\beta(n,x)$. Now define the quantities y_k $(k = N, N-1, \ldots, 1)$ by the recurrence

$$\begin{aligned} y_{N+2} &= y_{N+1} = 0 \\ y_k &= \alpha(k,x)y_{k+1} + \beta(k+1,x)y_{k+2} + c_k \qquad (k = N, N-1, \ldots, 1) \end{aligned} \tag{5.4.21}$$

If you solve equation (5.4.21) for c_k on the left, and then write out explicitly the sum (5.4.19), it will look (in part) like this:

$$\begin{aligned} f(x) = \cdots & \\ &+ [y_8 - \alpha(8,x)y_9 - \beta(9,x)y_{10}]F_8(x) \\ &+ [y_7 - \alpha(7,x)y_8 - \beta(8,x)y_9]F_7(x) \\ &+ [y_6 - \alpha(6,x)y_7 - \beta(7,x)y_8]F_6(x) \\ &+ [y_5 - \alpha(5,x)y_6 - \beta(6,x)y_7]F_5(x) \\ &+ \cdots \\ &+ [y_2 - \alpha(2,x)y_3 - \beta(3,x)y_4]F_2(x) \\ &+ [y_1 - \alpha(1,x)y_2 - \beta(2,x)y_3]F_1(x) \\ &+ [c_0 + \beta(1,x)y_2 - \beta(1,x)y_2]F_0(x) \end{aligned} \tag{5.4.22}$$

Notice that we have added and subtracted $\beta(1,x)y_2$ in the last line. If you examine the terms containing a factor of y_8 in (5.4.22), you will find that they sum to zero as a consequence of the recurrence relation (5.4.20); similarly for all the other y_k's down through y_2. The only surviving terms in (5.4.22) are

$$f(x) = \beta(1,x)F_0(x)y_2 + F_1(x)y_1 + F_0(x)c_0 \tag{5.4.23}$$

Equations (5.4.21) and (5.4.23) are *Clenshaw's recurrence formula* for doing the sum (5.4.19): You make one pass down through the y_k's using (5.4.21); when you have reached y_2 and y_1, you apply (5.4.23) to get the desired answer.

Clenshaw's recurrence as written above incorporates the coefficients c_k in a downward order, with k decreasing. At each stage, the effect of all previous c_k's is "remembered" as two coefficients that multiply the functions F_{k+1} and F_k (ultimately F_0 and F_1). If the functions F_k are small when k is large, *and* if the coefficients c_k are small when k is *small*, then the sum can be dominated by small F_k's. In this case, the remembered coefficients will involve a delicate cancellation and there can be a catastrophic loss of significance. An example would be to sum the trivial series

$$J_{15}(1) = 0 \times J_0(1) + 0 \times J_1(1) + \ldots + 0 \times J_{14}(1) + 1 \times J_{15}(1) \tag{5.4.24}$$

Here J_{15}, which is tiny, ends up represented as a canceling linear combination of J_0 and J_1, which are of order unity.

The solution in such cases is to use an alternative Clenshaw recurrence that incorporates the c_k's in an upward direction. The relevant equations are

$$y_{-2} = y_{-1} = 0 \tag{5.4.25}$$

$$y_k = \frac{1}{\beta(k+1,x)}[y_{k-2} - \alpha(k,x)y_{k-1} - c_k], \quad k = 0, 1, \ldots, N-1 \tag{5.4.26}$$

$$f(x) = c_N F_N(x) - \beta(N,x)F_{N-1}(x)y_{N-1} - F_N(x)y_{N-2} \tag{5.4.27}$$

The rare case where equations (5.4.25) – (5.4.27) should be used instead of equations (5.4.21) and (5.4.23) can be detected automatically by testing whether the operands in the first sum in (5.4.23) are opposite in sign and nearly equal in magnitude. Other than in this special case, Clenshaw's recurrence is always stable, independent of whether the recurrence for the functions F_k is stable in the upward or downward direction.

5.4.3 Parallel Evaluation of Linear Recurrence Relations

When desirable, linear recurrence relations can be evaluated with a lot of parallelism. Consider the general first-order linear recurrence relation

$$u_j = a_j + b_{j-1}u_{j-1}, \qquad j = 2, 3, \ldots, n \tag{5.4.28}$$

with initial value $u_1 = a_1$. To parallelize the recurrence, we can employ the powerful general strategy of *recursive doubling*. Write down equation (5.4.28) for $2j$ and for $2j-1$:

$$\begin{aligned} u_{2j} &= a_{2j} + b_{2j-1}u_{2j-1} \\ u_{2j-1} &= a_{2j-1} + b_{2j-2}u_{2j-2} \end{aligned} \tag{5.4.29}$$

Substitute the second of these equations into the first to eliminate u_{2j-1} and get

$$u_{2j} = (a_{2j} + a_{2j-1}b_{2j-1}) + (b_{2j-2}b_{2j-1})u_{2j-2} \tag{5.4.30}$$

This is a new recurrence of the same form as (5.4.28) but over only the even u_j, and hence involving only $n/2$ terms. Clearly we can continue this process recursively, halving the number of terms in the recurrence at each stage, until we are left with a recurrence of length 1 or 2 that we can do explicitly. Each time we finish a subpart of the recursion, we fill in the odd terms in the recurrence, using the second equation in (5.4.29). In practice, it's even easier than it sounds. The total number of operations is the same as for serial evaluation, but they are done in about $\log_2 n$ parallel steps.

There is a variant of recursive doubling, called *cyclic reduction*, that can be implemented with a straightforward iteration loop instead of a recursive procedure [6]. Here we start by writing down the recurrence (5.4.28) for all adjacent terms u_j and u_{j-1} (not just the even ones, as before). Eliminating u_{j-1}, just as in equation (5.4.30), gives

$$u_j = (a_j + a_{j-1}b_{j-1}) + (b_{j-2}b_{j-1})u_{j-2} \tag{5.4.31}$$

which is a first-order recurrence with new coefficients a'_j and b'_j. Repeating this process gives successive formulas for u_j in terms of $u_{j-2}, u_{j-4}, u_{j-8}, \ldots$. The procedure terminates when we reach u_{j-n} (for n a power of 2), which is zero for all j. Thus the last step gives u_j equal to the last set of a'_j's.

In cyclic reduction, the length of the vector u_j that is updated at each stage does not decrease by a factor of 2 at each stage, but rather only decreases from $\sim n$ to $\sim n/2$ during all $\log_2 n$ stages. Thus the total number of operations carried out is $O(n \log n)$, as opposed to $O(n)$ for recursive doubling. Whether this is important depends on the details of the computer's architecture.

Second-order recurrence relations can also be parallelized. Consider the second-order recurrence relation

$$y_j = a_j + b_{j-2}y_{j-1} + c_{j-2}y_{j-2}, \qquad j = 3, 4, \ldots, n \tag{5.4.32}$$

with initial values

$$y_1 = a_1, \qquad y_2 = a_2 \tag{5.4.33}$$

With this numbering scheme, you supply coefficients $a_1, \ldots, a_n$, $b_1, \ldots, b_{n-2}$, and $c_1, \ldots, c_{n-2}$. Rewrite the recurrence relation in the form [6]

$$\begin{pmatrix} y_j \\ y_{j+1} \end{pmatrix} = \begin{pmatrix} 0 \\ a_{j+1} \end{pmatrix} + \begin{pmatrix} 0 & 1 \\ c_{j-1} & b_{j-1} \end{pmatrix} \begin{pmatrix} y_{j-1} \\ y_j \end{pmatrix}, \qquad j = 2, \ldots, n-1 \tag{5.4.34}$$

that is,

$$\mathbf{u}_j = \mathbf{a}_j + \mathbf{b}_{j-1} \cdot \mathbf{u}_{j-1}, \qquad j = 2, \ldots, n-1 \tag{5.4.35}$$

where

$$\mathbf{u}_j = \begin{pmatrix} y_j \\ y_{j+1} \end{pmatrix}, \qquad \mathbf{a}_j = \begin{pmatrix} 0 \\ a_{j+1} \end{pmatrix}, \qquad \mathbf{b}_{j-1} = \begin{pmatrix} 0 & 1 \\ c_{j-1} & b_{j-1} \end{pmatrix} \tag{5.4.36}$$

and

$$\mathbf{u}_1 = \mathbf{a}_1 = \begin{pmatrix} y_1 \\ y_2 \end{pmatrix} = \begin{pmatrix} a_1 \\ a_2 \end{pmatrix} \tag{5.4.37}$$

This is a first-order recurrence relation for the vectors $\mathbf{u}_j$ and can be solved by either of the algorithms described above. The only difference is that the multiplications are matrix multiplications with the 2×2 matrices $\mathbf{b}_j$. After the first recursive call, the zeros in $\mathbf{a}$ and $\mathbf{b}$ are lost, so we have to write the routine for general two-dimensional vectors and matrices. Note that this algorithm does not avoid the potential instability problems associated with second-order recurrences that were discussed in §5.4.1. Also note that the algorithm generalizes in the obvious way to higher-order recurrences: An nth-order recurrence can be written as a first-order recurrence involving vectors and matrices of dimension n.

CITED REFERENCES AND FURTHER READING:

Abramowitz, M., and Stegun, I.A. 1964, *Handbook of Mathematical Functions* (Washington: National Bureau of Standards); reprinted 1968 (New York: Dover); online at `http://www.nr.com/aands`, pp. xiii, 697.[1]

Gautschi, W. 1967, "Computational Aspects of Three-Term Recurrence Relations," *SIAM Review*, vol. 9, pp. 24–82.[2]

Lakshmikantham, V., and Trigiante, D. 1988, *Theory of Difference Equations: Numerical Methods and Applications* (San Diego: Academic Press).[3]

Acton, F.S. 1970, *Numerical Methods That Work*; 1990, corrected edition (Washington, DC: Mathematical Association of America), pp. 20ff.[4]

Clenshaw, C.W. 1962, *Mathematical Tables*, vol. 5, National Physical Laboratory (London: H.M. Stationery Office).[5]

Dahlquist, G., and Bjorck, A. 1974, *Numerical Methods* (Englewood Cliffs, NJ: Prentice-Hall); reprinted 2003 (New York: Dover), §4.4.3, p. 111.

Goodwin, E.T. (ed.) 1961, *Modern Computing Methods*, 2nd ed. (New York: Philosophical Library), p. 76.

Hockney, R.W., and Jesshope, C.R. 1988, *Parallel Computers 2: Architecture, Programming, and Algorithms* (Bristol and Philadelphia: Adam Hilger), §5.2.4 and §5.4.2.[6]

5.5 Complex Arithmetic

Since C++ has a built-in class `complex`, you can generally let the compiler and the class library take care of complex arithmetic for you. Generally, but not always. For a program with only a small number of complex operations, you may want to code these yourself, in-line. Or, you may find that your compiler is not up to snuff: It is disconcertingly common to encounter complex operations that produce overflows or underflows when both the complex operands and the complex result are perfectly representable. This occurs, we think, because software companies mistake the implementation of complex arithmetic for a completely trivial task, not requiring any particular finesse.

Actually, complex arithmetic is not *quite* trivial. Addition and subtraction are done in the obvious way, performing the operation separately on the real and imaginary parts of the operands. Multiplication can also be done in the obvious way, with four multiplications, one addition, and one subtraction:

$$(a + ib)(c + id) = (ac - bd) + i(bc + ad) \tag{5.5.1}$$

(the addition sign before the i doesn't count; it just separates the real and imaginary parts notationally). But it is sometimes faster to multiply via

$$(a + ib)(c + id) = (ac - bd) + i[(a + b)(c + d) - ac - bd] \tag{5.5.2}$$

which has only three multiplications (ac, bd, $(a+b)(c+d)$), plus two additions and three subtractions. The total operations count is higher by two, but multiplication is a slow operation on some machines.

While it is true that intermediate results in equations (5.5.1) and (5.5.2) can overflow even when the final result is representable, this happens only when the final

answer is on the edge of representability. Not so for the complex modulus, if you or your compiler is misguided enough to compute it as

$$|a + ib| = \sqrt{a^2 + b^2} \qquad \text{(bad!)} \tag{5.5.3}$$

whose intermediate result will overflow if either a or b is as large as the square root of the largest representable number (e.g., 10^{19} as compared to 10^{38}). The right way to do the calculation is

$$|a + ib| = \begin{cases} |a|\sqrt{1 + (b/a)^2} & |a| \geq |b| \\ |b|\sqrt{1 + (a/b)^2} & |a| < |b| \end{cases} \tag{5.5.4}$$

Complex division should use a similar trick to prevent avoidable overflow, underflow, or loss of precision:

$$\frac{a + ib}{c + id} = \begin{cases} \dfrac{[a + b(d/c)] + i[b - a(d/c)]}{c + d(d/c)} & |c| \geq |d| \\ \dfrac{[a(c/d) + b] + i[b(c/d) - a]}{c(c/d) + d} & |c| < |d| \end{cases} \tag{5.5.5}$$

Of course you should calculate repeated subexpressions, like c/d or d/c, only once.

Complex square root is even more complicated, since we must both guard intermediate results and also enforce a chosen branch cut (here taken to be the negative real axis). To take the square root of $c + id$, first compute

$$w \equiv \begin{cases} 0 & c = d = 0 \\ \sqrt{|c|}\sqrt{\dfrac{1 + \sqrt{1 + (d/c)^2}}{2}} & |c| \geq |d| \\ \sqrt{|d|}\sqrt{\dfrac{|c/d| + \sqrt{1 + (c/d)^2}}{2}} & |c| < |d| \end{cases} \tag{5.5.6}$$

Then the answer is

$$\sqrt{c + id} = \begin{cases} 0 & w = 0 \\ w + i\left(\dfrac{d}{2w}\right) & w \neq 0, c \geq 0 \\ \dfrac{|d|}{2w} + iw & w \neq 0, c < 0, d \geq 0 \\ \dfrac{|d|}{2w} - iw & w \neq 0, c < 0, d < 0 \end{cases} \tag{5.5.7}$$

CITED REFERENCES AND FURTHER READING:

Midy, P., and Yakovlev, Y. 1991, "Computing Some Elementary Functions of a Complex Variable," *Mathematics and Computers in Simulation*, vol. 33, pp. 33–49.

Knuth, D.E. 1997, *Seminumerical Algorithms*, 3rd ed., vol. 2 of *The Art of Computer Programming* (Reading, MA: Addison-Wesley) [see solutions to exercises 4.2.1.16 and 4.6.4.41].

5.6 Quadratic and Cubic Equations

The roots of simple algebraic equations can be viewed as being functions of the equations' coefficients. We are taught these functions in elementary algebra. Yet, surprisingly many people don't know the right way to solve a quadratic equation with two real roots, or to obtain the roots of a cubic equation.

There are two ways to write the solution of the *quadratic equation*

$$ax^2 + bx + c = 0 \tag{5.6.1}$$

with real coefficients a, b, c, namely

$$x = \frac{-b \pm \sqrt{b^2 - 4ac}}{2a} \tag{5.6.2}$$

and

$$x = \frac{2c}{-b \pm \sqrt{b^2 - 4ac}} \tag{5.6.3}$$

If you use *either* (5.6.2) *or* (5.6.3) to get the two roots, you are asking for trouble: If either a or c (or both) is small, then one of the roots will involve the subtraction of b from a very nearly equal quantity (the discriminant); you will get that root very inaccurately. The correct way to compute the roots is

$$q \equiv -\frac{1}{2}\left[b + \operatorname{sgn}(b)\sqrt{b^2 - 4ac}\right] \tag{5.6.4}$$

Then the two roots are

$$x_1 = \frac{q}{a} \qquad \text{and} \qquad x_2 = \frac{c}{q} \tag{5.6.5}$$

If the coefficients a, b, c, are complex rather than real, then the above formulas still hold, except that in equation (5.6.4) the sign of the square root should be chosen so as to make

$$\operatorname{Re}(b^* \sqrt{b^2 - 4ac}) \geq 0 \tag{5.6.6}$$

where Re denotes the real part and asterisk denotes complex conjugation.

Apropos of quadratic equations, this seems a convenient place to recall that the inverse hyperbolic functions $\sinh^{-1}$ and $\cosh^{-1}$ are in fact just logarithms of solutions to such equations

$$\sinh^{-1}(x) = \ln\left(x + \sqrt{x^2 + 1}\right) \tag{5.6.7}$$

$$\cosh^{-1}(x) = \pm\ln\left(x + \sqrt{x^2 - 1}\right) \tag{5.6.8}$$

Equation (5.6.7) is numerically robust for $x \geq 0$. For negative x, use the symmetry $\sinh^{-1}(-x) = -\sinh^{-1}(x)$. Equation (5.6.8) is of course valid only for $x \geq 1$.

For the *cubic equation*

$$x^3 + ax^2 + bx + c = 0 \tag{5.6.9}$$

with real or complex coefficients a, b, c, first compute

$$Q \equiv \frac{a^2 - 3b}{9} \qquad \text{and} \qquad R \equiv \frac{2a^3 - 9ab + 27c}{54} \tag{5.6.10}$$

If Q and R are real (always true when a, b, c are real) *and* $R^2 < Q^3$, then the cubic equation has three real roots. Find them by computing

$$\theta = \arccos(R/\sqrt{Q^3}) \tag{5.6.11}$$

in terms of which the three roots are

$$\begin{aligned} x_1 &= -2\sqrt{Q}\cos\left(\frac{\theta}{3}\right) - \frac{a}{3} \\ x_2 &= -2\sqrt{Q}\cos\left(\frac{\theta + 2\pi}{3}\right) - \frac{a}{3} \\ x_3 &= -2\sqrt{Q}\cos\left(\frac{\theta - 2\pi}{3}\right) - \frac{a}{3} \end{aligned} \tag{5.6.12}$$

(This equation first appears in Chapter VI of François Viète's treatise "De emendatione," published in 1615!)

Otherwise, compute

$$A = -\left[R + \sqrt{R^2 - Q^3}\right]^{1/3} \tag{5.6.13}$$

where the sign of the square root is chosen to make

$$\text{Re}(R^* \sqrt{R^2 - Q^3}) \geq 0 \tag{5.6.14}$$

(asterisk again denoting complex conjugation). If Q and R are both real, equations (5.6.13) – (5.6.14) are equivalent to

$$A = -\text{sgn}(R)\left[|R| + \sqrt{R^2 - Q^3}\right]^{1/3} \tag{5.6.15}$$

where the positive square root is assumed. Next compute

$$B = \begin{cases} Q/A & (A \neq 0) \\ 0 & (A = 0) \end{cases} \tag{5.6.16}$$

in terms of which the three roots are

$$x_1 = (A + B) - \frac{a}{3} \tag{5.6.17}$$

(the single real root when a, b, c are real) and

$$\begin{aligned} x_2 &= -\frac{1}{2}(A + B) - \frac{a}{3} + i\frac{\sqrt{3}}{2}(A - B) \\ x_3 &= -\frac{1}{2}(A + B) - \frac{a}{3} - i\frac{\sqrt{3}}{2}(A - B) \end{aligned} \tag{5.6.18}$$

(in that same case, a complex-conjugate pair). Equations (5.6.13) – (5.6.16) are arranged both to minimize roundoff error and also (as pointed out by A.J. Glassman) to ensure that no choice of branch for the complex cube root can result in the spurious loss of a distinct root.

If you need to solve many cubic equations with only slightly different coefficients, it is more efficient to use Newton's method (§9.4).

CITED REFERENCES AND FURTHER READING:

Weast, R.C. (ed.) 1967, *Handbook of Tables for Mathematics*, 3rd ed. (Cleveland: The Chemical Rubber Co.), pp. 130–133.

Pachner, J. 1983, *Handbook of Numerical Analysis Applications* (New York: McGraw-Hill), §6.1.

McKelvey, J.P. 1984, "Simple Transcendental Expressions for the Roots of Cubic Equations," *American Journal of Physics*, vol. 52, pp. 269–270; see also vol. 53, p. 775, and vol. 55, pp. 374–375.

5.7 Numerical Derivatives

Imagine that you have a procedure that computes a function $f(x)$, and now you want to compute its derivative $f'(x)$. Easy, right? The definition of the derivative, the limit as $h \to 0$ of

$$f'(x) \approx \frac{f(x+h) - f(x)}{h} \tag{5.7.1}$$

practically suggests the program: Pick a small value h; evaluate $f(x+h)$; you probably have $f(x)$ already evaluated, but if not, do it too; finally, apply equation (5.7.1). What more needs to be said?

Quite a lot, actually. Applied uncritically, the above procedure is almost guaranteed to produce inaccurate results. Applied properly, it can be the right way to compute a derivative only when the function f is *fiercely* expensive to compute; when you already have invested in computing $f(x)$; and when, therefore, you want to get the derivative in no more than a single additional function evaluation. In such a situation, the remaining issue is to choose h properly, an issue we now discuss.

There are two sources of error in equation (5.7.1), truncation error and roundoff error. The truncation error comes from higher terms in the Taylor series expansion,

$$f(x+h) = f(x) + hf'(x) + \tfrac{1}{2}h^2 f''(x) + \tfrac{1}{6}h^3 f'''(x) + \cdots \tag{5.7.2}$$

whence

$$\frac{f(x+h) - f(x)}{h} = f' + \frac{1}{2}hf'' + \cdots \tag{5.7.3}$$

The roundoff error has various contributions. First there is roundoff error in h: Suppose, by way of an example, that you are at a point $x = 10.3$ and you blindly choose $h = 0.0001$. Neither $x = 10.3$ nor $x + h = 10.30001$ is a number with an exact representation in binary; each is therefore represented with some fractional error characteristic of the machine's floating-point format, ϵ_m, whose value in single precision may be $\sim 10^{-7}$. The error in the *effective* value of h, namely the difference between $x + h$ and x as represented in the machine, is therefore on the order of $\epsilon_m x$,

which implies a fractional error in h of order $\sim \epsilon_m x/h \sim 10^{-2}$! By equation (5.7.1), this immediately implies at least the same large fractional error in the derivative.

We arrive at Lesson 1: Always choose h so that $x+h$ and x differ by an exactly representable number. This can usually be accomplished by the program steps

$$\begin{aligned} \texttt{temp} &= x + h \\ h &= \texttt{temp} - x \end{aligned} \tag{5.7.4}$$

Some optimizing compilers, and some computers whose floating-point chips have higher internal accuracy than is stored externally, can foil this trick; if so, it is usually enough to declare `temp` as `volatile`, or else to call a dummy function `donothing(temp)` between the two equations (5.7.4). This forces `temp` into and out of addressable memory.

With h an "exact" number, the roundoff error in equation (5.7.1) is approximately $e_r \sim \epsilon_f |f(x)/h|$. Here ϵ_f is the fractional accuracy with which f is computed; for a simple function this may be comparable to the machine accuracy, $\epsilon_f \approx \epsilon_m$, but for a complicated calculation with additional sources of inaccuracy it may be larger. The truncation error in equation (5.7.3) is on the order of $e_t \sim |hf''(x)|$. Varying h to minimize the sum $e_r + e_t$ gives the optimal choice of h,

$$h \sim \sqrt{\frac{\epsilon_f f}{f''}} \approx \sqrt{\epsilon_f}\, x_c \tag{5.7.5}$$

where $x_c \equiv (f/f'')^{1/2}$ is the "curvature scale" of the function f or the "characteristic scale" over which it changes. In the absence of any other information, one often assumes $x_c = x$ (except near $x = 0$, where some other estimate of the typical x scale should be used).

With the choice of equation (5.7.5), the fractional accuracy of the computed derivative is

$$(e_r + e_t)/|f'| \sim \sqrt{\epsilon_f}(ff''/f'^2)^{1/2} \sim \sqrt{\epsilon_f} \tag{5.7.6}$$

Here the last order-of-magnitude equality assumes that f, f', and f'' all share the same characteristic length scale, which is usually the case. One sees that the simple finite difference equation (5.7.1) gives *at best* only the square root of the machine accuracy ϵ_m.

If you can afford two function evaluations for each derivative calculation, then it is significantly better to use the symmetrized form

$$f'(x) \approx \frac{f(x+h) - f(x-h)}{2h} \tag{5.7.7}$$

In this case, by equation (5.7.2), the truncation error is $e_t \sim h^2 f'''$. The roundoff error e_r is about the same as before. The optimal choice of h, by a short calculation analogous to the one above, is now

$$h \sim \left(\frac{\epsilon_f f}{f'''}\right)^{1/3} \sim (\epsilon_f)^{1/3} x_c \tag{5.7.8}$$

and the fractional error is

$$(e_r + e_t)/|f'| \sim (\epsilon_f)^{2/3} f^{2/3} (f''')^{1/3}/f' \sim (\epsilon_f)^{2/3} \tag{5.7.9}$$

which will typically be an order of magnitude (single precision) or two orders of magnitude (double precision) *better* than equation (5.7.6). We have arrived at Lesson 2: Choose h to be *the correct* power of ϵ_f or ϵ_m times a characteristic scale x_c.

You can easily derive the correct powers for other cases [1]. For a function of two dimensions, for example, and the mixed derivative formula

$$\frac{\partial^2 f}{\partial x \partial y} = \frac{[f(x+h,y+h) - f(x+h,y-h)] - [f(x-h,y+h) - f(x-h,y-h)]}{4h^2} \tag{5.7.10}$$

the correct scaling is $h \sim \epsilon_f^{1/4} x_c$.

It is disappointing, certainly, that no simple finite difference formula like equation (5.7.1) or (5.7.7) gives an accuracy comparable to the machine accuracy ϵ_m, or even the lower accuracy to which f is evaluated, ϵ_f. Are there no better methods?

Yes, there are. All, however, involve exploration of the function's behavior over scales comparable to x_c, plus some assumption of smoothness, or analyticity, so that the high-order terms in a Taylor expansion like equation (5.7.2) have some meaning. Such methods also involve multiple evaluations of the function f, so their increased accuracy must be weighed against increased cost.

The general idea of "Richardson's deferred approach to the limit" is particularly attractive. For numerical integrals, that idea leads to so-called Romberg integration (for review, see §4.3). For derivatives, one seeks to extrapolate, to $h \to 0$, the result of finite difference calculations with smaller and smaller finite values of h. By the use of Neville's algorithm (§3.2), one uses each new finite difference calculation to produce both an extrapolation of higher order and also extrapolations of previous, lower, orders but with smaller scales h. Ridders [2] has given a nice implementation of this idea; the following program, `dfridr`, is based on his algorithm, modified by an improved termination criterion. Input to the routine is a function f (called `func`), a position x, and a *largest* stepsize `h` (more analogous to what we have called x_c above than to what we have called h). Output is the returned value of the derivative and an estimate of its error, `err`.

dfridr.h

```
template<class T>
Doub dfridr(T &func, const Doub x, const Doub h, Doub &err)
```
Returns the derivative of a function `func` at a point `x` by Ridders' method of polynomial extrapolation. The value `h` is input as an estimated initial stepsize; it need not be small, but rather should be an increment in `x` over which `func` changes substantially. An estimate of the error in the derivative is returned as `err`.
```
{
    const Int ntab=10;                         Sets maximum size of tableau.
    const Doub con=1.4, con2=(con*con);        Stepsize decreased by CON at each iteration.
    const Doub big=numeric_limits<Doub>::max();
    const Doub safe=2.0;                       Return when error is SAFE worse than the
    Int i,j;                                       best so far.
    Doub errt,fac,hh,ans;
    MatDoub a(ntab,ntab);
    if (h == 0.0) throw("h must be nonzero in dfridr.");
    hh=h;
    a[0][0]=(func(x+hh)-func(x-hh))/(2.0*hh);
    err=big;
    for (i=1;i<ntab;i++) {
```
Successive columns in the Neville tableau will go to smaller stepsizes and higher orders of extrapolation.
```
        hh /= con;
        a[0][i]=(func(x+hh)-func(x-hh))/(2.0*hh);      Try new, smaller stepsize.
        fac=con2;
```

```
        for (j=1;j<=i;j++) {          Compute extrapolations of various orders, requiring
            a[j][i]=(a[j-1][i]*fac-a[j-1][i-1])/(fac-1.0);      no new function eval-
            fac=con2*fac;                                        uations.
            errt=MAX(abs(a[j][i]-a[j-1][i]),abs(a[j][i]-a[j-1][i-1]));
            The error strategy is to compare each new extrapolation to one order lower, both
            at the present stepsize and the previous one.
            if (errt <= err) {        If error is decreased, save the improved answer.
                err=errt;
                ans=a[j][i];
            }
        }
        if (abs(a[i][i]-a[i-1][i-1]) >= safe*err) break;
        If higher order is worse by a significant factor SAFE, then quit early.
    }
    return ans;
}
```

In dfridr, the number of evaluations of func is typically 6 to 12, but is allowed to be as great as 2×NTAB. As a function of input h, it is typical for the accuracy to get *better* as h is made larger, until a sudden point is reached where nonsensical extrapolation produces an early return with a large error. You should therefore choose a fairly large value for h but monitor the returned value err, decreasing h if it is not small. For functions whose characteristic x scale is of order unity, we typically take h to be a few tenths.

Besides Ridders' method, there are other possible techniques. If your function is fairly smooth, and you know that you will want to evaluate its derivative many times at arbitrary points in some interval, then it makes sense to construct a Chebyshev polynomial approximation to the function in that interval, and to evaluate the derivative directly from the resulting Chebyshev coefficients. This method is described in §5.8 – §5.9, following.

Another technique applies when the function consists of data that is tabulated at equally spaced intervals, and perhaps also noisy. One might then want, at each point, to least-squares *fit* a polynomial of some degree M, using an additional number n_L of points to the left and some number n_R of points to the right of each desired x value. The estimated derivative is then the derivative of the resulting fitted polynomial. A very efficient way to do this construction is via Savitzky-Golay smoothing filters, which will be discussed later, in §14.9. There we will give a routine for getting filter coefficients that not only construct the fitting polynomial but, in the accumulation of a single sum of data points times filter coefficients, evaluate it as well. In fact, the routine given, savgol, has an argument ld that determines which derivative of the fitted polynomial is evaluated. For the first derivative, the appropriate setting is ld=1, and the value of the derivative is the accumulated sum divided by the sampling interval h.

CITED REFERENCES AND FURTHER READING:

Dennis, J.E., and Schnabel, R.B. 1983, *Numerical Methods for Unconstrained Optimization and Nonlinear Equations*; reprinted 1996 (Philadelphia: S.I.A.M.), §5.4 – §5.6.[1]

Ridders, C.J.F. 1982, "Accurate computation of $F'(x)$ and $F'(x)F''(x)$," *Advances in Engineering Software*, vol. 4, no. 2, pp. 75–76.[2]

5.8 Chebyshev Approximation

The Chebyshev polynomial of degree n is denoted $T_n(x)$ and is given by the explicit formula

$$T_n(x) = \cos(n \arccos x) \tag{5.8.1}$$

This may look trigonometric at first glance (and there is in fact a close relation between the Chebyshev polynomials and the discrete Fourier transform); however, (5.8.1) can be combined with trigonometric identities to yield explicit expressions for $T_n(x)$ (see Figure 5.8.1):

$$\begin{aligned} T_0(x) &= 1 \\ T_1(x) &= x \\ T_2(x) &= 2x^2 - 1 \\ T_3(x) &= 4x^3 - 3x \\ T_4(x) &= 8x^4 - 8x^2 + 1 \\ &\cdots \\ T_{n+1}(x) &= 2xT_n(x) - T_{n-1}(x) \quad n \geq 1. \end{aligned} \tag{5.8.2}$$

(There also exist inverse formulas for the powers of x in terms of the T_n's — see, e.g., [1].)

The Chebyshev polynomials are orthogonal in the interval $[-1, 1]$ over a weight $(1 - x^2)^{-1/2}$. In particular,

$$\int_{-1}^{1} \frac{T_i(x)T_j(x)}{\sqrt{1 - x^2}} dx = \begin{cases} 0 & i \neq j \\ \pi/2 & i = j \neq 0 \\ \pi & i = j = 0 \end{cases} \tag{5.8.3}$$

The polynomial $T_n(x)$ has n zeros in the interval $[-1, 1]$, and they are located at the points

$$x = \cos\left(\frac{\pi(k + \frac{1}{2})}{n}\right) \qquad k = 0, 1, \ldots, n - 1 \tag{5.8.4}$$

In this same interval there are $n + 1$ extrema (maxima and minima), located at

$$x = \cos\left(\frac{\pi k}{n}\right) \qquad k = 0, 1, \ldots, n \tag{5.8.5}$$

At all of the maxima $T_n(x) = 1$, while at all of the minima $T_n(x) = -1$; it is precisely this property that makes the Chebyshev polynomials so useful in polynomial approximation of functions.

The Chebyshev polynomials satisfy a discrete orthogonality relation as well as the continuous one (5.8.3): If $x_k (k = 0, \ldots, m - 1)$ are the m zeros of $T_m(x)$ given by (5.8.4), and if $i, j < m$, then

$$\sum_{k=0}^{m-1} T_i(x_k)T_j(x_k) = \begin{cases} 0 & i \neq j \\ m/2 & i = j \neq 0 \\ m & i = j = 0 \end{cases} \tag{5.8.6}$$

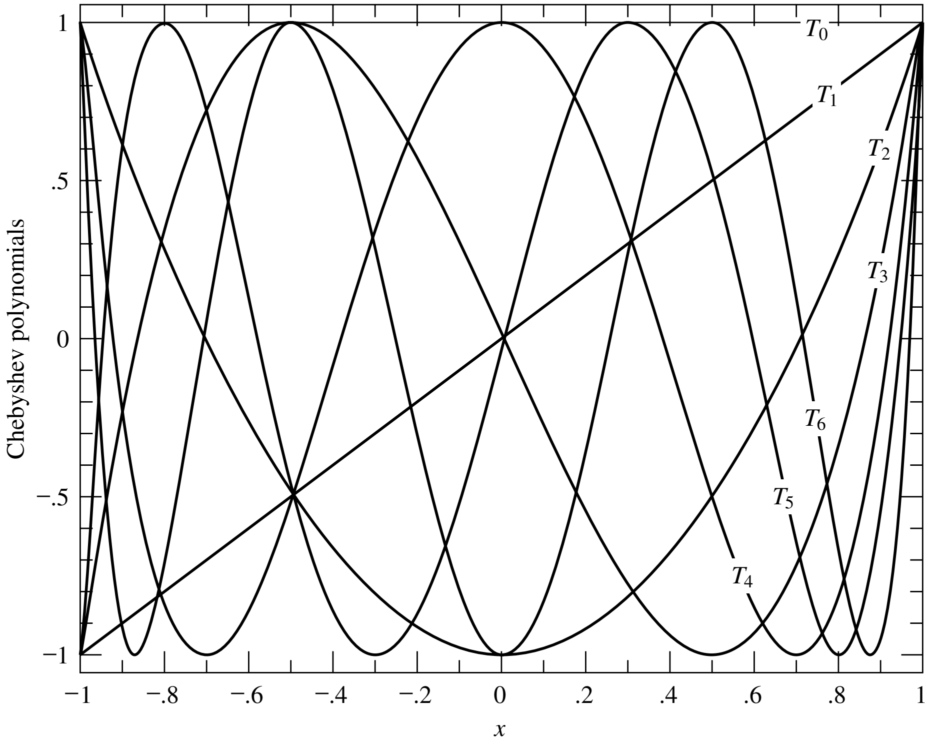

Figure 5.8.1. Chebyshev polynomials $T_0(x)$ through $T_6(x)$. Note that T_j has j roots in the interval $(-1, 1)$ and that all the polynomials are bounded between ± 1.

It is not too difficult to combine equations (5.8.1), (5.8.4), and (5.8.6) to prove the following theorem: If $f(x)$ is an arbitrary function in the interval $[-1, 1]$, and if N coefficients c_j, $j = 0, \ldots, N-1$, are defined by

$$\begin{aligned} c_j &= \frac{2}{N} \sum_{k=0}^{N-1} f(x_k) T_j(x_k) \\ &= \frac{2}{N} \sum_{k=0}^{N-1} f\left[\cos\left(\frac{\pi(k+\frac{1}{2})}{N}\right)\right] \cos\left(\frac{\pi j(k+\frac{1}{2})}{N}\right) \end{aligned} \tag{5.8.7}$$

then the approximation formula

$$f(x) \approx \left[\sum_{k=0}^{N-1} c_k T_k(x)\right] - \frac{1}{2} c_0 \tag{5.8.8}$$

is *exact* for x equal to all of the N zeros of $T_N(x)$.

For a fixed N, equation (5.8.8) is a polynomial in x that approximates the function $f(x)$ in the interval $[-1, 1]$ (where all the zeros of $T_N(x)$ are located). Why is this particular approximating polynomial better than any other one, exact on some other set of N points? The answer is *not* that (5.8.8) is necessarily more accurate

than some other approximating polynomial of the same order N (for some specified definition of "accurate"), but rather that (5.8.8) can be truncated to a polynomial of *lower* degree $m \ll N$ in a very graceful way, one that *does* yield the "most accurate" approximation of degree m (in a sense that can be made precise). Suppose N is so large that (5.8.8) is virtually a perfect approximation of $f(x)$. Now consider the truncated approximation

$$f(x) \approx \left[\sum_{k=0}^{m-1} c_k T_k(x)\right] - \frac{1}{2}c_0 \tag{5.8.9}$$

with the same c_j's, computed from (5.8.7). Since the $T_k(x)$'s are all bounded between ± 1, the difference between (5.8.9) and (5.8.8) can be no larger than the sum of the neglected c_k's ($k = m, \ldots, N-1$). In fact, if the c_k's are rapidly decreasing (which is the typical case), then the error is dominated by $c_m T_m(x)$, an oscillatory function with $m + 1$ equal extrema distributed smoothly over the interval $[-1, 1]$. This smooth spreading out of the error is a very important property: The Chebyshev approximation (5.8.9) is very nearly the same polynomial as that holy grail of approximating polynomials the *minimax polynomial*, which (among all polynomials of the same degree) has the smallest maximum deviation from the true function $f(x)$. The minimax polynomial is very difficult to find; the Chebyshev approximating polynomial is almost identical and is very easy to compute!

So, given some (perhaps difficult) means of computing the function $f(x)$, we now need algorithms for implementing (5.8.7) and (after inspection of the resulting c_k's and choice of a truncating value m) evaluating (5.8.9). The latter equation then becomes an easy way of computing $f(x)$ for all subsequent time.

The first of these tasks is straightforward. A generalization of equation (5.8.7) that is here implemented is to allow the range of approximation to be between two arbitrary limits a and b, instead of just -1 to 1. This is effected by a change of variable

$$y \equiv \frac{x - \frac{1}{2}(b+a)}{\frac{1}{2}(b-a)} \tag{5.8.10}$$

and by the approximation of $f(x)$ by a Chebyshev polynomial in y.

It will be convenient for us to group a number of functions related to Chebyshev polynomials into a single object, even though discussion of their specifics is spread out over §5.8 – §5.11:

chebyshev.h

```
struct Chebyshev {
Object for Chebyshev approximation and related methods.
    Int n,m;                        Number of total, and truncated, coefficients.
    VecDoub c;
    Doub a,b;                       Approximation interval.

    Chebyshev(Doub func(Doub), Doub aa, Doub bb, Int nn);
    Constructor. Approximate the function func in the interval [aa,bb] with nn terms.
    Chebyshev(VecDoub &cc, Doub aa, Doub bb)
        : n(cc.size()), m(n), c(cc), a(aa), b(bb) {}
    Constructor from previously computed coefficients.
    Int setm(Doub thresh) {while (m>1 && abs(c[m-1])<thresh) m--; return m;}
    Set m, the number of coefficients after truncating to an error level thresh, and return the
    value set.
```

```
    Doub eval(Doub x, Int m);
    inline Doub operator() (Doub x) {return eval(x,m);}
    Return a value for the Chebyshev fit, either using the stored m or else overriding it.

    Chebyshev derivative();          See §5.9.
    Chebyshev integral();

    VecDoub polycofs(Int m);         See §5.10.
    inline VecDoub polycofs() {return polycofs(m);}
    Chebyshev(VecDoub &pc);          See §5.11.

};
```

The first constructor, the one with an arbitrary function `func` as its first argument, calculates and saves `nn` Chebyshev coefficients that approximate `func` in the range `aa` to `bb`. (You can ignore for now the second constructor, which simply makes a `Chebyshev` object from already-calculated data.) Let us also note the method `setm`, which provides a quick way to truncate the Chebyshev series by (in effect) deleting, from the right, all coefficients smaller in magnitude than some threshold `thresh`.

chebyshev.h

```
Chebyshev::Chebyshev(Doub func(Doub), Doub aa, Doub bb, Int nn=50)
    : n(nn), m(nn), c(n), a(aa), b(bb)
```

Chebyshev fit: Given a function `func`, lower and upper limits of the interval [a,b], compute and save `nn` coefficients of the Chebyshev approximation such that $\mathtt{func}(x) \approx [\sum_{k=0}^{\mathtt{nn}-1} c_k T_k(y)] - c_0/2$, where y and x are related by (5.8.10). This routine is intended to be called with moderately large n (e.g., 30 or 50), the array of c's subsequently to be truncated at the smaller value m such that c_m and subsequent elements are negligible.

```
{
    const Doub pi=3.141592653589793;
    Int k,j;
    Doub fac,bpa,bma,y,sum;
    VecDoub f(n);
    bma=0.5*(b-a);
    bpa=0.5*(b+a);
    for (k=0;k<n;k++) {                 We evaluate the function at the n points required
        y=cos(pi*(k+0.5)/n);                by (5.8.7).
        f[k]=func(y*bma+bpa);
    }
    fac=2.0/n;
    for (j=0;j<n;j++) {                 Now evaluate (5.8.7).
        sum=0.0;
        for (k=0;k<n;k++)
            sum += f[k]*cos(pi*j*(k+0.5)/n);
        c[j]=fac*sum;
    }
}
```

If you find that the constructor's execution time is dominated by the calculation of N^2 cosines, rather than by the N evaluations of your function, then you should look ahead to §12.3, especially equation (12.4.16), which shows how fast cosine transform methods can be used to evaluate equation (5.8.7).

Now that we have the Chebyshev coefficients, how do we evaluate the approximation? One could use the recurrence relation of equation (5.8.2) to generate values for $T_k(x)$ from $T_0 = 1, T_1 = x$, while also accumulating the sum of (5.8.9). It is better to use Clenshaw's recurrence formula (§5.4), effecting the two processes simultaneously. Applied to the Chebyshev series (5.8.9), the recurrence is

$$\begin{aligned} d_{m+1} &\equiv d_m \equiv 0 \\ d_j &= 2x d_{j+1} - d_{j+2} + c_j \qquad j = m-1, m-2, \dots, 1 \\ f(x) &\equiv d_0 = x d_1 - d_2 + \tfrac{1}{2} c_0 \end{aligned} \tag{5.8.11}$$

chebyshev.h

```
Doub Chebyshev::eval(Doub x, Int m)
```

Chebyshev evaluation: The Chebyshev polynomial $\sum_{k=0}^{m-1} c_k T_k(y) - c_0/2$ is evaluated at a point $y = [x - (b + a)/2]/[(b - a)/2]$, and the result is returned as the function value.

```
{
    Doub d=0.0,dd=0.0,sv,y,y2;
    Int j;
    if ((x-a)*(x-b) > 0.0) throw("x not in range in Chebyshev::eval");
    y2=2.0*(y=(2.0*x-a-b)/(b-a));            Change of variable.
    for (j=m-1;j>0;j--) {                    Clenshaw's recurrence.
        sv=d;
        d=y2*d-dd+c[j];
        dd=sv;
    }
    return y*d-dd+0.5*c[0];                  Last step is different.
}
```

The method `eval` has an argument for specifying how many leading coefficients `m` should be used in the evaluation. If you simply want to use a stored value of `m` that was set by a previous call to `setm` (or, by hand, by you), then you can use the `Chebyshev` object as a functor. For example,

```
Chebyshev approxfunc(func,0.,1.,50);
approxfunc.setm(1.e-8);
...
y = approxfunc(x);
```

If we are approximating an *even* function on the interval $[-1, 1]$, its expansion will involve only even Chebyshev polynomials. It is wasteful to construct a `Chebyshev` object with all the odd coefficients zero [2]. Instead, using the half-angle identity for the cosine in equation (5.8.1), we get the relation

$$T_{2n}(x) = T_n(2x^2 - 1) \tag{5.8.12}$$

Thus we can construct a more efficient `Chebyshev` object for even functions simply by replacing the function's argument x by $2x^2 - 1$, and likewise when we evaluate the Chebyshev approximation.

An odd function will have an expansion involving only odd Chebyshev polynomials. It is best to rewrite it as an expansion for the function $f(x)/x$, which involves only even Chebyshev polynomials. This has the added benefit of giving accurate values for $f(x)/x$ near $x = 0$. Don't try to construct the series by evaluating $f(x)/x$ numerically, however. Rather, the coefficients c'_n for $f(x)/x$ can be found from those for $f(x)$ by recurrence:

$$\begin{aligned} c'_{N+1} &= 0 \\ c'_{n-1} &= 2c_n - c'_{n+1}, \qquad n = N-1, N-3, \dots \end{aligned} \tag{5.8.13}$$

Equation (5.8.13) follows from the recurrence relation in equation (5.8.2).

If you insist on evaluating an odd Chebyshev series, the efficient way is to once again to replace x by $y = 2x^2 - 1$ as the argument of your function. Now, however,

you must also change the last formula in equation (5.8.11) to be

$$f(x) = x[(2y-1)d_1 - d_2 + c_0] \tag{5.8.14}$$

and change the corresponding line in `eval`.

5.8.1 Chebyshev and Exponential Convergence

Since first mentioning *truncation error* in §1.1, we have seen many examples of algorithms with an adjustable order, say M, such that the truncation error decreases as the Mth power of something. Examples include most of the interpolation methods in Chapter 3 and most of the quadrature methods in Chapter 4. In these examples there is also another parameter, N, which is the number of points at which a function will be evaluated.

We have many times warned that "higher order does not necessarily give higher accuracy." That remains good advice when N is held fixed while M is increased. However, a recently emerging theme in many areas of scientific computation is the use of methods that allow, in very special cases, M and N to be increased *together*, with the result that errors not only do decrease with higher order, but decrease exponentially!

The common thread in almost all of these relatively new methods is the remarkable fact that *infinitely smooth* functions become *exponentially* well determined by N sample points as N is increased. Thus, mere power-law convergence may be just a consequence of either (i) functions that are not smooth enough, or (ii) endpoint effects.

We already saw several examples of this in Chapter 4. In §4.1 we pointed out that high-order quadrature rules can have interior weights of unity, just like the trapezoidal rule; all of the "high-orderness" is obtained by a proper treatment near the boundaries. In §4.5 we further saw that variable transformations that push the boundaries off to infinity produce rapidly converging quadrature algorithms. In §4.5.1 we in fact proved exponential convergence, as a consequence of the Euler-Maclaurin formula. Then in §4.6 we remarked on the fact that the convergence of Gaussian quadratures could be exponentially rapid (an example, in the language above, of increasing M and N simultaneously).

Chebyshev approximation can be exponentially convergent for a different (though related) reason: Smooth *periodic* functions avoid endpoint effects by not having endpoints at all! Chebyshev approximation can be viewed as mapping the x interval $[-1, 1]$ onto the angular interval $[0, \pi]$ (cf. equations 5.8.4 and 5.8.5) in such a way that any infinitely smooth function on the interval $[-1, 1]$ becomes an infinitely smooth, even, periodic function on $[0, 2\pi]$. Figure 5.8.2 shows the idea geometrically. By projecting the abscissas onto a semicircle, a half-period is produced. The other half-period is obtained by reflection, or could be imagined as the result of projecting the function onto an identical lower semicircle. The zeros of the Chebyshev polynomial, or nodes of a Chebyshev approximation, are equally spaced on the circle, where the Chebyshev polynomial itself is a cosine function (cf. equation 5.8.1). This illustrates the close connection between Chebyshev approximation and periodic functions on the circle; in Chapter 12, we will apply the discrete Fourier transform to such functions in an almost equivalent way (§12.4.2).

The reason that Chebyshev works so well (and also why Gaussian quadratures work so well) is thus seen to be intimately related to the special way that the the

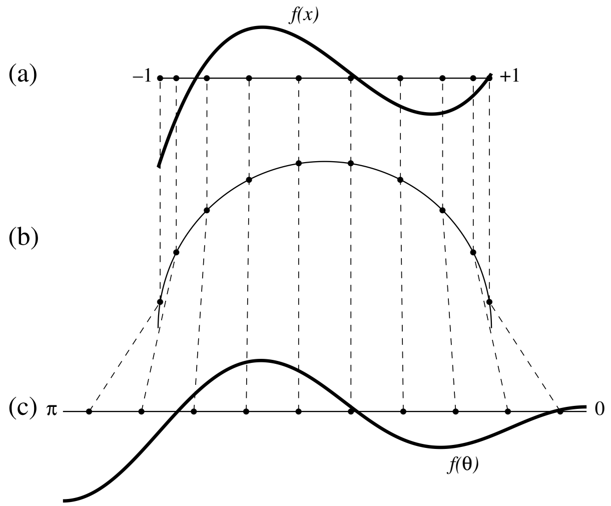

Figure 5.8.2. Geometrical construction showing how Chebyshev approximation is related to periodic functions. A smooth function on the interval is plotted in (a). In (b), the abscissas are mapped to a semicircle. In (c), the semicircle is unrolled. Because of the semicircle's vertical tangents, the function is now nearly constant at the endpoints. In fact, if reflected into the interval $[\pi, 2\pi]$, it is a smooth, even, periodic function on $[0, 2\pi]$.

sample points are bunched up near the endpoints of the interval. Any function that is bounded on the interval will have a convergent Chebyshev approximation as $N \to \infty$, even if there are nearby poles in the complex plane. For functions that are not infinitely smooth, the actual rate of convergence depends on the smoothness of the function: the more deriviatives that are bounded, the greater the convergence rate. For the special case of a C^∞ function, the convergence is exponential. In §3.0, in connection with polynomial interpolation, we mentioned the other side of the coin: equally spaced samples on the interval are about the *worst* possible geometry and often lead to ill-conditioned problems.

Use of the sampling theorem (§4.5, §6.9, §12.1, §13.11) is often closely associated with exponentially convergent methods. We will return to many of the concepts of exponentially convergent methods when we discuss spectral methods for partial differential equations in §20.7.

CITED REFERENCES AND FURTHER READING:

Arfken, G. 1970, *Mathematical Methods for Physicists*, 2nd ed. (New York: Academic Press), p. 631.[1]

Clenshaw, C.W. 1962, *Mathematical Tables*, vol. 5, National Physical Laboratory (London: H.M. Stationery Office).[2]

Goodwin, E.T. (ed.) 1961, *Modern Computing Methods*, 2nd ed. (New York: Philosophical Library), Chapter 8.

Dahlquist, G., and Bjorck, A. 1974, *Numerical Methods* (Englewood Cliffs, NJ: Prentice-Hall); reprinted 2003 (New York: Dover), §4.4.1, p. 104.

Johnson, L.W., and Riess, R.D. 1982, *Numerical Analysis*, 2nd ed. (Reading, MA: Addison-Wesley), §6.5.2, p. 334.

Carnahan, B., Luther, H.A., and Wilkes, J.O. 1969, *Applied Numerical Methods* (New York: Wiley), §1.10, p. 39.

5.9 Derivatives or Integrals of a Chebyshev-Approximated Function

If you have obtained the Chebyshev coefficients that approximate a function in a certain range (e.g., from chebft in §5.8), then it is a simple matter to transform them to Chebyshev coefficients corresponding to the derivative or integral of the function. Having done this, you can evaluate the derivative or integral just as if it were a function that you had Chebyshev-fitted *ab initio*.

The relevant formulas are these: If c_i, $i = 0, \ldots, m-1$ are the coefficients that approximate a function f in equation (5.8.9), C_i are the coefficients that approximate the indefinite integral of f, and c'_i are the coefficients that approximate the derivative of f, then

$$C_i = \frac{c_{i-1} - c_{i+1}}{2i} \qquad (i > 0) \tag{5.9.1}$$

$$c'_{i-1} = c'_{i+1} + 2i\,c_i \qquad (i = m-1, m-2, \ldots, 1) \tag{5.9.2}$$

Equation (5.9.1) is augmented by an arbitrary choice of C_0, corresponding to an arbitrary constant of integration. Equation (5.9.2), which is a recurrence, is started with the values $c'_m = c'_{m-1} = 0$, corresponding to no information about the $m+1$st Chebyshev coefficient of the original function f.

Here are routines for implementing equations (5.9.1) and (5.9.2). Each returns a new Chebyshev object on which you can setm, call eval, or use directly as a functor.

chebyshev.h

```
Chebyshev Chebyshev::derivative()
```
Return a new Chebyshev object that approximates the derivative of the existing function over the same range [a,b].
```
{
    Int j;
    Doub con;
    VecDoub cder(n);
    cder[n-1]=0.0;                              n-1 and n-2 are special cases.
    cder[n-2]=2*(n-1)*c[n-1];
    for (j=n-2;j>0;j--)                         Equation (5.9.2).
        cder[j-1]=cder[j+1]+2*j*c[j];
    con=2.0/(b-a);
    for (j=0;j<n;j++) cder[j] *= con;           Normalize to the interval b-a.
    return Chebyshev(cder,a,b);
}
```

chebyshev.h

```
Chebyshev Chebyshev::integral()
```
Return a new Chebyshev object that approximates the indefinite integral of the existing function over the same range [a,b]. The constant of integration is set so that the integral vanishes at a.
```
{
    Int j;
    Doub sum=0.0,fac=1.0,con;
    VecDoub cint(n);
    con=0.25*(b-a);                          Factor that normalizes to the interval b-a.
    for (j=1;j<n-1;j++) {
        cint[j]=con*(c[j-1]-c[j+1])/j;       Equation (5.9.1).
        sum += fac*cint[j];                  Accumulates the constant of integration.
        fac = -fac;                          Will equal ±1.
    }
    cint[n-1]=con*c[n-2]/(n-1);              Special case of (5.9.1) for n-1.
```

```
    sum += fac*cint[n-1];
    cint[0]=2.0*sum;                    Set the constant of integration.
    return Chebyshev(cint,a,b);
}
```

5.9.1 Clenshaw-Curtis Quadrature

Since a smooth function's Chebyshev coefficients c_i decrease rapidly, generally exponentially, equation (5.9.1) is often quite efficient as the basis for a quadrature scheme. As described above, the `Chebyshev` object can be used to compute the integral $\int_a^x f(x)dx$ when many different values of x in the range $a \le x \le b$ are needed. If only the single definite integral $\int_a^b f(x)dx$ is required, then instead use the simpler formula, derived from equation (5.9.1),

$$\int_a^b f(x)dx = (b-a)\left[\frac{1}{2}c_0 - \frac{1}{3}c_2 - \frac{1}{15}c_4 - \cdots - \frac{1}{(2k+1)(2k-1)}c_{2k} - \cdots\right] \quad (5.9.3)$$

where the c_i's are as returned by `chebft`. The series can be truncated when c_{2k} becomes negligible, and the first neglected term gives an error estimate.

This scheme is known as *Clenshaw-Curtis quadrature* [1]. It is often combined with an adaptive choice of N, the number of Chebyshev coefficients calculated via equation (5.8.7), which is also the number of function evaluations of $f(x)$. If a modest choice of N does not give a sufficiently small c_{2k} in equation (5.9.3), then a larger value is tried. In this adaptive case, it is even better to replace equation (5.8.7) by the so-called "trapezoidal" or Gauss-Lobatto (§4.6) variant,

$$c_j = \frac{2}{N}\sum_{k=0}^{N}{}'' f\left[\cos\left(\frac{\pi k}{N}\right)\right]\cos\left(\frac{\pi j k}{N}\right) \qquad j = 0,\ldots,N-1 \quad (5.9.4)$$

where (N.B.!) the two primes signify that the first and last terms in the sum are to be multiplied by $1/2$. If N is doubled in equation (5.9.4), then half of the new function evaluation points are identical to the old ones, allowing the previous function evaluations to be reused. This feature, plus the analytic weights and abscissas (cosine functions in 5.9.4), often give Clenshaw-Curtis quadrature an edge over high-order adaptive Gaussian quadrature (cf. §4.6.4), which the method otherwise resembles.

If your problem forces you to large values of N, you should be aware that equation (5.9.4) can be evaluated rapidly, and simultaneously for all the values of j, by a fast cosine transform. (See §12.3, especially equation 12.4.11. We already remarked that the nontrapezoidal form (5.8.7) can also be done by fast cosine methods, cf. equation 12.4.16.)

CITED REFERENCES AND FURTHER READING:

Goodwin, E.T. (ed.) 1961, *Modern Computing Methods*, 2nd ed. (New York: Philosophical Library), pp. 78–79.

Clenshaw, C.W., and Curtis, A.R. 1960, "A Method for Numerical Integration on an Automatic Computer," *Numerische Mathematik*, vol. 2, pp. 197–205.[1]

5.10 Polynomial Approximation from Chebyshev Coefficients

You may well ask after reading the preceding two sections: Must I store and evaluate my Chebyshev approximation as an array of Chebyshev coefficients for a transformed variable y?

Can't I convert the c_k's into actual polynomial coefficients in the original variable x and have an approximation of the following form?

$$f(x) \approx \sum_{k=0}^{m-1} g_k x^k, \qquad a \le x \le b \tag{5.10.1}$$

Yes, you can do this (and we will give you the algorithm to do it), but we caution you against it: Evaluating equation (5.10.1), where the coefficient g's reflect an underlying Chebyshev approximation, usually requires more significant figures than evaluation of the Chebyshev sum directly (as by `eval`). This is because the Chebyshev polynomials themselves exhibit a rather delicate cancellation: The leading coefficient of $T_n(x)$, for example, is 2^{n-1}; other coefficients of $T_n(x)$ are even bigger; yet they all manage to combine into a polynomial that lies between ± 1. *Only* when m is no larger than 7 or 8 should you contemplate writing a Chebyshev fit as a direct polynomial, and even in those cases you should be willing to tolerate two or so significant figures less accuracy than the roundoff limit of your machine.

You get the g's in equation (5.10.1) in two steps. First, use the member function `polycofs` in `Chebyshev` to output a set of polynomial coefficients equivalent to the stored c_k's (that is, with the range $[a,b]$ scaled to $[-1,1]$). Second, use the routine `pcshft` to transform the coefficients so as to map the range back to $[a,b]$. The two required routines are listed here:

chebyshev.h

```
VecDoub Chebyshev::polycofs(Int m)
```

Polynomial coefficients from a Chebyshev fit. Given a coefficient array `c[0..n-1]`, this routine returns a coefficient array `d[0..n-1]` such that $\sum_{k=0}^{n-1} d_k y^k = \sum_{k=0}^{n-1} c_k T_k(y) - c_0/2$. The method is Clenshaw's recurrence (5.8.11), but now applied algebraically rather than arithmetically.

```
{
    Int k,j;
    Doub sv;
    VecDoub d(m),dd(m);
    for (j=0;j<m;j++) d[j]=dd[j]=0.0;
    d[0]=c[m-1];
    for (j=m-2;j>0;j--) {
        for (k=m-j;k>0;k--) {
            sv=d[k];
            d[k]=2.0*d[k-1]-dd[k];
            dd[k]=sv;
        }
        sv=d[0];
        d[0] = -dd[0]+c[j];
        dd[0]=sv;
    }
    for (j=m-1;j>0;j--) d[j]=d[j-1]-dd[j];
    d[0] = -dd[0]+0.5*c[0];
    return d;
}
```

pcshft.h

```
void pcshft(Doub a, Doub b, VecDoub_IO &d)
```

Polynomial coefficient shift. Given a coefficient array `d[0..n-1]`, this routine generates a coefficient array `g[0..n-1]` such that $\sum_{k=0}^{n-1} d_k y^k = \sum_{k=0}^{n-1} g_k x^k$, where x and y are related by (5.8.10), i.e., the interval $-1 < y < 1$ is mapped to the interval $a < x < b$. The array g is returned in `d`.

```
{
    Int k,j,n=d.size();
    Doub cnst=2.0/(b-a), fac=cnst;
    for (j=1;j<n;j++) {            First we rescale by the factor const...
        d[j] *= fac;
        fac *= cnst;
    }
    cnst=0.5*(a+b);                ...which is then redefined as the desired shift.
```

```
    for (j=0;j<=n-2;j++)
        for (k=n-2;k>=j;k--)
            d[k] -= cnst*d[k+1];
}
```

We accomplish the shift by synthetic division, a miracle of high-school algebra.

CITED REFERENCES AND FURTHER READING:

Acton, F.S. 1970, *Numerical Methods That Work*; 1990, corrected edition (Washington, DC: Mathematical Association of America), pp. 59, 182–183 [synthetic division].

5.11 Economization of Power Series

One particular application of Chebyshev methods, the *economization of power series*, is an occasionally useful technique, with a flavor of getting something for nothing.

Suppose that you know how to compute a function by the use of a convergent power series, for example,

$$f(x) \equiv \frac{1}{2} - \frac{x}{4} + \frac{x^2}{8} - \frac{x^3}{16} + \cdots \tag{5.11.1}$$

(This function is actually just $1/(x+2)$, but pretend you don't know that.) You might be doing a problem that requires evaluating the series many times in some particular interval, say $[0,1]$. Everything is fine, except that the series requires a large number of terms before its error (approximated by the first neglected term, say) is tolerable. In our example, with $x = 1$, it takes about 30 terms before the first neglected term is $< 10^{-9}$.

Notice that because of the large exponent in x^{30}, the error is *much smaller* than 10^{-9} everywhere in the interval except at the very largest values of x. This is the feature that allows "economization": If we are willing to let the error elsewhere in the interval rise to about the same value that the first neglected term has at the extreme end of the interval, then we can replace the 30-term series by one that is significantly shorter.

Here are the steps for doing this:

1. Compute enough coefficients of the power series to get accurate function values everywhere in the range of interest.
2. Change variables from x to y, as in equation (5.8.10), to map the x interval into $-1 \le y \le 1$.
3. Find the Chebyshev series (like equation 5.8.8) that exactly equals your truncated power series.
4. Truncate this Chebyshev series to a smaller number of terms, using the coefficient of the first neglected Chebyshev polynomial as an estimate of the error.
5. Convert back to a polynomial in y.
6. Change variables back to x.

We already have tools for all of the steps, except for steps 2 and 3. Step 2 is exactly the inverse of the routine `pcshft` (§5.10), which mapped a polynomial from y (in the interval $[-1,1]$) to x (in the interval $[a,b]$). But since equation (5.8.10) is a linear relation between x and y, one can also use `pcshft` for the inverse. The inverse of

`pcshft(`a`,`b`,d,n)`

turns out to be (you can check this)

pcshft.h

```
void ipcshft(Doub a, Doub b, VecDoub_IO &d) {
    pcshft(-(2.+b+a)/(b-a),(2.-b-a)/(b-a),d);
}
```

Step 3 requires a new `Chebyshev` constructor, one that computes Chebyshev coefficients from a vector of polynomial coefficients. The following code accomplishes this. The algorithm is based on constructing the polynomial by the technique of §5.3 starting with the highest coefficient `d[n-1]` and using the recurrence of equation (5.8.2) written in the form

$$\begin{aligned} xT_0 &= T_1 \\ xT_n &= \tfrac{1}{2}(T_{n+1} + T_{n-1}), \quad n \geq 1. \end{aligned} \tag{5.11.2}$$

The only subtlety is to multiply the coefficient of T_0 by 2 since it gets used with a factor 1/2 in equation (5.8.8).

chebyshev.h

```
Chebyshev::Chebyshev(VecDoub &d)
    : n(d.size()), m(n), c(n), a(-1.), b(1.)
```
Inverse of routine `polycofs` in `Chebyshev`: Given an array of polynomial coefficients `d[0..n-1]`, construct an equivalent `Chebyshev` object.
```
{
    c[n-1]=d[n-1];
    c[n-2]=2.0*d[n-2];
    for (Int j=n-3;j>=0;j--) {
        c[j]=2.0*d[j]+c[j+2];
        for (Int i=j+1;i<n-2;i++) {
                c[i] = (c[i]+c[i+2])/2;
        }
        c[n-2] /= 2;
        c[n-1] /= 2;
    }
}
```

Putting them all together, steps 2 through 6 will look something like this (starting with a vector `powser` of power series coefficients):

```
    ipcshft(a,b,powser);
    Chebyshev cpowser(powser);
    cpowser.setm(1.e-9);
    VecDoub d=cpowser.polycofs();
    pcshft(a,b,d);
```

In our example, by the way, the number of terms required for 10^{-9} accuracy is reduced from 30 to 9. Replacing a 30-term polynomial with a 9-term polynomial without any loss of accuracy — that does seem to be getting something for nothing. Is there some magic in this technique? Not really. The 30-term polynomial defined a function $f(x)$. Equivalent to economizing the series, we could instead have evaluated $f(x)$ at enough points to construct its Chebyshev approximation in the interval of interest, by the methods of §5.8. We would have obtained just the same lower-order polynomial. The principal lesson is that the rate of convergence of Chebyshev coefficients has nothing to do with the rate of convergence of power series coefficients; and it is the *former* that dictates the number of terms needed in a polynomial approximation. A function might have a *divergent* power series in some region of interest, but if the function itself is well-behaved, it will have perfectly good polynomial approximations. These can be found by the methods of §5.8, but *not* by economization of series. There is slightly less to economization of series than meets the eye.

CITED REFERENCES AND FURTHER READING:

Acton, F.S. 1970, *Numerical Methods That Work*; 1990, corrected edition (Washington, DC: Mathematical Association of America), Chapter 12.

5.12 Padé Approximants

A *Padé approximant*, so called, is that rational function (of a specified order) whose power series expansion agrees with a given power series to the highest possible order. If the rational function is

$$R(x) \equiv \frac{\displaystyle\sum_{k=0}^{M} a_k x^k}{1 + \displaystyle\sum_{k=1}^{N} b_k x^k} \tag{5.12.1}$$

then $R(x)$ is said to be a Padé approximant to the series

$$f(x) \equiv \sum_{k=0}^{\infty} c_k x^k \tag{5.12.2}$$

if

$$R(0) = f(0) \tag{5.12.3}$$

and also

$$\left.\frac{d^k}{dx^k} R(x)\right|_{x=0} = \left.\frac{d^k}{dx^k} f(x)\right|_{x=0}, \qquad k = 1, 2, \ldots, M + N \tag{5.12.4}$$

Equations (5.12.3) and (5.12.4) furnish $M + N + 1$ equations for the unknowns $a_0, \ldots, a_M$ and $b_1, \ldots, b_N$. The easiest way to see what these equations are is to equate (5.12.1) and (5.12.2), multiply both by the denominator of equation (5.12.1), and equate all powers of x that have either a's or b's in their coefficients. If we consider only the special case of a diagonal rational approximation, $M = N$ (cf. §3.4), then we have $a_0 = c_0$, with the remaining a's and b's satisfying

$$\sum_{m=1}^{N} b_m c_{N-m+k} = -c_{N+k}, \qquad k = 1, \ldots, N \tag{5.12.5}$$

$$\sum_{m=0}^{k} b_m c_{k-m} = a_k, \qquad k = 1, \ldots, N \tag{5.12.6}$$

(note, in equation 5.12.1, that $b_0 = 1$). To solve these, start with equations (5.12.5), which are a set of linear equations for all the unknown b's. Although the set is in the form of a Toeplitz matrix (compare equation 2.8.8), experience shows that the equations are frequently close to singular, so that one should not solve them by the methods of §2.8, but rather by full LU decomposition. Additionally, it is a good idea to refine the solution by iterative improvement (method `mprove` in §2.5) [1].

Once the b's are known, then equation (5.12.6) gives an explicit formula for the unknown a's, completing the solution.

Padé approximants are typically used when there is some unknown underlying function $f(x)$. We suppose that you are able somehow to compute, perhaps by laborious analytic expansions, the values of $f(x)$ and a few of its derivatives at $x = 0$: $f(0)$, $f'(0)$, $f''(0)$, and so on. These are of course the first few coefficients in the power series expansion of $f(x)$; but they are not necessarily getting small, and you have no idea where (or whether) the power series is convergent.

By contrast with techniques like Chebyshev approximation (§5.8) or economization of power series (§5.11) that only condense the information that you already know about a function, Padé approximants can give you genuinely new information about your function's values.

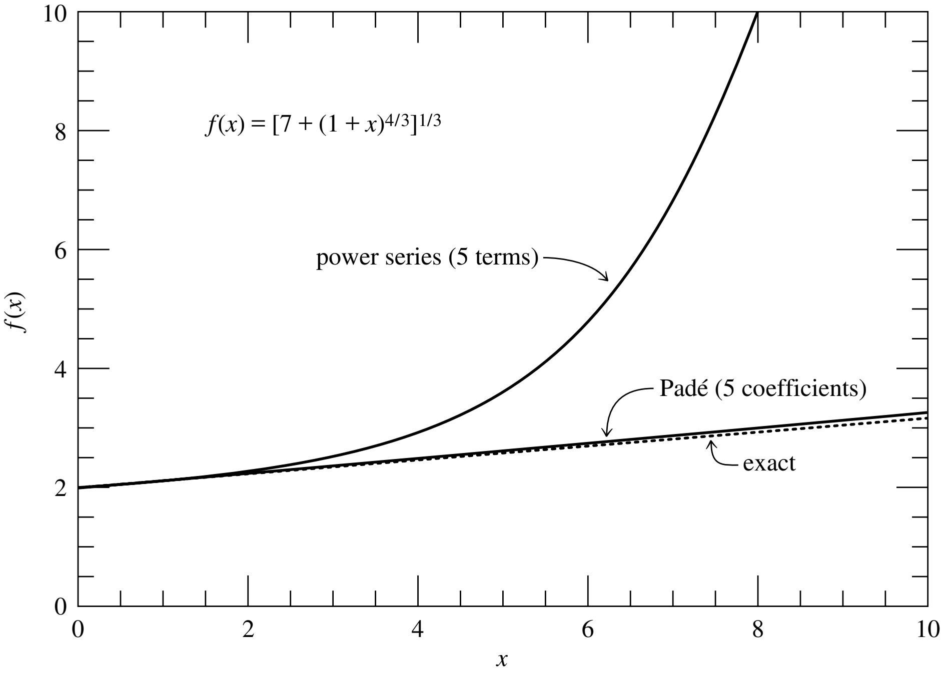

Figure 5.12.1. The five-term power series expansion and the derived five-coefficient Padé approximant for a sample function $f(x)$. The full power series converges only for $x < 1$. Note that the Padé approximant maintains accuracy far outside the radius of convergence of the series.

It is sometimes quite mysterious how well this can work. (Like other mysteries in mathematics, it relates to *analyticity*.) An example will illustrate.

Imagine that, by extraordinary labors, you have ground out the first five terms in the power series expansion of an unknown function $f(x)$,

$$f(x) \approx 2 + \frac{1}{9}x + \frac{1}{81}x^2 - \frac{49}{8748}x^3 + \frac{175}{78732}x^4 + \cdots \tag{5.12.7}$$

(It is not really necessary that you know the coefficients in exact rational form — numerical values are just as good. We here write them as rationals to give you the impression that they derive from some side analytic calculation.) Equation (5.12.7) is plotted as the curve labeled "power series" in Figure 5.12.1. One sees that for $x \gtrsim 4$ it is dominated by its largest, quartic, term.

We now take the five coefficients in equation (5.12.7) and run them through the routine `pade` listed below. It returns five rational coefficients, three a's and two b's, for use in equation (5.12.1) with $M = N = 2$. The curve in the figure labeled "Padé" plots the resulting rational function. Note that both solid curves derive from the *same* five original coefficient values.

To evaluate the results, we need *Deus ex machina* (a useful fellow, when he is available) to tell us that equation (5.12.7) is in fact the power series expansion of the function

$$f(x) = [7 + (1 + x)^{4/3}]^{1/3} \tag{5.12.8}$$

which is plotted as the dotted curve in the figure. This function has a branch point at $x = -1$, so its power series is convergent only in the range $-1 < x < 1$. In most of the range shown in the figure, the series is divergent, and the value of its truncation to five terms is rather meaningless. Nevertheless, those five terms, converted to a Padé approximant, give a remarkably good representation of the function up to at least $x \sim 10$.

Why does this work? Are there not other functions with the same first five terms in their power series but completely different behavior in the range (say) $2 < x < 10$? Indeed there are. Padé approximation has the uncanny knack of picking the function *you had in mind* from among all the possibilities. *Except when it doesn't!* That is the downside of Padé approximation: It is uncontrolled. There is, in general, no way to tell how accurate it is, or how far out in x it can usefully be extended. It is a powerful, but in the end still mysterious, technique.

Here is the routine that returns a `Ratfn` rational function object that is the Padé approximant to a set of power series coefficients that you provide. Note that the routine is specialized to the case $M = N$. You can then use the `Ratfn` object directly as a functor, or else read out its coefficients by hand (§5.1).

pade.h

```
Ratfn pade(VecDoub_I &cof)
Given cof[0..2*n], the leading terms in the power series expansion of a function, solve the
linear Padé equations to return a Ratfn object that embodies a diagonal rational function
approximation to the same function.
{
    const Doub BIG=1.0e99;
    Int j,k,n=(cof.size()-1)/2;
    Doub sum;
    MatDoub q(n,n),qlu(n,n);
    VecInt indx(n);
    VecDoub x(n),y(n),num(n+1),denom(n+1);
    for (j=0;j<n;j++) {                      Set up matrix for solving.
        y[j]=cof[n+j+1];
        for (k=0;k<n;k++) q[j][k]=cof[j-k+n];
    }
    LUdcmp lu(q);                            Solve by LU decomposition and backsubstitu-
    lu.solve(y,x);                               tion, with iterative improvement.
    for (j=0;j<4;j++) lu.mprove(y,x);
    for (k=0;k<n;k++) {                      Calculate the remaining coefficients.
        for (sum=cof[k+1],j=0;j<=k;j++) sum -= x[j]*cof[k-j];
        y[k]=sum;
    }
    num[0] = cof[0];
    denom[0] = 1.;
    for (j=0;j<n;j++) {                      Copy answers to output.
        num[j+1]=y[j];
        denom[j+1] = -x[j];
    }
    return Ratfn(num,denom);
}
```

CITED REFERENCES AND FURTHER READING:

Ralston, A. and Wilf, H.S. 1960, *Mathematical Methods for Digital Computers* (New York: Wiley), p. 14.

Cuyt, A., and Wuytack, L. 1987, *Nonlinear Methods in Numerical Analysis* (Amsterdam: North-Holland), Chapter 2.

Graves-Morris, P.R. 1979, in *Padé Approximation and Its Applications*, Lecture Notes in Mathematics, vol. 765, L. Wuytack, ed. (Berlin: Springer).[1]

5.13 Rational Chebyshev Approximation

In §5.8 and §5.10 we learned how to find good polynomial approximations to a given function $f(x)$ in a given interval $a \le x \le b$. Here, we want to generalize the task to find

good approximations that are rational functions (see §5.1). The reason for doing so is that, for some functions and some intervals, the optimal rational function approximation is able to achieve substantially higher accuracy than the optimal polynomial approximation with the same number of coefficients. This must be weighed against the fact that finding a rational function approximation is not as straightforward as finding a polynomial approximation, which, as we saw, could be done elegantly via Chebyshev polynomials.

Let the desired rational function $R(x)$ have a numerator of degree m and denominator of degree k. Then we have

$$R(x) \equiv \frac{p_0 + p_1 x + \cdots + p_m x^m}{1 + q_1 x + \cdots + q_k x^k} \approx f(x) \qquad \text{for } a \le x \le b \tag{5.13.1}$$

The unknown quantities that we need to find are $p_0, \ldots, p_m$ and $q_1, \ldots, q_k$, that is, $m + k + 1$ quantities in all. Let $r(x)$ denote the deviation of $R(x)$ from $f(x)$, and let r denote its maximum absolute value,

$$r(x) \equiv R(x) - f(x) \qquad r \equiv \max_{a \le x \le b} |r(x)| \tag{5.13.2}$$

The ideal *minimax* solution would be that choice of p's and q's that minimizes r. Obviously there is *some* minimax solution, since r is bounded below by zero. How can we find it, or a reasonable approximation to it?

A first hint is furnished by the following fundamental theorem: If $R(x)$ is nondegenerate (has no common polynomial factors in numerator and denominator), then there is a unique choice of p's and q's that minimizes r; for this choice, $r(x)$ has $m + k + 2$ extrema in $a \le x \le b$, *all of magnitude* r *and with alternating sign.* (We have omitted some technical assumptions in this theorem. See Ralston [1] for a precise statement.) We thus learn that the situation with rational functions is quite analogous to that for minimax polynomials: In §5.8 we saw that the error term of an nth-order approximation, with $n + 1$ Chebyshev coefficients, was generally dominated by the first neglected Chebyshev term, namely T_{n+1}, which itself has $n + 2$ extrema of equal magnitude and alternating sign. So, here, the number of rational coefficients, $m + k + 1$, plays the same role of the number of polynomial coefficients, $n + 1$.

A different way to see why $r(x)$ should have $m + k + 2$ extrema is to note that $R(x)$ can be made exactly equal to $f(x)$ at any $m + k + 1$ points x_i. Multiplying equation (5.13.1) by its denominator gives the equations

$$p_0 + p_1 x_i + \cdots + p_m x_i^m = f(x_i)(1 + q_1 x_i + \cdots + q_k x_i^k) \qquad i = 0, 1, \ldots, m + k \tag{5.13.3}$$

This is a set of $m + k + 1$ linear equations for the unknown p's and q's, which can be solved by standard methods (e.g., LU decomposition). If we choose the x_i's to all be in the interval (a, b), then there will generically be an extremum between each chosen x_i and x_{i+1}, plus also extrema where the function goes out of the interval at a and b, for a total of $m + k + 2$ extrema. For arbitrary x_i's, the extrema will not have the same magnitude. The theorem says that, for one particular choice of x_i's, the magnitudes can be beaten down to the identical, minimal, value of r.

Instead of making $f(x_i)$ and $R(x_i)$ equal at the points x_i, one can instead force the residual $r(x_i)$ to any desired values y_i by solving the linear equations

$$p_0 + p_1 x_i + \cdots + p_m x_i^m = [f(x_i) - y_i](1 + q_1 x_i + \cdots + q_k x_i^k) \qquad i = 0, 1, \ldots, m + k \tag{5.13.4}$$

In fact, if the x_i's are chosen to be the extrema (not the zeros) of the minimax solution, then the equations satisfied will be

$$p_0 + p_1 x_i + \cdots + p_m x_i^m = [f(x_i) \pm r](1 + q_1 x_i + \cdots + q_k x_i^k) \qquad i = 0, 1, \ldots, m + k + 1 \tag{5.13.5}$$

where the $\pm$ alternates for the alternating extrema. Notice that equation (5.13.5) is satisfied at $m + k + 2$ extrema, while equation (5.13.4) was satisfied only at $m + k + 1$ arbitrary points. How can this be? The answer is that r in equation (5.13.5) is an additional unknown, so that

the number of both equations and unknowns is $m + k + 2$. True, the set is mildly nonlinear (in r), but in general it is still perfectly soluble by methods that we will develop in Chapter 9.

We thus see that, given only the *locations* of the extrema of the minimax rational function, we can solve for its coefficients and maximum deviation. Additional theorems, leading up to the so-called *Remes algorithms* [1], tell how to converge to these locations by an iterative process. For example, here is a (slightly simplified) statement of *Remes' Second Algorithm*: (1) Find an initial rational function with $m + k + 2$ extrema x_i (not having equal deviation). (2) Solve equation (5.13.5) for new rational coefficients and r. (3) Evaluate the resulting $R(x)$ to find its actual extrema (which will not be the same as the guessed values). (4) Replace each guessed value with the nearest actual extremum of the same sign. (5) Go back to step 2 and iterate to convergence. Under a broad set of assumptions, this method will converge. Ralston [1] fills in the necessary details, including how to find the initial set of x_i's.

Up to this point, our discussion has been textbook standard. We now reveal ourselves as heretics. We don't much like the elegant Remes algorithm. Its two nested iterations (on r in the nonlinear set 5.13.5, and on the new sets of x_i's) are finicky and require a lot of special logic for degenerate cases. Even more heretical, we doubt that compulsive searching for the *exactly best*, equal deviation approximation is worth the effort — except perhaps for those few people in the world whose business it is to find optimal approximations that get built into compilers and microcode.

When we use rational function approximation, the goal is usually much more pragmatic: Inside some inner loop we are evaluating some function a zillion times, and we want to speed up its evaluation. Almost never do we need this function to the last bit of machine accuracy. Suppose (heresy!) we use an approximation whose error has $m + k + 2$ extrema whose deviations differ by a factor of 2. The theorems on which the Remes algorithms are based guarantee that the perfect minimax solution will have extrema somewhere within this factor of 2 range — forcing down the higher extrema will cause the lower ones to rise, until all are equal. So our "sloppy" approximation is in fact within a fraction of a least significant bit of the minimax one.

That is good enough for us, especially when we have available a very robust method for finding the so-called "sloppy" approximation. Such a method is the least-squares solution of overdetermined linear equations by singular value decomposition (§2.6 and §15.4). We proceed as follows: First, solve (in the least-squares sense) equation (5.13.3), not just for $m + k + 1$ values of x_i, but for a significantly larger number of x_i's, spaced approximately like the zeros of a high-order Chebyshev polynomial. This gives an initial guess for $R(x)$. Second, tabulate the resulting deviations, find the mean absolute deviation, call it r, and then solve (again in the least-squares sense) equation (5.13.5) with r fixed and the $\pm$ chosen to be the sign of the observed deviation at each point x_i. Third, repeat the second step a few times.

You can spot some Remes orthodoxy lurking in our algorithm: The equations we solve are trying to bring the deviations not to zero, but rather to plus-or-minus some consistent value. However, we dispense with keeping track of actual extrema, and we solve only linear equations at each stage. One additional trick is to solve a *weighted* least-squares problem, where the weights are chosen to beat down the largest deviations fastest.

Here is a function implementing these ideas. Notice that the only calls to the function `fn` occur in the initial filling of the table `fs`. You could easily modify the code to do this filling outside of the routine. It is not even necessary that your abscissas `xs` be exactly the ones that we use, though the quality of the fit will deteriorate if you do not have several abscissas between each extremum of the (underlying) minimax solution. The function returns a `Ratfn` object that you can subsequently use as a functor, or from which you can extract the stored coefficients.

```
Ratfn ratlsq(Doub fn(const Doub), const Doub a, const Doub b, const Int mm,
    const Int kk, Doub &dev)
```
ratlsq.h

Returns a rational function approximation to the function `fn` in the interval `(a, b)`. Input quantities `mm` and `kk` specify the order of the numerator and denominator, respectively. The maximum absolute deviation of the approximation (insofar as is known) is returned as `dev`.

```
{
    const Int NPFAC=8,MAXIT=5;
    const Doub BIG=1.0e99,PIO2=1.570796326794896619;
```

```
    Int i,it,j,ncof=mm+kk+1,npt=NPFAC*ncof;
    Number of points where function is evaluated, i.e., fineness of the mesh.
    Doub devmax,e,hth,power,sum;
    VecDoub bb(npt),coff(ncof),ee(npt),fs(npt),wt(npt),xs(npt);
    MatDoub u(npt,ncof);
    Ratfn ratbest(coff,mm+1,kk+1);
    dev=BIG;
    for (i=0;i<npt;i++) {                  Fill arrays with mesh abscissas and function values.
        if (i < (npt/2)-1) {
            hth=PIO2*i/(npt-1.0);          At each end, use formula that minimizes round-
            xs[i]=a+(b-a)*SQR(sin(hth));      off sensitivity.
        } else {
            hth=PIO2*(npt-i)/(npt-1.0);
            xs[i]=b-(b-a)*SQR(sin(hth));
        }
        fs[i]=fn(xs[i]);
        wt[i]=1.0;                         In later iterations we will adjust these weights to
        ee[i]=1.0;                            combat the largest deviations.
    }
    e=0.0;
    for (it=0;it<MAXIT;it++) {             Loop over iterations.
        for (i=0;i<npt;i++) {              Set up the "design matrix" for the least-squares
            power=wt[i];                      fit.
            bb[i]=power*(fs[i]+SIGN(e,ee[i]));
            Key idea here: Fit to fn(x)+e where the deviation is positive, to fn(x)-e where
            it is negative. Then e is supposed to become an approximation to the equal-ripple
            deviation.
            for (j=0;j<mm+1;j++) {
                u[i][j]=power;
                power *= xs[i];
            }
            power = -bb[i];
            for (j=mm+1;j<ncof;j++) {
                power *= xs[i];
                u[i][j]=power;
            }
        }
        SVD svd(u);                        Singular value decomposition.
        svd.solve(bb,coff);
        In especially singular or difficult cases, one might here edit the singular values, replacing
        small values by zero in w[0..ncof-1].
        devmax=sum=0.0;
        Ratfn rat(coff,mm+1,kk+1);
        for (j=0;j<npt;j++) {              Tabulate the deviations and revise the weights.
            ee[j]=rat(xs[j])-fs[j];
            wt[j]=abs(ee[j]);              Use weighting to emphasize most deviant points.
            sum += wt[j];
            if (wt[j] > devmax) devmax=wt[j];
        }
        e=sum/npt;                         Update e to be the mean absolute deviation.
        if (devmax <= dev) {               Save only the best coefficient set found.
            ratbest = rat;
            dev=devmax;
        }
        cout << " ratlsq iteration= " << it;
        cout << "  max error= " << setw(10) << devmax << endl;
    }
    return ratbest;
}
```

Figure 5.13.1 shows the discrepancies for the first five iterations of `ratlsq` when it is applied to find the $m = k = 4$ rational fit to the function $f(x) = \cos x/(1 + e^x)$ in the interval $(0, \pi)$. One sees that after the first iteration, the results are virtually as good as the

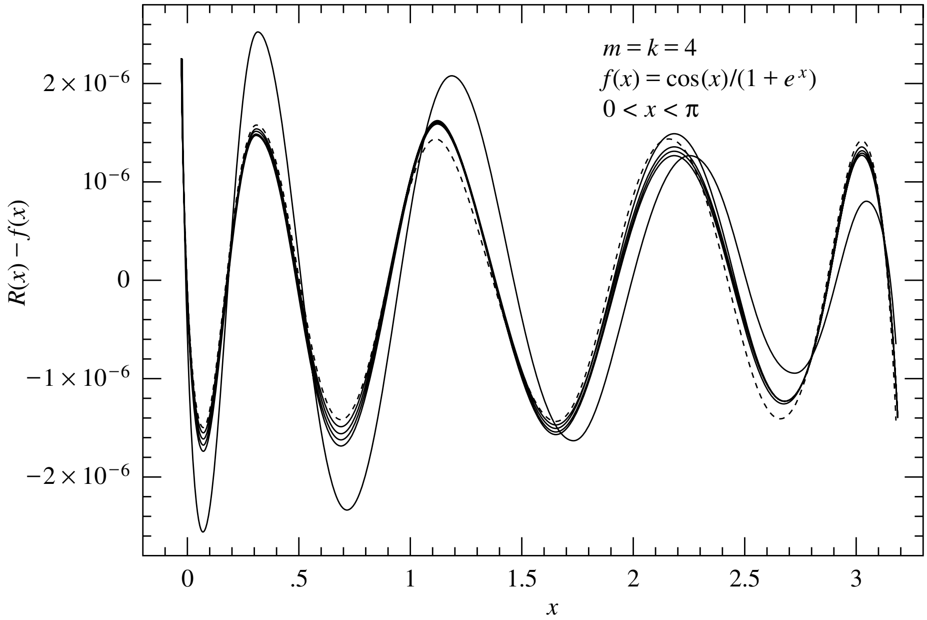

Figure 5.13.1. Solid curves show deviations $r(x)$ for five successive iterations of the routine `ratlsq` for an arbitrary test problem. The algorithm does not converge to exactly the minimax solution (shown as the dotted curve). But, after one iteration, the discrepancy is a small fraction of the last significant bit of accuracy.

minimax solution. The iterations do not converge in the order that the figure suggests. In fact, it is the second iteration that is best (has smallest maximum deviation). The routine `ratlsq` accordingly returns the best of its iterations, not necessarily the last one; there is no advantage in doing more than five iterations.

CITED REFERENCES AND FURTHER READING:

Ralston, A. and Wilf, H.S. 1960, *Mathematical Methods for Digital Computers* (New York: Wiley), Chapter 13.[1]

5.14 Evaluation of Functions by Path Integration

In computer programming, the technique of choice is not necessarily the most efficient, or elegant, or fastest executing one. Instead, it may be the one that is quick to implement, general, and easy to check.

One sometimes needs only a few, or a few thousand, evaluations of a special function, perhaps a complex-valued function of a complex variable, that has many different parameters, or asymptotic regimes, or both. Use of the usual tricks (series, continued fractions, rational function approximations, recurrence relations, and so forth) may result in a patchwork program with tests and branches to different formulas. While such a program may be highly efficient in execution, it is often not the shortest way to the answer from a standing start.

A different technique of considerable generality is direct integration of a function's defining differential equation — an *ab initio* integration for each desired function value — along a path in the complex plane if necessary. While this may at first seem like swatting a fly with a golden brick, it turns out that when you already have the brick, and the fly is asleep right under it, all you have to do is let it fall!

As a specific example, let us consider the complex hypergeometric function ${}_2F_1(a, b, c; z)$, which is defined as the analytic continuation of the so-called hypergeometric series,

$$
\begin{aligned}
{}_2F_1(a, b, c; z) = 1 &+ \frac{ab}{c}\frac{z}{1!} + \frac{a(a+1)b(b+1)}{c(c+1)}\frac{z^2}{2!} + \cdots \\
&+ \frac{a(a+1)\ldots(a+j-1)b(b+1)\ldots(b+j-1)}{c(c+1)\ldots(c+j-1)}\frac{z^j}{j!} + \cdots
\end{aligned}
\tag{5.14.1}
$$

The series converges only within the unit circle $|z| < 1$ (see [1]), but one's interest in the function is often not confined to this region.

The hypergeometric function ${}_2F_1$ is a solution (in fact *the* solution that is regular at the origin) of the hypergeometric differential equation, which we can write as

$$
z(1-z)F'' = abF - [c - (a+b+1)z]F' \tag{5.14.2}
$$

Here prime denotes d/dz. One can see that the equation has regular singular points at $z = 0, 1$, and ∞. Since the desired solution is regular at $z = 0$, the values 1 and ∞ will in general be branch points. If we want ${}_2F_1$ to be a single-valued function, we must have a branch cut connecting these two points. A conventional position for this cut is along the positive real axis from 1 to ∞, though we may wish to keep open the possibility of altering this choice for some applications.

Our golden brick consists of a collection of routines for the integration of sets of ordinary differential equations, which we will develop in detail later, in Chapter 17. For now, we need only a high-level, "black-box" routine that integrates such a set from initial conditions at one value of a (real) independent variable to final conditions at some other value of the independent variable, while automatically adjusting its internal stepsize to maintain some specified accuracy. That routine is called `Odeint` and, in one particular invocation, it calculates its individual steps with a sophisticated Bulirsch-Stoer technique.

Suppose that we know values for F and its derivative F' at some value z_0, and that we want to find F at some other point z_1 in the complex plane. The straight-line path connecting these two points is parametrized by

$$
z(s) = z_0 + s(z_1 - z_0) \tag{5.14.3}
$$

with s a real parameter. The differential equation (5.14.2) can now be written as a set of two first-order equations,

$$
\begin{aligned}
\frac{dF}{ds} &= (z_1 - z_0)F' \\
\frac{dF'}{ds} &= (z_1 - z_0)\left(\frac{abF - [c - (a+b+1)z]F'}{z(1-z)}\right)
\end{aligned}
\tag{5.14.4}
$$

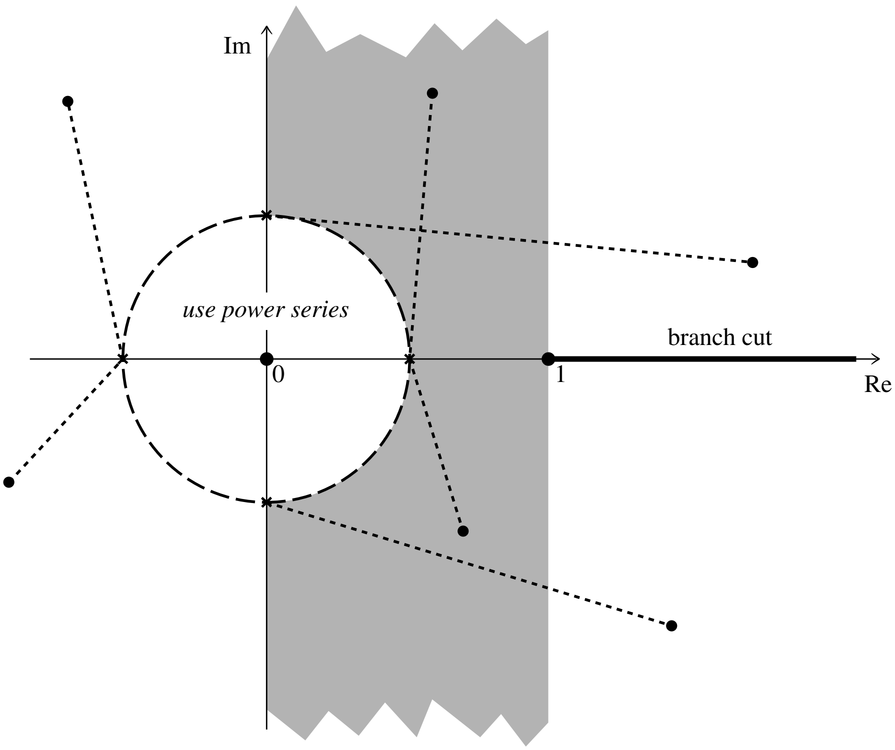

Figure 5.14.1. Complex plane showing the singular points of the hypergeometric function, its branch cut, and some integration paths from the circle $|z| = 1/2$ (where the power series converges rapidly) to other points in the plane.

to be integrated from $s = 0$ to $s = 1$. Here F and F' are to be viewed as two independent complex variables. The fact that prime means d/dz can be ignored; it will emerge as a consequence of the first equation in (5.14.4). Moreover, the real and imaginary parts of equation (5.14.4) define a set of four *real* differential equations, with independent variable s. The complex arithmetic on the right-hand side can be viewed as mere shorthand for how the four components are to be coupled. It is precisely this point of view that gets passed to the routine `Odeint`, since it knows nothing of either complex functions or complex independent variables.

It remains only to decide where to start, and what path to take in the complex plane, to get to an arbitrary point z. This is where consideration of the function's singularities, and the adopted branch cut, enter. Figure 5.14.1 shows the strategy that we adopt. For $|z| \leq 1/2$, the series in equation (5.14.1) will in general converge rapidly, and it makes sense to use it directly. Otherwise, we integrate along a straight-line path from one of the starting points $(\pm 1/2, 0)$ or $(0, \pm 1/2)$. The former choices are natural for $0 < \text{Re}(z) < 1$ and $\text{Re}(z) < 0$, respectively. The latter choices are used for $\text{Re}(z) > 1$, above and below the branch cut; the purpose of starting away from the real axis in these cases is to avoid passing too close to the singularity at $z = 1$ (see Figure 5.14.1). The location of the branch cut is *defined* by the fact that our adopted strategy never integrates across the real axis for $\text{Re}(z) > 1$.

An implementation of this algorithm is given in §6.13 as the routine `hypgeo`.

A number of variants on the procedure described thus far are possible and easy to program. If successively called values of z are close together (with identical values of a, b, and c), then you can save the state vector (F, F') and the corresponding value of z on each call, and use these as starting values for the next call. The incremental integration may then take only one or two steps. Avoid integrating across the branch cut unintentionally: The function value will be "correct," but not the one you want.

Alternatively, you may wish to integrate to some position z by a dog-leg path that *does* cross the real axis $\mathrm{Re}(z) > 1$, as a means of *moving* the branch cut. For example, in some cases you might want to integrate from $(0, 1/2)$ to $(3/2, 1/2)$, and go from there to any point with $\mathrm{Re}(z) > 1$ — with either sign of $\mathrm{Im}z$. (If you are, for example, finding roots of a function by an iterative method, you do not want the integration for nearby values to take different paths around a branch point. If it does, your root-finder will see discontinuous function values and will likely not converge correctly!)

In any case, be aware that a loss of numerical accuracy can result if you integrate through a region of large function value on your way to a final answer where the function value is small. (For the hypergeometric function, a particular case of this is when a and b are both large and positive, with c and $x \gtrsim 1$.) In such cases, you'll need to find a better dog-leg path.

The general technique of evaluating a function by integrating its differential equation in the complex plane can also be applied to other special functions. For example, the complex Bessel function, Airy function, Coulomb wave function, and Weber function are all special cases of the *confluent hypergeometric function*, with a differential equation similar to the one used above (see, e.g., [1] §13.6, for a table of special cases). The confluent hypergeometric function has no singularities at finite z: That makes it easy to integrate. However, its essential singularity at infinity means that it can have, along some paths and for some parameters, highly oscillatory or exponentially decreasing behavior: That makes it hard to integrate. Some case-by-case judgment (or experimentation) is therefore required.

CITED REFERENCES AND FURTHER READING:

Abramowitz, M., and Stegun, I.A. 1964, *Handbook of Mathematical Functions* (Washington: National Bureau of Standards); reprinted 1968 (New York: Dover); online at `http://www.nr.com/aands`.[1]

Special Functions

CHAPTER 6

6.0 Introduction

There is nothing particularly special about a *special function*, except that some person in authority or a textbook writer (not the same thing!) has decided to bestow the moniker. Special functions are sometimes called *higher transcendental functions* (higher than what?) or *functions of mathematical physics* (but they occur in other fields also) or *functions that satisfy certain frequently occurring second-order differential equations* (but not all special functions do). One might simply call them "useful functions" and let it go at that. The choice of which functions to include in this chapter is highly arbitrary.

Commercially available program libraries contain many special function routines that are intended for users who will have no idea what goes on inside them. Such state-of-the-art black boxes are often very messy things, full of branches to completely different methods depending on the value of the calling arguments. Black boxes have, or should have, careful control of accuracy, to some stated uniform precision in all regimes.

We will not be quite so fastidious in our examples, in part because we want to illustrate techniques from Chapter 5, and in part because we *want* you to understand what goes on in the routines presented. Some of our routines have an accuracy parameter that can be made as small as desired, while others (especially those involving polynomial fits) give only a certain stated accuracy, one that we believe is serviceable (usually, but not always, close to double precision). We do *not* certify that the routines are perfect black boxes. We do hope that, if you ever encounter trouble in a routine, you will be able to diagnose and correct the problem on the basis of the information that we have given.

In short, the special function routines of this chapter are meant to be used — we use them all the time — but we also want you to learn from their inner workings.

CITED REFERENCES AND FURTHER READING:

Abramowitz, M., and Stegun, I.A. 1964, *Handbook of Mathematical Functions* (Washington: National Bureau of Standards); reprinted 1968 (New York: Dover); online at `http://www.nr.com/aands`.

Andrews, G.E., Askey, R., and Roy, R. 1999, *Special Functions* (Cambridge, UK: Cambridge University Press).

Thompson, W.J. 1997, *Atlas for Computing Mathematical Functions* (New York: Wiley-Interscience).

Spanier, J., and Oldham, K.B. 1987, *An Atlas of Functions* (Washington: Hemisphere Pub. Corp.).

Wolfram, S. 2003, *The Mathematica Book*, 5th ed. (Champaign, IL: Wolfram Media).

Hart, J.F., et al. 1968, *Computer Approximations* (New York: Wiley).

Hastings, C. 1955, *Approximations for Digital Computers* (Princeton: Princeton University Press).

Luke, Y.L. 1975, *Mathematical Functions and Their Approximations* (New York: Academic Press).

6.1 Gamma Function, Beta Function, Factorials, Binomial Coefficients

The gamma function is defined by the integral

$$\Gamma(z) = \int_0^\infty t^{z-1} e^{-t} \, dt \tag{6.1.1}$$

When the argument z is an integer, the gamma function is just the familiar factorial function, but offset by 1:

$$n! = \Gamma(n+1) \tag{6.1.2}$$

The gamma function satisfies the recurrence relation

$$\Gamma(z+1) = z\,\Gamma(z) \tag{6.1.3}$$

If the function is known for arguments $z > 1$ or, more generally, in the half complex plane $\text{Re}(z) > 1$, it can be obtained for $z < 1$ or $\text{Re}\,(z) < 1$ by the reflection formula

$$\Gamma(1-z) = \frac{\pi}{\Gamma(z)\sin(\pi z)} = \frac{\pi z}{\Gamma(1+z)\sin(\pi z)} \tag{6.1.4}$$

Notice that $\Gamma(z)$ has a pole at $z = 0$ and at all negative integer values of z.

There are a variety of methods in use for calculating the function $\Gamma(z)$ numerically, but none is quite as neat as the approximation derived by Lanczos [1]. This scheme is entirely specific to the gamma function, seemingly plucked from thin air. We will not attempt to derive the approximation, but only state the resulting formula: For certain choices of rational γ and integer N, and for certain coefficients $c_1, c_2, \ldots, c_N$, the gamma function is given by

$$\begin{aligned}\Gamma(z+1) = {} & (z+\gamma+\tfrac{1}{2})^{z+\frac{1}{2}} e^{-(z+\gamma+\frac{1}{2})} \\ & \times \sqrt{2\pi}\left[c_0 + \frac{c_1}{z+1} + \frac{c_2}{z+2} + \cdots + \frac{c_N}{z+N} + \epsilon\right] \qquad (z > 0)\end{aligned} \tag{6.1.5}$$

You can see that this is a sort of take-off on Stirling's approximation, but with a series of corrections that take into account the first few poles in the left complex plane. The constant c_0 is very nearly equal to 1. The error term is parametrized by

ϵ. For $N = 14$, and a certain set of c's and γ (calculated by P. Godfrey), the error is smaller than $|\epsilon| < 10^{-15}$. Even more impressive is the fact that, with these same constants, the formula (6.1.5) applies for the *complex* gamma function, *everywhere in the half complex plane Re* $z > 0$, achieving almost the same accuracy as on the real line.

It is better to implement $\ln \Gamma(x)$ than $\Gamma(x)$, since the latter will overflow at quite modest values of x. Often the gamma function is used in calculations where the large values of $\Gamma(x)$ are divided by other large numbers, with the result being a perfectly ordinary value. Such operations would normally be coded as subtraction of logarithms. With (6.1.5) in hand, we can compute the logarithm of the gamma function with two calls to a logarithm and a few dozen arithmetic operations. This makes it not much more difficult than other built-in functions that we take for granted, such as $\sin x$ or e^x:

gamma.h

```
Doub gammln(const Doub xx) {
Returns the value ln[Γ(xx)] for xx > 0.
    Int j;
    Doub x,tmp,y,ser;
    static const Doub cof[14]={57.1562356658629235,-59.5979603554754912,
    14.1360979747417471,-0.491913816097620199,.339946499848118887e-4,
    .465236289270485756e-4,-.983744753048795646e-4,.158088703224912494e-3,
    -.210264441724104883e-3,.217439618115212643e-3,-.164318106536763890e-3,
    .844182239838527433e-4,-.261908384015814087e-4,.368991826595316234e-5};
    if (xx <= 0) throw("bad arg in gammln");
    y=x=xx;
    tmp = x+5.24218750000000000;        Rational 671/128.
    tmp = (x+0.5)*log(tmp)-tmp;
    ser = 0.999999999999997092;
    for (j=0;j<14;j++) ser += cof[j]/++y;
    return tmp+log(2.5066282746310005*ser/x);
}
```

How shall we write a routine for the factorial function $n!$? Generally the factorial function will be called for small integer values, and in most applications the same integer value will be called for many times. It is obviously inefficient to call `exp(gammln(n+1.))` for each required factorial. Better is to initialize a static table on the first call, and do a fast lookup on subsequent calls. The fixed size 171 for the table is because 170! is representable as an IEEE double precision value, but 171! overflows. It is also sometimes useful to know that factorials up to 22! have exact double precision representations (52 bits of mantissa, not counting powers of two that are absorbed into the exponent), while 23! and above are represented only approximately.

gamma.h

```
Doub factrl(const Int n) {
Returns the value n! as a floating-point number.
    static VecDoub a(171);
    static Bool init=true;
    if (init) {
        init = false;
        a[0] = 1.;
        for (Int i=1;i<171;i++) a[i] = i*a[i-1];
    }
    if (n < 0 || n > 170) throw("factrl out of range");
    return a[n];
}
```

More useful in practice is a function returning the log of a factorial, which doesn't have overflow issues. The size of the table of logarithms is whatever you can afford in space and initialization time. The value NTOP = 2000 should be increased if your integer arguments are often larger.

gamma.h

```
Doub factln(const Int n) {
Returns ln(n!).
    static const Int NTOP=2000;
    static VecDoub a(NTOP);
    static Bool init=true;
    if (init) {
        init = false;
        for (Int i=0;i<NTOP;i++) a[i] = gammln(i+1.);
    }
    if (n < 0) throw("negative arg in factln");
    if (n < NTOP) return a[n];
    return gammln(n+1.);                    Out of range of table.
}
```

The binomial coefficient is defined by

$$\binom{n}{k} = \frac{n!}{k!(n-k)!} \qquad 0 \le k \le n \tag{6.1.6}$$

A routine that takes advantage of the tables stored in `factrl` and `factln` is

gamma.h

```
Doub bico(const Int n, const Int k) {
Returns the binomial coefficient (n k) as a floating-point number.
    if (n<0 || k<0 || k>n) throw("bad args in bico");
    if (n<171) return floor(0.5+factrl(n)/(factrl(k)*factrl(n-k)));
    return floor(0.5+exp(factln(n)-factln(k)-factln(n-k)));
    The floor function cleans up roundoff error for smaller values of n and k.
}
```

If your problem requires a series of related binomial coefficients, a good idea is to use recurrence relations, for example,

$$\begin{aligned}\binom{n+1}{k} &= \frac{n+1}{n-k+1}\binom{n}{k} = \binom{n}{k} + \binom{n}{k-1} \\ \binom{n}{k+1} &= \frac{n-k}{k+1}\binom{n}{k}\end{aligned} \tag{6.1.7}$$

Finally, turning away from the combinatorial functions with integer-valued arguments, we come to the beta function,

$$B(z,w) = B(w,z) = \int_0^1 t^{z-1}(1-t)^{w-1}\,dt \tag{6.1.8}$$

which is related to the gamma function by

$$B(z,w) = \frac{\Gamma(z)\Gamma(w)}{\Gamma(z+w)} \tag{6.1.9}$$

hence

```
Doub beta(const Doub z, const Doub w) {
Returns the value of the beta function B(z,w).
    return exp(gammln(z)+gammln(w)-gammln(z+w));
}
```
gamma.h

CITED REFERENCES AND FURTHER READING:

Abramowitz, M., and Stegun, I.A. 1964, *Handbook of Mathematical Functions* (Washington: National Bureau of Standards); reprinted 1968 (New York: Dover); online at http://www.nr.com/aands, Chapter 6.

Lanczos, C. 1964, "A Precision Approximation of the Gamma Function," *SIAM Journal on Numerical Analysis*, ser. B, vol. 1, pp. 86–96.[1]

6.2 Incomplete Gamma Function and Error Function

The incomplete gamma function is defined by

$$P(a,x) \equiv \frac{\gamma(a,x)}{\Gamma(a)} \equiv \frac{1}{\Gamma(a)} \int_0^x e^{-t} t^{a-1} dt \qquad (a > 0) \tag{6.2.1}$$

It has the limiting values

$$P(a,0) = 0 \qquad \text{and} \qquad P(a,\infty) = 1 \tag{6.2.2}$$

The incomplete gamma function $P(a,x)$ is monotonic and (for a greater than one or so) rises from "near-zero" to "near-unity" in a range of x centered on about $a-1$, and of width about $\sqrt{a}$ (see Figure 6.2.1).

The complement of $P(a,x)$ is also confusingly called an incomplete gamma function,

$$Q(a,x) \equiv 1 - P(a,x) \equiv \frac{\Gamma(a,x)}{\Gamma(a)} \equiv \frac{1}{\Gamma(a)} \int_x^\infty e^{-t} t^{a-1} dt \qquad (a > 0) \tag{6.2.3}$$

It has the limiting values

$$Q(a,0) = 1 \qquad \text{and} \qquad Q(a,\infty) = 0 \tag{6.2.4}$$

The notations $P(a,x), \gamma(a,x)$, and $\Gamma(a,x)$ are standard; the notation $Q(a,x)$ is specific to this book.

There is a series development for $\gamma(a,x)$ as follows:

$$\gamma(a,x) = e^{-x} x^a \sum_{n=0}^{\infty} \frac{\Gamma(a)}{\Gamma(a+1+n)} x^n \tag{6.2.5}$$

One does not actually need to compute a new $\Gamma(a+1+n)$ for each n; one rather uses equation (6.1.3) and the previous coefficient.

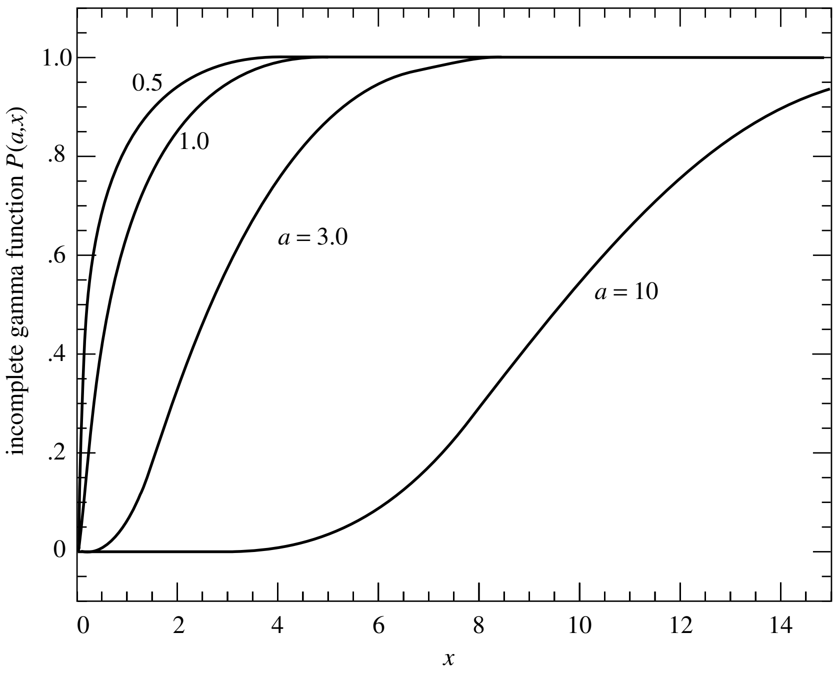

Figure 6.2.1. The incomplete gamma function $P(a,x)$ for four values of a.

A continued fraction development for $\Gamma(a,x)$ is

$$\Gamma(a,x) = e^{-x}x^a\left(\frac{1}{x+}\,\frac{1-a}{1+}\,\frac{1}{x+}\,\frac{2-a}{1+}\,\frac{2}{x+}\cdots\right) \qquad (x>0) \qquad (6.2.6)$$

It is computationally better to use the even part of (6.2.6), which converges twice as fast (see §5.2):

$$\Gamma(a,x) = e^{-x}x^a\left(\frac{1}{x+1-a-}\,\frac{1\cdot(1-a)}{x+3-a-}\,\frac{2\cdot(2-a)}{x+5-a-}\cdots\right) \qquad (x>0) \qquad (6.2.7)$$

It turns out that (6.2.5) converges rapidly for x less than about $a+1$, while (6.2.6) or (6.2.7) converges rapidly for x greater than about $a+1$. In these respective regimes each requires at most a few times $\sqrt{a}$ terms to converge, and this many only near $x = a$, where the incomplete gamma functions are varying most rapidly. For moderate values of a, less than 100, say, (6.2.5) and (6.2.7) together allow evaluation of the function for all x. An extra dividend is that we never need to compute a function value near zero by subtracting two nearly equal numbers.

Some applications require $P(a,x)$ and $Q(a,x)$ for much larger values of a, where both the series and the continued fraction are inefficient. In this regime, however, the integrand in equation (6.2.1) falls off sharply in both directions from its peak, within a few times $\sqrt{a}$. An efficient procedure is to evaluate the integral directly, with a single step of high-order Gauss-Legendre quadrature (§4.6) extending from x just far enough into the nearest tail to achieve negligible values of the integrand. Actually it is "half a step," because we need the dense abscissas only near x, not far out on the tail where the integrand is effectively zero.

We package the various incomplete gamma parts into an object `Gamma`. The only persistent state is the value `gln`, which is set to $\Gamma(a)$ for the most recent call to $P(a,x)$ or $Q(a,x)$. This is useful when you need a different normalization convention, for example $\gamma(a,x)$ or $\Gamma(a,x)$ in equations (6.2.1) or (6.2.3).

incgammabeta.h

```
struct Gamma : Gauleg18 {
Object for incomplete gamma function. Gauleg18 provides coefficients for Gauss-Legendre
quadrature.
    static const Int ASWITCH=100;          When to switch to quadrature method.
    static const Doub EPS;                 See end of struct for initializations.
    static const Doub FPMIN;
    Doub gln;

    Doub gammp(const Doub a, const Doub x) {
    Returns the incomplete gamma function P(a,x).
        if (x < 0.0 || a <= 0.0) throw("bad args in gammp");
        if (x == 0.0) return 0.0;
        else if ((Int)a >= ASWITCH) return gammpapprox(a,x,1); Quadrature.
        else if (x < a+1.0) return gser(a,x);         Use the series representation.
        else return 1.0-gcf(a,x);           Use the continued fraction representation.
    }

    Doub gammq(const Doub a, const Doub x) {
    Returns the incomplete gamma function Q(a,x) ≡ 1 − P(a,x).
        if (x < 0.0 || a <= 0.0) throw("bad args in gammq");
        if (x == 0.0) return 1.0;
        else if ((Int)a >= ASWITCH) return gammpapprox(a,x,0); Quadrature.
        else if (x < a+1.0) return 1.0-gser(a,x);     Use the series representation.
        else return gcf(a,x);               Use the continued fraction representation.
    }

    Doub gser(const Doub a, const Doub x) {
    Returns the incomplete gamma function P(a,x) evaluated by its series representation.
    Also sets ln Γ(a) as gln. User should not call directly.
        Doub sum,del,ap;
        gln=gammln(a);
        ap=a;
        del=sum=1.0/a;
        for (;;) {
            ++ap;
            del *= x/ap;
            sum += del;
            if (fabs(del) < fabs(sum)*EPS) {
                return sum*exp(-x+a*log(x)-gln);
            }
        }
    }

    Doub gcf(const Doub a, const Doub x) {
    Returns the incomplete gamma function Q(a,x) evaluated by its continued fraction rep-
    resentation. Also sets ln Γ(a) as gln. User should not call directly.
        Int i;
        Doub an,b,c,d,del,h;
        gln=gammln(a);
        b=x+1.0-a;                         Set up for evaluating continued fraction
        c=1.0/FPMIN;                           by modified Lentz's method (§5.2)
        d=1.0/b;                               with b0 = 0.
        h=d;
        for (i=1;;i++) {                   Iterate to convergence.
            an = -i*(i-a);
            b += 2.0;
            d=an*d+b;
```

```
            if (fabs(d) < FPMIN) d=FPMIN;
            c=b+an/c;
            if (fabs(c) < FPMIN) c=FPMIN;
            d=1.0/d;
            del=d*c;
            h *= del;
            if (fabs(del-1.0) <= EPS) break;
        }
        return exp(-x+a*log(x)-gln)*h;          Put factors in front.
    }

    Doub gammpapprox(Doub a, Doub x, Int psig) {
    Incomplete gamma by quadrature. Returns P(a,x) or Q(a,x), when psig is 1 or 0,
    respectively. User should not call directly.
        Int j;
        Doub xu,t,sum,ans;
        Doub a1 = a-1.0, lna1 = log(a1), sqrta1 = sqrt(a1);
        gln = gammln(a);
        Set how far to integrate into the tail:
        if (x > a1) xu = MAX(a1 + 11.5*sqrta1, x + 6.0*sqrta1);
        else xu = MAX(0.,MIN(a1 - 7.5*sqrta1, x - 5.0*sqrta1));
        sum = 0;
        for (j=0;j<ngau;j++) {                          Gauss-Legendre.
            t = x + (xu-x)*y[j];
            sum += w[j]*exp(-(t-a1)+a1*(log(t)-lna1));
        }
        ans = sum*(xu-x)*exp(a1*(lna1-1.)-gln);
        return (psig?(ans>0.0? 1.0-ans:-ans):(ans>=0.0? ans:1.0+ans));
    }

    Doub invgammp(Doub p, Doub a);
    Inverse function on x of P(a,x). See §6.2.1.

};
const Doub Gamma::EPS = numeric_limits<Doub>::epsilon();
const Doub Gamma::FPMIN = numeric_limits<Doub>::min()/EPS;
```

Remember that since `Gamma` is an object, you have to declare an instance of it before you can use its member functions. We habitually write

```
    Gamma gam;
```

as a global declaration, and then call `gam.gammp` or `gam.gammq` as needed. The structure `Gauleg18` just contains the abscissas and weights for the Gauss-Legendre quadrature.

incgammabeta.h

```
struct Gauleg18 {
Abscissas and weights for Gauss-Legendre quadrature.
    static const Int ngau = 18;
    static const Doub y[18];
    static const Doub w[18];
};
const Doub Gauleg18::y[18] = {0.0021695375159141994,
0.011413521097787704,0.027972308950302116,0.051727015600492421,
0.082502225484340941, 0.12007019910960293,0.16415283300752470,
0.21442376986779355, 0.27051082840644336, 0.33199876341447887,
0.39843234186401943, 0.46931971407375483, 0.54413605556657973,
0.62232745288031077, 0.70331500465597174, 0.78649910768313447,
0.87126389619061517, 0.95698180152629142};
const Doub Gauleg18::w[18] = {0.0055657196642445571,
0.012915947284065419,0.020181515297735382,0.027298621498568734,
0.034213810770299537,0.040875750923643261,0.047235083490265582,
0.053244713977759692,0.058860144245324798,0.064039797355015485,
```

```
0.068745323835736408,0.072941885005653087,0.076598410645870640,
0.079687828912071670,0.082187266704339706,0.084078218979661945,
0.085346685739338721,0.085983275670394821};
```

6.2.1 Inverse Incomplete Gamma Function

In many statistical applications one needs the inverse of the incomplete gamma function, that is, the value x such that $P(a,x) = p$, for a given value $0 \leq p \leq 1$. Newton's method works well if we can devise a good-enough initial guess. In fact, this is a good place to use Halley's method (see §9.4), since the second derivative (that is, the first derivative of the integrand) is easy to compute.

For $a > 1$, we use an initial guess that derives from §26.2.22 and §26.4.17 in reference [1]. For $a \leq 1$, we first roughly approximate $P_a \equiv P(a,1)$:

$$P_a \equiv P(a,1) \approx 0.253a + 0.12a^2, \qquad 0 \leq a \leq 1 \tag{6.2.8}$$

and then solve for x in one or the other of the (rough) approximations:

$$P(a,x) \approx \begin{cases} P_a x^a, & x < 1 \\ P_a + (1 - P_a)(1 - e^{1-x}), & x \geq 1 \end{cases} \tag{6.2.9}$$

An implementation is

incgammabeta.h

```
Doub Gamma::invgammp(Doub p, Doub a) {
Returns x such that P(a,x) = p for an argument p between 0 and 1.
    Int j;
    Doub x,err,t,u,pp,lna1,afac,a1=a-1;
    const Doub EPS=1.e-8;                      Accuracy is the square of EPS.
    gln=gammln(a);
    if (a <= 0.) throw("a must be pos in invgammap");
    if (p >= 1.) return MAX(100.,a + 100.*sqrt(a));
    if (p <= 0.) return 0.0;
    if (a > 1.) {                              Initial guess based on reference [1].
        lna1=log(a1);
        afac = exp(a1*(lna1-1.)-gln);
        pp = (p < 0.5)? p : 1. - p;
        t = sqrt(-2.*log(pp));
        x = (2.30753+t*0.27061)/(1.+t*(0.99229+t*0.04481)) - t;
        if (p < 0.5) x = -x;
        x = MAX(1.e-3,a*pow(1.-1./(9.*a)-x/(3.*sqrt(a)),3));
    } else {                                   Initial guess based on equations (6.2.8)
        t = 1.0 - a*(0.253+a*0.12);                and (6.2.9).
        if (p < t) x = pow(p/t,1./a);
        else x = 1.-log(1.-(p-t)/(1.-t));
    }
    for (j=0;j<12;j++) {
        if (x <= 0.0) return 0.0;              x too small to compute accurately.
        err = gammp(a,x) - p;
        if (a > 1.) t = afac*exp(-(x-a1)+a1*(log(x)-lna1));
        else t = exp(-x+a1*log(x)-gln);
        u = err/t;
        x -= (t = u/(1.-0.5*MIN(1.,u*((a-1.)/x - 1))));        Halley's method.
        if (x <= 0.) x = 0.5*(x + t);          Halve old value if x tries to go negative.
        if (fabs(t) < EPS*x ) break;
    }
    return x;
}
```

6.2.2 Error Function

The error function and complementary error function are special cases of the incomplete gamma function and are obtained moderately efficiently by the above procedures. Their definitions are

$$\mathrm{erf}(x) = \frac{2}{\sqrt{\pi}} \int_0^x e^{-t^2} dt \tag{6.2.10}$$

and

$$\mathrm{erfc}(x) \equiv 1 - \mathrm{erf}(x) = \frac{2}{\sqrt{\pi}} \int_x^\infty e^{-t^2} dt \tag{6.2.11}$$

The functions have the following limiting values and symmetries:

$$\mathrm{erf}(0) = 0 \qquad \mathrm{erf}(\infty) = 1 \qquad \mathrm{erf}(-x) = -\mathrm{erf}(x) \tag{6.2.12}$$

$$\mathrm{erfc}(0) = 1 \qquad \mathrm{erfc}(\infty) = 0 \qquad \mathrm{erfc}(-x) = 2 - \mathrm{erfc}(x) \tag{6.2.13}$$

They are related to the incomplete gamma functions by

$$\mathrm{erf}(x) = P\left(\frac{1}{2}, x^2\right) \qquad (x \geq 0) \tag{6.2.14}$$

and

$$\mathrm{erfc}(x) = Q\left(\frac{1}{2}, x^2\right) \qquad (x \geq 0) \tag{6.2.15}$$

A faster calculation takes advantage of an approximation of the form

$$\mathrm{erfc}(z) \approx t \; \exp[-z^2 + \mathcal{P}(t)], \qquad z > 0 \tag{6.2.16}$$

where

$$t \equiv \frac{2}{2+z} \tag{6.2.17}$$

and $\mathcal{P}(t)$ is a polynomial for $0 \leq t \leq 1$ that can be found by Chebyshev methods (§5.8). As with `Gamma`, implementation is by an object that also includes the inverse function, here an inverse for both erf and erfc. Halley's method is again used for the inverses (as suggested by P.J. Acklam).

erf.h

```
struct Erf {
```
Object for error function and related functions.
```
    static const Int ncof=28;
    static const Doub cof[28];            Initialization at end of struct.

    inline Doub erf(Doub x) {
```
Return erf(x) for any x.
```
        if (x >=0.) return 1.0 - erfccheb(x);
        else return erfccheb(-x) - 1.0;
    }

    inline Doub erfc(Doub x) {
```
Return erfc(x) for any x.
```
        if (x >= 0.) return erfccheb(x);
        else return 2.0 - erfccheb(-x);
    }
```

```
    Doub erfccheb(Doub z){
```
Evaluate equation (6.2.16) using stored Chebyshev coefficients. User should not call directly.
```
        Int j;
        Doub t,ty,tmp,d=0.,dd=0.;
        if (z < 0.) throw("erfccheb requires nonnegative argument");
        t = 2./(2.+z);
        ty = 4.*t - 2.;
        for (j=ncof-1;j>0;j--) {
            tmp = d;
            d = ty*d - dd + cof[j];
            dd = tmp;
        }
        return t*exp(-z*z + 0.5*(cof[0] + ty*d) - dd);
    }

    Doub inverfc(Doub p) {
```
Inverse of complementary error function. Returns x such that $\mathrm{erfc}(x) = p$ for argument p between 0 and 2.
```
        Doub x,err,t,pp;
        if (p >= 2.0) return -100.;          Return arbitrary large pos or neg value.
        if (p <= 0.0) return 100.;
        pp = (p < 1.0)? p : 2. - p;
        t = sqrt(-2.*log(pp/2.));            Initial guess:
        x = -0.70711*((2.30753+t*0.27061)/(1.+t*(0.99229+t*0.04481)) - t);
        for (Int j=0;j<2;j++) {
            err = erfc(x) - pp;
            x += err/(1.12837916709551257*exp(-SQR(x))-x*err);  Halley.
        }
        return (p < 1.0? x : -x);
    }

    inline Doub inverf(Doub p) {return inverfc(1.-p);}
```
Inverse of the error function. Returns x such that $\mathrm{erf}(x) = p$ for argument p between -1 and 1.

```
};

const Doub Erf::cof[28] = {-1.3026537197817094, 6.4196979235649026e-1,
    1.9476473204185836e-2,-9.561514786808631e-3,-9.46595344482036e-4,
    3.66839497852761e-4,4.2523324806907e-5,-2.0278578112534e-5,
    -1.624290004647e-6,1.303655835580e-6,1.5626441722e-8,-8.5238095915e-8,
    6.529054439e-9,5.059343495e-9,-9.91364156e-10,-2.27365122e-10,
    9.6467911e-11, 2.394038e-12,-6.886027e-12,8.94487e-13, 3.13092e-13,
    -1.12708e-13,3.81e-16,7.106e-15,-1.523e-15,-9.4e-17,1.21e-16,-2.8e-17};
```

A lower-order Chebyshev approximation produces a very concise routine, though with only about single precision accuracy:

erf.h
```
Doub erfcc(const Doub x)
```
Returns the complementary error function erfc(x) with fractional error everywhere less than 1.2×10^{-7}.
```
{
    Doub t,z=fabs(x),ans;
    t=2./(2.+z);
    ans=t*exp(-z*z-1.26551223+t*(1.00002368+t*(0.37409196+t*(0.09678418+
        t*(-0.18628806+t*(0.27886807+t*(-1.13520398+t*(1.48851587+
        t*(-0.82215223+t*0.17087277)))))))));
    return (x >= 0.0 ? ans : 2.0-ans);
}
```

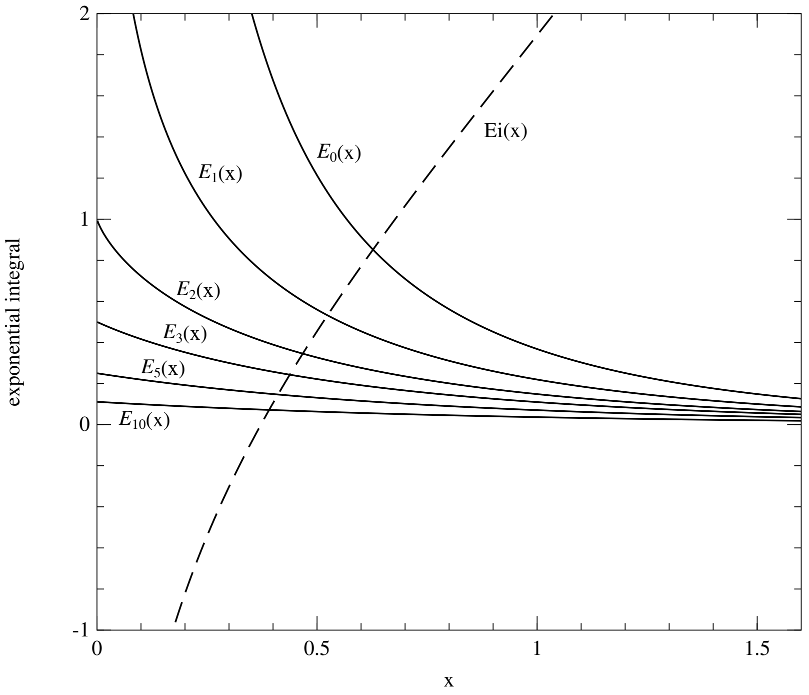

Figure 6.3.1. Exponential integrals $E_n(x)$ for $n = 0, 1, 2, 3, 5$, and 10, and the exponential integral $\mathrm{Ei}(x)$.

CITED REFERENCES AND FURTHER READING:

Abramowitz, M., and Stegun, I.A. 1964, *Handbook of Mathematical Functions* (Washington: National Bureau of Standards); reprinted 1968 (New York: Dover); online at `http://www.nr.com/aands`, Chapters 6, 7, and 26.[1]

Pearson, K. (ed.) 1951, *Tables of the Incomplete Gamma Function* (Cambridge, UK: Cambridge University Press).

6.3 Exponential Integrals

The standard definition of the exponential integral is

$$E_n(x) = \int_1^\infty \frac{e^{-xt}}{t^n} dt, \qquad x > 0, \quad n = 0, 1, \ldots \tag{6.3.1}$$

The function defined by the principal value of the integral

$$\mathrm{Ei}(x) = -\int_{-x}^\infty \frac{e^{-t}}{t}\, dt = \int_{-\infty}^x \frac{e^t}{t}\, dt, \qquad x > 0 \tag{6.3.2}$$

is also called an exponential integral. Note that $\mathrm{Ei}(-x)$ is related to $-E_1(x)$ by analytic continuation. Figure 6.3.1 plots these functions for representative values of their parameters.

The function $E_n(x)$ is a special case of the incomplete gamma function

$$E_n(x) = x^{n-1}\Gamma(1-n, x) \tag{6.3.3}$$

We can therefore use a similar strategy for evaluating it. The continued fraction — just equation (6.2.6) rewritten — converges for all $x > 0$:

$$E_n(x) = e^{-x}\left(\frac{1}{x+}\,\frac{n}{1+}\,\frac{1}{x+}\,\frac{n+1}{1+}\,\frac{2}{x+}\cdots\right) \tag{6.3.4}$$

We use it in its more rapidly converging even form,

$$E_n(x) = e^{-x}\left(\frac{1}{x+n-}\,\frac{1\cdot n}{x+n+2-}\,\frac{2(n+1)}{x+n+4-}\cdots\right) \tag{6.3.5}$$

The continued fraction only really converges fast enough to be useful for $x \gtrsim 1$. For $0 < x \lesssim 1$, we can use the series representation

$$E_n(x) = \frac{(-x)^{n-1}}{(n-1)!}[-\ln x + \psi(n)] - \sum_{\substack{m=0\\ m\neq n-1}}^{\infty} \frac{(-x)^m}{(m-n+1)m!} \tag{6.3.6}$$

The quantity $\psi(n)$ here is the digamma function, given for integer arguments by

$$\psi(1) = -\gamma, \qquad \psi(n) = -\gamma + \sum_{m=1}^{n-1}\frac{1}{m} \tag{6.3.7}$$

where $\gamma = 0.5772156649\ldots$ is Euler's constant. We evaluate the expression (6.3.6) in order of ascending powers of x:

$$\begin{aligned} E_n(x) = &-\left[\frac{1}{(1-n)} - \frac{x}{(2-n)\cdot 1} + \frac{x^2}{(3-n)(1\cdot 2)} - \cdots + \frac{(-x)^{n-2}}{(-1)(n-2)!}\right] \\ &+ \frac{(-x)^{n-1}}{(n-1)!}[-\ln x + \psi(n)] - \left[\frac{(-x)^n}{1\cdot n!} + \frac{(-x)^{n+1}}{2\cdot(n+1)!} + \cdots\right] \end{aligned} \tag{6.3.8}$$

The first square bracket is omitted when $n = 1$. This method of evaluation has the advantage that, for large n, the series converges before reaching the term containing $\psi(n)$. Accordingly, one needs an algorithm for evaluating $\psi(n)$ only for small n, $n \lesssim 20$ – 40. We use equation (6.3.7), although a table lookup would improve efficiency slightly.

Amos [1] presents a careful discussion of the truncation error in evaluating equation (6.3.8) and gives a fairly elaborate termination criterion. We have found that simply stopping when the last term added is smaller than the required tolerance works about as well.

Two special cases have to be handled separately:

$$\begin{aligned} E_0(x) &= \frac{e^{-x}}{x} \\ E_n(0) &= \frac{1}{n-1}, \qquad n > 1 \end{aligned} \tag{6.3.9}$$

The routine expint allows fast evaluation of $E_n(x)$ to any accuracy EPS within the reach of your machine's precision for floating-point numbers. The only modification required for increased accuracy is to supply Euler's constant with enough significant digits. Wrench [2] can provide you with the first 328 digits if necessary!

expint.h

```
Doub expint(const Int n, const Doub x)
Evaluates the exponential integral E_n(x).
{
    static const Int MAXIT=100;
    static const Doub EULER=0.577215664901533,
        EPS=numeric_limits<Doub>::epsilon(),
        BIG=numeric_limits<Doub>::max()*EPS;
        Here MAXIT is the maximum allowed number of iterations; EULER is Euler's constant
        γ; EPS is the desired relative error, not smaller than the machine precision; BIG is a
        number near the largest representable floating-point number.
    Int i,ii,nm1=n-1;
    Doub a,b,c,d,del,fact,h,psi,ans;
    if (n < 0 || x < 0.0 || (x==0.0 && (n==0 || n==1)))
        throw("bad arguments in expint");
    if (n == 0) ans=exp(-x)/x;                        Special case.
    else {
        if (x == 0.0) ans=1.0/nm1;                    Another special case.
        else {
            if (x > 1.0) {                            Lentz's algorithm (§5.2).
                b=x+n;
                c=BIG;
                d=1.0/b;
                h=d;
                for (i=1;i<=MAXIT;i++) {
                    a = -i*(nm1+i);
                    b += 2.0;
                    d=1.0/(a*d+b);                    Denominators cannot be zero.
                    c=b+a/c;
                    del=c*d;
                    h *= del;
                    if (abs(del-1.0) <= EPS) {
                        ans=h*exp(-x);
                        return ans;
                    }
                }
                throw("continued fraction failed in expint");
            } else {                                  Evaluate series.
                ans = (nm1!=0 ? 1.0/nm1 : -log(x)-EULER);     Set first term.
                fact=1.0;
                for (i=1;i<=MAXIT;i++) {
                    fact *= -x/i;
                    if (i != nm1) del = -fact/(i-nm1);
                    else {
                        psi = -EULER;                 Compute ψ(n).
                        for (ii=1;ii<=nm1;ii++) psi += 1.0/ii;
                        del=fact*(-log(x)+psi);
                    }
                    ans += del;
                    if (abs(del) < abs(ans)*EPS) return ans;
                }
                throw("series failed in expint");
            }
        }
    }
    return ans;
}
```

A good algorithm for evaluating Ei is to use the power series for small x and the asymptotic series for large x. The power series is

$$\mathrm{Ei}(x) = \gamma + \ln x + \frac{x}{1 \cdot 1!} + \frac{x^2}{2 \cdot 2!} + \cdots \tag{6.3.10}$$

where γ is Euler's constant. The asymptotic expansion is

$$\mathrm{Ei}(x) \sim \frac{e^x}{x}\left(1 + \frac{1!}{x} + \frac{2!}{x^2} + \cdots\right) \tag{6.3.11}$$

The lower limit for the use of the asymptotic expansion is approximately $|\ln \mathrm{EPS}|$, where EPS is the required relative error.

expint.h

```
Doub ei(const Doub x) {
```
Computes the exponential integral $\mathrm{Ei}(x)$ for $x > 0$.
```
    static const Int MAXIT=100;
    static const Doub EULER=0.577215664901533,
        EPS=numeric_limits<Doub>::epsilon(),
        FPMIN=numeric_limits<Doub>::min()/EPS;
```
Here MAXIT is the maximum number of iterations allowed; EULER is Euler's constant γ; EPS is the relative error, or absolute error near the zero of Ei at $x = 0.3725$; FPMIN is a number close to the smallest representable floating-point number.
```
    Int k;
    Doub fact,prev,sum,term;
    if (x <= 0.0) throw("Bad argument in ei");
    if (x < FPMIN) return log(x)+EULER;       Special case: Avoid failure of convergence
                                                  test because of underflow.
    if (x <= -log(EPS)) {
        sum=0.0;                              Use power series.
        fact=1.0;
        for (k=1;k<=MAXIT;k++) {
            fact *= x/k;
            term=fact/k;
            sum += term;
            if (term < EPS*sum) break;
        }
        if (k > MAXIT) throw("Series failed in ei");
        return sum+log(x)+EULER;
    } else {                                  Use asymptotic series.
        sum=0.0;                              Start with second term.
        term=1.0;
        for (k=1;k<=MAXIT;k++) {
            prev=term;
            term *= k/x;
            if (term < EPS) break;
```
Since final sum is greater than one, term itself approximates the relative error.
```
            if (term < prev) sum += term;     Still converging: Add new term.
            else {
                sum -= prev;                  Diverging: Subtract previous term and
                break;                            exit.
            }
        }
        return exp(x)*(1.0+sum)/x;
    }
}
```

CITED REFERENCES AND FURTHER READING:

Stegun, I.A., and Zucker, R. 1974, "Automatic Computing Methods for Special Functions. II. The Exponential Integral $E_n(x)$," *Journal of Research of the National Bureau of Standards*,

vol. 78B, pp. 199–216; 1976, "Automatic Computing Methods for Special Functions. III. The Sine, Cosine, Exponential Integrals, and Related Functions," *op. cit.*, vol. 80B, pp. 291–311.

Amos D.E. 1980, "Computation of Exponential Integrals," *ACM Transactions on Mathematical Software*, vol. 6, pp. 365–377[1]; also vol. 6, pp. 420–428.

Abramowitz, M., and Stegun, I.A. 1964, *Handbook of Mathematical Functions* (Washington: National Bureau of Standards); reprinted 1968 (New York: Dover); online at `http://www.nr.com/aands`, Chapter 5.

Wrench J.W. 1952, "A New Calculation of Euler's Constant," *Mathematical Tables and Other Aids to Computation*, vol. 6, p. 255.[2]

6.4 Incomplete Beta Function

The incomplete beta function is defined by

$$I_x(a,b) \equiv \frac{B_x(a,b)}{B(a,b)} \equiv \frac{1}{B(a,b)} \int_0^x t^{a-1}(1-t)^{b-1}dt \qquad (a,b>0) \tag{6.4.1}$$

It has the limiting values

$$I_0(a,b) = 0 \qquad I_1(a,b) = 1 \tag{6.4.2}$$

and the symmetry relation

$$I_x(a,b) = 1 - I_{1-x}(b,a) \tag{6.4.3}$$

If a and b are both rather greater than one, then $I_x(a,b)$ rises from "near-zero" to "near-unity" quite sharply at about $x = a/(a+b)$. Figure 6.4.1 plots the function for several pairs (a,b).

The incomplete beta function has a series expansion

$$I_x(a,b) = \frac{x^a(1-x)^b}{aB(a,b)} \left[1 + \sum_{n=0}^{\infty} \frac{B(a+1,n+1)}{B(a+b,n+1)} x^{n+1} \right] \tag{6.4.4}$$

but this does not prove to be very useful in its numerical evaluation. (Note, however, that the beta functions in the coefficients can be evaluated for each value of n with just the previous value and a few multiplies, using equations 6.1.9 and 6.1.3.)

The continued fraction representation proves to be much more useful:

$$I_x(a,b) = \frac{x^a(1-x)^b}{aB(a,b)} \left[\frac{1}{1+} \frac{d_1}{1+} \frac{d_2}{1+} \cdots \right] \tag{6.4.5}$$

where

$$\begin{aligned} d_{2m+1} &= -\frac{(a+m)(a+b+m)x}{(a+2m)(a+2m+1)} \\ d_{2m} &= \frac{m(b-m)x}{(a+2m-1)(a+2m)} \end{aligned} \tag{6.4.6}$$

This continued fraction converges rapidly for $x < (a+1)/(a+b+2)$, except when a and b are both large, when it can take $O(\sqrt{\min(a,b)})$ iterations. For $x >$

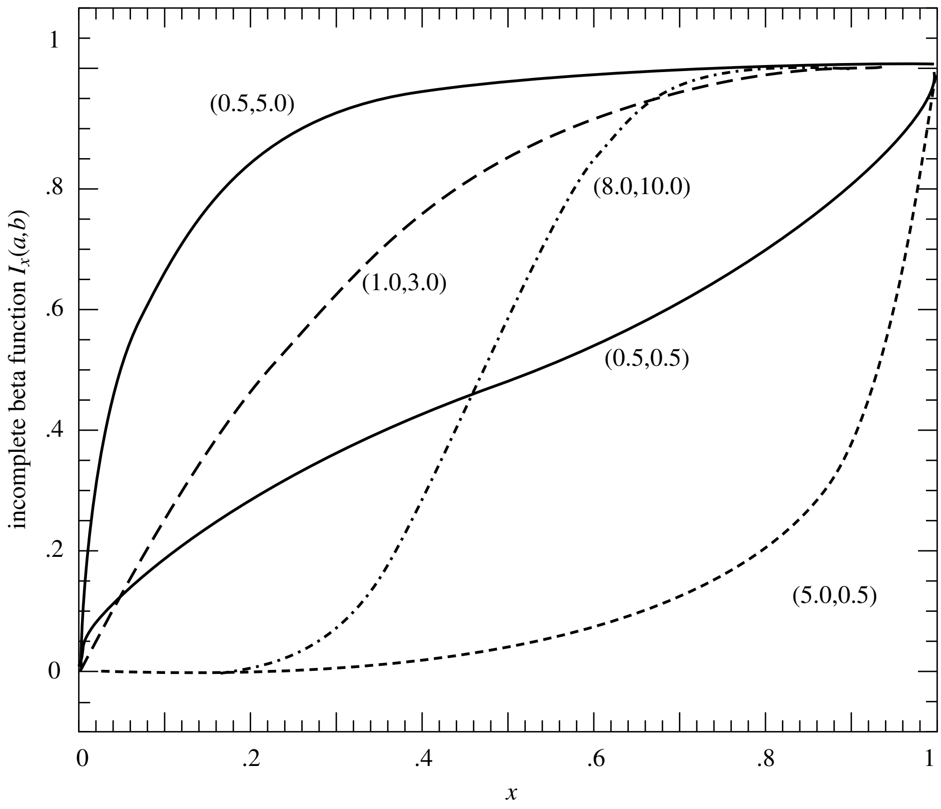

Figure 6.4.1. The incomplete beta function $I_x(a,b)$ for five different pairs of (a,b). Notice that the pairs $(0.5, 5.0)$ and $(5.0, 0.5)$ are symmetrically related as indicated in equation (6.4.3).

$(a+1)/(a+b+2)$ we can just use the symmetry relation (6.4.3) to obtain an equivalent computation in which the convergence is again rapid. Our computational strategy is thus very similar to that used in `Gamma`: We use the continued fraction except when a and b are both large, in which case we do a single step of high-order Gauss-Legendre quadrature.

Also as in `Gamma`, we code an inverse function using Halley's method. When a and b are both ≥ 1, the initial guess comes from §26.5.22 in reference [1]. When either is less than 1, the guess comes from first crudely approximating

$$\int_0^1 t^{a-1}(1-t)^{b-1}dt \approx \frac{1}{a}\left(\frac{a}{a+b}\right)^a + \frac{1}{b}\left(\frac{b}{a+b}\right)^b \equiv S \tag{6.4.7}$$

which comes from breaking the integral at $t = a/(a+b)$ and ignoring one factor in the integrand on each side of the break. We then write

$$I_x(a,b) \approx \begin{cases} x^a/(Sa) & x \leq a/(a+b) \\ (1-x)^b/(Sb) & x > a/(a+b) \end{cases} \tag{6.4.8}$$

and solve for x in the respective regimes. While crude, this is good enough to get well within the basin of convergence in all cases.

incgammabeta.h

```
struct Beta : Gauleg18 {
```
Object for incomplete beta function. Gauleg18 provides coefficients for Gauss-Legendre quadrature.
```
    static const Int SWITCH=3000;          When to switch to quadrature method.
    static const Doub EPS, FPMIN;          See end of struct for initializations.

    Doub betai(const Doub a, const Doub b, const Doub x) {
```
Returns incomplete beta function $I_x(a,b)$ for positive a and b, and x between 0 and 1.
```
        Doub bt;
        if (a <= 0.0 || b <= 0.0) throw("Bad a or b in routine betai");
        if (x < 0.0 || x > 1.0) throw("Bad x in routine betai");
        if (x == 0.0 || x == 1.0) return x;
        if (a > SWITCH && b > SWITCH) return betaiapprox(a,b,x);
        bt=exp(gammln(a+b)-gammln(a)-gammln(b)+a*log(x)+b*log(1.0-x));
        if (x < (a+1.0)/(a+b+2.0)) return bt*betacf(a,b,x)/a;
        else return 1.0-bt*betacf(b,a,1.0-x)/b;
    }

    Doub betacf(const Doub a, const Doub b, const Doub x) {
```
Evaluates continued fraction for incomplete beta function by modified Lentz's method (§5.2). User should not call directly.
```
        Int m,m2;
        Doub aa,c,d,del,h,qab,qam,qap;
        qab=a+b;                            These q's will be used in factors that
        qap=a+1.0;                              occur in the coefficients (6.4.6).
        qam=a-1.0;
        c=1.0;                              First step of Lentz's method.
        d=1.0-qab*x/qap;
        if (fabs(d) < FPMIN) d=FPMIN;
        d=1.0/d;
        h=d;
        for (m=1;m<10000;m++) {
            m2=2*m;
            aa=m*(b-m)*x/((qam+m2)*(a+m2));
            d=1.0+aa*d;                     One step (the even one) of the recur-
            if (fabs(d) < FPMIN) d=FPMIN;       rence.
            c=1.0+aa/c;
            if (fabs(c) < FPMIN) c=FPMIN;
            d=1.0/d;
            h *= d*c;
            aa = -(a+m)*(qab+m)*x/((a+m2)*(qap+m2));
            d=1.0+aa*d;                     Next step of the recurrence (the odd
            if (fabs(d) < FPMIN) d=FPMIN;       one).
            c=1.0+aa/c;
            if (fabs(c) < FPMIN) c=FPMIN;
            d=1.0/d;
            del=d*c;
            h *= del;
            if (fabs(del-1.0) <= EPS) break;    Are we done?
        }
        return h;
    }

    Doub betaiapprox(Doub a, Doub b, Doub x) {
```
Incomplete beta by quadrature. Returns $I_x(a,b)$. User should not call directly.
```
        Int j;
        Doub xu,t,sum,ans;
        Doub a1 = a-1.0, b1 = b-1.0, mu = a/(a+b);
        Doub lnmu=log(mu),lnmuc=log(1.-mu);
        t = sqrt(a*b/(SQR(a+b)*(a+b+1.0)));
        if (x > a/(a+b)) {                  Set how far to integrate into the tail:
            if (x >= 1.0) return 1.0;
            xu = MIN(1.,MAX(mu + 10.*t, x + 5.0*t));
        } else {
```

```
        if (x <= 0.0) return 0.0;
        xu = MAX(0.,MIN(mu - 10.*t, x - 5.0*t));
    }
    sum = 0;
    for (j=0;j<18;j++) {
```
Gauss-Legendre.
```
        t = x + (xu-x)*y[j];
        sum += w[j]*exp(a1*(log(t)-lnmu)+b1*(log(1-t)-lnmuc));
    }
    ans = sum*(xu-x)*exp(a1*lnmu-gammln(a)+b1*lnmuc-gammln(b)+gammln(a+b));
    return ans>0.0? 1.0-ans : -ans;
}

Doub invbetai(Doub p, Doub a, Doub b) {
```
Inverse of incomplete beta function. Returns x such that $I_x(a,b) = p$ for argument p between 0 and 1.
```
    const Doub EPS = 1.e-8;
    Doub pp,t,u,err,x,al,h,w,afac,a1=a-1.,b1=b-1.;
    Int j;
    if (p <= 0.) return 0.;
    else if (p >= 1.) return 1.;
    else if (a >= 1. && b >= 1.) {
```
Set initial guess. See text.
```
        pp = (p < 0.5)? p : 1. - p;
        t = sqrt(-2.*log(pp));
        x = (2.30753+t*0.27061)/(1.+t*(0.99229+t*0.04481)) - t;
        if (p < 0.5) x = -x;
        al = (SQR(x)-3.)/6.;
        h = 2./(1./(2.*a-1.)+1./(2.*b-1.));
        w = (x*sqrt(al+h)/h)-(1./(2.*b-1)-1./(2.*a-1.))*(al+5./6.-2./(3.*h));
        x = a/(a+b*exp(2.*w));
    } else {
        Doub lna = log(a/(a+b)), lnb = log(b/(a+b));
        t = exp(a*lna)/a;
        u = exp(b*lnb)/b;
        w = t + u;
        if (p < t/w) x = pow(a*w*p,1./a);
        else x = 1. - pow(b*w*(1.-p),1./b);
    }
    afac = -gammln(a)-gammln(b)+gammln(a+b);
    for (j=0;j<10;j++) {
        if (x == 0. || x == 1.) return x;
```
a or b too small for accurate calculation.
```
        err = betai(a,b,x) - p;
        t = exp(a1*log(x)+b1*log(1.-x) + afac);
        u = err/t;
```
Halley:
```
        x -= (t = u/(1.-0.5*MIN(1.,u*(a1/x - b1/(1.-x)))));
        if (x <= 0.) x = 0.5*(x + t);
```
Bisect if x tries to go neg or > 1.
```
        if (x >= 1.) x = 0.5*(x + t + 1.);
        if (fabs(t) < EPS*x && j > 0) break;
    }
    return x;
}

};
const Doub Beta::EPS = numeric_limits<Doub>::epsilon();
const Doub Beta::FPMIN = numeric_limits<Doub>::min()/EPS;
```

CITED REFERENCES AND FURTHER READING:

Abramowitz, M., and Stegun, I.A. 1964, *Handbook of Mathematical Functions* (Washington: National Bureau of Standards); reprinted 1968 (New York: Dover); online at `http://www.nr.com/aands`, Chapters 6 and 26.[1]

Pearson, E., and Johnson, N. 1968, *Tables of the Incomplete Beta Function* (Cambridge, UK: Cambridge University Press).

6.5 Bessel Functions of Integer Order

This section presents practical algorithms for computing various kinds of Bessel functions of integer order. In §6.6 we deal with fractional order. Actually, the more complicated routines for fractional order work fine for integer order too. For integer order, however, the routines in this section are simpler and faster.

For any real ν, the Bessel function $J_\nu(x)$ can be defined by the series representation

$$J_\nu(x) = \left(\frac{1}{2}x\right)^\nu \sum_{k=0}^{\infty} \frac{(-\frac{1}{4}x^2)^k}{k!\Gamma(\nu+k+1)} \tag{6.5.1}$$

The series converges for all x, but it is not computationally very useful for $x \gg 1$.

For ν *not* an integer, the Bessel function $Y_\nu(x)$ is given by

$$Y_\nu(x) = \frac{J_\nu(x)\cos(\nu\pi) - J_{-\nu}(x)}{\sin(\nu\pi)} \tag{6.5.2}$$

The right-hand side goes to the correct limiting value $Y_n(x)$ as ν goes to some integer n, but this is also not computationally useful.

For arguments $x < \nu$, both Bessel functions look qualitatively like simple power laws, with the asymptotic forms for $0 < x \ll \nu$

$$\begin{aligned} J_\nu(x) &\sim \frac{1}{\Gamma(\nu+1)}\left(\frac{1}{2}x\right)^\nu \qquad \nu \geq 0 \\ Y_0(x) &\sim \frac{2}{\pi}\ln(x) \\ Y_\nu(x) &\sim -\frac{\Gamma(\nu)}{\pi}\left(\frac{1}{2}x\right)^{-\nu} \qquad \nu > 0 \end{aligned} \tag{6.5.3}$$

For $x > \nu$, both Bessel functions look qualitatively like sine or cosine waves whose amplitude decays as $x^{-1/2}$. The asymptotic forms for $x \gg \nu$ are

$$\begin{aligned} J_\nu(x) &\sim \sqrt{\frac{2}{\pi x}}\cos\left(x - \frac{1}{2}\nu\pi - \frac{1}{4}\pi\right) \\ Y_\nu(x) &\sim \sqrt{\frac{2}{\pi x}}\sin\left(x - \frac{1}{2}\nu\pi - \frac{1}{4}\pi\right) \end{aligned} \tag{6.5.4}$$

In the transition region where $x \sim \nu$, the typical amplitudes of the Bessel functions are on the order

$$\begin{aligned} J_\nu(\nu) &\sim \frac{2^{1/3}}{3^{2/3}\Gamma(\frac{2}{3})}\frac{1}{\nu^{1/3}} \sim \frac{0.4473}{\nu^{1/3}} \\ Y_\nu(\nu) &\sim -\frac{2^{1/3}}{3^{1/6}\Gamma(\frac{2}{3})}\frac{1}{\nu^{1/3}} \sim -\frac{0.7748}{\nu^{1/3}} \end{aligned} \tag{6.5.5}$$

which holds asymptotically for large ν. Figure 6.5.1 plots the first few Bessel functions of each kind.

The Bessel functions satisfy the recurrence relations

$$J_{n+1}(x) = \frac{2n}{x}J_n(x) - J_{n-1}(x) \tag{6.5.6}$$

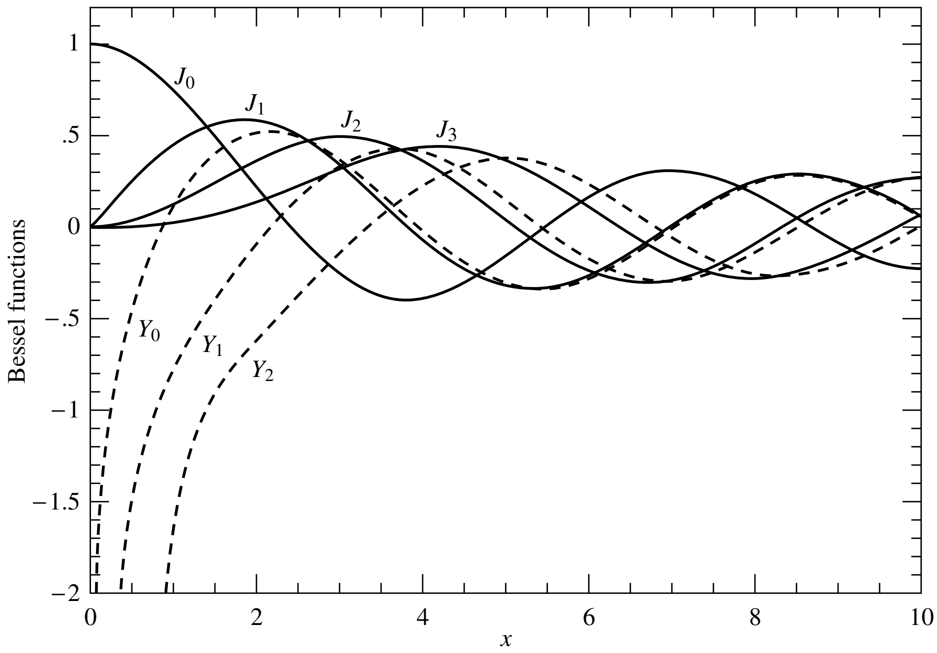

Figure 6.5.1. Bessel functions $J_0(x)$ through $J_3(x)$ and $Y_0(x)$ through $Y_2(x)$.

and

$$Y_{n+1}(x) = \frac{2n}{x} Y_n(x) - Y_{n-1}(x) \tag{6.5.7}$$

As already mentioned in §5.4, only the second of these, (6.5.7), is stable in the direction of increasing n for $x < n$. The reason that (6.5.6) is unstable in the direction of increasing n is simply that it is *the same recurrence* as (6.5.7): A small amount of "polluting" Y_n introduced by roundoff error will quickly come to swamp the desired J_n, according to equation (6.5.3).

A practical strategy for computing the Bessel functions of integer order divides into two tasks: first, how to compute J_0, J_1, Y_0, and Y_1; and second, how to use the recurrence relations stably to find other J's and Y's. We treat the first task first.

For x between zero and some arbitrary value (we will use the value 8), approximate $J_0(x)$ and $J_1(x)$ by rational functions in x. Likewise approximate by rational functions the "regular part" of $Y_0(x)$ and $Y_1(x)$, defined as

$$Y_0(x) - \frac{2}{\pi} J_0(x) \ln(x) \qquad \text{and} \qquad Y_1(x) - \frac{2}{\pi}\left[J_1(x)\ln(x) - \frac{1}{x}\right] \tag{6.5.8}$$

For $8 < x < \infty$, use the approximating forms ($n = 0, 1$)

$$J_n(x) = \sqrt{\frac{2}{\pi x}}\left[P_n\left(\frac{8}{x}\right)\cos(X_n) - Q_n\left(\frac{8}{x}\right)\sin(X_n)\right] \tag{6.5.9}$$

$$Y_n(x) = \sqrt{\frac{2}{\pi x}}\left[P_n\left(\frac{8}{x}\right)\sin(X_n) + Q_n\left(\frac{8}{x}\right)\cos(X_n)\right] \tag{6.5.10}$$

where

$$X_n \equiv x - \frac{2n+1}{4}\pi \tag{6.5.11}$$

and where P_0, P_1, Q_0, and Q_1 are each polynomials in their arguments, for $0 < 8/x < 1$. The P's are even polynomials, the Q's odd.

In the routines below, the various coefficients were calculated in multiple precision so as to achieve full double precision in the relative error. (In the neighborhood of the zeros of the functions, it is the absolute error that is double precision.) However, because of roundoff, evaluating the approximations can lead to a loss of up to two significant digits.

One additional twist: The rational approximation for $0 < x < 8$ is actually computed in the form [1]

$$J_0(x) = (x^2 - x_0^2)(x^2 - x_1^2)\frac{r(x^2)}{s(x^2)} \tag{6.5.12}$$

and similarly for J_1, Y_0 and Y_1. Here x_0 and x_1 are the two zeros of J_0 in the interval, and r and s are polynomials. The polynomial $r(x^2)$ has alternating signs. Writing it in terms of $64 - x^2$ makes all the signs the same and reduces roundoff error. For the approximations (6.5.9) and (6.5.10), our coefficients are similar but not identical to those given by Hart [2].

The functions J_0, J_1, Y_0, and Y_1 share a lot of code, so we package them as a single object `Bessjy`. The routines for higher J_n and Y_n are also member functions, with implementations discussed below. All the numerical coefficients are declared in `Bessjy` but defined (as a long list of constants) separately; the listing is in a Webnote [3].

bessel.h

```
struct Bessjy {
    static const Doub xj00,xj10,xj01,xj11,twoopi,pio4;
    static const Doub j0r[7],j0s[7],j0pn[5],j0pd[5],j0qn[5],j0qd[5];
    static const Doub j1r[7],j1s[7],j1pn[5],j1pd[5],j1qn[5],j1qd[5];
    static const Doub y0r[9],y0s[9],y0pn[5],y0pd[5],y0qn[5],y0qd[5];
    static const Doub y1r[8],y1s[8],y1pn[5],y1pd[5],y1qn[5],y1qd[5];
    Doub nump,denp,numq,denq,y,z,ax,xx;

    Doub j0(const Doub x) {
    Returns the Bessel function J0(x) for any real x.
        if ((ax=abs(x)) < 8.0) {          Direct rational function fit.
            rat(x,j0r,j0s,6);
            return nump*(y-xj00)*(y-xj10)/denp;
        } else {                          Fitting function (6.5.9).
            asp(j0pn,j0pd,j0qn,j0qd,1.);
            return sqrt(twoopi/ax)*(cos(xx)*nump/denp-z*sin(xx)*numq/denq);
        }
    }

    Doub j1(const Doub x) {
    Returns the Bessel function J1(x) for any real x.
        if ((ax=abs(x)) < 8.0) {          Direct rational approximation.
            rat(x,j1r,j1s,6);
            return x*nump*(y-xj01)*(y-xj11)/denp;
        } else {                          Fitting function (6.5.9).
            asp(j1pn,j1pd,j1qn,j1qd,3.);
            Doub ans=sqrt(twoopi/ax)*(cos(xx)*nump/denp-z*sin(xx)*numq/denq);
            return x > 0.0 ? ans : -ans;
        }
    }
```

```
    Doub y0(const Doub x) {
    Returns the Bessel function Y0(x) for positive x.
        if (x < 8.0) {                  Rational function approximation of (6.5.8).
            Doub j0x = j0(x);
            rat(x,y0r,y0s,8);
            return nump/denp+twoopi*j0x*log(x);
        } else {                        Fitting function (6.5.10).
            ax=x;
            asp(y0pn,y0pd,y0qn,y0qd,1.);
            return sqrt(twoopi/x)*(sin(xx)*nump/denp+z*cos(xx)*numq/denq);
        }
    }

    Doub y1(const Doub x) {
    Returns the Bessel function Y1(x) for positive x.
        if (x < 8.0) {                  Rational function approximation of (6.5.8).
            Doub j1x = j1(x);
            rat(x,y1r,y1s,7);
            return x*nump/denp+twoopi*(j1x*log(x)-1.0/x);
        } else {                        Fitting function (6.5.10).
            ax=x;
            asp(y1pn,y1pd,y1qn,y1qd,3.);
            return sqrt(twoopi/x)*(sin(xx)*nump/denp+z*cos(xx)*numq/denq);
        }
    }

    Doub jn(const Int n, const Doub x);
    Returns the Bessel function Jn(x) for any real x and integer n ≥ 0.

    Doub yn(const Int n, const Doub x);
    Returns the Bessel function Yn(x) for any positive x and integer n ≥ 0.

    void rat(const Doub x, const Doub *r, const Doub *s, const Int n) {
    Common code: Evaluates rational approximation.
        y = x*x;
        z=64.0-y;
        nump=r[n];
        denp=s[n];
        for (Int i=n-1;i>=0;i--) {
            nump=nump*z+r[i];
            denp=denp*y+s[i];
        }
    }

    void asp(const Doub *pn, const Doub *pd, const Doub *qn, const Doub *qd,
    Common code: Evaluates asymptotic approximation.
        const Doub fac) {
        z=8.0/ax;
        y=z*z;
        xx=ax-fac*pio4;
        nump=pn[4];
        denp=pd[4];
        numq=qn[4];
        denq=qd[4];
        for (Int i=3;i>=0;i--) {
            nump=nump*y+pn[i];
            denp=denp*y+pd[i];
            numq=numq*y+qn[i];
            denq=denq*y+qd[i];
        }
    }
};
```

We now turn to the second task, namely, how to use the recurrence formulas (6.5.6) and (6.5.7) to get the Bessel functions $J_n(x)$ and $Y_n(x)$ for $n \geq 2$. The latter of these is straightforward, since its upward recurrence is always stable:

bessel.h

```
Doub Bessjy::yn(const Int n, const Doub x)
Returns the Bessel function Yn(x) for any positive x and integer n ≥ 0.
{
    Int j;
    Doub by,bym,byp,tox;
    if (n==0) return y0(x);
    if (n==1) return y1(x);
    tox=2.0/x;
    by=y1(x);                        Starting values for the recurrence.
    bym=y0(x);
    for (j=1;j<n;j++) {              Recurrence (6.5.7).
        byp=j*tox*by-bym;
        bym=by;
        by=byp;
    }
    return by;
}
```

The cost of this algorithm is the calls to y1 and y0 (which generate a call to each of j1 and j0), plus $O(n)$ operations in the recurrence.

For $J_n(x)$, things are a bit more complicated. We can start the recurrence upward on n from J_0 and J_1, but it will remain stable only while n does not exceed x. This is, however, just fine for calls with large x and small n, a case that occurs frequently in practice.

The harder case to provide for is that with $x < n$. The best thing to do here is to use Miller's algorithm (see discussion preceding equation 5.4.16), applying the recurrence *downward* from some arbitrary starting value and making use of the upward-unstable nature of the recurrence to put us *onto* the correct solution. When we finally arrive at J_0 or J_1 we are able to normalize the solution with the sum (5.4.16) accumulated along the way.

The only subtlety is in deciding at how large an n we need start the downward recurrence so as to obtain a desired accuracy by the time we reach the n that we really want. If you play with the asymptotic forms (6.5.3) and (6.5.5), you should be able to convince yourself that the answer is to start larger than the desired n by an additive amount of order $[\text{constant} \times n]^{1/2}$, where the square root of the constant is, very roughly, the number of significant figures of accuracy.

The above considerations lead to the following function.

bessel.h

```
Doub Bessjy::jn(const Int n, const Doub x)
Returns the Bessel function Jn(x) for any real x and integer n ≥ 0.
{
    const Doub ACC=160.0;                         ACC determines accuracy.
    const Int IEXP=numeric_limits<Doub>::max_exponent/2;
    Bool jsum;
    Int j,k,m;
    Doub ax,bj,bjm,bjp,dum,sum,tox,ans;
    if (n==0) return j0(x);
    if (n==1) return j1(x);
    ax=abs(x);
    if (ax*ax <= 8.0*numeric_limits<Doub>::min()) return 0.0;
    else if (ax > Doub(n)) {                      Upwards recurrence from J0 and J1.
        tox=2.0/ax;
```

```
        bjm=j0(ax);
        bj=j1(ax);
        for (j=1;j<n;j++) {
            bjp=j*tox*bj-bjm;
            bjm=bj;
            bj=bjp;
        }
        ans=bj;
    } else {                                Downward recurrence from an even m here
        tox=2.0/ax;                             computed.
        m=2*((n+Int(sqrt(ACC*n)))/2);
        jsum=false;                         jsum will alternate between false and true;
        bjp=ans=sum=0.0;                        when it is true, we accumulate in sum
        bj=1.0;                                 the even terms in (5.4.16).
        for (j=m;j>0;j--) {                 The downward recurrence.
            bjm=j*tox*bj-bjp;
            bjp=bj;
            bj=bjm;
            dum=frexp(bj,&k);
            if (k > IEXP) {                 Renormalize to prevent overflows.
                bj=ldexp(bj,-IEXP);
                bjp=ldexp(bjp,-IEXP);
                ans=ldexp(ans,-IEXP);
                sum=ldexp(sum,-IEXP);
            }
            if (jsum) sum += bj;            Accumulate the sum.
            jsum=!jsum;                     Change false to true or vice versa.
            if (j == n) ans=bjp;            Save the unnormalized answer.
        }
        sum=2.0*sum-bj;                     Compute (5.4.16)
        ans /= sum;                         and use it to normalize the answer.
    }
    return x < 0.0 && (n & 1) ? -ans : ans;
}
```

The function `ldexp`, used above, is a standard C and C++ library function for scaling the binary exponent of a number.

6.5.1 Modified Bessel Functions of Integer Order

The modified Bessel functions $I_n(x)$ and $K_n(x)$ are equivalent to the usual Bessel functions J_n and Y_n evaluated for purely imaginary arguments. In detail, the relationship is

$$\begin{aligned} I_n(x) &= (-i)^n J_n(ix) \\ K_n(x) &= \frac{\pi}{2} i^{n+1} [J_n(ix) + iY_n(ix)] \end{aligned} \tag{6.5.13}$$

The particular choice of prefactor and of the linear combination of J_n and Y_n to form K_n are simply choices that make the functions real-valued for real arguments x.

For small arguments $x \ll n$, both $I_n(x)$ and $K_n(x)$ become, asymptotically, simple powers of their arguments

$$\begin{aligned} I_n(x) &\approx \frac{1}{n!}\left(\frac{x}{2}\right)^n \qquad n \geq 0 \\ K_0(x) &\approx -\ln(x) \\ K_n(x) &\approx \frac{(n-1)!}{2}\left(\frac{x}{2}\right)^{-n} \qquad n > 0 \end{aligned} \tag{6.5.14}$$

These expressions are virtually identical to those for $J_n(x)$ and $Y_n(x)$ in this region, except for the factor of $-2/\pi$ difference between $Y_n(x)$ and $K_n(x)$. In the region $x \gg n$, however, the modified functions have quite different behavior than the Bessel functions,

$$\begin{aligned} I_n(x) &\approx \frac{1}{\sqrt{2\pi x}}\exp(x) \\ K_n(x) &\approx \frac{\pi}{\sqrt{2\pi x}}\exp(-x) \end{aligned} \tag{6.5.15}$$

The modified functions evidently have exponential rather than sinusoidal behavior for large arguments (see Figure 6.5.2). Rational approximations analogous to those for the J and Y Bessel functions are efficient for computing I_0, I_1, K_0, and K_1. The corresponding routines are packaged as an object Bessik. The routines are similar to those in [1], although different in detail. (All the constants are again listed in a Webnote [3].)

bessel.h

```
struct Bessik {
    static const Doub i0p[14],i0q[5],i0pp[5],i0qq[6];
    static const Doub i1p[14],i1q[5],i1pp[5],i1qq[6];
    static const Doub k0pi[5],k0qi[3],k0p[5],k0q[3],k0pp[8],k0qq[8];
    static const Doub k1pi[5],k1qi[3],k1p[5],k1q[3],k1pp[8],k1qq[8];
    Doub y,z,ax,term;

    Doub i0(const Doub x) {
    Returns the modified Bessel function I0(x) for any real x.
        if ((ax=abs(x)) < 15.0) {          Rational approximation.
            y = x*x;
            return poly(i0p,13,y)/poly(i0q,4,225.-y);
        } else {                           Rational approximation with e^x/sqrt(x) factored out.
            z=1.0-15.0/ax;
            return exp(ax)*poly(i0pp,4,z)/(poly(i0qq,5,z)*sqrt(ax));
        }
    }

    Doub i1(const Doub x) {
    Returns the modified Bessel function I1(x) for any real x.
        if ((ax=abs(x)) < 15.0) {          Rational approximation.
            y=x*x;
            return x*poly(i1p,13,y)/poly(i1q,4,225.-y);
        } else {                           Rational approximation with e^x/sqrt(x) factored out.
            z=1.0-15.0/ax;
            Doub ans=exp(ax)*poly(i1pp,4,z)/(poly(i1qq,5,z)*sqrt(ax));
            return x > 0.0 ? ans : -ans;
        }
    }

    Doub k0(const Doub x) {
    Returns the modified Bessel function K0(x) for positive real x.
        if (x <= 1.0) {                    Use two rational approximations.
            z=x*x;
            term = poly(k0pi,4,z)*log(x)/poly(k0qi,2,1.-z);
            return poly(k0p,4,z)/poly(k0q,2,1.-z)-term;
        } else {                           Rational approximation with e^-x/sqrt(x) factored
            z=1.0/x;                           out.
            return exp(-x)*poly(k0pp,7,z)/(poly(k0qq,7,z)*sqrt(x));
        }
    }
```

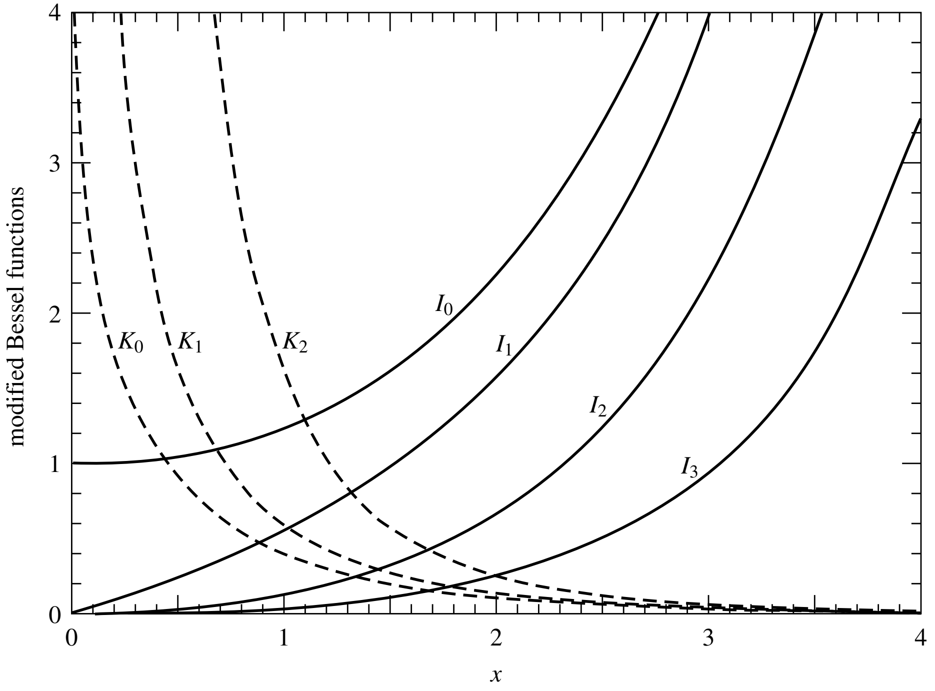

Figure 6.5.2. Modified Bessel functions $I_0(x)$ through $I_3(x)$, and $K_0(x)$ through $K_2(x)$.

```
Doub k1(const Doub x) {
```
Returns the modified Bessel function $K_1(x)$ for positive real x.
```
    if (x <= 1.0) {                    Use two rational approximations.
        z=x*x;
        term = poly(k1pi,4,z)*log(x)/poly(k1qi,2,1.-z);
        return x*(poly(k1p,4,z)/poly(k1q,2,1.-z)+term)+1./x;
    } else {                           Rational approximation with e^-x/sqrt(x) factored
        z=1.0/x;                       out.
        return exp(-x)*poly(k1pp,7,z)/(poly(k1qq,7,z)*sqrt(x));
    }
}

Doub in(const Int n, const Doub x);
```
Returns the modified Bessel function $I_n(x)$ for any real x and $n \geq 0$.
```
Doub kn(const Int n, const Doub x);
```
Returns the modified Bessel function $K_n(x)$ for positive x and $n \geq 0$.
```
inline Doub poly(const Doub *cof, const Int n, const Doub x) {
```
Common code: Evaluate a polynomial.
```
    Doub ans = cof[n];
    for (Int i=n-1;i>=0;i--) ans = ans*x+cof[i];
    return ans;
  }
};
```

The recurrence relation for $I_n(x)$ and $K_n(x)$ is the same as that for $J_n(x)$ and $Y_n(x)$ provided that ix is substituted for x. This has the effect of changing a sign in the relation,

$$I_{n+1}(x) = -\left(\frac{2n}{x}\right) I_n(x) + I_{n-1}(x)$$
$$K_{n+1}(x) = +\left(\frac{2n}{x}\right) K_n(x) + K_{n-1}(x) \qquad (6.5.16)$$

These relations are always *unstable* for upward recurrence. For K_n, itself growing, this presents no problem. The implementation is

bessel.h

```
Doub Bessik::kn(const Int n, const Doub x)
Returns the modified Bessel function K_n(x) for positive x and n ≥ 0.
{
    Int j;
    Doub bk,bkm,bkp,tox;
    if (n==0) return k0(x);
    if (n==1) return k1(x);
    tox=2.0/x;
    bkm=k0(x);                      Upward recurrence for all x...
    bk=k1(x);
    for (j=1;j<n;j++) {             ...and here it is.
        bkp=bkm+j*tox*bk;
        bkm=bk;
        bk=bkp;
    }
    return bk;
}
```

For I_n, the strategy of downward recursion is required once again, and the starting point for the recursion may be chosen in the same manner as for the routine `Bessjy::jn`. The only fundamental difference is that the normalization formula for $I_n(x)$ has an alternating minus sign in successive terms, which again arises from the substitution of ix for x in the formula used previously for J_n:

$$1 = I_0(x) - 2I_2(x) + 2I_4(x) - 2I_6(x) + \cdots \qquad (6.5.17)$$

In fact, we prefer simply to normalize with a call to `i0`.

bessel.h

```
Doub Bessik::in(const Int n, const Doub x)
Returns the modified Bessel function I_n(x) for any real x and n ≥ 0.
{
    const Doub ACC=200.0;                    ACC determines accuracy.
    const Int IEXP=numeric_limits<Doub>::max_exponent/2;
    Int j,k;
    Doub bi,bim,bip,dum,tox,ans;
    if (n==0) return i0(x);
    if (n==1) return i1(x);
    if (x*x <= 8.0*numeric_limits<Doub>::min()) return 0.0;
    else {
        tox=2.0/abs(x);
        bip=ans=0.0;
        bi=1.0;
        for (j=2*(n+Int(sqrt(ACC*n)));j>0;j--) {      Downward recurrence.
            bim=bip+j*tox*bi;
            bip=bi;
            bi=bim;
            dum=frexp(bi,&k);
            if (k > IEXP) {                  Renormalize to prevent overflows.
                ans=ldexp(ans,-IEXP);
                bi=ldexp(bi,-IEXP);
                bip=ldexp(bip,-IEXP);
```

```
            }
            if (j == n) ans=bip;
        }
        ans *= i0(x)/bi;                    Normalize with bessi0.
        return x < 0.0 && (n & 1) ? -ans : ans;
    }
}
```

The function ldexp, used above, is a standard C and C++ library function for scaling the binary exponent of a number.

CITED REFERENCES AND FURTHER READING:

Abramowitz, M., and Stegun, I.A. 1964, *Handbook of Mathematical Functions* (Washington: National Bureau of Standards); reprinted 1968 (New York: Dover); online at http://www.nr.com/aands, Chapter 9.

Carrier, G.F., Krook, M. and Pearson, C.E. 1966, *Functions of a Complex Variable* (New York: McGraw-Hill), pp. 220ff.

SPECFUN, 2007+, at http://www.netlib.org/specfun.[1]

Hart, J.F., et al. 1968, *Computer Approximations* (New York: Wiley), §6.8, p. 141.[2]

Numerical Recipes Software 2007, "Coefficients Used in the Bessjy and Bessik Objects," *Numerical Recipes Webnote No. 7*, at http://www.nr.com/webnotes?7 [3]

6.6 Bessel Functions of Fractional Order, Airy Functions, Spherical Bessel Functions

Many algorithms have been proposed for computing Bessel functions of fractional order numerically. Most of them are, in fact, not very good in practice. The routines given here are rather complicated, but they can be recommended wholeheartedly.

6.6.1 Ordinary Bessel Functions

The basic idea is *Steed's method*, which was originally developed [1] for Coulomb wave functions. The method calculates J_ν, J'_ν, Y_ν, and Y'_ν simultaneously, and so involves four relations among these functions. Three of the relations come from two continued fractions, one of which is complex. The fourth is provided by the Wronskian relation

$$W \equiv J_\nu Y'_\nu - Y_\nu J'_\nu = \frac{2}{\pi x} \tag{6.6.1}$$

The first continued fraction, CF1, is defined by

$$\begin{aligned} f_\nu \equiv \frac{J'_\nu}{J_\nu} &= \frac{\nu}{x} - \frac{J_{\nu+1}}{J_\nu} \\ &= \frac{\nu}{x} - \frac{1}{2(\nu+1)/x -}\;\frac{1}{2(\nu+2)/x -}\cdots \end{aligned} \tag{6.6.2}$$

You can easily derive it from the three-term recurrence relation for Bessel functions: Start with equation (6.5.6) and use equation (5.4.18). Forward evaluation of the continued fraction by one of the methods of §5.2 is essentially equivalent to backward recurrence of the recurrence relation. The rate of convergence of CF1 is determined by the position of the *turning point* $x_{tp} = \sqrt{\nu(\nu+1)} \approx \nu$, beyond which the Bessel functions become oscillatory. If $x \lesssim x_{tp}$,

convergence is very rapid. If $x \gtrsim x_{\rm tp}$, then each iteration of the continued fraction effectively increases ν by one until $x \lesssim x_{\rm tp}$; thereafter rapid convergence sets in. Thus the number of iterations of CF1 is of order x for large x. In the routine `besseljy` we set the maximum allowed number of iterations to 10,000. For larger x, you can use the usual asymptotic expressions for Bessel functions.

One can show that the sign of J_ν is the same as the sign of the denominator of CF1 once it has converged.

The complex continued fraction CF2 is defined by

$$p + iq \equiv \frac{J'_\nu + iY'_\nu}{J_\nu + iY_\nu} = -\frac{1}{2x} + i + \frac{i}{x}\,\frac{(1/2)^2 - \nu^2}{2(x+i)+}\,\frac{(3/2)^2 - \nu^2}{2(x+2i)+}\cdots \tag{6.6.3}$$

(We sketch the derivation of CF2 in the analogous case of modified Bessel functions in the next subsection.) This continued fraction converges rapidly for $x \gtrsim x_{\rm tp}$, while convergence fails as $x \to 0$. We have to adopt a special method for small x, which we describe below. For x not too small, we can ensure that $x \gtrsim x_{\rm tp}$ by a stable recurrence of J_ν and J'_ν downward to a value $\nu = \mu \lesssim x$, thus yielding the ratio f_μ at this lower value of ν. This is the stable direction for the recurrence relation. The initial values for the recurrence are

$$J_\nu = \text{arbitrary}, \qquad J'_\nu = f_\nu J_\nu, \tag{6.6.4}$$

with the sign of the arbitrary initial value of J_ν chosen to be the sign of the denominator of CF1. Choosing the initial value of J_ν very small minimizes the possibility of overflow during the recurrence. The recurrence relations are

$$\begin{aligned} J_{\nu-1} &= \frac{\nu}{x} J_\nu + J'_\nu \\ J'_{\nu-1} &= \frac{\nu-1}{x} J_{\nu-1} - J_\nu \end{aligned} \tag{6.6.5}$$

Once CF2 has been evaluated at $\nu = \mu$, then with the Wronskian (6.6.1) we have enough relations to solve for all four quantities. The formulas are simplified by introducing the quantity

$$\gamma \equiv \frac{p - f_\mu}{q} \tag{6.6.6}$$

Then

$$J_\mu = \pm\left(\frac{W}{q + \gamma(p - f_\mu)}\right)^{1/2} \tag{6.6.7}$$

$$J'_\mu = f_\mu J_\mu \tag{6.6.8}$$

$$Y_\mu = \gamma J_\mu \tag{6.6.9}$$

$$Y'_\mu = Y_\mu\left(p + \frac{q}{\gamma}\right) \tag{6.6.10}$$

The sign of J_μ in (6.6.7) is chosen to be the same as the sign of the initial J_ν in (6.6.4).

Once all four functions have been determined at the value $\nu = \mu$, we can find them at the original value of ν. For J_ν and J'_ν, simply scale the values in (6.6.4) by the ratio of (6.6.7) to the value found after applying the recurrence (6.6.5). The quantities Y_ν and Y'_ν can be found by starting with the values in (6.6.9) and (6.6.10) and using the stable upward recurrence

$$Y_{\nu+1} = \frac{2\nu}{x} Y_\nu - Y_{\nu-1} \tag{6.6.11}$$

together with the relation

$$Y'_\nu = \frac{\nu}{x} Y_\nu - Y_{\nu+1} \tag{6.6.12}$$

Now turn to the case of small x, when CF2 is not suitable. Temme [2] has given a good method of evaluating Y_ν and $Y_{\nu+1}$, and hence Y'_ν from (6.6.12), by series expansions that

accurately handle the singularity as $x \to 0$. The expansions work only for $|\nu| \leq 1/2$, and so now the recurrence (6.6.5) is used to evaluate f_ν at a value $\nu = \mu$ in this interval. Then one calculates J_μ from

$$J_\mu = \frac{W}{Y'_\mu - Y_\mu f_\mu} \tag{6.6.13}$$

and J'_μ from (6.6.8). The values at the original value of ν are determined by scaling as before, and the Y's are recurred up as before.

Temme's series are

$$Y_\nu = -\sum_{k=0}^{\infty} c_k g_k \qquad Y_{\nu+1} = -\frac{2}{x}\sum_{k=0}^{\infty} c_k h_k \tag{6.6.14}$$

Here

$$c_k = \frac{(-x^2/4)^k}{k!} \tag{6.6.15}$$

while the coefficients g_k and h_k are defined in terms of quantities p_k, q_k, and f_k that can be found by recursion:

$$\begin{aligned}
g_k &= f_k + \frac{2}{\nu}\sin^2\left(\frac{\nu\pi}{2}\right) q_k\\
h_k &= -k g_k + p_k\\
p_k &= \frac{p_{k-1}}{k-\nu}\\
q_k &= \frac{q_{k-1}}{k+\nu}\\
f_k &= \frac{k f_{k-1} + p_{k-1} + q_{k-1}}{k^2 - \nu^2}
\end{aligned} \tag{6.6.16}$$

The initial values for the recurrences are

$$\begin{aligned}
p_0 &= \frac{1}{\pi}\left(\frac{x}{2}\right)^{-\nu}\Gamma(1+\nu)\\
q_0 &= \frac{1}{\pi}\left(\frac{x}{2}\right)^{\nu}\Gamma(1-\nu)\\
f_0 &= \frac{2}{\pi}\frac{\nu\pi}{\sin\nu\pi}\left[\cosh\sigma\,\Gamma_1(\nu) + \frac{\sinh\sigma}{\sigma}\ln\left(\frac{2}{x}\right)\Gamma_2(\nu)\right]
\end{aligned} \tag{6.6.17}$$

with

$$\begin{aligned}
\sigma &= \nu\ln\left(\frac{2}{x}\right)\\
\Gamma_1(\nu) &= \frac{1}{2\nu}\left[\frac{1}{\Gamma(1-\nu)} - \frac{1}{\Gamma(1+\nu)}\right]\\
\Gamma_2(\nu) &= \frac{1}{2}\left[\frac{1}{\Gamma(1-\nu)} + \frac{1}{\Gamma(1+\nu)}\right]
\end{aligned} \tag{6.6.18}$$

The whole point of writing the formulas in this way is that the potential problems as $\nu \to 0$ can be controlled by evaluating $\nu\pi/\sin\nu\pi$, $\sinh\sigma/\sigma$, and Γ_1 carefully. In particular, Temme gives Chebyshev expansions for $\Gamma_1(\nu)$ and $\Gamma_2(\nu)$. We have rearranged his expansion for Γ_1 to be explicitly an even series in ν for more efficient evaluation, as explained in §5.8.

Because J_ν, Y_ν, J'_ν, and Y'_ν are all calculated simultaneously, a single `void` function sets them all. You then grab those that you need directly from the object. Alternatively, the functions `jnu` and `ynu` can be used. (We've omitted similar helper functions for the derivatives, but you can easily add them.) The object `Bessel` contains various other methods that will be discussed below.

The routines assume $\nu \geq 0$. For negative ν you can use the reflection formulas

$$\begin{aligned} J_{-\nu} &= \cos \nu\pi \; J_\nu - \sin \nu\pi \; Y_\nu \\ Y_{-\nu} &= \sin \nu\pi \; J_\nu + \cos \nu\pi \; Y_\nu \end{aligned} \tag{6.6.19}$$

The routine also assumes $x > 0$. For $x < 0$, the functions are in general complex but expressible in terms of functions with $x > 0$. For $x = 0$, Y_ν is singular. The complex arithmetic is carried out explicitly with real variables.

besselfrac.h

```
struct Bessel {
Object for Bessel functions of arbitrary order ν, and related functions.
    static const Int NUSE1=7, NUSE2=8;
    static const Doub c1[NUSE1],c2[NUSE2];
    Doub xo,nuo;                                Saved x and ν from last call.
    Doub jo,yo,jpo,ypo;                         Set by besseljy.
    Doub io,ko,ipo,kpo;                         Set by besselik.
    Doub aio,bio,aipo,bipo;                     Set by airy.
    Doub sphjo,sphyo,sphjpo,sphypo;             Set by sphbes.
    Int sphno;

    Bessel() : xo(9.99e99), nuo(9.99e99), sphno(-9999) {}
    Default constructor. No arguments.

    void besseljy(const Doub nu, const Doub x);
    Calculate Bessel functions Jν(x) and Yν(x) and their derivatives.
    void besselik(const Doub nu, const Doub x);
    Calculate Bessel functions Iν(x) and Kν(x) and their derivatives.

    Doub jnu(const Doub nu, const Doub x) {
    Simple interface returning Jν(x).
        if (nu != nuo || x != xo) besseljy(nu,x);
        return jo;
    }
    Doub ynu(const Doub nu, const Doub x) {
    Simple interface returning Yν(x).
        if (nu != nuo || x != xo) besseljy(nu,x);
        return yo;
    }
    Doub inu(const Doub nu, const Doub x) {
    Simple interface returning Iν(x).
        if (nu != nuo || x != xo) besselik(nu,x);
        return io;
    }
    Doub knu(const Doub nu, const Doub x) {
    Simple interface returning Kν(x).
        if (nu != nuo || x != xo) besselik(nu,x);
        return ko;
    }

    void airy(const Doub x);
    Calculate Airy functions Ai(x) and Bi(x) and their derivatives.
    Doub airy_ai(const Doub x);
    Simple interface returning Ai(x).
    Doub airy_bi(const Doub x);
    Simple interface returning Bi(x).

    void sphbes(const Int n, const Doub x);
    Calculate spherical Bessel functions jn(x) and yn(x) and their derivatives.
    Doub sphbesj(const Int n, const Doub x);
    Simple interface returning jn(x).
    Doub sphbesy(const Int n, const Doub x);
    Simple interface returning yn(x).
```

```
    inline Doub chebev(const Doub *c, const Int m, const Doub x) {
    Utility used by besseljy and besselik, evaluates Chebyshev series.
        Doub d=0.0,dd=0.0,sv;
        Int j;
        for (j=m-1;j>0;j--) {
            sv=d;
            d=2.*x*d-dd+c[j];
            dd=sv;
        }
        return x*d-dd+0.5*c[0];
    }
};

const Doub Bessel::c1[7] = {-1.142022680371168e0,6.5165112670737e-3,
    3.087090173086e-4,-3.4706269649e-6,6.9437664e-9,3.67795e-11,
    -1.356e-13};
const Doub Bessel::c2[8] = {1.843740587300905e0,-7.68528408447867e-2,
    1.2719271366546e-3,-4.9717367042e-6,-3.31261198e-8,2.423096e-10,
    -1.702e-13,-1.49e-15};
```

The code listing for `Bessel::besseljy` is in a Webnote [4].

6.6.2 Modified Bessel Functions

Steed's method does not work for modified Bessel functions because in this case CF2 is purely imaginary and we have only three relations among the four functions. Temme [3] has given a normalization condition that provides the fourth relation.

The Wronskian relation is

$$W \equiv I_\nu K'_\nu - K_\nu I'_\nu = -\frac{1}{x} \tag{6.6.20}$$

The continued fraction CF1 becomes

$$f_\nu \equiv \frac{I'_\nu}{I_\nu} = \frac{\nu}{x} + \frac{1}{2(\nu+1)/x+}\ \frac{1}{2(\nu+2)/x+}\cdots \tag{6.6.21}$$

To get CF2 and the normalization condition in a convenient form, consider the sequence of confluent hypergeometric functions

$$z_n(x) = U(\nu + 1/2 + n, 2\nu + 1, 2x) \tag{6.6.22}$$

for fixed ν. Then

$$K_\nu(x) = \pi^{1/2}(2x)^\nu e^{-x} z_0(x) \tag{6.6.23}$$

$$\frac{K_{\nu+1}(x)}{K_\nu(x)} = \frac{1}{x}\left[\nu + \frac{1}{2} + x + \left(\nu^2 - \frac{1}{4}\right)\frac{z_1}{z_0}\right] \tag{6.6.24}$$

Equation (6.6.23) is the standard expression for K_ν in terms of a confluent hypergeometric function, while equation (6.6.24) follows from relations between contiguous confluent hypergeometric functions (equations 13.4.16 and 13.4.18 in Ref. [5]). Now the functions z_n satisfy the three-term recurrence relation (equation 13.4.15 in Ref. [5])

$$z_{n-1}(x) = b_n z_n(x) + a_{n+1} z_{n+1} \tag{6.6.25}$$

with

$$\begin{aligned} b_n &= 2(n+x) \\ a_{n+1} &= -[(n+1/2)^2 - \nu^2] \end{aligned} \tag{6.6.26}$$

Following the steps leading to equation (5.4.18), we get the continued fraction CF2

$$\frac{z_1}{z_0} = \frac{1}{b_1+}\ \frac{a_2}{b_2+}\cdots \tag{6.6.27}$$

from which (6.6.24) gives $K_{\nu+1}/K_\nu$ and thus K'_ν/K_ν.

Temme's normalization condition is that

$$\sum_{n=0}^{\infty} C_n z_n = \left(\frac{1}{2x}\right)^{\nu+1/2} \tag{6.6.28}$$

where

$$C_n = \frac{(-1)^n}{n!}\frac{\Gamma(\nu+1/2+n)}{\Gamma(\nu+1/2-n)} \tag{6.6.29}$$

Note that the C_n's can be determined by recursion:

$$C_0 = 1, \qquad C_{n+1} = -\frac{a_{n+1}}{n+1}C_n \tag{6.6.30}$$

We use the condition (6.6.28) by finding

$$S = \sum_{n=1}^{\infty} C_n \frac{z_n}{z_0} \tag{6.6.31}$$

Then

$$z_0 = \left(\frac{1}{2x}\right)^{\nu+1/2} \frac{1}{1+S} \tag{6.6.32}$$

and (6.6.23) gives K_ν.

Thompson and Barnett [6] have given a clever method of doing the sum (6.6.31) simultaneously with the forward evaluation of the continued fraction CF2. Suppose the continued fraction is being evaluated as

$$\frac{z_1}{z_0} = \sum_{n=0}^{\infty} \Delta h_n \tag{6.6.33}$$

where the increments Δh_n are being found by, e.g., Steed's algorithm or the modified Lentz's algorithm of §5.2. Then the approximation to S keeping the first N terms can be found as

$$S_N = \sum_{n=1}^{N} Q_n \Delta h_n \tag{6.6.34}$$

Here

$$Q_n = \sum_{k=1}^{n} C_k q_k \tag{6.6.35}$$

and q_k is found by recursion from

$$q_{k+1} = (q_{k-1} - b_k q_k)/a_{k+1} \tag{6.6.36}$$

starting with $q_0 = 0$, $q_1 = 1$. For the case at hand, approximately three times as many terms are needed to get S to converge as are needed simply for CF2 to converge.

To find K_ν and $K_{\nu+1}$ for small x we use series analogous to (6.6.14):

$$K_\nu = \sum_{k=0}^{\infty} c_k f_k \qquad K_{\nu+1} = \frac{2}{x}\sum_{k=0}^{\infty} c_k h_k \tag{6.6.37}$$

Here

$$\begin{aligned}
c_k &= \frac{(x^2/4)^k}{k!} \\
h_k &= -kf_k + p_k \\
p_k &= \frac{p_{k-1}}{k-\nu} \\
q_k &= \frac{q_{k-1}}{k+\nu} \\
f_k &= \frac{kf_{k-1} + p_{k-1} + q_{k-1}}{k^2 - \nu^2}
\end{aligned} \tag{6.6.38}$$

The initial values for the recurrences are

$$\begin{aligned}
p_0 &= \frac{1}{2}\left(\frac{x}{2}\right)^{-\nu} \Gamma(1+\nu) \\
q_0 &= \frac{1}{2}\left(\frac{x}{2}\right)^{\nu} \Gamma(1-\nu) \\
f_0 &= \frac{\nu\pi}{\sin\nu\pi}\left[\cosh\sigma\,\Gamma_1(\nu) + \frac{\sinh\sigma}{\sigma}\ln\left(\frac{2}{x}\right)\Gamma_2(\nu)\right]
\end{aligned} \tag{6.6.39}$$

Both the series for small x, and CF2 and the normalization relation (6.6.28) require $|\nu| \le 1/2$. In both cases, therefore, we recurse I_ν down to a value $\nu = \mu$ in this interval, find K_μ there, and recurse K_ν back up to the original value of ν.

The routine assumes $\nu \ge 0$. For negative ν use the reflection formulas

$$\begin{aligned}
I_{-\nu} &= I_\nu + \frac{2}{\pi}\sin(\nu\pi)\,K_\nu \\
K_{-\nu} &= K_\nu
\end{aligned} \tag{6.6.40}$$

Note that for large x, $I_\nu \sim e^x$ and $K_\nu \sim e^{-x}$, and so these functions will overflow or underflow. It is often desirable to be able to compute the scaled quantities $e^{-x} I_\nu$ and $e^x K_\nu$. Simply omitting the factor e^{-x} in equation (6.6.23) will ensure that all four quantities will have the appropriate scaling. If you also want to scale the four quantities for small x when the series in equation (6.6.37) are used, you must multiply each series by e^x.

As with `besseljy`, you can either call the `void` function `besselik`, and then retrieve the function and/or derivative values from the object, or else just call `inu` or `knu`.

The code listing for `Bessel::besselik` is in a Webnote [4].

6.6.3 Airy Functions

For positive x, the Airy functions are defined by

$$\mathrm{Ai}(x) = \frac{1}{\pi}\sqrt{\frac{x}{3}}\,K_{1/3}(z) \tag{6.6.41}$$

$$\mathrm{Bi}(x) = \sqrt{\frac{x}{3}}\,[I_{1/3}(z) + I_{-1/3}(z)] \tag{6.6.42}$$

where

$$z = \frac{2}{3}x^{3/2} \tag{6.6.43}$$

By using the reflection formula (6.6.40), we can convert (6.6.42) into the computationally more useful form

$$\mathrm{Bi}(x) = \sqrt{x}\left[\frac{2}{\sqrt{3}}I_{1/3}(z) + \frac{1}{\pi}K_{1/3}(z)\right] \tag{6.6.44}$$

so that Ai and Bi can be evaluated with a single call to `besselik`.

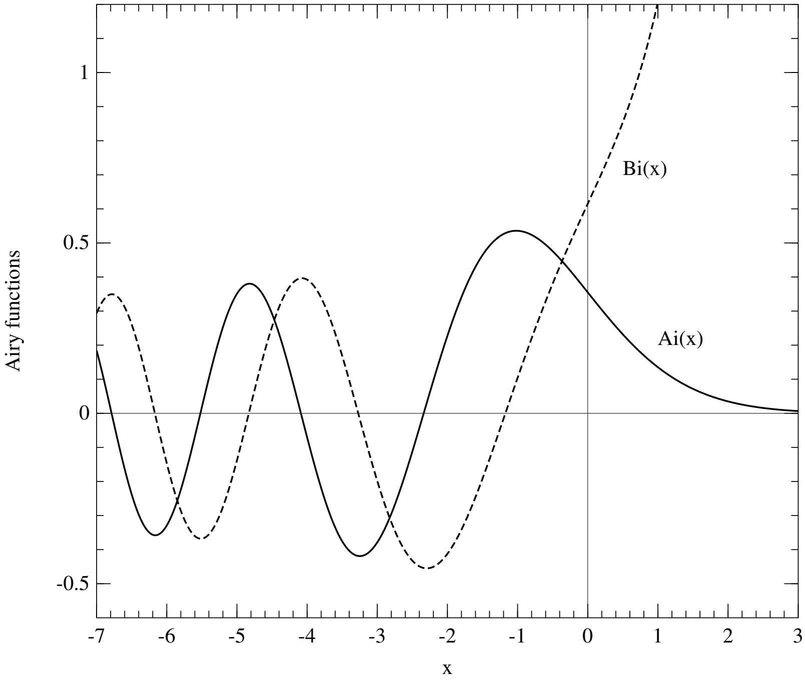

Figure 6.6.1. Airy functions $\text{Ai}(x)$ and $\text{Bi}(x)$.

The derivatives should not be evaluated by simply differentiating the above expressions because of possible subtraction errors near $x = 0$. Instead, use the equivalent expressions

$$\begin{aligned}
\text{Ai}'(x) &= -\frac{x}{\pi\sqrt{3}} K_{2/3}(z) \\
\text{Bi}'(x) &= x\left[\frac{2}{\sqrt{3}} I_{2/3}(z) + \frac{1}{\pi} K_{2/3}(z)\right]
\end{aligned} \tag{6.6.45}$$

The corresponding formulas for negative arguments are

$$\begin{aligned}
\text{Ai}(-x) &= \frac{\sqrt{x}}{2}\left[J_{1/3}(z) - \frac{1}{\sqrt{3}} Y_{1/3}(z)\right] \\
\text{Bi}(-x) &= -\frac{\sqrt{x}}{2}\left[\frac{1}{\sqrt{3}} J_{1/3}(z) + Y_{1/3}(z)\right] \\
\text{Ai}'(-x) &= \frac{x}{2}\left[J_{2/3}(z) + \frac{1}{\sqrt{3}} Y_{2/3}(z)\right] \\
\text{Bi}'(-x) &= \frac{x}{2}\left[\frac{1}{\sqrt{3}} J_{2/3}(z) - Y_{2/3}(z)\right]
\end{aligned} \tag{6.6.46}$$

besselfrac.h

```
void Bessel::airy(const Doub x) {
```

Sets aio, bio, aipo, and bipo, respectively, to the Airy functions $\text{Ai}(x)$, $\text{Bi}(x)$ and their derivatives $\text{Ai}'(x)$, $\text{Bi}'(x)$.

```
    static const Doub PI=3.141592653589793238,
        ONOVRT=0.577350269189626,THR=1./3.,TWOTHR=2.*THR;
    Doub absx,rootx,z;
    absx=abs(x);
    rootx=sqrt(absx);
    z=TWOTHR*absx*rootx;
```

```
    if (x > 0.0) {
        besselik(THR,z);
        aio = rootx*ONOVRT*ko/PI;
        bio = rootx*(ko/PI+2.0*ONOVRT*io);
        besselik(TWOTHR,z);
        aipo = -x*ONOVRT*ko/PI;
        bipo = x*(ko/PI+2.0*ONOVRT*io);
    } else if (x < 0.0) {
        besseljy(THR,z);
        aio = 0.5*rootx*(jo-ONOVRT*yo);
        bio = -0.5*rootx*(yo+ONOVRT*jo);
        besseljy(TWOTHR,z);
        aipo = 0.5*absx*(ONOVRT*yo+jo);
        bipo = 0.5*absx*(ONOVRT*jo-yo);
    } else {                          Case x = 0.
        aio=0.355028053887817;
        bio=aio/ONOVRT;
        aipo = -0.258819403792807;
        bipo = -aipo/ONOVRT;
    }
}

Doub Bessel::airy_ai(const Doub x) {
Simple interface returning Ai(x).
    if (x != xo) airy(x);
    return aio;
}
Doub Bessel::airy_bi(const Doub x) {
Simple interface returning Bi(x).
    if (x != xo) airy(x);
    return bio;
}
```

6.6.4 Spherical Bessel Functions

For integer n, spherical Bessel functions are defined by

$$
\begin{aligned}
j_n(x) &= \sqrt{\frac{\pi}{2x}} J_{n+\frac{1}{2}}(x) \\
y_n(x) &= \sqrt{\frac{\pi}{2x}} Y_{n+\frac{1}{2}}(x)
\end{aligned}
\tag{6.6.47}
$$

They can be evaluated by a call to besseljy, and the derivatives can safely be found from the derivatives of equation (6.6.47).

Note that in the continued fraction CF2 in (6.6.3) just the first term survives for $\nu = 1/2$. Thus one can make a very simple algorithm for spherical Bessel functions along the lines of besseljy by always recursing j_n down to $n = 0$, setting p and q from the first term in CF2, and then recursing y_n up. No special series is required near $x = 0$. However, besseljy is already so efficient that we have not bothered to provide an independent routine for spherical Bessels.

besselfrac.h

```
void Bessel::sphbes(const Int n, const Doub x) {
Sets sphjo, sphyo, sphjpo, and sphypo, respectively, to the spherical Bessel functions j_n(x),
y_n(x), and their derivatives j'_n(x), y'_n(x) for integer n (which is saved as sphno).
    const Doub RTPIO2=1.253314137315500251;
    Doub factor,order;
    if (n < 0 || x <= 0.0) throw("bad arguments in sphbes");
    order=n+0.5;
    besseljy(order,x);
    factor=RTPIO2/sqrt(x);
    sphjo=factor*jo;
```

```
    sphyo=factor*yo;
    sphjpo=factor*jpo-sphjo/(2.*x);
    sphypo=factor*ypo-sphyo/(2.*x);
    sphno = n;
}

Doub Bessel::sphbesj(const Int n, const Doub x) {
Simple interface returning j_n(x).
    if (n != sphno || x != xo) sphbes(n,x);
    return sphjo;
}
Doub Bessel::sphbesy(const Int n, const Doub x) {
Simple interface returning y_n(x).
    if (n != sphno || x != xo) sphbes(n,x);
    return sphyo;
}
```

CITED REFERENCES AND FURTHER READING:

Barnett, A.R., Feng, D.H., Steed, J.W., and Goldfarb, L.J.B. 1974, "Coulomb Wave Functions for All Real η and ρ," *Computer Physics Communications*, vol. 8, pp. 377–395.[1]

Temme, N.M. 1976, "On the Numerical Evaluation of the Ordinary Bessel Function of the Second Kind," *Journal of Computational Physics*, vol. 21, pp. 343–350[2]; 1975, *op. cit.*, vol. 19, pp. 324–337.[3]

Numerical Recipes Software 2007, "Bessel Function Implementations," *Numerical Recipes Webnote No. 8*, at http://www.nr.com/webnotes?8 [4]

Abramowitz, M., and Stegun, I.A. 1964, *Handbook of Mathematical Functions* (Washington: National Bureau of Standards); reprinted 1968 (New York: Dover); online at http://www.nr.com/aands, Chapter 10.[5]

Thompson, I.J., and Barnett, A.R. 1987, "Modified Bessel Functions $I_\nu(z)$ and $K_\nu(z)$ of Real Order and Complex Argument, to Selected Accuracy," *Computer Physics Communications*, vol. 47, pp. 245–257.[6]

Barnett, A.R. 1981, "An Algorithm for Regular and Irregular Coulomb and Bessel functions of Real Order to Machine Accuracy," *Computer Physics Communications*, vol. 21, pp. 297–314.

Thompson, I.J., and Barnett, A.R. 1986, "Coulomb and Bessel Functions of Complex Arguments and Order," *Journal of Computational Physics*, vol. 64, pp. 490–509.

6.7 Spherical Harmonics

Spherical harmonics occur in a large variety of physical problems, for example, whenever a wave equation, or Laplace's equation, is solved by separation of variables in spherical coordinates. The spherical harmonic $Y_{lm}(\theta,\phi)$, $-l \le m \le l$, is a function of the two coordinates θ,ϕ on the surface of a sphere.

The spherical harmonics are orthogonal for different l and m, and they are normalized so that their integrated square over the sphere is unity:

$$\int_0^{2\pi} d\phi \int_{-1}^{1} d(\cos\theta) Y^*_{l'm'}(\theta,\phi) Y_{lm}(\theta,\phi) = \delta_{l'l}\delta_{m'm} \tag{6.7.1}$$

Here the asterisk denotes complex conjugation.

Mathematically, the spherical harmonics are related to *associated Legendre polynomials* by the equation

$$Y_{lm}(\theta,\phi) = \sqrt{\frac{2l+1}{4\pi}\frac{(l-m)!}{(l+m)!}}\,P_l^m(\cos\theta)e^{im\phi} \tag{6.7.2}$$

By using the relation

$$Y_{l,-m}(\theta,\phi) = (-1)^m Y_{lm}^*(\theta,\phi) \tag{6.7.3}$$

we can always relate a spherical harmonic to an associated Legendre polynomial with $m \geq 0$. With $x \equiv \cos\theta$, these are defined in terms of the ordinary Legendre polynomials (cf. §4.6 and §5.4) by

$$P_l^m(x) = (-1)^m(1-x^2)^{m/2}\frac{d^m}{dx^m}P_l(x) \tag{6.7.4}$$

Be careful: There are alternative normalizations for the associated Legendre polynomials and alternative sign conventions.

The first few associated Legendre polynomials, and their corresponding normalized spherical harmonics, are

$P_0^0(x) =$	1	$Y_{00} =$	$\sqrt{\frac{1}{4\pi}}$
$P_1^1(x) =$	$-(1-x^2)^{1/2}$	$Y_{11} =$	$-\sqrt{\frac{3}{8\pi}}\sin\theta e^{i\phi}$
$P_1^0(x) =$	x	$Y_{10} =$	$\sqrt{\frac{3}{4\pi}}\cos\theta$
$P_2^2(x) =$	$3(1-x^2)$	$Y_{22} =$	$\frac{1}{4}\sqrt{\frac{15}{2\pi}}\sin^2\theta e^{2i\phi}$
$P_2^1(x) =$	$-3(1-x^2)^{1/2}x$	$Y_{21} =$	$-\sqrt{\frac{15}{8\pi}}\sin\theta\cos\theta e^{i\phi}$
$P_2^0(x) =$	$\frac{1}{2}(3x^2-1)$	$Y_{20} =$	$\sqrt{\frac{5}{4\pi}}(\frac{3}{2}\cos^2\theta - \frac{1}{2})$

(6.7.5)

There are many bad ways to evaluate associated Legendre polynomials numerically. For example, there are explicit expressions, such as

$$P_l^m(x) = \frac{(-1)^m(l+m)!}{2^m m!(l-m)!}(1-x^2)^{m/2}\left[1 - \frac{(l-m)(m+l+1)}{1!(m+1)}\left(\frac{1-x}{2}\right) + \frac{(l-m)(l-m-1)(m+l+1)(m+l+2)}{2!(m+1)(m+2)}\left(\frac{1-x}{2}\right)^2 - \cdots\right] \tag{6.7.6}$$

where the polynomial continues up through the term in $(1-x)^{l-m}$. (See [1] for this and related formulas.) This is not a satisfactory method because evaluation of the polynomial involves delicate cancellations between successive terms, which alternate in sign. For large l, the individual terms in the polynomial become very much larger than their sum, and all accuracy is lost.

In practice, (6.7.6) can be used only in single precision (32-bit) for l up to 6 or 8, and in double precision (64-bit) for l up to 15 or 18, depending on the precision required for the answer. A more robust computational procedure is therefore desirable, as follows.

The associated Legendre functions satisfy numerous recurrence relations, tabulated in [1,2]. These are recurrences on l alone, on m alone, and on both l and m simultaneously. Most of the recurrences involving m are unstable, and so are dangerous for numerical work. The following recurrence on l is, however, stable (compare 5.4.1):

$$(l-m)P_l^m = x(2l-1)P_{l-1}^m - (l+m-1)P_{l-2}^m \tag{6.7.7}$$

Even this recurrence is useful only for moderate l and m, since the P_l^m's themselves grow rapidly with l and quickly overflow. The spherical harmonics by contrast remain bounded — after all, they are normalized to unity (eq. 6.7.1). It is exactly the square-root factor in equation (6.7.2) that balances the divergence. So the right function to use in the recurrence relation is the renormalized associated Legendre function,

$$\widetilde{P}_l^m = \sqrt{\frac{2l+1}{4\pi}\frac{(l-m)!}{(l+m)!}}\,P_l^m \tag{6.7.8}$$

Then the recurrence relation (6.7.7) becomes

$$\widetilde{P}_l^m = \sqrt{\frac{4l^2-1}{l^2-m^2}}\left[x\widetilde{P}_{l-1}^m - \sqrt{\frac{(l-1)^2-m^2}{4(l-1)^2-1}}\,\widetilde{P}_{l-2}^m\right] \tag{6.7.9}$$

We start the recurrence with the closed-form expression for the $l=m$ function,

$$\widetilde{P}_m^m = (-1)^m\sqrt{\frac{2m+1}{4\pi(2m)!}}\,(2m-1)!!\,(1-x^2)^{m/2} \tag{6.7.10}$$

(The notation $n!!$ denotes the product of all *odd* integers less than or equal to n.) Using (6.7.9) with $l=m+1$, and setting $\widetilde{P}_{m-1}^m = 0$, we find

$$\widetilde{P}_{m+1}^m = x\sqrt{2m+3}\,\widetilde{P}_m^m \tag{6.7.11}$$

Equations (6.7.10) and (6.7.11) provide the two starting values required for (6.7.9) for general l.

The function that implements this is

plegendre.h

```
Doub plegendre(const Int l, const Int m, const Doub x) {
```

Computes the renormalized associated Legendre polynomial $\widetilde{P}_l^m(x)$, equation (6.7.8). Here m and l are integers satisfying $0 \le m \le l$, while x lies in the range $-1 \le x \le 1$.

```
    static const Doub PI=3.141592653589793;
    Int i,ll;
    Doub fact,oldfact,pll,pmm,pmmp1,omx2;
    if (m < 0 || m > l || abs(x) > 1.0)
        throw("Bad arguments in routine plgndr");
    pmm=1.0;
```

Compute $\widetilde{P}_m^m$.

```
    if (m > 0) {
        omx2=(1.0-x)*(1.0+x);
        fact=1.0;
        for (i=1;i<=m;i++) {
            pmm *= omx2*fact/(fact+1.0);
            fact += 2.0;
        }
    }
    pmm=sqrt((2*m+1)*pmm/(4.0*PI));
```

```
    if (m & 1)
        pmm=-pmm;
    if (l == m)
        return pmm;
    else {                          Compute P̃^m_{m+1}.
        pmmp1=x*sqrt(2.0*m+3.0)*pmm;
        if (l == (m+1))
            return pmmp1;
        else {                      Compute P̃^m_l, l > m + 1.
            oldfact=sqrt(2.0*m+3.0);
            for (ll=m+2;ll<=l;ll++) {
                fact=sqrt((4.0*ll*ll-1.0)/(ll*ll-m*m));
                pll=(x*pmmp1-pmm/oldfact)*fact;
                oldfact=fact;
                pmm=pmmp1;
                pmmp1=pll;
            }
            return pll;
        }
    }
}
```

Sometimes it is convenient to have the functions with the standard normalization, as defined by equation (6.7.4). Here is a routine that does this. Note that it will overflow for $m \gtrsim 80$, or even sooner if $l \gg m$.

```
Doub plgndr(const Int l, const Int m, const Doub x)                    plegendre.h
```

Computes the associated Legendre polynomial $P_l^m(x)$, equation (6.7.4). Here m and l are integers satisfying $0 \le m \le l$, while x lies in the range $-1 \le x \le 1$. These functions will overflow for $m \gtrsim 80$.

```
{
    const Doub PI=3.141592653589793238;
    if (m < 0 || m > l || abs(x) > 1.0)
        throw("Bad arguments in routine plgndr");
    Doub prod=1.0;
    for (Int j=l-m+1;j<=l+m;j++)
        prod *= j;
    return sqrt(4.0*PI*prod/(2*l+1))*plegendre(l,m,x);
}
```

6.7.1 Fast Spherical Harmonic Transforms

Any smooth function on the surface of a sphere can be written as an expansion in spherical harmonics. Suppose the function can be well-approximated by truncating the expansion at $l = l_{\max}$:

$$\begin{aligned} f(\theta_i, \phi_j) &= \sum_{l=0}^{l_{\max}} \sum_{m=-l}^{m=l} a_{lm} Y_{lm}(\theta_i, \phi_j) \\ &= \sum_{l=0}^{l_{\max}} \sum_{m=-l}^{m=l} a_{lm} \widetilde{P}_l^m(\cos\theta_i) e^{im\phi_j} \end{aligned} \tag{6.7.12}$$

Here we have written the function evaluated at one of N_θ sample points θ_i and one of N_ϕ sample points ϕ_j. The total number of sample points is $N = N_\theta N_\phi$. In applications, typically $N_\theta \sim N_\phi \sim \sqrt{N}$. Since the total number of spherical harmonics in the sum (6.7.12) is $l_{\max}^2$, we also have $l_{\max} \sim \sqrt{N}$.

How many operations does it take to evaluate the sum (6.7.12)? Direct evaluation of $l_{\max}^2$ terms at N sample points is an $O(N^2)$ process. You might try to speed this up by choosing the sample points ϕ_j to be equally spaced in angle and doing the sum over m by an FFT. Each FFT is $O(N_\phi \ln N_\phi)$, and you have to do $O(N_\theta l_{\max})$ of them, for a total of $O(N^{3/2} \ln N)$ operations, which is some improvement. A simple rearrangement [3-5] gives an even better way: Interchange the order of summation

$$\sum_{l=0}^{l_{\max}} \sum_{m=-l}^{l} \longleftrightarrow \sum_{m=-l_{\max}}^{l_{\max}} \sum_{l=|m|}^{l_{\max}} \tag{6.7.13}$$

so that

$$f(\theta_i, \phi_j) = \sum_{m=-l_{\max}}^{l_{\max}} q_m(\theta_i) e^{im\phi_j} \tag{6.7.14}$$

where

$$q_m(\theta_i) = \sum_{l=|m|}^{l_{\max}} a_{lm} \widetilde{P}_l^m(\cos\theta_i) \tag{6.7.15}$$

Evaluating the sum in (6.7.15) is $O(l_{\max})$, and one must do this for $O(l_{\max} N_\theta)$ q_m's, so the total work is $O(N^{3/2})$. To evaluate equation (6.7.14) by an FFT at fixed θ_i is $O(N_\phi \ln N_\phi)$. There are N_θ FFTs to be done, for a total operations count of $O(N \ln N)$, which is negligible in comparison. So the total algorithm is $O(N^{3/2})$. Note that you can evaluate equation (6.7.14) either by precomputing and storing the $\widetilde{P}_l^m$'s using the recurrence relation (6.7.9), or by Clenshaw's method (§5.4).

What about inverting the transform (6.7.12)? The formal inverse for the expansion of a continuous function $f(\theta, \phi)$ follows from the orthonormality of the Y_{lm}'s, equation (6.7.1),

$$a_{lm} = \int \sin\theta \, d\theta \, d\phi \, f(\theta, \phi) e^{-im\phi} \widetilde{P}_l^m(\cos\theta) \tag{6.7.16}$$

For the discrete case, where we have a sampled function, the integral becomes a quadrature:

$$a_{lm} = \sum_{i,j} w(\theta_i) f(\theta_i, \phi_j) e^{-im\phi_j} \widetilde{P}_l^m(\cos\theta_i) \tag{6.7.17}$$

Here $w(\theta_i)$ are the quadrature weights. In principle we could consider weights that depend on ϕ_j as well, but in practice we do the ϕ quadrature by an FFT, so the weights are unity. A good choice for the weights for an equi-angular grid in θ is given in Ref. [3], Theorem 3. Another possibility is to use Gaussian quadrature for the θ integral. In this case, you choose the sample points so that the $\cos\theta_i$'s are the abscissas returned by `gauleg` and the $w(\theta_i)$'s are the corresponding weights. The best way to organize the calculation is to first do the FFTs, computing

$$g_m(\theta_i) = \sum_j f(\theta_i, \phi_j) e^{-im\phi_j} \tag{6.7.18}$$

Then

$$a_{lm} = \sum_i w(\theta_i) g_m(\theta_i) \widetilde{P}_l^m(\cos\theta_i) \tag{6.7.19}$$

You can verify that the operations count is dominated by equation (6.7.19) and scales as $O(N^{3/2})$ once again. In a real calculation, you should exploit all the symmetries that let you reduce the workload, such as $g_{-m} = g_m^*$ and $\widetilde{P}_l^m[\cos(\pi - \theta)] = (-1)^{l+m}\widetilde{P}_l^m(\cos\theta)$.

Very recently, algorithms for fast Legendre transforms have been developed, similar in spirit to the FFT [3,6,7]. Theoretically, they reduce the forward and inverse spherical harmonic transforms to $O(N \log^2 N)$ problems. However, current implementations [8] are not much faster than the $O(N^{3/2})$ methods above for $N \sim 500$, and there are stability and accuracy problems that require careful attention [9]. Stay tuned!

CITED REFERENCES AND FURTHER READING:

Magnus, W., and Oberhettinger, F. 1949, *Formulas and Theorems for the Functions of Mathematical Physics* (New York: Chelsea), pp. 54ff.[1]

Abramowitz, M., and Stegun, I.A. 1964, *Handbook of Mathematical Functions* (Washington: National Bureau of Standards); reprinted 1968 (New York: Dover); online at `http://www.nr.com/aands`, Chapter 8.[2]

Driscoll, J.R., and Healy, D.M. 1994, "Computing Fourier Transforms and Convolutions on the 2–sphere," *Advances in Applied Mathematics*, vol. 15, pp. 202–250.[3]

Muciaccia, P.F., Natoli, P., and Vittorio, N. 1997, "Fast Spherical Harmonic Analysis: A Quick Algorithm for Generating and/or Inverting Full-Sky, High-Resolution Cosmic Microwave Background Anisotropy Maps," *Astrophysical Journal*, vol. 488, pp. L63–66.[4]

Oh, S.P., Spergel, D.N., and Hinshaw, G. 1999, "An Efficient Technique to Determine the Power Spectrum from Cosmic Microwave Background Sky Maps," *Astrophysical Journal*, vol. 510, pp. 551–563, Appendix A.[5]

Healy, D.M., Rockmore, D., Kostelec, P.J., and Moore, S. 2003, "FFTs for the 2-Sphere: Improvements and Variations," *Journal of Fourier Analysis and Applications*, vol. 9, pp. 341–385.[6]

Potts, D., Steidl, G., and Tasche, M. 1998, " Fast and Stable Algorithms for Discrete Spherical Fourier Transforms," *Linear Algebra and Its Applications*, vol. 275-276, pp. 433–450.[7]

Moore, S., Healy, D.M., Rockmore, D., and Kostelec, P.J. 2007+, *SpharmonicKit*. Software at `http://www.cs.dartmouth.edu/~geelong/sphere`.[8]

Healy, D.M., Kostelec, P.J., and Rockmore, D. 2004, "Towards Safe and Effective High-Order Legendre Transforms with Applications to FFTs for the 2-Sphere," *Advances in Computational Mathematics*, vol. 21, pp. 59–105.[9]

6.8 Fresnel Integrals, Cosine and Sine Integrals

6.8.1 Fresnel Integrals

The two Fresnel integrals are defined by

$$C(x) = \int_0^x \cos\left(\frac{\pi}{2}t^2\right) dt, \qquad S(x) = \int_0^x \sin\left(\frac{\pi}{2}t^2\right) dt \tag{6.8.1}$$

and are plotted in Figure 6.8.1.

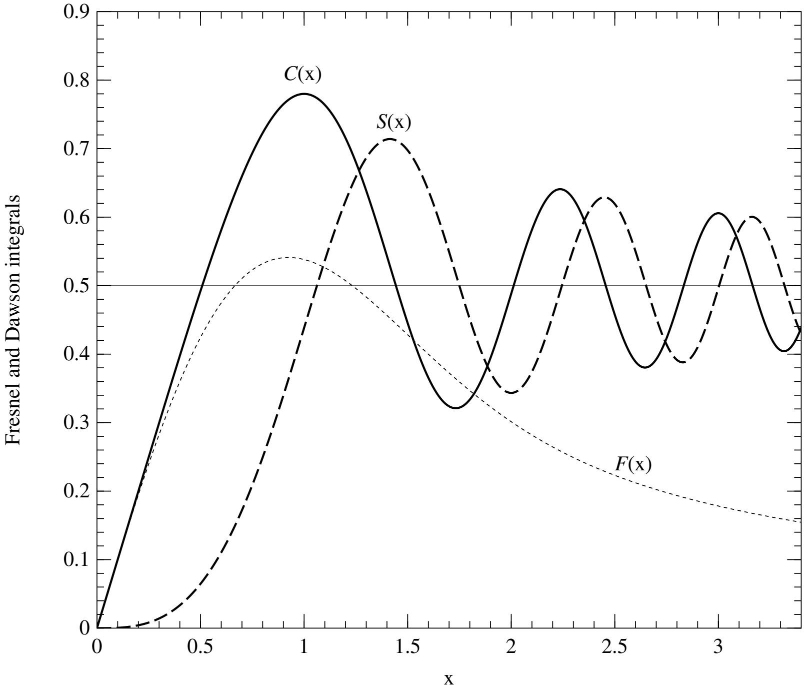

Figure 6.8.1. Fresnel integrals $C(x)$ and $S(x)$ (§6.8), and Dawson's integral $F(x)$ (§6.9).

The most convenient way of evaluating these functions to arbitrary precision is to use power series for small x and a continued fraction for large x. The series are

$$C(x) = x - \left(\frac{\pi}{2}\right)^2 \frac{x^5}{5\cdot 2!} + \left(\frac{\pi}{2}\right)^4 \frac{x^9}{9\cdot 4!} - \cdots$$
$$S(x) = \left(\frac{\pi}{2}\right) \frac{x^3}{3\cdot 1!} - \left(\frac{\pi}{2}\right)^3 \frac{x^7}{7\cdot 3!} + \left(\frac{\pi}{2}\right)^5 \frac{x^{11}}{11\cdot 5!} - \cdots \tag{6.8.2}$$

There is a complex continued fraction that yields both $S(x)$ and $C(x)$ simultaneously:

$$C(x) + iS(x) = \frac{1+i}{2}\,\text{erf}\, z, \qquad z = \frac{\sqrt{\pi}}{2}(1-i)x \tag{6.8.3}$$

where

$$e^{z^2}\,\text{erfc}\, z = \frac{1}{\sqrt{\pi}}\left(\frac{1}{z+}\;\frac{1/2}{z+}\;\frac{1}{z+}\;\frac{3/2}{z+}\;\frac{2}{z+}\cdots\right)$$
$$= \frac{2z}{\sqrt{\pi}}\left(\frac{1}{2z^2+1-}\;\frac{1\cdot 2}{2z^2+5-}\;\frac{3\cdot 4}{2z^2+9-}\cdots\right) \tag{6.8.4}$$

In the last line we have converted the "standard" form of the continued fraction to its "even" form (see §5.2), which converges twice as fast. We must be careful not to evaluate the alternating series (6.8.2) at too large a value of x; inspection of the terms shows that $x = 1.5$ is a good point to switch over to the continued fraction.

Note that for large x

$$C(x) \sim \frac{1}{2} + \frac{1}{\pi x}\sin\left(\frac{\pi}{2}x^2\right), \qquad S(x) \sim \frac{1}{2} - \frac{1}{\pi x}\cos\left(\frac{\pi}{2}x^2\right) \tag{6.8.5}$$

Thus the precision of the routine `frenel` may be limited by the precision of the library routines for sine and cosine for large x.

frenel.h

```
Complex frenel(const Doub x) {
Computes the Fresnel integrals S(x) and C(x) for all real x. C(x) is returned as the real part
of cs and S(x) as the imaginary part.
    static const Int MAXIT=100;
    static const Doub PI=3.141592653589793238, PIBY2=(PI/2.0), XMIN=1.5,
        EPS=numeric_limits<Doub>::epsilon(),
        FPMIN=numeric_limits<Doub>::min(),
        BIG=numeric_limits<Doub>::max()*EPS;
        Here MAXIT is the maximum number of iterations allowed; EPS is the relative error;
        FPMIN is a number near the smallest representable floating-point number; BIG is a
        number near the machine overflow limit; and XMIN is the dividing line between using
        the series and continued fraction.
    Bool odd;
    Int k,n;
    Doub a,ax,fact,pix2,sign,sum,sumc,sums,term,test;
    Complex b,cc,d,h,del,cs;
    if ((ax=abs(x)) < sqrt(FPMIN)) {           Special case: Avoid failure of convergence
        cs=ax;                                     test because of underflow.
    } else if (ax <= XMIN) {                   Evaluate both series simultaneously.
        sum=sums=0.0;
        sumc=ax;
        sign=1.0;
        fact=PIBY2*ax*ax;
        odd=true;
        term=ax;
        n=3;
        for (k=1;k<=MAXIT;k++) {
            term *= fact/k;
            sum += sign*term/n;
            test=abs(sum)*EPS;
            if (odd) {
                sign = -sign;
                sums=sum;
                sum=sumc;
            } else {
                sumc=sum;
                sum=sums;
            }
            if (term < test) break;
            odd=!odd;
            n += 2;
        }
        if (k > MAXIT) throw("series failed in frenel");
        cs=Complex(sumc,sums);
    } else {                                   Evaluate continued fraction by modified
        pix2=PI*ax*ax;                             Lentz's method (§5.2).
        b=Complex(1.0,-pix2);
        cc=BIG;
        d=h=1.0/b;
        n = -1;
        for (k=2;k<=MAXIT;k++) {
            n += 2;
            a = -n*(n+1);
            b += 4.0;
            d=1.0/(a*d+b);                     Denominators cannot be zero.
            cc=b+a/cc;
            del=cc*d;
            h *= del;
            if (abs(real(del)-1.0)+abs(imag(del)) <= EPS) break;
        }
```

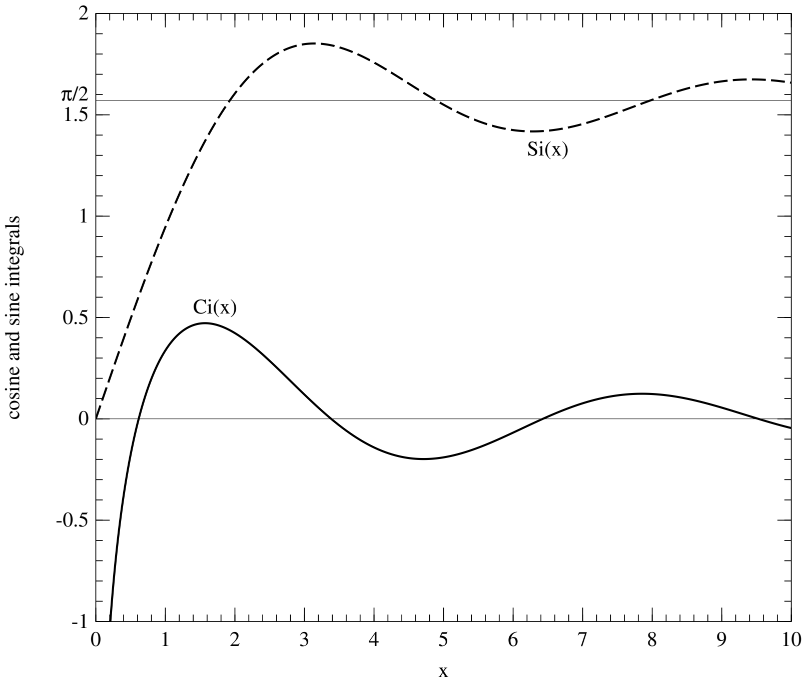

Figure 6.8.2. Sine and cosine integrals $\mathrm{Si}(x)$ and $\mathrm{Ci}(x)$.

```
        if (k > MAXIT) throw("cf failed in frenel");
        h *= Complex(ax,-ax);
        cs=Complex(0.5,0.5)
            *(1.0-Complex(cos(0.5*pix2),sin(0.5*pix2))*h);
    }
    if (x < 0.0) cs = -cs;                    Use antisymmetry.
    return cs;
}
```

6.8.2 Cosine and Sine Integrals

The cosine and sine integrals are defined by

$$\begin{aligned}\mathrm{Ci}(x) &= \gamma + \ln x + \int_0^x \frac{\cos t - 1}{t}\,dt \\ \mathrm{Si}(x) &= \int_0^x \frac{\sin t}{t}\,dt\end{aligned} \tag{6.8.6}$$

and are plotted in Figure 6.8.2. Here $\gamma \approx 0.5772\ldots$ is Euler's constant. We only need a way to calculate the functions for $x > 0$, because

$$\mathrm{Si}(-x) = -\mathrm{Si}(x), \qquad \mathrm{Ci}(-x) = \mathrm{Ci}(x) - i\pi \tag{6.8.7}$$

Once again we can evaluate these functions by a judicious combination of power

series and complex continued fraction. The series are

$$\begin{aligned} \mathrm{Si}(x) &= x - \frac{x^3}{3 \cdot 3!} + \frac{x^5}{5 \cdot 5!} - \cdots \\ \mathrm{Ci}(x) &= \gamma + \ln x + \left(-\frac{x^2}{2 \cdot 2!} + \frac{x^4}{4 \cdot 4!} - \cdots \right) \end{aligned} \tag{6.8.8}$$

The continued fraction for the exponential integral $E_1(ix)$ is

$$\begin{aligned} E_1(ix) &= -\mathrm{Ci}(x) + i[\mathrm{Si}(x) - \pi/2] \\ &= e^{-ix} \left(\frac{1}{ix+} \, \frac{1}{1+} \, \frac{1}{ix+} \, \frac{2}{1+} \, \frac{2}{ix+} \cdots \right) \\ &= e^{-ix} \left(\frac{1}{1+ix-} \, \frac{1^2}{3+ix-} \, \frac{2^2}{5+ix-} \cdots \right) \end{aligned} \tag{6.8.9}$$

The "even" form of the continued fraction is given in the last line and converges twice as fast for about the same amount of computation. A good crossover point from the alternating series to the continued fraction is $x = 2$ in this case. As for the Fresnel integrals, for large x the precision may be limited by the precision of the sine and cosine routines.

cisi.h

```
Complex cisi(const Doub x) {
```
Computes the cosine and sine integrals $\mathrm{Ci}(x)$ and $\mathrm{Si}(x)$. The function $\mathrm{Ci}(x)$ is returned as the real part of cs, and $\mathrm{Si}(x)$ as the imaginary part. $\mathrm{Ci}(0)$ is returned as a large negative number and no error message is generated. For $x < 0$ the routine returns $\mathrm{Ci}(-x)$ and you must supply the $-i\pi$ yourself.
```
    static const Int MAXIT=100;                     Maximum number of iterations allowed.
    static const Doub EULER=0.577215664901533, PIBY2=1.570796326794897,
        TMIN=2.0, EPS=numeric_limits<Doub>::epsilon(),
        FPMIN=numeric_limits<Doub>::min()*4.0,
        BIG=numeric_limits<Doub>::max()*EPS;
```
Here EULER is Euler's constant γ; PIBY2 is $\pi/2$; TMIN is the dividing line between using the series and continued fraction; EPS is the relative error, or absolute error near a zero of $\mathrm{Ci}(x)$; FPMIN is a number close to the smallest representable floating-point number; and BIG is a number near the machine overflow limit.
```
    Int i,k;
    Bool odd;
    Doub a,err,fact,sign,sum,sumc,sums,t,term;
    Complex h,b,c,d,del,cs;
    if ((t=abs(x)) == 0.0) return -BIG;             Special case.
    if (t > TMIN) {                                 Evaluate continued fraction by modified
        b=Complex(1.0,t);                              Lentz's method (§5.2).
        c=Complex(BIG,0.0);
        d=h=1.0/b;
        for (i=1;i<MAXIT;i++) {
            a= -i*i;
            b += 2.0;
            d=1.0/(a*d+b);                          Denominators cannot be zero.
            c=b+a/c;
            del=c*d;
            h *= del;
            if (abs(real(del)-1.0)+abs(imag(del)) <= EPS) break;
        }
        if (i >= MAXIT) throw("cf failed in cisi");
        h=Complex(cos(t),-sin(t))*h;
        cs= -conj(h)+Complex(0.0,PIBY2);
    } else {                                        Evaluate both series simultaneously.
```

```
        if (t < sqrt(FPMIN)) {                    Special case: Avoid failure of convergence
            sumc=0.0;                                 test because of underflow.
            sums=t;
        } else {
            sum=sums=sumc=0.0;
            sign=fact=1.0;
            odd=true;
            for (k=1;k<=MAXIT;k++) {
                fact *= t/k;
                term=fact/k;
                sum += sign*term;
                err=term/abs(sum);
                if (odd) {
                    sign = -sign;
                    sums=sum;
                    sum=sumc;
                } else {
                    sumc=sum;
                    sum=sums;
                }
                if (err < EPS) break;
                odd=!odd;
            }
            if (k > MAXIT) throw("maxits exceeded in cisi");
        }
        cs=Complex(sumc+log(t)+EULER,sums);
    }
    if (x < 0.0) cs = conj(cs);
    return cs;
}
```

CITED REFERENCES AND FURTHER READING:

Stegun, I.A., and Zucker, R. 1976, "Automatic Computing Methods for Special Functions. III. The Sine, Cosine, Exponential integrals, and Related Functions," *Journal of Research of the National Bureau of Standards*, vol. 80B, pp. 291–311; 1981, "Automatic Computing Methods for Special Functions. IV. Complex Error Function, Fresnel Integrals, and Other Related Functions," *op. cit.*, vol. 86, pp. 661–686.

Abramowitz, M., and Stegun, I.A. 1964, *Handbook of Mathematical Functions* (Washington: National Bureau of Standards); reprinted 1968 (New York: Dover); online at `http://www.nr.com/aands`, Chapters 5 and 7.

6.9 Dawson's Integral

Dawson's Integral $F(x)$ is defined by

$$F(x) = e^{-x^2} \int_0^x e^{t^2}\, dt \tag{6.9.1}$$

See Figure 6.8.1 for a graph of the function. The function can also be related to the complex error function by

$$F(z) = \frac{i\sqrt{\pi}}{2} e^{-z^2} \left[1 - \text{erfc}(-iz)\right]. \tag{6.9.2}$$

A remarkable approximation for $F(z)$, due to Rybicki [1], is

$$F(z) = \lim_{h \to 0} \frac{1}{\sqrt{\pi}} \sum_{n \text{ odd}} \frac{e^{-(z-nh)^2}}{n} \tag{6.9.3}$$

What makes equation (6.9.3) unusual is that its accuracy increases *exponentially* as h gets small, so that quite moderate values of h (and correspondingly quite rapid convergence of the series) give very accurate approximations.

We will discuss the theory that leads to equation (6.9.3) later, in §13.11, as an interesting application of Fourier methods. Here we simply implement a routine for real values of x based on the formula.

It is first convenient to shift the summation index to center it approximately on the maximum of the exponential term. Define n_0 to be the even integer nearest to x/h, and $x_0 \equiv n_0 h$, $x' \equiv x - x_0$, and $n' \equiv n - n_0$, so that

$$F(x) \approx \frac{1}{\sqrt{\pi}} \sum_{n'=-N \atop n' \text{ odd}}^{N} \frac{e^{-(x'-n'h)^2}}{n'+n_0} \tag{6.9.4}$$

where the approximate equality is accurate when h is sufficiently small and N is sufficiently large. The computation of this formula can be greatly speeded up if we note that

$$e^{-(x'-n'h)^2} = e^{-x'^2} e^{-(n'h)^2} \left(e^{2x'h}\right)^{n'} \tag{6.9.5}$$

The first factor is computed once, the second is an array of constants to be stored, and the third can be computed recursively, so that only two exponentials need be evaluated. Advantage is also taken of the symmetry of the coefficients $e^{-(n'h)^2}$ by breaking up the summation into positive and negative values of n' separately.

In the following routine, the choices $h = 0.4$ and $N = 11$ are made. Because of the symmetry of the summations and the restriction to odd values of n, the limits on the `for` loops are 0 to 5. The accuracy of the result in this version is about 2×10^{-7}. In order to maintain relative accuracy near $x = 0$, where $F(x)$ vanishes, the program branches to the evaluation of the power series [2] for $F(x)$, for $|x| < 0.2$.

dawson.h

```
Doub dawson(const Doub x) {
Returns Dawson's integral F(x) = exp(-x^2) ∫_0^x exp(t^2)dt for any real x.
    static const Int NMAX=6;
    static VecDoub c(NMAX);
    static Bool init = true;
    static const Doub H=0.4, A1=2.0/3.0, A2=0.4, A3=2.0/7.0;
    Int i,n0;                          Flag is true if we need to initialize, else false.
    Doub d1,d2,e1,e2,sum,x2,xp,xx,ans;
    if (init) {
        init=false;
        for (i=0;i<NMAX;i++) c[i]=exp(-SQR((2.0*i+1.0)*H));
    }
    if (abs(x) < 0.2) {                Use series expansion.
        x2=x*x;
        ans=x*(1.0-A1*x2*(1.0-A2*x2*(1.0-A3*x2)));
    } else {                           Use sampling theorem representation.
        xx=abs(x);
        n0=2*Int(0.5*xx/H+0.5);
        xp=xx-n0*H;
        e1=exp(2.0*xp*H);
```

```
        e2=e1*e1;
        d1=n0+1;
        d2=d1-2.0;
        sum=0.0;
        for (i=0;i<NMAX;i++,d1+=2.0,d2-=2.0,e1*=e2)
            sum += c[i]*(e1/d1+1.0/(d2*e1));
        ans=0.5641895835*SIGN(exp(-xp*xp),x)*sum;          Constant is 1/√π.
    }
    return ans;
}
```

Other methods for computing Dawson's integral are also known [2,3].

CITED REFERENCES AND FURTHER READING:

Rybicki, G.B. 1989, "Dawson's Integral and The Sampling Theorem," *Computers in Physics*, vol. 3, no. 2, pp. 85–87.[1]

Cody, W.J., Pociorek, K.A., and Thatcher, H.C. 1970, "Chebyshev Approximations for Dawson's Integral," *Mathematics of Computation*, vol. 24, pp. 171–178.[2]

McCabe, J.H. 1974, "A Continued Fraction Expansion, with a Truncation Error Estimate, for Dawson's Integral," *Mathematics of Computation*, vol. 28, pp. 811–816.[3]

6.10 Generalized Fermi-Dirac Integrals

The generalized Fermi-Dirac integral is defined as

$$F_k(\eta,\theta) = \int_0^\infty \frac{x^k(1+\frac{1}{2}\theta x)^{1/2}}{e^{x-\eta}+1}\,dx \tag{6.10.1}$$

It occurs, for example, in astrophysical applications with θ nonnegative and arbitrary η. In condensed matter physics one usually has the simpler case of $\theta = 0$ and omits the "generalized" from the name of the function. The important values of k are $-1/2$, 1/2, 3/2, and 5/2, but we'll consider arbitrary values greater than -1. Watch out for an alternative definition that multiplies the integral by $1/\Gamma(k+1)$.

For $\eta \ll -1$ and $\eta \gg 1$ there are useful series expansions for these functions (see, e.g., [1]). These give, for example,

$$\begin{aligned} F_{1/2}(\eta,\theta) &\to \frac{1}{\sqrt{2\theta}}e^{\eta}e^{1/\theta}K_1\left(\frac{1}{\theta}\right), \qquad \eta\to-\infty \\ F_{1/2}(\eta,\theta) &\to \frac{1}{2\sqrt{2}}\eta^{3/2}\frac{y\sqrt{1+y^2}-\sinh^{-1}y}{(\sqrt{1+y^2}-1)^{3/2}}, \qquad \eta\to\infty \end{aligned} \tag{6.10.2}$$

Here y is defined by

$$1+y^2 = (1+\eta\theta)^2 \tag{6.10.3}$$

It is the middle range of η values that is difficult to handle.

For $\theta = 0$, Macleod [2] has given Chebyshev expansions accurate to 10^{-16} for the four important k values, covering all η values. In this case, one need look no further for an algorithm. Goano [3] handles arbitrary k for $\theta = 0$. For nonzero θ,

it is reasonable to compute the functions by direct integration, using variable transformation to get rapidly converging quadratures [4]. (Of course, this works also for $\theta = 0$, but is not as efficient.) The usual transformation $x = \exp(t - e^{-t})$ handles the singularity at $x = 0$ and the exponential fall off at large x (cf. equation 4.5.14). For $\eta \gtrsim 15$, it is better to split the integral into two regions, $[0, \eta]$ and $[\eta, \eta + 60]$. (The contribution beyond $\eta + 60$ is negligible.) Each of these integrals can then be done with the DE rule. Between 60 and 500 function evaluations give full double precision, larger η requiring more function evaluations. A more efficient strategy would replace the quadrature by a series expansion for large η.

In the implementation below, note how `operator()` is overloaded to define both a function of one variable (for `Trapzd`) and a function of two variables (for `DErule`). Note also the syntax

```
Trapzd<Fermi> s(*this,a,b);
```

for declaring a `Trapzd` object inside the `Fermi` object itself.

fermi.h

```
struct Fermi {
    Doub kk,etaa,thetaa;
    Doub operator() (const Doub t);
    Doub operator() (const Doub x, const Doub del);
    Doub val(const Doub k, const Doub eta, const Doub theta);
};

Doub Fermi::operator() (const Doub t) {
```
Integrand for trapezoidal quadrature of generalized Fermi-Dirac integral with transformation $x = \exp(t - e^{-t})$.
```
    Doub x;
    x=exp(t-exp(-t));
    return x*(1.0+exp(-t))*pow(x,kk)*sqrt(1.0+thetaa*0.5*x)/
        (exp(x-etaa)+1.0);
}

Doub Fermi::operator() (const Doub x, const Doub del) {
```
Integrand for DE rule quadrature of generalized Fermi-Dirac integral.
```
    if (x < 1.0)
        return pow(del,kk)*sqrt(1.0+thetaa*0.5*x)/(exp(x-etaa)+1.0);
    else
        return pow(x,kk)*sqrt(1.0+thetaa*0.5*x)/(exp(x-etaa)+1.0);
}

Doub Fermi::val(const Doub k, const Doub eta, const Doub theta)
```
Computes the generalized Fermi-Dirac integral $F_k(\eta, \theta)$, where $k > -1$ and $\theta \geq 0$. The accuracy is approximately the square of the parameter `EPS`. `NMAX` limits the total number of quadrature steps.
```
{
    const Doub EPS=3.0e-9;
    const Int NMAX=11;
    Doub a,aa,b,bb,hmax,olds,sum;
    kk=k;                          Load the arguments into the member variables
    etaa=eta;                         for use in the function evaluations.
    thetaa=theta;
    if (eta <= 15.0) {
        a=-4.5;                    Set limits for x = exp(t - e^-t) mapping.
        b=5.0;
        Trapzd<Fermi> s(*this,a,b);
        for (Int i=1;i<=NMAX;i++) {
            sum=s.next();
            if (i > 3)             Test for convergence.
                if (abs(sum-olds) <= EPS*abs(olds))
```

```
                    return sum;
            olds=sum;                     Save value for next convergence test.
        }
    }
    else {
        a=0.0;                            Set limits for DE rule.
        b=eta;
        aa=eta;
        bb=eta+60.0;
        hmax=4.3;                         Big enough to handle negative k or large η.
        DErule<Fermi> s(*this,a,b,hmax);
        DErule<Fermi> ss(*this,aa,bb,hmax);
        for (Int i=1;i<=NMAX;i++) {
            sum=s.next()+ss.next();
            if (i > 3)
                if (abs(sum-olds) <= EPS*abs(olds))
                    return sum;
            olds=sum;
        }
    }
    throw("no convergence in fermi");
    return 0.0;
}
```

You get values of the Fermi-Dirac functions by declaring a `Fermi` object:

```
Fermi ferm;
```

and then making repeated calls to the `val` function:

```
ans=ferm.val(k,eta,theta);
```

Other quadrature methods exist for these functions [5-7]. A reasonably efficient method [8] involves trapezoidal quadrature with "pole correction," but it is restricted to $\theta \lesssim 0.2$. Generalized Bose-Einstein integrals can also be computed by the DE rule or the methods in these references.

CITED REFERENCES AND FURTHER READING:

Cox, J.P., and Giuli, R.T. 1968, *Principles of Stellar Structure* (New York: Gordon and Breach), vol. II, §24.7.[1]

Macleod, A.J. 1998, "Fermi-Dirac Functions of Order $-1/2$, $1/2$, $3/2$, $5/2$," *ACM Transactions on Mathematical Software*, vol. 24, pp. 1–12. (Algorithm 779, available from netlib.)[2]

Goano, M. 1995, "Computation of the Complete and Incomplete Fermi-Dirac Integral," *ACM Transactions on Mathematical Software*, vol. 21, pp. 221–232. (Algorithm 745, available from netlib.)[3]

Natarajan, A., and Kumar, N.M. 1993, "On the Numerical Evaluation of the Generalised Fermi-Dirac Integrals," *Computer Physics Communications*, vol. 76, pp. 48–50.[4]

Pichon, B. 1989, "Numerical Calculation of the Generalized Fermi-Dirac Integrals," *Computer Physics Communications*, vol. 55, pp. 127–136.[5]

Sagar, R.P. 1991, "A Gaussian Quadrature for the Calculation of Generalized Fermi-Dirac Integrals," *Computer Physics Communications*, vol. 66, pp. 271–275.[6]

Gautschi, W. 1992, "On the Computation of Generalized Fermi-Dirac and Bose-Einstein Integrals," *Computer Physics Communications*, vol. 74, pp. 233–238.[7]

Mohankumar, N., and Natarajan, A. 1996, "A Note on the Evaluation of the Generalized Fermi-Dirac Integral," *Astrophysical Journal*, vol. 458, pp. 233–235.[8]

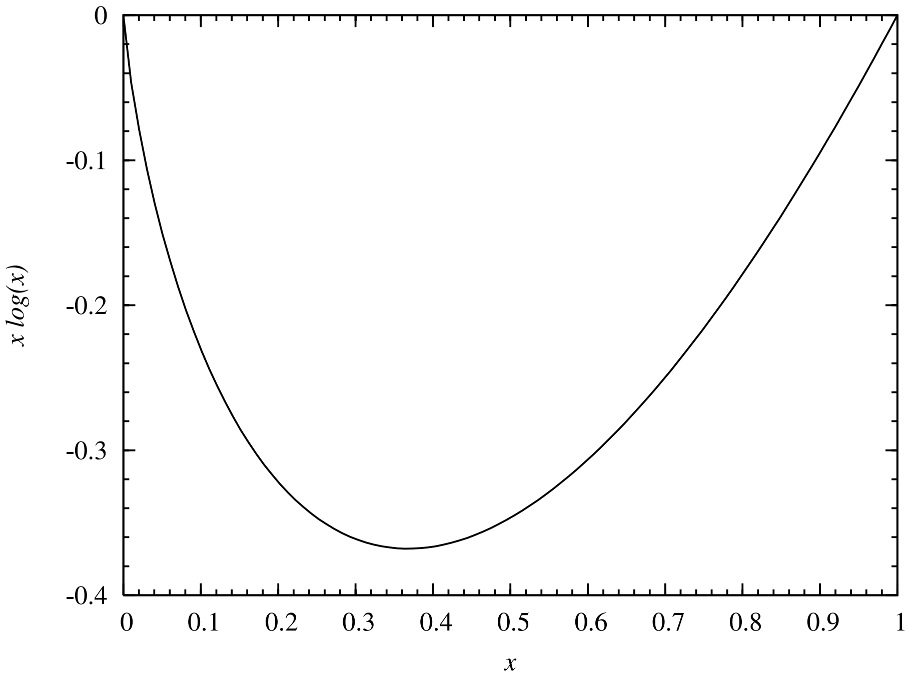

Figure 6.11.1. The function $x \log(x)$ is shown for $0 < x < 1$. Although nearly invisible, an essential singularity at $x = 0$ makes this function tricky to invert.

6.11 Inverse of the Function x log(x)

The function

$$y(x) = x \log(x) \tag{6.11.1}$$

and its inverse function $x(y)$ occur in a number of statistical and information theoretical contexts. Obviously $y(x)$ is nonsingular for all positive x, and easy to evaluate. For x between 0 and 1, it is negative, with a single minimum at $(x, y) = (e^{-1}, -e^{-1})$. The function has the value 0 at $x = 1$, and it has the value 0 as its limit at $x = 0$, since the linear factor x easily dominates the singular logarithm.

Computing the inverse function $x(y)$ is, however, not so easy. (We will need this inverse in §6.14.12.) From the appearance of Figure 6.11.1, it might seem easy to invert the function on its left branch, that is, return a value x between 0 and e^{-1} for every value y between 0 and $-e^{-1}$. However, the lurking logarithmic singularity at $x = 0$ causes difficulties for many methods that you might try.

Polynomial fits work well over any range of y that is less than a decade or so (e.g., from 0.01 to 0.1), but fail badly if you demand high fractional precision extending all the way to $y = 0$.

What about Newton's method? We write

$$\begin{aligned} f(x) &\equiv x \log(x) - y \\ f'(x) &= 1 + \log(x) \end{aligned} \tag{6.11.2}$$

giving the iteration

$$x_{i+1} = x_i - \frac{x_i \log(x_i) - y}{1 + \log(x_i)} \tag{6.11.3}$$

This doesn't work. The problem is not with its rate of convergence, which is of course quadratic for any finite y *if* we start close enough to the solution (see §9.4). The problem is that the region in which it converges at all is very small, especially as $y \to 0$. So, if we don't already have a good approximation as we approach the singularity, we are sunk.

If we change variables, we can get different (not computationally equivalent) versions of Newton's method. For example, let

$$u \equiv \log(x), \qquad x = e^u \tag{6.11.4}$$

Newton's method in u looks like this:

$$\begin{aligned} f(u) &= ue^u - y \\ f'(u) &= (1+u)e^u \\ u_{i+1} &= u_i - \frac{u_i - e^{-u_i}y}{1+u_i} \end{aligned} \tag{6.11.5}$$

But it turns out that iteration (6.11.5) is no better than (6.11.3).

The observation that leads to a good solution is that, since its log term varies only slowly, $y = x\log(x)$ is only very modestly curved *when it is plotted in log-log coordinates*. (Actually it is the negative of y that is plotted, since log-log coordinates require positive quantities.) Algebraically, we rewrite equation (6.11.1) as

$$(-y) = (-u)e^u \tag{6.11.6}$$

(with u as defined above) and take logarithms, giving

$$\log(-y) = u + \log(-u) \tag{6.11.7}$$

This leads to the Newton formulas,

$$\begin{aligned} f(u) &= u + \log(-u) - \log(-y) \\ f'(u) &= \frac{u+1}{u} \\ u_{i+1} &= u_i + \frac{u_i}{u_i+1}\left[\log\left(\frac{y}{u_i}\right) - u_i\right] \end{aligned} \tag{6.11.8}$$

It turns out that the iteration (6.11.8) converges quadratically over quite a broad region of initial guesses. For $-0.2 < y < 0$, you can just choose -10 (for example) as a fixed initial guess. When $-0.2 < y < -e^{-1}$, one can use the Taylor series expansion around $x = e^{-1}$,

$$y(x - e^{-1}) = -e^{-1} + \tfrac{1}{2}e(x - e^{-1})^2 + \cdots \tag{6.11.9}$$

which yields

$$x \approx e^{-1} - \sqrt{2e^{-1}(y + e^{-1})} \tag{6.11.10}$$

With these initial guesses, (6.11.8) never takes more than six iterations to converge to double precision accuracy, and there is just one log and a few arithmetic operations per iteration. The implementation looks like this:

ksdist.h

```
Doub invxlogx(Doub y) {
```
For negative y, $0 > y > -e^{-1}$, return x such that $y = x\log(x)$. The value returned is always the smaller of the two roots and is in the range $0 < x < e^{-1}$.
```
    const Doub ooe = 0.367879441171442322;
    Doub t,u,to=0.;
    if (y >= 0. || y <= -ooe) throw("no such inverse value");
     if (y < -0.2) u = log(ooe-sqrt(2*ooe*(y+ooe)));   First approximation by inverse
    else u = -10.;                                         of Taylor series.
    do {                                                 See text for derivation.
        u += (t=(log(y/u)-u)*(u/(1.+u)));
        if (t < 1.e-8 && abs(t+to)<0.01*abs(t)) break;
        to = t;
    } while (abs(t/u) > 1.e-15);
    return exp(u);
}
```

6.12 Elliptic Integrals and Jacobian Elliptic Functions

Elliptic integrals occur in many applications, because any integral of the form

$$\int R(t,s)\,dt \tag{6.12.1}$$

where R is a rational function of t and s, and s is the square root of a cubic or quartic polynomial in t, can be evaluated in terms of elliptic integrals. Standard references [1] describe how to carry out the reduction, which was originally done by Legendre. Legendre showed that only three basic elliptic integrals are required. The simplest of these is

$$I_1 = \int_y^x \frac{dt}{\sqrt{(a_1+b_1t)(a_2+b_2t)(a_3+b_3t)(a_4+b_4t)}} \tag{6.12.2}$$

where we have written the quartic s^2 in factored form. In standard integral tables [2], one of the limits of integration is always a zero of the quartic, while the other limit lies closer than the next zero, so that there is no singularity within the interval. To evaluate I_1, we simply break the interval $[y,x]$ into subintervals, each of which either begins or ends on a singularity. The tables, therefore, need only distinguish the eight cases in which each of the four zeros (ordered according to size) appears as the upper or lower limit of integration. In addition, when one of the b's in (6.12.2) tends to zero, the quartic reduces to a cubic, with the largest or smallest singularity moving to $\pm\infty$; this leads to eight more cases (actually just special cases of the first eight). The 16 cases in total are then usually tabulated in terms of Legendre's standard elliptic integral of the first kind, which we will define below. By a change of the variable of integration t, the zeros of the quartic are mapped to standard locations on the real axis. Then only two dimensionless parameters are needed to tabulate Legendre's integral. However, the symmetry of the original integral (6.12.2) under permutation of the roots is concealed in Legendre's notation. We will get back to Legendre's notation below. But first, here is a better approach:

Carlson [3] has given a new definition of a standard elliptic integral of the first kind,

$$R_F(x, y, z) = \frac{1}{2}\int_0^\infty \frac{dt}{\sqrt{(t+x)(t+y)(t+z)}} \tag{6.12.3}$$

where x, y, and z are nonnegative and at most one is zero. By standardizing the range of integration, he retains permutation symmetry for the zeros. (Weierstrass' canonical form also has this property.) Carlson first shows that when x or y is a zero of the quartic in (6.12.2), the integral I_1 can be written in terms of R_F in a form that is symmetric under permutation of the *remaining* three zeros. In the general case, when neither x nor y is a zero, two such R_F functions can be combined into a single one by an *addition theorem*, leading to the fundamental formula

$$I_1 = 2R_F(U_{12}^2, U_{13}^2, U_{14}^2) \tag{6.12.4}$$

where

$$U_{ij} = (X_i X_j Y_k Y_m + Y_i Y_j X_k X_m)/(x - y) \tag{6.12.5}$$

$$X_i = (a_i + b_i x)^{1/2}, \qquad Y_i = (a_i + b_i y)^{1/2} \tag{6.12.6}$$

and i, j, k, m is any permutation of $1, 2, 3, 4$. A short-cut in evaluating these expressions is

$$\begin{aligned} U_{13}^2 &= U_{12}^2 - (a_1 b_4 - a_4 b_1)(a_2 b_3 - a_3 b_2) \\ U_{14}^2 &= U_{12}^2 - (a_1 b_3 - a_3 b_1)(a_2 b_4 - a_4 b_2) \end{aligned} \tag{6.12.7}$$

The U's correspond to the three ways of pairing the four zeros, and I_1 is thus manifestly symmetric under permutation of the zeros. Equation (6.12.4) therefore reproduces all 16 cases when one limit is a zero, and also includes the cases when neither limit is a zero.

Thus Carlson's function allows arbitrary ranges of integration and arbitrary positions of the branch points of the integrand relative to the interval of integration. To handle elliptic integrals of the second and third kinds, Carlson defines the standard integral of the third kind as

$$R_J(x, y, z, p) = \frac{3}{2}\int_0^\infty \frac{dt}{(t+p)\sqrt{(t+x)(t+y)(t+z)}} \tag{6.12.8}$$

which is symmetric in x, y, and z. The degenerate case when two arguments are equal is denoted

$$R_D(x, y, z) = R_J(x, y, z, z) \tag{6.12.9}$$

and is symmetric in x and y. The function R_D replaces Legendre's integral of the second kind. The degenerate form of R_F is denoted

$$R_C(x, y) = R_F(x, y, y) \tag{6.12.10}$$

It embraces logarithmic, inverse circular, and inverse hyperbolic functions.

Carlson [4-7] gives integral tables in terms of the exponents of the linear factors of the quartic in (6.12.1). For example, the integral where the exponents are $(\frac{1}{2}, \frac{1}{2}, -\frac{1}{2}, -\frac{3}{2})$ can be expressed as a single integral in terms of R_D; it accounts for 144 separate cases in Gradshteyn and Ryzhik [2]!

Refer to Carlson's papers [3-8] for some of the practical details in reducing elliptic integrals to his standard forms, such as handling complex-conjugate zeros.

Turn now to the numerical evaluation of elliptic integrals. The traditional methods [9] are Gauss or Landen transformations. *Descending* transformations decrease the modulus k of the Legendre integrals toward zero, and *increasing* transformations increase it toward unity. In these limits the functions have simple analytic expressions. While these methods converge quadratically and are quite satisfactory for integrals of the first and second kinds, they generally lead to loss of significant figures in certain regimes for integrals of the third kind. Carlson's algorithms [10,11], by contrast, provide a unified method for all three kinds with no significant cancellations.

The key ingredient in these algorithms is the *duplication theorem*:

$$R_F(x,y,z) = 2R_F(x+\lambda, y+\lambda, z+\lambda) = R_F\left(\frac{x+\lambda}{4}, \frac{y+\lambda}{4}, \frac{z+\lambda}{4}\right) \tag{6.12.11}$$

where

$$\lambda = (xy)^{1/2} + (xz)^{1/2} + (yz)^{1/2} \tag{6.12.12}$$

This theorem can be proved by a simple change of variable of integration [12]. Equation (6.12.11) is iterated until the arguments of R_F are nearly equal. For equal arguments we have

$$R_F(x,x,x) = x^{-1/2} \tag{6.12.13}$$

When the arguments are close enough, the function is evaluated from a fixed Taylor expansion about (6.12.13) through fifth-order terms. While the iterative part of the algorithm is only linearly convergent, the error ultimately decreases by a factor of $4^6 = 4096$ for each iteration. Typically only two or three iterations are required, perhaps six or seven if the initial values of the arguments have huge ratios. We list the algorithm for R_F here, and refer you to Carlson's paper [10] for the other cases.

Stage 1: For $n = 0, 1, 2, \ldots$ compute

$$\mu_n = (x_n + y_n + z_n)/3$$
$$X_n = 1 - (x_n/\mu_n), \quad Y_n = 1 - (y_n/\mu_n), \quad Z_n = 1 - (z_n/\mu_n)$$
$$\epsilon_n = \max(|X_n|, |Y_n|, |Z_n|)$$

If $\epsilon_n <$ tol, go to Stage 2; else compute

$$\lambda_n = (x_n y_n)^{1/2} + (x_n z_n)^{1/2} + (y_n z_n)^{1/2}$$
$$x_{n+1} = (x_n + \lambda_n)/4, \quad y_{n+1} = (y_n + \lambda_n)/4, \quad z_{n+1} = (z_n + \lambda_n)/4$$

and repeat this stage.

Stage 2: Compute

$$E_2 = X_n Y_n - Z_n^2, \quad E_3 = X_n Y_n Z_n$$
$$R_F = (1 - \tfrac{1}{10}E_2 + \tfrac{1}{14}E_3 + \tfrac{1}{24}E_2^2 - \tfrac{3}{44}E_2 E_3)/(\mu_n)^{1/2}$$

In some applications the argument p in R_J or the argument y in R_C is negative, and the Cauchy principal value of the integral is required. This is easily handled by using the formulas

$$R_J(x,y,z,p) = [(\gamma - y)R_J(x,y,z,\gamma) - 3R_F(x,y,z) + 3R_C(xz/y, p\gamma/y)]/(y-p) \tag{6.12.14}$$

where

$$\gamma \equiv y + \frac{(z-y)(y-x)}{y-p} \tag{6.12.15}$$

is positive if p is negative, and

$$R_C(x,y) = \left(\frac{x}{x-y}\right)^{1/2} R_C(x-y, -y) \tag{6.12.16}$$

The Cauchy principal value of R_J has a zero at some value of $p < 0$, so (6.12.14) will give some loss of significant figures near the zero.

elliptint.h

```
Doub rf(const Doub x, const Doub y, const Doub z) {
```

Computes Carlson's elliptic integral of the first kind, $R_F(x,y,z)$. x, y, and z must be nonnegative, and at most one can be zero.

```
    static const Doub ERRTOL=0.0025, THIRD=1.0/3.0,C1=1.0/24.0, C2=0.1,
        C3=3.0/44.0, C4=1.0/14.0;
    static const Doub TINY=5.0*numeric_limits<Doub>::min(),
        BIG=0.2*numeric_limits<Doub>::max();
    Doub alamb,ave,delx,dely,delz,e2,e3,sqrtx,sqrty,sqrtz,xt,yt,zt;
    if (MIN(MIN(x,y),z) < 0.0 || MIN(MIN(x+y,x+z),y+z) < TINY ||
        MAX(MAX(x,y),z) > BIG) throw("invalid arguments in rf");
    xt=x;
    yt=y;
    zt=z;
    do {
        sqrtx=sqrt(xt);
        sqrty=sqrt(yt);
        sqrtz=sqrt(zt);
        alamb=sqrtx*(sqrty+sqrtz)+sqrty*sqrtz;
        xt=0.25*(xt+alamb);
        yt=0.25*(yt+alamb);
        zt=0.25*(zt+alamb);
        ave=THIRD*(xt+yt+zt);
        delx=(ave-xt)/ave;
        dely=(ave-yt)/ave;
        delz=(ave-zt)/ave;
    } while (MAX(MAX(abs(delx),abs(dely)),abs(delz)) > ERRTOL);
    e2=delx*dely-delz*delz;
    e3=delx*dely*delz;
    return (1.0+(C1*e2-C2-C3*e3)*e2+C4*e3)/sqrt(ave);
}
```

A value of 0.0025 for the error tolerance parameter gives full double precision (16 significant digits). Since the error scales as ϵ_n^6, we see that 0.08 would be adequate for single precision (7 significant digits), but would save at most two or three more iterations. Since the coefficients of the sixth-order truncation error are different for the other elliptic functions, these values for the error tolerance should be set to 0.04 (single precision) or 0.0012 (double precision) in the algorithm for R_C, and 0.05 or 0.0015 for R_J and R_D. As well as being an algorithm in its own right for certain combinations of elementary functions, the algorithm for R_C is used repeatedly in the computation of R_J.

The C++ implementations test the input arguments against two machine-dependent constants, TINY and BIG, to ensure that there will be no underflow or overflow during the computation. You can always extend the range of admissible argument values by using the homogeneity relations (6.12.22), below.

elliptint.h

```
Doub rd(const Doub x, const Doub y, const Doub z) {
```

Computes Carlson's elliptic integral of the second kind, $R_D(x,y,z)$. x and y must be nonnegative, and at most one can be zero. z must be positive.

```
    static const Doub ERRTOL=0.0015, C1=3.0/14.0, C2=1.0/6.0, C3=9.0/22.0,
        C4=3.0/26.0, C5=0.25*C3, C6=1.5*C4;
    static const Doub TINY=2.0*pow(numeric_limits<Doub>::max(),-2./3.),
        BIG=0.1*ERRTOL*pow(numeric_limits<Doub>::min(),-2./3.);
    Doub alamb,ave,delx,dely,delz,ea,eb,ec,ed,ee,fac,sqrtx,sqrty,
        sqrtz,sum,xt,yt,zt;
    if (MIN(x,y) < 0.0 || MIN(x+y,z) < TINY || MAX(MAX(x,y),z) > BIG)
        throw("invalid arguments in rd");
    xt=x;
    yt=y;
    zt=z;
    sum=0.0;
    fac=1.0;
    do {
        sqrtx=sqrt(xt);
```

```
        sqrty=sqrt(yt);
        sqrtz=sqrt(zt);
        alamb=sqrtx*(sqrty+sqrtz)+sqrty*sqrtz;
        sum += fac/(sqrtz*(zt+alamb));
        fac=0.25*fac;
        xt=0.25*(xt+alamb);
        yt=0.25*(yt+alamb);
        zt=0.25*(zt+alamb);
        ave=0.2*(xt+yt+3.0*zt);
        delx=(ave-xt)/ave;
        dely=(ave-yt)/ave;
        delz=(ave-zt)/ave;
    } while (MAX(MAX(abs(delx),abs(dely)),abs(delz)) > ERRTOL);
    ea=delx*dely;
    eb=delz*delz;
    ec=ea-eb;
    ed=ea-6.0*eb;
    ee=ed+ec+ec;
    return 3.0*sum+fac*(1.0+ed*(-C1+C5*ed-C6*delz*ee)
        +delz*(C2*ee+delz*(-C3*ec+delz*C4*ea)))/(ave*sqrt(ave));
}
```

elliptint.h

```
Doub rj(const Doub x, const Doub y, const Doub z, const Doub p) {
```

Computes Carlson's elliptic integral of the third kind, $R_J(x, y, z, p)$. x, y, and z must be nonnegative, and at most one can be zero. p must be nonzero. If $p < 0$, the Cauchy principal value is returned.

```
    static const Doub ERRTOL=0.0015, C1=3.0/14.0, C2=1.0/3.0, C3=3.0/22.0,
        C4=3.0/26.0, C5=0.75*C3, C6=1.5*C4, C7=0.5*C2, C8=C3+C3;
    static const Doub TINY=pow(5.0*numeric_limits<Doub>::min(),1./3.),
        BIG=0.3*pow(0.2*numeric_limits<Doub>::max(),1./3.);
    Doub a,alamb,alpha,ans,ave,b,beta,delp,delx,dely,delz,ea,eb,ec,ed,ee,
        fac,pt,rcx,rho,sqrtx,sqrty,sqrtz,sum,tau,xt,yt,zt;
    if (MIN(MIN(x,y),z) < 0.0 || MIN(MIN(x+y,x+z),MIN(y+z,abs(p))) < TINY
        || MAX(MAX(x,y),MAX(z,abs(p))) > BIG) throw("invalid arguments in rj");
    sum=0.0;
    fac=1.0;
    if (p > 0.0) {
        xt=x;
        yt=y;
        zt=z;
        pt=p;
    } else {
        xt=MIN(MIN(x,y),z);
        zt=MAX(MAX(x,y),z);
        yt=x+y+z-xt-zt;
        a=1.0/(yt-p);
        b=a*(zt-yt)*(yt-xt);
        pt=yt+b;
        rho=xt*zt/yt;
        tau=p*pt/yt;
        rcx=rc(rho,tau);
    }
    do {
        sqrtx=sqrt(xt);
        sqrty=sqrt(yt);
        sqrtz=sqrt(zt);
        alamb=sqrtx*(sqrty+sqrtz)+sqrty*sqrtz;
        alpha=SQR(pt*(sqrtx+sqrty+sqrtz)+sqrtx*sqrty*sqrtz);
        beta=pt*SQR(pt+alamb);
        sum += fac*rc(alpha,beta);
        fac=0.25*fac;
        xt=0.25*(xt+alamb);
        yt=0.25*(yt+alamb);
```

```
        zt=0.25*(zt+alamb);
        pt=0.25*(pt+alamb);
        ave=0.2*(xt+yt+zt+pt+pt);
        delx=(ave-xt)/ave;
        dely=(ave-yt)/ave;
        delz=(ave-zt)/ave;
        delp=(ave-pt)/ave;
    } while (MAX(MAX(abs(delx),abs(dely)),
        MAX(abs(delz),abs(delp))) > ERRTOL);
    ea=delx*(dely+delz)+dely*delz;
    eb=delx*dely*delz;
    ec=delp*delp;
    ed=ea-3.0*ec;
    ee=eb+2.0*delp*(ea-ec);
    ans=3.0*sum+fac*(1.0+ed*(-C1+C5*ed-C6*ee)+eb*(C7+delp*(-C8+delp*C4))
        +delp*ea*(C2-delp*C3)-C2*delp*ec)/(ave*sqrt(ave));
    if (p <= 0.0) ans=a*(b*ans+3.0*(rcx-rf(xt,yt,zt)));
    return ans;
}
```

elliptint.h

```
Doub rc(const Doub x, const Doub y) {
```

Computes Carlson's degenerate elliptic integral, $R_C(x,y)$. x must be nonnegative and y must be nonzero. If $y < 0$, the Cauchy principal value is returned.

```
    static const Doub ERRTOL=0.0012, THIRD=1.0/3.0, C1=0.3, C2=1.0/7.0,
        C3=0.375, C4=9.0/22.0;
    static const Doub TINY=5.0*numeric_limits<Doub>::min(),
        BIG=0.2*numeric_limits<Doub>::max(), COMP1=2.236/sqrt(TINY),
        COMP2=SQR(TINY*BIG)/25.0;
    Doub alamb,ave,s,w,xt,yt;
    if (x < 0.0 || y == 0.0 || (x+abs(y)) < TINY || (x+abs(y)) > BIG ||
        (y<-COMP1 && x > 0.0 && x < COMP2)) throw("invalid arguments in rc");
    if (y > 0.0) {
        xt=x;
        yt=y;
        w=1.0;
    } else {
        xt=x-y;
        yt= -y;
        w=sqrt(x)/sqrt(xt);
    }
    do {
        alamb=2.0*sqrt(xt)*sqrt(yt)+yt;
        xt=0.25*(xt+alamb);
        yt=0.25*(yt+alamb);
        ave=THIRD*(xt+yt+yt);
        s=(yt-ave)/ave;
    } while (abs(s) > ERRTOL);
    return w*(1.0+s*s*(C1+s*(C2+s*(C3+s*C4))))/sqrt(ave);
}
```

At times you may want to express your answer in Legendre's notation. Alternatively, you may be given results in that notation and need to compute their values with the programs given above. It is a simple matter to transform back and forth. The *Legendre elliptic integral of the first kind* is defined as

$$F(\phi,k) \equiv \int_0^{\phi} \frac{d\theta}{\sqrt{1-k^2\sin^2\theta}} \tag{6.12.17}$$

The *complete elliptic integral of the first kind* is given by

$$K(k) \equiv F(\pi/2,k) \tag{6.12.18}$$

In terms of R_F,

$$F(\phi,k) = \sin\phi R_F(\cos^2\phi, 1-k^2\sin^2\phi, 1)$$
$$K(k) = R_F(0, 1-k^2, 1) \tag{6.12.19}$$

The *Legendre elliptic integral of the second kind* and the *complete elliptic integral of the second kind* are given by

$$\begin{aligned} E(\phi,k) &\equiv \int_0^\phi \sqrt{1-k^2\sin^2\theta}\, d\theta \\ &= \sin\phi R_F(\cos^2\phi, 1-k^2\sin^2\phi, 1) \\ &\quad - \tfrac{1}{3}k^2\sin^3\phi R_D(\cos^2\phi, 1-k^2\sin^2\phi, 1) \\ E(k) &\equiv E(\pi/2,k) = R_F(0,1-k^2,1) - \tfrac{1}{3}k^2 R_D(0,1-k^2,1) \end{aligned} \tag{6.12.20}$$

Finally, the *Legendre elliptic integral of the third kind* is

$$\begin{aligned} \Pi(\phi,n,k) &\equiv \int_0^\phi \frac{d\theta}{(1+n\sin^2\theta)\sqrt{1-k^2\sin^2\theta}} \\ &= \sin\phi R_F(\cos^2\phi, 1-k^2\sin^2\phi, 1) \\ &\quad - \tfrac{1}{3}n\sin^3\phi R_J(\cos^2\phi, 1-k^2\sin^2\phi, 1, 1+n\sin^2\phi) \end{aligned} \tag{6.12.21}$$

(Note that this sign convention for n is opposite that of Abramowitz and Stegun [13], and that their $\sin\alpha$ is our k.)

```
Doub ellf(const Doub phi, const Doub ak) {
```
elliptint.h

Legendre elliptic integral of the first kind $F(\phi,k)$, evaluated using Carlson's function R_F. The argument ranges are $0 \le \phi \le \pi/2$, $0 \le k\sin\phi \le 1$.

```
    Doub s=sin(phi);
    return s*rf(SQR(cos(phi)),(1.0-s*ak)*(1.0+s*ak),1.0);
}
```

```
Doub elle(const Doub phi, const Doub ak) {
```
elliptint.h

Legendre elliptic integral of the second kind $E(\phi,k)$, evaluated using Carlson's functions R_D and R_F. The argument ranges are $0 \le \phi \le \pi/2$, $0 \le k\sin\phi \le 1$.

```
    Doub cc,q,s;
    s=sin(phi);
    cc=SQR(cos(phi));
    q=(1.0-s*ak)*(1.0+s*ak);
    return s*(rf(cc,q,1.0)-(SQR(s*ak))*rd(cc,q,1.0)/3.0);
}
```

```
Doub ellpi(const Doub phi, const Doub en, const Doub ak) {
```
elliptint.h

Legendre elliptic integral of the third kind $\Pi(\phi,n,k)$, evaluated using Carlson's functions R_J and R_F. (Note that the sign convention on n is opposite that of Abramowitz and Stegun.) The ranges of ϕ and k are $0 \le \phi \le \pi/2$, $0 \le k\sin\phi \le 1$.

```
    Doub cc,enss,q,s;
    s=sin(phi);
    enss=en*s*s;
    cc=SQR(cos(phi));
    q=(1.0-s*ak)*(1.0+s*ak);
    return s*(rf(cc,q,1.0)-enss*rj(cc,q,1.0,1.0+enss)/3.0);
}
```

Carlson's functions are homogeneous of degree $-\frac{1}{2}$ and $-\frac{3}{2}$, so

$$\begin{aligned} R_F(\lambda x, \lambda y, \lambda z) &= \lambda^{-1/2} R_F(x, y, z) \\ R_J(\lambda x, \lambda y, \lambda z, \lambda p) &= \lambda^{-3/2} R_J(x, y, z, p) \end{aligned} \tag{6.12.22}$$

Thus, to express a Carlson function in Legendre's notation, permute the first three arguments into ascending order, use homogeneity to scale the third argument to be 1, and then use equations (6.12.19) – (6.12.21).

6.12.1 Jacobian Elliptic Functions

The Jacobian elliptic function sn is defined as follows: Instead of considering the elliptic integral

$$u(y, k) \equiv u = F(\phi, k) \tag{6.12.23}$$

consider the *inverse* function

$$y = \sin\phi = \mathrm{sn}(u, k) \tag{6.12.24}$$

Equivalently,

$$u = \int_0^{\mathrm{sn}} \frac{dy}{\sqrt{(1-y^2)(1-k^2y^2)}} \tag{6.12.25}$$

When $k = 0$, sn is just sin. The functions cn and dn are defined by the relations

$$\mathrm{sn}^2 + \mathrm{cn}^2 = 1, \qquad k^2\mathrm{sn}^2 + \mathrm{dn}^2 = 1 \tag{6.12.26}$$

The routine given below actually takes $m_c \equiv k_c^2 = 1 - k^2$ as an input parameter. It also computes all three functions sn, cn, and dn since computing all three is no harder than computing any one of them. For a description of the method, see [9].

elliptint.h

```
void sncndn(const Doub uu, const Doub emmc, Doub &sn, Doub &cn, Doub &dn) {
```

Returns the Jacobian elliptic functions $\mathrm{sn}(u, k_c)$, $\mathrm{cn}(u, k_c)$, and $\mathrm{dn}(u, k_c)$. Here uu $= u$, while emmc $= k_c^2$.

```
    static const Doub CA=1.0e-8;        The accuracy is the square of CA.
    Bool bo;
    Int i,ii,l;
    Doub a,b,c,d,emc,u;
    VecDoub em(13),en(13);
    emc=emmc;
    u=uu;
    if (emc != 0.0) {
        bo=(emc < 0.0);
        if (bo) {
            d=1.0-emc;
            emc /= -1.0/d;
            u *= (d=sqrt(d));
        }
        a=1.0;
        dn=1.0;
        for (i=0;i<13;i++) {
            l=i;
            em[i]=a;
            en[i]=(emc=sqrt(emc));
            c=0.5*(a+emc);
            if (abs(a-emc) <= CA*a) break;
            emc *= a;
```

```
            a=c;
        }
        u *= c;
        sn=sin(u);
        cn=cos(u);
        if (sn != 0.0) {
            a=cn/sn;
            c *= a;
            for (ii=l;ii>=0;ii--) {
                b=em[ii];
                a *= c;
                c *= dn;
                dn=(en[ii]+a)/(b+a);
                a=c/b;
            }
            a=1.0/sqrt(c*c+1.0);
            sn=(sn >= 0.0 ? a : -a);
            cn=c*sn;
        }
        if (bo) {
            a=dn;
            dn=cn;
            cn=a;
            sn /= d;
        }
    } else {
        cn=1.0/cosh(u);
        dn=cn;
        sn=tanh(u);
    }
}
```

CITED REFERENCES AND FURTHER READING:

Erdélyi, A., Magnus, W., Oberhettinger, F., and Tricomi, F.G. 1953, *Higher Transcendental Functions*, Vol. II, (New York: McGraw-Hill).[1]

Gradshteyn, I.S., and Ryzhik, I.W. 1980, *Table of Integrals, Series, and Products* (New York: Academic Press).[2]

Carlson, B.C. 1977, "Elliptic Integrals of the First Kind," *SIAM Journal on Mathematical Analysis*, vol. 8, pp. 231–242.[3]

Carlson, B.C. 1987, "A Table of Elliptic Integrals of the Second Kind," *Mathematics of Computation*, vol. 49, pp. 595–606[4]; 1988, "A Table of Elliptic Integrals of the Third Kind," *op. cit.*, vol. 51, pp. 267–280[5]; 1989, "A Table of Elliptic Integrals: Cubic Cases," *op. cit.*, vol. 53, pp. 327–333[6]; 1991, "A Table of Elliptic Integrals: One Quadratic Factor," *op. cit.*, vol. 56, pp. 267–280.[7]

Carlson, B.C., and FitzSimons, J. 2000, "Reduction Theorems for Elliptic Integrands with the Square Root of Two Quadratic Factors," *Journal of Computational and Applied Mathematics*, vol. 118, pp. 71–85.[8]

Bulirsch, R. 1965, "Numerical Calculation of Elliptic Integrals and Elliptic Functions," *Numerische Mathematik*, vol. 7, pp. 78–90; 1965, *op. cit.*, vol. 7, pp. 353–354; 1969, *op. cit.*, vol. 13, pp. 305–315.[9]

Carlson, B.C. 1979, "Computing Elliptic Integrals by Duplication," *Numerische Mathematik*, vol. 33, pp. 1–16.[10]

Carlson, B.C., and Notis, E.M. 1981, "Algorithms for Incomplete Elliptic Integrals," *ACM Transactions on Mathematical Software*, vol. 7, pp. 398–403.[11]

Carlson, B.C. 1978, "Short Proofs of Three Theorems on Elliptic Integrals," *SIAM Journal on Mathematical Analysis*, vol. 9, p. 524–528.[12]

Abramowitz, M., and Stegun, I.A. 1964, *Handbook of Mathematical Functions* (Washington: National Bureau of Standards); reprinted 1968 (New York: Dover); online at `http://www.nr.com/aands`, Chapter 17.[13]

Mathews, J., and Walker, R.L. 1970, *Mathematical Methods of Physics*, 2nd ed. (Reading, MA: W.A. Benjamin/Addison-Wesley), pp. 78–79.

6.13 Hypergeometric Functions

As was discussed in §5.14, a fast, general routine for the the complex hypergeometric function ${}_2F_1(a,b,c;z)$ is difficult or impossible. The function is defined as the analytic continuation of the hypergeometric series

$$
\begin{aligned}
{}_2F_1(a,b,c;z) = 1 &+ \frac{ab}{c}\frac{z}{1!} + \frac{a(a+1)b(b+1)}{c(c+1)}\frac{z^2}{2!} + \cdots \\
&+ \frac{a(a+1)\ldots(a+j-1)b(b+1)\ldots(b+j-1)}{c(c+1)\ldots(c+j-1)}\frac{z^j}{j!} + \cdots
\end{aligned}
\tag{6.13.1}
$$

This series converges only within the unit circle $|z| < 1$ (see [1]), but one's interest in the function is not confined to this region.

Section 5.14 discussed the method of evaluating this function by direct path integration in the complex plane. We here merely list the routines that result.

Implementation of the function `hypgeo` is straightforward and is described by comments in the program. The machinery associated with Chapter 17's routine for integrating differential equations, `Odeint`, is only minimally intrusive and need not even be completely understood: Use of `Odeint` requires one function call to the constructor, with a prescribed format for the derivative routine `Hypderiv`, followed by a call to the `integrate` method.

The function `hypgeo` will fail, of course, for values of z too close to the singularity at 1. (If you need to approach this singularity, or the one at ∞, use the "linear transformation formulas" in §15.3 of [1].) Away from $z = 1$, and for moderate values of a, b, c, it is often remarkable how few steps are required to integrate the equations. A half-dozen is typical.

hypgeo.h

```
Complex hypgeo(const Complex &a, const Complex &b,const Complex &c,
    const Complex &z)
```
Complex hypergeometric function ${}_2F_1$ for complex a, b, c, and z, by direct integration of the hypergeometric equation in the complex plane. The branch cut is taken to lie along the real axis, Re $z > 1$.
```
{
    const Doub atol=1.0e-14,rtol=1.0e-14;       Accuracy parameters.
    Complex ans,dz,z0,y[2];
    VecDoub yy(4);
    if (norm(z) <= 0.25) {                      Use series...
        hypser(a,b,c,z,ans,y[1]);
        return ans;
    }
    ...or pick a starting point for the path integration.
    else if (real(z) < 0.0) z0=Complex(-0.5,0.0);
    else if (real(z) <= 1.0) z0=Complex(0.5,0.0);
```

```
    else z0=Complex(0.0,imag(z) >= 0.0 ? 0.5 : -0.5);
    dz=z-z0;
    hypser(a,b,c,z0,y[0],y[1]);                Get starting function and derivative.
    yy[0]=real(y[0]);
    yy[1]=imag(y[0]);
    yy[2]=real(y[1]);
    yy[3]=imag(y[1]);
    Hypderiv d(a,b,c,z0,dz);                   Set up the functor for the derivatives.
    Output out;                                Suppress output in Odeint.
    Odeint<StepperBS<Hypderiv> > ode(yy,0.0,1.0,atol,rtol,0.1,0.0,out,d);
```
The arguments to `Odeint` are the vector of independent variables, the starting and ending values of the dependent variable, the accuracy parameters, an initial guess for the stepsize, a minimum stepsize, and the names of the output object and the derivative object. The integration is performed by the Bulirsch-Stoer stepping routine.
```
    ode.integrate();
    y[0]=Complex(yy[0],yy[1]);
    return y[0];
}
```

hypgeo.h
```
void hypser(const Complex &a, const Complex &b, const Complex &c,
    const Complex &z, Complex &series, Complex &deriv)
```
Returns the hypergeometric series ${}_2F_1$ and its derivative, iterating to machine accuracy. For $|z| \le 1/2$ convergence is quite rapid.
```
{
    deriv=0.0;
    Complex fac=1.0;
    Complex temp=fac;
    Complex aa=a;
    Complex bb=b;
    Complex cc=c;
    for (Int n=1;n<=1000;n++) {
        fac *= ((aa*bb)/cc);
        deriv += fac;
        fac *= ((1.0/n)*z);
        series=temp+fac;
        if (series == temp) return;
        temp=series;
        aa += 1.0;
        bb += 1.0;
        cc += 1.0;
    }
    throw("convergence failure in hypser");
}
```

hypgeo.h
```
struct Hypderiv {
```
Functor to compute derivatives for the hypergeometric equation; see text equation (5.14.4).
```
    Complex a,b,c,z0,dz;
    Hypderiv(const Complex &aa, const Complex &bb,
        const Complex &cc, const Complex &z00,
        const Complex &dzz) : a(aa),b(bb),c(cc),z0(z00),dz(dzz) {}
    void operator() (const Doub s, VecDoub_I &yy, VecDoub_O &dyyds) {
        Complex z,y[2],dyds[2];
        y[0]=Complex(yy[0],yy[1]);
        y[1]=Complex(yy[2],yy[3]);
        z=z0+s*dz;
        dyds[0]=y[1]*dz;
        dyds[1]=(a*b*y[0]-(c-(a+b+1.0)*z)*y[1])*dz/(z*(1.0-z));
        dyyds[0]=real(dyds[0]);
        dyyds[1]=imag(dyds[0]);
        dyyds[2]=real(dyds[1]);
        dyyds[3]=imag(dyds[1]);
    }
};
```

CITED REFERENCES AND FURTHER READING:

Abramowitz, M., and Stegun, I.A. 1964, *Handbook of Mathematical Functions* (Washington: National Bureau of Standards); reprinted 1968 (New York: Dover); online at `http://www.nr.com/aands`.[1]

6.14 Statistical Functions

Certain special functions get frequent use because of their relation to common univariate statistical distributions, that is, probability densities in a single variable. In this section we survey a number of such common distributions in a unified way, giving, in each case, routines for computing the probability density function $p(x)$; the cumulative density function or *cdf*, written $P(< x)$; and the inverse of the cumulative density function $x(P)$. The latter function is needed for finding the values of x associated with specified *percentile points* or *quantiles* in significance tests, for example, the 0.5%, 5%, 95% or 99.5% points.

The emphasis of this section is on defining and computing these statistical functions. Section §7.3 is a related section that discusses how to generate random deviates from the distributions discussed here. We defer discussion of the actual use of these distributions in statistical tests to Chapter 14.

6.14.1 Normal (Gaussian) Distribution

If x is drawn from a *normal distribution* with mean μ and standard deviation σ, then we write

$$x \sim \mathrm{N}(\mu, \sigma), \qquad \sigma > 0$$
$$p(x) = \frac{1}{\sqrt{2\pi}\sigma} \exp\left(-\frac{1}{2}\left[\frac{x-\mu}{\sigma}\right]^2\right) \tag{6.14.1}$$

with $p(x)$ the probability density function. Note the special use of the notation "$\sim$" in this section, which can be read as "is drawn from a distribution." The variance of the distribution is, of course, σ^2.

The cumulative distribution function is the probability of a value $\leq x$. For the normal distribution, this is given in terms of the complementary error function by

$$\text{cdf} \equiv P(< x) \equiv \int_{-\infty}^{x} p(x')dx' = \frac{1}{2}\text{erfc}\left(-\frac{1}{\sqrt{2}}\left[\frac{x-\mu}{\sigma}\right]\right) \tag{6.14.2}$$

The inverse cdf can thus be calculated in terms of the inverse of erfc,

$$x(P) = \mu - \sqrt{2}\sigma\,\text{erfc}^{-1}(2P) \tag{6.14.3}$$

The following structure implements the above relations.

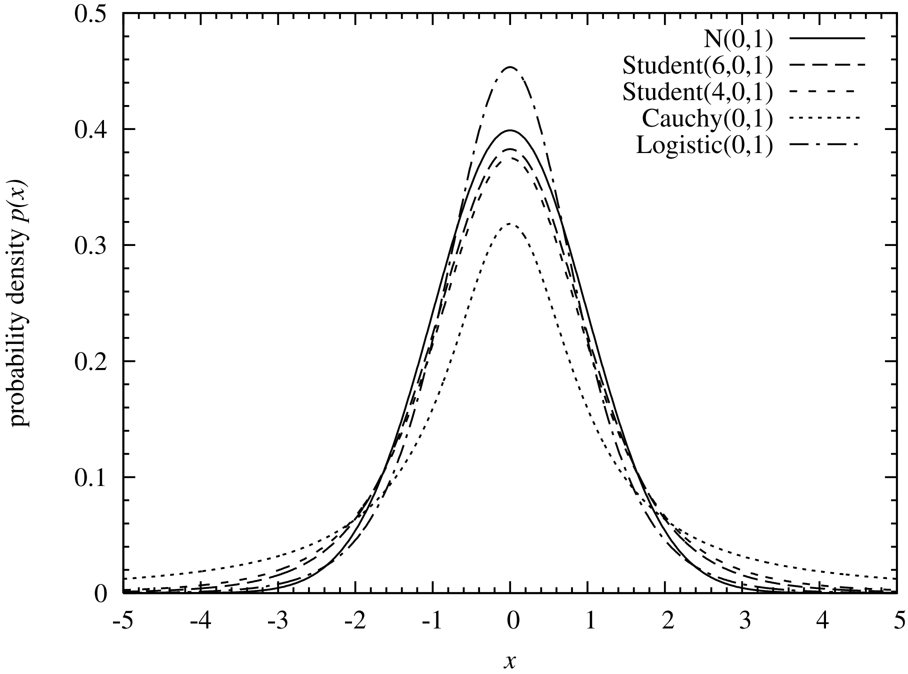

Figure 6.14.1. Examples of centrally peaked distributions that are symmetric on the real line. Any of these can substitute for the normal distribution either as an approximation or in applications such as robust estimation. They differ largely in the decay rate of their tails.

erf.h

```
struct Normaldist : Erf {
Normal distribution, derived from the error function Erf.
    Doub mu, sig;
    Normaldist(Doub mmu = 0., Doub ssig = 1.) : mu(mmu), sig(ssig) {
    Constructor. Initialize with μ and σ. The default with no arguments is N(0,1).
        if (sig <= 0.) throw("bad sig in Normaldist");
    }
    Doub p(Doub x) {
    Return probability density function.
        return (0.398942280401432678/sig)*exp(-0.5*SQR((x-mu)/sig));
    }
    Doub cdf(Doub x) {
    Return cumulative distribution function.
        return 0.5*erfc(-0.707106781186547524*(x-mu)/sig);
    }
    Doub invcdf(Doub p) {
    Return inverse cumulative distribution function.
        if (p <= 0. || p >= 1.) throw("bad p in Normaldist");
        return -1.41421356237309505*sig*inverfc(2.*p)+mu;
    }
};
```

We will use the conventions of the above code for all the distributions in this section. A distribution's parameters (here, μ and σ) are set by the constructor and then referenced as needed by the member functions. The density function is always `p()`, the cdf is `cdf()`, and the inverse cdf is `invcdf()`. We will generally check the arguments of probability functions for validity, since many program bugs can show up as, e.g., a probability out of the range [0, 1].

6.14.2 Cauchy Distribution

Like the normal distribution, the *Cauchy distribution* is a centrally peaked, symmetric distribution with a parameter μ that specifies its center and a parameter σ that specifies its width. *Unlike* the normal distribution, the Cauchy distribution has tails that decay very slowly at infinity, as $|x|^{-2}$, so slowly that moments higher than the zeroth moment (the area under the curve) don't even exist. The parameter μ is therefore, strictly speaking, not the mean, and the parameter σ is not, technically, the standard deviation. But these two parameters substitute for those moments as measures of central position and width.

The defining probability density is

$$x \sim \text{Cauchy}(\mu, \sigma), \qquad \sigma > 0$$
$$p(x) = \frac{1}{\pi\sigma}\left(1 + \left[\frac{x-\mu}{\sigma}\right]^2\right)^{-1} \tag{6.14.4}$$

If $x \sim \text{Cauchy}(0,1)$, then also $1/x \sim \text{Cauchy}(0,1)$ and also $(ax+b)^{-1} \sim \text{Cauchy}(-b/a, 1/a)$.

The cdf is given by

$$\text{cdf} \equiv P(<x) \equiv \int_{-\infty}^{x} p(x')dx' = \frac{1}{2} + \frac{1}{\pi}\arctan\left(\frac{x-\mu}{\sigma}\right) \tag{6.14.5}$$

The inverse cdf is given by

$$x(P) = \mu + \sigma\tan\left(\pi[P - \tfrac{1}{2}]\right) \tag{6.14.6}$$

Figure 6.14.1 shows Cauchy(0, 1) as compared to the normal distribution N(0, 1), as well as several other similarly shaped distributions discussed below.

The Cauchy distribution is sometimes called the *Lorentzian distribution*.

distributions.h

```
struct Cauchydist {
Cauchy distribution.
    Doub mu, sig;
    Cauchydist(Doub mmu = 0., Doub ssig = 1.) : mu(mmu), sig(ssig) {
    Constructor. Initialize with μ and σ. The default with no arguments is Cauchy(0, 1).
        if (sig <= 0.) throw("bad sig in Cauchydist");
    }
    Doub p(Doub x) {
    Return probability density function.
        return 0.318309886183790671/(sig*(1.+SQR((x-mu)/sig)));
    }
    Doub cdf(Doub x) {
    Return cumulative distribution function.
        return 0.5+0.318309886183790671*atan2(x-mu,sig);
    }
    Doub invcdf(Doub p) {
    Return inverse cumulative distribution function.
        if (p <= 0. || p >= 1.) throw("bad p in Cauchydist");
        return mu + sig*tan(3.14159265358979324*(p-0.5));
    }
};
```

6.14.3 Student-t Distribution

A generalization of the Cauchy distribution is the Student-t distribution, named for the early 20th century statistician William Gosset, who published under the name "Student" because his employer, Guinness Breweries, required him to use a pseudonym. Like the Cauchy distribution, the Student-t distribution has power-law tails at infinity, but it has an additional parameter ν that specifies how rapidly they decay, namely as $|t|^{-(\nu+1)}$. When ν is an integer, the number of convergent moments, including the zeroth, is thus ν.

The defining probability density (conventionally written in a variable t instead of x) is

$$t \sim \text{Student}(\nu, \mu, \sigma), \qquad \nu > 0,\ \sigma > 0$$
$$p(t) = \frac{\Gamma(\frac{1}{2}[\nu+1])}{\Gamma(\frac{1}{2}\nu)\sqrt{\nu\pi}\sigma}\left(1 + \frac{1}{\nu}\left[\frac{t-\mu}{\sigma}\right]^2\right)^{-\frac{1}{2}(\nu+1)} \tag{6.14.7}$$

The Cauchy distribution is obtained in the case $\nu = 1$. In the opposite limit, $\nu \to \infty$, the normal distribution is obtained. In pre-computer days, this was the basis of various approximation schemes for the normal distribution, now all generally irrelevant. Figure 6.14.1 shows examples of the Student-t distribution for $\nu = 1$ (Cauchy), $\nu = 4$, and $\nu = 6$. The approach to the normal distribution is evident.

The mean of Student(ν, μ, σ) is (by symmetry) μ. The variance is not σ^2, but rather

$$\text{Var}\{\text{Student}(\nu, \mu, \sigma)\} = \frac{\nu}{\nu - 2}\sigma^2 \tag{6.14.8}$$

For additional moments, and other properties, see [1].

The cdf is given by an incomplete beta function. If we let

$$x \equiv \frac{\nu}{\nu + \left(\frac{t-\mu}{\sigma}\right)^2} \tag{6.14.9}$$

then

$$\text{cdf} \equiv P(<t) \equiv \int_{-\infty}^{t} p(t')dt' = \begin{cases} \frac{1}{2} I_x(\frac{1}{2}\nu, \frac{1}{2}), & t \le \mu \\ 1 - \frac{1}{2} I_x(\frac{1}{2}\nu, \frac{1}{2}), & t > \mu \end{cases} \tag{6.14.10}$$

The inverse cdf is given by an inverse incomplete beta function (see code below for the exact formulation).

In practice, the Student-t cdf is the above form is rarely used, since most statistical tests using Student-t are double-sided. Conventionally, the two-tailed function $A(t|\nu)$ is defined (only) for the case $\mu = 0$ and $\sigma = 1$ by

$$A(t|\nu) \equiv \int_{-t}^{+t} p(t')dt' = 1 - I_x(\tfrac{1}{2}\nu, \tfrac{1}{2}) \tag{6.14.11}$$

with x as given above. The statistic $A(t|\nu)$ is notably used in the test of whether two observed distributions have the same mean. The code below implements both equations (6.14.10) and (6.14.11), as well as their inverses.

incgammabeta.h

```
struct Studenttdist : Beta {
```
Student-t distribution, derived from the beta function Beta.
```
    Doub nu, mu, sig, np, fac;
    Studenttdist(Doub nnu, Doub mmu = 0., Doub ssig = 1.)
    : nu(nnu), mu(mmu), sig(ssig) {
```
Constructor. Initialize with ν, μ and σ. The default with one argument is Student$(\nu, 0, 1)$.
```
        if (sig <= 0. || nu <= 0.) throw("bad sig,nu in Studentdist");
        np = 0.5*(nu + 1.);
        fac = gammln(np)-gammln(0.5*nu);
    }
    Doub p(Doub t) {
```
Return probability density function.
```
        return exp(-np*log(1.+SQR((t-mu)/sig)/nu)+fac)
            /(sqrt(3.14159265358979324*nu)*sig);
    }
    Doub cdf(Doub t) {
```
Return cumulative distribution function.
```
        Doub p = 0.5*betai(0.5*nu, 0.5, nu/(nu+SQR((t-mu)/sig)));
        if (t >= mu) return 1. - p;
        else return p;
    }
    Doub invcdf(Doub p) {
```
Return inverse cumulative distribution function.
```
        if (p <= 0. || p >= 1.) throw("bad p in Studentdist");
        Doub x = invbetai(2.*MIN(p,1.-p), 0.5*nu, 0.5);
        x = sig*sqrt(nu*(1.-x)/x);
        return (p >= 0.5? mu+x : mu-x);
    }
    Doub aa(Doub t) {
```
Return the two-tailed cdf $A(t|\nu)$.
```
        if (t < 0.) throw("bad t in Studentdist");
        return 1.-betai(0.5*nu, 0.5, nu/(nu+SQR(t)));
    }
    Doub invaa(Doub p) {
```
Return the inverse, namely t such that $p = A(t|\nu)$.
```
        if (p < 0. || p >= 1.) throw("bad p in Studentdist");
        Doub x = invbetai(1.-p, 0.5*nu, 0.5);
        return sqrt(nu*(1.-x)/x);
    }
};
```

6.14.4 Logistic Distribution

The *logistic distribution* is another symmetric, centrally peaked distribution that can be used instead of the normal distribution. Its tails decay exponentially, but still much more slowly than the normal distribution's "exponent of the square."

The defining probability density is

$$p(y) = \frac{e^{-y}}{(1+e^{-y})^2} = \frac{e^{y}}{(1+e^{y})^2} = \tfrac{1}{4}\operatorname{sech}^2\left(\tfrac{1}{2}y\right) \tag{6.14.12}$$

The three forms are algebraically equivalent, but, to avoid overflows, it is wise to use the negative and positive exponential forms for positive and negative values of y, respectively.

The variance of the distribution (6.14.12) turns out to be $\pi^2/3$. Since it is convenient to have parameters μ and σ with the conventional meanings of mean and standard deviation, equation (6.14.12) is often replaced by the *standardized logistic*

distribution,

$$x \sim \text{Logistic}(\mu, \sigma), \qquad \sigma > 0$$
$$p(x) = \frac{\pi}{4\sqrt{3}\sigma}\,\text{sech}^2\left(\frac{\pi}{2\sqrt{3}}\left[\frac{x-\mu}{\sigma}\right]\right) \tag{6.14.13}$$

which implies equivalent forms using the positive and negative exponentials (see code below).

The cdf is given by

$$\text{cdf} \equiv P(<x) \equiv \int_{-\infty}^{x} p(x')dx' = \left[1 + \exp\left(-\frac{\pi}{\sqrt{3}}\left[\frac{x-\mu}{\sigma}\right]\right)\right]^{-1} \tag{6.14.14}$$

The inverse cdf is given by

$$x(P) = \mu + \frac{\sqrt{3}}{\pi}\sigma \log\left(\frac{P}{1-P}\right) \tag{6.14.15}$$

distributions.h

```
struct Logisticdist {
Logistic distribution.
    Doub mu, sig;
    Logisticdist(Doub mmu = 0., Doub ssig = 1.) : mu(mmu), sig(ssig) {
    Constructor. Initialize with μ and σ. The default with no arguments is Logistic(0, 1).
        if (sig <= 0.) throw("bad sig in Logisticdist");
    }
    Doub p(Doub x) {
    Return probability density function.
        Doub e = exp(-abs(1.81379936423421785*(x-mu)/sig));
        return 1.81379936423421785*e/(sig*SQR(1.+e));
    }
    Doub cdf(Doub x) {
    Return cumulative distribution function.
        Doub e = exp(-abs(1.81379936423421785*(x-mu)/sig));
        if (x >= mu) return 1./(1.+e);          Because we used abs to control over-
        else return e/(1.+e);                      flow, we now have two cases.
    }
    Doub invcdf(Doub p) {
    Return inverse cumulative distribution function.
        if (p <= 0. || p >= 1.) throw("bad p in Logisticdist");
        return mu + 0.551328895421792049*sig*log(p/(1.-p));
    }
};
```

The logistic distribution is cousin to the *logit transformation* that maps the open unit interval $0 < p < 1$ onto the real line $-\infty < u < \infty$ by the relation

$$u = \log\left(\frac{p}{1-p}\right) \tag{6.14.16}$$

Back when a book of tables and a slide rule were a statistician's working tools, the logit transformation was used to approximate processes on the interval by analytically simpler processes on the real line. A uniform distribution on the interval maps by the logit transformation to a logistic distribution on the real line. With the computer's ability to calculate distributions on the interval directly (beta distributions, for example), that motivation has vanished.

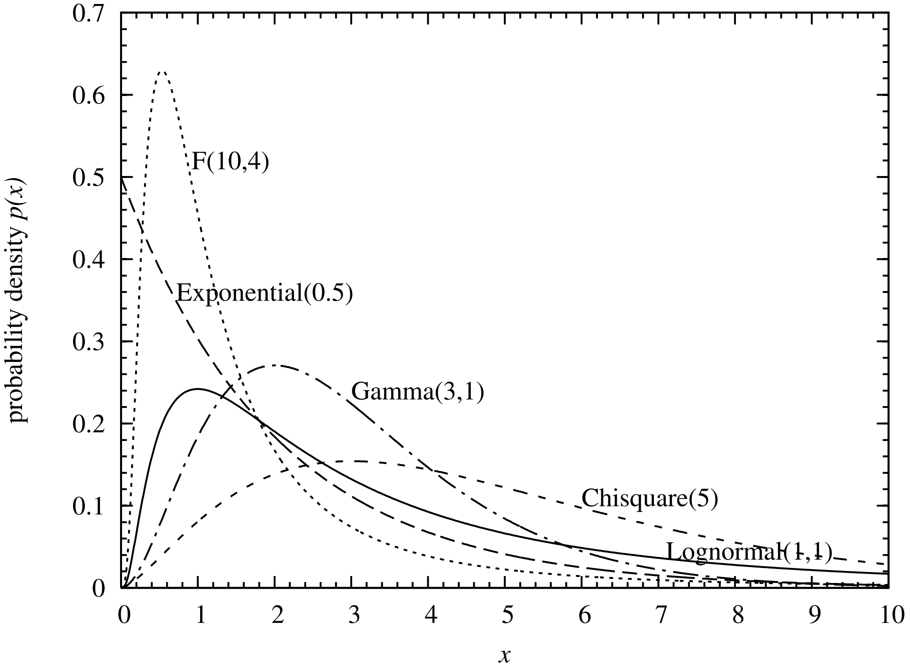

Figure 6.14.2. Examples of common distributions on the half-line $x > 0$.

Another cousin is the *logistic equation*,

$$\frac{dy}{dt} \propto y(y_{\max} - y) \tag{6.14.17}$$

a differential equation describing the growth of some quantity y, starting off as an exponential but reaching, asymptotically, a value $y_{\max}$. The solution of this equation is identical, up to a scaling, to the cdf of the logistic distribution.

6.14.5 Exponential Distribution

With the *exponential distribution* we now turn to common distribution functions defined on the positive real axis $x \geq 0$. Figure 6.14.2 shows examples of several of the distributions that we will discuss. The exponential is the simplest of them all. It has a parameter β that can control its width (in inverse relationship), but its mode is always at zero:

$$\begin{aligned} x &\sim \text{Exponential}(\beta), & \beta > 0 \\ p(x) &= \beta \exp(-\beta x), & x > 0 \end{aligned} \tag{6.14.18}$$

$$\text{cdf} \equiv P(< x) \equiv \int_0^x p(x')dx' = 1 - \exp(-\beta x) \tag{6.14.19}$$

$$x(P) = -\frac{1}{\beta}\log(1 - P) \tag{6.14.20}$$

The mean and standard deviation of the exponential distribution are both $1/\beta$. The median is $\log(2)/\beta$. Reference [1] has more to say about the exponential distribution than you would ever think possible.

distributions.h

```
struct Expondist {
Exponential distribution.
    Doub bet;
    Expondist(Doub bbet) : bet(bbet) {
    Constructor. Initialize with β.
        if (bet <= 0.) throw("bad bet in Expondist");
    }
    Doub p(Doub x) {
    Return probability density function.
        if (x < 0.) throw("bad x in Expondist");
        return bet*exp(-bet*x);
    }
    Doub cdf(Doub x) {
    Return cumulative distribution function.
        if (x < 0.) throw("bad x in Expondist");
        return 1.-exp(-bet*x);
    }
    Doub invcdf(Doub p) {
    Return inverse cumulative distribution function.
        if (p < 0. || p >= 1.) throw("bad p in Expondist");
        return -log(1.-p)/bet;
    }
};
```

6.14.6 Weibull Distribution

The Weibull distribution generalizes the exponential distribution in a way that is often useful in hazard, survival, or reliability studies. When the lifetime (time to failure) of an item is exponentially distributed, there is a constant probability per unit time that an item will fail, if it has not already done so. That is,

$$\text{hazard} \equiv \frac{p(x)}{P(>x)} \propto \text{constant} \tag{6.14.21}$$

Exponentially lived items don't age; they just keep rolling the same dice until, one day, their number comes up. In many other situations, however, it is observed that an item's hazard (as defined above) does change with time, say as a power law,

$$\frac{p(x)}{P(>x)} \propto x^{\alpha-1}, \qquad \alpha > 0 \tag{6.14.22}$$

The distribution that results is the Weibull distribution, named for Swedish physicist Waloddi Weibull, who used it as early as 1939. When $\alpha > 1$, the hazard increases with time, as for components that wear out. When $0 < \alpha < 1$, the hazard decreases with time, as for components that experience "infant mortality."

We say that

$$x \sim \text{Weibull}(\alpha, \beta) \qquad \text{iff} \qquad y \equiv \left(\frac{x}{\beta}\right)^{\alpha} \sim \text{Exponential}(1) \tag{6.14.23}$$

The probability density is

$$p(x) = \left(\frac{\alpha}{\beta}\right)\left(\frac{x}{\beta}\right)^{\alpha-1} e^{-(x/\beta)^{\alpha}}, \qquad x > 0 \tag{6.14.24}$$

The cdf is

$$\text{cdf} \equiv P(<x) \equiv \int_0^x p(x')dx' = 1 - e^{-(x/\beta)^\alpha} \tag{6.14.25}$$

The inverse cdf is

$$x(P) = \beta\,[-\log(1-P)]^{1/\alpha} \tag{6.14.26}$$

For $0 < \alpha < 1$, the distribution has an infinite (but integrable) cusp at $x = 0$ and is monotonically decreasing. The exponential distribution is the case of $\alpha = 1$. When $\alpha > 1$, the distribution is zero at $x = 0$ and has a single maximum at the value $x = \beta\,[(\alpha-1)/\alpha]^{1/\alpha}$.

The mean and variance are given by

$$\begin{aligned} \mu &= \beta\,\Gamma(1+\alpha^{-1}) \\ \sigma^2 &= \beta^2 \left\{\Gamma(1+2\alpha^{-1}) - \left[\Gamma(1+\alpha^{-1})\right]^2\right\} \end{aligned} \tag{6.14.27}$$

With correct normalization, equation (6.14.22) becomes

$$\text{hazard} \equiv \frac{p(x)}{P(>x)} = \left(\frac{\alpha}{\beta}\right)\left(\frac{x}{\beta}\right)^{\alpha-1} \tag{6.14.28}$$

6.14.7 Lognormal Distribution

Many processes that live on the positive x-axis are naturally approximated by normal distributions on the "log(x)-axis," that is, for $-\infty < \log(x) < \infty$. A simple, but important, example is the multiplicative random walk, which starts at some positive value x_0, and then generates new values by a recurrence like

$$x_{i+1} = \begin{cases} x_i(1+\epsilon) & \text{with probability 0.5} \\ x_i/(1+\epsilon) & \text{with probability 0.5} \end{cases} \tag{6.14.29}$$

Here ϵ is some small, fixed, constant.

These considerations motivate the definition

$$x \sim \text{Lognormal}(\mu,\sigma) \qquad \text{iff} \qquad u \equiv \frac{\log(x)-\mu}{\sigma} \sim \text{N}(0,1) \tag{6.14.30}$$

or the equivalent definition

$$\begin{aligned} &x \sim \text{Lognormal}(\mu,\sigma), \qquad \sigma > 0 \\ &p(x) = \frac{1}{\sqrt{2\pi}\sigma x} \exp\left(-\frac{1}{2}\left[\frac{\log(x)-\mu}{\sigma}\right]^2\right), \qquad x > 0 \end{aligned} \tag{6.14.31}$$

Note the required extra factor of x^{-1} in front of the exponential: The density that is "normal" is $p(\log x)d\log x$.

While μ and σ are the mean and standard deviation in $\log x$ space, they are *not* so in x space. Rather,

$$\begin{aligned} \text{Mean}\{\text{Lognormal}(\mu,\sigma)\} &= e^{\mu+\frac{1}{2}\sigma^2} \\ \text{Var}\{\text{Lognormal}(\mu,\sigma)\} &= e^{2\mu}\,e^{\sigma^2}(e^{\sigma^2}-1) \end{aligned} \tag{6.14.32}$$

The cdf is given by

$$\text{cdf} \equiv P(< x) \equiv \int_0^x p(x')dx' = \frac{1}{2}\text{erfc}\left(-\frac{1}{\sqrt{2}}\left[\frac{\log(x) - \mu}{\sigma}\right]\right) \tag{6.14.33}$$

The inverse to the cdf involves the inverse complementary error function,

$$x(P) = \exp[\mu - \sqrt{2}\sigma\ \text{erfc}^{-1}(2P)] \tag{6.14.34}$$

erf.h

```
struct Lognormaldist : Erf {
Lognormal distribution, derived from the error function Erf.
    Doub mu, sig;
    Lognormaldist(Doub mmu = 0., Doub ssig = 1.) : mu(mmu), sig(ssig) {
        if (sig <= 0.) throw("bad sig in Lognormaldist");
    }
    Doub p(Doub x) {
    Return probability density function.
        if (x < 0.) throw("bad x in Lognormaldist");
        if (x == 0.) return 0.;
        return (0.398942280401432678/(sig*x))*exp(-0.5*SQR((log(x)-mu)/sig));
    }
    Doub cdf(Doub x) {
    Return cumulative distribution function.
        if (x < 0.) throw("bad x in Lognormaldist");
        if (x == 0.) return 0.;
        return 0.5*erfc(-0.707106781186547524*(log(x)-mu)/sig);
    }
    Doub invcdf(Doub p) {
    Return inverse cumulative distribution function.
        if (p <= 0. || p >= 1.) throw("bad p in Lognormaldist");
        return exp(-1.41421356237309505*sig*inverfc(2.*p)+mu);
    }
};
```

Multiplicative random walks like (6.14.29) and lognormal distributions are key ingredients in the economic theory of efficient markets, leading to (among many other results) the celebrated *Black-Scholes formula* for the probability distribution of the price of an investment after some elapsed time τ. A key piece of the Black-Scholes derivation is implicit in equation (6.14.32): If an investment's average return is zero (which may be true in the limit of zero risk), then its price cannot simply be a widening lognormal distribution with fixed μ and increasing σ, for its expected value would then diverge to infinity! The actual Black-Scholes formula thus defines both how σ increases with time (basically as $\tau^{1/2}$) *and* how μ correspondingly decreases with time, so as to keep the overall mean under control. A simplified version of the Black-Scholes formula can be written as

$$S(\tau) \sim S(0) \times \text{Lognormal}\left(r\tau - \tfrac{1}{2}\sigma^2\tau,\ \sigma\sqrt{\tau}\right) \tag{6.14.35}$$

where $S(\tau)$ is the price of a stock at time τ, r is its expected (annualized) rate of return, and σ is now redefined to be the stock's (annualized) volatility. The definition of volatility is that, for small values of τ, the fractional variance of the stock's price is $\sigma^2\tau$. You can check that (6.14.35) has the desired expectation value $E[S(\tau)] = S(0)$, for all τ, if $r = 0$. A good reference is [3].

6.14.8 Chi-Square Distribution

The *chi-square* (or χ^2) distribution has a single parameter $\nu > 0$ that controls both the location and width of its peak. In most applications ν is an integer and is referred to as the *number of degrees of freedom* (see §14.3).

The defining probability density is

$$\chi^2 \sim \text{Chisquare}(\nu), \qquad \nu > 0$$
$$p(\chi^2)d\chi^2 = \frac{1}{2^{\frac{1}{2}\nu}\Gamma(\frac{1}{2}\nu)}(\chi^2)^{\frac{1}{2}\nu-1}\exp\left(-\tfrac{1}{2}\chi^2\right)d\chi^2, \qquad \chi^2 > 0 \tag{6.14.36}$$

where we have written the differentials $d\chi^2$ merely to emphasize that χ^2, not χ, is to be viewed as the independent variable.

The mean and variance are given by

$$\begin{aligned}\text{Mean}\{\text{Chisquare}(\nu)\} &= \nu \\ \text{Var}\{\text{Chisquare}(\nu)\} &= 2\nu\end{aligned} \tag{6.14.37}$$

When $\nu \geq 2$ there is a single mode at $\chi^2 = \nu - 2$.

The chi-square distribution is actually just a special case of the gamma distribution, below, so its cdf is given by an incomplete gamma function $P(a,x)$,

$$\text{cdf} \equiv P(<\chi^2) \equiv P(\chi^2|\nu) \equiv \int_0^{\chi^2} p(\chi^{2\prime})d\chi^{2\prime} = P\left(\frac{\nu}{2},\frac{\chi^2}{2}\right) \tag{6.14.38}$$

One frequently also sees the complement of the cdf, which can be calculated either from the incomplete gamma function $P(a,x)$, or from its complement $Q(a,x)$ (often more accurate if P is very close to 1):

$$Q(\chi^2|\nu) \equiv 1 - P(\chi^2|n) = 1 - P\left(\frac{\nu}{2},\frac{\chi^2}{2}\right) \equiv Q\left(\frac{\nu}{2},\frac{\chi^2}{2}\right) \tag{6.14.39}$$

The inverse cdf is given in terms of the function that is the inverse of $P(a,x)$ on its second argument, which we here denote $P^{-1}(a,p)$:

$$x(P) = 2P^{-1}\left(\frac{\nu}{2},P\right) \tag{6.14.40}$$

incgammabeta.h

```
struct Chisqdist : Gamma {
```
χ^2 distribution, derived from the gamma function `Gamma`.
```
    Doub nu,fac;
    Chisqdist(Doub nnu) : nu(nnu) {
```
Constructor. Initialize with ν.
```
        if (nu <= 0.) throw("bad nu in Chisqdist");
        fac = 0.693147180559945309*(0.5*nu)+gammln(0.5*nu);
    }
    Doub p(Doub x2) {
```
Return probability density function.
```
        if (x2 <= 0.) throw("bad x2 in Chisqdist");
        return exp(-0.5*(x2-(nu-2.)*log(x2))-fac);
    }
    Doub cdf(Doub x2) {
```

```
Return cumulative distribution function.
        if (x2 < 0.) throw("bad x2 in Chisqdist");
        return gammp(0.5*nu,0.5*x2);
    }
    Doub invcdf(Doub p) {
    Return inverse cumulative distribution function.
        if (p < 0. || p >= 1.) throw("bad p in Chisqdist");
        return 2.*invgammp(p,0.5*nu);
    }
};
```

6.14.9 Gamma Distribution

The *gamma distribution* is defined by

$$\begin{aligned} x &\sim \text{Gamma}(\alpha, \beta), & \alpha > 0,\ \beta > 0 \\ p(x) &= \frac{\beta^\alpha}{\Gamma(\alpha)} x^{\alpha-1} e^{-\beta x}, & x > 0 \end{aligned} \tag{6.14.41}$$

The exponential distribution is the special case with $\alpha = 1$. The chi-square distribution is the special case with $\alpha = \nu/2$ and $\beta = 1/2$.

The mean and variance are given by,

$$\begin{aligned} \text{Mean}\{\text{Gamma}(\alpha, \beta)\} &= \alpha/\beta \\ \text{Var}\{\text{Gamma}(\alpha, \beta)\} &= \alpha/\beta^2 \end{aligned} \tag{6.14.42}$$

When $\alpha \geq 1$ there is a single mode at $x = (\alpha - 1)/\beta$.

Evidently, the cdf is the incomplete gamma function

$$\text{cdf} \equiv P(< x) \equiv \int_0^x p(x')dx' = P\,(\alpha, \beta x) \tag{6.14.43}$$

while the inverse cdf is given in terms of the inverse of $P(a, x)$ on its second argument by

$$x(P) = \frac{1}{\beta} P^{-1}\,(\alpha, P) \tag{6.14.44}$$

incgammabeta.h

```
struct Gammadist : Gamma {
Gamma distribution, derived from the gamma function Gamma.
    Doub alph, bet, fac;
    Gammadist(Doub aalph, Doub bbet = 1.) : alph(aalph), bet(bbet) {
    Constructor. Initialize with α and β.
        if (alph <= 0. || bet <= 0.) throw("bad alph,bet in Gammadist");
        fac = alph*log(bet)-gammln(alph);
    }
    Doub p(Doub x) {
    Return probability density function.
        if (x <= 0.) throw("bad x in Gammadist");
        return exp(-bet*x+(alph-1.)*log(x)+fac);
    }
    Doub cdf(Doub x) {
    Return cumulative distribution function.
        if (x < 0.) throw("bad x in Gammadist");
        return gammp(alph,bet*x);
    }
```

```
    Doub invcdf(Doub p) {
    Return inverse cumulative distribution function.
        if (p < 0. || p >= 1.) throw("bad p in Gammadist");
        return invgammp(p,alph)/bet;
    }
};
```

6.14.10 F-Distribution

The *F-distribution* is parameterized by two positive values ν_1 and ν_2, usually (but not always) integers.

The defining probability density is

$$\begin{aligned} F &\sim \mathrm{F}(\nu_1,\nu_2), \qquad \nu_1>0,\ \nu_2>0 \\ p(F) &= \frac{\nu_1^{\frac{1}{2}\nu_1}\nu_2^{\frac{1}{2}\nu_2}}{B(\frac{1}{2}\nu_1,\frac{1}{2}\nu_2)}\frac{F^{\frac{1}{2}\nu_1-1}}{(\nu_2+\nu_1F)^{(\nu_1+\nu_2)/2}}, \qquad F>0 \end{aligned} \tag{6.14.45}$$

where $B(a,b)$ denotes the beta function. The mean and variance are given by

$$\begin{aligned} \mathrm{Mean}\{\mathrm{F}(\nu_1,\nu_2)\} &= \frac{\nu_2}{\nu_2-2}, \qquad \nu_2>2 \\ \mathrm{Var}\{\mathrm{F}(\nu_1,\nu_2)\} &= \frac{2\nu_2^2(\nu_1+\nu_2-2)}{\nu_1(\nu_2-2)^2(\nu_2-4)}, \qquad \nu_2>4 \end{aligned} \tag{6.14.46}$$

When $\nu_1 \geq 2$ there is a single mode at

$$F = \frac{\nu_2(\nu_1-2)}{\nu_1(\nu_2+2)} \tag{6.14.47}$$

.

For fixed ν_1, if $\nu_2 \to \infty$, the F-distribution becomes a chi-square distribution, namely

$$\lim_{\nu_2\to\infty} \mathrm{F}(\nu_1,\nu_2) \cong \frac{1}{\nu_1}\mathrm{Chisquare}(\nu_1) \tag{6.14.48}$$

where "$\cong$" means "are identical distributions."

The F-distribution's cdf is given in terms of the incomplete beta function $I_x(a,b)$ by

$$\mathrm{cdf} \equiv P(<x) \equiv \int_0^x p(x')dx' = I_{\nu_1F/(\nu_2+\nu_1F)}\left(\tfrac{1}{2}\nu_1,\tfrac{1}{2}\nu_2\right) \tag{6.14.49}$$

while the inverse cdf is given in terms of the inverse of $I_x(a,b)$ on its subscript argument by

$$\begin{aligned} u &\equiv I_p^{-1}\left(\tfrac{1}{2}\nu_1,\tfrac{1}{2}\nu_2\right) \\ x(P) &= \frac{\nu_2 u}{\nu_1(1-u)} \end{aligned} \tag{6.14.50}$$

A frequent use of the F-distribution is to test whether two observed samples have the same variance.

incgammabeta.h

```
struct Fdist : Beta {
F distribution, derived from the beta function Beta.
    Doub nu1,nu2;
    Doub fac;
    Fdist(Doub nnu1, Doub nnu2) : nu1(nnu1), nu2(nnu2) {
    Constructor. Initialize with ν1 and ν2.
        if (nu1 <= 0. || nu2 <= 0.) throw("bad nu1,nu2 in Fdist");
        fac = 0.5*(nu1*log(nu1)+nu2*log(nu2))+gammln(0.5*(nu1+nu2))
            -gammln(0.5*nu1)-gammln(0.5*nu2);
    }
    Doub p(Doub f) {
    Return probability density function.
        if (f <= 0.) throw("bad f in Fdist");
        return exp((0.5*nu1-1.)*log(f)-0.5*(nu1+nu2)*log(nu2+nu1*f)+fac);
    }
    Doub cdf(Doub f) {
    Return cumulative distribution function.
        if (f < 0.) throw("bad f in Fdist");
        return betai(0.5*nu1,0.5*nu2,nu1*f/(nu2+nu1*f));
    }
    Doub invcdf(Doub p) {
    Return inverse cumulative distribution function.
        if (p <= 0. || p >= 1.) throw("bad p in Fdist");
        Doub x = invbetai(p,0.5*nu1,0.5*nu2);
        return nu2*x/(nu1*(1.-x));
    }
};
```

6.14.11 Beta Distribution

The *beta distribution* is defined on the unit interval $0 < x < 1$ by

$$x \sim \text{Beta}(\alpha, \beta), \qquad \alpha > 0,\ \beta > 0$$
$$p(x) = \frac{1}{B(\alpha, \beta)} x^{\alpha-1}(1-x)^{\beta-1}, \qquad 0 < x < 1 \tag{6.14.51}$$

The mean and variance are given by

$$\text{Mean}\{\text{Beta}(\alpha, \beta)\} = \frac{\alpha}{\alpha + \beta}$$
$$\text{Var}\{\text{Beta}(\alpha, \beta)\} = \frac{\alpha\beta}{(\alpha+\beta)^2(\alpha+\beta+1)} \tag{6.14.52}$$

When $\alpha > 1$ and $\beta > 1$, there is a single mode at $(\alpha - 1)/(\alpha + \beta - 2)$. When $\alpha < 1$ and $\beta < 1$, the distribution function is "U-shaped" with a minimum at this same value. In other cases there is neither a maximum nor a minimum.

In the limit that β becomes large as α is held fixed, all the action in the beta distribution shifts toward $x = 0$, and the density function takes the shape of a gamma distribution. More precisely,

$$\lim_{\beta\to\infty} \beta\,\text{Beta}(\alpha, \beta) \cong \text{Gamma}(\alpha, 1) \tag{6.14.53}$$

The cdf is the incomplete beta function

$$\text{cdf} \equiv P(< x) \equiv \int_0^x p(x')dx' = I_x(\alpha, \beta) \tag{6.14.54}$$

while the inverse cdf is given in terms of the inverse of $I_x(\alpha,\beta)$ on its subscript argument by

$$x(P) = I_p^{-1}(\alpha,\beta) \tag{6.14.55}$$

incgammabeta.h

```
struct Betadist : Beta {
Beta distribution, derived from the beta function Beta.
    Doub alph, bet, fac;
    Betadist(Doub aalph, Doub bbet) : alph(aalph), bet(bbet) {
    Constructor. Initialize with α and β.
        if (alph <= 0. || bet <= 0.) throw("bad alph,bet in Betadist");
        fac = gammln(alph+bet)-gammln(alph)-gammln(bet);
    }
    Doub p(Doub x) {
    Return probability density function.
        if (x <= 0. || x >= 1.) throw("bad x in Betadist");
        return exp((alph-1.)*log(x)+(bet-1.)*log(1.-x)+fac);
    }
    Doub cdf(Doub x) {
    Return cumulative distribution function.
        if (x < 0. || x > 1.) throw("bad x in Betadist");
        return betai(alph,bet,x);
    }
    Doub invcdf(Doub p) {
    Return inverse cumulative distribution function.
        if (p < 0. || p > 1.) throw("bad p in Betadist");
        return invbetai(p,alph,bet);
    }
};
```

6.14.12 Kolmogorov-Smirnov Distribution

The *Kolmogorov-Smirnov* or *KS* distribution, defined for positive z, is key to an important statistical test that is discussed in §14.3. Its probability density function does not directly enter into the test and is virtually never even written down. What one typically needs to compute is the cdf, denoted $P_{KS}(z)$, or its complement, $Q_{KS}(z) \equiv 1 - P_{KS}(z)$.

The cdf $P_{KS}(z)$ is defined by the series

$$P_{KS}(z) = 1 - 2\sum_{j=1}^{\infty}(-1)^{j-1}\exp(-2j^2z^2) \tag{6.14.56}$$

or by the equivalent series (nonobviously so!)

$$P_{KS}(z) = \frac{\sqrt{2\pi}}{z}\sum_{j=1}^{\infty}\exp\left(-\frac{(2j-1)^2\pi^2}{8z^2}\right) \tag{6.14.57}$$

Limiting values are what you'd expect for cdf's named "P" and "Q":

$$\begin{aligned} P_{KS}(0) &= 0 \qquad & P_{KS}(\infty) &= 1 \\ Q_{KS}(0) &= 1 \qquad & Q_{KS}(\infty) &= 0 \end{aligned} \tag{6.14.58}$$

Both of the series (6.14.56) and (6.14.57) are convergent for all $z > 0$. Moreover, for any z, one or the other series converges extremely rapidly, requiring no more

than three terms to get to IEEE double precision fractional accuracy. A good place to switch from one series to the other is at $z \approx 1.18$. This renders the KS functions computable by a single exponential and a small number of arithmetic operations (see code below).

Getting the inverse functions $P_{KS}^{-1}(P)$ and $Q_{KS}^{-1}(Q)$, which return a value of z from a P or Q value, is a little trickier. For $Q \lesssim 0.3$ (that is, $P \gtrsim 0.7$), an iteration based on (6.14.56) works nicely:

$$\begin{aligned} x_0 &\equiv 0 \\ x_{i+1} &= \tfrac{1}{2}Q + x_i^4 - x_i^9 + x_i^{16} - x_i^{25} + \cdots \\ z(Q) &= \sqrt{-\tfrac{1}{2}\log(x_\infty)} \end{aligned} \tag{6.14.59}$$

For $x \lesssim 0.06$ you only need the first two powers of x_i.

For larger values of Q, that is, $P \lesssim 0.7$, the number of powers of x required quickly becomes excessive. A useful approach is to write (6.14.57) as

$$\begin{aligned} y\log(y) &= -\frac{\pi P^2}{8}\left(1 + y^4 + y^{12} + \cdots + y^{2j(j-1)} + \cdots\right)^{-1} \\ z(P) &= \frac{\pi/2}{\sqrt{-\log(y)}} \end{aligned} \tag{6.14.60}$$

If we can get a good enough initial guess for y, we can solve the first equation in (6.14.60) by a variant of Halley's method: Use values of y from the *previous* iteration on the right-hand side of (6.14.60), and use Halley only for the $y\log(y)$ piece, so that the first and second derivatives are analytically simple functions.

A good initial guess is obtained by using the inverse function to $y\log(y)$ (the function `invxlogx` in §6.11) with the argument $-\pi P^2/8$. The number of iterations within the `invxlogx` function and the Halley loop is never more than half a dozen in each, often less. Code for the KS functions and their inverses follows.

ksdist.h

```
struct KSdist {
Kolmogorov-Smirnov cumulative distribution functions and their inverses.
    Doub pks(Doub z) {
    Return cumulative distribution function.
        if (z < 0.) throw("bad z in KSdist");
        if (z == 0.) return 0.;
        if (z < 1.18) {
            Doub y = exp(-1.23370055013616983/SQR(z));
            return 2.25675833419102515*sqrt(-log(y))
                *(y + pow(y,9) + pow(y,25) + pow(y,49));
        } else {
            Doub x = exp(-2.*SQR(z));
            return 1. - 2.*(x - pow(x,4) + pow(x,9));
        }
    }
    Doub qks(Doub z) {
    Return complementary cumulative distribution function.
        if (z < 0.) throw("bad z in KSdist");
        if (z == 0.) return 1.;
        if (z < 1.18) return 1.-pks(z);
        Doub x = exp(-2.*SQR(z));
        return 2.*(x - pow(x,4) + pow(x,9));
    }
    Doub invqks(Doub q) {
```

Return inverse of the complementary cumulative distribution function.

```
    Doub y,logy,yp,x,xp,f,ff,u,t;
    if (q <= 0. || q > 1.) throw("bad q in KSdist");
    if (q == 1.) return 0.;
    if (q > 0.3) {
        f = -0.392699081698724155*SQR(1.-q);
        y = invxlogx(f);                          Initial guess.
        do {
            yp = y;
            logy = log(y);
            ff = f/SQR(1.+ pow(y,4)+ pow(y,12));
            u = (y*logy-ff)/(1.+logy);       Newton's method correction.
            y = y - (t=u/MAX(0.5,1.-0.5*u/(y*(1.+logy))));  Halley.
        } while (abs(t/y)>1.e-15);
        return 1.57079632679489662/sqrt(-log(y));
    } else {
        x = 0.03;
        do {                                      Iteration (6.14.59).
            xp = x;
            x = 0.5*q+pow(x,4)-pow(x,9);
            if (x > 0.06) x += pow(x,16)-pow(x,25);
        } while (abs((xp-x)/x)>1.e-15);
        return sqrt(-0.5*log(x));
    }
}
Doub invpks(Doub p) {return invqks(1.-p);}
```

Return inverse of the cumulative distribution function.

```
};
```

6.14.13 Poisson Distribution

The eponymous *Poisson distribution* was derived by Poisson in 1837. It applies to a process where discrete, uncorrelated events occur at some mean rate per unit time. If, for a given period, λ is the mean expected number of events, then the probability distribution of seeing exactly k events, $k \geq 0$, can be written as

$$k \sim \text{Poisson}(\lambda), \qquad \lambda > 0$$
$$p(k) = \frac{1}{k!}\lambda^k e^{-\lambda}, \qquad k = 0, 1, \ldots \tag{6.14.61}$$

Evidently $\sum_k p(k) = 1$, since the k-dependent factors in (6.14.61) are just the series expansion of e^{λ}.

The mean and variance of Poisson(λ) are both λ. There is a single mode at $k = \lfloor \lambda \rfloor$, that is, at λ rounded down to an integer.

The Poisson distribution's cdf is an incomplete gamma function $Q(a, x)$,

$$P_\lambda(< k) = Q(k, \lambda) \tag{6.14.62}$$

Since k is discrete, $P_\lambda(< k)$ is of course different from $P_\lambda(\leq k)$, the latter being given by

$$P_\lambda(\leq k) = Q(k + 1, \lambda) \tag{6.14.63}$$

Some particular values are

$$P_\lambda(< 0) = 0 \qquad P_\lambda(< 1) = e^{-\lambda} \qquad P_\lambda(< \infty) = 1 \tag{6.14.64}$$

Some other relations involving the incomplete gamma functions $Q(a,x)$ and $P(a,x)$ are

$$P_\lambda(\geq k) = P(k,\lambda) = 1 - Q(k,\lambda)$$
$$P_\lambda(> k) = P(k+1,\lambda) = 1 - Q(k+1,\lambda) \tag{6.14.65}$$

Because of the discreteness in k, the inverse of the cdf must be defined with some care: Given a value P, we define $k_\lambda(P)$ as the integer such that

$$P_\lambda(< k) \leq P < P_\lambda(\leq k) \tag{6.14.66}$$

In the interest of conciseness, the code below cheats a little bit and allows the right-hand $<$ to be $\leq$. If you may be supplying P's that are *exact* $P_\lambda(< k)$'s, then you will need to check both $k_\lambda(P)$ as returned, and $k_\lambda(P)+1$. (This will essentially never happen for "round" P's like 0.95, 0.99, etc.)

incgammabeta.h

```
struct Poissondist : Gamma {
Poisson distribution, derived from the gamma function Gamma.
    Doub lam;
    Poissondist(Doub llam) : lam(llam) {
    Constructor. Initialize with λ.
        if (lam <= 0.) throw("bad lam in Poissondist");
    }
    Doub p(Int n) {
    Return probability density function.
        if (n < 0) throw("bad n in Poissondist");
        return exp(-lam + n*log(lam) - gammln(n+1.));
    }
    Doub cdf(Int n) {
    Return cumulative distribution function.
        if (n < 0) throw("bad n in Poissondist");
        if (n == 0) return 0.;
        return gammq((Doub)n,lam);
    }
    Int invcdf(Doub p) {
    Given argument P, return integer n such that P(< n) ≤ P ≤ P(< n + 1).
        Int n,nl,nu,inc=1;
        if (p <= 0. || p >= 1.) throw("bad p in Poissondist");
        if (p < exp(-lam)) return 0;
        n = (Int)MAX(sqrt(lam),5.);               Starting guess near peak of density.
        if (p < cdf(n)) {                         Expand interval until we bracket.
            do {
                n = MAX(n-inc,0);
                inc *= 2;
            } while (p < cdf(n));
            nl = n; nu = n + inc/2;
        } else {
            do {
                n += inc;
                inc *= 2;
            } while (p > cdf(n));
            nu = n; nl = n - inc/2;
        }
        while (nu-nl>1) {                         Now contract the interval by bisection.
            n = (nl+nu)/2;
            if (p < cdf(n)) nu = n;
            else nl = n;
        }
        return nl;
    }
};
```

6.14.14 Binomial Distribution

Like the Poisson distribution, the *binomial distribution* is a discrete distribution over $k \geq 0$. It has two parameters, $n \geq 1$, the "sample size" or maximum value for which k can be nonzero; and p, the "event probability" (not to be confused with $p(k)$, the probability of a particular k). We write

$$k \sim \text{Binomial}(n, p), \qquad n \geq 1,\ 0 < p < 1$$
$$p(k) = \binom{n}{k} p^k (1-p)^{n-k}, \qquad k = 0, 1, \ldots, n \tag{6.14.67}$$

where $\binom{n}{k}$ is, of course, the binomial coefficient.

The mean and variance are given by

$$\begin{aligned} \text{Mean}\{\text{Binomial}(n, p)\} &= np \\ \text{Var}\{\text{Binomial}(n, p)\} &= np(1-p) \end{aligned} \tag{6.14.68}$$

There is a single mode at the value k that satisfies

$$(n+1)p - 1 < k \leq (n+1)p \tag{6.14.69}$$

The distribution is symmetrical iff $p = \frac{1}{2}$. Otherwise it has positive skewness for $p < \frac{1}{2}$ and negative for $p > \frac{1}{2}$. Many additional properties are described in [2].

The Poisson distribution is obtained from the binomial distribution in the limit $n \to \infty$, $p \to 0$ with the np remaining finite. More precisely,

$$\lim_{n\to\infty} \text{Binomial}(n, \lambda/n) \cong \text{Poisson}(\lambda) \tag{6.14.70}$$

The binomial distribution's cdf can be computed from the incomplete beta function $I_x(a, b)$,

$$P(<k) = 1 - I_p(k, n-k+1) \tag{6.14.71}$$

so we also have (analogously to the Poisson distribution)

$$\begin{aligned} P(\leq k) &= 1 - I_p(k+1, n-k) \\ P(>k) &= I_p(k+1, n-k) \\ P(\geq k) &= I_p(k, n-k+1) \end{aligned} \tag{6.14.72}$$

Some particular values are

$$P(<0) = 0 \qquad P(<[n+1]) = 1 \tag{6.14.73}$$

The inverse cdf is defined exactly as for the Poisson distribution, above, and with the same small warning about the code.

incgammabeta.h

```
struct Binomialdist : Beta {
Binomial distribution, derived from the beta function Beta.
    Int n;
    Doub pe, fac;
    Binomialdist(Int nn, Doub ppe) : n(nn), pe(ppe) {
    Constructor. Initialize with n (sample size) and p (event probability).
        if (n <= 0 || pe <= 0. || pe >= 1.) throw("bad args in Binomialdist");
```

```
        fac = gammln(n+1.);
    }
    Doub p(Int k) {
    Return probability density function.
        if (k < 0) throw("bad k in Binomialdist");
        if (k > n) return 0.;
        return exp(k*log(pe)+(n-k)*log(1.-pe)
            +fac-gammln(k+1.)-gammln(n-k+1.));
    }
    Doub cdf(Int k) {
    Return cumulative distribution function.
        if (k < 0) throw("bad k in Binomialdist");
        if (k == 0) return 0.;
        if (k > n) return 1.;
        return 1. - betai((Doub)k,n-k+1.,pe);
    }
    Int invcdf(Doub p) {
    Given argument P, return integer n such that P(< n) ≤ P ≤ P(< n + 1).
        Int k,kl,ku,inc=1;
        if (p <= 0. || p >= 1.) throw("bad p in Binomialdist");
        k = MAX(0,MIN(n,(Int)(n*pe)));          Starting guess near peak of density.
        if (p < cdf(k)) {                        Expand interval until we bracket.
            do {
                k = MAX(k-inc,0);
                inc *= 2;
            } while (p < cdf(k));
            kl = k; ku = k + inc/2;
        } else {
            do {
                k = MIN(k+inc,n+1);
                inc *= 2;
            } while (p > cdf(k));
            ku = k; kl = k - inc/2;
        }
        while (ku-kl>1) {                        Now contract the interval by bisection.
            k = (kl+ku)/2;
            if (p < cdf(k)) ku = k;
            else kl = k;
        }
        return kl;
    }
};
```

CITED REFERENCES AND FURTHER READING:

Johnson, N.L. and Kotz, S. 1970, *Continuous Univariate Distributions*, 2 vols. (Boston: Houghton Mifflin).[1]

Johnson, N.L. and Kotz, S. 1969, *Discrete Distributions* (Boston: Houghton Mifflin).[2]

Gelman, A., Carlin, J.B., Stern, H.S., and Rubin, D.B. 2003, *Bayesian Data Analysis*, 2nd ed. (Boca Raton, FL: Chapman & Hall/CRC), Appendix A.

Lyuu, Y-D. 2002, *Financial Engineering and Computation* (Cambridge, UK: Cambridge University Press).[3]

Random Numbers

CHAPTER 7

7.0 Introduction

It may seem perverse to use a computer, that most precise and deterministic of all machines conceived by the human mind, to produce "random" numbers. More than perverse, it may seem to be a conceptual impossibility. After all, any program produces output that is entirely predictable, hence not truly "random."

Nevertheless, practical computer "random number generators" are in common use. We will leave it to philosophers of the computer age to resolve the paradox in a deep way (see, e.g., Knuth [1] §3.5 for discussion and references). One sometimes hears computer-generated sequences termed *pseudo-random*, while the word *random* is reserved for the output of an intrinsically random physical process, like the elapsed time between clicks of a Geiger counter placed next to a sample of some radioactive element. We will not try to make such fine distinctions.

A working definition of randomness in the context of computer-generated sequences is to say that the deterministic program that produces a random sequence should be different from, and — in all measurable respects — statistically uncorrelated with, the computer program that *uses* its output. In other words, any two different random number generators ought to produce statistically the same results when coupled to your particular applications program. If they don't, then at least one of them is not (from your point of view) a good generator.

The above definition may seem circular, comparing, as it does, one generator to another. However, there exists a large body of random number generators that mutually do satisfy the definition over a very, very broad class of applications programs. And it is also found empirically that statistically identical results are obtained from random numbers produced by physical processes. So, because such generators are known to exist, we can leave to the philosophers the problem of defining them.

The pragmatic point of view is thus that randomness is in the eye of the beholder (or programmer). What is random enough for one application may not be random enough for another. Still, one is not entirely adrift in a sea of incommensurable applications programs: There is an accepted list of statistical tests, some sensible and some merely enshrined by history, that on the whole do a very good job of ferreting out any nonrandomness that is likely to be detected by an applications program (in this case, yours). Good random number generators ought to pass all of these tests,

or at least the user had better be aware of any that they fail, so that he or she will be able to judge whether they are relevant to the case at hand.

For references on this subject, the one to turn to first is Knuth [1]. Be cautious about any source earlier than about 1995, since the field progressed enormously in the following decade.

CITED REFERENCES AND FURTHER READING:

Knuth, D.E. 1997, *Seminumerical Algorithms*, 3rd ed., vol. 2 of *The Art of Computer Programming* (Reading, MA: Addison-Wesley), Chapter 3, especially §3.5.[1]

Gentle, J.E. 2003, *Random Number Generation and Monte Carlo Methods*, 2nd ed. (New York: Springer).

7.1 Uniform Deviates

Uniform deviates are just random numbers that lie within a specified range, typically 0.0 to 1.0 for floating-point numbers, or 0 to $2^{32} - 1$ or $2^{64} - 1$ for integers. Within the range, any one number is just as likely as any other. They are, in other words, what you probably think "random numbers" are. However, we want to distinguish uniform deviates from other sorts of random numbers, for example, numbers drawn from a normal (Gaussian) distribution of specified mean and standard deviation. These other sorts of deviates are almost always generated by performing appropriate operations on one or more uniform deviates, as we will see in subsequent sections. So, a reliable source of random uniform deviates, the subject of this section, is an essential building block for any sort of stochastic modeling or Monte Carlo computer work.

The state of the art for generating uniform deviates has advanced considerably in the last decade and now begins to resemble a mature field. It is now reasonable to expect to get "perfect" deviates in no more than a dozen or so arithmetic or logical operations per deviate, and fast, "good enough" deviates in many fewer operations than that. Three factors have all contributed to the field's advance: first, new mathematical algorithms; second, better understanding of the practical pitfalls; and, third, standardization of programming languages in general, and of integer arithmetic in particular — and especially the universal availability of unsigned 64-bit arithmetic in C and C++. It may seem ironic that something as down-in-the-weeds as this last factor can be so important. But, as we will see, it really is.

The greatest lurking danger for a user today is that many out-of-date and inferior methods remain in general use. Here are some traps to watch for:

- Never use a generator principally based on a *linear congruential generator* (LCG) or a *multiplicative linear congruential generator* (MLCG). We say more about this below.
- Never use a generator with a period less than $\sim 2^{64} \approx 2 \times 10^{19}$, or any generator whose period is undisclosed.
- Never use a generator that warns against using its low-order bits as being completely random. That was good advice once, but it now indicates an obsolete algorithm (usually a LCG).

- Never use the built-in generators in the C and C++ languages, especially `rand` and `srand`. These have no standard implementation and are often badly flawed.

If all scientific papers whose results are in doubt because of one or more of the above traps were to disappear from library shelves, there would be a gap on each shelf about as big as your fist.

You may also want to watch for indications that a generator is overengineered, and therefore wasteful of resources:

- Avoid generators that take more than (say) two dozen arithmetic or logical operations to generate a 64-bit integer or double precision floating result.
- Avoid using generators (over-)designed for serious cryptographic use.
- Avoid using generators with period $> 10^{100}$. You *really* will never need it, and, above some minimum bound, the period of a generator has little to do with its quality.

Since we have told you what to avoid from the past, we should immediately follow with the received wisdom of the present:

An acceptable random generator must combine at least two (ideally, unrelated) methods. The methods combined should evolve independently and share no state. The combination should be by simple operations that do not produce results less random than their operands.

If you don't want to read the rest of this section, then use the following code to generate all the uniform deviates you'll ever need. This is our suspenders-and-belt, full-body-armor, never-any-doubt generator;* and, it also meets the above guidelines for avoiding wasteful, overengineered methods. (The fastest generators that we recommend, below, are only $\sim 2.5 \times$ faster, even when their code is copied inline into an application.)

ran.h
```
struct Ran {
```
Implementation of the highest quality recommended generator. The constructor is called with an integer seed and creates an instance of the generator. The member functions `int64`, `doub`, and `int32` return the next values in the random sequence, as a variable type indicated by their names. The period of the generator is $\approx 3.138 \times 10^{57}$.
```
    Ullong u,v,w;
    Ran(Ullong j) : v(4101842887655102017LL), w(1) {
```
Constructor. Call with any integer seed (except value of `v` above).
```
        u = j ^ v; int64();
        v = u; int64();
        w = v; int64();
    }
    inline Ullong int64() {
```

*"What about the $1000 reward?" some long-time readers may wonder. That is a tale in itself: Two decades ago, the first edition of *Numerical Recipes* included a flawed random number generator. (Forgive us, we were young!) In the second edition, in a misguided attempt to buy back some credibility, we offered a prize of $1000 to the "first reader who convinces us" that that edition's best generator was in any way flawed. No one ever won that prize (`ran2` is a sound generator within its stated limits). We did learn, however, that many people don't understand what constitutes a statistical proof. Multiple claimants over the years have submitted claims based on one of two fallacies: (1) finding, after much searching, some particular seed that makes the first few random values seem unusual, or (2) finding, after some millions of trials, a statistic that, just that once, is as unlikely as a part in a million. In the interests of our own sanity, we are not offering any rewards in this edition. And the previous offer is hereby revoked.

```
    Return 64-bit random integer. See text for explanation of method.
        u = u * 2862933555777941757LL + 7046029254386353087LL;
        v ^= v >> 17; v ^= v << 31; v ^= v >> 8;
        w = 4294957665U*(w & 0xffffffff) + (w >> 32);
        Ullong x = u ^ (u << 21); x ^= x >> 35; x ^= x << 4;
        return (x + v) ^ w;
    }
    inline Doub doub() { return 5.42101086242752217E-20 * int64(); }
    Return random double-precision floating value in the range 0. to 1.
    inline Uint int32() { return (Uint)int64(); }
    Return 32-bit random integer.
};
```

The basic premise here is that a random generator, because it maintains internal state between calls, should be an object, a `struct`. You can declare more than one instance of it (although it is hard to think of a reason for doing so), and different instances will in no way interact.

The constructor `Ran()` takes a single integer argument, which becomes the seed for the sequence generated. Different seeds generate (for all practical purposes) completely different sequences. Once constructed, an instance of `Ran` offers several different formats for random output. To be specific, suppose you have created an instance by the declaration

```
Ran myran(17);
```

where `myran` is now the name of this instance, and 17 is its seed. Then, the function `myran.int64()` returns a random 64-bit unsigned integer; the function `myran.int32()` returns an unsigned 32-bit integer; and the function `myran.doub()` returns a double-precision floating value in the range 0.0 to 1.0. You can intermix calls to these functions as you wish. You can use *any* returned random bits for any purpose. If you need a random integer between 1 and `n` (inclusive), say, then the expression `1 + myran.int64() % (n-1)` is perfectly OK (though there are faster idioms than the use of `%`).

In the rest of this section, we briefly review some history (the rise and fall of the LCG), then give details on some of the algorithmic methods that go into a good generator, and on how to combine those methods. Finally, we will give some further recommended generators, additional to `Ran` above.

7.1.1 Some History

With hindsight, it seems clear that the whole field of random number generation was mesmerized, for far too long, by the simple recurrence equation

$$I_{j+1} = aI_j + c \pmod{m} \tag{7.1.1}$$

Here m is called the *modulus*, a is a positive integer called the *multiplier*, and c (which may be zero) is nonnegative integer called the *increment*. For $c \neq 0$, equation (7.1.1) is called a linear congruential generator (LCG). When $c = 0$, it is sometimes called a multiplicative LCG or MLCG.

The recurrence (7.1.1) must eventually repeat itself, with a period that is obviously no greater than m. If m, a, and c are properly chosen, then the period will be of maximal length, i.e., of length m. In that case, all possible integers between 0 and $m - 1$ occur at some point, so any initial "seed" choice of I_0 is as good as any other:

The sequence just takes off from that point, and successive values I_j are the returned "random" values.

The idea of LCGs goes back to the dawn of computing, and they were widely used in the 1950s and thereafter. The trouble in paradise first began to be noticed in the mid-1960s (e.g., [1]): If k random numbers at a time are used to plot points in k-dimensional space (with each coordinate between 0 and 1), then the points will not tend to "fill up" the k-dimensional space, but rather will lie on $(k-1)$-dimensional "planes." There will be *at most* about $m^{1/k}$ such planes. If the constants m and a are not very carefully chosen, there will be *many fewer than that.* The number m was usually close to the machine's largest representable integer, often $\sim 2^{32}$. So, for example, the number of planes on which triples of points lie in three-dimensional space can be no greater than about the cube root of 2^{32}, about 1600. You might well be focusing attention on a physical process that occurs in a small fraction of the total volume, so that the discreteness of the planes can be very pronounced.

Even worse, many early generators happened to make particularly bad choices for m and a. One infamous such routine, RANDU, with $a = 65539$ and $m = 2^{31}$, was widespread on IBM mainframe computers for many years, and widely copied onto other systems. One of us recalls as a graduate student producing a "random" plot with only 11 planes and being told by his computer center's programming consultant that he had misused the random number generator: "We guarantee that each number is random individually, but we don't guarantee that more than one of them is random." That set back our graduate education by at least a year!

LCGs and MLCGs have additional weaknesses: When m is chosen as a power of 2 (e.g., RANDU), then the low-order bits generated are hardly random at all. In particular, the least significant bit has a period of at most 2, the second at most 4, the third at most 8, and so on. But, if you don't choose m as a power of 2 (in fact, choosing m prime is generally a good thing), then you generally need access to double-length registers to do the multiplication and modulo functions in equation (7.1.1). These were often unavailable in computers of the time (and usually still are).

A lot of effort subsequently went into "fixing" these weaknesses. An elegant number-theoretical test of m and a, the *spectral test*, was developed to characterize the density of planes in arbitrary dimensional space. (See [2] for a recent review that includes graphical renderings of some of the appallingly poor generators that were used historically, and also [3].) *Schrage's method* [4] was invented to do the multiplication $a\,I_j$ with only 32-bit arithmetic for m as large as $2^{32}-1$, but, unfortunately, only for certain a's, not always the best ones. The review by Park and Miller [5] gives a good contemporary picture of LCGs in their heyday.

Looking back, it seems clear that the field's long preoccupation with LCGs was somewhat misguided. There is no technological reason that the better, non-LCG, generators of the last decade could not have been discovered decades earlier, nor any reason that the impossible dream of an elegant "single algorithm" generator could not also have been abandoned much earlier (in favor of the more pragmatic patchwork in combined generators). As we will explain below, LCGs and MLCGs can still be useful, but only in carefully controlled situations, and with due attention to their manifest weaknesses.

7.1.2 Recommended Methods for Use in Combined Generators

Today, there are at least a dozen plausible algorithms that deserve serious consideration for use in random generators. Our selection of a few is motivated by aesthetics as much as mathematics. We like algorithms with few and fast operations, with foolproof initialization, and with state small enough to keep in registers or first-level cache (if the compiler and hardware are able to do so). This means that we tend to avoid otherwise fine algorithms whose state is an array of some length, despite the relative simplicity with which such algorithms can achieve truly humongous periods. For overviews of broader sets of methods, see [6] and [7].

To be recommendable for use in a combined generator, we require a method to be understood theoretically to some degree, and to pass a reasonably broad suite of empirical tests (or, if it fails, have weaknesses that are well characterized). Our minimal theoretical standard is that the period, the set of returned values, and the set of valid initializations should be completely understood. As a minimal empirical standard, we have used the second release (2003) of Marsaglia's whimsically named Diehard battery of statistical tests [8].* An alternative test suite, NIST-STS [9], might be used instead, or in addition.

Simply requiring a combined generator to pass Diehard or NIST-STS is not an acceptably stringent test. These suites make only $\sim 10^7$ calls to the generator, whereas a user program might make 10^{12} or more. Much more meaningful is to require that each method in a combined generator separately pass the chosen suite. Then the combination generator (if correctly constructed) should be vastly better than any one component. In the tables below, we use the symbol "$\divideontimes$" to indicate that a method passes the Diehard tests by itself. (For 64-bit quantities, the statement is that the 32 high and low bits each pass.) Correspondingly, the words "can be used as random," below, do not imply perfect randomness, but only a minimum level for quick-and-dirty applications where a better, combined, generator is just not needed.

We turn now to specific methods, starting with methods that use 64-bit unsigned arithmetic (what we call `Ullong`, that is, `unsigned long long` in the Linux/Unix world, or `unsigned __int64` on planet Microsoft).

(A) 64-bit Xorshift Method. This generator was discovered and characterized by Marsaglia [10]. In just three XORs and three shifts (generally fast operations) it produces a full period of $2^{64} - 1$ on 64 bits. (The missing value is zero, which perpetuates itself and must be avoided.) High and low bits pass Diehard. A generator can use either the three-line update rule, below, that starts with <<, or the rule that starts with >>. (The two update rules produce different sequences, related by bit reversal.)

$$
\begin{array}{ll}
\text{state:} & x \text{ (unsigned 64-bit)} \\
\text{initialize:} & x \neq 0 \\
\text{update:} & x \leftarrow x \wedge (x \;\texttt{>>}\; a_1), \\
& x \leftarrow x \wedge (x \;\texttt{<<}\; a_2), \\
& x \leftarrow x \wedge (x \;\texttt{>>}\; a_3); \\
\quad\text{or} & x \leftarrow x \wedge (x \;\texttt{<<}\; a_1), \\
& x \leftarrow x \wedge (x \;\texttt{>>}\; a_2),
\end{array}
$$

*Be sure that you use a version of Diehard that includes the so-called "Gorilla Test."

$$x \leftarrow x \wedge (x \ll a_3);$$

can use as random:	x (all bits) ⁎
can use in bit mix:	x (all bits)
can improve by:	output 64-bit MLCG successor
period:	$2^{64} - 1$

Here is a very brief outline of the theory behind these generators: Consider the 64 bits of the integer as components in a vector of length 64, in a linear space where addition and multiplication are done modulo 2. Noting that XOR ($\wedge$) is the same as addition, each of the three lines in the updating can be written as the action of a 64×64 matrix on a vector, where the matrix is all zeros except for ones on the diagonal, and on exactly one super- or subdiagonal (corresponding to << or >>). Denote this matrix as $\mathbf{S}_k$, where k is the shift argument (positive for left-shift, say, and negative for right-shift). Then, one full step of updating (three lines of the updating rule, above) corresponds to multiplication by the matrix $\mathbf{T} \equiv \mathbf{S}_{k_3}\mathbf{S}_{k_2}\mathbf{S}_{k_1}$.

One next needs to find triples of integers (k_1, k_2, k_3), for example $(21, -35, 4)$, that give the full $M \equiv 2^{64} - 1$ period. Necessary and sufficient conditions are that $\mathbf{T}^M = \mathbf{1}$ (the identity matrix) and that $\mathbf{T}^N \neq \mathbf{1}$ for these seven values of N: $M/6700417$, $M/65537$, $M/641$, $M/257$, $M/17$, $M/5$, and $M/3$, that is, M divided by each of its seven distinct prime factors. The required large powers of $\mathbf{T}$ are readily computed by successive squarings, requiring only on the order of 64^4 operations. With this machinery, one can find full-period triples (k_1, k_2, k_3) by exhaustive search, at a reasonable cost.

Brent [11] has pointed out that the 64-bit xorshift method produces, at each bit position, a sequence of bits that is identical to one produced by a certain linear feedback shift register (LFSR) on 64 bits. (We will learn more about LFSRs in §7.5.) The xorshift method thus potentially has some of the same strengths and weaknesses as an LFSR. Mitigating this, however, is the fact that the primitive polynomial equivalent of a typical xorshift generator has many nonzero terms, giving it better statistical properties than LFSR generators based, for example, on primitive trinomials. In effect, the xorshift generator is a way to step simultaneously 64 nontrivial one-bit LFSR registers, using only six fast, 64-bit operations. There are other ways of making fast steps on LFSRs, and combining the output of more than one such generator [12,13], but none as simple as the xorshift method.

While each bit position in an xorshift generator has the same recurrence, and therefore the same sequence with period $2^{64} - 1$, the method guarantees offsets to each sequence such that all nonzero 64-bit words are produced *across* the bit positions during one complete cycle (as we just saw).

A selection of full-period triples is tabulated in [10]. Only a small fraction of full-period triples actually produce generators that pass Diehard. Also, a triple may pass in its <<-first version, and fail in its >>-first version, or vice versa. Since the two versions produce simply bit-reversed sequences, a failure of either sense must obviously be considered a failure of both (and a weakness in Diehard). The following recommended parameter sets pass Diehard for both the << and >> rules. The sets near the top of the list may be slightly superior to the sets near the bottom. The column labeled ID assigns an identification string to each recommended generator that we will refer to later.

ID	a_1	a_2	a_3
A1	21	35	4
A2	20	41	5
A3	17	31	8
A4	11	29	14
A5	14	29	11
A6	30	35	13
A7	21	37	4
A8	21	43	4
A9	23	41	18

It is easy to design a test that the xorshift generator fails if used by itself. Each bit at step $i + 1$ depends on at most 8 bits of step i, so some simple logical combinations of the two timesteps (and appropriate masks) will show immediate nonrandomness. Also, when the state passes though a value with only small numbers of 1 bits, as it must eventually do (so-called states of *low Hamming weight*), it will take longer than expected to recover. Nevertheless, used in combination, the xorshift generator is an exceptionally powerful and useful method. Much grief could have been avoided had it, instead of LCGs, been discovered in 1949!

(B) Multiply with Carry (MWC) with Base $b = 2^{32}$. Also discovered by Marsaglia, the *base* b of an MWC generator is most conveniently chosen to be a power of 2 that is half the available word length (i.e., $b = 32$ for 64-bit words). The MWC is then defined by its *multiplier* a.

state:	x (unsigned 64-bit)
initialize:	$1 \le x \le 2^{32} - 1$
update:	$x \leftarrow a\ (x\ \texttt{\&}\ [2^{32} - 1]) + (x\ \texttt{>>}\ 32)$
can use as random:	x (low 32 bits) ⁕
can use in bit mix:	x (all 64 bits)
can improve by:	output 64-bit xorshift successor to 64 bit x
period:	$(2^{32}a - 2)/2$ (a prime)

An MWC generator with parameters b and a is related theoretically [14] to, though not identical to, an LCG with modulus $m = ab - 1$ and multiplier a. It is easy to find values of a that make m a prime, so we get, in effect, the benefit of a prime modulus using only power-of-two modular arithmetic. It is not possible to choose a to give the maximal period m, but if a is chosen to make both m and $(m - 1)/2$ prime, then the period of the MCG is $(m - 1)/2$, almost as good. A fraction of candidate a's thus chosen passes the standard statistical test suites; a spectral test [14] is a promising development, but we have not made use of it here.

Although only the low b bits of the state x can be taken as algorithmically random, there is considerable randomness in all the bits of x that represent the product ab. This is very convenient in a combined generator, allowing the entire state x to be used as a component. In fact, the first two recommended a's below give ab so close to 2^{64} (within about 2 ppm) that the high bits of x actually pass Diehard. (This is a good example of how any test suite can fail to find small amounts of highly nonrandom behavior, in this case as many as 8000 missing values in the top 32 bits.)

Apart from this kind of consideration, the values below are recommended with no particular ordering.

ID	a
B1	4294957665
B2	4294963023
B3	4162943475
B4	3947008974
B5	3874257210
B6	2936881968
B7	2811536238
B8	2654432763
B9	1640531364

(C) LCG Modulo 2^{64}. Why in the world do we include this generator after vilifying it so thoroughly above? For the parameters given (which strongly pass the spectral test), its high 32 bits almost, but don't quite, pass Diehard, and its low 32 bits are a complete disaster. Yet, as we will see when we discuss the construction of combined generators, there is still a niche for it to fill. The recommended multipliers a below have good spectral characteristics [15].

state:	x (unsigned 64-bit)
initialize:	any value
update:	$x \leftarrow ax + c \pmod{2^{64}}$
can use as random:	x (high 32 bits, with caution)
can use in bit mix:	x (high 32 bits)
can improve by:	output 64-bit xorshift successor
period:	2^{64}

ID	a	c (any odd value ok)
C1	3935559000370003845	2691343689449507681
C2	3202034522624059733	4354685564936845319
C3	2862933555777941757	7046029254386353087

(D) MLCG Modulo 2^{64}. As for the preceding one, the useful role for this generator is strictly limited. The low bits are highly nonrandom. The recommended multipliers have good spectral characteristics (some from [15]).

state:	x (unsigned 64-bit)
initialize:	$x \neq 0$
update:	$x \leftarrow ax \pmod{2^{64}}$
can use as random:	x (high 32 bits, with caution)
can use in bit mix:	x (high 32 bits)
can improve by:	output 64-bit xorshift successor
period:	2^{62}

ID	a
D1	2685821657736338717
D2	7664345821815920749
D3	4768777513237032717
D4	1181783497276652981
D5	702098784532940405

(E) MLCG with $m \gg 2^{32}$, m Prime. When 64-bit unsigned arithmetic is available, the MLCGs with prime moduli and large multipliers of good spectral character are decent 32-bit generators. Their main liability is that the 64-bit multiply and 64-bit remainder operations are quite expensive for the mere 32 (or so) bits of the result.

state:	x (unsigned 64-bit)	
initialize:	$1 \le x \le m-1$	
update:	$x \leftarrow ax \pmod{m}$	
can use as random:	$x \quad (1 \le x \le m-1)$ or low 32 bits	⁎
can use in bit mix:	(same)	
period:	$m-1$	

The parameter values below were kindly computed for us by P. L'Ecuyer. The multipliers are about the best that can be obtained for the prime moduli, close to powers of 2, shown. Although the recommended use is for only the low 32 bits (which all pass Diehard), you can see that (depending on the modulus) as many as 43 reasonably good bits can be obtained for the cost of the 64-bit multiply and remainder operations.

ID	m	a
E1	$2^{39}-7 = 549755813881$	10014146
E2		30508823
E3		25708129
E4	$2^{41}-21 = 2199023255531$	5183781
E5		1070739
E6		6639568
E7	$2^{42}-11 = 4398046511093$	1781978
E8		2114307
E9		1542852
E10	$2^{43}-57 = 8796093022151$	2096259
E11		2052163
E12		2006881

(F) MLCG with $m \gg 2^{32}$, m Prime, and $a(m-1) \approx 2^{64}$. A variant, for use in combined generators, is to choose m and a to make $a(m-1)$ as close as possible to 2^{64}, while still requiring that m be prime and that a pass the spectral test. The purpose of this maneuver is to make ax a 64-bit value with good randomness in its high bits, for use in combined generators. The expense of the multiply and remainder operations is still the big liability, however. The low 32 bits of x are not significantly less random than those of the previous MLCG generators E1–E12.

state:	x (unsigned 64-bit)
initialize:	$1 \le x \le m-1$
update:	$x \leftarrow ax \pmod m$
can use as random:	$x \quad (1 \le x \le m-1)$ or low 32 bits ⁎
can use in bit mix:	ax (but don't use both ax and x) ⁎
can improve by:	output 64-bit xorshift successor of ax
period:	$m-1$

ID	m	a
F1	$1148 \times 2^{32} + 11 = 4930622455819$	3741260
F2	$1264 \times 2^{32} + 9 = 5428838662153$	3397916
F3	$2039 \times 2^{32} + 3 = 8757438316547$	2106408

7.1.3 How to Construct Combined Generators

While the construction of combined generators is an art, it should be informed by underlying mathematics. Rigorous theorems about combined generators are usually possible only when the generators being combined are algorithmically related; but that in itself is usually a bad thing to do, on the general principle of "don't put all your eggs in one basket." So, one is left with guidelines and rules of thumb.

The methods being combined should be independent of one another. They must share no state (although their initializations are allowed to derive from some convenient common seed). They should have different, incommensurate, periods. And, ideally, they should "look like" each other algorithmically as little as possible. This latter criterion is where some art necessarily enters.

The output of the combination generator should in no way perturb the independent evolution of the individual methods, nor should the operations effecting combination have any side effects.

The methods should be combined by binary operations whose output is no less random than one input if the other input is held fixed. For 32- or 64-bit unsigned arithmetic, this in practice means that only the $+$ and $\wedge$ operators can be used. As an example of a forbidden operator, consider multiplication: If one operand is a power of 2, then the product will end in trailing zeros, no matter how random is the other operand.

All bit positions in the combined output should depend on high-quality bits from at least two methods, and may also depend on lower-quality bits from additional methods. In the tables above, the bits labeled "can use as random" are considered high quality; those labeled "can use in bit mix" are considered low quality, unless they also pass a statistical suite such as Diehard.

There is one further trick at our disposal, the idea of using a method as a *successor relation* instead of as a generator in its own right. Each of the methods described above is a mapping from some 64-bit state x_i to a unique successor state x_{i+1}. For a method to pass a good statistical test suite, it must have no detectable correlations between a state and its successor. If, in addition, the method has period 2^{64} or $2^{64}-1$, then all values (except possibly zero) occur exactly once as successor states.

Suppose we take the output of a generator, say C1 above, with period 2^{64}, and run it through generator A6, whose period is $2^{64}-1$, as a successor relation. This is conveniently denoted by "A6(C1)," which we will call a *composed* generator. Note that the composed output is emphatically *not* fed back into the state of C1, which

continues unperturbed. The composed generator A6(C1) has the period of C1, not, unfortunately, the product of the two periods. But its random mapping of C1's output values effectively fixes C1's problems with short-period low bits. (The better so if the form of A6 with left-shift first is used.) And, A6(C1) will also fix A6's weakness that a bit depends only on a few bits of the previous state. We will thus consider a carefully constructed composed generator as being a combined generator, on a par with direct combining via $+$ or $\wedge$.

Composition is inferior to direct combining in that it costs almost as much but does not increase the size of the state or the length of the period. It is superior to direct combining in its ability to mix widely differing bit positions. In the previous example we would not have accepted A6+C1 as a combined generator, because the low bits of C1 are so poor as to add little value to the combination; but A6(C1) has no such liability, and much to recommend it. In the preceding summary tables of each method, we have indicated recommended combinations for composed generators in the table entries, "can improve by."

We can now completely describe the generator in Ran, above, by the pseudo-equation,

$$\texttt{Ran} = [\mathrm{A1}_l(\mathrm{C3}) + \mathrm{A3}_r] \wedge \mathrm{B1} \qquad (7.1.2)$$

that is, the combination and/or composition of four different generators. For the methods A1 and A3, the subscripts l and r denote whether a left- or right-shift operation is done first. The period of Ran is the least common multiple of the periods of C3, A3, and B1.

The simplest and fastest generator that we can readily recommend is

$$\texttt{Ranq1} \equiv \mathrm{D1}(\mathrm{A1}_r) \qquad (7.1.3)$$

implemented as

ran.h

```
struct Ranq1 {
```

Recommended generator for everyday use. The period is $\approx 1.8 \times 10^{19}$. Calling conventions same as Ran, above.

```
    Ullong v;
    Ranq1(Ullong j) : v(4101842887655102017LL) {
        v ^= j;
        v = int64();
    }
    inline Ullong int64() {
        v ^= v >> 21; v ^= v << 35; v ^= v >> 4;
        return v * 2685821657736338717LL;
    }
    inline Doub doub() { return 5.42101086242752217E-20 * int64(); }
    inline Uint int32() { return (Uint)int64(); }
};
```

Ranq1 generates a 64-bit random integer in 3 shifts, 3 xors, and one multiply, or a double floating value in one additional multiply. Its method is concise enough to go easily inline in an application. It has a period of "only" 1.8×10^{19}, so it should not be used by an application that makes more than $\sim 10^{12}$ calls. With that restriction, we think that Ranq1 will do just fine for 99.99% of all user applications, and that Ran can be reserved for the remaining 0.01%.

If the "short" period of Ranq1 bothers you (which it shouldn't), you can instead use

$$\texttt{Ranq2} \equiv \mathrm{A3}_r \wedge \mathrm{B1} \qquad (7.1.4)$$

whose period is 8.5×10^{37}.

ran.h

```
struct Ranq2 {
```
Backup generator if Ranq1 has too short a period and Ran is too slow. The period is $\approx 8.5 \times 10^{37}$. Calling conventions same as Ran, above.
```
    Ullong v,w;
    Ranq2(Ullong j) : v(4101842887655102017LL), w(1) {
        v ^= j;
        w = int64();
        v = int64();
    }
    inline Ullong int64() {
        v ^= v >> 17; v ^= v << 31; v ^= v >> 8;
        w = 4294957665U*(w & 0xffffffff) + (w >> 32);
        return v ^ w;
    }
    inline Doub doub() { return 5.42101086242752217E-20 * int64(); }
    inline Uint int32() { return (Uint)int64(); }
};
```

7.1.4 Random Hashes and Random Bytes

Every once in a while, you want a random sequence H_i whose values you can visit or revisit in any order of i's. That is to say, you want a *random hash* of the integers i, one that passes serious tests for randomness, even for very ordered sequences of i's. In the language already developed, you want a generator that has no state at all and is built entirely of successor relationships, starting with the value i.

An example that easily passes the Diehard test is

$$\texttt{Ranhash} \equiv \mathrm{A2}_l(\mathrm{D3}(\mathrm{A7}_r(\mathrm{C1}(i)))) \qquad (7.1.5)$$

Note the alternation between successor relations that utilize 64-bit multiplication and ones using shifts and XORs.

ran.h

```
struct Ranhash {
```
High-quality random hash of an integer into several numeric types.
```
    inline Ullong int64(Ullong u) {
```
Returns hash of u as a 64-bit integer.
```
        Ullong v = u * 3935559000370003845LL + 2691343689449507681LL;
        v ^= v >> 21; v ^= v << 37; v ^= v >> 4;
        v *= 4768777513237032717LL;
        v ^= v << 20; v ^= v >> 41; v ^= v << 5;
        return  v;
    }
    inline Uint int32(Ullong u)
```
Returns hash of u as a 32-bit integer.
```
        { return (Uint)(int64(u) & 0xffffffff) ; }
    inline Doub doub(Ullong u)
```
Returns hash of u as a double-precision floating value between 0. and 1.
```
        { return 5.42101086242752217E-20 * int64(u); }
};
```

Since Ranhash has no state, it has no constructor. You just call its int64(i) function, or any of its other functions, with your value of i whenever you want.

Random Bytes. In a different set of circumstances, you may want to generate random integers a byte at a time. You can of course pull bytes out of any of the above

recommended combination generators, since they are constructed to be equally good on all bits. The following code, added to any of the generators above, augments them with an `int8()` method. (Be sure to initialize `bc` to zero in the constructor.)

```
Ullong breg;
Int bc;
inline unsigned char int8() {
    if (bc--) return (unsigned char)(breg >>= 8);
    breg = int64();
    bc = 7;
    return (unsigned char)breg;
}
```

If you want a more byte-oriented, though not necessarily faster, algorithm, an interesting one — in part because of its interesting history — is Rivest's RC4, used in many Internet applications. RC4 was originally a proprietary algorithm of RSA, Inc., but it was protected simply as a trade secret and not by either patent or copyright. The result was that when the secret was breached, by an anonymous posting to the Internet in 1994, RC4 became, in almost all respects, public property. The name RC4 is still protectable, and is a trademark of RSA. So, to be scrupulous, we give the following implementation another name, `Ranbyte`.

ran.h

```
struct Ranbyte {
Generator for random bytes using the algorithm generally known as RC4.
    Int s[256],i,j,ss;
    Uint v;
    Ranbyte(Int u) {
    Constructor. Call with any integer seed.
        v = 2244614371U ^ u;
        for (i=0; i<256; i++) {s[i] = i;}
        for (j=0, i=0; i<256; i++) {
            ss = s[i];
            j = (j + ss + (v >> 24)) & 0xff;
            s[i] = s[j]; s[j] = ss;
            v = (v << 24) | (v >> 8);
        }
        i = j = 0;
        for (Int k=0; k<256; k++) int8();
    }
    inline unsigned char int8() {
    Returns next random byte in the sequence.
        i = (i+1) & 0xff;
        ss = s[i];
        j = (j+ss) & 0xff;
        s[i] = s[j]; s[j] = ss;
        return (unsigned char)(s[(s[i]+s[j]) & 0xff]);
    }
    Uint int32() {
    Returns a random 32-bit integer constructed from 4 random bytes. Slow!
        v = 0;
        for (int k=0; k<4; k++) {
            i = (i+1) & 0xff;
            ss = s[i];
            j = (j+ss) & 0xff;
            s[i] = s[j]; s[j] = ss;
            v = (v << 8) | s[(s[i]+s[j]) & 0xff];
        }
        return v;
    }
```

```
    Doub doub() {
    Returns a random double-precision floating value between 0. and 1. Slow!!
        return 2.32830643653869629E-10 * ( int32() +
               2.32830643653869629E-10 * int32() );
    }
};
```

Notice that there is a lot of overhead in starting up an instance of `Ranbyte`, so you should not create instances inside loops that are executed many times. The methods that return 32-bit integers, or double floating-point values, are *slow* in comparison to the other generators above, but are provided in case you want to use `Ranbyte` as a test substitute for another, perhaps questionable, generator.

If you find any nonrandomness at all in `Ranbyte`, don't tell us. But there are several national cryptological agencies that might, or might not, want to talk to you!

7.1.5 Faster Floating-Point Values

The steps above that convert a 64-bit integer to a double-precision floating-point value involves both a nontrivial type conversion and a 64-bit floating multiply. They are performance bottlenecks. One can instead directly move the random bits into the right place in the `double` word with `union` structure, a mask, and some 64-bit logical operations; but in our experience this is not significantly faster.

To generate faster floating-point values, if that is an absolute requirement, we need to bend some of our design rules. Here is a variant of "Knuth's subtractive generator," which is a so-called *lagged Fibonacci generator* on a circular list of 55 values, with lags 24 and 55. Its interesting feature is that new values are generated directly as floating point, by the floating-point subtraction of two previous values.

ran.h

```
struct Ranfib {
Implements Knuth's subtractive generator using only floating operations. See text for cautions.
    Doub dtab[55], dd;
    Int inext, inextp;
    Ranfib(Ullong j) : inext(0), inextp(31) {
    Constructor. Call with any integer seed. Uses Ranq1 to initialize.
        Ranq1 init(j);
        for (int k=0; k<55; k++) dtab[k] = init.doub();
    }
    Doub doub() {
    Returns random double-precision floating value between 0. and 1.
        if (++inext == 55) inext = 0;
        if (++inextp == 55) inextp = 0;
        dd = dtab[inext] - dtab[inextp];
        if (dd < 0) dd += 1.0;
        return (dtab[inext] = dd);
    }
    inline unsigned long int32()
    Returns random 32-bit integer. Recommended only for testing purposes.
        { return (unsigned long)(doub() * 4294967295.0);}
};
```

The `int32` method is included merely for testing, or incidental use. Note also that we use `Ranq1` to initialize `Ranfib`'s table of 55 random values. See earlier editions of Knuth or *Numerical Recipes* for a (somewhat awkward) way to do the initialization purely internally.

`Ranfib` fails the Diehard "birthday test," which is able to discern the simple relation among the three values at lags 0, 24, and 55. Aside from that, it is a good,

but not great, generator, with speed as its principal recommendation.

7.1.6 Timing Results

Timings depend so intimately on highly specific hardware and compiler details, that it is hard to know whether a single set of tests is of any use at all. This is especially true of combined generators, because a good compiler, or a CPU with sophisticated instruction look-ahead, can interleave and pipeline the operations of the individual methods, up to the final combination operations. Also, as we write, desktop computers are in transition from 32 bits to 64, which will affect the timing of 64-bit operations. So, you ought to familiarize yourself with C's "`clock_t clock(void)`" facility and run your own experiments.

That said, the following tables give typical results for routines in this section, normalized to a 3.4 GHz Pentium CPU, vintage 2004. The units are 10^6 returned values per second. Large numbers are better.

Generator	`int64()`	`doub()`	`int8()`
`Ran`	19	10	51
`Ranq1`	39	13	59
`Ranq2`	32	12	58
`Ranfib`		24	
`Ranbyte`			43

The `int8()` timings for `Ran`, `Ranq1`, and `Ranq2` refer to versions augmented as indicated above.

7.1.7 When You Have Only 32-Bit Arithmetic

Our best advice is: Get a better compiler! But if you seriously must live in a world with only unsigned 32-bit arithmetic, then here are some options. None of these individually pass Diehard.

(G) 32-Bit Xorshift RNG

state:	x (unsigned 32-bit)
initialize:	$x \neq 0$
update:	$x \leftarrow x \wedge (x \gg b_1)$,
	$x \leftarrow x \wedge (x \ll b_2)$,
	$x \leftarrow x \wedge (x \gg b_3)$;
or	$x \leftarrow x \wedge (x \ll b_1)$,
	$x \leftarrow x \wedge (x \gg b_2)$,
	$x \leftarrow x \wedge (x \ll b_3)$;
can use as random:	x (32 bits, with caution)
can use in bit mix:	x (32 bits)
can improve by:	output 32-bit MLCG successor
period:	$2^{32} - 1$

ID	b_1	b_2	b_3
G1	13	17	5
G2	7	13	3
G3	9	17	6
G4	6	13	5
G5	9	21	2
G6	17	15	5
G7	3	13	7
G8	5	13	6
G9	12	21	5

(H) MWC with Base $b = 2^{16}$

state:	x, y (unsigned 32-bit)
initialize:	$1 \le x, y \le 2^{16} - 1$
update:	$x \leftarrow a\ (x\ \texttt{\&}\ [2^{16} - 1]) + (x\ \texttt{>>}\ 16)$
	$y \leftarrow b\ (y\ \texttt{\&}\ [2^{16} - 1]) + (y\ \texttt{>>}\ 16)$
can use as random:	$(x\ \texttt{<<}\ 16) + y$
can use in bit mix:	same, or (with caution) x or y
can improve by:	output 32-bit xorshift successor
period:	$(2^{16}a - 2)(2^{16}b - 2)/4$ (product of two primes)

ID	a	b
H1	62904	41874
H2	64545	34653
H3	34653	64545
H4	57780	55809
H5	48393	57225
H6	63273	33378

(I) LCG Modulo 2^{32}

state:	x (unsigned 32-bit)
initialize:	any value
update:	$x \leftarrow ax + c \pmod{2^{32}}$
can use as random:	not recommended
can use in bit mix:	not recommended
can improve by:	output 32-bit xorshift successor
period:	2^{32}

ID	a	c (any odd ok)
I1	1372383749	1289706101
I2	2891336453	1640531513
I3	2024337845	797082193
I4	32310901	626627237
I5	29943829	1013904223

(J) MLCG Modulo 2^{32}

state:	x (unsigned 32-bit)
initialize:	$x \neq 0$
update:	$x \leftarrow ax \pmod{2^{32}}$
can use as random:	not recommended
can use in bit mix:	not recommended
can improve by:	output 32-bit xorshift successor
period:	2^{30}

ID	a
J1	1597334677
J2	741103597
J3	1914874293
J4	990303917
J5	747796405

A high-quality, if somewhat slow, combined generator is

$$\texttt{Ranlim32} \equiv [\mathrm{G3}_l(\mathrm{I2}) + \mathrm{G1}_r] \wedge [\mathrm{G6}_l(\mathrm{H6}_b) + \mathrm{H5}_b] \qquad (7.1.6)$$

implemented as

ran.h

```
struct Ranlim32 {
```
High-quality random generator using only 32-bit arithmetic. Same conventions as Ran. Period $\approx 3.11 \times 10^{37}$. Recommended only when 64-bit arithmetic is not available.
```
    Uint u,v,w1,w2;
    Ranlim32(Uint j) : v(2244614371U), w1(521288629U), w2(362436069U) {
        u = j ^ v; int32();
        v = u; int32();
    }
    inline Uint int32() {
        u = u * 2891336453U + 1640531513U;
        v ^= v >> 13; v ^= v << 17; v ^= v >> 5;
        w1 = 33378 * (w1 & 0xffff) + (w1 >> 16);
        w2 = 57225 * (w2 & 0xffff) + (w2 >> 16);
        Uint x = u ^ (u << 9); x ^= x >> 17; x ^= x << 6;
        Uint y = w1 ^ (w1 << 17); y ^= y >> 15; y ^= y << 5;
        return (x + v) ^ (y + w2);
    }
    inline Doub doub() { return 2.32830643653869629E-10 * int32(); }
    inline Doub truedoub() {
        return 2.32830643653869629E-10 * ( int32() +
        2.32830643653869629E-10 * int32() );
    }
};
```

Note that the doub() method returns floating-point numbers with only 32 bits of precision. For full precision, use the slower truedoub() method.

CITED REFERENCES AND FURTHER READING:

Gentle, J.E. 2003, *Random Number Generation and Monte Carlo Methods*, 2nd ed. (New York: Springer), Chapter 1.

Marsaglia, G 1968, "Random Numbers Fall Mainly in the Planes", *Proceedings of the National Academy of Sciences*, vol. 61, pp. 25–28.[1]

Entacher, K. 1997, "A Collection of Selected Pseudorandom Number Generators with Linear Structures", Technical Report No. 97, Austrian Center for Parallel Computation, University of Vienna. [Available on the Web at multiple sites.][2]

Knuth, D.E. 1997, *Seminumerical Algorithms*, 3rd ed., vol. 2 of *The Art of Computer Programming* (Reading, MA: Addison-Wesley).[3]

Schrage, L. 1979, "A More Portable Fortran Random Number Generator," *ACM Transactions on Mathematical Software*, vol. 5, pp. 132–138.[4]

Park, S.K., and Miller, K.W. 1988, "Random Number Generators: Good Ones Are Hard to Find," *Communications of the ACM*, vol. 31, pp. 1192–1201.[5]

L'Ecuyer, P. 1997 "Uniform Random Number Generators: A Review," *Proceedings of the 1997 Winter Simulation Conference*, Andradóttir, S. et al., eds. (Piscataway, NJ: IEEE).[6]

Marsaglia, G. 1999, "Random Numbers for C: End, at Last?", posted 1999 January 20 to sci.stat.math.[7]

Marsaglia, G. 2003, "Diehard Battery of Tests of Randomness v0.2 beta," 2007+ at `http://www.cs.hku.hk/~diehard/`.[8]

Rukhin, A. et al. 2001, "A Statistical Test Suite for Random and Pseudorandom Number Generators", NIST Special Publication 800-22 (revised to May 15, 2001).[9]

Marsaglia, G. 2003, "Xorshift RNGs", *Journal of Statistical Software*, vol. 8, no. 14, pp. 1-6.[10]

Brent, R.P. 2004, "Note on Marsaglia's Xorshift Random Number Generators", *Journal of Statistical Software*, vol. 11, no. 5, pp. 1-5.[11]

L'Ecuyer, P. 1996, "Maximally Equidistributed Combined Tausworthe Generators," *Mathematics of Computation*, vol. 65, pp. 203-213.[12]

L'Ecuyer, P. 1999, "Tables of Maximally Equidistributed Combined LSFR Generators," *Mathematics of Computation*, vol. 68, pp. 261-269.[13]

Couture, R. and L'Ecuyer, P. 1997, "Distribution Properties of Multiply-with-Carry Random Number Generators," *Mathematics of Computation*, vol. 66, pp. 591-607.[14]

L'Ecuyer, P. 1999, "Tables of Linear Congruential Generators of Different Sizes and Good Lattice Structure", *Mathematics of Computation*, vol. 68, pp. 249-260.[15]

7.2 Completely Hashing a Large Array

We introduced the idea of a random hash or *hash function* in §7.1.4. Once in a while we might want a hash function that operates not on a single word, but on an entire array of length M. Being perfectionists, we want every single bit in the hashed output array to depend on every single bit in the given input array. One way to achieve this is to borrow structural concepts from algorithms as unrelated as the Data Encryption Standard (DES) and the Fast Fourier Transform (FFT)!

DES, like its progenitor cryptographic system LUCIFER, is a so-called "block product cipher" [1]. It acts on 64 bits of input by iteratively applying (16 times, in fact) a kind of highly nonlinear bit-mixing function. Figure 7.2.1 shows the flow of information in DES during this mixing. The function g, which takes 32 bits into 32 bits, is called the "cipher function." Meyer and Matyas [1] discuss the importance of the cipher function being nonlinear, as well as other design criteria.

DES constructs its cipher function g from an intricate set of bit permutations and table lookups acting on short sequences of consecutive bits. For our purposes, a different function g that can be rapidly computed in a high-level computer language is preferable. Such a function probably weakens the algorithm cryptographically. Our purposes are not, however, cryptographic: We want to find the fastest g, and the smallest number of iterations of the mixing procedure in Figure 7.2.1, such that our output random sequence passes the tests that are customarily applied to random number generators. The resulting algorithm is not DES, but rather a kind of "pseudo-DES," better suited to the purpose at hand.

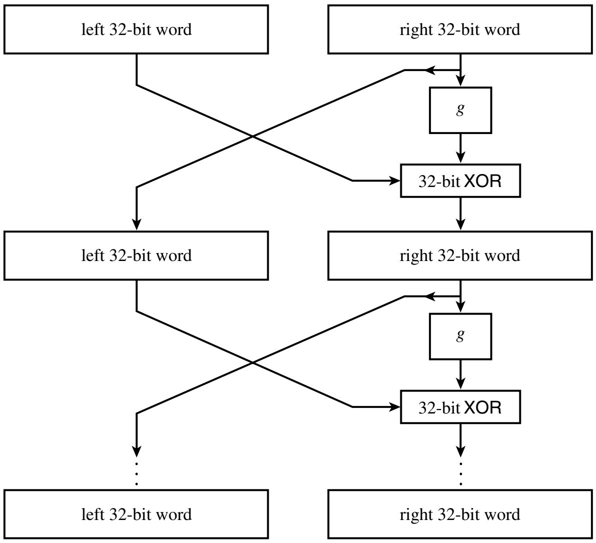

Figure 7.2.1. The Data Encryption Standard (DES) iterates a nonlinear function g on two 32-bit words, in the manner shown here (after Meyer and Matyas [1]).

Following the criterion mentioned above, that g should be nonlinear, we must give the integer multiply operation a prominent place in g. Confining ourselves to multiplying 16-bit operands into a 32-bit result, the general idea of g is to calculate the three distinct 32-bit products of the high and low 16-bit input half-words, and then to combine these, and perhaps additional fixed constants, by fast operations (e.g., add or exclusive-or) into a single 32-bit result.

There are only a limited number of ways of effecting this general scheme, allowing systematic exploration of the alternatives. Experimentation and tests of the randomness of the output lead to the sequence of operations shown in Figure 7.2.2. The few new elements in the figure need explanation: The values C_1 and C_2 are fixed constants, chosen randomly with the constraint that they have exactly 16 1-bits and 16 0-bits; combining these constants via exclusive-or ensures that the overall g has no bias toward 0- or 1-bits. The "reverse half-words" operation in Figure 7.2.2 turns out to be essential; otherwise, the very lowest and very highest bits are not properly mixed by the three multiplications.

It remains to specify the smallest number of iterations N_{it} that we can get away with. For purposes of this section, we recommend $N_{it} = 2$. We have not found any statistical deviation from randomness in sequences of up to 10^9 random deviates derived from this scheme. However, we include C_1 and C_2 constants for $N_{it} \leq 4$.

```
void psdes(Uint &lword, Uint &rword) {
```
hashall.h

Pseudo-DES hashing of the 64-bit word `(lword,rword)`. Both 32-bit arguments are returned hashed on all bits.

```
    const int NITER=2;
    static const Uint c1[4]={
        0xbaa96887L, 0x1e17d32cL, 0x03bcdc3cL, 0x0f33d1b2L};
    static const Uint c2[4]={
        0x4b0f3b58L, 0xe874f0c3L, 0x6955c5a6L, 0x55a7ca46L};
    Uint i,ia,ib,iswap,itmph=0,itmpl=0;
    for (i=0;i<NITER;i++) {
```
Perform `niter` iterations of DES logic, using a simpler (noncryptographic) nonlinear function instead of DES's.

```
        ia = (iswap=rword) ^ c1[i];
        itmpl = ia & 0xffff;
        itmph = ia >> 16;
```
The bit-rich constants `c1` and (below) `c2` guarantee lots of nonlinear mixing.

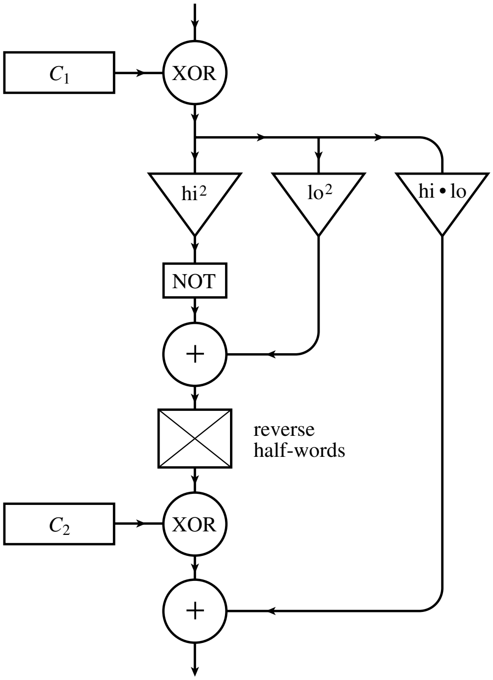

Figure 7.2.2. The nonlinear function g used by the routine `psdes`.

```
        ib=itmpl*itmpl+ ~(itmph*itmph);
        rword = lword ^ (((ia = (ib >> 16) |
            ((ib & 0xffff) << 16)) ^ c2[i])+itmpl*itmph);
        lword = iswap;
    }
}
```

Thus far, this doesn't seem to have much to do with completely hashing a large array. However, `psdes` gives us a building block, a routine for mutually hashing two arbitrary 32-bit integers. We now turn to the FFT concept of the *butterfly* to extend the hash to a whole array.

The butterfly is a particular algorithmic construct that applies to an array of length N, a power of 2. It brings every element into mutual communication with every other element in about $N \log_2 N$ operations. A useful metaphor is to imagine that one array element has a disease that infects any other element with which it has contact. Then the butterfly has two properties of interest here: (i) After its $\log_2 N$ stages, everyone has the disease. Furthermore, (ii) after j stages, 2^j elements are infected; there is never an "eye of the needle" or "necking down" of the communication path.

The butterfly is very simple to describe: In the first stage, every element in the first half of the array mutually communicates with its corresponding element in the second half of the array. Now recursively do this same thing to each of the halves, and so on. We can see by induction that every element now has a communication path to every other one: Obviously it works when $N = 2$. And if it works for N, it must also work for $2N$, because the first step gives every element a communication path into both its own and the other half of the array, after which it has, by assumption, a path everywhere.

We need to modify the butterfly slightly, so that our array size M does not have to be a power of 2. Let N be the next larger power of 2. We do the butterfly on the (virtual) size N, ignoring any communication with nonexistent elements larger than M. This, by itself, doesn't do the job, because the later elements in the first $N/2$ were not able to "infect" the second $N/2$ (and similarly at later recursive levels). However, if we do one extra communication between

elements of the first $N/2$ and second $N/2$ at the very end, then all missing communication paths are restored by traveling through the first $N/2$ elements.

The third line in the following code is an idiom that sets n to the next larger power of 2 greater or equal to m, a miniature masterpiece due to S.E. Anderson [2]. If you look closely, you'll see that it is itself a sort of butterfly, but now on bits!

hashall.h

```
void hashall(VecUint &arr) {
Replace the array arr by a same-sized hash, all of whose bits depend on all of the bits in arr.
Uses psdes for the mutual hash of two 32-bit words.
    Int m=arr.size(), n=m-1;
    n|=n>>1; n|=n>>2; n|=n>>4; n|=n>>8; n|=n>>16; n++;
    Incredibly, n is now the next power of 2 ≥ m.
    Int nb=n,nb2=n>>1,j,jb;
    if (n<2) throw("size must be > 1");
    while (nb > 1) {
        for (jb=0;jb<n-nb+1;jb+=nb)
            for (j=0;j<nb2;j++)
                if (jb+j+nb2 < m) psdes(arr[jb+j],arr[jb+j+nb2]);
        nb = nb2;
        nb2 >>= 1;
    }
    nb2 = n>>1;
    if (m != n) for (j=nb2;j<m;j++) psdes(arr[j],arr[j-nb2]);
    Final mix needed only if m is not a power of 2.
}
```

CITED REFERENCES AND FURTHER READING:

Meyer, C.H. and Matyas, S.M. 1982, *Cryptography: A New Dimension in Computer Data Security* (New York: Wiley).[1]

Zonst, A.E. 2000, *Understanding the FFT*, 2nd revised ed. (Titusville, FL: Citrus Press).

Anderson, S.E. 2005, "Bit Twiddling Hacks," 2007+ at http://graphics.stanford.edu/~seander/bithacks.html .[2]

Data Encryption Standard, 1977 January 15, Federal Information Processing Standards Publication, number 46 (Washington: U.S. Department of Commerce, National Bureau of Standards).

Guidelines for Implementing and Using the NBS Data Encryption Standard, 1981 April 1, Federal Information Processing Standards Publication, number 74 (Washington: U.S. Department of Commerce, National Bureau of Standards).

7.3 Deviates from Other Distributions

In §7.1 we learned how to generate random deviates with a uniform probability between 0 and 1, denoted U(0, 1). The probability of generating a number between x and $x + dx$ is

$$p(x)dx = \begin{cases} dx & 0 \le x < 1 \\ 0 & \text{otherwise} \end{cases} \tag{7.3.1}$$

and we write

$$x \sim \mathrm{U}(0,1) \tag{7.3.2}$$

As in §6.14, the symbol $\sim$ can be read as "is drawn from the distribution."

In this section, we learn how to generate random deviates drawn from other probability distributions, including all of those discussed in §6.14. Discussion of specific distributions is interleaved with the discussion of the general methods used.

7.3.1 Exponential Deviates

Suppose that we generate a uniform deviate x and then take some prescribed function of it, $y(x)$. The probability distribution of y, denoted $p(y)dy$, is determined by the fundamental transformation law of probabilities, which is simply

$$|p(y)dy| = |p(x)dx| \tag{7.3.3}$$

or

$$p(y) = p(x)\left|\frac{dx}{dy}\right| \tag{7.3.4}$$

As an example, take

$$y(x) = -\ln(x) \tag{7.3.5}$$

with $x \sim \mathrm{U}(0,1)$. Then

$$p(y)dy = \left|\frac{dx}{dy}\right| dy = e^{-y}dy \tag{7.3.6}$$

which is the exponential distribution with unit mean, Exponential (1), discussed in §6.14.5. This distribution occurs frequently in real life, usually as the distribution of waiting times between independent Poisson-random events, for example the radioactive decay of nuclei. You can also easily see (from 7.3.6) that the quantity y/β has the probability distribution $\beta e^{-\beta y}$, so

$$y/\beta \sim \text{Exponential}\,(\beta) \tag{7.3.7}$$

We can thus generate exponential deviates at a cost of about one uniform deviate, plus a logarithm, per call.

deviates.h

```
struct Expondev : Ran {
Structure for exponential deviates.
    Doub beta;
    Expondev(Doub bbeta, Ullong i) : Ran(i), beta(bbeta) {}
    Constructor arguments are β and a random sequence seed.
    Doub dev() {
    Return an exponential deviate.
        Doub u;
        do u = doub(); while (u == 0.);
        return -log(u)/beta;
    }
};
```

Our convention here and in the rest of this section is to derive the class for each kind of deviate from the uniform generator class `Ran`. We use the constructor to set the distribution's parameters and set the initial seed for the generator. We then provide a method `dev()` that returns a random deviate from the distribution.

7.3.2 Transformation Method in General

Let's see what is involved in using the above *transformation method* to generate some arbitrary desired distribution of y's, say one with $p(y) = f(y)$ for some positive function f whose integral is 1. According to (7.3.4), we need to solve the differential equation

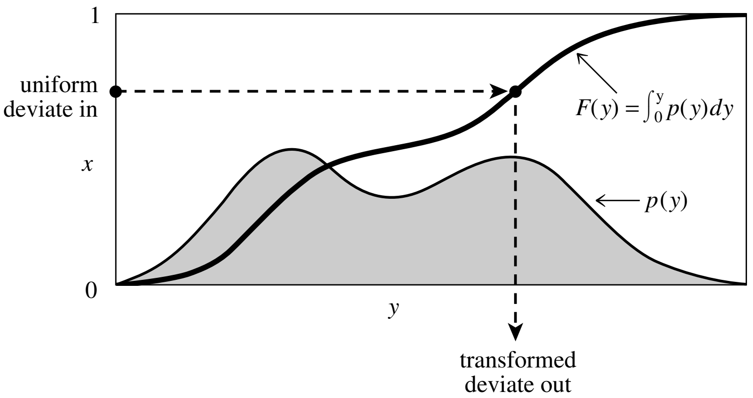

Figure 7.3.1. Transformation method for generating a random deviate y from a known probability distribution $p(y)$. The indefinite integral of $p(y)$ must be known and invertible. A uniform deviate x is chosen between 0 and 1. Its corresponding y on the definite-integral curve is the desired deviate.

$$\frac{dx}{dy} = f(y) \tag{7.3.8}$$

But the solution of this is just $x = F(y)$, where $F(y)$ is the indefinite integral of $f(y)$. The desired transformation that takes a uniform deviate into one distributed as $f(y)$ is therefore

$$y(x) = F^{-1}(x) \tag{7.3.9}$$

where F^{-1} is the inverse function to F. Whether (7.3.9) is feasible to implement depends on whether the *inverse function of the integral of f(y)* is itself feasible to compute, either analytically or numerically. Sometimes it is, and sometimes it isn't.

Incidentally, (7.3.9) has an immediate geometric interpretation: Since $F(y)$ is the area under the probability curve to the left of y, (7.3.9) is just the prescription: Choose a uniform random x, then find the value y that has that fraction x of probability area to its left, and return the value y. (See Figure 7.3.1.)

7.3.3 Logistic Deviates

Deviates from the logistic distribution, as discussed in §6.14.4, are readily generated by the transformation method, using equation (6.14.15). The cost is again dominated by one uniform deviate, and a logarithm, per logistic deviate.

```
struct Logisticdev : Ran {                                                    deviates.h
Structure for logistic deviates.
    Doub mu,sig;
    Logisticdev(Doub mmu, Doub ssig, Ullong i) : Ran(i), mu(mmu), sig(ssig) {}
    Constructor arguments are μ, σ, and a random sequence seed.
    Doub dev() {
    Return a logistic deviate.
        Doub u;
        do u = doub(); while (u*(1.-u) == 0.);
        return mu + 0.551328895421792050*sig*log(u/(1.-u));
    }
};
```

7.3.4 Normal Deviates by Transformation (Box-Muller)

Transformation methods generalize to more than one dimension. If $x_1, x_2, \ldots$ are random deviates with a *joint* probability distribution $p(x_1, x_2, \ldots)dx_1 dx_2 \ldots$, and if $y_1, y_2, \ldots$ are each functions of all the x's (same number of y's as x's), then the joint probability distribution of the y's is

$$p(y_1, y_2, \ldots)dy_1 dy_2 \ldots = p(x_1, x_2, \ldots)\left|\frac{\partial(x_1, x_2, \ldots)}{\partial(y_1, y_2, \ldots)}\right| dy_1 dy_2 \ldots \tag{7.3.10}$$

where $|\partial(\quad)/\partial(\quad)|$ is the Jacobian determinant of the x's with respect to the y's (or the reciprocal of the Jacobian determinant of the y's with respect to the x's).

An important historical example of the use of (7.3.10) is the *Box-Muller* method for generating random deviates with a normal (Gaussian) distribution (§6.14.1):

$$p(y)dy = \frac{1}{\sqrt{2\pi}} e^{-y^2/2} dy \tag{7.3.11}$$

Consider the transformation between two uniform deviates on (0,1), x_1, x_2, and two quantities y_1, y_2,

$$\begin{aligned} y_1 &= \sqrt{-2\ln x_1}\cos 2\pi x_2 \\ y_2 &= \sqrt{-2\ln x_1}\sin 2\pi x_2 \end{aligned} \tag{7.3.12}$$

Equivalently we can write

$$\begin{aligned} x_1 &= \exp\left[-\frac{1}{2}(y_1^2 + y_2^2)\right] \\ x_2 &= \frac{1}{2\pi}\arctan\frac{y_2}{y_1} \end{aligned} \tag{7.3.13}$$

Now the Jacobian determinant can readily be calculated (try it!):

$$\frac{\partial(x_1, x_2)}{\partial(y_1, y_2)} = \begin{vmatrix} \frac{\partial x_1}{\partial y_1} & \frac{\partial x_1}{\partial y_2} \\ \frac{\partial x_2}{\partial y_1} & \frac{\partial x_2}{\partial y_2} \end{vmatrix} = -\left[\frac{1}{\sqrt{2\pi}} e^{-y_1^2/2}\right]\left[\frac{1}{\sqrt{2\pi}} e^{-y_2^2/2}\right] \tag{7.3.14}$$

Since this is the product of a function of y_2 alone and a function of y_1 alone, we see that each y is independently distributed according to the normal distribution (7.3.11).

One further trick is useful in applying (7.3.12). Suppose that, instead of picking uniform deviates x_1 and x_2 in the unit square, we instead pick v_1 and v_2 as the ordinate and abscissa of a random point inside the unit circle around the origin. Then the sum of their squares, $R^2 \equiv v_1^2 + v_2^2$, is a uniform deviate, which can be used for x_1, while the angle that (v_1, v_2) defines with respect to the v_1-axis can serve as the random angle $2\pi x_2$. What's the advantage? It's that the cosine and sine in (7.3.12) can now be written as $v_1/\sqrt{R^2}$ and $v_2/\sqrt{R^2}$, obviating the trigonometric function calls! (In the next section we will generalize this trick considerably.)

Code for generating normal deviates by the Box-Muller method follows. Consider it for pedagogical use only, because a significantly faster method for generating normal deviates is coming, below, in §7.3.9.

deviates.h

```
struct Normaldev_BM : Ran {
Structure for normal deviates.
    Doub mu,sig;
    Doub storedval;
    Normaldev_BM(Doub mmu, Doub ssig, Ullong i)
    : Ran(i), mu(mmu), sig(ssig), storedval(0.) {}
    Constructor arguments are μ, σ, and a random sequence seed.
    Doub dev() {
    Return a normal deviate.
        Doub v1,v2,rsq,fac;
        if (storedval == 0.) {              We don't have an extra deviate handy, so
            do {
                v1=2.0*doub()-1.0;          pick two uniform numbers in the square ex-
                v2=2.0*doub()-1.0;              tending from -1 to +1 in each direction,
                rsq=v1*v1+v2*v2;            see if they are in the unit circle,
            } while (rsq >= 1.0 || rsq == 0.0);          or try again.
            fac=sqrt(-2.0*log(rsq)/rsq);    Now make the Box-Muller transformation to
            storedval = v1*fac;                 get two normal deviates.  Return one and
            return mu + sig*v2*fac;             save the other for next time.
        } else {                            We have an extra deviate handy,
            fac = storedval;
            storedval = 0.;
            return mu + sig*fac;            so return it.
        }
    }
};
```

7.3.5 Rayleigh Deviates

The *Rayleigh distribution* is defined for positive z by

$$p(z)dz = z\exp\left(-\tfrac{1}{2}z^2\right)dz \qquad (z > 0) \tag{7.3.15}$$

Since the indefinite integral can be done analytically, and the result easily inverted, a simple transformation method from a uniform deviate x results:

$$z = \sqrt{-2\ln x}, \quad x \sim \mathrm{U}(0,1) \tag{7.3.16}$$

A Rayleigh deviate z can also be generated from two normal deviates y_1 and y_2 by

$$z = \sqrt{y_1^2 + y_2^2}, \quad y_1, y_2 \sim \mathrm{N}(0,1) \tag{7.3.17}$$

Indeed, the relation between equations (7.3.16) and (7.3.17) is immediately evident in the equation for the Box-Muller method, equation (7.3.12), if we square and sum that method's two normal deviates y_1 and y_2.

7.3.6 Rejection Method

The *rejection method* is a powerful, general technique for generating random deviates whose distribution function $p(x)dx$ (probability of a value occurring between x and $x + dx$) is known and computable. The rejection method does not require that the cumulative distribution function (indefinite integral of $p(x)$) be readily computable, much less the inverse of that function — which was required for the transformation method in the previous section.

The rejection method is based on a simple geometrical argument (Figure 7.3.2):

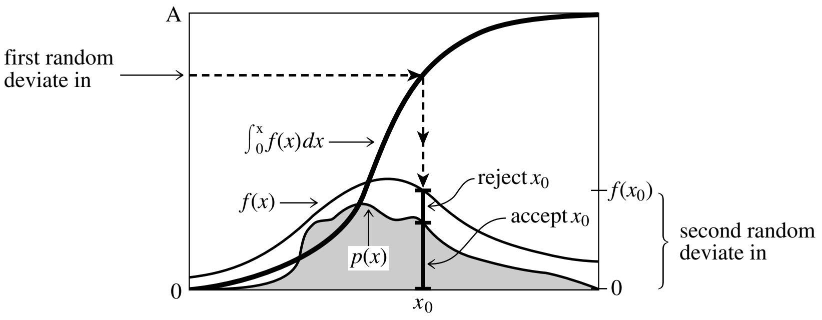

Figure 7.3.2. Rejection method for generating a random deviate x from a known probability distribution $p(x)$ that is everywhere less than some other function $f(x)$. The transformation method is first used to generate a random deviate x of the distribution f (compare Figure 7.3.1). A second uniform deviate is used to decide whether to accept or reject that x. If it is rejected, a new deviate of f is found, and so on. The ratio of accepted to rejected points is the ratio of the area under p to the area between p and f.

Draw a graph of the probability distribution $p(x)$ that you wish to generate, so that the area under the curve in any range of x corresponds to the desired probability of generating an x in that range. If we had some way of choosing a random point *in two dimensions*, with uniform probability in the *area* under your curve, then the x value of that random point would have the desired distribution.

Now, on the same graph, draw any other curve $f(x)$ that has finite (not infinite) area and lies everywhere *above* your original probability distribution. (This is always possible, because your original curve encloses only unit area, by definition of probability.) We will call this $f(x)$ the *comparison function*. Imagine now that you have some way of choosing a random point in two dimensions that is uniform in the area under the comparison function. Whenever that point lies outside the area under the original probability distribution, we will *reject* it and choose another random point. Whenever it lies inside the area under the original probability distribution, we will *accept* it.

It should be obvious that the accepted points are uniform in the accepted area, so that their x values have the desired distribution. It should also be obvious that the fraction of points rejected just depends on the ratio of the area of the comparison function to the area of the probability distribution function, not on the details of shape of either function. For example, a comparison function whose area is less than 2 will reject fewer than half the points, even if it approximates the probability function very badly at some values of x, e.g., remains finite in some region where $p(x)$ is zero.

It remains only to suggest how to choose a uniform random point in two dimensions under the comparison function $f(x)$. A variant of the transformation method (§7.3) does nicely: Be sure to have chosen a comparison function whose indefinite integral is known analytically, and is also analytically invertible to give x as a function of "area under the comparison function to the left of x." Now pick a uniform deviate between 0 and A, where A is the total area under $f(x)$, and use it to get a corresponding x. Then pick a uniform deviate between 0 and $f(x)$ as the y value for the two-dimensional point. Finally, accept or reject according to whether it is respectively less than or greater than $p(x)$.

So, to summarize, the rejection method for some given $p(x)$ requires that one find, once and for all, some reasonably good comparison function $f(x)$. Thereafter,

each deviate generated requires two uniform random deviates, one evaluation of f (to get the coordinate y) and one evaluation of p (to decide whether to accept or reject the point x, y). Figure 7.3.1 illustrates the whole process. Then, of course, this process may need to be repeated, on the average, A times before the final deviate is obtained.

7.3.7 Cauchy Deviates

The "further trick" described following equation (7.3.14) in the context of the Box-Muller method is now seen to be a rejection method for getting trigonometric functions of a uniformly random angle. If we combine this with the explicit formula, equation (6.14.6), for the inverse cdf of the Cauchy distribution (see §6.14.2), we can generate Cauchy deviates quite efficiently.

deviates.h

```
struct Cauchydev : Ran {
Structure for Cauchy deviates.
    Doub mu,sig;
    Cauchydev(Doub mmu, Doub ssig, Ullong i) : Ran(i), mu(mmu), sig(ssig) {}
    Constructor arguments are μ, σ, and a random sequence seed.
    Doub dev() {
    Return a Cauchy deviate.
        Doub v1,v2;
        do {                                   Find a random point in the unit semicircle.
            v1=2.0*doub()-1.0;
            v2=doub();
        } while (SQR(v1)+SQR(v2) >= 1. || v2 == 0.);
        return mu + sig*v1/v2;                 Ratio of its coordinates is the tangent of a
    }                                              random angle.
};
```

7.3.8 Ratio-of-Uniforms Method

In finding Cauchy deviates, we took the ratio of two uniform deviates chosen to lie within the unit circle. If we generalize to shapes other than the unit circle, and combine it with the principle of the rejection method, a powerful variant emerges. Kinderman and Monahan [1] showed that deviates of virtually *any* probability distribution $p(x)$ can be generated by the following rather amazing prescription:

- Construct the region in the (u, v) plane bounded by $0 \le u \le [p(v/u)]^{1/2}$.
- Choose two deviates, u and v, that lie uniformly in this region.
- Return v/u as the deviate.

Proof: We can represent the ordinary rejection method by the equation in the (x, p) plane,

$$p(x)dx = \int_{p'=0}^{p'=p(x)} dp'dx \tag{7.3.18}$$

Since the integrand is 1, we are justified in sampling uniformly in (x, p') as long as p' is within the limits of the integral (that is, $0 < p' < p(x)$). Now make the change of variable

$$\begin{aligned} \frac{v}{u} &= x \\ u^2 &= p \end{aligned} \tag{7.3.19}$$

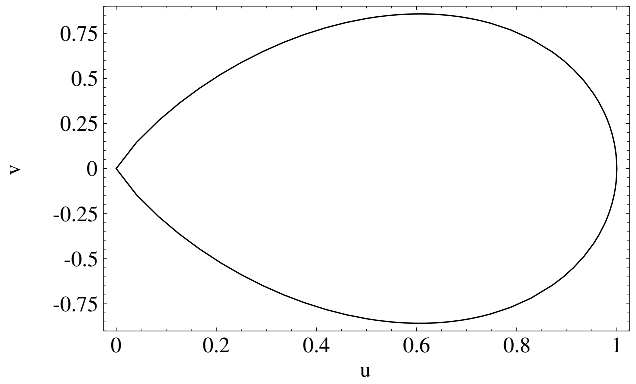

Figure 7.3.3. Ratio-of-uniforms method. The interior of this teardrop shape is the acceptance region for the normal distribution: If a random point is chosen inside this region, then the ratio v/u will be a normal deviate.

Then equation (7.3.18) becomes

$$p(x)dx = \int_{p'=0}^{p'=p(x)} dp'dx = \int_{u=0}^{u=\sqrt{p(x)}} \frac{\partial(p,x)}{\partial(u,v)} du\,dv = 2\int_{u=0}^{u=\sqrt{p(v/u)}} du\,dv \tag{7.3.20}$$

because (as you can work out) the Jacobian determinant is the constant 2. Since the new integrand is constant, uniform sampling in (u, v) with the limits indicated for u is equivalent to the rejection method in (x, p).

The above limits on u very often define a region that is "teardrop" shaped. To see why, note that the locii of constant $x = v/u$ are radial lines. Along each radial, the acceptance region goes from the origin to a point where $u^2 = p(x)$. Since most probability distributions go to zero for both large and small x, the acceptance region accordingly shrinks toward the origin along radials, producing a teardrop. Of course, it is the exact shape of this teardrop that matters. Figure 7.3.3 shows the shape of the acceptance region for the case of the normal distribution.

Typically this *ratio-of-uniforms* method is used when the desired region can be closely bounded by a rectangle, parallelogram, or some other shape that is easy to sample uniformly. Then, we go from sampling the easy shape to sampling the desired region by rejection of points outside the desired region.

An important adjunct to the ratio-of-uniforms method is the idea of a *squeeze*. A squeeze is any easy-to-compute shape that tightly bounds the region of acceptance of a rejection method, either from the inside or from the outside. Best of all is when you have squeezes on both sides. Then you can immediately reject points that are outside the outer squeeze and immediately accept points that are inside the inner squeeze. Only when you have the bad luck of drawing a point between the two squeezes do you actually have to do the more lengthy computation of comparing with the actual rejection boundary. Squeezes are useful both in the ordinary rejection method and in the ratio-of-uniforms method.

7.3.9 Normal Deviates by Ratio-of-Uniforms

Leva [2] has given an algorithm for normal deviates that uses the ratio-of-uniforms method with great success. He uses quadratic curves to provide both inner

and outer squeezes that hug the desired region in the (u, v) plane (Figure 7.3.3). Only about 1% of the time is it necessary to calculate an exact boundary (requiring a logarithm).

The resulting code looks so simple and "un-transcendental" that it may be hard to believe that exact normal deviates are generated. But they are!

deviates.h

```
struct Normaldev : Ran {
Structure for normal deviates.
    Doub mu,sig;
    Normaldev(Doub mmu, Doub ssig, Ullong i)
    : Ran(i), mu(mmu), sig(ssig){}
    Constructor arguments are μ, σ, and a random sequence seed.
    Doub dev() {
    Return a normal deviate.
        Doub u,v,x,y,q;
        do {
            u = doub();
            v = 1.7156*(doub()-0.5);
            x = u - 0.449871;
            y = abs(v) + 0.386595;
            q = SQR(x) + y*(0.19600*y-0.25472*x);
        } while (q > 0.27597
            && (q > 0.27846 || SQR(v) > -4.*log(u)*SQR(u)));
        return mu + sig*v/u;
    }
};
```

Note that the `while` clause makes use of C's (and C++'s) guarantee that logical expressions are evaluated conditionally: If the first operand is sufficient to determine the outcome, the second is not evaluated at all. With these rules, the logarithm is evaluated only when `q` is between 0.27597 and 0.27846.

On average, each normal deviate uses 2.74 uniform deviates. By the way, even though the various constants are given only to six digits, the method is exact (to full double precision). Small perturbations of the bounding curves are of no consequence. The accuracy is implicit in the (rare) evaluations of the exact boundary.

7.3.10 Gamma Deviates

The distribution $\text{Gamma}(\alpha, \beta)$ was described in §6.14.9. The β parameter enters only as a scaling,

$$\text{Gamma}(\alpha, \beta) \cong \frac{1}{\beta}\text{Gamma}(\alpha, 1) \tag{7.3.21}$$

(Translation: To generate a $\text{Gamma}(\alpha, \beta)$ deviate, generate a $\text{Gamma}(\alpha, 1)$ deviate and divide it by β.)

If α is a small positive integer, a fast way to generate $x \sim \text{Gamma}(\alpha, 1)$ is to use the fact that it is distributed as the waiting time to the αth event in a Poisson random process of unit mean. Since the time between two consecutive events is just the exponential distribution Exponential (1), you can simply add up α exponentially distributed waiting times, i.e., logarithms of uniform deviates. Even better, since the sum of logarithms is the logarithm of the product, you really only have to compute the product of a uniform deviates and then take the log. Because this is such a special case, however, we don't include it in the code below.

When $\alpha < 1$, the gamma distribution's density function is not bounded, which is inconvenient. However, it turns out [4] that if

$$y \sim \text{Gamma}(\alpha + 1, 1), \qquad u \sim \text{Uniform}(0, 1) \tag{7.3.22}$$

then

$$yu^{1/\alpha} \sim \text{Gamma}(\alpha, 1) \tag{7.3.23}$$

We will use this in the code below.

For $\alpha > 1$, Marsaglia and Tsang [5] give an elegant rejection method based on a simple transformation of the gamma distribution combined with a squeeze. After transformation, the gamma distribution can be bounded by a Gaussian curve whose area is never more than 5% greater than that of the gamma curve. The cost of a gamma deviate is thus only a little more than the cost of the normal deviate that is used to sample the comparison function. The following code gives the precise formulation; see the original paper for a full explanation.

deviates.h

```
struct Gammadev : Normaldev {
Structure for gamma deviates.
    Doub alph, oalph, bet;
    Doub a1,a2;
    Gammadev(Doub aalph, Doub bbet, Ullong i)
    : Normaldev(0.,1.,i), alph(aalph), oalph(aalph), bet(bbet) {
    Constructor arguments are α, β, and a random sequence seed.
        if (alph <= 0.) throw("bad alph in Gammadev");
        if (alph < 1.) alph += 1.;
        a1 = alph-1./3.;
        a2 = 1./sqrt(9.*a1);
    }
    Doub dev() {
    Return a gamma deviate by the method of Marsaglia and Tsang.
        Doub u,v,x;
        do {
            do {
                x = Normaldev::dev();
                v = 1. + a2*x;
            } while (v <= 0.);
            v = v*v*v;
            u = doub();
        } while (u > 1. - 0.331*SQR(SQR(x)) &&
            log(u) > 0.5*SQR(x) + a1*(1.-v+log(v)));   Rarely evaluated.
        if (alph == oalph) return a1*v/bet;
        else {                                          Case where α < 1, per Ripley.
            do u=doub(); while (u == 0.);
            return pow(u,1./oalph)*a1*v/bet;
        }
    }
};
```

There exists a sum rule for gamma deviates. If we have a set of independent deviates y_i with possibly different α_i's, but sharing a common value of β,

$$y_i \sim \text{Gamma}(\alpha_i, \beta) \tag{7.3.24}$$

then their sum is also a gamma deviate,

$$y \equiv \sum_i y_i \sim \text{Gamma}(\alpha_T, \beta), \qquad \alpha_T = \sum_i \alpha_i \tag{7.3.25}$$

If the α_i's are integers, you can see how this relates to the discussion of Poisson waiting times above.

7.3.11 Distributions Easily Generated by Other Deviates

From normal, gamma and uniform deviates, we get a bunch of other distributions for free. Important: When you are going to combine their results, be sure that all distinct instances of `Normaldist`, `Gammadist`, and `Ran` have different random seeds! (`Ran` and its derived classes are sufficiently robust that seeds $i, i+1, \ldots$ are fine.)

Chi-Square Deviates (cf. §6.14.8)

This one is easy:

$$\text{Chisquare}(\nu) \cong \text{Gamma}\left(\frac{\nu}{2}, \frac{1}{2}\right) \cong 2\,\text{Gamma}\left(\frac{\nu}{2}, 1\right) \tag{7.3.26}$$

Student-t Deviates (cf. §6.14.3)

Deviates from the Student-t distribution can be generated by a method very similar to the Box-Muller method. The analog of equation (7.3.12) is

$$y = \sqrt{\nu(u_1^{-2/\nu} - 1)}\cos 2\pi u_2 \tag{7.3.27}$$

If u_1 and u_2 are independently uniform, U(0, 1), then

$$y \sim \text{Student}(\nu, 0, 1) \tag{7.3.28}$$

or

$$\mu + \sigma y \sim \text{Student}(\nu, \mu, \sigma) \tag{7.3.29}$$

Unfortunately, you can't do the Box-Muller trick of getting two deviates at a time, because the Jacobian determinant analogous to equation (7.3.14) does not factorize. You might want to use the polar method anyway, just to get $\cos 2\pi u_2$, but its advantage is now not so large.

An alternative method uses the quotients of normal and gamma deviates. If we have

$$x \sim \text{N}(0, 1), \qquad y \sim \text{Gamma}\left(\frac{\nu}{2}, \frac{1}{2}\right) \tag{7.3.30}$$

then

$$x\sqrt{\nu/y} \sim \text{Student}(\nu, 0, 1) \tag{7.3.31}$$

Beta Deviates (cf. §6.14.11)

If

$$x \sim \text{Gamma}(\alpha, 1), \qquad y \sim \text{Gamma}(\beta, 1) \tag{7.3.32}$$

then

$$\frac{x}{x+y} \sim \text{Beta}(\alpha, \beta) \tag{7.3.33}$$

F-Distribution Deviates (cf. §6.14.10)

If

$$x \sim \text{Beta}(\tfrac{1}{2}\nu_1, \tfrac{1}{2}\nu_2) \tag{7.3.34}$$

(see equation 7.3.33), then

$$\frac{\nu_2 x}{\nu_1(1-x)} \sim \text{F}(\nu_1, \nu_2) \tag{7.3.35}$$

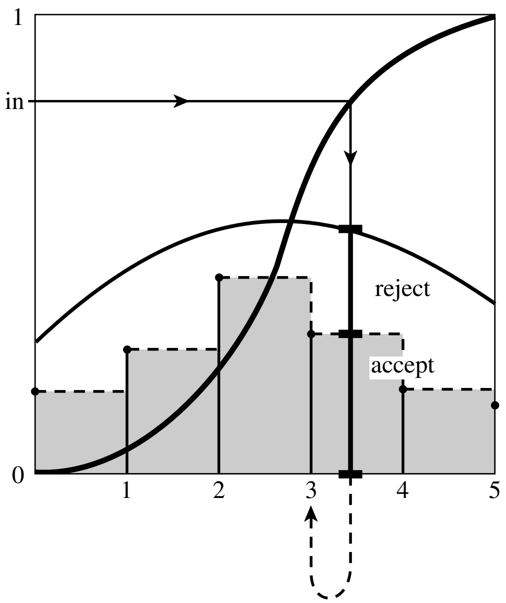

Figure 7.3.4. Rejection method as applied to an integer-valued distribution. The method is performed on the step function shown as a dashed line, yielding a real-valued deviate. This deviate is rounded down to the next lower integer, which is output.

7.3.12 Poisson Deviates

The Poisson distribution, Poisson(λ), previously discussed in §6.14.13, is a discrete distribution, so its deviates will be integers, k. To use the methods already discussed, it is convenient to convert the Poisson distribution into a continuous distribution by the following trick: Consider the finite probability $p(k)$ as being spread out uniformly into the interval from k to $k+1$. This defines a continuous distribution $q_\lambda(k)dk$ given by

$$q_\lambda(k)dk = \frac{\lambda^{\lfloor k \rfloor} e^{-\lambda}}{\lfloor k \rfloor !} dk \tag{7.3.36}$$

where $\lfloor k \rfloor$ represents the largest integer $\leq k$. If we now use a rejection method, or any other method, to generate a (noninteger) deviate from (7.3.36), and then take the integer part of that deviate, it will be as if drawn from the discrete Poisson distribution. (See Figure 7.3.4.) This trick is general for any integer-valued probability distribution. Instead of the "floor" operator, one can equally well use "ceiling" or "nearest" — anything that spreads the probability over a unit interval.

For λ large enough, the distribution (7.3.36) is qualitatively bell-shaped (albeit with a bell made out of small, square steps). In that case, the ratio-of-uniforms method works well. It is not hard to find simple inner and outer squeezes in the (u, v) plane of the form $v^2 = Q(u)$, where $Q(u)$ is a simple polynomial in u. The only trick is to allow a big enough gap between the squeezes to enclose the true, jagged, boundaries for all values of λ. (Look ahead to Figure 7.3.5 for a similar example.)

For intermediate values of λ, the jaggedness is so large as to render squeezes impractical, but the ratio-of-uniforms method, unadorned, still works pretty well.

For small λ, we can use an idea similar to that mentioned above for the gamma distribution in the case of integer a. When the sum of independent exponential

deviates first exceeds λ, their number (less 1) is a Poisson deviate k. Also, as explained for the gamma distribution, we can multiply uniform deviates from U(0, 1) instead of adding deviates from Exponential (1).

These ideas produce the following routine.

deviates.h

```
struct Poissondev : Ran {
Structure for Poisson deviates.
    Doub lambda, sqlam, loglam, lamexp, lambold;
    VecDoub logfact;
    Int swch;
    Poissondev(Doub llambda, Ullong i) : Ran(i), lambda(llambda),
        logfact(1024,-1.), lambold(-1.) {}
    Constructor arguments are λ and a random sequence seed.
    Int dev() {
    Return a Poisson deviate using the most recently set value of λ.
        Doub u,u2,v,v2,p,t,lfac;
        Int k;
        if (lambda < 5.) {                      Will use product of uniforms method.
            if (lambda != lambold) lamexp=exp(-lambda);
            k = -1;
            t=1.;
            do {
                ++k;
                t *= doub();
            } while (t > lamexp);
        } else {                                Will use ratio-of-uniforms method.
            if (lambda != lambold) {
                sqlam = sqrt(lambda);
                loglam = log(lambda);
            }
            for (;;) {
                u = 0.64*doub();
                v = -0.68 + 1.28*doub();
                if (lambda > 13.5) {            Outer squeeze for fast rejection.
                    v2 = SQR(v);
                    if (v >= 0.) {if (v2 > 6.5*u*(0.64-u)*(u+0.2)) continue;}
                    else {if (v2 > 9.6*u*(0.66-u)*(u+0.07)) continue;}
                }
                k = Int(floor(sqlam*(v/u)+lambda+0.5));
                if (k < 0) continue;
                u2 = SQR(u);
                if (lambda > 13.5) {            Inner squeeze for fast acceptance.
                    if (v >= 0.) {if (v2 < 15.2*u2*(0.61-u)*(0.8-u)) break;}
                    else {if (v2 < 6.76*u2*(0.62-u)*(1.4-u)) break;}
                }
                if (k < 1024) {
                    if (logfact[k] < 0.) logfact[k] = gammln(k+1.);
                    lfac = logfact[k];
                } else lfac = gammln(k+1.);
                p = sqlam*exp(-lambda + k*loglam - lfac);     Only when we must.
                if (u2 < p) break;
            }
        }
        lambold = lambda;
        return k;
    }
    Int dev(Doub llambda) {
    Reset λ and then return a Poisson deviate.
        lambda = llambda;
        return dev();
    }
};
```

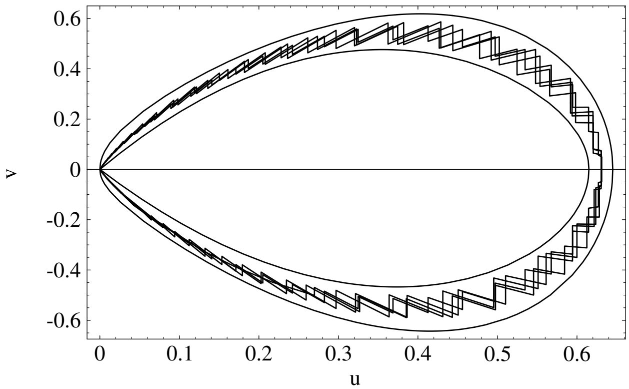

Figure 7.3.5. Ratio-of-uniforms method as applied to the generation of binomial deviates. Points are chosen randomly in the (u, v)-plane. The smooth curves are inner and outer squeezes. The jagged curves correspond to various binomial distributions with $n > 64$ and $np > 30$. An evaluation of the binomial probability is required only when the random point falls between the smooth curves.

In the regime $\lambda > 13.5$, the above code uses about 3.3 uniform deviates per output Poisson deviate and does 0.4 evaluations of the exact probability (costing an exponential and, for large k, a call to `gammln`).

`Poissondev` is slightly faster if you draw many deviates with the same value λ, using the `dev` function with no arguments, than if you vary λ on each call, using the one-argument overloaded form of `dev` (which is provided for just that purpose). The difference is just an extra exponential ($\lambda < 5$) or square root and logarithm ($\lambda \geq 5$). Note also the object's table of previously computed log-factorials. If your λ's are as large as $\sim 10^3$, you might want to make the table larger.

7.3.13 Binomial Deviates

The generation of binomial deviates $k \sim \text{Binomial}(n, p)$ involves many of the same ideas as for Poisson deviates. The distribution is again integer-valued, so we use the same trick to convert it into a stepped continuous distribution. We can always restrict attention to the case $p \leq 0.5$, since the distribution's symmetries let us trivially recover the case $p > 0.5$.

When $n > 64$ and $np > 30$, we use the ratio-of-uniforms method, with squeezes shown in Figure 7.3.5. The cost is about 3.2 uniform deviates, plus 0.4 evaluations of the exact probability, per binomial deviate.

It would be foolish to waste much thought on the case where $n > 64$ and $np < 30$, because it is so easy simply to tabulate the cdf, say for $0 \leq k < 64$, and then loop over k's until the right one is found. (A bisection search, implemented below, is even better.) With a cdf table of length 64, the neglected probability at the end of the table is never larger than $\sim 10^{-20}$. (At 10^9 deviates per second, you could run 3000 years before losing a deviate.)

What is left is the interesting case $n < 64$, which we will explore in some detail, because it demonstrates the important concept of *bit-parallel random comparison*.

Analogous to the methods for gamma deviates with small integer a and for Poisson deviates with small λ, is this direct method for binomial deviates: Generate n uniform deviates in U(0, 1). Count the number of them $< p$. Return the count as

$k \sim \text{Binomial}(n, p)$. Indeed this is essentially the definition of a binomial process!

The problem with the direct method is that it seems to require n uniform deviates, even when the mean value of k is much smaller. Would you be surprised if we told you that for $n \leq 64$ you can achieve the same goal with at most *seven* 64-bit uniform deviates, on average? Here is how.

Expand $p < 1$ into its first 5 bits, plus a residual,

$$p = b_1 2^{-1} + b_2 2^{-2} + \cdots + b_5 2^{-5} + p_r 2^{-5} \qquad (7.3.37)$$

where each b_i is 0 or 1, and $0 \leq p_r \leq 1$.

Now imagine that you have generated and stored 64 uniform U(0, 1) deviates, and that the 64-bit word P displays just the first bit of each of the 64. Compare each bit of P to b_1. If the bits are the same, then we don't yet know whether that uniform deviate is less than or greater than p. But if the bits are *different*, then we know that the generator is less than p (in the case that $b_1 = 1$) or greater than p (in the case that $b_1 = 0$). If we keep a mask of "known" versus "unknown" cases, we can do these comparisons in a bit-parallel manner by bitwise logical operations (see code below to learn how). Now move on to the second bit, b_2, in the same way. At each stage we change half the remaining unknowns to knowns. After five stages (for $n = 64$) there will be two remaining unknowns, on average, each of which we finish off by generating a new uniform and comparing it to p_r. (This requires a loop through the 64 bits; but since C++ has no bitwise "popcount" operation, we are stuck doing such a loop anyway. If you can do popcounts, you may be better off just doing more stages until the unknowns mask is zero.)

The trick is that the bits used in the five stages are not actually the leading five bits of 64 generators, they are just five independent 64-bit random integers. The number five was chosen because it minimizes $64 \times 2^{-j} + j$, the expected number of deviates needed.

So, the code for binomial deviates ends up with three separate methods: bit-parallel direct, cdf lookup (by bisection), and squeezed ratio-of-uniforms.

deviates.h

```
struct Binomialdev : Ran {
Structure for binomial deviates.
    Doub pp,p,pb,expnp,np,glnp,plog,pclog,sq;
    Int n,swch;
    Ullong uz,uo,unfin,diff,rltp;
    Int pbits[5];
    Doub cdf[64];
    Doub logfact[1024];
    Binomialdev(Int nn, Doub ppp, Ullong i) : Ran(i), pp(ppp), n(nn) {
    Constructor arguments are n, p, and a random sequence seed.
        Int j;
        pb = p = (pp <= 0.5 ? pp : 1.0-pp);
        if (n <= 64) {                          Will use bit-parallel direct method.
            uz=0;
            uo=0xffffffffffffffffLL;
            rltp = 0;
            for (j=0;j<5;j++) pbits[j] = 1 & ((Int)(pb *= 2.));
            pb -= floor(pb);                    Leading bits of p (above) and remaining
            swch = 0;                               fraction.
        } else if (n*p < 30.) {                 Will use precomputed cdf table.
            cdf[0] = exp(n*log(1-p));
            for (j=1;j<64;j++) cdf[j] =  cdf[j-1] + exp(gammln(n+1.)
                -gammln(j+1.)-gammln(n-j+1.)+j*log(p)+(n-j)*log(1.-p));
            swch = 1;
```

```
        } else {                                    Will use ratio-of-uniforms method.
            np = n*p;
            glnp=gammln(n+1.);
            plog=log(p);
            pclog=log(1.-p);
            sq=sqrt(np*(1.-p));
            if (n < 1024) for (j=0;j<=n;j++) logfact[j] = gammln(j+1.);
            swch = 2;
        }
    }
    Int dev() {
    Return a binomial deviate.
        Int j,k,kl,km;
        Doub y,u,v,u2,v2,b;
        if (swch == 0) {
            unfin = uo;                             Mark all bits as "unfinished."
            for (j=0;j<5;j++) {                     Compare with first five bits of p.
                diff = unfin & (int64()^(pbits[j]? uo : uz));   Mask of diff.
                if (pbits[j]) rltp |= diff;         Set bits to 1, meaning ran < p.
                else rltp = rltp & ~diff;           Set bits to 0, meaning ran > p.
                unfin = unfin & ~diff;              Update unfinished status.
            }
            k=0;                                    Now we just count the events.
            for (j=0;j<n;j++) {
                if (unfin & 1) {if (doub() < pb) ++k;}      Clean up unresolved cases,
                else {if (rltp & 1) ++k;}                   or use bit answer.
                unfin >>= 1;
                rltp >>= 1;
            }
        } else if (swch == 1) {                     Use stored cdf.
            y = doub();
            kl = -1;
            k = 64;
            while (k-kl>1) {
                km = (kl+k)/2;
                if (y < cdf[km]) k = km;
                else kl = km;
            }
        } else {                                    Use ratio-of-uniforms method.
            for (;;) {
                u = 0.645*doub();
                v = -0.63 + 1.25*doub();
                v2 = SQR(v);
                Try squeeze for fast rejection:
                if (v >= 0.) {if (v2 > 6.5*u*(0.645-u)*(u+0.2)) continue;}
                else {if (v2 > 8.4*u*(0.645-u)*(u+0.1)) continue;}
                k = Int(floor(sq*(v/u)+np+0.5));
                if (k < 0) continue;
                u2 = SQR(u);
                Try squeeze for fast acceptance:
                if (v >= 0.) {if (v2 < 12.25*u2*(0.615-u)*(0.92-u)) break;}
                else {if (v2 < 7.84*u2*(0.615-u)*(1.2-u)) break;}
                b = sq*exp(glnp+k*plog+(n-k)*pclog      Only when we must.
                    - (n < 1024 ? logfact[k]+logfact[n-k]
                        : gammln(k+1.)+gammln(n-k+1.)));
                if (u2 < b) break;
            }
        }
        if (p != pp) k = n - k;
        return k;
    }
};
```

If you are in a situation where you are drawing only one or a few deviates each for many different values of n and/or p, you'll need to restructure the code so that n and p can be changed without creating a new instance of the object and without reinitializing the underlying `Ran` generator.

7.3.14 When You Need Greater Speed

In particular situations you can cut some corners to gain greater speed. Here are some suggestions.

- All of the algorithms in this section can be speeded up significantly by using `Ranq1` in §7.1 instead of `Ran`. We know of no reason not to do this. You can gain some further speed by coding `Ranq1`'s algorithm inline, thus eliminating the function calls.
- If you are using `Poissondev` or `Binomialdev` with large values of λ or n, then the above codes revert to calling `gammln`, which is slow. You can instead increase the length of the stored tables.
- For Poisson deviates with $\lambda < 20$, you may want to use a stored table of cdfs combined with bisection to find the value of k. The code in `Binomialdev` shows how to do this.
- If your need is for binomial deviates with small n, you can easily modify the code in `Binomialdev` to get multiple deviates ($\sim 64/n$, in fact) from each execution of the bit-parallel code.
- Do you need exact deviates, or would an approximation do? If your distribution of interest can be approximated by a normal distribution, consider substituting `Normaldev`, above, especially if you also code the uniform random generation inline.
- If you sum exactly 12 uniform deviates $U(0,1)$ and then subtract 6, you get a pretty good approximation of a normal deviate $N(0,1)$. This is definitely slower then `Normaldev` (not to mention less accurate) on a general-purpose CPU. However, there are reported to be some special-purpose signal processing chips in which all the operations can be done with integer arithmetic and in parallel.

See Gentle [3], Ripley [4], Devroye [6], Bratley [7], and Knuth [8] for many additional algorithms.

CITED REFERENCES AND FURTHER READING:

Kinderman, A.J. and Monahan, J.F 1977, "Computer Generation of Random Variables Using the Ratio of Uniform Deviates," *ACM Transactions on Mathematical Software*, vol. 3, pp. 257–260.[1]

Leva, J.L. 1992. "A Fast Normal Random Number Generator," *ACM Transactions on Mathematical Software*, vol. 18, no. 4, pp. 449-453.[2]

Gentle, J.E. 2003, *Random Number Generation and Monte Carlo Methods*, 2nd ed. (New York: Springer), Chapters 4–5.[3]

Ripley, B.D. 1987, *Stochastic Simulation* (New York: Wiley).[4]

Marsaglia, G. and Tsang W-W. 2000, "A Simple Method for Generating Gamma Variables," *ACM Transactions on Mathematical Software*, vol. 26, no. 3, pp. 363–372.[5]

Devroye, L. 1986, *Non-Uniform Random Variate Generation* (New York: Springer).[6]

Bratley, P., Fox, B.L., and Schrage, E.L. 1983, *A Guide to Simulation*, 2nd ed. (New York: Springer).[7].

Knuth, D.E. 1997, *Seminumerical Algorithms*, 3rd ed., vol. 2 of *The Art of Computer Programming* (Reading, MA: Addison-Wesley), pp. 125ff.[8]

7.4 Multivariate Normal Deviates

A multivariate random deviate of dimension M is a point in M-dimensional space. Its coordinates are a vector, each of whose M components are random — but not, in general, independently so, or identically distributed. The special case of *multivariate normal deviates* is defined by the multidimensional Gaussian density function

$$N(\mathbf{x} \mid \boldsymbol{\mu}, \boldsymbol{\Sigma}) = \frac{1}{(2\pi)^{M/2} \det(\boldsymbol{\Sigma})^{1/2}} \exp[-\tfrac{1}{2}(\mathbf{x} - \boldsymbol{\mu}) \cdot \boldsymbol{\Sigma}^{-1} \cdot (\mathbf{x} - \boldsymbol{\mu})] \qquad (7.4.1)$$

where the parameter $\boldsymbol{\mu}$ is a vector that is the mean of the distribution, and the parameter $\boldsymbol{\Sigma}$ is a symmetrical, positive-definite matrix that is the distribution's covariance.

There is a quite general way to construct a vector deviate $\mathbf{x}$ with a specified covariance $\boldsymbol{\Sigma}$ and mean $\boldsymbol{\mu}$, starting with a vector $\mathbf{y}$ of independent random deviates of zero mean and unit variance: First, use Cholesky decomposition (§2.9) to factor $\boldsymbol{\Sigma}$ into a left triangular matrix $\mathbf{L}$ times its transpose,

$$\boldsymbol{\Sigma} = \mathbf{L}\mathbf{L}^T \qquad (7.4.2)$$

This is always possible because $\boldsymbol{\Sigma}$ is positive-definite, and you need do it only once for each distinct $\boldsymbol{\Sigma}$ of interest. Next, whenever you want a new deviate $\mathbf{x}$, fill $\mathbf{y}$ with independent deviates of unit variance and then construct

$$\mathbf{x} = \mathbf{L}\mathbf{y} + \boldsymbol{\mu} \qquad (7.4.3)$$

The proof is straightforward, with angle brackets denoting expectation values: Since the components y_i are independent with unit variance, we have

$$\langle \mathbf{y} \otimes \mathbf{y} \rangle = \mathbf{1} \qquad (7.4.4)$$

where $\mathbf{1}$ is the identity matrix. Then,

$$\begin{aligned} \langle (\mathbf{x} - \boldsymbol{\mu}) \otimes (\mathbf{x} - \boldsymbol{\mu}) \rangle &= \langle (\mathbf{L}\mathbf{y}) \otimes (\mathbf{L}\mathbf{y}) \rangle \\ &= \left\langle \mathbf{L}(\mathbf{y} \otimes \mathbf{y})\mathbf{L}^T \right\rangle = \mathbf{L} \langle \mathbf{y} \otimes \mathbf{y} \rangle \mathbf{L}^T \\ &= \mathbf{L}\mathbf{L}^T = \boldsymbol{\Sigma} \end{aligned} \qquad (7.4.5)$$

As general as this procedure is, it is, however, rarely useful for anything except multivariate *normal* deviates. The reason is that while the components of $\mathbf{x}$ indeed have the right mean and covariance structure, their detailed distribution is not anything "nice." The x_i's are linear combinations of the y_i's, and, in general, a linear combination of random variables is distributed as a complicated convolution of their individual distributions.

For Gaussians, however, we do have "nice." All linear combinations of normal deviates are themselves normally distributed, and completely defined by their mean and covariance structure. Thus, if we always fill the components of $\mathbf{y}$ with normal deviates,

$$y_i \sim \mathrm{N}(0,1) \tag{7.4.6}$$

then the deviate (7.4.3) will be distributed according to equation (7.4.1).

Implementation is straightforward, since the `Cholesky` structure both accomplishes the decomposition and provides a method for doing the matrix multiplication efficiently, taking advantage of $\mathbf{L}$'s triangular structure. The generation of normal deviates is inline for efficiency, identical to `Normaldev` in §7.3.

multinormaldev.h

```
struct Multinormaldev : Ran {
Structure for multivariate normal deviates.
    Int mm;
    VecDoub mean;
    MatDoub var;
    Cholesky chol;
    VecDoub spt, pt;

    Multinormaldev(Ullong j, VecDoub &mmean, MatDoub &vvar) :
    Ran(j), mm(mmean.size()), mean(mmean), var(vvar), chol(var),
    spt(mm), pt(mm) {
    Constructor. Arguments are the random generator seed, the (vector) mean, and the (ma-
    trix) covariance. Cholesky decomposition of the covariance is done here.
        if (var.ncols() != mm || var.nrows() != mm) throw("bad sizes");
    }

    VecDoub &dev() {
    Return a multivariate normal deviate.
        Int i;
        Doub u,v,x,y,q;
        for (i=0;i<mm;i++) {                Fill a vector of independent normal deviates.
            do {
                u = doub();
                v = 1.7156*(doub()-0.5);
                x = u - 0.449871;
                y = abs(v) + 0.386595;
                q = SQR(x) + y*(0.19600*y-0.25472*x);
            } while (q > 0.27597
                && (q > 0.27846 || SQR(v) > -4.*log(u)*SQR(u)));
            spt[i] = v/u;
        }
        chol.elmult(spt,pt);                Apply equation (7.4.3).
        for (i=0;i<mm;i++) {pt[i] += mean[i];}
        return pt;
    }

};
```

7.4.1 Decorrelating Multiple Random Variables

Although not directly related to the generation of random deviates, this is a convenient place to point out how Cholesky decomposition can be used in the reverse manner, namely to find linear combinations of correlated random variables that have no correlation. In this application we are given a vector $\mathbf{x}$ whose components have a known covariance $\mathbf{\Sigma}$ and mean $\boldsymbol{\mu}$. Decomposing $\mathbf{\Sigma}$ as in equation (7.4.2), we assert

that

$$\mathbf{y} = \mathbf{L}^{-1}(\mathbf{x} - \boldsymbol{\mu}) \tag{7.4.7}$$

has uncorrelated components, each of unit variance. Proof:

$$\begin{aligned}\langle \mathbf{y} \otimes \mathbf{y} \rangle &= \left\langle (\mathbf{L}^{-1}[\mathbf{x} - \boldsymbol{\mu}]) \otimes (\mathbf{L}^{-1}[\mathbf{x} - \boldsymbol{\mu}]) \right\rangle \\ &= \mathbf{L}^{-1} \left\langle (\mathbf{x} - \boldsymbol{\mu}) \otimes (\mathbf{x} - \boldsymbol{\mu}) \right\rangle \mathbf{L}^{-1T} \\ &= \mathbf{L}^{-1} \boldsymbol{\Sigma} \mathbf{L}^{-1T} = \mathbf{L}^{-1}\mathbf{L}\mathbf{L}^{T}\mathbf{L}^{-1T} = \mathbf{1}\end{aligned} \tag{7.4.8}$$

Be aware that this linear combination is not unique. In fact, once you have obtained a vector $\mathbf{y}$ of uncorrelated components, you can perform any rotation on it and still have uncorrelated components. In particular, if $\mathbf{K}$ is an orthogonal matrix, so that

$$\mathbf{K}^T\mathbf{K} = \mathbf{K}\mathbf{K}^T = \mathbf{1} \tag{7.4.9}$$

then

$$\langle (\mathbf{K}\mathbf{y}) \otimes (\mathbf{K}\mathbf{y}) \rangle = \mathbf{K} \langle \mathbf{y} \otimes \mathbf{y} \rangle \mathbf{K}^T = \mathbf{K}\mathbf{K}^T = \mathbf{1} \tag{7.4.10}$$

A common (though slower) alternative to Cholesky decomposition is to use the Jacobi transformation (§11.1) to decompose $\boldsymbol{\Sigma}$ as

$$\boldsymbol{\Sigma} = \mathbf{V} \operatorname{diag}(\sigma_i^2) \mathbf{V}^T \tag{7.4.11}$$

where $\mathbf{V}$ is the orthogonal matrix of eigenvectors, and the σ_i's are the standard deviations of the (new) uncorrelated variables. Then $\mathbf{V}\operatorname{diag}(\sigma_i)$ plays the role of $\mathbf{L}$ in the proofs above.

Section §16.1.1 discusses some further applications of Cholesky decomposition relating to multivariate random variables.

7.5 Linear Feedback Shift Registers

A *linear feedback shift register* (LFSR) consists of a *state vector* and a certain kind of *update rule*. The state vector is often the set of bits in a 32- or 64-bit word, but it can sometimes be a set of words in an array. To qualify as an LFSR, the update rule must generate a *linear* combination of the bits (or words) in the current state, and then shift that result onto one end of the state vector. The oldest value, at the other end of the state vector, falls off and is gone. The output of an LFSR consists of the sequence of new bits (or words) as they are shifted in.

For single bits, "linear" means arithmetic modulo 2, which is the same as using the logical XOR operation for $+$ and the logical AND operation for $\times$. It is convenient, however, to write equations using the arithmetic notation. So, for an LFSR of length n, the words in the paragraph above translate to

$$\begin{aligned} a_1' &= \left(\sum_{j=1}^{n-1} c_j a_j \right) + a_n \\ a_i' &= a_{i-1}, \qquad i = 2, \ldots, n \end{aligned} \tag{7.5.1}$$

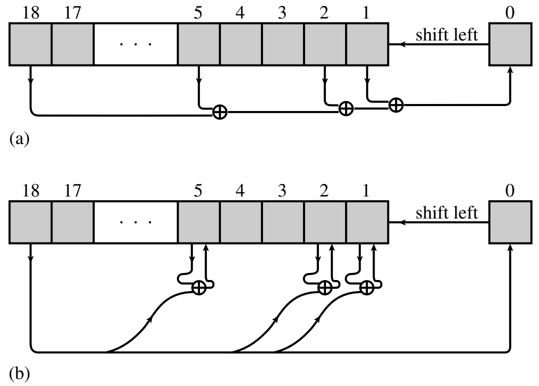

Figure 7.5.1. Two related methods for obtaining random bits from a shift register and a primitive polynomial modulo 2. (a) The contents of selected taps are combined by XOR (addition modulo 2), and the result is shifted in from the right. This method is easiest to implement in hardware. (b) Selected bits are modified by XOR with the leftmost bit, which is then shifted in from the right. This method is easiest to implement in software.

Here $\mathbf{a}'$ is the new state vector, derived from $\mathbf{a}$ by the update rule as shown. The reason for singling out a_n in the first line above is that its coefficient c_n must be $\equiv 1$. Otherwise, the LFSR wouldn't be of length n, but only of length up to the last nonzero coefficient in the c_j's.

There is also a reason for numbering the bits (henceforth we consider only the case of a vector of bits, not of words) starting with 1 rather than the more comfortable 0. The mathematical properties of equation (7.5.1) derive from the properties of the polynomials over the integers modulo 2. The polynomial associated with (7.5.1) is

$$P(x) = x^n + c_{n-1}x^{n-1} + \cdots + c_2x^2 + c_1x + 1 \tag{7.5.2}$$

where each of the c_i's has the value 0 or 1. So, c_0, like c_n, exists but is implicitly $\equiv 1$. There are several notations for describing specific polynomials like (7.5.2). One is to simply list the values i for which c_i is nonzero (by convention including c_n and c_0). So the polynomial

$$x^{18} + x^5 + x^2 + x + 1 \tag{7.5.3}$$

is abbreviated as

$$(18, 5, 2, 1, 0) \tag{7.5.4}$$

Another, when a value of n (here 18), and $c_n = c_0 = 1$, is assumed, is to construct a "serial number" from the binary word $c_{n-1}c_{n-1}\cdots c_2c_1$ (by convention now excluding c_n and c_0). For (7.5.3) this would be 19, that is, $2^4 + 2^1 + 2^0$. The nonzero c_i's are often referred to as an LFSR's *taps*.

Figure 7.5.1(a) illustrates how the polynomial (7.5.3) and (7.5.4) looks as an update process on a register of 18 bits. Bit 0 is the temporary where a bit that is to become the new bit 1 is computed.

The maximum period of an LFSR of n bits, before its output starts repeating, is $2^n - 1$. This is because the maximum number of distinct states is 2^n, but the special vector with all bits zero simply repeats itself with period 1. If you pick a random polynomial $P(x)$, then the generator you construct will usually not be full-period. A

fraction of polynomials over the integers modulo 2 are *irreducible*, meaning that they can't be factored. A fraction of the irreducible polynomials are *primitive*, meaning that they generate maximum period LFSRs. For example, the polynomial $x^2 + 1 = (x + 1)(x + 1)$ is not irreducible, so it is not primitive. (Remember to do arithmetic on the coefficients mod 2.) The polynomial $x^4 + x^3 + x^2 + x + 1$ is irreducible, but it turns out not to be primitive. The polynomial $x^4 + x + 1$ is both irreducible and primitive.

Maximum period LFSRs are often used as sources of random bits in hardware devices, because logic like that shown in Figure 7.5.1(a) requires only a few gates and can be made to run extremely fast. There is not much of a niche for LFSRs in software applications, because implementing equation (7.5.1) in code requires at least two full-word logical operations for each nonzero c_i, and all this work produces a meager one bit of output. We call this "Method I." A better software approach, "Method II," is not obviously an LFSR at all, but it turns out to be mathematically equivalent to one. It is shown in Figure 7.5.1(b). In code, this is implemented from a primitive polynomial as follows:

Let `maskp` and `maskn` be two bit masks,

$$\begin{aligned} \texttt{maskp} &\equiv (0 \;\cdots\; 0 \;\; c_{n-1} \;\; c_{n-2} \;\cdots\; c_2 \;\; c_1) \\ \texttt{maskn} &\equiv (0 \;\cdots\; 1 \;\; 0 \;\; 0 \;\cdots\; 0 \;\; 0) \end{aligned} \tag{7.5.5}$$

Then, a word $\mathbf{a}$ is updated by

```
if (a & maskn) a = ((a ^ maskp) << 1) | 1;
else a <<= 1;
```
(7.5.6)

You should work through the above prescription to see that it is identical to what is shown in the figure. The output of this update (still only one bit) can be taken as `(a & maskn)`, or for that matter any fixed bit in `a`.

LFSRs (either Method I or Method II) are sometimes used to get random m-bit words by concatenating the output bits from m consecutive updates (or, equivalently for Method I, grabbing the low-order m bits of state after every m updates). This is generally a bad idea, because the resulting words usually fail some standard statistical tests for randomness. It is especially a bad idea if m and $2^n - 1$ are not relatively prime, in which case the method does not even give all m-bit words uniformly.

Next, we'll develop a bit of theory to see the relation between Method I and Method II, and this will lead us to a routine for testing whether any given polynomial (expressed as a bit string of c_i's) is primitive. But, for now, if you only need a table of some primitive polynomials go get going, one is provided on the next page.

Since the update rule (7.5.1) is linear, it can be written as a matrix $\mathbf{M}$ that multiplies from the left a column vector of bits $\mathbf{a}$ to produce an updated state $\mathbf{a}'$. (Note that the low-order bits of $\mathbf{a}$ start at the top of the column vector.) One can readily read off

$$\mathbf{M} = \begin{bmatrix} c_1 & c_2 & \dots & c_{n-2} & c_{n-1} & 1 \\ 1 & 0 & \dots & 0 & 0 & 0 \\ 0 & 1 & \dots & 0 & 0 & 0 \\ \vdots & \vdots & & \vdots & \vdots & \vdots \\ 0 & 0 & \dots & 1 & 0 & 0 \\ 0 & 0 & \dots & 0 & 1 & 0 \end{bmatrix} \tag{7.5.7}$$

Some Primitive Polynomials Modulo 2 (after Watson [1])	
(1, 0)	(51, 6, 3, 1, 0)
(2, 1, 0)	(52, 3, 0)
(3, 1, 0)	(53, 6, 2, 1, 0)
(4, 1, 0)	(54, 6, 5, 4, 3, 2, 0)
(5, 2, 0)	(55, 6, 2, 1, 0)
(6, 1, 0)	(56, 7, 4, 2, 0)
(7, 1, 0)	(57, 5, 3, 2, 0)
(8, 4, 3, 2, 0)	(58, 6, 5, 1, 0)
(9, 4, 0)	(59, 6, 5, 4, 3, 1, 0)
(10, 3, 0)	(60, 1, 0)
(11, 2, 0)	(61, 5, 2, 1, 0)
(12, 6, 4, 1, 0)	(62, 6, 5, 3, 0)
(13, 4, 3, 1, 0)	(63, 1, 0)
(14, 5, 3, 1, 0)	(64, 4, 3, 1, 0)
(15, 1, 0)	(65, 4, 3, 1, 0)
(16, 5, 3, 2, 0)	(66, 8, 6, 5, 3, 2, 0)
(17, 3, 0)	(67, 5, 2, 1, 0)
(18, 5, 2, 1, 0)	(68, 7, 5, 1, 0)
(19, 5, 2, 1, 0)	(69, 6, 5, 2, 0)
(20, 3, 0)	(70, 5, 3, 1, 0)
(21, 2, 0)	(71, 5, 3, 1, 0)
(22, 1, 0)	(72, 6, 4, 3, 2, 1, 0)
(23, 5, 0)	(73, 4, 3, 2, 0)
(24, 4, 3, 1, 0)	(74, 7, 4, 3, 0)
(25, 3, 0)	(75, 6, 3, 1, 0)
(26, 6, 2, 1, 0)	(76, 5, 4, 2, 0)
(27, 5, 2, 1, 0)	(77, 6, 5, 2, 0)
(28, 3, 0)	(78, 7, 2, 1, 0)
(29, 2, 0)	(79, 4, 3, 2, 0)
(30, 6, 4, 1, 0)	(80, 7, 5, 3, 2, 1, 0)
(31, 3, 0)	(81, 4 0)
(32, 7, 5, 3, 2, 1, 0)	(82, 8, 7, 6, 4, 1, 0)
(33, 6, 4, 1, 0)	(83, 7, 4, 2, 0)
(34, 7, 6, 5, 2, 1, 0)	(84, 8, 7, 5, 3, 1, 0)
(35, 2, 0)	(85, 8, 2, 1, 0)
(36, 6, 5, 4, 2, 1, 0)	(86, 6, 5, 2, 0)
(37, 5, 4, 3, 2, 1, 0)	(87, 7, 5, 1, 0)
(38, 6, 5, 1, 0)	(88, 8, 5, 4, 3, 1, 0)
(39, 4, 0)	(89, 6, 5, 3, 0)
(40, 5, 4 3, 0)	(90, 5, 3, 2, 0)
(41, 3, 0)	(91, 7, 6, 5, 3, 2, 0)
(42, 5, 4, 3, 2, 1, 0)	(92, 6, 5, 2, 0)
(43, 6, 4, 3, 0)	(93, 2, 0)
(44, 6, 5, 2, 0)	(94, 6, 5, 1, 0)
(45, 4, 3, 1, 0)	(95, 6, 5, 4, 2, 1, 0)
(46, 8, 5, 3, 2, 1, 0)	(96, 7, 6, 4, 3, 2, 0)
(47, 5, 0)	(97, 6, 0)
(48, 7, 5, 4, 2, 1, 0)	(98, 7, 4, 3, 2, 1, 0)
(49, 6, 5, 4, 0)	(99, 7, 5, 4, 0)
(50, 4, 3, 2, 0)	(100, 8, 7, 2, 0)

What are the conditions on $\mathbf{M}$ that give a full-period generator, and thereby prove that the polynomial with coefficients c_i is primitive? Evidently we must have

$$\mathbf{M}^{(2^n-1)} = \mathbf{1} \tag{7.5.8}$$

where $\mathbf{1}$ is the identity matrix. This states that the period, or some multiple of it, is $2^n - 1$. But the only possible such multiples are integers that divide $2^n - 1$. To rule these out, and ensure a full period, we need only check that

$$\mathbf{M}^{q_k} \neq \mathbf{1}, \qquad q_k \equiv (2^n - 1)/f_k \tag{7.5.9}$$

for every prime factor f_k of $2^n - 1$. (This is exactly the logic behind the tests of the matrix $\mathbf{T}$ that we described, but did not justify, in §7.1.2.)

It may at first sight seem daunting to compute the humongous powers of $\mathbf{M}$ in equations (7.5.8) and (7.5.9). But, by the method of repeated squaring of $\mathbf{M}$, each such power takes only about n (a number like 32 or 64) matrix multiplies. And, since all the arithmetic is done modulo 2, there is no possibility of overflow! The conditions (7.5.8) and (7.5.9) are in fact an efficient way to test a polynomial for primitiveness. The following code implements the test. Note that you must customize the constants in the constructor for your choice of n (called `N` in the code), in particular the prime factors of $2^n - 1$. The case $n = 32$ is shown. Other than that customization, the code as written is valid for $n \leq 64$. The input to the test is the "serial number," as defined above following equation (7.5.4), of the polynomial to be tested. After declaring an instance of the `Primpolytest` structure, you can repeatedly call its `test()` method to test multiple polynomials. To make `Primpolytest` entirely self-contained, matrices are implemented as linear arrays, and the structure builds from scratch the few matrix operations that it needs. This is inelegant, but effective.

primpolytest.h

```
struct Primpolytest {
Test polynomials over the integers mod 2 for primitiveness.
    Int N, nfactors;
    VecUllong factors;
    VecInt t,a,p;

    Primpolytest() : N(32), nfactors(5), factors(nfactors), t(N*N),
        a(N*N), p(N*N) {
        Constructor. The constants are specific to 32-bit LFSRs.
        Ullong factordata[5] = {3,5,17,257,65537};
        for (Int i=0;i<nfactors;i++) factors[i] = factordata[i];
    }

    Int ispident() {                          Utility to test if p is the identity matrix.
        Int i,j;
        for (i=0; i<N; i++) for (j=0; j<N; j++) {
            if (i == j) { if (p[i*N+j] != 1) return 0; }
            else {if (p[i*N+j] != 0) return 0; }
        }
        return 1;
    }

    void mattimeseq(VecInt &a, VecInt &b) {   Utility for a *= b on matrices a and b.
        Int i,j,k,sum;
        VecInt tmp(N*N);
        for (i=0; i<N; i++) for (j=0; j<N; j++) {
            sum = 0;
            for (k=0; k<N; k++) sum += a[i*N+k] * b[k*N+j];
            tmp[i*N+j] = sum & 1;
        }
        for (k=0; k<N*N; k++) a[k] = tmp[k];
    }

    void matpow(Ullong n) {                   Utility for matrix p = a^n by successive
        Int k;                                    squares.
```

```
        for (k=0; k<N*N; k++) p[k] = 0;
        for (k=0; k<N; k++) p[k*N+k] = 1;
        while (1) {
            if (n & 1) mattimeseq(p,a);
            n >>= 1;
            if (n == 0) break;
            mattimeseq(a,a);
        }
    }

    Int test(Ullong n) {
```
Main test routine. Returns 1 if the polynomial with serial number `n` (see text) is primitive, 0 otherwise.
```
        Int i,k,j;
        Ullong pow, tnm1, nn = n;
        tnm1 = ((Ullong)1 << N) - 1;
        if (n > (tnm1 >> 1)) throw("not a polynomial of degree N");
        for (k=0; k<N*N; k++) t[k] = 0;          Construct the update matrix in t.
        for (i=1; i<N; i++) t[i*N+(i-1)] = 1;
        j=0;
        while (nn) {
            if (nn & 1) t[j] = 1;
            nn >>= 1;
            j++;
        }
        t[N-1] = 1;
        for (k=0; k<N*N; k++) a[k] = t[k];       Test that t^tnm1 is the identity matrix.
        matpow(tnm1);
        if (ispident() != 1) return 0;
        for (i=0; i<nfactors; i++) {             Test that the t to the required submulti-
            pow = tnm1/factors[i];                   ple powers is not the identity matrix.
            for (k=0; k<N*N; k++) a[k] = t[k];
            matpow(pow);
            if (ispident() == 1) return 0;
        }
        return 1;
    }
};
```

It is straightforward to generalize this method to $n > 64$ or to prime moduli p other than 2. If $p^n > 2^{64}$, you'll need a multiword binary representation of the integers $p^n - 1$ and its quotients with its prime factors, so that `matpow` can still find powers by successive squares. Note that the computation time scales roughly as $O(n^4)$, so $n = 64$ is fast, while $n = 1024$ would be rather a long calculation.

Some random primitive polynomials for $n = 32$ bits (giving their serial numbers as decimal values) are 2046052277, 1186898897, 221421833, 55334070, 1225518245, 216563424, 1532859853, 1735381519, 2049267032, 1363072601, and 130420448. Some random ones for $n = 64$ bits are 9267739486094806 34, 3195735403700392248, 4407129700254524327, 2564575827068603 11, 5017679982664373343, and 1723461400905116882.

Given a matrix $\mathbf{M}$ that satisfies equations (7.5.8) and (7.5.9), there are some related matrices that also satisfy those relations. An example is the inverse of $\mathbf{M}$, which you can easily verify as

$$\mathbf{M}^{-1} = \begin{bmatrix} 0 & 1 & 0 & \dots & 0 & 0 \\ 0 & 0 & 1 & \dots & 0 & 0 \\ \vdots & \vdots & \vdots & & \vdots & \vdots \\ 0 & 0 & 0 & \dots & 0 & 1 \\ 1 & c_1 & c_2 & \dots & c_{n-2} & c_{n-1} \end{bmatrix} \qquad (7.5.10)$$

This is the update rule that backs up a state $\mathbf{a}'$ to its predecessor state $\mathbf{a}$. You can easily convert (7.5.10) to a prescription analogous to equation (7.5.1) or to Figure 7.5.1(a).

Another matrix satisfying the relations that guarantee a full period is the transpose of the

inverse (or inverse of the transpose) of **M**,

$$\left(\mathbf{M}^{-1}\right)^T = \begin{bmatrix} 0 & 0 & \dots & 0 & 0 & 1 \\ 1 & 0 & \dots & 0 & 0 & c_1 \\ 0 & 1 & \dots & 0 & 0 & c_2 \\ \vdots & \vdots & & \vdots & \vdots & \vdots \\ 0 & 0 & \dots & 1 & 0 & c_{n-2} \\ 0 & 0 & \dots & 0 & 1 & c_{n-1} \end{bmatrix} \qquad (7.5.11)$$

Surprise! This is exactly Method II, as also shown in Figure 7.5.1(b). (Work it out.)

Even more specifically, the sequence of bits output by a Method II LFSR based on a primitive polynomial $P(x)$ is identical to the sequence output by a Method I LFSR that uses the *reciprocal polynomial* $x^n P(1/x)$. The proof is a bit beyond our scope, but it is essentially because the matrix **M** and its transpose are both roots of the characteristic polynomial, equation (7.5.2), while the inverse matrix $\mathbf{M}^{-1}$ and its transpose are both roots of the reciprocal polynomial. The reciprocal polynomial, as you can easily check from the definition, just swaps the positions of nonzero coefficients end-to-end. For example, the reciprocal polynomial of equation (7.5.3) is $(18, 17, 16, 13, 1)$. If a polynomial is primitive, so is its reciprocal.

Try this experiment: Run a Method II generator for a while. Then take n consecutive bits of its output (from its highest bit, say) and put them into a Method I shift register as initialization (low bit the most recent one). Now step the two methods together, using the reciprocal polynomial in the Method I. You'll get identical output from the two generators.

CITED REFERENCES AND FURTHER READING:

Knuth, D.E. 1997, *Seminumerical Algorithms*, 3rd ed., vol. 2 of *The Art of Computer Programming* (Reading, MA: Addison-Wesley), pp. 30ff.

Horowitz, P., and Hill, W. 1989, *The Art of Electronics*, 2nd ed. (Cambridge, UK: Cambridge University Press), §9.32 – §9.37.

Tausworthe, R.C. 1965, "Random Numbers Generated by Linear Recurrence Modulo Two," *Mathematics of Computation*, vol. 19, pp. 201–209.

Watson, E.J. 1962, "Primitive Polynomials (Mod 2)," *Mathematics of Computation*, vol. 16, pp. 368–369.[1]

7.6 Hash Tables and Hash Memories

It's a strange dream. You're in a kind of mailroom whose walls are lined with numbered pigeonhole boxes. A man, Mr. Hacher, sits at a table. You are standing. There is an in-basket mounted on the wall. Your job is to take letters from the in-basket and sort them into the pigeonholes.

But how? The letters are addressed by name, while the pigeonholes are only numbered. That is where Mr. Hacher comes in. You show him each letter, and he immediately tells you its pigeonhole number. He always gives the same number for the same name, while different names always get different numbers (and therefore unique pigeonholes).

Over time, as the number of addressees grows, there are fewer and fewer empty boxes until, finally, none at all. This is not a problem as long as letters arrive only for existing boxholders. But one day, you spot a new name on an envelope. With trepidation you put it in front of Mr. Hacher . . . and you wake up!

Mr. Hacher and his table are a *hash table*. A hash table behaves as if it keeps a running ledger of all the *hash keys* (the addressee names) that it has ever seen, assigns a unique number to each, and is able to look through all the names for every new query, either returning the same number as before (for a repeat key) or, for a new key, assigning a new one. There is usually also an option to erase a key.

The goal in implementing a hash table is to make all these functions take only a few computer operations each, not even $O(\log N)$. That is quite a trick, if you think about it. Even if you somehow maintain an ordered or alphabetized list of keys, it will still take $O(\log N)$ operations to find a place in the list, by bisection, say. The big idea behind hash tables is the use of random number techniques (§7.1) to map a hash key to a pseudo-random integer between 0 and $N - 1$, where N is the total number of pigeonholes. Here we definitely want pseudo-random and not random integers, because the same key must produce the same integer each time.

In first approximation, ideally much of the time, that initial pseudo-random integer, called the output of the *hash function*, or (for short) the key's *hash*, is what the hash table puts out, i.e., the number given out by Mr. Hacher. However, it is possible that, by chance, two keys have the same hash; in fact this becomes increasingly probable as the number of distinct keys approaches N, and a certainty when N is exceeded (the *pigeonhole principle*). The implementation of a hash table therefore requires a *collision strategy* that ensures that unique integers are returned, even for (different) keys that have the same hash.

Many vendors' implementations of the C++'s Standard Template Library (STL) provide a hash table as the class `hash_map`. Unfortunately, at this writing, `hash_map` is not a part of the actual STL standard, and the quality of vendor implementations is also quite variable. We therefore here implement our own; thereby we can both learn more about the principles involved and build in some specific features that will be useful later in this book (for example §21.8 and §21.6).

7.6.1 Hash Function Object

By a hash function object we mean a structure that combines a hashing algorithm (as in §7.1) with the "glue" needed to make a hash table. The object should map an arbitrary key type `keyT`, which itself may be a structure containing multiple data values, into (for our implementation) a pseudo-random 64-bit integer. All the hash function object really needs to know about `keyT` is its length in bytes, that is, `sizeof(keyT)`, since it doesn't care how those bytes are used, only that they are part of the key to be hashed. We therefore give the hash function object a constructor that tells it how many bytes to hash; and we let it access a key by a `void` pointer to the key's address. Thus the object can access those bytes any way it wants.

As a first example of a hash function object, let's just put a wrapper around the hash function algorithm of §7.1.4. This is quite efficient when `sizeof(keyT)` $= 4$ or 8.

hash.h

```
struct Hashfn1 {
Example of an object encapsulating a hash function for use by the class Hashmap.
    Ranhash hasher;                          The actual hash function.
    Int n;                                   Size of key in bytes.
    Hashfn1(Int nn) : n(nn) {}               Constructor just saves key size.
    Ullong fn(const void *key) {             Function that returns hash from key.
        Uint *k;
        Ullong *kk;
```

```
        switch (n) {
            case 4:
                k = (Uint *)key;
                return hasher.int64(*k);        Return 64-bit hash of 32-bit key.
            case 8:
                kk = (Ullong *)key;\
                return hasher.int64(*kk);       Return 64-bit hash of 64-bit key.
            default:
                throw("Hashfn1 is for 4 or 8 byte keys only.");
        }
    }
};
```

(Since n is constant for the life of the object, it's a bit inefficient to be testing it on every call; you should edit out the unnecessary code when you know n in advance.)

More generally, a hash function object can be designed to work on arbitrary sized keys by incorporating them into a final hash value a byte at a time. There is a trade-off between speed and degree-of-randomness. Historically, hash functions have favored speed, with simple incorporation rules like

$$\begin{aligned} h_0 &= \text{some fixed constant} \\ h_i &= (m\, h_{i-1} \text{ op } k_i) \mod 2^{32} \qquad (i = 1 \dots K) \end{aligned} \tag{7.6.1}$$

Here k_i is the ith byte of the key ($1 \le i \le K$), m is a multiplier with popular values that include 33, 63689, and $2^{16} + 2^6 - 1$ (doing the multiplication by shifts and adds in the first and third cases), and "op" is either addition or bitwise XOR. You get the mod function for free when you use 32-bit unsigned integer arithmetic. However, since 64-bit arithmetic is fast on modern machines, we think that the days of small multipliers, or many operations changing only a few bits at a time, are over. We favor hash functions that can pass good tests for randomness. (When you know a lot about your keys, it is possible to design hash functions that are even *better* than random, but that is beyond our scope here.)

A hash function object may also do some initialization (of tables, etc.) when it is created. Unlike a random number generator, however, it may not store any history-dependent state between calls, because it must return the same hash for the same key every time. Here is an example of a self-contained hash function object for keys of any length. This is the hash function object that we will use below.

hash.h

```
struct Hashfn2 {
```

Another example of an object encapsulating a hash function, allowing arbitrary fixed key sizes or variable-length null terminated strings. The hash function algorithm is self-contained.

```
    static Ullong hashfn_tab[256];
    Ullong h;
    Int n;                                  Size of key in bytes, when fixed size.
    Hashfn2(Int nn) : n(nn) {
        if (n == 1) n = 0;                  Null terminated string key signaled by n = 0
                                                or 1.
        h = 0x544B2FBACAAF1684LL;
        for (Int j=0; j<256; j++) {         Length 256 lookup table is initialized with
            for (Int i=0; i<31; i++) {          values from a 64-bit Marsaglia generator
                h = (h >>  7) ^ h;              stepped 31 times between each.
                h = (h << 11) ^ h;
                h = (h >> 10) ^ h;
            }
            hashfn_tab[j] = h;
        }
    }
```

```
    Ullong fn(const void *key) {              Function that returns hash from key.
        Int j;
        char *k = (char *)key;                Cast the key pointer to char pointer.
        h=0xBB40E64DA205B064LL;
        j=0;
        while (n ? j++ < n : *k) {            Fixed length or else until null.
            h = (h * 7664345821815920749LL) ^ hashfn_tab[(unsigned char)(*k)];
            k++;
        }
        return h;
    }
};
Ullong Hashfn2::hashfn_tab[256];              Defines storage for the lookup table.
```

The method used is basically equation (7.6.1), but (i) with a large constant that is known to be a good multiplier for a linear congruential random number generator mod 2^{64}, and, more importantly, (ii) a table lookup that substitutes a random (but fixed) 64-bit value for every byte value in $0 \dots 255$. Note also the tweak that allows `Hashfn2` to be used either for fixed length key types (call constructor with $\texttt{n} > 1$) or with null terminated byte arrays of variable length (call constructor with $\texttt{n} = 0$ or 1).

7.6.2 Hash Table

By *hash table* we mean an object with the functionality of Mr. Hacher (and his table) in the dream, namely to turn arbitrary keys into unique integers in a specified range. Let's dive right in. In outline, the `Hashtable` object is

hash.h

```
template<class keyT, class hfnT> struct Hashtable {
Instantiate a hash table, with methods for maintaining a one-to-one correspondence between
arbitrary keys and unique integers in a specified range.
    Int nhash, nmax, nn, ng;
    VecInt htable, next, garbg;
    VecUllong thehash;
    hfnT hash;                          An instance of a hash function object.
    Hashtable(Int nh, Int nv);
    Constructor. Arguments are size of hash table and max number of stored elements (keys).

    Int iget(const keyT &key);          Return integer for a previously set key.
    Int iset(const keyT &key);          Return unique integer for a new key.
    Int ierase(const keyT &key);        Erase a key.
    Int ireserve();                     Reserve an integer (with no key).
    Int irelinquish(Int k);             Un-reserve an integer.
};

template<class keyT, class hfnT>
Hashtable<keyT,hfnT>::Hashtable(Int nh, Int nv):
Constructor. Set nhash, the size of the hash table, and nmax, the maximum number of elements
(keys) that can be accommodated. Allocate arrays appropriately.
    hash(sizeof(keyT)), nhash(nh), nmax(nv), nn(0), ng(0),
    htable(nh), next(nv), garbg(nv), thehash(nv) {
    for (Int j=0; j<nh; j++) { htable[j] = -1; }      Signifies empty.
}
```

A `Hashtable` object is templated by two class names: the class of the key (which may be as simple as `int` or as complicated as a multiply derived class) and the class of the hash function object (e.g., `Hashfn1` or `Hashfn2`, above). Note how the hash function object is automatically created using the size of `keyT`, so the user is not responsible for knowing this value. If you are going to use variable length, null

terminated byte arrays as keys, then the type of keyT is char, not char*; see §7.6.5 for an example.

The hash table object is created from two integer parameters. The most important one is nm, the maximum number of objects that can be stored — in the dream, the number of pigeonholes in the room. For now, suppose that the second parameter, nh, has the same value as nm.

The overall scheme is to convert arbitrary keys into integers in the range 0 ... nh-1 that index into the array htable, by taking the output of the hash function modulo nh. That array's indexed element contains either -1, meaning "empty," or else an index in the range 0...nm-1 that points into the arrays thehash and next. (For a computer science flavor one could do this with list elements linked by pointers, but in the spirit of numerical computation, we will use arrays; both ways are about equally efficient.)

An element in thehash contains the 64-bit hash of whatever key was previously assigned to that index. We will take the identity of two hashes as being positive proof that their keys were identical. Of course this is not really true. There is a probability of $2^{-64} \sim 5 \times 10^{-20}$ of two keys giving identical hashes by chance. To guarantee error-free performance, a hash table must in fact store the actual key, not just the hash; but for our purposes we will accept the very small chance that two elements might get confused. (Don't use these routines if you are typically storing more than a billion elements in a single hash table. But you already knew that!)

This 10^{-20} coincidence is *not* what is meant by *hash collision*. Rather, hash collisions occur when two hashes yield the same value modulo nh, so that they point to the same element in htable. That is not at all unusual, and we must provide for handling it. Elements in the array next contain values that index back into thehash and next, i.e., form a linked list. So, when two or more keys have landed on the same value i, $0 \le i <$ nh, and we want to retrieve a particular one of them, it will either be in the location thehash[i], or else in the (hopefully short) list that starts there and is linked by next[i], next[next[i]], and so forth.

We can now say more about the value that should be initially specified for the parameter nh. For a full table with all nm values assigned, the linked lists attached to each element of htable have lengths that are Poisson distributed with a mean $\lambda \equiv$ nm/nh. Thus, large λ (nh too small) implies a lot of list traversal, while small λ (nh too large) implies wasted space in htable. Conventional wisdom is to choose $\lambda \sim 0.75$, in which case (assuming a good hash function) 47% of htable will be empty, 67% of the nonempty elements will have lists of length one (i.e., you get the correct key on the first try), and the mean number of indirections (steps in traversing the next pointers) is 0.42. For $\lambda = 1$, that is, nh = nm, the values are 37% table empty, 58% first try hits, and 0.58 mean indirections. So, in this general range, any choice is basically fine. The general formulas are

$$\begin{aligned} \text{empty fraction} &= P_\lambda(0) = e^{-\lambda} \\ \text{first try hits} &= P_\lambda(1)/[1 - P_\lambda(0)] = \frac{\lambda e^{-\lambda}}{1 - e^{-\lambda}} \\ \text{mean indirections} &= \sum_{j=2}^{\infty} \frac{(j-1) P_\lambda(j)}{1 - P_\lambda(0)} = \frac{e^{-\lambda} - 1 + \lambda}{1 - e^{-\lambda}} \end{aligned} \tag{7.6.2}$$

where $P_\lambda(j)$ is the Poisson probability function.

Now to the implementations within Hashtable. The simplest to understand is the "get" function, which returns an index value only if the key was previously "set," and returns -1 (by convention) if it was not. Our data structure is designed to make this as fast as possible.

hash.h

```
template<class keyT, class hfnT>
Int Hashtable<keyT,hfnT>::iget(const keyT &key) {
Returns integer in 0..nmax-1 corresponding to key, or −1 if no such key was previously stored.
    Int j,k;
    Ullong pp = hash.fn(&key);                          Get 64-bit hash
    j = (Int)(pp % nhash);                              and map it into the hash table.
    for (k = htable[j]; k != -1; k = next[k]) {         Traverse linked list until an ex-
        if (thehash[k] == pp) {                             act match is found.
            return k;
        }
    }
    return -1;                                          Key was not previously stored.
}
```

A language subtlety to be noted is that iget receives key as a const reference, and then passes its *address*, namely &key, to the hash function object. C++ allows this, because the hash function object's void pointer argument is itself declared as const.

The routine that "sets" a key is slightly more complicated. If the key has previously been set, we want to return the same value as the first time. If it hasn't been set, we initialize the necessary links for the future.

hash.h

```
template<class keyT, class hfnT>
Int Hashtable<keyT,hfnT>::iset(const keyT &key) {
Returns integer in 0..nmax-1 that will henceforth correspond to key. If key was previously set,
return the same integer as before.
    Int j,k,kprev;
    Ullong pp = hash.fn(&key);                  Get 64-bit hash
    j = (Int)(pp % nhash);                      and map it into the hash table.
    if (htable[j] == -1) {                      Key not in table. Find a free integer, either
        k = ng ? garbg[--ng] : nn++ ;               new or previously erased.
        htable[j] = k;
    } else {                                    Key might be in table. Traverse list.
        for (k = htable[j]; k != -1; k = next[k]) {
            if (thehash[k] == pp) {
                return k;                       Yes. Return previous value.
            }
            kprev = k;
        }
        k = ng ? garbg[--ng] : nn++ ;           No. Get new integer.
        next[kprev] = k;
    }
    if (k >= nmax) throw("storing too many values");
    thehash[k] = pp;                            Store the key at the new or previous integer.
    next[k] = -1;
    return k;
}
```

A word here about garbage collection. When a key is erased (by the routine immediately below), we want to make its integer available to future "sets," so that nmax keys can always be stored. This is very easy to implement if we allocate a garbage array (garbg) and use it as a last-in first-out stack of available integers. The set routine above always checks this stack when it needs a new integer. (By the way, had we designed Hashtable with list elements linked by pointers, instead of

arrays, efficient garbage collection would have been more difficult to implement; see Stroustrop [1].)

hash.h

```
template<class keyT, class hfnT>
Int Hashtable<keyT,hfnT>::ierase(const keyT &key) {
```
Erase a key, returning the integer in 0..nmax-1 erased, or −1 if the key was not previously set.
```
    Int j,k,kprev;
    Ullong pp = hash.fn(&key);
    j = (Int)(pp % nhash);
    if (htable[j] == -1) return -1;        Key not previously set.
    kprev = -1;
    for (k = htable[j]; k != -1; k = next[k]) {
        if (thehash[k] == pp) {            Found key. Splice linked list around it.
            if (kprev == -1) htable[j] = next[k];
            else next[kprev] = next[k];
            garbg[ng++] = k;               Add k to garbage stack as an available integer.
            return k;
        }
        kprev = k;
    }
    return -1;                             Key not previously set.
}
```

Finally, Hashtable has routines that reserve and relinquish integers in the range 0 to nmax. When an integer is reserved, it is guaranteed not to be used by the hash table. Below, we'll use this feature as a convenience in constructing a hash memory that can store more than one element under a single key.

hash.h

```
template<class keyT, class hfnT>
Int Hashtable<keyT,hfnT>::ireserve() {
```
Reserve an integer in 0..nmax-1 so that it will not be used by set(), and return its value.
```
    Int k = ng ? garbg[--ng] : nn++ ;
    if (k >= nmax) throw("reserving too many values");
    next[k] = -2;
    return k;
}

template<class keyT, class hfnT>
Int Hashtable<keyT,hfnT>::irelinquish(Int k) {
```
Return to the pool an index previously reserved by reserve(), and return it, or return −1 if it was not previously reserved.
```
    if (next[k] != -2) {return -1;}
    garbg[ng++] = k;
    return k;
}
```

7.6.3 Hash Memory

The Hashtable class, above, implements Mr. Hacher's task. Building on it, we next implement *your* job in the dream, namely to do the actual storage and retrieval of arbitrary objects by arbitrary keys. This is termed a *hash memory*.

When you store into an ordinary computer memory, the value of anything previously stored there is overwritten. If you want your hash memory to behave the same way, then a hash memory class, Hash, derived from Hashtable, is almost trivial to write. The class is templated by three structure types: keyT for the key type; elT for the type of the element that is stored in the hash memory; and hfnT, as before, for the object that encapsulates the hash function of your choice.

hash.h

```
template<class keyT, class elT, class hfnT>
struct Hash : Hashtable<keyT, hfnT> {
```
Extend the Hashtable class with storage for elements of type elT, and provide methods for storing, retrieving. and erasing elements. key is passed by address in all methods.
```
    using Hashtable<keyT,hfnT>::iget;
    using Hashtable<keyT,hfnT>::iset;
    using Hashtable<keyT,hfnT>::ierase;
    vector<elT> els;

    Hash(Int nh, Int nm) : Hashtable<keyT, hfnT>(nh, nm), els(nm) {}
```
Same constructor syntax as Hashtable.
```
    void set(const keyT &key, const elT &el)
```
Store an element el.
```
        {els[iset(key)] = el;}

    Int get(const keyT &key, elT &el) {
```
Retrieve an element into el. Returns 0 if no element is stored under key, or 1 for success.
```
        Int ll = iget(key);
        if (ll < 0) return 0;
        el = els[ll];
        return 1;
    }

    elT& operator[] (const keyT &key) {
```
Store or retrieve an element using subscript notation for its key. Returns a reference that can be used as an l-value.
```
        Int ll = iget(key);
        if (ll < 0) {
            ll = iset(key);
            els[ll] = elT();
        }
        return els[ll];
    }

    Int count(const keyT &key) {
```
Return the number of elements stored under key, that is, either 0 or 1.
```
        Int ll = iget(key);
        return (ll < 0 ? 0 : 1);
    }

    Int erase(const keyT &key) {
```
Erase an element. Returns 1 for success, or 0 if no element is stored under key.
```
        return (ierase(key) < 0 ? 0 : 1);
    }
};
```

The `operator[]` method, above, is intended for two distinct uses. First, it implements an intuitive syntax for storing and retrieving elements, e.g.,

`myhash[` *some-key* `]` = *rhs*

for storing, and

lhs = `myhash[` *some-key* `]`

for retrieving. Note, however, that a small inefficiency is introduced, namely a superfluous call to `get` when an element is set for the first time. Second, the method returns a non-`const` reference that cannot only be used as an l-value, but also be pointed to, as in

some-pointer = &myhash[*some-key*]

Now the stored element can be referenced through the pointer, possibly multiple times, without any additional overhead of key lookup. This can be an important gain in efficiency in some applications. Of course you can also use the `set` and `get` methods directly.

7.6.4 Hash Multimap Memory

Next turn to the case where you want to be able to store *more than one* element under the same key. If ordinary computer memory behaved this way, you could set a variable to a series of values and have it remember all of them! Obviously this is a somewhat more complicated an extension of `Hashtable` than was `Hash`. We will call it `Mhash`, where the M stands for "multivalued" or "multimap." One requirement is to provide a convenient syntax for retrieving multiple values of a single key, one at a time. We do this by the functions `getinit` and `getnext`. Also, in `Mhash`, below, `nmax` now means the maximum number of *values* that can be stored, not the number of keys, which may in general be smaller.

The code, with comments, should be understandable without much additional explanation. We use the `reserve` and `relinquish` features of `Hashtable` so as to have a common numbering system for all stored elements, both the first instance of a key (which `Hashtable` must know about) and subsequent instances of the same key (which are invisible to `Hashtable` but managed by `Mhash` through the linked list `nextsis`).

hash.h

```
template<class keyT, class elT, class hfnT>
struct Mhash : Hashtable<keyT,hfnT> {
```
Extend the `Hashtable` class with storage for elements of type `elT`, allowing more than one element to be stored under a single key.
```
    using Hashtable<keyT,hfnT>::iget;
    using Hashtable<keyT,hfnT>::iset;
    using Hashtable<keyT,hfnT>::ierase;
    using Hashtable<keyT,hfnT>::ireserve;
    using Hashtable<keyT,hfnT>::irelinquish;
    vector<elT> els;
    VecInt nextsis;                      Links to next sister element under a single key.
    Int nextget;
    Mhash(Int nh, Int nm);               Same constructor syntax as Hashtable.
    Int store(const keyT &key, const elT &el);    Store an element under key.
    Int erase(const keyT &key, const elT &el);    Erase a specified element under key.
    Int count(const keyT &key);          Count elements stored under key.
    Int getinit(const keyT &key);        Prepare to retrieve elements from key.
    Int getnext(elT &el);                Retrieve next element specified by getinit.
};

template<class keyT, class elT, class hfnT>
Mhash<keyT,elT,hfnT>::Mhash(Int nh, Int nm)
    : Hashtable<keyT, hfnT>(nh, nm), nextget(-1), els(nm), nextsis(nm) {
    for (Int j=0; j<nm; j++) {nextsis[j] = -2;}   Initialize to "empty".
}

template<class keyT, class elT, class hfnT>
Int Mhash<keyT,elT,hfnT>::store(const keyT &key, const elT &el) {
```
Store an element `el` under `key`. Return index in `0..nmax-1`, giving the storage location utilized.
```
    Int j,k;
    j = iset(key);                       Find root index for this key.
    if (nextsis[j] == -2) {              It is the first object with this key.
```

```
        els[j] = el;
        nextsis[j] = -1;                    −1 means it is the terminal element.
        return j;
    } else {
        while (nextsis[j] != -1) {j = nextsis[j];}    Traverse the tree.
        k = ireserve();                     Get a new index and link it into the list.
        els[k] = el;
        nextsis[j] = k;
        nextsis[k] = -1;
        return k;
    }
}

template<class keyT, class elT, class hfnT>
Int Mhash<keyT,elT,hfnT>::erase(const keyT &key, const elT &el) {
```

Erase an element `el` previously stored under `key`. Return 1 for success, or 0 if no matching element is found. Note: The == operation must be defined for the type `elT`.

```
    Int j = -1,kp = -1,kpp = -1;
    Int k = iget(key);
    while (k >= 0) {
        if (j < 0 && el == els[k]) j = k;       Save index of matching el as j.
        kpp = kp;
        kp = k;
        k=nextsis[k];
    }
    if (j < 0) return 0;                        No matching el found.
    if (kpp < 0) {                              The element el was unique under key.
        ierase(key);
        nextsis[j] = -2;
    } else {                                    Patch the list.
        if (j != kp) els[j] = els[kp];          Overwrite j with the terminal element
        nextsis[kpp] = -1;                          and then shorten the list.
        irelinquish(kp);
        nextsis[kp] = -2;
    }
    return 1;                                   Success.
}

template<class keyT, class elT, class hfnT>
Int Mhash<keyT,elT,hfnT>::count(const keyT &key) {
```

Return the number of elements stored under `key`, 0 if none.

```
    Int next, n = 1;
    if ((next = iget(key)) < 0) return 0;
    while ((next = nextsis[next]) >= 0)  {n++;}
    return n;
}

template<class keyT, class elT, class hfnT>
Int Mhash<keyT,elT,hfnT>::getinit(const keyT &key) {
```

Initialize `nextget` so that it points to the first element stored under `key`. Return 1 for success, or 0 if no such element.

```
    nextget = iget(key);
    return ((nextget < 0)? 0 : 1);
}

template<class keyT, class elT, class hfnT>
Int Mhash<keyT,elT,hfnT>::getnext(elT &el) {
```

If `nextget` points validly, copy its element into `el`, update `nextget` to the next element with the same key, and return 1. Otherwise, do not modify `el`, and return 0.

```
    if (nextget < 0) {return 0;}
    el = els[nextget];
    nextget = nextsis[nextget];
    return 1;
}
```

The methods getinit and getnext are designed to be used in code like this, where myhash is a variable of type Mhash:

```
Retrieve all elements el stored under a single key and do something with them.
if (myhash.getinit(&key)) {
    while (myhash.getnext(el)) {
        Here use the returned element el.
    }
}
```

7.6.5 Usage Examples

Having exposed in such detail the inner workings of the Hash and Mhash classes, we may have left the impression that these are difficult to use. Quite the contrary. Here's a piece code that declares a hash memory for integers, and then stores the birth years of some personages:

```
Hash<string,Int,Hashfn2> year(1000,1000);

year[string("Marie Antoinette")] = 1755;
year[string("Ludwig van Beethoven")] = 1770;
year[string("Charles Babbage")] = 1791;
```

As declared, year can hold up to 1000 entries. We use the C++ string class as the key type. If we want to know how old Marie was when Charles was born, we can write,

```
Int diff = year[string("Charles Babbage")] - year[string("Marie Antoinette")];
cout << diff << '\n';
```

which prints "36".

Instead of using the C++ string class, you can, if you must, use null terminated C strings as keys, like this:

```
Hash<char,Int,Hashfn2> yearc(1000,1000);
yearc["Charles Babbage"[0]] = 1791;
```

This works because Hashfn2 has a special tweak, mentioned above, for key types that are apparently one byte long. Note the required use of [0] to send only the first byte of the C string; but that byte is passed by address, so Hashfn2 knows where to find the rest of the string. (The syntax yearc[*"Charles Babbage"] is equivalent, also sending the first byte.)

Suppose we want to go the other direction, namely store the names of people into a hash memory indexed by birth year. Since more than one person may be born in a single year, we want to use a hash multimap memory, Mhash:

```
Mhash<Int,string,Hashfn2> person(1000,1000);

person.store(1775, string("Jane Austen"));
person.store(1791, string("Charles Babbage"));
person.store(1767, string("Andrew Jackson"));
person.store(1791, string("James Buchanan"));
person.store(1767, string("John Quincy Adams"));
person.store(1770, string("Ludwig van Beethoven"));
person.store(1791, string("Samuel Morse"));
person.store(1755, string("Marie Antoinette"));
```

It doesn't matter, of course, the order in which we put the names into the hash. Here is a piece of code to loop over years, printing the people born in that year:

```
string str;
for (Int i=1750;i<1800;i++) {
    if (person.getinit(i)) {
        cout << '\n' << "born in " << i << ":\n";
        while (person.getnext(str)) cout << str.data() << '\n';
    }
}
```

which gives as output

```
born in 1755:
Marie Antoinette

born in 1767:
Andrew Jackson
John Quincy Adams

born in 1770:
Ludwig van Beethoven

born in 1775:
Jane Austen

born in 1791:
Charles Babbage
James Buchanan
Samuel Morse
```

Notice that we could *not* have used null terminated C strings in this example, because C++ does not regard them as *first-class objects* that can be stored as elements of a vector. When you are using `Hash` or `Mhash` with strings, you will usually be better off using the C++ `string` class.

In §21.2 and §21.8 we will make extensive use of both the `Hash` and `Mhash` classes and almost all their member functions; look there for further usage examples.

By the way, Mr. Hacher's name is from the French *hacher*, meaning "to mince or hash."

CITED REFERENCES AND FURTHER READING:

Stroustrup, B. 1997, *The C++ Programming Language*, 3rd ed. (Reading, MA: Addison-Wesley), §17.6.2.[1]

Knuth, D.E. 1997, *Sorting and Searching*, 3rd ed., vol. 3 of *The Art of Computer Programming* (Reading, MA: Addison-Wesley), §6.4–§6.5.

Vitter, J.S., and Chen, W-C. 1987, *Design and Analysis of Coalesced Hashing* (New York: Oxford University Press).

7.7 Simple Monte Carlo Integration

Inspirations for numerical methods can spring from unlikely sources. "Splines" first were flexible strips of wood used by draftsmen. "Simulated annealing" (we shall

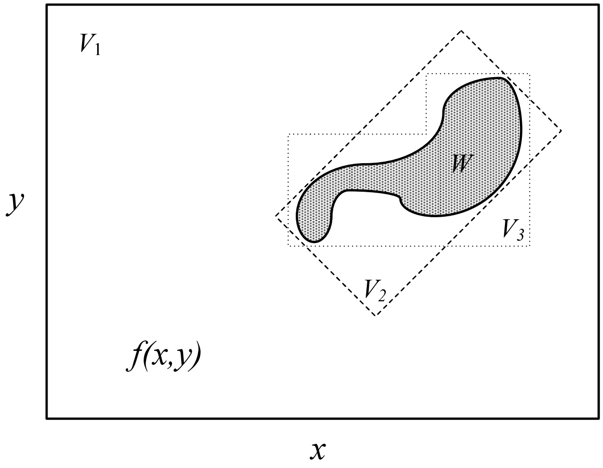

Figure 7.7.1. Monte Carlo integration of a function $f(x, y)$ in a region W. Random points are chosen within an area V that includes W and that can easily be sampled uniformly. Of the three possible V's shown, V_1 is a poor choice because W occupies only a small fraction of its area, while V_2 and V_3 are better choices.

see in §10.12) is rooted in a thermodynamic analogy. And who does not feel at least a faint echo of glamor in the name "Monte Carlo method"?

Suppose that we pick N random points, uniformly distributed in a multidimensional volume V. Call them $x_0, \ldots, x_{N-1}$. Then the basic theorem of Monte Carlo integration estimates the integral of a function f over the multidimensional volume,

$$\int f\, dV \approx V \langle f \rangle \pm V \sqrt{\frac{\langle f^2 \rangle - \langle f \rangle^2}{N}} \qquad (7.7.1)$$

Here the angle brackets denote taking the arithmetic mean over the N sample points,

$$\langle f \rangle \equiv \frac{1}{N} \sum_{i=0}^{N-1} f(x_i) \qquad \langle f^2 \rangle \equiv \frac{1}{N} \sum_{i=0}^{N-1} f^2(x_i) \qquad (7.7.2)$$

The "plus-or-minus" term in (7.7.1) is a one standard deviation error estimate for the integral, not a rigorous bound; further, there is no guarantee that the error is distributed as a Gaussian, so the error term should be taken only as a rough indication of probable error.

Suppose that you want to integrate a function g over a region W that is not easy to sample randomly. For example, W might have a very complicated shape. No problem. Just find a region V that *includes* W and that *can* easily be sampled, and then define f to be equal to g for points in W and equal to zero for points outside of W (but still inside the sampled V). You want to try to make V enclose W as closely as possible, because the zero values of f will increase the error estimate term of (7.7.1). And well they should: Points chosen outside of W have no information content, so the effective value of N, the number of points, is reduced. The error estimate in (7.7.1) takes this into account.

Figure 7.7.1 shows three possible regions V that might be used to sample a complicated region W. The first, V_1, is obviously a poor choice. A good choice, V_2,

can be sampled by picking a pair of uniform deviates (s, t) and then mapping them into (x, y) by a linear transformation. Another good choice, V_3, can be sampled by, first, using a uniform deviate to choose between the left and right rectangular subregions (in proportion to their respective areas!) and, then, using two more deviates to pick a point inside the chosen rectangle.

Let's create an object that embodies the general scheme described. (We will discuss the implementing code later.) The general idea is to create an `MCintegrate` object by providing (as constructor arguments) the following items:

- a vector `xlo` of lower limits of the coordinates for the rectangular box to be sampled
- a vector `xhi` of upper limits of the coordinates for the rectangular box to be sampled
- a vector-valued function `funcs` that returns as its components one or more functions that we want to integrate simultaneously
- a boolean function that returns whether a point is in the (possibly complicated) region W that we want to integrate; the point will already be within the region V defined by `xlo` and `xhi`
- a mapping function to be discussed below, or `NULL` if there is no mapping function or if your attention span is too short
- a seed for the random number generator

The object `MCintegrate` has this structure.

mcintegrate.h

```
struct MCintegrate {
Object for Monte Carlo integration of one or more functions in an ndim-dimensional region.
    Int ndim,nfun,n;                   Number of dimensions, functions, and points sampled.
    VecDoub ff,fferr;                  Answers: The integrals and their standard errors.
    VecDoub xlo,xhi,x,xx,fn,sf,sferr;
    Doub vol;                          Volume of the box V.

    VecDoub (*funcsp)(const VecDoub &);         Pointers to the user-supplied functions.
    VecDoub (*xmapp)(const VecDoub &);
    Bool (*inregionp)(const VecDoub &);
    Ran ran;                                    Random number generator.

    MCintegrate(const VecDoub &xlow, const VecDoub &xhigh,
    VecDoub funcs(const VecDoub &), Bool inregion(const VecDoub &),
    VecDoub xmap(const VecDoub &), Int ranseed);
    Constructor. The arguments are in the order described in the itemized list above.

    void step(Int nstep);
    Sample an additional nstep points, accumulating the various sums.

    void calcanswers();
    Calculate answers ff and fferr using the current sums.
};
```

The member function `step` adds sample points, the number of which is given by its argument. The member function `calcanswers` updates the vectors `ff` and `fferr`, which contain respectively the estimated Monte Carlo integrals of the functions and the errors on these estimates. You can examine these values, and then, if you want, call `step` and `calcanswers` again to further reduce the errors.

A worked example will show the underlying simplicity of the method. Suppose that we want to find the weight and the position of the center of mass of an object of

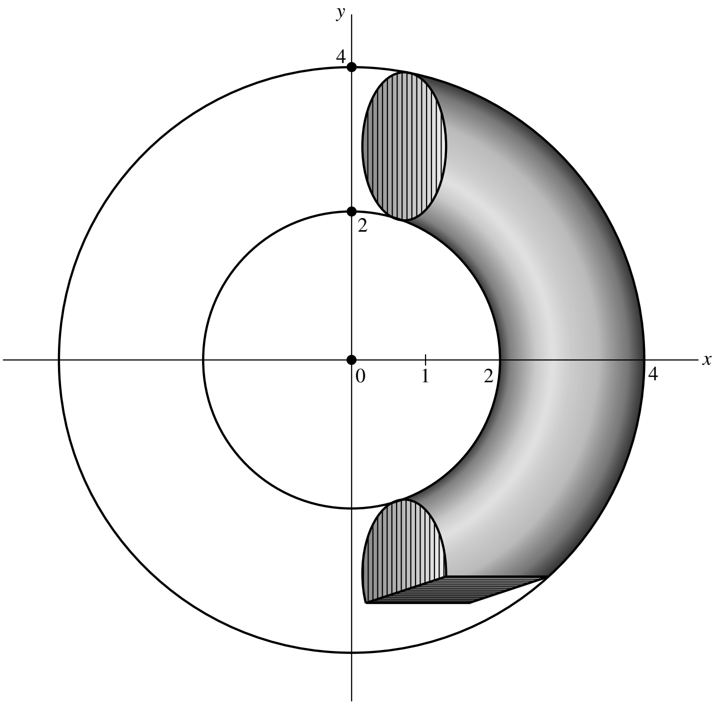

Figure 7.7.2. Example of Monte Carlo integration (see text). The region of interest is a piece of a torus, bounded by the intersection of two planes. The limits of integration of the region cannot easily be written in analytically closed form, so Monte Carlo is a useful technique.

complicated shape, namely the intersection of a torus with the faces of a large box. In particular, let the object be defined by the three simultaneous conditions:

$$z^2 + \left(\sqrt{x^2 + y^2} - 3\right)^2 \leq 1 \qquad (7.7.3)$$

(torus centered on the origin with major radius = 3, minor radius = 1)

$$x \geq 1 \qquad y \geq -3 \qquad (7.7.4)$$

(two faces of the box; see Figure 7.7.2). Suppose for the moment that the object has a constant density $\rho = 1$.

We want to estimate the following integrals over the interior of the complicated object:

$$\int \rho\, dx\, dy\, dz \qquad \int x\rho\, dx\, dy\, dz \qquad \int y\rho\, dx\, dy\, dz \qquad \int z\rho\, dx\, dy\, dz \qquad (7.7.5)$$

The coordinates of the center of mass will be the ratio of the latter three integrals (linear moments) to the first one (the weight).

To use the `MCintegrate` object, we first write functions that describe the integrands and the region of integration W inside the box V.

mcintegrate.h

```
VecDoub torusfuncs(const VecDoub &x) {
Return the integrands in equation (7.7.5), with ρ = 1.
    Doub den = 1.;
    VecDoub f(4);
    f[0] = den;
    for (Int i=1;i<4;i++) f[i] = x[i-1]*den;
    return f;
}

Bool torusregion(const VecDoub &x) {
Return the inequality (7.7.3).
    return SQR(x[2])+SQR(sqrt(SQR(x[0])+SQR(x[1]))-3.) <= 1.;
}
```

The code to actually do the integration is now quite simple,

```
VecDoub xlo(3), xhi(3);
xlo[0] = 1.; xhi[0] = 4.;
xlo[1] = -3.; xhi[1] = 4.;
xlo[2] = -1.; xhi[2] = 1.;
MCintegrate mymc(xlo,xhi,torusfuncs,torusregion,NULL,10201);
mymc.step(1000000);
mymc.calcanswers();
```

Here we've specified the box V by `xlo` and `xhi`, created an instance of `MCintegrate`, sampled a million times, and updated the answers `mymc.ff` and `mymc.fferr`, which can be accessed for printing or another use.

7.7.1 Change of Variables

A change of variable can often be extremely worthwhile in Monte Carlo integration. Suppose, for example, that we want to evaluate the same integrals, but for a piece of torus whose density is a strong function of z, in fact varying according to

$$\rho(x, y, z) = e^{5z} \tag{7.7.6}$$

One way to do this is, in `torusfuncs`, simply to replace the statement

```
Doub den = 1.;
```

by the statement

```
Doub den = exp(5.*x[2]);
```

This will work, but it is not the best way to proceed. Since (7.7.6) falls so rapidly to zero as z decreases (down to its lower limit -1), most sampled points contribute almost nothing to the sum of the weight or moments. These points are effectively wasted, almost as badly as those that fall outside of the region W. A change of variable, exactly as in the transformation methods of §7.3, solves this problem. Let

$$ds = e^{5z} dz \qquad \text{so that} \qquad s = \tfrac{1}{5}e^{5z}, \quad z = \tfrac{1}{5}\ln(5s) \tag{7.7.7}$$

Then $\rho dz = ds$, and the limits $-1 < z < 1$ become $.00135 < s < 29.682$.

The `MCintegrate` object knows that you might want to do this. If it sees an argument `xmap` that is not `NULL`, it will assume that the sampling region defined by `xlo` and `xhi` is not in physical space, but rather needs to be mapped into physical space before either the functions or the region boundary are calculated. Thus, to effect our change of variable, we *don't* need to modify `torusfuncs` or `torusregion`, but we *do* need to modify `xlo` and `xhi`, as well as supply the following function for the argument `xmap`:

mcintegrate.h

```
VecDoub torusmap(const VecDoub &s) {
Return the mapping from s to z defined by the last equation in (7.7.7), mapping the other coordinates by the identity map.
    VecDoub xx(s);
    xx[2] = 0.2*log(5.*s[2]);
    return xx;
}
```

Code for the actual integration now looks like this:

```
    VecDoub slo(3), shi(3);
    slo[0] = 1.; shi[0] = 4.;
    slo[1] = -3.; shi[1] = 4.;
    slo[2] = 0.2*exp(5.*(-1.)); shi[2] = 0.2*exp(5.*(1.));
    MCintegrate mymc2(slo,shi,torusfuncs,torusregion,torusmap,10201);
    mymc2.step(1000000);
    mymc2.calcanswers();
```

If you think for a minute, you will realize that equation (7.7.7) was useful only because the part of the integrand that we wanted to eliminate (e^{5z}) was both integrable analytically and had an integral that could be analytically inverted. (Compare §7.3.2.) In general these properties will not hold. Question: What then? Answer: Pull out of the integrand the "best" factor that *can* be integrated and inverted. The criterion for "best" is to try to reduce the remaining integrand to a function that is as close as possible to constant.

The limiting case is instructive: If you manage to make the integrand f *exactly* constant, and if the region V, of known volume, *exactly* encloses the desired region W, then the average of f that you compute will be exactly its constant value, and the error estimate in equation (7.7.1) will exactly vanish. You will, in fact, have done the integral exactly, and the Monte Carlo numerical evaluations are superfluous. So, backing off from the extreme limiting case, *to the extent* that you are able to make f approximately constant by change of variable, and *to the extent* that you can sample a region only slightly larger than W, you will increase the accuracy of the Monte Carlo integral. This technique is generically called *reduction of variance* in the literature.

The fundamental disadvantage of simple Monte Carlo integration is that its accuracy increases only as the square root of N, the number of sampled points. If your accuracy requirements are modest, or if your computer is large, then the technique is highly recommended as one of great generality. In §7.8 and §7.9 we will see that there are techniques available for "breaking the square root of N barrier" and achieving, at least in some cases, higher accuracy with fewer function evaluations.

There should be nothing surprising in the implementation of `MCintegrate`. The constructor stores pointers to the user functions, makes an otherwise superfluous call to `funcs` just to find out the size of returned vector, and then sizes the sum and answer vectors accordingly. The `step` and `calcanswer` methods implement exactly equations (7.7.1) and (7.7.2).

mcintegrate.h

```
MCintegrate::MCintegrate(const VecDoub &xlow, const VecDoub &xhigh,
    VecDoub funcs(const VecDoub &), Bool inregion(const VecDoub &),
    VecDoub xmap(const VecDoub &), Int ranseed)
    : ndim(xlow.size()), n(0), xlo(xlow), xhi(xhigh), x(ndim), xx(ndim),
    funcsp(funcs), xmapp(xmap), inregionp(inregion), vol(1.), ran(ranseed) {
    if (xmapp) nfun = funcs(xmapp(xlo)).size();
    else nfun = funcs(xlo).size();
    ff.resize(nfun);
    fferr.resize(nfun);
```

```
    fn.resize(nfun);
    sf.assign(nfun,0.);
    sferr.assign(nfun,0.);
    for (Int j=0;j<ndim;j++) vol *= abs(xhi[j]-xlo[j]);
}

void MCintegrate::step(Int nstep) {
    Int i,j;
    for (i=0;i<nstep;i++) {
        for (j=0;j<ndim;j++)
            x[j] = xlo[j]+(xhi[j]-xlo[j])*ran.doub();
        if (xmapp) xx = (*xmapp)(x);
        else xx = x;
        if ((*inregionp)(xx)) {
            fn = (*funcsp)(xx);
            for (j=0;j<nfun;j++) {
                sf[j] += fn[j];
                sferr[j] += SQR(fn[j]);
            }
        }
    }
    n += nstep;
}

void MCintegrate::calcanswers(){
    for (Int j=0;j<nfun;j++) {
        ff[j] = vol*sf[j]/n;
        fferr[j] = vol*sqrt((sferr[j]/n-SQR(sf[j]/n))/n);
    }
}
```

CITED REFERENCES AND FURTHER READING:

Robert, C.P., and Casella, G. 2006, *Monte Carlo Statistical Methods*, 2nd ed. (New York: Springer)

Sobol', I.M. 1994, *A Primer for the Monte Carlo Method* (Boca Raton, FL: CRC Press).

Hammersley, J.M., and Handscomb, D.C. 1964, *Monte Carlo Methods* (London: Methuen).

Gentle, J.E. 2003, *Random Number Generation and Monte Carlo Methods*, 2nd ed. (New York: Springer), Chapter 7.

Shreider, Yu. A. (ed.) 1966, *The Monte Carlo Method* (Oxford: Pergamon).

Kalos, M.H., and Whitlock, P.A. 1986, *Monte Carlo Methods: Volume 1: Basics* (New York: Wiley).

7.8 Quasi- (that is, Sub-) Random Sequences

We have just seen that choosing N points uniformly randomly in an n-dimensional space leads to an error term in Monte Carlo integration that decreases as $1/\sqrt{N}$. In essence, each new point sampled adds linearly to an accumulated sum that will become the function average, and also linearly to an accumulated sum of squares that will become the variance (equation 7.7.2). The estimated error comes from the square root of this variance, hence the power $N^{-1/2}$.

Just because this square-root convergence is familiar does not, however, mean that it is inevitable. A simple counterexample is to choose sample points that lie on a Cartesian grid, and to sample each grid point exactly once (in whatever order).

The Monte Carlo method thus becomes a deterministic quadrature scheme — albeit a simple one — whose fractional error decreases at least as fast as N^{-1} (even faster if the function goes to zero smoothly at the boundaries of the sampled region or is periodic in the region).

The trouble with a grid is that one has to decide *in advance* how fine it should be. One is then committed to completing all of its sample points. With a grid, it is not convenient to "sample *until*" some convergence or termination criterion is met. One might ask if there is not some intermediate scheme, some way to pick sample points "at random," yet spread out in some self-avoiding way, avoiding the chance clustering that occurs with uniformly random points.

A similar question arises for tasks other than Monte Carlo integration. We might want to search an n-dimensional space for a point where some (locally computable) condition holds. Of course, for the task to be computationally meaningful, there had better be continuity, so that the desired condition will hold in some finite n-dimensional neighborhood. We may not know a priori how large that neighborhood is, however. We want to "sample *until*" the desired point is found, moving smoothly to finer scales with increasing samples. Is there any way to do this that is better than uncorrelated, random samples?

The answer to the above question is "yes." Sequences of n-tuples that fill n-space more uniformly than uncorrelated random points are called *quasi-random sequences*. That term is somewhat of a misnomer, since there is nothing "random" about quasi-random sequences: They are cleverly crafted to be, in fact, *sub*random. The sample points in a quasi-random sequence are, in a precise sense, "maximally avoiding" of each other.

A conceptually simple example is *Halton's sequence* [1]. In one dimension, the jth number H_j in the sequence is obtained by the following steps: (i) Write j as a number in base b, where b is some prime. (For example, $j = 17$ in base $b = 3$ is 122.) (ii) Reverse the digits and put a radix point (i.e., a decimal point base b) in front of the sequence. (In the example, we get 0.221 base 3.) The result is H_j. To get a sequence of n-tuples in n-space, you make each component a Halton sequence with a different prime base b. Typically, the first n primes are used.

It is not hard to see how Halton's sequence works: Every time the number of digits in j increases by one place, j's digit-reversed fraction becomes a factor of b finer-meshed. Thus the process is one of filling in all the points on a sequence of finer and finer Cartesian grids — and in a kind of maximally spread-out order on each grid (since, e.g., the most rapidly changing digit in j controls the *most* significant digit of the fraction).

Other ways of generating quasi-random sequences have been proposed by Faure, Sobol', Niederreiter, and others. Bratley and Fox [2] provide a good review and references, and discuss a particularly efficient variant of the Sobol' [3] sequence suggested by Antonov and Saleev [4]. It is this Antonov-Saleev variant whose implementation we now discuss.

The Sobol' sequence generates numbers between zero and one directly as binary fractions of length w bits, from a set of w special binary fractions, V_i, $i = 1, 2, \dots, w$, called *direction numbers*. In Sobol's original method, the jth number X_j is generated by XORing (bitwise exclusive or) together the set of V_i's satisfying the criterion on i, "the ith bit of j is nonzero." As j increments, in other words, different ones of the V_i's flash in and out of X_j on different time scales. V_1 alternates between being present and absent most quickly, while

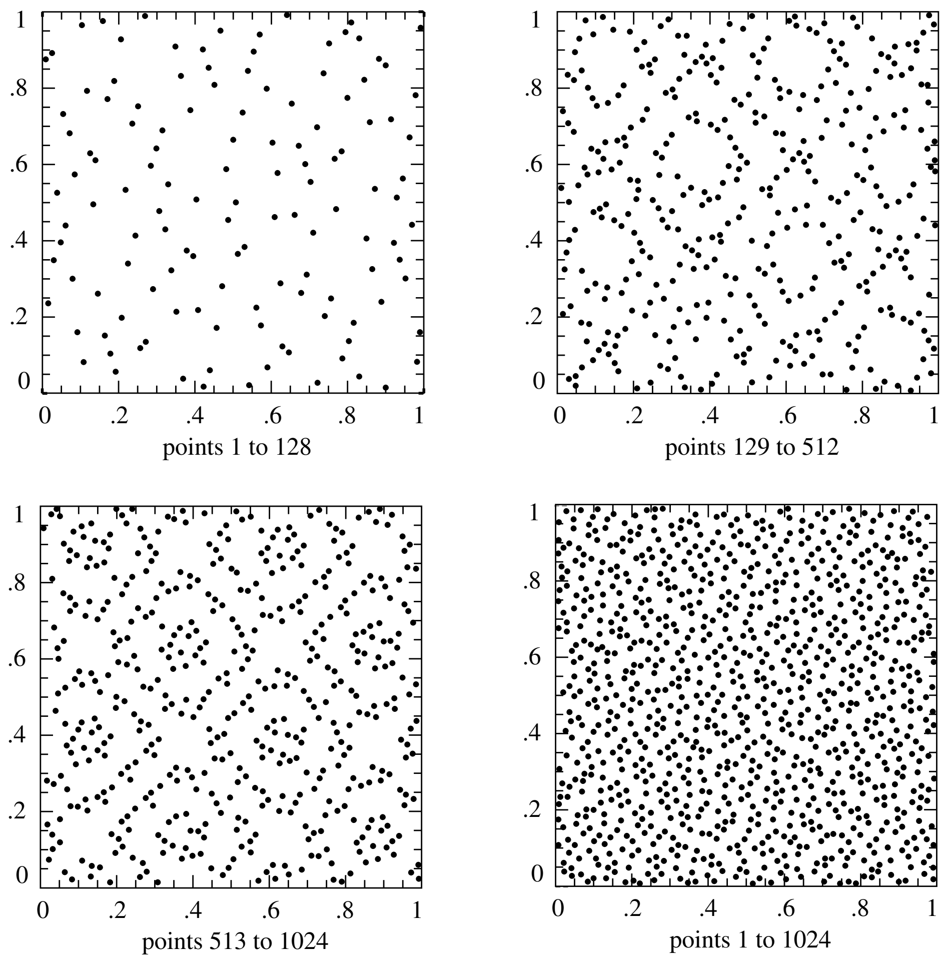

Figure 7.8.1. First 1024 points of a two-dimensional Sobol' sequence. The sequence is generated number-theoretically, rather than randomly, so successive points at any stage "know" how to fill in the gaps in the previously generated distribution.

V_k goes from present to absent (or vice versa) only every 2^{k-1} steps.

Antonov and Saleev's contribution was to show that instead of using the bits of the integer j to select direction numbers, one could just as well use the bits of the *Gray code* of j, $G(j)$. (For a quick review of Gray codes, look at §22.3.)

Now $G(j)$ and $G(j+1)$ differ in exactly one bit position, namely in the position of the rightmost zero bit in the binary representation of j (adding a leading zero to j if necessary). A consequence is that the $j+1$st Sobol'-Antonov-Saleev number can be obtained from the jth by XORing it with *a single* V_i, namely with i the position of the rightmost zero bit in j. This makes the calculation of the sequence very efficient, as we shall see.

Figure 7.8.1 plots the first 1024 points generated by a two-dimensional Sobol' sequence. One sees that successive points do "know" about the gaps left previously, and keep filling them in, hierarchically.

We have deferred to this point a discussion of how the direction numbers V_i are generated. Some nontrivial mathematics is involved in that, so we will content ourselves with a cookbook summary only: Each different Sobol' sequence (or component of an n-dimensional

Degree	Primitive Polynomials Modulo 2*
1	0 (i.e., $x + 1$)
2	1 (i.e., $x^2 + x + 1$)
3	1, 2 (i.e., $x^3 + x + 1$ and $x^3 + x^2 + 1$)
4	1, 4 (i.e., $x^4 + x + 1$ and $x^4 + x^3 + 1$)
5	2, 4, 7, 11, 13, 14
6	1, 13, 16, 19, 22, 25
7	1, 4, 7, 8, 14, 19, 21, 28, 31, 32, 37, 41, 42, 50, 55, 56, 59, 62
8	14, 21, 22, 38, 47, 49, 50, 52, 56, 67, 70, 84, 97, 103, 115, 122
9	8, 13, 16, 22, 25, 44, 47, 52, 55, 59, 62, 67, 74, 81, 82, 87, 91, 94, 103, 104, 109, 122, 124, 137, 138, 143, 145, 152, 157, 167, 173, 176, 181, 182, 185, 191, 194, 199, 218, 220, 227, 229, 230, 234, 236, 241, 244, 253
10	4, 13, 19, 22, 50, 55, 64, 69, 98, 107, 115, 121, 127, 134, 140, 145, 152, 158, 161, 171, 181, 194, 199, 203, 208, 227, 242, 251, 253, 265, 266, 274, 283, 289, 295, 301, 316, 319, 324, 346, 352, 361, 367, 382, 395, 398, 400, 412, 419, 422, 426, 428, 433, 446, 454, 457, 472, 493, 505, 508

*Expressed as a decimal integer whose binary representation gives the coefficients, from the highest to lowest power of x. Only the internal terms are represented — the highest-order term and the constant term always have coefficient 1.

sequence) is based on a different primitive polynomial over the integers modulo 2, that is, a polynomial whose coefficients are either 0 or 1, and which generates a maximal length shift register sequence. (Primitive polynomials modulo 2 were used in §7.5 and are further discussed in §22.4.) Suppose P is such a polynomial, of degree q,

$$P = x^q + a_1 x^{q-1} + a_2 x^{q-2} + \cdots + a_{q-1} x + 1 \qquad (7.8.1)$$

Define a sequence of integers M_i by the q-term recurrence relation,

$$M_i = 2a_1 M_{i-1} \oplus 2^2 a_2 M_{i-2} \oplus \cdots \oplus 2^{q-1} M_{i-q+1} a_{q-1} \oplus (2^q M_{i-q} \oplus M_{i-q}) \qquad (7.8.2)$$

Here bitwise XOR is denoted by $\oplus$. The starting values for this recurrence are that $M_1, \ldots, M_q$ can be arbitrary odd integers less than $2, \ldots, 2^q$, respectively. Then, the direction numbers V_i are given by

$$V_i = M_i / 2^i \qquad i = 1, \ldots, w \qquad (7.8.3)$$

The table above lists all primitive polynomials modulo 2 with degree $q \leq 10$. Since the coefficients are either 0 or 1, and since the coefficients of x^q and of 1 are predictably 1, it is convenient to denote a polynomial by its middle coefficients taken as the bits of a binary number (higher powers of x being more significant bits). The table uses this convention.

Turn now to the implementation of the Sobol' sequence. Successive calls to the function `sobseq` (after a preliminary initializing call) return successive points in an n-dimensional Sobol' sequence based on the first n primitive polynomials in the table. As given, the routine is initialized for maximum n of 6 dimensions, and for a word length w of 30 bits. These parameters can be altered by changing `MAXBIT` ($\equiv w$) and `MAXDIM`, and by adding more initializing data to the arrays `ip` (the primitive polynomials from the table above), `mdeg` (their degrees), and `iv` (the starting values for the recurrence, equation 7.8.2). A second table, on the next page, elucidates the initializing data in the routine.

Initializing Values Used in sobseq					
Degree	Polynomial	Starting Values			
1	0	1	(3)	(5)	(15) ...
2	1	1	1	(7)	(11) ...
3	1	1	3	7	(5) ...
3	2	1	3	3	(15) ...
4	1	1	1	3	13 ...
4	4	1	1	5	9 ...
Parenthesized values are not freely specifiable, but are forced by the required recurrence for this degree.					

sobseq.h

```
void sobseq(const Int n, VecDoub_O &x)
```

When n is negative, internally initializes a set of MAXBIT direction numbers for each of MAXDIM different Sobol' sequences. When n is positive (but ≤MAXDIM), returns as the vector x[0..n-1] the next values from n of these sequences. (n must not be changed between initializations.)

```
{
    const Int MAXBIT=30,MAXDIM=6;
    Int j,k,l;
    Uint i,im,ipp;
    static Int mdeg[MAXDIM]={1,2,3,3,4,4};
    static Uint in;
    static VecUint ix(MAXDIM);
    static NRvector<Uint*> iu(MAXBIT);
    static Uint ip[MAXDIM]={0,1,1,2,1,4};
    static Uint iv[MAXDIM*MAXBIT]=
        {1,1,1,1,1,1,3,1,3,3,1,1,5,7,7,3,3,5,15,11,5,15,13,9};
    static Doub fac;

    if (n < 0) {                                        Initialize, don't return a vector.
        for (k=0;k<MAXDIM;k++) ix[k]=0;
        in=0;
        if (iv[0] != 1) return;
        fac=1.0/(1 << MAXBIT);
        for (j=0,k=0;j<MAXBIT;j++,k+=MAXDIM) iu[j] = &iv[k];
        To allow both 1D and 2D addressing.
        for (k=0;k<MAXDIM;k++) {
            for (j=0;j<mdeg[k];j++) iu[j][k] <<= (MAXBIT-1-j);
            Stored values only require normalization.
            for (j=mdeg[k];j<MAXBIT;j++) {              Use the recurrence to get other values.
                ipp=ip[k];
                i=iu[j-mdeg[k]][k];
                i ^= (i >> mdeg[k]);
                for (l=mdeg[k]-1;l>=1;l--) {
                    if (ipp & 1) i ^= iu[j-l][k];
                    ipp >>= 1;
                }
                iu[j][k]=i;
            }
        }
    } else {                                            Calculate the next vector in the sequence.
        im=in++;
        for (j=0;j<MAXBIT;j++) {                        Find the rightmost zero bit.
            if (!(im & 1)) break;
            im >>= 1;
        }
```

```
        if (j >= MAXBIT) throw("MAXBIT too small in sobseq");
        im=j*MAXDIM;
        for (k=0;k<MIN(n,MAXDIM);k++) {          XOR the appropriate direction num-
            ix[k] ^= iv[im+k];                       ber into each component of the
            x[k]=ix[k]*fac;                          vector and convert to a floating
        }                                            number.
    }
}
```

How good is a Sobol' sequence, anyway? For Monte Carlo integration of a smooth function in n dimensions, the answer is that the fractional error will decrease with N, the number of samples, as $(\ln N)^n/N$, i.e., almost as fast as $1/N$. As an example, let us integrate a function that is nonzero inside a torus (doughnut) in three-dimensional space. If the major radius of the torus is R_0 and the minor radius is r_0, the minor radial coordinate r is defined by

$$r = \left([(x^2+y^2)^{1/2} - R_0]^2 + z^2\right)^{1/2} \tag{7.8.4}$$

Let us try the function

$$f(x,y,z) = \begin{cases} 1+\cos\left(\dfrac{\pi r^2}{r_0^2}\right) & r < r_0 \\ 0 & r \geq r_0 \end{cases} \tag{7.8.5}$$

which can be integrated analytically in cylindrical coordinates, giving

$$\iiint dx\,dy\,dz\; f(x,y,z) = 2\pi^2 r_0^2 R_0 \tag{7.8.6}$$

With parameters $R_0 = 0.6$, $r_0 = 0.3$, we did 100 successive Monte Carlo integrations of equation (7.8.4), sampling uniformly in the region $-1 < x,y,z < 1$, for the two cases of uncorrelated random points and the Sobol' sequence generated by the routine `sobseq`. Figure 7.8.2 shows the results, plotting the r.m.s. average error of the 100 integrations as a function of the number of points sampled. (For any *single* integration, the error of course wanders from positive to negative, or vice versa, so a logarithmic plot of fractional error is not very informative.) The thin, dashed curve corresponds to uncorrelated random points and shows the familiar $N^{-1/2}$ asymptotics. The thin, solid gray curve shows the result for the Sobol' sequence. The logarithmic term in the expected $(\ln N)^3/N$ is readily apparent as curvature in the curve, but the asymptotic N^{-1} is unmistakable.

To understand the importance of Figure 7.8.2, suppose that a Monte Carlo integration of f with 1% accuracy is desired. The Sobol' sequence achieves this accuracy in a few thousand samples, while pseudo-random sampling requires nearly 100,000 samples. The ratio would be even greater for higher desired accuracies.

A different, not quite so favorable, case occurs when the function being integrated has hard (discontinuous) boundaries inside the sampling region, for example the function that is one inside the torus and zero outside,

$$f(x,y,z) = \begin{cases} 1 & r < r_0 \\ 0 & r \geq r_0 \end{cases} \tag{7.8.7}$$

where r is defined in equation (7.8.4). Not by coincidence, this function has the same analytic integral as the function of equation (7.8.5), namely $2\pi^2 r_0^2 R_0$.

The carefully hierarchical Sobol' sequence is based on a set of Cartesian grids, but the boundary of the torus has no particular relation to those grids. The result is that it is essentially random whether sampled points in a thin layer at the surface of the torus, containing on the order of $N^{2/3}$ points, come out to be inside or outside the torus. The square root law, applied to this thin layer, gives $N^{1/3}$ fluctuations in the sum, or $N^{-2/3}$ fractional error in the Monte Carlo integral. One sees this behavior verified in Figure 7.8.2 by the thicker gray curve. The

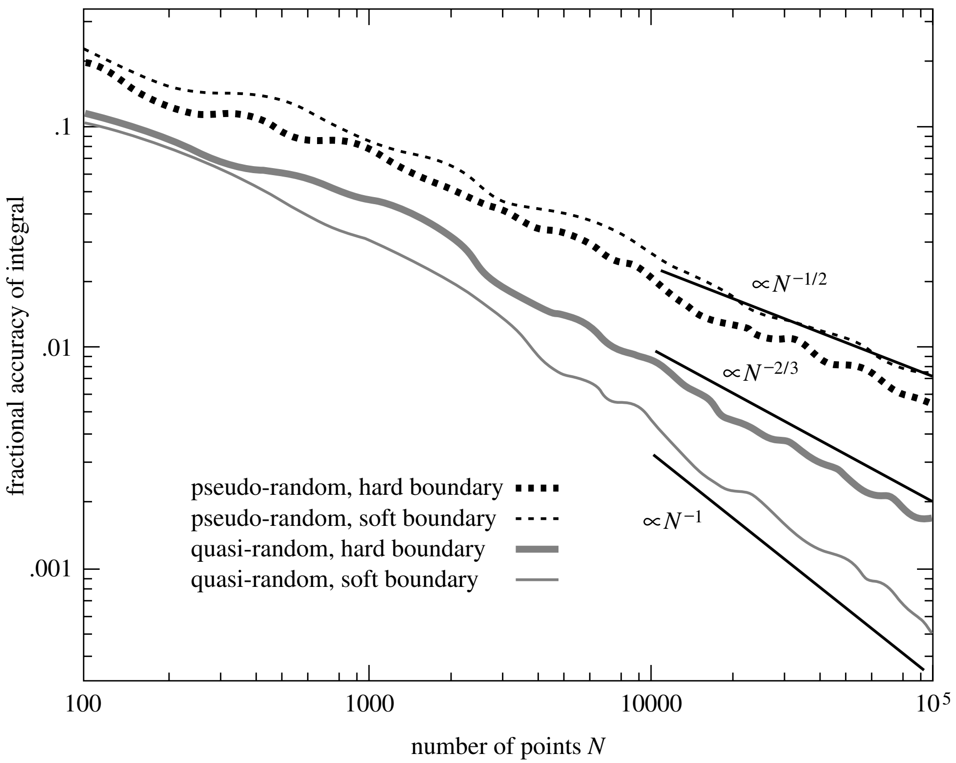

Figure 7.8.2. Fractional accuracy of Monte Carlo integrations as a function of number of points sampled, for two different integrands and two different methods of choosing random points. The quasi-random Sobol' sequence converges much more rapidly than a conventional pseudo-random sequence. Quasi-random sampling does better when the integrand is smooth ("soft boundary") than when it has step discontinuities ("hard boundary"). The curves shown are the r.m.s. averages of 100 trials.

thicker dashed curve in Figure 7.8.2 is the result of integrating the function of equation (7.8.7) using independent random points. While the advantage of the Sobol' sequence is not quite so dramatic as in the case of a smooth function, it can nonetheless be a significant factor ($\sim$5) even at modest accuracies like 1%, and greater at higher accuracies.

Note that we have not provided the routine `sobseq` with a means of starting the sequence at a point other than the beginning, but this feature would be easy to add. Once the initialization of the direction numbers `iv` has been done, the jth point can be obtained directly by XORing together those direction numbers corresponding to nonzero bits in the Gray code of j, as described above.

7.8.1 The Latin Hypercube

We mention here the unrelated technique of *Latin square* or *Latin hypercube* sampling, which is useful when you must sample an N-dimensional space *exceedingly* sparsely, at M points. For example, you may want to test the crashworthiness of cars as a simultaneous function of four different design parameters, but with a budget of only three expendable cars. (The issue is not whether this is a good plan — it isn't — but rather how to make the best of the situation!)

The idea is to partition each design parameter (dimension) into M segments, so that the whole space is partitioned into M^N cells. (You can choose the segments

in each dimension to be equal or unequal, according to taste.) With four parameters and three cars, for example, you end up with $3 \times 3 \times 3 \times 3 = 81$ cells.

Next, choose M cells to contain the sample points by the following algorithm: Randomly choose one of the M^N cells for the first point. Now eliminate all cells that agree with this point on *any* of its parameters (that is, cross out all cells in the same row, column, etc.), leaving $(M-1)^N$ candidates. Randomly choose one of these, eliminate new rows and columns, and continue the process until there is only one cell left, which then contains the final sample point.

The result of this construction is that *each* design parameter will have been tested in *every one* of its subranges. If the response of the system under test is dominated by *one* of the design parameters (the *main effect*), that parameter will be found with this sampling technique. On the other hand, if there are important *interaction effects* among different design parameters, then the Latin hypercube gives no particular advantage. Use with care.

There is a large field in statistics that deals with *design of experiments*. A brief pedagogical introduction is [5].

CITED REFERENCES AND FURTHER READING:

Halton, J.H. 1960, "On the Efficiency of Certain Quasi-Random Sequences of Points in Evaluating Multi-dimensional Integrals," *Numerische Mathematik*, vol. 2, pp. 84–90.[1]

Bratley P., and Fox, B.L. 1988, "Implementing Sobol's Quasirandom Sequence Generator," *ACM Transactions on Mathematical Software*, vol. 14, pp. 88–100.[2]

Lambert, J.P. 1988, "Quasi-Random Sequences in Numerical Practice," in *Numerical Mathematics — Singapore 1988*, ISNM vol. 86, R.P. Agarwal, Y.M. Chow, and S.J. Wilson, eds. (Basel: Birkhäuser), pp. 273–284.

Niederreiter, H. 1988, "Quasi-Monte Carlo Methods for Multidimensional Numerical Integration." in *Numerical Integration III*, ISNM vol. 85, H. Brass and G. Hämmerlin, eds. (Basel: Birkhäuser), pp. 157–171.

Sobol', I.M. 1967, "On the Distribution of Points in a Cube and the Approximate Evaluation of Integrals," *USSR Computational Mathematics and Mathematical Physics*, vol. 7, no. 4, pp. 86–112.[3]

Antonov, I.A., and Saleev, V.M 1979, "An Economic Method of Computing lp_t Sequences," *USSR Computational Mathematics and Mathematical Physics*, vol. 19, no. 1, pp. 252–256.[4]

Dunn, O.J., and Clark, V.A. 1974, *Applied Statistics: Analysis of Variance and Regression* (New York, Wiley) [discusses Latin Square].

Czitrom, V. 1999, "One-Factor-at-a-Time Versus Designed Experiments," *The American Statistician*, vol. 53, pp. 126–131, online at `http://www.amstat.org/publications/tas/czitrom.pdf`.[5]

7.9 Adaptive and Recursive Monte Carlo Methods

This section discusses more advanced techniques of Monte Carlo integration. As examples of the use of these techniques, we include two rather different, fairly sophisticated, multidimensional Monte Carlo codes: `vegas` [1,2], and `miser` [4]. The techniques that we discuss all fall under the general rubric of *reduction of variance* (§7.7), but are otherwise quite distinct.

7.9.1 Importance Sampling

The use of *importance sampling* was already implicit in equations (7.7.6) and (7.7.7). We now return to it in a slightly more formal way. Suppose that an integrand f can be written as the product of a function h that is almost constant times another, positive, function g. Then its integral over a multidimensional volume V is

$$\int f\,dV = \int (f/g)\,g\,dV = \int h\,g\,dV \tag{7.9.1}$$

In equation (7.7.7) we interpreted equation (7.9.1) as suggesting a change of variable to G, the indefinite integral of g. That made $g\,dV$ a perfect differential. We then proceeded to use the basic theorem of Monte Carlo integration, equation (7.7.1). A more general interpretation of equation (7.9.1) is that we can integrate f by instead sampling h — not, however, with uniform probability density dV, but rather with nonuniform density $g\,dV$. In this second interpretation, the first interpretation follows as the special case, where the *means* of generating the nonuniform sampling of $g\,dV$ is via the transformation method, using the indefinite integral G (see §7.3).

More directly, one can go back and generalize the basic theorem (7.7.1) to the case of nonuniform sampling: Suppose that points x_i are chosen within the volume V with a probability density p satisfying

$$\int p\,dV = 1 \tag{7.9.2}$$

The generalized fundamental theorem is that the integral of any function f is estimated, using N sample points $x_0, \ldots, x_{N-1}$, by

$$I \equiv \int f\,dV = \int \frac{f}{p}\,p\,dV \approx \left\langle \frac{f}{p} \right\rangle \pm \sqrt{\frac{\langle f^2/p^2\rangle - \langle f/p\rangle^2}{N}} \tag{7.9.3}$$

where angle brackets denote arithmetic means over the N points, exactly as in equation (7.7.2). As in equation (7.7.1), the "plus-or-minus" term is a one standard deviation error estimate. Notice that equation (7.7.1) is in fact the special case of equation (7.9.3), with $p = \text{constant} = 1/V$.

What is the best choice for the sampling density p? Intuitively, we have already seen that the idea is to make $h = f/p$ as close to constant as possible. We can be more rigorous by focusing on the numerator inside the square root in equation (7.9.3), which is the variance per sample point. Both angle brackets are themselves Monte Carlo estimators of integrals, so we can write

$$S \equiv \left\langle \frac{f^2}{p^2} \right\rangle - \left\langle \frac{f}{p} \right\rangle^2 \approx \int \frac{f^2}{p^2}\,p\,dV - \left[\int \frac{f}{p}\,p\,dV\right]^2 = \int \frac{f^2}{p}\,dV - \left[\int f\,dV\right]^2 \tag{7.9.4}$$

We now find the optimal p subject to the constraint equation (7.9.2) by the functional variation

$$0 = \frac{\delta}{\delta p}\left(\int \frac{f^2}{p}\,dV - \left[\int f\,dV\right]^2 + \lambda \int p\,dV\right) \tag{7.9.5}$$

with λ a Lagrange multiplier. Note that the middle term does not depend on p. The variation (which comes inside the integrals) gives $0 = -f^2/p^2 + \lambda$ or

$$p = \frac{|f|}{\sqrt{\lambda}} = \frac{|f|}{\int |f|\,dV} \tag{7.9.6}$$

where λ has been chosen to enforce the constraint (7.9.2).

If f has one sign in the region of integration, then we get the obvious result that the optimal choice of p — if one can figure out a practical way of effecting the sampling — is that it be proportional to $|f|$. Then the variance is reduced to zero. Not so obvious, but seen

to be true, is the fact that $p \propto |f|$ is optimal even if f takes on both signs. In that case the variance per sample point (from equations 7.9.4 and 7.9.6) is

$$S = S_{\text{optimal}} = \left(\int |f|\, dV \right)^2 - \left(\int f\, dV \right)^2 \qquad (7.9.7)$$

One curiosity is that one can add a constant to the integrand to make it all of one sign, since this changes the integral by a known amount, constant$\times V$. Then, the optimal choice of p always gives zero variance, that is, a perfectly accurate integral! The resolution of this seeming paradox (already mentioned at the end of §7.7) is that perfect knowledge of p in equation (7.9.6) requires perfect knowledge of $\int |f| dV$, which is tantamount to already knowing the integral you are trying to compute!

If your function f takes on a known constant value in most of the volume V, it is certainly a good idea to add a constant so as to make that value zero. Having done that, the accuracy attainable by importance sampling depends in practice not on how small equation (7.9.7) is, but rather on how small is equation (7.9.4) for an *implementable* p, likely only a crude approximation to the ideal.

7.9.2 Stratified Sampling

The idea of *stratified sampling* is quite different from importance sampling. Let us expand our notation slightly and let $\langle\langle f \rangle\rangle$ denote the true average of the function f over the volume V (namely the integral divided by V), while $\langle f \rangle$ denotes as before the simplest (uniformly sampled) Monte Carlo *estimator* of that average:

$$\langle\langle f \rangle\rangle \equiv \frac{1}{V} \int f\, dV \qquad \langle f \rangle \equiv \frac{1}{N} \sum_i f(x_i) \qquad (7.9.8)$$

The variance of the estimator, $\text{Var}(\langle f \rangle)$, which measures the square of the error of the Monte Carlo integration, is asymptotically related to the variance of the function, $\text{Var}(f) \equiv \langle\langle f^2 \rangle\rangle - \langle\langle f \rangle\rangle^2$, by the relation

$$\text{Var}(\langle f \rangle) = \frac{\text{Var}(f)}{N} \qquad (7.9.9)$$

(compare equation 7.7.1).

Suppose we divide the volume V into two equal, disjoint subvolumes, denoted a and b, and sample $N/2$ points in each subvolume. Then another estimator for $\langle\langle f \rangle\rangle$, different from equation (7.9.8), which we denote $\langle f \rangle'$, is

$$\langle f \rangle' \equiv \tfrac{1}{2} \left(\langle f \rangle_a + \langle f \rangle_b \right) \qquad (7.9.10)$$

in other words, the mean of the sample averages in the two half-regions. The variance of estimator (7.9.10) is given by

$$\begin{aligned} \text{Var}\left(\langle f \rangle'\right) &= \frac{1}{4} \left[\text{Var}(\langle f \rangle_a) + \text{Var}(\langle f \rangle_b) \right] \\ &= \frac{1}{4} \left[\frac{\text{Var}_a(f)}{N/2} + \frac{\text{Var}_b(f)}{N/2} \right] \\ &= \frac{1}{2N} \left[\text{Var}_a(f) + \text{Var}_b(f) \right] \end{aligned} \qquad (7.9.11)$$

Here $\text{Var}_a(f)$ denotes the variance of f in subregion a, that is, $\langle\langle f^2 \rangle\rangle_a - \langle\langle f \rangle\rangle_a^2$, and correspondingly for b.

From the definitions already given, it is not difficult to prove the relation

$$\text{Var}(f) = \tfrac{1}{2} \left[\text{Var}_a(f) + \text{Var}_b(f) \right] + \tfrac{1}{4} \left(\langle\langle f \rangle\rangle_a - \langle\langle f \rangle\rangle_b \right)^2 \qquad (7.9.12)$$

(In physics, this formula for combining second moments is the "parallel axis theorem.") Comparing equations (7.9.9), (7.9.11), and (7.9.12), one sees that the stratified (into two subvolumes) sampling gives a variance that is never larger than the simple Monte Carlo case — and smaller whenever the means of the stratified samples, $\langle\langle f\rangle\rangle_a$ and $\langle\langle f\rangle\rangle_b$, are different.

We have not yet exploited the possibility of sampling the two subvolumes with *different numbers* of points, say N_a in subregion a and $N_b \equiv N - N_a$ in subregion b. Let us do so now. Then the variance of the estimator is

$$\text{Var}\left(\langle f\rangle'\right) = \frac{1}{4}\left[\frac{\text{Var}_a(f)}{N_a} + \frac{\text{Var}_b(f)}{N - N_a}\right] \tag{7.9.13}$$

which is minimized (one can easily verify) when

$$\frac{N_a}{N} = \frac{\sigma_a}{\sigma_a + \sigma_b} \tag{7.9.14}$$

Here we have adopted the shorthand notation $\sigma_a \equiv [\text{Var}_a(f)]^{1/2}$, and correspondingly for b. If N_a satisfies equation (7.9.14), then equation (7.9.13) reduces to

$$\text{Var}\left(\langle f\rangle'\right) = \frac{(\sigma_a + \sigma_b)^2}{4N} \tag{7.9.15}$$

Equation (7.9.15) reduces to equation (7.9.9) if $\text{Var}(f) = \text{Var}_a(f) = \text{Var}_b(f)$, in which case stratifying the sample makes no difference.

A standard way to generalize the above result is to consider the volume V divided into more than two equal subregions. One can readily obtain the result that the optimal allocation of sample points among the regions is to have the number of points in each region j proportional to σ_j (that is, the square root of the variance of the function f in that subregion). In spaces of high dimensionality (say $d \gtrsim 4$) this is not in practice very useful, however. Dividing a volume into K segments along each dimension implies K^d subvolumes, typically much too large a number when one contemplates estimating all the corresponding σ_j's.

7.9.3 Mixed Strategies

Importance sampling and stratified sampling seem, at first sight, inconsistent with each other. The former concentrates sample points where the magnitude of the integrand $|f|$ is largest, the latter where the variance of f is largest. How can both be right?

The answer is that (like so much else in life) it all depends on what you know and how well you know it. Importance sampling depends on already knowing some approximation to your integral, so that you are able to generate random points x_i with the desired probability density p. To the extent that your p is not ideal, you are left with an error that decreases only as $N^{-1/2}$. Things are particularly bad if your p is far from ideal in a region where the integrand f is changing rapidly, since then the sampled function $h = f/p$ will have a large variance. Importance sampling works by smoothing the values of the sampled function h and is effective only to the extent that you succeed in this.

Stratified sampling, by contrast, does not necessarily require that you know anything about f. Stratified sampling works by smoothing out the fluctuations of the *number* of points in subregions, not by smoothing the values of the points. The simplest stratified strategy, dividing V into N equal subregions and choosing one point randomly in each subregion, already gives a method whose error decreases asymptotically as N^{-1}, much faster than $N^{-1/2}$. (Note that quasi-random numbers, §7.8, are another way of smoothing fluctuations in the density of points, giving nearly as good a result as the "blind" stratification strategy.)

However, "asymptotically" is an important caveat: For example, if the integrand is negligible in all but a single subregion, then the resulting one-sample integration is all but useless. Information, even very crude, allowing importance sampling to put many points in the active subregion would be much better than blind stratified sampling.

Stratified sampling really comes into its own if you have some way of estimating the variances, so that you can put unequal numbers of points in different subregions, according

to (7.9.14) or its generalizations, *and* if you can find a way of dividing a region into a practical number of subregions (notably *not* K^d with large dimension d), while yet significantly reducing the variance of the function in each subregion compared to its variance in the full volume. Doing this requires a lot of knowledge about f, though different knowledge from what is required for importance sampling.

In practice, importance sampling and stratified sampling are not incompatible. In many, if not most, cases of interest, the integrand f is small everywhere in V except for a small fractional volume of "active regions." In these regions the magnitude of $|f|$ and the standard deviation $\sigma = [\text{Var}(f)]^{1/2}$ are comparable in size, so both techniques will give about the same concentration of points. In more sophisticated implementations, it is also possible to "nest" the two techniques, so that, e.g., importance sampling on a crude grid is followed by stratification within each grid cell.

7.9.4 Adaptive Monte Carlo: VEGAS

The VEGAS algorithm, invented by Peter Lepage [1,2], is widely used for multidimensional integrals that occur in elementary particle physics. VEGAS is primarily based on importance sampling, but it also does some stratified sampling if the dimension d is small enough to avoid K^d explosion (specifically, if $(K/2)^d < N/2$, with N the number of sample points). The basic technique for importance sampling in VEGAS is to construct, adaptively, a multidimensional weight function g that is *separable*,

$$p \propto g(x, y, z, \ldots) = g_x(x) g_y(y) g_z(z) \ldots \tag{7.9.16}$$

Such a function avoids the K^d explosion in two ways: (i) It can be stored in the computer as d separate one-dimensional functions, each defined by K tabulated values, say — so that $K \times d$ replaces K^d. (ii) It can be sampled as a probability density by consecutively sampling the d one-dimensional functions to obtain coordinate vector components $(x, y, z, \ldots)$.

The optimal separable weight function can be shown to be [1]

$$g_x(x) \propto \left[\int dy \int dz \ldots \frac{f^2(x, y, z, \ldots)}{g_y(y) g_z(z) \ldots} \right]^{1/2} \tag{7.9.17}$$

(and correspondingly for $y, z, \ldots$). Notice that this reduces to $g \propto |f|$ (7.9.6) in one dimension. Equation (7.9.17) immediately suggests VEGAS' adaptive strategy: Given a set of g-functions (initially all constant, say), one samples the function f, accumulating not only the overall estimator of the integral, but also the Kd estimators (K subdivisions of the independent variable in each of d dimensions) of the right-hand side of equation (7.9.17). These then determine improved g functions for the next iteration.

When the integrand f is concentrated in one, or at most a few, regions in d-space, then the weight function g's quickly become large at coordinate values that are the projections of these regions onto the coordinate axes. The accuracy of the Monte Carlo integration is then enormously enhanced over what simple Monte Carlo would give.

The weakness of VEGAS is the obvious one: To the extent that the projection of the function f onto individual coordinate directions is uniform, VEGAS gives no concentration of sample points in those dimensions. The worst case for VEGAS, e.g., is an integrand that is concentrated close to a body diagonal line, e.g., one from $(0, 0, 0, \ldots)$ to $(1, 1, 1, \ldots)$. Since this geometry is completely nonseparable, VEGAS can give no advantage at all. More generally, VEGAS may not do well when the integrand is concentrated in one-dimensional (or higher) curved trajectories (or hypersurfaces), unless these happen to be oriented close to the coordinate directions.

The routine `vegas` that follows is essentially Lepage's standard version, minimally modified to conform to our conventions. (We thank Lepage for permission to reproduce the program here.) For consistency with other versions of the VEGAS algorithm in circulation, we have preserved original variable names. The parameter `NDMX` is what we have called K, the maximum number of increments along each axis; `MXDIM` is the maximum value of d; some other parameters are explained in the comments.

The `vegas` routine performs m = `itmx` statistically independent evaluations of the desired integral, each with N = `ncall` function evaluations. While statistically independent, these iterations do assist each other, since each one is used to refine the sampling grid for the next one. The results of all iterations are combined into a single best answer, and its estimated error, by the relations

$$I_{\text{best}} = \sum_{i=0}^{m-1} \frac{I_i}{\sigma_i^2} \Bigg/ \sum_{i=0}^{m-1} \frac{1}{\sigma_i^2} \qquad \sigma_{\text{best}} = \left(\sum_{i=0}^{m-1} \frac{1}{\sigma_i^2} \right)^{-1/2} \tag{7.9.18}$$

Also returned is the quantity

$$\chi^2/m \equiv \frac{1}{m-1} \sum_{i=0}^{m-1} \frac{(I_i - I_{\text{best}})^2}{\sigma_i^2} \tag{7.9.19}$$

If this is significantly larger than 1, then the results of the iterations are statistically inconsistent, and the answers are suspect.

Here is the interface to `vegas`. (The full code is given in [3].)

```
void vegas(VecDoub_I &regn, Doub fxn(VecDoub_I &, const Doub), const Int init,
    const Int ncall, const Int itmx, const Int nprn, Doub &tgral, Doub &sd,
    Doub &chi2a) {
```

Performs Monte Carlo integration of a user-supplied `ndim`-dimensional function `fxn` over a rectangular volume specified by `regn[0..2*ndim-1]`, a vector consisting of `ndim` "lower left" coordinates of the region followed by `ndim` "upper right" coordinates. The integration consists of `itmx` iterations, each with approximately `ncall` calls to the function. After each iteration the grid is refined; more than 5 or 10 iterations are rarely useful. The input flag `init` signals whether this call is a new start or a subsequent call for additional iterations (see comments in the code). The input flag `nprn` (normally 0) controls the amount of diagnostic output. Returned answers are `tgral` (the best estimate of the integral), `sd` (its standard deviation), and `chi2a` (χ^2 per degree of freedom, an indicator of whether consistent results are being obtained). See text for further details.

The input flag `init` can be used to advantage. One might have a call with `init=0, ncall=1000, itmx=5` immediately followed by a call with `init=1, ncall=100000, itmx=1`. The effect would be to develop a sampling grid over five iterations of a small number of samples, then to do a single high accuracy integration on the optimized grid.

To use `vegas` for the torus example discussed in §7.7 (the density integrand only, say), the function `fxn` would be

```
Doub torusfunc(const VecDoub &x, const Doub wgt) {
    Doub den = exp(5.*x[2]);
    if (SQR(x[2])+SQR(sqrt(SQR(x[0])+SQR(x[1]))-3.) <= 1.) return den;
    else return 0.;
}
```

and the `main` code would be

```
    Doub tgral, sd, chi2a;
    VecDoub regn(6);
    regn[0] = 1.; regn[3] = 4.;
    regn[1] = -3.; regn[4] = 4.;
    regn[2] = -1.; regn[5] = 1.;
    vegas(regn,torusfunc,0,10000,10,0,tgral,sd,chi2a);
    vegas(regn,torusfunc,1,900000,1,0,tgral,sd,chi2a);
```

Note that the user-supplied integrand function, `fxn`, has an argument `wgt` in addition to the expected evaluation point `x`. In most applications you ignore `wgt` inside the function. Occasionally, however, you may want to integrate some additional function or functions along with the principal function f. The integral of any such function g can be estimated by

$$I_g = \sum_i w_i g(\mathbf{x}) \tag{7.9.20}$$

where the w_i's and $\mathbf{x}$'s are the arguments `wgt` and `x`, respectively. It is straightforward to accumulate this sum inside your function `fxn` and to pass the answer back to your main program via global variables. Of course, $g(\mathbf{x})$ had better resemble the principal function f to some degree, since the sampling will be optimized for f.

The full listing of `vegas` is given in a Webnote [3].

7.9.5 Recursive Stratified Sampling

The problem with stratified sampling, we have seen, is that it may not avoid the K^d explosion inherent in the obvious, Cartesian, tessellation of a d-dimensional volume. A technique called *recursive stratified sampling* [4] attempts to do this by successive bisections of a volume, not along all d dimensions, but rather along only one dimension at a time. The starting points are equations (7.9.10) and (7.9.13), applied to bisections of successively smaller subregions.

Suppose that we have a quota of N evaluations of the function f and want to evaluate $\langle f\rangle'$ in the rectangular parallelepiped region $R = (\mathbf{x}_a, \mathbf{x}_b)$. (We denote such a region by the two coordinate vectors of its diagonally opposite corners.) First, we allocate a fraction p of N toward exploring the variance of f in R: We sample pN function values uniformly in R and accumulate the sums that will give the d different pairs of variances corresponding to the d different coordinate directions along which R can be bisected. In other words, in pN samples, we estimate Var (f) in each of the regions resulting from a possible bisection of R,

$$\begin{aligned} R_{ai} &\equiv (\mathbf{x}_a, \mathbf{x}_b - \tfrac{1}{2}\mathbf{e}_i \cdot (\mathbf{x}_b - \mathbf{x}_a)\mathbf{e}_i) \\ R_{bi} &\equiv (\mathbf{x}_a + \tfrac{1}{2}\mathbf{e}_i \cdot (\mathbf{x}_b - \mathbf{x}_a)\mathbf{e}_i, \mathbf{x}_b) \end{aligned} \tag{7.9.21}$$

Here $\mathbf{e}_i$ is the unit vector in the ith coordinate direction, $i = 1, 2, \ldots, d$.

Second, we inspect the variances to find the most favorable dimension i to bisect. By equation (7.9.15), we could, for example, choose that i for which the sum of the square roots of the variance estimators in regions R_{ai} and R_{bi} is minimized. (Actually, as we will explain, we do something slightly different.)

Third, we allocate the remaining $(1-p)N$ function evaluations between the regions R_{ai} and R_{bi}. If we used equation (7.9.15) to choose i, we should do this allocation according to equation (7.9.14).

We now have two parallelepipeds, each with its own allocation of function evaluations for estimating the mean of f. Our "RSS" algorithm now shows itself to be *recursive*: To evaluate the mean in each region, we go back to the sentence beginning "First,..." in the paragraph above equation (7.9.21). (Of course, when the allocation of points to a region falls below some number, we resort to simple Monte Carlo rather than continue with the recursion.)

Finally, we combine the means and also estimated variances of the two subvolumes using equation (7.9.10) and the first line of equation (7.9.11).

This completes the RSS algorithm in its simplest form. Before we describe some additional tricks under the general rubric of "implementation details," we need to return briefly to equations (7.9.13) – (7.9.15) and derive the equations that we actually use instead of these. The right-hand side of equation (7.9.13) applies the familiar scaling law of equation (7.9.9) twice, once to a and again to b. This would be correct if the estimates $\langle f\rangle_a$ and $\langle f\rangle_b$ were each made by simple Monte Carlo, with uniformly random sample points. However, the two estimates of the mean are in fact made recursively. Thus, there is no reason to expect equation (7.9.9) to hold. Rather, we might substitute for equation (7.9.13) the relation,

$$\text{Var}\left(\langle f\rangle'\right) = \frac{1}{4}\left[\frac{\text{Var}_a(f)}{N_a^\alpha} + \frac{\text{Var}_b(f)}{(N-N_a)^\alpha}\right] \tag{7.9.22}$$

where α is an unknown constant ≥ 1 (the case of equality corresponding to simple Monte Carlo). In that case, a short calculation shows that Var $\left(\langle f\rangle'\right)$ is minimized when

$$\frac{N_a}{N} = \frac{\text{Var}_a(f)^{1/(1+\alpha)}}{\text{Var}_a(f)^{1/(1+\alpha)} + \text{Var}_b(f)^{1/(1+\alpha)}} \tag{7.9.23}$$

and that its minimum value is

$$\text{Var}\left(\langle f\rangle'\right) \propto \left[\text{Var}_a\left(f\right)^{1/(1+\alpha)} + \text{Var}_b\left(f\right)^{1/(1+\alpha)}\right]^{1+\alpha} \tag{7.9.24}$$

Equations (7.9.22) – (7.9.24) reduce to equations (7.9.13) – (7.9.15) when $\alpha = 1$. Numerical experiments to find a self-consistent value for α find that $\alpha \approx 2$. That is, when equation (7.9.23) with $\alpha = 2$ is used recursively to allocate sample opportunities, the observed variance of the RSS algorithm goes approximately as N^{-2}, while any other value of α in equation (7.9.23) gives a poorer fall-off. (The sensitivity to α is, however, not very great; it is not known whether $\alpha = 2$ is an analytically justifiable result or only a useful heuristic.)

The principal difference between `miser`'s implementation and the algorithm as described thus far lies in how the variances on the right-hand side of equation (7.9.23) are estimated. We find empirically that it is somewhat more robust to use the square of the difference of maximum and minimum sampled function values, instead of the genuine second moment of the samples. This estimator is of course increasingly biased with increasing sample size; however, equation (7.9.23) uses it only to compare two subvolumes (a and b) having approximately equal numbers of samples. The "max minus min" estimator proves its worth when the preliminary sampling yields only a single point, or a small number of points, in active regions of the integrand. In many realistic cases, these are indicators of nearby regions of even greater importance, and it is useful to let them attract the greater sampling weight that "max minus min" provides.

A second modification embodied in the code is the introduction of a "dithering parameter," `dith`, whose nonzero value causes subvolumes to be divided not exactly down the middle, but rather into fractions 0.5±`dith`, with the sign of the ± randomly chosen by a built-in random number routine. Normally `dith` can be set to zero. However, there is a large advantage in taking `dith` to be nonzero if some special symmetry of the integrand puts the active region exactly at the midpoint of the region, or at the center of some power-of-two submultiple of the region. One wants to avoid the extreme case of the active region being evenly divided into 2^d abutting corners of a d-dimensional space. A typical nonzero value of `dith`, on those occasions when it is useful, might be 0.1. Of course, when the dithering parameter is nonzero, we must take the differing sizes of the subvolumes into account; the code does this through the variable `fracl`.

One final feature in the code deserves mention. The RSS algorithm uses a single set of sample points to evaluate equation (7.9.23) in all d directions. At the bottom levels of the recursion, the number of sample points can be quite small. Although rare, it can happen that in one direction all the samples are in one half of the volume; in that case, that direction is ignored as a candidate for bifurcation. Even more rare is the possibility that all of the samples are in one half of the volume in *all* directions. In this case, a random direction is chosen. If this happens too often in your application, then you should increase `MNPT` (see line `if (jb == -1)`... in the code).

Note that `miser`, as given, returns as `ave` an estimate of the average function value $\langle\langle f\rangle\rangle$, not the integral of f over the region. The routine `vegas`, adopting the other convention, returns as `tgral` the integral. The two conventions are of course trivially related, by equation (7.9.8), since the volume V of the rectangular region is known.

The interface to the `miser` routine is this:

```
void miser(Doub func(VecDoub_I &), VecDoub_I &regn, const Int npts,
    const Doub dith, Doub &ave, Doub &var) {
```

Monte Carlo samples a user-supplied `ndim`-dimensional function `func` in a rectangular volume specified by `regn[0..2*ndim-1]`, a vector consisting of `ndim` "lower-left" coordinates of the region followed by `ndim` "upper-right" coordinates. The function is sampled a total of `npts` times, at locations determined by the method of recursive stratified sampling. The mean value of the function in the region is returned as `ave`; an estimate of the statistical uncertainty of `ave` (square of standard deviation) is returned as `var`. The input parameter `dith` should normally be set to zero, but can be set to (e.g.) 0.1 if `func`'s active region falls on the boundary of a power-of-two subdivision of `region`.

Implementing code for the torus problem in §7.7 is

```
Doub torusfunc(const VecDoub &x) {
    Doub den = exp(5.*x[2]);
    if (SQR(x[2])+SQR(sqrt(SQR(x[0])+SQR(x[1]))-3.) <= 1.) return den;
    else return 0.;
}
```

and the main code is

```
    Doub ave, var, tgral, sd, vol = 3.*7.*2.;
    regn[0] = 1.; regn[3] = 4.;
    regn[1] = -3.; regn[4] = 4.;
    regn[2] = -1.; regn[5] = 1.;
    miser(torusfunc,regn,1000000,0.,ave,var);
    tgral = ave*vol;
    sd = sqrt(var)*vol;
```

(Actually, `miser` is not particularly well-suited to this problem.)

The complete listing of `miser` is given in a Webnote [5]. The `miser` routine calls the short function `ranpt` to get a random point within a specified d-dimensional region. The version of `ranpt` in the Webnote makes consecutive calls to a uniform random number generator and does the obvious scaling. One can easily modify `ranpt` to generate its points via the quasi-random routine `sobseq` (§7.8). We find that `miser` with `sobseq` can be considerably more accurate than `miser` with uniform random deviates. Since the use of RSS and the use of quasi-random numbers are completely separable, however, we have not made the code given here dependent on `sobseq`. A similar remark might be made regarding importance sampling, which could in principle be combined with RSS. (One could in principle combine `vegas` and `miser`, although the programming would be intricate.)

CITED REFERENCES AND FURTHER READING:

Hammersley, J.M. and Handscomb, D.C. 1964, *Monte Carlo Methods* (London: Methuen).

Kalos, M.H. and Whitlock, P.A. 1986, *Monte Carlo Methods* (New York: Wiley).

Bratley, P., Fox, B.L., and Schrage, E.L. 1983, *A Guide to Simulation*, 2nd ed. (New York: Springer).

Lepage, G.P. 1978, "A New Algorithm for Adaptive Multidimensional Integration," *Journal of Computational Physics*, vol. 27, pp. 192–203.[1]

Lepage, G.P. 1980, "VEGAS: An Adaptive Multidimensional Integration Program," Publication CLNS-80/447, Cornell University.[2]

Numerical Recipes Software 2007, "Complete VEGAS Code Listing," *Numerical Recipes Webnote No. 9*, at `http://www.nr.com/webnotes?9` [3]

Press, W.H., and Farrar, G.R. 1990, "Recursive Stratified Sampling for Multidimensional Monte Carlo Integration," *Computers in Physics*, vol. 4, pp. 190–195.[4]

Numerical Recipes Software 2007, "Complete Miser Code Listing," *Numerical Recipes Webnote No. 10*, at `http://www.nr.com/webnotes?10` [5]

Sorting and Selection

CHAPTER 8

8.0 Introduction

This chapter almost doesn't belong in a book on *numerical* methods: Sorting and selection are bread-and-butter topics in the standard computer science curriculum. However, some review of the techniques for sorting, from the perspective of scientific computing, will prove useful in subsequent chapters. We can develop some standard interfaces for later use, and also illustrate the usefulness of *templates* in object-oriented programming.

In conjunction with numerical work, sorting is frequently necessary when data (either experimental or numerically generated) are being processed. One has tables or lists of numbers, representing one or more independent (or *control*) variables, and one or more dependent (or *measured*) variables. One may wish to arrange these data, in various circumstances, in order by one or another of these variables. Alternatively, one may simply wish to identify the *median* value or the *upper quartile* value of one of the lists of values. (These kinds of values are generically called *quantiles*.) This task, closely related to sorting, is called *selection*.

Here, more specifically, are the tasks that this chapter will deal with:

- Sort, i.e., rearrange, an array of numbers into numerical order.
- Rearrange an array into numerical order while performing the corresponding rearrangement of one or more additional arrays, so that the correspondence between elements in all arrays is maintained.
- Given an array, prepare an *index table* for it, i.e., a table of pointers telling which number array element comes first in numerical order, which second, and so on.
- Given an array, prepare a *rank table* for it, i.e., a table telling what is the numerical rank of the first array element, the second array element, and so on.
- Select the Mth largest element from an array.
- Select the Mth largest value, or estimate arbitrary quantile values, from a data stream in one pass (i.e., without storing the stream for later processing).
- Given a bunch of equivalence relations, organize them into equivalence classes.

For the basic task of sorting N elements, the best algorithms require on the

order of several times $N \log_2 N$ operations. The algorithm inventor tries to reduce the constant in front of this estimate to as small a value as possible. Two of the best algorithms are *Quicksort* (§8.2), invented by the inimitable C.A.R. Hoare, and *Heapsort* (§8.3), invented by J.W.J. Williams.

For large N (say > 1000), Quicksort is faster, on most machines, by a factor of 1.5 or 2; it requires a bit of extra memory, however, and is a moderately complicated program. Heapsort is a true "sort in place," and is somewhat more compact to program and therefore a bit easier to modify for special purposes. On balance, we recommend Quicksort because of its speed, but we implement both routines.

For small N one does better to use an algorithm whose operation count goes as a higher, i.e., poorer, power of N, if the constant in front is small enough. For $N < 20$, roughly, the method of *straight insertion* (§8.1) is concise and fast enough. We include it with some trepidation: It is an N^2 algorithm, whose potential for misuse (by using it for too large an N) is great. The resultant waste of computer resource can be so awesome that we were tempted not to include any N^2 routine at all. We *will* draw the line, however, at the inefficient N^2 algorithm, beloved of elementary computer science texts, called *bubble sort*. If you know what bubble sort is, wipe it from your mind; if you don't know, make a point of never finding out!

For $N < 50$, roughly, *Shell's method* (§8.1), only slightly more complicated to program than straight insertion, is competitive with the more complicated Quicksort on many machines. This method goes as $N^{3/2}$ in the worst case, but is usually faster.

See Refs. [1,2] for further information on the subject of sorting, and for detailed references to the literature.

CITED REFERENCES AND FURTHER READING:

Knuth, D.E. 1997, *Sorting and Searching*, 3rd ed., vol. 3 of *The Art of Computer Programming* (Reading, MA: Addison-Wesley).[1]

Sedgewick, R. 1998, *Algorithms in C*, 3rd ed. (Reading, MA: Addison-Wesley), Chapters 8–13.[2]

8.1 Straight Insertion and Shell's Method

Straight insertion is an N^2 routine and should be used only for small N, say < 20.

The technique is exactly the one used by experienced card players to sort their cards: Pick out the second card and put it in order with respect to the first; then pick out the third card and insert it into the sequence among the first two; and so on until the last card has been picked out and inserted.

sort.h

```
template<class T>
void piksrt(NRvector<T> &arr)
```

Sort an array arr[0..n-1] into ascending numerical order, by straight insertion. arr is replaced on output by its sorted rearrangement.

```
{
    Int i,j,n=arr.size();
    T a;
    for (j=1;j<n;j++) {                    Pick out each element in turn.
        a=arr[j];
```

```
        i=j;
        while (i > 0 && arr[i-1] > a) {       Look for the place to insert it.
            arr[i]=arr[i-1];
            i--;
        }
        arr[i]=a;                             Insert it.
    }
}
```

Notice the use of a template in order to make the routine general for any type of NRvector, including both VecInt and VecDoub. The only thing required of the elements of type T in the vector is that they have an assignment operator and a > relation. (We will generally assume that the relations <, >, and == all exist.) If you try to sort a vector of elements without these properties, the compiler will complain, so you can't go wrong.

It is a matter of taste whether to template on the element type, as above, or on the vector itself, as

```
template<class T>
void piksrt(T &arr)
```

This would seem more general, since it will work for any type T that has a subscript operator [], not just NRvectors. However, it also requires that T have some method for getting at the type of its elements, necessary for the declaration of the variable a. If T follows the conventions of STL containers, then that declaration can be written

```
T::value_type a;
```

but if it doesn't, then you can find yourself lost at C.

What if you also want to rearrange an array brr at the same time as you sort arr? Simply move an element of brr whenever you move an element of arr:

sort.h

```
template<class T, class U>
void piksr2(NRvector<T> &arr, NRvector<U> &brr)
Sort an array arr[0..n-1] into ascending numerical order, by straight insertion, while making
the corresponding rearrangement of the array brr[0..n-1].
{
    Int i,j,n=arr.size();
    T a;
    U b;
    for (j=1;j<n;j++) {                       Pick out each element in turn.
        a=arr[j];
        b=brr[j];
        i=j;
        while (i > 0 && arr[i-1] > a) {       Look for the place to insert it.
            arr[i]=arr[i-1];
            brr[i]=brr[i-1];
            i--;
        }
        arr[i]=a;                             Insert it.
        brr[i]=b;
    }
}
```

Note that the types of arr and brr are separately templated, so they don't have to be the same.

Don't generalize this technique to the rearrangement of a larger number of arrays by sorting on one of them. Instead see §8.4.

8.1.1 Shell's Method

This is actually a variant on straight insertion, but a very powerful variant indeed. The rough idea, e.g., for the case of sorting 16 numbers $n_0 \dots n_{15}$, is this: First sort, by straight insertion, each of the 8 groups of 2 (n_0, n_8), (n_1, n_9), ..., (n_7, n_{15}). Next, sort each of the 4 groups of 4 (n_0, n_4, n_8, n_{12}), ..., $(n_3, n_7, n_{11}, n_{15})$. Next sort the 2 groups of 8 records, beginning with $(n_0, n_2, n_4, n_6, n_8, n_{10}, n_{12}, n_{14})$. Finally, sort the whole list of 16 numbers.

Of course, only the *last* sort is *necessary* for putting the numbers into order. So what is the purpose of the previous partial sorts? The answer is that the previous sorts allow numbers efficiently to filter up or down to positions close to their final resting places. Therefore, the straight insertion passes on the final sort rarely have to go past more than a "few" elements before finding the right place. (Think of sorting a hand of cards that are already almost in order.)

The spacings between the numbers sorted on each pass through the data (8,4,2,1 in the above example) are called the *increments*, and a Shell sort is sometimes called a *diminishing increment sort*. There has been a lot of research into how to choose a good set of increments, but the optimum choice is not known. The set $\dots, 8, 4, 2, 1$ is in fact not a good choice, especially for N a power of 2. A much better choice is the sequence

$$(3^k - 1)/2, \dots, 40, 13, 4, 1 \tag{8.1.1}$$

which can be generated by the recurrence

$$i_0 = 1, \qquad i_{k+1} = 3i_k + 1, \quad k = 0, 1, \dots \tag{8.1.2}$$

It can be shown (see [1]) that for this sequence of increments the number of operations required in all is of order $N^{3/2}$ for the worst possible ordering of the original data. For "randomly" ordered data, the operations count goes approximately as $N^{1.25}$, at least for $N < 60000$. For $N > 50$, however, Quicksort is generally faster.

sort.h

```
template<class T>
void shell(NRvector<T> &a, Int m=-1)
```

Sort an array `a[0..n-1]` into ascending numerical order by Shell's method (diminishing increment sort). `a` is replaced on output by its sorted rearrangement. Normally, the optional argument `m` should be omitted, but if it is set to a positive value, then only the first `m` elements of `a` are sorted.

```
{
    Int i,j,inc,n=a.size();
    T v;
    if (m>0) n = MIN(m,n);              Use optional argument.
    inc=1;                              Determine the starting increment.
    do {
        inc *= 3;
        inc++;
    } while (inc <= n);
    do {                                Loop over the partial sorts.
        inc /= 3;
        for (i=inc;i<n;i++) {           Outer loop of straight insertion.
            v=a[i];
            j=i;
            while (a[j-inc] > v) {      Inner loop of straight insertion.
                a[j]=a[j-inc];
                j -= inc;
                if (j < inc) break;
            }
```

```
            a[j]=v;
        }
    } while (inc > 1);
}
```

CITED REFERENCES AND FURTHER READING:

Knuth, D.E. 1997, *Sorting and Searching*, 3rd ed., vol. 3 of *The Art of Computer Programming* (Reading, MA: Addison-Wesley), §5.2.1.[1]

Sedgewick, R. 1998, *Algorithms in C*, 3rd ed. (Reading, MA: Addison-Wesley), Chapter 8.

8.2 Quicksort

Quicksort is, on most machines, on average, for large N, the fastest known sorting algorithm. It is a "partition-exchange" sorting method: A "partitioning element" a is selected from the array. Then, by pairwise exchanges of elements, the original array is partitioned into two subarrays. At the end of a round of partitioning, the element a is in its final place in the array. All elements in the left subarray are $\leq$ a, while all elements in the right subarray are $\geq$ a. The process is then repeated on the left and right subarrays independently, and so on.

The partitioning process is carried out by selecting some element, say the leftmost, as the partitioning element a. Scan a pointer up the array until you find an element $>$ a, and then scan another pointer down from the end of the array until you find an element $<$ a. These two elements are clearly out of place for the final partitioned array, so exchange them. Continue this process until the pointers cross. This is the right place to insert a, and that round of partitioning is done. The question of the best strategy when an element is equal to the partitioning element is subtle; see Sedgewick [1] for a discussion. (Answer: You should stop and do an exchange.)

For speed of execution, we don't implement Quicksort using recursion. Thus the algorithm requires an auxiliary array of storage, of length $2 \log_2 N$, which it uses as a push-down stack for keeping track of the pending subarrays. When a subarray has gotten down to some size M, it becomes faster to sort it by straight insertion (§8.1), so we will do this. The optimal setting of M is machine-dependent, but $M = 7$ is not too far wrong. Some people advocate leaving the short subarrays unsorted until the end, and then doing one giant insertion sort at the end. Since each element moves at most seven places, this is just as efficient as doing the sorts immediately, and saves on the overhead. However, on modern machines with a cache hierarchy, there is increased overhead when dealing with a large array all at once. We have not found any advantage in saving the insertion sorts till the end.

As already mentioned, Quicksort's *average* running time is fast, but its *worst case* running time can be very slow: For the worst case it is, in fact, an N^2 method! And for the most straightforward implementation of Quicksort it turns out that the worst case is achieved for an input array that is already in order! This ordering of the input array might easily occur in practice. One way to avoid this is to use a little random number generator to choose a random element as the partitioning element. Another is to use instead the median of the first, middle, and last elements of the current subarray.

The great speed of Quicksort comes from the simplicity and efficiency of its inner loop. Simply adding one unnecessary test (for example, a test that your pointer has not moved off the end of the array) can almost double the running time! One avoids such unnecessary tests by placing "sentinels" at either end of the subarray being partitioned. The leftmost sentinel is $\leq$ a, the rightmost $\geq$ a. With the "median-of-three" selection of a partitioning element, we can use the two elements that were not the median to be the sentinels for that subarray.

Our implementation closely follows [1]:

sort.h

```
template<class T>
void sort(NRvector<T> &arr, Int m=-1)
```

Sort an array arr[0..n-1] into ascending numerical order using the Quicksort algorithm. arr is replaced on output by its sorted rearrangement. Normally, the optional argument m should be omitted, but if it is set to a positive value, then only the first m elements of arr are sorted.

```
{
    static const Int M=7, NSTACK=64;
```

Here M is the size of subarrays sorted by straight insertion and NSTACK is the required auxiliary storage.

```
    Int i,ir,j,k,jstack=-1,l=0,n=arr.size();
    T a;
    VecInt istack(NSTACK);
    if (m>0) n = MIN(m,n);                  Use optional argument.
    ir=n-1;
    for (;;) {                              Insertion sort when subarray small enough.
        if (ir-l < M) {
            for (j=l+1;j<=ir;j++) {
                a=arr[j];
                for (i=j-1;i>=l;i--) {
                    if (arr[i] <= a) break;
                    arr[i+1]=arr[i];
                }
                arr[i+1]=a;
            }
            if (jstack < 0) break;
            ir=istack[jstack--];            Pop stack and begin a new round of parti-
            l=istack[jstack--];                 tioning.
        } else {
            k=(l+ir) >> 1;                  Choose median of left, center, and right el-
            SWAP(arr[k],arr[l+1]);              ements as partitioning element a. Also
            if (arr[l] > arr[ir]) {             rearrange so that a[l] ≤ a[l+1] ≤ a[ir].
                SWAP(arr[l],arr[ir]);
            }
            if (arr[l+1] > arr[ir]) {
                SWAP(arr[l+1],arr[ir]);
            }
            if (arr[l] > arr[l+1]) {
                SWAP(arr[l],arr[l+1]);
            }
            i=l+1;                          Initialize pointers for partitioning.
            j=ir;
            a=arr[l+1];                     Partitioning element.
            for (;;) {                      Beginning of innermost loop.
                do i++; while (arr[i] < a);         Scan up to find element > a.
                do j--; while (arr[j] > a);         Scan down to find element < a.
                if (j < i) break;           Pointers crossed. Partitioning complete.
                SWAP(arr[i],arr[j]);        Exchange elements.
            }                               End of innermost loop.
            arr[l+1]=arr[j];                Insert partitioning element.
            arr[j]=a;
            jstack += 2;
```

Push pointers to larger subarray on stack; process smaller subarray immediately.

```
            if (jstack >= NSTACK) throw("NSTACK too small in sort.");
            if (ir-i+1 >= j-l) {
                istack[jstack]=ir;
                istack[jstack-1]=i;
                ir=j-1;
            } else {
                istack[jstack]=j-1;
                istack[jstack-1]=l;
                l=i;
            }
        }
    }
}
```

As usual, you can move any other arrays around at the same time as you sort arr. At the risk of being repetitious:

sort.h

```
template<class T, class U>
void sort2(NRvector<T> &arr, NRvector<U> &brr)
```

Sort an array arr[0..n-1] into ascending order using Quicksort, while making the corresponding rearrangement of the array brr[0..n-1].

```
{
    const Int M=7,NSTACK=64;
    Int i,ir,j,k,jstack=-1,l=0,n=arr.size();
    T a;
    U b;
    VecInt istack(NSTACK);
    ir=n-1;
    for (;;) {                          Insertion sort when subarray small enough.
        if (ir-l < M) {
            for (j=l+1;j<=ir;j++) {
                a=arr[j];
                b=brr[j];
                for (i=j-1;i>=l;i--) {
                    if (arr[i] <= a) break;
                    arr[i+1]=arr[i];
                    brr[i+1]=brr[i];
                }
                arr[i+1]=a;
                brr[i+1]=b;
            }
            if (jstack < 0) break;
            ir=istack[jstack--];        Pop stack and begin a new round of parti-
            l=istack[jstack--];             tioning.
        } else {
            k=(l+ir) >> 1;              Choose median of left, center, and right el-
            SWAP(arr[k],arr[l+1]);          ements as partitioning element a. Also
            SWAP(brr[k],brr[l+1]);          rearrange so that a[l] ≤ a[l+1] ≤ a[ir].
            if (arr[l] > arr[ir]) {
                SWAP(arr[l],arr[ir]);
                SWAP(brr[l],brr[ir]);
            }
            if (arr[l+1] > arr[ir]) {
                SWAP(arr[l+1],arr[ir]);
                SWAP(brr[l+1],brr[ir]);
            }
            if (arr[l] > arr[l+1]) {
                SWAP(arr[l],arr[l+1]);
                SWAP(brr[l],brr[l+1]);
            }
            i=l+1;                      Initialize pointers for partitioning.
            j=ir;
            a=arr[l+1];                 Partitioning element.
```

```
            b=brr[l+1];
            for (;;) {                          Beginning of innermost loop.
                do i++; while (arr[i] < a);         Scan up to find element > a.
                do j--; while (arr[j] > a);         Scan down to find element < a.
                if (j < i) break;               Pointers crossed. Partitioning complete.
                SWAP(arr[i],arr[j]);            Exchange elements of both arrays.
                SWAP(brr[i],brr[j]);
            }                                   End of innermost loop.
            arr[l+1]=arr[j];                    Insert partitioning element in both arrays.
            arr[j]=a;
            brr[l+1]=brr[j];
            brr[j]=b;
            jstack += 2;
            Push pointers to larger subarray on stack; process smaller subarray immediately.
            if (jstack >= NSTACK) throw("NSTACK too small in sort2.");
            if (ir-i+1 >= j-l) {
                istack[jstack]=ir;
                istack[jstack-1]=i;
                ir=j-1;
            } else {
                istack[jstack]=j-1;
                istack[jstack-1]=l;
                l=i;
            }
        }
    }
}
```

You could, in principle, rearrange any number of additional arrays along with `brr`, but this is inefficient if the number of such arrays is larger than one. The preferred technique is to make use of an index table, as described in §8.4.

CITED REFERENCES AND FURTHER READING:

Sedgewick, R. 1978, "Implementing Quicksort Programs," *Communications of the ACM*, vol. 21, pp. 847–857.[1]

8.3 Heapsort

Heapsort is slower than Quicksort by a constant factor. It is so beautiful that we sometimes use it anyway, just for the sheer joy of it. (However, we don't recommend that you do this if your employer is paying for efficient code.) Heapsort is a true "in-place" sort, requiring no auxiliary storage. It is an $N \log_2 N$ algorithm, not only on average, but also for the worst case order of input data. In fact, its worst case is only 20 % or so worse than its average running time.

It is beyond our scope to give a complete exposition on the theory of Heapsort. We mention the general principles, then refer you to the references [1,2]; or you can analyze the program yourself, if you want to understand the details.

A set of N numbers a_j, $j = 0, \ldots, N-1$, is said to form a "heap" if it satisfies the relation

$$a_{(j-1)/2} \geq a_j \quad \text{for} \quad 0 \leq (j-1)/2 < j < N \tag{8.3.1}$$

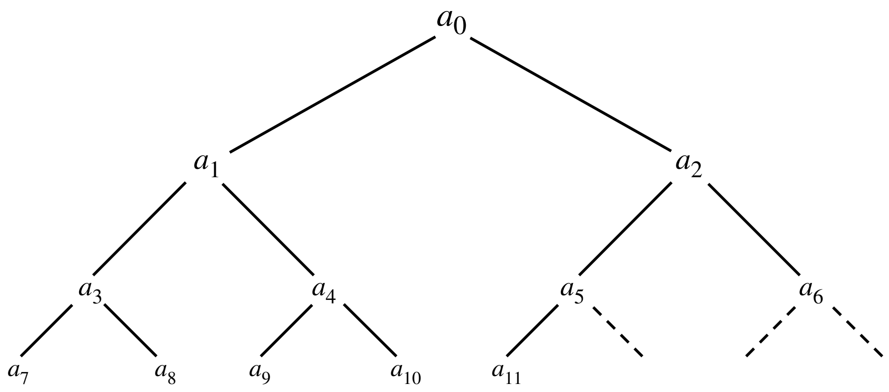

Figure 8.3.1. Ordering implied by a "heap," here of 12 elements. Elements connected by an upward path are sorted with respect to one another, but there is not necessarily any ordering among elements related only "laterally."

Here the division in $j/2$ means "integer divide," i.e., is an exact integer or else is rounded down to the closest integer. Definition (8.3.1) will make sense if you think of the numbers a_i as being arranged in a binary tree, with the top, "boss," node being a_0; the two "underling" nodes being a_1 and a_2; *their* four underling nodes being a_3 through a_6; etc. (See Figure 8.3.1.) In this form, a heap has every "supervisor" greater than or equal to its two "supervisees," down through the levels of the hierarchy.

If you have managed to rearrange your array into an order that forms a heap, then sorting it is very easy: You pull off the "top of the heap," which will be the largest element yet unsorted. Then you "promote" to the top of the heap its largest underling. Then you promote *its* largest underling, and so on. The process is like what happens (or is supposed to happen) in a large corporation when the chairman of the board retires. You then repeat the whole process by retiring the new chairman of the board. Evidently the whole thing is an $N \log_2 N$ process, since each retiring chairman leads to $\log_2 N$ promotions of underlings.

Well, how do you arrange the array into a heap in the first place? The answer is again a "sift-up" process like corporate promotion. Imagine that the corporation starts out with $N/2$ employees on the production line, but with no supervisors. Now a supervisor is hired to supervise two workers. If he is less capable than one of his workers, that one is promoted in his place, and he joins the production line. After supervisors are hired, then supervisors of supervisors are hired, and so on up the corporate ladder. Each employee is brought in at the top of the tree, but then immediately sifted down, with more capable workers promoted until their proper corporate level has been reached.

In the Heapsort implementation, the same sift-up code can be used for the initial creation of the heap and for the subsequent retirement-and-promotion phase. One execution of the Heapsort function represents the entire life-cycle of a giant corporation: $N/2$ workers are hired; $N/2$ potential supervisors are hired; there is a sifting up in the ranks, a sort of super Peter Principle: In due course, each of the original employees gets promoted to chairman of the board.

sort.h

```
namespace hpsort_util {
    template<class T>
    void sift_down(NRvector<T> &ra, const Int l, const Int r)
```
Carry out the sift-down on element ra(l) to maintain the heap structure. l and r determine the "left" and "right" range of the sift-down.
```
    {
        Int j,jold;
        T a;
        a=ra[l];
        jold=l;
        j=2*l+1;
        while (j <= r) {
            if (j < r && ra[j] < ra[j+1]) j++;     Compare to the better underling.
            if (a >= ra[j]) break;                 Found a's level. Terminate the sift-
            ra[jold]=ra[j];                            down. Otherwise, demote a and
            jold=j;                                    continue.
            j=2*j+1;
        }
        ra[jold]=a;                                Put a into its slot.
    }
}

template<class T>
void hpsort(NRvector<T> &ra)
```
Sort an array ra[0..n-1] into ascending numerical order using the Heapsort algorithm. ra is replaced on output by its sorted rearrangement.
```
{
    Int i,n=ra.size();
    for (i=n/2-1; i>=0; i--)
```
The index i, which here determines the "left" range of the sift-down, i.e., the element to be sifted down, is decremented from n/2-1 down to 0 during the "hiring" (heap creation) phase.
```
        hpsort_util::sift_down(ra,i,n-1);
    for (i=n-1; i>0; i--) {
```
Here the "right" range of the sift-down is decremented from n-2 down to 0 during the "retirement-and-promotion" (heap selection) phase.
```
        SWAP(ra[0],ra[i]);                   Clear a space at the end of the array, and retire
        hpsort_util::sift_down(ra,0,i-1);    the top of the heap into it.
    }
}
```

CITED REFERENCES AND FURTHER READING:

Knuth, D.E. 1997, *Sorting and Searching*, 3rd ed., vol. 3 of *The Art of Computer Programming* (Reading, MA: Addison-Wesley), §5.2.3.[1]

Sedgewick, R. 1998, *Algorithms in C*, 3rd ed. (Reading, MA: Addison-Wesley), Chapter 11.[2]

8.4 Indexing and Ranking

The concept of *keys* plays a prominent role in the management of data files. A data *record* in such a file may contain several items, or *fields*. For example, a record in a file of weather observations may have fields recording time, temperature, and wind velocity. When we sort the records, we must decide which of these fields we want to be brought into sorted order. The other fields in a record just come along for the ride and will not, in general, end up in any particular order. The field on which the sort is performed is called the *key* field.

For a data file with many records and many fields, the actual movement of N records into the sorted order of their keys K_i, $i = 0, \ldots, N-1$, can be a daunting task. Instead, one can construct an *index table* I_j, $j = 0, \ldots, N-1$, such that the smallest K_i has $i = I_0$, the second smallest has $i = I_1$, and so on up to the largest K_i with $i = I_{N-1}$. In other words, the array

$$K_{I_j} \quad j = 0, 1, \ldots, N-1 \tag{8.4.1}$$

is in sorted order when indexed by j. When an index table is available, one need not move records from their original order. Further, different index tables can be made from the same set of records, indexing them to different keys.

The algorithm for constructing an index table is straightforward: Initialize the index array with the integers from 0 to $N-1$; then perform the Quicksort algorithm, moving the elements around *as if* one were sorting the keys. The integer that initially numbered the smallest key thus ends up in the number one position, and so on.

The concept of an index table maps particularly nicely into an object, say `Indexx`. The constructor takes a vector `arr` as its argument; it stores an index table to `arr`, leaving `arr` unmodified. Subsequently, the method `sort` can be invoked to rearrange `arr`, or any other vector, into the sorted order of `arr`. `Indexx` is not a templated class, since the stored index table does not depend on the type of vector that is indexed. However, it does need a templated constructor.

sort.h

```
struct Indexx {
    Int n;
    VecInt indx;

    template<class T> Indexx(const NRvector<T> &arr) {
    Constructor. Calls index and stores an index to the array arr[0..n-1].
        index(&arr[0],arr.size());
    }
    Indexx() {}                        Empty constructor. See text.

    template<class T> void sort(NRvector<T> &brr) {
    Sort an array brr[0..n-1] into the order defined by the stored index. brr is replaced on
    output by its sorted rearrangement.
        if (brr.size() != n) throw("bad size in Index sort");
        NRvector<T> tmp(brr);
        for (Int j=0;j<n;j++) brr[j] = tmp[indx[j]];
    }

    template<class T> inline const T & el(NRvector<T> &brr, Int j) const {
    This function, and the next, return the element of brr that would be in sorted position j
    according to the stored index. The vector brr is not changed.
        return brr[indx[j]];
    }
    template<class T> inline T & el(NRvector<T> &brr, Int j) {
    Same, but return an l-value.
        return brr[indx[j]];
    }

    template<class T> void index(const T *arr, Int nn);
    This does the actual work of indexing. Normally not called directly by the user, but see
    text for exceptions.

    void rank(VecInt_O &irank) {
    Returns a rank table, whose jth element is the rank of arr[j], where arr is the vector
    originally indexed. The smallest arr[j] has rank 0.
        irank.resize(n);
```

```
        for (Int j=0;j<n;j++) irank[indx[j]] = j;
    }

};

template<class T>
void Indexx::index(const T *arr, Int nn)
```

Indexes an array arr[0..nn-1], i.e., resizes and sets indx[0..nn-1] such that arr[indx[j]] is in ascending order for j = $0, 1, \ldots$,nn-1. Also sets member value n. The input array arr is not changed.

```
{
    const Int M=7,NSTACK=64;
    Int i,indxt,ir,j,k,jstack=-1,l=0;
    T a;
    VecInt istack(NSTACK);
    n = nn;
    indx.resize(n);
    ir=n-1;
    for (j=0;j<n;j++) indx[j]=j;
    for (;;) {
        if (ir-l < M) {
            for (j=l+1;j<=ir;j++) {
                indxt=indx[j];
                a=arr[indxt];
                for (i=j-1;i>=l;i--) {
                    if (arr[indx[i]] <= a) break;
                    indx[i+1]=indx[i];
                }
                indx[i+1]=indxt;
            }
            if (jstack < 0) break;
            ir=istack[jstack--];
            l=istack[jstack--];
        } else {
            k=(l+ir) >> 1;
            SWAP(indx[k],indx[l+1]);
            if (arr[indx[l]] > arr[indx[ir]]) {
                SWAP(indx[l],indx[ir]);
            }
            if (arr[indx[l+1]] > arr[indx[ir]]) {
                SWAP(indx[l+1],indx[ir]);
            }
            if (arr[indx[l]] > arr[indx[l+1]]) {
                SWAP(indx[l],indx[l+1]);
            }
            i=l+1;
            j=ir;
            indxt=indx[l+1];
            a=arr[indxt];
            for (;;) {
                do i++; while (arr[indx[i]] < a);
                do j--; while (arr[indx[j]] > a);
                if (j < i) break;
                SWAP(indx[i],indx[j]);
            }
            indx[l+1]=indx[j];
            indx[j]=indxt;
            jstack += 2;
            if (jstack >= NSTACK) throw("NSTACK too small in index.");
            if (ir-i+1 >= j-l) {
                istack[jstack]=ir;
                istack[jstack-1]=i;
                ir=j-1;
            } else {
```

```
                istack[jstack]=j-1;
                istack[jstack-1]=l;
                l=i;
            }
        }
    }
}
```

A typical use of Indexx might be to rearrange three vectors (not necessarily of the same type) into the sorted order defined by one of them:

```
Indexx arrindex(arr);
arrindex.sort(arr);
arrindex.sort(brr);
arrindex.sort(crr);
```

The generalization to any other number of arrays is obvious.

The Indexx object also provides a method el (for "element") to access any vector in arr-sorted order without actually modifying that vector (or, for that matter, arr). In other words, after we index arr, say by

```
Indexx arrindex(arr);
```

we can address an element in brr that corresponds to the jth element of a *virtually sorted* arr as simply arrindex.el(brr,j). Neither arr nor brr are altered from their original state. el is provided in two versions, so that it can be both an l-value (on the left-hand side of an assignment) and an r-value (in an expression).

As an aside, the reason that the internal workhorse index uses a pointer, not a vector, for its argument is so that it can be used (purists would say misused) in other situations, such as indexing one row in a matrix. That is also the reason for providing an additional, empty, constructor. If you want to index nn consecutive elements sitting around somewhere, pointed to by ptr, you write

```
Indexx myhack;
myhack.index(ptr,nn);
```

A *rank table* is different from an index table. A rank table's jth entry gives the rank of the jth element of the original array of keys, ranging from 0 (if that element was the smallest) to $N-1$ (if that element was the largest). One can easily construct a rank table from an index table. Indeed, you might already have noticed the method rank in Indexx that returns just such a table, stored as a vector.

Figure 8.4.1 summarizes the concepts discussed in this section.

8.5 Selecting the Mth Largest

Selection is sorting's austere sister. (Say *that* five times quickly!) Where sorting demands the rearrangement of an entire data array, selection politely asks for a single returned value: What is the kth smallest (or, equivalently, the $m = N-1-k$th largest) element out of N elements? (In this convention, used throughout this section, k takes on values $k = 0, 1, \ldots, N-1$, so $k = 0$ is the smallest array element and $k = N-1$ the largest.) The fastest methods for selection do, unfortunately, rearrange the array for their own computational purposes, typically putting all smaller elements

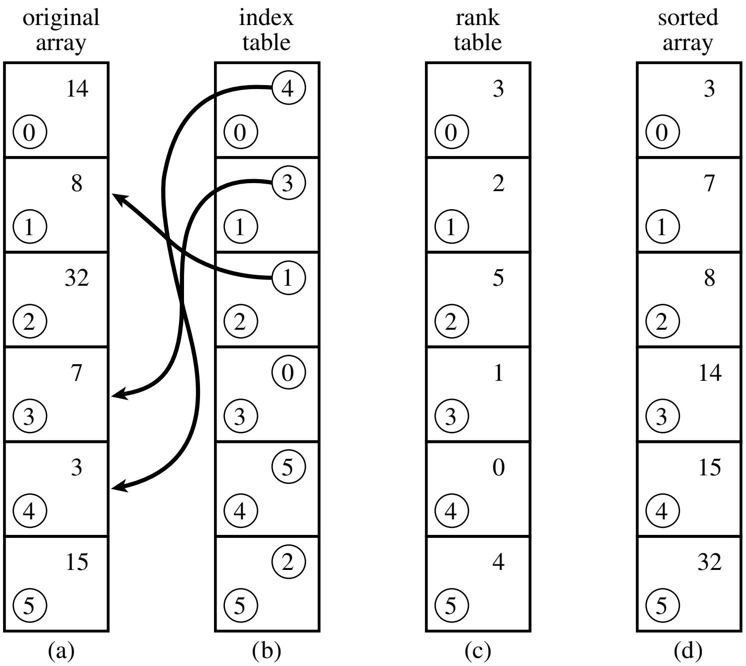

Figure 8.4.1. (a) An unsorted array of six numbers. (b) Index table whose entries are pointers to the elements of (a) in ascending order. (c) Rank table whose entries are the ranks of the corresponding elements of (a). (d) Sorted array of the elements in (a).

to the left of the kth, all larger elements to the right, and scrambling the order within each subset. This side effect is at best innocuous, at worst downright inconvenient. When an array is very long, so that making a scratch copy of it is taxing on memory, one turns to *in-place* algorithms without side effects, which are slower but leave the original array undisturbed.

The most common use of selection is in the statistical characterization of a set of data. One often wants to know the median element in an array (quantile $p = 1/2$) or the top and bottom quartile elements (quantile $p = 1/4$, $3/4$). When N is odd, the exact definition of the median is that it is the kth element, with $k = (N-1)/2$. When N is even, statistics books define the median as the arithmetic mean of the elements $k = N/2 - 1$ and $k = N/2$ (that is, $N/2$ from the bottom and $N/2$ from the top). If you embrace such formality, you must perform two separate selections to find these elements. (If you do the first selection by a partition method, see below, you can do the second by a single pass through $N/2$ elements in the right partition, looking for the smallest element.) For $N > 100$ we usually use $k = N/2$ as the median element, formalists be damned.

A variant on selection for large data sets is *single-pass selection*, where we have a stream of input values, each of which we get to see only once. We want to be able to report at any time, say after N values, the kth smallest (or largest) value seen so far, or, equivalently, the quantile value for some p. We will describe two approaches: If we care only about the smallest (or largest) M values, for fixed M, so that $0 \le k < M$, then there are good algorithms that require only M storage. On the other hand, if we can tolerate an approximate answer, then there are efficient

algorithms that can report at any time a good *estimate* of the p-quantile value for any p, $0 < p < 1$. That is to say, we will get not the exact kth smallest element, $k = pN$, of the N that have gone by, but something very close to it — and without requiring N storage or having to know p in advance.

The fastest general method for selection, allowing rearrangement, is *partitioning*, exactly as was done in the Quicksort algorithm (§8.2). Selecting a "random" partition element, one marches through the array, forcing smaller elements to the left, larger elements to the right. As in Quicksort, it is important to optimize the inner loop, using "sentinels" (§8.2) to minimize the number of comparisons. For sorting, one would then proceed to further partition both subsets. For selection, we can ignore one subset and attend only to the one that contains our desired kth element. Selection by partitioning thus does not need a stack of pending operations, and its operations count scales as N rather than as $N \log N$ (see [1]). Comparison with `sort` in §8.2 should make the following routine obvious.

sort.h

```
template<class T>
T select(const Int k, NRvector<T> &arr)
```
Given `k` in `[0..n-1]` returns an array value from `arr[0..n-1]` such that `k` array values are less than or equal to the one returned. The input array will be rearranged to have this value in location `arr[k]`, with all smaller elements moved to `arr[0..k-1]` (in arbitrary order) and all larger elements in `arr[k+1..n-1]` (also in arbitrary order).
```
{
    Int i,ir,j,l,mid,n=arr.size();
    T a;
    l=0;
    ir=n-1;
    for (;;) {
        if (ir <= l+1) {                    Active partition contains 1 or 2 elements.
            if (ir == l+1 && arr[ir] < arr[l])        Case of 2 elements.
                SWAP(arr[l],arr[ir]);
            return arr[k];
        } else {
            mid=(l+ir) >> 1;                Choose median of left, center, and right el-
            SWAP(arr[mid],arr[l+1]);            ements as partitioning element a. Also
            if (arr[l] > arr[ir])               rearrange so that arr[l] ≤ arr[l+1],
                SWAP(arr[l],arr[ir]);           arr[ir] ≥ arr[l+1].
            if (arr[l+1] > arr[ir])
                SWAP(arr[l+1],arr[ir]);
            if (arr[l] > arr[l+1])
                SWAP(arr[l],arr[l+1]);
            i=l+1;                          Initialize pointers for partitioning.
            j=ir;
            a=arr[l+1];                     Partitioning element.
            for (;;) {                      Beginning of innermost loop.
                do i++; while (arr[i] < a);       Scan up to find element > a.
                do j--; while (arr[j] > a);       Scan down to find element < a.
                if (j < i) break;           Pointers crossed. Partitioning complete.
                SWAP(arr[i],arr[j]);
            }                               End of innermost loop.
            arr[l+1]=arr[j];                Insert partitioning element.
            arr[j]=a;
            if (j >= k) ir=j-1;             Keep active the partition that contains the
            if (j <= k) l=i;                    kth element.
        }
    }
}
```

If you don't want your array `arr` to be rearranged, then you will want to make

a scratch copy before calling `select`, e.g.,

```
VecDoub brr(arr);
```

The reason for not doing this internally in `select` is because you may wish to call `select` with a variety of different values `k`, and it would be wasteful to copy the vector anew each time; instead, just let `brr` keep getting rearranged.

8.5.1 Tracking the M Largest in a Single Pass

Of course `select` should not be used for the trivial cases of finding the largest, or smallest, element in an array. Those cases, you code by hand as simple `for` loops.

There are also efficient ways to code the case where k is bounded by some fixed M, modest in comparison to N, so that memory of order M is not burdensome. Indeed, N may not even be known: You may have a stream of incoming data values and be called upon at any time to provide a list of the M largest values seen so far.

A good approach to this case is to use the method of Heapsort (§8.3), maintaining a heap of the M largest values. The advantage of the heap structure, as opposed to a linear array of length M, is that at most $\log M$, rather than M, operations are required every time a new data value is processed.

The object `Heapselect` has a constructor, by which you specify M, an "`add`" method that assimilates a new data value, and a "`report`" method for getting the kth largest seen so far. Note that the initial cost of a report is $O(M \log M)$, because we need to sort the heap; but you can then get all values of k at no extra cost, until you do the next `add`. A special case is that getting the $M-1$st largest is always cheap, since it is at the top of the heap; so if you have a single favorite value of k, it is best to choose M with $M-1=k$.

sort.h

```
struct Heapselect {
Object for tracking the m largest values seen thus far in a stream of values.
    Int m,n,srtd;
    VecDoub heap;

    Heapselect(Int mm) : m(mm), n(0), srtd(0), heap(mm,1.e99) {}
    Constructor. The argument is the number of largest values to track.

    void add(Doub val) {
    Assimilate a new value from the stream.
        Int j,k;
        if (n<m) {                                Heap not yet filled.
            heap[n++] = val;
            if (n==m) sort(heap);                 Create initial heap by overkill!
        } else {
            if (val > heap[0]) {                  Put it on the heap?
                heap[0]=val;
                for (j=0;;) {                     Sift down.
                    k=(j << 1) + 1;
                    if (k > m-1) break;
                    if (k != (m-1) && heap[k] > heap[k+1]) k++;
                    if (heap[j] <= heap[k]) break;
                    SWAP(heap[k],heap[j]);
                    j=k;
                }
            }
            n++;
        }
        srtd = 0;                                 Mark heap as "unsorted".
    }
```

```
    Doub report(Int k) {
    Return the kth largest value seen so far. k=0 returns the largest value seen, k=1 the second
    largest, ... , k=m-1 the last position tracked. Also, k must be less than the number of
    previous values assimilated.
        Int mm = MIN(n,m);
        if (k > mm-1) throw("Heapselect k too big");
        if (k == m-1) return heap[0];              Always free, since top of heap.
        if (! srtd) { sort(heap); srtd = 1; } Otherwise, need to sort the heap.
        return heap[mm-1-k];
    }
};
```

8.5.2 Single-Pass Estimation of Arbitrary Quantiles

The data values fly by in a stream. You get to look at each value only once, and do a constant-time process on it (meaning that you can't take longer and longer to process later and later data values). Also, you have only a fixed amount of storage memory. From time to time you want to know the median value (or 95th percentile value, or arbitrary p-quantile value) of the data that you have seen thus far. How do you do this?

Evidently, with the conditions stated, you'll have to tolerate an approximate answer, since an exact answer must require unbounded storage and (perhaps) unlimited processing. If you think that "binning" is somehow the answer, you are right. But it is not immediately obvious how to choose the bins, since you have to see a potentially unlimited amount of data before you can tell for sure how its values are distributed.

Chambers et al. [2] have given a robust, and extremely fast, algorithm, which they call *IQ agent*, that adaptively adjusts a set of bins so that they converge to the data values of specified quantile p-values. The general idea (see Figure 8.5.1) is to accumulate incoming data into batches, then to update a stored, piecewise linear, cumulative distribution function (cdf) by adding a batch's cdf and then interpolating back to a fixed set of p-values. Arbitrary requested quantile values ("incremental quantiles," or "IQs," hence the algorithm's name) can be obtained at any time by linear interpolation on the stored cdf. Batching allows the program to be very efficient, with an (amortized) cost of only a small number of operations per new data value. The batching is done transparently to the user.

Similar to `Heapselect`, the `IQagent` object has `add` and `report` methods, the latter now taking a value for p as its argument. In the implementation below, we use a batch size of `nbuf=1000` but do an early update step with a partial batch whenever a quantile is requested. With these parameters, you should therefore request quantile information no more frequently than after every few `nbuf` data values, at which point you can request as many different values of p as you want before continuing. The alternative is to remove the call to `update` from `report`, in which case you'll get fast, but constant, answers, changing only after each regular batch update.

`IQagent` uses internally a general purpose set of 251 p-values that includes integer percentile points from 10 to 90, and a logarithmically spaced set of smaller and larger values spanning 10^{-6} to $1 - 10^{-6}$. Other p-values that you request are obtained by interpolation. Of course you cannot get meaningful tail quantiles for small values of p until at least several times $1/p$ data values have been processed. Before that, the program will simply report the smallest value previously seen (or largest value previously seen, for $p \to 1$).

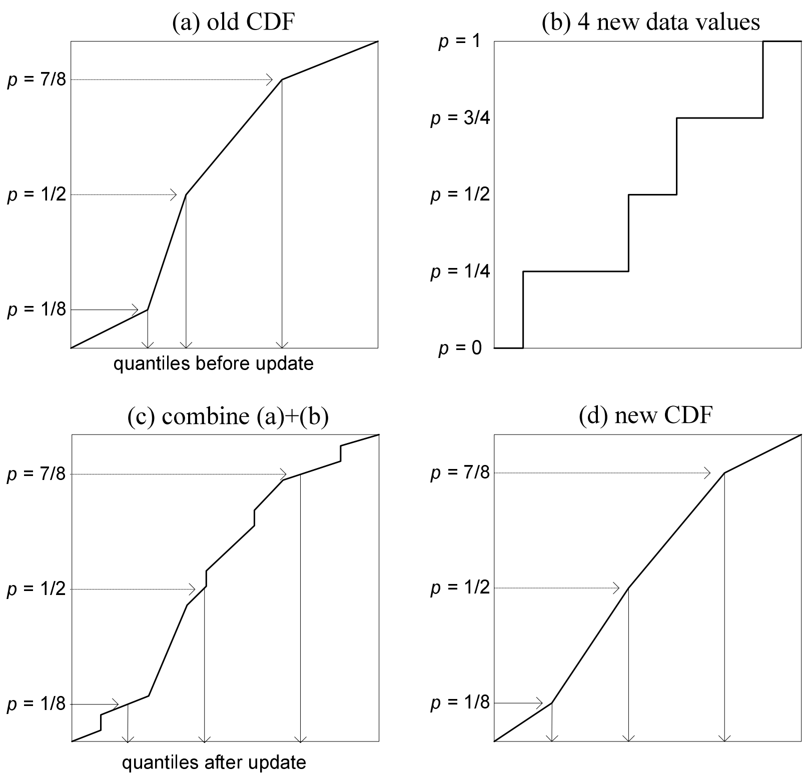

Figure 8.5.1. Algorithm for updating a piecewise linear cumulative distribution function (cdf). (a) The cdf is represented by quantile values at a fixed set of p-values (here, just 3). (b) A batch of new data values (here, just 4) define a stepwise constant cdf. (c) The two cdfs are summed. New data steps are small in proportion to the new batch size versus number of data values previously processed. (d) The new cdf representation is obtained by interpolating the fixed p-values to (c).

iqagent.h

```
struct IQagent {
```
Object for estimating arbitrary quantile values from a continuing stream of data values.
```
    static const Int nbuf = 1000;
```
Batch size. You may $\times 10$ if you expect $> 10^6$ data values.
```
    Int nq, nt, nd;
    VecDoub pval,dbuf,qile;
    Doub q0, qm;

    IQagent() : nq(251), nt(0), nd(0), pval(nq), dbuf(nbuf),
    qile(nq,0.), q0(1.e99), qm(-1.e99) {
```
Constructor. No arguments.
```
        for (Int j=85;j<=165;j++) pval[j] = (j-75.)/100.;
```
Set general purpose array of p-values ranging from 10^{-6} to $1-10^{-6}$. You can change this if you want:
```
        for (Int j=84;j>=0;j--) {
            pval[j] = 0.87191909*pval[j+1];
            pval[250-j] = 1.-pval[j];
        }
    }

    void add(Doub datum) {
```
Assimilate a new value from the stream.
```
        dbuf[nd++] = datum;
        if (datum < q0) {q0 = datum;}
        if (datum > qm) {qm = datum;}
```

```
        if (nd == nbuf) update();               Time for a batch update.
    }

    void update() {
    Batch update, as shown in Figure 8.5.1. This function is called by add or report and
    should not be called directly by the user.
        Int jd=0,jq=1,iq;
        Doub target, told=0., tnew=0., qold, qnew;
        VecDoub newqile(nq);                    Will be new quantiles after update.
        sort(dbuf,nd);
        qold = qnew = qile[0] = newqile[0] = q0;       Set lowest and highest to min
        qile[nq-1] = newqile[nq-1] = qm;                  and max values seen so far,
        pval[0] = min(0.5/(nt+nd),0.5*pval[1]);           and set compatible p-values.
        pval[nq-1] = max(1.-0.5/(nt+nd),0.5*(1.+pval[nq-2]));
        for (iq=1;iq<nq-1;iq++) {               Main loop over target p-values for inter-
            target = (nt+nd)*pval[iq];              polation.
            if (tnew < target) for (;;) {
                Here's the guts: We locate a succession of abscissa-ordinate pairs (qnew,tnew)
                that are the discontinuities of value or slope in Figure 8.5.1(c), breaking to
                perform an interpolation as we cross each target.
                if (jq < nq && (jd >= nd || qile[jq] < dbuf[jd])) {
                    Found slope discontinuity from old CDF.
                    qnew = qile[jq];
                    tnew = jd + nt*pval[jq++];
                    if (tnew >= target) break;
                } else {                        Found value discontinuity from batch data
                    qnew = dbuf[jd];                CDF.
                    tnew = told;
                    if (qile[jq]>qile[jq-1]) tnew += nt*(pval[jq]-pval[jq-1])
                        *(qnew-qold)/(qile[jq]-qile[jq-1]);
                    jd++;
                    if (tnew >= target) break;
                    told = tnew++;
                    qold = qnew;
                    if (tnew >= target) break;
                }
                told = tnew;
                qold = qnew;
            }                                   Break to here and perform the new interpolation.
            if (tnew == told) newqile[iq] = 0.5*(qold+qnew);
            else newqile[iq] = qold + (qnew-qold)*(target-told)/(tnew-told);
            told = tnew;
            qold = qnew;
        }
        qile = newqile;
        nt += nd;
        nd = 0;
    }

    Doub report(Doub p) {
    Return estimated p-quantile for the data seen so far. (E.g., p = 0.5 for median.)
        Doub q;
        if (nd > 0) update();                   You may want to remove this line. See text.
        Int jl=0,jh=nq-1,j;
        while (jh-jl>1) {                       Locate place in table by bisection.
            j = (jh+jl)>>1;
            if (p > pval[j]) jl=j;
            else jh=j;
        }
        j = jl;                                 Interpolate.
        q =  qile[j] + (qile[j+1]-qile[j])*(p-pval[j])/(pval[j+1]-pval[j]);
        return MAX(qile[0],MIN(qile[nq-1],q));
    }
};
```

How accurate is the IQ agent algorithm, as compared, say, to storing all N data values in an array A and then reporting the "exact" quantile $A_{\lfloor pN \rfloor}$? There are several sources of error, all of which you can control by modifying parameters in `IQagent`. (We think that the default parameters will work just fine for almost all users.) First, there is interpolation error: The desired cdf is represented by a piecewise linear function between `nq=251` stored values. For typical distributions, this limits the accuracy to three or four significant figures. We find it hard to believe that anyone needs to know a median, e.g., more accurately than this, but if you do, then you can increase the density of p-values in the regions of interest.

Second, there are statistical errors. One way to characterize these is to ask what value j has A_j closest to the quantile reported by IQ agent, and then how small is $|j - pN|$ as a fraction of $[Np(1-p)]^{1/2}$, the accuracy inherent in your finite sample size N. If this fraction is $\lesssim 1$, then the estimate is "good enough," meaning that no method can do substantially better at estimating the population quantiles given your sample.

With the default parameters, and for reasonably behaved distributions, `IQagent` passes this test for $N \lesssim 10^6$. For larger N, the statistical error becomes significant (though still generally smaller than the interpolation error, above). You can, however, decrease it by increasing the batch size, `nbuf`. Larger is always better, if you have the memory and can tolerate the logarithmic increase in the cost per point of the sort.

Although the accuracy of `IQagent` is not guaranteed by a provable bound, the algorithm is fast, robust, and highly recommended. For other approaches to incremental quantile estimation, including some that do give provable bounds (but have other issues), see [3,4] and references cited therein.

8.5.3 Other Uses for Incremental Quantile Estimation

Incremental quantile estimation provides a useful way to histogram data into variable-size bins that each contain the same number of points, without knowing in advance the bin boundaries: First, throw N data values at an `IQagent` object. Next, choose a number of bins m, and define

$$p_i \equiv \frac{i}{m}, \qquad i = 0, \ldots, m \tag{8.5.1}$$

Finally, if q_i is the quantile value at p_i, plot the ith bin from q_i to q_{i+1} with a height

$$h_i = N \frac{p_{i+1} - p_i}{q_{i+1} - q_i}, \qquad i = 0, \ldots, m-1 \tag{8.5.2}$$

A different application concerns the monitoring of quantile values for changes. For example, you might be producing widgets with a parameter T whose tolerance is $T \pm \delta T$, and you want an early warning if the observed values of T at the 5th and 95th percentiles start to drift.

The `IQagent` object is easily modified for such applications. Simply change the line `nt += nd` to `nt = my_constant`, where `my_constant` is the number of past widgets that you want to average over. (More precisely, the number corresponding to one e-fold of weight decrease in an exponentially decreasing average over all past production.) Now, the stored cdf combines with a new batch of data with a constant, not an increasing, weight, and you can look for changes over time in any desired quantiles.

8.5.4 In-Place Selection

In-place, nondestructive, selection is conceptually simple, but it requires a lot of bookkeeping, and it is correspondingly slow. The general idea is to pick some number M of elements at random, to sort them, and then to make a pass through the array *counting* how many elements fall in each of the $M+1$ intervals defined by these elements. The kth largest will fall in one such interval — call it the "live" interval. One then does a second round, first picking M random elements in the live interval, and then determining which of the new, finer, $M+1$ intervals all presently live elements fall into. And so on, until the kth element is finally localized within a single array of size M, at which point direct selection is possible.

How shall we pick M? The number of rounds, $\log_M N = \log_2 N/\log_2 M$, will be smaller if M is larger; but the work to locate each element among $M+1$ subintervals will be larger, scaling as $\log_2 M$ for bisection, say. Each round requires looking at all N elements, if only to find those that are still alive, while the bisections are dominated by the N that occur in the first round. Minimizing $O(N \log_M N) + O(N \log_2 M)$ thus yields the result

$$M \sim 2^{\sqrt{\log_2 N}} \tag{8.5.3}$$

The square root of the logarithm is so slowly varying that secondary considerations of machine timing become important. We use $M = 64$ as a convenient constant value.

Further discussion, and code, is in a Webnote [5].

CITED REFERENCES AND FURTHER READING:

Sedgewick, R. 1998, *Algorithms in C*, 3rd ed. (Reading, MA: Addison-Wesley), pp. 126ff.[1]

Knuth, D.E. 1997, *Sorting and Searching*, 3rd ed., vol. 3 of *The Art of Computer Programming* (Reading, MA: Addison-Wesley).

Chambers, J.M., James, D.A., Lambert, D., and Vander Wiel, S. 2006, "Monitoring Networked Applications with Incremental Quantiles," *Statistical Science*, vol. 21.[2]

Tieney, L. 1983, "A Space-efficient Recursive Procedure for Estimating a Quantile of an Unknown Distribution," *SIAM Journal on Scientific and Statistical Computing*, vol. 4, pp. 706-711.[3]

Liechty, J.C., Lin, D.K.J, and McDermott, J.P. 2003, "Single-Pass Low-Storage Arbitrary Quantile Estimation for Massive Datasets," *Statistics and Computing*, vol. 13, pp. 91–100.[4]

Numerical Recipes Software 2007, "Code Listing for Selip," *Numerical Recipes Webnote No. 11*, at `http://www.nr.com/webnotes?11` [5]

8.6 Determination of Equivalence Classes

A number of techniques for sorting and searching relate to data structures whose details are beyond the scope of this book, for example, trees, linked lists, etc. These structures and their manipulations are the bread and butter of computer science, as distinct from numerical analysis, and there is no shortage of books on the subject.

In working with experimental data, we have found that one particular such manipulation, namely the determination of equivalence classes, arises sufficiently often to justify inclusion here.

The problem is this: There are N "elements" (or "data points" or whatever), numbered $0, \ldots, N-1$. You are given pairwise information about whether the elements are in the same *equivalence class* of "sameness," by whatever criterion happens to be of interest. For example, you may have a list of facts like: "Element 3 and element 7 are in the same class; element 19 and element 4 are in the same class; element 7 and element 12 are in the same class, ..." Alternatively, you may have a procedure, given the numbers of two elements j and k, for deciding whether they are in the same class or different classes. (Recall that an equivalence relation can be anything satisfying the *RST properties*: reflexive, symmetric, transitive. This is compatible with any intuitive definition of "sameness.")

The desired output is an assignment to each of the N elements of an equivalence class number, such that two elements are in the same class if and only if they are assigned the same class number.

Efficient algorithms work like this: Let $F(j)$ be the class or "family" number of element j. Start off with each element in its own family, so that $F(j) = j$. The array $F(j)$ can be interpreted as a tree structure, where $F(j)$ denotes the parent of j. If we arrange for each family to be its own tree, disjoint from all the other "family trees," then we can label each family (equivalence class) by its most senior great-great-...grandparent. The detailed topology of the tree doesn't matter at all, as long as we graft each related element onto it *somewhere*.

Therefore, we process each elemental datum "j is equivalent to k" by (i) tracking j up to its highest ancestor; (ii) tracking k up to its highest ancestor; and (iii) giving j to k as a new parent, or vice versa (it makes no difference). After processing all the relations, we go through all the elements j and reset their $F(j)$'s to their highest possible ancestors, which then label the equivalence classes.

The following routine, based on Knuth [1], assumes that there are `m` elemental pieces of information, stored in two arrays of length `m`, `lista,listb`, the interpretation being that `lista[j]` and `listb[j]`, `j=0...m-1`, are the numbers of two elements that (we are thus told) are related.

eclass.h

```
void eclass(VecInt_O &nf, VecInt_I &lista, VecInt_I &listb)
```

Given `m` equivalences between pairs of `n` individual elements in the form of the input arrays `lista[0..m-1]` and `listb[0..m-1]`, this routine returns in `nf[0..n-1]` the number of the equivalence class of each of the `n` elements, integers between `0` and `n-1` (not all such integers used).

```
{
    Int l,k,j,n=nf.size(),m=lista.size();
    for (k=0;k<n;k++) nf[k]=k;              Initialize each element its own class.
    for (l=0;l<m;l++) {                     For each piece of input information...
        j=lista[l];
        while (nf[j] != j) j=nf[j];         Track first element up to its ancestor.
        k=listb[l];
        while (nf[k] != k) k=nf[k];         Track second element up to its ancestor.
        if (j != k) nf[j]=k;                If they are not already related, make them
                                                so.
    }
    for (j=0;j<n;j++)                       Final sweep up to highest ancestors.
        while (nf[j] != nf[nf[j]]) nf[j]=nf[nf[j]];
}
```

Alternatively, we may be able to construct a boolean function `equiv(j,k)` that returns a value `true` if elements `j` and `k` are related, or `false` if they are not. Then we want to loop over all pairs of elements to get the complete picture. D. Eardley has devised a clever way of doing this while simultaneously sweeping the tree up to high ancestors in a manner that keeps it current and obviates most of the final sweep phase:

eclass.h

```
void eclazz(VecInt_O &nf, Bool equiv(const Int, const Int))
```

Given a user-supplied boolean function `equiv` that tells whether a pair of elements, each in the range `0...n-1`, are related, return in `nf[0..n-1]` equivalence class numbers for each element.

```
{
    Int kk,jj,n=nf.size();
    nf[0]=0;
    for (jj=1;jj<n;jj++) {                  Loop over first element of all pairs.
```

```
        nf[jj]=jj;
        for (kk=0;kk<jj;kk++) {                 Loop over second element of all pairs.
            nf[kk]=nf[nf[kk]];                  Sweep it up this much.
            if (equiv(jj+1,kk+1)) nf[nf[nf[kk]]]=jj;
            Good exercise for the reader to figure out why this much ancestry is necessary!
        }
    }
    for (jj=0;jj<n;jj++) nf[jj]=nf[nf[jj]];        Only this much sweeping is needed
}                                                      finally.
```


CITED REFERENCES AND FURTHER READING:

Knuth, D.E. 1997, *Fundamental Algorithms*, 3rd ed., vol. 1 of *The Art of Computer Programming* (Reading, MA: Addison-Wesley), §2.3.3.[1]

Sedgewick, R. 1998, *Algorithms in C*, 3rd ed. (Reading, MA: Addison-Wesley), Chapter 30.

CHAPTER 9

Root Finding and Nonlinear Sets of Equations

9.0 Introduction

We now consider that most basic of tasks, solving equations numerically. While most equations are born with both a right-hand side and a left-hand side, one traditionally moves all terms to the left, leaving

$$f(x) = 0 \tag{9.0.1}$$

whose solution or solutions are desired. When there is only one independent variable, the problem is *one-dimensional*, namely to find the root or roots of a function.

With more than one independent variable, more than one equation can be satisfied simultaneously. You likely once learned the *implicit function theorem*, which (in this context) gives us the hope of satisfying N equations in N unknowns simultaneously. Note that we have only hope, not certainty. A nonlinear set of equations may have no (real) solutions at all. Contrariwise, it may have more than one solution. The implicit function theorem tells us that "generically" the solutions will be distinct, pointlike, and separated from each other. If, however, life is so unkind as to present you with a nongeneric, i.e., degenerate, case, then you can get a continuous family of solutions. In vector notation, we want to find one or more N-dimensional solution vectors $\mathbf{x}$ such that

$$\mathbf{f}(\mathbf{x}) = \mathbf{0} \tag{9.0.2}$$

where $\mathbf{f}$ is the N-dimensional vector-valued function whose components are the individual equations to be satisfied simultaneously.

Don't be fooled by the apparent notational similarity of equations (9.0.2) and (9.0.1). Simultaneous solution of equations in N dimensions is *much* more difficult than finding roots in the one-dimensional case. The principal difference between one and many dimensions is that, in one dimension, it is possible to bracket or "trap" a root between bracketing values, and then hunt it down like a rabbit. In multidimensions, you can never be sure that the root is there at all until you have found it.

Except in linear problems, root finding invariably proceeds by iteration, and this is equally true in one or in many dimensions. Starting from some approximate trial solution, a useful algorithm will improve the solution until some predetermined convergence criterion is satisfied. For smoothly varying functions, good algorithms will always converge, *provided* that the initial guess is good enough. Indeed one can even determine in advance the rate of convergence of most algorithms.

It cannot be overemphasized, however, how crucially success depends on having a good first guess for the solution, especially for multidimensional problems. This crucial beginning usually depends on analysis rather than numerics. Carefully crafted initial estimates reward you not only with reduced computational effort, but also with understanding and increased self-esteem. Hamming's motto, "the purpose of computing is insight, not numbers," is particularly apt in the area of finding roots. You should repeat this motto aloud whenever your program converges, with sixteen-digit accuracy, to the wrong root of a problem, or whenever it fails to converge because there is actually *no* root, or because there is a root but your initial estimate was not sufficiently close to it.

"This talk of insight is all very well, but what do I actually do?" For one-dimensional root finding, it is possible to give some straightforward answers: You should try to get some idea of what your function looks like before trying to find its roots. If you need to mass-produce roots for many different functions, then you should at least know what some typical members of the ensemble look like. Next, you should always bracket a root, that is, know that the function changes sign in an identified interval, before trying to converge to the root's value.

Finally (this is advice with which some daring souls might disagree, but we give it nonetheless) never let your iteration method get outside of the best bracketing bounds obtained at any stage. We will see below that some pedagogically important algorithms, such as the *secant method* or *Newton-Raphson*, can violate this last constraint and are thus not recommended unless certain fixups are implemented.

Multiple roots, or very close roots, are a real problem, especially if the multiplicity is an even number. In that case, there may be no readily apparent sign change in the function, so the notion of bracketing a root — and maintaining the bracket — becomes difficult. We are hard-liners: We nevertheless insist on bracketing a root, even if it takes the minimum-searching techniques of Chapter 10 to determine whether a tantalizing dip in the function really does cross zero. (You can easily modify the simple golden section routine of §10.2 to return early if it detects a sign change in the function. And, if the minimum of the function is exactly zero, then you have found a *double* root.)

As usual, we want to discourage you from using routines as black boxes without understanding them. However, as a guide to beginners, here are some reasonable starting points:

- Brent's algorithm in §9.3 is the method of choice to find a bracketed root of a general one-dimensional function, when you cannot easily compute the function's derivative. Ridders' method (§9.2) is concise, and a close competitor.
- When you can compute the function's derivative, the routine `rtsafe` in §9.4, which combines the Newton-Raphson method with some bookkeeping on the bounds, is recommended. Again, you must first bracket your root. If you can easily compute *two* derivatives, then Halley's method (§9.4.2) is often worth a try.

- Roots of polynomials are a special case. Laguerre's method, in §9.5, is recommended as a starting point. Beware: Some polynomials are ill-conditioned!
- Finally, for multidimensional problems, the only elementary method is Newton-Raphson (§9.6), which works *very* well if you can supply a good first guess of the solution. Try it. Then read the more advanced material in §9.7 for some more complicated, but globally more convergent, alternatives.

The routines in this chapter require that you input the function whose roots you seek. For maximum flexibility, the routines typically will accept either a function or a functor (see §1.3.3).

9.0.1 Graphical Search for Roots

It never hurts to *look at your function*, especially if you encounter any difficulty in finding its roots blindly. If you are thus hunting roots "by eye," it is useful to have a routine that repeatedly plots a function to the screen, accepting user-supplied lower and upper limits for x, automatically scaling y, and making zero crossings visible. The following routine, or something like it, can occasionally save you a lot of grief.

scrsho.h

```
template<class T>
void scrsho(T &fx) {
```

Graph the function or functor fx over the prompted-for interval x1,x2. Query for another plot until the user signals satisfaction.

```
    const Int RES=500;                    Number of function evaluations for each plot.
    const Doub XLL=75., XUR=525., YLL=250., YUR=700.;        Corners of plot, in points.
    char *plotfilename = tmpnam(NULL);
    VecDoub xx(RES), yy(RES);
    Doub x1,x2;
    Int i;
    for (;;) {
        Doub ymax = -9.99e99, ymin = 9.99e99, del;
        cout << endl << "Enter x1 x2 (x1=x2 to stop):" << endl;
        cin >> x1 >> x2;                  Query for another plot, quit if x1=x2.
        if (x1==x2) break;
        for (i=0;i<RES;i++) {             Evaluate the function at equal intervals. Find
            xx[i] = x1 + i*(x2-x1)/(RES-1.);       the largest and smallest values.
            yy[i] = fx(xx[i]);
            if (yy[i] > ymax) ymax=yy[i];
            if (yy[i] < ymin) ymin=yy[i];
        }
        del = 0.05*((ymax-ymin)+(ymax==ymin ? abs(ymax) : 0.));
```

Plot commands, following, are in PSplot syntax (§22.1). You can substitute commands for your favorite plotting package.

```
        PSpage pg(plotfilename);
        PSplot plot(pg,XLL,XUR,YLL,YUR);
        plot.setlimits(x1,x2,ymin-del,ymax+del);
        plot.frame();
        plot.autoscales();
        plot.lineplot(xx,yy);
        if (ymax*ymin < 0.) plot.lineseg(x1,0.,x2,0.);
        plot.display();
    }
    remove(plotfilename);
}
```

CITED REFERENCES AND FURTHER READING:

Stoer, J., and Bulirsch, R. 2002, *Introduction to Numerical Analysis*, 3rd ed. (New York: Springer), Chapter 5.

Acton, F.S. 1970, *Numerical Methods That Work*; 1990, corrected edition (Washington, DC: Mathematical Association of America), Chapters 2, 7, and 14.

Deuflhard, P. 2004, *Newton Methods for Nonlinear Problems* (Berlin: Springer).

Ralston, A., and Rabinowitz, P. 1978, *A First Course in Numerical Analysis*, 2nd ed.; reprinted 2001 (New York: Dover), Chapter 8.

Householder, A.S. 1970, *The Numerical Treatment of a Single Nonlinear Equation* (New York: McGraw-Hill).

9.1 Bracketing and Bisection

We will say that a root is *bracketed* in the interval (a, b) if $f(a)$ and $f(b)$ have opposite signs. If the function is continuous, then at least one root must lie in that interval (the *intermediate value theorem*). If the function is discontinuous, but bounded, then instead of a root there might be a step discontinuity that crosses zero (see Figure 9.1.1). For numerical purposes, that might as well be a root, since the behavior is indistinguishable from the case of a continuous function whose zero crossing occurs in between two "adjacent" floating-point numbers in a machine's finite-precision representation. Only for functions with singularities is there the possibility that a bracketed root is not really there, as for example

$$f(x) = \frac{1}{x - c} \tag{9.1.1}$$

Some root-finding algorithms (e.g., bisection in this section) will readily converge to c in (9.1.1). Luckily there is not much possibility of your mistaking c, or any number x close to it, for a root, since mere evaluation of $|f(x)|$ will give a very large, rather than a very small, result.

If you are given a function in a black box, there is no sure way of bracketing its roots, or even of determining that it has roots. If you like pathological examples, think about the problem of locating the two real roots of equation (3.0.1), which dips below zero only in the ridiculously small interval of about $x = \pi \pm 10^{-667}$.

In the next chapter we will deal with the related problem of bracketing a function's minimum. There it is possible to give a procedure that always succeeds; in essence, "Go downhill, taking steps of increasing size, until your function starts back uphill." There is no analogous procedure for roots. The procedure "go downhill until your function changes sign," can be foiled by a function that has a simple extremum. Nevertheless, if you are prepared to deal with a "failure" outcome, this procedure is often a good first start; success is usual if your function has opposite signs in the limit $x \to \pm\infty$.

roots.h

```
template <class T>
Bool zbrac(T &func, Doub &x1, Doub &x2)
```

Given a function or functor `func` and an initial guessed range `x1` to `x2`, the routine expands the range geometrically until a root is bracketed by the returned values `x1` and `x2` (in which case `zbrac` returns `true`) or until the range becomes unacceptably large (in which case `zbrac` returns `false`).

```
{
    const Int NTRY=50;
    const Doub FACTOR=1.6;
```

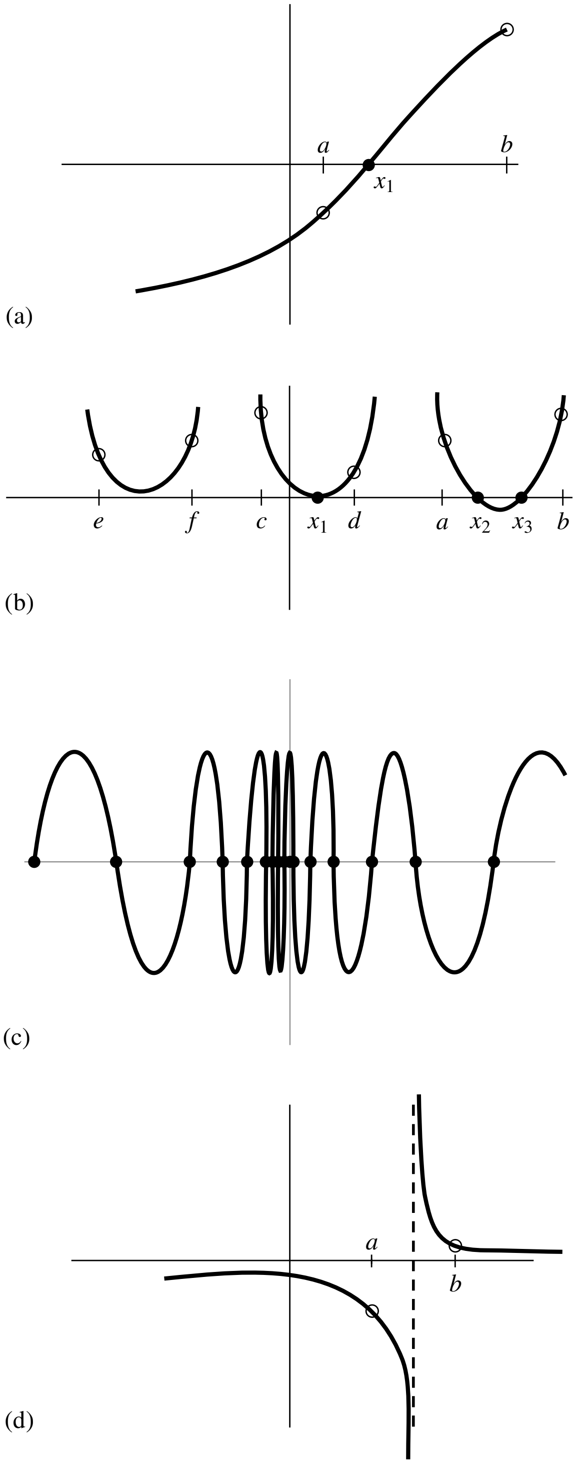

Figure 9.1.1. Some situations encountered while root finding: (a) an isolated root x_1 bracketed by two points a and b at which the function has opposite signs; (b) there is not necessarily a sign change in the function near a double root (in fact, there is not necessarily a root!); (c) a pathological function with many roots; in (d) the function has opposite signs at points a and b, but the points bracket a singularity, not a root.

```
    if (x1 == x2) throw("Bad initial range in zbrac");
    Doub f1=func(x1);
    Doub f2=func(x2);
    for (Int j=0;j<NTRY;j++) {
        if (f1*f2 < 0.0) return true;
        if (abs(f1) < abs(f2))
            f1=func(x1 += FACTOR*(x1-x2));
        else
            f2=func(x2 += FACTOR*(x2-x1));
    }
    return false;
}
```

Alternatively, you might want to "look inward" on an initial interval, rather than "look outward" from it, asking if there are any roots of the function $f(x)$ in the interval from x_1 to x_2 when a search is carried out by subdivision into n equal intervals. The following function calculates brackets for distinct intervals that each contain one or more roots.

roots.h

```
template <class T>
void zbrak(T &fx, const Doub x1, const Doub x2, const Int n, VecDoub_O &xb1,
    VecDoub_O &xb2, Int &nroot)
```

Given a function or functor `fx` defined on the interval `[x1,x2]`, subdivide the interval into `n` equally spaced segments, and search for zero crossings of the function. `nroot` will be set to the number of bracketing pairs found. If it is positive, the arrays `xb1[0..nroot-1]` and `xb2[0..nroot-1]` will be filled sequentially with any bracketing pairs that are found. On input, these vectors may have any size, including zero; they will be resized to $\geq$ `nroot`.

```
{
    Int nb=20;
    xb1.resize(nb);
    xb2.resize(nb);
    nroot=0;
    Doub dx=(x2-x1)/n;                Determine the spacing appropriate to the mesh.
    Doub x=x1;
    Doub fp=fx(x1);
    for (Int i=0;i<n;i++) {           Loop over all intervals
        Doub fc=fx(x += dx);
        if (fc*fp <= 0.0) {           If a sign change occurs, then record values for the
            xb1[nroot]=x-dx;              bounds.
            xb2[nroot++]=x;
            if(nroot == nb) {
                VecDoub tempvec1(xb1),tempvec2(xb2);
                xb1.resize(2*nb);
                xb2.resize(2*nb);
                for (Int j=0; j<nb; j++) {
                    xb1[j]=tempvec1[j];
                    xb2[j]=tempvec2[j];
                }
                nb *= 2;
            }
        }
        fp=fc;
    }
}
```

9.1.1 Bisection Method

Once we know that an interval contains a root, several classical procedures are available to refine it. These proceed with varying degrees of speed and sure-

ness toward the answer. Unfortunately, the methods that are guaranteed to converge plod along most slowly, while those that rush to the solution in the best cases can also dash rapidly to infinity without warning if measures are not taken to avoid such behavior.

The *bisection method* is one that cannot fail. It is thus not to be sneered at as a method for otherwise badly behaved problems. The idea is simple. Over some interval the function is known to pass through zero because it changes sign. Evaluate the function at the interval's midpoint and examine its sign. Use the midpoint to replace whichever limit has the same sign. After each iteration the bounds containing the root decrease by a factor of two. If after n iterations the root is known to be within an interval of size ϵ_n, then after the next iteration it will be bracketed within an interval of size

$$\epsilon_{n+1} = \epsilon_n/2 \tag{9.1.2}$$

neither more nor less. Thus, we know in advance the number of iterations required to achieve a given tolerance in the solution,

$$n = \log_2 \frac{\epsilon_0}{\epsilon} \tag{9.1.3}$$

where ϵ_0 is the size of the initially bracketing interval and ϵ is the desired ending tolerance.

Bisection *must* succeed. If the interval happens to contain more than one root, bisection will find one of them. If the interval contains no roots and merely straddles a singularity, it will converge on the singularity.

When a method converges as a factor (less than 1) times the previous uncertainty to the first power (as is the case for bisection), it is said to converge *linearly*. Methods that converge as a higher power,

$$\epsilon_{n+1} = \text{constant} \times (\epsilon_n)^m \qquad m > 1 \tag{9.1.4}$$

are said to converge superlinearly. In other contexts, "linear" convergence would be termed "exponential" or "geometrical." That is not too bad at all: Linear convergence means that successive significant figures are won linearly with computational effort.

It remains to discuss practical criteria for convergence. It is crucial to keep in mind that only a finite set of floating point values have exact computer representations. While your function might analytically pass through zero, it is probable that its computed value is never zero, for any floating-point argument. One must decide what accuracy on the root is attainable: Convergence to within 10^{-10} in absolute value is reasonable when the root lies near 1 but certainly unachievable if the root lies near 10^{26}. One might thus think to specify convergence by a relative (fractional) criterion, but this becomes unworkable for roots near zero. To be most general, the routines below will require you to specify an absolute tolerance, such that iterations continue until the interval becomes smaller than this tolerance in absolute units. Often you may wish to take the tolerance to be $\epsilon(|x_1|+|x_2|)/2$, where ϵ is the machine precision and x_1 and x_2 are the initial brackets. When the root lies near zero you ought to consider carefully what reasonable tolerance means for your function. The following routine quits after 50 bisections in any event, with $2^{-50} \approx 10^{-15}$.

roots.h

```
template <class T>
Doub rtbis(T &func, const Doub x1, const Doub x2, const Doub xacc) {
```
Using bisection, return the root of a function or functor `func` known to lie between `x1` and `x2`. The root will be refined until its accuracy is ±`xacc`.
```
    const Int JMAX=50;                    Maximum allowed number of bisections.
    Doub dx,xmid,rtb;
    Doub f=func(x1);
    Doub fmid=func(x2);
    if (f*fmid >= 0.0) throw("Root must be bracketed for bisection in rtbis");
    rtb = f < 0.0 ? (dx=x2-x1,x1) : (dx=x1-x2,x2);        Orient the search so that f>0
    for (Int j=0;j<JMAX;j++) {                                lies at x+dx.
        fmid=func(xmid=rtb+(dx *= 0.5));                  Bisection loop.
        if (fmid <= 0.0) rtb=xmid;
        if (abs(dx) < xacc || fmid == 0.0) return rtb;
    }
    throw("Too many bisections in rtbis");
}
```

9.2 Secant Method, False Position Method, and Ridders' Method

For functions that are smooth near a root, the methods known respectively as *false position* (or *regula falsi*) and the *secant method* generally converge faster than bisection. In both of these methods the function is assumed to be approximately linear in the local region of interest, and the next improvement in the root is taken as the point where the approximating line crosses the axis. After each iteration, one of the previous boundary points is discarded in favor of the latest estimate of the root.

The *only* difference between the methods is that secant retains the most recent of the prior estimates (Figure 9.2.1; this requires an arbitrary choice on the first iteration), while false position retains that prior estimate for which the function value has the opposite sign from the function value at the current best estimate of the root, so that the two points continue to bracket the root (Figure 9.2.2). Mathematically, the secant method converges more rapidly near a root of a sufficiently continuous function. Its order of convergence can be shown to be the "golden ratio" 1.618 ..., so that

$$\lim_{k\to\infty} |\epsilon_{k+1}| \approx \text{const} \times |\epsilon_k|^{1.618} \tag{9.2.1}$$

The secant method has, however, the disadvantage that the root does not necessarily remain bracketed. For functions that are *not* sufficiently continuous, the algorithm can therefore not be guaranteed to converge: Local behavior might send it off toward infinity.

False position, since it sometimes keeps an older rather than newer function evaluation, has a lower order of convergence. Since the newer function value will *sometimes* be kept, the method is often superlinear, but estimation of its exact order is not so easy.

Here are sample implementations of these two related methods. While these methods are standard textbook fare, *Ridders' method*, described below, or *Brent's method*, described in the next section, are almost always better choices. Figure 9.2.3 shows the behavior of the secant and false-position methods in a difficult situation.

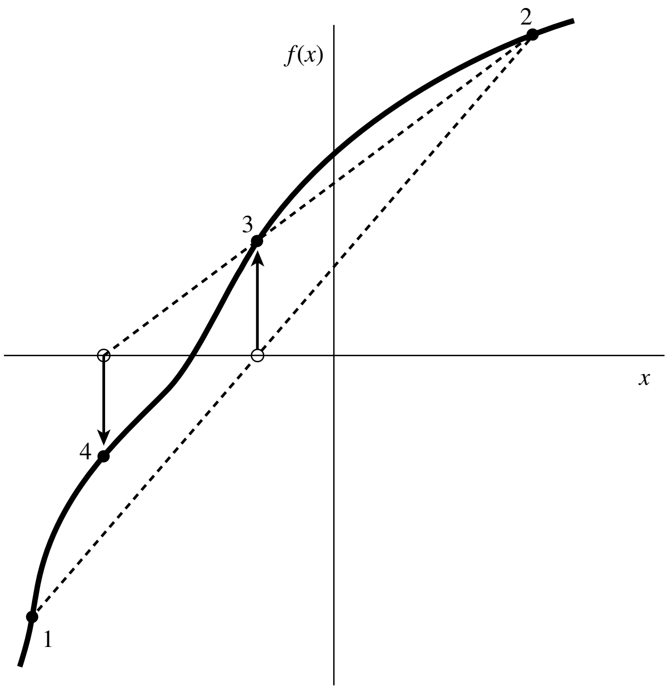

Figure 9.2.1. Secant method. Extrapolation or interpolation lines (dashed) are drawn through the two most recently evaluated points, whether or not they bracket the function. The points are numbered in the order that they are used.

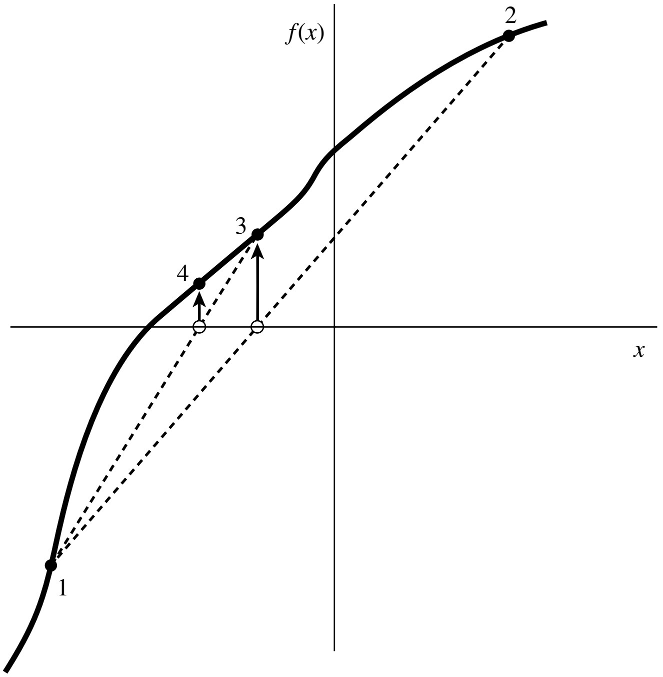

Figure 9.2.2. False-position method. Interpolation lines (dashed) are drawn through the most recent points *that bracket the root*. In this example, point 1 thus remains "active" for many steps. False position converges less rapidly than the secant method, but it is more certain.

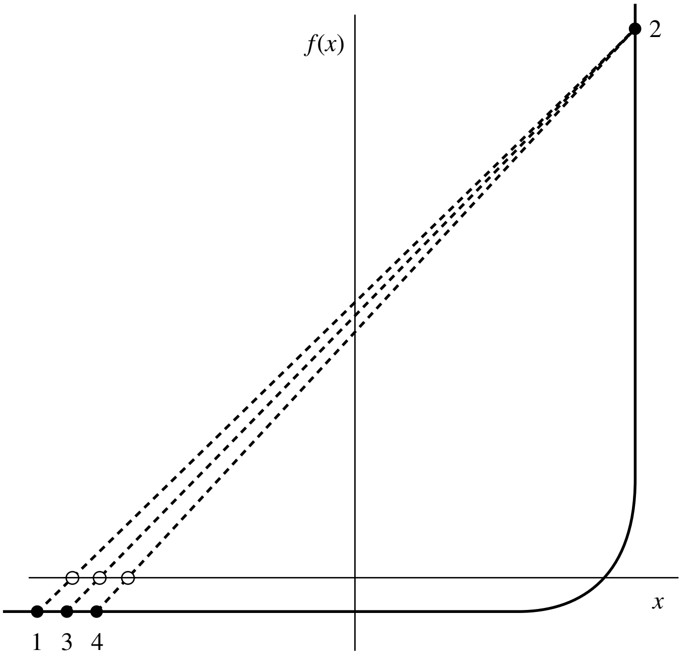

Figure 9.2.3. Example where both the secant and false-position methods will take many iterations to arrive at the true root. This function would be difficult for many other root-finding methods.

roots.h

```
template <class T>
Doub rtflsp(T &func, const Doub x1, const Doub x2, const Doub xacc) {
```
Using the false-position method, return the root of a function or functor func known to lie between x1 and x2. The root is refined until its accuracy is ±xacc.
```
    const Int MAXIT=30;                 Set to the maximum allowed number of iterations.
    Doub xl,xh,del;
    Doub fl=func(x1);
    Doub fh=func(x2);                   Be sure the interval brackets a root.
    if (fl*fh > 0.0) throw("Root must be bracketed in rtflsp");
    if (fl < 0.0) {                     Identify the limits so that xl corresponds to the low
        xl=x1;                              side.
        xh=x2;
    } else {
        xl=x2;
        xh=x1;
        SWAP(fl,fh);
    }
    Doub dx=xh-xl;
    for (Int j=0;j<MAXIT;j++) {         False-position loop.
        Doub rtf=xl+dx*fl/(fl-fh);      Increment with respect to latest value.
        Doub f=func(rtf);
        if (f < 0.0) {                  Replace appropriate limit.
            del=xl-rtf;
            xl=rtf;
            fl=f;
        } else {
            del=xh-rtf;
            xh=rtf;
            fh=f;
        }
        dx=xh-xl;
        if (abs(del) < xacc || f == 0.0) return rtf;      Convergence.
```

```
    }
    throw("Maximum number of iterations exceeded in rtflsp");
}
```

roots.h

```
template <class T>
Doub rtsec(T &func, const Doub x1, const Doub x2, const Doub xacc) {
```

Using the secant method, return the root of a function or functor `func` thought to lie between `x1` and `x2`. The root is refined until its accuracy is ±`xacc`.

```
    const Int MAXIT=30;              Maximum allowed number of iterations.
    Doub xl,rts;
    Doub fl=func(x1);
    Doub f=func(x2);
    if (abs(fl) < abs(f)) {          Pick the bound with the smaller function value as
        rts=x1;                          the most recent guess.
        xl=x2;
        SWAP(fl,f);
    } else {
        xl=x1;
        rts=x2;
    }
    for (Int j=0;j<MAXIT;j++) {      Secant loop.
        Doub dx=(xl-rts)*f/(f-fl);   Increment with respect to latest value.
        xl=rts;
        fl=f;
        rts += dx;
        f=func(rts);
        if (abs(dx) < xacc || f == 0.0) return rts;      Convergence.
    }
    throw("Maximum number of iterations exceeded in rtsec");
}
```

9.2.1 Ridders' Method

A powerful variant on false position is due to Ridders [1]. When a root is bracketed between x_1 and x_2, Ridders' method first evaluates the function at the midpoint $x_3 = (x_1 + x_2)/2$. It then factors out that unique exponential function that turns the residual function into a straight line. Specifically, it solves for a factor e^Q that gives

$$f(x_1) - 2f(x_3)e^Q + f(x_2)e^{2Q} = 0 \tag{9.2.2}$$

This is a quadratic equation in e^Q, which can be solved to give

$$e^Q = \frac{f(x_3) + \text{sign}[f(x_2)]\sqrt{f(x_3)^2 - f(x_1)f(x_2)}}{f(x_2)} \tag{9.2.3}$$

Now the false-position method is applied, not to the values $f(x_1)$, $f(x_3)$, $f(x_2)$, but to the values $f(x_1)$, $f(x_3)e^Q$, $f(x_2)e^{2Q}$, yielding a new guess for the root, x_4. The overall updating formula (incorporating the solution 9.2.3) is

$$x_4 = x_3 + (x_3 - x_1)\frac{\text{sign}[f(x_1) - f(x_2)]f(x_3)}{\sqrt{f(x_3)^2 - f(x_1)f(x_2)}} \tag{9.2.4}$$

Equation (9.2.4) has some very nice properties. First, x_4 is guaranteed to lie in the interval (x_1, x_2), so the method never jumps out of its brackets. Second, the convergence of successive applications of equation (9.2.4) is *quadratic*, that is, $m = 2$

in equation (9.1.4). Since each application of (9.2.4) requires two function evaluations, the actual order of the method is $\sqrt{2}$, not 2; but this is still quite respectably superlinear: The number of significant digits in the answer approximately *doubles* with each two function evaluations. Third, taking out the function's "bend" via exponential (that is, ratio) factors, rather than via a polynomial technique (e.g., fitting a parabola), turns out to give an extraordinarily robust algorithm. In both reliability and speed, Ridders' method is generally competitive with the more highly developed and better established (but more complicated) method of van Wijngaarden, Dekker, and Brent, which we next discuss.

roots.h

```
template <class T>
Doub zriddr(T &func, const Doub x1, const Doub x2, const Doub xacc) {
```
Using Ridders' method, return the root of a function or functor `func` known to lie between `x1` and `x2`. The root will be refined to an approximate accuracy `xacc`.
```
    const Int MAXIT=60;
    Doub fl=func(x1);
    Doub fh=func(x2);
    if ((fl > 0.0 && fh < 0.0) || (fl < 0.0 && fh > 0.0)) {
        Doub xl=x1;
        Doub xh=x2;
        Doub ans=-9.99e99;                      Any highly unlikely value, to simplify logic
                                                   below.
        for (Int j=0;j<MAXIT;j++) {
            Doub xm=0.5*(xl+xh);
            Doub fm=func(xm);                   First of two function evaluations per it-
                                                   eration.
            Doub s=sqrt(fm*fm-fl*fh);
            if (s == 0.0) return ans;
            Doub xnew=xm+(xm-xl)*((fl >= fh ? 1.0 : -1.0)*fm/s);    Updating formula.
            if (abs(xnew-ans) <= xacc) return ans;
            ans=xnew;
            Doub fnew=func(ans);                Second of two function evaluations per
                                                   iteration.
            if (fnew == 0.0) return ans;
            if (SIGN(fm,fnew) != fm) {          Bookkeeping to keep the root bracketed
                                                   on next iteration.
                xl=xm;
                fl=fm;
                xh=ans;
                fh=fnew;
            } else if (SIGN(fl,fnew) != fl) {
                xh=ans;
                fh=fnew;
            } else if (SIGN(fh,fnew) != fh) {
                xl=ans;
                fl=fnew;
            } else throw("never get here.");
            if (abs(xh-xl) <= xacc) return ans;
        }
        throw("zriddr exceed maximum iterations");
    }
    else {
        if (fl == 0.0) return x1;
        if (fh == 0.0) return x2;
        throw("root must be bracketed in zriddr.");
    }
}
```

CITED REFERENCES AND FURTHER READING:

Ralston, A., and Rabinowitz, P. 1978, *A First Course in Numerical Analysis*, 2nd ed.; reprinted 2001 (New York: Dover), §8.3.

Ostrowski, A.M. 1966, *Solutions of Equations and Systems of Equations*, 2nd ed. (New York: Academic Press), Chapter 12.

Ridders, C.J.F. 1979, "A New Algorithm for Computing a Single Root of a Real Continuous Function," *IEEE Transactions on Circuits and Systems*, vol. CAS-26, pp. 979–980.[1]

9.3 Van Wijngaarden-Dekker-Brent Method

While secant and false position formally converge faster than bisection, one finds in practice pathological functions for which bisection converges more rapidly. These can be choppy, discontinuous functions, or even smooth functions if the second derivative changes sharply near the root. Bisection always halves the interval, while secant and false position can sometimes spend many cycles slowly pulling distant bounds closer to a root. Ridders' method does a much better job, but it too can sometimes be fooled. Is there a way to combine superlinear convergence with the sureness of bisection?

Yes. We can keep track of whether a supposedly superlinear method is actually converging the way it is supposed to, and, if it is not, we can intersperse bisection steps so as to guarantee *at least* linear convergence. This kind of super-strategy requires attention to bookkeeping detail, and also careful consideration of how roundoff errors can affect the guiding strategy. Also, we must be able to determine reliably when convergence has been achieved.

An excellent algorithm that pays close attention to these matters was developed in the 1960s by van Wijngaarden, Dekker, and others at the Mathematical Center in Amsterdam, and later improved by Brent [1]. For brevity, we refer to the final form of the algorithm as *Brent's method*. The method is *guaranteed* (by Brent) to converge, so long as the function can be evaluated within the initial interval known to contain a root.

Brent's method combines root bracketing, bisection, and *inverse quadratic interpolation* to converge from the neighborhood of a zero crossing. While the false-position and secant methods assume approximately linear behavior between two prior root estimates, inverse quadratic interpolation uses three prior points to fit an inverse quadratic function (x as a quadratic function of y) whose value at $y = 0$ is taken as the next estimate of the root x. Of course one must have contingency plans for what to do if the root falls outside of the brackets. Brent's method takes care of all that. If the three point pairs are $[a, f(a)]$, $[b, f(b)]$, $[c, f(c)]$, then the interpolation formula (cf. equation 3.2.1) is

$$x = \frac{[y - f(a)][y - f(b)]c}{[f(c) - f(a)][f(c) - f(b)]} + \frac{[y - f(b)][y - f(c)]a}{[f(a) - f(b)][f(a) - f(c)]} + \frac{[y - f(c)][y - f(a)]b}{[f(b) - f(c)][f(b) - f(a)]} \tag{9.3.1}$$

Setting y to zero gives a result for the next root estimate, which can be written as

$$x = b + P/Q \tag{9.3.2}$$

where, in terms of

$$R \equiv f(b)/f(c), \qquad S \equiv f(b)/f(a), \qquad T \equiv f(a)/f(c) \tag{9.3.3}$$

we have

$$P = S\,[T(R-T)(c-b)-(1-R)(b-a)]$$
$$Q = (T-1)(R-1)(S-1) \tag{9.3.4}$$

In practice b is the current best estimate of the root and P/Q ought to be a "small" correction. Quadratic methods work well only when the function behaves smoothly; they run the serious risk of giving very bad estimates of the next root or causing machine failure by an inappropriate division by a very small number ($Q \approx 0$). Brent's method guards against this problem by maintaining brackets on the root and checking where the interpolation would land before carrying out the division. When the correction P/Q would not land within the bounds, or when the bounds are not collapsing rapidly enough, the algorithm takes a bisection step. Thus, Brent's method combines the sureness of bisection with the speed of a higher-order method when appropriate. We recommend it as the method of choice for general one-dimensional root finding where a function's values only (and not its derivative or functional form) are available.

roots.h

```
template <class T>
Doub zbrent(T &func, const Doub x1, const Doub x2, const Doub tol)
```

Using Brent's method, return the root of a function or functor `func` known to lie between `x1` and `x2`. The root will be refined until its accuracy is `tol`.

```
{
    const Int ITMAX=100;                      Maximum allowed number of iterations.
    const Doub EPS=numeric_limits<Doub>::epsilon();
    Machine floating-point precision.
    Doub a=x1,b=x2,c=x2,d,e,fa=func(a),fb=func(b),fc,p,q,r,s,tol1,xm;
    if ((fa > 0.0 && fb > 0.0) || (fa < 0.0 && fb < 0.0))
        throw("Root must be bracketed in zbrent");
    fc=fb;
    for (Int iter=0;iter<ITMAX;iter++) {
        if ((fb > 0.0 && fc > 0.0) || (fb < 0.0 && fc < 0.0)) {
            c=a;                              Rename a, b, c and adjust bounding interval
            fc=fa;                                d.
            e=d=b-a;
        }
        if (abs(fc) < abs(fb)) {
            a=b;
            b=c;
            c=a;
            fa=fb;
            fb=fc;
            fc=fa;
        }
        tol1=2.0*EPS*abs(b)+0.5*tol;          Convergence check.
        xm=0.5*(c-b);
        if (abs(xm) <= tol1 || fb == 0.0) return b;
        if (abs(e) >= tol1 && abs(fa) > abs(fb)) {
            s=fb/fa;                          Attempt inverse quadratic interpolation.
            if (a == c) {
                p=2.0*xm*s;
                q=1.0-s;
            } else {
                q=fa/fc;
                r=fb/fc;
                p=s*(2.0*xm*q*(q-r)-(b-a)*(r-1.0));
                q=(q-1.0)*(r-1.0)*(s-1.0);
            }
            if (p > 0.0) q = -q;              Check whether in bounds.
            p=abs(p);
```

```
            Doub min1=3.0*xm*q-abs(tol1*q);
            Doub min2=abs(e*q);
            if (2.0*p < (min1 < min2 ? min1 : min2)) {
                e=d;                          Accept interpolation.
                d=p/q;
            } else {
                d=xm;                         Interpolation failed, use bisection.
                e=d;
            }
        } else {                              Bounds decreasing too slowly, use bisection.
            d=xm;
            e=d;
        }
        a=b;                                  Move last best guess to a.
        fa=fb;
        if (abs(d) > tol1)                    Evaluate new trial root.
            b += d;
        else
            b += SIGN(tol1,xm);
            fb=func(b);
    }
    throw("Maximum number of iterations exceeded in zbrent");
}
```

CITED REFERENCES AND FURTHER READING:

Brent, R.P. 1973, *Algorithms for Minimization without Derivatives* (Englewood Cliffs, NJ: Prentice-Hall); reprinted 2002 (New York: Dover), Chapters 3, 4.[1]

Forsythe, G.E., Malcolm, M.A., and Moler, C.B. 1977, *Computer Methods for Mathematical Computations* (Englewood Cliffs, NJ: Prentice-Hall), §7.2.

9.4 Newton-Raphson Method Using Derivative

Perhaps the most celebrated of all one-dimensional root-finding routines is *Newton's method*, also called the *Newton-Raphson method*. Joseph Raphson (1648–1715) was a contemporary of Newton who independently invented the method in 1690, some 20 years after Newton did, but some 20 years before Newton actually published it. This method is distinguished from the methods of previous sections by the fact that it requires the evaluation of both the function $f(x)$ *and* the derivative $f'(x)$, at arbitrary points x. The Newton-Raphson formula consists geometrically of extending the tangent line at a current point x_i until it crosses zero, then setting the next guess x_{i+1} to the abscissa of that zero crossing (see Figure 9.4.1). Algebraically, the method derives from the familiar Taylor series expansion of a function in the neighborhood of a point,

$$f(x+\delta) \approx f(x) + f'(x)\delta + \frac{f''(x)}{2}\delta^2 + \cdots \tag{9.4.1}$$

For small enough values of δ, and for well-behaved functions, the terms beyond linear are unimportant, hence $f(x+\delta) = 0$ implies

$$\delta = -\frac{f(x)}{f'(x)} \tag{9.4.2}$$

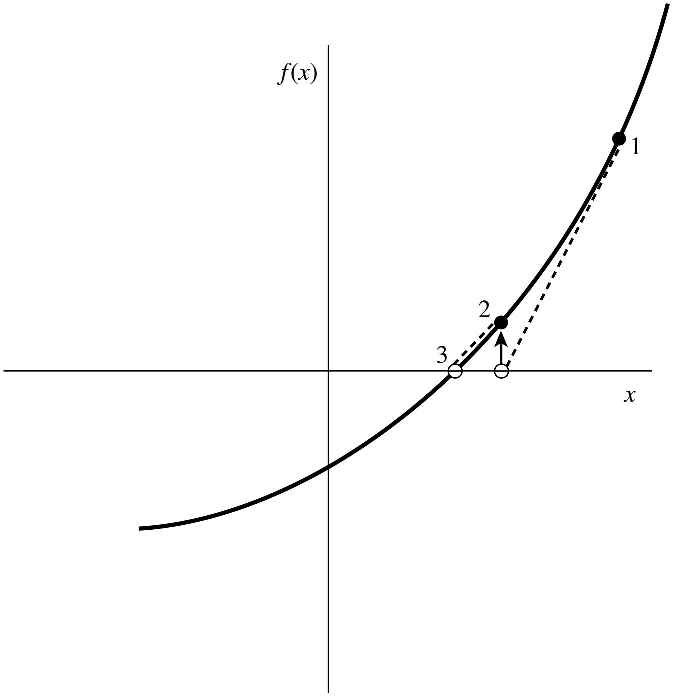

Figure 9.4.1. Newton's method extrapolates the local derivative to find the next estimate of the root. In this example it works well and converges quadratically.

Newton-Raphson is not restricted to one dimension. The method readily generalizes to multiple dimensions, as we shall see in §9.6 and §9.7, below.

Far from a root, where the higher-order terms in the series *are* important, the Newton-Raphson formula can give grossly inaccurate, meaningless corrections. For instance, the initial guess for the root might be so far from the true root as to let the search interval include a local maximum or minimum of the function. This can be death to the method (see Figure 9.4.2). If an iteration places a trial guess near such a local extremum, so that the first derivative nearly vanishes, then Newton-Raphson sends its solution off to limbo, with vanishingly small hope of recovery. Figure 9.4.3 demonstrates another possible pathology.

Why is Newton-Raphson so powerful? The answer is its rate of convergence: Within a small distance ϵ of x, the function and its derivative are approximately

$$\begin{aligned} f(x+\epsilon) &= f(x) + \epsilon f'(x) + \epsilon^2 \frac{f''(x)}{2} + \cdots, \\ f'(x+\epsilon) &= f'(x) + \epsilon f''(x) + \cdots \end{aligned} \tag{9.4.3}$$

By the Newton-Raphson formula,

$$x_{i+1} = x_i - \frac{f(x_i)}{f'(x_i)} \tag{9.4.4}$$

so that

$$\epsilon_{i+1} = \epsilon_i - \frac{f(x_i)}{f'(x_i)} \tag{9.4.5}$$

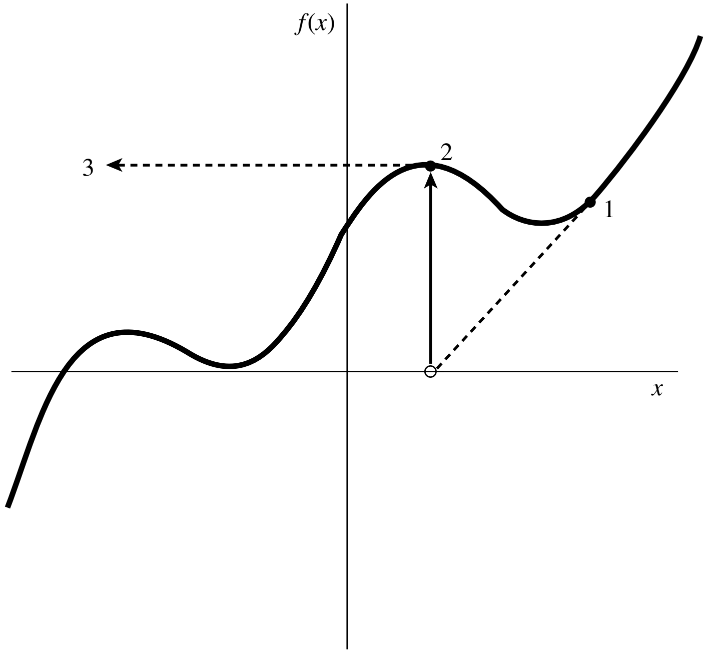

Figure 9.4.2. Unfortunate case where Newton's method encounters a local extremum and shoots off to outer space. Here bracketing bounds, as in `rtsafe`, would save the day.

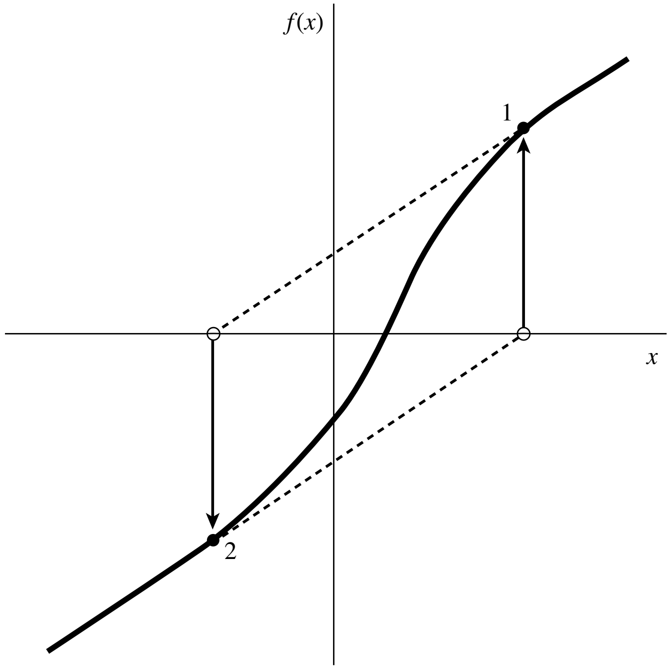

Figure 9.4.3. Unfortunate case where Newton's method enters a nonconvergent cycle. This behavior is often encountered when the function f is obtained, in whole or in part, by table interpolation. With a better initial guess, the method would have succeeded.

When a trial solution x_i differs from the true root by ϵ_i, we can use (9.4.3) to express $f(x_i)$, $f'(x_i)$ in (9.4.4) in terms of ϵ_i and derivatives at the root itself. The result is a recurrence relation for the deviations of the trial solutions

$$\epsilon_{i+1} = -\epsilon_i^2 \frac{f''(x)}{2f'(x)} \tag{9.4.6}$$

Equation (9.4.6) says that Newton-Raphson converges *quadratically* (cf. equation 9.2.3). Near a root, the number of significant digits approximately *doubles* with each step. This very strong convergence property makes Newton-Raphson the method of choice for any function whose derivative can be evaluated efficiently, and whose derivative is continuous and nonzero in the neighborhood of a root.

Even where Newton-Raphson is rejected for the early stages of convergence (because of its poor global convergence properties), it is very common to "polish up" a root with one or two steps of Newton-Raphson, which can multiply by two or four its number of significant figures.

For an efficient realization of Newton-Raphson, the user provides a routine that evaluates both $f(x)$ and its first derivative $f'(x)$ at the point x. The Newton-Raphson formula can also be applied using a numerical difference to approximate the true local derivative,

$$f'(x) \approx \frac{f(x+dx) - f(x)}{dx} \tag{9.4.7}$$

This is not, however, a recommended procedure for the following reasons: (i) You are doing two function evaluations per step, so *at best* the superlinear order of convergence will be only $\sqrt{2}$. (ii) If you take dx too small, you will be wiped out by roundoff, while if you take it too large, your order of convergence will be only linear, no better than using the *initial* evaluation $f'(x_0)$ for all subsequent steps. Therefore, Newton-Raphson with numerical derivatives is (in one dimension) always dominated by Brent's method (§9.3). (In multidimensions, where there is a paucity of available methods, Newton-Raphson with numerical derivatives must be taken more seriously. See §9.6 – §9.7.)

The following routine invokes a user-supplied structure that supplies the function value and the derivative. The function value is returned in the usual way as a functor by overloading `operator()`, while the derivative is returned by the `df` function in the structure. For example, to find a root of the Bessel function $J_0(x)$ (derivative $-J_1(x)$) you would have a structure like

```
struct Funcd {
    Bessjy bess;
    Doub operator() (const Doub x) {
        return bess.j0(x);
    }
    Doub df(const Doub x) {
        return -bess.j1(x);
    }
};
```

(While you can use any name for `Funcd`, the name `df` is fixed.) Your code should then create an instance of this structure and pass it to `rtnewt`:

```
Funcd fx;
Doub root=rtnewt(fx,x1,x2,xacc);
```

The routine rtnewt includes input bounds on the root x1 and x2 simply to be consistent with previous root-finding routines: Newton does not adjust bounds, and works only on local information at the point x. The bounds are used only to pick the midpoint as the first guess, and to reject the solution if it wanders outside of the bounds.

roots.h

```
template <class T>
Doub rtnewt(T &funcd, const Doub x1, const Doub x2, const Doub xacc) {
```

Using the Newton-Raphson method, return the root of a function known to lie in the interval [x1, x2]. The root will be refined until its accuracy is known within ±xacc. funcd is a user-supplied struct that returns the function value as a functor and the first derivative of the function at the point x as the function df (see text).

```
    const Int JMAX=20;                      Set to maximum number of iterations.
    Doub rtn=0.5*(x1+x2);                   Initial guess.
    for (Int j=0;j<JMAX;j++) {
        Doub f=funcd(rtn);
        Doub df=funcd.df(rtn);
        Doub dx=f/df;
        rtn -= dx;
        if ((x1-rtn)*(rtn-x2) < 0.0)
            throw("Jumped out of brackets in rtnewt");
        if (abs(dx) < xacc) return rtn;     Convergence.
    }
    throw("Maximum number of iterations exceeded in rtnewt");
}
```

While Newton-Raphson's global convergence properties are poor, it is fairly easy to design a fail-safe routine that utilizes a combination of bisection and Newton-Raphson. The hybrid algorithm takes a bisection step whenever Newton-Raphson would take the solution out of bounds, or whenever Newton-Raphson is not reducing the size of the brackets rapidly enough.

roots.h

```
template <class T>
Doub rtsafe(T &funcd, const Doub x1, const Doub x2, const Doub xacc) {
```

Using a combination of Newton-Raphson and bisection, return the root of a function bracketed between x1 and x2. The root will be refined until its accuracy is known within ±xacc. funcd is a user-supplied struct that returns the function value as a functor and the first derivative of the function at the point x as the function df (see text).

```
    const Int MAXIT=100;                    Maximum allowed number of iterations.
    Doub xh,xl;
    Doub fl=funcd(x1);
    Doub fh=funcd(x2);
    if ((fl > 0.0 && fh > 0.0) || (fl < 0.0 && fh < 0.0))
        throw("Root must be bracketed in rtsafe");
    if (fl == 0.0) return x1;
    if (fh == 0.0) return x2;
    if (fl < 0.0) {                         Orient the search so that f(xl) < 0.
        xl=x1;
        xh=x2;
    } else {
        xh=x1;
        xl=x2;
    }
    Doub rts=0.5*(x1+x2);                   Initialize the guess for root,
    Doub dxold=abs(x2-x1);                  the "stepsize before last,"
    Doub dx=dxold;                          and the last step.
    Doub f=funcd(rts);
    Doub df=funcd.df(rts);
    for (Int j=0;j<MAXIT;j++) {             Loop over allowed iterations.
        if ((((rts-xh)*df-f)*((rts-xl)*df-f) > 0.0)     Bisect if Newton out of range,
            || (abs(2.0*f) > abs(dxold*df))) {          or not decreasing fast enough.
```

```
            dxold=dx;
            dx=0.5*(xh-xl);
            rts=xl+dx;
            if (xl == rts) return rts;         Change in root is negligible.
        } else {                               Newton step acceptable. Take it.
            dxold=dx;
            dx=f/df;
            Doub temp=rts;
            rts -= dx;
            if (temp == rts) return rts;
        }
        if (abs(dx) < xacc) return rts;        Convergence criterion.
        Doub f=funcd(rts);
        Doub df=funcd.df(rts);
        The one new function evaluation per iteration.
        if (f < 0.0)                           Maintain the bracket on the root.
            xl=rts;
        else
            xh=rts;
    }
    throw("Maximum number of iterations exceeded in rtsafe");
}
```

For many functions, the derivative $f'(x)$ often converges to machine accuracy before the function $f(x)$ itself does. When that is the case one need not subsequently update $f'(x)$. This shortcut is recommended only when you confidently understand the generic behavior of your function, but it speeds computations when the derivative calculation is laborious. (Formally, this makes the convergence only linear, but if the derivative isn't changing anyway, you can do no better.)

9.4.1 Newton-Raphson and Fractals

An interesting sidelight to our repeated warnings about Newton-Raphson's unpredictable global convergence properties — its very rapid local convergence notwithstanding — is to investigate, for some particular equation, the set of starting values from which the method does, or doesn't, converge to a root.

Consider the simple equation

$$z^3 - 1 = 0 \tag{9.4.8}$$

whose single real root is $z = 1$, but which also has complex roots at the other two cube roots of unity, $\exp(\pm 2\pi i/3)$. Newton's method gives the iteration

$$z_{j+1} = z_j - \frac{z_j^3 - 1}{3z_j^2} \tag{9.4.9}$$

Up to now, we have applied an iteration like equation (9.4.9) only for real starting values z_0, but in fact all of the equations in this section also apply in the complex plane. We can therefore map out the complex plane into regions from which a starting value z_0, iterated in equation (9.4.9), will, or won't, converge to $z = 1$. Naively, we might expect to find a "basin of convergence" somehow surrounding the root $z = 1$. We surely do not expect the basin of convergence to fill the whole plane, because the plane must also contain regions that converge to each of the two complex roots. In fact, by symmetry, the three regions must have identical shapes. Perhaps they will be three symmetric 120° wedges, with one root centered in each?

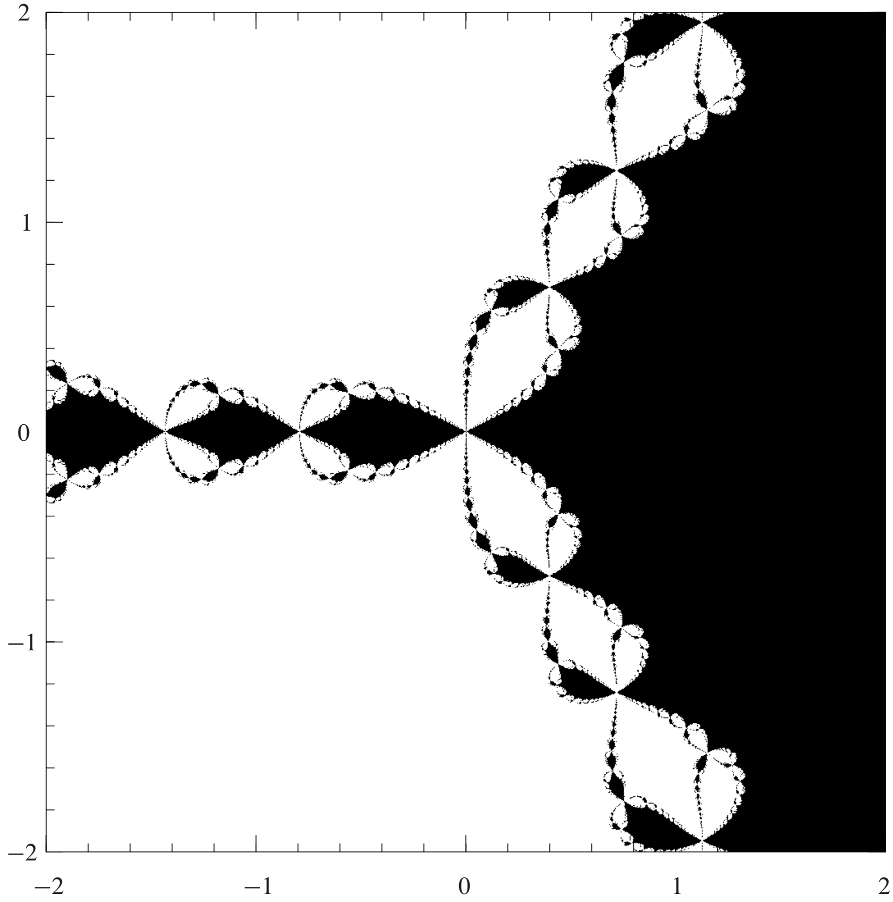

Figure 9.4.4. The complex z-plane with real and imaginary components in the range $(-2, 2)$. The black region is the set of points from which Newton's method converges to the root $z = 1$ of the equation $z^3 - 1 = 0$. Its shape is fractal.

Now take a look at Figure 9.4.4, which shows the result of a numerical exploration. The basin of convergence does indeed cover $1/3$ the area of the complex plane, but its boundary is highly irregular — in fact, *fractal*. (A fractal, so called, has self-similar structure that repeats on all scales of magnification.) How does this fractal emerge from something as simple as Newton's method and an equation as simple as (9.4.8)? The answer is already implicit in Figure 9.4.2, which showed how, on the real line, a local extremum causes Newton's method to shoot off to infinity. Suppose one is *slightly* removed from such a point. Then one might be shot off not to infinity, but — by luck — right into the basin of convergence of the desired root. But that means that in the neighborhood of an extremum there must be a tiny, perhaps distorted, copy of the basin of convergence — a kind of "one-bounce away" copy. Similar logic shows that there can be "two-bounce" copies, "three-bounce" copies, and so on. A fractal thus emerges.

Notice that, for equation (9.4.8), almost the whole real axis is in the domain of convergence for the root $z = 1$. We say "almost" because of the peculiar discrete points on the negative real axis whose convergence is indeterminate (see figure). What happens if you start Newton's method from one of these points? (Try it.)

9.4.2 Halley's Method

Edmund Halley (1656–1742) was a contemporary and close friend of Newton. His contribution to root finding was to extend Newton's method to use information from the next term in the (as we would now say it) Taylor series, the second derivative $f''(x)$. Omitting a straightforward derivation, the update formula (9.4.4) now becomes

$$x_{i+1} = x_i - \frac{f(x_i)}{f'(x_i)\left(1 - \frac{f(x_i)f''(x_i)}{2f'(x_i)^2}\right)} \tag{9.4.10}$$

You can see that the update scheme is essentially Newton-Raphson, but with an extra, hopefully small, correction term in the denominator.

It only makes sense to use Halley's method when it is easy to calculate $f''(x_i)$, often from pieces of functions that are already being used in calculating $f(x_i)$ and $f'(x_i)$. Otherwise, you might just as well do another step of ordinary Newton-Raphson. Halley's method converges cubically; in the final convergence each iteration *triples* the number of significant digits. But two steps of Newton-Raphson *quadruple* that number.

There is no reason to think that the basin of convergence of Halley's method is generally larger than Newton's; more often it is probably smaller. So don't look to Halley's method for better convergence in the large.

Nevertheless, when you *can* get a second derivative almost for free, you can often usefully shave an iteration or two off Newton-Raphson by something like this,

$$x_{i+1} = x_i - \frac{f(x_i)}{f'(x_i)} \bigg/ \max\left[0.8, \min\left(1.2, 1 - \frac{f(x_i)f''(x_i)}{2f'(x_i)^2}\right)\right] \tag{9.4.11}$$

the idea being to limit the influence of the higher-order correction, so that it gets used only in the endgame. We have already used Halley's method in just this fashion in §6.2, §6.4, and §6.14.

CITED REFERENCES AND FURTHER READING:

Acton, F.S. 1970, *Numerical Methods That Work*; 1990, corrected edition (Washington, DC: Mathematical Association of America), Chapter 2.

Ralston, A., and Rabinowitz, P. 1978, *A First Course in Numerical Analysis*, 2nd ed.; reprinted 2001 (New York: Dover), §8.4.

Ortega, J., and Rheinboldt, W. 1970, *Iterative Solution of Nonlinear Equations in Several Variables* (New York: Academic Press); reprinted 2000 (Philadelphia: S.I.A.M.).

Mandelbrot, B.B. 1983, *The Fractal Geometry of Nature* (San Francisco: W.H. Freeman).

Peitgen, H.-O., and Saupe, D. (eds.) 1988, *The Science of Fractal Images* (New York: Springer).

9.5 Roots of Polynomials

Here we give a few methods for finding roots of polynomials. These will serve for most practical problems involving polynomials of low-to-moderate degree or for well-conditioned polynomials of higher degree. Not as well appreciated as it ought to be is the fact that some polynomials are exceedingly ill-conditioned. The tiniest

changes in a polynomial's coefficients can, in the worst case, send its roots sprawling all over the complex plane. (An infamous example due to Wilkinson is detailed by Acton [1].)

Recall that a polynomial of degree n will have n roots. The roots can be real or complex, and they might not be distinct. If the coefficients of the polynomial are real, then complex roots will occur in pairs that are conjugate; i.e., if $x_1 = a + bi$ is a root, then $x_2 = a - bi$ will also be a root. When the coefficients are complex, the complex roots need not be related.

Multiple roots, or closely spaced roots, produce the most difficulty for numerical algorithms (see Figure 9.5.1). For example, $P(x) = (x - a)^2$ has a double real root at $x = a$. However, we cannot bracket the root by the usual technique of identifying neighborhoods where the function changes sign, nor will slope-following methods such as Newton-Raphson work well, because both the function and its derivative vanish at a multiple root. Newton-Raphson *may* work, but slowly, since large roundoff errors can occur. When a root is known in advance to be multiple, then special methods of attack are readily devised. Problems arise when (as is generally the case) we do not know in advance what pathology a root will display.

9.5.1 Deflation of Polynomials

When seeking several or all roots of a polynomial, the total effort can be significantly reduced by the use of *deflation*. As each root r is found, the polynomial is factored into a product involving the root and a reduced polynomial of degree one less than the original, i.e., $P(x) = (x - r)Q(x)$. Since the roots of Q are exactly the remaining roots of P, the effort of finding additional roots decreases, because we work with polynomials of lower and lower degree as we find successive roots. Even more important, with deflation we can avoid the blunder of having our iterative method converge twice to the same (nonmultiple) root instead of separately to two different roots.

Deflation, which amounts to synthetic division, is a simple operation that acts on the array of polynomial coefficients. The concise code for synthetic division by a monomial factor was given in §5.1. You can deflate complex roots either by converting that code to complex data type, or else — in the case of a polynomial with real coefficients but possibly complex roots — by deflating by a quadratic factor,

$$[x - (a + ib)]\,[x - (a - ib)] = x^2 - 2ax + (a^2 + b^2) \tag{9.5.1}$$

The routine `poldiv` in §5.1 can be used to divide the polynomial by this factor.

Deflation must, however, be utilized with care. Because each new root is known with only finite accuracy, errors creep into the determination of the coefficients of the successively deflated polynomial. Consequently, the roots can become more and more inaccurate. It matters a lot whether the inaccuracy creeps in stably (plus or minus a few multiples of the machine precision at each stage) or unstably (erosion of successive significant figures until the results become meaningless). Which behavior occurs depends on just how the root is divided out. *Forward deflation*, where the new polynomial coefficients are computed in the order from the highest power of x down to the constant term, was illustrated in §5.1. This turns out to be stable if the root of smallest absolute value is divided out at each stage. Alternatively, one can do *backward deflation*, where new coefficients are computed in order from the constant

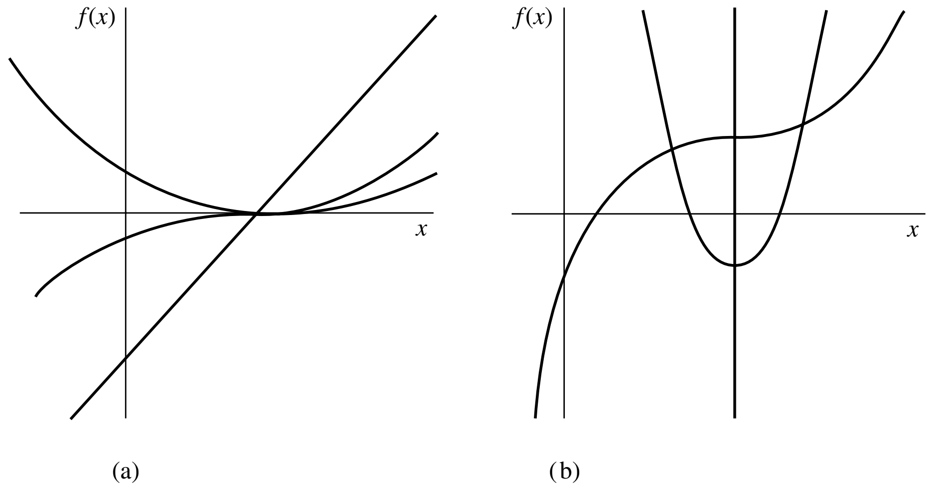

Figure 9.5.1. (a) Linear, quadratic, and cubic behavior at the roots of polynomials. Only under high magnification (b) does it become apparent that the cubic has one, not three, roots, and that the quadratic has two roots rather than none.

term up to the coefficient of the highest power of x. This is stable if the remaining root of *largest* absolute value is divided out at each stage.

A polynomial whose coefficients are interchanged "end-to-end," so that the constant becomes the highest coefficient, etc., has its roots mapped into their reciprocals. (Proof: Divide the whole polynomial by its highest power x^n and rewrite it as a polynomial in $1/x$.) The algorithm for backward deflation is therefore virtually identical to that of forward deflation, except that the original coefficients are taken in reverse order and the reciprocal of the deflating root is used. Since we will use forward deflation below, we leave to you the exercise of writing a concise coding for backward deflation (as in §5.1). For more on the stability of deflation, consult [2].

To minimize the impact of increasing errors (even stable ones) when using deflation, it is advisable to treat roots of the successively deflated polynomials as only *tentative* roots of the original polynomial. One then *polishes* these tentative roots by taking them as initial guesses that are to be re-solved for, using the *nondeflated* original polynomial P. Again you must beware lest two deflated roots are inaccurate enough that, under polishing, they both converge to the same undeflated root; in that case you gain a spurious root multiplicity and lose a distinct root. This is detectable, since you can compare each polished root for equality to previous ones from distinct tentative roots. When it happens, you are advised to deflate the polynomial just once (and for this root only), then again polish the tentative root, or use Maehly's procedure (see equation 9.5.29 below).

Below we say more about techniques for polishing real and complex-conjugate tentative roots. First, let's get back to overall strategy.

There are two schools of thought about how to proceed when faced with a polynomial of real coefficients. One school says to go after the easiest quarry, the real, distinct roots, by the same kinds of methods that we have discussed in previous sections for general functions, i.e., trial-and-error bracketing followed by a safe Newton-

Raphson as in `rtsafe`. Sometimes you are *only* interested in real roots, in which case the strategy is complete. Otherwise, you then go after quadratic factors of the form (9.5.1) by any of a variety of methods. One such is Bairstow's method, which we will discuss below in the context of root polishing. Another is Muller's method, which we here briefly discuss.

9.5.2 Muller's Method

Muller's method generalizes the secant method but uses quadratic interpolation among three points instead of linear interpolation between two. Solving for the zeros of the quadratic allows the method to find complex pairs of roots. Given *three* previous guesses for the root x_{i-2}, x_{i-1}, x_i, and the values of the polynomial $P(x)$ at those points, the next approximation x_{i+1} is produced by the following formulas,

$$\begin{aligned} q &\equiv \frac{x_i - x_{i-1}}{x_{i-1} - x_{i-2}} \\ A &\equiv qP(x_i) - q(1+q)P(x_{i-1}) + q^2 P(x_{i-2}) \\ B &\equiv (2q+1)P(x_i) - (1+q)^2 P(x_{i-1}) + q^2 P(x_{i-2}) \\ C &\equiv (1+q)P(x_i) \end{aligned} \tag{9.5.2}$$

followed by

$$x_{i+1} = x_i - (x_i - x_{i-1}) \frac{2C}{B \pm \sqrt{B^2 - 4AC}} \tag{9.5.3}$$

where the sign in the denominator is chosen to make its absolute value or modulus as large as possible. You can start the iterations with any three values of x that you like, e.g., three equally spaced values on the real axis. Note that you must allow for the possibility of a complex denominator, and subsequent complex arithmetic, in implementing the method.

Muller's method is sometimes also used for finding complex zeros of analytic functions (not just polynomials) in the complex plane, for example in the IMSL routine ZANLY [3].

9.5.3 Laguerre's Method

The second school regarding overall strategy happens to be the one to which we belong. That school advises you to use one of a very small number of methods that will converge (though with greater or lesser efficiency) to all types of roots: real, complex, single, or multiple. Use such a method to get tentative values for all n roots of your nth degree polynomial. Then go back and polish them as you desire.

Laguerre's method is by far the most straightforward of these general, complex methods. It does require complex arithmetic, even while converging to real roots; however, for polynomials with all real roots, it is guaranteed to converge to a root from any starting point. For polynomials with some complex roots, little is theoretically proved about the method's convergence. Much empirical experience, however, suggests that nonconvergence is extremely unusual and, further, can almost always be fixed by a simple scheme to break a nonconverging limit cycle. (This is implemented in our routine below.) An example of a polynomial that requires this cycle-breaking scheme is one of high degree ($\gtrsim 20$), with all its roots just outside of

the complex unit circle, approximately equally spaced around it. When the method converges on a simple complex zero, it is known that its convergence is third order.

In some instances the complex arithmetic in the Laguerre method is no disadvantage, since the polynomial itself may have complex coefficients.

To motivate (although not rigorously derive) the Laguerre formulas we can note the following relations between the polynomial and its roots and derivatives:

$$P_n(x) = (x - x_0)(x - x_1)\dots(x - x_{n-1}) \tag{9.5.4}$$

$$\ln|P_n(x)| = \ln|x - x_0| + \ln|x - x_1| + \dots + \ln|x - x_{n-1}| \tag{9.5.5}$$

$$\frac{d\ln|P_n(x)|}{dx} = +\frac{1}{x - x_0} + \frac{1}{x - x_1} + \dots + \frac{1}{x - x_{n-1}} = \frac{P_n'}{P_n} \equiv G \tag{9.5.6}$$

$$\begin{aligned} -\frac{d^2\ln|P_n(x)|}{dx^2} &= +\frac{1}{(x - x_0)^2} + \frac{1}{(x - x_1)^2} + \dots + \frac{1}{(x - x_{n-1})^2} \\ &= \left[\frac{P_n'}{P_n}\right]^2 - \frac{P_n''}{P_n} \equiv H \end{aligned} \tag{9.5.7}$$

Starting from these relations, the Laguerre formulas make what Acton [1] nicely calls "a rather drastic set of assumptions": The root x_0 that we seek is assumed to be located some distance a from our current guess x, while *all other roots* are assumed to be located at a distance b,

$$x - x_0 = a, \quad x - x_i = b, \qquad i = 1, 2, \dots, n - 1 \tag{9.5.8}$$

Then we can express (9.5.6) and (9.5.7) as

$$\frac{1}{a} + \frac{n-1}{b} = G \tag{9.5.9}$$

$$\frac{1}{a^2} + \frac{n-1}{b^2} = H \tag{9.5.10}$$

which yields as the solution for a

$$a = \frac{n}{G \pm \sqrt{(n-1)(nH - G^2)}} \tag{9.5.11}$$

where the sign should be taken to yield the largest magnitude for the denominator. Since the factor inside the square root can be negative, a can be complex. (A more rigorous justification of equation 9.5.11 is in [4].)

The method operates iteratively: For a trial value x, calculate a by equation (9.5.11). Then use $x - a$ as the next trial value. Continue until a is sufficiently small.

The following routine implements the Laguerre method to find one root of a given polynomial of degree `m`, whose coefficients can be complex. As usual, the first coefficient, `a[0]`, is the constant term, while `a[m]` is the coefficient of the highest power of x. The routine implements a simplified version of an elegant stopping criterion due to Adams [5], which neatly balances the desire to achieve full machine accuracy, on the one hand, with the danger of iterating forever in the presence of roundoff error, on the other.

roots_poly.h

```
void laguer(VecComplex_I &a, Complex &x, Int &its) {
```

Given the m+1 complex coefficients a[0..m] of the polynomial $\sum_{i=0}^{m} a[i]x^i$, and given a complex value x, this routine improves x by Laguerre's method until it converges, within the achievable roundoff limit, to a root of the given polynomial. The number of iterations taken is returned as its.

```
    const Int MR=8,MT=10,MAXIT=MT*MR;
    const Doub EPS=numeric_limits<Doub>::epsilon();
```

Here EPS is the estimated fractional roundoff error. We try to break (rare) limit cycles with MR different fractional values, once every MT steps, for MAXIT total allowed iterations.

```
    static const Doub frac[MR+1]=
        {0.0,0.5,0.25,0.75,0.13,0.38,0.62,0.88,1.0};
```

Fractions used to break a limit cycle.

```
    Complex dx,x1,b,d,f,g,h,sq,gp,gm,g2;
    Int m=a.size()-1;
    for (Int iter=1;iter<=MAXIT;iter++) {    Loop over iterations up to allowed maximum.
        its=iter;
        b=a[m];
        Doub err=abs(b);
        d=f=0.0;
        Doub abx=abs(x);
        for (Int j=m-1;j>=0;j--) {           Efficient computation of the polynomial and
            f=x*f+d;                             its first two derivatives. f stores P''/2.
            d=x*d+b;
            b=x*b+a[j];
            err=abs(b)+abx*err;
        }
        err *= EPS;
```

Estimate of roundoff error in evaluating polynomial.

```
        if (abs(b) <= err) return;           We are on the root.
        g=d/b;                               The generic case: Use Laguerre's formula.
        g2=g*g;
        h=g2-2.0*f/b;
        sq=sqrt(Doub(m-1)*(Doub(m)*h-g2));
        gp=g+sq;
        gm=g-sq;
        Doub abp=abs(gp);
        Doub abm=abs(gm);
        if (abp < abm) gp=gm;
        dx=MAX(abp,abm) > 0.0 ? Doub(m)/gp : polar(1+abx,Doub(iter));
        x1=x-dx;
        if (x == x1) return;                 Converged.
        if (iter % MT != 0) x=x1;
        else x -= frac[iter/MT]*dx;
```

Every so often we take a fractional step, to break any limit cycle (itself a rare occurrence).

```
    }
    throw("too many iterations in laguer");
```

Very unusual; can occur only for complex roots. Try a different starting guess.

```
}
```

Here is a driver routine that calls laguer in succession for each root, performs the deflation, optionally polishes the roots by the same Laguerre method — if you are not going to polish in some other way — and finally sorts the roots by their real parts. (We will use this routine in Chapter 13.)

roots_poly.h

```
void zroots(VecComplex_I &a, VecComplex_O &roots, const Bool &polish)
```

Given the m+1 complex coefficients a[0..m] of the polynomial $\sum_{i=0}^{m} a(i)x^i$, this routine successively calls laguer and finds all m complex roots in roots[0..m-1]. The boolean variable polish should be input as true if polishing (also by Laguerre's method) is desired, false if the roots will be subsequently polished by other means.

```
{
    const Doub EPS=1.0e-14;                  A small number.
```

```
    Int i,its;
    Complex x,b,c;
    Int m=a.size()-1;
    VecComplex ad(m+1);
    for (Int j=0;j<=m;j++) ad[j]=a[j];        Copy of coefficients for successive deflation.
    for (Int j=m-1;j>=0;j--) {                Loop over each root to be found.
        x=0.0;                                Start at zero to favor convergence to small-
        VecComplex ad_v(j+2);                     est remaining root, and return the root.
        for (Int jj=0;jj<j+2;jj++) ad_v[jj]=ad[jj];
        laguer(ad_v,x,its);
        if (abs(imag(x)) <= 2.0*EPS*abs(real(x)))
            x=Complex(real(x),0.0);
        roots[j]=x;
        b=ad[j+1];                            Forward deflation.
        for (Int jj=j;jj>=0;jj--) {
            c=ad[jj];
            ad[jj]=b;
            b=x*b+c;
        }
    }
    if (polish)
        for (Int j=0;j<m;j++)                 Polish the roots using the undeflated coeffi-
            laguer(a,roots[j],its);               cients.
    for (Int j=1;j<m;j++) {                   Sort roots by their real parts by straight in-
        x=roots[j];                               sertion.
        for (i=j-1;i>=0;i--) {
            if (real(roots[i]) <= real(x)) break;
            roots[i+1]=roots[i];
        }
        roots[i+1]=x;
    }
}
```

9.5.4 Eigenvalue Methods

The eigenvalues of a matrix $\mathbf{A}$ are the roots of the "characteristic polynomial" $P(x) = \det[\mathbf{A} - x\mathbf{I}]$. However, as we will see in Chapter 11, root finding is not generally an efficient way to find eigenvalues. Turning matters around, we can use the more efficient eigenvalue methods that are discussed in Chapter 11 to find the roots of arbitrary polynomials. You can easily verify (see, e.g., [6]) that the characteristic polynomial of the special $m \times m$ *companion matrix*

$$\mathbf{A} = \begin{pmatrix} -\frac{a_{m-1}}{a_m} & -\frac{a_{m-2}}{a_m} & \cdots & -\frac{a_1}{a_m} & -\frac{a_0}{a_m} \\ 1 & 0 & \cdots & 0 & 0 \\ 0 & 1 & \cdots & 0 & 0 \\ \vdots & & & & \vdots \\ 0 & 0 & \cdots & 1 & 0 \end{pmatrix} \tag{9.5.12}$$

is equivalent to the general polynomial

$$P(x) = \sum_{i=0}^{m} a_i x^i \tag{9.5.13}$$

If the coefficients a_i are real, rather than complex, then the eigenvalues of $\mathbf{A}$ can be found using the routine `Unsymmeig` in §11.6 – §11.7 (see discussion there). This

method, implemented in the routine zrhqr following, is typically about a factor 2 slower than zroots (above). However, for some classes of polynomials, it is a more robust technique, largely because of the fairly sophisticated convergence methods embodied in Unsymmeig. If your polynomial has real coefficients, and you are having trouble with zroots, then zrhqr is a recommended alternative.

zrhqr.h

```
void zrhqr(VecDoub_I &a, VecComplex_O &rt)
```

Find all the roots of a polynomial with real coefficients, $\sum_{i=0}^{m} a(i)x^i$, given the coefficients a[0..m]. The method is to construct an upper Hessenberg matrix whose eigenvalues are the desired roots and then use the routine Unsymmeig. The roots are returned in the complex vector rt[0..m-1], sorted in descending order by their real parts.

```
{
    Int m=a.size()-1;
    MatDoub hess(m,m);
    for (Int k=0;k<m;k++) {                 Construct the matrix.
        hess[0][k] = -a[m-k-1]/a[m];
        for (Int j=1;j<m;j++) hess[j][k]=0.0;
        if (k != m-1) hess[k+1][k]=1.0;
    }
    Unsymmeig h(hess, false, true);         Find its eigenvalues.
    for (Int j=0;j<m;j++)
        rt[j]=h.wri[j];
}
```

9.5.5 Other Sure-Fire Techniques

The *Jenkins-Traub method* has become practically a standard in black-box polynomial root finders, e.g., in the IMSL library [3]. The method is too complicated to discuss here, but is detailed, with references to the primary literature, in [4].

The *Lehmer-Schur algorithm* is one of a class of methods that isolate roots in the complex plane by generalizing the notion of one-dimensional bracketing. It is possible to determine efficiently whether there are any polynomial roots within a circle of given center and radius. From then on it is a matter of bookkeeping to hunt down all the roots by a series of decisions regarding where to place new trial circles. Consult [1] for an introduction.

9.5.6 Techniques for Root Polishing

Newton-Raphson works very well for real roots once the neighborhood of a root has been identified. The polynomial and its derivative can be efficiently simultaneously evaluated as in §5.1. For a polynomial of degree n with coefficients c[0]...c[n], the following segment of code carries out one cycle of Newton-Raphson:

```
p=c[n]*x+c[n-1];
p1=c[n];
for(i=n-2;i>=0;i--) {
    p1=p+p1*x;
    p=c[i]+p*x;
}
if (p1 == 0.0) throw("derivative should not vanish");
x -= p/p1;
```

Once all real roots of a polynomial have been polished, one must polish the complex roots, either directly or by looking for quadratic factors.

Direct polishing by Newton-Raphson is straightforward for complex roots if the above code is converted to complex data types. With real polynomial coefficients, note that your starting guess (tentative root) *must* be off the real axis, otherwise you will never get off that axis — and may get shot off to infinity by a minimum or maximum of the polynomial.

For real polynomials, the alternative means of polishing complex roots (or, for that matter, double real roots) is *Bairstow's method*, which seeks quadratic factors. The advantage of going after quadratic factors is that it avoids all complex arithmetic. Bairstow's method seeks a quadratic factor that embodies the two roots $x = a \pm ib$, namely

$$x^2 - 2ax + (a^2 + b^2) \equiv x^2 + Bx + C \tag{9.5.14}$$

In general, if we divide a polynomial by a quadratic factor, there will be a linear remainder

$$P(x) = (x^2 + Bx + C)Q(x) + Rx + S. \tag{9.5.15}$$

Given B and C, R and S can be readily found, by polynomial division (§5.1). We can consider R and S to be adjustable functions of B and C, and they will be zero if the quadratic factor is a divisor of $P(x)$.

In the neighborhood of a root, a first-order Taylor series expansion approximates the variation of R, S with respect to small changes in B, C:

$$R(B + \delta B, C + \delta C) \approx R(B, C) + \frac{\partial R}{\partial B}\delta B + \frac{\partial R}{\partial C}\delta C \tag{9.5.16}$$

$$S(B + \delta B, C + \delta C) \approx S(B, C) + \frac{\partial S}{\partial B}\delta B + \frac{\partial S}{\partial C}\delta C \tag{9.5.17}$$

To evaluate the partial derivatives, consider the derivative of (9.5.15) with respect to C. Since $P(x)$ is a fixed polynomial, it is independent of C, hence

$$0 = (x^2 + Bx + C)\frac{\partial Q}{\partial C} + Q(x) + \frac{\partial R}{\partial C}x + \frac{\partial S}{\partial C} \tag{9.5.18}$$

which can be rewritten as

$$-Q(x) = (x^2 + Bx + C)\frac{\partial Q}{\partial C} + \frac{\partial R}{\partial C}x + \frac{\partial S}{\partial C} \tag{9.5.19}$$

Similarly, $P(x)$ is independent of B, so differentiating (9.5.15) with respect to B gives

$$-xQ(x) = (x^2 + Bx + C)\frac{\partial Q}{\partial B} + \frac{\partial R}{\partial B}x + \frac{\partial S}{\partial B} \tag{9.5.20}$$

Now note that equation (9.5.19) matches equation (9.5.15) in form. Thus if we perform a second synthetic division of $P(x)$, i.e., a division of $Q(x)$ by the same quadratic factor, yielding a remainder $R_1x + S_1$, then

$$\frac{\partial R}{\partial C} = -R_1 \qquad \frac{\partial S}{\partial C} = -S_1 \tag{9.5.21}$$

To get the remaining partial derivatives, evaluate equation (9.5.20) at the two roots of the quadratic, x_+ and x_-. Since

$$Q(x_\pm) = R_1x_\pm + S_1 \tag{9.5.22}$$

we get

$$\frac{\partial R}{\partial B}x_+ + \frac{\partial S}{\partial B} = -x_+(R_1x_+ + S_1) \tag{9.5.23}$$

$$\frac{\partial R}{\partial B}x_- + \frac{\partial S}{\partial B} = -x_-(R_1x_- + S_1) \tag{9.5.24}$$

Solve these two equations for the partial derivatives, using

$$x_+ + x_- = -B \qquad x_+ x_- = C \tag{9.5.25}$$

and find

$$\frac{\partial R}{\partial B} = BR_1 - S_1 \qquad \frac{\partial S}{\partial B} = CR_1 \tag{9.5.26}$$

Bairstow's method now consists of using Newton-Raphson in two dimensions (which is actually the subject of the *next* section) to find a simultaneous zero of R and S. Synthetic division is used twice per cycle to evaluate R, S and their partial derivatives with respect to B, C. Like one-dimensional Newton-Raphson, the method works well in the vicinity of a root pair (real or complex), but it can fail miserably when started at a random point. We therefore recommend it only in the context of polishing tentative complex roots.

qroot.h

```
void qroot(VecDoub_I &p, Doub &b, Doub &c, const Doub eps)
```

Given n+1 coefficients p[0..n] of a polynomial of degree n, and trial values for the coefficients of a quadratic factor x*x+b*x+c, improve the solution until the coefficients b,c change by less than eps. The routine poldiv in §5.1 is used.

```
{
    const Int ITMAX=20;                     At most ITMAX iterations.
    const Doub TINY=1.0e-14;
    Doub sc,sb,s,rc,rb,r,dv,delc,delb;
    Int n=p.size()-1;
    VecDoub d(3),q(n+1),qq(n+1),rem(n+1);
    d[2]=1.0;
    for (Int iter=0;iter<ITMAX;iter++) {
        d[1]=b;
        d[0]=c;
        poldiv(p,d,q,rem);
        s=rem[0];                           First division, r,s.
        r=rem[1];
        poldiv(q,d,qq,rem);
        sb = -c*(rc = -rem[1]);             Second division, partial r,s with respect to
        rb = -b*rc+(sc = -rem[0]);              c.
        dv=1.0/(sb*rc-sc*rb);               Solve 2x2 equation.
        delb=(r*sc-s*rc)*dv;
        delc=(-r*sb+s*rb)*dv;
        b += (delb=(r*sc-s*rc)*dv);
        c += (delc=(-r*sb+s*rb)*dv);
        if ((abs(delb) <= eps*abs(b) || abs(b) < TINY)
            && (abs(delc) <= eps*abs(c) || abs(c) < TINY)) {
            return;                         Coefficients converged.
        }
    }
    throw("Too many iterations in routine qroot");
}
```

We have already remarked on the annoyance of having two tentative roots collapse to one value under polishing. You are left not knowing whether your polishing procedure has lost a root, or whether there *is* actually a double root, which was split only by roundoff errors in your previous deflation. One solution is deflate-and-repolish; but deflation is what we are trying to avoid at the polishing stage. An alternative is *Maehly's procedure*. Maehly pointed out that the derivative of the reduced polynomial

$$P_j(x) \equiv \frac{P(x)}{(x-x_0)\cdots(x-x_{j-1})} \tag{9.5.27}$$

can be written as

$$P'_j(x) = \frac{P'(x)}{(x - x_0)\cdots(x - x_{j-1})} - \frac{P(x)}{(x - x_0)\cdots(x - x_{j-1})} \sum_{i=0}^{j-1}(x - x_i)^{-1} \quad (9.5.28)$$

Hence one step of Newton-Raphson, taking a guess x_k into a new guess x_{k+1}, can be written as

$$x_{k+1} = x_k - \frac{P(x_k)}{P'(x_k) - P(x_k)\sum_{i=0}^{j-1}(x_k - x_i)^{-1}} \quad (9.5.29)$$

This equation, if used with i ranging over the roots already polished, will prevent a tentative root from spuriously hopping to another one's true root. It is an example of so-called *zero suppression* as an alternative to true deflation.

Muller's method, which was described above, can also be a useful adjunct at the polishing stage.

CITED REFERENCES AND FURTHER READING:

Acton, F.S. 1970, *Numerical Methods That Work*; 1990, corrected edition (Washington, DC: Mathematical Association of America), Chapter 7.[1]

Peters G., and Wilkinson, J.H. 1971, "Practical Problems Arising in the Solution of Polynomial Equations," *Journal of the Institute of Mathematics and Its Applications*, vol. 8, pp. 16–35.[2]

IMSL Math/Library Users Manual (Houston: IMSL Inc.), see 2007+, `http://www.vni.com/products/imsl`.[3]

Ralston, A., and Rabinowitz, P. 1978, *A First Course in Numerical Analysis*, 2nd ed.; reprinted 2001 (New York: Dover), §8.9–8.13.[4]

Adams, D.A. 1967, "A Stopping Criterion for Polynomial Root Finding," *Communications of the ACM*, vol. 10, pp. 655–658.[5]

Johnson, L.W., and Riess, R.D. 1982, *Numerical Analysis*, 2nd ed. (Reading, MA: Addison-Wesley), §4.4.3.[6]

Henrici, P. 1974, *Applied and Computational Complex Analysis*, vol. 1 (New York: Wiley).

Stoer, J., and Bulirsch, R. 2002, *Introduction to Numerical Analysis*, 3rd ed. (New York: Springer), §5.5 – §5.9.

9.6 Newton-Raphson Method for Nonlinear Systems of Equations

We make an extreme, but wholly defensible, statement: There are *no* good, general methods for solving systems of more than one nonlinear equation. Furthermore, it is not hard to see why (very likely) there *never will be* any good, general methods: Consider the case of two dimensions, where we want to solve simultaneously

$$\begin{aligned} f(x, y) &= 0 \\ g(x, y) &= 0 \end{aligned} \quad (9.6.1)$$

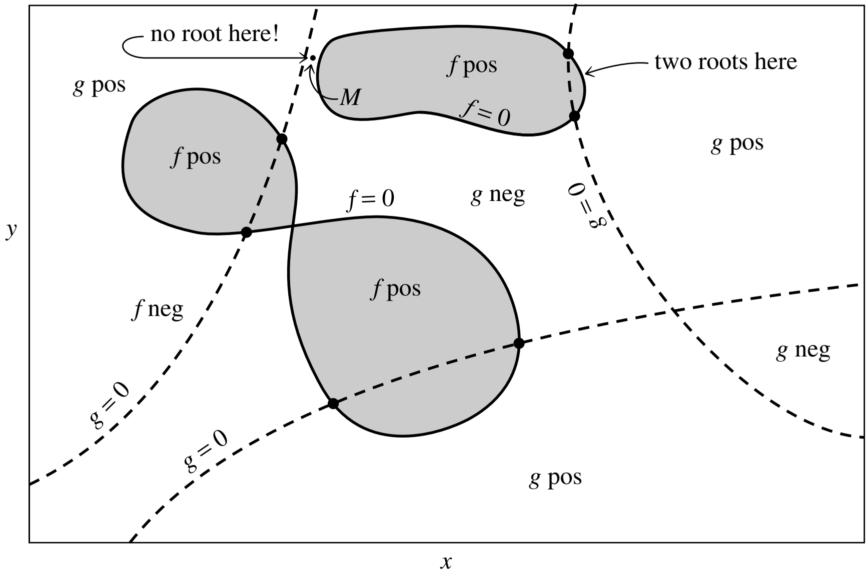

Figure 9.6.1. Solution of two nonlinear equations in two unknowns. Solid curves refer to $f(x, y)$, dashed curves to $g(x, y)$. Each equation divides the (x, y)-plane into positive and negative regions, bounded by zero curves. The desired solutions are the intersections of these unrelated zero curves. The number of solutions is a priori unknown.

The functions f and g are two arbitrary functions, each of which has zero contour lines that divide the (x, y)-plane into regions where their respective function is positive or negative. These zero contour boundaries are of interest to us. The solutions that we seek are those points (if any) that are common to the zero contours of f and g (see Figure 9.6.1). Unfortunately, the functions f and g have, in general, no relation to each other at all! There is nothing special about a common point from either f's point of view, or from g's. In order to find all common points, which are the solutions of our nonlinear equations, we will (in general) have to do neither more nor less than map out the full zero contours of both functions. Note further that the zero contours will (in general) consist of an unknown number of disjoint closed curves. How can we ever hope to know when we have found all such disjoint pieces?

For problems in more than two dimensions, we need to find points mutually common to N unrelated zero-contour hypersurfaces, each of dimension $N - 1$. You see that root finding becomes virtually impossible without insight! You will almost always have to use additional information, specific to your particular problem, to answer such basic questions as, "Do I expect a unique solution?" and "Approximately where?" Acton [1] has a good discussion of some of the particular strategies that can be tried.

In this section we discuss the simplest multidimensional root-finding method, Newton-Raphson. This method gives a very efficient means of converging to a root, if you have a sufficiently good initial guess. It can also spectacularly fail to converge, indicating (though not proving) that your putative root does not exist nearby. In §9.7 we discuss more sophisticated implementations of the Newton-Raphson method, which try to improve on Newton-Raphson's poor global convergence. A multidimensional generalization of the secant method, called Broyden's method, is also

discussed in §9.7.

A typical problem gives N functional relations to be zeroed, involving variables $x_i, i = 0, 1, \ldots, N-1$:

$$F_i(x_0, x_1, \ldots, x_{N-1}) = 0 \qquad i = 0, 1, \ldots, N-1. \tag{9.6.2}$$

We let $\mathbf{x}$ denote the entire vector of values x_i and $\mathbf{F}$ denote the entire vector of functions F_i. In the neighborhood of $\mathbf{x}$, each of the functions F_i can be expanded in Taylor series:

$$F_i(\mathbf{x} + \delta\mathbf{x}) = F_i(\mathbf{x}) + \sum_{j=0}^{N-1} \frac{\partial F_i}{\partial x_j} \delta x_j + O(\delta\mathbf{x}^2). \tag{9.6.3}$$

The matrix of partial derivatives appearing in equation (9.6.3) is the *Jacobian* matrix $\mathbf{J}$:

$$J_{ij} \equiv \frac{\partial F_i}{\partial x_j}. \tag{9.6.4}$$

In matrix notation equation (9.6.3) is

$$\mathbf{F}(\mathbf{x} + \delta\mathbf{x}) = \mathbf{F}(\mathbf{x}) + \mathbf{J} \cdot \delta\mathbf{x} + O(\delta\mathbf{x}^2). \tag{9.6.5}$$

By neglecting terms of order $\delta\mathbf{x}^2$ and higher and by setting $\mathbf{F}(\mathbf{x} + \delta\mathbf{x}) = 0$, we obtain a set of linear equations for the corrections $\delta\mathbf{x}$ that move each function closer to zero simultaneously, namely

$$\mathbf{J} \cdot \delta\mathbf{x} = -\mathbf{F}. \tag{9.6.6}$$

Matrix equation (9.6.6) can be solved by LU decomposition as described in §2.3. The corrections are then added to the solution vector,

$$\mathbf{x}_{\text{new}} = \mathbf{x}_{\text{old}} + \delta\mathbf{x} \tag{9.6.7}$$

and the process is iterated to convergence. In general it is a good idea to check the degree to which both functions and variables have converged. Once either reaches machine accuracy, the other won't change.

The following routine `mnewt` performs `ntrial` iterations starting from an initial guess at the solution vector `x[0..n-1]`. Iteration stops if either the sum of the magnitudes of the functions F_i is less than some tolerance `tolf`, or the sum of the absolute values of the corrections to δx_i is less than some tolerance `tolx`. `mnewt` calls a user-supplied function with the fixed name `usrfun`, which must provide the function values $\mathbf{F}$ and the Jacobian matrix $\mathbf{J}$. (The more sophisticated methods later in this chapter will have a more versatile interface.) If $\mathbf{J}$ is difficult to compute analytically, you can try having `usrfun` invoke the routine `NRfdjac` of §9.7 to compute the partial derivatives by finite differences. You should not make `ntrial` too big; rather, inspect to see what is happening before continuing for some further iterations.

mnewt.h

```
void usrfun(VecDoub_I &x, VecDoub_O &fvec, MatDoub_O &fjac);

void mnewt(const Int ntrial, VecDoub_IO &x, const Doub tolx, const Doub tolf) {
Given an initial guess x[0..n-1] for a root in n dimensions, take ntrial Newton-Raphson steps
to improve the root. Stop if the root converges in either summed absolute variable increments
tolx or summed absolute function values tolf.
    Int i,n=x.size();
    VecDoub p(n),fvec(n);
    MatDoub fjac(n,n);
    for (Int k=0;k<ntrial;k++) {
        usrfun(x,fvec,fjac);            User function supplies function values at x in
        Doub errf=0.0;                      fvec and Jacobian matrix in fjac.
        for (i=0;i<n;i++) errf += abs(fvec[i]);     Check function convergence.
        if (errf <= tolf) return;
        for (i=0;i<n;i++) p[i] = -fvec[i];      Right-hand side of linear equations.
        LUdcmp alu(fjac);               Solve linear equations using LU decomposition.
        alu.solve(p,p);
        Doub errx=0.0;                  Check root convergence.
        for (i=0;i<n;i++) {             Update solution.
            errx += abs(p[i]);
            x[i] += p[i];
        }
        if (errx <= tolx) return;
    }
    return;
}
```

9.6.1 Newton's Method versus Minimization

In the next chapter, we will find that there *are* efficient general techniques for finding a minimum of a function of many variables. Why is that task (relatively) easy, while multidimensional root finding is often quite hard? Isn't minimization equivalent to finding a zero of an N-dimensional gradient vector, which is not so different from zeroing an N-dimensional function? No! The components of a gradient vector are not independent, arbitrary functions. Rather, they obey so-called integrability conditions that are highly restrictive. Put crudely, you can always find a minimum by sliding downhill on a single surface. The test of "downhillness" is thus one-dimensional. There is no analogous conceptual procedure for finding a multidimensional root, where "downhill" must mean simultaneously downhill in N separate function spaces, thus allowing a multitude of trade-offs as to how much progress in one dimension is worth compared with progress in another.

It might occur to you to carry out multidimensional root finding by collapsing all these dimensions into one: Add up the sums of squares of the individual functions F_i to get a master function F that (i) is positive-definite and (ii) has a global minimum of zero exactly at all solutions of the original set of nonlinear equations. Unfortunately, as you will see in the next chapter, the efficient algorithms for finding minima come to rest on global and local minima indiscriminately. You will often find, to your great dissatisfaction, that your function F has a great number of local minima. In Figure 9.6.1, for example, there is likely to be a local minimum wherever the zero contours of f and g make a close approach to each other. The point labeled M is such a point, and one sees that there are no nearby roots.

However, we will now see that sophisticated strategies for multidimensional root finding can in fact make use of the idea of minimizing a master function F, by *combining* it with Newton's method applied to the full set of functions F_i. While such methods can still occasionally fail by coming to rest on a local minimum of F,

they often succeed where a direct attack via Newton's method alone fails. The next section deals with these methods.

CITED REFERENCES AND FURTHER READING:

Acton, F.S. 1970, *Numerical Methods That Work*; 1990, corrected edition (Washington, DC: Mathematical Association of America), Chapter 14.[1]

Ostrowski, A.M. 1966, *Solutions of Equations and Systems of Equations*, 2nd ed. (New York: Academic Press).

Ortega, J., and Rheinboldt, W. 1970, *Iterative Solution of Nonlinear Equations in Several Variables* (New York: Academic Press); reprinted 2000 (Philadelphia: S.I.A.M.).

9.7 Globally Convergent Methods for Nonlinear Systems of Equations

We have seen that Newton's method for solving nonlinear equations has an unfortunate tendency to wander off into the wild blue yonder if the initial guess is not sufficiently close to the root. A *global* method [1] would be one that converges to a solution from almost any starting point. Such global methods do exist for minimization problems; an example is the quasi-Newton method that we will describe in §10.9. In this section we will develop an algorithm that is an analogous quasi-Newton method for multidimensional root finding. Alas, while it is better behaved than Newton's method, it is still not truly global.

What the method *does* do is combine the rapid local convergence of Newton's method with a higher-level strategy that guarantees at least *some* progress at each step — either toward an actual root (usually), or else, hopefully rarely, toward the situation labeled "no root here!" in Figure 9.6.1. In the latter case, the method recognizes the problem and signals failure. By contrast, Newton's method can bounce around forever, and you are never sure whether or not to quit.

Recall our discussion of §9.6: The Newton step for the set of equations

$$\mathbf{F}(\mathbf{x}) = 0 \tag{9.7.1}$$

is

$$\mathbf{x}_{\text{new}} = \mathbf{x}_{\text{old}} + \delta\mathbf{x} \tag{9.7.2}$$

where

$$\delta\mathbf{x} = -\mathbf{J}^{-1} \cdot \mathbf{F} \tag{9.7.3}$$

Here $\mathbf{J}$ is the Jacobian matrix. How do we decide whether to accept the Newton step $\delta\mathbf{x}$? A reasonable strategy is to require that the step decrease $|\mathbf{F}|^2 = \mathbf{F} \cdot \mathbf{F}$. This is the same requirement we would impose if we were trying to minimize

$$f = \tfrac{1}{2}\mathbf{F} \cdot \mathbf{F} \tag{9.7.4}$$

(The $\frac{1}{2}$ is for later convenience.) Every solution to (9.7.1) minimizes (9.7.4), but there may be local minima of (9.7.4) that are not solutions to (9.7.1). Thus, as already mentioned, simply applying one of our minimum-finding algorithms from Chapter 10 to (9.7.4) is *not* a good idea.

To develop a better strategy, note that the Newton step (9.7.3) is a *descent direction* for f:

$$\nabla f \cdot \delta \mathbf{x} = (\mathbf{F} \cdot \mathbf{J}) \cdot (-\mathbf{J}^{-1} \cdot \mathbf{F}) = -\mathbf{F} \cdot \mathbf{F} < 0 \tag{9.7.5}$$

Thus our strategy is quite simple: We always first try the full Newton step, because once we are close enough to the solution we will get quadratic convergence. However, we check at each iteration that the proposed step reduces f. If not, we *backtrack* along the Newton direction until we have an acceptable step. Because the Newton step is a descent direction for f, we are guaranteed to find an acceptable step by backtracking. We will discuss the backtracking algorithm in more detail below.

Note that this method minimizes f only "incidentally," either by taking Newton steps designed to bring $\mathbf{F}$ to zero, or by backtracking along such a step. The method is *not* equivalent to minimizing f directly by taking Newton steps designed to bring ∇f to zero. While the method can nevertheless still fail by converging to a local minimum of f that is not a root (as in Figure 9.6.1), this is quite rare in real applications. The routine `newt` below will warn you if this happens. The only remedy is to try a new starting point.

9.7.1 Line Searches and Backtracking

When we are not close enough to the minimum of f, taking the full Newton step $\mathbf{p} = \delta\mathbf{x}$ need not decrease the function; we may move too far for the quadratic approximation to be valid. All we are guaranteed is that *initially* f decreases as we move in the Newton direction. So the goal is to move to a new point $\mathbf{x}_{\text{new}}$ along the *direction* of the Newton step $\mathbf{p}$, but not necessarily all the way:

$$\mathbf{x}_{\text{new}} = \mathbf{x}_{\text{old}} + \lambda \mathbf{p}, \qquad 0 < \lambda \le 1 \tag{9.7.6}$$

The aim is to find λ so that $f(\mathbf{x}_{\text{old}} + \lambda\mathbf{p})$ has decreased sufficiently. Until the early 1970s, standard practice was to choose λ so that $\mathbf{x}_{\text{new}}$ exactly minimizes f in the direction $\mathbf{p}$. However, we now know that it is extremely wasteful of function evaluations to do so. A better strategy is as follows: Since $\mathbf{p}$ is always the Newton direction in our algorithms, we first try $\lambda = 1$, the full Newton step. This will lead to quadratic convergence when $\mathbf{x}$ is sufficiently close to the solution. However, if $f(\mathbf{x}_{\text{new}})$ does not meet our acceptance criteria, we *backtrack* along the Newton direction, trying a smaller value of λ, until we find a suitable point. Since the Newton direction is a descent direction, we are guaranteed to decrease f for sufficiently small λ.

What should the criterion for accepting a step be? It is *not* sufficient to require merely that $f(\mathbf{x}_{\text{new}}) < f(\mathbf{x}_{\text{old}})$. This criterion can fail to converge to a minimum of f in one of two ways. First, it is possible to construct a sequence of steps satisfying this criterion with f decreasing too slowly relative to the step lengths. Second, one can have a sequence where the step lengths are too small relative to the initial rate of decrease of f. (For examples of such sequences, see [2], p. 117.)

A simple way to fix the first problem is to require the *average* rate of decrease of f to be at least some fraction α of the *initial* rate of decrease $\nabla f \cdot \mathbf{p}$:

$$f(\mathbf{x}_{\text{new}}) \le f(\mathbf{x}_{\text{old}}) + \alpha \nabla f \cdot (\mathbf{x}_{\text{new}} - \mathbf{x}_{\text{old}}) \tag{9.7.7}$$

Here the parameter α satisfies $0 < \alpha < 1$. We can get away with quite small values of α; $\alpha = 10^{-4}$ is a good choice.

The second problem can be fixed by requiring the rate of decrease of f at $\mathbf{x}_{\text{new}}$ to be greater than some fraction β of the rate of decrease of f at $\mathbf{x}_{\text{old}}$. In practice, we will not need to impose this second constraint because our backtracking algorithm will have a built-in cutoff to avoid taking steps that are too small.

Here is the strategy for a practical backtracking routine: Define

$$g(\lambda) \equiv f(\mathbf{x}_{\text{old}} + \lambda\mathbf{p}) \tag{9.7.8}$$

so that

$$g'(\lambda) = \nabla f \cdot \mathbf{p} \tag{9.7.9}$$

If we need to backtrack, then we model g with the most current information we have and choose λ to minimize the model. We start with $g(0)$ and $g'(0)$ available. The first step is always the Newton step, $\lambda = 1$. If this step is not acceptable, we have available $g(1)$ as well. We can therefore model $g(\lambda)$ as a quadratic:

$$g(\lambda) \approx [g(1) - g(0) - g'(0)]\lambda^2 + g'(0)\lambda + g(0) \tag{9.7.10}$$

Taking the derivative of this quadratic, we find that it is a minimum when

$$\lambda = -\frac{g'(0)}{2[g(1) - g(0) - g'(0)]} \tag{9.7.11}$$

Since the Newton step failed, one can show that $\lambda \lesssim \frac{1}{2}$ for small α. We need to guard against too small a value of λ, however. We set $\lambda_{\min} = 0.1$.

On second and subsequent backtracks, we model g as a cubic in λ, using the previous value $g(\lambda_1)$ and the second most recent value $g(\lambda_2)$:

$$g(\lambda) = a\lambda^3 + b\lambda^2 + g'(0)\lambda + g(0) \tag{9.7.12}$$

Requiring this expression to give the correct values of g at λ_1 and λ_2 gives two equations that can be solved for the coefficients a and b:

$$\begin{bmatrix} a \\ b \end{bmatrix} = \frac{1}{\lambda_1 - \lambda_2} \begin{bmatrix} 1/\lambda_1^2 & -1/\lambda_2^2 \\ -\lambda_2/\lambda_1^2 & \lambda_1/\lambda_2^2 \end{bmatrix} \cdot \begin{bmatrix} g(\lambda_1) - g'(0)\lambda_1 - g(0) \\ g(\lambda_2) - g'(0)\lambda_2 - g(0) \end{bmatrix} \tag{9.7.13}$$

The minimum of the cubic (9.7.12) is at

$$\lambda = \frac{-b + \sqrt{b^2 - 3ag'(0)}}{3a} \tag{9.7.14}$$

We enforce that λ lie between $\lambda_{\max} = 0.5\lambda_1$ and $\lambda_{\min} = 0.1\lambda_1$.

The routine has two additional features, a minimum step length `alamin` and a maximum step length `stpmax`. `lnsrch` will also be used in the quasi-Newton minimization routine `dfpmin` in the next section.

roots_multidim.h

```
template <class T>
void lnsrch(VecDoub_I &xold, const Doub fold, VecDoub_I &g, VecDoub_IO &p,
VecDoub_O &x, Doub &f, const Doub stpmax, Bool &check, T &func) {
```

Given an n-dimensional point `xold[0..n-1]`, the value of the function and gradient there, `fold` and `g[0..n-1]`, and a direction `p[0..n-1]`, finds a new point `x[0..n-1]` along the direction `p` from `xold` where the function or functor `func` has decreased "sufficiently." The new function value is returned in `f`. `stpmax` is an input quantity that limits the length of the steps so that you do not try to evaluate the function in regions where it is undefined or subject to overflow. `p` is usually the Newton direction. The output quantity `check` is `false` on a normal exit. It is `true` when `x` is too close to `xold`. In a minimization algorithm, this usually signals convergence and can be ignored. However, in a zero-finding algorithm the calling program should check whether the convergence is spurious.

```
    const Doub ALF=1.0e-4, TOLX=numeric_limits<Doub>::epsilon();
```

`ALF` ensures sufficient decrease in function value; `TOLX` is the convergence criterion on Δx.

```
    Doub a,alam,alam2=0.0,alamin,b,disc,f2=0.0;
    Doub rhs1,rhs2,slope=0.0,sum=0.0,temp,test,tmplam;
    Int i,n=xold.size();
    check=false;
    for (i=0;i<n;i++) sum += p[i]*p[i];
    sum=sqrt(sum);
    if (sum > stpmax)
        for (i=0;i<n;i++)
```

```
            p[i] *= stpmax/sum;                         Scale if attempted step is too big.
    for (i=0;i<n;i++)
        slope += g[i]*p[i];
    if (slope >= 0.0) throw("Roundoff problem in lnsrch.");
    test=0.0;                                           Compute λ_min.
    for (i=0;i<n;i++) {
        temp=abs(p[i])/MAX(abs(xold[i]),1.0);
        if (temp > test) test=temp;
    }
    alamin=TOLX/test;
    alam=1.0;                                           Always try full Newton step first.
    for (;;) {                                          Start of iteration loop.
        for (i=0;i<n;i++) x[i]=xold[i]+alam*p[i];
        f=func(x);
        if (alam < alamin) {                            Convergence on Δx. For zero find-
            for (i=0;i<n;i++) x[i]=xold[i];                 ing, the calling program should
            check=true;                                     verify the convergence.
            return;
        } else if (f <= fold+ALF*alam*slope) return;    Sufficient function decrease.
        else {                                          Backtrack.
            if (alam == 1.0)
                tmplam = -slope/(2.0*(f-fold-slope));   First time.
            else {                                      Subsequent backtracks.
                rhs1=f-fold-alam*slope;
                rhs2=f2-fold-alam2*slope;
                a=(rhs1/(alam*alam)-rhs2/(alam2*alam2))/(alam-alam2);
                b=(-alam2*rhs1/(alam*alam)+alam*rhs2/(alam2*alam2))/(alam-alam2);
                if (a == 0.0) tmplam = -slope/(2.0*b);
                else {
                    disc=b*b-3.0*a*slope;
                    if (disc < 0.0) tmplam=0.5*alam;
                    else if (b <= 0.0) tmplam=(-b+sqrt(disc))/(3.0*a);
                    else tmplam=-slope/(b+sqrt(disc));
                }
                if (tmplam>0.5*alam)
                    tmplam=0.5*alam;                    λ ≤ 0.5λ_1.
            }
        }
        alam2=alam;
        f2 = f;
        alam=MAX(tmplam,0.1*alam);                      λ ≥ 0.1λ_1.
    }                                                   Try again.
}
```

9.7.2 Globally Convergent Newton Method

Using the above results on backtracking, here is the globally convergent Newton routine `newt` that uses `lnsrch`. A feature of `newt` is that you need not supply the Jacobian matrix analytically; the routine will attempt to compute the necessary partial derivatives of $\mathbf{F}$ by finite differences in the routine `NRfdjac`. This routine uses some of the techniques described in §5.7 for computing numerical derivatives. Of course, you can always replace `NRfdjac` with a routine that calculates the Jacobian analytically if this is easy for you to do.

The routine requires a user-supplied function or functor that computes the vector of functions to be zeroed. Its declaration as a function is

```
VecDoub vecfunc(VecDoub_I x);
```

(The name `vecfunc` is arbitrary.) The declaration as a functor is similar.

roots_multidim.h

```
template <class T>
void newt(VecDoub_IO &x, Bool &check, T &vecfunc) {
```

Given an initial guess x[0..n-1] for a root in n dimensions, find the root by a globally convergent Newton's method. The vector of functions to be zeroed, called fvec[0..n-1] in the routine below, is returned by the user-supplied function or functor vecfunc (see text). The output quantity check is false on a normal return and true if the routine has converged to a local minimum of the function fmin defined below. In this case try restarting from a different initial guess.

```
    const Int MAXITS=200;
    const Doub TOLF=1.0e-8,TOLMIN=1.0e-12,STPMX=100.0;
    const Doub TOLX=numeric_limits<Doub>::epsilon();
```

Here MAXITS is the maximum number of iterations; TOLF sets the convergence criterion on function values; TOLMIN sets the criterion for deciding whether spurious convergence to a minimum of fmin has occurred; STPMX is the scaled maximum step length allowed in line searches; and TOLX is the convergence criterion on $\delta\mathbf{x}$.

```
    Int i,j,its,n=x.size();
    Doub den,f,fold,stpmax,sum,temp,test;
    VecDoub g(n),p(n),xold(n);
    MatDoub fjac(n,n);
    NRfmin<T> fmin(vecfunc);                    Set up NRfmin object.
    NRfdjac<T> fdjac(vecfunc);                  Set up NRfdjac object.
    VecDoub &fvec=fmin.fvec;                    Make an alias to simplify coding.
    f=fmin(x);                                  fvec is also computed by this call.
    test=0.0;                                   Test for initial guess being a root. Use
    for (i=0;i<n;i++)                               more stringent test than simply TOLF.
        if (abs(fvec[i]) > test) test=abs(fvec[i]);
    if (test < 0.01*TOLF) {
        check=false;
        return;
    }
    sum=0.0;
    for (i=0;i<n;i++) sum += SQR(x[i]);         Calculate stpmax for line searches.
    stpmax=STPMX*MAX(sqrt(sum),Doub(n));
    for (its=0;its<MAXITS;its++) {              Start of iteration loop.
        fjac=fdjac(x,fvec);
```

If analytic Jacobian is available, you can replace the struct NRfdjac below with your own struct.

```
        for (i=0;i<n;i++) {                     Compute ∇f for the line search.
            sum=0.0;
            for (j=0;j<n;j++) sum += fjac[j][i]*fvec[j];
            g[i]=sum;
        }
        for (i=0;i<n;i++) xold[i]=x[i];         Store x,
        fold=f;                                 and f.
        for (i=0;i<n;i++) p[i] = -fvec[i];      Right-hand side for linear equations.
        LUdcmp alu(fjac);                       Solve linear equations by LU decompo-
        alu.solve(p,p);                             sition.
        lnsrch(xold,fold,g,p,x,f,stpmax,check,fmin);
```

lnsrch returns new $\mathbf{x}$ and f. It also calculates fvec at the new $\mathbf{x}$ when it calls fmin.

```
        test=0.0;                               Test for convergence on function values.
        for (i=0;i<n;i++)
            if (abs(fvec[i]) > test) test=abs(fvec[i]);
        if (test < TOLF) {
            check=false;
            return;
        }
        if (check) {                            Check for gradient of f zero, i.e., spu-
            test=0.0;                               rious convergence.
            den=MAX(f,0.5*n);
            for (i=0;i<n;i++) {
                temp=abs(g[i])*MAX(abs(x[i]),1.0)/den;
                if (temp > test) test=temp;
            }
```

```
            check=(test < TOLMIN);
            return;
        }
        test=0.0;                              Test for convergence on δx.
        for (i=0;i<n;i++) {
            temp=(abs(x[i]-xold[i]))/MAX(abs(x[i]),1.0);
            if (temp > test) test=temp;
        }
        if (test < TOLX)
            return;
    }
    throw("MAXITS exceeded in newt");
}
```

roots_multidim.h

```
template <class T>
struct NRfdjac {
```
Computes forward-difference approximation to Jacobian.
```
    const Doub EPS;                    Set to approximate square root of the machine pre-
    T &func;                              cision.
    NRfdjac(T &funcc) : EPS(1.0e-8),func(funcc) {}
```
Initialize with user-supplied function or functor that returns the vector of functions to be zeroed.
```
    MatDoub operator() (VecDoub_I &x, VecDoub_I &fvec) {
```
Returns the Jacobian array `df[0..n-1][0..n-1]`. On input, `x[0..n-1]` is the point at which the Jacobian is to be evaluated and `fvec[0..n-1]` is the vector of function values at the point.
```
        Int n=x.size();
        MatDoub df(n,n);
        VecDoub xh=x;
        for (Int j=0;j<n;j++) {
            Doub temp=xh[j];
            Doub h=EPS*abs(temp);
            if (h == 0.0) h=EPS;
            xh[j]=temp+h;                  Trick to reduce finite-precision error.
            h=xh[j]-temp;
            VecDoub f=func(xh);
            xh[j]=temp;
            for (Int i=0;i<n;i++)          Forward difference formula.
                df[i][j]=(f[i]-fvec[i])/h;
        }
        return df;
    }
};
```

roots_multidim.h

```
template <class T>
struct NRfmin {
```
Returns $f = \frac{1}{2}\mathbf{F}\cdot\mathbf{F}$. Also stores value of $\mathbf{F}$ in `fvec`.
```
    VecDoub fvec;
    T &func;
    Int n;
    NRfmin(T &funcc) : func(funcc){}
```
Initialize with user-supplied function or functor that returns the vector of functions to be zeroed.
```
    Doub operator() (VecDoub_I &x) {
```
Returns f at `x`, and stores $\mathbf{F}(\mathbf{x})$ in `fvec`.
```
        n=x.size();
        Doub sum=0;
        fvec=func(x);
        for (Int i=0;i<n;i++) sum += SQR(fvec[i]);
        return 0.5*sum;
    }
};
```

The routine newt assumes that the typical values of all components of $\mathbf{x}$ and of $\mathbf{F}$ are of order unity, and it can fail if this assumption is badly violated. You should rescale the variables by their typical values before invoking newt if this problem occurs.

9.7.3 Multidimensional Secant Methods: Broyden's Method

Newton's method as implemented above is quite powerful, but it still has several disadvantages. One drawback is that the Jacobian matrix is needed. In many problems analytic derivatives are unavailable. If function evaluation is expensive, then the cost of finite difference determination of the Jacobian can be prohibitive.

Just as the quasi-Newton methods to be discussed in §10.9 provide cheap approximations for the Hessian matrix in minimization algorithms, there are quasi-Newton methods that provide cheap approximations to the Jacobian for zero finding. These methods are often called *secant methods*, since they reduce to the secant method (§9.2) in one dimension (see, e.g., [2]). The best of these methods still seems to be the first one introduced, *Broyden's method* [3].

Let us denote the approximate Jacobian by $\mathbf{B}$. Then the ith quasi-Newton step $\delta\mathbf{x}_i$ is the solution of

$$\mathbf{B}_i \cdot \delta\mathbf{x}_i = -\mathbf{F}_i \tag{9.7.15}$$

where $\delta\mathbf{x}_i = \mathbf{x}_{i+1} - \mathbf{x}_i$ (cf. equation 9.7.3). The quasi-Newton or secant condition is that $\mathbf{B}_{i+1}$ satisfy

$$\mathbf{B}_{i+1} \cdot \delta\mathbf{x}_i = \delta\mathbf{F}_i \tag{9.7.16}$$

where $\delta\mathbf{F}_i = \mathbf{F}_{i+1} - \mathbf{F}_i$. This is the generalization of the one-dimensional secant approximation to the derivative, $\delta F/\delta x$. However, equation (9.7.16) does not determine $\mathbf{B}_{i+1}$ uniquely in more than one dimension.

Many different auxiliary conditions to pin down $\mathbf{B}_{i+1}$ have been explored, but the best-performing algorithm in practice results from Broyden's formula. This formula is based on the idea of getting $\mathbf{B}_{i+1}$ by making the least change to $\mathbf{B}_i$ consistent with the secant equation (9.7.16). Broyden showed that the resulting formula is

$$\mathbf{B}_{i+1} = \mathbf{B}_i + \frac{(\delta\mathbf{F}_i - \mathbf{B}_i \cdot \delta\mathbf{x}_i) \otimes \delta\mathbf{x}_i}{\delta\mathbf{x}_i \cdot \delta\mathbf{x}_i} \tag{9.7.17}$$

You can easily check that $\mathbf{B}_{i+1}$ satisfies (9.7.16).

Early implementations of Broyden's method used the Sherman-Morrison formula, equation (2.7.2), to invert equation (9.7.17) analytically,

$$\mathbf{B}_{i+1}^{-1} = \mathbf{B}_i^{-1} + \frac{(\delta\mathbf{x}_i - \mathbf{B}_i^{-1} \cdot \delta\mathbf{F}_i) \otimes \delta\mathbf{x}_i \cdot \mathbf{B}_i^{-1}}{\delta\mathbf{x}_i \cdot \mathbf{B}_i^{-1} \cdot \delta\mathbf{F}_i} \tag{9.7.18}$$

Then, instead of solving equation (9.7.3) by, e.g., LU decomposition, one determined

$$\delta\mathbf{x}_i = -\mathbf{B}_i^{-1} \cdot \mathbf{F}_i \tag{9.7.19}$$

by matrix multiplication in $O(N^2)$ operations. The disadvantage of this method is that it cannot easily be embedded in a globally convergent strategy, for which the gradient of equation (9.7.4) requires $\mathbf{B}$, not $\mathbf{B}^{-1}$,

$$\nabla(\tfrac{1}{2}\mathbf{F} \cdot \mathbf{F}) \simeq \mathbf{B}^T \cdot \mathbf{F} \tag{9.7.20}$$

Accordingly, we implement the update formula in the form (9.7.17).

However, we can still preserve the $O(N^2)$ solution of (9.7.3) by using QR decomposition (§2.10) instead of LU decomposition. The reason is that because of the special form of equation (9.7.17), the QR decomposition of $\mathbf{B}_i$ can be updated into the QR decomposition of $\mathbf{B}_{i+1}$ in $O(N^2)$ operations (§2.10). All we need is an initial approximation $\mathbf{B}_0$ to start the ball rolling. It is often acceptable to start simply with the identity matrix, and then allow $O(N)$

updates to produce a reasonable approximation to the Jacobian. We prefer to spend the first N function evaluations on a finite difference approximation to initialize $\mathbf{B}$ via a call to NRfdjac.

Since $\mathbf{B}$ is not the exact Jacobian, we are not guaranteed that $\delta\mathbf{x}$ is a descent direction for $f = \frac{1}{2}\mathbf{F}\cdot\mathbf{F}$ (cf. equation 9.7.5). Thus the line search algorithm can fail to return a suitable step if $\mathbf{B}$ wanders far from the true Jacobian. In this case, we reinitialize $\mathbf{B}$ by another call to NRfdjac.

Like the secant method in one dimension, Broyden's method converges superlinearly once you get close enough to the root. Embedded in a global strategy, it is almost as robust as Newton's method, and often needs far fewer function evaluations to determine a zero. Note that the final value of $\mathbf{B}$ is *not* always close to the true Jacobian at the root, even when the method converges.

The routine broydn, given below, is very similar to newt in organization. The principal differences are the use of QR decomposition instead of LU, and the updating formula instead of directly determining the Jacobian. The remarks at the end of newt about scaling the variables apply equally to broydn.

roots_multidim.h

```
template <class T>
void broydn(VecDoub_IO &x, Bool &check, T &vecfunc) {
```

Given an initial guess x[0..n-1] for a root in n dimensions, find the root by Broyden's method embedded in a globally convergent strategy. The vector of functions to be zeroed, called fvec[0..n-1] in the routine below, is returned by the user-supplied function or functor vecfunc. The routines NRfdjac and NRfmin from newt are used. The output quantity check is false on a normal return and true if the routine has converged to a local minimum of the function fmin or if Broyden's method can make no further progress. In this case try restarting from a different initial guess.

```
    const Int MAXITS=200;
    const Doub EPS=numeric_limits<Doub>::epsilon();
    const Doub TOLF=1.0e-8, TOLX=EPS, STPMX=100.0, TOLMIN=1.0e-12;
```

Here MAXITS is the maximum number of iterations; EPS is the machine precision; TOLF is the convergence criterion on function values; TOLX is the convergence criterion on $\delta\mathbf{x}$; STPMX is the scaled maximum step length allowed in line searches; and TOLMIN is used to decide whether spurious convergence to a minimum of fmin has occurred.

```
    Bool restrt,skip;
    Int i,its,j,n=x.size();
    Doub den,f,fold,stpmax,sum,temp,test;
    VecDoub fvcold(n),g(n),p(n),s(n),t(n),w(n),xold(n);
    QRdcmp *qr;
    NRfmin<T> fmin(vecfunc);                    Set up NRfmin object.
    NRfdjac<T> fdjac(vecfunc);                  Set up NRfdjac object.
    VecDoub &fvec=fmin.fvec;                    Make an alias to simplify coding.
    f=fmin(x);                                  The vector fvec is also computed by this
                                                   call.
    test=0.0;                                   Test for initial guess being a root. Use more
    for (i=0;i<n;i++)                              stringent test than simply TOLF.
        if (abs(fvec[i]) > test) test=abs(fvec[i]);
    if (test < 0.01*TOLF) {
        check=false;
        return;
    }
    for (sum=0.0,i=0;i<n;i++) sum += SQR(x[i]);      Calculate stpmax for line searches.
    stpmax=STPMX*MAX(sqrt(sum),Doub(n));
    restrt=true;                                Ensure initial Jacobian gets computed.
    for (its=1;its<=MAXITS;its++) {             Start of iteration loop.
        if (restrt) {                           Initialize or reinitialize Jacobian and QR de-
            qr=new QRdcmp(fdjac(x,fvec));          compose it.
            if (qr->sing) throw("singular Jacobian in broydn");
        } else {                                Carry out Broyden update.
            for (i=0;i<n;i++) s[i]=x[i]-xold[i];        s = δx.
            for (i=0;i<n;i++) {                         t = R · s.
                for (sum=0.0,j=i;j<n;j++) sum += qr->r[i][j]*s[j];
                t[i]=sum;
            }
            skip=true;
```

```
        for (i=0;i<n;i++) {                          w = δF − B·s.
            for (sum=0.0,j=0;j<n;j++) sum += qr->qt[j][i]*t[j];
            w[i]=fvec[i]-fvcold[i]-sum;
            if (abs(w[i]) >= EPS*(abs(fvec[i])+abs(fvcold[i]))) skip=false;
            Don't update with noisy components of w.
            else w[i]=0.0;
        }
        if (!skip) {
            qr->qtmult(w,t);                         t = Q^T · w.
            for (den=0.0,i=0;i<n;i++) den += SQR(s[i]);
            for (i=0;i<n;i++) s[i] /= den;           Store s/(s·s) in s.
            qr->update(t,s);                         Update R and Q^T.
            if (qr->sing) throw("singular update in broydn");
        }
    }
    qr->qtmult(fvec,p);
    for (i=0;i<n;i++)                    Right-hand side for linear equations is −Q^T · F.
        p[i] = -p[i];
    for (i=n-1;i>=0;i--) {               Compute ∇f ≈ (Q·R)^T · F for the line search.
        for (sum=0.0,j=0;j<=i;j++) sum -= qr->r[j][i]*p[j];
        g[i]=sum;
    }
    for (i=0;i<n;i++) {                  Store x and F.
        xold[i]=x[i];
        fvcold[i]=fvec[i];
    }
    fold=f;                              Store f.
    qr->rsolve(p,p);                     Solve linear equations.
    lnsrch(xold,fold,g,p,x,f,stpmax,check,fmin);
    lnsrch returns new x and f. It also calculates fvec at the new x when it calls fmin.

    test=0.0;                            Test for convergence on function values.
    for (i=0;i<n;i++)
        if (abs(fvec[i]) > test) test=abs(fvec[i]);
    if (test < TOLF) {
        check=false;
        delete qr;
        return;
    }
    if (check) {                         True if line search failed to find a new x.
        if (restrt) {                    Failure; already tried reinitializing the Jacobian.
            delete qr;
            return;
        } else {
            test=0.0;                    Check for gradient of f zero, i.e., spurious con-
            den=MAX(f,0.5*n);                vergence.
            for (i=0;i<n;i++) {
                temp=abs(g[i])*MAX(abs(x[i]),1.0)/den;
                if (temp > test) test=temp;
            }
            if (test < TOLMIN) {
                delete qr;
                return;
            }
            else restrt=true;            Try reinitializing the Jacobian.
        }
    } else {                             Successful step; will use Broyden update for next
        restrt=false;                        step.
        test=0.0;                        Test for convergence on δx.
        for (i=0;i<n;i++) {
            temp=(abs(x[i]-xold[i]))/MAX(abs(x[i]),1.0);
            if (temp > test) test=temp;
        }
```

```
            if (test < TOLX) {
                delete qr;
                return;
            }
        }
    }
    throw("MAXITS exceeded in broydn");
}
```

9.7.4 More Advanced Implementations

One of the principal ways that the methods described so far can fail is if **J** (in Newton's method) or **B** in (Broyden's method) becomes singular or nearly singular, so that $\delta\mathbf{x}$ cannot be determined. If you are lucky, this situation will not occur very often in practice. Methods developed so far to deal with this problem involve monitoring the condition number of **J** and perturbing **J** if singularity or near singularity is detected. This is most easily implemented if the QR decomposition is used instead of LU in Newton's method (see [2] for details). Our personal experience is that, while such an algorithm can solve problems where **J** is exactly singular and the standard Newton method fails, it is occasionally less robust on other problems where LU decomposition succeeds. Clearly implementation details involving roundoff, underflow, etc., are important here and the last word is yet to be written.

Our global strategies both for minimization and zero finding have been based on line searches. Other global algorithms, such as the *hook step* and *dogleg step* methods, are based instead on the *model-trust region* approach, which is related to the Levenberg-Marquardt algorithm for nonlinear least squares (§15.5). While somewhat more complicated than line searches, these methods have a reputation for robustness even when starting far from the desired zero or minimum [2].

CITED REFERENCES AND FURTHER READING:

Deuflhard, P. 2004, *Newton Methods for Nonlinear Problems* (Berlin: Springer).[1]

Dennis, J.E., and Schnabel, R.B. 1983, *Numerical Methods for Unconstrained Optimization and Nonlinear Equations*; reprinted 1996 (Philadelphia: S.I.A.M.).[2]

Broyden, C.G. 1965, "A Class of Methods for Solving Nonlinear Simultaneous Equations," *Mathematics of Computation*, vol. 19, pp. 577–593.[3]

CHAPTER 10

Minimization or Maximization of Functions

10.0 Introduction

In a nutshell: You are given a single function f that depends on one or more independent variables. You want to find the value of those variables where f takes on a maximum or a minimum value. You can then calculate what value of f is achieved at the maximum or minimum. The tasks of maximization and minimization are trivially related to each other, since one person's function f could just as well be another's $-f$. The computational desiderata are the usual ones: Do it quickly, cheaply, and in small memory. Often the computational effort is dominated by the cost of evaluating f (and also perhaps its partial derivatives with respect to all variables, if the chosen algorithm requires them). In such cases the desiderata are sometimes replaced by the simple surrogate: Evaluate f as few times as possible.

An extremum (maximum or minimum point) can be either *global* (truly the highest or lowest function value) or *local* (the highest or lowest in a finite neighborhood and not on the boundary of that neighborhood). (See Figure 10.0.1.) Finding a global extremum is, in general, a very difficult problem. Two standard heuristics are widely used: (i) Find local extrema starting from widely varying starting values of the independent variables (perhaps chosen quasi-randomly, as in §7.8), and then pick the most extreme of these (if they are not all the same); or (ii) perturb a local extremum by taking a finite amplitude step away from it, and then see if your routine returns you to a better point, or "always" to the same one. More recently, so-called *simulated annealing methods* (§10.12) have demonstrated important successes on a variety of global extremization problems.

Our chapter title could just as well be *optimization*, which is the usual name for this very large field of numerical research. The importance ascribed to the various tasks in this field depends strongly on the particular interests of whom you talk to. Economists, and some engineers, are particularly concerned with *constrained optimization*, where there are a priori limitations on the allowed values of independent variables. For example, the production of wheat in the United States must be a non-

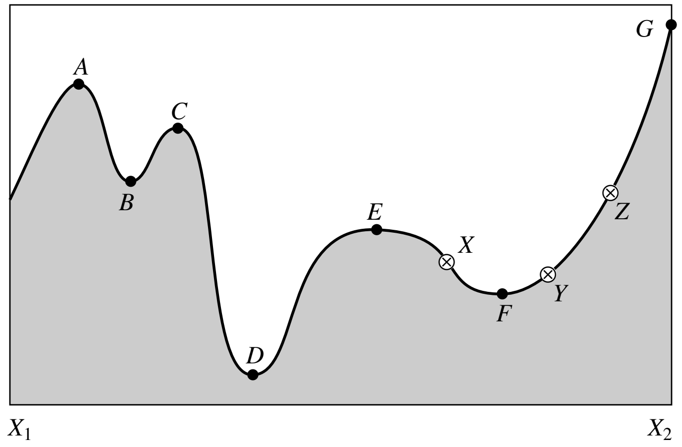

Figure 10.0.1. Extrema of a function in an interval. Points A, C, and E are local, but not global, maxima. Points B and F are local, but not global, minima. The global maximum occurs at G, which is on the boundary of the interval so that the derivative of the function need not vanish there. The global minimum is at D. At point E, derivatives higher than the first vanish, a situation that can cause difficulty for some algorithms. The points X, Y, and Z are said to *bracket* the minimum F, since Y is less than both X and Z.

negative number. One particularly well-developed area of constrained optimization is *linear programming*, where both the function to be optimized and the constraints happen to be linear functions of the independent variables. Sections 10.10 and 10.11, which are otherwise somewhat disconnected from the rest of the material that we have chosen to include in this chapter, discuss the two major approaches to such problems, the so-called *simplex algorithm* and *interior-point methods*.

Two other sections, §10.12 and §10.13, also lie outside of our main thrust, but for a different reasons. As mentioned, §10.12 discusses so-called *annealing methods*. These are stochastic, rather than deterministic, algorithms. Annealing methods have solved some problems previously thought to be practically insoluble: They address directly the problem of finding global extrema in the presence of large numbers of undesired local extrema. Section 10.13 discusses a different kind of minimization, namely that of path length along a directed graph by the technique known as *dynamic programming*. This will prove important later, in Chapter 16.

The other sections in this chapter constitute a selection of established algorithms for unconstrained minimization. (For definiteness, we will henceforth regard the optimization problem as that of minimization.) These sections are connected, with later ones depending on earlier ones. If you are just looking for the one “perfect” algorithm to solve your particular application, you may feel that we are telling you more than you want to know. Unfortunately, there is *no* perfect optimization algorithm. This is a case where we strongly urge you to try more than one method in comparative fashion. However, here are some guidelines:

For one-dimensional minimization (minimize a function of one variable), you must choose between methods that need only evaluations of the function, and methods that also require evalutations of the function’s derivative. The latter are typically more powerful, but not always enough so as to compensate for the additional calculations of derivatives. We can easily construct examples favoring one approach or

the other.

- For one-dimensional minimization *without* calculation of the derivative, first bracket the minimum as described in §10.2, and then use *Brent's method* as described in §10.3. If your function has a discontinuous second (or lower) derivative, then the parabolic interpolations of Brent's method are of no advantage, and you might wish to use the simplest form of *golden section search*, as described in §10.2.
- For one-dimensional minimization *with* calculation of the derivative, §10.4 supplies a variant of Brent's method that makes limited use of the first derivative information. We shy away from the alternative of using derivative information to construct high-order interpolating polynomials. In our experience, the improvement in convergence very near a smooth, analytic minimum does not make up for the tendency of polynomials sometimes to give wildly wrong interpolations at early stages, especially for functions that may have sharp, "exponential" features.

For the multidimensional case, where you want to minimize a function of two or more variables, the analog of the derivative is the gradient, a vector quantity. You now have three options: compute the gradients using your function's known analytic form, compute the gradients by taking finite differences of computed function values, or don't compute the gradients at all. You also get to choose between methods that require storage of order N^2 and those that require only of order N, where N is the number of dimensions. For moderate values of N this is not a serious constraint; but if N is itself the number of points in a two- or three-dimensional grid, then N^2 storage may be prohibitive.

- For minimization without gradients, the *downhill simplex method* due to Nelder and Mead, discussed in §10.5, is slow, but sure. (This use of the word "simplex" is not to be confused with the simplex method of linear programming.) The method just crawls downhill in a straightforward fashion that makes almost no special assumptions about your function. While this can be extremely slow, it can also be extremely robust. Not to be overlooked is the fact that the code is concise and completely self-contained: a general N-dimensional minimization program in under 100 program lines! The storage requirement is of order N^2, and derivative calculations are not required.
- When your function has some smoothness to it, but you still don't want to compute gradients, turn to *direction set methods*, of which *Powell's method* is the prototype (§10.7). Powell's method requires a one-dimensional minimization subalgorithm such as Brent's method (see above). Storage is of order N^2. Direction set methods are much faster than the downhill simplex method. But keep reading for, possibly, an even better alternative.

Now the case where you are willing to calculate gradients from your function's known analytic form:

- *Conjugate gradient methods*, as typified by the *Fletcher-Reeves algorithm* and the closely related and probably superior *Polak-Ribiere algorithm* are widely used. Conjugate gradient methods require only of order a few times N storage, derivative calculations, and one-dimensional subminimization. Turn to §10.8 for detailed discussion and implementation.
- *Quasi-Newton* or *variable metric* methods are typified by the *Davidon-Fletcher-*

Powell (DFP) algorithm (sometimes referred to just as the *Fletcher-Powell* method) or the closely related *Broyden-Fletcher-Goldfarb-Shanno (BFGS)* algorithm. These methods require of order N^2 storage, derivative calculations, and one-dimensional subminimization. Details are in §10.9. Our personal experience is that the quasi-Newton methods dominate the conjugate gradient methods (if you can afford the storage), but there are probably applications where the reverse is true.

Finally, the case where the method uses gradients, but you are willing to let them be calculated by extra function evaluations (and finite differences):

- In our experience, the quasi-Newton (variable metric) methods work very well in this case, so much so that they can be significantly more efficient than Powell's method on suitable problems. In §10.9 we give an implementation that (almost) hides the gradient calculation completely. Thus quasi-Newton is effectively our first choice of method both when you are willing to calculate gradients, and when you are not willing!

You can now proceed to scale the peaks (and/or plumb the depths) of practical optimization.

CITED REFERENCES AND FURTHER READING:

Dennis, J.E., and Schnabel, R.B. 1983, *Numerical Methods for Unconstrained Optimization and Nonlinear Equations*; reprinted 1996 (Philadelphia: S.I.A.M.).

Polak, E. 1971, *Computational Methods in Optimization* (New York: Academic Press).

Gill, P.E., Murray, W., and Wright, M.H. 1981, *Practical Optimization* (New York: Academic Press).

Acton, F.S. 1970, *Numerical Methods That Work*; 1990, corrected edition (Washington, DC: Mathematical Association of America), Chapter 17.

Jacobs, D.A.H. (ed.) 1977, *The State of the Art in Numerical Analysis* (London: Academic Press), Chapter III.1.

Brent, R.P. 1973, *Algorithms for Minimization without Derivatives* (Englewood Cliffs, NJ: Prentice-Hall); reprinted 2002 (New York: Dover).

Dahlquist, G., and Bjorck, A. 1974, *Numerical Methods* (Englewood Cliffs, NJ: Prentice-Hall); reprinted 2003 (New York: Dover), Chapter 10.

10.1 Initially Bracketing a Minimum

What does it mean to *bracket* a minimum? A root of a function is known to be bracketed by a pair of points, a and b, when the function has opposite sign at those two points. A minimum, by contrast, is known to be bracketed only when there is a *triplet* of points, $a < b < c$ (or $c < b < a$), such that $f(b)$ is less than both $f(a)$ and $f(c)$. In this case we know that the function (if it is smooth) has a minimum in the interval (a, c).

We consider the initial bracketing of a minimum to be an essential part of any one-dimensional minimization. There are some one-dimensional algorithms that do not require a rigorous initial bracketing. However, we would *never* trade the secure feeling of *knowing* that a minimum is "in there somewhere" for the dubious reduction of function evaluations that these nonbracketing routines may promise. Please bracket your minima (or, for that matter, your zeros) before isolating them!

There is not much theory as to how to do this bracketing. Obviously you want to step downhill. But how far? We like to take larger and larger steps, starting with some (wild?) initial guess and then increasing the stepsize at each step either by a constant factor, or else by the result of a parabolic extrapolation of the preceding points that is designed to take us to the extrapolated turning point. It doesn't much matter if the steps get big. After all, we are stepping downhill, so we already have the left and middle points of the bracketing triplet. We just need to take a big enough step to stop the downhill trend and get a high third point.

Here is our standard routine, the function `bracket`. It appears in the structure `Bracketmethod` that serves as the base class for all the one-dimensional minimization methods we give in this chapter.

mins.h

```
struct Bracketmethod {
Base class for one-dimensional minimization routines. Provides a routine to bracket a minimum
and several utility functions.
    Doub ax,bx,cx,fa,fb,fc;
    template <class T>
    void bracket(const Doub a, const Doub b, T &func)
    Given a function or functor func, and given distinct initial points ax and bx, this routine
    searches in the downhill direction (defined by the function as evaluated at the initial points)
    and returns new points ax, bx, cx that bracket a minimum of the function. Also returned
    are the function values at the three points, fa, fb, and fc.
    {
        const Doub GOLD=1.618034,GLIMIT=100.0,TINY=1.0e-20;
        Here GOLD is the default ratio by which successive intervals are magnified and GLIMIT
        is the maximum magnification allowed for a parabolic-fit step.
        ax=a; bx=b;
        Doub fu;
        fa=func(ax);
        fb=func(bx);
        if (fb > fa) {                       Switch roles of a and b so that we can go
            SWAP(ax,bx);                         downhill in the direction from a to b.
            SWAP(fb,fa);
        }
        cx=bx+GOLD*(bx-ax);                  First guess for c.
        fc=func(cx);
        while (fb > fc) {                    Keep returning here until we bracket.
            Doub r=(bx-ax)*(fb-fc);          Compute u by parabolic extrapolation from
            Doub q=(bx-cx)*(fb-fa);              a, b, c. TINY is used to prevent any pos-
            Doub u=bx-((bx-cx)*q-(bx-ax)*r)/         sible division by zero.
                (2.0*SIGN(MAX(abs(q-r),TINY),q-r));
            Doub ulim=bx+GLIMIT*(cx-bx);
            We won't go farther than this. Test various possibilities:
            if ((bx-u)*(u-cx) > 0.0) {       Parabolic u is between b and c: try it.
                fu=func(u);
                if (fu < fc) {               Got a minimum between b and c.
                    ax=bx;
                    bx=u;
                    fa=fb;
                    fb=fu;
                    return;
                } else if (fu > fb) {        Got a minimum between between a and u.
                    cx=u;
                    fc=fu;
                    return;
                }
                u=cx+GOLD*(cx-bx);           Parabolic fit was no use. Use default mag-
                fu=func(u);                      nification.
            } else if ((cx-u)*(u-ulim) > 0.0) {        Parabolic fit is between c and
                fu=func(u);                                its allowed limit.
```

```
            if (fu < fc) {
                shft3(bx,cx,u,u+GOLD*(u-cx));
                shft3(fb,fc,fu,func(u));
            }
        } else if ((u-ulim)*(ulim-cx) >= 0.0) {    Limit parabolic u to maximum
            u=ulim;                                    allowed value.
            fu=func(u);
        } else {                                 Reject parabolic u, use default magnifica-
            u=cx+GOLD*(cx-bx);                       tion.
            fu=func(u);
        }
        shft3(ax,bx,cx,u);                       Eliminate oldest point and continue.
        shft3(fa,fb,fc,fu);
    }
}
inline void shft2(Doub &a, Doub &b, const Doub c)
Utility function used in this structure or others derived from it.
{
    a=b;
    b=c;
}
inline void shft3(Doub &a, Doub &b, Doub &c, const Doub d)
{
    a=b;
    b=c;
    c=d;
}
inline void mov3(Doub &a, Doub &b, Doub &c, const Doub d, const Doub e,
    const Doub f)
{
    a=d; b=e; c=f;
}
};
```

(Because of the housekeeping involved in moving around three or four points and their function values, the above program ends up looking deceptively formidable. That is true of several other programs in this chapter as well. The underlying ideas, however, are quite simple.)

10.2 Golden Section Search in One Dimension

Recall how the bisection method finds roots of functions in one dimension (§9.1): The root is supposed to have been bracketed in an interval (a, b). One then evaluates the function at an intermediate point x and obtains a new, smaller bracketing interval, either (a, x) or (x, b). The process continues until the bracketing interval is acceptably small. It is optimal to choose x to be the midpoint of (a, b) so that the decrease in the interval length is maximized when the function is as uncooperative as it can be, i.e., when the luck of the draw forces you to take the bigger bisected segment.

There is a precise, though slightly subtle, translation of these considerations to the minimization problem. The analog of bisection is to choose a new point x, either between a and b or between b and c. Suppose, to be specific, that we make the latter choice. Then we evaluate $f(x)$. If $f(b) < f(x)$, then the new bracketing triplet of points is (a, b, x); contrariwise, if $f(b) > f(x)$, then the new bracketing triplet

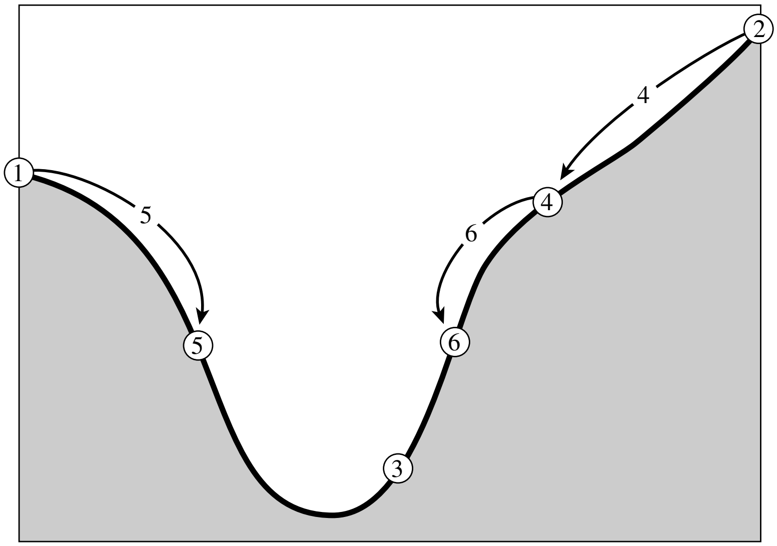

Figure 10.2.1. Successive bracketing of a minimum. The minimum is originally bracketed by points 1,3,2. The function is evaluated at 4, which replaces 2; then at 5, which replaces 1; then at 6, which replaces 4. The rule at each stage is to keep a center point that is lower than the two outside points. After the steps shown, the minimum is bracketed by points 5,3,6.

is (b, x, c). In all cases, the middle point of the new triplet is the abscissa whose ordinate is the best minimum achieved so far; see Figure 10.2.1. We continue the process of bracketing until the distance between the two outer points of the triplet is tolerably small.

How small is "tolerably" small? For a minimum located at a value b, you might naively think that you will be able to bracket it in as small a range as $(1-\epsilon)b < b < (1+\epsilon)b$, where ϵ is your computer's floating-point precision, a number like 10^{-7} (for `float`) or 2×10^{-16} (for `double`). Not so! In general, the shape of your function $f(x)$ near b will be given by Taylor's theorem,

$$f(x) \approx f(b) + \tfrac{1}{2} f''(b)(x-b)^2 \tag{10.2.1}$$

The second term will be negligible compared to the first (that is, will be a factor ϵ smaller and will act just like zero when added to it) whenever

$$|x-b| < \sqrt{\epsilon}|b| \sqrt{\frac{2\,|f(b)|}{b^2 f''(b)}} \tag{10.2.2}$$

The reason for writing the right-hand side in this way is that, for most functions, the final square root is a number of order unity. Therefore, as a rule of thumb, it is hopeless to ask for a bracketing interval of width less than $\sqrt{\epsilon}$ times its central value, a fractional width of only about 10^{-4} (single precision) or 10^{-8} (double precision). Knowing this inescapable fact will save you a lot of useless bisections!

The minimum-finding routines of this chapter will often call for a user-supplied argument `tol`, and return with an abscissa whose fractional precision is about $\pm$`tol` (bracketing interval of fractional size about 2×`tol`). Unless you have a better estimate for the right-hand side of equation (10.2.2), you should set `tol` equal to (or

not much less than) the square root of your machine's floating-point precision, since smaller values will gain you nothing.

It remains to decide on a strategy for choosing the new point x, given (a, b, c). Suppose that b is a fraction w of the way between a and c, i.e.,

$$\frac{b-a}{c-a} = w \qquad \frac{c-b}{c-a} = 1-w \tag{10.2.3}$$

Also suppose that our next trial point x is an additional fraction z beyond b,

$$\frac{x-b}{c-a} = z \tag{10.2.4}$$

Then the next bracketing segment will either be of length $w+z$ relative to the current one, or else of length $1-w$. If we want to minimize the worst case possibility, then we will choose z to make these equal, namely

$$z = 1 - 2w \tag{10.2.5}$$

We see at once that the new point is the symmetric point to b in the original interval, namely with $|b-a|$ equal to $|x-c|$. This implies that the point x lies in the larger of the two segments (z is positive only if $w < 1/2$).

But where in the larger segment? Where did the value of w itself come from? Presumably from the previous stage of applying our same strategy. Therefore, if z is chosen to be optimal, then so was w before it. This *scale similarity* implies that x should be the same fraction of the way from b to c (if that is the bigger segment) as was b from a to c, in other words,

$$\frac{z}{1-w} = w \tag{10.2.6}$$

Equations (10.2.5) and (10.2.6) give the quadratic equation

$$w^2 - 3w + 1 = 0 \qquad \text{yielding} \qquad w = \frac{3-\sqrt{5}}{2} \approx 0.38197 \tag{10.2.7}$$

In other words, the optimal bracketing interval (a, b, c) has its middle point b a fractional distance 0.38197 from one end (say a), and 0.61803 from the other end (say b). These fractions are those of the so-called *golden mean* or *golden section*, whose supposedly aesthetic properties hark back to the ancient Pythagoreans. This optimal method of function minimization, the analog of the bisection method for finding zeros, is thus called the *golden section search*, which can be summarized as follows:

Given, at each stage, a bracketing triplet of points, the next point to be tried is that which is a fraction 0.38197 into the larger of the two intervals (measuring from the central point of the triplet). If you start out with a bracketing triplet whose segments are not in the golden ratios, the procedure of choosing successive points at the golden mean point of the larger segment will quickly converge you to the proper self-replicating ratios.

The golden section search guarantees that each new function evaluation will (after self-replicating ratios have been achieved) bracket the minimum to an interval just 0.61803 times the size of the preceding interval. This is comparable to, but not

quite as good as, the 0.50000 that holds when finding roots by bisection. Note that the convergence is *linear* (in the language of Chapter 9), meaning that successive significant figures are won linearly with additional function evaluations. In the next section we will give a superlinear method, in which the rate at which successive significant figures are liberated increases with each successive function evaluation.

To use the golden section search, you need statements like the following:

```
Golden golden;
golden.bracket(a,b,func);
xmin=golden.minimize(func);
```

The value of the function at the minimum is available in `golden.fmin`. If you want to specify a function tolerance different from the default value of `3.0e-8`, simply override the default value in the constructor:

```
tol = ...
Golden golden(tol);
```

If you want to use a specified bracket as the initial condition, omit the call to `bracket` and set the bracket explicitly:

```
golden.ax = ...; golden.bx = ...; golden.cx = ...;
```

Here is the routine:

mins.h

```
struct Golden : Bracketmethod {
Golden section search for minimum.
    Doub xmin,fmin;
    const Doub tol;
    Golden(const Doub toll=3.0e-8) : tol(toll) {}
    template <class T>
    Doub minimize(T &func)
    Given a function or functor f, and given a bracketing triplet of abscissas ax, bx, cx (such
    that bx is between ax and cx, and f(bx) is less than both f(ax) and f(cx)), this routine
    performs a golden section search for the minimum, isolating it to a fractional precision of
    about tol. The abscissa of the minimum is returned as xmin, and the function value at
    the minimum is returned as min, the returned function value.
    {
        const Doub R=0.61803399,C=1.0-R;    The golden ratios.
        Doub x1,x2;
        Doub x0=ax;                          At any given time we will keep track of four
        Doub x3=cx;                              points, x0,x1,x2,x3.
        if (abs(cx-bx) > abs(bx-ax)) {       Make x0 to x1 the smaller segment,
            x1=bx;
            x2=bx+C*(cx-bx);                 and fill in the new point to be tried.
        } else {
            x2=bx;
            x1=bx-C*(bx-ax);
        }
        Doub f1=func(x1);                    The initial function evaluations. Note that
        Doub f2=func(x2);                        we never need to evaluate the function
        while (abs(x3-x0) > tol*(abs(x1)+abs(x2))) {   at the original endpoints.
            if (f2 < f1) {                   One possible outcome,
                shft3(x0,x1,x2,R*x2+C*x3);   its housekeeping,
                shft2(f1,f2,func(x2));       and a new function evaluation.
            } else {                         The other outcome,
                shft3(x3,x2,x1,R*x1+C*x0);
                shft2(f2,f1,func(x1));       and its new function evaluation.
            }
        }                                    Back to see if we are done.
        if (f1 < f2) {                       We are done. Output the best of the two
            xmin=x1;                             current values.
```

```
            fmin=f1;
        } else {
            xmin=x2;
            fmin=f2;
        }
        return xmin;
    }
};
```

10.3 Parabolic Interpolation and Brent's Method in One Dimension

We already tipped our hand about the desirability of parabolic interpolation in the `bracket` routine of §10.1, but it is now time to be more explicit. A golden section search is designed to handle, in effect, the worst possible case of function minimization, with the uncooperative minimum hunted down and cornered like a scared rabbit. But why assume the worst? If the function is nicely parabolic near to the minimum — surely the generic case for sufficiently smooth functions — then the parabola fitted through any three points ought to take us in a single leap to the minimum, or at least very near to it (see Figure 10.3.1). Since we want to find an abscissa rather than an ordinate, the procedure is technically called *inverse parabolic interpolation*.

The formula for the abscissa x that is the minimum of a parabola through three points $f(a)$, $f(b)$, and $f(c)$ is

$$x = b - \frac{1}{2}\frac{(b-a)^2[f(b)-f(c)] - (b-c)^2[f(b)-f(a)]}{(b-a)[f(b)-f(c)] - (b-c)[f(b)-f(a)]} \qquad (10.3.1)$$

as you can easily derive. This formula fails only if the three points are collinear, in which case the denominator is zero (the minimum of the parabola is infinitely far away). Note, however, that (10.3.1) is as happy jumping to a parabolic maximum as to a minimum. No minimization scheme that depends solely on (10.3.1) is likely to succeed in practice.

The exacting task is to invent a scheme that relies on a sure-but-slow technique, like golden section search, when the function is not cooperative, but that switches over to (10.3.1) when the function allows. The task is nontrivial for several reasons, including these: (i) The housekeeping needed to avoid unnecessary function evaluations in switching between the two methods can be complicated. (ii) Careful attention must be given to the "endgame," where the function is being evaluated very near to the roundoff limit of equation (10.2.2). (iii) The scheme for detecting a cooperative versus noncooperative function must be very robust.

Brent's method [1] is up to the task in all particulars. At any particular stage, it is keeping track of six function points (not necessarily all distinct), a, b, u, v, w and x, defined as follows: The minimum is bracketed between a and b; x is the point with the very least function value found so far (or the most recent one in case of a tie); w is the point with the second least function value; v is the previous value of w; and u is the point at which the function was evaluated most recently. Also appearing in the algorithm is the point x_m, the midpoint between a and b; however, the function is not evaluated there.

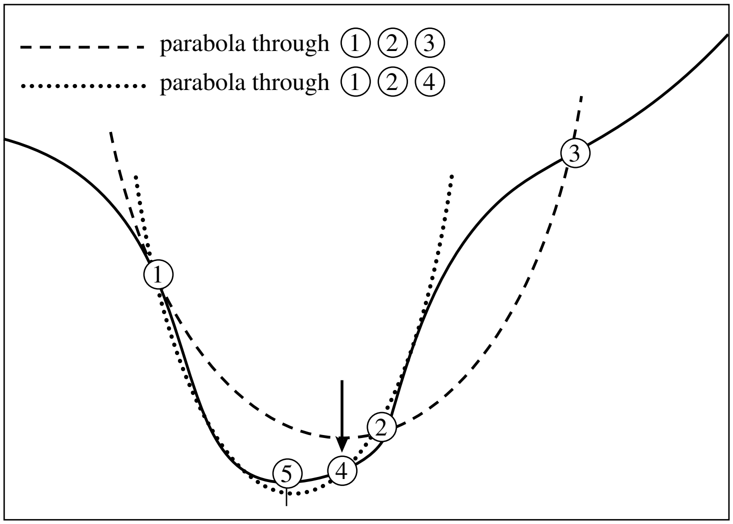

Figure 10.3.1. Convergence to a minimum by inverse parabolic interpolation. A parabola (dashed line) is drawn through the three original points 1,2,3 on the given function (solid line). The function is evaluated at the parabola's minimum, 4, which replaces point 3. A new parabola (dotted line) is drawn through points 1,4,2. The minimum of this parabola is at 5, which is close to the minimum of the function.

You can read the code below to understand the method's logical organization. Mention of a few general principles here may, however, be helpful: Parabolic interpolation is attempted, fitting through the points x, v, and w. To be acceptable, the parabolic step must (i) fall within the bounding interval (a, b), and (ii) imply a movement from the best current value x that is *less* than half the movement of the *step before last*. This second criterion ensures that the parabolic steps are actually converging to something, rather than, say, bouncing around in some nonconvergent limit cycle. In the worst possible case, where the parabolic steps are acceptable but useless, the method will approximately alternate between parabolic steps and golden sections, converging in due course by virtue of the latter. The reason for comparing to the step *before* last seems essentially heuristic: Experience shows that it is better not to "punish" the algorithm for a single bad step if it can make it up on the next one.

Another principle exemplified in the code is never to evaluate the function less than a distance `tol` from a point already evaluated (or from a known bracketing point). The reason is that, as we saw in equation (10.2.2), there is simply no information content in doing so: The function will differ from the value already evaluated only by an amount of order the roundoff error. Therefore, in the code below you will find several tests and modifications of a potential new point, imposing this restriction. This restriction also interacts subtly with the test for "doneness," which the method takes into account.

A typical ending configuration for Brent's method is that a and b are $2 \times x \times$ `tol` apart, with x (the best abscissa) at the midpoint of a and b, and therefore fractionally accurate to $\pm$`tol`.

The calling sequence for `Brent` is exactly analogous to that of `Golden` in the previous section. Indulge us a final reminder that `tol` should generally be no smaller than the square root of your machine's floating-point precision.

mins.h

```
struct Brent : Bracketmethod {
```
Brent's method to find a minimum.
```
    Doub xmin,fmin;
    const Doub tol;
    Brent(const Doub toll=3.0e-8) : tol(toll) {}
    template <class T>
    Doub minimize(T &func)
```
Given a function or functor f, and given a bracketing triplet of abscissas ax, bx, cx (such that bx is between ax and cx, and f(bx) is less than both f(ax) and f(cx)), this routine isolates the minimum to a fractional precision of about tol using Brent's method. The abscissa of the minimum is returned as xmin, and the function value at the minimum is returned as min, the returned function value.
```
    {
        const Int ITMAX=100;
        const Doub CGOLD=0.3819660;
        const Doub ZEPS=numeric_limits<Doub>::epsilon()*1.0e-3;
```
Here ITMAX is the maximum allowed number of iterations; CGOLD is the golden ratio; and ZEPS is a small number that protects against trying to achieve fractional accuracy for a minimum that happens to be exactly zero.
```
        Doub a,b,d=0.0,etemp,fu,fv,fw,fx;
        Doub p,q,r,tol1,tol2,u,v,w,x,xm;
        Doub e=0.0;                                   This will be the distance moved on
                                                          the step before last.
        a=(ax < cx ? ax : cx);                        a and b must be in ascending order,
        b=(ax > cx ? ax : cx);                            but input abscissas need not be.
        x=w=v=bx;                                     Initializations...
        fw=fv=fx=func(x);
        for (Int iter=0;iter<ITMAX;iter++) {          Main program loop.
            xm=0.5*(a+b);
            tol2=2.0*(tol1=tol*abs(x)+ZEPS);
            if (abs(x-xm) <= (tol2-0.5*(b-a))) {      Test for done here.
                fmin=fx;
                return xmin=x;
            }
            if (abs(e) > tol1) {                      Construct a trial parabolic fit.
                r=(x-w)*(fx-fv);
                q=(x-v)*(fx-fw);
                p=(x-v)*q-(x-w)*r;
                q=2.0*(q-r);
                if (q > 0.0) p = -p;
                q=abs(q);
                etemp=e;
                e=d;
                if (abs(p) >= abs(0.5*q*etemp) || p <= q*(a-x)
                        || p >= q*(b-x))
                    d=CGOLD*(e=(x >= xm ? a-x : b-x));
```
The above conditions determine the acceptability of the parabolic fit. Here we take the golden section step into the larger of the two segments.
```
                else {
                    d=p/q;                            Take the parabolic step.
                    u=x+d;
                    if (u-a < tol2 || b-u < tol2)
                        d=SIGN(tol1,xm-x);
                }
            } else {
                d=CGOLD*(e=(x >= xm ? a-x : b-x));
            }
            u=(abs(d) >= tol1 ? x+d : x+SIGN(tol1,d));
            fu=func(u);
```
This is the one function evaluation per iteration.
```
            if (fu <= fx) {                           Now decide what to do with our func-
                if (u >= x) a=x; else b=x;                tion evaluation.
                shft3(v,w,x,u);                       Housekeeping follows:
                shft3(fv,fw,fx,fu);
```

```
            } else {
                if (u < x) a=u; else b=u;
                if (fu <= fw || w == x) {
                    v=w;
                    w=u;
                    fv=fw;
                    fw=fu;
                } else if (fu <= fv || v == x || v == w) {
                    v=u;
                    fv=fu;
                }
            }                                       Done with housekeeping. Back for
        }                                               another iteration.
        throw("Too many iterations in brent");
    }
};
```

CITED REFERENCES AND FURTHER READING:

Brent, R.P. 1973, *Algorithms for Minimization without Derivatives* (Englewood Cliffs, NJ: Prentice-Hall); reprinted 2002 (New York: Dover), Chapter 5.[1]

Forsythe, G.E., Malcolm, M.A., and Moler, C.B. 1977, *Computer Methods for Mathematical Computations* (Englewood Cliffs, NJ: Prentice-Hall), §8.2.

10.4 One-Dimensional Search with First Derivatives

Here we want to accomplish precisely the same goal as in the previous section, namely to isolate a functional minimum that is bracketed by the triplet of abscissas (a, b, c), but utilizing an additional capability to compute the function's first derivative as well as its value.

In principle, we might simply search for a zero of the derivative, ignoring the function value information, using a root finder like `rtflsp` or `zbrent` (§9.2 – §9.3). It doesn't take long to reject *that* idea: How do we distinguish maxima from minima? Where do we go from initial conditions where the derivatives on one or both of the outer bracketing points indicate that "downhill" is in the direction *out* of the bracketed interval?

We don't want to give up our strategy of maintaining a rigorous bracket on the minimum at all times. The only way to keep such a bracket is to update it using function (not derivative) information, with the central point in the bracketing triplet always that with the lowest function value. Therefore, the role of the derivatives can only be to help us choose new trial points within the bracket.

One school of thought is to "use everything you've got": Compute a polynomial of relatively high order (cubic or above) that agrees with some number of previous function and derivative evaluations. For example, there is a unique cubic that agrees with function and derivative at two points, and one can jump to the interpolated minimum of that cubic (if there is a minimum within the bracket). Suggested by Davidon and others, formulas for this tactic are given in [1].

We like to be more conservative than this. Once superlinear convergence sets in, it hardly matters whether its order is moderately lower or higher. In practical

problems that we have met, most function evaluations are spent in getting globally close enough to the minimum for superlinear convergence to commence. So we are more worried about all the funny "stiff" things that high-order polynomials can do (cf. Figure 3.0.1(b)), and about their sensitivities to roundoff error.

This leads us to use derivative information only as follows: The sign of the derivative at the central point of the bracketing triplet (a, b, c) indicates uniquely whether the next test point should be taken in the interval (a, b) or in the interval (b, c). The value of this derivative and of the derivative at the second-best-so-far point are extrapolated to zero by the secant method (inverse linear interpolation), which by itself is superlinear of order 1.618. (The golden mean again: See [1], p. 57.) We impose the same sort of restrictions on this new trial point as in Brent's method. If the trial point must be rejected, we *bisect* the interval under scrutiny.

Yes, we are fuddy-duddies when it comes to making flamboyant use of derivative information in one-dimensional minimization. But we have met too many functions whose computed "derivatives" *don't* integrate up to the function value and *don't* accurately point the way to the minimum, usually because of roundoff errors, sometimes because of truncation error in the method of derivative evaluation.

You will see that the following routine is closely modeled on `Brent` in the previous section. One difference is in the input to the routine. Whereas `Brent` takes either a function or functor argument, `Dbrent` takes only a functor. The functor returns not only the function value, by overloading `operator()`, but also the derivative as the member function `df`. For example, here's how you would code the function x^2:

```
struct Funcd {                    Name Funcd is arbitrary.
    Doub operator() (const Doub x) {
        return x*x;
    }
    Doub df(const Doub x) {
        return 2.0*x;
    }
};
```

To invoke `Dbrent`, you need statements like the following:

```
Dbrent dbrent;
Funcd f;
dbrent.bracket(a,b,f);
xmin=dbrent.minimize(f);
```

The value of the function at the minimum is available in `dbrent.fmin` as usual. Here is the routine:

mins.h
```
struct Dbrent : Bracketmethod {
Brent's method to find a minimum, modified to use derivatives.
    Doub xmin,fmin;
    const Doub tol;
    Dbrent(const Doub toll=3.0e-8) : tol(toll) {}
    template <class T>
    Doub minimize(T &funcd)
    Given a functor funcd that computes a function and also its derivative function df, and
    given a bracketing triplet of abscissas ax, bx, cx [such that bx is between ax and cx, and
    f(bx) is less than both f(ax) and f(cx)], this routine isolates the minimum to a fractional
    precision of about tol using a modification of Brent's method that uses derivatives. The
    abscissa of the minimum is returned as xmin, and the minimum function value is returned
    as min, the returned function value.
    {
        const Int ITMAX=100;
```

```
const Doub ZEPS=numeric_limits<Doub>::epsilon()*1.0e-3;
Bool ok1,ok2;                              Will be used as flags for whether pro-
Doub a,b,d=0.0,d1,d2,du,dv,dw,dx,e=0.0;    posed steps are acceptable or not.
Doub fu,fv,fw,fx,olde,tol1,tol2,u,u1,u2,v,w,x,xm;
```

Comments following will point out only differences from the routine in `Brent`. Read that routine first.

```
a=(ax < cx ? ax : cx);
b=(ax > cx ? ax : cx);
x=w=v=bx;
fw=fv=fx=funcd(x);
dw=dv=dx=funcd.df(x);                       All our housekeeping chores are dou-
for (Int iter=0;iter<ITMAX;iter++) {            bled by the necessity of moving
    xm=0.5*(a+b);                               aorund derivative values as well
    tol1=tol*abs(x)+ZEPS;                       as function values.
    tol2=2.0*tol1;
    if (abs(x-xm) <= (tol2-0.5*(b-a))) {
        fmin=fx;
        return xmin=x;
    }
    if (abs(e) > tol1) {
        d1=2.0*(b-a);                       Initialize these d's to an out-of-bracket
        d2=d1;                                  value.
        if (dw != dx) d1=(w-x)*dx/(dx-dw);      Secant method with one point.
        if (dv != dx) d2=(v-x)*dx/(dx-dv);      And the other.
        Which of these two estimates of d shall we take? We will insist that they be
        within the bracket, and on the side pointed to by the derivative at x:
        u1=x+d1;
        u2=x+d2;
        ok1 = (a-u1)*(u1-b) > 0.0 && dx*d1 <= 0.0;
        ok2 = (a-u2)*(u2-b) > 0.0 && dx*d2 <= 0.0;
        olde=e;                             Movement on the step before last.
        e=d;
        if (ok1 || ok2) {                   Take only an acceptable d, and if
            if (ok1 && ok2)                         both are acceptable, then take
                d=(abs(d1) < abs(d2) ? d1 : d2);    the smallest one.
            else if (ok1)
                d=d1;
            else
                d=d2;
            if (abs(d) <= abs(0.5*olde)) {
                u=x+d;
                if (u-a < tol2 || b-u < tol2)
                    d=SIGN(tol1,xm-x);
            } else {                        Bisect, not golden section.
                d=0.5*(e=(dx >= 0.0 ? a-x : b-x));
                Decide which segment by the sign of the derivative.
            }
        } else {
            d=0.5*(e=(dx >= 0.0 ? a-x : b-x));
        }
    } else {
        d=0.5*(e=(dx >= 0.0 ? a-x : b-x));
    }
    if (abs(d) >= tol1) {
        u=x+d;
        fu=funcd(u);
    } else {
        u=x+SIGN(tol1,d);
        fu=funcd(u);
        if (fu > fx) {                      If the minimum step in the downhill
            fmin=fx;                            direction takes us uphill, then
            return xmin=x;                      we are done.
        }
```

```
            }
            du=funcd.df(u);                          Now all the housekeeping, sigh.
            if (fu <= fx) {
                if (u >= x) a=x; else b=x;
                mov3(v,fv,dv,w,fw,dw);
                mov3(w,fw,dw,x,fx,dx);
                mov3(x,fx,dx,u,fu,du);
            } else {
                if (u < x) a=u; else b=u;
                if (fu <= fw || w == x) {
                    mov3(v,fv,dv,w,fw,dw);
                    mov3(w,fw,dw,u,fu,du);
                } else if (fu < fv || v == x || v == w) {
                    mov3(v,fv,dv,u,fu,du);
                }
            }
        }
        throw("Too many iterations in routine dbrent");
    }
};
```

CITED REFERENCES AND FURTHER READING:

Acton, F.S. 1970, *Numerical Methods That Work*; 1990, corrected edition (Washington, DC: Mathematical Association of America), pp. 55; 454–458.[1]

Brent, R.P. 1973, *Algorithms for Minimization without Derivatives* (Englewood Cliffs, NJ: Prentice-Hall); reprinted 2002 (New York: Dover), p. 78.

10.5 Downhill Simplex Method in Multidimensions

With this section we begin consideration of multidimensional minimization, that is, finding the minimum of a function of more than one independent variable. This section stands apart from those that follow, however: All of the algorithms after this section will make explicit use of a one-dimensional minimization algorithm as a part of their computational strategy. This section implements an entirely self-contained strategy, in which one-dimensional minimization does not figure.

The *downhill simplex method* is due to Nelder and Mead [1]. The method requires only function evaluations, not derivatives. It is not very efficient in terms of the number of function evaluations that it requires. Powell's method (§10.7) or the DFP method with finite differences (§10.9) is almost surely faster in all likely applications. However, the downhill simplex method may frequently be the *best* method to use if the figure of merit is "get something working quickly" for a problem whose computational burden is small.

The method has a geometrical naturalness about it that makes it delightful to describe or work through:

A *simplex* is the geometrical figure consisting, in N dimensions, of $N+1$ points (or vertices) and all their interconnecting line segments, polygonal faces, etc. In two dimensions, a simplex is a triangle. In three dimensions, it is a tetrahedron, not necessarily the regular tetrahedron. (The *simplex method* of linear programming, described in §10.10, also makes use of the geometrical concept of a simplex. Otherwise

it is completely unrelated to the algorithm that we are describing in this section.) In general we are only interested in simplexes that are nondegenerate, i.e., that enclose a finite inner N-dimensional volume. If any point of a nondegenerate simplex is taken as the origin, then the N other points define vector directions that span the N-dimensional vector space.

In one-dimensional minimization, it was possible to bracket a minimum, so that the success of a subsequent isolation was guaranteed. Alas! There is no analogous procedure in multidimensional space. For multidimensional minimization, the best we can do is give our algorithm a starting guess, that is, an N-vector of independent variables as the first point to try. The algorithm is then supposed to make its own way downhill through the unimaginable complexity of an N-dimensional topography, until it encounters a (local, at least) minimum.

The downhill simplex method must be started not just with a single point, but with $N + 1$ points, defining an initial simplex. If you think of one of these points (it matters not which) as being your initial starting point $\mathbf{P}_0$, then you can take the other N points to be

$$\mathbf{P}_i = \mathbf{P}_0 + \Delta \mathbf{e}_i \tag{10.5.1}$$

where the $\mathbf{e}_i$'s are N unit vectors, and where Δ is a constant that is your guess of the problem's characteristic length scale. (Or, you could have different Δ_i's for each vector direction.)

The downhill simplex method now takes a series of steps, most steps just moving the point of the simplex where the function is largest ("highest point") through the opposite face of the simplex to a lower point. These steps are called reflections, and they are constructed to conserve the volume of the simplex (and hence maintain its nondegeneracy). When it can do so, the method expands the simplex in one or another direction to take larger steps. When it reaches a "valley floor," the method contracts itself in the transverse direction and tries to ooze down the valley. If there is a situation where the simplex is trying to "pass through the eye of a needle," it contracts itself in all directions, pulling itself in around its lowest (best) point. The routine name `amoeba` is intended to be descriptive of this kind of behavior; the basic moves are summarized in Figure 10.5.1.

Termination criteria can be delicate in any multidimensional minimization routine. Without bracketing, and with more than one independent variable, we no longer have the option of requiring a certain tolerance for a single independent variable. We typically can identify one "cycle" or "step" of our multidimensional algorithm. It is then possible to terminate when the vector distance moved in that step is fractionally smaller in magnitude than some tolerance `tol`. Alternatively, we could require that the decrease in the function value in the terminating step be fractionally smaller than some tolerance `ftol`. Note that while `tol` should not usually be smaller than the square root of the machine precision, it is perfectly appropriate to let `ftol` be of order the machine precision (or perhaps slightly larger so as not to be confused by roundoff).

Note well that either of the above criteria might be fooled by a single anomalous step that, for one reason or another, failed to get anywhere. Therefore, it is frequently a good idea to *restart* a multidimensional minimization routine at a point where it claims to have found a minimum. For this restart, you should reinitialize any ancillary input quantities. In the downhill simplex method, for example, you should reinitialize N of the $N + 1$ vertices of the simplex again by equation (10.5.1), with

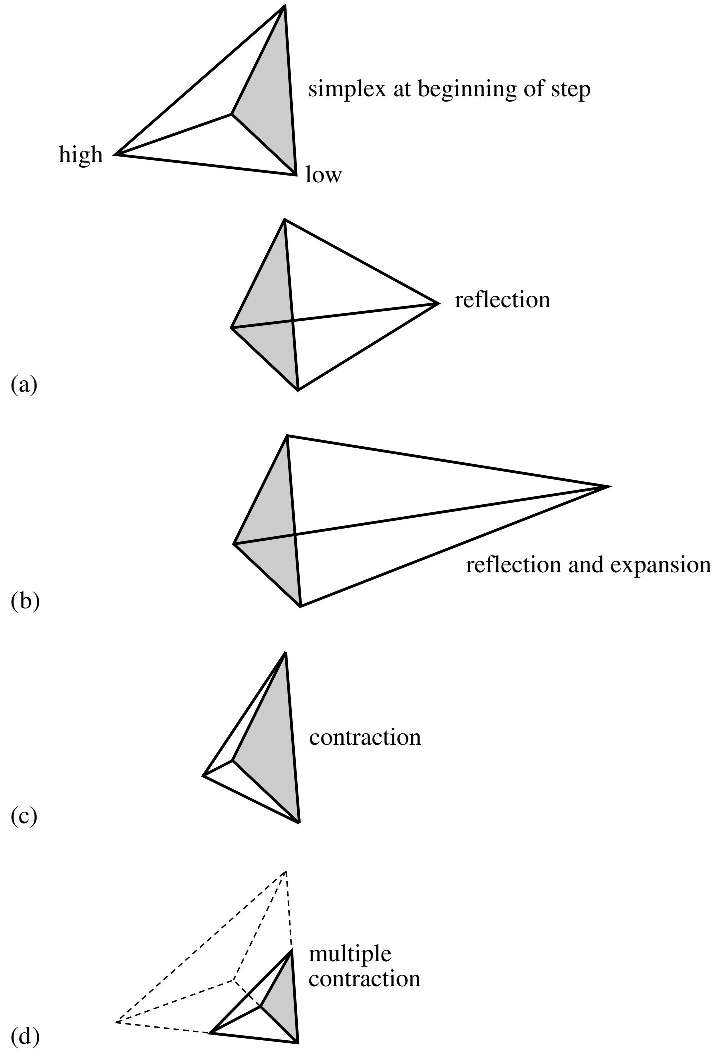

Figure 10.5.1. Possible outcomes for a step in the downhill simplex method. The simplex at the beginning of the step, here a tetrahedron, is shown, top. The simplex at the end of the step can be any one of (a) a reflection away from the high point, (b) a reflection and expansion away from the high point, (c) a contraction along one dimension from the high point, or (d) a contraction along all dimensions toward the low point. An appropriate sequence of such steps will always converge to a minimum of the function.

$\mathbf{P}_0$ being one of the vertices of the claimed minimum.

Restarts should never be very expensive; your algorithm did, after all, converge to the restart point once, and now you are starting the algorithm already there.

The routine below has three different user interfaces. The simplest requires you to supply the initial simplex as in equation (10.5.1):

```
Amoeba am(ftol);
VecDoub point = ...; Doub del = ...;
pmin=am.minimize(point,del,func);
```

The value of the function at the minimum is available in `am.fmin`.

Second, you can use equation (10.5.1) with a vector of increments Δ_i:

```
    VecDoub dels = ...;
    pmin=am.minimize(point,dels,func);
```

Lastly, you can provide the simplex as an $(N+1) \times N$ matrix whose rows are the coordinates of each vertex:

```
    MatDoub p = ...;
    pmin=am.minimize(p,func);
```

Consider, then, our N-dimensional amoeba:

amoeba.h

```
struct Amoeba {
Multidimensional minimization by the downhill simplex method of Nelder and Mead.
    const Doub ftol;
    Int nfunc;                  The number of function evaluations.
    Int mpts;
    Int ndim;
    Doub fmin;                  Function value at the minimum.
    VecDoub y;                  Function values at the vertices of the simplex.
    MatDoub p;                  Current simplex.
    Amoeba(const Doub ftoll) : ftol(ftoll) {}
    The constructor argument ftoll is the fractional convergence tolerance to be achieved in
    the function value (n.b.!).
    template <class T>
    VecDoub minimize(VecDoub_I &point, const Doub del, T &func)
    Multidimensional minimization of the function or functor func(x), where x[0..ndim-1]
    is a vector in ndim dimensions, by the downhill simplex method of Nelder and Mead.
    The initial simplex is specified as in equation (10.5.1) by a point[0..ndim-1] and a
    constant displacement del along each coordinate direction. Returned is the location of the
    minimum.
    {
        VecDoub dels(point.size(),del);
        return minimize(point,dels,func);
    }
    template <class T>
    VecDoub minimize(VecDoub_I &point, VecDoub_I &dels, T &func)
    Alternative interface that takes different displacements dels[0..ndim-1] in different di-
    rections for the initial simplex.
    {
        Int ndim=point.size();
        MatDoub pp(ndim+1,ndim);
        for (Int i=0;i<ndim+1;i++) {
            for (Int j=0;j<ndim;j++)
                pp[i][j]=point[j];
            if (i !=0 ) pp[i][i-1] += dels[i-1];
        }
        return minimize(pp,func);
    }
    template <class T>
    VecDoub minimize(MatDoub_I &pp, T &func)
    Most general interface: initial simplex specified by the matrix pp[0..ndim][0..ndim-1].
    Its ndim+1 rows are ndim-dimensional vectors that are the vertices of the starting simplex.
    {
        const Int NMAX=5000;            Maximum allowed number of function evalua-
        const Doub TINY=1.0e-10;            tions.
        Int ihi,ilo,inhi;
        mpts=pp.nrows();
        ndim=pp.ncols();
        VecDoub psum(ndim),pmin(ndim),x(ndim);
        p=pp;
        y.resize(mpts);
        for (Int i=0;i<mpts;i++) {
            for (Int j=0;j<ndim;j++)
                x[j]=p[i][j];
            y[i]=func(x);
```

```
    }
    nfunc=0;
    get_psum(p,psum);
    for (;;) {
        ilo=0;
```
First we must determine which point is the highest (worst), next-highest, and lowest (best), by looping over the points in the simplex.
```
        ihi = y[0]>y[1] ? (inhi=1,0) : (inhi=0,1);
        for (Int i=0;i<mpts;i++) {
            if (y[i] <= y[ilo]) ilo=i;
            if (y[i] > y[ihi]) {
                inhi=ihi;
                ihi=i;
            } else if (y[i] > y[inhi] && i != ihi) inhi=i;
        }
        Doub rtol=2.0*abs(y[ihi]-y[ilo])/(abs(y[ihi])+abs(y[ilo])+TINY);
```
Compute the fractional range from highest to lowest and return if satisfactory.
```
        if (rtol < ftol) {            If returning, put best point and value in slot 0.
            SWAP(y[0],y[ilo]);
            for (Int i=0;i<ndim;i++) {
                SWAP(p[0][i],p[ilo][i]);
                pmin[i]=p[0][i];
            }
            fmin=y[0];
            return pmin;
        }
        if (nfunc >= NMAX) throw("NMAX exceeded");
        nfunc += 2;
```
Begin a new iteration. First extrapolate by a factor −1 through the face of the simplex across from the high point, i.e., reflect the simplex from the high point.
```
        Doub ytry=amotry(p,y,psum,ihi,-1.0,func);
        if (ytry <= y[ilo])
```
Gives a result better than the best point, so try an additional extrapolation by a factor 2.
```
            ytry=amotry(p,y,psum,ihi,2.0,func);
        else if (ytry >= y[inhi]) {
```
The reflected point is worse than the second-highest, so look for an intermediate lower point, i.e., do a one-dimensional contraction.
```
            Doub ysave=y[ihi];
            ytry=amotry(p,y,psum,ihi,0.5,func);
            if (ytry >= ysave) {          Can't seem to get rid of that high point.
                for (Int i=0;i<mpts;i++) {      Better contract around the lowest
                    if (i != ilo) {             (best) point.
                        for (Int j=0;j<ndim;j++)
                            p[i][j]=psum[j]=0.5*(p[i][j]+p[ilo][j]);
                        y[i]=func(psum);
                    }
                }
                nfunc += ndim;         Keep track of function evaluations.
                get_psum(p,psum);      Recompute psum.
            }
        } else --nfunc;                Correct the evaluation count.
    }                                  Go back for the test of doneness and the next
                                           iteration.
}
inline void get_psum(MatDoub_I &p, VecDoub_O &psum)
```
Utility function.
```
{
    for (Int j=0;j<ndim;j++) {
        Doub sum=0.0;
        for (Int i=0;i<mpts;i++)
            sum += p[i][j];
        psum[j]=sum;
    }
}
```

```
    template <class T>
    Doub amotry(MatDoub_IO &p, VecDoub_O &y, VecDoub_IO &psum,
        const Int ihi, const Doub fac, T &func)
```
Helper function: Extrapolates by a factor fac through the face of the simplex across from the high point, tries it, and replaces the high point if the new point is better.
```
    {
        VecDoub ptry(ndim);
        Doub fac1=(1.0-fac)/ndim;
        Doub fac2=fac1-fac;
        for (Int j=0;j<ndim;j++)
            ptry[j]=psum[j]*fac1-p[ihi][j]*fac2;
        Doub ytry=func(ptry);           Evaluate the function at the trial point.
        if (ytry < y[ihi]) {            If it's better than the highest, then replace the
            y[ihi]=ytry;                    highest.
            for (Int j=0;j<ndim;j++) {
                psum[j] += ptry[j]-p[ihi][j];
                p[ihi][j]=ptry[j];
            }
        }
        return ytry;
    }
};
```


CITED REFERENCES AND FURTHER READING:

Nelder, J.A., and Mead, R. 1965, "A Simplex Method for Function Minimization," *Computer Journal*, vol. 7, pp. 308–313.[1]

Yarbro, L.A., and Deming, S.N. 1974, "Selection and Preprocessing of Factors for Simplex Optimization," *Analytica Chimica Acta*, vol. 73, pp. 391–398.

Jacoby, S.L.S, Kowalik, J.S., and Pizzo, J.T. 1972, *Iterative Methods for Nonlinear Optimization Problems* (Englewood Cliffs, NJ: Prentice-Hall).

10.6 Line Methods in Multidimensions

We know (§10.2 – §10.4) how to minimize a function of one variable. If we start at a point $\mathbf{P}$ in N-dimensional space, and proceed from there in some vector direction $\mathbf{n}$, then any function of N variables $f(\mathbf{P})$ can be minimized along the line $\mathbf{n}$ by our one-dimensional methods. One can dream up various multidimensional minimization methods that consist of sequences of such line minimizations. Different methods will differ only by how, at each stage, they choose the next direction $\mathbf{n}$ to try. The minimization methods in the next two sections fall under this general schema of successive line minimizations. (The quasi-Newton algorithm in §10.9 does not need very accurate line minimizations. Accordingly, it uses the approximate line minimization routine, `lnsrch` from §9.7.1.)

In this section we provide the line minimization routine `linmin` as part of the base class `Linemethod` from which we will derive the minimization methods in the next two sections. These minimization routines regard `linmin` as a black-box subalgorithm, whose definition is

> `linmin`: Given as input the vectors $\mathbf{P}$ and $\mathbf{n}$, and the function f, find the scalar λ that minimizes $f(\mathbf{P} + \lambda\mathbf{n})$. Replace $\mathbf{P}$ by $\mathbf{P} + \lambda\mathbf{n}$. Replace $\mathbf{n}$ by $\lambda\mathbf{n}$. Done.

Since we will want to use linmin with methods whose choice of successive directions does not involve explicit computation of the function's gradient, linmin itself cannot use gradient information. Later, in §10.8, we will consider a method that does use gradient information. Accordingly, there we provide a routine dlinmin that makes use of this information to reduce the total computational burden.

The obvious way to implement linmin is to use the methods of one-dimensional minimization described in §10.2 – §10.4, but to rewrite the programs of those sections so that their bookkeeping is done on vector-valued points **P** (all lying along a given direction **n**) rather than scalar-valued abscissas x. That straightforward task produces long routines densely populated with "for(k=0;k<n;k++)" loops.

As an alternative, we can simply reuse the one-dimensional minimization routines by constructing a functor F1dim, which gives the value of your function, say func, along the line going through the point p in the direction xi. The function linmin calls our familiar one-dimensional routine Brent (§10.4) and instructs it to minimize F1dim. The routine linmin communicates with F1dim "over the head" of Brent through the constructor, our usual C++ idiom.

mins_ndim.h

```
template <class T>
struct Linemethod {
Base class for line-minimization algorithms. Provides the line-minimization routine linmin.
    VecDoub p;
    VecDoub xi;
    T &func;
    Int n;
    Linemethod(T &funcc) : func(funcc) {}
    Constructor argument is the user-supplied function or functor to be minimized.
    Doub linmin()
    Line-minimization routine. Given an n-dimensional point p[0..n-1] and an n-dimensional
    direction xi[0..n-1], moves and resets p to where the function or functor func(p) takes on
    a minimum along the direction xi from p, and replaces xi by the actual vector displacement
    that p was moved. Also returns the value of func at the returned location p. This is actually
    all accomplished by calling the routines bracket and minimize of Brent.
    {
        Doub ax,xx,xmin;
        n=p.size();
        F1dim<T> f1dim(p,xi,func);
        ax=0.0;                                Initial guess for brackets.
        xx=1.0;
        Brent brent;
        brent.bracket(ax,xx,f1dim);
        xmin=brent.minimize(f1dim);
        for (Int j=0;j<n;j++) {                Construct the vector results to return.
            xi[j] *= xmin;
            p[j] += xi[j];
        }
        return brent.fmin;
    }
};
```

mins_ndim.h

```
template <class T>
struct F1dim {
Must accompany linmin in Linemethod.
    const VecDoub &p;
    const VecDoub &xi;
    Int n;
    T &func;
    VecDoub xt;
```

```
    F1dim(VecDoub_I &pp, VecDoub_I &xii, T &funcc) : p(pp),
        xi(xii), n(pp.size()), func(funcc), xt(n) {}
```
Constructor takes as inputs an n-dimensional point p[0..n-1] and an n-dimensional direction xi[0..n-1] from linmin, as well as the function or functor that takes a vector argument.
```
    Doub operator() (const Doub x)
```
Functor returning value of the given function along a one-dimensional line.
```
    {
        for (Int j=0;j<n;j++)
            xt[j]=p[j]+x*xi[j];
        return func(xt);
    }
};
```

10.7 Direction Set (Powell's) Methods in Multidimensions

With a routine for line minimization in hand, you might think of this simple method for general multidimensional minimization: Take the unit vectors $\mathbf{e}_0, \mathbf{e}_1, \ldots \mathbf{e}_{N-1}$ as a *set of directions*. Using linmin, move along the first direction to its minimum, then *from there* along the second direction to *its* minimum, and so on, cycling through the whole set of directions as many times as necessary, until the function stops decreasing.

This simple method is actually not too bad for many functions. Even more interesting is why it *is* bad, i.e., very inefficient, for some other functions. Consider a function of two dimensions whose contour map (level lines) happens to define a long, narrow valley at some angle to the coordinate basis vectors (see Figure 10.7.1). Then the only way "down the length of the valley" going along the basis vectors at each stage is by a series of many tiny steps. More generally, in N dimensions, if the function's second derivatives are much larger in magnitude in some directions than in others, then many cycles through all N basis vectors will be required in order to get anywhere. This condition is not all that unusual; according to Murphy's Law, you should count on it.

Obviously what we need is a better set of directions than the $\mathbf{e}_i$'s. All *direction set methods* consist of prescriptions for updating the set of directions as the method proceeds, attempting to come up with a set that either (i) includes some very good directions that will take us far along narrow valleys, or else (more subtly) (ii) includes some number of "noninterfering" directions with the special property that minimization along one is not "spoiled" by subsequent minimization along another, so that interminable cycling through the set of directions can be avoided.

10.7.1 Conjugate Directions

This concept of "noninterfering" directions, more conventionally called *conjugate directions*, is worth making mathematically explicit.

First, note that if we minimize a function along some direction $\mathbf{u}$, then the gradient of the function must be perpendicular to $\mathbf{u}$ at the line minimum; if not, then there would still be a nonzero directional derivative along $\mathbf{u}$.

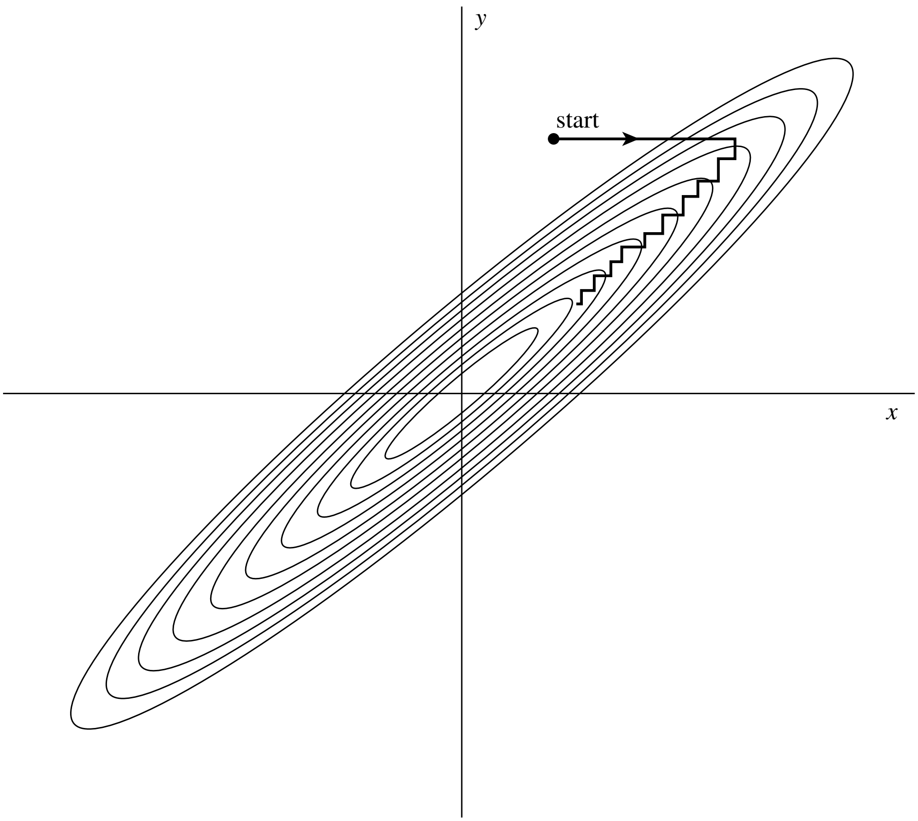

Figure 10.7.1. Successive minimizations along coordinate directions in a long, narrow "valley" (shown as contour lines). Unless the valley is optimally oriented, this method is extremely inefficient, taking many tiny steps to get to the minimum, crossing and re-crossing the principal axis.

Next take some particular point $\mathbf{P}$ as the origin of the coordinate system with coordinates $\mathbf{x}$. Then any function f can be approximated by its Taylor series

$$\begin{aligned} f(\mathbf{x}) &= f(\mathbf{P}) + \sum_i \frac{\partial f}{\partial x_i} x_i + \frac{1}{2} \sum_{i,j} \frac{\partial^2 f}{\partial x_i \partial x_j} x_i x_j + \cdots \\ &\approx c - \mathbf{b} \cdot \mathbf{x} + \frac{1}{2} \mathbf{x} \cdot \mathbf{A} \cdot \mathbf{x} \end{aligned} \tag{10.7.1}$$

where

$$c \equiv f(\mathbf{P}) \qquad \mathbf{b} \equiv -\nabla f|_{\mathbf{P}} \qquad [\mathbf{A}]_{ij} \equiv \left. \frac{\partial^2 f}{\partial x_i \partial x_j} \right|_{\mathbf{P}} \tag{10.7.2}$$

The matrix $\mathbf{A}$ whose components are the second partial derivative matrix of the function is called the *Hessian matrix* of the function at $\mathbf{P}$.

In the approximation of (10.7.1), the gradient of f is easily calculated as

$$\nabla f = \mathbf{A} \cdot \mathbf{x} - \mathbf{b} \tag{10.7.3}$$

(This implies that the gradient will vanish — the function will be at an extremum — at a value of $\mathbf{x}$ obtained by solving $\mathbf{A} \cdot \mathbf{x} = \mathbf{b}$. We will return to this idea in §10.9!)

How does the gradient ∇f *change* as we move along some direction? Evidently

$$\delta(\nabla f) = \mathbf{A} \cdot (\delta \mathbf{x}) \tag{10.7.4}$$

Suppose that we have moved along some direction $\mathbf{u}$ to a minimum and now propose to move along some new direction $\mathbf{v}$. The condition that motion along $\mathbf{v}$ not *spoil* our minimization along $\mathbf{u}$ is just that the gradient stay perpendicular to $\mathbf{u}$, i.e., that the change in the gradient be perpendicular to $\mathbf{u}$. By equation (10.7.4) this is just

$$0 = \mathbf{u} \cdot \delta(\nabla f) = \mathbf{u} \cdot \mathbf{A} \cdot \mathbf{v} \tag{10.7.5}$$

When (10.7.5) holds for two vectors $\mathbf{u}$ and $\mathbf{v}$, they are said to be *conjugate*. When the relation holds pairwise for all members of a set of vectors, they are said to be a conjugate set. If you do successive line minimizations of a function along a conjugate set of directions, then you don't need to redo any of those directions (unless, of course, you spoil things by minimizing along a direction that they are *not* conjugate to).

A triumph for a direction set method is to come up with a set of N linearly independent, mutually conjugate directions. Then, one pass of N line minimizations will put it exactly at the minimum of a quadratic form like (10.7.1). For functions f that are not exactly quadratic forms, it won't be exactly at the minimum, but repeated cycles of N line minimizations will in due course converge *quadratically* to the minimum.

10.7.2 Powell's Quadratically Convergent Method

Powell first discovered a direction set method that does produce N mutually conjugate directions. Here is how it goes: Initialize the set of directions $\mathbf{u}_i$ to the basis vectors,

$$\mathbf{u}_i = \mathbf{e}_i \qquad i = 0, \ldots, N-1 \tag{10.7.6}$$

Now repeat the following sequence of steps ("basic procedure") until your function stops decreasing:

- Save your starting position as $\mathbf{P}_0$.
- For $i = 0, \ldots, N-1$, move $\mathbf{P}_i$ to the minimum along direction $\mathbf{u}_i$ and call this point $\mathbf{P}_{i+1}$.
- For $i = 0, \ldots, N-2$, set $\mathbf{u}_i \leftarrow \mathbf{u}_{i+1}$.
- Set $\mathbf{u}_{N-1} \leftarrow \mathbf{P}_N - \mathbf{P}_0$.
- Move $\mathbf{P}_N$ to the minimum along direction $\mathbf{u}_{N-1}$ and call this point $\mathbf{P}_0$.

Powell, in 1964, showed that, for a quadratic form like (10.7.1), k iterations of the above basic procedure produce a set of directions $\mathbf{u}_i$ whose last k members are mutually conjugate. Therefore, N iterations of the basic procedure, amounting to $N(N+1)$ line minimizations in all, will exactly minimize a quadratic form. Brent [1] gives proofs of these statements in accessible form.

Unfortunately, there is a problem with Powell's quadratically convergent algorithm. The procedure of throwing away, at each stage, $\mathbf{u}_0$ in favor of $\mathbf{P}_N - \mathbf{P}_0$ tends to produce sets of directions that "fold up on each other" and become linearly dependent. Once this happens, the procedure finds the minimum of the function f only over a subspace of the full N-dimensional case; in other words, it gives the wrong answer. Therefore, the algorithm must not be used in the form given above.

There are a number of ways to fix up the problem of linear dependence in Powell's algorithm, among them:

1. You can reinitialize the set of directions $\mathbf{u}_i$ to the basis vectors $\mathbf{e}_i$ after every N or $N+1$ iterations of the basic procedure. This produces a serviceable method, which we commend to you if quadratic convergence is important for your application (i.e., if your functions are close to quadratic forms and if you desire high accuracy).

2. Brent points out that the set of directions can equally well be reset to the columns of any orthogonal matrix. Rather than throw away the information on conjugate directions already built up, he resets the direction set to calculated principal directions of the matrix $\mathbf{A}$ (which he gives a procedure for determining). The calculation is essentially a singular value decomposition algorithm (see §2.6). Brent has a number of other cute tricks up his sleeve, and his modification of Powell's method is probably the best presently known. Consult [1] for a detailed description and listing of the program. Unfortunately it is rather too elaborate for us to include here.

3. You can give up the property of quadratic convergence in favor of a more heuristic scheme (due to Powell) that tries to find a few good directions along narrow valleys instead of N necessarily conjugate directions. This is the method that we now implement. (It is also the version of Powell's method given in Acton [2], from which parts of the following discussion are drawn.)

10.7.3 Discarding the Direction of Largest Decrease

The fox and the grapes: Now that we are going to give up the property of quadratic convergence, was it so important after all? That depends on the function that you are minimizing. Some applications produce functions with long, twisty valleys. Quadratic convergence is of no particular advantage to a program that must slalom down the length of a valley floor that twists one way and another (and another, and another, ... — there are N dimensions!). Along the long direction, a quadratically convergent method is trying to extrapolate to the minimum of a parabola that just isn't (yet) there while the conjugacy of the $N-1$ transverse directions keeps getting spoiled by the twists.

Sooner or later, however, we do arrive at an approximately ellipsoidal minimum (cf. equation 10.7.1 when $\mathbf{b}$, the gradient, is zero). Then, depending on how much accuracy we require, a method with quadratic convergence can save us several times N^2 extra line minimizations, since quadratic convergence *doubles* the number of significant figures at each iteration.

The basic idea of our now-modified Powell's method is still to take $\mathbf{P}_N - \mathbf{P}_0$ as a new direction; it is, after all, the average direction moved in after trying all N possible directions. For a valley whose long direction is twisting slowly, this direction is likely to give us a good run along the new long direction. The change is to discard the old direction along which the function f made its *largest decrease*. This seems paradoxical, since that direction was the *best* of the previous iteration. However, it is also likely to be a major component of the new direction that we are adding, so dropping it gives us the best chance of avoiding a buildup of linear dependence.

There are a couple of exceptions to this basic idea. Sometimes it is better *not* to add a new direction at all. Define

$$f_0 \equiv f(\mathbf{P}_0) \qquad f_N \equiv f(\mathbf{P}_N) \qquad f_E \equiv f(2\mathbf{P}_N - \mathbf{P}_0) \tag{10.7.7}$$

Here f_E is the function value at an "extrapolated" point somewhat further along the proposed new direction. Also define Δf to be the magnitude of the largest decrease along one particular direction of the present basic procedure iteration. (Δf is a positive number.) Then:

1. If $f_E \geq f_0$, then keep the old set of directions for the next basic procedure, because the average direction $\mathbf{P}_N - \mathbf{P}_0$ is all played out.

2. If $2\,(f_0 - 2f_N + f_E)\,[(f_0 - f_N) - \Delta f]^2 \geq (f_0 - f_E)^2 \Delta f$, then keep the old set of directions for the next basic procedure, because either (i) the decrease along the average direction was not primarily due to any single direction's decrease, or (ii) there is a substantial second derivative along the average direction and we seem to be near to the bottom of its minimum.

The following routine implements Powell's method in the version just described. In the routine, `xi` is the matrix whose columns are the set of directions $\mathbf{n}_i$; otherwise the correspondence of notation should be self-evident. If the function to be minimized is provided as a functor `Func`

```
struct Func {
    Doub operator()(VecDoub_I &x);
};
```

then the normal calling sequence for `Powell` looks something like this:

```
VecDoub p = ...;
Func func;
Powell<Func> powell(func);
p=powell.minimize(p);           OK to overwrite initial guess.
```

The function value at the minimum is available as `powell.fret`.

If, on the other hand, the function to be minimized is provided as a normal C++ function,

```
Doub func(VecDoub_I &x);
```

then the constructor call looks like this instead:

```
Powell<Doub (VecDoub_I &)> powell(func);
```

Note that the constructor takes an optional argument that specifies the function tolerance for the minimization.

mins_ndim.h

```
template <class T>
struct Powell : Linemethod<T> {
Multidimensional minimization by Powell's method.
    Int iter;
    Doub fret;                      Value of the function at the minimum.
    using Linemethod<T>::func;      Variables from a templated base class are not auto-
    using Linemethod<T>::linmin;        matically inherited.
    using Linemethod<T>::p;
    using Linemethod<T>::xi;
    const Doub ftol;
    Powell(T &func, const Doub ftoll=3.0e-8) : Linemethod<T>(func),
        ftol(ftoll) {}
    Constructor arguments are func, the function or functor to be minimized, and an optional
    argument ftoll, the fractional tolerance in the function value such that failure to decrease
    by more than this amount on one iteration signals doneness.
    VecDoub minimize(VecDoub_I &pp)
    Minimization of a function or functor n variables. Input consists of an initial starting point
    pp[0..n-1]. The initial matrix ximat[0..n-1][0..n-1], whose columns contain the initial
    set of directions, is set to the n unit vectors. Returned is the best point found, at which
    point fret is the minimum function value and iter is the number of iterations taken.
```

```
    {
        Int n=pp.size();
        MatDoub ximat(n,n,0.0);
        for (Int i=0;i<n;i++) ximat[i][i]=1.0;
        return minimize(pp,ximat);
    }
    VecDoub minimize(VecDoub_I &pp, MatDoub_IO &ximat)
```
Alternative interface: Input consists of the initial starting point pp[0..n-1] and an initial matrix ximat[0..n-1][0..n-1], whose columns contain the initial set of directions. On output ximat is the then-current direction set.
```
    {
        const Int ITMAX=200;            Maximum allowed iterations.
        const Doub TINY=1.0e-25;        A small number.
        Doub fptt;
        Int n=pp.size();
        p=pp;
        VecDoub pt(n),ptt(n);
        xi.resize(n);
        fret=func(p);
        for (Int j=0;j<n;j++) pt[j]=p[j];  Save the initial point.
        for (iter=0;;++iter) {
            Doub fp=fret;
            Int ibig=0;
            Doub del=0.0;               Will be the biggest function decrease.
            for (Int i=0;i<n;i++) {     In each iteration, loop over all directions in the set.
                for (Int j=0;j<n;j++) xi[j]=ximat[j][i];    Copy the direction,
                fptt=fret;
                fret=linmin();                  minimize along it,
                if (fptt-fret > del) {          and record it if it is the largest decrease
                    del=fptt-fret;                  so far.
                    ibig=i+1;
                }
            }                                   Here comes the termination criterion:
            if (2.0*(fp-fret) <= ftol*(abs(fp)+abs(fret))+TINY) {
                return p;
            }
            if (iter == ITMAX) throw("powell exceeding maximum iterations.");
            for (Int j=0;j<n;j++) {             Construct the extrapolated point and the
                ptt[j]=2.0*p[j]-pt[j];              average direction moved. Save the
                xi[j]=p[j]-pt[j];                   old starting point.
                pt[j]=p[j];
            }
            fptt=func(ptt);                     Function value at extrapolated point.
            if (fptt < fp) {
                Doub t=2.0*(fp-2.0*fret+fptt)*SQR(fp-fret-del)-del*SQR(fp-fptt);
                if (t < 0.0) {
                    fret=linmin();              Move to the minimum of the new direc-
                    for (Int j=0;j<n;j++) {         tion, and save the new direction.
                        ximat[j][ibig-1]=ximat[j][n-1];
                        ximat[j][n-1]=xi[j];
                    }
                }
            }
        }
    }
};
```

CITED REFERENCES AND FURTHER READING:

Brent, R.P. 1973, *Algorithms for Minimization without Derivatives* (Englewood Cliffs, NJ: Prentice-Hall); reprinted 2002 (New York: Dover), Chapter 7.[1]

Acton, F.S. 1970, *Numerical Methods That Work*; 1990, corrected edition (Washington, DC: Mathematical Association of America), pp. 464–467.[2]

Jacobs, D.A.H. (ed.) 1977, *The State of the Art in Numerical Analysis* (London: Academic Press), pp. 259–262.

10.8 Conjugate Gradient Methods in Multidimensions

Consider now the case where you are able to calculate, at a given N-dimensional point $\mathbf{P}$, not just the value of a function $f(\mathbf{P})$ but also the gradient (vector of first partial derivatives) $\nabla f(\mathbf{P})$.

A rough counting argument will show how advantageous it is to use the gradient information: Suppose that the function f is roughly approximated as a quadratic form, as above in equation (10.7.1),

$$f(\mathbf{x}) \approx c - \mathbf{b} \cdot \mathbf{x} + \frac{1}{2} \mathbf{x} \cdot \mathbf{A} \cdot \mathbf{x} \tag{10.8.1}$$

Then the number of unknown parameters in f is equal to the number of free parameters in $\mathbf{A}$ and $\mathbf{b}$, which is $\frac{1}{2}N(N+1)$, which we see to be of order N^2. Changing any one of these parameters can move the location of the minimum. Therefore, we should not expect to be able to *find* the minimum until we have collected an equivalent information content, of order N^2 numbers.

In the direction set methods of §10.7, we collected the necessary information by making on the order of N^2 separate line minimizations, each requiring "a few" (but sometimes a *big* few!) function evaluations. Now, each evaluation of the gradient will bring us N new components of information. If we use them wisely, we should need to make only of order N separate line minimizations. That is in fact the case for the algorithms in this section and the next.

A factor of N improvement in computational speed is not necessarily implied. As a rough estimate, we might imagine that the calculation of *each component* of the gradient takes about as long as evaluating the function itself. In that case there will be of order N^2 equivalent function evaluations both with and without gradient information. Even if the advantage is not of order N, however, it is nevertheless quite substantial: (i) Each calculated component of the gradient will typically save not just one function evaluation, but a number of them, equivalent to, say, a whole line minimization. (ii) There is often a high degree of redundancy in the formulas for the various components of a function's gradient. When this is so, especially when there is also redundancy with the calculation of the function, the calculation of the gradient may cost significantly less than N function evaluations.

A common beginner's error is to assume that any reasonable way of incorporating gradient information should be about as good as any other. This line of thought leads to the following *not-very-good* algorithm, the *steepest descent method*:

> Steepest Descent: Start at a point $\mathbf{P}_0$. As many times as needed, move from point $\mathbf{P}_i$ to the point $\mathbf{P}_{i+1}$ by minimizing along the line from $\mathbf{P}_i$ in the direction of the local downhill gradient $-\nabla f(\mathbf{P}_i)$.

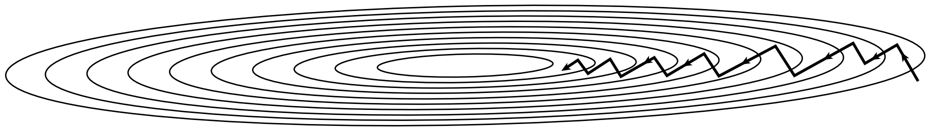

(a)

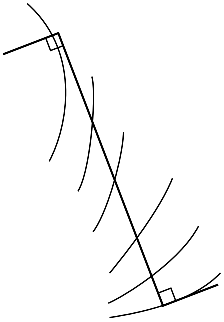

(b)

Figure 10.8.1. (a) Steepest descent method in a long, narrow "valley." While more efficient than the strategy of Figure 10.7.1, steepest descent is nonetheless an inefficient strategy, taking many steps to reach the valley floor. (b) Magnified view of one step: A step starts off in the local gradient direction, perpendicular to the contour lines, and traverses a straight line until a local minimum is reached, where the traverse is parallel to the local contour lines.

The problem with the steepest descent method (which, incidentally, goes back to Cauchy), is similar to the problem that was shown in Figure 10.7.1. The method will perform many small steps in going down a long, narrow valley, even if the valley is a perfect quadratic form. You might have hoped that, say in two dimensions, your first step would take you to the valley floor, the second step directly down the long axis; but remember that the new gradient at the minimum point of any line minimization is perpendicular to the direction just traversed. Therefore, with the steepest descent method, you *must* make a right angle turn, which does *not*, in general, take you to the minimum. (See Figure 10.8.1.)

Just as in the discussion that led up to equation (10.7.5), we really want a way of proceeding not down the new gradient, but rather in a direction that is somehow constructed to be *conjugate* to the old gradient, and, insofar as possible, to all previous directions traversed. Methods that accomplish this construction are called *conjugate gradient* methods.

In §2.7 we discussed the conjugate gradient method as a technique for solving linear algebraic equations by minimizing a quadratic form. That formalism can also be applied to the problem of minimizing a function *approximated* by the quadratic form (10.8.1). Recall that, starting with an arbitrary initial vector $\mathbf{g}_0$ and letting $\mathbf{h}_0 = \mathbf{g}_0$, the conjugate gradient method constructs two sequences of vectors from the recurrence

$$\mathbf{g}_{i+1} = \mathbf{g}_i - \lambda_i \mathbf{A} \cdot \mathbf{h}_i \qquad \mathbf{h}_{i+1} = \mathbf{g}_{i+1} + \gamma_i \mathbf{h}_i \qquad i = 0, 1, 2, \ldots \tag{10.8.2}$$

The vectors satisfy the orthogonality and conjugacy conditions

$$\mathbf{g}_i \cdot \mathbf{g}_j = 0 \qquad \mathbf{h}_i \cdot \mathbf{A} \cdot \mathbf{h}_j = 0 \qquad \mathbf{g}_i \cdot \mathbf{h}_j = 0 \qquad j < i \tag{10.8.3}$$

The scalars λ_i and γ_i are given by

$$\lambda_i = \frac{\mathbf{g}_i \cdot \mathbf{g}_i}{\mathbf{h}_i \cdot \mathbf{A} \cdot \mathbf{h}_i} = \frac{\mathbf{g}_i \cdot \mathbf{h}_i}{\mathbf{h}_i \cdot \mathbf{A} \cdot \mathbf{h}_i} \tag{10.8.4}$$

$$\gamma_i = \frac{\mathbf{g}_{i+1} \cdot \mathbf{g}_{i+1}}{\mathbf{g}_i \cdot \mathbf{g}_i} \tag{10.8.5}$$

Equations (10.8.2) – (10.8.5) are simply equations (2.7.32) – (2.7.35) for a symmetric $\mathbf{A}$ in a new notation. (A self-contained derivation of these results in the context of function minimization is given by Polak [1].)

Now suppose that we knew the Hessian matrix $\mathbf{A}$ in equation (10.8.1). Then we could use the construction (10.8.2) to find successively conjugate directions $\mathbf{h}_i$ along which to line-minimize. After N such, we would efficiently have arrived at the minimum of the quadratic form. But we don't know $\mathbf{A}$.

Here is a remarkable theorem to save the day: Suppose we happen to have $\mathbf{g}_i = -\nabla f(\mathbf{P}_i)$, for some point $\mathbf{P}_i$, where f is of the form (10.8.1). Suppose also that we proceed from $\mathbf{P}_i$ along the direction $\mathbf{h}_i$ to the local minimum of f located at some point $\mathbf{P}_{i+1}$ and then set $\mathbf{g}_{i+1} = -\nabla f(\mathbf{P}_{i+1})$. Then, this $\mathbf{g}_{i+1}$ is the same vector as would have been constructed by equation (10.8.2). (And we have constructed it without knowledge of $\mathbf{A}$!)

Proof: By equation (10.7.3), $\mathbf{g}_i = -\mathbf{A} \cdot \mathbf{P}_i + \mathbf{b}$ and

$$\mathbf{g}_{i+1} = -\mathbf{A} \cdot (\mathbf{P}_i + \lambda \mathbf{h}_i) + \mathbf{b} = \mathbf{g}_i - \lambda \mathbf{A} \cdot \mathbf{h}_i \tag{10.8.6}$$

with λ chosen to take us to the line minimum. But at the line minimum $\mathbf{h}_i \cdot \nabla f = -\mathbf{h}_i \cdot \mathbf{g}_{i+1} = 0$. This latter condition is easily combined with (10.8.6) to solve for λ. The result is exactly the expression (10.8.4). But with this value of λ, (10.8.6) is the same as (10.8.2), q.e.d.

We have, then, the basis of an algorithm that requires neither knowledge of the Hessian matrix $\mathbf{A}$ nor even the storage necessary to store such a matrix. A sequence of directions $\mathbf{h}_i$ is constructed, using only line minimizations, evaluations of the gradient vector, and an auxiliary vector to store the latest in the sequence of $\mathbf{g}$'s.

The algorithm described so far is the original Fletcher-Reeves version of the conjugate gradient algorithm. Later, Polak and Ribiere introduced one tiny, but sometimes significant, change. They proposed using the form

$$\gamma_i = \frac{(\mathbf{g}_{i+1} - \mathbf{g}_i) \cdot \mathbf{g}_{i+1}}{\mathbf{g}_i \cdot \mathbf{g}_i} \tag{10.8.7}$$

instead of equation (10.8.5). "Wait," you say, "aren't they equal by the orthogonality conditions (10.8.3)?" They are equal for exact quadratic forms. In the real world, however, your function is not exactly a quadratic form. Arriving at the supposed minimum of the quadratic form, you may still need to proceed for another set of iterations. There is some evidence [2] that the Polak-Ribiere formula accomplishes the transition to further iterations more gracefully: When it runs out of steam, it tends to reset $\mathbf{h}$ to be down the local gradient, which is equivalent to beginning the conjugate gradient procedure anew.

The following routine implements the Polak-Ribiere variant, which we recommend; but changing one program line, as shown, will give you Fletcher-Reeves. The

routine presumes the existence of a functor (not a function) that returns the function value by overloading `operator()` and also provides a function to set the vector gradient `df[0..n-1]` evaluated at the input point `p`. Here's an example for the function $x_0^2 + x_1^2$:

```
struct Funcd {                                    Name Funcd is arbitrary.
    Doub operator() (VecDoub_I &x)
    {
        return x[0]*x[0]+x[1]*x[1];
    }
    void df(VecDoub_I &x, VecDoub_O &deriv)       Name df is fixed.
    {
        deriv[0]=2.0*x[0];
        deriv[1]=2.0*x[1];
    }
};
```

To use `frprmn`, you need statements like the following:

```
Funcd funcd;
Frprmn<Funcd> frprmn(funcd);
VecDoub p = ...;
p=frprmn.minimize(p);             OK to overwrite initial guess.
```

The function value at the minimum is available as `frprmn.fret`. Note that the constructor takes an optional argument that specifies the function tolerance for the minimization.

The routine calls `linmin` to do the line minimizations. As already discussed, you may wish to use a modified version of `linmin` that uses `Dbrent` instead of `Brent`, i.e., that uses the gradient in doing the line minimizations. See note below (§10.8.1).

mins_ndim.h

```
template <class T>
struct Frprmn : Linemethod<T> {
Multidimensional minimization by the Fletcher-Reeves-Polak-Ribiere method.
    Int iter;
    Doub fret;                                 Value of the function at the minimum.
    using Linemethod<T>::func;                 Variables from a templated base class
    using Linemethod<T>::linmin;                  are not automatically inherited.
    using Linemethod<T>::p;
    using Linemethod<T>::xi;
    const Doub ftol;
    Frprmn(T &funcd, const Doub ftoll=3.0e-8) : Linemethod<T>(funcd),
        ftol(ftoll) {}
    Constructor arguments are funcd, the function or functor to be minimized, and an optional
    argument ftoll, the fractional tolerance in the function value such that failure to decrease
    by more than this amount on one iteration signals doneness.
    VecDoub minimize(VecDoub_I &pp)
    Given a starting point pp[0..n-1], performs the minimization on a function whose value
    and gradient are provided by a functor funcd (see text).
    {
        const Int ITMAX=200;
        const Doub EPS=1.0e-18;
        const Doub GTOL=1.0e-8;
        Here ITMAX is the maximum allowed number of iterations; EPS is a small number to
        rectify the special case of converging to exactly zero function value; and GTOL is the
        convergence criterion for the zero gradient test.
        Doub gg,dgg;
        Int n=pp.size();                       Initializations.
        p=pp;
        VecDoub g(n),h(n);
```

```
        xi.resize(n);
        Doub fp=func(p);
        func.df(p,xi);
        for (Int j=0;j<n;j++) {
            g[j] = -xi[j];
            xi[j]=h[j]=g[j];
        }
        for (Int its=0;its<ITMAX;its++) {      Loop over iterations.
            iter=its;
            fret=linmin();                     Next statement is one possible return:
            if (2.0*abs(fret-fp) <= ftol*(abs(fret)+abs(fp)+EPS))
                return p;
            fp=fret;
            func.df(p,xi);
            Doub test=0.0;                     Test for convergence on zero gradient.
            Doub den=MAX(fp,1.0);
            for (Int j=0;j<n;j++) {
                Doub temp=abs(xi[j])*MAX(abs(p[j]),1.0)/den;
                if (temp > test) test=temp;
            }
            if (test < GTOL) return p;         The other possible return.
            dgg=gg=0.0;
            for (Int j=0;j<n;j++) {
                gg += g[j]*g[j];
//              dgg += xi[j]*xi[j];            This statement for Fletcher-Reeves.
                dgg += (xi[j]+g[j])*xi[j];     This statement for Polak-Ribiere.
            }
            if (gg == 0.0)                     Unlikely. If gradient is exactly zero, then
                return p;                          we are already done.
            Doub gam=dgg/gg;
            for (Int j=0;j<n;j++) {
                g[j] = -xi[j];
                xi[j]=h[j]=g[j]+gam*h[j];
            }
        }
        throw("Too many iterations in frprmn");
    }
};
```

10.8.1 Note on Line Minimization Using Derivatives

Kindly reread §10.6. We here want to do the same thing, but using derivative information in performing the line minimization. Simply replace all occurrences of `Linemethod` in `Frprmn` with `Dlinemethod`. The routine `Dlinemethod` is exactly the same as `Linemethod` except that `Brent` is replaced by `Dbrent` and `F1dim` by `Df1dim`:

mins_ndim.h

```
template <class T>
struct Dlinemethod {
Base class for line-minimization algorithms using derivative information. Provides the line-
minimization routine linmin.
    VecDoub p;
    VecDoub xi;
    T &func;
    Int n;
    Dlinemethod(T &funcc) : func(funcc) {}
    Constructor argument is the user-supplied function or functor to be minimized.
    Doub linmin()
    Line-minimization routine. Given an n-dimensional point p[0..n-1] and an n-dimensional
    direction xi[0..n-1], moves and resets p to where the function or functor func(p) takes on
```

a minimum along the direction xi from p, and replaces xi by the actual vector displacement that p was moved. Also returns the value of func at the returned location p. All of this is actually accomplished by calling the routines bracket and minimize of Dbrent.

```
    {
        Doub ax,xx,xmin;
        n=p.size();
        Df1dim<T> df1dim(p,xi,func);
        ax=0.0;                                  Initial guess for brackets.
        xx=1.0;
        Dbrent dbrent;
        dbrent.bracket(ax,xx,df1dim);
        xmin=dbrent.minimize(df1dim);
        for (Int j=0;j<n;j++) {                  Construct the vector results to return.
            xi[j] *= xmin;
            p[j] += xi[j];
        }
        return dbrent.fmin;
    }
};
```

mins_ndim.h

```
template <class T>
struct Df1dim {
```

Must accompany linmin in Dlinemethod.

```
    const VecDoub &p;
    const VecDoub &xi;
    Int n;
    T &funcd;
    VecDoub xt;
    VecDoub dft;
    Df1dim(VecDoub_I &pp, VecDoub_I &xii, T &funcdd) : p(pp),
        xi(xii), n(pp.size()), funcd(funcdd), xt(n), dft(n) {}
```

Constructor takes as inputs an n-dimensional point p[0..n-1] and an n-dimensional direction xi[0..n-1] from linmin, as well as the functor funcd.

```
    Doub operator()(const Doub x)
```

Functor returning value of the given function along a one-dimensional line.

```
    {
        for (Int j=0;j<n;j++)
            xt[j]=p[j]+x*xi[j];
        return funcd(xt);
    }
    Doub df(const Doub x)
```

Returns the derivative along the line.

```
    {
        Doub df1=0.0;
        funcd.df(xt,dft);                 Dbrent always evaluates the derivative at the
        for (Int j=0;j<n;j++)                 same value as the function, so xt is un-
            df1 += dft[j]*xi[j];              changed.
        return df1;
    }
};
```

CITED REFERENCES AND FURTHER READING:

Polak, E. 1971, *Computational Methods in Optimization* (New York: Academic Press), §2.3.[1]

Jacobs, D.A.H. (ed.) 1977, *The State of the Art in Numerical Analysis* (London: Academic Press), Chapter III.1.7 (by K.W. Brodlie).[2]

Stoer, J., and Bulirsch, R. 2002, *Introduction to Numerical Analysis*, 3rd ed. (New York: Springer), §8.7.

10.9 Quasi-Newton or Variable Metric Methods in Multidimensions

The goal of *quasi-Newton* methods, which are also called *variable metric* methods, is not different from the goal of conjugate gradient methods: to accumulate information from successive line minimizations so that N such line minimizations lead to the exact minimum of a quadratic form in N dimensions. In that case, the method will also be quadratically convergent for more general smooth functions.

Both quasi-Newton and conjugate gradient methods require that you are able to compute your function's gradient, or first partial derivatives, at arbitrary points. The quasi-Newton approach differs from the conjugate gradient in the way that it stores and updates the information that is accumulated. Instead of requiring intermediate storage on the order of N, the number of dimensions, it requires a matrix of size $N \times N$. Generally, for any moderate N, this hardly matters.

On the other hand, there is not, as far as we know, any overwhelming advantage that the quasi-Newton methods hold over the conjugate gradient techniques, except perhaps a historical one. Developed somewhat earlier, and more widely propagated, the quasi-Newton methods have by now developed a wider constituency of satisfied users. Likewise, some fancier implementations of quasi-Newton methods (going beyond the scope of this book; see below) have been developed to a greater level of sophistication on issues like the minimization of roundoff error, handling of special conditions, and so on.

Quasi-Newton methods come in two main flavors. One is the *Davidon-Fletcher-Powell (DFP)* algorithm (sometimes referred to as simply *Fletcher-Powell*). The other goes by the name *Broyden-Fletcher-Goldfarb-Shanno (BFGS)*. The BFGS and DFP schemes differ only in details of their roundoff error, convergence tolerances, and similar "dirty" issues that are outside of our scope [1,2]. However, it has become generally recognized that, empirically, the BFGS scheme is superior in these details. We will implement BFGS in this section.

As before, we imagine that our arbitrary function $f(\mathbf{x})$ can be locally approximated by the quadratic form of equation (10.8.1). We don't, however, have any information about the values of the quadratic form's parameters $\mathbf{A}$ and $\mathbf{b}$, except insofar as we can glean such information from our function evaluations and line minimizations.

The basic idea of the quasi-Newton method is to build up, iteratively, a good approximation to the inverse Hessian matrix $\mathbf{A}^{-1}$, that is, to construct a sequence of matrices $\mathbf{H}_i$ with the property

$$\lim_{i\to\infty} \mathbf{H}_i = \mathbf{A}^{-1} \tag{10.9.1}$$

Even better if the limit is achieved after N iterations instead of ∞.

The reason that these methods are called *quasi-Newton* can now be explained. Consider finding a minimum by using Newton's method to search for a zero of the gradient of the function. Near the current point $\mathbf{x}_i$, we have to second order

$$f(\mathbf{x}) = f(\mathbf{x}_i) + (\mathbf{x}-\mathbf{x}_i)\cdot\nabla f(\mathbf{x}_i) + \tfrac{1}{2}(\mathbf{x}-\mathbf{x}_i)\cdot\mathbf{A}\cdot(\mathbf{x}-\mathbf{x}_i) \tag{10.9.2}$$

so

$$\nabla f(\mathbf{x}) = \nabla f(\mathbf{x}_i) + \mathbf{A}\cdot(\mathbf{x}-\mathbf{x}_i) \tag{10.9.3}$$

In Newton's method we set $\nabla f(\mathbf{x}) = 0$ to determine the next iteration point:

$$\mathbf{x} - \mathbf{x}_i = -\mathbf{A}^{-1} \cdot \nabla f(\mathbf{x}_i) \tag{10.9.4}$$

The left-hand side is the finite step we need to take to get to the exact minimum; the right-hand side is known once we have accumulated an accurate $\mathbf{H} \approx \mathbf{A}^{-1}$.

The "quasi" in quasi-Newton is because we don't use the actual Hessian matrix of f, but instead use our current approximation of it. This is often *better* than using the true Hessian. We can understand this paradoxical result by considering the *descent directions* of f at $\mathbf{x}_i$. These are the directions $\mathbf{p}$ along which f decreases: $\nabla f \cdot \mathbf{p} < 0$. For the Newton direction (10.9.4) to be a descent direction, we must have

$$\nabla f(\mathbf{x}_i) \cdot (\mathbf{x} - \mathbf{x}_i) = -(\mathbf{x} - \mathbf{x}_i) \cdot \mathbf{A} \cdot (\mathbf{x} - \mathbf{x}_i) < 0 \tag{10.9.5}$$

which is true if $\mathbf{A}$ is positive-definite. In general, far from a minimum, we have no guarantee that the Hessian is positive-definite. Taking the actual Newton step with the real Hessian can move us to points where the function is *increasing* in value. The idea behind quasi-Newton methods is to start with a positive-definite, symmetric approximation to $\mathbf{A}$ (usually the unit matrix) and build up the approximating $\mathbf{H}_i$'s in such a way that the matrix $\mathbf{H}_i$ remains positive-definite and symmetric. Far from the minimum, this guarantees that we always move in a downhill direction. Close to the minimum, the updating formula approaches the true Hessian and we enjoy the quadratic convergence of Newton's method.

When we are not close enough to the minimum, taking the full Newton step $\mathbf{p}$ even with a positive-definite $\mathbf{A}$ need not decrease the function; we may move too far for the quadratic approximation to be valid. All we are guaranteed is that *initially* f decreases as we move in the Newton direction. Once again we can use the backtracking strategy described in §9.7 to choose a step along the *direction* of the Newton step $\mathbf{p}$, but not necessarily all the way.

We won't rigorously derive the DFP algorithm for taking $\mathbf{H}_i$ into $\mathbf{H}_{i+1}$; you can consult [3] for clear derivations. Following Brodlie (in [2]), we will give the following heuristic motivation of the procedure.

Subtracting equation (10.9.4) at $\mathbf{x}_{i+1}$ from that same equation at $\mathbf{x}_i$ gives

$$\mathbf{x}_{i+1} - \mathbf{x}_i = \mathbf{A}^{-1} \cdot (\nabla f_{i+1} - \nabla f_i) \tag{10.9.6}$$

where $\nabla f_j \equiv \nabla f(\mathbf{x}_j)$. Having made the step from $\mathbf{x}_i$ to $\mathbf{x}_{i+1}$, we might reasonably want to require that the new approximation $\mathbf{H}_{i+1}$ satisfy (10.9.6) as if it were actually $\mathbf{A}^{-1}$, that is,

$$\mathbf{x}_{i+1} - \mathbf{x}_i = \mathbf{H}_{i+1} \cdot (\nabla f_{i+1} - \nabla f_i) \tag{10.9.7}$$

We might also imagine that the updating formula should be of the form $\mathbf{H}_{i+1} = \mathbf{H}_i + \text{correction}$.

What "objects" are around out of which to construct a correction term? Most notable are the two vectors $\mathbf{x}_{i+1} - \mathbf{x}_i$ and $\nabla f_{i+1} - \nabla f_i$, and there is also $\mathbf{H}_i$. There are not infinitely many natural ways of making a matrix out of these objects, especially if (10.9.7) must hold! One such way, the *DFP updating formula*, is

$$\begin{aligned}\mathbf{H}_{i+1} = \mathbf{H}_i &+ \frac{(\mathbf{x}_{i+1} - \mathbf{x}_i) \otimes (\mathbf{x}_{i+1} - \mathbf{x}_i)}{(\mathbf{x}_{i+1} - \mathbf{x}_i) \cdot (\nabla f_{i+1} - \nabla f_i)} \\ &- \frac{[\mathbf{H}_i \cdot (\nabla f_{i+1} - \nabla f_i)] \otimes [\mathbf{H}_i \cdot (\nabla f_{i+1} - \nabla f_i)]}{(\nabla f_{i+1} - \nabla f_i) \cdot \mathbf{H}_i \cdot (\nabla f_{i+1} - \nabla f_i)}\end{aligned} \tag{10.9.8}$$

where $\otimes$ denotes the "outer" or "direct" product of two vectors, a matrix: The ij component of $\mathbf{u} \otimes \mathbf{v}$ is $u_i v_j$. (You might want to verify that 10.9.8 does indeed satisfy 10.9.7.)

The *BFGS updating formula* is exactly the same, but with one additional term,

$$\cdots + [(\nabla f_{i+1} - \nabla f_i) \cdot \mathbf{H}_i \cdot (\nabla f_{i+1} - \nabla f_i)]\, \mathbf{u} \otimes \mathbf{u} \tag{10.9.9}$$

where $\mathbf{u}$ is defined as the vector

$$\mathbf{u} \equiv \frac{(\mathbf{x}_{i+1} - \mathbf{x}_i)}{(\mathbf{x}_{i+1} - \mathbf{x}_i) \cdot (\nabla f_{i+1} - \nabla f_i)} - \frac{\mathbf{H}_i \cdot (\nabla f_{i+1} - \nabla f_i)}{(\nabla f_{i+1} - \nabla f_i) \cdot \mathbf{H}_i \cdot (\nabla f_{i+1} - \nabla f_i)} \tag{10.9.10}$$

(You might also verify that this satisfies 10.9.7.)

You will have to take on faith — or else consult [3] for details of — the "deep" result that equation (10.9.8), with or without (10.9.9), does in fact converge to $\mathbf{A}^{-1}$ in N steps, if f is a quadratic form.

Here now is the routine `dfpmin` that implements the quasi-Newton method and uses `lnsrch` from §9.7. As mentioned at the end of `newt` in §9.7, this algorithm can fail if your variables are badly scaled. You must provide a functor with the same format as the one for `frprmn` in §10.8 to calculate the function and its gradient.

quasinewton.h

```
template <class T>
void dfpmin(VecDoub_IO &p, const Doub gtol, Int &iter, Doub &fret, T &funcd)
```
Given a starting point `p[0..n-1]`, the Broyden-Fletcher-Goldfarb-Shanno variant of Davidon-Fletcher-Powell minimization is performed on a function whose value and gradient are provided by a functor `funcd` (see text in §10.8). The convergence requirement on zeroing the gradient is input as `gtol`. Returned quantities are `p[0..n-1]` (the location of the minimum), `iter` (the number of iterations that were performed), and `fret` (the minimum value of the function). The routine `lnsrch` is called to perform approximate line minimizations.
```
{
    const Int ITMAX=200;
    const Doub EPS=numeric_limits<Doub>::epsilon();
    const Doub TOLX=4*EPS,STPMX=100.0;
```
Here `ITMAX` is the maximum allowed number of iterations; `EPS` is the machine precision; `TOLX` is the convergence criterion on x values; and `STPMX` is the scaled maximum step length allowed in line searches.
```
    Bool check;
    Doub den,fac,fad,fae,fp,stpmax,sum=0.0,sumdg,sumxi,temp,test;
    Int n=p.size();
    VecDoub dg(n),g(n),hdg(n),pnew(n),xi(n);
    MatDoub hessin(n,n);
    fp=funcd(p);                                  Calculate starting function value and gra-
    funcd.df(p,g);                                    dient,
    for (Int i=0;i<n;i++) {                       and initialize the inverse Hessian to the
        for (Int j=0;j<n;j++) hessin[i][j]=0.0;       unit matrix.
        hessin[i][i]=1.0;
        xi[i] = -g[i];                            Initial line direction.
        sum += p[i]*p[i];
    }
    stpmax=STPMX*MAX(sqrt(sum),Doub(n));
    for (Int its=0;its<ITMAX;its++) {             Main loop over the iterations.
        iter=its;
        lnsrch(p,fp,g,xi,pnew,fret,stpmax,check,funcd);
```
The new function evaluation occurs in `lnsrch`; save the function value in `fp` for the next line search. It is usually safe to ignore the value of `check`.
```
        fp=fret;
        for (Int i=0;i<n;i++) {
            xi[i]=pnew[i]-p[i];                   Update the line direction,
```

```
            p[i]=pnew[i];                       and the current point.
        }
        test=0.0;                               Test for convergence on Δx.
        for (Int i=0;i<n;i++) {
            temp=abs(xi[i])/MAX(abs(p[i]),1.0);
            if (temp > test) test=temp;
        }
        if (test < TOLX)
            return;
        for (Int i=0;i<n;i++) dg[i]=g[i];       Save the old gradient,
        funcd.df(p,g);                          and get the new gradient.
        test=0.0;                               Test for convergence on zero gradient.
        den=MAX(fret,1.0);
        for (Int i=0;i<n;i++) {
            temp=abs(g[i])*MAX(abs(p[i]),1.0)/den;
            if (temp > test) test=temp;
        }
        if (test < gtol)
            return;
        for (Int i=0;i<n;i++)                   Compute difference of gradients,
            dg[i]=g[i]-dg[i];
        for (Int i=0;i<n;i++) {                 and difference times current matrix.
            hdg[i]=0.0;
            for (Int j=0;j<n;j++) hdg[i] += hessin[i][j]*dg[j];
        }
        fac=fae=sumdg=sumxi=0.0;                Calculate dot products for the denomi-
        for (Int i=0;i<n;i++) {                     nators.
            fac += dg[i]*xi[i];
            fae += dg[i]*hdg[i];
            sumdg += SQR(dg[i]);
            sumxi += SQR(xi[i]);
        }
        if (fac > sqrt(EPS*sumdg*sumxi)) {      Skip update if fac not sufficiently posi-
            fac=1.0/fac;                            tive.
            fad=1.0/fae;
            The vector that makes BFGS different from DFP:
            for (Int i=0;i<n;i++) dg[i]=fac*xi[i]-fad*hdg[i];
            for (Int i=0;i<n;i++) {             The BFGS updating formula:
                for (Int j=i;j<n;j++) {
                    hessin[i][j] += fac*xi[i]*xi[j]
                        -fad*hdg[i]*hdg[j]+fae*dg[i]*dg[j];
                    hessin[j][i]=hessin[i][j];
                }
            }
        }
        for (Int i=0;i<n;i++) {                 Now calculate the next direction to go,
            xi[i]=0.0;
            for (Int j=0;j<n;j++) xi[i] -= hessin[i][j]*g[j];
        }
    }                                           and go back for another iteration.
    throw("too many iterations in dfpmin");
}
```

Quasi-Newton methods like `dfpmin` work well with the approximate line minimization done by `lnsrch`. The routines `Powell` (§10.7) and `Frprmn` (§10.8), however, need more accurate line minimization, which is carried out by the routine `linmin` in `Linemethod` or `Dlinemethod`.

10.9.1 Quasi-Newton Methods Without Derivatives

In using Newton's method to find a zero of a function in multidimensions, we saw in §9.7 that one can use finite differences to calculate the partial derivatives

instead of providing them analytically. Similarly, dfpmin very often succeeds when the gradient is calculated with finite differences. In our experience, this method often involves less total computation than one of the other methods that avoids analytic derivatives, such as Powell.

To use this idea, all you need to do is supply a suitable functor to dfpmin, which remains unchanged. Here is the code, which is very similar to that of Fdjac in §9.7:

quasinewton.h

```
template <class T>
struct Funcd {
    Doub EPS;                       Set to approximate square root of the machine pre-
    T &func;                            cision.
    Doub f;
    Funcd(T &funcc) : EPS(1.0e-8), func(funcc) {}
    Doub operator() (VecDoub_I &x)
    {
        return f=func(x);
    }

    void df(VecDoub_I &x, VecDoub_O &df)
    {
        Int n=x.size();
        VecDoub xh=x;
        Doub fold=f;
        for (Int j=0;j<n;j++) {
            Doub temp=x[j];
            Doub h=EPS*abs(temp);
            if (h == 0.0) h=EPS;
            xh[j]=temp+h;           Trick to reduce finite-precision error.
            h=xh[j]-temp;
            Doub fh=operator()(xh);
            xh[j]=temp;
            df[j]=(fh-fold)/h;
        }
    }
};
```

10.9.2 Advanced Implementations of Variable Metric Methods

Although rare, it can conceivably happen that roundoff errors cause the matrix $\mathbf{H}_i$ to become nearly singular or non-positive-definite. This can be serious, because the supposed search directions might then not lead downhill, and because nearly singular $\mathbf{H}_i$'s tend to give subsequent $\mathbf{H}_i$'s that are also nearly singular.

There is a simple fix for this rare problem, the same as was mentioned in §10.5: In case of any doubt, you should *restart* the algorithm at the claimed minimum point and see if it goes anywhere. Simple, but not very elegant. Modern implementations of quasi-Newton methods deal with the problem in a more sophisticated way.

Instead of building up an approximation to $\mathbf{A}^{-1}$, it is possible to build up an approximation of $\mathbf{A}$ itself. Then, instead of calculating the left-hand side of (10.9.4) directly, one solves the set of linear equations

$$\mathbf{A}\cdot(\mathbf{x}-\mathbf{x}_i) = -\nabla f(\mathbf{x}_i) \tag{10.9.11}$$

At first glance this seems like a bad idea, since solving (10.9.11) is a process of order N^3 — and anyway, how does this help the roundoff problem? The trick is not to store $\mathbf{A}$ but rather a triangular decomposition of $\mathbf{A}$, its *Cholesky decomposition* (cf. §2.9). The updating formula used for the Cholesky decomposition of $\mathbf{A}$ is of order N^2 and can be arranged to guarantee that the matrix remains positive-definite and nonsingular, even in the presence of finite roundoff. This method is due to Gill and Murray [1,2].

CITED REFERENCES AND FURTHER READING:

Dennis, J.E., and Schnabel, R.B. 1983, *Numerical Methods for Unconstrained Optimization and Nonlinear Equations*; reprinted 1996 (Philadelphia: S.I.A.M.).[1]

Jacobs, D.A.H. (ed.) 1977, *The State of the Art in Numerical Analysis* (London: Academic Press), Chapter III.1, §3 – §6 (by K. W. Brodlie).[2]

Polak, E. 1971, *Computational Methods in Optimization* (New York: Academic Press), pp. 56ff.[3]

Acton, F.S. 1970, *Numerical Methods That Work*; 1990, corrected edition (Washington, DC: Mathematical Association of America), pp. 467–468.

10.10 Linear Programming: The Simplex Method

The subject of *linear programming*, sometimes called *linear optimization*, concerns itself with the following problem: For n independent variables $x_1, \ldots, x_n$, *minimize* the function

$$\zeta = c_1 x_1 + c_2 x_2 + \cdots + c_n x_n \tag{10.10.1}$$

subject to the nonnegativity conditions

$$x_1 \geq 0, \quad x_2 \geq 0, \quad \ldots \quad x_n \geq 0 \tag{10.10.2}$$

and simultaneously subject to m additional constraints of the form

$$a_{i1} x_1 + a_{i2} x_2 + \cdots + a_{in} x_n \leq b_i \tag{10.10.3}$$

or

$$a_{i1} x_1 + a_{i2} x_2 + \cdots + a_{in} x_n = b_i \tag{10.10.4}$$

Here $i = 1, \ldots, m$. Note that an inequality with a $\geq$ can be converted to a $\leq$ by multiplying by -1. Some formulations of linear programming require you to write all the constraints with the b's nonnegative and separately treat the $\geq$ and $\leq$ constraints. We will use the above formulation, with either sign of b_i, instead. However, it is still useful to refer to the inequalities with $b_i \leq 0$ as "$\geq$ inequalities" (which they would be with $b_i \geq 0$), since, as we shall see, they enter the problem in a different way from the $\leq$ inequalities.

There is no particular significance in the number of constraints m being less than, equal to, or greater than the number of unknowns n. Also, note that there is no special significance to minimizing ζ in equation (10.10.1): We can convert a maximizing problem to a minimizing problem by changing the signs of all the c's. The solution $x_1, \ldots, x_n$ is the same, and the required maximum is the negative of the minimum ζ found.

A set of values $x_1, \ldots, x_n$ that satisfies the constraints (10.10.2) – (10.10.4) is called a *feasible vector*. The function that we are trying to minimize is called the *objective function*. The feasible vector that minimizes the objective function is called the *optimal feasible vector*. An optimal feasible vector can fail to exist for two distinct reasons: (i) There are *no* feasible vectors, i.e., the given constraints

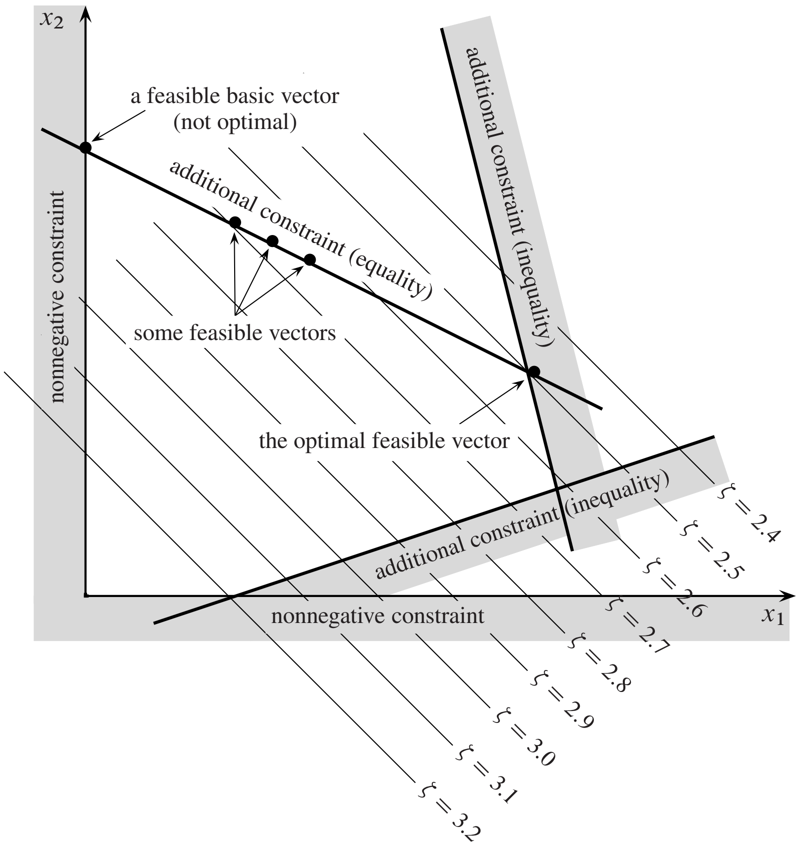

Figure 10.10.1. Basic concepts of linear programming. The case of only two independent variables, x_1, x_2, is shown. The linear function ζ, to be minimized, is represented by its contour lines. Nonnegativity constraints require x_1 and x_2 to be positive. Additional constraints may restrict the solution to regions (inequality constraints) or to surfaces of lower dimensionality (equality constraints). Feasible vectors satisfy all constraints. Feasible basic vectors also lie on the boundary of the allowed region. The simplex method steps among feasible basic vectors until the optimal feasible vector is found.

are incompatible, or (ii) there is no minimum, i.e., there is a direction in n-space where one or more of the variables can be taken to infinity while still satisfying the constraints, giving an unbounded value for the objective function. Figure 10.10.1 summarizes some of the terminology thus far.

As you see, the subject of linear programming is surrounded by notational and terminological thickets. Both of these thorny defenses are lovingly cultivated by a coterie of stern acolytes who have devoted themselves to the field. Actually, the basic ideas of linear programming are quite simple. Avoiding the shrubbery, let's elucidate the basics by means of a couple of specific examples; it should then be quite obvious how to generalize.

Why is linear programming so important? (i) Because "nonnegativity" is the usual constraint on any variable x_i that represents the tangible amount of some physical commodity, like guns, butter, dollars, units of vitamin E, food calories, kilowatt hours, mass, etc. Hence equation (10.10.2). (ii) Because one is often interested in additive (linear) limitations or bounds imposed by man or nature: minimum nutritional

requirement, maximum affordable cost, maximum on available labor or capital, minimum tolerable level of voter approval, etc. Hence equations (10.10.3) – (10.10.4). (iii) Because the function that one wants to optimize may be linear, or else may at least be approximated by a linear function — since that is the problem that linear programming *can* solve. Hence equation (10.10.1). For a short, semipopular survey of linear programming applications, see Bland [1].

10.10.1 Fundamental Theorem of Linear Optimization

Imagine that we start with a full n-dimensional space of candidate vectors. Then (in our mind's eye, at least) we carve away the regions that are eliminated in turn by each imposed constraint. Since the constraints are linear, every boundary introduced by this process is a plane, or rather a hyperplane. Equality constraints of the form (10.10.4) force the feasible region onto hyperplanes of smaller dimension, while inequalities simply divide the then-feasible region into allowed and nonallowed pieces.

When all the constraints are imposed, either we are left with some feasible region or else there are no feasible vectors. Since the feasible region is bounded by hyperplanes, it is geometrically a kind of convex polyhedron or simplex (cf. §10.5). If there is a feasible region, can the optimal feasible vector be somewhere in its interior, away from the boundaries? No, because the objective function is linear. This means that it always has a nonzero vector gradient. This, in turn, means that we could always decrease the objective function by running down the gradient until we hit a boundary wall.

The boundary of any geometrical region has one less dimension than its interior. Therefore, we can now run down the gradient projected into the boundary wall until we reach an edge of that wall. We can then run down that edge, and so on, down through whatever number of dimensions, until we finally arrive at a point, a *vertex* of the original simplex. Since this point has all n of its coordinates defined, it must be the solution of n simultaneous *equalities* drawn from the original set of equalities and inequalities (10.10.2) – (10.10.4).

Points that are feasible vectors and that satisfy n of the original constraints as equalities, including the nonnegativity constraints, are termed *feasible basic vectors*. If $n > m$, then a feasible basic vector has *at least* $n - m$ of its components equal to zero, since at least that many of the constraints (10.10.2) will be needed to make up the total of n. Put the other way, *at most* m components of a feasible basic vector are nonzero.

Put together the two preceding paragraphs and you have the *Fundamental Theorem of Linear Optimization*: If an optimal feasible vector exists, then there is a feasible basic vector that is optimal. (Didn't we warn you about the terminological thicket?)

The importance of the fundamental theorem is that it reduces the optimization problem to a "combinatorial" problem, that of determining which n constraints (out of the $m + n$ constraints in 10.10.2 – 10.10.4) should be satisfied by the optimal feasible vector. We have only to keep trying different combinations, and computing the objective function for each trial, until we find the best.

Doing this blindly would take halfway to forever. The *simplex method*, first published by Dantzig in 1948 (see [2]), is a way of organizing the procedure so that (i) a series of combinations is tried for which the objective function decreases at each step, and (ii) the optimal feasible vector is reached after a number of iterations that

is almost always no larger than of order m or n, whichever is larger. An interesting mathematical sidelight is that this second property, although known empirically ever since the simplex method was devised, was not proved to be true until the 1982 work of Stephen Smale. (For a contemporary account, see [3].)

10.10.2 Writing the General Problem in Standard Form

There is a standard form for linear programming problems, and we have to learn how to write a general problem like (10.10.1) – (10.10.4) in this standard form. For definiteness, consider the problem

$$\text{Minimize} \quad \zeta = -40x_1 - 60x_2 \tag{10.10.5}$$

with the x's nonnegative and also with

$$2x_1 + x_2 \le 70 \tag{10.10.6}$$
$$x_1 + x_2 \ge 40 \tag{10.10.7}$$
$$x_1 + 3x_2 = 90 \tag{10.10.8}$$

First, we rewrite the inequalities as equalities. We do this by adding to the problem so-called *slack variables* $x_{n+1}, x_{n+2}, \ldots$ In our example, equations (10.10.6) and (10.10.7) become

$$2x_1 + x_2 + x_3 = 70 \tag{10.10.9}$$
$$-x_1 - x_2 + x_4 = -40 \tag{10.10.10}$$

(A slack variable like x_4 for a $\ge$ inequality is sometimes called a *surplus* variable.) Requiring the slack variables to be nonnegative makes these equalities equivalent to the original inequalities. Once they are introduced, you treat slack variables on an equal footing with the original variables x_i; then, at the very end, you just ignore them. The simplex solution for each slack variable is simply the amount by which the original inequality is satisfied.

The key idea in the simplex method is to start with a feasible basic vector and make a sequence of exchanges between basic and nonbasic variables. At each step the vector stays feasible (satisfies the constraints), and the objective function decreases (or at least does not increase).

How do we find an initial feasible basic vector to start the procedure? Suppose that our example were changed so that equations (10.10.7) and (10.10.8) were both $\le$ inequalities, like (10.10.6). Then, after introducing slack variables, we would have

$$2x_1 + x_2 + x_3 = 70 \tag{10.10.11}$$
$$x_1 + x_2 + x_4 = 40 \tag{10.10.12}$$
$$x_1 + 3x_2 + x_5 = 90 \tag{10.10.13}$$

In this case it is easy to write down a feasible basic vector: Set the original variables x_1 and x_2 to zero and take $(x_3, x_4, x_5) = (70, 40, 90)$. Here $n = 2$ of the constraints, namely $x_1 \ge 0$, $x_2 \ge 0$, are satisfied as equalities, while $m = 3$ components of the feasible basic vector are nonzero. The variables (x_3, x_4, x_5) are called *basic variables*, while the variables that are zero, (x_1, x_2), are called *nonbasic variables*. Note

that if we write equations (10.10.11) – (10.10.13) as a 3×5 matrix equation, then the last three columns of the matrix, corresponding to the slack variables (x_3, x_4, x_5), form a 3×3 unit matrix.

So $\le$ constraints are easy. But how do we handle constraints like equations (10.10.7) and (10.10.8)? The trick is again to invent new variables called *artificial variables*. We rewrite equation (10.10.8) as

$$x_1 + 3x_2 + x_5 = 90 \qquad (10.10.14)$$

Now equations (10.10.9), (10.10.10), and (10.10.14) are almost in the form to give us an easy initial feasible basic vector by setting $x_1 = x_2 = 0$. The obstacle is equation (10.10.10), which would give a negative value for x_4. We have to precede the actual simplex procedure by a preliminary procedure, called *phase one* of the simplex method, to find an initial feasible vector. (The actual optimization is called *phase two*.)

In phase one, we replace our objective function (10.10.5) by a so-called *auxiliary objective function*,

$$\zeta' \equiv -x_4 \qquad (10.10.15)$$

We now perform the simplex method on the auxiliary objective function (10.10.15) with the constraints (10.10.9), (10.10.10), and (10.10.14), starting with the basis given by $x_1 = x_2 = 0$. The variable x_4 starts off negative (at -40). Minimizing the function (10.10.15) drives x_4 toward satisfying $x_4 \ge 0$, the condition for feasibility. In fact, we don't even have to solve phase one all the way to the exact minimum. As we do the exchanges between variables during this phase, we continually redefine the auxiliary objective function at each iteration to be minus the sum of all negative basic variables. As soon as all the basic variables are nonnegative, we are done with phase one.

And what if the first phase *doesn't* drive the auxiliary objective function to a negative value (i.e., all basic variables nonnegative)? That signals that there is *no* initial feasible basic vector, i.e., that the constraints given to us are inconsistent among themselves. Report that fact, and you are done.

An artificial variable in an equality constraint is an example of a *zero variable*, a variable that must vanish in the optimal solution. Typically the way a zero variable gets to be zero is by being nonbasic in the optimal solution. So we can precede phase one with a "phase zero" in which we exchange each zero variable out of the basis.

One last piece of jargon: Slack and artificial variables are often called *logical* variables, to distinguish them from the original independent variables, which are sometimes called *structural* variables.

10.10.3 The Simplex Method: A Worked Example

The easiest way to describe the actual simplex procedure is with a worked example. We write the general linear programming problem in the following form: Minimize the objective function

$$\zeta = \mathbf{c} \cdot \mathbf{x} = c_1 x_1 + c_2 x_2 + \cdots + c_n x_n \qquad (10.10.16)$$

subject to the constraints

$$\mathbf{A} \cdot \mathbf{x} = \mathbf{b} \qquad (10.10.17)$$

and

$$x_i \geq 0, \qquad i = 1, \ldots, n + m \tag{10.10.18}$$

Here we assume that we started with an $m \times n$ matrix of constraint coefficients given by equations like (10.10.3) and (10.10.4). We then added slack variables to the inequality constraints and artificial variables to the equality constraints so that the constraint matrix is now the $m \times (n + m)$ matrix $\mathbf{A}$. The last m columns form an $m \times m$ identity matrix. Note that the coefficients of the slack variables are taken to be $+1$, so that an original $\geq$ inequality will have a negative right-hand side. For our example given in equations (10.10.5) – (10.10.8), transformed as in equations (10.10.9), (10.10.10), and (10.10.14), the matrix $\mathbf{A}$ has five columns:

$$\mathbf{a}_1 = \begin{pmatrix} 2 \\ -1 \\ 1 \end{pmatrix} \quad \mathbf{a}_2 = \begin{pmatrix} 1 \\ -1 \\ 3 \end{pmatrix} \quad \mathbf{a}_3 = \begin{pmatrix} 1 \\ 0 \\ 0 \end{pmatrix} \quad \mathbf{a}_4 = \begin{pmatrix} 0 \\ 1 \\ 0 \end{pmatrix} \quad \mathbf{a}_5 = \begin{pmatrix} 0 \\ 0 \\ 1 \end{pmatrix} \tag{10.10.19}$$

The right-hand side and the objective function coefficients are

$$\mathbf{b} = \begin{pmatrix} 70 \\ -40 \\ 90 \end{pmatrix} \qquad \mathbf{c} = (-40, -60, 0, 0, 0)^T \tag{10.10.20}$$

We partition the matrix $\mathbf{A}$ into two submatrices,

$$\mathbf{A} = \left[\mathbf{A}_B \mid \mathbf{A}_N\right] \tag{10.10.21}$$

where we have permuted the columns corresponding to the basic variables to be in $\mathbf{A}_B$, while the nonbasic columns are in $\mathbf{A}_N$. In our example, the initial basic variables are (x_3, x_4, x_5) and the initial basis $\mathbf{A}_B$ is the unit matrix composed of the last three columns of $\mathbf{A}$, $\mathbf{a}_3$, $\mathbf{a}_4$ and $\mathbf{a}_5$. A basic solution of $\mathbf{A} \cdot \mathbf{x} = \mathbf{b}$ consists of a set of basic and nonbasic variables $[\mathbf{x}_B \mid \mathbf{x}_N]$ with $\mathbf{x}_N = 0$. In our example, initially $\mathbf{x}_N = (x_1, x_2) = 0$. The basic solution satisfies $\mathbf{A}_B \cdot \mathbf{x}_B = \mathbf{b}$, or $\mathbf{x}_B = \mathbf{A}_B^{-1} \cdot \mathbf{b}$.

To derive the simplex method, we need one simple equation: how a basic vector changes as a nonbasic variable (i.e., one that is zero) becomes nonzero. This corresponds to starting at a vertex of the simplex and sliding along an edge toward another vertex. Suppose the variable x_k is the one increasing from zero. The constraint equation $\mathbf{A} \cdot \mathbf{x} = \mathbf{A}_B \cdot \mathbf{x}_B = \mathbf{b}$ becomes

$$\mathbf{A}_B \mathbf{x}'_B + \mathbf{a}_k x_k = \mathbf{b} \tag{10.10.22}$$

since only x_k is nonzero among the nonbasic variables. Multiplying this equation by $\mathbf{A}_B^{-1}$ gives

$$\mathbf{x}'_B = \mathbf{A}_B^{-1} \cdot \mathbf{b} - x_k \mathbf{A}_B^{-1} \cdot \mathbf{a}_k = \mathbf{x}_B - x_k \mathbf{A}_B^{-1} \cdot \mathbf{a}_k \tag{10.10.23}$$

The first application of equation (10.10.23) is to the idea of a *reduced cost*. The coefficient c_i in the objective function (10.10.16) is sometimes called the cost of variable x_i, because it represents the cost of having x_i amount of quantity i in the objective function. The simplex method requires instead the cost of *changing* a variable that is zero (not in the basis) to a nonzero value. If the initial value of the objective function is $\mathbf{c} \cdot \mathbf{x}_B = \mathbf{c}_B \cdot \mathbf{x}_B$ and the final value is $\mathbf{c} \cdot (\mathbf{x}'_B + x_k \mathbf{e}_k)$, where $\mathbf{e}_k$

is a unit vector, then using equation (10.10.23) you find that the difference is $x_k u_k$, where the reduced cost of x_k is given by

$$u_k = c_k - \mathbf{a}_k \cdot \mathbf{y}, \qquad \mathbf{y} \equiv (\mathbf{A}_B^{-1})^T \cdot \mathbf{c}_B \tag{10.10.24}$$

Note that if $u_k < 0$, you can make the value of the objective function smaller by bringing x_k into the basis (making it nonzero).

The simplex procedure consists of the following steps:

1. Find a feasible basis (phase 1)
2. Compute the reduced costs (10.10.24) for all x_k not in the basis.
3. If $u_k \geq 0$ for all k, the solution is optimal: No exchange will improve things. Otherwise, choose k corresponding to the most negative u_k as the entering column.
4. Choose the leaving column i from the *minimum ratio test* (motivated below): Compute
$$\mathbf{x}_B = \mathbf{A}_B^{-1} \cdot \mathbf{b}, \qquad \mathbf{w} = \mathbf{A}_B^{-1} \cdot \mathbf{a}_k \tag{10.10.25}$$
For each component $w_i > 0$, compute the ratio x_B^i / w_i. Choose i that corresponds to the smallest such ratio. (If no $w_i > 0$, the objective function is unbounded. Exit and report this.)
5. Exchange columns i and k and go back to step 2.

The minimum ratio test is the second application of equation (10.10.23), which can be written as

$$(x_B^i)' = x_B^i - x_k w_i \tag{10.10.26}$$

where w_i is defined in equation (10.10.25). For each $w_i > 0$, x_B^i decreases as x_k increases from zero. The minimum ratio test selects the i corresponding to the first x_B^i to hit zero, while the other basis variables are still positive. The idea is to allow x_k to be as big as possible so that the objective function is reduced as much as possible by bringing it into the basis.

Let's see how this applies to our example. We start with phase zero, where we remove the zero variable x_5 from the basis. Suppose we choose x_2 to be the incoming variable (x_1 would work fine, too). Using x_2, we find for the new basis and its inverse

$$\mathbf{A}_B = \begin{pmatrix} 1 & 0 & 1 \\ 0 & 1 & -1 \\ 0 & 0 & 3 \end{pmatrix} \qquad \mathbf{A}_B^{-1} = \begin{pmatrix} 1 & 0 & -\frac{1}{3} \\ 0 & 1 & \frac{1}{3} \\ 0 & 0 & \frac{1}{3} \end{pmatrix} \tag{10.10.27}$$

The new basic solution is

$$\mathbf{x}_B = \begin{pmatrix} x_3 \\ x_4 \\ x_2 \end{pmatrix} = \mathbf{A}_B^{-1} \cdot \mathbf{b} = \begin{pmatrix} 40 \\ -10 \\ 30 \end{pmatrix} \tag{10.10.28}$$

The solution (10.10.28) is not feasible because x_4 is negative. We enter phase one, with $\zeta' = \overline{\mathbf{c}} \cdot \mathbf{x} = -x_4$, i.e.,

$$\overline{\mathbf{c}}_B = \begin{pmatrix} 0 \\ -1 \\ 0 \end{pmatrix} \tag{10.10.29}$$

Here the order of elements corresponds to the order (x_3, x_4, x_2) for the basic variables. We compute the reduced costs from equation (10.10.24). Only $k = 1$ is relevant, since x_5 is never allowed to re-enter the basis (zero variable). We find

$$u_1 = -\mathbf{a}_1 \cdot (\mathbf{A}_B^{-1})^T \cdot \overline{\mathbf{c}}_B = -\tfrac{2}{3} \qquad (10.10.30)$$

which is negative, confirming that x_1 should enter. For the minimum ratio test to determine which variable leaves, we need the quantity

$$\mathbf{A}_B^{-1} \cdot \mathbf{a}_1 = \begin{pmatrix} \frac{5}{3} \\ -\frac{2}{3} \\ \frac{1}{3} \end{pmatrix} \qquad (10.10.31)$$

So the ratios of the elements in equation (10.10.28) to those in equation (10.10.31) are

$$\frac{40}{5/3} = 24, \qquad \frac{-10}{-2/3} = 15, \qquad \frac{30}{1/3} = 90 \qquad (10.10.32)$$

The middle ratio is the minimum, so x_4 goes out. (Note that in phase one we relax the requirement that $w_i > 0$, since we haven't yet made all the variables nonnegative.)

Now the basic variables are (x_3, x_1, x_2). Proceeding as before, we find

$$\mathbf{A}_B = \begin{pmatrix} 1 & 2 & 1 \\ 0 & -1 & -1 \\ 0 & 1 & 3 \end{pmatrix} \qquad \mathbf{A}_B^{-1} = \begin{pmatrix} 1 & \frac{5}{2} & \frac{1}{2} \\ 0 & -\frac{3}{2} & -\frac{1}{2} \\ 0 & \frac{1}{2} & \frac{1}{2} \end{pmatrix} \qquad (10.10.33)$$

and

$$\mathbf{x}_B = \begin{pmatrix} x_3 \\ x_1 \\ x_2 \end{pmatrix} = \mathbf{A}_B^{-1} \cdot \mathbf{b} = \begin{pmatrix} 15 \\ 15 \\ 25 \end{pmatrix} \qquad (10.10.34)$$

All the variables are positive, so the basis is feasible and we enter phase two, with $\mathbf{c}_B = (0, -40, -60)^T$. We find the reduced cost $u_4 = -30$, so x_4 re-enters the basis. The minimum ratio test (10.10.25) gives a minimum for the term involving x_3, so the next basis is (x_4, x_1, x_2). The basic solution turns out to be $(6, 24, 22)$. When we compute the reduced cost u_3 for this basis, it is positive, so we are done. The minimum occurs at $x_1 = 24$, $x_2 = 22$, and the minimum value, obtained by substitution in the objective function, is -2280. The meaning of $x_4 = 6$ is that the inequality (10.10.7) is satisfied by 6. The other two constraints are satisfied as equalities.

The graphical interpretation of the solution procedure is shown in Figure 10.10.2. The initial basic vector is at the origin. We first proceed to the vertex A, which puts us on the line where the equality (10.10.8) is satisfied. This is not a feasible point, since we are on the wrong side of the line Y. So we move along the line X to the vertex B, which is now feasible. Finally we move to vertex C, which is the minimum value of the objective function.

10.10.4 Degeneracy

Nonbasic variables in a basic feasible solution are all zero. If any *basic* variable is zero, we say the basis is *degenerate*. Geometrically, this situation corresponds in n dimensions to having more than n hyperplanes intersect at a vertex. Degeneracy can cause problems in the simplex method. Consider the simple case when three

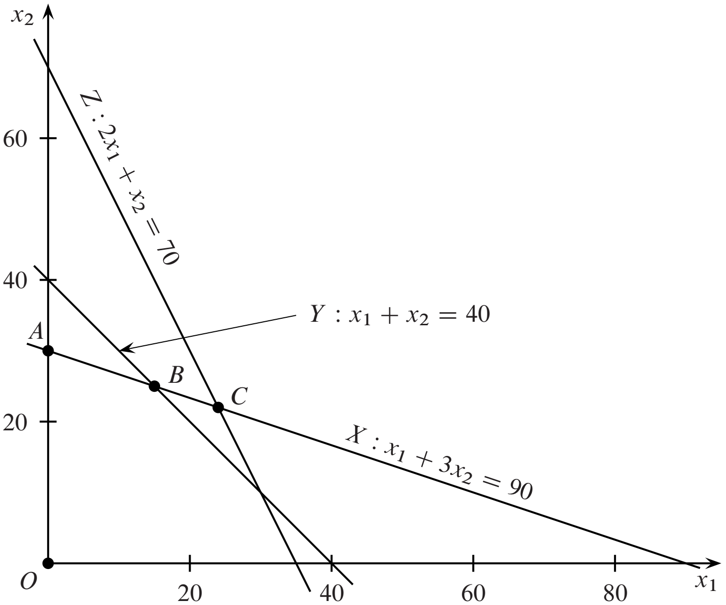

Figure 10.10.2. Graphical interpretation of the simplex solution of the problem (10.10.5) – (10.10.8). The initial basic vector is at the origin O. To satisfy the equality (10.10.8), the first step moves to A, on the line X. This is not yet a feasible point, since it is on the wrong side of the line Y. The next move is to B, which is feasible. We enter phase two, and find that we can reduce the objective function by moving to C. No further moves are possible, so we are done. Note that the figure is really the projection of a five-dimensional simplex onto the x_1-x_2 plane.

lines intersect at a point in two dimensions. Only two of the lines are necessary to define the vertex. When the leaving variable is chosen, it can correspond to the third direction at the vertex. On making this change in the basis, the objective function doesn't improve. You can see this algebraically from equation (10.10.26): If $x_B^i = 0$ and $w^i > 0$, then a step size of zero is required for the new variable x_k. Clearly special measures need to be taken.

Degeneracy allows the possibility of *cycling*, where you keep exchanging the same set of vectors in and out of the basis without making any progress. In practice, however, cycling is almost never a problem. More common in very degenerate problems is *stalling*, where you spend a long time making exchanges before finally leaving the vertex.

10.10.5 Sparseness and Stability

If you examine the operations carried out during each simplex step, you see that a key ingredient is to solve equations of the form $\mathbf{A}_B \cdot \mathbf{x} = \mathbf{b}$ and similar equations with $\mathbf{A}_B^T$. We know that a good method for doing this, absent other considerations, is to use the LU decomposition of $\mathbf{A}_B$ (cf. §2.3), since we can use partial pivoting to maintain stability. Decomposing $\mathbf{A}_B$ afresh each step is expensive, but since successive bases differ only by the replacement of a single column, one can use techniques analogous to the Sherman-Morrison formula (§2.7) to update $\mathbf{L}$ and $\mathbf{U}$.

However, most linear programming problems that occur in practice have a constraint matrix $\mathbf{A}$ that is very sparse. It is crucial to take advantage of this sparseness in the linear algebra procedures that make up each simplex step, since real-life prob-

lems can involve many thousands or even millions of constraints and variables. Standard LU decomposition with partial pivoting to maintain stability is not desirable, because it leads to excessive *fill-in*, that is, the generation of nonzero matrix elements where there were zeros before. Instead, one chooses the pivot element by a trade-off between stability and sparsity. A popular strategy is based on the *Markowitz criterion* [4]. Here the pivot is a nonzero with the smallest product of the number of other nonzeros in its row and its column. Empirically, the Markowitz criterion works about as well as any other general strategy for minimizing fill-in. In linear programming applications, one also generally needs to impose some kind of stability criterion. In *threshold partial pivoting*, no pivot is less than α times the largest element in its row, where α is a parameter between zero and one. A typical value is $\alpha \sim 0.1$; $\alpha = 1$ gives straight partial pivoting.

The first stable updating procedure for sparse LU decomposition was given by Bartels and Golub [5,6]. This procedure updates $\mathbf{L}$ and $\mathbf{U}$ when a column is replaced in $\mathbf{A}_B$. A good sparse LU algorithm that includes the Bartels-Golub update is that of [7]. It is freely available in the software package LUSOL as part of [8], and we use it in our pedagogical implementation described below.

There are newer methods for the LU decomposition and the update procedure. According to [9], the best of these is probably that of [10].

10.10.6 State-of-the-Art Simplex Codes

A high-quality implementation of the simplex method will have a number of features that we have not discussed so far.

- It will implement more than one variant of the simplex method. In addition to the algorithm we have described, the *primal algorithm*, it will typically also implement the so-called *dual algorithm* (duality is discussed in §10.11). The number of iterations can be significantly reduced by the appropriate use of more than one algorithm.
- It will accept multiple input formats for the specification of the problem, with suitable error checking.
- It will preprocess the problem, with the aim of reducing its size and improving its numerical properties. Many complex problems are inadvertently specified in reducible form.
- It will have multiple options for scaling the problem. As with solving linear equations, no universal scaling algorithm is known for linear programming.
- It will have multiple options for starting the iteration, including several procedures for phase 1.
- It will have several pricing strategies (procedures for selecting the incoming variable). These go by names like multiple pricing, Devex, and steepest edge.
- It will have multiple pivot methods (variants of the ratio test) for the outgoing variable.
- It will handle bounded variables, that is, variables that satisfy a requirement $l_i \le x_i \le u_i$ instead of $x_i \ge 0$. It is possible to handle such bounds using slack variables, but that increases the size of the matrix $\mathbf{A}$. A slight generalization of the algorithm we have described allows you to handle bounded variables directly.
- It will have efficient sparse matrix algorithms.

All these issues are thoroughly discussed in [9].

There are several excellent public domain simplex implementations that incorporate most of the above items. These include CLP [11], GLPK [12] and lp_solve [8]. If you are planning to do any serious LP solving, you should definitely explore these options. It may even be worthwhile to invest in a commercial LP solver. For more information on all these options, see [13]. But first look at the next section, where *interior-point methods* are discussed. It appears now that for many very big problems (but not all), interior-point methods can out-perform simplex methods [14-16]. Even for moderate-sized problems, an interior-point method could be your best choice.

The simplex codes mentioned above are large software efforts, with many thousands of lines of code. They are quite formidable if you want to study how the simplex algorithm works and play around with various options. Accordingly, in a Webnote [17], we give a pedagogical implementation of the simplex method. Even though it uses reasonably good sparse matrix algebra, it is slower than the public domain implementations by one to two orders of magnitude on problems with $\sim 10^4$ variables. If you don't care about the simplex algorithm and you just want a simple method to get your problem solved quickly without getting a public domain code up and running, take a look at our pedagogical interior-point code in the next section.

10.10.7 Other Topics Briefly Mentioned

Problems where the objective function and/or one or more of the constraints are replaced by expressions nonlinear in the variables are called *nonlinear programming problems*. The literature on such problems is vast, but outside our scope. The special case of quadratic expressions is called *quadratic programming*. Optimization problems where the variables take on only integer values are called *integer programming* problems, a special case of *discrete optimization* generally. Section 10.12 looks at a particular kind of discrete optimization problem.

CITED REFERENCES AND FURTHER READING:

Bland, R.G. 1981, "The Allocation of Resources by Linear Programming," *Scientific American*, vol. 244 (June), pp. 126–144.[1]

Dantzig, G.B. 1963, *Linear Programming and Extensions* (Princeton, NJ: Princeton University Press).[2]

Kolata, G. 1982, "Mathematician Solves Simplex Problem," *Science*, vol. 217, p. 39.[3]

Markowitz, H.M. 1957, "The Elimination Form of the Inverse and Its Application to Linear Programming," *Management Science*, vol. 3, pp. 255–269.[4]

Bartels. R.H., and Golub, G.H. 1969, "The Simplex Method of Linear Programming Using the LU Decomposition," *Communications of the ACM*, vol. 12, pp. 266–268.[5]

Bartels, R.H. 1971, "A Stabilization of the Simplex Method," *Numerische Mathematik*, vol. 16, pp. 414–434.[6]

Gill, P.E., Murray, W., Saunders, M.A. and Wright, M.H. 1987, "Maintaining LU Factors of a General Sparse Matrix," *Linear Algebra and Its Applications*, vol. 88/89, pp. 239–270.[7]

lp_solve, `http://groups.yahoo.com/group/lp_solve`.[8]

Maros, I. 2003, *Computational Techniques of the Simplex Method* (Boston: Kluwer).[9]

Suhl, U., and Suhl, L. 1990, "Computing Sparse LU Factorizations for Large-Scale Linear Programming Bases," *ORSA Journal on Computing*, vol. 2, pp. 325–335; 1993, "A Fast LU Update for Linear Programming," *Annals of Operations Research*, vol. 43, pp. 33–47.[10]

CLP, `http://www.coin-or.org/Clp`.[11]

GLPK (GNU Linear Programming Kit), `http://www.gnu.org/software/glpk`.[12]

Linear Programming Frequently Asked Questions, `http://www-unix.mcs.anl.gov/otc/Guide/faq/linear-programming-faq.html`.[13]

Lustig, I.J., Marsten, R.E., and Shanno, D.F. 1994, "Interior Point Methods: Computational State of the Art," *ORSA Journal on Computing*, vol. 6, pp. 1–14. See also Commentaries and Rejoinder, pp. 15–36.[14]

Wright, S.J. 1997, *Primal-Dual Interior-Point Methods* (Philadelphia: S.I.A.M.).[15]

Bixby, R.E. 2002, "Solving Real-World Linear Programs: A Decade and More of Progress," *Operations Research*, vol. 50, pp. 3–15.[16]

Stoer, J., and Bulirsch, R. 2002, *Introduction to Numerical Analysis*, 3rd ed. (New York: Springer), §4.10.

Nemhauser, G.L., Rinnooy Kan, A.H.G., and Todd, M.J. (eds.) 1989, *Optimization* (Amsterdam: North-Holland).

Gill, P.E., Murray, W., and Wright, M.H. 1991, *Numerical Linear Algebra and Optimization*, vol. 1 (Redwood City, CA: Addison-Wesley), Chapters 7–8.

Ignizio, J.P., and Cavalier, T.M. 1994, *Linear Programming* (Englewood Cliffs, NJ: Prentice-Hall). [Undergraduate text]

Vanderbei, R.J. 2001, *Linear Programming: Foundations and Extensions*, 2nd ed. (Boston: Kluwer). [Undergraduate/graduate text]

Chvátal, V. 1983, *Linear Programming* (New York: Freeman). [Undergraduate text]

Gass, S.I. 1985, *Linear Programming: Methods and Applications*, 5th ed. (New York: McGraw-Hill), reprinted 2003 (New York: Dover). [Undergraduate text]

Murty, K.G. 1983, *Linear Programming* (New York: Wiley). [Undergraduate text]

Numerical Recipes Software 2007, "Routine Implementing the Simplex Method," *Numerical Recipes Webnote No. 12*, at `http://www.nr.com/webnotes?12` [17]

10.11 Linear Programming: Interior-Point Methods

As we mentioned in §10.10, the worst-case number of iterations for the simplex method is an exponential function of n. (The worst case occurs for $m = n$.) The average number of iterations, however, is a small multiple of m. For a long time it was not known if there was another algorithm for linear programming that would be bounded by, for example, some polynomial in n. In 1979, Khachian published a new algorithm [1], the *ellipsoid method*, that is in fact polynomial in n. Disappointingly, however, in practical implementations it was much slower than the simplex method.

In 1984, the field was electrified by Karmarkar's paper [2] describing an *interior-point* method. Not only was it polynomial in n, but he claimed it solved large LP problems significantly faster than the simplex method. This claim turned out to be somewhat exaggerated, but in the frenzy of activity over the next decade interior-point algorithms were developed that *do* solve many problems much faster than the simplex method, especially very large problems. Ironically, the rivalry between the two algorithms led to improvements of about a factor of 100 in the simplex method itself over the same period. We give some recommendations on which method to use at the end of this section.

Originally, interior-point methods traversed the interior of the feasible region, homing in on the optimal vertex. So-called infeasible interior-point methods follow

a path in the interior of the nonnegative region, $x_i \geq 0$, $i = 1, \ldots, n$, but possibly through the infeasible region.

To understand how interior-point methods work, we need to develop some more theory about linear programming, in particular about *duality*.

10.11.1 Dual Problem

As we saw in §10.10, any LP problem can be written in standard form:

$$\boxed{\begin{array}{ll} \text{minimize} & \mathbf{c} \cdot \mathbf{x} \\ \text{subject to} & \mathbf{A} \cdot \mathbf{x} = \mathbf{b} \\ & \mathbf{x} \geq 0 \end{array}} \tag{10.11.1}$$

Here slack variables have been appended to the x's to write all inequality constraints as equalities, but no other logical variables have been added. This is called the *primal* problem. Recall that if the constraints in (10.11.1) are satisfied, we say that $\mathbf{x}$ is a feasible point.

The *dual* problem corresponding to (10.11.1) interchanges the roles of variables and constraints: Corresponding to the m constraints is a set of variables $(y_1, \ldots, y_m)$ determined by

$$\boxed{\begin{array}{ll} \text{maximize} & \mathbf{b} \cdot \mathbf{y} \\ \text{subject to} & \mathbf{A}^T \cdot \mathbf{y} \leq \mathbf{c} \\ & \mathbf{y} \text{ free} \end{array}} \tag{10.11.2}$$

Here "free" means unconstrained. Most of the textbooks mentioned at the end of the previous section discuss exactly how to go from the primal problem to its dual. For a hint, see §16.5.2, where a different primal-dual problem is discussed. After that, a particularly clear discussion is in [3]. Note how the constraint matrix for the dual problem is the transpose of the matrix for the primal problem. Forming the dual of the dual simply takes you back to the primal problem.

The dual problem (10.11.2) can be rewritten by adding slack variables $(z_1, \ldots, z_n)$:

$$\boxed{\begin{array}{ll} \text{maximize} & \mathbf{b} \cdot \mathbf{y} \\ \text{subject to} & \mathbf{A}^T \cdot \mathbf{y} + \mathbf{z} = \mathbf{c} \\ & \mathbf{z} \geq 0, \quad \mathbf{y} \text{ free} \end{array}} \tag{10.11.3}$$

If $(\mathbf{y}, \mathbf{z})$ satisfy the constraints in (10.11.3), we say that they are *dual feasible*. For a good introduction to the meaning of the relation between primal and dual problems, see [4].

The *weak duality theorem* asserts that the value of the dual objective function provides a lower bound to the value of the objective function if they are each evaluated at feasible points. Proof: $\mathbf{b} \cdot \mathbf{y} = \mathbf{y} \cdot \mathbf{A} \cdot \mathbf{x} = \mathbf{x} \cdot \mathbf{A}^T \cdot \mathbf{y} \leq \mathbf{x} \cdot \mathbf{c}$. The difference

$$\mathbf{c} \cdot \mathbf{x} - \mathbf{b} \cdot \mathbf{y} \tag{10.11.4}$$

is called the *duality gap*. If the primal is unbounded (the objective can be made arbitrarily negative), then the dual must be infeasible, and vice versa. Moreover, we

have the *strong duality theorem*: If either the primal or the dual has a finite optimal solution, so does the other, and $\mathbf{c} \cdot \mathbf{x} = \mathbf{b} \cdot \mathbf{y}$ for the optimal solution.

There is a further important relation between the primal and dual variables at optimality. Consider a particular x_j and the corresponding $z_j = (\mathbf{c} - \mathbf{A}^T \cdot \mathbf{y})_j$. The *Karush-Kuhn-Tucker complementarity condition* says that they can't both be strictly greater than zero: At least one must be equal to zero. In other words,

$$x_j \, z_j = 0, \quad j = 1, \dots, n \tag{10.11.5}$$

Adopting the convention that an uppercase letter denotes a matrix with the corresponding lowercase vector along the diagonal, we can write equation (10.11.5) alternatively as

$$\mathbf{X} \cdot \mathbf{Z} \cdot \mathbf{e} = 0, \qquad \mathbf{X} = \text{diag}(x_1, \dots, x_n), \quad \mathbf{Z} = \text{diag}(z_1, \dots, z_n), \quad \mathbf{e} = (1, \dots, 1) \tag{10.11.6}$$

Since each x_j and z_j is nonnegative, equation (10.11.5) is equivalent to $\mathbf{x} \cdot \mathbf{z} = 0$. In fact, this result holds in both directions: The *complementary slackness theorem* says that feasible solutions are optimal if and only if $\mathbf{x} \cdot \mathbf{z} = 0$. It is easy to show that for feasible solutions, $\mathbf{x} \cdot \mathbf{z}$ is simply equal to the duality gap (10.11.4).

Note that complementary slackness allows the possibility that both x_j and z_j might be zero at an optimal solution. *Strict complementarity* is the property that exactly one of these quantities is zero for all j. The *Goldman-Tucker theorem* says that if the primal and dual are feasible, there exists a strictly complementary pair of optimal solutions. As we'll see, interior-point methods find such a solution.

10.11.2 The KKT Conditions

Linear programming is a special case of general constrained optimization, where one wants to minimize some function $f(\mathbf{x})$ subject to constraints. The general optimality conditions are called the Karush-Kuhn-Tucker or KKT conditions. Specialized to the LP problem (10.11.1), the KKT conditions are

$$\begin{aligned} \mathbf{A} \cdot \mathbf{x} &= \mathbf{b} \qquad & \mathbf{x} &\geq 0 \\ \mathbf{A}^T \cdot \mathbf{y} + \mathbf{z} &= \mathbf{c} \qquad & \mathbf{z} &\geq 0 \\ \mathbf{X} \cdot \mathbf{Z} \cdot \mathbf{e} &= 0 \qquad & \mathbf{y} &\text{ free} \end{aligned} \tag{10.11.7}$$

Note that these are exactly the conditions that follow from strong duality and complementarity. Later we will see how to derive these conditions directly using Lagrange multipliers to handle the constraints.

The KKT conditions (10.11.7) are necessary and sufficient for $\mathbf{x}$ to be an optimal solution of (10.11.1). Moreover, they are necessary and sufficient for $(\mathbf{x}, \mathbf{y}, \mathbf{z})$ to solve the primal and dual problems (10.11.1) and (10.11.2). In this case, we call $(\mathbf{x}, \mathbf{y}, \mathbf{z})$ a *primal-dual solution*. Primal-dual interior-point methods solve the equations (10.11.7) in such a way that the inequalities are satisfied strictly at every iteration, that is, $\mathbf{x}, \mathbf{z} > 0$.

The equations are solved using a variant of Newton's method. Recall that if we define the vector of equations in (10.11.7) as

$$\mathbf{F}(\mathbf{v}) = \begin{bmatrix} \mathbf{A} \cdot \mathbf{x} - \mathbf{b} \\ \mathbf{A}^T \cdot \mathbf{y} + \mathbf{z} - \mathbf{c} \\ \mathbf{X} \cdot \mathbf{Z} \cdot \mathbf{e} \end{bmatrix} = 0 \tag{10.11.8}$$

where $\mathbf{v}$ is shorthand for $(\mathbf{x}, \mathbf{y}, \mathbf{z})$, then Newton's method determines the update $\Delta\mathbf{v}$ to the current point by solving

$$\mathbf{J} \cdot \Delta\mathbf{v} = -\mathbf{F} \tag{10.11.9}$$

Here $\mathbf{J}$ is the Jacobian matrix of $\mathbf{F}$ (see §9.7). A full step with this value of $\Delta\mathbf{v}$ is usually not allowed because it would violate the condition $(\mathbf{x}, \mathbf{z}) \geq 0$. So the new iterate is chosen from a line search along the Newton direction:

$$\mathbf{v}_{\text{new}} = \mathbf{v}_{\text{old}} + \alpha\Delta\mathbf{v}, \qquad \alpha \in (0, 1] \tag{10.11.10}$$

You choose $\alpha = 1$ if possible; otherwise, you choose the maximum α that preserves nonnegativity.

Note the importance of keeping the nonnegative variables strictly positive at all times: The Newton equation for $x_j z_j = 0$ is $x_j \Delta z_j + z_j \Delta x_j = -x_j z_j$. Suppose z_j is zero. Then the Newton equation becomes $x_j \Delta z_j = 0$, or $\Delta z_j = 0$. So z_j remains zero if it ever becomes zero. The algorithm can never recover. Of course, one should also expect difficulties if any variable gets "too close" to zero.

This simple damped Newton's method is not a practical algorithm, because too often the allowed stepsize becomes very small ($\alpha \ll 1$). There are two important modifications that are crucial to producing a viable algorithm:

- Change the search direction so that it aims toward the "center" of the nonnegative region. The idea is to allow larger steps before one of the variables would become negative.
- Don't allow the variables to come "too close" to the boundary of the nonnegative region. As discussed above, little progress tends to be made from such points.

10.11.3 The Central Path

One way to bias the search direction away from boundary is to arrange for all the complementarity pairs $x_j z_j$ to converge to zero at the same rate, say $x_j z_j = \tau$, where $\tau \to 0$ during the iterations. In other words, modify the last equation in (10.11.8) so that the system becomes

$$\mathbf{F}(\mathbf{v}) = \begin{bmatrix} \mathbf{A} \cdot \mathbf{x} - \mathbf{b} \\ \mathbf{A}^T \cdot \mathbf{y} + \mathbf{z} - \mathbf{c} \\ \mathbf{X} \cdot \mathbf{Z} \cdot \mathbf{e} \end{bmatrix} = \begin{bmatrix} 0 \\ 0 \\ \tau\mathbf{e} \end{bmatrix} \tag{10.11.11}$$

The set of solutions $\mathbf{v}(\tau)$ to equations (10.11.11) defines the *central path*. Primal-dual algorithms take steps toward points on the central path with $\tau > 0$. During the iterations, $\tau \to 0$ and the central path homes in on the optimal solution.

If you plot the central path in the hyperspace of $(\mathbf{x}, \mathbf{z})$ coordinates, it's some contorted line that doesn't look central to anything. However, if you plot it in coordinates $(x_1 z_1, x_2 z_2, \ldots)$, you see that it is equidistant from all the coordinate surfaces, with the optimal solution at the origin. When the current iterate is close to the central path, the next iteration can make a large step toward the optimal solution. When the current iterate is close to one of the boundaries, a good algorithm makes the next iteration get close to the central path again.

10.11.4 Path-Following Methods

Path-following methods don't just aim steps in the direction of the central path; they explicitly attempt to stay close to it. These methods are currently the most successful interior-point methods. In primal-dual methods, the duality gap (10.11.4), which is equal to $\mathbf{x} \cdot \mathbf{z}$ for feasible points, provides a figure-of-merit for how close one is to the optimal solution. Accordingly, we set

$$\mu = \mathbf{x} \cdot \mathbf{z}/n, \qquad \tau = \mu\delta, \quad \delta \in [0, 1] \tag{10.11.12}$$

The quantity δ is called the *centering parameter*, while μ is called the *duality measure*. If $\delta = 1$, the Newton step calculated from (10.11.11) is in a *centering direction*, toward a point at which each product $x_j z_j$ is equal to the average value μ defined in (10.11.12). On the other hand, the value $\delta = 0$ defines the Newton step for the original system (10.11.8). Good algorithms use intermediate values to trade off between improving centrality and reducing μ.

Methods that keep δ close to 1, so that unit steps ($\alpha = 1$) stay close to the central path, are called *short-step* methods. Methods that allow small values of δ are called *long-step* methods (less conservative choices of δ). There is an interesting gap between theory and practice between the methods. Short-step methods have been proved to converge in $O(\sqrt{n} \log \frac{1}{\epsilon})$ iterations, where ϵ is the desired tolerance. Long-step methods take $O(n \log \frac{1}{\epsilon})$ iterations, according to theory. Yet in practice short-step methods take hopelessly small steps, while long-step methods provide practical algorithms.

This is a somewhat academic discussion anyway. Real-life examples take many fewer than $O(\sqrt{n})$ iterations — a few dozen is typical for large problems.

10.11.5 Barrier Methods

Introducing a "penalty" function is a standard technique to enforce a constraint in general optimization problems. For example, to enforce the condition $\mathbf{x} \geq 0$, consider the logarithmic penalty function

$$\sum_{j=1}^{n} \log x_j \tag{10.11.13}$$

If any $x_j \to 0$, this function tends to $-\infty$. So instead of trying to minimize $\mathbf{c} \cdot \mathbf{x}$ in the standard primal problem (10.11.1), consider minimizing

$$\mathbf{c} \cdot \mathbf{x} - \tau \sum_{j=1}^{n} \log x_j \tag{10.11.14}$$

If one takes the limit $\tau \to 0$ after the minimization, we expect this to be equivalent to the original problem.

Equation (10.11.14) is called a *logarithmic barrier function*. It defines a family of nonlinear objective functions that gives the solution to the original problem as the parameter $\tau \to 0$.

The power of the barrier function idea is that it lets us handle the constraint $\mathbf{x} \geq 0$ with calculus. To minimize (10.11.14) subject to the constraint $\mathbf{A} \cdot \mathbf{x} = \mathbf{b}$, introduce a Lagrange multiplier $-\mathbf{y}$ and extremize the Lagrangian

$$L = \mathbf{c} \cdot \mathbf{x} - \tau \sum_{j=1}^{n} \log x_j - \mathbf{y} \cdot (\mathbf{A} \cdot \mathbf{x} - \mathbf{b}) \tag{10.11.15}$$

The optimality conditions $\nabla_x L = 0$ and $\nabla_y L = 0$ give

$$\begin{aligned} \mathbf{A} \cdot \mathbf{x} &= \mathbf{b} \\ \mathbf{A}^T \cdot \mathbf{y} + \tau \mathbf{X}^{-1} \cdot \mathbf{e} &= \mathbf{c} \end{aligned} \tag{10.11.16}$$

Define the vector

$$\mathbf{z} = \tau \mathbf{X}^{-1} \cdot \mathbf{e}, \quad \text{i.e.,} \quad z_j = \tau / x_j \tag{10.11.17}$$

Then equation (10.11.16) becomes

$$\begin{aligned} \mathbf{A} \cdot \mathbf{x} &= \mathbf{b} \\ \mathbf{A}^T \cdot \mathbf{y} + \mathbf{z} &= \mathbf{c} \\ \mathbf{X} \cdot \mathbf{Z} \cdot \mathbf{e} &= \tau \mathbf{e} \end{aligned} \tag{10.11.18}$$

These are exactly the equations (10.11.11) defining the central path, and they reduce to the KKT conditions (10.11.7) if we set τ to zero.

Note that equation (10.11.16) can be used to define an algorithm, the *primal interior-point method*, that doesn't depend on $\mathbf{z}$. Similarly, by starting with a logarithmic barrier function for the dual objective function, one can derive a purely dual method that doesn't involve $\mathbf{x}$. In practice, These methods are not competitive with the primal-dual methods.

Originally the logarithmic barrier function idea played an important role in motivating interior-point methods. More recently, the viewpoint has shifted to emphasize the importance of τ as defining the centering property of the algorithm rather than being simply a parameter to enforce the nonnegativity constraint.

10.11.6 A Primal-Dual Infeasible Interior-Point Algorithm

Let's pull all the pieces together now to define the algorithm. Write equation (10.11.11) for the new iterate:

$$\begin{aligned} \mathbf{A} \cdot (\mathbf{x} + \Delta\mathbf{x}) - \mathbf{b} &= 0 \\ \mathbf{A}^T \cdot (\mathbf{y} + \Delta\mathbf{y}) + \mathbf{z} + \Delta\mathbf{z} - \mathbf{c} &= 0 \\ (\mathbf{X} + \Delta\mathbf{X}) \cdot (\mathbf{Z} + \Delta\mathbf{Z}) \cdot \mathbf{e} &= \tau \mathbf{e} \end{aligned} \tag{10.11.19}$$

Drop the quadratic term $\Delta\mathbf{X} \cdot \Delta\mathbf{Z} \cdot \mathbf{e}$ and get

$$\begin{bmatrix} \mathbf{A} & \mathbf{0} & \mathbf{0} \\ \mathbf{0} & \mathbf{A}^T & \mathbf{1} \\ \mathbf{Z} & \mathbf{0} & \mathbf{X} \end{bmatrix} \begin{bmatrix} \Delta\mathbf{x} \\ \Delta\mathbf{y} \\ \Delta\mathbf{z} \end{bmatrix} = \begin{bmatrix} -\mathbf{r}_p \\ -\mathbf{r}_d \\ \tau\mathbf{e} - \mathbf{X} \cdot \mathbf{Z} \cdot \mathbf{e} \end{bmatrix} \tag{10.11.20}$$

where the primal and dual residuals are defined by

$$\begin{aligned} \mathbf{r}_p &= \mathbf{A} \cdot \mathbf{x} - \mathbf{b} \\ \mathbf{r}_d &= \mathbf{A}^T \cdot \mathbf{y} + \mathbf{z} - \mathbf{c} \end{aligned} \tag{10.11.21}$$

Equation (10.11.20) is simply the Newton equation (10.11.9) for (10.11.11). Note that the only nonlinearity comes from the innocuous looking quadratic term for complementary slackness. Yet it's exactly what leads to all the difficulty!

Since $\mathbf{X}$ is a diagonal positive-definite matrix, we can trivially invert it and use the last equation in (10.11.20) to eliminate $\Delta\mathbf{z}$ from the second equation. Interchanging the order of the variables $\Delta\mathbf{x}$ and $\Delta\mathbf{y}$, we get

$$\begin{bmatrix} \mathbf{0} & \mathbf{A} \\ \mathbf{A}^T & -\mathbf{X}^{-1}\cdot\mathbf{Z} \end{bmatrix} \begin{bmatrix} \Delta\mathbf{y} \\ \Delta\mathbf{x} \end{bmatrix} = \begin{bmatrix} -\mathbf{r}_p \\ \mathbf{z} - \tau\mathbf{X}^{-1}\cdot\mathbf{e} - \mathbf{r}_d \end{bmatrix} \tag{10.11.22}$$

Similarly, since $-\mathbf{X}^{-1}\cdot\mathbf{Z}$ is easily invertible, we can use the second equation in (10.11.22) to eliminate $\Delta\mathbf{x}$ from the first. This gives

$$\mathbf{A}\cdot(\mathbf{X}\cdot\mathbf{Z}^{-1})\cdot\mathbf{A}^T\cdot\Delta\mathbf{y} = -\mathbf{r}_p + \mathbf{A}\cdot(\mathbf{x} - \tau\mathbf{Z}^{-1}\cdot\mathbf{e} - \mathbf{X}\cdot\mathbf{Z}^{-1}\cdot\mathbf{r}_d) \tag{10.11.23}$$

These are called the *normal equations*, by analogy with the normal equations that occur in least-squares problems (cf. 15.4.10). The predecessor equations in (10.11.22) are called the *augmented equations*. Note that the matrix on the left-hand side of the normal equations is symmetric and positive-definite, except for some delicacy as $\mathbf{x}$ and $\mathbf{z} \to 0$. This suggests solving them with some version of the Cholesky decomposition (§2.9).

Once $\Delta\mathbf{y}$ is determined from the normal equations, the second equation in (10.11.20) gives $\Delta\mathbf{z}$:

$$\Delta\mathbf{z} = -\mathbf{A}^T\cdot\Delta\mathbf{y} - \mathbf{r}_d \tag{10.11.24}$$

Finally, the third equation in (10.11.20) gives $\Delta\mathbf{x}$:

$$\Delta\mathbf{x} = -\mathbf{X}\cdot\mathbf{Z}^{-1}\cdot\Delta\mathbf{z} + \tau\mathbf{Z}^{-1}\cdot\mathbf{e} - \mathbf{x} \tag{10.11.25}$$

In a feasible interior-point method, an initial point is somehow found in the feasible region, that is, with $\mathbf{r}_p = \mathbf{r}_d = 0$ and $(\mathbf{x},\mathbf{z}) > 0$. Then equations (10.11.23) – (10.11.25) are solved with $\mathbf{r}_p$ and $\mathbf{r}_d$ set to zero. The consensus now is that it is not necessary to do this. It is much easier to choose a point that may be infeasible initially, and allow the iterations to converge toward a feasible point. As explained above, it is still crucial to maintain nonnegativity, however.

Equation (10.11.23) contains three contributions to the step $\Delta\mathbf{y}$ and hence to $\Delta\mathbf{x}$ and $\Delta\mathbf{z}$. First there are the terms that involve $\mathbf{r}_p$ and $\mathbf{r}_d$, which drive the solution toward feasibility. Then there is the term independent of τ. It drives the solution toward optimality. In the literature, this term is called the *affine scaling* term, because there is a geometric interpretation of its effect in terms of a linear scaling of variables. Finally there is the term proportional to τ, which is the centering term.

Here is the framework for a simple primal-dual infeasible interior-point method:

1. Choose an initial nonnegative point.
2. If the infeasibilities $\mathbf{r}_p$ and $\mathbf{r}_d$ and the complementarity gap $\mathbf{x}\cdot\mathbf{z}$ are below the desired tolerance, exit. Otherwise, continue.
3. Set the value of τ from equation (10.11.12). A value of $\delta \approx 0.02$ works well.
4. Compute the direction of the step $(\Delta\mathbf{x}, \Delta\mathbf{y}, \Delta\mathbf{z})$ from equations (10.11.23) – (10.11.25). The solution of the normal equations is done in two steps: factorization of the matrix to some easily invertible form, followed by solution using this factorization.
5. Determine the maximum stepsizes that do not allow the variables to become negative. Separate stepsizes can be determined for the primal and dual variables:

$$\begin{aligned}\mathbf{x}_{\text{new}} &= \mathbf{x}_{\text{old}} + \alpha_p \Delta\mathbf{x} \\ \mathbf{y}_{\text{new}} &= \mathbf{y}_{\text{old}} + \alpha_d \Delta\mathbf{y} \\ \mathbf{z}_{\text{new}} &= \mathbf{z}_{\text{old}} + \alpha_d \Delta\mathbf{z}\end{aligned} \tag{10.11.26}$$

where α_p and α_d are initially chosen to be the largest values that keep all components of $\mathbf{x}_{\text{new}}$ and $\mathbf{z}_{\text{new}}$ nonnegative but no larger than unity. Then reduce the values of α_p and α_d by a safety factor σ. A conservative choice is $\sigma = 0.9$, but $\sigma = 0.99995$ works for many problems.

6. Go back to step 2 for the next iteration.

Since in real-life linear programming the constraint matrix $\mathbf{A}$ is sparse, the code must take advantage of this. The various matrix products such as $\mathbf{A}\cdot\mathbf{x}$, $\mathbf{A}^T\cdot\mathbf{y}$ and $\mathbf{A}\cdot(\mathbf{X}\cdot\mathbf{Z}^{-1})\cdot\mathbf{A}^T$ should be computed efficiently. More important, the factorization and backsubstitution involved in solving the normal equations must use a suitable sparse matrix Cholesky decomposition. The factorization step in fact dominates the running time of the algorithm. Our implementation uses the relatively simple package LDL [5], combined with the package AMD [6] to compute an ordering (permutation) of the matrix that minimizes fill-in during the factorization. Both of these packages are freely available. Note that LDL has to be modified to deal with the singularities that occur as the diagonal matrix elements $x_j/z_j \to 0$. It is sufficient to modify the line of code in LDL that tests for a diagonal element equal to zero to something like

```
if (D[k] < 1.0e-40)
    D[k] = 1.0e128;
```

This has the effect of setting the corresponding variable to zero, which is the desired behavior. Here is our interface `NRldl.h` to these packages. The full implementation is given in a Webnote [13].

interior.h

```
extern "C" {
    #include "ldl.h"
    #include "amd.h"
}

struct NRldl {
```
Interface between Numerical Recipes routine `intpt` and the required external packages LDL and AMD.
```
    Doub Info [AMD_INFO];
    Int lnz,n,nz;
    VecInt PP,PPinv,PPattern,LLnz,LLp,PParent,FFlag,*LLi;
    VecDoub YY,DD,*LLx;
    Doub *Ax, *Lx, *B, *D, *X, *Y;
    Int *Ai, *Ap, *Li, *Lp, *P, *Pinv, *Flag,*Pattern, *Lnz, *Parent;
    NRldl(NRsparseMat &adat);
```
Constructor only needs `adat` to have been declared with appropriate dimensions.
```
    void order();
```
AMD ordering and LDL symbolic factorization. Only needs nonzero pattern of `adat`, not actual values.
```
    void factorize();
```
Numerical factorization of matrix.
```
    void solve(VecDoub_O &y,VecDoub &rhs);
```
Solves for `y` given `rhs`. Can be invoked multiple times after a single call to `factorize`.
```
    ~NRldl();
};
```

Here is a simple implementation of the interior-point algorithm. Although it is a pedagogical code, it is actually quite powerful — better than the pedagogical

simplex code of the previous section. Below we will explain what would be required to turn this code into a serious implementation.

interior.h

```
Doub dotprod(VecDoub_I &x, VecDoub_I &y)
```
Compute the dot product of two vectors, $\mathbf{x} \cdot \mathbf{y}$.
```
{
    Doub sum=0.0;
    for (Int i=0;i<x.size();i++)
        sum += x[i]*y[i];
    return sum;
}
```

```
Int intpt(const NRsparseMat &a, VecDoub_I &b, VecDoub_I &c, VecDoub_O &x)
```
Interior-point method for linear programming. On input a contains the coefficient matrix for the constraints in the form $\mathbf{A} \cdot \mathbf{x} = \mathbf{b}$. The right-hand side of the constraints is input in b[0..m-1]. The coefficients of the objective function to be minimized, $\mathbf{c} \cdot \mathbf{x}$, are input in c[0..n-1]. Note that c should generally be padded with zeros corresponding to the slack variables that extend the number of columns to be n. The function returns 0 if an optimal solution is found; 1 if the problem is infeasible; 2 if the dual problem is infeasible, i.e., if the problem is unbounded or perhaps infeasible; and 3 if the number of iterations is exceeded. The solution is returned in x[0..n-1].
```
{
    const Int MAXITS=200;                   Maximum iterations.
    const Doub EPS=1.0e-6;                  Tolerance for optimality and feasibility.
    const Doub SIGMA=0.9;                   Stepsize reduction factor (conservative choice).
    const Doub DELTA=0.02;                  Factor to set centrality parameter μ.
    const Doub BIG=numeric_limits<Doub>::max();
    Int i,j,iter,status;
    Int m=a.nrows;
    Int n=a.ncols;
    VecDoub y(m),z(n),ax(m),aty(n),rp(m),rd(n),d(n),dx(n),dy(m),dz(n),
        rhs(m),tempm(m),tempn(n);
    NRsparseMat at=a.transpose();           Compute A^T.
    ADAT adat(a,at);                        Setup for A·D·A^T, where D = X·Z^-1.
    NRldl solver(adat.ref());               Initialize interface to LDL package.
    solver.order();                         AMD ordering and LDL symbolic factorization.
    Doub rpfact=1.0+sqrt(dotprod(b,b));     Compute factors for convergence test.
    Doub rdfact=1.0+sqrt(dotprod(c,c));
    for (j=0;j<n;j++) {                     Initial point.
        x[j]=1000.0;
        z[j]=1000.0;
    }
    for (i=0;i<m;i++) {
        y[i]=1000.0;
    }
    Doub normrp_old=BIG;
    Doub normrd_old=BIG;
    cout << setw(4) << "iter" << setw(12) << "Primal obj." << setw(9) <<
        "||r_p||" << setw(13) << "Dual obj." << setw(11) << "||r_d||" <<
        setw(13) << "duality gap" << setw(16) << "normalized gap" << endl;
    cout << scientific << setprecision(4);
    for (iter=0;iter<MAXITS;iter++) {       Start of main loop.
        ax=a.ax(x);                         Compute normalized residuals r_p and r_d.
        for (i=0;i<m;i++)
            rp[i]=ax[i]-b[i];
        Doub normrp=sqrt(dotprod(rp,rp))/rpfact;
        aty=at.ax(y);
        for (j=0;j<n;j++)
            rd[j]=aty[j]+z[j]-c[j];
        Doub normrd=sqrt(dotprod(rd,rd))/rdfact;
        Doub gamma=dotprod(x,z);            Duality gap is x·z for feasible points.
        Doub mu=DELTA*gamma/n;              Choice of μ.
        Doub primal_obj=dotprod(c,x);       Print current iteration.
```

```
        Doub dual_obj=dotprod(b,y);
        Doub gamma_norm=gamma/(1.0+abs(primal_obj));
         cout << setw(3) << iter << setw(12) << primal_obj << setw(12) <<
            normrp << setw(12) << dual_obj << setw(12) << normrd << setw(12)
            << gamma << setw(12) << gamma_norm<<endl;
        if (normrp < EPS && normrd < EPS && gamma_norm < EPS)
            return status=0;                Optimal solution found.
        if (normrp > 1000*normrp_old && normrp > EPS)
            return status=1;                Primal infeasible.
        if (normrd > 1000*normrd_old && normrd > EPS)
            return status=2;                Dual infeasible.
        for (j=0;j<n;j++)                   Compute step directions. First form matrix
            d[j]=x[j]/z[j];                     A · X · Z^-1 · A^T.
        adat.updateD(d);
        solver.factorize();                 Factorize matrix.
        for (j=0;j<n;j++)                   Form right-hand side.
            tempn[j]=x[j]-mu/z[j]-d[j]*rd[j];
        tempm=a.ax(tempn);
        for (i=0;i<m;i++)
            rhs[i]=-rp[i]+tempm[i];
        solver.solve(dy,rhs);               Solve for dy.
        tempn=at.ax(dy);                    Solve for dz.
        for (j=0;j<n;j++)
            dz[j]=-tempn[j]-rd[j];
        for (j=0;j<n;j++)                   Solve for dx.
            dx[j]=-d[j]*dz[j]+mu/z[j]-x[j];
        Doub alpha_p=1.0;                   Find step length.
        for (j=0;j<n;j++)
            if (x[j]+alpha_p*dx[j] < 0.0)
                alpha_p=-x[j]/dx[j];
        Doub alpha_d=1.0;
        for (j=0;j<n;j++)
            if (z[j]+alpha_d*dz[j] < 0.0)
                alpha_d=-z[j]/dz[j];
        alpha_p = MIN(alpha_p*SIGMA,1.0);
        alpha_d = MIN(alpha_d*SIGMA,1.0);
        for (j=0;j<n;j++) {                 Step to new point.
            x[j]+=alpha_p*dx[j];
            z[j]+=alpha_d*dz[j];
        }
        for (i=0;i<m;i++)
            y[i]+=alpha_d*dy[i];
        normrp_old=normrp;                  Update norms.
        normrd_old=normrd;
    }
    return status=3;                        Maximum iterations exceeded.
}
```

10.11.7 Practical Interior-Point Codes

There are a number of important features that would be needed to turn the above simple implementation into a state-of-the-art code.

- Initial point. Choosing a good starting point cuts down the number of iterations required. A good algorithm is described in [7].
- Preprocessing. As for the simplex method, preprocessing can often reduce the size of the problem.
- Scaling. A badly scaled problem can lead to numerical difficulties.
- Handling bounded variables. Suppose that instead of the requirement $\mathbf{x} \geq 0$ the variables are *bounded*:

$$\mathbf{l} \le \mathbf{x} \le \mathbf{u} \tag{10.11.27}$$

Here, for simplicity, we have written the vectors of lower and upper bounds, $\mathbf{l}$ and $\mathbf{u}$, as being of length n. In practice, only some of the variables $\mathbf{x}$ may have bounds. One way to deal with bounds is to add them to the system $\mathbf{A} \cdot \mathbf{x} = \mathbf{b}$ with slack variables in the usual way. However, this increases the dimension of the matrix $\mathbf{A}$. There is a simpler way to proceed. First, lower bounds of the form $l_j \le x_j$ can be handled by a simple shift: $x'_j = x_j - l_j \ge 0$. Making this replacement everywhere allows the problem to be solved as before, and then you simply undo the shift to get the solution in terms of the original x_j. So without loss of generality we can assume all the bounds are of the form

$$0 \le \mathbf{x} \le \mathbf{u} \tag{10.11.28}$$

If we introduce slack variables $\mathbf{s}$ and dual slack variables $\mathbf{w}$, the optimality conditions are

$$\begin{aligned} \mathbf{A} \cdot \mathbf{x} &= \mathbf{b} \\ \mathbf{x} + \mathbf{s} &= \mathbf{u} \\ \mathbf{A}^T \cdot \mathbf{y} + \mathbf{z} - \mathbf{w} &= \mathbf{c} \\ \mathbf{X} \cdot \mathbf{Z} \cdot \mathbf{e} &= 0 \\ \mathbf{S} \cdot \mathbf{W} \cdot \mathbf{e} &= 0 \end{aligned} \tag{10.11.29}$$

with $\mathbf{x}$, $\mathbf{s}$, $\mathbf{z}$, and $\mathbf{w}$ all nonnegative. It is simple to change the right-hand sides of the last two equations in (10.11.29) to $\tau \mathbf{e}$ and apply Newton's method as for equation (10.11.11). You find that the equations to be solved are very similar in form to equations (10.11.23) – (10.11.25).

- Predictor-corrector. Most of the time spent in an iteration goes into the factorization of the matrix in the normal equations. Given the factorization, the solve step is relatively cheap. Mehotra's predictor-corrector method [7] takes advantage of this by using an extra solve step per iteration to improve the overall efficiency of the algorithm.

  Recall that in going from equation (10.11.19) to equation (10.11.20) we dropped the term $\Delta\mathbf{X} \cdot \Delta\mathbf{Z} \cdot \mathbf{e}$. The idea in Mehotra's method is to first take a predictor step that solves equation (10.11.20), but with the τ term omitted. The values of $(\Delta\mathbf{x}, \Delta\mathbf{y}, \Delta\mathbf{z})$ obtained are used to estimate $\Delta\mathbf{X} \cdot \Delta\mathbf{Z} \cdot \mathbf{e}$. Then the corrector step solves for an additional set $(\Delta\mathbf{x}, \Delta\mathbf{y}, \Delta\mathbf{z})$ from equation (10.11.20) with the right-hand side replaced by

$$\begin{bmatrix} 0 \\ 0 \\ \tau\mathbf{e} - \Delta\mathbf{X} \cdot \Delta\mathbf{Z} \cdot \mathbf{e} \end{bmatrix} \tag{10.11.30}$$

The value of τ in equation (10.11.30) is set differently from equation (10.11.12). First $\hat{\mu}$ is computed using the predictor step:

$$\hat{\mu} = (\mathbf{x} + \alpha_p \Delta\mathbf{x}) \cdot (\mathbf{z} + \alpha_d \Delta\mathbf{z})/n \tag{10.11.31}$$

Here α_p and α_d are the largest values that maintain nonnegativity, but no larger than unity. (No safety factor is used.) Then τ is set as

$$\tau = \left(\frac{\hat{\mu}}{\mu}\right)^2 \hat{\mu} \tag{10.11.32}$$

where μ is computed using the starting values of $\mathbf{x}$ and $\mathbf{z}$ as in equation (10.11.12). This heuristic choice makes τ small when the predictor step gives a large decrease in complementarity, and large otherwise.

The total step is the sum of the predictor and corrector steps. The cutdown factor from α_p or α_d equal to unity is calculated from a heuristic procedure described in [7] or [8].

Gondzio [9,10] has developed an extension to the predictor-corrector algorithm that incorporates higher-order corrections when they can improve the efficiency.

- Better sparse matrix algebra. While AMD is a good general-purpose choice for an ordering algorithm, LDL is a good but basic sparse Cholesky routine, chosen mainly for its simplicity and availability. More powerful algorithms are known and are starting to become publicly available.

  One of the problems with the normal equations is that the matrix can be quite dense, even when $\mathbf{A}$ itself is rather sparse. This has motivated algorithms that solve the augmented equations (10.11.22) directly. On some problems, this leads to significant savings. A good implementation will provide both alternatives.

  Solving the equations can become numerically delicate, especially as the optimal point is approached. Good implementations will use some form of iterative refinement to preserve accuracy.

- Crossover to the simplex method. Often the convergence of the interior-point algorithm slows down near the optimal point. By switching to a simplex method with a basis that is presumably close to optimal, one can get rapid convergence to the answer. This feature has the additional benefit that the optimal point is given in terms of basis vectors, whereas the interior-point solution never actually attains zeros for any $\mathbf{x}$'s. Some kinds of post-analysis need the actual basis.

  Interestingly, using a previously-found solution as the initial point for a "nearby" problem seldom helps much for interior-point methods. The reason is that interior-point methods don't make good progress from a point near the boundary. The simplex method, by contrast, generally converges much more rapidly with a "warm start." A good strategy for solving a sequence of closely related problems is therefore interior-point with crossover to an optimal basis for the first one, then simplex with a warm start for the remainder.

There are several codes that are free for noncommercial use and that give full implementations of interior-point methods. We particularly like PCx (in C with Fortran sparse algebra routines) [11] and HOPDM (in Fortran) [10]. For a discussion of more options, including commercial codes, see [12].

So which should you use: a simplex or an interior-point code? If you have only our codes, use the interior-point one. If you have a production implementation of either algorithm, it will probably suffice. If you are solving many large problems, however, you should have both so you can use the optimum algorithm in each case. If you are solving a large problem for the first time, there is a lot to be said for using an interior-point code. There are fewer choices to make to get almost optimal performance. By contrast, finding which particular choices of components in a simplex method give optimal performance can involve a lot of experimentation. Your first try will usually not be as good as a default interior-point code. And often the

interior-point code will beat all simplex variants.

CITED REFERENCES AND FURTHER READING:

Khachian, L. 1979, *Doklady Academiia Nauk SSSR*, vol. 244, pp. 191–194. English translation: "A Polynomial Time Algorithm in Linear Programming," *Soviet Mathematics Doklady*, vol. 20, pp. 191–194.[1]

Karmarkar, N. 1984, "A New Polynomial-time Algorithm for Linear Programming," *Combinatorica* vol. 4, pp. 373–395.[2]

Maros, I. 2003, *Computational Techniques of the Simplex Method* (Boston: Kluwer).[3]

Nazareth, J.L. 2004, *An Optimization Primer* (New York: Springer).[4]

LDL, `http://www.cise.ufl.edu/research/sparse`.[5]

AMD, `http://www.cise.ufl.edu/research/sparse`. See also Amestoy, P.R., Enseeiht-Irit, Davis, T.A., and Duff, I.S. 2004, "AMD, an Approximate Minimum Degree Ordering Algorithm." *ACM Transactions on Mathematical Software* vol. 30, pp. 381–388.[6]

Mehrotra, S. 1992, "On the Implementation of a Primal-dual Interior Point Method," *SIAM Journal on Optimization* vol. 2, pp. 575–601.[7]

Wright, S.J. 1997, *Primal-Dual Interior-Point Methods* (Philadelphia: S.I.A.M.).[8]

Gondzio, J. 1996, "Multiple Centrality Corrections in a Primal-dual Method for Linear Programming," *Computational Optimization and Applications* vol. 6, pp. 137–156.[9]

HOPDM, `http://www.maths.ed.ac.uk/~gondzio/software/hopdm.html`.[10]

PCX, `http://www-fp.mcs.anl.gov/otc/Tools/PCx`.[11]

Linear Programming Frequently Asked Questions, `http://www-unix.mcs.anl.gov/otc/Guide/faq/linear-programming-faq.html`.[12]

Vanderbei, R.J. 2001, *Linear Programming: Foundations and Extensions*, 2nd ed. (Boston: Kluwer). [Undergraduate/graduate text]

Numerical Recipes Software 2007, "Interface to AMD and LDL Packages," *Numerical Recipes Webnote No. 13*, at `http://www.nr.com/webnotes?13` [13]

10.12 Simulated Annealing Methods

The *method of simulated annealing* [1,2] is a technique that has attracted significant attention as suitable for optimization problems of large scale, especially ones where a desired global extremum is hidden among many poorer, local extrema. For practical purposes, simulated annealing has effectively "solved" the famous *traveling salesman problem* of finding the shortest cyclical itinerary for a traveling salesman who must visit each of N cities in turn. (Other practical methods have also been found.) The method has also been used successfully for designing complex integrated circuits: The arrangement of several hundred thousand circuit elements on a tiny silicon substrate is optimized so as to minimize interference among their connecting wires [3,4]. Surprisingly, the implementation of the algorithm is relatively simple.

Notice that the two applications cited are both examples of *combinatorial minimization*. There is an objective function to be minimized, as usual, but the space over which that function is defined is not simply the N-dimensional space of N continuously variable parameters. Rather, it is a discrete, but very large, configuration space, like the set of possible orders of cities, or the set of possible allocations of silicon

"real estate" blocks to circuit elements. The number of elements in the configuration space is factorially large, so that they cannot be explored exhaustively. Furthermore, since the set is discrete, we are deprived of any notion of "continuing downhill in a favorable direction." The concept of "direction" may not have any meaning in the configuration space.

Below, we will also discuss how to use simulated annealing methods for spaces with continuous control parameters, like those of §10.5 – §10.9. This application is actually more complicated than the combinatorial one, since the familiar problem of "long, narrow valleys" again asserts itself. Simulated annealing, as we will see, tries "random" steps; but in a long, narrow valley, almost all random steps are uphill! Some additional finesse is therefore required.

At the heart of the method of simulated annealing is an analogy with thermodynamics, specifically with the way that liquids freeze and crystallize or metals cool and anneal. At high temperatures, the molecules of a liquid move freely with respect to one another. If the liquid is cooled slowly, thermal mobility is lost. The atoms are often able to line themselves up and form a pure crystal that is completely ordered over a distance up to billions of times the size of an individual atom in all directions. This crystal is the state of minimum energy for this system. The amazing fact is that, for slowly cooled systems, nature is able to find this minimum energy state. In fact, if a liquid metal is cooled quickly or "quenched," it does not reach this state but rather ends up in a polycrystalline or amorphous state having somewhat higher energy.

So the essence of the process is *slow* cooling, allowing ample time for the redistribution of the atoms as they lose mobility. This is the technical definition of *annealing*, and it is essential for ensuring that a low energy state will be achieved.

Although the analogy is not perfect, there is a sense in which all of the minimization algorithms thus far in this chapter correspond to rapid cooling or quenching. In all cases, we have gone greedily for the quick, nearby solution: From the starting point, go immediately downhill as far as you can go. This, as often remarked above, leads to a local, but not necessarily a global, minimum. Nature's own minimization algorithm is based on quite a different procedure. The so-called Boltzmann probability distribution,

$$\text{Prob}\,(E) \sim \exp(-E/kT) \tag{10.12.1}$$

expresses the idea that a system in thermal equilibrium at temperature T has its energy probabilistically distributed among all different energy states E. Even at low temperature, there is a chance, albeit a very small one, of a system being in a high energy state. Therefore, there is a corresponding chance for the system to get out of a local energy minimum in favor of finding a better, more global one. The quantity k (Boltzmann's constant) is a constant of nature that relates temperature to energy. In other words, the system sometimes goes *uphill* as well as downhill; but the lower the temperature, the less likely is any significant uphill excursion.

In 1953, Metropolis and coworkers [5] first incorporated these kinds of principles into numerical calculations. Offered a succession of options, a simulated thermodynamic system was assumed to change its configuration from energy E_1 to energy E_2 with probability $p = \exp[-(E_2 - E_1)/kT]$. Notice that if $E_2 < E_1$, this probability is greater than unity; in such cases the change is arbitrarily assigned a probability $p = 1$, i.e., the system *always* took such an option. This general scheme, of always taking a downhill step while *sometimes* taking an uphill step, has come to be known as the Metropolis algorithm.

To make use of the Metropolis algorithm for other than thermodynamic systems, one must provide the following elements:

1. A description of possible system configurations.
2. A generator of random changes in the configuration; these changes are the "options" presented to the system.
3. An objective function E (analog of energy) whose minimization is the goal of the procedure.
4. A control parameter T (analog of temperature) and an *annealing schedule* that tells how it is lowered from high to low values, e.g., after how many random changes in configuration is each downward step in T taken, and how large is that step. The meaning of "high" and "low" in this context, and the assignment of a schedule, may require physical insight and/or trial-and-error experiments.

We will return to these ideas in §15.8, with a more rigorous discussion of *Markov chain Monte Carlo* and the *Metropolis-Hastings algorithm*.

10.12.1 Combinatorial Minimization: The Traveling Salesman

A concrete illustration is provided by the traveling salesman problem. The proverbial seller visits N cities with given positions (x_i, y_i), returning finally to his or her city of origin. Each city is to be visited only once, and the route is to be made as short as possible. This problem belongs to a class known as *NP-complete* problems, whose computation time for an *exact* solution increases with N as exp(const. $\times N$), becoming rapidly prohibitive in cost as N increases. The traveling salesman problem also belongs to a class of minimization problems for which the objective function E has many local minima. In practical cases, it is often enough to be able to choose from these a minimum that, even if not absolute, cannot be significantly improved upon. The annealing method manages to achieve this, while limiting its calculations to scale as a small power of N.

As a problem in simulated annealing, the traveling salesman problem is handled as follows:

1. *Configuration.* The cities are numbered $i = 0 \ldots N-1$ and each has coordinates (x_i, y_i). A configuration is a permutation of the number $0 \ldots N-1$, interpreted as the order in which the cities are visited.
2. *Rearrangements.* An efficient set of moves has been suggested by Lin [6]. The moves consist of two types: (i) A section of path is removed and then replaced with the same cities running in the opposite order; or (ii) a section of path is removed and then replaced in between two cities on another, randomly chosen, part of the path.
3. *Objective function.* In the simplest form of the problem, E is taken just as the total length of the journey,

$$E = L \equiv \sum_{i=0}^{N-1} \sqrt{(x_i - x_{i+1})^2 + (y_i - y_{i+1})^2} \tag{10.12.2}$$

with the convention that point N is identified with point 0. To illustrate the flexibility of the method, however, we can add the following additional wrinkle: Suppose that the salesman has an irrational fear of flying over the Missis-

sippi River. In that case, we would assign each city a parameter μ_i, equal to $+1$ if it is east of the Mississippi and -1 if it is west, and take the objective function to be

$$E = \sum_{i=0}^{N-1} \left[\sqrt{(x_i - x_{i+1})^2 + (y_i - y_{i+1})^2} + \lambda(\mu_i - \mu_{i+1})^2 \right] \qquad (10.12.3)$$

A penalty 4λ is thereby assigned to any river crossing. The algorithm now finds the shortest path that avoids crossings. The relative importance that it assigns to length of path versus river crossings is determined by our choice of λ. Figure 10.12.1 shows the results obtained. Clearly, this technique can be generalized to include many conflicting goals in the minimization.

4. *Annealing schedule.* This requires experimentation. We first generate some random rearrangements, and use them to determine the range of values of ΔE that will be encountered from move to move. Choosing a starting value for the parameter T that is considerably larger than the largest ΔE normally encountered, we proceed downward in multiplicative steps each amounting to a 10% decrease in T. We hold each new value of T constant for, say, $100N$ reconfigurations, or for $10N$ successful reconfigurations, whichever comes first. When efforts to reduce E further become sufficiently discouraging, we stop.

In a Webnote [7], we give a complete implementation of the above ideas for the traveling salesman problem, using the Metropolis algorithm.

10.12.2 Continuous Minimization by Simulated Annealing

The basic ideas of simulated annealing are also applicable to optimization problems with continuous N-dimensional control spaces, e.g., finding the (ideally, global) minimum of some function $f(\mathbf{x})$, in the presence of many local minima, where $\mathbf{x}$ is an N-dimensional vector. The four elements required by the Metropolis procedure are now as follows: The value of f is the objective function. The system state is the point $\mathbf{x}$. The control parameter T is, as before, something like a temperature, with an annealing schedule by which it is gradually reduced. And there must be a generator of random changes in the configuration, that is, a procedure for taking a random step from $\mathbf{x}$ to $\mathbf{x} + \Delta\mathbf{x}$.

The last of these elements is the most problematical. The literature to date [8-12] describes several different schemes for choosing $\Delta\mathbf{x}$, none of which, in our view, inspires complete confidence. The problem is one of efficiency: A generator of random changes is inefficient if, *when local downhill moves exist*, it nevertheless almost always proposes an uphill move. A good generator, we think, should not become inefficient in narrow valleys, nor should it become more and more inefficient as convergence to a minimum is approached. Except possibly for [8], all of the schemes that we have seen are inefficient in one or both of these situations.

Our own way of doing simulated annealing minimization on continuous control spaces is to use a modification of the downhill simplex method (§10.5). Complete code for this is given in a Webnote [9]. The technique amounts to replacing the single point $\mathbf{x}$ as a description of the system state by a simplex of $N + 1$ points. The "moves" are the same as described in §10.5, namely reflections, expansions, and contractions of the simplex. The implementation of the Metropolis procedure is slightly subtle: We *add* a positive, logarithmically distributed random variable, proportional

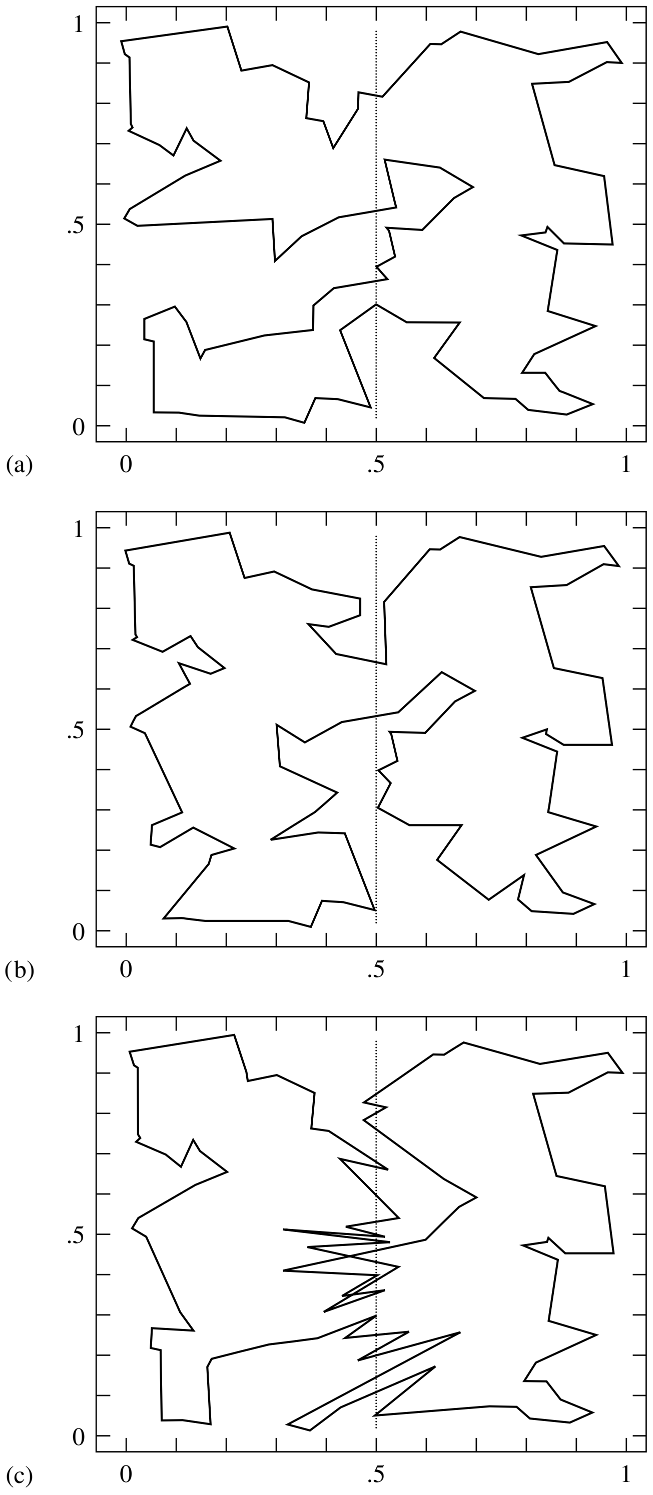

Figure 10.12.1. Traveling salesman problem solved by simulated annealing. The (nearly) shortest path among 100 randomly positioned cities is shown in (a). The dotted line is a river, but there is no penalty in crossing. In (b) the river-crossing penalty is made large, and the solution restricts itself to the minimum number of crossings, two. In (c) the penalty has been made negative: The salesman is actually a smuggler who crosses the river on the flimsiest excuse!

to the temperature T, to the stored function value associated with every vertex of the simplex, and we *subtract* a similar random variable from the function value of every new point that is tried as a replacement point. Like the ordinary Metropolis procedure, this method always accepts a true downhill step, but sometimes accepts an uphill one. In the limit $T \to 0$, this algorithm reduces exactly to the downhill simplex method and converges to a local minimum.

At a finite value of T, the simplex expands to a scale that approximates the size of the region that can be reached at this temperature, and then executes a stochastic, tumbling Brownian motion within that region, sampling new, approximately random, points as it does so. The efficiency with which a region is explored is independent of its narrowness (for an ellipsoidal valley, the ratio of its principal axes) and orientation. If the temperature is reduced sufficiently slowly, it becomes highly likely that the simplex will shrink into that region containing the lowest relative minimum encountered.

As in all applications of simulated annealing, there can be quite a lot of problem-dependent subtlety in the phrase "sufficiently slowly"; success or failure is quite often determined by the choice of annealing schedule. Here are some possibilities worth trying:

- Reduce T to $(1-\epsilon)T$ after every m moves, where ϵ/m is determined by experiment.
- Budget a total of K moves, and reduce T after every m moves to a value $T = T_0(1-k/K)^\alpha$, where k is the cumulative number of moves thus far, and α is a constant, say 1, 2, or 4. The optimal value for α depends on the statistical distribution of relative minima of various depths. Larger values of α spend more iterations at lower temperature.
- After every m moves, set T to β times $f_1 - f_b$, where β is an experimentally determined constant of order 1, f_1 is the smallest function value currently represented in the simplex, and f_b is the best function ever encountered. However, never reduce T by more than some fraction γ at a time.

Another strategic question is whether to do an occasional *restart*, where a vertex of the simplex is discarded in favor of the "best-ever" point. (You must be sure that the best-ever point is not one of the other vertices of the simplex when you do this!) We have found problems for which restarts — every time the temperature has decreased by a factor of 3, say — are highly beneficial; we have found other problems for which restarts have no positive, or a somewhat negative, effect.

There is not yet enough practical experience with the method of simulated annealing to say definitively what its place among optimization methods is. The method has several extremely attractive features that are rather unique when compared with other optimization techniques.

First, it is not "greedy," in the sense that it is not easily fooled by the quick payoff achieved by falling into unfavorable local minima. Provided that sufficiently general reconfigurations are given, it wanders freely among local minima of depth less than about T. As T is lowered, the number of such minima qualifying for frequent visits is gradually reduced.

Second, configuration decisions tend to proceed in a logical order. Changes that cause the greatest energy differences are sifted over when the control parameter T is large. These decisions become more permanent as T is lowered, and attention

then shifts more to smaller refinements in the solution. For example, in the traveling salesman problem with the Mississippi River twist, if λ is large, a decision to cross the Mississippi only twice is made at high T, while the specific routes on each side of the river are determined only at later stages.

The analogies to thermodynamics may be pursued to a greater extent than we have done here. Quantities analogous to specific heat and entropy may be defined, and these can be useful in monitoring the progress of the algorithm toward an acceptable solution [1].

CITED REFERENCES AND FURTHER READING:

Salamon, P., Sibani, P., and Frost, R. 2002, *Facts, Conjectures, and Improvements for Simulated Annealing* (New York: SIAM Press).

van Laarhoven, P.J.M., and Aarts, E.H.L. 1987, *Simulated Annealing: Theory and Applications* (Berlin: Springer).

Kirkpatrick, S., Gelatt, C.D., and Vecchi, M.P. 1983, "Optimization by Simulated Annealing," *Science*, vol. 220, pp. 671–680.[1]

Kirkpatrick, S. 1984, "Optimization by Simulated Annealing: Quantitative Studies," *Journal of Statistical Physics*, vol. 34, pp. 975–986.[2]

Vecchi, M.P. and Kirkpatrick, S. 1983, "Global Wiring by Simulated Annealing," *IEEE Transactions on Computer Aided Design*, vol. CAD-2, pp. 215–222.[3]

Otten, R.H.J.M., and van Ginneken, L.P.P.P. 1989, *The Annealing Algorithm* (Boston: Kluwer) [contains many references to the literature].[4]

Metropolis, N., Rosenbluth, A., Rosenbluth, M., Teller A., and Teller, E. 1953, "Equations of State Calculations by Fast Computing Machines," *Journal of Chemical Physics*, vol. 21, pp. 1087–1092.[5]

Lin, S. 1965, "Computer Solutions of the Traveling Salesman Problem," *Bell System Technical Journal*, vol. 44, pp. 2245–2269.[6]

Numerical Recipes Software 2007, "Code Implementation for the Traveling Salesman Problem," *Numerical Recipes Webnote No. 14*, at `http://www.nr.com/webnotes?14` [7]

Vanderbilt, D., and Louie, S.G. 1984, "A Monte Carlo Simulated Annealing Approach to Optimization over Continuous Variables," *Journal of Computational Physics*, vol. 56, pp. 259–271.[8]

Numerical Recipes Software 2007, "Code for Minimization with Simulated Annealing," *Numerical Recipes Webnote No. 15*, at `http://www.nr.com/webnotes?15` [9]

Bohachevsky, I.O., Johnson, M.E., and Stein, M.L. 1986, "Generalized Simulated Annealing for Function Optimization," *Technometrics*, vol. 28, pp. 209–217.[10]

Corana, A., Marchesi, M., Martini, C., and Ridella, S. 1987, "Minimizing Multimodal Functions of Continuous Variables with the Simulated Annealing Algorithm," *ACM Transactions on Mathematical Software*, vol. 13, pp. 262–280.[11]

Bélisle, C.J.P., Romeijn, H.E., and Smith, R.L. 1990, "Hide and Seek: A Simulated Annealing Algorithm for Global Optimization," Technical Report 90–25, Department of Industrial and Operations Engineering, University of Michigan.[12]

Christofides, N., Mingozzi, A., Toth, P., and Sandi, C. (eds.) 1979, *Combinatorial Optimization* (London and New York: Wiley-Interscience) [not simulated annealing, but other topics and algorithms].

10.13 Dynamic Programming

Dynamic programming, or *DP*, is an optimization technique that applies when a known sequence of choices, each with a cost or benefit, is to be made and one wants

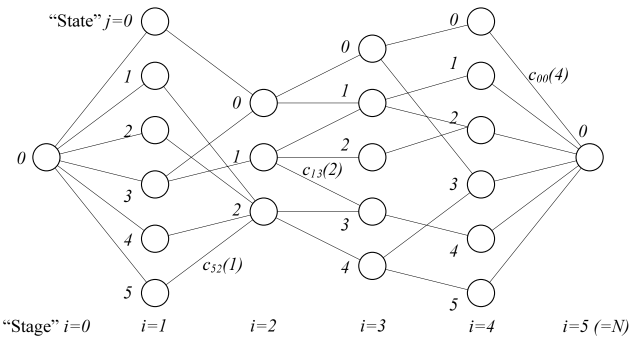

Figure 10.13.1. Canonical dynamical programming problem. It is desired to find the lowest cost path from a starting state to an ending state through $N-1$ intermediate *stages*. Each stage is characterized by a set of *states* (not necessarily the same at each stage). An allowed edge between state j in stage i and state k in stage $i+1$ has a cost denoted $c_{jk}(i)$ (not all labeled in the figure).

to minimize the total cost, or maximize the total benefit, after the sequence has been traversed. More specifically, a problem that is amenable to dynamic programming can be broken up into an ordered series of discrete *stages*, and within each stage a set of discrete *states*. These stages and states form a directed graph (see Figure 10.13.1) that we want to traverse from a given starting state ($i = 0$) to a given ending state ($i = N$). Allowed decisions that take one from state j in stage i to state k in stage $i+1$ are edges in the graph. Their cost is denoted $c_{jk}(i)$. Without any loss of generality, one can connect all the states in stage i to all the states in stage $i+1$, but with $c_{jk}(i) = \infty$ for forbidden paths.

Computer science is rich in graph-theoretic problems and algorithms, but only a few of these are within the scope of this book. Dynamic programming is one of these because its basic idea is very simple and its applications are very broad. It is important that you be able to recognize a problem amenable to DP when you see one. In particular, we will use several of the concepts in this section later, in §16.2, when we discuss the estimation of states from probabilistic data, including probabilistic decoding algorithms.

The key idea of dynamic programming is called the Bellman, Dijkstra, or Viterbi algorithm, depending on the field of training of the caller. As shown in Figure 10.13.2, the idea is that one can do a single sweep of a stage-ordered graph from left to right, labeling each vertex by the single number that is the cost of the *best* way of having reached it. (Henceforth we'll take the canonical DP problem as a cost-minimization problem; if your problem is instead a benefit-maximization problem, just use the negative of your benefits as costs.)

When the end state is reached, the global minimum cost of getting to it becomes known. Now, in a single backward pass, we can read off exactly what set of decisions led to this global minimum, by reconstructing which predecessor state was the one actually in the chain that led to the best result. Arriving back at the starting state, our solution is complete.

The art of DP involves, in many cases, the clever organization of the problem to minimize the number of states at each stage, so as to avoid the "curse of dimensionality" (a phrase first used by Bellman in exactly this context). Sometimes the order of the stages is not chronological at all, but merely reflects the decomposition of a

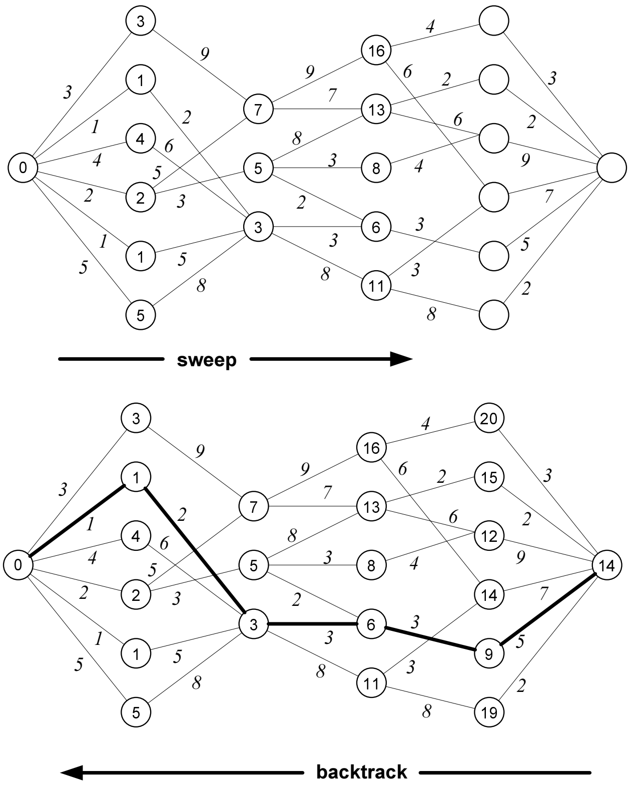

Figure 10.13.2. Two snapshots during the solution of a DP problem by the Bellman-Dijkstra-Viterbi algorithm. Edge costs are given as shown. Top: During the rightward sweep (here not yet complete) each state is labeled by the minimum cost to reach it, as determined solely by the labels of the preceding stage and the connecting edge costs. Bottom: After the rightward sweep is complete, the unique set of edges that produce the global minimum is found by one pass of backtracking.

problem into a convenient form for DP.

Here is a function embodying the Bellman-Dijkstra-Viterbi algorithm. You should consider this function more a precise statement of the algorithm than a production DP code. For example, it simply pushes off to the user function `cost()` the important issue of how to retrieve efficiently the (usually) sparse set of allowed edges that have finite costs. (You might want to consider a hash memory. See §7.6.) Also, this routine loops explicitly over all combinations of states j and k, the origin and destination states in going from stage i to $i+1$. If you have a problem big enough to need some kind of sparse lookup, then you'll want to change these explicit loops accordingly.

dynpro.h

```
VecInt dynpro(const VecInt &nstate,
    Doub cost(Int jj, Int kk, Int ii)) {
    Given the vector nstate whose integer values are the number of states in each stage (1
    for the first and last stages), and given a function cost(j,k,i) that returns the cost of
    moving between state j of stage i and state k of stage i+1, this routine returns a vector
    of the same length as nstate containing the state numbers of the lowest cost path. States
    number from 0, and the first and last components of the returned vector will thus always
    be 0.
    const Doub BIG = 1.e99;
```

```
    static const Doub EPS=numeric_limits<Doub>::epsilon();
    Int i, j ,k, nstage = nstate.size() - 1;
    Doub a,b;
    VecInt answer(nstage+1);
    if (nstate[0] != 1 || nstate[nstage] != 1)
        throw("One state allowed in first and last stages.");
    Doub **best = new Doub*[nstage+1];          Allocate array-of-arrays for storing scores.
    best[0] = new Doub[nstate[0]];
    best[0][0] = 0.;
    for (i=1; i<=nstage; i++) {                 Forward sweep through stages.
        best[i] = new Doub[nstate[i]];
        for (k=0; k<nstate[i]; k++) {
            b = BIG;
            for (j=0; j<nstate[i-1]; j++) {     Find predecessor giving min cost.
                if ((a = best[i-1][j] + cost(j,k,i-1)) < b) b = a;
            }
            best[i][k] = b;
        }
    }
    answer[nstage] = answer[0] = 0;
    for (i=nstage-1; i>0; i--) {                Backtracking pass.
        k = answer[i+1];
        b = best[i+1][k];
        for (j=0; j<nstate[i]; j++) {           Find a predecessor that gave min.
            Doub temp = best[i][j] + cost(j,k,i);
            if (fabs(b - temp) <= EPS*fabs(temp)) break;
        }
        answer[i] = j;
    }
    for (i=nstage; i>=0; i--) delete [] best[i];    Cleanup storage.
    delete [] best;
    return answer;
}
```

10.13.1 Example: Order of Matrix Multiplication

Suppose we have five matrices to multiply, so as to get a result $\mathbf{T}$,

$$\mathbf{T} = \mathbf{ABCDE} \tag{10.13.1}$$

The matrices may all have different shapes, as long as the number of columns of a matrix is the same as the number of rows of the matrix immediately to its right. Matrix multiplication is associative, and we can do the multiplies in any order we want; but the total number of scalar multiplications can be quite different, depending on which order is chosen. You should be able to see this in the following figure:

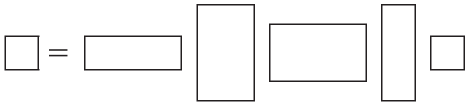

What we want to minimize is the total number of scalar multiplications. In this example, a good choice of "stage" is just how many matrix multiplications have been performed. So the stages and states might look something like this:

Stage 0	Stage 1	Stage 2	Stage 3	Stage 4
**ABCDE**	**(AB)CDE**	**(ABC)DE**	**(ABCD)E**	**(ABCDE)**
	**A(BC)DE**	**(AB)(CD)E**	**(ABC)(DE)**	
	**AB(CD)E**	**(AB)C(DE)**	**(AB)(CDE)**	
	**ABC(DE)**	**A(BCD)E**	**A(BCDE)**	
		**A(BC)(DE)**		
		**AB(CDE)**		

Here parentheses group matrix factors that are already fully multiplied (i.e., they are, by now, a single matrix). We will leave it to you to connect the states by allowed edges, and to calculate the cost of each edge in terms of the dimensions of the various matrices.

So how could we have done this example *wrong*? We might have identified states with all possible ways of parenthesizing **ABCDE**, including, for example, **A(B(CD)E)**. That is unnecessary, because only the outermost parentheses matter: A matrix state doesn't care about the exact path taken to reach it, as long as its factors were multiplied in some associative order. The power of DP is realized when, at every stage, many histories collapse to a (relatively) small number of states, which can then be taken as ignorant of their past history.

10.13.2 Example: DNA Sequence Alignment

DNA sequences of different organisms, at one time identical in a common ancestor, can diverge over time by the deletion, insertion, or substitution of bases in one or the other organism's sequence. It is desired to find the best match between two given sequences. In finding the best match, we are allowed to insert gaps in either sequence; but in the end we will be assessed a penalty for gap positions, a penalty for mismatches, and a reward for matches.

For example [2], before matching, we might have the two sequences

G	A	A	T	T	C	A	G	T	T	A
G	G	A	T	C	G	A				

A possible match might be

G	*A*	A	T	T	C	A	G	T	T	A
G	*G*	A		T	C		G			A

for which we would earn six rewards, less one mismatch penalty (shown italic) and four gap penalties. (We will consider all rewards and penalties as having positive or zero values, with the accounting done by *adding* rewards and *subtracting* penalties.)

Needleman and Wunsch [1] first pointed out that this problem is amenable to solution by DP, allowing *all possible* matchings to be scored, and the highest-scoring one identified. The clever idea is to form a two-dimensional array with the two sequences defining the columns and rows. In the above example, this looks like

		G	A	A	T	T	C	A	G	T	T	A
	0											
G												
G												
A												
T												
C												
G												
A												

A matching consists of a path through the (initially) empty boxes in the above tableau, starting in the box labeled by a zero and moving, in each step, either one box right, one box down, or diagonally down and right. A right or down move corresponds to using up a letter in the first or second sequence (respectively) without using up one in the second or first (respective) sequence. Therefore it corresponds to inserting a gap and incurs a gap penalty. A diagonal move corresponds to pairing a new character in each sequence. Therefore, it incurs either the matching reward, if the two sequences match, or a mismatch penalty, if they don't.

Also useful is to distinguish right or down moves between two boxes in the bordering rows or columns (respectively) of the table from right or down moves in the interior of the table. The former kind don't open gaps in either sequence, but merely allow the sequences to shift with respect to one another. So we might assess a smaller, or zero, penalty for these overall "skews."

Now, characteristic of dynamic programming, you just fill in the boxes with the total score of the *best* way to reach that box, either from above it, from its left, or from the upper-left diagonal neighbor. A score is computed, naturally, by taking the score of a predecessor box and then adding the reward (or subtracting the penalty) associated with the move. Starting at the upper left, there are always boxes ready to be filled in as you work your way, by rows or columns, to the lower right.

Also characteristic of dynamic programming, when you are done filling in all the boxes, you do a backtrack pass: Start at the lower right box in the table. Now figure out the path back through the table that contributed to that best score. (It may not be unique.) Finally, translate that path into the series of letters and gaps that it implies.

A straightforward implementation of this is the following routine. Note that the matching reward is normalized to one per matched character, while the penalties for mismatches, gaps, and overall skews are input arguments. You can set all three to zero in most cases. If you set a nonzero mismatch penalty, however, you will probably also want to have a gap penalty, since otherwise the program will always avoid a mismatch by creating two gaps, one in each string.

The routine's output includes a summary string showing where the matches, mismatches, and gaps occur. For the example above, the summary string is

```
=!= == = =
```

(You can change the symbols used to your liking.)

You can modify the program in various other ways. For example, you might want to have a bigger penalty for initially opening a gap than for extending it once

opened. This requires a more complicated logic in the initial filling of the cost table. As long as you are able to fill each box with the cost of the *best* way to reach it, then the logic of dynamic programming will still apply.

stringalign.h

```
void stringalign(char *ain, char *bin, Doub mispen, Doub gappen,
    Doub skwpen, char *aout, char *bout, char *summary) {
```
Given null terminated input strings ain and bin, and given penalties mispen, gappen, and skwpen, respectively, for mismatches, interior gaps, and gaps before/after either string, set null terminated output strings aout, bout, and summary as the aligned versions of the input strings, and a summary string. User must supply storage for the output strings of size equal to the sum of the two input strings.
```
    Int i,j,k;
    Doub dn,rt,dg;                          Cost of down, right, and diagonal moves.
    Int ia = strlen(ain), ib = strlen(bin);
    MatDoub cost(ia+1,ib+1);                Cost table, as illustrated in the text.
```
First we fill in the cost table.
```
    cost[0][0] = 0.;
    for (i=1;i<=ia;i++) cost[i][0] = cost[i-1][0] + skwpen;
    for (i=1;i<=ib;i++) cost[0][i] = cost[0][i-1] + skwpen;
    for (i=1;i<=ia;i++) for (j=1;j<=ib;j++) {
        dn = cost[i-1][j] + ((j == ib)? skwpen : gappen);
        rt = cost[i][j-1] + ((i == ia)? skwpen : gappen);
        dg = cost[i-1][j-1] + ((ain[i-1] == bin[j-1])? -1. : mispen);
        cost[i][j] = MIN(MIN(dn,rt),dg);
    }
```
Next, we do the backtrack pass, writing the output (backward, however).
```
    i=ia; j=ib; k=0;
    while (i > 0 || j > 0) {
        dn = rt = dg = 9.99e99;             Any large value will do.
        if (i>0) dn = cost[i-1][j] + ((j==ib)? skwpen : gappen);
        if (j>0) rt = cost[i][j-1] + ((i==ia)? skwpen : gappen);
        if (i>0 && j>0) dg = cost[i-1][j-1] +
            ((ain[i-1] == bin[j-1])? -1. : mispen);
        if (dg <= MIN(dn,rt)) {             Diagonal move produces either match or in-
            aout[k] = ain[i-1];                 equality.
            bout[k] = bin[j-1];
            summary[k++] = ((ain[i-1] == bin[j-1])? '=' : '!');
            i--; j--;
        }
        else if (dn < rt) {                 Down move produces a gap in the B string.
            aout[k] = ain[i-1];
            bout[k] = ' ';
            summary[k++] = ' ';
            i--;
        }
        else {                              Right move produces a gap in the A string.
            aout[k] = ' ';
            bout[k] = bin[j-1];
            summary[k++] = ' ';
            j--;
        }
    }
```
Finally, reverse the output strings.
```
    for (i=0;i<k/2;i++) {
        SWAP(aout[i],aout[k-1-i]);
        SWAP(bout[i],bout[k-1-i]);
        SWAP(summary[i],summary[k-1-i]);
    }
    aout[k] = bout[k] = summary[k] = 0;     Don't forget the terminating nulls!
}
```

Various modifications of the Needleman-Wunsch method are also in use, most

notably the generalization by Smith and Waterman [3]. There are also a number of heuristic methods for identifying sequence similarity, with names like BLAST, FASTA, BLAT, etc. The field is highly developed, so you should use the routine above only pedagogically.

CITED REFERENCES AND FURTHER READING:

Cybenko, G. 1997, "Dynamic Programming: A Discrete Calculus of Variations," *IEEE Computational Science and Engineering*, vol. 4, no. 1, pp. 92-97.

Bertsekas, 2001, *Dynamic Programming and Optimal Control*, 2nd ed., 2 vols. (Belmont, MA: Athena Scientific).

Hillier, F.S. and Lieberman, G.J. 2002, *Introduction to Operations Research*, 7th ed. (New York: McGraw-Hill).

Needleman, S.B. and Wunsch, C.D. 1970, "A General Method Applicable to the Search for Similarities in the Amino Acid Sequence of Two Proteins," *Journal of Molecular Biology*, vol. 48, pp. 443-453.[1]

Rouchka, E.C. 2001, "Dynamic Programming," at multiple Web sites.[2]

Smith, T.F. and Waterman, M.S. 1981, "Identification of Common Molecular Subsequences," *Journal of Molecular Biology*, vol. 147, pp. 195-197.[3]

Eigensystems

CHAPTER 11

11.0 Introduction

An $N \times N$ matrix $\mathbf{A}$ is said to have an *eigenvector* $\mathbf{x}$ and corresponding *eigenvalue* λ if

$$\mathbf{A} \cdot \mathbf{x} = \lambda \mathbf{x} \tag{11.0.1}$$

Obviously any multiple of an eigenvector $\mathbf{x}$ will also be an eigenvector, but we won't consider such multiples as being distinct eigenvectors. (The zero vector is not considered to be an eigenvector at all.) Evidently (11.0.1) can hold only if

$$\det |\mathbf{A} - \lambda \mathbf{1}| = 0 \tag{11.0.2}$$

which, if expanded out, is an Nth degree polynomial in λ whose roots are the eigenvalues. This proves that there are always N (not necessarily distinct) eigenvalues. Equal eigenvalues coming from multiple roots are called *degenerate*. Root searching in the characteristic equation (11.0.2) is usually a very poor computational method for finding eigenvalues. We will learn much better ways in this chapter, as well as efficient ways for finding corresponding eigenvectors.

The above two equations also prove that every one of the N eigenvalues has a (not necessarily distinct) corresponding eigenvector: If λ is set to an eigenvalue, then the matrix $\mathbf{A} - \lambda \mathbf{1}$ is singular, and we know that every singular matrix has at least one nonzero vector in its nullspace (see §2.6.1).

If you add $\tau \mathbf{x}$ to both sides of (11.0.1), you will easily see that the eigenvalues of any matrix can be changed or *shifted* by an additive constant τ by adding to the matrix that constant times the identity matrix. The eigenvectors are unchanged by this shift. Shifting, as we will see, is an important part of many algorithms for computing eigenvalues. We see also that there is no special significance to a zero eigenvalue. Any eigenvalue can be shifted to zero, or any zero eigenvalue can be shifted away from zero.

11.0.1 Definitions and Basic Facts

A matrix is called *symmetric* if it is equal to its transpose,

$$\mathbf{A} = \mathbf{A}^T \qquad \text{or} \qquad a_{ij} = a_{ji} \tag{11.0.3}$$

It is called *Hermitian* or *self-adjoint* if it equals the complex conjugate of its transpose (its *Hermitian conjugate*, denoted by "†")

$$\mathbf{A} = \mathbf{A}^{\dagger} \qquad \text{or} \qquad a_{ij} = a_{ji}* \tag{11.0.4}$$

It is termed *orthogonal* if its transpose equals its inverse,

$$\mathbf{A}^T \cdot \mathbf{A} = \mathbf{A} \cdot \mathbf{A}^T = \mathbf{1} \tag{11.0.5}$$

and *unitary* if its Hermitian conjugate equals its inverse. Finally, a matrix is called *normal* if it *commutes* with its Hermitian conjugate,

$$\mathbf{A} \cdot \mathbf{A}^{\dagger} = \mathbf{A}^{\dagger} \cdot \mathbf{A} \tag{11.0.6}$$

For real matrices, Hermitian means the same as symmetric, unitary means the same as orthogonal, and *both* of these distinct classes are normal.

The reason that Hermitian is an important concept has to do with eigenvalues. The eigenvalues of a Hermitian matrix are all real. In particular, the eigenvalues of a real symmetric matrix are all real. Contrariwise, the eigenvalues of a real nonsymmetric matrix may include real values, but may also include pairs of complex-conjugate values; and the eigenvalues of a complex matrix that is not Hermitian will in general be complex.

The reason that normal is an important concept has to do with the eigenvectors. The eigenvectors of a normal matrix with nondegenerate (i.e., distinct) eigenvalues are complete and orthogonal, spanning the N-dimensional vector space. For a normal matrix with degenerate eigenvalues, we have the additional freedom of replacing the eigenvectors corresponding to a degenerate eigenvalue by linear combinations of themselves. Using this freedom, we can always perform Gram-Schmidt orthogonalization (consult any linear algebra text) and *find* a set of eigenvectors that are complete and orthogonal, just as in the nondegenerate case. The matrix whose columns are an orthonormal set of eigenvectors is evidently unitary. A special case is that the matrix of eigenvectors of a real symmetric matrix is orthogonal, since the eigenvectors of that matrix are all real.

When a matrix is not normal, as typified by any random, nonsymmetric, real matrix, then in general we cannot find *any* orthonormal set of eigenvectors, nor even any pairs of eigenvectors that are orthogonal (except perhaps by rare chance). While the N nonorthonormal eigenvectors will "usually" span the N-dimensional vector space, they do not always do so; that is, the eigenvectors are not always complete. Such a matrix is said to be *defective*.

11.0.2 Left and Right Eigenvectors

While the eigenvectors of a nonnormal matrix are not particularly orthogonal among themselves, they *do* have an orthogonality relation with a different set of vectors, which we must now define. Up to now our eigenvectors have been column vectors that are multiplied to the right of a matrix $\mathbf{A}$, as in (11.0.1). These, more explicitly, are termed *right eigenvectors*. We could also, however, try to find row vectors, which multiply $\mathbf{A}$ to the left and satisfy

$$\mathbf{x} \cdot \mathbf{A} = \lambda \mathbf{x} \tag{11.0.7}$$

These are called *left eigenvectors*. By taking the transpose of equation (11.0.7), we see that every left eigenvector is the transpose of a right eigenvector *of the transpose of* $\mathbf{A}$. Now, by comparing to (11.0.2) and using the fact that the determinant of a matrix equals the determinant of its transpose, we also see that the left and right eigen*values* of $\mathbf{A}$ are identical.

If the matrix $\mathbf{A}$ is symmetric, then the left and right eigenvectors are just transposes of each other, that is, they have the same numerical components. Likewise, if the matrix is self-adjoint, the left and right eigenvectors are Hermitian conjugates of each other. For the general nonnormal case, however, we have the following calculation: Let $\mathbf{X}_R$ be the matrix formed by columns from the right eigenvectors and $\mathbf{X}_L$ be the matrix formed by rows from the left eigenvectors. Then (11.0.1) and (11.0.7) can be rewritten as

$$\mathbf{A} \cdot \mathbf{X}_R = \mathbf{X}_R \cdot \mathrm{diag}(\lambda_0 \ldots \lambda_{N-1}) \qquad \mathbf{X}_L \cdot \mathbf{A} = \mathrm{diag}(\lambda_0 \ldots \lambda_{N-1}) \cdot \mathbf{X}_L \quad (11.0.8)$$

Multiplying the first of these equations on the left by $\mathbf{X}_L$, the second on the right by $\mathbf{X}_R$, and subtracting the two, gives

$$(\mathbf{X}_L \cdot \mathbf{X}_R) \cdot \mathrm{diag}(\lambda_0 \ldots \lambda_{N-1}) = \mathrm{diag}(\lambda_0 \ldots \lambda_{N-1}) \cdot (\mathbf{X}_L \cdot \mathbf{X}_R) \qquad (11.0.9)$$

This says that the matrix of dot products of the left and right eigenvectors commutes with the diagonal matrix of eigenvalues. But the only matrices that commute with a diagonal matrix *of distinct elements* are themselves diagonal. Thus, if the eigenvalues are nondegenerate, each left eigenvector is orthogonal to all right eigenvectors except its corresponding one, and vice versa. By choice of normalization, the dot products of corresponding left and right eigenvectors can always be made unity for any matrix with nondegenerate eigenvalues.

If some eigenvalues are degenerate, then either the left or the right eigenvectors corresponding to a degenerate eigenvalue must be linearly combined among themselves to achieve orthogonality with the right or left ones, respectively. This can always be done by a procedure akin to Gram-Schmidt orthogonalization. The normalization can then be adjusted to give unity for the nonzero dot products between corresponding left and right eigenvectors. If the dot product of corresponding left and right eigenvectors is zero at this stage, then you have a case where the eigenvectors are incomplete! Note that incomplete eigenvectors can occur only where there are degenerate eigenvalues, but they do not always occur in such cases (in fact, they never occur for the class of "normal" matrices). See [1] for a clear discussion.

In both the degenerate and nondegenerate cases, the final normalization to unity of all nonzero dot products produces the result: The matrix whose rows are left eigenvectors is the inverse matrix of the matrix whose columns are right eigenvectors, *if the inverse exists*. When it does exist, equations (11.0.8) and (11.0.9) imply the useful factorizations

$$\mathbf{A} = \mathbf{X}_R \cdot \mathrm{diag}(\lambda_0 \ldots \lambda_{N-1}) \cdot \mathbf{X}_L \quad \text{and} \quad \mathrm{diag}(\lambda_0 \ldots \lambda_{N-1}) = \mathbf{X}_L \cdot \mathbf{A} \cdot \mathbf{X}_R \quad (11.0.10)$$

11.0.3 Diagonalization of a Matrix

From equation (11.0.10) and the fact that $\mathbf{X}_L$ and $\mathbf{X}_R$ are matrix inverses, we get

$$\mathbf{X}_R^{-1} \cdot \mathbf{A} \cdot \mathbf{X}_R = \mathrm{diag}(\lambda_0 \ldots \lambda_{N-1}) \qquad (11.0.11)$$

This is a particular case of a *similarity transform* of the matrix $\mathbf{A}$,

$$\mathbf{A} \quad \rightarrow \quad \mathbf{Z}^{-1} \cdot \mathbf{A} \cdot \mathbf{Z} \tag{11.0.12}$$

for some transformation matrix $\mathbf{Z}$. Similarity transformations play a crucial role in the computation of eigenvalues, because they leave the eigenvalues of a matrix unchanged. This is easily seen from

$$\begin{aligned} \det\left|\mathbf{Z}^{-1} \cdot \mathbf{A} \cdot \mathbf{Z} - \lambda \mathbf{1}\right| &= \det\left|\mathbf{Z}^{-1} \cdot (\mathbf{A} - \lambda \mathbf{1}) \cdot \mathbf{Z}\right| \\ &= \det|\mathbf{Z}| \ \det|\mathbf{A} - \lambda \mathbf{1}| \ \det\left|\mathbf{Z}^{-1}\right| \\ &= \det|\mathbf{A} - \lambda \mathbf{1}| \end{aligned} \tag{11.0.13}$$

Equation (11.0.11) shows that any matrix with complete eigenvectors (which includes all normal matrices and "most" random nonnormal ones) can be diagonalized by a similarity transformation, that the columns of the transformation matrix that effects the diagonalization are the right eigenvectors, and that the rows of its inverse are the left eigenvectors.

For real symmetric matrices, the eigenvectors are real and orthonormal, so the transformation matrix is orthogonal. The similarity transformation is then also an *orthogonal transformation* of the form

$$\mathbf{A} \quad \rightarrow \quad \mathbf{Z}^{T} \cdot \mathbf{A} \cdot \mathbf{Z} \tag{11.0.14}$$

While real nonsymmetric matrices can be diagonalized in their usual case of complete eigenvectors, the transformation matrix is not necessarily real. It turns out, however, that a real similarity transformation can "almost" do the job. It can reduce the matrix down to a form with little two-by-two blocks along the diagonal and all other elements zero. Each two-by-two block corresponds to a complex-conjugate pair of complex eigenvalues. We will see this idea exploited in some routines given later in the chapter.

The "grand strategy" of virtually all modern eigensystem routines is to nudge the matrix $\mathbf{A}$ toward diagonal form by a sequence of similarity transformations,

$$\begin{aligned} \mathbf{A} \quad &\rightarrow \quad \mathbf{P}_1^{-1} \cdot \mathbf{A} \cdot \mathbf{P}_1 \quad \rightarrow \quad \mathbf{P}_2^{-1} \cdot \mathbf{P}_1^{-1} \cdot \mathbf{A} \cdot \mathbf{P}_1 \cdot \mathbf{P}_2 \\ &\rightarrow \quad \mathbf{P}_3^{-1} \cdot \mathbf{P}_2^{-1} \cdot \mathbf{P}_1^{-1} \cdot \mathbf{A} \cdot \mathbf{P}_1 \cdot \mathbf{P}_2 \cdot \mathbf{P}_3 \quad \rightarrow \quad \text{etc.} \end{aligned} \tag{11.0.15}$$

If we get all the way to diagonal form, then the eigenvectors are the columns of the accumulated transformation

$$\mathbf{X}_R = \mathbf{P}_1 \cdot \mathbf{P}_2 \cdot \mathbf{P}_3 \cdot \ldots \tag{11.0.16}$$

Sometimes we do not want to go all the way to diagonal form. For example, if we are interested only in eigenvalues, not eigenvectors, it is enough to transform the matrix $\mathbf{A}$ to be triangular, with all elements below (or above) the diagonal zero. In this case the diagonal elements are already the eigenvalues, as you can see by mentally evaluating (11.0.2) using expansion by minors.

There are two rather different sets of techniques for implementing the grand strategy (11.0.15). It turns out that they work rather well in combination, so most modern eigensystem routines use both. The first set of techniques constructs individual $\mathbf{P}_i$'s as explicit "atomic" transformations designed to perform specific tasks,

for example zeroing a particular off-diagonal element (Jacobi transformation, §11.1), or a whole particular row or column (Householder transformation, §11.3; elimination method, §11.6). In general, a finite sequence of these simple transformations cannot completely diagonalize a matrix. There are then two choices: either use the finite sequence of transformations to go most of the way (e.g., to some special form like *tridiagonal* or *Hessenberg*; see §11.3 and §11.6 below) and follow up with the second set of techniques about to be mentioned; or else iterate the finite sequence of simple transformations over and over until the deviation of the matrix from diagonal is negligibly small. This latter approach is conceptually simplest, so we will discuss it in the next section; however, for N greater than ~ 10, it is computationally inefficient by a roughly constant factor ~ 5.

The second set of techniques, called *factorization methods*, is more subtle. Suppose that the matrix $\mathbf{A}$ can be factored into a left factor $\mathbf{F}_L$ and a right factor $\mathbf{F}_R$. Then

$$\mathbf{A} = \mathbf{F}_L \cdot \mathbf{F}_R \qquad \text{or equivalently} \qquad \mathbf{F}_L^{-1} \cdot \mathbf{A} = \mathbf{F}_R \tag{11.0.17}$$

If we now multiply back together the factors in the reverse order and use the second equation in (11.0.17), we get

$$\mathbf{F}_R \cdot \mathbf{F}_L = \mathbf{F}_L^{-1} \cdot \mathbf{A} \cdot \mathbf{F}_L \tag{11.0.18}$$

which we recognize as having effected a similarity transformation on $\mathbf{A}$ with the transformation matrix being $\mathbf{F}_L$! In §11.4 and §11.7 we will discuss the *QR method* that exploits this idea.

Factorization methods also do not converge exactly in a finite number of transformations. But the better ones do converge rapidly and reliably, and, when following an appropriate initial reduction by simple similarity transformations, they are generally the methods of choice.

11.0.4 "Eigenpackages of Canned Eigenroutines"

You have probably gathered by now that the solution of eigensystems is a fairly complicated business. It is. It is one of the few subjects covered in this book for which we do *not* recommend that you avoid canned routines. On the contrary, the purpose of this chapter is precisely to give you some appreciation of what is going on inside such canned routines, so that you can make intelligent choices about using them, and intelligent diagnoses when something goes wrong.

You will find that almost all canned routines in use nowadays trace their ancestry back to routines published in Wilkinson and Reinsch's *Handbook for Automatic Computation, Vol. II, Linear Algebra* [2]. This excellent reference, containing papers by a number of authors, is the Bible of the field. A public-domain implementation of the *Handbook* routines in Fortran is the EISPACK set of programs [3]. The routines in this chapter are translations of either the *Handbook* or EISPACK routines, so understanding these will take you a lot of the way toward understanding those canonical packages.

The successor to EISPACK is LAPACK [4], which also includes the linear algebra routines of LINPACK. This is a Fortran package in which a lot of attention has been paid to efficient execution on modern machines. A C translation is available as CLAPACK.

IMSL [5] and NAG [6] each provide proprietary implementations, in Fortran and C, of what are essentially the *Handbook* routines.

A good "eigenpackage" will provide separate routines, or separate paths through sequences of routines, for the following desired calculations:

- all eigenvalues and no eigenvectors
- all eigenvalues and some corresponding eigenvectors
- all eigenvalues and all corresponding eigenvectors

The purpose of these distinctions is to save compute time and storage; it is wasteful to calculate eigenvectors that you don't need. Often one is interested only in the eigenvectors corresponding to the largest few eigenvalues, or largest few in magnitude, or few that are negative. The method usually used to calculate "some" eigenvectors is typically more efficient than calculating all eigenvectors if you desire fewer than about a quarter of the eigenvectors.

A good eigenpackage also provides separate paths for each of the above calculations for each of the following special forms of the matrix:

- real, symmetric, tridiagonal
- real, symmetric, banded (only a small number of sub- and superdiagonals are nonzero)
- real, symmetric
- real, nonsymmetric
- complex, Hermitian
- complex, non-Hermitian

Again, the purpose of these distinctions is to save time and storage by using the *least* general routine that will serve in any particular application.

In this chapter, as a bare introduction, we give good routines for the following paths:

- all eigenvalues and eigenvectors of a real symmetric, tridiagonal matrix (§11.4)
- all eigenvalues and eigenvectors of a real symmetric, matrix (§11.1 – §11.4)
- all eigenvalues and eigenvectors of a complex Hermitian matrix (§11.5)
- all eigenvalues and eigenvectors of a real nonsymmetric matrix (§11.6 – §11.7)

We also discuss, in §11.8, how to obtain some eigenvectors of general matrices by the method of inverse iteration.

11.0.5 Generalized and Nonlinear Eigenvalue Problems

Many eigenpackages also deal with the so-called *generalized eigenproblem* [7],

$$\mathbf{A} \cdot \mathbf{x} = \lambda \mathbf{B} \cdot \mathbf{x} \tag{11.0.19}$$

where $\mathbf{A}$ and $\mathbf{B}$ are both matrices. Most such problems, where $\mathbf{B}$ is nonsingular, can be handled by the equivalent

$$(\mathbf{B}^{-1} \cdot \mathbf{A}) \cdot \mathbf{x} = \lambda \mathbf{x} \tag{11.0.20}$$

Often $\mathbf{A}$ and $\mathbf{B}$ are symmetric and $\mathbf{B}$ is positive-definite. The matrix $\mathbf{B}^{-1} \cdot \mathbf{A}$ in (11.0.20) is not symmetric, but we can recover a symmetric eigenvalue problem by using the Cholesky decomposition $\mathbf{B} = \mathbf{L} \cdot \mathbf{L}^T$ of §2.9. Multiplying equation (11.0.19) by $\mathbf{L}^{-1}$, we get

$$\mathbf{C} \cdot (\mathbf{L}^T \cdot \mathbf{x}) = \lambda (\mathbf{L}^T \cdot \mathbf{x}) \tag{11.0.21}$$

where

$$\mathbf{C} = \mathbf{L}^{-1} \cdot \mathbf{A} \cdot (\mathbf{L}^{-1})^T \tag{11.0.22}$$

The matrix $\mathbf{C}$ is symmetric and its eigenvalues are the same as those of the original problem (11.0.19); its eigenfunctions are $\mathbf{L}^T \cdot \mathbf{x}$. The efficient way to form $\mathbf{C}$ is first to solve the equation

$$\mathbf{Y} \cdot \mathbf{L}^T = \mathbf{A} \tag{11.0.23}$$

for the lower triangle of the matrix $\mathbf{Y}$. Then solve

$$\mathbf{L} \cdot \mathbf{C} = \mathbf{Y} \tag{11.0.24}$$

for the lower triangle of the symmetric matrix $\mathbf{C}$.

Another generalization of the standard eigenvalue problem is to problems nonlinear in the eigenvalue λ, for example,

$$(\mathbf{A}\lambda^2 + \mathbf{B}\lambda + \mathbf{C}) \cdot \mathbf{x} = 0 \tag{11.0.25}$$

This can be turned into a linear problem by introducing an additional unknown eigenvector $\mathbf{y}$ and solving the $2N \times 2N$ eigensystem

$$\begin{pmatrix} 0 & \mathbf{1} \\ -\mathbf{A}^{-1} \cdot \mathbf{C} & -\mathbf{A}^{-1} \cdot \mathbf{B} \end{pmatrix} \cdot \begin{pmatrix} \mathbf{x} \\ \mathbf{y} \end{pmatrix} = \lambda \begin{pmatrix} \mathbf{x} \\ \mathbf{y} \end{pmatrix} \tag{11.0.26}$$

This technique generalizes to higher-order polynomials in λ. A polynomial of degree M produces a linear $MN \times MN$ eigensystem (see [8]).

11.0.6 Relation to Singular Value Decomposition

The factorization of a matrix $\mathbf{A}$ by the use of its eigenvectors and eigenvalues, equation (11.0.10), seems similar to singular value decomposition (SVD), as was discussed in §2.6. Is it the same thing? In general, no. A first obvious difference is that SVD is not restricted to square matrices, while eigendecomposition is. But what if $\mathbf{A}$ is square? Are the two decompositions then identical?

In general, still no. The difference has to do with what is orthogonal to what. If for a square matrix $\mathbf{A}$ we write the two decompositions (cf. equation 2.6.1 or 2.6.4 and equation 11.0.10),

$$\mathbf{A} = \mathbf{U} \cdot \text{diag}(w_0 \dots w_{N-1}) \cdot \mathbf{V}^T = \mathbf{X}_R \cdot \text{diag}(\lambda_0 \dots \lambda_{N-1}) \cdot \mathbf{X}_L \tag{11.0.27}$$

then for SVD the columns of $\mathbf{U}$ are mutually orthonormal, as are the columns of $\mathbf{V}$. There is no particular orthonormality *between* $\mathbf{U}$ and $\mathbf{V}$. For the eigendecomposition, the situation is the reverse: The rows of $\mathbf{X}_L$ are orthogonal to the columns of $\mathbf{X}_R$ (except for those corresponding to the same eigenvalue), but there is no particular orthogonality among the rows or columns of $\mathbf{X}_L$ or the rows or columns of $\mathbf{X}_R$. The two decompositions in equation (11.0.27) are just, in general, different!

However, the difference disappears when $\mathbf{A}$ is symmetric (or, if complex, Hermitian). In that case, equation (11.0.27) becomes

$$\mathbf{A} = \mathbf{V} \cdot \text{diag}(w_0 \dots w_{N-1}) \cdot \mathbf{V}^T = \mathbf{X}_R \cdot \text{diag}(\lambda_0 \dots \lambda_{N-1}) \cdot \mathbf{X}_R^T \tag{11.0.28}$$

and the fact that each decomposition is unique implies

$$\mathbf{V} = \mathbf{U} = \mathbf{X}_R = \mathbf{X}_L^T \tag{11.0.29}$$

and

$$\lambda_i = w_i, \qquad i = 0, \ldots, N-1 \tag{11.0.30}$$

That is, the (left and right) eigenvectors are the columns of any of the matrices listed in equation (11.0.29), and the corresponding eigenvalues and singular values are identical.

From a general matrix $\mathbf{A}$, not necessarily even square, one can form the two symmetric matrices $\mathbf{A}^T \cdot \mathbf{A}$ and $\mathbf{A} \cdot \mathbf{A}^T$. You can work out from equation (11.0.27) that the eigenvalues of either of these two matrices are squares of singular values of $\mathbf{A}$. However, this doesn't tell you about the eigenvalues of $\mathbf{A}$: The matrix whose eigenvalues are the squares of the eigenvalues of $\mathbf{A}$ is the unrelated matrix $\mathbf{A} \cdot \mathbf{A}$, not $\mathbf{A}^T \cdot \mathbf{A}$ or $\mathbf{A} \cdot \mathbf{A}^T$.

CITED REFERENCES AND FURTHER READING:

Stoer, J., and Bulirsch, R. 2002, *Introduction to Numerical Analysis*, 3rd ed. (New York: Springer), Chapter 6.[1]

Wilkinson, J.H., and Reinsch, C. 1971, *Linear Algebra*, vol. II of *Handbook for Automatic Computation* (New York: Springer).[2]

Smith, B.T., et al. 1976, *Matrix Eigensystem Routines — EISPACK Guide*, 2nd ed., vol. 6 of Lecture Notes in Computer Science (New York: Springer).[3]

Anderson, E., et al. 1999, LAPACK User's Guide, 3rd ed. (Philadelphia: S.I.A.M.). Online with software at 2007+, `http://www.netlib.org/lapack`.[4]

IMSL Math/Library Users Manual (Houston: IMSL Inc.), see 2007+, `http://www.vni.com/products/imsl`.[5]

NAG Fortran Library (Oxford, UK: Numerical Algorithms Group), see 2007+, `http://www.nag.co.uk`, Chapter F02.[6]

Golub, G.H., and Van Loan, C.F. 1996, *Matrix Computations*, 3rd ed. (Baltimore: Johns Hopkins University Press), §7.7.[7]

Wilkinson, J.H. 1965, *The Algebraic Eigenvalue Problem* (New York: Oxford University Press).[8]

Acton, F.S. 1970, *Numerical Methods That Work*; 1990, corrected edition (Washington, DC: Mathematical Association of America), Chapter 13.

Horn, R.A., and Johnson, C.R. 1985, *Matrix Analysis* (Cambridge: Cambridge University Press).

11.1 Jacobi Transformations of a Symmetric Matrix

The Jacobi method consists of a sequence of orthogonal similarity transformations of the form of equation (11.0.15). Each transformation (a *Jacobi rotation*) is just a plane rotation designed to annihilate one of the off-diagonal matrix elements. Successive transformations undo previously set zeros, but the off-diagonal elements nevertheless get smaller and smaller, until the matrix is diagonal to machine precision. Accumulating the product of the transformations as you go gives the matrix of

eigenvectors, equation (11.0.16), while the elements of the final diagonal matrix are the eigenvalues.

The Jacobi method is absolutely foolproof for all real symmetric matrices. In particular, it returns the small eigenvalues with better relative accuracy than methods that first reduce the matrix to tridiagonal form. For matrices of order greater than about 10, however, the algorithm is slower, by a significant constant factor, than the QR method we shall give in §11.4. However, the Jacobi algorithm is much simpler than the more efficient methods. We thus recommend it for matrices of moderate order, where expense is not a major consideration.

The basic Jacobi rotation $\mathbf{P}_{pq}$ is a matrix of the form

$$\mathbf{P}_{pq} = \begin{bmatrix} 1 & & & & & & \\ & \cdots & & & & & \\ & & c & \cdots & s & & \\ & & \vdots & 1 & \vdots & & \\ & & -s & \cdots & c & & \\ & & & & & \cdots & \\ & & & & & & 1 \end{bmatrix} \tag{11.1.1}$$

Here all the diagonal elements are unity except for the two elements c in rows (and columns) p and q. All off-diagonal elements are zero except the two elements s and $-s$. The numbers c and s are the cosine and sine of a rotation angle ϕ, so $c^2+s^2 = 1$.

A plane rotation such as (11.1.1) is used to transform the matrix $\mathbf{A}$ according to

$$\mathbf{A}' = \mathbf{P}_{pq}^T \cdot \mathbf{A} \cdot \mathbf{P}_{pq} \tag{11.1.2}$$

Now, $\mathbf{P}_{pq}^T \cdot \mathbf{A}$ changes only rows p and q of $\mathbf{A}$, while $\mathbf{A} \cdot \mathbf{P}_{pq}$ changes only columns p and q. Notice that the subscripts p and q do not denote components of $\mathbf{P}_{pq}$, but rather label which kind of rotation the matrix is, i.e., which rows and columns it affects. Thus the changed elements of $\mathbf{A}$ in (11.1.2) are only in rows p and q, and columns p and q, as indicated below:

$$\mathbf{A}' = \begin{bmatrix} & & a'_{0p} & & a'_{0q} & & \\ & & \vdots & & \vdots & & \\ a'_{p0} & \cdots & a'_{pp} & \cdots & a'_{pq} & \cdots & a'_{p,n-1} \\ & & \vdots & & \vdots & & \\ a'_{q0} & \cdots & a'_{qp} & \cdots & a'_{qq} & \cdots & a'_{q,n-1} \\ & & \vdots & & \vdots & & \\ & & a'_{n-1,p} & & a'_{n-1,q} & & \end{bmatrix} \tag{11.1.3}$$

Multiplying out equation (11.1.2) and using the symmetry of $\mathbf{A}$, we get the explicit formulas

$$\left.\begin{aligned} a'_{rp} &= ca_{rp} - sa_{rq} \\ a'_{rq} &= ca_{rq} + sa_{rp} \end{aligned}\right\} \quad r \neq p,\ r \neq q \tag{11.1.4}$$

$$a'_{pp} = c^2 a_{pp} + s^2 a_{qq} - 2sca_{pq} \tag{11.1.5}$$

$$a'_{qq} = s^2 a_{pp} + c^2 a_{qq} + 2sca_{pq} \tag{11.1.6}$$

$$a'_{pq} = (c^2 - s^2)a_{pq} + sc(a_{pp} - a_{qq}) \tag{11.1.7}$$

The idea of the Jacobi method is to try to zero the off-diagonal elements by a series of plane rotations. Accordingly, to set $a'_{pq} = 0$, equation (11.1.7) gives the following expression for the rotation angle ϕ:

$$\theta \equiv \cot 2\phi \equiv \frac{c^2 - s^2}{2sc} = \frac{a_{qq} - a_{pp}}{2a_{pq}} \tag{11.1.8}$$

If we let $t \equiv s/c$, the definition of θ can be rewritten

$$t^2 + 2t\theta - 1 = 0 \tag{11.1.9}$$

The smaller root of this equation corresponds to a rotation angle less than $\pi/4$ in magnitude; this choice at each stage gives the most stable reduction. Using the form of the quadratic formula with the discriminant in the denominator, we can write this smaller root as

$$t = \frac{\text{sgn}(\theta)}{|\theta| + \sqrt{\theta^2 + 1}} \tag{11.1.10}$$

If θ is so large that θ^2 would overflow on the computer, we set $t = 1/(2\theta)$. It now follows that

$$c = \frac{1}{\sqrt{t^2 + 1}} \tag{11.1.11}$$

$$s = tc \tag{11.1.12}$$

When we actually use equations (11.1.4) – (11.1.7) numerically, we rewrite them to minimize roundoff error. Equation (11.1.7) is replaced by

$$a'_{pq} = 0 \tag{11.1.13}$$

The idea in the remaining equations is to set the new quantity equal to the old quantity plus a small correction. Thus we can use (11.1.7) and (11.1.13) to eliminate a_{qq} from (11.1.5), giving

$$a'_{pp} = a_{pp} - ta_{pq} \tag{11.1.14}$$

Similarly,

$$a'_{qq} = a_{qq} + ta_{pq} \tag{11.1.15}$$

$$a'_{rp} = a_{rp} - s(a_{rq} + \tau a_{rp}) \tag{11.1.16}$$

$$a'_{rq} = a_{rq} + s(a_{rp} - \tau a_{rq}) \tag{11.1.17}$$

where τ ($= \tan\phi/2$) is defined by

$$\tau \equiv \frac{s}{1 + c} \tag{11.1.18}$$

One can see the convergence of the Jacobi method by considering the sum of the squares of the off-diagonal elements

$$S = \sum_{r \neq s} |a_{rs}|^2 \tag{11.1.19}$$

Equations (11.1.4) – (11.1.7) imply that

$$S' = S - 2|a_{pq}|^2 \tag{11.1.20}$$

(Since the transformation is orthogonal, the sum of the squares of the diagonal elements increases correspondingly by $2|a_{pq}|^2$.) The sequence of S's thus decreases monotonically. Since the sequence is bounded below by zero, and since we can choose a_{pq} to be whatever element we want, the sequence can be made to converge to zero.

Eventually one obtains a matrix $\mathbf{D}$ that is diagonal to machine precision. The diagonal elements give the eigenvalues of the original matrix $\mathbf{A}$, since

$$\mathbf{D} = \mathbf{V}^T \cdot \mathbf{A} \cdot \mathbf{V} \tag{11.1.21}$$

where

$$\mathbf{V} = \mathbf{P}_1 \cdot \mathbf{P}_2 \cdot \mathbf{P}_3 \cdots \tag{11.1.22}$$

the $\mathbf{P}_i$'s being the successive Jacobi rotation matrices. The columns of $\mathbf{V}$ are the eigenvectors (since $\mathbf{A} \cdot \mathbf{V} = \mathbf{V} \cdot \mathbf{D}$). They can be computed by applying

$$\mathbf{V}' = \mathbf{V} \cdot \mathbf{P}_i \tag{11.1.23}$$

at each stage of calculation, where initially $\mathbf{V}$ is the identity matrix. In detail, equation (11.1.23) is

$$\begin{aligned} v'_{rs} &= v_{rs} \qquad (s \neq p,\ s \neq q) \\ v'_{rp} &= c v_{rp} - s v_{rq} \\ v'_{rq} &= s v_{rp} + c v_{rq} \end{aligned} \tag{11.1.24}$$

We rewrite these equations in terms of τ as in equations (11.1.16) and (11.1.17) to minimize roundoff.

The only remaining question is the strategy one should adopt for the order in which the elements are to be annihilated. Jacobi's original algorithm of 1846 searched the whole upper triangle at each stage and set the largest off-diagonal element to zero. This is a reasonable strategy for hand calculation, but it is prohibitive on a computer since the search alone makes each Jacobi rotation a process of order N^2 instead of N.

A better strategy for our purposes is the *cyclic Jacobi method*, where one annihilates elements in strict order. For example, one can simply proceed down the rows: $\mathbf{P}_{01}, \mathbf{P}_{02}, ..., \mathbf{P}_{0,n-1}$; then $\mathbf{P}_{12}, \mathbf{P}_{13}$, etc. One can show that convergence is generally quadratic for either the original or the cyclic Jacobi method, for nondegenerate eigenvalues. One such set of $n(n-1)/2$ Jacobi rotations is called a *sweep*.

The program below, based on the implementations in [1,2], uses two further refinements:

- In the first three sweeps, we carry out the pq rotation only if $|a_{pq}| > \epsilon$ for some threshold value

$$\epsilon = \frac{1}{5}\frac{S_0}{n^2} \tag{11.1.25}$$

where S_0 is the sum of the off-diagonal moduli,

$$S_0 = \sum_{r<s} |a_{rs}| \tag{11.1.26}$$

- After four sweeps, if $|a_{pq}| \ll |a_{pp}|$ and $|a_{pq}| \ll |a_{qq}|$, we set $|a_{pq}| = 0$ and skip the rotation. The criterion used in the comparison is $|a_{pq}| < 10^{-(D+2)}|a_{pp}|$, where D is the number of significant decimal digits on the machine, and similarly for $|a_{qq}|$.

Typical matrices require six to ten sweeps to achieve convergence, or $3n^2$ to $5n^2$ Jacobi rotations. Each rotation requires of order $8n$ floating-point operations, so the total labor is of order $24n^3$ to $40n^3$ operations. Calculation of the eigenvectors as well as the eigenvalues changes the operation count from $8n$ to $12n$ per rotation, which is only a 50% overhead.

The following routine implements the Jacobi method. Simply create a `Jacobi` object using your symmetric matrix `a[0..n-1] [0..n-1]`:

```
Jacobi jac(a);
```

The vector `d[0..n-1]` then contains the eigenvalues of `a`. During the computation, it contains the current diagonal of `a`. The matrix `v[0..n-1][0..n-1]` outputs the normalized eigenvector belonging to `d[k]` in column `k`. The parameter `nrot` is the number of Jacobi rotations that were needed to achieve convergence.

eigen_sym.h

```
struct Jacobi {
```
Computes all eigenvalues and eigenvectors of a real symmetric matrix by Jacobi's method.
```
    const Int n;
    MatDoub a,v;
    VecDoub d;
    Int nrot;
    const Doub EPS;

    Jacobi(MatDoub_I &aa) : n(aa.nrows()), a(aa), v(n,n), d(n), nrot(0),
        EPS(numeric_limits<Doub>::epsilon())
```
Computes all eigenvalues and eigenvectors of a real symmetric matrix `a[0..n-1][0..n-1]`. On output, `d[0..n-1]` contains the eigenvalues of `a` sorted into descending order, while `v[0..n-1][0..n-1]` is a matrix whose columns contain the corresponding normalized eigenvectors. `nrot` contains the number of Jacobi rotations that were required. Only the upper triangle of `a` is accessed.
```
    {
        Int i,j,ip,iq;
        Doub tresh,theta,tau,t,sm,s,h,g,c;
        VecDoub b(n),z(n);
        for (ip=0;ip<n;ip++) {                      Initialize to the identity matrix.
            for (iq=0;iq<n;iq++) v[ip][iq]=0.0;
            v[ip][ip]=1.0;
        }
        for (ip=0;ip<n;ip++) {                      Initialize b and d to the diagonal
            b[ip]=d[ip]=a[ip][ip];                      of a.
            z[ip]=0.0;                              This vector will accumulate terms
        }                                               of the form ta_pq as in equa-
        for (i=1;i<=50;i++) {                           tion (11.1.14).
            sm=0.0;
            for (ip=0;ip<n-1;ip++) {                Sum magnitude of off-diagonal
                for (iq=ip+1;iq<n;iq++)                 elements.
                    sm += abs(a[ip][iq]);
            }
            if (sm == 0.0) {                        The normal return, which relies
                eigsrt(d,&v);                           on quadratic convergence to
                return;                                 machine underflow.
            }
            if (i < 4)
                tresh=0.2*sm/(n*n);                 On the first three sweeps...
```

```
            else
                tresh=0.0;                              ...thereafter.
            for (ip=0;ip<n-1;ip++) {
                for (iq=ip+1;iq<n;iq++) {
                    g=100.0*abs(a[ip][iq]);
                    After four sweeps, skip the rotation if the off-diagonal element is small.
                    if (i > 4 && g <= EPS*abs(d[ip]) && g <= EPS*abs(d[iq]))
                            a[ip][iq]=0.0;
                    else if (abs(a[ip][iq]) > tresh) {
                        h=d[iq]-d[ip];
                        if (g <= EPS*abs(h))
                            t=(a[ip][iq])/h;            t = 1/(2θ)
                        else {
                            theta=0.5*h/(a[ip][iq]);    Equation (11.1.10).
                            t=1.0/(abs(theta)+sqrt(1.0+theta*theta));
                            if (theta < 0.0) t = -t;
                        }
                        c=1.0/sqrt(1+t*t);
                        s=t*c;
                        tau=s/(1.0+c);
                        h=t*a[ip][iq];
                        z[ip] -= h;
                        z[iq] += h;
                        d[ip] -= h;
                        d[iq] += h;
                        a[ip][iq]=0.0;
                        for (j=0;j<ip;j++)              Case of rotations 0 ≤ j < p.
                            rot(a,s,tau,j,ip,j,iq);
                        for (j=ip+1;j<iq;j++)           Case of rotations p < j < q.
                            rot(a,s,tau,ip,j,j,iq);
                        for (j=iq+1;j<n;j++)            Case of rotations q < j < n.
                            rot(a,s,tau,ip,j,iq,j);
                        for (j=0;j<n;j++)
                            rot(v,s,tau,j,ip,j,iq);
                        ++nrot;
                    }
                }
            }
            for (ip=0;ip<n;ip++) {
                b[ip] += z[ip];
                d[ip]=b[ip];                            Update d with the sum of ta_pq,
                z[ip]=0.0;                              and reinitialize z.
            }
        }
        throw("Too many iterations in routine jacobi");
    }
    inline void rot(MatDoub_IO &a, const Doub s, const Doub tau, const Int i,
        const Int j, const Int k, const Int l)
    {
        Doub g=a[i][j];
        Doub h=a[k][l];
        a[i][j]=g-s*(h+g*tau);
        a[k][l]=h+s*(g-h*tau);
    }
};
```

Note that the above routine assumes that underflows are set to zero. On machines where this is not true, the program must be modified. See §1.5.4 and/or find out about the `fesetenv` (Linux) or `__controlfp` (Microsoft) functions.

The Jacobi method does not order the eigenvalues itself. We incorporate the following routine to sort the eigenvalues into descending order. The same routine is used in `Symmeig` in the next section. (The method, straight insertion, is N^2 rather

than $N \log N$; but since you have just done an N^3 procedure to get the eigenvalues, you can afford yourself this little indulgence.)

eigen_sym.h

```
void eigsrt(VecDoub_IO &d, MatDoub_IO *v=NULL)
```

Given the eigenvalues `d[0..n-1]` and (optionally) the eigenvectors `v[0..n-1][0..n-1]` as determined by `Jacobi` (§11.1) or `tqli` (§11.4), this routine sorts the eigenvalues into descending order and rearranges the columns of `v` correspondingly. The method is straight insertion.

```
{
    Int k;
    Int n=d.size();
    for (Int i=0;i<n-1;i++) {
        Doub p=d[k=i];
        for (Int j=i;j<n;j++)
            if (d[j] >= p) p=d[k=j];
        if (k != i) {
            d[k]=d[i];
            d[i]=p;
            if (v != NULL)
                for (Int j=0;j<n;j++) {
                    p=(*v)[j][i];
                    (*v)[j][i]=(*v)[j][k];
                    (*v)[j][k]=p;
                }
        }
    }
}
```

CITED REFERENCES AND FURTHER READING:

Golub, G.H., and Van Loan, C.F. 1996, *Matrix Computations*, 3rd ed. (Baltimore: Johns Hopkins University Press), §8.4.

Smith, B.T., et al. 1976, *Matrix Eigensystem Routines — EISPACK Guide*, 2nd ed., vol. 6 of Lecture Notes in Computer Science (New York: Springer).[1]

Wilkinson, J.H., and Reinsch, C. 1971, *Linear Algebra*, vol. II of *Handbook for Automatic Computation* (New York: Springer).[2]

11.2 Real Symmetric Matrices

As already mentioned, the optimum strategy in most cases for finding eigenvalues and eigenvectors is, first, to reduce the matrix to a simple form, only then beginning an iterative procedure. For symmetric matrices, the preferred simple form is tridiagonal.

Here is a routine based on this strategy that finds all eigenvalues and eigenvectors of a real symmetric matrix. It is typically a factor of about five faster than the Jacobi routine of the previous section. The implementations of the functions `tred2` and `tqli` that reduce the matrix to tridiagonal form and then find the eigensystem are discussed in the next two sections.

There are two user interfaces, implemented as two constructors. The first constructor is the usual one:

```
Symmeig s(a);
```

It returns the eigenvalues of a in descending order in `s.d[0..n-1]`. The normalized eigenvector corresponding to `d[k]` is in the matrix column `s.z[0..n-1][k]`. Setting the default argument to `false` suppresses the computation of the eigenvectors:

```
Symmeig s(a,false);
```

If you already have a matrix in tridiagonal form, you use the other constructor, which accepts the diagonal and subdiagonal of the matrix as vectors:

```
Symmeig s(d,e);
```

Again, you can suppress the computation of eigenvectors by setting the default argument to `false`.

Here is the routine:

eigen_sym.h

```
struct Symmeig {
Computes all eigenvalues and eigenvectors of a real symmetric matrix by reduction to tridiagonal
form followed by QL iteration.
    Int n;
    MatDoub z;
    VecDoub d,e;
    Bool yesvecs;

    Symmeig(MatDoub_I &a, Bool yesvec=true) : n(a.nrows()), z(a), d(n),
        e(n), yesvecs(yesvec)
    Computes all eigenvalues and eigenvectors of a real symmetric matrix a[0..n-1][0..n-1]
    by reduction to tridiagonal form followed by QL iteration. On output, d[0..n-1] contains
    the eigenvalues of a sorted into descending order, while z[0..n-1][0..n-1] is a matrix
    whose columns contain the corresponding normalized eigenvectors. If yesvecs is input as
    true (the default), then the eigenvectors are computed. If yesvecs is input as false, only
    the eigenvalues are computed.
    {
        tred2();                    Reduction to tridiagonal form; see §11.3.
        tqli();                     Eigensystem of tridiagonal matrix; see §11.4.
        sort();
    }
    Symmeig(VecDoub_I &dd, VecDoub_I &ee, Bool yesvec=true) :
        n(dd.size()), d(dd), e(ee), z(n,n,0.0), yesvecs(yesvec)
    Computes all eigenvalues and (optionally) eigenvectors of a real, symmetric, tridiagonal
    matrix by QL iteration. On input, dd[0..n-1] contains the diagonal elements of the tridi-
    agonal matrix. The vector ee[0..n-1] inputs the subdiagonal elements of the tridiagonal
    matrix, with ee[0] arbitrary. Output is the same as the constructor above.
    {
        for (Int i=0;i<n;i++) z[i][i]=1.0;
        tqli();
        sort();
    }
    void sort() {
        if (yesvecs)
            eigsrt(d,&z);
        else
            eigsrt(d);
    }
    void tred2();
    void tqli();
    Doub pythag(const Doub a, const Doub b);
};
```

11.3 Reduction of a Symmetric Matrix to Tridiagonal Form: Givens and Householder Reductions

The previous section outlined the grand strategy of (i) reduction to tridiagonal form, followed by (ii) finding the eigenvalues and eigenvectors of a tridiagonal matrix. In this section we implement the first of these steps.

11.3.1 Givens Method

The *Givens reduction* is a modification of the Jacobi method. Instead of trying to reduce the matrix all the way to diagonal form, we are content to stop when the matrix is tridiagonal. This allows the procedure to be carried out *in a finite number of steps*, unlike the Jacobi method, which requires iteration to convergence.

For the Givens method, we choose the rotation angle in equation (11.1.1) so as to zero an element that is *not* at one of the four "corners," i.e., not a_{pp}, a_{pq}, or a_{qq} in equation (11.1.3). Specifically, we first choose $\mathbf{P}_{12}$ to annihilate a_{20} (and, by symmetry, a_{02}). Then we choose $\mathbf{P}_{13}$ to annihilate a_{30}. In general, we choose the sequence

$$\mathbf{P}_{12}, \mathbf{P}_{13}, \dots, \mathbf{P}_{1,n-1}; \mathbf{P}_{23}, \dots, \mathbf{P}_{2,n-1}; \dots ; \mathbf{P}_{n-2,n-1}$$

where $\mathbf{P}_{jk}$ annihilates $a_{k,j-1}$. The method works because elements such as a'_{rp} and a'_{rq}, with $r \neq p \; r \neq q$, are linear combinations of the old quantities a_{rp} and a_{rq}, by equation (11.1.4). Thus, if a_{rp} and a_{rq} have already been set to zero, they remain zero as the reduction proceeds. Evidently, of order $n^2/2$ rotations are required, and the number of multiplications in a straightforward implementation is of order $4n^3/3$, not counting those for keeping track of the product of the transformation matrices, required for the eigenvectors.

The Householder method, to be discussed next, is just as stable as the Givens reduction and it is a factor of two more efficient, so the Givens method is not generally used. However, the Givens reduction can be reformulated to reduce the number of operations by a factor of two, and also avoid the necessity of taking square roots [1]. This appears to make the algorithm competitive with the Householder reduction. Unfortunately, this "fast Givens" reduction has to be monitored to avoid overflows, and the variables have to be periodically rescaled. There does not seem to be any compelling reason to prefer the Givens reduction over the Householder method.

11.3.2 Householder Method

The Householder algorithm reduces an $n \times n$ symmetric matrix $\mathbf{A}$ to tridiagonal form by $n-2$ orthogonal transformations. Each transformation annihilates the required part of a whole column and whole corresponding row. The basic ingredient is a Householder matrix $\mathbf{P}$, which has the form

$$\mathbf{P} = \mathbf{1} - 2\mathbf{w} \cdot \mathbf{w}^T \qquad (11.3.1)$$

where $\mathbf{w}$ is a real vector with $|\mathbf{w}|^2 = 1$. (In the present notation, the *outer* or matrix product of two vectors, $\mathbf{a}$ and $\mathbf{b}$, is written $\mathbf{a} \cdot \mathbf{b}^T$, while the *inner* or scalar product

of the vectors is written as $\mathbf{a}^T \cdot \mathbf{b}$.) The matrix $\mathbf{P}$ is orthogonal, because

$$\begin{aligned} \mathbf{P}^2 &= (\mathbf{1} - 2\mathbf{w}\cdot\mathbf{w}^T)\cdot(\mathbf{1} - 2\mathbf{w}\cdot\mathbf{w}^T) \\ &= \mathbf{1} - 4\mathbf{w}\cdot\mathbf{w}^T + 4\mathbf{w}\cdot(\mathbf{w}^T\cdot\mathbf{w})\cdot\mathbf{w}^T \\ &= \mathbf{1} \end{aligned} \tag{11.3.2}$$

Therefore $\mathbf{P} = \mathbf{P}^{-1}$. But $\mathbf{P}^T = \mathbf{P}$, and so $\mathbf{P}^T = \mathbf{P}^{-1}$, proving orthogonality.

Rewrite $\mathbf{P}$ as

$$\mathbf{P} = \mathbf{1} - \frac{\mathbf{u}\cdot\mathbf{u}^T}{H} \tag{11.3.3}$$

where the scalar H is

$$H \equiv \tfrac{1}{2}\,|\mathbf{u}|^2 \tag{11.3.4}$$

and $\mathbf{u}$ can now be any vector. Suppose $\mathbf{x}$ is the vector composed of the first column of $\mathbf{A}$. Choose

$$\mathbf{u} = \mathbf{x} \mp |\mathbf{x}|\mathbf{e}_0 \tag{11.3.5}$$

where $\mathbf{e}_0$ is the unit vector $[1, 0, \ldots, 0]^T$ and the choice of signs will be made later. Then

$$\begin{aligned} \mathbf{P}\cdot\mathbf{x} &= \mathbf{x} - \frac{\mathbf{u}}{H}\cdot(\mathbf{x} \mp |\mathbf{x}|\mathbf{e}_0)^T\cdot\mathbf{x} \\ &= \mathbf{x} - \frac{2\mathbf{u}\cdot(|\mathbf{x}|^2 \mp |\mathbf{x}|x_0)}{2|\mathbf{x}|^2 \mp 2|\mathbf{x}|x_0} \\ &= \mathbf{x} - \mathbf{u} \\ &= \pm|\mathbf{x}|\mathbf{e}_0 \end{aligned} \tag{11.3.6}$$

This shows that the Householder matrix $\mathbf{P}$ acts on a given vector $\mathbf{x}$ to zero all its elements except the first one.

To reduce a symmetric matrix $\mathbf{A}$ to tridiagonal form, we choose the vector $\mathbf{x}$ for the first Householder matrix to be the lower $n-1$ elements of column 0. Then the lower $n-2$ elements will be zeroed:

$$\begin{aligned} \mathbf{P}_1\cdot\mathbf{A} &= \left[\begin{array}{c|cccc} 1 & 0 & 0 & \cdots & 0 \\ \hline 0 & & & & \\ 0 & & & & \\ \vdots & & {}^{(n-1)}\mathbf{P}_1 & & \\ 0 & & & & \end{array}\right]\cdot\left[\begin{array}{c|cccc} a_{00} & a_{01} & a_{02} & \cdots & a_{0,n-1} \\ \hline a_{10} & & & & \\ a_{20} & & & & \\ \vdots & & & \text{irrelevant} & \\ a_{n-1,0} & & & & \end{array}\right] \\ &= \left[\begin{array}{c|cccc} a_{00} & a_{01} & a_{02} & \cdots & a_{0,n-1} \\ \hline k & & & & \\ 0 & & & & \\ \vdots & & & \text{irrelevant} & \\ 0 & & & & \end{array}\right] \end{aligned} \tag{11.3.7}$$

Here we have written the matrices in partitioned form, with ${}^{(n-1)}\mathbf{P}$ denoting a Householder matrix with dimensions $(n-1) \times (n-1)$. The quantity k is simply plus or minus the magnitude of the vector $[a_{10}, \ldots, a_{n-1,0}]^T$.

The complete orthogonal transformation is now

$$\mathbf{A}' = \mathbf{P} \cdot \mathbf{A} \cdot \mathbf{P} = \left[\begin{array}{c|ccc} a_{00} & k & 0 \cdots & 0 \\ \hline k & & & \\ 0 & & & \\ \vdots & & \text{irrelevant} & \\ 0 & & & \end{array}\right] \tag{11.3.8}$$

We have used the fact that $\mathbf{P}^T = \mathbf{P}$.

Now choose the vector $\mathbf{x}$ for the second Householder matrix to be the bottom $n-2$ elements of column 1, and from it construct

$$\mathbf{P}_2 \equiv \left[\begin{array}{cc|ccc} 1 & 0 & 0 & \cdots & 0 \\ 0 & 1 & 0 & \cdots & 0 \\ \hline 0 & 0 & & & \\ \vdots & \vdots & & {}^{(n-2)}\mathbf{P}_2 & \\ 0 & 0 & & & \end{array}\right] \tag{11.3.9}$$

The identity block in the upper-left corner ensures that the tridiagonalization achieved in the first step will not be spoiled by this one, while the $(n-2)$-dimensional Householder matrix ${}^{(n-2)}\mathbf{P}_2$ creates one additional row and column of the tridiagonal output. Clearly, a sequence of $n-2$ such transformations will reduce the matrix $\mathbf{A}$ to tridiagonal form.

Instead of actually carrying out the matrix multiplications in $\mathbf{P} \cdot \mathbf{A} \cdot \mathbf{P}$, we compute a vector

$$\mathbf{p} \equiv \frac{\mathbf{A} \cdot \mathbf{u}}{H} \tag{11.3.10}$$

Then

$$\begin{aligned} \mathbf{A} \cdot \mathbf{P} &= \mathbf{A} \cdot \left(1 - \frac{\mathbf{u} \cdot \mathbf{u}^T}{H}\right) = \mathbf{A} - \mathbf{p} \cdot \mathbf{u}^T \\ \mathbf{A}' &= \mathbf{P} \cdot \mathbf{A} \cdot \mathbf{P} = \mathbf{A} - \mathbf{p} \cdot \mathbf{u}^T - \mathbf{u} \cdot \mathbf{p}^T + 2K\mathbf{u} \cdot \mathbf{u}^T \end{aligned}$$

where the scalar K is defined by

$$K = \frac{\mathbf{u}^T \cdot \mathbf{p}}{2H} \tag{11.3.11}$$

If we write

$$\mathbf{q} \equiv \mathbf{p} - K\mathbf{u} \tag{11.3.12}$$

then we have

$$\mathbf{A}' = \mathbf{A} - \mathbf{q} \cdot \mathbf{u}^T - \mathbf{u} \cdot \mathbf{q}^T \tag{11.3.13}$$

This is the computationally useful formula.

Following [2], the routine for Householder reduction given below actually starts in the column $n-1$ of $\mathbf{A}$, not column 0 as in the explanation above. In detail, the equations are as follows: At stage m $(m = 1, 2, \ldots, n-2)$, the vector $\mathbf{u}$ has the form

$$\mathbf{u}^T = [a_{i0}, a_{i1}, \ldots, a_{i,i-2},\ a_{i,i-1} \pm \sqrt{\sigma},\ 0, \ldots, 0] \tag{11.3.14}$$

Here

$$i \equiv n - m = n - 1, n - 2, \ldots, 2 \tag{11.3.15}$$

and the quantity σ ($|x|^2$ in our earlier notation) is

$$\sigma = (a_{i0})^2 + \cdots + (a_{i,i-1})^2 \tag{11.3.16}$$

Choose the sign of $\sqrt{\sigma}$ in (11.3.14) to be the same as the sign of $a_{i,i-1}$ to lessen roundoff error.

Variables are thus computed in the following order: $\sigma, \mathbf{u}, H, \mathbf{p}, K, \mathbf{q}, \mathbf{A}'$. At any stage m, $\mathbf{A}$ is tridiagonal in its last $m - 1$ rows and columns.

No extra storage arrays are needed for the intermediate results. At stage m, the vectors $\mathbf{p}$ and $\mathbf{q}$ are nonzero only in elements $0, \ldots, i$ (recall that $i = n - m$), while $\mathbf{u}$ is nonzero only in elements $0, \ldots, i - 1$. The elements of the vector `e` are being determined in the order $n - 1, n - 2, \ldots$, so we can store $\mathbf{p}$ in the elements of `e` not already determined. The vector $\mathbf{q}$ can overwrite $\mathbf{p}$ once $\mathbf{p}$ is no longer needed. We store $\mathbf{u}$ in row i of `a` and $\mathbf{u}/H$ in column i of `a`. Once the reduction is complete, we compute the matrices $\mathbf{Q}_j$ using the quantities $\mathbf{u}$ and $\mathbf{u}/H$ that have been stored in `a`. Since $\mathbf{Q}_j$ is an identity matrix from row and column $n - j$ on, we only need compute its elements up to row and column $n - j - 1$. These can overwrite the $\mathbf{u}$'s and $\mathbf{u}/H$'s in the corresponding rows and columns of `a`, which are no longer required for subsequent $\mathbf{Q}$'s.

The routine `tred2`, given below, includes one further refinement. If the quantity σ is zero or "small" at any stage, one can skip the corresponding transformation. A simple criterion, such as

$$\sigma < \frac{\text{smallest positive number representable on machine}}{\text{machine precision}}$$

would be fine most of the time. A more careful criterion is actually used. At stage i, define the quantity

$$\epsilon = \sum_{k=0}^{i-1} |a_{ik}| \tag{11.3.17}$$

If $\epsilon = 0$ to machine precision, we skip the transformation. Otherwise we redefine

$$a_{ik} \quad \text{becomes} \quad a_{ik}/\epsilon \tag{11.3.18}$$

and use the scaled variables for the transformation. (A Householder transformation depends only on the ratios of the elements.)

If the eigenvectors of the final tridiagonal matrix are found (for example, by the routine in the next section), then the eigenvectors of $\mathbf{A}$ can be obtained by applying the accumulated transformation

$$\mathbf{Q} = \mathbf{P}_1 \cdot \mathbf{P}_2 \cdots \mathbf{P}_{n-2} \tag{11.3.19}$$

to those eigenvectors. We therefore form $\mathbf{Q}$ by recursion after all the $\mathbf{P}$'s have been determined:

$$\begin{aligned} \mathbf{Q}_{n-2} &= \mathbf{P}_{n-2} \\ \mathbf{Q}_j &= \mathbf{P}_j \cdot \mathbf{Q}_{j+1}, \qquad j = n-3, \ldots, 1 \\ \mathbf{Q} &= \mathbf{Q}_1 \end{aligned} \tag{11.3.20}$$

Input for the routine below is the real symmetric matrix $\mathbf{A}$ stored in the matrix `z[0..n-1][0..n-1]`. On output, `z` contains the elements of the orthogonal matrix $\mathbf{Q}$. The vector `d[0..n-1]` is set to the diagonal elements of the tridiagonal matrix $\mathbf{A}'$, while the vector `e[0..n-1]` is set to the off-diagonal elements in its components `1` through `n-1`, with `e[0]=0`.

Note that when dealing with a matrix whose elements vary over many orders of magnitude, it is desirable that the matrix be permuted, insofar as possible, so that the smaller elements are in the top left-hand corner. This is because the reduction is performed starting from the bottom right-hand corner, and a mixture of small and large elements there can lead to considerable rounding errors.

In the limit of large n, the operation count of the Householder reduction is $4n^3/3$ for eigenvalues only, and $8n^3/3$ for both eigenvalues and eigenvectors. The routine `tred2` is designed for use with the routine `tqli` of the next section. `tqli` finds the eigenvalues and eigenvectors of a symmetric tridiagonal matrix. For many years, the combination of `tred2` and `tqli` was the most efficient known technique for finding all the eigenvalues and eigenvectors (or just all the eigenvalues) of a real symmetric matrix. For moderate-sized matrices, it is still competitive with newer, more complicated methods.

eigen_sym.h

```
void Symmeig::tred2()
```

Householder reduction of a real symmetric matrix `z[0..n-1][0..n-1]`. (The input matrix $\mathbf{A}$ to `Symmeig` is stored in `z`.) On output, `z` is replaced by the orthogonal matrix $\mathbf{Q}$ effecting the transformation. `d[0..n-1]` contains the diagonal elements of the tridiagonal matrix and `e[0..n-1]` the off-diagonal elements, with `e[0]=0`. If `yesvecs` is `false`, so that only eigenvalues will subsequently be determined, several statements are omitted, in which case `z` contains no useful information on output.

```
{
    Int l,k,j,i;
    Doub scale,hh,h,g,f;
    for (i=n-1;i>0;i--) {
        l=i-1;
        h=scale=0.0;
        if (l > 0) {
            for (k=0;k<i;k++)
                scale += abs(z[i][k]);
            if (scale == 0.0)                 Skip transformation.
                e[i]=z[i][l];
            else {
                for (k=0;k<i;k++) {
                    z[i][k] /= scale;         Use scaled a's for transformation.
                    h += z[i][k]*z[i][k];     Form σ in h.
                }
                f=z[i][l];
                g=(f >= 0.0 ? -sqrt(h) : sqrt(h));
                e[i]=scale*g;
                h -= f*g;                     Now h is equation (11.3.4).
                z[i][l]=f-g;                  Store u in row i of z.
                f=0.0;
                for (j=0;j<i;j++) {
                    if (yesvecs)              Store u/H in column i of z.
                        z[j][i]=z[i][j]/h;
                    g=0.0;                    Form an element of A·u in g.
                    for (k=0;k<j+1;k++)
                        g += z[j][k]*z[i][k];
                    for (k=j+1;k<i;k++)
                        g += z[k][j]*z[i][k];
                    e[j]=g/h;                 Form element of p in temporarily unused
                                                 element of e.
                    f += e[j]*z[i][j];
                }
                hh=f/(h+h);                   Form K, equation (11.3.11).
                for (j=0;j<i;j++) {           Form q and store in e overwriting p.
                    f=z[i][j];
                    e[j]=g=e[j]-hh*f;
                    for (k=0;k<j+1;k++)       Reduce z, equation (11.3.13).
```

```
                    z[j][k] -= (f*e[k]+g*z[i][k]);
                }
            }
        } else
            e[i]=z[i][l];
        d[i]=h;
    }
    if (yesvecs) d[0]=0.0;
    e[0]=0.0;
    for (i=0;i<n;i++) {                          Begin accumulation of transformation ma-
        if (yesvecs) {                               trices.
            if (d[i] != 0.0) {                   This block skipped when i=0.
                for (j=0;j<i;j++) {
                    g=0.0;
                    for (k=0;k<i;k++)            Use u and u/H stored in z to form P·Q.
                        g += z[i][k]*z[k][j];
                    for (k=0;k<i;k++)
                        z[k][j] -= g*z[k][i];
                }
            }
            d[i]=z[i][i];
            z[i][i]=1.0;                         Reset row and column of z to identity
            for (j=0;j<i;j++) z[j][i]=z[i][j]=0.0;   matrix for next iteration.
        } else {
            d[i]=z[i][i];                        Only this statement remains.
        }
    }
}
```


CITED REFERENCES AND FURTHER READING:

Golub, G.H., and Van Loan, C.F. 1996, *Matrix Computations*, 3rd ed. (Baltimore: Johns Hopkins University Press), §5.1.[1]

Smith, B.T., et al. 1976, *Matrix Eigensystem Routines — EISPACK Guide*, 2nd ed., vol. 6 of Lecture Notes in Computer Science (New York: Springer).

Wilkinson, J.H., and Reinsch, C. 1971, *Linear Algebra*, vol. II of *Handbook for Automatic Computation* (New York: Springer).[2]

11.4 Eigenvalues and Eigenvectors of a Tridiagonal Matrix

We now turn to the second step in the grand strategy outlined in §11.2, namely computing the eigenvectors and eigenvalues of a tridiagonal matrix.

11.4.1 Evaluation of the Characteristic Polynomial

Once our original real symmetric matrix has been reduced to tridiagonal form, one possible way to determine its eigenvalues is to find the roots of the characteristic polynomial $p_n(\lambda)$ directly. The characteristic polynomial of a tridiagonal matrix can be evaluated for any trial value of λ by an efficient recursion relation (see [1], for example). The polynomials of lower degree produced during the recurrence form a Sturmian sequence that can be used to localize the eigenvalues to intervals on the

real axis. A root-finding method such as bisection or Newton's method can then be employed to refine the intervals. The corresponding eigenvectors can then be found by inverse iteration (see §11.8).

Procedures based on these ideas can be found in [2,3]. If, however, more than a small fraction of all the eigenvalues and eigenvectors is required, then the factorization method next considered is much more efficient.

11.4.2 The QR and QL Algorithms

The basic idea behind the QR algorithm is that any real matrix can be decomposed in the form

$$\mathbf{A} = \mathbf{Q} \cdot \mathbf{R} \tag{11.4.1}$$

where $\mathbf{Q}$ is orthogonal and $\mathbf{R}$ is upper triangular. For a general matrix, the decomposition is constructed by applying Householder transformations to annihilate successive columns of $\mathbf{A}$ below the diagonal (see §2.10).

Now consider the matrix formed by writing the factors in (11.4.1) in the opposite order:

$$\mathbf{A}' = \mathbf{R} \cdot \mathbf{Q} \tag{11.4.2}$$

Since $\mathbf{Q}$ is orthogonal, equation (11.4.1) gives $\mathbf{R} = \mathbf{Q}^T \cdot \mathbf{A}$. Thus equation (11.4.2) becomes

$$\mathbf{A}' = \mathbf{Q}^T \cdot \mathbf{A} \cdot \mathbf{Q} \tag{11.4.3}$$

We see that $\mathbf{A}'$ is an orthogonal transformation of $\mathbf{A}$.

You can verify that a QR transformation preserves the following properties of a matrix: symmetry, tridiagonal form, and Hessenberg form (to be defined in §11.6).

There is nothing special about choosing one of the factors of $\mathbf{A}$ to be upper triangular; one could equally well make it lower triangular. This is called the QL algorithm, since

$$\mathbf{A} = \mathbf{Q} \cdot \mathbf{L} \tag{11.4.4}$$

where $\mathbf{L}$ is lower triangular. (The standard, but confusing, nomenclature R and L stands for whether the *right* or *left* of the matrix is nonzero.)

Recall that in the Householder reduction to tridiagonal form in §11.3, we started in column $n-1$ of the original matrix. To minimize roundoff, we then exhorted you to put the biggest elements of the matrix in the lower right-hand corner, if you can. If we now wish to diagonalize the resulting tridiagonal matrix, the QL algorithm will have smaller roundoff than the QR algorithm, so we shall use QL henceforth.

The QL algorithm consists of a *sequence* of orthogonal transformations:

$$\begin{aligned} \mathbf{A}_s &= \mathbf{Q}_s \cdot \mathbf{L}_s \\ \mathbf{A}_{s+1} &= \mathbf{L}_s \cdot \mathbf{Q}_s \qquad (= \mathbf{Q}_s^T \cdot \mathbf{A}_s \cdot \mathbf{Q}_s) \end{aligned} \tag{11.4.5}$$

The following (nonobvious!) theorem is the basis of the algorithm for a general matrix $\mathbf{A}$: (i) If $\mathbf{A}$ has eigenvalues of different absolute value $|\lambda_i|$, then $\mathbf{A}_s \to$ [lower triangular form] as $s \to \infty$. The eigenvalues appear on the diagonal in increasing order of absolute magnitude. (ii) If $\mathbf{A}$ has an eigenvalue $|\lambda_i|$ of multiplicity p, $\mathbf{A}_s \to$ [lower triangular form] as $s \to \infty$, except for a diagonal block matrix of

order p, whose eigenvalues $\to \lambda_i$. The proof of this theorem is fairly lengthy; see, for example, [4].

The workload in the QL algorithm is $O(n^3)$ per iteration for a general matrix, which is prohibitive. However, the workload is only $O(n)$ per iteration for a tridiagonal matrix and $O(n^2)$ for a Hessenberg matrix, which makes it highly efficient on these forms.

In this section we are concerned only with the case where $\mathbf{A}$ is a real, symmetric, tridiagonal matrix. All the eigenvalues λ_i are thus real. According to the theorem, if any λ_i has a multiplicity p, then there must be at least $p-1$ zeros on the sub- and superdiagonals. Thus the matrix can be split into submatrices that can be diagonalized separately, and the complication of diagonal blocks that can arise in the general case is irrelevant.

In the proof of the theorem quoted above, one finds that in general a superdiagonal element converges to zero like

$$a_{ij}^{(s)} \sim \left(\frac{\lambda_i}{\lambda_j}\right)^s \tag{11.4.6}$$

Although $\lambda_i < \lambda_j$, convergence can be slow if λ_i is close to λ_j. Convergence can be accelerated by the technique of *shifting*: If k is any constant, then $\mathbf{A} - k\mathbf{1}$ has eigenvalues $\lambda_i - k$. If we decompose

$$\mathbf{A}_s - k_s\mathbf{1} = \mathbf{Q}_s \cdot \mathbf{L}_s \tag{11.4.7}$$

so that

$$\begin{aligned} \mathbf{A}_{s+1} &= \mathbf{L}_s \cdot \mathbf{Q}_s + k_s\mathbf{1} \\ &= \mathbf{Q}_s^T \cdot \mathbf{A}_s \cdot \mathbf{Q}_s \end{aligned} \tag{11.4.8}$$

then the convergence is determined by the ratio

$$\frac{\lambda_i - k_s}{\lambda_j - k_s} \tag{11.4.9}$$

The idea is to choose the shift k_s at each stage to maximize the rate of convergence. A good choice for the shift initially would be k_s close to λ_0, the smallest eigenvalue. Then the first row of off-diagonal elements would tend rapidly to zero. However, λ_0 is not usually known a priori. A very effective strategy in practice (although there is no proof that it is optimal) is to compute the eigenvalues of the leading 2×2 diagonal submatrix of $\mathbf{A}$. Then set k_s equal to the eigenvalue closer to a_{00}.

More generally, suppose you have already found r eigenvalues of $\mathbf{A}$. Then you can *deflate* the matrix by crossing out the first r rows and columns, leaving

$$\mathbf{A} = \begin{bmatrix} 0 & & \cdots & \cdots & & & 0 \\ & \cdots & & & & & \\ & & 0 & & & & \\ \vdots & & & d_r & e_r & & \vdots \\ \vdots & & & e_r & d_{r+1} & & \\ & & & & & \cdots & & 0 \\ & & & & & & d_{n-2} & e_{n-2} \\ 0 & & \cdots & & & 0 & e_{n-2} & d_{n-1} \end{bmatrix} \tag{11.4.10}$$

Choose k_s equal to the eigenvalue of the leading 2×2 submatrix that is closer to d_r. One can show that the convergence of the algorithm with this strategy is generally cubic (and at worst quadratic for degenerate eigenvalues). This rapid convergence is what makes the algorithm so attractive.

Note that with shifting, the eigenvalues no longer necessarily appear on the diagonal in order of increasing absolute magnitude. The routine `eigsrt` (§11.1) can be used if required.

As we mentioned earlier, the QL decomposition of a general matrix is effected by a sequence of Householder transformations. For a tridiagonal matrix, however, it is more efficient to use plane rotations $\mathbf{P}_{pq}$. One uses the sequence $\mathbf{P}_{01}, \mathbf{P}_{12}, \dots, \mathbf{P}_{n-2,n-1}$ to annihilate the elements $a_{01}, a_{12}, \dots, a_{n-2,n-1}$. By symmetry, the subdiagonal elements $a_{10}, a_{21}, \dots, a_{n-1,n-2}$ will be annihilated too. Thus each $\mathbf{Q}_s$ is a product of plane rotations:

$$\mathbf{Q}_s^T = \mathbf{P}_1^{(s)} \cdot \mathbf{P}_2^{(s)} \cdots \mathbf{P}_{n-1}^{(s)} \tag{11.4.11}$$

where $\mathbf{P}_i$ annihilates $a_{i-1,i}$. Note that it is $\mathbf{Q}^T$ in equation (11.4.11), not $\mathbf{Q}$, because we defined $\mathbf{L} = \mathbf{Q}^T \cdot \mathbf{A}$.

11.4.3 QL Algorithm with Implicit Shifts

The algorithm as described so far can be very successful. However, when the elements of $\mathbf{A}$ differ widely in order of magnitude, subtracting a large k_s from the diagonal elements can lead to loss of accuracy for the small eigenvalues. This difficulty is avoided by the QL algorithm with *implicit shifts*. The implicit QL algorithm is mathematically equivalent to the original QL algorithm, but the computation does not require $k_s\mathbf{1}$ to be actually subtracted from $\mathbf{A}$.

The algorithm is based on the following lemma: If $\mathbf{A}$ is a symmetric nonsingular matrix and $\mathbf{B} = \mathbf{Q}^T \cdot \mathbf{A} \cdot \mathbf{Q}$, where $\mathbf{Q}$ is orthogonal and $\mathbf{B}$ is tridiagonal with positive off-diagonal elements, then $\mathbf{Q}$ and $\mathbf{B}$ are fully determined when the last row of $\mathbf{Q}^T$ is specified. Proof: Let $\mathbf{q}_i^T$ denote the row vector i of the matrix $\mathbf{Q}^T$. Then $\mathbf{q}_i$ is the column vector i of the matrix $\mathbf{Q}$. The relation $\mathbf{B} \cdot \mathbf{Q}^T = \mathbf{Q}^T \cdot \mathbf{A}$ can be written

$$\begin{bmatrix} \beta_0 & \gamma_0 & & & & \\ \alpha_1 & \beta_1 & \gamma_1 & & & \\ & & \vdots & & & \\ & & & \alpha_{n-2} & \beta_{n-2} & \gamma_{n-2} \\ & & & & \alpha_{n-1} & \beta_{n-1} \end{bmatrix} \cdot \begin{bmatrix} \mathbf{q}_0^T \\ \mathbf{q}_1^T \\ \vdots \\ \mathbf{q}_{n-2}^T \\ \mathbf{q}_{n-1}^T \end{bmatrix} = \begin{bmatrix} \mathbf{q}_0^T \\ \mathbf{q}_1^T \\ \vdots \\ \mathbf{q}_{n-2}^T \\ \mathbf{q}_{n-1}^T \end{bmatrix} \cdot \mathbf{A} \tag{11.4.12}$$

Row $n-1$ of this matrix equation is

$$\alpha_{n-1}\mathbf{q}_{n-2}^T + \beta_{n-1}\mathbf{q}_{n-1}^T = \mathbf{q}_{n-1}^T \cdot \mathbf{A} \tag{11.4.13}$$

Since $\mathbf{Q}$ is orthogonal,

$$\mathbf{q}_{n-1}^T \cdot \mathbf{q}_m = \delta_{n-1,m} \tag{11.4.14}$$

Thus, if we postmultiply equation (11.4.13) by $\mathbf{q}_{n-1}$, we find

$$\beta_{n-1} = \mathbf{q}_{n-1}^T \cdot \mathbf{A} \cdot \mathbf{q}_{n-1} \tag{11.4.15}$$

which is known since $\mathbf{q}_{n-1}$ is known. Then equation (11.4.13) gives

$$\alpha_{n-1}\mathbf{q}_{n-2}^T = \mathbf{z}_{n-2}^T \tag{11.4.16}$$

where

$$\mathbf{z}_{n-2}^T \equiv \mathbf{q}_{n-1}^T \cdot \mathbf{A} - \beta_{n-1}\mathbf{q}_{n-1}^T \tag{11.4.17}$$

is known. Therefore

$$\alpha_{n-1}^2 = \mathbf{z}_{n-2}^T \mathbf{z}_{n-2}, \tag{11.4.18}$$

or

$$\alpha_{n-1} = |\mathbf{z}_{n-2}| \tag{11.4.19}$$

and

$$\mathbf{q}_{n-2}^T = \mathbf{z}_{n-2}^T/\alpha_{n-1} \tag{11.4.20}$$

(where α_{n-1} is nonzero by hypothesis). Similarly, one can show by induction that if we know $\mathbf{q}_{n-1}, \mathbf{q}_{n-2}, \dots, \mathbf{q}_{n-j}$ and the α's, β's, and γ's up to level $n-j$, one can determine the quantities at level $n-(j+1)$.

To apply the lemma in practice, suppose one can somehow find a tridiagonal matrix $\bar{\mathbf{A}}_{s+1}$ such that

$$\bar{\mathbf{A}}_{s+1} = \bar{\mathbf{Q}}_s^T \cdot \bar{\mathbf{A}}_s \cdot \bar{\mathbf{Q}}_s \tag{11.4.21}$$

where $\bar{\mathbf{Q}}_s^T$ is orthogonal and has the same last row as $\mathbf{Q}_s^T$ in the original QL algorithm. Then $\bar{\mathbf{Q}}_s = \mathbf{Q}_s$ and $\bar{\mathbf{A}}_{s+1} = \mathbf{A}_{s+1}$.

Now, in the original algorithm, from equation (11.4.11) we see that the last row of $\mathbf{Q}_s^T$ is the same as the last row of $\mathbf{P}_{n-1}^{(s)}$. But recall that $\mathbf{P}_{n-1}^{(s)}$ is a plane rotation designed to annihilate the $(n-2, n-1)$ element of $\mathbf{A}_s - k_s\mathbf{1}$. A simple calculation using the expression (11.1.1) shows that it has parameters

$$c = \frac{d_{n-1} - k_s}{\sqrt{e_{n-1}^2 + (d_{n-1} - k_s)^2}}, \qquad s = \frac{-e_{n-2}}{\sqrt{e_{n-1}^2 + (d_{n-1} - k_s)^2}} \tag{11.4.22}$$

The matrix $\mathbf{P}_{n-1}^{(s)} \cdot \mathbf{A}_s \cdot \mathbf{P}_{n-1}^{(s)T}$ is tridiagonal with two extra elements:

$$\begin{bmatrix} \cdots & & & & & \\ & \times & \times & \times & & \\ & & \times & \times & \times & \mathbf{x} \\ & & & \times & \times & \times \\ & & & \mathbf{x} & \times & \times \end{bmatrix} \tag{11.4.23}$$

We must now reduce this to tridiagonal form with an orthogonal matrix whose last row is $[0, 0, \dots, 0, 1]$ so that the last row of $\bar{\mathbf{Q}}_s^T$ will stay equal to $\mathbf{P}_{n-1}^{(s)}$. This can be done by a sequence of Householder or Givens transformations. For the special form of the matrix (11.4.23), Givens is better. We rotate in the plane $(n-3, n-2)$ to annihilate the $(n-3, n-1)$ element. [By symmetry, the $(n-1, n-3)$ element will also be zeroed.] This leaves us with a tridiagonal form except for the extra elements $(n-4, n-2)$ and $(n-2, n-4)$. We annihilate these with a rotation in the $(n-4, n-3)$-plane, and so on. Thus a sequence of $n-2$ Givens rotations is required. The result is that

$$\mathbf{Q}_s^T = \bar{\mathbf{Q}}_s^T = \bar{\mathbf{P}}_1^{(s)} \cdot \bar{\mathbf{P}}_2^{(s)} \cdots \bar{\mathbf{P}}_{n-2}^{(s)} \cdot \mathbf{P}_{n-1}^{(s)} \tag{11.4.24}$$

where the $\bar{\mathbf{P}}$'s are the Givens rotations and $\mathbf{P}_{n-1}$ is the same plane rotation as in the original algorithm. Then equation (11.4.21) gives the next iterate of $\mathbf{A}$. Note that the shift k_s enters implicitly through the parameters (11.4.22).

The following routine tqli ("Tridiagonal QL Implicit"), based algorithmically on the implementations in [2,3], works extremely well in practice. The number of iterations for the first few eigenvalues might be four or five, say, but meanwhile the off-diagonal elements in the lower right-hand corner have been reduced too. The later eigenvalues are liberated with very little work. The average number of iterations per eigenvalue is typically 1.3–1.6. The operation count per iteration is $O(n)$, with a fairly large effective coefficient, say $\sim 20n$. The total operation count for the diagonalization is then very roughly $\sim 20n \times (1.3\text{–}1.6)n \sim 30n^2$. If the eigenvectors are required, the statements indicated by comments are included and there is an additional, much larger, workload of about $6n^3$ operations.

eigen_sym.h

```
void Symmeig::tqli()
```

QL algorithm with implicit shifts to determine the eigenvalues and (optionally) the eigenvectors of a real, symmetric, tridiagonal matrix, or of a real symmetric matrix previously reduced by tred2 (§11.3). On input, d[0..n-1] contains the diagonal elements of the tridiagonal matrix. On output, it returns the eigenvalues. The vector e[0..n-1] inputs the subdiagonal elements of the tridiagonal matrix, with e[0] arbitrary. On output e is destroyed. If the eigenvectors of a tridiagonal matrix are desired, the matrix z[0..n-1][0..n-1] is input as the identity matrix. If the eigenvectors of a matrix that has been reduced by tred2 are required, then z is input as the matrix output by tred2. In either case, column k of z returns the normalized eigenvector corresponding to d[k].

```
{
    Int m,l,iter,i,k;
    Doub s,r,p,g,f,dd,c,b;
    const Doub EPS=numeric_limits<Doub>::epsilon();
    for (i=1;i<n;i++) e[i-1]=e[i];                      Convenient to renumber the el-
    e[n-1]=0.0;                                             ements of e.
    for (l=0;l<n;l++) {
        iter=0;
        do {
            for (m=l;m<n-1;m++) {                       Look for a single small subdi-
                dd=abs(d[m])+abs(d[m+1]);                   agonal element to split the
                if (abs(e[m]) <= EPS*dd) break;             matrix.
            }
            if (m != l) {
                if (iter++ == 30) throw("Too many iterations in tqli");
                g=(d[l+1]-d[l])/(2.0*e[l]);             Form shift.
                r=pythag(g,1.0);
                g=d[m]-d[l]+e[l]/(g+SIGN(r,g));         This is d_m - k_s.
                s=c=1.0;
                p=0.0;
                for (i=m-1;i>=l;i--) {                  A plane rotation as in the origi-
                    f=s*e[i];                               nal QL, followed by Givens
                    b=c*e[i];                               rotations to restore tridiag-
                    e[i+1]=(r=pythag(f,g));                 onal form.
                    if (r == 0.0) {                     Recover from underflow.
                        d[i+1] -= p;
                        e[m]=0.0;
                        break;
                    }
                    s=f/r;
                    c=g/r;
                    g=d[i+1]-p;
                    r=(d[i]-g)*s+2.0*c*b;
                    d[i+1]=g+(p=s*r);
                    g=c*r-b;
                    if (yesvecs) {
                        for (k=0;k<n;k++) {             Form eigenvectors.
                            f=z[k][i+1];
                            z[k][i+1]=s*z[k][i]+c*f;
```

```
                        z[k][i]=c*z[k][i]-s*f;
                    }
                }
            }
            if (r == 0.0 && i >= l) continue;
            d[l] -= p;
            e[l]=g;
            e[m]=0.0;
        }
    } while (m != l);
}
}

Doub Symmeig::pythag(const Doub a, const Doub b) {
```
Computes $(a^2 + b^2)^{1/2}$ without destructive underflow or overflow.
```
    Doub absa=abs(a), absb=abs(b);
    return (absa > absb ? absa*sqrt(1.0+SQR(absb/absa)) :
        (absb == 0.0 ? 0.0 : absb*sqrt(1.0+SQR(absa/absb))));
}
```

11.4.4 Newer Methods

There are two newer algorithms for tridiagonal symmetric systems that are generally more efficient than the QL method, especially for large matrices. The first is the *divide-and-conquer method* [5]. This method divides the tridiagonal matrix into two halves, solves the eigenproblems in each of the two halves, and then stitches the two solutions together to generate the solution of the original problem. The method is applied recursively, with the QL method used once the matrices are sufficiently small. The method is implemented in LAPACK as `dstevd` and is about 2.5 times faster than the QL method for large matrices.

The fastest method of all for the vast majority of matrices is the *MRRR algorithm* (Multiple Relatively Robust Representations) [6]. As we will see in §11.8, inverse iteration can determine the eigenvectors of a tridiagonal matrix in $O(n^2)$ operations. However, clustered eigenvalues lead to eigenvectors that are not properly orthogonal to one another. Using a procedure like Gram-Schmidt to orthogonalize the vectors is $O(n^3)$. The MRRR algorithm is a sophisticated version of inverse iteration that is $O(n^2)$ without requiring Gram-Schmidt. An implementation is available in LAPACK as `dstegr`.

CITED REFERENCES AND FURTHER READING:

Acton, F.S. 1970, *Numerical Methods That Work*; 1990, corrected edition (Washington, DC: Mathematical Association of America), pp. 331–335.[1]

Wilkinson, J.H., and Reinsch, C. 1971, *Linear Algebra*, vol. II of *Handbook for Automatic Computation* (New York: Springer).[2]

Smith, B.T., et al. 1976, *Matrix Eigensystem Routines — EISPACK Guide*, 2nd ed., vol. 6 of Lecture Notes in Computer Science (New York: Springer).[3]

Stoer, J., and Bulirsch, R. 2002, *Introduction to Numerical Analysis*, 3rd ed. (New York: Springer), §6.6.4.[4]

Cuppen, J.J.M. 1981, "A Divide-and-Conquer Method for the Symmetric Tridiagonal Eigenproblem," *Numerische Mathematik*, vol. 36, pp. 177–195.[5]

Dhillon, I.S., and Parlett, B.N. 2004, "Multiple Representations to Compute Orthogonal Eigenvectors of Symmetric Tridiagonal Matrices," *Linear Algebra and Its Applications*, vol. 387, pp. 1–28.[6]

11.5 Hermitian Matrices

The complex analog of a real symmetric matrix is a Hermitian matrix, satisfying equation (11.0.4). Jacobi transformations can be used to find eigenvalues and eigenvectors, as can Householder reduction to tridiagonal form followed by QL iteration. Complex versions of the previous routines `jacobi`, `tred2`, and `tqli` are quite analogous to their real counterparts. For working routines, consult [1,2].

An alternative, using the routines in this book, is to convert the Hermitian problem to a real symmetric one: If $\mathbf{C} = \mathbf{A} + i\mathbf{B}$ is a Hermitian matrix, then the $n \times n$ complex eigenvalue problem

$$(\mathbf{A} + i\mathbf{B}) \cdot (\mathbf{u} + i\mathbf{v}) = \lambda(\mathbf{u} + i\mathbf{v}) \tag{11.5.1}$$

is equivalent to the $2n \times 2n$ real problem

$$\begin{bmatrix} \mathbf{A} & -\mathbf{B} \\ \mathbf{B} & \mathbf{A} \end{bmatrix} \cdot \begin{bmatrix} \mathbf{u} \\ \mathbf{v} \end{bmatrix} = \lambda \begin{bmatrix} \mathbf{u} \\ \mathbf{v} \end{bmatrix} \tag{11.5.2}$$

Note that the $2n \times 2n$ matrix in (11.5.2) is symmetric: $\mathbf{A}^T = \mathbf{A}$ and $\mathbf{B}^T = -\mathbf{B}$ if $\mathbf{C}$ is Hermitian.

Corresponding to a given eigenvalue λ, the vector

$$\begin{bmatrix} -\mathbf{v} \\ \mathbf{u} \end{bmatrix} \tag{11.5.3}$$

is also an eigenvector, as you can verify by writing out the two matrix equations implied by (11.5.2). Thus, if $\lambda_0, \lambda_1, \ldots, \lambda_{n-1}$ are the eigenvalues of $\mathbf{C}$, then the $2n$ eigenvalues of the augmented problem (11.5.2) are $\lambda_0, \lambda_0, \lambda_1, \lambda_1, \ldots, \lambda_{n-1}, \lambda_{n-1}$; each, in other words, is repeated twice. The eigenvectors are pairs of the form $\mathbf{u} + i\mathbf{v}$ and $i(\mathbf{u} + i\mathbf{v})$; that is, they are the same up to an inessential phase. Thus we solve the augmented problem (11.5.2) and choose one eigenvalue and eigenvector from each pair. These give the eigenvalues and eigenvectors of the original matrix $\mathbf{C}$.

Working with the augmented matrix requires a factor of two more storage than the original complex matrix. In principle, a complex algorithm is also a factor of two more efficient in computer time than is the solution of the augmented problem. In practice, most complex implementations do not achieve this factor unless they are written entirely in real arithmetic. (Good library routines always do this.)

CITED REFERENCES AND FURTHER READING:

Wilkinson, J.H., and Reinsch, C. 1971, *Linear Algebra*, vol. II of *Handbook for Automatic Computation* (New York: Springer).[1]

Smith, B.T., et al. 1976, *Matrix Eigensystem Routines — EISPACK Guide*, 2nd ed., vol. 6 of Lecture Notes in Computer Science (New York: Springer).[2]

11.6 Real Nonsymmetric Matrices

The algorithms for symmetric matrices given in the preceding sections are highly satisfactory in practice. By contrast, it is impossible to design equally satisfactory

algorithms for the nonsymmetric case. There are two reasons for this. First, the eigenvalues of a nonsymmetric matrix can be very sensitive to small changes in the matrix elements. Second, the matrix itself can be defective, so that there is no complete set of eigenvectors. We emphasize that these difficulties are intrinsic properties of certain nonsymmetric matrices, and no numerical procedure can "cure" them. The best we can hope for are procedures that don't exacerbate such problems.

The presence of rounding error can only make the situation worse. With finite-precision arithmetic, one cannot even design a foolproof algorithm to determine whether a given matrix is defective or not. Thus current algorithms generally *try* to find a *complete* set of eigenvectors and rely on the user to inspect the results. If any eigenvectors are almost parallel, the matrix is probably defective.

The strategy for finding the eigensystem of a general matrix parallels that of the symmetric case. First we reduce the matrix to a simpler form, and then we perform an iterative procedure on the simplified matrix. The simpler structure we use here is called *Hessenberg* form, defined later in this section. The user interface to the routine is very simple. The declaration

```
Unsymmeig h(a);
```

computes all eigenvalues and eigenvectors of the matrix a. The eigenvalues are stored in the complex vector h.wri and the corresponding eigenvectors in the columns of the matrix h.zz. If h.wri[i] is real, the real eigenvector is in h.zz[0..n-1][i]. For complex eigenvalues, if h.wri[i] has a positive imaginary part, then the complex-conjugate eigenvalue is in h.wri[i+1]. Only the eigenvector corresponding to h.wri[i] is returned, with the real part in h.zz[0..n-1][i] and the imaginary part in h.zz[0..n-1][i+1]. The eigenvector corresponding to h.wri[i+1] is simply the complex conjugate of this one.

Optional arguments allow you to compute only the eigenvalues, or to input a matrix already in Hessenberg form:

```
Unsymmeig h(a,false);          Only eigenvalues computed.
Unsymmeig h(a,true,true);      Both eigenvalues and eigenvectors, Hessenberg matrix.
```

Here is the routine. The implementations of the various components are discussed in the rest of this section and the next.

eigen_unsym.h

```
struct Unsymmeig {
Computes all eigenvalues and eigenvectors of a real nonsymmetric matrix by reduction to Hessenberg form followed by QR iteration.
    Int n;
    MatDoub a,zz;
    VecComplex wri;
    VecDoub scale;                  Stores scaling from balance.
    VecInt perm;                    Stores permutation from elmhes.
    Bool yesvecs,hessen;

    Unsymmeig(MatDoub_I &aa, Bool yesvec=true, Bool hessenb=false) :
        n(aa.nrows()), a(aa), zz(n,n,0.0), wri(n), scale(n,1.0), perm(n),
        yesvecs(yesvec), hessen(hessenb)
    Computes all eigenvalues and (optionally) eigenvectors of a real nonsymmetric matrix
    a[0..n-1][0..n-1] by reduction to Hessenberg form followed by QR iteration. If yesvecs
    is input as true (the default), then the eigenvectors are computed. Otherwise, only the
    eigenvalues are computed. If hessen is input as false (the default), the matrix is first
    reduced to Hessenberg form. Otherwise it is assumed that the matrix is already in Hessen-
    berg from. On output, wri[0..n-1] contains the eigenvalues of a sorted into descending
    order, while zz[0..n-1][0..n-1] is a matrix whose columns contain the corresponding
```

eigenvectors. For a complex eigenvalue, only the eigenvector corresponding to the eigenvalue with a positive imaginary part is stored, with the real part in zz[0..n-1][i] and the imaginary part in h.zz[0..n-1][i+1]. The eigenvectors are not normalized.

```
    {
        balance();
        if (!hessen) elmhes();
        if (yesvecs) {
            for (Int i=0;i<n;i++)          Initialize to unit matrix.
                zz[i][i]=1.0;
            if (!hessen) eltran();
            hqr2();
            balbak();
            sortvecs();
        } else {
            hqr();
            sort();
        }
    }
    void balance();
    void elmhes();
    void eltran();
    void hqr();
    void hqr2();
    void balbak();
    void sort();
    void sortvecs();
};
```

11.6.1 Balancing

The sensitivity of eigenvalues to rounding errors during the execution of some algorithms can be reduced by the procedure of *balancing*. The errors in the eigensystem found by a numerical procedure are generally proportional to the Euclidean norm of the matrix, that is, to the square root of the sum of the squares of the elements. The idea of balancing is to use similarity transformations to make corresponding rows and columns of the matrix have comparable norms, thus reducing the overall norm of the matrix while leaving the eigenvalues unchanged. A symmetric matrix is already balanced.

Balancing is a procedure with of order N^2 operations. Thus, the time taken by the procedure balance, given below, should never be significant compared to the total time required to find the eigenvalues. It is therefore recommended that you *always* balance nonsymmetric matrices. It never hurts, and it can substantially improve the accuracy of the eigenvalues computed for a badly balanced matrix.

The actual algorithm used is due to Osborne, as discussed in [1]. It consists of a sequence of similarity transformations by diagonal matrices $\mathbf{D}$. To avoid introducing rounding errors during the balancing process, the elements of $\mathbf{D}$ are restricted to be exact powers of the radix base employed for floating-point arithmetic (i.e., 2 for all modern machines, but 16 for some historical mainframe architectures). The output is a matrix that is balanced in the norm given by summing the absolute magnitudes of the matrix elements. This is more efficient than using the Euclidean norm, and equally effective: A large reduction in one norm implies a large reduction in the other.

Note that if the off-diagonal elements of any row or column of a matrix are all zero, then the diagonal element is an eigenvalue. If the eigenvalue happens to be ill-conditioned (sensitive to small changes in the matrix elements), it will have

relatively large errors when determined by the routine hqr (§11.7). Had we merely inspected the matrix beforehand, we could have determined the isolated eigenvalue exactly and then deleted the corresponding row and column from the matrix. You should consider whether such a pre-inspection might be useful in your application. (For symmetric matrices, the routines we gave will determine isolated eigenvalues accurately in all cases.)

The routine balance keeps track of the scale factors used in the balancing. If you are computing eigenvectors as well as eigenvalues, then the accumulated similarity transformation of the original matrix is undone by applying these scale factors in the routine balbak.

```
void Unsymmeig::balance()                                                  eigen_unsym.h
```
Given a matrix a[0..n-1][0..n-1], this routine replaces it by a balanced matrix with identical eigenvalues. A symmetric matrix is already balanced and is unaffected by this procedure.
```
{
    const Doub RADIX = numeric_limits<Doub>::radix;
    Bool done=false;
    Doub sqrdx=RADIX*RADIX;
    while (!done) {
        done=true;
        for (Int i=0;i<n;i++) {                    Calculate row and column norms.
            Doub r=0.0,c=0.0;
            for (Int j=0;j<n;j++)
                if (j != i) {
                    c += abs(a[j][i]);
                    r += abs(a[i][j]);
                }
            if (c != 0.0 && r != 0.0) {            If both are nonzero,
                Doub g=r/RADIX;
                Doub f=1.0;
                Doub s=c+r;
                while (c<g) {                      find the integer power of the machine
                    f *= RADIX;                        radix that comes closest to balanc-
                    c *= sqrdx;                        ing the matrix.
                }
                g=r*RADIX;
                while (c>g) {
                    f /= RADIX;
                    c /= sqrdx;
                }
                if ((c+r)/f < 0.95*s) {
                    done=false;
                    g=1.0/f;
                    scale[i] *= f;
                    for (Int j=0;j<n;j++) a[i][j] *= g;   Apply similarity transforma-
                    for (Int j=0;j<n;j++) a[j][i] *= f;       tion.
                }
            }
        }
    }
}
```

```
void Unsymmeig::balbak()                                                   eigen_unsym.h
```
Forms the eigenvectors of a real nonsymmetric matrix by back transforming those of the corresponding balanced matrix determined by balance.
```
{
    for (Int i=0;i<n;i++)
        for (Int j=0;j<n;j++)
            zz[i][j] *= scale[i];
}
```

11.6.2 Reduction to Hessenberg Form

An *upper Hessenberg* matrix has zeros everywhere below the diagonal except for the first subdiagonal row. For example, in the 6×6 case, the nonzero elements are

$$\begin{bmatrix} \times & \times & \times & \times & \times & \times \\ \times & \times & \times & \times & \times & \times \\ & \times & \times & \times & \times & \times \\ & & \times & \times & \times & \times \\ & & & \times & \times & \times \\ & & & & \times & \times \end{bmatrix}$$

By now you should be able to tell at a glance that such a structure can be achieved by a sequence of Householder transformations, each one zeroing the required elements in a column of the matrix. Householder reduction to Hessenberg form is in fact an accepted technique. An alternative, however, is a procedure analogous to Gaussian elimination with pivoting. We will use this elimination procedure since it is about a factor of two more efficient than the Householder method, and also since we want to teach you the method. It is possible to construct matrices for which the Householder reduction, being orthogonal, is stable and elimination is not, but such matrices are extremely rare in practice.

Straight Gaussian elimination is not a similarity transformation of the matrix. Accordingly, the actual elimination procedure used is slightly different. We proceed in a series of stages $r = 1, 2, \ldots, N-2$. Before the rth stage, the original matrix $\mathbf{A} \equiv \mathbf{A}_1$ has become $\mathbf{A}_r$, which is upper Hessenberg up to, but not including, row and column $r-1$. The rth stage then consists of the following sequence of operations:

- Find the element of maximum magnitude in column $r-1$ below the diagonal. If it is zero, skip the next two "bullets" and the stage is done. Otherwise, suppose the maximum element was in row r'.
- Interchange rows r' and r. This is the pivoting procedure. To make the permutation a similarity transformation, also interchange columns r' and r.
- For $i = r+1, r+2, \ldots, N-1$, compute the multiplier

$$n_{ir} \equiv \frac{a_{i,r-1}}{a_{r,r-1}}$$

  Subtract n_{ir} times row r from row i. To make the elimination a similarity transformation, also *add* n_{ir} times column i to column r.

A total of $N-2$ such stages are required.

When the magnitudes of the matrix elements vary over many orders, you should try to rearrange the matrix so that the largest elements are in the top left-hand corner. This reduces the roundoff error, since the reduction proceeds from left to right.

The routine `elmhes` keeps track of the permutations applied during the elimination. If you are computing eigenvectors, then the accumulated similarity transformation is applied to the eigenvectors by the routine `eltran`, which includes any necessary permutations. The operation count is about $5N^3/3$ for large N.

```
void Unsymmeig::elmhes()
```
eigen_unsym.h

Reduction to Hessenberg form by the elimination method. Replaces the real nonsymmetric matrix a[0..n-1][0..n-1] by an upper Hessenberg matrix with identical eigenvalues. Recommended, but not required, is that this routine be preceded by balance. On output, the Hessenberg matrix is in elements a[i][j] with i $\leq$ j+1. Elements with i $>$ j+1 are to be thought of as zero, but are returned with random values.

```
{
    for (Int m=1;m<n-1;m++) {                  m is called r in the text.
        Doub x=0.0;
        Int i=m;
        for (Int j=m;j<n;j++) {                Find the pivot.
            if (abs(a[j][m-1]) > abs(x)) {
                x=a[j][m-1];
                i=j;
            }
        }
        perm[m]=i;                             Store permutation.
        if (i != m) {                          Interchange rows and columns.
            for (Int j=m-1;j<n;j++) SWAP(a[i][j],a[m][j]);
            for (Int j=0;j<n;j++) SWAP(a[j][i],a[j][m]);
        }
        if (x != 0.0) {                        Carry out the elimination.
            for (i=m+1;i<n;i++) {
                Doub y=a[i][m-1];
                if (y != 0.0) {
                    y /= x;
                    a[i][m-1]=y;
                    for (Int j=m;j<n;j++) a[i][j] -= y*a[m][j];
                    for (Int j=0;j<n;j++) a[j][m] += y*a[j][i];
                }
            }
        }
    }
}
```

```
void Unsymmeig::eltran()
```
eigen_unsym.h

This routine accumulates the stabilized elementary similarity transformations used in the reduction to upper Hessenberg form by elmhes. The multipliers that were used in the reduction are obtained from the lower triangle (below the subdiagonal) of a. The transformations are permuted according to the permutations stored in perm by elmhes.

```
{
    for (Int mp=n-2;mp>0;mp--) {
        for (Int k=mp+1;k<n;k++)
            zz[k][mp]=a[k][mp-1];
        Int i=perm[mp];
        if (i != mp) {
            for (Int j=mp;j<n;j++) {
                zz[mp][j]=zz[i][j];
                zz[i][j]=0.0;
            }
            zz[i][mp]=1.0;
        }
    }
}
```

CITED REFERENCES AND FURTHER READING:

Wilkinson, J.H., and Reinsch, C. 1971, *Linear Algebra*, vol. II of *Handbook for Automatic Computation* (New York: Springer).[1]

Smith, B.T., et al. 1976, *Matrix Eigensystem Routines — EISPACK Guide*, 2nd ed., vol. 6 of Lecture Notes in Computer Science (New York: Springer).[2]

Stoer, J., and Bulirsch, R. 2002, *Introduction to Numerical Analysis*, 3rd ed. (New York: Springer), §6.5.4.[3]

11.7 The QR Algorithm for Real Hessenberg Matrices

To complete the strategy for real, nonsymmetric matrices that was laid out in §11.6, we need to compute the eigenvalues and eigenvectors of a real Hessenberg matrix. Recall the following relations for the QR algorithm with shifts:

$$\mathbf{Q}_s \cdot (\mathbf{A}_s - k_s \mathbf{1}) = \mathbf{R}_s \tag{11.7.1}$$

where $\mathbf{Q}$ is orthogonal and $\mathbf{R}$ is upper triangular, and

$$\begin{aligned} \mathbf{A}_{s+1} &= \mathbf{R}_s \cdot \mathbf{Q}_s^T + k_s \mathbf{1} \\ &= \mathbf{Q}_s \cdot \mathbf{A}_s \cdot \mathbf{Q}_s^T \end{aligned} \tag{11.7.2}$$

The QR transformation preserves the upper Hessenberg form of the original matrix $\mathbf{A} \equiv \mathbf{A}_1$, and the workload on such a matrix is $O(n^2)$ per iteration as opposed to $O(n^3)$ on a general matrix. As $s \to \infty$, $\mathbf{A}_s$ converges to a form where the eigenvalues are either isolated on the diagonal or are eigenvalues of a 2×2 submatrix on the diagonal.

As we pointed out in §11.4, shifting is essential for rapid convergence. A key difference here is that a nonsymmetric real matrix can have complex eigenvalues. This means that good choices for the shifts k_s may be complex, apparently necessitating complex arithmetic.

Complex arithmetic can be avoided, however, by a clever trick. This trick, plus a detailed description of how the QR algorithm is used, is described in a Webnote [1].

The operation count for the QR algorithm for Hessenberg matrices is $\sim 10k^2$ per iteration, where k is the current size of the matrix. The typical average number of iterations per eigenvalue is about two, so the total operation count for all the eigenvalues is $\sim 10n^3$. The total operation count for both eigenvalues and eigenvectors is $\sim 25n^3$.

The routines `hqr` for the eigenvalues only, and `hqr2`, which computes both eigenvalues and eigenvectors, are given in full in a Webnote [2], along with a few `Unsymmeig` utility routines not already listed. The implementations are based algorithmically on the above description, in turn following the implementations in [3,4].

CITED REFERENCES AND FURTHER READING:

Numerical Recipes Software 2007, "Description of the QR Algorithm for Hessenberg Matrices," *Numerical Recipes Webnote No. 16*, at `http://www.nr.com/webnotes?16` [1]

Numerical Recipes Software 2007, "Implementations in Unsymmeig," *Numerical Recipes Webnote No. 17*, at `http://www.nr.com/webnotes?17` [2]

Wilkinson, J.H., and Reinsch, C. 1971, *Linear Algebra*, vol. II of *Handbook for Automatic Computation* (New York: Springer).[3]

Golub, G.H., and Van Loan, C.F. 1996, *Matrix Computations*, 3rd ed. (Baltimore: Johns Hopkins University Press), §7.5.

Smith, B.T., et al. 1976, *Matrix Eigensystem Routines — EISPACK Guide*, 2nd ed., vol. 6 of Lecture Notes in Computer Science (New York: Springer).[4]

11.8 Improving Eigenvalues and/or Finding Eigenvectors by Inverse Iteration

The basic idea behind inverse iteration is quite simple. Let $\mathbf{y}$ be the solution of the linear system

$$(\mathbf{A} - \tau\mathbf{1}) \cdot \mathbf{y} = \mathbf{b} \tag{11.8.1}$$

where $\mathbf{b}$ is a random vector and τ is close to some eigenvalue λ of $\mathbf{A}$. Then the solution $\mathbf{y}$ will be close to the eigenvector corresponding to λ. The procedure can be iterated: Replace $\mathbf{b}$ by $\mathbf{y}$ and solve for a new $\mathbf{y}$, which will be even closer to the true eigenvector.

We can see why this works by expanding both $\mathbf{y}$ and $\mathbf{b}$ as linear combinations of the eigenvectors $\mathbf{x}_j$ of $\mathbf{A}$:

$$\mathbf{y} = \sum_j \alpha_j \mathbf{x}_j \qquad \mathbf{b} = \sum_j \beta_j \mathbf{x}_j \tag{11.8.2}$$

Then (11.8.1) gives

$$\sum_j \alpha_j (\lambda_j - \tau)\mathbf{x}_j = \sum_j \beta_j \mathbf{x}_j \tag{11.8.3}$$

so that

$$\alpha_j = \frac{\beta_j}{\lambda_j - \tau} \tag{11.8.4}$$

and

$$\mathbf{y} = \sum_j \frac{\beta_j \mathbf{x}_j}{\lambda_j - \tau} \tag{11.8.5}$$

If τ is close to λ_n, say, then provided β_n is not accidentally too small, $\mathbf{y}$ will be approximately $\mathbf{x}_n$, up to a normalization. Moreover, each iteration of this procedure gives another power of $\lambda_j - \tau$ in the denominator of (11.8.5). Thus the convergence is rapid for well-separated eigenvalues.

Suppose at the kth stage of iteration we are solving the equation

$$(\mathbf{A} - \tau_k\mathbf{1}) \cdot \mathbf{y} = \mathbf{b}_k \tag{11.8.6}$$

where $\mathbf{b}_k$ and τ_k are our current guesses for some eigenvector and eigenvalue of interest (let's say $\mathbf{x}_n$ and λ_n). Normalize $\mathbf{b}_k$ so that $\mathbf{b}_k \cdot \mathbf{b}_k = 1$. The exact eigenvector and eigenvalue satisfy

$$\mathbf{A} \cdot \mathbf{x}_n = \lambda_n \mathbf{x}_n \tag{11.8.7}$$

so

$$(\mathbf{A} - \tau_k\mathbf{1}) \cdot \mathbf{x}_n = (\lambda_n - \tau_k)\mathbf{x}_n \tag{11.8.8}$$

Since $\mathbf{y}$ of (11.8.6) is an improved approximation to $\mathbf{x}_n$, we normalize it and set

$$\mathbf{b}_{k+1} = \frac{\mathbf{y}}{|\mathbf{y}|} \tag{11.8.9}$$

We get an improved estimate of the eigenvalue by substituting our improved guess $\mathbf{y}$ for $\mathbf{x}_n$ in (11.8.8). By (11.8.6), the left-hand side is $\mathbf{b}_k$, so, calling λ_n our new value τ_{k+1}, we find

$$\tau_{k+1} = \tau_k + \frac{1}{\mathbf{b}_k \cdot \mathbf{y}} \tag{11.8.10}$$

While the above formulas look simple enough, in practice the implementation can be quite tricky. The first question to be resolved is *when* to use inverse iteration. Most of the computational load occurs in solving the linear system (11.8.6). Thus a possible strategy is first to reduce the matrix $\mathbf{A}$ to a special form that allows easy solution of (11.8.6). Tridiagonal form for symmetric matrices or Hessenberg for nonsymmetric are the obvious choices. The tridiagonal form can be solved in $O(N)$ operations, the Hessenberg form in $O(N^2)$ operations. If you then apply inverse iteration to generate all the eigenvectors, this gives an $O(N^2)$ method for tridiagonal matrices. The problem is that closely spaced eigenvalues lead to eigenvectors that are not properly orthogonal to one another. Using a procedure like Gram-Schmidt to orthogonalize the vectors is $O(n^3)$, and not entirely satisfactory anyway. Accordingly, inverse iteration is generally used when one already has good eigenvalues and wants only a few selected eigenvectors.

You can write a simple inverse iteration routine yourself using LU decomposition to solve (11.8.6). You can decide whether to use the general LU algorithm we gave in Chapter 2 or whether to take advantage of tridiagonal or Hessenberg form. Note that, since the linear system (11.8.6) is nearly singular, you must be careful to use a version of LU decomposition like that in §2.3 which replaces a zero pivot with a very small number.

We have chosen not to give a general inverse iteration routine in this book, because it is quite cumbersome to take account of all the cases that can arise. Routines are given, for example, in [1-3]. If you use these, or write your own routine, you may appreciate the following pointers.

One starts by supplying an initial value τ_0 for the eigenvalue λ_n of interest. Choose a random normalized vector $\mathbf{b}_0$ as the initial guess for the eigenvector $\mathbf{x}_n$, and solve (11.8.6). The new vector $\mathbf{y}$ is bigger than $\mathbf{b}_0$ by a "growth factor" $|\mathbf{y}|$, which ideally should be large. Equivalently, the change in the eigenvalue, which by (11.8.10) is essentially $1/\,|\mathbf{y}|$, should be small. The following cases can arise:

- If the growth factor is too small initially, then we assume we have made a "bad" choice of random vector. This can happen not just because of a small β_n in (11.8.5), but also in the case of a defective matrix, when (11.8.5) does not even apply (see, e.g., [1] or [4] for details). We go back to the beginning and choose a new initial vector.
- The change $|\mathbf{b}_1 - \mathbf{b}_0|$ might be less than some tolerance ϵ. We can use this as a criterion for stopping, iterating until it is satisfied, with a maximum of 5–10 iterations, say.
- After a few iterations, if $|\mathbf{b}_{k+1} - \mathbf{b}_k|$ is not decreasing rapidly enough, we can try updating the eigenvalue according to (11.8.10). If $\tau_{k+1} = \tau_k$ to machine accuracy, we are not going to improve the eigenvector much more and can quit. Otherwise start another cycle of iterations with the new eigenvalue.

The reason we do not update the eigenvalue at every step is that when we solve the linear system (11.8.6) by LU decomposition, we can save the decomposition if

τ_k is fixed (assuming we are working with the full matrix). We only need to do the backsubstitution step each time we update $\mathbf{b}_k$. The number of iterations we decide to do with a fixed τ_k is a trade-off between the quadratic convergence but $O(N^3)$ workload for updating τ_k at each step and the linear convergence but $O(N^2)$ load for keeping τ_k fixed. If you have determined the eigenvalue by one of the routines given earlier in the chapter, it is probably correct to machine accuracy anyway, and you can omit updating it.

There are two different pathologies that can arise during inverse iteration. The first is multiple or closely spaced roots. This is more often a problem with symmetric matrices. Inverse iteration will find only one eigenvector for a given initial guess τ_0. A good strategy is to perturb the last few significant digits in τ_0 and then repeat the iteration. Usually this provides an independent eigenvector. Special steps generally have to be taken to ensure orthogonality of the linearly independent eigenvectors, whereas the Jacobi and QL algorithms automatically yield orthogonal eigenvectors even in the case of multiple eigenvalues.

The second problem, peculiar to nonsymmetric matrices, is the defective case. Unless one makes a "good" initial guess, the growth factor is small. Moreover, iteration does not improve matters. In this case, the remedy is to choose random initial vectors, solve (11.8.6) once, and quit as soon as *any* vector gives an acceptably large growth factor. Typically only a few trials are necessary.

One further complication in the nonsymmetric case is that a real matrix can have complex-conjugate pairs of eigenvalues. You will then have to use complex arithmetic to solve (11.8.6) for the complex eigenvectors. For any moderate-sized (or larger) nonsymmetric matrix, our recommendation is to avoid inverse iteration in favor of a QR method like `Unsymmeig`.

A good discussion of these and other problems with inverse iteration is given in [5]. As discussed in §11.4.4, for symmetric tridiagonal matrices, the MRRR algorithm is a sophisticated version of inverse iteration that avoids all these problems.

CITED REFERENCES AND FURTHER READING:

Acton, F.S. 1970, *Numerical Methods That Work*; 1990, corrected edition (Washington, DC: Mathematical Association of America).

Wilkinson, J.H., and Reinsch, C. 1971, *Linear Algebra*, vol. II of *Handbook for Automatic Computation* (New York: Springer), p. 418.[1]

Smith, B.T., et al. 1976, *Matrix Eigensystem Routines — EISPACK Guide*, 2nd ed., vol. 6 of Lecture Notes in Computer Science (New York: Springer).[2]

Anderson, E., et al. 1999, LAPACK User's Guide, 3rd ed. (Philadelphia: S.I.A.M.). Online with software at 2007+, `http://www.netlib.org/lapack`.[3]

Stoer, J., and Bulirsch, R. 2002, *Introduction to Numerical Analysis*, 3rd ed. (New York: Springer), §6.6.3.[4]

Dhillon, I.S. 1998, "Current Inverse Iteration Software Can Fail," *BIT Numerical Mathematics*, vol. 38, pp. 685–704.[5]

Fast Fourier Transform

CHAPTER 12

12.0 Introduction

A very large class of important computational problems falls under the general rubric of *Fourier transform methods* or *spectral methods*. For some of these problems, the Fourier transform is simply an efficient computational tool for accomplishing certain common manipulations of data. In other cases, we have problems for which the Fourier transform (or the related *power spectrum*) is itself of intrinsic interest. These two kinds of problems share a common methodology.

Historically, Fourier and spectral methods have been considered a part of "signal processing," rather than "numerical analysis" proper. There is really no justification for such a distinction. Fourier methods are commonplace in research and we will not treat them as specialized or arcane. However, we realize that many users have had relatively less experience with this field than with, say, differential equations or numerical integration. Therefore our summary of analytical results will be more complete. Numerical algorithms, per se, begin in §12.2. Various applications of Fourier transform methods are discussed in Chapter 13.

A physical process can be described either in the *time domain*, by the values of some quantity h as a function of time t, e.g., $h(t)$, or else in the *frequency domain*, where the process is specified by giving its amplitude H (generally a complex number indicating phase also) as a function of frequency f, that is, $H(f)$, with $-\infty < f < \infty$. For many purposes it is useful to think of $h(t)$ and $H(f)$ as being two different *representations* of the same function. One goes back and forth between these two representations by means of the *Fourier transform* equations,

$$\begin{aligned} H(f) &= \int_{-\infty}^{\infty} h(t) e^{2\pi i f t}\, dt \\ h(t) &= \int_{-\infty}^{\infty} H(f) e^{-2\pi i f t}\, df \end{aligned} \tag{12.0.1}$$

If t is measured in seconds, then f in equation (12.0.1) is in cycles per second, or Hertz (the unit of frequency). However, the equations work with other units, too. If h is a function of position x (in meters), H will be a function of inverse wavelength

(cycles per meter), and so on. If you are trained as a physicist or mathematician, you are probably more used to using *angular frequency* ω, which is given in *radians* per second. The relation between ω and f, $H(\omega)$ and $H(f)$, is

$$\omega \equiv 2\pi f \qquad H(\omega) \equiv [H(f)]_{f=\omega/2\pi} \tag{12.0.2}$$

and equation (12.0.1) looks like this:

$$\begin{aligned} H(\omega) &= \int_{-\infty}^{\infty} h(t)e^{i\omega t}\,dt \\ h(t) &= \frac{1}{2\pi}\int_{-\infty}^{\infty} H(\omega)e^{-i\omega t}\,d\omega \end{aligned} \tag{12.0.3}$$

We were raised on the ω-convention, but we changed! There are fewer factors of 2π to remember if you use the f-convention, especially when we get to discretely sampled data in §12.1.

From equation (12.0.1) it is evident at once that Fourier transformation is a *linear* operation. The transform of the sum of two functions is equal to the sum of the transforms. The transform of a constant times a function is that same constant times the transform of the function.

In the time domain, the function $h(t)$ may happen to have one or more special symmetries. It might be *purely real* or *purely imaginary* or it might be *even*, $h(t) = h(-t)$, or *odd*, $h(t) = -h(-t)$. In the frequency domain, these symmetries lead to relationships between $H(f)$ and $H(-f)$. The following table gives the correspondence between symmetries in the two domains:

If ...	then ...
$h(t)$ is real	$H(-f) = [H(f)]^*$
$h(t)$ is imaginary	$H(-f) = -[H(f)]^*$
$h(t)$ is even	$H(-f) = H(f)$ [i.e., $H(f)$ is even]
$h(t)$ is odd	$H(-f) = -H(f)$ [i.e., $H(f)$ is odd]
$h(t)$ is real and even	$H(f)$ is real and even
$h(t)$ is real and odd	$H(f)$ is imaginary and odd
$h(t)$ is imaginary and even	$H(f)$ is imaginary and even
$h(t)$ is imaginary and odd	$H(f)$ is real and odd

In subsequent sections we shall see how to use these symmetries to increase computational efficiency.

Here are some other elementary properties of the Fourier transform. (We'll use the "$\Longleftrightarrow$" symbol to indicate transform pairs.) If

$$h(t) \Longleftrightarrow H(f) \tag{12.0.4}$$

is such a pair, then other transform pairs are

$$h(at) \Longleftrightarrow \frac{1}{|a|}H(\frac{f}{a}) \qquad \text{time scaling} \tag{12.0.5}$$

$$\frac{1}{|b|}h(\frac{t}{b}) \Longleftrightarrow H(bf) \qquad \text{frequency scaling} \tag{12.0.6}$$

$$h(t - t_0) \Longleftrightarrow H(f)\, e^{2\pi i f t_0} \qquad \text{time shifting} \tag{12.0.7}$$

$$h(t)\, e^{-2\pi i f_0 t} \Longleftrightarrow H(f - f_0) \qquad \text{frequency shifting} \tag{12.0.8}$$

With two functions $h(t)$ and $g(t)$, and their corresponding Fourier transforms $H(f)$ and $G(f)$, we can form two combinations of special interest. The *convolution* of the two functions, denoted $g * h$, is defined by

$$g * h \equiv \int_{-\infty}^{\infty} g(\tau)h(t - \tau)\, d\tau \tag{12.0.9}$$

Note that $g * h$ is a function in the time domain and that $g * h = h * g$. It turns out that the function $g * h$ is one member of a simple transform pair,

$$g * h \Longleftrightarrow G(f)H(f) \qquad \text{convolution theorem} \tag{12.0.10}$$

In other words, the Fourier transform of the convolution is just the product of the individual Fourier transforms.

The *correlation* of two functions, denoted $\text{Corr}(g, h)$, is defined by

$$\text{Corr}(g, h) \equiv \int_{-\infty}^{\infty} g(\tau + t)h(\tau)\, d\tau \tag{12.0.11}$$

The correlation is a function of t, which is called the *lag*. It therefore lies in the time domain, and it turns out to be one member of the transform pair:

$$\text{Corr}(g, h) \Longleftrightarrow G(f)H^*(f) \qquad \text{correlation theorem} \tag{12.0.12}$$

[More generally, the second member of the pair is $G(f)H(-f)$, but we are restricting ourselves to the usual case in which g and h are real functions, so we take the liberty of setting $H(-f) = H^*(f)$.] This result shows that multiplying the Fourier transform of one function by the complex conjugate of the Fourier transform of the other gives the Fourier transform of their correlation. The correlation of a function with itself is called its *autocorrelation*. In this case (12.0.12) becomes the transform pair

$$\text{Corr}(g, g) \Longleftrightarrow |G(f)|^2 \qquad \text{Wiener-Khinchin theorem} \tag{12.0.13}$$

The *total power* in a signal is the same whether we compute it in the time domain or in the frequency domain. This result is known as *Parseval's theorem*:

$$\text{total power} \equiv \int_{-\infty}^{\infty} |h(t)|^2\, dt = \int_{-\infty}^{\infty} |H(f)|^2\, df \tag{12.0.14}$$

Frequently one wants to know "how much power" is contained in the frequency interval between f and $f + df$. In such circumstances, one does not usually distinguish between positive and negative f, but rather regards f as varying from 0 ("zero frequency" or D.C.) to $+\infty$. In such cases, one defines the *one-sided power spectral density (PSD)* of the function h as

$$P_h(f) \equiv |H(f)|^2 + |H(-f)|^2 \qquad 0 \le f < \infty \tag{12.0.15}$$

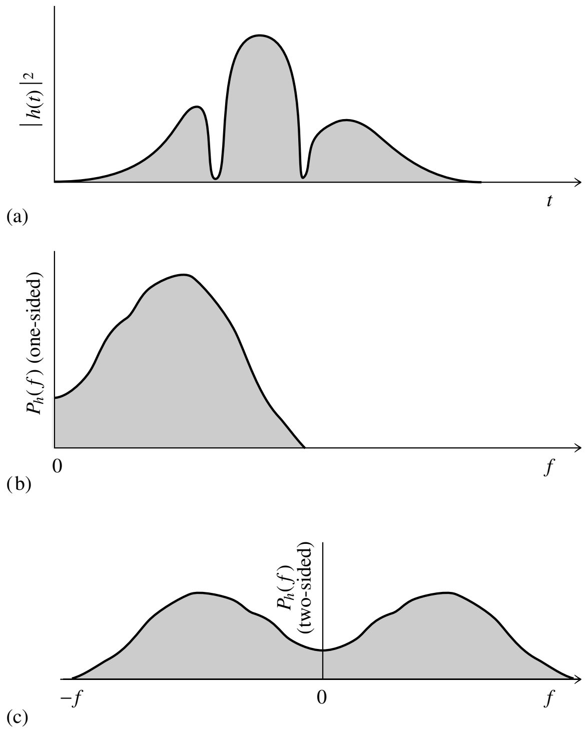

Figure 12.0.1. Normalizations of one- and two-sided power spectra. The area under the square of the function, (a), equals the area under its one-sided power spectrum at positive frequencies, (b), and also equals the area under its two-sided power spectrum at positive and negative frequencies, (c).

so that the total power is just the integral of $P_h(f)$ from $f = 0$ to $f = \infty$. When the function $h(t)$ is real, the two terms in (12.0.15) are equal, so $P_h(f) = 2\,|H(f)|^2$. Be warned that one occasionally sees PSDs defined without this factor two. These, strictly speaking, are called *two-sided power spectral densities*, but some books are not careful about stating whether one- or two-sided is to be assumed. We will always use the one-sided density given by equation (12.0.15). Figure 12.0.1 contrasts the two conventions.

If the function $h(t)$ goes endlessly from $-\infty < t < \infty$, then its total power and power spectral density will, in general, be infinite. Of interest then is the *(one- or two-sided) power spectral density per unit time*. This is computed by taking a long, but finite, stretch of the function $h(t)$, computing its PSD [that is, the PSD of a function that equals $h(t)$ in the finite stretch but is zero everywhere else], and then dividing the resulting PSD by the length of the stretch used. Parseval's theorem in this case states that the integral of the one-sided PSD-per-unit-time over positive frequency is equal to the mean square amplitude of the signal $h(t)$.

You might well worry about how the PSD-per-unit-time, which is a function of frequency f, converges as one evaluates it using longer and longer stretches of data. This interesting question is the content of the subject of "power spectrum estimation" and will be considered below in §13.4 – §13.7. A crude answer for now is, the PSD-per-unit-time converges to finite values at all frequencies *except* those where $h(t)$ has a discrete sine-wave (or cosine-wave) component of finite amplitude. At

those frequencies, it becomes a delta-function, i.e., a sharp spike, whose width gets narrower and narrower, but whose area converges to be the mean square amplitude of the discrete sine or cosine component at that frequency.

We have by now stated all of the analytical formalism that we will need in this chapter, with one exception: In computational work, especially with experimental data, we are almost never given a continuous function $h(t)$ to work with, but are given, rather, a list of measurements of $h(t_i)$ for a discrete set of t_i's. The profound implications of this seemingly trivial fact are the subject of §12.1.

12.0.1 Higher-Order Statistics

The Wiener-Khinchin theorem, equation (12.0.13), along with the definition (12.0.11), shows that the power spectrum of a function is fully equivalent to the function's *two-point statistic*, that is, the expectation value of the product of the function at two different points separated by t. One can correspondingly define *higher-order statistics* in both the time and Fourier domains. For example, a function's *three-point correlation* is

$$\text{Corr3}(g, g, g) \equiv \int_{-\infty}^{\infty} g(\tau)g(\tau + t_1)g(\tau + t_2)\, d\tau \tag{12.0.16}$$

a function of the two variables t_1 and t_2. The two-dimensional Fourier transform (§12.5) of equation (12.0.16) over t_1 and t_2 is called the *bispectrum*, a function of two frequencies f_1 and f_2.

Higher-order statistics, including the bispectrum, can make visible non-Gaussian and nonlinear phenomena to which two-point statistics (and thus power spectra) are blind. However, they have the disadvantages of being often difficult to interpret and, because of the high powers of the signal that enter, highly susceptible to noise. On these grounds, we advise caution. Useful, if sometimes overly enthusiastic, references are [1,2,3].

CITED REFERENCES AND FURTHER READING:

Bracewell, R.N. 1999, *The Fourier Transform and Its Applications*, 3rd ed. (New York: McGraw-Hill)

Folland, G.B. 1992, *Fourier Analysis and Its Applications* (Pacific Grove, CA: Wadsworth & Brooks).

James, J.F. 2002, *A Student's Guide to Fourier Transforms*, 2nd ed. (Cambridge, UK: Cambridge University Press)

Elliott, D.F., and Rao, K.R. 1982, *Fast Transforms: Algorithms, Analyses, Applications* (New York: Academic Press).

Brillinger, D., and Rosenblatt, M. 1967, "Computation and Intepretation of kth Order Spectra," in B. Harris, ed., *Spectral Analysis of Time Signals* (New York: Wiley).[1]

Mendel, J.M. 1991, "Tutorial on Higher-Order Statistics (Spectra) in Signal Processing and System Theory: Theoretical Results and Some Applications," *Proceedings of the IEEE*, vol. 79, pp. 278–305.[2]

Nikias, C.L., and Petropulu, A.P. 1993, *Higher-Order Spectra Analysis* (New Jersey: Prentice-Hall).[3]

12.1 Fourier Transform of Discretely Sampled Data

In the most common situations, function $h(t)$ is sampled (that is, its value is recorded) at evenly spaced intervals in time. Let Δ denote the time interval between consecutive samples, so that the sequence of sampled values is

$$h_n = h(n\Delta) \qquad n = \ldots, -3, -2, -1, 0, 1, 2, 3, \ldots \tag{12.1.1}$$

The reciprocal of the time interval Δ is called the *sampling rate*; if Δ is measured in seconds, for example, then the sampling rate is the number of samples recorded per second.

12.1.1 Sampling Theorem and Aliasing

For any sampling interval Δ, there is also a special frequency f_c, called the *Nyquist critical frequency*, given by

$$f_c \equiv \frac{1}{2\Delta} \tag{12.1.2}$$

If a sine wave of the Nyquist critical frequency is sampled at its positive peak value, then the next sample will be at its negative trough value, the sample after that at the positive peak again, and so on. Expressed otherwise: *Critical sampling of a sine wave is two sample points per cycle.* One frequently chooses to measure time in units of the sampling interval Δ. In this case, the Nyquist critical frequency is just the constant 1/2.

The Nyquist critical frequency is important for two related, but distinct, reasons. One is good news, and the other bad news. First the good news. It is the remarkable fact known as the *sampling theorem*: If a continuous function $h(t)$, sampled at an interval Δ, happens to be *bandwidth limited* to frequencies smaller in magnitude than f_c, i.e., if $H(f) = 0$ for all $|f| \geq f_c$, then the function $h(t)$ is *completely determined* by its samples h_n. In fact, $h(t)$ is given explicitly by the formula

$$h(t) = \Delta \sum_{n=-\infty}^{+\infty} h_n \frac{\sin[2\pi f_c(t - n\Delta)]}{\pi(t - n\Delta)} \tag{12.1.3}$$

This is a remarkable theorem for many reasons, among them that it shows that the "information content" of a bandwidth limited function is, in some sense, infinitely smaller than that of a general continuous function. Fairly often, one is dealing with a signal that is known on physical grounds to be bandwidth limited (or at least approximately bandwidth limited). For example, the signal may have passed through a physical component with a known, finite frequency response. In this case, the sampling theorem tells us that the entire information content of the signal can be recorded by sampling it at a rate Δ^{-1} equal to twice the maximum frequency passed by the amplifier (cf. equation 12.1.2).

Now the bad news. The bad news concerns the effect of sampling a continuous function that is *not* bandwidth limited to less than the Nyquist critical frequency. In that case, it turns out that all of the power spectral density that lies outside of

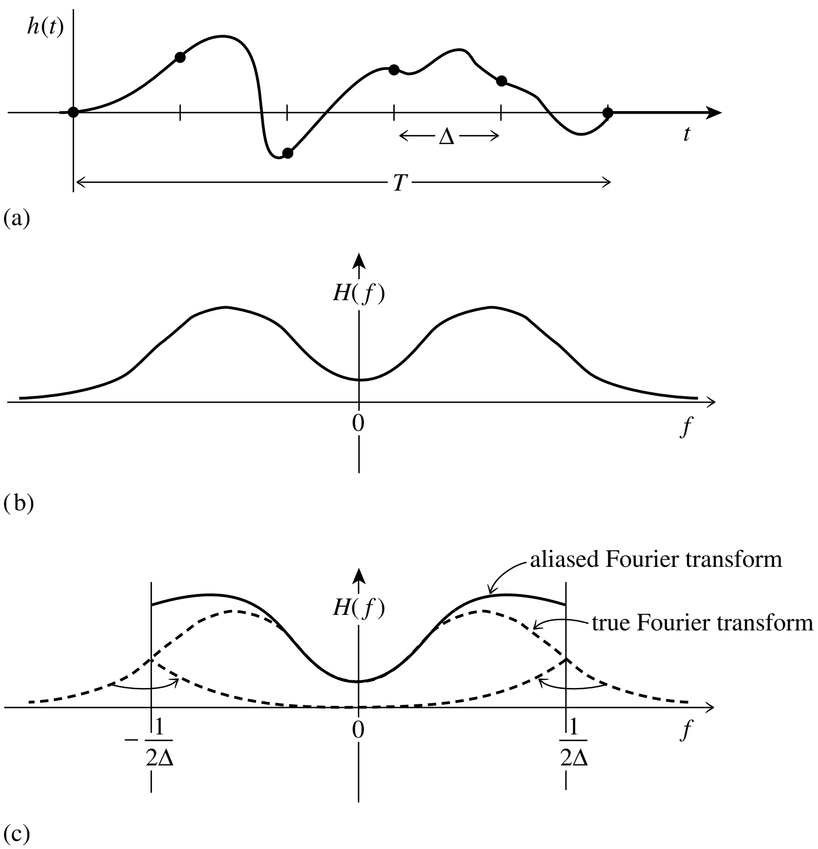

Figure 12.1.1. The continuous function shown in (a) is nonzero only for a finite interval of time T. It follows that its Fourier transform, whose modulus is shown schematically in (b), is not bandwidth limited but has finite amplitude for all frequencies. If the original function is sampled with a sampling interval Δ, as in (a), then the Fourier transform (c) is defined only between plus and minus the Nyquist critical frequency. Power outside that range is folded over or "aliased" into the range. The effect can be eliminated only by low-pass filtering the original function *before sampling*.

the frequency range $-f_c < f < f_c$ is spuriously moved into that range. This phenomenon is called *aliasing*. Any frequency component outside of the frequency range $(-f_c, f_c)$ is *aliased* (falsely translated) into that range by the very act of discrete sampling. You can readily convince yourself that two waves $\exp(2\pi i f_1 t)$ and $\exp(2\pi i f_2 t)$ give the same samples at an interval Δ if and only if f_1 and f_2 differ by a multiple of $1/\Delta$, which is just the width in frequency of the range $(-f_c, f_c)$. There is little that you can do to remove aliased power once you have discretely sampled a signal. The way to overcome aliasing is to (i) know the natural bandwidth limit of the signal — or else enforce a known limit by analog filtering of the continuous signal, and then (ii) sample at a rate sufficiently rapid to give at least two points per cycle of the highest frequency present. Figure 12.1.1 illustrates these considerations.

To put the best face on this, we can take the alternative point of view: If a continuous function has been competently sampled, then, when we come to estimate its Fourier transform from the discrete samples, we can *assume* (or rather we *might as well* assume) that its Fourier transform is equal to zero outside of the frequency range

in between $-f_c$ and f_c. Then we look to the Fourier transform to tell whether the continuous function *has* been competently sampled (aliasing effects minimized). We do this by looking to see whether the Fourier transform is already approaching zero as the frequency approaches f_c from below or $-f_c$ from above. If, on the contrary, the transform is going toward some finite value, then chances are that components outside of the range have been folded back over onto the critical range.

12.1.2 Discrete Fourier Transform

We now estimate the Fourier transform of a function from a finite number of its sampled points. Suppose that we have N consecutive sampled values,

$$h_k \equiv h(t_k), \qquad t_k \equiv k\Delta, \qquad k = 0, 1, 2, \ldots, N-1 \tag{12.1.4}$$

so that the sampling interval is Δ. To make things simpler, let us also suppose that N is even. If the function $h(t)$ is nonzero only in a finite interval of time, then that whole interval of time is supposed to be contained in the range of the N points given. Alternatively, if the function $h(t)$ goes on forever, then the sampled points are supposed to be at least "typical" of what $h(t)$ looks like at all other times.

With N numbers of input, we will evidently be able to produce no more than N independent numbers of output. So, instead of trying to estimate the Fourier transform $H(f)$ at all values of f in the range $-f_c$ to f_c, let us seek estimates only at the discrete values

$$f_n \equiv \frac{n}{N\Delta}, \qquad n = -\frac{N}{2}, \ldots, \frac{N}{2} \tag{12.1.5}$$

The extreme values of n in (12.1.5) correspond exactly to the lower and upper limits of the Nyquist critical frequency range. If you are really on the ball, you will have noticed that there are $N+1$, not N, values of n in (12.1.5); it will turn out that the two extreme values of n are not independent (in fact they are equal), but all the others are. This reduces the count to N.

The remaining step is to approximate the integral in (12.0.1) by a discrete sum:

$$H(f_n) = \int_{-\infty}^{\infty} h(t)e^{2\pi i f_n t}\,dt \approx \sum_{k=0}^{N-1} h_k\, e^{2\pi i f_n t_k}\Delta = \Delta \sum_{k=0}^{N-1} h_k\, e^{2\pi i k n/N} \tag{12.1.6}$$

Here equations (12.1.4) and (12.1.5) have been used in the final equality. The final summation in equation (12.1.6) is called the *discrete Fourier transform* of the N points h_k. Let us denote it by H_n,

$$H_n \equiv \sum_{k=0}^{N-1} h_k\, e^{2\pi i k n/N} \tag{12.1.7}$$

The discrete Fourier transform maps N complex numbers (the h_k's) into N complex numbers (the H_n's). It does not depend on any dimensional parameter, such as the time scale Δ. The relation (12.1.6) between the discrete Fourier transform of a set of numbers and their continuous Fourier transform when they are viewed as samples of a continuous function sampled at an interval Δ can be rewritten as

$$H(f_n) \approx \Delta H_n \tag{12.1.8}$$

where f_n is given by (12.1.5).

Up to now we have taken the view that the index n in (12.1.7) varies from $-N/2$ to $N/2$ (cf. 12.1.5). You can easily see, however, that (12.1.7) is periodic in n, with period N. Therefore, $H_{-n} = H_{N-n}$, $n = 1, 2, \ldots$. With this conversion in mind, one generally lets the n in H_n vary from 0 to $N-1$ (one complete period). Then n and k (in h_k) vary exactly over the same range, so the mapping of N numbers into N numbers is manifest. When this convention is followed, you must remember that zero frequency corresponds to $n = 0$ and positive frequencies $0 < f < f_c$ correspond to values $1 \le n \le N/2 - 1$, while negative frequencies $-f_c < f < 0$ correspond to $N/2 + 1 \le n \le N - 1$. The value $n = N/2$ corresponds to *both* $f = f_c$ and $f = -f_c$.

The discrete Fourier transform has symmetry properties almost exactly the same as the continuous Fourier transform. For example, all the symmetries in the table following equation (12.0.3) hold if we read h_k for $h(t)$, H_n for $H(f)$, and H_{N-n} for $H(-f)$. (Likewise, "even" and "odd" in time refer to whether the values h_k at k and $N-k$ are identical or the negative of each other.)

The formula for the discrete *inverse* Fourier transform, which recovers the set of h_k's exactly from the H_n's is

$$h_k = \frac{1}{N} \sum_{n=0}^{N-1} H_n \, e^{-2\pi i k n/N} \tag{12.1.9}$$

Notice that the only differences between (12.1.9) and (12.1.7) are (i) changing the sign in the exponential, and (ii) dividing the answer by N. This means that a routine for calculating discrete Fourier transforms can also, with slight modification, calculate the inverse transforms.

The discrete form of Parseval's theorem is

$$\sum_{k=0}^{N-1} |h_k|^2 = \frac{1}{N} \sum_{n=0}^{N-1} |H_n|^2 \tag{12.1.10}$$

There are also discrete analogs to the convolution and correlation theorems (equations 12.0.10 and 12.0.12), but we shall defer them to §13.1 and §13.2, respectively.

CITED REFERENCES AND FURTHER READING:

Brigham, E.O. 1974, *The Fast Fourier Transform* (Englewood Cliffs, NJ: Prentice-Hall).

James, J.F. 2002, *A Student's Guide to Fourier Transforms*, 2nd ed. (Cambridge, UK: Cambridge University Press)

Elliott, D.F., and Rao, K.R. 1982, *Fast Transforms: Algorithms, Analyses, Applications* (New York: Academic Press).

12.2 Fast Fourier Transform (FFT)

How much computation is involved in computing the discrete Fourier transform (12.1.7) of N points? For many years, until the mid-1960s, the standard answer was this: Define W as the complex number

$$W \equiv e^{2\pi i/N} \tag{12.2.1}$$

Then (12.1.7) can be written as

$$H_n = \sum_{k=0}^{N-1} W^{nk} h_k \tag{12.2.2}$$

In other words, the vector of h_k's is multiplied by a matrix whose (n,k)th element is the constant W to the power $n \times k$. The matrix multiplication produces a vector result whose components are the H_n's. This matrix multiplication evidently requires N^2 complex multiplications, plus a smaller number of operations to generate the required powers of W. So, the discrete Fourier transform appears to be an $O(N^2)$ process. These appearances are deceiving! The discrete Fourier transform can, in fact, be computed in $O(N \log_2 N)$ operations with an algorithm called the *fast Fourier transform*, or *FFT*. The difference between $N \log_2 N$ and N^2 is immense. With $N = 10^8$, for example, it is a factor of several million, comparable to the ratio of one second to one month. The existence of an FFT algorithm became generally known only in the mid-1960s, from the work of J.W. Cooley and J.W. Tukey. Retrospectively, we now know (see [1]) that efficient methods for computing the DFT had been independently discovered, and in some cases implemented, by as many as a dozen individuals, starting with Gauss in 1805!

One "rediscovery" of the FFT, that of Danielson and Lanczos in 1942, provides one of the clearest derivations of the algorithm. Danielson and Lanczos showed that a discrete Fourier transform of length N can be rewritten as the sum of two discrete Fourier transforms, each of length $N/2$. One of the two is formed from the even-numbered points of the original N, the other from the odd-numbered points. The proof is simply this:

$$\begin{aligned} F_k &= \sum_{j=0}^{N-1} e^{2\pi ijk/N} f_j \\ &= \sum_{j=0}^{N/2-1} e^{2\pi ik(2j)/N} f_{2j} + \sum_{j=0}^{N/2-1} e^{2\pi ik(2j+1)/N} f_{2j+1} \\ &= \sum_{j=0}^{N/2-1} e^{2\pi ikj/(N/2)} f_{2j} + W^k \sum_{j=0}^{N/2-1} e^{2\pi ikj/(N/2)} f_{2j+1} \\ &= F_k^e + W^k F_k^o \end{aligned} \tag{12.2.3}$$

In the last line, W is the same complex constant as in (12.2.1), F_k^e denotes the kth component of the Fourier transform of length $N/2$ formed from the even components of the original f_j's, while F_k^o is the corresponding transform of length $N/2$ formed from the odd components. Notice also that k in the last line of (12.2.3) varies from 0 to N, not just to $N/2$. Nevertheless, the transforms F_k^e and F_k^o are periodic in k with length $N/2$. So each is repeated through two cycles to obtain F_k.

The wonderful thing about the *Danielson-Lanczos lemma* is that it can be used recursively. Having reduced the problem of computing F_k to that of computing F_k^e and F_k^o, we can do the same reduction of F_k^e to the problem of computing the transform of *its* $N/4$ even-numbered input data and $N/4$ odd-numbered data. In other words, we can define F_k^{ee} and F_k^{eo} to be the discrete Fourier transforms of the

points that are respectively even-even and even-odd on the successive subdivisions of the data.

Although there are ways of treating other cases, by far the easiest case is the one in which the original N is an integer power of 2. In fact, we categorically recommend that you *only* use FFTs with N a power of 2. If the length of your data set is not a power of 2, pad it with zeros up to the next power of 2. (We will give more sophisticated suggestions in subsequent sections below.) With this restriction on N, it is evident that we can continue applying the Danielson-Lanczos lemma until we have subdivided the data all the way down to transforms of length one. What is the Fourier transform of length one? It is just the identity operation that copies its one input number into its one output slot! In other words, for every pattern of $\log_2 N$ e's and o's, there is a one-point transform that is just one of the input numbers f_n,

$$F_k^{eoeeoeo\cdots oee} = f_n \qquad \text{for some } n \tag{12.2.4}$$

(Of course this one-point transform actually does not depend on k, since it is periodic in k with period 1.)

The next trick is to figure out which value of n corresponds to which pattern of e's and o's in equation (12.2.4). The answer is: Reverse the pattern of e's and o's, then let $e = 0$ and $o = 1$, and you will have, *in binary*, the value of n. Do you see why it works? It is because the successive subdivisions of the data into even and odd are tests of successive low-order (least significant) bits of n. This idea of *bit reversal* can be exploited in a very clever way that, along with the Danielson-Lanczos lemma, makes FFTs practical: Suppose we take the original vector of data f_j and rearrange it into bit-reversed order (see Figure 12.2.1), so that the individual numbers are in the order not of j, but of the number obtained by bit reversing j. Then the bookkeeping on the recursive application of the Danielson-Lanczos lemma becomes extraordinarily simple. The points as given are the one-point transforms. We combine adjacent pairs to get two-point transforms, then combine adjacent pairs of pairs to get four-point transforms, and so on, until the first and second halves of the whole data set are combined into the final transform. Each combination takes of order N operations, and there are evidently $\log_2 N$ combinations, so the whole algorithm is of order $N \log_2 N$ (assuming, as is the case, that the process of sorting into bit-reversed order is no greater in order than $N \log_2 N$).

This, then, is the structure of an FFT algorithm: It has two sections. The first section sorts the data into bit-reversed order. Luckily this takes no additional storage, since it involves only swapping pairs of elements. (If k_1 is the bit reverse of k_2, then k_2 is the bit reverse of k_1.) The second section has an outer loop that is executed $\log_2 N$ times and calculates, in turn, transforms of length $2, 4, 8, \ldots, N$. This series of operations is often called a *butterfly*. For each stage of the process, two nested inner loops range over the subtransforms already computed and the elements of each transform, implementing the Danielson-Lanczos lemma. The operation is made more efficient by restricting external calls for trigonometric sines and cosines to the outer loop, where they are made only $\log_2 N$ times. Computation of the sines and cosines of multiple angles is through simple recurrence relations in the inner loops (cf. 5.4.6).

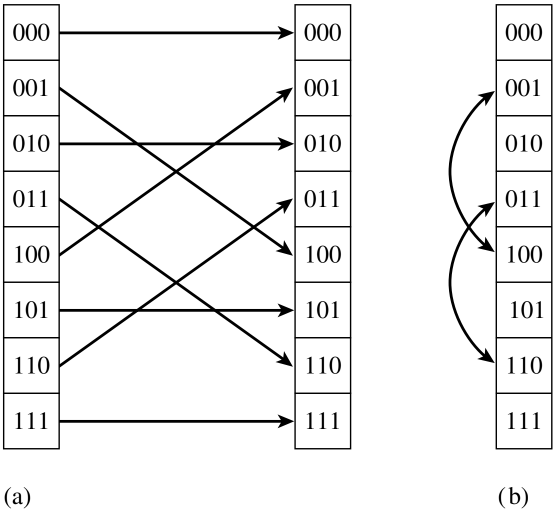

Figure 12.2.1. Reordering an array (here of length 8) by bit reversal, (a) between two arrays, versus (b) in place. Bit-reversal reordering is a necessary part of the fast Fourier transform (FFT) algorithm.

12.2.1 Bare FFT Routine and Helper Interfaces

Experience convinces us that a good way to package the FFT is as (i) a bare routine with a minimal interface, plus also (ii) a small set of interface objects that make it easier to get data in and out of the bare routine. The bare FFT routine given below is based on one originally written by N.M. Brenner. The input quantities are the number of complex data points n ($=N$), a pointer to the data array (data[0..2*n-1]), and isign, which is set to either ± 1 and is the sign of i in the exponential of equation (12.1.7). When isign is set to -1, the routine thus calculates the inverse transform (12.1.9) — except that it does not multiply by the normalizing factor $1/N$ that appears in that equation. You do that yourself. We test to be sure that n is a power of 2 by the C++ idiom n&(n-1), which is zero only if n is, in binary, 1 followed by any number of zeros.

Notice that the argument n is the number of *complex* data points. The actual length of the Doub array (data[0..2*n-1]) is 2n, with each complex value occupying two consecutive locations. In other words, data[0] is the real part of f_0, data[1] is the imaginary part of f_0, and so on up to data[2*n-2], which is the real part of f_{N-1}, and data[2*n-1], which is the imaginary part of f_{N-1}.

The FFT routine gives back the F_n's packed in exactly the same fashion, as n complex numbers. The real and imaginary parts of the zero frequency component F_0 are in data[0] and data[1]; the smallest nonzero positive frequency has real and imaginary parts in data[2] and data[3]; the smallest (in magnitude) nonzero negative frequency has real and imaginary parts in data[2*n-2] and data[2*n-1]. Positive frequencies increasing in magnitude are stored in the real-imaginary pairs data[4], data[5] up to data[n-2], data[n-1]. Negative frequencies of increasing magnitude are stored in data[2*n-4], data[2*n-3] down to data[n+2], data[n+3]. Finally, the pair data[n], data[n+1] contains the real and imaginary parts of the one aliased point that contains the most positive and the most negative frequencies. You should try to develop a familiarity with this storage arrangement of

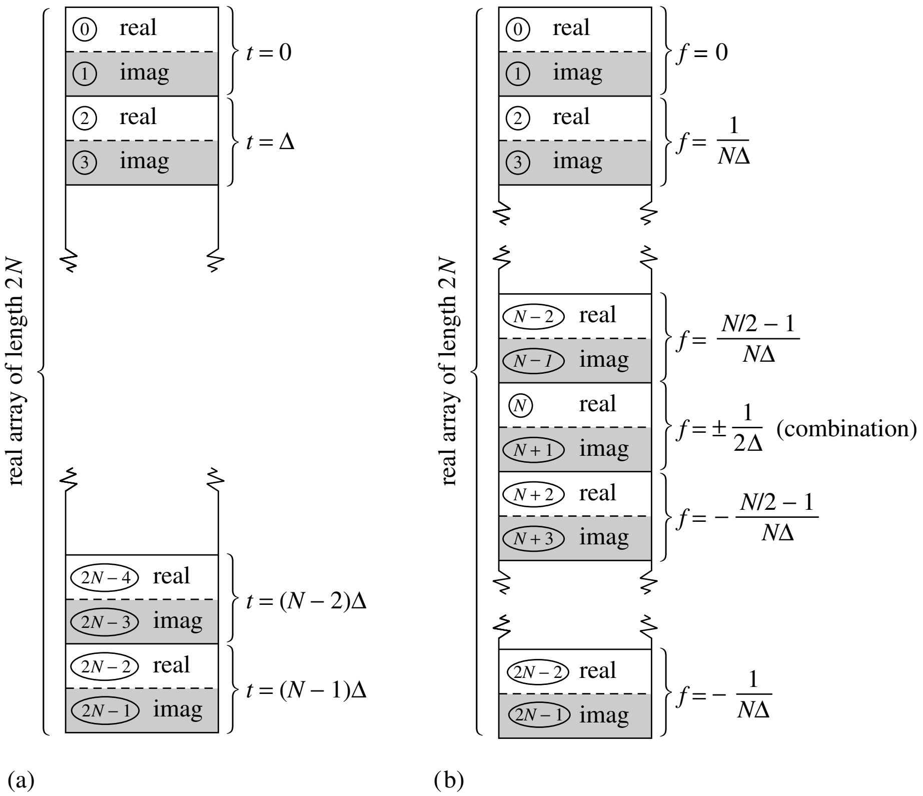

Figure 12.2.2. Input and output arrays for FFT. (a) The input array contains N (a power of 2) complex time samples in a real array of length $2N$, with real and imaginary parts alternating. (b) The output array contains the complex Fourier spectrum at N values of frequency. Real and imaginary parts again alternate. The array starts with zero frequency, works up to the most positive frequency (which is aliased with the most negative frequency). Negative frequencies follow, from the second-most negative up to the frequency just below zero.

complex spectra, also shown in Figure 12.2.2, since it is the practical standard.

fourier.h

```
void four1(Doub *data, const Int n, const Int isign) {
```

Replaces `data[0..2*n-1]` by its discrete Fourier transform, if `isign` is input as 1; or replaces `data[0..2*n-1]` by `n` times its inverse discrete Fourier transform, if `isign` is input as −1. `data` is a complex array of length `n` stored as a real array of length `2*n`. `n` must be an integer power of 2.

```
    Int nn,mmax,m,j,istep,i;
    Doub wtemp,wr,wpr,wpi,wi,theta,tempr,tempi;
    if (n<2 || n&(n-1)) throw("n must be power of 2 in four1");
    nn = n << 1;
    j = 1;
    for (i=1;i<nn;i+=2) {                          This is the bit-reversal section of the
        if (j > i) {                                   routine.
            SWAP(data[j-1],data[i-1]);             Exchange the two complex numbers.
            SWAP(data[j],data[i]);
        }
        m=n;
        while (m >= 2 && j > m) {
            j -= m;
```

```
            m >>= 1;
        }
        j += m;
    }
    Here begins the Danielson-Lanczos section of the routine.
    mmax=2;
    while (nn > mmax) {                           Outer loop executed log2 n times.
        istep=mmax << 1;
        theta=isign*(6.28318530717959/mmax);      Initialize the trigonometric recurrence.
        wtemp=sin(0.5*theta);
        wpr = -2.0*wtemp*wtemp;
        wpi=sin(theta);
        wr=1.0;
        wi=0.0;
        for (m=1;m<mmax;m+=2) {                   Here are the two nested inner loops.
            for (i=m;i<=nn;i+=istep) {
                j=i+mmax;                         This is the Danielson-Lanczos for-
                tempr=wr*data[j-1]-wi*data[j];       mula:
                tempi=wr*data[j]+wi*data[j-1];
                data[j-1]=data[i-1]-tempr;
                data[j]=data[i]-tempi;
                data[i-1] += tempr;
                data[i] += tempi;
            }
            wr=(wtemp=wr)*wpr-wi*wpi+wr;          Trigonometric recurrence.
            wi=wi*wpr+wtemp*wpi+wi;
        }
        mmax=istep;
    }
}
```

For an interface at a slightly higher level, we can overload the bare `four1` with equivalent functions that input and output `data` as either a `VecDoub` of length $2N$ or a `VecComplex` of length N:

fourier.h

```
void four1(VecDoub_IO &data, const Int isign) {
Overloaded interface to four1. Replaces the vector data, a complex vector of length N stored
as a real vector of twice that length, by its discrete Fourier transform, with components in
wraparound order, if isign is 1; or by N times the inverse Fourier transform, if isign is −1.
    four1(&data[0],data.size()/2,isign);
}

void four1(VecComplex_IO &data, const Int isign) {
Overloaded interface to four1. Replaces the vector data, a complex vector of length N stored
as such, by its discrete Fourier transform, with components in wraparound order, if isign is 1;
or by N times the inverse Fourier transform, if isign is −1.
    four1((Doub*)(&data[0]),data.size(),isign);
}
```

In these overloaded versions, however, you are still responsible for decoding on your own the wraparound order. To get an interface that takes care of that for you, we can define an object `WrapVecDoub` that creates a real vector (or binds a reference to an existing one) and then defines methods for addressing the vector as if it were a complex vector of half the size. Since the `WrapVecDoub` object also knows about wraparound periodicity, you can access frequencies either by subscripts `[0..n-1]` or `[-n/2..n/2-1]`, or, for that matter, any `n` consecutive complex components. The object has a conversion operator to `VecDoub`, so you can (e.g.) send it directly to `four1`.

fourier.h

```
struct WrapVecDoub {
```
Object for accessing a VecDoub as if it were a complex vector of half the length, with wraparound periodicity.
```
    VecDoub vvec;                         Used when data are stored internally.
    VecDoub &v;
    Int n, mask;

    WrapVecDoub(const Int nn) : vvec(nn), v(vvec), n(nn/2),
    mask(n-1) {validate();}
```
Constructor. Declare a new vector with nn real components (half as many complex).
```
    WrapVecDoub(VecDoub &vec) : v(vec), n(vec.size()/2),
    mask(n-1) {validate();}
```
Constructor. Bind the data in an existing vector vec for access as if complex.
```
    void validate() {if (n&(n-1)) throw("vec size must be power of 2");}

    inline Complex& operator[] (Int i) {return (Complex &)v[(i&mask) << 1];}
```
Reduce any integer i to the periodic range [0..n] and return that complex component. Can also be an l-value.
```
    inline Doub& real(Int i) {return v[(i&mask) << 1];}
```
As above, but return the real part only. Can also be an l-value.
```
    inline Doub& imag(Int i) {return v[((i&mask) << 1)+1];}
```
As above, but return the imaginary part only. Can also be an l-value.
```
    operator VecDoub&() {return v;}
```
Conversion operator. Allows a WrapVecDoub object to be sent to any function that expects a VecDoub.
```
};
```

Here are some sample lines of code (not a useful program) that show how WrapVecDoub can be used:

```
    Int j,n=256;                              256 complex components, e.g.
    VecDoub dat(2*n);                         A real vector to hold them.
    WrapVecDoub data1(dat), data2(2*n);       Examples of the two constructors.
    for (j=0;j<n;j++) {                       Loop over complex components.
        data1[j] = Complex(... , ...);        Set a complex value directly,
        data2.real(j) = ... ;                 or set real and imag separately.
        data2.imag(j) = ... ;
    }
    four1(data1,1);                           Invokes four1(VecDoub&,Int) through the
    four1(data2,1);                               conversion operator.
    for (j=-n/2;j<n/2;j++) {                  Can address negative frequencies directly!
        ... = data1.real(j);                  Get real part of component j.
        ... = data2[j];                       Get component as a complex value.
    }
```

12.2.2 Decomposing the FFT for Parallelism

It is possible to decompose the calculation of an FFT of size N into a set of smaller FFTs that can be done independently of one another. This can be useful either to achieve parallelism or to allow more versatile memory management. The basic idea is to address the input array as if it were a two-dimensional array of size $m \times M$, where $N = mM$ and N, m, and M are all powers of 2. Then the components of f can be addressed as

$$f[Jm + j], \qquad 0 \le j < m,\ 0 \le J < M \tag{12.2.5}$$

where the j index changes more rapidly, the J index more slowly, and brackets denote C++ subscripts.

What we want to compute is the FFT of the original array of length N, which we can write as

$$F[kM+K] \equiv \sum_{j,J} e^{2\pi i(kM+K)(Jm+j)/(Mm)} f(Jm+j), \qquad 0 \le k < m,\ 0 \le K < M \tag{12.2.6}$$

You can see that the indices k and K address the desired result (FFT of the original array), with K varying more rapidly.

From equation (12.2.6) it is easy to verify the identity

$$F[kM+K] = \sum_j \left\{ e^{2\pi ijk/m} \left[e^{2\pi ijK/(Mm)} \left(\sum_J e^{2\pi iJK/M} f(Jm+j) \right) \right] \right\} \tag{12.2.7}$$

But this, reading it from the innermost operation outward, is just the method that we need:

- For each value of j form a vector of input values whose components vary with J, $0 \le J < M$. This is basically a transpose operation.
- FFT each such vector. (OK to do in parallel.) Notationally, the J index now becomes a K index.
- Multiply each component by a phase factor $\exp[2\pi ijK/(Mm)]$.
- Rearrange the data so that they are accessible as a set of vectors whose components vary with j, $0 \le j < m$, another transpose operation.
- FFT each such vector. (OK to do in parallel.) The j index now becomes a k index.
- The answer is now available as $F[kM+K]$. It takes a third transpose operation to get it back into the desired order (with k varying most rapidly).

Even though two FFTs are performed on each element, the operations count is about the same as for the ordinary FFT: The first set of FFTs scales as $N \log M$, the second set as $N \log m$, and the total is thus $N \log(Mn) = N \log N$.

For further discussion, see [2], where the above is called the *six-step framework*. You can easily eliminate the first two of the three transpose operations by writing a new `four1` routine with an additional *stride* argument, specifying the constant increment between logically "next" components. The stride will be m for the first set of FFTs, and 1 for the second set. An algorithm very similar to this is called the *four-step framework*. See [2,3] for more details.

Related decompositions, called *zoom transforms*, can be used to get an approximation to the spectrum of a long data stream, at high resolution in only certain frequency bands. See [4-6].

12.2.3 Other FFT Algorithms

We should mention that there are a number of variants on the basic FFT algorithm given above. As we have seen, that algorithm first rearranges the input elements into bit-reverse order, then builds up the output transform in $\log_2 N$ iterations. In the literature, this sequence is called a *decimation-in-time* or *Cooley-Tukey* FFT

algorithm. It is also possible to derive FFT algorithms that first go through a set of $\log_2 N$ iterations on the input data, and rearrange the *output* values into bit-reverse order. These are called *decimation-in-frequency* or *Sande-Tukey* FFT algorithms. For some applications, such as convolution (§13.1), one takes a data set into the Fourier domain and then, after some manipulation, back out again. In these cases it is possible to avoid all bit reversing. You use a decimation-in-frequency algorithm (without its bit reversing) to get into the "scrambled" Fourier domain, do your operations there, and then use an inverse algorithm (without *its* bit reversing) to get back to the time domain. While elegant in principle, this procedure does not in practice save much computation time, since the bit reversals represent only a small fraction of an FFT's operations count, and since most useful operations in the frequency domain require a knowledge of which points correspond to which frequencies.

Another class of FFTs subdivides the initial data set of length N not all the way down to the trivial transform of length 1, but rather only down to some other small power of 2, for example $N = 4$, *base-4 FFTs*, or $N = 8$, *base-8 FFTs*. These small transforms are then done by small sections of highly optimized coding that take advantage of special symmetries of that particular small N. For example, for $N = 4$, the trigonometric sines and cosines that enter are all ± 1 or 0, so many multiplications are eliminated, leaving largely additions and subtractions. These can be faster than simpler FFTs by some significant, but not overwhelming, factor, e.g., 20 or 30%.

There are also FFT algorithms for data sets of length N not a power of 2. They work by using relations analogous to the Danielson-Lanczos lemma to subdivide the initial problem into successively smaller problems, not by factors of 2, but by whatever small prime factors happen to divide N. The larger that the largest prime factor of N is, the worse this method works. If N is prime, then no subdivision is possible, and the user (whether he knows it or not) is taking a *slow* Fourier transform, of order N^2 instead of order $N \log_2 N$. Our advice is to stay clear of such FFT implementations, with perhaps one class of exceptions, the *Winograd Fourier transform algorithms*. Winograd algorithms are in some ways analogous to the base-4 and base-8 FFTs. Winograd has derived highly optimized codings for taking small-N discrete Fourier transforms, e.g., for $N = 2, 3, 4, 5, 7, 8, 11, 13, 16$. The algorithms also use a different and clever way of combining the subfactors. The method involves a reordering of the data both before the hierarchical processing and after it, but it allows a significant reduction in the number of multiplications in the algorithm. For some especially favorable values of N, the Winograd algorithms can be significantly (e.g., up to a factor of 2) faster than the simpler FFT algorithms of the nearest integer power of 2. This advantage in speed, however, must be weighed against the considerably more complicated data indexing involved in these transforms, and the fact that the Winograd transform cannot be done "in place."

Finally, an interesting class of transforms for doing convolutions quickly is number-theoretic transforms [7,8]. These schemes replace floating-point arithmetic with integer arithmetic modulo some large prime $N+1$, and the Nth root of 1 by the modulo arithmetic equivalent. Strictly speaking, these are not *Fourier* transforms at all, but the properties are quite similar and computational speed can be far superior. On the other hand, their use is somewhat restricted to quantities like correlations and convolutions since the transform itself is not easily interpretable as a "frequency" spectrum.

CITED REFERENCES AND FURTHER READING:

Brigham, E.O. 1974, *The Fast Fourier Transform* (Englewood Cliffs, NJ: Prentice-Hall).[1]

Nussbaumer, H.J. 1982, *Fast Fourier Transform and Convolution Algorithms* (New York: Springer).

Elliott, D.F., and Rao, K.R. 1982, *Fast Transforms: Algorithms, Analyses, Applications* (New York: Academic Press).

Walker, J.S. 1996, *Fast Fourier Transforms*, 2nd ed. (Boca Raton, FL: CRC Press)

Bloomfield, P. 1976, *Fourier Analysis of Time Series – An Introduction* (New York: Wiley).

Van Loan, C. 1992, *Computational Frameworks for the Fast Fourier Transform* (Philadelphia: S.I.A.M.).[2]

Press, W.H., Teukolsky, S.A., Vetterling, W.T., and Flannery, B.P. 1996, *Numerical Recipes in Fortran 90: The Art of Parallel Scientific Computing* (Cambridge, UK: Cambridge University Press), §22.4.[3]

Yip, P.C.Y. 1976, "Some Aspects of the Zoom Transform," *IEEE Transactions on Computers*, vol. C-25, pp. 287–296.[4]

Hung, E.K.L. 1981, "A Multiresolution Sampled-Data Spectrum Analyzer for a Detection System," *IEEE Transactions on Acoustics, Speech and Signal Processing*, vol. ASSP-29, pp. 163–170.[5]

de Wild, R., Nieuwkerk, L.R., and van Sinttruyen, J.S. 1987, "Method for Partial Spectrum Computation," *IEE Proceedings F (Radar and Signal Processing)*, vol. 134, pp. 659–666[6]

Beauchamp, K.G. 1984, *Applications of Walsh Functions and Related Functions* (New York: Academic Press) [non-Fourier transforms].

Pollard, J.M. 1971, "The Fast Fourier Transform in a Finite Field," *Mathematics of Computation*, vol. 25, pp. 365–374.[7]

McClellan, J.H., and Rader, C.M. 1979, *Number Theory in Digital Signal Processing* (New York: Prentice-Hall).[8]

Heideman, M.T., Johnson, D.H., and Burris, C.S. 1984, "Gauss and the History of the Fast Fourier Transform," *IEEE ASSP Magazine*, pp. 14–21 (October).

12.3 FFT of Real Functions

It happens frequently that the data whose FFT is desired consist of real-valued samples $f_j,\ \ j = 0 \dots N-1$. To use `four1`, we put these into a complex array with all imaginary parts set to zero. The resulting transform $F_n,\ \ n = 0 \dots N-1$ satisfies $(F_{N-n})^* = F_n$. Since this complex-valued array has real values for F_0 and $F_{N/2}$, and $(N/2)-1$ other independent values $F_1 \dots F_{N/2-1}$, it has the same $2(N/2-1)+2 = N$ "degrees of freedom" as the original, real data set. However, the use of the full complex FFT algorithm for real data is inefficient, both in execution time and in storage required. You would think that there is a better way.

Actually, there are *two* better ways.

12.3.1 Transform of Two Real Functions Simultaneously

The first better way is "mass production": Pack two separate real functions into the input array in such a way that their individual transforms can be separated from the result. This may remind you of a one-cent sale, at which you are coerced to purchase two of an item when you only need one. However, remember that for correlations and convolutions the Fourier transforms of two functions are involved, and this is a handy way to do them both at once.

Here is how to exploit the symmetry of the FFT to handle two real functions at once: Pack the two data arrays as the real and imaginary parts, respectively, of the complex input array of `four1` and take the transform. This gives

$$H_n = \sum_j e^{2\pi ijn/N}(f_j + ig_j) \tag{12.3.1}$$

Now look at the $N - n$ component, and take its complex conjugate,

$$(H_{N-n})^* = \left(\sum_j e^{2\pi ij(N-n)/N}(f_j + ig_j)\right)^* = \sum_j e^{2\pi ijn/N}(f_j - ig_j) \tag{12.3.2}$$

where we have used $f_j^* = f_j$ and $g_j^* = g_j$. Now, adding and subtracting equations (12.3.1) and (12.3.2) gives

$$H_n + H_{N-n}^* = 2F_n, \qquad H_n - H_{N-n}^* = 2iG_n \tag{12.3.3}$$

Equations (12.3.3) with $n = 0, 1, \ldots, N/2$ easily yield the independent (zero and positive frequency) components of the two desired transforms F_n and G_n. Note that F_0, G_0, $F_{N/2}$, and $G_{N/2}$ are real (using $H_0 = H_N$), but that the other values are, in general, complex.

What about the reverse process? This is even easier. Using the symmetries $F_{N-n} = F_n^*$ and $G_{N-n} = G_n^*$, form $F_n + iG_n$ for $0 \le n < N$. Now take the inverse FFT. The real and imaginary parts of the resulting complex array are the two desired real functions.

The only potential drawback of this method occurs if f and g are very different in scale. Then roundoff error can cause the smaller function's FFT to be inaccurate.

12.3.2 FFT of a Single Real Function

To implement the second method, which allows us to perform the FFT of a *single* real function without redundancy, we split the data set in half, thereby forming two real arrays of half the size. We can apply the method above to these two, but of course the result will not be the transform of the original data. It will be a schizophrenic combination of two transforms, each of which has half of the information we need. Fortunately, this schizophrenia is treatable. It works like this:

The right way to split the original data is to take the even-numbered f_j as one data set, and the odd-numbered f_j as the other. The beauty of this is that we can take the original real array and treat it as a complex array h_j of half the length. The first data set is the real part of this array, and the second is the imaginary part, just as was described above. No repacking is required. In other words, $h_j = f_{2j} + if_{2j+1}$, $j = 0, \ldots, N/2 - 1$. We submit this to `four1`, and it gives back a complex array $H_n = F_n^e + iF_n^o$, $n = 0, \ldots, N/2 - 1$ with

$$\begin{aligned} F_n^e &= \sum_{k=0}^{N/2-1} f_{2k}\, e^{2\pi ikn/(N/2)} \\ F_n^o &= \sum_{k=0}^{N/2-1} f_{2k+1}\, e^{2\pi ikn/(N/2)} \end{aligned} \tag{12.3.4}$$

The previous discussion tells us how to separate the two transforms F_n^e and F_n^o out of H_n. How do you work them into the transform F_n of the original data set f_j? Simply glance back at equation (12.2.3):

$$F_n = F_n^e + e^{2\pi i n/N} F_n^o \qquad n = 0, \ldots, N-1 \tag{12.3.5}$$

Expressed directly in terms of the transform H_n of our real (masquerading as complex) data set, the result is

$$F_n = \frac{1}{2}(H_n + H_{N/2-n}^*) - \frac{i}{2}(H_n - H_{N/2-n}^*)e^{2\pi i n/N} \qquad n = 0, \ldots, N-1 \tag{12.3.6}$$

A few remarks:

- Since $F_{N-n}^* = F_n$, there is no point in saving the entire spectrum. The positive frequency half is sufficient and can be stored in the same array as the original data. The operation can, in fact, be done in place.
- Even so, we need values H_n, $n = 0, \ldots, N/2$, whereas `four1` gives only the values $n = 0, \ldots, N/2-1$. Symmetry to the rescue, $H_{N/2} = H_0$.
- The values F_0 and $F_{N/2}$ are real and independent. In order to actually get the entire F_n in the original array space, it is convenient to put $F_{N/2}$ into the imaginary part of F_0.
- Despite its complicated form, the process above is invertible. First peel $F_{N/2}$ out of F_0. Then construct

$$\begin{aligned} F_n^e &= \tfrac{1}{2}(F_n + F_{N/2-n}^*) \\ F_n^o &= \tfrac{1}{2}e^{-2\pi i n/N}(F_n - F_{N/2-n}^*) \qquad n = 0, \ldots, N/2-1 \end{aligned} \tag{12.3.7}$$

  and use `four1` to find the inverse transform of $H_n = F_n^{(1)} + iF_n^{(2)}$. Surprisingly, the actual algebraic steps are virtually identical to those of the forward transform.

Here is a representation of what we have said:

fourier.h

```
void realft(VecDoub_IO &data, const Int isign) {
```

Calculates the Fourier transform of a set of n real-valued data points. Replaces these data (which are stored in array `data[0..n-1]`) by the positive frequency half of their complex Fourier transform. The real-valued first and last components of the complex transform are returned as elements `data[0]` and `data[1]`, respectively. n must be a power of 2. This routine also calculates the inverse transform of a complex data array if it is the transform of real data. (Result in this case must be multiplied by 2/n.)

```
    Int i,i1,i2,i3,i4,n=data.size();
    Doub c1=0.5,c2,h1r,h1i,h2r,h2i,wr,wi,wpr,wpi,wtemp;
    Doub theta=3.141592653589793238/Doub(n>>1);     Initialize the recurrence.
    if (isign == 1) {
        c2 = -0.5;
        four1(data,1);                              The forward transform is here.
    } else {
        c2=0.5;                                     Otherwise set up for an inverse trans-
        theta = -theta;                                 form.
    }
    wtemp=sin(0.5*theta);
    wpr = -2.0*wtemp*wtemp;
    wpi=sin(theta);
    wr=1.0+wpr;
    wi=wpi;
```

```
    for (i=1;i<(n>>2);i++) {                          Case i=0 done separately below.
        i2=1+(i1=i+i);
        i4=1+(i3=n-i1);
        h1r=c1*(data[i1]+data[i3]);                   The two separate transforms are sep-
        h1i=c1*(data[i2]-data[i4]);                       arated out of data.
        h2r= -c2*(data[i2]+data[i4]);
        h2i=c2*(data[i1]-data[i3]);
        data[i1]=h1r+wr*h2r-wi*h2i;                   Here they are recombined to form
        data[i2]=h1i+wr*h2i+wi*h2r;                       the true transform of the origi-
        data[i3]=h1r-wr*h2r+wi*h2i;                       nal real data.
        data[i4]= -h1i+wr*h2i+wi*h2r;
        wr=(wtemp=wr)*wpr-wi*wpi+wr;                  The recurrence.
        wi=wi*wpr+wtemp*wpi+wi;
    }
    if (isign == 1) {
        data[0] = (h1r=data[0])+data[1];              Squeeze the first and last data to-
        data[1] = h1r-data[1];                            gether to get them all within the
    } else {                                              original array.
        data[0]=c1*((h1r=data[0])+data[1]);
        data[1]=c1*(h1r-data[1]);
        four1(data,-1);                               This is the inverse transform for the
    }                                                     case isign=-1.
}
```

You can't use `WrapVecDoub` (§12.2) to access the output of `realft` as complex values; it assumes a wraparound order that is not valid when we are storing only the positive part of the spectrum. An even simpler trick, however, is to define an inline function

```
inline Complex* Cmplx(VecDoub &d) {return (Complex *)&d[0];}
```

and then write things like

```
    Cmplx(data)[k] = Complex(... , ...);
    cvalue = Cmplx(data)[k];
```

when you want to set or get the kth complex value in `data`, viewed as a complex array. (You are still responsible for separating the two real values stored in the first complex component, however.)

CITED REFERENCES AND FURTHER READING:

Brigham, E.O. 1974, *The Fast Fourier Transform* (Englewood Cliffs, NJ: Prentice-Hall), §10-10.

Sorensen, H.V., Jones, D.L., Heideman, M.T., and Burris, C.S. 1987, "Real-Valued Fast Fourier Transform Algorithms," *IEEE Transactions on Acoustics, Speech, and Signal Processing*, vol. ASSP-35, pp. 849–863.

Hockney, R.W. 1971, in *Methods in Computational Physics*, vol. 9 (New York: Academic Press).

Russ, J.C. 2002, *The Image Processing Handbook*, 4th ed. (Boca Raton, FL: CRC Press)

Clarke, R.J. 1985, *Transform Coding of Images*, (Reading, MA: Addison-Wesley).

Gonzalez, R.C., and Woods, R.E. 1992, *Digital Image Processing*, 2nd ed. (Reading, MA: Addison-Wesley).

12.4 Fast Sine and Cosine Transforms

Among their other uses, the Fourier transforms of functions can be used to solve differential equations (see §20.4). The most common boundary conditions for the solutions are (i)

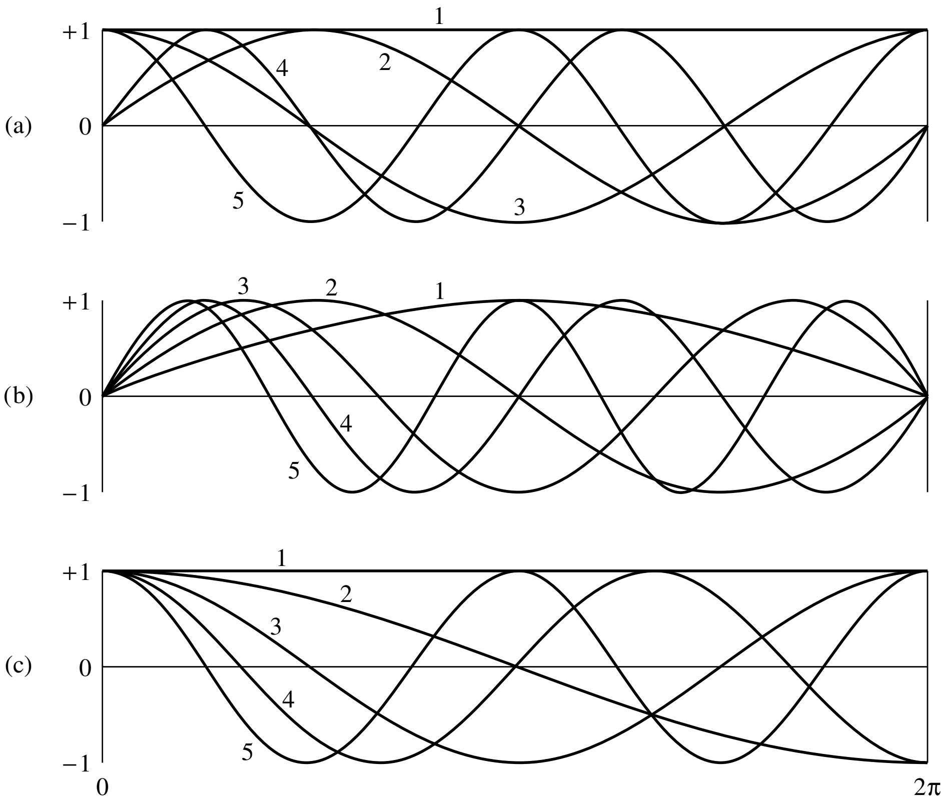

Figure 12.4.1. Basis functions used by the Fourier transform (a), sine transform (b), and cosine transform (c) are plotted. The first five basis functions are shown in each case. (For the Fourier transform, the real and imaginary parts of the basis functions are both shown.) While some basis functions occur in more than one transform, the basis sets are distinct. For example, the sine transform functions labeled (1), (3), (5) are not present in the Fourier basis. Any of the three sets can expand any function in the interval shown; however, the sine or cosine transform best expands functions matching the boundary conditions of the respective basis functions, namely zero function values for sine, zero derivatives for cosine.

they have the value zero at the boundaries, or (ii) their derivatives are zero at the boundaries. In the first instance, the natural transform to use is the *sine transform*, while in the second, one of several variations of the *cosine transform* is a natural choice.

12.4.1 Sine Transform

The sine transform is given by

$$F_k = \sum_{j=1}^{N-1} f_j \sin(\pi jk/N) \tag{12.4.1}$$

where $f_j,\ j = 0, \ldots, N-1$ is the data array, and $f_0 \equiv 0$.

At first blush this appears to be simply the imaginary part of the discrete Fourier transform. However, the argument of the sine differs by a factor of two from the value that would make this so. The sine transform uses *sines only* as a complete set of functions in the interval from 0 to 2π, and, as we shall see, the cosine transform uses *cosines only*. By contrast, the normal FFT uses both sines and cosines, but only half as many of each. (See Figure 12.4.1.)

The expression (12.4.1) can be "force-fit" into a form that allows its calculation via the FFT. The idea is to extend the given function rightward past its last tabulated value. We extend

the data to twice their length in such a way as to make them an *odd* function about $j = N$, with $f_N = 0$,

$$f_{2N-j} \equiv -f_j \qquad j = 0, \dots, N-1 \tag{12.4.2}$$

Consider the FFT of this extended function:

$$F_k = \sum_{j=0}^{2N-1} f_j e^{2\pi ijk/(2N)} \tag{12.4.3}$$

The half of this sum from $j = N$ to $j = 2N - 1$ can be rewritten with the substitution $j' = 2N - j$,

$$\begin{aligned}\sum_{j=N}^{2N-1} f_j e^{2\pi ijk/(2N)} &= \sum_{j'=1}^{N} f_{2N-j'} e^{2\pi i(2N-j')k/(2N)} \\ &= -\sum_{j'=0}^{N-1} f_{j'} e^{-2\pi ij'k/(2N)}\end{aligned} \tag{12.4.4}$$

so that

$$\begin{aligned}F_k &= \sum_{j=0}^{N-1} f_j \left[e^{2\pi ijk/(2N)} - e^{-2\pi ijk/(2N)}\right] \\ &= 2i \sum_{j=0}^{N-1} f_j \sin(\pi jk/N)\end{aligned} \tag{12.4.5}$$

Thus, up to a factor $2i$ we get the sine transform from the FFT of the extended function.

This method introduces a factor of two inefficiency into the computation by extending the data. This inefficiency shows up in the FFT output, which has zeros for the real part of every element of the transform. For a one-dimensional problem, the factor of two may be bearable, especially in view of the simplicity of the method. When we work with partial differential equations in two or three dimensions, though, the factor becomes four or eight, so efforts to eliminate the inefficiency are well rewarded.

From the original real data array f_j we will construct an auxiliary array y_j and apply to it the routine `realft`. The output will then be used to construct the desired transform. For the sine transform of data f_j, $j = 1, \dots, N-1$, the auxiliary array is

$$\begin{aligned}y_0 &= 0 \\ y_j &= \sin(j\pi/N)(f_j + f_{N-j}) + \tfrac{1}{2}(f_j - f_{N-j}) \qquad j = 1, \dots, N-1\end{aligned} \tag{12.4.6}$$

This array is of the same dimension as the original. Notice that the first term is symmetric about $j = N/2$ and the second is antisymmetric. Consequently, when `realft` is applied to y_j, the result has real parts R_k and imaginary parts I_k given by

$$\begin{aligned}R_k &= \sum_{j=0}^{N-1} y_j \cos(2\pi jk/N) \\ &= \sum_{j=1}^{N-1} (f_j + f_{N-j}) \sin(j\pi/N) \cos(2\pi jk/N) \\ &= \sum_{j=0}^{N-1} 2f_j \sin(j\pi/N) \cos(2\pi jk/N)\end{aligned}$$

$$= \sum_{j=0}^{N-1} f_j \left[\sin \frac{(2k+1)j\pi}{N} - \sin \frac{(2k-1)j\pi}{N} \right]$$

$$= F_{2k+1} - F_{2k-1} \tag{12.4.7}$$

$$I_k = \sum_{j=0}^{N-1} y_j \sin(2\pi jk/N)$$

$$= \sum_{j=1}^{N-1} (f_j - f_{N-j}) \frac{1}{2} \sin(2\pi jk/N)$$

$$= \sum_{j=0}^{N-1} f_j \sin(2\pi jk/N)$$

$$= F_{2k} \tag{12.4.8}$$

Therefore, F_k can be determined as follows:

$$F_{2k} = I_k \qquad F_{2k+1} = F_{2k-1} + R_k \qquad k = 0, \dots, (N/2 - 1) \tag{12.4.9}$$

The even terms of F_k are thus determined very directly. The odd terms require a recursion, the starting point of which follows from setting $k = 0$ in equation (12.4.9) and using $F_1 = -F_{-1}$:

$$F_1 = \tfrac{1}{2} R_0 \tag{12.4.10}$$

The implementing program is

fourier.h

```
void sinft(VecDoub_IO &y) {
Calculates the sine transform of a set of n real-valued data points stored in array y[0..n-1].
The number n must be a power of 2. On exit, y is replaced by its transform. This program,
without changes, also calculates the inverse sine transform, but in this case the output array
should be multiplied by 2/n.
    Int j,n=y.size();
    Doub sum,y1,y2,theta,wi=0.0,wr=1.0,wpi,wpr,wtemp;
    theta=3.141592653589793238/Doub(n);          Initialize the recurrence.
    wtemp=sin(0.5*theta);
    wpr= -2.0*wtemp*wtemp;
    wpi=sin(theta);
    y[0]=0.0;
    for (j=1;j<(n>>1)+1;j++) {
        wr=(wtemp=wr)*wpr-wi*wpi+wr;      Calculate the sine for the auxiliary array.
        wi=wi*wpr+wtemp*wpi+wi;           The cosine is needed to continue the recurrence.
        y1=wi*(y[j]+y[n-j]);              Construct the auxiliary array.
        y2=0.5*(y[j]-y[n-j]);
        y[j]=y1+y2;                       Terms j and N - j are related.
        y[n-j]=y1-y2;
    }
    realft(y,1);                          Transform the auxiliary array.
    y[0]*=0.5;                            Initialize the sum used for odd terms below.
    sum=y[1]=0.0;
    for (j=0;j<n-1;j+=2) {
        sum += y[j];
        y[j]=y[j+1];                      Even terms determined directly.
        y[j+1]=sum;                       Odd terms determined by this running sum.
    }
}
```

The sine transform, curiously, is its own inverse. If you apply it twice, you get the original data, but multiplied by a factor of $N/2$.

12.4.2 Cosine Transform

The other common boundary condition for differential equations is that the derivative of the function is zero at the boundary. In this case, the natural transform is the *cosine* transform. There are several possible ways of defining the transform. Each can be thought of as resulting from a different way of extending a given array to create an even array of double the length, and/or from whether the extended array contains $2N-1$, $2N$, or some other number of points. In practice, only two of the numerous possibilities are useful, so we will restrict ourselves to just these two.

The first form of the cosine transform uses $N+1$ data points:

$$F_k = \frac{1}{2}[f_0 + (-1)^k f_N] + \sum_{j=1}^{N-1} f_j \cos(\pi jk/N) \tag{12.4.11}$$

It results from extending the given array to an even array about $j = N$, with

$$f_{2N-j} = f_j, \qquad j = 0, \ldots, N-1 \tag{12.4.12}$$

If you substitute this extended array into equation (12.4.3) and follow steps analogous to those leading up to equation (12.4.5), you will find that the Fourier transform is just twice the cosine transform (12.4.11). Another way of thinking about the formula (12.4.11) is to notice that it is the Chebyshev-Gauss-Lobatto quadrature formula (see §4.6), often used in Clenshaw-Curtis adaptive quadrature (see §5.9, equation 5.9.4).

Once again the transform can be computed without the factor of two inefficiency. In this case the auxiliary function is

$$y_j = \tfrac{1}{2}(f_j + f_{N-j}) - \sin(j\pi/N)(f_j - f_{N-j}) \qquad j = 0, \ldots, N-1 \tag{12.4.13}$$

Instead of equation (12.4.9), `realft` now gives

$$F_{2k} = R_k \qquad F_{2k+1} = F_{2k-1} + I_k \qquad k = 0, \ldots, (N/2-1) \tag{12.4.14}$$

The starting value for the recursion for odd k in this case is

$$F_1 = \frac{1}{2}(f_0 - f_N) + \sum_{j=1}^{N-1} f_j \cos(j\pi/N) \tag{12.4.15}$$

This sum does not appear naturally among the R_k and I_k, and so we accumulate it during the generation of the array y_j.

Once again this transform is its own inverse, and so the following routine works for both directions of the transformation. Note that although this form of the cosine transform has $N+1$ input and output values, it passes an array only of length N to `realft`.

fourier.h

```
void cosft1(VecDoub_IO &y) {
```

Calculates the cosine transform of a set `y[0..n]` of real-valued data points. The transformed data replace the original data in array `y`. `n` must be a power of 2. This program, without changes, also calculates the inverse cosine transform, but in this case the output array should be multiplied by `2/n`.

```
    const Doub PI=3.141592653589793238;
    Int j,n=y.size()-1;
    Doub sum,y1,y2,theta,wi=0.0,wpi,wpr,wr=1.0,wtemp;
    VecDoub yy(n);                          Need array of length n, not n+1, for realft.
    theta=PI/n;                             Initialize the recurrence.
    wtemp=sin(0.5*theta);
    wpr = -2.0*wtemp*wtemp;
    wpi=sin(theta);
    sum=0.5*(y[0]-y[n]);
    yy[0]=0.5*(y[0]+y[n]);
    for (j=1;j<n/2;j++) {
```

```
        wr=(wtemp=wr)*wpr-wi*wpi+wr;        Carry out the recurrence.
        wi=wi*wpr+wtemp*wpi+wi;
        y1=0.5*(y[j]+y[n-j]);               Calculate the auxiliary function.
        y2=(y[j]-y[n-j]);
        yy[j]=y1-wi*y2;                     The values for j and N - j are related.
        yy[n-j]=y1+wi*y2;
        sum += wr*y2;                       Carry along this sum for later use in unfold-
    }                                           ing the transform.
    yy[n/2]=y[n/2];                         y[n/2] unchanged.
    realft(yy,1);                           Calculate the transform of the auxiliary func-
    for (j=0;j<n;j++) y[j]=yy[j];               tion.
    y[n]=y[1];
    y[1]=sum;                               sum is the value of F_1 in equation (12.4.15).
    for (j=3;j<n;j+=2) {
        sum += y[j];                        Equation (12.4.14).
        y[j]=sum;
    }
}
```

The second important form of the cosine transform is defined by

$$F_k = \sum_{j=0}^{N-1} f_j \cos \frac{\pi k(j+\frac{1}{2})}{N} \tag{12.4.16}$$

with inverse

$$f_j = \frac{2}{N} \sum_{k=0}^{N-1}{}' F_k \cos \frac{\pi k(j+\frac{1}{2})}{N} \tag{12.4.17}$$

Here the prime on the summation symbol means that the term for $k = 0$ has a coefficient of $\frac{1}{2}$ in front. This form arises by extending the given data, defined for $j = 0, \ldots, N-1$, to $j = N, \ldots, 2N-1$ in such a way that they are even about the point $N - \frac{1}{2}$ and periodic. (They are therefore also even about $j = -\frac{1}{2}$.) The form (12.4.17) is related to Gauss-Chebyshev quadrature (see equation 4.6.19), to Chebyshev approximation (§5.8, equation 5.8.7), and Clenshaw-Curtis quadrature (§5.9).

This form of the cosine transform is useful when solving differential equations on "staggered" grids, where the variables are centered midway between mesh points. It is also the standard form in the field of data compression and image processing.

The auxiliary function used in this case is similar to equation (12.4.13):

$$y_j = \frac{1}{2}(f_j + f_{N-j-1}) + \sin\frac{\pi(j+\frac{1}{2})}{N}(f_j - f_{N-j-1}) \qquad j = 0, \ldots, N-1 \tag{12.4.18}$$

Carrying out the steps similar to those used to get from (12.4.6) to (12.4.9), we find

$$F_{2k} = \cos\frac{\pi k}{N} R_k - \sin\frac{\pi k}{N} I_k \tag{12.4.19}$$

$$F_{2k-1} = \sin\frac{\pi k}{N} R_k + \cos\frac{\pi k}{N} I_k + F_{2k+1} \tag{12.4.20}$$

Note that equation (12.4.20) gives

$$F_{N-1} = \tfrac{1}{2} R_{N/2} \tag{12.4.21}$$

Thus the even components are found directly from (12.4.19), while the odd components are found by recursing (12.4.20) down from $k = N/2 - 1$, using (12.4.21) to start.

Since the transform is not self-inverting, we have to reverse the above steps to find the inverse. Here is the routine:

fourier.h

```
void cosft2(VecDoub_IO &y, const Int isign) {
```

Calculates the "staggered" cosine transform of a set y[0..n-1] of real-valued data points. The transformed data replace the original data in array y. n must be a power of 2. Set isign to $+1$ for a transform, and to -1 for an inverse transform. For an inverse transform, the output array should be multiplied by $2/\mathrm{n}$.

```
    const Doub PI=3.141592653589793238;
    Int i,n=y.size();
    Doub sum,sum1,y1,y2,ytemp,theta,wi=0.0,wi1,wpi,wpr,wr=1.0,wr1,wtemp;
    theta=0.5*PI/n;                         Initialize the recurrences.
    wr1=cos(theta);
    wi1=sin(theta);
    wpr = -2.0*wi1*wi1;
    wpi=sin(2.0*theta);
    if (isign == 1) {                       Forward transform.
        for (i=0;i<n/2;i++) {
            y1=0.5*(y[i]+y[n-1-i]);         Calculate the auxiliary function.
            y2=wi1*(y[i]-y[n-1-i]);
            y[i]=y1+y2;
            y[n-1-i]=y1-y2;
            wr1=(wtemp=wr1)*wpr-wi1*wpi+wr1;    Carry out the recurrence.
            wi1=wi1*wpr+wtemp*wpi+wi1;
        }
        realft(y,1);                        Transform the auxiliary function.
        for (i=2;i<n;i+=2) {                Even terms.
            wr=(wtemp=wr)*wpr-wi*wpi+wr;
            wi=wi*wpr+wtemp*wpi+wi;
            y1=y[i]*wr-y[i+1]*wi;
            y2=y[i+1]*wr+y[i]*wi;
            y[i]=y1;
            y[i+1]=y2;
        }
        sum=0.5*y[1];                       Initialize recurrence for odd terms
        for (i=n-1;i>0;i-=2) {                  with (1/2)R_{N/2}.
            sum1=sum;                       Carry out recurrence for odd terms.
            sum += y[i];
            y[i]=sum1;
        }
    } else if (isign == -1) {               Inverse transform.
        ytemp=y[n-1];
        for (i=n-1;i>2;i-=2)                Form difference of odd terms.
            y[i]=y[i-2]-y[i];
        y[1]=2.0*ytemp;
        for (i=2;i<n;i+=2) {                Calculate R_k and I_k.
            wr=(wtemp=wr)*wpr-wi*wpi+wr;
            wi=wi*wpr+wtemp*wpi+wi;
            y1=y[i]*wr+y[i+1]*wi;
            y2=y[i+1]*wr-y[i]*wi;
            y[i]=y1;
            y[i+1]=y2;
        }
        realft(y,-1);
        for (i=0;i<n/2;i++) {               Invert auxiliary array.
            y1=y[i]+y[n-1-i];
            y2=(0.5/wi1)*(y[i]-y[n-1-i]);
            y[i]=0.5*(y1+y2);
            y[n-1-i]=0.5*(y1-y2);
            wr1=(wtemp=wr1)*wpr-wi1*wpi+wr1;
            wi1=wi1*wpr+wtemp*wpi+wi1;
        }
    }
}
```

An alternative way of implementing this algorithm is to form an auxiliary function by

copying the even elements of f_j into the first $N/2$ locations, and the odd elements into the next $N/2$ elements in reverse order. However, it is not easy to implement the alternative algorithm without a temporary storage array and we prefer the above in-place algorithm.

Finally, we mention that there exist fast cosine transforms for small N that do not rely on an auxiliary function or use an FFT routine. Instead, they carry out the transform directly, often coded in hardware for fixed N of small dimension [1].

CITED REFERENCES AND FURTHER READING:

Walker, J.S. 1996, *Fast Fourier Transforms*, 2nd ed. (Boca Raton, FL: CRC Press)

Rao, K.R. and Yip, P. 1990, *Discrete Cosine Transform: Algorithms, Advantages, Applications* (San Diego, CA: Academic Press)

Hou, H.S. 1987, "A Fast, Recursive Algorithm for Computing the Discrete Cosine Transform," *IEEE Transactions on Acoustics, Speech, and Signal Processing*, vol. ASSP-35, pp. 1455–1461 [see for additional references].

Chen, W., Smith, C.H., and Fralick, S.C. 1977, "A Fast Computational Algorithm for the Discrete Cosine Transform," *IEEE Transactions on Communications*, vol. COM-25, pp. 1004–1009.[1]

12.5 FFT in Two or More Dimensions

Given a complex function $h(k_1, k_2)$ defined over the two-dimensional grid $0 \le k_1 \le N_1 - 1,\ 0 \le k_2 \le N_2 - 1$, we can define its two-dimensional discrete Fourier transform as a complex function $H(n_1, n_2)$, defined over the same grid,

$$H(n_1, n_2) \equiv \sum_{k_2=0}^{N_2-1} \sum_{k_1=0}^{N_1-1} \exp(2\pi i k_2 n_2/N_2)\ \exp(2\pi i k_1 n_1/N_1)\ h(k_1, k_2) \tag{12.5.1}$$

By pulling the "subscripts 2" exponential outside of the sum over k_1, or by reversing the order of summation and pulling the "subscripts 1" outside of the sum over k_2, we can see instantly that the two-dimensional FFT can be computed by taking one-dimensional FFTs sequentially on each index of the original function. Symbolically,

$$\begin{aligned} H(n_1, n_2) &= \text{FFT-on-index-1 (FFT-on-index-2 } [h(k_1, k_2)]) \\ &= \text{FFT-on-index-2 (FFT-on-index-1 } [h(k_1, k_2)]) \end{aligned} \tag{12.5.2}$$

For this to be practical, of course, both N_1 and N_2 should be some efficient length for an FFT, usually a power of 2. Programming a two-dimensional FFT, using (12.5.2) with a one-dimensional FFT routine, is a bit clumsier than it seems at first. Because the one-dimensional routine requires that its input be in consecutive order as a one-dimensional complex array, you find that you are endlessly copying things out of the multidimensional input array and then copying things back into it. This is not recommended technique. Rather, you should use a multidimensional FFT routine, such as the one we give below.

The generalization of (12.5.1) to more than two dimensions, say to L dimensions, is evidently

$$H(n_1,\dots,n_L) \equiv \sum_{k_L=0}^{N_L-1} \cdots \sum_{k_1=0}^{N_1-1} \exp(2\pi i k_L n_L/N_L) \times \cdots \times \exp(2\pi i k_1 n_1/N_1)\; h(k_1,\dots,k_L) \tag{12.5.3}$$

where n_1 and k_1 range from 0 to N_1-1, ... , and n_L and k_L range from 0 to N_L-1. How many calls to a one-dimensional FFT are in (12.5.3)? Quite a few! For each value of $k_1, k_2, \dots, k_{L-1}$ you FFT to transform the L index. Then for each value of $k_1, k_2, \dots, k_{L-2}$ and n_L you FFT to transform the $L-1$ index. And so on. It is best to rely on someone else having done the bookkeeping for once and for all.

The inverse transforms of (12.5.1) or (12.5.3) are just what you would expect them to be: Change the i's in the exponentials to $-i$'s, and put an overall factor of $1/(N_1 \times \cdots \times N_L)$ in front of the whole thing. Most other features of multidimensional FFTs are also analogous to features already discussed in the one-dimensional case:

- Frequencies are arranged in wraparound order in the transform, but now for each separate dimension.
- The input data are also treated as if they were wrapped around. If they are discontinuous across this periodic identification (in any dimension), then the spectrum will have some excess power at high frequencies because of the discontinuity. The fix, if you care, is to remove multidimensional linear trends.
- If you are doing spatial filtering and are worried about wraparound effects, then you need to zero-pad all around the border of the multidimensional array. However, be sure to notice how costly zero-padding is in multidimensional transforms. If you use too thick a zero-pad, you are going to waste a *lot* of storage, especially in three or more dimensions!
- Aliasing occurs as always if sufficient bandwidth limiting does not exist along one or more of the dimensions of the transform.

The routine `fourn` that we furnish herewith is a descendant of one written by N.M. Brenner. It requires as input (i) a vector, telling the length of the array in each dimension, e.g., $(32, 64)$ (note that these lengths *must all* be powers of 2, and are the numbers of *complex* values in each direction); (ii) the usual scalar equal to ± 1 indicating whether you want the transform or its inverse; and, finally, (iii) the array of data. The number of dimensions is determined from the length of the vector in (i).

A few words about the data array: `fourn` accesses it as a one-dimensional array of real numbers, that is, `data[0..`$(2N_1N_2\dots N_L)$`-1]`, of length equal to twice the product of the lengths of the L dimensions. It assumes that the array represents an L-dimensional complex array, with individual components ordered as follows: (i) each complex value occupies two sequential locations, a real part followed by an imaginary; (ii) the first subscript changes least rapidly as one goes through the array; the last subscript changes most rapidly (that is, "store by rows," the C++ norm). Figure 12.5.1 illustrates the format of the output array.

As for `four1` earlier, we find it useful to give a bare form of the routine, where the data array is passed as a pointer, and then an overloaded function that passes the data array (by reference) as a `VecDoub`.

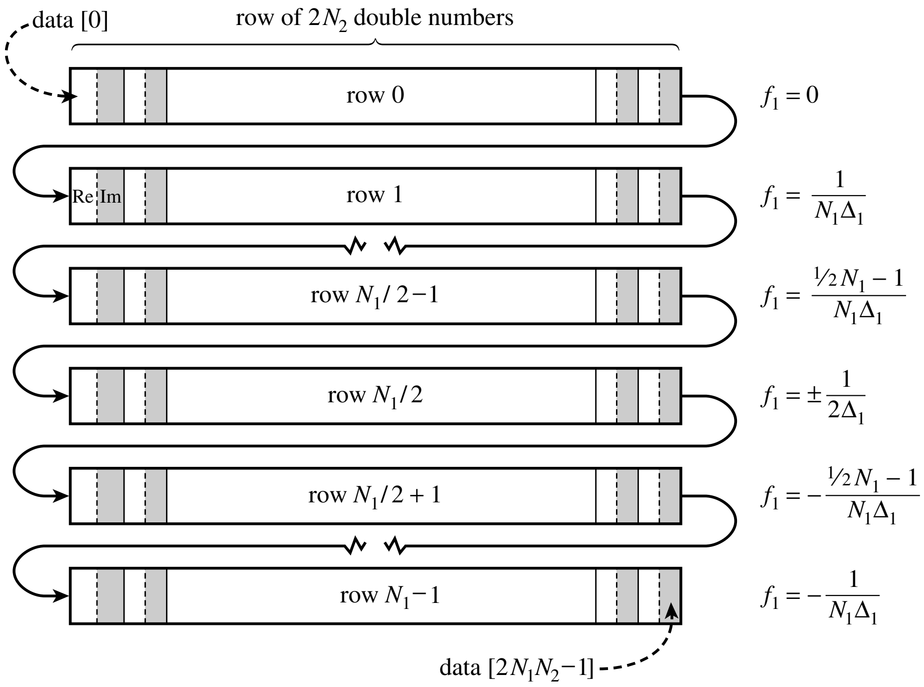

Figure 12.5.1. Storage arrangement of frequencies in the output $H(f_1, f_2)$ of a two-dimensional FFT. The input data is a two-dimensional $N_1 \times N_2$ array $h(t_1, t_2)$ (stored by columns of complex numbers). The output is also stored by complex columns. Each column corresponds to a particular value of f_2, as shown in the figure. Within each column, the arrangement of frequencies f_1 is exactly as shown in Figure 12.2.2. Δ_1 and Δ_2 are the sampling intervals in the 1 and 2 directions, respectively. The total number of (real) array elements is $2N_1N_2$. The program `fourn` can also do more than two dimensions, and the storage arrangement generalizes in the obvious way.

fourier_ndim.h

```
void fourn(Doub *data, VecInt_I &nn, const Int isign) {
```

Replaces `data` by its `ndim`-dimensional discrete Fourier transform, if `isign` is input as 1. `nn[0..ndim-1]` is an integer array containing the lengths of each dimension (number of complex values), which must all be powers of 2. `data` is a real array of length twice the product of these lengths, in which the data are stored as in a multidimensional complex array: real and imaginary parts of each element are in consecutive locations, and the rightmost index of the array increases most rapidly as one proceeds along `data`. For a two-dimensional array, this is equivalent to storing the array by rows. If `isign` is input as -1, `data` is replaced by its inverse transform times the product of the lengths of all dimensions.

```
    Int idim,i1,i2,i3,i2rev,i3rev,ip1,ip2,ip3,ifp1,ifp2;
    Int ibit,k1,k2,n,nprev,nrem,ntot=1,ndim=nn.size();
    Doub tempi,tempr,theta,wi,wpi,wpr,wr,wtemp;
    for (idim=0;idim<ndim;idim++) ntot *= nn[idim];   Total no. of complex values.
    if (ntot<2 || ntot&(ntot-1)) throw("must have powers of 2 in fourn");
    nprev=1;
    for (idim=ndim-1;idim>=0;idim--) {                Main loop over the dimensions.
        n=nn[idim];
        nrem=ntot/(n*nprev);
        ip1=nprev << 1;
        ip2=ip1*n;
        ip3=ip2*nrem;
        i2rev=0;
        for (i2=0;i2<ip2;i2+=ip1) {                    This is the bit-reversal sec-
            if (i2 < i2rev) {                              tion of the routine.
```

```
                for (i1=i2;i1<i2+ip1-1;i1+=2) {
                    for (i3=i1;i3<ip3;i3+=ip2) {
                        i3rev=i2rev+i3-i2;
                        SWAP(data[i3],data[i3rev]);
                        SWAP(data[i3+1],data[i3rev+1]);
                    }
                }
            }
            ibit=ip2 >> 1;
            while (ibit >= ip1 && i2rev+1 > ibit) {
                i2rev -= ibit;
                ibit >>= 1;
            }
            i2rev += ibit;
        }
        ifp1=ip1;                                   Here begins the Danielson-
        while (ifp1 < ip2) {                            Lanczos section of the
            ifp2=ifp1 << 1;                             routine.
            theta=isign*6.28318530717959/(ifp2/ip1);   Initialize for the trigonomet-
            wtemp=sin(0.5*theta);                       ric recurrence.
            wpr= -2.0*wtemp*wtemp;
            wpi=sin(theta);
            wr=1.0;
            wi=0.0;
            for (i3=0;i3<ifp1;i3+=ip1) {
                for (i1=i3;i1<i3+ip1-1;i1+=2) {
                    for (i2=i1;i2<ip3;i2+=ifp2) {
                        k1=i2;                      Danielson-Lanczos formula:
                        k2=k1+ifp1;
                        tempr=wr*data[k2]-wi*data[k2+1];
                        tempi=wr*data[k2+1]+wi*data[k2];
                        data[k2]=data[k1]-tempr;
                        data[k2+1]=data[k1+1]-tempi;
                        data[k1] += tempr;
                        data[k1+1] += tempi;
                    }
                }
                wr=(wtemp=wr)*wpr-wi*wpi+wr;        Trigonometric recurrence.
                wi=wi*wpr+wtemp*wpi+wi;
            }
            ifp1=ifp2;
        }
        nprev *= n;
    }
}

void fourn(VecDoub_IO &data, VecInt_I &nn, const Int isign) {
Overloaded version for the case where data is of type VecDoub.
    fourn(&data[0],nn,isign);
}
```

CITED REFERENCES AND FURTHER READING:

Nussbaumer, H.J. 1982, *Fast Fourier Transform and Convolution Algorithms* (New York: Springer).

12.6 Fourier Transforms of Real Data in Two and Three Dimensions

Two-dimensional FFTs are particularly important in the field of image processing. An image is usually represented as a two-dimensional array of pixel intensities, real (and usually positive) numbers. One commonly desires to filter high, or low, frequency spatial components from an image; or to convolve or deconvolve the image with some instrumental point spread function. Use of the FFT is by far the most efficient technique.

In three dimensions, a common use of the FFT is to solve Poisson's equation for a potential (e.g., electromagnetic or gravitational) on a three-dimensional lattice that represents the discretization of three-dimensional space. Here the source terms (mass or charge distribution) and the desired potentials are also real. In two and three dimensions, with large arrays, memory is often at a premium. It is therefore important to perform the FFTs, insofar as possible, on the data "in place." We want a routine with functionality similar to the multidimensional FFT routine `fourn` (§12.5), but which operates on real, not complex, input data. We give such a routine in this section. The development is analogous to that of §12.3 leading to the one-dimensional routine `realft`. (You might wish to review that material at this point, particularly equation 12.3.6.)

It is convenient to think of the independent variables $n_1, \ldots, n_L$ in equation (12.5.3) as representing an L-dimensional vector $\vec{n}$ in wave-number space, with values on the lattice of integers. The transform $H(n_1, \ldots, n_L)$ is then denoted $H(\vec{n})$.

It is easy to see that the transform $H(\vec{n})$ is periodic in each of its L dimensions. Specifically, if $\vec{P}_1, \vec{P}_2, \vec{P}_3, \ldots$ denote the vectors $(N_1, 0, 0, \ldots)$, $(0, N_2, 0, \ldots)$, $(0, 0, N_3, \ldots)$, and so forth, then

$$H(\vec{n} \pm \vec{P}_j) = H(\vec{n}) \qquad j = 1, \ldots, L \tag{12.6.1}$$

Equation (12.6.1) holds for any input data, real or complex. When the data are real, we have the additional symmetry

$$H(-\vec{n}) = H(\vec{n})^* \tag{12.6.2}$$

Equations (12.6.1) and (12.6.2) imply that the full transform can be trivially obtained from the subset of lattice values $\vec{n}$ that have

$$\begin{aligned} 0 \le n_1 &\le N_1 - 1 \\ 0 \le n_2 &\le N_2 - 1 \\ &\cdots \\ 0 \le n_L &\le \frac{N_L}{2} \end{aligned} \tag{12.6.3}$$

In fact, this set of values is overcomplete, because there are additional symmetry relations among the transform values that have $n_L = 0$ and $n_L = N_L/2$. However, these symmetries are complicated and their use becomes extremely confusing. Therefore, we will compute our FFT on the lattice subset of equation (12.6.3), even though this requires a small amount of extra storage for the answer, i.e., the transform

is not *quite* "in place." (Although an in-place transform is in fact possible, we have found it virtually impossible to explain to any user how to unscramble its output, i.e., where to find the real and imaginary components of the transform at some particular frequency!)

As a "bare" routine, we will implement the multidimensional real Fourier transform for the three-dimensional case $L = 3$, with the input data stored as a real three-dimensional array `data[0..nn1-1][0..nn2-1][0..nn3-1]`. This scheme will allow two-dimensional data to be processed with effectively no loss of efficiency simply by choosing `nn1` = 1. (Note that it must be the *first* dimension that is set to 1.) We also provide more convenient overloaded functions whose input data are stored as a `Mat3DDoub` (for three-dimensional data) or as a `MatDoub` (for two-dimensional data).

The output spectrum comes back packaged, logically at least, as a *complex* three-dimensional array that we can call `spec[0..nn1-1][0..nn2-1][0..nn3/2]` (cf. equation 12.6.3). In the first two of its three dimensions, the respective frequency values f_1 or f_2 are stored in wraparound order, that is, with zero frequency in the first index value, the smallest positive frequency in the second index value, the smallest *negative* frequency in the *last* index value, and so on (cf. the discussion leading up to routines `four1` and `fourn`). The third of the three dimensions returns only the positive half of the frequency spectrum. Figure 12.6.1 shows the logical storage scheme. The returned portion of the complex output spectrum is shown as the unshaded part of the lower figure.

The physical, as opposed to logical, packaging of the output spectrum is necessarily a bit different from the logical packaging, because, counting components, `spec` doesn't quite fit into `data`. The subscript range `spec[0..nn1-1][0..nn2-1][0..nn3/2-1]` is returned in the input array `data[0..nn1-1][0..nn2-1][0..nn3-1]`, with the correspondence

$$\begin{aligned}\mathrm{Re}(\texttt{spec[i1][i2][i3]}) &= \texttt{data[i1][i2][2*i3]}\\ \mathrm{Im}(\texttt{spec[i1][i2][i3]}) &= \texttt{data[i1][i2][2*i3+1]}\end{aligned} \tag{12.6.4}$$

The remaining "plane" of values, `spec[0..nn1-1][0..nn2-1][nn3/2]`, is returned in the two-dimensional `MatDoub` array `speq[0..nn1-1][0..2*nn2-1]`, with the correspondence

$$\begin{aligned}\mathrm{Re}(\texttt{spec[i1][i2][nn3/2]}) &= \texttt{speq[i1][2*i2]}\\ \mathrm{Im}(\texttt{spec[i1][i2][nn3/2]}) &= \texttt{speq[i1][2*i2+1]}\end{aligned} \tag{12.6.5}$$

Note that `speq` contains only frequency components whose third component f_3 is at the Nyquist critical frequency $\pm f_c$. In some applications these values will in fact be ignored or set to zero, since they are intrinsically aliased between positive and negative frequencies.

With this much introduction, the implementing procedure, called `rlft3`, is something of an anticlimax. Look in the innermost loop in the procedure, and you will see equation (12.3.6) implemented on the *last* transform index. The case of `i3=0` is coded separately, to account for the fact that `speq` is to be filled instead of overwriting the input array of data. The three enclosing `for` loops (indices `i2`, `i3`, and `i1`, from inside to outside) could in fact be done in any order — their actions all commute. We chose the order shown because of the following considerations: (i)

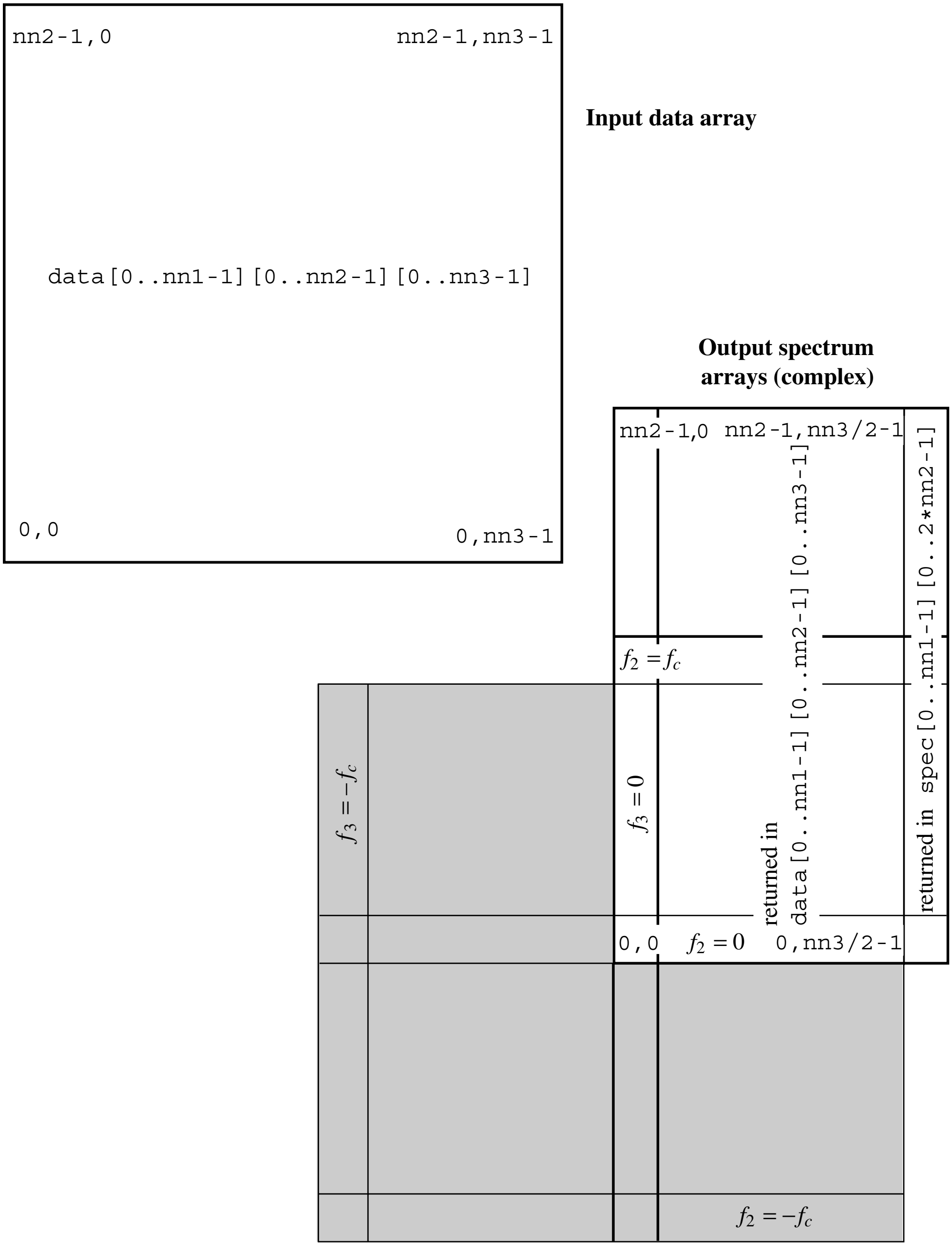

Figure 12.6.1. Input and output data arrangements for `rlft3`. All arrays shown are presumed to have a first (leftmost) dimension of range `[0..nn1-1]`, coming out of the page. The input data array is a real three-dimensional array `data[0..nn1-1][0..nn2-1][0..nn3-1]`. (For two-dimensional data, one sets `nn1` $=$ 1.) The output data can be viewed as a single complex array with dimensions `[0..nn1-1][0..nn2-1][0..nn3/2]` (cf. equation 12.6.3), corresponding to the frequency components f_1 and f_2 being stored in wraparound order, but only positive f_3 values being stored (others being obtainable by symmetry). The output data are actually returned mostly in the input array `data`, but partly stored in the real array `speq[0..nn1-1][0..2*nn2-1]`. See text for details.

i3 should not be the inner loop, because if it is, then the recurrence relations on wr and wi become burdensome. (ii) On modern processors with a cache hierarchy, i1 should be the outer loop, because (with C++ order of array storage) this results in the array data, which might be very large, being accessed in block sequential order.

Keep in mind that all the computing in rlft3 is negligible, by a logarithmic factor, compared with the actual work of computing the associated complex FFT, done in the routine fourn. Complex operations are carried out explicitly in terms of real and imaginary parts. The routine rlft3 is based on an earlier routine by G.B. Rybicki. As previously, it is convenient to provide a bare routine, and then more convenient overloaded functions. The overload for three-dimensional data inputs the data as a Mat3DDoub, with speq a MatDoub. The overload for two-dimensional data inputs the data as a MatDoub, with speq a VecDoub.

fourier_ndim.h

```
void rlft3(Doub *data, Doub *speq, const Int isign,
    const Int nn1, const Int nn2, const Int nn3) {
```

Given a three-dimensional real array data[0..nn1-1][0..nn2-1][0..nn3-1] (where nn1 = 1 for the case of a logically two-dimensional array), this routine returns (for isign=1) the complex fast Fourier transform as two complex arrays: On output, data contains the zero and positive frequency values of the third frequency component, while speq[0..nn1-1][0..2*nn2-1] contains the Nyquist critical frequency values of the third frequency component. First (and second) frequency components are stored for zero, positive, and negative frequencies, in standard wraparound order. See text for description of how complex values are arranged. For isign=-1, the inverse transform (times nn1*nn2*nn3/2 as a constant multiplicative factor) is performed, with output data (viewed as a real array) deriving from input data (viewed as complex) and speq. For inverse transforms on data not generated first by a forward transform, make sure the complex input data array satisfies property (12.6.2). The dimensions nn1, nn2, nn3 must always be integer powers of 2.

```
    Int i1,i2,i3,j1,j2,j3,k1,k2,k3,k4;
    Doub theta,wi,wpi,wpr,wr,wtemp;
    Doub c1,c2,h1r,h1i,h2r,h2i;
    VecInt nn(3);
    VecDoubp spq(nn1);
    for (i1=0;i1<nn1;i1++) spq[i1] = speq + 2*nn2*i1;
    c1 = 0.5;
    c2 = -0.5*isign;
    theta = isign*(6.28318530717959/nn3);
    wtemp = sin(0.5*theta);
    wpr = -2.0*wtemp*wtemp;
    wpi = sin(theta);
    nn[0] = nn1;
    nn[1] = nn2;
    nn[2] = nn3 >> 1;
    if (isign == 1) {                          Case of forward transform.
        fourn(data,nn,isign);                  Here is where most all of the com-
        k1=0;                                     pute time is spent.
        for (i1=0;i1<nn1;i1++)                 Extend data periodically into speq.
            for (i2=0,j2=0;i2<nn2;i2++,k1+=nn3) {
                spq[i1][j2++]=data[k1];
                spq[i1][j2++]=data[k1+1];
            }
    }
    for (i1=0;i1<nn1;i1++) {
        j1=(i1 != 0 ? nn1-i1 : 0);
```

Zero frequency is its own reflection, otherwise locate corresponding negative frequency in wraparound order.

```
        wr=1.0;                                Initialize trigonometric recurrence.
        wi=0.0;
        for (i3=0;i3<=(nn3>>1);i3+=2) {
            k1=i1*nn2*nn3;
            k3=j1*nn2*nn3;
```

```
            for (i2=0;i2<nn2;i2++,k1+=nn3) {
                if (i3 == 0) {                          Equation (12.3.6).
                    j2=(i2 != 0 ? ((nn2-i2)<<1) : 0);
                    h1r=c1*(data[k1]+spq[j1][j2]);
                    h1i=c1*(data[k1+1]-spq[j1][j2+1]);
                    h2i=c2*(data[k1]-spq[j1][j2]);
                    h2r= -c2*(data[k1+1]+spq[j1][j2+1]);
                    data[k1]=h1r+h2r;
                    data[k1+1]=h1i+h2i;
                    spq[j1][j2]=h1r-h2r;
                    spq[j1][j2+1]=h2i-h1i;
                } else {
                    j2=(i2 != 0 ? nn2-i2 : 0);
                    j3=nn3-i3;
                    k2=k1+i3;
                    k4=k3+j2*nn3+j3;
                    h1r=c1*(data[k2]+data[k4]);
                    h1i=c1*(data[k2+1]-data[k4+1]);
                    h2i=c2*(data[k2]-data[k4]);
                    h2r= -c2*(data[k2+1]+data[k4+1]);
                    data[k2]=h1r+wr*h2r-wi*h2i;
                    data[k2+1]=h1i+wr*h2i+wi*h2r;
                    data[k4]=h1r-wr*h2r+wi*h2i;
                    data[k4+1]= -h1i+wr*h2i+wi*h2r;
                }
            }
            wr=(wtemp=wr)*wpr-wi*wpi+wr;            Do the recurrence.
            wi=wi*wpr+wtemp*wpi+wi;
        }
    }
    if (isign == -1) fourn(data,nn,isign);      Case of reverse transform.
}

void rlft3(Mat3DDoub_IO &data, MatDoub_IO &speq, const Int isign) {
Overloaded version for three-dimensional data. When isign is 1, replace data and spec by
data's three-dimensional FFT. When isign is −1, the inverse transform (times one-half the
product of data's dimensions) is performed. See comments in version above.
    if (speq.nrows() != data.dim1() || speq.ncols() != 2*data.dim2())
        throw("bad dims in rlft3");
    rlft3(&data[0][0][0],&speq[0][0],isign,data.dim1(),data.dim2(),data.dim3());
}

void rlft3(MatDoub_IO &data, VecDoub_IO &speq, const Int isign) {
Overloaded version for two-dimensional data. When isign is 1, replace data and spec by data's
two-dimensional FFT. When isign is −1, the inverse transform (times one-half the product of
data's dimensions) is performed. See comments in version above.
    if (speq.size() != 2*data.nrows()) throw("bad dims in rlft3");
    rlft3(&data[0][0],&speq[0],isign,1,data.nrows(),data.ncols());
}
```

As in earlier sections of this chapter, we can use a bit of C++ trickery to access the output Fourier components (logical array spec) more easily. We define two overloaded helper functions (the first of which is identical to the definition in section §12.3)

```
inline Complex* Cmplx(VecDoub &d) {return (Complex *)&d[0];}
inline Complex* Cmplx(Doub *d) {return (Complex *)d;}
```

Now suppose that data is two-dimensional with input dimensions nx and ny. Then, on output, a complex frequency component (i, j), with $0 \le \mathtt{i} \le \mathtt{nx}/2$ and $0 \le \mathtt{j} \le \mathtt{ny}/2 - 1$, can be accessed as

```
    Cmplx(data[i])[j]
```

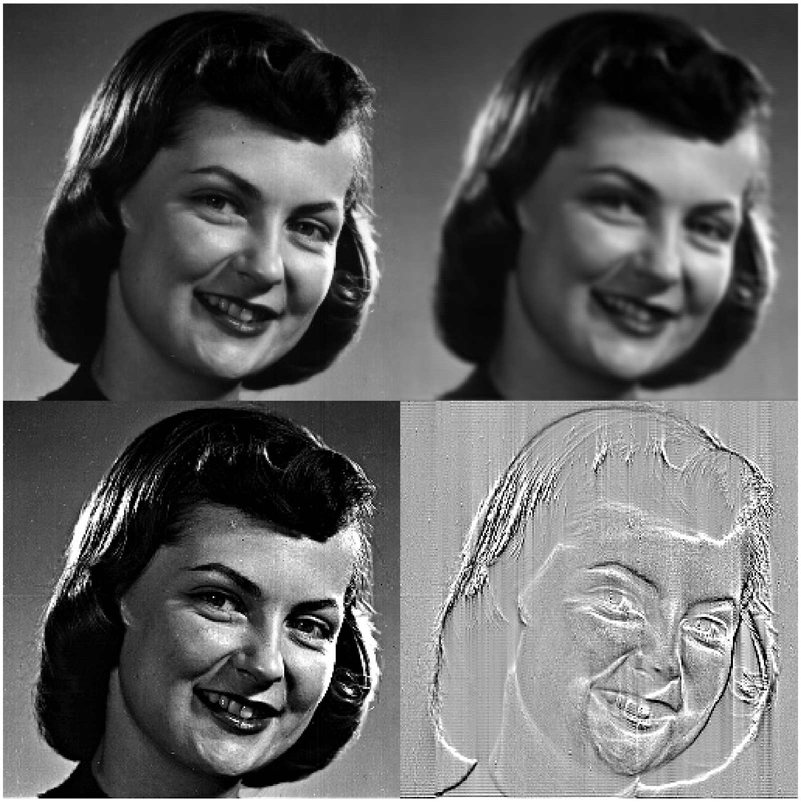

Figure 12.6.2. Fourier processing of an image. Upper left: Original image. Upper right: Blurred by low-pass filtering. Lower left: Sharpened by enhancing high frequency components. Lower right: Magnitude of the derivative operator as computed in Fourier space.

Yes, the right parenthesis really is between the subscripts! The corresponding negative (wraparound) frequencies are at

```
Cmplx(data[nx-i])[j]
```

but now with $1 \leq \texttt{i} \leq \texttt{nx}/2 - 1$. The Nyquist critical values $\texttt{j} = \texttt{ny}/2$ can be accessed as

```
Cmplx(speq)[i]
```

for $0 \leq \texttt{i} \leq \texttt{nx}/2$ and

```
Cmplx(speq)[nx-i]
```

for $1 \leq \texttt{i} \leq \texttt{nx}/2 - 1$. If you don't understand how this all works, a useful exercise is to locate each of these expressions in Figure 12.6.1. All of the above expressions can be l-values as well as r-values.

Figure 12.6.2 shows a test image* and three examples of processing with `rlft3` (using the overloaded function for two-dimensional data). The first example is a simple low-pass filter. A sharp image becomes blurry when its high-frequency spatial

*We are inordinately fond of this 1950s vintage IEEE test image, despite the fact that many readers have urged us to use instead the historically important "Lenna" image from the early 1970s. See [1] for an interesting recounting of the history. "Lenna," a strategically cropped Playboy centerfold, is also said to be the source of the term "discreet Fourier transform."

components are suppressed by the factor (here) max $(1 - 6f^2/f_c^2, 0)$. The second example is a sharpening filter where high frequencies are enhanced. Code for producing this image looks something like this:

rlft3_sharpen.h

```
Int i, j, nx=256, ny=256;                          Image is 256 × 256.
MatDoub data(nx,ny);
VecDoub speq(2*nx);
Doub fac;
...                                                Here we would fill data with the image.
rlft3(data,speq,1);                                Forward transform.
for (i=0;i<nx/2;i++) for (j=0;j<ny/2;j++) {        Loop over all frequencies except Nyquist.
        fac = 1.+3.*sqrt(SQR(i*2./nx)+SQR(j*2./ny));
        Cmplx(data[i])[j] *= fac;
        if (i>0) Cmplx(data[nx-i])[j] *= fac;      Negative (wraparound) frequencies.
}
for (j=0;j<ny/2;j++) {                             Loop over frequencies where i is Nyquist.
    fac = 1.+3.*sqrt(1.+SQR(j*2./ny));
    Cmplx(data[nx/2])[j] *= fac;
}
for (i=0;i<nx/2;i++) {                             Loop over frequencies where j is Nyquist.
    fac = 1.+3.*sqrt(SQR(i*2./nx)+1.);
    Cmplx(speq)[i] *= fac;
    if (i>0) Cmplx(speq)[nx-i] *= fac;             Wraparound.
}
Cmplx(speq)[nx/2] *= (1.+3.*sqrt(2.));             Both i and j are Nyquist.
rlft3(data,speq,-1);                               Reverse transform.
```

The third example is a derivative filter, where a Fourier component at frequency (f_x, f_y) is multiplied by $2\pi i(f_x^2 + f_y^2)^{1/2}$, and the resulting intensities are then linearly mapped into an appropriate range.

To extend `rlft3` to four dimensions, you simply add an additional (outer) nested `for` loop in `i0`, analogous to the present `i1`. (Modifying the routine to do an *arbitrary* number of dimensions, as in `fourn`, is a good programming exercise for the reader.)

CITED REFERENCES AND FURTHER READING:

Brigham, E.O. 1974, *The Fast Fourier Transform* (Englewood Cliffs, NJ: Prentice-Hall).

Swartztrauber, P. N. 1986, "Symmetric FFTs," *Mathematics of Computation*, vol. 47, pp. 323–346.

Hutchinson, J. 2001, in *IEEE Professional Communication Society Newsletter*, vol. 45, no. 3. See also `http://www.lenna.org`.[1]

12.7 External Storage or Memory-Local FFTs

Sometime in your life, you might have to compute the Fourier transform of a *really large* data set, larger than the size of your computer's physical memory. In such a case, the data will be stored on some external medium, such as magnetic or optical disk. Needed is an algorithm that makes some manageable number of sequential passes through the external data, processing it on the fly and outputting intermediate results to other external media, which can be read on subsequent passes.

In fact, an algorithm of just this description was developed by Singleton [1] very soon after the discovery of the FFT. The algorithm requires four sequential storage devices, each

capable of holding half of the input data. The first half of the input data is initially on one device, the second half on another.

Singleton's algorithm is based on the observation that it is possible to bit reverse 2^M values by the following sequence of operations: On the first pass, values are read alternately from the two input devices, and written to a single output device (until it holds half the data), and then to the other output device. On the second pass, the output devices become input devices, and vice versa. Now, we copy *two* values from the first device, then *two* values from the second, writing them (as before) first to fill one output device, then to fill a second. Subsequent passes read 4, 8, etc., input values at a time. After completion of pass $M-1$, the data are in bit-reverse order.

Singleton's next observation is that it is possible to alternate the passes of essentially this bit-reversal technique with passes that implement one stage of the Danielson-Lanczos combination formula (12.2.3). The scheme, roughly, is this: One starts as before with half the input data on one device and half on another. In the first pass, one complex value is read from each input device. Two combinations are formed, and one is written to each of two output devices. After this "computing" pass, the devices are rewound, and a "permutation" pass is performed, where groups of values are read from the first input device and alternately written to the first and second output devices; when the first input device is exhausted, the second is similarly processed. This sequence of computing and permutation passes is repeated $M-K-1$ times, where 2^K is the size of internal buffer available to the program. The second phase of the computation consists of a final K computation passes. What distinguishes the second phase from the first is that, now, the permutations are local enough to do in place during the computation. There are thus no separate permutation passes in the second phase. In all, there are $2M-K-2$ passes through the data.

An implementation of Singleton's algorithm, `fourfs`, based on reference [1], is given in a Webnote [2].

For one-dimensional data, Singleton's algorithm produces output in exactly the same order as a standard FFT (e.g., `four1`). For multidimensional data, the output is in *transpose* order rather than in the conventional C++ array order output by `fourn`. That is, in scanning through the data, it is the leftmost array index that cycles most quickly, then the second leftmost, and so on. This peculiarity, which is intrinsic to the method, is generally only a minor inconvenience. For convolutions, one simply computes the component-by-component product of two transforms in their nonstandard arrangement, and then does an inverse transform on the result. Note that, if the lengths of the different dimensions are not all the same, then you must reverse the order of the values in `nn[0..ndim-1]` (thus giving the dimensions of the transpose-order output array) before performing the inverse transform. Note also that, just like `fourn`, performing a transform and then an inverse results in multiplying the original data by the product of the lengths of all dimensions.

We leave it as an exercise for the reader to figure out how to reorder `fourfs`'s output into normal order, taking additional passes through the externally stored data. We doubt that such reordering is ever really needed.

You will likely want to modify `fourfs` to fit your particular application. For example, as written, `KBF` $\equiv 2^K$ plays the dual role of being the size of the internal buffers, and the record size of the unformatted reads and writes. The latter role limits its size to that allowed by your machine's I/O facility. It is a simple matter to perform multiple reads for a much larger `KBF`, thus reducing the number of passes by a few.

Another modification of `fourfs` would be for the case where your virtual memory machine has sufficient address space, but not sufficient physical memory, to do an efficient FFT by the conventional algorithm (whose memory references are extremely nonlocal). In that case, you will need to replace the reads, writes, and rewinds by mappings of the arrays `afa`, `afb`, and `afc` into your address space. In other words, these arrays are replaced by references to a single data array, with offsets that get modified wherever `fourfs` performs an I/O operation. The resulting algorithm will have its memory references local within blocks of size `KBF`. Execution speed is thereby sometimes increased enormously, albeit at the cost of requiring twice as much virtual memory as an in-place FFT.

CITED REFERENCES AND FURTHER READING:

Singleton, R.C. 1967, "A Method for Computing the Fast Fourier Transform with Auxiliary Memory and Limited High-speed Storage," *IEEE Transactions on Audio and Electroacoustics*, vol. AU-15, pp. 91–97.[1]

Numerical Recipes Software 2007, "Code for External or Memory-Local Fourier Transform," *Numerical Recipes Webnote No. 18*, at `http://www.nr.com/webnotes?18` [2]

Oppenheim, A.V., Schafer, R.W., and Buck, J.R. 1999, *Discrete-Time Signal Processing*, 2nd ed. (Englewood Cliffs, NJ: Prentice-Hall), Chapter 9.

CHAPTER 13

Fourier and Spectral Applications

13.0 Introduction

Fourier methods have revolutionized fields of science and engineering, from astronomy to medical imaging, from seismology to spectroscopy. In this chapter, we present some of the basic applications of Fourier and spectral methods that have made these revolutions possible.

Say the word "Fourier" to a numericist, and the response, as if by Pavlovian conditioning, will likely be "FFT." Indeed, the wide application of Fourier methods must be credited principally to the existence of the fast Fourier transform. Better mousetraps move over: If you speed up *any* nontrivial algorithm by a factor of a million or so, the world will beat a path toward finding useful applications for it. The most direct applications of the FFT are to the convolution or deconvolution of data (§13.1), correlation and autocorrelation (§13.2), optimal filtering (§13.3), power spectrum estimation (§13.4), and the computation of Fourier integrals (§13.9).

As important as they are, however, FFT methods are not the be-all and end-all of spectral analysis. Section 13.5 is a brief introduction to the field of time-domain digital filters. In the spectral domain, one limitation of the FFT is that it always represents a function's Fourier transform as a polynomial in $z = \exp(2\pi i f \Delta)$ (cf. equation 12.1.7). Sometimes, processes have spectra whose shapes are not well represented by this form. An alternative form, which allows the spectrum to have poles in z, is used in the techniques of linear prediction (§13.6) and maximum entropy spectral estimation (§13.7).

Another significant limitation of all FFT methods is that they require the input data to be sampled at evenly spaced intervals. For irregularly or incompletely sampled data, other (albeit slower) methods are available, as discussed in §13.8.

So-called wavelet methods inhabit a representation of function space that is neither in the temporal nor in the spectral domain, but rather somewhere in-between. Section 13.10 is an introduction to this subject. Finally, §13.11 is an excursion into the numerical use of the Fourier sampling theorem.

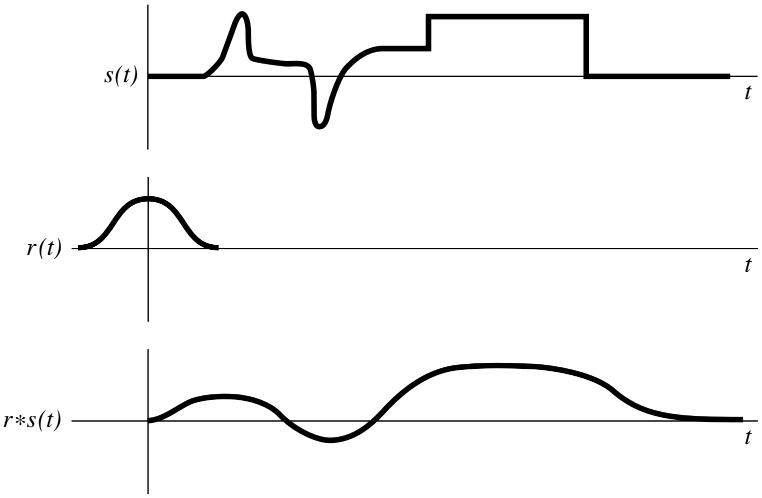

Figure 13.1.1. Example of the convolution of two functions. A signal $s(t)$ is convolved with a response function $r(t)$. Since the response function is broader than some features in the original signal, these are "washed out" in the convolution. In the absence of any additional noise, the process can be reversed by deconvolution.

13.1 Convolution and Deconvolution Using the FFT

We have defined the *convolution* of two functions for the continuous case in equation (12.0.9), and have given the *convolution theorem* as equation (12.0.10). The theorem says that the Fourier transform of the convolution of two functions is equal to the product of their individual Fourier transforms. Now, we want to deal with the discrete case. We will mention first the context in which convolution is a useful procedure, and then discuss how to compute it efficiently using the FFT.

The convolution of two functions $r(t)$ and $s(t)$, denoted $r * s$, is mathematically equal to their convolution in the opposite order, $s * r$. Nevertheless, in most applications the two functions have quite different meanings and characters. One of the functions, say s, is typically a signal or data stream, which goes on indefinitely in time (or in whatever the appropriate independent variable may be). The other function r is a "response function," typically a peaked function that falls to zero in both directions from its maximum. The effect of convolution is to smear the signal $s(t)$ in time according to the recipe provided by the response function $r(t)$, as shown in Figure 13.1.1. In particular, a spike or delta-function of unit area in s which occurs at some time t_0 is supposed to be smeared into the shape of the response function itself, but translated from time 0 to time t_0 as $r(t - t_0)$.

In the discrete case, the signal $s(t)$ is represented by its sampled values at equal time intervals s_j. The response function is also a discrete set of numbers r_k, with the following interpretation: r_0 tells what multiple of the input signal in one channel (one particular value of j) is copied into the identical output channel (same value of j); r_1 tells what multiple of input signal in channel j is additionally copied into output channel $j + 1$; r_{-1} tells the multiple that is copied into channel $j - 1$; and so on for both positive and negative values of k in r_k. Figure 13.1.2 illustrates the situation.

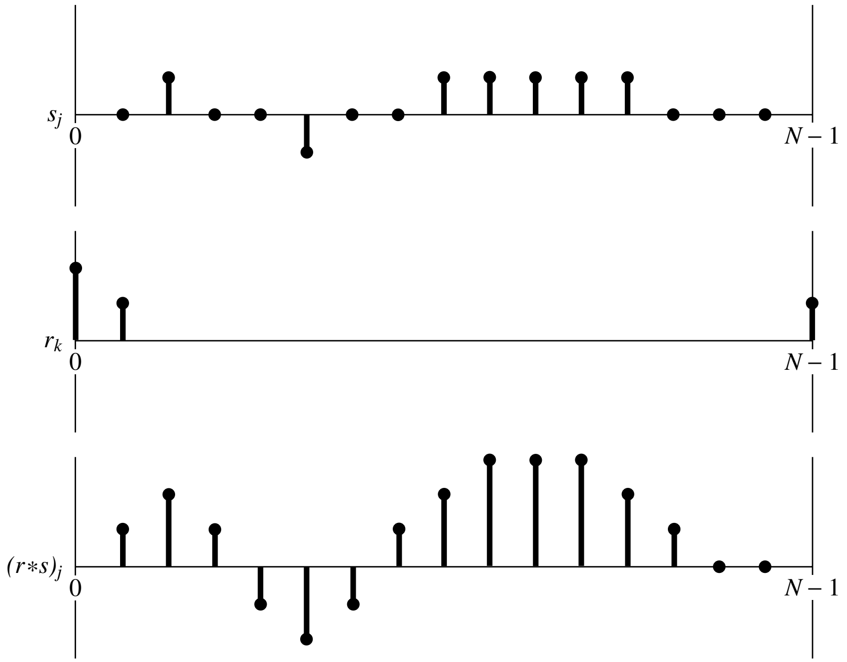

Figure 13.1.2. Convolution of discretely sampled functions. Note how the response function for negative times is wrapped around and stored at the extreme right end of the array r_k.

Example: A response function with $r_0 = 1$ and all other r_k's equal to zero is just the identity filter. Convolution of a signal with this response function gives identically the signal. Another example is the response function with $r_{14} = 1.5$ and all other r_k's equal to zero. This produces convolved output that is the input signal multiplied by 1.5 and delayed by 14 sample intervals.

Evidently, we have just described in words the following definition of discrete convolution with a response function of finite duration M:

$$(r * s)_j \equiv \sum_{k=-M/2+1}^{M/2} s_{j-k}\, r_k \tag{13.1.1}$$

If a discrete response function is nonzero only in some range $-M/2 < k \leq M/2$, where M is a sufficiently large even integer, then the response function is called a *finite impulse response (FIR)*, and its *duration* is M. (Notice that we are defining M as the number of nonzero *values* of r_k; these values span a time interval of $M - 1$ sampling times.) In most practical circumstances the case of finite M is the case of interest, either because the response really has a finite duration, or because we choose to truncate it at some point and approximate it by a finite-duration response function.

The *discrete convolution theorem* is this: If a signal s_j is *periodic* with period N, so that it is completely determined by the N values $s_0, \ldots, s_{N-1}$, then its discrete convolution with a response function *of finite duration* N is a member of the discrete Fourier transform pair,

$$\sum_{k=-N/2+1}^{N/2} s_{j-k}\, r_k \quad \Longleftrightarrow \quad S_n R_n \tag{13.1.2}$$

Here S_n $(n = 0, \ldots, N-1)$ is the discrete Fourier transform of the values s_j $(j = 0, \ldots, N-1)$, while R_n $(n = 0, \ldots, N-1)$ is the discrete Fourier transform of the values r_k $(k = 0, \ldots, N-1)$. These values of r_k are the same as for the range $k = -N/2+1, \ldots, N/2$, but in wraparound order, exactly as was described at the end of §12.2.

13.1.1 Treatment of End Effects by Zero Padding

The discrete convolution theorem presumes a set of two circumstances that are not universal. First, it assumes that the input signal is periodic, whereas real data often either go forever without repetition or else consist of one nonperiodic stretch of finite length. Second, the convolution theorem takes the duration of the response to be the same as the period of the data; they are both N. We need to work around these two constraints.

The second is very straightforward. Almost always, one is interested in a response function whose duration M is much shorter than the length of the data set N. In this case, you simply extend the response function to length N by padding it with zeros, i.e., define $r_k = 0$ for $M/2 \le k \le N/2$ and also for $-N/2 + 1 \le\le -M/2 + 1$. Dealing with the first constraint is more challenging. Since the convolution theorem rashly assumes that the data are periodic, it will falsely "pollute" the first output channel $(r * s)_0$ with some wrapped-around data from the far end of the data stream s_{N-1}, s_{N-2}, etc. (See Figure 13.1.3.) So, we need to set up a buffer zone of zero-padded values at the end of the s_j vector, in order to make this pollution zero. How many zero values do we need in this buffer? Exactly as many as the most negative index for which the response function is nonzero. For example, if r_{-3} is nonzero while $r_{-4}, r_{-5}, \ldots$ are all zero, then we need three zero pads at the end of the data: $s_{N-3} = s_{N-2} = s_{N-1} = 0$. These zeros will protect the first output channel $(r*s)_0$ from wraparound pollution. It should be obvious that the second output channel $(r * s)_1$ and subsequent ones will also be protected by these same zeros. Let K denote the number of padding zeros, so that the last actual input data point is s_{N-K-1}.

What now about pollution of the very *last* output channel? Since the data now end with s_{N-K-1}, the last output channel of interest is $(r * s)_{N-K-1}$. This channel can be polluted by wraparound from input channel s_0 unless the number K is also large enough to take care of the most positive index k for which the response function r_k is nonzero. For example, if r_0 through r_6 are nonzero, while $r_7, r_8 \ldots$ are all zero, then we need at least $K = 6$ padding zeros at the end of the data: $s_{N-6} = \ldots = s_{N-1} = 0$.

To summarize — we need to pad the data with a number of zeros *on one end* equal to the maximum positive duration *or* maximum negative duration of the response function, *whichever is larger*. (For a symmetric response function of duration M, you will need only $M/2$ zero pads.) Combining this operation with the padding of the response r_k described above, we effectively insulate the data from artifacts of undesired periodicity. Figure 13.1.4 illustrates matters.

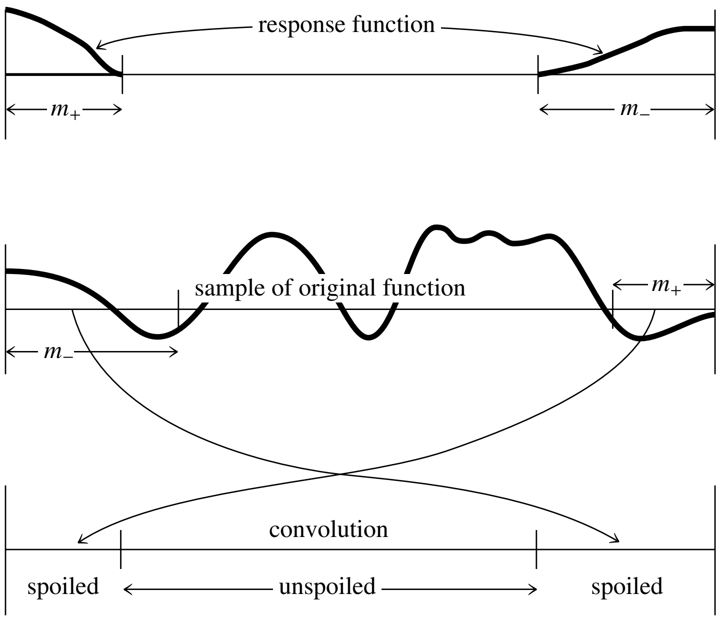

Figure 13.1.3. The wraparound problem in convolving finite segments of a function. Not only must the response function wrap be viewed as cyclic, but so must the sampled original function. Therefore, a portion at each end of the original function is erroneously wrapped around by convolution with the response function.

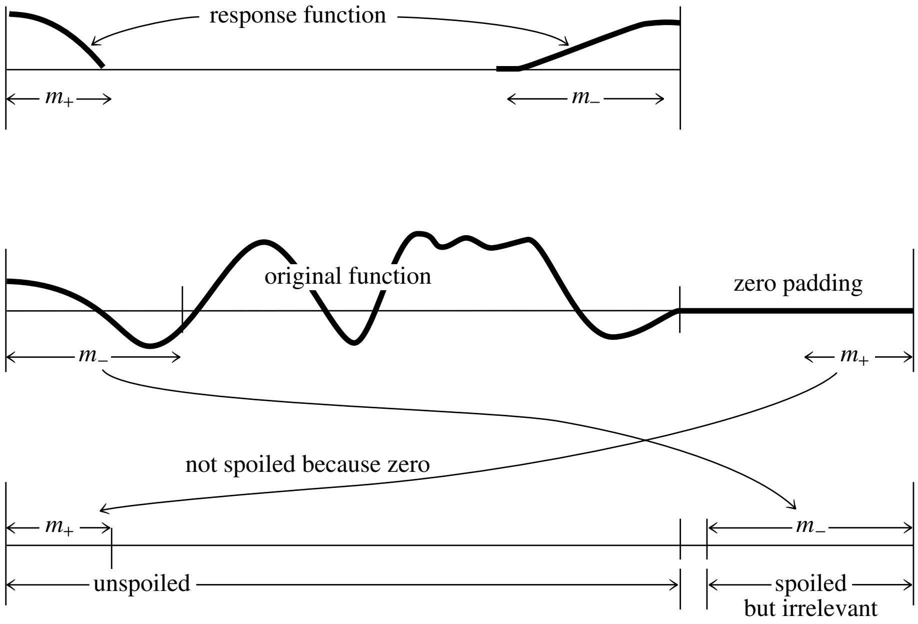

Figure 13.1.4. Zero-padding as solution to the wraparound problem. The original function is extended by zeros, serving a dual purpose: When the zeros wrap around, they do not disturb the true convolution; and while the original function wraps around onto the zero region, that region can be discarded.

13.1.2 Use of FFT for Convolution

The data, complete with zero-padding, are now a set of real numbers s_j, $j = 0, \ldots, N-1$, and the response function is zero-padded out to duration N and arranged in wraparound order. (Generally this means that a large contiguous section of the r_k's, in the middle of that array, is zero, with nonzero values clustered at the two extreme ends of the array.) You now compute the discrete convolution as follows: Use the FFT algorithm to compute the discrete Fourier transform of s and of r. Multiply the two transforms together component-by-component, remembering that the transforms consist of complex numbers. Then use the FFT algorithm to take the inverse discrete Fourier transform of the products. The answer is the convolution $r * s$.

What about *deconvolution*? Deconvolution is the process of *undoing* the smearing in a data set that has occurred under the influence of a known response function, for example, because of the known effect of a less-than-perfect measuring apparatus. The defining equation of deconvolution is the same as that for convolution, namely (13.1.1), except now the left-hand side is taken to be known and (13.1.1) is to be considered as a set of N linear equations for the unknown quantities s_j. Solving these simultaneous linear equations in the time domain of (13.1.1) is unrealistic in most cases, but the FFT renders the problem almost trivial. Instead of multiplying the transform of the signal and response to get the transform of the convolution, we just divide the transform of the (known) convolution by the transform of the response to get the transform of the deconvolved signal.

This procedure can go wrong *mathematically* if the transform of the response function is exactly zero for some value R_n, so that we can't divide by it. This indicates that the original convolution has truly lost all information at that one frequency, so that a reconstruction of that frequency component is not possible. You should be aware, however, that apart from mathematical problems, the process of deconvolution has other practical shortcomings. The process is generally quite sensitive to noise in the input data, and to the accuracy to which the response function r_k is known. Perfectly reasonable attempts at deconvolution can sometimes produce nonsense for these reasons. In such cases you may want to make use of the additional process of *optimal filtering*, which is discussed in §13.3.

Here is our routine for convolution and deconvolution, using the FFT as implemented in `realft` (§12.3). The data are assumed to be stored in a `VecDoub` array `data[0..n-1]`, with `n` an integer power of 2. The response function is assumed to be stored in wraparound order in a `VecDoub` array `respns[0..m-1]`. The value of `m` can be any *odd* integer less than or equal to `n`, since the first thing the program does is to recopy the response function into the appropriate wraparound order in an array of length `n`. The answer is provided in `ans`, which is also used as working space.

convlv.h

```
void convlv(VecDoub_I &data, VecDoub_I &respns, const Int isign,
    VecDoub_O &ans) {
```

Convolves or deconvolves a real data set `data[0..n-1]` (including any user-supplied zero padding) with a response function `respns[0..m-1]`, where `m` is an odd integer $\leq$ `n`. The response function must be stored in wraparound order: The first half of the array `respns` contains the impulse response function at positive times, while the second half of the array contains the impulse response function at negative times, counting down from the highest element `respns[m-1]`. On input `isign` is $+1$ for convolution, -1 for deconvolution. The answer is returned in `ans[0..n-1]`. `n` must be an integer power of 2.

```
    Int i,no2,n=data.size(),m=respns.size();
```

```
    Doub mag2,tmp;
    VecDoub temp(n);
    temp[0]=respns[0];
    for (i=1;i<(m+1)/2;i++) {                Put respns in array of length n.
        temp[i]=respns[i];
        temp[n-i]=respns[m-i];
    }
    for (i=(m+1)/2;i<n-(m-1)/2;i++)         Pad with zeros.
        temp[i]=0.0;
    for (i=0;i<n;i++)
        ans[i]=data[i];
    realft(ans,1);                           FFT both arrays.
    realft(temp,1);
    no2=n>>1;
    if (isign == 1) {
        for (i=2;i<n;i+=2) {                 Multiply FFTs to convolve.
            tmp=ans[i];
            ans[i]=(ans[i]*temp[i]-ans[i+1]*temp[i+1])/no2;
            ans[i+1]=(ans[i+1]*temp[i]+tmp*temp[i+1])/no2;
        }
        ans[0]=ans[0]*temp[0]/no2;
        ans[1]=ans[1]*temp[1]/no2;
    } else if (isign == -1) {
        for (i=2;i<n;i+=2) {                 Divide FFTs to deconvolve.
            if ((mag2=SQR(temp[i])+SQR(temp[i+1])) == 0.0)
                throw("Deconvolving at response zero in convlv");
            tmp=ans[i];
            ans[i]=(ans[i]*temp[i]+ans[i+1]*temp[i+1])/mag2/no2;
            ans[i+1]=(ans[i+1]*temp[i]-tmp*temp[i+1])/mag2/no2;
        }
        if (temp[0] == 0.0 || temp[1] == 0.0)
            throw("Deconvolving at response zero in convlv");
        ans[0]=ans[0]/temp[0]/no2;
        ans[1]=ans[1]/temp[1]/no2;
    } else throw("No meaning for isign in convlv");
    realft(ans,-1);                          Inverse transform back to time domain.
}
```

13.1.3 Convolving or Deconvolving Very Large Data Sets

If your data set is so long that you do not want to fit it into memory all at once, then you must break it up into sections and convolve each section separately. Now, however, the treatment of end effects is a bit different. You have to worry not only about spurious wraparound effects, but also about the fact that the ends of each section of data *should* have been influenced by data at the nearby ends of the immediately preceding and following sections of data, but were not so influenced since only one section of data is in the machine at a time.

There are two, related, standard solutions to this problem. Both are fairly obvious, so with a few words of description here, you ought to be able to implement them for yourself. The first solution is called the *overlap-save method*. In this technique you pad only the very beginning of the data with enough zeros to avoid wraparound pollution. After this initial padding, you forget about zero-padding altogether. Bring in a section of data and convolve or deconvolve it. Then throw out the points at each end that are polluted by wraparound end effects. Output only the remaining good points in the middle. Now bring in the next section of data, but not all new data. The first points in each new section overlap the last points from the preceding section of data. The sections must be overlapped sufficiently so that the polluted output points

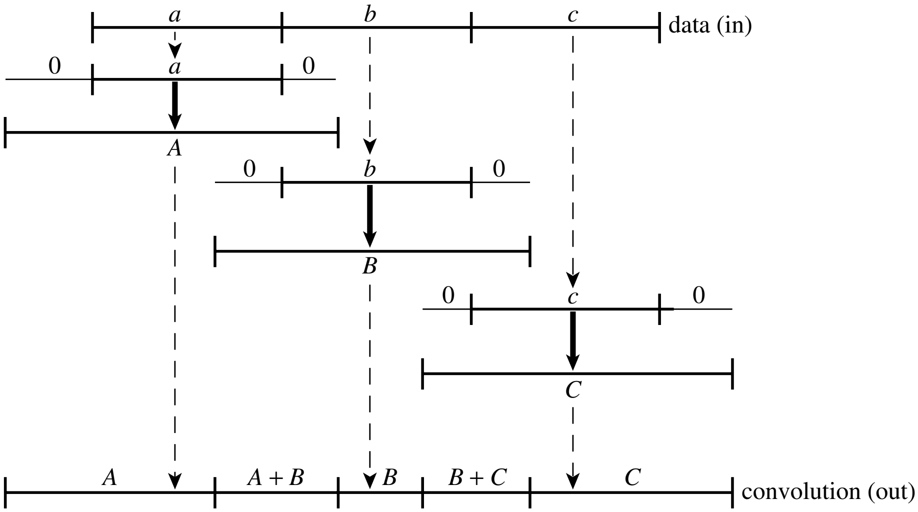

Figure 13.1.5. The overlap-add method for convolving a response with a very long signal. The signal data are broken up into smaller pieces. Each is zero-padded at both ends and convolved (denoted by bold arrows in the figure). Finally the pieces are added back together, including the overlapping regions formed by the zero-pads.

at the end of one section are recomputed as the first of the unpolluted output points from the subsequent section. With a bit of thought you can easily determine how many points to overlap and save.

The second solution, called the *overlap-add method*, is illustrated in Figure 13.1.5. Here you *don't* overlap the input data. Each section of data is disjoint from the others and is used exactly once. However, you carefully zero-pad it at both ends so that there is no wraparound ambiguity in the output convolution or deconvolution. Now you overlap *and add* these sections of output. Thus, an output point near the end of one section will have the response due to the input points at the beginning of the next section of data properly added in to it, and likewise for an output point near the beginning of a section, *mutatis mutandis*.

Even when computer memory is available, there is some slight gain in computing speed in segmenting a long data set, since the FFTs' $N \log_2 N$ is slightly slower than linear in N. However, the log term is so slowly varying that you will often be much happier to avoid the bookkeeping complexities of the overlap-add or overlap-save methods: If it is practical to do so, just cram the whole data set into memory and FFT away. Then you will have more time for the finer things in life, some of which are described in succeeding sections of this chapter.

CITED REFERENCES AND FURTHER READING:

Nussbaumer, H.J. 1982, *Fast Fourier Transform and Convolution Algorithms* (New York: Springer).

Elliott, D.F., and Rao, K.R. 1982, *Fast Transforms: Algorithms, Analyses, Applications* (New York: Academic Press).

Brigham, E.O. 1974, *The Fast Fourier Transform* (Englewood Cliffs, NJ: Prentice-Hall), Chapter 13.

13.2 Correlation and Autocorrelation Using the FFT

Correlation is the close mathematical cousin of convolution. It is in some ways simpler, however, because the two functions that go into a correlation are not as conceptually distinct as were the data and response functions that entered into convolution. Rather, in correlation, the functions are represented by different, but generally similar, data sets. We investigate their "correlation," by comparing them both directly superposed, and with one of them shifted left or right.

We have already defined in equation (12.0.11) the correlation between two continuous functions $g(t)$ and $h(t)$, which is denoted $\text{Corr}(g,h)$, and is a function of *lag* t. We will occasionally show this time dependence explicitly, with the rather awkward notation $\text{Corr}(g,h)(t)$. The correlation will be large at some value of t if the first function (g) is a close copy of the second (h) but lags it in time by t, i.e., if the first function is shifted to the right of the second. Likewise, the correlation will be large for some negative value of t if the first function *leads* the second, i.e., is shifted to the left of the second. The relation that holds when the two functions are interchanged is

$$\text{Corr}(g,h)(t) = \text{Corr}(h,g)(-t) \tag{13.2.1}$$

The discrete correlation of two sampled functions g_k and h_k, each periodic with period N, is defined by

$$\text{Corr}(g,h)_j \equiv \sum_{k=0}^{N-1} g_{j+k} h_k \tag{13.2.2}$$

The *discrete correlation theorem* says that this discrete correlation of two real functions g and h is one member of the discrete Fourier transform pair

$$\text{Corr}(g,h)_j \iff G_k H_k^* \tag{13.2.3}$$

where G_k and H_k are the discrete Fourier transforms of g_j and h_j, and the asterisk denotes complex conjugation. This theorem makes the same presumptions about the functions as those encountered for the discrete convolution theorem.

We can compute correlations using the FFT as follows: FFT the two data sets, multiply one resulting transform by the complex conjugate of the other, and inverse transform the product. The result (call it r_k) will formally be a complex vector of length N. However, it will turn out to have all its imaginary parts zero since the original data sets were both real. The components of r_k are the values of the correlation at different lags, with positive and negative lags stored in the by-now familiar wraparound order: The correlation at zero lag is in r_0, the first component; the correlation at lag 1 is in r_1, the second component; the correlation at lag -1 is in r_{N-1}, the last component; etc.

Just as in the case of convolution we have to consider end effects, since our data will not, in general, be periodic as intended by the correlation theorem. Here again, we can use zero-padding. If you are interested in the correlation for lags as large as $\pm K$, then you must append a buffer zone of K zeros at the end of both input data sets. If you want all possible lags from N data points (not a usual thing), then you will need to pad the data with an equal number of zeros; this is the extreme case. So here is the program:

```
void correl(VecDoub_I &data1, VecDoub_I &data2, VecDoub_O &ans) {
```
correl.h

Computes the correlation of two real data sets data1[0..n-1] and data2[0..n-1] (including any user-supplied zero padding). n must be an integer power of 2. The answer is returned in ans[0..n-1] stored in wraparound order, i.e., correlations at increasingly negative lags are in ans[n-1] on down to ans[n/2], while correlations at increasingly positive lags are in ans[0] (zero lag) on up to ans[n/2-1]. Sign convention of this routine: if data1 lags data2, i.e., is shifted to the right of it, then ans will show a peak at positive lags.

```
    Int no2,i,n=data1.size();
    Doub tmp;
    VecDoub temp(n);
    for (i=0;i<n;i++) {
        ans[i]=data1[i];
        temp[i]=data2[i];
    }
    realft(ans,1);                          Transform both data vectors.
    realft(temp,1);
    no2=n>>1;                               Normalization for inverse FFT.
    for (i=2;i<n;i+=2) {                    Multiply to find FFT of their correlation.
        tmp=ans[i];
        ans[i]=(ans[i]*temp[i]+ans[i+1]*temp[i+1])/no2;
        ans[i+1]=(ans[i+1]*temp[i]-tmp*temp[i+1])/no2;
    }
    ans[0]=ans[0]*temp[0]/no2;
    ans[1]=ans[1]*temp[1]/no2;
    realft(ans,-1);                         Inverse transform gives correlation.
}
```

The *discrete autocorrelation* of a sampled function g_j is just the discrete correlation of the function with itself. Obviously this is always symmetric with respect to positive and negative lags. Feel free to use the above routine correl to obtain autocorrelations, simply calling it with the same data vector in both arguments. If the inefficiency bothers you, you can edit the program so that only one call is made to realft for the forward transform.

CITED REFERENCES AND FURTHER READING:

Brigham, E.O. 1974, *The Fast Fourier Transform* (Englewood Cliffs, NJ: Prentice-Hall), §13–2.

13.3 Optimal (Wiener) Filtering with the FFT

There are a number of other tasks in numerical processing that are routinely handled with Fourier techniques. One of these is filtering for the removal of noise from a "corrupted" signal. The particular situation we consider is this: There is some underlying, uncorrupted signal $u(t)$ that we want to measure. The measurement process is imperfect, however, and what comes out of our measurement device is a corrupted signal $c(t)$. The signal $c(t)$ may be less than perfect in either or both of two respects. First, the apparatus may not have a perfect delta-function response, so that the true signal $u(t)$ is convolved with (smeared out by) some known response function $r(t)$ to give a smeared signal $s(t)$,

$$s(t) = \int_{-\infty}^{\infty} r(t-\tau)u(\tau)\,d\tau \quad \text{or} \quad S(f) = R(f)U(f) \tag{13.3.1}$$

where S, R, U are the Fourier transforms of s, r, u, respectively. Second, the measured signal $c(t)$ may contain an additional component of noise $n(t)$,

$$c(t) = s(t) + n(t) \tag{13.3.2}$$

We already know how to deconvolve the effects of the response function r in the absence of any noise (§13.1); we just divide $C(f)$ by $R(f)$ to get a deconvolved signal. We now want to treat the analogous problem when noise is present. Our task is to find the *optimal filter*, $\phi(t)$ or $\Phi(f)$, which, when applied to the measured signal $c(t)$ or $C(f)$ and then deconvolved by $r(t)$ or $R(f)$, produces a signal $\widetilde{u}(t)$ or $\widetilde{U}(f)$ that is as close as possible to the uncorrupted signal $u(t)$ or $U(f)$. In other words, we will estimate the true signal U by

$$\widetilde{U}(f) = \frac{C(f)\Phi(f)}{R(f)} \tag{13.3.3}$$

In what sense is $\widetilde{U}$ to be close to U? We ask that they be *close in the least-square sense*

$$\int_{-\infty}^{\infty} |\widetilde{u}(t) - u(t)|^2 \, dt = \int_{-\infty}^{\infty} \left|\widetilde{U}(f) - U(f)\right|^2 \, df \quad \text{is minimized.} \tag{13.3.4}$$

Substituting equations (13.3.3) and (13.3.2), the right-hand side of (13.3.4) becomes

$$\begin{aligned} &\int_{-\infty}^{\infty} \left| \frac{[S(f) + N(f)]\Phi(f)}{R(f)} - \frac{S(f)}{R(f)} \right|^2 df \\ &= \int_{-\infty}^{\infty} |R(f)|^{-2} \left\{ |S(f)|^2 \, |1 - \Phi(f)|^2 + |N(f)|^2 \, |\Phi(f)|^2 \right\} df \end{aligned} \tag{13.3.5}$$

The signal S and the noise N are *uncorrelated*, so their cross product, when integrated over frequency f, gave zero. (This is practically the *definition* of what we mean by noise!) Obviously (13.3.5) will be a minimum if and only if the integrand is minimized with respect to $\Phi(f)$ at every value of f. Let us search for such a solution where $\Phi(f)$ is a real function. Differentiating with respect to Φ and setting the result equal to zero gives

$$\Phi(f) = \frac{|S(f)|^2}{|S(f)|^2 + |N(f)|^2} \tag{13.3.6}$$

This is the formula for the optimal filter $\Phi(f)$.

Notice that equation (13.3.6) involves S, the smeared signal, and N, the noise. The two of these add up to be C, the measured signal. Equation (13.3.6) does not contain U, the "true" signal. This makes for an important simplification: The optimal filter can be determined independently of the determination of the deconvolution function that relates S and U.

To determine the optimal filter from equation (13.3.6) we need some way of separately estimating $|S|^2$ and $|N|^2$. There is no way to do this from the measured signal C alone without some other information, or some assumption or guess. Luckily, the extra information is often easy to obtain. For example, we can sample a long

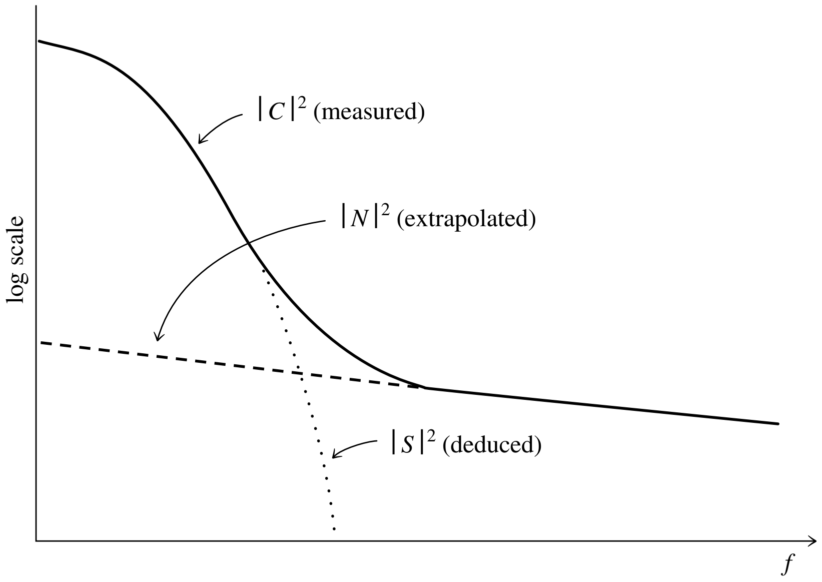

Figure 13.3.1. Optimal (Wiener) filtering. The power spectrum of signal plus noise shows a signal peak added to a noise tail. The tail is extrapolated back into the signal region as a "noise model." Subtracting gives the "signal model." The models need not be accurate for the method to be useful. A simple algebraic combination of the models gives the optimal filter (see text).

stretch of data $c(t)$ and plot its power spectral density using equations (12.0.15), (12.1.8), and (12.1.5). This quantity is proportional to the sum $|S|^2 + |N|^2$, so we have

$$|S(f)|^2 + |N(f)|^2 \approx P_c(f) = |C(f)|^2 \qquad 0 \le f < f_c \tag{13.3.7}$$

(More sophisticated methods of estimating the power spectral density will be discussed in §13.4 and §13.7, but the estimation above is almost always good enough for the optimal filter problem.) The resulting plot (see Figure 13.3.1) will often immediately show the spectral signature of a signal sticking up above a continuous noise spectrum. The noise spectrum may be flat, or tilted, or smoothly varying; it doesn't matter, as long as we can guess a reasonable hypothesis as to what it is. Draw a smooth curve through the noise spectrum, extrapolating it into the region dominated by the signal as well. Now draw a smooth curve through the signal plus noise power. The difference between these two curves is your smooth "model" of the signal power. The quotient of your model of signal power to your model of signal plus noise power is the optimal filter $\Phi(f)$. [Extend it to negative values of f by the formula $\Phi(-f) = \Phi(f)$.] Notice that $\Phi(f)$ will be close to unity where the noise is negligible, and close to zero where the noise is dominant. That is how it does its job! The intermediate dependence given by equation (13.3.6) just turns out to be the optimal way of going in between these two extremes.

Because the optimal filter results from a minimization problem, the quality of the results obtained by optimal filtering differs from the true optimum by an amount that is *second order* in the precision to which the optimal filter is determined. In other words, even a fairly crudely determined optimal filter (sloppy, say, at the 10% level) can give excellent results when it is applied to data. That is why the separation

of the measured signal C into signal and noise components S and N can usefully be done "by eye" from a crude plot of power spectral density. All of this may give you thoughts about iterating the procedure we have just described. For example, after designing a filter with response $\Phi(f)$ and using it to make a respectable guess at the signal $\widetilde{U}(f) = \Phi(f)C(f)/R(f)$, you might turn about and regard $\widetilde{U}(f)$ as a fresh new signal that you could improve even further with the same filtering technique. Don't waste your time on this line of thought. The scheme converges to a signal of $S(f) = 0$. Converging iterative methods do exist; this just isn't one of them.

You can use the routine `four1` (§12.2) or `realft` (§12.3) to FFT your data when you are constructing an optimal filter. To apply the filter to your data, you can use the methods described in §13.1. The specific routine `convlv` is not needed for optimal filtering, since your filter is constructed in the frequency domain to begin with. If you are also deconvolving your data with a known response function, however, you can modify `convlv` to multiply by your optimal filter just before it takes the inverse Fourier transform.

CITED REFERENCES AND FURTHER READING:

Rabiner, L.R., and Gold, B. 1975, *Theory and Application of Digital Signal Processing* (Englewood Cliffs, NJ: Prentice-Hall).

Nussbaumer, H.J. 1982, *Fast Fourier Transform and Convolution Algorithms* (New York: Springer).

Elliott, D.F., and Rao, K.R. 1982, *Fast Transforms: Algorithms, Analyses, Applications* (New York: Academic Press).

13.4 Power Spectrum Estimation Using the FFT

In the previous section we "informally" estimated the power spectral density of a function $c(t)$ by taking the modulus-squared of the discrete Fourier transform of some finite, sampled stretch of it. In this section we'll do roughly the same thing, but with considerably greater attention to details. This attention will uncover some surprises.

The first detail is the normalization of the power spectrum (or *power spectral density* or *PSD*). In general, there is *some* relation of proportionality between a measure of the squared amplitude of the function and a measure of the amplitude of the PSD. Unfortunately, there are several different conventions for describing the normalization in each domain, and many opportunities for getting wrong the relationship between the two domains. Suppose that our function $c(t)$ is sampled at N points to produce values $c_0 \dots c_{N-1}$, and that these points span a range of time T, that is, $T = (N-1)\Delta$, where Δ is the sampling interval. Then here are several different descriptions of the total power:

$$\sum_{j=0}^{N-1} |c_j|^2 \equiv \text{sum squared amplitude} \tag{13.4.1}$$

$$\frac{1}{T}\int_0^T |c(t)|^2\, dt \approx \frac{1}{N}\sum_{j=0}^{N-1} |c_j|^2 \equiv \text{mean squared amplitude} \tag{13.4.2}$$

$$\int_0^T |c(t)|^2 \, dt \approx \Delta \sum_{j=0}^{N-1} |c_j|^2 \equiv \text{time-integral squared amplitude} \tag{13.4.3}$$

PSD estimators, as we shall see, have an even greater variety. In this section, we consider a class of them that give estimates at discrete values of frequency f_i, where i will range over integer values. In the next section, we will learn about a different class of estimators that produce estimates that are continuous functions of frequency f. Even if it is agreed always to relate the PSD normalization to a particular description of the function normalization (e.g., 13.4.2), there are at least the following possibilities: The PSD is

- defined for discrete positive, zero, and negative frequencies, and its sum over these is the function mean squared amplitude
- defined for zero and discrete positive frequencies only, and its sum over these is the function mean squared amplitude
- defined in the Nyquist interval from $-f_c$ to f_c, where $f_c = 1/(2\Delta)$, and its integral over this range is the function mean squared amplitude
- defined from 0 to f_c, and its integral over this range is the function mean squared amplitude (it *never* makes sense to integrate the PSD of a sampled function outside of the Nyquist interval $-f_c$ and f_c since, according to the sampling theorem, power there will have been aliased into the Nyquist interval).

It is hopeless to define enough notation to distinguish all possible combinations of normalizations. In what follows, we use the notation $P(f)$ to mean *any* of the above PSDs, stating in each instance how the particular $P(f)$ is normalized. Beware the inconsistent notation in the literature.

The method of power spectrum estimation used in the previous section is a simple version of an estimator called, historically, the *periodogram*. If we take an N-point sample of the function $c(t)$ at equal intervals and use the FFT to compute its discrete Fourier transform

$$C_k = \sum_{j=0}^{N-1} c_j \, e^{2\pi i jk/N} \qquad k = 0, \ldots, N-1 \tag{13.4.4}$$

then the periodogram estimate of the power spectrum is defined at $N/2 + 1$ frequencies as

$$\begin{aligned} P(0) &= P(f_0) = \frac{1}{N^2} |C_0|^2 \\ P(f_k) &= \frac{1}{N^2} \left[|C_k|^2 + |C_{N-k}|^2 \right] \qquad k = 1, 2, \ldots, \left(\frac{N}{2} - 1 \right) \\ P(f_c) &= P(f_{N/2}) = \frac{1}{N^2} \left| C_{N/2} \right|^2 \end{aligned} \tag{13.4.5}$$

where f_k is defined only for the zero and positive frequencies

$$f_k \equiv \frac{k}{N\Delta} = 2 f_c \frac{k}{N} \qquad k = 0, 1, \ldots, \frac{N}{2} \tag{13.4.6}$$

By Parseval's theorem, equation (12.1.10), we see immediately that equation (13.4.5) is normalized so that the sum of the $N/2+1$ values of P is equal to the mean squared amplitude of the function c_j.

Here is an object (Spectreg for "spectrum register") that implements equations (13.4.4) – (13.4.6). Its constructor takes an integer argument M that defines both the number of data points, $2M \equiv N$, and $M+1$, the number of frequencies in the estimate between 0 and f_c, inclusive. M must be a power of 2. Spectreg has some other features that we will build on below when we learn about window functions and variance reduction. For now, the function window that appears should be defined as returning the constant 1, that is,

```
Doub window(Int j,Int n) {return 1.;}
```

spectrum.h

```
struct Spectreg {
Object for accumulating power spectrum estimates from one or more segments of data.
    Int m,m2,nsum;
    VecDoub specsum, wksp;

    Spectreg(Int em) : m(em), m2(2*m), nsum(0), specsum(m+1,0.), wksp(m2) {
    Constructor. Sets M, such that data segments will have length 2M, and the spectrum will
    be estimated at M + 1 frequencies.
        if (m & (m-1)) throw("m must be power of 2");
    }

    template<class D>
    void adddataseg(VecDoub_I &data, D &window) {
    Process a data segment of length 2M using the window function, which can be either a
    bare function or a functor.
        Int i;
        Doub w,fac,sumw = 0.;
        if (data.size() != m2) throw("wrong size data segment");
        for (i=0;i<m2;i++) {          Load the data.
            w = window(i,m2);
            wksp[i] = w*data[i];
            sumw += SQR(w);
        }
        fac = 2./(sumw*m2);
        realft(wksp,1);                Take its Fourier transform.
        specsum[0] += 0.5*fac*SQR(wksp[0]);
        for (i=1;i<m;i++) specsum[i] += fac*(SQR(wksp[2*i])+SQR(wksp[2*i+1]));
        specsum[m] += 0.5*fac*SQR(wksp[1]);
        nsum++;
    }

    VecDoub spectrum() {
    Return power spectrum estimates as a vector. You can instead just access specsum directly,
    and divide by nsum.
        VecDoub spec(m+1);
        if (nsum == 0) throw("no data yet");
        for (Int i=0;i<=m;i++) spec[i] = specsum[i]/nsum;
        return spec;
    }

    VecDoub frequencies() {
    Return vector of frequencies (in units of 1/Δ) at which estimates are made.
        VecDoub freq(m+1);
        for (Int i=0;i<=m;i++) freq[i] = i*0.5/m;
        return freq;
    }
};
```

The naive use of Spectreg would be as follows: Declare an instance with a power-of-two value of M. Call adddataseg to process a vector of data, length $2M$. Call spectrum and frequencies to get, respectively, the PSD estimates and the frequencies at which they are made (in units of $1/\Delta$).

Before we rush to use Spectreg, however, we must now ask this question: In what sense is the periodogram estimate (13.4.5) a "true" estimator of the power spectrum of the underlying function $c(t)$? You can find the answer treated in considerable detail in the literature cited (see, e.g., [1] for an introduction). Here is a summary.

First, is the *expectation value* of the periodogram estimate equal to the power spectrum, i.e., is the estimator correct (unbiased) on average? Well, yes and no. We wouldn't really expect one of the $P(f_k)$'s to equal the continuous $P(f)$ at *exactly* f_k, since f_k is supposed to be representative of a whole frequency "bin" extending from halfway from the preceding discrete frequency to halfway to the next one. We *should* be expecting the $P(f_k)$ to be some kind of average of $P(f)$ over a narrow window function centered on its f_k. For the periodogram estimate (13.4.6), that window function, as a function of s the frequency offset *in bins*, is

$$W(s) = \frac{1}{N^2}\left[\frac{\sin(\pi s)}{\sin(\pi s/N)}\right]^2 \tag{13.4.7}$$

Notice that $W(s)$ has oscillatory lobes but, apart from these, falls off only about as $W(s) \approx (\pi s)^{-2}$. This is not a very rapid fall-off, and it results in significant *leakage* (that is the technical term) from one frequency to another in the periodogram estimate. Notice also that $W(s)$ happens to be zero for s equal to a nonzero integer. This means that if the function $c(t)$ is a pure sine wave of frequency exactly equal to one of the f_k's, then there will be *no* leakage to adjacent f_k's. But this is not the characteristic case! If the frequency is, say, one-third of the way between two adjacent f_k's, then the leakage will extend *well* beyond those two adjacent bins. The solution to the problem of leakage is called *data windowing*, and we will discuss it below.

Turn now to another question about the periodogram estimate. What is the variance of that estimate as N goes to infinity? In other words, as we take more sampled points from the original function (either sampling a longer stretch of data at the same sampling rate, or else by resampling the same stretch of data with a faster sampling rate), then how much more accurate do the estimates P_k become? The unpleasant answer is that the periodogram estimates *do not become more accurate at all!* In fact, the variance of the periodogram estimate at a frequency f_k is always equal to the square of its expectation value at that frequency. In other words, the standard deviation is always 100% of the value, independent of N!

How can this be? Where did all the information go as we added points? It all went into producing estimates at a greater number of discrete frequencies f_k. If we sample a longer run of data using the same sampling rate, then the Nyquist critical frequency f_c is unchanged, but we now have finer frequency resolution (more f_k's) within the Nyquist frequency interval; alternatively, if we sample the same length of data with a finer sampling interval, then our frequency resolution is unchanged, but the Nyquist range now extends up to a higher frequency. In neither case do the additional samples reduce the variance of any one particular frequency's estimated PSD.

You don't have to live with PSD estimates with 100% standard deviations, however. You simply have to know some techniques for reducing the variance of the

estimates. Here are two techniques that are very nearly identical mathematically, though different in implementation. The first is to compute a periodogram estimate with finer discrete frequency spacing than you really need, and then to sum the periodogram estimates at K consecutive discrete frequencies to get one "smoother" estimate at the mid-frequency of those K. The variance of that summed estimate will be smaller than the estimate itself by a factor of exactly $1/K$, i.e., the standard deviation will be smaller than 100% by a factor $1/\sqrt{K}$. Thus, to estimate the power spectrum at $M+1$ discrete frequencies between 0 and f_c inclusive, you begin by taking the FFT of $2MK$ points (which number had better be an integer power of 2!). You then take the modulus square of the resulting coefficients, add positive and negative frequency pairs, and divide by $(2MK)^2$, all according to equation (13.4.5) with $N = 2MK$. Finally, you "bin" the results into summed (not averaged) groups of K. This procedure is very easy to program, so we will not bother to give a routine for it. The reason that you sum, rather than average, K consecutive points is so that your final PSD estimate will preserve the normalization property that the sum of its $M+1$ values equals the mean square value of the function.

A second technique for estimating the PSD at $M+1$ discrete frequencies in the range 0 to f_c is to partition the original sampled data into K segments each of $2M$ consecutive sampled points. Each segment is separately FFT'd to produce a periodogram estimate (equation 13.4.5 with $N \equiv 2M$). Finally, the K periodogram estimates are averaged at each frequency. It is this final averaging that reduces the variance of the estimate by a factor K (standard deviation by $\sqrt{K}$). This second technique is computationally more efficient than the first technique above by a modest factor, since it is logarithmically more efficient to take many shorter FFTs than one longer one. The principal advantage of the second technique, however, is that only $2M$ data points are manipulated at a single time, not $2KM$ as in the first technique. This means that the second technique is the natural choice for processing long runs of data, as from a real-time device or slow storage.

In fact, you may already have noticed, the object `Spectreg` implements this second technique. If you call `adddataseg` K times, with a different vector of $2M$ data points each time, then the result returned by `spectrum` is the average of the K periodograms. However, we should *still* not rush to use `Spectreg`. We need first to return to the matters of leakage and data windowing that were brought up after equation (13.4.7) above.

13.4.1 Data Windowing

The purpose of data windowing is to modify equation (13.4.7), which expresses the relation between the spectral estimate P_k at a discrete frequency and the actual underlying continuous spectrum $P(f)$ at nearby frequencies. In general, the spectral power in one "bin" k contains leakage from frequency components that are actually s bins away, where s is the independent variable in equation (13.4.7). There is, as we pointed out, quite substantial leakage even from moderately large values of s. Note that s is not an integer, in general, because actual frequencies can have any real value.

When we select a run of N sampled points for periodogram spectral estimation, we are in effect multiplying an infinite run of sampled data c_j by a window function in time, one that is zero except during the total sampling time $N\Delta$ and is unity during that time. In other words, the data are windowed by a square window function. By the convolution theorem (12.0.10; but interchanging the roles of f and t), the Fourier

transform of the product of the data with this square window function is equal to the convolution of the data's Fourier transform with the window's Fourier transform. In fact, equation (13.4.7) is nothing more than the square of the discrete Fourier transform of the unity window function.

$$W(s) = \frac{1}{N^2}\left[\frac{\sin(\pi s)}{\sin(\pi s/N)}\right]^2 = \frac{1}{N^2}\left|\sum_{k=0}^{N-1} e^{2\pi i s k/N}\right|^2 \tag{13.4.8}$$

The reason for the leakage at large values of s is that the square window function turns on and off so rapidly. Its Fourier transform has substantial components at high frequencies. To remedy this situation, we can multiply the input data c_j, $j = 0, \ldots, N-1$ by a window function w_j that changes more gradually from zero to a maximum and then back to zero as j ranges from 0 to N. In this case, the equations for the periodogram estimator (13.4.4 – 13.4.5) become

$$D_k \equiv \sum_{j=0}^{N-1} c_j w_j\, e^{2\pi i j k/N} \qquad k = 0, \ldots, N-1 \tag{13.4.9}$$

$$\begin{aligned} P(0) = P(f_0) &= \frac{1}{W_{ss}}\left|D_0\right|^2 \\ P(f_k) &= \frac{1}{W_{ss}}\left[\left|D_k\right|^2 + \left|D_{N-k}\right|^2\right] \qquad k = 1, 2, \ldots, \left(\frac{N}{2}-1\right) \\ P(f_c) = P(f_{N/2}) &= \frac{1}{W_{ss}}\left|D_{N/2}\right|^2 \end{aligned} \tag{13.4.10}$$

where W_{ss} stands for "window squared and summed,"

$$W_{ss} \equiv N \sum_{j=0}^{N-1} w_j^2 \tag{13.4.11}$$

and f_k is given by (13.4.6). The more general form of (13.4.7) can now be written in terms of the window function w_j as

$$W(s) = \frac{1}{W_{ss}}\left|\sum_{k=0}^{N-1} e^{2\pi i s k/N} w_k\right|^2 \tag{13.4.12}$$

There is a lot of perhaps unnecessary lore about the choice of a window function, and practically every function that rises from zero to a peak and then falls again has been named after someone. A few of the more common (also shown in Figure 13.4.1) are

$$w_j = 1 - \left|\frac{j - \frac{1}{2}N}{\frac{1}{2}N}\right| \equiv \text{Bartlett window} \tag{13.4.13}$$

(the "Parzen window" is a smoother, but similarly shaped, functional form)

$$w_j = \frac{1}{2}\left[1 - \cos\left(\frac{2\pi j}{N}\right)\right] \equiv \text{Hann window} \tag{13.4.14}$$

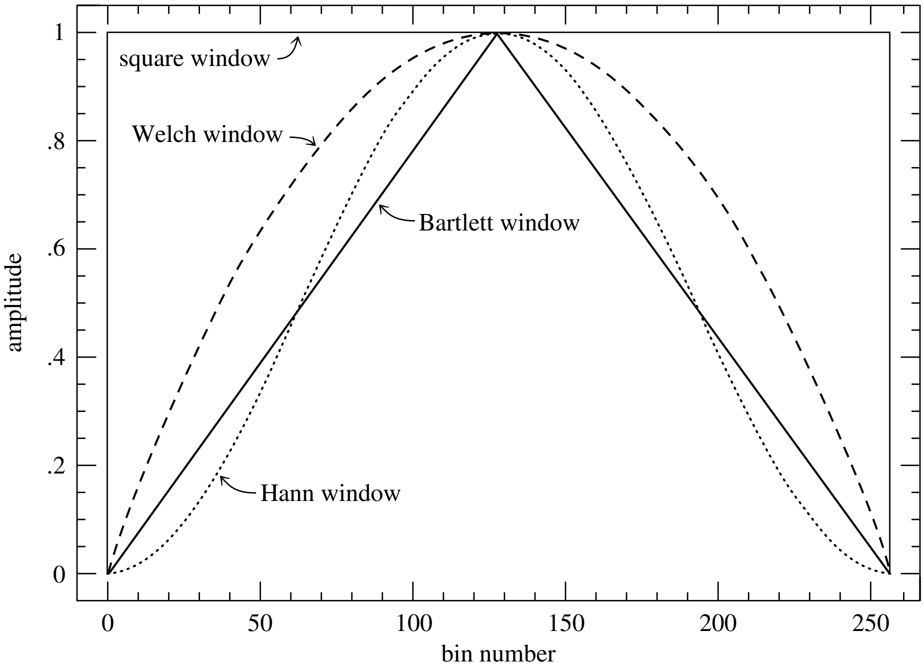

Figure 13.4.1. Window functions commonly used in FFT power spectral estimation. The data segment, here of length 256, is multiplied (bin by bin) by the window function before the FFT is computed. The square window, which is equivalent to no windowing, is least recommended. The Welch and Bartlett windows are good choices.

(the "Hamming window" is similar but does not go exactly to zero at the ends)

$$w_j = 1 - \left(\frac{j - \frac{1}{2}N}{\frac{1}{2}N} \right)^2 \equiv \text{Welch window} \qquad (13.4.15)$$

We are inclined to follow Welch in recommending that you use either (13.4.13) or (13.4.15) in practical work. However, at the level of the discussion thus far, there is *little difference* between any of these (or similar) window functions. Their difference lies in subtle trade-offs among the various figures of merit that can be used to describe the narrowness or peakedness of the spectral leakage functions computed by (13.4.12). These figures of merit have such names as: *highest sidelobe level (dB), sidelobe fall-off (dB per octave), equivalent noise bandwidth (bins), 3-dB bandwidth (bins), scallop loss (dB), and worst-case process loss (dB).* Roughly speaking, the principal trade-off is between making the central peak as narrow as possible versus making the tails of the distribution fall off as rapidly as possible. For details, see, e.g., [2]. Figure 13.4.2 plots the leakage amplitudes for several windows already discussed.

There is a particular lore about window functions that rise smoothly from zero to unity in the first small fraction (say 10%) of the data, then stay at unity until the last small fraction (again say 10%) of the data, during which the window function falls smoothly back to zero. These windows will squeeze a little bit of extra narrowness out of the main lobe of the leakage function (never as much as a factor of two,

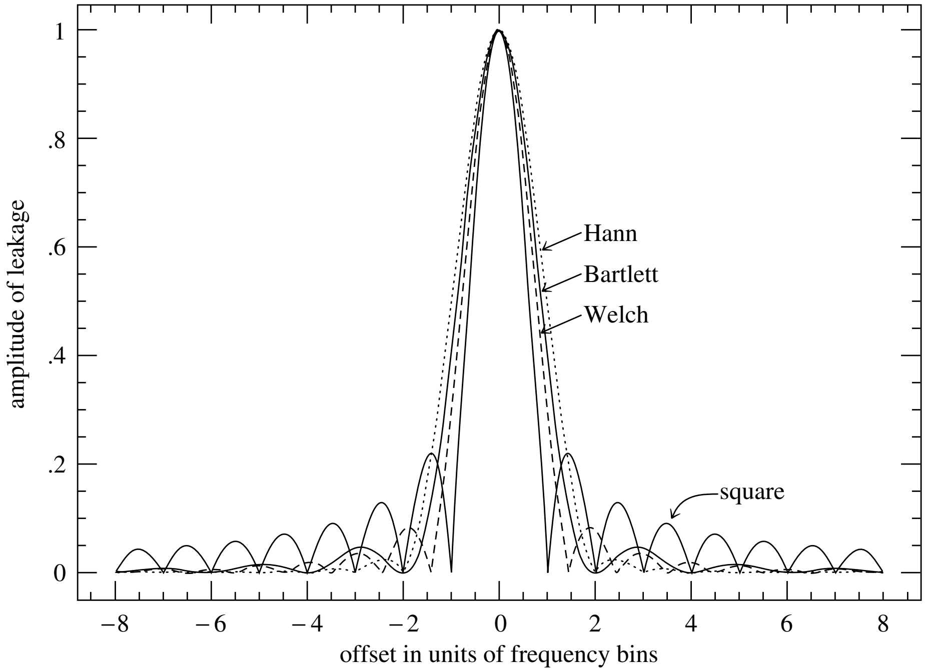

Figure 13.4.2. Leakage functions for the window functions of Figure 13.4.1. A signal whose frequency is actually located at zero offset "leaks" into neighboring bins with the amplitude shown. The purpose of windowing is to reduce the leakage at large offsets, where square (no) windowing has large sidelobes. Offset can have a fractional value, since the actual signal frequency can be located between two frequency bins of the FFT.

however), but trade this off by widening the leakage tail by a significant factor (e.g., the reciprocal of 10%, a factor of ten). If we distinguish between the *width* of a window (number of samples for which it is at its maximum value) and its *rise/fall time* (number of samples during which it rises and falls); and if we distinguish between the *FWHM* (full width to half maximum value) of the leakage function's main lobe and the *leakage width* (full width that contains half of the spectral power that is not contained in the main lobe), then these quantities are related roughly by

$$\text{(FWHM in bins)} \approx \frac{N}{\text{(window width)}} \tag{13.4.16}$$

$$\text{(leakage width in bins)} \approx \frac{N}{\text{(window rise/fall time)}} \tag{13.4.17}$$

For the windows given above in (13.4.13) – (13.4.15), the effective window widths and the effective window rise/fall times are both of order $\frac{1}{2}N$. Generally speaking, we feel that the advantages of windows whose rise and fall times are only small fractions of the data length are minor or nonexistent, and we avoid using them. One sometimes hears it said that flat-topped windows "throw away less of the data," but we will show you two better ways of dealing with that problem, namely by overlapping data segments or by multitaper methods.

Now, at last, we really *are* ready to use the `Spectreg` object. First, choose a window function. The templating in `Spectreg` allows it to accept either a bare

function or a functor. Use the former if your window function is fast to compute, or the latter if you want to precompute and store a more complicated window function, or one with auxiliary parameters. Examples are

spectrum.h

```
Doub square(Int j,Int n) {return 1.;}                        Don't use this!

Doub bartlett(Int j,Int n) {return 1.-abs(2.*j/(n-1.)-1.);}   Use this,

Doub welch(Int j,Int n) {return 1.-SQR(2.*j/(n-1.)-1.);}      or this...

struct Hann {
...or this. This is an example of a functor.
    Int nn;
    VecDoub win;
    Hann(Int n) : nn(n), win(n) {
    Constructor. Compute and store the window function in a table.
        Doub twopi = 8.*atan(1.);
        for (Int i=0;i<nn;i++) win[i] = 0.5*(1.-cos(i*twopi/(nn-1.)));
    }
    Doub operator() (Int j, Int n) {
    Make it a functor, able to return values as if it were a function.
        if (n != nn) throw("incorrect n for this Hann");
        return win[j];
    }
};
```

Second, pick a value of M and declare a `Spectreg` object. Third, process K data segments, each of length $2M$. The larger K, the more accurate your answer. Fourth, get the PSD estimate at $M + 1$ frequencies by a call to the `spectrum` method (or, to avoid copying vectors, directly from the `specsum` member vector).

13.4.2 Overlapping Data Segments

We introduced window functions to mitigate leakage, a major problem, but in doing so we have created a new problem, luckily minor, which we now address. All good window functions, because they approach zero at their endpoints, deweight, and in effect throw away, valid data. A consequence is that, for any number of data segments K each of length $2M$, the variance of the PSD estimate is somewhat larger with a good window function than with the (bad) square window.

Sometimes you are not limited in the number of data points, but rather by the computer resources to process them. For example, the data may be pouring out of a real-time device at a high rate. In such a situation, data deweighting is not an issue. You should use `Spectreg` as already described, accumulating as many data segments as you need to obtain the desired accuracy. Indeed, this gives you the smallest variance estimate per computer operation.

More often, however, you are limited in the total number of data points, and you want to get the smallest variance estimate from them, but without giving up the low-leakage benefit of windowing. In this situation it turns out to be optimal, or nearly optimal, to overlap the segments by one-half of their length. The first and second sets of M points become segment number 1 (length $2M$ as usual); the second and third sets of M points become segment number 2; and so on, up to segment number K, which is made of the Kth and $K+1$st sets of M points. The total number of sampled points is therefore $(K+1)M$, just over half as many as with nonoverlapping segments. The reduction in the variance is not a full factor

of K, since the segments are not statistically independent. It can be shown that the variance is instead reduced by a factor of about $9K/11$ [3]. This is, however, significantly better than the reduction of about $K/2$ that would have resulted if the same *number* of data points were segmented without overlapping.

Here is an object Spectolap, derived from Spectreg as a base class, that implements the overlap method. As far as the user is concerned, the only difference is that the adddataseg method now requires a data segment of length M, not $2M$.

spectrum.h

```
struct Spectolap : Spectreg {
Object for power spectral estimation using overlapping data segments. The user sends non-
overlapping segments of length M, which are processed in pairs of length 2M, with overlap.

    Int first;
    VecDoub fullseg;

    Spectolap(Int em) : Spectreg(em), first(1), fullseg(2*em) {}
    Constructor. Sets M.

    template<class D>
    void adddataseg(VecDoub_I &data, D &window) {
    Process a data segment of length M using the window function, which can be either a bare
    function or a functor.
        Int i;
        if (data.size() != m) throw("wrong size data segment");
        if (first) {                              First segment is just stored.
            for (i=0;i<m;i++) fullseg[i+m] = data [i];
            first = 0;
        } else {                                  Subsequent segments are processed.
            for (i=0;i<m;i++) {
                fullseg[i] = fullseg[i+m];
                fullseg[i+m] = data [i];
            }
            Spectreg::adddataseg(fullseg,window);    Base class method, the data length
        }                                                is 2M.
    }

    template<class D>
    void addlongdata(VecDoub_I &data, D &window) {
    Process a long vector of data as overlapping segments each of length 2M.
        Int i, k, noff, nt=data.size(), nk=(nt-1)/m;
        Doub del = nk > 1 ? (nt-m2)/(nk-1.) : 0.;        Target separation.
        if (nt < m2) throw("data length too short");
        for (k=0;k<nk;k++) {                      Process nk overlapping segments.
            noff = (Int)(k*del+0.5);              Offset is nearest integer.
            for (i=0;i<m2;i++) fullseg[i] = data[noff+i];
            Spectreg::adddataseg(fullseg,window);
        }
    }
};
```

The method addlongdata in Spectolap is provided to deal with another common situation: You want to estimate the PSD at $M + 1$ frequencies (as usual), but your data are in a long vector that is not necessarily a multiple of M, or $2M$, or a power of 2. Here we are assuming that the length of your data vector, N_{tot}, is much larger than $2M$. The problem is not that the number of segments K is small, but rather that K is not an integer. Overlapping data segments provide a nifty fix: We start with the next-larger integer number of segments, and then squeeze them together just a bit, like an accordion, until they exactly fit into N_{tot}. In other words,

we overlap them by slightly *more* than half of their length, to get an exact fit.

Here is our plain-vanilla recommendation for PSD estimation when your N_{tot} data points are not taxing the size of memory: Pick M, a power of 2, such that estimates at $M + 1$ frequencies between 0 and f_c (inclusive) are enough. Don't be too greedy on M, because the fractional standard deviation of your estimates will on the order of $(M/N_{tot})^{1/2}$. Then do

```
Int ntot=..., m=...;
VecDoub data(ntot), psd(m), freq(m);
...
Spectolap myspec(m);
myspec.addlongdata(data,bartlett);
psd = myspec.spectrum()
freq = myspec.frequencies()
```

13.4.3 Multitaper Methods and Slepian Functions

Multitaper methods provide a principled approach to the trade-off between (very) low leakage and minimizing the variance of the PSD estimate. If the leakage profiles in Figure 13.4.2 are acceptable to you (and see also Figure 13.4.4 below), then you don't need to read this section. In some applications, however, minimizing leakage is the whole game. For example, you may be looking for very weak spectral signals, either line or continuum, that can be masked by leakage from nearby strong lines. Or, you may be interested in the tail of a spectrum at high frequencies, which can be spuriously dominated by leakage from lower frequencies.

You have to give something to get something. Here, you have to accept a (small) broadening of the main lobe of the leakage function $W(s)$ in order to (greatly) suppress leakage outside of the main lobe. Broadening the main lobe is equivalent to giving up some frequency resolution. We can parameterize this by a value j_{res}. The goal is to minimize leakage for $|s| > j_{res}$, measured in bins, in exchange for which we are willing to have leakage near unity for any $|s| < j_{res}$. Typical values of j_{res} might be in the range of 2 to 10. (We'll see that larger values are not necessary.)

There are two key ideas in *multitaper methods*, somewhat independent of each other, originating in the work of Slepian [4]. The first idea is that, for a given data length N and choice j_{res}, one can actually solve for the *best possible* weights w_j, meaning the ones that make the leakage smallest among all possible choices. The beautiful and nonobvious answer (see [5]) is that the vector of optimal weights is the eigenvector corresponding to the smallest eigenvalue of the symmetric tridiagonal matrix with diagonal elements

$$\frac{1}{4}\left[N^2 - (N-1-2j)^2 \cos\left(\frac{2\pi j_{res}}{N}\right)\right], \qquad j = 0, \dots, N-1 \tag{13.4.18}$$

and off-diagonal elements

$$-\tfrac{1}{2} j(N-j), \qquad j = 1, \dots, N-1 \tag{13.4.19}$$

The second key idea is that the next few eigenvectors of this same matrix are also pretty good window functions. And because they are orthogonal to the first eigenvector (and each other), they give statistically independent estimates, which can be averaged together to decrease the variance of the final answer. Let k_T (for "taper") denote the number of such estimates that are averaged. Figure 13.4.3 shows the first five window functions (eigenvectors number $k = 0, \dots, k_T - 1$) for the case $j_{res} = 3$, $N = 1024$. The functions (actually discrete sequences) obtained as eigenvectors of equations (13.4.18) and (13.4.19) are called *Slepian functions* or *discrete prolate spheroidal sequences (dpss)*. You can see that larger values of k pick up the information in data regions that were deweighted in the first eigenvector $k = 0$. (You may have thought that window functions needed to be positive, but there is actually no such restriction in any of the discussion above.)

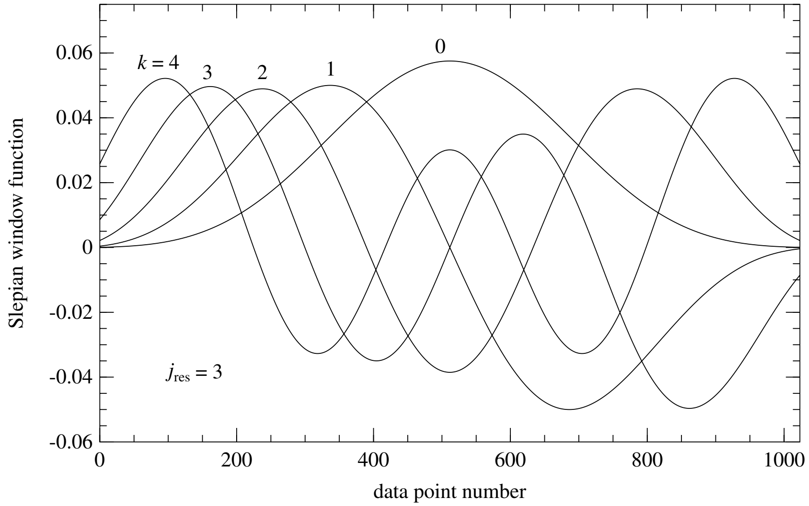

Figure 13.4.3. Slepian taper (window) functions for the case $j_{\text{res}} = 3$, $k = 0, 1, 2, 3, 4$, $N = 1024$. Combining the power spectrum estimates of different k's uses, effectively, more of the data segment and decreases the variance.

The reason that you can't continue this indefinitely, using eigenvectors corresponding to larger and larger eigenvalues (increasing k_T), is that the leakage of the kth window function increases rather rapidly with k. Only $k_T < 2j_{\text{res}}$ values are worth considering at all, and only for $k_T \lesssim j_{\text{res}}$ is the leakage really tiny, which was, after all, the whole point. In Figure 13.4.3 you can already guess that $k = 3$ and 4 are going to have rather poor leakage properties, because they noticeably don't go to zero at their endpoints. Figure 13.4.4 shows the leakage function $W(s)$ for a variety of window functions, including those we met previously, now plotted on a logarithmic scale. Window functions shown as shaded have leakage so large as to be ruled out almost categorically; the square window is notable in this group. You can see how the main lobe of the Slepian functions extends almost exactly out to j_{res}, and that the suppression of the sidelobes of the lowest eigenvectors (e.g., Slepian 3,0 and 3,1) is really quite remarkable.

Here is an object, again derived from `Spectreg` as a base class, for estimating the PSD using the multitaper method with Slepian window functions. As in the base class, the method `adddataseg` accepts data segments of length $2M$, but it now adds to the average the result of the first k_T tapers of resolution j_{res}. Values for M, j_{res} and k_T are set in the constructor.

spectrum.h

```
struct Slepian  : Spectreg {
Object for power spectral estimation using the multitaper method with Slepian tapers.
    Int jres, kt;
    MatDoub dpss;                          Table of Slepians.
    Doub p,pp,d,dd;
    Slepian(Int em, Int jjres, Int kkt)
    : Spectreg(em), jres(jjres), kt(kkt), dpss(kkt,2*em) {
    Constructor sets M (same meaning as previously), jres, and kT, see text.
        if (jres < 1 || kt >= 2*jres) throw("kt too big or jres too small");
        filltable();
    }
    void filltable();                      Implementation in next subsection.
    void renorm(Int n) {
    Utility used by filltable.
        p = ldexp(p,n); pp = ldexp(pp,n); d = ldexp(d,n); dd = ldexp(dd,n);
    }
    struct Slepwindow {
    Captive functor will be sent to the base class as a window function.
        Int k;
```

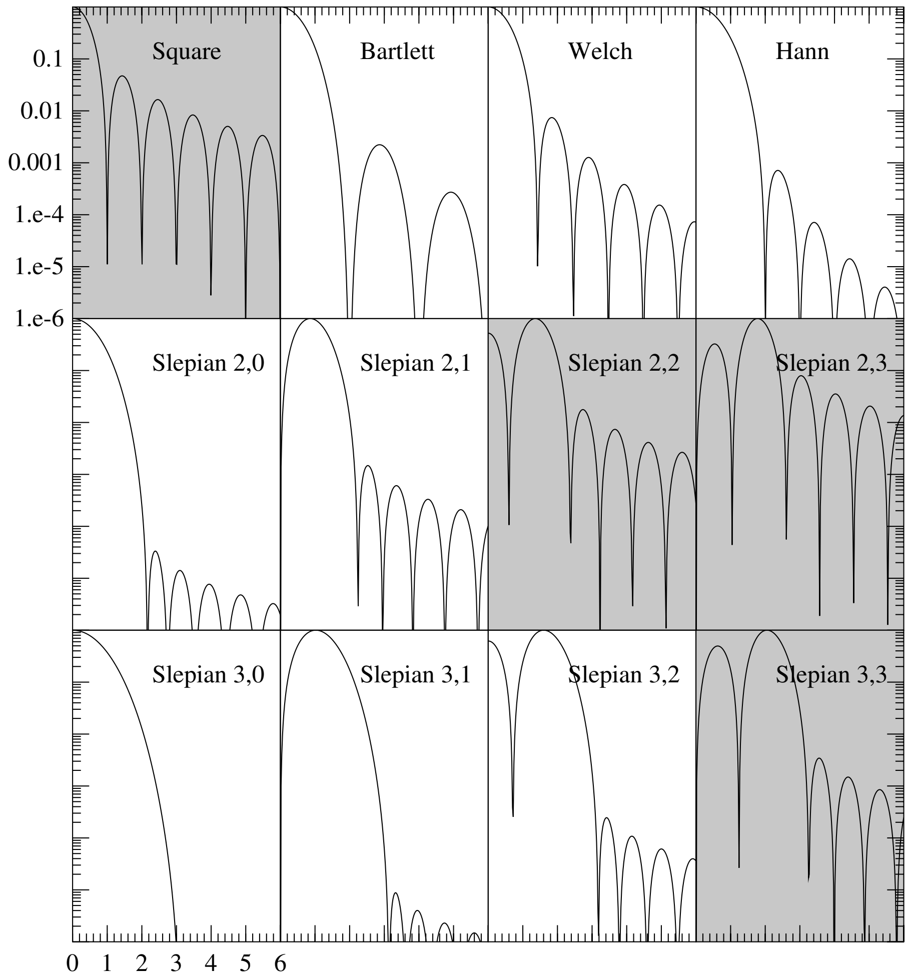

Figure 13.4.4. Leakage function $W(s)$ for various window functions. The top row is essentially the same as Figure 13.4.2, but squared (to get power) and plotted logarithmically. The second and third rows are examples of Slepian functions, identified by j_{res}, k values. Small k values have exceedingly small leakage for $|s| > j_{\text{res}}$; but as k increases, so does the leakage. Shaded functions have unacceptably large leakage and are not recommended.

```
        MatDoub &dps;
        Slepwindow(Int kkt, MatDoub &dpss) : k(kkt), dps(dpss) {}
        Doub operator() (Int j, Int n) {return dps[k][j];}
    };

    void adddataseg(VecDoub_I &data) {
    Process a data segment of length 2M using k_T tapers.
        Int k;
        if (data.size() != m2) throw("wrong size data segment");
        for (k=0;k<kt;k++) {                    Loop over tapers, initializing the functor
            Slepwindow window(k,dpss);              separately for each.
            Spectreg::adddataseg(data,window);
        }
    }
};
```

We discuss the body of `filltable`, where the Slepian functions are actually computed, below.

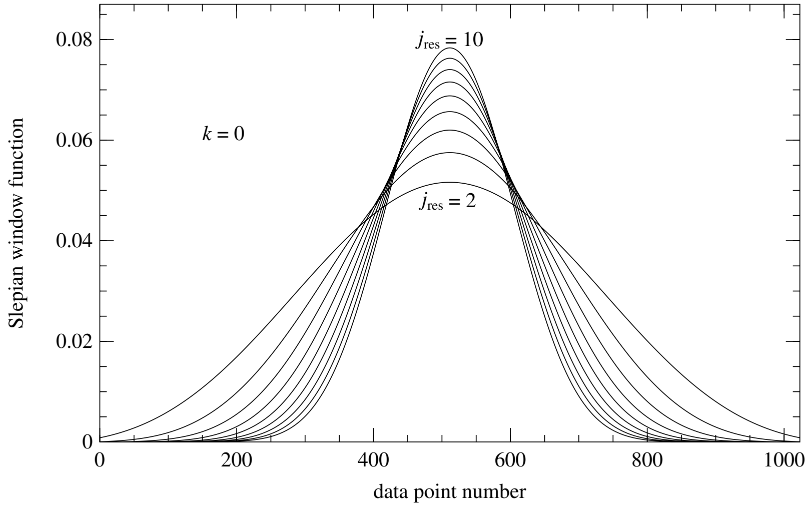

Figure 13.4.5. Slepian taper (window) functions for $k = 0$ (smallest eigenvalue) and $j_{res} = 2, 3, \ldots, 10$, with $N = 1024$. Any of these, used by itself, is a good choice for the overlapping data segment method; see text.

First, a few words about the use and misuse of multitaper methods.

The Slepian multitaper method is fundamentally about low leakage. The fact that it can reduce the variance a bit by taking $k_T > 1$ is only a secondary consideration, because there are better ways to achieve the latter goal, for example by overlapping data segments. It follows that you should never need to take j_{res} or k_T very large, greater than 10, say. Your logical path for choosing parameters should be something like this: Leakage suppression of the Slepian functions is so amazingly good that you can get to any plausible desired level for the first few eigenvectors with modest j_{res}. Find that value, and the largest acceptable value for k_T. The frequency resolution is now j_{res}, measured in bins. You now pick M to get the physical frequency resolution that you actually need,

$$f_{res} = \frac{j_{res}}{2M\Delta} \tag{13.4.20}$$

(compare equation 13.4.6). Don't be too greedy, or you will produce an unacceptably large variance. Now, if you have N_{tot} data points, you process $N_{tot}/(2M)$ separate data segments using `adddataseg`.

It would be misguided to increase j_{res} to a large value just to increase k_T for the purpose of variance reduction. The reason is that, for a fixed desired physical frequency resolution, you will need to increase M in proportion, and thus decrease your number of separate data segments, also in proportion. You thus gain nothing in variance reduction, and (potentially) lose greatly in leakage.

If squeezing down the variance by the last little bit is important, then you might consider using only the first Slepian function for a given j_{res}, and then using overlapping data segments. You can code this using `Spectolap` and `Slepian` as models. As j_{res} increases, the optimal spacing of overlapped segments decreases, as you can intuit from the narrowing central peaks in Figure 13.4.5. A spacing of $0.7N/\sqrt{j_{res} + 0.3}$, that is, overlap of $N - 0.7N/\sqrt{j_{res} + 0.3}$, should be about right.

13.4.4 Computation of the Slepian Functions

We want to find the first few eigenvectors and eigenvalues of the tridiagonal matrix, equations (13.4.18) and (13.4.19). For $N \gg 1$ (always our situation), the eigenvalues are well separated and approximately a function of j_{res} only. A good starting approximation for the

smallest eigenvalue λ_0 is

$$\lambda_0 \approx 1.5692 j_{\text{res}} - 0.10859 - 0.068762/j_{\text{res}}, \qquad j_{\text{res}} \geq 1 \tag{13.4.21}$$

and a similar approximation for the spacing of the first two eigenvalues is

$$\lambda_1 - \lambda_0 \approx 3.1387 j_{\text{res}} - 0.47276 - 0.20273/j_{\text{res}}, \qquad j_{\text{res}} \geq 1 \tag{13.4.22}$$

With these hints, a workable strategy is to find the eigenvalues as roots of the characteristic polynomial, using Newton's method. As starting guesses, we use equations (13.4.21) and (13.4.22), and subsequently linear interpolation on λ_{k-1} and λ_k, to estimate λ_{k+1}. There exists a straightforward recurrence relation that evaluates the characteristic polynomial of a tridiagonal system and its first derivative simultaneously (see [6]), and more than three or four iterations are seldom required. Once the eigenvalue is in hand, the eigenvector is obtained by setting one component arbitrarily, solving the tridiagonal system for the other components, and then renormalizing the solution. The code uses an algebraically equivalent form for equation (13.4.18) that is less susceptible to roundoff error.

spectrum.h

```
void Slepian::filltable() {
Calculate Slepian functions and store in table.
    const Doub EPS = 1.e-10, PI = 4.*atan(1.);
    Doub xx,xnew,xold,sw,ppp,ddd,sum,bet,ssub,ssup,*u;
    Int i,j,k,nl;
    VecDoub dg(m2),dgg(m2),gam(m2),sup(m2-1),sub(m2-1);
    sw = 2.*SQR(sin(jres*PI/m2));
    dg[0] = 0.25*(2*m2+sw*SQR(m2-1.)-1.);          Set up the tridiagonal matrix.
    for (i=1;i<m2;i++) {
        dg[i] = 0.25*(sw*SQR(m2-1.-2*i)+(2*(m2-i)-1.)*(2*i+1.));
        sub[i-1] = sup[i-1] = -i*(Doub)(m2-i)/2.;
    }
    xx = -0.10859 - 0.068762/jres + 1.5692*jres;   Eigenvalue first guess
    xold = xx + 0.47276 + 0.20273/jres - 3.1387*jres;
    for (k=0; k<kt; k++) {                    Loop over number of desired eigenvalues.
        u = &dpss[k][0];                      Point output vector into table.
        for (i=0;i<20;i++) {                  Loop over iterations of Newton's method.
            pp = 1.;
            p = dg[0] - xx;
            dd = 0.;
            d = -1.;
            for (j=1; j<m2; j++) {            Recurrence evaluates polynomial and deriva-
                ppp = pp; pp = p;                 tive.
                ddd = dd; dd = d;
                p = pp*(dg[j]-xx) - ppp*SQR(sup[j-1]);
                d = -pp + dd*(dg[j]-xx) - ddd*SQR(sup[j-1]);
                if (abs(p)>1.e30) renorm(-100);
                else if (abs(p)<1.e-30) renorm(100);
            }
            xnew = xx - p/d;                      Newton's method.
            if (abs(xx-xnew) < EPS*abs(xnew)) break;
            xx = xnew;
        }
        xx = xnew - (xold - xnew);
        xold = xnew;
        for (i=0;i<m2;i++) dgg[i] = dg[i] - xnew;     Subtract eigenvalue from matrix
        nl = m2/3;                                        diagonal. Then, set one com-
        dgg[nl] = 1.;                                     ponent (saving current val-
        ssup = sup[nl]; ssub = sub[nl-1];                 ues).
        u[0] = sup[nl] = sub[nl-1] = 0.;
        bet = dgg[0];                             Begin tridiagonal solution.
        for (i=1; i<m2; i++) {
            gam[i] = sup[i-1]/bet;
            bet = dgg[i] - sub[i-1]*gam[i];
            u[i] = ((i==nl? 1. : 0.) - sub[i-1]*u[i-1])/bet;
```

```
        }
        for (i=m2-2; i>=0; i--) u[i] -= gam[i+1]*u[i+1];
        sup[nl] = ssup; sub[nl-1] = ssub;      Restore saved values.
        sum = 0.;                              Renormalize and fix sign convention.
        for (i=0; i<m2; i++) sum += SQR(u[i]);
        sum = (u[3] > 0.)? sqrt(sum) : -sqrt(sum);
        for (i=0; i<m2; i++) u[i] /= sum;
    }
}
```

CITED REFERENCES AND FURTHER READING:

Oppenheim, A.V., Schafer, R.W., and Buck, J.R. 1999, *Discrete-Time Signal Processing*, 2nd ed. (Englewood Cliffs, NJ: Prentice-Hall).[1]

Harris, F.J. 1978, "On the Use of Windows for Harmonic Analysis with the Discrete Fourier Transform," *Proceedings of the IEEE*, vol. 66, pp. 51–83.[2]

Welch, P.D. 1967, "The Use of Fast Fourier Transform for the Estimation of Power Spectra: A Method Based on Time Averaging Over Short, Modified Periodograms," *IEEE Transactions on Audio and Electroacoustics*, vol. AU-15, pp. 70–73.[3]

Slepian, D. 1976, "Prolate Spheroidal Wave Functions, Fourier Analysis, and Uncertainty — V: The Discrete Case," *Bell System Technical Journal*, vol. 57, pp. 1371-1430.[4]

Percival, D.B., and Walden, A.T. 1993, *Spectral Analysis for Physical Applications: Multitaper and Conventional Univariate Techniques* (Cambridge, UK: Cambridge University Press).[5]

Acton, F.S. 1970, *Numerical Methods That Work*; 1990, corrected edition (Washington, DC: Mathematical Association of America), pp. 331–334.[6]

Elliott, D.F., and Rao, K.R. 1982, *Fast Transforms: Algorithms, Analyses, Applications* (New York: Academic Press).

13.5 Digital Filtering in the Time Domain

Suppose that you have a signal that you want to filter digitally. For example, perhaps you want to apply *high-pass* or *low-pass* filtering to eliminate noise at low or high frequencies respectively; or perhaps the interesting part of your signal lies only in a certain frequency band, so that you need a *bandpass* filter. Or, if your measurements are contaminated by 60 Hz power-line interference, you may need a *notch filter* to remove only a narrow band around that frequency. This section speaks particularly about the case in which you have chosen to do such filtering in the time domain.

Before continuing, we hope you will reconsider this choice. Remember how convenient it is to filter in the Fourier domain. You just take your whole data record, FFT it, multiply the FFT output by a filter function $\mathcal{H}(f)$, and then do an inverse FFT to get back a filtered data set in time domain. Here is some additional background on the Fourier technique that you will want to take into account.

- Remember that you must define your filter function $\mathcal{H}(f)$ for both positive and negative frequencies, and that the magnitude of the frequency extremes is always the Nyquist frequency $1/(2\Delta)$, where Δ is the sampling interval. The magnitude of the smallest nonzero frequencies in the FFT is $\pm 1/(N\Delta)$, where N is the number of (complex) points in the FFT. The positive and negative frequencies to which this filter are applied are arranged in wraparound order.
- If the measured data are real, and you want the filtered output also to be real, then your arbitrary filter function should obey $\mathcal{H}(-f) = \mathcal{H}(f)^*$. You can arrange this most easily by picking an $\mathcal{H}$ that is real and even in f.

- If your chosen $\mathcal{H}(f)$ has sharp vertical edges in it, then the *impulse response* of your filter (the output arising from a short impulse as input) will have damped "ringing" at frequencies corresponding to these edges. There is nothing wrong with this, but if you don't like it, then pick a smoother $\mathcal{H}(f)$. To get a first-hand look at the impulse response of your filter, just take the inverse FFT of your $\mathcal{H}(f)$. If you smooth all edges of the filter function over some number k of points, then the impulse response function of your filter will have a span on the order of a fraction $1/k$ of the whole data record.
- If your data set is too long to FFT all at once, then break it up into segments of any convenient size, as long as they are much longer than the impulse response function of the filter. Use zero-padding, if necessary.
- You should probably remove any trend from the data, by subtracting from it a straight line through the first and last points (i.e., make the first and last points equal to zero). If you are segmenting the data, then you can pick overlapping segments and use only the middle section of each, comfortably distant from edge effects.
- A digital filter is said to be *causal* or *physically realizable* if its output for a particular timestep depends only on inputs at that particular timestep or earlier. It is said to be *acausal* if its output can depend on both earlier and later inputs. Filtering in the Fourier domain is, in general, acausal, since the data are processed "in a batch," without regard to time ordering. Don't let this bother you! Acausal filters can generally give superior performance (e.g., less dispersion of phases, sharper edges, less asymmetric impulse response functions). People use causal filters not because they are better, but because some situations just don't allow access to out-of-time-order data. Time domain filters can, in principle, be either causal or acausal, but they are most often used in applications where physical realizability is a constraint. For this reason we will restrict ourselves to the causal case in what follows.

If you are still favoring time-domain filtering after all we have said, it is probably because you have a real-time application for which you must process a continuous data stream and wish to output filtered values at the same rate as you receive raw data. Otherwise, it may be that the quantity of data to be processed is so large that you can afford only a very small number of floating operations on each data point and cannot afford even a modest-sized FFT (with a number of floating operations per data point several times the logarithm of the number of points in the data set or segment).

13.5.1 Linear Filters

The most general linear filter takes a sequence x_k of input points and produces a sequence y_n of output points by the formula

$$y_n = \sum_{k=0}^{M} c_k \, x_{n-k} + \sum_{j=0}^{N-1} d_j \, y_{n-j-1} \tag{13.5.1}$$

Here the $M+1$ coefficients c_k and the N coefficients d_j are fixed and define the filter response. The filter (13.5.1) produces each new output value from the current and M previous input values, and from its own N previous output values. If $N = 0$, so that there is no second sum in (13.5.1), then the filter is called *nonrecursive* or *finite impulse response (FIR)*. If $N \neq 0$, then it is called *recursive* or *infinite impulse response (IIR)*. (The term "IIR" connotes only that such filters are *capable* of having infinitely long impulse responses, not that their impulse response is necessarily long in a particular application. Typically the response of an IIR filter will drop off exponentially at late times, rapidly becoming negligible.)

The relation between the c_k's and d_j's and the filter response function $\mathcal{H}(f)$ is

$$\mathcal{H}(f) = \frac{\sum\limits_{k=0}^{M} c_k e^{-2\pi i k (f\Delta)}}{1 - \sum\limits_{j=0}^{N-1} d_j e^{-2\pi i (j+1)(f\Delta)}} \tag{13.5.2}$$

where Δ is, as usual, the sampling interval. The Nyquist interval corresponds to $f\Delta$ between $-1/2$ and $1/2$. For FIR filters the denominator of (13.5.2) is just unity.

Equation (13.5.2) tells how to determine $\mathcal{H}(f)$ from the c's and d's. To design a filter, though, we need a way of doing the inverse, getting a suitable set of c's and d's — as small a set as possible, to minimize the computational burden — from a desired $\mathcal{H}(f)$. Entire books are devoted to this issue. Like many other "inverse problems," it has no all-purpose solution. One clearly has to make compromises, since $\mathcal{H}(f)$ is a full continuous function, while the short list of c's and d's represents only a few adjustable parameters. The subject of digital filter design concerns itself with the various ways of making these compromises. We cannot hope to give any sort of complete treatment of the subject, only sketch a couple of basic techniques to get you started. For further details, consult the specialized books (see references).

13.5.2 FIR (Nonrecursive) Filters

When the denominator in (13.5.2) is unity, the right-hand side is just a discrete Fourier transform. The transform is easily invertible, giving the desired small number of c_k coefficients in terms of the same small number of values of $\mathcal{H}(f_i)$ at some discrete frequencies f_i. This fact, however, is not very useful. The reason is that, for values of c_k computed in this way, $\mathcal{H}(f)$ will tend to oscillate wildly in between the discrete frequencies where it is pinned down to specific values.

A better strategy, and one that is the basis of several formal methods in the literature, is this: Start by pretending that you are willing to have a relatively large number of filter coefficients, that is, a relatively large value of M. Then $\mathcal{H}(f)$ can be fixed to desired values on a relatively fine mesh, and the M coefficients c_k, $k = 0, \ldots, M-1$ can be found by an FFT. Next, truncate (set to zero) most of the c_k's, leaving nonzero only the first, say K $(c_0, c_1, \ldots, c_{K-1})$ and last $K-1$ $(c_{M-K+1}, \ldots, c_{M-1})$. The last few c_k's are filter coefficients at *negative lag*, because of the wraparound property of the FFT. But we don't want coefficients at negative lag. Thereforea, we cyclically shift the array of c_k's, to bring everything to positive lag. (This corresponds to introducing a time delay into the filter.) Do this by copying the c_k's into a new array of length M in the following order:

$$(c_{M-K+1}, \ldots, c_{M-1},\ c_0,\ c_1, \ldots, c_{K-1},\ 0,\ 0, \ldots, 0) \tag{13.5.3}$$

To see if your truncation is acceptable, take the FFT of the array (13.5.3), giving an approximation to your original $\mathcal{H}(f)$. You will generally want to compare the *modulus* $|\mathcal{H}(f)|$ to your original function, since the time delay will have introduced complex phases into the filter response.

If the new filter function is acceptable, then you are done and have a set of $2K-1$ filter coefficients. If it is not acceptable, then you can either (i) increase K and try again, or (ii) do something fancier to improve the acceptability for the same K. An example of something fancier is to modify the magnitudes (but not the phases) of the unacceptable $\mathcal{H}(f)$ to bring it more in line with your ideal, and then to FFT to get new c_k's. Once again set to zero all but the first $2K-1$ values of these (no need to cyclically shift since you have preserved the time-delaying phases), and then inverse transform to get a new $\mathcal{H}(f)$, which will often be more acceptable. You can iterate this procedure. Note, however, that the procedure will not converge if your requirements for acceptability are more stringent than your $2K-1$ coefficients can handle.

The key idea, in other words, is to iterate between the space of coefficients and the space of functions $\mathcal{H}(f)$, until a Fourier conjugate pair that satisfies the imposed constraints *in both spaces* is found. A more formal technique for this kind of iteration is the *Remes exchange algorithm*, which produces the best Chebyshev approximation to a given desired frequency response with a fixed number of filter coefficients (cf. §5.13).

13.5.3 IIR (Recursive) Filters

Recursive filters, whose output at a given time depends both on the current and previous inputs and on previous outputs, can generally have performance that is superior to nonrecursive filters with the same total number of coefficients (or same number of floating operations

per input point). The reason is fairly clear by inspection of (13.5.2): A nonrecursive filter has a frequency response that is a polynomial in the variable $1/z$, where

$$z \equiv e^{2\pi i (f\Delta)} \tag{13.5.4}$$

By contrast, a recursive filter's frequency response is a *rational function* in $1/z$. The class of rational functions is especially good at fitting functions with sharp edges or narrow features, and most desired filter functions are in this category.

Nonrecursive filters are always stable. If you turn off the sequence of incoming x_i's, then after no more than M steps the sequence of y_j's produced by (13.5.1) will also turn off. Recursive filters, feeding as they do on their own output, are not necessarily stable. If the coefficients d_j are badly chosen, a recursive filter can have exponentially growing, so-called *homogeneous*, modes, which become huge even after the input sequence has been turned off. This is not good. The problem of designing recursive filters, therefore, is not just an inverse problem; it is an inverse problem with an additional stability constraint.

How do you tell if the filter (13.5.1) is stable for a given set of c_k and d_j coefficients? Stability depends only on the d_j's. The filter is stable if and only if all N complex roots of the *characteristic polynomial* equation

$$z^N - \sum_{j=0}^{N-1} d_j z^{(N-1)-j} = 0 \tag{13.5.5}$$

are inside the unit circle, i.e., satisfy

$$|z| \le 1 \tag{13.5.6}$$

The various methods for constructing stable recursive filters again form a subject area for which you will need more specialized books. One very useful technique, however, is the *bilinear transformation method*. For this topic we define a new variable w that reparametrizes the frequency f,

$$w \equiv \tan[\pi(f\Delta)] = i\left(\frac{1 - e^{2\pi i(f\Delta)}}{1 + e^{2\pi i(f\Delta)}}\right) = i\left(\frac{1-z}{1+z}\right) \tag{13.5.7}$$

Don't be fooled by the i's in (13.5.7). This equation maps real frequencies f into real values of w. In fact, it maps the Nyquist interval $-\frac{1}{2} < f\Delta < \frac{1}{2}$ onto the real w-axis $-\infty < w < +\infty$. The inverse equation to (13.5.7) is

$$z = e^{2\pi i(f\Delta)} = \frac{1 + iw}{1 - iw} \tag{13.5.8}$$

In reparametrizing f, w also reparametrizes z, of course. Therefore, the condition for stability (13.5.5) – (13.5.6) can be rephrased in terms of w: If the filter response $\mathcal{H}(f)$ is written as a function of w, then the filter is stable if and only if the poles of the filter function (zeros of its denominator) are all in the upper half complex plane,

$$\mathrm{Im}(w) \ge 0 \tag{13.5.9}$$

The idea of the bilinear transformation method is that instead of specifying your desired $\mathcal{H}(f)$, you specify only its desired modulus square, $|\mathcal{H}(f)|^2 = \mathcal{H}(f)\mathcal{H}(f)^* = \mathcal{H}(f)\mathcal{H}(-f)$. Pick this to be approximated by some rational function in w^2. Then find all the poles of this function in the w complex plane. Every pole in the lower half-plane will have a corresponding pole in the upper half-plane, by symmetry. The idea is to form a product of only the factors with good poles, ones in the upper half-plane. This product is your *stably realizable* $\mathcal{H}(f)$. Now substitute equation (13.5.7) to write the function as a rational function in z and compare with equation (13.5.2) to read off the c's and d's.

The procedure becomes clearer when we go through an example. Suppose we want to design a simple bandpass filter, whose lower cutoff frequency corresponds to a value $w = a$,

and whose upper cutoff frequency corresponds to a value $w = b$, with a and b both positive numbers. A simple rational function that accomplishes this is

$$|\mathcal{H}(f)|^2 = \left(\frac{w^2}{w^2 + a^2}\right)\left(\frac{b^2}{w^2 + b^2}\right) \tag{13.5.10}$$

This function does not have a very sharp cutoff, but it is illustrative of the more general case. To obtain sharper edges, one could take the function (13.5.10) to some positive integer power, or, equivalently, run the data sequentially through some number of copies of the filter that we will obtain from (13.5.10).

The poles of (13.5.10) are evidently at $w = \pm ia$ and $w = \pm ib$. Therefore the stably realizable $\mathcal{H}(f)$ is

$$\mathcal{H}(f) = \left(\frac{w}{w - ia}\right)\left(\frac{ib}{w - ib}\right) = \frac{\left(\frac{1-z}{1+z}\right)b}{\left[\left(\frac{1-z}{1+z}\right) - a\right]\left[\left(\frac{1-z}{1+z}\right) - b\right]} \tag{13.5.11}$$

We put the i in the numerator of the second factor in order to end up with real-valued coefficients. If we multiply out all the denominators, (13.5.11) can be rewritten in the form

$$\mathcal{H}(f) = \frac{-\frac{b}{(1+a)(1+b)} + \frac{b}{(1+a)(1+b)}z^{-2}}{1 - \frac{(1+a)(1-b)+(1-a)(1+b)}{(1+a)(1+b)}z^{-1} + \frac{(1-a)(1-b)}{(1+a)(1+b)}z^{-2}} \tag{13.5.12}$$

from which one reads off the filter coefficients for equation (13.5.1),

$$\begin{aligned}
c_0 &= -\frac{b}{(1+a)(1+b)} \\
c_1 &= 0 \\
c_2 &= \frac{b}{(1+a)(1+b)} \\
d_0 &= \frac{(1+a)(1-b) + (1-a)(1+b)}{(1+a)(1+b)} \\
d_1 &= -\frac{(1-a)(1-b)}{(1+a)(1+b)}
\end{aligned} \tag{13.5.13}$$

This completes the design of the bandpass filter.

Sometimes you can figure out how to construct directly a rational function in w for $\mathcal{H}(f)$, rather than having to start with its modulus square. The function that you construct has to have its poles only in the upper half-plane, for stability. It should also have the property of going into its own complex conjugate if you substitute $-w$ for w, so that the filter coefficients will be real.

For example, here is a function for a notch filter, designed to remove only a narrow frequency band around some fiducial frequency $w = w_0$, where w_0 is a positive number,

$$\begin{aligned}
\mathcal{H}(f) &= \left(\frac{w - w_0}{w - w_0 - i\epsilon w_0}\right)\left(\frac{w + w_0}{w + w_0 - i\epsilon w_0}\right) \\
&= \frac{w^2 - w_0^2}{(w - i\epsilon w_0)^2 - w_0^2}
\end{aligned} \tag{13.5.14}$$

In (13.5.14), the parameter ϵ is a small positive number that is the desired width of the notch, as

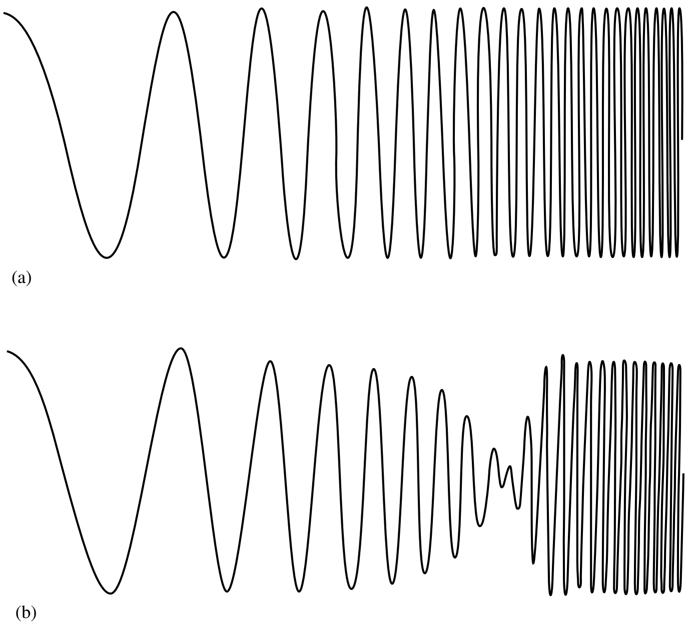

Figure 13.5.1. (a) A "chirp," or signal whose frequency increases continuously with time. (b) Same signal after it has passed through the notch filter (13.5.15). The parameter ϵ is here 0.2.

a fraction of w_0. Going through the algebra of substituting z for w gives the filter coefficients

$$
\begin{aligned}
c_0 &= \frac{1+w_0^2}{(1+\epsilon w_0)^2+w_0^2} \\
c_1 &= -2\frac{1-w_0^2}{(1+\epsilon w_0)^2+w_0^2} \\
c_2 &= \frac{1+w_0^2}{(1+\epsilon w_0)^2+w_0^2} \\
d_0 &= 2\frac{1-\epsilon^2 w_0^2-w_0^2}{(1+\epsilon w_0)^2+w_0^2} \\
d_1 &= -\frac{(1-\epsilon w_0)^2+w_0^2}{(1+\epsilon w_0)^2+w_0^2}
\end{aligned}
\qquad (13.5.15)
$$

Figure 13.5.1 shows the results of using a filter of the form (13.5.15) on a "chirp" input signal, one that glides upward in frequency, crossing the notch frequency along the way.

While the bilinear transformation may seem very general, its applications are limited by some features of the resulting filters. The method is good at getting the general shape of the desired filter, and good where "flatness" is a desired goal. However, the nonlinear mapping between w and f makes it difficult to design to a desired shape for a cutoff, and may move cutoff frequencies (defined by a certain number of dB) from their desired places. Consequently, practitioners of the art of digital filter design reserve the bilinear transformation for specific situations, and arm themselves with a variety of other tricks. We suggest that you do likewise, as your projects demand.

CITED REFERENCES AND FURTHER READING:

Hamming, R.W. 1983, *Digital Filters*, 2nd ed. (Englewood Cliffs, NJ: Prentice-Hall).

Antoniou, A. 1979, *Digital Filters: Analysis and Design* (New York: McGraw-Hill).

Parks, T.W., and Burrus, C.S. 1987, *Digital Filter Design* (New York: Wiley).

Oppenheim, A.V., Schafer, R.W., and Buck, J.R. 1999, *Discrete-Time Signal Processing*, 2nd ed. (Englewood Cliffs, NJ: Prentice-Hall).

Rice, J.R. 1964, *The Approximation of Functions* (Reading, MA: Addison-Wesley); also 1969, *op. cit.*, Vol. 2.

Rabiner, L.R., and Gold, B. 1975, *Theory and Application of Digital Signal Processing* (Englewood Cliffs, NJ: Prentice-Hall).

13.6 Linear Prediction and Linear Predictive Coding

We begin with a very general formulation that will allow us to make connections to various special cases. Let $\{y'_\alpha\}$ be a set of measured values for some underlying set of true values of a quantity y, denoted $\{y_\alpha\}$, related to these true values by the addition of random noise,

$$y'_\alpha = y_\alpha + n_\alpha \tag{13.6.1}$$

(compare equation 13.3.2, with a somewhat different notation). Our use of a Greek subscript to index the members of the set is meant to indicate that the data points are not necessarily equally spaced along a line, or even ordered; they might be "random" points in three-dimensional space, for example. Now, suppose we want to construct the "best" estimate of the true value of some particular point $y_\star$ as a linear combination of the known, noisy, values. Writing

$$y_\star = \sum_\alpha d_{\star\alpha} y'_\alpha + x_\star \tag{13.6.2}$$

we want to find coefficients $d_{\star\alpha}$ that minimize, in some way, the *discrepancy* $x_\star$. The coefficients $d_{\star\alpha}$ have a "star" subscript to indicate that they depend on the choice of point $y_\star$. Later, we might want to let $y_\star$ be one of the existing y_α's. In that case, our problem becomes one of optimal filtering or estimation, closely related to the discussion in §13.3. On the other hand, we might want $y_\star$ to be a completely new point. In that case, our problem will be one of *linear prediction*.

A natural way to minimize the discrepancy $x_\star$ is in the statistical mean square sense. If angle brackets denote statistical averages, then we seek $d_{\star\alpha}$'s that minimize

$$\begin{aligned}\langle x_\star^2 \rangle &= \left\langle \left[\sum_\alpha d_{\star\alpha}(y_\alpha + n_\alpha) - y_\star \right]^2 \right\rangle \\ &= \sum_{\alpha\beta} (\langle y_\alpha y_\beta \rangle + \langle n_\alpha n_\beta \rangle) d_{\star\alpha} d_{\star\beta} - 2 \sum_\alpha \langle y_\star y_\alpha \rangle\, d_{\star\alpha} + \langle y_\star^2 \rangle\end{aligned} \tag{13.6.3}$$

Here we have used the fact that noise is uncorrelated with signal, e.g., $\langle n_\alpha y_\beta \rangle = 0$. The quantities $\langle y_\alpha y_\beta \rangle$ and $\langle y_\star y_\alpha \rangle$ describe the autocorrelation structure of the underlying data. We have already seen an analogous expression, (13.2.2), for the case of equally spaced data points on a line; we will meet correlation several times again in its statistical sense in Chapters 14 and 15. The quantities $\langle n_\alpha n_\beta \rangle$ describe the autocorrelation properties of the noise. Often, for point-to-point uncorrelated

noise, we have $\langle n_\alpha n_\beta \rangle = \langle n_\alpha^2 \rangle \delta_{\alpha\beta}$. It is convenient to think of the various correlation quantities as comprising matrices and vectors,

$$\phi_{\alpha\beta} \equiv \langle y_\alpha y_\beta \rangle \qquad \phi_{\star\alpha} \equiv \langle y_\star y_\alpha \rangle \qquad \eta_{\alpha\beta} \equiv \langle n_\alpha n_\beta \rangle \text{ or } \langle n_\alpha^2 \rangle \delta_{\alpha\beta} \tag{13.6.4}$$

Setting the derivative of equation (13.6.3) with respect to the $d_{\star\alpha}$'s equal to zero, one readily obtains the set of linear equations

$$\sum_\beta \left[\phi_{\alpha\beta} + \eta_{\alpha\beta}\right] d_{\star\beta} = \phi_{\star\alpha} \tag{13.6.5}$$

If we write the solution as a matrix inverse, then the estimation equation (13.6.2) becomes, omitting the minimized discrepancy $x_\star$,

$$y_\star \approx \sum_{\alpha\beta} \phi_{\star\alpha} \left[\phi_{\mu\nu} + \eta_{\mu\nu}\right]^{-1}_{\alpha\beta} y'_\beta \tag{13.6.6}$$

From equations (13.6.3) and (13.6.5) one can also calculate the expected mean square value of the discrepancy at its minimum, denoted $\langle x_\star^2 \rangle_0$,

$$\langle x_\star^2 \rangle_0 = \langle y_\star^2 \rangle - \sum_\beta d_{\star\beta}\phi_{\star\beta} = \langle y_\star^2 \rangle - \sum_{\alpha\beta} \phi_{\star\alpha} \left[\phi_{\mu\nu} + \eta_{\mu\nu}\right]^{-1}_{\alpha\beta} \phi_{\star\beta} \tag{13.6.7}$$

Although the notation is now different, equations (13.6.6) and (13.6.7) are close relatives to equations (3.7.14) and (3.7.15), which we exhibited without proof in connection with kriging interpolation. (See also §13.6.3, below.)

A final general result tells how much the mean square discrepancy $\langle x_\star^2 \rangle$ is increased if we use the estimation equation (13.6.2) not with the best values $d_{\star\beta}$, but with some other values $\widehat{d}_{\star\beta}$. The above equations then imply

$$\langle x_\star^2 \rangle = \langle x_\star^2 \rangle_0 + \sum_{\alpha\beta} (\widehat{d}_{\star\alpha} - d_{\star\alpha}) \left[\phi_{\alpha\beta} + \eta_{\alpha\beta}\right] (\widehat{d}_{\star\beta} - d_{\star\beta}) \tag{13.6.8}$$

Since the second term is a pure quadratic form, we see that the increase in the discrepancy is only second order in any error made in estimating the $d_{\star\beta}$'s.

13.6.1 Connection to Optimal Filtering

If we change "star" to a Greek index, say γ, then the above formulas describe optimal filtering, generalizing the discussion of §13.3. One sees, for example, that if the noise amplitudes n_α go to zero, so likewise do the noise autocorrelations $\eta_{\alpha\beta}$, and, canceling a matrix times its inverse, equation (13.6.6) simply becomes $y_\gamma = y'_\gamma$. Another special case occurs if the matrices $\phi_{\alpha\beta}$ and $\eta_{\alpha\beta}$ are diagonal. In that case, equation (13.6.6) becomes

$$y_\gamma = \frac{\phi_{\gamma\gamma}}{\phi_{\gamma\gamma} + \eta_{\gamma\gamma}} y'_\gamma \tag{13.6.9}$$

which is readily recognizable as equation (13.3.6) with $S^2 \to \phi_{\gamma\gamma}$, $N^2 \to \eta_{\gamma\gamma}$. What is going on is this: For the case of equally spaced data points, and in the Fourier domain, autocorrelations become simply squares of Fourier amplitudes (Wiener-Khinchin theorem, equation 12.0.13), and the optimal filter can be constructed algebraically, as equation (13.6.9), without inverting any matrix.

More generally, in the time domain, or any other domain, an optimal filter (one that minimizes the square of the discrepancy from the underlying true value in the presence of measurement noise) can be constructed by estimating the autocorrelation matrices $\phi_{\alpha\beta}$ and $\eta_{\alpha\beta}$, and applying equation (13.6.6) with $\star \to \gamma$. (Equation 13.6.8 is in fact the basis for the §13.3's statement that even crude optimal filtering can be quite effective.)

13.6.2 Linear Prediction

Classical *linear prediction* specializes to the case where the data points y_β are equally spaced along a line, y_i, $i = 0, 1, \ldots, N-1$, and we want to use M consecutive values of y_i to predict an $M + 1$st. Stationarity is assumed. That is, the autocorrelation $\langle y_j y_k \rangle$ is assumed to depend only on the difference $|j - k|$, and not on j or k individually, so that the autocorrelation ϕ has only a single index,

$$\phi_j \equiv \langle y_i y_{i+j} \rangle \approx \frac{1}{N-j} \sum_{i=0}^{N-j-1} y_i y_{i+j} \tag{13.6.10}$$

Here, the approximate equality shows one way to use the actual data set values to estimate the autocorrelation components. (In fact, there is a better way to make these estimates; see below.) In the situation described, the estimation equation (13.6.2) is

$$y_n = \sum_{j=0}^{M-1} d_j y_{n-j-1} + x_n \tag{13.6.11}$$

(compare equation 13.5.1) and equation (13.6.5) becomes the set of M equations for the M unknown d_j's, now called the *linear prediction (LP) coefficients*,

$$\sum_{j=0}^{M-1} \phi_{|j-k-1|} d_j = \phi_k \qquad (k = 1, \ldots, M) \tag{13.6.12}$$

Notice that while noise is not explicitly included in the equations, it is properly accounted for, *if* it is point-to-point uncorrelated: ϕ_0, as estimated by equation (13.6.10) using *measured* values y'_i, actually estimates the diagonal part of $\phi_{\alpha\alpha} + \eta_{\alpha\alpha}$, above. The mean square discrepancy $\langle x_n^2 \rangle$ is estimated by equation (13.6.7) as

$$\langle x_n^2 \rangle = \phi_0 - \phi_1 d_0 - \phi_2 d_1 - \cdots - \phi_M d_{M-1} \tag{13.6.13}$$

To use linear prediction, we first compute the d_j's, using equations (13.6.10) and (13.6.12). We then calculate equation (13.6.13) or, more concretely, apply (13.6.11) to the known record to get an idea of how large are the discrepancies x_i. If the discrepancies are small, then we can continue applying (13.6.11) right on into the future, imagining the unknown "future" discrepancies x_i to be zero. In this application, (13.6.11) is a kind of extrapolation formula. In many situations, this extrapolation turns out to be vastly more powerful than any kind of simple polynomial extrapolation. (By the way, you should not confuse the terms "linear prediction" and "linear extrapolation"; the general functional form used by linear prediction is *much* more complex than a straight line, or even a low-order polynomial!)

However, to achieve its full usefulness, linear prediction must be constrained in one additional respect: One must take additional measures to guarantee its *stability*. Equation (13.6.11) is a special case of the general linear filter (13.5.1). The condition that (13.6.11) be stable as a linear predictor is precisely that given in equations (13.5.5) and (13.5.6), namely that the characteristic polynomial

$$z^N - \sum_{j=0}^{N-1} d_j z^{(N-1)-j} = 0 \tag{13.6.14}$$

have all N of its roots inside the unit circle

$$|z| \le 1 \tag{13.6.15}$$

There is no guarantee that the coefficients produced by equation (13.6.12) will have this property. If the data contain many oscillations without any particular trend toward increasing or decreasing amplitude, then the complex roots of (13.6.14) will generally all be rather close to the unit circle. The finite length of the data set will cause some of these roots to be inside the unit circle, others outside. In some applications, where the resulting instabilities are slowly growing and the linear prediction is not pushed too far, it is best to use the "unmassaged" LP coefficients that come directly out of (13.6.12). For example, one might be extrapolating to fill a short gap in a data set; then one might extrapolate both forward across the gap and backward from the data beyond the gap. If the two extrapolations agree tolerably well, then instability is not a problem.

When instability *is* a problem, you have to "massage" the LP coefficients. You do this by (i) solving (numerically) equation (13.6.14) for its N complex roots; (ii) moving the roots to where you think they ought to be inside or on the unit circle; and (iii) reconstituting the now-modified LP coefficients. You may think that step (ii) sounds a little vague. It is. There is no "best" procedure. If you think that your signal is truly a sum of undamped sine and cosine waves (perhaps with incommensurate periods), then you will want simply to move each root z_i onto the unit circle

$$z_i \rightarrow z_i / |z_i| \tag{13.6.16}$$

In other circumstances it may seem appropriate to reflect a bad root across the unit circle

$$z_i \rightarrow 1/z_i^* \tag{13.6.17}$$

This alternative has the property that it preserves the amplitude of the output of (13.6.11) when it is driven by a sinusoidal set of x_i's. It assumes that (13.6.12) has correctly identified the spectral width of a resonance, but only slipped up on identifying its time sense so that signals that should be damped as time proceeds end up growing in amplitude. The choice between (13.6.16) and (13.6.17) sometimes might as well be based on voodoo. We prefer (13.6.17).

Also magical is the choice of M, the number of LP coefficients to use. You should choose M to be as small as works for you, that is, you should choose it by experimenting with your data. Try $M = 5, 10, 20, 40$. If you need larger M's than this, be aware that the procedure of "massaging" all those complex roots is quite sensitive to roundoff error. Double precision is crucial.

Linear prediction is especially successful at extrapolating signals that are smooth and oscillatory, though not necessarily periodic. In such cases, linear prediction often extrapolates accurately through *many cycles* of the signal. By contrast, polynomial extrapolation in general becomes seriously inaccurate after at most a cycle or two. A prototypical example of a signal that can successfully be linearly predicted is the height of ocean tides, for which the fundamental 12-hour period is modulated in phase and amplitude over the course of the month and year, and for which local hydrodynamic effects may make even one cycle of the curve look rather different in shape from a sine wave.

We already remarked that equation (13.6.10) is not necessarily the best way to estimate the covariances ϕ_k from the data set. In fact, results obtained from linear prediction are remarkably sensitive to exactly how the ϕ_k's are estimated. One particularly good method is due to Burg [1], and involves a recursive procedure for increasing the order M by one unit at a time, at each stage re-estimating the coefficients d_j, $j = 0, \ldots, M-1$ so as to minimize the residual in equation (13.6.13). Although further discussion of the Burg method is beyond our scope here, the method is implemented in the following routine [1,2] for estimating the LP coefficients d_j of a data set.

linpredict.h

```
void memcof(VecDoub_I &data, Doub &xms, VecDoub_O &d) {
```
Given a real vector of data[0..n-1], this routine returns m linear prediction coefficients as d[0..m-1], and returns the mean square discrepancy as xms.
```
    Int k,j,i,n=data.size(),m=d.size();
    Doub p=0.0;
    VecDoub wk1(n),wk2(n),wkm(m);
    for (j=0;j<n;j++) p += SQR(data[j]);
    xms=p/n;
    wk1[0]=data[0];
    wk2[n-2]=data[n-1];
    for (j=1;j<n-1;j++) {
        wk1[j]=data[j];
        wk2[j-1]=data[j];
    }
    for (k=0;k<m;k++) {
        Doub num=0.0,denom=0.0;
        for (j=0;j<(n-k-1);j++) {
            num += (wk1[j]*wk2[j]);
            denom += (SQR(wk1[j])+SQR(wk2[j]));
        }
        d[k]=2.0*num/denom;
        xms *= (1.0-SQR(d[k]));
        for (i=0;i<k;i++)
            d[i]=wkm[i]-d[k]*wkm[k-1-i];
```
The algorithm is recursive, building up the answer for larger and larger values of m until the desired value is reached. At this point in the algorithm, one could return the vector d and scalar xms for a set of LP coefficients with k (rather than m) terms.
```
        if (k == m-1)
            return;
        for (i=0;i<=k;i++) wkm[i]=d[i];
        for (j=0;j<(n-k-2);j++) {
            wk1[j] -= (wkm[k]*wk2[j]);
            wk2[j]=wk2[j+1]-wkm[k]*wk1[j+1];
        }
    }
    throw("never get here in memcof");
}
```

Here are procedures for rendering the LP coefficients stable (if you choose to do so) and for extrapolating a data set by linear prediction, using the original or massaged LP coefficients. The routine zroots (§9.5) is used to find all complex roots of a polynomial.

linpredict.h

```
void fixrts(VecDoub_IO &d) {
```
Given the LP coefficients d[0..m-1], this routine finds all roots of the characteristic polynomial (13.6.14), reflects any roots that are outside the unit circle back inside, and then returns a modified set of coefficients d[0..m-1].
```
    Bool polish=true;
    Int i,j,m=d.size();
    VecComplex a(m+1),roots(m);
    a[m]=1.0;
    for (j=0;j<m;j++)                    Set up complex coefficients for polynomial root
        a[j]= -d[m-1-j];                     finder.
    zroots(a,roots,polish);              Find all the roots.
    for (j=0;j<m;j++)                    Look for a root outside the unit circle, and reflect
        if (abs(roots[j]) > 1.0)             it back inside.
            roots[j]=1.0/conj(roots[j]);
    a[0]= -roots[0];                     Now reconstruct the polynomial coefficients,
    a[1]=1.0;
    for (j=1;j<m;j++) {                  by looping over the roots
        a[j+1]=1.0;
        for (i=j;i>=1;i--)               and synthetically multiplying.
            a[i]=a[i-1]-roots[j]*a[i];
        a[0]= -roots[j]*a[0];
    }
    for (j=0;j<m;j++)                    The polynomial coefficients are guaranteed to be
        d[m-1-j] = -real(a[j]);              real, so we need only return the real part as
}                                            new LP coefficients.
```

linpredict.h

```
void predic(VecDoub_I &data, VecDoub_I &d, VecDoub_O &future) {
```
Given data[0..ndata-1], and given the data's LP coefficients d[0..m-1], this routine applies equation (13.6.11) to predict the next nfut data points, which it returns in the array future[0..nfut-1]. Note that the routine references only the last m values of data, as initial values for the prediction.
```
    Int k,j,ndata=data.size(),m=d.size(),nfut=future.size();
    Doub sum,discrp;
    VecDoub reg(m);
    for (j=0;j<m;j++) reg[j]=data[ndata-1-j];
    for (j=0;j<nfut;j++) {
        discrp=0.0;
        This is where you would put in a known discrepancy if you were reconstructing a func-
        tion by linear predictive coding rather than extrapolating a function by linear prediction.
        See text.
        sum=discrp;
        for (k=0;k<m;k++) sum += d[k]*reg[k];
        for (k=m-1;k>=1;k--) reg[k]=reg[k-1];      [If you want to implement circular
        future[j]=reg[0]=sum;                          arrays, you can avoid this shift-
    }                                                  ing of coefficients.]
}
```

13.6.3 Removing the Bias in Linear Prediction

You might expect that the sum of the d_j's in equation (13.6.11) (or, more generally, in equation 13.6.2) should be 1, so that, e.g., adding a constant to all the data points y_i yields a prediction that is increased by the same constant. However, the d_j's do not sum to 1 but, in general, to a value slightly less than one. This fact re-

veals a subtle point, that the estimator of classical linear prediction is not *unbiased*, even though it does minimize the mean square discrepancy. At any place where the measured autocorrelation does not imply a better estimate, the equations of linear prediction tend to predict a value that tends toward zero.

Sometimes, that is just what you want. If the process that generates the y_i's in fact has zero mean, then zero is the best guess absent other information. At other times, however, this behavior is unwarranted. If you have data that show only small variations around a positive value, you don't want linear predictions that droop toward zero.

Often it is a workable approximation to subtract the mean off your data set, perform the linear prediction, and then add the mean back. This procedure contains the germ of the correct solution; but the simple arithmetic mean is not quite the correct constant to subtract. In fact, an unbiased estimator is obtained by subtracting from every data point an autocorrelation-weighted mean defined by [3,4]

$$\bar{y} \equiv \sum_{\beta} [\phi_{\mu\nu} + \eta_{\mu\nu}]^{-1}_{\alpha\beta}\, y_\beta \Bigg/ \sum_{\alpha\beta} [\phi_{\mu\nu} + \eta_{\mu\nu}]^{-1}_{\alpha\beta} \tag{13.6.18}$$

With this subtraction, the sum of the LP coefficients should be unity, up to roundoff and differences in how the ϕ_k's are estimated.

Equations (3.7.14) and (3.7.15), given in connection with kriging, are in fact exactly equivalent to equations (13.6.6) and (13.6.7) if the mean (13.6.18) is used to remove the estimator bias. To prove this, start by writing the inverse of the matrix (3.7.13) in the obvious partitioned form (e.g., using equation 2.7.23).

13.6.4 Linear Predictive Coding (LPC)

A different, though related, method to which the formalism above can be applied is the "compression" of a sampled signal so that it can be stored more compactly. The original form should be *exactly* recoverable from the compressed version. Obviously, compression can be accomplished only if there is redundancy in the signal. Equation (13.6.11) describes one kind of redundancy: It says that the signal, except for a small discrepancy, is predictable from its previous values and from a small number of LP coefficients. Compression of a signal by the use of (13.6.11) is thus called *linear predictive coding*, or *LPC*.

The basic idea of LPC (in its simplest form) is to record as a compressed file (i) the number of LP coefficients M; (ii) their M values, e.g., as obtained by `memcof`; (iii) the first M data points; and then (iv) for each subsequent data point only its residual discrepancy x_i (equation 13.6.1). When you are creating the compressed file, you find the residual by applying (13.6.1) to the previous M points, subtracting the sum from the actual value of the current point. When you are reconstructing the original file, you add the residual back in, at the point indicated in the routine `predic`.

It may not be obvious why there is any compression at all in this scheme. After all, we are storing one value of residual per data point! Why not just store the original data point? The answer depends on the relative sizes of the numbers involved. The residual is obtained by subtracting two very nearly equal numbers (the data and the linear prediction). Therefore, the discrepancy typically has only a very small number of nonzero bits. These can be stored in a compressed file. How do you do it in a high-level language? A rudimentary approach would be to scale your data to have

integer values, say between $+1000000$ and -1000000 (supposing that you need six significant figures). Now modify equation (13.6.11) by enclosing the sum term in an "integer part of" operator. The discrepancy will now, by definition, be an integer. Experiment with different values of M to find LP coefficients that make the range of the discrepancy as small as you can. If you can get to within a range of ± 127 (and in our experience this is not at all difficult), then you can write it to a file as a single byte. This is a compression factor of 4, compared to 4-byte integer or floating formats.

Notice that the LP coefficients are computed using the *quantized* data, and that the discrepancy is also quantized, i.e., quantization is done both outside and inside the LPC loop. If you are careful in following this prescription, then, apart from the initial quantization of the data, you will not introduce even a single bit of roundoff error into the compression-reconstruction process: While the evaluation of the sum in (13.6.11) may have roundoff errors, the residual that you store is the value that, when added back to the sum, gives *exactly* the original (quantized) data value. Notice also that you do not need to massage the LP coefficients for stability; by adding the residual back in to each point, you never depart from the original data, so instabilities cannot grow. There is therefore no need for `fixrts`, above.

Look at §22.5 to learn about *Huffman coding*, which will further compress the residuals by taking advantage of the fact that smaller values of discrepancy will occur more often than larger values. A very primitive version of Huffman coding would be this: If most of the discrepancies are in the range ± 127, but an occasional one is outside, then reserve the value 127 to mean "out of range," and then record on the file (immediately following the 127) a full-word value of the out-of-range discrepancy. Section 22.5 explains how to do much better.

There are many variant procedures that all fall under the rubric of LPC:

- If the spectral character of the data is time-variable, then it is best not to use a single set of LP coefficients for the whole data set, but rather to partition the data into segments, computing and storing different LP coefficients for each segment.
- If the data are really well characterized by their LP coefficients, and you can tolerate some small amount of error, then don't bother storing all of the residuals. Just do linear prediction until you are outside of tolerances, and then reinitialize (using M sequential stored residuals) and continue predicting.
- In some applications, most notably speech synthesis, one cares only about the spectral content of the reconstructed signal, not the relative phases. In this case, one need not store any starting values at all, but only the LP coefficients for each segment of the data. The output is reconstructed by driving these coefficients with initial conditions consisting of all zeros except for one nonzero spike. A speech synthesizer chip may have of order 10 LP coefficients, which change perhaps 20 to 50 times per second.
- Some people believe that it is interesting to analyze a signal by LPC, even when the residuals x_i are *not* small. The x_i's are then interpreted as the underlying "input signal" that, when filtered through the all-poles filter defined by the LP coefficients (see §13.7), produces the observed "output signal." LPC reveals simultaneously, it is said, the nature of the filter *and* the particular input that is driving it. We are skeptical of these applications; the literature, however, is full of extravagant claims.

CITED REFERENCES AND FURTHER READING:

Childers, D.G. (ed.) 1978, *Modern Spectrum Analysis* (New York: IEEE Press), especially the paper by J. Makhoul, "Linear Prediction: A Tutorial Review," reprinted from *Proceedings of the IEEE*, vol. 63, p. 561, 1975.

Burg, J.P. 1968, "A New Analysis Technique for Time Series Data," reprinted in Childers, 1978.[1]

Anderson, N. 1974, "On the Calculation of Filter Coefficients for Maximum Entropy Spectral Analysis," *Geophysics*, vol. 39, pp. 69–72, reprinted in Childers, 1978.[2]

Cressie, N. 1991, "Geostatistical Analysis of Spatial Data," in *Spatial Statistics and Digital Image Analysis* (Washington: National Academy Press).[3]

Press, W.H., and Rybicki, G.B. 1992, "Interpolation, Realization, and Reconstruction of Noisy, Irregularly Sampled Data," *Astrophysical Journal*, vol. 398, pp. 169–176.[4]

13.7 Power Spectrum Estimation by the Maximum Entropy (All-Poles) Method

The FFT is not the only way to estimate the power spectrum of a process, nor is it necessarily the best way for all purposes. To see how one might devise another method, let us enlarge our view for a moment, so that it includes not only real frequencies in the Nyquist interval $-f_c < f < f_c$, but also the entire complex frequency plane. From that vantage point, let us transform the complex f-plane to a new plane, called the *z-transform plane* or *z-plane*, by the relation

$$z \equiv e^{2\pi i f \Delta} \tag{13.7.1}$$

where Δ is, as usual, the sampling interval in the time domain. Notice that the Nyquist interval on the real axis of the f-plane maps one-to-one onto the unit circle in the complex z-plane.

If we now compare (13.7.1) to equations (13.4.4) and (13.4.6), we see that the FFT power spectrum estimate (13.4.5) for any real sampled function $c_k \equiv c(t_k)$ can be written, except for normalization convention, as

$$P(f) = \left| \sum_{k=-N/2}^{N/2-1} c_k z^k \right|^2 \tag{13.7.2}$$

Of course, (13.7.2) is not the *true* power spectrum of the underlying function $c(t)$, but only an estimate. We can see in two related ways why the estimate is not likely to be exact. First, in the time domain, the estimate is based on only a finite range of the function $c(t)$, which may, for all we know, have continued from $t = -\infty$ to ∞. Second, in the z-plane of equation (13.7.2), the finite Laurent series offers, in general, only an approximation to a general analytic function of z. In fact, a formal expression for representing "true" power spectra (up to normalization) is

$$P(f) = \left| \sum_{k=-\infty}^{\infty} c_k z^k \right|^2 \tag{13.7.3}$$

This is an infinite Laurent series that depends on an infinite number of values c_k. Equation (13.7.2) is just one kind of analytic approximation to the analytic function of z represented by (13.7.3), the kind, in fact, that is implicit in the use of FFTs to estimate power spectra by periodogram methods. It goes under several names, including *direct method, all-zero model,* and *moving average (MA) model*. The term "all-zero" in particular refers to the fact that the model spectrum can have zeros in the z-plane, but not poles.

If we look at the problem of approximating (13.7.3) more generally, it seems clear that we could do a better job with a rational function, one with a series of type (13.7.2) in both the

numerator and the denominator. Less obviously, it turns out that there are some advantages in an approximation whose free parameters all lie in the *denominator*, namely,

$$P(f) \approx \frac{1}{\left|\sum_{k=-M/2}^{M/2} b_k z^k\right|^2} = \frac{a_0}{\left|1 + \sum_{k=1}^{M} a_k z^k\right|^2} \tag{13.7.4}$$

Here the second equality brings in a new set of coefficients a_k's, which can be determined from the b_k's using the fact that z lies on the unit circle. The b_k's can be thought of as being determined by the condition that power series expansion of (13.7.4) agree with the first $M + 1$ terms of (13.7.3). In practice, as we shall see, one determines the b_k's or a_k's by another method.

The differences between the approximations (13.7.2) and (13.7.4) are not just cosmetic. They are approximations of very different character. Most notable is the fact that (13.7.4) can have *poles*, corresponding to infinite power spectral density, on the unit z-circle, i.e., at real frequencies in the Nyquist interval. Such poles can provide an accurate representation for underlying power spectra that have sharp, discrete "lines" or delta-functions. By contrast, (13.7.2) can have only zeros, not poles, at real frequencies in the Nyquist interval, and must thus attempt to fit sharp spectral features with, essentially, a polynomial. The approximation (13.7.4) goes under several names: *all-poles model, maximum entropy method (MEM), autoregressive model (AR)*. We need only find out how to compute the coefficients a_0 and the a_k's from a data set, so that we can actually use (13.7.4) to obtain spectral estimates.

A pleasant surprise is that we already know how! Look at equation (13.6.11) for linear prediction. Compare it with linear filter equations (13.5.1) and (13.5.2), and you will see that, viewed as a filter that takes input x's into output y's, linear prediction has a filter function

$$\mathcal{H}(f) = \frac{1}{1 - \sum_{j=0}^{N-1} d_j z^{-(j+1)}} \tag{13.7.5}$$

Thus, the power spectrum of the y's should be equal to the power spectrum of the x's multiplied by $|\mathcal{H}(f)|^2$. Now let us think about what the spectrum of the input x's is, when they are residual discrepancies from linear prediction. Although we will not prove it formally, it is intuitively believable that the x's are independently random and therefore have a flat (white noise) spectrum. (Roughly speaking, any residual correlations left in the x's would have allowed a more accurate linear prediction, and would have been removed.) The overall normalization of this flat spectrum is just the mean square amplitude of the x's. But this is exactly the quantity computed in equation (13.6.13) and returned by the routine `memcof` as `xms`. Thus, the coefficients a_0 and a_k in equation (13.7.4) are related to the LP coefficients returned by `memcof` simply by

$$a_0 = \texttt{xms} \qquad a_k = -\texttt{d}(k-1), \quad k = 1, \ldots, M \tag{13.7.6}$$

There is also another way to describe the relation between the a_k's and the autocorrelation components ϕ_k. The Wiener-Khinchin theorem (12.0.13) says that the Fourier transform of the autocorrelation is equal to the power spectrum. In z-transform language, this Fourier transform is just a Laurent series in z. The equation that is to be satisfied by the coefficients in equation (13.7.4) is thus

$$\frac{a_0}{\left|1 + \sum_{k=1}^{M} a_k z^k\right|^2} \approx \sum_{j=-M}^{M} \phi_j z^j \tag{13.7.7}$$

The approximately equal sign in (13.7.7) has a somewhat special interpretation. It means that the series expansion of the left-hand side is supposed to agree with the right-hand side term-by-term from z^{-M} to z^M. Outside this range of terms, the right-hand side is obviously zero, while the left-hand side will still have nonzero terms. Notice that M, the number of

coefficients in the approximation on the left-hand side, can be any integer up to N, the total number of autocorrelations available. (In practice, one often chooses M much smaller than N.) M is called the *order* or *number of poles* of the approximation.

Whatever the chosen value of M, the series expansion of the left-hand side of (13.7.7) defines a certain sort of *extrapolation* of the autocorrelation function to lags larger than M, in fact even to lags larger than N, i.e., *larger than the run of data can actually measure*. It turns out that this particular extrapolation can be shown to have, among all possible extrapolations, the maximum *entropy* in a definable information-theoretic sense. Hence the name *maximum entropy method*, or MEM. The maximum entropy property has caused MEM to acquire a certain "cult" popularity; one sometimes hears that it gives an intrinsically "better" estimate than is given by other methods. Don't believe it. MEM has the very cute property of being able to fit sharp spectral features, but there is nothing else magical about its power spectrum estimates.

The operations count in `memcof` scales as the product of N (the number of data points) and M (the desired order of the MEM approximation). If M were chosen to be as large as N, then the method would be much slower than the $N \log N$ FFT methods of the previous section. In practice, however, one usually wants to limit the order (or number of poles) of the MEM approximation to a few times the number of sharp spectral features that one desires it to fit. With this restricted number of poles, the method will smooth the spectrum somewhat, but this is often a desirable property. While exact values depend on the application, one might take $M = 10$ or 20 or 50 for $N = 1000$ or 10000. In that case, MEM estimation is not much slower than FFT estimation.

We feel obliged to warn you that `memcof` can be a bit quirky at times. If the number of poles or number of data points is too large, roundoff error can be a problem, even in double precision. With "peaky" data (i.e., data with extremely sharp spectral features), the algorithm may suggest split peaks even at modest orders, and the peaks may shift with the phase of the sine wave. Also, with noisy input functions, if you choose too high an order, you will find spurious peaks galore! Some experts recommend the use of this algorithm in conjunction with more conservative methods, like periodograms, to help choose the correct model order and to avoid getting too fooled by spurious spectral features. MEM can be finicky, but it can also do remarkable things. We recommend that you try it out, cautiously, on your own problems. We now turn to the evaluation of the MEM spectral estimate from its coefficients.

The MEM estimation (13.7.4) is a function of continuously varying frequency f. There is no special significance to specific equally spaced frequencies as there was in the FFT case. In fact, since the MEM estimate may have very sharp spectral features, one wants to be able to evaluate it on a very fine mesh near to those features, but perhaps only more coarsely farther away from them. Here is a function that, given the coefficients already computed, evaluates (13.7.4) and returns the estimated power spectrum as a function of $f\Delta$ (the frequency times the sampling interval). Of course, $f\Delta$ should lie in the Nyquist range between $-1/2$ and $1/2$.

```
Doub evlmem(const Doub fdt, VecDoub_I &d, const Doub xms)                    linpredict.h
```
Given `d[0..m-1]` and `xms` as returned by `memcof`, this function returns the power spectrum estimate $P(f)$ as a function of `fdt` $= f\Delta$.
```
{
    Int i;
    Doub sumr=1.0,sumi=0.0,wr=1.0,wi=0.0,wpr,wpi,wtemp,theta;

    Int m=d.size();
    theta=6.28318530717959*fdt;
    wpr=cos(theta);                    Set up for recurrence relations.
    wpi=sin(theta);
    for (i=0;i<m;i++) {                Loop over the terms in the sum.
        wr=(wtemp=wr)*wpr-wi*wpi;
        wi=wi*wpr+wtemp*wpi;
        sumr -= d[i]*wr;               These accumulate the denominator of (13.7.4).
        sumi -= d[i]*wi;
    }
    return xms/(sumr*sumr+sumi*sumi);
}
```

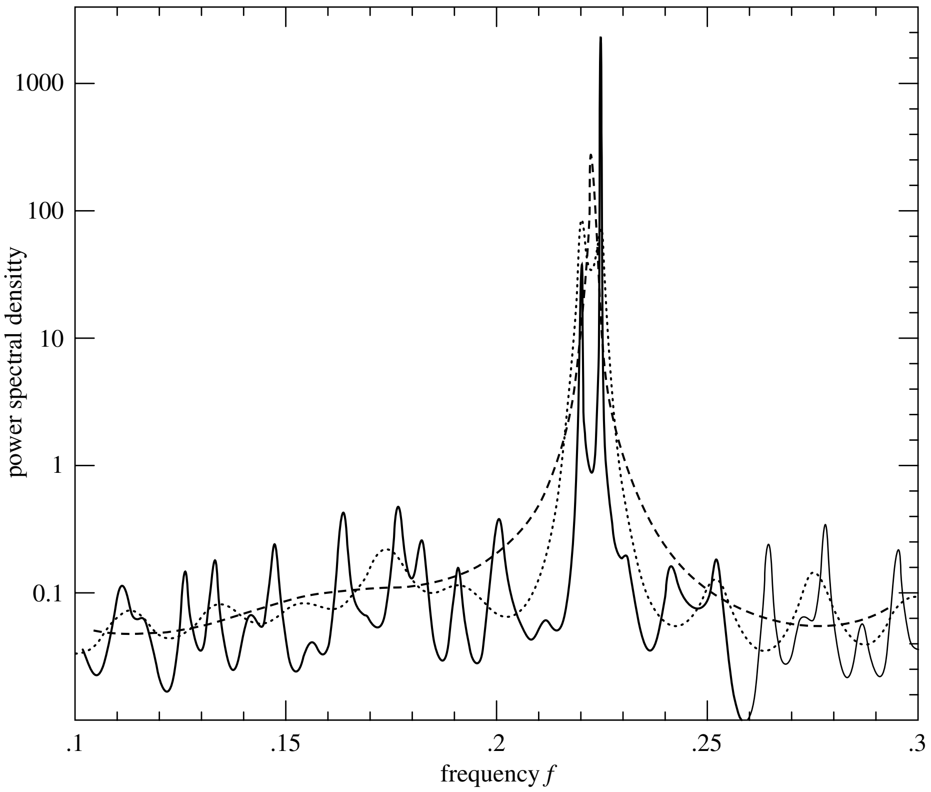

Figure 13.7.1. Sample output of maximum entropy spectral estimation. The input signal consists of 512 samples of the sum of two sinusoids of very nearly the same frequency, plus white noise with about equal power. Shown is an expanded portion of the full Nyquist frequency interval (which would extend from zero to 0.5). The dashed spectral estimate uses 20 poles; the dotted, 40; the solid, 150. With the larger number of poles, the method can resolve the distinct sinusoids, but the flat noise background is beginning to show spurious peaks. (Note logarithmic scale.)

Be sure to evaluate $P(f)$ on a fine enough grid to *find* any narrow features that may be there! Such narrow features, if present, can contain virtually all of the power in the data. You might also wish to know how the $P(f)$ produced by the routines `memcof` and `evlmem` is normalized with respect to the mean square value of the input data vector. The answer is

$$\int_{-1/2}^{1/2} P(f\Delta)d(f\Delta) = 2\int_{0}^{1/2} P(f\Delta)d(f\Delta) = \text{mean square value of data} \qquad (13.7.8)$$

Sample spectra produced by the routines `memcof` and `evlmem` are shown in Figure 13.7.1.

CITED REFERENCES AND FURTHER READING:

Childers, D.G. (ed.) 1978, *Modern Spectrum Analysis* (New York: IEEE Press), Chapter II.

Kay, S.M., and Marple, S.L. 1981, "Spectrum Analysis: A Modern Perspective," *Proceedings of the IEEE*, vol. 69, pp. 1380–1419.

13.8 Spectral Analysis of Unevenly Sampled Data

Thus far, we have been dealing exclusively with evenly sampled data,

$$h_n = h(n\Delta) \qquad n = \ldots, -3, -2, -1, 0, 1, 2, 3, \ldots \tag{13.8.1}$$

where Δ is the sampling interval, whose reciprocal is the sampling rate. Recall also (§12.1) the significance of the Nyquist critical frequency

$$f_c \equiv \frac{1}{2\Delta} \tag{13.8.2}$$

as codified by the sampling theorem: A sampled data set like equation (13.8.1) contains *complete* information about all spectral components for a signal $h(t)$ containing only frequencies below the Nyquist frequency, and scrambled or *aliased* information about any signals containing frequencies larger than the Nyquist frequency. The sampling theorem thus defines both the attractiveness and the limitation of any analysis of an evenly spaced data set.

There are situations, however, where evenly spaced data cannot be obtained. A common case is where instrumental dropouts occur, so that data are obtained only on a (not consecutive integer) subset of equation (13.8.1), the so-called *missing data* problem. Another case, common in observational sciences like astronomy, is that the observer cannot completely control the time of the observations, but must simply accept a certain dictated set of t_i's.

There are some obvious ways to get from unevenly spaced t_i's to evenly spaced ones, as in equation (13.8.1). Interpolation is one way: Lay down a grid of evenly spaced times on your data and interpolate values onto that grid; then use FFT methods. In the missing data problem, you only have to interpolate on missing data points. If a lot of consecutive points are missing, you might as well just set them to zero, or perhaps "clamp" the value at the last measured point. However, the experience of practitioners of such interpolation techniques *is not reassuring*. Generally speaking, such techniques perform poorly. Long gaps in the data, for example, often produce a spurious bulge of power at low frequencies (wavelengths comparable to gaps).

A completely different method of spectral analysis for unevenly sampled data, one that mitigates these difficulties and has some other very desirable properties, was developed by Lomb [1], based in part on earlier work by Barning [2] and Vaníček [3], and additionally elaborated by Scargle [4]. The Lomb method (as we will call it) evaluates data, and sines and cosines, only at times t_i that are actually measured. Suppose that there are N data points $h_i \equiv h(t_i)$, $i = 0, \ldots, N-1$. Then first find the mean and variance of the data by the usual formulas,

$$\bar{h} \equiv \frac{1}{N}\sum_{i=0}^{N-1} h_i \qquad \sigma^2 \equiv \frac{1}{N-1}\sum_{i=0}^{N-1}(h_i - \bar{h})^2 \tag{13.8.3}$$

Now, the Lomb *normalized periodogram* (spectral power as a function of angular frequency $\omega \equiv 2\pi f > 0$) is defined by

$$P_N(\omega) \equiv \frac{1}{2\sigma^2}\left\{ \frac{\left[\sum_j (h_j - \bar{h})\cos\omega(t_j - \tau)\right]^2}{\sum_j \cos^2\omega(t_j - \tau)} + \frac{\left[\sum_j (h_j - \bar{h})\sin\omega(t_j - \tau)\right]^2}{\sum_j \sin^2\omega(t_j - \tau)} \right\} \tag{13.8.4}$$

Here τ is defined by the relation

$$\tan(2\omega\tau) = \frac{\sum_j \sin 2\omega t_j}{\sum_j \cos 2\omega t_j} \tag{13.8.5}$$

The constant τ is a kind of offset that makes $P_N(\omega)$ completely independent of shifting all the t_i's by any constant. Lomb shows that this particular choice of offset has another, deeper, effect: It makes equation (13.8.4) identical to the equation that one would obtain if one

estimated the harmonic content of a data set, at a given frequency ω, by linear least-squares fitting to the model

$$h(t) = A\cos\omega t + B\sin\omega t \tag{13.8.6}$$

This fact gives some insight into why the method can give results superior to FFT methods: It weights the data on a "per-point" basis instead of on a "per-time interval" basis, when uneven sampling can render the latter seriously in error.

A very common occurrence is that the measured data points h_i are the sum of a periodic signal and independent (white) Gaussian noise. If we are trying to determine the presence or absence of such a periodic signal, we want to be able to give a quantitative answer to the question, "How significant is a peak in the spectrum $P_N(\omega)$?" In this question, the null hypothesis is that the data values are independent Gaussian random values. A very nice property of the Lomb normalized periodogram is that the viability of the null hypothesis can be tested fairly rigorously, as we now discuss.

The word "normalized" refers to the factor σ^2 in the denominator of equation (13.8.4). Scargle [4] shows that with this normalization, at any particular ω and *in the case of the null hypothesis*, $P_N(\omega)$ has an exponential probability distribution with unit mean. In other words, the probability that $P_N(\omega)$ will be between some positive z and $z + dz$ is $\exp(-z)dz$. It readily follows that, if we scan some M *independent* frequencies, the probability that none give values larger than z is $(1 - e^{-z})^M$. So

$$P(> z) \equiv 1 - (1 - e^{-z})^M \tag{13.8.7}$$

is the false-alarm probability of the null hypothesis, that is, the *significance level* of any peak in $P_N(\omega)$ that we do see. A small value for the false-alarm probability indicates a highly significant periodic signal.

To evaluate this significance, we need to know M. After all, the more frequencies we look at, the less significant is some one modest bump in the spectrum. (Look long enough, find anything!) A typical procedure will be to plot $P_N(\omega)$ as a function of many closely spaced frequencies in some large frequency range. How many of these are independent?

Before answering, let us first see how accurately we need to know M. The interesting region is where the significance is a small (significant) number, $\ll 1$. There, equation (13.8.7) can be series expanded to give

$$P(> z) \approx Me^{-z} \tag{13.8.8}$$

We see that the significance scales linearly with M. Practical significance levels are numbers like 0.05, 0.01, 0.001, etc. An error of even $\pm 50\%$ in the estimated significance is often tolerable, since quoted significance levels are typically spaced apart by factors of 5 or 10. So our estimate of M need not be very accurate.

Horne and Baliunas [5] give results from extensive Monte Carlo experiments for determining M in various cases. In general, M depends on the number of frequencies sampled, the number of data points N, and their detailed spacing. It turns out that M is very nearly equal to N when the data points are approximately equally spaced and when the sampled frequencies "fill" (oversample) the frequency range from 0 to the Nyquist frequency f_c (equation 13.8.2). Further, the value of M is not importantly different for random spacing of the data points than for equal spacing. When a larger frequency range than the Nyquist range is sampled, M increases proportionally. About the only case where M differs significantly from the case of evenly spaced points is when the points are closely clumped, say into groups of three; then (as one would expect) the number of independent frequencies is reduced by a factor of about 3.

The program `period`, below, calculates an effective value for M based on the above rough-and-ready rules and assumes that there is no important clumping. This will be adequate for most purposes. In any particular case, if it really matters, it is not too difficult to compute a better value of M by simple Monte Carlo: Holding fixed the number of data points and their locations t_i, generate synthetic data sets of Gaussian (normal) deviates, find the largest values of $P_N(\omega)$ for each such data set (using the accompanying program), and fit the resulting distribution for M in equation (13.8.7).

Figure 13.8.1 shows the results of applying the method as discussed so far. In the upper figure, the data points are plotted against time. Their number is $N = 100$, and their distribution in t is Poisson random. There is certainly no sinusoidal signal evident to the eye. The

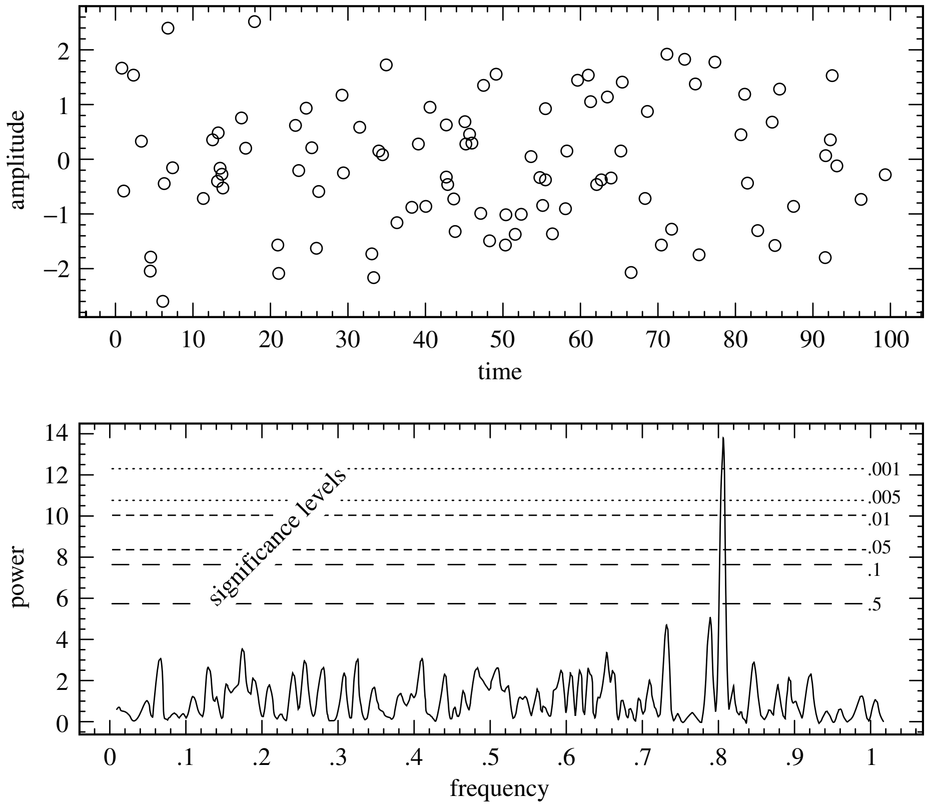

Figure 13.8.1. Example of the Lomb algorithm in action. The 100 data points (upper figure) are at random times between 0 and 100. Their sinusoidal component is readily uncovered (lower figure) by the algorithm, at a significance level $p < 0.001$. If the 100 data points had been evenly spaced at unit interval, the Nyquist critical frequency would have been 0.5. Note that, for these unevenly spaced points, there is no visible aliasing into the Nyquist range.

lower figure plots $P_N(\omega)$ against frequency $f = \omega/2\pi$. The Nyquist critical frequency that would obtain if the points were evenly spaced is at $f = f_c = 0.5$. Since we have searched up to about twice that frequency, and oversampled the f's to the point where successive values of $P_N(\omega)$ vary smoothly, we take $M = 2N$. The horizontal dashed and dotted lines are (respectively from bottom to top) significance levels 0.5, 0.1, 0.05, 0.01, 0.005, and 0.001. One sees a highly significant peak at a frequency of 0.81. That is in fact the frequency of the sine wave that is present in the data. (You will have to take our word for this!)

Note that two other peaks approach but do not exceed the 50% significance level; that is about what one might expect by chance. It is also worth commenting on the fact that the significant peak was found (correctly) *above the Nyquist frequency* and without any significant aliasing down into the Nyquist interval! That would not be possible for evenly spaced data. It is possible here because the randomly spaced data have *some* points spaced much closer than the "average" sampling rate, and these remove ambiguity from any aliasing.

Implementation of the normalized periodogram in code is straightforward, with, however, a few points to be kept in mind. We are dealing with a *slow* algorithm. Typically, for N data points, we may wish to examine on the order of $2N$ or $4N$ frequencies. Each combination of frequency and data point has, in equations (13.8.4) and (13.8.5), not just a few adds or multiplies, but four calls to trigonometric functions; the operations count can easily reach several hundred times N^2. It is highly desirable — in fact results in a factor 4 speedup — to replace these trigonometric calls by recurrences. That is possible only if the sequence of

frequencies examined is a linear sequence. Since such a sequence is probably what most users would want anyway, we have built this into the implementation.

At the end of this section we describe a way to evaluate equations (13.8.4) and (13.8.5) — approximately, but to any desired degree of approximation — by a fast method [6] whose operation count goes only as $N \log N$. This faster method should be used for long data sets.

The lowest independent frequency f to be examined is the inverse of the span of the input data, $\max_i(t_i) - \min_i(t_i) \equiv T$. This is the frequency such that the data can include one complete cycle. In subtracting off the data's mean, equation (13.8.4) already assumed that you are not interested in the data's zero frequency piece — which is just that mean value. In an FFT method, higher independent frequencies would be integer multiples of $1/T$. Because we are interested in the statistical significance of any peak that may occur, however, we had better (over-)sample more finely than at interval $1/T$, so that sample points lie close to the top of any peak. Thus, the accompanying program includes an oversampling parameter, called `ofac`; a value `ofac` $\gtrsim 4$ might be typical in use. We also want to specify how high in frequency to go, say f_{hi}. One guide to choosing f_{hi} is to compare it with the Nyquist frequency f_c that would obtain if the N data points were evenly spaced over the same span T, that is, $f_c = N/(2T)$. The accompanying program includes an input parameter `hifac`, defined as f_{hi}/f_c. The number of different frequencies N_P returned by the program is then given by

$$N_P = \frac{\mathtt{ofac} \times \mathtt{hifac}}{2} N \tag{13.8.9}$$

(You have to remember to dimension the output arrays to at least this size.)

The trigonometric recurrences should be done in double precision even if you convert the rest of the routine to single precision. The code embodies a few tricks with trigonometric identities, to decrease roundoff errors. If you are an aficionado of such things, you can puzzle it out. A final detail is that equation (13.8.7) will fail because of roundoff error if z is too large; but equation (13.8.8) is fine in this regime.

period.h

```
void period(VecDoub_I &x, VecDoub_I &y, const Doub ofac, const Doub hifac,
    VecDoub_O &px, VecDoub_O &py, Int &nout, Int &jmax, Doub &prob) {
```

Given `n` data points with abscissas `x[0..n-1]` (which need not be equally spaced) and ordinates `y[0..n-1]`, and given a desired oversampling factor `ofac` (a typical value being 4 or larger), this routine fills array `px[0..nout-1]` with an increasing sequence of frequencies (not angular frequencies) up to `hifac` times the "average" Nyquist frequency, and fills array `py[0..nout-1]` with the values of the Lomb normalized periodogram at those frequencies. The arrays `x` and `y` are not altered. The vectors `px` and `py` are resized to `nout` (eq. 13.8.9) if their initial size is less than this; otherwise, only their first `nout` components are filled. The routine also returns `jmax` such that `py[jmax]` is the maximum element in `py`, and `prob`, an estimate of the significance of that maximum against the hypothesis of random noise. A small value of `prob` indicates that a significant periodic signal is present.

```
    const Doub TWOPI=6.283185307179586476;
    Int i,j,n=x.size(),np=px.size();
    Doub ave,c,cc,cwtau,effm,expy,pnow,pymax,s,ss,sumc,sumcy,sums,sumsh,
        sumsy,swtau,var,wtau,xave,xdif,xmax,xmin,yy,arg,wtemp;
    VecDoub wi(n),wpi(n),wpr(n),wr(n);
    nout=Int(0.5*ofac*hifac*n);
    if (np < nout) {px.resize(nout); py.resize(nout);}
    avevar(y,ave,var);                          Get mean and variance of the input data.
    if (var == 0.0) throw("zero variance in period");
    xmax=xmin=x[0];                             Go through data to get the range of abscis-
    for (j=0;j<n;j++) {                             sas.
        if (x[j] > xmax) xmax=x[j];
        if (x[j] < xmin) xmin=x[j];
    }
    xdif=xmax-xmin;
    xave=0.5*(xmax+xmin);
    pymax=0.0;
    pnow=1.0/(xdif*ofac);                       Starting frequency.
    for (j=0;j<n;j++) {                         Initialize values for the trigonometric recur-
        arg=TWOPI*((x[j]-xave)*pnow);               rences at each data point.
        wpr[j]= -2.0*SQR(sin(0.5*arg));
```

```
        wpi[j]=sin(arg);
        wr[j]=cos(arg);
        wi[j]=wpi[j];
    }
    for (i=0;i<nout;i++) {                  Main loop over the frequencies to be evalu-
        px[i]=pnow;                             ated.
        sumsh=sumc=0.0;                     First, loop over the data to get τ and related
        for (j=0;j<n;j++) {                     quantities.
            c=wr[j];
            s=wi[j];
            sumsh += s*c;
            sumc += (c-s)*(c+s);
        }
        wtau=0.5*atan2(2.0*sumsh,sumc);
        swtau=sin(wtau);
        cwtau=cos(wtau);
        sums=sumc=sumsy=sumcy=0.0;          Then, loop over the data again to get the
        for (j=0;j<n;j++) {                     periodogram value.
            s=wi[j];
            c=wr[j];
            ss=s*cwtau-c*swtau;
            cc=c*cwtau+s*swtau;
            sums += ss*ss;
            sumc += cc*cc;
            yy=y[j]-ave;
            sumsy += yy*ss;
            sumcy += yy*cc;
            wr[j]=((wtemp=wr[j])*wpr[j]-wi[j]*wpi[j])+wr[j];    Update the trigono-
            wi[j]=(wi[j]*wpr[j]+wtemp*wpi[j])+wi[j];             metric recurrences.
        }
        py[i]=0.5*(sumcy*sumcy/sumc+sumsy*sumsy/sums)/var;
        if (py[i] >= pymax) pymax=py[jmax=i];
        pnow += 1.0/(ofac*xdif);            The next frequency.
    }
    expy=exp(-pymax);                       Evaluate statistical significance of the max-
    effm=2.0*nout/ofac;                         imum.
    prob=effm*expy;
    if (prob > 0.01) prob=1.0-pow(1.0-expy,effm);
}
```

13.8.1 Fast Computation of the Lomb Periodogram

We here show how equations (13.8.4) and (13.8.5) can be calculated — approximately, but to any desired precision — with an operation count only of order $N_P \log N_P$. The method uses the FFT, but it is in no sense an FFT periodogram of the data. It is an actual evaluation of equations (13.8.4) and (13.8.5), the Lomb normalized periodogram, with exactly that method's strengths and weaknesses. This fast algorithm, due to Press and Rybicki [6], makes feasible the application of the Lomb method to data sets at least as large as 10^6 points; it is already faster than straightforward evaluation of equations (13.8.4) and (13.8.5) for data sets as small as 60 or 100 points.

Notice that the trigonometric sums that occur in equations (13.8.5) and (13.8.4) can be reduced to four simpler sums. If we define

$$S_h \equiv \sum_{j=0}^{N-1} (h_j - \bar{h}) \sin(\omega t_j) \qquad C_h \equiv \sum_{j=0}^{N-1} (h_j - \bar{h}) \cos(\omega t_j) \tag{13.8.10}$$

and

$$S_2 \equiv \sum_{j=0}^{N-1} \sin(2\omega t_j) \qquad C_2 \equiv \sum_{j=0}^{N-1} \cos(2\omega t_j) \tag{13.8.11}$$

then

$$\begin{aligned}
\sum_{j=0}^{N-1}(h_j-\bar{h})\cos\omega(t_j-\tau) &= C_h\cos\omega\tau + S_h\sin\omega\tau \\
\sum_{j=0}^{N-1}(h_j-\bar{h})\sin\omega(t_j-\tau) &= S_h\cos\omega\tau - C_h\sin\omega\tau \\
\sum_{j=0}^{N-1}\cos^2\omega(t_j-\tau) &= \frac{N}{2}+\frac{1}{2}C_2\cos(2\omega\tau)+\frac{1}{2}S_2\sin(2\omega\tau) \\
\sum_{j=0}^{N-1}\sin^2\omega(t_j-\tau) &= \frac{N}{2}-\frac{1}{2}C_2\cos(2\omega\tau)-\frac{1}{2}S_2\sin(2\omega\tau)
\end{aligned} \tag{13.8.12}$$

Now notice that *if* the t_js *were* evenly spaced, then the four quantities S_h, C_h, S_2, and C_2 could be evaluated by two complex FFTs, and the results could then be substituted back through equation (13.8.12) to evaluate equations (13.8.5) and (13.8.4). The problem is therefore only to evaluate equations (13.8.10) and (13.8.11) for unevenly spaced data.

Interpolation, or rather reverse interpolation — we will here call it *extirpolation* — provides the key. Interpolation, as classically understood, uses several function values on a regular mesh to construct an accurate approximation at an arbitrary point. Extirpolation, just the opposite, *replaces* a function value at an arbitrary point by several function values on a regular mesh, doing this in such a way that sums over the mesh are an accurate approximation to sums over the original arbitrary point.

It is not hard to see that the weight functions for extirpolation are identical to those for interpolation. Suppose that the function $h(t)$ to be extirpolated is known only at the discrete (unevenly spaced) points $h(t_i) \equiv h_i$, and that the function $g(t)$ (which will be, e.g., $\cos\omega t$) can be evaluated anywhere. Let $\hat{t}_k$ be a sequence of evenly spaced points on a regular mesh. Then Lagrange interpolation (§3.2) gives an approximation of the form

$$g(t) \approx \sum_k w_k(t)g(\hat{t}_k) \tag{13.8.13}$$

where $w_k(t)$ are interpolation weights. Now let us evaluate a sum of interest by the following scheme:

$$\sum_{j=0}^{N-1} h_j g(t_j) \approx \sum_{j=0}^{N-1} h_j\left[\sum_k w_k(t_j)g(\hat{t}_k)\right] = \sum_k\left[\sum_{j=0}^{N-1} h_j w_k(t_j)\right]g(\hat{t}_k) \equiv \sum_k \hat{h}_k\, g(\hat{t}_k) \tag{13.8.14}$$

Here $\hat{h}_k \equiv \sum_j h_j w_k(t_j)$. Notice that equation (13.8.14) replaces the original sum by one on the regular mesh. Notice also that the accuracy of equation (13.8.13) depends only on the fineness of the mesh with respect to the function g and has nothing to do with the spacing of the points t_j or the function h; therefore, the accuracy of equation (13.8.14) also has this property.

The general outline of the fast evaluation method is therefore this: (i) Choose a mesh size large enough to accommodate some desired oversampling factor, and large enough to have several extirpolation points per half-wavelength of the highest frequency of interest. (ii) Extirpolate the values h_i onto the mesh and take the FFT; this gives S_h and C_h in equation (13.8.10). (iii) Extirpolate the constant values 1 onto another mesh, and take its FFT; this, with some manipulation, gives S_2 and C_2 in equation (13.8.11). (iv) Evaluate equations (13.8.12), (13.8.5), and (13.8.4), in that order.

There are several other tricks involved in implementing this algorithm efficiently. You can figure most out from the code, but we will mention the following points: (a) A nice way to get transform values at frequencies 2ω instead of ω is to stretch the time-domain data by a factor 2, and then wrap it to double-cover the original length. (This trick goes back to Tukey.) In the program, this appears as a modulo function. (b) Trigonometric identities are used to get from the left-hand side of equation (13.8.5) to the various needed trigonometric functions

of $\omega\tau$. C++ identifiers like, e.g., cwt and hs2wt represent quantities like, e.g., $\cos\omega\tau$ and $\frac{1}{2}\sin(2\omega\tau)$. (c) The function spread does extirpolation onto the M most nearly centered mesh points around an arbitrary point; its turgid code evaluates coefficients of the Lagrange interpolating polynomials, in an efficient manner.

fasper.h

```
void fasper(VecDoub_I &x, VecDoub_I &y, const Doub ofac, const Doub hifac,
    VecDoub_O &px, VecDoub_O &py, Int &nout, Int &jmax, Doub &prob) {
```

Given n data points with abscissas x[0..n-1] (which need not be equally spaced) and ordinates y[0..n-1], and given a desired oversampling factor ofac (a typical value being 4 or larger), this routine fills array px[0..nout-1] with an increasing sequence of frequencies (not angular frequencies) up to hifac times the “average” Nyquist frequency, and fills array py[0..nout-1] with the values of the Lomb normalized periodogram at those frequencies. The arrays x and y are not altered. The vectors px and py are resized to nout (eq. 13.8.9) if their initial size is less than this; otherwise, only their first nout components are filled. The routine also returns jmax such that py[jmax] is the maximum element in py, and prob, an estimate of the significance of that maximum against the hypothesis of random noise. A small value of prob indicates that a significant periodic signal is present.

```
    const Int MACC=4;
    Int j,k,nwk,nfreq,nfreqt,n=x.size(),np=px.size();
    Doub ave,ck,ckk,cterm,cwt,den,df,effm,expy,fac,fndim,hc2wt,hs2wt,
        hypo,pmax,sterm,swt,var,xdif,xmax,xmin;
    nout=Int(0.5*ofac*hifac*n);
    nfreqt=Int(ofac*hifac*n*MACC);              Size the FFT as next power of 2 above
    nfreq=64;                                        nfreqt.
    while (nfreq < nfreqt) nfreq <<= 1;
    nwk=nfreq << 1;
    if (np < nout) {px.resize(nout); py.resize(nout);}
    avevar(y,ave,var);                           Compute the mean, variance, and range
    if (var == 0.0) throw("zero variance in fasper");     of the data.
    xmin=x[0];
    xmax=xmin;
    for (j=1;j<n;j++) {
        if (x[j] < xmin) xmin=x[j];
        if (x[j] > xmax) xmax=x[j];
    }
    xdif=xmax-xmin;
    VecDoub wk1(nwk,0.);                         Zero the workspaces.
    VecDoub wk2(nwk,0.);
    fac=nwk/(xdif*ofac);
    fndim=nwk;
    for (j=0;j<n;j++) {                          Extirpolate the data into the workspaces.
        ck=fmod((x[j]-xmin)*fac,fndim);
        ckk=2.0*(ck++);
        ckk=fmod(ckk,fndim);
        ++ckk;
        spread(y[j]-ave,wk1,ck,MACC);
        spread(1.0,wk2,ckk,MACC);
    }
    realft(wk1,1);                               Take the Fast Fourier Transforms.
    realft(wk2,1);
    df=1.0/(xdif*ofac);
    pmax = -1.0;
    for (k=2,j=0;j<nout;j++,k+=2) {              Compute the Lomb value for each fre-
        hypo=sqrt(wk2[k]*wk2[k]+wk2[k+1]*wk2[k+1]);       quency.
        hc2wt=0.5*wk2[k]/hypo;
        hs2wt=0.5*wk2[k+1]/hypo;
        cwt=sqrt(0.5+hc2wt);
        swt=SIGN(sqrt(0.5-hc2wt),hs2wt);
        den=0.5*n+hc2wt*wk2[k]+hs2wt*wk2[k+1];
        cterm=SQR(cwt*wk1[k]+swt*wk1[k+1])/den;
        sterm=SQR(cwt*wk1[k+1]-swt*wk1[k])/(n-den);
        px[j]=(j+1)*df;
        py[j]=(cterm+sterm)/(2.0*var);
```

```
        if (py[j] > pmax) pmax=py[jmax=j];
    }
    expy=exp(-pmax);                              Estimate significance of largest peak value.
    effm=2.0*nout/ofac;
    prob=effm*expy;
    if (prob > 0.01) prob=1.0-pow(1.0-expy,effm);
}
```

fasper.h

```
void spread(const Doub y, VecDoub_IO &yy, const Doub x, const Int m) {
```

Given an array yy[0..n-1], extirpolate (spread) a value y into m actual array elements that best approximate the "fictional" (i.e., possibly noninteger) array element number x. The weights used are coefficients of the Lagrange interpolating polynomial.

```
    static Int nfac[11]={0,1,1,2,6,24,120,720,5040,40320,362880};
    Int ihi,ilo,ix,j,nden,n=yy.size();
    Doub fac;
    if (m > 10) throw("factorial table too small in spread");
    ix=Int(x);
    if (x == Doub(ix)) yy[ix-1] += y;
    else {
        ilo=MIN(MAX(Int(x-0.5*m),0),Int(n-m));
        ihi=ilo+m;
        nden=nfac[m];
        fac=x-ilo-1;
        for (j=ilo+1;j<ihi;j++) fac *= (x-j-1);
        yy[ihi-1] += y*fac/(nden*(x-ihi));
        for (j=ihi-1;j>ilo;j--) {
            nden=(nden/(j-ilo))*(j-ihi);
            yy[j-1] += y*fac/(nden*(x-j));
        }
    }
}
```

CITED REFERENCES AND FURTHER READING:

Lomb, N.R. 1976, "Least-Squares Frequency Analysis of Unequally Spaced Data," *Astrophysics and Space Science*, vol. 39, pp. 447–462.[1]

Barning, F.J.M. 1963, "The Numerical Analysis of the Light-Curve of 12 Lacertae," *Bulletin of the Astronomical Institutes of the Netherlands*, vol. 17, pp. 22–28.[2]

Vaníček, P. 1971, "Further Development and Properties of the Spectral Analysis by Least Squares," *Astrophysics and Space Science*, vol. 12, pp. 10–33.[3]

Scargle, J.D. 1982, "Studies in Astronomical Time Series Analysis II. Statistical Aspects of Spectral Analysis of Unevenly Sampled Data," *Astrophysical Journal*, vol. 263, pp. 835–853.[4]

Horne, J.H., and Baliunas, S.L. 1986, "A Prescription for Period Analysis of Unevenly Sampled Time Series," *Astrophysical Journal*, vol. 302, pp. 757–763.[5]

Press, W.H. and Rybicki, G.B. 1989, "Fast Algorithm for Spectral Analysis of Unevenly Sampled Data," *Astrophysical Journal*, vol. 338, pp. 277–280.[6]

13.9 Computing Fourier Integrals Using the FFT

Not uncommonly, one wants to calculate accurate numerical values for integrals of the form

$$I = \int_a^b e^{i\omega t} h(t) dt \ , \tag{13.9.1}$$

or the equivalent real and imaginary parts

$$I_c = \int_a^b \cos(\omega t) h(t) dt \qquad I_s = \int_a^b \sin(\omega t) h(t) dt \ , \tag{13.9.2}$$

and one wants to evaluate this integral for many different values of ω. In cases of interest, $h(t)$ is often a smooth function, but it is not necessarily periodic in $[a, b]$, nor does it necessarily go to zero at a or b. While it seems intuitively obvious that the *force majeure* of the FFT ought to be applicable to this problem, doing so turns out to be a surprisingly subtle matter, as we will now see.

Let us first approach the problem naively, to see where the difficulty lies. Divide the interval $[a, b]$ into M subintervals, where M is a large integer, and define

$$\Delta \equiv \frac{b-a}{M}, \quad t_j \equiv a + j\Delta, \quad h_j \equiv h(t_j), \quad j = 0, \ldots, M \tag{13.9.3}$$

Notice that $h_0 = h(a)$ and $h_M = h(b)$, and that there are $M + 1$ values h_j. We can approximate the integral I by a sum,

$$I \approx \Delta \sum_{j=0}^{M-1} h_j \exp(i\omega t_j) \tag{13.9.4}$$

which is at any rate first-order accurate. (If we centered the h_j's and the t_j's in the intervals, we could be accurate to second order.) Now, for certain values of ω and M, the sum in equation (13.9.4) can be made into a discrete Fourier transform, or DFT, and evaluated by the fast Fourier transform (FFT) algorithm. In particular, we can choose M to be an integer power of 2 and define a set of special ω's by

$$\omega_m \Delta \equiv \frac{2\pi m}{M} \tag{13.9.5}$$

where m has the values $m = 0, 1, \ldots, M/2 - 1$. Then equation (13.9.4) becomes

$$I(\omega_m) \approx \Delta e^{i\omega_m a} \sum_{j=0}^{M-1} h_j e^{2\pi i m j/M} = \Delta e^{i\omega_m a} [\text{DFT}(h_0 \ldots h_{M-1})]_m \tag{13.9.6}$$

Equation (13.9.6), while simple and clear, is emphatically *not recommended* for use: It is likely to give wrong answers!

The problem lies in the oscillatory nature of the integral (13.9.1). If $h(t)$ is at all smooth, and if ω is large enough to imply several cycles in the interval $[a, b]$ — in fact, ω_m in equation (13.9.5) gives exactly m cycles — then the value of I is typically very small, so small that it is easily swamped by first-order, or even (with centered values) second-order, truncation error. Furthermore, the characteristic "small parameter" that occurs in the error term is not $\Delta/(b-a) = 1/M$, as it would be if the integrand were not oscillatory, but $\omega\Delta$, which can be as large as π for ω's within the Nyquist interval of the DFT (cf. equation 13.9.5). The result is that equation (13.9.6) becomes systematically inaccurate as ω increases.

It is a sobering exercise to implement equation (13.9.6) for an integral that can be done analytically and to see just how bad it is. We recommend that you try it.

Let us therefore turn to a more sophisticated treatment. Given the sampled points h_j, we can approximate the function $h(t)$ everywhere in the interval $[a, b]$ by interpolation on nearby h_j's. The simplest case is linear interpolation, using the two nearest h_j's, one to the left and one to the right. A higher-order interpolation, e.g., would be cubic interpolation, using two points to the left and two to the right — except in the first and last subintervals, where we must interpolate with three h_j's on one side, one on the other.

The formulas for such interpolation schemes are (piecewise) polynomial in the independent variable t, but with coefficients that are of course linear in the function values h_j.

Although one does not usually think of it in this way, interpolation can be viewed as approximating a function by a sum of kernel functions (which depend only on the interpolation scheme) times sample values (which depend only on the function). Let us write

$$h(t) \approx \sum_{j=0}^{M} h_j \, \psi\left(\frac{t-t_j}{\Delta}\right) + \sum_{j=\text{endpoints}} h_j \, \varphi_j\left(\frac{t-t_j}{\Delta}\right) \tag{13.9.7}$$

Here $\psi(s)$ is the kernel function of an interior point: It is zero for s sufficiently negative or sufficiently positive and becomes nonzero only when s is in the range where the h_j multiplying it is actually used in the interpolation. We always have $\psi(0) = 1$ and $\psi(m) = 0$, $m = \pm1, \pm2, \dots$, since interpolation right on a sample point should give the sampled function value. For linear interpolation, $\psi(s)$ is piecewise linear, rises from 0 to 1 for s in $(-1, 0)$, and falls back to 0 for s in $(0, 1)$. For higher-order interpolation, $\psi(s)$ is made up piecewise of segments of Lagrange interpolation polynomials. It has discontinuous derivatives at integer values of s, where the pieces join, because the set of points used in the interpolation changes discretely.

As already remarked, the subintervals closest to a and b require different (noncentered) interpolation formulas. This is reflected in equation (13.9.7) by the second sum, with the special endpoint kernels $\varphi_j(s)$. Actually, for reasons that will become clearer below, we have included *all* the points in the *first* sum (with kernel ψ), so the φ_j's are actually differences between true endpoint kernels and the interior kernel ψ. It is a tedious, but straightforward, exercise to write down all the $\varphi_j(s)$'s for any particular order of interpolation, each one consisting of differences of Lagrange interpolating polynomials spliced together piecewise.

Now apply the integral operator $\int_a^b dt \exp(i\omega t)$ to both sides of equation (13.9.7), interchange the sums and integral, and make the changes of variable $s = (t - t_j)/\Delta$ in the first sum and $s = (t - a)/\Delta$ in the second sum. The result is

$$I \approx \Delta e^{i\omega a}\left[W(\theta) \sum_{j=0}^{M} h_j e^{ij\theta} + \sum_{j=\text{endpoints}} h_j \alpha_j(\theta)\right] \tag{13.9.8}$$

Here $\theta \equiv \omega\Delta$, and the functions $W(\theta)$ and $\alpha_j(\theta)$ are defined by

$$W(\theta) \equiv \int_{-\infty}^{\infty} ds \, e^{i\theta s} \psi(s) \tag{13.9.9}$$

$$\alpha_j(\theta) \equiv \int_{-\infty}^{\infty} ds \, e^{i\theta s} \varphi_j(s - j) \tag{13.9.10}$$

The key point is that equations (13.9.9) and (13.9.10) can be evaluated, analytically, once and for all, for any given interpolation scheme. Then equation (13.9.8) is an algorithm for applying "endpoint corrections" to a sum that (as we will see) can be done using the FFT, giving a result with high-order accuracy.

We will consider only interpolations that are left-right symmetric. Then symmetry implies

$$\varphi_{M-j}(s) = \varphi_j(-s) \qquad \alpha_{M-j}(\theta) = e^{i\theta M} \alpha_j^*(\theta) = e^{i\omega(b-a)} \alpha_j^*(\theta) \tag{13.9.11}$$

where $*$ denotes complex conjugation. Also, $\psi(s) = \psi(-s)$ implies that $W(\theta)$ is real.

Turn now to the first sum in equation (13.9.8), which we want to do by FFT methods. To do so, choose some N that is an integer power of 2 with $N \geq M+1$. (Note that M need not be a power of 2, so $M = N-1$ is allowed.) If $N > M+1$, define $h_j \equiv 0$, $M+1 < j \leq N-1$, i.e., "zero-pad" the array of h_j's so that j takes on the range $0 \leq j \leq N-1$. Then the sum can be done as a DFT for the special values $\omega = \omega_n$ given by

$$\omega_n \Delta \equiv \frac{2\pi n}{N} \equiv \theta \qquad n = 0, 1, \dots, \frac{N}{2} - 1 \tag{13.9.12}$$

For fixed M, the larger N is chosen, the finer the sampling in frequency space. The value M, on the other hand, determines the *highest* frequency sampled, since Δ decreases with increasing M (equation 13.9.3), and the largest value of $\omega\Delta$ is always just under π (equation 13.9.12). In general it is advantageous to oversample by *at least* a factor of 4, i.e., $N > 4M$ (see below). We can now rewrite equation (13.9.8) in its final form as

$$\begin{aligned} I(\omega_n) = \Delta e^{i\omega_n a}\Big\{ & W(\theta)[\text{DFT}(h_0 \dots h_{N-1})]_n \\ & + \alpha_0(\theta)h_0 + \alpha_1(\theta)h_1 + \alpha_2(\theta)h_2 + \alpha_3(\theta)h_3 + \dots \\ & + e^{i\omega(b-a)}\left[\alpha_0^*(\theta)h_M + \alpha_1^*(\theta)h_{M-1} + \alpha_2^*(\theta)h_{M-2} + \alpha_3^*(\theta)h_{M-3} + \dots\right]\Big\} \end{aligned} \tag{13.9.13}$$

For cubic (or lower) polynomial interpolation, at most the terms explicitly shown above are nonzero; the ellipses (...) can therefore be ignored, and we need explicit forms only for the functions $W, \alpha_0, \alpha_1, \alpha_2, \alpha_3$, calculated with equations (13.9.9) and (13.9.10). We have worked these out for you, in the trapezoidal (second-order) and cubic (fourth-order) cases. Here are the results, along with the first few terms of their power series expansions for small θ:

Trapezoidal order:

$$W(\theta) = \frac{2(1-\cos\theta)}{\theta^2} \approx 1 - \frac{1}{12}\theta^2 + \frac{1}{360}\theta^4 - \frac{1}{20160}\theta^6$$

$$\begin{aligned} \alpha_0(\theta) &= -\frac{(1-\cos\theta)}{\theta^2} + i\frac{(\theta - \sin\theta)}{\theta^2} \\ &\approx -\frac{1}{2} + \frac{1}{24}\theta^2 - \frac{1}{720}\theta^4 + \frac{1}{40320}\theta^6 + i\theta\left(\frac{1}{6} - \frac{1}{120}\theta^2 + \frac{1}{5040}\theta^4 - \frac{1}{362880}\theta^6\right) \end{aligned}$$

$$\alpha_1 = \alpha_2 = \alpha_3 = 0$$

Cubic order:

$$W(\theta) = \left(\frac{6+\theta^2}{3\theta^4}\right)(3 - 4\cos\theta + \cos 2\theta) \approx 1 - \frac{11}{720}\theta^4 + \frac{23}{15120}\theta^6$$

$$\begin{aligned} \alpha_0(\theta) &= \frac{(-42+5\theta^2) + (6+\theta^2)(8\cos\theta - \cos 2\theta)}{6\theta^4} + i\frac{(-12\theta + 6\theta^3) + (6+\theta^2)\sin 2\theta}{6\theta^4} \\ &\approx -\frac{2}{3} + \frac{1}{45}\theta^2 + \frac{103}{15120}\theta^4 - \frac{169}{226800}\theta^6 + i\theta\left(\frac{2}{45} + \frac{2}{105}\theta^2 - \frac{8}{2835}\theta^4 + \frac{86}{467775}\theta^6\right) \end{aligned}$$

$$\begin{aligned} \alpha_1(\theta) &= \frac{14(3-\theta^2) - 7(6+\theta^2)\cos\theta}{6\theta^4} + i\frac{30\theta - 5(6+\theta^2)\sin\theta}{6\theta^4} \\ &\approx \frac{7}{24} - \frac{7}{180}\theta^2 + \frac{5}{3456}\theta^4 - \frac{7}{259200}\theta^6 + i\theta\left(\frac{7}{72} - \frac{1}{168}\theta^2 + \frac{11}{72576}\theta^4 - \frac{13}{5987520}\theta^6\right) \end{aligned}$$

$$\begin{aligned} \alpha_2(\theta) &= \frac{-4(3-\theta^2) + 2(6+\theta^2)\cos\theta}{3\theta^4} + i\frac{-12\theta + 2(6+\theta^2)\sin\theta}{3\theta^4} \\ &\approx -\frac{1}{6} + \frac{1}{45}\theta^2 - \frac{5}{6048}\theta^4 + \frac{1}{64800}\theta^6 + i\theta\left(-\frac{7}{90} + \frac{1}{210}\theta^2 - \frac{11}{90720}\theta^4 + \frac{13}{7484400}\theta^6\right) \end{aligned}$$

$$\begin{aligned} \alpha_3(\theta) &= \frac{2(3-\theta^2) - (6+\theta^2)\cos\theta}{6\theta^4} + i\frac{6\theta - (6+\theta^2)\sin\theta}{6\theta^4} \\ &\approx \frac{1}{24} - \frac{1}{180}\theta^2 + \frac{5}{24192}\theta^4 - \frac{1}{259200}\theta^6 + i\theta\left(\frac{7}{360} - \frac{1}{840}\theta^2 + \frac{11}{362880}\theta^4 - \frac{13}{29937600}\theta^6\right) \end{aligned}$$

The program dftcor, below, implements the endpoint corrections for the cubic case. Given input values of ω, Δ, a, b, and an array with the eight values $h_0, \ldots, h_3, h_{M-3}, \ldots, h_M$, it returns the real and imaginary parts of the endpoint corrections in equation (13.9.13), and the factor $W(\theta)$. The code is turgid, but only because the formulas above are complicated. The formulas have cancellations to high powers of θ. It is therefore necessary to compute the right-hand sides in double precision, even when the corrections are desired only to single precision. It is also necessary to use the series expansion for small values of θ. The optimal cross-over value of θ depends on your machine's wordlength, but you can always find it experimentally as the largest value where the two methods give identical results to machine precision.

dftintegrate.h

```
void dftcor(const Doub w, const Doub delta, const Doub a, const Doub b,
    VecDoub_I &endpts, Doub &corre, Doub &corim, Doub &corfac) {
```

For an integral approximated by a discrete Fourier transform, this routine computes the correction factor that multiplies the DFT and the endpoint correction to be added. Input is the angular frequency w, stepsize delta, lower and upper limits of the integral a and b, while the array endpts contains the first 4 and last 4 function values. The correction factor $W(\theta)$ is returned as corfac, while the real and imaginary parts of the endpoint correction are returned as corre and corim.

```
    Doub a0i,a0r,a1i,a1r,a2i,a2r,a3i,a3r,arg,c,cl,cr,s,sl,sr,t,t2,t4,t6,
        cth,ctth,spth2,sth,sth4i,stth,th,th2,th4,tmth2,tth4i;
    th=w*delta;
    if (a >= b || th < 0.0e0 || th > 3.1416e0)
        throw("bad arguments to dftcor");
    if (abs(th) < 5.0e-2) {                 Use series.
        t=th;
        t2=t*t;
        t4=t2*t2;
        t6=t4*t2;
        corfac=1.0-(11.0/720.0)*t4+(23.0/15120.0)*t6;
        a0r=(-2.0/3.0)+t2/45.0+(103.0/15120.0)*t4-(169.0/226800.0)*t6;
        a1r=(7.0/24.0)-(7.0/180.0)*t2+(5.0/3456.0)*t4-(7.0/259200.0)*t6;
        a2r=(-1.0/6.0)+t2/45.0-(5.0/6048.0)*t4+t6/64800.0;
        a3r=(1.0/24.0)-t2/180.0+(5.0/24192.0)*t4-t6/259200.0;
        a0i=t*(2.0/45.0+(2.0/105.0)*t2-(8.0/2835.0)*t4+(86.0/467775.0)*t6);
        a1i=t*(7.0/72.0-t2/168.0+(11.0/72576.0)*t4-(13.0/5987520.0)*t6);
        a2i=t*(-7.0/90.0+t2/210.0-(11.0/90720.0)*t4+(13.0/7484400.0)*t6);
        a3i=t*(7.0/360.0-t2/840.0+(11.0/362880.0)*t4-(13.0/29937600.0)*t6);
    } else {                                Use trigonometric formulas.
        cth=cos(th);
        sth=sin(th);
        ctth=cth*cth-sth*sth;
        stth=2.0e0*sth*cth;
        th2=th*th;
        th4=th2*th2;
        tmth2=3.0e0-th2;
        spth2=6.0e0+th2;
        sth4i=1.0/(6.0e0*th4);
        tth4i=2.0e0*sth4i;
        corfac=tth4i*spth2*(3.0e0-4.0e0*cth+ctth);
        a0r=sth4i*(-42.0e0+5.0e0*th2+spth2*(8.0e0*cth-ctth));
        a0i=sth4i*(th*(-12.0e0+6.0e0*th2)+spth2*stth);
        a1r=sth4i*(14.0e0*tmth2-7.0e0*spth2*cth);
        a1i=sth4i*(30.0e0*th-5.0e0*spth2*sth);
        a2r=tth4i*(-4.0e0*tmth2+2.0e0*spth2*cth);
        a2i=tth4i*(-12.0e0*th+2.0e0*spth2*sth);
        a3r=sth4i*(2.0e0*tmth2-spth2*cth);
        a3i=sth4i*(6.0e0*th-spth2*sth);
    }
    cl=a0r*endpts[0]+a1r*endpts[1]+a2r*endpts[2]+a3r*endpts[3];
    sl=a0i*endpts[0]+a1i*endpts[1]+a2i*endpts[2]+a3i*endpts[3];
    cr=a0r*endpts[7]+a1r*endpts[6]+a2r*endpts[5]+a3r*endpts[4];
    sr= -a0i*endpts[7]-a1i*endpts[6]-a2i*endpts[5]-a3i*endpts[4];
```

```
    arg=w*(b-a);
    c=cos(arg);
    s=sin(arg);
    corre=cl+c*cr-s*sr;
    corim=sl+s*cr+c*sr;
}
```

Since the use of `dftcor` can be confusing, we also give an illustrative program `dftint` that uses `dftcor` to compute equation (13.9.1) for general a, b, ω, and $h(t)$. Several points within this program bear mentioning: The constants `M` and `NDFT` correspond to M and N in the above discussion. On successive calls, we recompute the Fourier transform only if a or b or $h(t)$ has changed.

Since `dftint` is designed to work for any value of ω satisfying $\omega\Delta < \pi$, not just the special values returned by the DFT (equation 13.9.12), we do polynomial interpolation of degree `MPOL` on the DFT spectrum. You should be warned that a large factor of oversampling ($N \gg M$) is required for this interpolation to be accurate. After interpolation, we add the endpoint corrections from `dftcor`, which can be evaluated for any ω.

While `dftcor` is good at what it does, the routine `dftint` is illustrative only. It is not a general-purpose program, because it does not adapt its parameters `M`, `NDFT`, `MPOL` or its interpolation scheme to any particular function $h(t)$. You will have to experiment with your own application.

dftintegrate.h

```
void dftint(Doub func(const Doub), const Doub a, const Doub b, const Doub w,
    Doub &cosint, Doub &sinint) {
```

Example program illustrating how to use the routine `dftcor`. The user supplies an external function `func` that returns the quantity $h(t)$. The routine then returns $\int_a^b \cos(\omega t)h(t)\,dt$ as `cosint` and $\int_a^b \sin(\omega t)h(t)\,dt$ as `sinint`.

```
    static Int init=0;
    static Doub (*funcold)(const Doub);
    static Doub aold = -1.e30,bold = -1.e30,delta;
    const Int M=64,NDFT=1024,MPOL=6;
```

The values of `M`, `NDFT`, and `MPOL` are merely illustrative and should be optimized for your particular application. `M` is the number of subintervals, `NDFT` is the length of the FFT (a power of 2), and `MPOL` is the degree of polynomial interpolation used to obtain the desired frequency from the FFT.

```
    const Doub TWOPI=6.283185307179586476;
    Int j,nn;
    Doub c,cdft,corfac,corim,corre,en,s,sdft;
    static VecDoub data(NDFT),endpts(8);
    VecDoub cpol(MPOL),spol(MPOL),xpol(MPOL);
    if (init != 1 || a != aold || b != bold || func != funcold) {
```

Do we need to initialize, or is only ω changed?

```
        init=1;
        aold=a;
        bold=b;
        funcold=func;
        delta=(b-a)/M;
        for (j=0;j<M+1;j++)                    Load the function values into the data
            data[j]=func(a+j*delta);               array.
        for (j=M+1;j<NDFT;j++)                 Zero-pad the rest of the data array.
            data[j]=0.0;
        for (j=0;j<4;j++) {                    Load the endpoints.
            endpts[j]=data[j];
            endpts[j+4]=data[M-3+j];
        }
        realft(data,1);
```

`realft` returns the unused value corresponding to $\omega_{N/2}$ in `data[1]`. We actually want this element to contain the imaginary part corresponding to ω_0, which is zero.

```
        data[1]=0.0;
    }
```

Now interpolate on the DFT result for the desired frequency. If the frequency is an ω_n,

i.e., the quantity en is an integer, then cdft=data[2*en-2], sdft=data[2*en-1], and you could omit the interpolation.

```
    en=w*delta*NDFT/TWOPI+1.0;
    nn=MIN(MAX(Int(en-0.5*MPOL+1.0),1),NDFT/2-MPOL+1);     Leftmost point for the
    for (j=0;j<MPOL;j++,nn++) {                                 interpolation.
        cpol[j]=data[2*nn-2];
        spol[j]=data[2*nn-1];
        xpol[j]=nn;
    }
    cdft = Poly_interp(xpol,cpol,MPOL).interp(en);
    sdft = Poly_interp(xpol,spol,MPOL).interp(en);
    dftcor(w,delta,a,b,endpts,corre,corim,corfac);             Now get the endpoint cor-
    cdft *= corfac;                                                rection and the mul-
    sdft *= corfac;                                                tiplicative factor W(θ).
    cdft += corre;
    sdft += corim;
    c=delta*cos(w*a);                                   Finally multiply by Δ and exp(iωa).
    s=delta*sin(w*a);
    cosint=c*cdft-s*sdft;
    sinint=s*cdft+c*sdft;
}
```

Sometimes one is interested only in the discrete frequencies ω_m of equation (13.9.5), the ones that have integral numbers of periods in the interval $[a,b]$. For smooth $h(t)$, the value of I tends to be much smaller in magnitude at these ω's than at values in between, since the integral half-periods tend to cancel precisely. (That is why one must oversample for interpolation to be accurate: $I(\omega)$ is oscillatory with small magnitude near the ω_m's.) If you want these ω_m's without messy (and possibly inaccurate) interpolation, you have to set N to a multiple of M (compare equations 13.9.5 and 13.9.12). In the method implemented above, however, N must be at least $M+1$, so the smallest such multiple is $2M$, resulting in a factor $\sim$2 unnecessary computing. Alternatively, one can derive a formula like equation (13.9.13), but with the last function sample $h_M = h(b)$ omitted from the DFT, but included entirely in the endpoint correction for h_M. Then one can set $M = N$ (an integer power of 2) and get the special frequencies of equation (13.9.5) with no additional overhead. The modified formula is

$$\begin{aligned} I(\omega_m) = \Delta e^{i\omega_m a}\Big\{&W(\theta)\,[\text{DFT}(h_0 \ldots h_{M-1})]_m \\ &+ \alpha_0(\theta)h_0 + \alpha_1(\theta)h_1 + \alpha_2(\theta)h_2 + \alpha_3(\theta)h_3 \\ &+ e^{i\omega(b-a)}\left[A(\theta)h_M + \alpha_1^*(\theta)h_{M-1} + \alpha_2^*(\theta)h_{M-2} + \alpha_3^*(\theta)h_{M-3}\right]\Big\} \end{aligned} \tag{13.9.14}$$

where $\theta \equiv \omega_m \Delta$ and $A(\theta)$ is given by

$$A(\theta) = -\alpha_0(\theta) \tag{13.9.15}$$

for the trapezoidal case, or

$$\begin{aligned} A(\theta) &= \frac{(-6 + 11\theta^2) + (6 + \theta^2)\cos 2\theta}{6\theta^4} - i\,\text{Im}[\alpha_0(\theta)] \\ &\approx \frac{1}{3} + \frac{1}{45}\theta^2 - \frac{8}{945}\theta^4 + \frac{11}{14175}\theta^6 - i\,\text{Im}[\alpha_0(\theta)] \end{aligned} \tag{13.9.16}$$

for the cubic case.

Factors like $W(\theta)$ arise naturally whenever one calculates Fourier coefficients of smooth functions, and they are sometimes called attenuation factors [1]. However, the endpoint corrections are equally important in obtaining accurate values of integrals. Narasimhan and Karthikeyan [2] have given a formula that is algebraically equivalent to our trapezoidal formula. However, their formula requires the evaluation of *two* FFTs, which is unnecessary. The basic idea used here goes back at least to Filon [3] in 1928 (before the FFT!). He used Simpson's rule (quadratic interpolation). Since this interpolation is not left-right symmetric, two Fourier transforms are required. An alternative algorithm for equation (13.9.14) has

been given by Lyness in [4]; for related references, see [5]. To our knowledge, the cubic-order formulas derived here have not previously appeared in the literature.

Calculating Fourier transforms when the range of integration is $(-\infty, \infty)$ can be tricky. If the function falls off reasonably quickly at infinity, you can split the integral at a large enough value of t. For example, the integration to $+\infty$ can be written

$$\begin{aligned} \int_a^\infty e^{i\omega t} h(t)\,dt &= \int_a^b e^{i\omega t} h(t)\,dt + \int_b^\infty e^{i\omega t} h(t)\,dt \\ &= \int_a^b e^{i\omega t} h(t)\,dt - \frac{h(b)e^{i\omega b}}{i\omega} + \frac{h'(b)e^{i\omega b}}{(i\omega)^2} - \cdots \end{aligned} \tag{13.9.17}$$

The splitting point b must be chosen large enough that the remaining integral over (b, ∞) is small. Successive terms in its asymptotic expansion are found by integrating by parts. The integral over (a, b) can be done using `dftint`. You keep as many terms in the asymptotic expansion as you can easily compute. See [6] for some examples of this idea. More powerful methods, which work well for long-tailed functions but which do not use the FFT, are described in [7-9].

CITED REFERENCES AND FURTHER READING:

Stoer, J., and Bulirsch, R. 2002, *Introduction to Numerical Analysis*, 3rd ed. (New York: Springer), §2.3.4.[1]

Narasimhan, M.S. and Karthikeyan, M. 1984, "Evaluation of Fourier Integrals Using a FFT with Improved Accuracy and Its Applications," *IEEE Transactions on Antennas and Propagation*, vol. 32, pp. 404–408.[2]

Filon, L.N.G. 1928, "On a Quadrature Formula for Trigonometric Integrals," *Proceedings of the Royal Society of Edinburgh*, vol. 49, pp. 38–47.[3]

Giunta, G. and Murli, A. 1987, "A Package for Computing Trigonometric Fourier Coefficients Based on Lyness's Algorithm," *ACM Transactions on Mathematical Software*, vol. 13, pp. 97–107.[4]

Lyness, J.N. 1987, in *Numerical Integration*, P. Keast and G. Fairweather, eds. (Dordrecht: Reidel).[5]

Pantis, G. 1975, "The Evaluation of Integrals with Oscillatory Integrands," *Journal of Computational Physics*, vol. 17, pp. 229–233.[6]

Blakemore, M., Evans, G.A., and Hyslop, J. 1976, "Comparison of Some Methods for Evaluating Infinite Range Oscillatory Integrals," *Journal of Computational Physics*, vol. 22, pp. 352–376.[7]

Lyness, J.N., and Kaper, T.J. 1987, "Calculating Fourier Transforms of Long Tailed Functions," *SIAM Journal on Scientific and Statistical Computing*, vol. 8, pp. 1005–1011.[8]

Thakkar, A.J., and Smith, V.H. 1975, "A Strategy for the Numerical Evaluation of Fourier Sine and Cosine Transforms to Controlled Accuracy," *Computer Physics Communications*, vol. 10, pp. 73–79.[9]

13.10 Wavelet Transforms

Like the fast Fourier transform (FFT), the discrete wavelet transform (DWT) is a fast, linear operation that operates on a data vector whose length is an integer power of 2, transforming it into a numerically different vector of the same length. Also like the FFT, the wavelet transform is invertible and in fact orthogonal — the inverse transform, when viewed as a big matrix, is simply the transpose of the transform.

Both FFT and DWT, therefore, can be viewed as a rotation in function space, from the input space (or time) domain, where the basis functions are the unit vectors $\mathbf{e}_i$, or Dirac delta functions in the continuum limit, to a different domain. For the FFT, this new domain has basis functions that are the familiar sines and cosines. In the wavelet domain, the basis functions are somewhat more complicated and have the fanciful names "mother functions" and "wavelets."

Of course there are an infinity of possible bases for function space, almost all of them uninteresting! What makes the wavelet basis interesting is that, *unlike* sines and cosines, individual wavelet functions are quite localized in space; simultaneously, *like* sines and cosines, individual wavelet functions are quite localized in frequency or (more precisely) characteristic scale. As we will see below, the particular kind of dual localization achieved by wavelets renders large classes of functions and operators sparse, or sparse to some high accuracy, when transformed into the wavelet domain. Analogously with the Fourier domain, where a class of computations, like convolutions, becomes computationally fast, there is a large class of computations — those that can take advantage of sparsity — that becomes computationally fast in the wavelet domain [1].

Unlike sines and cosines, which define a unique Fourier transform, there is not one single unique set of wavelets; in fact, there are infinitely many possible sets. Roughly, the different sets of wavelets make different trade-offs between how compactly they are localized in space, how smooth they are, and whether they have any special boundary conditions. (There are further fine distinctions.)

13.10.1 Daubechies Wavelet Filter Coefficients

A particular set of wavelets is specified by a particular set of numbers, called *wavelet filter coefficients*. Here, we will largely restrict ourselves to wavelet filters in a class discovered by Daubechies [2]. This class includes members ranging from highly localized to highly smooth. The simplest (and most localized) member, often called *DAUB4*, has only four coefficients, $c_0, \ldots, c_3$. For the moment we specialize to this case for ease of notation.

Consider the following transformation matrix acting on a column vector of data to its right:

$$\begin{bmatrix}
c_0 & c_1 & c_2 & c_3 & & & & & & & \\
c_3 & -c_2 & c_1 & -c_0 & & & & & & & \\
 & & c_0 & c_1 & c_2 & c_3 & & & & & \\
 & & c_3 & -c_2 & c_1 & -c_0 & & & & & \\
\vdots & \vdots & & & & & \ddots & & & & \\
 & & & & & & & c_0 & c_1 & c_2 & c_3 \\
 & & & & & & & c_3 & -c_2 & c_1 & -c_0 \\
c_2 & c_3 & & & & & & & & c_0 & c_1 \\
c_1 & -c_0 & & & & & & & & c_3 & -c_2
\end{bmatrix} \tag{13.10.1}$$

Here blank entries signify zeroes. Note the structure of this matrix. The first row convolves four consecutive data points with the filter coefficients $c_0 \ldots, c_3$; likewise, the third, fifth, and other odd rows. If the even rows followed this pattern, offset by one, then the matrix would be a circulant, that is, an ordinary convolution that could be done by FFT methods. (Note how the last two rows wrap around like convolutions

with periodic boundary conditions.) Instead of convolving with $c_0, \ldots, c_3$, however, the even rows perform a different convolution, with coefficients $c_3, -c_2, c_1, -c_0$. The action of the matrix, overall, is thus to perform two related convolutions, then to decimate each of them by half (throw away half the values), and interleave the remaining halves.

It is useful to think of the filter $c_0, \ldots, c_3$ as being a smoothing filter, call it H, something like a moving average of four points. Then, because of the minus signs, the filter $c_3, -c_2, c_1, -c_0$, call it G, is *not* a smoothing filter. (In signal processing contexts, H and G are called *quadrature mirror filters* [3].) In fact, the c's are chosen so as to make G yield, insofar as possible, a *zero* response to a sufficiently smooth data vector. This is done by requiring the sequence $c_3, -c_2, c_1, -c_0$ to have a certain number of vanishing moments. When this is the case for p moments (starting with the zeroth), a set of wavelets is said to satisfy an "approximation condition of order p." This results in the output of H, decimated by half, accurately representing the data's "smooth" information. The output of G, also decimated, is referred to as the data's "detail" information [4].

For such a characterization to be useful, it must be possible to reconstruct the original data vector of length N from its $N/2$ smooth or s-components and its $N/2$ detail or d-components. That is effected by requiring the matrix (13.10.1) to be orthogonal, so that its inverse is just the transposed matrix

$$\begin{bmatrix} c_0 & c_3 & & & \cdots & & & & c_2 & c_1 \\ c_1 & -c_2 & & & \cdots & & & & c_3 & -c_0 \\ c_2 & c_1 & c_0 & c_3 & & & & & & \\ c_3 & -c_0 & c_1 & -c_2 & & & & & & \\ & & & & \ddots & & & & & \\ & & & & & c_2 & c_1 & c_0 & c_3 & \\ & & & & & c_3 & -c_0 & c_1 & -c_2 & \\ & & & & & & & c_2 & c_1 & c_0 & c_3 \\ & & & & & & & c_3 & -c_0 & c_1 & -c_2 \end{bmatrix} \tag{13.10.2}$$

One sees immediately that matrix (13.10.2) is inverse to matrix (13.10.1) if and only if these two equations hold,

$$\begin{aligned} c_0^2 + c_1^2 + c_2^2 + c_3^2 &= 1 \\ c_2 c_0 + c_3 c_1 &= 0 \end{aligned} \tag{13.10.3}$$

If additionally we require the approximation condition of order $p = 2$, then two additional relations are required,

$$\begin{aligned} c_3 - c_2 + c_1 - c_0 &= 0 \\ 0c_3 - 1c_2 + 2c_1 - 3c_0 &= 0 \end{aligned} \tag{13.10.4}$$

Equations (13.10.3) and (13.10.4) are four equations for the four unknowns $c_0, \ldots, c_3$, first recognized and solved by Daubechies. The unique solution (up to a left-right reversal) is

$$\begin{aligned} c_0 &= (1+\sqrt{3})/4\sqrt{2} \qquad & c_1 &= (3+\sqrt{3})/4\sqrt{2} \\ c_2 &= (3-\sqrt{3})/4\sqrt{2} \qquad & c_3 &= (1-\sqrt{3})/4\sqrt{2} \end{aligned} \tag{13.10.5}$$

In fact, DAUB4 is only the most compact of a sequence of wavelet sets: If we had six coefficients instead of four, there would be three orthogonality requirements in equation (13.10.3) (with offsets of zero, two, and four), and we could require the vanishing of $p = 3$ moments in equation (13.10.4). In this case, DAUB6, the solution coefficients can also be expressed in closed form,

$$
\begin{aligned}
c_0 &= (1+\sqrt{10}+\sqrt{5+2\sqrt{10}})/16\sqrt{2} & c_1 &= (5+\sqrt{10}+3\sqrt{5+2\sqrt{10}})/16\sqrt{2}\\
c_2 &= (10-2\sqrt{10}+2\sqrt{5+2\sqrt{10}})/16\sqrt{2} & c_3 &= (10-2\sqrt{10}-2\sqrt{5+2\sqrt{10}})/16\sqrt{2}\\
c_4 &= (5+\sqrt{10}-3\sqrt{5+2\sqrt{10}})/16\sqrt{2} & c_5 &= (1+\sqrt{10}-\sqrt{5+2\sqrt{10}})/16\sqrt{2}
\end{aligned}
\tag{13.10.6}
$$

For higher p, the coefficients are available only numerically, e.g., tabulated in [5] or [6]. (We use some of these below.) The number of coefficients increases by two each time p is increased by one.

13.10.2 Discrete Wavelet Transform

We have not yet defined the discrete wavelet transform (DWT), but we are almost there: The DWT consists of applying a wavelet coefficient matrix like (13.10.1) *hierarchically*, first to the full data vector of length N, then to the "smooth" vector of length $N/2$, then to the "smooth-smooth" vector of length $N/4$, and so on until only a trivial number of "smooth-. . .-smooth" components (usually 2 or 4) remain. The procedure is sometimes called a *pyramidal algorithm* [4], for obvious reasons. The output of the DWT consists of these remaining components and all the "detail" components that were accumulated along the way. A diagram should make the procedure clear:

$$
\begin{bmatrix} y_0\\ y_1\\ y_2\\ y_3\\ y_4\\ y_5\\ y_6\\ y_7\\ y_8\\ y_9\\ y_{10}\\ y_{11}\\ y_{12}\\ y_{13}\\ y_{14}\\ y_{15} \end{bmatrix}
\xrightarrow{13.10.1}
\begin{bmatrix} s_0\\ d_0\\ s_1\\ d_1\\ s_2\\ d_2\\ s_3\\ d_3\\ s_4\\ d_4\\ s_5\\ d_5\\ s_6\\ d_6\\ s_7\\ d_7 \end{bmatrix}
\xrightarrow{\text{permute}}
\left[\begin{array}{c} s_0\\ s_1\\ s_2\\ s_3\\ s_4\\ s_5\\ s_6\\ s_7\\ \hline d_0\\ d_1\\ d_2\\ d_3\\ d_4\\ d_5\\ d_6\\ d_7 \end{array}\right]
\xrightarrow{13.10.1}
\left[\begin{array}{c} S_0\\ D_0\\ S_1\\ D_1\\ S_2\\ D_2\\ S_3\\ D_3\\ \hline d_0\\ d_1\\ d_2\\ d_3\\ d_4\\ d_5\\ d_6\\ d_7 \end{array}\right]
\xrightarrow{\text{permute}}
\left[\begin{array}{c} S_0\\ S_1\\ S_2\\ S_3\\ \hline D_0\\ D_1\\ D_2\\ D_3\\ \hline d_0\\ d_1\\ d_2\\ d_3\\ d_4\\ d_5\\ d_6\\ d_7 \end{array}\right]
\xrightarrow{\text{etc.}}
\left[\begin{array}{c} \mathcal{S}_0\\ \mathcal{S}_1\\ \hline \mathcal{D}_0\\ \mathcal{D}_1\\ \hline D_0\\ D_1\\ D_2\\ D_3\\ \hline d_0\\ d_1\\ d_2\\ d_3\\ d_4\\ d_5\\ d_6\\ d_7 \end{array}\right]
\tag{13.10.7}
$$

If the length of the data vector were a higher power of 2, there would be more stages of applying (13.10.1) (or any other wavelet coefficients) and permuting. The endpoint will always be a vector with two $\mathcal{S}$'s and a hierarchy of $\mathcal{D}$'s, D's, d's,

etc. Notice that once d's are generated, they simply propagate through to all subsequent stages.

A value d_i of any level is termed a "wavelet coefficient" of the original data vector; the final values $\mathcal{S}_0, \mathcal{S}_1$ should strictly be called "mother-function coefficients," although the term "wavelet coefficients" is often used loosely for both d's and final $\mathcal{S}$'s. Since the full procedure is a composition of orthogonal linear operations, the whole DWT is itself an orthogonal linear operator.

To invert the DWT, one simply reverses the procedure, starting with the smallest level of the hierarchy and working (in equation 13.10.7) from right to left. The inverse matrix (13.10.2) is of course used instead of the matrix (13.10.1).

As already noted, the matrices (13.10.1) and (13.10.2) embody periodic ("wraparound") boundary conditions on the data vector. One normally accepts this as a minor inconvenience: The last few wavelet coefficients at each level of the hierarchy are affected by data from both ends of the data vector. By circularly shifting the matrix (13.10.1) $N/2$ columns to the left, one can symmetrize the wraparound; but this does not eliminate it. It is in fact possible to eliminate the wraparound completely by altering the coefficients in the first and last few rows of (13.10.1), giving an orthogonal matrix that is purely band-diagonal. This variant can be useful when, e.g., the data vary by many orders of magnitude from one end of the data vector to the other. We discuss it in §13.10.5, below.

Here is a DWT routine, `wt1`, that performs the pyramidal algorithm (or its inverse if `isign` is negative) on some data vector `a[0..n-1]`. Successive applications of the wavelet filter, and accompanying permutations, are performed by the object `wlet`, of class `Wavelet`, to be described below. The routine `wt1` also provides for the possibility of preconditioning and postconditioning steps, which we won't need until a later subsection.

wavelet.h

```
void wt1(VecDoub_IO &a, const Int isign, Wavelet &wlet)
One-dimensional discrete wavelet transform. This routine implements the pyramid algorithm,
replacing a[0..n-1] by its wavelet transform (for isign=1), or performing the inverse operation
(for isign=-1). Note that n MUST be an integer power of 2. The object wlet, of type Wavelet,
is the underlying wavelet filter. Examples of Wavelet types are Daub4, Daubs, and Daub4i.
{
    Int nn, n=a.size();
    if (n < 4) return;
    if (isign >= 0) {                                    Wavelet transform.
        wlet.condition(a,n,1);
        for (nn=n;nn>=4;nn>>=1) wlet.filt(a,nn,isign);
        Start at largest hierarchy, and work toward smallest.
    } else {
        for (nn=4;nn<=n;nn<<=1) wlet.filt(a,nn,isign);
        Start at smallest hierarchy, and work toward largest.
        wlet.condition(a,n,-1);
    }
}
```

The `Wavelet` class is an "abstract base class," meaning that it is really only a promise that specific wavelets that derive from it will contain a method called `filt`, the actual wavelet filter. `Wavelet` also provides a default, null, pre- and post-conditioning method. The class `Daub4` is derived from `Wavelet` and is intended for use with `wt1`. Its `filt` method implements the matrices (13.10.1) and (13.10.2), along with the permutation shown in (13.10.7).

wavelet.h

```
struct Wavelet {
    virtual void filt(VecDoub_IO &a, const Int n, const Int isign) = 0;
    virtual void condition(VecDoub_IO &a, const Int n, const Int isign) {}
};

struct Daub4 : Wavelet {
    void filt(VecDoub_IO &a, const Int n, const Int isign) {
    Applies the Daubechies 4-coefficient wavelet filter to data vector a[0..n-1] (for isign=1)
    or applies its transpose (for isign=-1). Used hierarchically by routines wt1 and wtn.
        const Doub C0=0.4829629131445341, C1=0.8365163037378077,
        C2=0.2241438680420134, C3=-0.1294095225512603;
        Int nh,i,j;
        if (n < 4) return;
        VecDoub wksp(n);
        nh = n >> 1;
        if (isign >= 0) {                                Apply filter.
            for (i=0,j=0;j<n-3;j+=2,i++) {
                wksp[i] = C0*a[j]+C1*a[j+1]+C2*a[j+2]+C3*a[j+3];
                wksp[i+nh] = C3*a[j]-C2*a[j+1]+C1*a[j+2]-C0*a[j+3];
            }
            wksp[i] = C0*a[n-2]+C1*a[n-1]+C2*a[0]+C3*a[1];
            wksp[i+nh] = C3*a[n-2]-C2*a[n-1]+C1*a[0]-C0*a[1];
        } else {                                         Apply transpose filter.
            wksp[0] = C2*a[nh-1]+C1*a[n-1]+C0*a[0]+C3*a[nh];
            wksp[1] = C3*a[nh-1]-C0*a[n-1]+C1*a[0]-C2*a[nh];
            for (i=0,j=2;i<nh-1;i++) {
                wksp[j++] = C2*a[i]+C1*a[i+nh]+C0*a[i+1]+C3*a[i+nh+1];
                wksp[j++] = C3*a[i]-C0*a[i+nh]+C1*a[i+1]-C2*a[i+nh+1];
            }
        }
        for (i=0;i<n;i++) a[i]=wksp[i];
    }
};
```

For larger sets of wavelet coefficients, the wraparound of the last rows or columns is a programming inconvenience. An efficient implementation would handle the wraparounds as special cases, outside of the main loop. For now, we will content ourselves with a more general scheme involving some extra arithmetic at run-time.

The following class, Daubs, takes an integer argument n in its constructor and creates a wavelet object with the filter DAUBn. Slightly better than "Hobson's choice," you can choose $n = 4$, 12, or 20. For other values of n you will need to add additional coefficient tables (e.g., from [6]).

wavelet.h

```
struct Daubs : Wavelet {
Structure for initializing and using the DAUBn wavelet filter for any n whose coefficients are
provided (here n = 4, 12, 20).
    Int ncof,ioff,joff;
    VecDoub cc,cr;
    static Doub c4[4],c12[12],c20[20];
    Daubs(Int n) : ncof(n), cc(n), cr(n) {
        Int i;
        ioff = joff = -(n >> 1);
        // ioff = -2; joff = -n + 2;        Alternative centering. (Used by Daub4, above.)
        if (n == 4) for (i=0; i<n; i++) cc[i] = c4[i];
        else if (n == 12) for (i=0; i<n; i++) cc[i] = c12[i];
        else if (n == 20) for (i=0; i<n; i++) cc[i] = c20[i];
        else throw("n not yet implemented in Daubs");
        Doub sig = -1.0;
        for (i=0; i<n; i++) {
            cr[n-1-i]=sig*cc[i];
            sig = -sig;
```

```
        }
    }
    void filt(VecDoub_IO &a, const Int n, const Int isign); See below.
};

Doub Daubs::c4[4]=
    {0.4829629131445341,0.8365163037378079,
    0.2241438680420134,-0.1294095225512604};
Doub Daubs::c12[12]=
    {0.111540743350, 0.494623890398, 0.751133908021,
    0.315250351709,-0.226264693965,-0.129766867567,
    0.097501605587, 0.027522865530,-0.031582039318,
    0.000553842201, 0.004777257511,-0.001077301085};
Doub Daubs::c20[20]=
    {0.026670057901, 0.188176800078, 0.527201188932,
    0.688459039454, 0.281172343661,-0.249846424327,
    -0.195946274377, 0.127369340336, 0.093057364604,
    -0.071394147166,-0.029457536822, 0.033212674059,
    0.003606553567,-0.010733175483, 0.001395351747,
    0.001992405295,-0.000685856695,-0.000116466855,
    0.000093588670,-0.000013264203};
```

There is some arbitrariness in how the wavelets at each hierarchical stage are centered over the data they act on. `Daubs` implements one popular choice, with another shown in commented code. Consult the literature if this matters to you (it rarely does).

The implementation of `Daubs::filt()` is straightforward:

wavelet.h

```
void Daubs::filt(VecDoub_IO &a, const Int n, const Int isign) {
Applies the previously initialized Daubn wavelet filter to data vector a[0..n-1] (for isign = 1)
or applies its transpose (for isign = -1). Used hierarchically by routines wt1 and wtn.
    Doub ai,ai1;
    Int i,ii,j,jf,jr,k,n1,ni,nj,nh,nmod;
    if (n < 4) return;
    VecDoub wksp(n);
    nmod = ncof*n;                      A positive constant equal to zero mod n.
    n1 = n-1;                           Mask of all bits, since n a power of 2.
    nh = n >> 1;
    for (j=0;j<n;j++) wksp[j]=0.0;
    if (isign >= 0) {                   Apply filter.
        for (ii=0,i=0;i<n;i+=2,ii++) {
            ni = i+1+nmod+ioff;         Pointer to be incremented and wrapped around.
            nj = i+1+nmod+joff;
            for (k=0;k<ncof;k++) {
                jf = n1 & (ni+k+1);     We use "bitwise and" to wrap around the
                jr = n1 & (nj+k+1);         pointers.
                wksp[ii] += cc[k]*a[jf];
                wksp[ii+nh] += cr[k]*a[jr];
            }
        }
    } else {                            Apply transpose filter.
        for (ii=0,i=0;i<n;i+=2,ii++) {
            ai = a[ii];
            ai1 = a[ii+nh];
            ni = i+1+nmod+ioff;         See comments above.
            nj = i+1+nmod+joff;
            for (k=0;k<ncof;k++) {
                jf = n1 & (ni+k+1);
                jr = n1 & (nj+k+1);
                wksp[jf] += cc[k]*ai;
                wksp[jr] += cr[k]*ai1;
            }
```

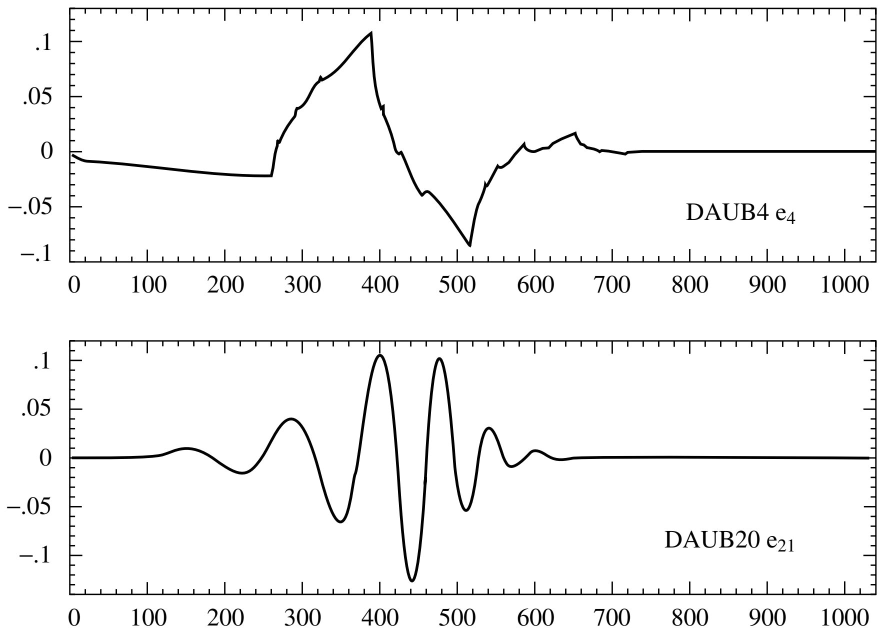

Figure 13.10.1. Wavelet functions, that is, single basis functions from the wavelet families DAUB4 and DAUB20. A complete, orthonormal wavelet basis consists of scalings and translations of either one of these functions. DAUB4 has an infinite number of cusps; DAUB20 would show similar behavior in a higher derivative.

```
        }
    }
    for (j=0;j<n;j++) a[j] = wksp[j];          Copy the results back from workspace.
}
```

13.10.3 What Do Wavelets Look Like?

We are now in a position to actually see some wavelets. To do so, we simply run unit vectors through any of the above discrete wavelet transforms, with `isign` negative so that the inverse transform is performed. Figure 13.10.1 shows the DAUB4 wavelet that is the inverse DWT of a unit vector in component 4 of a vector of length 1024, and also the DAUB20 wavelet that is the inverse of component 21. (One needs to go to a later hierarchical level for DAUB20 to avoid a wavelet with a wrapped-around tail.) Other unit vectors would give wavelets with the same shapes but different positions and scales.

One sees that both DAUB4 and DAUB20 have wavelets that are continuous. DAUB20 wavelets also have higher continuous derivatives. DAUB4 has the peculiar property that its derivative exists only *almost* everywhere. Examples of where it fails to exist are the points $p/2^n$, where p and n are integers; at such points, DAUB4 is left differentiable, but not right differentiable! This kind of discontinuity — at least in some derivative — is a necessary feature of wavelets with compact support, like the Daubechies series. For every increase in the number of wavelet coefficients by two, the Daubechies wavelets gain about *half* a derivative of continuity. (But not exactly half; the actual orders of regularity are irrational numbers!)

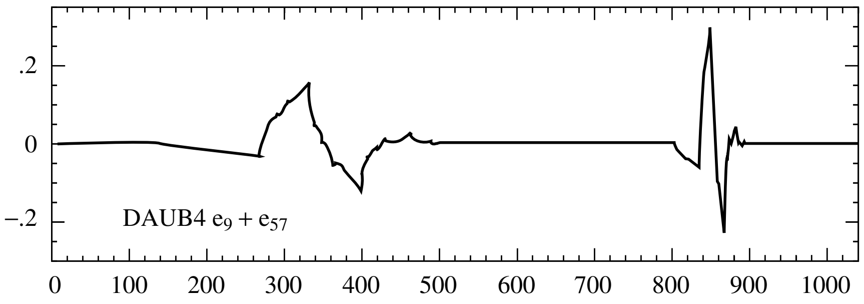

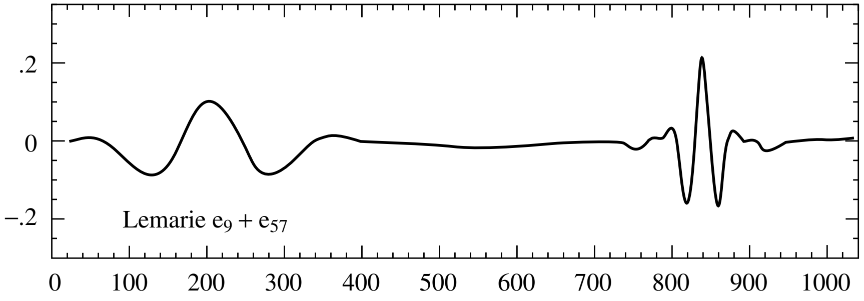

Figure 13.10.2. More wavelets, here generated from the sum of two unit vectors, $\mathbf{e}_9 + \mathbf{e}_{57}$, which are in different hierarchical levels of scale, and also at different spatial positions. DAUB4 wavelets (top) are defined by a filter in coordinate space (equation 13.10.5), while Lemarie wavelets (bottom) are defined by a filter most easily written in Fourier space (equation 13.10.14).

Note that the fact that wavelets are not smooth does not prevent their having exact representations for some smooth functions, as demanded by their approximation order p. The continuity of a wavelet is not the same as the continuity of functions that a set of wavelets can represent. For example, DAUB4 can represent (piecewise) linear functions of arbitrary slope: In the correct linear combinations, the cusps all cancel out. Every increase of two in the number of coefficients allows one higher order of polynomial to be exactly represented.

Figure 13.10.2 shows the result of performing the inverse DWT on the input vector $\mathbf{e}_9 + \mathbf{e}_{57}$, again for the two different particular wavelets. Since 9 lies early in the hierarchical range of 8–15, that wavelet lies on the left side of the picture. Since 57 lies in a later (smaller-scale) hierarchy, it is a narrower wavelet; in the range of 32–63 it is toward the end, so it lies on the right side of the picture. Note that smaller-scale wavelets are taller, so as to have the same squared integral.

13.10.4 Wavelet Filters in the Fourier Domain

The Fourier transform of a set of filter coefficients c_j is given by

$$H(\omega) = \sum_j c_j e^{ij\omega} \qquad (13.10.8)$$

Here H is a function periodic in 2π, and it has the same meaning as before: It is the wavelet filter, now written in the Fourier domain. A very useful fact is that the orthogonality conditions for the c's (e.g., equation 13.10.3 above) collapse to two

simple relations in the Fourier domain,

$$\tfrac{1}{2}|H(0)|^2 = 1 \tag{13.10.9}$$

and

$$\tfrac{1}{2}\left[|H(\omega)|^2 + |H(\omega+\pi)|^2\right] = 1 \tag{13.10.10}$$

Likewise, the approximation condition of order p (e.g., equation 13.10.4 above) has a simple formulation, requiring that $H(\omega)$ have a pth order zero at $\omega = \pi$, or (equivalently)

$$H^{(m)}(\pi) = 0 \qquad m = 0, 1, \dots, p-1 \tag{13.10.11}$$

It is thus relatively straightforward to invent wavelet sets in the Fourier domain. You simply invent a function $H(\omega)$ satisfying equations (13.10.9) – (13.10.11). To find the actual c_j's applicable to a data (or s-component) vector of length N, and with periodic wraparound as in matrices (13.10.1) and (13.10.2), you invert equation (13.10.8) by the discrete Fourier transform

$$c_j = \frac{1}{N}\sum_{k=0}^{N-1} H(2\pi\frac{k}{N})e^{-2\pi ijk/N} \tag{13.10.12}$$

The quadrature mirror filter G (reversed c_j's with alternating signs), incidentally, has the Fourier representation

$$G(\omega) = e^{-i\omega}H^*(\omega+\pi) \tag{13.10.13}$$

where the asterisk denotes complex conjugation.

In general, the above procedure will *not* produce wavelet filters with compact support. In other words, all N of the c_j's, $j = 0, \dots, N-1$ will in general be nonzero (though they may be rapidly decreasing in magnitude). The Daubechies wavelets, or other wavelets with compact support, are specially chosen so that $H(\omega)$ is a trigonometric polynomial with only a small number of Fourier components, guaranteeing that there will be only a small number of nonzero c_j's.

On the other hand, there is sometimes no particular reason to demand compact support. Giving it up in fact allows the ready construction of relatively smoother wavelets (higher values of p). Even without compact support, the convolutions implicit in the matrix (13.10.1) can be done efficiently by FFT methods.

Lemarie's wavelet (see [4]) has $p = 4$, does not have compact support, and is defined by the choice of $H(\omega)$,

$$H(\omega) = \left[2(1-u)^4\frac{315 - 420u + 126u^2 - 4u^3}{315 - 420v + 126v^2 - 4v^3}\right]^{1/2} \tag{13.10.14}$$

where

$$u \equiv \sin^2\frac{\omega}{2} \qquad v \equiv \sin^2\omega \tag{13.10.15}$$

It is beyond our scope to explain where equation (13.10.14) comes from. An informal description is that the quadrature mirror filter $G(\omega)$ deriving from equation (13.10.14) has the property that it gives identically zero when applied to any function whose odd-numbered samples are equal to the cubic spline interpolation of its even-numbered samples. Since this class of functions includes many very smooth members, it follows that $H(\omega)$ does a good job of truly selecting a function's smooth information content. Sample Lemarie wavelets are shown in Figure 13.10.2.

13.10.5 Daubechies Wavelets on the Interval

The discrete wavelet transforms that we have seen thus far are periodic and thus "live on a circle.". Wavelets close to one edge of the data vector have tails that wrap around to the other edge. Said differently, some components of a discrete wavelet transform depend on data values at both ends of the data vector.

Most of the time, this periodicity is merely something between a curiosity and a minor nuisance, exactly like the discrete Fourier transform's similar periodicity. Similar simple workarounds (e.g., zero-padding of the data) apply. Occasionally, however, the wraparound can produce undesirable effects, for example when the data differ by orders of magnitude at the two ends, or are smooth at one end but unsmooth at the other.

By modifying the coefficients of the wavelet filters near the two ends of the data vector, it is possible to produce wavelets that utilize only local data at each edge, that is, wavelets that "live on the interval" instead of on the circle. For such wavelets, the orthogonal matrix analogous to (13.10.1) is purely band-diagonal, and is identical to (13.10.1) except for modifications in the first and last few rows. Various such constructions have been proposed. Our favorite is that of [7].

One wrinkle needs to be mentioned: We would hope that those modified rows of the new matrix that are "detail filters" have the property of giving exactly zero when applied to smooth polynomial sequences like $1, 1, 1, 1, 1$ or $1, 2, 3, 4, 5$. Indeed, all the period wavelets previously discussed have this property. Alas, this condition, plus orthogonality, imposes too many constraints on the coefficients, and is unachievable. It turns out, however, that a simple linear preconditioning of the first and last few data points (that is, replacing the values by linear combinations of themselves) restores the desired property. The preconditioning is done only once in the transform, *not* at every pyramidal level. This need for preconditioning (with a corresponding postconditioning for the inverse) is the reason that our `Wavelet` abstract class has a method named `condition`. Finally we get to use it in a nontrivial way!

Here is an implementation of DAUB4 wavelets on the interval as a class derived from `Wavelet`, compatible for use in `wt1`. The ugliness of the code reflects only the large number of new coefficients that must be provided. If you want to implement higher DAUBn's on the interval, you'll need even more coefficients, as found in [6] or [5].

wavelet.h

```
struct Daub4i : Wavelet {
    void filt(VecDoub_IO &a, const Int n, const Int isign) {
    Applies the Cohen-Daubechies-Vial 4-coefficient wavelet on the interval filter to data vector
    a[0..n-1] (for isign=1) or applies its transpose (for isign=-1). Used hierarchically by
    routines wt1 and wtn.
        const Doub C0=0.4829629131445341, C1=0.8365163037378077,
            C2=0.2241438680420134, C3=-0.1294095225512603;
        const Doub R00=0.603332511928053,R01=0.690895531839104,
            R02=-0.398312997698228,R10=-0.796543516912183,R11=0.546392713959015,
            R12=-0.258792248333818,R20=0.0375174604524466,R21=0.457327659851769,
            R22=0.850088102549165,R23=0.223820356983114,R24=-0.129222743354319,
            R30=0.0100372245644139,R31=0.122351043116799,R32=0.227428111655837,
            R33=-0.836602921223654,R34=0.483012921773304,R43=0.443149049637559,
            R44=0.767556669298114,R45=0.374955331645687,R46=0.190151418429955,
            R47=-0.194233407427412,R53=0.231557595006790,R54=0.401069519430217,
            R55=-0.717579999353722,R56=-0.363906959570891,R57=0.371718966535296,
            R65=0.230389043796969,R66=0.434896997965703,R67=0.870508753349866,
            R75=-0.539822500731772,R76=0.801422961990337,R77=-0.257512919478482;
```

```
        Int nh,i,j;
        if (n < 8) return;
        VecDoub wksp(n);
        nh = n >> 1;
        if (isign >= 0) {
            wksp[0]  = R00*a[0]+R01*a[1]+R02*a[2];
            wksp[nh] = R10*a[0]+R11*a[1]+R12*a[2];
            wksp[1] = R20*a[0]+R21*a[1]+R22*a[2]+R23*a[3]+R24*a[4];
            wksp[nh+1] = R30*a[0]+R31*a[1]+R32*a[2]+R33*a[3]+R34*a[4];
            for (i=2,j=3;j<n-4;j+=2,i++) {
                wksp[i] = C0*a[j]+C1*a[j+1]+C2*a[j+2]+C3*a[j+3];
                wksp[i+nh] = C3*a[j]-C2*a[j+1]+C1*a[j+2]-C0*a[j+3];
            }
            wksp[nh-2] = R43*a[n-5]+R44*a[n-4]+R45*a[n-3]+R46*a[n-2]+R47*a[n-1];
            wksp[n-2] = R53*a[n-5]+R54*a[n-4]+R55*a[n-3]+R56*a[n-2]+R57*a[n-1];
            wksp[nh-1] = R65*a[n-3]+R66*a[n-2]+R67*a[n-1];
            wksp[n-1] = R75*a[n-3]+R76*a[n-2]+R77*a[n-1];
        } else {
            wksp[0] = R00*a[0]+R10*a[nh]+R20*a[1]+R30*a[nh+1];
            wksp[1] = R01*a[0]+R11*a[nh]+R21*a[1]+R31*a[nh+1];
            wksp[2] = R02*a[0]+R12*a[nh]+R22*a[1]+R32*a[nh+1];
            if (n == 8) {
                wksp[3] = R23*a[1]+R33*a[5]+R43*a[2]+R53*a[6];
                wksp[4] = R24*a[1]+R34*a[5]+R44*a[2]+R54*a[6];
            } else {
                wksp[3] = R23*a[1]+R33*a[nh+1]+C0*a[2]+C3*a[nh+2];
                wksp[4] = R24*a[1]+R34*a[nh+1]+C1*a[2]-C2*a[nh+2];
                wksp[n-5] = C2*a[nh-3]+C1*a[n-3]+R43*a[nh-2]+R53*a[n-2];
                wksp[n-4] = C3*a[nh-3]-C0*a[n-3]+R44*a[nh-2]+R54*a[n-2];
            }
            for (i=2,j=5;i<nh-3;i++) {
                wksp[j++] = C2*a[i]+C1*a[i+nh]+C0*a[i+1]+C3*a[i+nh+1];
                wksp[j++] = C3*a[i]-C0*a[i+nh]+C1*a[i+1]-C2*a[i+nh+1];
            }
            wksp[n-3] = R45*a[nh-2]+R55*a[n-2]+R65*a[nh-1]+R75*a[n-1];
            wksp[n-2] = R46*a[nh-2]+R56*a[n-2]+R66*a[nh-1]+R76*a[n-1];
            wksp[n-1] = R47*a[nh-2]+R57*a[n-2]+R67*a[nh-1]+R77*a[n-1];
        }
        for (i=0;i<n;i++) a[i]=wksp[i];
    }
    void condition(VecDoub_IO &a, const Int n, const Int isign) {
        Doub t0,t1,t2,t3;
        if (n < 4) return;
        if (isign >= 0) {
            t0 = 0.324894048898962*a[0]+0.0371580151158803*a[1];
            t1 = 1.00144540498130*a[1];
            t2 = 1.08984305289504*a[n-2];
            t3 = -0.800813234246437*a[n-2]+2.09629288435324*a[n-1];
            a[0]=t0; a[1]=t1; a[n-2]=t2; a[n-1]=t3;
        } else {
            t0 = 3.07792649138669*a[0]-0.114204567242137*a[1];
            t1 = 0.998556681198888*a[1];
            t2 = 0.917563310922261*a[n-2];
            t3 = 0.350522032550918*a[n-2]+0.477032578540915*a[n-1];
            a[0]=t0; a[1]=t1; a[n-2]=t2; a[n-1]=t3;
        }
    }
};
```

Do you really need wavelets on the interval, instead of ordinary, periodic wavelets? Occasionally, yes. If you look ahead to Figure 13.10.6, which is a graphical display of two-dimensional wavelet coefficients, you can see the difference between

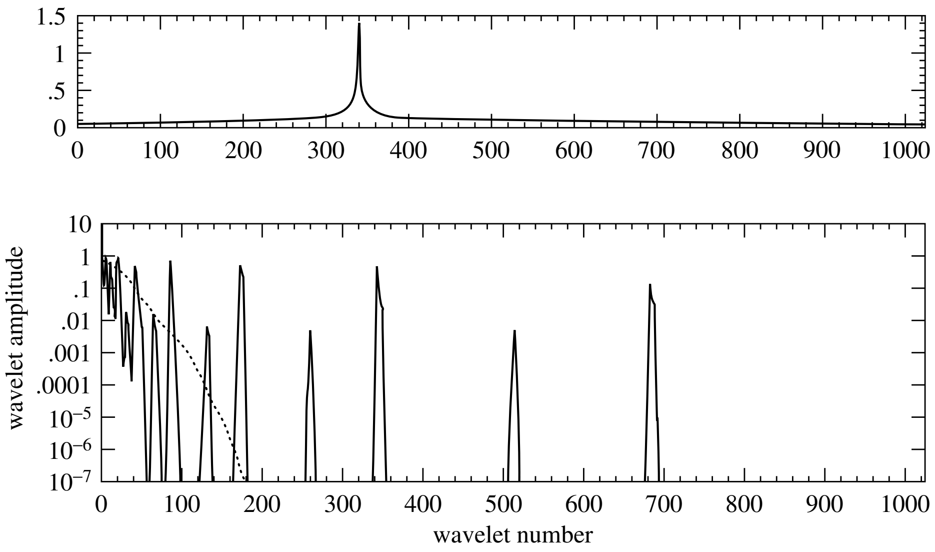

Figure 13.10.3. Top: Arbitrary test function, with cusp, sampled on a vector of length 1024. Bottom: Absolute value of the 1024 wavelet coefficients produced by the discrete wavelet transform of the function. Note log scale. The dotted curve plots the same amplitudes when sorted by decreasing size. One sees that only 130 out of 1024 coefficients are larger than 10^{-4} (or larger than about 10^{-5} times the largest coefficient, whose value is ~ 10).

allowing and suppressing wraparound.

13.10.6 Truncated Wavelet Approximations

Most of the usefulness of wavelets rests on the fact that wavelet transforms can usefully be severely truncated, that is, turned into sparse expansions. The case of Fourier transforms is different: FFTs are ordinarily used without truncation, to compute fast convolutions, for example. This works because the convolution operator is particularly simple in the Fourier basis. There are not, however, any standard mathematical operations that are especially simple in the wavelet basis.

To see how truncation works, consider the simple example shown in Figure 13.10.3. The upper panel shows an arbitrarily chosen test function, smooth except for a square-root cusp, sampled onto a vector of length 2^{10}. The bottom panel (solid curve) shows, on a log scale, the absolute value of the vector's components after it has been run through the DAUB4 discrete wavelet transform. One notes, from right to left, the different levels of hierarchy, 512–1023, 256–511, 128–255, etc. Within each level, the wavelet coefficients are nonnegligible only very near the location of the cusp, or very near the left and right boundaries of the hierarchical range (edge effects).

The dotted curve in the lower panel of Figure 13.10.3 plots the same amplitudes as the solid curve, but sorted into decreasing order of size. One can read off, for example, that the 130th largest wavelet coefficient has an amplitude less than 10^{-5} of the largest coefficient, whose magnitude is ~ 10 (power or square integral ratio less than 10^{-10}). Thus, the example function can be represented quite accurately by only 130, rather than 1024, coefficients — the remaining ones being set to zero.

Note that this kind of truncation makes the vector sparse, but still of logical length 1024. It is very important that vectors in wavelet space be truncated according to the *amplitude* of the components, not their position in the vector. Keeping the first 256 components of the vector (all levels of the hierarchy except the last two) would give an extremely poor, and jagged, approximation to the function. When you compress a function with wavelets, you have to record both the values *and the positions* of the nonzero coefficients.

Generally, compact (and therefore unsmooth) wavelets are better for lower accuracy approximations and for functions with discontinuities (like edges). Smooth (and therefore noncompact) wavelets are better for achieving high numerical accuracy. This makes compact wavelets a good choice for image compression, for example, while it makes smooth wavelets best for fast solution of integral equations.

In real applications of wavelets to compression, components are not starkly "kept" or "discarded." Rather, components may be kept with a varying number of bits of accuracy, depending on their magnitude. The JPEG-2000 image compression standard utilizes wavelets in such a manner.

13.10.7 Wavelet Transform in Multidimensions

A wavelet transform of a d-dimensional array is most easily obtained by transforming the array sequentially on its first index (for all values of its other indices), then on its second, and so on. Each transformation corresponds to multiplication by an orthogonal matrix $\mathbf{M}$. Because (illustrating the case $d = 2$)

$$\sum_j M_{nj}\left(\sum_i M_{mi} a_{ij}\right) = \sum_i M_{mi}\left(\sum_j M_{nj} a_{ij}\right) \qquad (13.10.16)$$

the result is independent of the order in which the indices were transformed. The situation is exactly like that for multidimensional FFTs. A routine for effecting the multidimensional DWT can thus be modeled on a multidimensional FFT routine like `fourn`:

wavelet.h

```
void wtn(VecDoub_IO &a, VecInt_I &nn, const Int isign, Wavelet &wlet)
Replaces a by its ndim-dimensional discrete wavelet transform, if isign is input as 1. Here
nn[0..ndim-1] is an integer array containing the lengths of each dimension (number of real
values), which must all be powers of 2. a is a real array of length equal to the product of these
lengths, in which the data are stored as in a multidimensional real array. If isign is input as
−1, a is replaced by its inverse wavelet transform. The object wlet, of type Wavelet, is the
underlying wavelet filter. Examples of Wavelet types are Daub4, Daubs, and Daub4i.
{
    Int idim,i1,i2,i3,k,n,nnew,nprev=1,nt,ntot=1;
    Int ndim=nn.size();
    for (idim=0;idim<ndim;idim++) ntot *= nn[idim];
    if (ntot&(ntot-1)) throw("all lengths must be powers of 2 in wtn");
    for (idim=0;idim<ndim;idim++) {              Main loop over the dimensions.
        n=nn[idim];
        VecDoub wksp(n);
        nnew=n*nprev;
        if (n > 4) {
            for (i2=0;i2<ntot;i2+=nnew) {
                for (i1=0;i1<nprev;i1++) {
                    for (i3=i1+i2,k=0;k<n;k++,i3+=nprev) wksp[k]=a[i3];
                    Copy the relevant row or column or etc. into workspace.
                    if (isign >= 0) {            Do one-dimensional wavelet transform.
```

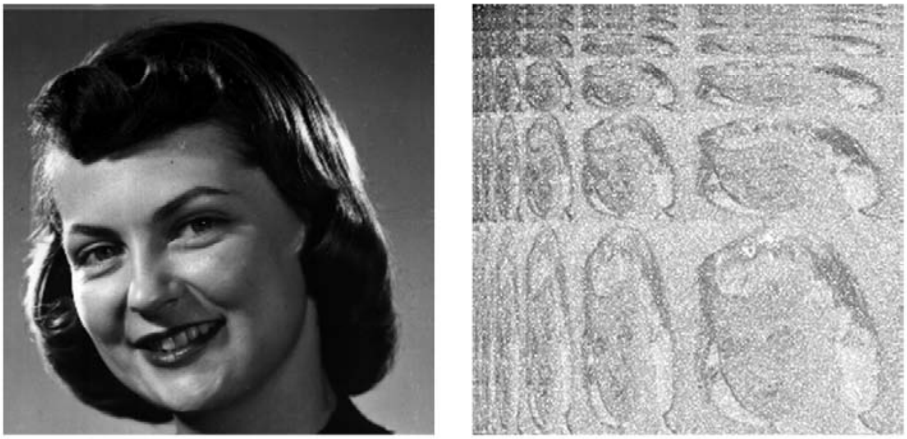

(a) (b)

Figure 13.10.4. (a) Two-dimensional array of intensities (i.e., a photograph) and (b) its two-dimensional discrete wavelet transform. Darker pixels represent wavelet components that are larger in magnitude, on a logarithmic scale. Wavelets number from the upper-left corner, where the "smooth" information content is encoded.

```
                    wlet.condition(wksp,n,1);
                    for(nt=n;nt>=4;nt >>= 1) wlet.filt(wksp,nt,isign);
                } else {                     Or inverse transform.
                    for(nt=4;nt<=n;nt <<= 1) wlet.filt(wksp,nt,isign);
                    wlet.condition(wksp,n,-1);
                }
                for (i3=i1+i2,k=0;k<n;k++,i3+=nprev) a[i3]=wksp[k];
                Copy back from workspace.
            }
        }
    }
    nprev=nnew;
  }
}
```

Here, as before, `wlet` is a `Wavelet` object that embodies a particular wavelet filter and (if required) pre-conditioner.

Figure 13.10.4 shows a sample image and its wavelet transform, represented graphically.

13.10.8 Compression of Images

An immediate application of the multidimensional transform `wtn` is to image compression. The overall procedure is to take the wavelet transform of a digitized image, and then to "allocate bits" among the wavelet coefficients in some highly nonuniform, optimized, manner. As already mentioned, large wavelet coefficients get quantized accurately, while small coefficients are quantized coarsely with only a bit or two — or else are truncated completely. If the resulting quantization levels are still statistically nonuniform, they may then be further compressed by a technique like Huffman coding (§22.5).

While a more detailed description of the "back end" of this process, namely the quantization and coding of the image, is beyond our scope, it is quite straightforward

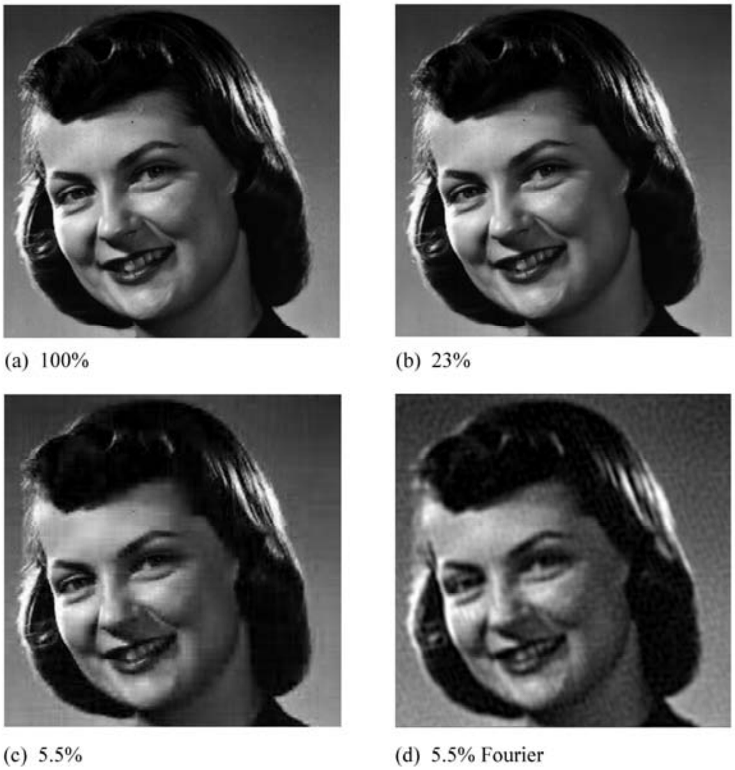

Figure 13.10.5. (a) IEEE test image, 256 × 256 pixels with 8-bit grayscale. (b) The image is transformed into the wavelet basis; 77% of its wavelet components are set to zero (those of smallest magnitude); it is then reconstructed from the remaining 23%. (c) Same as (b), but 94.5% of the wavelet components are deleted. (d) Same as (c), but the Fourier transform is used instead of the wavelet transform. Wavelet coefficients are better than the Fourier coefficients at preserving relevant details.

to demonstrate the "front-end" wavelet encoding with a simple truncation: We keep (with full accuracy) all wavelet coefficients larger than some threshold, and we delete (set to zero) all smaller wavelet coefficients. We can then adjust the threshold to vary the fraction of preserved coefficients.

Figure 13.10.5 shows a sequence of images that differ in the number of wavelet coefficients that have been kept. The original picture (a), which is an official IEEE test image, has 256 by 256 pixels with an 8-bit grayscale. The two reproductions following are reconstructed with 23% (b) and 5.5% (c) of the 65536 wavelet coefficients. The latter image illustrates the kind of compromises made by the truncated wavelet representation. High-contrast edges (the model's right cheek and hair highlights, e.g.) are maintained at a relatively high resolution, while low-contrast areas (the model's left eye and cheek, e.g.) are washed out into what amounts to large constant pixels. Figure 13.10.5(d) is the result of performing the identical procedure with Fourier, instead of wavelet, transforms: The figure is reconstructed from the 5.5% of 65536 real Fourier components having the largest magnitudes. One sees that, since sines and cosines are nonlocal, the resolution is uniformly poor across the picture; also, the deletion of any components produces a mottled "ringing" everywhere. (Practical Fourier image compression schemes therefore break up an image

into small blocks of pixels, 16×16, say, and do rather elaborate smoothing across block boundaries when the image is reconstructed.)

Viewers will sometimes choose (b) over (a), in Figure 13.10.5, as the superior image. The reason is that a "little bit" of wavelet compression has the effect of *denoising* the image. See [8] for a rigorous development.

13.10.9 Fast Solution of Linear Systems

There are interesting applications of wavelets to linear algebra. The basic idea [1] is to think of an integral operator (that is, a large matrix) as a digital image. Suppose that the operator compresses well under a two-dimensional wavelet transform, i.e., that a large fraction of its wavelet coefficients are so small as to be negligible. Then any linear system involving the operator becomes a sparse system in the wavelet basis. In other words, to solve

$$\mathbf{A} \cdot \mathbf{x} = \mathbf{b} \tag{13.10.17}$$

we first wavelet-transform the operator $\mathbf{A}$ and the right-hand side $\mathbf{b}$ by

$$\widetilde{\mathbf{A}} \equiv \mathbf{W} \cdot \mathbf{A} \cdot \mathbf{W}^T, \qquad \widetilde{\mathbf{b}} \equiv \mathbf{W} \cdot \mathbf{b} \tag{13.10.18}$$

where $\mathbf{W}$ represents the one-dimensional wavelet transform, then solve

$$\widetilde{\mathbf{A}} \cdot \widetilde{\mathbf{x}} = \widetilde{\mathbf{b}} \tag{13.10.19}$$

and finally transform to the answer by the inverse wavelet transform

$$\mathbf{x} = \mathbf{W}^T \cdot \widetilde{\mathbf{x}} \tag{13.10.20}$$

(Note that the routine `wtn` does the complete transformation of $\mathbf{A}$ into $\widetilde{\mathbf{A}}$.)

A typical integral operator that compresses well into wavelets has arbitrary (or even nearly singular) elements near its main diagonal, but becomes smooth away from the diagonal. An example might be

$$A_{ij} = \begin{cases} -1 & \text{if } i = j \\ |i-j|^{-1/2} & \text{otherwise} \end{cases} \tag{13.10.21}$$

Figure 13.10.6 shows a graphical representation of the wavelet transform of this matrix, where i and j range over $0 \ldots 255$, using the DAUB4 wavelet, both in its conventional, periodic, implementation, and its modified form on the interval. Elements larger in magnitude than 10^{-3} times the maximum element are shown as black pixels, while elements between 10^{-3} and 10^{-6} are shown in gray. White pixels are $< 10^{-6}$. The indices i and j each number from the lower left.

In the figure, one sees the hierarchical decomposition into power-of-two sized blocks. At the edges or corners of the various blocks, one sees edge effects caused by the wraparound wavelet boundary conditions. Apart from edge effects, within each block, the nonnegligible elements are concentrated along the block diagonals. This is a statement that, for this type of linear operator, a wavelet is coupled mainly to near neighbors in its own hierarchy (square blocks along the main diagonal) and near neighbors in other hierarchies (rectangular blocks off the diagonal).

The number of nonnegligible elements in a matrix like that in Figure 13.10.6 scales only as N, the linear size of the matrix; as a rough rule of thumb it is about

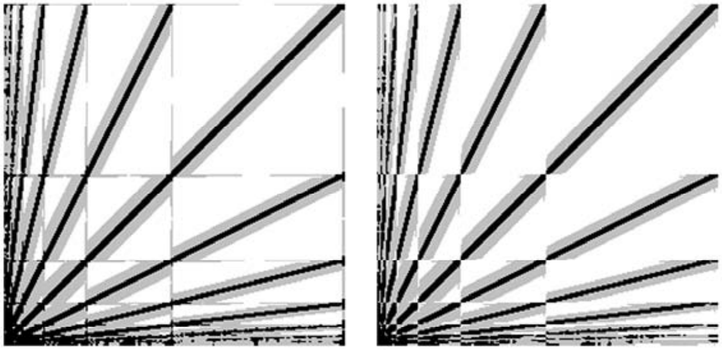

(a) (b)

Figure 13.10.6. Wavelet transform of a 256×256 matrix, represented graphically. The original matrix has a discontinuous cusp along its diagonal, decaying smoothly away on both sides of the diagonal. In wavelet basis, the matrix becomes sparse: Components larger than 10^{-3} are shown as black, components larger than 10^{-6} as gray, and smaller-magnitude components are white. The matrix indices i and j number from the lower left. (a) Ordinary DAUB4 (periodic) is used. (b) Modified DAUB4 on the interval is used, eliminating wraparound artifacts and producing a more regular pattern of significant components.

$10N \log_{10}(1/\epsilon)$, where ϵ is the truncation level, e.g., 10^{-6}. For a 2000 by 2000 matrix, then, the matrix is sparse by a factor on the order of 30.

Various numerical schemes can be used to solve sparse linear systems of this "hierarchically band-diagonal" form. Beylkin, Coifman, and Rokhlin [1] make the interesting observations that (1) the product of two such matrices is itself hierarchically band-diagonal (truncating, of course, newly generated elements that are smaller than the predetermined threshold ϵ); and, moreover, that (2) the product can be formed in order N operations.

Fast matrix multiplication enables finding the matrix inverse by Schultz's (or Hotelling's) method; see §2.5.

Other schemes are also possible for fast solution of hierarchically band-diagonal forms. For example, one can use the conjugate gradient method, implemented in §2.7 as `linbcg`.

CITED REFERENCES AND FURTHER READING:

Daubechies, I. 1992, *Wavelets* (Philadelphia: S.I.A.M.).

Strang, G. 1989, "Wavelets and Dilation Equations: A Brief Introduction," *SIAM Review*, vol. 31, pp. 614–627.

Beylkin, G., Coifman, R., and Rokhlin, V. 1991, "Fast Wavelet Transforms and Numerical Algorithms," *Communications on Pure and Applied Mathematics*, vol. 44, pp. 141–183.[1]

Daubechies, I. 1988, "Orthonormal Bases of Compactly Supported Wavelets," *Communications on Pure and Applied Mathematics*, vol. 41, pp. 909–996.[2]

Vaidyanathan, P.P. 1990, "Multirate Digital Filters, Filter Banks, Polyphase Networks, and Applications," *Proceedings of the IEEE*, vol. 78, pp. 56–93.[3]

Mallat, S.G. 1989, "A Theory for Multiresolution Signal Decomposition: The Wavelet Representation," *IEEE Transactions on Pattern Analysis and Machine Intelligence*, vol. 11, pp. 674–693.[4]

Cohen, A. 1993, "Tables for Wavelet Filters Adapted to Life on an Interval," multiple Web sites; mirrored at `http://www.nr.com/contrib`.[5]

Brewster, M.E. and Beylkin, G. 1994, tables from "Double Precision Wavelet Transform Library," mirrored at http://www.nr.com/contrib.[6]

Cohen, A., Daubechies, I., and Vial, P. 1993, "Wavelets on the Interval and Fast Wavelet Transforms," *Applied and Computational Harmonic Analysis*, vol. 1, pp. 54–81.[7]

Donoho, D. and Johnstone, I.M. 1994, "Ideal Spatial Adaptation via Wavelet Shrinkage," *Biometrika*, vol. 81, no. 3, pp. 425–455.[8]

13.11 Numerical Use of the Sampling Theorem

We have met the sampling theorem before, in §4.5 (in relation to the accuracy of the trapezoidal rule for integration); in §6.9, where we implemented an approximating formula for Dawson's integral due to Rybicki, and in §12.1, where we first saw it in a Fourier context. Now that we have become Fourier sophisticates, we can readily supply a derivation of the formula in §6.9 and illustrate the use of the sampling theorem as a purely numerical tool. Our discussion is identical to Rybicki [1].

For our present purposes, the sampling theorem is most conveniently stated as follows: Consider an arbitrary function $g(t)$ and the grid of sampling points $t_n = \alpha + nh$, where n ranges over the integers and α is a constant that allows an arbitrary shift of the sampling grid. We then write

$$g(t) = \sum_{n=-\infty}^{\infty} g(t_n) \operatorname{sinc} \frac{\pi}{h}(t - t_n) + e(t) \tag{13.11.1}$$

where $\operatorname{sinc} x \equiv \sin x / x$. The summation over the sampling points is called the *sampling representation* of $g(t)$, and $e(t)$ is its error term. The sampling theorem asserts that the sampling representation is exact, that is, $e(t) \equiv 0$, if the Fourier transform of $g(t)$,

$$G(\omega) = \int_{-\infty}^{\infty} g(t) e^{i\omega t}\, dt \tag{13.11.2}$$

vanishes identically for $|\omega| \ge \pi/h$.

When can sampling representations be used to advantage for the approximate numerical computation of functions? In order that the error term be small, the Fourier transform $G(\omega)$ must be sufficiently small for $|\omega| \ge \pi/h$. On the other hand, in order for the summation in (13.11.1) to be approximated by a reasonably small number of terms, the function $g(t)$ itself should be very small outside of a fairly limited range of values of t. Thus we are led to two conditions to be satisfied in order that (13.11.1) be useful numerically: Both the function $g(t)$ and its Fourier transform $G(\omega)$ must rapidly approach zero for large values of their respective arguments.

Unfortunately, these two conditions are mutually antagonistic — the Uncertainty Principle in quantum mechanics. There exist strict limits on how rapidly the simultaneous approach to zero can be in both arguments. According to a theorem of Hardy [2], if $g(t) = O(e^{-t^2})$ as $|t| \to \infty$ and $G(\omega) = O(e^{-\omega^2/4})$ as $|\omega| \to \infty$, then $g(t) \equiv Ce^{-t^2}$, where C is a constant. This can be interpreted as saying that, of all functions, the Gaussian is the most rapidly decaying in both t and ω, and in this sense is the "best" function to be expressed numerically as a sampling representation.

Let us then write for the Gaussian $g(t) = e^{-t^2}$,

$$e^{-t^2} = \sum_{n=-\infty}^{\infty} e^{-t_n^2} \operatorname{sinc} \frac{\pi}{h}(t - t_n) + e(t) \tag{13.11.3}$$

The error $e(t)$ depends on the parameters h and α as well as on t, but it is sufficient for the present purposes to state the bound,

$$|e(t)| < e^{-(\pi/2h)^2} \tag{13.11.4}$$

which can be understood simply as the order of magnitude of the Fourier transform of the Gaussian at the point where it "spills over" into the region $|\omega| > \pi/h$.

When the summation in (13.11.3) is approximated by one with finite limits, say from $N_0 - N$ to $N_0 + N$, where N_0 is the integer nearest to $-\alpha/h$, there is a further truncation error. However, if N is chosen so that $N > \pi/(2h^2)$, the truncation error in the summation is less than the bound given by (13.11.4), and, since this bound is an overestimate, we shall continue to use it for (13.11.3) as well. The truncated summation gives a remarkably accurate representation for the Gaussian even for moderate values of N. For example, $|e(t)| < 5\times10^{-5}$ for $h = 1/2$ and $N = 7$; $|e(t)| < 2\times10^{-10}$ for $h = 1/3$ and $N = 15$; and $|e(t)| < 7\times10^{-18}$ for $h = 1/4$ and $N = 25$.

One may ask, what is the point of such a numerical representation for the Gaussian, which can be computed so easily and quickly as an exponential? The answer is that many transcendental functions can be expressed as an integral involving the Gaussian, and by substituting (13.11.3) one can often find excellent approximations to the integrals as a sum over elementary functions.

Let us consider as an example the function $w(z)$ of the complex variable $z = x + iy$, related to the complex error function by

$$w(z) = e^{-z^2}\,\mathrm{erfc}(-iz) \tag{13.11.5}$$

having the integral representation

$$w(z) = \frac{1}{\pi i}\int_C \frac{e^{-t^2}\,dt}{t - z} \tag{13.11.6}$$

where the contour C extends from $-\infty$ to ∞, passing below z (see, e.g., [3]). Many methods exist for the evaluation of this function (e.g., [4]). Substituting the sampling representation (13.11.3) into (13.11.6) and performing the resulting elementary contour integrals, we obtain

$$w(z) \approx \frac{1}{\pi i}\sum_{n=-\infty}^{\infty} he^{-t_n^2}\,\frac{1 - (-1)^n e^{-\pi i(\alpha - z)/h}}{t_n - z} \tag{13.11.7}$$

where we now omit the error term. One should note that there is no singularity as $z \to t_m$ for some $n = m$, but a special treatment of the mth term will be required in this case (for example, by power series expansion).

An alternative form of equation (13.11.7) can be found by expressing the complex exponential in (13.11.7) in terms of trigonometric functions and using the sampling representation (13.11.3) with z replacing t. This yields

$$w(z) \approx e^{-z^2} + \frac{1}{\pi i}\sum_{n=-\infty}^{\infty} he^{-t_n^2}\,\frac{1 - (-1)^n \cos\pi(\alpha - z)/h}{t_n - z} \tag{13.11.8}$$

This form is particularly useful in obtaining Re $w(z)$ when $|y| \ll 1$. Note that in evaluating (13.11.7) the complex exponential inside the summation is a constant and needs to be evaluated only once; a similar comment holds for the cosine in (13.11.8).

There are a variety of formulas that can now be derived from either equation (13.11.7) or (13.11.8) by choosing particular values of α. Eight interesting choices are $\alpha = 0$, x, iy, or z, plus the values obtained by adding $h/2$ to each of these. Since the error bound (13.11.3) assumed a real value of α, the choices involving a complex α are useful only if the imaginary part of z is not too large. This is not the place to catalog all 16 possible formulas, and we give only two particular cases that show some of the important features.

First of all let $\alpha = 0$ in equation (13.11.8), which yields,

$$w(z) \approx e^{-z^2} + \frac{1}{\pi i}\sum_{n=-\infty}^{\infty} he^{-(nh)^2}\,\frac{1 - (-1)^n \cos(\pi z/h)}{nh - z} \tag{13.11.9}$$

This approximation is good over the entire z-plane. As stated previously, one has to treat the case where one denominator becomes small by expansion in a power series. Formulas for

the case $\alpha = 0$ were discussed briefly in [5]. They are similar, but not identical, to formulas derived by Chiarella and Reichel [6], using the method of Goodwin [7].

Next, let $\alpha = z$ in (13.11.7), which yields

$$w(z) \approx e^{-z^2} - \frac{2}{\pi i} \sum_{n \text{ odd}} \frac{e^{-(z-nh)^2}}{n} \tag{13.11.10}$$

the sum being over all odd integers (positive and negative). Note that we have made the substitution $n \to -n$ in the summation. This formula is simpler than (13.11.9) and contains half the number of terms, but its error is worse if y is large. Equation (13.11.10) is the source of the approximation formula (6.9.3) for Dawson's integral, used in §6.9.

CITED REFERENCES AND FURTHER READING:

Rybicki, G.B. 1989, "Dawson's Integral and The Sampling Theorem," *Computers in Physics*, vol. 3, no. 2, pp. 85–87.[1]

Hardy, G.H. 1933, "A Theorem Concerning Fourier Transforms," *Journal of the London Mathematical Society*, vol. 8, pp. 227–231.[2]

Abramowitz, M., and Stegun, I.A. 1964, *Handbook of Mathematical Functions* (Washington: National Bureau of Standards); reprinted 1968 (New York: Dover); online at `http://www.nr.com/aands`.[3]

Gautschi, W. 1970, "Efficient Computation of the Complex Error function," *SIAM Journal on Numerical Analysis*, vol. 7, pp. 187–198.[4]

Armstrong, B.H., and Nicholls, R.W. 1972, *Emission, Absorption and Transfer of Radiation in Heated Atmospheres* (New York: Pergamon).[5]

Chiarella, C., and Reichel, A. 1968, "On the Evaluation of Integrals Related to the Error Function," *Mathematics of Computation*, vol. 22, pp. 137–143.[6]

Goodwin, E.T. 1949, "The Evaluation of Integrals of the Form $\int_{-x}^{+x} f(x)e^{-x^2}dx$," *Proceedings of the Cambridge Philosophical Society*, vol. 45, pp. 241–245.[7]

CHAPTER 14

Statistical Description of Data

14.0 Introduction

In this chapter and the next, the concept of *data* enters the discussion more prominently than before.

Data consist of numbers, of course. But these numbers are given to the computer, not produced by it. These are numbers to be treated with considerable respect, neither to be tampered with, nor subjected to a computational process whose character you do not completely understand. You are well advised to acquire a reverence for data, rather different from the "sporty" attitude that is sometimes allowable, or even commendable, in other numerical tasks.

The analysis of data inevitably involves some trafficking with the field of *statistics*, that wonderful gray area that is not quite a branch of mathematics — and just as surely not quite a branch of science. In the following sections, you will repeatedly encounter the following paradigm, usually called a *tail test* or *p-value test*:

- apply some formula to the data to compute "a statistic"
- compute where the value of that statistic falls in a probability distribution that is computed on the basis of some "null hypothesis"
- if it falls in a very unlikely spot, way out on a tail of the distribution, conclude that the null hypothesis is *false* for your data set

If a statistic falls in a *reasonable* part of the distribution, you must not make the mistake of concluding that the null hypothesis is "verified" or "proved." That is the curse of statistics, that it can never prove things, only disprove them! At best, you can substantiate a hypothesis by ruling out, statistically, a whole long list of competing hypotheses, every one that has ever been proposed. After a while your adversaries and competitors will give up trying to think of alternative hypotheses, or else they will grow old and die, and *then your hypothesis will become accepted*. Sounds crazy, we know, but that's how science works!*

In this book we make a somewhat arbitrary distinction between data analysis procedures that are *model-independent* and those that are *model-dependent*. In the

*"Science advances one funeral at a time." —Max Planck (attributed)

former category, we include so-called *descriptive statistics* that characterize a data set in general terms: its mean, variance, and so on. We also include statistical tests that seek to establish the "sameness" or "differentness" of two or more data sets, or that seek to establish and measure a degree of *correlation* between two data sets. These subjects are discussed in this chapter.

In the other category, model-dependent statistics, we lump the whole subject of fitting data to a theory, parameter estimation, least-squares fits, and so on. Those subjects are introduced in Chapter 15.

Section 14.1 deals with so-called *measures of central tendency*, the moments of a distribution, the median and mode. In §14.2 we learn to test whether different data sets are drawn from distributions with different values of these measures of central tendency. This leads naturally, in §14.3, to the more general question of whether two distributions can be shown to be (significantly) different.

In §14.4 – §14.7, we deal with *measures of association* for two distributions. We want to determine whether two variables are "correlated" or "dependent" on one another. If they are, we want to characterize the degree of correlation in some simple ways. The distinction between parametric and nonparametric (rank) methods is emphasized. Information-theoretic methods are discussed in §14.7. Section 14.9 introduces the concept of data smoothing, and discusses the particular case of Savitzky-Golay smoothing filters.

This chapter draws mathematically on the material on special functions that was presented in Chapter 6, especially §6.1 – §6.4 and §6.14. You may wish, at this point, to review those sections.

Bayesian methods make little appearance in this chapter, but become more prominent in the two chapters following this one.

CITED REFERENCES AND FURTHER READING:

Bevington, P.R., and Robinson, D.K. 2002, *Data Reduction and Error Analysis for the Physical Sciences*, 3rd ed. (New York: McGraw-Hill).

Taylor, J.R. 1997, *An Introduction to Error Analysis*, 2nd ed. (Sausalito, CA: University Science Books).

Devore, J.L. 2003, *Probability and Statistics for Engineering and the Sciences*, 6th ed. (Belmont, CA: Duxbury Press).

Wall, J.V., and Jenkins, C.R. 2003, *Practical Statistics for Astronomers* (Cambridge, UK: Cambridge University Press).

Lupton, R. 1993, *Statistics in Theory and Practice* (Princeton, NJ: Princeton University Press).

14.1 Moments of a Distribution: Mean, Variance, Skewness, and So Forth

When a set of values has a sufficiently strong central tendency, that is, a tendency to cluster around some particular value, then it may be useful to characterize the set by a few numbers that are related to its *moments*, the sums of integer powers of the values.

Best known is the *mean* of the values $x_0, \ldots, x_{N-1}$,

$$\overline{x} = \frac{1}{N} \sum_{j=0}^{N-1} x_j \tag{14.1.1}$$

which estimates the value around which central clustering occurs. Note the use of an overbar to denote the mean; angle brackets are an equally common notation, e.g., $\langle x \rangle$. You should be aware that the mean is not the only available estimator of this quantity, nor is it necessarily the best one. For values drawn from a probability distribution with very broad "tails," the mean may converge poorly, or not at all, as the number of sampled points is increased. Alternative estimators, the *median* and the *mode*, are mentioned at the end of this section.

Having characterized a distribution's central value, one conventionally next characterizes its "width" or "variability" around that value. Here again, more than one measure is available. Most common is the *variance*,

$$\text{Var}(x_0 \ldots x_{N-1}) = \frac{1}{N-1} \sum_{j=0}^{N-1} (x_j - \overline{x})^2 \tag{14.1.2}$$

or its square root, the *standard deviation*,

$$\sigma(x_0 \ldots x_{N-1}) = \sqrt{\text{Var}(x_0 \ldots x_{N-1})} \tag{14.1.3}$$

Equation (14.1.2) estimates the mean squared deviation of x from its mean value. There is a long story about why the denominator of (14.1.2) is $N-1$ instead of N. If you have never heard that story, you should consult any good statistics text. Here we will be content to note that the $N-1$ *should* be changed to N if you are ever in the situation of measuring the variance of a distribution whose mean $\overline{x}$ is known a priori rather than being estimated from the data. (We might also comment that if the difference between N and $N-1$ ever matters to you, then you are probably up to no good anyway — e.g., trying to substantiate a questionable hypothesis with marginal data.)

If we calculate equation (14.1.1) many times with different sets of sampled data (each set having N values), the values $\overline{x}$ will themselves have a standard deviation. This is called the *standard error* of the estimated mean $\overline{x}$. When the underlying distribution is Gaussian, it is given approximately by $\sigma/\sqrt{N}$. Correspondingly, there is a standard error of the estimated variance, equation (14.1.2), which is approximately $\sigma^2\sqrt{2/N}$, and a standard error for the estimated σ, equation (14.1.3), which is approximately $\sigma/\sqrt{2N}$.

As the mean depends on the first moment of the data, so do the variance and standard deviation depend on the second moment. It is not uncommon, in real life, to be dealing with a distribution whose second moment does not exist (i.e., is infinite). In this case, the variance or standard deviation is useless as a measure of the data's width around its central value: The values obtained from equations (14.1.2) or (14.1.3) will not converge with increased numbers of points, nor show any consistency from data set to data set drawn from the same distribution. This can occur even when the width of the peak looks, by eye, perfectly finite. A more robust estimator

of the width is the *average deviation* or *mean absolute deviation*, defined by

$$\text{ADev}(x_0 \dots x_{N-1}) = \frac{1}{N} \sum_{j=0}^{N-1} \left| x_j - \overline{x} \right| \tag{14.1.4}$$

One often substitutes the sample median x_{med} for $\overline{x}$ in equation (14.1.4). For any fixed sample, the median in fact minimizes the mean absolute deviation.

Statisticians have historically sniffed at the use of (14.1.4) instead of (14.1.2), since the absolute value brackets in (14.1.4) are "nonanalytic" and make theorem-proving more difficult. In recent years, however, the fashion has changed, and the subject of *robust estimation* (meaning, estimation for broad distributions with significant numbers of "outlier" points) has become a popular and important one. Higher moments, or statistics involving higher powers of the input data, are almost always less robust than lower moments or statistics that involve only linear sums or (the lowest moment of all) counting.

That being the case, the *skewness* or *third moment*, and the *kurtosis* or *fourth moment* should be used with caution or, better yet, not at all.

The skewness characterizes the degree of asymmetry of a distribution around its mean. While the mean, standard deviation, and average deviation are *dimensional* quantities, that is, have the same units as the measured quantities x_j, the skewness is conventionally defined in such a way as to make it *nondimensional*. It is a pure number that characterizes only the shape of the distribution. The usual definition is

$$\text{Skew}(x_0 \dots x_{N-1}) = \frac{1}{N} \sum_{j=0}^{N-1} \left[\frac{x_j - \overline{x}}{\sigma} \right]^3 \tag{14.1.5}$$

where $\sigma = \sigma(x_0 \dots x_{N-1})$ is the distribution's standard deviation (14.1.3). A positive value of skewness signifies a distribution with an asymmetric tail extending out toward more positive x; a negative value signifies a distribution whose tail extends out toward more negative x (see Figure 14.1.1).

Of course, any set of N measured values is likely to give a nonzero value for (14.1.5), even if the underlying distribution is in fact symmetrical (has zero skewness). For (14.1.5) to be meaningful, we need to have some idea of *its* standard error. Unfortunately, that depends on the shape of the underlying distribution, and rather critically on its tails! For the idealized case of a normal (Gaussian) distribution, the standard error of (14.1.5) is approximately $\sqrt{15/N}$ when $\overline{x}$ is the true mean and $\sqrt{6/N}$ when it is estimated by the sample mean, (14.1.1). (Yes, using the sample mean is likely to give a more accurate estimate than using the true mean!) In real life it is good practice to believe in skewnesses only when they are several or many times as large as this.

The kurtosis is also a nondimensional quantity. It measures the relative peakedness or flatness of a distribution. Relative to what? A normal distribution! What else? A distribution with positive kurtosis is termed *leptokurtic*; the outline of the Matterhorn is an example. A distribution with negative kurtosis is termed *platykurtic*; the outline of a loaf of bread is an example. (See Figure 14.1.1.) And, as you no doubt expect, an in-between distribution is termed *mesokurtic*.

The conventional definition of the kurtosis is

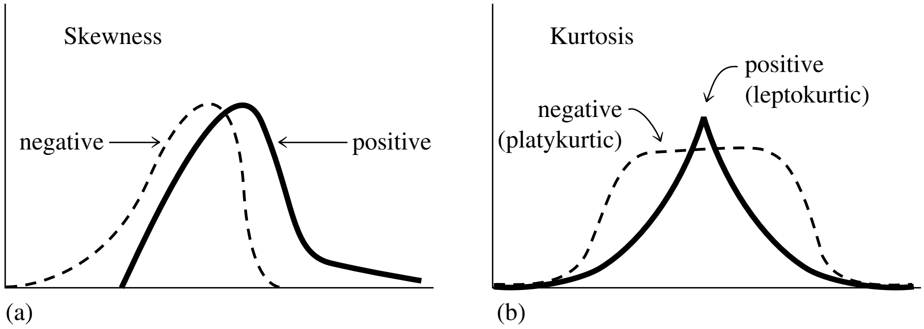

Figure 14.1.1. Distributions whose third and fourth moments are significantly different from a normal (Gaussian) distribution. (a) Skewness or third moment. (b) Kurtosis or fourth moment.

$$\mathrm{Kurt}(x_0 \ldots x_{N-1}) = \left\{ \frac{1}{N} \sum_{j=0}^{N-1} \left[\frac{x_j - \overline{x}}{\sigma} \right]^4 \right\} - 3 \tag{14.1.6}$$

where the -3 term makes the value zero for a normal distribution.

The standard error of (14.1.6) as an estimator of the kurtosis of an underlying normal distribution is $\sqrt{96/N}$ when σ is the true standard deviation, and $\sqrt{24/N}$ when it is the sample estimate (14.1.3). (Yes, you are better off using the sample variance.) However, the kurtosis depends on such a high moment that there are many real-life distributions for which the standard deviation of (14.1.6) as an estimator is effectively infinite.

Calculation of the quantities defined in this section is perfectly straightforward. Many textbooks use the binomial theorem to expand out the definitions into sums of various powers of the data, e.g., the familiar

$$\mathrm{Var}(x_0 \ldots x_{N-1}) = \frac{1}{N-1} \left[\left(\sum_{j=0}^{N-1} x_j^2 \right) - N\overline{x}^2 \right] \approx \overline{x^2} - \overline{x}^2 \tag{14.1.7}$$

but this can magnify the roundoff error by a large factor and is generally unjustifiable in terms of computing speed. A clever way to minimize roundoff error, especially for large samples, is to use the *corrected two-pass algorithm* [1]: First calculate $\overline{x}$, then calculate $\mathrm{Var}(x_0 \ldots x_{N-1})$ by

$$\mathrm{Var}(x_0 \ldots x_{N-1}) = \frac{1}{N-1} \left\{ \sum_{j=0}^{N-1} (x_j - \overline{x})^2 - \frac{1}{N} \left[\sum_{j=0}^{N-1} (x_j - \overline{x}) \right]^2 \right\} \tag{14.1.8}$$

The second sum would be zero if $\overline{x}$ were exact, but otherwise it does a good job of correcting the roundoff error in the first term.

moment.h

```
void moment(VecDoub_I &data, Doub &ave, Doub &adev, Doub &sdev, Doub &var,
    Doub &skew, Doub &curt) {
```

Given an array of `data[0..n-1]`, this routine returns its mean `ave`, average deviation `adev`, standard deviation `sdev`, variance `var`, skewness `skew`, and kurtosis `curt`.

```
    Int j,n=data.size();
    Doub ep=0.0,s,p;
```

```
    if (n <= 1) throw("n must be at least 2 in moment");
    s=0.0;                                    First pass to get the mean.
    for (j=0;j<n;j++) s += data[j];
    ave=s/n;
    adev=var=skew=curt=0.0;                   Second pass to get the first (absolute), sec-
    for (j=0;j<n;j++) {                           ond, third, and fourth moments of the
        adev += abs(s=data[j]-ave);               deviation from the mean.
        ep += s;
        var += (p=s*s);
        skew += (p *= s);
        curt += (p *= s);
    }
    adev /= n;
    var=(var-ep*ep/n)/(n-1);                  Corrected two-pass formula.
    sdev=sqrt(var);                           Put the pieces together according to the con-
    if (var != 0.0) {                             ventional definitions.
        skew /= (n*var*sdev);
        curt=curt/(n*var*var)-3.0;
    } else throw("No skew/kurtosis when variance = 0 (in moment)");
}
```

14.1.1 Semi-Invariants

The mean and variance of independent random variables are additive: If x and y are drawn independently from two, possibly different, probability distributions, then

$$\overline{(x+y)} = \overline{x} + \overline{y} \qquad \mathrm{Var}(x+y) = \mathrm{Var}(x) + \mathrm{Var}(x) \tag{14.1.9}$$

Higher moments are not, in general, additive. However, certain combinations of them, called *semi-invariants*, are in fact additive. If the centered moments of a distribution are denoted M_k,

$$M_k \equiv \left\langle (x_i - \overline{x})^k \right\rangle \tag{14.1.10}$$

so that, e.g., $M_2 = \mathrm{Var}(x)$, then the first few semi-invariants, denoted I_k, are given by

$$\begin{gathered} I_2 = M_2 \qquad I_3 = M_3 \qquad I_4 = M_4 - 3M_2^2 \\ I_5 = M_5 - 10M_2M_3 \qquad I_6 = M_6 - 15M_2M_4 - 10M_3^2 + 30M_2^3 \end{gathered} \tag{14.1.11}$$

Notice that the skewness and kurtosis, equations (14.1.5) and (14.1.6), are simple powers of the semi-invariants,

$$\mathrm{Skew}(x) = I_3/I_2^{3/2} \qquad \mathrm{Kurt}(x) = I_4/I_2^2 \tag{14.1.12}$$

A Gaussian distribution has all its semi-invariants higher than I_2 equal to zero. A Poisson distribution has all of its semi-invariants equal to its mean. For more details, see [2].

14.1.2 Median and Mode

The median of a probability distribution function $p(x)$ is the value x_{med} for which larger and smaller values of x are equally probable:

$$\int_{-\infty}^{x_{\mathrm{med}}} p(x)\,dx = \frac{1}{2} = \int_{x_{\mathrm{med}}}^{\infty} p(x)\,dx \tag{14.1.13}$$

The median of a distribution is estimated from a sample of values $x_0, \ldots, x_{N-1}$ by finding that value x_i which has equal numbers of values above it and below it. Of course, this is not possible when N is even. In that case it is conventional to

estimate the median as the mean of the unique *two* central values. If the values x_j, $j = 0, \dots, N-1$, are sorted into ascending (or, for that matter, descending) order, then the formula for the median is

$$x_{\text{med}} = \begin{cases} x_{(N-1)/2}, & N \text{ odd} \\ \frac{1}{2}(x_{(N/2)-1} + x_{N/2}), & N \text{ even} \end{cases} \tag{14.1.14}$$

If a distribution has a strong central tendency, so that most of its area is under a single peak, then the median is an estimator of the central value. It is a more robust estimator than the mean is: The median fails as an estimator only if the area in the tails is large, while the mean fails if the first moment of the tails is large; it is easy to construct examples where the first moment of the tails is large even though their area is negligible.

To find the median of a set of values, one can proceed by sorting the set and then applying (14.1.14). This is a process of order $N \log N$. You might rightly think that this is wasteful, since it yields much more information than just the median (e.g., the upper and lower quartile points, the deciles, etc.). In fact, we saw in §8.5 that the element $x_{(N-1)/2}$ can be located in of order N operations. Consult that section for routines, including a method for getting a good approximation to the median in a single pass through the data.

The *mode* of a probability distribution function $p(x)$ is the value of x where it takes on a maximum value. The mode is useful primarily when there is a single, sharp maximum, in which case it estimates the central value. Occasionally, a distribution will be *bimodal*, with two relative maxima; then one may wish to know the two modes individually. Note that, in such cases, the mean and median are not very useful, since they will give only a "compromise" value between the two peaks.

CITED REFERENCES AND FURTHER READING:

Bevington, P.R., and Robinson, D.K. 2002, *Data Reduction and Error Analysis for the Physical Sciences*, 3rd ed. (New York: McGraw-Hill), Chapter 1.

Spiegel, M.R., Schiller, J., and Srinivasan, R.A. 2000, *Schaum's Outline of Theory and Problem of Probability and Statistics*, 2nd ed. (New York: McGraw-Hill).

Stuart, A., and Ord, J.K. 1994, *Kendall's Advanced Theory of Statistics*, 6th ed. (London: Edward Arnold) [previous eds. published as Kendall, M., and Stuart, A., *The Advanced Theory of Statistics*], vol. 1, §10.15

Norusis, M.J. 2006, *SPSS 14.0 Guide to Data Analysis* (Englewood Cliffs, NJ: Prentice-Hall).

Chan, T.F., Golub, G.H., and LeVeque, R.J. 1983, "Algorithms for Computing the Sample Variance: Analysis and Recommendations," *American Statistician*, vol. 37, pp. 242–247.[1]

Cramér, H. 1946, *Mathematical Methods of Statistics* (Princeton, NJ: Princeton University Press), §15.10.[2]

14.2 Do Two Distributions Have the Same Means or Variances?

Not uncommonly we want to know whether two distributions have the same mean. For example, a first set of measured values may have been gathered before

some event, a second set after it. We want to know whether the event, a "treatment" or a "change in a control parameter," made a difference.

Our first thought is to ask "how many standard deviations" one sample mean is from the other. That number may in fact be a useful thing to know. It does relate to the strength or "importance" of a difference of means *if that difference is genuine*. However, by itself, it says nothing about whether the difference *is* genuine, that is, statistically significant. A difference of means can be very small compared to the standard deviation, and yet very significant, if the number of data points is large. Conversely, a difference may be moderately large but not significant, if the data are sparse. We will be meeting these distinct concepts of *strength* and *significance* several times in the next few sections.

A quantity that measures the significance of a difference of means is not the number of standard deviations that they are apart, but the number of so-called *standard errors* that they are apart. The standard error of a set of values measures the accuracy with which the sample mean estimates the population (or "true") mean. Typically the standard error is equal to the sample's standard deviation divided by the square root of the number of points in the sample.

14.2.1 Student's t-Test for Significantly Different Means

Applying the concept of standard error, the conventional statistic for measuring the significance of a difference of means is termed *Student's t*. When the two distributions are thought to have the same variance, but possibly different means, then Student's t is computed as follows: First, estimate the standard error of the difference of the means, s_D, from the "pooled variance" by the formula

$$s_D = \sqrt{\frac{\sum_{i \in A}(x_i - \overline{x}_A)^2 + \sum_{i \in B}(x_i - \overline{x}_B)^2}{N_A + N_B - 2}\left(\frac{1}{N_A} + \frac{1}{N_B}\right)} \tag{14.2.1}$$

where each sum is over the points in one sample, the first or second; each mean likewise refers to one sample or the other; and N_A and N_B are the numbers of points in the first and second samples, respectively. Second, compute t by

$$t = \frac{\overline{x}_A - \overline{x}_B}{s_D} \tag{14.2.2}$$

Third, evaluate the p-value or significance of this value of t for Student's distribution with $N_A + N_B - 2$ degrees of freedom, by equation (6.14.11).

The p-value is a number between zero and one. It is the probability that $|t|$ could be this large or larger just by chance, for distributions with equal means. Therefore, a small numerical value of the p-value (0.01 or 0.001) means that the observed difference is "very significant." The function $A(t|\nu)$ in equation (6.14.11) is one minus the p-value.

As a routine, we have

```
void ttest(VecDoub_I &data1, VecDoub_I &data2, Doub &t, Doub &prob)
```
stattests.h

Given the arrays `data1[0..n1-1]` and `data2[0..n2-1]`, returns Student's t as `t`, and its p-value as `prob`, small values of `prob` indicating that the arrays have significantly different means. The data arrays are assumed to be drawn from populations with the same true variance.

```
{
    Beta beta;
```

```
    Doub var1,var2,svar,df,ave1,ave2;
    Int n1=data1.size(), n2=data2.size();
    avevar(data1,ave1,var1);
    avevar(data2,ave2,var2);
    df=n1+n2-2;                                     Degrees of freedom.
    svar=((n1-1)*var1+(n2-1)*var2)/df;              Pooled variance.
    t=(ave1-ave2)/sqrt(svar*(1.0/n1+1.0/n2));
    prob=beta.betai(0.5*df,0.5,df/(df+t*t));        See equation (6.14.11).
}
```

which makes use of the following routine for computing the mean and variance of a set of numbers,

moment.h

```
void avevar(VecDoub_I &data, Doub &ave, Doub &var) {
Given array data[0..n-1], returns its mean as ave and its variance as var.
    Doub s,ep;
    Int j,n=data.size();
    ave=0.0;
    for (j=0;j<n;j++) ave += data[j];
    ave /= n;
    var=ep=0.0;
    for (j=0;j<n;j++) {
        s=data[j]-ave;
        ep += s;
        var += s*s;
    }
    var=(var-ep*ep/n)/(n-1);            Corrected two-pass formula (14.1.8).
}
```

The next case to consider is where the two distributions have significantly different variances, but we nevertheless want to know if their means are the same or different. (A treatment for baldness has caused some patients to *lose* all their hair and turned others into werewolves, but we want to know if it helps cure baldness *on the average*!) Be suspicious of the unequal-variance t-test: If two distributions have very different variances, then they may also be substantially different in shape; in that case, the difference of the means may not be a particularly useful thing to know.

To find out whether the two data sets have variances that are significantly different, you use the *F-test*, described later on in this section.

The relevant statistic for the unequal-variance t-test is

$$t = \frac{\overline{x}_A - \overline{x}_B}{[\mathrm{Var}(x_A)/N_A + \mathrm{Var}(x_B)/N_B]^{1/2}} \tag{14.2.3}$$

This statistic is distributed *approximately* as Student's t with a number of degrees of freedom equal to

$$\frac{\left[\dfrac{\mathrm{Var}(x_A)}{N_A} + \dfrac{\mathrm{Var}(x_B)}{N_B}\right]^2}{\dfrac{\left[\mathrm{Var}(x_A)/N_A\right]^2}{N_A - 1} + \dfrac{\left[\mathrm{Var}(x_B)/N_B\right]^2}{N_B - 1}} \tag{14.2.4}$$

Expression (14.2.4) is in general not an integer, but equation (6.14.11) doesn't care.

The routine is

```
void tutest(VecDoub_I &data1, VecDoub_I &data2, Doub &t, Doub &prob) {
```
stattests.h

Given the arrays data1[0..n1-1] and data2[0..n2-1], this routine returns Student's t as t, and its p-value as prob, small values of prob indicating that the arrays have significantly different means. The data arrays are allowed to be drawn from populations with unequal variances.

```
    Beta beta;
    Doub var1,var2,df,ave1,ave2;
    Int n1=data1.size(), n2=data2.size();
    avevar(data1,ave1,var1);
    avevar(data2,ave2,var2);
    t=(ave1-ave2)/sqrt(var1/n1+var2/n2);
    df=SQR(var1/n1+var2/n2)/(SQR(var1/n1)/(n1-1)+SQR(var2/n2)/(n2-1));
    prob=beta.betai(0.5*df,0.5,df/(df+SQR(t)));
}
```

Our final example of a Student's t-test is the case of *paired samples*. Here we imagine that much of the variance in *both* samples is due to effects that are point-by-point identical in the two samples. For example, we might have two job candidates who have each been rated by the same ten members of a hiring committee. We want to know if the means of the ten scores differ significantly. We first try ttest above, and obtain a value of prob that is not especially significant (e.g., > 0.05). But perhaps the significance is being washed out by the tendency of some committee members always to give high scores and others always to give low scores, which increases the apparent variance and thus decreases the significance of any difference in the means. We thus try the paired-sample formulas,

$$\text{Cov}(x_A, x_B) \equiv \frac{1}{N-1} \sum_{i=0}^{N-1} (x_{Ai} - \overline{x}_A)(x_{Bi} - \overline{x}_B) \tag{14.2.5}$$

$$s_D = \left[\frac{\text{Var}(x_A) + \text{Var}(x_B) - 2\text{Cov}(x_A, x_B)}{N} \right]^{1/2} \tag{14.2.6}$$

$$t = \frac{\overline{x}_A - \overline{x}_B}{s_D} \tag{14.2.7}$$

where N is the number in each sample (number of pairs). Notice that it is important that a particular value of i label the corresponding points in each sample, that is, the ones that are paired. The p-value for the t statistic in (14.2.7) is evaluated for $N - 1$ degrees of freedom.

The routine is

```
void tptest(VecDoub_I &data1, VecDoub_I &data2, Doub &t, Doub &prob) {
```
stattests.h

Given the paired arrays data1[0..n-1] and data2[0..n-1], this routine returns Student's t for paired data as t, and its p-value as prob, small values of prob indicating a significant difference of means.

```
    Beta beta;
    Int j, n=data1.size();
    Doub var1,var2,ave1,ave2,sd,df,cov=0.0;
    avevar(data1,ave1,var1);
    avevar(data2,ave2,var2);
    for (j=0;j<n;j++) cov += (data1[j]-ave1)*(data2[j]-ave2);
    cov /= (df=n-1);
    sd=sqrt((var1+var2-2.0*cov)/n);
    t=(ave1-ave2)/sd;
    prob=beta.betai(0.5*df,0.5,df/(df+t*t));
}
```

14.2.2 F-Test for Significantly Different Variances

The *F-test* tests the hypothesis that two samples have different variances by trying to reject the null hypothesis that their variances are actually consistent. The statistic F is the ratio of one variance to the other, so values either $\gg 1$ or $\ll 1$ will indicate very significant differences. The distribution of F in the null case is given in equation (6.14.49), which is evaluated using the routine `betai`. In the most common case, we are willing to disprove the null hypothesis (of equal variances) by either very large or very small values of F, so the correct p-value is *two-tailed*, the sum of two incomplete beta functions. It turns out, by equation (6.4.3), that the two tails are always equal; we need compute only one, and double it. Occasionally, when the null hypothesis is strongly viable, the identity of the two tails can become confused, giving an indicated probability greater than one. Changing the probability to two minus itself correctly exchanges the tails. These considerations and equation (6.4.3) give the routine

stattests.h

```
void ftest(VecDoub_I &data1, VecDoub_I &data2, Doub &f, Doub &prob) {
```

Given the arrays `data1[0..n1-1]` and `data2[0..n2-1]`, this routine returns the value of `f`, and its p-value as `prob`. Small values of `prob` indicate that the two arrays have significantly different variances.

```
    Beta beta;
    Doub var1,var2,ave1,ave2,df1,df2;
    Int n1=data1.size(), n2=data2.size();
    avevar(data1,ave1,var1);
    avevar(data2,ave2,var2);
    if (var1 > var2) {                  Make F the ratio of the larger variance to the smaller
        f=var1/var2;                        one.
        df1=n1-1;
        df2=n2-1;
    } else {
        f=var2/var1;
        df1=n2-1;
        df2=n1-1;
    }
    prob = 2.0*beta.betai(0.5*df2,0.5*df1,df2/(df2+df1*f));
    if (prob > 1.0) prob=2.-prob;
}
```


CITED REFERENCES AND FURTHER READING:

Spiegel, M.R., Schiller, J., and Srinivasan, R.A. 2000, *Schaum's Outline of Theory and Problem of Probability and Statistics*, 2nd ed. (New York: McGraw-Hill).

Lupton, R. 1993, *Statistics in Theory and Practice* (Princeton, NJ: Princeton University Press), Chapter 9.

Devore, J.L. 2003, *Probability and Statistics for Engineering and the Sciences*, 6th ed. (Belmont, CA: Duxbury Press), Chapters 7–8.

Norusis, M.J. 2006, *SPSS 14.0 Guide to Data Analysis* (Englewood Cliffs, NJ: Prentice-Hall).

14.3 Are Two Distributions Different?

Given two sets of data, we can generalize the questions asked in the previous section and ask the single question: Are the two sets drawn from the same distribution function, or from different distribution functions? Equivalently, in proper

statistical language, "Can we disprove, to a certain required level of significance, the null hypothesis that two data sets are drawn from the same population distribution function?" Disproving the null hypothesis in effect proves that the data sets are from different distributions. Failing to disprove the null hypothesis, on the other hand, only shows that the data sets can be *consistent* with a single distribution function. One can never *prove* that two data sets come from a single distribution, since, e.g., no practical amount of data can distinguish between two distributions that differ only by one part in 10^{10}.

Proving that two distributions are different, or showing that they are consistent, is a task that comes up all the time in many areas of research: Are the visible stars distributed uniformly in the sky? (That is, is the distribution of stars as a function of declination — position in the sky — the same as the distribution of sky area as a function of declination?) Are educational patterns the same in Brooklyn as in the Bronx? (That is, are the distributions of people as a function of last-grade-attended the same?) Do two brands of fluorescent lights have the same distribution of burn-out times? Is the incidence of chicken pox the same for first-born, second-born, third-born children, etc.?

These four examples illustrate the four combinations arising from two different dichotomies: (1) The data are either continuous or binned. (2) Either we wish to compare one data set to a known distribution, or we wish to compare two equally unknown data sets. The data sets on fluorescent lights and on stars are continuous, since we can be given lists of individual burnout times or of stellar positions. The data sets on chicken pox and educational level are binned, since we are given tables of numbers of events in discrete categories: first-born, second-born, etc.; or 6th grade, 7th grade, etc. Stars and chicken pox, on the other hand, share the property that the null hypothesis is a known distribution (distribution of area in the sky, or incidence of chicken pox in the general population). Fluorescent lights and educational level involve the comparison of two equally unknown data sets (the two brands, or Brooklyn and the Bronx).

One can always turn continuous data into binned data, by grouping the events into specified ranges of the continuous variable(s): declinations between 0 and 10 degrees, 10 and 20, 20 and 30, etc. Binning involves a loss of information, however. Also, there is often considerable arbitrariness as to how the bins should be chosen. Along with many other investigators, we prefer to avoid unnecessary binning of data.

The accepted test for differences between binned distributions is the *chi-square test*. For continuous data as a function of a single variable, the most generally accepted test is the *Kolmogorov-Smirnov test*. We consider each in turn.

14.3.1 Chi-Square Test

Suppose that N_i is the number of events observed in the ith bin, and that n_i is the number expected according to some known distribution. Note that the N_i's are integers, while the n_i's may not be. Then the chi-square statistic is

$$\chi^2 = \sum_i \frac{(N_i - n_i)^2}{n_i} \tag{14.3.1}$$

where the sum is over all bins. A large value of χ^2 indicates that the null hypothesis (that the N_i's are drawn from the population represented by the n_i's) is

rather unlikely.

Any term j in (14.3.1) with $0 = n_j = N_j$ should be omitted from the sum. A term with $n_j = 0,\ N_j \neq 0$ gives an infinite χ^2, as it should, since in this case the N_i's cannot possibly be drawn from the n_i's!

The *chi-square probability function* $Q(\chi^2|\nu)$ is an incomplete gamma function, and was already discussed in §6.14 (see equation 6.14.38). Strictly speaking, $Q(\chi^2|\nu)$ is the probability that the sum of the squares of ν random *normal* variables of unit variance (and zero mean) will be greater than χ^2. The terms in the sum (14.3.1) are not exactly the squares of a normal variable. However, if the number of events in each bin is large ($\gg 1$), then the normal distribution is approximately achieved and the chi-square probability function is a good approximation to the distribution of (14.3.1) in the case of the null hypothesis. Its use to estimate the p-value significance of the chi-square test is standard (but see §14.3.2).

The appropriate value of ν, the number of degrees of freedom, bears some additional discussion. If the data are collected with the model n_i's fixed — that is, not later renormalized to fit the total observed number of events ΣN_i — then ν equals the number of bins N_B. (Note that this is *not* the total number of *events*!) Much more commonly, the n_i's are normalized after the fact so that their sum equals the sum of the N_i's. In this case, the correct value for ν is $N_B - 1$, and the model is said to have one constraint (knstrn=1 in the program below). If the model that gives the n_i's has additional free parameters that were adjusted after the fact to agree with the data, then each of these additional "fitted" parameters decreases ν (and increases knstrn) by one additional unit.

We have, then, the following program:

stattests.h

```
void chsone(VecDoub_I &bins, VecDoub_I &ebins, Doub &df,
    Doub &chsq, Doub &prob, const Int knstrn=1) {
```

Given the array bins[0..nbins-1] containing the observed numbers of events, and an array ebins[0..nbins-1] containing the expected numbers of events, and given the number of constraints knstrn (normally one), this routine returns (trivially) the number of degrees of freedom df, and (nontrivially) the chi-square chsq and the p-value prob. A small value of prob indicates a significant difference between the distributions bins and ebins. Note that bins and ebins are both double arrays, although bins will normally contain integer values.

```
    Gamma gam;
    Int j,nbins=bins.size();
    Doub temp;
    df=nbins-knstrn;
    chsq=0.0;
    for (j=0;j<nbins;j++) {
        if (ebins[j]<0.0 || (ebins[j]==0. && bins[j]>0.))
            throw("Bad expected number in chsone");
        if (ebins[j]==0.0 && bins[j]==0.0) {
            --df;                                 No data means one less degree of free-
        } else {                                      dom.
            temp=bins[j]-ebins[j];
            chsq += temp*temp/ebins[j];
        }
    }
    prob=gam.gammq(0.5*df,0.5*chsq);         Chi-square probability function. See §6.2.
}
```

Next we consider the case of comparing *two* binned data sets. Let R_i be the number of events in bin i for the first data set and S_i the number of events in the same bin i for the second data set. Then the chi-square statistic is

$$\chi^2 = \sum_i \frac{(R_i - S_i)^2}{R_i + S_i} \tag{14.3.2}$$

Comparing (14.3.2) to (14.3.1), you should note that the denominator of (14.3.2) is *not* just the average of R_i and S_i (which would be an estimator of n_i in 14.3.1). Rather, it is twice the average, the sum. The reason is that each term in a chi-square sum is supposed to approximate the square of a normally distributed quantity with unit variance. The variance of the difference of two normal quantities is the sum of their individual variances, not the average.

If the data were collected in such a way that the sum of the R_i's is necessarily equal to the sum of S_i's, then the number of degrees of freedom is equal to one less than the number of bins, $N_B - 1$ (that is, knstrn $= 1$), the usual case. If this requirement were absent, then the number of degrees of freedom would be N_B. Example: A birdwatcher wants to know whether the distribution of sighted birds as a function of species is the same this year as last. Each bin corresponds to one species. If the birdwatcher takes his data to be the first 1000 birds that he saw in each year, then the number of degrees of freedom is $N_B - 1$. If he takes his data to be all the birds he saw on a random sample of days, the same days in each year, then the number of degrees of freedom is N_B (knstrn $= 0$). In this latter case, note that he is also testing whether the birds were more numerous overall in one year or the other: That is the extra degree of freedom. Of course, any additional constraints on the data set lower the number of degrees of freedom (i.e., increase knstrn to *more positive* values) in accordance with their number.

The program is

stattests.h

```
void chstwo(VecDoub_I &bins1, VecDoub_I &bins2, Doub &df,
    Doub &chsq, Doub &prob, const Int knstrn=1) {
```

Given the arrays bins1[0..nbins-1] and bins2[0..nbins-1], containing two sets of binned data, and given the number of constraints knstrn (normally 1 or 0), this routine returns the number of degrees of freedom df, the chi-square chsq, and the p-value prob. A small value of prob indicates a significant difference between the distributions bins1 and bins2. Note that bins1 and bins2 are both double arrays, although they will normally contain integer values.

```
    Gamma gam;
    Int j,nbins=bins1.size();
    Doub temp;
    df=nbins-knstrn;
    chsq=0.0;
    for (j=0;j<nbins;j++)
        if (bins1[j] == 0.0 && bins2[j] == 0.0)
            --df;                                   No data means one less degree of free-
        else {                                          dom.
            temp=bins1[j]-bins2[j];
            chsq += temp*temp/(bins1[j]+bins2[j]);
        }
    prob=gam.gammq(0.5*df,0.5*chsq);            Chi-square probability function. See §6.2.
}
```

Equation (14.3.2) and the routine chstwo both apply to the case where the total number of data points is the same in the two binned sets, or to the case where any difference in the totals is part of what is being tested for. For intentionally unequal sample sizes, the formula analogous to (14.3.2) is

$$\chi^2 = \sum_i \frac{(\sqrt{S/R}\,R_i - \sqrt{R/S}\,S_i)^2}{R_i + S_i} \tag{14.3.3}$$

where

$$R \equiv \sum_i R_i \qquad S \equiv \sum_i S_i \tag{14.3.4}$$

are the respective numbers of data points. It is straightforward to make the corresponding change in chstwo. The fact that R_i and S_i occur in the denominator of equation (14.3.3) with equal weights may seem unintuitive, but the following heuristic derivation shows how this comes about: In the null hypothesis that R_i and S_i are drawn from the same distribution, we can estimate the probability associated with bin i as

$$\widehat{p}_i = \frac{R_i + S_i}{R + S} \tag{14.3.5}$$

The expected number of counts is thus

$$\widehat{R}_i = R\,\widehat{p}_i \qquad \text{and} \qquad \widehat{S}_i = S\,\widehat{p}_i \tag{14.3.6}$$

and the chi-square statistic summing over all observations is

$$\chi^2 = \sum_i \frac{(R_i - \widehat{R}_i)^2}{\widehat{R}_i} + \sum_i \frac{(S_i - \widehat{S}_i)^2}{\widehat{S}_i} \tag{14.3.7}$$

Substituting equations (14.3.6) and (14.3.5) into equation (14.3.7) gives, after some algebra, exactly equation (14.3.3). Although there are $2N_B$ terms in equation (14.3.7), the number of degrees of freedom is actually $N_B - 1$ (minus any additional constraints), the same as equation (14.3.2), because we implicitly estimated $N_B + 1$ parameters, the $\widehat{p}_i$'s and the ratio of the two sample sizes. This number of degrees of freedom must thus be subtracted from the original $2N_B$.

For three or more samples, see equation (14.4.3) and related discussion.

14.3.2 Chi-Square with Small Numbers of Counts

When a significant fraction of bins have small numbers of counts ($\lesssim$ 10, say), then the χ^2 statistics (14.3.1), (14.3.2), and (14.3.3) are not well approximated by a chi-square probability function. Let us quantify this problem and suggest some remedies.

Consider first equation (14.3.1). In the null hypothesis, the count in an individual bin, N_i, is a Poisson deviate of mean n_i, so it occurs with probability

$$p(N_i|n_i) = \exp(-n_i)\frac{n_i^{N_i}}{N_i!} \tag{14.3.8}$$

(cf. equation 6.14.61). We can calculate the mean μ and variance σ^2 of the term $(N_i - n_i)^2/n_i$ by evaluating the appropriate expectation values. There are various analytical ways to do this. The sums, and the answers, are

$$\begin{aligned}
\mu &= \sum_{N_i=0}^{\infty} p(N_i|n_i)\,\frac{(N_i - n_i)^2}{n_i} = 1 \\
\sigma^2 &= \left\{ \sum_{N_i=0}^{\infty} p(N_i|n_i) \left[\frac{(N_i - n_i)^2}{n_i} \right]^2 \right\} - \mu^2 = 2 + \frac{1}{n_i}
\end{aligned} \tag{14.3.9}$$

Now we can see what the problem is: Equation (14.3.9) says that each term in (14.3.1) adds, on average, 1 to the value of the χ^2 statistic, and slightly more than 2 to its variance. But

the variance of the chi-square probability function is *exactly* twice its mean (equation 6.14.37). If a significant fraction of n_i's are small, then quite probable values of the χ^2 statistic will appear to lie farther out on the tail than they actually are, so that the null hypothesis may be rejected even when it is true.

Several approximate remedies are possible. One is simply to rescale the observed χ^2 statistic so as to "fix" its variance, an idea due to Lucy [1]. If we define

$$Y^2 \equiv \nu + \sqrt{\frac{2\nu}{2\nu + \sum_i n_i^{-1}}}\left(\chi^2 - \nu\right) \tag{14.3.10}$$

where ν is the number of degrees of freedom (see discussion above), then Y^2 is asymptotically approximated by the chi-square probability function even when many n_i's are small. The basic idea in (14.3.10) is to subtract off the mean, rescale the difference from the mean, and then add back the mean. Lucy [1] also defines a similar Z^2 statistic by rescaling not the χ^2 sum of all the terms, but the terms individually, using equation (14.3.9) separately for each.

Another possibility, valid when ν is large, is to use the central limit theorem directly. From its mean and standard deviation, we now know that the χ^2 statistic must be approximately the normal distribution,

$$\chi^2 \sim \mathrm{N}\left(\nu, \left[2\nu + \sum_i n_i^{-1}\right]^{1/2}\right) \tag{14.3.11}$$

We can then obtain p-values from equation (6.14.2), computing a complementary error function. (The p-value is one minus that cdf.)

The same ideas go through in the case of two binned data sets, with counts R_i and S_i, and total numbers of counts R and S (equation 14.3.3, with equation 14.3.2 as the special case with $R = S$). Now, in the null hypothesis, and glossing over some technical issues beyond our scope, we can think of $T_i \equiv R_i + S_i$ as being fixed, while R_i is a random variable drawn from the binomial distribution

$$R_i \sim \text{Binomial}\left(T_i, \frac{R}{R+S}\right) \tag{14.3.12}$$

(see equation 6.14.67). Calculating moments over the binomial distribution, one can obtain as analogs of equations (14.3.9)

$$\begin{aligned} \mu &= 1 \\ \sigma^2 &= 2 + \left[\frac{(R+S)^2}{RS} - 6\right]\frac{1}{R_i + S_i} \end{aligned} \tag{14.3.13}$$

Notice that, now, depending on the values of R and S, the variance can be either greater or less than its nominal value 2, and that it is less than 2 for the case $R = S$. The formulas (14.3.9) and (14.3.13) are originally due to Haldane [2] (see also [3]).

Summing over i, one obtains the analogs of equations (14.3.10) and (14.3.11) simply by the replacement,

$$\sum_i n_i^{-1} \longrightarrow \left[\frac{(R+S)^2}{RS} - 6\right]\sum_i \frac{1}{R_i + S_i} \tag{14.3.14}$$

In fact, equation (14.3.9) is a limiting form of equation (14.3.13) in just the same limit that Poisson is a limiting form of binomial, namely

$$S \to \infty, \qquad \frac{R}{R+S} S_i \to n_i, \qquad R_i \to N_i \tag{14.3.15}$$

There are also other ways of treating small-number counts, including the likelihood ratio test [4], the *modified Neyman* χ^2 [5], and the *chi-square-gamma* statistic [5].

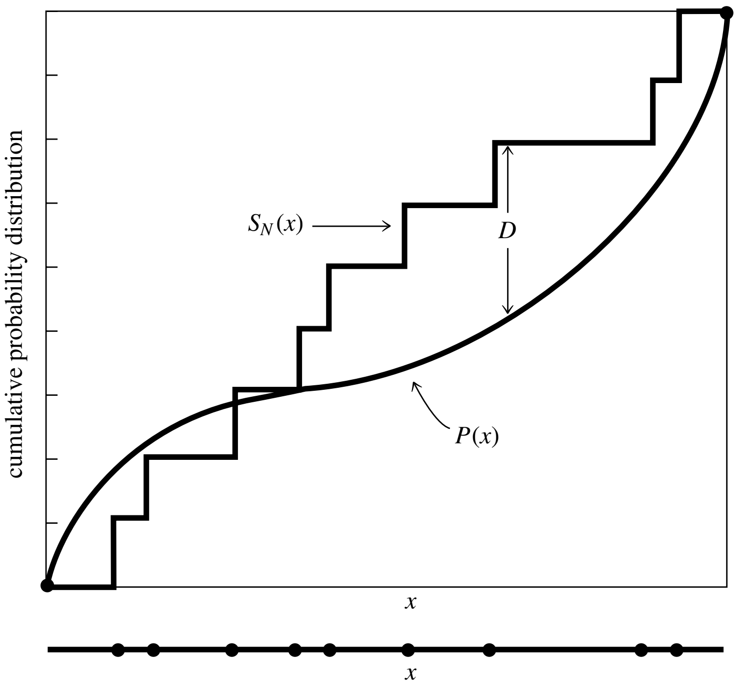

Figure 14.3.1. Kolmogorov-Smirnov statistic D. A measured distribution of values in x (shown as N dots on the lower abscissa) is to be compared with a theoretical distribution whose cumulative probability distribution is plotted as $P(x)$. A step-function cumulative probability distribution $S_N(x)$ is constructed, one that rises an equal amount at each measured point. D is the greatest distance between the two cumulative distributions.

14.3.3 Kolmogorov-Smirnov Test

The Kolmogorov-Smirnov (or *K–S*) test is applicable to unbinned distributions that are functions of a single independent variable, that is, to data sets where each data point can be associated with a single number (lifetime of each lightbulb when it burns out, or declination of each star). In such cases, the list of data points can be easily converted to an unbiased estimator $S_N(x)$ of the *cumulative* distribution function of the probability distribution from which it was drawn: If the N events are located at values x_i, $i = 0, \ldots, N-1$, then $S_N(x)$ is the function giving the fraction of data points to the left of a given value x. This function is obviously constant between consecutive (i.e., sorted into ascending order) x_i's and jumps by the same constant $1/N$ at each x_i. (See Figure 14.3.1.)

Different distribution functions, or sets of data, give different cumulative distribution function estimates by the above procedure. However, all cumulative distribution functions agree at the smallest allowable value of x (where they are zero) and at the largest allowable value of x (where they are unity). (The smallest and largest values might of course be $\pm\infty$.) So it is the behavior between the largest and smallest values that distinguishes distributions.

One can think of any number of statistics to measure the overall difference between two cumulative distribution functions: the absolute value of the area between them, for example, or their integrated mean square difference. The Kolmogorov-Smirnov D is a particularly simple measure: It is defined as the *maximum value*

of the absolute difference between two cumulative distribution functions. Thus, for comparing one data set's $S_N(x)$ to a known cumulative distribution function $P(x)$, the K–S statistic is

$$D = \max_{-\infty<x<\infty} |S_N(x) - P(x)| \tag{14.3.16}$$

while for comparing two different cumulative distribution functions $S_{N_1}(x)$ and $S_{N_2}(x)$, the K–S statistic is

$$D = \max_{-\infty<x<\infty} \left|S_{N_1}(x) - S_{N_2}(x)\right| \tag{14.3.17}$$

What makes the K–S statistic useful is that *its* distribution in the case of the null hypothesis (data sets drawn from the same distribution) can be calculated, at least to a useful approximation, thus giving the p-value significance of any observed nonzero value of D. A central feature of the K–S test is that it is invariant under reparametrization of x; in other words, you can locally slide or stretch the x-axis in Figure 14.3.1, and the maximum distance D remains unchanged. For example, you will get the same significance using x as using $\log x$.

The function that enters into the calculation of the p-value was discussed previously in §6.14, was defined in equations (6.14.56) and (6.14.57), and was implemented in the object `KSdist`. In terms of the function Q_{KS}, the p-value of an observed value of D (as a disproof of the null hypothesis that the distributions are the same) is given approximately [6] by the formula

$$\text{Probability}\ (D > \text{observed}\ ) = Q_{KS}\left(\left[\sqrt{N_e} + 0.12 + 0.11/\sqrt{N_e}\right] D\right) \tag{14.3.18}$$

where N_e is the effective number of data points, $N_e = N$ for the case (14.3.16) of one distribution, and

$$N_e = \frac{N_1 N_2}{N_1 + N_2} \tag{14.3.19}$$

for the case (14.3.17) of two distributions, where N_1 is the number of data points in the first distribution and N_2 the number in the second.

The nature of the approximation involved in (14.3.18) is that it becomes asymptotically accurate as the N_e becomes large, but is already quite good for $N_e \geq 4$, as small a number as one might ever actually use. (See [6].)

Here is the routine for the case of one distribution:

kstests.h

```
void ksone(VecDoub_IO &data, Doub func(const Doub), Doub &d, Doub &prob)
```
Given an array `data[0..n-1]`, and given a user-supplied function of a single variable `func` that is a cumulative distribution function ranging from 0 (for smallest values of its argument) to 1 (for largest values of its argument), this routine returns the K–S statistic `d` and the p-value `prob`. Small values of `prob` show that the cumulative distribution function of `data` is significantly different from `func`. The array `data` is modified by being sorted into ascending order.
```
{
    Int j,n=data.size();
    Doub dt,en,ff,fn,fo=0.0;
    KSdist ks;
    sort(data);                 If the data are already sorted into as-
                                    cending order, then this call can be
                                    omitted.
    en=n;
    d=0.0;
    for (j=0;j<n;j++) {         Loop over the sorted data points.
        fn=(j+1)/en;            Data's c.d.f. after this step.
```

```
        ff=func(data[j]);                          Compare to the user-supplied function.
        dt=MAX(abs(fo-ff),abs(fn-ff));             Maximum distance.
        if (dt > d) d=dt;
        fo=fn;
    }
    en=sqrt(en);
    prob=ks.qks((en+0.12+0.11/en)*d);              Compute p-value.
}
```

While the K-S statistic is intended for use with a continuous distribution, it can also be used for a discrete distribution. In this case, it can be shown that the test is conservative, that is, the statistic returned is no larger than in the continuous case. If you allow discrete variables in the case of two distributions, you have to consider how to deal with ties. The standard way to handle ties is to combine all the tied data points and add them to the cdf at once (see, e.g., [7]). This refinement is included in the routine kstwo.

kstests.h

```
void kstwo(VecDoub_IO &data1, VecDoub_IO &data2, Doub &d, Doub &prob)
```

Given an array data1[0..n1-1], and an array data2[0..n2-1], this routine returns the K–S statistic d and the p-value prob for the null hypothesis that the data sets are drawn from the same distribution. Small values of prob show that the cumulative distribution function of data1 is significantly different from that of data2. The arrays data1 and data2 are modified by being sorted into ascending order.

```
{
    Int j1=0,j2=0,n1=data1.size(),n2=data2.size();
    Doub d1,d2,dt,en1,en2,en,fn1=0.0,fn2=0.0;
    KSdist ks;
    sort(data1);
    sort(data2);
    en1=n1;
    en2=n2;
    d=0.0;
    while (j1 < n1 && j2 < n2) {                     If we are not done...
        if ((d1=data1[j1]) <= (d2=data2[j2]))        Next step is in data1.
            do
                fn1=++j1/en1;
            while (j1 < n1 && d1 == data1[j1]);
        if (d2 <= d1)                                Next step is in data2.
            do
                fn2=++j2/en2;
            while (j2 < n2 && d2 == data2[j2]);
        if ((dt=abs(fn2-fn1)) > d) d=dt;
    }
    en=sqrt(en1*en2/(en1+en2));
    prob=ks.qks((en+0.12+0.11/en)*d);                Compute p-value.
}
```

14.3.4 Variants on the K–S Test

The sensitivity of the K–S test to deviations from a cumulative distribution function $P(x)$ is not independent of x. In fact, the K–S test tends to be most sensitive around the median value, where $P(x) = 0.5$, and less sensitive at the extreme ends of the distribution, where $P(x)$ is near 0 or 1. The reason is that the difference $|S_N(x) - P(x)|$ does not, in the null hypothesis, have a probability distribution that is independent of x. Rather, its variance is proportional to $P(x)[1 - P(x)]$, which is largest at $P = 0.5$. Since the K–S statistic (14.3.16) is the maximum difference over all x of two cumulative distribution functions, a deviation that might be statistically significant at *its own* value of x gets compared to the expected chance deviation at $P = 0.5$ and is thus discounted. A result is that, while the K–S test is good at

finding *shifts* in a probability distribution, especially changes in the median value, it is not always so good at finding *spreads*, which more affect the tails of the probability distribution, and which may leave the median unchanged.

One way of increasing the power of the K–S statistic out on the tails is to replace D (equation 14.3.16) by a so-called *stabilized* or *weighted* statistic [8-10], for example the *Anderson-Darling statistic*,

$$D^* = \max_{-\infty<x<\infty} \frac{|S_N(x) - P(x)|}{\sqrt{P(x)[1-P(x)]}} \tag{14.3.20}$$

Unfortunately, there is no simple formula analogous to equation (14.3.18) for this statistic, although Noé [11] gives a computational method using a recursion relation and provides a graph of numerical results. There are many other possible similar statistics, for example

$$D^{**} = \int_{P=0}^{1} \frac{[S_N(x) - P(x)]^2}{P(x)[1-P(x)]} dP(x) \tag{14.3.21}$$

which is also discussed by Anderson and Darling (see [9]).

Another approach, which we prefer as simpler and more direct, is due to Kuiper [12,13]. We already mentioned that the standard K–S test is invariant under reparametrizations of the variable x. An even more general symmetry, which guarantees equal sensitivities at all values of x, is to wrap the x-axis around into a circle (identifying the points at $\pm\infty$), and to look for a statistic that is now invariant under all shifts and parametrizations on the circle. This allows, for example, a probability distribution to be "cut" at some central value of x and the left and right halves to be interchanged, without altering the statistic or its significance.

Kuiper's statistic, defined as

$$V = D_+ + D_- = \max_{-\infty<x<\infty} [S_N(x) - P(x)] + \max_{-\infty<x<\infty} [P(x) - S_N(x)] \tag{14.3.22}$$

is the sum of the maximum distance of $S_N(x)$ *above and below* $P(x)$. You should be able to convince yourself that this statistic has the desired invariance on the circle: Sketch the indefinite integral of two probability distributions defined on the circle as a function of angle around the circle, as the angle goes through several times 360°. If you change the starting point of the integration, D_+ and D_- change individually, but their sum is constant.

Furthermore, there is a simple formula for the asymptotic distribution of the statistic V, directly analogous to equations (14.3.18) – (14.3.19). Let

$$Q_{KP}(\lambda) = 2\sum_{j=1}^{\infty}(4j^2\lambda^2 - 1)e^{-2j^2\lambda^2} \tag{14.3.23}$$

which is monotonic and satisfies

$$Q_{KP}(0) = 1 \qquad Q_{KP}(\infty) = 0 \tag{14.3.24}$$

In terms of this function the p-value is [6]

$$\text{Probability } (V > \text{observed }) = Q_{KP}\left(\left[\sqrt{N_e} + 0.155 + 0.24/\sqrt{N_e}\right] V\right) \tag{14.3.25}$$

Here N_e is N in the one-sample case or is given by equation (14.3.19) in the case of two samples.

Of course, Kuiper's test is ideal for any problem originally defined on a circle, for example, to test whether the distribution in longitude of something agrees with some theory, or whether two somethings have different distributions in longitude. (See also [14].)

We will leave to you the coding of routines analogous to `ksone`, `kstwo`, and `KSdist::qks`. (For $\lambda < 0.4$, don't try to do the sum 14.3.23. Its value is 1, to 7 figures, but the series can require many terms to converge, and loses accuracy to roundoff.)

Two final cautionary notes: First, we should mention that all varieties of the K–S test lack the ability to discriminate some kinds of distributions. A simple example is a probability distribution with a narrow "notch" within which the probability falls to zero. Such a distribution is of course ruled out by the existence of even one data point within the notch, but, because of its cumulative nature, a K–S test would require many data points in the notch before signaling a discrepancy.

Second, we should note that, if you estimate any parameters from a data set (e.g., a mean and variance), then the distribution of the K–S statistic D for a cumulative distribution function $P(x)$ that *uses the estimated parameters* is no longer given by equation (14.3.18). In general, you will have to determine the new distribution yourself, e.g., by Monte Carlo methods.

CITED REFERENCES AND FURTHER READING:

Devore, J.L. 2003, *Probability and Statistics for Engineering and the Sciences*, 6th ed. (Belmont, CA: Duxbury Press), Chapter 14.

Lupton, R. 1993, *Statistics in Theory and Practice* (Princeton, NJ: Princeton University Press), Chapter 14.

Lucy, L.B. 2000, "Hypothesis Testing for Meagre Data Sets," *Monthly Notices of the Royal Astronomical Society*, vol. 318, pp. 92–100.[1]

Haldane, J.B.S. 1937, "The Exact Value of the Moments of the Distribution of χ^2, Used as a Test of Goodness of Fit, When Expectations Are Small," *Biometrika*, vol. 29, pp. 133–143.[2]

Read, T.R.C., and Cressie, N.A.C. 1988, *Goodness-of-Fit Statistics for Discrete Multivariate Data* (New York: Springer), pp. 140–144.[3]

Baker, S., and Cousins, R.D. 1984, "Clarification of the Use of Chi-Square and Likelihood Functions in Fits to Histograms," *Nuclear Instruments and Methods in Physics Research*, vol. 221, pp. 437–442.[4]

Mighell, K.J. 1999, "Parameter Estimation in Astronomy with Poisson-Distributed Data. I.The χ^2_γ Statistic," *Astrophysical Journal*, vol. 518, pp. 380–393[5]

Stephens, M.A. 1970, "Use of Kolmogorov-Smirnov, Cramer-von Mises and Related Statistics without Extensive Tables," *Journal of the Royal Statistical Society*, ser. B, vol. 32, pp. 115–122.[6]

Hollander, M., and Wolfe, D.A. 1999, *Nonparametric Statistical Methods*, 2nd ed. (New York: Wiley), p. 183.[7]

Anderson, T.W., and Darling, D.A. 1952, "Asymptotic Theory of Certain Goodness of Fit Criteria Based on Stochastic Processes," *Annals of Mathematical Statistics*, vol. 23, pp. 193–212.[8]

Darling, D.A. 1957, "The Kolmogorov-Smirnov, Cramer-von Mises Tests," *Annals of Mathematical Statistics*, vol. 28, pp. 823–838.[9]

Michael, J.R. 1983, "The Stabilized Probability Plot," *Biometrika*, vol. 70, no. 1, pp. 11–17.[10]

Noé, M. 1972, "The Calculation of Distributions of Two-Sided Kolmogorov-Smirnov Type Statistics," *Annals of Mathematical Statistics*, vol. 43, pp. 58–64.[11]

Kuiper, N.H. 1962, "Tests Concerning Random Points on a Circle," *Proceedings of the Koninklijke Nederlandse Akademie van Wetenschappen*, ser. A., vol. 63, pp. 38–47.[12]

Stephens, M.A. 1965, "The Goodness-of-Fit Statistic V_n: Distribution and Significance Points," *Biometrika*, vol. 52, pp. 309–321.[13]

Fisher, N.I., Lewis, T., and Embleton, B.J.J. 1987, *Statistical Analysis of Spherical Data* (New York: Cambridge University Press).[14]

14.4 Contingency Table Analysis of Two Distributions

In this section and the next three sections, we deal with *measures of association* for two distributions. The situation is this: Each data point has two or more different quantities associated with it, and we want to know whether knowledge of one quantity gives us any demonstrable advantage in predicting the value of another quantity. In many cases, one variable will be an "independent" or "control" variable, and another will be a "dependent" or "measured" variable. Then, we want to know if the latter variable *is* in fact dependent on or *associated* with the former variable. If it is, we want to have some quantitative measure of the strength of the association. One often hears this loosely stated as the question of whether two variables are *correlated* or *uncorrelated*, but we will reserve those terms for a particular kind of association (linear, or at least monotonic), as discussed in §14.5 and §14.6.

Notice that, as in previous sections, the different concepts of significance and strength appear: The association between two distributions may be very significant even if that association is weak — if the quantity of data is large enough.

It is useful to distinguish among some different kinds of variables, with different categories forming a loose hierarchy.

- A variable is called *nominal* if its values are the members of some unordered set. For example, "state of residence" is a nominal variable that (in the U.S.) takes on one of 50 values; in astrophysics, "type of galaxy" is a nominal variable with the three values "spiral," "elliptical," and "irregular."
- A variable is termed *ordinal* if its values are the members of a discrete, but ordered, set. Examples are grade in school, planetary order from the Sun (Mercury = 1, Venus = 2, ...), and number of offspring. There need not be any concept of "equal metric distance" between the values of an ordinal variable, only that they be intrinsically ordered.
- We will call a variable *continuous* if its values are real numbers, as are times, distances, temperatures, etc. (Social scientists sometimes distinguish between *interval* and *ratio* continuous variables, but we do not find that distinction very compelling.)

A continuous variable can always be made into an ordinal one by binning it into ranges. If we choose to ignore the ordering of the bins, then we can turn it into a nominal variable. Nominal variables constitute the lowest type of the hierarchy, and therefore the most general. For example, a set of *several* continuous or ordinal variables can be turned, if crudely, into a single nominal variable, by coarsely binning each variable and then taking each distinct combination of bin assignments as a single nominal value. When multidimensional data are sparse, this is often the only sensible way to proceed.

The remainder of this section will deal with measures of association between *nominal* variables. For any pair of nominal variables, the data can be displayed as a *contingency table*, a table whose rows are labeled by the values of one nominal variable, whose columns are labeled by the values of the other nominal variable, and whose entries are nonnegative integers giving the number of observed events for each combination of row and column (see Figure 14.4.1). The analysis of association between nominal variables is thus called *contingency table analysis* or *cross-*

	0. red	1. green	. . .	
0. male	# of red males N_{00}	# of green males N_{01}	. . .	# of males $N_{0\cdot}$
1. female	# of red females N_{10}	# of green females N_{11}	. . .	# of females $N_{1\cdot}$
⋮	⋮	⋮	. . .	⋮
	# of red $N_{\cdot 0}$	# of green $N_{\cdot 1}$	. . .	total # N

Figure 14.4.1. Example of a contingency table for two nominal variables, here sex and color. The row and column marginals (totals) are shown. The variables are "nominal," i.e., the order in which their values are listed is arbitrary and does not affect the result of the contingency table analysis. If the ordering of values has some intrinsic meaning, then the variables are "ordinal" or "continuous," and correlation techniques (§14.5 – §14.6) can be utilized.

tabulation analysis.

The remainder of this section gives an approach, based on the chi-square statistic, that does a good job of characterizing the significance of association but is only so-so as a measure of the strength (principally because its numerical values have no very direct interpretations). We will return to contingency table analysis in §14.7 with an approach, based on the information-theoretic concept of *entropy*, that will say little about the significance of association (use chi-square for that!) but is capable of very elegantly characterizing the strength of an association already known to be significant.

14.4.1 Measures of Association Based on Chi-Square

Some notation first: Let N_{ij} denote the number of events that occur with the first variable x taking on its ith value and the second variable y taking on its jth value. Let N denote the total number of events, the sum of all the N_{ij}'s. Let $N_{i\cdot}$ denote the number of events for which the first variable x takes on its ith value regardless of the value of y; $N_{\cdot j}$ is the number of events with the jth value of y regardless of x. So we have

$$\begin{aligned} N_{i\cdot} &= \sum_j N_{ij} \qquad N_{\cdot j} = \sum_i N_{ij} \\ N &= \sum_i N_{i\cdot} = \sum_j N_{\cdot j} \end{aligned} \tag{14.4.1}$$

In other words, "dot" is a placeholder that means, "sum over the missing index". $N_{\cdot j}$ and $N_{i\cdot}$ are sometimes called the *row and column totals* or *marginals*, but we will use these terms cautiously since we can never keep straight which are the rows and which are the columns!

The null hypothesis is that the two variables x and y have no association. In this case, the probability of a particular value of x given a particular value of y should be the same as the probability of that value of x regardless of y. Therefore, in the null hypothesis, the expected number for any N_{ij}, which we will denote n_{ij}, can be calculated from only the row and column totals,

$$\frac{n_{ij}}{N_{\cdot j}} = \frac{N_{i\cdot}}{N} \qquad \text{which implies} \qquad n_{ij} = \frac{N_{i\cdot}N_{\cdot j}}{N} \tag{14.4.2}$$

Notice that if a column or row total is zero, then the expected number for all the entries in that column or row is also zero; in that case, the never-occurring bin of x or y should simply be removed from the analysis.

The chi-square statistic is now given by equation (14.3.1), which, in the present case, is summed over all entries in the table:

$$\chi^2 = \sum_{i,j} \frac{(N_{ij} - n_{ij})^2}{n_{ij}} \tag{14.4.3}$$

The number of degrees of freedom is equal to the number of entries in the table (product of its row size and column size) minus the number of constraints that have arisen from our use of the data themselves to determine the n_{ij}. Each row total and column total is a constraint, except that this overcounts by one, since the total of the column totals and the total of the row totals both equal N, the total number of data points. Therefore, if the table is of size I by J, the number of degrees of freedom is $IJ - I - J + 1$. Equation (14.4.3), along with the chi-square probability function (§6.2), now give the significance of an association between the variables x and y. Incidentally, the two-sample chi-square test for equality of distributions, equation (14.3.3), is a special case of equation (14.4.3) with $J = 2$ and with the y variable simply a label distinguishing the two samples.

Suppose there is a significant association. How do we quantify its strength, so that (e.g.) we can compare the strength of one association with another? The idea here is to find some reparametrization of χ^2 that maps it into some convenient interval, like 0 to 1, where the result is not dependent on the quantity of data that we happen to sample, but rather depends only on the underlying population from which the data were drawn. There are several different ways of doing this. Two of the more common are called *Cramer's V* and the *contingency coefficient C*.

The formula for Cramer's V is

$$V = \sqrt{\frac{\chi^2}{N \min(I-1, J-1)}} \tag{14.4.4}$$

where I and J are again the numbers of rows and columns, and N is the total number of events. Cramer's V has the pleasant property that it lies between zero and one inclusive, equals zero when there is no association, and equals one only when the association is perfect: All the events in any row lie in one unique column, and vice

versa. (In chess parlance, no two rooks, placed on a nonzero table entry, can capture each other.)

In the case of $I = J = 2$, Cramer's V is also referred to as the *phi* statistic.

The contingency coefficient C is defined as

$$C = \sqrt{\frac{\chi^2}{\chi^2 + N}} \tag{14.4.5}$$

It also lies between zero and one, but (as is apparent from the formula) it can never achieve the upper limit. While it can be used to compare the strength of association of two tables with the same I and J, its upper limit depends on I and J. Therefore it can never be used to compare tables of different sizes.

The trouble with both Cramer's V and the contingency coefficient C is that, when they take on values in between their extremes, there is no very direct interpretation of what that value means. For example, you are in Las Vegas, and a friend tells you that there is a small, but significant, association between the color of a croupier's eyes and the occurrence of red and black on his roulette wheel. Cramer's V is about 0.028, your friend tells you. You know what the usual odds against you are (because of the green zero and double zero on the wheel). Is this association sufficient for you to make money? Don't ask us! For a measure of association that is directly applicable to gambling, look at §14.7.

stattests.h

```
void cntab(MatInt_I &nn, Doub &chisq, Doub &df, Doub &prob, Doub &cramrv,
    Doub &ccc)
```

Given a two-dimensional contingency table in the form of an array `nn[0..ni-1][0..nj-1]` of integers, this routine returns the chi-square `chisq`, the number of degrees of freedom `df`, the p-value `prob` (small values indicating a significant association), and two measures of association, Cramer's V (`cramrv`) and the contingency coefficient C (`ccc`).

```
{
    const Doub TINY=1.0e-30;                A small number.
    Gamma gam;
    Int i,j,nnj,nni,minij,ni=nn.nrows(),nj=nn.ncols();
    Doub sum=0.0,expctd,temp;
    VecDoub sumi(ni),sumj(nj);
    nni=ni;                                 Number of rows...
    nnj=nj;                                 ...and columns.
    for (i=0;i<ni;i++) {                    Get the row totals.
        sumi[i]=0.0;
        for (j=0;j<nj;j++) {
            sumi[i] += nn[i][j];
            sum += nn[i][j];
        }
        if (sumi[i] == 0.0) --nni;          Eliminate any zero rows by reducing the num-
    }                                           ber.
    for (j=0;j<nj;j++) {                    Get the column totals.
        sumj[j]=0.0;
        for (i=0;i<ni;i++) sumj[j] += nn[i][j];
        if (sumj[j] == 0.0) --nnj;          Eliminate any zero columns.
    }
    df=nni*nnj-nni-nnj+1;                   Corrected number of degrees of freedom.
    chisq=0.0;
    for (i=0;i<ni;i++) {                    Do the chi-square sum.
        for (j=0;j<nj;j++) {
            expctd=sumj[j]*sumi[i]/sum;
            temp=nn[i][j]-expctd;
            chisq += temp*temp/(expctd+TINY);       Here TINY guarantees that any
        }                                               eliminated row or column will
                                                        not contribute to the sum.
```

```
    }
    prob=gam.gammq(0.5*df,0.5*chisq);              Chi-square probability function.
    minij = nni < nnj ? nni-1 : nnj-1;
    cramrv=sqrt(chisq/(sum*minij));
    ccc=sqrt(chisq/(chisq+sum));
}
```

CITED REFERENCES AND FURTHER READING:

Agresti, A. 2002, *Categorical Data Analysis*, 2nd ed. (New York: Wiley).

Mickey, R.M., Dunn, O.J., and Clark, V.A. 2004, *Applied Statistics: Analysis of Variance and Regression*, 3rd ed. (New York: Wiley).

Norusis, M.J. 2006, *SPSS 14.0 Guide to Data Analysis* (Englewood Cliffs, NJ: Prentice-Hall).

14.5 Linear Correlation

We next turn to measures of association between variables that are ordinal or continuous, rather than nominal. Most widely used is the *linear correlation coefficient*. For pairs of quantities (x_i, y_i), $i = 0, \ldots, N-1$, the linear correlation coefficient r (also called the product-moment correlation coefficient, or *Pearson's* r) is given by the formula

$$r = \frac{\sum_i (x_i - \overline{x})(y_i - \overline{y})}{\sqrt{\sum_i (x_i - \overline{x})^2}\sqrt{\sum_i (y_i - \overline{y})^2}} \tag{14.5.1}$$

where, as usual, $\overline{x}$ is the mean of the x_i's and $\overline{y}$ is the mean of the y_i's.

The value of r lies between -1 and 1, inclusive. It takes on a value of 1, termed "complete positive correlation," when the data points lie on a perfect straight line with positive slope, with x and y increasing together. The value 1 holds independent of the magnitude of the slope. If the data points lie on a perfect straight line with negative slope, y decreasing as x increases, then r has the value -1; this is called "complete negative correlation." A value of r near zero indicates that the variables x and y are *uncorrelated*.

When a correlation is known to be significant, r is one conventional way of summarizing its strength. In fact, the value of r can be translated into a statement about what residuals (root-mean-square deviations) are to be expected if the data are fitted to a straight line by the least-squares method (see §15.2, especially equation 15.2.13). Unfortunately, r is a rather poor statistic for deciding *whether* an observed correlation is statistically significant and/or whether one observed correlation is significantly stronger than another. The reason is that r is ignorant of the individual distributions of x and y, so there is no universal way to compute its distribution in the case of the null hypothesis.

About the only general statement that can be made is this: If the null hypothesis is that x and y are uncorrelated, and if the distributions for x and y each have enough convergent moments ("tails" die off sufficiently rapidly), and if N is large (typically

> 500), then r is distributed approximately normally, with a mean of zero and a standard deviation of $1/\sqrt{N}$. In that case, the (double-sided) significance of the correlation, that is, the probability that $|r|$ should be larger than its observed value in the null hypothesis, is

$$\operatorname{erfc}\left(\frac{|r|\sqrt{N}}{\sqrt{2}}\right) \tag{14.5.2}$$

where erfc(x) is the complementary error function, equation (6.2.10), computed by the routines `Erf.erfc` or `erfcc` of §6.2. A small value of (14.5.2) indicates that the two distributions are significantly correlated. (See expression 14.5.9 below for a more accurate test.)

Most statistics books try to go beyond (14.5.2) and give additional statistical tests that can be made using r. In almost all cases, however, these tests are valid only for a very special class of hypotheses, namely that the distributions of x and y jointly form a *binormal* or *two-dimensional Gaussian* distribution around their mean values, with joint probability density

$$p(x,y)\,dxdy = \text{const.} \times \exp\left[-\tfrac{1}{2}(a_{00}x^2 - 2a_{01}xy + a_{11}y^2)\right]\,dxdy \tag{14.5.3}$$

where a_{00}, a_{01}, and a_{11} are arbitrary constants. For this distribution r has the value

$$r = -\frac{a_{01}}{\sqrt{a_{00}a_{11}}} \tag{14.5.4}$$

There are occasions when (14.5.3) may be known to be a good model of the data. There may be other occasions when we are willing to take (14.5.3) as at least a rough-and-ready guess, since many two-dimensional distributions do resemble a binormal distribution, (that is, a two-dimensional Gaussian) at least not too far out on their tails. In either situation, we can use (14.5.3) to go beyond (14.5.2) in any of several directions:

First, we can allow for the possibility that the number N of data points is not large. Here, it turns out that the statistic

$$t = r\sqrt{\frac{N-2}{1-r^2}} \tag{14.5.5}$$

is distributed in the null case (of no correlation) like Student's t-distribution with $\nu = N - 2$ degrees of freedom, whose two-sided significance level is given by $1 - A(t|\nu)$ (equation 6.14.11) [1]. As N becomes large, this significance and (14.5.2) become asymptotically the same, so that one never does worse by using (14.5.5), even if the binormal assumption is not well substantiated.

Second, when N is only moderately large (≥ 10), we can compare whether the difference of two significantly nonzero r's, e.g., from different experiments, is itself significant. In other words, we can quantify whether a change in some control variable significantly alters an existing correlation between two other variables. This is done by using *Fisher's z-transformation* to associate each measured r with a corresponding z:

$$z = \frac{1}{2}\ln\left(\frac{1+r}{1-r}\right) \tag{14.5.6}$$

Then, each z is approximately normally distributed with a mean value

$$\overline{z} = \frac{1}{2}\left[\ln\left(\frac{1+r_{\text{true}}}{1-r_{\text{true}}}\right) + \frac{r_{\text{true}}}{N-1}\right] \tag{14.5.7}$$

where r_{true} is the actual or population value of the correlation coefficient, and with a standard deviation

$$\sigma(z) \approx \frac{1}{\sqrt{N-3}} \tag{14.5.8}$$

Equations (14.5.7) and (14.5.8), when they are valid, give several useful statistical tests [1]. For example, the significance level at which a measured value of r differs from some hypothesized value r_{true} is given by

$$\text{erfc}\left(\frac{|z-\overline{z}|\sqrt{N-3}}{\sqrt{2}}\right) \tag{14.5.9}$$

where z and $\overline{z}$ are given by (14.5.6) and (14.5.7), with small values of (14.5.9) indicating a significant difference. (Setting $\overline{z} = 0$ makes expression 14.5.9 a more accurate replacement for expression 14.5.2 above.) Similarly, the significance of a difference between two measured correlation coefficients r_1 and r_2 is

$$\text{erfc}\left(\frac{|z_1-z_2|}{\sqrt{2}\sqrt{\frac{1}{N_1-3}+\frac{1}{N_2-3}}}\right) \tag{14.5.10}$$

where z_1 and z_2 are obtained from r_1 and r_2 using (14.5.6), and where N_1 and N_2 are, respectively, the number of data points in the measurement of r_1 and r_2.

All of the significances above are two-sided. If you wish to disprove the null hypothesis in favor of a one-sided hypothesis, such as that $r_1 > r_2$ (where the sense of the inequality was decided *a priori*), then (i) if your measured r_1 and r_2 have the *wrong* sense, you have failed to demonstrate your one-sided hypothesis, but (ii) if they have the right ordering, you can multiply the significances given above by 0.5, which makes them more significant.

But keep in mind: These interpretations of the r statistic can be completely meaningless if the joint probability distribution of your variables x and y is too different from a binormal distribution.

```
void pearsn(VecDoub_I &x, VecDoub_I &y, Doub &r, Doub &prob, Doub &z)
```
stattests.h

Given two arrays `x[0..n-1]` and `y[0..n-1]`, this routine computes their correlation coefficient r (returned as `r`), the p-value at which the null hypothesis of zero correlation is disproved (`prob` whose small value indicates a significant correlation), and Fisher's z (returned as `z`), whose value can be used in further statistical tests as described above.

```
{
    const Doub TINY=1.0e-20;                    Will regularize the unusual case of
    Beta beta;                                      complete correlation.
    Int j,n=x.size();
    Doub yt,xt,t,df;
    Doub syy=0.0,sxy=0.0,sxx=0.0,ay=0.0,ax=0.0;
    for (j=0;j<n;j++) {                         Find the means.
        ax += x[j];
        ay += y[j];
    }
```

```
    ax /= n;
    ay /= n;
    for (j=0;j<n;j++) {                         Compute the correlation coefficient.
        xt=x[j]-ax;
        yt=y[j]-ay;
        sxx += xt*xt;
        syy += yt*yt;
        sxy += xt*yt;
    }
    r=sxy/(sqrt(sxx*syy)+TINY);
    z=0.5*log((1.0+r+TINY)/(1.0-r+TINY));       Fisher's z transformation.
    df=n-2;
    t=r*sqrt(df/((1.0-r+TINY)*(1.0+r+TINY)));   Equation (14.5.5).
    prob=beta.betai(0.5*df,0.5,df/(df+t*t));    Student's t probability.
    // prob=erfcc(abs(z*sqrt(n-1.0))/1.4142136);
    For large n, this easier computation of prob, using the short routine erfcc, would give
    approximately the same value.
}
```


CITED REFERENCES AND FURTHER READING:

Taylor, J.R. 1997, *An Introduction to Error Analysis*, 2nd ed. (Sausalito, CA: University Science Books), Chapter 9.

Mickey, R.M., Dunn, O.J., and Clark, V.A. 2004, *Applied Statistics: Analysis of Variance and Regression*, 3rd ed. (New York: Wiley).

Devore, J.L. 2003, *Probability and Statistics for Engineering and the Sciences*, 6th ed. (Belmont, CA: Duxbury Press), Chapter 12.

Hoel, P.G. 1971, *Introduction to Mathematical Statistics*, 4th ed. (New York: Wiley), Chapter 7.

Korn, G.A., and Korn, T.M. 1968, *Mathematical Handbook for Scientists and Engineers*, 2nd rev. ed., reprinted 2000 (New York: Dover), §19.7.

Norusis, M.J. 2006, *SPSS 14.0 Guide to Data Analysis* (Englewood Cliffs, NJ: Prentice-Hall).

Stuart, A., and Ord, J.K. 1994, *Kendall's Advanced Theory of Statistics*, 6th ed. (London: Edward Arnold) [previous eds. published as Kendall, M., and Stuart, A., *The Advanced Theory of Statistics*], §16.28 and §16.33.[1]

14.6 Nonparametric or Rank Correlation

It is precisely the uncertainty in interpreting the significance of the linear correlation coefficient r that leads us to the important concepts of *nonparametric* or *rank correlation*. As before, we are given N pairs of measurements (x_i, y_i). Before, difficulties arose because we did not necessarily know the probability distribution function from which the x_i's or y_i's were drawn.

The key concept of nonparametric correlation is this: If we replace the value of each x_i by the value of its *rank* among all the other x_i's in the sample, that is, $1, 2, 3, \ldots, N$, then the resulting list of numbers will be drawn from a perfectly known distribution function, namely uniformly from the integers between 1 and N, inclusive. Better than uniformly, in fact, since if the x_i's are all distinct, then each integer will occur precisely once. If some of the x_i's have identical values, it is conventional to assign to all these "ties" the mean of the ranks that they would have had if their values had been slightly different. This *midrank* will sometimes be an integer,

sometimes a half-integer. In all cases the sum of all assigned ranks will be the same as the sum of the integers from 1 to N, namely $\frac{1}{2}N(N+1)$.

Of course we do exactly the same procedure for the y_i's, replacing each value by its rank among the other y_i's in the sample.

Now we are free to invent statistics for detecting correlation between uniform sets of integers between 1 and N, keeping in mind the possibility of ties in the ranks. There is, of course, some loss of information in replacing the original numbers by ranks. We could construct some rather artificial examples where a correlation could be detected parametrically (e.g., in the linear correlation coefficient r) but could not be detected nonparametrically. Such examples are very rare in real life, however, and the slight loss of information in ranking is a small price to pay for a very major advantage: When a correlation is demonstrated to be present nonparametrically, then it is really there! (That is, to a certainty level that depends on the significance chosen.) Nonparametric correlation is more robust than linear correlation, more resistant to unplanned defects in the data, in the same sort of sense that the median is more robust than the mean. For more on the concept of robustness, see §15.7.

As always in statistics, some particular choices of a statistic have already been invented for us and consecrated, if not beatified, by popular use. We will discuss two, the *Spearman rank-order correlation coefficient* (r_s), and *Kendall's tau* (τ).

14.6.1 Spearman Rank-Order Correlation Coefficient

Let R_i be the rank of x_i among the other x's and S_i be the rank of y_i among the other y's, with ties being assigned the appropriate midrank as described above. Then the rank-order correlation coefficient is defined to be the linear correlation coefficient of the ranks, namely,

$$r_s = \frac{\sum_i (R_i - \overline{R})(S_i - \overline{S})}{\sqrt{\sum_i (R_i - \overline{R})^2}\sqrt{\sum_i (S_i - \overline{S})^2}} \tag{14.6.1}$$

The significance of a nonzero value of r_s is tested by computing

$$t = r_s\sqrt{\frac{N-2}{1-r_s^2}} \tag{14.6.2}$$

which is distributed approximately as Student's distribution with $N-2$ degrees of freedom. A key point is that this approximation does not depend on the original distribution of the x's and y's; it is always the same approximation, and always pretty good.

It turns out that r_s is closely related to another conventional measure of nonparametric correlation, the so-called *sum squared difference of ranks*, defined as

$$D = \sum_{i=0}^{N-1} (R_i - S_i)^2 \tag{14.6.3}$$

(This D is sometimes denoted D^{**}, where the asterisks are used to indicate that ties are treated by midranking.)

When there are no ties in the data, the exact relation between D and r_s is

$$r_s = 1 - \frac{6D}{N^3 - N} \tag{14.6.4}$$

When there are ties, the exact relation is slightly more complicated: Let f_k be the number of ties in the kth group of ties among the R_i's, and let g_m be the number of ties in the mth group of ties among the S_i's. Then it turns out that

$$r_s = \frac{1 - \frac{6}{N^3 - N}\left[D + \frac{1}{12}\sum_k (f_k^3 - f_k) + \frac{1}{12}\sum_m (g_m^3 - g_m)\right]}{\left[1 - \frac{\sum_k (f_k^3 - f_k)}{N^3 - N}\right]^{1/2}\left[1 - \frac{\sum_m (g_m^3 - g_m)}{N^3 - N}\right]^{1/2}} \tag{14.6.5}$$

holds exactly. Notice that if all the f_k's and all the g_m's are equal to one, meaning that there are no ties, then equation (14.6.5) reduces to equation (14.6.4).

In (14.6.2) we gave a t-statistic that tests the significance of a nonzero r_s. It is also possible to test the significance of D directly. The expectation value of D in the null hypothesis of uncorrelated data sets is

$$\overline{D} = \frac{1}{6}(N^3 - N) - \frac{1}{12}\sum_k (f_k^3 - f_k) - \frac{1}{12}\sum_m (g_m^3 - g_m) \tag{14.6.6}$$

its variance is

$$\text{Var}(D) = \frac{(N-1)N^2(N+1)^2}{36}\left[1 - \frac{\sum_k (f_k^3 - f_k)}{N^3 - N}\right]\left[1 - \frac{\sum_m (g_m^3 - g_m)}{N^3 - N}\right] \tag{14.6.7}$$

and it is approximately normally distributed, so that the significance level is a complementary error function (cf. equation 14.5.2). Of course, (14.6.2) and (14.6.7) are not independent tests, but simply variants of the same test. In the program that follows, we calculate both the significance level obtained by using (14.6.2) and the significance level obtained by using (14.6.7); their discrepancy will give you an idea of how good the approximations are. You will also notice that we break off the task of assigning ranks (including tied midranks) into a separate function, `crank`.

stattests.h

```
void spear(VecDoub_I &data1, VecDoub_I &data2, Doub &d, Doub &zd, Doub &probd,
    Doub &rs, Doub &probrs)
```

Given two data arrays, `data1[0..n-1]` and `data2[0..n-1]`, this routine returns their sum squared difference of ranks as D, the number of standard deviations by which D deviates from its null-hypothesis expected value as `zd`, the two-sided p-value of this deviation as `probd`, Spearman's rank correlation r_s as `rs`, and the two-sided p-value of its deviation from zero as `probrs`. The external routines `crank` (below) and `sort2` (§8.2) are used. A small value of either `probd` or `probrs` indicates a significant correlation (`rs` positive) or anticorrelation (`rs` negative).

```
{
    Beta bet;
    Int j,n=data1.size();
    Doub vard,t,sg,sf,fac,en3n,en,df,aved;
    VecDoub wksp1(n),wksp2(n);
    for (j=0;j<n;j++) {
        wksp1[j]=data1[j];
        wksp2[j]=data2[j];
    }
    sort2(wksp1,wksp2);             Sort each of the data arrays, and convert the en-
    crank(wksp1,sf);                    tries to ranks. The values sf and sg return
    sort2(wksp2,wksp1);                 the sums ∑(f_k^3 − f_k) and ∑(g_m^3 − g_m),
    crank(wksp2,sg);                    respectively.
    d=0.0;
    for (j=0;j<n;j++)               Sum the squared difference of ranks.
```

```
        d += SQR(wksp1[j]-wksp2[j]);
    en=n;
    en3n=en*en*en-en;
    aved=en3n/6.0-(sf+sg)/12.0;                        Expectation value of D,
    fac=(1.0-sf/en3n)*(1.0-sg/en3n);
    vard=((en-1.0)*en*en*SQR(en+1.0)/36.0)*fac;        and variance of D give
    zd=(d-aved)/sqrt(vard);                            number of standard devia-
    probd=erfcc(abs(zd)/1.4142136);                        tions and p-value.
    rs=(1.0-(6.0/en3n)*(d+(sf+sg)/12.0))/sqrt(fac);    Rank correlation coefficient,
    fac=(rs+1.0)*(1.0-rs);
    if (fac > 0.0) {
        t=rs*sqrt((en-2.0)/fac);                       and its t-value,
        df=en-2.0;
        probrs=bet.betai(0.5*df,0.5,df/(df+t*t));      give its p-value.
    } else
        probrs=0.0;
}
```

```
void crank(VecDoub_IO &w, Doub &s)
```
stattests.h

Given a sorted array `w[0..n-1]`, replaces the elements by their rank, including midranking of ties, and returns as `s` the sum of $f^3 - f$, where f is the number of elements in each tie.

```
{
    Int j=1,ji,jt,n=w.size();
    Doub t,rank;
    s=0.0;
    while (j < n) {
        if (w[j] != w[j-1]) {                  Not a tie.
            w[j-1]=j;
            ++j;
        } else {                               A tie:
            for (jt=j+1;jt<=n && w[jt-1]==w[j-1];jt++);      How far does it go?
            rank=0.5*(j+jt-1);                 This is the mean rank of the tie,
            for (ji=j;ji<=(jt-1);ji++)         so enter it into all the tied entries,
                w[ji-1]=rank;
            t=jt-j;
            s += (t*t*t-t);                    and update s.
            j=jt;
        }
    }
    if (j == n) w[n-1]=n;                      If the last element was not tied, this is its
}                                                  rank.
```

14.6.2 Kendall's Tau

Kendall's τ is even more nonparametric than Spearman's r_s or D. Instead of using the numerical difference of ranks, it uses only the relative ordering of ranks: higher in rank, lower in rank, or the same in rank. But in that case we don't even have to rank the data! Ranks will be higher, lower, or the same if and only if the values are larger, smaller, or equal, respectively. On balance, we prefer r_s as being the more straightforward nonparametric test, but both statistics are in general use. In fact, τ and r_s are very strongly correlated and, in most applications, are effectively the same test.

To define τ, we start with the N data points (x_i, y_i). Now consider all $\frac{1}{2}N(N-1)$ *pairs* of data points, where a data point cannot be paired with itself, and where the points in either order count as one pair. We call a pair *concordant* if the relative ordering of the ranks of the two x's (or for that matter the two x's themselves) is the same as the relative ordering of the ranks of the two y's (or for that matter the two y's themselves). We call a pair *discordant* if the relative ordering of the ranks of the

two x's is opposite from the relative ordering of the ranks of the two y's. If there is a tie in either the ranks of the two x's or the ranks of the two y's, then we don't call the pair either concordant or discordant. If the tie is in the x's, we will call the pair an "extra y pair." If the tie is in the y's, we will call the pair an "extra x pair." If the tie is in both the x's and the y's, we don't call the pair anything at all. Are you still with us?

Kendall's τ is now the following simple combination of these various counts:

$$\tau = \frac{\text{concordant} - \text{discordant}}{\sqrt{\text{concordant} + \text{discordant} + \text{extra-}y}\ \sqrt{\text{concordant} + \text{discordant} + \text{extra-}x}} \tag{14.6.8}$$

You can easily convince yourself that this must lie between 1 and -1, and that it takes on the extreme values only for complete rank agreement or complete rank reversal, respectively.

More important, Kendall has worked out, from the combinatorics, the approximate distribution of τ in the null hypothesis of no association between x and y. In this case, τ is approximately normally distributed, with zero expectation value and a variance of

$$\text{Var}(\tau) = \frac{4N + 10}{9N(N-1)} \tag{14.6.9}$$

The following program proceeds according to the above description, and therefore loops over all pairs of data points. Beware: This is an $O(N^2)$ algorithm, unlike the algorithm for r_s, whose dominant sort operations are of order $N \log N$. If you are routinely computing Kendall's τ for data sets of more than a few thousand points, you may be in for some serious computing. If, however, you are willing to bin your data into a moderate number of bins, then read on.

stattests.h

```
void kendl1(VecDoub_I &data1, VecDoub_I &data2, Doub &tau, Doub &z, Doub &prob)
```

Given data arrays `data1[0..n-1]` and `data2[0..n-1]`, this program returns Kendall's τ as `tau`, its number of standard deviations from zero as `z`, and its two-sided p-value as `prob`. Small values of `prob` indicate a significant correlation (`tau` positive) or anticorrelation (`tau` negative).

```
{
    Int is=0,j,k,n2=0,n1=0,n=data1.size();
    Doub svar,aa,a2,a1;
    for (j=0;j<n-1;j++) {                       Loop over first member of pair,
        for (k=j+1;k<n;k++) {                   and second member.
            a1=data1[j]-data1[k];
            a2=data2[j]-data2[k];
            aa=a1*a2;
            if (aa != 0.0) {                    Neither array has a tie.
                ++n1;
                ++n2;
                aa > 0.0 ? ++is : --is;
            } else {                            One or both arrays have ties.
                if (a1 != 0.0) ++n1;            An "extra x" event.
                if (a2 != 0.0) ++n2;            An "extra y" event.
            }
        }
    }
    tau=is/(sqrt(Doub(n1))*sqrt(Doub(n2)));     Equation (14.6.8).
    svar=(4.0*n+10.0)/(9.0*n*(n-1.0));          Equation (14.6.9).
    z=tau/sqrt(svar);
    prob=erfcc(abs(z)/1.4142136);               p-value.
}
```

Sometimes it happens that there are only a few possible values each for x and y. In that case, the data can be recorded as a contingency table (see §14.4) that gives the number of data points for each contingency of x and y.

Spearman's rank-order correlation coefficient is not a very natural statistic under these circumstances, since it assigns to each x and y bin a not-very-meaningful midrank value and then totals up vast numbers of identical rank differences. Kendall's tau, on the other hand, with its simple counting, remains quite natural. Furthermore, its $O(N^2)$ algorithm is no longer a problem, since we can arrange for it to loop over pairs of contingency table entries (each containing many data points) instead of over pairs of data points. This is implemented in the program that follows.

Note that Kendall's tau can be applied only to contingency tables where both variables are *ordinal*, i.e., well-ordered, and that it looks specifically for monotonic correlations, not for arbitrary associations. These two properties make it less general than the methods of §14.4, which applied to *nominal*, i.e., unordered, variables and arbitrary associations.

Comparing `kendl1` above with `kendl2` below, you will see that we have changed a number of variables from `int` to `double`. This is because the number of events in a contingency table might be sufficiently large as to cause overflows in some of the integer arithmetic, while the number of individual data points in a list could not possibly be that large (for an $O(N^2)$ routine!).

```
void kendl2(MatDoub_I &tab, Doub &tau, Doub &z, Doub &prob)                stattests.h
```

Given a two-dimensional table `tab[0..i-1][0..j-1]`, such that `tab[k][l]` contains the number of events falling in bin `k` of one variable and bin `l` of another, this program returns Kendall's τ as `tau`, its number of standard deviations from zero as `z`, and its two-sided p-value as `prob`. Small values of `prob` indicate a significant correlation (`tau` positive) or anticorrelation (`tau` negative) between the two variables. Although `tab` is a `double` array, it will normally contain integral values.

```
{
    Int k,l,nn,mm,m2,m1,lj,li,kj,ki,i=tab.nrows(),j=tab.ncols();
    Doub svar,s=0.0,points,pairs,en2=0.0,en1=0.0;
    nn=i*j;                             Total number of entries in contingency table.
    points=tab[i-1][j-1];
    for (k=0;k<=nn-2;k++) {             Loop over entries in table,
        ki=(k/j);                       decoding a row,
        kj=k-j*ki;                      and a column.
        points += tab[ki][kj];          Increment the total count of events.
        for (l=k+1;l<=nn-1;l++) {       Loop over other member of the pair,
            li=l/j;                     decoding its row
            lj=l-j*li;                  and column.
            mm=(m1=li-ki)*(m2=lj-kj);
            pairs=tab[ki][kj]*tab[li][lj];
            if (mm != 0) {              Not a tie.
                en1 += pairs;
                en2 += pairs;
                s += (mm > 0 ? pairs : -pairs);         Concordant, or discordant.
            } else {
                if (m1 != 0) en1 += pairs;
                if (m2 != 0) en2 += pairs;
            }
        }
    }
    tau=s/sqrt(en1*en2);
    svar=(4.0*points+10.0)/(9.0*points*(points-1.0));
    z=tau/sqrt(svar);
    prob=erfcc(abs(z)/1.4142136);
}
```

CITED REFERENCES AND FURTHER READING:

Lupton, R. 1993, *Statistics in Theory and Practice* (Princeton, NJ: Princeton University Press), Chapter 13.

Lehmann, E.L. 1975, *Nonparametrics: Statistical Methods Based on Ranks* (San Francisco: Holden-Day); reprinted 2006 (New York: Springer).

Hollander, M., and Wolfe, D.A. 1999, *Nonparametric Statistical Methods*, 2nd ed. (New York: Wiley).

Downie, N.M., and Heath, R.W. 1965, *Basic Statistical Methods*, 2nd ed. (New York: Harper & Row), pp. 206–209.

Norusis, M.J. 2006, *SPSS 14.0 Guide to Data Analysis* (Englewood Cliffs, NJ: Prentice-Hall).

14.7 Information-Theoretic Properties of Distributions

In this section we return to nominal distributions, that is to say, to distributions with discrete outcomes that have no meaningful ordering. Information theory [1-3] provides a different, and sometimes very useful, perspective on the nature of such a distribution $\mathbf{p}$ with outcomes i, $0 \le i \le I-1$, and associated probabilities p_i, and on the relation between two or more such distributions. We develop that perspective in this section, starting with a review of some key concepts.

14.7.1 Entropy of a Distribution

Suppose that we make M sequential, independent draws from a distribution $\mathbf{p}$, thus generating a *message* that describes the outcomes, an M-vector of integers i_j, each in the range $0 \le i_j \le I-1$, with $j = 0, \ldots, M-1$. We want to send the message to a waiting confederate, but we first want to compress it (that is, *encode* it) into the most parsimonious format, say into the smallest possible number of bits, B. We can calculate a lower bound on B by equating 2^B, the number of possible different compressed messages, to a statistical estimate of the number of likely input messages. That equation, in the limit of M becoming very large, is

$$2^B \approx \frac{M!}{\prod_i (Mp_i)!} \tag{14.7.1}$$

The rationale for the right-hand side is that our message will contain very nearly Mp_i occurrences of the integer i for each i, so the count of messages will be very nearly the number of ways that we can arrange M objects of I types, with Mp_i of them identical for each type i. Taking the logarithm of equation (14.7.1), using Stirling's approximation on the factorials, and keeping only terms that scale as fast as M, we readily obtain

$$B \approx -M \sum_{i=0}^{I-1} p_i \log_2 p_i \equiv M\, H_2(\mathbf{p}) \tag{14.7.2}$$

where $H_2(\mathbf{p})$ is called the *entropy (in bits)* of the distribution $\mathbf{p}$, a terminology borrowed from statistical physics. The subscript 2 is to remind us that the logarithm has

base 2. We can also define an entropy with base e,

$$H(\mathbf{p}) \equiv -\sum_{i=0}^{I-1} p_i \ln p_i = -(\ln 2) \sum_{i=0}^{I-1} p_i \log_2 p_i = (\ln 2)\, H_2(\mathbf{p}) \tag{14.7.3}$$

If $H_2(\mathbf{p})$ is measured in *bits*, then $H(\mathbf{p})$ will be measured in *nats*, with 1 nat = 1.4427 bits. In evaluating (14.7.3), note that

$$\lim_{p \to 0} p \ln p = 0 \tag{14.7.4}$$

The value $H(\mathbf{p})$ lies between 0 and $\ln I$. It is zero only when one of the p_i's is one, all the others zero.

Although we derived B as a lower bound, a central result of information theory is that, in the limit of large M, one can find codes that actually achieve that bound. (Arithmetic coding, described in §22.6, is an example of such a code.) Heuristically, one can interpret equation (14.7.2) as saying that it takes, on average, $-\log_2 p_i$ bits (a positive number, since $p_i < 1$) to encode an outcome i. Thus, the compressed message size is M times the expectation of $-\log_2 p_i$ over outcomes occurring with probability p_i.

Yet a different view of entropy arises if we consider the game of "twenty questions," where by repeated yes/no questions you try to eliminate all except one correct possibility for an unknown object. Better yet, let us consider a generalization of the game, where you are allowed to ask multiple choice questions as well as binary (yes/no) ones. The categories in your multiple choice questions are supposed to be mutually exclusive and exhaustive (as are "yes" and "no").

The value to you of an answer increases with the number of possibilities that it eliminates. More specifically, an answer that eliminates all except a fraction p of the remaining possibilities can be assigned a value $-\ln p$. The purpose of the logarithm is to make the value additive, since, e.g., one question that eliminates all but 1/6 of the possibilities is considered as good as two questions that, in sequence, reduce the number by factors 1/2 and 1/3.

So that is the value of an answer; but what is the value of a question? If there are I possible answers to the question and the fraction of possibilities consistent with answer i is p_i, then the value of the question is the expectation value of the value of the answer, which is just $-\sum_i p_i \ln p_i$ or $H(\mathbf{p})$, as above.

As already mentioned, the entropy is zero only if one of the p_i's is unity, with all the others zero. In this case, the question is valueless, since its answer is preordained. $H(\mathbf{p})$ takes on its maximum value when all the p_i's are equal, in which case the question is sure to eliminate all but a fraction $1/I$ of the remaining possibilities.

A third, still different, view of entropy comes from thinking about bets (or, more politely, "investments"). A *fair bet* on an outcome i of probability p_i is one that has a payoff $o_i = 1/p_i$. This is the unique payoff (per unit wagered) for which, in the long run, the bettor will neither win nor lose, since in expectation value

$$\langle o_i \rangle = p_i o_i = 1 \tag{14.7.5}$$

Suppose you have the opportunity to bet repeatedly on a game offering fair bets on each outcome. This is not very interesting as a money-making proposition. But suppose that you are *clairvoyant* and can know in advance the outcome of each play

(although you cannot affect that outcome). Now you're in business! You always put your money on the winning choice of i. How much money can you make?

Since your profit on each (sure thing!) wager scales multiplicatively with your accumulated wealth, the appropriate figure of merit is the the average *doubling rate*, or, equivalently, *e-folding rate*, at which you can increase your capital. Since you always win, but can't control the outcome, this is given by

$$W \equiv \langle \ln o_i \rangle = \langle -\ln p_i \rangle = -\sum_i p_i \ln p_i = H(\mathbf{p}) \tag{14.7.6}$$

In other words, the entropy of a distribution is the e-folding rate of capital for a fair game about which you have perfect predictive information. While this may seem fanciful, we will see in §14.7.3 how it generalizes to the more realistic case where you have only imperfect, perhaps very small, predictive information.

14.7.2 Kullback-Leibler Distance

Back in the context of message compression, suppose that events occur with a distribution $\mathbf{p}$, that is, p_i, $0 \le i \le I-1$, but we try to compress the message of their outcomes with a code that is optimized for some other distribution $\mathbf{q}$, that is, q_i, $0 \le i \le I-1$. Our code therefore takes about $-\log_2 q_i$ bits, or $-\ln q_i$ nats, to encode outcome i, and the average compressed length per outcome is

$$-\sum_i p_i \ln q_i = H(\mathbf{p}) + \sum_i p_i \ln \frac{p_i}{q_i} \equiv H(\mathbf{p}) + D(\mathbf{p}\|\mathbf{q}) \tag{14.7.7}$$

The quantity

$$D(\mathbf{p}\|\mathbf{q}) \equiv \sum_i p_i \ln \frac{p_i}{q_i} \tag{14.7.8}$$

is called the *Kullback-Leibler distance* between $\mathbf{p}$ and $\mathbf{q}$, also called the *relative entropy* between the two distributions. We can easily prove that it is nonnegative, since

$$-D(\mathbf{p}\|\mathbf{q}) = \sum_i p_i \ln\left(\frac{q_i}{p_i}\right) \le \sum_i p_i \left(\frac{q_i}{p_i} - 1\right) = 1 - 1 = 0 \tag{14.7.9}$$

where the inequality follows from the fact that

$$\ln w \le w - 1 \tag{14.7.10}$$

(Of course we already knew it had to be nonnegative, because we knew that $H(\mathbf{p})$ was the *smallest* possible compressed message size for the distribution $\mathbf{p}$.) The Kullback-Leibler distance between two distributions is zero only when the two distributions are identical. The Kullback-Leibler distance between any distribution $\mathbf{p}$ and the uniform distribution $\mathbf{U}$ is just the difference between the entropy of $\mathbf{p}$ and the maximum possible entropy $\ln I$, that is,

$$H(\mathbf{p}) + D(\mathbf{p}\|\mathbf{U}) = \ln I \tag{14.7.11}$$

This is illustrated in Figure 14.7.1. Just like entropy, the Kullback-Leibler distance is measured in bits or nats, depending on whether the logarithms are taken base 2 or e, respectively.

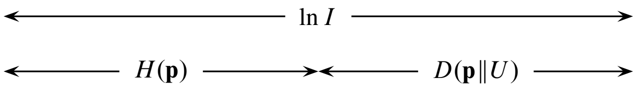

Figure 14.7.1. Relation between the entropy of a distribution $\mathbf{p}$, its Kullback-Leibler distance to the uniform distribution $\mathbf{U}$, and its maximum possible entropy $\ln I$.

Notice that the Kullback-Leibler distance is not symmetric, nor (it turns out) does it satisfy the triangle inequality. So it is not a true metric distance. It is, however, a useful measure of the degree by which some "target" distribution $\mathbf{q}$ differs from some "base" distribution $\mathbf{p}$. We now give a couple of examples of where it naturally occurs.

Example 1. Suppose that we are seeing events drawn from the distribution $\mathbf{p}$, but we want to rule out an alternative hypothesis that they are drawn from $\mathbf{q}$. We might do this by computing a likelihood ratio,

$$\mathcal{L} = \frac{p(\text{Data}|\mathbf{p})}{p(\text{Data}|\mathbf{q})} = \prod_{\text{data}} \frac{p_i}{q_i} \tag{14.7.12}$$

and rejecting the alternative hypothesis $\mathbf{q}$ if this ratio is larger than some large number, say 10^6. (In the above shorthand notation, the product over "data" means that we substitute for i in each factor the particular outcome of that factor's individual data event.) Taking the logarithm of equation (14.7.12), you can easily see that, under hypothesis $\mathbf{p}$, the average increase in $\ln \mathcal{L}$ per data event is just $D(\mathbf{p}\|\mathbf{q})$. In other words, the Kullback-Leibler distance is the expected log-likelihood with which a false hypothesis $\mathbf{q}$ can be rejected, per event. As we might expect, this has something to do with "how different" $\mathbf{q}$ is from $\mathbf{p}$.

As a Bayesian aside, the reason that the above likelihood test is unsatisfyingly asymmetric is that, without the notion of a prior, we have no way to treat hypotheses $\mathbf{p}$ and $\mathbf{q}$ democratically. But suppose that $p(\mathbf{p})$ is the prior probability of $\mathbf{p}$, so that $p(\mathbf{q}) = 1 - p(\mathbf{p})$ is the prior for $\mathbf{q}$. Then the Bayes odds ratio on the two hypotheses is

$$\text{O.R.} = \frac{p(\mathbf{p}|\text{Data})}{p(\mathbf{q}|\text{Data})} = \frac{p(\text{Data}|\mathbf{p})\, p(\mathbf{p})}{p(\text{Data}|\mathbf{q})\, p(\mathbf{q})} = \frac{p(\mathbf{p})}{p(\mathbf{q})} \prod_{\text{data}} \frac{p_i}{q_i} \tag{14.7.13}$$

The figure of merit is now the expected increase in ln(O.R.) if $\mathbf{p}$ is true, *minus* the expected increase (that is, *plus* the expected decrease) if $\mathbf{q}$ is true, which can readily be seen to be

$$p(\mathbf{p})\, D(\mathbf{p}\|\mathbf{q}) + p(\mathbf{q})\, D(\mathbf{q}\|\mathbf{p}) \tag{14.7.14}$$

per data event, which has the appropriate symmetry. We can use expression (14.7.14) to estimate how many data events we will need on average to distinguish between two distributions. Notice that in the case of a uniform ("noninformative") prior, $p(\mathbf{p}) = p(\mathbf{q}) = 0.5$, we get just the symmetrized average of the two Kullback-Leibler distances.

Example 2. Meanwhile, back at the racetrack where we are offered payoffs of o_i on events with probability p_i, $\sum_i p_i = 1$, we want to work out the best way to divide our capital across all the possible outcomes i of each race. Suppose we bet a fraction b_i on outcome i. Analgously to equation (14.7.6), we want to maximize the

average e-folding rate,

$$W = \langle \ln(b_i o_i) \rangle = \sum_i p_i \ln(b_i o_i) \tag{14.7.15}$$

subject to the constraint

$$\sum_i b_i = 1 \tag{14.7.16}$$

An easy calculation (using a Lagrange multiplier to impose the constraint) gives the result that the maximum occurs for

$$b_i = p_i \tag{14.7.17}$$

completely independent of the values o_i! This remarkable result is called *proportional betting*, or sometimes *Kelly's formula* [4].

In practice, the distribution $\mathbf{p}$ is imperfectly known, both to you and to the bookie at the track. Suppose that you estimate the outcome probabilities as $\mathbf{q}$, while the bookie's estimate is $\mathbf{r}$. If the bookie is feeling generous, he offers payoffs that are fair bets according to his estimate,

$$o_i = 1/r_i \tag{14.7.18}$$

while you place proportional bets with $b_i = q_i$. Your e-folding rate is now

$$W = \langle \ln(b_i o_i) \rangle = \sum_i p_i \ln \frac{q_i}{r_i} = D(\mathbf{p}\|\mathbf{r}) - D(\mathbf{p}\|\mathbf{q}) \tag{14.7.19}$$

This will be positive if and only if your estimate of the probabilities is better than the bookie's, that is, closer as measured by the Kullback-Leibler distance. Betting, in other words, is a competition between you and the bookie over who can better estimate the true odds.

A more realistic variant is to assume that the bookie offers payoffs of only some fraction $f < 1$ of his reciprocal probability estimates. Then (you can work out), you can win only if

$$D(\mathbf{p}\|\mathbf{r}) - D(\mathbf{p}\|\mathbf{q}) > -\ln f \tag{14.7.20}$$

14.7.3 Conditional Entropy and Mutual Information

We now want to look at the association of two variables. Let us return to the guessing game that was discussed in §14.7.1. Suppose we are deciding what question to ask next in the game and have to choose between two candidates, or possibly want to ask both in one order or another. Suppose that one question, x, has I possible answers, labeled by i, and that the other question, y, has J possible answers, labeled by j. Then the possible outcomes of asking both questions form a contingency table whose entries are the joint outcome probabilities p_{ij}, normalized by

$$\sum_{i=0}^{I-1} \sum_{j=0}^{J-1} p_{ij} \equiv \sum_{i,j} p_{ij} = 1 \tag{14.7.21}$$

We use the same "dot" notation as in §14.4 to denote the row and column sums, so that $p_{i\cdot}$ is the probability of outcome i asking question x only, while $p_{\cdot j}$ is the probability of outcome j asking question y only. The entropies of the questions x and y are thus, respectively,

$$H(x) = -\sum_i p_{i\cdot} \ln p_{i\cdot} \qquad H(y) = -\sum_j p_{\cdot j} \ln p_{\cdot j} \tag{14.7.22}$$

The entropy of the two questions together is

$$H(x,y) = -\sum_{i,j} p_{ij} \ln p_{ij} \tag{14.7.23}$$

Now what is the entropy of the question y *given* x (that is, if x is asked first)? It is the expectation value over the answers to x of the entropy of the restricted y distribution that lies in a single column of the contingency table (corresponding to the x answer):

$$H(y|x) = -\sum_i p_{i\cdot} \sum_j \frac{p_{ij}}{p_{i\cdot}} \ln \frac{p_{ij}}{p_{i\cdot}} = -\sum_{i,j} p_{ij} \ln \frac{p_{ij}}{p_{i\cdot}} \tag{14.7.24}$$

Correspondingly, the entropy of x given y is

$$H(x|y) = -\sum_j p_{\cdot j} \sum_i \frac{p_{ij}}{p_{\cdot j}} \ln \frac{p_{ij}}{p_{\cdot j}} = -\sum_{i,j} p_{ij} \ln \frac{p_{ij}}{p_{\cdot j}} \tag{14.7.25}$$

We can readily prove that the entropy of y given x is never more than the entropy of y alone, i.e., that asking x first can only reduce the usefulness of asking y (in which case the two variables are *associated*):

$$\begin{aligned} H(y|x) - H(y) &= -\sum_{i,j} p_{ij} \ln \frac{p_{ij}/p_{i\cdot}}{p_{\cdot j}} \\ &= \sum_{i,j} p_{ij} \ln \frac{p_{\cdot j}\, p_{i\cdot}}{p_{ij}} \\ &\le \sum_{i,j} p_{ij} \left(\frac{p_{\cdot j}\, p_{i\cdot}}{p_{ij}} - 1 \right) \\ &= \sum_{i,j} p_{i\cdot} p_{\cdot j} - \sum_{i,j} p_{ij} \\ &= 1 - 1 = 0 \end{aligned} \tag{14.7.26}$$

Quantities like $H(x|y)$ or $H(y|x)$ are called *conditional entropies*. You can easily show that

$$H(x,y) = H(x) + H(y|x) = H(y) + H(x|y) \tag{14.7.27}$$

sometimes called the *chain rule for entropies*. It immediately follows that

$$H(x) - H(x|y) = H(y) - H(y|x) \equiv I(x,y) \tag{14.7.28}$$

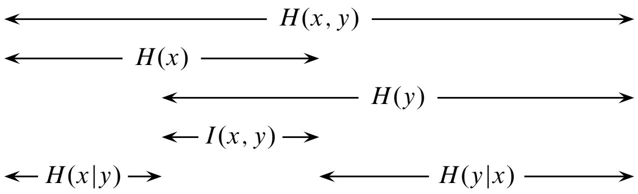

Figure 14.7.2. Relations among the entropies, conditional entropies, and mutual information of two variables. The quantities shown as segment lengths are always positive.

a quantity called the *mutual information* between x and y, given explicitly by

$$I(x, y) = \sum_{i,j} p_{ij} \ln\left(\frac{p_{ij}}{p_{i\cdot}p_{\cdot j}}\right) \tag{14.7.29}$$

Notice that the mutual information is symmetrical, $I(x, y) = I(y, x)$.

Figure 14.7.2 provides a handy way to visualize the additive relations and inequalities among the quantities discussed. As before, all the quantities are measured in bits or nats. Using mutual information, one can make statements like this about the degree of association of two variables: "The variables have information (entropy) 6.5 and 4.2 bits, respectively. However, their mutual information is 3.8 bits, so together they provide only 6.9 bits of information."

As a more detailed example, let us go back to the racetrack one last time. Suppose that you have some *side information* relevant to the outcome, but not completely predictive. That is, x is the random variable of which outcome i wins, while y is a random variable whose value j you know. Instead of a simple set of probabilities p_i, we now have a contingency table of joint outcomes, p_{ij}. How should you bet, and what is your expected e-folding rate?

First, we need to generalize equation (14.7.17). Suppose that b_{ij} is the fraction of assets that we bet on outcome i when our side information has the value j. There are now J separate constraints,

$$\sum_i b_{ij} = 1, \qquad 0 \le j \le J-1 \tag{14.7.30}$$

For simplicity, let us take the case where the payoffs are for fair bets (but without the side information), $o_i = 1/p_{i\cdot}$. Then we want to maximize

$$W = \left\langle \ln \frac{b_{ij}}{p_{i\cdot}} \right\rangle = \sum_{i,j} p_{ij} \ln \frac{b_{ij}}{p_{i\cdot}} \tag{14.7.31}$$

A simple calculation, now with J distinct Lagrange multipliers, gives the result,

$$b_{ij} = \frac{p_{ij}}{p_{\cdot j}} \tag{14.7.32}$$

This is again proportional betting, except that it is now conditioned on the value j that is known to us. Substituting equation (14.7.32) into (14.7.31) gives

$$W = \sum_{i,j} p_{ij} \ln\left(\frac{p_{ij}}{p_{i\cdot}p_{\cdot j}}\right) = I(x, y) \tag{14.7.33}$$

We see that the expected e-folding rate is exactly the mutual information between x and y. In other words, we can make money if and only if our side information y has nonzero mutual information with the outcome x. As in equation (14.7.20), you can easily work out more realistic cases where the payouts are not fair bets, or are based on inexact estimates of the true probabilities. A special case of equation (14.7.33) is when the side information y predicts the outcome x *perfectly*. Then, $I(x,y) = H(x) = H(y) = H(x,y)$ and we recover exactly equation (14.7.6).

14.7.4 Uncertainty Coefficients

By analogy with the various coefficients of correlation discussed earlier in this chapter, one sometimes sees *uncertainty coefficients* defined from the various entropies defined above (and in Figure 14.7.2). The uncertainty coefficient of y with respect to x, denoted $U(y|x)$, is defined by

$$U(y|x) \equiv \frac{H(y) - H(y|x)}{H(y)} \tag{14.7.34}$$

This measure lies between 0 and 1, with the value 0 indicating that x and y have no association and the value 1 indicating that knowledge of x completely predicts y. For in-between values, $U(y|x)$ gives the fraction of y's entropy $H(y)$ that is lost if x is already known. In our game of "twenty questions," $U(y|x)$ is the fractional loss in the utility of question y if question x is to be asked first.

If we wish to view x as the dependent variable and y as the independent one, then interchanging x and y we can of course define the dependency of x on y,

$$U(x|y) \equiv \frac{H(x) - H(x|y)}{H(x)} \tag{14.7.35}$$

If we want to treat x and y symmetrically, then the useful combination turns out to be

$$U(x,y) \equiv 2\left[\frac{H(y) + H(x) - H(x,y)}{H(x) + H(y)}\right] \tag{14.7.36}$$

If the two variables are completely independent, then $H(x,y) = H(x) + H(y)$, so (14.7.36) vanishes. If the two variables are completely dependent, then $H(x) = H(y) = H(x,y)$, so (14.7.35) equals unity. You can easily show that

$$U(x,y) = \frac{H(x)U(x|y) + H(y)U(y|x)}{H(x) + H(y)} \tag{14.7.37}$$

that is, that the symmetrical measure is just a weighted average of the two asymmetrical measures (14.7.34) and (14.7.35), weighted by the entropy of each variable separately.

Generally we find the entropy measures themselves, in bits or nats, more useful than the uncertainty coefficients derived from them.

CITED REFERENCES AND FURTHER READING:

Shannon, C.E., and Weaver, W. 1949, *The Mathematical Theory of Communication*, reprinted 1998 (Urbana, IL: University of Illinois Press).[1]

Cover, T.M., and Thomas, J.A. 1991, *Elements of Information Theory* (New York: Wiley). [2]

MacKay, D.J.C. 2003, *Information Theory, Inference, and Learning Algorithms* (Cambridge, UK: Cambridge University Press). [3]

Kelly, J. 1956, "A New Interpretation of Information Rate," *Bell System Technical Journal*, vol. 35, pp. 917–926. [4]

14.8 Do Two-Dimensional Distributions Differ?

We here discuss a useful generalization of the K–S test (§14.3) to *two-dimensional* distributions. This generalization is due to Fasano and Franceschini [1], a variant on an earlier idea due to Peacock [2].

In a two-dimensional distribution, each data point is characterized by an (x, y) pair of values. An example near to our hearts is that each of the 19 neutrinos that were detected from Supernova 1987A is characterized by a time t_i and by an energy E_i (see [3]). We might wish to know whether these measured pairs (t_i, E_i), $i = 0 \dots 18$ are consistent with a theoretical model that predicts neutrino flux as a function of both time and energy — that is, a two-dimensional probability distribution in the (x, y) [here, (t, E)] plane. That would be a one-sample test. Or, given two sets of neutrino detections, from two comparable detectors, we might want to know whether they are compatible with each other, a two-sample test.

In the spirit of the tried-and-true one-dimensional K–S test, we want to range over the (x, y)-plane in search of some kind of maximum *cumulative* difference between two two-dimensional distributions. Unfortunately, cumulative probability distribution is not well-defined in more than one dimension! Peacock's insight was that a good surrogate is the *integrated probability in each of four natural quadrants* around a given point (x_i, y_i), namely the total probabilities (or fraction of data) in $(x > x_i, y > y_i)$, $(x < x_i, y > y_i)$, $(x < x_i, y < y_i)$, $(x > x_i, y < y_i)$. The two-dimensional K–S statistic D is now taken to be the maximum difference (ranging both over data points and over quadrants) of the corresponding integrated probabilities. When comparing two data sets, the value of D may depend on which data set is ranged over. In that case, define an effective D as the average of the two values obtained. If you are confused at this point about the exact definition of D, don't fret; the accompanying computer routines amount to a precise algorithmic definition.

Figure 14.8.1 gives a feeling for what is going on. The 65 triangles and 35 squares seem to have somewhat different distributions in the plane. The dotted lines are centered on the triangle that maximizes the D statistic; the maximum occurs in the upper-left quadrant. That quadrant contains only 0.12 of all the triangles, but it contains 0.56 of all the squares. The value of D is thus 0.44. Is this statistically significant?

Even for fixed sample sizes, it is unfortunately not rigorously true that the distribution of D in the null hypothesis is independent of the shape of the two-dimensional distribution. In this respect the two-dimensional K–S test is not as natural as its one-dimensional parent. However, extensive Monte Carlo integrations have shown that the distribution of the two-dimensional D is *very nearly* identical for even quite different distributions, as long as they have the same coefficient of correlation r, defined in the usual way by equation (14.5.1). In their paper, Fasano and Franceschini tabulate Monte Carlo results for (what amounts to) the distribution of D as a function

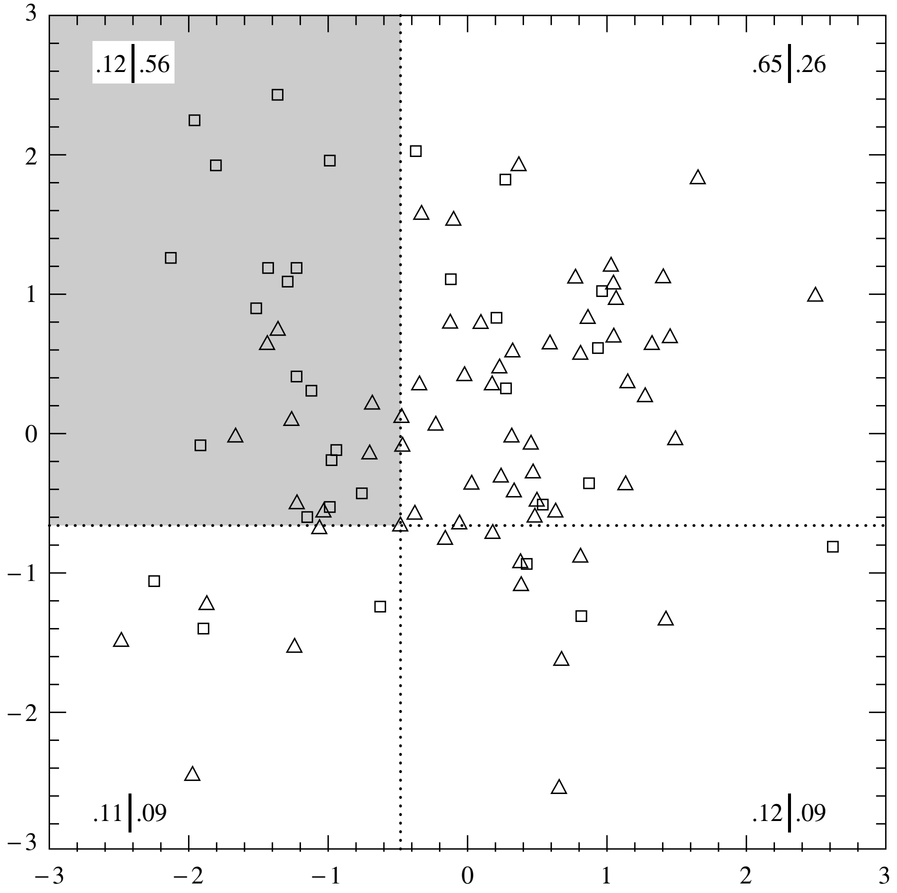

Figure 14.8.1. Two-dimensional distributions of 65 triangles and 35 squares. The two-dimensional K–S test finds that point one of whose quadrants (shown by dotted lines) maximizes the difference between fraction of triangles and fraction of squares. Then, equation (14.8.1) indicates whether the difference is statistically significant, i.e., whether the triangles and squares must have different underlying distributions.

of (of course) D, sample size N, and coefficient of correlation r. Analyzing their results, one finds that the significance levels for the two-dimensional K–S test can be summarized by the simple, though approximate, formulas

$$\text{Probability}\ (D > \text{observed}\ ) = Q_{KS}\left(\frac{\sqrt{N}\,D}{1+\sqrt{1-r^2}(0.25-0.75/\sqrt{N})}\right) \tag{14.8.1}$$

for the one-sample case, and the same for the two-sample case, but with

$$N = \frac{N_1 N_2}{N_1 + N_2}. \tag{14.8.2}$$

The above formulas are accurate enough when $N \gtrsim 20$, and when the indicated probability (significance level) is less than (more significant than) 0.20 or so. When the indicated probability is > 0.20, its value may not be accurate, but the

implication that the data and model (or two data sets) are not significantly different is certainly correct. Notice that in the limit of $r \to 1$ (perfect correlation), equations (14.8.1) and (14.8.2) reduce to equations (14.3.18) and (14.3.19): The two-dimensional data lie on a perfect straight line, and the two-dimensional K–S test becomes a one-dimensional K–S test.

The significance level for the data in Figure 14.8.1, by the way, is about 0.001. This establishes to a near-certainty that the triangles and squares were drawn from different distributions. (As in fact they were.)

Of course, if you do not want to rely on the Monte Carlo experiments embodied in equation (14.8.1), you can do your own: Generate a lot of synthetic data sets from your model, each one with the same number of points as the real data set. Compute D for each synthetic data set, using the accompanying computer routines (but ignoring their calculated probabilities), and count what fraction of the time these synthetic D's exceed the D from the real data. That fraction is your significance.

One disadvantage of the two-dimensional tests, by comparison with their one-dimensional progenitors, is that the two-dimensional tests require of order N^2 operations: Two nested loops of order N take the place of an $N \log N$ sort. For desktop computers, this restricts the usefulness of the tests to N less than several thousand.

We now give computer implementations. The one-sample case is embodied in the routine `ks2d1s` (that is, two dimensions, one sample). This routine calls a straightforward utility routine `quadct` to count points in the four quadrants, and it calls a user-supplied routine `quadvl` that must be capable of returning the integrated probability of an analytic model in each of four quadrants around an arbitrary (x, y) point. A trivial sample `quadvl` is shown; realistic `quadvl`s can be quite complicated, often incorporating numerical quadratures over analytic two-dimensional distributions.

kstests_2d.h

```
void ks2d1s(VecDoub_I &x1, VecDoub_I &y1, void quadvl(const Doub, const Doub,
    Doub &, Doub &, Doub &, Doub &), Doub &d1, Doub &prob)
```

Two-dimensional Kolmogorov-Smirnov test of one sample against a model. Given the x and y coordinates of `n1` data points in arrays `x1[0..n1-1]` and `y1[0..n1-1]`, and given a user-supplied function `quadvl` that exemplifies the model, this routine returns the two-dimensional K-S statistic as `d1`, and its p-value as `prob`. Small values of `prob` show that the sample is significantly different from the model. Note that the test is slightly distribution-dependent, so `prob` is only an estimate.

```
{
    Int j,n1=x1.size();
    Doub dum,dumm,fa,fb,fc,fd,ga,gb,gc,gd,r1,rr,sqen;
    KSdist ks;
    d1=0.0;
    for (j=0;j<n1;j++) {                        Loop over the data points.
        quadct(x1[j],y1[j],x1,y1,fa,fb,fc,fd);
        quadvl(x1[j],y1[j],ga,gb,gc,gd);
        if (fa > ga) fa += 1.0/n1;
        if (fb > gb) fb += 1.0/n1;
        if (fc > gc) fc += 1.0/n1;
        if (fd > gd) fd += 1.0/n1;
        d1=MAX(d1,abs(fa-ga));
        d1=MAX(d1,abs(fb-gb));
        d1=MAX(d1,abs(fc-gc));
        d1=MAX(d1,abs(fd-gd));
        For both the sample and the model, the distribution is integrated in each of four
        quadrants, and the maximum difference is saved.
    }
    pearsn(x1,y1,r1,dum,dumm);                  Get the linear correlation coefficient r1.
```

```
    sqen=sqrt(Doub(n1));
    rr=sqrt(1.0-r1*r1);
    Estimate the probability using the K-S probability function.
    prob=ks.qks(d1*sqen/(1.0+rr*(0.25-0.75/sqen)));
}
```

kstests_2d.h

```
void quadct(const Doub x, const Doub y, VecDoub_I &xx, VecDoub_I &yy, Doub &fa,
    Doub &fb, Doub &fc, Doub &fd)
```

Given an origin (x, y), and an array of nn points with coordinates xx[0..nn-1] and yy[0..nn-1], count how many of them are in each quadrant around the origin, and return the normalized fractions. Quadrants are labeled alphabetically, counterclockwise from the upper right. Used by ks2d1s and ks2d2s.

```
{
    Int k,na,nb,nc,nd,nn=xx.size();
    Doub ff;
    na=nb=nc=nd=0;
    for (k=0;k<nn;k++) {
        if (yy[k] == y && xx[k] == x) continue;
        if (yy[k] > y)
            xx[k] > x ? ++na : ++nb;
        else
            xx[k] > x ? ++nd : ++nc;
    }
    ff=1.0/nn;
    fa=ff*na;
    fb=ff*nb;
    fc=ff*nc;
    fd=ff*nd;
}
```

quadvl.h

```
void quadvl(const Doub x, const Doub y, Doub &fa, Doub &fb, Doub &fc, Doub &fd)
```

This is a sample of a user-supplied routine to be used with ks2d1s. In this case, the model distribution is uniform inside the square $-1 < x < 1$, $-1 < y < 1$. In general, this routine should return, for any point (x, y), the fraction of the total distribution in each of the four quadrants around that point. The fractions, fa, fb, fc, and fd, must add up to 1. Quadrants are alphabetical, counterclockwise from the upper right.

```
{
    Doub qa,qb,qc,qd;
    qa=MIN(2.0,MAX(0.0,1.0-x));
    qb=MIN(2.0,MAX(0.0,1.0-y));
    qc=MIN(2.0,MAX(0.0,x+1.0));
    qd=MIN(2.0,MAX(0.0,y+1.0));
    fa=0.25*qa*qb;
    fb=0.25*qb*qc;
    fc=0.25*qc*qd;
    fd=0.25*qd*qa;
}
```

The routine ks2d2s is the two-sample case of the two-dimensional K–S test. It also calls quadct, pearsn, and KSdist::qks. Being a two-sample test, it does not need an analytic model.

kstests_2d.h

```
void ks2d2s(VecDoub_I &x1, VecDoub_I &y1, VecDoub_I &x2, VecDoub_I &y2, Doub &d,
    Doub &prob)
```

Two-dimensional Kolmogorov-Smirnov test on two samples. Given the x and y coordinates of the first sample as n1 values in arrays x1[0..n1-1] and y1[0..n1-1], and likewise for the second sample, n2 values in arrays x2 and y2, this routine returns the two-dimensional, two-sample K-S statistic as d, and its p-value as prob. Small values of prob show that the two samples are significantly different. Note that the test is slightly distribution-dependent, so prob is only an estimate.

```
{
    Int j,n1=x1.size(),n2=x2.size();
    Doub d1,d2,dum,dumm,fa,fb,fc,fd,ga,gb,gc,gd,r1,r2,rr,sqen;
    KSdist ks;
    d1=0.0;
    for (j=0;j<n1;j++) {                    First, use points in the first sample as origins.
        quadct(x1[j],y1[j],x1,y1,fa,fb,fc,fd);
        quadct(x1[j],y1[j],x2,y2,ga,gb,gc,gd);
        if (fa > ga) fa += 1.0/n1;
        if (fb > gb) fb += 1.0/n1;
        if (fc > gc) fc += 1.0/n1;
        if (fd > gd) fd += 1.0/n1;
        d1=MAX(d1,abs(fa-ga));
        d1=MAX(d1,abs(fb-gb));
        d1=MAX(d1,abs(fc-gc));
        d1=MAX(d1,abs(fd-gd));
    }
    d2=0.0;
    for (j=0;j<n2;j++) {                    Then, use points in the second sample as
        quadct(x2[j],y2[j],x1,y1,fa,fb,fc,fd);          origins.
        quadct(x2[j],y2[j],x2,y2,ga,gb,gc,gd);
        if (ga > fa) ga += 1.0/n1;
        if (gb > fb) gb += 1.0/n1;
        if (gc > fc) gc += 1.0/n1;
        if (gd > fd) gd += 1.0/n1;
        d2=MAX(d2,abs(fa-ga));
        d2=MAX(d2,abs(fb-gb));
        d2=MAX(d2,abs(fc-gc));
        d2=MAX(d2,abs(fd-gd));
    }
    d=0.5*(d1+d2);                          Average the K-S statistics.
    sqen=sqrt(n1*n2/Doub(n1+n2));
    pearsn(x1,y1,r1,dum,dumm);              Get the linear correlation coefficient for each
    pearsn(x2,y2,r2,dum,dumm);                  sample.
    rr=sqrt(1.0-0.5*(r1*r1+r2*r2));
    Estimate the probability using the K-S probability function.
    prob=ks.qks(d*sqen/(1.0+rr*(0.25-0.75/sqen)));
}
```

CITED REFERENCES AND FURTHER READING:

Fasano, G. and Franceschini, A. 1987, "A Multidimensional Version of the Kolmogorov-Smirnov Test," *Monthly Notices of the Royal Astronomical Society*, vol. 225, pp. 155–170.[1]

Peacock, J.A. 1983, "Two-Dimensional Goodness-of-Fit Testing in Astronomy," *Monthly Notices of the Royal Astronomical Society*, vol. 202, pp. 615–627.[2]

Spergel, D.N., Piran, T., Loeb, A., Goodman, J., and Bahcall, J.N. 1987, "A Simple Model for Neutrino Cooling of the LMC Supernova," *Science*, vol. 237, pp. 1471–1473.[3]

14.9 Savitzky-Golay Smoothing Filters

In §13.5 we learned something about the construction and application of digital filters, but little guidance was given on *which particular* filter to use. That, of course, depends on what you want to accomplish by filtering. One obvious use for *low-pass* filters is to smooth noisy data.

The premise of data smoothing is that one is measuring a variable that is both slowly varying and also corrupted by random noise. Then it can sometimes be useful

to replace each data point by some kind of local average of surrounding data points. Since nearby points measure very nearly the same underlying value, averaging can reduce the level of noise without (much) biasing the value obtained.

We must comment editorially that the smoothing of data lies in a murky area, beyond the fringe of some better-posed, and therefore more highly recommended, techniques that are discussed elsewhere in this book. If you are fitting data to a parametric model, for example (see Chapter 15), it is almost always better to use raw data than to use data that have been pre-processed by a smoothing procedure. Another alternative to blind smoothing is so-called "optimal" or Wiener filtering, as discussed in §13.3 and more generally in §13.6. Data smoothing is probably most justified when it is used simply as a graphical technique, to guide the eye through a forest of data points all with large error bars, or as a means of making initial *rough* estimates of simple parameters from a graph.

In this section we discuss a particular type of low-pass filter, well-adapted for data smoothing, and termed variously *Savitzky-Golay* [1], *least-squares* [2], or *DISPO* (Digital Smoothing Polynomial) [3] filters. Rather than having their properties defined in the Fourier domain and then translated to the time domain, Savitzky-Golay filters derive directly from a particular formulation of the data smoothing problem in the time domain, as we will now see. Savitzky-Golay filters were initially (and are still often) used to render visible the relative widths and heights of spectral lines in noisy spectrometric data.

Recall that a digital filter is applied to a series of equally spaced data values $f_i \equiv f(t_i)$, where $t_i \equiv t_0 + i\Delta$ for some constant sample spacing Δ and $i = \ldots -2, -1, 0, 1, 2, \ldots$. We have seen (§13.5) that the simplest type of digital filter (the nonrecursive or finite impulse response filter) replaces each data value f_i by a linear combination g_i of itself and some number of nearby neighbors,

$$g_i = \sum_{n=-n_L}^{n_R} c_n f_{i+n} \tag{14.9.1}$$

Here n_L is the number of points used "to the left" of a data point i, i.e., earlier than it, while n_R is the number used to the right, i.e., later. A so-called *causal* filter would have $n_R = 0$.

As a starting point for understanding Savitzky-Golay filters, consider the simplest possible averaging procedure: For some fixed $n_L = n_R$, compute each g_i as the average of the data points from f_{i-n_L} to f_{i+n_R}. This is sometimes called *moving window averaging* and corresponds to equation (14.9.1) with constant $c_n = 1/(n_L + n_R + 1)$. If the underlying function is constant, or is changing linearly with time (increasing or decreasing), then no bias is introduced into the result. Higher points at one end of the averaging interval are on the average balanced by lower points at the other end. A bias is introduced, however, if the underlying function has a nonzero second derivative. At a local maximum, for example, moving window averaging always reduces the function value. In the spectrometric application, a narrow spectral line has its height reduced and its width increased. Since these parameters are themselves of physical interest, the bias introduced is distinctly undesirable.

Note, however, that moving window averaging does preserve the area under a spectral line, which is its zeroth moment, and also (if the window is symmetric with $n_L = n_R$) its mean position in time, which is its first moment. What is violated is

the second moment, equivalent to the line width.

The idea of Savitzky-Golay filtering is to find filter coefficients c_n that preserve higher moments. Equivalently, the idea is to approximate the underlying function within the moving window not by a constant (whose estimate is the average), but by a polynomial of higher order, typically quadratic or quartic: For each point f_i, we least-squares fit a polynomial to all $n_L + n_R + 1$ points in the moving window, and then set g_i to be the value of that polynomial at position i. (If you are not familiar with least-squares fitting, you might want to look ahead to Chapter 15.) We make no use of the value of the polynomial at any other point. When we move on to the next point f_{i+1}, we do a whole new least-squares fit using a shifted window.

All these least-squares fits would be laborious if done as described. Luckily, since the process of least-squares fitting involves only a linear matrix inversion, the coefficients of a fitted polynomial are themselves linear in the values of the data. That means that we can do all the fitting in advance, for fictitious data consisting of all zeros except for a single 1, and then do the fits on the real data just by taking linear combinations. This is the key point, then: There are particular sets of filter coefficients c_n for which equation (14.9.1) "automatically" accomplishes the process of polynomial least-squares fitting inside a moving window.

To derive such coefficients, consider how g_0 might be obtained: We want to fit a polynomial of degree M in i, namely $a_0 + a_1 i + \cdots + a_M i^M$, to the values $f_{-n_L}, \ldots, f_{n_R}$. Then g_0 will be the value of that polynomial at $i = 0$, namely a_0. The design matrix for this problem (§15.4) is

$$A_{ij} = i^j \qquad i = -n_L, \ldots, n_R, \quad j = 0, \ldots, M \tag{14.9.2}$$

and the normal equations for the vector of a_j's in terms of the vector of f_i's is in matrix notation

$$(\mathbf{A}^T \cdot \mathbf{A}) \cdot \mathbf{a} = \mathbf{A}^T \cdot \mathbf{f} \qquad \text{or} \qquad \mathbf{a} = (\mathbf{A}^T \cdot \mathbf{A})^{-1} \cdot (\mathbf{A}^T \cdot \mathbf{f}) \tag{14.9.3}$$

We also have the specific forms

$$\left\{\mathbf{A}^T \cdot \mathbf{A}\right\}_{ij} = \sum_{k=-n_L}^{n_R} A_{ki} A_{kj} = \sum_{k=-n_L}^{n_R} k^{i+j} \tag{14.9.4}$$

and

$$\left\{\mathbf{A}^T \cdot \mathbf{f}\right\}_j = \sum_{k=-n_L}^{n_R} A_{kj} f_k = \sum_{k=-n_L}^{n_R} k^j f_k \tag{14.9.5}$$

Since the coefficient c_n is the component a_0 when $\mathbf{f}$ is replaced by the unit vector $\mathbf{e}_n$, $-n_L \le n < n_R$, we have

$$c_n = \left\{(\mathbf{A}^T \cdot \mathbf{A})^{-1} \cdot (\mathbf{A}^T \cdot \mathbf{e}_n)\right\}_0 = \sum_{m=0}^{M} \left\{(\mathbf{A}^T \cdot \mathbf{A})^{-1}\right\}_{0m} n^m \tag{14.9.6}$$

Equation (14.9.6) says that we need only one row of the inverse matrix. (Numerically we can get this by LU decomposition with only a single backsubstitution.)

The function `savgol`, below, implements equation (14.9.6). As input, it takes the parameters `nl` $= n_L$, `nr` $= n_R$, and `m` $= M$ (the desired order). Also input

M	n_L	n_R	Sample Savitzky-Golay Coefficients										
2	2	2				−0.086	0.343	0.486	0.343	−0.086			
2	3	1			−0.143	0.171	0.343	0.371	0.257				
2	4	0		0.086	−0.143	−0.086	0.257	0.886					
2	5	5	−0.084	0.021	0.103	0.161	0.196	0.207	0.196	0.161	0.103	0.021	−0.084
4	4	4		0.035	−0.128	0.070	0.315	0.417	0.315	0.070	−0.128	0.035	
4	5	5	0.042	−0.105	−0.023	0.140	0.280	0.333	0.280	0.140	−0.023	−0.105	0.042

is np, the physical length of the output array c, and a parameter ld that for data fitting should be zero. In fact, ld specifies which coefficient among the a_i's should be returned, and we are here interested in a_0. For another purpose, namely the computation of numerical derivatives (already mentioned in §5.7), the useful choice is ld ≥ 1. With ld $= 1$, for example, the filtered first derivative is the convolution (14.9.1) divided by the stepsize Δ. For ld $= k > 1$, the array c must be multiplied by $k!$ to give derivative coefficients. For derivatives, one usually wants m $= 4$ or larger.

savgol.h

```
void savgol(VecDoub_O &c, const Int np, const Int nl, const Int nr,
    const Int ld, const Int m)
```

Returns in c[0..np-1], in wraparound order (N.B.!) consistent with the argument respns in routine convlv, a set of Savitzky-Golay filter coefficients. nl is the number of leftward (past) data points used, while nr is the number of rightward (future) data points, making the total number of data points used nl $+$ nr $+ 1$. ld is the order of the derivative desired (e.g., ld $= 0$ for smoothed function. For the derivative of order k, you must multiply the array c by $k!$.) m is the order of the smoothing polynomial, also equal to the highest conserved moment; usual values are m $= 2$ or m $= 4$.

```
{
    Int j,k,imj,ipj,kk,mm;
    Doub fac,sum;
    if (np < nl+nr+1 || nl < 0 || nr < 0 || ld > m || nl+nr < m)
        throw("bad args in savgol");
    VecInt indx(m+1);
    MatDoub a(m+1,m+1);
    VecDoub b(m+1);
    for (ipj=0;ipj<=(m << 1);ipj++) {        Set up the normal equations of the desired
        sum=(ipj ? 0.0 : 1.0);                   least-squares fit.
        for (k=1;k<=nr;k++) sum += pow(Doub(k),Doub(ipj));
        for (k=1;k<=nl;k++) sum += pow(Doub(-k),Doub(ipj));
        mm=MIN(ipj,2*m-ipj);
        for (imj = -mm;imj<=mm;imj+=2) a[(ipj+imj)/2][(ipj-imj)/2]=sum;
    }
    LUdcmp alud(a);                           Solve them: LU decomposition.
    for (j=0;j<m+1;j++) b[j]=0.0;
    b[ld]=1.0;
    Right-hand side vector is unit vector, depending on which derivative we want.
    alud.solve(b,b);                          Get one row of the inverse matrix.
    for (kk=0;kk<np;kk++) c[kk]=0.0;          Zero the output array (it may be bigger than
    for (k = -nl;k<=nr;k++) {                    number of coefficients).
        sum=b[0];                             Each Savitzky-Golay coefficient is the dot
        fac=1.0;                                 product of powers of an integer with the
        for (mm=1;mm<=m;mm++) sum += b[mm]*(fac *= k);    inverse matrix row.
        kk=(np-k) % np;                       Store in wraparound order.
        c[kk]=sum;
    }
}
```

As output, savgol returns the coefficients c_n, for $-n_L \le n \le n_R$. These are stored in c in "wraparound order"; that is, c_0 is in c[0], c_{-1} is in c[1], and so on for further negative indices. The value c_1 is stored in c[np-1], c_2 in c[np-2], and so on for positive indices. This order may seem arcane, but it is the natural one where causal filters have nonzero coefficients in low array elements of c. It is also the order required by the function convlv in §13.1, which can be used to apply the digital filter to a data set.

The table on the previous page shows some typical output from savgol. For orders 2 and 4, the coefficients of Savitzky-Golay filters with several choices of n_L and n_R are shown. The central column is the coefficient applied to the data f_i in obtaining the smoothed g_i. Coefficients to the left are applied to earlier data, to the right, to later. The coefficients always add (within roundoff error) to unity. One sees that, as befits a smoothing operator, the coefficients always have a central positive lobe, but with smaller, outlying corrections of both positive and negative sign. In practice, the Savitzky-Golay filters are most useful for much larger values of n_L and n_R, since these few-point formulas can accomplish only a relatively small amount of smoothing.

Figure 14.9.1 shows a numerical experiment using a 33-point smoothing filter, that is, $n_L = n_R = 16$. The upper panel shows a test function, constructed to have six "bumps" of varying widths, all of height 8 units. To this function Gaussian white noise of unit variance has been added. (The test function without noise is shown as the dotted curves in the center and lower panels.) The widths of the bumps (full width at half of maximum, or FWHM) are 140, 43, 24, 17, 13, and 10, respectively.

The middle panel of Figure 14.9.1 shows the result of smoothing by a moving window average. One sees that the window of width 33 does quite a nice job of smoothing the broadest bump, but that the narrower bumps suffer considerable loss of height and increase of width. The underlying signal (dotted) is very badly represented.

The lower panel shows the result of smoothing with a Savitzky-Golay filter of the identical width and degree $M = 4$. One sees that the heights and widths of the bumps are quite extraordinarily preserved. A trade-off is that the broadest bump is less smoothed. That is because the central positive lobe of the Savitzky-Golay filter coefficients fills only a fraction of the full 33-point width. As a rough guideline, best results are obtained when the full width of the degree 4 Savitzky-Golay filter is between 1 and 2 times the FWHM of desired features in the data. (References [3] and [4] give additional practical hints.)

Figure 14.9.2 shows the result of smoothing the same noisy "data" with broader Savitzky-Golay filters of three different orders. Here we have $n_L = n_R = 32$ (65-point filter) and $M = 2, 4, 6$. One sees that, when the bumps are too narrow with respect to the filter size, even the Savitzky-Golay filter must at some point give out. The higher-order filter manages to track narrower features, but at the cost of less smoothing on broad features.

To summarize: Within limits, Savitzky-Golay filtering does manage to provide smoothing without loss of resolution. It does this by assuming that relatively distant data points have some significant redundancy that can be used to reduce the level of noise. The specific nature of the assumed redundancy is that the underlying function should be locally well-fitted by a polynomial. When this is true, as it is for smooth line profiles not too much narrower than the filter width, the performance of

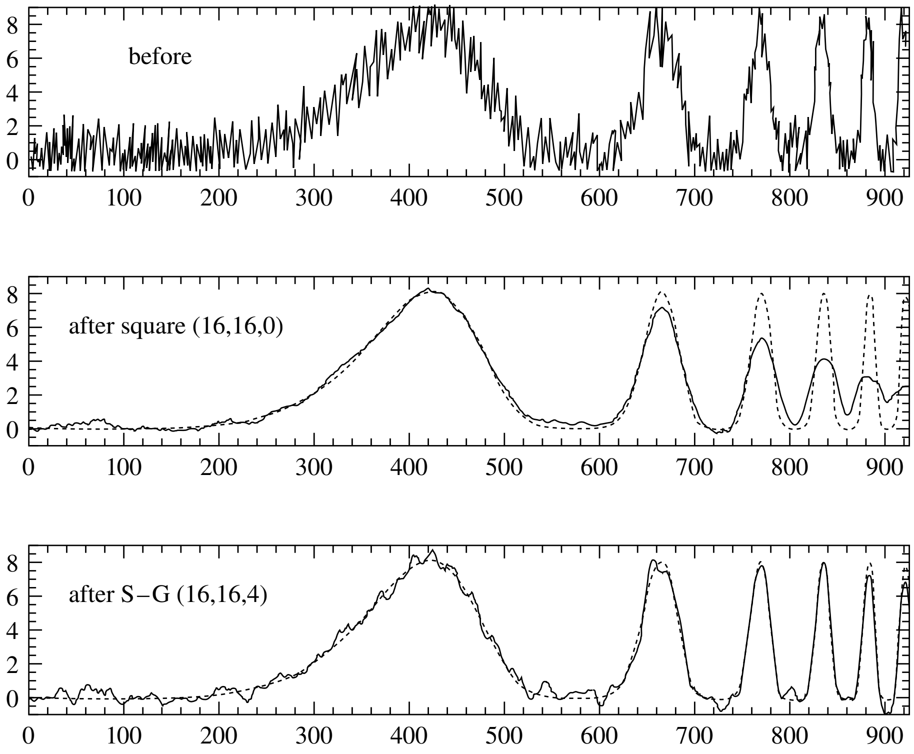

Figure 14.9.1. Top: Synthetic noisy data consisting of a sequence of progressively narrower bumps and additive Gaussian white noise. Center: Result of smoothing the data by a simple moving window average. The window extends 16 points leftward and rightward, for a total of 33 points. Note that narrow features are broadened and suffer corresponding loss of amplitude. The dotted curve is the underlying function used to generate the synthetic data. Bottom: Result of smoothing the data by a Savitzky-Golay smoothing filter (of degree 4) using the same 33 points. While there is less smoothing of the broadest feature, narrower features have their heights and widths preserved.

Savitzky-Golay filters can be spectacular. When it is not true, these filters have no compelling advantage over other classes of smoothing filter coefficients.

A last remark concerns irregularly sampled data, where the values f_i are not uniformly spaced in time. The obvious generalization of Savitzky-Golay filtering would be to do a least-squares fit within a moving window around each data point, one containing a fixed number of data points to the left (n_L) and right (n_R). Because of the irregular spacing, however, there is no way to obtain universal filter coefficients applicable to more than one data point. One must instead do the actual least-squares fits for each data point. This becomes computationally burdensome for larger n_L, n_R, and M.

As a cheap alternative, one can simply pretend that the data points *are* equally spaced. This amounts to virtually shifting, within each moving window, the data points to equally spaced positions. Such a shift introduces the equivalent of an additional source of noise into the function values. In those cases where smoothing is useful, this noise will often be much smaller than the noise already present. Specifically, if the location of the points is approximately random within the window, then a rough

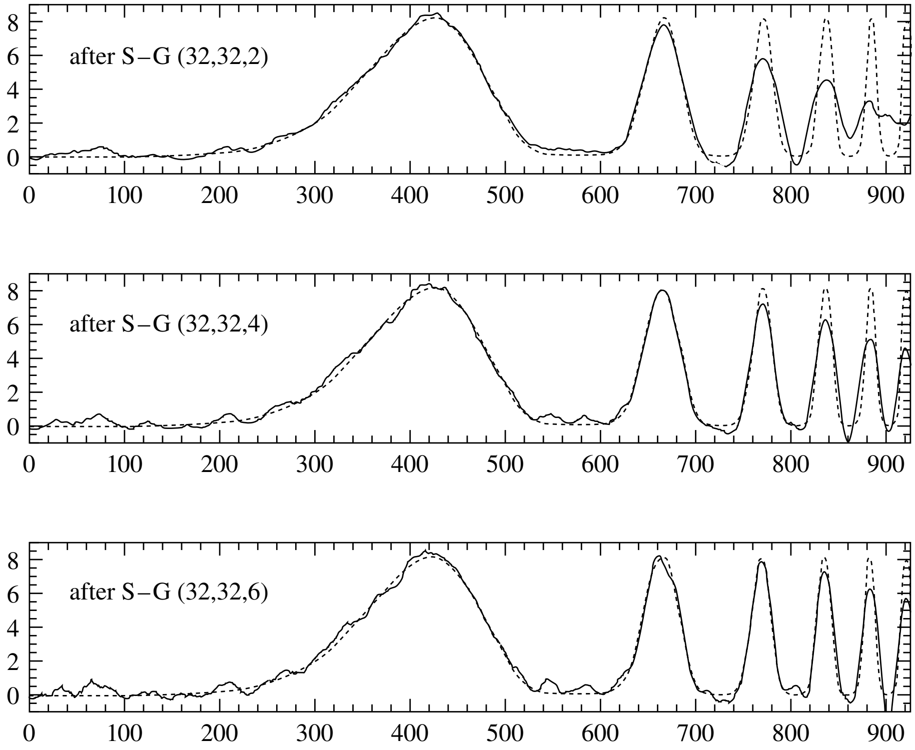

Figure 14.9.2. Result of applying wider 65-point Savitzky-Golay filters to the same data set as in Figure 14.9.1. Top: degree 2. Center: degree 4. Bottom: degree 6. All of these filters are inoptimally broad for the resolution of the narrow features. Higher-order filters do best at preserving feature heights and widths, but do less smoothing on broader features.

criterion is this: If the change in f across the full width of the $N = n_L + n_R + 1$ point window is less than $\sqrt{N/2}$ times the measurement noise on a single point, then the cheap method can be used.

CITED REFERENCES AND FURTHER READING:

Savitzky A., and Golay, M.J.E. 1964, "Smoothing and Differentiation of Data by Simplified Least Squares Procedures," *Analytical Chemistry*, vol. 36, pp. 1627–1639.[1]

Hamming, R.W. 1983, *Digital Filters*, 2nd ed. (Englewood Cliffs, NJ: Prentice-Hall).[2]

Ziegler, H. 1981, "Properties of Digital Smoothing Polynomial (DISPO) Filters," *Applied Spectroscopy*, vol. 35, pp. 88–92.[3]

Bromba, M.U.A., and Ziegler, H. 1981, "Application Hints for Savitzky-Golay Digital Smoothing Filters," *Analytical Chemistry*, vol. 53, pp. 1583–1586.[4]

Modeling of Data

CHAPTER 15

15.0 Introduction

Given a set of observations, one often wants to condense and summarize the data by fitting it to a *model* that depends on adjustable parameters. Sometimes the model is simply a convenient class of functions, such as polynomials or Gaussians, and the fit supplies the appropriate coefficients. Other times, the model's parameters come from some underlying theory that the data are supposed to satisfy; examples are rate coefficients in a complex network of chemical reactions or orbital elements of a binary star. Modeling can also be used as a kind of constrained interpolation, where you want to extend a few data points into a continuous function, but with some underlying idea of what that function should look like.

One very general approach has the following paradigm: You choose or design a *figure-of-merit function* (merit function, for short) that measures the agreement between the data and the model with a particular choice of parameters. In frequentist statistics, the merit function is conventionally arranged so that small values represent close agreement. Bayesians choose as their merit function the probability of the parameters given the data (or often its logarithm) so that larger values represent closer agreement.

In either case, the parameters of the model are then adjusted to find a happy extremum in the merit function, yielding *best-fit parameters*. The adjustment process is thus a problem in minimization in many dimensions. This optimization was the subject of Chapter 10; however, there exist special, more efficient, methods that are specific to modeling, and we will discuss these in this chapter.

There are important issues that go beyond the mere finding of best-fit parameters. Data are generally not exact. They are subject to *measurement errors* (called *noise* in the context of signal processing). Thus, typical data never exactly fit the model that is being used, even when that model is correct. We need the means to assess whether or not the model is appropriate, that is, we need to test the *goodness-of-fit* against some useful statistical standard.

We usually also need to know the accuracy with which parameters are determined by the data set. In frequentist terms, we need to know the standard errors of the best-fit parameters. Alternatively, in Bayesian language, we want to find not just the peak of the joint parameter probability distribution, but the whole distribution.

Or we at least want to be able to sample from that distribution, typically by Markov chain Monte Carlo, as we will discuss at length in §15.8.

It is not uncommon in fitting data to discover that the merit function is not unimodal, with a single minimum. In some cases, we may be interested in global rather than local questions. Not, "how good is this fit?" but rather, "how sure am I that there is not a *very much better* fit in some corner of parameter space?" As we have seen in Chapter 10, especially §10.12, this kind of problem is generally quite difficult to solve.

The important message is that fitting of parameters is not the end-all of model parameter estimation. To be genuinely useful, a fitting procedure should provide (i) parameters, (ii) error estimates on the parameters or a way to sample from their probability distribution, and (iii) a statistical measure of goodness-of-fit. When the third item suggests that the model is an unlikely match to the data, then items (i) and (ii) are probably worthless. Unfortunately, many practitioners of parameter estimation never proceed beyond item (i). They deem a fit acceptable if a graph of data and model "looks good." This approach is known as *chi-by-eye*. Luckily, its practitioners get what they deserve.

15.0.1 Basic Bayes

Because the discussion in this and subsequent chapters will move freely between frequentist and Bayesian methods, this is a good place to compare these two powerfully useful ways of thinking. In §14.0, when we discussed tail, or p-value, tests, we were adopting a frequentist viewpoint. The central frequentist idea is that, given the details of a null hypothesis, there is an implied population (that is, probability distribution) of possible data sets. If the assumed null hypothesis is correct, then the actual, measured, data set is drawn from that population. (We expand on this in §15.6.) It then makes sense to ask questions about how "frequently" some aspect of the measured data occurs in the population. If the answer is "very infrequently," then the hypothesis is rejected. The frequentist viewpoint avoids questions like, "what is the *probability* that this hypothesis is true?" because its focus is on the distribution of data sets, not hypotheses. Indeed, whether by dogma or merely benign neglect, it eschews the machinery needed to handle the concept of a probability distribution of hypotheses.

That machinery is Bayes' theorem, which follows from the standard axioms of probability. Bayes' theorem relates the conditional probabilities of two events, say A and B:

$$P(A|B) = P(A)\,\frac{P(B|A)}{P(B)} \tag{15.0.1}$$

Here $P(A|B)$ is the probability of A *given* that B has occurred, and similarly for $P(B|A)$, while $P(A)$ and $P(B)$ are unconditional probabilities.

Bayesians allow a broader set of uses for probabilities than frequentists. To a Bayesian, $P(A|B)$ is a measure of the degree of plausibility of A (given B) on a scale ranging from zero to one. In this broader view, A and B need not be repeatable events; they can indeed be propositions or hypotheses. In equation (15.0.1), A might be a hypothesis and B might be some data, so that $P(A|B)$ expresses the probability of a hypothesis, given the data. The equations of probability theory thus become a set of consistent rules for conducting inference [1,2]. Interestingly, this viewpoint was

universal before the 20th century. The Bernoullis (both of them), Laplace, Gauss, Legendre, and Poisson, among others, made little or no distinction between inference and probability. An opposing frequentist view, that these concepts should be kept separate, became explicit only with the work of Fisher, Box, Kendall, Neyman, and Pearson (among others), much later.

Since plausibility is itself always conditioned on some, perhaps unarticulated, set of assumptions, all Bayesian probabilities are viewed as conditional on some collective background information I. Suppose H is some hypothesis. Even before there exist any explicit data, a Bayesian can assign to H some degree of plausibility $P(H|I)$, called the "Bayesian prior." Now, when some data D_1 comes along, Bayes theorem tells how to reassess the plausibility of H,

$$P(H|D_1 I) = P(H|I)\frac{P(D_1|HI)}{P(D_1|I)} \tag{15.0.2}$$

The factor in the numerator on the right of equation (15.0.2) is calculable as the probability of a data set *given* the hypothesis (comparable to "likelihood" as we will define it in §15.1). The denominator, called the "prior predictive probability" of the data, is in this case merely a normalization constant that can be calculated by the requirement that the probability of all hypotheses should sum to unity. (In other Bayesian contexts, the prior predictive probabilities of two qualitatively different models can be used to assess their relative plausibility.)

If some additional data D_2 come along tomorrow, we can further refine our estimate of H's probability, as

$$P(H|D_2 D_1 I) = P(H|D_1 I)\frac{P(D_2|HD_1 I)}{P(D_2|D_1 I)} \tag{15.0.3}$$

Using the product rule for probabilities, $P(AB|C) = P(A|C)P(B|AC)$, we find that equations (15.0.2) and (15.0.3) imply

$$P(H|D_2 D_1 I) = P(H|I)\frac{P(D_2 D_1|HI)}{P(D_2 D_1|I)} \tag{15.0.4}$$

which shows that we would have gotten the same answer if all the data $D_1 D_2$ had been taken together.

We might wonder, before we adopt the laws of probability as our calculus of inference and thus become Bayesians, whether there are any other alternatives. The answer is, basically, no. Cox [3] showed that making a small number of very reasonable assumptions about "degree of belief" leads inevitably to the axioms of probability, and thus the application of Bayes theorem to the evaluation of hypotheses, given data. Either you become a Bayesian or else you must live in a world with no general calculus of inference.

CITED REFERENCES AND FURTHER READING:

Bevington, P.R., and Robinson, D.K. 2002, *Data Reduction and Error Analysis for the Physical Sciences*, 3rd ed. (New York: McGraw-Hill), Chapters 6–11.

Devore, J.L. 2003, *Probability and Statistics for Engineering and the Sciences*, 6th ed. (Belmont, CA: Duxbury Press), Chapters 12–13.

Brownlee, K.A. 1965, *Statistical Theory and Methodology*, 2nd ed. (New York: Wiley).

Martin, B.R. 1971, *Statistics for Physicists* (New York: Academic Press).

Gelman, A., Carlin, J.B., Stern, H.S., and Rubin, D.B. 2004, *Bayesian Data Analysis*, 2nd ed. (Boca Raton, FL: Chapman & Hall/CRC).

Sivia, D.S. 1996, *Data Analysis: A Bayesian Tutorial* (Oxford, UK: Oxford University Press).

Jaynes, E.T. 1976, in *Foundations of Probability Theory, Statistical Inference, and Statistical Theories of Science*, W.L. Harper and C.A. Hooker, eds. (Dordrecht: Reidel).[1]

Jaynes, E.T. 1985, in *Maximum-Entropy and Bayesian Methods in Inverse Problems*, C.R. Smith and W.T. Grandy, Jr., eds. (Dordrecht: Reidel).[2]

Cox, R.T. 1946, "Probability, Frequency, and Reasonable Expectation," *American Journal of Physics*, vol. 14, pp. 1–13.[3]

15.1 Least Squares as a Maximum Likelihood Estimator

Suppose that we are fitting N data points (x_i, y_i), $i = 0, \ldots, N-1$, to a model that has M adjustable parameters a_j, $j = 0, \ldots, M-1$. The model predicts a functional relationship between the measured independent and dependent variables,

$$y(x) = y(x|a_0 \ldots a_{M-1}) \tag{15.1.1}$$

where the notation indicates dependence on the parameters explicitly on the right-hand side, following the vertical bar.

What, exactly, do we want to minimize to get fitted values for the a_j's? The first thing that comes to mind is the familiar least-squares fit,

$$\text{minimize over } a_0 \ldots a_{M-1}: \qquad \sum_{i=0}^{N-1} [y_i - y(x_i|a_0 \ldots a_{M-1})]^2 \tag{15.1.2}$$

But where does this come from? What general principles is it based on?

To answer these questions, let us start by asking, "*Given a particular set of parameters*, what is the probability that the observed data set should have occurred?" If the y_i's take on continuous values, the probability will always be zero unless we add the phrase, "...plus or minus some small, fixed Δy on each data point." So let's always take this phrase as understood. If the probability of obtaining the data set is too small, then we can conclude that the parameters under consideration are "unlikely" to be right. Conversely, our intuition tells us that the data set should not be too improbable for the correct choice of parameters.

To be more quantitative, suppose that each data point y_i has a measurement error that is independently random and distributed as a normal (Gaussian) distribution around the "true" model $y(x)$. And suppose that the standard deviations σ of these normal distributions are the same for all points. Then the probability of the data set is the product of the probabilities of each point:

$$P(\text{data} \mid \text{model}) \propto \prod_{i=0}^{N-1} \left\{ \exp\left[-\frac{1}{2} \left(\frac{y_i - y(x_i)}{\sigma} \right)^2 \right] \Delta y \right\} \tag{15.1.3}$$

Notice that there is a factor Δy in each term in the product.

If we are Bayesians, we proceed by invoking Bayes' theorem, in the form

$$P(\text{model} \mid \text{data}) \propto P(\text{data} \mid \text{model})\, P(\text{model}) \tag{15.1.4}$$

where $P(\text{model}) = P(a_0 \ldots a_{M-1})$ is our prior probability distribution on all models. As often as not, we take a constant, *noninformative* prior. The most probable model, then, is the one that maximizes equation (15.1.3) or, equivalently, minimizes the negative of its logarithm,

$$\left[\sum_{i=0}^{N-1} \frac{[y_i - y(x_i)]^2}{2\sigma^2}\right] - N \log \Delta y \tag{15.1.5}$$

Since N, σ, and Δy are all constants, minimizing this equation is equivalent to minimizing (15.1.2).

If we are frequentists, we must get to the same destination by a more tortuous path (as is so often the case when Bayesian and frequentist methods coincide). We are not allowed to think about the notion of probability as applied to parameter sets, because, for frequentists, there is no statistical universe of models from which the parameters are drawn. We instead substitute a dictum: We identify the probability of the data given the parameters (which is computable as above), as the *likelihood* of the parameters given the data. This identification is entirely based on intuition. It has no formal mathematical basis in and of itself. Parameters derived in this way are called *maximum likelihood estimators*.

What we see is that least-squares fitting gives an answer that is both (i) the most probable parameter set in the Bayesian sense, assuming a flat prior, and (ii) the maximum likelihood estimate of the fitted parameters, in both cases *if* the measurement errors are independent and normally distributed with constant standard deviation. Notice that we made no assumption about the linearity or nonlinearity of the model $y(x|a_0 \ldots)$ in its parameters $a_0 \ldots a_{M-1}$. Just below, we will relax our assumption of constant standard deviations and obtain the very similar formulas for what is called "chi-square fitting" or "weighted least-squares fitting." First, however, let us discuss further our very stringent assumption of a normal distribution.

For a hundred years or so, mathematical statisticians have been in love with the fact that the probability distribution of the sum of a very large number of very small random deviations almost always converges to a normal distribution. (For precise statements of this *central limit theorem*, consult [1] or other standard works on mathematical statistics.) This infatuation tended to focus interest away from the fact that, for real data, the normal distribution is often rather poorly realized, if it is realized at all. We are often taught, rather casually, that, on average, measurements will fall within $\pm\sigma$ of the true value 68% of the time, within $\pm 2\sigma$ 95% of the time, and within $\pm 3\sigma$ 99.7% of the time. Extending this, one would expect a measurement to be off by $\pm 20\sigma$ only one time out of 2×10^{88}. We all know that "glitches" are much more likely than *that*!

In some instances, the deviations from a normal distribution are easy to understand and quantify. For example, in measurements obtained by counting events, the measurement errors are usually distributed as a Poisson distribution, whose cumulative probability function was already discussed in §6.2. When the number of counts going into one data point is large, the Poisson distribution converges toward

a Gaussian. However, the convergence is not uniform when measured in fractional accuracy. The more standard deviations out on the tail of the distribution, the larger the number of counts must be before a value close to the Gaussian is realized. The sign of the effect is always the same: The Gaussian predicts that "tail" events are much less likely than they actually (by Poisson) are. This causes such events, when they occur, to skew a least-squares fit much more than they ought.

Other times, the deviations from a normal distribution are not so easy to understand in detail. Experimental points are occasionally just *way off*. Perhaps the power flickered during a point's measurement, or someone kicked the apparatus, or someone wrote down a wrong number. Points like this are called *outliers*. They can easily turn a least-squares fit on otherwise adequate data into nonsense. Their probability of occurrence in the assumed Gaussian model is so small that the maximum likelihood estimator is willing to distort the whole curve to try to bring them, mistakenly, into line.

The subject of *robust statistics* deals with cases where the normal or Gaussian model is a bad approximation, or cases where outliers are important. We will discuss robust methods briefly in §15.7. All the sections between this one and that one assume, one way or the other, a Gaussian model for the measurement errors in the data. It it quite important that you keep the limitations of that model in mind, even as you use the very useful methods that follow from assuming it.

Finally, note that our discussion of measurement errors has been limited to *statistical* errors, the kind that will average away if we only take enough data. Measurements are also susceptible to *systematic* errors that will not go away with any amount of averaging. For example, the calibration of a metal meter stick might depend on its temperature. If we take all our measurements at the same wrong temperature, then no amount of averaging or numerical processing will correct for this unrecognized systematic error.

15.1.1 Chi-Square Fitting

We considered the chi-square statistic once before, in §14.3. Here it arises in a slightly different context.

If each data point (x_i, y_i) has its own, known standard deviation σ_i, then equation (15.1.3) is modified only by putting a subscript i on the symbol σ. That subscript also propagates docilely into (15.1.5), so that the maximum likelihood estimate of the model parameters (and also the Bayesian most probable parameter set) is obtained by minimizing the quantity

$$\chi^2 \equiv \sum_{i=0}^{N-1} \left(\frac{y_i - y(x_i | a_0 \dots a_{M-1})}{\sigma_i} \right)^2 \tag{15.1.6}$$

called the "chi-square."

To whatever extent the measurement errors actually *are* normally distributed, the quantity χ^2 is correspondingly a sum of N squares of normally distributed quantities, each normalized to unit variance. Once we have adjusted the $a_0 \dots a_{M-1}$ to minimize the value of χ^2, the terms in the sum are not all statistically independent. For models that are linear in the a's, however, it turns out that the probability distribution for different values of χ^2 at its minimum can nevertheless be derived analytically, and is the *chi-square distribution for $N - M$ degrees of freedom*. We

learned how to compute this probability function using the incomplete gamma function in §6.2. In particular, equation (6.14.39) gives the probability Q that the chi-square should exceed a particular value χ^2 by chance, where $\nu = N - M$ is the *number of degrees of freedom*. The quantity Q, or its complement $P \equiv 1 - Q$, is frequently tabulated in appendices to statistics books, or it can be computed as $P =$ `Chisqdist(`ν`).invcdf(`χ^2`)` by the routine in §6.14.8. It is quite common, and usually not too wrong, to assume that the chi-square distribution holds even for models that are not strictly linear in the a's.

This computed probability gives a quantitative measure for the goodness-of-fit of the model. If Q is a very small probability for some particular data set, then the apparent discrepancies are unlikely to be chance fluctuations. Much more probably either (i) the model is wrong — can be statistically rejected, or (ii) someone has lied to you about the size of the measurement errors σ_i — they are really larger than stated.

While above we were quick to poke fun at the frequentist's foundations for maximum likelihood estimation (or lack thereof), we must now take aim at strict Bayesians: There are no good fully Bayesian methods for assessing goodness-of-fit, that is, for comparing the probability of a best-fit model to that of a nonspecific alternative hypothesis like "the model is wrong." The problem is that the strict application of Bayes theorem requires either (i) a comparison between two well-posed hypotheses (the *odds ratio*), or (ii) a normalization of the probability of the best-fit model against an integral of such probabilities over all possible models (the *normalizing constant*). In most situations neither of these is available. Sensible Bayesians usually fall back to p-value tail statistics like chi-square probability when they really need to know if a model is wrong.

Another important point is that the chi-square probability Q does not directly measure the credibility of the assumption that the measurement errors are normally distributed. It assumes they are. In most, but not all, cases, however, the effect of nonnormal errors is to create an abundance of outlier points. These decrease the probability Q, so that we can add another possible, though less definitive, conclusion to the above list: (iii) the measurement errors may not be normally distributed.

Possibility (iii) is fairly common, and also fairly benign. It is for this reason that reasonable experimenters are often rather tolerant of low probabilities Q. It is not uncommon to deem acceptable on equal terms any models with, say, $Q > 0.001$. This is not as sloppy as it sounds: Truly *wrong* models will often be rejected with vastly smaller values of Q, 10^{-18}, say. However, if day-in and day-out you find yourself accepting models with $Q \sim 10^{-3}$, you really should track down the cause.

If you happen to know the actual distribution law of your measurement errors, then you might wish to *Monte Carlo simulate* some data sets drawn from a particular model, cf. §7.3 – §7.4. You can then subject these synthetic data sets to your actual fitting procedure, so as to determine both the probability distribution of the χ^2 statistic and also the accuracy with which your model parameters are reproduced by the fit. We discuss this further in §15.6. The technique is very general, but it can also be slow.

At the opposite extreme, it sometimes happens that the probability Q is too large, too near to 1, literally too good to be true! Nonnormal measurement errors cannot in general produce this disease, since the normal distribution is about as "compact" as a distribution can be. Almost always, the cause of too good a chi-

square fit is that the experimenter, in a "fit" of conservativism, has *overestimated* his or her measurement errors. Very rarely, too good a chi-square signals actual fraud, data that have been "fudged" to fit the model.

A rule of thumb is that a "typical" value of χ^2 for a "moderately" good fit is $\chi^2 \approx \nu$. More precise is the statement that the χ^2 statistic has a mean ν and a standard deviation $\sqrt{2\nu}$ and, asymptotically for large ν, becomes normally distributed.

In some cases the uncertainties associated with a set of measurements are not known in advance, and considerations related to χ^2 fitting are used to derive a value for σ. If we assume that all measurements have the same standard deviation, $\sigma_i = \sigma$, and that the model does fit well, then we can proceed by first assigning an arbitrary constant σ to all points, next fitting for the model parameters by minimizing χ^2, and finally recomputing

$$\sigma^2 = \sum_{i=0}^{N-1} [y_i - y(x_i)]^2/(N-M) \qquad (15.1.7)$$

Obviously, this approach prohibits an independent assessment of goodness-of-fit, a fact occasionally missed by its adherents. When, however, the measurement error is not known, this approach at least allows *some* kind of error bar to be assigned to the points.

If we take the derivative of equation (15.1.6) with respect to the parameters a_k, we obtain equations that must hold at the chi-square minimum:

$$0 = \sum_{i=0}^{N-1} \left(\frac{y_i - y(x_i)}{\sigma_i^2}\right)\left(\frac{\partial y(x_i|\ldots a_k \ldots)}{\partial a_k}\right) \qquad k = 0, \ldots, M-1 \qquad (15.1.8)$$

Equation (15.1.8) is, in general, a set of M nonlinear equations for the M unknown a_k. Various of the procedures described subsequently in this chapter derive from (15.1.8) and its specializations.

CITED REFERENCES AND FURTHER READING:

Lupton, R. 1993, *Statistics in Theory and Practice* (Princeton, NJ: Princeton University Press), Chapters 10–11.[1]

Devore, J.L. 2003, *Probability and Statistics for Engineering and the Sciences*, 6th ed. (Belmont, CA: Duxbury Press), Chapter 6.

Gelman, A., Carlin, J.B., Stern, H.S., and Rubin, D.B. 2004, *Bayesian Data Analysis*, 2nd ed. (Boca Raton, FL: Chapman & Hall/CRC), Chapter 8.

15.2 Fitting Data to a Straight Line

A concrete example will make the considerations of the previous section more meaningful. We consider the problem of fitting a set of N data points (x_i, y_i) to a straight-line model

$$y(x) = y(x|a,b) = a + bx \qquad (15.2.1)$$

This problem is often called *linear regression*, a terminology that originated, long ago, in the social sciences. We assume that the uncertainty σ_i associated with each

measurement y_i is known, and that the x_i's (values of the dependent variable) are known exactly.

To measure how well the model agrees with the data, we use the chi-square merit function (15.1.6), which in this case is

$$\chi^2(a,b) = \sum_{i=0}^{N-1} \left(\frac{y_i - a - bx_i}{\sigma_i} \right)^2 \tag{15.2.2}$$

If the measurement errors are normally distributed, then this merit function will give maximum likelihood parameter estimations of a and b; if the errors are not normally distributed, then the estimations are not maximum likelihood but may still be useful in a practical sense. In §15.7, we will treat the case where outlier points are so numerous as to render the χ^2 merit function useless.

Equation (15.2.2) is minimized to determine a and b. At its minimum, derivatives of $\chi^2(a,b)$ with respect to a, b vanish:

$$\begin{aligned} 0 &= \frac{\partial \chi^2}{\partial a} = -2 \sum_{i=0}^{N-1} \frac{y_i - a - bx_i}{\sigma_i^2} \\ 0 &= \frac{\partial \chi^2}{\partial b} = -2 \sum_{i=0}^{N-1} \frac{x_i(y_i - a - bx_i)}{\sigma_i^2} \end{aligned} \tag{15.2.3}$$

These conditions can be rewritten in a convenient form if we define the following sums:

$$\begin{aligned} S &\equiv \sum_{i=0}^{N-1} \frac{1}{\sigma_i^2} \qquad S_x \equiv \sum_{i=0}^{N-1} \frac{x_i}{\sigma_i^2} \qquad S_y \equiv \sum_{i=0}^{N-1} \frac{y_i}{\sigma_i^2} \\ S_{xx} &\equiv \sum_{i=0}^{N-1} \frac{x_i^2}{\sigma_i^2} \qquad S_{xy} \equiv \sum_{i=0}^{N-1} \frac{x_i y_i}{\sigma_i^2} \end{aligned} \tag{15.2.4}$$

With these definitions (15.2.3) becomes

$$\begin{aligned} aS + bS_x &= S_y \\ aS_x + bS_{xx} &= S_{xy} \end{aligned} \tag{15.2.5}$$

The solution of these two equations in two unknowns is calculated as

$$\begin{aligned} \Delta &\equiv SS_{xx} - (S_x)^2 \\ a &= \frac{S_{xx}S_y - S_x S_{xy}}{\Delta} \\ b &= \frac{SS_{xy} - S_x S_y}{\Delta} \end{aligned} \tag{15.2.6}$$

Equation (15.2.6) gives the solution for the best-fit model parameters a and b.

We are not done, however. We must estimate the probable uncertainties in the estimates of a and b, since obviously the measurement errors in the data must introduce some uncertainty in the determination of those parameters. If the data are

independent, then each contributes its own bit of uncertainty to the parameters. Consideration of propagation of errors shows that the variance σ_f^2 in the value of any function will be

$$\sigma_f^2 = \sum_{i=0}^{N-1} \sigma_i^2 \left(\frac{\partial f}{\partial y_i} \right)^2 \tag{15.2.7}$$

For the straight line, the derivatives of a and b with respect to y_i can be directly evaluated from the solution:

$$\begin{aligned} \frac{\partial a}{\partial y_i} &= \frac{S_{xx} - S_x x_i}{\sigma_i^2 \Delta} \\ \frac{\partial b}{\partial y_i} &= \frac{S x_i - S_x}{\sigma_i^2 \Delta} \end{aligned} \tag{15.2.8}$$

Summing over the points as in (15.2.7), we get

$$\begin{aligned} \sigma_a^2 &= S_{xx}/\Delta \\ \sigma_b^2 &= S/\Delta \end{aligned} \tag{15.2.9}$$

which are the variances in the estimates of a and b, respectively. We will see in §15.6 that an additional number is also needed to characterize properly the probable uncertainty of the parameter estimation. That number is the *covariance* of a and b, and (as we will see below) is given by

$$\text{Cov}(a, b) = -S_x/\Delta \tag{15.2.10}$$

The coefficient of correlation between the uncertainty in a and the uncertainty in b, which is a number between -1 and 1, follows from (15.2.10) (compare equation 14.5.1),

$$r_{ab} = \frac{-S_x}{\sqrt{S S_{xx}}} \tag{15.2.11}$$

A positive value of r_{ab} indicates that the errors in a and b are likely to have the same sign, while a negative value indicates the errors are anticorrelated, likely to have opposite signs.

We are *still* not done. We must estimate the goodness-of-fit of the data to the model. Absent this estimate, we have not the slightest indication that the parameters a and b in the model have any meaning at all! The probability Q that a value of chi-square as *poor* as the value (15.2.2) should occur by chance is

$$Q = 1 - \texttt{Chisqdist}(N-2)\texttt{.invcdf}\,(\chi^2) \tag{15.2.12}$$

Here `Chisqdist` is our object embodying the chi-square distribution function (see §6.14.8) and `invcdf` is its inverse cumulative distribution function. If Q is larger than, say, 0.1, then the goodness-of-fit is believable. If it is larger than, say, 0.001, then the fit *may* be acceptable if the errors are nonnormal or have been moderately underestimated. If Q is less than 0.001, then the model and/or estimation procedure can rightly be called into question. In this latter case, turn to §15.7 to proceed further.

If you do not know the individual measurement errors of the points σ_i, and are proceeding (dangerously) to use equation (15.1.7) for estimating these errors, then

here is the procedure for estimating the probable uncertainties of the parameters a and b: Set $\sigma_i \equiv 1$ in all equations through (15.2.6), and multiply σ_a and σ_b, as obtained from equation (15.2.9), by the additional factor $\sqrt{\chi^2/(N-2)}$, where χ^2 is computed by (15.2.2) using the fitted parameters a and b. As discussed above, this procedure is equivalent to *assuming* a good fit, so you get no independent goodness-of-fit probability Q.

In §14.5 we promised a relation between the linear correlation coefficient r (equation 14.5.1) and a goodness-of-fit measure, χ^2 (equation 15.2.2). For unweighted data (all $\sigma_i = 1$), that relation is

$$\chi^2 = (1-r^2)\sum_{i=0}^{N-1}(y_i - \overline{y})^2 \tag{15.2.13}$$

For data with varying errors σ_i, the above equations remain valid if the sums in equations (15.2.13) and (14.5.1) are weighted by $1/\sigma_i^2$.

The following object, `Fitab`, carries out exactly the operations that we have discussed. You call its constructor either with, or without, errors σ_i. If the σ_i's are known, the calculations exactly correspond to the formulas above. However, when σ_i's are unavailable, the routine *assumes* equal values of σ for each point and *assumes* a good fit, as discussed in §15.1.

The formulas (15.2.6) are susceptible to roundoff error. Accordingly, we rewrite them as follows: Define

$$t_i = \frac{1}{\sigma_i}\left(x_i - \frac{S_x}{S}\right), \qquad i = 0, 1, \ldots, N-1 \tag{15.2.14}$$

and

$$S_{tt} = \sum_{i=0}^{N-1} t_i^2 \tag{15.2.15}$$

Then, as you can verify by direct substitution,

$$b = \frac{1}{S_{tt}}\sum_{i=0}^{N-1}\frac{t_i y_i}{\sigma_i} \tag{15.2.16}$$

$$a = \frac{S_y - S_x b}{S} \tag{15.2.17}$$

$$\sigma_a^2 = \frac{1}{S}\left(1 + \frac{S_x^2}{S S_{tt}}\right) \tag{15.2.18}$$

$$\sigma_b^2 = \frac{1}{S_{tt}} \tag{15.2.19}$$

$$\mathrm{Cov}(a,b) = -\frac{S_x}{S S_{tt}} \tag{15.2.20}$$

$$r_{ab} = \frac{\mathrm{Cov}(a,b)}{\sigma_a \sigma_b} \tag{15.2.21}$$

fitab.h

```
struct Fitab {
```
Object for fitting a straight line $y = a + bx$ to a set of points (x_i, y_i), with or without available errors σ_i. Call one of the two constructors to calculate the fit. The answers are then available as the variables a, b, siga, sigb, chi2, and either q or sigdat.

```
    Int ndata;
    Doub a, b, siga, sigb, chi2, q, sigdat;          Answers.
    VecDoub_I &x, &y, &sig;

    Fitab(VecDoub_I &xx, VecDoub_I &yy, VecDoub_I &ssig)
    : ndata(xx.size()), x(xx), y(yy), sig(ssig), chi2(0.), q(1.), sigdat(0.)  {
```
Constructor. Given a set of data points x[0..ndata-1], y[0..ndata-1] with individual standard deviations sig[0..ndata-1], sets a,b and their respective probable uncertainties siga and sigb, the chi-square chi2, and the goodness-of-fit probability q (that the fit would have χ^2 this large or larger).

```
        Gamma gam;
        Int i;
        Doub ss=0.,sx=0.,sy=0.,st2=0.,t,wt,sxoss;
        b=0.0;                                       Accumulate sums ...
        for (i=0;i<ndata;i++) {
            wt=1.0/SQR(sig[i]);                      ...with weights
            ss += wt;
            sx += x[i]*wt;
            sy += y[i]*wt;
        }
        sxoss=sx/ss;
        for (i=0;i<ndata;i++) {
            t=(x[i]-sxoss)/sig[i];
            st2 += t*t;
            b += t*y[i]/sig[i];
        }
        b /= st2;                                    Solve for a, b, σa, and σb.
        a=(sy-sx*b)/ss;
        siga=sqrt((1.0+sx*sx/(ss*st2))/ss);
        sigb=sqrt(1.0/st2);                          Calculate χ2.
        for (i=0;i<ndata;i++) chi2 += SQR((y[i]-a-b*x[i])/sig[i]);
        if (ndata>2) q=gam.gammq(0.5*(ndata-2),0.5*chi2);      Equation (15.2.12).
    }

    Fitab(VecDoub_I &xx, VecDoub_I &yy)
    : ndata(xx.size()), x(xx), y(yy), sig(xx), chi2(0.), q(1.), sigdat(0.) {
```
Constructor. As above, but without known errors (sig is not used). The uncertainties siga and sigb are estimated by assuming equal errors for all points, and that a straight line is a good fit. q is returned as 1.0, the normalization of chi2 is to unit standard deviation on all points, and sigdat is set to the estimated error of each point.

```
        Int i;
        Doub ss,sx=0.,sy=0.,st2=0.,t,sxoss;
        b=0.0;                                       Accumulate sums ...
        for (i=0;i<ndata;i++) {
            sx += x[i];                              ...without weights.
            sy += y[i];
        }
        ss=ndata;
        sxoss=sx/ss;
        for (i=0;i<ndata;i++) {
            t=x[i]-sxoss;
            st2 += t*t;
            b += t*y[i];
        }
        b /= st2;                                    Solve for a, b, σa, and σb.
        a=(sy-sx*b)/ss;
        siga=sqrt((1.0+sx*sx/(ss*st2))/ss);
        sigb=sqrt(1.0/st2);                          Calculate χ2.
        for (i=0;i<ndata;i++) chi2 += SQR(y[i]-a-b*x[i]);
```

```
        if (ndata > 2) sigdat=sqrt(chi2/(ndata-2));
        siga *= sigdat;
        sigb *= sigdat;
    }
};
```

For unweighted data evaluate typical sig using chi2, and adjust the standard deviations.

CITED REFERENCES AND FURTHER READING:

Bevington, P.R., and Robinson, D.K. 2002, *Data Reduction and Error Analysis for the Physical Sciences*, 3rd ed. (New York: McGraw-Hill), Chapter 6.

Devore, J.L. 2003, *Probability and Statistics for Engineering and the Sciences*, 6th ed. (Belmont, CA: Duxbury Press), Chapter 12.

15.3 Straight-Line Data with Errors in Both Coordinates

If experimental data are subject to measurement error not only in the y_i's, but also in the x_i's, then the task of fitting a straight-line model

$$y(x) = a + bx \tag{15.3.1}$$

is considerably harder. It is straightforward to write down the χ^2 merit function for this case,

$$\chi^2(a,b) = \sum_{i=0}^{N-1} \frac{(y_i - a - bx_i)^2}{\sigma_{y\,i}^2 + b^2\sigma_{x\,i}^2} \tag{15.3.2}$$

where $\sigma_{x\,i}$ and $\sigma_{y\,i}$ are, respectively, the x and y standard deviations for the ith point. The weighted sum of variances in the denominator of equation (15.3.2) can be understood both as the variance in the direction of the smallest χ^2 between each data point and the line with slope b, and also as the variance of the linear combination $y_i - a - bx_i$ of two random variables x_i and y_i,

$$\mathrm{Var}(y_i - a - bx_i) = \mathrm{Var}(y_i) + b^2\mathrm{Var}(x_i) = \sigma_{y\,i}^2 + b^2\sigma_{x\,i}^2 \equiv 1/w_i \tag{15.3.3}$$

The sum of the square of N random variables, each normalized by its variance, is thus chi-square distributed.

We want to minimize equation (15.3.2) with respect to a and b. Unfortunately, the occurrence of b in the denominator of equation (15.3.2) makes the resulting equation for the slope $\partial\chi^2/\partial b = 0$ nonlinear. However, the corresponding condition for the intercept, $\partial\chi^2/\partial a = 0$, is still linear and yields

$$a = \left[\sum_i w_i(y_i - bx_i)\right] \Big/ \sum_i w_i \tag{15.3.4}$$

where the w_i's are defined by equation (15.3.3). A reasonable strategy, now, is to use the machinery of Chapter 10 (e.g., a `Brent` object) for minimizing a general one-dimensional function to minimize with respect to b while using equation (15.3.4) at each stage to ensure that the minimum with respect to b is also minimized with respect to a.

Because of the finite error bars on the x_i's, the minimum χ^2 as a function of b will be finite, though usually large, when b equals infinity (line of infinite slope). The angle $\theta \equiv$

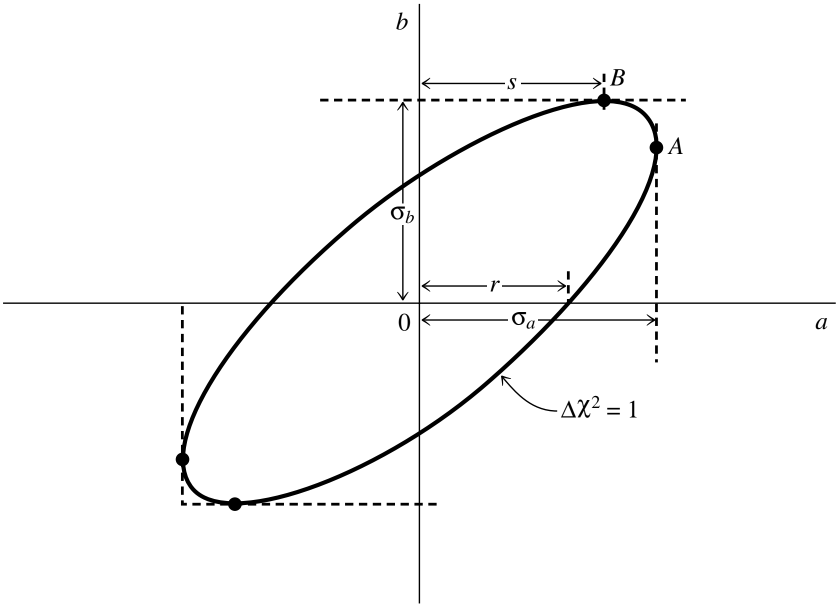

Figure 15.3.1. Standard errors for the parameters a and b. The point B can be found by varying the slope b while simultaneously minimizing the intercept a. This gives the standard error σ_b and also the value s. The standard error σ_a can then be found by the geometric relation $\sigma_a^2 = s^2 + r^2$.

arctan b is thus more suitable as a parametrization of slope than b itself. The value of χ^2 will then be periodic in θ with period π (not 2π!). If any data points have very small σ_y's but moderate or large σ_x's, then it is also possible to have a maximum in χ^2 near zero slope, $\theta \approx 0$. In that case, there can sometimes be two χ^2 minima, one at positive slope and the other at negative. Only one of these is the correct global minimum. It is therefore important to have a good starting guess for b (or θ). Our strategy, implemented below, is to scale the y_i's so as to have variance equal to the x_i's, and then to do a conventional (as in §15.2) linear fit with weights derived from the (scaled) sum $\sigma_{y\,i}^2 + \sigma_{x\,i}^2$. This yields a good starting guess for b if the data are even *plausibly* related to a straight-line model.

Finding the standard errors σ_a and σ_b on the parameters a and b is more complicated. We will see in §15.6 that, in appropriate circumstances, the standard errors in a and b are the respective projections onto the a- and b-axes of the "confidence region boundary" where χ^2 takes on a value one greater than its minimum, $\Delta\chi^2 = 1$. In the linear case of §15.2, these projections follow from the Taylor series expansion

$$\Delta\chi^2 \approx \frac{1}{2}\left[\frac{\partial^2\chi^2}{\partial a^2}(\Delta a)^2 + \frac{\partial^2\chi^2}{\partial b^2}(\Delta b)^2\right] + \frac{\partial^2\chi^2}{\partial a\,\partial b}\Delta a\,\Delta b \tag{15.3.5}$$

Because of the present nonlinearity in b, however, analytic formulas for the second derivatives are quite unwieldy; more important, the lowest-order term frequently gives a poor approximation to $\Delta\chi^2$. Our strategy is therefore to find the roots of $\Delta\chi^2 = 1$ numerically, by adjusting the value of the slope b away from the minimum. In the program below, the general root finder `zbrent` is used. It may occur that there are no roots at all — for example, if all error bars are so large that all the data points are compatible with each other. It is important, therefore, to make some effort at bracketing a putative root before refining it (cf. §9.1).

Because a is minimized at each stage of varying b, successful numerical root finding leads to a value of Δa that minimizes χ^2 for the value of Δb that gives $\Delta\chi^2 = 1$. This (see Figure 15.3.1) directly gives the tangent projection of the confidence region onto the b-axis, and thus σ_b. It does not, however, give the tangent projection of the confidence region onto

the a-axis. In the figure, we have found the point labeled B; to find σ_a we need to find the point A. Geometry to the rescue: To the extent that the confidence region is approximated by an ellipse, you can prove (see figure) that $\sigma_a^2 = r^2 + s^2$. The value of s is known from having found the point B. The value of r follows from equations (15.3.2) and (15.3.3) applied at the χ^2 minimum (point O in the figure), giving

$$r^2 = 1 \Big/ \sum_i w_i \tag{15.3.6}$$

Actually, since b can go through infinity, this whole procedure makes more sense in (a, θ) space than in (a, b) space. That is, in fact, how the following program works. Since it is conventional, however, to return standard errors for a and b, not a and θ, we finally use the relation

$$\sigma_b = \sigma_\theta / \cos^2 \theta \tag{15.3.7}$$

We caution that if b and its standard error are both large, so that the confidence region actually includes infinite slope, then the standard error σ_b is not very meaningful. The functor `Chixy` is normally called only by the routine `Fitexy`. However, if you want, you can yourself explore the confidence region by making repeated calls to `Chixy` (whose argument is an angle θ, not a slope b), after a single initializing call to `Fitexy`.

Be aware that the literature on the seemingly straightforward subject of this section is generally confusing and sometimes plain wrong. Deming's [1] early treatment is sound, but its reliance on Taylor expansions gives inaccurate error estimates. References [2-4] are reliable, more recent, general treatments with critiques of earlier work. York [5] and Reed [6] usefully discuss the simple case of a straight line as treated here, but the latter paper has some errors, corrected in [7]. All this commotion has attracted the Bayesians [8-10], who have still different points of view.

A final caution, repeated from §15.0, is that if the goodness-of-fit is not acceptable (returned probability is too small), the standard errors σ_a and σ_b are surely not believable. In dire circumstances, you might try scaling all your x and y error bars by a constant factor until the probability is acceptable (0.5, say), to get more plausible values for σ_a and σ_b.

Implementing code is given in a Webnote [11].

CITED REFERENCES AND FURTHER READING:

Deming, W.E. 1943, *Statistical Adjustment of Data* (New York: Wiley), reprinted 1964 (New York: Dover).[1]

Jefferys, W.H. 1980, "On the Method of Least Squares," *Astronomical Journal*, vol. 85, pp. 177–181; see also vol. 95, p. 1299 (1988).[2]

Jefferys, W.H. 1981, "On the Method of Least Squares — Part Two," *Astronomical Journal*, vol. 86, pp. 149–155; see also vol. 95, p. 1300 (1988).[3]

Lybanon, M. 1984, "A Better Least-Squares Method When Both Variables Have Uncertainties," *American Journal of Physics*, vol. 52, pp. 22–26.[4]

York, D. 1966, "Least-Squares Fitting of a Straight Line," *Canadian Journal of Physics*, vol. 44, pp. 1079–1086.[5]

Reed, B.C. 1989, "Linear Least-Squares Fits with Error in Both Coordinates," *American Journal of Physics*, vol. 57, pp. 642–646; see also vol. 58, p. 189, and vol. 58, p. 1209.[6]

Reed, B.C. 1992, "Linear Least-squares Fits with Errors in Both Coordinates. II: Comments on Parameter Variances," *American Journal of Physics*, vol. 60, pp. 59–62.[7]

Zellner, A. 1971, *An Introduction to Bayesian Inference in Econometrics* (New York: Wiley); reprinted 1987 (Malabar, FL: R. E. Krieger).[8]

Gull, S.F. 1989, in *Maximum Entropy and Bayesian Methods*, J. Skilling, ed. (Boston: Kluwer).[9]

Jaynes, E.T. 1991, in *Maximum-Entropy and Bayesian Methods, Proceedings of the 10th International Workshop*, W.T. Grandy, Jr., and L.H. Schick, eds. (Boston: Kluwer).[10]

Macdonald, J.R., and Thompson, W.J. 1992, "Least-Squares Fitting When Both Variables Contain Errors: Pitfalls and Possibilities," *American Journal of Physics*, vol. 60, pp. 66–73.

Numerical Recipes Software 2007, "Code Implementation for Fitexy," *Numerical Recipes Webnote No. 19*, at http://www.nr.com/webnotes?19 [11]

15.4 General Linear Least Squares

An immediate generalization of §15.2 is to fit a set of data points (x_i, y_i) to a model that is not just a linear combination of 1 and x (namely $a + bx$), but rather a linear combination of *any* M specified functions of x. For example, the functions could be $1, x, x^2, \dots, x^{M-1}$, in which case their general linear combination,

$$y(x) = a_0 + a_1 x + a_2 x^2 + \cdots + a_{M-1} x^{M-1} \tag{15.4.1}$$

is a polynomial of degree $M - 1$. Or, the functions could be sines and cosines, in which case their general linear combination is a Fourier series. The general form of this kind of model is

$$y(x) = \sum_{k=0}^{M-1} a_k X_k(x) \tag{15.4.2}$$

where the quantities $X_0(x), \dots, X_{M-1}(x)$ are arbitrary fixed functions of x, called the *basis functions*.

Note that the functions $X_k(x)$ can be wildly nonlinear functions of x. In this discussion, "linear" refers only to the model's dependence on its *parameters* a_k.

For these linear models we generalize the discussion of the previous section by defining a merit function

$$\chi^2 = \sum_{i=0}^{N-1} \left[\frac{y_i - \sum_{k=0}^{M-1} a_k X_k(x_i)}{\sigma_i} \right]^2 \tag{15.4.3}$$

As before, σ_i is the measurement error (standard deviation) of the ith data point, presumed to be known. If the measurement errors are not known, they may all (as discussed at the end of §15.1) be set to the constant value $\sigma = 1$.

Once again, we will pick as best parameters those that minimize χ^2. There are several different techniques available for finding this minimum. Two are particularly useful, and we will discuss both in this section. To introduce them and elucidate their relationship, we need some notation.

Let $\mathbf{A}$ be a matrix whose $N \times M$ components are constructed from the M basis functions evaluated at the N abscissas x_i, and from the N measurement errors σ_i, by the prescription

$$A_{ij} = \frac{X_j(x_i)}{\sigma_i} \tag{15.4.4}$$

The matrix $\mathbf{A}$ is called the *design matrix* of the fitting problem. Notice that in general $\mathbf{A}$ has more rows than columns, $N \geq M$, since there must be more data points than model parameters to be solved for. (You can fit a straight line to two points, but not a very meaningful quintic!) The design matrix is shown schematically in Figure 15.4.1.

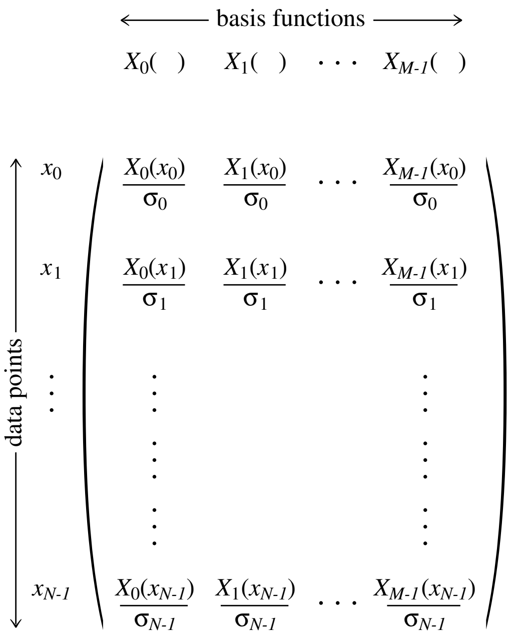

Figure 15.4.1. Design matrix $\mathbf{A}$ for the least-squares fit of a linear combination of M basis functions to N data points. The matrix elements involve the basis functions evaluated at the values of the independent variable at which measurements are made and the standard deviations of the measured dependent variable. The measured values of the dependent variable do not enter the design matrix.

Also define a vector $\mathbf{b}$ of length N by

$$b_i = \frac{y_i}{\sigma_i} \tag{15.4.5}$$

and denote the M vector whose components are the parameters to be fitted, $a_0, \dots, a_{M-1}$, by $\mathbf{a}$.

15.4.1 Solution by Use of the Normal Equations

The minimum of (15.4.3) occurs where the derivative of χ^2 with respect to all M parameters a_k vanishes. Specializing equation (15.1.8) to the case of the model (15.4.2), this condition yields the M equations

$$0 = \sum_{i=0}^{N-1} \frac{1}{\sigma_i^2} \left[y_i - \sum_{j=0}^{M-1} a_j X_j(x_i) \right] X_k(x_i) \qquad k = 0, \dots, M-1 \tag{15.4.6}$$

Interchanging the order of summations, we can write (15.4.6) as the matrix equation

$$\sum_{j=0}^{M-1} \alpha_{kj} a_j = \beta_k \tag{15.4.7}$$

where

$$\alpha_{kj} = \sum_{i=0}^{N-1} \frac{X_j(x_i)X_k(x_i)}{\sigma_i^2} \qquad \text{or, equivalently,} \qquad \boldsymbol{\alpha} = \mathbf{A}^T \cdot \mathbf{A} \tag{15.4.8}$$

an $M \times M$ matrix, and

$$\beta_k = \sum_{i=0}^{N-1} \frac{y_i X_k(x_i)}{\sigma_i^2} \qquad \text{or, equivalently,} \qquad \boldsymbol{\beta} = \mathbf{A}^T \cdot \mathbf{b} \tag{15.4.9}$$

a vector of length M.

The equations (15.4.6) or (15.4.7) are called the *normal equations* of the least-squares problem. They can be solved for the vector of parameters **a** by the standard methods of Chapter 2, notably LU decomposition and backsubstitution, Choleksy decomposition, or Gauss-Jordan elimination. In matrix form, the normal equations can be written as either

$$\boldsymbol{\alpha} \cdot \mathbf{a} = \boldsymbol{\beta} \qquad \text{or as} \qquad \left(\mathbf{A}^T \cdot \mathbf{A}\right) \cdot \mathbf{a} = \mathbf{A}^T \cdot \mathbf{b} \tag{15.4.10}$$

The inverse matrix $\mathbf{C} \equiv \boldsymbol{\alpha}^{-1}$, called the covariance matrix, is closely related to the probable (or, more precisely, *standard*) uncertainties of the estimated parameters **a**. To estimate these uncertainties, consider that

$$a_j = \sum_{k=0}^{M-1} \boldsymbol{\alpha}_{jk}^{-1} \beta_k = \sum_{k=0}^{M-1} C_{jk} \left[\sum_{i=0}^{N-1} \frac{y_i X_k(x_i)}{\sigma_i^2} \right] \tag{15.4.11}$$

and that the variance associated with the estimate a_j can be found as in (15.2.7) from

$$\sigma^2(a_j) = \sum_{i=0}^{N-1} \sigma_i^2 \left(\frac{\partial a_j}{\partial y_i} \right)^2 \tag{15.4.12}$$

Note that α_{jk} is independent of y_i, so that

$$\frac{\partial a_j}{\partial y_i} = \sum_{k=0}^{M-1} C_{jk} X_k(x_i)/\sigma_i^2 \tag{15.4.13}$$

Consequently, we find that

$$\sigma^2(a_j) = \sum_{k=0}^{M-1} \sum_{l=0}^{M-1} C_{jk} C_{jl} \left[\sum_{i=0}^{N-1} \frac{X_k(x_i) X_l(x_i)}{\sigma_i^2} \right] \tag{15.4.14}$$

The final term in brackets is just the matrix $\boldsymbol{\alpha}$. Since this is the matrix inverse of **C**, (15.4.14) reduces immediately to

$$\sigma^2(a_j) = C_{jj} \tag{15.4.15}$$

In other words, the diagonal elements of **C** are the variances (squared uncertainties) of the fitted parameters **a**. It should not surprise you to learn that the off-diagonal elements C_{jk} are the covariances between a_j and a_k (cf. 15.2.10); but we shall defer discussion of these to §15.6.

We will now give a routine that implements the above formulas for the general linear least-squares problem, by the method of normal equations. Since we wish to compute not only the solution vector **a** but also the covariance matrix **C**, it is most convenient to use Gauss-Jordan elimination (routine `gaussj` of §2.1) to perform the linear algebra. The operation count in this application is no larger than that for LU decomposition. If you have no need for the covariance matrix, however, you can save a factor of 3 on the linear algebra by switching to LU decomposition, without computation of the matrix inverse. In theory, since $\mathbf{A}^T \cdot \mathbf{A}$ is positive-definite, Cholesky decomposition is the most efficient way to solve the normal equations. However, in practice, most of the computing time is spent in looping over the data to form the equations, and Gauss-Jordan is quite adequate.

We need to warn you that the solution of a least-squares problem directly from the normal equations is rather susceptible to roundoff error, because the condition number of the matrix $\boldsymbol{\alpha}$ is the square of the condition number of $\mathbf{A}$. An alternative, and preferred, technique involves QR decomposition (§2.10, §11.4, and §11.7) of the design matrix $\mathbf{A}$. This is essentially what we did at the end of §15.2 for fitting data to a straight line, but without invoking all the machinery of QR to derive the necessary formulas. Later in this section, we will discuss other difficulties in the least-squares problem, for which the cure is *singular value decomposition* (SVD), of which we give an implementation. It turns out that SVD also fixes the roundoff problem, so it is our recommended technique for all but "easy" least-squares problems. It is for these easy problems that the following routine, which solves the normal equations, is intended.

The object `Fitlin`, below, has one "value-added feature" that can be quite useful in practical work: Frequently it is a matter of art to decide which parameters a_k in a model should be fit from the data set, and which should be held constant at fixed values, for example values predicted by a theory or measured in a previous experiment. One wants, therefore, to have a convenient means for "freezing" and "unfreezing" the parameters a_k. In the following code, the total number of parameters a_k is denoted `ma` (called M above) and is deduced from the size of the vector that is returned by the user-supplied fitting function routine. The `Fitlin` object maintains a boolean array `ia[0..ma-1]`. Components that are `false` indicate that you want the corresponding elements of the parameter vector `a[0..ma-1]` to be held fixed at their input values. Components that are `true` indicate parameters that should be fitted for. On output, any frozen parameters will have their variances, and all their covariances, set to zero in the covariance matrix.

```
struct Fitlin {                                                              fitlin.h
Object for general linear least-squares fitting by solving the normal equations, also including
the ability to hold specified parameters at fixed, specified values. Call constructor to bind data
vectors and fitting functions. Then call any combination of hold, free, and fit as often as
desired. fit sets the output quantities a, covar, and chisq.
    Int ndat, ma;
    VecDoub_I &x,&y,&sig;
    VecDoub (*funcs)(const Doub);
    VecBool ia;

    VecDoub a;                          Output values. a is the vector of fitted coefficients,
    MatDoub covar;                          covar is its covariance matrix, and chisq is the
    Doub chisq;                             value of χ^2 for the fit.

    Fitlin(VecDoub_I &xx, VecDoub_I &yy, VecDoub_I &ssig, VecDoub funks(const Doub))
```

```
: ndat(xx.size()), x(xx), y(yy), sig(ssig), funcs(funks) {
```

Constructor. Binds references to the data arrays xx, yy, and ssig, and to a user-supplied function funks(x) that returns a VecDoub containing ma basis functions evaluated at $x =$ x. Initializes all parameters as free (not held).

```
    ma = funcs(x[0]).size();
    a.resize(ma);
    covar.resize(ma,ma);
    ia.resize(ma);
    for (Int i=0;i<ma;i++) ia[i] = true;
}

void hold(const Int i, const Doub val) {ia[i]=false; a[i]=val;}
void free(const Int i) {ia[i]=true;}
```

Optional functions for holding a parameter, identified by a value i in the range $0,\ldots,$ma-1, fixed at the value val, or for freeing a parameter that was previously held fixed. hold and free may be called for any number of parameters before calling fit to calculate best-fit values for the remaining (not held) parameters, and the process may be repeated multiple times. Alternatively, you can set the boolean vector ia directly, before calling fit.

```
void fit() {
```

Solve the normal equations for χ^2 minimization to fit for some or all of the coefficients a[0..ma-1] of a function that depends linearly on a, $y = \sum_i$ $a_i \times$ $funks_i(x)$. Set answer values for a[0..ma-1], $\chi^2 =$ chisq, and the covariance matrix covar[0..ma-1][0..ma-1]. (Parameters held fixed by calls to hold will return zero covariances.)

```
    Int i,j,k,l,m,mfit=0;
    Doub ym,wt,sum,sig2i;
    VecDoub afunc(ma);
    for (j=0;j<ma;j++) if (ia[j]) mfit++;
    if (mfit == 0) throw("lfit: no parameters to be fitted");
    MatDoub temp(mfit,mfit,0.),beta(mfit,1,0.);
    for (i=0;i<ndat;i++) {              Loop over data to accumulate coefficients of
        afunc = funcs(x[i]);                the normal equations.
        ym=y[i];
        if (mfit < ma) {                Subtract off dependences on known pieces
            for (j=0;j<ma;j++)              of the fitting function.
                if (!ia[j]) ym -= a[j]*afunc[j];
        }
        sig2i=1.0/SQR(sig[i]);
        for (j=0,l=0;l<ma;l++) {        Set up matrix and r.h.s. for matrix inversion.
            if (ia[l]) {
                wt=afunc[l]*sig2i;
                for (k=0,m=0;m<=l;m++)
                    if (ia[m]) temp[j][k++] += wt*afunc[m];
                beta[j++][0] += ym*wt;
            }
        }
    }
    for (j=1;j<mfit;j++) for (k=0;k<j;k++) temp[k][j]=temp[j][k];
    gaussj(temp,beta);                  Matrix solution.
    for (j=0,l=0;l<ma;l++) if (ia[l]) a[l]=beta[j++][0];
```

Spread the solution to appropriate positions in a, and evaluate χ^2 of the fit.

```
    chisq=0.0;
    for (i=0;i<ndat;i++) {
        afunc = funcs(x[i]);
        sum=0.0;
        for (j=0;j<ma;j++) sum += a[j]*afunc[j];
        chisq += SQR((y[i]-sum)/sig[i]);
    }
    for (j=0;j<mfit;j++) for (k=0;k<mfit;k++) covar[j][k]=temp[j][k];
    for (i=mfit;i<ma;i++)               Rearrange covariance matrix into the correct
        for (j=0;j<i+1;j++) covar[i][j]=covar[j][i]=0.0;      order.
    k=mfit-1;
    for (j=ma-1;j>=0;j--) {
```

```
            if (ia[j]) {
                for (i=0;i<ma;i++) SWAP(covar[i][k],covar[i][j]);
                for (i=0;i<ma;i++) SWAP(covar[k][i],covar[j][i]);
                k--;
            }
        }
    }
};
```

Typical use of `Fitlin` will look something like this:

```
const Int npts=...
VecDoub xx(npts),yy(npts),ssig(npts);
...
Fitlin myfit(xx,yy,ssig,cubicfit);
myfit.fit();
```

where (in this example) `cubicfit` is a user-supplied function that might look like this:

```
VecDoub cubicfit(const Doub x) {
    VecDoub ans(4);
    ans[0] = 1.;
    for (Int i=1;i<4;i++) ans[i] = x*ans[i-1];
    return ans;
}
```

15.4.2 Solution by Use of Singular Value Decomposition

In some applications, the normal equations are perfectly adequate for linear least-squares problems. However, in many other cases the normal equations are very close to singular. A zero pivot element may be encountered during the solution of the linear equations (e.g., in `gaussj`), in which case you get no solution at all. Or a very small pivot may occur, in which case you typically get fitted parameters a_k with very large magnitudes that are delicately (and unstably) balanced to cancel out almost precisely when the fitted function is evaluated.

Why does this commonly occur? A mathematical reason is that the condition number of the matrix $\boldsymbol{\alpha}$ is the square of the condition number of $\mathbf{A}$. But an additional physical reason is that, more often than experimenters would like to admit, data do not clearly distinguish between two or more of the basis functions provided. If two such functions, or two different combinations of functions, happen to fit the data about equally well — or equally badly — then the matrix $\boldsymbol{\alpha}$, unable to distinguish between them, neatly folds up its tent and becomes singular. There is a certain mathematical irony in the fact that least-squares problems are *both* overdetermined (number of data points greater than number of parameters) *and* underdetermined (ambiguous combinations of parameters exist); but that is how it frequently is. The ambiguities can be extremely hard to notice a priori in complicated problems.

Enter singular value decomposition (SVD). This would be a good time for you to review the material in §2.6, which we will not repeat here. In the case of an overdetermined system, SVD produces a solution that is the best approximation in the least-squares sense, cf. equation (2.6.10). That is exactly what we want. In the case of an underdetermined system, SVD produces a solution whose values (for us, the a_k's) are smallest in the least-squares sense, cf. equation (2.6.8). That is also what we want: When some combination of basis functions is irrelevant to the fit, that

combination will be driven down to a small, innocuous, value, rather than pushed up to delicately canceling infinities.

In terms of the design matrix **A** (equation 15.4.4) and the vector **b** (equation 15.4.5), minimization of χ^2 in (15.4.3) can be written as

$$\text{find} \quad \mathbf{a} \quad \text{that minimizes} \quad \chi^2 = |\mathbf{A}\cdot\mathbf{a} - \mathbf{b}|^2 \tag{15.4.16}$$

Comparing to equation (2.6.9), we see that this is precisely the problem that routines in the SVD object are designed to solve. The solution, which is given by equation (2.6.12), can be rewritten as follows: If **U** and **V** enter the SVD decomposition of **A** according to equation (2.6.1), as computed by SVD, then let the vectors $\mathbf{U}_{(i)}$ $i = 0,\dots,M-1$ denote the *columns* of **U** (each one a vector of length N), and let the vectors $\mathbf{V}_{(i)}$ $i = 0,\dots,M-1$ denote the *columns* of **V** (each one a vector of length M). Then the solution (2.6.12) of the least-squares problem (15.4.16) can be written as

$$\mathbf{a} = \sum_{i=0}^{M-1}\left(\frac{\mathbf{U}_{(i)}\cdot\mathbf{b}}{w_i}\right)\mathbf{V}_{(i)} \tag{15.4.17}$$

where the w_i are, as in §2.6, the singular values calculated by SVD.

Equation (15.4.17) says that the fitted parameters **a** are linear combinations of the columns of **V**, with coefficients obtained by forming dot products of the columns of **U** with the weighted data vector (15.4.5). Though it is beyond our scope to prove here, it turns out that the standard (loosely, "probable") errors in the fitted parameters are also linear combinations of the columns of **V**. In fact, equation (15.4.17) can be written in a form displaying these errors as

$$\mathbf{a} = \left[\sum_{i=0}^{M-1}\left(\frac{\mathbf{U}_{(i)}\cdot\mathbf{b}}{w_i}\right)\mathbf{V}_{(i)}\right] \pm \frac{1}{w_0}\mathbf{V}_{(0)} \pm \cdots \pm \frac{1}{w_{M-1}}\mathbf{V}_{(M-1)} \tag{15.4.18}$$

Here each $\pm$ is followed by a standard deviation. The amazing fact is that, decomposed in this fashion, the standard deviations are all mutually independent (uncorrelated). Therefore they can be added together in root-mean-square fashion. What is going on is that the vectors $\mathbf{V}_{(i)}$ are the principal axes of the error ellipsoid of the fitted parameters **a** (see §15.6).

It follows that the variance in the estimate of a parameter a_j is given by

$$\sigma^2(a_j) = \sum_{i=0}^{M-1}\frac{1}{w_i^2}[\mathbf{V}_{(i)}]_j^2 = \sum_{i=0}^{M-1}\left(\frac{V_{ji}}{w_i}\right)^2 \tag{15.4.19}$$

whose result should be identical to (15.4.14). As before, you should not be surprised at the formula for the covariances, here given without proof,

$$\text{Cov}(a_j, a_k) = \sum_{i=0}^{M-1}\left(\frac{V_{ji}V_{ki}}{w_i^2}\right) \tag{15.4.20}$$

We introduced this subsection by noting that the normal equations can fail by encountering a zero pivot. We have not yet, however, mentioned how SVD overcomes this problem. The answer is: If any singular value w_i is zero, its reciprocal

in equation (15.4.18) should be set to zero, not infinity. (Compare the discussion preceding equation 2.6.7.) This corresponds to adding to the fitted parameters **a** a *zero* multiple, rather than some random large multiple, of any linear combination of basis functions that are degenerate in the fit. It is a good thing to do!

Moreover, if a singular value w_i is nonzero but very small, you should also define *its* reciprocal to be zero, since its apparent value is probably an artifact of roundoff error, not a meaningful number. A plausible answer to the question "how small is small?" is to edit in this fashion all singular values whose ratio to the largest singular value is less than N times the machine precision ϵ. (This is a more conservative recommendation than the default in §2.6, which scales as $N^{1/2}$.)

There is another reason for editing even *additional* singular values, ones large enough that roundoff error is not a question. Singular value decomposition allows you to identify linear combinations of variables that just happen not to contribute much to reducing the χ^2 of your data set. Editing these can sometimes reduce the probable error errors on your coefficients quite significantly, while increasing the minimum χ^2 only negligibly. We will learn more about identifying and treating such cases in §15.6.

Generally speaking, we recommend that you always use SVD techniques instead of using the normal equations. SVD's only significant disadvantage is that it requires extra storage of order $N \times M$ for the design matrix and its decomposition. Storage is also required for the $M \times M$ matrix $\mathbf{V}$, but this is instead of the same-sized matrix for the coefficients of the normal equations. SVD can be significantly slower than solving the normal equations; however, its great advantage, that it (theoretically) *cannot fail*, more than makes up for the speed disadvantage.

The following object, `Fitsvd`, has an interface almost identical to `Fitlin`, above. An additional optional parameter in the constructor sets the threshold for editing singular values.

fitsvd.h

```
struct Fitsvd {
```
Object for general linear least-squares fitting using singular value decomposition. Call constructor to bind data vectors and fitting functions. Then call `fit`, which sets the output quantities `a`, `covar`, and `chisq`.

```
    Int ndat, ma;
    Doub tol;
    VecDoub_I *x,&y,&sig;
    VecDoub (*funcs)(const Doub);
    VecDoub a;
    MatDoub covar;
    Doub chisq;
```
(Why is `x` a pointer? Explained in §15.4.4.)

Output values. `a` is the vector of fitted coefficients, `covar` is its covariance matrix, and `chisq` is the value of χ^2 for the fit.

```
    Fitsvd(VecDoub_I &xx, VecDoub_I &yy, VecDoub_I &ssig,
    VecDoub funks(const Doub), const Doub TOL=1.e-12)
    : ndat(yy.size()), x(&xx), xmd(NULL), y(yy), sig(ssig),
    funcs(funks), tol(TOL) {}
```
Constructor. Binds references to the data arrays `xx`, `yy`, and `ssig`, and to a user-supplied function `funks(x)` that returns a `VecDoub` containing `ma` basis functions evaluated at $x =$ `x`. If `TOL` is positive, it is the threshold (relative to the largest singular value) for discarding small singular values. If it is ≤ 0, the default value in `SVD` is used.

```
    void fit() {
```
Solve by singular value decomposition the χ^2 minimization that fits for the coefficients `a[0..ma-1]` of a function that depends linearly on `a`, $y = \sum_i \mathtt{a}_i \times \mathtt{funks}_i(x)$. Set answer values for `a[0..ma-1]`, `chisq` $= \chi^2$, and the covariance matrix `covar[0..ma-1][0..ma-1]`.

```
        Int i,j,k;
        Doub tmp,thresh,sum;
        if (x) ma = funcs((*x)[0]).size();
        else ma = funcsmd(row(*xmd,0)).size();          (Discussed in §15.4.4.)
        a.resize(ma);
        covar.resize(ma,ma);
        MatDoub aa(ndat,ma);
        VecDoub b(ndat),afunc(ma);
        for (i=0;i<ndat;i++) {                          Accumulate coefficients of the
            if (x) afunc=funcs((*x)[i]);                    design matrix.
            else afunc=funcsmd(row(*xmd,i));            (Discussed in §15.4.4.)
            tmp=1.0/sig[i];
            for (j=0;j<ma;j++) aa[i][j]=afunc[j]*tmp;
            b[i]=y[i]*tmp;
        }
        SVD svd(aa);                                    Singular value decomposition.
        thresh = (tol > 0. ? tol*svd.w[0] : -1.);
        svd.solve(b,a,thresh);                          Solve for the coefficients.
        chisq=0.0;                                      Evaluate chi-square.
        for (i=0;i<ndat;i++) {
            sum=0.;
            for (j=0;j<ma;j++) sum += aa[i][j]*a[j];
            chisq += SQR(sum-b[i]);
        }
        for (i=0;i<ma;i++) {                            Sum contributions to covariance
            for (j=0;j<i+1;j++) {                           matrix (15.4.20).
                sum=0.0;
                for (k=0;k<ma;k++) if (svd.w[k] > svd.tsh)
                    sum += svd.v[i][k]*svd.v[j][k]/SQR(svd.w[k]);
                covar[j][i]=covar[i][j]=sum;
            }
        }

    }
```

From here on, code for multidimensional fits, to be discussed in §15.4.4.

```
    MatDoub_I *xmd;
    VecDoub (*funcsmd)(VecDoub_I &);

    Fitsvd(MatDoub_I &xx, VecDoub_I &yy, VecDoub_I &ssig,
    VecDoub funks(VecDoub_I &), const Doub TOL=1.e-12)
    : ndat(yy.size()), x(NULL), xmd(&xx), y(yy), sig(ssig),
    funcsmd(funks), tol(TOL) {}
```

Constructor for multidimensional fits. Exactly the same as the previous constructor, except that `xx` is now a matrix whose rows are the multidimensional data points and `funks` is now a function of a multidimensional data point (as a `VecDoub`).

```
    VecDoub row(MatDoub_I &a, const Int i) {
```

Utility. Returns the row of a `MatDoub` as a `VecDoub`.

```
        Int j,n=a.ncols();
        VecDoub ans(n);
        for (j=0;j<n;j++) ans[j] = a[i][j];
        return ans;
    }
};
```

For degenerate or nearly degenerate problems, if you want to try different singular value thresholds, you call the `Fitsvd` constructor once. Then, as many times as you want, "reach in" and increase `tol`, then call `fit` again and examine the resulting value of `chisq` (and optionally also the covariance matrix). Keep going as long as `chisq` does not increase by too much. To learn what is "too much," see §15.6; but a few $\times 0.1$ is almost always OK.

15.4.3 Examples

Be aware that some apparently nonlinear problems can be expressed so that they are linear. For example, an exponential model with two parameters a and b,

$$y(x) = a \exp(-bx) \tag{15.4.21}$$

can be rewritten as

$$\log[y(x)] = c - bx \tag{15.4.22}$$

which is linear in its parameters c and b. (Of course you must be aware that such transformations do not exactly take Gaussian errors into Gaussian errors.)

Also watch out for "nonparameters," as in

$$y(x) = a \exp(-bx + d) \tag{15.4.23}$$

Here the parameters a and d are, in fact, indistinguishable. This is a good example of where the normal equations will be exactly singular, and where SVD will find a zero singular value. SVD will then make a least-squares choice for setting a balance between a and d (or, rather, their equivalents in the linear model derived by taking the logarithms). However — and this is true whenever SVD gives back a zero singular value — you are better advised to figure out analytically where the degeneracy is among your basis functions, and then make appropriate deletions in the basis set.

We already gave an example of a user-supplied fitting-function routine, `cubicfit`, above. Here are two further examples. First, we trivially generalize `cubicfit` for polynomials of an arbitrary, preset, degree:

```
Int fpoly_np = 10;                       Global variable for the degree plus one.        fit_examples.h

VecDoub fpoly(const Doub x) {
Fitting routine for a polynomial of degree fpoly_np-1.
    Int j;
    VecDoub p(fpoly_np);
    p[0]=1.0;
    for (j=1;j<fpoly_np;j++) p[j]=p[j-1]*x;
    return p;
}
```

The second example is slightly less trivial. It is used to fit Legendre polynomials up to some order `fleg_nl` to a data set. (Note that, for most uses, the data should satisfy $-1 \le x \le 1$.)

```
Int fleg_nl = 10;                        Global variable for the degree plus one.        fit_examples.h

VecDoub fleg(const Doub x) {
Fitting routine for an expansion with nl Legendre polynomials, evaluated using the recurrence
relation as in §5.4.
    Int j;
    Doub twox,f2,f1,d;
    VecDoub pl(fleg_nl);
    pl[0]=1.;
    pl[1]=x;
    if (fleg_nl > 2) {
        twox=2.*x;
        f2=x;
        d=1.;
        for (j=2;j<fleg_nl;j++) {
```

```
            f1=d++;
            f2+=twox;
            pl[j]=(f2*pl[j-1]-f1*pl[j-2])/d;
        }
    }
    return pl;
}
```

15.4.4 Multidimensional Fits

If you are measuring a single variable y as a function of more than one variable — say, a *vector* of variables $\mathbf{x}$ — then your basis functions will be functions of a vector, $X_0(\mathbf{x}), \ldots, X_{M-1}(\mathbf{x})$. The χ^2 merit function is now

$$\chi^2 = \sum_{i=0}^{N-1} \left[\frac{y_i - \sum_{k=0}^{M-1} a_k X_k(\mathbf{x}_i)}{\sigma_i} \right]^2 \tag{15.4.24}$$

All of the preceding discussion goes through unchanged, with x replaced by $\mathbf{x}$. In fact, we anticipated this in the coding of Fitsvd, above, which can do multidimensional general linear fits as easily as one-dimensional. Here is how:

A second, overloaded, constructor in Fitsvd substitutes a matrix xx for what was previously a vector. The rows of the matrix are the ndat data points. The number of columns is the dimensionality of the space (that is, of $\mathbf{x}$). Similarly, the user-supplied function funks now takes a vector argument, an $\mathbf{x}$. A simple example (fitting a quadratic function to data in two dimensions) might be

```
VecDoub quadratic2d(VecDoub_I &xx) {
    VecDoub ans(6);
    Doub x=xx[0], y=xx[1];
    ans[0] = 1;
    ans[1] = x; ans[2] = y;
    ans[3] = x*x; ans[4] = x*y; ans[5] = y*y;
    return ans;
}
```

Be sure that the argument of your user function has exactly the type "VecDoub_I &" (and not, for example, "VecDoub &" or "VecDoub_I"), since strict C++ compilers are picky about this.

The two constructors in Fitsvd communicate to fit whether data points are one-dimensional or multidimensional by setting either xmd or x to NULL. This explains the oddity that x was bound to the user data as a pointer, while y and sig were bound as references. (Yes, we know this is a bit of a hack!)

CITED REFERENCES AND FURTHER READING:

Bevington, P.R., and Robinson, D.K. 2002, *Data Reduction and Error Analysis for the Physical Sciences*, 3rd ed. (New York: McGraw-Hill), Chapter 7.

Lupton, R. 1993, *Statistics in Theory and Practice* (Princeton, NJ: Princeton University Press), Chapter 11.

Lawson, C.L., and Hanson, R. 1974, *Solving Least Squares Problems* (Englewood Cliffs, NJ: Prentice-Hall); reprinted 1995 (Philadelphia: S.I.A.M.).

Monahan, J.F. 2001, *Numerical Methods of Statistics* (Cambridge, UK: Cambridge University Press), Chapter 5.

Forsythe, G.E., Malcolm, M.A., and Moler, C.B. 1977, *Computer Methods for Mathematical Computations* (Englewood Cliffs, NJ: Prentice-Hall), Chapter 9.

Gelman, A., Carlin, J.B., Stern, H.S., and Rubin, D.B. 2004, *Bayesian Data Analysis*, 2nd ed. (Boca Raton, FL: Chapman & Hall/CRC), Chapter 14.

15.5 Nonlinear Models

We now consider fitting when the model depends *nonlinearly* on the set of M unknown parameters $a_k, k = 0, 1, \ldots, M-1$. We use the same approach as in previous sections, namely to define a χ^2 merit function and determine best-fit parameters by its minimization. With nonlinear dependences, however, the minimization must proceed iteratively. Given trial values for the parameters, we develop a procedure that improves the trial solution. The procedure is then repeated until χ^2 stops (or effectively stops) decreasing.

How is this problem different from the general nonlinear function minimization problem already dealt with in Chapter 10? Superficially, not at all. Sufficiently close to the minimum, we expect the χ^2 function to be well approximated by a quadratic form, which we can write as

$$\chi^2(\mathbf{a}) \approx \gamma - \mathbf{d} \cdot \mathbf{a} + \tfrac{1}{2}\mathbf{a} \cdot \mathbf{D} \cdot \mathbf{a} \tag{15.5.1}$$

where $\mathbf{d}$ is an M-vector and $\mathbf{D}$ is an $M \times M$ matrix. (Compare equation 10.8.1.) If the approximation is a good one, we know how to jump from the current trial parameters $\mathbf{a}_{\text{cur}}$ to the minimizing ones $\mathbf{a}_{\text{min}}$ in a single leap, namely

$$\mathbf{a}_{\text{min}} = \mathbf{a}_{\text{cur}} + \mathbf{D}^{-1} \cdot \left[-\nabla\chi^2(\mathbf{a}_{\text{cur}})\right] \tag{15.5.2}$$

(Compare equation 10.9.4.)

On the other hand, (15.5.1) might be a poor local approximation to the shape of the function that we are trying to minimize at $\mathbf{a}_{\text{cur}}$. In that case, about all we can do is take a step down the gradient, as in the steepest descent method (§10.8). In other words,

$$\mathbf{a}_{\text{next}} = \mathbf{a}_{\text{cur}} - \text{constant} \times \nabla\chi^2(\mathbf{a}_{\text{cur}}) \tag{15.5.3}$$

where the constant is small enough not to exhaust the downhill direction.

To use (15.5.2) or (15.5.3), we must be able to compute the gradient of the χ^2 function at any set of parameters $\mathbf{a}$. To use (15.5.2) we also need the matrix $\mathbf{D}$, which is the second derivative matrix (Hessian matrix) of the χ^2 merit function, at any $\mathbf{a}$.

Now, this is the crucial difference from Chapter 10: There, we had no way of directly evaluating the Hessian matrix. We were given only the ability to evaluate the function to be minimized and (in some cases) its gradient. Therefore, we had to resort to iterative methods *not just* because our function was nonlinear, *but also* in order to build up information about the Hessian matrix. Sections 10.9 and 10.8 concerned themselves with two different techniques for building up this information.

Here, life is much simpler. We *know* exactly the form of χ^2, since it is based on a model function that we ourselves have specified. Therefore, the Hessian matrix is known to us. Thus we are free to use (15.5.2) whenever we care to do so. The only reason to use (15.5.3) will be failure of (15.5.2) to improve the fit, signaling failure of (15.5.1) as a good local approximation.

15.5.1 Calculation of the Gradient and Hessian

The model to be fitted is

$$y = y(x|\mathbf{a}) \tag{15.5.4}$$

and the χ^2 merit function is

$$\chi^2(\mathbf{a}) = \sum_{i=0}^{N-1} \left[\frac{y_i - y(x_i|\mathbf{a})}{\sigma_i} \right]^2 \tag{15.5.5}$$

The gradient of χ^2 with respect to the parameters $\mathbf{a}$, which will be zero at the χ^2 minimum, has components

$$\frac{\partial \chi^2}{\partial a_k} = -2 \sum_{i=0}^{N-1} \frac{[y_i - y(x_i|\mathbf{a})]}{\sigma_i^2} \frac{\partial y(x_i|\mathbf{a})}{\partial a_k} \qquad k = 0, 1, \ldots, M-1 \tag{15.5.6}$$

Taking an additional partial derivative gives

$$\frac{\partial^2 \chi^2}{\partial a_k \partial a_l} = 2 \sum_{i=0}^{N-1} \frac{1}{\sigma_i^2} \left[\frac{\partial y(x_i|\mathbf{a})}{\partial a_k} \frac{\partial y(x_i|\mathbf{a})}{\partial a_l} - [y_i - y(x_i|\mathbf{a})] \frac{\partial^2 y(x_i|\mathbf{a})}{\partial a_l \partial a_k} \right] \tag{15.5.7}$$

It is conventional to remove the factors of 2 by defining

$$\beta_k \equiv -\frac{1}{2} \frac{\partial \chi^2}{\partial a_k} \qquad \alpha_{kl} \equiv \frac{1}{2} \frac{\partial^2 \chi^2}{\partial a_k \partial a_l} \tag{15.5.8}$$

making $\boldsymbol{\alpha} = \frac{1}{2}\mathbf{D}$ in equation (15.5.2), in terms of which that equation can be rewritten as the set of linear equations:

$$\sum_{l=0}^{M-1} \alpha_{kl}\, \delta a_l = \beta_k \tag{15.5.9}$$

This set is solved for the increments δa_l that, added to the current approximation, give the next approximation. In the context of least squares, the matrix $\boldsymbol{\alpha}$, equal to one-half times the Hessian matrix, is usually called the *curvature matrix*.

Equation (15.5.3), the steepest descent formula, translates to

$$\delta a_l = \text{constant} \times \beta_l \tag{15.5.10}$$

Note that the components α_{kl} of the Hessian matrix (15.5.7) depend both on the first derivatives and on the second derivatives of the basis functions with respect to their parameters. Some treatments proceed to ignore the second derivative without comment. We will ignore it also, but only *after* a few comments.

Second derivatives occur because the gradient (15.5.6) already has a dependence on $\partial y/\partial a_k$, and so the next derivative simply must contain terms involving $\partial^2 y/\partial a_l \partial a_k$. The second derivative term can be dismissed when it is zero (as in the linear case of equation 15.4.8) or small enough to be negligible when compared to the term involving the first derivative. It also has an additional possibility of being ignorably small in practice: The term multiplying the second derivative in equation

(15.5.7) is $[y_i - y(x_i|\mathbf{a})]$. For a successful model, this term should just be the random measurement error of each point. This error can have either sign, and should in general be uncorrelated with the model. Therefore, the second derivative terms tend to cancel out when summed over i.

Inclusion of the second derivative term can in fact be destabilizing if the model fits badly or is contaminated by outlier points that are unlikely to be offset by compensating points of opposite sign. From this point on, we will always use as the definition of α_{kl} the formula

$$\alpha_{kl} = \sum_{i=0}^{N-1} \frac{1}{\sigma_i^2} \left[\frac{\partial y(x_i|\mathbf{a})}{\partial a_k} \frac{\partial y(x_i|\mathbf{a})}{\partial a_l} \right] \tag{15.5.11}$$

This expression more closely resembles its linear cousin (15.4.8). You should understand that minor (or even major) fiddling with $\boldsymbol{\alpha}$ has no effect at all on what final set of parameters $\mathbf{a}$ is reached, but affects only the iterative route that is taken in getting there. The condition at the χ^2 minimum, that $\beta_k = 0$ for all k, is independent of how $\boldsymbol{\alpha}$ is defined.

15.5.2 Levenberg-Marquardt Method

Marquardt [1] put forth an elegant method, related to an earlier suggestion of Levenberg, for varying smoothly between the extremes of the inverse-Hessian method (15.5.9) and the steepest descent method (15.5.10). The latter method is used far from the minimum, switching continuously to the former as the minimum is approached. This *Levenberg-Marquardt method* (also called the *Marquardt method*) works very well in practice if you can guess plausible starting guesses for your parameters. It has become a standard nonlinear least-squares routine.

The method is based on two elementary, but important, insights. Consider the "constant" in equation (15.5.10). What should it be, even in order of magnitude? What sets its scale? There is no information about the answer in the gradient. That tells only the slope, not how far that slope extends. Marquardt's first insight is that the components of the Hessian matrix, even if they are not usable in any precise fashion, give *some* information about the order-of-magnitude scale of the problem.

The quantity χ^2 is nondimensional, i.e., is a pure number; this is evident from its definition (15.5.5). On the other hand, β_k has the dimensions of $1/a_k$, which may well be dimensional, i.e., have units like cm^{-1}, or kilowatt-hours, or whatever. (In fact, each component of β_k can have different dimensions!) The constant of proportionality between β_k and δa_k must therefore have the dimensions of a_k^2. Scan the components of $\boldsymbol{\alpha}$ and you see that there is only one obvious quantity with these dimensions, and that is $1/\alpha_{kk}$, the reciprocal of the diagonal element. So that must set the scale of the constant. But that scale might itself be too big. So let's divide the constant by some (nondimensional) fudge factor λ, with the possibility of setting $\lambda \gg 1$ to cut down the step. In other words, replace equation (15.5.10) by

$$\delta a_l = \frac{1}{\lambda \alpha_{ll}} \beta_l \qquad \text{or} \qquad \lambda\, \alpha_{ll}\, \delta a_l = \beta_l \tag{15.5.12}$$

It is necessary that α_{ll} be positive, but this is guaranteed by definition (15.5.11) — another reason for adopting that equation.

Marquardt's second insight is that equations (15.5.12) and (15.5.9) can be combined if we define a new matrix α' by the following prescription:

$$\begin{aligned} \alpha'_{jj} &\equiv \alpha_{jj}(1+\lambda) \\ \alpha'_{jk} &\equiv \alpha_{jk} \qquad (j \neq k) \end{aligned} \tag{15.5.13}$$

and then replace both (15.5.12) and (15.5.9) by

$$\sum_{l=0}^{M-1} \alpha'_{kl}\, \delta a_l = \beta_k \tag{15.5.14}$$

When λ is very large, the matrix α' is forced into being *diagonally dominant*, so equation (15.5.14) goes over to be identical to (15.5.12). On the other hand, as λ approaches zero, equation (15.5.14) goes over to (15.5.9).

Given an initial guess for the set of fitted parameters $\mathbf{a}$, the recommended Marquardt recipe is as follows:

- Compute $\chi^2(\mathbf{a})$.
- Pick a modest value for λ, say $\lambda = 0.001$.
- (†) Solve the linear equations (15.5.14) for $\delta\mathbf{a}$ and evaluate $\chi^2(\mathbf{a}+\delta\mathbf{a})$.
- If $\chi^2(\mathbf{a}+\delta\mathbf{a}) \geq \chi^2(\mathbf{a})$, *increase* λ by a factor of 10 (or any other substantial factor) and go back to (†).
- If $\chi^2(\mathbf{a}+\delta\mathbf{a}) < \chi^2(\mathbf{a})$, *decrease* λ by a factor of 10, update the trial solution $\mathbf{a} \leftarrow \mathbf{a}+\delta\mathbf{a}$, and go back to (†).

Also necessary is a condition for stopping. Iterating to convergence (to machine accuracy or to the roundoff limit) is generally wasteful and unnecessary since the minimum is at best only a statistical estimate of the parameters $\mathbf{a}$. As we will see in §15.6, a change in the parameters that changes χ^2 by an amount $\ll 1$ is *never* statistically meaningful.

Furthermore, it is not uncommon to find the parameters wandering around near the minimum in a flat valley of complicated topography. The reason is that Marquardt's method generalizes the method of normal equations (§15.4); hence it has the same problem as that method with regard to near-degeneracy of the minimum. Outright failure by a zero pivot is possible, but unlikely. More often, a small pivot will generate a large correction that is then rejected, the value of λ being then increased. For sufficiently large λ, the matrix $\boldsymbol{\alpha}'$ is positive-definite and can have no small pivots. Thus the method does tend to stay away from zero pivots, but at the cost of a tendency to wander around doing steepest descent in very unsteep degenerate valleys.

These considerations suggest that, in practice, one might as well stop iterating after a few occurrences of χ^2 decreasing by a negligible amount, say either less than 0.001 absolutely or (in case roundoff prevents that being reached) fractionally. Don't stop after a step where χ^2 *increases* more than trivially: That only shows that λ has not yet adjusted itself optimally.

Once the acceptable minimum has been found, one wants to set $\lambda = 0$ and compute the matrix

$$\mathbf{C} \equiv \boldsymbol{\alpha}^{-1} \tag{15.5.15}$$

which, as before, is the estimated covariance matrix of the standard errors in the fitted parameters $\mathbf{a}$ (see next section).

The following object, Fitmrq, implements Marquardt's method for nonlinear parameter estimation. The user interface is intentionally very close to that of Fitlin in §15.4. In particular, the feature of being able to freeze or unfreeze chosen parameters is available here, too.

One difference from Fitlin is that you have to supply an initial guess for the parameters **a**. Now *that* is a can of worms! When you are fitting for parameters that enter highly nonlinearly, there is no reason in the world that the χ^2 surface should have only a single minimum. Marquardt's method embodies no magical insight into finding the global minimum; it's just a downhill search. Often, it should be the endgame strategy for fitting parameters, preceded by perhaps cruder, and likely problem-specific, methods for getting into the right general basin of convergence.

Another difference between Fitmrq and Fitlin is the format of the user-supplied function funks. Since Fitmrq needs both function and gradient values, funks is now coded as a void function returning answers through arguments passed by reference. An example is given below. You call Fitmrq's constructor once, to bind your data vectors and function. Then (after any optional calls to hold or free) you call fit, which sets values for a, chisq, and covar. The curvature matrix alpha is also available. Note that the original vector of parameter guesses that you send to the constructor is not modified; rather, the answer is returned in a.

fitmrq.h

```
struct Fitmrq {
```
Object for nonlinear least-squares fitting by the Levenberg-Marquardt method, also including the ability to hold specified parameters at fixed, specified values. Call constructor to bind data vectors and fitting functions and to input an initial parameter guess. Then call any combination of hold, free, and fit as often as desired. fit sets the output quantities a, covar, alpha, and chisq.
```
    static const Int NDONE=4, ITMAX=1000;       Convergence parameters.
    Int ndat, ma, mfit;
    VecDoub_I &x,&y,&sig;
    Doub tol;
    void (*funcs)(const Doub, VecDoub_I &, Doub &, VecDoub_O &);
    VecBool ia;
    VecDoub a;                  Output values. a is the vector of fitted coefficients,
    MatDoub covar;                 covar is its covariance matrix, alpha is the cur-
    MatDoub alpha;                 vature matrix, and chisq is the value of χ^2 for
    Doub chisq;                    the fit.

    Fitmrq(VecDoub_I &xx, VecDoub_I &yy, VecDoub_I &ssig, VecDoub_I &aa,
    void funks(const Doub, VecDoub_I &, Doub &, VecDoub_O &), const Doub
    TOL=1.e-3) : ndat(xx.size()), ma(aa.size()), x(xx), y(yy), sig(ssig),
    tol(TOL), funcs(funks), ia(ma), alpha(ma,ma), a(aa), covar(ma,ma) {
```
Constructor. Binds references to the data arrays xx, yy, and ssig, and to a user-supplied function funks that calculates the nonlinear fitting function and its derivatives. Also inputs the initial parameters guess aa (which is copied, not modified) and an optional convergence tolerance TOL. Initializes all parameters as free (not held).
```
        for (Int i=0;i<ma;i++) ia[i] = true;
    }

    void hold(const Int i, const Doub val) {ia[i]=false; a[i]=val;}
    void free(const Int i) {ia[i]=true;}
```
Optional functions for holding a parameter, identified by a value i in the range 0,...,ma-1, fixed at the value val, or for freeing a parameter that was previously held fixed. hold and free may be called for any number of parameters before calling fit to calculate best-fit values for the remaining (not held) parameters, and the process may be repeated multiple times.
```
    void fit() {
```

Iterate to reduce the χ^2 of a fit between a set of data points x[0..ndat-1], y[0..ndat-1] with individual standard deviations sig[0..ndat-1], and a nonlinear function that depends on ma coefficients a[0..ma-1]. When χ^2 is no longer decreasing, set best-fit values for the parameters a[0..ma-1], and chisq = χ^2, covar[0..ma-1][0..ma-1], and alpha[0..ma-1][0..ma-1]. (Parameters held fixed will return zero covariances.)

```
    Int j,k,l,iter,done=0;
    Doub alamda=.001,ochisq;
    VecDoub atry(ma),beta(ma),da(ma);
    mfit=0;
    for (j=0;j<ma;j++) if (ia[j]) mfit++;
    MatDoub oneda(mfit,1), temp(mfit,mfit);
    mrqcof(a,alpha,beta);             Initialization.
    for (j=0;j<ma;j++) atry[j]=a[j];
    ochisq=chisq;
    for (iter=0;iter<ITMAX;iter++) {
        if (done==NDONE) alamda=0.;          Last pass. Use zero alamda.
        for (j=0;j<mfit;j++) {        Alter linearized fitting matrix, by augmenting di-
            for (k=0;k<mfit;k++) covar[j][k]=alpha[j][k];    agonal elements.
            covar[j][j]=alpha[j][j]*(1.0+alamda);
            for (k=0;k<mfit;k++) temp[j][k]=covar[j][k];
            oneda[j][0]=beta[j];
        }
        gaussj(temp,oneda);           Matrix solution.
        for (j=0;j<mfit;j++) {
            for (k=0;k<mfit;k++) covar[j][k]=temp[j][k];
            da[j]=oneda[j][0];
        }
        if (done==NDONE) {            Converged. Clean up and return.
            covsrt(covar);
            covsrt(alpha);
            return;
        }
        for (j=0,l=0;l<ma;l++)        Did the trial succeed?
            if (ia[l]) atry[l]=a[l]+da[j++];
        mrqcof(atry,covar,da);
        if (abs(chisq-ochisq) < MAX(tol,tol*chisq)) done++;
        if (chisq < ochisq) {         Success, accept the new solution.
            alamda *= 0.1;
            ochisq=chisq;
            for (j=0;j<mfit;j++) {
                for (k=0;k<mfit;k++) alpha[j][k]=covar[j][k];
                    beta[j]=da[j];
            }
            for (l=0;l<ma;l++) a[l]=atry[l];
        } else {                      Failure, increase alamda.
            alamda *= 10.0;
            chisq=ochisq;
        }
    }
    throw("Fitmrq too many iterations");
}
```

```
void mrqcof(VecDoub_I &a, MatDoub_O &alpha, VecDoub_O &beta) {
```

Used by fit to evaluate the linearized fitting matrix alpha, and vector beta as in (15.5.8), and to calculate χ^2.

```
    Int i,j,k,l,m;
    Doub ymod,wt,sig2i,dy;
    VecDoub dyda(ma);
    for (j=0;j<mfit;j++) {            Initialize (symmetric) alpha, beta.
        for (k=0;k<=j;k++) alpha[j][k]=0.0;
        beta[j]=0.;
    }
```

```
        chisq=0.;
        for (i=0;i<ndat;i++) {          Summation loop over all data.
            funcs(x[i],a,ymod,dyda);
            sig2i=1.0/(sig[i]*sig[i]);
            dy=y[i]-ymod;
            for (j=0,l=0;l<ma;l++) {
                if (ia[l]) {
                    wt=dyda[l]*sig2i;
                    for (k=0,m=0;m<l+1;m++)
                        if (ia[m]) alpha[j][k++] += wt*dyda[m];
                    beta[j++] += dy*wt;
                }
            }
            chisq += dy*dy*sig2i;       And find χ².
        }
        for (j=1;j<mfit;j++)            Fill in the symmetric side.
            for (k=0;k<j;k++) alpha[k][j]=alpha[j][k];
    }

    void covsrt(MatDoub_IO &covar) {
```

Expand in storage the covariance matrix covar, so as to take into account parameters that are being held fixed. (For the latter, return zero covariances.)

```
        Int i,j,k;
        for (i=mfit;i<ma;i++)
            for (j=0;j<i+1;j++) covar[i][j]=covar[j][i]=0.0;
        k=mfit-1;
        for (j=ma-1;j>=0;j--) {
            if (ia[j]) {
                for (i=0;i<ma;i++) SWAP(covar[i][k],covar[i][j]);
                for (i=0;i<ma;i++) SWAP(covar[k][i],covar[j][i]);
                k--;
            }
        }
    }

};
```

15.5.3 Example

The following function `fgauss` is an example of a user-supplied function `funks`. Used with `Fitmrq`, it fits for the model

$$y(x) = \sum_{k=0}^{K-1} B_k \exp\left[-\left(\frac{x - E_k}{G_k}\right)^2\right] \tag{15.5.16}$$

which is a sum of K Gaussians, each with a variable position, amplitude, and width. We store the parameters in the order $B_0, E_0, G_0, B_1, E_1, G_1, \ldots, B_{K-1}, E_{K-1}, G_{K-1}$.

fit_examples.h

```
void fgauss(const Doub x, VecDoub_I &a, Doub &y, VecDoub_O &dyda) {
```

$y(x;a)$ is the sum of na/3 Gaussians (15.5.16). The amplitude, center, and width of the Gaussians are stored in consecutive locations of a: a[$3k$] $= B_k$, a[$3k+1$] $= E_k$, a[$3k+2$] $=$ G_k, $k = 0, ..., $ na$/3 - 1$. The dimensions of the arrays are a[0..na-1], dyda[0..na-1].

```
    Int i,na=a.size();
    Doub fac,ex,arg;
    y=0.;
    for (i=0;i<na-1;i+=3) {
        arg=(x-a[i+1])/a[i+2];
        ex=exp(-SQR(arg));
```

```
            fac=a[i]*ex*2.*arg;
            y += a[i]*ex;
            dyda[i]=ex;
            dyda[i+1]=fac/a[i+2];
            dyda[i+2]=fac*arg/a[i+2];
        }
}
```

15.5.4 More Advanced Methods for Nonlinear Least Squares

You will need more capability than Fitmrq can supply if either (i) it is converging too slowly, or (ii) it is converging to a local minimum that is not the one you want. Several options are available.

NL2SOL [3] is a highly regarded nonlinear least-squares implementation with many advanced features. For example, it keeps the second-derivative term we dropped in the Levenberg-Marquardt method whenever it would be better to do so, a so-called *full Newton-type* method.

A different variant on the Levenberg-Marquardt algorithm is to implement it as a model-trust region method for minimization (see §9.7 and ref. [2]) applied to the special case of a least-squares function. A code of this kind due to Moré [4] can be found in MINPACK [5].

CITED REFERENCES AND FURTHER READING:

Bevington, P.R., and Robinson, D.K. 2002, *Data Reduction and Error Analysis for the Physical Sciences*, 3rd ed. (New York: McGraw-Hill), Chapter 8.

Monahan, J.F. 2001, *Numerical Methods of Statistics* (Cambridge, UK: Cambridge University Press), Chapters 5–9.

Seber, G.A.F., and Wild, C.J. 2003, *Nonlinear Regression* (Hoboken, NJ: Wiley).

Gelman, A., Carlin, J.B., Stern, H.S., and Rubin, D.B. 2004, *Bayesian Data Analysis*, 2nd ed. (Boca Raton, FL: Chapman & Hall/CRC).

Jacobs, D.A.H. (ed.) 1977, *The State of the Art in Numerical Analysis* (London: Academic Press), Chapter III.2 (by J.E. Dennis).

Marquardt, D.W. 1963, *Journal of the Society for Industrial and Applied Mathematics*, vol. 11, pp. 431–441.[1]

Dennis, J.E., and Schnabel, R.B. 1983, *Numerical Methods for Unconstrained Optimization and Nonlinear Equations*; reprinted 1996 (Philadelphia: S.I.A.M.).[2]

Dennis, J.E., Gay, D.M, and Welsch, R.E. 1981, "An Adaptive Nonlinear Least-Squares Algorithm," *ACM Transactions on Mathematical Software*, vol. 7, pp. 348–368; *op. cit.*, pp. 369–383.[3].

Moré, J.J. 1977, in *Numerical Analysis*, Lecture Notes in Mathematics, vol. 630, G.A. Watson, ed. (Berlin: Springer), pp. 105–116.[4]

Moré, J.J., Garbow, B.S., and Hillstrom, K.E. 1980, *User Guide for MINPACK-1*, Argonne National Laboratory Report ANL-80-74.[5]

15.6 Confidence Limits on Estimated Model Parameters

Several times already in this chapter we have made statements about the standard errors, or uncertainties, in a set of M estimated parameters $\mathbf{a}$. We have given some formulas for computing standard deviations or variances of individual parameters (equations 15.2.9, 15.4.15, and 15.4.19), as well as some formulas for covariances between pairs of parameters (equation 15.2.10; remark following equation 15.4.15; equation 15.4.20; equation 15.5.15).

In this section, we want to be more explicit regarding the precise meaning of these quantitative uncertainties, and to give further information about how quantitative confidence limits on fitted parameters can be estimated. The subject can get somewhat technical, and even somewhat confusing, so we will try to make precise statements, even when they must be offered without proof.

Figure 15.6.1 shows the conceptual scheme of an experiment that "measures" a set of parameters. There is some underlying true set of parameters $\mathbf{a}_{\text{true}}$ that are known to Mother Nature but hidden from the experimenter. These true parameters are statistically realized, along with random measurement errors, as a measured data set, which we will symbolize as $\mathcal{D}_{(0)}$. The data set $\mathcal{D}_{(0)}$ *is* known to the experimenter. He or she fits the data to a model by χ^2 minimization or some other technique and obtains measured, i.e., fitted, values for the parameters, which we here denote $\mathbf{a}_{(0)}$.

Because measurement errors have a random component, $\mathcal{D}_{(0)}$ is not a unique realization of the true parameters $\mathbf{a}_{\text{true}}$. Rather, there are infinitely many other realizations of the true parameters as "hypothetical data sets" each of which *could* have been the one measured, but happened not to be. Let us symbolize these by $\mathcal{D}_{(1)}, \mathcal{D}_{(2)}, \ldots$. Each one, had it been realized, would have given a slightly different set of fitted parameters, $\mathbf{a}_{(1)}, \mathbf{a}_{(2)}, \ldots$, respectively. These parameter sets $\mathbf{a}_{(i)}$ therefore occur with some probability distribution in the M-dimensional space of all possible parameter sets $\mathbf{a}$. The actual measured set $\mathbf{a}_{(0)}$ is one member drawn from this distribution.

Even more interesting than the probability distribution of $\mathbf{a}_{(i)}$ would be the distribution of the difference $\mathbf{a}_{(i)} - \mathbf{a}_{\text{true}}$. This distribution differs from the former one by a translation that puts Mother Nature's true value at the origin. If we knew *this* distribution, we would know everything that there is to know about the quantitative uncertainties in our experimental measurement $\mathbf{a}_{(0)}$.

So the name of the game is to find some way of estimating or approximating the probability distribution of $\mathbf{a}_{(i)} - \mathbf{a}_{\text{true}}$ without knowing $\mathbf{a}_{\text{true}}$ and without having available to us an infinite universe of hypothetical data sets.

15.6.1 Monte Carlo Simulation of Synthetic Data Sets

Although the measured parameter set $\mathbf{a}_{(0)}$ is not the true one, let us consider a fictitious world in which it *was* the true one. Since we hope that our measured parameters are not *too* wrong, we hope that that fictitious world is not too different from the actual world with parameters $\mathbf{a}_{\text{true}}$. In particular, let us hope — no, let us *assume* — that the shape of the probability distribution $\mathbf{a}_{(i)} - \mathbf{a}_{(0)}$ in the fictitious world is the same, or very nearly the same, as the shape of the probability distribution

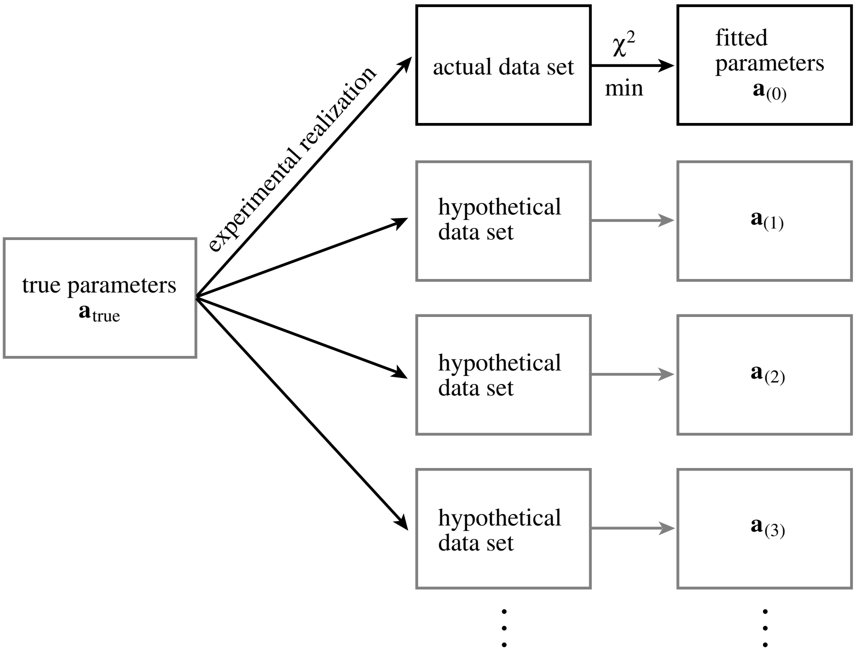

Figure 15.6.1. A statistical universe of data sets from an underlying model. True parameters $\mathbf{a}_{\text{true}}$ are realized in a data set, from which fitted (observed) parameters $\mathbf{a}_{(0)}$ are obtained. If the experiment were repeated many times, new data sets and new values of the fitted parameters would be obtained.

$\mathbf{a}_{(i)} - \mathbf{a}_{\text{true}}$ in the real world. Notice that we are not assuming that $\mathbf{a}_{(0)}$ and $\mathbf{a}_{\text{true}}$ are equal; they are certainly not. We are only assuming that the way in which random errors enter the experiment and data analysis does not vary rapidly as a function of $\mathbf{a}_{\text{true}}$, so that $\mathbf{a}_{(0)}$ can serve as a reasonable surrogate.

Now, often, the distribution of $\mathbf{a}_{(i)} - \mathbf{a}_{(0)}$ in the fictitious world *is* within our power to calculate (see Figure 15.6.2). If we know something about the process that generated our data, given an assumed set of parameters $\mathbf{a}_{(0)}$, then we can usually figure out how to *simulate* our own sets of "synthetic" realizations of these parameters as "synthetic data sets." The procedure is to draw random numbers from appropriate distributions (cf. §7.3 – §7.4) so as to mimic our best understanding of the underlying process and measurement errors in our apparatus. With such random draws, we construct data sets with exactly the same numbers of measured points, and precisely the same values of all control (independent) variables, as our actual data set $\mathcal{D}_{(0)}$. Let us call these simulated data sets $\mathcal{D}^S_{(1)}, \mathcal{D}^S_{(2)}, \dots$. By construction, these are supposed to have exactly the same statistical relationship to $\mathbf{a}_{(0)}$ as the $\mathcal{D}_{(i)}$'s have to $\mathbf{a}_{\text{true}}$. (For the case where you don't know enough about what you are measuring to do a credible job of simulating it, see below.)

Next, for each $\mathcal{D}^S_{(j)}$, perform exactly the same procedure for estimation of parameters, e.g., χ^2 minimization, as was performed on the actual data to get the parameters $\mathbf{a}_{(0)}$, giving simulated measured parameters $\mathbf{a}^S_{(1)}, \mathbf{a}^S_{(2)}, \dots$. Each simulated measured parameter set yields a point $\mathbf{a}^S_{(i)} - \mathbf{a}_{(0)}$. Simulate enough data sets and enough derived simulated measured parameters, and you map out the desired probability distribution in M dimensions.

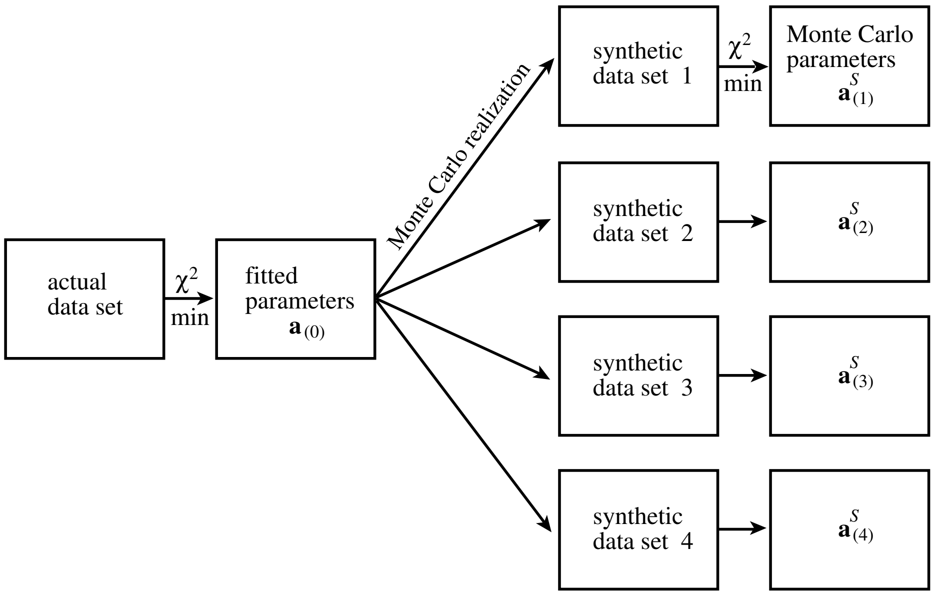

Figure 15.6.2. Monte Carlo simulation of an experiment. The fitted parameters from an actual experiment are used as surrogates for the true parameters. Computer-generated random numbers are used to simulate many synthetic data sets. Each of these is analyzed to obtain its fitted parameters. The distribution of these fitted parameters around the (known) surrogate true parameters is thus studied.

In fact, the ability to do *Monte Carlo simulations* in this fashion has revolutionized many fields of modern experimental science. Not only is one able to characterize the errors of parameter estimation in a very precise way; one can also try out on the computer different methods of parameter estimation, or different data reduction techniques, and seek to minimize the uncertainty of the result according to any desired criteria. Offered the choice between mastery of a five-foot shelf of analytical statistics books and middling ability at performing statistical Monte Carlo simulations, we would surely choose to have the latter skill.

15.6.2 Quick-and-Dirty Monte Carlo: The Bootstrap Method

Here is a powerful technique that can often be used when you don't know enough about the underlying process, or the nature of your measurement errors, to do a credible Monte Carlo simulation. Suppose that your data set consists of *N independent and identically distributed* (or *iid*) "data points." Each data point probably consists of several numbers, e.g., one or more control variables (uniformly distributed, say, in the range that you have decided to measure) and one or more associated measured values (each distributed however Mother Nature chooses). "Iid" means that the sequential order of the data points is not of consequence to the process that you are using to get the fitted parameters $\mathbf{a}$. For example, a χ^2 sum like (15.5.5) does not care in what order the points are added. Even simpler examples are the mean value of a measured quantity and the mean of some function of the measured quantities.

The *bootstrap method* [1] uses the actual data set $\mathcal{D}^S_{(0)}$, with its N data points, to generate any number of synthetic data sets $\mathcal{D}^S_{(1)}, \mathcal{D}^S_{(2)}, \ldots$, also with N data points. The procedure is simply to draw N data points at a time *with replacement* from the set $\mathcal{D}^S_{(0)}$. Because of the replacement, you do not simply get back your original data set each time. You get sets in which a random fraction of the original points, typically $\sim 1/e \approx 37\%$, are replaced by *duplicated* original points. Now, exactly as in the previous discussion, you subject these data sets to the same estimation procedure as was performed on the actual data, giving a set of simulated measured parameters $\mathbf{a}^S_{(1)}, \mathbf{a}^S_{(2)}, \ldots$. These will be distributed around $\mathbf{a}_{(0)}$ in close to the same way that $\mathbf{a}_{(0)}$ is distributed around $\mathbf{a}_{\text{true}}$.

Sounds like getting something for nothing, doesn't it? In fact, it took a while for the bootstrap method to become accepted by statisticians. By now, however, enough theorems have been proved to render the bootstrap reputable (see [2] for references). The basic idea behind the bootstrap is that the actual data set, viewed as a probability distribution consisting of delta functions at the measured values, is in most cases the best — or only — available estimator of the underlying probability distribution. It takes courage, but one can often simply use *that* distribution as the basis for Monte Carlo simulations.

Watch out for cases where the bootstrap's iid assumption is violated. For example, if you have made measurements at evenly spaced intervals of some control variable, then you can *usually* get away with pretending that these are iid uniformly distributed over the measured range. However, some estimators of $\mathbf{a}$ (e.g., ones involving Fourier methods) might be particularly sensitive to all the points on a grid being present. In that case, the bootstrap is going to give a wrong distribution. Also watch out for estimators that look at anything like small-scale clumpiness within the N data points, or estimators that sort the data and look at sequential differences. Obviously the bootstrap will fail on these, too. (The theorems justifying the method are still true, but some of their technical assumptions are violated by these examples.)

For a large class of problems, however, the bootstrap does yield easy, *very quick*, Monte Carlo estimates of the errors in an estimated parameter set.

15.6.3 Confidence Limits

Rather than present all details of the probability distribution of errors in parameter estimation, it is common practice to summarize the distribution in the form of *confidence limits*. The full probability distribution is a function defined on the M-dimensional space of parameters $\mathbf{a}$. A *confidence region* (or *confidence interval*) is just a region of that M-dimensional space (hopefully a small region) that contains a certain (hopefully large) percentage of the total probability distribution. You point to a confidence region and say, e.g., "there is a 99% chance that the true parameter values fall within this region around the measured value."

It is worth emphasizing that you, the experimenter, get to pick both the *confidence level* (99% in the above example) and the shape of the confidence region. The only requirement is that your region does include the stated percentage of probability. Certain percentages are, however, customary in scientific usage: 68.3% (the lowest confidence worthy of quoting), 90%, 95.4%, 99%, and 99.73%. Higher confidence levels are conventionally "ninety-nine point nine … nine." As for shape, obviously you want a region that is compact and reasonably centered on your mea-

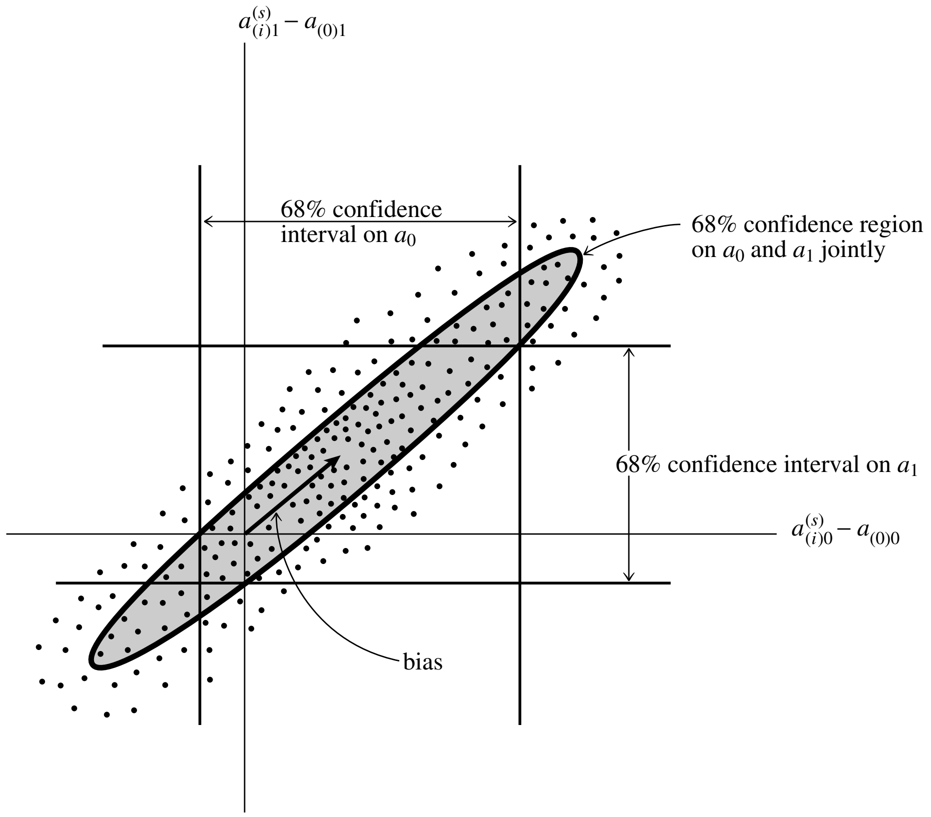

Figure 15.6.3. Confidence intervals in one and two dimensions. The same fraction of measured points (here 68%) lies (i) between the two vertical lines, (ii) between the two horizontal lines, (iii) within the ellipse.

surement $\mathbf{a}_{(0)}$, since the whole purpose of a confidence limit is to inspire confidence in that measured value. In one dimension, the convention is to use a line segment centered on the measured value; in higher dimensions, ellipses or ellipsoids are most frequently used.

You might suspect, correctly, that the numbers 68.3%, 95.4%, and 99.73%, and the use of ellipsoids, have some connection with a normal distribution. That is true historically, but not always relevant nowadays. In general, the probability distribution of the parameters will not be normal, and the above numbers, used as levels of confidence, are purely matters of convention.

Figure 15.6.3 sketches a possible probability distribution for the case $M = 2$. Shown are three different confidence regions that might usefully be given, all at the same confidence level. The two vertical lines enclose a band (horizontal interval) that represents the 68% confidence interval for the variable a_0 without regard to the value of a_1. Similarly the horizontal lines enclose a 68% confidence interval for a_1. The ellipse shows a 68% confidence interval for a_0 and a_1 jointly. Notice that to enclose the same probability as the two bands, the ellipse must necessarily extend outside of both of them (a point we will return to below).

15.6.4 Constant Chi-Square Boundaries as Confidence Limits

When the method used to estimate the parameters $\mathbf{a}_{(0)}$ is chi-square minimization, as in the previous sections of this chapter, then there is a natural choice for the shape of confidence intervals, whose use is almost universal. For the observed data set $\mathcal{D}_{(0)}$, the value of χ^2 is a minimum at $\mathbf{a}_{(0)}$. Call this minimum value $\chi^2_{\min}$. If the vector $\mathbf{a}$ of parameter values is perturbed away from $\mathbf{a}_{(0)}$, then χ^2 increases. The region within which χ^2 increases by no more than a set amount $\Delta\chi^2$ defines some M-dimensional confidence region around $\mathbf{a}_{(0)}$. If $\Delta\chi^2$ is set to be a large number, this will be a big region; if it is small, it will be small. Somewhere in between there will be choices of $\Delta\chi^2$ that cause the region to contain, variously, 68%, 90%, etc., of probability distribution for $\mathbf{a}$'s, as defined above. These regions are taken as the confidence regions for the parameters $\mathbf{a}_{(0)}$.

Very frequently one is interested not in the full M-dimensional confidence region, but in individual confidence regions for some smaller number ν of parameters. For example, one might be interested in the confidence interval of each parameter taken separately (the bands in Figure 15.6.3), in which case $\nu = 1$. In that case, the natural confidence regions in the ν-dimensional subspace of the M-dimensional parameter space are the *projections* of the M-dimensional regions defined by fixed $\Delta\chi^2$ into the ν-dimensional spaces of interest. In Figure 15.6.4, for the case $M = 2$, we show regions corresponding to several values of $\Delta\chi^2$. The one-dimensional confidence interval in a_1 corresponding to the region bounded by $\Delta\chi^2 = 1$ lies between the lines A and A'.

Note that it is the projection of the higher-dimensional region on the lower-dimension space that is used, not the intersection. The intersection would be the band between Z and Z'. It is *never* used. It is shown in the figure only for the purpose of making this cautionary point, that it should not be confused with the projection.

15.6.5 Probability Distribution of Parameters in the Normal Case

You may be wondering why we have, in this section up to now, made no connection at all with the error estimates that come out of the χ^2 fitting procedure, most notably the covariance matrix C_{ij}. The reason is this: χ^2 minimization is a useful means for estimating parameters even if the measurement errors are not normally distributed. While normally distributed errors are required if the χ^2 parameter estimate is to be a maximum likelihood estimator (§15.1), one is often willing to give up that property in return for the relative convenience of the χ^2 procedure. Only in extreme cases, i.e., measurement error distributions with very large "tails," is χ^2 minimization abandoned in favor of more robust techniques, as will be discussed in §15.7.

However, the formal covariance matrix that comes out of a χ^2 minimization has a clear quantitative interpretation only if (or to the extent that) the measurement errors actually are normally distributed. In the case of *non*normal errors, you are "allowed"

- to fit for parameters by minimizing χ^2
- to use a contour of constant $\Delta\chi^2$ as the boundary of your confidence region
- to use Monte Carlo simulation or detailed analytic calculation in determining

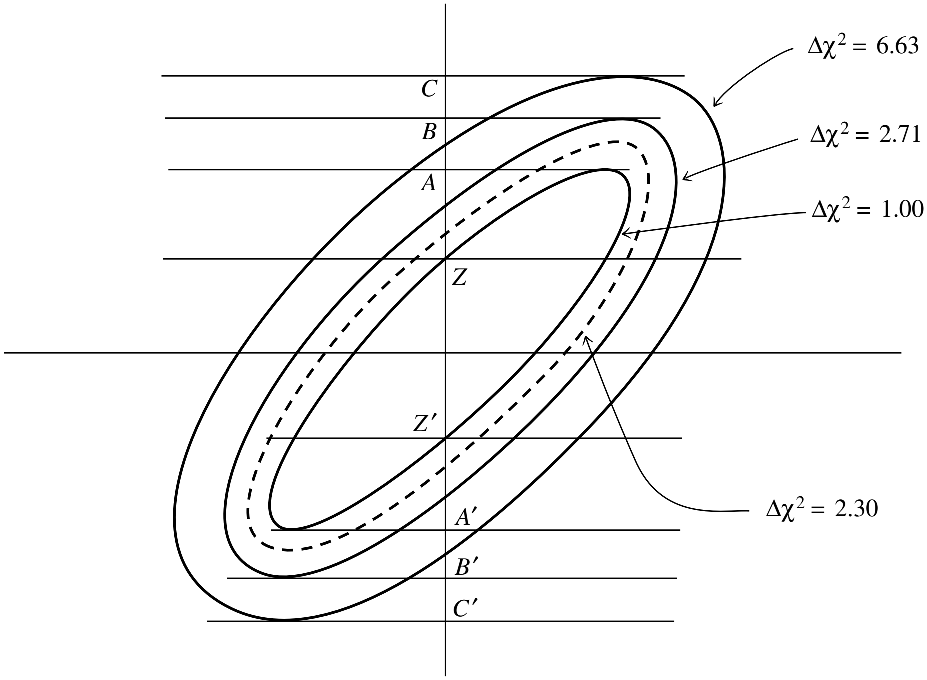

Figure 15.6.4. Confidence region ellipses corresponding to values of chi-square larger than the fitted minimum. The solid curves, with $\Delta\chi^2 = 1.00, 2.71, 6.63$, project onto one-dimensional intervals AA', BB', CC'. These intervals — not the ellipses themselves — contain 68.3%, 90%, and 99% of normally distributed data. The ellipse that contains 68.3% of normally distributed data is shown dashed and has $\Delta\chi^2 = 2.30$. For additional numerical values, see the table on p. 815.

which contour $\Delta\chi^2$ is the correct one for your desired confidence level

- to give the covariance matrix C_{ij} as the "formal covariance matrix of the fit."

You are *not* allowed

- to use formulas that we now give for the case of normal errors, which establish quantitative relationships among $\Delta\chi^2$, C_{ij}, and the confidence level.

Here are the key theorems that hold when (i) the measurement errors are normally distributed, and either (ii) the model is linear in its parameters or (iii) the sample size is large enough that the uncertainties in the fitted parameters $\mathbf{a}$ do not extend outside a region in which the model could be replaced by a suitable linearized model. [Note that condition (iii) does not preclude your use of a nonlinear routine like `Fitmrq` to *find* the fitted parameters.]

Theorem A. $\chi^2_{\min}$ is distributed as a chi-square distribution with $N - M$ degrees of freedom, where N is the number of data points and M is the number of fitted parameters. This is the basic theorem that lets you evaluate the goodness-of-fit of the model, as discussed above in §15.1. We list it first to remind you that unless the goodness-of-fit is credible, the whole estimation of parameters is suspect.

Theorem B. If $\mathbf{a}^S_{(j)}$ is drawn from the universe of simulated data sets with actual parameters $\mathbf{a}_{(0)}$, then the probability distribution of $\delta\mathbf{a} \equiv \mathbf{a}^S_{(j)} - \mathbf{a}_{(0)}$ is the multivariate normal distribution

$$P(\delta\mathbf{a})\ da_0 \ldots da_{M-1} = \text{const.} \times \exp\left(-\tfrac{1}{2}\delta\mathbf{a}\cdot\boldsymbol{\alpha}\cdot\delta\mathbf{a}\right)\ da_0 \ldots da_{M-1}$$

where $\boldsymbol{\alpha}$ is the curvature matrix defined in equation (15.5.8).

Theorem C. If $\mathbf{a}^S_{(j)}$ is drawn from the universe of simulated data sets with actual parameters $\mathbf{a}_{(0)}$, then the quantity $\Delta\chi^2 \equiv \chi^2(\mathbf{a}_{(j)}) - \chi^2(\mathbf{a}_{(0)})$ is distributed as a chi-square distribution with M degrees of freedom. Here the χ^2's are all evaluated using the fixed (actual) data set $\mathcal{D}_{(0)}$. This theorem makes the connection between particular values of $\Delta\chi^2$ and the fraction of the probability distribution that they enclose as an M-dimensional region, i.e., the confidence level of the M-dimensional confidence region.

Theorem D. Suppose that $\mathbf{a}^S_{(j)}$ is drawn from the universe of simulated data sets (as above); that its first ν components $a_0, \ldots, a_{\nu-1}$ are held fixed; and that its remaining $M - \nu$ components are varied so as to minimize χ^2. Call this minimum value χ^2_ν. Then $\Delta\chi^2_\nu \equiv \chi^2_\nu - \chi^2_{\min}$ is distributed as a chi-square distribution with ν degrees of freedom. If you consult Figure 15.6.4, you will see that this theorem connects the *projected* $\Delta\chi^2$ region with a confidence level. In the figure, a point that is held fixed in a_1 and allowed to vary in a_0 minimizing χ^2 will seek out the ellipse whose top or bottom edge is tangent to the line of constant a_1, and is therefore the line that projects it onto the smaller-dimensional space.

As a first example, let us consider the case $\nu = 1$, where we want to find the confidence interval of a single parameter, say a_0. Notice that the chi-square distribution with $\nu = 1$ degree of freedom is the same distribution as that of the square of a single normally distributed quantity. Thus $\Delta\chi^2_\nu < 1$ occurs 68.3% of the time (1-σ for the normal distribution), $\Delta\chi^2_\nu < 4$ occurs 95.4% of the time (2-σ for the normal distribution), $\Delta\chi^2_\nu < 9$ occurs 99.73% of the time (3-σ for the normal distribution), etc. In this manner you find the $\Delta\chi^2_\nu$ that corresponds to your desired confidence level. (Additional values are given in the table on the next page.)

Let $\delta\mathbf{a}$ be a change in the parameters whose first component is arbitrary, δa_0, but the rest of whose components are chosen to minimize the $\Delta\chi^2$. Then Theorem D applies. The value of $\Delta\chi^2$ is given in general by

$$\Delta\chi^2 = \delta\mathbf{a}\cdot\boldsymbol{\alpha}\cdot\delta\mathbf{a} \tag{15.6.1}$$

which follows from equation (15.5.8) applied at $\chi^2_{\min}$ where $\beta_k = 0$. Since $\delta\mathbf{a}$ by hypothesis minimizes χ^2 in all but its zeroth component, components 1 through $M-1$ of the normal equations (15.5.9) continue to hold. Therefore, the solution of (15.5.9) is

$$\delta\mathbf{a} = \boldsymbol{\alpha}^{-1}\cdot\begin{pmatrix} c \\ 0 \\ \vdots \\ 0 \end{pmatrix} = \mathbf{C}\cdot\begin{pmatrix} c \\ 0 \\ \vdots \\ 0 \end{pmatrix} \tag{15.6.2}$$

where c is one arbitrary constant that we get to adjust to make (15.6.1) give the desired left-hand value. Plugging (15.6.2) into (15.6.1) and using the fact that $\mathbf{C}$ and $\boldsymbol{\alpha}$ are inverse matrices of one another, we get

$$c = \delta a_0 / C_{00} \qquad \text{and} \qquad \Delta\chi^2_\nu = (\delta a_0)^2 / C_{00} \tag{15.6.3}$$

or

$\Delta\chi^2$ as a Function of Confidence Level p and Number of Parameters of Interest ν						
	ν					
p	1	2	3	4	5	6
68.27%	1.00	2.30	3.53	4.72	5.89	7.04
90%	2.71	4.61	6.25	7.78	9.24	10.6
95.45%	4.00	6.18	8.02	9.72	11.3	12.8
99%	6.63	9.21	11.3	13.3	15.1	16.8
99.73%	9.00	11.8	14.2	16.3	18.2	20.1
99.99%	15.1	18.4	21.1	23.5	25.7	27.9

$$\delta a_0 = \pm\sqrt{\Delta\chi^2_\nu}\,\sqrt{C_{00}} \tag{15.6.4}$$

At last! A relation between the confidence interval $\pm\delta a_0$ and the formal standard error $\sigma_0 \equiv \sqrt{C_{00}}$. Not unreasonably, we find that the 68% confidence interval is $\pm\sigma_0$, the 95% confidence interval is $\pm 2\sigma_0$, etc.

These considerations hold not just for the individual parameters a_i, but also for any linear combination of them: If

$$b \equiv \sum_{k=0}^{M-1} c_i a_i = \mathbf{c}\cdot\mathbf{a} \tag{15.6.5}$$

then the 68% confidence interval on b is

$$\delta b = \pm\sqrt{\mathbf{c}\cdot\mathbf{C}\cdot\mathbf{c}} \tag{15.6.6}$$

However, these simple, normal-sounding numerical relationships do *not* hold in the case $\nu > 1$ [3]. In particular, $\Delta\chi^2 = 1$ is not the boundary, nor does it project onto the boundary, of a 68.3% confidence region when $\nu > 1$. If you want to calculate not confidence intervals in one parameter, but confidence ellipses in two parameters jointly, or ellipsoids in three, or higher, then you must follow the following prescription for implementing Theorems C and D above:

- Let ν be the number of fitted parameters whose joint confidence region you wish to display, $\nu \le M$. Call these parameters the "parameters of interest."
- Let p be the confidence limit desired, e.g., $p = 0.68$ or $p = 0.95$.
- Find Δ (i.e., $\Delta\chi^2$) such that the probability of a chi-square variable with ν degrees of freedom being less than Δ is p. For some useful values of p and ν, Δ is given in the table above. For other values, you can use the `invcdf` method of the `Chisqdist` object in §6.14.8 with p as the argument.
- Take the $M \times M$ covariance matrix $\mathbf{C} = \boldsymbol{\alpha}^{-1}$ of the chi-square fit. Copy the intersection of the ν rows and columns corresponding to the parameters of interest into a $\nu \times \nu$ matrix denoted $\mathbf{C}_{\text{proj}}$.
- Invert the matrix $\mathbf{C}_{\text{proj}}$. (In the one-dimensional case this was just taking the reciprocal of the element C_{00}.)

- The equation for the elliptical boundary of your desired confidence region in the ν-dimensional subspace of interest is

$$\Delta = \delta\mathbf{a}' \cdot \mathbf{C}_{\text{proj}}^{-1} \cdot \delta\mathbf{a}' \tag{15.6.7}$$

where $\delta\mathbf{a}'$ is the ν-dimensional vector of parameters of interest.

If you are confused at this point, you may find it helpful to compare Figure 15.6.4 and the table on the previous page considering the case $M = 2$ with $\nu = 1$ and $\nu = 2$. You should be able to verify the following statements: (i) The horizontal band between C and C' contains 99% of the probability distribution, so it is a confidence limit on a_1 alone at this level of confidence. (ii) Ditto the band between B and B' at the 90% confidence level. (iii) The dashed ellipse, labeled by $\Delta\chi^2 = 2.30$, contains 68.3% of the probability distribution, so it is a confidence region for a_0 and a_1 jointly, at this level of confidence.

Another point of possible confusion might also be worth airing here. In §15.1.1, when we discussed the use of χ^2 as a goodness-of-fit statistic, we mentioned that a "moderately good" fit could have a χ^2 value that differed by as much as $\pm\sqrt{2\nu}$ from its expected value ν (now the total number of degrees of freedom $N - M$, not ν as used above). Indeed, the suggested tail probability that embodies this advice is $Q = 1 - \texttt{Chisqdist}(\nu)\texttt{.invcdf}\,(\chi^2)$. Yet, in the discussion above, we seem to be saying that small changes in χ^2, as little as ± 1 or ± 2.71 (see table on the previous page), are significant. Can both statements be true?

Yes. In §15.1.1 we were considering the variation in χ^2 over a population of hypothetical data sets with the same parameter values, $\mathbf{a}_{\text{true}}$ (cf. Figure 15.6.1). These values vary by typically $\pm\sqrt{2\nu}$. By contrast, in the discussion above, we took a single data set and held it fixed. We then asked, essentially as an exercise in propagation of errors, how much uncertainty in the fitted parameter values $\mathbf{a}_0$ was generated by the uncertainties in the data. One way to see that these are quite different concepts is to think about how they should each scale with N, the number of data points. As N gets large, χ^2 scales as N, while its variation over hypothetical data sets scales as $N^{1/2}$, essentially a random walk. Now imagine $\mathbf{a}$ varying around its fitted value $\mathbf{a}_0$ by a small amount, $\mathbf{a} = \mathbf{a}_0 + \delta\mathbf{a}$. The change in χ^2 scales with the number of terms in the sum, N, and quadratically with distance from the minimum,

$$\delta\chi^2 \propto N(\delta\mathbf{a})^2 \tag{15.6.8}$$

As the number of data points increases, we reasonably expect the parameters to become more accurately determined, scaling as

$$\delta\mathbf{a} \propto N^{-1/2} \tag{15.6.9}$$

Combining these two equations, we find that $\delta\chi^2$ for the minimum significant change in parameters $\delta\mathbf{a}$ scales as N^0, that is, as a constant. In fact, Theorems B and C above tell us that this is not just reasonable expectation on our part; it is actually true.

15.6.6 Confidence Limits from Singular Value Decomposition

When you have obtained your χ^2 fit by singular value decomposition (§15.4), the information about the fit's formal errors comes packaged in a somewhat different,

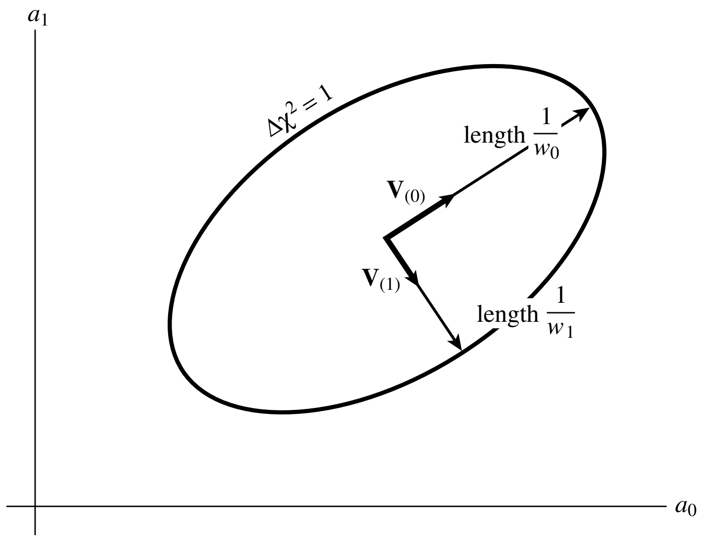

Figure 15.6.5. Relation of the confidence region ellipse $\Delta\chi^2 = 1$ to quantities computed by singular value decomposition. The vectors $\mathbf{V}_{(i)}$ are unit vectors along the principal axes of the confidence region. The semi-axes have lengths equal to the reciprocal of the singular values w_i. If the axes are all scaled by some constant factor α, $\Delta\chi^2$ is scaled by the factor α^2.

but generally more convenient, form. The columns of the matrix $\mathbf{V}$ are an orthonormal set of M vectors that are the principal axes of the $\Delta\chi^2 =$ constant ellipsoids. We denote the columns as $\mathbf{V}_{(0)} \ldots \mathbf{V}_{(M-1)}$. The lengths of those axes are inversely proportional to the corresponding singular values $w_0 \ldots w_{M-1}$; see Figure 15.6.5. The boundaries of the ellipsoids are thus given by

$$\Delta\chi^2 = w_0^2(\mathbf{V}_{(0)} \cdot \delta\mathbf{a})^2 + \cdots + w_{M-1}^2(\mathbf{V}_{(M-1)} \cdot \delta\mathbf{a})^2 \tag{15.6.10}$$

which is the justification for writing equation (15.4.18) above. Keep in mind that it is *much* easier to plot an ellipsoid given a list of its vector principal axes than given its matrix quadratic form: Loop over points $\mathbf{z}$ on a unit sphere in any desired way (e.g., by latitude and longitude) and plot the mapped points

$$\delta\mathbf{a} = \sqrt{\Delta\chi^2}\sum_i \frac{1}{w_i}(\mathbf{z} \cdot \mathbf{V}_{(i)})\mathbf{V}_{(i)} \tag{15.6.11}$$

The formula for the covariance matrix $\mathbf{C}$ in terms of the columns $\mathbf{V}_{(i)}$ is

$$\mathbf{C} = \sum_{i=0}^{M-1} \frac{1}{w_i^2}\mathbf{V}_{(i)} \otimes \mathbf{V}_{(i)} \tag{15.6.12}$$

or, in components,

$$C_{jk} = \sum_{i=0}^{M-1} \frac{1}{w_i^2} V_{ji} V_{ki} \tag{15.6.13}$$

A method for plotting error ellipses (2-dimensions) or ellipsoids (3-dimensions) from the covariance matrix $\mathbf{C}$ directly, not using its principal axes, is described in §16.1.1.

CITED REFERENCES AND FURTHER READING:

Davison, A.C., and Hinkley, D.V. 1997, *Bootstrap Methods and Their Application* (New York: Cambridge University Press).

Efron, B. 1982, *The Jackknife, the Bootstrap, and Other Resampling Plans* (Philadelphia: S.I.A.M.).[1]

Efron, B., and Tibshirani, R. 1993, *An Introduction to the Bootstrap* (Boca Raton, FL: CRC Press).[2]

Lupton, R. 1993, *Statistics in Theory and Practice* (Princeton, NJ: Princeton University Press), Chapters 10–11.

Avni, Y. 1976, "Energy Spectra of X-Ray Clusters of Galaxies," *Astrophysical Journal*, vol. 210, pp. 642–646.[3]

Lampton, M., Margon, M., and Bowyer, S. 1976, "Parameter Estimation in X-ray Astronomy," *Astrophysical Journal*, vol. 208, pp. 177–190.

Brownlee, K.A. 1965, *Statistical Theory and Methodology*, 2nd ed. (New York: Wiley).

Martin, B.R. 1971, *Statistics for Physicists* (New York: Academic Press).

15.7 Robust Estimation

The concept of *robustness* has been mentioned in passing several times already. In §14.1 we noted that the median was a more robust estimator of central value than the mean; in §14.6 it was mentioned that rank correlation is more robust than linear correlation. The concept of outlier points as exceptions to a Gaussian model for experimental error was discussed in §15.1.

The term "robust" was coined in statistics by G.E.P. Box in 1953. Various definitions of greater or lesser mathematical rigor are possible for the term, but in general, referring to a statistical estimator, it means "insensitive to small departures from the idealized assumptions for which the estimator is optimized" [1,2,3]. The word "small" can have two different interpretations, both important: either fractionally small departures for all data points, or else fractionally large departures for a small number of data points. It is the latter interpretation, leading to the notion of outlier points, that is generally the most stressful for statistical procedures.

Statisticians have developed various sorts of robust statistical estimators. Many, if not most, can be grouped into one of three categories.

M-estimates follow from maximum likelihood arguments very much as equations (15.1.6) and (15.1.8) followed from equation (15.1.3). M-estimates are usually the most relevant class for model fitting, that is, estimation of parameters. We therefore consider these estimates in some detail below.

L-estimates are "linear combinations of order statistics." These are most applicable to estimations of central value and central tendency, though they can occasionally be applied to some problems in estimation of parameters. Two "typical" L-estimates will give you the general idea. They are (i) the median, and (ii) *Tukey's trimean*, defined as the weighted average of the first, second, and third quartile points in a distribution, with weights 1/4, 1/2, and 1/4, respectively.

R-estimates are estimates based on rank tests. For example, the equality or inequality of two distributions can be estimated by the *Wilcoxon test* of computing the mean rank of one distribution in a combined sample of both distribu-

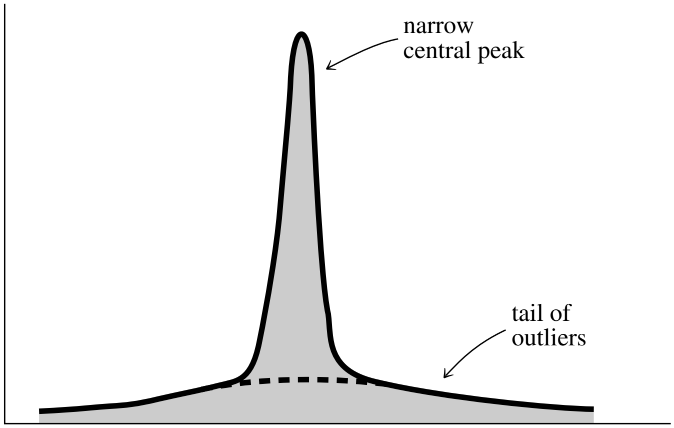

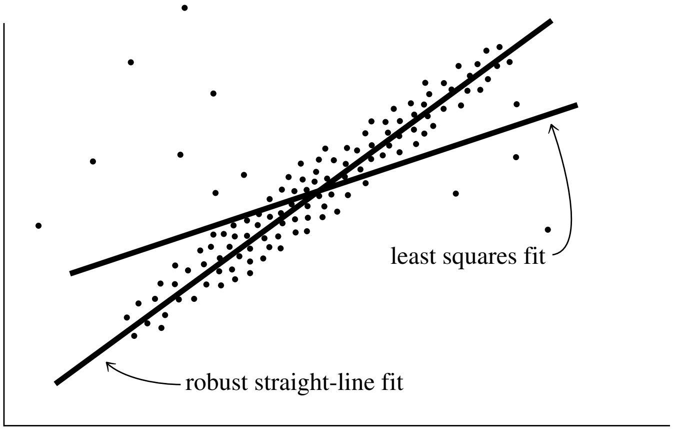

Figure 15.7.1. Examples where robust statistical methods are desirable: (a) A one-dimensional distribution with a tail of outliers; statistical fluctuations in these outliers can prevent accurate determination of the position of the central peak. (b) A distribution in two dimensions fitted to a straight line; nonrobust techniques such as least-squares fitting can have undesired sensitivity to outlying points.

tions. The Kolmogorov-Smirnov statistic (equation 14.3.17) and the Spearman rank-order correlation coefficient (14.6.1) are R-estimates in essence, if not always by formal definition.

Some other kinds of robust techniques, coming from the fields of optimal control and filtering rather than from the field of mathematical statistics, are mentioned at the end of this section. Some examples where robust statistical methods are desirable are shown in Figure 15.7.1.

15.7.1 Estimation of Parameters by Local M-Estimates

Suppose we know that our measurement errors are not normally distributed. Then, in deriving a maximum likelihood formula for the estimated parameters $\mathbf{a}$ in a model $y(x|\mathbf{a})$, we would write instead of equation (15.1.3)

$$P = \prod_{i=0}^{N-1} \{\exp[-\rho(y_i, y\{x_i|\mathbf{a}\})]\,\Delta y\} \tag{15.7.1}$$

where the function ρ is the negative logarithm of the probability density. Taking the logarithm of (15.7.1) analogously with (15.1.5), we find that we want to minimize the expression

$$\sum_{i=0}^{N-1} \rho(y_i, y\{x_i|\mathbf{a}\}) \tag{15.7.2}$$

Very often, it is the case that the function ρ depends not independently on its two arguments, measured y_i and predicted $y(x_i)$, but only on their difference, at least if scaled by some weight factors σ_i that we are able to assign to each point. In this case the M-estimate is said to be *local*, and we can replace (15.7.2) by the prescription

$$\text{minimize over } \mathbf{a} \qquad \sum_{i=0}^{N-1} \rho\left(\frac{y_i - y(x_i|\mathbf{a})}{\sigma_i}\right) \tag{15.7.3}$$

where the function $\rho(z)$ is a function of a single variable $z \equiv [y_i - y(x_i)]/\sigma_i$.

If we now define the derivative of $\rho(z)$ to be a function $\psi(z)$,

$$\psi(z) \equiv \frac{d\rho(z)}{dz} \tag{15.7.4}$$

then the generalization of (15.1.8) to the case of a general M-estimate is

$$0 = \sum_{i=0}^{N-1} \frac{1}{\sigma_i}\psi\left(\frac{y_i - y(x_i)}{\sigma_i}\right)\left(\frac{\partial y(x_i|\mathbf{a})}{\partial a_k}\right) \qquad k = 0, \ldots, M-1 \tag{15.7.5}$$

If you compare (15.7.3) to (15.1.3) and (15.7.5) to (15.1.8), you see at once that the specialization for normally distributed errors is

$$\rho(z) = \tfrac{1}{2}z^2 \qquad \psi(z) = z \qquad \text{(normal)} \tag{15.7.6}$$

If the errors are distributed as a *double* or *two-sided exponential*, namely

$$\text{Prob}\{y_i - y(x_i)\} \sim \exp\left(-\left|\frac{y_i - y(x_i)}{\sigma_i}\right|\right) \tag{15.7.7}$$

then, by contrast,

$$\rho(x) = |z| \qquad \psi(z) = \text{sgn}(z) \qquad \text{(double exponential)} \tag{15.7.8}$$

Comparing to equation (15.7.3), we see that in this case the maximum likelihood estimator is obtained by minimizing the *mean absolute deviation*, rather than the mean square deviation. Here the tails of the distribution, although exponentially decreasing, are asymptotically much larger than any corresponding Gaussian.

A distribution with even more extensive — and therefore sometimes even more realistic — tails is the *Cauchy* or *Lorentzian* distribution,

$$\text{Prob}\{y_i - y(x_i)\} \sim \frac{1}{1 + \frac{1}{2}\left(\frac{y_i - y(x_i)}{\sigma_i}\right)^2} \tag{15.7.9}$$

This implies

$$\rho(z) = \log\left(1 + \frac{1}{2}z^2\right) \qquad \psi(z) = \frac{z}{1 + \frac{1}{2}z^2} \qquad \text{(Lorentzian)} \qquad (15.7.10)$$

Notice that the ψ function occurs as a weighting function in the generalized normal equations (15.7.5). For normally distributed errors, equation (15.7.6) says that the more deviant the points, the greater the weight. By contrast, when tails are somewhat more prominent, as in (15.7.7), then (15.7.8) says that all deviant points get the same relative weight, with only the sign information used. Finally, when the tails are even larger, (15.7.10) says the ψ increases with deviation, then starts *decreasing*, so that very deviant points — the true outliers — are not counted at all in the estimation of the parameters.

This general idea, that the weight given individual points should first increase with deviation, then decrease, motivates some additional prescriptions for ψ that do not especially correspond to standard, textbook probability distributions. Two examples are

Andrew's sine

$$\psi(z) = \begin{cases} \sin(z/c) & |z| < c\pi \\ 0 & |z| > c\pi \end{cases} \qquad (15.7.11)$$

If the measurement errors happen to be normal after all, with standard deviations σ_i, then it can be shown that the optimal value for the constant c is $c = 2.1$.

Tukey's biweight

$$\psi(z) = \begin{cases} z(1 - z^2/c^2)^2 & |z| < c \\ 0 & |z| > c \end{cases} \qquad (15.7.12)$$

where the optimal value of c for normal errors is $c = 6.0$.

15.7.2 Numerical Calculation of M-Estimates

To fit a model by means of an M-estimate, you first decide which M-estimate you want, that is, which matching pair ρ, ψ you want to use. We rather like (15.7.8) or (15.7.10).

You then have to make an unpleasant choice between two fairly difficult problems. Either find the solution of the nonlinear set of M equations (15.7.5), or else minimize the single function in M variables (15.7.3).

Notice that the function (15.7.8) has a discontinuous ψ and a discontinuous derivative for ρ. Such discontinuities frequently wreak havoc on both general nonlinear equation solvers and general function minimizing routines. You might now think of rejecting (15.7.8) in favor of (15.7.10), which is smoother. However, you will find that the latter choice is also bad news for many general equation solving or minimization routines: Small changes in the fitted parameters can drive $\psi(z)$ off its peak into one or the other of its asymptotically small regimes. Therefore, different terms in the equation spring into or out of action (almost as bad as analytic discontinuities).

Don't despair. If your computer is fast enough, or if your patience is great enough, this is an excellent application for the downhill simplex minimization algorithm exemplified in `Amoeba` (§10.5) or `Amebsa` (§10.12). Those algorithms make

no assumptions about continuity; they just ooze downhill and will work for virtually any sane choice of the function ρ.

It is very much to your (patience) advantage to find good starting values, however. Often this is done by first fitting the model by the standard χ^2 (nonrobust) techniques, e.g., as described in §15.4 or §15.5. The fitted parameters thus obtained are then used as starting values in `Amoeba`, now using the robust choice of ρ and minimizing the expression (15.7.3).

15.7.3 Fitting a Line by Minimizing Absolute Deviation

Occasionally there is a special case that happens to be much easier than is suggested by the general strategy outlined above. The case of equations (15.7.7) – (15.7.8), when the model is a simple straight line,

$$y(x|a,b) = a + bx \tag{15.7.13}$$

and where the weights σ_i are all equal, happens to be such a case. The problem is precisely the robust version of the problem posed in equation (15.2.1) above, namely fit a straight line through a set of data points. The merit function to be minimized is

$$\sum_{i=0}^{N-1} |y_i - a - bx_i| \tag{15.7.14}$$

rather than the χ^2 given by equation (15.2.2).

The key simplification is based on the following fact: The median c_M of a set of numbers c_i is also the value that minimizes the sum of the absolute deviations

$$\sum_i |c_i - c_M|$$

(Proof: Differentiate the above expression with respect to c_M and set it to zero.)

It follows that, for fixed b, the value of a that minimizes (15.7.14) is

$$a = \text{median}\,\{y_i - bx_i\} \tag{15.7.15}$$

Equation (15.7.5) for the parameter b is

$$0 = \sum_{i=0}^{N-1} x_i \,\text{sgn}(y_i - a - bx_i) \tag{15.7.16}$$

(where sgn(0) is to be interpreted as zero). If we replace a in this equation by the implied function $a(b)$ of (15.7.15), then we are left with an equation in a single variable that can be solved by bracketing and bisection, as described in §9.1. (In fact, it is dangerous to use any fancier method of root finding, because of the discontinuities in equation 15.7.16.)

Here is an object that does all this. It calls `select` (§8.5) to find the median. The bracketing and bisection are built into the routine, as is the linear fit that generates the initial guesses for a and b.

fitmed.h

```
struct Fitmed {
```
Object for fitting a straight line $y = a + bx$ to a set of points (x_i, y_i), by the criterion of least absolute deviations. Call the constructor to calculate the fit. The answers are then available as the variables a, b, and abdev (the mean absolute deviation of the points from the line).

```
    Int ndata;
    Doub a, b, abdev;                                    Answers.
    VecDoub_I &x, &y;

    Fitmed(VecDoub_I &xx, VecDoub_I &yy) : ndata(xx.size()), x(xx), y(yy) {
```
Constructor. Given a set of data points xx[0..ndata-1], yy[0..ndata-1], sets a, b, and abdev.

```
        Int j;
        Doub b1,b2,del,f,f1,f2,sigb,temp;
        Doub sx=0.0,sy=0.0,sxy=0.0,sxx=0.0,chisq=0.0;
        for (j=0;j<ndata;j++) {          As a first guess for a and b, we will find the
            sx += x[j];                      least-squares fitting line.
            sy += y[j];
            sxy += x[j]*y[j];
            sxx += SQR(x[j]);
        }
        del=ndata*sxx-sx*sx;
        a=(sxx*sy-sx*sxy)/del;           Least-squares solutions.
        b=(ndata*sxy-sx*sy)/del;
        for (j=0;j<ndata;j++)
            chisq += (temp=y[j]-(a+b*x[j]),temp*temp);
        sigb=sqrt(chisq/del);            The standard deviation will give some idea of
        b1=b;                                how big an iteration step to take.
        f1=rofunc(b1);
        if (sigb > 0.0) {
            b2=b+SIGN(3.0*sigb,f1);      Guess bracket as 3-σ away, in the downhill di-
            f2=rofunc(b2);                   rection known from f1.
            if (b2 == b1) {
                abdev /= ndata;
                return;
            }
            while (f1*f2 > 0.0) {        Bracketing.
                b=b2+1.6*(b2-b1);
                b1=b2;
                f1=f2;
                b2=b;
                f2=rofunc(b2);
            }
            sigb=0.01*sigb;
            while (abs(b2-b1) > sigb) {
                b=b1+0.5*(b2-b1);        Bisection.
                if (b == b1 || b == b2) break;
                f=rofunc(b);
                if (f*f1 >= 0.0) {
                    f1=f;
                    b1=b;
                } else {
                    f2=f;
                    b2=b;
                }
            }
        }
        abdev /= ndata;
    }

    Doub rofunc(const Doub b) {
```
Evaluates the right-hand side of equation (15.7.16) for a given value of b.

```
        const Doub EPS=numeric_limits<Doub>::epsilon();
        Int j;
        Doub d,sum=0.0;
```

```
        VecDoub arr(ndata);
        for (j=0;j<ndata;j++) arr[j]=y[j]-b*x[j];
        if ((ndata & 1) == 1) {
            a=select((ndata-1)>>1,arr);
        } else {
            j=ndata >> 1;
            a=0.5*(select(j-1,arr)+select(j,arr));
        }
        abdev=0.0;
        for (j=0;j<ndata;j++) {
            d=y[j]-(b*x[j]+a);
            abdev += abs(d);
            if (y[j] != 0.0) d /= abs(y[j]);
            if (abs(d) > EPS) sum += (d >= 0.0 ? x[j] : -x[j]);
        }
        return sum;
    }
};
```

15.7.4 Other Robust Techniques

Sometimes you may have a priori knowledge about the probable values and probable uncertainties of some parameters that you are trying to estimate from a data set. In such cases you may want to perform a fit that takes this advance information properly into account, neither completely freezing a parameter at a predetermined value (as in `Fitlin` §15.4) nor completely leaving it to be determined by the data set. The formalism for doing this is called "use of a priori covariances."

A related problem occurs in signal processing and control theory, where it is sometimes desired to "track" (i.e., maintain an estimate of) a time-varying signal in the presence of noise. If the signal is known to be characterized by some number of parameters that vary only slowly, then the formalism of *Kalman filtering* tells how the incoming raw measurements of the signal should be processed to produce best parameter estimates as a function of time. For example, if the signal is a frequency-modulated sine wave, then the slowly varying parameter might be the instantaneous frequency. The Kalman filter for this case is called a *phase-locked loop* and is implemented in the circuitry of modern radio receivers [4,5].

CITED REFERENCES AND FURTHER READING:

Huber, P.J. 1981, *Robust Statistics* (New York: Wiley).[1]

Maronna, R., Martin, D., and Yohai, V. 2006, *Robust Statistics: Theory and Methods* (Hoboken, NJ: Wiley).[2]

Launer, R.L., and Wilkinson, G.N. (eds.) 1979, *Robustness in Statistics* (New York: Academic Press).[3]

Sayed, A.H. 2003, *Fundamentals of Adaptive Filtering* (New York: Wiley-IEEE).[4]

Harvey, A.C. 1989, *Forecasting, Structural Time Series Models and the Kalman Filter* (Cambridge, UK: Cambridge University Press).[5]

15.8 Markov Chain Monte Carlo

In this section and the next we redress somewhat the imbalance, at this point, between frequentist and Bayesian methods of modeling. Like Monte Carlo integra-

tion, *Markov chain Monte Carlo* or *MCMC* is a random sampling method. Unlike Monte Carlo integration, however, the goal of MCMC is not to sample a multidimensional region uniformly. Rather, the goal is to visit a point $\mathbf{x}$ with a probability proportional to some given distribution function $\pi(\mathbf{x})$. The distribution $\pi(\mathbf{x})$ is not quite a probability, because it is not necessarily normalized to have unity integral over the sampled region; but it is proportional to a probability.

Why would we want to sample a distribution in this way? The answer is that Bayesian methods, often implemented using MCMC, provide a powerful way of estimating the parameters of a model and their degree of uncertainty. A typical case is that there is a given set of data $\mathbf{D}$, and that we are able to calculate the probability of the data set *given* the values of the model parameters $\mathbf{x}$, that is, $P(\mathbf{D}|\mathbf{x})$. If we assume a prior $P(\mathbf{x})$, then Bayes' theorem says that the (posterior) probability of the model is proportional to $\pi(\mathbf{x}) \equiv P(\mathbf{D}|\mathbf{x})P(\mathbf{x})$, but with an unknown normalizing constant. Because of this unknown constant, $\pi(\mathbf{x})$ is not a normalized probability density. But if we can sample from it, we can estimate any quantity of interest, for example its mean or variance. Indeed, we can readily recover a normalized probability density by observing how often we sample a given volume $d\mathbf{x}$. Often even more useful, we can observe the distribution of any single component or set of components of the vector $\mathbf{x}$, equivalent to *marginalizing* (i.e., integrating over) the other components.

We could in principle obtain all the same information by ordinary Monte Carlo integration over the region of interest, computing the value of $\pi(\mathbf{x}_i)$ at every (uniformly) sampled point $\mathbf{x}_i$. The huge advantage of MCMC is that it "automatically" puts its sample points preferentially where $\pi(\mathbf{x})$ is large (in fact, in direct proportion). In a high-dimensional space, or where $\pi(\mathbf{x})$ is expensive to compute, this can be advantageous by many orders of magnitude.

Two insights, originally due to Metropolis and colleagues in the early 1950s, lead to feasible MCMC methods. The first is the idea that we should try to sample $\pi(\mathbf{x})$ not via unrelated, independent points, but rather by a *Markov chain*, a sequence of points $\mathbf{x}_0, \mathbf{x}_1, \mathbf{x}_2, \ldots$ that, while locally correlated, can be shown to eventually visit every point $\mathbf{x}$ in proportion to $\pi(\mathbf{x})$, the *ergodic* property. Here the word "Markov" means that each point $\mathbf{x}_i$ is chosen from a distribution that depends only on the value of the immediately preceding point $\mathbf{x}_{i-1}$. In other words, the chain has memory extending only to one previous point and is completely defined by a transition probability function of two variables $p(\mathbf{x}_i|\mathbf{x}_{i-1})$, the probability with which $\mathbf{x}_i$ is picked given a previous point $\mathbf{x}_{i-1}$.

The second insight is that if $p(\mathbf{x}_i|\mathbf{x}_{i-1})$ is chosen to satisfy the *detailed balance equation*,

$$\pi(\mathbf{x}_1)p(\mathbf{x}_2|\mathbf{x}_1) = \pi(\mathbf{x}_2)p(\mathbf{x}_1|\mathbf{x}_2) \tag{15.8.1}$$

then (up to some technical conditions) the Markov chain will in fact sample $\pi(\mathbf{x})$ ergodically. This amazing fact is worthy of some contemplation. Equation (15.8.1) expresses the idea of *physical equilibrium* in the reversible transition

$$\mathbf{x}_1 \longleftrightarrow \mathbf{x}_2 \tag{15.8.2}$$

That is, if $\mathbf{x}_1$ and $\mathbf{x}_2$ occur in proportion to $\pi(\mathbf{x}_1)$ and $\pi(\mathbf{x}_2)$, respectively, then the overall transition rates in each direction, each the product of a population density and a transition probability, are the same. To see that this might have something to do with the Markov chain being ergodic, integrate both sides of equation (15.8.1) with

respect to $\mathbf{x}_1$:

$$\int p(\mathbf{x}_2|\mathbf{x}_1)\pi(\mathbf{x}_1)\,d\mathbf{x}_1 = \pi(\mathbf{x}_2)\int p(\mathbf{x}_1|\mathbf{x}_2)\,d\mathbf{x}_1 = \pi(\mathbf{x}_2) \tag{15.8.3}$$

The left-hand side of equation (15.8.3) is the probability of $\mathbf{x}_2$, computed by integrating over all possible values of $\mathbf{x}_1$ with the corresponding transition probability. The right-hand side is seen to be the desired $\pi(\mathbf{x}_2)$. So equation (15.8.3) says that if $\mathbf{x}_1$ is drawn from π, then *so is its successor* in the Markov chain, $\mathbf{x}_2$.

We also need to show that the equilibrium distribution is rapidly approached from any starting point $\mathbf{x}_0$. While the formal proof is beyond our scope, a heuristic proof is to recognize that, because of ergodicity, even very unlikely values $\mathbf{x}_0$ will be visited by the equilibrium Markov chain once in a great while. Since the chain has no past memory, choosing any such point as a starting point $\mathbf{x}_0$ is equivalent to just picking up the equilibrium distribution chain at that particular point in time, q.e.d. In practice we need to recognize that when we start from a very unlikely point, successor points will themselves be quite unlikely until we rejoin a more probable part of the distribution. There is thus a need to *burn-in* an MCMC chain by stepping through, and discarding, a certain number of points $\mathbf{x}_i$. Below, we discuss how to determine the length of the burn-in.

We can gain a better understanding the nature of the approach to π using concepts from §11.0 and (in the next chapter) §16.3. Heuristically, let us pretend that the states x_i are discrete. Then $p(x_j|x_i) \equiv P_{ij}$ is a transition matrix satisfying equation (16.3.1). The discussion following equation (16.3.4) shows that the matrix $\mathbf{P}^T$ must have at least one unity eigenvalue. In fact, the vector $\boldsymbol{\pi}$ (the discrete form of the distribution $\pi(\mathbf{x})$) is an eigenvector of $\mathbf{P}^T$ with unity eigenvalue, by equation (15.8.3).

Can there be eigenvalues with magnitude greater than unity? No. Suppose to the contrary that $\lambda > 1$ is the largest eigenvalue, with eigenvector $\mathbf{v}$. Then, repeatedly applying $\mathbf{P}^T$,

$$\lim_{n\to\infty} \quad (\mathbf{P}^T)^n \cdot \mathbf{v} = \lambda^n \mathbf{v} \to \infty \times \mathbf{v} \tag{15.8.4}$$

Any starting distribution that contains even a tiny piece of $\mathbf{v}$ (always possible to arrange) will be driven to have values either < 0 or > 1, which is impossible. Hence it must be that $\lambda \le 1$.

From an arbitrary starting distribution $\mathbf{u}$, repeated steps of $\mathbf{P}^T$ must thus converge to $\boldsymbol{\pi}$ geometrically, with a rate that is asymptotically the magnitude of the second-largest eigenvalue, which will be < 1 if $\boldsymbol{\pi}$ is the unique equilibrium distribution. If the second eigenvalue is small, the distribution $p(x_j|x_i)$ is said to be *rapidly mixing*.

Obviously missing from this discussion, and beyond our scope, is a discussion of degenerate eigenvalues (related to the question of uniqueness) and a continuous, rather than discrete, treatment. In practice, one rarely knows enough about $\mathbf{P}$ to compute useful bounds on the second eigenvalue a priori.

15.8.1 Metropolis-Hastings Algorithm

Unless we can find a transition probability function $p(\mathbf{x}_2|\mathbf{x}_1)$ that satisfies the detailed balance equation (15.8.1), we have no way to proceed. Luckily, Hastings [1], generalizing Metropolis' work, has given a very general prescription:

Pick a *proposal distribution* $q(\mathbf{x}_2|\mathbf{x}_1)$. This can be pretty much anything you want, as long as a succession of steps generated by it can, in principle, reach everywhere in the region of interest. For example, $q(\mathbf{x}_2|\mathbf{x}_1)$ might be a multivariate normal distribution centered on $\mathbf{x}_1$.

Now, to generate a step starting at $\mathbf{x}_1$, first generate a *candidate point* $\mathbf{x}_{2c}$ by drawing from the proposal distribution. Second, calculate an *acceptance probability* $\alpha(\mathbf{x}_1, \mathbf{x}_{2c})$ by the formula

$$\alpha(\mathbf{x}_1, \mathbf{x}_{2c}) = \min\left(1, \frac{\pi(\mathbf{x}_{2c})\, q(\mathbf{x}_1|\mathbf{x}_{2c})}{\pi(\mathbf{x}_1)\, q(\mathbf{x}_{2c}|\mathbf{x}_1)}\right) \tag{15.8.5}$$

Finally, with probability $\alpha(\mathbf{x}_1, \mathbf{x}_{2c})$, accept the candidate point and set $\mathbf{x}_2 = \mathbf{x}_{2c}$; otherwise reject it and leave the point unchanged (that is, $\mathbf{x}_2 = \mathbf{x}_1$). The net result of this process is a transition probability,

$$p(\mathbf{x}_2|\mathbf{x}_1) = q(\mathbf{x}_2|\mathbf{x}_1)\,\alpha(\mathbf{x}_1, \mathbf{x}_2), \qquad (\mathbf{x}_2 \neq \mathbf{x}_1) \tag{15.8.6}$$

To see how this satisfies detailed balance, first multiply equation (15.8.5) by the denominator in the second argument of the min function. Then write down the identical equation, but exchange $\mathbf{x}_1$ and $\mathbf{x}_2$. From these pieces, one writes,

$$\begin{aligned}\pi(\mathbf{x}_1)\, q(\mathbf{x}_2|\mathbf{x}_1)\, \alpha(\mathbf{x}_1, \mathbf{x}_2) &= \min[\pi(\mathbf{x}_1)\, q(\mathbf{x}_2|\mathbf{x}_1),\ \pi(\mathbf{x}_2)\, q(\mathbf{x}_1|\mathbf{x}_2)] \\ &= \min[\pi(\mathbf{x}_2)\, q(\mathbf{x}_1|\mathbf{x}_2),\ \pi(\mathbf{x}_1)\, q(\mathbf{x}_2|\mathbf{x}_1)] \\ &= \pi(\mathbf{x}_2)\, q(\mathbf{x}_1|\mathbf{x}_2)\, \alpha(\mathbf{x}_2, \mathbf{x}_1)\end{aligned} \tag{15.8.7}$$

which, using equation (15.8.6), can be seen to be exactly the detailed balance equation (15.8.1).

It is often possible to choose the proposal distribution $q(\mathbf{x}_2|\mathbf{x}_1)$ in such a way as to simplify equation (15.8.5). For example, if $q(\mathbf{x}_2|\mathbf{x}_1)$ depends only on the absolute difference $|\mathbf{x}_1 - \mathbf{x}_2|$, as in the case of a normal distribution with fixed covariance, then the ratio $q(\mathbf{x}_1|\mathbf{x}_{2c})/q(\mathbf{x}_{2c}|\mathbf{x}_1)$ is just 1. Another case that occurs frequently is when, for some component x of $\mathbf{x}$, $q(x_{2c}|x_1)$ is lognormally distributed with a mode at x_1. In that case the ratio for this component is x_{2c}/x_1 (cf. equation 6.14.31).

15.8.2 Gibbs Sampler

An important special case of the Metropolis-Hastings algorithm is the *Gibbs sampler*. (Historically, the Gibbs sampler was developed independently of Metropolis-Hastings, see [2,5], but we discuss it here in a unified framework.) The Gibbs sampler is based on the fact that a multivariate distribution is uniquely determined by the set of all of its full conditional distributions; but if you don't know what this means, just read on anyway.

A *full conditional distribution* of $\pi(\mathbf{x})$ is obtained by holding all of the components of $\mathbf{x}$ constant *except one* (call it x), and then sampling as a function of x alone. In other words, it is the distribution that you see when you "drill through" $\pi(\mathbf{x})$ along a coordinate direction, and with fixed values of all the other coordinates. We'll denote a full conditional distribution by the notation $\pi(x \mid \mathbf{x}^-)$, where $\mathbf{x}^-$ means "values of all the coordinates except one." (To keep the notation readable, we are suppressing an index i that would tell which component of $\mathbf{x}$ is x.)

Suppose that we construct a Metropolis-Hastings chain that allows only the one coordinate x to vary. Then equation (15.8.5) would look like this:

$$\alpha(x_1, x_{2c}|\mathbf{x}^-) = \min\left(1, \frac{\pi(x_{2c}|\mathbf{x}^-)\, q(x_1|x_{2c}, \mathbf{x}^-)}{\pi(x_1|\mathbf{x}^-)\, q(x_{2c}|x_1, \mathbf{x}^-)}\right) \tag{15.8.8}$$

Now let's pick as our proposal distribution,

$$q(x_2|x_1, \mathbf{x}^-) = \pi(x_2|\mathbf{x}^-) \tag{15.8.9}$$

Look what happens: The second argument of the min function becomes 1, so the acceptance probability α is also 1. In other words, if we propose a value x_2 from the full conditional distribution $\pi(x_2|\mathbf{x}^-)$, we can always accept it. The advantage is obvious. The disadvantage is that the full conditional distribution *must* be properly normalized as a probability distribution — otherwise how could we use it as a transition probability? Thus, we will usually need to calculate (either analytically or by numerical integration) the normalizing constant

$$\int \pi(x|\mathbf{x}^-)dx \tag{15.8.10}$$

for every $\mathbf{x}^-$ of interest, and we will need to have a practical algorithm for drawing x_2 from the thus-normalized distribution. Note that these one-dimensional normalizing constants are *much* easier to compute than would be the multidimensional normalizing constant for the whole distribution $\pi(\mathbf{x})$.

The full Gibbs sampler operates as follows: Cycle through each component of $\mathbf{x}$ in turn. (A fixed cyclical order is usually used, but choosing a component randomly each time is also fine.) For each component, hold all the other components fixed and draw a new value x from the full conditional distribution $\pi(x \mid \mathbf{x}^-)$ of all possible values of that component. (This is where you might have to do a numerical integral at each step.) Set the component to the new value and go on to the next component.

You can see that the Gibbs sampler is "more global" than the regular Metropolis-Hastings algorithm. At each step, a component of $\mathbf{x}$ gets reset to a value completely independent of its previous value (independent, at least, in the conditional distribution). If we tried to get behavior like this with regular Metropolis-Hastings, by proposing really big multivariate normal steps, say, we would get nowhere, since the steps would be almost always rejected!

On the other hand, the need to draw from a normalized conditional distribution can be a real killer in terms of computational workload. Gibbs sampling can be recommended enthusiastically when the components of $\mathbf{x}$ have discrete, not continuous, values, and not too many possible values for each component. In that case the normalization is just a sum over not-too-many terms, and the Gibbs sampler can be very efficient. For the case of continuous variables, you are probably better off with regular Metropolis-Hastings, unless your particular problem admits to some fast, tricky way of getting the normalizations.

Don't confuse the Gibbs sampler with the tactic of doing regular Metropolis-Hastings steps along one component at a time. For the latter, we restrict the proposal distribution to proposing a change in a single component, either randomly chosen or else cycling through all the components in a regular order. This is sometimes useful if it lets us compute $\pi(\mathbf{x})$ more efficiently (e.g., using saved pieces from the previous calculation on components that have not changed). What makes this *not* Gibbs is that we calculate an acceptance probability in the regular way, with equation (15.8.5) and the full distribution $\pi(\mathbf{x})$, which need not be normalized.

15.8.3 MCMC: A Worked Example

A number of practical details regarding MCMC are best discussed in the context of a worked example:

> *At the beginning of an experiment, events occur Poisson randomly with a mean rate λ_1, but only every k_1th event is recorded. Then, at time t_c, the mean rate changes to λ_2, but now only every k_2th event is recorded. We are given the times $t_0, \ldots, t_{N-1}$ of the N recorded events. Oh, by the way, the values λ_1, λ_2, k_1, k_2, and t_c are all unknown. We want to find them.*

Let's decompose the separate parts of the calculation into separate objects. First we need an object that represents the point $\mathbf{x}$. Although we've been discussing $\mathbf{x}$ as if it were a vector, it can actually be a mixture of continuous, discrete, boolean, or any other kind of variable. In our example we have both continuous and discrete variables.

mcmc.h

```
struct State {
Worked MCMC example: Structure containing the components of x.
    Doub lam1, lam2;                λ1 and λ2
    Doub tc;                        tc
    Int k1, k2;                     k1 and k2
    Doub plog;                      Set to log P by Plog, below.

    State(Doub la1, Doub la2, Doub t, Int kk1, Int kk2) :
        lam1(la1), lam2(la2), tc(t), k1(kk1), k2(kk2) {}
    State() {};
};
```

The constructor is used to set initial values. (The `plog` variable is not part of $\mathbf{x}$, but it will be used later.)

Next, we need an object for calculating $\pi(\mathbf{x}) = P(\mathbf{D}|\mathbf{x})$, the probability of the data given the parameters. For our example, we need to use a couple of facts about Poisson processes: If a Poisson process has a rate λ, then the waiting time to the kth event is distributed as Gamma(k, λ), that is,

$$p(\tau|k,\lambda) = \frac{\lambda^k}{(k-1)!}\tau^{k-1}e^{-\lambda\tau} \tag{15.8.11}$$

where $\tau = t_{i+k} - t_i$. (Compare equation 6.14.41, and also §7.3.10.) The exponential distribution is a special case with $k = 1$. Further, probabilities for non-overlapping intervals such as $t_{i+k} - t_i$ and $t_{i+2k} - t_{i+k}$ are independent. It follows that, for our example,

$$P(\mathbf{D}|\mathbf{x}) = \prod_{t_i \le t_c} p(t_{i+1} - t_i \,|\, k_1, \lambda_1) \times \prod_{t_i > t_c} p(t_{i+1} - t_i \,|\, k_2, \lambda_2) \tag{15.8.12}$$

where $p(\tau|k,\lambda)$ is as given in (15.8.11), and where t_i is now the ith *recorded* time. (In the words following equation 15.8.11, t_i was the ith event whether recorded or not.)

Actually, as the amount of data gets large, $P(\mathbf{D}|\mathbf{x})$ is likely to over- or underflow, so it is best to calculate $\log P$. It is important to make this calculation as

efficient as possible, because it will be done at every step. Particularly important is to minimize the amount of looping over all the data points. In our example, if you take the logarithm of equations (15.8.11) and (15.8.12), you'll see that the individual t_i's enter into $\log P$ only as a sum of intervals and sum of log of intervals, less than and greater than t_c. An efficient way to proceed is thus to digest the data once and store two cumulative sums. Then, given a value t_c, we can find our place in the table of sums by bisection and read off the left and right sums directly. There is thus no loop over the data at all! Life is rarely so good, but when it is, then *carpe diem*. The resulting object looks like this:

mcmc.h

```
struct Plog {
Functor that calculates log P of a State.
    VecDoub &dat;                                Bind to data vector.
    Int ndat;
    VecDoub stau, slogtau;

    Plog(VecDoub &data) : dat(data), ndat(data.size()),
    stau(ndat), slogtau(ndat) {
    Constructor. Digest the data vector for subsequent fast calculation of log P. The data are
    assumed to be sorted in ascending order.
        Int i;
        stau[0] = slogtau[0] = 0.;
        for (i=1;i<ndat;i++) {
            stau[i] = dat[i]-dat[0];             Equal to sum of intervals.
            slogtau[i] = slogtau[i-1] + log(dat[i]-dat[i-1]);
        }
    }

    Doub operator() (State &s) {
    Return log P of s, and also set s.plog.
        Int i,ilo,ihi,n1,n2;
        Doub st1,st2,stl1,stl2, ans;
        ilo = 0;
        ihi = ndat-1;
        while (ihi-ilo>1) {                      Bisection to find where is t_c in the data.
            i = (ihi+ilo) >> 1;
            if (s.tc > dat[i]) ilo=i;
            else ihi=i;
        }
        n1 = ihi;
        n2 = ndat-1-ihi;
        st1 = stau[ihi];
        st2 = stau[ndat-1]-st1;
        stl1 = slogtau[ihi];
        stl2 = slogtau[ndat-1]-stl1;
        Equations (15.8.11) and (15.8.12):
        ans =  n1*(s.k1*log(s.lam1)-factln(s.k1-1))+(s.k1-1)*stl1-s.lam1*st1;
        ans += n2*(s.k2*log(s.lam2)-factln(s.k2-1))+(s.k2-1)*stl2-s.lam2*st2;
        return (s.plog = ans);
    }
};
```

The `Plog` object is the only place that the data enter, and they enter only through the constructor. All other parts of the calculation see the data only through the calculation of $\log P$.

Next we come to the proposal generator, which we call `Proposal`. It doesn't have any contact with the data, or with $\log P$. All it needs to know about is the domain of $\mathbf{x}$ (that is, `State`). It is worth thinking hard about the proposal gener-

ator. Although "almost any" generator will work in theory, a poor generator will take longer than the age of the universe to converge, while a good, *rapidly mixing* generator can go like lightning. This is where MCMC starts becoming an art.

Our example is designed to furnish an illustration of this in the interaction between the λ parameters and their corresponding k's. The mean rate of recorded counts is λ/k. Since λ is a continuous variable, we will be proposing relatively small changes in it at each step. Since k is discrete, there is no such thing as a small change, especially when k is small.

If we naively write a generator that proposes random independent changes in λ and k, then, after we have settled down to roughly the right value of λ/k, essentially all proposals for changing k will be rejected. The reason is that the acceptable step in λ required for a change in k from 1 to 2 (say) is so large (doubling λ) that our generator will pick it only, say, every billion years! If we are not smart enough to recognize this problem ahead of time, we can find it experimentally by inspecting the Markov chain as it evolves and noting the proposals to change k are never accepted.

A solution in our case is to have two kinds of steps. The first changes λ (by a small amount) and keeps k fixed. The second changes k and λ, keeping λ/k fixed. We choose randomly between the two kinds of steps, mostly choosing the first kind.

The general issue here is what to do when $\pi(\mathbf{x})$ defines some highly correlated directions among the components in $\mathbf{x}$. If you can recognize these directions, your proposal generator should, at least sometimes, generate proposals along them. Otherwise, it will have to propose very small steps, if they are ever to be accepted. In our example, this latter choice was made impossible by the discreteness in k, forcing us to diagnose and confront the issue directly. So, although `Proposal` doesn't directly have to know about $\log P$, *you* may need a qualitative understanding of $\log P$ when you design `Proposal`.

Since only `Proposal` knows the algorithm by which a proposal is generated, this object must also calculate, and return, the ratio $q(\mathbf{x}_1|\mathbf{x}_{2c})/q(\mathbf{x}_{2c}|\mathbf{x}_1)$, which is needed in equation (15.8.5). Here is an example that proposes small lognormal steps for the variables λ_1, λ_2, and t_c, or else proposes incrementing k_1 and k_2 by 1, 0, or -1, with corresponding changes in the λ's as described above.

mcmc.h

```
struct Proposal {
Functor implementing the proposal distribution.
    Normaldev gau;
    Doub logstep;

    Proposal(Int ranseed, Doub lstep) : gau(0.,1.,ranseed), logstep(lstep) {}

    void operator() (const State &s1, State &s2, Doub &qratio) {
    Given state s1, set state s2 to a proposed candidate. Also set qratio to q(s1|s2)/q(s2|s1).

        Doub r=gau.doub();
        if (r < 0.9) {                          Lognormal steps holding the k's constant.
            s2.lam1 = s1.lam1 * exp(logstep*gau.dev());
            s2.lam2 = s1.lam2 * exp(logstep*gau.dev());
            s2.tc = s1.tc * exp(logstep*gau.dev());
            s2.k1 = s1.k1;
            s2.k2 = s1.k2;
            qratio = (s2.lam1/s1.lam1)*(s2.lam2/s1.lam2)*(s2.tc/s1.tc);
            Factors for lognormal steps.
        } else {                                Steps that change k1 and/or k2.
            r=gau.doub();
```

```
            if (s1.k1>1) {
                if (r<0.5) s2.k1 = s1.k1;
                else if (r<0.75) s2.k1 = s1.k1 + 1;
                else s2.k1 = s1.k1 - 1;
            } else {                            k1 = 1 requires special treatment.
                if (r<0.75) s2.k1 = s1.k1;
                else s2.k1 = s1.k1 + 1;
            }
            s2.lam1 = s2.k1*s1.lam1/s1.k1;
            r=gau.doub();                       Now all the same for k2.
            if (s1.k2>1) {
                if (r<0.5) s2.k2 = s1.k2;
                else if (r<0.75) s2.k2 = s1.k2 + 1;
                else s2.k2 = s1.k2 - 1;
            } else {
                if (r<0.75) s2.k2 = s1.k2;
                else s2.k2 = s1.k2 + 1;
            }
            s2.lam2 = s2.k2*s1.lam2/s1.k2;
            s2.tc = s1.tc;
            qratio = 1.;
        }
    }
};
```

(We use the convenient fact that since Normaldev is derived from Ran, it contains both normal and uniform random number generators.)

How shall we set logstep, the size of the proposed lognormal step? A rule of thumb for proposals like this with an adjustable scale is that the average acceptance probability ought to be roughly between 0.1 and 0.4. If it is very much smaller, then decrease the step size parameter; if it is much larger, then increase the step size parameter. In our example, the value logstep $= 0.01$ (i.e., proposed changes on the order of $\pm 1\%$) gives good results.

Finally, there is a function that takes a specified number of steps, implementing equation (15.8.5). This short piece of code is about the only "universal" part of MCMC; it has no persistent state and gets all the information it needs via the State, Plog, and Proposal structures. As we have seen, these are all problem-dependent and benefit from cleverness and special tricks.

mcmc.h

```
Doub mcmcstep(Int m, State &s, Plog &plog, Proposal &propose) {
Take m MCMC steps, starting with (and updating) s.
    State sprop;                                Storage for candidate.
    Doub qratio,alph,ran;
    Int accept=0;
    plog(s);
    for (Int i=0;i<m;i++) {                     Loop over steps.
        propose(s,sprop,qratio);
        alph = min(1.,qratio*exp(plog(sprop)-s.plog));      Equation (15.8.5).
        ran = propose.gau.doub();
        if (ran < alph) {                       Accept the candidate.
            s = sprop;
            plog(s);
            accept++;
        }
    }
    return accept/Doub(m);
}
```

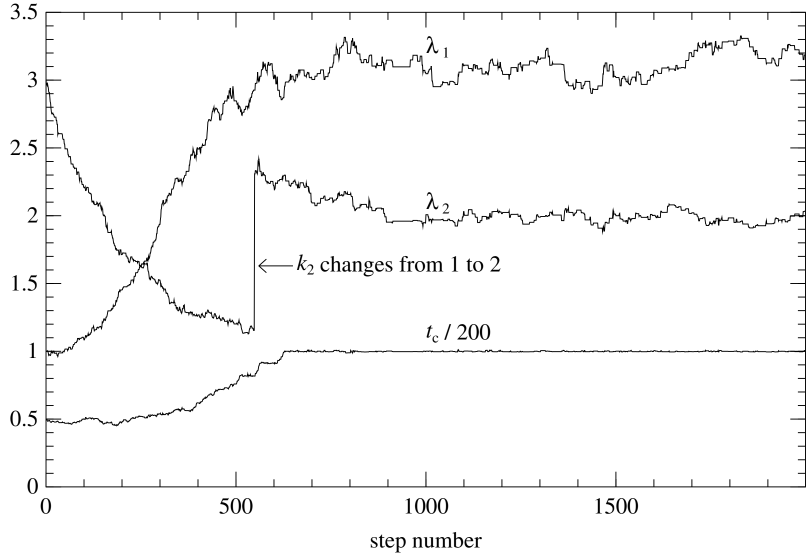

Figure 15.8.1. Evolution of model parameters λ_1, λ_2, and t_c as a function of Markov chain Monte Carlo step. In this example, the burn-in time is seen to be ~ 1000 steps, after which the Markov chain explores the equilibrium distribution.

Let's try it all out. We'll assume $N = 1000$ data points t_i and start $\mathbf{x}$ with the values $\lambda_1 = 1$, $\lambda_2 = 3$, $t_c = 100$, and $k_1 = k_2 = 1$. (Secretly, we know that the data were generated using actual values $3, 2, 200, 1, 2$, respectively.) The random seed is 10102, and the lognormal stepsize is 0.01. We'll take 1000 steps of burn-in, and thereafter store values after every 10 steps. Driver code in `main` for this run is

```
VecDoub times(1000);
...                                    Fill the vector times here.
State s(1.,3.,100.,1,1);
Plog plog(times);
Proposal propose(10102,.01);
for (i=0;i<1000;i++) accept = mcmcstep(1,s,plog,propose);   Burn-in.
for (i=0;i<10000;i++) {                                     Production.
    accept = mcmcstep(10,s,plog,propose);
    ...                                Save values, increment averages, etc., here.
}
```

Figure 15.8.1 shows the evolution of the parameters λ_1, λ_2 and t_c. During burn-in, you can see the parameters heading toward equilibrium, mostly monotonically, but with the exception of λ_2, which goes rapidly toward the value 1, with the value $k = 1$. These values indeed replicate the mean rate of the recorded data. Only when it is near convergence (around step 560), does the model discover that the t_i's greater than t_c don't actually fit an exponential distribution ($k_2 = 1$) but *do* fit a gamma distribution with the same mean rate, but with $k_2 = 2$ (the correct answer). Had we not provided `Proposal` with a step that tests for this, we would likely have converged to a wrong answer. More precisely, we would have produced a model whose true burn-in time was, unknowably, a figurative billion years.

Figure 15.8.2 shows how λ_1 and λ_2 distribute themselves during 10^5 steps after step 1000. This is the payoff of MCMC: We learn not just about most likely parameter values, but also details about how well the parameters are determined by this

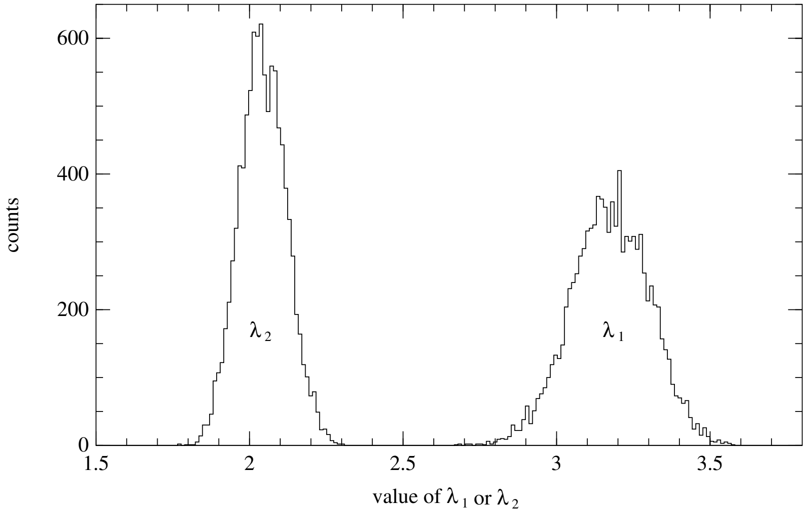

Figure 15.8.2. After burn-in, the MCMC model is run for an additional 10^5 steps. Parameter values are saved every 10 steps, giving the histograms shown for parameters λ_1 and λ_2. These histograms represent the inferred parameter values and their uncertainties. The model data were generated with parameters $\lambda_1 = 3$ and $\lambda_2 = 2$. The inferred values for this particular sample of 1000 data points are seen to be about as accurate as should be expected from their uncertainties.

particular data set. We could also have shown any joint distribution of interest, or computed any average quantity, for example

$$\langle \lambda_1 \rangle = \frac{1}{n-k} \sum_{i=k}^{n-1} (\lambda_1)_i \tag{15.8.13}$$

Here $k = 1000$ is the number of burn-in steps, which we reject; $n = k + 10^5$ is the number of steps that are averaged; and $(\lambda_1)_i$ denotes the value of λ_1 at the ith step. Sums like (15.8.13) are called *ergodic averages*.

Some remarks should be made about equation (15.8.13): One is allowed to average all steps, even though successive steps are not independent samples of $\pi(\mathbf{x})$. One would also be allowed to include in the average only every mth step, where you choose m to be greater than some empirically observed correlation time in the Markov chain. The latter is sometimes recommended as a means of estimating the standard error of $\langle \lambda_1 \rangle$, just as in Monte Carlo integration. (Compare equations 7.7.1 and 7.7.2.) Warning: Doing this in the context of a finite data set is often associated with conceptual error. While it is true that as $n \to \infty$, equation (15.8.13) does converge to a precise value, it is *not* true that this value has anything to do with the actual (population) value of λ_1. Rather, it is just the best apparent (sample) value of λ_1 for this particular data set. The relation of this apparent value to the actual value has nothing to do with the standard error of $\langle \lambda_1 \rangle$, but is instead indicated by the width of the distribution of all the $(\lambda_1)_i$'s.

Figure 15.8.2 illustrates this point well. By running the model for a long time, we could achieve beautifully precise distributions that have extremely well-converged means. But they would not be centered on the (here secretly known) true values 3 and 2. You should run an MCMC model only (i) long enough to be sure (or sure

enough) that there was sufficient burn-in, and (ii) long enough to characterize distributions well enough that the error in mean quantities of interest is reasonably small compared with the observed dispersion of those quantities in the Markov chain.

15.8.4 Other Aspects of MCMC

We have said little about how to determine the necessary length of burn-in, other than to advise you to *look at the output* (which is always a good idea). There is in fact a large literature on this subject [2-4]. A number of so-called *convergence diagnostic* tools have been developed. The problem is that, even when you use these tools, you really must *look at the output* anyway; so their added value is often not large. It is always a good idea to have the length of burn-in be *at least* 1 or 2% of the total length of your run, which will be determined by the accuracy that you need in estimating model parameters. Keep in mind that it is easy to construct scary examples of distributions $\pi(\mathbf{x})$ full of false convergence traps. The more you know about your distribution, the better off you will be.

Multiple, independent Markov chains can be run to explore a single distribution $\pi(\mathbf{x})$. On a single processor the only reason to do this would be to meet some unusual need for independent samples of the distribution. However, on machines with multiple processors, this is a natural way of achieving efficient parallelization.

We have assumed that the number of dimensions in $\mathbf{x}$ is fixed. It is possible to have models, however, in which the number of fitted parameters is itself a variable. These *variable dimension models* require special care in the design of proposal distributions that can step between different numbers of dimensions. See the paper by Phillips and Smith in [2] for an introduction.

15.8.5 Importance Sampling and MCMC

In §7.9 we noted that the error in Monte Carlo integration could be reduced by importance sampling, where we write (in the notation of this section)

$$I \equiv \int f(\mathbf{x})\,d\mathbf{x} = \int \frac{f(\mathbf{x})}{p(\mathbf{x})}\,p(\mathbf{x})\,d\mathbf{x} \tag{15.8.14}$$

for an aptly chosen p. We saw that the ideal p would (i) resemble f in functional form, cf. equation (7.9.6), and (ii) admit to a good method for sampling uniformly over $p(\mathbf{x})\,d\mathbf{x}$.

You might think that MCMC provides a great general-purpose way to sample over any p and thus make importance sampling easy to implement in all cases. Unfortunately, no. The problem, once again (as for the Gibbs sampler), is the normalizing constant. MCMC's great virtue is that it samples over a distribution $\pi(\mathbf{x})$ without requiring that it be normalized. If you ask what normalized probability distribution $p(\mathbf{x})$ is actually being sampled over, it is of course

$$p(\mathbf{x}) = \frac{\pi(\mathbf{x})}{\int \pi(\mathbf{x})d\mathbf{x}} \tag{15.8.15}$$

Equation (15.8.14) then becomes

$$I \equiv \int f(\mathbf{x})\,d\mathbf{x} = \int \pi(\mathbf{x})\,d\mathbf{x} \;\times\; \int \frac{f(\mathbf{x})}{\pi(\mathbf{x})}\,p(\mathbf{x})\,d\mathbf{x} \tag{15.8.16}$$

The differential $p(\mathbf{x})\,d\mathbf{x}$ can be sampled over by MCMC, knowing only $\pi(\mathbf{x})$; no problem. The $\pi(\mathbf{x})$ in the denominator of the integrand can also be readily computed. But we have, in general, no easy way to calculate that pesky normalizing constant, $\int \pi(\mathbf{x})\,d\mathbf{x}$.

Sometimes, though not often, you can construct a function $\pi(\mathbf{x})$ that both resembles f and also can be integrated analytically, so that the normalizing constant is knowable. Then, yes, by all means use MCMC to sample $\pi(\mathbf{x})$. In this case the idea of recording only every mth step, after choosing m large enough so that the points thus chosen are independent samples, is not a bad idea after all. In fact you'll have to do this if you expect to use the error estimate in equation (7.9.3) as written.

Finally, if the integral that you really want is

$$J \equiv \frac{\int f(\mathbf{x})\pi(\mathbf{x})\,d\mathbf{x}}{\int \pi(\mathbf{x})\,d\mathbf{x}} = \int f(\mathbf{x})\,p(\mathbf{x})\,d\mathbf{x} \qquad (15.8.17)$$

with $f(\mathbf{x})$ and $\pi(\mathbf{x})$ both known (and $p(\mathbf{x})$ only implied), then MCMC is exactly what you need. It provides uniform samples over $p(\mathbf{x})d\mathbf{x}$, and no calculation of a normalizing constant is needed.

CITED REFERENCES AND FURTHER READING:

Hastings, W.K. 1970, "Monte Carlo Sampling Methods Using Markov Chains and Their Applications," *Biometrika*, vol. 57, pp. 97–109.[1]

Gilks, W.R., Richardson, S., and Spiegelhalter, D.J., eds. 1996, *Markov Chain Monte Carlo in Practice* (Boca Raton, FL: Chapman & Hall/CRC), especially Chapter 1.[2]

Gamerman, D. 1997, *Markov Chain Monte Carlo: Stochastic Simulation for Bayesian Inference* (London: Chapman & Hall). [3]

Neal, R.M. 1993, "Probabilistic Inference Using Markov Chain Monte Carlo Methods," *Technical Report CRG-TR-93-1*, Department of Computer Science, University of Toronto. Available at `http://www.cs.toronto.edu/~radford/ftp/review.pdf`. [4]

Casella, G., and George, E.I. 1992, "Explaining the Gibbs Sampler," *American Statistician*, vol. 46, no. 3, pp. 167–174.[5]

Tanner, M.A. 2005, *Tools for Statistical Inference: Methods for the Exploration of Posterior Distributions and Likelihood Functions*, 3rd ed. (New York: Springer).

Liu, J.S. 2002, *Monte Carlo Strategies in Scientific Computing* (New York: Springer).

Beichl, I., and Sullivan, F. (eds.) 2006, *Computing in Science and Engineering*, special issue on Monte Carlo Methods, vol. 8, no. 2 (March/April), pp. 7–47.

15.9 Gaussian Process Regression

Some types of statistical models do not depend on knowing (or guessing) parameterized functional forms, and thus lie outside of the parameter-fitting paradigm that has thus far occupied our attention. As an alternative to assuming that our data have some functional form, we can assume that they have some statistical property. A common example is to assume that the data, viewed as an entire set, is drawn from some multivariate normal (Gaussian) distribution in a high-dimensional space. That distribution is allowed to have a complicated correlation structure: The individual data points are *not* assumed to be independent. We can then ask, *given* the

data points that we observe, what are the most probable values for other quantities of interest, for example the values of variables at points other than the ones measured. Of course, as previously, we are also encouraged to ask not just about the most probable values, but about the whole distribution around the most probable values. This general scheme is called *Gaussian process regression*.

We have already met examples of Gaussian process regression twice before in this book, though under different names. In §3.7 we discussed *kriging* as a multidimensional interpolation technique. Later, in §13.6, we discussed *linear prediction*, mostly in the context of one-dimensional data such as time series. Here we can usefully merge some of the ideas in those two sections.

As we presented it in §3.7, kriging was an interpolation, not a fitting, technique. This was evident from the facts that (i) the interpolated function output by the `Krig` object went exactly through the measured data points, and (ii) we never discussed how to input measurement errors. However, the `Krig` object's constructor did have an argument `err`, introduced with the mysterious remark that you should leave it set to `NULL` until you read §15.9. Well, here we are!

We did incorporate measurement errors in §13.6, although they were there called *noise*. In particular, equations (13.6.6) and (13.6.7) can be used (after some change of notation and algebraic manipulation) to derive the appropriate generalization of equations (3.7.14) and (3.7.15) to the case where the measurements y_i, $i = 0, \ldots, N-1$, have errors characterized by some covariance matrix $\mathbf{\Sigma}$. In most cases $\mathbf{\Sigma}$ will be simply a diagonal matrix with elements σ_i^2, the squares of the individual errors. The answers are

$$\widehat{y}_* = \mathbf{V}_* \cdot (\mathbf{V} - \mathbf{\Sigma}')^{-1} \cdot \mathbf{Y} \tag{15.9.1}$$

and

$$\mathrm{Var}(\widehat{y}_*) = \mathbf{V}_* \cdot (\mathbf{V} - \mathbf{\Sigma}')^{-1} \cdot \mathbf{V}_* \tag{15.9.2}$$

where

$$\mathbf{\Sigma}' \equiv \begin{pmatrix} \mathbf{\Sigma} & 0 \\ 0 & 0 \end{pmatrix} \tag{15.9.3}$$

That is, we simply subtract $\mathbf{\Sigma}$ (suitably augmented by bordering zeros) from $\mathbf{V}$ (equation 3.7.13) before inverting the matrix. The argument `err`, input as the σ_i's (not squared), does this for the case of diagonal measurement errors. Note that `err` has type `Doub*`. If your errors are stored in a `VecDoub`, then you'll send `&err[0]` to the `Krig` constructor. (Sorry about this hack. The purpose was to make `NULL` a possible default value.)

So, no new code is needed in this section. In `Krig`, you already have a serviceable multidimensional Gaussian process regression fitting routine, all ready to go.

When you are fitting, rather than interpolating, it is a good idea to pay more attention to the choice of variogram model than we did in §3.7. While for simple applications there is nothing wrong with the power-law model implemented in the `Powvargram` object

$$v(r) = \alpha r^{\beta} \tag{15.9.4}$$

several other models are widely used. These include the *exponential model*,

$$v(r) = b[1 - \exp(-r/a)] \tag{15.9.5}$$

the *spherical model*,

$$v(r) = \begin{cases} b\left(\frac{3}{2}\frac{r}{a} - \frac{1}{2}\frac{r^3}{a^3}\right) & 0 \le r \le a \\ b & a \le r \end{cases} \tag{15.9.6}$$

and various anisotropic models for which $v(\mathbf{r})$ is not just a function of the magnitude r. See [1,2] for derivations and examples.

We should also mention the so-called *nugget effect*, though, in our opinion, its name vastly outshines its utility. If $v(\mathbf{r})$ does not go to zero as $\mathbf{r} \to 0$, but instead goes to some constant v_0, then the resulting variogram describes a distribution that decorrelates by some finite amount in an infinitesimal distance. That is, if you find a gold nugget at location $\mathbf{x}$, there is no certainty that you'll find another one at location $\mathbf{x} + \delta\mathbf{x}$, no matter how small you make $\delta\mathbf{x}$. Some practitioners deem it desirable to allow for a nonzero nugget effect, allowing nonzero values of v_0 when they empirically fit $v(r)$ from a data set. That seems debatable to us; but in deference to such opinion we have given the `Powvargram` constructor an otherwise undocumented argument, `nug`, for feeding in the value v_0 of your choice. (We draw the line at actually fitting for such a parameter!)

Beyond debatable, and actually incorrect, however, is to confuse the nugget effect with the effect of measurement error. They seem superficially similar: Measurement error also decorrelates measured values, even at arbitrarily small distances (even zero). Conceptually, and mathematically, however, they are different. Referring to equation (3.7.13), a nugget effect adds a constant positive value to all the *off-diagonal* v_{ij}'s. Measurement errors, on the other hand, subtract (not necessarily constant) negative values from the *diagonal* v_{ii}'s. These actions do not have equivalent effects on equations (3.7.14) and (3.7.15). This can readily be seen in Figure 15.9.1, which may also help elucidate the difference between kriging interpolation and kriging fitting. Only panel (d) in the figure shows a correct use of kriging for data with measurement errors, that is, kriging fitting with errors σ_i. Panels (b) and (c) show the results of kriging interpolation with and without a nugget effect. One sees that even with a positive nugget, the interpolated curve goes exactly through the data points, which is incorrect when measurement errors are significant. The legitimate use of kriging interpolation (as in §3.7) is for smooth functions that are "exactly" known at scattered points. Kriging fits using σ_i's (this section) are for data with errors.

CITED REFERENCES AND FURTHER READING:

Cressie, N. 1991, *Statistics for Spatial Data* (New York: Wiley).[1]

Wackernagel, H. 1998, *Multivariate Geostatistics*, 2nd ed. (Berlin: Springer).[2]

Isaaks, E.H., and Srivastava, R.M. 1989, *Applied Geostatistics* (New York: Oxford University Press).

Rasmussen, C.E., and Williams, C.K.I. 2006, *Gaussian Processes for Machine Learning* (Cambridge, MA: MIT Press).

Rybicki, G.B., and Press, W.H. 1992, "Interpolation, Realization, and Reconstruction of Noisy, Irregularly Sampled Data," *Astrophys. J.*, vol. 398, pp. 169–176.

Deutsch, C.V., and Journel, A.G. 1992, *GSLIB: Geostatistical Software Library and User's Guide* (New York: Oxford University Press).

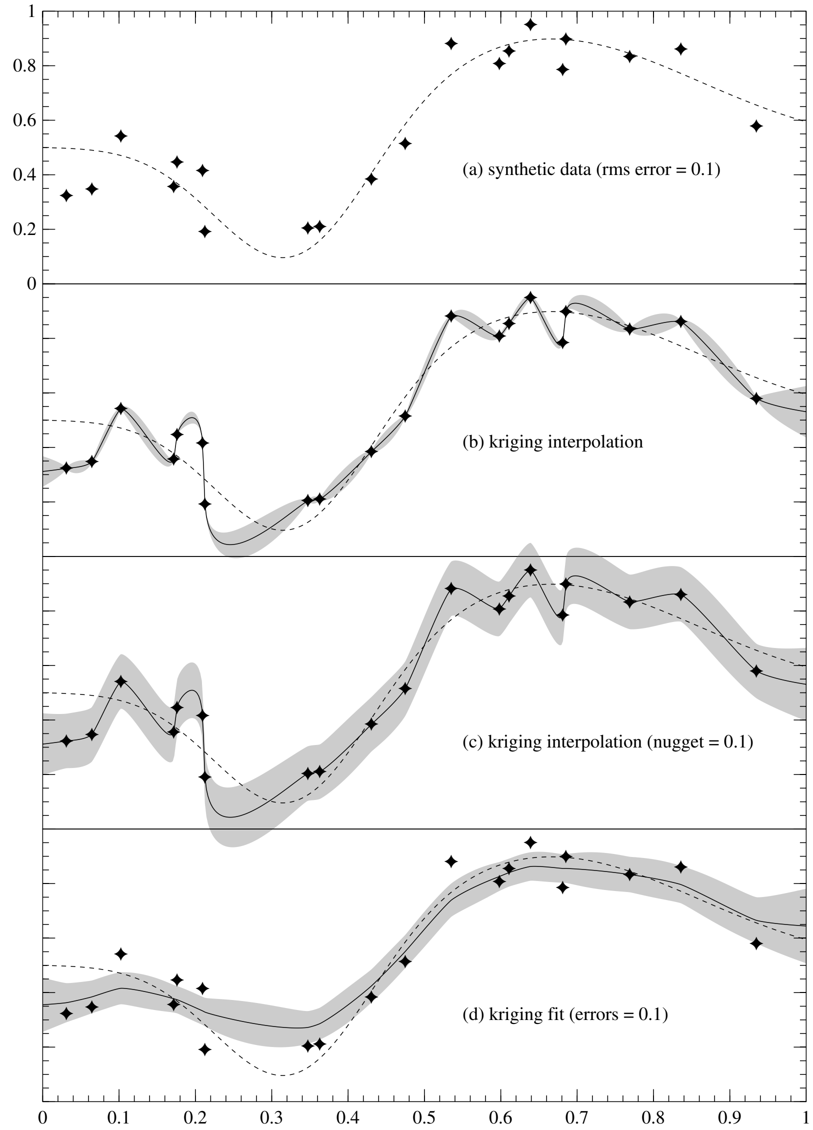

Figure 15.9.1. One-dimensional examples of interpolation and fitting by kriging. (a) Synthetic data points generated from the known curve (dashed line) with Gaussian errors (r.m.s. magnitude 0.1). (b) Result of kriging interpolation. Equation (3.7.14) is plotted as the solid line, while the 1-σ estimated interpolation errors (3.7.15) are shown as the shaded band. The interpolation error is seen to be meaningless for data with measurement errors. (c) Same as (b), but with a nugget effect of 0.1. (d) Result of kriging fit (equations 15.9.1 and 15.9.2) using the actual measurement errors. This is the correct use of kriging for data with errors.

CHAPTER 16

Classification and Inference

16.0 Introduction

This chapter groups together a selection of computational techniques whose common feature is that they treat problems of classification and inference on complex models. Given substantially more space, the chapter might have been expanded to be a more complete survey of *machine learning*; but at its present length, it cannot pretend to be such. (A few general references are given below.)

Classification and inference, in a loose sense, are also the goals of many of the purely statistical methods that we already discussed in Chapters 14 and 15, and the line between such techniques and machine learning is a fuzzy one. This chapter's topics tend to have one or both of two characteristics: the underlying model (i) has discrete or combinatorial aspects that distinguish it from "classical" statistical methods, and/or (ii) has empirical or heuristic aspects that make exact statistical treatments unattainable.

There is a list of important topics, related to those in this chapter, that we would have wanted to include if only they could have been reduced to suitable length. *Bayesian networks* is at the top of this list. Section 16.0.1 gives an example of the kind of problem that a Bayesian network can solve. Other significant topics that we must omit include

- genetic algorithms
- neural nets
- kernel methods more general than those discussed in §16.5

16.0.1 Bayesian networks

These are sometimes called *Bayes nets*, *Bayesian learning networks*, or *belief networks*. Here we want only to give a flavor of the method, so that you will know when to consult the references below.

A Bayesian network consists of nodes, each of which can have a value. The values can be {true,false}, or a set of possibilities like {low, medium, high}, or an integer. Figure 16.0.1 shows an example where all the nodes have true/false values.

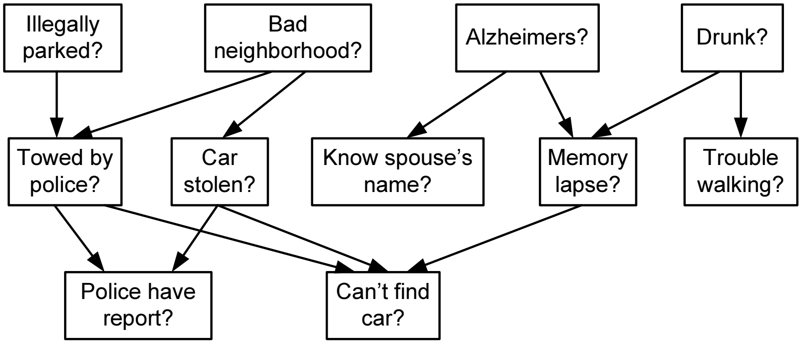

Figure 16.0.1. Example of a Bayesian network. Evidence about any node can be propagated to give probabilistic conclusions about any other node.

Each node in a network has a set of *prior probabilities* or *priors* that give the likelihood of its values absent any additional evidence. If a node has one or more *parents*, then its priors are conditioned on the values of the parents. For example, referring to the Figure, we might have

P(Illegally-Parked $=$ true)
0.20

Bad-N'hood	P(Car-Stolen $=$ true | Bad-N'hood)
true	0.05
false	0.001

Alzheimers	Drunk	P(Memory-Lapse $=$ true | Alzheimers, Drunk)
true	true	0.999
true	false	0.95
false	true	0.50
false	false	0.01

And so on, for all the other nodes.

Things get interesting when we have some evidence to assimilate into the network. For example, you might be coming out of a bar in a bad neighborhood, walking with some difficulty, and be unable to find your car. Is it stolen? The Bayesian network theory gives algorithms for propagating information both up (from "Can't find car?") and down (from "Bad neighborhood?") to get new *posterior* estimates for the probabilities at all nodes, including, here, "Car stolen?" You can also compute in advance the value of new evidence. For example, how much would it help to call the police and see if there is a police report of a towed or recovered, stolen, car?

For more than this brief taste, see [1-3].

CITED REFERENCES AND FURTHER READING:

Hastie, T., Tibshirani, R., and Friedman, J.H. 2003, *The Elements of Statistical Learning* (Berlin: Springer).

Duda, R.O., Hart, P.E., and Stork, D.G. 2000, *Pattern Classification*, 2nd ed. (New York: Wiley).

Witten, I.H., and Frank, E. 2005, *Data Mining: Practical Machine Learning Tools and Techniques*, 2nd ed. (San Francisco: Morgan Kaufmann).

Mitchell, T.M. 1997, *Machine Learning* (New York: McGraw-Hill).

Vapnik, V. 1998, *Statistical Learning Theory* (New York: Wiley).

Russell, S., and Norvig, P. 2002, *Artificial Intelligence: A Modern Approach*, 2nd ed. (Upper Saddle River, NJ: Prentice-Hall).

Haykin, S. 1998, *Neural Networks: A Comprehensive Foundation*, 2nd ed. (Upper Saddle River, NJ: Prentice-Hall).

Bishop, C.M. 1996, *Neural Networks for Pattern Recognition* (New York: Oxford University Press).

Korb, K.B., and Nicholson, A.E. 2004, *Bayesian Artificial Intelligence* (Boca Raton, FL: Chapman & Hall/CRC).[1]

Neapolitan, R.E. 1990, *Probabilistic Reasoning in Expert Systems* (New York: Wiley).[2]

Jensen, F.V. 2001, *Bayesian Networks and Decision Graphs* (New York: Springer).[3]

16.1 Gaussian Mixture Models and k-Means Clustering

Gaussian mixture models, so called, are one of the simplest examples of classification by *unsupervised learning*. They are also one of the simplest examples where solution by the *EM (expectation-maximization) algorithm* proves highly successful.

Here is the setup: You are given N data points in an M-dimensional space, usually with M in the range one to a few (say, three or four dimensions, tops). You want to "fit" the data, in this special sense: Find a set of K multivariate Gaussian distributions that best represents the observed distribution of data points. The number K is fixed in advance but the means and covariances of the distributions are unknown,

What makes the exercise "unsupervised" is that you are *not told* which of the N data points come from which of the K Gaussians. Indeed, one of the desired *outputs* is, for each data point n, an estimate of the probability that it came from distribution number k. This probability is denoted $P(k|n)$ or p_{nk}, where (using a zero-based counting scheme) $0 \le k < K$ and $0 \le n < N$. The matrix p_{nk} is sometimes called the *responsibility matrix*, because its entries indicate how much "responsibility" component k has for data point n.

Thus, given the data points, say as an $N \times M$ matrix whose rows are vectors of length M, there are a whole bunch of parameters that we want to estimate:

$$\begin{aligned}
&\boldsymbol{\mu}_k && \text{(the } K \text{ means, each a vector of length } M\text{)} \\
&\boldsymbol{\Sigma}_k && \text{(the } K \text{ covariance matrices, each of size } M \times M\text{)} \\
P(k|n) \equiv\ &p_{nk} && \text{(the } K \text{ probabilities for each of } N \text{ data points)}
\end{aligned} \qquad (16.1.1)$$

We will also get some additional estimates as by-products: $P(k)$ denotes the fraction of all data points in component k, that is, the probability that a data point chosen at random is in k; $P(\mathbf{x})$ denotes the probability (actually a probability density) of finding a data point at some position $\mathbf{x}$, where $\mathbf{x}$ is the M-dimensional position vector; and $\mathcal{L}$ denotes the overall likelihood of the estimated parameter set.

In fact, $\mathcal{L}$ is the key to the whole problem. $\mathcal{L}$ is defined, as usual, as proportional to the probability of the data set, *given* all the fitted parameters. We find the best values for the parameters by maximizing the likelihood $\mathcal{L}$. You can also think of this as maximizing the posterior probability of the parameters, given uniform or very broad priors.

Let's work backward from $\mathcal{L}$. Since the data points are (assumed) independent, $\mathcal{L}$ is the product of the probabilities of finding a point at each observed position $\mathbf{x}_n$,

$$\mathcal{L} = \prod_n P(\mathbf{x}_n) \tag{16.1.2}$$

We can split $P(\mathbf{x}_n)$ into its contribution from each of the K Gaussians and write

$$P(\mathbf{x}_n) = \sum_k N(\mathbf{x}_n \,|\, \boldsymbol{\mu}_k, \boldsymbol{\Sigma}_k) P(k) \tag{16.1.3}$$

where $N(\mathbf{x} \,|\, \boldsymbol{\mu}, \boldsymbol{\Sigma})$ is the multivariate Gaussian density,

$$N(\mathbf{x} \,|\, \boldsymbol{\mu}, \boldsymbol{\Sigma}) = \frac{1}{(2\pi)^{M/2} \det(\boldsymbol{\Sigma})^{1/2}} \exp[-\tfrac{1}{2}(\mathbf{x} - \boldsymbol{\mu}) \cdot \boldsymbol{\Sigma}^{-1} \cdot (\mathbf{x} - \boldsymbol{\mu})] \tag{16.1.4}$$

$P(\mathbf{x}_n)$ is sometimes called the *mixture weight* of the data point $\mathbf{x}_n$. We can "take apart" $P(\mathbf{x}_n)$ into its K individual contributions, giving the individual probabilities

$$p_{nk} \equiv P(k|n) = \frac{N(\mathbf{x}_n \,|\, \boldsymbol{\mu}_k, \boldsymbol{\Sigma}_k) P(k)}{P(\mathbf{x}_n)} \tag{16.1.5}$$

Equations (16.1.2) through (16.1.5) are a prescription for calculating $\mathcal{L}$ and the p_{nk}'s, given the data, and given values for the $\boldsymbol{\mu}_k$'s, $\boldsymbol{\Sigma}_k$'s, and $P(k)$. In the language of the EM algorithm, this is called an *expectation step* or *E-step*.

But how do we get the $\boldsymbol{\mu}_k$'s, $\boldsymbol{\Sigma}_k$'s, and $P(k)$?

Suppose we *knew* the p_{nk}'s. A familiar theorem for the one-dimensional Gaussian distribution is that the maximum likelihood estimate of its mean is just the arithmetic mean of a set of points drawn from it. This theorem straightforwardly generalizes to yield maximum likelihood estimates for the means, and covariance matrices, of multivariate Gaussians. A further small generalization is that, since we know only probabilistically whether a particular point is drawn from a particular Gaussian, we should count only the appropriate fraction p_{nk} of each point. These considerations result in the following maximum likelihood estimates:

$$\begin{aligned}
\hat{\boldsymbol{\mu}}_k &= \sum_n p_{nk} \mathbf{x}_n \Big/ \sum_n p_{nk} \\
\hat{\boldsymbol{\Sigma}}_k &= \sum_n p_{nk} (\mathbf{x}_n - \hat{\boldsymbol{\mu}}_k) \otimes (\mathbf{x}_n - \hat{\boldsymbol{\mu}}_k) \Big/ \sum_n p_{nk}
\end{aligned} \tag{16.1.6}$$

and, in a similar vein,

$$\hat{P}(k) = \frac{1}{N} \sum_n p_{nk} \tag{16.1.7}$$

"Hats" here denote estimators; however, this is a notational nicety that we will henceforth ignore. Equations (16.1.6) and (16.1.7) are the so-called *maximization step* or *M-step* of the EM algorithm.

What we have motivated thus far is that *right at* the maximum likelihood solution, both the E-step and the M-step relations will hold. That is, the maximum likelihood parameters are a stationary point for both E-steps and M-steps. The power of the EM algorithm derives from the more powerful theorem (beyond our scope to prove here) that, starting from *any* parameter values, an iteration of E-step followed by an M-step will increase the likelihood value $\mathcal{L}$; and that repeated iterations will converge to (at least a local) likelihood maximum. Often, happily, the convergence is to the global maximum.

The EM algorithm, in brief, is thus

- Guess starting values for the $\boldsymbol{\mu}_k$'s, $\boldsymbol{\Sigma}_k$'s, and fractions $P(k)$.
- Repeat: An E-step to get new p_{nk}'s and new $\mathcal{L}$, followed by an M-step to get new $\boldsymbol{\mu}_k$'s, $\boldsymbol{\Sigma}_k$'s, and $P(k)$.
- Quit when the value of $\mathcal{L}$ is no longer changing.

One important practical detail is that the values of the Gaussian density function will often be so small as to underflow to zero. It is therefore important to work with logarithms of these densities, rather than the densities themselves, e.g.,

$$\log N(\mathbf{x} \mid \boldsymbol{\mu}, \boldsymbol{\Sigma}) = -\tfrac{1}{2}(\mathbf{x}-\boldsymbol{\mu})\cdot\boldsymbol{\Sigma}^{-1}\cdot(\mathbf{x}-\boldsymbol{\mu}) - \frac{M}{2}\log(2\pi) - \frac{1}{2}\log\det(\boldsymbol{\Sigma}) \qquad (16.1.8)$$

A problem arises with equation (16.1.3), where we need to take the sum of quantities, all of which may be so small as to underflow if ever reconstructed from their logarithms. The solution to this problem is the so-called *log-sum-exp* formula,

$$\log\left(\sum_i \exp(z_i)\right) = z_{\max} + \log\left(\sum_i \exp(z_i - z_{\max})\right) \qquad (16.1.9)$$

where the z_i's are the logarithms that we are using to represent small quantities and $z_{\max}$ is their maximum. Equation (16.1.9) guarantees that at least one exponentiation won't underflow, and that any that do could have been neglected anyway.

Figure 16.1.1 shows an example of how the EM algorithm converges to a solution with 1000 two-dimensional data points and four components. As the number of data points increases, the topography of the likelihood space gets smoother, with fewer local minima, so that it becomes more and more likely that the global maximum will be found (as in this case).

You should always inspect an EM solution for reasonableness. If you are getting hung up on an unacceptable local maximum, one strategy is to do a series of independent runs, using K randomly chosen data points as the starting means in each case. (Be sure that you don't duplicate a data point in the starting guesses.) Then pick the best one, i.e., the one that converges to the largest log-likelihood.

Here is a structure that implements the EM algorithm for Gaussian mixture models, given only the data points and initial estimates of the means $\boldsymbol{\mu}_k$. The constructor sets the problem up, and does one initial E-step and M-step. Thereafter, the user alternately calls the `estep()` and `mstep()` routines, until convergence is achieved, as signaled by the return value of `estep()`, the change in log-likelihood, becoming sufficiently small (say, 10^{-6}). The results are then available in the structure members `means`, `resp`, `frac`, and `sig`.

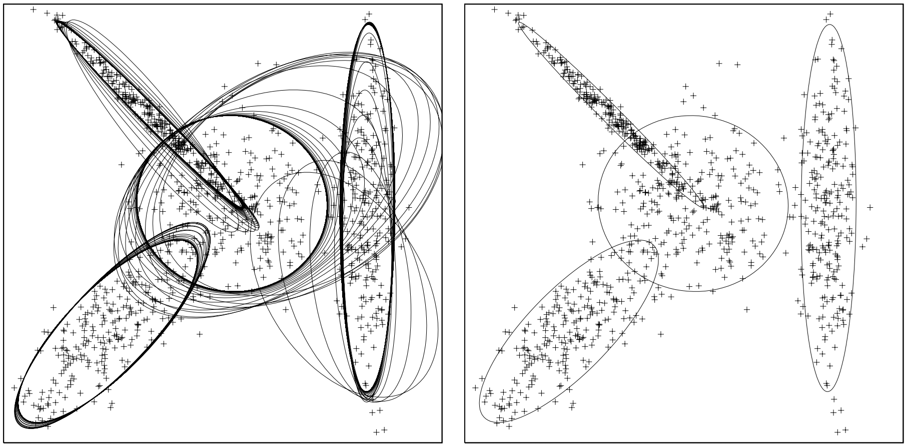

Figure 16.1.1. Example of a Gaussian mixture model in $M = 2$ dimensions, with $N = 1000$ data points and $K = 4$ components. Left: Evolution of the estimated means and covariances, shown as 2-sigma ellipses. The ellipses are plotted after each iteration of an E-step and M-step (see text). Right: The converged result. The leftmost two components converged rapidly. The rightmost component took about 10 iterations to get to near-convergence; only after it had done so did the central component shrink down to a converged result.

gaumixmod.h

```
struct preGaumixmod {
For nonwizards, this is basically a typedev of Mat_mm as an mm×mm matrix.  For wizards, what is
going on is that we need to set a static variable mmstat before defining Mat_mm, and this must
happen before the Gaumixmod constructor is invoked.
    static Int mmstat;
    struct Mat_mm : MatDoub {Mat_mm() : MatDoub(mmstat,mmstat) {} };
    preGaumixmod(Int mm) {mmstat = mm;}
};
Int preGaumixmod::mmstat = -1;

struct Gaumixmod : preGaumixmod {
Solve for a Gaussian mixture model from a set of data points and initial guesses of k means.
    Int nn, kk, mm;                   Nos. of data points, components, and dimensions.
    MatDoub data, means, resp;        Local copies of x_n's, μ_k's, and the p_nk's.
    VecDoub frac, lndets;             P(k)'s and log det Σ_k's.
    vector<Mat_mm> sig;               Σ_k's
    Doub loglike;                     log ℒ.
    Gaumixmod(MatDoub &ddata, MatDoub &mmeans) : preGaumixmod(ddata.ncols()),
    nn(ddata.nrows()), kk(mmeans.nrows()), mm(mmstat), data(ddata), means(mmeans),
    resp(nn,kk), frac(kk), lndets(kk), sig(kk) {
    Constructor. Arguments are the data points (as rows in a matrix) and initial guesses for
    the means (also as rows in a matrix).
        Int i,j,k;
        for (k=0;k<kk;k++) {
            frac[k] = 1./kk;              Uniform prior on P(k).
            for (i=0;i<mm;i++) {
                for (j=0;j<mm;j++) sig[k][i][j] = 0.;
                sig[k][i][i] = 1.0e-10;   See text at end of this section.
            }
        }
        estep();                          Perform one initial E-step and M-step. User
        mstep();                              is responsible for calling additional steps
    }                                         until convergence is obtained.
    Doub estep() {
    Perform one E-step of the EM algorithm.
        Int k,m,n;
```

```
        Doub tmp,sum,max,oldloglike;
        VecDoub u(mm),v(mm);
        oldloglike = loglike;
        for (k=0;k<kk;k++) {                    Outer loop for computing the p_nk's.
            Cholesky choltmp(sig[k]);           Decompose Σ_k in the outer loop.
            lndets[k] = choltmp.logdet();
            for (n=0;n<nn;n++) {                Inner loop for p_nk's.
                for (m=0;m<mm;m++) u[m] = data[n][m]-means[k][m];
                choltmp.elsolve(u,v);           Solve L · v = u.
                for (sum=0.,m=0; m<mm; m++) sum += SQR(v[m]);
                resp[n][k] = -0.5*(sum + lndets[k]) + log(frac[k]);
            }
        }
```

At this point we have unnormalized logs of the p_{nk}'s. We need to normalize using log-sum-exp and compute the log-likelihood.

```
        loglike = 0;
        for (n=0;n<nn;n++) {                    Separate normalization for each n.
            max = -99.9e99;                     Log-sum-exp trick begins here.
            for (k=0;k<kk;k++) if (resp[n][k] > max) max = resp[n][k];
            for (sum=0.,k=0; k<kk; k++) sum += exp(resp[n][k]-max);
            tmp = max + log(sum);
            for (k=0;k<kk;k++) resp[n][k] = exp(resp[n][k] - tmp);
            loglike +=tmp;
        }
        return loglike - oldloglike;            When abs of this is small, then we have
    }                                               converged.
    void mstep() {
```

Perform one M-step of the EM algorithm.

```
        Int j,n,k,m;
        Doub wgt,sum;
        for (k=0;k<kk;k++) {
            wgt=0.;
            for (n=0;n<nn;n++) wgt += resp[n][k];
            frac[k] = wgt/nn;                   Equation (16.1.7).
            for (m=0;m<mm;m++) {
                for (sum=0.,n=0; n<nn; n++) sum += resp[n][k]*data[n][m];
                means[k][m] = sum/wgt;          Equation (16.1.6).
                for (j=0;j<mm;j++) {
                    for (sum=0.,n=0; n<nn; n++) {
                        sum += resp[n][k]*
                            (data[n][m]-means[k][m])*(data[n][j]-means[k][j]);
                    }
                    sig[k][m][j] = sum/wgt;     Equation (16.1.6).
                }
            }
        }
    }
};
```

About the only place that Gaumixmod can fail algorithmically (as distinct from converging to a poor, local, solution) is by encountering a zero or negative diagonal element in the Cholesky decomposition. As a result, all sins tend to appear, sometimes confusingly, as exceptions at that point in the code. If you are getting such exceptions, here are some possibilities:

- You have duplicated vectors in your initial guesses for the μ_k's.
- One or more of your μ_k's is so distant from all data points that it is not "attracting" enough of them to solve for the parameters of its component. Try using random data points as starting guesses, or reduce K.
- You may just have too few data points N to support a nondegenerate model with K components. Reduce K or get more data!

- Rarely, you might want to change the constant `1.0e-10` that initializes the diagonal components of $\mathbf{\Sigma}_k$ in the code. (See discussion under "K-Means Clustering," below.)
- You can reduce the number of parameters in $\mathbf{\Sigma}$, as we now discuss.

Occasionally data are too sparse, or too noisy, to give meaningful results for all the components of the covariance matrices $\mathbf{\Sigma}_k$. In such cases, you can impose simpler covariance models by changing the re-estimation formulas for $\mathbf{\Sigma}$ in equation (16.1.6). One step of simplification is to make $\mathbf{\Sigma}$ diagonal, while still allowing different variances for the different dimensions. The re-estimation formula for the diagonal components of $\mathbf{\Sigma}_k$ is then

$$(\widehat{\mathbf{\Sigma}}_k)_{mm} = \sum_n p_{nk}[(\mathbf{x}_n)_m - (\widehat{\boldsymbol{\mu}}_k)_m]^2 \Big/ \sum_n p_{nk} \tag{16.1.10}$$

where subscripts m indicate that particular component of the vector. Set nondiagonal components of $\mathbf{\Sigma}_k$ to zero.

Even more drastic, we can replace $\mathbf{\Sigma}_k$ by a single scalar (that is, spherical) variance by using the re-estimation formula

$$(\widehat{\mathbf{\Sigma}}_k) = \mathbf{1} \times \left(\sum_n p_{nk} |\mathbf{x}_n - \widehat{\boldsymbol{\mu}}_k|^2 \Big/ \sum_n p_{nk} \right) \tag{16.1.11}$$

where $\mathbf{1}$ is the identity matrix.

We have not coded these options in `Gaumixmod`, but they are easy to add.

16.1.1 A Note on the Use of Cholesky Decomposition

It is worth remarking briefly on the use of Cholesky decomposition (§2.9) in this and similar manipulations of multivariate Gaussians.

In the `Gaumixmod` routine above, we need a way of inverting the covariance matrices — or, more precisely, an efficient way to compute expressions like $\mathbf{y}\cdot\mathbf{\Sigma}^{-1}\cdot\mathbf{y}$. Because the covariance matrix $\mathbf{\Sigma}$ is symmetric and positive-definite, the Cholesky decomposition, which has fewer operations than other methods, can be used, giving

$$\mathbf{\Sigma} = \mathbf{L} \cdot \mathbf{L}^T \tag{16.1.12}$$

where $\mathbf{L}$ is a lower triangular matrix, implying

$$Q = \mathbf{y} \cdot \mathbf{\Sigma}^{-1} \cdot \mathbf{y} = \left|\mathbf{L}^{-1} \cdot \mathbf{y}\right|^2 \tag{16.1.13}$$

Since $\mathbf{L}$ is triangular, $\mathbf{L}^{-1} \cdot \mathbf{y}$ can be obtained efficiently by backsubstitution.

Another very convenient use for the decomposition (16.1.12) is in the mundane task of drawing error ellipses, as in Figure 16.1.1 (or, similarly, error ellipsoids in three dimensions). The locus of points $\mathbf{x}$ that are one standard deviation ("1-sigma") away from the mean $\boldsymbol{\mu}$ is given by

$$1 = (\mathbf{x} - \boldsymbol{\mu}) \cdot \mathbf{\Sigma}^{-1} \cdot (\mathbf{x} - \boldsymbol{\mu}) \quad \Rightarrow \quad \left|\mathbf{L}^{-1} \cdot (\mathbf{x} - \boldsymbol{\mu})\right| = 1 \tag{16.1.14}$$

Now suppose that $\mathbf{z}$ is a point on the unit circle (two dimensions) or unit sphere (three dimensions). Then, by substitution into equation (16.1.14), you can easily see that

$$\mathbf{x} = \mathbf{L} \cdot \mathbf{z} + \boldsymbol{\mu} \tag{16.1.15}$$

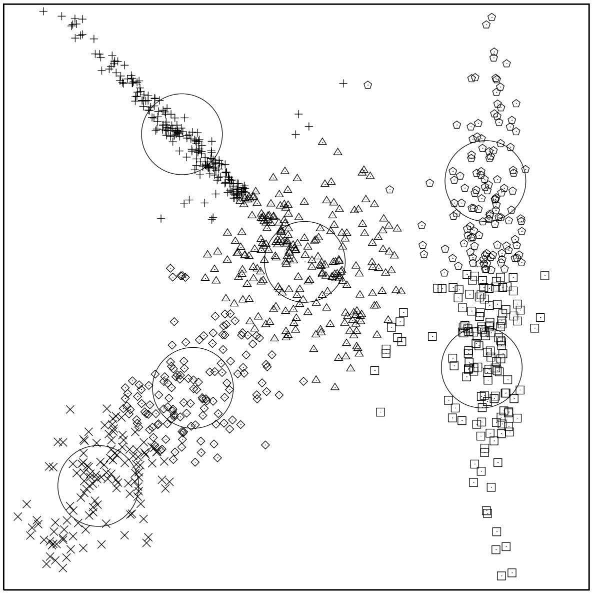

Figure 16.1.2. Output of k-means clustering as applied to the same data as Figure 16.1.1, with $K = 6$ components. The final assignments are shown as different plotted symbols. The centers of the large circles are the locations of the final means. (The radius of those circles is arbitrary, for visibility only.) Unlike Gaussian mixture modeling, k-means clustering can't split a point probabilistically between two components, so many points from the Gaussian at the upper left are mistakenly assigned to the central component. Also, k-means clustering needs more than one component to model a Gaussian with a large aspect ratio, because it clusters by radial distance.

is a point on the 1-sigma locus. Going around the unit circle in $\mathbf{z}$, and using the mapping (16.1.15), gives the desired ellipse. Put a constant 2 in front of the $\mathbf{L}$ in (16.1.15) for 2-sigma ellipses, and so forth.

We already remarked, in §7.4, on the closely related use of Cholesky decomposition to generate multivariate Gaussian deviates from a given covariance matrix.

16.1.2 K-Means Clustering

One interesting simplification of Gaussian mixture modeling has an independent history and is known as *k-means clustering*. We forget about the $\boldsymbol{\Sigma}_k$ covariances matrices completely, and we forget about probabilistic assignments of data points to components. Instead, each data point gets assigned to one (and only one) of the K components.

The E-step is simply: Assign each data point $\mathbf{x}_n$ to the component k whose mean $\boldsymbol{\mu}_k$ it is closest to, by Euclidean distance.

The M-step is simply: For all k, re-estimate the mean $\boldsymbol{\mu}_k$ as the average of data points $\mathbf{x}_n$ assigned to component k.

The convergence criterion is: Stop when an E-step doesn't change the assignment of any data point (in which case the M-step would also produce unchanged $\boldsymbol{\mu}_k$'s).

Interestingly, convergence is guaranteed — you can't get into an infinite loop of, say, shifting a point back and forth between two components. Despite its simplicity, k-means clustering can be quite useful: It is very fast, and it converges very

rapidly. It can be used as a method to reduce a large number of data points to a much smaller number of "centers," which can then be used as starting points for more sophisticated methods.

For example, you might use k-means clustering to get starting values for a Gaussian mixture model that has difficulty converging to a good, global maximum. If K is the ultimate number of components that you want, you might use k-means to get down to $\sim 3 \times K$ components, then (repeatedly) randomly select K of these as starting guesses for the Gaussian model.

Be alert to the fact that k-means clustering has an intrinsically "spherical" view of the world, because of its Euclidean "nearest-to" assignments. If you have components that might have big aspect ratios, be sure to set K large enough so that these can be represented by several different centers. Figure 16.1.2 shows the same input data as Figure 16.1.1, now clustered by k-means. The Gaussians at the lower left and right have broken up into two centers each. The Gaussian at the upper left is only a single component, because it has had many of its points misclassified into the central component. (A Gaussian mixture model would have assigned those points probabilistically to both components.)

Code for k-means classification is similar to, but much shorter than, the previous code for a Gaussian mixture model:

kmeans.h

```
struct Kmeans {
```
Solve for a k-means clustering model from a set of data points and initial guesses of the means. Output is a set of means and an assignment of each data point to one component.
```
    Int nn, mm, kk, nchg;
    MatDoub data, means;
    VecInt assign, count;
    Kmeans(MatDoub &ddata, MatDoub &mmeans) : nn(ddata.nrows()), mm(ddata.ncols()),
    kk(mmeans.nrows()), data(ddata), means(mmeans), assign(nn), count(kk) {
```
Constructor. Arguments are the data points (as rows in a matrix), and initial guesses for the means (also as rows in a matrix).
```
        estep();
        mstep();
    }
```
Perform one initial E-step and M-step. User is responsible for calling additional steps until convergence is obtained.
```
    Int estep() {
```
Perform one E-step.
```
        Int k,m,n,kmin;
        Doub dmin,d;
        nchg = 0;
        for (k=0;k<kk;k++) count[k] = 0;
        for (n=0;n<nn;n++) {
            dmin = 9.99e99;
            for (k=0;k<kk;k++) {
                for (d=0.,m=0; m<mm; m++) d += SQR(data[n][m]-means[k][m]);
                if (d < dmin) {dmin = d; kmin = k;}
            }
            if (kmin != assign[n]) nchg++;
            assign[n] = kmin;
            count[kmin]++;
        }
        return nchg;
    }
    void mstep() {
```
Perform one M-step.
```
        Int n,k,m;
        for (k=0;k<kk;k++) for (m=0;m<mm;m++) means[k][m] = 0.;
        for (n=0;n<nn;n++) for (m=0;m<mm;m++) means[assign[n]][m] += data[n][m];
        for (k=0;k<kk;k++) {
```

```
            if (count[k] > 0) for (m=0;m<mm;m++) means[k][m] /= count[k];
        }
    }
};
```

Incidentally, k-means clustering is not only a simplification of Gaussian mixture models; it is actually a limiting case. If the $\mathbf{\Sigma}_k$ matrices are all held fixed as

$$\mathbf{\Sigma}_k = \epsilon\, \mathbf{1} \tag{16.1.16}$$

with ϵ infinitesimal and $\mathbf{1}$ the identity matrix, then the component k with mean closest to $\mathbf{x}_n$ will be assigned all of the responsibility p_{nk} for that n. The re-estimation of the $\boldsymbol{\mu}_k$'s then is identical to k-means clustering. The theorem that proves that the EM algorithm converges for Gaussian mixtures can easily be modified to prove the convergence of k-means clustering. (Basically, there is a hidden log-likelihood function that can be shown to increase at each step.)

Indeed, we can now explain the obscure constant `1.0e-10` in the initialization part of `Gaumixmod`: It is a value for ϵ that makes that routine's *first* E-step, M-step iteration be one of k-means clustering.

CITED REFERENCES AND FURTHER READING:

McLachlan, G. and Peel, D. 2000, *Finite Mixture Models* (New York: Wiley).

Moore, A.W. 2004, "Clustering with Gaussian Mixtures," at `http://www.cs.cmu.edu/~awm`.

Dempster, A.P., Laird, N.M., and Rubin, D.B. 1977, "Maximum Likelihood from Incomplete Data via the EM Algorithm," *Journal of the Royal Statistical Society*, Series B, vol. 39, pp. 1-38. [The original paper on EM methods.]

Tanner, M.A. 2005, *Tools for Statistical Inference: Methods for the Exploration of Posterior Distributions and Likelihood Functions*, 3rd ed. (New York: Springer).

16.2 Viterbi Decoding

In this section we discuss models with discrete states, and how to use data to estimate what state a model is in, or what succession of states it traverses by allowed transitions. By *state*, we mean some discrete condition that can be characterized as a node on a directed graph like that in Figure 16.2.1. By *transition*, we mean moving along one of the directed edges of the graph. If you want to characterize a continuous variable in the context of this section, you need to define a set of discrete bins for its possible values, and make these the states.

The setup we describe is slightly more general than its close cousin, the directed graph of *stages and states* that defined the dynamic programming (DP) problem in §10.13. For some applications, the estimation problem of interest does live on a graph that has states and stages, exactly like DP; but for other applications, we need a general directed graph. We'll consider both types below.

Historically, problems involving the estimation of states have arisen in diverse, and often noncommunicating, fields. There are often multiple names for single concepts. (We saw this previously in the Bellman-Dijkstra-Viterbi algorithm for DP.) This history also makes it hard to give, in this section, a unified treatment with a single narrative. A more practical approach is to go through a couple of examples from different fields, and then, afterward, make some comparisons and give some advice.

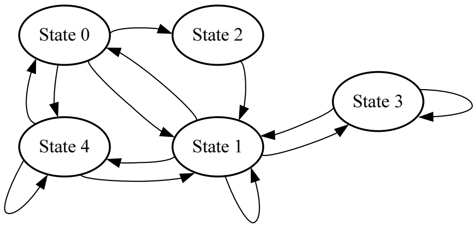

Figure 16.2.1. Graph of a dynamical system with five states. Allowed transitions are shown by arrows.

16.2.1 Error-Correcting Codes and Soft-Decision Decoding

An (N, K) *binary block code* is a list of 2^K binary *codewords*, each of length $N > K$ bits, designed to send a K-bit message in such a way that it can be received correctly even if one or more of the N bits arrive garbled (that is, 0 instead of 1, or vice versa). Two simple examples are shown below. In these particular cases, the message bits are the initial bits of the codeword, but that need not be true in general. Assigning any permutation of the codewords to message words is, effectively, the same code; likewise an arbitrary permutation of the bits in all the codewords (permuting bit-columns in the table).

(6,3) Shortened Hamming	
message	codeword
000	000000
001	001110
010	010101
011	011011
100	100011
101	101101
110	110110
111	111000

(7,4) Hamming	
message	codeword
0000	0000000
0001	0001011
0010	0010111
0011	0011100
0100	0100110
0101	0101101
0110	0110001
0111	0111010
1000	1000101
1001	1001110
1010	1010010
1011	1011001
1100	1100011
1101	1101000
1110	1110100
1111	1111111

Both of the codes shown have the property that their *Hamming distance* is 3. This means that all pairs of codewords differ in at least three bits. This is the property of the code that makes it "error correcting on one bit." If you receive a codeword with one of its bits wrong, then (i) it will not be in the above table, and (ii) there will be a unique codeword in the table that differs from it in one bit position. So, trying each bit position in turn, you can figure out what was the intended codeword.

A longer code, with a larger Hamming distance d, can be error correcting for more than one garbled bit, in fact for $(d-1)/2$ bits (rounding down). An (N, K) code can have d as large as $N - K$. However, trying all possible corrections until you find a valid codeword is a very poor decode strategy!

For so-called *linear codes* it is possible to construct a *parity-check matrix* $\mathbf{P}$ with the property that multiplying it by the vector of received bits (and doing all

arithmetic modulo 2) gives a vector, the so-called *syndrome*, that is either all zeros (indicating that the received bits are ok) or else it uniquely corresponds to a mask (termed a *coset leader*) that tells which bits need correcting. So this error-correction algorithm, called *syndrome decoding*, can be summarized as:

- multiply the received bits by the parity-check matrix to get the syndrome,
- do a table lookup of the syndrome to get the coset leader, and
- XOR the coset leader with the received bits to get a valid codeword.

For example, the parity check matrix for the (7, 4) Hamming code, above, is

$$\mathbf{P} = \begin{pmatrix} 1 & 1 & 1 & 0 & 1 & 0 & 0 \\ 0 & 1 & 1 & 1 & 0 & 1 & 0 \\ 1 & 0 & 1 & 1 & 0 & 0 & 1 \end{pmatrix} \tag{16.2.1}$$

(Convert codewords to column vectors by reading from left to right.) The lookup table relating syndrome to coset leader is

syndrome	coset leader
000	0000000
001	0000001
010	0000010
011	0001000
100	0000100
101	1000000
110	0100000
111	0010000

This particular code is called a *perfect code* because the number of syndromes exactly equals the number of coset leaders with one nonzero bit, plus 1 for the zero syndrome, a numerological coincidence. There are very few perfect codes, because there are very few sets of integers satisfying

$$1 + \binom{N}{1} + \cdots + \binom{N}{e} = 2^{N-K} \tag{16.2.2}$$

where e is the number of bits corrected. Probably the most nontrivial perfect code is the *Golay code*, with $N = 23$, $K = 12$, and $e = 3$. (Check out the numerology yourself.)

It's no big deal if a code is not perfect. It just means that there are some extra syndromes that correct *some* errors of more than e bits, but not enough to correct all such errors. You include these extra syndromes in the table, and run the algorithm exactly as already described. However, if a code is too far from perfect, you are wasting syndromes without gaining more bits of sure correction.

In practical applications, N and K are larger than these examples. For example, the lowest level of error correction on an audio compact disk (CD) is a $(28, 24)$ *Reed-Solomon* (RS) code, which can correct $e = 2$ bits. (On a CD, bits of the output codewords from many consecutive blocks are then interleaved and further protected by an RS(32,28) code.) Reed-Solomon codes are typically decoded by a more efficient process than syndrome decoding, using the so-called *Berlekamp-Massey* algorithm.

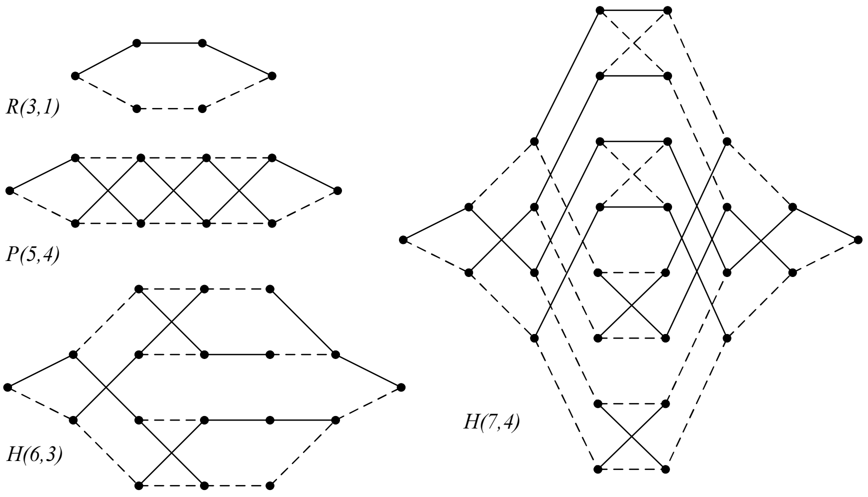

Figure 16.2.2. Trellises associated with four binary codes. The graph is traversed from left to right. A zero is output when any dotted edge is traversed, a one for a solid edge. Every path yields a valid codeword.

Now take a deep breath. Everything we have discussed thus far is what is called *hard-decision decoding* (HDD), meaning that hard decisions are made as to whether each incoming bit is a 1 or 0, with the error-correction algorithm acting on the resulting, possibly garbled, codeword. Virtually all coding theory utilized HDD until the early 1970s. Then came the giant leap forward with the recognition by multiple practitioners that Viterbi's 1967 decoding algorithm (an independent rediscovery of Bellman-Dijkstra, we might now say) could utilize "soft" data about each bit as easily as hard.

To understand *soft-decision decoding* (SDD), let us first note that every binary code can be represented by the kind of stage/state graph that we met in dynamic programming (§10.13), which, in the present context, is called a *trellis*. Figure 16.2.2 shows the trellises for the two codes given explicitly above, as well as for the schoolbook examples of a repetition code ("tell me three times") and a parity code. The latter, with $d = 1$, is error detecting, but not error correcting.

Although arrows are not shown, the trellis is traversed from left to right. Any such path on the trellis generates a valid codeword. A zero bit is emitted when a dotted edge is traversed, a one bit for a solid edge. You encode message bits by deciding whether to branch up or down, when you have a choice. Notice that you don't get such a choice at every stage: "Forced" edges generate precisely the extra codeword bits that the code's redundancy requires.

Although every code has a trellis, it is not so easy to find the *minimal trellis*, the one that has the fewest possible states at its maximum expansion. MacKay [1] gives a brief introduction; many additional references are in [3].

The first great idea behind soft-decision decoding is that we don't need to decide whether an incoming bit is a 0 or 1. Rather, we just need to assign a probability to each possibility (summing to unity, of course). For example, a bit's value may be determined by whether an instantaneous voltage is positive or negative — but the voltage measurement has some Gaussian spread of errors. If the voltage is many standard deviations positive, or negative, then the respective probabilities are very

close to one or zero; but if the voltage is only $t = 0.5$ (say) standard deviations away from zero, we may want to assign a probability of 0.6915 to one more favored outcome, and 0.3085 to the less favored, since

$$\frac{1}{\sqrt{2\pi}}\int_{-\infty}^{0.5} e^{-z^2/2}dz \approx 0.6915 \tag{16.2.3}$$

(By the end of this section we will be more sophisticated about the notion of assigning probabilities to transitions.)

The second great idea is that the problem of finding the maximum likelihood path through a trellis — that is, the path with the maximum product of the probabilities at each stage — is just a dynamic programming problem, where the cost of traversing an edge whose probability is p is taken as $-\log(p)$, a positive number, since $0 \leq p \leq 1$. The minimum cost path, with this metric, is the maximum likelihood path. In each stage, all the 0 edges (dotted lines in the figure) get one probability, and all the 1 edges (solid lines) get its complement. The edge probabilities can, and in general will, vary with each bit received (that is, from stage to stage), since the noise and path loss can vary with time.

Take these two ideas together and you have *soft-decision decoding using the Viterbi algorithm*.

The following tableau decodes one codeword for the shortened Hamming (6, 3) code given above. In this example, five of the six bits are received fairly unambiguously, while one bit (the second) is seen to be rather ambiguous. Nevertheless, the algorithm treats all bits equally "softly." Reviewing §10.13 as necessary, you should be able to see where all the numbers in the tableau come from, as well as how the darker path (which is the final "hard" decision for the codeword 011011) is obtained by backtracking. Given the appropriate cost function, the routine `dynpro` in §10.13 does exactly this calculation.

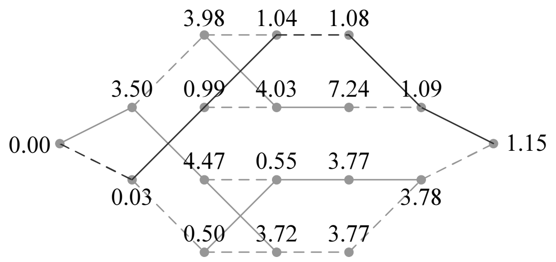

Prob(0) =	.97	.62	.04	.96	.01	.06
Prob(1) =	.03	.38	.96	.04	.99	.94
–log[Prob(0)] =	0.03	0.47	3.22	0.04	4.61	2.81
–log[Prob(1)] =	3.50	0.96	0.05	3.21	0.01	0.06

You might have the "bright idea" of converting the final minimum path length, 1.15 in the above example, into a probability by taking its negative exponential. The result is 0.3166. Does this mean that you'll get the right codeword only 31% of the time? No! Go stand in the corner! You are confusing likelihood with probability. The likelihoods of all eight codewords (not found by the DP algorithm, but computed exhaustively) are, for this example,

codeword	likelihood
000000	0.000014
001110	0.001372
010101	0.000006
011011	0.316126
100011	0.000665
101101	0.000007
110110	0.000001
111000	0.000006

You can see that the likelihoods don't add up to 1, and that the favored path wins over any competitor by a large factor. (Bayesians: We know that this paragraph is making you break out in a rash. We are on your side, and will have more to say about this in §16.3.4.)

In the above example, the second bit was merely ambiguous. What if it had instead been really wrong, indicating, say, 0.99 probability of having a value zero? No problem. Since the underlying code is one-bit error correcting, the DP algorithm will readily decide to traverse the unlikely single edge, since the alternative would be to traverse two or more unlikely edges on other bits. However, if we made the second bit incorrect with probability 0.999999, the algorithm would "correct" two other bits instead, which, under the circumstances, would be the best decision.

You can see that it is not meaningful to say exactly how many bits e a soft-decision decode algorithm can correct. It just makes the best choice determined by the probabilities. As another example, we might consider the simple parity code shown in Figure 16.2.2. With a hard-decision decode, parity does not give enough information to correct a single bit. With a soft-decision algorithm, however, the parity bit can cast the deciding vote if some other bit is wavering too close to an ambiguous 50% probability level.

Soft-decision decoding algorithms are available for essentially all codes in use today, including Reed-Solomon codes and the important *turbo codes* [2] that are beyond our scope. Some important applications (e.g., *trellis coded modulation*), use short trellises whose end states loop around to become identified with their start states. The Viterbi algorithm is applied to long sequences of input symbols that loop through the trellis many times. In trellis coded modulation, the symbols being (softly) decoded are not single bits, but locations in the complex phase plane that comprise a carefully chosen *constellation* centered at the origin (for example, a hexagonal lattice).

CITED REFERENCES AND FURTHER READING:

Lin, S. and Costello, D.J. 2004, *Error Control Coding*, 2nd ed. (Upper Saddle River, NJ: Pearson-Prentice Hall).

Blahut, R.E. 2002, *Algebraic Codes for Data Transmission* (Cambridge, UK: Cambridge University Press).

MacKay, D.J.C. 2003, *Information Theory, Inference, and Learning Algorithms* (Cambridge, UK: Cambridge University Press).[1]

Schlegel, C. and Perez, L. 2000, *Trellis and Turbo Coding*, (Piscataway, NJ: IEEE Press).[2]

"Special Issue on Codes and Complexity", 1996, *IEEE Transactions on Information Theory*, vol. 42, no. 6, pp. 1649-2064.[3]

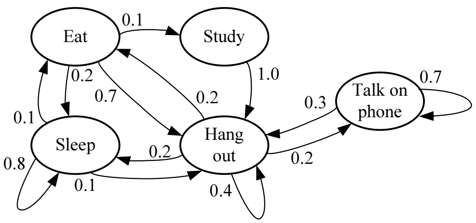

Figure 16.3.1. Example of a Markov model. Transitions occur between states along the directed edges shown. Each outgoing edge is labeled by its probability. The sum of the probabilities on the outgoing edges from each state is 1. This model is called "Teen Life."

16.3 Markov Models and Hidden Markov Modeling

Trellises, like those in §16.2, are directed graphs without any loops, so a path that begins at the leftmost node inevitably ends, after a finite number of stages, at the rightmost node. The more general *Markov model* lives on a graph that can have loops (as in Figure 16.2.1), so some paths can continue indefinitely. Indeed, as a convention, one can add a self-loop (a directed edge connecting a state to itself) to any state that would otherwise have "no way out." Then, all paths can be continued indefinitely, even those whose fate is to remain stuck in a single state.

To turn such a directed graph into a Markov model (also known as a *Markov chain* or *first-order Markov process*), we simply label all of its edges with *transition probabilities*, such that the sum of probabilities over the outgoing edges from each node is 1. Figure 16.3.1 shows an example, a Markov model with five states, that we call "Teen Life."

A single realization of a Markov model is a random path that moves from state to state according to the model's probabilities. These are conveniently organized into a *transition matrix* $\mathbf{A}$ whose element A_{ij} is the probability associated with an $i \to j$ transition, that is, the probability of moving to state j, given state i as the starting point. A valid transition matrix satisfies

$$0 \le A_{ij} \le 1 \qquad \text{and} \qquad \sum_j A_{ij} = 1 \tag{16.3.1}$$

The transition matrix for Teen Life (Figure 16.3.1) is

$$\mathbf{A} = \begin{pmatrix} 0 & 0.7 & 0.1 & 0 & 0.2 \\ 0.2 & 0.4 & 0 & 0.2 & 0.2 \\ 0 & 1.0 & 0 & 0 & 0 \\ 0 & 0.3 & 0 & 0.7 & 0 \\ 0.1 & 0.1 & 0 & 0 & 0.8 \end{pmatrix} \tag{16.3.2}$$

where the states are numbered in the order (Eat, Hang, Study, Talk, and Sleep).

A routine for generating a realization of a Markov model from its $M \times M$ transition matrix, using the `Ran` structure of §7.1 to get random numbers, is straightforward.

markovgen.h

```
void markovgen(const MatDoub_I &atrans, VecInt_O &out, Int istart=0,
    Int seed=1) {
```
Generate a realization of an M-state Markov model, given its $M \times M$ transition matrix atrans. The vector out is filled with integers in the range $0 \dots M-1$. The starting state is the optional argument istart (defaults to 0). seed is an optional argument that sets the seed of the random number generator.
```
    Int i, ilo, ihi, ii, j, m = atrans.nrows(), n = out.size();
    MatDoub cum(atrans);                Temporary matrix to hold cumulative probabilities.
    Doub r;
    Ran ran(seed);                      Use the random number generator Ran.
    if (m != atrans.ncols()) throw("transition matrix must be square");
    for (i=0; i<m; i++) {               Fill cum and die if clearly not a transition matrix.
        for (j=1; j<m; j++) cum[i][j] += cum[i][j-1];
        if (abs(cum[i][m-1]-1.) > 0.01)
            throw("transition matrix rows must sum to 1");
    }
    j = istart;                         The current state is kept in j.
    out[0] = j;
    for (ii=1; ii<n; ii++) {            Main loop.
        r = ran.doub()/cum[j][m-1];     Slightly-off normalization gets corrected here.
        ilo = 0;
        ihi = m;
        while (ihi-ilo > 1) {           Use bisection to find location among the cumu-
            i = (ihi+ilo) >> 1;             lative probabilities.
            if (r>cum[j][i-1]) ilo = i;
            else ihi = i;
        }
        out[ii] = j = ilo;              Set new current state.
    }
}
```

What makes the transition matrix a matrix, and not just a table of probabilities, is its connection to *ensembles* of realizations of the corresponding Markov model. An ensemble can be characterized by the components of a *population vector* $\mathbf{s}_t$ whose components give the number of models in each state at time t. (Here and below, we use t as an integer, discrete time variable. On a trellis it would be called a *stage* instead of a *time*.) For Teen Life, we can give names to the components of $\mathbf{s}_t$ corresponding to the states: (E, H, S, T, Z).

If all the models in the ensemble are evolved by one timestep (one transition), then the population vector $\mathbf{s}_t$ turns into $\mathbf{s}_{t+1}$ by the matrix multiplication

$$\mathbf{s}_{t+1} = \mathbf{A}^T \, \mathbf{s}_t \tag{16.3.3}$$

The transpose operation is needed only because two common conventions are at odds: The time order $i \to j$ for A_{ij} versus the left matrix multiplication of a column vector (whose implicit time ordering is "from" the second index "to" the first). Given a matrix, you can easily tell whether it is intended to be an $\mathbf{A}$ or an $\mathbf{A}^T$, by whether, respectively, its rows or columns sum to unit probability.

Note that we can evolve more than one step at a time, by precomputing powers of $\mathbf{A}^T$. So, $\mathbf{s}_{t+n} = (\mathbf{A}^T)^n \, \mathbf{s}_t$, for example.

Every Markov model has at least one equilibrium distribution of states that remains unaffected when multiplied by $\mathbf{A}^T$. To prove this, we write

$$\mathbf{A}^T \, \mathbf{s}_e = \mathbf{s}_e \qquad \Longleftrightarrow \qquad (\mathbf{A}^T - \mathbf{1}) \, \mathbf{s}_e = \mathbf{0} \tag{16.3.4}$$

where $\mathbf{s}_e$ is the sought-after equilibrium state. Equation (16.3.4) can hold if and only if $\mathbf{A}^T - \mathbf{1}$ is a singular matrix. Since the columns of $\mathbf{A}^T$ all sum to 1, the columns

of $\mathbf{A}^T - \mathbf{1}$ all sum to zero, and hence are linearly dependent, q.e.d. Equivalently, we have proved that $\mathbf{A}^T$ has at least one eigenvalue equal to 1. The corresponding eigenvector is an equilibrium distribution of states. If there is only one eigenvalue equal to 1, the equilibrium is unique. For the Teen Life model, there is one eigenvalue of 1, and the corresponding eigenvector (normalized so that its components sum to unity) is approximately $(0.099, 0.297, 0.001, 0.198, 0.395)$. (This teenager spends about 39.5% of his/her time sleeping, 19.8% talking on the phone, 0.1% studying, and so forth.)

Do almost all starting distributions converge to a unique equilibrium, in which case the model is said to be *ergodic*? Not necessarily. Two things can go wrong. First, if there is more than one eigenvalue equal to 1, a model will converge to some different linear combination of the corresponding eigenvectors for different starting distributions. Such models are said to fail the test of *irreducibility*. Second, the model may have a periodic limit cycle, so that, for most starting distributions, it doesn't converge at all. Such models are said to fail the test of *aperiodicity*. The theorem (and vocabulary test) is: Irreducibility and aperiodicity imply ergodicity.

One way to diagnose these conditions is to perform successive squarings of the matrix $\mathbf{A}^T$ to take it to a very high power, say 2^{32}. This requires $O(32\,M^3)$ operations for a model with M states. (While there are more sophisticated methods, none scale better than M^3.) If all M columns in the result are converging to identical vectors, then there is just one eigenvalue (unity), and all starting distributions will converge to its eigenvector (which is in fact the repeated column vector). The model is then ergodic.

Otherwise, locate any rows in the power matrix that are zero, and cross out those rows and their corresponding columns. (These are states that become permanently unpopulated as the model evolves.) Then, check to see if the remaining columns are all eigenvectors with unit eigenvalue. You can do the test by multiplying each such column by the original $\mathbf{A}^T$. If all columns pass the test, then there are multiple equilibria, but all starting distributions will converge to some combination of them. If any column fails the test, then the model has a periodic limit cycle. There are still equilibria, given by the eigenvectors of unit eigenvalue, but a starting state must be very special to evolve to one. Indeed, such states are a set of measure zero, and we can say that the equilibria are *unstable*.

A simple example with multiple equilibria and periodic limit cycles is the transition matrix

$$\mathbf{A}' = \begin{pmatrix} 0 & 1.0 & 0 & 0 & 0 \\ 1.0 & 0 & 0 & 0 & 0 \\ 0 & 0.7 & 0 & 0.3 & 0 \\ 0 & 0 & 0 & 0 & 1.0 \\ 0 & 0 & 0 & 1.0 & 0 \end{pmatrix} \tag{16.3.5}$$

corresponding to the graph

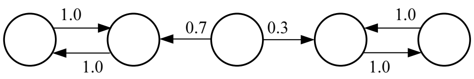

$\mathbf{A}'^T$ has two eigenvectors with unit eigenvalue (you can guess them from the graph): $(0.5, 0.5, 0, 0, 0)$ and $(0, 0, 0, 0.5, 0.5)$. However, $\mathbf{A}'^T$ to the power 2^{32} does not have

these as any of its columns, but is rather

$$(\mathbf{A}'^T)^{2^{32}} = \begin{pmatrix} 1.0 & 0 & 0.7 & 0 & 0 \\ 0 & 1.0 & 0 & 0 & 0 \\ 0 & 0 & 0 & 0 & 0 \\ 0 & 0 & 0 & 1.0 & 0 \\ 0 & 0 & 0.3 & 0 & 1.0 \end{pmatrix} \tag{16.3.6}$$

thus showing that the model has only unstable equilibria. (The little identity matrix blocks are merely artifacts of the limit cycles having period 2, while 2^{32} is even. In general, you will get some other pattern.)

Successive squaring is a poor way to get the equilibrium distribution for a model that is known (or guessed) to have a single stable equilibrium. A better way, since we already know the eigenvalue, is inverse iteration. Just solve the equation

$$(\mathbf{A}^T - \mathbf{1})\,\mathbf{s}_e = \mathbf{b} \tag{16.3.7}$$

by LU decomposition (§2.3), with the right-hand vector $\mathbf{b} = (1, 1, \ldots, 1)$. If your solver complains about the zero pivot instead of substituting a small value for it (which is what we want for this application), then use the matrix $(\mathbf{A}^T - 0.999999 \times \mathbf{1})$ instead. In either case, you will want to renormalize $\mathbf{s}_e$, to make, e.g., its components have unit sum.

You can test for multiple equilibria by perturbing the right-hand side vector and seeing if you get the same $\mathbf{s}_e$. If you do have multiple equilibria, it is probably time to turn to the methods of Chapter 11 and calculate $\mathbf{A}^T$'s eigenvalues and eigenvectors directly.

That is all (in fact, more) than we want to tell about Markov models in general. We turn now to the real business at hand, which is to estimate statistically the state of a "hidden" Markov model, given only partial or imperfect information.

16.3.1 Hidden Markov Models

In a *hidden Markov model* (HMM), we don't get to observe the state of the model directly. Rather, whenever it is in any state i (one of M states), it emits a *symbol* k, chosen probabilistically from a set of K symbols. The probability of emitting symbol number k from state number i is denoted by

$$b_i(k) \equiv P(\text{symbol } k \mid \text{state } i) \qquad (0 \le i < M, \quad 0 \le k < K) \tag{16.3.8}$$

with the normalization condition

$$\sum_{k=0}^{K-1} b_i(k) = 1 \qquad (0 \le i < M) \tag{16.3.9}$$

Thus, when the model evolves through N timesteps, the *hidden states* are a vector of integers,

$$\mathbf{s} = \{s_t\} = (s_0, s_1, \ldots s_{N-1}) \tag{16.3.10}$$

each in the range $0 \le s_i < M$, while the *observations* or *data* are a vector of integers,

$$\mathbf{y} = \{y_t\} = (y_0, y_1, \ldots y_{N-1}) \tag{16.3.11}$$

each in the range $0 \le y_i < K$.

For the Teen Life example, here is a table of symbols and their probabilities of being emitted from each state, in response to the repeated parental query, "What are you doing?":

			$i = 0$	1	2	3	4
k	symbol	meaning	Eat	Hang	Study	Talk	Sleep
0	o	[silence]	0.2	0.2	0	0.3	0.5
1	s	"I'm studying!"	0	0	1.0	0.2	0
2	b	"I'm busy!"	0	0.6	0	0.4	0
3	g	[grunt]	0.8	0.2	0	0.1	0
4	z	[snore]	0	0	0	0	0.5

The key point is that the emitted symbols give only incomplete, or garbled, state information (e.g., the claim of studying when actually talking on the phone). A state can emit more than one possible symbol, and a symbol can be emitted by more than one possible state. Nevertheless, our goal is to make the best statistical reconstruction of the vector $\mathbf{s}$ from $\mathbf{y}$.

More specifically, at each time t we want to estimate

$$P_t(i) \equiv P(s_t = i \mid \mathbf{y}) \qquad (16.3.12)$$

the probability that the actual state of the system is i at time t, given all the data. (If the word "probability" in this context bothers you, you may think of it as a likelihood.)

Let $\alpha_t(i)$ be defined for $t = 0 \ldots N-1$ and $i = 0 \ldots M-1$ as the probability of the observed data up to time t (that is, $y_0 \ldots y_t$), *given* that we are in state i at time t. Since we are not specifying any of the previous states, we must sum over all possible paths that get to state i at time t. Thus,

$$\begin{aligned} \alpha_0(i) &= b_i(y_0) \\ \alpha_t(i) &= \sum_{i_0, i_i, \ldots, i_{t-1}} b_{i_0}(y_0)\; A_{i_0 i_1} b_{i_1}(y_1)\; \ldots\; A_{i_{t-1} i} b_i(y_t) \qquad (1 \le t < N) \end{aligned} \qquad (16.3.13)$$

In other words, each transition contributes to the product both a transition probability and a symbol probability, and we sum over all possible combinations of previous states, that is, all possible values of $i_0, i_1, \ldots, i_{t-1}$, each in the range $0 \ldots M-1$.

Since $\alpha_t(i)$ is the probability of data given state, it can also be interpreted as the likelihood of the state, given the data; or, if we are Bayesians (and we are!), as the unnormalized posterior probability of being in state i, which can be normalized simply by dividing by $\sum_i \alpha_t(i)$. However, equation (16.3.13) seems useless for a big and a little reason. Big: It has exponentially many terms to evaluate. Little: it uses only part of the data (the data earlier in time) to estimate the state i at time t. It is what is called a *forward estimate*.

Amazingly, both problems are easy to fix. It is not hard to see that the $\alpha_t(i)$'s

satisfy a recurrence relation that advances them all one step in t:

$$\alpha_{t+1}(j) = \sum_{i=0}^{M-1} \alpha_t(i) A_{ij} b_j(y_{t+1}) \qquad (0 \le j < M) \quad \text{for} \quad t = 0, \dots, N-2 \tag{16.3.14}$$

One step of this recurrence takes only $O(M^2)$ operations, so the whole table of $\alpha_t(i)$'s can be computed in $O(NM^2)$.

To fix the second problem, we come at the issue from "the other end of time." Let $\beta_t(i)$ be defined for $t = N-1\dots0$ and $i = 0\dots M-1$ as the probability of the *future* observed data $(y_{t+1}, y_{t+2}, \dots y_{N-1})$ again *given* that we are in state i at time t. Analogously to the α's, we have

$$\beta_t(i) = \sum_{i_{t+1},\dots,i_{N-1}} A_{i i_{t+1}} b_{i_{t+1}}(y_{t+1}) \; \dots \; A_{i_{N-2} i_{N-1}} b_{i_{N-1}}(y_{N-1}) \tag{16.3.15}$$

with the special case $\beta_{N-1}(i) = 1$. (Because there are no data to the future of $t = N-1$, those data's probability is by definition 1.) In the formula for the $\beta_t(i)$'s there is a factor in the product for each future transition probability and each future symbol probability (fixing the symbols to be the actual y's of course). Just as for the α's, the β's can be interpreted as likelihoods, or unnormalized posterior probabilities. And, wonderfully, equation (16.3.15) can be solved by a *backward* recurrence,

$$\beta_{t-1}(i) = \sum_{j=0}^{M-1} A_{ij} b_j(y_t) \beta_t(j) \qquad (0 \le i < M) \quad \text{for} \quad t = N-1, \dots, 1 \tag{16.3.16}$$

Calculating all the β's for $t = N-1, N-2, \dots, 0$ is called a *backward estimate*.

Now here is the big payoff: From the definitions of the α's and β's, the product $\alpha_t(i)\beta_t(i)$ is the unnormalized posterior probability of state i at time t given *all* the data. If we normalize it by dividing by

$$\mathcal{L}_t = \sum_{i=0}^{M-1} \alpha_t(i)\beta_t(i) \tag{16.3.17}$$

we get the desired estimate of the probability of each separate state at each separate time,

$$P_t(i) = \frac{\alpha_t(i)\beta_t(i)}{\mathcal{L}_t} \tag{16.3.18}$$

Further, it follows from the definitions (16.3.13) and (16.3.15) that $\mathcal{L}_t$ is actually independent of t, so we can omit the subscript t and calculate it only once. (In practice, one often renormalizes at each time t for greater numerical stability, however.) The value of $\mathcal{L}$ can be interpreted as the probability (or likelihood) of the whole data set, given the parameters of the model.

Equations (16.3.14) and (16.3.16), taken together, are called the *forward-backward algorithm* for state estimation in a hidden Markov model.

Translating HMM into code, we start with a structure that will hold the various quantities that come into play, and its constructor. You construct an `HMM` structure by specifying a transition probability matrix $\mathbf{A}$ (N.B.: not $\mathbf{A}^T$), a symbol probability matrix $b_{ik} \equiv b_i(k)$, and a vector of observed data $\mathbf{y}$.

hmm.h

```
struct HMM {
Structure for a hidden Markov model and its methods.
    MatDoub a, b;                           Transition matrix and symbol probability matrix.
    VecInt obs;                             Observed data.
    Int fbdone;
    Int mstat, nobs, ksym;                  Number of states, observations, and symbols.
    Int lrnrm;
    MatDoub alpha, beta, pstate;            Matrices α, β, and P_i(t).
    VecInt arnrm, brnrm;
    Doub BIG, BIGI, lhood;
    HMM(MatDoub_I &aa, MatDoub_I &bb, VecInt_I &obs);    Constructor; see below.
    void forwardbackward();                 HMM state estimation.
    void baumwelch();                       HMM parameter re-estimation.
    Doub loglikelihood() {return log(lhood)+lrnrm*log(BIGI);}
    Returns the log-likelihood computed by forwardbackward().
};

HMM::HMM(MatDoub_I &aa, MatDoub_I &bb, VecInt_I &obss) :
    a(aa), b(bb), obs(obss), fbdone(0),
    mstat(a.nrows()), nobs(obs.size()), ksym(b.ncols()),
    alpha(nobs,mstat), beta(nobs,mstat), pstate(nobs,mstat),
    arnrm(nobs), brnrm(nobs), BIG(1.e20), BIGI(1./BIG)  {
    Constructor. Input are the transition matrix aa, the symbol probability matrix bb, and the
    observed vector of symbols obss. Local copies are made, so the input quantities need not
    be preserved by the calling program.
    Int i,j,k;
    Doub sum;
    Although space constraints make us generally stingy about printing code for checking input,
    we will save you a lot of grief by doing so in this case. If you get “matrix not normalized”
    errors, you probably have your matrix transposed. Note that normalization errors <1% are
    silently fixed.
    if (a.ncols() != mstat) throw("transition matrix not square");
    if (b.nrows() != mstat) throw("symbol prob matrix wrong size");
    for (i=0; i<nobs; i++) {
        if (obs[i] < 0 || obs[i] >= ksym) throw("bad data in obs");
    }
    for (i=0; i<mstat; i++) {
        sum = 0.;
        for (j=0; j<mstat; j++) sum += a[i][j];
        if (abs(sum - 1.) > 0.01) throw("transition matrix not normalized");
        for (j=0; j<mstat; j++) a[i][j] /= sum;
    }
    for (i=0; i<mstat; i++) {
        sum = 0.;
        for (k=0; k<ksym; k++) sum += b[i][k];
        if (abs(sum - 1.) > 0.01) throw("symbol prob matrix not normalized");
        for (k=0; k<ksym; k++) b[i][k] /= sum;
    }
}
```

Now, to actually do the forward-backward estimation, you call the function `forwardbackward`. This fills the matrix `pstate`, so that $\texttt{pstate}_{ti} = P_t(i)$. It also sets the internal variables `lhood` and `lrnrm` so that the function `loglikelihood` returns the logarithm of $\mathcal{L}$. Don't be surprised at how large in magnitude this (negative) number can be. The probability of any *particular* data set of more than trivial length is astronomically small!

In the following code, the quantities `BIG`, `BIGI`, `arnrm`, `brnrm`, and `lrnrm` all relate to dealing with values that would far underflow an ordinary floating format. The basic idea is to renormalize as necessary, keeping track of the accumulated number of renormalizations. At the end, when an α, a β, and an $\mathcal{L}$ are combined, probability values of reasonable magnitude result.

```
void HMM::forwardbackward() {                                                          hmm.h
```
HMM forward-backward algorithm. Using the stored a, b, and obs matrices, the matrices alpha, beta, and pstate are calculated. The latter is the state estimation of the model, given the data.
```
    Int i,j,t;
    Doub sum,asum,bsum;
    for (i=0; i<mstat; i++) alpha[0][i] = b[i][obs[0]];
    arnrm[0] = 0;
    for (t=1; t<nobs; t++) {                Forward pass.
        asum = 0;
        for (j=0; j<mstat; j++) {
            sum = 0.;
            for (i=0; i<mstat; i++) sum += alpha[t-1][i]*a[i][j]*b[j][obs[t]];
            alpha[t][j] = sum;
            asum += sum;
        }
        arnrm[t] = arnrm[t-1];              Renormalize the α's as necessary to avoid under-
        if (asum < BIGI) {                      flow, keeping track of how many renormal-
            ++arnrm[t];                         izations for each α.
            for (j=0; j<mstat; j++) alpha[t][j] *= BIG;
        }
    }
    for (i=0; i<mstat; i++) beta[nobs-1][i] = 1.;
    brnrm[nobs-1] = 0;
    for (t=nobs-2; t>=0; t--) {             Backward pass.
        bsum = 0.;
        for (i=0; i<mstat; i++) {
            sum = 0.;
            for (j=0; j<mstat; j++) sum += a[i][j]*b[j][obs[t+1]]*beta[t+1][j];
            beta[t][i] = sum;
            bsum += sum;
        }
        brnrm[t] = brnrm[t+1];
        if (bsum < BIGI) {                  Similarly, renormalize the β's as necessary.
            ++brnrm[t];
            for (j=0; j<mstat; j++) beta[t][j] *= BIG;
        }
    }
    lhood = 0.;                             Overall likelihood is lhood with lnorm renormal-
    for (i=0; i<mstat; i++) lhood += alpha[0][i]*beta[0][i];          izations.
    lrnrm = arnrm[0] + brnrm[0];
    while (lhood < BIGI) {lhood *= BIG; lrnrm++;}
    for (t=0; t<nobs; t++) {                Get state probabilities from α's and β's.
        sum = 0.;
        for (i=0; i<mstat; i++) sum += (pstate[t][i] = alpha[t][i]*beta[t][i]);
```
The next line is an equivalent calculation of sum. But we'd rather have the normalization of the $P_i(t)$'s be more immune to roundoff error. Hence we do the above sum for each value of t.
```
        // sum = lhood*pow(BIGI, lrnrm - arnrm[t] - brnrm[t]);
        for (i=0; i<mstat; i++) pstate[t][i] /= sum;
    }
    fbdone = 1;                             Flag prevents misuse of baumwelch(), later.
}
```

You may be wondering how well forwardbackward is able to do at predicting the hidden states of Teen Life, given just a long string of output symbols. If we take the prediction to be the state with the highest probability at each time, then this is correct about 78% of the time. Another 17% of the time, the correct state has the second-highest probability, often when the top two probabilities are nearly equal. It is an important property of HMMs that the output is not only a prediction, but also a quantitative assessment of how "sure" the model is of that prediction.

16.3.2 Some Variations on HMM

HMM state estimation with the forward-backward algorithm is a very flexible formalism, and many variants are possible. For example, in decoding codes on a trellis, as we did above, the symbols 0 or 1 are emitted not by the states, but by the transitions between the states. If we want to use HMM for that problem (we will say more about this below), we must replace $b_i(k)$ with $b_{ij}(k)$, the probability of emitting symbol k in a transition from state i to state j. The forward and backward recurrences now become

$$\begin{aligned}\alpha_{t+1}(j) &= \sum_{i=0}^{M-1} \alpha_t(i) A_{ij} b_{ij}(y_{t+1}) \\ \beta_{t-1}(i) &= \sum_{j=0}^{M-1} A_{ij} b_{ij}(y_t) \beta_t(j)\end{aligned} \tag{16.3.19}$$

and we start off the α's with the special rule $\alpha_0(i) = 1$, since (like the case of $\beta_{N-1}(i)$ previously) the probability of the data is 1 before there are any data.

Another variant case is where one or more intermediate states are known exactly. In that case, one or more of the sums over $i_0, i_1, \ldots, i_{t-1}$ in equation (16.3.13) is left out, and the corresponding index on an A and b gets replaced by the known state number. If you track through how this affects the recurrence equation (16.3.14), you'll see that the new procedure is

- calculate the α's forward to, and including, a known state;
- zero all the α values at that time except for that of the known state;
- don't renormalize anything (though you feel tempted to do so); and
- continue forward with the α's for the next timestep.

Proceed similarly for the β's.

The opposite variant is where you have *missing data*, meaning that for some values of t there is no observation of the symbol $\mathbf{y}_t$. In this case, all you need to do is to make a special case for the symbol probability,

$$b_i(y_t) \equiv 1, \qquad (0 \le i < M) \qquad t \in \{\text{missing}\} \tag{16.3.20}$$

meaning that, regardless of state i, the probability of observing the data (meaning no data) at time t is unity. Now proceed as usual to calculate the state probabilities. If you then want to *reconstruct* the missing data, you can calculate its posterior probabilities,

$$P(y_t = k \mid \mathbf{y}) = \sum_{i=0}^{M-1} P_i(t) b_i(k) = \sum_{i=0}^{M-1} \frac{\alpha_i(t)\beta_i(t)}{\mathcal{L}} b_i(k) \qquad t \in \{\text{missing}\} \tag{16.3.21}$$

16.3.3 Bayesian Re-Estimation of the Model Parameters

This is magical. The probability that we were in state i at time t is $\alpha_t(i)\beta_t(i)/\mathcal{L}$. What is the probability, given the data $\mathbf{y}$, that a given transition, say between time

t and time $t+1$, was a transition between state i and state j? We write, applying various of the laws of probability,

$$
\begin{aligned}
P(s_t = i, s_{t+1} = j \mid \mathbf{y})& \\
&= P(s_{t+1} = j \mid \mathbf{y}, s_t = i) P(s_t = i \mid \mathbf{y}) \\
&= \frac{P(\mathbf{y} \mid s_{t+1} = j, s_t = i) P(s_{t+1} = j \mid s_t = i)}{\sum_j P(\mathbf{y} \mid s_{t+1} = j, s_t = i) P(s_{t+1} = j \mid s_t = i)} P(s_t = i \mid \mathbf{y}) \\
&= \frac{[\alpha_t(i) b_j(y_{t+1}) \beta_{t+1}(j)][A_{ij}]}{\sum_j [\alpha_t(i) b_j(y_{t+1}) \beta_{t+1}(j)][A_{ij}]} \frac{[\alpha_t(i)\beta_t(i)]}{\mathcal{L}} \\
&= \frac{\alpha_t(i) A_{ij} b_j(y_{t+1}) \beta_{t+1}(j)}{\mathcal{L}}
\end{aligned}
\tag{16.3.22}
$$

Note how the sum over j in the denominator disappears by the recurrence (16.3.16) for $\beta_t(i)$.

So, for a long run of data, we can compute the fraction of the time that a state i transitions to state j as the estimated number of $i \rightarrow j$ transitions divided by the estimated number of i states,

$$
\widehat{A}_{ij} = \frac{\sum_t \alpha_t(i) A_{ij} b_j(y_{t+1}) \beta_{t+1}(j)}{\sum_t \alpha_t(i) \beta_t(i)}
\tag{16.3.23}
$$

noting that the $\mathcal{L}$'s cancel out. The reason for calling this quotient $\widehat{A}_{ij}$ is that it is a *re-estimation* of the transition probability A_{ij}. The corresponding re-estimation of the symbol probability matrix $b_i(k)$ is the fraction of all i states that emit a symbol k, namely

$$
\widehat{b}_i(k) = \frac{\sum_t \delta(y_t, k) \alpha_t(i) \beta_t(i)}{\sum_t \alpha_t(i) \beta_t(i)}
\tag{16.3.24}
$$

where $\delta(j, k)$ is 1 if $j = k$, zero otherwise.

You might think that this process is somehow circular, or that re-estimating A_{ij} and $b_i(k)$ in this fashion only introduces noise that degrades the model. Far from it! Baum and Welch first showed that replacing A_{ij} by $\widehat{A}_{ij}$ and $b_j(k)$ by $\widehat{b}_j(k)$, and then recalculating the probabilities of each state at each time by the forward-backward algorithm, always *increases* $\mathcal{L}$, the overall likelihood of the model. It is, in fact, an EM algorithm (cf. §16.1, and see below). You can continue this cycle of estimating states (forward-backward) and re-estimating model probabilities (Baum-Welch), obtaining further increases in $\mathcal{L}$, until convergence to a maximum is achieved. Equations (16.3.23) and (16.3.24) are known as *Baum-Welch re-estimation*.

So the magic is this: We began by estimating states in a *known* hidden Markov model. We now see that, *just from the data*, we can get not only an estimate of the states, but also an estimate of the model itself, that is, the transition probabilities and symbol probabilities. Like any iterative process, this works best if we have a good initial guess. But it will often converge to a good model from a fairly random initial guess. (You should not start with exactly uniform probabilities, because that creates a symmetry that the iteration finds hard to break.)

The code is straightforward. The updating of $b_i(k)$ comes almost for free as a byproduct of computing the denominator in the update for A_{ij}. Like the forward-backward algorithm, Baum-Welch re-estimation takes $O(NM^2)$ operations.

hmm.h

```
void HMM::baumwelch() {
```

Baum-Welch re-estimation of the stored matrices a and b, using the data obs and the matrices alpha and beta as computed by forwardbackward() (which must be called first). The previous values of a and b are overwritten.

```
    Int i,j,k,t;
    Doub num,denom,term;
    MatDoub bnew(mstat,ksym);
    Doub powtab[10];                    Fill table of powers of BIGI.
    for (i=0; i<10; i++) powtab[i] = pow(BIGI,i-6);
    if (fbdone != 1) throw("must do forwardbackward first");
    for (i=0; i<mstat; i++) {           Looping over i, get denominators and new b.
        denom = 0.;
        for (k=0; k<ksym; k++) bnew[i][k] = 0.;
        for (t=0; t<nobs-1; t++) {
            term = (alpha[t][i]*beta[t][i]/lhood)
                * powtab[arnrm[t] + brnrm[t] - lrnrm + 6];
            denom += term;
            bnew[i][obs[t]] += term;
        }
        for (j=0; j<mstat; j++) {       Inner loop over j gets elements of a.
            num = 0.;
            for (t=0; t<nobs-1; t++) {
                num += alpha[t][i]*b[j][obs[t+1]]*beta[t+1][j]
                    * powtab[arnrm[t] + brnrm[t+1] - lrnrm + 6]/lhood;
            }
            a[i][j] *= (num/denom);
        }
        for (k=0; k<ksym; k++) bnew[i][k] /= denom;
    }
    b = bnew;
    fbdone = 0;                         Don't let this routine be called again until forward-
}                                           backward() has been called.
```

You must always precede a call to baumwelch by a call to forwardbackward, since the latter updates the α and β tables. Also, as you alternate calls to the two functions, you monitor convergence by the value of the log-likelihood calculated by forwardbackward.

Be aware that convergence can be excruciatingly slow! The references describe methods by which convergence can be accelerated in some cases. Common difficulties are when a rare state is not correctly captured by the model, or when the model thinks that there are two states, with nearly identical transition probabilities, when there is really only one. If you have even a plausible guess for the transition probability matrix, you should use it to start. There are many applications where you shouldn't use re-estimation at all: If you have a pretty good model to start with, just use it (via forwardbackward) to analyze your data, and don't even think about re-estimating.

The Baum-Welch re-estimation algorithm, which dates from the mid-1960s, was generalized in the mid-1970s by Dempster, Laird, and Rubin, as the *expectation-maximization (EM) algorithm*, with a variety of applications to problems with missing or censored data. (An example is the Gaussian mixture model in §16.1.) In this more general language, the forward-backward algorithm is an E-step, while Baum-Welch is an M-step. Alas, one small cloud in an otherwise bright sky is that the maximum of $\mathcal{L}$ achieved by multiple EM iterations is only guaranteed to be a local, not a global, maximum.

HMM has found wide application in speech recognition, gene sequence comparison, financial models, and a variety of other fields. The references give specifics.

16.3.4 Comparing the Viterbi Algorithm with HMM

It is important to understand the similarities and differences between the Viterbi algorithm and hidden Markov modeling (its forward-backward algorithm in particular).

When we discussed the Viterbi algorithm in the context of decoding, we made the implicit assumption that a 1 bit was a priori as likely as a 0 bit. It is straightforward to generalize the Viterbi algorithm to include an arbitrary a priori transition probability A_{ij}, just like HMM. In that case, the probability factor on each edge (whose negative logarithm is the edge cost) is the product of two terms, again just like HMM, $A_{ij} b_{ij}(k)$, where now $b_{ij}(k)$ is the probability of observing the observed symbol k given that an $i \rightarrow j$ transition occurred.

We discussed Baum-Welch parameter re-estimation for HMMs in some detail. Re-estimation of the parameters in a Viterbi model, often called *Viterbi training*, is analogous. Take the most probable path output by the algorithm (or ensemble of paths collected from the decodes of many codewords). Count the number of $i \rightarrow j$ transitions seen along these paths and the numbers of each symbol k seen for each pair i, j. Now re-estimate A_{ij} and $b_{ij}(k)$ by the obvious normalizations of these counts.

The Viterbi algorithm and the forward estimation part of the forward-backward algorithm are structurally very similar. In both cases, we sweep forward in time (or by stages) and assign a likelihood (or posterior probability) to each node, based on the data already seen. The difference is that Viterbi assigns to a node the probability of the *single best path* that reaches it, while forward-backward assigns the sum of probabilities over *all possible paths* to that node. Indeed the Viterbi algorithm is sometimes called the *min-sum algorithm* while forward-backward is referred to as the *sum-product algorithm*, just to highlight this distinction. (In the context of coding theory, the forward-backward algorithm is also sometimes called the *BCJR* or *Bahl-Cocke-Jelinek-Raviv* algorithm. In other contexts it is sometimes called *belief propagation*.)

The backward passes of the two algorithms have somewhat different structures. For Viterbi, the backward pass simply consolidates the information about the single most probable path that is already implicit in the node labeling. For forward-backward, as we have seen, the backward pass is needed to get posterior probabilities for each node that use all the data, both ahead of and behind any time t.

If you think you have a choice between using the Viterbi algorithm or using HMM, you should probably think again. Most problems clearly favor only one or the other method. If your desired output is a valid *path* on the graph, then HMM won't do: It might yield a set of highly probable nodes that just don't lie on any single path. For example, you might have the first half of one codeword and the second half of another, with no graph edge connecting the two halves. That is why decoding theory usually starts in the world of Viterbi (although, in some more complicated constructs, it can end up with a foot in each world).

On the other hand, if you care about which *nodes* are visited, then HMM is most likely what you want. In fact, Viterbi can give very poor results. The most probable path is often *very improbable* when compared to the sum of all paths that lead through a particular node, one possibly not on the most probable path. Or, another way of describing this, there may be exponentially many paths with not-too-different probabilities, so the node probabilities are determined by the statistics of where they

all channel, not by which one path happens to have the highest probability.

It is very easy to "mine" HMM for alternative possibilities, since it yields seemingly every possible posterior probability that you might want to know. It is quite difficult to get anything from the Viterbi algorithm other than the single most probable path. That is because the enumeration of all possible paths is vastly harder than the enumeration of all possible nodes; the Bellman-Dijkstra-Viterbi algorithm is exquisitely good at keeping only the information that it needs. Data structures for finding more than one probable path rapidly become very complex.

Finally, we must take aim at the myth, occasionally heard, that the Viterbi algorithm, as a pure maximum likelihood (ML) estimate, is somehow "less Bayesian" than HMM. In fact, HMM is also a pure ML estimate if you look only at the state i with the largest $\alpha_t(i)\beta_t(i)$ at each time t, neither normalizing its value nor looking at any other i's. But you are then ignoring a wealth of useful information about the other possible states. (This, in part, is why you should get with the Bayesian program!) We think that both HMM and Viterbi are in fact Bayesian to the core. If there were good ways to enumerate all the other paths and their likelihoods, we would not hesitate to normalize the best-path likelihood and call it a posterior probability. It is only because of the difficulty of this enumeration that it is possible to keep the Viterbi algorithm's Bayesian character "in the closet"; and there is no advantage, that we can see, in doing so.

CITED REFERENCES AND FURTHER READING:

Hsu, H.P. 1997, *Schaum's Outline of Theory and Problems of Probability, Random Variables, and Random Processes* (New York: McGraw-Hill).

Häggström, O. 2002, *Finite Markov Chains and Algorithmic Applications* (Cambridge, UK: Cambridge University Press).

Norris, J.R. 1998, *Markov Chains*, Cambridge Series in Statistical and Probabilistic Mathematics (Cambridge, UK: Cambridge University Press).

MacDonald, I.L. and Zucchini, W. 1997, *Hidden Markov and Other Models for Discrete-Valued Time Series* (Boca Raton, FL: Chapman & Hall/CRC).

McLachlan, G.J. and Krishnan, T. 1996, *The EM Algorithm and Extensions* (New York: Wiley).

Rabiner, L. 1989, "A Tutorial on Hidden Harkov Models and Selected Applications in Speech Recognition," *Proceedings of the IEEE*, vol. 77, no. 2, pp. 257-286. [Review article on the use of HMMs in speech recognition.]

Eddy, S.R. 1998, "Profile Hidden Markov Models," *Bioinformatics*, vol. 14, pp. 755-763. [Review article on the use of HMMs in genetics.]

16.4 Hierarchical Clustering by Phylogenetic Trees

Hierarchical clustering is a type of *unsupervised learning*: We seek algorithms that figure out how to cluster an unordered set of input data without ever being given any training data with the "right answer." As the name implies, the output of a hierarchical clustering algorithm is a bunch of fully nested sets. The smallest sets are the individual data elements. The largest set is the whole data set. Intermediate

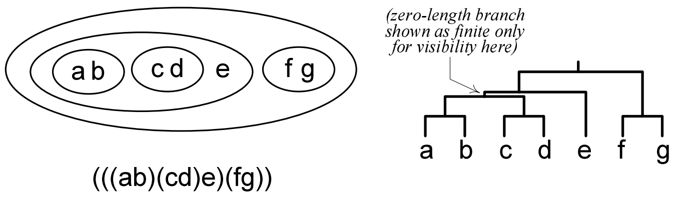

Figure 16.4.1. Representations of hierarchical classification. Top left: Diagram showing fully nested sets. Bottom left: Equivalent parenthesized expression. Right: Binary tree (with possibly zero branch lengths).

sets are nested; that is, the intersection of any two sets is either the null set, or else the smaller of the two sets.

What you need to get started with hierarchical clustering is either a set of *sequences*, or else a *distance matrix*. *Character-based methods* start with n sequences each m characters long (for example, DNA bases or protein amino acids). A toy example might be the $n = 16$ sequences of $m = 12$ characters,

0.	CGGTTGGGAGCT	8.	GCGCGGTGCAGC
1.	AGGTCGTGAGGT	9.	AGGCGGTGCGGG
2.	TGGTTGGGGTTT	10.	GGGCGGGGCGGG
3.	TGGGTGCGAGTT	11.	GGGCGCTGCGGG
4.	ACGTTTGGGTGA	12.	GGACGGAGGCTG
5.	AAGGTTGGGGAA	13.	GGGTGGGAGCTG
6.	GTCTTTCGGGTG	14.	AGGAGGCTGATG
7.	CACTTGCGGGGG	15.	TGGCGGATGATG

It is probably not immediately obvious that these sequences were generated from a balanced five-level binary tree, with GGGGGGGGGGGG at the root, and with each daughter node having two random mutations from her parent. We will see below the extent to which some of the algorithms that we discuss can figure this out from the data. A realistic case likely would have significantly longer sequences than this, and fewer mean mutations per character; the number of sequences might be either more, or fewer, than this toy.

The alternative starting point is with an $n \times n$ matrix d_{ij} of distances between all pairs of your n data points, which might now be sequences, points in N-dimensional space, or whatever. You are responsible for making sure that the distance matrix satisfies four conditions:

$$\begin{aligned} d_{ij} &\geq 0 && \text{(positivity)} \\ d_{ii} &= 0 && \text{(zero self-distance)} \\ d_{ij} &= d_{ji} && \text{(symmetry)} \\ d_{ik} &\leq d_{ij} + d_{jk} && \text{(triangle inequality)} \end{aligned} \tag{16.4.1}$$

for all i, j, k. We'll discuss below how to get distances from sequences, if that's the way you want to go.

Figure 16.4.1 shows three representations of the same hierarchical clustering of seven data elements. The two representations on the left are self-explanatory. The one on the right, the binary tree, takes explaining on one point: If, in the set diagram, (ab), (cd), and (e) are clustered "democratically," then why does the binary tree

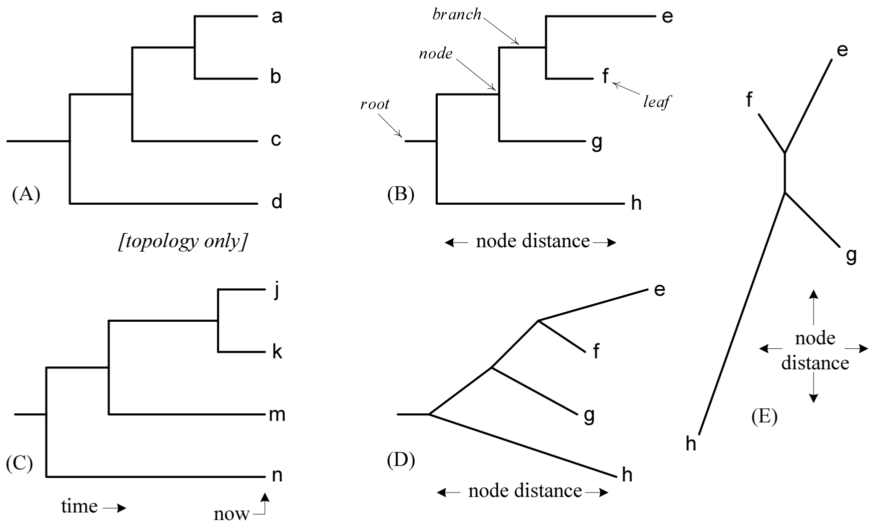

Figure 16.4.2. Types of trees. A *cladogram* (A) has arbitrary branch lengths. Only the topology is intended to be represented. A *phylogram* or *phylogenetic tree* (B) is an additive tree, where the distance between any two nodes/leaves is the sum of lengths of the horizontal connecting branches. An *ultrametric tree* (C) is an additive tree with the property that any node has the same distance to all of its leaves (as when all lengths represent time). Tree (D) is an alternative way of drawing tree (B). Again, only horizontal distances are significant. In an *unrooted tree* (E), line lengths represent distances independent of orientation. The tree (E) is the unrooted depiction of (B) or (D).

select (e) arbitrarily as the descendant of a higher node, instead of having three equal descendants from a common node?

The answer is just convention. Binary trees are the adopted common language of hierarchical clustering because (i) they emerge naturally from the concept of mutation events in biology, (ii) they are somewhat easier to represent in a computer than more general trees, and, (iii) they are often easier to prove theorems about. Our binary trees will almost always have the concept of *branch length*, a representation of some measure of difference between a parent node and its child. When we need to connect up democratically some number of nodes greater than two, we do it with zero-length branches. A convention is to view all topologies of nodes so connected as being equivalent.

16.4.1 Tree Basics

Figure 16.4.2 shows several ways of drawing binary trees and introduces some further terminology. The data points are *leaves*, meaning that they are generally taken as terminal nodes on the tree. Trees are often drawn either sideways (root left, leaves right) or upside down (root top, leaves bottom) by comparison with their arboreal namesake whose roots are on the bottom, leaves on top — at least for most trees that we see! A tree without meaningful branch lengths is usually called a *cladogram*. These have a rich historical tradition in pre-molecular biology but are viewed with alarm by most bioscientists today. A tree with meaningful branch lengths representing *distances* (in some metric) between nodes and their children, or between leaves, is called a *phylogram* or *phylogenetic tree*. (However, some authors use the terms cladogram and phylogram interchangeably, while some others use the words merely

to distinguish different drawing styles.)

To a mathematician, a phylogenetic tree is an *additive tree*, meaning that the tree's path lengths induce a *distance metric* between any two leaves, namely the sum of path lengths up and down that connect the two leaves through their unique closest common ancestor. In real situations, the data we are given often are not exactly represented by an additive distance metric. Thus, the problem of hierarchical clustering amounts to finding a way of projecting such data onto the set of all additive trees in a useful, or statistically justifiable, way.

Given an additive tree, it is easy to compute its distance matrix d_{ij}, defined now as the matrix of all distances between pairs of leaf nodes. But what about the reverse? Given some symmetric matrix d_{ij}, is it possible to determine whether there exists an additive tree that instantiates it? Yes. One answer is the *four-point condition* for additive trees: Given four distinct leaves i, j, k, l, an additive tree exists if and only if

$$d_{ij} + d_{kl} \le \max(d_{ik} + d_{jl},\ d_{il} + d_{jk}) \tag{16.4.2}$$

for all choices of i, j, k, l. In words, this is equivalent to the statement: For all distinct i, j, k, l, there is a tie for the maximum of the three sums of the form $d_{ij} + d_{kl}$. Later, when we discuss the neighbor-joining method, we will have a more practical algorithmic answer.

As Figure 16.4.2 illustrates, a tree can either be *rooted* or *unrooted*. An unrooted tree can always be rooted arbitrarily, by choosing any branch, grasping its midpoint between your thumb and forefinger, and then shaking the tree so that all the other branches drop downward into place. (We could give a much more mathematical description, but it would not add any clarity.) Some hierarchical clustering algorithms yield rooted trees, where the root has some meaning with respect to the data; others yield unrooted trees, although they may be drawn as if rooted, simply as a graphical convention. It's important to keep track of which kind of algorithm you are using.

You may wonder why the data points must always be leaves (terminal nodes). Might not some data points actually be good ancestors of others? The answer is again a mixture of history and convention: If the leaves are observed living taxa, then they are by definition alive today. If "today" represents terminal nodes, then they are by definition leaves. What makes this a mere convention is that we can always connect a leaf to an ancestor node by a zero-length branch, so that ancestor-versus-living-taxon becomes a distinction without a difference. A benefit of this convention is that we always know in advance how many internal nodes will be generated by n data points: $n - 1$ if the tree is rooted, or $n - 2$ for an unrooted tree, independent of the tree's topology. (If this isn't obvious, then draw a few pictures.)

If path length denotes, literally, evolutionary time, then a phylogenetic tree has the additional property of being *ultrametric* (refer to Figure 16.4.2). Ultrametric trees are defined as additive trees with the property that the distance from any node to all of its descendant leaves is the same for all such leaves. Clearly this is the case if all the leaves have the same "time distance" from their common ancestor. In the early 1960s, it was proposed that accepted mutation rates might be close enough to constant that, at the molecular level, actual evolutionary data might be close to ultrametric, i.e., that there was a "molecular clock." For most cases, this is no longer believed to be true. For example, mutation rates in *E. coli* have been found to vary by two orders of magnitude. Ultrametric trees are important mathematically, as we will see, but almost never realistic in their own right.

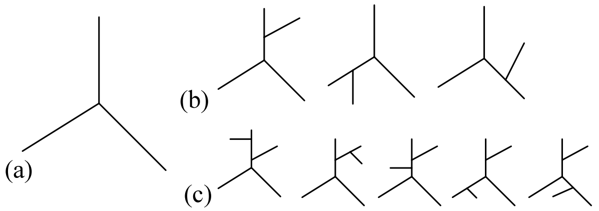

Figure 16.4.3. Tree counting. (a) There is just one unrooted tree with three leaves. (b) There are three ways to add a fourth leaf. (c) For each of the trees in (b), there are five ways to add a fifth leaf. Continuing to add leaves, the number of trees with n leaves is $1 \times 3 \times 5 \times \cdots \times (2n-5)$.

The test, analogous to equation (16.4.2), for whether a given distance matrix is ultrametric is the *three-point condition*,

$$d_{ij} \le \max(d_{ik}, d_{jk}) \tag{16.4.3}$$

for all distinct i, j, k. Equivalently, in words: Among the three distances connecting three distinct leaves, there is a tie for the maximum. Here too there is a more practical algorithmic answer, which we will mention later.

There are a *lot* of possible ways to draw a tree that connects n leaves. As Figure 16.4.3 illustrates, the number of distinct, unrooted, possibilities is

$$N_{\text{trees}}(n) = 1 \times 3 \times 5 \times \cdots \times (2n-5) \equiv (2n-5)!! \tag{16.4.4}$$

The fact that this expression grows super-exponentially with n creates a dilemma in the field of computational phylogenetics: An algorithm that requires an explicit enumeration of, or explicit search over, all possible trees can be useful only for small values of n. Thus, $N_{\text{trees}}(10) \approx 2 \times 10^6$, is easy, but $N_{\text{trees}}(20) \approx 2 \times 10^{20}$ is practically impossible.

16.4.2 Strategies for the Hierarchical Clustering Problem

If you are starting with a set of sequences, then, schematically, the goal of a character-based method is to find the best of all possible trees, given the data, for some definition of "best":

$$(\text{sequences}) \xrightarrow[\ ]{\text{search for ``best'' tree}} (\text{tree}) \tag{16.4.5}$$

The two most common definitions of "best" are *maximum parsimony* and *maximum likelihood*, both of which we will say more about below [1]. Character methods are generally limited by the super-exponential explosion in the number of trees. Although the exhaustive search can be limited to some extent, for example by heuristics of various kinds, its long shadow can never be avoided entirely.

Alternatively, if you are starting with a distance matrix, the problem is to find the additive tree whose induced distance matrix (by branch lengths up and down) is closest to d_{ij}, according to some criterion of closeness. This is also an exponentially difficult problem. In practice, however, distance methods almost always use fast heuristic methods that, while provably exact only for the unrealistic case where d_{ij}

already comes from an ultrametric or additive tree, actually work pretty well for distance matrices encountered in practice. In other words, the adopted scheme is

$$\begin{pmatrix}\text{distance}\\ \text{matrix}\end{pmatrix} \xrightarrow[\ ]{\substack{\text{ultrametric tree}\\ \text{heuristic}}} (\text{tree}) \tag{16.4.6}$$

or

$$\begin{pmatrix}\text{distance}\\ \text{matrix}\end{pmatrix} \xrightarrow[\ ]{\substack{\text{additive tree}\\ \text{heuristic}}} (\text{tree}) \tag{16.4.7}$$

The ability of these fast heuristic methods to give "pretty good" solutions to NP-hard problems is remarkable, and only partially understood [2].

The most widely used heuristics are all *agglomerative methods*, meaning that they start by connecting individual data points into small clusters, then connect those clusters, and so forth. Common ultrametric-tree heuristic methods are *UPGMA*, *WPGMA*, *single linkage clustering*, and *complete linkage clustering*. The most widely used additive-tree heuristic method — and probably the most widely used phylogenetic clustering method overall — is the *neighbor-joining (NJ)* method [6]. We will discuss, and implement, all the mentioned heuristic methods below.

There are a few, less-well-developed, distance-based methods that avoid heuristics by finding provably error-bounded methods for transforming an arbitrary distance matrix into the matrix of an additive tree, then exactly constructing the resulting tree [3,4],

$$\begin{pmatrix}\text{distance}\\ \text{matrix}\end{pmatrix} \xrightarrow[\ ]{\substack{\text{find nearby}\\ \text{additive}}} \begin{pmatrix}\text{additive}\\ \text{matrix}\end{pmatrix} \xrightarrow[\ ]{\substack{\text{exact}\\ \text{construction}}} (\text{tree}) \tag{16.4.8}$$

Though more rigorous than the heuristic methods, there is little evidence that these methods produce better results [5].

Evidently, one can always turn a character-based problem into a distance-based one by defining a distance on character sequences,

$$(\text{sequences}) \xrightarrow[\ ]{\substack{\text{define a}\\ \text{distance}}} \begin{pmatrix}\text{distance}\\ \text{matrix}\end{pmatrix} \tag{16.4.9}$$

and then continuing with schemes (16.4.6) or (16.4.7).

The obvious distance between two sequences is their *Hamming distance* $H(i, j)$, which is defined as the number of character positions in which sequence i differs from sequence j, an integer between 0 and m. However, when you are given not just the data, but also a statistical model defining how it was generated (i.e., "evolved"), there is often a *corrected distance transformation* that will give better tree reconstructions [2]. For example, the popular *Cavender-Felsenstein* model (whose discussion is beyond our scope) has the corrected distance transformation

$$d_{ij} = -\tfrac{1}{2}\log\left(1 - 2H(i, j)/m\right) \tag{16.4.10}$$

This expression can be used directly when sequences are long enough, or mutation probabilities small enough, that the argument of the logarithm is never negative. If your data produce a negative argument, then a standard workaround is to use a multiple (1× or 2×) of the largest computable d_{ij} for all uncomputable d_{ij}'s. Corrected distance transformations also exist for general Markov models.

Corrected distance transformations have the defining (and desirable) property that as the sequence length increases, the matrix of observed corrected distances will converge to the distance matrix of an additive tree. (This is not true for the uncorrected Hamming distance, incidentally.) In such a case, the power of an additive-tree heuristic method like neighbor joining is much less mysterious. Corrected distance transformations thus provide a statistical justification for the use of the neighbor-joining method.

16.4.3 Implementation of Agglomerative Methods

The general scheme of an agglomerative method is, first, to initialize n active clusters, each containing one data point, and, second, to repeat the following operations exactly $n-2$ times:

- Find the two active clusters that are closest by some prescribed distance measure.
- Create a new active cluster that combines the two.
- Connect the new cluster, as parent, to the two closest clusters, as children, with some prescription for the two branch lengths.
- Delete the two children from the active list.
- Compute, by some prescription, distances from the new cluster to the active clusters that remain.

Each repetition of these steps reduces the active cluster list by exactly one (one addition, two deletions), so after $n-2$ repetitions there will be exactly two active clusters. You connect these either by a single branch (unrooted case), or by creating a root node between them (rooted case) with some prescription as to the two root branch lengths.

As we now turn to implementing phylogenetic tree routines, a few words of caution are in order. Hamming's dictum, that the purpose of computing is insight, not numbers, applies here: Much of the value of a phylogenetic tree program lies in its graphics and user interface, both areas outside of our scope. If you are working with any significant quantity of real data, you probably want to use a sophisticated package. As we write, PAUP (Phylogenetic Analysis Using Parsimony) [7] is the most widely used commercial package, both for maximum parsimony trees and also for the various heuristic methods. PHYLIP (Phylogeny Inference Package) [8] is a free package for smaller trees ($\lesssim 20$ taxa). TreeView is a widely used, free, program used for drawing trees in various formats. A user guide to the use of these and other programs is [9]. If the insight you desire lies in algorithmics, not production data, then you may read on.

Here is an abstract base class that implements the general agglomerative method, leaving the various "prescriptions" to be specified by particular derived classes, which we give later.

phylo.h

```
struct Phylagglomnode {
Node for phylogenetic tree.
    Int mo,ldau,rdau,nel;              Pointers up and down; no. of elements.
    Doub modist,dep,seq;               Branch length to parent. See text re. dep and
                                           seq.
};

struct Phylagglom{
Abstract base class for constructing an agglomerative phylogenetic tree.
```

```
Int n, root, fsroot;                    No. of data points, root node, forced root.
Doub seqmax, depmax;                    Max. values of seq, dep over the tree.
vector<Phylagglomnode> t;               The tree.
virtual void premin(MatDoub &d, VecInt &nextp) = 0;
Function called before minimum search.
virtual Doub dminfn(MatDoub &d, Int i, Int j) = 0;
Distance function to be minimized
virtual Doub dbranchfn(MatDoub &d, Int i, Int j) = 0;
Branch length, node i to mother (j is sister).
virtual Doub dnewfn(MatDoub &d, Int k, Int i, Int j, Int ni, Int nj) = 0;
Distance function for newly constructed nodes.
virtual void drootbranchfn(MatDoub &d, Int i, Int j, Int ni, Int nj,
Doub &bi, Doub &bj) = 0;
Sets branch lengths to the final root node.
Int comancestor(Int leafa, Int leafb);        See text discussion of NJ.
Phylagglom(const MatDoub &dist, Int fsr = -1)
    : n(dist.nrows()), fsroot(fsr), t(2*n-1) {}
    Constructor is always called by a derived class.

void makethetree(const MatDoub &dist) {
Routine that actually constructs the tree, called by the constructor of a derived class.
    Int i, j, k, imin, jmin, ncurr, node, ntask;
    Doub dd, dmin;
    MatDoub d(dist);                    Matrix d is initialized with dist.
    VecInt tp(n), nextp(n), prevp(n), tasklist(2*n+1);
    VecDoub tmp(n);
    for (i=0;i<n;i++) {                 Initializations on leaf elements.
        nextp[i] = i+1;                 nextp and prevp are for looping on the distance
        prevp[i] = i-1;                     matrix even as it becomes sparse.
        tp[i] = i;                      tp points from a distance matrix row to a tree
        t[i].ldau = t[i].rdau = -1;         element.
        t[i].nel = 1;
    }
    prevp[0] = nextp[n-1] = -1;         Signifying end of loop.
    ncurr = n;
    for (node = n; node < 2*n-2; node++) {    Main loop!
        premin(d,nextp);                Any calculations needed before min finding.
        dmin = 9.99e99;
        for (i=0; i>=0; i=nextp[i]) {             Find i, j pair with min distance.
            if (tp[i] == fsroot) continue;
            for (j=nextp[i]; j>=0; j=nextp[j]) {
                if (tp[j] == fsroot) continue;
                if ((dd = dminfn(d,i,j)) < dmin) {
                    dmin = dd;
                    imin = i; jmin = j;
                }
            }
        }
        i = imin; j = jmin;
        t[tp[i]].mo = t[tp[j]].mo = node;         Now set properties of the parent
        t[tp[i]].modist = dbranchfn(d,i,j);           and children.
        t[tp[j]].modist = dbranchfn(d,j,i);
        t[node].ldau = tp[i];
        t[node].rdau = tp[j];
        t[node].nel = t[tp[i]].nel + t[tp[j]].nel;
        for (k=0; k>=0; k=nextp[k]) {             Get new-node distances.
            tmp[k] = dnewfn(d,k,i,j,t[tp[i]].nel,t[tp[j]].nel);
        }
        for (k=0; k>=0; k=nextp[k]) d[i][k] = d[k][i] = tmp[k];
        tp[i] = node;                 New node replaces child i in dist. matrix, while child
        if (prevp[j] >= 0) nextp[prevp[j]] = nextp[j];    j gets patched around.
        if (nextp[j] >= 0) prevp[nextp[j]] = prevp[j];
        ncurr--;
    }                                   End of main loop.
```

```
        i = 0; j = nextp[0];                    Set properties of the root node.
        root = node;
        t[tp[i]].mo = t[tp[j]].mo = t[root].mo = root;
        drootbranchfn(d,i,j,t[tp[i]].nel,t[tp[j]].nel,
            t[tp[i]].modist,t[tp[j]].modist);
        t[root].ldau = tp[i];
        t[root].rdau = tp[j];
        t[root].modist = t[root].dep = 0.;
        t[root].nel = t[tp[i]].nel + t[tp[j]].nel;
        We now traverse the tree computing seq and dep, hints for where to plot nodes in a
        two-dimensional representation. See text.
        ntask = 0;
        seqmax = depmax = 0.;
        tasklist[ntask++] = root;
        while (ntask > 0) {
            i = tasklist[--ntask];
            if (i >= 0) {                       Meaning, process going down the tree.
                t[i].dep = t[t[i].mo].dep + t[i].modist;
                if (t[i].dep > depmax) depmax = t[i].dep;
                if (t[i].ldau < 0) {            A leaf node.
                    t[i].seq = seqmax++;
                } else {                        Not a leaf node.
                    tasklist[ntask++] = -i-1;
                    tasklist[ntask++] = t[i].ldau;
                    tasklist[ntask++] = t[i].rdau;
                }
            } else {                            Meaning, process coming up the tree.
                i = -i-1;
                t[i].seq = 0.5*(t[t[i].ldau].seq + t[t[i].rdau].seq);
            }
        }
    }
};
```

The Phylagglom structure creates a tree of Phylagglomnodes. Each node carries pointers to its mother and two daughters, and knows its number of elements (original data points), branch length to its mother, and two floating values dep and seq, which we now explain: The final while block in makethetree() does a depth-first traversal of the finished tree. When it reaches a node in the downward direction, it sets dep to the sum of branch lengths to the root node. The variable dep is thus a hint as to where to plot the node in the depth direction. When the traversal reaches a leaf, it sets seq to a sequential numbering of leaves. When it reaches an internal node in the upward direction, it sets its seq value to the average of the seq values of its two children. The value of seq thus becomes a hint as to where to plot a node perpendicular to the depth direction. If you plot nodes by dep and seq, then plotted branches won't cross each other.

Looking at the nested loops, you can see that makethetree() is $O(n^3)$ in time. Actually, it is straightforward to reduce this to $O(n^2)$: With some extra bookkeeping, you can keep the distances in a structure that allows the shortest to be found without an n^2 search. We have not coded this, just to keep the code shorter and simpler.

16.4.4 Algorithms That Are Exact for Ultrametric Trees

Given a distance matrix that is exactly ultrametric, all of the agglomerative algorithms that we now discuss will (modulo some technical details) reconstruct its tree exactly. The reason that we need more than one such algorithm is because their behaviors can be somewhat different on realistic, nonultrametric, data, in the general

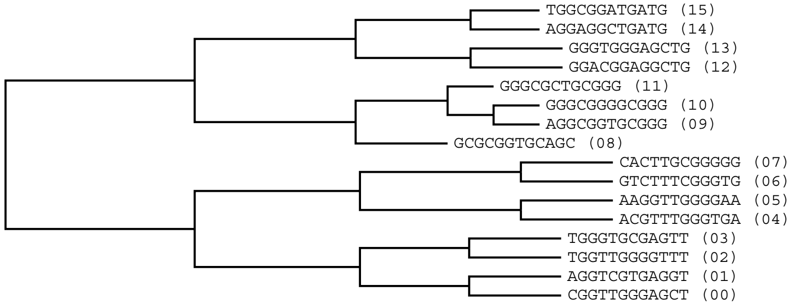

Figure 16.4.4. Example of WPGMA agglomerative clustering on a toy problem. Strings were mutated hierarchically from GGGGGGGGGGGG to produce the input data. WPGMA and related methods (UPGMA, SLC, CLC) yield perfect results for perfectly ultrametric input data, but can deviate badly when that assumption is violated. In this example, however, it does quite well.

scheme of (16.4.6). The different algorithms are distinguished by their prescriptions for computing the distances to new nodes.

The *weighted pair group method using arithmetic averages (WPGMA)* uses this prescription: If a new cluster k is formed from old clusters i and j, then the distance from k to another active cluster p is

$$d_{pk} = d_{kp} = \frac{1}{2}(d_{pi} + d_{pj}) \tag{16.4.11}$$

that is, just the average of distances to the two children.

Implementing code, as a class derived from `Phylagglom`, is

phylo.h

```
struct Phylo_wpgma : Phylagglom {
Derived class implementing the WPGMA method. Only need to define functions that are virtual
in Phylagglom.
    void premin(MatDoub &d, VecInt &nextp) {}          No pre-min calculations.
    Doub dminfn(MatDoub &d, Int i, Int j) {return d[i][j];}
    Doub dbranchfn(MatDoub &d, Int i, Int j) {return 0.5*d[i][j];}
    Doub dnewfn(MatDoub &d, Int k, Int i, Int j, Int ni, Int nj) {
        return 0.5*(d[i][k]+d[j][k]);}                 New-node distance is average.
    void drootbranchfn(MatDoub &d, Int i, Int j, Int ni, Int nj,
        Doub &bi, Doub &bj) {bi = bj = 0.5*d[i][j];}
    Phylo_wpgma(const MatDoub &dist) : Phylagglom(dist)
        {makethetree(dist);}                           This call actually makes the tree.
};
```

Figure 16.4.4 shows the result of applying the WPGMA method to the toy data at the beginning of this section, using the Hamming distance as the distance metric. You can see that the tree captures almost all of the correct underlying topology, erring only in its pairing of 09 and 10 and thus missing the correct pairings 08-09 and 10-11.

The *unweighted pair group method using arithmetic averages (UPGMA)* uses

$$d_{pk} = d_{kp} = \frac{n_i d_{pi} + n_j d_{pj}}{n_i + n_j} \tag{16.4.12}$$

Although, paradoxically, UPGMA looks "weighted" while WPGMA looks "unweighted," the names derive from the fact that the UPGMA formula is equivalent

to an *un*weighted average of distances to all of a node's descendant leaves. The UPGMA method is the most widely used of the ultrametric heuristic methods.

Implementing code is

phylo.h

```
struct Phylo_upgma : Phylagglom {
Derived class implementing the UPGMA method. Only need to define functions that are virtual
in Phylagglom.
    void premin(MatDoub &d, VecInt &nextp) {}            No pre-min calculations.
    Doub dminfn(MatDoub &d, Int i, Int j) {return d[i][j];}
    Doub dbranchfn(MatDoub &d, Int i, Int j) {return 0.5*d[i][j];}
    Doub dnewfn(MatDoub &d, Int k, Int i, Int j, Int ni, Int nj) {
        return (ni*d[i][k] + nj*d[j][k]) / (ni+nj);}     Distance is weighted.
    void drootbranchfn(MatDoub &d, Int i, Int j, Int ni, Int nj,
        Doub &bi, Doub &bj) {bi = bj = 0.5*d[i][j];}
    Phylo_upgma(const MatDoub &dist) : Phylagglom(dist)
        {makethetree(dist);}                             This call actually makes the
};                                                           tree.
```

The *single linkage clustering method* and the *complete linkage clustering method* use, respectively, the minimum and maximum distances to the two children,

$$\begin{aligned} d_{pk} = d_{kp} &= \min(d_{pi}, d_{pj}) \qquad \text{(single linkage)} \\ d_{pk} = d_{kp} &= \max(d_{pi}, d_{pj}) \qquad \text{(complete linkage)} \end{aligned} \tag{16.4.13}$$

Implementing code is

phylo.h

```
struct Phylo_slc : Phylagglom {
Derived class implementing the single linkage clustering method.
    void premin(MatDoub &d, VecInt &nextp) {}      No pre-min calculations.
    Doub dminfn(MatDoub &d, Int i, Int j) {return d[i][j];}
    Doub dbranchfn(MatDoub &d, Int i, Int j) {return 0.5*d[i][j];}
    Doub dnewfn(MatDoub &d, Int k, Int i, Int j, Int ni, Int nj) {
        return MIN(d[i][k],d[j][k]);}             New-node distance is min of children.
    void drootbranchfn(MatDoub &d, Int i, Int j, Int ni, Int nj,
        Doub &bi, Doub &bj) {bi = bj = 0.5*d[i][j];}
    Phylo_slc(const MatDoub &dist) : Phylagglom(dist)
        {makethetree(dist);}                        This call actually makes the tree.
};

struct Phylo_clc : Phylagglom {
Derived class implementing the complete linkage clustering method.
    void premin(MatDoub &d, VecInt &nextp) {}          No pre-min calculations.
    Doub dminfn(MatDoub &d, Int i, Int j) {return d[i][j];}
    Doub dbranchfn(MatDoub &d, Int i, Int j) {return 0.5*d[i][j];}
    Doub dnewfn(MatDoub &d, Int k, Int i, Int j, Int ni, Int nj) {
        return MAX(d[i][k],d[j][k]);}             New-node distance is max of children.
    void drootbranchfn(MatDoub &d, Int i, Int j, Int ni, Int nj,
        Doub &bi, Doub &bj) {bi = bj = 0.5*d[i][j];}
    Phylo_clc(const MatDoub &dist) : Phylagglom(dist)
        {makethetree(dist);}                      This call actually makes the tree.
};
```

16.4.5 Neighbor Joining: Exact for Additive Trees

Saitou and Nei's *neighbor-joining method (NJ)* [6] is an agglomerative method with the remarkable property that it exactly reconstructs any additive tree, given that tree's distance matrix (again modulo some technical details). NJ is probably the most widely used agglomerative method, and perhaps the most widely used method

for phylogenetic tree construction overall. Real biological trees are almost never close enough to ultrametric to give UPGMA a significant advantage over NJ, so NJ is likely the method, among the fast heuristic methods, that you will want to try first.

The prescriptions for treating NJ within the framework of `Phyloagglom` are slightly more complicated than for the ultrametric heuristics. At each stage of forming a new cluster, we compute an auxiliary quantity,

$$u_i \equiv \frac{1}{n_a - 2} \sum_{j \neq i} d_{ij} \tag{16.4.14}$$

where the sum is over active clusters, whose number is n_a. Then, we find not the minimum distance, per se, but the minimum of the expression

$$d_{ij} - u_i - u_j \tag{16.4.15}$$

When we connect clusters i and j to form a new node k, the branch lengths from i to k and from j to k are

$$\begin{aligned} d_{ik} &= \tfrac{1}{2}(d_{ij} + u_i - u_j) \\ d_{jk} &= \tfrac{1}{2}(d_{ij} + u_j - u_i) \end{aligned} \tag{16.4.16}$$

Finally, the distance between new node k and another node p is

$$d_{pk} = d_{kp} = \tfrac{1}{2}(d_{pi} + d_{pj} - d_{ij}) \tag{16.4.17}$$

(You can now see why `Phylagglom` was coded with some features that were not exercised by the ultrametric heuristics.)

phylo.h

```
struct Phylo_nj : Phylagglom {
Derived class implementing the neighbor joining (NJ) method.
    VecDoub u;
    void premin(MatDoub &d, VecInt &nextp) {
    Before finding the minimum we (re-)calculate the u's.
        Int i,j,ncurr = 0;
        Doub sum;
        for (i=0; i>=0; i=nextp[i]) ncurr++;           Count live entries.
        for (i=0; i>=0; i=nextp[i]) {                  Compute u[i].
            sum = 0.;
            for (j=0; j>=0; j=nextp[j]) if (i != j) sum += d[i][j];
            u[i] = sum/(ncurr-2);
        }
    }
    Doub dminfn(MatDoub &d, Int i, Int j) {
        return d[i][j] - u[i] - u[j];                  NJ finds min of this.
    }
    Doub dbranchfn(MatDoub &d, Int i, Int j) {
        return 0.5*(d[i][j]+u[i]-u[j]);                NJ setting for branch lengths.
    }
    Doub dnewfn(MatDoub &d, Int k, Int i, Int j, Int ni, Int nj) {
        return 0.5*(d[i][k] + d[j][k] - d[i][j]);      NJ new distances.
    }
    void drootbranchfn(MatDoub &d, Int i, Int j, Int ni, Int nj,
    Doub &bi, Doub &bj) {
        Since NJ is unrooted, it is a matter of taste how to assign branch lengths to the root.
        This prescription plots aesthetically.
        bi = d[i][j]*(nj - 1 + 1.e-15)/(ni + nj -2 + 2.e-15);
```

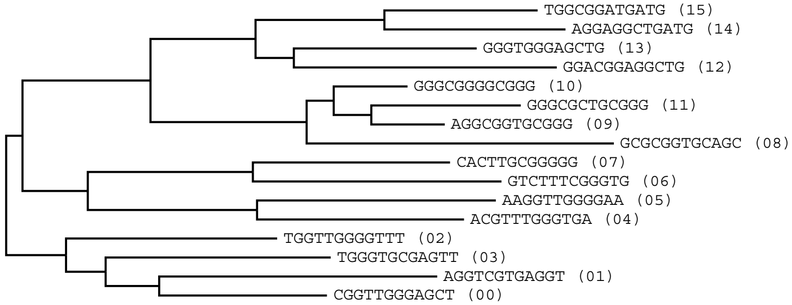

Figure 16.4.5. Same data as previous figure, clustered by the neighbor-joining (NJ) method. The method yields perfect results when the input data are the metric of an additive tree (which these data are not). While displayed here as if rooted, the NJ method outputs an unrooted tree (see next figure).

```
        bj = d[i][j]*(ni - 1 + 1.e-15)/(ni + nj -2 + 2.e-15);
    }
    Phylo_nj(const MatDoub &dist, Int fsr = -1)
        : Phylagglom(dist,fsr), u(n) {makethetree(dist);}
};
```

Computing the u_i's is here coded as an $O(n^2)$ process, but it is repeated $O(n)$ times, so it adds $O(n^3)$ to the workload. It is straightforward to make this $O(n^2)$, in line with the best coding for the rest of the tree construction. When you compute the u_i's, most distances have not changed; you just need to correct those that have. We have not coded this, just to keep the code concise.

It is important to keep in mind that the neighbor-joining method intrinsically produces an *unrooted* tree, regardless of how the graphical output may be drawn. Figure 16.4.5 shows the tree produced by the above code, run on the same toy example as above. It is clear by inspection that, if we want to root the tree at all, we will likely do so at some different point than the one drawn. It is for just this reason that Phylo_nj's constructor has an optional integer argument for specifying a node as an immediate daughter to a "forced" root. (You can't specify a new root by its node number, because it doesn't exist yet.) Also, since you may not know how Phyloagglom has numbered its internal nodes, there is a method that returns the node number of an internal node, given two leaves that have it as their first common ancestor.

phylo.h

```
Int Phylagglom::comancestor(Int leafa, Int leafb) {
Given the node numbers of two leaves, return the node number of their first common ancestor.
    Int i, j;
    for (i = leafa; i != root; i = t[i].mo) {
        for (j = leafb; j != root; j = t[j].mo) if (i == j) break;
        if (i == j) break;
    }
    return i;
}
```

Figure 16.4.6 shows the result of rerooting the tree of Figure 16.4.5 to the common ancestor of leaves 08 and 15. The recovered topology is now seen to be almost identical to that recovered by WPGMA, except for one additional mistake in not giving 02 and 03 a common parent.

The two figures, 16.4.5 and 16.4.6, were produced by lines of code like

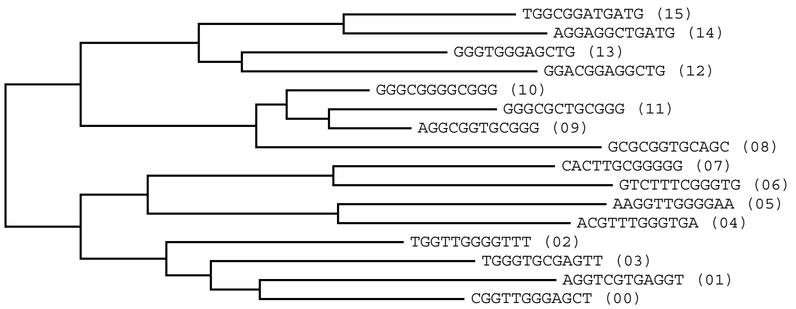

Figure 16.4.6. Same (NJ) tree as previous figure, but displayed with a different root, so as to produce a more balanced tree.

```
Mat_DP dist(n,n);
...
Phylo_nj mytree(dist);
Int i = mytree.comancestor(8,15);
Phylo_nj myrerootedtree(dist,i);
```

Although an unlikely application, you can use neighbor joining to test whether a given distance matrix is additive: Construct the NJ tree, and then see if its induced (branch length) distance matrix is the same as the one you started with. Analogously, you can use any of the ultrametric heuristic methods to test whether a distance matrix is ultrametric.

To view a tree produced by `Phyagglom`, you can use the following routine to produce an output file in the so-called "Newick" or "New Hampshire" format. Most tree viewing programs (e.g., TreeView) can read such a file. Alternatively, see Webnote [10] for a routine to convert a `Phyagglom` to PostScript graphics directly.

```
void newick(Phylagglom &p, MatChar str, char *filename) {
```
phylo.h

Output a phylogenetic tree p in the "Newick" or "New Hampshire" standard format. Text labels for the leaf nodes are input as the rows of `str`, each a null terminated string. The output file name is specified by `filename`.

```
    FILE *OUT = fopen(filename,"wb");
    Int i, s, ntask = 0, n = p.n, root = p.root;
    VecInt tasklist(2*n+1);
    tasklist[ntask++] = (1 << 16) + root;
    while (ntask-- > 0) {                         Depth-first traversal of the tree.
        s = tasklist[ntask] >> 16;                Code indicating context.
        i = tasklist[ntask] & 0xffff;             Node number to be processed.
        if (s == 1 || s == 2) {                   Left or right dau, down.
            tasklist[ntask++] = ((s+2) << 16) + p.t[i].mo;
            if (p.t[i].ldau >= 0) {
                fprintf(OUT,"(");
                tasklist[ntask++] = (2 << 16) + p.t[i].rdau;
                tasklist[ntask++] = (1 << 16) + p.t[i].ldau;
            }
            else fprintf(OUT,"%s:%f",&str[i][0],p.t[i].modist);
        }
        else if (s == 3) {if (ntask > 0) fprintf(OUT,",\n");}   Left dau up.
        else if (s == 4) {                                      Right dau up.
            if (i == root) fprintf(OUT,");\n");
            else fprintf(OUT,"):%f",p.t[i].modist);
        }
    }
    fclose(OUT);
}
```

16.4.6 Maximum Likelihood and Maximum Parsimony

Both methods that we now discuss are character-based. If you have a problem that starts with a distance matrix, you can skip this section.

Maximum likelihood (or its Bayesian equivalent, maximum posterior probability) *sounds* like a good idea for phylogenetic inference, but it has two crippling disabilities:

First, its exact solution requires looping over (more or less) all possible trees, so it must confront a super-exponential increase of workload with n, the number of data points.

Second, since you need to compute the probability of each tree, given the terminal node (leaf) data, you need to have a precise statistical model of how trees are generated, i.e., how evolution works. While there are various such models, with varying degrees of support by empirical data, no such model can convincingly claim to be a universal truth. Under different models, maximum likelihood will produce different trees.

There are heuristic methods, e.g. *quartet puzzling* [11], that finesse, to some extent, the first disability, at the price of yielding "solutions" that are not necessasrily the absolute global optimum. However, the combination of both disabilities generally makes maximum likelihood a method of last resort.

Maximum parsimony shares maximum likelihood's first disability, but not its second. Since in many situations it has proved itself to be an accurate and robust method [5], a lot of work has been done on heuristics that can overcome its workload limitations, again at the price of yielding only approximate solutions, and with significant success.

The basic idea of maximum parsimony is very simple: Consider all trees that have the observed data as their leaves. By "all trees" we mean not just all tree topologies, but rather all actual trees with interior nodes that are fully labeled by posited ancestor sequences. Now, for each such tree, define its branch lengths to be the Hamming distances between parent and child. For example, if a child's sequence differs from its parent's in two character positions, then their connecting branch has length two. The parsimony score for a tree is the sum of all of its branch lengths. The maximum parsimony tree is the tree with the smallest parsimony score.

It turns out that one subpart of this search can be done in a computationally efficient way. The *small parsimony problem* is: Given the *topology* of a tree over the observed leaves, find the maximum parsimony way to assign sequences to all the internal nodes. *Fitch's algorithm*, which is beyond our scope to describe, solves this in $O(n\,m\,k)$ time, where m is the length of the sequences and k is the number of possible values for each character (e.g., $k = 4$ for DNA bases) [1].

The hard part of maximum parsimony is therefore the search over topologies. When n is less than around 17, exhaustive search is possible. For larger n, various techniques including random addition order, branch swapping, hill climbing, branch and bound, simulated annealing, and genetic algorithms are used singly and in combination. In general, these can give a result with only a local maximum parsimony; but the results are often very good [1,5]. Unfortunately, the details are all beyond our allowed scope here. PAUP [7] and TNT [12,13] both implement sophisticated maximum parsimony searches.

CITED REFERENCES AND FURTHER READING:

Felsenstein, J. 2004, *Inferring Phylogenies* (Sunderland, MA: Sinauer).[1]

Warnow, T. 1996, "Some Combinatorial Optimization Problems in Phylogenetics," in *Proceedings of the International Colloquium on Combinatorics and Graph Theory*, eds. A. Gyarfas, L. Lovasz, L.A. Szekely, Bolyai Society Mathematical Studies, vol. 7, (Budapest: Bolyai Society).[2]

Agarwala, R. et al. 1999, "On the Approximability of Numerical Taxonomy," *SIAM Journal of Computing*, vol. 28, no. 3, pp. 1073-1085.[3]

Cohen, J. and Farach M. 1997, in *Proceedings of the 8th Annual ACM-SIAM Symposium on Discrete Algorithms SODA '97* (Philadelphia: S.I.A.M.).[4]

Rice, K. and Warnow, T. 1997, "Parsimony Is Hard to Beat," in *Computing and Combinatorics*, 3rd Annual International Conference COCOON '97, T. Jiang and D.T. Lee, eds. (New York: Springer).[5]

Saitou, N. and Nei, M. 1987, "The Neighbor-joining Method: A New Method for Reconstructing Phylogenetic Trees," *Molecular Biology and Evolution*, vol. 4, no. 4, pp. 406-425; see also *op. cit.*, vol. 5, no. 6, pp. 729-731.[6]

Swofford, D. 2004+, *PAUP: Phylogenetic Analysis Using Parsimony and Other Methods*, version 4, at `http://paup.csit.fsu.edu/`.[7]

Felsenstein, J. 2003+, *PHYLIP: Phylogeny Inference Package*, version 3.6, at `http://evolution.genetics.washington.edu/phylip.html`.[8]

Hall, B.G. 2004, *Phylogenetic Trees Made Easy: A How-To Manual*, 2nd ed. (Sunderland, MA: Sinauer).[9]

Numerical Recipes Software 2007, "Code for Rendering a Phylagglom Tree in Simple PostScript," *Numerical Recipes Webnote No. 28*, at `http://www.nr.com/webnotes?28` [10]

Strimmer, K. and von Haeseler, A. 1996, "Quartet Puzzling: A Quartet Maximum Likelihood Method for Reconstructing Tree Topologies," *Molecular Biology and Evolution*, vol. 13, no. 7, pp. 964-969.[11]

Goloboff, P.A. 1999, "Analyzing Large Data Sets in Reasonable Times: Solutions for Composite Optima," *Cladistics*, vol. 15, pp. 415-428; also see `http://www.cladistics.com`.[12]

Nixon, K.C. 1999, "The Parsimony Ratchet, a New Method for Rapid Parsimony Analysis," *Cladistics*, vol. 15, pp. 407-414.[13]

16.5 Support Vector Machines

The *support vector machine* or *SVM*, first described by Vapnik and collaborators in 1992 [1], has rapidly established itself as a powerful algorithmic approach to the problem of *classification* within the larger context known as *supervised learning*. SVMs are no more "machines" than are Turing "machines"; the use of the word is inherited from that part of computer science long known as "machine learning." A number of classification problems whose solutions were previously dominated by neural nets and more complicated methods have been found to be straightforwardly solvable by SVMs [2]. Moreover, SVMs are generally easier to implement than are neural nets; and it is generally easier to intuit what SVMs "think they are doing" than for neural nets, which are famous for their opaqueness.

In the supervised learning problem of classification, we are given a set of *training data* consisting of m points,

$$(\mathbf{x}_i, y_i) \qquad i = 1, \ldots, m \tag{16.5.1}$$

Each $\mathbf{x}_i$ is a *feature vector* in n dimensions (say) that describes the data point, while each corresponding y_i has the value ± 1, indicating whether that data point is in $(+1)$ or out of (-1) the set that we want to learn to recognize. We desire a *decision rule*, in the form of a function $f(\mathbf{x})$ whose sign predicts the value of y, not just for the data in the training set, but also for new values of $\mathbf{x}$ never before seen.

For some applications, the feature vector $\mathbf{x}$ truly lives in the continuous space $\mathbf{R}^n$. However, you are allowed to be creative in mapping your problem into this framework: In many applications, the feature vector will be a binary vector that encodes the presence or absence of many "features" (hence its name). For example, the feature vector describing a DNA sequence of length p could have $n = 4p$ dimensions, with each base position using four dimensions, and having the value one in one of the four (depending on whether it is A, C, G, or T), zero in the others.

16.5.1 Special Case of Linearly Separable Data

One can understand SVMs conceptually as a series of generalizations from an idealized, and rather unrealistic, starting point, We discuss these generalizations sequentially in the rest of this section. The starting point is the special case of *linearly separable data*. In this case, we are told (by an oracle?) that there exists a hyperplane in n dimensions, that is, an $n-1$ dimensional surface defined by the equation

$$f(\mathbf{x}) \equiv \mathbf{w}\cdot\mathbf{x} + b = 0 \tag{16.5.2}$$

that completely separates the training data. In other words, all the training points with $y_i = 1$ lie on one side of the hyperplane (and thus have $f(\mathbf{x}_i) > 0$), while all the training points with $y_i = -1$ lie on the other side (and have $f(\mathbf{x}_i) < 0$). All we have to do is find $\mathbf{w}$ (a normal vector to the hyperplane) and b (an offset). Then $f(\mathbf{x})$ in equation (16.5.2) will be the decision rule.

Actually, we can do better than this. In general, more than one hyperplane will separate linearly separable data. Let's pick the hyperplane that has the largest *margin*, i.e., maximizes the perpendicular distance to points nearest to the hyperplane on both sides. Specifically, given any hyperplane that separates the data, we can always scale $\mathbf{w}$ by a constant and adjust b appropriately, to make

$$\begin{aligned} \mathbf{w}\cdot\mathbf{x}_i + b &\ge +1 \qquad \text{when } y_i = +1 \\ \mathbf{w}\cdot\mathbf{x}_i + b &\le -1 \qquad \text{when } y_i = -1 \end{aligned} \tag{16.5.3}$$

These equations represent parallel bounding hyperplanes that separate the data (see Figure 16.5.1), a structure whimsically called a *fat plane*. With a bit of analytical geometry, you can easily convince yourself that the perpendicular distance between the bounding hyperplanes (twice the margin) is

$$2 \times \text{margin} = 2(\mathbf{w}\cdot\mathbf{w})^{-1/2} \tag{16.5.4}$$

Also note that both cases of equation (16.5.3) can be summarized as the single equation

$$y_i(\mathbf{w}\cdot\mathbf{x}_i + b) \ge 1 \tag{16.5.5}$$

What we see is that the fattest fat plane, also known as the *maximum margin SVM*, can be found by solving a particular problem in quadratic programming:

$$\boxed{\begin{array}{ll} \text{minimize:} & \frac{1}{2}\mathbf{w}\cdot\mathbf{w} \\ \text{subject to:} & y_i(\mathbf{w}\cdot\mathbf{x}_i + b) \ge 1 \qquad i = 1,\ldots,m \end{array}} \tag{16.5.6}$$

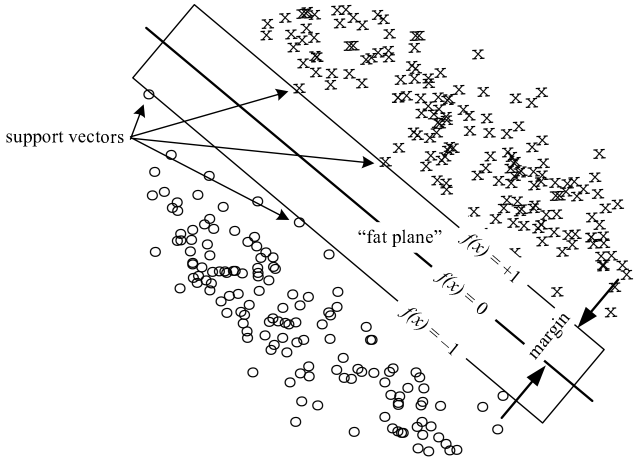

Figure 16.5.1. Support vector machine (SVM) in the idealized case of linearly separable data. We want to classify regions of the plane as containing x's or o's. The "fat plane" defined by $-1 \leq f(x) \leq 1$ is chosen to maximize the margin (shown). At such a maximum, a small number of points, the "support vectors," will lie on the bounding planes.

Note that we minimize $\mathbf{w} \cdot \mathbf{w}$ instead of equivalently maximizing its reciprocal. The factor of $1/2$ merely simplifies some algebra, later.

General methods for solving quadratic programming problems like the above are discussed in [3,4]. Later in this section, we discuss a method specialized for some SVMs. For now, consider the solution of (16.5.6) and similar problems as an available "black box."

At a solution of (16.5.6), some (usually a small number) of the data points must lie exactly on one or the other bounding hyperplane, because, otherwise, the fat plane could have been made fatter. These data points, with $f(\mathbf{x}) = \pm 1$, are called the *support vectors* of the solution. However, despite the fact that support vector machines were originally named after these support vectors, they don't play much of a role in the more realistic generalizations that we will soon discuss.

16.5.2 Primal and Dual Problems in Quadratic Programming

The first of our promised generalizations may at first sight seem a puzzling direction to go, since it consists merely of replacing one quadratic programming problem with another. We will see later, however, that this replacement has profound consequences.

The general problem in quadratic programming, known as the *primal* problem, can be stated as

$$\boxed{\begin{aligned} &\text{minimize:} && f(\mathbf{w}) \\ &\text{subject to:} && g_j(\mathbf{w}) \leq 0 \\ & && h_k(\mathbf{w}) = 0 \end{aligned}} \qquad (16.5.7)$$

where $f(\mathbf{w})$ is quadratic in $\mathbf{w}$; $g(\mathbf{w})$ and $h(\mathbf{w})$ are affine in $\mathbf{w}$ (i.e., linear plus a constant); and j and k index, respectively, the sets of inequality and equality constraints.

Every primal problem has a *dual* problem, which can be thought of as an alternative way of solving the primal problem (cf. §10.11.1). To get from the primal to the dual, one writes a Lagrangian that incorporates both the quadratic form, and — with Lagrange multipliers — all the constraints, namely,

$$\mathcal{L} \equiv \tfrac{1}{2} f(\mathbf{w}) + \sum_j \alpha_j g_j(\mathbf{w}) + \sum_k \beta_k h_k(\mathbf{w}) \tag{16.5.8}$$

One then writes this subset of conditions for an extremum:

$$\frac{\partial \mathcal{L}}{\partial w_i} = 0, \qquad \frac{\partial \mathcal{L}}{\partial \beta_k} = 0, \tag{16.5.9}$$

and uses the resulting equations algebraically to eliminate $\mathbf{w}$ from $\mathcal{L}$, in favor of $\boldsymbol{\alpha}$ and $\boldsymbol{\beta}$ (where we now write the α_j's and β_k's as vectors). Call the result, the reduced Lagrangian, $\mathcal{L}(\boldsymbol{\alpha}, \boldsymbol{\beta})$. Then the important result, which follows from the so-called *strong duality* and *Kuhn-Tucker* theorems (cf. §10.11.1), is that the solution of the following dual problem is equivalent to the original primal problem:

$$\boxed{\begin{array}{lll} \text{maximize:} & \mathcal{L}(\boldsymbol{\alpha}, \boldsymbol{\beta}) & \\ \text{subject to:} & \alpha_j \geq 0 & \text{for all } j \end{array}} \tag{16.5.10}$$

In fact, this result is more general than quadratic programming and is true, roughly speaking, for any convex $f(\mathbf{w})$. Furthermore, if $\widehat{\mathbf{w}}$ is the optimal solution of the primal problem, and $\widehat{\boldsymbol{\alpha}}, \widehat{\boldsymbol{\beta}}$ are the optimal solutions of the dual problem, we have

$$\begin{aligned} f(\widehat{\mathbf{w}}) &= \mathcal{L}(\widehat{\boldsymbol{\alpha}}, \widehat{\boldsymbol{\beta}}) \\ \widehat{\alpha}_j \, g_j(\widehat{\mathbf{w}}) &= 0 \qquad \text{for all } j \end{aligned} \tag{16.5.11}$$

The latter condition is called the *Karush-Kuhn-Tucker complementarity condition*. It says that at least one of $\widehat{\alpha}_j$ and $g_j(\widehat{\mathbf{w}})$ must be zero for each j. (We previously met the linear case in equation 10.11.5.) This means that, from the solution of the dual problem, you can instantly identify inequality constraints in the primal problem that are "pinned" against their limit, namely those with nonzero $\widehat{\alpha}_j$'s in the solution of the dual.

16.5.3 Dual Formulation of the Maximum Margin SVM

The above procedure is readily performed on the quadratic programming problem (16.5.6) for the maximum margin SVM. There are no β_k's, since there are no equality constraints. The Lagrangian (16.5.8) is

$$\mathcal{L} = \tfrac{1}{2}\mathbf{w}\cdot\mathbf{w} + \sum_i \alpha_i [1 - y_i(\mathbf{w}\cdot\mathbf{x}_i + b)] \tag{16.5.12}$$

The conditions for an extremum are

$$0 = \frac{\partial \mathcal{L}}{\partial \mathbf{w}} = \mathbf{w} - \sum_i \alpha_i y_i \mathbf{x}_i \quad \Longrightarrow \quad \widehat{\mathbf{w}} = \sum_i \widehat{\alpha}_i y_i \mathbf{x}_i \tag{16.5.13}$$

and

$$0 = \frac{\partial \mathcal{L}}{\partial b} = \sum_i \alpha_i y_i \tag{16.5.14}$$

Substituting equations (16.5.13) and (16.5.14) back into (16.5.12) gives the reduced Lagrangian

$$\begin{aligned} \mathcal{L}(\boldsymbol{\alpha}) &= \sum_j \alpha_j - \tfrac{1}{2} \sum_{j,k} \alpha_j y_j (\mathbf{x}_j \cdot \mathbf{x}_k) y_k \alpha_k \\ &\equiv \mathbf{e} \cdot \boldsymbol{\alpha} - \tfrac{1}{2} \boldsymbol{\alpha} \cdot \text{diag}(\mathbf{y}) \cdot \mathbf{G} \cdot \text{diag}(\mathbf{y}) \cdot \boldsymbol{\alpha} \end{aligned} \tag{16.5.15}$$

In the second form of the above equation we introduce some convenient matrix notation: $\mathbf{e}$ is the vector whose components are all unity, diag denotes a diagonal matrix formed from a vector in the obvious way, and $\mathbf{G}$ is the *Gram matrix* of dot products of all the $\mathbf{x}_j$'s,

$$G_{ij} \equiv \mathbf{x}_i \cdot \mathbf{x}_j \tag{16.5.16}$$

Remember that subscripts on $\mathbf{x}$ don't indicate components, but rather index which data point is referenced.

The dual problem, in toto, thus turns out to be

$$\boxed{\begin{array}{lll} \text{minimize:} & \tfrac{1}{2} \boldsymbol{\alpha} \cdot \text{diag}(\mathbf{y}) \cdot \mathbf{G} \cdot \text{diag}(\mathbf{y}) \cdot \boldsymbol{\alpha} - \mathbf{e} \cdot \boldsymbol{\alpha} & \\ \text{subject to:} & \alpha_i > 0 & \text{for all } i \\ & \boldsymbol{\alpha} \cdot \mathbf{y} = 0 & \text{(from 16.5.14)} \end{array}} \tag{16.5.17}$$

We also have the Karush-Kuhn-Tucker relation,

$$\widehat{\alpha}_i \left[y_i (\widehat{\mathbf{w}} \cdot \mathbf{x}_i + b) - 1 \right] = 0 \tag{16.5.18}$$

Equation (16.5.13) tells how to get the optimal solution $\widehat{\mathbf{w}}$ of the primal problem from the solution $\widehat{\boldsymbol{\alpha}}$ of the dual. Equation (16.5.18) is then used to get $\widehat{b}$: Find *any* nonzero α_i, then, with the corresponding y_i, $\mathbf{x}_i$, and $\widehat{\mathbf{w}}$, solve the above relation for $\widehat{b}$. Alternatively, one can average out some roundoff error by taking a weighted average of α_i's,

$$\widehat{b} = \sum_i \widehat{\alpha}_i (y_i - \widehat{\mathbf{w}} \cdot \mathbf{x}_i) \Big/ \sum_i \alpha_i \tag{16.5.19}$$

Finally, the decision rule is $f(\mathbf{x}) = \widehat{\mathbf{w}} \cdot \mathbf{x} + \widehat{b}$.

A few observations will become important later:

- Data points with nonzero $\widehat{\alpha}_i$ satisfy the constraints as equalities, i.e., they are support vectors.
- The only place that the data $\mathbf{x}_i$'s appear in (16.5.17) is in the Gram matrix $\mathbf{G}$.
- The only part of the calculation that scales with n (the dimensionality of the feature vector) is computing the components of the Gram matrix.
- All other parts of the calculation scale with m, the number of data points.

Thus, in going from primal to dual, we have substituted for a problem that scales (mostly) with the dimensionality of the feature matrix a problem that scales (mostly) with the number of data points. This might seem odd, because it makes problems with huge numbers of data points difficult. However, it makes easy, as we will soon see, problems with moderate amounts of data but *huge* feature vectors. This is in fact the regime where SVMs really shine.

16.5.4 The 1-Norm Soft-Margin SVM and Its Dual

The next important generalization is to relax the unrealistic assumption that there exists a hyperplane that separates the training data, i.e., get rid of the "oracle." We do this by introducing a so-called slack variable ξ_i for each data point $\mathbf{x}_i$. If the data point is one that *can* be separated by a fat plane, then $\xi_i = 0$. If it *can't* be, then $\xi_i > 0$ is the amount of the discrepancy, expressed by the modified inequality

$$y_i(\mathbf{w} \cdot \mathbf{x}_i + b) \geq 1 - \xi_i \tag{16.5.20}$$

We must of course build in an inducement for the optimization to make the ξ_i's as small as possible, zero whenever possible. We thus have a trade-off between making the ξ_i's small and making the fat plane fat. In other words, we now have a problem that requires not only optimization, but also *regularization*, in the same sense as the discussion in §18.4. In the notation of equation (18.4.12), our quadratic forms ($\mathbf{w} \cdot \mathbf{w}$ or $\mathcal{L}$) are examples of $\mathcal{A}$'s. We need to invent a regularizing operator $\mathcal{B}$ that expresses our hopes for the ξ_i's, and then minimize $\mathcal{A} + \lambda\mathcal{B}$, instead of just $\mathcal{A}$ alone. As we vary λ in the range $0 < \lambda < \infty$, we explore a regularization trade-off curve.

The *1-norm soft-margin SVM* adopts, as the name indicates, a linear sum of the (positive) ξ_i's as its regularization operator. The primal problem is thus

$$\begin{array}{ll} \text{minimize:} & \frac{1}{2}\mathbf{w} \cdot \mathbf{w} + \lambda \sum_i \xi_i \\ \text{subject to:} & \xi_i \geq 0, \\ & y_i(\mathbf{w} \cdot \mathbf{x}_i + b) \geq 1 - \xi_i \qquad i = 1, \ldots, m \end{array} \tag{16.5.21}$$

A possible variant is the *2-norm soft-margin SVM*, where the regularization term would be $\sum_i {\xi_i}^2$; however, this gives somewhat more complicated equations, so we will put it beyond our scope here.

Along the trade-off curve $0 < \lambda < \infty$, we vary from a a solution that prefers a *really fat* fat plane (no matter how many points are inside, or on the wrong side, of it) to a solution that is so miserly in allowing discrepancies that it settles for a fat plane with hardly any margin at all. The former is less accurate on the training data but possibly more robust on new data; the latter is as accurate as possible on the training data but possibly fragile (and less accurate) on new data. As in Chapter 19, the choice of λ is a design trade-off that you have to make. (However, we give you some guidance, below.)

Importantly, *any* nonnegative value of λ allows there to be *some* solution, whether the data are linearly separable or not. You can see this by noting that $\mathbf{w} = 0$ is always a feasible (but not optimal) solution of (16.5.21) for sufficiently large positive ξ_i's, no matter what the value of λ. If there is a feasible solution, there must, of course, be an optimal solution.

The very astute reader might notice that λ here seems to have the opposite qualitative sense from the λ's in Chapter 19. Specifically, $\lambda \to 0$ (here) gives the "softer," more robust, solution, while in Chapter 19 it is $\lambda \to \infty$ that, in a similar way, favors a priori smoothness. The reason for this switch is that the quadratic program (16.5.21) becomes the quadratic program (16.5.6) in the limit $\lambda \to \infty$, not 0. This is because there are no ξ_i's in the constraints in (16.5.6), so (16.5.21) must, in

the limit, force them to zero, requiring infinite λ. Correspondingly, as λ approaches zero, the ξ_i's become unconstrained. So the regularization term here indeed does act with the opposite sense from Chapter 19, because of the way it acts through the constraints, not the main functional.

Curiously, the dual to the 1-norm soft-margin SVM turns out to be *almost* identical to the dual of the (unrealistic) maximum margin SVM (16.5.17). Omitting details of the calculation, the result is

$$\boxed{\begin{array}{lll} \text{minimize:} & \frac{1}{2}\boldsymbol{\alpha}\cdot\text{diag}(\mathbf{y})\cdot\mathbf{G}\cdot\text{diag}(\mathbf{y})\cdot\boldsymbol{\alpha}-\mathbf{e}\cdot\boldsymbol{\alpha} & \\ \text{subject to:} & 0\le\alpha_i\le\lambda & \text{for all } i \\ & \alpha\cdot\mathbf{y}=0 & \end{array}} \tag{16.5.22}$$

That is, the only difference is that there is now a constraining *upper* bound of λ on α_i in addition to the *lower* bound of zero. (This kind of constraint is called a *box constraint*.)

The formula for $\widehat{\mathbf{w}}$ is unchanged from equation (16.5.13), while the Karush-Kuhn-Tucker conditions now become

$$\begin{aligned} (\widehat{\alpha}_i-\lambda)\widehat{\xi}_i &= 0 \\ \widehat{\alpha}_i\left[y_i(\widehat{\mathbf{w}}\cdot\mathbf{x}_i+\widehat{b})-1+\widehat{\xi}_i\right] &= 0 \end{aligned} \tag{16.5.23}$$

We see that, except for rare degenerate cases of double zeros,

$$\begin{array}{rcl} \widehat{\alpha}_i=0 & \Longleftrightarrow & \text{data point } i \text{ on correct side of fat plane} \\ 0<\widehat{\alpha}_i<\lambda & \Longleftrightarrow & \text{data point } i \text{ exactly on fat plane boundary (a support vector)} \\ \widehat{\alpha}_i=\lambda & \Longleftrightarrow & \text{data point } i \text{ inside, or on wrong side, of fat plane} \end{array} \tag{16.5.24}$$

Here again we see that, as we reduce λ toward zero, pinning more and more α_i's at the value λ, we get solutions with increasing numbers of "wrong" points, but fatter fat planes.

The roundoff-averaged estimator for $\widehat{b}$, analogous to equation (16.5.19), is

$$\widehat{b}=\sum_i\widehat{\alpha}_i(\lambda-\widehat{\alpha}_i)(y_i-\widehat{\mathbf{w}}\cdot\mathbf{x}_i)\Big/\sum_i\widehat{\alpha}_i(\lambda-\widehat{\alpha}_i) \tag{16.5.25}$$

Although the linear assumption (that is, using hyperplanes to separate the data) is still somewhat restrictive, the model defined by (16.5.22) does have some practical utility in problems where there is some reason to believe that the response is (at least somewhat) linear in the components of the feature vector. But that is far from the end of the story.

16.5.5 The Kernel Trick

Finally, we get to the generalization that gives SVMs their real power. Imagine an embedding function $\boldsymbol{\varphi}$ that maps n-dimensional feature vectors, in some manner, into a much higher N-dimensional space,

$$\mathbf{x}\quad(n\text{-dimensional})\quad\longrightarrow\quad\boldsymbol{\varphi}(\mathbf{x})\quad(N\text{-dimensional}) \tag{16.5.26}$$

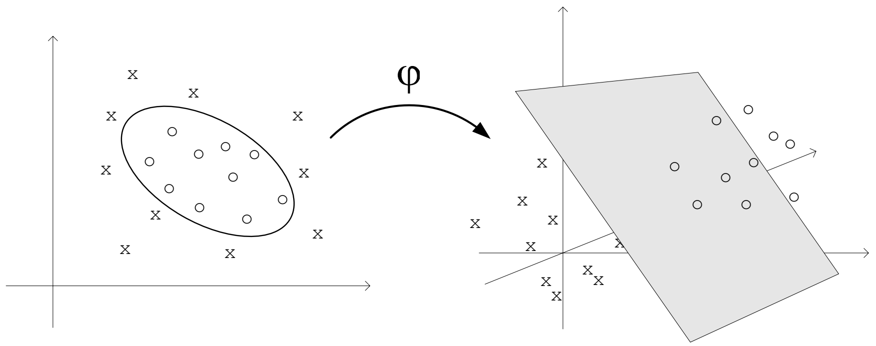

Figure 16.5.2. When feature vectors are mapped from a lower-dimensional space (here 2) to a higher-dimensional embedding space (here 3), nonlinear separation surfaces can become well approximated by linear ones. In practice, *very* high-dimensional embedding spaces are used, but they enter the SVM calculation only implicitly, through the "kernel trick."

The basic idea, as shown in Figure 16.5.2, is that a very nonlinear separating surface in the n-dimensional space might map into (or be well approximated by) a linear hyperplane in the N-dimensional space.

To see why this might work, consider this mapping from two to five dimensions:

$$(x_0, x_1) \quad \overset{\varphi}{\longrightarrow} \quad (x_0^2, x_0 x_1, x_1^2, x_0, x_1) \tag{16.5.27}$$

With this mapping, a decision rule $f(\mathbf{x})$ that is constructed as linear in the embedding space becomes general enough to include all linear and quadratic forms (lines, ellipses, hyperbolas) in the original feature space, namely,

$$f(\mathbf{x}) = F[\boldsymbol{\varphi}(\mathbf{x})] \equiv \mathbf{W} \cdot \boldsymbol{\varphi}(\mathbf{x}) + B \tag{16.5.28}$$

where we are using uppercase letters for quantities in the embedding space. Although $N = 5$ in this example, it might instead have a value like a million or a billion (we'll see how this works in a minute).

Give our data, how do we find $\mathbf{W}$ and B in the embedding space? Let's try exactly as before, but just in the higher-dimensional space. The primal problem (compare to equation 16.5.21) is

$$\begin{array}{|ll|}\hline \text{minimize:} & \frac{1}{2}\mathbf{W}\cdot\mathbf{W} + \lambda \sum_i \Xi_i \\ \text{subject to:} & \Xi_i \ge 0, \\ & y_i(\mathbf{W}\cdot\boldsymbol{\varphi}(\mathbf{x}_i) + B) \ge 1 - \Xi_i \qquad i = 1, \dots, m \\ \hline \end{array} \tag{16.5.29}$$

Uh-oh! This is a quadratic programming problem in a million- or billion-dimensional space, not likely to be tractable on your ordinary desktop computer.

What about the dual problem? It turns out to be

$$\begin{array}{|ll|}\hline \text{minimize:} & \frac{1}{2}\boldsymbol{\alpha}\cdot\text{diag}(\mathbf{y})\cdot\mathbf{K}\cdot\text{diag}(\mathbf{y})\cdot\boldsymbol{\alpha} - \mathbf{e}\cdot\boldsymbol{\alpha} \\ \text{subject to:} & 0 \le \alpha_i \le \lambda \qquad \text{for all } i \\ & \alpha\cdot\mathbf{y} = 0 \\ \hline \end{array} \tag{16.5.30}$$

This is exactly the same as (16.5.22), except that the Gram matrix G_{ij} has been replaced by the so-called *kernel* K_{ij},

$$K_{ij} \equiv K(\mathbf{x}_i, \mathbf{x}_j) \equiv \boldsymbol{\varphi}(\mathbf{x}_i) \cdot \boldsymbol{\varphi}(\mathbf{x}_j) \tag{16.5.31}$$

Well, this is progress. The quadratic programming problem (16.5.30) is no harder than the original problem (16.5.22)! Both live in a space of dimension m, the number of data points, and both get fed a fixed matrix, precalculated from the data: G_{ij} in one case, K_{ij} in the other.

We have succeeded in maneuvering the "curse of dimensionality" into a very tight corner, namely the calculation of just the m^2 values K_{ij}. Now we annihilate it entirely with *the kernel trick*:

The "trick" is that we never really need to know the mapping $\boldsymbol{\varphi}(\mathbf{x})$ at all. All we need is a way of computing a kernel K_{ij} that *could* have come from some mapping $\boldsymbol{\varphi}(\mathbf{x})$, that is, a matrix of size $m \times m$ with the mathematical properties of an inner product space in higher dimension. We already know one possible kernel, namely the Gram matrix G_{ij}. Here are some provable properties of kernel functions $K(\mathbf{x}_i, \mathbf{x}_j)$ in general:

- $K_{ij} = K(\mathbf{x}_i, \mathbf{x}_j)$ must be symmetric in i and j and must have nonnegative eigenvalues (Mercer's theorem).
- Any multinomial combination of kernel functions is a kernel function. That is, you can freely combine kernel functions by multiplication, addition, and scaling by a constant.
- $K(\boldsymbol{\varphi}(\mathbf{x}_i), \boldsymbol{\varphi}(\mathbf{x}_j))$ is a kernel if $K(,)$ is one, for any $\boldsymbol{\varphi}$. This generalizes the original idea of the embedding space.
- $K(\mathbf{x}_i, \mathbf{x}_j) = g(\mathbf{x}_i)g(\mathbf{x}_j)$ is always a kernel, for any function g.

Once you settle on a kernel and solve the quadratic programming problem (16.5.30), then your final decision rule for any new feature vector $\mathbf{x}$ is

$$f(\mathbf{x}) = \sum_i \widehat{\alpha}_i y_i K(\mathbf{x}_i, \mathbf{x}) + \widehat{b} \tag{16.5.32}$$

where (again using the averaging trick)

$$\widehat{b} = \sum_i \widehat{\alpha}_i (\lambda - \widehat{\alpha}_i)[y_i - \sum_j \widehat{\alpha}_j y_j K(\mathbf{x}_j, \mathbf{x}_i)] \Big/ \sum_i \widehat{\alpha}_i (\lambda - \widehat{\alpha}_i) \tag{16.5.33}$$

While the construction of the ideal kernel for any particular problem can involve some art, some very generic kernels turn out to be quite powerful in solving real-world problems. Often you can just try a few of these and pick the one that works best. The following are good ones to try first:

$$\begin{aligned}
\text{linear:} \quad & K(\mathbf{x}_i, \mathbf{x}_j) = \mathbf{x}_i \cdot \mathbf{x}_j \\
\text{power:} \quad & K(\mathbf{x}_i, \mathbf{x}_j) = (\mathbf{x}_i \cdot \mathbf{x}_j)^d, \qquad 2 \le d \le 20 \text{ (say)} \\
\text{polynomial:} \quad & K(\mathbf{x}_i, \mathbf{x}_j) = (a\,\mathbf{x}_i \cdot \mathbf{x}_j + b)^d \\
\text{sigmoid:} \quad & K(\mathbf{x}_i, \mathbf{x}_j) = \tanh(a\,\mathbf{x}_i \cdot \mathbf{x}_j + b) \\
\text{Gaussian radial basis function:} \quad & K(\mathbf{x}_i, \mathbf{x}_j) = \exp(-\tfrac{1}{2}|\mathbf{x}_i - \mathbf{x}_j|^2/\sigma^2)
\end{aligned} \tag{16.5.34}$$

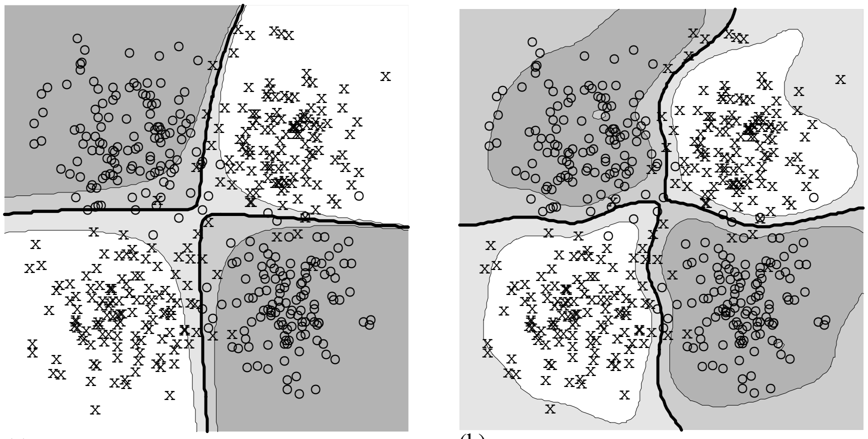

(a) (b)

Figure 16.5.3. SVMs that "learn" to partition the plane. The input data are drawn from four two-dimensional Gaussians, slightly overlapping, with diagonally opposite ones given the same label (x or o). Heavy solid lines are the decision rule surfaces $f(\mathbf{x}) = 0$ derived by the SVMs. Lighter lines show $f(\mathbf{x}) = \pm 1$. (a) Polynomial kernel with $d = 8$. (b) Gaussian radial basis function kernel.

See §2.3 of [5] for additional standard kernels. Chapter 13 of [5] describes many specialized kernels, e.g., for comparing strings or passages of text, for image recognition, and for a number of other applications.

Figure 16.5.3 shows a test example using both a polynomial kernel with $d = 8$ and a Gaussian radial basis kernel. It is characteristic of the Gaussian kernel that it is more influenced by local nearest neighbor effects (which may be good or bad, depending on the application), while polynomial kernels seek smoother, more global solutions.

Although it is beyond our scope in this section, we should mention that the kernel trick is applicable not only to SVMs (that is, to algorithms based on separating hyperplanes), but also to a number of other pattern recognition algorithms, for example principal component analysis (PCA) and the Fisher discriminant algorithm. See [5] and [6] for extensive treatments of these *kernel-based learning algorithms*.

16.5.6 Some Practical Advice on SVMs

The Gaussian radial basis function kernel is very popular, because it has only one adjustable parameter, σ, and it is easy to guess a first value to try, namely any characteristic distance between nearby points in the feature space. As mentioned, the Gaussian kernel classifies to some degree by the local neighborhood consensus.

For the polynomial kernels, start by choosing a and b to make $a\,\mathbf{x}_i \cdot \mathbf{x}_j + b$ lie between ± 1 for all i and j. The power d has a (very rough) interpretation as how many different features you want the comparison to "mix." That is, $d = 1$ (linear) partitions the space by one feature at at time; $d = 2$ looks at pairs of features simultaneously, and so on. Also very roughly, the difference between power and polynomial is whether you want to consider only exactly d features at a time (power), or all combinations of d or fewer features (polynomial). These interpretations should not be taken too seriously, however. Specifically, larger d is not always better.

We have not said much about how to choose λ, the regularization parameter.

Try $\lambda = 1$ first, then try increasing and decreasing it by factors of 10. There is typically a broad plateau, as a function of $\log_{10}(\lambda)$, where the precise value of λ doesn't matter much. There is some belief that $\langle \mathbf{x}_i \cdot \mathbf{x}_j \rangle^{-1}$ or $\langle K(\mathbf{x}_i, \mathbf{x}_j)\rangle^{-1}$, where angle brackets denote averages over all i, j pairs, are good starting guesses; but for properly scaled kernels these should not be too different from unity in any case.

As you vary λ and repeatedly solve the quadratic program, look at the fraction of α_i's that are pinned at zero, pinned at λ, or floating between these limits. A good profile will often have the biggest fraction at zero, a smaller (but not necessarily much smaller) fraction at λ, and the smallest fraction in between (see equation 16.5.24 for interpretation). These fractions are also often good indicators for adjusting parameters in your kernel. Naturally you will also be looking at the fraction of your training data that is predicted correctly, that is, has $y_i f(\mathbf{x}_i) > 0$.

Below, we will give a short, self-contained program for finding the solution to SVMs; but for anything other than small problems you will want to use a more sophisticated software package. There are many tricks and shortcuts that can speed the solution of an SVM relative to the general problem of quadratic programming — good SVM packages take advantage of these. For example, a good package should take advantage of sparseness in the feature vectors to save on computation. Our favorite package is Thorsten Joachims' *SVMlight* [7], available for free on the Web. *Gist* [8] is another popular free implementation. The Web site cited in [2] has a page with links to a wide variety of SVM software.

16.5.7 The Mangasarian-Musicant Variant and Its Solution by SOR

Mangasarian and Musicant [9,10] have suggested a very slight variant of equation (16.5.21), and its kernel generalization, that has the interesting property that it can be solved, quite compactly, by the method of successive overrelaxation (SOR; see §20.5.1). In particular, a complete SVM solution program using SOR is less than 100 lines long. We discuss this *M-M variant* here, and implement it in code, just because of its brevity. We have used this code for problems of up to several thousand data points, with feature vectors of length several hundred. Such problems take seconds to solve on a desktop machine. For larger problems, our advice is that you use the more efficient specialized packages [7,8]. *SVMlight*, for example, is typically about an order of magnitude faster than the code we give below.

The primal problem in the 1-norm soft-margin form of the M-M variant is

$$\boxed{\begin{aligned} &\text{minimize:} && \tfrac{1}{2}\mathbf{w}\cdot\mathbf{w} + b^2 + \lambda\sum_i \xi_i \\ &\text{subject to:} && \xi_i \ge 0, \\ & && y_i(\mathbf{w}\cdot\mathbf{x}_i + b) \ge 1 - \xi_i \qquad i = 1,\dots,m \end{aligned}} \tag{16.5.35}$$

The only difference from (16.5.21) is that a term b^2 has been added to the functional that is minimized. On its face, this should have the effect of slightly favoring hyperplanes closer to the origin, all else being equal, an innocuous (albeit arbitrary) change. The real purpose of the b^2 term, however, is its algebraic effect when we calculate the dual problem:

$$\boxed{\begin{array}{ll}\text{minimize:} & \frac{1}{2}\boldsymbol{\alpha}\cdot\text{diag}(\mathbf{y})\cdot(\mathbf{G}+\mathbf{e}\otimes\mathbf{e})\cdot\text{diag}(\mathbf{y})\cdot\boldsymbol{\alpha}-\mathbf{e}\cdot\boldsymbol{\alpha}\\ \text{subject to:} & 0\le\alpha_i\le\lambda \qquad \text{for all } i\end{array}} \tag{16.5.36}$$

Aside from an extra term $\mathbf{e}\otimes\mathbf{e}$ (the matrix of all ones) now added to the Gram matrix, the main change from (16.5.21) is that the equality constraint is gone! This renders the solution *much* more tractable numerically. The dual problem also now has a simpler expression for $\widehat{b}$,

$$\widehat{b} = \sum_i \widehat{\alpha}_i y_i \tag{16.5.37}$$

(As before, $\widehat{\mathbf{w}}$ is computed from 16.5.13.)

When we do the kernel trick, the only change in (16.5.36) is to change G_{ij} to K_{ij}. Equation (16.5.37) still holds, but $\widehat{b}$ is actually superfluous since the decision rule can be written directly as

$$f(\mathbf{x}) = \sum_i \widehat{\alpha}_i y_i [K(\mathbf{x}_i, \mathbf{x}) + 1] \tag{16.5.38}$$

Mangasarian and Musicant have shown that the solution of the M-M variant SVM is often identical to the solution of the the standard 1-norm soft-margin SVM (albeit with a different value of λ) and is almost never significantly different. What is quite different, however, is that (16.5.36) and its kernel version can be solved by the following, linearly convergent, relaxation procedure:

- Define $\mathbf{M} \equiv \text{diag}(\mathbf{y})\cdot(\mathbf{K}+\mathbf{e}\otimes\mathbf{e})\cdot\text{diag}(\mathbf{y})$.
- Initialize all the α_i's to zero.
- Repeat *ad libitum* the relaxation replacement, for $i = 1, 2, \dots, m$,

$$\alpha_i \;\leftarrow\; \mathcal{P}\left[\alpha_i - \omega\frac{1}{M_{ii}}\left(\sum_j M_{ij}\alpha_j - 1\right)\right] \tag{16.5.39}$$

Here $\mathcal{P}$ is the projection operator that just puts α back into its allowed range. [Note the similarity to the method of projection onto convex sets (POCS) in §19.5.2.]

$$\mathcal{P} = \begin{cases} 0, & \alpha < 0 \\ \alpha, & 0 \le \alpha \le \lambda \\ \lambda, & \alpha > \lambda \end{cases} \tag{16.5.40}$$

The constant ω is the overrelaxation parameter, exactly as in §20.5.1. You pick it in the range $0 < \omega < 2$. In our experience, the convergence rate does not depend sensitively on ω. If you don't have a better idea, take $\omega = 1.3$.

Our implementation begins with a virtual class that defines the interface to a kernel function,

svm.h

```
struct Svmgenkernel {
```
Virtual class that defines what a kernel structure needs to provide.
```
    Int m, kcalls;            No. of data points; counter for kernel calls.
    MatDoub ker;              Locally stored kernel matrix.
    VecDoub_I &y;             Must provide reference to the y_i's.
    MatDoub_I &data;          Must provide reference to the x_i's.
```

```
    Svmgenkernel(VecDoub_I &yy, MatDoub_I &ddata)
        : m(yy.size()),kcalls(0),ker(m,m),y(yy),data(ddata) {}
```
Every kernel structure must provide a `kernel` function that returns the kernel for arbitrary feature vectors.
```
    virtual Doub kernel(const Doub *xi, const Doub *xj) = 0;
    inline Doub kernel(Int i, Doub *xj) {return kernel(&data[i][0],xj);}
```
Every kernel structure's constructor must call `fill` to fill the `ker` matrix.
```
    void fill() {
        Int i,j;
        for (i=0;i<m;i++) for (j=0;j<=i;j++) {
            ker[i][j] = ker[j][i] = kernel(&data[i][0],&data[j][0]);
        }
    }
};
```

Basically, a kernel structure is required to provide references to the data (the $\mathbf{x}_i$'s) and the $\mathbf{y}_i$'s, a matrix of kernel values for all pairs of data points, and two forms of the kernel function: one with two arbitrary feature vectors as arguments, and another with one argument a data point and the other an arbitrary feature vector. Here are three examples of kernels, for three of the standard kernels in equation (16.5.34), built on the above `Svmgenkernel`.

svm.h

```
struct Svmlinkernel : Svmgenkernel {
```
Kernel structure for the linear kernel, the dot product of two feature vectors (with overall means of each component subtracted).
```
    Int n;
    VecDoub mu;
    Svmlinkernel(MatDoub_I &ddata, VecDoub_I &yy)
```
Constructor is called with the $m \times n$ data matrix, and the vector of y_i's, length m.
```
        : Svmgenkernel(yy,ddata), n(data.ncols()), mu(n) {
        Int i,j;
        for (j=0;j<n;j++) mu[j] = 0.;
        for (i=0;i<m;i++) for (j=0;j<n;j++) mu[j] += data[i][j];
        for (j=0;j<n;j++) mu[j] /= m;
        fill();
    }
    Doub kernel(const Doub *xi, const Doub *xj) {
        Doub dott = 0.;
        for (Int k=0; k<n; k++) dott += (xi[k]-mu[k])*(xj[k]-mu[k]);
        return dott;
    }
};

struct Svmpolykernel : Svmgenkernel {
```
Kernel structure for the polynomial kernel.
```
    Int n;
    Doub a, b, d;
    Svmpolykernel(MatDoub_I &ddata, VecDoub_I &yy, Doub aa, Doub bb, Doub dd)
```
Constructor is called with the $m \times n$ data matrix, the vector of y_i's, length m, and the constants a, b, and d.
```
        : Svmgenkernel(yy,ddata), n(data.ncols()), a(aa), b(bb), d(dd) {fill();}
    Doub kernel(const Doub *xi, const Doub *xj) {
        Doub dott = 0.;
        for (Int k=0; k<n; k++) dott += xi[k]*xj[k];
        return pow(a*dott+b,d);
    }
};

struct Svmgausskernel : Svmgenkernel {
```
Kernel structure for the Gaussian radial basis function kernel.
```
    Int n;
```

```
    Doub sigma;
    Svmgausskernel(MatDoub_I &ddata, VecDoub_I &yy, Doub ssigma)
```
Constructor is called with the $m \times n$ data matrix, the vector of y_i's, length m, and the constant σ.
```
        : Svmgenkernel(yy,ddata), n(data.ncols()), sigma(ssigma) {fill();}
    Doub kernel(const Doub *xi, const Doub *xj) {
        Doub dott = 0.;
        for (Int k=0; k<n; k++) dott += SQR(xi[k]-xj[k]);
        return exp(-0.5*dott/(sigma*sigma));
    }
};
```

The above is all prefatory to the SVM solution structure. You declare an instance of Svm with your kernel as the argument. It then makes available three functions: relax performs one "group" of relaxation steps and returns the norm of how much change in $\boldsymbol{\alpha}$ has occurred. (We define "group" below.) You call relax repeatedly, with λ and ω as arguments, until the returned value is small enough: 10^{-3} or 10^{-4} is usually plenty. Then (and only then) you may repeatedly call either of two forms of predict, which returns the decision rule $f(\mathbf{x})$. One form of predict returns the prediction for data points, the other for arbitrary new feature vectors. If you want to examine the α_i's, or count how many are pinned at 0 or λ, you can examine the vector alph.

svm.h

```
struct Svm {
```
Class for solving SVM problems by the SOR method.
```
    Svmgenkernel &gker;                  Reference bound to user's kernel (and data).
    Int m, fnz, fub, niter;
    VecDoub alph, alphold;               Vectors of α's before and after a step.
    Ran ran;                             Random number generator.
    Bool alphinit;
    Doub dalph;                          Change in norm of the α's in one step.
    Svm(Svmgenkernel &inker) : gker(inker), m(gker.y.size()),
        alph(m), alphold(m), ran(21), alphinit(false) {}
```
Constructor binds the user's kernel and allocates storage.
```
    Doub relax(Doub lambda, Doub om) {
```
Perform one group of relaxation steps: a single step over all the α's, and multiple steps over only the interior α's.
```
        Int iter,j,jj,k,kk;
        Doub sum;                        Index when α's are sorted by value.
        VecDoub pinsum(m);               Stored sums over noninterior variables.
        if (alphinit == false) {         Start all α's at 0.
            for (j=0; j<m; j++) alph[j] = 0.;
            alphinit = true;
        }
        alphold = alph;                  Save old α's.
```
Here begins the relaxation pass over all the α's.
```
        Indexx x(alph);                  Sort α's, then find first nonzero one.
        for (fnz=0; fnz<m; fnz++) if (alph[x.indx[fnz]] != 0.) break;
        for (j=fnz; j<m-2; j++) {            Randomly permute all the nonzero α's.
            k = j + (ran.int32() % (m-j));
            SWAP(x.indx[j],x.indx[k]);
        }
        for (jj=0; jj<m; jj++) {             Main loop over α's.
            j = x.indx[jj];
            sum = 0.;
            for (kk=fnz; kk<m; kk++) {       Sums start with first nonzero.
                k = x.indx[kk];
                sum += (gker.ker[j][k] + 1.)*gker.y[k]*alph[k];
            }
            alph[j] = alph[j] - (om/(gker.ker[j][j]+1.))*(gker.y[j]*sum-1.);
```

```
            alph[j] = MAX(0.,MIN(lambda,alph[j]));          Projection operator.
            if (jj < fnz && alph[j]) SWAP(x.indx[--fnz],x.indx[jj]);
        }                               (Above) Make an α active if it becomes nonzero.
```
Here begins the relaxation passes over the interior α's.
```
        Indexx y(alph);                                 Sort. Identify interior α's.
        for (fnz=0; fnz<m; fnz++) if (alph[y.indx[fnz]] != 0.) break;
        for (fub=fnz; fub<m; fub++) if (alph[y.indx[fub]] == lambda) break;
        for (j=fnz; j<fub-2; j++) {                     Permute.
            k = j + (ran.int32() % (fub-j));
            SWAP(y.indx[j],y.indx[k]);
        }
        for (jj=fnz; jj<fub; jj++) {                    Compute sums over pinned α's just
            j = y.indx[jj];                                 once.
            sum = 0.;
            for (kk=fub; kk<m; kk++) {
                k = y.indx[kk];
                sum += (gker.ker[j][k] + 1.)*gker.y[k]*alph[k];
            }
            pinsum[jj] = sum;
        }
        niter = MAX(Int(0.5*(m+1.0)*(m-fnz+1.0)/(SQR(fub-fnz+1.0))),1);
```
Calculate a number of iterations that will take about half as long as the full pass just completed.
```
        for (iter=0; iter<niter; iter++) {              Main loop over α's.
            for (jj=fnz; jj<fub; jj++) {
                j = y.indx[jj];
                sum = pinsum[jj];
                for (kk=fnz; kk<fub; kk++) {
                    k = y.indx[kk];
                    sum += (gker.ker[j][k] + 1.)*gker.y[k]*alph[k];
                }
                alph[j] = alph[j] - (om/(gker.ker[j][j]+1.))*(gker.y[j]*sum-1.);
                alph[j] = MAX(0.,MIN(lambda,alph[j]));
            }
        }
        dalph = 0.;                                     Return change in norm of α vector.
        for (j=0;j<m;j++) dalph += SQR(alph[j]-alphold[j]);
        return sqrt(dalph);
    }
    Doub predict(Int k) {
```
Call only after convergence via repeated calls to `relax`. Returns the decision rule $f(\mathbf{x})$ for data point `k`.
```
        Doub sum = 0.;
        for (Int j=0; j<m; j++) sum += alph[j]*gker.y[j]*(gker.ker[j][k]+1.0);
        return sum;
    }
    Doub predict(Doub *x) {
```
Call only after convergence via repeated calls to `relax`. Returns the decision rule $f(\mathbf{x})$ for an arbitrary feature vector.
```
        Doub sum = 0.;
        for (Int j=0; j<m; j++) sum += alph[j]*gker.y[j]*(gker.kernel(j,x)+1.0);
        return sum;
    }
};
```

Although the enforced brevity doesn't allow for *too* many optimizing tricks, `Svm` does have a couple that bear mentioning:

First, each call to the `relax` routine performs, as previously mentioned, a group of relaxations. Specifically, it does one full relaxation pass over all the α_i's, and then multiple passes over only the "interior" α_i's, i.e., those that are not pinned at either 0 or λ. These passes are typically *much* faster than the full pass, since most variables

are typically pinned. To realize the gain, sums over pinned variables that don't vary are computed only once at the beginning of these multiple passes. The number of such passes is calculated dynamically so as to take about half as long as the full pass just taken.

Second, before each pass (both the full and interior), the order of the variables is randomized by a permutation generated from a `Ran` object (§7.1). This randomization speeds up the convergence by as much as an order of magnitude.

CITED REFERENCES AND FURTHER READING:

Boser, B.E., Guyon, I.M., and Vapnik, V.N. 1992, in D. Haussler, ed., *Proceedings of the 5th Annual ACM Workshop on Computational Learning Theory* (New York: ACM Press).[1]

Christianini, N. and Shawe-Taylor, J. 2000, *An Introduction to Support Vector Machines* (Cambridge, U.K.: Cambridge University Press); related Web site `http://www.support-vector.net`.[2]

Bazaraa, M.S., Sherali, H.D., and Shetty, C.M. 2006, *Nonlinear Programming: Theory and Algorithms*, 3rd ed. (Hoboken, NJ: Wiley).[3]

den Hertog, D. 1994, *Interior Point Approach to Linear, Quadratic and Convex Programming: Algorithms and Complexity* (Dordrecht: Kluwer).[4]

Schölkopf, B. and Smola, A.J. 2002, *Learning with Kernels: Support Vector Machines, Regularization, Optimization, and Beyond* (Cambridge, MA: MIT Press).[5]

Shawe-Taylor, J. and Christianini, N. 2004, *Kernel Methods for Pattern Analysis* (Cambridge, UK: Cambridge University Press).[6]

Vapnik, V. 1998, *Statistical Learning Theory* (New York: Wiley).

Joachims, T. 1999–, *SVMlight, Implementing Support Vector Machines in C*, at `http://svmlight.joachims.org`.[7]

Noble, W.S. and Pavlidis, P. 1999–, *Gist: Software Tools for Support Vector Machine Classification and for Kernel Principal Components Analysis*, at `http://microarray.cpmc.columbia.edu/gist`.[8]

Mangasarian, O.L. and Musicant, D.R. 1999, "Successive Overrelaxation for Support Vector Machines," *IEEE Transactions on Neural Networks*, vol. 10, no. 5, p. 1032.[9]

Mangasarian, O.L. and Musicant, D.R. 2001, in *Complementarity: Applications, Algorithms and Extensions*, M.C. Ferris, O.L. Mangasarian and J.-S. Pang, eds. (Dordrecht: Kluwer) pp. 233-251.[10]

Integration of Ordinary Differential Equations

CHAPTER 17

17.0 Introduction

Problems involving ordinary differential equations (ODEs) can always be reduced to the study of sets of first-order differential equations. For example the second-order equation

$$\frac{d^2y}{dx^2} + q(x)\frac{dy}{dx} = r(x) \tag{17.0.1}$$

can be rewritten as two first-order equations,

$$\begin{aligned}\frac{dy}{dx} &= z(x)\\ \frac{dz}{dx} &= r(x) - q(x)z(x)\end{aligned} \tag{17.0.2}$$

where z is a new variable. This exemplifies the procedure for an arbitrary ODE. The usual choice for the new variables is to let them be just derivatives of each other (and of the original variable). Occasionally, it is useful to incorporate into their definition some other factors in the equation, or some powers of the independent variable, for the purpose of mitigating singular behavior that could result in overflows or increased roundoff error. Let common sense be your guide: If you find that the original variables are smooth in a solution, while your auxiliary variables are doing crazy things, then figure out why and choose different auxiliary variables.

The generic problem in ordinary differential equations is thus reduced to the study of a set of N coupled *first-order* differential equations for the functions y_i, $i = 0, 1, \ldots, N-1$, having the general form

$$\frac{dy_i(x)}{dx} = f_i(x, y_0, \ldots, y_{N-1}), \qquad i = 0, \ldots, N-1 \tag{17.0.3}$$

where the functions f_i on the right-hand side are known.

A problem involving ODEs is not completely specified by its equations. Even more crucial in determining how to attack the problem numerically is the nature of

the problem's boundary conditions. Boundary conditions are algebraic conditions on the values of the functions y_i in (17.0.3). In general they can be satisfied at discrete specified points, but do not hold between those points, i.e., are not preserved automatically by the differential equations. Boundary conditions can be as simple as requiring that certain variables have certain numerical values, or as complicated as a set of nonlinear algebraic equations among the variables.

Usually, it is the nature of the boundary conditions that determines which numerical methods will be feasible. Boundary conditions divide into two broad categories.

- In *initial value problems* all the y_i are given at some starting value x_s, and it is desired to find the y_i's at some final point x_f, or at some discrete list of points (for example, at tabulated intervals).
- In *two-point boundary value problems*, on the other hand, boundary conditions are specified at more than one x. Typically, some of the conditions will be specified at x_s and the remainder at x_f.

This chapter will consider exclusively the initial value problem, deferring two-point boundary value problems, which are generally more difficult, to Chapter 18.

The underlying idea of any routine for solving the initial value problem is always this: Rewrite the dy's and dx's in (17.0.3) as finite steps Δy and Δx, and multiply the equations by Δx. This gives algebraic formulas for the change in the functions when the independent variable x is "stepped" by one "stepsize" Δx. In the limit of making the stepsize very small, a good approximation to the underlying differential equation is achieved. Literal implementation of this procedure results in *Euler's method* (equation 17.1.1, below), which is, however, *not* recommended for any practical use. Euler's method is conceptually important, however; one way or another, practical methods all come down to this same idea: Add small increments to your functions corresponding to derivatives (right-hand sides of the equations) multiplied by stepsizes.

In this chapter we consider three major types of practical numerical methods for solving initial value problems for ODEs:

- Runge-Kutta methods
- Richardson extrapolation and its particular implementation as the Bulirsch-Stoer method
- predictor-corrector methods, also known as multistep methods.

A brief description of each of these types follows.

1. *Runge-Kutta* methods propagate a solution over an interval by combining the information from several Euler-style steps (each involving one evaluation of the right-hand f's), and then using the information obtained to match a Taylor series expansion up to some higher order.

2. *Richardson extrapolation* uses the powerful idea of extrapolating a computed result to the value that *would* have been obtained if the stepsize had been very much smaller than it actually was. In particular, extrapolation to zero stepsize is the desired goal. The first practical ODE integrator that implemented this idea was developed by Bulirsch and Stoer, and so extrapolation methods are often called Bulirsch-Stoer methods.

3. *Predictor-corrector* methods or *multistep methods* store the solution along the way, and use those results to extrapolate the solution one step advanced; they

then correct the extrapolation using derivative information at the new point. These are best for very smooth functions.

Runge-Kutta used to be what you used when (i) you didn't know any better, or (ii) you had an intransigent problem where Bulirsch-Stoer was failing, or (iii) you had a trivial problem where computational efficiency was of no concern. However, advances in Runge-Kutta methods, particularly the development of higher-order methods, have made Runge-Kutta competitive with the other methods in many cases. Runge-Kutta succeeds virtually always; it is usually the fastest method when evaluating f_i is cheap and the accuracy requirement is not ultra-stringent ($\lesssim 10^{-10}$), or in general when moderate accuracy ($\lesssim 10^{-5}$) is required. Predictor-corrector methods have a relatively high overhead and so come into their own only when evaluating f_i is expensive. However, for many smooth problems, they are computationally more efficient than Runge-Kutta. In recent years, Bulirsch-Stoer has been replacing predictor-corrector in many applications, but it is too soon to say that predictor-corrector is dominated in all cases. However, it appears that only rather sophisticated predictor-corrector routines are competitive. Accordingly, we have chosen *not* to give an implementation of predictor-corrector in this book. We discuss predictor-corrector further in §17.6, so that you can use a packaged routine knowledgeably should you encounter a suitable problem. In our experience, the relatively simple Runge-Kutta and Bulirsch-Stoer routines we give are adequate for most problems.

Each of the three types of methods can be organized to monitor internal consistency. This allows numerical errors, which are inevitably introduced into the solution, to be controlled by automatic (*adaptive*) changing of the fundamental stepsize. We always recommend that adaptive stepsize control be implemented, and we will do so below.

In general, all three types of methods can be applied to any initial value problem. Each comes with its own set of debits and credits that must be understood before it is used.

Section 17.5 of this chapter treats the subject of *stiff equations*, relevant both to ordinary differential equations and also to partial differential equations (Chapter 20).

17.0.1 Organization of the Routines in This Chapter

We have organized the routines in this chapter into three nested levels, enabling modularity and sharing common code wherever possible.

The highest level is the *driver* object, which starts and stops the integration, stores intermediate results, and generally acts as an interface with the user. There is nothing canonical about our driver object, `Odeint`. You should consider it to be an example, and you can customize it for your particular application.

The next level down is a *stepper* object. The stepper oversees the actual incrementing of the independent variable x. It knows how to call the underlying *algorithm* routine. It may reject the result, set a smaller stepsize, and call the algorithm routine again, until compatibility with a predetermined accuracy criterion has been achieved. The stepper's fundamental task is to take the largest stepsize consistent with specified performance. Only when this is accomplished does the true power of an algorithm come to light.

All our steppers are derived from a base object called `StepperBase`: `StepperDopr5` and `StepperDopr853` (two Runge-Kutta routines), `StepperBS` and `StepperStoerm` (two Bulirsch-Stoer routines), and `StepperRoss` and `StepperSIE`

(for so-called stiff equations).

Standing apart from the stepper, but interacting with it at the same level, is an Output object. This is basically a container into which the stepper writes the output of the integration, but it has some intelligence of its own: It can save, or not save, intermediate results according to several different prescriptions that are specified by its constructor. In particular, it has the option to provide so-called dense output, that is, output at user-specified intermediate points without loss of efficiency.

The lowest or "nitty-gritty" level is the piece we call the *algorithm* routine. This implements the basic formulas of the method, starts with dependent variables y_i at x, and calculates new values of the dependent variables at the value $x + h$. The algorithm routine also yields some information about the quality of the solution after the step. The routine is dumb, however, in that it is unable to make any adaptive decision about whether the solution is of acceptable quality. Each algorithm routine is implemented as a member function dy() in its corresponding stepper object.

17.0.2 The Odeint Object

It is a real time saver to have a single high-level interface to what are otherwise quite diverse methods. We use the Odeint driver for a variety of problems, notably including garden-variety ODEs or sets of ODEs, and definite integrals (augmenting the methods of Chapter 4). The Odeint driver is templated on the stepper. This means that you can usually change from one ODE method to another in just a few keystrokes. For example, changing from the Dormand-Prince fifth-order Runge-Kutta method to Bulirsch-Stoer is as simple as changing the template parameter from StepperDopr5 to StepperBS.

The Odeint constructor simply initializes a bunch of things, including a call to the stepper constructor. The meat is in the integrate routine, which repeatedly invokes the step routine of the stepper to advance the solution from x_1 to x_2. It also calls the functions of the Output object to save the results at appropriate points.

odeint.h
```
template<class Stepper>
struct Odeint {
```
Driver for ODE solvers with adaptive stepsize control. The template parameter should be one of the derived classes of StepperBase defining a particular integration algorithm.
```
    static const Int MAXSTP=50000;                Take at most MAXSTP steps.
    Doub EPS;
    Int nok;
    Int nbad;
    Int nvar;
    Doub x1,x2,hmin;
    bool dense;                                   true if dense output requested by
    VecDoub y,dydx;                                  out.
    VecDoub &ystart;
    Output &out;
    typename Stepper::Dtype &derivs;              Get the type of derivs from the
    Stepper s;                                       stepper.
    Int nstp;
    Doub x,h;
    Odeint(VecDoub_IO &ystartt,const Doub xx1,const Doub xx2,
        const Doub atol,const Doub rtol,const Doub h1,
        const Doub hminn,Output &outt,typename Stepper::Dtype &derivss);
```
Constructor sets everything up. The routine integrates starting values ystart[0..nvar-1] from xx1 to xx2 with absolute tolerance atol and relative tolerance rtol. The quantity h1 should be set as a guessed first stepsize, hmin as the minimum allowed stepsize (can be zero). An Output object out should be input to control the saving of intermediate values.

On output, nok and nbad are the number of good and bad (but retried and fixed) steps taken, and ystart is replaced by values at the end of the integration interval. derivs is the user-supplied routine (function or functor) for calculating the right-hand side derivative.

```
    void integrate();                          Does the actual integration.
};

template<class Stepper>
Odeint<Stepper>::Odeint(VecDoub_IO &ystartt, const Doub xx1, const Doub xx2,
    const Doub atol, const Doub rtol, const Doub h1, const Doub hminn,
    Output &outt,typename Stepper::Dtype &derivss) : nvar(ystartt.size()),
    y(nvar),dydx(nvar),ystart(ystartt),x(xx1),nok(0),nbad(0),
    x1(xx1),x2(xx2),hmin(hminn),dense(outt.dense),out(outt),derivs(derivss),
    s(y,dydx,x,atol,rtol,dense) {
    EPS=numeric_limits<Doub>::epsilon();
    h=SIGN(h1,x2-x1);
    for (Int i=0;i<nvar;i++) y[i]=ystart[i];
    out.init(s.neqn,x1,x2);
}

template<class Stepper>
void Odeint<Stepper>::integrate() {
    derivs(x,y,dydx);
    if (dense)                                 Store initial values.
        out.out(-1,x,y,s,h);
    else
        out.save(x,y);
    for (nstp=0;nstp<MAXSTP;nstp++) {
        if ((x+h*1.0001-x2)*(x2-x1) > 0.0)
            h=x2-x;                            If stepsize can overshoot, decrease.
        s.step(h,derivs);                      Take a step.
        if (s.hdid == h) ++nok; else ++nbad;
        if (dense)
            out.out(nstp,x,y,s,s.hdid);
        else
            out.save(x,y);
        if ((x-x2)*(x2-x1) >= 0.0) {           Are we done?
            for (Int i=0;i<nvar;i++) ystart[i]=y[i];     Update ystart.
            if (out.kmax > 0 && abs(out.xsave[out.count-1]-x2) > 100.0*abs(x2)*EPS)
                out.save(x,y);                 Make sure last step gets saved.
            return;                            Normal exit.
        }
        if (abs(s.hnext) <= hmin) throw("Step size too small in Odeint");
        h=s.hnext;
    }
    throw("Too many steps in routine Odeint");
}
```

The Odeint object doesn't know in advance which specific stepper object it will be instantiated with. It does, however, rely on the fact that the stepper object will be derived from, and thus have the methods in, this StepperBase object, which serves as the base class for all subsequent ODE algorithms in this chapter:

```
struct StepperBase {                                                stepper.h
Base class for all ODE algorithms.
    Doub &x;
    Doub xold;                     Used for dense output.
    VecDoub &y,&dydx;
    Doub atol,rtol;
    bool dense;
    Doub hdid;                     Actual stepsize accomplished by the step routine.
    Doub hnext;                    Stepsize predicted by the controller for the next step.
```

```
    Doub EPS;
    Int n,neqn;                    neqn = n except for StepperStoerm.
    VecDoub yout,yerr;             New value of y and error estimate.
    StepperBase(VecDoub_IO &yy, VecDoub_IO &dydxx, Doub &xx, const Doub atoll,
        const Doub rtoll, bool dens) : x(xx),y(yy),dydx(dydxx),atol(atoll),
        rtol(rtoll),dense(dens),n(y.size()),neqn(n),yout(n),yerr(n) {}
        Input to the constructor are the dependent variable vector y[0..n-1] and its derivative
        dydx[0..n-1] at the starting value of the independent variable x. Also input are the
        absolute and relative tolerances, atol and rtol, and the boolean dense, which is true
        if dense output is required.
};
```

17.0.3 The Output Object

Output is controlled by the various constructors in the Output structure. The default constructor, with no arguments, suppresses all output. The constructor with argument nsave provides *dense output* provided nsave > 0. This means output at values of x of your choosing, not necessarily the natural places that the stepper method would land. The output points are nsave $+ 1$ uniformly spaced points including x1 and x2. If nsave ≤ 0, output is saved at every integration step, that is, only at the points where the stepper happens to land. While most of your needs should be met by these options, you should find it easy to modify Output for your particular application.

odeint.h

```
struct Output {
Structure for output from ODE solver such as odeint.
    Int kmax;                   Current capacity of storage arrays.
    Int nvar;
    Int nsave;                  Number of intervals to save at for dense output.
    bool dense;                 true if dense output requested.
    Int count;                  Number of values actually saved.
    Doub x1,x2,xout,dxout;
    VecDoub xsave;              Results stored in the vector xsave[0..count-1] and the
    MatDoub ysave;                  matrix ysave[0..nvar-1][0..count-1].
    Output() : kmax(-1),dense(false),count(0) {}
    Default constructor gives no output.
    Output(const Int nsavee) : kmax(500),nsave(nsavee),count(0),xsave(kmax) {
    Constructor provides dense output at nsave equally spaced intervals. If nsave ≤ 0, output
    is saved only at the actual integration steps.
        dense = nsave > 0 ? true : false;
    }
    void init(const Int neqn, const Doub xlo, const Doub xhi) {
    Called by Odeint constructor, which passes neqn, the number of equations, xlo, the starting
    point of the integration, and xhi, the ending point.
        nvar=neqn;
        if (kmax == -1) return;
        ysave.resize(nvar,kmax);
        if (dense) {
            x1=xlo;
            x2=xhi;
            xout=x1;
            dxout=(x2-x1)/nsave;
        }
    }
    void resize() {
    Resize storage arrays by a factor of two, keeping saved data.
        Int kold=kmax;
        kmax *= 2;
        VecDoub tempvec(xsave);
```

```
        xsave.resize(kmax);
        for (Int k=0; k<kold; k++)
            xsave[k]=tempvec[k];
        MatDoub tempmat(ysave);
        ysave.resize(nvar,kmax);
        for (Int i=0; i<nvar; i++)
            for (Int k=0; k<kold; k++)
                ysave[i][k]=tempmat[i][k];
    }
    template <class Stepper>
    void save_dense(Stepper &s, const Doub xout, const Doub h) {
```
Invokes dense_out function of stepper routine to produce output at xout. Normally called by out rather than directly. Assumes that xout is between xold and xold+h, where the stepper must keep track of xold, the location of the previous step, and x=xold+h, the current step.
```
        if (count == kmax) resize();
        for (Int i=0;i<nvar;i++)
            ysave[i][count]=s.dense_out(i,xout,h);
        xsave[count++]=xout;
    }
    void save(const Doub x, VecDoub_I &y) {
```
Saves values of current x and y.
```
        if (kmax <= 0) return;
        if (count == kmax) resize();
        for (Int i=0;i<nvar;i++)
            ysave[i][count]=y[i];
        xsave[count++]=x;
    }
    template <class Stepper>
    void out(const Int nstp,const Doub x,VecDoub_I &y,Stepper &s,const Doub h) {
```
Typically called by Odeint to produce dense output. Input variables are nstp, the current step number, the current values of x and y, the stepper s, and the stepsize h. A call with nstp=-1 saves the initial values. The routine checks whether x is greater than the desired output point xout. If so, it calls save_dense.
```
        if (!dense)
            throw("dense output not set in Output!");
        if (nstp == -1) {
            save(x,y);
            xout += dxout;
        } else {
            while ((x-xout)*(x2-x1) > 0.0) {
                save_dense(s,xout,h);
                xout += dxout;
            }
        }
    }
};
```

17.0.4 A Quick-Start Example

Before we dive deep into the pros and cons of the different stepper types (the meat of this chapter), let's see how to code the solution of an actual problem. Suppose we want to solve Van der Pol's equation, which when written in first-order form is

$$\begin{aligned} y_0' &= y_1 \\ y_1' &= [(1-y_0^2)y_1 - y_0]/\epsilon \end{aligned} \tag{17.0.4}$$

First encapsulate (17.0.4) in a functor (see §1.3.3). Using a functor instead of a bare function gives you the opportunity to pass other information to the function,

such as the values of fixed parameters. Every stepper class in this chapter is accordingly templated on the type of the functor defining the right-hand side derivatives. For our example, the right-hand side functor looks like:

```
struct rhs_van {
    Doub eps;
    rhs_van(Doub epss) : eps(epss) {}
    void operator() (const Doub x, VecDoub_I &y, VecDoub_O &dydx) {
        dydx[0]= y[1];
        dydx[1]=((1.0-y[0]*y[0])*y[1]-y[0])/eps;
    }
};
```

The key thing is the line beginning `void operator()`: It *always* should have this form, with the definition of `dydx` following. Here we have chosen to specify ϵ as a parameter in the constructor so that the main program can easily pass a specific value to the right-hand side. Alternatively, you could have omitted the constructor, relying on the compiler-supplied default constructor, and hard-coded a value of ϵ in the routine. Note, of course, that there is nothing special about the name `rhs_van`.

We will integrate from 0 to 2 with initial conditions $y_0 = 2$, $y_1 = 0$ and with $\epsilon = 10^{-3}$. Then your main program will have declarations like the following:

```
const Int nvar=2;
const Doub atol=1.0e-3, rtol=atol, h1=0.01, hmin=0.0, x1=0.0, x2=2.0;
VecDoub ystart(nvar);
ystart[0]=2.0;
ystart[1]=0.0;
Output out(20);                          Dense output at 20 points plus x1.
rhs_van d(1.0e-3);                       Declare d as a rhs_van object.
Odeint<StepperDopr5<rhs_van> > ode(ystart,x1,x2,atol,rtol,h1,hmin,out,d);
ode.integrate();
```

Note how the `Odeint` object is templated on the stepper, which in turn is templated on the derivative object, `rhs_van` in this case. The space between the two closing angle brackets is necessary; otherwise the compiler parses >> as the right-shift operator!

The number of good steps taken is available in `ode.nok` and the number of bad steps in `ode.nbad`. The output, which is equally spaced, can be printed by statements like

```
for (Int i=0;i<out.count;i++)
    cout << out.xsave[i] << " " << out.ysave[0][i] << " " <<
        out.ysave[1][i] << endl;
```

You can alternatively save output at the actual integration steps by the declaration

```
Output out(-1);
```

or suppress all saving of output with

```
Output out;
```

In this case, the solution values at the endpoint are available in `ystart[0]` and `ystart[1]`, overwriting the starting values.

CITED REFERENCES AND FURTHER READING:

Gear, C.W. 1971, *Numerical Initial Value Problems in Ordinary Differential Equations* (Englewood Cliffs, NJ: Prentice-Hall).

Acton, F.S. 1970, *Numerical Methods That Work*; 1990, corrected edition (Washington, DC: Mathematical Association of America), Chapter 5.

Stoer, J., and Bulirsch, R. 2002, *Introduction to Numerical Analysis*, 3rd ed. (New York: Springer), Chapter 7.

Hairer, E., Nørsett, S.P., and Wanner, G. 1993, *Solving Ordinary Differential Equations I. Nonstiff Problems*, 2nd ed. (New York: Springer)

Hairer, E., Nørsett, S.P., and Wanner, G. 1996, *Solving Ordinary Differential Equations II. Stiff and Differential-Algebraic Problems*, 2nd ed. (New York: Springer)

Lambert, J. 1973, *Computational Methods in Ordinary Differential Equations* (New York: Wiley).

Lapidus, L., and Seinfeld, J. 1971, *Numerical Solution of Ordinary Differential Equations* (New York: Academic Press).

17.1 Runge-Kutta Method

The formula for the Euler method is

$$y_{n+1} = y_n + hf(x_n, y_n) \tag{17.1.1}$$

which advances a solution from x_n to $x_{n+1} \equiv x_n + h$. The formula is unsymmetrical: It advances the solution through an interval h, but uses derivative information only at the beginning of that interval (see Figure 17.1.1). That means (and you can verify by expansion in power series) that the step's error is only one power of h smaller than the correction, i.e., $O(h^2)$ added to (17.1.1).

There are several reasons that Euler's method is not recommended for practical use, among them, (i) the method is not very accurate when compared to other, fancier, methods run at the equivalent stepsize, and (ii) neither is it very stable (see §17.5 below).

Consider, however, the use of a step like (17.1.1) to take a "trial" step to the midpoint of the interval. Then use the values of both x and y at that midpoint to compute the "real" step across the whole interval. Figure 17.1.2 illustrates the idea. In equations,

$$\begin{aligned} k_1 &= hf(x_n, y_n) \\ k_2 &= hf\left(x_n + \tfrac{1}{2}h, y_n + \tfrac{1}{2}k_1\right) \\ y_{n+1} &= y_n + k_2 + O(h^3) \end{aligned} \tag{17.1.2}$$

As indicated in the error term, this symmetrization cancels out the first-order error term, making the method *second order*. [A method is conventionally called nth order if its error term is $O(h^{n+1})$.] In fact, (17.1.2) is called the *second-order Runge-Kutta* or *midpoint* method.

We needn't stop there. There are many ways to evaluate the right-hand side $f(x, y)$ that all agree to first order, but that have different coefficients of higher-order error terms. Adding up the right combination of these, we can eliminate the error terms order by order. That is the basic idea of the Runge-Kutta method. Abramowitz and Stegun [1] and Gear [2] give various specific formulas that derive from this basic idea. By far the most often used is the classical *fourth-order Runge-Kutta formula*,

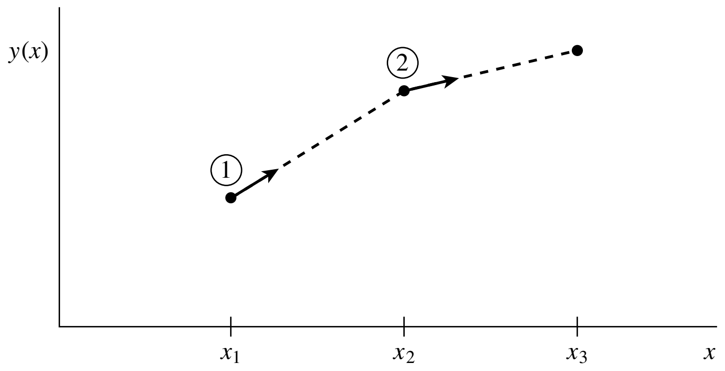

Figure 17.1.1. Euler's method. In this simplest (and least accurate) method for integrating an ODE, the derivative at the starting point of each interval is extrapolated to find the next function value. The method has first-order accuracy.

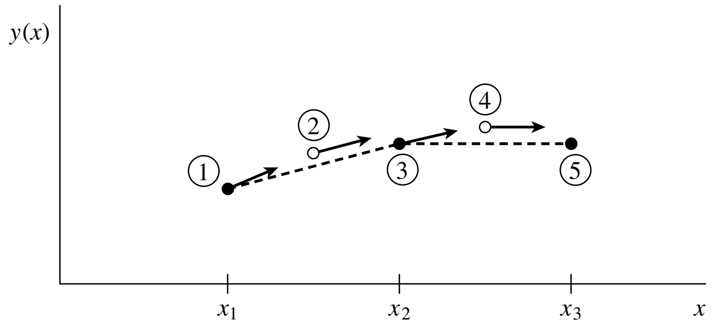

Figure 17.1.2. Midpoint method. Second-order accuracy is obtained by using the initial derivative at each step to find a point halfway across the interval, then using the midpoint derivative across the full width of the interval. In the figure, filled dots represent final function values, while open dots represent function values that are discarded once their derivatives have been calculated and used.

which has a certain sleekness of organization about it:

$$\begin{aligned}
k_1 &= hf(x_n, y_n) \\
k_2 &= hf(x_n + \tfrac{1}{2}h, y_n + \tfrac{1}{2}k_1) \\
k_3 &= hf(x_n + \tfrac{1}{2}h, y_n + \tfrac{1}{2}k_2) \\
k_4 &= hf(x_n + h, y_n + k_3) \\
y_{n+1} &= y_n + \tfrac{1}{6}k_1 + \tfrac{1}{3}k_2 + \tfrac{1}{3}k_3 + \tfrac{1}{6}k_4 + O(h^5)
\end{aligned} \tag{17.1.3}$$

The fourth-order Runge-Kutta method requires four evaluations of the right-hand side per step h (see Figure 17.1.3). This will be superior to the midpoint method (17.1.2) *if* at least twice as large a step is possible with (17.1.3) for the same accuracy. Is that so? The answer is: often, perhaps even usually, but surely not always! This takes us back to a central theme, namely that *high order* does not always mean *high accuracy*. The statement "fourth-order Runge-Kutta is generally superior to second-order" is a true one, but as much a statement about the kind of problems that people solve as a statement about strict mathematics.

For many scientific users, fourth-order Runge-Kutta is not just the first word

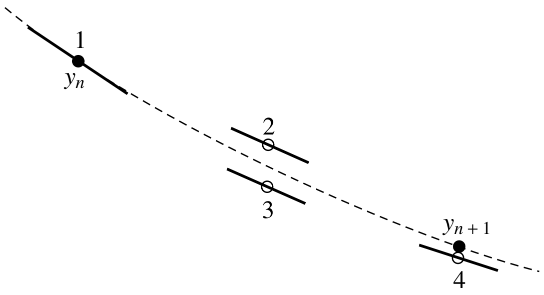

Figure 17.1.3. Fourth-order Runge-Kutta method. In each step the derivative is evaluated four times: once at the initial point, twice at trial midpoints, and once at a trial endpoint. From these derivatives the final function value (shown as a filled dot) is calculated. (See text for details.)

on ODE integrators, but the last word as well. In fact, you can get pretty far on this old workhorse, especially if you combine it with an adaptive stepsize algorithm. Keep in mind, however, that the old workhorse's last trip may well be to take you to the poorhouse: Newer Runge-Kutta methods are *much* more efficient, and Bulirsch-Stoer or predictor-corrector methods can be even more efficient for problems where very high accuracy is a requirement. Those methods are the high-strung racehorses. Runge-Kutta is for ploughing the fields. However, even the old workhorse is more nimble with new horseshoes. In §17.2 we will give a modern implementation of a Runge-Kutta method that is quite competitive as long as very high accuracy is not required. An excellent discussion of the pitfalls in constructing a good Runge-Kutta code is given in [3].

Here is the routine `rk4` for carrying out one classical Runge-Kutta step on a set of `n` differential equations. This routine is completely separate from the various stepper routines introduced in the previous section and given in the rest of the chapter. It is meant for only the most trivial applications. You input the values of the independent variables, and you get out new values that are stepped by a stepsize `h` (which can be positive or negative). You will notice that the routine requires you to supply not only function `derivs` for calculating the right-hand side, but also values of the derivatives at the starting point. Why not let the routine call `derivs` for this first value? The answer will become clear only in the next section, but in brief is this: This call may not be your only one with these starting conditions. You may have taken a previous step with too large a stepsize, and this is your replacement. In that case, you do not want to call `derivs` unnecessarily at the start. Note that the routine that follows has, therefore, only three calls to `derivs`.

```
void rk4(VecDoub_I &y, VecDoub_I &dydx, const Doub x, const Doub h,
    VecDoub_O &yout, void derivs(const Doub, VecDoub_I &, VecDoub_O &))
```
rk4.h

Given values for the variables `y[0..n-1]` and their derivatives `dydx[0..n-1]` known at `x`, use the fourth-order Runge-Kutta method to advance the solution over an interval `h` and return the incremented variables as `yout[0..n-1]`. The user supplies the routine `derivs(x,y,dydx)`, which returns derivatives `dydx` at `x`.

```
{
    Int n=y.size();
    VecDoub dym(n),dyt(n),yt(n);
    Doub hh=h*0.5;
    Doub h6=h/6.0;
    Doub xh=x+hh;
```

```
    for (Int i=0;i<n;i++) yt[i]=y[i]+hh*dydx[i];              First step.
    derivs(xh,yt,dyt);                                        Second step.
    for (Int i=0;i<n;i++) yt[i]=y[i]+hh*dyt[i];
    derivs(xh,yt,dym);                                        Third step.
    for (Int i=0;i<n;i++) {
        yt[i]=y[i]+h*dym[i];
        dym[i] += dyt[i];
    }
    derivs(x+h,yt,dyt);                                       Fourth step.
    for (Int i=0;i<n;i++)                                     Accumulate increments with
        yout[i]=y[i]+h6*(dydx[i]+dyt[i]+2.0*dym[i]);              proper weights.
}
```

The Runge-Kutta method treats every step in a sequence of steps in an identical manner. Prior behavior of a solution is not used in its propagation. This is mathematically proper, since any point along the trajectory of an ordinary differential equation can serve as an initial point. The fact that all steps are treated identically also makes it easy to incorporate Runge-Kutta into relatively simple "driver" schemes.

CITED REFERENCES AND FURTHER READING:

Abramowitz, M., and Stegun, I.A. 1964, *Handbook of Mathematical Functions* (Washington: National Bureau of Standards); reprinted 1968 (New York: Dover); online at `http://www.nr.com/aands`, §25.5.[1]

Gear, C.W. 1971, *Numerical Initial Value Problems in Ordinary Differential Equations* (Englewood Cliffs, NJ: Prentice-Hall), Chapter 2.[2]

Shampine, L.F., and Watts, H.A. 1977, "The Art of Writing a Runge-Kutta Code, Part I," in *Mathematical Software III*, J.R. Rice, ed. (New York: Academic Press), pp. 257–275; 1979, "The Art of Writing a Runge-Kutta Code. II," *Applied Mathematics and Computation*, vol. 5, pp. 93–121.[3]

17.2 Adaptive Stepsize Control for Runge-Kutta

A good ODE integrator should exert some adaptive control over its own progress, making frequent changes in its stepsize. Usually the purpose of this adaptive stepsize control is to achieve some predetermined accuracy in the solution with minimum computational effort. Many small steps should tiptoe through treacherous terrain, while a few great strides should speed through smooth uninteresting countryside. The resulting gains in efficiency are not mere tens of percents or factors of two; they can sometimes be factors of ten, a hundred, or more. Sometimes accuracy may be demanded not directly in the solution itself, but in some related conserved quantity that can be monitored.

Implementation of adaptive stepsize control requires that the stepping algorithm signal information about its performance, most important, an estimate of its truncation error. In this section we will learn how such information can be obtained. Obviously, the calculation of this information will add to the computational overhead, but the investment will generally be repaid handsomely.

With fourth-order Runge-Kutta, the most straightforward technique by far is *step doubling* (see, e.g., [1]). We take each step twice, once as a full step, then,

independently, as two half-steps (see Figure 17.2.1). How much overhead is this, say in terms of the number of evaluations of the right-hand sides? Each of the three separate Runge-Kutta steps in the procedure requires 4 evaluations, but the single and double sequences share a starting point, so the total is 11. This is to be compared not to 4, but to 8 (the two half-steps), since — stepsize control aside — we are achieving the accuracy of the smaller (half-) stepsize. The overhead cost is therefore a factor 1.375. What does it buy us?

Let us denote the exact solution for an advance from x to $x + 2h$ by $y(x + 2h)$ and the two approximate solutions by y_1 (one step $2h$) and y_2 (two steps each of size h). Since the basic method is fourth order, the true solution and the two numerical approximations are related by

$$\begin{aligned} y(x + 2h) &= y_1 + (2h)^5 \phi + O(h^6) + \dots \\ y(x + 2h) &= y_2 + 2(h^5)\phi + O(h^6) + \dots \end{aligned} \tag{17.2.1}$$

where, to order h^5, the value ϕ remains constant over the step. [Taylor series expansion tells us the ϕ is a number whose order of magnitude is $y^{(5)}(x)/5!$.] The first expression in (17.2.1) involves $(2h)^5$ since the stepsize is $2h$, while the second expression involves $2(h^5)$ since the error on each step is $h^5\phi$. The difference between the two numerical estimates is a convenient indicator of truncation error,

$$\Delta \equiv y_2 - y_1 \tag{17.2.2}$$

It is this difference that we shall endeavor to keep to a desired degree of accuracy, neither too large nor too small. We do this by adjusting h.

It might also occur to you that, ignoring terms of order h^6 and higher, we can solve the two equations in (17.2.1) to improve our numerical estimate of the true solution $y(x + 2h)$, namely,

$$y(x + 2h) = y_2 + \frac{\Delta}{15} + O(h^6) \tag{17.2.3}$$

This estimate is accurate to *fifth order*, one order higher than the original Runge-Kutta steps (Richardson extrapolation again!). However, we can't have our cake and eat it too: (17.2.3) may be fifth-order accurate, but we have no way of monitoring *its* truncation error. Higher order is not always higher accuracy! Use of (17.2.3) rarely does harm, but we have no way of directly knowing whether it is doing any good. Therefore we should use Δ as the error estimate and take as "gravy" any additional accuracy gain derived from (17.2.3). In the technical literature, use of a procedure like (17.2.3) is called "local extrapolation."

Step doubling has been superseded by a more efficient stepsize adjustment algorithm based on *embedded Runge-Kutta formulas*, originally invented by Merson and popularized in a method of Fehlberg. An interesting fact about Runge-Kutta formulas is that for orders M higher than four, more than M function evaluations are required. This accounts for the popularity of the classical fourth-order method: It seems to give the most bang for the buck. However, Fehlberg discovered a fifth-order method with six function evaluations where another combination of the six functions gives a fourth-order method. The difference between the two estimates of $y(x + h)$ can then be used as an estimate of the truncation error to adjust the

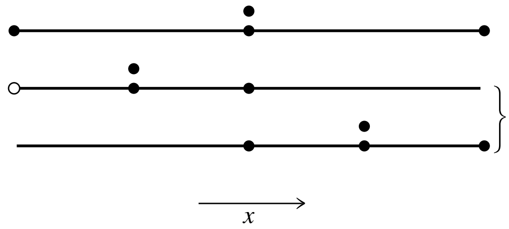

Figure 17.2.1. Step doubling as a means for adaptive stepsize control in fourth-order Runge-Kutta. Points where the derivative is evaluated are shown as filled circles. The open circle represents the same derivatives as the filled circle immediately above it, so the total number of evaluations is 11 per two steps. Comparing the accuracy of the big step with the two small steps gives a criterion for adjusting the stepsize on the next step, or for rejecting the current step as inaccurate.

stepsize. Since Fehlberg's original formula, many other embedded Runge-Kutta formulas have been found.

As an aside, the general question of how many function evaluations are required for a Runge-Kutta method of a given order is still open. Order 5 requires 6 function evaluations, order 6 requires 7, order 7 requires 9, order 8 requires 11. It is known that for order $M \geq 8$, at least $M + 3$ evaluations are required. The highest order explicitly constructed method so far is order 10, with 17 evaluations. The calculation of the coefficients of these high-order methods is very complicated.

We will spend most of this section setting up an efficient fifth-order Runge-Kutta method, coded in the routine `StepperDopr5`. This will allow us to explore the various issues that have to be dealt with in any Runge-Kutta scheme. However, ultimately you should not use this routine except for low accuracy requirements ($\lesssim 10^{-3}$) or trivial problems. Use the more efficient higher-order Runge-Kutta code `StepperDopr853` or the Bulirsch-Stoer code `StepperBS`.

The general form of a fifth-order Runge-Kutta formula is

$$\begin{aligned} k_1 &= hf(x_n, y_n) \\ k_2 &= hf(x_n + c_2 h, y_n + a_{21} k_1) \\ &\cdots \\ k_6 &= hf(x_n + c_6 h, y_n + a_{61} k_1 + \cdots + a_{65} k_5) \\ y_{n+1} &= y_n + b_1 k_1 + b_2 k_2 + b_3 k_3 + b_4 k_4 + b_5 k_5 + b_6 k_6 + O(h^6) \end{aligned} \qquad (17.2.4)$$

The embedded fourth-order formula is

$$y^*_{n+1} = y_n + b^*_1 k_1 + b^*_2 k_2 + b^*_3 k_3 + b^*_4 k_4 + b^*_5 k_5 + b^*_6 k_6 + O(h^5) \qquad (17.2.5)$$

and so the error estimate is

$$\Delta \equiv y_{n+1} - y^*_{n+1} = \sum_{i=1}^{6} (b_i - b^*_i) k_i \qquad (17.2.6)$$

The particular values of the various constants that we favor are those found by Dormand and Prince [2] and given in the table on the next page. These give a more efficient method than Fehlberg's original values, with better error properties.

We said that the Dormand-Prince method needs six function evaluations per step, yet the table on the next page shows seven and the sums in equations (17.2.5) and (17.2.6) should really go up to $i = 7$. What's going on? The idea is to use

Dormand-Prince 5(4) Parameters for Embedded Runga-Kutta Method									
i	c_i	a_{ij}						b_i	b_i^*
1								$\frac{35}{384}$	$\frac{5179}{57600}$
2	$\frac{1}{5}$	$\frac{1}{5}$						0	0
3	$\frac{3}{10}$	$\frac{3}{40}$	$\frac{9}{40}$					$\frac{500}{1113}$	$\frac{7571}{16695}$
4	$\frac{4}{5}$	$\frac{44}{45}$	$-\frac{56}{15}$	$\frac{32}{9}$				$\frac{125}{192}$	$\frac{393}{640}$
5	$\frac{8}{9}$	$\frac{19372}{6561}$	$-\frac{25360}{2187}$	$\frac{64448}{6561}$	$-\frac{212}{729}$			$-\frac{2187}{6784}$	$-\frac{92097}{339200}$
6	1	$\frac{9017}{3168}$	$-\frac{355}{33}$	$\frac{46732}{5247}$	$\frac{49}{176}$	$-\frac{5103}{18656}$		$\frac{11}{84}$	$\frac{187}{2100}$
7	1	$\frac{35}{384}$	0	$\frac{500}{1113}$	$\frac{125}{192}$	$-\frac{2187}{6784}$	$\frac{11}{84}$	0	$\frac{1}{40}$
$j =$		1	2	3	4	5	6		

y_{n+1} itself to provide a seventh stage. Because $f(x_n + h, y_{n+1})$ has to be evaluated anyway to start the next step, this costs nothing (unless the step is rejected because the error is too big). This trick is called FSAL (first-same-as-last). You can see in the table that the coefficients in the last row are the same as the b_i column.

Now that we know, at least approximately, what our error is, we need to consider how to keep it within desired bounds. We require

$$|\Delta| = |y_{n+1} - y_{n+1}^*| \le \texttt{scale} \tag{17.2.7}$$

where

$$\texttt{scale} = \texttt{atol} + |y|\,\texttt{rtol} \tag{17.2.8}$$

Here `atol` is the absolute error tolerance and `rtol` is the relative error tolerance. (Practical detail: In a code, you use $\max(|y_n|, |y_{n+1}|)$ for $|y|$ in the above formula in case one of them is close to zero.)

Our notation hides the fact that Δ is actually a vector of desired accuracies, Δ_i, one for each equation in the set of ODEs. In practice one takes some norm of the vector Δ. While taking the maximum component value is fine (i.e., rescaling the stepsize according to the needs of the "worst-offender" equation), we will use the usual Euclidean norm. Also, while `atol` and `rtol` could be different for each component of y, we will take them as constant. So define

$$\texttt{err} = \sqrt{\frac{1}{N}\sum_{i=0}^{N-1}\left(\frac{\Delta_i}{\texttt{scale}_i}\right)^2} \tag{17.2.9}$$

and accept the step if $\texttt{err} \le 1$, otherwise reject it.

What is the relation between the scaled error `err` and h? According to (17.2.4) – (17.2.5), Δ scales as h^5 and hence so does `err`. If we take a step h_1 and produce an error $\texttt{err}_1$, therefore, the step h_0 that *would have given* some other value $\texttt{err}_0$ is readily estimated as

$$h_0 = h_1 \left|\frac{\texttt{err}_0}{\texttt{err}_1}\right|^{1/5} \tag{17.2.10}$$

Let $\texttt{err}_0$ denote the desired error, which is 1 in an efficient integration. Then

equation (17.2.10) is used in two ways: If $\mathtt{err}_1$ is larger than 1 in magnitude, the equation tells how much to decrease the stepsize *when we retry the present (failed) step*. If $\mathtt{err}_1$ is smaller than 1, on the other hand, then the equation tells how much we can safely increase the stepsize *for the next step*. Local extrapolation means that we use the fifth-order value y_{n+1}, even though the error estimate actually applies to the fourth-order value y^*_{n+1}.

How is the quantity err related to some looser prescription like "get a solution good to one part in 10^6"? That can be a subtle question, and it depends on exactly what your application is. You may be dealing with a set of equations whose dependent variables differ enormously in magnitude. In that case, you probably want to use fractional errors, $\mathtt{atol} = 0$, $\mathtt{rtol} = \epsilon$, where ϵ is the number like 10^{-6} or whatever. On the other hand, you may have oscillatory functions that pass through zero but are bounded by some maximum values. In that case you probably want to set $\mathtt{atol} = \mathtt{rtol} = \epsilon$. This latter choice is the safest in general, and should usually be your first choice.

Here is a more technical point. The error criteria mentioned thus far are "local," in that they bound the error of each step individually. In some applications you may be unusually sensitive about a "global" accumulation of errors, from beginning to end of the integration and in the worst possible case where the errors all are presumed to add with the same sign. Then, the smaller the stepsize h, the *more steps* between your starting and ending values of x. In such a case you might want to set scale proportional to h, typically to something like

$$\mathtt{scale} = \epsilon h \times \mathtt{dydx[i]} \tag{17.2.11}$$

This enforces fractional accuracy ϵ not on the values of y but (much more stringently) on the *increments* to those values at each step. But now look back at (17.2.10). The exponent $1/5$ is no longer correct: When the stepsize is reduced from a too-large value, the new predicted value h_1 will fail to meet the desired accuracy when scale is also altered to this new h_1 value. Instead of $1/5$, we must scale by the exponent $1/4$ for things to work out.

Error control that tries to constrain the global error by setting the scale factor proportional to h is called "error per unit step," as opposed to the original "error per step" method. As a point of principle, controlling the global error by controlling the local error is very difficult. The global error at any point is the sum of the global error up to the start of the last step plus the local error of that step. This cumulative nature of the global error means it depends on things that cannot always be controlled, like stability properties of the differential equation. Accordingly, we recommend the straightforward "error per step" method in most cases. If you want to estimate the global error of your solution, you have to integrate again with a reduced tolerance and use the change in the solution as an estimate of the global error. This works *if* the stepsize controller produces errors roughly proportional to the tolerance, which is not always guaranteed.

Because our error estimates are not exact, but only accurate to the leading order in h, we are advised to put in a safety factor S that is a few percent smaller than unity. Equation (17.2.10) (with $\mathtt{err}_0 = 1$ and the subscripts $1 \to n$ and $0 \to n+1$) is thus replaced by

$$h_{n+1} = S h_n \left(\frac{1}{\mathtt{err}_n} \right)^{1/5} \tag{17.2.12}$$

Moreover, experience shows that it is not wise to let the stepsize increase or decrease too fast, and not to let the stepsize increase at all if the previous step was rejected. In `StepperDopr5`, the stepsize cannot increase by more than a factor of 10 nor decrease by more than a factor of 5 in a single step.

17.2.1 PI Stepsize Control

One situation in which the above stepsize controller has difficulty is when the stepsize is being limited by the stability properties of the integration method, rather than the accuracy of the individual steps. (We will see more about this in §17.5 on stiff differential equations.) The stepsize increases slowly as successive steps are accepted, until the method becomes unstable. The controller responds to the sudden increase in the error by cutting the stepsize drastically, and the cycle repeats itself. Similar problems can occur when the solution to the differential equation enters a region with drastically different behavior than the previous region. A long sequence of alternating accepted and rejected steps ensues. Since rejected steps are expensive, it is worth improving the stepsize control.

The most effective way to do this seems to be to use ideas from *control theory*. The integration routine and the differential equation play the role of the *process*, like a chemical plant manufacturing a product. The stepsize h is the input and the error estimate `err` is the output. (The numerical solution is also output, but it is not used for stepsize control.) The *controller* is the stepsize control algorithm. It tries to hold the error at the prescribed tolerance by varying the stepsize. Deriving an improved stepsize controller from control theory ideas is beyond our scope here, so we will introduce some basic concepts and then refer you to the literature for derivations and a fuller explanation [6-8].

The standard stepsize controller (17.2.12), when expressed in the language of control theory, is known as an *integrating controller*, with $\log h$ as the discrete control variable. This means that the control variable is obtained by "integrating" the control error signal. It is well known in control theory that more stable control can be achieved by adding an additional term *proportional* to the control error. This is called a PI controller, where the P stands for proportional feedback and the I for integral feedback. Instead of (17.2.12), the resulting algorithm takes the simple form

$$h_{n+1} = S h_n \mathtt{err}_n^{-\alpha} \mathtt{err}_{n-1}^{\beta} \tag{17.2.13}$$

Typically α and β should be scaled as $1/k$, where k is the exponent of h in `err` ($k = 5$ for a fifth-order method). Setting $\alpha = 1/k$, $\beta = 0$ recovers the classical controller (17.2.12). Nonzero β improves the stability but loses some efficiency for "easy" parts of the solution. A good compromise [6] is to set

$$\beta \approx 0.4/k, \qquad \alpha \approx 0.7/k = 1/k - 0.75\beta \tag{17.2.14}$$

17.2.2 Dense Output

Adaptive stepsize control means the algorithm marches along producing y values at x's that it chooses itself. What if you want output at values that you specify? The simplest option is just to integrate from one desired output point to the next. But if you specify a lot of output points, this is inefficient: The code has to take steps

based on where you want output, rather than the "natural" stepsizes it would like to choose. High-order methods like to take large steps for smooth solutions, so the problem is especially acute in this case.

The solution is to find an *interpolation* method that uses information produced during the integration and is of an order comparable to the order of the method so that full accuracy of the solution is preserved. This is called providing a *dense output* method.

For example, any method has available y and $dy/dx = f$ at the beginning and end of the step. These four quantities specify a cubic interpolating polynomial:

$$y(x_n+\theta h) = (1-\theta)y_n+\theta y_{n+1}+\theta(\theta-1)[(1-2\theta)(y_{n+1}-y_n)+(\theta-1)hf_n+\theta hf_{n+1}] \tag{17.2.15}$$

where $0 \le \theta \le 1$. Evaluating this polynomial at any θ in the interval gives a value of y that is third-order accurate, as you can verify by Taylor expansion in h. (Equation 17.2.15 is an example of *Hermite interpolation*, which uses both function and derivative values.)

We are interested, however, in integration methods with order higher than three, so higher-order dense output formulas are needed. The general approach for Runge-Kutta methods is to regard the b_i coefficients in (17.2.4) as polynomials in θ instead of constants. This defines a continuous solution,

$$y(x_n + \theta h) = y_n + b_1(\theta)k_1 + b_2(\theta)k_2 + b_3(\theta)k_3 + b_4(\theta)k_4 + b_5(\theta)k_5 + b_6(\theta)k_6 \tag{17.2.16}$$

and we require the polynomials $b_i(\theta)$ to approximate the true solution to the required order. Equation (17.2.15) is a special case of this.

The Dormand-Prince fifth-order method allows dense output of order four without any further function evaluations. This is usually sufficient: The number of steps to get to a typical point scales as $1/h$, so the global error at that point is typically $O(h^5)$ (fourth order). (Fifth-order dense output, needed, for example, for full accuracy in $y'(x_n + \theta h)$, turns out to need two extra function evaluations per step.) `StepperDopr5` contains a dense output option based on the formulas in [3] as simplified in [4].

Dense output simplifies problems where you don't know in advance how far to integrate. You want to locate the position x_c where some condition is satisfied. Examples include integrating the equations of stellar structure out from the center of the star until the pressure goes to zero at the surface, or the study of limit cycles when one integrates until the solution reaches the Poincaré section for the first time. Write the condition as finding the zero of some function:

$$g(x, y_i(x)) = 0 \tag{17.2.17}$$

Monitor g in the output routine. When g changes sign between two steps, use the dense output routine to supply function values to your favorite root-finding routine, such as bisection or Newton's method.

17.2.3 Implementation

Here follows the implementation of the fifth-order Dormand-Prince method.

stepperdopr5.h

```
template <class D>
struct StepperDopr5 : StepperBase {
```
Dormand-Prince fifth-order Runge-Kutta step with monitoring of local truncation error to ensure accuracy and adjust stepsize.
```
    typedef D Dtype;                        Make the type of derivs available to odeint.
    VecDoub k2,k3,k4,k5,k6;
    VecDoub rcont1,rcont2,rcont3,rcont4,rcont5;
    VecDoub dydxnew;
    StepperDopr5(VecDoub_IO &yy, VecDoub_IO &dydxx, Doub &xx,
        const Doub atoll, const Doub rtoll, bool dens);
    void step(const Doub htry,D &derivs);
    void dy(const Doub h,D &derivs);
    void prepare_dense(const Doub h,D &derivs);
    Doub dense_out(const Int i, const Doub x, const Doub h);
    Doub error();
    struct Controller {
        Doub hnext,errold;
        bool reject;
        Controller();
        bool success(const Doub err, Doub &h);
    };
    Controller con;
};
```

The constructor simply invokes the base class instructor and initializes variables:

stepperdopr5.h

```
template <class D>
StepperDopr5<D>::StepperDopr5(VecDoub_IO &yy,VecDoub_IO &dydxx,Doub &xx,
    const Doub atoll,const Doub rtoll,bool dens) :
    StepperBase(yy,dydxx,xx,atoll,rtoll,dens), k2(n),k3(n),k4(n),k5(n),k6(n),
    rcont1(n),rcont2(n),rcont3(n),rcont4(n),rcont5(n),dydxnew(n) {
```
Input to the constructor are the dependent variable y[0..n-1] and its derivative dydx[0..n-1] at the starting value of the independent variable x. Also input are the absolute and relative tolerances, atol and rtol, and the boolean dense, which is true if dense output is required.
```
    EPS=numeric_limits<Doub>::epsilon();
}
```

The step method is the actual stepper. It attempts a step, invokes the controller to decide whether to accept the step or try again with a smaller stepsize, and sets up the coefficients in case dense output is needed between x and $x + h$.

stepperdopr5.h

```
template <class D>
void StepperDopr5<D>::step(const Doub htry,D &derivs) {
```
Attempts a step with stepsize htry. On output, y and x are replaced by their new values, hdid is the stepsize that was actually accomplished, and hnext is the estimated next stepsize.
```
    Doub h=htry;                            Set stepsize to the initial trial value.
    for (;;) {
        dy(h,derivs);                       Take a step.
        Doub err=error();                   Evaluate accuracy.
        if (con.success(err,h)) break;      Step rejected. Try again with reduced h set
        if (abs(h) <= abs(x)*EPS)               by controller.
            throw("stepsize underflow in StepperDopr5");
    }
    if (dense)                              Step succeeded. Compute coefficients for dense
        prepare_dense(h,derivs);                output.
    dydx=dydxnew;                           Reuse last derivative evaluation for next step.
    y=yout;
    xold=x;                                 Used for dense output.
    x += (hdid=h);
    hnext=con.hnext;
}
```

The algorithm routine dy does the six steps plus the seventh FSAL step, and computes y_{n+1} and the error Δ.

stepperdopr5.h

```
template <class D>
void StepperDopr5<D>::dy(const Doub h,D &derivs) {
Given values for n variables y[0..n-1] and their derivatives dydx[0..n-1] known at x, use the
fifth-order Dormand-Prince Runge-Kutta method to advance the solution over an interval h and
store the incremented variables in yout[0..n-1]. Also store an estimate of the local truncation
error in yerr using the embedded fourth-order method.
    static const Doub c2=0.2,c3=0.3,c4=0.8,c5=8.0/9.0,a21=0.2,a31=3.0/40.0,
    a32=9.0/40.0,a41=44.0/45.0,a42=-56.0/15.0,a43=32.0/9.0,a51=19372.0/6561.0,
    a52=-25360.0/2187.0,a53=64448.0/6561.0,a54=-212.0/729.0,a61=9017.0/3168.0,
    a62=-355.0/33.0,a63=46732.0/5247.0,a64=49.0/176.0,a65=-5103.0/18656.0,
    a71=35.0/384.0,a73=500.0/1113.0,a74=125.0/192.0,a75=-2187.0/6784.0,
    a76=11.0/84.0,e1=71.0/57600.0,e3=-71.0/16695.0,e4=71.0/1920.0,
    e5=-17253.0/339200.0,e6=22.0/525.0,e7=-1.0/40.0;
    VecDoub ytemp(n);
    Int i;
    for (i=0;i<n;i++)                                   First step.
        ytemp[i]=y[i]+h*a21*dydx[i];
    derivs(x+c2*h,ytemp,k2);                            Second step.
    for (i=0;i<n;i++)
        ytemp[i]=y[i]+h*(a31*dydx[i]+a32*k2[i]);
    derivs(x+c3*h,ytemp,k3);                            Third step.
    for (i=0;i<n;i++)
        ytemp[i]=y[i]+h*(a41*dydx[i]+a42*k2[i]+a43*k3[i]);
    derivs(x+c4*h,ytemp,k4);                            Fourth step.
    for (i=0;i<n;i++)
        ytemp[i]=y[i]+h*(a51*dydx[i]+a52*k2[i]+a53*k3[i]+a54*k4[i]);
    derivs(x+c5*h,ytemp,k5);                            Fifth step.
    for (i=0;i<n;i++)
        ytemp[i]=y[i]+h*(a61*dydx[i]+a62*k2[i]+a63*k3[i]+a64*k4[i]+a65*k5[i]);
    Doub xph=x+h;
    derivs(xph,ytemp,k6);                               Sixth step.
    for (i=0;i<n;i++)                                   Accumulate increments with proper weights.
        yout[i]=y[i]+h*(a71*dydx[i]+a73*k3[i]+a74*k4[i]+a75*k5[i]+a76*k6[i]);
    derivs(xph,yout,dydxnew);                           Will also be first evaluation for next step.
    for (i=0;i<n;i++) {
        Estimate error as difference between fourth- and fifth-order methods.
        yerr[i]=h*(e1*dydx[i]+e3*k3[i]+e4*k4[i]+e5*k5[i]+e6*k6[i]+e7*dydxnew[i]);
    }
}
```

The routine prepare_dense uses the coefficients of [4] to set up the dense output quantities. Our coding of the dense output is closely based on that of the Fortran code DOPRI5 of [5].

stepperdopr5.h

```
template <class D>
void StepperDopr5<D>::prepare_dense(const Doub h,D &derivs) {
Store coefficients of interpolating polynomial for dense output in rcont1...rcont5.
    VecDoub ytemp(n);
    static const Doub d1=-12715105075.0/11282082432.0,
    d3=87487479700.0/32700410799.0, d4=-10690763975.0/1880347072.0,
    d5=701980252875.0/199316789632.0, d6=-1453857185.0/822651844.0,
    d7=69997945.0/29380423.0;
    for (Int i=0;i<n;i++) {
        rcont1[i]=y[i];
        Doub ydiff=yout[i]-y[i];
        rcont2[i]=ydiff;
        Doub bspl=h*dydx[i]-ydiff;
        rcont3[i]=bspl;
        rcont4[i]=ydiff-h*dydxnew[i]-bspl;
```

```
        rcont5[i]=h*(d1*dydx[i]+d3*k3[i]+d4*k4[i]+d5*k5[i]+d6*k6[i]+
            d7*dydxnew[i]);
    }
}
```

The next routine, dense_out, uses the coefficients stored by the previous routine to evaluate the solution at an arbitrary point.

stepperdopr5.h

```
template <class D>
Doub StepperDopr5<D>::dense_out(const Int i,const Doub x,const Doub h) {
```

Evaluate interpolating polynomial for y[i] at location x, where xold $\leq$ x $\leq$ xold + h.

```
    Doub s=(x-xold)/h;
    Doub s1=1.0-s;
    return rcont1[i]+s*(rcont2[i]+s1*(rcont3[i]+s*(rcont4[i]+s1*rcont5[i])));
}
```

The error routine converts Δ into the scaled quantity err.

stepperdopr5.h

```
template <class D>
Doub StepperDopr5<D>::error() {
```

Use yerr to compute norm of scaled error estimate. A value less than one means the step was successful.

```
    Doub err=0.0,sk;
    for (Int i=0;i<n;i++) {
        sk=atol+rtol*MAX(abs(y[i]),abs(yout[i]));
        err += SQR(yerr[i]/sk);
    }
    return sqrt(err/n);
}
```

Finally, the controller tests whether err ≤ 1 and adjusts the stepsize. The default setting is beta $= 0$ (no PI control). Set beta to 0.04 or 0.08 to turn on PI control.

stepperdopr5.h

```
template <class D>
StepperDopr5<D>::Controller::Controller() : reject(false), errold(1.0e-4) {}
```

Step size controller for fifth-order Dormand-Prince method.

```
template <class D>
bool StepperDopr5<D>::Controller::success(const Doub err,Doub &h) {
```

Returns true if err ≤ 1, false otherwise. If step was successful, sets hnext to the estimated optimal stepsize for the next step. If the step failed, reduces h appropriately for another try.

```
    static const Doub beta=0.0,alpha=0.2-beta*0.75,safe=0.9,minscale=0.2,
        maxscale=10.0;
```

Set beta to a nonzero value for PI control. beta $= 0.04$–0.08 is a good default.

```
    Doub scale;
    if (err <= 1.0) {                         Step succeeded. Compute hnext.
        if (err == 0.0)
            scale=maxscale;
        else {                                PI control if beta ≠ 0.
            scale=safe*pow(err,-alpha)*pow(errold,beta);
            if (scale<minscale) scale=minscale;    Ensure minscale ≤ hnext/h ≤ maxscale.
            if (scale>maxscale) scale=maxscale;
        }
        if (reject)                           Don't let step increase if last one was re-
            hnext=h*MIN(scale,1.0);               jected.
        else
            hnext=h*scale;
        errold=MAX(err,1.0e-4);               Bookkeeping for next call.
        reject=false;
        return true;
    } else {                                  Truncation error too large, reduce stepsize.
```

```
            scale=MAX(safe*pow(err,-alpha),minscale);
            h *= scale;
            reject=true;
            return false;
        }
}
```

A warning: Don't be too greedy in specifying atol and rtol. The punishment for excessive greediness is interesting and worthy of Gilbert and Sullivan's *Mikado*: The routine can always achieve an apparent *zero* error by making the stepsize so small that quantities of order hy' add to quantities of order y as if they were zero. Then the routine chugs happily along taking infinitely many infinitesimal steps and never changing the dependent variables one iota. (On a supercomputer, you guard against this catastrophic loss of your time allocation by signaling on abnormally small stepsizes or on the dependent variable vector remaining unchanged from step to step. On a desktop computer, you guard against it by not taking too long a lunch hour while the program is running.)

17.2.4 Dopr853 — An Eighth-Order Method

Once you understand the above implementation of StepperDopr5, then you have the framework for essentially any Runge-Kutta method. For production work, we recommend that you use the following method, StepperDopr853. It is again a Dormand-Prince embedded method, this time of eighth order that uses 12 function evaluations. The original version used a sixth-order embedded method for error estimation. However, it turned out that the error estimation was not robust in certain circumstances because the last evaluation point happened not to be used in computing the error. Accordingly, Hairer, Nörsett, and Wanner [5] constructed both fifth-order and third-order embedded methods that use the last point. Then the error is estimated as

$$\mathtt{err} = \mathtt{err}_5 \frac{\mathtt{err}_5}{\sqrt{(\mathtt{err}_3)^2 + 0.01(\mathtt{err}_5)^2}} \tag{17.2.18}$$

Most of the time, $\mathtt{err}_5 \ll \mathtt{err}_3$, so $\mathtt{err} = O(h^8)$. If the estimation breaks down so that either $\mathtt{err}_3$ gets small or $\mathtt{err}_5$ gets large, then err will still give a reasonable basis for stepsize control. This method has worked well in practice and is the basis for the "853" in the name of the method.

For an eighth-order method we would like seventh-order dense output. It turns out this requires three more function evaluations. Our coding of the dense output follows closely the Fortran implementation of [5]. Since the code is somewhat lengthy, but basically similar to StepperDopr5, we give it as StepperDopr853 in a Webnote [9].

CITED REFERENCES AND FURTHER READING:

Gear, C.W. 1971, *Numerical Initial Value Problems in Ordinary Differential Equations* (Englewood Cliffs, NJ: Prentice-Hall).[1]

Dormand, J.R, and Prince, P.J. 1980, "A Family of Embedded Runge-Kutta Formulae," *Journal of Computational and Applied Mathematics*, vol. 6, pp. 19–26.[2]

Shampine, L.F., and Watts, H.A. 1977, "The Art of Writing a Runge-Kutta Code, Part I," in *Mathematical Software III*, J.R. Rice, ed. (New York: Academic Press), pp. 257–275; 1979, "The Art of Writing a Runge-Kutta Code. II," *Applied Mathematics and Computation*, vol. 5, pp. 93–121.

Forsythe, G.E., Malcolm, M.A., and Moler, C.B. 1977, *Computer Methods for Mathematical Computations* (Englewood Cliffs, NJ: Prentice-Hall).

Dormand, J.R, and Prince, P.J. 1986, “Runge-Kutta Triples,” *Computers and Mathematics with Applications*, vol. 12A, pp. 1007–1017.[3]

Shampine, L.F. 1986, “Some Practical Runge-Kutta Formulas,” *Mathematics of Computation*, vol. 46, pp. 135–150.[4]

Hairer, E., Nørsett, S.P., and Wanner, G. 1993, *Solving Ordinary Differential Equations I. Nonstiff Problems*, 2nd ed. (New York: Springer). Fortran codes at `http://www.unige.ch/~hairer/software.html`.[5]

Gustafsson, K. 1991, “Control Theoretic Techniques for Stepsize Selection in Explicit Runge-Kutta Methods,” *ACM Transactions on Mathematical Software*, vol. 17, pp. 533-554.[6]

Hairer, E., Nørsett, S.P., and Wanner, G. 1996, *Solving Ordinary Differential Equations II. Stiff and Differential-Algebraic Problems*, 2nd ed. (New York: Springer), p. 28.[7]

Söderlind, G. 2003, “Digital Filters in Adaptive Time-stepping,” *ACM Transactions on Mathematical Software*, vol. 29, pp. 1–26.[8]

Numerical Recipes Software 2007, “Routine Implementing an Eighth-order Runge-Kutta Method,” *Numerical Recipes Webnote No. 20*, at `http://www.nr.com/webnotes?20` [9]

17.3 Richardson Extrapolation and the Bulirsch-Stoer Method

The techniques in this section are for differential equations containing smooth functions. With just three caveats, we believe that the Bulirsch-Stoer method, discussed here, is the best-known way to obtain high accuracy solutions to ordinary differential equations with minimal computational effort. The caveats are these:

- If you have a nonsmooth problem, for example, a differential equation whose right-hand side involves a function that is evaluated by table look-up and interpolation, go back to Runge-Kutta with an adaptive stepsize choice. That method does an excellent job of feeling its way through rocky or discontinuous terrain. It is also an excellent choice for a quick-and-dirty, low accuracy solution of a set of equations.
- The techniques in this section are not particularly good for differential equations that have singular points *inside* the interval of integration. A regular solution must tiptoe very carefully across such points. Runge-Kutta with adaptive stepsize can sometimes effect this; more generally, there are special techniques available for such problems, beyond our scope here but touched on in §18.6.
- There *may* be a few problems that are both very smooth and have right-hand sides that are very expensive to evaluate, for which predictor-corrector methods, discussed in §17.6, are the methods of choice.

The methods in this section involve three key ideas. The first is *Richardson's deferred approach to the limit*, which we already met in §4.3 on Romberg integration. The idea is to consider the final answer of a numerical calculation as itself being an analytic function (if a complicated one) of an adjustable parameter like the stepsize h. That analytic function can be probed by performing the calculation with various values of h, *none* of them being necessarily small enough to yield the accuracy that we desire. When we know enough about the function, we *fit* it to some analytic form

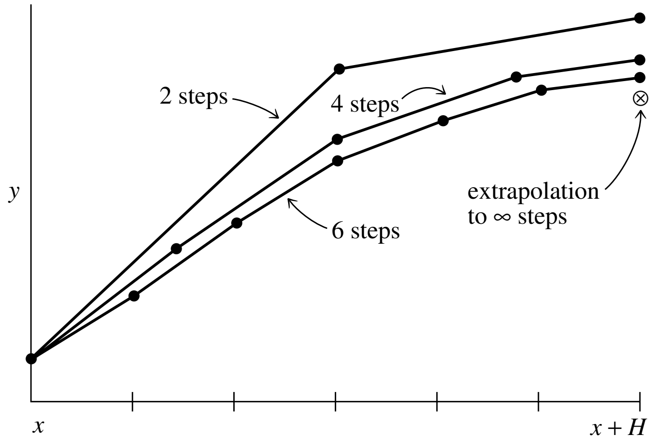

Figure 17.3.1. Richardson extrapolation as used in the Bulirsch-Stoer method. A large interval H is spanned by different sequences of finer and finer substeps. Their results are extrapolated to an answer that is supposed to correspond to infinitely fine substeps. In the Bulirsch-Stoer method, the integrations are done by the modified midpoint method, and the extrapolation technique is polynomial extrapolation.

and then *evaluate* it at that mythical and golden point $h = 0$ (see Figure 17.3.1). Richardson extrapolation is a method for turning straw into gold! (Lead into gold for alchemist readers.)

The second idea has to do with what kind of fitting function is used. Bulirsch and Stoer first recognized the strength of *rational function extrapolation* in Richardson-type applications. That strength is to break the shackles of the power series and its limited radius of convergence, out only to the distance of the first pole in the complex plane. Rational function fits can remain good approximations to analytic functions even after the various terms in powers of h all have comparable magnitudes. In other words, h can be so large as to make the whole notion of the "order" of the method meaningless — and the method can still work superbly. Nevertheless, more recent experience suggests that for smooth problems straightforward polynomial extrapolation is slightly more efficient than rational function extrapolation. (This may tell us more about the kinds of problems used for tests than about the methods themselves.) In any event, we will adopt polynomial extrapolation as our default. You might wish at this point to review §3.2, where polynomial function extrapolation was already discussed.

The third idea is to to use an integration method whose error function is strictly *even*, allowing the rational function or polynomial approximation to be in terms of the variable h^2 instead of just h. We will expand on this idea in the next subsection, on the modified midpoint method.

Put these ideas together and you have the *Bulirsch-Stoer method* [1]. A single Bulirsch-Stoer step takes us from x to $x + H$, where H is supposed to be quite a large — not at all infinitesimal — distance. That single step is a grand leap consisting of many (e.g., dozens to hundreds) substeps of the modified midpoint method, which are then extrapolated to zero stepsize.

17.3.1 Modified Midpoint Method

The *modified midpoint method* advances a vector of dependent variables $y(x)$ from a point x to a point $x + H$ by a sequence of n substeps each of size h,

$$h = H/n \tag{17.3.1}$$

In principle, one could use the modified midpoint method in its own right as an ODE integrator. In practice, the method finds its most important application as a part of the more powerful Bulirsch-Stoer technique,.

The number of right-hand side evaluations required by the modified midpoint method is $n + 1$. The formulas for the method are

$$\begin{aligned} z_0 &\equiv y(x) \\ z_1 &= z_0 + hf(x, z_0) \\ z_{m+1} &= z_{m-1} + 2hf(x + mh, z_m) \qquad \text{for} \quad m = 1, 2, \ldots, n-1 \\ y(x + H) \approx y_n &\equiv \tfrac{1}{2}[z_n + z_{n-1} + hf(x + H, z_n)] \end{aligned} \tag{17.3.2}$$

Here the z's are intermediate approximations that march along in steps of h, while y_n is the final approximation to $y(x + H)$. The method is basically a "centered difference" or "midpoint" method (compare equation 17.1.2), except at the first and last points. Those give the qualifier "modified."

The modified midpoint method is a second-order method, like (17.1.2), but with the advantage of requiring (asymptotically for large n) only one derivative evaluation per step h instead of the two required by second-order Runge-Kutta.

The usefulness of the modified midpoint method to the Bulirsch-Stoer technique (§17.3) derives from a "deep" result about equations (17.3.2), due to Gragg. It turns out that the error of (17.3.2), expressed as a power series in h, the stepsize, contains only *even* powers of h,

$$y_n - y(x + H) = \sum_{i=1}^{\infty} \alpha_i h^{2i} \tag{17.3.3}$$

where H is held constant but h changes by varying n in (17.3.1). The importance of this even power series is that, if we play our usual tricks of combining steps to knock out higher-order error terms, we can gain *two* orders at a time!

For example, suppose n is even, and let $y_{n/2}$ denote the result of applying (17.3.1) and (17.3.2) with half as many steps, $n \to n/2$. Then the estimate

$$y(x + H) \approx \frac{4y_n - y_{n/2}}{3} \tag{17.3.4}$$

is *fourth-order* accurate, the same as fourth-order Runge-Kutta, but requires only about 1.5 derivative evaluations per step h instead of Runge-Kutta's four evaluations. Don't be too anxious to implement (17.3.4), since we will soon do even better.

Now would be a good time to look back at the routine `qsimp` in §4.2, and especially to compare equation (4.2.4) with equation (17.3.4) above. You will see that the transition in Chapter 4 to the idea of Richardson extrapolation, as embodied in Romberg integration of §4.3, is exactly analogous to the transition in going from this section to the next one.

A routine that implements the modified midpoint method will be given as part of the implementation of `StepperBS`, in the `dy` member function.

17.3.2 The Bulirsch-Stoer Method

Consider attempting to cross the interval H using the modified midpoint method with increasing values of n, the number of substeps. Bulirsch and Stoer originally proposed the sequence

$$n = 2, 4, 6, 8, 12, 16, 24, 32, 48, 64, 96, \ldots, [n_j = 2n_{j-2}], \ldots \tag{17.3.5}$$

More recent work by Deuflhard [2,3] suggests that the sequence

$$n = 2, 4, 6, 8, 10, 12, 14, \ldots, [n_j = 2(j+1)], \ldots \tag{17.3.6}$$

is usually more efficient. For each step, we do not know in advance how far up this sequence we will go. After each successive n is tried, a polynomial extrapolation is attempted. That extrapolation gives both extrapolated values and error estimates. If the errors are not satisfactory, we go higher in n. If they are satisfactory, we go on to the next step and begin anew with $n = 2$.

Of course there must be some upper limit, beyond which we conclude that there is some obstacle in our path in the interval H, so that we must reduce H rather than just subdivide it more finely. Moreover, precision loss sets in if we choose too fine a subdivision. In the implementations below, the maximum number of n's to be tried is called `KMAXX`. We usually take this equal to 8; the eighth value of the sequence (17.3.6) is 16, so this is the maximum number of subdivisions of H that we allow.

We enforce error control, as in the Runge-Kutta method, by monitoring internal consistency and adapting the stepsize to match a prescribed bound on the local truncation error. Each new result from the sequence of modified midpoint integrations allows a tableau like that in equation (3.2.2) to be extended by one additional set of diagonals. Write the tableau as a lower triangular matrix:

$$\begin{array}{llll} T_{00} & & & \\ T_{10} & T_{11} & & \\ T_{20} & T_{21} & T_{22} & \\ \cdots & \cdots & \cdots & \cdots \end{array} \tag{17.3.7}$$

Here $T_{k0} = y_k$, where y_k is $y(x_n + H)$ computed with the stepsize $h_k = H/n_k$. Neville's algorithm, equation (3.2.3), with P replaced by T, $x_i = h_i^2$, and $x = 0$, can be written

$$T_{k,j+1} = T_{kj} + \frac{T_{kj} - T_{k-1,j}}{(n_k/n_{k-j})^2 - 1} \qquad j = 0, 1, \ldots, k-1 \tag{17.3.8}$$

Each new stepsize h_i starts a new row in the tableau, and then the polynomial extrapolation fills the rest of the row. Each new element in the tableau comes from the two closest elements in the previous column. Elements in the same column have the same order, and T_{kk}, the last element in each row, is the highest-order approximation with that stepsize. The difference between the last two elements in a row is taken as the (conservative) error estimate. How should we use this error estimate to adjust the stepsize? A good strategy was originally proposed by Deuflhard [2,3]. We will use a modified version [4], next described.

17.3.3 Stepsize Control Algorithm for Bulirsch-Stoer

The elements in the tableau are actually vectors corresponding to the vector y of dependent variables. Accordingly, define

$$\mathtt{err}_k = \|T_{kk} - T_{k,k-1}\| \tag{17.3.9}$$

where the norm is the same scaled norm used in equation (17.2.9). Error control is enforced by requiring $\mathtt{err}_k \le 1$.

Now T_{kk} is of order $2k+2$ and $T_{k,k-1}$ is of order $2k$, which is therefore the order of $\mathtt{err}_k$. In other words,

$$\mathtt{err}_k \sim H^{2k+1} \tag{17.3.10}$$

Thus a simple estimate of a new stepsize H_k to obtain convergence in a fixed column k would be (cf. equation 17.2.12)

$$H_k = H S_1 \left(\frac{S_2}{\mathtt{err}_k}\right)^{1/(2k+1)} \tag{17.3.11}$$

where S_1 and S_2 are safety factors smaller than one.

Which column k should we aim to achieve convergence in? Let's compare the work required for different k. Suppose A_k is the work to obtain row k of the extrapolation tableau. Assume the work is dominated by the cost of evaluating the functions defining the right-hand sides of the differential equations. For n_k subdivisions in H, the number of function evaluations can be found from the recurrence

$$\begin{aligned} A_0 &= n_0 + 1 \\ A_{k+1} &= A_k + n_{k+1} \end{aligned} \tag{17.3.12}$$

The work per unit step to get column k is therefore

$$W_k = \frac{A_k}{H_k} \tag{17.3.13}$$

The optimal column index is the one that minimizes W_k. The strategy is to set a target k for the next step, and then choose the stepsize from (17.3.11) to try to get convergence (i.e., $\mathtt{err}_k \le 1$) for that value of k on the next step.

In practice, you compute the extrapolation tableau (17.3.7) row by row, but only test for convergence within an *order window* between $k-1$ and $k+1$. The rationale for the order window is that if convergence appears to occur before column $k-1$, it is often spurious, resulting from some fortuitously small error estimate in the extrapolation. On the other hand, if you need to go beyond $k+1$ to obtain convergence, your local model of the convergence behavior is obviously not very good and you need to cut the stepsize and reestablish it.

Here are the steps:

- Test for convergence in column $k-1$: If $\mathtt{err}_{k-1} \le 1$, accept $T_{k-1,k-1}$. Set the new target as

$$k_{\text{new}} = \begin{cases} k-2 & \text{if } W_{k-2} < 0.8 W_{k-1} \text{ (order decrease)} \\ k & \text{if } W_{k-1} < 0.9 W_{k-2} \text{ (order increase)} \\ k-1 & \text{otherwise} \end{cases} \tag{17.3.14}$$

Set the corresponding stepsize as

$$H_{\text{new}} = \begin{cases} H_{k_{\text{new}}} & \text{if } k_{\text{new}} = k-1 \text{ or } k-2 \\ H_{k-1} \dfrac{A_k}{A_{k-1}} & \text{if } k_{\text{new}} = k \end{cases} \tag{17.3.15}$$

The idea behind the last formula is that you can't set $H_{\text{new}} = H_k$ because you're stopping the integration in row $k-1$ so you don't compute H_k. However, since k is supposedly optimal, $W_k \approx W_{k-1}$, which gives the last formula for H_{new}.

- If $\mathtt{err}_{k-1} > 1$: Check if you can expect convergence by row $k+1$ by estimating what $\mathtt{err}_{k+1}$ will be. Assuming one is in the asymptotic regime, one can show that

$$\mathtt{err}_k \approx \left(\frac{n_0}{n_k}\right)^2 \mathtt{err}_{k-1} \tag{17.3.16}$$

and hence that $\mathtt{err}_{k+1}$ will be greater than one if approximately

$$\mathtt{err}_{k-1} > \left(\frac{n_k}{n_0}\right)^2 \left(\frac{n_{k+1}}{n_0}\right)^2 \tag{17.3.17}$$

If this condition is satisfied, reject the step and restart with k_{new} and H_{new} chosen according to (17.3.14) and (17.3.15).

- If (17.3.17) is not satisfied, compute the next row of the tableau (i.e., for the target value of k) and see if convergence is attained for column k. Thus, if $\mathtt{err}_k \le 1$, accept the step and continue with

$$k_{\text{new}} = \begin{cases} k-1 & \text{if } W_{k-1} < 0.8W_k \text{ (order decrease)} \\ k+1 & \text{if } W_k < 0.9W_{k-1} \text{ (order increase)} \\ k & \text{otherwise} \end{cases} \tag{17.3.18}$$

Set the corresponding stepsize as

$$H_{\text{new}} = \begin{cases} H_{k_{\text{new}}} & \text{if } k_{\text{new}} = k \text{ or } k-1 \\ H_k \dfrac{A_{k+1}}{A_k} & \text{if } k_{\text{new}} = k+1 \end{cases} \tag{17.3.19}$$

- If $\mathtt{err}_k > 1$, check if you can expect convergence by the next row. Analogously to (17.3.17), check if

$$\mathtt{err}_k > \left(\frac{n_{k+1}}{n_0}\right)^2 \tag{17.3.20}$$

If this condition is satisfied, reject the step and restart with k_{new} and H_{new} chosen according to (17.3.18) and (17.3.19).

- If (17.3.17) is not satisfied, compute row $k+1$ of the tableau. If $\mathtt{err}_{k+1} \le 1$, accept the step. Set the new target with the following prescription:

$$\begin{array}{ll} k_{\text{new}} = k & \\ \text{if } W_{k-1} < 0.8W_k & k_{\text{new}} = k-1 \text{ (order decrease)} \\ \text{if } W_{k+1} < 0.9W_{k_{\text{new}}} & k_{\text{new}} = k+1 \text{ (order increase)} \end{array} \tag{17.3.21}$$

The stepsize is set as in (17.3.19).

- If $\mathtt{err}_{k+1} > 1$, reject the step. Restart with k_{new} and H_{new} chosen according to (17.3.18) and (17.3.19).

Two important refinements to this strategy are

- After a step is rejected, the order and stepsize are not allowed to increase.
- H_{new} computed from equation (16.4.5) is not allowed to change too rapidly in one step. It is restricted by

$$\frac{F}{S_4} \le \frac{H_{\text{new}}}{H} \le \frac{1}{F} \qquad F \equiv S_3^{1/(2k+1)} \tag{17.3.22}$$

The default values of the parameters are $S_3 = 0.02$, $S_4 = 4$.

For the first step, the target k is estimated crudely from the requested precision, but the step is accepted if the error is small enough for any smaller k. For the last step, the stepsize is decreased to be the length of the remaining integration interval, so a similar increase in the order window is allowed.

17.3.4 Dense Output

The basic Bulirsch-Stoer step H is typically much larger than in Runge-Kutta methods because of the high orders invoked, so a dense output option is even more important. Our implementation once again is based closely on the coding in [4], which is based on [5].

A dense output algorithm turns out to be possible only for certain stepsize sequences, for example increasing by fours:

$$n = 2, 6, 10, 14, 18, 22, 26, 30, \dots \tag{17.3.23}$$

The idea is to do Hermite interpolation using the function and derivative values at the beginning and end of the step. These are supplemented with values of the function and its derivatives at the midpoint obtained by extrapolation of values saved during the integration.

The error of the Hermite interpolation needs to be monitored. If it is too big, the step is rejected and the stepsize reduced appropriately. The error estimate of the interpolation is also used if necessary to limit the size of the next step after a successful step.

17.3.5 Implementation

The use of StepperBS is exactly the same as the use of the Runge-Kutta routines. For example, to solve the problem at the end of §17.2, everything is exactly the same except the line

```
Odeint<StepperDopr5<rhs_van> > ode(ystart,x1,x2,atol,rtol,h1,hmin,out,d);
```

is replaced by

```
Odeint<StepperBS<rhs_van> > ode(ystart,x1,x2,atol,rtol,h1,hmin,out,d);
```

The object StepperBS implements a Bulirsch-Stoer step. Some of its functions are declared virtual because the algorithm StepperStoerm will be implemented as a derived class from it in the next section, and these functions will be overridden. As with StepperDopr5, the class is templated on the functor class defining the right-hand side derivatives.

stepperbs.h

```
template <class D>
struct StepperBS : StepperBase {
Bulirsch-Stoer step with monitoring of local truncation error to ensure accuracy and adjust
stepsize.
    typedef D Dtype;                   Make the type of derivs available to odeint.
    static const Int KMAXX=8,IMAXX=KMAXX+1;
    KMAXX is the maximum number of rows used in the extrapolation.
    Int k_targ;                        Optimal row number for convergence.
    VecInt nseq;                       Stepsize sequence.
    VecInt cost;                       A_k.
    MatDoub table;                     Extrapolation tableau.
    VecDoub dydxnew;
    Int mu;                            Used for dense output.
    MatDoub coeff;                     Coefficients used in extrapolation tableau.
    VecDoub errfac;                    Used to compute dense interpolation error.
    MatDoub ysave;                     ysave and fsave store values and derivatives to be
    MatDoub fsave;                         used for dense output.
    VecInt ipoint;                     Keeps track of where values are stored in fsave.
    VecDoub dens;                      Stores quantities for dense interpolating polynomial.
    StepperBS(VecDoub_IO &yy, VecDoub_IO &dydxx, Doub &xx, const Doub atol,
```

```
        const Doub rtol, bool dens);
    void step(const Doub htry,D &derivs);
    virtual void dy(VecDoub_I &y, const Doub htot, const Int k, VecDoub_O &yend,
        Int &ipt, D &derivs);
    void polyextr(const Int k, MatDoub_IO &table, VecDoub_IO &last);
    virtual void prepare_dense(const Doub h,VecDoub_I &dydxnew, VecDoub_I &ysav,
        VecDoub_I &scale, const Int k, Doub &error);
    virtual Doub dense_out(const Int i,const Doub x,const Doub h);
    virtual void dense_interp(const Int n, VecDoub_IO &y, const Int imit);
};
```

Detailed implementations of the member functions are given in a Webnote [6].

CITED REFERENCES AND FURTHER READING:

Stoer, J., and Bulirsch, R. 2002, *Introduction to Numerical Analysis*, 3rd ed. (New York: Springer), §7.2.14.[1]

Gear, C.W. 1971, *Numerical Initial Value Problems in Ordinary Differential Equations* (Englewood Cliffs, NJ: Prentice-Hall), §6.1.4 and §6.2.

Deuflhard, P. 1983, "Order and Stepsize Control in Extrapolation Methods," *Numerische Mathematik*, vol. 41, pp. 399–422.[2]

Deuflhard, P. 1985, "Recent Progress in Extrapolation Methods for Ordinary Differential Equations," *SIAM Review*, vol. 27, pp. 505–535.[3]

Hairer, E., Nørsett, S.P., and Wanner, G. 1993, *Solving Ordinary Differential Equations I. Nonstiff Problems*, 2nd ed. (New York: Springer). Fortran codes at http://www.unige.ch/~hairer/software.html.[4]

Hairer, E., and Ostermann, A. 1990, "Dense Output for Extrapolation Methods," *Numerische Mathematik*, vol. 58, pp. 419–439.[5]

Numerical Recipes Software 2007, "StepperBS Implementations," *Numerical Recipes Webnote No. 21*, at http://www.nr.com/webnotes?21 [6]

17.4 Second-Order Conservative Equations

Usually when you have a system of high-order differential equations to solve it is best to reformulate them as a system of first-order equations, as discussed in §17.0. There is a particular class of equations that occurs quite frequently in practice where you can gain about a factor of two in efficiency by differencing the equations directly. The equations are second-order systems where the derivative does not appear on the right-hand side:

$$y'' = f(x, y), \qquad y(x_0) = y_0, \qquad y'(x_0) = z_0 \tag{17.4.1}$$

As usual, y can denote a vector of values.

Stoermer's rule, dating back to 1907, has been a popular method for discretizing such systems. With $h = H/m$ we have

$$\begin{aligned} y_1 &= y_0 + h[z_0 + \tfrac{1}{2}hf(x_0, y_0)] \\ y_{k+1} - 2y_k + y_{k-1} &= h^2 f(x_0 + kh, y_k), \qquad k = 1, \dots, m-1 \\ z_m &= (y_m - y_{m-1})/h + \tfrac{1}{2}hf(x_0 + H, y_m) \end{aligned} \tag{17.4.2}$$

Here z_m is $y'(x_0 + H)$. Henrici showed how to rewrite equations (17.4.2) to reduce roundoff error by using the quantities $\Delta_k \equiv y_{k+1} - y_k$. Start with

$$\begin{aligned} \Delta_0 &= h[z_0 + \tfrac{1}{2}hf(x_0, y_0)] \\ y_1 &= y_0 + \Delta_0 \end{aligned} \tag{17.4.3}$$

Then, for $k = 1, \ldots, m-1$, set

$$\begin{aligned} \Delta_k &= \Delta_{k-1} + h^2 f(x_0 + kh, y_k) \\ y_{k+1} &= y_k + \Delta_k \end{aligned} \qquad (17.4.4)$$

Finally compute the derivative from

$$z_m = \Delta_{m-1}/h + \tfrac{1}{2} h f(x_0 + H, y_m) \qquad (17.4.5)$$

Gragg again showed that the error series for equations (17.4.3) – (17.4.5) contains only even powers of h, and so the method is a logical candidate for extrapolation à la Bulirsch-Stoer.

Here is the StepperStoerm routine:

stepperstoerm.h

```
template <class D>
struct StepperStoerm : StepperBS<D> {
```
Stoermer's rule for integrating $y'' = f(x, y)$ for a system of equations.
```
    using StepperBS<D>::x; using StepperBS<D>::xold; using StepperBS<D>::y;
    using StepperBS<D>::dydx; using StepperBS<D>::dense; using StepperBS<D>::n;
    using StepperBS<D>::KMAXX; using StepperBS<D>::IMAXX; using StepperBS<D>::nseq;
    using StepperBS<D>::cost; using StepperBS<D>::mu; using StepperBS<D>::errfac;
    using StepperBS<D>::ysave; using StepperBS<D>::fsave;
    using StepperBS<D>::dens; using StepperBS<D>::neqn;
    MatDoub ysavep;
    StepperStoerm(VecDoub_IO &yy, VecDoub_IO &dydxx, Doub &xx,
        const Doub atol, const Doub rtol, bool dens);
    void dy(VecDoub_I &y, const Doub htot, const Int k, VecDoub_O &yend,
        Int &ipt,D &derivs);
    void prepare_dense(const Doub h,VecDoub_I &dydxnew, VecDoub_I &ysav,
        VecDoub_I &scale, const Int k, Doub &error);
    Doub dense_out(const Int i,const Doub x,const Doub h);
    void dense_interp(const Int n, VecDoub_IO &y, const Int imit);
};
```

Because the base class StepperBS is templated on the derivs class, the derived class StepperStoerm does not automatically inherit its member variables. This is the reason for the using declarations.

Note that in order to reuse the StepperBS code and make StepperStoerm a derived class, the arrays y and dydx are of length $2n$ for a system of n second-order equations. The values of y are stored in the first n elements of y, while the first derivatives are stored in the second n elements. The right-hand side f is stored in the first n elements of the array dydx, which therefore actually contains y''; the second n elements are unused.

The constructor has to redefine the costs A_k because there are half the number of function evaluations per step compared with the midpoint method:

stepperstoerm.h

```
template <class D>
StepperStoerm<D>::StepperStoerm(VecDoub_IO &yy, VecDoub_IO &dydxx, Doub &xx,
    const Doub atoll,const Doub rtoll, bool dens)
    : StepperBS<D>(yy,dydxx,xx,atoll,rtoll,dens),ysavep(IMAXX,n/2) {
```
Constructor. On input, y[0..n-1] contains y in its first n/2 elements and y' in its second n/2 elements, all evaluated at x. The vector dydx[0..n-1] contains the right-hand side function f (also evaluated at x) in its first n/2 elements. Its second n/2 elements are not referenced. Also input are the absolute and relative tolerances, atol and rtol, and the boolean dense, which is true if dense output is required.
```
    neqn=n/2;                                  Number of equations.
    cost[0]=nseq[0]/2+1;                       Redefine cost: half as many function evalu-
    for (Int k=0;k<KMAXX;k++)                      ations as Bulirsch-Stoer.
        cost[k+1]=cost[k]+nseq[k+1]/2;
    for (Int i=0; i<2*IMAXX+1; i++) {          Coefficients for interpolation error are differ-
        Int ip7=i+7;                               ent too.
        Doub fac=1.5/ip7;
        errfac[i]=fac*fac*fac;
        Doub e = 0.5*sqrt(Doub(i+1)/ip7);
```

```
        for (Int j=0; j<=i; j++) {
            errfac[i] *= e/(j+1);
        }
    }
}
```

Here is the routine dy that implements Stoermer's rule:

stepperstoerm.h

```
template <class D>
void StepperStoerm<D>::dy(VecDoub_I &y, const Doub htot, const Int k,
    VecDoub_O &yend, Int &ipt, D &derivs) {
```

Stoermer step. Inputs are y, H, and k. The output is returned as yend[0..2n-1]. The counter ipt keeps track of saving the right-hand sides in the correct locations for dense output.

```
    VecDoub ytemp(n);
    Int nstep=nseq[k];
    Doub h=htot/nstep;                      Stepsize this trip.
    Doub h2=2.0*h;
    for (Int i=0;i<neqn;i++) {              First step.
        ytemp[i]=y[i];
        Int ni=neqn+i;
        ytemp[ni]=y[ni]+h*dydx[i];
    }
    Doub xnew=x;
    Int nstp2=nstep/2;
    for (Int nn=1;nn<=nstp2;nn++) {         General step.
        if (dense && nn == (nstp2+1)/2) {
            for (Int i=0;i<neqn;i++) {
                ysavep[k][i]=ytemp[neqn+i];
                ysave[k][i]=ytemp[i]+h*ytemp[neqn+i];
            }
        }
        for (Int i=0;i<neqn;i++)
            ytemp[i] += h2*ytemp[neqn+i];
        xnew += h2;
        derivs(xnew,ytemp,yend);            Store derivatives temporarily in yend.
        if (dense && abs(nn-(nstp2+1)/2) < k+1) {
            ipt++;
            for (Int i=0;i<neqn;i++)
                fsave[ipt][i]=yend[i];
        }
        if (nn != nstp2) {
            for (Int i=0;i<neqn;i++)
                ytemp[neqn+i] += h2*yend[i];
        }
    }
    for (Int i=0;i<neqn;i++) {              Last step.
        Int ni=neqn+i;
        yend[ni]=ytemp[ni]+h*yend[i];
        yend[i]=ytemp[i];
    }
}
```

The dense output routines are in a Webnote [1].

CITED REFERENCES AND FURTHER READING:

Deuflhard, P. 1985, "Recent Progress in Extrapolation Methods for Ordinary Differential Equations," *SIAM Review*, vol. 27, pp. 505–535.

Hairer, E., Nørsett, S.P., and Wanner, G. 1993, *Solving Ordinary Differential Equations I. Nonstiff Problems*, 2nd ed. (New York: Springer). Fortran codes at http://www.unige.ch/~hairer/software.html.

Numerical Recipes Software 2007, "Dense Output for Stoermer's Rule," *Numerical Recipes Webnote No. 22*, at http://www.nr.com/webnotes?22 [1]

17.5 Stiff Sets of Equations

As soon as one deals with more than one first-order differential equation, the possibility of a *stiff* set of equations arises. Stiffness typically occurs in a problem where there are two or more very different scales of the independent variable on which the dependent variables are changing. For example, consider the following set of equations [1]:

$$\begin{aligned} u' &= 998u + 1998v \\ v' &= -999u - 1999v \end{aligned} \tag{17.5.1}$$

with boundary conditions

$$u(0) = 1 \qquad v(0) = 0 \tag{17.5.2}$$

By means of the transformation

$$u = 2y - z \qquad v = -y + z \tag{17.5.3}$$

we find the solution

$$\begin{aligned} u &= 2e^{-x} - e^{-1000x} \\ v &= -e^{-x} + e^{-1000x} \end{aligned} \tag{17.5.4}$$

If we integrated the system (17.5.1) with any of the methods given so far in this chapter, the presence of the e^{-1000x} term would require a stepsize $h \ll 1/1000$ for the method to be stable (the reason for this is explained below). This is so even though the e^{-1000x} term is completely negligible in determining the values of u and v as soon as one is away from the origin (see Figure 17.5.1).

This is the generic disease of stiff equations: We are required to follow the variation in the solution on the shortest length scale to maintain the stability of the integration, even though accuracy requirements allow a much larger stepsize.

To see how we might cure this problem, consider the single equation

$$y' = -cy \tag{17.5.5}$$

where $c > 0$ is a constant. The explicit (or *forward*) Euler scheme for integrating this equation with stepsize h is

$$y_{n+1} = y_n + hy'_n = (1 - ch)y_n \tag{17.5.6}$$

The method is called explicit because the new value y_{n+1} is given explicitly in terms of the old value y_n. Clearly the method is unstable if $h > 2/c$, for then $|y_n| \to \infty$ as $n \to \infty$ even though the solution of (17.5.5) is bounded.

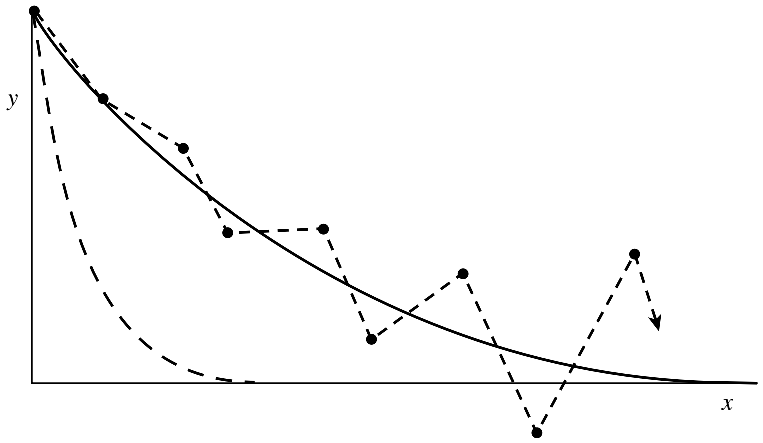

Figure 17.5.1. Example of an instability encountered in integrating a stiff equation (schematic). Here it is supposed that the equation has two solutions, shown as solid and dashed lines. Although the initial conditions are such as to give the solid solution, the stability of the integration (shown as the unstable dotted sequence of segments) is determined by the more rapidly varying dashed solution, even after that solution has effectively died away to zero. Implicit integration methods are the cure.

The simplest cure is to resort to *implicit* differencing, where the right-hand side is evaluated at the *new* y location. In this case, we get the *backward Euler* scheme:

$$y_{n+1} = y_n + hy'_{n+1} \tag{17.5.7}$$

or

$$y_{n+1} = \frac{y_n}{1 + ch} \tag{17.5.8}$$

The method is absolutely stable: Even as $h \to \infty$, $y_{n+1} \to 0$, which is in fact the correct solution of the differential equation. If we think of x as representing time, then the implicit method converges to the true equilibrium solution (i.e., the solution at late times) for large stepsizes. This nice feature of implicit methods holds only for linear systems, but even in the general case implicit methods give better stability. Of course, we give up *accuracy* in following the evolution toward equilibrium if we use large stepsizes, but we maintain *stability*.

These considerations can easily be generalized to sets of linear equations with constant coefficients:

$$\mathbf{y}' = -\mathbf{C} \cdot \mathbf{y} \tag{17.5.9}$$

Consider first the usual case where the matrix $\mathbf{C}$ can be diagonalized by a similarity transformation (cf. eqn. 11.0.11)

$$\mathbf{T} \cdot \mathbf{C} \cdot \mathbf{T}^{-1} = \text{diag}(\lambda_0 \dots \lambda_{N-1}) \tag{17.5.10}$$

where λ_i are the eigenvalues of $\mathbf{C}$. If we define the vector $\mathbf{z}(x)$ by $\mathbf{z} = \mathbf{T}^{-1} \cdot \mathbf{y}(x)$, then equation (17.5.9) becomes

$$\mathbf{z}' = -\text{diag}(\lambda_0 \dots \lambda_{N-1}) \cdot \mathbf{z} \tag{17.5.11}$$

This is a set of N independent equations for the components of $\mathbf{z}$ with solution

$$\mathbf{z} = \text{diag}(e^{-\lambda_0 x} \dots e^{-\lambda_{N-1} x}) \cdot \mathbf{z}_0 \tag{17.5.12}$$

Thus the solution of the original equation is

$$\mathbf{y} = \mathbf{T} \cdot \text{diag}(e^{-\lambda_0 x} \ldots e^{-\lambda_{N-1} x}) \cdot \mathbf{T}^{-1} \cdot \mathbf{y}_0 \tag{17.5.13}$$

We will be interested in the *stable* solutions, that is, those that decay as $x \to \infty$. (This notion can be made more rigorous by considering *Liapunov stability*, the idea that if $\mathbf{y}_0$ is small then $\mathbf{y}$ is small for all $x > 0$.) From equation (17.5.13) we see that the criterion for stable solutions is

$$\text{Re}\,\lambda_i > 0 \qquad i = 0, \ldots, N-1 \tag{17.5.14}$$

What if the matrix $\mathbf{C}$ in equation (17.5.9) cannot be diagonalized? Then it can always be transformed to so-called Jordan canonical form, which is the "closest" it can come to being made diagonal. Using this form, one can show that criterion (17.5.14) is still the stability criterion [2].

Now consider solving equation (17.5.9) by explicit differencing as in equation (17.5.6):

$$\mathbf{y}_{n+1} = (\mathbf{1} - \mathbf{C}h) \cdot \mathbf{y}_n \tag{17.5.15}$$

and so

$$\mathbf{y}_n = (\mathbf{1} - \mathbf{C}h)^n \cdot \mathbf{y}_0 \tag{17.5.16}$$

If $\mathbf{C}$ can be diagonalized, it has a complete set of eigenvectors $\{\boldsymbol{\xi}_i\}$ that can be used as a basis to expand $\mathbf{y}_0$:

$$\mathbf{y}_0 = \sum_{i=0}^{N-1} \alpha_i \boldsymbol{\xi}_i \tag{17.5.17}$$

Substituting this expansion in equation (17.5.16) gives

$$\mathbf{y}_n = \sum_{i=0}^{N-1} \alpha_i (1 - h\lambda_i)^n \boldsymbol{\xi}_i \tag{17.5.18}$$

If the original differential equation is stable, we require the difference scheme to be stable in that it must have bounded solutions, that is, $\mathbf{y}_n$ must be bounded as $n \to \infty$. From equation (17.5.18) we see that stability of the difference scheme requires

$$|1 - h\lambda_i| < 1 \qquad i = 0, \ldots, N-1 \tag{17.5.19}$$

If the λ_i are all real, then since they are positive for the differential equation to be stable, the stability criterion for the difference scheme is

$$h < \frac{2}{\lambda_{\max}} \tag{17.5.20}$$

where $\lambda_{\max}$ is the largest eigenvalue of $\mathbf{C}$.

As usual, if $\mathbf{C}$ cannot be diagonalized and so does not have a complete set of eigenvectors, then by working with the Jordan canonical form one can show the same result.

Consider now implicit differencing, which gives

$$\mathbf{y}_{n+1} = \mathbf{y}_n + h\mathbf{y}'_{n+1} \tag{17.5.21}$$

or

$$\mathbf{y}_{n+1} = (\mathbf{1} + \mathbf{C}h)^{-1} \cdot \mathbf{y}_n \tag{17.5.22}$$

Criterion (17.5.19) becomes

$$|1 + h\lambda_i|^{-1} < 1 \qquad i = 0, \ldots, N-1 \tag{17.5.23}$$

which is satisfied for all h — the method is stable for all stepsizes. The penalty we pay for this stability is that we are required to invert a matrix at each step.

Not all equations are linear with constant coefficients, unfortunately! For the system

$$\mathbf{y}' = \mathbf{f}(\mathbf{y}) \tag{17.5.24}$$

implicit differencing gives

$$\mathbf{y}_{n+1} = \mathbf{y}_n + h\mathbf{f}(\mathbf{y}_{n+1}) \tag{17.5.25}$$

In general this is some nasty set of nonlinear equations that has to be solved iteratively at each step. Suppose we try linearizing the equations, as in Newton's method:

$$\mathbf{y}_{n+1} = \mathbf{y}_n + h\left[\mathbf{f}(\mathbf{y}_n) + \left.\frac{\partial \mathbf{f}}{\partial \mathbf{y}}\right|_{\mathbf{y}_n} \cdot (\mathbf{y}_{n+1} - \mathbf{y}_n)\right] \tag{17.5.26}$$

Here $\partial\mathbf{f}/\partial\mathbf{y}$ is the matrix of the partial derivatives of the right-hand side (the Jacobian matrix). Rearrange equation (17.5.26) into the form

$$\mathbf{y}_{n+1} = \mathbf{y}_n + h\left[\mathbf{1} - h\frac{\partial \mathbf{f}}{\partial \mathbf{y}}\right]^{-1} \cdot \mathbf{f}(\mathbf{y}_n) \tag{17.5.27}$$

If h is not too big, only one iteration of Newton's method may be accurate enough to solve equation (17.5.25) using equation (17.5.27). In other words, at each step we have to invert the matrix

$$\mathbf{1} - h\frac{\partial \mathbf{f}}{\partial \mathbf{y}} \tag{17.5.28}$$

to find $\mathbf{y}_{n+1}$. Solving implicit methods by linearization is called a "semi-implicit" method, so equation (17.5.27) is the *semi-implicit Euler method.* It is not guaranteed to be stable, but it usually is, because the behavior is locally similar to the case of a constant matrix $\mathbf{C}$ described above.

So far we have dealt only with implicit methods that are first-order accurate. While these are very robust, most problems will benefit from higher-order methods. There are three important classes of higher-order methods for stiff systems:

- Generalizations of the Runge-Kutta method. These consist of implicit methods, where nonlinear equations are solved by Newton iteration at each step, and semi-implicit methods that solve linear equations analogous to (17.5.27). These semi-implicit methods are often called Rosenbrock methods. The first good implementation of these ideas was by Kaps and Rentrop, and so these methods are also called Kaps-Rentrop methods.
- Generalizations of the Bulirsch-Stoer method, which extrapolate a semi-implicit sequence of integrations to zero stepsize.
- Predictor-corrector methods, most of which are descendants of Gear's backward differentiation method.

We shall give implementations of Rosenbrock and extrapolation methods. An example of a good implicit Runge-Kutta code in Fortran is RADAU [3], while several stiff predictor-corrector–type Fortran codes (LSODE, DEBDF, VODE and MEBDF) are available from Netlib [5].

Here is an important point: *It is absolutely crucial to scale your variables properly when integrating stiff problems with automatic stepsize adjustment.* As in our nonstiff routines, you will be asked to supply absolute and relative tolerances `atol` and `rtol`. In stiff problems, there are often strongly decreasing pieces of the solution that you are not particularly interested in following once they are small. Thus you should almost never integrate with a pure relative error criterion by setting `atol` $= 0$. A good default choice is `atol` $=$ `rtol`, or sometimes a few orders of magnitude smaller.

One final warning: Solving stiff problems can sometimes lead to catastrophic precision loss. Double precision is often a requirement, not an option.

17.5.1 Rosenbrock Methods

These methods have the advantage of being relatively simple to understand and implement. For moderate accuracies (tolerances of order 10^{-4}–10^{-5}) and moderate-sized systems ($N \lesssim 10$), they are competitive with the more complicated algorithms. For more stringent parameters, Rosenbrock methods remain reliable; they merely become less efficient than competitors like the semi-implicit extrapolation method (see below).

A Rosenbrock method seeks a solution of the form

$$\mathbf{y}(x_0 + h) = \mathbf{y}_0 + \sum_{i=1}^{s} b_i \mathbf{k}_i \qquad (17.5.29)$$

where the corrections $\mathbf{k}_i$ are found by solving s linear equations that generalize the structure in (17.5.27):

$$(\mathbf{1} - \gamma h \mathbf{f}') \cdot \mathbf{k}_i = h\mathbf{f}\left(\mathbf{y}_0 + \sum_{j=1}^{i-1} \alpha_{ij}\mathbf{k}_j\right) + h\mathbf{f}' \cdot \sum_{j=1}^{i-1} \gamma_{ij}\mathbf{k}_j, \qquad i = 1, \ldots, s \qquad (17.5.30)$$

Here we denote the Jacobian matrix by $\mathbf{f}'$. The coefficients γ, b_i, α_{ij}, and γ_{ij} are fixed constants independent of the problem. If $\gamma = \gamma_{ij} = 0$, this is simply a Runge-Kutta scheme. Equations (17.5.30) can be solved successively for $\mathbf{k}_1, \mathbf{k}_2, \ldots$.

To minimize the matrix-vector multiplications on the right-hand side of (17.5.30), rewrite the equations in terms of quantities

$$\mathbf{g}_i = \sum_{j=1}^{i-1} \gamma_{ij}\mathbf{k}_j + \gamma \mathbf{k}_i \qquad (17.5.31)$$

The equations then take the form (for four stages as an example)

$$\begin{aligned}
(\mathbf{1}/\gamma h - \mathbf{f}') \cdot \mathbf{g}_1 &= \mathbf{f}(\mathbf{y}_0) \\
(\mathbf{1}/\gamma h - \mathbf{f}') \cdot \mathbf{g}_2 &= \mathbf{f}(\mathbf{y}_0 + a_{21}\mathbf{g}_1) + c_{21}\mathbf{g}_1/h \\
(\mathbf{1}/\gamma h - \mathbf{f}') \cdot \mathbf{g}_3 &= \mathbf{f}(\mathbf{y}_0 + a_{31}\mathbf{g}_1 + a_{32}\mathbf{g}_2) + (c_{31}\mathbf{g}_1 + c_{32}\mathbf{g}_2)/h \\
(\mathbf{1}/\gamma h - \mathbf{f}') \cdot \mathbf{g}_4 &= \mathbf{f}(\mathbf{y}_0 + a_{41}\mathbf{g}_1 + a_{42}\mathbf{g}_2 + a_{43}\mathbf{g}_3) + (c_{41}\mathbf{g}_1 + c_{42}\mathbf{g}_2 + c_{43}\mathbf{g}_3)/h
\end{aligned} \qquad (17.5.32)$$

Here a_{ij} and c_{ij} can be expressed in terms of α_{ij} and γ_{ij}.

Note that systems where the right-hand side $\mathbf{f}(\mathbf{y}, x)$ depends explicitly on x can be handled by adding x to the list of dependent variables so that the system to be solved is

$$\begin{pmatrix} \mathbf{y} \\ x \end{pmatrix}' = \begin{pmatrix} \mathbf{f} \\ 1 \end{pmatrix} \qquad (17.5.33)$$

In the routine given below, we have explicitly carried out this replacement for you, so the routines can handle right-hand sides of the form $\mathbf{f}(\mathbf{y}, x)$ without any special effort on your part.

Crucial to the success of a stiff integration scheme is an automatic stepsize adjustment algorithm. Kaps and Rentrop [6] discovered an *embedded* or Runge-Kutta-Fehlberg method as described in §17.2: Two estimates of the form (17.5.29) are computed, the "real" one $\mathbf{y}$ and a lower-order estimate $\hat{\mathbf{y}}$ with different coefficients $\hat{b}_i, i = 1, \ldots, \hat{s}$, where $\hat{s} < s$ but the $\mathbf{k}_i$ are the same. The difference between $\mathbf{y}$ and $\hat{\mathbf{y}}$ leads to an estimate of the local truncation error, which can then be used for stepsize control. Kaps and Rentrop showed that the smallest value of s for which embedding is possible is $s = 4$, $\hat{s} = 3$, leading to a fourth-order method. By a suitable choice of parameters, only three function evaluations are needed for the four stages in each step.

In recent years, Kaps-Rentrop has lost favor to so-called *stiffly stable* methods, an implementation of which we give here as the routine StepperRoss (Rosenbrock Stiffly Stable), based on the Fortran routine RODAS [3]. It is also a fourth-order method with a third-order embedded method for stepsize control. Despite having six stages with six function evaluations, the enhanced stability makes it significantly more efficient than the Kaps-Rentrop method. Moreover, it has a simple dense output feature.

As with the earlier stepper routines in this chapter, you have to provide a functor for derivs, the right-hand side routine. In the structure you now must also supply a function called jacobian that returns $\mathbf{f}'$ and $\partial\mathbf{f}/\partial x$ as functions of x and $\mathbf{y}$. If x does not occur explicitly on the right-hand side, then dfdx will be zero. Usually the Jacobian matrix will be available to you by analytic differentiation of the right-hand side $\mathbf{f}$. If not, your routine will have to compute it by numerical differencing with appropriate increments $\Delta\mathbf{y}$. We will give an example of a complete derivative and jacobian structure at the end of this subsection.

The class StepperRoss uses a set of constants, which are provided by deriving the class from a class Ross_constants. This latter class is listed in a Webnote [4]. Here is the declaration of StepperRoss:

stepperross.h

```
template <class D>
struct StepperRoss : StepperBase, Ross_constants {
```

Fourth-order stiffly stable Rosenbrock step for integrating stiff ODEs, with monitoring of local truncation error to adjust stepsize.

```
    typedef D Dtype;                    Make the type of derivs available to odeint.
    MatDoub dfdy;                       f'
    VecDoub dfdx;                       ∂f/∂x
    VecDoub k1,k2,k3,k4,k5,k6;
    VecDoub cont1,cont2,cont3,cont4;
    MatDoub a;
    StepperRoss(VecDoub_IO &yy, VecDoub_IO &dydxx, Doub &xx, const Doub atoll,
        const Doub rtoll, bool dens);
    void step(const Doub htry,D &derivs);
    void dy(const Doub h,D &derivs);
    void prepare_dense(const Doub h,VecDoub_I &dydxnew);
    Doub dense_out(const Int i, const Doub x, const Doub h);
    Doub error();
    struct Controller {
        Doub hnext;
        bool reject;
        bool first_step;                first_step, errold, and hold are used by
        Doub errold;                        the predictive controller.
        Doub hold;
        Controller();
        bool success(Doub err, Doub &h);
    };
    Controller con;
};
```

The implementation will seem very familiar if you've looked at StepperDopr5, the explicit Runge-Kutta routine. Note that in the algorithm routine dy of StepperRoss, the linear equations (17.5.32) are solved by first computing the LU decomposition of the matrix

$\mathbf{1}/\gamma h - \mathbf{f}'$ using the routine LUdcmp. Then the six $\mathbf{g}_i$ are found by backsubstitution of the six different right-hand sides using the routine solve in LUdcmp. Thus each step of the integration requires one call to jacobian and six calls to derivs (one call outside dy and five calls inside). The evaluation of the Jacobian matrix is roughly equivalent to N evaluations of the right-hand side $\mathbf{f}$ (although it can often be less than this, especially if commonality of code can be exploited). Thus this scheme involves about $N + 6$ function evaluations per step. Note that if N is large and the Jacobian matrix is sparse, you should replace the LU decomposition by a suitable sparse matrix procedure.

stepperross.h

```
template <class D>
StepperRoss<D>::StepperRoss(VecDoub_IO &yy, VecDoub_IO &dydxx, Doub &xx,
    const Doub atoll,const Doub rtoll, bool dens) :
    StepperBase(yy,dydxx,xx,atoll,rtoll,dens),dfdy(n,n),dfdx(n),k1(n),k2(n),
    k3(n),k4(n),k5(n),k6(n),cont1(n),cont2(n),cont3(n),cont4(n),a(n,n) {
```
Input to the constructor are the dependent variable y[0..n-1] and its derivative dydx[0..n-1] at the starting value of the independent variable x. Also input are the absolute and relative tolerances, atol and rtol, and the boolean dense, which is true if dense output is required.
```
    EPS=numeric_limits<Doub>::epsilon();
}
template <class D>
void StepperRoss<D>::step(const Doub htry,D &derivs) {
```
Attempts a step with stepsize htry. On output, y and x are replaced by their new values, hdid is the stepsize that was actually accomplished, and hnext is the estimated next stepsize.
```
    VecDoub dydxnew(n);
    Doub h=htry;                              Set stepsize to the initial trial value.
    derivs.jacobian(x,y,dfdx,dfdy);           Compute the Jacobian and ∂f/∂x.
    for (;;) {
        dy(h,derivs);                         Take a step.
        Doub err=error();                     Evaluate accuracy.
        if (con.success(err,h)) break;        Step rejected. Try again with reduced h set
        if (abs(h) <= abs(x)*EPS)                 by controller.
            throw("stepsize underflow in StepperRoss");
    }
    derivs(x+h,yout,dydxnew);                 Step succeeded.
    if (dense)                                Compute coefficients for dense output.
        prepare_dense(h,dydxnew);
    dydx=dydxnew;                             Reuse last derivative evaluation for next step.
    y=yout;
    xold=x;                                   Used for dense output.
    x += (hdid=h);
    hnext=con.hnext;
}
template<class D>
void StepperRoss<D>::dy(const Doub h,D &derivs) {
```
Given values for n variables y[0..n-1] and their derivatives dydx[0..n-1] known at x, use the fourth-order stiffly stable Rosenbrock method to advance the solution over an interval h and store the incremented variables in yout[0..n-1]. Also store an estimate of the local truncation error in yerr using the embedded third-order method.
```
    VecDoub ytemp(n),dydxnew(n);
    Int i;
    for (i=0;i<n;i++) {                       Set up the matrix 1/γh − f'.
        for (Int j=0;j<n;j++) a[i][j] = -dfdy[i][j];
        a[i][i] += 1.0/(gam*h);
    }
    LUdcmp alu(a);                            LU decomposition of the matrix.
    for (i=0;i<n;i++)                         Set up right-hand side for g1.
        ytemp[i]=dydx[i]+h*d1*dfdx[i];
     alu.solve(ytemp,k1);                     Solve for g1.
    for (i=0;i<n;i++)                         Compute intermediate values of y.
        ytemp[i]=y[i]+a21*k1[i];
    derivs(x+c2*h,ytemp,dydxnew);             Compute dydx at the intermediate values.
    for (i=0;i<n;i++)                         Set up right-hand side for g2.
        ytemp[i]=dydxnew[i]+h*d2*dfdx[i]+c21*k1[i]/h;
```

```
    alu.solve(ytemp,k2);                         Solve for g_2.
    for (i=0;i<n;i++)                            Compute intermediate values of y.
        ytemp[i]=y[i]+a31*k1[i]+a32*k2[i];
    derivs(x+c3*h,ytemp,dydxnew);                Compute dydx at the intermediate values.
    for (i=0;i<n;i++)                            Set up right-hand side for g_3.
        ytemp[i]=dydxnew[i]+h*d3*dfdx[i]+(c31*k1[i]+c32*k2[i])/h;
    alu.solve(ytemp,k3);                         Solve for g_3.
    for (i=0;i<n;i++)                            Compute intermediate values of y.
        ytemp[i]=y[i]+a41*k1[i]+a42*k2[i]+a43*k3[i];
    derivs(x+c4*h,ytemp,dydxnew);                Compute dydx at the intermediate values.
    for (i=0;i<n;i++)                            Set up right-hand side for g_4.
        ytemp[i]=dydxnew[i]+h*d4*dfdx[i]+(c41*k1[i]+c42*k2[i]+c43*k3[i])/h;
    alu.solve(ytemp,k4);                         Solve for g_4.
    for (i=0;i<n;i++)                            Compute intermediate values of y.
        ytemp[i]=y[i]+a51*k1[i]+a52*k2[i]+a53*k3[i]+a54*k4[i];
    Doub xph=x+h;
    derivs(xph,ytemp,dydxnew);                   Compute dydx at the intermediate values.
    for (i=0;i<n;i++)                            Set up right-hand side for g_5.
        k6[i]=dydxnew[i]+(c51*k1[i]+c52*k2[i]+c53*k3[i]+c54*k4[i])/h;
    alu.solve(k6,k5);                            Solve for g_5.
    for (i=0;i<n;i++)                            Compute the embedded solution.
        ytemp[i] += k5[i];
    derivs(xph,ytemp,dydxnew);                   Last derivative evaluation.
    for (i=0;i<n;i++)                            Compute the solution and the error.
        k6[i]=dydxnew[i]+(c61*k1[i]+c62*k2[i]+c63*k3[i]+c64*k4[i]+c65*k5[i])/h;
    alu.solve(k6,yerr);
    for (i=0;i<n;i++)
        yout[i]=ytemp[i]+yerr[i];
}
template <class D>
void StepperRoss<D>::prepare_dense(const Doub h,VecDoub_I &dydxnew) {
Store coefficients of interpolating polynomial for dense output in cont1...cont4.
    for (Int i=0;i<n;i++) {
        cont1[i]=y[i];
        cont2[i]=yout[i];
        cont3[i]=d21*k1[i]+d22*k2[i]+d23*k3[i]+d24*k4[i]+d25*k5[i];
        cont4[i]=d31*k1[i]+d32*k2[i]+d33*k3[i]+d34*k4[i]+d35*k5[i];
    }
}
template <class D>
Doub StepperRoss<D>::dense_out(const Int i,const Doub x,const Doub h) {
Evaluate interpolating polynomial for y[i] at location x, where xold ≤ x ≤ xold + h.
    Doub s=(x-xold)/h;
    Doub s1=1.0-s;
    return cont1[i]*s1+s*(cont2[i]+s1*(cont3[i]+s*cont4[i]));
}
template <class D>
Doub StepperRoss<D>::error() {
Use yerr to compute norm of scaled error estimate. A value less than one means the step was
successful.
    Doub err=0.0,sk;
    for (Int i=0;i<n;i++) {
        sk=atol+rtol*MAX(abs(y[i]),abs(yout[i]));
        err += SQR(yerr[i]/sk);
    }
    return sqrt(err/n);
}
```

Stepsize control depends on the fact that

$$\begin{aligned} \mathbf{y}_{\text{exact}} &= \mathbf{y} + O(h^5) \\ \mathbf{y}_{\text{exact}} &= \hat{\mathbf{y}} + O(h^4) \end{aligned} \tag{17.5.34}$$

Thus

$$|\mathbf{y} - \hat{\mathbf{y}}| = O(h^4) \tag{17.5.35}$$

Referring back to the steps leading from equation (17.2.4) to equation (17.2.12), we see that the new stepsize should be chosen as in equation (17.2.12) but with the exponent 1/5 replaced by 1/4. Also, experience shows that it is wise to prevent too large a stepsize change in one step, otherwise we will probably have to undo the large change in the next step. We adopt 0.2 and 6 as the maximum allowed decrease and increase of h in one step.

Methods for integrating stiff equations do not suffer from the stability limitations that led to the PI controller of §17.2.1. However, stiff problems often need a rapid decrease in stepsize even when the previous step is successful. Also, sometimes the effective order of the method can be lower than the simple Taylor series prediction. Gustafsson [7] has proposed a *predictive controller* that does a good job of dealing with these problems. The resulting formula is

$$h_{n+1} = S h_n \left(\frac{1}{\mathtt{err}_n}\right)^{1/4} \frac{h_n}{h_{n-1}} \left(\frac{\mathtt{err}_{n-1}}{\mathtt{err}_n}\right)^{1/4} \tag{17.5.36}$$

It is used only when a step is accepted.

stepperross.h

```
template <class D>
StepperRoss<D>::Controller::Controller() : reject(false), first_step(true) {}
```
Step size controller for fourth-order Rosenbrock method.
```
template <class D>
bool StepperRoss<D>::Controller::success(Doub err, Doub &h) {
```
Returns true if err ≤ 1, false otherwise. If step was successful, sets hnext to the estimated optimal stepsize for the next step. If the step failed, reduces h appropriately for another try.
```
    static const Doub safe=0.9,fac1=5.0,fac2=1.0/6.0;
    Doub fac=MAX(fac2,MIN(fac1,pow(err,0.25)/safe));
    Doub hnew=h/fac;                     Ensure 1/fac1 <= hnew/h <= 1/fac2.
    if (err <= 1.0) {                    Step succeeded.
        if (!first_step) {               Predictive control.
            Doub facpred=(hold/h)*pow(err*err/errold,0.25)/safe;
            facpred=MAX(fac2,MIN(fac1,facpred));
            fac=MAX(fac,facpred);
            hnew=h/fac;
        }
        first_step=false;
        hold=h;
        errold=MAX(0.01,err);
        if (reject)                      Don't let step increase if last one was rejected.
            hnew=(h >= 0.0 ? MIN(hnew,h) : MAX(hnew,h));
        hnext=hnew;
        reject=false;
        return true;
    } else {                             Truncation error too large, reduce stepsize.
        h=hnew;
        reject=true;
        return false;
    }
}
```

As an example of how StepperRoss is used, one can solve the system

$$\begin{aligned} y_0' &= -.013y_0 - 1000y_0y_2 \\ y_1' &= -2500y_1y_2 \\ y_2' &= -.013y_0 - 1000y_0y_2 - 2500y_1y_2 \end{aligned} \tag{17.5.37}$$

with initial conditions

$$y_0(0) = 1, \qquad y_1(0) = 1, \qquad y_2(0) = 0 \tag{17.5.38}$$

(This is test problem D4 in [8].) We integrate the system up to $x = 50$ with an initial stepsize of $h = 2.9 \times 10^{-4}$ using `Odeint`. We set `atol` $=$ `rtol` $= 10^{-5}$. The right-hand side routine for this problem is given below. Even though the ratio of largest to smallest decay constants for this problem is around 10^6, `StepperRoss` succeeds in integrating this set in only 11 steps with 67 function evaluations. By contrast, the explicit Runge-Kutta routine `StepperDopr5` requires almost 60,000 steps and over 400,000 function evaluations!

```
struct rhs {
    void operator() (const Doub x, VecDoub_I &y, VecDoub_O &dydx) {
        dydx[0] = -0.013*y[0]-1000.0*y[0]*y[2];
        dydx[1] = -2500.0*y[1]*y[2];
        dydx[2] = -0.013*y[0]-1000.0*y[0]*y[2]-2500.0*y[1]*y[2];
    }
    void jacobian(const Doub x, VecDoub_I &y, VecDoub_O &dfdx,
        MatDoub_O &dfdy) {
        Int n=y.size();
        for (Int i=0;i<n;i++) dfdx[i]=0.0;
        dfdy[0][0] = -0.013-1000.0*y[2];
        dfdy[0][1] = 0.0;
        dfdy[0][2] = -1000.0*y[0];
        dfdy[1][0] = 0.0;
        dfdy[1][1] = -2500.0*y[2];
        dfdy[1][2] = -2500.0*y[1];
        dfdy[2][0] = -0.013-1000.0*y[2];
        dfdy[2][1] = -2500.0*y[2];
        dfdy[2][2] = -1000.0*y[0]-2500.0*y[1];
    }
};
```

17.5.2 Semi-Implicit Extrapolation Method

The Bulirsch-Stoer method, which discretizes the differential equation using the modified midpoint rule, does not work for stiff problems. For many years, successful extrapolation-type routines for stiff equations were based on an algorithm of Bader and Deuflhard [9]. This algorithm uses a semi-implicit version of the midpoint method that has an even error series.

Not long afterward, however, Deuflhard [10] investigated a semi-implicit version of the Euler method, equation (17.5.27). This does not have an even error series. Nevertheless, it turns out that for high precision, using this method as the basis of an extrapolation scheme is even more efficient than using the semi-implicit midpoint rule. (Some theoretical insight into this behavior is provided in §VI.5 of [3].) Since `StepperRoss` is generally satisfactory for low precision, this method is a good companion. We give it as `StepperSie` ("Semi-Implicit Euler").

The basic equation of the method is equation (17.5.27) rewritten in the form

$$\left[\mathbf{1}/h - \frac{\partial \mathbf{f}}{\partial \mathbf{y}}\right] \cdot (\mathbf{y}_{n+1} - \mathbf{y}_n) = \mathbf{f}(\mathbf{y}_n) \tag{17.5.39}$$

A sequence of stepsizes $h_i = H/n_i$ is used with this equation to advance the solution a distance H. The linear equations are solved with LU decomposition. Polynomial extrapolation is used as in the original Bulirsch-Stoer method, except that in equation (17.3.8) the ratio of stepsizes is not squared because the error series is not even.

Instead of making the replacement (17.5.33) in the above formula, it turns out to be slightly better to add a single simplified Newton iteration of the fully implicit Euler step (17.5.25):

$$\mathbf{y}_{n+1} = \mathbf{y}_n + h\mathbf{f}(x_{n+1}, \mathbf{y}_{n+1}) \longrightarrow \left[\mathbf{1} - h\frac{\partial \mathbf{f}}{\partial \mathbf{y}}\right] \cdot (\mathbf{y}_{n+1} - \mathbf{y}_n) = h\mathbf{f}(x_{n+1}, \mathbf{y}_n) \tag{17.5.40}$$

This costs an extra function evaluation but avoids the computation of $\partial \mathbf{f}/\partial x$. In the code, we leave $\partial \mathbf{f}/\partial x$ as an argument of the `jacobian` function for compatibility with `StepperRoss`, but it is not used.

Another difference from StepperRoss is that the Jacobian does not have to be exact. Its main role is to ensure stability, not accuracy. Accordingly, the code has a test to see when the Jacobian needs to be recomputed.

Differences from StepperBS include

- The default stepsize sequence is

$$n = 2, 3, 4, 6, 8, 12, 16, 24, 32, 48, 64, \ldots, [n_j = 2n_{j-2}], \ldots \tag{17.5.41}$$

- The work per unit step now includes the cost of Jacobian evaluations as well as function evaluations. We count one Jacobian evaluation as equivalent to five function evaluations by default, but it could be as large as N, the number of equations. The work per unit step also includes the cost of the LU decomposition and the backsubstitutions, each set by default to the cost of a function evaluation.
- Several checks for instability are included. If the estimated error $\mathtt{err}_k$ starts increasing with k during a step, the step is restarted with the stepsize reduced by a factor of two. Similarly, a stability test is made for $k = 0, 1$ during the Euler step and the step is rejected if the test is failed. You could add a test for failure of the LU decomposition and similarly reduce the stepsize if that happened.

The routine, which is based on the Fortran routine SEULEX [3], next follows.

17.5.3 Implementation of Semi-Implicit Extrapolation Method

The routine StepperSie is an excellent routine for all stiff problems, competitive with the best Gear-type routines. StepperRoss is often better in execution time for moderate N and $\epsilon \lesssim 10^{-5}$. The detailed implementation is listed in a Webnote [11].

CITED REFERENCES AND FURTHER READING:

Gear, C.W. 1971, *Numerical Initial Value Problems in Ordinary Differential Equations* (Englewood Cliffs, NJ: Prentice-Hall).[1]

Hairer, E., Nørsett, S.P., and Wanner, G. 1993, *Solving Ordinary Differential Equations I. Nonstiff Problems*, 2nd ed. (New York: Springer). Fortran codes at http://www.unige.ch/~hairer/software.html.[2]

Hairer, E., Nørsett, S.P., and Wanner, G. 1996, *Solving Ordinary Differential Equations II. Stiff and Differential-Algebraic Problems*, 2nd ed. (New York: Springer).[3]

Numerical Recipes Software 2007, "Constants for Stiffly Stable Rosenbrock Method," *Numerical Recipes Webnote No. 23*, at http://www.nr.com/webnotes?23 [4]

Netlib: http://www.netlib.org/.[5]

Kaps, P., and Rentrop, P. 1979, "Generalized Runge-Kutta Methods of Order Four with Stepsize Control for Stiff Ordinary Differential Equations," *Numerische Mathematik*, vol. 33, pp. 55–68.[6]

Gustafsson, K. 1994, "Control Theoretic Techniques for Stepsize Selection in Implicit Runge-Kutta Methods," *ACM Transactions on Mathematical Software*, vol. 20, pp. 496-517.[7]

Enright, W.H., and Pryce, J.D. 1987, "Two FORTRAN Packages for Assessing Initial Value Methods," *ACM Transactions on Mathematical Software*, vol. 13, pp. 1–27.[8]

Bader, G., and Deuflhard, P. 1983, "A Semi-Implicit Mid-Point Rule for Stiff Systems of Ordinary Differential Equations," *Numerische Mathematik*, vol. 41, pp. 373–398.[9]

Deuflhard, P. 1985, "Recent Progress in Extrapolation Methods for Ordinary Differential Equations," *SIAM Review*, vol. 27, pp. 505–535.[10]

Numerical Recipes Software 2007, "StepperSie Implementation," *Numerical Recipes Webnote No. 24*, at http://www.nr.com/webnotes?24 [11]

Deuflhard, P. 1983, "Order and Stepsize Control in Extrapolation Methods," *Numerische Mathematik*, vol. 41, pp. 399–422.

Enright, W.H., Hull, T.E., and Lindberg, B. 1975, "Comparing Numerical Methods for Stiff Systems of ODE's," *BIT*, vol. 15, pp. 10–48.

Wanner, G. 1988, in *Numerical Analysis 1987*, Pitman Research Notes in Mathematics, vol. 170, D.F. Griffiths and G.A. Watson, eds. (Harlow, Essex, UK: Longman Scientific and Technical).

Stoer, J., and Bulirsch, R. 2002, *Introduction to Numerical Analysis*, 3rd ed. (New York: Springer).

17.6 Multistep, Multivalue, and Predictor-Corrector Methods

The terms "multistep" and "multivalue" describe two different ways of implementing essentially the same integration technique for ODEs. Predictor-corrector is a particular subcategory of these methods — in fact, the most widely used. Accordingly, the name predictor-corrector is often loosely used to denote all these methods.

We suspect that predictor-corrector integrators have had their day, and that they are no longer the method of choice for most problems in ODEs. For high-precision applications, or applications where evaluations of the right-hand sides are expensive, Bulirsch-Stoer dominates. For convenience, or for moderate precision, adaptive-stepsize Runge-Kutta dominates. Predictor-corrector methods have been, we think, squeezed out in the middle. There is possibly only one exceptional case: high-precision solution of very smooth equations with very complicated right-hand sides, as we will describe later.

Nevertheless, these methods have had a long historical run. Textbooks are full of information on them, and there are a lot of standard ODE programs around that are based on predictor-corrector methods. Many capable researchers have a lot of experience with predictor-corrector routines, and they see no reason to make a precipitous change of habit. It is not a bad idea for you to be familiar with the principles involved, and even with the sorts of bookkeeping details that are the bane of these methods. Otherwise, there will be a big surprise in store when you first have to fix a problem in a predictor-corrector routine.

Let us first consider the multistep approach. Think about how integrating an ODE is different from finding the integral of a function: For a function, the integrand has a known dependence on the independent variable x and can be evaluated at will. For an ODE, the "integrand" is the right-hand side, which depends both on x and on the dependent variables y. Thus, to advance the solution of $y' = f(x, y)$ from x_n to x, we have

$$y(x) = y_n + \int_{x_n}^{x} f(x', y)\, dx' \tag{17.6.1}$$

In a single-step method like Runge-Kutta or Bulirsch-Stoer, the value y_{n+1} at x_{n+1} depends only on y_n. In a multistep method, we approximate $f(x, y)$ by a polynomial passing through *several* previous points $x_n, x_{n-1}, \ldots$ and possibly also through x_{n+1}. The result of evaluating the integral (17.6.1) at $x = x_{n+1}$ is then of the form

$$y_{n+1} = y_n + h(\beta_0 y'_{n+1} + \beta_1 y'_n + \beta_2 y'_{n-1} + \beta_3 y'_{n-2} + \cdots) \tag{17.6.2}$$

where y'_n denotes $f(x_n, y_n)$, and so on. If $\beta_0 = 0$, the method is explicit; otherwise it is implicit. The order of the method depends on how many previous steps we use to get each new value of y.

Consider how we might solve an implicit formula of the form (17.6.2) for y_{n+1}. Two methods suggest themselves: *functional iteration* and *Newton's method.* In functional iteration, we take some initial guess for y_{n+1}, insert it into the right-hand side of (17.6.2) to get an updated value of y_{n+1}, insert this updated value back into the right-hand side, and continue iterating. But how are we to get an initial guess for y_{n+1}? Easy! Just use some *explicit* formula of the same form as (17.6.2). This is called the *predictor step*. In the predictor step we are essentially *extrapolating* the polynomial fit to the derivative from the previous points to the new point x_{n+1} and then doing the integral (17.6.1) in a Simpson-like manner from x_n to x_{n+1}. The subsequent Simpson-like integration, using the prediction step's value of y_{n+1} to *interpolate* the derivative, is called the *corrector step*. The difference between the predicted and corrected function values supplies information on the local truncation error that can be used to control accuracy and to adjust stepsize.

If one corrector step is good, aren't many better? Why not use each corrector as an improved predictor and iterate to convergence on each step? Answer: Even if you had a *perfect* predictor, the step would still be accurate only to the finite order of the corrector. This incurable error term is on the same order as that which your iteration is supposed to cure, so you are at best changing only the coefficient in front of the error term by a fractional amount. So dubious an improvement is certainly not worth the effort. Your extra effort would be better spent in taking a smaller stepsize.

As described so far, you might think it desirable or necessary to predict several intervals ahead at each step, then to use all these intervals, with various weights, in a Simpson-like corrector step. That is not a good idea. Extrapolation is the least stable part of the procedure, and it is desirable to minimize its effect. Therefore, the integration steps of a predictor-corrector method are overlapping, each one involving several stepsize intervals h, but extending just one such interval farther than the previous ones. Only that one extended interval is extrapolated by each predictor step.

The most popular predictor-corrector methods are probably the Adams-Bashforth-Moulton schemes, which have good stability properties. The Adams-Bashforth part is the predictor. For example, the third-order case is

$$\text{predictor:} \qquad y_{n+1} = y_n + \frac{h}{12}(23y'_n - 16y'_{n-1} + 5y'_{n-2}) + O(h^4) \qquad (17.6.3)$$

Here information at the current point x_n, together with the two previous points x_{n-1} and x_{n-2} (assumed equally spaced), is used to predict the value y_{n+1} at the next point, x_{n+1}. The Adams-Moulton part is the corrector. The third-order case is

$$\text{corrector:} \qquad y_{n+1} = y_n + \frac{h}{12}(5y'_{n+1} + 8y'_n - y'_{n-1}) + O(h^4) \qquad (17.6.4)$$

Without the trial value of y_{n+1} from the predictor step to insert on the right-hand side, the corrector would be a nasty implicit equation for y_{n+1}. (Despite the names, these formulas are actually all due to Adams.)

There are actually three separate processes occurring in a predictor-corrector method: the predictor step, which we call P; the evaluation of the derivative y'_{n+1} from the latest value of y, which we call E; and the corrector step, which we call C.

In this notation, iterating m times with the corrector (a practice we inveighed against earlier) would be written P(EC)^m. One also has the choice of finishing with a C or an E step. The lore is that a final E is superior, so the strategy usually recommended is PECE.

Notice that a PC method with a fixed number of iterations (say, one) is an explicit method. When we fix the number of iterations in advance, the final value of y_{n+1} can be written as some complicated function of known quantities. Thus fixed iteration PC methods lose the strong stability properties of implicit methods and *should only be used for nonstiff problems*.

For stiff problems we *must* use an implicit method if we want to avoid having tiny stepsizes. (Not all implicit methods are good for stiff problems, but fortunately some good ones such as the Gear formulas are known.) We then appear to have two choices for solving the implicit equations: functional iteration to convergence, or Newton iteration. However, it turns out that for stiff problems functional iteration will not even converge unless we use tiny stepsizes, no matter how close our prediction is! Thus Newton iteration is usually an essential part of a multistep stiff solver. For convergence, Newton's method doesn't particularly care what the stepsize is, as long as the prediction is accurate enough.

Multistep methods, as we have described them so far, suffer from two serious difficulties when one tries to implement them:

- Since the formulas require results from equally spaced steps, adjusting the stepsize is difficult.
- Starting and stopping present problems. For starting, we need the initial values plus several previous steps to prime the pump. Stopping is a problem because equal steps are unlikely to land directly on the desired termination point.

Older implementations of PC methods have various cumbersome ways of dealing with these problems. For example, they might use Runge-Kutta to start and stop. Changing the stepsize requires considerable bookkeeping to do some kind of interpolation procedure. Fortunately, both these drawbacks disappear with the multivalue approach.

For multivalue methods (also called Nordsieck methods), the basic data available to the integrator are the first few terms of the Taylor series expansion of the solution at the current point x_n. The aim is to advance the solution and obtain the expansion coefficients at the next point x_{n+1}. This is in contrast to multistep methods, where the data are the values of the solution at $x_n, x_{n-1}, \dots$. We'll illustrate the idea by considering a four-value method, for which the basic data are

$$\mathbf{y}_n \equiv \begin{pmatrix} y_n \\ hy'_n \\ (h^2/2)y''_n \\ (h^3/6)y'''_n \end{pmatrix} \tag{17.6.5}$$

It is also conventional to scale the derivatives with the powers of $h = x_{n+1} - x_n$ as shown. Note that here we use the vector notation $\mathbf{y}$ to denote the solution and its first few derivatives at a point, not the fact that we are solving a system of equations with many components y.

In terms of the data in (17.6.5), we can approximate the value of the solution y

at some point x:

$$y(x) = y_n + (x - x_n)y'_n + \frac{(x - x_n)^2}{2} y''_n + \frac{(x - x_n)^3}{6} y'''_n \tag{17.6.6}$$

Set $x = x_{n+1}$ in equation (17.6.6) to get an approximation to y_{n+1}. Differentiate equation (17.6.6) and set $x = x_{n+1}$ to get an approximation to y'_{n+1}, and similarly for y''_{n+1} and y'''_{n+1}. Call the resulting approximation $\widetilde{\mathbf{y}}_{n+1}$, where the tilde is a reminder that all we have done so far is a polynomial extrapolation of the solution and its derivatives; we have not yet used the differential equation. You can easily verify that

$$\widetilde{\mathbf{y}}_{n+1} = \mathbf{B} \cdot \mathbf{y}_n \tag{17.6.7}$$

where the matrix $\mathbf{B}$ is

$$\mathbf{B} = \begin{pmatrix} 1 & 1 & 1 & 1 \\ 0 & 1 & 2 & 3 \\ 0 & 0 & 1 & 3 \\ 0 & 0 & 0 & 1 \end{pmatrix} \tag{17.6.8}$$

We now write the actual approximation to $\mathbf{y}_{n+1}$ that we will use by adding a correction to $\widetilde{\mathbf{y}}_{n+1}$:

$$\mathbf{y}_{n+1} = \widetilde{\mathbf{y}}_{n+1} + \alpha \mathbf{r} \tag{17.6.9}$$

Here $\mathbf{r}$ will be a fixed vector of numbers, in the same way that $\mathbf{B}$ is a fixed matrix. We fix α by requiring that the differential equation

$$y'_{n+1} = f(x_{n+1}, y_{n+1}) \tag{17.6.10}$$

be satisfied. The second of the equations in (17.6.9) is

$$h y'_{n+1} = h \widetilde{y}'_{n+1} + \alpha r_1 \tag{17.6.11}$$

and this will be consistent with (17.6.10) provided

$$r_1 = 1, \qquad \alpha = h f(x_{n+1}, y_{n+1}) - h \widetilde{y}'_{n+1} \tag{17.6.12}$$

The values of r_0, r_2, and r_3 are free for the inventor of a given four-value method to choose. Different choices give different orders of method (i.e., through what order in h the final expression 17.6.9 actually approximates the solution) and different stability properties.

An interesting result, not obvious from our presentation, is that multivalue and multistep methods are entirely equivalent. In other words, the value y_{n+1} given by a multivalue method with given $\mathbf{B}$ and $\mathbf{r}$ is exactly the same value given by some multistep method with given β's in equation (17.6.2). For example, it turns out that the Adams-Bashforth formula (17.6.3) corresponds to a four-value method with $r_0 = 0$, $r_2 = 3/4$, and $r_3 = 1/6$. The method is explicit because $r_0 = 0$. The Adams-Moulton method (17.6.4) corresponds to the implicit four-value method with $r_0 = 5/12$, $r_2 = 3/4$, and $r_3 = 1/6$. Implicit multivalue methods are solved the same way as implicit multistep methods: either by a predictor-corrector approach using an explicit method for the predictor, or by Newton iteration for stiff systems.

Why go to all the trouble of introducing a whole new method that turns out to be equivalent to a method you already knew? The reason is that multivalue methods allow an easy solution to the two difficulties we mentioned above in actually implementing multistep methods.

Consider first the question of stepsize adjustment. To change stepsize from h to h' at some point x_n, simply multiply the components of $\mathbf{y}_n$ in (17.6.5) by the appropriate powers of h'/h, and you are ready to continue to $x_n + h'$.

Multivalue methods also allow a relatively easy change in the *order* of the method: Simply change $\mathbf{r}$. The usual strategy for this is first to determine the new stepsize with the current order from the error estimate. Then check what stepsize would be predicted using an order one greater and one smaller than the current order. Choose the order that allows you to take the biggest next step. Being able to change order also allows an easy solution to the starting problem: Simply start with a first-order method and let the order automatically increase to the appropriate level.

For moderate accuracy requirements, the most efficient choice is almost always a Runge-Kutta routine like `StepperDopr853`. For high accuracy, `StepperBS` is both robust and efficient. For very smooth functions, a variable-order PC method can invoke very high orders. If the right-hand side of the equation is relatively complicated, so that the expense of evaluating it outweighs the bookkeeping expense, then the best PC packages can outperform Bulirsch-Stoer on such problems. As you can imagine, however, such a variable-stepsize, variable-order method is not trivial to program. If you suspect that your problem is suitable for this treatment, we recommend the use of a packaged PC routine. For further details consult [1-3].

Our prediction is that, as extrapolation methods like Bulirsch-Stoer continue to gain sophistication, they will eventually beat out PC methods in *all* applications. We are willing, however, to be corrected.

CITED REFERENCES AND FURTHER READING:

Gear, C.W. 1971, *Numerical Initial Value Problems in Ordinary Differential Equations* (Englewood Cliffs, NJ: Prentice-Hall), Chapter 9.[1]

Shampine, L.F., and Gordon, M.K. 1975, *Computer Solution of Ordinary Differential Equations. The Initial Value Problem.* (San Francisco: W.H Freeman).[2]

Hairer, E., Nørsett, S.P., and Wanner, G. 1993, *Solving Ordinary Differential Equations I. Nonstiff Problems*, 2nd ed. (New York: Springer).[3]

Acton, F.S. 1970, *Numerical Methods That Work*; 1990, corrected edition (Washington, DC: Mathematical Association of America), Chapter 5.

Kahaner, D., Moler, C., and Nash, S. 1989, *Numerical Methods and Software* (Englewood Cliffs, NJ: Prentice-Hall), Chapter 8.

Stoer, J., and Bulirsch, R. 2002, *Introduction to Numerical Analysis*, 3rd ed. (New York: Springer), Chapter 7.

17.7 Stochastic Simulation of Chemical Reaction Networks

We are so used to thinking of chemical (or nuclear) reaction networks as implying sets of continuous differential equations, that it takes an effort to remember

their underlying discrete, atomic, nature. To give an example, we have all learned to translate a set of reactions like

$$\begin{aligned} A + X &\xrightarrow{k_0} 2X \\ X + Y &\xrightarrow{k_1} 2Y \\ Y &\xrightarrow{k_2} B \end{aligned} \tag{17.7.1}$$

into a set of differential equations (*rate equations*) governing the concentrations of each species,

$$\begin{aligned} \frac{d[A]}{dt} &= -k_0[A][X] \equiv -a_0 \\ \frac{d[X]}{dt} &= k_0[A][X] - k_1[X][Y] \equiv a_0 - a_1 \\ \frac{d[Y]}{dt} &= k_1[X][Y] - k_2[Y] \equiv a_1 - a_2 \\ \frac{d[B]}{dt} &= k_2[Y] \equiv a_2 \end{aligned} \tag{17.7.2}$$

where a_0, a_1, and a_2 are respectively the rates of the three reactions in equation (17.7.1).

Increasingly in biological applications, however, one is faced with situations where the actual numbers of reacting molecules is so small that discreteness effects and fluctuations become important. In such cases, one needs to replace continuum concentrations like $[X]$ and $[Y]$ with actual numbers of molecular species. The equations (17.7.2) now become meaningless. What we need to do is directly simulate the discrete reactions in (17.7.1), assigning a sequence of stochastically generated times, and corresponding discrete changes in species numbers, to the occurrences of each reaction. This task is known as *stochastic simulation*, from the original work of Gillespie [1]. Stochastic simulation is a remarkably simple, and elegant, technique. Like many powerful tools, it can be both used and misused, as we will discuss.

Before we get to the details, it is useful to formalize some aspects of the structure of equations (17.7.1) and (17.7.2). In general, we have M reactions occurring among N species. Each reaction $j = 0, \ldots, M-1$, has an instantaneous rate, denoted a_j. In the discrete case, $1/a_j$ is the mean time until the next occurrence of reaction j, if no other reaction happens first. An important point is that each rate a_j depends only on the numbers of those species on the left-hand side of reaction j, its *reactants*. Define a *reactant matrix* λ_{ij} by

$$\lambda_{ij} = \begin{cases} 1 & \text{if species } S_i \text{ is an input to reaction } j \\ 0 & \text{otherwise} \end{cases} \tag{17.7.3}$$

On the output side (*products*), each set of reactions j is characterized by a *state change matrix* ν_{ij} whose i, j component is the net change in the number of species S_i due to one occurrence of reaction j. (The jth column of this matrix is often called the *state change vector* for reaction j.) In terms of these quantities, the conventional

rate equations, like (17.7.2), can be written in general as

$$\frac{d[S_i]}{dt} = \sum_{j=0}^{M-1} \nu_{ij}\, a_j(\{[S_k]\}), \quad k \text{ s.t. } \lambda_{kj} \neq 0, \quad i = 0,\ldots,N-1 \tag{17.7.4}$$

But back to the discrete case: At an instant in time, if we know all the S_i's, we can compute all the rates a_j. Since rates are additive, the total rate at which *something* will happen is

$$a_{\text{tot}} = \sum_{j=0}^{M-1} a_j \tag{17.7.5}$$

Moreover, because the system is assumed to be "memoryless" (except for the S_j's) and "well-mixed," the probability distribution of times to this next occurrence of *some* reaction j must be exponentially distributed (like radioactive decay). Furthermore, given that *some* reaction occurs, it is easy to state what is the probability distribution of *which* reaction it is: It will be reaction j with probability a_j/a_{tot}.

This is all there is, conceptually, to stochastic simulation. The rest is just implementation details, including some clever tricks to speed up the calculation. The steps in the so-called *direct method* are

- From all the S_i's, compute all the a_j's and a_{tot}.
- Draw a random number U_1, uniform in [0, 1], and compute the time τ to the next reaction by

$$\tau = \frac{1}{a_{\text{tot}}} \log\left(\frac{1}{U_1}\right) \tag{17.7.6}$$

  (This generates an exponential distribution; cf. §7.3.)
- Draw a second uniform random number U_2 in [0, 1] and find the smallest k such that

$$\sum_{j=0}^{k} a_j > a_{\text{tot}} U_2 \tag{17.7.7}$$

  A value k will thus be chosen with probability a_k/a_{tot}.
- Increment the time t by τ.
- Update each S_i by adding the value ν_{ik}.
- Go back to the first step.

17.7.1 Speeding Up the Direct Method

We can speed up the direct method, first, by identifying all steps that are (naively) of $O(M)$ or $O(N)$ and finding ways to make them $O(1)$ (or maybe log); and second, by hand-crafting the inner loop of the program to have the fastest possible execution. The second of these tasks is very important and can make or break a stochastic simulation code's performance; but, unfortunately, it is very machine-, compiler-, and problem-dependent, so it is outside our scope here.

As for the first task, we first note that realistic reaction networks of any size almost always have very sparse reactant and state change matrices: Reactions generally involve only one or two reactants and produce at most a few products. Therefore, it is important to use some kind of sparse matrix structure for the matrices that occur.

When ν_{ij} is stored sparsely, for example, the updating step is reduced from $O(N)$ to $O(1)$.

Next, we note that most a_j's are unchanged after each reaction occurs. After a reaction k, for example, the only a_j's that need to be recomputed are those with reactants (inputs) that were changed by a nonzero entry in the kth column of the ν_{jk} matrix. A way to formalize this is by a *dependency graph* or *dependency matrix* G, whose component G_{jk} is nonzero only if reaction k changes a species that is an input to reaction j. With a moment of thought, you will figure out that the matrix G can be obtained by the logical matrix multiplication of λ^T and ν, namely

$$G_{ij} = \bigcup_k \lambda_{ki} \cap \nu_{kj} \tag{17.7.8}$$

where $\cup$ denotes logical-or, $\cap$ denotes logical-and, and the C convention of "true iff nonzero" is assumed. Now, after each reaction j, we only update the a_i's indicated by the jth column of G_{ij}. Of course we must also store G in a sparse format.

Finally, there is the question of how to speed up the choice of which reaction to update, equation (17.7.7), which can be at worst $O(M)$. Here there are two schools of thought. The one that we implement below, following advice in [3], takes advantage of the fact that for many, if not most, actual applications, a small number of reactions ($\ll M$) dominate the reaction rates. If we arrange the order of the a_j's in equation (17.7.7) with these dominant reactions first, then it can take, on average, only O(1) tests to select the next reaction. In [3], it is suggested to do preliminary runs to find which reactions dominate. We prefer the more transparent alternative, implemented below, of just letting frequent reactions adaptively bubble up in a priority list.

The other school of thought, called the *next reaction method* [2], is discussed separately, below. It cleverly changes $O(M)$ to something like $O(\log M)$, even in the most unfavorable case. However, the number of operations in the inner program loop is significantly larger than for the (optimized, as above) direct method. Which method is fastest is very likely problem- and implementation-dependent.

For the modest test case illustrated, namely the set of three reactions (17.7.1), most of the optimizations illustrated in the following code are unnecessary, and likely counterproductive. However, the intent is to be illustrative of what a code for larger problems would look like.

stochsim.h

```
struct Stochsim {
Object for stochastic simulation of a set of chemical reactions.
    VecDoub s;                                  Vector of species numbers.
    VecDoub a;                                  Vector of rates.
    MatDoub instate, outstate;
    NRvector<NRsparseCol> outchg, depend;       Sparse matrices ν_ij and G_ij
    VecInt pr;                                  Priority list.
    Doub t, asum;
    Ran ran;
    typedef Doub(Stochsim::*rateptr)();         Obscure C++ used to create a vec-
    rateptr *dispatch;                              tor dispatch of function point-
                                                    ers to the rate functions.
    // begin user section
    Replace this section, using as a template the example (17.7.1) shown here, by the particulars
    of your reaction network. If you have a large number of reactions, you will want to generate
    the matrices instate and outstate externally, and pass them as globals (or read them
    here).
    static const Int mm=3;                      Set number of reactions.
    static const Int nn=4;                      Set number of species.
```

```
Doub k0,k1,k2;                                Declare any rate constants needed.
Doub rate0() {return k0*s[0]*s[1];}           Your rate functions go here.
Doub rate1() {return k1*s[1]*s[2];}
Doub rate2() {return k2*s[2];}
void describereactions () {
```
You provide a function with this name that sets any constants that you have defined and sets the instate and outstate matrices to describe your reactions.
```
    k0 = 0.01;
    k1 = .1;
    k2 = 1.;
    Doub indat[] = {                          The reactant matrix λ_ij.
        1.,0.,0.,
        1.,1.,0.,
        0.,1.,1.,
        0.,0.,0.
    };
    instate = MatDoub(nn,mm,indat);
    Doub outdat[] = {                         The state change matrix ν_ij.
        -1.,0.,0.,
        1.,-1.,0.,
        0.,1.,-1.,
        0.,0.,1.
    };
    outstate = MatDoub(nn,mm,outdat);
    dispatch[0] = &Stochsim::rate0;           You must also point the dispatch ta-
    dispatch[1] = &Stochsim::rate1;              ble entries to the correct rate
    dispatch[2] = &Stochsim::rate2;              functions.
}
// end user section

Stochsim(VecDoub &sinit, Int seed=1)
```
Constructor. Input initial species numbers and an optional random seed.
```
: s(sinit), a(mm,0.), outchg(mm), depend(mm), pr(mm), t(0.),
asum(0.), ran(seed), dispatch(new rateptr[mm]) {
    Int i,j,k,d;
    describereactions();
    sparmatfill(outchg,outstate);
    MatDoub dep(mm,mm);
    for (i=0;i<mm;i++) for (j=0;j<mm;j++) {  Logical matrix multiply calculates the
        d = 0;                                   dependency matrix.
        for (k=0;k<nn;k++) d = d || (instate[k][i] && outstate[k][j]);
        dep[i][j] = d;
    }
    sparmatfill(depend,dep);
    for (i=0;i<mm;i++) {                      Calculate all initial rates.
        pr[i] = i;
        a[i] = (this->*dispatch[i])();
        asum += a[i];
    }
}
~Stochsim() {delete [] dispatch;}

Doub step() {
```
Take a single stochastic step (one reaction) and return the new time.
```
    Int i,n,m,k=0;
    Doub tau,atarg,sum,anew;
    if (asum == 0.) {t *= 2.; return t;}     Rare: All reactions have stopped ex-
    tau = -log(ran.doub())/asum;                 actly, so double the time until
    atarg = ran.doub()*asum;                     the user notices!
    sum = a[pr[0]];
    while (sum < atarg) sum += a[pr[++k]];    Equation (17.7.7).
    m = pr[k];
    if (k > 0) SWAP(pr[k],pr[k-1]);           Move reaction up on the priority list.
    if (k == mm-1) asum = sum;                Free update of asum fixes accumu-
                                                 lated roundoff.
```

```
        n = outchg[m].nvals;
        for (i=0;i<n;i++) {                          Apply state change vector.
            k = outchg[m].row_ind[i];
            s[k] += outchg[m].val[i];
        }
        n = depend[m].nvals;
        for (i=0;i<n;i++) {                          Recalculate rates required by depen-
            k = depend[m].row_ind[i];                    dency matrix.
            anew = (this->*dispatch[k])();
            asum += (anew - a[k]);
            a[k] = anew;
        }
        if (t*asum < 0.1)                            Rare: Rates heading toward zero.
            for (asum=0.,i=0;i<mm;i++) asum += a[i];      Better recalculate asum.
        return (t += tau);
    }
};
```

Note that Stochsim uses some arcane C++ syntax ("array of pointers to member functions") in connection with the identifier dispatch. The underlying idea is simple, and important: We want to jump directly to the appropriate user-supplied rate function, as directed by an integer index. There are various ways of coding this, but what you *don't* want to have is a long chain of if tests that would be $O(M)$ instead of $O(1)$. (Perhaps we should believe that C's switch statement is always properly implemented by compilers as a fast table dispatch, but we don't.)

The utility routine that constructs a sparse matrix out of a full one is this (cf. §2.7):

stochsim.h

```
void sparmatfill(NRvector<NRsparseCol> &sparmat, MatDoub &fullmat) {
Utility that fills a sparse matrix from a full one. See §2.7.
    Int n,m,nz,nn=fullmat.nrows(),mm=fullmat.ncols();
    if (sparmat.size() != mm) throw("bad sizes");
    for (m=0;m<mm;m++) {
        for (nz=n=0;n<nn;n++) if (fullmat[n][m]) nz++;
        sparmat[m].resize(nn,nz);
        for (nz=n=0;n<nn;n++) if (fullmat[n][m]) {
            sparmat[m].row_ind[nz] = n;
            sparmat[m].val[nz++] = fullmat[n][m];
        }
    }
}
```

As a cultural note, the system (17.7.1) is not just any old chemical reaction network, but is actually a form of the *Lotka-Volterra* equation, discovered independently by Alfred J. Lotka and Vito Volterra in 1925–1926. In fact, it's not originally a chemical reaction network at all, but a set of relationships intended to model predator-prey relationships. The first equation says, roughly, that rabbits (X) eat grass (A) to produce more rabbits. The second says that foxes (Y) eat rabbits (X) to produce more foxes. The third says that foxes don't live forever. (For some reason, rabbits do live forever in this model, unless they are eaten by foxes.)

Figure 17.7.1 shows an example of the system's evolution, starting with initial conditions $A = 150$, $X = Y = 10$, and $B = 0$. One sees two cycles of prey population growth, with predator growth following, and then a collapse of both populations. After the second cycle, by a fluctuation, the predator population goes to zero, from which it cannot recover. At the end of the evolution shown, the prey population is starting to recover; but it is not a happy ending, because, by now, the food supply is running out. The world of stochastic simulation is a harsh one. Stochastic ef-

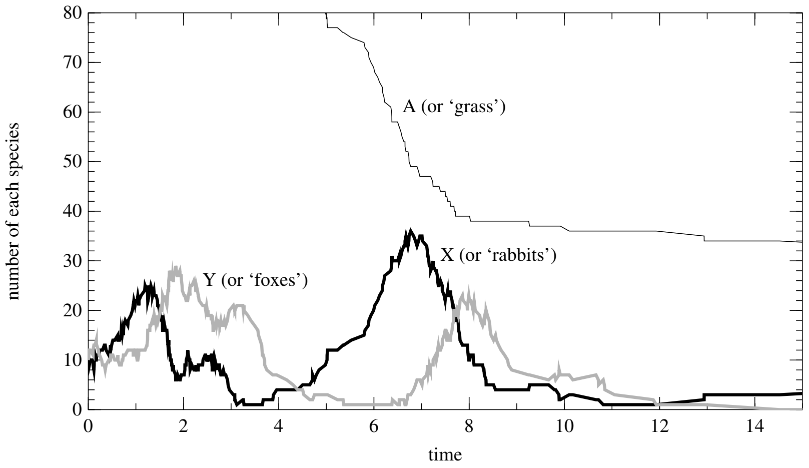

Figure 17.7.1. Evolution of the reaction network (17.7.1). This network evolves by the Lotka-Volterra equations, originally developed as a model for predator-prey interactions. Stochastic effects are important; with different random seeds, different time histories occur.

fects are genuinely dominant in this example: Exactly the same equations and initial conditions, but with a different random seed, give entirely different evolutions.

17.7.2 Next Reaction Method

The *next reaction method* [2] starts by computing not a single reaction time, equation (17.7.6), but rather a separate next reaction time for each reaction j,

$$\tau_j = \frac{1}{a_j} \log\left(\frac{1}{U_j}\right) \tag{17.7.9}$$

where the U_j's are independent uniform random deviates in $[0, 1]$. These times are all stored in a heap (see §8.3), so that the smallest value can be easily accessed at the top of the heap (call it k). The following steps are now repeatedly executed:

- Do reaction k and update the affected S_i's (using the matrix ν_{ik}). Increment time t by τ_k.
- Compute a next time for reaction k (using equation 17.7.9 and adding t) and store it on the heap.
- For every affected reaction j (as determined by a nonzero entry G_{jk}), correct its stored next time by the formula

$$\tau_j \leftarrow \frac{a_{j,\text{old}}}{a_{j,\text{new}}}(\tau_j - t) + t \tag{17.7.10}$$

  This is called *time reuse*. In effect, it reuses the random deviate U_j that originally generated τ_j, but it corrects the resulting time prediction for an intermediate step function change in a_j. Sounds dodgy, we know, but it is probabilistically sound.
- Get the heap back into order by bubbling elements up or down as required. This is where the complexity of the inner loop gets increased, to as much as $O(\log M)$.

Unquestionably, one can construct reaction networks for which the next reaction method is considerably faster than the optimized direct method. However, networks dominated by a small number of fast reactions are so common in practice that this performance advantage should not be assumed a priori [3].

17.7.3 Practical Advice

Don't ever use a stochastic simulation method — of any flavor — unless your problem is genuinely stochastic. Instead, use the deterministic rate equations (17.7.4) with a good stiff equation solver like StepperSie in §17.5. Such solvers are not limited by the rate of the fastest reaction, and will frequently be orders of magnitude faster than any stochastic method. (We are reliably informed that an unconscionable number of CPU hours are wasted by misguided researchers who think that the stochastic simulation method is an all-purpose tool for reaction networks.)

Just to show you how easy this is, here is how you would do the Lotka-Volterra problem (17.7.2) by integrating the equations directly. First encode the right-hand side $\mathbf{f}$ and the Jacobian of the right-hand side in a structure. (The (i, j) element of the Jacobian is $\partial f_i / \partial y_j$.)

```
struct rhs {
    Doub k0,k1,k2;
    rhs(Doub kk0, Doub kk1, Doub kk2) : k0(kk0),k1(kk1),k2(kk2) {}
    void operator() (const Doub x, VecDoub_I &y, VecDoub_O &dydx) {
        dydx[0]= -k0*y[0]*y[1];
        dydx[1]= k0*y[0]*y[1]-k1*y[1]*y[2];
        dydx[2]= k1*y[1]*y[2]-k2*y[2];
        dydx[3]= k2*y[2];
    }
    void jacobian(const Doub x, VecDoub_I &y, VecDoub_O &dfdx,
          MatDoub_O &dfdy) {
        Int n=y.size();
        for (Int i=0;i<n;i++) dfdx[i]=0.0;
        dfdy[0][0] = -k0*y[1];
        dfdy[0][1] = -k0*y[0];
        dfdy[0][2] = 0.0;
        dfdy[0][3] = 0.0;
        dfdy[1][0] = k0*y[1];
        dfdy[1][1] = k0*y[0]-k1*y[2];
        dfdy[1][2] = -k1*y[1];
        dfdy[1][3] = 0.0;
        dfdy[2][0] = 0.0;
        dfdy[2][1] = k1*y[2];
        dfdy[2][2] = k1*y[1]-k2;
        dfdy[2][3] = 0.0;
        dfdy[3][0] = 0.0;
        dfdy[3][1] = 0.0;
        dfdy[3][2] = k2;
        dfdy[3][3] = 0.0;
    }
};
```

Next set the parameters for Odeint, for example

```
const Int n=4;
Doub rtol=1.0e-7,atol=1.0e-4*rtol,h1=1.0e-6,hmin=0.0,x1=0.0,x2=15.0;
VecDoub ystart(n);
ystart[0]=150.0;
ystart[1]=10.0;
```

```
ystart[2]=10.0;
ystart[3]=0.0;
Output out(100);                          Output at 100 uniform points
rhs d(0.01,0.1,1.0);                      Declare d as a rhs object.
Odeint<StepperSIE<rhs> > ode(ystart,x1,x2,atol,rtol,h1,hmin,out,d);
ode.integrate();
```

Note how the values of k_0, k_1, and k_2 are passed as arguments in the constructor call that declares d. These particular values don't make the system of equations particularly stiff, so you could use a standard integrator. However, this is not true in general for real-world examples.

The output, which is equally spaced, can be printed by statements like

```
for (Int i=0;i<out.count;i++)
    cout << out.xsave[i] << " " << out.ysave[0][i] << " " <<
        out.ysave[1][i] << " " << out.ysave[2][i] << endl;
```

If your network's fastest reactions are not stochastic, but there are some slower reactions where stochastic effects are important, then look into so-called hybrid methods (e.g., [4]).

CITED REFERENCES AND FURTHER READING:

Gillespie, D.T. 1976, "A General Method for Numerically Simulating the Stochastic Time Evolution of Coupled Chemical Reactions," *Journal of Computational Physics*, vol. 11, pp. 403–434.[1]

Gibson, M.A., and Bruck, J. 2000, "Efficient Exact Stochastic Simulation of Chemical Systems with Many Species and Many Channels," *Journal of Physical Chemistry A*, vol. 104, pp. 1876–1889.[2]

Cao, Y., Li, H., and Petzold, L. 2004, "Efficient Formulation of the Stochastic Simulation Algorithm for Chemically Reacting Systems," *Journal of Chemical Physics*, vol. 121, pp. 4059–4067.[3]

Salis, H., and Kaznessis, Y. 2005, "Accurate Hybrid Stochastic Simulation of a System of Coupled Chemical or Biochemical Reactions," *Journal of Chemical Physics*, vol. 122, art. 054103.[4]

CHAPTER 18

Two-Point Boundary Value Problems

18.0 Introduction

When ordinary differential equations are required to satisfy boundary conditions at more than one value of the independent variable, the resulting problem is called a *two-point boundary value problem.* As the terminology indicates, the most common case by far is where boundary conditions are supposed to be satisfied at two points — usually the starting and ending values of the integration. However, the phrase "two-point boundary value problem" is also used loosely to include more complicated cases, e.g., where some conditions are specified at endpoints, others at interior (usually singular) points.

The crucial distinction between initial value problems (Chapter 17) and two-point boundary value problems (this chapter) is that in the former case we are able to start an acceptable solution at its beginning (initial values) and just march it along by numerical integration to its end (final values), while in the present case the boundary conditions at the starting point do not determine a unique solution to start with — and a "random" choice among the solutions that satisfy these (incomplete) starting boundary conditions is almost certain *not* to satisfy the boundary conditions at the other specified point(s).

It should not surprise you that iteration is in general required to meld these spatially scattered boundary conditions into a single global solution of the differential equations. For this reason, two-point boundary value problems require considerably more effort to solve than do initial value problems. You have to integrate your differential equations over the interval of interest, or perform an analogous "relaxation" procedure (see below), at least several, and sometimes very many, times. Only in the special case of linear differential equations can you say in advance just how many such iterations will be required.

The "standard" two-point boundary value problem has the following form: We desire the solution to a set of N coupled first-order ordinary differential equations, satisfying n_1 boundary conditions at the starting point x_1 and a remaining set of $n_2 = N - n_1$ boundary conditions at the final point x_2. (Recall that all differential equations of order higher than first can be written as coupled sets of first-order

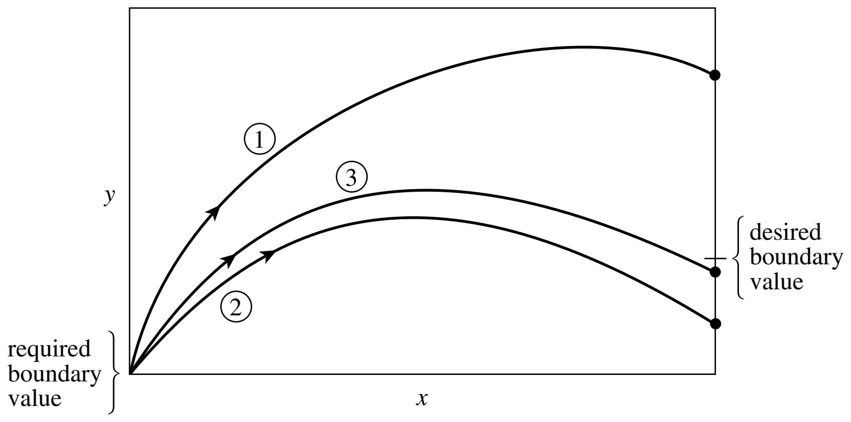

Figure 18.0.1. Shooting method (schematic). Trial integrations that satisfy the boundary condition at one endpoint are "launched." The discrepancies from the desired boundary condition at the other endpoint are used to adjust the starting conditions, until boundary conditions at both endpoints are ultimately satisfied.

equations; cf. §17.0.)

The differential equations are

$$\frac{dy_i(x)}{dx} = g_i(x, y_0, y_1, \ldots, y_{N-1}) \qquad i = 0, 1, \ldots, N-1 \tag{18.0.1}$$

At x_1, the solution is supposed to satisfy

$$B_{1j}(x_1, y_0, y_1, \ldots, y_{N-1}) = 0 \qquad j = 0, \ldots, n_1 - 1 \tag{18.0.2}$$

while at x_2, it is supposed to satisfy

$$B_{2k}(x_2, y_0, y_1, \ldots, y_{N-1}) = 0 \qquad k = 0, \ldots, n_2 - 1 \tag{18.0.3}$$

There are two distinct classes of numerical methods for solving two-point boundary value problems. In the *shooting method* (§18.1) we choose values for all of the dependent variables at one boundary. These values must be consistent with any boundary conditions for *that* boundary, but otherwise are arranged to depend on arbitrary free parameters whose values we initially "randomly" guess. We then integrate the ODEs by initial value methods, arriving at the other boundary (and/or any interior points with boundary conditions specified). In general, we find discrepancies from the desired boundary values there. Now we have a multidimensional root-finding problem, as was treated in §9.6 and §9.7: Find the adjustment of the free parameters at the starting point that zeros the discrepancies at the other boundary point(s). If we liken integrating the differential equations to following the trajectory of a shot from gun to target, then picking the initial conditions corresponds to aiming (see Figure 18.0.1). The shooting method provides a systematic approach to taking a set of "ranging" shots that allow us to improve our "aim" systematically.

As another variant of the shooting method (§18.2), we can guess unknown free parameters at both ends of the domain, integrate the equations to a common midpoint, and seek to adjust the guessed parameters so that the solution joins "smoothly" at the fitting point. In all shooting methods, trial solutions satisfy the differential

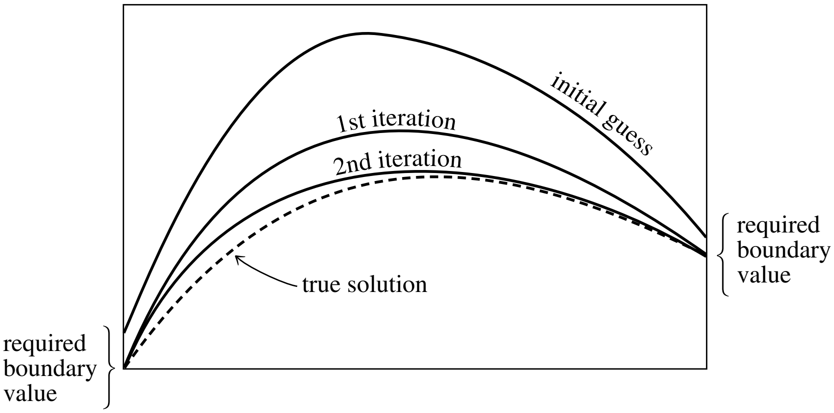

Figure 18.0.2. Relaxation method (schematic). An initial solution is guessed that approximately satisfies the differential equation and boundary conditions. An iterative process adjusts the function to bring it into close agreement with the true solution.

equations "exactly" (or as exactly as we care to make our numerical integration), but the trial solutions come to satisfy the required boundary conditions only after the iterations are finished.

Relaxation methods use a different approach. The differential equations are replaced by finite difference equations on a mesh of points that covers the range of the integration. A trial solution consists of values for the dependent variables at each mesh point, *not* satisfying the desired finite difference equations, nor necessarily even satisfying the required boundary conditions. The iteration, now called *relaxation*, consists of adjusting all the values on the mesh so as to bring them into successively closer agreement with the finite difference equations and, simultaneously, with the boundary conditions (see Figure 18.0.2). For example, if the problem involves three coupled equations and a mesh of 100 points, we must guess and improve 300 variables representing the solution.

With all this adjustment, you may be surprised that relaxation is ever an efficient method, but (for the right problems) it really is! Relaxation works better than shooting when the boundary conditions are especially delicate or subtle, or where they involve complicated algebraic relations that cannot easily be solved in closed form. Relaxation works best when the solution is smooth and not highly oscillatory. Such oscillations would require many grid points for accurate representation. The number and position of required points may not be known a priori. Shooting methods are usually preferred in such cases, because their variable stepsize integrations adjust naturally to a solution's peculiarities.

Relaxation methods are often preferred when the ODEs have extraneous solutions that, while not appearing in the final solution satisfying all boundary conditions, may wreak havoc on the initial value integrations required by shooting. The typical case is that of trying to maintain a dying exponential in the presence of growing exponentials.

Good initial guesses are the secret of efficient relaxation methods. Often one has to solve a problem many times, each time with a slightly different value of some parameter. In that case, the previous solution is usually a good initial guess when the

parameter is changed, and relaxation will work well.

Until you have enough experience to make your own judgment between the two methods, you might wish to follow the advice of your authors, who are notorious computer gunslingers: We always shoot first, and only then relax.

18.0.1 Problems Reducible to the Standard Boundary Problem

There are two important problems that can be reduced to the standard boundary value problem described by equations (18.0.1) – (18.0.3). The first is the *eigenvalue problem for differential equations*. Here the right-hand side of the system of differential equations depends on a parameter λ,

$$\frac{dy_i(x)}{dx} = g_i(x, y_0, \dots, y_{N-1}, \lambda) \tag{18.0.4}$$

and one has to satisfy $N + 1$ boundary conditions instead of just N. The problem is overdetermined and in general there is no solution for arbitrary values of λ. For certain special values of λ, the eigenvalues, equation (18.0.4) does have a solution.

We reduce this problem to the standard case by introducing a new dependent variable

$$y_N \equiv \lambda \tag{18.0.5}$$

and another differential equation

$$\frac{dy_N}{dx} = 0 \tag{18.0.6}$$

An example of this trick is given in §18.4.

The other case that can be put in the standard form is a *free boundary problem*. Here only one boundary abscissa x_1 is specified, while the other boundary x_2 is to be determined so that the system (18.0.1) has a solution satisfying a total of $N + 1$ boundary conditions. Here we again add an extra constant dependent variable:

$$y_N \equiv x_2 - x_1 \tag{18.0.7}$$

$$\frac{dy_N}{dx} = 0 \tag{18.0.8}$$

We also define a new *independent* variable t by setting

$$x - x_1 \equiv t y_N, \qquad 0 \le t \le 1 \tag{18.0.9}$$

The system of $N + 1$ differential equations for dy_i/dt is now in the standard form, with t varying between the known limits 0 and 1.

CITED REFERENCES AND FURTHER READING:

Keller, H.B. 1968, *Numerical Methods for Two-Point Boundary-Value Problems*; reprinted 1991 (New York: Dover).

Kippenhan, R., Weigert, A., and Hofmeister, E. 1968, in *Methods in Computational Physics*, vol. 7 (New York: Academic Press), pp. 129ff.

Eggleton, P.P. 1971, "The Evolution of Low Mass Stars," *Monthly Notices of the Royal Astronomical Society*, vol. 151, pp. 351–364.

London, R.A., and Flannery, B.P. 1982, "Hydrodynamics of X-Ray Induced Stellar Winds," *Astrophysical Journal*, vol. 258, pp. 260–269.

Stoer, J., and Bulirsch, R. 2002, *Introduction to Numerical Analysis*, 3rd ed. (New York: Springer), §7.3 – §7.4.

18.1 The Shooting Method

In this section we discuss "pure" shooting, where the integration proceeds from x_1 to x_2, and we try to match boundary conditions at the end of the integration. In the next section, we describe shooting to an intermediate fitting point, where the solution to the equations and boundary conditions is found by launching "shots" from both sides of the interval and trying to match continuity conditions at some intermediate point.

Our implementation of the shooting method exactly implements multidimensional, globally convergent Newton-Raphson (§9.7). It seeks to zero n_2 functions of n_2 variables. The functions are obtained by integrating N differential equations from x_1 to x_2. Let us see how this works.

At the starting point x_1 there are N starting values y_i to be specified, but subject to n_1 conditions. Therefore there are $n_2 = N - n_1$ *freely specifiable* starting values. Let us imagine that these freely specifiable values are the components of a vector $\mathbf{V}$ that lives in a vector space of dimension n_2. Then you, the user, knowing the functional form of the boundary conditions (18.0.2), can write a function or functor that generates a complete set of N starting values $\mathbf{y}$, satisfying the boundary conditions at x_1, from an arbitrary vector value of $\mathbf{V}$ in which there are no restrictions on the n_2 component values. In other words, (18.0.2) converts to a prescription

$$y_i(x_1) = y_i(x_1; V_0, \ldots, V_{n_2-1}) \qquad i = 0, \ldots, N-1 \tag{18.1.1}$$

In the routine `Shoot` below, the function or functor that implements (18.1.1) will be called `load`, but you can pass it as an argument to the routine with any name of your choosing.

Notice that the components of $\mathbf{V}$ might be exactly the values of certain "free" components of $\mathbf{y}$, with the other components of $\mathbf{y}$ determined by the boundary conditions. Alternatively, the components of $\mathbf{V}$ might parametrize the solutions that satisfy the starting boundary conditions in some other convenient way. Boundary conditions often impose algebraic relations among the y_i, rather than specific values for each of them. Using some auxiliary set of parameters often makes it easier to "solve" the boundary relations for a consistent set of y_i's. It makes no difference which way you go, as long as your vector space of $\mathbf{V}$'s generates (through 18.1.1) all allowed starting vectors $\mathbf{y}$.

Given a particular $\mathbf{V}$, a particular $\mathbf{y}(x_1)$ is thus generated. It can then be turned into a $\mathbf{y}(x_2)$ by integrating the ODEs to x_2 as an initial value problem (e.g., using Chapter 17's `Odeint`). Now, at x_2, let us define a *discrepancy vector* $\mathbf{F}$, also of dimension n_2, whose components measure how far we are from satisfying the n_2

boundary conditions at x_2 (18.0.3). Simplest of all is just to use the right-hand sides of (18.0.3),

$$F_k = B_{2k}(x_2, \mathbf{y}) \qquad k = 0, \ldots, n_2 - 1 \tag{18.1.2}$$

As in the case of $\mathbf{V}$, however, you can use any other convenient parametrization, as long as your space of $\mathbf{F}$'s spans the space of possible discrepancies from the desired boundary conditions, with all components of $\mathbf{F}$ equal to zero if and only if the boundary conditions at x_2 are satisfied. Below, you will be asked to supply a user-written function or functor that uses (18.0.3) to convert an N-vector of ending values $\mathbf{y}(x_2)$ into an n_2-vector of discrepancies $\mathbf{F}$. Inside `Shoot`, this function is called `score`.

Now, as far as Newton-Raphson is concerned, we are nearly in business. We want to find a vector value of $\mathbf{V}$ that zeros the vector value of $\mathbf{F}$. We do this by invoking the globally convergent Newton's method implemented in the routine `newt` of §9.7. Recall that the heart of Newton's method involves solving the set of n_2 linear equations

$$\mathbf{J} \cdot \delta\mathbf{V} = -\mathbf{F} \tag{18.1.3}$$

and then adding the correction back,

$$\mathbf{V}^{\text{new}} = \mathbf{V}^{\text{old}} + \delta\mathbf{V} \tag{18.1.4}$$

In (18.1.3), the Jacobian matrix $\mathbf{J}$ has components given by

$$J_{ij} = \frac{\partial F_i}{\partial V_j} \tag{18.1.5}$$

It is not feasible to compute these partial derivatives analytically. Rather, each requires a *separate* integration of the N ODEs, followed by the evaluation of

$$\frac{\partial F_i}{\partial V_j} \approx \frac{F_i(V_0, \ldots, V_j + \Delta V_j, \ldots) - F_i(V_0, \ldots, V_j, \ldots)}{\Delta V_j} \tag{18.1.6}$$

This is done automatically for you in the functor `NRfdjac` that comes with `newt`. The only input to `newt` that you have to provide is the routine `vecfunc` that calculates $\mathbf{F}$ by integrating the ODEs. Here is the appropriate routine, a functor called `Shoot`, that is to be passed as the actual argument in `newt`:

shoot.h
```
template <class L, class R, class S>
struct Shoot {
```
Functor for use with `newt` to solve a two-point boundary value problem by shooting.
```
    Int nvar;                          Number of coupled ODEs.
    Doub x1,x2;                        Start and end points.
    L &load;                           Supplies initial values for ODEs from v[0..n2-1].
    R &d;                              Supplies derivative information to the ODE integrator.
    S &score;                          Returns the n2 functions that ought to be zero to satisfy
    Doub atol,rtol;                        the boundary conditions at x2.
    Doub h1,hmin;
    VecDoub y;
    Shoot(Int nvarr, Doub xx1, Doub xx2, L &loadd, R &dd, S &scoree) :
        nvar(nvarr), x1(xx1), x2(xx2), load(loadd), d(dd),
        score(scoree), atol(1.0e-14), rtol(atol), hmin(0.0), y(nvar) {}
```
Routine for use with `newt` to solve a two-point boundary value problem for `nvar` coupled ODEs by shooting from `x1` to `x2`. Initial values for the `nvar` ODEs at `x1` are generated from the `n2` input coefficients `v[0..n2-1]`, using the user-supplied routine `load`.
```
    VecDoub operator() (VecDoub_I &v) {
```
This is the functor used by `newt`. It integrates the ODEs to `x2` using an eighth-order Runge-Kutta method with absolute and relative tolerances `atol` and `rtol`, initial stepsize `h1`, and

minimum stepsize hmin. At x2 it calls the user-supplied routine score and returns the n2 functions that ought to be zero. newt uses a globally convergent Newton's method to adjust the values of v until the returned functions are in fact zero.

```
        h1=(x2-x1)/100.0;
        y=load(x1,v);
        Output out;                  No output generated by Odeint.
        Odeint<StepperDopr853<R> > integ(y,x1,x2,atol,rtol,h1,hmin,out,d);
        integ.integrate();
        return score(x2,y);
    }
};
```

Note that Shoot is templated on the load, right-hand side for Odeint, and score routines. In practice, you will almost always want to write these as functors rather than functions. This makes communicating the various parameters in the problem easy — just pass them as parameters in the constructors.

For some problems the initial stepsize ΔV might depend sensitively upon the initial conditions. It is straightforward to alter load to compute a suggested stepsize h1 as a member variable and feed it fist to Shoot and hence to NRfdjac when the Shoot object is passed to newt.

A complete cycle of the shooting method thus requires $n_2 + 1$ integrations of the N coupled ODEs: one integration to evaluate the current degree of mismatch, and n_2 for the partial derivatives. Each new cycle requires a new round of $n_2 + 1$ integrations. This illustrates the enormous extra effort involved in solving two-point boundary value problems compared with initial value problems.

If the differential equations are *linear*, then only one complete cycle is required, since (18.1.3) – (18.1.4) should take us right to the solution. A second round can be useful, however, in mopping up some (never all) of the roundoff error.

As given here, Shoot uses the high-efficiency eighth-order Runge-Kutta method of §17.2 to integrate the ODEs, but any of the other methods of Chapter 17 could just as well be used.

You, the user, must supply Shoot with: (i) a function or functor load(x1,v) that returns the n-vector y[0..n-1] (satisfying the starting boundary conditions, of course), given the freely specifiable variables of v[0..n2-1] at the initial point x1; (ii) a function or functor score(x2,y) that returns the discrepancy vector f[0..n2-1] of the ending boundary conditions, given the vector y[0..n-1] at the endpoint x2; (iii) a starting vector v[0..n2-1]; (iv) a function or functor, called d in the routine, for the ODE integration; and other obvious parameters as described in the header comment above.

In §18.4 we give a sample program illustrating how to use Shoot.

CITED REFERENCES AND FURTHER READING:

Acton, F.S. 1970, *Numerical Methods That Work*; 1990, corrected edition (Washington, DC: Mathematical Association of America).

Keller, H.B. 1968, *Numerical Methods for Two-Point Boundary-Value Problems*; reprinted 1991 (New York: Dover).

18.2 Shooting to a Fitting Point

The shooting method described in §18.1 tacitly assumed that the "shots" would be able to traverse the entire domain of integration, even at the early stages of convergence to a correct solution. In some problems it can happen that, for very wrong starting conditions, an initial solution can't even get from x_1 to x_2 without encountering some incalculable, or catastrophic, result. For example, the argument of a square root might go negative, causing the numerical code to crash. Simple shooting would be stymied.

A different, but related, case is where the endpoints are both singular points of the set of ODEs. One frequently needs to use special methods to integrate near the singular points, analytic asymptotic expansions, for example. In such cases it is feasible to integrate in the direction *away* from a singular point, using the special method to get through the first little bit and then reading off "initial" values for further numerical integration. However, it is generally not feasible to integrate *into* a singular point. Usually the desired boundary condition is that one wants a regular solution at the singular point, but integrating into a singularity is guaranteed to pick out a singular solution, which by definition is growing as one integrates inward. Any small numerical inaccuracy will include some admixture of the "wrong" solution, which grows and swamps the desired solution.

The solution to the above-mentioned difficulties is *shooting to a fitting point*. Instead of integrating from x_1 to x_2, we integrate first from x_1 to some point x_f that is *between* x_1 and x_2; and second from x_2 (in the opposite direction) to x_f.

If (as before) the number of boundary conditions imposed at x_1 is n_1, and the number imposed at x_2 is n_2, then there are n_2 freely specifiable starting values at x_1 and n_1 freely specifiable starting values at x_2. (If you are confused by this, go back to §18.1.) We can therefore define an n_2-vector $\mathbf{V}^{(1)}$ of starting parameters at x_1 and a prescription `load1(x1,v1)` for mapping $\mathbf{V}^{(1)}$ into a $\mathbf{y}$ that satisfies the boundary conditions at x_1:

$$y_i(x_1) = y_i(x_1; V_0^{(1)}, \ldots, V_{n_2-1}^{(1)}) \qquad i = 0, \ldots, N-1 \tag{18.2.1}$$

Likewise we can define an n_1-vector $\mathbf{V}^{(2)}$ of starting parameters at x_2 and a prescription `load2(x2,v2)` for mapping $\mathbf{V}^{(2)}$ into a $\mathbf{y}$ that satisfies the boundary conditions at x_2:

$$y_i(x_2) = y_i(x_2; V_0^{(2)}, \ldots, V_{n_1-1}^{(2)}) \qquad i = 0, \ldots, N-1 \tag{18.2.2}$$

We thus have a total of N freely adjustable parameters in the combination of $\mathbf{V}^{(1)}$ and $\mathbf{V}^{(2)}$. The N conditions that must be satisfied are that there be agreement in N components of $\mathbf{y}$ at x_f between the values obtained integrating from one side and from the other,

$$y_i(x_f; \mathbf{V}^{(1)}) = y_i(x_f; \mathbf{V}^{(2)}) \qquad i = 0, \ldots, N-1 \tag{18.2.3}$$

In some problems, the N matching conditions can be better described (physically, mathematically, or numerically) by using N different functions F_i, $i = 0 \ldots N-1$, each possibly depending on the N components y_i. In those cases, (18.2.3) is replaced by

$$F_i[\mathbf{y}(x_f; \mathbf{V}^{(1)})] = F_i[\mathbf{y}(x_f; \mathbf{V}^{(2)})] \qquad i = 0, \ldots, N-1 \tag{18.2.4}$$

In the program below, a user-supplied function or functor, called `score(xf,y)` in the routine, is supposed to map an input N-vector $\mathbf{y}$ into an output N-vector $\mathbf{F}$. In most cases, you can simply use the identity mapping $\mathbf{F} = \mathbf{y}$.

Shooting to a fitting point uses globally convergent Newton-Raphson exactly as in §18.1. Comparing closely with the routine `Shoot` of the previous section, you should have no difficulty in understanding the following routine `Shootf`. The main differences in use are that you have to supply both `load1` and `load2`. Also, in the calling program you must supply initial guesses for `v1[0..n2-1]` and `v2[0..n1-1]`. Once again, a sample program illustrating shooting to a fitting point is given in §18.4.

shootf.h

```
template <class L1, class L2, class R, class S>
struct Shootf {
```
Functor for use with `newt` to solve a two-point boundary value problem by shooting to a fitting point.
```
    Int nvar,n2;                    nvar is the number of coupled ODEs.
    Doub x1,x2,xf;                  Start and end points and fitting point.
    L1 &load1;                      load1 and load2 supply initial values for the ODEs.
    L2 &load2;
    R &d;                           Supplies derivative information to the ODE integrator.
    S &score;                       Computes the mismatch of the solutions at the fitting
                                        point.
    Doub atol,rtol;
    Doub h1,hmin;
    VecDoub y,f1,f2;
    Shootf(Int nvarr, Int nn2,  Doub xx1, Doub xx2, Doub xxf, L1 &loadd1,
        L2 &loadd2, R &dd, S &scoree) : nvar(nvarr), n2(nn2), x1(xx1),
        x2(xx2), xf(xxf), load1(loadd1), load2(loadd2), d(dd),
        score(scoree), atol(1.0e-14), rtol(atol), hmin(0.0), y(nvar),
        f1(nvar), f2(nvar) {}
```
Routine for use with `newt` to solve a two-point boundary value problem for `nvar` coupled ODEs by shooting from `x1` and `x2` to a fitting point `xf`. Initial values for the `nvar` ODEs at `x1` are generated from the `n2` coefficients `v1` and the user-supplied routine `load1`. Likewise, those at `x2` are from the `n1=nvar-n2` coefficients `v2`, using `load2`. The coefficients `v1` and `v2` should be stored in a single array `v[0..nvar-1]` in the main program with `v1` in `v[0..n2-1]` and `v2` in `v[n2..nvar-1]`.
```
    VecDoub operator() (VecDoub_I &v) {
```
This is the functor used by `newt`. It integrates the ODEs to `xf` using an eighth-order Runge-Kutta method with absolute and relative tolerances `atol` and `rtol`, initial stepsize `h1`, and minimum stepsize `hmin`. At `xf` it calls the user-supplied routine `score` to evaluate the `nvar` functions `f1` and `f2` that ought to match at `xf`. The differences are returned on output. `newt` uses a globally convergent Newton's method to adjust the values of `v` until the differences are zero. A user-supplied function or functor `d` supplies derivative information to the ODE integrator (see Chapter 17).
```
        VecDoub v2(nvar-n2,&v[n2]);
        h1=(x2-x1)/100.0;
        y=load1(x1,v);              Path from x1 to xf with best trial values v1.
        Output out;                 No output generated by Odeint.
        Odeint<StepperDopr853<R> > integ1(y,x1,xf,atol,rtol,h1,hmin,out,d);
        integ1.integrate();
        f1=score(xf,y);
        y=load2(x2,v2);             Path from x2 to xf with best trial values v2.
        Odeint<StepperDopr853<R> > integ2(y,x2,xf,atol,rtol,h1,hmin,out,d);
        integ2.integrate();
        f2=score(xf,y);
        for (Int i=0;i<nvar;i++) f1[i] -= f2[i];
        return f1;
    }
};
```

There are boundary value problems where even shooting to a fitting point fails

— the integration interval has to be partitioned by several fitting points with the solution being matched at each such point. For more details see [1].

CITED REFERENCES AND FURTHER READING:

Acton, F.S. 1970, *Numerical Methods That Work*; 1990, corrected edition (Washington, DC: Mathematical Association of America).

Keller, H.B. 1968, *Numerical Methods for Two-Point Boundary-Value Problems*; reprinted 1991 (New York: Dover).

Stoer, J., and Bulirsch, R. 2002, *Introduction to Numerical Analysis*, 3rd ed. (New York: Springer), §7.3.5 – §7.3.6.[1]

18.3 Relaxation Methods

In *relaxation methods* we replace ODEs by approximate *finite difference equations* (FDEs) on a grid or mesh of points that spans the domain of interest. As a typical example, we could replace a general first-order differential equation

$$\frac{dy}{dx} = g(x, y) \tag{18.3.1}$$

with an algebraic equation relating function values at two points $k, k-1$:

$$y_k - y_{k-1} - (x_k - x_{k-1})\, g\left[\tfrac{1}{2}(x_k + x_{k-1}), \tfrac{1}{2}(y_k + y_{k-1})\right] = 0 \tag{18.3.2}$$

The form of the FDE in (18.3.2) illustrates the idea, but not uniquely: There are many ways to turn the ODE into an FDE. When the problem involves N coupled first-order ODEs represented by FDEs on a mesh of M points, a solution consists of values for N dependent functions given at each of the M mesh points, or $N \times M$ variables in all. The relaxation method determines the solution by starting with a guess and improving it, iteratively. As the iterations improve the solution, the result is said to *relax* to the true solution.

While several iteration schemes are possible, for most problems our old standby, multidimensional Newton's method, works well. The method produces a matrix equation that must be solved, but the matrix takes a special, "block diagonal" form that allows it to be inverted far more economically both in time and storage than would be possible for a general matrix of size $(MN) \times (MN)$. Since MN can easily be several thousand or more, this is crucial for the feasibility of the method.

Our implementation couples at most pairs of points, as in equation (18.3.2). More points can be coupled, but then the method becomes more complex. We will provide enough background so that you can write a more general scheme if you have the patience to do so.

Let us develop a general set of algebraic equations that represent the ODEs by FDEs. The ODE problem is exactly identical to that expressed in equations (18.0.1) – (18.0.3), where we had N coupled first-order equations that satisfy n_1 boundary conditions at one end of the interval and $n_2 = N - n_1$ boundary conditions at the other. We first define a mesh or grid by a set of $k = 0, 1, ..., M-1$ points at which we supply values for the independent variable x_k. In particular, x_0 is the initial boundary and x_{M-1} is the final boundary. We use the notation $\mathbf{y}_k$ to refer to the entire set of dependent variables $y_0, y_1, \dots, y_{N-1}$ at point x_k. At an arbitrary point k in the middle of the mesh, we approximate the set of N first-order ODEs by algebraic relations of the form

$$0 = \mathbf{E}_k \equiv \mathbf{y}_k - \mathbf{y}_{k-1} - (x_k - x_{k-1})\mathbf{g}_k(x_k, x_{k-1}, \mathbf{y}_k, \mathbf{y}_{k-1}), \quad k = 1, 2, \dots, M-1 \tag{18.3.3}$$

The notation signifies that $\mathbf{g}_k$ can be evaluated using information from both points $k, k-1$. The FDEs labeled by $\mathbf{E}_k$ provide N equations coupling $2N$ variables at points $k, k-1$. There

are $M - 1$ points, $k = 1, 2, \ldots, M - 1$, at which difference equations of the form (18.3.3) apply. Thus the FDEs provide a total of $(M - 1)N$ equations for the MN unknowns. The remaining N equations come from the boundary conditions.

At the first boundary we have

$$0 = \mathbf{E}_0 \equiv \mathbf{B}(x_0, \mathbf{y}_0) \tag{18.3.4}$$

while at the second boundary

$$0 = \mathbf{E}_M \equiv \mathbf{C}(x_{M-1}, \mathbf{y}_{M-1}) \tag{18.3.5}$$

The vectors $\mathbf{E}_0$ and $\mathbf{B}$ have only n_1 nonzero components, corresponding to the n_1 boundary conditions at x_0. It will turn out to be useful to take these nonzero components to be the *last* n_1 components. In other words, $E_{j,0} \neq 0$ only for $j = n_2, n_2 + 1, \ldots, N - 1$. At the other boundary, only the first n_2 components of $\mathbf{E}_M$ and $\mathbf{C}$ are nonzero: $E_{j,M} \neq 0$ only for $j = 0, 1, \ldots, n_2 - 1$.

The "solution" of the FDE problem in (18.3.3) – (18.3.5) consists of a set of variables $y_{j,k}$, the values of the N variables y_j at the M points x_k. The algorithm we describe below requires an initial guess for the $y_{j,k}$. We then determine increments $\Delta y_{j,k}$ such that $y_{j,k} + \Delta y_{j,k}$ is an improved approximation to the solution.

Equations for the increments are developed by expanding the FDEs in first-order Taylor series with respect to small changes $\Delta\mathbf{y}_k$. At an interior point, $k = 1, 2, \ldots, M-1$, this gives

$$\mathbf{E}_k(\mathbf{y}_k + \Delta\mathbf{y}_k, \mathbf{y}_{k-1} + \Delta\mathbf{y}_{k-1}) \approx \mathbf{E}_k(\mathbf{y}_k, \mathbf{y}_{k-1}) + \sum_{n=0}^{N-1} \frac{\partial \mathbf{E}_k}{\partial y_{n,k-1}} \Delta y_{n,k-1} + \sum_{n=0}^{N-1} \frac{\partial \mathbf{E}_k}{\partial y_{n,k}} \Delta y_{n,k} \tag{18.3.6}$$

For a solution we want the updated value $\mathbf{E}(\mathbf{y} + \Delta\mathbf{y})$ to be zero, so the general set of equations at an interior point can be written in matrix form as

$$\sum_{n=0}^{N-1} S_{j,n} \Delta y_{n,k-1} + \sum_{n=N}^{2N-1} S_{j,n} \Delta y_{n-N,k} = -E_{j,k}, \quad j = 0, 1, \ldots, N - 1 \tag{18.3.7}$$

where

$$S_{j,n} = \frac{\partial E_{j,k}}{\partial y_{n,k-1}}, \quad S_{j,n+N} = \frac{\partial E_{j,k}}{\partial y_{n,k}}, \quad n = 0, 1, \ldots, N - 1 \tag{18.3.8}$$

The quantity $S_{j,n}$ is an $N \times 2N$ matrix at each point k. Each interior point thus supplies a block of N equations coupling $2N$ corrections to the solution variables at the points $k, k - 1$.

Similarly, the algebraic relations at the boundaries can be expanded in a first-order Taylor series for increments that improve the solution. Since $\mathbf{E}_0$ depends only on $\mathbf{y}_0$, we find at the first boundary

$$\sum_{n=0}^{N-1} S_{j,n} \Delta y_{n,0} = -E_{j,0}, \quad j = n_2, n_2 + 1, \ldots, N - 1 \tag{18.3.9}$$

where

$$S_{j,n} = \frac{\partial E_{j,0}}{\partial y_{n,0}}, \quad n = 0, 1, \ldots, N - 1 \tag{18.3.10}$$

At the second boundary,

$$\sum_{n=0}^{N-1} S_{j,n} \Delta y_{n,M-1} = -E_{j,M}, \quad j = 0, 1, \ldots, n_2 - 1 \tag{18.3.11}$$

where

$$S_{j,n} = \frac{\partial E_{j,M}}{\partial y_{n,M-1}}, \quad n = 0, 1, \ldots, N - 1 \tag{18.3.12}$$

```
X X X X X                                V B
X X X X X                                V B
X X X X X                                V B
X X X X X X X X X X                      V B
X X X X X X X X X X                      V B
X X X X X X X X X X                      V B
X X X X X X X X X X                      V B
X X X X X X X X X X                      V B
          X X X X X X X X X X            V B
          X X X X X X X X X X            V B
          X X X X X X X X X X            V B
          X X X X X X X X X X            V B
          X X X X X X X X X X            V B
                    X X X X X X X X X X  V B
                    X X X X X X X X X X  V B
                    X X X X X X X X X X  V B
                    X X X X X X X X X X  V B
                    X X X X X X X X X X  V B
                              X X X X X  V B
                              X X X X X  V B
```

Figure 18.3.1. Matrix structure of a set of linear finite difference equations (FDEs) with boundary conditions imposed at both endpoints. Here X represents a coefficient of the FDEs, V represents a component of the unknown solution vector, and B is a component of the known right-hand side. Empty spaces represent zeros. The matrix equation is to be solved by a special form of Gaussian elimination. (See text for details.)

We thus have in equations (18.3.7) – (18.3.12) a set of linear equations to be solved for the corrections $\Delta\mathbf{y}$, iterating until the corrections are sufficiently small. The equations have a special structure, because each $S_{j,n}$ couples only points $k, k-1$. Figure 18.3.1 illustrates the typical structure of the complete matrix equation for the case of five variables and four mesh points, with three boundary conditions at the first boundary and two at the second. The 3×5 block of nonzero entries in the top left-hand corner of the matrix comes from the boundary condition $S_{j,n}$ at point $k = 0$. The next three 5×10 blocks are the $S_{j,n}$ at the interior points, coupling variables at mesh points (2,1), (3,2), and (4,3). Finally we have the block corresponding to the second boundary condition.

We can solve equations (18.3.7) – (18.3.12) for the increments $\Delta\mathbf{y}$ using a form of Gaussian elimination that exploits the special structure of the matrix to minimize the total number of operations, and that minimizes storage of matrix coefficients by packing the elements in a special blocked structure. (You might wish to review Chapter 2, especially §2.2, if you are unfamiliar with the steps involved in Gaussian elimination.) Recall that Gaussian elimination consists of manipulating the equations by elementary operations such as dividing rows of coefficients by a common factor to produce unity in diagonal elements, and adding appropriate multiples of other rows to produce zeros below the diagonal. Here we take advantage of the block structure by performing a bit more reduction than in pure Gaussian elimination, so that the storage of coefficients is minimized. Figure 18.3.2 shows the form that we wish to achieve by elimination, just prior to the backsubstitution step. Only a small subset of the reduced $MN \times MN$ matrix elements needs to be stored as the elimination progresses. Once the matrix elements reach the stage in Figure 18.3.2, the solution follows quickly by a backsubstitution procedure.

Furthermore, the entire procedure, except the backsubstitution step, operates only on one block of the matrix at a time. The procedure contains four types of operations: (1) partial reduction to zero of certain elements of a block using results from a previous step; (2) elimination of the square structure of the remaining block elements such that the square section contains unity along the diagonal, and zero in off-diagonal elements; (3) storage of the

1			X	X																V	B
	1		X	X																V	B
		1	X	X																V	B
			1					X	X											V	B
				1				X	X											V	B
					1			X	X											V	B
						1		X	X											V	B
							1	X	X											V	B
								1					X	X						V	B
									1				X	X						V	B
										1			X	X						V	B
											1		X	X						V	B
												1	X	X						V	B
													1					X	X	V	B
														1				X	X	V	B
															1			X	X	V	B
																1		X	X	V	B
																	1	X	X	V	B
																		1		V	B
																			1	V	B

Figure 18.3.2. Target structure of the Gaussian elimination. Once the matrix of Figure 18.3.1 has been reduced to this form, the solution follows quickly by backsubstitution.

(a)	D	D	D	A	A	V	A
	D	D	D	A	A	V	A
	D	D	D	A	A	V	A
(b)	1	0	0	S	S	V	S
	0	1	0	S	S	V	S
	0	0	1	S	S	V	S

Figure 18.3.3. Reduction process for the first (upper-left) block of the matrix in Figure 18.3.1. (a) Original form of the block, (b) final form. (See text for explanation.)

remaining nonzero coefficients for use in later steps; and (4) backsubstitution. We illustrate the steps schematically by figures.

Consider the block of equations describing corrections available from the initial boundary conditions. We have n_1 equations for N unknown corrections. We wish to transform the first block so that its left-hand $n_1 \times n_1$ square section becomes unity along the diagonal and zero in off-diagonal elements. Figure 18.3.3 shows the original and final forms of the first block of the matrix. In the figure we designate matrix elements that are subject to diagonalization by "D" and elements that will be altered by "A"; in the final block, elements that are stored are labeled by "S." We get from start to finish by selecting in turn n_1 "pivot" elements from among the first n_1 columns, normalizing the pivot row so that the value of the "pivot" element is unity, and adding appropriate multiples of this row to the remaining rows so that they contain zeros in the pivot column. In its final form, the reduced block expresses values for the corrections to the first n_1 variables at mesh point 0 in terms of values for the remaining n_2 unknown corrections at point 0, i.e., we now know what the first n_1 elements are in terms of the remaining n_2 elements. We store only the final set of n_2 nonzero columns from the initial block, plus the column for the altered right-hand side of the matrix equation.

We must emphasize here an important detail of the method. To exploit the reduced storage allowed by operating on blocks, it is essential that the ordering of columns in the s matrix of derivatives be such that pivot elements can be found among the first n_1 rows of

(a)	1	0	0	S	S						V	S
	0	1	0	S	S						V	S
	0	0	1	S	S						V	S
	Z	Z	Z	D	D	D	D	D	A	A	V	A
	Z	Z	Z	D	D	D	D	D	A	A	V	A
	Z	Z	Z	D	D	D	D	D	A	A	V	A
	Z	Z	Z	D	D	D	D	D	A	A	V	A
	Z	Z	Z	D	D	D	D	D	A	A	V	A

(b)	1	0	0	S	S						V	S
	0	1	0	S	S						V	S
	0	0	1	S	S						V	S
	0	0	0	1	0	0	0	0	S	S	V	S
	0	0	0	0	1	0	0	0	S	S	V	S
	0	0	0	0	0	1	0	0	S	S	V	S
	0	0	0	0	0	0	1	0	S	S	V	S
	0	0	0	0	0	0	0	1	S	S	V	S

Figure 18.3.4. Reduction process for intermediate blocks of the matrix in Figure 18.3.1. (a) Original form, (b) final form. (See text for explanation.)

the matrix. This means that the n_1 boundary conditions at the first point must contain some dependence on the first `j=0,1,...,`$n_1 - 1$ dependent variables, `y[j][0]`. If not, then the original square $n_1 \times n_1$ subsection of the first block will appear to be singular, and the method will fail. Alternatively, we would have to allow the search for pivot elements to involve all N columns of the block, and this would require column swapping and far more bookkeeping. The code provides a simple method of reordering the variables, i.e., the columns of the `s` matrix, so that this can be done easily. End of important detail.

Next consider the block of N equations representing the FDEs that describe the relation between the $2N$ corrections at points 1 and 0. The elements of that block, together with results from the previous step, are illustrated in Figure 18.3.4. Note that by adding suitable multiples of rows from the first block we can reduce to zero the first n_1 columns of the block (labeled by "Z"), and, to do so, we will need to alter only the columns from n_1 to $N - 1$ and the vector element on the right-hand side. Of the remaining columns we can diagonalize a square subsection of $N \times N$ elements, labeled by "D" in the figure. In the process we alter the final set of n_2 columns, denoted "A" in the figure. The second half of the figure shows the block when we finish operating on it, with the stored $n_2 \times N$ elements labeled by "S."

If we operate on the next set of equations corresponding to the FDEs coupling corrections at points 2 and 1, we see that the state of available results and new equations exactly reproduces the situation described in the previous paragraph. Thus, we can carry out those steps again for each block in turn through block $M - 1$. Finally on block M we encounter the remaining boundary conditions.

Figure 18.3.5 shows the final block of n_2 FDEs relating the N corrections for variables at mesh point $M - 1$, together with the result of reducing the previous block. Again, we can first use the prior results to zero the first n_1 columns of the block. Now, when we diagonalize the remaining square section, we strike gold: We get values for the final n_2 corrections at mesh point $M - 1$.

With the final block reduced, the matrix has the desired form shown previously in Figure 18.3.2, and the matrix is ripe for backsubstitution. Starting with the bottom row and working up toward the top, at each stage we can simply determine one unknown correction in terms of known quantities.

The object `Solvde` organizes the steps described above. The principal procedures used in the algorithm are performed by functions called internally by `Solvde`. The function `red` eliminates leading columns of the `s` matrix using results from prior blocks. `pinvs` diagonalizes the square subsection of `s` and stores unreduced coefficients. `bksub` carries out the

(a)	0	0	0	1	0	0	0	0	S	S	V	S
	0	0	0	0	1	0	0	0	S	S	V	S
	0	0	0	0	0	1	0	0	S	S	V	S
	0	0	0	0	0	0	1	0	S	S	V	S
	0	0	0	0	0	0	0	1	S	S	V	S
						Z	Z	Z	D	D	V	A
						Z	Z	Z	D	D	V	A
(b)	0	0	0	1	0	0	0	0	S	S	V	S
	0	0	0	0	1	0	0	0	S	S	V	S
	0	0	0	0	0	1	0	0	S	S	V	S
	0	0	0	0	0	0	1	0	S	S	V	S
	0	0	0	0	0	0	0	1	S	S	V	S
						0	0	0	1	0	V	S
						0	0	0	0	1	V	S

Figure 18.3.5. Reduction process for the last (lower-right) block of the matrix in Figure 18.3.1. (a) Original form, (b) final form. (See text for explanation.)

backsubstitution step. The user of `Solvde` must understand the calling arguments, as described below, and supply an object `Difeq`, called by `Solvde`, with a method `smatrix` that evaluates the `s` matrix for each block.

Most of the arguments in the constructor call to `Solvde` have already been described, but some require discussion. On input, array `y[j][k]` contains the initial guess for the solution, with `j` labeling the dependent variables at mesh points `k`. The problem involves `ne` FDEs spanning points `k=0,..., m-1`. `nb` boundary conditions apply at the first point `k=0`. The array `indexv[j]` establishes the correspondence between columns of the `s` matrix; equations (18.3.8), (18.3.10), and (18.3.12); and the dependent variables. As described above, it is essential that the `nb` boundary conditions at `k=0` involve the dependent variables referenced by the first `nb` columns of the `s` matrix. Thus, columns `j` of the `s` matrix can be ordered by the user in `Difeq` to refer to derivatives with respect to the dependent variable `indexv[j]`.

The function only attempts `itmax` correction cycles before returning, even if the solution has not converged. The parameters `conv`, `slowc`, and `scalv` relate to convergence. Each inversion of the matrix produces corrections for `ne` variables at `m` mesh points. We want these to become vanishingly small as the iterations proceed, but we must define a measure for the size of corrections. This error "norm" is very problem-specific, so the user might wish to rewrite this section of the code as appropriate. In the program below we compute a value for the average correction `err` by summing the absolute value of all corrections, weighted by a scale factor appropriate to each type of variable:

$$\mathtt{err} = \frac{1}{\mathtt{m} \times \mathtt{ne}} \sum_{\mathtt{k=0}}^{\mathtt{m-1}} \sum_{\mathtt{j=0}}^{\mathtt{ne-1}} \frac{|\Delta Y\mathtt{[j][k]}|}{\mathtt{scalv[j]}} \qquad (18.3.13)$$

When `err` $\leq$ `conv`, the method has converged. Note that the user gets to supply an array `scalv` that measures the typical size of each variable.

Obviously, if `err` is large, we are far from a solution, and perhaps it is a bad idea to believe that the corrections generated from a first-order Taylor series are accurate. The number `slowc` modulates the application of corrections. After each iteration we apply only a fraction of the corrections found by matrix inversion:

$$Y\mathtt{[j][k]} \rightarrow Y\mathtt{[j][k]} + \frac{\mathtt{slowc}}{\max(\mathtt{slowc},\mathtt{err})} \Delta Y\mathtt{[j][k]} \qquad (18.3.14)$$

Thus, when `err` $>$ `slowc` only a fraction of the corrections are used, but when `err` $\leq$ `slowc` the entire correction gets applied.

As already mentioned, the constructor initializes the array `y[0..ne-1][0..m-1]` in `Solvde` with the trial solution. Internally, workspace arrays `c[0..ne-1][0..ne-nb][0..m]`, `s[0..ne-1][0..2*ne]` are allocated. The array `c` is the blockbuster: It stores the unreduced elements of the matrix built up for the backsubstitution step. If there are `m` mesh points, then there will be `m+1` blocks, each requiring `ne` rows and `ne-nb+1` columns. Although large, this is small compared with $(\mathtt{ne} \times \mathtt{m})^2$ elements required for the whole matrix if we did not break it into blocks.

We now describe the workings of the user-supplied object `Difeq`. The constructor can be used to pass problem-specific information from your main program. The object must contain a method `smatrix` with the following declaration:

```
void smatrix(const Int k, const Int k1, const Int k2, const Int jsf,
    const Int is1, const Int isf, VecInt_I &indexv, MatDoub_O &s,
    MatDoub_I &y);
```

As the declaration shows, the only information passed from `Difeq` to `Solvde` is the matrix of derivatives `s[0..ne-1][0..2*ne]`; all other arguments are input to `smatrix` and should not be altered. `k` indicates the current mesh point, or block number. `k1`, `k2` label the first and last points in the mesh. If $\mathtt{k} = \mathtt{k1}$ or $\mathtt{k} > \mathtt{k2}$, the block involves the boundary conditions at the first or final point; otherwise the block acts on FDEs coupling variables at points `k-1`, `k`.

The convention on storing information into the array `s[i][j]` follows that used in equations (18.3.8), (18.3.10), and (18.3.12): Rows `i` label equations and columns `j` refer to derivatives with respect to dependent variables in the solution. Recall that each equation will depend on the `ne` dependent variables at either one or two points. Thus, `j` runs from `0` to either `ne-1` or `2*ne-1`. The column ordering for dependent variables at each point must agree with the list supplied in `indexv[j]`. Thus, for a block not at a boundary, the first column multiplies ΔY(`l=indexv[0],k-1`), and the column `ne` multiplies ΔY(`l=indexv[0],k`). The parameters `is1`, `isf` give the numbers of the starting and final *rows* that need to be filled in the `s` matrix for this block. `jsf` labels the column in which the difference equations $E_{j,k}$ of equations (18.3.3) – (18.3.5) are stored. Thus, $-$`s[i][jsf]` is the vector on the right-hand side of the matrix. The reason for the minus sign is that `smatrix` supplies the actual difference equation, $E_{j,k}$, not its negative. Note that `Solvde` supplies a value for `jsf` such that the difference equation is put in the column *just after* all derivatives in the `s` matrix. Thus, `smatrix` expects to find values entered into `s[i][j]` for rows $\mathtt{is1} \le \mathtt{i} \le \mathtt{isf}$ and $0 \le \mathtt{j} \le \mathtt{jsf}$.

Finally, the quantities `s[0..nsi-1][0..nsj-1]` and `y[0..nyj-1][0..nyk-1]` supply `smatrix` with storage for `s` and the values of the solution variables `y` for this iteration. An example of how to use this routine is given in the next section.

Detailed implementing code for `Solvde` is given in a Webnote [1], many ideas in which are due to Eggleton [2].

18.3.1 "Algebraically Difficult" Sets of Differential Equations

Relaxation methods allow you to take advantage of an additional opportunity that, while not obvious, can speed up some calculations enormously. It is not necessary that the set of variables $y_{j,k}$ correspond exactly with the dependent variables of the original differential equations. They can be related to those variables through algebraic equations. Obviously, it is necessary only that the solution variables allow us to *evaluate* the functions $y, g, \mathbf{B}, \mathbf{C}$ that are used to construct the FDEs from the ODEs. In some problems, g depends on functions of y that are known only implicitly, so that iterative solutions are necessary to evaluate functions in the ODEs. Often one can dispense with this "internal" nonlinear problem by defining a new set of variables from which both y, g and the boundary conditions can be obtained directly. A typical example occurs in physical problems where the equations require the solution of a complex equation of state that can be expressed in more convenient terms using variables other than the original dependent variables in the ODE. While this approach is analogous to performing an *analytic* change of variables directly on the original ODEs, such an analytic transformation might be prohibitively complicated. The change of variables in the relaxation method is easy and requires no analytic manipulations.

CITED REFERENCES AND FURTHER READING:

Numerical Recipes Software 2007, "Solvde Implementation," *Numerical Recipes Webnote No. 25*, at `http://www.nr.com/webnotes?25` [1]

Eggleton, P.P. 1971, "The Evolution of Low Mass Stars," *Monthly Notices of the Royal Astronomical Society*, vol. 151, pp. 351–364.[2]

Keller, H.B. 1968, *Numerical Methods for Two-Point Boundary-Value Problems*; reprinted 1991 (New York: Dover).

Kippenhan, R., Weigert, A., and Hofmeister, E. 1968, in *Methods in Computational Physics*, vol. 7 (New York: Academic Press), pp. 129ff.

18.4 A Worked Example: Spheroidal Harmonics

The best way to understand the algorithms of the previous sections is to see them employed to solve an actual problem. As a sample problem, we have selected the computation of spheroidal harmonics. (The more common name is spheroidal angle functions, but we prefer the explicit reminder of the kinship with spherical harmonics.) We will show how to find spheroidal harmonics, first by the method of relaxation (§18.3), and then by the methods of shooting (§18.1) and shooting to a fitting point (§18.2).

Spheroidal harmonics typically arise when certain partial differential equations are solved by separation of variables in spheroidal coordinates. They satisfy the following differential equation on the interval $-1 \leq x \leq 1$:

$$\frac{d}{dx}\left[(1-x^2)\frac{dS}{dx}\right] + \left(\lambda - c^2x^2 - \frac{m^2}{1-x^2}\right)S = 0 \qquad (18.4.1)$$

Here m is an integer, c is the "oblateness parameter," and λ is the eigenvalue. Despite the notation, c^2 can be positive or negative. For $c^2 > 0$, the functions are called "prolate," while if $c^2 < 0$ they are called "oblate." The equation has singular points at $x = \pm 1$ and is to be solved subject to the boundary conditions that the solution be regular at $x = \pm 1$. Only for certain values of λ, the eigenvalues, will this be possible.

If we consider first the spherical case, where $c = 0$, we recognize the differential equation for Legendre functions $P_n^m(x)$. In this case the eigenvalues are $\lambda_{mn} = n(n+1)$, $n = m, m+1, \ldots$. The integer n labels successive eigenvalues for fixed m: When $n = m$ we have the lowest eigenvalue, and the corresponding eigenfunction has no nodes in the interval $-1 < x < 1$; when $n = m+1$ we have the next eigenvalue, and the eigenfunction has one node inside $(-1, 1)$; and so on.

A similar situation holds for the general case $c^2 \neq 0$. We write the eigenvalues of (18.4.1) as $\lambda_{mn}(c)$ and the eigenfunctions as $S_{mn}(x;c)$. For fixed m, $n = m, m+1, \ldots$ labels the successive eigenvalues.

The computation of $\lambda_{mn}(c)$ and $S_{mn}(x;c)$ traditionally has been quite difficult. Complicated recurrence relations, power series expansions, etc., can be found in [1-3]. Cheap computing makes evaluation by direct solution of the differential equation quite feasible.

The first step is to investigate the behavior of the solution near the singular

points $x = \pm 1$. Substituting a power series expansion of the form

$$S = (1 \pm x)^{\alpha} \sum_{k=0}^{\infty} a_k (1 \pm x)^k \tag{18.4.2}$$

in equation (18.4.1), we find that the regular solution has $\alpha = m/2$. (Without loss of generality we can take $m \geq 0$ since $m \to -m$ is a symmetry of the equation.) We get an equation that is numerically more tractable if we factor out this behavior. Accordingly we set

$$S = (1 - x^2)^{m/2} y \tag{18.4.3}$$

We then find from (18.4.1) that y satisfies the equation

$$(1 - x^2)\frac{d^2 y}{dx^2} - 2(m+1)x\frac{dy}{dx} + (\mu - c^2 x^2)y = 0 \tag{18.4.4}$$

where

$$\mu \equiv \lambda - m(m+1) \tag{18.4.5}$$

Both equations (18.4.1) and (18.4.4) are invariant under the replacement $x \to -x$. Thus the functions S and y must also be invariant, except possibly for an overall scale factor. (Since the equations are linear, a constant multiple of a solution is also a solution.) Because the solutions will be normalized, the scale factor can only be ± 1. If $n - m$ is odd, there are an odd number of zeros in the interval $(-1, 1)$. Thus we must choose the antisymmetric solution $y(-x) = -y(x)$, which has a zero at $x = 0$. Conversely, if $n - m$ is even, we must have the symmetric solution. Thus

$$y_{mn}(-x) = (-1)^{n-m} y_{mn}(x) \tag{18.4.6}$$

and similarly for S_{mn}.

The boundary conditions on (18.4.4) require that y be regular at $x = \pm 1$. In other words, near the endpoints the solution takes the form

$$y = a_0 + a_1(1 - x^2) + a_2(1 - x^2)^2 + \cdots \tag{18.4.7}$$

Substituting this expansion in equation (18.4.4) and letting $x \to 1$, we find that

$$a_1 = -\frac{\mu - c^2}{4(m+1)} a_0 \tag{18.4.8}$$

Equivalently,

$$y'(1) = \frac{\mu - c^2}{2(m+1)} y(1) \tag{18.4.9}$$

A similar equation holds at $x = -1$ with a minus sign on the right-hand side. The irregular solution has a different relation between function and derivative at the endpoints.

Instead of integrating the equation from -1 to 1, we can exploit the symmetry (18.4.6) to integrate from 0 to 1. The boundary condition at $x = 0$ is

$$\begin{aligned} y(0) &= 0, \quad n - m \text{ odd} \\ y'(0) &= 0, \quad n - m \text{ even} \end{aligned} \tag{18.4.10}$$

A third boundary condition comes from the fact that any constant multiple of a solution y is a solution. We can thus *normalize* the solution. We adopt the normalization that the function S_{mn} has the same limiting behavior as P_n^m at $x = 1$:

$$\lim_{x \to 1} (1 - x^2)^{-m/2} S_{mn}(x;c) = \lim_{x \to 1} (1 - x^2)^{-m/2} P_n^m(x) \tag{18.4.11}$$

Various normalization conventions in the literature are tabulated by Flammer [1].

Imposing three boundary conditions for the second-order equation (18.4.4) turns it into an eigenvalue problem for λ or equivalently for μ. We write it in the standard form by setting

$$y_0 = y \tag{18.4.12}$$

$$y_1 = y' \tag{18.4.13}$$

$$y_2 = \mu \tag{18.4.14}$$

Then

$$y_0' = y_1 \tag{18.4.15}$$

$$y_1' = \frac{1}{1 - x^2} \left[2x(m+1) y_1 - (y_2 - c^2 x^2) y_0 \right] \tag{18.4.16}$$

$$y_2' = 0 \tag{18.4.17}$$

The boundary condition at $x = 0$ in this notation is

$$\begin{aligned} y_0 &= 0, \quad n - m \ \text{odd} \\ y_1 &= 0, \quad n - m \ \text{even} \end{aligned} \tag{18.4.18}$$

At $x = 1$ we have two conditions:

$$y_1 = \frac{y_2 - c^2}{2(m+1)} y_0 \tag{18.4.19}$$

$$y_0 = \lim_{x \to 1} (1 - x^2)^{-m/2} P_n^m(x) = \frac{(-1)^m (n+m)!}{2^m m! (n-m)!} \equiv \gamma \tag{18.4.20}$$

We are now ready to illustrate the use of the methods of previous sections on this problem.

18.4.1 Relaxation

If we just want a few isolated values of λ or S, shooting is probably the quickest method. However, if we want values for a large sequence of values of c, relaxation is better. Relaxation rewards a good initial guess with rapid convergence, and the previous solution should be a good initial guess if c is changed only slightly.

For simplicity, we choose a uniform grid on the interval $0 \le x \le 1$. For a total of M mesh points, we have

$$h = \frac{1}{M - 1} \tag{18.4.21}$$

$$x_k = kh, \qquad k = 0, 1, \ldots, M - 1 \tag{18.4.22}$$

At interior points $k = 1, 2, \ldots, M-1$, equation (18.4.15) gives

$$E_{0,k} = y_{0,k} - y_{0,k-1} - \frac{h}{2}(y_{1,k} + y_{1,k-1}) \tag{18.4.23}$$

Equation (18.4.16) gives

$$\begin{aligned} E_{1,k} &= y_{1,k} - y_{1,k-1} - \beta_k \\ &\times \left[\frac{(x_k + x_{k-1})(m+1)(y_{1,k} + y_{1,k-1})}{2} - \alpha_k \frac{(y_{0,k} + y_{0,k-1})}{2} \right] \end{aligned} \tag{18.4.24}$$

where

$$\alpha_k = \frac{y_{2,k} + y_{2,k-1}}{2} - \frac{c^2(x_k + x_{k-1})^2}{4} \tag{18.4.25}$$

$$\beta_k = \frac{h}{1 - \frac{1}{4}(x_k + x_{k-1})^2} \tag{18.4.26}$$

Finally, equation (18.4.17) gives

$$E_{2,k} = y_{2,k} - y_{2,k-1} \tag{18.4.27}$$

Now recall that the matrix of partial derivatives $S_{i,j}$ of equation (18.3.8) is defined so that i labels the equation and j the variable. In our case, j runs from 0 to 2 for y_j at $k-1$ and from 3 to 5 for y_j at k. Thus equation (18.4.23) gives

$$\begin{aligned} S_{0,0} &= -1, & S_{0,1} &= -\frac{h}{2}, & S_{0,2} &= 0 \\ S_{0,3} &= 1, & S_{0,4} &= -\frac{h}{2}, & S_{0,5} &= 0 \end{aligned} \tag{18.4.28}$$

Similarly equation (18.4.24) yields

$$\begin{aligned} S_{1,0} &= \alpha_k \beta_k / 2, & S_{1,1} &= -1 - \beta_k (x_k + x_{k-1})(m+1)/2, \\ S_{1,2} &= \beta_k (y_{0,k} + y_{0,k-1})/4, & S_{1,3} &= S_{1,0}, \\ S_{1,4} &= 2 + S_{1,1}, & S_{1,5} &= S_{1,2} \end{aligned} \tag{18.4.29}$$

while from equation (18.4.27) we find

$$\begin{aligned} S_{2,0} &= 0, & S_{2,1} &= 0, & S_{2,2} &= -1 \\ S_{2,3} &= 0, & S_{2,4} &= 0, & S_{2,5} &= 1 \end{aligned} \tag{18.4.30}$$

At $x = 0$ we have the boundary condition

$$E_{2,0} = \begin{cases} y_{0,0}, & n-m \ \text{odd} \\ y_{1,0}, & n-m \ \text{even} \end{cases} \tag{18.4.31}$$

Recall the convention adopted in the `solvde` routine that for one boundary condition at $k = 0$ only $S_{2,j}$ can be nonzero. Also, j takes on the values 3 to 5 since the boundary condition involves only y_k, not y_{k-1}. Accordingly, the only nonzero values of $S_{2,j}$ at $x = 0$ are

$$\begin{aligned} S_{2,3} &= 1, & n-m \ \text{odd} \\ S_{2,4} &= 1, & n-m \ \text{even} \end{aligned} \tag{18.4.32}$$

At $x = 1$ we have

$$E_{0,M} = y_{1,M-1} - \frac{y_{2,M-1} - c^2}{2(m+1)} y_{0,M-1} \tag{18.4.33}$$

$$E_{1,M} = y_{0,M-1} - \gamma \tag{18.4.34}$$

Thus

$$S_{0,3} = -\frac{y_{2,M-1} - c^2}{2(m+1)}, \qquad S_{0,4} = 1, \qquad S_{0,5} = -\frac{y_{0,M-1}}{2(m+1)} \tag{18.4.35}$$

$$S_{1,3} = 1, \qquad S_{1,4} = 0, \qquad S_{1,5} = 0 \tag{18.4.36}$$

Here now is the sample program that implements the above algorithm. We need a `main` program, `sfroid`, that calls the routine `Solvde`, and we must supply the object `Difeq` to be passed to `Solvde`. For simplicity we choose an equally spaced mesh of `m` = 41 points, that is, $h = .025$. As we shall see, this gives good accuracy for the eigenvalues up to moderate values of $n - m$.

Since the boundary condition at $x = 0$ does not involve y_0 if $n - m$ is even, we have to use the `indexv` feature of `Solvde`. Recall that the value of `indexv[j]` describes which column of `s[i][j]` the variable `y[j]` has been put in. If $n - m$ is even, we need to interchange the columns for y_0 and y_1 so that there is not a zero pivot element in `s[i][j]`.

The program prompts for values of m and n. It then computes an initial guess for y based on the Legendre function P_n^m. It next prompts for c^2, solves for y, prompts for c^2, solves for y using the previous values as an initial guess, and so on.

```
Int main_sfroid(void)                                                    sfroid.h
```
Sample program using `Solvde`. Computes eigenvalues of spheroidal harmonics $S_{mn}(x;c)$ for $m \ge 0$ and $n \ge m$. In the program, m is `mm`, c^2 is `c2`, and γ of equation (18.4.20) is `anorm`.

```
{
    const Int M=40,MM=4;
    const Int NE=3,NB=1,NYJ=NE,NYK=M+1;
    Int mm=3,n=5,mpt=M+1;
    VecInt indexv(NE);
    VecDoub x(M+1),scalv(NE);
    MatDoub y(NYJ,NYK);
    Int itmax=100;
    Doub c2[]={16.0,20.0,-16.0,-20.0};
    Doub conv=1.0e-14,slowc=1.0,h=1.0/M;
    if ((n+mm & 1) != 0) {                          No interchanges necessary.
        indexv[0]=0;
        indexv[1]=1;
        indexv[2]=2;
    } else {                                        Interchange y0 and y1.
        indexv[0]=1;
        indexv[1]=0;
        indexv[2]=2;
    }
    Doub anorm=1.0;                                 Compute γ.
    if (mm != 0) {
        Doub q1=n;
        for (Int i=1;i<=mm;i++) anorm = -0.5*anorm*(n+i)*(q1--/i);
    }
    for (Int k=0;k<M;k++) {                         Initial guess.
        x[k]=k*h;
        Doub fac1=1.0-x[k]*x[k];
        Doub fac2=exp((-mm/2.0)*log(fac1));
```

```
        y[0][k]=plgndr(n,mm,x[k])*fac2;                       P_n^m from §6.7.
        Doub deriv = -((n-mm+1)*plgndr(n+1,mm,x[k])-          Derivative of P_n^m from a recur-
            (n+1)*x[k]*plgndr(n,mm,x[k]))/fac1;                   rence relation.
        y[1][k]=mm*x[k]*y[0][k]/fac1+deriv*fac2;
        y[2][k]=n*(n+1)-mm*(mm+1);
    }
    x[M]=1.0;                                                 Initial guess at x = 1 done sep-
    y[0][M]=anorm;                                                arately.
    y[2][M]=n*(n+1)-mm*(mm+1);
    y[1][M]=y[2][M]*y[0][M]/(2.0*(mm+1.0));
    scalv[0]=abs(anorm);                                      Set scaling.
    scalv[1]=(y[1][M] > scalv[0] ? y[1][M] : scalv[0]);
    scalv[2]=(y[2][M] > 1.0 ? y[2][M] : 1.0);
    for (Int j=0;j<MM;j++) {
        Difeq difeq(mm,n,mpt,h,c2[j],anorm,x);                Set up Difeq object.
        Solvde solvde(itmax,conv,slowc,scalv,indexv,NB,y,difeq);
        cout << endl << " m = " << setw(3) << mm;
        cout << "  n = " << setw(3) << n << "  c**2 = ";
        cout << fixed << setprecision(3) << setw(7) << c2[j];
        cout << " lamda = " << setprecision(6) << (y[2][0]+mm*(mm+1));
        cout << endl;                                         Return for another value of c^2.
    }
    return 0;
}
```

difeq.h

```
struct Difeq {
Provides matrix s for Solvde.
    const Int &mm,&n,&mpt;                                 These variables are defined in sfroid.
    const Doub &h,&c2,&anorm;
    const VecDoub &x;
    Difeq(const Int &mmm, const Int &nn, const Int &mptt, const Doub &hh,
        const Doub &cc2, const Doub &anormm, VecDoub_I &xx) : mm(mmm),
        n(nn), mpt(mptt), h(hh), c2(cc2), anorm(anormm), x(xx) {}

    void smatrix(const Int k, const Int k1, const Int k2, const Int jsf,
        const Int is1, const Int isf, VecInt_I &indexv, MatDoub_O &s,
        MatDoub_I &y)
    Returns matrix s for solvde.
    {
        Doub temp,temp1,temp2;

        if (k == k1) {                                     Boundary condition at first point.
            if ((n+mm & 1) != 0) {
                s[2][3+indexv[0]]=1.0;                     Equation (18.4.32).
                s[2][3+indexv[1]]=0.0;
                s[2][3+indexv[2]]=0.0;
                s[2][jsf]=y[0][0];                         Equation (18.4.31).
            } else {
                s[2][3+indexv[0]]=0.0;                     Equation (18.4.32).
                s[2][3+indexv[1]]=1.0;
                s[2][3+indexv[2]]=0.0;
                s[2][jsf]=y[1][0];                         Equation (18.4.31).
            }
        } else if (k > k2-1) {                             Boundary conditions at last point.
            s[0][3+indexv[0]] = -(y[2][mpt-1]-c2)/(2.0*(mm+1.0));          (18.4.35).
            s[0][3+indexv[1]]=1.0;
            s[0][3+indexv[2]] = -y[0][mpt-1]/(2.0*(mm+1.0));
            s[0][jsf]=y[1][mpt-1]-(y[2][mpt-1]-c2)*y[0][mpt-1]/            (18.4.33).
                (2.0*(mm+1.0));
            s[1][3+indexv[0]]=1.0;                         Equation (18.4.36).
            s[1][3+indexv[1]]=0.0;
            s[1][3+indexv[2]]=0.0;
```

```
            s[1][jsf]=y[0][mpt-1]-anorm;            Equation (18.4.34).
        } else {                                    Interior point.
            s[0][indexv[0]] = -1.0;                 Equation (18.4.28).
            s[0][indexv[1]] = -0.5*h;
            s[0][indexv[2]]=0.0;
            s[0][3+indexv[0]]=1.0;
            s[0][3+indexv[1]] = -0.5*h;
            s[0][3+indexv[2]]=0.0;
            temp1=x[k]+x[k-1];
            temp=h/(1.0-temp1*temp1*0.25);
            temp2=0.5*(y[2][k]+y[2][k-1])-c2*0.25*temp1*temp1;
            s[1][indexv[0]]=temp*temp2*0.5;         Equation (18.4.29).
            s[1][indexv[1]] = -1.0-0.5*temp*(mm+1.0)*temp1;
            s[1][indexv[2]]=0.25*temp*(y[0][k]+y[0][k-1]);
            s[1][3+indexv[0]]=s[1][indexv[0]];
            s[1][3+indexv[1]]=2.0+s[1][indexv[1]];
            s[1][3+indexv[2]]=s[1][indexv[2]];
            s[2][indexv[0]]=0.0;                    Equation (18.4.30).
            s[2][indexv[1]]=0.0;
            s[2][indexv[2]] = -1.0;
            s[2][3+indexv[0]]=0.0;
            s[2][3+indexv[1]]=0.0;
            s[2][3+indexv[2]]=1.0;
            s[0][jsf]=y[0][k]-y[0][k-1]-0.5*h*(y[1][k]+y[1][k-1]);      (18.4.23).
            s[1][jsf]=y[1][k]-y[1][k-1]-temp*((x[k]+x[k-1])             (18.4.24).
                *0.5*(mm+1.0)*(y[1][k]+y[1][k-1])-temp2
                *0.5*(y[0][k]+y[0][k-1]));
            s[2][jsf]=y[2][k]-y[2][k-1];            Equation (18.4.27).
        }
    }
};
```

You can run the program and check it against values of $\lambda_{mn}(c)$ given in the tables at the back of Flammer's book [1] or in Table 21.1 of Abramowitz and Stegun [2]. Typically it converges in about three iterations. The table below gives a few comparisons.

Selected Output of sfroid

m	n	c^2	λ_{exact}	$\lambda_{\texttt{sfroid}}$
2	2	0.1	6.01427	6.01427
		1.0	6.14095	6.14095
		4.0	6.54250	6.54253
2	5	1.0	30.4361	30.4372
		16.0	36.9963	37.0135
4	11	−1.0	131.560	131.554

18.4.2 Shooting

To solve the same problem via shooting (§18.1), we supply a functor `Rhs` that implements equations (18.4.15) – (18.4.17). We will integrate the equations over the range $-1 \le x \le 0$. We provide the functor `Load`, which sets the eigenvalue y_2 to its current best estimate, `v[0]`. It also sets the boundary values of y_0 and y_1 using equations (18.4.20) and (18.4.19) (with a minus sign corresponding to $x = -1$). Note that the boundary condition is actually applied a distance `dx` from the boundary

to avoid having to evaluate y_1' right on the boundary. The functor `Score` follows from equation (18.4.18).

sphoot.h

```
struct Rhs {
```
Evaluates derivatives for `Odeint`.
```
    Int m;
    Doub c2;
    Rhs(Int mm, Doub cc2) : m(mm), c2(cc2) {}
```
Constructor gets parameters from `main`.
```
    void operator() (const Doub x, VecDoub_I &y, VecDoub_O &dydx)
    {
        dydx[0]=y[1];
        dydx[1]=(2.0*x*(m+1.0)*y[1]-(y[2]-c2*x*x)*y[0])/(1.0-x*x);
        dydx[2]=0.0;
    }
};

struct Load {
```
Supplies starting values for integration at $x = -1 + dx$.
```
    Int n,m;
    Doub gmma,c2,dx;
    VecDoub y;
    Load(Int nn, Int mm, Doub gmmaa, Doub cc2, Doub dxx) : n(nn), m(mm),
        gmma(gmmaa), c2(cc2), dx(dxx), y(3) {}
```
Constructor gets parameters from `main`.
```
    VecDoub operator() (const Doub x1, VecDoub_I &v)
    {
        Doub y1 = ((n-m & 1) != 0 ? -gmma : gmma);
        y[2]=v[0];
        y[1] = -(y[2]-c2)*y1/(2*(m+1));
        y[0]=y1+y[1]*dx;
        return y;
    }
};

struct Score {
```
Computes amount by which boundary condition at $x = 0$ is violated.
```
    Int n,m;
    VecDoub f;
    Score(Int nn, Int mm) : n(nn), m(mm), f(1) {}
```
Constructor gets parameters from `main`.
```
    VecDoub operator() (const Doub xf, VecDoub_I &y)
    {
        f[0]=((n-m & 1) != 0 ? y[0] : y[1]);
        return f;
    }
};

Int main_sphoot(void) {
```
Sample program using `Shoot`. Computes eigenvalues of spheroidal harmonics $S_{mn}(x;c)$ for $m \geq 0$ and $n \geq m$. Note how the functor `vecfunc` for `newt` is provided by `Shoot` (§18.1).
```
    const Int N2=1,MM=3;
    Bool check;
    VecDoub v(N2);
    Int j,m=3,n=5;
    Doub c2[]={1.5,-1.5,0.0};
    Int nvar=3;                          Number of equations.
    Doub dx=1.0e-8;                      Avoid evaluating derivatives exactly at x = -1.
    for (j=0;j<MM;j++) {
        Doub gmma=1.0;                       Compute γ of equation (18.4.20).
        Doub q1=n;
        for (Int i=1;i<=m;i++) gmma *= -0.5*(n+i)*(q1--/i);
        v[0]=n*(n+1)-m*(m+1)+c2[j]/2.0;      Initial guess for eigenvalue.
```

```
        Doub x1= -1.0+dx;                    Set range of integration.
        Doub x2=0.0;
        Load load(n,m,gmma,c2[j],dx);        Set up Load, Rhs, and Score objects ...
        Rhs d(m,c2[j]);
        Score score(n,m);                    ... use them to set up Shoot object ...
        Shoot<Load,Rhs,Score> shoot(nvar,x1,x2,load,d,score);
        newt(v,check,shoot);                 ... and use it to find v that zeros vector f in
        if (check) {                             Score.
            cout << "shoot failed; bad initial guess" << endl;
        } else {
            cout << "    " << "mu(m,n)" << endl;
            cout << fixed << setprecision(6);
            cout << setw(12) << v[0] << endl;
        }
    }
    return 0;
}
```

18.4.3 Shooting to a Fitting Point

For variety we illustrate `Shootf` from §18.2 by integrating over the whole range $-1 + dx \leq x \leq 1 - dx$, with the fitting point chosen to be at $x = 0$. The routine `Rhsfpt` is identical to `Rhs` for `Shoot` since we are integrating the same equation. Now, however, there are two load routines. The functor `Load1` for $x = -1$ is essentially identical to `Load` above. At $x = 1$, `Load2` sets the function value y_0 and the eigenvalue y_2 to their best current estimates, `v2[0]` and `v2[1]`, respectively. If you quite sensibly make your initial guess of the eigenvalue the same in the two intervals, then `v1[0]` will stay equal to `v2[1]` during the iteration. The functor `Score` computes the degree of mismatch of the three function values at the fitting point.

sphfpt.h

```
struct Rhsfpt {
    Int m;
    Doub c2;
    Rhsfpt(Int mm, Doub cc2) : m(mm), c2(cc2) {}
    void operator() (const Doub x, VecDoub_I &y, VecDoub_O &dydx)
    {
        dydx[0]=y[1];
        dydx[1]=(2.0*x*(m+1.0)*y[1]-(y[2]-c2*x*x)*y[0])/(1.0-x*x);
        dydx[2]=0.0;
    }
};

struct Load1 {
Supplies starting values for integration at x = -1 + dx.
    Int n,m;
    Doub gmma,c2,dx;
    VecDoub y;
    Load1(Int nn, Int mm, Doub gmmaa, Doub cc2, Doub dxx) : n(nn), m(mm),
        gmma(gmmaa), c2(cc2), dx(dxx), y(3) {}
    VecDoub operator() (const Doub x1, VecDoub_I &v1)
    {
        Doub y1 = ((n-m & 1) != 0 ? -gmma : gmma);
        y[2]=v1[0];
        y[1] = -(y[2]-c2)*y1/(2*(m+1));
        y[0]=y1+y[1]*dx;
        return y;
    }
};
```

```
struct Load2 {
```
Supplies starting values for integration at $x = 1 - dx$.
```
    Int m;
    Doub c2;
    VecDoub y;
    Load2(Int mm, Doub cc2) : m(mm), c2(cc2), y(3) {}
    VecDoub operator() (const Doub x2, VecDoub_I &v2)
    {
        y[2]=v2[1];
        y[0]=v2[0];
        y[1]=(y[2]-c2)*y[0]/(2*(m+1));
        return y;
    }
};

struct Score {
```
Computes the mismatch of the solutions at the fitting point $x = 0$.
```
    VecDoub f;
    Score() : f(3) {}
    VecDoub operator() (const Doub xf, VecDoub_I &y)
    {
        for (Int i=0;i<3;i++) f[i]=y[i];
        return f;
    }
};

Int main_sphfpt(void) {
```
Sample program using `Shootf`. Computes eigenvalues of spheroidal harmonics $S_{mn}(x;c)$ for $m \geq 0$ and $n \geq m$. Note how the functor `vecfunc` for `newt` is provided by `Shootf` (§18.2). The routine `Rhsfpt` is the same as `Rhs` for `sphoot`.
```
    const Int N1=2,N2=1,NTOT=N1+N2,MM=3;
    Bool check;
    VecDoub v(NTOT);
    Int j,m=3,n=5,n2=N2;
    Doub c2[]={1.5,-1.5,0.0};
    Int nvar=NTOT;                          Number of equations.
    Doub dx=1.0e-8;                         Avoid evaluating derivatives exactly at x = ±1.
    for (j=0;j<MM;j++) {
        Doub gmma=1.0;                      Compute γ of equation (18.4.20).
        Doub q1=n;
        for (Int i=1;i<=m;i++) gmma *= -0.5*(n+i)*(q1--/i);
        v[0]=n*(n+1)-m*(m+1)+c2[j]/2.0;Initial guess for eigenvalue and function value.
        v[2]=v[0];
        v[1]=gmma*(1.0-(v[2]-c2[j])*dx/(2*(m+1)));
        Doub x1= -1.0+dx;                   Set range of integration.
        Doub x2=1.0-dx;
        Doub xf=0.0;                        Fitting point.
        Load1 load1(n,m,gmma,c2[j],dx);Set up Load1, Load2, Rhsfpt, and Score
        Load2 load2(m,c2[j]);                   objects ...
        Rhsfpt d(m,c2[j]);
        Score score;
        Shootf<Load1,Load2,Rhsfpt,Score> shootf(nvar,n2,x1,x2,xf,load1,
            load2,d,score);                 ... use them to set up Shootf object ...
        newt(v,check,shootf);               ... and use it to find v that zeros vector f in
        if (check) {                            Score.
            cout << "shootf failed; bad initial guess" << endl;
        } else {
            cout << "    " << "mu(m,n)" << endl;
            cout << fixed << setprecision(6);
            cout << setw(12) << v[0] << endl;
        }
    }
    return 0;
}
```

CITED REFERENCES AND FURTHER READING:

Flammer, C. 1957, *Spheroidal Wave Functions* (Stanford, CA: Stanford University Press); reprinted 2005 (New York: Dover).[1]

Abramowitz, M., and Stegun, I.A. 1964, *Handbook of Mathematical Functions* (Washington: National Bureau of Standards); reprinted 1968 (New York: Dover); online at `http://www.nr.com/aands`, §21.[2]

Morse, P.M., and Feshbach, H. 1953, *Methods of Theoretical Physics*, Part II (New York: McGraw-Hill), pp. 1502ff.[3]

18.5 Automated Allocation of Mesh Points

In relaxation problems, you have to choose values for the independent variable at the mesh points. This is called *allocating* the grid or mesh. The usual procedure is to pick a plausible set of values and, if it works, to be content. If it doesn't work, increasing the number of points usually cures the problem.

If we know ahead of time where our solutions will be rapidly varying, we can put more grid points there and less elsewhere. Alternatively, we can solve the problem first on a uniform mesh and then examine the solution to see where we should add more points. We then repeat the solution with the improved grid. The object of the exercise is to allocate points in such a way as to represent the solution accurately.

It is also possible to automate the allocation of mesh points, so that it is done "dynamically" during the relaxation process. This powerful technique not only improves the accuracy of the relaxation method, but also (as we will see in the next section) allows internal singularities to be handled in quite a neat way. Here we learn how to accomplish the automatic allocation.

We want to focus attention on the independent variable x and consider two alternative reparametrizations of it. The first, we term q; this is just the coordinate corresponding to the mesh points themselves, so that $q = 0$ at $\mathtt{k} = 0$, $q = 1$ at $\mathtt{k} = 1$, and so on. Between any two mesh points we have $\Delta q = 1$. In the change of independent variable in the ODEs from x to q,

$$\frac{d\mathbf{y}}{dx} = \mathbf{g} \tag{18.5.1}$$

becomes

$$\frac{d\mathbf{y}}{dq} = \mathbf{g}\frac{dx}{dq} \tag{18.5.2}$$

In terms of q, equation (18.5.2) as an FDE might be written

$$\mathbf{y}_k - \mathbf{y}_{k-1} - \frac{1}{2}\left[\left(\mathbf{g}\frac{dx}{dq}\right)_k + \left(\mathbf{g}\frac{dx}{dq}\right)_{k-1}\right] = 0 \tag{18.5.3}$$

or some related version. Note that dx/dq should accompany $\mathbf{g}$. The transformation between x and q depends only on the *Jacobian* dx/dq. Its reciprocal dq/dx is proportional to the density of mesh points.

Now, given the function $\mathbf{y}(x)$, or its approximation at the current stage of relaxation, we are supposed to have some idea of how we want to specify the density of mesh points. For example, we might want dq/dx to be larger where $\mathbf{y}$ is changing rapidly, or near to the boundaries, or both. In fact, we can probably make up a formula for what we would like dq/dx to be proportional to. The problem is that we do not know the proportionality constant. That is, the formula that we might invent would not have the correct integral over the whole range of x so as to make q vary from 0 to $M-1$, according to its definition. To solve this problem we introduce a second reparametrization $Q(q)$, where Q is a new independent

variable. The relation between Q and q is taken to be *linear*, so that a mesh spacing formula for dQ/dx differs only in its unknown proportionality constant. A linear relation implies

$$\frac{d^2Q}{dq^2} = 0 \tag{18.5.4}$$

or, expressed in the usual manner as coupled first-order equations,

$$\frac{dQ(x)}{dq} = \psi \qquad \frac{d\psi}{dq} = 0 \tag{18.5.5}$$

where ψ is a new intermediate variable. We add these two equations to the set of ODEs being solved.

Completing the prescription, we add a third ODE that is just our desired mesh-density function, namely

$$\phi(x) = \frac{dQ}{dx} = \frac{dQ}{dq}\frac{dq}{dx} \tag{18.5.6}$$

where $\phi(x)$ is chosen by us. Written in terms of the mesh variable q, this equation is

$$\frac{dx}{dq} = \frac{\psi}{\phi(x)} \tag{18.5.7}$$

Notice that $\phi(x)$ should be chosen to be positive-definite, so that the density of mesh points is everywhere positive. Otherwise (18.5.7) can have a zero in its denominator.

To use automated mesh spacing, you add the three ODEs (18.5.5) and (18.5.7) to your set of equations, i.e., to the array `y[j][k]`. Now x becomes a dependent variable! Q and ψ also become new dependent variables. Normally, evaluating ϕ requires little extra work since it will be composed from pieces of the g's that exist anyway. The automated procedure allows one to investigate quickly how the numerical results might be affected by various strategies for mesh spacing. (A special case occurs if the desired mesh spacing function Q can be found analytically, i.e., dQ/dx is directly integrable. Then, you need to add only two equations, those in 18.5.5, and two new variables x, ψ.)

As an example of a typical strategy for implementing this scheme, consider a system with one dependent variable $y(x)$. We could set

$$dQ = \frac{dx}{\Delta} + \frac{|d \ln y|}{\delta} \tag{18.5.8}$$

or

$$\phi(x) = \frac{dQ}{dx} = \frac{1}{\Delta} + \left|\frac{dy/dx}{y\delta}\right| \tag{18.5.9}$$

where Δ and δ are constants that we choose. The first term would give a uniform spacing in x if it alone were present. The second term forces more grid points to be used where y is changing rapidly. The constants act to make every logarithmic change in y of an amount δ about as "attractive" to a grid point as a change in x of amount Δ. You adjust the constants according to taste. Other strategies are possible, such as a logarithmic spacing in x, where dx in the first term is replaced with $d \ln x$.

CITED REFERENCES AND FURTHER READING:

Eggleton, P.P. 1971, "The Evolution of Low Mass Stars," *Monthly Notices of the Royal Astronomical Society*, vol. 151, pp. 351–364.

Kippenhan, R., Weigert, A., and Hofmeister, E. 1968, in *Methods in Computational Physics*, vol. 7 (New York: Academic Press), pp. 129ff.

18.6 Handling Internal Boundary Conditions or Singular Points

Singularities can occur in the interiors of two-point boundary value problems. Typically, there is a point x_s at which a derivative must be evaluated by an expression of the form

$$S(x_s) = \frac{N(x_s, \mathbf{y})}{D(x_s, \mathbf{y})} \tag{18.6.1}$$

where the denominator $D(x_s, \mathbf{y}) = 0$. In physical problems with finite answers, singular points usually come with their own cure: Where $D \to 0$, there the physical solution $\mathbf{y}$ must be such as to make $N \to 0$ simultaneously, in such a way that the ratio takes on a meaningful value. This constraint on the solution $\mathbf{y}$ is often called a *regularity condition*. The condition that $D(x_s, \mathbf{y})$ satisfy some special constraint at x_s is entirely analogous to an extra boundary condition, an algebraic relation among the dependent variables that must hold at a point.

We discussed a related situation earlier, in §18.2, when we described the "fitting point method" to handle the task of integrating equations with singular behavior at the boundaries. In those problems you are unable to integrate from one side of the domain to the other. However, the ODEs do have well-behaved derivatives and solutions in the neighborhood of the singularity, so it is readily possible to integrate away from the point. Both the relaxation method and the method of shooting to a fitting point handle such problems easily. Also, in those problems the presence of singular behavior served to isolate some special boundary values that had to be satisfied to solve the equations.

The difference here is that we are concerned with singularities arising at intermediate points, where the location of the singular point depends on the solution, so is not known a priori. Consequently, we face a circular task: The singularity prevents us from finding a numerical solution, but we need a numerical solution to find its location. Such singularities are also associated with selecting a special value for some variable that allows the solution to satisfy the regularity condition at the singular point. Thus, internal singularities take on aspects of being internal boundary conditions.

One way of handling internal singularities is to treat the problem as a free boundary problem, as discussed at the end of §18.0. Suppose, as a simple example, we consider the equation

$$\frac{dy}{dx} = \frac{N(x, y)}{D(x, y)} \tag{18.6.2}$$

where N and D are required to pass through zero at some unknown point x_s. We add the equation

$$z \equiv x_s - x_1 \qquad \frac{dz}{dx} = 0 \tag{18.6.3}$$

where x_s is the unknown location of the singularity, and change the independent variable to t by setting

$$x - x_1 = tz, \qquad 0 \le t \le 1 \tag{18.6.4}$$

The boundary conditions at $t = 1$ become

$$N(x, y) = 0, \qquad D(x, y) = 0 \tag{18.6.5}$$

Use of an adaptive mesh as discussed in the previous section is another way to overcome the difficulties of an internal singularity. For the problem (18.6.2), we add the mesh spacing equations

$$\frac{dQ}{dq} = \psi \tag{18.6.6}$$

$$\frac{d\psi}{dq} = 0 \tag{18.6.7}$$

with a simple mesh spacing function that maps x uniformly into q, where q runs from 0 to $M-1$, with M the number of mesh points:

$$Q(x) = x - x_1, \qquad \frac{dQ}{dx} = 1 \tag{18.6.8}$$

Having added three first-order differential equations, we must also add their corresponding boundary conditions. If there were no singularity, these could simply be

$$\text{at } q = 0: \qquad x = x_1, \quad Q = 0 \tag{18.6.9}$$

$$\text{at } q = M-1: \quad x = x_2 \tag{18.6.10}$$

and a total of N values y_i specified at $q = 0$. In this case the problem is essentially an initial value problem with all boundary conditions specified at x_1 and the mesh spacing function is superfluous.

However, in the actual case at hand we impose the conditions

$$\text{at } q = 0: \qquad x = x_1, \qquad Q = 0 \tag{18.6.11}$$

$$\text{at } q = M-1: \quad N(x, y) = 0, \quad D(x, y) = 0 \tag{18.6.12}$$

and $N-1$ values y_i at $q = 0$. The "missing" y_i is to be adjusted, in other words, so as to make the solution go through the singular point in a regular (zero-over-zero) rather than irregular (finite-over-zero) manner. Notice also that these boundary conditions do not directly impose a value for x_2, which becomes an adjustable parameter that the code varies in an attempt to match the regularity condition.

In this example the singularity occurred at a boundary, and the complication arose because the location of the boundary was unknown. In other problems we might wish to continue the integration beyond the internal singularity. For the example given above, we could simply integrate the ODEs to the singular point, and then as a separate problem recommence the integration from the singular point on as far we care to go. However, in other cases the singularity occurs internally, but does not completely determine the problem: There are still some more boundary conditions to be satisfied further along in the mesh. Such cases present no difficulty in principle, but do require some adaptation of the relaxation code given in §18.3. In effect, all you need to do is to add a "special" block of equations at the mesh point where the internal boundary conditions occur, and do the proper bookkeeping.

Figure 18.6.1 illustrates a concrete example where the overall problem contains five equations with two boundary conditions at the first point, one "internal" boundary condition, and two final boundary conditions. The figure shows the structure of the overall matrix equations along the diagonal in the vicinity of the special block. In the middle of the domain, blocks typically involve five equations (rows) in ten unknowns (columns). For each block prior to the special block, the initial boundary conditions provided enough information to zero the first two columns of the blocks. The five FDEs eliminate five more columns, and the final three columns need to be stored for the backsubstitution step (as described in §18.3). To handle the extra condition, we break the normal cycle and add a special block with only one equation: the internal boundary condition. This effectively reduces the required storage of unreduced coefficients by one column for the rest of the grid, and allows us to reduce to zero the first three columns of subsequent blocks. The functions `red`, `pinvs`, and `bksub` can readily handle these cases with minor recoding, but each problem makes for a special case, and you will have to make the modifications as required.

CITED REFERENCES AND FURTHER READING:

London, R.A., and Flannery, B.P. 1982, "Hydrodynamics of X-Ray Induced Stellar Winds," *Astrophysical Journal*, vol. 258, pp. 260–269.

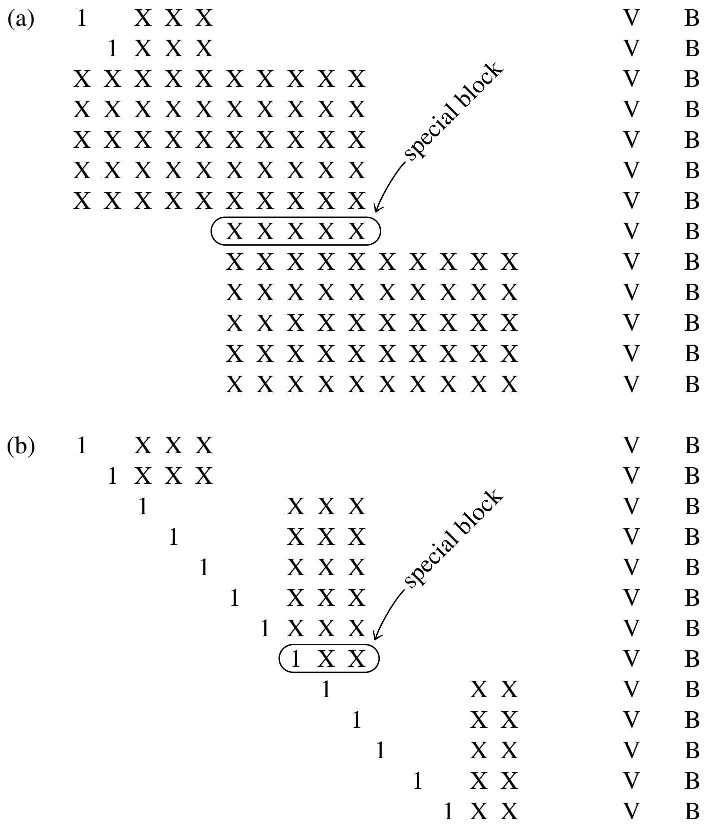

Figure 18.6.1. FDE matrix structure with an internal boundary condition. The internal condition introduces a special block. (a) Original form, compare with Figure 18.3.1; (b) final form, compare with Figure 18.3.2.

Integral Equations and Inverse Theory

CHAPTER 19

19.0 Introduction

Many people, otherwise numerically knowledgable, imagine that the numerical solution of integral equations must be an extremely arcane topic, since, until recently, it was almost never treated in numerical analysis textbooks. Actually there is a large and growing literature on the numerical solution of integral equations, including several good monographs [1-3]. One reason for the sheer volume of this activity is that there are many different kinds of equations, each with many different possible pitfalls; often many different algorithms have been proposed to deal with a single case.

There is a close correspondence between linear integral equations, which specify linear, integral relations among functions in an infinite-dimensional function space, and plain old linear equations, which specify analogous relations among vectors in a finite-dimensional vector space. Because this correspondence lies at the heart of most computational algorithms, it is worth making it explicit as we recall how integral equations are classified.

Fredholm equations involve definite integrals with fixed upper and lower limits. An *inhomogeneous Fredholm equation of the first kind* has the form

$$g(t) = \int_a^b K(t,s)\, f(s)\, ds \tag{19.0.1}$$

Here $f(t)$ is the unknown function to be solved for, while $g(t)$ is a known "right-hand side." (In integral equations, for some odd reason, the familiar "right-hand side" is conventionally written on the left!) The function of two variables, $K(t,s)$, is called the *kernel*. Equation (19.0.1) is analogous to the matrix equation

$$\mathbf{K} \cdot \mathbf{f} = \mathbf{g} \tag{19.0.2}$$

whose solution is $\mathbf{f} = \mathbf{K}^{-1} \cdot \mathbf{g}$, where $\mathbf{K}^{-1}$ is the matrix inverse. Like equation (19.0.2), equation (19.0.1) has a unique solution whenever g is nonzero (the homogeneous case with $g = 0$ is almost never useful) and K is invertible. However, as we shall see, this latter condition is as often the exception as the rule.

The analog of the finite-dimensional eigenvalue problem

$$(\mathbf{K} - \sigma\mathbf{1}) \cdot \mathbf{f} = \mathbf{g} \tag{19.0.3}$$

is called a *Fredholm equation of the second kind*, usually written

$$f(t) = \lambda \int_a^b K(t,s) f(s)\, ds + g(t) \tag{19.0.4}$$

Again, the notational conventions do not exactly correspond: λ in equation (19.0.4) is $1/\sigma$ in (19.0.3), while $\mathbf{g}$ is $-g/\lambda$. If g (or $\mathbf{g}$) is zero, then the equation is said to be *homogeneous*. If the kernel $K(t,s)$ is bounded, then, like equation (19.0.3), equation (19.0.4) has the property that its homogeneous form has solutions for at most a denumerably infinite set $\lambda = \lambda_n$, $n = 1, 2, \ldots$, the *eigenvalues*. The corresponding solutions $f_n(t)$ are the *eigenfunctions*. The eigenvalues are real if the kernel is symmetric.

In the *inhomogeneous* case of nonzero g (or $\mathbf{g}$), equations (19.0.3) and (19.0.4) are soluble *except* when λ (or σ) is an eigenvalue — because the integral operator (or matrix) is singular then. In integral equations this dichotomy is called *the Fredholm alternative*.

Fredholm equations of the first kind are often extremely ill-conditioned. Applying the kernel to a function is generally a smoothing operation, so the solution, which requires inverting the operator, will be extremely sensitive to small changes or errors in the input. Smoothing often actually loses information, and there is no way to get it back in an inverse operation. Specialized methods have been developed for such equations, which are often called *inverse problems*. In general, a method must augment the information given with some prior knowledge of the nature of the solution. This prior knowledge is then used, in one way or another, to restore lost information. We will introduce such techniques in §19.4.

Inhomogeneous Fredholm equations of the second kind are much less often ill-conditioned. Equation (19.0.4) can be rewritten as

$$\int_a^b [K(t,s) - \sigma\delta(t-s)] f(s)\, ds = -\sigma g(t) \tag{19.0.5}$$

where $\delta(t-s)$ is a Dirac delta function (and where we have changed from λ to its reciprocal σ for clarity). If σ is large enough in magnitude, then equation (19.0.5) is, in effect, diagonally dominant and thus well-conditioned. Only if σ is small do we go back to the ill-conditioned case.

Homogeneous Fredholm equations of the second kind are likewise not particularly ill-posed. If K is a smoothing operator, then it will map many f's to zero, or near-zero; there will thus be a large number of degenerate or nearly degenerate eigenvalues around $\sigma = 0$ ($\lambda \to \infty$), but this will cause no particular computational difficulties. In fact, we can now see that the magnitude of σ needed to rescue the inhomogeneous equation (19.0.5) from an ill-conditioned fate is generally much *less* than that required for diagonal dominance. Since the σ term shifts all eigenvalues, it is enough that it be large enough to shift a smoothing operator's forest of near-zero eigenvalues away from zero, so that the resulting operator becomes invertible (except, of course, at the discrete eigenvalues).

Volterra equations are a special case of Fredholm equations with $K(t,s) = 0$ for $s > t$. Chopping off the unnecessary part of the integration, Volterra equations are written in a form where the upper limit of integration is the independent variable t. The *Volterra equation of the first kind*,

$$g(t) = \int_a^t K(t,s) f(s)\, ds \tag{19.0.6}$$

has as its analog the matrix equation (now written out in components)

$$\sum_{j=0}^{k} K_{kj} f_j = g_k \tag{19.0.7}$$

Comparing with equation (19.0.2), we see that the Volterra equation corresponds to a matrix $\mathbf{K}$ that is lower (i.e., left) triangular, with zero entries above the diagonal. As we know from Chapter 2, such matrix equations are trivially soluble by forward substitution. Techniques for solving Volterra equations are similarly straightforward. When experimental measurement noise does not dominate, Volterra equations of the first kind tend *not* to be ill-conditioned; the upper limit to the integral introduces a sharp step that conveniently spoils any smoothing properties of the kernel.

The Volterra equation of the second kind is written

$$f(t) = \int_a^t K(t,s) f(s)\, ds + g(t) \tag{19.0.8}$$

whose matrix analog is the equation

$$(\mathbf{K} - \mathbf{1}) \cdot \mathbf{f} = \mathbf{g} \tag{19.0.9}$$

with $\mathbf{K}$ lower triangular. The reason there is no λ in these equations is that (i) in the inhomogeneous case (nonzero g) it can be absorbed into K, while (ii) in the homogeneous case ($g = 0$), it is a theorem that Volterra equations of the second kind with bounded kernels have no eigenvalues with square-integrable eigenfunctions.

We have specialized our definitions to the case of linear integral equations. In a nonlinear version of equation (19.0.1) or (19.0.6), instead of $K(t,s) f(s)$ the integrand would be $K(t,s,f(s))$. A nonlinear version of equation (19.0.4) or (19.0.8) would have an integrand $K(t,s,f(t),f(s))$. Nonlinear Fredholm equations are considerably more complicated than their linear counterparts. Fortunately, they do not occur as frequently in practice and we shall by and large ignore them in this chapter. By contrast, solving nonlinear Volterra equations usually involves only a slight modification of the algorithm for linear equations, as we shall see.

Almost all methods for solving integral equations numerically make use of *quadrature rules*, frequently Gaussian quadratures. This would be a good time for you to go back and review §4.6, especially the advanced material toward the end of that section.

In the sections that follow, we first discuss Fredholm equations of the second kind with smooth kernels (§19.1). Nontrivial quadrature rules come into the discussion, but we will be dealing with well-conditioned systems of equations. We then return to Volterra equations (§19.2), and find that simple and straightforward methods are generally satisfactory for these equations.

In §19.3 we discuss how to proceed in the case of singular kernels, focusing largely on Fredholm equations (both first and second kinds). Singularities require special quadrature rules, but they are also sometimes blessings in disguise, since they can spoil a kernel's smoothing and make problems well-conditioned.

In §19.4 – §19.7 we face up to the issues of inverse problems. Section 19.4 is an introduction to this large subject.

We should note here that wavelet transforms, already discussed in §13.10, are applicable not only to data compression and signal processing, but can also be used to transform some classes of integral equations into sparse linear problems that allow fast solution. You may wish to review §13.10 as part of reading this chapter.

Some subjects, such as *integro-differential equations*, we must simply declare to be beyond our scope. For a review of methods for integro-differential equations, see Brunner [4].

It should go without saying that this one short chapter can only barely touch on a few of the most basic methods involved in this complicated subject.

CITED REFERENCES AND FURTHER READING:

Delves, L.M., and Mohamed, J.L. 1985, *Computational Methods for Integral Equations* (Cambridge, UK: Cambridge University Press).[1]

Linz, P. 1985, *Analytical and Numerical Methods for Volterra Equations* (Philadelphia: S.I.A.M.).[2]

Atkinson, K.E. 1976, *A Survey of Numerical Methods for the Solution of Fredholm Integral Equations of the Second Kind* (Philadelphia: S.I.A.M.).[3]

Brunner, H. 1988, in *Numerical Analysis 1987*, Pitman Research Notes in Mathematics vol. 170, D.F. Griffiths and G.A. Watson, eds. (Harlow, Essex, UK: Longman Scientific and Technical), pp. 18–38.[4]

Smithies, F. 1958, *Integral Equations* (Cambridge, UK: Cambridge University Press).

Kanwal, R.P. 1971, *Linear Integral Equations* (New York: Academic Press).

Green, C.D. 1969, *Integral Equation Methods* (New York: Barnes & Noble).

19.1 Fredholm Equations of the Second Kind

We desire a numerical solution for $f(t)$ in the equation

$$f(t) = \lambda \int_a^b K(t,s) f(s)\, ds + g(t) \tag{19.1.1}$$

The method we describe, a very basic one, is called the *Nystrom method*. It requires the choice of some approximate *quadrature rule*:

$$\int_a^b y(s)\, ds = \sum_{j=0}^{N-1} w_j\, y(s_j) \tag{19.1.2}$$

Here the set $\{w_j\}$ are the weights of the quadrature rule, while the N points $\{s_j\}$ are the abscissas.

What quadrature rule should we use? It is certainly possible to solve integral equations with low-order quadrature rules like the extended trapezoidal or Simpson's

rules. We will see, however, that the solution method involves $O(N^3)$ operations, and so the most efficient methods tend to use high-order quadrature rules to keep N as small as possible. For smooth, nonsingular problems, nothing beats Gaussian quadrature (e.g., Gauss-Legendre quadrature, §4.6). (For nonsmooth or singular kernels, see §19.3.)

Delves and Mohamed [1] investigated methods more complicated than the Nystrom method. For straightforward Fredholm equations of the second kind, they concluded "...the clear winner of this contest has been the Nystrom routine ... with the N-point Gauss-Legendre rule. This routine is extremely simple.... Such results are enough to make a numerical analyst weep."

If we apply the quadrature rule (19.1.2) to equation (19.1.1), we get

$$f(t) = \lambda \sum_{j=0}^{N-1} w_j K(t, s_j) f(s_j) + g(t) \tag{19.1.3}$$

Evaluate equation (19.1.3) at the quadrature points:

$$f(t_i) = \lambda \sum_{j=0}^{N-1} w_j K(t_i, s_j) f(s_j) + g(t_i) \tag{19.1.4}$$

Let f_i be the vector $f(t_i)$, g_i the vector $g(t_i)$, K_{ij} the matrix $K(t_i, s_j)$, and define

$$\widetilde{K}_{ij} = K_{ij} w_j \tag{19.1.5}$$

Then, in matrix notation, equation (19.1.4) becomes

$$(\mathbf{1} - \lambda \widetilde{\mathbf{K}}) \cdot \mathbf{f} = \mathbf{g} \tag{19.1.6}$$

This is a set of N linear algebraic equations in N unknowns that can be solved by standard triangular decomposition techniques (§2.3) — that is where the $O(N^3)$ operations count comes in. The solution is usually well-conditioned, unless λ is very close to an eigenvalue.

Having obtained the solution at the quadrature points $\{t_i\}$, how do you get the solution at some other point t? You do *not* simply use polynomial interpolation. This destroys all the accuracy you have worked so hard to achieve. Nystrom's key observation was that you should use equation (19.1.3) as an interpolatory formula, maintaining the accuracy of the solution.

Our routine for solving linear Fredholm equations of the second kind is coded as the object `Fred2`. The constructor sets up equation (19.1.6) and then solves it by LU decomposition with `LUdcmp`. The Gauss-Legendre quadrature is implemented by first getting the weights and abscissas with a call to `gauleg`. The routine `Fred2` requires that you provide an external function or functor that returns $g(t)$ and another that returns λK_{ij}. It then computes the solution f at the quadrature points in the member variable `f`. It also stores the quadrature points and weights. These are used by the member function `fredin` to carry out the Nystrom interpolation of equation (19.1.3) and return the value of f at any point in the interval $[a, b]$.

To be sure the usage is clear, here is the calling sequence when you have coded the external routines as functions:

```
Doub g(const Doub t) { ... }
Doub ak(const Doub t, const Doub s) { ... }
    ...
Fred2<Doub (Doub), Doub (Doub,Doub)> fred2(a,b,n,g,ak);
Doub ans=fred2.fredin(x);
```

If the external routines are functors `Gfunc` and `Kernel`, say, then the declarations are

```
Gfunc g;                              This could have arguments if you like.
Kernel ak;
Fred2<Gfunc, Kernel> fred2(a,b,n,g,ak);
```

Here is the routine:

fred2.h

```
template <class G, class K>
struct Fred2 {
Solves a linear Fredholm equation of the second kind.
    const Doub a,b;
    const Int n;
    G &g;
    K &ak;
    VecDoub t,f,w;
    Fred2(const Doub aa, const Doub bb, const Int nn, G &gg, K &akk) :
        a(aa), b(bb), n(nn), g(gg), ak(akk), t(n), f(n), w(n)
    Quantities a and b are input as the limits of integration. The quantity n is the number of
    points to use in the Gaussian quadrature. g and ak are user-supplied functions or functors
    that respectively return g(t) and λK(t,s). This constructor computes arrays t[0..n-1]
    and f[0..n-1] containing the abscissas t_i of the Gaussian quadrature and the solution f
    at these abscissas. Also computed is the array w[0..n-1] of Gaussian weights for use with
    the Nystrom interpolation routine fredin.
    {
        MatDoub omk(n,n);
        gauleg(a,b,t,w);            Replace gauleg with another routine if not using
        for (Int i=0;i<n;i++) {         Gauss-Legendre quadrature.
            for (Int j=0;j<n;j++)   Form 1 − λK̃.
                omk[i][j]=Doub(i == j)-ak(t[i],t[j])*w[j];
            f[i]=g(t[i]);
        }
        LUdcmp alu(omk);            Solve linear equations.
        alu.solve(f,f);
    }

    Doub fredin(const Doub x)
    Given arrays t[0..n-1] and w[0..n-1] containing the abscissas and weights of the Gauss-
    ian quadrature, and given the solution array f[0..n-1], this function returns the value of
    f at x using the Nystrom interpolation formula.
    {
        Doub sum=0.0;
        for (Int i=0;i<n;i++) sum += ak(x,t[i])*w[i]*f[i];
        return g(x)+sum;
    }
};
```

One disadvantage of a method based on Gaussian quadrature is that there is no simple way to obtain an estimate of the error in the result. The best practical method is to increase N by 50%, say, and treat the difference between the two estimates as a conservative estimate of the error in the result obtained with the larger value of N.

Turn now to solutions of the homogeneous equation. If we set $\lambda = 1/\sigma$ and $\mathbf{g} = 0$, then equation (19.1.6) becomes a standard eigenvalue equation,

$$\widetilde{\mathbf{K}} \cdot \mathbf{f} = \sigma \mathbf{f} \tag{19.1.7}$$

which we can solve with any convenient matrix eigenvalue routine (see Chapter 11). Note that if our original problem had a symmetric kernel, then the matrix $\mathbf{K}$ would be symmetric. However, since the weights w_j are not equal for most quadrature rules, the matrix $\widetilde{\mathbf{K}}$ (equation 19.1.5) is not symmetric. The matrix eigenvalue problem is much easier for symmetric matrices, and so we should restore the symmetry if possible. Provided the weights are positive (which they are for Gaussian quadrature), we can define the diagonal matrix $\mathbf{D} = \text{diag}(w_j)$ and its square root, $\mathbf{D}^{1/2} = \text{diag}(\sqrt{w_j})$. Then equation (19.1.7) becomes

$$\mathbf{K} \cdot \mathbf{D} \cdot \mathbf{f} = \sigma \mathbf{f}$$

Multiplying by $\mathbf{D}^{1/2}$, we get

$$\left(\mathbf{D}^{1/2} \cdot \mathbf{K} \cdot \mathbf{D}^{1/2}\right) \cdot \mathbf{h} = \sigma \mathbf{h} \tag{19.1.8}$$

where $\mathbf{h} = \mathbf{D}^{1/2} \cdot \mathbf{f}$. Equation (19.1.8) is now in the form of a symmetric eigenvalue problem.

Solution of equations (19.1.7) or (19.1.8) will in general give N eigenvalues, where N is the number of quadrature points used. For square-integrable kernels, these will provide good approximations to the lowest N eigenvalues of the integral equation. Kernels of *finite rank* (also called *degenerate* or *separable* kernels) have only a finite number of nonzero eigenvalues (possibly none). You can diagnose this situation by a cluster of eigenvalues σ that are zero to machine precision. The number of nonzero eigenvalues will stay constant as you increase N to improve their accuracy. Some care is required here: A nondegenerate kernel can have an infinite number of eigenvalues that have an accumulation point at $\sigma = 0$. You distinguish the two cases by the behavior of the solution as you increase N. If you suspect a degenerate kernel, you will usually be able to solve the problem by analytic techniques described in all the textbooks.

CITED REFERENCES AND FURTHER READING:

Delves, L.M., and Mohamed, J.L. 1985, *Computational Methods for Integral Equations* (Cambridge, UK: Cambridge University Press).[1]

Atkinson, K.E. 1976, *A Survey of Numerical Methods for the Solution of Fredholm Integral Equations of the Second Kind* (Philadelphia: S.I.A.M.).

19.2 Volterra Equations

Let us now turn to Volterra equations, of which our prototype is the Volterra equation of the second kind,

$$f(t) = \int_a^t K(t,s) f(s)\, ds + g(t) \tag{19.2.1}$$

Most algorithms for Volterra equations march out from $t = a$, building up the solution as they go. In this sense they resemble not only forward substitution (as discussed in §19.0), but also initial value problems for ordinary differential equations. In fact, many algorithms for ODEs have counterparts for Volterra equations.

The simplest way to proceed is to solve the equation on a mesh with uniform spacing:

$$t_i = a + ih, \quad i = 0, 1, \ldots, N, \qquad h \equiv \frac{b-a}{N} \tag{19.2.2}$$

To do so, we must choose a quadrature rule. For a uniform mesh, the simplest scheme is the trapezoidal rule, equation (4.1.11):

$$\int_a^{t_i} K(t_i, s) f(s)\, ds = h\left(\tfrac{1}{2}K_{i0} f_0 + \sum_{j=1}^{i-1} K_{ij} f_j + \tfrac{1}{2}K_{ii} f_i\right) \tag{19.2.3}$$

Thus the trapezoidal method for equation (19.2.1) is

$$\begin{aligned} f_0 &= g_0 \\ (1 - \tfrac{1}{2}hK_{ii}) f_i &= h\left(\tfrac{1}{2}K_{i0} f_0 + \sum_{j=1}^{i-1} K_{ij} f_j\right) + g_i, \qquad i = 1, \ldots, N \end{aligned} \tag{19.2.4}$$

(For a Volterra equation of the first kind, the leading 1 on the left would be absent, and g would have opposite sign, with corresponding straightforward changes in the rest of the discussion.)

Equation (19.2.4) is an explicit prescription that gives the solution in $O(N^2)$ operations. Unlike Fredholm equations, it is not necessary to solve a system of linear equations. Volterra equations thus usually involve less work than the corresponding Fredholm equations, which, as we have seen, do involve the inversion of, sometimes large, linear systems.

The efficiency of solving Volterra equations is somewhat counterbalanced by the fact that *systems* of these equations occur more frequently in practice. If we interpret equation (19.2.1) as a *vector* equation for the vector of m functions $f(t)$, then the kernel $K(t, s)$ is an $m \times m$ matrix. Equation (19.2.4) must now also be understood as a vector equation. For each i, we have to solve the $m \times m$ set of linear algebraic equations by Gaussian elimination.

The routine `voltra` below implements this algorithm. You must supply an external function or functor that returns the kth function of the vector $g(t)$ at the point t and another that returns the (k, l) element of the matrix $K(t, s)$ at (t, s). The routine `voltra` then returns the vector $f(t)$ at the regularly spaced points t_i.

voltra.h

```
template <class G, class K>
void voltra(const Doub t0, const Doub h, G &g, K &ak, VecDoub_O &t, MatDoub_O &f)
```

Solves a set of `m` linear Volterra equations of the second kind using the extended trapezoidal rule. On input, `t0` is the starting point of the integration and `h` is the stepsize. `g(k,t)` is a user-supplied function or functor that returns $g_k(t)$, while `ak(k,l,t,s)` is another user-supplied function or functor that returns the (k, l) element of the matrix $K(t, s)$. The solution is returned in `f[0..m-1][0..n-1]`, with the corresponding abscissas in `t[0..n-1]`, where `n-1` is the number of steps to be taken. The value of `m` is determined from the row-dimension of the solution matrix `f`.

```
{
    Int m=f.nrows();
```

```
    Int n=f.ncols();
    VecDoub b(m);
    MatDoub a(m,m);
    t[0]=t0;
    for (Int k=0;k<m;k++) f[k][0]=g(k,t[0]);        Initialize.
    for (Int i=1;i<n;i++) {                         Take a step h.
        t[i]=t[i-1]+h;
        for (Int k=0;k<m;k++) {
            Doub sum=g(k,t[i]);                     Accumulate right-hand side of linear
            for (Int l=0;l<m;l++) {                     equations in sum.
                sum += 0.5*h*ak(k,l,t[i],t[0])*f[l][0];
                for (Int j=1;j<i;j++)
                    sum += h*ak(k,l,t[i],t[j])*f[l][j];
                if (k == l)                         Left-hand side goes in matrix a.
                    a[k][l]=1.0-0.5*h*ak(k,l,t[i],t[i]);
                else
                    a[k][l] = -0.5*h*ak(k,l,t[i],t[i]);
            }
            b[k]=sum;
        }
        LUdcmp alu(a);                              Solve linear equations.
        alu.solve(b,b);
        for (Int k=0;k<m;k++) f[k][i]=b[k];
    }
}
```

For nonlinear Volterra equations, equation (19.2.4) holds with the product $K_{ii} f_i$ replaced by $K_{ii}(f_i)$, and similarly for the other two products of K's and f's. Thus, for each i we solve a nonlinear equation for f_i with a known right-hand side. Newton's method (§9.4 or §9.6) with an initial guess of f_{i-1} usually works very well provided the stepsize is not too big.

Higher-order methods for solving Volterra equations are, in our opinion, not as important as for Fredholm equations, since Volterra equations are relatively easy to solve. However, there is an extensive literature on the subject. Several difficulties arise. First, any method that achieves higher order by operating on several quadrature points simultaneously will need a special method to get started, when values at the first few points are not yet known.

Second, stable quadrature rules can give rise to unexpected instabilities in integral equations. For example, suppose we try to replace the trapezoidal rule in the algorithm above with Simpson's rule. Simpson's rule naturally integrates over an interval $2h$, so we easily get the function values at the even mesh points. For the odd mesh points, we could try appending one panel of the trapezoidal rule. But to which end of the integration should we append it? We could do one step of the trapezoidal rule followed by all Simpson's rule, or Simpson's rule with one step of the trapezoidal rule at the end. Surprisingly, the former scheme is unstable, while the latter is fine!

A simple approach that can be used with the trapezoidal method given above is Richardson extrapolation: Compute the solution with stepsizes h and $h/2$. Then, assuming the error scales with h^2, compute

$$f_E = \frac{4f(h/2) - f(h)}{3} \tag{19.2.5}$$

This procedure can be repeated as with Romberg integration.

The general consensus is that the best of the higher-order methods is the *block-by-block method* (see [1]). Another important topic is the use of variable stepsize

methods, which are much more efficient if there are sharp features in K or f. Variable stepsize methods are quite a bit more complicated than their counterparts for differential equations; we refer you to the literature [1,2] for a discussion.

You should also be on the lookout for singularities in the integrand. If you find them, look to §19.3 for additional ideas.

CITED REFERENCES AND FURTHER READING:

Linz, P. 1985, *Analytical and Numerical Methods for Volterra Equations* (Philadelphia: S.I.A.M.).[1]

Delves, L.M., and Mohamed, J.L. 1985, *Computational Methods for Integral Equations* (Cambridge, UK: Cambridge University Press).[2]

19.3 Integral Equations with Singular Kernels

Many integral equations have singularities in either the kernel or the solution or both. A simple quadrature method will show poor convergence with N if such singularities are ignored. There is sometimes art in how singularities are best handled.

We start with a few straightforward suggestions:

1. Integrable singularities can often be removed by a change of variable. For example, the singular behavior $K(t,s) \sim s^{1/2}$ or $s^{-1/2}$ near $s = 0$ can be removed by the transformation $z = s^{1/2}$. Note that we are assuming that the singular behavior is confined to K, whereas the quadrature actually involves the product $K(t,s)f(s)$, and it is this product that must be "fixed." Ideally, you must deduce the singular nature of the product before you try a numerical solution, and take the appropriate action. Commonly, however, a singular kernel does *not* produce a singular solution $f(t)$. (The highly singular kernel $K(t,s) = \delta(t-s)$ is simply the identity operator, for example.)

2. If $K(t,s)$ can be factored as $w(s)\bar{K}(t,s)$, where $w(s)$ is singular and $\bar{K}(t,s)$ is smooth, then a Gaussian quadrature based on $w(s)$ as a weight function will work well. Even if the factorization is only approximate, the convergence is often improved dramatically. All you have to do is replace `gauleg` in the routine `fred2` by another quadrature routine. Section 4.6 explained how to construct such quadratures; or you can find tabulated abscissas and weights in the standard references [1,2]. You must of course supply $\bar{K}$ instead of K.

This method is a special case of the *product Nystrom method* [3,4], where one factors out a singular term $p(t,s)$ depending on both t and s from K and constructs suitable weights for its Gaussian quadrature. The calculations in the general case are quite cumbersome, because the weights depend on the chosen $\{t_i\}$ as well as the form of $p(t,s)$.

We prefer to implement the product Nystrom method on a uniform grid, with a quadrature scheme that generalizes the extended Simpson's 3/8 rule (equation 4.1.5) to arbitrary weight functions. We discuss this in the subsections below.

3. Special quadrature formulas are also useful when the kernel is not strictly singular, but is "almost" so. One example is when the kernel is concentrated near $t = s$ on a scale much smaller than the scale on which the solution $f(t)$ varies. In that case, a quadrature formula can be based on locally approximating $f(s)$ by a polynomial or spline, while calculating the first few *moments* of the kernel $K(t,s)$ at the tabulation points t_i. In such a scheme the narrow width of the kernel becomes an asset, rather than a liability: The quadrature becomes exact as the width of the kernel goes to zero.

4. An infinite range of integration is also a form of singularity. Truncating the range at a large finite value should be used only as a last resort. If the kernel goes rapidly to zero, then a Gauss-Laguerre [$w \sim \exp(-\alpha s)$] or Gauss-Hermite [$w \sim \exp(-s^2)$] quadrature should work well. Long-tailed functions often succumb to the transformation

$$s = \frac{2\alpha}{z+1} - \alpha \tag{19.3.1}$$

which maps $0 < s < \infty$ to $1 > z > -1$ so that Gauss-Legendre integration can be used. Here $\alpha > 0$ is a constant that you adjust to improve the convergence.

5. A common situation in practice is that $K(t,s)$ is singular along the diagonal line $t = s$. Here the Nystrom method fails completely because the kernel gets evaluated at (t_i, s_i). *Subtraction of the singularity* is one possible cure:

$$\begin{aligned}\int_a^b K(t,s)f(s)\,ds &= \int_a^b K(t,s)[f(s)-f(t)]\,ds + \int_a^b K(t,s)f(t)\,ds \\ &= \int_a^b K(t,s)[f(s)-f(t)]\,ds + r(t)f(t)\end{aligned} \tag{19.3.2}$$

where $r(t) = \int_a^b K(t,s)\,ds$ is computed analytically or numerically. If the first term on the right-hand side is now regular, we can use the Nystrom method. Instead of equation (19.1.4), we get

$$f_i = \lambda \sum_{\substack{j=0 \\ j \neq i}}^{N-1} w_j K_{ij}[f_j - f_i] + \lambda r_i f_i + g_i \tag{19.3.3}$$

Sometimes the subtraction process must be repeated before the kernel is completely regularized. See [3] for details. (And read on for a different, we think better, way to handle diagonal singularities.)

19.3.1 Quadrature on a Uniform Mesh with Arbitrary Weight

It is possible in general to find n-point linear quadrature rules that approximate the integral of a function $f(x)$, times an arbitrary weight function $w(x)$, over an arbitrary range of integration (a,b), as the sum of weights times n evenly spaced values of the function $f(x)$, say at $x = kh, (k+1)h, \dots, (k+n-1)h$. The general scheme for deriving such quadrature rules is to write down the n linear equations that must be satisfied if the quadrature rule is to be exact for the n functions $f(x) = \text{const}, x, x^2, \dots, x^{n-1}$, and then solve these for the coefficients. This can be done analytically, once and for all, if the moments of the weight function over the same range of integration,

$$W_n \equiv \frac{1}{h^n}\int_a^b x^n w(x)dx \tag{19.3.4}$$

are assumed to be known. Here the prefactor h^{-n} is chosen to make W_n scale as h if (as in the usual case) $b-a$ is proportional to h.

Carrying out this prescription for the four-point case gives the result

$$\begin{aligned}&\int_a^b w(x)f(x)dx = \\ &\quad \tfrac{1}{6}f(kh)\big[(k+1)(k+2)(k+3)W_0 - (3k^2+12k+11)W_1 + 3(k+2)W_2 - W_3\big] \\ &\quad + \tfrac{1}{2}f([k+1]h)\big[-k(k+2)(k+3)W_0 + (3k^2+10k+6)W_1 - (3k+5)W_2 + W_3\big] \\ &\quad + \tfrac{1}{2}f([k+2]h)\big[k(k+1)(k+3)W_0 - (3k^2+8k+3)W_1 + (3k+4)W_2 - W_3\big] \\ &\quad + \tfrac{1}{6}f([k+3]h)\big[-k(k+1)(k+2)W_0 + (3k^2+6k+2)W_1 - 3(k+1)W_2 + W_3\big]\end{aligned} \tag{19.3.5}$$

While the terms in brackets superficially appear to scale as k^2, there is typically cancellation at both $O(k^2)$ and $O(k)$.

Equation (19.3.5) can be specialized to various choices of (a,b). The obvious choice is $a = kh$, $b = (k+3)h$, in which case we get a four-point quadrature rule that generalizes

Simpson's 3/8 rule (equation 4.1.5). In fact, we can recover this special case by setting $w(x) = 1$, in which case (19.3.4) becomes

$$W_n = \frac{h}{n+1}[(k+3)^{n+1} - k^{n+1}] \tag{19.3.6}$$

The four terms in square brackets in equation (19.3.5) each become independent of k, and (19.3.5) in fact reduces to

$$\int_{kh}^{(k+3)h} f(x)dx = \frac{3h}{8}f(kh) + \frac{9h}{8}f([k+1]h) + \frac{9h}{8}f([k+2]h) + \frac{3h}{8}f([k+3]h) \tag{19.3.7}$$

Back to the case of general $w(x)$, some other choices for a and b are also useful. For example, we may want to choose (a, b) to be $([k+1]h, [k+3]h)$ or $([k+2]h, [k+3]h)$, allowing us to finish off an extended rule whose number of intervals is not a multiple of three, without loss of accuracy: The integral will be estimated using the four values $f(kh), \ldots, f([k+3]h)$. Even more useful is to choose (a, b) to be $([k+1]h, [k+2]h)$, thus using four points to integrate a centered single interval. These weights, when sewed together into an extended formula, give quadrature schemes that have smooth coefficients, i.e., without the Simpson-like 2, 4, 2, 4, 2 alternation. (In fact, this was the technique that we used to derive equation 4.1.14, which you may now wish to re-examine.)

All these rules are of the same order as the extended Simpson's rule, that is, exact for $f(x)$ a cubic polynomial. Rules of lower order, if desired, are similarly obtained. The three-point formula is

$$\begin{aligned}\int_a^b w(x)f(x)dx = {} & \tfrac{1}{2}f(kh)\big[(k+1)(k+2)W_0 - (2k+3)W_1 + W_2\big] \\ & + f([k+1]h)\big[-k(k+2)W_0 + 2(k+1)W_1 - W_2\big] \\ & + \tfrac{1}{2}f([k+2]h)\big[k(k+1)W_0 - (2k+1)W_1 + W_2\big]\end{aligned} \tag{19.3.8}$$

Here the simple special case is to take $w(x) = 1$, so that

$$W_n = \frac{h}{n+1}[(k+2)^{n+1} - k^{n+1}] \tag{19.3.9}$$

Then equation (19.3.8) becomes Simpson's rule,

$$\int_{kh}^{(k+2)h} f(x)dx = \frac{h}{3}f(kh) + \frac{4h}{3}f([k+1]h) + \frac{h}{3}f([k+2]h) \tag{19.3.10}$$

For nonconstant weight functions $w(x)$, however, equation (19.3.8) gives rules of one order less than Simpson, since they do not benefit from the extra symmetry of the constant case.

The two-point formula is simply

$$\int_{kh}^{(k+1)h} w(x)f(x)dx = f(kh)[(k+1)W_0 - W_1] + f([k+1]h)[-kW_0 + W_1] \tag{19.3.11}$$

Here is a routine `Wwghts` that uses the above formulas to compute an extended N-point quadrature rule for the interval $(a, b) = (0, [N-1]h)$. Input to `Wwghts` is a user-supplied object called `quad` in the routine. This object must contain a function `kermom`, which is called to get the first four *indefinite-integral* moments of $w(x)$, namely

$$F_m(y) \equiv \int^y s^m w(s)ds \qquad m = 0, 1, 2, 3 \tag{19.3.12}$$

(The lower limit is arbitrary and can be chosen for convenience.) Cautionary note: When called with $N < 4$, `Wwghts` returns a rule of lower order than Simpson; you should structure your problem to avoid this.

fred_singular.h

```
template <class Q>
struct Wwghts {
```
Constructs weights for the n-point equal-interval quadrature from 0 to (n−1)h of a function $f(x)$ times an arbitrary (possibly singular) weight function $w(x)$. The indefinite-integral moments $F_n(y)$ of $w(x)$ are provided by the user-supplied function kermom in the quad object.
```
    Doub h;
    Int n;
    Q &quad;
    VecDoub wghts;
    Wwghts(Doub hh, Int nn, Q &q) : h(hh), n(nn), quad(q), wghts(n) {}
```
Constructor arguments are h, n, and the user-supplied quad object.
```
    VecDoub weights()
```
This function returns the weights in wghts[0..n-1].
```
    {
        Int k;
        Doub fac;
        Doub hi=1.0/h;
        for (Int j=0;j<n;j++)                 Zero all the weights so we can sum into
            wghts[j]=0.0;                        them.
        if (n >= 4) {                         Use highest available order.
            VecDoub wold(4),wnew(4),w(4);
            wold=quad.kermom(0.0);            Evaluate indefinite integrals at lower end.
            Doub b=0.0;                       For another problem, you might change
                                                 this lower limit.
            for (Int j=0;j<n-3;j++) {
                Doub c=j;                     This is called k in equation (19.3.5).
                Doub a=b;                     Set upper and lower limits for this step.
                b=a+h;
                if (j == n-4) b=(n-1)*h;      Last interval: Go all the way to end.
                wnew=quad.kermom(b);
                for (fac=1.0,k=0;k<4;k++,fac*=hi)           Equation (19.3.4).
                    w[k]=(wnew[k]-wold[k])*fac;
                wghts[j] += (((c+1.0)*(c+2.0)*(c+3.0)*w[0]   Equation (19.3.5).
                    -(11.0+c*(12.0+c*3.0))*w[1]+3.0*(c+2.0)*w[2]-w[3])/6.0);
                wghts[j+1] += ((-c*(c+2.0)*(c+3.0)*w[0]
                    +(6.0+c*(10.0+c*3.0))*w[1]-(3.0*c+5.0)*w[2]+w[3])*0.5);
                wghts[j+2] += ((c*(c+1.0)*(c+3.0)*w[0]
                    -(3.0+c*(8.0+c*3.0))*w[1]+(3.0*c+4.0)*w[2]-w[3])*0.5);
                wghts[j+3] += ((-c*(c+1.0)*(c+2.0)*w[0]
                    +(2.0+c*(6.0+c*3.0))*w[1]-3.0*(c+1.0)*w[2]+w[3])/6.0);
                for (k=0;k<4;k++) wold[k]=wnew[k];    Reset lower limits for moments.
            }
        } else if (n == 3) {                  Lower-order cases; not recommended.
            VecDoub wold(3),wnew(3),w(3);
            wold=quad.kermom(0.0);
            wnew=quad.kermom(h+h);
            w[0]=wnew[0]-wold[0];
            w[1]=hi*(wnew[1]-wold[1]);
            w[2]=hi*hi*(wnew[2]-wold[2]);
            wghts[0]=w[0]-1.5*w[1]+0.5*w[2];
            wghts[1]=2.0*w[1]-w[2];
            wghts[2]=0.5*(w[2]-w[1]);
        } else if (n == 2) {
            VecDoub wold(2),wnew(2),w(2);
            wold=quad.kermom(0.0);
            wnew=quad.kermom(h);
            wghts[0]=wnew[0]-wold[0]-(wghts[1]=hi*(wnew[1]-wold[1]));
        }
        return wghts;
    }
};
```

We will now give an example of how to apply Wwghts to a singular integral equation.

19.3.2 Worked Example: A Diagonally Singular Kernel

As a particular example, consider the integral equation

$$f(x) + \int_0^{\pi} K(x,y) f(y) dy = \sin x \tag{19.3.13}$$

with the (arbitrarily chosen) nasty kernel

$$K(x,y) = \cos x \cos y \times \begin{cases} -\ln(x-y) & y < x \\ \sqrt{y-x} & y \ge x \end{cases} \tag{19.3.14}$$

which has a logarithmic singularity on the left of the diagonal, combined with a square-root discontinuity on the right.

The first step is to do (analytically, in this case) the required moment integrals over the singular part of the kernel, equation (19.3.12). Since these integrals are done at a fixed value of x, we can use x as the lower limit. For any specified value of y, the required indefinite integral is then either

$$F_m(y;x) = \int_x^y s^m (s-x)^{1/2} ds = \int_0^{y-x} (x+t)^m t^{1/2} dt \qquad \text{if } y > x \tag{19.3.15}$$

or

$$F_m(y;x) = -\int_x^y s^m \ln(x-s) ds = \int_0^{x-y} (x-t)^m \ln t \, dt \qquad \text{if } y < x \tag{19.3.16}$$

(where a change of variable has been made in the second equality in each case). Doing these integrals analytically (e.g., using a symbolic integration package), we package the resulting formulas in the function `kermom` in the following routine, `Quad_matrix`. Note that `w`$(j+1)$ returns $F_j(y;x)$. The constructor of `Quad_matrix` calls `Wwghts` to get the quadrature weights and then constructs the quadrature matrix.

fred_singular.h

```
struct Quad_matrix {
```
Constructs in `a[0..n-1][0..n-1]` the quadrature matrix for an example Fredholm equation of the second kind.
```
    Int n;
    Doub x;                              Communicates with kermom.
    Quad_matrix(MatDoub_O &a) : n(a.nrows())
```
The constructor obtains the quadrature weights that integrate the singular part of the kernel via calls to `Wwghts`. It then sums the weights with the nonsingular part of the kernel to obtain the quadrature matrix.
```
    {
        const Doub PI=3.14159263589793238;
        VecDoub wt(n);
        Doub h=PI/(n-1);
        Wwghts<Quad_matrix> w(h,n,*this);
        for (Int j=0;j<n;j++) {
            x=j*h;                       Set x for kermom.
            wt=w.weights();
            Doub cx=cos(x);              Part of nonsingular kernel.
            for (Int k=0;k<n;k++)        Put together all the pieces of the kernel.
                a[j][k]=wt[k]*cx*cos(k*h);
            ++a[j][j];                   For equations of the second kind, there is a diagonal
        }                                    piece independent of h.
    }
    VecDoub kermom(const Doub y)
```
Returns `w[0..m-1]`, the first `m` indefinite-integral moments of one row of the singular part of the kernel. (For this example, `m` is hard-wired to be 4.) The input variable `y` labels the column, while the member variable `x` is the row. We can take `x` as the lower limit of integration. Thus, we return the moment integrals either purely to the left or purely to the right of the diagonal.
```
    {
```

```
        Doub d,df,clog,x2,x3,x4,y2;
        VecDoub w(4);
        if (y >= x) {
            d=y-x;
            df=2.0*sqrt(d)*d;
            w[0]=df/3.0;
            w[1]=df*(x/3.0+d/5.0);
            w[2]=df*((x/3.0 + 0.4*d)*x + d*d/7.0);
            w[3]=df*(((x/3.0 + 0.6*d)*x + 3.0*d*d/7.0)*x+d*d*d/9.0);
        } else {
            x3=(x2=x*x)*x;
            x4=x2*x2;
            y2=y*y;
            d=x-y;
            w[0]=d*((clog=log(d))-1.0);
            w[1] = -0.25*(3.0*x+y-2.0*clog*(x+y))*d;
            w[2]=(-11.0*x3+y*(6.0*x2+y*(3.0*x+2.0*y))
                +6.0*clog*(x3-y*y2))/18.0;
            w[3]=(-25.0*x4+y*(12.0*x3+y*(6.0*x2+y*
                (4.0*x+3.0*y)))+12.0*clog*(x4-(y2*y2)))/48.0;
        }
        return w;
    }
};
```

Finally, we solve the linear system for any particular right-hand side, here $\sin x$.

fred_singular.h

```
Int main_fredex(void)
```

This sample program shows how to solve a Fredholm equation of the second kind using the product Nystrom method and a quadrature rule especially constructed for a particular, singular, kernel.

```
{
    const Int N=40;                     Here the size of the grid is specified.
    const Doub PI=3.141592653589793238;
    VecDoub g(N);
    MatDoub a(N,N);
    Quad_matrix qmx(a);                 Make the quadrature matrix; all the action is here.
    LUdcmp alu(a);                      Decompose the matrix.
    for (Int j=0;j<N;j++)               Construct the right-hand side, here sin x.
        g[j]=sin(j*PI/(N-1));
    alu.solve(g,g);                     Backsubstitute.
    for (Int j=0;j<N;j++) {             Write out the solution.
        Doub x=j*PI/(N-1);
        cout << fixed << setprecision(2) << setw(6) << (j+1);
        cout << setprecision(6) << setw(13) << x << setw(13) << g[j] << endl;
    }
    return 0;
}
```

With $N = 40$, this program gives accuracy at about the 10^{-5} level. The accuracy increases as N^4 (as it should for our Simpson-order quadrature scheme) *despite* the highly singular kernel. Figure 19.3.1 shows the solution obtained, also plotting the solution for smaller values of N, which are themselves seen to be remarkably faithful. Notice that the solution is smooth, even though the kernel is singular, a common occurrence.

CITED REFERENCES AND FURTHER READING:

Abramowitz, M., and Stegun, I.A. 1964, *Handbook of Mathematical Functions* (Washington: National Bureau of Standards); reprinted 1968 (New York: Dover); online at http://www.nr.com/aands.[1]

Stroud, A.H., and Secrest, D. 1966, *Gaussian Quadrature Formulas* (Englewood Cliffs, NJ: Prentice-Hall).[2]

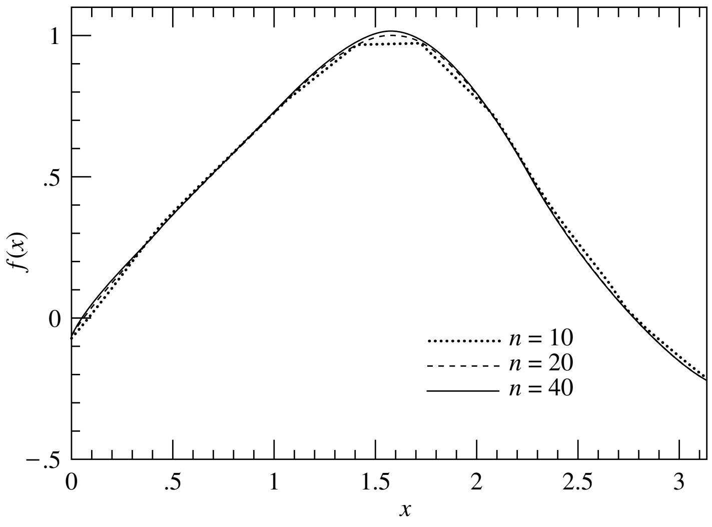

Figure 19.3.1. Solution of the example integral equation (19.3.14) with grid sizes $N = 10$, 20, and 40. The tabulated solution values have been connected by straight lines; in practice, one would interpolate a small N solution more smoothly.

Delves, L.M., and Mohamed, J.L. 1985, *Computational Methods for Integral Equations* (Cambridge, UK: Cambridge University Press).[3]

Atkinson, K.E. 1976, *A Survey of Numerical Methods for the Solution of Fredholm Integral Equations of the Second Kind* (Philadelphia: S.I.A.M.).[4]

19.4 Inverse Problems and the Use of A Priori Information

Later discussion will be facilitated by some preliminary mention of a couple of mathematical points. Suppose that $\mathbf{u}$ is an "unknown" vector that we plan to determine by some minimization principle. Let $\mathcal{A}[\mathbf{u}] > 0$ and $\mathcal{B}[\mathbf{u}] > 0$ be two positive functionals of $\mathbf{u}$, so that we can try to determine $\mathbf{u}$ by either

$$\text{minimize:} \quad \mathcal{A}[\mathbf{u}] \qquad \text{or} \qquad \text{minimize:} \quad \mathcal{B}[\mathbf{u}] \tag{19.4.1}$$

(Of course these will generally give different answers for $\mathbf{u}$.) As another possibility, now suppose that we want to minimize $\mathcal{A}[\mathbf{u}]$ subject to the *constraint* that $\mathcal{B}[\mathbf{u}]$ have some particular value, say b. The method of Lagrange multipliers gives the variation

$$\frac{\delta}{\delta \mathbf{u}}\left\{\mathcal{A}[\mathbf{u}] + \lambda_1(\mathcal{B}[\mathbf{u}] - b)\right\} = \frac{\delta}{\delta \mathbf{u}}\left(\mathcal{A}[\mathbf{u}] + \lambda_1\mathcal{B}[\mathbf{u}]\right) = 0 \tag{19.4.2}$$

where λ_1 is a Lagrange multiplier. Notice that b is absent in the second equality, since it doesn't depend on $\mathbf{u}$.

Next, suppose that we change our minds and decide to minimize $\mathcal{B}[\mathbf{u}]$ subject to the constraint that $\mathcal{A}[\mathbf{u}]$ have a particular value, a. Instead of equation (19.4.2) we

have

$$\frac{\delta}{\delta \mathbf{u}}\left\{\mathcal{B}[\mathbf{u}]+\lambda_2(\mathcal{A}[\mathbf{u}]-a)\right\}=\frac{\delta}{\delta \mathbf{u}}\left(\mathcal{B}[\mathbf{u}]+\lambda_2\mathcal{A}[\mathbf{u}]\right)=0 \tag{19.4.3}$$

with, this time, λ_2 the Lagrange multiplier. Multiplying equation (19.4.3) by the constant $1/\lambda_2$, and identifying $1/\lambda_2$ with λ_1, we see that the actual variations are exactly the same in the two cases. Both cases will yield the same one-parameter family of solutions, say $\mathbf{u}(\lambda_1)$. As λ_1 varies from 0 to ∞, the solution $\mathbf{u}(\lambda_1)$ varies along a so-called *trade-off curve* between the problem of minimizing $\mathcal{A}$ and the problem of minimizing $\mathcal{B}$. Any solution along this curve can equally well be thought of as either (i) a minimization of $\mathcal{A}$ for some constrained value of $\mathcal{B}$, or (ii) a minimization of $\mathcal{B}$ for some constrained value of $\mathcal{A}$, or (iii) a weighted minimization of the sum $\mathcal{A}+\lambda_1\mathcal{B}$.

The second preliminary point has to do with *degenerate* minimization principles. In the example above, now suppose that $\mathcal{A}[\mathbf{u}]$ has the particular form

$$\mathcal{A}[\mathbf{u}]=|\mathbf{A}\cdot\mathbf{u}-\mathbf{c}|^2 \tag{19.4.4}$$

for some matrix $\mathbf{A}$ and vector $\mathbf{c}$. If $\mathbf{A}$ has fewer rows than columns, or if $\mathbf{A}$ is square but degenerate (has a nontrivial nullspace; see §2.6, especially Figure 2.6.1), then minimizing $\mathcal{A}[\mathbf{u}]$ will *not* give a unique solution for $\mathbf{u}$. (To see why, review §15.4, and note that for a "design matrix" $\mathbf{A}$ with fewer rows than columns, the matrix $\mathbf{A}^T\cdot\mathbf{A}$ in the normal equations 15.4.10 is degenerate.) *However*, if we add any multiple λ times a nondegenerate quadratic form $\mathcal{B}[\mathbf{u}]$, for example $\mathbf{u}\cdot\mathbf{H}\cdot\mathbf{u}$ with $\mathbf{H}$ a positive-definite matrix, then minimization of $\mathcal{A}[\mathbf{u}]+\lambda\mathcal{B}[\mathbf{u}]$ *will* lead to a unique solution for $\mathbf{u}$. (The sum of two quadratic forms is itself a quadratic form, with the second piece guaranteeing nondegeneracy.)

We can combine these two points, for this conclusion: When a quadratic minimization principle is combined with a quadratic constraint, and both are positive, only *one* of the two need be nondegenerate for the overall problem to be well-posed. We are now equipped to face the subject of inverse problems.

19.4.1 The Inverse Problem with Zeroth-Order Regularization

Suppose that $u(x)$ is some unknown or underlying (u stands for both unknown and underlying!) physical process, which we hope to determine by a set of N measurements c_i, $i=0,1,\ldots,N-1$. The relation between $u(x)$ and the c_i's is that each c_i measures a (hopefully distinct) aspect of $u(x)$ through its own linear response kernel r_i, and with its own measurement error n_i. In other words,

$$c_i \equiv s_i+n_i=\int r_i(x)u(x)dx+n_i \tag{19.4.5}$$

(compare this to equations 13.3.1 and 13.3.2). Within the assumption of linearity, this is quite a general formulation. The c_i's might approximate values of $u(x)$ at certain locations x_i, in which case $r_i(x)$ would have the form of a more or less narrow instrumental response centered around $x=x_i$. Or, the c_i's might "live" in an entirely different function space from $u(x)$, measuring different Fourier components of $u(x)$, for example.

The *inverse problem* is, given the c_i's, the $r_i(x)$'s, and perhaps some information about the errors n_i such as their covariance matrix,

$$S_{ij} \equiv \text{Covar}[n_i, n_j] \tag{19.4.6}$$

how do we find a good statistical estimator of $u(x)$, call it $\widehat{u}(x)$?

It should be obvious that this is an ill-posed problem. After all, how can we reconstruct a whole function $\widehat{u}(x)$ from only a finite number of discrete values c_i? Yet, whether formally or informally, we do this all the time in science. We routinely measure "enough points" and then "draw a curve through them." In doing so, we are making some assumptions, either about the underlying function $u(x)$, or about the nature of the response functions $r_i(x)$, or both. Our purpose now is to formalize these assumptions, and to extend our abilities to cases where the measurements and underlying function live in quite different function spaces. (How do you "draw a curve" through a scattering of Fourier coefficients?)

We can't really want every point x of the function $\widehat{u}(x)$. We do want some large number M of discrete points x_μ, $\mu = 0, 1, \ldots, M-1$, where M is sufficiently large, and the x_μ's are sufficiently evenly spaced, that neither $u(x)$ nor $r_i(x)$ varies much between any x_μ and $x_{\mu+1}$. (Here and following we will use Greek letters like μ to denote values in the space of the underlying process, and Roman letters like i to denote values of immediate observables.) For such a dense set of x_μ's, we can replace equation (19.4.5) by a quadrature like

$$c_i = \sum_\mu R_{i\mu} u(x_\mu) + n_i \tag{19.4.7}$$

where the $N \times M$ matrix $\mathbf{R}$ has components

$$R_{i\mu} \equiv r_i(x_\mu)(x_{\mu+1} - x_{\mu-1})/2 \tag{19.4.8}$$

(or any other simple quadrature — it rarely matters which). We will view equations (19.4.5) and (19.4.7) as being equivalent for practical purposes.

How do you solve a set of equations like equation (19.4.7) for the unknown $u(x_\mu)$'s? Here is a bad way, but one that contains the germ of some correct ideas: Form a χ^2 measure of how well a model $u(x)$ agrees with the measured data,

$$\begin{aligned} \chi^2 &= \sum_{i=0}^{N-1} \sum_{j=0}^{N-1} \left[c_i - \sum_{\mu=0}^{M-1} R_{i\mu} u(x_\mu) \right] S_{ij}^{-1} \left[c_j - \sum_{\mu=0}^{M-1} R_{j\mu} u(x_\mu) \right] \\ &\approx \sum_{i=0}^{N-1} \left[\frac{c_i - \sum_{\mu=0}^{M-1} R_{i\mu} u(x_\mu)}{\sigma_i} \right]^2 \end{aligned} \tag{19.4.9}$$

(compare with equation 15.1.6). Here $\mathbf{S}^{-1}$ is the inverse of the covariance matrix, and the approximate equality holds if you can neglect the off-diagonal covariances, with $\sigma_i \equiv (\text{Covar}[i, i])^{1/2}$.

Now you can use the method of singular value decomposition (SVD) in §15.4 to find the vector $\mathbf{u}$ that minimizes equation (19.4.9). Don't try to use the method of normal equations; since M is greater than N, they will be singular, as we already discussed. The SVD process will thus surely find a large number of zero singular

values, indicative of a highly nonunique solution. Among the infinity of degenerate solutions (most of them badly behaved with arbitrarily large $u(x_\mu)$'s) SVD will select the one of them, call it $\hat{\mathbf{u}}$, with the smallest norm $|\hat{\mathbf{u}}|$ in the sense of

$$\sum_\mu [\hat{u}(x_\mu)]^2 \quad \text{a minimum} \tag{19.4.10}$$

(look at Figure 2.6.1). This solution is often called the *principal solution*. It is a limiting case of what is called *zeroth-order regularization*, corresponding to minimizing the sum of the two positive functionals

$$\hat{\mathbf{u}} \text{ minimizes:} \qquad \chi^2[\mathbf{u}] + \lambda(\mathbf{u} \cdot \mathbf{u}) \tag{19.4.11}$$

in the limit of small λ. Below, we will learn how to do such minimizations, as well as more general ones, without the ad hoc use of SVD.

What happens if we determine $\hat{\mathbf{u}}$ by equation (19.4.11) with a non-infinitesimal value of λ? First, note that if $M \gg N$ (many more unknowns than equations), then $\mathbf{u}$ will often have enough freedom to be able to make χ^2 (equation 19.4.9) quite unrealistically small, if not zero. In the language of §15.1, the number of degrees of freedom $\nu = N - M$, which is approximately the expected value of χ^2 when ν is large, is being driven down to zero (and, not meaningfully, beyond). Yet, we know that for the *true* underlying function $u(x)$, which has no adjustable parameters, the number of degrees of freedom and the expected value of χ^2 should be about $\nu \approx N$.

Increasing λ pulls the solution away from minimizing χ^2 in favor of minimizing $\hat{\mathbf{u}} \cdot \hat{\mathbf{u}}$. From the preliminary discussion above, we can view this as minimizing $\hat{\mathbf{u}} \cdot \hat{\mathbf{u}}$ subject to the *constraint* that χ^2 have some constant nonzero value. A popular choice, in fact, is to find that value of λ which yields $\chi^2 = N$, that is, to get about as much extra regularization as a plausible value of χ^2 dictates. The resulting $\hat{u}(x)$ is called *the solution of the inverse problem with zeroth-order regularization*.

The value N is actually a surrogate for any value drawn from a Gaussian distribution with mean N and standard deviation $(2N)^{1/2}$ (the asymptotic χ^2 distribution). One might equally plausibly try two values of λ, one giving $\chi^2 = N + (2N)^{1/2}$, the other $N - (2N)^{1/2}$.

Zeroth-order regularization, though dominated by better methods, demonstrates most of the basic ideas that are used in inverse problem theory. In general, there are two positive functionals, call them $\mathcal{A}$ and $\mathcal{B}$. The first, $\mathcal{A}$, measures something like the agreement of a model to the data (e.g., χ^2), or sometimes a related quantity like the "sharpness" of the mapping between the solution and the underlying function. When $\mathcal{A}$ by itself is minimized, the agreement or sharpness becomes very good (often impossibly good), but the solution becomes unstable, wildly oscillating, or in other ways unrealistic, reflecting that $\mathcal{A}$ alone typically defines a highly degenerate minimization problem.

That is where $\mathcal{B}$ comes in. It measures something like the "smoothness" of the desired solution, or sometimes a related quantity that parametrizes the stability of the solution with respect to variations in the data, or sometimes a quantity reflecting a priori judgments about the likelihood of a solution. $\mathcal{B}$ is called the *stabilizing functional* or *regularizing operator*. In any case, minimizing $\mathcal{B}$ by itself is supposed to give a solution that is "smooth" or "stable" or "likely" — and that has nothing at all to do with the measured data.

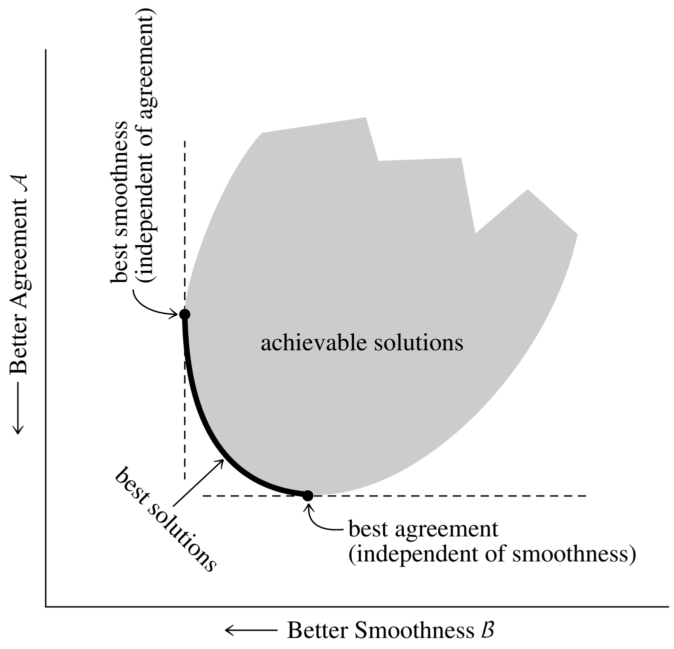

Figure 19.4.1. Almost all inverse problem methods involve a trade-off between two optimizations: agreement between data and solution, or "sharpness" of mapping between true and estimated solutions (here denoted $\mathscr{A}$), and smoothness or stability of the solution (here denoted $\mathscr{B}$). Among all possible solutions, shown here schematically as the shaded region, those on the boundary connecting the unconstrained minimum of $\mathscr{A}$ and the unconstrained minimum of $\mathscr{B}$ are the "best" solutions, in the sense that every other solution is dominated by at least one solution on the curve.

The single central idea in inverse theory is the prescription

$$\text{minimize:} \quad \mathscr{A} + \lambda \mathscr{B} \tag{19.4.12}$$

for various values of $0 < \lambda < \infty$ along the so-called trade-off curve (see Figure 19.4.1), and then to settle on a "best" value of λ by one or another criterion, ranging from fairly objective (e.g., making $\chi^2 = N$) to entirely subjective. Successful methods, several of which we will now describe, differ as to their choices of $\mathscr{A}$ and $\mathscr{B}$, as to whether the prescription (19.4.12) yields linear or nonlinear equations, as to their recommended method for selecting a final λ, and as to their practicality for computer-intensive two-dimensional problems like image processing.

Equation (19.4.12) has a natural Bayesian interpretation that gives some additional insight. Given the data points $\mathbf{c}$ and measurements $\mathbf{u}$, we can use Bayes' law, equation (15.0.1), to write

$$P(\mathbf{u}|\mathbf{c}, I) \propto P(\mathbf{c}|\mathbf{u}, I) P(\mathbf{u}|I) \tag{19.4.13}$$

where $P(\mathbf{u}|I)$ is the Bayesian prior on $\mathbf{u}$ before we see any data, given any background information I. Often, we can usefully write the right-hand side as the product of two exponentials, that is,

$$P(\mathbf{c}|\mathbf{u}, I) \equiv e^{-\mathscr{A}(\mathbf{c},\mathbf{u})}, \qquad P(\mathbf{u}|I) \equiv e^{-\lambda \mathscr{B}(\mathbf{u})} \tag{19.4.14}$$

For example, if the measurement errors are distributed as a multivariate Gaussian, then equation (19.4.9) implies

$$\mathcal{A} = \frac{1}{2}\chi^2(\mathbf{c}, \mathbf{u}) \tag{19.4.15}$$

while a Bayesian prior expressing the belief that $\mathbf{u}$ should not have wild, large-amplitude oscillations might be captured by the multivariate Gaussian prior

$$\mathcal{B} = \lambda(\mathbf{u} \cdot \mathbf{u}) \tag{19.4.16}$$

Maximizing $P(\mathbf{u}|\mathbf{c}, I)$ to find the most probable $\hat{\mathbf{u}}$ is now exactly equivalent to equation (19.4.11). The constant λ is now merely a convenient parameterization for the narrowness of the Gaussian prior. It acts as a trade-off parameter, exactly as described above. In subsequent sections we will learn how to devise more sophisticated *smoothness priors*. In several cases these will be positive-definite quadratic forms in $\mathbf{u}$; those cases correspond to more complicated multivariate Gaussian priors on $\mathbf{u}$. In §19.7 we will meet a prior that is not exactly Gaussian, but rather based on the concept of *entropy*.

Within the Bayesian framework, you can do more than just solve for the most likely model $\hat{\mathbf{u}}$. For example, you can use Markov chain Monte Carlo (§15.8) to sample from the distribution of $\mathbf{u}$'s given the observed data.

CITED REFERENCES AND FURTHER READING:

Craig, I.J.D., and Brown, J.C. 1986, *Inverse Problems in Astronomy* (Bristol, UK: Adam Hilger).

Twomey, S. 1977, *Introduction to the Mathematics of Inversion in Remote Sensing and Indirect Measurements* (Amsterdam: Elsevier).

Tikhonov, A.N., and Arsenin, V.Y. 1977, *Solutions of Ill-Posed Problems* (New York: Wiley).

Tikhonov, A.N., and Goncharsky, A.V. (eds.) 1987, *Ill-Posed Problems in the Natural Sciences* (Moscow: MIR).

Parker, R.L. 1977, "Understanding Inverse Theory," *Annual Review of Earth and Planetary Science*, vol. 5, pp. 35–64.

Frieden, B.R. 1975, in *Picture Processing and Digital Filtering*, T.S. Huang, ed. (New York: Springer).

Tarantola, A. 1995, *Inverse Problem Theory and Methods for Model Parameter Estimation* (Philadelphia: S.I.A.M.). Also available at `http://www.ipgp.jussieu.fr/~tarantola/Files/Professional/SIAM`.

Baumeister, J. 1987, *Stable Solution of Inverse Problems* (Braunschweig, Germany: Friedr. Vieweg) [mathematically oriented].

Titterington, D.M. 1985, "General Structure of Regularization Procedures in Image Reconstruction," *Astronomy and Astrophysics*, vol. 144, pp. 381–387.

Jeffrey, W., and Rosner, R. 1986, "On Strategies for Inverting Remote Sensing Data," *Astrophysical Journal*, vol. 310, pp. 463–472.

19.5 Linear Regularization Methods

What we will call *linear regularization* is also called the *Phillips-Twomey method* [1,2], the *constrained linear inversion method* [3], the *method of regularization* [4],

and *Tikhonov-Miller regularization* [5-7]. (It probably has other names also, since it is so obviously a good idea.) In its simplest form, the method is an immediate generalization of zeroth-order regularization (equation 19.4.11, above). As before, the functional $\mathcal{A}$ is taken to be the χ^2 deviation, equation (19.4.9), but the functional $\mathcal{B}$ is replaced by more sophisticated measures of smoothness that derive from first or higher derivatives.

For example, suppose that your a priori belief is that a credible $u(x)$ is not too different from a constant. Then a reasonable functional to minimize is

$$\mathcal{B} \propto \int [\widehat{u}'(x)]^2 dx \propto \sum_{\mu=0}^{M-2} [\widehat{u}_\mu - \widehat{u}_{\mu+1}]^2 \tag{19.5.1}$$

since it is nonnegative and equal to zero only when $\widehat{u}(x)$ is constant. Here $\widehat{u}_\mu \equiv \widehat{u}(x_\mu)$, and the second equality (proportionality) assumes that the x_μ's are uniformly spaced. We can write the second form of $\mathcal{B}$ as

$$\mathcal{B} = |\mathbf{B} \cdot \widehat{\mathbf{u}}|^2 = \widehat{\mathbf{u}} \cdot (\mathbf{B}^T \cdot \mathbf{B}) \cdot \widehat{\mathbf{u}} \equiv \widehat{\mathbf{u}} \cdot \mathbf{H} \cdot \widehat{\mathbf{u}} \tag{19.5.2}$$

where $\widehat{\mathbf{u}}$ is the vector of components $\widehat{u}_\mu$, $\mu = 0, \ldots, M-1$; $\mathbf{B}$ is the $(M-1) \times M$ first difference matrix

$$\mathbf{B} = \begin{pmatrix} -1 & 1 & 0 & 0 & 0 & 0 & 0 & \cdots & 0 \\ 0 & -1 & 1 & 0 & 0 & 0 & 0 & \cdots & 0 \\ \vdots & & & & \ddots & & & & \vdots \\ 0 & \cdots & 0 & 0 & 0 & 0 & -1 & 1 & 0 \\ 0 & \cdots & 0 & 0 & 0 & 0 & 0 & -1 & 1 \end{pmatrix} \tag{19.5.3}$$

and $\mathbf{H}$ is the $M \times M$ matrix

$$\mathbf{H} = \mathbf{B}^T \cdot \mathbf{B} = \begin{pmatrix} 1 & -1 & 0 & 0 & 0 & 0 & 0 & \cdots & 0 \\ -1 & 2 & -1 & 0 & 0 & 0 & 0 & \cdots & 0 \\ 0 & -1 & 2 & -1 & 0 & 0 & 0 & \cdots & 0 \\ \vdots & & & & \ddots & & & & \vdots \\ 0 & \cdots & 0 & 0 & 0 & -1 & 2 & -1 & 0 \\ 0 & \cdots & 0 & 0 & 0 & 0 & -1 & 2 & -1 \\ 0 & \cdots & 0 & 0 & 0 & 0 & 0 & -1 & 1 \end{pmatrix} \tag{19.5.4}$$

Note that $\mathbf{B}$ has one fewer row than column. It follows that the symmetric $\mathbf{H}$ is degenerate; it has exactly one zero eigenvalue corresponding to the *value* of a constant function, any one of which makes $\mathcal{B}$ exactly zero.

If, just as in §15.4, we write

$$A_{i\mu} \equiv R_{i\mu}/\sigma_i \qquad b_i \equiv c_i/\sigma_i \tag{19.5.5}$$

then, using equation (19.4.9), the minimization principle (19.4.12) is

$$\text{minimize:} \quad \mathcal{A} + \lambda\mathcal{B} = |\mathbf{A} \cdot \widehat{\mathbf{u}} - \mathbf{b}|^2 + \lambda \widehat{\mathbf{u}} \cdot \mathbf{H} \cdot \widehat{\mathbf{u}} \tag{19.5.6}$$

This can readily be reduced to a linear set of *normal equations*, just as in §15.4: The components $\widehat{u}_\mu$ of the solution satisfy the set of M equations in M unknowns,

$$\sum_\rho \left[\left(\sum_i A_{i\mu} A_{i\rho} \right) + \lambda H_{\mu\rho} \right] \widehat{u}_\rho = \sum_i A_{i\mu} b_i \qquad \mu = 0, 1, \ldots, M-1 \tag{19.5.7}$$

or, in vector notation,

$$(\mathbf{A}^T \cdot \mathbf{A} + \lambda \mathbf{H}) \cdot \widehat{\mathbf{u}} = \mathbf{A}^T \cdot \mathbf{b} \tag{19.5.8}$$

Equations (19.5.7) or (19.5.8) can be solved by the standard techniques of Chapter 2, e.g., LU decomposition. The usual warnings about normal equations being ill-conditioned do not apply, since the whole purpose of the λ term is to cure that same ill-conditioning. Note, however, that the λ term *by itself* is ill-conditioned, since it does not select a preferred constant value. You hope your data can at least do *that*!

Although inversion of the matrix $(\mathbf{A}^T \cdot \mathbf{A} + \lambda \mathbf{H})$ is not generally the best way to solve for $\widehat{\mathbf{u}}$, let us digress to write the solution to equation (19.5.8) schematically as

$$\widehat{\mathbf{u}} = \left(\frac{1}{\mathbf{A}^T \cdot \mathbf{A} + \lambda \mathbf{H}} \cdot \mathbf{A}^T \cdot \mathbf{A} \right) \mathbf{A}^{-1} \cdot \mathbf{b} \qquad \text{(schematic only!)} \tag{19.5.9}$$

where the identity matrix in the form $\mathbf{A} \cdot \mathbf{A}^{-1}$ has been inserted. This is schematic not only because the matrix inverse is fancifully written as a denominator, but also because, in general, the inverse matrix $\mathbf{A}^{-1}$ does not exist. However, it is illuminating to compare equation (19.5.9) with equation (13.3.6) for optimal or Wiener filtering, or with equation (13.6.6) for general linear prediction. (The concepts of §15.9 are also related.) One sees that $\mathbf{A}^T \cdot \mathbf{A}$ plays the role of S^2, the signal power or autocorrelation, while $\lambda \mathbf{H}$ plays the role of N^2, the noise power or autocorrelation. The term in parentheses in equation (19.5.9) is something like an optimal filter, whose effect is to pass the ill-posed inverse $\mathbf{A}^{-1} \cdot \mathbf{b}$ through unmodified when $\mathbf{A}^T \cdot \mathbf{A}$ is sufficiently large, but to suppress it when $\mathbf{A}^T \cdot \mathbf{A}$ is small.

The above choices of $\mathbf{B}$ and $\mathbf{H}$ are only the simplest in an obvious sequence of derivatives. If your a priori belief is that a *linear* function is a good approximation to $u(x)$, then minimize

$$\mathcal{B} \propto \int [\widehat{u}''(x)]^2 dx \propto \sum_{\mu=0}^{M-3} [-\widehat{u}_\mu + 2\widehat{u}_{\mu+1} - \widehat{u}_{\mu+2}]^2 \tag{19.5.10}$$

implying

$$\mathbf{B} = \begin{pmatrix} -1 & 2 & -1 & 0 & 0 & 0 & 0 & \cdots & 0 \\ 0 & -1 & 2 & -1 & 0 & 0 & 0 & \cdots & 0 \\ \vdots & & & & \ddots & & & & \vdots \\ 0 & \cdots & 0 & 0 & 0 & -1 & 2 & -1 & 0 \\ 0 & \cdots & 0 & 0 & 0 & 0 & -1 & 2 & -1 \end{pmatrix} \tag{19.5.11}$$

and

$$\mathbf{H} = \mathbf{B}^T \cdot \mathbf{B} = \begin{pmatrix} 1 & -2 & 1 & 0 & 0 & 0 & 0 & \cdots & 0 \\ -2 & 5 & -4 & 1 & 0 & 0 & 0 & \cdots & 0 \\ 1 & -4 & 6 & -4 & 1 & 0 & 0 & \cdots & 0 \\ 0 & 1 & -4 & 6 & -4 & 1 & 0 & \cdots & 0 \\ \vdots & & & & \ddots & & & & \vdots \\ 0 & \cdots & 0 & 1 & -4 & 6 & -4 & 1 & 0 \\ 0 & \cdots & 0 & 0 & 1 & -4 & 6 & -4 & 1 \\ 0 & \cdots & 0 & 0 & 0 & 1 & -4 & 5 & -2 \\ 0 & \cdots & 0 & 0 & 0 & 0 & 1 & -2 & 1 \end{pmatrix} \tag{19.5.12}$$

This **H** has two zero eigenvalues, corresponding to the two undetermined parameters of a linear function.

If your a priori belief is that a *quadratic* function is preferable, then minimize

$$\mathcal{B} \propto \int [\hat{u}'''(x)]^2 dx \propto \sum_{\mu=0}^{M-4} [-\hat{u}_\mu + 3\hat{u}_{\mu+1} - 3\hat{u}_{\mu+2} + \hat{u}_{\mu+3}]^2 \tag{19.5.13}$$

with

$$\mathbf{B} = \begin{pmatrix} -1 & 3 & -3 & 1 & 0 & 0 & 0 & \cdots & 0 \\ 0 & -1 & 3 & -3 & 1 & 0 & 0 & \cdots & 0 \\ \vdots & & & & \ddots & & & & \vdots \\ 0 & \cdots & 0 & 0 & -1 & 3 & -3 & 1 & 0 \\ 0 & \cdots & 0 & 0 & 0 & -1 & 3 & -3 & 1 \end{pmatrix} \tag{19.5.14}$$

and now

$$\mathbf{H} = \begin{pmatrix} 1 & -3 & 3 & -1 & 0 & 0 & 0 & 0 & 0 & \cdots & 0 \\ -3 & 10 & -12 & 6 & -1 & 0 & 0 & 0 & 0 & \cdots & 0 \\ 3 & -12 & 19 & -15 & 6 & -1 & 0 & 0 & 0 & \cdots & 0 \\ -1 & 6 & -15 & 20 & -15 & 6 & -1 & 0 & 0 & \cdots & 0 \\ 0 & -1 & 6 & -15 & 20 & -15 & 6 & -1 & 0 & \cdots & 0 \\ \vdots & & & & & \ddots & & & & & \vdots \\ 0 & \cdots & 0 & -1 & 6 & -15 & 20 & -15 & 6 & -1 & 0 \\ 0 & \cdots & 0 & 0 & -1 & 6 & -15 & 20 & -15 & 6 & -1 \\ 0 & \cdots & 0 & 0 & 0 & -1 & 6 & -15 & 19 & -12 & 3 \\ 0 & \cdots & 0 & 0 & 0 & 0 & -1 & 6 & -12 & 10 & -3 \\ 0 & \cdots & 0 & 0 & 0 & 0 & 0 & -1 & 3 & -3 & 1 \end{pmatrix} \tag{19.5.15}$$

(We'll leave the calculation of cubics and above to the compulsive reader.)

Notice that you can regularize with "closeness to a differential equation," if you want. Just pick **B** to be the appropriate sum of finite difference operators (the coefficients can depend on x) and calculate $\mathbf{H} = \mathbf{B}^T \cdot \mathbf{B}$. You don't need to know the values of your boundary conditions, since **B** can have fewer rows than columns, as above; hopefully, your data will determine them. Of course, if you do know some boundary conditions, you can build these into **B**, too.

With all the proportionality signs above, you may have lost track of what actual value of λ to try first. A simple trick for at least getting "on the map" is to first try

$$\lambda = \mathrm{Tr}(\mathbf{A}^T \cdot \mathbf{A}) / \mathrm{Tr}(\mathbf{H}) \tag{19.5.16}$$

where Tr is the trace of the matrix (sum of diagonal components). This choice will tend to make the two parts of the minimization have comparable weights, and you can adjust from there.

As for what is the "correct" value of λ, an objective criterion, if you know your errors σ_i with reasonable accuracy, is to make χ^2 (that is, $|\mathbf{A} \cdot \hat{\mathbf{u}} - \mathbf{b}|^2$) equal to N, the number of measurements. We remarked above on the twin acceptable

choices $N \pm (2N)^{1/2}$. A subjective criterion is to pick any value that you like in the range $0 < \lambda < \infty$, depending on your relative degree of belief in the a priori and a posteriori evidence. (Yes, people actually do that.) The problem with being a rigorous Bayesian at this stage is that rarely, if ever, is your understanding of the prior so complete as to give a firm, objective, value for λ, and, as we pointed out in §15.1.1, purely Bayesian methods for assessing goodness-of-fit are largely nonexistent.

19.5.1 Two-Dimensional Problems and Iterative Methods

Up to now our notation has been indicative of a one-dimensional problem, finding $\widehat{u}(x)$ or $\widehat{u}_\mu = \widehat{u}(x_\mu)$. However, all of the discussion easily generalizes to the problem of estimating a two-dimensional set of unknowns $\widehat{u}_{\mu\kappa}$, $\mu = 0, \ldots, M-1$, $\kappa = 0, \ldots, K-1$, corresponding, say, to the pixel intensities of a measured image. In this case, equation (19.5.8) is still the one we want to solve.

In image processing, it is usual to have the same number of input pixels in a measured "raw" or "dirty" image as desired "clean" pixels in the processed output image, so the matrices $\mathbf{R}$ and $\mathbf{A}$ (equation 19.5.5) are square and of size $MK \times MK$. $\mathbf{A}$ is typically too large to represent as a full matrix, but often it is either (i) sparse, with coefficients blurring an underlying pixel (i,j) only into measurements ($i \pm$ few, $j \pm$ few), or (ii) translationally invariant, so that $A_{(i,j)(\mu,\nu)} = A(i-\mu, j-\nu)$. Both of these situations lead to tractable problems.

In the case of translational invariance, fast Fourier transforms (FFTs) are the obvious method of choice. The general linear relation between underlying function and measured values (19.4.7) now becomes a discrete convolution like equation (13.1.1). If $\mathbf{k}$ denotes a two-dimensional wave vector, then the two-dimensional FFT takes us back and forth between the transform pairs

$$A(i-\mu, j-\nu) \Longleftrightarrow \widetilde{\mathbf{A}}(\mathbf{k}) \qquad b_{(i,j)} \Longleftrightarrow \widetilde{b}(\mathbf{k}) \qquad \widehat{u}_{(i,j)} \Longleftrightarrow \widetilde{u}(\mathbf{k}) \tag{19.5.17}$$

We also need a regularization or smoothing operator $\mathbf{B}$ and the derived $\mathbf{H} = \mathbf{B}^T \cdot \mathbf{B}$. One popular choice for $\mathbf{B}$ is the five-point finite difference approximation of the Laplacian operator, that is, the difference between the value of each point and the average of its four Cartesian neighbors. In Fourier space, this choice implies

$$\begin{aligned} \widetilde{B}(\mathbf{k}) &\propto \sin^2(\pi k_1/M)\sin^2(\pi k_2/K) \\ \widetilde{H}(\mathbf{k}) &\propto \sin^4(\pi k_1/M)\sin^4(\pi k_2/K) \end{aligned} \tag{19.5.18}$$

In Fourier space, equation (19.5.7) is merely algebraic, with solution

$$\widetilde{u}(\mathbf{k}) = \frac{\widetilde{A}^*(\mathbf{k})\widetilde{b}(\mathbf{k})}{|\widetilde{A}(\mathbf{k})|^2 + \lambda \widetilde{H}(\mathbf{k})} \tag{19.5.19}$$

where the asterisk denotes complex conjugation. You can make use of the FFT routines for real data in §12.6.

Turn now to the case where $\mathbf{A}$ is not translationally invariant. Direct solution of (19.5.8) is now hopeless, since the matrix $\mathbf{A}$ is just too large. We need some kind of iterative scheme.

One way to proceed is to use the full machinery of the conjugate gradient method in §10.8 to find the minimum of $\mathcal{A} + \lambda\mathcal{B}$, equation (19.5.6). Of the various methods in Chapter 10, the conjugate gradient is the unique best choice because (i) it does not require storage of a Hessian matrix, which would be infeasible here, and (ii) it does exploit gradient information, which we can readily compute: The gradient of equation (19.5.6) is

$$\nabla(\mathcal{A} + \lambda\mathcal{B}) = 2[(\mathbf{A}^T \cdot \mathbf{A} + \lambda\mathbf{H}) \cdot \hat{\mathbf{u}} - \mathbf{A}^T \cdot \mathbf{b}] \tag{19.5.20}$$

(cf. 19.5.8). Evaluation of both the function and the gradient should of course take advantage of the sparsity of $\mathbf{A}$, for example using the methods `ax` and `atx` in the `NRsparseMat` object in §2.7.5. We will discuss the conjugate gradient technique further in §19.7, in the context of the (nonlinear) maximum entropy method. Some of that discussion can apply here as well.

The conjugate gradient method notwithstanding, application of the unsophisticated steepest descent method (see §10.8) can sometimes produce useful results, particularly when combined with projections onto convex sets (see below). If the solution after k iterations is denoted $\hat{\mathbf{u}}^{(k)}$, then after $k+1$ iterations we have

$$\hat{\mathbf{u}}^{(k+1)} = [\mathbf{1} - \epsilon(\mathbf{A}^T \cdot \mathbf{A} + \lambda\mathbf{H})] \cdot \hat{\mathbf{u}}^{(k)} + \epsilon\mathbf{A}^T \cdot \mathbf{b} \tag{19.5.21}$$

Here ϵ is a parameter that dictates how far to move in the downhill gradient direction. The method converges when ϵ is small enough, in particular satisfying

$$0 < \epsilon < \frac{2}{\text{max eigenvalue}\,(\mathbf{A}^T \cdot \mathbf{A} + \lambda\mathbf{H})} \tag{19.5.22}$$

There exist complicated schemes for finding optimal values or sequences for ϵ, see [7]; or, one can adopt an experimental approach, evaluating (19.5.6) to be sure that downhill steps are in fact being taken.

In those image processing problems where the final measure of success is somewhat subjective (e.g., "how good does the picture look?"), iteration (19.5.21) sometimes produces significantly improved images long before convergence is achieved. This probably accounts for much of its use, since its mathematical convergence is extremely slow. In fact, (19.5.21) can be used with $\mathbf{H} = 0$, in which case the solution is not regularized at all, and full convergence would be disastrous! This is called *Van Cittert's method* and goes back to the 1930s. A number of iterations the order of 10^3 is not uncommon [7].

19.5.2 Deterministic Constraints: Projections onto Convex Sets

A set of possible underlying functions (or images) $\{\hat{\mathbf{u}}\}$ is said to be *convex* if, for any two elements $\hat{\mathbf{u}}_a$ and $\hat{\mathbf{u}}_b$ in the set, all the linearly interpolated combinations

$$(1-\eta)\hat{\mathbf{u}}_a + \eta\hat{\mathbf{u}}_b \qquad 0 \le \eta \le 1 \tag{19.5.23}$$

are also in the set. Many *deterministic constraints* that one might want to impose on the solution $\hat{\mathbf{u}}$ to an inverse problem in fact define convex sets, for example:

- positivity
- compact support (i.e., zero value outside of a certain region)
- known bounds (i.e., $u_L(x) \leq \widehat{u}(x) \leq u_U(x)$ for specified functions u_L and u_U).

(In this last case, the bounds might be related to an initial estimate and its error bars, e.g., $\widehat{u}_0(x) \pm \gamma\sigma(x)$, where γ is of order 1 or 2.) Notice that these, and similar, constraints can be either in the image space, or in the Fourier transform space, or (in fact) in the space of any linear transformation of $\widehat{\mathbf{u}}$.

If C_i is a convex set, then $\mathcal{P}_i$ is called a *nonexpansive projection operator* onto that set if (i) $\mathcal{P}_i$ leaves unchanged any $\widehat{\mathbf{u}}$ already in C_i, and (ii) $\mathcal{P}_i$ maps any $\widehat{\mathbf{u}}$ outside C_i to the *closest* element of C_i, in the sense that

$$|\mathcal{P}_i\widehat{\mathbf{u}} - \widehat{\mathbf{u}}| \leq |\widehat{\mathbf{u}}_a - \widehat{\mathbf{u}}| \quad \text{for all } \widehat{\mathbf{u}}_a \text{ in } C_i \tag{19.5.24}$$

While this definition sounds complicated, examples are very simple: A nonexpansive projection onto the set of positive $\widehat{\mathbf{u}}$'s is "set all negative components of $\widehat{\mathbf{u}}$ equal to zero." A nonexpansive projection onto the set of $\widehat{u}(x)$'s bounded by $u_L(x) \leq \widehat{u}(x) \leq u_U(x)$ is "set all values less than the lower bound equal to that bound, and set all values greater than the upper bound equal to *that* bound." A nonexpansive projection onto functions with compact support is "zero the values outside of the region of support."

The usefulness of these definitions is the following remarkable theorem: Let C be the intersection of m convex sets $C_1, C_2, \ldots, C_m$. Then the iteration

$$\widehat{\mathbf{u}}^{(k+1)} = (\mathcal{P}_1\mathcal{P}_2\cdots\mathcal{P}_m)\widehat{\mathbf{u}}^{(k)} \tag{19.5.25}$$

will converge to C from all starting points, as $k \to \infty$. Also, if C is empty (there is no intersection), then the iteration will have no limit point. Application of this theorem is called the *method of projections onto convex sets* or sometimes *POCS* [7].

A generalization of the POCS theorem is that the $\mathcal{P}_i$'s can be replaced by a set of $\mathcal{T}_i$'s,

$$\mathcal{T}_i \equiv \mathbf{1} + \beta_i(\mathcal{P}_i - \mathbf{1}) \qquad 0 < \beta_i < 2 \tag{19.5.26}$$

A well-chosen set of β_i's can accelerate the convergence to the intersection set C.

Some inverse problems can be completely solved by iteration (19.5.25) alone! For example, a problem that occurs in both astronomical imaging and X-ray diffraction work is to recover an image given only the *modulus* of its Fourier transform (equivalent to its power spectrum or autocorrelation) and not the *phase*. Here two convex sets can be utilized: the set of all positive images, and the set of all images with zero intensity outside of some specified region. A third set, the set of all images whose Fourier transform has the specified modulus to within specified error bounds, is not convex: It is an annulus in the complex plane for each Fourier component. (Of course FFTs are used to get in and out of Fourier space each time the Fourier constraint is imposed.) The POCS iteration (19.5.25) that cycles among all three sets, imposing each constraint in turn, is thus not guaranteed (by the POCS theorem) to converge; it can get stuck in *traps* [8]. However, it often does work.

The specific application of POCS to constraints (not necessarily all convex) alternately in the spatial and Fourier domains is known as the *Gerchberg-Saxton* algorithm [9]. While this algorithm is nonexpansive, and is frequently convergent

in practice, it does not converge in all cases [8,10]. In the phase-retrieval problem just mentioned, the algorithm often gets stuck in traps for many iterations. After as many as 10^4 to 10^5 iterations, sudden, dramatic improvements may occur. In principle, some traps may be permanent, requiring more interventional "unsticking" procedures; see [8,11]. The uniqueness of the solution is also not well understood, although for two-dimensional images of reasonable complexity it is believed to be unique. The use of nonconvex sets in an iteration like (19.5.25) is called the *method of generalized projections*.

Deterministic constraints can be incorporated, via projection operators, into iterative methods of linear regularization. In particular, rearranging terms somewhat, we can write the iteration (19.5.21) as

$$\widehat{\mathbf{u}}^{(k+1)} = (\mathbf{1} - \epsilon\lambda\mathbf{H}) \cdot \widehat{\mathbf{u}}^{(k)} + \epsilon\mathbf{A}^T \cdot (\mathbf{b} - \mathbf{A} \cdot \widehat{\mathbf{u}}^{(k)}) \qquad (19.5.27)$$

If the iteration is modified by the insertion of projection operators at each step

$$\widehat{\mathbf{u}}^{(k+1)} = (\mathcal{P}_1\mathcal{P}_2 \cdots \mathcal{P}_m)[(\mathbf{1} - \epsilon\lambda\mathbf{H}) \cdot \widehat{\mathbf{u}}^{(k)} + \epsilon\mathbf{A}^T \cdot (\mathbf{b} - \mathbf{A} \cdot \widehat{\mathbf{u}}^{(k)})] \qquad (19.5.28)$$

(or, instead of $\mathcal{P}_i$'s, the $\mathcal{T}_i$ operators of equation 19.5.26), then it can be shown that the convergence condition (19.5.22) is unmodified, and the iteration will converge to minimize the quadratic functional (19.5.6) subject to the desired nonlinear deterministic constraints. See [7] for references to more sophisticated, and faster converging, iterations along these lines.

CITED REFERENCES AND FURTHER READING:

Phillips, D.L. 1962, "A Technique for the Numerical Solution of Certain Integral Equations of the First Kind," *Journal of the Association for Computing Machinery*, vol. 9, pp. 84–97.[1]

Twomey, S. 1963, "On the Numerical Solution of Fredholm Integral Equations of the First Kind by the Inversion of the Linear System Produced by Quadrature," *Journal of the Association for Computing Machinery*, vol. 10, pp. 97–101.[2]

Twomey, S. 1977, *Introduction to the Mathematics of Inversion in Remote Sensing and Indirect Measurements* (Amsterdam: Elsevier).[3]

Craig, I.J.D., and Brown, J.C. 1986, *Inverse Problems in Astronomy* (Bristol, UK: Adam Hilger).[4]

Tikhonov, A.N., and Arsenin, V.Y. 1977, *Solutions of Ill-Posed Problems* (New York: Wiley).[5]

Tikhonov, A.N., and Goncharsky, A.V. (eds.) 1987, *Ill-Posed Problems in the Natural Sciences* (Moscow: MIR).

Miller, K. 1970, "Least Squares Methods for Ill-Posed Problems with a Prescribed Bound," *SIAM Journal on Mathematical Analysis*, vol. 1, pp. 52–74.[6]

Schafer, R.W., Mersereau, R.M., and Richards, M.A. 1981, "Constrained Iterative Restoration Algorithm," *Proceedings of the IEEE*, vol. 69, pp. 432–450.

Biemond, J., Lagendijk, R.L., and Mersereau, R.M. 1990, "Iterative Methods for Image Deblurring," *Proceedings of the IEEE*, vol. 78, pp. 856–883.[7]

Sezan, M.I. 1992, "An Overview of Convex Projections Theory and Its Application to Image Recovery Problems," *Ultramicroscopy*, vol. 40, pp. 55–67.[8]

Gerchberg, R.W., and Saxton, W.O. 1972, "A Practical Algorithm for the Determination of Phase from Image and Diffraction Plane Pictures," *Optik*, vol. 35, pp. 237–246.[9]

Fienup, J.R. 1982, "Phase Retrieval Algorithms: A Comparison," *Applied Optics*, vol. 15, pp. 2758–2769.[10]

Fienup, J.R., and Wackerman, C.C. 1986, "Phase-Retrieval Stagnation Problems and Solutions," *Journal of the Optical Society of America A*, vol. 3, pp. 1897–1907.[11]

19.6 Backus-Gilbert Method

The *Backus-Gilbert method* [1,2], also known as the *optimally localized average (OLA)* method (see, e.g., [3] or [4] for summaries) differs from other regularization methods in the nature of its functionals $\mathcal{A}$ and $\mathcal{B}$. For $\mathcal{B}$, the method seeks to maximize the *stability* of the solution $\widehat{u}(x)$ rather than, in the first instance, its smoothness. That is,

$$\mathcal{B} \equiv \text{Var}[\widehat{u}(x)] \tag{19.6.1}$$

is used as a measure of how much the solution $\widehat{u}(x)$ varies as the data vary within their measurement errors. Note that this variance is not the expected deviation of $\widehat{u}(x)$ from the true $u(x)$ — that will be constrained by $\mathcal{A}$ — but rather measures the expected experiment-to-experiment scatter among estimates $\widehat{u}(x)$ if the whole experiment were to be repeated many times.

For $\mathcal{A}$ the Backus-Gilbert method looks at the relationship between the solution $\widehat{u}(x)$ and the true function $u(x)$, and seeks to make the mapping between these as close to the identity map as possible in the limit of error-free data. The method is linear, so the relationship between $\widehat{u}(x)$ and $u(x)$ can be written as

$$\widehat{u}(x) = \int \widehat{\delta}(x, x')u(x')dx' \tag{19.6.2}$$

for some so-called *resolution function* or *averaging kernel* $\widehat{\delta}(x, x')$. The Backus-Gilbert method seeks to minimize the width or *spread* of $\widehat{\delta}$ (that is, maximize the resolving power). $\mathcal{A}$ is chosen to be some positive measure of the spread.

While Backus-Gilbert's philosophy is thus rather different from that of Phillips-Twomey and related methods, in practice the differences between the methods are less than one might think. A *stable* solution is almost inevitably bound to be *smooth*: The wild, unstable oscillations that result from an unregularized solution are always exquisitely sensitive to small changes in the data. Likewise, making $\widehat{u}(x)$ close to $u(x)$ inevitably will bring error-free data into agreement with the model. Thus $\mathcal{A}$ and $\mathcal{B}$ play roles closely analogous to their corresponding roles in the previous two sections.

The principal advantage of the Backus-Gilbert formulation is that it gives good control over just those properties that it seeks to measure, namely stability and resolving power. Moreover, in the Backus-Gilbert method, the choice of λ (playing its usual role of compromise between $\mathcal{A}$ and $\mathcal{B}$) is conventionally made, or at least can easily be made, *before* any actual data are processed. One's uneasiness at making a post hoc, and therefore potentially subjectively biased, choice of λ is thus removed. Backus-Gilbert is often recommended as the method of choice for designing and predicting the performance of experiments that require data inversion.

Let's see how this all works. Starting with equation (19.4.5),

$$c_i \equiv s_i + n_i = \int r_i(x)u(x)dx + n_i \tag{19.6.3}$$

and building in linearity from the start, we seek a set of *inverse response kernels* $q_i(x)$ such that

$$\widehat{u}(x) = \sum_i q_i(x)c_i \tag{19.6.4}$$

is the desired estimator of $u(x)$. It is useful to define the integrals of the response kernels for each data point,

$$R_i \equiv \int r_i(x)dx \tag{19.6.5}$$

Substituting equation (19.6.4) into equation (19.6.3), and comparing with equation (19.6.2), we see that

$$\widehat{\delta}(x, x') = \sum_i q_i(x) r_i(x') \tag{19.6.6}$$

We can require this averaging kernel to have unit area at every x, giving

$$1 = \int \widehat{\delta}(x, x')dx' = \sum_i q_i(x) \int r_i(x')dx' = \sum_i q_i(x) R_i \equiv \mathbf{q}(x) \cdot \mathbf{R} \tag{19.6.7}$$

where $\mathbf{q}(x)$ and $\mathbf{R}$ are each vectors of length N, the number of measurements.

Standard propagation of errors, and equation (19.6.1), give

$$\mathcal{B} = \text{Var}[\widehat{u}(x)] = \sum_i \sum_j q_i(x) S_{ij} q_j(x) = \mathbf{q}(x) \cdot \mathbf{S} \cdot \mathbf{q}(x) \tag{19.6.8}$$

where S_{ij} is the covariance matrix (equation 19.4.6). If one can neglect off-diagonal covariances (as when the errors on the c_i's are independent), then $S_{ij} = \delta_{ij}\sigma_i^2$ is diagonal.

We now need to define a measure of the width or spread of $\widehat{\delta}(x, x')$ at each value of x. While many choices are possible, Backus and Gilbert choose the second moment of its square. This measure becomes the functional $\mathcal{A}$,

$$\begin{aligned} \mathcal{A} \equiv w(x) &= \int (x' - x)^2 [\widehat{\delta}(x, x')]^2 dx' \\ &= \sum_i \sum_j q_i(x) W_{ij}(x) q_j(x) \equiv \mathbf{q}(x) \cdot \mathbf{W}(x) \cdot \mathbf{q}(x) \end{aligned} \tag{19.6.9}$$

where we have here used equation (19.6.6) and defined the *spread matrix* $\mathbf{W}(x)$ by

$$W_{ij}(x) \equiv \int (x' - x)^2 r_i(x') r_j(x') dx' \tag{19.6.10}$$

The functions $q_i(x)$ are now determined by the minimization principle

$$\text{minimize:} \quad \mathcal{A} + \lambda\mathcal{B} = \mathbf{q}(x) \cdot \left[\mathbf{W}(x) + \lambda\mathbf{S}\right] \cdot \mathbf{q}(x) \tag{19.6.11}$$

subject to the constraint (19.6.7) that $\mathbf{q}(x) \cdot \mathbf{R} = 1$.

The solution of equation (19.6.11) is

$$\mathbf{q}(x) = \frac{[\mathbf{W}(x) + \lambda\mathbf{S}]^{-1} \cdot \mathbf{R}}{\mathbf{R} \cdot [\mathbf{W}(x) + \lambda\mathbf{S}]^{-1} \cdot \mathbf{R}} \tag{19.6.12}$$

(Reference [4] gives an accessible proof.) For any particular data set $\mathbf{c}$ (set of measurements c_i), the solution $\widehat{u}(x)$ is thus

$$\widehat{u}(x) = \frac{\mathbf{c} \cdot [\mathbf{W}(x) + \lambda\mathbf{S}]^{-1} \cdot \mathbf{R}}{\mathbf{R} \cdot [\mathbf{W}(x) + \lambda\mathbf{S}]^{-1} \cdot \mathbf{R}} \tag{19.6.13}$$

(Don't let this notation mislead you into inverting the full matrix $\mathbf{W}(x) + \lambda\mathbf{S}$. You only need to solve the linear system $(\mathbf{W}(x) + \lambda\mathbf{S}) \cdot \mathbf{y} = \mathbf{R}$ for the vector $\mathbf{y}$, and then substitute $\mathbf{y}$ into both the numerators and denominators of 19.6.12 or 19.6.13.)

Equations (19.6.12) and (19.6.13) have a completely different character from the linearly regularized solutions to (19.5.7) and (19.5.8). The vectors and matrices in (19.6.12) all have size N, the number of measurements. There is no discretization of the underlying variable x, so M does not come into play at all. One solves a different $N \times N$ set of linear equations for each desired value of x. By contrast, in (19.5.8), one solves an $M \times M$ linear set, but only once. In general, the computational burden of repeatedly solving linear systems makes the Backus-Gilbert method unsuitable for other than one-dimensional problems.

How does one choose λ within the Backus-Gilbert scheme? As already mentioned, you can (in some cases *should*) make the choice *before* you see any actual data. For a given trial value of λ, and for a sequence of x's, use equation (19.6.12) to calculate $\mathbf{q}(x)$; then use equation (19.6.6) to plot the resolution functions $\widehat{\delta}(x, x')$ as a function of x'. These plots will exhibit the amplitude with which different underlying values x' contribute to the point $\widehat{u}(x)$ of your estimate. For the same value of λ, also plot the function $\sqrt{\mathrm{Var}[\widehat{u}(x)]}$ using equation (19.6.8). (You need an estimate of your measurement covariance matrix for this.)

As you change λ you will see very explicitly the trade-off between resolution and stability. Pick the value that meets your needs. You can even choose λ to be a function of x, $\lambda = \lambda(x)$, in equations (19.6.12) and (19.6.13), should you desire to do so. (This is one benefit of solving a separate set of equations for each x.) For the chosen value or values of λ, you now have a quantitative understanding of your inverse solution procedure. This can prove invaluable if — once you are processing real data — you need to judge whether a particular feature, a spike or jump for example, is genuine, and/or is actually resolved. The Backus-Gilbert method has found particular success among geophysicists, who use it to obtain information about the structure of the Earth (e.g., density run with depth) from seismic travel time data.

CITED REFERENCES AND FURTHER READING:

Backus, G.E., and Gilbert, F. 1968, "The Resolving Power of Gross Earth Data," *Geophysical Journal of the Royal Astronomical Society*, vol. 16, pp. 169–205.[1]

Backus, G.E., and Gilbert, F. 1970, "Uniqueness in the Inversion of Inaccurate Gross Earth Data," *Philosophical Transactions of the Royal Society of London A*, vol. 266, pp. 123–192.[2]

Parker, R.L. 1977, "Understanding Inverse Theory," *Annual Review of Earth and Planetary Science*, vol. 5, pp. 35–64.[3]

Loredo, T.J., and Epstein, R.I. 1989, "Analyzing Gamma-Ray Burst Spectral Data," *Astrophysical Journal*, vol. 336, pp. 896–919.[4]

19.7 Maximum Entropy Image Restoration

We must first comment in passing that the connection between maximum entropy inverse methods, considered here, and maximum entropy spectral estimation, discussed in §13.7, is rather distant. For practical purposes, the two techniques, though both named *maximum entropy method* or *MEM*, are unrelated. On the other

hand, what we discuss here has a close connection to the discussion of entropy in §14.7.

The entropy of a physical system in some macroscopic state, usually denoted S, is the logarithm of the number of microscopically distinct configurations that all have the same macroscopic observables (i.e., consistent with the observed macroscopic state). Actually, we will find it useful to denote the *negative* of the entropy, also called the *negentropy*, by $H \equiv -S$ (a notation that goes back to Boltzmann). In situations where there is reason to believe that the a priori probabilities of the *microscopic* configurations are all the same (these situations are called *ergodic*), the Bayesian prior $P(\mathbf{u}|I)$ for a *macroscopic* state with entropy S is proportional to $\exp(S)$ or $\exp(-H)$.

MEM uses this concept to assign a prior probability to any given underlying function $\mathbf{u}$. This very general idea is applicable to much more than image restoration [1,2]. For definiteness, however, we consider that application only. Suppose [3-5] that the measurement of luminance in each pixel in an image is quantized to (in some units) an integer value. Let

$$U = \sum_{\mu=0}^{M-1} u_\mu \tag{19.7.1}$$

be the total number of luminance quanta in the whole image. Then we can base our "prior" on the notion that each luminance quantum has an equal a priori chance of being in any pixel. (See [6] for a more abstract justification of this idea.) The number of ways of getting a particular configuration $\mathbf{u}$ is

$$\frac{U!}{u_0!u_1!\cdots u_{M-1}!} \propto \exp\left[-\sum_\mu u_\mu \ln(u_\mu/U) + \tfrac{1}{2}\left(\ln U - \sum_\mu \ln u_\mu\right)\right] \tag{19.7.2}$$

Here the left side can be understood as the number of distinct orderings of all the luminance quanta, divided by the numbers of equivalent reorderings within each pixel, while the right side follows by Stirling's approximation to the factorial function. Taking the negative of the logarithm, and neglecting terms of order $\log U$ in the presence of terms of order U, we get the negentropy

$$H(\mathbf{u}) = \sum_{\mu=0}^{M-1} u_\mu \ln(u_\mu/U) \tag{19.7.3}$$

As discussed for equations (19.4.13) – (19.4.15), we now seek to maximize

$$P(\mathbf{u}|\mathbf{c}, I) \propto \exp\left[-\tfrac{1}{2}\chi^2\right] \exp[-H(\mathbf{u})] \tag{19.7.4}$$

or, equivalently,

$$\text{minimize:} \quad -\ln\left[\,P(\mathbf{u}|\mathbf{c}, I)\,\right] = \tfrac{1}{2}\chi^2[\mathbf{u}] + H(\mathbf{u}) = \tfrac{1}{2}\chi^2[\mathbf{u}] + \sum_{\mu=0}^{M-1} u_\mu \ln(u_\mu/U) \tag{19.7.5}$$

This ought to remind you of equation (19.4.11), or equation (19.5.6), or in fact any of our previous minimization principles along the lines of $\mathcal{A}+\lambda\mathcal{B}$, where $\lambda\mathcal{B} = H(\mathbf{u})$ is a regularizing operator. Where is λ? We need to put it in for exactly the reason discussed following equation (19.4.11): Degenerate inversions are likely to be able to achieve unrealistically small values of χ^2. We need an adjustable parameter to bring χ^2 into its expected narrow statistical range of $N \pm (2N)^{1/2}$. The discussion at the beginning of §19.4 showed that it makes no difference which term we attach the λ to. For consistency in notation, we absorb a factor 2 into λ and put it on the entropy term. (Another way to see the necessity of an undetermined λ factor is to note that it is necessary if our minimization principle is to be invariant under changing the units in which $\mathbf{u}$ is quantized, e.g., if an 8-bit analog-to-digital converter is replaced by a 12-bit one.) We can now also put "hats" back to indicate that this is the procedure for obtaining our chosen statistical estimator:

$$\hat{\mathbf{u}} \text{ minimizes:} \quad \mathcal{A}+\lambda\mathcal{B} = \chi^2[\mathbf{u}] + \lambda H(\mathbf{u}) = \chi^2[\mathbf{u}] + \lambda \sum_{\mu=0}^{M-1} u_\mu \ln(u_\mu) \quad (19.7.6)$$

(Formally, we might also add a second Lagrange multiplier $\lambda' U$, to constrain the total intensity U to be constant.)

It is not hard to see that the negentropy, $H(\mathbf{u})$, is in fact a regularizing operator, similar to $\mathbf{u}\cdot\mathbf{u}$ (equation 19.4.11) or $\mathbf{u}\cdot\mathbf{H}\cdot\mathbf{u}$ (equation 19.5.6). The following of its properties are noteworthy:

1. When U is held constant, $H(\mathbf{u})$ is minimized for $\hat{u}_\mu = U/M =$ constant, so it smooths in the sense of trying to achieve a constant solution, similar to equation (19.5.4). The fact that the constant solution is a minimum follows from the fact that the second derivative of $u \ln u$ is positive.
2. Unlike equation (19.5.4), however, $H(\hat{\mathbf{u}})$ is *local*, in the sense that it does not difference neighboring pixels. It simply sums some function f, here

$$f(u) = u \ln u \qquad (19.7.7)$$

over all pixels; it is invariant, in fact, under a complete scrambling of the pixels in an image. This form implies that $H(\mathbf{u})$ is not seriously increased by the occurrence of a small number of very bright pixels (point sources) embedded in a low-intensity smooth background.
3. $H(\mathbf{u})$ goes to infinite slope as any one pixel goes to zero. This causes it to enforce positivity of the image, without the necessity of additional deterministic constraints.
4. The biggest difference between $H(\mathbf{u})$ and the other regularizing operators that we have met is that $H(\mathbf{u})$ is not a quadratic functional of $\mathbf{u}$, so the equations obtained by varying equation (19.7.6) are *nonlinear*. This fact is itself worthy of some additional discussion.

Nonlinear equations are harder to solve than linear equations. For image processing, however, the large number of equations usually dictates an iterative solution procedure, even for linear equations, so the practical effect of the nonlinearity is somewhat mitigated. Below, we will summarize some of the methods that are successfully used for MEM inverse problems.

For some problems, notably the problem in radio-astronomy of image recovery from an incomplete set of Fourier coefficients, the superior performance of MEM

inversion can be, in part, traced to the nonlinearity of $H(\mathbf{u})$. One way to see this [3] is to consider the limit of perfect measurements $\sigma_i \to 0$. In this case, the χ^2 term in the minimization principle (19.7.6) gets replaced by a set of constraints, each with its own Lagrange multiplier, requiring agreement between model and data; that is,

$$\hat{\mathbf{u}} \text{ minimizes:} \qquad \sum_j \lambda_j \left[c_j - \sum_\mu R_{j\mu} u_\mu \right] + H(\mathbf{u}) \tag{19.7.8}$$

(cf. equation 19.4.7). Setting the formal derivative with respect to u_μ to zero gives

$$\frac{\partial H}{\partial u_\mu} = f'(u_\mu) = \sum_j \lambda_j R_{j\mu} \tag{19.7.9}$$

or defining a function G as the inverse function of f',

$$u_\mu = G\left(\sum_j \lambda_j R_{j\mu} \right) \tag{19.7.10}$$

This solution is only formal, since the λ_j's must be found by requiring that equation (19.7.10) satisfy all the constraints built into equation (19.7.8). However, equation (19.7.10) does show the crucial fact that if G is *linear*, then the solution $\hat{\mathbf{u}}$ contains *only* a linear combination of basis functions $R_{j\mu}$ corresponding to actual measurements j. This is equivalent to setting unmeasured c_j's to zero. Notice that the principal solution obtained from equation (19.4.11) in fact has a linear G.

In the problem of incomplete Fourier image reconstruction, the typical $R_{j\mu}$ has the form $\exp(-2\pi i \mathbf{k}_j \cdot \mathbf{x}_\mu)$, where $\mathbf{x}_\mu$ is a two-dimensional vector in the image space and $\mathbf{k}_\mu$ is a two-dimensional wave vector. If an image contains strong point sources, then the effect of setting unmeasured c_j's to zero is to produce sidelobe ripples throughout the image plane. These ripples can mask any actual extended, low-intensity image features lying between the point sources. If, however, the slope of G is smaller for small values of its argument and larger for large values, then ripples in low-intensity portions of the image are relatively suppressed, while strong point sources will be relatively sharpened ("superresolution"). This behavior on the slope of G is equivalent to requiring $f'''(u) < 0$. For $f(u) = u \ln u$, we in fact have $f'''(u) = -1/u^2 < 0$.

In more picturesque language, the nonlinearity acts to "create" nonzero values for the unmeasured c_i's, so as to suppress the low-intensity ripple and sharpen the point sources.

19.7.1 Is MEM Really Magical?

How unique is the negentropy functional (19.7.3)? Recall that that equation is based on the assumption that luminance elements are a priori distributed over the pixels uniformly. If we instead had some other preferred a priori image in mind, one with pixel intensities m_μ, then it is easy to show that the negentropy becomes

$$H(\mathbf{u}) = \sum_{\mu=0}^{M-1} u_\mu \ln(u_\mu / m_\mu) + \text{constant} \tag{19.7.11}$$

(the constant can then be ignored). All the rest of the discussion then goes through.

More fundamentally, and despite statements by zealots to the contrary [5], there is actually nothing universal about the functional form $f(u) = u \ln u$. In some other physical situations (for example, the entropy of an electromagnetic field in the limit of many photons per mode, as in radio-astronomy) the physical negentropy functional is actually $f(u) = -\ln u$ (see [3] for other examples). In general, the question, "Entropy of what?" is not uniquely answerable in any particular situation. (See reference [7] for an attempt at articulating a more general principle that reduces to one or another entropy functional under appropriate circumstances.)

The four numbered properties summarized above, plus the desirable sign for nonlinearity, $f'''(u) < 0$, are all as true for the function $f(u) = -\ln u$ as for $f(u) = u \ln u$. In fact, these properties are shared by a nonlinear function as simple as $f(u) = -\sqrt{u}$, which has no information-theoretic justification at all (no logarithms!). MEM reconstructions of test images using any of these entropy forms are virtually indistinguishable [3].

By all available evidence, MEM seems to be neither more nor less than one usefully nonlinear version of the general regularization scheme $\mathcal{A} + \lambda\mathcal{B}$ that we have by now considered in many forms. Its peculiarities become strengths when applied to the reconstruction from incomplete Fourier data of images that are expected to be dominated by very bright point sources, but which also contain interesting low-intensity, extended sources. For images of some other character, there is no reason to suppose that MEM methods will generally dominate other regularization schemes, either ones already known or yet to be invented.

19.7.2 Algorithms for MEM

The goal is to find the vector $\hat{\mathbf{u}}$ that minimizes $\mathcal{A} + \lambda\mathcal{B}$ where in the notation of equations (19.5.5), (19.5.6), and (19.7.7),

$$\mathcal{A} = |\mathbf{b} - \mathbf{A} \cdot \mathbf{u}|^2 \qquad \mathcal{B} = \sum_\mu f(u_\mu) \tag{19.7.12}$$

Compared with a "general" minimization problem, we have the advantage that we can compute the gradients and the second partial derivative matrices (Hessian matrices) explicitly,

$$\begin{aligned} \nabla\mathcal{A} &= 2(\mathbf{A}^T \cdot \mathbf{A} \cdot \mathbf{u} - \mathbf{A}^T \cdot \mathbf{b}) & \frac{\partial^2 \mathcal{A}}{\partial u_\mu \partial u_\rho} &= [2\mathbf{A}^T \cdot \mathbf{A}]_{\mu\rho} \\ [\nabla\mathcal{B}]_\mu &= f'(u_\mu) & \frac{\partial^2 \mathcal{B}}{\partial u_\mu \partial u_\rho} &= \delta_{\mu\rho} f''(u_\mu) \end{aligned} \tag{19.7.13}$$

It is important to note that while $\mathcal{A}$'s second partial derivative matrix cannot be stored (its size is the square of the number of pixels), it can be applied to any vector by first applying $\mathbf{A}$, then $\mathbf{A}^T$. In the case of reconstruction from incomplete Fourier data, or in the case of convolution with a translation invariant point spread function, these applications will typically involve several FFTs. Likewise, the calculation of the gradient $\nabla\mathcal{A}$ will involve FFTs in the application of $\mathbf{A}$ and $\mathbf{A}^T$.

While some success has been achieved with the classical conjugate gradient method (§10.8), it is often found that the nonlinearity in $f(u) = u \ln u$ causes problems. Attempted steps that give $\mathbf{u}$ with even one negative value must be cut in magnitude, sometimes so severely as to slow the solution to a crawl. The underlying

problem is that the conjugate gradient method develops its information about the inverse of the Hessian matrix a bit at a time, while changing its location in the search space. When a nonlinear function is quite different from a pure quadratic form, the old information becomes obsolete before it gets usefully exploited.

Skilling and collaborators [4,5,8,9] developed a complicated but highly successful scheme, wherein a minimum is repeatedly sought not along a single search direction, but in a small- (typically three-) dimensional subspace, spanned by vectors that are calculated anew at each landing point. The subspace basis vectors are chosen in such a way as to avoid directions leading to negative values. One of the most successful choices is the three-dimensional subspace spanned by the vectors with components given by

$$\begin{aligned} e_\mu^{(1)} &= u_\mu[\nabla\mathcal{A}]_\mu \\ e_\mu^{(2)} &= u_\mu[\nabla\mathcal{B}]_\mu \\ e_\mu^{(3)} &= \frac{u_\mu \sum_\rho (\partial^2\mathcal{A}/\partial u_\mu \partial u_\rho) u_\rho [\nabla\mathcal{B}]_\rho}{\sqrt{\sum_\rho u_\rho \left([\nabla\mathcal{B}]_\rho\right)^2}} - \frac{u_\mu \sum_\rho (\partial^2\mathcal{A}/\partial u_\mu \partial u_\rho) u_\rho [\nabla\mathcal{A}]_\rho}{\sqrt{\sum_\rho u_\rho \left([\nabla\mathcal{A}]_\rho\right)^2}} \end{aligned} \tag{19.7.14}$$

(In these equations there is no sum over μ.) The form of the $\mathbf{e}^{(3)}$ has some justification if one views dot products as occurring in a space with the metric $g_{\mu\nu} = \delta_{\mu\nu}/u_\mu$, chosen to make zero values "far away"; see [4].

Within the three-dimensional subspace, the three-component gradient and nine-component Hessian matrix are computed by projection from the large space, and the minimum in the subspace is estimated by (trivially) solving three simultaneous linear equations, as in §10.9, equation (10.9.4). The size of a step $\Delta\mathbf{u}$ is required to be limited by the inequality

$$\sum_\mu (\Delta u_\mu)^2/u_\mu < (0.1 \text{ to } 0.5)U \tag{19.7.15}$$

Because the gradient directions $\nabla\mathcal{A}$ and $\nabla\mathcal{B}$ are separately available, it is possible to combine the minimum search with a simultaneous adjustment of λ so as finally to satisfy the desired constraint. There are various further tricks employed.

A less general, but in practice often equally satisfactory, approach is due to Cornwell and Evans [10]. Here, noting that $\mathcal{B}$'s Hessian (second partial derivative) matrix is diagonal, one asks whether there is a useful diagonal approximation to $\mathcal{A}$'s Hessian, namely $2\mathbf{A}^T \cdot \mathbf{A}$. If Λ_μ denotes the diagonal components of such an approximation, then a useful step in $\mathbf{u}$ would be

$$\Delta u_\mu = -\frac{1}{\Lambda_\mu + \lambda f''(u_\mu)}(\nabla\mathcal{A} + \lambda\nabla\mathcal{B}) \tag{19.7.16}$$

(again compare equation 10.9.4). Even more extreme, one might seek an approximation with constant diagonal elements, $\Lambda_\mu = \Lambda$, so that

$$\Delta u_\mu = -\frac{1}{\Lambda + \lambda f''(u_\mu)}(\nabla\mathcal{A} + \lambda\nabla\mathcal{B}) \tag{19.7.17}$$

Since $\mathbf{A}^T \cdot \mathbf{A}$ has something of the nature of a doubly convolved point spread function, and since in real cases one often has a point spread function with a sharp

central peak, even the more extreme of these approximations is often fruitful. One starts with a rough estimate of Λ obtained from the $A_{i\mu}$'s, e.g.,

$$\Lambda \sim \left\langle \sum_i [A_{i\mu}]^2 \right\rangle \tag{19.7.18}$$

An accurate value is not important, since in practice Λ is adjusted adaptively: If Λ is too large, then equation (19.7.17)'s steps will be too small (that is, larger steps in the same direction will produce even greater decrease in $\mathcal{A} + \lambda\mathcal{B}$). If Λ is too small, then attempted steps will land in an unfeasible region (negative values of u_μ) or will result in an increased $\mathcal{A} + \lambda\mathcal{B}$. There is an obvious similarity between the adjustment of Λ here and the Levenberg-Marquardt method of §15.5; this should not be too surprising, since MEM is closely akin to the problem of nonlinear least-squares fitting. Reference [10] also discusses how the value of $\Lambda + \lambda f''(u_\mu)$ can be used to adjust the Lagrange multiplier λ so as to converge to the desired value of χ^2.

All practical MEM algorithms are found to require on the order of 30 to 50 iterations to converge. This convergence behavior is not now understood in any fundamental way.

19.7.3 "Bayesian" versus "Historic" Maximum Entropy

Several generalizations of the basic maximum entropy image restoration technique go under the rubric "Bayesian" to distinguish them from the previous "historic" methods. See [11] for details and references. (Our view, of course, is that all the methods are about equally Bayesian, as discussed in §19.4.)

- Better priors: We already noted that the entropy functional (equation 19.7.7) is invariant under scrambling all pixels and has no notion of smoothness. The so-called "intrinsic correlation function" (ICF) model (reference [11], where it is called "New MaxEnt") is similar enough to the entropy functional to allow similar algorithms, but it makes the values of neighboring pixels correlated, enforcing smoothness.
- Better estimation of λ: Above we chose λ to bring χ^2 into its expected narrow statistical range of $N \pm (2N)^{1/2}$. This in effect overestimates χ^2, however, since some effective number γ of parameters are being "fitted" in doing the reconstruction. A Bayesian approach leads to a self-consistent estimate of this γ and an objectively better choice for λ.

CITED REFERENCES AND FURTHER READING:

Gzyl, H. 1995, *The Method of Maximum Entropy* (Singapore: World Scientific).[1]

Wu, N. 1997, *The Maximum Entropy Method* (Berlin: Springer).[2]

Narayan, R., and Nityananda, R. 1986, "Maximum Entropy Image Restoration in Astronomy," *Annual Review of Astronomy and Astrophysics*, vol. 24, pp. 127–170.[3]

Skilling, J., and Bryan, R.K. 1984, "Maximum Entropy Image Reconstruction: General Algorithm," *Monthly Notices of the Royal Astronomical Society*, vol. 211, pp. 111–124.[4]

Burch, S.F., Gull, S.F., and Skilling, J. 1983, "Image Restoration by a Powerful Maximum Entropy Method," *Computer Vision, Graphics and Image Processing*, vol. 23, pp. 113–128.[5]

Skilling, J. 1989, in *Maximum Entropy and Bayesian Methods*, J. Skilling, ed. (Boston: Kluwer).[6]

Frieden, B.R. 1983, "Unified Theory for Estimating Frequency-of-Occurrence Laws and Optical Objects," *Journal of the Optical Society of America*, vol. 73, pp. 927–938.[7]

Skilling, J., and Gull, S.F. 1985, in *Maximum-Entropy and Bayesian Methods in Inverse Problems*, C.R. Smith and W.T. Grandy, Jr., eds. (Dordrecht: Reidel).[8]

Skilling, J. 1986, in *Maximum Entropy and Bayesian Methods in Applied Statistics*, J.H. Justice, ed. (Cambridge, UK: Cambridge University Press).[9]

Cornwell, T.J., and Evans, K.F. 1985, "A Simple Maximum Entropy Deconvolution Algorithm," *Astronomy and Astrophysics*, vol. 143, pp. 77–83.[10]

Gull, S.F. 1989, in *Maximum Entropy and Bayesian Methods*, J. Skilling, ed. (Boston: Kluwer).[11]

CHAPTER 20

Partial Differential Equations

20.0 Introduction

The numerical treatment of partial differential equations (PDEs) is, by itself, a vast subject. Partial differential equations are at the heart of many, if not most, computer analyses or simulations of continuous physical systems, such as fluids, electromagnetic fields, the human body, and so on. The intent of this chapter is to give the briefest possible useful introduction. Ideally, there would be an entire second volume of *Numerical Recipes* dealing with partial differential equations alone. (The references [1-4] provide, of course, available alternatives.)

Mathematicians like to classify the partial differential equations that typically occur in applications into three categories, *hyperbolic, parabolic,* and *elliptic*, on the basis of their *characteristics*, or curves of information propagation. The prototypical example of a hyperbolic equation is the one-dimensional *wave* equation

$$\frac{\partial^2 u}{\partial t^2} = v^2 \frac{\partial^2 u}{\partial x^2} \tag{20.0.1}$$

where $v = \text{constant}$ is the velocity of wave propagation. The prototypical parabolic equation is the *diffusion* equation

$$\frac{\partial u}{\partial t} = \frac{\partial}{\partial x}\left(D \frac{\partial u}{\partial x}\right) \tag{20.0.2}$$

where D is the diffusion coefficient. The prototypical elliptic equation is the *Poisson* equation

$$\frac{\partial^2 u}{\partial x^2} + \frac{\partial^2 u}{\partial y^2} = \rho(x, y) \tag{20.0.3}$$

where the source term ρ is given. If the source term is equal to zero, the equation is *Laplace's equation*.

From a computational point of view, the classification into these three canonical types is not very meaningful — or at least not as important as some other essential distinctions. Equations (20.0.1) and (20.0.2) both define *initial value* or *Cauchy* problems: If information on u (perhaps including time derivative information) is given at some initial time t_0 for all x, then the equations describe how $u(x, t)$ propagates itself forward in time. In other words, equations (20.0.1) and (20.0.2) describe

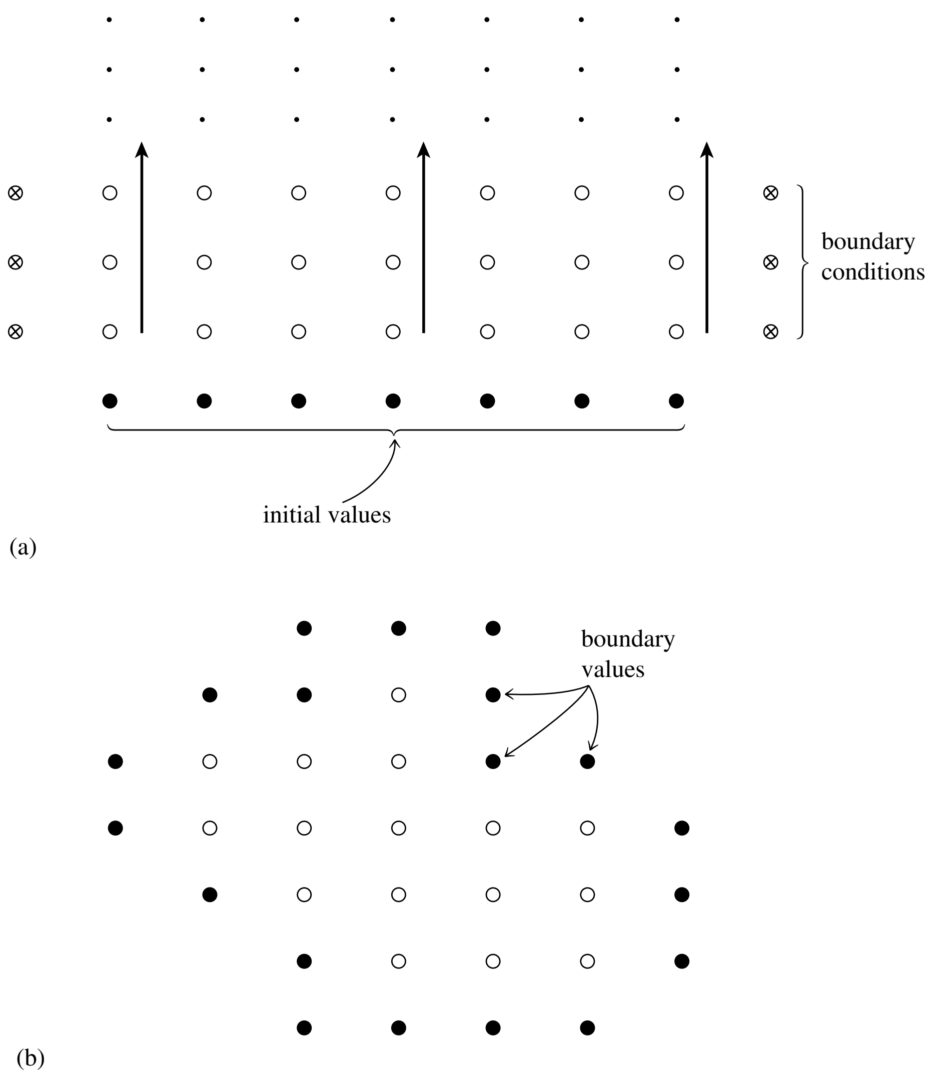

Figure 20.0.1. Initial value problem (a) and boundary value problem (b) are contrasted. In (a), initial values are given on one "time slice," and it is desired to advance the solution in time, computing successive rows of open dots in the direction shown by the arrows. Boundary conditions at the left and right edges of each row (⊗) must also be supplied, but only one row at a time. Only one, or a few, previous rows need be maintained in memory. In (b), boundary values are specified around the edge of a grid, and an iterative process is employed to find the values of all the internal points (open circles). All grid points must be maintained in memory.

time evolution. The goal of a numerical code should be to track that time evolution with some desired accuracy.

By contrast, equation (20.0.3) directs us to find a single "static" function $u(x, y)$ that satisfies the equation within some (x, y) region of interest, and that — one must also specify — has some desired behavior on the boundary of the region of interest. These problems are called *boundary value problems* (see Figure 20.0.1). In general it is not possible stably to just "integrate in from the boundary" in the same sense

that an initial value problem can be "integrated forward in time." Therefore, the goal of a numerical code is somehow to converge on the correct solution everywhere at once.

This, then, is the most important classification from a computational point of view: Is the problem at hand an *initial value* (time evolution) problem? or is it a *boundary value* (static solution) problem? Figure 20.0.1 emphasizes the distinction. Notice that while the italicized terminology is standard, the terminology in parentheses is a much better description of the dichotomy from a computational perspective. The subclassification of initial value problems into parabolic and hyperbolic is much less important because (i) many actual problems are of a mixed type, and (ii) as we will see, most hyperbolic problems get parabolic pieces mixed into them by the time one is discussing practical computational schemes.

20.0.1 Initial Value Problems

An initial value problem is defined by answers to the following questions:

- What are the dependent variables to be propagated forward in time?
- What is the evolution equation for each variable? Usually the evolution equations will all be coupled, with more than one dependent variable appearing on the right-hand side of each equation.
- What is the highest time derivative that occurs in each variable's evolution equation? If possible, this time derivative should be put alone on the equation's left-hand side. Not only the value of a variable, but also the value of all its time derivatives — up to the highest one — must be specified to define the evolution.
- What special equations (boundary conditions) govern the evolution in time of points on the boundary of the spatial region of interest? Examples: *Dirichlet conditions* specify the values of the boundary points as a function of time; *Neumann conditions* specify the values of the normal gradients on the boundary; *outgoing wave boundary conditions* are just what they say.

Sections 20.1 – 20.3 of this chapter deal with initial value problems of several different forms. We make no pretense of completeness, but rather hope to convey a certain amount of generalizable information through a few carefully chosen model examples. These examples will illustrate an important point: One's principal *computational* concern must be the *stability* of the algorithm. Many reasonable-looking algorithms for initial value problems just don't work — they are numerically unstable.

20.0.2 Boundary Value Problems

The questions that define a boundary value problem are

- What are the variables?
- What equations are satisfied in the interior of the region of interest?
- What equations are satisfied by points on the boundary of the region of interest? (Here Dirichlet and Neumann conditions are possible choices for elliptic second-order equations, but more complicated boundary conditions can also be encountered.)

In contrast to initial value problems, stability is relatively easy to achieve for boundary value problems. Thus, the *efficiency* of the algorithms, both in computa-

tional load and storage requirements, becomes the principal concern.

Because all the conditions on a boundary value problem must be satisfied "simultaneously," these problems usually boil down, at least conceptually, to the solution of large numbers of simultaneous algebraic equations. When such equations are nonlinear, they are usually solved by linearization and iteration; so without much loss of generality we can view the problem as being the solution of special, large linear sets of equations.

As an example, one that we will refer to in §20.4 – §20.6 as our "model problem," let us consider the solution of equation (20.0.3) by the *finite difference method*. We represent the function $u(x, y)$ by its values at the discrete set of points

$$\begin{aligned} x_j &= x_0 + j\Delta, \qquad & j &= 0, 1, ..., J \\ y_l &= y_0 + l\Delta, \qquad & l &= 0, 1, ..., L \end{aligned} \tag{20.0.4}$$

where Δ is the *grid spacing*. From now on, we will write $u_{j,l}$ for $u(x_j, y_l)$ and $\rho_{j,l}$ for $\rho(x_j, y_l)$. For (20.0.3) we substitute a finite difference representation (see Figure 20.0.2),

$$\frac{u_{j+1,l} - 2u_{j,l} + u_{j-1,l}}{\Delta^2} + \frac{u_{j,l+1} - 2u_{j,l} + u_{j,l-1}}{\Delta^2} = \rho_{j,l} \tag{20.0.5}$$

or, equivalently,

$$u_{j+1,l} + u_{j-1,l} + u_{j,l+1} + u_{j,l-1} - 4u_{j,l} = \Delta^2 \rho_{j,l} \tag{20.0.6}$$

To write this system of linear equations in matrix form we need to make a vector out of u. Let us number the two dimensions of grid points in a single one-dimensional sequence by defining

$$i \equiv j(L+1) + l \qquad \text{for} \qquad j = 0, 1, ..., J, \qquad l = 0, 1, ..., L \tag{20.0.7}$$

In other words, i increases most rapidly along the columns representing y values. Equation (20.0.6) now becomes

$$u_{i+L+1} + u_{i-(L+1)} + u_{i+1} + u_{i-1} - 4u_i = \Delta^2 \rho_i \tag{20.0.8}$$

This equation holds only at the interior points $j = 1, 2, ..., J-1; l = 1, 2, ..., L-1$.

The points where

$$\begin{aligned} j &= 0 \qquad && [\text{i.e., } i = 0, ..., L] \\ j &= J \qquad && [\text{i.e., } i = J(L+1), ..., J(L+1)+L] \\ l &= 0 \qquad && [\text{i.e., } i = 0, L+1, ..., J(L+1)] \\ l &= L \qquad && [\text{i.e., } i = L, L+1+L, ..., J(L+1)+L] \end{aligned} \tag{20.0.9}$$

are boundary points where either u or its derivative has been specified. If we pull all this "known" information over to the right-hand side of equation (20.0.8), then the equation takes the form

$$\mathbf{A} \cdot \mathbf{u} = \mathbf{b} \tag{20.0.10}$$

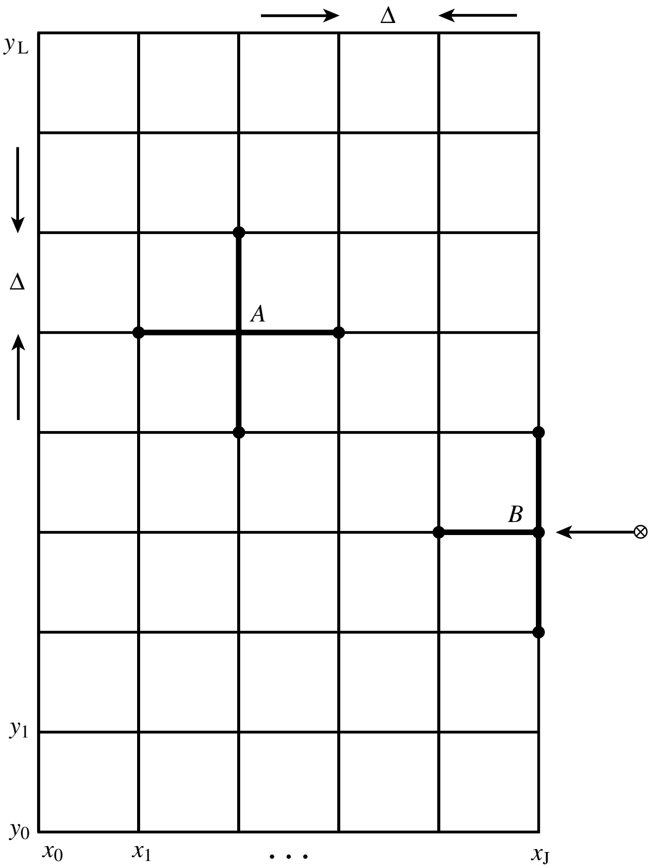

Figure 20.0.2. Finite difference representation of a second-order elliptic equation on a two-dimensional grid. The second derivatives at the point A are evaluated using the points to which A is shown connected. The second derivatives at point B are evaluated using the connected points and also using "right-hand side" boundary information, shown schematically as ⊗.

where **A** has the form shown in Figure 20.0.3. The matrix **A** is called "tridiagonal with fringes." A general linear second-order elliptic equation

$$a(x,y)\frac{\partial^2 u}{\partial x^2} + b(x,y)\frac{\partial u}{\partial x} + c(x,y)\frac{\partial^2 u}{\partial y^2} + d(x,y)\frac{\partial u}{\partial y} + e(x,y)\frac{\partial^2 u}{\partial x \partial y} + f(x,y)u = g(x,y) \tag{20.0.11}$$

will lead to a matrix of similar structure except that the nonzero entries will not be constants.

As a rough classification, there are three different approaches to the solution of equation (20.0.10), not all applicable in all cases: relaxation methods, "rapid" methods (e.g., Fourier methods), and direct matrix methods.

Relaxation methods make immediate use of the structure of the sparse matrix **A**. The matrix is split into two parts,

$$\mathbf{A} = \mathbf{E} - \mathbf{F} \tag{20.0.12}$$

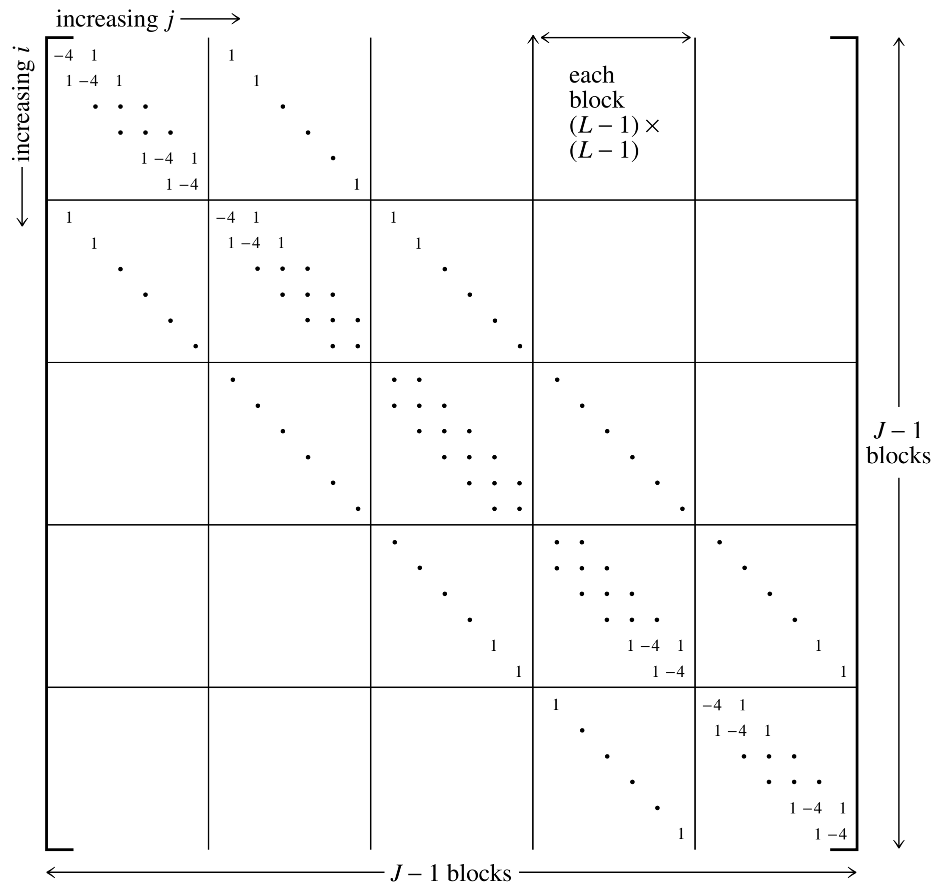

Figure 20.0.3. Matrix structure derived from a second-order elliptic equation (here equation 20.0.6). All elements not shown are zero. The matrix has diagonal blocks that are themselves tridiagonal, and sub- and superdiagonal blocks that are diagonal. This form is called "tridiagonal with fringes." A matrix this sparse would never be stored in its full form as shown here.

where $\mathbf{E}$ is easily invertible and $\mathbf{F}$ is the remainder. Then (20.0.10) becomes

$$\mathbf{E} \cdot \mathbf{u} = \mathbf{F} \cdot \mathbf{u} + \mathbf{b} \tag{20.0.13}$$

The relaxation method involves choosing an initial guess $\mathbf{u}^{(0)}$ and then solving successively for iterates $\mathbf{u}^{(r)}$ from

$$\mathbf{E} \cdot \mathbf{u}^{(r)} = \mathbf{F} \cdot \mathbf{u}^{(r-1)} + \mathbf{b} \tag{20.0.14}$$

Since $\mathbf{E}$ is chosen to be easily invertible, each iteration is fast. We will discuss relaxation methods in some detail in §20.5 and §20.6.

So-called rapid methods [5] apply for only a rather special class of equations: those with constant coefficients, or, more generally, those that are separable in the chosen coordinates. In addition, the boundaries must coincide with coordinate lines. This special class of equations is met quite often in practice. We defer detailed discussion to §20.4. Note, however, that the multigrid relaxation methods discussed in §20.6 can be faster than "rapid" methods.

Matrix methods attempt to solve the equation

$$\mathbf{A} \cdot \mathbf{x} = \mathbf{b} \tag{20.0.15}$$

directly. The degree to which this is practical depends very strongly on the exact structure of the matrix **A** for the problem at hand, so our discussion can go no farther than a few remarks and references at this point.

Sparseness of the matrix *must* be the guiding force. Otherwise the matrix problem becomes prohibitively large. For example, a problem on a 1000×1000 spatial grid would involve 10^6 unknown $u_{j,l}$'s, implying a $10^6 \times 10^6$ matrix **A** containing 10^{12} elements. A non-sparse $O(N^3)$ solution method would require $O(10^{18})$ operations.

As we discussed at the end of §2.7, if **A** is symmetric and positive-definite (as it usually is in elliptic problems), the conjugate gradient algorithm can be used. In practice, rounding error often spoils the effectiveness of the conjugate gradient algorithm for solving finite difference equations. However, it is useful when incorporated in methods that first rewrite the equations so that **A** is transformed to a matrix $\mathbf{A}'$ that is close to the identity matrix. The quadratic surface defined by the equations then has almost spherical contours, and the conjugate gradient algorithm works very well. In §2.7, in the routine `linbcg`, an analogous *preconditioner* was exploited for non-positive-definite problems with the more general biconjugate gradient method. There is a huge literature on the general subject of iterative methods to solve the sparse equations that typically arise in solving PDEs. Good places to start are [6-8].

Another class of matrix methods is the analyze-factorize-operate approach as described in §2.7.

Generally speaking, when you have the storage available to implement these methods — not nearly as much as the 10^{12} above, but usually much more than is required by relaxation methods — then you should consider doing so. Only multigrid relaxation methods (§20.6) are competitive with the best matrix methods. For grids larger than, say, 1000×1000, however, it is typically found that only relaxation methods, or "rapid" methods when they are applicable, are possible.

20.0.3 There Is More to Life than Finite Differencing

Besides finite differencing, there are other methods for solving PDEs. Most important are finite element, Monte Carlo, spectral, and variational methods. Unfortunately, we shall barely be able to do justice to finite differencing in this chapter, and we will give a brief introduction to spectral methods in §20.7. We shall not be able to discuss the other methods in this book. Finite element methods [9-11] are often preferred by practitioners in solid mechanics and structural engineering; these methods allow considerable freedom in putting computational elements where you want them, which is important when dealing with highly irregular geometries.

CITED REFERENCES AND FURTHER READING:

Ames, W.F. 1992, *Numerical Methods for Partial Differential Equations*, 3rd ed. (New York: Academic Press).[1]

Richtmyer, R.D., and Morton, K.W. 1967, *Difference Methods for Initial Value Problems*, 2nd ed. (New York: Wiley-Interscience); republished 1994 (Melbourne, FL: Krieger).[2]

Roache, P.J. 1998, *Computational Fluid Dynamics*, revised edition (Albuquerque: Hermosa).[3]

Thomas, J.W. 1995, *Numerical Partial Differential Equations: Finite Difference Methods* (New York: Springer).[4]

Dorr, F.W. 1970, "The Direct Solution of the Discrete Poisson Equation on a Rectangle," *SIAM Review*, vol. 12, pp. 248–263.[5]

Saad, Y. 2003, *Iterative Methods for Sparse Linear Systems*, 2nd ed. (Philadelphia: S.I.A.M.).[6]

Barrett, R., et al. 1993, *Templates for the Solution of Linear Systems: Building Blocks for Iterative Methods* (Philadelphia: S.I.A.M.).[7]

Greenbaum, A. 1997, *Iterative Methods for Solving Linear Systems* (Philadelphia: S.I.A.M.).[8]

Reddy, J.N. 2005, *An Introduction to the Finite Element Method*, 3rd ed. (New York: McGraw-Hill).[9]

Smith, I.M., and Griffiths, V. 2004, *Programming the Finite Element Method* (New York: Wiley).[10]

Zienkiewicz, O.C., Taylor, R.L., and Zhu, J.Z. 2005, *The Finite Element Method: Its Basis and Fundamentals*, 6th ed. (Oxford, UK: Elsevier Butterworth-Heinemann).[11]

20.1 Flux-Conservative Initial Value Problems

A large class of initial value (time-evolution) PDEs in one space dimension can be cast into the form of a *flux-conservative equation*,

$$\frac{\partial \mathbf{u}}{\partial t} = -\frac{\partial \mathbf{F}(\mathbf{u})}{\partial x} \tag{20.1.1}$$

where $\mathbf{u}$ and $\mathbf{F}$ are vectors, and where (in some cases) $\mathbf{F}$ may depend not only on $\mathbf{u}$ but also on spatial derivatives of $\mathbf{u}$. The vector $\mathbf{F}$ is called the *conserved flux*.

For example, the prototypical hyperbolic equation, the one-dimensional wave equation with constant velocity of propagation v,

$$\frac{\partial^2 u}{\partial t^2} = v^2 \frac{\partial^2 u}{\partial x^2} \tag{20.1.2}$$

can be rewritten as a set of two first-order equations:

$$\begin{aligned} \frac{\partial r}{\partial t} &= v\frac{\partial s}{\partial x} \\ \frac{\partial s}{\partial t} &= v\frac{\partial r}{\partial x} \end{aligned} \tag{20.1.3}$$

where

$$\begin{aligned} r &\equiv v\frac{\partial u}{\partial x} \\ s &\equiv \frac{\partial u}{\partial t} \end{aligned} \tag{20.1.4}$$

In this case, r and s become the two components of $\mathbf{u}$, and the flux is given by the linear matrix relation

$$\mathbf{F}(\mathbf{u}) = \begin{pmatrix} 0 & -v \\ -v & 0 \end{pmatrix} \cdot \mathbf{u} \tag{20.1.5}$$

(The physicist reader may recognize equations 20.1.3 as analogous to Maxwell's equations for one-dimensional propagation of electromagnetic waves.)

We will consider, in this section, a prototypical example of the general flux-conservative equation (20.1.1), namely the equation for a scalar u,

$$\frac{\partial u}{\partial t} = -v\frac{\partial u}{\partial x} \tag{20.1.6}$$

with v a constant. As it happens, we already know analytically that the general solution of this equation is a wave propagating in the positive x-direction,

$$u = f(x - vt) \tag{20.1.7}$$

where f is an arbitrary function. However, the numerical strategies that we develop will be equally applicable to the more general equations represented by (20.1.1). In some contexts, equation (20.1.6) is called an *advective* equation, because the quantity u is transported by a "fluid flow" with a velocity v.

How do we go about finite differencing equation (20.1.6) (or, analogously, 20.1.1)? The straightforward approach is to choose equally spaced points along both the t- and x-axes. Thus denote

$$\begin{aligned} x_j &= x_0 + j\Delta x, \qquad & j &= 0, 1, \dots, J \\ t_n &= t_0 + n\Delta t, \qquad & n &= 0, 1, \dots, N \end{aligned} \tag{20.1.8}$$

Let u_j^n denote $u(t_n, x_j)$. We have several choices for representing the time derivative term. The obvious way is to set

$$\left.\frac{\partial u}{\partial t}\right|_{j,n} = \frac{u_j^{n+1} - u_j^n}{\Delta t} + O(\Delta t) \tag{20.1.9}$$

This is called *forward Euler* differencing (cf. equation 17.1.1). While forward Euler is only first-order accurate in Δt, it has the advantage that one is able to calculate quantities at timestep $n+1$ in terms of only quantities known at timestep n. For the space derivative, we can use a second-order representation still using only quantities known at timestep n:

$$\left.\frac{\partial u}{\partial x}\right|_{j,n} = \frac{u_{j+1}^n - u_{j-1}^n}{2\Delta x} + O(\Delta x^2) \tag{20.1.10}$$

The resulting finite difference approximation to equation (20.1.6) is called the FTCS representation (forward time centered space),

$$\frac{u_j^{n+1} - u_j^n}{\Delta t} = -v\left(\frac{u_{j+1}^n - u_{j-1}^n}{2\Delta x}\right) \tag{20.1.11}$$

which can easily be rearranged to be a formula for u_j^{n+1} in terms of the other quantities. The FTCS scheme is illustrated in Figure 20.1.1. It's a fine example of an algorithm that is easy to derive, takes little storage, and executes quickly. Too bad it doesn't work! (See below.)

The FTCS representation is an *explicit* scheme. This means that u_j^{n+1} for each j can be calculated explicitly from the quantities that are already known. Later we

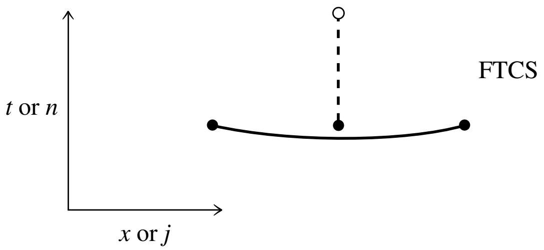

Figure 20.1.1. Representation of the forward time centered space (FTCS) differencing scheme. In this and subsequent figures, the open circle is the new point at which the solution is desired; filled circles are known points whose function values are used in calculating the new point; the solid lines connect points that are used to calculate spatial derivatives; the dashed lines connect points that are used to calculate time derivatives. The FTCS scheme is generally unstable for hyperbolic problems and cannot usually be used.

shall meet *implicit* schemes, which require us to solve implicit equations coupling the u_j^{n+1} for various j. (Explicit and implicit methods for ordinary differential equations were discussed in §17.5.) The FTCS algorithm is also an example of a *single-level* scheme, since only values at time level n have to be stored to find values at time level $n+1$.

20.1.1 von Neumann Stability Analysis

Unfortunately, equation (20.1.11) is of very limited usefulness. It is an *unstable* method, which can be used only (if at all) to study waves for a short fraction of one oscillation period. To find alternative methods with more general applicability, we introduce the *von Neumann stability analysis*.

The von Neumann analysis is local: Imagine that the coefficients of the difference equations are so slowly varying as to be considered constant in space and time. In that case, the independent solutions, or *eigenmodes*, of the difference equations are all of the form

$$u_j^n = \xi^n e^{ikj\Delta x} \tag{20.1.12}$$

where k is a real spatial wave number (which can have any value) and $\xi = \xi(k)$ is a complex number that depends on k. The key fact is that the time dependence of a single eigenmode is nothing more than successive integer powers of the complex number ξ. Therefore, the difference equations are unstable (have exponentially growing modes) if $|\xi(k)| > 1$ for *some* k. The number ξ is called the *amplification factor* at a given wave number k.

To find $\xi(k)$, simply substitute (20.1.12) back into (20.1.11). Divide by ξ^n to get

$$\xi(k) = 1 - i\frac{v\Delta t}{\Delta x}\sin k\Delta x \tag{20.1.13}$$

whose modulus is > 1 for *all* k; so the FTCS scheme is unconditionally unstable.

If the velocity v were a function of t and x, then we would write v_j^n in equation (20.1.11). In the von Neumann stability analysis we would still treat v as a constant, the idea being that for v slowly varying the analysis is local. In fact, even in the case of strictly constant v, the von Neumann analysis does not rigorously treat the end effects at $j = 0$ and $j = N$.

More generally, if the equation's right-hand side were nonlinear in u, then a von Neumann analysis would linearize by writing $u = u_0 + \delta u$, expanding to linear

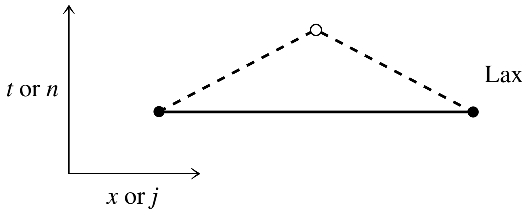

Figure 20.1.2. Representation of the Lax differencing scheme, as in the previous figure. The stability criterion for this scheme is the Courant condition.

order in δu. Assuming that the u_0 quantities already satisfy the difference equation exactly, the analysis would look for an unstable eigenmode of δu.

Despite its lack of rigor, the von Neumann method generally gives valid answers and is much easier to apply than more careful methods. We accordingly adopt it exclusively. (See, for example, [1] for a discussion of other methods of stability analysis.)

20.1.2 Lax Method

The instability in the FTCS method can be cured by a simple change due to Lax. One replaces the term u_j^n in the time derivative term by its average (Figure 20.1.2):

$$u_j^n \to \tfrac{1}{2}\left(u_{j+1}^n + u_{j-1}^n\right) \tag{20.1.14}$$

This turns (20.1.11) into

$$u_j^{n+1} = \frac{1}{2}\left(u_{j+1}^n + u_{j-1}^n\right) - \frac{v\Delta t}{2\Delta x}\left(u_{j+1}^n - u_{j-1}^n\right) \tag{20.1.15}$$

Substituting equation (20.1.12), we find for the amplification factor

$$\xi = \cos k\Delta x - i\frac{v\Delta t}{\Delta x}\sin k\Delta x \tag{20.1.16}$$

The stability condition $|\xi|^2 \le 1$ leads to the requirement

$$\frac{|v|\Delta t}{\Delta x} \le 1 \tag{20.1.17}$$

This is the famous Courant-Friedrichs-Lewy stability criterion, often called simply the *Courant condition*. Intuitively, the stability condition can be understood as follows (Figure 20.1.3): The quantity u_j^{n+1} in equation (20.1.15) is computed from information at points $j-1$ and $j+1$ at time n. In other words, x_{j-1} and x_{j+1} are the boundaries of the spatial region that is allowed to communicate information to u_j^{n+1}. Now recall that in the continuum wave equation, information actually propagates with a maximum velocity v. If the point u_j^{n+1} is outside of the shaded region in Figure 20.1.3, then it requires information from points more distant than the differencing scheme allows. Lack of that information gives rise to an instability. Therefore, Δt cannot be made too large.*

*Actually, this simple picture works only for hyperbolic equations with the order of the spatial differencing not higher than the order of the PDE. In general, the stability analysis determines the eigenvalues of a matrix. These eigenvalues correspond to the characteristic velocities of the difference scheme. Stability requires that all these velocities be greater than or equal to the characteristic velocities of the PDE.

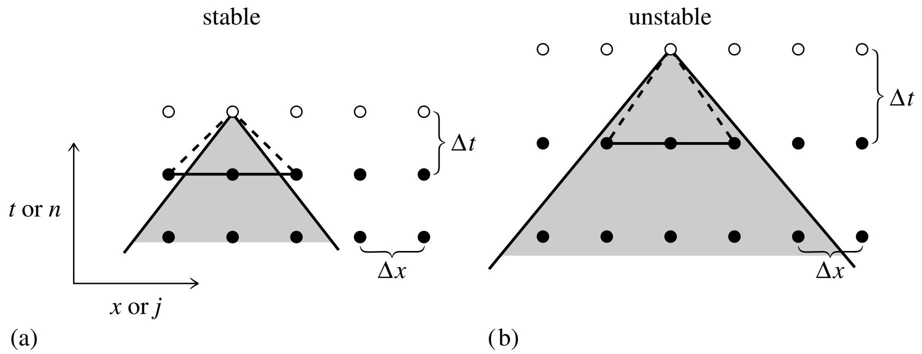

Figure 20.1.3. Courant condition for stability of a differencing scheme. The solution of a hyperbolic problem at a point depends on information within some domain of dependency to the past, shown here shaded. The differencing scheme (20.1.15) has its own domain of dependency determined by the choice of points on one time slice (shown as connected solid dots) whose values are used in determining a new point (shown connected by dashed lines). A differencing scheme is Courant stable if the differencing domain of dependency is larger than that of the PDEs, as in (a), and unstable if the relationship is the reverse, as in (b). For more complicated differencing schemes, the domain of dependency might not be determined simply by the outermost points.

The surprising result, that the simple replacement (20.1.14) stabilizes the FTCS scheme, is our first encounter with the fact that differencing PDEs is an art as much as a science. To see if we can demystify the art somewhat, let us compare the FTCS and Lax schemes by rewriting equation (20.1.15) so that it is in the form of equation (20.1.11) with a remainder term:

$$\frac{u_j^{n+1} - u_j^n}{\Delta t} = -v\left(\frac{u_{j+1}^n - u_{j-1}^n}{2\Delta x}\right) + \frac{1}{2}\left(\frac{u_{j+1}^n - 2u_j^n + u_{j-1}^n}{\Delta t}\right) \tag{20.1.18}$$

But this is exactly the FTCS representation of the equation

$$\frac{\partial u}{\partial t} = -v\frac{\partial u}{\partial x} + \frac{(\Delta x)^2}{2\Delta t}\nabla^2 u \tag{20.1.19}$$

where $\nabla^2 = \partial^2/\partial x^2$ in one dimension. We have, in effect, added a diffusion term to the equation, or, if you recall the form of the Navier-Stokes equation for viscous fluid flow, a dissipative term. The Lax scheme is thus said to have *numerical dissipation*, or *numerical viscosity*. We can see this also in the amplification factor. Unless $|v|\Delta t$ is exactly equal to Δx, $|\xi| < 1$ and the amplitude of the wave decreases spuriously.

Isn't a spurious decrease as bad as a spurious increase? No. The scales that we hope to study accurately are those that encompass many grid points, so that they have $k\Delta x \ll 1$. (The spatial wave number k is defined by equation 20.1.12.) For these scales, the amplification factor can be seen to be very close to one, in both the stable and unstable schemes. The stable and unstable schemes are therefore about equally accurate. For the unstable scheme, however, short scales with $k\Delta x \sim 1$, *which we are not interested in*, will blow up and swamp the interesting part of the solution. It is much better to have a stable scheme in which these short wavelengths die away innocuously. Both the stable and the unstable schemes are *inaccurate* for these short wavelengths, but the inaccuracy is of a tolerable character when the scheme is stable.

When the independent variable **u** is a vector, the von Neumann analysis is slightly more complicated. For example, we can consider equation (20.1.3), rewritten as

$$\frac{\partial}{\partial t}\begin{bmatrix} r \\ s \end{bmatrix} = \frac{\partial}{\partial x}\begin{bmatrix} vs \\ vr \end{bmatrix} \tag{20.1.20}$$

The Lax method for this equation is

$$\begin{aligned} r_j^{n+1} &= \frac{1}{2}(r_{j+1}^n + r_{j-1}^n) + \frac{v\Delta t}{2\Delta x}(s_{j+1}^n - s_{j-1}^n) \\ s_j^{n+1} &= \frac{1}{2}(s_{j+1}^n + s_{j-1}^n) + \frac{v\Delta t}{2\Delta x}(r_{j+1}^n - r_{j-1}^n) \end{aligned} \tag{20.1.21}$$

The von Neumann stability analysis now proceeds by assuming that the eigenmode is of the following (vector) form:

$$\begin{bmatrix} r_j^n \\ s_j^n \end{bmatrix} = \xi^n e^{ikj\Delta x}\begin{bmatrix} r^0 \\ s^0 \end{bmatrix} \tag{20.1.22}$$

Here the vector on the right-hand side is a constant (both in space and in time) eigenvector, and ξ is a complex number, as before. Substituting (20.1.22) into (20.1.21) and dividing by the power ξ^n, gives the homogeneous vector equation

$$\begin{bmatrix} (\cos k\Delta x) - \xi & i\dfrac{v\Delta t}{\Delta x}\sin k\Delta x \\ i\dfrac{v\Delta t}{\Delta x}\sin k\Delta x & (\cos k\Delta x) - \xi \end{bmatrix} \cdot \begin{bmatrix} r^0 \\ s^0 \end{bmatrix} = \begin{bmatrix} 0 \\ 0 \end{bmatrix} \tag{20.1.23}$$

This admits a solution only if the determinant of the matrix on the left vanishes, a condition easily shown to yield the two roots ξ,

$$\xi = \cos k\Delta x \pm i\frac{v\Delta t}{\Delta x}\sin k\Delta x \tag{20.1.24}$$

The stability condition is that both roots satisfy $|\xi| \le 1$. This again turns out to be simply the Courant condition (20.1.17).

20.1.3 Other Varieties of Error

Thus far we have been concerned with *amplitude error*, because of its intimate connection with the stability or instability of a differencing scheme. Other varieties of error are relevant when we shift our concern to accuracy, rather than stability.

Finite difference schemes for hyperbolic equations can exhibit dispersion, or *phase errors*. For example, equation (20.1.16) can be rewritten as

$$\xi = e^{-ik\Delta x} + i\left(1 - \frac{v\Delta t}{\Delta x}\right)\sin k\Delta x \tag{20.1.25}$$

An arbitrary initial wave packet is a superposition of modes with different k's. At each timestep the modes get multiplied by different phase factors (20.1.25), depending on their value of k. If $\Delta t = \Delta x/v$, then the exact solution for each mode of a wave packet $f(x - vt)$ is obtained if each mode gets multiplied by $\exp(-ik\Delta x)$. For this value of Δt, equation (20.1.25) shows that the finite difference solution gives

the exact analytic result. However, if $v\Delta t/\Delta x$ is not exactly 1, the phase relations of the modes can become hopelessly garbled and the wave packet disperses. Note from (20.1.25) that the dispersion becomes large as soon as the wavelength becomes comparable to the grid spacing Δx.

A third type of error is one associated with nonlinear hyperbolic equations and is therefore sometimes called *nonlinear instability*. For example, a piece of the Euler or Navier-Stokes equations for fluid flow looks like

$$\frac{\partial v}{\partial t} = -v\frac{\partial v}{\partial x} + \dots \tag{20.1.26}$$

The nonlinear term in v can cause a transfer of energy in Fourier space from long wavelengths to short wavelengths. This results in a wave profile steepening until a vertical profile or "shock" develops. Since the von Neumann analysis suggests that the stability can depend on $k\Delta x$, a scheme that was stable for shallow profiles can become unstable for steep profiles. This kind of difficulty arises in a differencing scheme where the cascade in Fourier space is halted at the shortest wavelength representable on the grid, that is, at $k \sim 1/\Delta x$. If energy simply accumulates in these modes, it eventually swamps the energy in the long wavelength modes of interest.

Nonlinear instability and shock formation are thus somewhat controlled by numerical viscosity such as that discussed in connection with equation (20.1.18) above. In some fluid problems, however, shock formation is not merely an annoyance, but an actual physical behavior of the fluid whose detailed study is a goal. Then, numerical viscosity alone may not be adequate or sufficiently controllable. This is a complicated subject that we discuss further in the subsection on fluid dynamics, below.

For wave equations, propagation errors (amplitude or phase) are usually most worrisome. For advective equations, on the other hand, *transport errors* are usually of greater concern. In the Lax scheme, equation (20.1.15), a disturbance in the advected quantity u at mesh point j propagates to mesh points $j+1$ and $j-1$ at the next timestep. In reality, however, if the velocity v is positive, then only mesh point $j+1$ should be affected.

The simplest way to model the transport properties "better" is to use *upwind differencing* (see Figure 20.1.4):

$$\frac{u_j^{n+1}-u_j^n}{\Delta t} = -v_j^n \begin{cases} \dfrac{u_j^n - u_{j-1}^n}{\Delta x}, & v_j^n > 0 \\[2ex] \dfrac{u_{j+1}^n - u_j^n}{\Delta x}, & v_j^n < 0 \end{cases} \tag{20.1.27}$$

Note that this scheme is only first-order, not second-order, accurate in the calculation of the spatial derivatives. So how can it be "better"? The answer is one that annoys the mathematicians: The goal of numerical simulations is not always "accuracy" in a strictly mathematical sense, but sometimes "fidelity" to the underlying physics in a sense that is looser and more pragmatic. In such contexts, some kinds of error are much more tolerable than others. Upwind differencing generally adds fidelity to problems where the advected variables are liable to undergo sudden changes of state, e.g., as they pass through shocks or other discontinuities. You will have to be guided by the specific nature of your own problem.

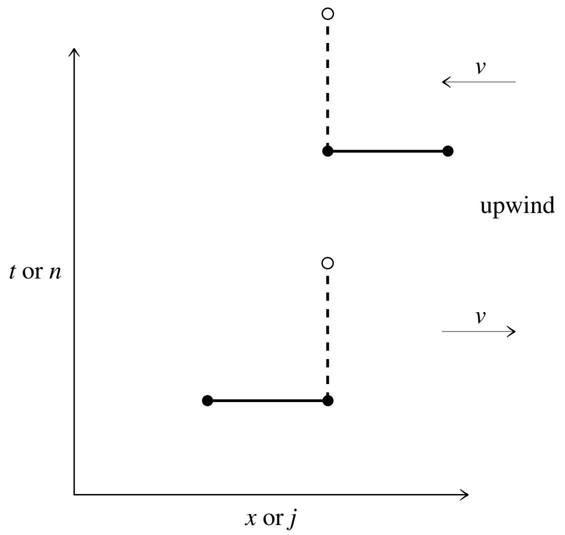

Figure 20.1.4. Representation of upwind differencing schemes. The upper scheme is stable when the advection constant v is negative, as shown; the lower scheme is stable when the advection constant v is positive, also as shown. The Courant condition must, of course, also be satisfied.

For the differencing scheme (20.1.27), the amplification factor (for constant v) is

$$\xi = 1 - \left|\frac{v\Delta t}{\Delta x}\right|(1 - \cos k\Delta x) - i\frac{v\Delta t}{\Delta x}\sin k\Delta x \tag{20.1.28}$$

$$|\xi|^2 = 1 - 2\left|\frac{v\Delta t}{\Delta x}\right|\left(1 - \left|\frac{v\Delta t}{\Delta x}\right|\right)(1 - \cos k\Delta x) \tag{20.1.29}$$

So the stability criterion $|\xi|^2 \le 1$ is (again) simply the Courant condition (20.1.17).

There are various ways of improving the accuracy of first-order upwind differencing. In the continuum equation, material originally a distance $v\Delta t$ away arrives at a given point after a time interval Δt. In the first-order method, the material always arrives from Δx away. If $v\Delta t \ll \Delta x$ (to insure accuracy), this can cause a large error. One way of reducing this error is to interpolate u between $j-1$ and j before transporting it. This gives effectively a second-order method. Various schemes for second-order upwind differencing are discussed and compared in [2,3].

20.1.4 Second-Order Accuracy in Time

When using a method that is first-order accurate in time but second-order accurate in space, one generally has to take $v\Delta t$ significantly smaller than Δx to achieve the desired accuracy, say, by at least a factor of 5. Thus the Courant condition is not actually the limiting factor with such schemes in practice. However, there are schemes that are second-order accurate in both space and time, and these can often be pushed right to their stability limit, with correspondingly smaller computation times.

For example, the *staggered leapfrog* method for the conservation equation (20.1.1) is defined as follows (Figure 20.1.5): Using the values of u^n at time t^n, compute the fluxes F_j^n. Then compute new values u^{n+1} using the time-centered

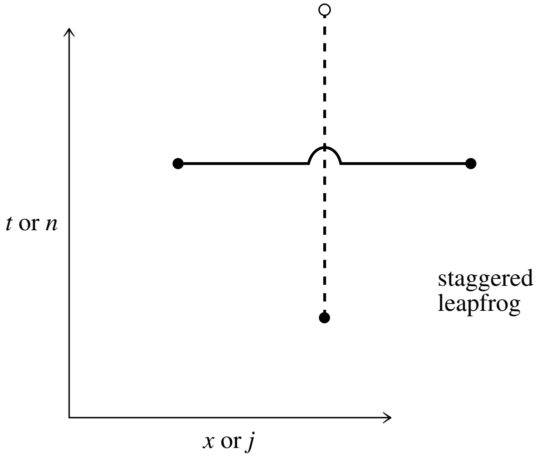

Figure 20.1.5. Representation of the staggered leapfrog differencing scheme. Note that information from two previous time slices is used in obtaining the desired point. This scheme is second-order accurate in both space and time.

values of the fluxes:

$$u_j^{n+1} - u_j^{n-1} = -\frac{\Delta t}{\Delta x}(F_{j+1}^n - F_{j-1}^n) \tag{20.1.30}$$

The name arises because the time levels in the time derivative term "leapfrog" over the time levels in the space derivative term. The method requires that u^{n-1} and u^n be stored to compute u^{n+1}.

For our simple model equation (20.1.6), staggered leapfrog takes the form

$$u_j^{n+1} - u_j^{n-1} = -\frac{v\Delta t}{\Delta x}(u_{j+1}^n - u_{j-1}^n) \tag{20.1.31}$$

The von Neumann stability analysis now gives a quadratic equation for ξ, rather than a linear one, because of the occurrence of three consecutive powers of ξ when the form (20.1.12) for an eigenmode is substituted into equation (20.1.31),

$$\xi^2 - 1 = -2i\xi\frac{v\Delta t}{\Delta x}\sin k\Delta x \tag{20.1.32}$$

whose solution is

$$\xi = -i\frac{v\Delta t}{\Delta x}\sin k\Delta x \pm \sqrt{1 - \left(\frac{v\Delta t}{\Delta x}\sin k\Delta x\right)^2} \tag{20.1.33}$$

Thus the Courant condition is again required for stability. In fact, $|\xi|^2 = 1$ in equation (20.1.33) for any $v\Delta t \le \Delta x$. This is the great advantage of the staggered leapfrog method: There is no amplitude dissipation.

Staggered leapfrog differencing of equations like (20.1.20) is most transparent

if the variables are centered on appropriate half-mesh points:

$$r^n_{j+1/2} \equiv v \left.\frac{\partial u}{\partial x}\right|^n_{j+1/2} = v \frac{u^n_{j+1} - u^n_j}{\Delta x}$$
$$s^{n+1/2}_j \equiv \left.\frac{\partial u}{\partial t}\right|^{n+1/2}_j = \frac{u^{n+1}_j - u^n_j}{\Delta t} \tag{20.1.34}$$

This is purely a notational convenience: We can think of the mesh on which r and s are defined as being twice as fine as the mesh on which the original variable u is defined. The leapfrog differencing of equation (20.1.20) is

$$\frac{r^{n+1}_{j+1/2} - r^n_{j+1/2}}{\Delta t} = \frac{s^{n+1/2}_{j+1} - s^{n+1/2}_j}{\Delta x}$$
$$\frac{s^{n+1/2}_j - s^{n-1/2}_j}{\Delta t} = v \frac{r^n_{j+1/2} - r^n_{j-1/2}}{\Delta x} \tag{20.1.35}$$

If you substitute equation (20.1.22) in equation (20.1.35), you will find that once again the Courant condition is required for stability, and that there is no amplitude dissipation when it is satisfied.

If we substitute equation (20.1.34) in equation (20.1.35), we find that equation (20.1.35) is equivalent to

$$\frac{u^{n+1}_j - 2u^n_j + u^{n-1}_j}{(\Delta t)^2} = v^2 \frac{u^n_{j+1} - 2u^n_j + u^n_{j-1}}{(\Delta x)^2} \tag{20.1.36}$$

This is just the "usual" second-order differencing of the wave equation (20.1.2). We see that it is a two-level scheme, requiring both u^n and u^{n-1} to obtain u^{n+1}. In equation (20.1.35), this shows up as both $s^{n-1/2}$ and r^n being needed to advance the solution.

For equations more complicated than our simple model equation, especially nonlinear equations, the leapfrog method usually becomes unstable when the gradients get large. The instability is related to the fact that odd and even mesh points are completely decoupled, like the black and white squares of a chess board, as shown in Figure 20.1.6. This mesh drifting instability is cured by coupling the two meshes through a numerical viscosity term, e.g., adding to the right side of (20.1.31) a small coefficient ($\ll 1$) times $u^n_{j+1} - 2u^n_j + u^n_{j-1}$. For more on stabilizing difference schemes by adding numerical dissipation, see, e.g., [4,5].

The *two-step Lax-Wendroff* scheme is a second-order in time method that avoids large numerical dissipation and mesh drifting. One defines intermediate values $u_{j+1/2}$ at the half-timesteps $t_{n+1/2}$ and the half-mesh points $x_{j+1/2}$. These are calculated by the Lax scheme:

$$u^{n+1/2}_{j+1/2} = \frac{1}{2}(u^n_{j+1} + u^n_j) - \frac{\Delta t}{\Delta x}(F^n_{j+1} - F^n_j) \tag{20.1.37}$$

Using these variables, one calculates the fluxes $F^{n+1/2}_{j+1/2}$. Then the updated values u^{n+1}_j are calculated by the properly centered expression

$$u^{n+1}_j = u^n_j - \frac{\Delta t}{\Delta x}\left(F^{n+1/2}_{j+1/2} - F^{n+1/2}_{j-1/2}\right) \tag{20.1.38}$$

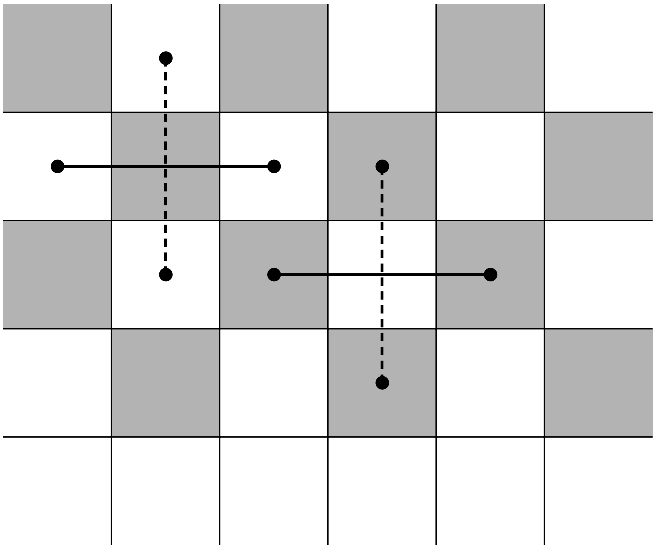

Figure 20.1.6. Origin of mesh drift instabilities in a staggered leapfrog scheme. If the mesh points are imagined to lie in the squares of a chess board, then white squares couple to themselves and black to themselves, but there is no coupling between white and black. The fix is to introduce a small diffusive mesh-coupling piece.

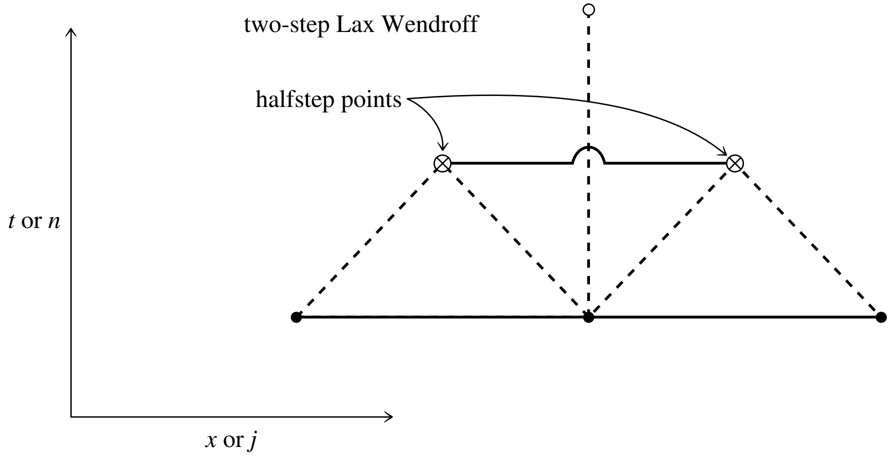

Figure 20.1.7. Representation of the two-step Lax-Wendroff differencing scheme. Two half-step points (⊗) are calculated by the Lax method. These, plus one of the original points, produce the new point via staggered leapfrog. Half-step points are used only temporarily and do not require storage allocation on the grid. This scheme is second-order accurate in both space and time.

The provisional values $u_{j+1/2}^{n+1/2}$ are now discarded. (See Figure 20.1.7.)

Let us investigate the stability of this method for our model advective equation, where $F = vu$. Substitute (20.1.37) in (20.1.38) to get

$$\begin{aligned} u_j^{n+1} = u_j^n - \alpha \Big[&\tfrac{1}{2}(u_{j+1}^n + u_j^n) - \tfrac{1}{2}\alpha(u_{j+1}^n - u_j^n) \\ &- \tfrac{1}{2}(u_j^n + u_{j-1}^n) + \tfrac{1}{2}\alpha(u_j^n - u_{j-1}^n) \Big] \end{aligned} \tag{20.1.39}$$

where

$$\alpha \equiv \frac{v\Delta t}{\Delta x} \tag{20.1.40}$$

Then

$$\xi = 1 - i\alpha \sin k\Delta x - \alpha^2(1 - \cos k\Delta x) \tag{20.1.41}$$

so

$$|\xi|^2 = 1 - \alpha^2(1 - \alpha^2)(1 - \cos k\Delta x)^2 \tag{20.1.42}$$

The stability criterion $|\xi|^2 \leq 1$ is therefore $\alpha^2 \leq 1$, or $v\Delta t \leq \Delta x$ as usual. Incidentally, you should not think that the Courant condition is the only stability requirement that ever turns up in PDEs. It keeps doing so in our model examples just because those examples are so simple in form. The method of analysis is, however, general.

Except when $\alpha = 1$, $|\xi|^2 < 1$ in (20.1.42), so some amplitude damping does occur. The effect is relatively small, however, for wavelengths large compared with the mesh size Δx. If we expand (20.1.42) for small $k\Delta x$, we find

$$|\xi|^2 = 1 - \alpha^2(1 - \alpha^2)\frac{(k\Delta x)^4}{4} + \cdots \tag{20.1.43}$$

The departure from unity occurs only at fourth order in k. This should be contrasted with equation (20.1.16) for the Lax method, which shows that

$$|\xi|^2 = 1 - (1 - \alpha^2)(k\Delta x)^2 + \cdots \tag{20.1.44}$$

for small $k\Delta x$.

In summary, our recommendation for initial value problems that can be cast in flux-conservative form, and especially problems related to the wave equation, is to use the staggered leapfrog method when possible. We have personally had better success with it than with the two-step Lax-Wendroff method. For problems sensitive to transport errors, upwind differencing or one of its refinements should be considered.

20.1.5 Fluid Dynamics with Shocks

As we alluded to earlier, the treatment of fluid dynamics problems with shocks has become a very complicated and very sophisticated subject. All we can attempt to do here is to guide you to some starting points in the literature.

There are basically three important general methods for handling shocks. The oldest and simplest method, invented by von Neumann and Richtmyer, is to add *artificial viscosity* to the equations, modeling the way Nature uses real viscosity to smooth discontinuities. A good starting point for trying out this method is the differencing scheme in §12.11 of [1]. This scheme is excellent for nearly all problems in one spatial dimension.

The second method combines a high-order differencing scheme that is accurate for smooth flows with a low-order scheme that is very dissipative and can smooth the shocks. Typically, various upwind differencing schemes are combined using weights chosen to zero the low-order scheme unless steep gradients are present, and also chosen to enforce various "monotonicity" constraints that prevent nonphysical oscillations from appearing in the numerical solution. References [2,3,6] are a good place to start with these methods.

The third, and potentially most powerful method, is Godunov's approach. Here one gives up the simple linearization inherent in finite differencing based on Taylor series and includes the nonlinearity of the equations explicitly. There is an analytic solution for the evolution of two uniform states of a fluid separated by a discontinuity, the Riemann shock problem. Godunov's idea was to approximate the fluid by a large number of cells of uniform states, and piece them together using the Riemann solution. There have been many generalizations of Godunov's approach, which are now called *high resolution shock capturing methods*. The most influential such algorithm has probably been the PPM method [7]. General discussions of high resolution shock capturing methods and other modern algorithms are given in [8-10].

CITED REFERENCES AND FURTHER READING:

Ames, W.F. 1992, *Numerical Methods for Partial Differential Equations*, 3rd ed. (New York: Academic Press), Chapter 4.

Richtmyer, R.D., and Morton, K.W. 1967, *Difference Methods for Initial Value Problems*, 2nd ed. (New York: Wiley-Interscience); republished 1994 (Melbourne, FL: Krieger).[1]

Centrella, J., and Wilson, J.R. 1984, "Planar Numerical Cosmology II: The Difference Equations and Numerical Tests," *Astrophysical Journal Supplement*, vol. 54, pp. 229–249, Appendix B.[2]

Hawley, J.F., Smarr, L.L., and Wilson, J.R. 1984, "A Numerical Study of Black Hole Accretion: II. Finite Differencing and Code Calibration," *Astrophysical Journal Supplement*, vol. 55, pp. 211–246, §2c.[3]

Kreiss, H.-O., and Busenhart, H. U. 2001, *Time-Dependent Partial Differential Equations and Their Numerical Solution* (Basel: Birkhäuser), pp. 49.[4]

Gustafsson, B., Kreiss, H.-O., and Oliger, J. 1995, *Time Dependent Problems and Difference Methods* (New York: Wiley), Ch. 2.[5]

Harten, A., Lax, P.D., and Van Leer, B. 1983, "On Upstream Differencing and Godunov-Type Schemes for Hyperbolic Conservation Laws," *SIAM Review*, vol. 25, pp. 36–61.[6]

Woodward, P., and Colella, P. 1984, "The Piecewise Parabolic Method (PPM) for Gasdynamical Simulations," *Journal of Computational Physics*, vol. 54, pp. 174–201; *op. cit.*, vol. 54, pp. 115–173.[7]

LeVeque, R.J. 2002, *Finite Volume Methods for Hyperbolic Problems* (Cambridge, UK: Cambridge University Press).[8]

LeVeque, R.J. 1992, *Numerical Methods for Conservation Laws*, 2nd ed. (Basel: Birkhäuser).[9]

Toro, E.F. 1997, *Riemann Solvers and Numerical Methods for Fluid Dynamics* (Berlin: Springer).[10]

20.2 Diffusive Initial Value Problems

Recall the model parabolic equation, the diffusion equation in one space dimension,

$$\frac{\partial u}{\partial t} = \frac{\partial}{\partial x}\left(D\frac{\partial u}{\partial x}\right) \tag{20.2.1}$$

where D is the diffusion coefficient. Actually, this equation is a flux-conservative equation of the form considered in the previous section, with

$$F = -D\frac{\partial u}{\partial x} \tag{20.2.2}$$

the flux in the x-direction. We will assume $D \geq 0$, otherwise equation (20.2.1) has physically unstable solutions: A small disturbance evolves to become more and more concentrated instead of dispersing. (Don't make the mistake of trying to find a stable differencing scheme for a problem whose underlying PDEs are themselves unstable!)

Even though (20.2.1) is of the form already considered, it is useful to consider it as a model in its own right. The particular form of flux (20.2.2), and its direct generalizations, occur quite frequently in practice. Moreover, we have already seen that numerical viscosity and artificial viscosity can introduce diffusive pieces like the right-hand side of (20.2.1) in many other situations.

Consider first the case when D is a constant. Then the equation

$$\frac{\partial u}{\partial t} = D \frac{\partial^2 u}{\partial x^2} \tag{20.2.3}$$

can be differenced in the obvious way:

$$\frac{u_j^{n+1} - u_j^n}{\Delta t} = D \left[\frac{u_{j+1}^n - 2u_j^n + u_{j-1}^n}{(\Delta x)^2} \right] \tag{20.2.4}$$

This is the FTCS scheme again, except that it is a second derivative that has been differenced on the right-hand side. But this makes a world of difference! The FTCS scheme was unstable for the hyperbolic equation; however, a quick calculation shows that the amplification factor for equation (20.2.4) is

$$\xi = 1 - \frac{4D\Delta t}{(\Delta x)^2} \sin^2 \left(\frac{k\Delta x}{2} \right) \tag{20.2.5}$$

The requirement $|\xi| \leq 1$ leads to the stability criterion

$$\frac{2D\Delta t}{(\Delta x)^2} \leq 1 \tag{20.2.6}$$

The physical interpretation of the restriction (20.2.6) is that the maximum allowed timestep is, up to a numerical factor, the diffusion time across a cell of width Δx.

More generally, the diffusion time τ across a spatial scale of size λ is of order

$$\tau \sim \frac{\lambda^2}{D} \tag{20.2.7}$$

Usually we are interested in modeling accurately the evolution of features with spatial scales $\lambda \gg \Delta x$. If we are limited to timesteps satisfying (20.2.6), we will need to evolve through of order $\lambda^2/(\Delta x)^2$ steps before things start to happen on the scale of interest. This number of steps is usually prohibitive. We must therefore find a stable way of taking timesteps comparable to, or perhaps — for accuracy — somewhat smaller than, the time scale of (20.2.7).

This goal poses an immediate "philosophical" question. Obviously the large timesteps that we propose to take are going to be woefully inaccurate for the small scales that we have decided not to be interested in. We want those scales to do something stable, "innocuous," and perhaps not too physically unreasonable. We

want to build this innocuous behavior into our differencing scheme. What should it be?

There are two different answers, each of which has its pros and cons. The first answer is to seek a differencing scheme that drives small-scale features to their *equilibrium* forms, e.g., satisfying equation (20.2.3) with the left-hand side set to zero. This answer generally makes the best physical sense; but, as we will see, it leads to a differencing scheme ("fully implicit") that is only *first-order* accurate in time for the scales that we are interested in. The second answer is to let small-scale features *maintain* their initial amplitudes, so that the evolution of the larger-scale features of interest takes place superposed with a kind of "frozen in" (though fluctuating) background of small-scale stuff. This answer gives a differencing scheme (Crank-Nicolson) that is *second-order* accurate in time. Toward the end of an evolution calculation, however, one might want to switch over to some steps of the other kind, to drive the small-scale stuff into equilibrium. Let us now see where these distinct differencing schemes come from.

Consider the following differencing of (20.2.3):

$$\frac{u_j^{n+1} - u_j^n}{\Delta t} = D\left[\frac{u_{j+1}^{n+1} - 2u_j^{n+1} + u_{j-1}^{n+1}}{(\Delta x)^2}\right] \tag{20.2.8}$$

This is exactly like the FTCS scheme (20.2.4), except that the spatial derivatives on the right-hand side are evaluated at timestep $n+1$. Schemes with this character are called *fully implicit* or *backward time*, by contrast with FTCS (which is called *fully explicit*). To solve equation (20.2.8), one has to solve a set of simultaneous linear equations at each timestep for the u_j^{n+1}. Fortunately, this is a simple problem because the system is tridiagonal: Just group the terms in equation (20.2.8) appropriately:

$$-\alpha u_{j-1}^{n+1} + (1+2\alpha)u_j^{n+1} - \alpha u_{j+1}^{n+1} = u_j^n, \qquad j = 1, 2 ... J-1 \tag{20.2.9}$$

where

$$\alpha \equiv \frac{D\Delta t}{(\Delta x)^2} \tag{20.2.10}$$

Supplemented by Dirichlet or Neumann boundary conditions at $j = 0$ and $j = J$, equation (20.2.9) is clearly a tridiagonal system, which can easily be solved at each timestep by the method of §2.4.

What is the behavior of (20.2.8) for very large timesteps? The answer is seen most clearly in (20.2.9), in the limit $\alpha \to \infty$ ($\Delta t \to \infty$). Dividing by α, we see that the difference equations are just the finite difference form of the equilibrium equation

$$\frac{\partial^2 u}{\partial x^2} = 0 \tag{20.2.11}$$

What about stability? The amplification factor for equation (20.2.8) is

$$\xi = \frac{1}{1 + 4\alpha \sin^2\left(\dfrac{k\Delta x}{2}\right)} \tag{20.2.12}$$

Clearly $|\xi| < 1$ for any stepsize Δt. The scheme is unconditionally stable. The details of the small-scale evolution from the initial conditions are obviously inaccurate

for large Δt. But, as advertised, the correct equilibrium solution is obtained. This is the characteristic feature of implicit methods.

Here, on the other hand, is how one gets to the second of our above philosophical answers, combining the stability of an implicit method with the accuracy of a method that is second order in both space and time. Simply form the average of the explicit and implicit FTCS schemes:

$$\frac{u_j^{n+1} - u_j^n}{\Delta t} = \frac{D}{2}\left[\frac{(u_{j+1}^{n+1} - 2u_j^{n+1} + u_{j-1}^{n+1}) + (u_{j+1}^n - 2u_j^n + u_{j-1}^n)}{(\Delta x)^2}\right] \tag{20.2.13}$$

Here both the left- and right-hand sides are centered at timestep $n + \frac{1}{2}$, so the method is second-order accurate in time as claimed. The amplification factor is

$$\xi = \frac{1 - 2\alpha \sin^2\left(\frac{k\Delta x}{2}\right)}{1 + 2\alpha \sin^2\left(\frac{k\Delta x}{2}\right)} \tag{20.2.14}$$

so the method is stable for any size Δt. This scheme is called the *Crank-Nicolson* scheme and is our recommended method for any simple diffusion problem (perhaps supplemented by a few fully implicit steps at the end). (See Figure 20.2.1.)

Now turn to some generalizations of the simple diffusion equation (20.2.3). Suppose first that the diffusion coefficient D is not constant, say $D = D(x)$. We can adopt either of two strategies. First, we can make an analytic change of variable

$$y = \int \frac{dx}{D(x)} \tag{20.2.15}$$

Then

$$\frac{\partial u}{\partial t} = \frac{\partial}{\partial x} D(x) \frac{\partial u}{\partial x} \tag{20.2.16}$$

becomes

$$\frac{\partial u}{\partial t} = \frac{1}{D(y)} \frac{\partial^2 u}{\partial y^2} \tag{20.2.17}$$

and we evaluate D at the appropriate y_j. Heuristically, the stability criterion (20.2.6) in an explicit scheme becomes

$$\Delta t \le \min_j \left[\frac{(\Delta y)^2}{2D_j^{-1}}\right] \tag{20.2.18}$$

Note that constant spacing Δy in y does not imply constant spacing in x.

An alternative method that does not require analytically tractable forms for D is simply to difference equation (20.2.16) as it stands, centering everything appropriately. Thus the FTCS method becomes

$$\frac{u_j^{n+1} - u_j^n}{\Delta t} = \frac{D_{j+1/2}(u_{j+1}^n - u_j^n) - D_{j-1/2}(u_j^n - u_{j-1}^n)}{(\Delta x)^2} \tag{20.2.19}$$

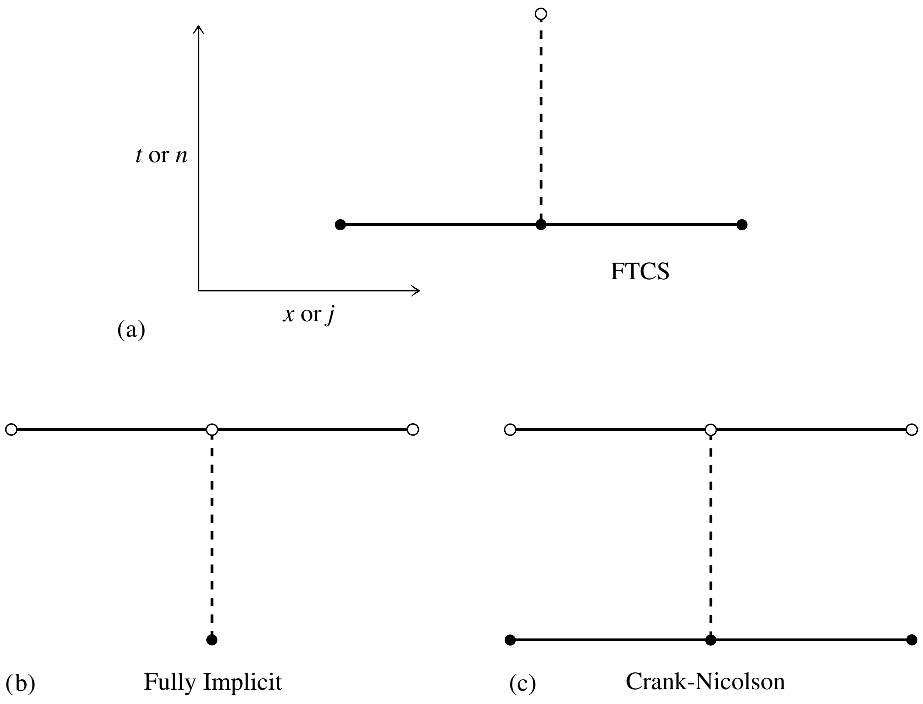

Figure 20.2.1. Three differencing schemes for diffusive problems (shown as in Figure 20.1.2). (a) Forward time centered space is first-order accurate but stable only for sufficiently small timesteps. (b) Fully implicit is stable for arbitrarily large timesteps but is still only first-order accurate. (c) Crank-Nicolson is second-order accurate and is usually stable for large timesteps.

where

$$D_{j+1/2} \equiv D(x_{j+1/2}) \tag{20.2.20}$$

and the heuristic stability criterion is

$$\Delta t \le \min_j \left[\frac{(\Delta x)^2}{2D_{j+1/2}} \right] \tag{20.2.21}$$

The Crank-Nicolson method can be generalized similarly.

The second complication one can consider is a nonlinear diffusion problem, for example where $D = D(u)$. Explicit schemes can be generalized in the obvious way. For example, in equation (20.2.19) write

$$D_{j+1/2} = \tfrac{1}{2}\left[D(u^n_{j+1}) + D(u^n_j)\right] \tag{20.2.22}$$

Implicit schemes are not as easy. The replacement (20.2.22) with $n \to n+1$ leaves us with a nasty set of coupled nonlinear equations to solve at each timestep. Often there is an easier way: If the form of $D(u)$ allows us to integrate

$$dz = D(u)du \tag{20.2.23}$$

analytically for $z(u)$, then the right-hand side of (20.2.1) becomes $\partial^2 z/\partial x^2$, which

we difference implicitly as

$$\frac{z_{j+1}^{n+1} - 2z_j^{n+1} + z_{j-1}^{n+1}}{(\Delta x)^2} \tag{20.2.24}$$

Now linearize each term on the right-hand side of equation (20.2.24), for example

$$\begin{aligned} z_j^{n+1} \equiv z(u_j^{n+1}) &= z(u_j^n) + (u_j^{n+1} - u_j^n) \left.\frac{\partial z}{\partial u}\right|_{j,n} \\ &= z(u_j^n) + (u_j^{n+1} - u_j^n) D(u_j^n) \end{aligned} \tag{20.2.25}$$

This reduces the problem to tridiagonal form again and in practice usually retains the stability advantages of fully implicit differencing.

20.2.1 Schrödinger Equation

Sometimes the physical problem being solved imposes constraints on the differencing scheme that we have not yet taken into account. For example, consider the time-dependent Schrödinger equation of quantum mechanics. This is basically a parabolic equation for the evolution of a complex quantity ψ. For the scattering of a wavepacket by a one-dimensional potential $V(x)$, the equation has the form

$$i\frac{\partial \psi}{\partial t} = -\frac{\partial^2 \psi}{\partial x^2} + V(x)\psi \tag{20.2.26}$$

(Here we have chosen units so that Planck's constant $\hbar = 1$ and the particle mass $m = 1/2$.) One is given the initial wavepacket, $\psi(x, t = 0)$, together with boundary conditions that $\psi \to 0$ at $x \to \pm\infty$. Suppose we content ourselves with first-order accuracy in time but want to use an implicit scheme, for stability. A slight generalization of (20.2.8) leads to

$$i\left[\frac{\psi_j^{n+1} - \psi_j^n}{\Delta t}\right] = -\left[\frac{\psi_{j+1}^{n+1} - 2\psi_j^{n+1} + \psi_{j-1}^{n+1}}{(\Delta x)^2}\right] + V_j \psi_j^{n+1} \tag{20.2.27}$$

for which

$$\xi = \frac{1}{1 + i\left[\frac{4\Delta t}{(\Delta x)^2}\sin^2\left(\frac{k\Delta x}{2}\right) + V_j \Delta t\right]} \tag{20.2.28}$$

This is unconditionally stable but unfortunately is not *unitary*. The underlying physical problem requires that the total probability of finding the particle somewhere remains unity. This is represented formally by the modulus-square norm of ψ remaining unity:

$$\int_{-\infty}^{\infty} |\psi|^2 dx = 1 \tag{20.2.29}$$

The initial wave function $\psi(x, 0)$ is normalized to satisfy (20.2.29). The Schrödinger equation (20.2.26) then guarantees that this condition is satisfied at all later times.

Let us write equation (20.2.26) in the form

$$i\frac{\partial \psi}{\partial t} = H\psi \tag{20.2.30}$$

where the operator H is

$$H = -\frac{\partial^2}{\partial x^2} + V(x) \tag{20.2.31}$$

The formal solution of equation (20.2.30) is

$$\psi(x,t) = e^{-iHt}\psi(x,0) \tag{20.2.32}$$

where the exponential of the operator is defined by its power series expansion.

The unstable explicit FTCS scheme approximates (20.2.32) as

$$\psi_j^{n+1} = (1 - iH\Delta t)\psi_j^n \tag{20.2.33}$$

where H is represented by a centered finite difference approximation in x. The stable implicit scheme (20.2.27) is, by contrast,

$$\psi_j^{n+1} = (1 + iH\Delta t)^{-1}\psi_j^n \tag{20.2.34}$$

These are both first-order accurate in time, as can be seen by expanding equation (20.2.32). However, neither operator in (20.2.33) or (20.2.34) is unitary.

The correct way to difference Schrödinger's equation [1,2] is to use *Cayley's form* for the finite difference representation of e^{-iHt}, which is second-order accurate *and* unitary:

$$e^{-iHt} \simeq \frac{1 - \frac{1}{2}iH\Delta t}{1 + \frac{1}{2}iH\Delta t} \tag{20.2.35}$$

In other words,

$$\left(1 + \tfrac{1}{2}iH\Delta t\right)\psi_j^{n+1} = \left(1 - \tfrac{1}{2}iH\Delta t\right)\psi_j^n \tag{20.2.36}$$

On replacing H by its finite difference approximation in x, we have a complex tridiagonal system to solve. The method is stable, unitary, and second-order accurate in space and time. In fact, it is simply the Crank-Nicolson method once again!

CITED REFERENCES AND FURTHER READING:

Thomas, J.W. 1995, *Numerical Partial Differential Equations: Finite Difference Methods* (New York: Springer).

Ames, W.F. 1992, *Numerical Methods for Partial Differential Equations*, 3rd ed. (New York: Academic Press), Chapter 2.

Goldberg, A., Schey, H.M., and Schwartz, J.L. 1967, "Computer-Generated Motion Pictures of One-Dimensional Quantum-Mechanical Transmission and Reflection Phenomena," *American Journal of Physics*, vol. 35, pp. 177–186.[1]

Galbraith, I., Ching, Y.S., and Abraham, E. 1984, "Two-Dimensional Time-Dependent Quantum-Mechanical Scattering Event," *American Journal of Physics*, vol. 52, pp. 60–68.[2]

20.3 Initial Value Problems in Multidimensions

The methods described in §20.1 and §20.2 for problems in $1 + 1$ dimension (one space and one time dimension) can easily be generalized to $N + 1$ dimensions. However, the computing power necessary to solve the resulting equations

grows extremely rapidly as the number of dimensions increases. If you have solved a one-dimensional problem with 100 spatial grid points, solving the two-dimensional version with 100×100 mesh points requires *at least* 100 times as much computing. You generally have to be content with very modest spatial resolution in multidimensional problems.

Indulge us in offering a bit of advice about the development and testing of multidimensional PDE codes: You should always first run your programs on *very small* grids, e.g., 8×8, even though the resulting accuracy is so poor as to be useless. When your program is all debugged and demonstrably stable, *then* you can increase the grid size to a reasonable one and start looking at the results. We have actually heard someone protest, "my program would be unstable for a crude grid, but I am sure the instability will go away on a larger grid." That is nonsense of a most pernicious sort, evidencing total confusion between accuracy and stability. In fact, new instabilities sometimes do show up on *larger* grids; but old instabilities never (in our experience) just go away.

Forced to live with modest grid sizes, some people recommend going to higher-order methods in an attempt to improve accuracy. This can be very dangerous. Unless the solution you are looking for is known to be smooth, and the high-order method you are using is known to be extremely stable, we do not recommend anything higher than second-order in time (for sets of first-order equations). For spatial differencing, we recommend the order of the underlying PDEs, perhaps allowing second-order spatial differencing for first-order-in-space PDEs. When you increase the order of a differencing method to greater than the order of the original PDEs, you introduce spurious solutions to the difference equations. This does not create a problem if they all happen to decay exponentially; otherwise you are going to see all hell break loose!

20.3.1 Lax Method for a Flux-Conservative Equation

As an example, we show now how to generalize the Lax method (20.1.15) to two dimensions for the conservation equation

$$\frac{\partial u}{\partial t} = -\nabla \cdot \mathbf{F} = -\left(\frac{\partial F_x}{\partial x} + \frac{\partial F_y}{\partial y}\right) \tag{20.3.1}$$

Use a spatial grid with

$$\begin{aligned} x_j &= x_0 + j\Delta \\ y_l &= y_0 + l\Delta \end{aligned} \tag{20.3.2}$$

We have chosen $\Delta x = \Delta y \equiv \Delta$ for simplicity. Then the Lax scheme is

$$\begin{aligned} u_{j,l}^{n+1} = {} & \frac{1}{4}(u_{j+1,l}^n + u_{j-1,l}^n + u_{j,l+1}^n + u_{j,l-1}^n) \\ & - \frac{\Delta t}{2\Delta}(F_{j+1,l}^n - F_{j-1,l}^n + F_{j,l+1}^n - F_{j,l-1}^n) \end{aligned} \tag{20.3.3}$$

Note that as an abbreviated notation F_{j+1} and F_{j-1} refer to F_x, while F_{l+1} and F_{l-1} refer to F_y.

Let us carry out a stability analysis for the model advective equation (analog of 20.1.6) with

$$F_x = v_x u, \qquad F_y = v_y u \tag{20.3.4}$$

This requires an eigenmode with two dimensions in space, though still only a simple dependence on powers of ξ in time:

$$u^n_{j,l} = \xi^n e^{ik_x j\Delta} e^{ik_y l\Delta} \tag{20.3.5}$$

Substituting in equation (20.3.3), we find

$$\xi = \tfrac{1}{2}(\cos k_x\Delta + \cos k_y\Delta) - i\alpha_x \sin k_x\Delta - i\alpha_y \sin k_y\Delta \tag{20.3.6}$$

where

$$\alpha_x = \frac{v_x\Delta t}{\Delta}, \qquad \alpha_y = \frac{v_y\Delta t}{\Delta} \tag{20.3.7}$$

The expression for $|\xi|^2$ can be manipulated into the form

$$\begin{aligned} |\xi|^2 = 1 - (\sin^2 k_x\Delta + \sin^2 k_y\Delta)\left[\tfrac{1}{2} - (\alpha_x^2 + \alpha_y^2)\right] \\ - \tfrac{1}{4}(\cos k_x\Delta - \cos k_y\Delta)^2 - (\alpha_y \sin k_x\Delta - \alpha_x \sin k_y\Delta)^2 \end{aligned} \tag{20.3.8}$$

The last two terms are negative, and so the stability requirement $|\xi|^2 \le 1$ becomes

$$\tfrac{1}{2} - (\alpha_x^2 + \alpha_y^2) \ge 0 \tag{20.3.9}$$

or

$$\Delta t \le \frac{\Delta}{\sqrt{2}(v_x^2 + v_y^2)^{1/2}} \tag{20.3.10}$$

This is an example of the general result for the N-dimensional Courant condition: If $|v|$ is the maximum propagation velocity in the problem, then

$$\Delta t \le \frac{\Delta}{\sqrt{N}|v|} \tag{20.3.11}$$

is the Courant condition.

20.3.2 Diffusion Equation in Multidimensions

Let us consider the two-dimensional diffusion equation,

$$\frac{\partial u}{\partial t} = D\left(\frac{\partial^2 u}{\partial x^2} + \frac{\partial^2 u}{\partial y^2}\right) \tag{20.3.12}$$

An explicit method, such as FTCS, can be generalized from the one-dimensional case in the obvious way. However, we have seen that diffusive problems are usually best treated implicitly. Suppose we try to implement the Crank-Nicolson scheme in two dimensions. This would give us

$$u^{n+1}_{j,l} = u^n_{j,l} + \tfrac{1}{2}\alpha\left(\delta_x^2 u^{n+1}_{j,l} + \delta_x^2 u^n_{j,l} + \delta_y^2 u^{n+1}_{j,l} + \delta_y^2 u^n_{j,l}\right) \tag{20.3.13}$$

Here

$$\alpha \equiv \frac{D\Delta t}{\Delta^2} \qquad \Delta \equiv \Delta x = \Delta y \tag{20.3.14}$$

$$\delta_x^2 u_{j,l}^n \equiv u_{j+1,l}^n - 2u_{j,l}^n + u_{j-1,l}^n \tag{20.3.15}$$

and similarly for $\delta_y^2 u_{j,l}^n$. This is certainly a viable scheme; the problem arises in solving the coupled linear equations. Whereas in one space dimension the system was tridiagonal, that is no longer true, though the matrix is still very sparse. One possibility is to use a suitable sparse matrix technique (see §2.7 and §20.0).

Another possibility, which we generally prefer, is a slightly different way of generalizing the Crank-Nicolson algorithm. It is still second-order accurate in time and space, and unconditionally stable, but the equations are easier to solve than (20.3.13). Called the *alternating-direction implicit method (ADI)*, this embodies the powerful concept of *operator splitting* or *time splitting*, about which we will say more below. Here, the idea is to divide each timestep into two steps of size $\Delta t/2$. In each substep, a different dimension is treated implicitly:

$$\begin{aligned} u_{j,l}^{n+1/2} &= u_{j,l}^n + \tfrac{1}{2}\alpha\left(\delta_x^2 u_{j,l}^{n+1/2} + \delta_y^2 u_{j,l}^n\right) \\ u_{j,l}^{n+1} &= u_{j,l}^{n+1/2} + \tfrac{1}{2}\alpha\left(\delta_x^2 u_{j,l}^{n+1/2} + \delta_y^2 u_{j,l}^{n+1}\right) \end{aligned} \tag{20.3.16}$$

The advantage of this method is that each substep requires only the solution of a simple tridiagonal system.

20.3.3 Operator Splitting Methods Generally

The basic idea of operator splitting, which is also called *time splitting* or *the method of fractional steps*, is this: Suppose you have an initial value equation of the form

$$\frac{\partial u}{\partial t} = \mathcal{L}u \tag{20.3.17}$$

where $\mathcal{L}$ is some operator. While $\mathcal{L}$ is not necessarily linear, suppose that it can at least be written as a linear sum of m pieces, which act additively on u,

$$\mathcal{L}u = \mathcal{L}_1 u + \mathcal{L}_2 u + \cdots + \mathcal{L}_m u \tag{20.3.18}$$

Finally, suppose that for *each* of the pieces, you already know a differencing scheme for updating the variable u from timestep n to timestep $n+1$, valid if that piece of the operator were the *only* one on the right-hand side. We will write these updatings symbolically as

$$\begin{aligned} u^{n+1} &= \mathcal{U}_1(u^n, \Delta t) \\ u^{n+1} &= \mathcal{U}_2(u^n, \Delta t) \\ &\cdots \\ u^{n+1} &= \mathcal{U}_m(u^n, \Delta t) \end{aligned} \tag{20.3.19}$$

Now, one form of operator splitting would be to get from n to $n+1$ by the

following sequence of updatings:

$$
\begin{aligned}
u^{n+(1/m)} &= \mathcal{U}_1(u^n, \Delta t) \\
u^{n+(2/m)} &= \mathcal{U}_2(u^{n+(1/m)}, \Delta t) \\
&\cdots \\
u^{n+1} &= \mathcal{U}_m(u^{n+(m-1)/m}, \Delta t)
\end{aligned}
\tag{20.3.20}
$$

For example, a combined advective-diffusion equation, such as

$$
\frac{\partial u}{\partial t} = -v\frac{\partial u}{\partial x} + D\frac{\partial^2 u}{\partial x^2} \tag{20.3.21}
$$

might profitably use an explicit scheme for the advective term combined with a Crank-Nicolson or other implicit scheme for the diffusion term.

The alternating-direction implicit (ADI) method, equation (20.3.16), is an example of operator splitting with a slightly different twist. Let us reinterpret (20.3.19) to have a different meaning: Let $\mathcal{U}_1$ now denote an updating method that includes algebraically *all* the pieces of the total operator $\mathcal{L}$, but which is desirably *stable* only for the $\mathcal{L}_1$ piece; likewise $\mathcal{U}_2, \ldots \mathcal{U}_m$. Then a method of getting from u^n to u^{n+1} is

$$
\begin{aligned}
u^{n+1/m} &= \mathcal{U}_1(u^n, \Delta t/m) \\
u^{n+2/m} &= \mathcal{U}_2(u^{n+1/m}, \Delta t/m) \\
&\cdots \\
u^{n+1} &= \mathcal{U}_m(u^{n+(m-1)/m}, \Delta t/m)
\end{aligned}
\tag{20.3.22}
$$

The timestep for each fractional step in (20.3.22) is now only $1/m$ of the full timestep, because each partial operation acts with all the terms of the original operator.

Equation (20.3.22) is usually, though not always, stable as a differencing scheme for the operator $\mathcal{L}$. In fact, as a rule of thumb, it is often sufficient to have stable $\mathcal{U}_i$'s only for the operator pieces having the highest number of spatial derivatives — the other $\mathcal{U}_i$'s can be *unstable* — to make the overall scheme stable!

It is at this point that we turn our attention from initial value problems to boundary value problems. These will occupy us for most of the remainder of the chapter.

CITED REFERENCES AND FURTHER READING:

Thomas, J.W. 1995, *Numerical Partial Differential Equations: Finite Difference Methods* (New York: Springer).

Ames, W.F. 1992, *Numerical Methods for Partial Differential Equations*, 3rd ed. (New York: Academic Press).

20.4 Fourier and Cyclic Reduction Methods for Boundary Value Problems

As discussed in §20.0, most boundary value problems (elliptic equations, for example) reduce to solving large sparse linear systems of the form

$$
\mathbf{A} \cdot \mathbf{u} = \mathbf{b} \tag{20.4.1}
$$

either once, for boundary value equations that are linear, or iteratively, for boundary value equations that are nonlinear.

Two important techniques lead to a "rapid" solution of equation (20.4.1) when the sparse matrix is of certain frequently occurring forms. The *Fourier transform method* is directly applicable when the equations have coefficients that are constant in space. The *cyclic reduction* method is somewhat more general; its applicability is related to the question of whether the equations are separable (in the sense of "separation of variables"). Both methods require the boundaries to coincide with the coordinate lines. Finally, for some problems, there is a powerful combination of these two methods called *FACR (Fourier analysis and cyclic reduction)*. We now consider each method in turn, using equation (20.0.3), with finite difference representation (20.0.6), as a model example. Generally speaking, the methods in this section are faster, when they apply, than the simpler relaxation methods discussed in §20.5, but they are not necessarily faster than the more complicated multigrid methods discussed in §20.6.

20.4.1 Fourier Transform Method

The discrete inverse Fourier transform in both x and y is

$$u_{jl} = \frac{1}{JL} \sum_{m=0}^{J-1} \sum_{n=0}^{L-1} \widehat{u}_{mn} e^{-2\pi ijm/J} e^{-2\pi iln/L} \tag{20.4.2}$$

This can be computed using the FFT independently in each dimension, or else all at once via the routine `fourn` of §12.5 or the routine `rlft3` of §12.6. Similarly,

$$\rho_{jl} = \frac{1}{JL} \sum_{m=0}^{J-1} \sum_{n=0}^{L-1} \widehat{\rho}_{mn} e^{-2\pi ijm/J} e^{-2\pi iln/L} \tag{20.4.3}$$

If we substitute expressions (20.4.2) and (20.4.3) in our model problem (20.0.6), we find

$$\widehat{u}_{mn} \left(e^{2\pi im/J} + e^{-2\pi im/J} + e^{2\pi in/L} + e^{-2\pi in/L} - 4 \right) = \widehat{\rho}_{mn} \Delta^2 \tag{20.4.4}$$

or

$$\widehat{u}_{mn} = \frac{\widehat{\rho}_{mn} \Delta^2}{2\left(\cos \dfrac{2\pi m}{J} + \cos \dfrac{2\pi n}{L} - 2 \right)} \tag{20.4.5}$$

Thus the strategy for solving equation (20.0.6) by FFT techniques is

- Compute $\widehat{\rho}_{mn}$ as the Fourier transform

$$\widehat{\rho}_{mn} = \sum_{j=0}^{J-1} \sum_{l=0}^{L-1} \rho_{jl}\, e^{2\pi imj/J} e^{2\pi inl/L} \tag{20.4.6}$$

- Compute $\widehat{u}_{mn}$ from equation (20.4.5).
- Compute u_{jl} by the inverse Fourier transform (20.4.2).

The above procedure is valid for periodic boundary conditions. In other words, the solution satisfies

$$u_{jl} = u_{j+J,l} = u_{j,l+L} \tag{20.4.7}$$

Next consider a Dirichlet boundary condition $u = 0$ on the rectangular boundary. Instead of the expansion (20.4.2), we now need an expansion in sine waves:

$$u_{jl} = \frac{2}{J}\frac{2}{L}\sum_{m=1}^{J-1}\sum_{n=1}^{L-1} \widehat{u}_{mn} \sin\frac{\pi jm}{J} \sin\frac{\pi ln}{L} \tag{20.4.8}$$

This satisfies the boundary conditions that $u = 0$ at $j = 0, J$ and at $l = 0, L$. If we substitute this expansion and the analogous one for ρ_{jl} into equation (20.0.6), we find that the solution procedure parallels that for periodic boundary conditions:

- Compute $\widehat{\rho}_{mn}$ by the sine transform

$$\widehat{\rho}_{mn} = \sum_{j=1}^{J-1}\sum_{l=1}^{L-1} \rho_{jl} \sin\frac{\pi jm}{J} \sin\frac{\pi ln}{L} \tag{20.4.9}$$

  (A fast sine transform algorithm was given in §12.3.)
- Compute $\widehat{u}_{mn}$ from the expression analogous to (20.4.5),

$$\widehat{u}_{mn} = \frac{\Delta^2 \widehat{\rho}_{mn}}{2\left(\cos\dfrac{\pi m}{J} + \cos\dfrac{\pi n}{L} - 2\right)} \tag{20.4.10}$$

- Compute u_{jl} by the inverse sine transform (20.4.8).

If we have inhomogeneous boundary conditions, for example $u = 0$ on all boundaries except $u = f(y)$ on the boundary $x = J\Delta$, we have to add to the above solution a solution u^H of the homogeneous equation

$$\frac{\partial^2 u}{\partial x^2} + \frac{\partial^2 u}{\partial y^2} = 0 \tag{20.4.11}$$

that satisfies the required boundary conditions. In the continuum case, this would be an expression of the form

$$u^H = \sum_n A_n \sinh\frac{n\pi x}{L\Delta} \sin\frac{n\pi y}{L\Delta} \tag{20.4.12}$$

where A_n would be found by requiring that $u = f(y)$ at $x = J\Delta$. In the discrete case, we have

$$u^H_{jl} = \frac{2}{L}\sum_{n=1}^{L-1} A_n \sinh\frac{\pi nj}{L} \sin\frac{\pi nl}{L} \tag{20.4.13}$$

If $f(y = l\Delta) \equiv f_l$, then we get A_n from the inverse formula

$$A_n = \frac{1}{\sinh(\pi nJ/L)} \sum_{l=1}^{L-1} f_l \sin\frac{\pi nl}{L} \tag{20.4.14}$$

The complete solution to the problem is

$$u = u_{jl} + u_{jl}^H \tag{20.4.15}$$

By adding appropriate terms of the form (20.4.12), we can handle inhomogeneous terms on any boundary surface.

A much simpler procedure for handling inhomogeneous terms is to note that whenever boundary terms appear on the left-hand side of (20.0.6), they can be taken over to the right-hand side since they are known. The effective source term is therefore ρ_{jl} plus a contribution from the boundary terms. To implement this idea formally, write the solution as

$$u = u' + u^B \tag{20.4.16}$$

where $u' = 0$ on the boundary, while u^B vanishes everywhere *except* on the boundary. There it takes on the given boundary value. In the above example, the only nonzero values of u^B would be

$$u^B_{J,l} = f_l \tag{20.4.17}$$

The model equation (20.0.3) becomes

$$\nabla^2 u' = -\nabla^2 u^B + \rho \tag{20.4.18}$$

or, in finite difference form,

$$\begin{aligned} &u'_{j+1,l} + u'_{j-1,l} + u'_{j,l+1} + u'_{j,l-1} - 4u'_{j,l} = \\ &\quad -(u^B_{j+1,l} + u^B_{j-1,l} + u^B_{j,l+1} + u^B_{j,l-1} - 4u^B_{j,l}) + \Delta^2 \rho_{j,l} \end{aligned} \tag{20.4.19}$$

All the u^B terms in equation (20.4.19) vanish except when the equation is evaluated at $j = J - 1$, where

$$u'_{J,l} + u'_{J-2,l} + u'_{J-1,l+1} + u'_{J-1,l-1} - 4u'_{J-1,l} = -f_l + \Delta^2 \rho_{J-1,l} \tag{20.4.20}$$

Thus the problem is now equivalent to the case of zero boundary conditions, except that one row of the source term is modified by the replacement

$$\Delta^2 \rho_{J-1,l} \to \Delta^2 \rho_{J-1,l} - f_l \tag{20.4.21}$$

The case of Neumann boundary conditions $\nabla u = 0$ is handled by the cosine expansion (12.4.11):

$$u_{jl} = \frac{2}{J}\frac{2}{L}\sum_{m=0}^{J}{}'' \sum_{n=0}^{L}{}'' \hat{u}_{mn} \cos\frac{\pi j m}{J} \cos\frac{\pi l n}{L} \tag{20.4.22}$$

Here the double prime notation means that the terms for $m = 0$ and $m = J$ should be multiplied by $\frac{1}{2}$, and similarly for $n = 0$ and $n = L$. Inhomogeneous terms $\nabla u = g$ can be again included by adding a suitable solution of the homogeneous equation, or more simply by taking boundary terms over to the right-hand side. For example, the condition

$$\frac{\partial u}{\partial x} = g(y) \qquad \text{at} \quad x = 0 \tag{20.4.23}$$

becomes

$$\frac{u_{1,l} - u_{-1,l}}{2\Delta} = g_l \tag{20.4.24}$$

where $g_l \equiv g(y = l\Delta)$. Once again we write the solution in the form (20.4.16), where now $\nabla u' = 0$ on the boundary. This time ∇u^B takes on the prescribed value on the boundary, but u^B vanishes everywhere except just *outside* the boundary. Thus equation (20.4.24) gives

$$u^B_{-1,l} = -2\Delta g_l \tag{20.4.25}$$

All the u^B terms in equation (20.4.19) vanish except when $j = 0$:

$$u'_{1,l} + u'_{-1,l} + u'_{0,l+1} + u'_{0,l-1} - 4u'_{0,l} = 2\Delta g_l + \Delta^2 \rho_{0,l} \tag{20.4.26}$$

Thus u' is the solution of a zero-gradient problem, with the source term modified by the replacement

$$\Delta^2 \rho_{0,l} \rightarrow \Delta^2 \rho_{0,l} + 2\Delta g_l \tag{20.4.27}$$

Sometimes Neumann boundary conditions are handled by using a staggered grid, with the u's defined midway between zone boundaries so that first derivatives are centered on the mesh points. You can solve such problems using similar techniques to those described above if you use the alternative form of the cosine transform, equation (12.4.17).

20.4.2 Cyclic Reduction

Evidently the FFT method works only when the original PDE has constant coefficients and boundaries that coincide with the coordinate lines. An alternative algorithm, which can be used on somewhat more general equations, is called *cyclic reduction (CR)*.

We illustrate cyclic reduction on the equation

$$\frac{\partial^2 u}{\partial x^2} + \frac{\partial^2 u}{\partial y^2} + b(y)\frac{\partial u}{\partial y} + c(y)u = g(x, y) \tag{20.4.28}$$

This form arises very often in practice from the Helmholtz or Poisson equations in polar, cylindrical, or spherical coordinate systems. More general separable equations are treated in [1].

The finite difference form of equation (20.4.28) can be written as a set of vector equations

$$\mathbf{u}_{j-1} + \mathbf{T} \cdot \mathbf{u}_j + \mathbf{u}_{j+1} = \mathbf{g}_j \Delta^2 \tag{20.4.29}$$

Here the index j comes from differencing in the x-direction, while the y-differencing (denoted by the index l previously) has been left in vector form. The matrix $\mathbf{T}$ has the form

$$\mathbf{T} = \mathbf{B} - 2\mathbf{1} \tag{20.4.30}$$

where the $2\mathbf{1}$ comes from the x-differencing and the matrix $\mathbf{B}$ from the y-differencing. The matrix $\mathbf{B}$, and hence $\mathbf{T}$, is tridiagonal with variable coefficients.

The CR method is derived by writing down three successive equations like (20.4.29):

$$\begin{aligned}
\mathbf{u}_{j-2} + \mathbf{T} \cdot \mathbf{u}_{j-1} + \mathbf{u}_j &= \mathbf{g}_{j-1} \Delta^2 \\
\mathbf{u}_{j-1} + \mathbf{T} \cdot \mathbf{u}_j + \mathbf{u}_{j+1} &= \mathbf{g}_j \Delta^2 \\
\mathbf{u}_j + \mathbf{T} \cdot \mathbf{u}_{j+1} + \mathbf{u}_{j+2} &= \mathbf{g}_{j+1} \Delta^2
\end{aligned} \tag{20.4.31}$$

Matrix-multiplying the middle equation by $-\mathbf{T}$ and then adding the three equations, we get

$$\mathbf{u}_{j-2} + \mathbf{T}^{(1)} \cdot \mathbf{u}_j + \mathbf{u}_{j+2} = \mathbf{g}_j^{(1)} \Delta^2 \tag{20.4.32}$$

This is an equation of the same form as (20.4.29), with

$$\begin{aligned} \mathbf{T}^{(1)} &= 2\mathbf{1} - \mathbf{T}^2 \\ \mathbf{g}_j^{(1)} &= \Delta^2(\mathbf{g}_{j-1} - \mathbf{T} \cdot \mathbf{g}_j + \mathbf{g}_{j+1}) \end{aligned} \tag{20.4.33}$$

After one level of CR, we have reduced the number of equations by a factor of two. Since the resulting equations are of the same form as the original equation, we can repeat the process. Taking the number of mesh points to be a power of 2 for simplicity, we finally end up with a single equation for the central line of variables:

$$\mathbf{T}^{(f)} \cdot \mathbf{u}_{J/2} = \Delta^2 \mathbf{g}_{J/2}^{(f)} - \mathbf{u}_0 - \mathbf{u}_J \tag{20.4.34}$$

Here we have moved $\mathbf{u}_0$ and $\mathbf{u}_J$ to the right-hand side because they are known boundary values. Equation (20.4.34) can be solved for $\mathbf{u}_{J/2}$ by the standard tridiagonal algorithm. The two equations at level $f-1$ involve $\mathbf{u}_{J/4}$ and $\mathbf{u}_{3J/4}$. The equation for $\mathbf{u}_{J/4}$ involves $\mathbf{u}_0$ and $\mathbf{u}_{J/2}$, both of which are known and hence can be solved by the usual tridiagonal routine. A similar result holds true at every stage, so we end up solving $J-1$ tridiagonal systems.

In practice, equations (20.4.33) should be rewritten to avoid numerical instability. For these and other practical details, refer to [2].

20.4.3 FACR Method

The *best* way to solve equations of the form (20.4.28), including the constant coefficient problem (20.0.3), is a combination of Fourier analysis and cyclic reduction, the FACR method [3-6]. If at the rth stage of CR we Fourier analyze the equations of the form (20.4.32) along y, that is, with respect to the suppressed vector index, we will have a tridiagonal system in the x-direction for each y-Fourier mode:

$$\widehat{u}_{j-2^r}^k + \lambda_k^{(r)} \widehat{u}_j^k + \widehat{u}_{j+2^r}^k = \Delta^2 g_j^{(r)k} \tag{20.4.35}$$

Here $\lambda_k^{(r)}$ is the eigenvalue of $\mathbf{T}^{(r)}$ corresponding to the kth Fourier mode. For the equation (20.0.3), we see from equation (20.4.5) that $\lambda_k^{(r)}$ will involve terms like $\cos(2\pi k/L) - 2$ raised to a power. Solve the tridiagonal systems for $\widehat{u}_j^k$ at the levels $j = 2^r, 2 \times 2^r, 4 \times 2^r, ..., J - 2^r$. Fourier synthesize to get the y-values on these x-lines. Then fill in the intermediate x-lines as in the original CR algorithm.

The trick is to choose the number of levels of CR so as to minimize the total number of arithmetic operations. One can show that for a typical case of a 128×128 mesh, the optimal level is $r = 2$; asymptotically, $r \to \log_2(\log_2 J)$.

A rough estimate of running times for these algorithms for equation (20.0.3) is as follows: The FFT method (in both x and y) and the CR method are roughly comparable. FACR with $r = 0$ (that is, FFT in one dimension and solve the tridiagonal equations by the usual algorithm in the other dimension) gives about a factor of two gain in speed. The optimal FACR with $r = 2$ gives another factor of two gain in speed.

CITED REFERENCES AND FURTHER READING:

Swartzrauber, P.N. 1977, "The Methods of Cyclic Reduction, Fourier Analysis and the FACR Algorithm for the Discrete Solution of Poisson's Equation on a Rectangle," *SIAM Review*, vol. 19, pp. 490–501.[1]

Buzbee, B.L, Golub, G.H., and Nielson, C.W. 1970, "On Direct Methods for Solving Poisson's Equation," *SIAM Journal on Numerical Analysis*, vol. 7, pp. 627–656; see also *op. cit.* vol. 11, pp. 753–763.[2]

Hockney, R.W. 1965, "A Fast Direct Solution of Poisson's Equation Using Fourier Analysis," *Journal of the Association for Computing Machinery*, vol. 12, pp. 95–113.[3]

Hockney, R.W. 1970, "The Potential Calculation and Some Applications," *Methods of Computational Physics*, vol. 9 (New York: Academic Press), pp. 135–211.[4]

Hockney, R.W., and Eastwood, J.W. 1981, *Computer Simulation Using Particles* (New York: McGraw-Hill), Chapter 6.[5]

Temperton, C. 1980, "On the FACR Algorithm for the Discrete Poisson Equation," *Journal of Computational Physics*, vol. 34, pp. 314–329.[6]

20.5 Relaxation Methods for Boundary Value Problems

As we mentioned in §20.0, relaxation methods involve splitting the sparse matrix that arises from finite differencing and then iterating until a solution is found.

There is another way of thinking about relaxation methods that is somewhat more physical. Suppose we wish to solve the elliptic equation

$$\mathcal{L}u = \rho \tag{20.5.1}$$

where $\mathcal{L}$ represents some elliptic operator and ρ is the source term. Rewrite the equation as a diffusion equation,

$$\frac{\partial u}{\partial t} = \mathcal{L}u - \rho \tag{20.5.2}$$

An initial distribution u *relaxes* to an equilibrium solution as $t \to \infty$. This equilibrium has all time derivatives vanishing. Therefore, it is the solution of the original elliptic problem (20.5.1). We see that all the machinery of §20.2, on diffusive initial value equations, can be brought to bear on the solution of boundary value problems by relaxation methods.

Let us apply this idea to our model problem (20.0.3). The diffusion equation is

$$\frac{\partial u}{\partial t} = \frac{\partial^2 u}{\partial x^2} + \frac{\partial^2 u}{\partial y^2} - \rho \tag{20.5.3}$$

If we use FTCS differencing (cf. equation 20.2.4), we get

$$u_{j,l}^{n+1} = u_{j,l}^n + \frac{\Delta t}{\Delta^2}\left(u_{j+1,l}^n + u_{j-1,l}^n + u_{j,l+1}^n + u_{j,l-1}^n - 4u_{j,l}^n\right) - \rho_{j,l}\Delta t \tag{20.5.4}$$

Recall from (20.2.6) that FTCS differencing is stable in one spatial dimension only if $\Delta t/\Delta^2 \le \frac{1}{2}$. In two dimensions this becomes $\Delta t/\Delta^2 \le \frac{1}{4}$. Suppose we try to take the largest possible timestep and set $\Delta t = \Delta^2/4$. Then equation (20.5.4) becomes

$$u_{j,l}^{n+1} = \frac{1}{4}\left(u_{j+1,l}^n + u_{j-1,l}^n + u_{j,l+1}^n + u_{j,l-1}^n\right) - \frac{\Delta^2}{4}\rho_{j,l} \tag{20.5.5}$$

Thus the algorithm consists of using the average of u at its four nearest-neighbor points on the grid (plus the contribution from the source). This procedure is then iterated until convergence.

This method is in fact a classical method with origins dating back to the last century, called *Jacobi's method* (not to be confused with the Jacobi method for eigenvalues). The method is not practical because it converges too slowly. However, it is the basis for understanding the modern methods, which are always compared with it.

Another classical method is the *Gauss-Seidel* method, which turns out to be important in multigrid methods (§20.6). Here we make use of updated values of u on the right-hand side of (20.5.5) as soon as they become available. In other words, the averaging is done "in place" instead of being "copied" from an earlier timestep to a later one. If we are proceeding along the rows, incrementing j for fixed l, we have

$$u_{j,l}^{n+1} = \frac{1}{4}\left(u_{j+1,l}^n + u_{j-1,l}^{n+1} + u_{j,l+1}^n + u_{j,l-1}^{n+1}\right) - \frac{\Delta^2}{4}\rho_{j,l} \tag{20.5.6}$$

This method is also slowly converging and only of theoretical interest when used by itself, but some analysis of it will be instructive.

Let us look at the Jacobi and Gauss-Seidel methods in terms of the matrix splitting concept. We change notation and call $\mathbf{u}$ "$\mathbf{x}$," to conform to standard matrix notation. To solve

$$\mathbf{A}\cdot\mathbf{x} = \mathbf{b} \tag{20.5.7}$$

we can consider splitting $\mathbf{A}$ as

$$\mathbf{A} = \mathbf{L} + \mathbf{D} + \mathbf{U} \tag{20.5.8}$$

where $\mathbf{D}$ is the diagonal part of $\mathbf{A}$, $\mathbf{L}$ is the lower triangle of $\mathbf{A}$ with zeros on the diagonal, and $\mathbf{U}$ is the upper triangle of $\mathbf{A}$ with zeros on the diagonal.

In the Jacobi method we write for the rth step of iteration

$$\mathbf{D}\cdot\mathbf{x}^{(r)} = -(\mathbf{L}+\mathbf{U})\cdot\mathbf{x}^{(r-1)} + \mathbf{b} \tag{20.5.9}$$

For our model problem (20.5.5), $\mathbf{D}$ is simply the identity matrix. The Jacobi method converges for matrices $\mathbf{A}$ that are "diagonally dominant" in a sense that can be made mathematically precise. For matrices arising from finite differencing, this condition is usually met.

What is the rate of convergence of the Jacobi method? A detailed analysis is beyond our scope, but here is some of the flavor: The matrix $-\mathbf{D}^{-1}\cdot(\mathbf{L}+\mathbf{U})$ is the *iteration matrix* that, apart from an additive term, maps one set of $\mathbf{x}$'s into the next. The iteration matrix has eigenvalues, each one of which reflects the factor by which the amplitude of a particular eigenmode of undesired residual is suppressed during one iteration. Evidently those factors had better all have modulus < 1 for the

relaxation to work at all! The rate of convergence of the method is set by the rate for the slowest-decaying eigenmode, i.e., the factor with the largest modulus. The modulus of this largest factor, therefore lying between 0 and 1, is called the *spectral radius* of the relaxation operator, denoted ρ_s.

The number of iterations r required to reduce the overall error by a factor 10^{-p} is thus estimated by

$$r \approx \frac{p \ln 10}{(-\ln \rho_s)} \tag{20.5.10}$$

In general, the spectral radius ρ_s goes asymptotically to the value 1 as the grid size J is increased, so that more iterations are required. For any given equation, grid geometry, *and boundary condition*, the spectral radius can, in principle, be computed analytically. For example, for equation (20.5.5) on a $J \times J$ grid with Dirichlet boundary conditions on all four sides, the asymptotic formula for large J turns out to be

$$\rho_s \simeq 1 - \frac{\pi^2}{2J^2} \tag{20.5.11}$$

The number of iterations r required to reduce the error by a factor of 10^{-p} is thus

$$r \simeq \frac{2pJ^2 \ln 10}{\pi^2} \simeq \frac{1}{2} pJ^2 \tag{20.5.12}$$

In other words, the number of iterations is proportional to the number of mesh points, J^2. Since 100×100 and larger problems are common, it is clear that the Jacobi method is only of theoretical interest.

The Gauss-Seidel method, equation (20.5.6), corresponds to the matrix decomposition

$$(\mathbf{L} + \mathbf{D}) \cdot \mathbf{x}^{(r)} = -\mathbf{U} \cdot \mathbf{x}^{(r-1)} + \mathbf{b} \tag{20.5.13}$$

The fact that $\mathbf{L}$ is on the left-hand side of the equation follows from the updating in place, as you can easily check if you write out (20.5.13) in components. One can show [1-3] that the spectral radius is just the square of the spectral radius of the Jacobi method. For our model problem, therefore,

$$\rho_s \simeq 1 - \frac{\pi^2}{J^2} \tag{20.5.14}$$

$$r \simeq \frac{pJ^2 \ln 10}{\pi^2} \simeq \frac{1}{4} pJ^2 \tag{20.5.15}$$

The factor of two improvement in the number of iterations over the Jacobi method still leaves the method impractical.

20.5.1 Successive Overrelaxation (SOR)

We get a better algorithm — one that was the standard algorithm until the 1970s — if we make an *overcorrection* to the value of $\mathbf{x}^{(r)}$ at the rth stage of Gauss-Seidel iteration, thus anticipating future corrections. Solve (20.5.13) for $\mathbf{x}^{(r)}$, add and subtract $\mathbf{x}^{(r-1)}$ on the right-hand side, and hence write the Gauss-Seidel method as

$$\mathbf{x}^{(r)} = \mathbf{x}^{(r-1)} - (\mathbf{L} + \mathbf{D})^{-1} \cdot [(\mathbf{L} + \mathbf{D} + \mathbf{U}) \cdot \mathbf{x}^{(r-1)} - \mathbf{b}] \tag{20.5.16}$$

The term in square brackets is just the residual vector $\xi^{(r-1)}$, so

$$\mathbf{x}^{(r)} = \mathbf{x}^{(r-1)} - (\mathbf{L} + \mathbf{D})^{-1} \cdot \xi^{(r-1)} \tag{20.5.17}$$

Now *overcorrect*, defining

$$\mathbf{x}^{(r)} = \mathbf{x}^{(r-1)} - \omega(\mathbf{L} + \mathbf{D})^{-1} \cdot \xi^{(r-1)} \tag{20.5.18}$$

Here ω is called the *overrelaxation parameter*, and the method is called *successive overrelaxation* (SOR).

The following theorems can be proved [1-3]:

- The method is convergent only for $0 < \omega < 2$. If $0 < \omega < 1$, we speak of *underrelaxation*.
- Under certain mathematical restrictions generally satisfied by matrices arising from finite differencing, only overrelaxation ($1 < \omega < 2$) can give faster convergence than the Gauss-Seidel method.
- If ρ_{Jacobi} is the spectral radius of the Jacobi iteration (so that the square of it is the spectral radius of the Gauss-Seidel iteration), then the *optimal* choice for ω is given by

$$\omega = \frac{2}{1 + \sqrt{1 - \rho_{\text{Jacobi}}^2}} \tag{20.5.19}$$

- For this optimal choice, the spectral radius for SOR is

$$\rho_{\text{SOR}} = \left(\frac{\rho_{\text{Jacobi}}}{1 + \sqrt{1 - \rho_{\text{Jacobi}}^2}} \right)^2 \tag{20.5.20}$$

As an application of the above results, consider our model problem for which ρ_{Jacobi} is given by equation (20.5.11). Then equations (20.5.19) and (20.5.20) give

$$\omega \simeq \frac{2}{1 + \pi/J} \tag{20.5.21}$$

$$\rho_{\text{SOR}} \simeq 1 - \frac{2\pi}{J} \qquad \text{for large} \quad J \tag{20.5.22}$$

Equation (20.5.10) gives for the number of iterations to reduce the initial error by a factor of 10^{-p},

$$r \simeq \frac{pJ \ln 10}{2\pi} \simeq \frac{1}{3} pJ \tag{20.5.23}$$

Comparing with equation (20.5.12) or (20.5.15), we see that optimal SOR requires of order J iterations, as opposed to of order J^2. Since J is typically 100 or larger, this makes a tremendous difference! Equation (20.5.23) leads to the mnemonic that three-figure accuracy ($p = 3$) requires a number of iterations equal to the number of mesh points along a side of the grid. For six-figure accuracy, we require about twice as many iterations.

How do we choose ω for a problem for which the answer is not known analytically? That is just the weak point of SOR! The advantages of SOR obtain only in a fairly narrow window around the correct value of ω. It is better to take ω slightly too large, rather than slightly too small, but best to get it right.

One way to choose ω is to map your problem approximately onto a known problem, replacing the coefficients in the equation by average values. Note, however, that the known problem must have the same grid size and boundary conditions as the actual problem. We give for reference purposes the value of ρ_{Jacobi} for our model problem on a rectangular $J \times L$ grid, allowing for the possibility that $\Delta x \neq \Delta y$:

$$\rho_{\text{Jacobi}} = \frac{\cos\dfrac{\pi}{J} + \left(\dfrac{\Delta x}{\Delta y}\right)^2 \cos\dfrac{\pi}{L}}{1 + \left(\dfrac{\Delta x}{\Delta y}\right)^2} \tag{20.5.24}$$

Equation (20.5.24) holds for homogeneous Dirichlet or Neumann boundary conditions. For periodic boundary conditions, make the replacement $\pi \to 2\pi$.

A second way, which is especially useful if you plan to solve many similar elliptic equations each time with slightly different coefficients, is to determine the optimum value ω empirically on the first equation and then use that value for the remaining equations. Various automated schemes for doing this and for "seeking out" the best values of ω are described in the literature.

While the matrix notation introduced earlier is useful for theoretical analyses, for practical implementation of the SOR algorithm we need explicit formulas. Consider a general second-order elliptic equation in x and y, finite differenced on a square as for our model equation. Corresponding to each row of the matrix $\mathbf{A}$ is an equation of the form

$$a_{j,l}u_{j+1,l} + b_{j,l}u_{j-1,l} + c_{j,l}u_{j,l+1} + d_{j,l}u_{j,l-1} + e_{j,l}u_{j,l} = f_{j,l} \tag{20.5.25}$$

For our model equation, we had $a = b = c = d = 1, e = -4$. The quantity f is proportional to the source term. The iterative procedure is defined by solving (20.5.25) for $u_{j,l}$:

$$u^*_{j,l} = \frac{1}{e_{j,l}}\left(f_{j,l} - a_{j,l}u_{j+1,l} - b_{j,l}u_{j-1,l} - c_{j,l}u_{j,l+1} - d_{j,l}u_{j,l-1}\right) \tag{20.5.26}$$

Then $u^{\text{new}}_{j,l}$ is a weighted average,

$$u^{\text{new}}_{j,l} = \omega u^*_{j,l} + (1-\omega)u^{\text{old}}_{j,l} \tag{20.5.27}$$

We calculate it as follows: The residual at any stage is

$$\xi_{j,l} = a_{j,l}u_{j+1,l} + b_{j,l}u_{j-1,l} + c_{j,l}u_{j,l+1} + d_{j,l}u_{j,l-1} + e_{j,l}u_{j,l} - f_{j,l} \tag{20.5.28}$$

and the SOR algorithm (20.5.18) or (20.5.27) is

$$u^{\text{new}}_{j,l} = u^{\text{old}}_{j,l} - \omega\frac{\xi_{j,l}}{e_{j,l}} \tag{20.5.29}$$

This formulation is very easy to program, and the norm of the residual vector $\xi_{j,l}$ can be used as a criterion for terminating the iteration.

Another practical point concerns the order in which mesh points are processed. The obvious strategy is simply to proceed in order down the rows (or columns).

Alternatively, suppose we divide the mesh into "odd" and "even" meshes, like the red and black squares of a checkerboard. Then equation (20.5.26) shows that the odd points depend only on the even mesh values, and vice versa. Accordingly, we can carry out one half-sweep updating the odd points, say, and then another half-sweep updating the even points with the new odd values. For the version of SOR implemented below, we shall adopt odd-even ordering.

The last practical point is that in practice the asymptotic rate of convergence in SOR is not attained until of order J iterations. The error often grows by a factor of 20 before convergence sets in. A trivial modification to SOR resolves this problem. It is based on the observation that, while ω is the optimum *asymptotic* relaxation parameter, it is not necessarily a good initial choice. In SOR with *Chebyshev acceleration*, one uses odd-even ordering and changes ω at each half-sweep according to the following prescription:

$$\begin{aligned}
\omega^{(0)} &= 1 \\
\omega^{(1/2)} &= 1/(1-\rho_{\text{Jacobi}}^2/2) \\
\omega^{(n+1/2)} &= 1/(1-\rho_{\text{Jacobi}}^2\omega^{(n)}/4), \qquad n = 1/2, 1, \ldots, \infty \\
\omega^{(\infty)} &\to \omega_{\text{optimal}}
\end{aligned} \tag{20.5.30}$$

The beauty of Chebyshev acceleration is that the norm of the error always decreases with each iteration. (This is the norm of the actual error in $u_{j,l}$. The norm of the residual $\xi_{j,l}$ need not decrease monotonically.) While the asymptotic rate of convergence is the same as ordinary SOR, there is never any excuse for not using Chebyshev acceleration to reduce the total number of iterations required.

Here we give a routine for SOR with Chebyshev acceleration.

sor.h
```
void sor(MatDoub_I &a, MatDoub_I &b, MatDoub_I &c, MatDoub_I &d, MatDoub_I &e,
    MatDoub_I &f, MatDoub_IO &u, const Doub rjac)
```
Successive overrelaxation solution of equation (20.5.25) with Chebyshev acceleration. a, b, c, d, e, and f are input as the coefficients of the equation, each dimensioned to the grid size [0..jmax-1][0..jmax-1]. u is input as the initial guess to the solution, usually zero, and returns with the final value. rjac is input as the spectral radius of the Jacobi iteration, or an estimate of it.
```
{
    const Int MAXITS=1000;
    const Doub EPS=1.0e-13;
    Doub anormf=0.0,omega=1.0;
    Int jmax=a.nrows();
    for (Int j=1;j<jmax-1;j++)
```
Compute initial norm of residual and terminate iterations when norm has been reduced by a factor EPS.
```
        for (Int l=1;l<jmax-1;l++)
            anormf += abs(f[j][l]);                Assumes initial u is zero.
    for (Int n=0;n<MAXITS;n++) {
        Doub anorm=0.0;
        Int jsw=1;
        for (Int ipass=0;ipass<2;ipass++) {        Odd-even ordering.
            Int lsw=jsw;
            for (Int j=1;j<jmax-1;j++) {
                for (Int l=lsw;l<jmax-1;l+=2) {
                    Doub resid=a[j][l]*u[j+1][l]+b[j][l]*u[j-1][l]
                        +c[j][l]*u[j][l+1]+d[j][l]*u[j][l-1]
                        +e[j][l]*u[j][l]-f[j][l];
```

```
                    anorm += abs(resid);
                    u[j][l] -= omega*resid/e[j][l];
                }
                lsw=3-lsw;
            }
            jsw=3-jsw;
            omega=(n == 0 && ipass == 0 ? 1.0/(1.0-0.5*rjac*rjac) :
                1.0/(1.0-0.25*rjac*rjac*omega));
        }
        if (anorm < EPS*anormf) return;
    }
    throw("MAXITS exceeded");
}
```

The main advantage of SOR is that it is very easy to program. Its main disadvantage is that it is still very inefficient on large problems.

20.5.2 ADI (Alternating-Direction Implicit) Method

The ADI method of §20.3 for diffusion equations can be turned into a relaxation method for elliptic equations [1-4]. In §20.3, we discussed ADI as a method for solving the time-dependent heat-flow equation

$$\frac{\partial u}{\partial t} = \nabla^2 u - \rho \tag{20.5.31}$$

By letting $t \to \infty$, one also gets an iterative method for solving the elliptic equation

$$\nabla^2 u = \rho \tag{20.5.32}$$

In either case, the operator splitting is of the form

$$\mathcal{L} = \mathcal{L}_x + \mathcal{L}_y \tag{20.5.33}$$

where $\mathcal{L}_x$ represents the differencing in x and $\mathcal{L}_y$ that in y.

For example, in our model problem (20.0.6) with $\Delta x = \Delta y = \Delta$, we have

$$\begin{aligned} \mathcal{L}_x u &= 2u_{j,l} - u_{j+1,l} - u_{j-1,l} \\ \mathcal{L}_y u &= 2u_{j,l} - u_{j,l+1} - u_{j,l-1} \end{aligned} \tag{20.5.34}$$

More complicated operators may be similarly split, but there is some art involved. A bad choice of splitting can lead to an algorithm that fails to converge. Usually one tries to base the splitting on the physical nature of the problem. We know for our model problem that an initial transient diffuses away, and we set up the x and y splitting to mimic diffusion in each dimension.

Having chosen a splitting, we difference the time-dependent equation (20.5.31) implicitly in two half-steps:

$$\begin{aligned} \frac{u^{n+1/2} - u^n}{\Delta t/2} &= -\frac{\mathcal{L}_x u^{n+1/2} + \mathcal{L}_y u^n}{\Delta^2} - \rho \\ \frac{u^{n+1} - u^{n+1/2}}{\Delta t/2} &= -\frac{\mathcal{L}_x u^{n+1/2} + \mathcal{L}_y u^{n+1}}{\Delta^2} - \rho \end{aligned} \tag{20.5.35}$$

(cf. equation 20.3.16). Here we have suppressed the spatial indices (j, l). In matrix notation, equations (20.5.35) are

$$(\mathbf{L}_x + r\mathbf{1}) \cdot \mathbf{u}^{n+1/2} = (r\mathbf{1} - \mathbf{L}_y) \cdot \mathbf{u}^n - \Delta^2 \rho \tag{20.5.36}$$

$$(\mathbf{L}_y + r\mathbf{1}) \cdot \mathbf{u}^{n+1} = (r\mathbf{1} - \mathbf{L}_x) \cdot \mathbf{u}^{n+1/2} - \Delta^2 \rho \tag{20.5.37}$$

where

$$r \equiv \frac{2\Delta^2}{\Delta t} \tag{20.5.38}$$

The matrices on the left-hand sides of equations (20.5.36) and (20.5.37) are tridiagonal (and usually positive-definite), so the equations can be solved by the standard tridiagonal algorithm. Given $\mathbf{u}^n$, one solves (20.5.36) for $\mathbf{u}^{n+1/2}$, substitutes on the right-hand side of (20.5.37), and then solves for $\mathbf{u}^{n+1}$. The key question is how to choose the iteration parameter r, the analog of a choice of timestep for an initial value problem.

As usual, the goal is to minimize the spectral radius of the iteration matrix. Although it is beyond our scope to go into details here, it turns out that, for the optimal choice of r, the ADI method has the same rate of convergence as SOR. The individual iteration steps in the ADI method are much more complicated than in SOR, so the ADI method would appear to be inferior. This is in fact true if we choose the same parameter r for every iteration step. However, it is possible to choose a *different* r for each step. If this is done optimally, then ADI is generally more efficient than SOR. We refer you to the literature [1-4] for details.

Our reason for not fully implementing ADI here is that, in most applications, it has been superseded by the multigrid methods described in the next section. Our advice is to use SOR for trivial problems (e.g., 30×30) or for solving a larger problem once only, where ease of programming outweighs expense of computer time. Occasionally, the sparse matrix methods of §2.7 are useful for solving a set of difference equations directly. For production solution of large elliptic problems, however, multigrid is now almost always the method of choice.

CITED REFERENCES AND FURTHER READING:

Hockney, R.W., and Eastwood, J.W. 1981, *Computer Simulation Using Particles* (New York: McGraw-Hill), Chapter 6.

Young, D.M. 1971, *Iterative Solution of Large Linear Systems* (New York: Academic Press); reprinted 2003 (New York: Dover).[1]

Stoer, J., and Bulirsch, R. 2002, *Introduction to Numerical Analysis*, 3rd ed. (New York: Springer), §8.3 – §8.6.[2]

Varga, R.S. 2000, *Matrix Iterative Analysis*, 2nd ed. (New York: Springer).[3]

Spanier, J. 1967, in *Mathematical Methods for Digital Computers, Volume 2* (New York: Wiley), Chapter 11.[4]

20.6 Multigrid Methods for Boundary Value Problems

Practical multigrid methods were first introduced in the 1970s by Brandt [1,2]. These methods can solve elliptic PDEs discretized on N grid points in $O(N)$ op-

erations. The “rapid” direct elliptic solvers discussed in §20.4 solve special kinds of elliptic equations in $O(N \log N)$ operations. The numerical coefficients in these estimates are such that multigrid methods are comparable to the rapid methods in execution speed. Unlike the rapid methods, however, the multigrid methods can solve general elliptic equations with nonconstant coefficients with hardly any loss in efficiency. Even nonlinear equations can be solved with comparable speed.

Unfortunately, there is not a single multigrid algorithm that solves all elliptic problems. Rather, there is a multigrid technique that provides the framework for solving these problems. You have to adjust the various components of the algorithm within this framework to solve your specific problem. We can only give a brief introduction to the subject here. In particular, we will give two sample multigrid routines, one linear and one nonlinear. By following these prototypes and by perusing the references [3-6], you should be able to develop routines to solve your own problems.

There are two related, but distinct, approaches to the use of multigrid techniques. The first, termed *the multigrid method*, is a means for speeding up the convergence of a traditional relaxation method, as defined by you on a grid of pre-specified fineness. In this case, you need to define your problem (e.g., evaluate its source terms) only on this grid. Other, coarser, grids defined by the method can be viewed as temporary computational adjuncts.

The second approach, termed (perhaps confusingly) *the full multigrid (FMG) method*, requires you to be able to define your problem on grids of various sizes (generally by discretizing the same underlying PDE into different-sized sets of finite difference equations). In this approach, the method obtains successive solutions on finer and finer grids. You can stop the solution either at a pre-specified fineness, or you can monitor the truncation error due to the discretization, quitting only when it is tolerably small.

In this section we will first discuss the multigrid method and then use the concepts developed to introduce the FMG method. The latter algorithm is the one that we implement in the accompanying programs.

20.6.1 From One-Grid, through Two-Grid, to Multigrid

The key idea of the multigrid method can be understood by considering the simplest case of a two-grid method. Suppose we are trying to solve the linear elliptic problem

$$\mathcal{L}u = f \tag{20.6.1}$$

where $\mathcal{L}$ is some linear elliptic operator and f is the source term. Discretize equation (20.6.1) on a uniform grid with mesh size h. Write the resulting set of linear algebraic equations as

$$\mathcal{L}_h u_h = f_h \tag{20.6.2}$$

Let $\widetilde{u}_h$ denote some approximate solution to equation (20.6.2). We will use the symbol u_h to denote the exact solution to the difference equations (20.6.2). Then the *error* in $\widetilde{u}_h$ or the *correction* is

$$v_h = u_h - \widetilde{u}_h \tag{20.6.3}$$

The *residual* or *defect* is

$$d_h = \mathcal{L}_h \widetilde{u}_h - f_h \tag{20.6.4}$$

(Beware: Some authors define residual as minus the defect, and there is not universal agreement about which of these two quantities 20.6.4 defines.) Since $\mathcal{L}_h$ is linear, the error satisfies

$$\mathcal{L}_h v_h = -d_h \tag{20.6.5}$$

At this point we need to make an approximation to $\mathcal{L}_h$ in order to find v_h. The classical iteration methods, such as Jacobi or Gauss-Seidel, do this by finding, at each stage, an approximate solution of the equation

$$\widehat{\mathcal{L}}_h \widehat{v}_h = -d_h \tag{20.6.6}$$

where $\widehat{\mathcal{L}}_h$ is a "simpler" operator than $\mathcal{L}_h$. For example, $\widehat{\mathcal{L}}_h$ is the diagonal part of $\mathcal{L}_h$ for Jacobi iteration, or the lower triangle for Gauss-Seidel iteration. The next approximation is generated by

$$\widetilde{u}_h^{\text{new}} = \widetilde{u}_h + \widehat{v}_h \tag{20.6.7}$$

Now consider, as an alternative, a completely different type of approximation for $\mathcal{L}_h$, one in which we "coarsify" rather than "simplify." That is, we form some appropriate approximation $\mathcal{L}_H$ of $\mathcal{L}_h$ on a coarser grid with mesh size H (we will always take $H = 2h$, but other choices are possible). The residual equation (20.6.5) is now approximated by

$$\mathcal{L}_H v_H = -d_H \tag{20.6.8}$$

Since $\mathcal{L}_H$ has smaller dimension, this equation will be easier to solve than equation (20.6.5). To define the defect d_H on the coarse grid, we need a *restriction operator* $\mathcal{R}$ that restricts d_h to the coarse grid:

$$d_H = \mathcal{R} d_h \tag{20.6.9}$$

The restriction operator is also called the *fine-to-coarse operator* or the *injection operator*. Once we have a solution $\widetilde{v}_H$ to equation (20.6.8), we need a *prolongation operator* $\mathcal{P}$ that prolongates or interpolates the correction to the fine grid:

$$\widetilde{v}_h = \mathcal{P} \widetilde{v}_H \tag{20.6.10}$$

The prolongation operator is also called the *coarse-to-fine operator* or the *interpolation operator*. Both $\mathcal{R}$ and $\mathcal{P}$ are chosen to be linear operators. Finally, the approximation $\widetilde{u}_h$ can be updated:

$$\widetilde{u}_h^{\text{new}} = \widetilde{u}_h + \widetilde{v}_h \tag{20.6.11}$$

One step of this *coarse-grid correction scheme* is thus:

Coarse-Grid Correction

- Compute the defect on the fine grid from (20.6.4).
- Restrict the defect by (20.6.9).
- Solve (20.6.8) exactly on the coarse grid for the correction.
- Interpolate the correction to the fine grid by (20.6.10).
- Compute the next approximation by (20.6.11).

Let's contrast the advantages and disadvantages of relaxation and the coarse-grid correction scheme. Consider the error v_h expanded into a discrete Fourier series. Call the components in the lower half of the frequency spectrum the *smooth components* and the high-frequency components the *nonsmooth components*. We have seen that relaxation becomes very slowly convergent in the limit $h \to 0$, i.e., when there are a large number of mesh points. The reason turns out to be that the smooth components are only slightly reduced in amplitude on each iteration. However, many relaxation methods reduce the amplitude of the nonsmooth components by large factors on each iteration: They are good *smoothing operators*.

For the two-grid iteration, on the other hand, components of the error with wavelengths $\lesssim 2H$ are not even representable on the coarse grid and so cannot be reduced to zero on this grid. But it is exactly these high-frequency components that can be reduced by relaxation on the fine grid! This leads us to combine the ideas of relaxation and coarse-grid correction:

Two-Grid Iteration

- Pre-smoothing: Compute $\bar{u}_h$ by applying $\nu_1 \geq 0$ steps of a relaxation method to $\widetilde{u}_h$.
- Coarse-grid correction: As above, using $\bar{u}_h$ to give $\bar{u}_h^{\text{new}}$.
- Post-smoothing: Compute $\widetilde{u}_h^{\text{new}}$ by applying $\nu_2 \geq 0$ steps of the relaxation method to $\bar{u}_h^{\text{new}}$.

It is only a short step from the above two-grid method to a multigrid method. Instead of solving the coarse-grid defect equation (20.6.8) exactly, we can get an approximate solution of it by introducing an even coarser grid and using the two-grid iteration method. If the convergence factor of the two-grid method is small enough, we will need only a few steps of this iteration to get a good enough approximate solution. We denote the number of such iterations by γ. Obviously we can apply this idea recursively down to some coarsest grid. There the solution is found easily, for example by direct matrix inversion or by iterating the relaxation scheme to convergence.

One iteration of a multigrid method, from finest grid to coarser grids and back to finest grid again, is called a *cycle*. The exact structure of a cycle depends on the value of γ, the number of two-grid iterations at each intermediate stage. The case $\gamma = 1$ is called a V-cycle, while $\gamma = 2$ is called a W-cycle (see Figure 20.6.1). These are the most important cases in practice.

Note that once more than two grids are involved, the pre-smoothing steps after the first one on the finest grid need an initial approximation for the error v. This should be taken to be zero.

20.6.2 Smoothing, Restriction, and Prolongation Operators

The most popular smoothing method, and the one you should try first, is Gauss-Seidel, since it usually leads to a good convergence rate. If we order the mesh points from 0 to $N-1$, then the Gauss-Seidel scheme is

$$u_i = -\Bigl(\sum_{\substack{j=0 \\ j\neq i}}^{N-1} L_{ij}u_j - f_i\Bigr)\frac{1}{L_{ii}} \qquad i = 0,\ldots,N-1 \tag{20.6.12}$$

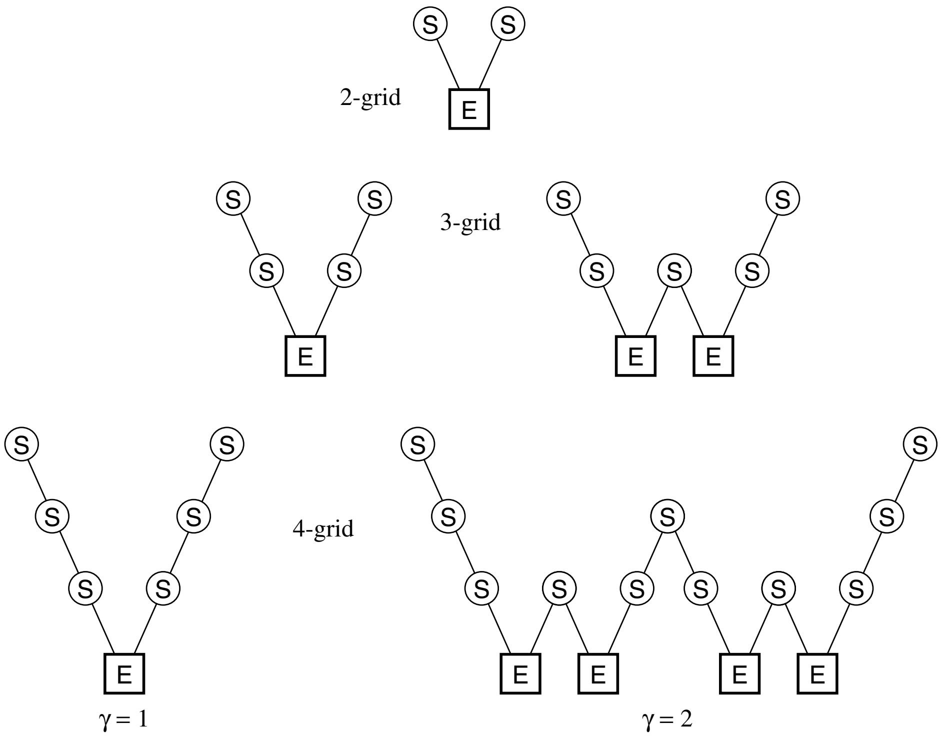

Figure 20.6.1. Structure of multigrid cycles. S denotes smoothing, while E denotes exact solution on the coarsest grid. Each descending line \ denotes restriction ($\mathcal{R}$) and each ascending line / denotes prolongation ($\mathcal{P}$). The finest grid is at the top level of each diagram. For the V-cycles ($\gamma = 1$) the E step is replaced by one two-grid iteration each time the number of grid levels is increased by one. For the W-cycles ($\gamma = 2$), each E step gets replaced by two two-grid iterations.

where new values of u are used on the right-hand side as they become available. The exact form of the Gauss-Seidel method depends on the ordering chosen for the mesh points. For typical second-order elliptic equations like our model problem equation (20.0.3), as differenced in equation (20.0.8), it is usually best to use red-black ordering, making one pass through the mesh updating the "even" points (like the red squares of a checkerboard) and another pass updating the "odd" points (the black squares). When quantities are more strongly coupled along one dimension than another, one should relax a whole line along that dimension simultaneously. Line relaxation for nearest-neighbor coupling involves solving a tridiagonal system, and so is still efficient. Relaxing odd and even lines on successive passes is called zebra relaxation and is usually preferred over simple line relaxation.

Note that SOR should *not* be used as a smoothing operator. The overrelaxation destroys the high-frequency smoothing that is so crucial for the multigrid method.

A succinct notation for the prolongation and restriction operators is to give their *symbol*. The symbol of $\mathcal{P}$ is found by considering v_H to be 1 at some mesh point (x, y), zero elsewhere, and then asking for the values of $\mathcal{P}v_H$. The most popular prolongation operator is simple bilinear interpolation. It gives nonzero values at the nine points $(x, y), (x + h, y), \ldots, (x - h, y - h)$, where the values are $1, \frac{1}{2}, \ldots, \frac{1}{4}$.

Its symbol is therefore

$$\begin{bmatrix} \frac{1}{4} & \frac{1}{2} & \frac{1}{4} \\ \frac{1}{2} & 1 & \frac{1}{2} \\ \frac{1}{4} & \frac{1}{2} & \frac{1}{4} \end{bmatrix} \tag{20.6.13}$$

The symbol of $\mathcal{R}$ is defined by considering v_h to be defined everywhere on the fine grid, and then asking what is $\mathcal{R}v_h$ at (x, y) as a linear combination of these values. The simplest possible choice for $\mathcal{R}$ is *straight injection*, which means simply filling each coarse-grid point with the value from the corresponding fine-grid point. Its symbol is "[1]." However, difficulties can arise in practice with this choice. It turns out that a safe choice for $\mathcal{R}$ is to make it the adjoint operator to $\mathcal{P}$. To define the adjoint, define the scalar product of two grid functions u_h and v_h for mesh size h as

$$\langle u_h | v_h \rangle_h \equiv h^2 \sum_{x,y} u_h(x, y) v_h(x, y) \tag{20.6.14}$$

Then the adjoint of $\mathcal{P}$, denoted $\mathcal{P}^\dagger$, is defined by

$$\langle u_H | \mathcal{P}^\dagger v_h \rangle_H = \langle \mathcal{P} u_H | v_h \rangle_h \tag{20.6.15}$$

Now take $\mathcal{P}$ to be bilinear interpolation, and choose $u_H = 1$ at (x, y), zero elsewhere. Set $\mathcal{P}^\dagger = \mathcal{R}$ in (20.6.15) and $H = 2h$. You will find that

$$(\mathcal{R}v_h)_{(x,y)} = \tfrac{1}{4} v_h(x, y) + \tfrac{1}{8} v_h(x + h, y) + \tfrac{1}{16} v_h(x + h, y + h) + \cdots \tag{20.6.16}$$

so that the symbol of $\mathcal{R}$ is

$$\begin{bmatrix} \frac{1}{16} & \frac{1}{8} & \frac{1}{16} \\ \frac{1}{8} & \frac{1}{4} & \frac{1}{8} \\ \frac{1}{16} & \frac{1}{8} & \frac{1}{16} \end{bmatrix} \tag{20.6.17}$$

Note the simple rule: The symbol of $\mathcal{R}$ is $\frac{1}{4}$ the transpose of the matrix defining the symbol of $\mathcal{P}$, equation (20.6.13). This rule is general whenever $\mathcal{R} = \mathcal{P}^\dagger$ and $H = 2h$.

The particular choice of $\mathcal{R}$ in (20.6.17) is called *full weighting*. Another popular choice for $\mathcal{R}$ is *half weighting*, "halfway" between full weighting and straight injection. Its symbol is

$$\begin{bmatrix} 0 & \frac{1}{8} & 0 \\ \frac{1}{8} & \frac{1}{2} & \frac{1}{8} \\ 0 & \frac{1}{8} & 0 \end{bmatrix} \tag{20.6.18}$$

A similar notation can be used to describe the difference operator $\mathcal{L}_h$. For example, the standard differencing of the model problem, equation (20.0.6), is represented by the *five-point difference star*

$$\mathcal{L}_h = \frac{1}{h^2} \begin{bmatrix} 0 & 1 & 0 \\ 1 & -4 & 1 \\ 0 & 1 & 0 \end{bmatrix} \tag{20.6.19}$$

If you are confronted with a new problem and you are not sure what $\mathcal{P}$ and $\mathcal{R}$ choices are likely to work well, here is a safe rule: Suppose m_p is the order of the

interpolation $\mathcal{P}$ (i.e., it interpolates polynomials of degree $m_p - 1$ exactly). Suppose m_r is the order of $\mathcal{R}$, and that $\mathcal{R}$ is the adjoint of some $\mathcal{P}$ (not necessarily the $\mathcal{P}$ you intend to use). Then, if m is the order of the differential operator $\mathcal{L}_h$, you should satisfy the inequality $m_p + m_r > m$. For example, bilinear interpolation and its adjoint, full weighting for Poisson's equation satisfy $m_p + m_r = 4 > m = 2$.

Of course the $\mathcal{P}$ and $\mathcal{R}$ operators should enforce the boundary conditions for your problem. The easiest way to do this is to rewrite the difference equation to have homogeneous boundary conditions by modifying the source term if necessary (cf. §20.4). Enforcing homogeneous boundary conditions simply requires the $\mathcal{P}$ operator to produce zeros at the appropriate boundary points. The corresponding $\mathcal{R}$ is then found by $\mathcal{R} = \mathcal{P}^\dagger$.

20.6.3 Full Multigrid Algorithm

So far we have described multigrid as an iterative scheme, where one starts with some initial guess on the finest grid and carries out enough cycles (V-cycles, W-cycles,...) to achieve convergence. This is the simplest way to use multigrid: Simply apply enough cycles until some appropriate convergence criterion is met. However, efficiency can be improved by using the *full multigrid algorithm* (FMG), also known as *nested iteration*.

Instead of starting with an arbitrary approximation on the finest grid (e.g., $u_h = 0$), the first approximation is obtained by interpolating from a coarse-grid solution:

$$u_h = \mathcal{P} u_H \tag{20.6.20}$$

The coarse-grid solution itself is found by a similar FMG process from even coarser grids. At the coarsest level, you start with the exact solution. Rather than proceed as in Figure 20.6.1, then, FMG gets to its solution by a series of increasingly tall "N's," each taller one probing a finer grid (see Figure 20.6.2).

Note that $\mathcal{P}$ in (20.6.20) need not be the same $\mathcal{P}$ used in the multigrid cycles. It should be at least of the same order as the discretization $\mathcal{L}_h$, but sometimes a higher-order operator leads to greater efficiency.

It turns out that you usually need one or at most two multigrid cycles at each level before proceeding down to the next finer grid. While there is theoretical guidance on the required number of cycles (e.g., [3]), you can easily determine it empirically. Fix the finest level and study the solution values as you increase the number of cycles per level. The asymptotic value of the solution is the exact solution of the difference equations. The difference between this exact solution and the solution for a small number of cycles is the iteration error. Now fix the number of cycles to be large, and vary the number of levels, i.e., the smallest value of h used. In this way you can estimate the truncation error for a given h. In your final production code, there is no point in using more cycles than you need to get the iteration error down to the size of the truncation error.

The simple multigrid iteration (cycle) needs the right-hand side f only at the finest level. FMG needs f at all levels. If the boundary conditions are homogeneous, you can use $f_H = \mathcal{R} f_h$. This prescription is not always safe for inhomogeneous boundary conditions. In that case it is better to discretize f on each coarse grid.

Note that the FMG algorithm produces the solution on all levels. It can therefore be combined with techniques like Richardson extrapolation.

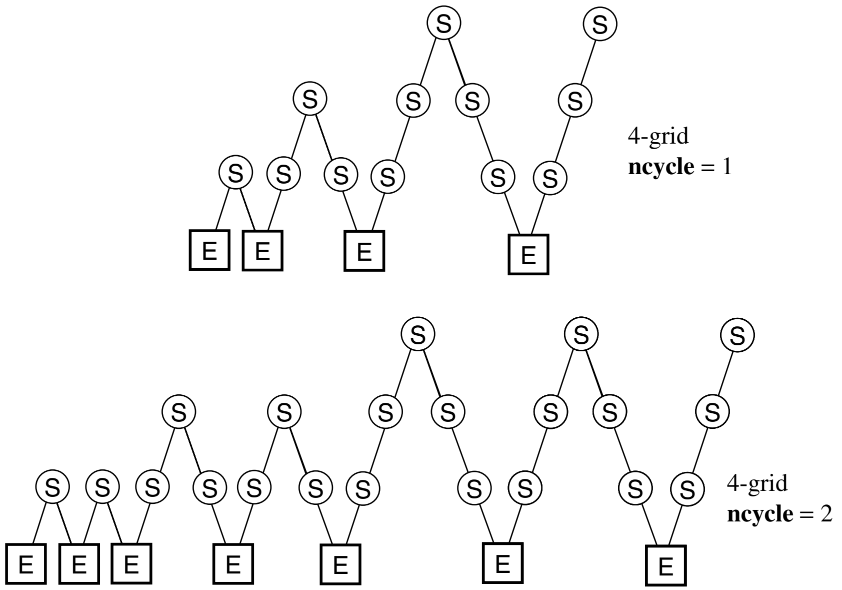

Figure 20.6.2. Structure of cycles for the full multigrid (FMG) method (notation as in Figure 20.6.1). This method starts on the coarsest grid, interpolates, and then refines (by "V's") the solution onto grids of increasing fineness.

We now give a routine `Mglin` that implements the full multigrid algorithm for a linear equation, the model problem (20.0.6). It uses red-black Gauss-Seidel as the smoothing operator, bilinear interpolation for $\mathcal{P}$, and half-weighting for $\mathcal{R}$. To change the routine to handle another linear problem, all you need do is modify the functions `relax`, `resid`, and `slvsml` appropriately. A feature of the routine is the dynamical allocation of storage for variables defined on the various grids.

mglin.h

```
struct Mglin {
```
Full multigrid algorithm for solution of linear elliptic equation, here the model problem (20.0.6) on a square domain of side 1, so that $\Delta = 1/(n-1)$.
```
    Int n,ng;
    MatDoub *uj,*uj1;
    NRvector<NRmatrix<Doub> *> rho;          Vector of pointers to ρ on each level.

    Mglin(MatDoub_IO &u, const Int ncycle) : n(u.nrows()), ng(0)
```
On input `u[0..n-1][0..n-1]` contains the right-hand side ρ, while on output it returns the solution. The dimension `n` must be of the form $2^j + 1$ for some integer j. (j is actually the number of grid levels used in the solution, called `ng` below.) `ncycle` is the number of V-cycles to be used at each level.
```
    {
        Int nn=n;
        while (nn >>= 1) ng++;
        if ((n-1) != (1 << ng))
            throw("n-1 must be a power of 2 in mglin.");
        nn=n;
        Int ngrid=ng-1;
        rho.resize(ng);
        rho[ngrid] = new MatDoub(nn,nn);      Allocate storage for r.h.s. on grid ng − 1,
        *rho[ngrid]=u;                        and fill it with the input r.h.s.
        while (nn > 3) {                      Similarly allocate storage and fill r.h.s. on
            nn=nn/2+1;                            all coarse grids by restricting from finer
                                                  grids.
```

```
        rho[--ngrid]=new MatDoub(nn,nn);
        rstrct(*rho[ngrid],*rho[ngrid+1]);
    }
    nn=3;
    uj=new MatDoub(nn,nn);
    slvsml(*uj,*rho[0]);                    Initial solution on coarsest grid.
    for (Int j=1;j<ng;j++) {                Nested iteration loop.
        nn=2*nn-1;
        uj1=uj;
        uj=new MatDoub(nn,nn);
        interp(*uj,*uj1);                   Interpolate from grid j-1 to next finer grid
        delete uj1;                             j.
        for (Int jcycle=0;jcycle<ncycle;jcycle++)      V-cycle loop.
            mg(j,*uj,*rho[j]);
    }
    u = *uj;                                               Return solution in u.
}

~Mglin()
```

Destructor deletes storage.

```
{
    if (uj != NULL) delete uj;
    for (Int j=0;j<ng;j++)
        if (rho[j] != NULL) delete rho[j];
}

void interp(MatDoub_O &uf, MatDoub_I &uc)
```

Coarse-to-fine prolongation by bilinear interpolation. If nf is the fine-grid dimension, the coarse-grid solution is input as uc[0..nc-1][0..nc-1], where nc $= \text{nf}/2 + 1$. The fine-grid solution is returned in uf[0..nf-1][0..nf-1].

```
{
    Int nf=uf.nrows();
    Int nc=nf/2+1;
    for (Int jc=0;jc<nc;jc++)               Do elements that are copies.
        for (Int ic=0;ic<nc;ic++) uf[2*ic][2*jc]=uc[ic][jc];
    for (Int jf=0;jf<nf;jf+=2)              Do even-numbered columns, interpolating ver-
        for (Int iif=1;iif<nf-1;iif+=2)         tically.
            uf[iif][jf]=0.5*(uf[iif+1][jf]+uf[iif-1][jf]);
    for (Int jf=1;jf<nf-1;jf+=2)            Do odd-numbered columns, interpolating hor-
        for (Int iif=0;iif<nf;iif++)            izontally.
            uf[iif][jf]=0.5*(uf[iif][jf+1]+uf[iif][jf-1]);
}

void addint(MatDoub_O &uf, MatDoub_I &uc, MatDoub_O &res)
```

Does coarse-to-fine interpolation and adds result to uf. If nf is the fine-grid dimension, the coarse-grid solution is input as uc[0..nc-1][0..nc-1], where nc $= \text{nf}/2 + 1$. The fine-grid solution is returned in uf[0..nf-1][0..nf-1]. res[0..nf-1][0..nf-1] is used for temporary storage.

```
{
    Int nf=uf.nrows();
    interp(res,uc);
    for (Int j=0;j<nf;j++)
        for (Int i=0;i<nf;i++)
            uf[i][j] += res[i][j];
}

void slvsml(MatDoub_O &u, MatDoub_I &rhs)
```

Solution of the model problem on the coarsest grid, where $h = \frac{1}{2}$. The right-hand side is input in rhs[0..2][0..2] and the solution is returned in u[0..2][0..2].

```
{
    Doub h=0.5;
    for (Int i=0;i<3;i++)
        for (Int j=0;j<3;j++)
            u[i][j]=0.0;
```

```
    u[1][1] = -h*h*rhs[1][1]/4.0;
}
```

```
void relax(MatDoub_IO &u, MatDoub_I &rhs)
```
Red-black Gauss-Seidel relaxation for model problem. Updates the current value of the solution u[0..n-1][0..n-1], using the right-hand side function rhs[0..n-1][0..n-1].

```
{
    Int n=u.nrows();
    Doub h=1.0/(n-1);
    Doub h2=h*h;
    for (Int ipass=0,jsw=1;ipass<2;ipass++,jsw=3-jsw) {      Red and black sweeps.
        for (Int j=1,isw=jsw;j<n-1;j++,isw=3-isw)
            for (Int i=isw;i<n-1;i+=2)                       Gauss-Seidel formula.
                u[i][j]=0.25*(u[i+1][j]+u[i-1][j]+u[i][j+1]
                    +u[i][j-1]-h2*rhs[i][j]);
    }
}
```

```
void resid(MatDoub_O &res, MatDoub_I &u, MatDoub_I &rhs)
```
Returns *minus* the residual for the model problem. Input quantities are u[0..n-1][0..n-1] and rhs[0..n-1][0..n-1], while res[0..n-1][0..n-1] is returned.

```
{
    Int n=u.nrows();
    Doub h=1.0/(n-1);
    Doub h2i=1.0/(h*h);
    for (Int j=1;j<n-1;j++)                                  Interior points.
        for (Int i=1;i<n-1;i++)
            res[i][j] = -h2i*(u[i+1][j]+u[i-1][j]+u[i][j+1]
                +u[i][j-1]-4.0*u[i][j])+rhs[i][j];
    for (Int i=0;i<n;i++)                                    Boundary points.
        res[i][0]=res[i][n-1]=res[0][i]=res[n-1][i]=0.0;
}
```

```
void rstrct(MatDoub_O &uc, MatDoub_I &uf)
```
Half-weighting restriction. If nc is the coarse-grid dimension, the fine-grid solution is input in uf[0..2*nc-2][0..2*nc-2].The coarse-grid solution obtained by restriction is returned in uc[0..nc-1][0..nc-1].

```
{
    Int nc=uc.nrows();
    Int ncc=2*nc-2;
    for (Int jf=2,jc=1;jc<nc-1;jc++,jf+=2) {                 Interior points.
        for (Int iif=2,ic=1;ic<nc-1;ic++,iif+=2) {
            uc[ic][jc]=0.5*uf[iif][jf]+0.125*(uf[iif+1][jf]+uf[iif-1][jf]
                +uf[iif][jf+1]+uf[iif][jf-1]);
        }
    }
    for (Int jc=0,ic=0;ic<nc;ic++,jc+=2) {                   Boundary points.
        uc[ic][0]=uf[jc][0];
        uc[ic][nc-1]=uf[jc][ncc];
    }
    for (Int jc=0,ic=0;ic<nc;ic++,jc+=2) {
        uc[0][ic]=uf[0][jc];
        uc[nc-1][ic]=uf[ncc][jc];
    }
}
```

```
void mg(Int j, MatDoub_IO &u, MatDoub_I &rhs)
```
Recursive multigrid iteration. On input, j is the current level, u is the current value of the solution, and rhs is the right-hand side. On output u contains the improved solution at the current level.

```
{
    const Int NPRE=1,NPOST=1;              Number of relaxation sweeps before and af-
    Int nf=u.nrows();                          ter the coarse-grid correction is computed.
    Int nc=(nf+1)/2;
```

```
        if (j == 0)                           Bottom of V: Solve on coarsest grid.
            slvsml(u,rhs);
        else {                                On downward stoke of the V.
            MatDoub res(nc,nc),v(nc,nc,0.0),temp(nf,nf);
            v is zero for initial guess in each relaxation.
            for (Int jpre=0;jpre<NPRE;jpre++)
                relax(u,rhs);                 Pre-smoothing.
            resid(temp,u,rhs);
            rstrct(res,temp);                 Restriction of the residual is the next r.h.s.
            mg(j-1,v,res);                    Recursive call for the coarse-grid correction.
            addint(u,v,temp);                 On upward stroke of V.
            for (Int jpost=0;jpost<NPOST;jpost++)
                relax(u,rhs);                 Post-smoothing.
        }
    }
};
```

The routine Mglin is written for clarity, not maximum efficiency, so that it is easy to modify. Several simple changes will speed up the execution time:

- The defect d_h vanishes identically at all black mesh points after a red-black Gauss-Seidel step. Thus $d_H = \mathcal{R} d_h$ for half-weighting reduces to simply copying half the defect from the fine grid to the corresponding coarse-grid point. The calls to resid followed by rstrct in the first part of the V-cycle can be replaced by a routine that loops only over the coarse grid, filling it with half the defect.
- Similarly, the quantity $\tilde{u}_h^{\text{new}} = \tilde{u}_h + \mathcal{P}\tilde{v}_H$ need not be computed at red mesh points, since they will immediately be redefined in the subsequent Gauss-Seidel sweep. This means that addint need only loop over black points.
- You can speed up relax in several ways. First, you can have a special form when the initial guess is zero. Next, you can store $h^2 f_h$ on the various grids and save a multiplication. Finally, it is possible to save an addition in the Gauss-Seidel formula by rewriting it with intermediate variables.
- For typical problems, Mglin with ncycle $= 1$ will return a solution with the iteration error bigger than the truncation error for the given size of h. To knock the error down to the size of the truncation error, you have to set ncycle $= 2$ or, more cheaply, NPRE $= 2$. A more efficient way turns out to be to use a higher-order $\mathcal{P}$ in (20.6.20) than the linear interpolation used in the V-cycle.

Implementing all the above features typically gives up to a factor of two improvement in execution time and is certainly worthwhile in a production code.

20.6.4 Nonlinear Multigrid: The FAS Algorithm

Now turn to solving a nonlinear elliptic equation, which we write symbolically as

$$\mathcal{L}(u) = 0 \qquad (20.6.21)$$

Any explicit source term has been moved to the left-hand side. Suppose equation (20.6.21) is suitably discretized:

$$\mathcal{L}_h(u_h) = 0 \qquad (20.6.22)$$

We will see below that in the multigrid algorithm we will have to consider equations where a nonzero right-hand side is generated during the course of the solution:

$$\mathcal{L}_h(u_h) = f_h \qquad (20.6.23)$$

One way of solving nonlinear problems with multigrid is to use Newton's method, which produces linear equations for the correction term at each iteration. We can then use linear multigrid to solve these equations. A great strength of the multigrid idea, however, is that it can be applied *directly* to nonlinear problems. All we need is a suitable *nonlinear* relaxation method to smooth the errors, plus a procedure for approximating corrections on coarser grids. This direct approach is Brandt's full approximation storage algorithm (FAS). No nonlinear equations need be solved, except perhaps on the coarsest grid.

To develop the nonlinear algorithm, suppose we have a relaxation procedure that can smooth the residual vector as we did in the linear case. Then we can seek a smooth correction v_h to solve (20.6.23):

$$\mathcal{L}_h(\widetilde{u}_h + v_h) = f_h \tag{20.6.24}$$

To find v_h, note that

$$\begin{aligned}\mathcal{L}_h(\widetilde{u}_h + v_h) - \mathcal{L}_h(\widetilde{u}_h) &= f_h - \mathcal{L}_h(\widetilde{u}_h)\\ &= -d_h\end{aligned} \tag{20.6.25}$$

The right-hand side is smooth after a few nonlinear relaxation sweeps. Thus we can transfer the left-hand side to a coarse grid:

$$\mathcal{L}_H(u_H) - \mathcal{L}_H(\mathcal{R}\widetilde{u}_h) = -\mathcal{R}d_h \tag{20.6.26}$$

that is, we solve

$$\mathcal{L}_H(u_H) = \mathcal{L}_H(\mathcal{R}\widetilde{u}_h) - \mathcal{R}d_h \tag{20.6.27}$$

on the coarse grid. (This is how nonzero right-hand sides appear.) Suppose the approximate solution is $\widetilde{u}_H$. Then the coarse-grid correction is

$$\widetilde{v}_H = \widetilde{u}_H - \mathcal{R}\widetilde{u}_h \tag{20.6.28}$$

and

$$\widetilde{u}_h^{\text{new}} = \widetilde{u}_h + \mathcal{P}(\widetilde{u}_H - \mathcal{R}\widetilde{u}_h) \tag{20.6.29}$$

Note that $\mathcal{P}\mathcal{R} \neq 1$ in general, so $\widetilde{u}_h^{\text{new}} \neq \mathcal{P}\widetilde{u}_H$. This is a key point: In equation (20.6.29), the interpolation error comes only from the correction, not from the full solution $\widetilde{u}_H$.

Equation (20.6.27) shows that one is solving for the full approximation u_H, not just the error as in the linear algorithm. This is the origin of the name FAS.

The FAS multigrid algorithm thus looks very similar to the linear multigrid algorithm. The only differences are that both the defect d_h and the relaxed approximation u_h have to be restricted to the coarse grid, where now it is equation (20.6.27) that is solved by recursive invocation of the algorithm. However, instead of implementing the algorithm this way, we will first describe the so-called *dual viewpoint*, which leads to a powerful alternative way of looking at the multigrid idea.

The dual viewpoint considers the *local truncation error*, defined as

$$\tau \equiv \mathcal{L}_h(u) - f_h \tag{20.6.30}$$

where u is the exact solution of the original continuum equation. If we rewrite this as

$$\mathcal{L}_h(u) = f_h + \tau \tag{20.6.31}$$

we see that τ can be regarded as the correction to f_h so that the solution of the fine-grid equation will be the exact solution u.

Now consider the *relative truncation error* τ_h, which is defined on the H-grid relative to the h-grid:

$$\tau_h \equiv \mathcal{L}_H(\mathcal{R}u_h) - \mathcal{R}\mathcal{L}_h(u_h) \tag{20.6.32}$$

Since $\mathcal{L}_h(u_h) = f_h$, this can be rewritten as

$$\mathcal{L}_H(u_H) = f_H + \tau_h \tag{20.6.33}$$

In other words, we can think of τ_h as the correction to f_H that makes the solution of the coarse-grid equation equal to the fine-grid solution. Of course we cannot compute τ_h, but we do have an approximation to it from using $\widetilde{u}_h$ in equation (20.6.32):

$$\tau_h \simeq \widetilde{\tau}_h \equiv \mathcal{L}_H(\mathcal{R}\widetilde{u}_h) - \mathcal{R}\mathcal{L}_h(\widetilde{u}_h) \tag{20.6.34}$$

Replacing τ_h by $\widetilde{\tau}_h$ in equation (20.6.33) gives

$$\mathcal{L}_H(u_H) = \mathcal{L}_H(\mathcal{R}\widetilde{u}_h) - \mathcal{R}d_h \tag{20.6.35}$$

which is just the coarse-grid equation (20.6.27)!

Thus we see that there are two complementary viewpoints for the relation between coarse and fine grids:

- Coarse grids are used to accelerate the convergence of the smooth components of the fine-grid residuals.
- Fine grids are used to compute correction terms to the coarse-grid equations, yielding fine-grid accuracy on the coarse grids.

One benefit of this new viewpoint is that it allows us to derive a natural stopping criterion for a multigrid iteration. Normally the criterion would be

$$\|d_h\| \leq \epsilon \tag{20.6.36}$$

and the question is how to choose ϵ. There is clearly no benefit in iterating beyond the point when the remaining error is dominated by the local truncation error τ. The computable quantity is $\widetilde{\tau}_h$. What is the relation between τ and $\widetilde{\tau}_h$? For the typical case of a second-order accurate differencing scheme,

$$\tau = \mathcal{L}_h(u) - \mathcal{L}_h(u_h) = h^2\tau_2(x,y) + \cdots \tag{20.6.37}$$

Assume the solution satisfies $u_h = u + h^2 u_2(x,y) + \cdots$. Then, assuming $\mathcal{R}$ is of high enough order that we can neglect its effect, equation (20.6.32) gives

$$\begin{aligned} \tau_h &\simeq \mathcal{L}_H(u + h^2u_2) - \mathcal{L}_h(u + h^2u_2) \\ &= \mathcal{L}_H(u) - \mathcal{L}_h(u) + h^2[\mathcal{L}'_H(u_2) - \mathcal{L}'_h(u_2)] + \cdots \\ &= (H^2 - h^2)\tau_2 + O(h^4) \end{aligned} \tag{20.6.38}$$

For the usual case of $H = 2h$, we therefore have

$$\tau \simeq \tfrac{1}{3}\tau_h \simeq \tfrac{1}{3}\widetilde{\tau}_h \tag{20.6.39}$$

The stopping criterion is thus equation (20.6.36) with

$$\epsilon = \alpha\|\widetilde{\tau}_h\|, \qquad \alpha \sim \tfrac{1}{3} \tag{20.6.40}$$

We have one remaining task before implementing our nonlinear multigrid algorithm: choosing a nonlinear relaxation scheme. Once again, your first choice should probably be the nonlinear Gauss-Seidel scheme. If the discretized equation (20.6.23) is written with some choice of ordering as

$$L_i(u_0,\ldots,u_{N-1}) = f_i, \qquad i = 0,\ldots,N-1 \tag{20.6.41}$$

then the nonlinear Gauss-Seidel schemes solves

$$L_i(u_0,\ldots,u_{i-1},u_i^{\text{new}},u_{i+1},\ldots,u_{N-1}) = f_i \tag{20.6.42}$$

for u_i^{new}. As usual, new u's replace old u's as soon as they have been computed. Often equation (20.6.42) is linear in u_i^{new}, since the nonlinear terms are discretized by means of

its neighbors. If this is not the case, we replace equation (20.6.42) by one step of a Newton iteration:

$$u_i^{\text{new}} = u_i^{\text{old}} - \frac{L_i(u_i^{\text{old}}) - f_i}{\partial L_i(u_i^{\text{old}})/\partial u_i} \tag{20.6.43}$$

For example, consider the simple nonlinear equation

$$\nabla^2 u + u^2 = \rho \tag{20.6.44}$$

In two-dimensional notation, we have

$$\mathcal{L}(u_{i,j}) = (u_{i+1,j} + u_{i-1,j} + u_{i,j+1} + u_{i,j-1} - 4u_{i,j})/h^2 + u_{i,j}^2 - \rho_{i,j} = 0 \tag{20.6.45}$$

Since

$$\frac{\partial \mathcal{L}}{\partial u_{i,j}} = -4/h^2 + 2u_{i,j} \tag{20.6.46}$$

the Newton Gauss-Seidel iteration is

$$u_{i,j}^{\text{new}} = u_{i,j} - \frac{\mathcal{L}(u_{i,j})}{-4/h^2 + 2u_{i,j}} \tag{20.6.47}$$

Here is a routine Mgfas that solves equation (20.6.44) using the full multigrid algorithm and the FAS scheme. Restriction and prolongation are done as in Mglin. We have included the convergence test based on equation (20.6.40). A successful multigrid solution of a problem should aim to satisfy this condition with the maximum number of V-cycles, maxcyc, equal to 1 or 2. The routine Mgfas uses the same functions interp and rstrct as Mglin.

mgfas.h

```
struct Mgfas {
```
Full multigrid algorithm for FAS solution of nonlinear elliptic equation, here equation (20.6.44) on a square domain of side 1, so that $h = 1/(n-1)$.
```
    Int n,ng;
    MatDoub *uj,*uj1;
    NRvector<NRmatrix<Doub> *> rho;          Vector of pointers to ρ on each level.

    Mgfas(MatDoub_IO &u, const Int maxcyc) : n(u.nrows()), ng(0)
```
On input u[0..n-1][0..n-1] contains the right-hand side ρ, while on output it returns the solution. The dimension n must be of the form $2^j + 1$ for some integer j. (j is actually the number of grid levels used in the solution, called ng below.) maxcyc is the maximum number of V-cycles to be used at each level.
```
    {
        Int nn=n;
        while (nn >>= 1) ng++;
        if ((n-1) != (1 << ng))
            throw("n-1 must be a power of 2 in mgfas.");
        nn=n;
        Int ngrid=ng-1;
        rho.resize(ng);
        rho[ngrid]=new MatDoub(nn,nn);       Allocate storage for r.h.s. on grid ng-1,
        *rho[ngrid]=u;                       and fill it with the input r.h.s.
        while (nn > 3) {                     Similarly allocate storage and fill r.h.s. by
            nn=nn/2+1;                           restriction on all coarse grids.
            rho[--ngrid]=new MatDoub(nn,nn);
            rstrct(*rho[ngrid],*rho[ngrid+1]);
        }
        nn=3;
        uj=new MatDoub(nn,nn);
        slvsm2(*uj,*rho[0]);                 Initial solution on coarsest grid.
        for (Int j=1;j<ng;j++) {             Nested iteration loop.
            nn=2*nn-1;
            uj1=uj;
            uj=new MatDoub(nn,nn);
```

```
        MatDoub temp(nn,nn);
        interp(*uj,*uj1);                  Interpolate from grid j-1 to next finer grid
        delete uj1;                            j.
        for (Int jcycle=0;jcycle<maxcyc;jcycle++) {      V-cycle loop.
            Doub trerr=1.0;                R.h.s. is dummy.
            mg(j,*uj,temp,rho,trerr);
            lop(temp,*uj);                 Form residual ||d_h||.
            matsub(temp,*rho[j],temp);
            Doub res=anorm2(temp);
            if (res < trerr) break;        No more V-cycles needed if residual small
        }                                      enough.
    }
    u = *uj;                               Return solution in u.
}

~Mgfas()
```
Destructor deletes storage.
```
{
    if (uj != NULL) delete uj;
    for (Int j=0;j<ng;j++)
        if (rho[j] != NULL) delete rho[j];
}

void matadd(MatDoub_I &a, MatDoub_I &b, MatDoub_O &c)
```
Matrix addition: Adds a[0..n-1][0..n-1] to b[0..n-1][0..n-1] and returns result in c[0..n-1][0..n-1].
```
{
    Int n=a.nrows();
    for (Int j=0;j<n;j++)
        for (Int i=0;i<n;i++)
            c[i][j]=a[i][j]+b[i][j];
}

void matsub(MatDoub_I &a, MatDoub_I &b, MatDoub_O &c)
```
Matrix subtraction: Subtracts b[0..n-1][0..n-1] from a[0..n-1][0..n-1] and returns result in c[0..n-1][0..n-1].
```
{
    Int n=a.nrows();
    for (Int j=0;j<n;j++)
        for (Int i=0;i<n;i++)
            c[i][j]=a[i][j]-b[i][j];
}

void slvsm2(MatDoub_O &u, MatDoub_I &rhs)
```
Solution of equation (20.6.44) on the coarsest grid, where $h = \frac{1}{2}$. The right-hand side is input in rhs[0..2][0..2] and the solution is returned in u[0..2][0..2].
```
{
    Doub h=0.5;
    for (Int i=0;i<3;i++)
        for (Int j=0;j<3;j++)
            u[i][j]=0.0;
    Doub fact=2.0/(h*h);
    Doub disc=sqrt(fact*fact+rhs[1][1]);
    u[1][1]= -rhs[1][1]/(fact+disc);
}

void relax2(MatDoub_IO &u, MatDoub_I &rhs)
```
Red-black Gauss-Seidel relaxation for equation (20.6.44). The current value of the solution u[0..n-1][0..n-1] is updated, using the right-hand side function rhs[0..n-1][0..n-1].
```
{
    Int n=u.nrows();
    Int jsw=1;
    Doub h=1.0/(n-1);
```

```
    Doub h2i=1.0/(h*h);
    Doub foh2 = -4.0*h2i;
    for (Int ipass=0;ipass<2;ipass++,jsw=3-jsw) {     Red and black sweeps.
        Int isw=jsw;
        for (Int j=1;j<n-1;j++,isw=3-isw) {
            for (Int i=isw;i<n-1;i+=2) {
                Doub res=h2i*(u[i+1][j]+u[i-1][j]+u[i][j+1]+u[i][j-1]-
                    4.0*u[i][j])+u[i][j]*u[i][j]-rhs[i][j];
                u[i][j] -= res/(foh2+2.0*u[i][j]);     Newton Gauss-Seidel formula.
            }
        }
    }
}

void rstrct(MatDoub_O &uc, MatDoub_I &uf)
```
Half-weighting restriction. If nc is the coarse-grid dimension, the fine-grid solution is input in uf[0..2*nc-2][0..2*nc-2]. The coarse-grid solution obtained by restriction is returned in uc[0..nc-1][0..nc-1].

```
{
    Int nc=uc.nrows();
    Int ncc=2*nc-2;
    for (Int jf=2,jc=1;jc<nc-1;jc++,jf+=2) {      Interior points.
        for (Int iif=2,ic=1;ic<nc-1;ic++,iif+=2) {
            uc[ic][jc]=0.5*uf[iif][jf]+0.125*(uf[iif+1][jf]+uf[iif-1][jf]
                +uf[iif][jf+1]+uf[iif][jf-1]);
        }
    }
    for (Int jc=0,ic=0;ic<nc;ic++,jc+=2) {        Boundary points.
        uc[ic][0]=uf[jc][0];
        uc[ic][nc-1]=uf[jc][ncc];
    }
    for (Int jc=0,ic=0;ic<nc;ic++,jc+=2) {
        uc[0][ic]=uf[0][jc];
        uc[nc-1][ic]=uf[ncc][jc];
    }
}

void lop(MatDoub_O &out, MatDoub_I &u)
```
Given u[0..n-1][0..n-1], returns $\mathcal{L}_h(\widetilde{u}_h)$ for eqn. (20.6.44) in out[0..n-1][0..n-1].

```
{
    Int n=u.nrows();
    Doub h=1.0/(n-1);
    Doub h2i=1.0/(h*h);
    for (Int j=1;j<n-1;j++)                        Interior points.
        for (Int i=1;i<n-1;i++)
            out[i][j]=h2i*(u[i+1][j]+u[i-1][j]+u[i][j+1]+u[i][j-1]-
                4.0*u[i][j])+u[i][j]*u[i][j];
    for (Int i=0;i<n;i++)                          Boundary points.
        out[i][0]=out[i][n-1]=out[0][i]=out[n-1][i]=0.0;
}

void interp(MatDoub_O &uf, MatDoub_I &uc)
```
Coarse-to-fine prolongation by bilinear interpolation. If nf is the fine-grid dimension, the coarse-grid solution is input as uc[0..nc-1][0..nc-1], where nc $= $ nf$/2+1$. The fine-grid solution is returned in uf[0..nf-1][0..nf-1].

```
{
    Int nf=uf.nrows();
    Int nc=nf/2+1;
    for (Int jc=0;jc<nc;jc++)              Do elements that are copies.
        for (Int ic=0;ic<nc;ic++) uf[2*ic][2*jc]=uc[ic][jc];
    for (Int jf=0;jf<nf;jf+=2)             Do even-numbered columns, interpolating ver-
        for (Int iif=1;iif<nf-1;iif+=2)        tically.
            uf[iif][jf]=0.5*(uf[iif+1][jf]+uf[iif-1][jf]);
    for (Int jf=1;jf<nf-1;jf+=2)           Do odd-numbered columns, interpolating hor-
                                               izontally.
```

```
        for (Int iif=0;iif<nf;iif++)
            uf[iif][jf]=0.5*(uf[iif][jf+1]+uf[iif][jf-1]);
}

Doub anorm2(MatDoub_I &a)
```

Returns the Euclidean norm of the matrix a[0..n-1][0..n-1].

```
{
    Doub sum=0.0;
    Int n=a.nrows();
    for (Int j=0;j<n;j++)
        for (Int i=0;i<n;i++)
            sum += a[i][j]*a[i][j];
    return sqrt(sum)/n;
}

void mg(const Int j, MatDoub_IO &u, MatDoub_I &rhs,
    NRvector<NRmatrix<Doub> *> &rho, Doub &trerr)
```

Recursive multigrid iteration. On input, j is the current level and u is the current value of the solution. For the first call on a given level, the right-hand side is zero, and the argument rhs is dummy. This is signaled by inputting trerr positive. Subsequent recursive calls supply a nonzero rhs as in equation (20.6.33). This is signaled by inputting trerr negative. rho is the vector of pointers to ρ on each level. On output u contains the improved solution at the current level. When the first call on a given level is made, the relative truncation error τ is returned in trerr.

```
{
    const Int NPRE=1,NPOST=1;
```

Number of relaxation sweeps before and after the coarse-grid correction is computed.

```
    const Doub ALPHA=0.33;              Relates the estimated truncation error to the
                                            norm of the residual.
    Doub dum=-1.0;
    Int nf=u.nrows();
    Int nc=(nf+1)/2;
    MatDoub temp(nf,nf);
    if (j == 0) {                       Bottom of V: Solve on coarsest grid.
        matadd(rhs,*rho[j],temp);
        slvsm2(u,temp);
    } else {                            On downward stoke of the V.
        MatDoub v(nc,nc),ut(nc,nc),tau(nc,nc),tempc(nc,nc);
        for (Int jpre=0;jpre<NPRE;jpre++) {     Pre-smoothing.
            if (trerr < 0.0) {
                matadd(rhs,*rho[j],temp);
                relax2(u,temp);
            }
            else
                relax2(u,*rho[j]);
        }
        rstrct(ut,u);                   $\mathcal{R}\tilde{u}_h$.
        v=ut;                           Make a copy in v.
        lop(tau,ut);                    $\mathcal{L}_H(\mathcal{R}\tilde{u}_h)$ stored temporarily in $\tilde{\tau}_h$.
        lop(temp,u);                    $\mathcal{L}_h(\tilde{u}_h)$.
        if (trerr < 0.0)                $\mathcal{L}_h(\tilde{u}_h) - f_h$.
            matsub(temp,rhs,temp);
        rstrct(tempc,temp);             $\mathcal{R}\mathcal{L}_h(\tilde{u}_h) - f_H$.
        matsub(tau,tempc,tau);          $\tilde{\tau}_h + f_H = \mathcal{L}_H(\mathcal{R}\tilde{u}_h) - \mathcal{R}\mathcal{L}_h(\tilde{u}_h) + f_H$.
        if (trerr > 0.0)
            trerr=ALPHA*anorm2(tau);    Estimate truncation error $\tau$.
        mg(j-1,v,tau,rho,dum);          Recursive call for the coarse-grid correction.
        matsub(v,ut,tempc);             On upward stroke of V, form $\tilde{u}_h^{\text{new}} = \tilde{u}_h +$
        interp(temp,tempc);                 $\mathcal{P}(\tilde{u}_H - \mathcal{R}\tilde{u}_h)$.
        matadd(u,temp,u);
        for (Int jpost=0;jpost<NPOST;jpost++) {     Post-smoothing.
            if (trerr < 0.0) {
                matadd(rhs,*rho[j],temp);
                relax2(u,temp);
            }
```

```
                else
                    relax2(u,*rho[j]);
            }
        }
    }
};
```

CITED REFERENCES AND FURTHER READING:

Brandt, A. 1977, "Multilevel Adaptive Solutions to Boundary-Value Problems," *Mathematics of Computation*, vol. 31, pp. 333–390.[1]

Brandt, A. 1982, in *Multigrid Methods*, W. Hackbusch and U. Trottenberg, eds. (Springer Lecture Notes in Mathematics No. 960) (New York: Springer).[2]

Hackbusch, W. 1985, *Multi-Grid Methods and Applications* (New York: Springer).[3]

Stuben, K., and Trottenberg, U. 1982, in *Multigrid Methods*, W. Hackbusch and U. Trottenberg, eds. (Springer Lecture Notes in Mathematics No. 960) (New York: Springer), pp. 1–176.[4]

Briggs, W.L., Henson, V.E., and McCormick, S. 2000, *A Multigrid Tutorial* (Cambridge, UK: Cambridge University Press).[5]

Trottenberg, U., Oosterlee, C.W., and Schuller, A. 2001, *Multigrid* (Cambridge, MA: Academic Press).[6]

McCormick, S., and Rude, U., eds. 2006, "Multigrid Computing," special issue of *Computing in Science and Engineering*, vol. 8, No. 6 (November/December), pp. 10–62.

Hackbusch, W., and Trottenberg, U. (eds.) 1991, *Multigrid Methods III* (Basel: Birkhäuser).

Wesseling, P. 1992, *An Introduction to Multigrid Methods* (New York: Wiley); corrected reprint 2004 (Philadelphia: R.T. Edwards).

20.7 Spectral Methods

Spectral methods are a very powerful tool for solving PDEs. When they can be used, they are the method of choice if you need high spatial resolution in multidimensions. For a second-order accurate finite difference code in three dimensions, increasing the resolution by a factor of 2 in each dimension requires eight times as many grid points, and improves the error typically by a factor of 4. In a spectral code, a similar increase in resolution often gives an improvement of a factor of 10^6. Even for one-dimensional problems, spectral methods will amaze you with their power and efficiency.

Spectral methods work well for smooth solutions. Discontinuities like shocks are bad — don't even try spectral methods. Even mild nonsmoothness (like a discontinuity in some high-order derivative of the solution) can spoil the convergence of spectral methods. (Actually, getting spectral methods to work with discontinuities and shocks is an active research area; see [1] for an introduction.)

The key difference between finite difference methods and spectral methods is that in finite difference methods you approximate the *equation* you are trying to solve, whereas in spectral methods you approximate the *solution* you are trying to find. While finite differencing replaces the continuum equation by an equation on grid points, a spectral method expresses the solution as a truncated expansion in a set

of basis functions:

$$f(x) \simeq f_N(x) = \sum_{n=0}^{N} a_n \phi_n(x) \tag{20.7.1}$$

Different choices of basis functions and methods of computing a_n give different flavors of spectral methods.

20.7.1 Example

We illustrate the idea of spectral methods with an example. Consider the one-sided wave equation (advective equation) in one dimension:

$$\frac{\partial u}{\partial t} = \frac{\partial u}{\partial x} \tag{20.7.2}$$

with periodic boundary conditions on $[0, 2\pi]$ and initial condition

$$u(t = 0, x) \;=\; f(x) \tag{20.7.3}$$

You get the analytic spectral solution by expanding u in a Fourier series,

$$u(t, x) = \sum_{n=-\infty}^{\infty} a_n(t) e^{inx} \tag{20.7.4}$$

Substituting this expansion into equation (20.7.2) gives

$$\frac{da_n}{dt} = ina_n \tag{20.7.5}$$

with solution

$$a_n(t) = a_n(0)e^{int} \tag{20.7.6}$$

You get $a_n(0)$ from the initial condition: Expand

$$f(x) = \sum_{n=-\infty}^{\infty} f_n e^{inx} \tag{20.7.7}$$

from which you see that

$$a_n(0) = f_n \tag{20.7.8}$$

For example, suppose

$$f(x) = \sin(\pi \cos x) \tag{20.7.9}$$

which gives the analytic solution

$$u(t, x) = \sin[\pi \cos(x + t)] \tag{20.7.10}$$

The spectral coefficients in the solution (20.7.4) are

$$\begin{aligned} a_n(0) &= \frac{1}{2\pi} \int_0^{2\pi} \sin(\pi \cos x) e^{-inx}\, dx \\ &= (-1)^{(n-1)/2} J_n(\pi), \qquad n \text{ odd} \end{aligned} \tag{20.7.11}$$

In a numerical version of this spectral solution, we would truncate the expansion at $n = N$. How well does $u_N(t, x)$ approximate the exact solution? One measure is the root-mean-square error,

$$
\begin{aligned}
L_2 &= \left[\frac{1}{2\pi} \int_0^{2\pi} |u(t,x) - u_N(t,x)|^2 \, dx \right]^{1/2} \\
&= \left[\frac{1}{2\pi} \int_0^{2\pi} \left| \sum_{|n|>N} a_n(0) e^{inx} e^{int} \right|^2 dx \right]^{1/2} \\
&= \left[\sum_{|n|>N} |a_n(0)|^2 \right]^{1/2} \qquad (20.7.12)
\end{aligned}
$$

Now $J_n(\pi)$ goes to zero exponentially as $n \to \infty$, so the error decreases *exponentially* with N for any $t \geq 0$. This is the key feature of a good spectral method, one you should always strive for. By contrast, a second-order finite difference method has an error that scales as $1/N^2$.

This exponential convergence of spectral methods sets in when one has resolved the main features of the solution. In the above example, the Bessel functions go rapidly to zero once $n \gtrsim \pi$, which corresponds to having about π basis functions per wavelength. On can show that this is a general property of spectral methods [2]. By contrast, second-order accurate finite differencing needs about 20 points per wavelength for 1% accuracy [2]. Moreover, once the solution is resolved, the accuracy improves much more quickly with spectral methods.

There are three properties of the functions e^{inx} that are crucial for this analytic spectral solution, which is just the separation of variables technique:

1. They are a complete set of basis functions.
2. Each basis function by itself obeys the boundary conditions.
3. They are eigenfunctions of the operator in the problem, d/dx.

As we'll see, only property 1 is essential for numerical spectral methods. Spectral methods are not limited to Fourier series — a wide choice of basis functions can be used.

20.7.2 Choice of Basis Functions

You can't simply use Fourier series as basis functions for all problems — it depends on the boundary conditions. Here is a recipe that will take care of 99% of cases you'll encounter:

- If the solution is periodic, use Fourier series.
- If the solution is not periodic and the domain is a square or a cube, or can be mapped to a rectangular region by a simple coordinate transformation, use Chebyshev polynomials along each dimension.
- If the domain is spherical, use spherical harmonics for the angles. In the radial direction, use Chebyshev polynomials for a spherical shell. For a sphere that includes the origin, use the radial basis functions in [8]. These incorporate the correct analytic behavior at the origin and are much better than other choices. They can also be used for cylindrical domains. If the domain is infinite, consult [9,10,4].

Expansions based on for example Chebyshev or Legendre polynomials have the property that their convergence rate is governed by the smoothness of the solution only, not the boundary conditions it satisfies. Fourier expansions, on the other hand, require periodic boundary conditions as well as smoothness for rapid convergence. (These properties are proved, e.g., in [2]. The key point is that basis functions whose convergence rate is independent of the boundary conditions are solutions of singular Sturm-Liouville equations.) It is this independence from the details of the boundary conditions that makes basis functions like Chebyshev polynomials "magical."

Another reason for the popularity of Chebyshev polynomials is that they are really just trigonometric functions whose argument θ has been mapped by $x = \cos\theta$:

$$T_n(x) = \cos(n\theta), \qquad x = \cos\theta \tag{20.7.13}$$

Thus an expansion in Chebyshev polynomials can be evaluated efficiently by the FFT. Moreover, the derivatives of such an expansion can also be evaluated by FFT techniques, as discussed below.

For spherical domains, spherical harmonics are products of Legendre functions in $\cos\theta$ and Fourier series in ϕ. Once again one gets exponential convergence for smooth functions.

20.7.3 Computing the Expansion Coefficients

How do we compute the a_n? There are three basic ways, which can be compared by considering the residual when the expansion (20.7.1) is substituted into the equation you are trying to solve:

1. *Tau method.* Here we require that the a_n be computed so that the boundary conditions are satisfied, and that the residual be orthogonal to as many of the basis functions as possible.
2. *Galerkin method.* In this case you combine the basis functions into a new set, each of which satifies the boundary conditions. Then make the residual orthogonal to as many of the new basis functions as possible. (This is essentially what you do when you separate variables in solving a PDE, as we did for equation 20.7.2. Usually you start with basis functions that already satisfy the boundary conditions individually.)
3. *Collocation* or *pseudospectral method.* As in the tau method, require the boundary conditions to be satisfied, but make the residual *zero* at a set of suitably chosen points.

As we will see, the pseudospectral method has an alternative interpretation that makes it very easy to use. Accordingly, we will only discuss this method, leaving the others to the references.

The big advantage of the pseudospectral method is that it is easy to implement for nonlinear problems. Instead of working with the spectral coefficients, as with the other two methods, you work with the values of the solution at the special grid points associated with the basis functions (typically, the Gaussian quadrature points). These are called the *collocation points.* Often we say we are working with the solution in *physical space* as opposed to in *spectral space*.

A pseudospectral method is an *interpolating* method: Think of the representation

$$y(x) = \sum_{n=0}^{N} a_n \phi_n(x) \tag{20.7.14}$$

as a polynomial that interpolates the solution. Require this interpolating polynomial to be exactly equal to the solution at the $N+1$ collocation points. If we do things right, then as $N \to \infty$, the errors in between the points tend to zero exponentially fast.

20.7.4 Spectral Methods and Gaussian Quadrature

Recall the formula for Gaussian quadrature (§4.6.1):

$$\int_a^b y(x)w(x)\,dx \approx \sum_{i=0}^{N} w_i y(x_i) \tag{20.7.15}$$

Here $w(x)$ is the so-called *weight function* that typically factors out some singular behavior of the integrand, leaving $y(x)$ as a smooth function. The formula is derived by choosing the $2N+2$ weights and abscissas, w_i and x_i, by requiring that the formula be exact for the polynomials $1, x, x^2, \dots, x^{2N+1}$. (Don't be confused by the notation: There is no direct relationship between w_i and $w(x)$.) As shown in §4.6, Gaussian quadrature is related to the orthogonal polynomials $\phi_n(x)$ with the given weight function:

$$\langle \phi_n | \phi_m \rangle \equiv \int_a^b \phi_n(x)\phi_m(x)w(x)\,dx = \delta_{mn} \tag{20.7.16}$$

The abscissas x_i turn out to be the $N+1$ roots of $\phi_{N+1}(x)$, and the weights w_i are given by equation (4.6.9).

We can use Gaussian quadrature to define the discrete inner product of two functions:

$$\langle f | g \rangle_{\mathrm{G}} \equiv \sum_{i=0}^{N} w_i f(x_i) g(x_i) \tag{20.7.17}$$

Here the subscript G stands for Gaussian.

An important property of Gaussian quadrature is the *discrete orthogonality relation*

$$\langle \phi_n | \phi_m \rangle_{\mathrm{G}} = \delta_{mn}, \qquad m+n \le 2N+1 \tag{20.7.18}$$

Proof: Equation (20.7.18) is the Gaussian quadrature version of equation (20.7.16). By assumption, the integrand $\phi_n(x)\phi_m(x)$ of equation (20.7.16) is a polynomial of degree $m+n \le 2N+1$. But Gaussian quadrature is arranged to integrate polynomials of degree $\le 2N+1$ exactly. QED.

Now suppose we approximate $y(x)$ by the pseudospectral interpolating polynomial

$$P_N(x) = \sum_{n=0}^{N} \bar{a}_n \phi_n(x) \tag{20.7.19}$$

where the collocation points are chosen to be the Gaussian quadrature points:

$$P_N(x_i) = y(x_i), \qquad i = 0, 1, \ldots, N \tag{20.7.20}$$

This is always possible, since the interpolating polynomial through $N+1$ points is a polynomial of degree N, and the functions up to $\phi_N(x)$ are a basis for such polynomials. The perhaps unexpected result is that the coefficients $\{\overline{a}_n\}$ of the expansion (20.7.19) are given *exactly* by the Gaussian quadrature

$$\overline{a}_n = \langle y|\phi_n\rangle_{\mathrm{G}} \tag{20.7.21}$$

To see this, take the discrete inner product of both sides of equation (20.7.19) with ϕ_m:

$$\langle P_N|\phi_m\rangle_{\mathrm{G}} = \sum_{n=0}^{N} \overline{a}_n \langle\phi_n|\phi_m\rangle_{\mathrm{G}} \tag{20.7.22}$$

If we use the discrete orthogonality relation (20.7.18), the right-hand side evaluates to $\overline{a}_m$. On the left-hand side, we can replace $P_N(x_i)$ in the Gaussian quadrature by $y(x_i)$ since P_N is the interpolating polynomial. Hence the result follows.

Now comes the key point. The actual spectral expansion of $y(x)$ is

$$y(x) = \sum_{n=0}^{\infty} a_n \phi_n(x) \tag{20.7.23}$$

where the *exact* spectral coefficients are

$$a_n = \langle y|\phi_n\rangle = \int_a^b y(x)\phi_n(x)w(x)\,dx \tag{20.7.24}$$

The pseudospectral expansion coefficients $\overline{a}_n$ are the exact expansion coefficients of $P_N(x)$, the interpolating polynomial (20.7.19). The relation between the exact spectral coefficients and the pseudospectral expansion coefficients follows from equation (20.7.21):

$$\begin{aligned}
\overline{a}_n &= \langle y|\phi_n\rangle_{\mathrm{G}} \\
&= \sum_{m=0}^{\infty} a_m \langle\phi_m|\phi_n\rangle_{\mathrm{G}} \qquad \text{(using equation 20.7.23)} \\
&= \sum_{m=0}^{N} a_m \langle\phi_m|\phi_n\rangle_{\mathrm{G}} + \sum_{m>N} a_m \langle\phi_m|\phi_n\rangle_{\mathrm{G}} \\
&= a_n + \sum_{m>N} a_m \langle\phi_m|\phi_n\rangle_{\mathrm{G}}
\end{aligned} \tag{20.7.25}$$

Thus, since for large N the exact spectral coefficients give an exponentially good approximation to $y(x)$, so do the pseudospectral coefficients. By the way, this is the reason for the name pseudospectral method: We use coefficients that are not the actual spectral coefficients, but are very close to them. From now on we won't bother to distinguish between the two sets of coefficients; we just write a_n for either a_n or $\overline{a}_n$.

The Gaussian quadrature collocation points, the roots of $\phi_{N+1}(x)$, all lie inside the interval (a,b), away from the endpoints. There is another version of Gaussian quadrature that includes the two endpoints of the interval. This is called Gauss-Lobatto quadrature, and the collocation points are the Gauss-Lobatto points (§4.6.4). These points are as effective as the ordinary Gaussian points, and are more convenient when you need to impose boundary conditions at the endpoints.

As a slight digression, you may be under the mistaken impression that the only advantage of Gaussian integration over integration with equally spaced points is that its degree of exactness is $2N+1$ as opposed to N, the maximum you can get with only the $N+1$ weights at your disposal. In fact, however, the main advantage of Gaussian integration is that it converges exponentially with N for smooth functions. You can see this explicitly from the above formulas by setting $m=0$ in equation (20.7.21):

$$\bar{a}_0 = \phi_0 \sum_{i=0}^{N} w_i y(x_i) \tag{20.7.26}$$

where ϕ_0 is a constant. But this converges exponentially to the expression given by equation (20.7.24):

$$a_0 = \phi_0 \int_a^b y(x)w(x)\,dx \tag{20.7.27}$$

as claimed.

How do Fourier series fit into this discussion? After all, the collocation points are equally spaced (usually $x_j = 2\pi j/N$, $j=0,\ldots,N-1$). But in fact these are the correct collocation points if we think of Fourier series as interpolating $y(x)$ by a *trigonometric* polynomial. The corresponding Gaussian quadrature (using the equally spaced points) is the midpoint rule, and the Gauss-Lobatto quadrature, which includes the endpoints, is the trapezoidal rule. The textbooks tell you that the midpoint and trapezoidal rules are low-order methods. This is true for arbitrary functions. But if you apply them to *periodic* functions (§5.8.1), or functions that go rapidly to zero at infinity (§4.5 and §13.11), they are in fact exponentially convergent, like any self-respecting Gaussian quadrature method should be.

20.7.5 Cardinal Functions

You can write *any* polynomial interpolation formula for a function $f(x)$ as

$$P_N(x) = \sum_{i=0}^{N} f(x_i)C_i(x) \tag{20.7.28}$$

where the $C_i(x)$ are called *cardinal functions*. They are polynomials of degree N that satisfy

$$C_i(x_j) = \delta_{ij} \tag{20.7.29}$$

i.e., $C_i(x)$ is 1 at the ith collocation point and 0 at all the others.

One explicit representation of cardinal functions comes from the formula for Lagrange interpolation (see equation 3.2.1):

$$C_i(x) = \prod_{\substack{j=0\\ j\neq i}}^{N} \frac{x-x_j}{x_i-x_j} \tag{20.7.30}$$

If you substitute this in equation (20.7.28), it is just the Lagrange interpolation formula. Each choice of basis functions implies a corresponding choice of collocation points x_j, and so a corresponding choice of cardinal functions by equation (20.7.30).

There are other equivalent ways of writing $C_i(x)$. For example, if $\phi_n(x)$ is a set of orthogonal polynomials, and the collocation points are the zeros of $\phi_{N+1}(x)$ (Gaussian quadrature points), then $C_i(x)$ is almost $\phi_{N+1}(x)$, except $\phi_{N+1}(x)$ vanishes at *all* the grid points. Since near $x = x_i$

$$\phi_{N+1}(x) = \phi_{N+1}(x_i) + (x - x_i)\phi'_{N+1}(x_i) + \cdots \tag{20.7.31}$$

we get the cardinal function by dividing out the zero at $x = x_i$:

$$C_i(x) = \frac{\phi_{N+1}(x)}{(x - x_i)\phi'_{N+1}(x_i)} \tag{20.7.32}$$

In practice you don't need to know any of the formulas like equations (20.7.30) or (20.7.32). The books in the references have formulas for the $C_i(x)$ for all the standard basis functions if you are curious. What you do need are the derivatives of the cardinal functions, the *differentiation matrices* (see below).

You might be nervous about using very high-order polynomial interpolation to represent your solution, especially if you've ever encountered the *Runge phenomenon*: If the grid points are *equally spaced*, then the error in $P_N(x)$ can tend to infinity as $N \to \infty$. What happens is that the error shows up near the endpoints of the interval — the middle is fine. The fix is to make the points more concentrated toward the endpoints, which is exactly what choosing the Gaussian points does. This is the same reason why Chebyshev approximation often works when polynomial approximation fails, as was discussed in §5.8.1.

20.7.6 Spectral vs. Grid Point Representation

Let's contrast the representations of the solution of

$$\mathcal{L}y = f \tag{20.7.33}$$

in spectral space and in physical space. Assume that $\mathcal{L}$ is a linear differential operator for simplicity.

**Spectral Space**	**Physical Space**
$y(x) = \sum_{n=0}^{N} a_n \phi_n(x)$	$y(x) = \sum_{j=0}^{N} y_j C_j(x)$
$\sum_{n=0}^{N} a_n \mathcal{L}\phi_n(x) = f(x)$	$\sum_{j=0}^{N} y_j \mathcal{L}C_j(x) = f(x)$

Impose at collocation points only:

$\sum_{n=0}^{N} a_n \mathcal{L}\phi_n(x_j) = f(x_j)$	$\sum_{j=0}^{N} y_j \mathcal{L}C_j(x_i) = f(x_i)$
i.e., $La = f$, where $L_{jn} = \mathcal{L}\phi_n(x_j)$	i.e., $L^{(c)}y = f$, where $L^{(c)}_{ij} = \mathcal{L}C_j(x_i)$

The two representations are related as follows: To go from grid point values to spectral coefficients you project $y(x)$ along each basis function:

$$\begin{aligned} a_i &= \langle \phi_i \mid y \rangle \\ &= \sum_j w_j \phi_i(x_j) y_j \qquad \text{(doing the integral by Gaussian quadrature)} \end{aligned} \tag{20.7.34}$$

That is,

$$\mathbf{a} = \mathbf{M} \cdot \mathbf{y}, \qquad \text{where} \quad M_{ij} = \phi_i(x_j)w_j \tag{20.7.35}$$

Thus the relation in spectral space $\mathbf{L} \cdot \mathbf{a} = \mathbf{f}$ becomes $\mathbf{L} \cdot \mathbf{M} \cdot \mathbf{y} = \mathbf{f}$. But in physical space $\mathbf{L}^{(c)} \cdot \mathbf{y} = \mathbf{f}$, so

$$\mathbf{L}^{(c)} = \mathbf{L} \cdot \mathbf{M} \tag{20.7.36}$$

with inverse

$$\mathbf{L} = \mathbf{L}^{(c)} \cdot \mathbf{M}^{-1} \tag{20.7.37}$$

Note also that equation (20.7.35) implies

$$\mathbf{y} = \mathbf{M}^{-1} \cdot \mathbf{a} \tag{20.7.38}$$

Since $y = \sum a_n \phi_n$, we see that $\mathbf{M}^{-1}$ is the matrix that sums the spectral series to get the grid point values, i.e.,

$$M^{-1}_{ij} = \phi_j(x_i) \tag{20.7.39}$$

You can check that these relations are all consistent:

$$\begin{aligned}
(\mathbf{M} \cdot \mathbf{M}^{-1})_{ij} &= \sum_k M_{ik} M^{-1}_{kj} \\
&= \sum_k [\phi_i(x_k)w_k][\phi_j(x_k)] \\
&= \langle \phi_i | \phi_j \rangle_{\mathrm{G}} \\
&= \delta_{ij} \qquad \text{(by discrete orthogonality)}
\end{aligned} \tag{20.7.40}$$

In practice, the transformations (20.7.35) and (20.7.38) are often done with FFTs for Fourier or Chebyshev basis functions if N is large. For simple programs, just do matrix multiplication.

20.7.7 Differentiation Matrices

We've seen above that the key ingredient in the pseudospectral method is to form

$$L^{(c)}_{ij} = \mathcal{L} C_j(x_i) \tag{20.7.41}$$

which involves taking derivatives of the cardinal functions at the collocation points. Consider the first derivative ∂_x. You then need the matrix

$$D^{(1)}_{ij} = \partial_x C_j(x_i) \tag{20.7.42}$$

This quantity can be computed ahead of time and stored. Then, to compute the vector of first derivatives at the grid points, just do a matrix multiplication:

$$\frac{\partial y}{\partial x} \quad \longleftrightarrow \quad \sum_{j=0}^{N} D^{(1)}_{ij} y_j \tag{20.7.43}$$

Similarly one can define the second derivative matrix $D^{(2)}_{ij}$, and so on.

The matrix multiplication in equation (20.7.43) requires $O(N^2)$ operations. For Fourier basis functions e^{ikx}, one can compute the derivative alternatively as follows:

$$\begin{aligned} y &\xrightarrow{\text{FFT}} a \\ a &\longrightarrow ika \\ ika &\xrightarrow{\text{inverse FFT}} y' \end{aligned}$$

For Chebyshev basis functions, there is a simple $O(N)$ recurrence relation in the middle step to get the coefficients for the derivative from the coefficients for the function (see equation 5.9.2). Thus the procedure is $O(N \log N)$. However, it is typically faster than the $O(N^2)$ matrix multiplication only for $N \gtrsim 16-128$, depending on the computer. So just use matrix multiplication for simple programs.

It is worth pointing out that this idea of using recurrence relations to evaluate operators in spectral space is much more general than the simple example of derivatives of Chebyshev functions. It is important for efficient production codes when the operators consist of derivatives times simple powers of the coordinates. See the references for details.

20.7.8 Computing Differentiation Matrices

There are several options for computing differentiation matrices:

1. Derive the formulas by differentiating the Lagrange polynomial representation (20.7.30).
2. Differentiate the basis function representation (20.7.32).
3. Look up the explicit formulas that have been derived for the various basis functions in books, e.g. Chapter 2 of [3].
4. Use the routine given below, based on the routine in [6]. This algorithm computes any order of differentiation matrix given only a set of collocation points $\{x_i\}$.

Obviously, the last choice is the easiest. However, it does have the potential drawback for high-precision work that roundoff error can be larger than necessary. If this is a problem, see [7].

weights.h

```
void weights(const Doub z, VecDoub_I &x, MatDoub_O &c)
```

Compute the differentiation matrices for pseudospectral collocation. Input are z, the location where the matrices are to be evaluated, and x[0..n], the set of n+1 grid points. On output, c[0..n][0..m] contains the weights at grid locations x[0..n] for derivatives of order 0..m. The element c[j][k] contains the weight to be applied to the function value at x[j] when the kth derivative is approximated by the set of $n+1$ collocation points x. Note that the elements of the zeroth derivative matrix are returned in c[0..n][0]. These are just the values of the cardinal functions, i.e., the weights for interpolation.

```
{
    Int n=c.nrows()-1;
    Int m=c.ncols()-1;
    Doub c1=1.0;
    Doub c4=x[0]-z;
    for (Int k=0;k<=m;k++)
        for (Int j=0;j<=n;j++)
            c[j][k]=0.0;
    c[0][0]=1.0;
    for (Int i=1;i<=n;i++) {
        Int mn=MIN(i,m);
```

```
        Doub c2=1.0;
        Doub c5=c4;
        c4=x[i]-z;
        for (Int j=0;j<i;j++) {
            Doub c3=x[i]-x[j];
            c2=c2*c3;
            if (j == i-1) {
                for (Int k=mn;k>0;k--)
                    c[i][k]=c1*(k*c[i-1][k-1]-c5*c[i-1][k])/c2;
                c[i][0]=-c1*c5*c[i-1][0]/c2;
            }
            for (Int k=mn;k>0;k--)
                c[j][k]=(c4*c[j][k]-k*c[j][k-1])/c3;
            c[j][0]=c4*c[j][0]/c3;
        }
        c1=c2;
    }
}
```

Typical usage of the weights routine to compute first- and second-order derivative matrices is

```
    VecDoub x(n);
    MatDoub c(n,3),d1(n,n),d2(n,n);
    for (j=0;j<n;j++)
        x[j]= ...
    for (i=0;i<n;i++) {
        weights(x[i],x,c);
        for (j=0;j<n;j++) {
            d1[i][j]=c[j][1];
            d2[i][j]=c[j][2];
        }
    }
```

20.7.9 A Note on Interpolation

Often you want to evaluate the solution at points that are not the collocation points. This requires an interpolation. To preserve the full spectral accuracy, you want to use all the information in the solution. However, it is not necessary to transform the solution to spectral space and then evaluate the representation (20.7.1) at the desired point, e.g., by Clenshaw's method. Just use the interpolation formula (20.7.28). A simple way to do this is to use the above routine, which will return the interpolation weights $C_i(x_k)$ for any set of target points x_k when m, the second dimension of c in the code, is zero. So interpolating to a set of points can again be done as a matrix multiplication.

20.7.10 Pseudospectral Collocation as a Finite Difference Method

Consider finite difference approximations for d/dx at the center of an equally spaced grid, for example

$$\begin{aligned} hf'(x) &= -\tfrac{1}{2}f(x-h) + \tfrac{1}{2}f(x+h) + O(h^2) \\ &= \tfrac{1}{12}f(x-2h) - \tfrac{2}{3}f(x-h) + \tfrac{2}{3}f(x+h) - \tfrac{1}{12}f(x+2h) + O(h^4) \\ &= \quad \ldots \end{aligned} \tag{20.7.44}$$

For centered differences like these, the limit as $N \to \infty$ of the weights (coefficients of f) is finite. But for one-sided approximations, or partially one-sided approximations, the weights diverge [5]. Since one has to use such approximations near the endpoints of the grid, it's not surprising that high-order finite difference approximations have large errors near the boundaries.

But suppose the grid points are not equally spaced. In particular, suppose they are closer together near the endpoints, like the Gaussian quadrature points. Then the finite difference approximation is convergent as $N \to \infty$.

The pseudospectral method gives the exact derivative of the interpolating polynomial that passes through the data at the $N + 1$ grid points. You would get the same result for a finite difference method that uses *all* $N + 1$ grid points, This follows from the uniqueness of the interpolating polynomial, a polynomial of degree N through all $N + 1$ points.

With this point of view, think of a pseudospectral method as a way to find high-order numerical approximations to derivatives at grid points. Then, just like finite difference methods, satisfy the equation you want to solve at the grid points.

20.7.11 Variable Coefficients and Nonlinearities

Suppose you have a term like $\sinh(x)\, y(x)$ in your equation. No need to expand $\sinh(x)$ in basis functions — just multiply $\sinh(x)$ by y at each collocation point. Similarly, nonlinear terms like y^2 are evaluated directly using the values at the collocation points. This is the big advantage over the tau and Galerkin methods — handling nonlinearities in physical space rather than spectral space is much easier.

20.7.12 A Worked Example

Here is a simple one-dimensional example, taken from Appendix B of [5]. Consider the equation

$$y'' + y' - 2y + 2 = 0, \qquad -1 \le x \le 1, \tag{20.7.45}$$

$$y(-1) = y(1) = 0 \tag{20.7.46}$$

The exact solution is

$$y(x) = 1 - \frac{e^x \sinh 2 + e^{-2x} \sinh 1}{\sinh 3} \tag{20.7.47}$$

Let's make an expansion in Chebyshev polynomials with $N = 4$:

$$y = \sum_{n=0}^{4} a_n T_n(x) \tag{20.7.48}$$

Choose the collocation points to be

$$x_i = -\cos\frac{i\pi}{4}, \qquad i = 0, \ldots, 4 \tag{20.7.49}$$

These are the Gauss-Lobatto points associated with Chebyshev polynomials, i.e., they include the endpoints. We always include the endpoints when we want to impose Dirichlet boundary conditions, that is, function values on the boundaries. Using

one of the methods for finding differentiation matrices, we get

$$[D^{(1)}y]_i = \begin{bmatrix} -\frac{11}{2} & 4+2\sqrt{2} & -2 & 4-2\sqrt{2} & -\frac{1}{2} \\ -1-\frac{1}{2}\sqrt{2} & \frac{1}{2}\sqrt{2} & \sqrt{2} & -\frac{1}{2}\sqrt{2} & 1-\frac{1}{2}\sqrt{2} \\ \frac{1}{2} & -\sqrt{2} & 0 & \sqrt{2} & -\frac{1}{2} \\ -1+\frac{1}{2}\sqrt{2} & \frac{1}{2}\sqrt{2} & -\sqrt{2} & -\frac{1}{2}\sqrt{2} & 1+\frac{1}{2}\sqrt{2} \\ \frac{1}{2} & -4+2\sqrt{2} & 2 & -4-2\sqrt{2} & \frac{11}{2} \end{bmatrix} \begin{bmatrix} y_0 \\ y_1 \\ y_2 \\ y_3 \\ y_4 \end{bmatrix} \tag{20.7.50}$$

and

$$[D^{(2)}y]_i = \begin{bmatrix} 17 & -20-6\sqrt{2} & 18 & -20+6\sqrt{2} & 5 \\ 5+3\sqrt{2} & -14 & 6 & -2 & 5-3\sqrt{2} \\ -1 & 4 & -6 & 4 & -1 \\ 5-3\sqrt{2} & -2 & 6 & -14 & 5+3\sqrt{2} \\ 5 & -20+6\sqrt{2} & 18 & -20-6\sqrt{2} & 17 \end{bmatrix} \begin{bmatrix} y_0 \\ y_1 \\ y_2 \\ y_3 \\ y_4 \end{bmatrix} \tag{20.7.51}$$

Requiring that the differential equation hold at the interior collocation points x_k, $k = 1, 2, 3$, uses the middle three rows of these matrices. Enforcing the boundary conditions $y_0 = y_4 = 0$ means we don't need the first and last columns. So equation (20.7.45) gives

$$\begin{bmatrix} -16+\frac{1}{2}\sqrt{2} & 6+\sqrt{2} & -2-\frac{1}{2}\sqrt{2} \\ 4-\sqrt{2} & -8 & 4+\sqrt{2} \\ -2+\frac{1}{2}\sqrt{2} & 6-\sqrt{2} & -16-\frac{1}{2}\sqrt{2} \end{bmatrix} \begin{bmatrix} y_1 \\ y_2 \\ y_3 \end{bmatrix} = \begin{bmatrix} -2 \\ -2 \\ -2 \end{bmatrix} \tag{20.7.52}$$

with solution

$$\begin{bmatrix} y_1 \\ y_2 \\ y_3 \end{bmatrix} = \begin{bmatrix} \frac{101}{350} + \frac{13}{350}\sqrt{2} \\ \frac{13}{25} \\ \frac{101}{350} - \frac{13}{350}\sqrt{2} \end{bmatrix} \tag{20.7.53}$$

The exact solution (20.7.47) gives for example $y(x = 0) = 0.52065$, compared with $y_2 = 0.52000$. Not bad for five grid points! The real point, however, is that the error is about 10^{-16} for $N = 16$. With a second-order finite difference scheme, the error would go down by only a factor of 10 or so with this increase in N.

20.7.13 Multidimensional Spectral Methods

For a time-dependent problem, the simplest approach is the *method of lines*. Expand the solution as

$$y(t, x) = \sum_j C_j(x) y_j(t) \tag{20.7.54}$$

where now the coefficients y_j are functions of time. Then

$$\left.\frac{\partial y}{\partial t}\right|_i = \dot{y}_i, \qquad \left.\frac{\partial y}{\partial x}\right|_i = \sum_j D^{(1)}_{ij} y_j, \quad \text{etc.} \tag{20.7.55}$$

You get a system of ODEs in t for the y_j, which you can solve in the standard way. Runge-Kutta is a good method to start with.

Problems with two or three spatial dimensions are usually handled by making expansions along each dimension separately:

$$u(x, y, z) = \sum_{ijk} u_{ijk} C_i(x) C_j(y) C_k(z) \tag{20.7.56}$$

Elliptic equations give simultaneous algebraic equations for the coefficients that are typically solved with iterative methods because of the large number of variables. See [11] for an example and references to the literature.

CITED REFERENCES AND FURTHER READING:

Hesthaven, J., Gottlieb, S., and Gottlieb, D. 2007, *Spectral Methods for Time-Dependent Problems* (New York: Cambridge University Press), Chapter 9.[1]

Gottlieb, D., and Orszag, S.A. 1977, *Numerical Analysis of Spectral Methods: Theory and Applications* (Philadelphia: S.I.A.M.).[2] [A classic, and still somewhat useful.]

Canuto, C., Hussaini, M.Y., Quarteroni, A., and Zang, T.A. 1988, *Spectral Methods in Fluid Dynamics* (Berlin: Springer).[3] [Standard reference for fluid dynamics applications, but applicable to other areas.]

Boyd, J.P. 2001, *Chebyshev and Fourier Spectral Methods*, 2nd ed. (New York: Dover Publications). Available at `http://www-personal.engin.umich.edu/~jpboyd`.[4] [Best single book: complete, and not too formal.]

Fornberg, B. 1996, *A Practical Guide to Pseudospectral Methods* (New York: Cambridge University Press).[5] [Good for getting started, but not for large-scale problems.]

Fornberg, B. 1998, "Calculation of Weights in Finite Difference Formulas," *SIAM Review* vol. 40, pp. 685–691.[6]

Baltensperger, R., and Trummer, M.R. 2003, "Spectral Differencing with a Twist," *SIAM Journal on Scientific Computing*, vol. 24, pp. 1465–1487.[7]

Matsushima, T., and Marcus, P.S. 1995, "A Spectral Method for Polar Coordinates," *Journal of Computational Physics* vol. 120, pp. 365–374.[8]

Matsushima, T., and Marcus, P.S. 1997, "A Spectral Method for Unbounded Domains," *Journal of Computational Physics* vol. 137, pp. 321–345.[9]

Rawitscher, G.H. 1991, "Accuracy Analysis of a Bessel Spectral Function Method for the Solution of Scattering Equations," *Journal of Computational Physics* vol. 94, pp. 81–101.[10]

Pfeiffer, H.P., Kidder, L.E., Scheel, M.A., and Teukolsky, S.A. 2003, "A Multidomain Spectral Method for Solving Elliptic Equations," *Computer Physics Communications*, vol. 152, pp. 253–273.[11]

Bjørhus, M. 1995, "The ODE Formulation of Hyperbolic PDEs Discretized by the Spectral Collocation Method," *SIAM Journal on Scientific Computing*, vol. 16, pp. 542–557. [Describes a good algorithm for hyperbolic equations.]

Computational Geometry

CHAPTER 21

21.0 Introduction

It is a safe bet that more computer cycles are expended on the formulas of computational geometry than on all other uses of computers put together. We include not just the computer's nominal CPU, of course, but also those other other, often vastly more powerful, CPUs hidden in the computer's graphics chipset, and in all the video entertainment and high-definition television boxes in the world.

Indeed, computational geometry, and the broader fields of computer graphics and computer vision in which it is embedded, have become central areas of computer science, supporting a huge industrial base of applied work and employment for computer scientists and program developers at all professional levels. It is impossible for us to do justice to this colossus in a single chapter. Yet, there are a number of elementary techniques from the field that ought to be in the repertory of any practicing computational scientist.

In this chapter we will build a body of methods sufficient to construct efficient Delaunay triangulations in two dimensions, and to use such triangulations for interpolating functions of two variables on an irregular grid, and other applications. In getting to this goal (and a bit beyond it) we will allow ourselves to be diverted into various other interesting, and often useful, topics, including:

- tree data structures for sets of points
- nearest-neighbor problems
- much about lines, triangles, and polygons
- spheres in n dimensions, and rotation matrices
- Voronoi and all that
- convex hulls
- minimum spanning trees
- finding intersecting objects

and more.

In the spirit of full disclosure, we must mention that our treatment of some of the most interesting topics in the above list will be restricted to the two-dimensional

case, even when the three-dimensional case may be equally relevant to computational science. The reason is simply one of space on the page. Three-dimensional algorithms are often more complex, have more special cases that must be treated, and generally result in codes that are too long for us to include. We have struggled to condense working, reasonably efficient, two-dimensional codes to an appropriate size for this chapter. You will be able to use these for two-dimensional problems, or you can mine them for understanding before seeking out three-dimensional solutions in the references.

An additional disclosure relates to our use of floating-point arithmetic, and our treatment of special cases of "exact" equality. Since floating-point numbers and their arithmetic are not exact, it usually does not make computational sense to test for cases of exact equality. However, historically, geometers have always distinguished, e.g., between a point being "inside" a triangle versus "on an edge" or "at a vertex." This has introduced a certain schizophrenia into the field. On the one hand (and especially before about 1990), practitioners have labored to create algorithms that use exact (integer) arithmetic, so that the traditional distinctions can be elegantly preserved. On the other hand (and especially after about 1990, when fast floating operations in special-purpose graphics processors started to be available), many of these niceties are no longer needed, and sloppiness at the level of "machine epsilon" can be tolerated in the interest of speed. In this chapter we are unapologetically in the sloppy camp. In boundary cases, our code is supposed to produce reasonable results, but not necessarily choose that specific reasonable result that you might think you want. Caveat emptor.

A less specific goal in this chapter is to give some of the "flavor" of the field of computational geometry. It is a flavor that deliciously combines elements of Euclid (pardon!) with elements of modern computer science and mathematics.

Some good general references are listed here.

CITED REFERENCES AND FURTHER READING:

de Berg, M., van Kreveld, M., Overmars, M., and Schwarzkopf, O. 2000, *Computational Geometry: Algorithms and Applications*, 2nd revised ed. (Berlin: Springer). [Best-selling text, especially strong on references to the published literature.]

O'Rourke, J. 1998, *Computational Geometry in C*, 2nd ed. (Cambridge, UK: Cambridge University Press). [Well written, with clear explanations and C code.]

Preparata, F.P. and Shamos, M.I. 1991, *Computational Geometry: An Introduction* (Berlin: Springer).

Schneider, P.J. and Eberly, D.H. 2003, *Geometric Tools for Computer Graphics* (San Francisco: Morgan Kaufmann). [Huge compendium of formulas and code.]

Bowyer, A. and Woodwark, J. 1983, *A Programmer's Geometry* (London: Butterworths). [Delightful classic, especially for those who get nostalgic at seeing Fortran printed in all uppercase.]

Glassner, A.S., ed. 1990, *Graphics Gems* (San Diego: Academic Press). [Series of books full of algorithmic tricks-of-the-trade.]

Arvo, J., ed. 1991, *Graphics Gems II* (San Diego: Academic Press).

Kirk, D., ed. 1992, *Graphics Gems III* (Cambridge, MA: Academic Press).

Heckbert, P.S., ed. 1994, *Graphics Gems IV* (Cambridge, MA: Academic Press).

Euclid, ca. 300BC, *Euclid's Elements*; reprinted 2002 (Santa Fe, NM: Green Lion Press).

21.1 Points and Boxes

A *point* $\mathbf{p}$ in a D-dimensional space is specified by its D Cartesian coordinates, $(x_0, x_1, \ldots, x_{D-1})$. Generally we will concern ourselves only with the cases $D = 2$ (points in a plane) and $D = 3$ (points in 3-space), but the concept is more general.

The representation in code follows just this paradigm. By eschewing special names for individual coordinates — like x, y, z — we keep the ability to loop easily over coordinates in D dimensions.

pointbox.h

```
template<Int DIM> struct Point {
Simple structure to represent a point in DIM dimensions.
    Doub x[DIM];                                          The coordinates.
    Point(const Point &p) {                               Copy constructor.
        for (Int i=0; i<DIM; i++) x[i] = p.x[i];
    }
    Point& operator= (const Point &p) {                   Assignment operator.
        for (Int i=0; i<DIM; i++) x[i] = p.x[i];
        return *this;
    }
    bool operator== (const Point &p) const {
        for (Int i=0; i<DIM; i++) if (x[i] != p.x[i]) return false;
        return true;
    }
    Point(Doub x0 = 0.0, Doub x1 = 0.0, Doub x2 = 0.0) {
        x[0] = x0;                               Constructor by coordinate values.  Arguments
        if (DIM > 1) x[1] = x1;                     beyond the required number are not used
        if (DIM > 2) x[2] = x2;                     and can be omitted.
        if (DIM > 3) throw("Point not implemented for DIM > 3");
    }
};
```

In the interest of concise code, the constructor above may pass some unnecessary default arguments of zero. You can easily clean this up if you care.

If we have two points $\mathbf{p}$ and $\mathbf{q}$, we can compute their distance d,

$$d = |\mathbf{p} - \mathbf{q}| = \left[\sum_{i=0}^{D-1} (p_i - q_i)^2\right]^{1/2} \tag{21.1.1}$$

where p_i and q_i are now the respective Cartesian coordinates for each point.

In code, we have

pointbox.h

```
template<Int DIM> Doub dist(const Point<DIM> &p, const Point<DIM> &q) {
Returns the distance between two points in DIM dimensions.
    Doub dd = 0.0;
    for (Int j=0; j<DIM; j++) dd += SQR(q.x[j]-p.x[j]);
    return sqrt(dd);
}
```

Note that `dist` is not a member of the class `Point`, but rather a freestanding function whose arguments are `Point`s. We will overload `dist` with other types of arguments, signifying other kinds of distances between objects.

21.1.1 Boxes

By a *box*, we mean a rectangle (for $D = 2$) or rectangular parallelepiped (for $D = 3$, a “brick” in other words) that is aligned with the coordinate axes. Boxes are

interesting because they can tessellate (that is, partition) D-dimensional space, and they can contain other objects. Indeed, every finite, extended object has a *bounding box*, which is the unique smallest box that contains it. One way to represent a box is by the points at two special, diagonally opposite, corners. The first point ("low") has coordinate values that are the minima on the surface of the box; the second point ("high") has coordinate values that are the maxima. All other corners of a box, it should be obvious, have coordinate values that are, dimension by dimension, either the value of "low" or the value of "high"; and all such permutations are corners, 2^D in all.

The code follows this description:

pointbox.h

```
template<Int DIM> struct Box {
Structure to represent a Cartesian box in DIM dimensions.
    Point<DIM> lo, hi;          Diagonally opposite corners (min of all coordinates and
    Box() {}                       max of all coordinates) are stored as two points.
    Box(const Point<DIM> &mylo, const Point<DIM> &myhi) : lo(mylo), hi(myhi) {}
};
```

Note that a copy constructor and assignment operator are not needed, since by default the two `Points` will be appropriately copied or assigned (one convenience of this representation).

A point can be either outside a box, inside it, or — in principle — on its surface. As mentioned in §21.0, we represent all coordinates as (approximate) floating-point numbers, not (exact) integers, so it would not be prudent to depend on any exact equalities of coordinate values or distances. We will be careful, therefore, not to put too much credence in the idea of the exact surface of a box; usually we'll consider the surface (should some exact equality happen to hold) as a part of the box's interior.

If a point is outside a box, then we define its distance from the box to be the distance to the nearest point on the surface of (or inside) the box. A glance at Figure 21.1.1 shows that this distance is the Pythagorean sum (that is, square root of sum of squares) of the distances from the point to some — but not all — of the hyperplanes that bound the box. The rule is that when a point has a coordinate that is greater than the corresponding max of the box, or less than the corresponding min, then *that* coordinate contributes to the sum. When the point has a coordinate between the max and min, then it does *not* contribute to the sum, since (along that coordinate) the shortest line can be perpendicular to the hyperplane. When a point is inside, or on the surface of, a box, we define its distance to the box to be zero.

These definitions of distance are embodied in the following code.

pointbox.h

```
template<Int DIM> Doub dist(const Box<DIM> &b, const Point<DIM> &p) {
If point p lies outside box b, the distance to the nearest point on b is returned. If p is inside b
or on its surface, zero is returned.
    Doub dd = 0;
    for (Int i=0; i<DIM; i++) {
        if (p.x[i]<b.lo.x[i]) dd += SQR(p.x[i]-b.lo.x[i]);
        if (p.x[i]>b.hi.x[i]) dd += SQR(p.x[i]-b.hi.x[i]);
    }
    return sqrt(dd);
}
```

Frequently we want to know if a point is inside or outside a box. The above `dist` routine can be used for this. A positive return means outside, zero means

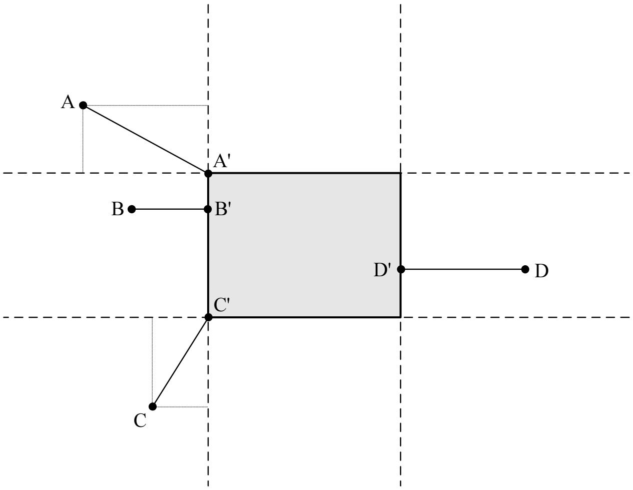

Figure 21.1.1. Distance from a point to a D-dimensional box. The general formula (as for lines AA' and CC') is a Pythagorean sum of D distances to the plane that includes the nearer side of the box. But when the point is between two such parallel planes (as for BB' and DD') then the corresponding coordinate is omitted from the sum.

inside. If inside-versus-outside, and not distance, is *all* you want to know, then some streamlining is possible: Replace dd by a Boolean variable, substitute logical-or's for the additions, and of course omit the square root. The logic remains otherwise the same.

21.1.2 Nodes for Binary Trees of Boxes

In the next section we will construct a *binary tree* of nested boxes, wherein each box is subdivided into, and linked to, two daughter boxes. Each box in the tree will also contain a list of points that lie inside the box. For use in such a task, we here give a structure, derived from the Box structure, but with additional variables that can point to a mother box, two daughter boxes, and the low and high indices on a list of points (designating the range of points inside the box). The constructor just sets all the values explicitly.

kdtree.h

```
template<Int DIM> struct Boxnode : Box<DIM> {
Node in a binary tree of boxes containing points. See text for details.
    Int mom, dau1, dau2, ptlo, pthi;
    Boxnode() {}
    Boxnode(Point<DIM> mylo, Point<DIM> myhi, Int mymom, Int myd1,
        Int myd2, Int myptlo, Int mypthi) :
        Box<DIM>(mylo, myhi), mom(mymom), dau1(myd1), dau2(myd2),
        ptlo(myptlo), pthi(mypthi) {}
};
```

21.2 KD Trees and Nearest-Neighbor Finding

Once, long ago, the term "kd tree" (or "k-D tree") was an abbreviation for "k-dimensional tree." However, the term has come to mean a very specific, and

useful, kind of tree structure for partitioning points, especially in small numbers of dimensions, like 2 or 3. A KD tree that contains N points can be constructed in $O(N \log N)$ time and $O(N)$ space. Once constructed, the KD tree facilitates such operations as finding a point's nearest neighbor in $O(\log N)$ time, or all nearest neighbors in $O(N \log N)$ time. KD trees were first described by Bentley [8] in 1975. Let's see how this works.

Start with a very large box, one that easily contains all possible points that are of interest. There is no penalty for making this *root box* humongous, so coordinates of $\pm 10^{99}$ are fine. Now generate a list of N points (of interest to your application) that lie inside the root box. The defining principles of a KD tree are

- Boxes are successively partitioned into two daughter boxes.
- Each partition is along the axis of one coordinate.
- The coordinates are used cyclically in successive partitions.
- In making the partition, the position of the "cut" is chosen to leave equal numbers of points on the two sides (or differing by one in the case of an odd number of points).

Within these principles, there are some arbitrary design choices to be made. In the implementation below, the partition "cut" goes exactly through one of the points (i.e., shares one of its coordinate values). This avoids a bit of extra bookkeeping incurred by other possible choices. Also, we terminate the tree when a box node contains either one or two points, avoiding the additional partitioning of two-point boxes into two one-point boxes. This choice is natural because the `Boxnode` structure already has pointers to two points (`ptlo` and `pthi`), and it reduces the total number of stored boxes by as much as 50%.

With these principles and design rules in mind, you can decode Figure 21.2.1, which shows a two-dimensional KD tree containing 1000 points. (As a bit of artistic license, the root box in the figure has been shrunk to just contain the points, instead of being off near infinity.)

Interestingly, given N, the number of points, it is possible to give an exact formula for the number of boxes generated by our KD tree partition rules. (This makes memory allocation for the tree very straightforward.) If $N_B(N)$ is the number of boxes needed for N points, then two obvious recurrence relations describe what happens in the initial partitioning of $2n$ points into n plus n, or $2n-1$ points into n plus $n-1$:

$$\begin{aligned} N_B(2n) &= 2N_B(n) + 1 \\ N_B(2n-1) &= N_B(n) + N_B(n-1) + 1 \end{aligned} \tag{21.2.1}$$

The $+1$ in both formulas refers to the additional mother box that "glues together" two daughter partial trees at each stage. The solution to these recurrences is

$$N_B(N) = \min(M-1,\ 2N - \tfrac{1}{2}M - 1) \tag{21.2.2}$$

where M is the smallest power of 2 greater or equal to N, that is,

$$M = 2^{\lceil \log_2 N \rceil} \tag{21.2.3}$$

(You can verify this solution by induction, working out the various possibilities of the min function. Or — much more fun — you can write a program to verify it numerically for $N < 10^9$, say.)

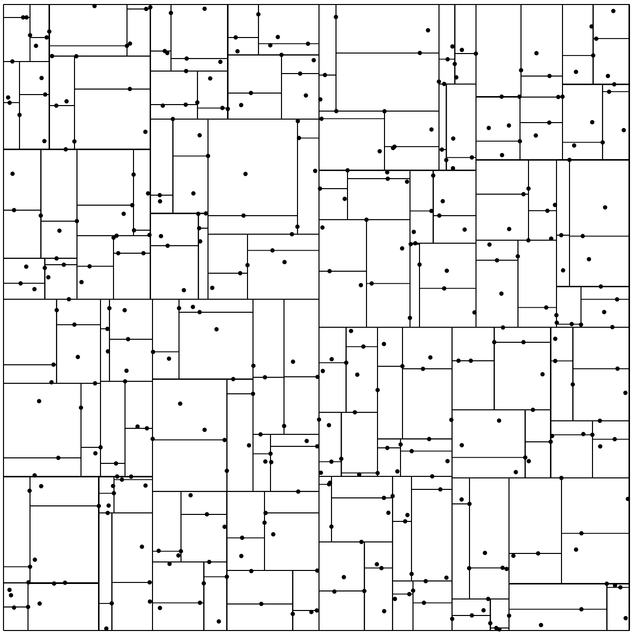

Figure 21.2.1. KD tree constructed from 1000 points in the plane. The first subdivision is visible as a full-height vertical line about halfway across the figure. The next subdivisions are horizontal lines, extending halfway across the figure. The subdivisions alternate between horizontal and vertical, and partition into (nearly) equal numbers of points at each stage. This tree terminates when there are either one or two points in a box (one of which is usually on the box boundary).

21.2.1 Implementation of the KD Tree

We implement the KD tree as a structure that gets built from a vector of `Points` and contains methods that embody the principal applications that we will discuss below, mainly various kinds of nearest-neighbor problems.

kdtree.h

```
template<Int DIM> struct KDtree {
Structure for implementing a KD tree.
    static const Doub BIG;                    Size of the root box, value set below.
    Int nboxes, npts;                         Number of boxes, number of points.
    vector< Point<DIM> > &ptss;               Reference to the vector of points in the KD tree.
    Boxnode<DIM> *boxes;                      The array of Boxnodes that form the tree.
    VecInt ptindx, rptindx;                   Index of points (see text), and reverse index.
    Doub *coords;                             Point coordinates rearranged contiguously.
    KDtree(vector< Point<DIM> > &pts);            Constructor.
    ~KDtree() {delete [] boxes;}
    Next, utility functions for use after the tree is constructed. See below.
    Doub disti(Int jpt, Int kpt);
    Int locate(Point<DIM> pt);
    Int locate(Int jpt);
    Next, applications that use the KD tree. See text.
    Int nearest(Int jpt);
    Int nearest(Point<DIM> pt);
    void nnearest(Int jpt, Int *nn, Doub *dn, Int n);
    static void sift_down(Doub *heap, Int *ndx, Int nn);     Used by nnearest.
    Int locatenear(Point<DIM> pt, Doub r, Int *list, Int nmax);
};

template<Int DIM> const Doub KDtree<DIM>::BIG(1.0e99);
```

Note that the KDtree structure keeps a reference to the vector of Points that created it. This is used in some of the applications and has the implication that the user should not modify the vector of points while its derived KD tree is in scope. The array coords is an internal representation of the points-vector that is used during the construction of the KD tree, and then is immediately returned to the memory pool.

What makes the KD tree fast to construct is the existence of fast partition algorithms, $O(N)$ in time, that not only find the median value in an array of N values, but also move all smaller values to one side of the array and all larger values to the other. We already met such an algorithm in §8.5, in the routine select. Here, we need a slight variant, selecti, that partitions an array of integers not by their values, but by using them to index a separate array of values that remain unaltered. Because we will want to partition subsegments of arrays, we pass all references to the arrays by address.

kdtree.h

```
Int selecti(const Int k, Int *indx, Int n, Doub *arr)
```

Permutes indx[0..n-1] to make arr[indx[0..k-1]] ≤ arr[indx[k]] ≤ arr[indx[k+1..n-1]]. The array arr is not modified. See comments in the routine select.

```
{
    Int i,ia,ir,j,l,mid;
    Doub a;

    l=0;
    ir=n-1;
    for (;;) {
        if (ir <= l+1) {
            if (ir == l+1 && arr[indx[ir]] < arr[indx[l]])
                SWAP(indx[l],indx[ir]);
            return indx[k];
        } else {
            mid=(l+ir) >> 1;
            SWAP(indx[mid],indx[l+1]);
            if (arr[indx[l]] > arr[indx[ir]]) SWAP(indx[l],indx[ir]);
            if (arr[indx[l+1]] > arr[indx[ir]]) SWAP(indx[l+1],indx[ir]);
            if (arr[indx[l]] > arr[indx[l+1]]) SWAP(indx[l],indx[l+1]);
            i=l+1;
            j=ir;
            ia = indx[l+1];
            a=arr[ia];
            for (;;) {
                do i++; while (arr[indx[i]] < a);
                do j--; while (arr[indx[j]] > a);
                if (j < i) break;
                SWAP(indx[i],indx[j]);
            }
            indx[l+1]=indx[j];
            indx[j]=ia;
            if (j >= k) ir=j-1;
            if (j <= k) l=i;
        }
    }
}
```

The basic strategy for constructing the KD tree is this: Set up an array of integers that index the N points (ptindx, below). Next, copy all the point coordinates into an array (coords) in which all the x_0 coordinates are contiguous, followed by all the x_1 coordinates, and so on through all the dimensions. Now use selecti to partition (and rearrange) the index of points according to the value of their x_0 co-

ordinates, with half the points on each side of the partition. These two halves, now viewed as separate arrays, contain the points in two new daughter boxes. Now partition each of them into half by the value of the x_1 coordinate. And so on, recursively, going through the coordinates cyclically.

The recursion is so simple that it is easy to code it as a simple "pending task list," thus avoiding the overhead of recursive function calls. A pending task consists of an index pointing to the box ready for further partitioning (the expectant mother, as it were) and a value that remembers which dimension is next to partition along. Because the tree is constructed "depth first," the task list never grows larger than the log of the total number of boxes. Every new daughter box is born with a pointer to its mother, and pointers to its beginning and end elements in the points index array `ptindx`. Although these elements will generally be permuted in subsequent partitionings, none will ever be moved out of the range specified when a daughter box is first created. That is why the whole process can be done in a single point-index array, with all boxes simply pointing into some subrange of that array.

The `KDtree` constructor, below, should now be straightforward to understand.

kdtree.h

```
template<Int DIM> KDtree<DIM>::KDtree(vector< Point<DIM> > &pts) :
ptss(pts), npts(pts.size()), ptindx(npts), rptindx(npts) {
Construct a KD tree from a vector of points.
    Int ntmp,m,k,kk,j,nowtask,jbox,np,tmom,tdim,ptlo,pthi;
    Int *hp;
    Doub *cp;
    Int taskmom[50], taskdim[50];                 Enough stack for 2^50 points!
    for (k=0; k<npts; k++) ptindx[k] = k;         Initialize the index of points.
    Calculate the number of boxes and allocate memory for them.
    m = 1;
    for (ntmp = npts; ntmp; ntmp >>= 1) {
        m <<= 1;
    }
    nboxes = 2*npts - (m >> 1);
    if (m < nboxes) nboxes = m;
    nboxes--;
    boxes = new Boxnode<DIM>[nboxes];
    Copy the point coordinates into a contiguous array.
    coords = new Doub[DIM*npts];
    for (j=0, kk=0; j<DIM; j++, kk += npts) {
        for (k=0; k<npts; k++) coords[kk+k] = pts[k].x[j];
    }
    Initialize the root box and put it on the task list for subdivision.
    Point<DIM> lo(-BIG,-BIG,-BIG), hi(BIG,BIG,BIG);      Syntax OK for 2-D too.
    boxes[0] = Boxnode<DIM>(lo, hi, 0, 0, 0, 0, npts-1);
    jbox = 0;
    taskmom[1] = 0;                         Which box.
    taskdim[1] = 0;                         Which dimension.
    nowtask = 1;
    while (nowtask) {                       Main loop over pending tasks.
        tmom = taskmom[nowtask];
        tdim = taskdim[nowtask--];
        ptlo = boxes[tmom].ptlo;
        pthi = boxes[tmom].pthi;
        hp = &ptindx[ptlo];                 Points to left end of subdivision.
        cp = &coords[tdim*npts];            Points to coordinate list for current dim.
        np = pthi - ptlo + 1;               Number of points in the subdivision.
        kk = (np-1)/2;                      Index of last point on left (boundary point).
        (void) selecti(kk,hp,np,cp);        Here is where all the work is done.
        Now create the daughters and push them onto the task list if they need further subdi-
        viding.
```

```
        hi = boxes[tmom].hi;
        lo = boxes[tmom].lo;
        hi.x[tdim] = lo.x[tdim] = coords[tdim*npts + hp[kk]];
        boxes[++jbox] = Boxnode<DIM>(boxes[tmom].lo,hi,tmom,0,0,ptlo,ptlo+kk);
        boxes[++jbox] = Boxnode<DIM>(lo,boxes[tmom].hi,tmom,0,0,ptlo+kk+1,pthi);
        boxes[tmom].dau1 = jbox-1;
        boxes[tmom].dau2 = jbox;
        if (kk > 1) {
            taskmom[++nowtask] = jbox-1;
            taskdim[nowtask] = (tdim+1) % DIM;
        }
        if (np - kk > 3) {
            taskmom[++nowtask] = jbox;
            taskdim[nowtask] = (tdim+1) % DIM;
        }
    }
    for (j=0; j<npts; j++) rptindx[ptindx[j]] = j;        Create reverse index.
    delete [] coords;                                      Don't need them anymore.
}
```

There are a small number of utility functions that are easy to provide. Although we generally prefer to have our distance (dist) functions be freestanding, it is useful to have a KDtree member routine that returns the distance between two points in a KD tree, referenced by their integer position in the underlying vector of points.

kdtree.h

```
template<Int DIM> Doub KDtree<DIM>::disti(Int jpt, Int kpt) {
Returns the distance between two points in the kdtree given their indices in the array of points,
but returns a large value if the points are identical.
    if (jpt == kpt) return BIG;
    else return dist(ptss[jpt], ptss[kpt]);
}
```

There is a special reason for returning BIG when the two points are identical: Later, when we find a point's nearest neighbor, we don't want the invariable answer to be "itself!"

Another simple function takes an arbitrary Point as the argument and returns the index of the box that uniquely contains it. In this function we first see an example of traversing the tree hierarchically, starting at the root box and then choosing only one of two daughter boxes at each stage. Also, by keeping track of which dimension is next to be partitioned on (jdim, below), we need only check one of the point's coordinates at each stage. Evidently, the whole process is $O(\log N)$ in time, since there can be only that many levels in the tree.

kdtree.h

```
template<Int DIM> Int KDtree<DIM>::locate(Point<DIM> pt) {
Given an arbitrary point pt, return the index of which kdtree box it is in.
    Int nb,d1,jdim;
    nb = jdim = 0;                          Start with the root box.
    while (boxes[nb].dau1) {                As far as possible down the tree.
        d1 = boxes[nb].dau1;
        if (pt.x[jdim] <= boxes[d1].hi.x[jdim]) nb=d1;
        else nb=boxes[nb].dau2;
        jdim = ++jdim % DIM;                Increment the dimension cyclically.
    }
    return nb;
}
```

The actual Box can be obtained from the returned integer, say j, by referencing boxes[j] in the KDtree, since Boxnode is a derived class of Box.

A very similar utility returns the index of the box that contains one of the points used to construct the KDtree. This is not necessarily the same box as the above routine would return, because of the possibility of multiple ties in coordinate values, in which case some tied points can lie on one side of the median partition and others on the other side.

kdtree.h
```
template<Int DIM> Int KDtree<DIM>::locate(Int jpt) {
Given the index of a point in the kdtree, return the index of which box it is in.
    Int nb,d1,jh;
    jh = rptindx[jpt];                   The reverse index tells where the point lies in the
    nb = 0;                                  index of points.
    while (boxes[nb].dau1) {
        d1 = boxes[nb].dau1;
        if (jh <= boxes[d1].pthi) nb=d1;
        else nb = boxes[nb].dau2;
    }
    return nb;
}
```

21.2.2 Applications of KD Trees

Most applications of KD trees make use of locality properties of its nested boxes. This is best seen in a few examples.

Suppose we want to know which of the N points in a KD tree is closest to an arbitrary point $\mathbf{p}$ (not necessarily one of the points in the tree). Without the tree, this is evidently a calculation that requires $O(N)$ operations, as we compare $\mathbf{p}$ to each candidate point in turn. However, if we have invested the $O(N \log N)$ operations required to construct the tree, then we can proceed in the following way. First, find the box in which $\mathbf{p}$ lies, and find the closest point in the tree that lies in that box. This takes $O(\log N)$ operations, as we saw above. The found point might in fact be the nearest neighbor (we don't know yet), but in any case its distance is now an upper bound on how far away the true nearest neighbor can be.

Second, traverse the tree by a depth-first recursion (exactly the way we did when we constructed the tree). As we encounter each new box, we check whether it could possibly contain a point closer than the nearest point found so far. Since we start with a point that is already pretty close (in the same box as $\mathbf{p}$), most boxes get rejected at this step. When a box is rejected, we *don't* need to open its daughter boxes, so a whole branch of the tree gets "pruned." On average, only about $O(\log N)$ boxes actually get opened, so the total work load to find the nearest point is $O(\log N)$.

If we are really interested in only a single point $\mathbf{p}$, then the "slow," $O(N)$, method would have been faster. But if we are repeating the operation for many different points $\mathbf{p}_i$, comparing to the same N points in the tree each time, then calling the following routine for each $\mathbf{p}_i$ in turn is a big win.

kdtree.h
```
template<Int DIM> Int KDtree<DIM>::nearest(Point<DIM> pt) {
Given an arbitrary location pt, return the index of the nearest point in the kdtree.
    Int i,k,nrst,ntask;
    Int task[50];                        Stack for boxes waiting to be opened.
    Doub dnrst = BIG, d;
    First stage, we find the nearest kdtree point in same box as pt.
    k = locate(pt);                      Which box is pt in?
    for (i=boxes[k].ptlo; i<=boxes[k].pthi; i++) {    Find nearest.
        d = dist(ptss[ptindx[i]],pt);
```

```
        if (d < dnrst) {
            nrst = ptindx[i];
            dnrst = d;
        }
    }
    Second stage, we traverse the tree opening only possibly better boxes.
    task[1] = 0;
    ntask = 1;
    while (ntask) {
        k = task[ntask--];
        if (dist(boxes[k],pt) < dnrst) {        Distance to closest point in box.
            if (boxes[k].dau1) {                If not an end node, put on task list.
                task[++ntask] = boxes[k].dau1;
                task[++ntask] = boxes[k].dau2;
            } else {                            Check the 1 or 2 points in the box.
                for (i=boxes[k].ptlo; i<=boxes[k].pthi; i++) {
                    d = dist(ptss[ptindx[i]],pt);
                    if (d < dnrst) {
                        nrst = ptindx[i];
                        dnrst = d;
                    }
                }
            }
        }
    }
    return nrst;
}
```

What if we want to know the nearest-neighbor point not of an arbitrary location, but of one of the points stored in the KD tree? The above routine won't do. If we send it a point in the tree, it will give the obvious result that the point is its own nearest neighbor! We need to modify the routine so as to use `disti` from `KDtree`, which defined a point's self-distance as being large, rather than small.

An additional useful feature is to find not the single nearest neighbor, but the n nearest neighbors for some specified $n < N - 1$. The trick here is to avoid making the algorithm $O(n \log N)$, which is what it would be if, for each candidate point, we compared the candidate to all n of the best points so far. A good way to proceed is with a heap structure, as described in §8.3 and used (for a very similar purpose) in the routine `hpsel` in §8.5. The work load then scales as $O(\log n \log N)$.

The following routine is coded so as to lose hardly any efficiency in the case $n = 1$ (find the single nearest neighbor) while using a heap structure in the case $n > 1$.

kdtree.h

```
template<Int DIM> void KDtree<DIM>::nnearest(Int jpt, Int *nn, Doub *dn, Int n)
Given the index jpt of a point in a kdtree, return a list nn[0..n-1] of the indices of the n
points in the tree nearest to point j, and a list dd[0..n-1] of their distances.
{
    Int i,k,ntask,kp;
    Int task[50];                       Stack for boxes to be opened.
    Doub d;
    if (n > npts-1) throw("too many neighbors requested");
    for (i=0; i<n; i++) dn[i] = BIG;
    Find smallest mother box with enough points to initialize the heap.
    kp = boxes[locate(jpt)].mom;
    while (boxes[kp].pthi - boxes[kp].ptlo < n) kp = boxes[kp].mom;
    Examine its points and save the n closest.
    for (i=boxes[kp].ptlo; i<=boxes[kp].pthi; i++) {
        if (jpt == ptindx[i]) continue;
        d = disti(ptindx[i],jpt);
```

```
        if (d < dn[0]) {
            dn[0] = d;
            nn[0] = ptindx[i];
            if (n>1) sift_down(dn,nn,n);     Maintain the heap structure.
        }
    }
    Now we traverse the tree opening only possibly better boxes.
    task[1] = 0;
    ntask = 1;
    while (ntask) {
        k = task[ntask--];
        if (k == kp) continue;              Don't redo the box used to initialize.
        if (dist(boxes[k],ptss[jpt]) < dn[0]) {
            if (boxes[k].dau1) {            If not an end node, put on task list.
                task[++ntask] = boxes[k].dau1;
                task[++ntask] = boxes[k].dau2;
            } else {                        Check the 1 or 2 points in the box.
                for (i=boxes[k].ptlo; i<=boxes[k].pthi; i++) {
                    d = disti(ptindx[i],jpt);
                    if (d < dn[0]) {
                        dn[0] = d;
                        nn[0] = ptindx[i];
                        if (n>1) sift_down(dn,nn,n);  Maintain the heap.
                    }
                }
            }
        }
    }
    return;
}
```

The following routine is used by the above for the sift-down process on the heap, differing from the `sift_down` used by `hpsort` (§8.3) only in its tailored interface for the present application, and the fact that it simultaneously rearranges two arrays, the distances (forming a heap) and the corresponding point numbers.

```
template<Int DIM> void KDtree<DIM>::sift_down(Doub *heap, Int *ndx, Int nn) {      kdtree.h
Fix heap[0..nn-1], whose first element (only) may be wrongly filed. Make a corresponding
permutation in ndx[0..nn-1]. The algorithm is identical to that used by sift_down in hpsort.
    Int n = nn - 1;
    Int j,jold,ia;
    Doub a;
    a = heap[0];
    ia = ndx[0];
    jold = 0;
    j = 1;
    while (j <= n) {
        if (j < n && heap[j] < heap[j+1]) j++;
        if (a >= heap[j]) break;
        heap[jold] = heap[j];
        ndx[jold] = ndx[j];
        jold = j;
        j = 2*j + 1;
    }
    heap[jold] = a;
    ndx[jold] = ia;
}
```

As a final illustrative example, here is how to find all points in a KD tree that lie within a specified radius r of some arbitrary location $\mathbf{p}$.

kdtree.h

```
template<Int DIM>
Int KDtree<DIM>::locatenear(Point<DIM> pt, Doub r, Int *list, Int nmax) {
Given a point pt and radius r, returns a value nret such that list[0..nret-1] is a list of all
kdtree points within a radius r of pt, up to a user-specified maximum of nmax points.
    Int k,i,nb,nbold,nret,ntask,jdim,d1,d2;
    Int task[50];
    nb = jdim = nret = 0;
    if (r < 0.0) throw("radius must be nonnegative");
    Find the smallest box that contains the "ball" of radius r.
    while (boxes[nb].dau1) {
        nbold = nb;
        d1 = boxes[nb].dau1;
        d2 = boxes[nb].dau2;
        Only need to check the dimension that divides the daughters.
        if (pt.x[jdim] + r <= boxes[d1].hi.x[jdim]) nb = d1;
        else if (pt.x[jdim] - r >= boxes[d2].lo.x[jdim]) nb = d2;
        jdim = ++jdim % DIM;
        if (nb == nbold) break;                   Neither daughter encloses the ball.
    }
    Now traverse the tree below the starting box only as needed.
    task[1] = nb;
    ntask = 1;
    while (ntask) {
        k = task[ntask--];
        if (dist(boxes[k],pt) > r) continue;      Box and ball are disjoint.
        if (boxes[k].dau1) {                      Expand box further when possible.
            task[++ntask] = boxes[k].dau1;
            task[++ntask] = boxes[k].dau2;
        } else {                                  Otherwise process points in the box.
            for (i=boxes[k].ptlo; i<=boxes[k].pthi; i++) {
                if (dist(ptss[ptindx[i]],pt) <= r && nret < nmax)
                    list[nret++] = ptindx[i];
                if (nret == nmax) return nmax;    Not enough space!
            }
        }
    }
    return nret;
}
```

You might wonder why the above routine doesn't also use the tree structure to find cases where a box lies entirely inside the "ball" of radius r, in which case it could add the box's points to the output list without further opening of its daughters. The improvement is potentially a factor of $O(\log n)$, where n is the typical number of neighbors returned. The resulting routine is slightly too long for us to include, however. A good exercise is to code this modification yourself. You'll see that it is harder to check whether a box is inside a ball than vice versa: You have to check all 2^D corners of the box, not just the diagonally opposite "low" and "high" ones.

CITED REFERENCES AND FURTHER READING:

Bentley, J.L. 1975, "Multidimensional Binary Search Trees Used for Associative Searching," *Communications of the ACM*, vol. 18, pp. 509–517.

de Berg, M., van Kreveld, M., Overmars, M., and Schwarzkopf, O. 2000, *Computational Geometry: Algorithms and Applications*, 2nd revised ed. (Berlin: Springer), §5.2.

Samet, H. 1990, *The Design and Analysis of Spatial Data Structures* (Reading, MA: Addison-Wesley).

21.3 Triangles in Two and Three Dimensions

Not since the time of Euclid has the lowly triangle attracted as much attention as it does today in computer graphics. Triangles and *triangulation* (the decomposition, or approximation, of complicated geometrical objects using only triangles) are at the heart of practically every computer-generated image.

Three points, call them **a**, **b**, **c**, define a triangle. They are its *vertices*. If the points are two-dimensional, the triangle lies in the two-dimensional plane. If the points have higher dimensionality, then the triangle floats in the corresponding D-dimensional space (most commonly $D = 3$). For now, consider only the former case, with $D = 2$, so that **a** has coordinates (a_0, a_1), and similarly for **b** and **c**.

Area. The area $\mathcal{A}(\mathbf{abc})$ of the triangle $\triangle\mathbf{abc}$ can be written in a number of equivalent ways, including

$$\begin{aligned} 2\mathcal{A}(\mathbf{abc}) &= \begin{vmatrix} a_0 & a_1 & 1 \\ b_0 & b_1 & 1 \\ c_0 & c_1 & 1 \end{vmatrix} \\ &= (\mathbf{b}-\mathbf{a})\times(\mathbf{c}-\mathbf{a}) = (b_0-a_0)(c_1-a_1)-(b_1-a_1)(c_0-a_0) \\ &= (\mathbf{c}-\mathbf{b})\times(\mathbf{a}-\mathbf{b}) = (c_0-b_0)(a_1-b_1)-(c_1-b_1)(a_0-b_0) \\ &= (\mathbf{a}-\mathbf{c})\times(\mathbf{b}-\mathbf{c}) = (a_0-c_0)(b_1-c_1)-(a_1-c_1)(b_0-c_0) \end{aligned} \tag{21.3.1}$$

Here $\times$ denotes the vector cross product, defined in two dimensions simply by

$$\mathbf{A}\times\mathbf{B} = A_0B_1 - B_1A_0 \qquad \text{(two dimensions only)} \tag{21.3.2}$$

Below, when we consider triangles in three dimensions, it will be the vector cross product forms in equation (21.3.1) that give a generalized formula for the area. Let us also note in passing that the formulas for area are separately *linear* in each of the six coordinates a_0, a_1, b_0, b_1, c_0, and c_1.

Equation (21.3.1) can yield a value that is positive, zero, or negative: The area is a *signed area*. By convention (embodied in equation 21.3.1), the area is positive if a traversal from **a** to **b** to **c** goes *counterclockwise (CCW)* around the triangle, and negative if it goes *clockwise (CW)*. The area is zero if and only if the three points are collinear, in which case the triangle is degenerate. (In the formulas that follow, we will generally assume the nondegenerate case.)

The absolute value $|\mathcal{A}|$ is the (unsigned) "area" of the triangle in the conventional geometrical sense. It can also be calculated directly from the side lengths d_{ab}, d_{bc}, and d_{ca} as follows:

$$|\mathcal{A}| = \sqrt{s(s-d_{ab})(s-d_{bc})(s-d_{ca})} \tag{21.3.3}$$

where s is half the perimeter,

$$s \equiv \tfrac{1}{2}(d_{ab}+d_{bc}+d_{ca}) \tag{21.3.4}$$

(Does it go without saying that you compute the side lengths by taking the coordinate differences and using the Pythagorean theorem?)

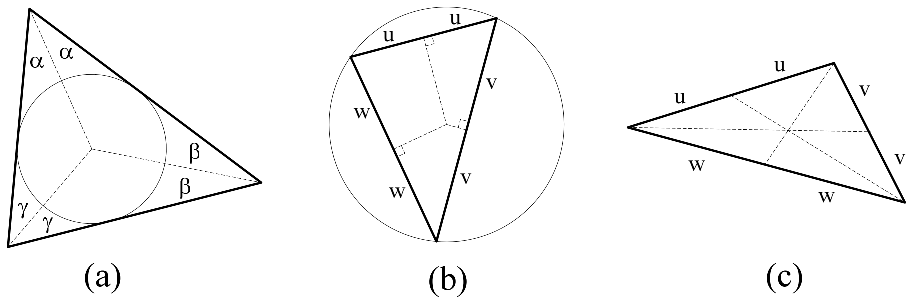

Figure 21.3.1. Three kinds of triangle centers. (a) Incircle and incenter; bisectors of the three vertex angles meet at the incenter. (b) Circumcircle and circumcenter; perpendicular bisectors of the edges meet at the circumcenter. (c) Centroid; lines from the edge midpoints to the opposite vertices meet at the centroid.

Related Circles. Every nondegenerate triangle has an *inscribed circle* or *incircle*, which is the largest circle that can be drawn inside the triangle. The incircle is tangent to all three sides of the triangle. Lines from its center, the *incenter*, to each vertex bisect the angle at that vertex (see Figure 21.3.1). If $\mathbf{q}$ is the incenter point, with coordinates (q_0, q_1), then its location is given by

$$q_i = \frac{1}{2s}(d_{bc}a_i + d_{ca}b_i + d_{ab}c_i) \qquad (i = 0, 1) \tag{21.3.5}$$

while its radius is given by

$$r_{\text{in}} = \left(\frac{(s - d_{ab})(s - d_{bc})(s - d_{ca})}{s}\right)^{1/2} \tag{21.3.6}$$

Every nondegenerate triangle also has a *circumscribed circle* or *circumcircle*, which is the unique circle that goes through its three vertices. Suppose $\mathbf{Q}$ is the *circumcenter* point, with coordinates (Q_0, Q_1). Let $[ba]_0$ and $[ba]_1$ denote the coordinate differences $b_0 - a_0$ and $b_1 - a_1$, respectively; and similarly for $[ca]_0$ and $[ca]_1$. Then, in 2×2 determinant form,

$$\begin{aligned} Q_0 &= a_0 + \frac{1}{2}\begin{vmatrix} ([ba]_0)^2 + ([ba]_1)^2 & [ba]_1 \\ ([ca]_0)^2 + ([ca]_1)^2 & [ca]_1 \end{vmatrix} \Big/ \begin{vmatrix} [ba]_0 & [ba]_1 \\ [ca]_0 & [ca]_1 \end{vmatrix} \\ Q_1 &= a_1 + \frac{1}{2}\begin{vmatrix} [ba]_0 & ([ba]_0)^2 + ([ba]_1)^2 \\ [ca]_0 & ([ca]_0)^2 + ([ca]_1)^2 \end{vmatrix} \Big/ \begin{vmatrix} [ba]_0 & [ba]_1 \\ [ca]_0 & [ca]_1 \end{vmatrix} \end{aligned} \tag{21.3.7}$$

The circumcenter is, by definition, the same distance from all three vertices. Therefore the radius of the circumcircle is

$$r_{\text{circum}} = \sqrt{(Q_0 - a_0)^2 + (Q_1 - a_1)^2} \tag{21.3.8}$$

where Q_0 and Q_1 are given above. (Obviously you can save the semi-final results in equation 21.3.7 for this computation, before adding a_0 or a_1.)

Later, in §21.6, we will be calculating a lot of circumcircles. We use the following simple definition of a structure `Circle`, and a routine `circumcircle()` that directly implements equations (21.3.7) and (21.3.8).

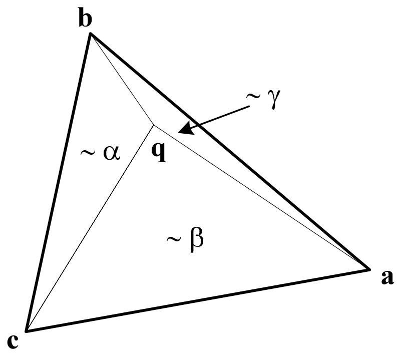

Figure 21.3.2. Any point in the plane **q** can be expressed as a linear combination of a triangle's three vertices. The coefficients (α, β, γ), called barycentric coordinates, sum to 1 and are proportional to the areas of $\triangle\mathbf{qbc}$, $\triangle\mathbf{qca}$, and $\triangle\mathbf{qab}$, respectively.

circumcircle.h

```
struct Circle {
    Point<2> center;
    Doub radius;
    Circle(const Point<2> &cen, Doub rad) : center(cen), radius(rad) {}
};

Circle circumcircle(Point<2> a, Point<2> b, Point<2> c) {
    Doub a0,a1,c0,c1,det,asq,csq,ctr0,ctr1,rad2;
    a0 = a.x[0] - b.x[0]; a1 = a.x[1] - b.x[1];
    c0 = c.x[0] - b.x[0]; c1 = c.x[1] - b.x[1];
    det = a0*c1 - c0*a1;
    if (det == 0.0) throw("no circle thru colinear points");
    det = 0.5/det;
    asq = a0*a0 + a1*a1;
    csq = c0*c0 + c1*c1;
    ctr0 = det*(asq*c1 - csq*a1);
    ctr1 = det*(csq*a0 - asq*c0);
    rad2 = ctr0*ctr0 + ctr1*ctr1;
    return Circle(Point<2>(ctr0 + b.x[0], ctr1 + b.x[1]), sqrt(rad2));
}
```

Centroid and Barycentric Coordinates. Distinct from both its incenter and its circumcenter is a triangle's centroid, or center of gravity, **M**. This point lies at the intersections of the lines drawn from each vertex to the midpoint of the opposite side. Its coordinates are simply the means of the coordinates of the vertices,

$$M_i = \tfrac{1}{3}(a_i + b_i + c_i) \qquad (i = 0, 1) \tag{21.3.9}$$

The centroid is also the point **M** where the areas $\mathcal{A}(\mathbf{abM})$, $\mathcal{A}(\mathbf{bcM})$, and $\mathcal{A}(\mathbf{caM})$ are all equal. In §21.7 we will be using a triangular mesh to interpolate a function. The significance of the centroid is that it is the point where a linearly interpolated function takes on the value that is the mean of the function values at the three vertices.

In fact, generalizing the idea of the centroid, any point **q** in the plane can be written as a linear combination of the three vertices **a**, **b**, **c**, with coefficients that sum to unity. These coefficients are called **q**'s *barycentric coordinates* and can be intuitively expressed in terms of the area formulas for triangles (see Figure 21.3.2). The equations are

$$\begin{aligned}
\mathbf{q} &= \alpha\mathbf{a} + \beta\mathbf{b} + \gamma\mathbf{c} \\
\alpha &= \mathcal{A}(\mathbf{bcq})/\mathcal{A}(\mathbf{abc}) \\
\beta &= \mathcal{A}(\mathbf{caq})/\mathcal{A}(\mathbf{abc}) \\
\gamma &= \mathcal{A}(\mathbf{abq})/\mathcal{A}(\mathbf{abc})
\end{aligned} \tag{21.3.10}$$

with, by construction,

$$\alpha + \beta + \gamma = 1 \tag{21.3.11}$$

The first line in equation (21.3.10) is thus equivalent to

$$\mathbf{q} = \mathbf{c} + \alpha(\mathbf{a} - \mathbf{c}) + \beta(\mathbf{b} - \mathbf{c}) \tag{21.3.12}$$

This can be viewed as the equation for a coordinate transformation, one that transforms from (α, β) coordinates to (q_0, q_1) coordinates. Evidently, since it is linear, its inverse — the formulas for α and β in equation (21.3.10) — must also be linear. But we knew this already, having remarked on the fact that the area formulas (21.3.1) are linear in all their coordinates, so linear in q_0 and q_1 in particular. Barycentric coordinates generalize to triangles in three or more dimensions in a useful way, as we will see below.

Note that α, β, or γ go to 1 as the point $\mathbf{q}$ approaches $\mathbf{a}$, $\mathbf{b}$, or $\mathbf{c}$, respectively; and that along any edge of the triangle (say $\overline{ab}$) the coefficient of the opposite vertex (here, γ) vanishes. The point $\mathbf{q}$ is inside the triangle $\triangle\mathbf{abc}$ if and only if α, β, and γ are all positive. In fact, this is a good way to test for a point's "insideness" in a triangle. (You can of course omit calculating the denominator area in this application.)

Barycentric coordinates are also useful when you want to pick a uniformly random point $\mathbf{q}$ inside $\triangle\mathbf{abc}$: First pick α and β as each uniformly random in $(0, 1)$. Next, if $\alpha + \beta > 1$, modify them both by $\alpha \leftarrow 1 - \alpha$ and $\beta \leftarrow 1 - \beta$. Finally, apply equation (21.3.12). The idea is that the first choice of α and β is random in the parallelogram spanned by two sides of the triangle; then, if it is on the wrong side of the diagonal, we move it to the correct side by a reflection.

21.3.1 Triangles in Three Dimensions

Our favorite triangle is still defined by the three points $\mathbf{a}$, $\mathbf{b}$, and $\mathbf{c}$, but these are now points in three dimensions, with coordinates (e.g., for $\mathbf{a}$) (a_0, a_1, a_2). The generalization of the signed area $\mathcal{A}$ (equation 21.3.1) is now a vector area $\vec{\mathcal{A}}$ whose direction is normal to the plane of the triangle and whose length is the area of the triangle. It is most easily written using a vector cross product, defined in three dimensions by

$$\begin{aligned}
\mathbf{A} \times \mathbf{B} &= \begin{vmatrix} \hat{\mathbf{e}}_0 & \hat{\mathbf{e}}_1 & \hat{\mathbf{e}}_2 \\ A_0 & A_1 & A_2 \\ B_0 & B_1 & B_2 \end{vmatrix} \\
&= (A_1 B_2 - A_2 B_1)\hat{\mathbf{e}}_0 + (A_2 B_0 - A_0 B_2)\hat{\mathbf{e}}_1 + (A_0 B_1 - A_1 B_0)\hat{\mathbf{e}}_2
\end{aligned} \tag{21.3.13}$$

where $\hat{\mathbf{e}}_0$, $\hat{\mathbf{e}}_1$, and $\hat{\mathbf{e}}_2$ are respectively the unit vectors $(1, 0, 0)$, $(0, 1, 0)$, and $(0, 0, 1)$. Then we have (cf. equation 21.3.1)

$$
\begin{aligned}
2\,\vec{\mathcal{A}}(\mathbf{abc}) &= (\mathbf{b}-\mathbf{a})\times(\mathbf{c}-\mathbf{a}) \\
&= (\mathbf{c}-\mathbf{b})\times(\mathbf{a}-\mathbf{b}) \\
&= (\mathbf{a}-\mathbf{c})\times(\mathbf{b}-\mathbf{c})
\end{aligned} \tag{21.3.14}
$$

You calculate the positive scalar area $\mathcal{A} \equiv |\vec{\mathcal{A}}|$ by the usual square-root sum of the squares of $\vec{\mathcal{A}}$'s three components; or you can instead use equation (21.3.3), with $d_{ab} = |\mathbf{a}-\mathbf{b}|$, etc.

Plane Defined by Triangle. A point $\mathbf{q}$ lies in the plane defined by $\triangle\mathbf{abc}$ if and only if the volume of the tetrahedron $\mathbf{abcq}$ is zero. The tetrahedral volume, in general, is given by

$$
\begin{aligned}
6\,\mathcal{V} &= \begin{vmatrix} a_0 & a_1 & a_2 & 1 \\ b_0 & b_1 & b_2 & 1 \\ c_0 & c_1 & c_2 & 1 \\ q_0 & q_1 & q_2 & 1 \end{vmatrix} \\
&= (\mathbf{b}-\mathbf{a})\cdot[(\mathbf{c}-\mathbf{a})\times(\mathbf{q}-\mathbf{a})] \\
&= (\mathbf{c}-\mathbf{a})\cdot[(\mathbf{q}-\mathbf{a})\times(\mathbf{b}-\mathbf{a})] \\
&= (\mathbf{q}-\mathbf{a})\cdot[(\mathbf{b}-\mathbf{a})\times(\mathbf{c}-\mathbf{a})]
\end{aligned} \tag{21.3.15}
$$

where "$\cdot$" signifies vector dot product. You can also cyclically permute $\mathbf{a}$, $\mathbf{b}$, and $\mathbf{c}$ in the above equation, for a seemingly infinite number of variations of the same formula!

The volume $\mathcal{V}$ is signed and is positive if $\triangle\mathbf{abc}$ is counterclockwise when viewed from outside (side away from $\mathbf{q}$), that is, the right-hand rule gives an outward-pointing normal.

The last form in equation (21.3.15) is particularly nice, because setting it to zero gives the equation satisfied by any point $\mathbf{q}$ in the plane defined by $\triangle\mathbf{abc}$:

$$
\mathbf{q}\cdot\mathbf{N} = D \tag{21.3.16}
$$

with

$$
\begin{aligned}
&\mathbf{N} = (\mathbf{b}-\mathbf{a})\times(\mathbf{c}-\mathbf{a}) \quad \text{(or cyclic permutation of } \mathbf{a}, \mathbf{b}, \mathbf{c}) \\
&D = \mathbf{a}\cdot\mathbf{N} \quad \text{(or, for that matter)} \quad = \mathbf{b}\cdot\mathbf{N} = \mathbf{c}\cdot\mathbf{N}
\end{aligned} \tag{21.3.17}
$$

We could also divide equation (21.3.16) by $|\mathbf{N}|$, in which case the vector on the left will be $\hat{\mathbf{N}} = \mathbf{N}/|\mathbf{N}|$, the unit vector normal to the plane, and $\hat{D} = D/|\mathbf{N}|$ will be the plane's distance from the origin.

With the same machinery, we can readily project any point $\mathbf{p}$ into a new point $\mathbf{b}'$ that lies in the plane of $\triangle\mathbf{abc}$:

$$
\mathbf{p} \longrightarrow \mathbf{p}' = \mathbf{p} + \frac{[(\mathbf{a}-\mathbf{p})\cdot\mathbf{N}]\mathbf{N}}{|\mathbf{N}|^2} \tag{21.3.18}
$$

where $\mathbf{N}$ is as above. For $\mathbf{a}$ in this formula, you can substitute $\mathbf{b}$, $\mathbf{c}$, or any other point in the plane.

We can project one triangle into the plane defined by another triangle by projecting its three points in turn. (This is a very common operation in rendering a triangulated three-dimensional model in the two-dimensional "camera plane" of your computer's screen.)

Barycentric Coordinates. Barycentric coordinates are valid in three dimensions for points $\mathbf{q}$ in the triangle's plane, and equation (21.3.10), in particular, still holds. To compute (α, β), one can in principle calculate the various $\mathcal{A}$'s from (21.3.14), but an easier equivalent calculation is

$$\begin{aligned}\alpha &= \frac{\mathbf{b}'^2(\mathbf{a}' \cdot \mathbf{q}') - (\mathbf{a}' \cdot \mathbf{b}')\,(\mathbf{b}' \cdot \mathbf{q}')}{\mathbf{a}'^2\mathbf{b}'^2 - (\mathbf{a}' \cdot \mathbf{b}')^2} \\ \beta &= \frac{\mathbf{a}'^2(\mathbf{b}' \cdot \mathbf{q}') - (\mathbf{a}' \cdot \mathbf{b}')\,(\mathbf{a}' \cdot \mathbf{q}')}{\mathbf{a}'^2\mathbf{b}'^2 - (\mathbf{a}' \cdot \mathbf{b}')^2}\end{aligned} \tag{21.3.19}$$

(compute identical denominators only once) where

$$\mathbf{a}' \equiv \mathbf{a} - \mathbf{c}, \qquad \mathbf{b}' \equiv \mathbf{b} - \mathbf{c}, \qquad \mathbf{q}' \equiv \mathbf{q} - \mathbf{c} \tag{21.3.20}$$

By the way, if $\mathbf{q}$ is not in the plane of $\triangle\mathbf{abc}$, you can still use equation (21.3.19). In that case, you get the (α, β) coordinates of the projected point in the plane. Also, notice what happens in the special case that $\triangle\mathbf{abc}$ is a right triangle, with right vertex $\mathbf{c}$, and with sides $\overline{ac}$ and $\overline{bc}$ of unit length, i.e., $d_{ac} = d_{bc} = 1$. Then the coordinate transformations, in both directions, are simply

$$\begin{aligned}\mathbf{q} &= \mathbf{c} + \alpha(\mathbf{a} - \mathbf{c}) + \beta(\mathbf{b} - \mathbf{c}) \\ [\alpha, \beta] &= [(\mathbf{a} - \mathbf{c}) \cdot (\mathbf{q} - \mathbf{c}), (\mathbf{b} - \mathbf{c}) \cdot (\mathbf{q} - \mathbf{c})]\end{aligned} \tag{21.3.21}$$

In other words, we project into an orthonormal coordinate system in the plane by a simple change of origin (to $\mathbf{c}$) and dot products with the two "axes" $\mathbf{a} - \mathbf{c}$ and $\mathbf{b} - \mathbf{c}$.

Frequently, barycentric coordinates are the coordinates of choice for operations in a plane in three dimensions that is (or can be) specified by a triangle. A trivial example is that we can test whether a projected point $\mathbf{p}'$ is inside or outside of $\triangle\mathbf{abc}$ by using equation (21.3.19) (or, if applicable, 21.3.21) to get α and β, and then checking that α, β, and $\gamma = 1 - \alpha - \beta$ are all positive.

Angle Between Two Triangles. The dihedral angle between two triangles (with a common edge, say) is the same as the angle between the normal vectors of the two triangles. The normal vectors are given by the vector area formula (21.3.14). The angle is best computed using equation (21.4.13), in the next section.

CITED REFERENCES AND FURTHER READING:

Bowyer, A. and Woodwark, J. 1983, *A Programmer's Geometry* (London: Butterworths), Chapter 4.

Schneider, P.J. and Eberly, D.H. 2003, *Geometric Tools for Computer Graphics* (San Francisco: Morgan Kaufmann), §3.5 and Appendix C.

López-López, F.J. 1992, "Triangles Revisited," in *Graphics Gems III*, Kirk, D., ed. (Cambridge, MA: Academic Press).

Glassner, A.S. 1990, "Useful 3D Geometry," in *Graphics Gems*, Glassner, A.S., ed. (San Diego: Academic Press).

21.4 Lines, Line Segments, and Polygons

A *line* is defined by any two points through which it passes. Call them **a** and **b**. As in §21.1, the points can be two-dimensional, if the domain of interest is a plane, or three-dimensional (or higher), if the line is embedded in a higher-dimensional space. For now, consider only the two-dimensional case.

Parametrically, any point **c** that lies on the line defined by **a** and **b** must be a linear combination of those two points. One way to write this is

$$\mathbf{c} = \mathbf{a} + s(\mathbf{b} - \mathbf{a}) \qquad (-\infty < s < \infty) \tag{21.4.1}$$

where s is a parameter along the line. The chosen normalization is to make $s = 0$ at **a** and $s = 1$ at **b**. The part of the line between **a** and **b** has $0 \le s \le 1$ and is a *line segment*, denoted $\overline{\mathbf{ab}}$. The whole line is denoted $\overleftrightarrow{\mathbf{ab}}$.

The easiest way to get the equation satisfied by all points **c** on the line $\overleftrightarrow{\mathbf{ab}}$ is to take the vector cross product of equation (21.4.1) with $(\mathbf{b} - \mathbf{a})$ on the right. Using the fact that the cross product of any vector with itself is zero, we get

$$\mathbf{c} \times (\mathbf{b} - \mathbf{a}) = \mathbf{a} \times \mathbf{b} \tag{21.4.2}$$

or writing out the components,

$$c_0(b_1 - a_1) - c_1(b_0 - a_0) = a_0 b_1 - a_1 b_0 \tag{21.4.3}$$

which is indeed a linear relation between the coordinates c_0 and c_1. While it is tempting to divide this equation by $b_0 - a_0$ to get an equation in that old familiar high school form "$y = mx + b$," one should often resist that temptation since, as written, equation (21.4.3) remains valid for the case of a vertical line, when $b_0 - a_0 = 0$.

Intersection of Two Lines. In the plane, two lines $\overleftrightarrow{\mathbf{ab}}$ and $\overleftrightarrow{\mathbf{xy}}$ most always intersect. We can solve for the point of intersection by equating the two lines' parametric forms,

$$\mathbf{a} + s(\mathbf{b} - \mathbf{a}) = \mathbf{x} - t(\mathbf{y} - \mathbf{x}) \tag{21.4.4}$$

and then solving the two equations (components 0 and 1) for the two unknowns s and t. The result is

$$\begin{aligned} s &= \frac{(\mathbf{x} - \mathbf{y}) \times (\mathbf{a} - \mathbf{x})}{(\mathbf{b} - \mathbf{a}) \times (\mathbf{x} - \mathbf{y})} = \frac{(x_0 - y_0)(a_1 - x_1) - (x_1 - y_1)(a_0 - x_0)}{(b_0 - a_0)(x_1 - y_1) - (b_1 - a_1)(x_0 - y_0)} \\ t &= \frac{(\mathbf{a} - \mathbf{x}) \times (\mathbf{b} - \mathbf{a})}{(\mathbf{b} - \mathbf{a}) \times (\mathbf{x} - \mathbf{y})} = \frac{(a_0 - x_0)(b_1 - a_1) - (a_1 - x_1)(b_0 - a_0)}{(b_0 - a_0)(x_1 - y_1) - (b_1 - a_1)(x_0 - y_0)} \end{aligned} \tag{21.4.5}$$

Of course, the special case of parallel lines with no intersection is indicated by the vanishing of the denominators.

All those cross products might make you think that equation (21.4.5) has a geometrical interpretation. Indeed so. In Figure 21.4.1, the lines intersect at **o**. Segment $\overline{\mathbf{xo}}$ is therefore just $\overline{\mathbf{xy}}$ scaled by t, while $\overline{\mathbf{ao}}$ is similarly $\overline{\mathbf{ab}}$ scaled by s. The area of $\triangle\mathbf{oxa}$ is therefore given (cf. equation 21.3.1) by

$$2\mathcal{A}(\mathbf{oxa}) = st\ (\mathbf{x} - \mathbf{y}) \times (\mathbf{a} - \mathbf{b}) \tag{21.4.6}$$

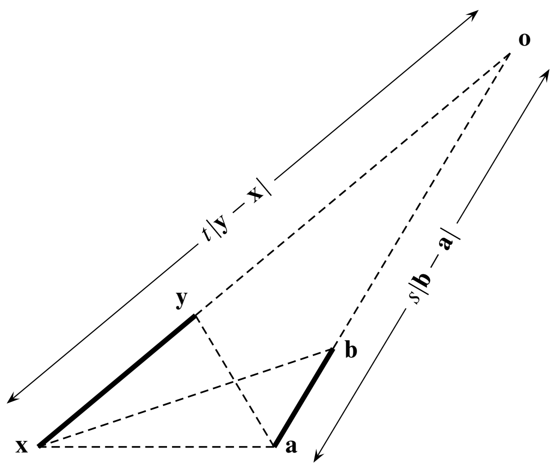

Figure 21.4.1. Geometrical construction that yields the intersection point of two lines in terms of ratios of triangle areas. See text for details.

By linearity of a triangle's area with its height (holding the base fixed), we also have

$$\mathcal{A}(\mathbf{oxa})/\mathcal{A}(\mathbf{yxa}) = t \qquad \mathcal{A}(\mathbf{oxa})/\mathcal{A}(\mathbf{bxa}) = s \tag{21.4.7}$$

Equation (21.4.5) follows immediately from these relations and equation (21.3.1).

Point-to-Line Distance. What is the perpendicular distance d from an arbitrary point $\mathbf{q}$ to the line $\overleftrightarrow{\mathbf{ab}}$ that passes through points $\mathbf{a}$ and $\mathbf{b}$? Evidently d is the height of $\triangle\mathbf{abq}$ when its base is the segment $\overline{\mathbf{ab}}$. Therefore, from the schoolbook "one-half base times height" formula,

$$d = \frac{2\mathcal{A}(\mathbf{abq})}{|\mathbf{a}-\mathbf{b}|} = \frac{(q_0-b_0)(a_1-b_1)-(q_1-b_1)(a_0-b_0)}{\sqrt{(a_0-b_0)^2+(a_1-b_1)^2}} \tag{21.4.8}$$

Note that d is signed, positive if it is to the left of the directed line from $\mathbf{a}$ to $\mathbf{b}$, negative if it is to the right, and a good segue to our next topic.

21.4.1 Line Segment Intersections and "Left-Of" Relations

You can use equation (21.4.5) to test whether two line segments, $\overline{\mathbf{ab}}$ and $\overline{\mathbf{xy}}$, intersect: Calculate s and t and then check if they are both in the range $(0, 1)$. (To keep our discussion brief, we won't say much here, or in what follows, about the various degenerate cases where s or t or both are exactly 0 or 1. These are straightforward to figure out if your application so demands.)

A related, but slightly different, approach is to use the fact that the formulas for triangle areas given in equation (21.3.1) are signed. This means that, as equivalent statements, we have

$$\mathcal{A}(\mathbf{abc}) > 0 \quad \Longleftrightarrow \quad \left[\begin{array}{l}\mathbf{c}\text{ is to the }\textit{left}\text{ of line }\overleftrightarrow{\mathbf{ab}}\\ \text{when it is traversed in}\\ \text{the direction from }\mathbf{a}\text{ to }\mathbf{b}\end{array}\right] \tag{21.4.9}$$

while $\mathcal{A}(\mathbf{abc}) < 0$ implies that $\mathbf{c}$ is to the right of the same line. We refer to either statement in (21.4.9) as a *left-of relation*.

A necessary and sufficient condition for two segments $\overline{\mathbf{ab}}$ and $\overline{\mathbf{xy}}$ to intersect is that **x** and **y** be on opposite sides of $\overleftrightarrow{\mathbf{ab}}$, *and* **a** and **b** be on opposite sides of $\overleftrightarrow{\mathbf{xy}}$. (We again omit discussion of the various special cases of collinearity.) This test, using the triangle area formulas in equation (21.3.1), involves evaluating four left-of relations, each computationally a cross product, which is just slightly more work than computing s and t (which share a denominator). However, you can sometimes use the same cross products, once computed, in other parts of your calculation. So, it is often a toss-up whether to use the "s, t" method or the "left-of" method — you should consider both.

Fig.	△abx	△aby	△xya	△xyb	Intersection	Hull
1	−	+	+	−	$\overline{ab} \times \overline{xy}$	□axby
2	+	−	−	+	$\overline{ab} \times \overline{xy}$	□aybx
3	+	−	−	−	$\overrightarrow{ab} \times \overline{xy}$	△ayx
4	−	+	−	−	$\overrightarrow{ba} \times \overline{xy}$	△byx
5	+	−	+	+	$\overrightarrow{ba} \times \overline{xy}$	△bxy
6	−	+	+	+	$\overrightarrow{ab} \times \overline{xy}$	△axy
7	−	−	−	+	$\overrightarrow{yx} \times \overline{ab}$	△yba
8	−	−	+	−	$\overrightarrow{xy} \times \overline{ab}$	△xba
9	+	+	−	+	$\overrightarrow{xy} \times \overline{ab}$	△xab
10	+	+	+	−	$\overrightarrow{yx} \times \overline{ab}$	△yab
11	−	−	−	−	external	□ayxb
12	+	+	−	−	external	□abxy
13	+	+	+	+	external	□abxy
14	−	−	+	+	external	□axyb
	−	+	−	+	Not possible!	
	+	−	+	−	Not possible!	

Table 21.4.1. Relationship between two line segments classified by the signs of the areas of various triangles. Refer to Figure 21.4.2 for an illustration of each case.

Table 21.4.1 enumerates the 16 cases that you get if you compute all four possible "left-of" tests for two line segments. (Actually, only 14 are geometrically possible!) The table classifies each possibility as to whether the segments intersect (intersection denoted by $\times$, not to be confused with vector cross product!), whether the unidirectional extension of one segment (a *ray*) intersects the other segment, or (lastly) whether an ordinary line intersection occurs external to both segments. Also shown for each case is the outer *hull* of the two segments (the smallest triangle or quadrilateral enclosing both segments) and how it is traversed in clockwise order. Figure 21.4.2 shows an example of each possibility.

You can use the table to find combinations that test for specific circumstances.

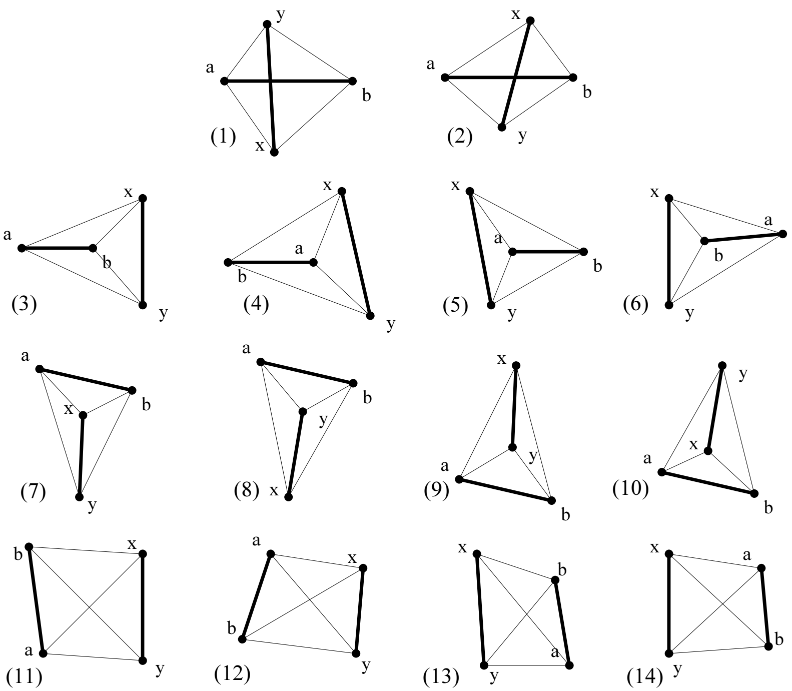

Figure 21.4.2. Two line segments, ab and xy, define four triangles ($\triangle abx$, $\triangle aby$, $\triangle xya$, and $\triangle xyb$), each of which can have positive or negative area. Of the 16 combinations of signs, 14 (shown here) correspond to to possible intersection relationships between the line segments or their extensions as rays.

For example, if you need a test for whether the ray $\overrightarrow{\mathbf{ab}}$ intersects the segment $\overline{\mathbf{xy}}$ (including the possibility of the segments intersecting), you examine rows 1, 2, 3, and 6 in the table and read off a test that involves just three left-of relations:

$$\mathcal{A}(\mathbf{abx})\mathcal{A}(\mathbf{aby}) < 0 \qquad \text{and} \qquad \mathcal{A}(\mathbf{aby})\mathcal{A}(\mathbf{xya}) > 0 \tag{21.4.10}$$

Of course there are exactly equivalent tests using s and t, for this example (with s and t as in equation 21.4.4) $s > 0$ and $0 < t < 1$.

Angle Between Two Vectors. Suppose that $\mathbf{U}$ and $\mathbf{V}$ are difference vectors along each of two lines, and that θ is the angle between the lines (measured from $\mathbf{U}$ to $\mathbf{V}$). In the previous notation, $\mathbf{U} = \mathbf{y} - \mathbf{x}$ and $\mathbf{V} = \mathbf{b} - \mathbf{a}$. Elementary vector analysis tells us that

$$\begin{aligned} \mathbf{U} \cdot \mathbf{V} &= U_0 V_0 + U_1 V_1 = |\mathbf{U}||\mathbf{V}|\cos(\theta) \\ \mathbf{U} \times \mathbf{V} &= U_0 V_1 - U_1 V_0 = |\mathbf{U}||\mathbf{V}|\sin(\theta) \end{aligned} \tag{21.4.11}$$

Many people try to get θ by using one or the other of the above relations, computing the vector norms and taking an inverse cosine or inverse sine. Big mistake! Not only are there quadrant ambiguities in the inverse trig functions, but there are also angles near the flat extrema of the sine and cosine functions where you can lose up to half of your significant figures in the answer. Not to mention the need to calculate square roots for the norms! The better approach is

$$\theta = \texttt{atan2}(\mathbf{U} \times \mathbf{V}, \mathbf{U} \cdot \mathbf{V}) = \text{atan2}(U_0 V_1 - U_1 V_0, U_0 V_0 + U_1 V_1) \tag{21.4.12}$$

where `atan2()` is C's or C++'s quadrant-sensitive arctangent function. That function allows either of its arguments to be zero and returns a value in the range $-\pi/2$ to $\pi/2$. (An identical function exists in Fortran and most other languages.)

21.4.2 Lines in Three Dimensions

The immediate generalization of equation (21.4.12) to three-dimensional space gives the angle between two 3-vectors,

$$\theta = \texttt{atan2}\,(|\mathbf{U} \times \mathbf{V}|, \mathbf{U} \cdot \mathbf{V}) \tag{21.4.13}$$

Note the occurrence of the modulus of the vector cross product, which requires taking a square root.

Brevity constraints allow us to say only a little more about lines in three-dimensional space. The parameterization

$$\mathbf{c} = \mathbf{a} + s\,\mathbf{v} \qquad\qquad (-\infty < s < \infty) \tag{21.4.14}$$

(equation 21.4.1 with $\mathbf{v} \equiv \mathbf{b} - \mathbf{a}$) still works, with $\mathbf{a}$, $\mathbf{v}$, and $\mathbf{c}$ now points in 3-space. The parameter s at which a line intersects a plane specified by $\mathbf{N}$ and D (see equation 21.3.16) is given by

$$s = \frac{D - \mathbf{a} \cdot \mathbf{N}}{\mathbf{v} \cdot \mathbf{N}} \tag{21.4.15}$$

with the denominator vanishing if the line is parallel to the plane.

The closest approach of a line to a point $\mathbf{q}$ occurs when

$$s = \frac{(\mathbf{q} - \mathbf{a}) \cdot \mathbf{v}}{|\mathbf{v}|^2} \tag{21.4.16}$$

You can also use this to see whether a line intersects a sphere in 3-space: Calculate the closest point on the line to the sphere's center, and then check if the distance is less than the sphere's radius (or compare squares of distances to avoid the square root).

Two lines, call them $\mathbf{a} + s\,\mathbf{v}$ and $\mathbf{x} + t\,\mathbf{u}$, will not, in general, share a common point; rather, they will be *skew* to one another. However, their points of closest approach can be calculated as [2]

$$s = \frac{\det\{(\mathbf{a} - \mathbf{x}), \mathbf{u}, \mathbf{u} \times \mathbf{v})\}}{|\mathbf{u} \times \mathbf{v}|^2} \qquad t = \frac{\det\{(\mathbf{a} - \mathbf{x}), \mathbf{v}, \mathbf{u} \times \mathbf{v})\}}{|\mathbf{u} \times \mathbf{v}|^2} \tag{21.4.17}$$

where det is the 3×3 determinant whose columns are the indicated 3-vectors. The denominator vanishes if the lines are parallel. If you really must check for an actual intersection, plug these values for s and t into the parametric forms for each line, and check whether the distance between two points thus obtained is less than some roundoff tolerance.

A common operation in computer graphics is to test whether a line intersects a triangle in three dimensions. To do this with methods already discussed, use equation (21.3.17) to get $\mathbf{N}$ and D for the triangle's plane. Then use equations (21.4.14) and (21.4.15) to get the line's intersection with that plane. Finally, use equation (21.3.19) to get the barycentric coordinates α and β of the intersection. If α, β, and $\gamma \equiv 1 - \alpha - \beta$ are all positive, then the intersection is inside the triangle. See [4,1] for various ways to streamline this procedure.

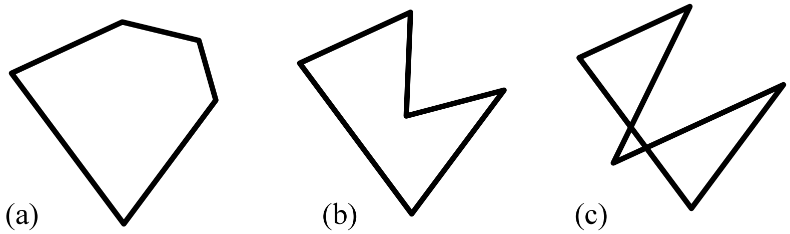

Figure 21.4.3. Polygons are classified as simple if they have no intersecting edge, as in (a) and (b), or complex (c) otherwise. Simple polygons are either convex (a) or concave (b).

21.4.3 Polygons

We define a *polygon* as a vector of N points (*vertices*), numbered from 0 to $N-1$, and the N directed line segments that connect them in cyclic order, that is 0 to 1, 1 to 2, ..., $N-2$ to $N-1$, and (importantly!) $N-1$ to 0. (In some formulas below we will use the convention that vertex N is to be taken as meaning vertex 0.)

We consider two polygons to be the same if they differ only by a cyclical renumbering of the points, so that all their line segments are the same. However, if we reverse the order of traversing the points, we consider the resulting polygon to be different. (For example, the sign of its area will change.) If the boundary of a region cannot be traversed by a single cyclical vector (e.g., the region between an outer square and an enclosed triangle), we don't call it a polygon; other conventions are of course also possible.

With the definition given, it is useful to classify a polygon as either *simple*, meaning that none of its N line segments intersect, or *complex* if there are one or more segment intersections. We classify simple polygons according to whether they are convex or concave. A convex polygon can be defined either by (i) all $N(N-1)/2$ line segments connecting two vertices lie in its interior (or on its boundary), or (ii) its exterior angles all have the same sign (zeros allowed). Whichever property is taken as the definition, the other becomes a theorem. Figure 21.4.3 shows examples of the three types.

For simple polygons, the sum of the exterior angles is always $\pm 2\pi$. That is, you turn through exactly one circle in driving around the polygon. If the polygon is concave, then the sign of the exterior angles must be taken into account when doing the sum. This is shown in Figure 21.4.4. The sign of the 2π is positive for a counterclockwise (CCW) traversal, negative for clockwise (CW).

Complex (that is, self-intersecting) polygons can also have exterior angles that sum to 2π, as the polygon in Figure 21.4.3(c), so the exterior angles do not provide, in general, any magical way of finding intersections. However, one small bit of magic does exist: If the exterior angles of a polygon are all of one sign (or zero), *and* if they sum to $\pm 2\pi$, then the polygon is both simple and convex. This provides a very rapid way to test for the simple-and-convex case, but it does not distinguish between simple-concave and complex polygons. Doing so requires a detailed check for intersecting edges (which we will implement in code below).

Winding Number. If you sit on a point $\mathbf{p}$ in the plane, and watch someone drive around a polygon, then they will drive around *you* some net integer number of times (with the usual sign convention, CCW being positive). This is the polygon's *winding number with respect to a point*. For simple polygons, the winding number is 1 for

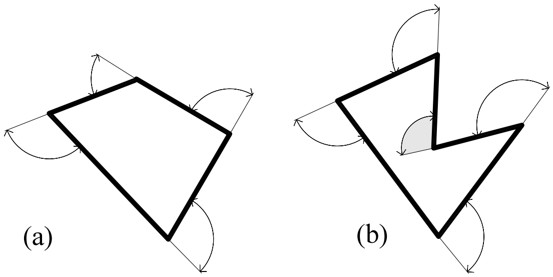

Figure 21.4.4. The exterior angles of simple polygons sum to one full circle. (a) If the polygon is convex, all the angles have the same sign. (b) If the polygon is concave, one or more angles (here, the shaded angle) has opposite sign.

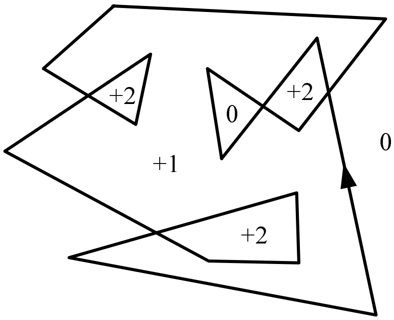

Figure 21.4.5. Complex polygon with different winding numbers (indicated by integers) around points in different regions. The total winding number of the polygon (sum of exterior angles divided by 2π) is 3, a value not realized in any single region. Note that interior regions can have winding number 0.

points inside a CCW polygon, -1 for points inside a CW polygon, and 0 for points outside. For complex polygons, however, there are no simple rules. Figure 21.4.5 shows a complicated case. Note that interior regions of a complex polygon can have winding number 0, so a point's winding number (alone) does not determine whether it is inside or outside a complex polygon. Note also that the sum of a polygon's exterior angles, divided by 2π, is not necessarily the winding number of that polygon with respect to any point in the plane.

Doubtless the *worst* way to compute a polygon's winding number with respect to a point $\mathbf{q}$ is to add up the N incremental angles between $\mathbf{q}$ and consecutive vertices $\mathbf{p}_i$ of the polygon, that is,

$$\text{W.N.}(\mathbf{q}) = \frac{1}{2\pi} \sum_{i=0}^{N-1} \angle(\mathbf{p}_{i+1}\mathbf{q}\mathbf{p}_i) \tag{21.4.18}$$

(with the usual convention $\mathbf{p}_N \equiv \mathbf{p}_0$). Even using the trick in equation (21.4.12) to get the angles, there is an enormous amount of unnecessary computation in this approach.

Instead, we can observe that if a polygon winds M times around $\mathbf{q}$, then its edges must cross any ray from $\mathbf{q}$ to infinity a net of exactly M times, where ray crossings in the CCW direction are counted as positive, CW as negative. In particular, if we take the ray to be the horizontal ray to the right of $\mathbf{q}$, we can immediately

reject edges that fail to cross the horizontal line that contains $\mathbf{q}$, and then check for the ray crossing (and its sign) with a single "left-of" test [5]. These ideas are embodied in the following routine.

polygon.h

```
Int polywind(const vector< Point<2> > &vt, const Point<2> &pt) {
Return the winding number of a polygon (specified by a vector of vertex points vt) around an
arbitrary point pt.
    Int i,np, wind = 0;
    Doub d0,d1,p0,p1,pt0,pt1;
    np = vt.size();
    pt0 = pt.x[0];
    pt1 = pt.x[1];
    p0 = vt[np-1].x[0];                 Save last vertex as "previous" to first.
    p1 = vt[np-1].x[1];
    for (i=0; i<np; i++) {              Loop over edges.
        d0 = vt[i].x[0];
        d1 = vt[i].x[1];
        if (p1 <= pt1) {
            if (d1 > pt1 &&             Upward-crossing edge. Is pt to its left?
                (p0-pt0)*(d1-pt1)-(p1-pt1)*(d0-pt0) > 0) wind++;
        }
        else {
            if (d1 <= pt1 &&            Downward-crossing edge. Is pt to its right?
                (p0-pt0)*(d1-pt1)-(p1-pt1)*(d0-pt0) < 0) wind--;
        }
        p0=d0;                          Current vertex becomes previous one.
        p1=d1;
    }
    return wind;
}
```

Is there a similarly efficient way to find the total winding number of a polygon $\mathbf{p}_i$ ($i = 0, \ldots, N-1$), that is, the sum of its exterior angles divided by 2π? Yes. Consider the derived polygon whose vertex points $\mathbf{q}_i$ are given by the vector differences

$$\mathbf{q}_i = \mathbf{p}_{i+1} - \mathbf{p}_i \qquad (i = 0, \ldots, N-1) \tag{21.4.19}$$

Then the winding number of this derived polygon *around the origin* is just the total winding number of the original polygon. (Draw a picture if this isn't immediately obvious to you.) The routine `polywind()` can be used for the computation.

Point Inside Polygon. How can you tell whether an arbitrary point $\mathbf{q}$ is inside or outside a polygon [5]? Let us first assume that your polygon is known to be simple. For simple polygons, two commonly used approaches are the "winding number method" and the "Jordan curve theorem method." However, when these are each implemented efficiently, they become virtually identical!

The winding number method is simply to compute the winding number of the polygon around the point (e.g., using `polywind()`, above). If the answer is ± 1, then the point is inside the polygon. If it is zero, it is outside. Any other answer indicates that the polygon wasn't simple as assumed.

The Jordan curve theorem method observes that any ray from the point to infinity will cross the polygon an odd number of times if the point is inside, or an even number of times if it is outside [Figure 21.4.6(a)]. If we implemented this in code, it would be almost identical to the code in `polywind` except for one detail: Instead of incrementing or decrementing a counter at each ray crossing (according to the direction of the crossing), we would always increment it. Then, at the end, we would

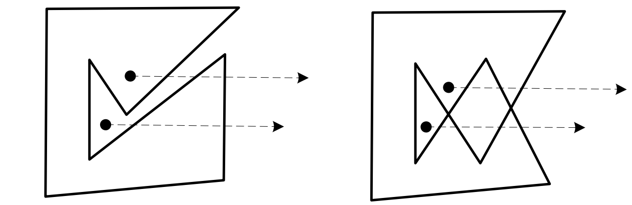

Figure 21.4.6. Is a point inside a polygon? (a) For a simple polygon, either the winding number, or the Jordan curve theorem (even or odd number of crossings of a ray) can be used. (b) For complex polygons, there is no simple test.

check whether the counter is even or odd. But if `polywind` as written returns 0, it *must* have encountered the same number of increments as decrements, hence an even number of crossings. And if it returns ± 1 (the only other possible value for a simple polygon), it must similarly have encountered an odd number. So the two methods are really the same.

What if your polygon is not simple? As Figure 21.4.6(b) illustrates, you are in deep waters. Both the winding number method and the Jordan curve theorem method will say that the upper point in the figure is inside the complex polygon shown, and this seems intuitively correct. However, both methods will say that the lower point is *outside* the polygon. Indeed, there are some self-consistent ways of defining "insideness" for complex polygons that make this the case. The result is so counterintuitive, however, as to be useless in most practical applications. It is generally better just to avoid using the idea of "insideness" with complex polygons.

Classification of Polygons. We are now in a position to combine several of the ideas already introduced into a function that classifies any polynomial as either simple or complex, and (if it is simple) whether it is convex or concave, and whether it is CCW (total winding number 1) or CW (total winding number -1).

```
Int ispolysimple(const vector< Point<2> > &vt) {
```
polygon.h

Classifies a polygon specified by a vector of vertex points `vt`. Returns 0 if the polygon is complex (has intersecting edges). Returns ± 1 if it is simple and convex. Returns ± 2 if it is simple and concave. The sign of the returned value indicates whether the polygon is CCW (+) or CW (−).

```
    Int i,ii,j,jj,np,schg=0,wind=0;          Initialize sign change and winding number.
    Doub p0,p1,d0,d1,pp0,pp1,dd0,dd1,t,tp,t1,t2,crs,crsp=0.0;
    np = vt.size();
    p0 = vt[0].x[0]-vt[np-1].x[0];
    p1 = vt[0].x[1]-vt[np-1].x[1];
    for (i=0,ii=1; i<np; i++,ii++) {         Loop over edges.
        if (ii == np) ii = 0;
        d0 = vt[ii].x[0]-vt[i].x[0];
        d1 = vt[ii].x[1]-vt[i].x[1];
        crs = p0*d1-p1*d0;                   Cross product at this vertex.
        if (crs*crsp < 0) schg = 1;          Sign change (i.e., concavity) found.
        if (p1 <= 0.0) {                     Winding number logic as in polywind.
            if (d1 > 0.0 && crs > 0.0) wind++;
        } else {
            if (d1 <= 0.0 && crs < 0.0) wind--;
        }
        p0=d0;
        p1=d1;
        if (crs != 0.0) crsp = crs;          Save previous cross product only if it has a
    }                                            sign!
```

```
    if (abs(wind) != 1) return 0;           Can already conclude polygon is complex.
    if (schg == 0) return (wind>0? 1 : -1);    Polygon is simple and convex.
    Drat, we've exhausted all the quick tricks and now have to check all pairs of edges for
    intersections:
    for (i=0,ii=1; i<np; i++,ii++) {
        if (ii == np) ii=0;
        d0 = vt[ii].x[0];
        d1 = vt[ii].x[1];
        p0 = vt[i].x[0];
        p1 = vt[i].x[1];
        tp = 0.0;
        for (j=i+1,jj=i+2; j<np; j++,jj++) {
            if (jj == np) {if (i==0) break; jj=0;}
            dd0 = vt[jj].x[0];
            dd1 = vt[jj].x[1];
            t = (dd0-d0)*(p1-d1) - (dd1-d1)*(p0-d0);
            if (t*tp <= 0.0 && j>i+1) {         First loop is only to compute starting tp,
                pp0 = vt[j].x[0];                   hence test on j.
                pp1 = vt[j].x[1];
                t1 = (p0-dd0)*(pp1-dd1) - (p1-dd1)*(pp0-dd0);
                t2 = (d0-dd0)*(pp1-dd1) - (d1-dd1)*(pp0-dd0);
                if (t1*t2 <= 0.0) return 0;     Found an intersection, so done.
            }
            tp = t;
        }
    }
    return (wind>0? 2 : -2);               No intersections found, so simple concave.
}
```

When `ispolysimple` finds that the quick indicators are not enough, and that it needs to check all pairs of edges for intersections, it does so by the obvious $O(N^2)$ method of two nested loops. For small N, say less than 10, this is likely as fast as any other strategy. If you are dealing with large numbers of large-N polygons, however, you will want to substitute a method with better scaling in N. One way, using the code from §21.8, would be to define a class for segments with a `collides()` method, then store the segments into a QO tree one at a time, looking for collisions at each step. (Don't forget that adjacent edges of a simple polynomial are allowed to "collide" at their shared vertex.)

Area of Polygons. As a next topic, let us turn to the *area* of a polygon. The (signed) area of a polygon is the sum of the areas of each of its regions weighted by that region's winding number. For simple polygons the area is thus what you would expect geometrically, except that its sign will be negative for a polygon traversed CW rather than CCW. (We previously saw this in the special case of triangles.) For a complex polygon like that shown in Figure 21.4.5, the answer is less intuitive (and generally less useful) since some regions, such as the interior region with winding number 0, are not counted at all, while others are counted (in this case) twice.

The great advantage of this definition of area, however, is that it results in a simple expression for the area that applies to both simple and complex polygons. Let x_i and y_i be, respectively, the 0 and 1 coordinates of the polygon's vertex $\mathbf{p}_i$, and let $\mathcal{A}$ be the polygon's area. Then, in three equivalent forms,

$$2\,\mathcal{A} = \sum_{i=0}^{N-1} x_i\,y_{i+1} - x_{i+1}\,y_i$$

$$= \sum_{i=0}^{N-1} (x_{i+1} + x_i)(y_{i+1} - y_i)$$
$$= \sum_{i=0}^{N-1} x_i (y_{i+1} - y_{i-1}) \qquad (21.4.20)$$

Evaluation of any of these forms takes just one loop over the polygon's vertices. (These formulas go back as far as Meister in 1769 and Gauss in 1795.)

Although we won't derive equation (21.4.20) in detail, the middle form does have an intuitive interpretation. It sums the areas of trapezoids each with two points on the y-axis ($x = 0$) at y_i and y_{i+1}, and with the other two points the points on the polygon at these y's. In going around the polygon, negative-area trapezoids subtract from positive-area ones so as to leave just the area inside.

Interestingly, there are very similar formulas for the x and y coordinates of the centroid or center-of-mass of an arbitrary polygon [3],

$$\overline{x} = \frac{1}{6} \sum_{i=0}^{N-1} (x_{i+1} + x_i)(x_i y_{i+1} - x_{i+1} y_i)$$
$$\overline{y} = \frac{1}{6} \sum_{i=0}^{N-1} (y_{i+1} + y_i)(x_i y_{i+1} - x_{i+1} y_i) \qquad (21.4.21)$$

Note the common subexpressions with equation (21.4.20), so that it is efficient to calculate the area and centroid position together.

Finally, a couple of polygon tidbits for your edification or amusement:

- If two simple polygons have the same area, then the first can be cut into a finite number of polygonal pieces that can be reassembled into the second. This is known as the Bolyai-Gerwien theorem. (The corresponding statement about polyhedra in three dimensions, "Hilbert's Third Problem," was proved false by Dehn in 1900.)
- The regular polygon with N sides is constructible with a compass and straightedge if the factorization of N contains only the factors 2, 3, 5, 17, 257, 257, and 65537 (whose odd members are the *Fermat primes*), with each odd factor occurring at most once. It is not known whether any other N-gons are also constructible; but, if so, then their N must contain a factor at least as large as $2^{2^{33}} + 1$. The product of the known Fermat primes, which is perforce the largest known constructible polygon with an odd number of sides, is $2^{32} - 1 = 4294967295$, a number well known to computer trolls as the largest positive 32-bit integer. Go figure.

CITED REFERENCES AND FURTHER READING:

Bowyer, A. and Woodwark, J. 1983, *A Programmer's Geometry* (London: Butterworths).

Schneider, P.J. and Eberly, D.H. 2003, *Geometric Tools for Computer Graphics* (San Francisco: Morgan Kaufmann), §11.1.2[1]

de Berg, M., van Kreveld, M., Overmars, M., and Schwarzkopf, O. 2000, *Computational Geometry: Algorithms and Applications*, 2nd revised ed. (Berlin: Springer), Chapter 2.

O'Rourke, J. 1998, *Computational Geometry in C*, 2nd ed. (Cambridge, UK: Cambridge University Press), §7.4.

Goldman, R. 1990, "Intersection of Two Lines in Three-Space," in *Graphics Gems*, Glassner, A.S., ed. (San Diego: Academic Press).[2]

Bashein, G. and Detmer, P.R. 1994, "Centroid of a Polygon," in *Graphics Gems IV*, Heckbert, P.S., ed. (Cambridge, MA: Academic Press).[3]

Sunday, D. 2007+, at `http://softsurfer.com/algorithm_archive.htm`.[4]

Haines, E. 1994, "Point in Polygon Strategies," in *Graphics Gems IV*, Heckbert, P.S., ed. (Cambridge, MA: Academic Press).[5]

Wikipedia 2007+, "Polygon," at `http://en.wikipedia.org`.

21.5 Spheres and Rotations

The surface of the Earth is called a 2-sphere by topologists, but a 3-sphere by geometers; so the term n-sphere is somewhat unclear. We'll say "sphere in n dimensions" to avoid any ambiguity. (For Earth, $n = 3$.) *Sphere* refers to the surface, *ball* to the interior volume.

A sphere of radius r in n dimensions, centered on the origin, is the locus of points for which

$$x_0^2 + \cdots + x_{n-1}^2 = r^2 \tag{21.5.1}$$

Points on the sphere in n dimensions can be specified by $n-1$ angular coordinates, roughly the analogs of latitude and longitude on the sphere in three dimensions,

$$\begin{aligned} x_0 &= r\cos\psi_0 \\ x_1 &= r\sin\psi_0\ \cos\psi_1 \\ &\cdots \\ x_{n-2} &= r\sin\psi_0\ \sin\psi_1\ \cdots\cos\psi_{n-2} \\ x_{n-1} &= r\sin\psi_0\ \sin\psi_1\ \cdots\sin\psi_{n-2} \end{aligned} \tag{21.5.2}$$

All the angles except the last have the range

$$0 \le \psi_i \le \pi, \qquad i = 0,\ldots,n-3 \tag{21.5.3}$$

i.e., are "latitude-like." The last angle is "longitude-like,"

$$0 \le \psi_{n-2} \le 2\pi \tag{21.5.4}$$

The surface area S_n of the sphere in n dimensions has a simple recurrence,

$$\begin{aligned} S_1 &= 2 && \text{(two points)} \\ S_2 &= 2\pi r && \text{(circumference of circle)} \\ S_n &= \frac{2\pi r^2}{n-2} S_{n-2}, && n > 2 \end{aligned} \tag{21.5.5}$$

The volume V_n of the n-dimensional ball is equal to r/n times the area of the enclosing sphere in n dimensions, and also has a simple recurrence,

$$\begin{aligned}
V_1 &= 2r && \text{(length of line)} \\
V_2 &= \pi r^2 && \text{(area of circle)} \\
&\dots \\
V_n &= \frac{r}{n} S_n = \frac{2\pi r^2}{n} V_{n-2}
\end{aligned} \tag{21.5.6}$$

Closed-form formulas require a gamma function,

$$\begin{aligned}
S_n &= \frac{2\pi^{n/2}}{\Gamma(\frac{1}{2}n)} r^{n-1} \\
V_n &= \frac{2\pi^{n/2}}{n\Gamma(\frac{1}{2}n)} r^n
\end{aligned} \tag{21.5.7}$$

As n becomes large, the ratio of the volume of a ball to the volume of the circumscribed (hyper-) cube rapidly becomes small,

$$\frac{V_n}{2^n} \to 0, \qquad n \to \infty \tag{21.5.8}$$

21.5.1 Picking a Random Point on the Sphere

You don't get a random point on the sphere in n dimensions by picking uniformly random values for the $n-1$ angles in equation (21.5.2), just as you don't get a random point on the Earth's surface by throwing darts at a Mercator map (or any other non-equal-area projection).

An elegant general method is to generate n independent, identically distributed, normal (Gaussian) deviates of zero mean, say $y_0, \dots, y_{n-1}$ (see §7.3), and then calculate a point $\mathbf{x}$ on the unit sphere in n dimensions by

$$\mathbf{x} = \frac{\mathbf{y}}{|\mathbf{y}|} \tag{21.5.9}$$

or, in other words,

$$x_i = y_i \Bigg/ \sqrt{\sum_{j=0}^{n-1} y_j^2} \tag{21.5.10}$$

This works because the spherically symmetric Gaussian distribution in n dimensions trivially factorizes into a product of independent one-dimensional Gaussians. If you want a random point inside the enclosed n-volume, generate an additional *uniform* random deviate u in [0, 1] and calculate the point's coordinates as

$$x_i = u^{1/n}\, y_i \Bigg/ \sqrt{\sum_{j=0}^{n-1} y_j^2} \tag{21.5.11}$$

You can of course scale to any other radius of sphere.

Faster special methods are available for the spheres in two, three, and four dimensions. For two dimensions, the circle, pick u_0 and u_1 uniform in $[-1, 1]$, rejecting choices for which $u_0^2 + u_1^2 > 1$. This picks a random point inside the unit circle. Now scale in the obvious way to get a point on the circle,

$$x_0 = \frac{u_0}{\sqrt{u_0^2 + u_1^2}}, \qquad x_1 = \frac{u_1}{\sqrt{u_0^2 + u_1^2}} \tag{21.5.12}$$

(We already discussed this method in §7.3, under Cauchy deviates.)

A faster method for three dimensions, also using only two random deviates, is due to Marsalgia [1]. Pick a point *inside* the unit circle (u_0, u_1) as above. Then a random point on the sphere in three dimensions is

$$\begin{aligned} x_0 &= 2u_0\sqrt{1 - u_0^2 - u_1^2} \\ x_1 &= 2u_1\sqrt{1 - u_0^2 - u_1^2} \\ x_2 &= 1 - 2(u_0^2 + u_1^2) \end{aligned} \tag{21.5.13}$$

For the sphere in four dimensions, pick two independent points *inside* the unit circle as above, (u_0, u_1) and (u_2, u_3). Then a random point on the sphere in four dimensions is [1]

$$\begin{aligned} x_0 &= u_0 \\ x_1 &= u_1 \\ x_2 &= u_2\sqrt{\frac{1 - u_0^2 - u_1^2}{u_2^2 + u_3^2}} \\ x_3 &= u_3\sqrt{\frac{1 - u_0^2 - u_1^2}{u_2^2 + u_3^2}} \end{aligned} \tag{21.5.14}$$

Unfortunately, there is no known generalization to higher dimensions.

21.5.2 Picking a Random Rotation Matrix

Don't confuse this with picking a point on a sphere. A rotation matrix $\mathbf{M}$ in n dimensions is an orthogonal $n \times n$ matrix. For a *proper rotation*, $\mathbf{M}$ must have determinant 1. The other possibility, determinant -1, represents an *improper rotation*, decomposable into a proper rotation followed by a reflection. The rotation matrix $\mathbf{M}$ maps any point $\mathbf{x}$ to a new point $\mathbf{x}'$ by

$$\mathbf{x}' = \mathbf{M} \cdot \mathbf{x} \tag{21.5.15}$$

A general method for picking a uniformly random rotation matrix is to fill an $n \times n$ matrix $\mathbf{G}$ with independent, identically distributed, normal (Gaussian) deviates of zero mean. Then, use `QRdcmp` in §2.10 to construct the QR decomposition, namely $\mathbf{G} = \mathbf{Q} \cdot \mathbf{R}$. Except for the possibility that it might have the wrong sign of determinant, the matrix $\mathbf{Q}$ is now a uniformly random rotation matrix. The method used in `QRdcmp` is to apply $n - 1$ Householder transformations, each of which is

a reflection with determinant -1. Thus, to get determinant 1, we do nothing to $\mathbf{Q}$ if n is odd; if n is even, we simply interchange any pair of rows in $\mathbf{Q}$, giving the final answer.

For large n the work of doing the decomposition scales as $O(n^3)$, which can be burdensome. For faster, but more complicated, methods, see [2,3].

Faster special methods are available for two and three dimensions. A random two-dimensional rotation matrix has components that are the sine and cosine of a random angle θ in $[0, 2\pi]$,

$$\begin{pmatrix} \cos\theta & \sin\theta \\ -\sin\theta & \cos\theta \end{pmatrix} \tag{21.5.16}$$

We get the components without trigonometric function calls by using (21.5.12) to find a random point on the unit circle and then taking $\cos\theta = x_0$ and $\sin\theta = x_1$.

In the case of three dimensions, a fast method is to use equation (21.5.14) to generate a random point on the sphere in four dimensions, and then to construct the 3×3 orthogonal matrix,

$$\begin{bmatrix} 1 - 2(x_1^2 + x_2^2) & 2(x_0x_1 - x_3x_2) & 2(x_0x_2 + x_3x_1) \\ 2(x_0x_1 + x_3x_2) & 1 - 2(x_0^2 + x_2^2) & 2(x_1x_2 - x_3x_0) \\ 2(x_0x_2 - x_3x_1) & 2(x_1x_2 + x_3x_0) & 1 - 2(x_0^2 + x_1^2) \end{bmatrix} \tag{21.5.17}$$

which will be uniformly random among all rotations [4,5].

CITED REFERENCES AND FURTHER READING:

Marsaglia, G. 1972, "Choosing a Point from the Surface of a Sphere," *Annals of Mathematical Statistics*, vol. 43, pp. 645–646.[1]

Genz, A. 2000, "Methods for Generating Random Orthogonal Matrices," in *Monte Carlo and Quasi-Monte Carlo Methods*, Proceedings of the Third International Conference on Monte Carlo and Quasi-Monte Carlo Methods in Scientific Computing (MCQMC98) (Berlin: Springer).[2]

Anderson, T.W., Olkin, I., and Underhill, L.G. 1987, "Generation of Random Orthogonal Matrices," *SIAM Journal on Scientific and Statistical Computing*, vol. 8, pp. 625–629.[3]

Shoemake, K. 1985, "Animating Rotation with Quaternion Curves," *Computer Graphics*, Proceedings of SIGGRAPH 1985, vol. 19, pp. 245–254.[4]

Shoemake, K. 1992, "Uniform Random Rotations," in *Graphics Gems III*, Kirk, D., ed. (Cambridge, MA: Academic Press), pp. 124–132.[5]

21.6 Triangulation and Delaunay Triangulation

We can informally define a *triangulation* of a set of N points in the plane as follows: Connect the given points by straight-line segments as many times as you can without any two segments crossing. When you can't connect any more, you have a triangulation. Obviously there are many triangulations of a given set of points. Figure 21.6.1 shows three triangulations of the same set of 50 points. Two are "random," where the informal definition was pretty much followed literally. The third one is a very special triangulation, called a Delaunay triangulation. In a sense that we will make more precise below, it is the triangulation whose triangles best avoid small angles and long sides.

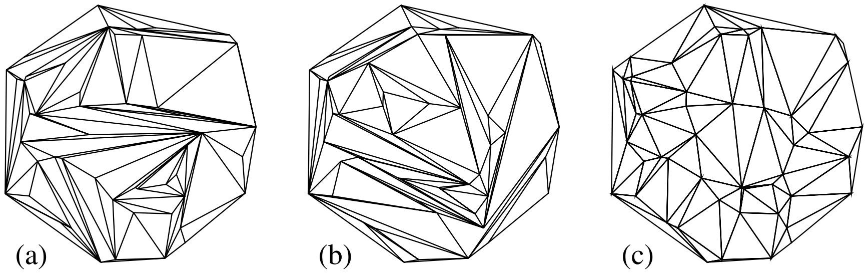

Figure 21.6.1. Three triangulations of the same 50 random points: (a) and (b) are "bad" (random) triangulations, while (c) is a "good" (Delaunay) triangulation. The number of lines and triangles is the same in each case.

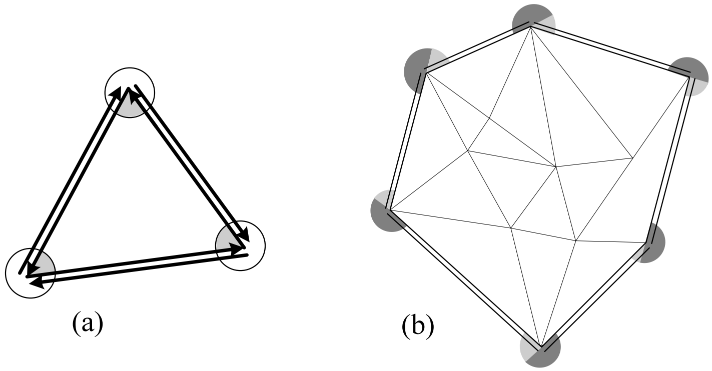

Figure 21.6.2. How to count lines and triangles in a triangulation. (a) Each triangle "uses up" $1/2$ of a point, and $3/2$ lines. (b) The n points in the convex hull "use up" $n/2 + 1$ points and $n/2$ lines.

All triangulations of a given set of points have the same outer boundary, called the *convex hull* of the point set. This should be evident, again informally, from the definition of triangulation: A line segment (edge) on the outer convex boundary can't interfere with any other actual or potential interior edges, so it will always be added before the stopping rule is reached. The number of points n (and also edges) in the convex hull is at least three, and can be as large as N, e.g., if the points all lie on a circle. (Here and below, we will ignore degenerate cases like "all points lie on a line.")

It is surprising, perhaps, that all triangulations of a given point set have the same number of lines (L) and triangles (T), given explicitly by the relations

$$\begin{aligned} L &= 3N - n - 3 \\ T &= 2N - n - 2 \end{aligned} \tag{21.6.1}$$

The proof, known to Gauss, is very easy if you consult Figure 21.6.2. Since the interior angles of a triangle sum to π radians, each triangle "uses up" half a point's worth of angle. It is useful to think about each line as being two half-lines, representing the two possible directions of traversal in clockwise triangles. Then, each triangle uses up three half-lines. We must separately account for the vertices on the convex hull, as follows: Each such point uses up π radians on its own (dark-shaded angles in the figure), plus (sum of the light-shaded exterior angles) 2π additional radians in going

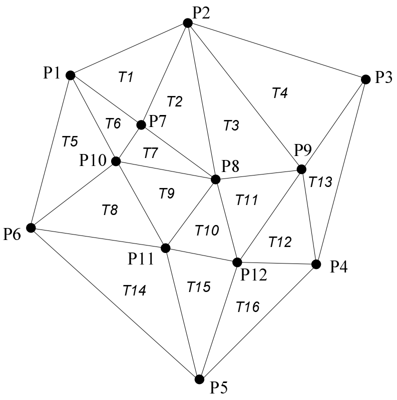

Figure 21.6.3. Example of a triangulation with $N = 12$, $n = 6$, $T = 16$, and $L = 27$, values that satisfy equation (21.6.1).

around the convex hull. These considerations give the relations

$$\begin{aligned} 2\pi N &= \pi T + \pi n + 2\pi \qquad &\text{(account for radians)} \\ 2L &= 3T + n &\text{(account for half-lines)} \end{aligned} \tag{21.6.2}$$

which can be rearranged to give equation (21.6.1). Figure 21.6.3 shows the triangulation of Figure 21.6.2(b) with its points and triangles enumerated.

21.6.1 Delaunay Triangulation

Boris Nikolaevich Delone (1890–1980), a Russian mathematician also celebrated as a rock climber, first published the ideas behind Delaunay triangulation in 1934. Since his paper was written in French, his name was transliterated so as to be pronounced (approximately) correctly by French speakers. A better English pronunciation might be “dyeh-LOAN-yeh.”

Delaunay triangulations have a number of remarkable properties and can be defined in various abstract ways. However, we’ll take as the definition one very concrete property, shown in Figure 21.6.4. Consider all triangulations in which four points, A, B, C, D, are the vertices of two back-to-back triangles. Then, one can get a different triangulation by deleting the common edge (BD in the figure) and replacing it by the other diagonal of the quadrilateral (AC in the figure). A Delaunay triangulation is defined as one that always chooses the diagonal that gives the *largest minimum angle* for the six interior angles in the two triangles. The edge shown as BD is thus *illegal* for a Delaunay triangulation, while AC is termed *legal*. Changing a triangulation from an illegal edge to a legal one is called an *edge flip*. When any two triangles have a common edge, exactly one configuration, unchanged or edge-flipped, is legal (unless all four points lie on a circle, in which case both are legal).

This “largest minimum angle” property is geometrically equivalent to other statements about the points A, B, C, D. One such statement is that the circumcircle of an illegal triangle, like ABD or BCD in part (a) of the figure, always contains another point, C or A, respectively. For a legal triangle, as in part (b) of the figure, this is never the case. One can use this as a starting point to prove the theorem:

- The circumcircle of any triangle in a Delaunay triangulation contains no other vertices.

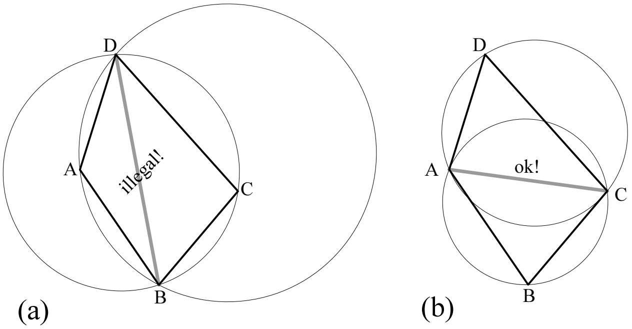

Figure 21.6.4. A Delaunay triangulation can be defined as one in which back-to-back triangles always have a larger minimum angle than they would have if their common edge were flipped to the other diagonal. Equivalently, any triangle's circumcircle does not contain another vertex. A so-called *edge flip* converts (a) to (b) in the figure.

Although the largest minimum angle property was defined locally, for one quadrilateral at a time, it can be shown to imply a remarkable global theorem:

- Among all triangulations of a set of points, a Delaunay triangulation has the largest minimum angles, as defined by sorting all angles from smallest to largest and comparing lexicographically to the angles of any other triangulation.

Comparing lexicographically means: First compare the smallest angle; if there is a winner, stop. If there is a tie on the smallest angle, compare the second-smallest angle. And so on.

Another theorem is

- Two vertices are connected by a Delaunay edge if and only if there is some circle that contains them and contains no other vertices.

If the points in a set have generic positions, meaning that no three are collinear and no four lie on the same circle, then the Delaunay triangulation exists and is unique; any method for constructing it will give the identical set of triangles.

You might wonder whether a Delaunay triangulation is also a *minimum weight* triangulation, defined as the triangulation with the smallest total of edge lengths. The answer is, in general, no. While minimum weight triangulations might be useful in applications, it is not even known whether they can be constructed in less than time that grows exponentially with N. Delaunay construction, on the other hand, is fast, $O(N \log N)$. So, in practice, Delaunay is what we've got!

So, how do we construct a Delaunay triangulation? Conceptually, we can start with *any* triangulation and then eliminate illegal edges, by edge flips, as long as possible. This must terminate in a Delaunay triangulation after a finite number of flips because (i) each flip changes and increases the lexicographic order in the list of angles, and (ii) there are only a finite number of possible triangulations. Although, as stated, this is not an efficient algorithm, it can be readily be turned into one, the so-called *randomized incremental algorithm* [1].

This algorithm, which we now implement, is "incremental" in that it adds points to the triangulation one at a time, maintaining a Delaunay triangulation at each stage. It is "randomized" in that the points are added in a random order. It turns out that the randomization (almost) guarantees $O(N \log N)$ expected time for the algorithm. (Without randomization, one could encounter pathological cases with

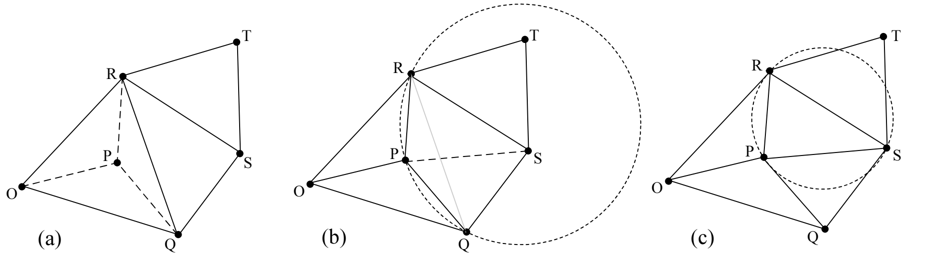

Figure 21.6.5. Steps in inserting a new point into a Delaunay triangulation. (a) Connect new point P to vertices of enclosing triangle. (b) Check enclosing triangle for illegal edges (here, replace QR by PS). (c) Recursively check any new triangles created that have P as a vertex. (Here, RS is legal, so we terminate.)

$O(N^2)$ running time.)

Figure 21.6.5 shows the procedure for adding a new point P that lies within an existing triangle. First, connect it to the vertices of the enclosing triangle. This creates three new triangles. (We exclude the special case where P is exactly on an existing line. More on this below.) Next, check whether the edges opposite P in the three new triangles are legal or illegal. If they are illegal, do edge flips. Each edge flip creates two new triangles with P as a vertex, and (therefore) with two edges opposite P that now also need to be checked for legality. So the process is recursive, but it never wanders away from P. That is the key point: The only edges that can be made illegal by inserting point a point P are edges opposite P in triangles that include P. The proof that the algorithm is $O(N \log N)$ uses this fact, and then bounds the average number of triangles of which P can be a vertex by relations like those of equation (21.6.1). (For details in the proof, see [2].)

Since, up to now, we only know how to add a point P that falls inside the triangulation, how can we get started? An easy way is to add three "fictitious" points to the set of points, forming a very large starting triangle that will enclose all the "real" points subsequently added. Then, at the very end, the fictitious points and all edges connecting to them are removed. Strictly speaking, the fictitious points must actually be treated as if at infinity (thus requiring special logic in the code whenever they are referenced). If their distance is merely finite, the constructed triangulation may be "not-quite" Delaunay. For example, its outer boundary (convex hull) could in unusual cases be left slightly concave, with small negative angles on the order of the diameter of the "real" point set divided by the distance to the "fictitious" points.

That's enough general information. We next get into the details.

21.6.2 Implementation Details

Since most readers skip a section with this title, this is a good place for us to confess a couple of dirty tricks in our Delaunay implementation, whose purpose is to keep the code and its explication to manageable length. If you need a bullet-proof Delaunay code, with no such tricks, a Web search will turn up several that are freely available. Our code is short and fast, and fine for its intended purpose; but it is approximate in two respects: First, we don't take the initial bounding triangle off to infinity (as we sanctimoniously advised, above). Instead, it lies at a distance of about `bigscale` (an adjustable parameter, default value 1000), measured in units of the bounding-box size of the set of points. Second, we don't provide for the special case, mentioned above, where the point being added falls on an existing edge (or does so within roundoff tolerance). For generic point locations, this should "never" happen; but in real

life it "always" happens, because users love to try test examples with points in regular patterns! When we detect this problem, we randomly alter the location of the offending point by a small fraction fuzz (another adjustable parameter, default 10^{-6}) of the bounding box dimensions.

A very important implementation detail, not yet discussed, is how to find in which existing triangle a new point lies. Conceptually, we might throw the triangles into a QO tree (§21.8), but this would not yield the desired $O(N \log N)$ behavior for our algorithm. A better solution, well established in the literature, is to maintain a tree structure of the descendants of any given triangle that ever existed in the construction. That is, starting with the huge "root" triangle, whenever a triangle is subdivided into three new triangles, we set pointers to its three daughters. And, when two triangles are lost in an edge flip, and two new triangles are created, we make the new triangles daughters of *both* of the lost triangles (even though each lost triangle contains only a part of each new one). In this scheme a triangle has two or three daughters at most, so we can easily reserve space for the pointers explicitly (i.e., no expandable linked lists needed).

With this structure, it is very fast to locate a point within the existing triangulation: Start at the root triangle, and recursively pick whichever daughter contains the point. When you reach a terminal node in the tree, you will have found a triangle in the current triangulation that contains the point. We thus need a structure for a "triangle element" or Triel:

delaunay.h

```
struct Triel {
Structure for an element in a descendancy tree of triangles, each having at most three daughters.
    Point<2> *pts;                  Pointer to the array of the points.
    Int p[3];                       The triangle's three vertices, always in CCW order.
    Int d[3];                       Pointers for up to three daughters.
    Int stat;                       Nonzero if this element is "live."
    void setme(Int a, Int b, Int c, Point<2> *ptss) {
    Set the data in a Triel.
        pts = ptss;
        p[0] = a; p[1] = b; p[2] = c;
        d[0] = d[1] = d[2] = -1;            The values -1 mean no daughters.
        stat = 1;                           Create as "live."
    }
    Int contains(Point<2> point) {
    Return 1 if point is in the triangle, 0 if on boundary, -1 if outside. (CCW triangle is
    assumed.)
        Doub d;
        Int i,j,ztest = 0;
        for (i=0; i<3; i++) {
            j = (i+1) %3;
            d = (pts[p[j]].x[0]-pts[p[i]].x[0])*(point.x[1]-pts[p[i]].x[1]) -
                (pts[p[j]].x[1]-pts[p[i]].x[1])*(point.x[0]-pts[p[i]].x[0]);
            if (d < 0.0) return -1;
            if (d == 0.0) ztest = 1;
        }
        return (ztest? 0 : 1);
    }
};
```

We create an big enough array of Triels at the start, and use integers to point to array elements. We've omitted any explicit constructor or assignment operators in Triel, since they are not needed for our use here. Be sure to add them if you use Triel in any other way.

We will need a way to do two other fast lookups: (1) Given a point and opposite edge in a triangle, find the fourth point in the quadrilateral, that is, the point (if any) on the other side of the given edge. (2) Given three points, find the index of their triangle (if it exists) in an array of Triel elements. Our strategy is to use hash memories (respectively called linehash and trihash) for these two functions. In particular, whenever we create a triangle (always CCW) with vertices A, B, C, we store an index pointing to each point under a specially constructed key,

$$\texttt{linehash}(h(B) - h(C)) \leftarrow A \qquad \text{(et cyc.)} \tag{21.6.3}$$

where the function h is a 64-bit hash function, and "et cyc." means do the same thing for the

other two cyclic permutations of A, B, C. The trick here is that, if we ever want to find the point on the other side of edge BC, we just look for a key $h(C) - h(B)$ ("negative" of the key in equation 21.6.3) in the hash table. The similar trick for storing and retrieving Triels is that, when we create a Triel at location j in the storage array, we set

$$\texttt{trihash}(h(A) \;\hat{}\; h(B) \;\hat{}\; h(C)) \leftarrow j \tag{21.6.4}$$

where ˆ is the XOR operation. Since this key is symmetric in A, B, C, we can find a triangle knowing its vertices in any order.

Since we are computing hash keys "by hand," we can signal the two hash memories to use a null (therefore fast) hash of their own. That, and a utility for determining whether a point is inside the circumcircle of three other points, are the following two code fragments:

delaunay.h

```
Doub incircle(Point<2> d, Point<2> a, Point<2> b, Point<2> c) {
Return positive, zero, or negative value if point d is respectively inside, on, or outside the circle
through points a, b, and c.
    Circle cc = circumcircle(a,b,c);        Routine defined in §21.3.
    Doub radd = SQR(d.x[0]-cc.center.x[0]) + SQR(d.x[1]-cc.center.x[1]);
    return (SQR(cc.radius) - radd);
}

struct Nullhash {
Null hash function. Use a key (assumed to be already hashed) as its own hash.
    Nullhash(Int nn) {}
    inline Ullong fn(const void *key) const { return *((Ullong *)key); }
};
```

These are all the preliminaries we need before declaring the Delaunay structure.

delaunay.h

```
struct Delaunay {
Structure for constructing a Delaunay triangulation from a given set of points.
    Int npts,ntri,ntree,ntreemax,opt;       Number of points, triangles, elements in the
    Doub delx,dely;                             Triel list, and maximum of same. Size
    vector< Point<2> > pts;                     of the bounding box.
    vector<Triel> thelist;                  The list of Triel elements.
    Hash<Ullong,Int,Nullhash> *linehash;    Create the hash memories with null hash
    Hash<Ullong,Int,Nullhash> *trihash;         function.
    Int *perm;
    Delaunay(vector<Point<2> > &pvec, Int options = 0);
    Construct the Delaunay triangulation from a vector of points. The variable options is used
    by some applications.
    Ranhash hashfn;                         The raw hash function.
    Doub interpolate(const Point<2> &p, const vector<Doub> &fnvals,
        Doub defaultval=0.0);
    The next four functions are explained in detail below.
    void insertapoint(Int r);
    Int whichcontainspt(const Point<2> &p, Int strict = 0);
    Int storetriangle(Int a, Int b, Int c);
    void erasetriangle(Int a, Int b, Int c, Int d0, Int d1, Int d2);
    static Uint jran;                       Random number counter.
    static const Doub fuzz, bigscale;
};
const Doub Delaunay::fuzz  = 1.0e-6;        Adjust if you wish. See text.
const Doub Delaunay::bigscale = 1000.0;     Adjust if you wish. See text.
Uint Delaunay::jran = 14921620;
```

The variable jran is used in conjunction with the hash function as a convenient random number generator. The function interpolate() is for the application of interpolating a function on an irregular mesh, to be discussed in §21.7. Everything else should become clear as we proceed.

The action starts with the constructor. We compute a bounding box for the set of points, construct and store the "huge" root triangle enclosing the points, create a random permuta-

tion to be the order in which points are added, and then (for the real work) call the function `insertapoint()` for each point in turn. After that there is just some cleanup housekeeping.

delaunay.h

```
Delaunay::Delaunay(vector< Point<2> > &pvec, Int options) :
    npts(pvec.size()), ntri(0), ntree(0), ntreemax(10*npts+1000),
    opt(options), pts(npts+3), thelist(ntreemax) {
Construct Delaunay triangulation from a vector of points pvec. If bit 0 in options is nonzero,
hash memories used in the construction are deleted. (Some applications may want to use them
and will set options to 1.)
    Int j;
    Doub xl,xh,yl,yh;
    linehash = new Hash<Ullong,Int,Nullhash>(6*npts+12,6*npts+12);
    trihash = new Hash<Ullong,Int,Nullhash>(2*npts+6,2*npts+6);
    perm = new Int[npts];                  Permutation for randomizing point order.
    xl = xh = pvec[0].x[0];                Copy points to local store and calculate their
    yl = yh = pvec[0].x[1];                    bounding box.
    for (j=0; j<npts; j++) {
        pts[j] = pvec[j];
        perm[j] = j;
        if (pvec[j].x[0] < xl) xl = pvec[j].x[0];
        if (pvec[j].x[0] > xh) xh = pvec[j].x[0];
        if (pvec[j].x[1] < yl) yl = pvec[j].x[1];
        if (pvec[j].x[1] > yh) yh = pvec[j].x[1];
    }
    delx = xh - xl;                        Store bounding box dimensions, then construct
    dely = yh - yl;                            the three fictitious points and store them.
    pts[npts] = Point<2>(0.5*(xl + xh), yh + bigscale*dely);
    pts[npts+1] = Point<2>(xl - 0.5*bigscale*delx,yl - 0.5*bigscale*dely);
    pts[npts+2] = Point<2>(xh + 0.5*bigscale*delx,yl - 0.5*bigscale*dely);
    storetriangle(npts,npts+1,npts+2);
    Create a random permutation:
    for (j=npts; j>0; j--) SWAP(perm[j-1],perm[hashfn.int64(jran++) % j]);
    for (j=0; j<npts; j++) insertapoint(perm[j]);      All the action is here!
    for (j=0; j<ntree; j++) {              Delete the huge root triangle and all of its con-
      if (thelist[j].stat > 0) {               necting edges.
            if (thelist[j].p[0] >= npts || thelist[j].p[1] >= npts ||
            thelist[j].p[2] >= npts) {
                thelist[j].stat = -1;
                ntri--;
            }
        }
    }
    if (!(opt & 1)) {                      Clean up, unless option bit says not to.
        delete [] perm;
        delete trihash;
        delete linehash;
    }
}
```

The guts of the algorithm as previously described are in `insertapoint()`. We first locate the triangle that contains the new point. (Failure here can mean only that the point lies on an existing line, in which case we fuzz the point's location, as we confessed above, and try again.) We store three new triangles and delete the old one. Then, we locate and fix any illegal edges, doing the recursion by a simple last-in-first-out stack of pending edges to check.

delaunay.h

```
void Delaunay::insertapoint(Int r) {
Add the point with index r incrementally to the Delaunay triangulation.
    Int i,j,k,l,s,tno,ntask,d0,d1,d2;
    Ullong key;
    Int tasks[50], taski[50], taskj[50];        Stacks (3 vertices) for legalizing edges.
    for (j=0; j<3; j++) {                       Find triangle containing point. Fuzz if it
        tno = whichcontainspt(pts[r],1);            lies on an edge.
        if (tno >= 0) break;                    The desired result: Point is OK.
```

```
        pts[r].x[0] += fuzz * delx * (hashfn.doub(jran++)-0.5);
        pts[r].x[1] += fuzz * dely * (hashfn.doub(jran++)-0.5);
    }
    if (j == 3) throw("points degenerate even after fuzzing");
    ntask = 0;
    i = thelist[tno].p[0]; j = thelist[tno].p[1]; k = thelist[tno].p[2];
```
The following line is relevant only when the indicated bit in opt is set. This feature is used by the convex hull application and causes any points already known to be interior to the convex hull to be omitted from the triangulation, saving time (but giving in an incomplete triangulation).
```
    if (opt & 2 && i < npts && j < npts && k < npts) return;
    d0 =storetriangle(r,i,j);                        Create three triangles and queue them
    tasks[++ntask] = r; taski[ntask] = i; taskj[ntask] = j;     for legal edge tests.
    d1 = storetriangle(r,j,k);
    tasks[++ntask] = r; taski[ntask] = j; taskj[ntask] = k;
    d2 = storetriangle(r,k,i);
    tasks[++ntask] = r; taski[ntask] = k; taskj[ntask] = i;
    erasetriangle(i,j,k,d0,d1,d2);                   Erase the old triangle.
    while (ntask) {                                  Legalize edges recursively.
        s=tasks[ntask]; i=taski[ntask]; j=taskj[ntask--];
        key = hashfn.int64(j) - hashfn.int64(i);       Look up fourth point.
        if ( ! linehash->get(key,l) ) continue;        Case of no triangle on other side.
        if (incircle(pts[l],pts[j],pts[s],pts[i]) > 0.0){    Needs legalizing?
            d0 = storetriangle(s,l,j);                 Create two new triangles
            d1 = storetriangle(s,i,l);
            erasetriangle(s,i,j,d0,d1,-1);             and erase old ones.
            erasetriangle(l,j,i,d0,d1,-1);
            key = hashfn.int64(i)-hashfn.int64(j);     Erase line in both directions.
            linehash->erase(key);
            key = 0 - key;                             Unsigned, hence binary minus.
            linehash->erase(key);
            Two new edges now need checking:
            tasks[++ntask] = s; taski[ntask] = l; taskj[ntask] = j;
            tasks[++ntask] = s; taski[ntask] = i; taskj[ntask] = l;
        }
    }
}
```

The only pieces left are the utility functions for finding the triangle that contains a point, and for storing and erasing a triangle. When we "erase" a triangle, we actually only mark it as inactive in the current triangulation, and we set its daughters in the descendancy tree, as already discussed.

```
Int Delaunay::whichcontainspt(const Point<2> &p, Int strict) {                delaunay.h
```
Given point p, return index in thelist of the triangle in the triangulation that contains it, or return −1 for failure. If strict is nonzero, require strict containment, otherwise allow the point to lie on an edge.
```
    Int i,j,k=0;
    while (thelist[k].stat <= 0) {            Descend in tree until reach a "live" triangle.
        for (i=0; i<3; i++) {                 Check up to three daughters.
            if ((j = thelist[k].d[i]) < 0) continue;      Daughter doesn't exist.
            if (strict) {
                if (thelist[j].contains(p) > 0) break;
            } else {                          Yes, descend on this branch.
                if (thelist[j].contains(p) >= 0) break;
            }
        }
        if (i == 3) return -1;                No daughters contain the point.
        k = j;                                Set new mother.
    }
    return k;                                 Normal return.
}
```

```
void Delaunay::erasetriangle(Int a, Int b, Int c, Int d0, Int d1, Int d2) {
Erase triangle abc in trihash and inactivate it in thelist after setting its daughters.
    Ullong key;
    Int j;
    key = hashfn.int64(a) ^ hashfn.int64(b) ^ hashfn.int64(c);
    if (trihash->get(key,j) == 0) throw("nonexistent triangle");
    trihash->erase(key);
    thelist[j].d[0] = d0; thelist[j].d[1] = d1; thelist[j].d[2] = d2;
    thelist[j].stat = 0;
    ntri--;
}

Int Delaunay::storetriangle(Int a, Int b, Int c) {
Store a triangle with vertices a, b, c in trihash. Store its points in linehash under keys to
opposite sides. Add it to thelist, returning its index there.
    Ullong key;
    thelist[ntree].setme(a,b,c,&pts[0]);
    key = hashfn.int64(a) ^ hashfn.int64(b) ^ hashfn.int64(c);
    trihash->set(key,ntree);
    key = hashfn.int64(b)-hashfn.int64(c);
    linehash->set(key,a);
    key = hashfn.int64(c)-hashfn.int64(a);
    linehash->set(key,b);
    key = hashfn.int64(a)-hashfn.int64(b);
    linehash->set(key,c);
    if (++ntree == ntreemax) throw("thelist is sized too small");
    ntri++;
    return (ntree-1);
}
```

You might wonder how to get the answer *out* of our `Delaunay` structure. We have not provided a function for this, because it so much depends on what you want to do with the answer. The general idea, however, is that you just loop through `thelist`[j] for $0 \le j < $ `nlist`. Each element is a `Triel`. If its value of `save` is ≤ 0, ignore it and go on. If it is 1, then the element represents a triangle in the final Delaunay triangulation. There should be `ntri` of these elements in all. The element's array `p[]` has integers that point to the triangle's three points in your vector of points. Several routines in the next section mine the `Delaunay` structure for points, edges, or triangles and can be used as template examples.

Figure 21.6.6 shows sample output for a Delaunay triangulation of 300 points.

CITED REFERENCES AND FURTHER READING:

Guibas, L.J., Knuth, D.E., and Sharir, M. 1992, "Randomized Incremental Construction of Delaunay and Voronoi Diagrams," *Algorithmica*, vol. 7, pp. 381–413.[1]

Lischinski, D. 1994, "Incremental Delaunay Triangulation," in *Graphics Gems IV*, Heckbert, P.S., ed. (Cambridge, MA: Academic Press). [Shows use of linked data structure instead of our use of hash memory.]

de Berg, M., van Kreveld, M., Overmars, M., and Schwarzkopf, O. 2000, *Computational Geometry: Algorithms and Applications*, 2nd revised ed. (Berlin: Springer), Chapter 9.[2]

O'Rourke, J. 1998, *Computational Geometry in C*, 2nd ed. (Cambridge, UK: Cambridge University Press), §5.3.

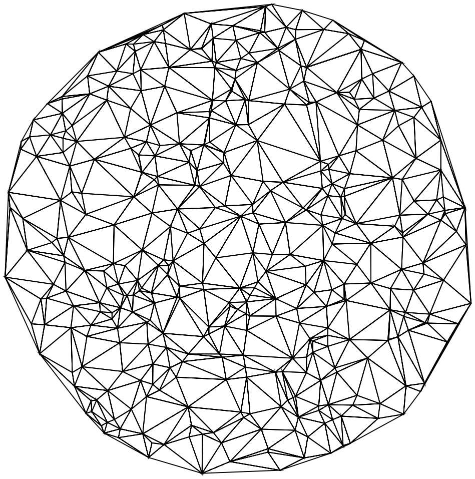

Figure 21.6.6. Delaunay triangulation of 300 points randomly chosen within a circle, as computed by the routines in this section.

21.7 Applications of Delaunay Triangulation

Emerging from the thicket of detail that was needed to implement the Delaunay triangulation, we are now ready to make use of it in several important applications. In this section we assume that you have a vector of points (say, `vecp`), and that you have invoked the code in §21.6 to construct a `Delaunay` structure. This usually means writing just the one line of code,

```
Delaunay mygrid(vecp);
```

So, what next?

21.7.1 Two-Dimensional Interpolation on an Irregular Grid

This is probably the most-asked-for algorithm that was missing from the previous two editions of *Numerical Recipes*. The basic setup is very simple. You are given a set of N points in the plane. You triangulate that set with a "good" triangulation, that is, one favoring short lines and big angles — in other words, Delaunay. You evaluate a function of interest at each of the points, and store the values in a vector (in the same order as the vector of points, of course).

Now it is easy to interpolate the function at a new point $\mathbf{p}$ that lies within the triangulation, that is, specifically, within the convex hull of your set of points. First, locate which triangle the point falls in. This takes only $O(\log N)$ operations if you use the `whichcontainspt()` method of the `Delaunay` structure. Then, linearly interpolate between the three function values at the three triangle vertices. The linear interpolation is uniquely defined, because (imagining your function plotted in the third dimension above the plane in which $\mathbf{p}$ lies) three points uniquely define a plane in three dimensions .

Constructively, the linear interpolation is easily done using barycentric coordinates as defined in equation (21.3.10), which in turn reduces to using the triangle area formula (equation 21.3.1) three times. Appropriately normalized, each barytropic coordinate value is exactly the weight of its corresponding vertex.

These ideas are implemented in the following function:

delaunay.h

```
Doub Delaunay::interpolate(const Point<2> &p,
const vector<Doub> &fnvals, Doub defaultval) {
Triangular grid interpolation of a function. Given an arbitrary point p and a vector of function values fnvals at the points that were used to construct the Delaunay structure, return the linearly interpolated function value in the triangle in which p lies. If p lies outside of the triangulation, instead return defaultval.
    Int n,i,j,k;
    Doub wgts[3];
    Int ipts[3];
    Doub sum, ans = 0.0;
    n = whichcontainspt(p);                        Locate the point in the triangulation.
    if (n < 0) return defaultval;                  Point outside of convex hull.
    for (i=0; i<3; i++) ipts[i] = thelist[n].p[i];
    for (i=0,j=1,k=2; i<3; i++,j++,k++) {          Calculate the barycentric coordinates, pro-
        if (j == 3) j=0;                               portional to the weights.
        if (k == 3) k=0;
        wgts[k]=(pts[ipts[j]].x[0]-pts[ipts[i]].x[0])*(p.x[1]-pts[ipts[i]].x[1])
            - (pts[ipts[j]].x[1]-pts[ipts[i]].x[1])*(p.x[0]-pts[ipts[i]].x[0]);
    }
    sum = wgts[0] + wgts[1] + wgts[2];             Normalization of the weights.
    if (sum == 0) throw("degenerate triangle");
    for (i=0; i<3; i++) ans += wgts[i]*fnvals[ipts[i]]/sum;     Linear interpolation.
    return ans;
}
```

Keep in mind that you should not expect high accuracy from linear interpolation. The interpolated function is piecewise linear, and continuous within the convex hull, but it has discontinuous derivatives in the direction perpendicular to the triangle's edges. On a triangle edge, it interpolates between the two function values at each end of the edge. You need a *lot* of triangles to get a reasonable representation of any function with much detailed structure.

21.7.2 Voronoi Diagrams

Around 1907, the Ukrainian mathematician Georgy Fedoseevich Voronoi revisited a problem that had been previously discussed by Dirichlet as early as 1850: Given N points, or *sites*, in the plane, each site $\mathbf{p}$ defines a region that is closer to $\mathbf{p}$ than to any of the other $N-1$ sites. That region is called $\mathbf{p}$'s *Voronoi region*. What are the properties of the Voronoi regions, and how can we construct them?

If you imagine that everyone in a city shops at the closest supermarket ("as the crow flies"), then the Voronoi regions map out the districts served by each supermarket. If you imagine that fires are simultaneously set at sites in a forest, and that they spread circularly at a fixed speed, then the Voronoi regions are the areas burned by each different fire.

Figure 21.7.1, an example of a *Voronoi diagram*, shows the Voronoi regions around 40 sites chosen randomly in the plane. Yes, the boundaries of the Voronoi regions are polygons, although possibly open and extending to infinity. It is obvious, in fact, that the boundary of a site $\mathbf{p}$'s Voronoi region must consist of line segments each lying on the perpendicular bisector of the line connecting $\mathbf{p}$ to some other site, say $\mathbf{q}_i$. That is because the perpendicular bisector is the locus of points equidistant from $\mathbf{p}$ and $\mathbf{q}_i$. So the real questions are, for a given $\mathbf{p}$, which are the $\mathbf{q}_i$'s that contribute boundary segments? and is there a fast way to compute their intersections (the *vertices* of the Voronoi diagram)?

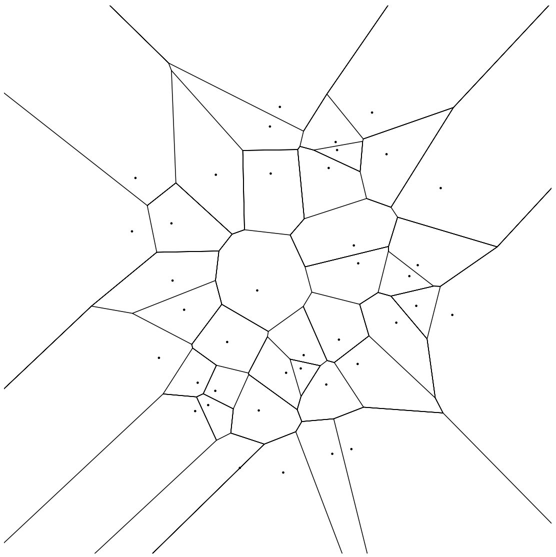

Figure 21.7.1. Voronoi diagram for 40 random sites. Each site has a Voronoi region, the area closer to it than to any other site. The boundaries of the Voronoi regions are straight-line segments that lie on the perpendicular bisectors between pairs of sites.

Remarkably, these questions are completely answered by the Delaunay triangulation of the Voronoi sites. (In fact, many texts start with the Voronoi diagram as fundamental, and then consider Delaunay triangulation as an application. We find it easier to go the other way.)

Some facts are

- Every edge in a site **p**'s Voronoi region boundary lies on the perpendicular bisector of a Delaunay edge that connects to **p**.
- In fact, every Delaunay edge corresponds to exactly one Voronoi edge, and vice versa.
- The vertices of the Voronoi diagram are exactly the circumcenters of the Delaunay triangles.
- The Voronoi diagram and the Delaunay triangulation are *dual graphs* (but don't worry if you don't know what this means)

Figure 21.7.2 shows the key ideas in the proof of the first two facts above. We already know that the boundary is made of *some* perpendicular bisector segments. We need to show that (i) every one of a point's Delaunay edges *does* contribute a segment, and (ii) lines drawn from that point to any other sites *don't* contribute any segments.

Part (a) of the figure shows a piece of Delaunay triangulation around site O. The perpendicular bisectors of OA and OC meet at the point X, which is therefore the center of the circle containing A, O, and C. The issue is whether the Delaunay edge OB can be "blocked" by the other two edges. Now, B must lie inside the circumcircle just mentioned; otherwise, the edge OB would have been an illegal edge when the Delaunay triangulation was constructed. But this means that the perpendicular bisector of OB, labeled UV, must "cut off the corner" at X. Thus it does contribute a segment to the boundary.

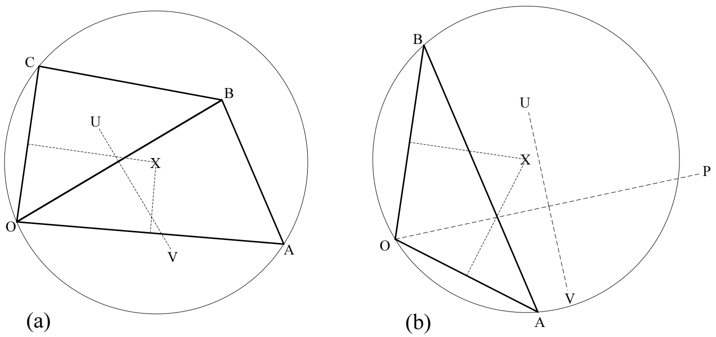

Figure 21.7.2. Key ideas in the proof that every Delaunay edge contributes exactly one Voronoi edge, lying on its perpendicular bisector. (a) Delaunay requires B to lie inside the circle AOC, hence its bisector must clip the corner inside X. (b) Delaunay requires any other site P to lie outside of the circle AOB, hence its bisector can't clip the corner inside X.

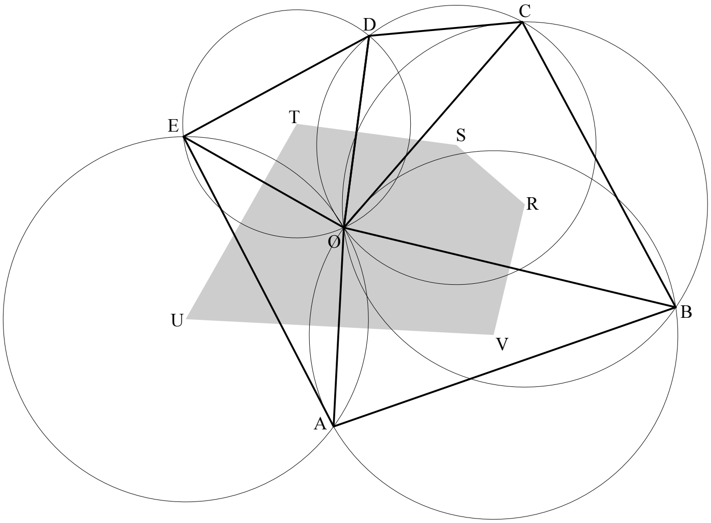

Figure 21.7.3. The circumcenters of the Delaunay triangles around a point O are the vertices of O's Voronoi region (shown as shaded), because the perpendicular bisectors of the Delaunay edges meet at these circumcenters. Notice that a Voronoi edge need not actually intersect the Delaunay edge with which it is associated, as SR and OC.

Part (b) of the figure shows a Delaunay triangle OAB whose edges OA and OB are contributing perpendicular bisector segments to the Voronoi boundary around O. Point P is some other site. Can it somehow worm its way in close enough to contribute a segment of its own bisector, between the other two? Evidently not: We know that the circumcircle of any Delaunay triangle contains no other sites. Since P must lie outside the circumcircle, its bisector UV can't cut off the corner at X.

The fact that the Voronoi vertices are the circumcenters of Delaunay triangles is an immediate consequence of the previous discussion (see Figure 21.7.3). The circumcenters are where the perpendicular bisectors of the edges meet. Since every Delaunay edge contributes a segment, every such circumcenter must be a vertex. Notice, however, that not every Delaunay triangle contains its own circumcenter (as OCD in the figure), so that a segment on the boundary of a Voronoi region need not

actually intersect the Delaunay edge with which it is associated (as RS and OC in the figure).

We can count the number of edges and vertices in a Voronoi diagram with N sites (n of which are on the convex hull) simply by knowing that its dual is the Delaunay triangulation and using equation (21.6.1). The number of Voronoi edges is thus L in that equation, while the number of Voronoi vertices is T. The number of Voronoi regions is by definition N. The unbounded Voronoi regions are exactly those whose points lie on the convex hull of the sites, so there are n of these. It turns out (not immediately obviously) that the average number of edges in one Voronoi region (averaged over all the sites) does not exceed six.

Turning to the implementing code, it is convenient to have a structure for holding Voronoi edges, and also their association with the site that they surround (as an integer pointer to a list of sites).

voronoi.h

```
struct Voredge {
```
Structure for an edge in a Voronoi diagram, containing its two endpoints and an integer pointer to the site of which it is a boundary.
```
    Point<2> p[2];
    Int nearpt;
    Voredge() {}
    Voredge(Point<2> pa, Point<2> pb, Int np) : nearpt(np) {
        p[0] = pa; p[1] = pb;
    }
};
```

Now it is straightforward to define a `Voronoi` structure, as a derived class of the `Delaunay` structure. The constructor creates a Delaunay triangulation of the sites, and then loops over the sites. For each, it first finds any one triangle with the site as a vertex, and then works its way around the site in a circle, navigating counterclockwise from one triangle to the next by looking up their common edge in the `linehash` hash memory. Each triangle's circumcenter is a Voronoi vertex, and a Voronoi edge is stored for each two consecutive circumcenters as the site is circumnavigated.

voronoi.h

```
struct Voronoi : Delaunay {
```
Structure for creating a Voronoi diagram, derived from the `Delaunay` structure.
```
    Int nseg;                            Number of edges in the diagram.
    VecInt trindx;                       Will index triangles.
    vector<Voredge> segs;                Will be array of all segments.
    Voronoi(vector< Point<2> > pvec);    Construct Voronoi diagram from array of points.
};

Voronoi::Voronoi(vector< Point<2> > pvec) :
    Delaunay(pvec,1), nseg(0), trindx(npts), segs(6*npts+12) {
```
Constructor for Voronoi diagram of a vector of sites `pvec`. Bit "1" sent to the `Delaunay` constructor tells it not to delete `linehash`.
```
    Int i,j,k,p,jfirst;
    Ullong key;
    Triel tt;
    Point<2> cc, ccp;                    Create a table so that, given a point num-
    for (j=0; j<ntree; j++) {                ber, we can find one triangle with it as
        if (thelist[j].stat <= 0) continue;  a vertex.
        tt = thelist[j];
        for (k=0; k<3; k++) trindx[tt.p[k]] = j;
    }                                    Now loop over the sites.
    for (p=0; p<npts; p++) {
```

```
        tt = thelist[trindx[p]];
        if (tt.p[0] == p) {i = tt.p[1]; j = tt.p[2];}       Get the vertices into canon-
        else if (tt.p[1] == p) {i = tt.p[2]; j = tt.p[0];}     ical order.
        else if (tt.p[2] == p) {i = tt.p[0]; j = tt.p[1];}
        else throw("triangle should contain p");
        jfirst = j;                              Save starting vertex and its circumcircle.
        ccp = circumcircle(pts[p],pts[i],pts[j]).center;
        while (1) {                 Go around CCW, find circumcenters and store segments.
            key = hashfn.int64(i) - hashfn.int64(p);
            if ( ! linehash->get(key,k) ) throw("Delaunay is incomplete");
            cc = circumcircle(pts[p],pts[k],pts[i]).center;
            segs[nseg++] = Voredge(ccp,cc,p);
            if (k == jfirst) break;          Circumnavigation completed. Normal way out.
            ccp = cc;
            j=i;
            i=k;
        }
    }
}
```

The result of the `Voronoi` construct is available by looping through the `segs` array from 0 to `nseg-1`. Each array element is a `Voredge` that stores the endpoints, and also the site number with which it is associated. Note that each segment appears twice in the list, with opposite sense, as it is associated in turn with the sites on its two sides.

If you read our confession about dirty tricks in the previous section, you'll want to keep in mind that the "open" Voronoi polygons are actually closed by segments that lie at a distance of order `bigscale` times the size of the bounding box of the sites. Those segments are included in `segs` but appear only once, since there is no site on their other side.

21.7.3 Other Applications

Nearest Neighbors, Again. A line segment that connects a point to its nearest neighbor among a set of points will be an edge in the set's Delaunay triangulation. Informal proof: The nearest neighbor obviously must contribute a boundary to the Voronoi diagram. Formal proof (using a theorem mentioned above): The circle whose diameter connects a point to its nearest neighbor can't contain any other points (they'd be closer than the nearest neighbor), so that diameter must be a Delaunay edge.

Since we can construct the Delaunay triangulation in $O(N \log N)$ time, it follows that we can use it to find all nearest neighbors of a set of N points in $O(N \log N)$ time. The process is as follows: (i) Construct `Delaunay`. (ii) For each point, circumnavigate it. (We saw how to do this in our implementation of `Voronoi`, above.) (iii) Pick the shortest of the edges with the point at one end.

Convex Hull. Sometimes you may need to know the convex hull of a set of points in the plane for some other application. Although it might seem wasteful to construct the whole Delaunay triangulation just to get the hull, doing so is not too bad a method. Better efficiency can be achieved by ignoring, during the triangulation, points that are found to be already inside interior triangles. To sort the edges into the order of a CCW polygon, we create a `nextpt` table of edge destinations as we go, then chain through it once to output the vertices of the convex hull in proper order.

```
struct Convexhull : Delaunay {
```
delaunay.h

Structure for constructing the convex hull of a set of points in the plane. After construction, nhull is the number of points in the hull, and hullpoints[0..nhull-1] are integers pointing to points in the vector pvec that are in the hull, in CCW order.

```
    Int nhull;
    Int *hullpts;
    Convexhull(vector< Point<2> > pvec);          Construct from a vector of points.
};
```

```
Convexhull::Convexhull(vector< Point<2> > pvec) : Delaunay(pvec,2), nhull(0) {
```
Constructor for convex hull of a vector of points pvec. Bit "2" sent to the Delaunay constructor tells it to ignore interior points when it can, for extra speed.

```
    Int i,j,k,pstart;
    vector<Int> nextpt(npts);
    for (j=0; j<ntree; j++) {                      Triangles with stat= −1 may contain
        if (thelist[j].stat != -1) continue;           hull segments.
        for (i=0,k=1; i<3; i++,k++) {              Need two valid points to qualify.
            if (k == 3) k=0;
            if (thelist[j].p[i] < npts && thelist[j].p[k] < npts) break;
        }
        if (i==3) continue;                        Case where failed to qualify.
        ++nhull;                          Yes! Put its other end in the lookup table, and save
        nextpt[(pstart = thelist[j].p[k])] = thelist[j].p[i];   its value in case it's
    }                                                           the last one we find.
    if (nhull == 0) throw("no hull segments found");
    hullpts = new Int[nhull];                      Now we know how many, can allocate.
    j=0;                                           One chain through the lookup table, start-
    i = hullpts[j++] = pstart;                         ing with pstart, gives the answer.
    while ((i=nextpt[i]) != pstart) hullpts[j++] = i;
}
```

Largest Empty Circle Problem. The largest empty circle whose center lies (strictly) inside the convex hull of a set of points in the plane has its center on a Voronoi vertex. So, you can find it by looping through the Voronoi vertices, calculating the radius of the largest circle centered on each one, and taking the maximum of these. Even better, loop through the Delaunay triangles, calculate the circumcenter of each, and pick the one with the largest circumradius (since Delaunay circumcenters are exactly Voronoi vertices). Think of yourself as finding the best location for a fast-food restaurant within the (convex) city limits, one that best avoids all the other fast-food restaurants.

Avoiding Obstacles. If you want to navigate around the plane staying as far as possible from a set of points, your path will be along the edges of a Voronoi diagram. Think of yourself as a fighter pilot avoiding enemy radars.

Minimum Spanning Tree. The *minimum spanning tree* (sometimes *Euclidean minimum spanning tree*) is the set of line segments of shortest total length that connect N points (see, e.g., Figure 21.7.4). Think of it as the highway map of the cheapest highway system that lets you visit all N cities. It is topologically a tree (i.e., has no loops) because if it did have a loop, you could save highway money by deleting one of the loop's segments.

The important theorem is: The minimum spanning tree is a subset of the Delaunay edges. You might think this isn't very useful, since it doesn't tell you *which* subset. Fortunately, there is a fast algorithm, *Kruskal's algorithm*, for doing the construction. The basic idea is to sort all the Delaunay edges by length, and then add them one at a time to the growing tree, in order from smallest to largest.

Your tree starts off growing in multiple disconnected components, but when

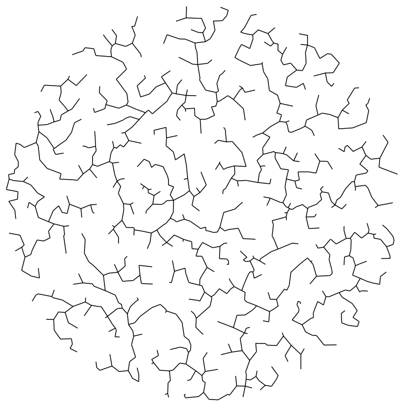

Figure 21.7.4. Minimum spanning tree of 1001 points at random locations within a circle. The tree is composed of 1000 segments that connect all the points with the shortest total length and is a subset of the Delaunay triangulation of the same points.

you have added exactly $N-1$ segments, it will be a single piece, and the answer. There is only one catch: As you add segments, you must not add a segment if both its ends are already in the same component (else it would form a loop). So, you have to maintain an "equivalence class" relation for each vertex, making it equivalent to all other vertices in its connected component. We already know how to do this efficiently, as in §8.6's routine `eclass`. In the code below, there is a similar logic of sweeping up pointers to single "mother" representatives. Do this properly, and the method is $O(N \log N)$.

Kruskal's algorithm is a so-called *greedy algorithm*, since it just takes the best edge at each step willy nilly. It is rare for a greedy algorithm to yield the true global optimum; but this is the happy case where it does.

delaunay.h

```
struct Minspantree : Delaunay {
```
Structure for constructing the minimum spanning tree of a set of points in the plane. After construction, `nspan` is the number of segments (always = `npts`−1), and `minsega[0..nspan-1]` and `minsegb[0..nspan-1]` contain integers pointing to points in the vector `pvec` that are the two ends of each segment.
```
    Int nspan;
    VecInt minsega, minsegb;            Allocate arrays for the output.
    Minspantree(vector< Point<2> > pvec);
};

Minspantree::Minspantree(vector< Point<2> > pvec) :
    Delaunay(pvec,0), nspan(npts-1), minsega(nspan), minsegb(nspan) {
```
Constructor for the minimum spanning tree of a vector of points `pvec`. The `Delaunay` constructor gives the triangulation. We need only find the correct subset of edges.
```
    Int i,j,k,jj,kk,m,tmp,nline,n = 0;
    Triel tt;
    nline = ntri + npts -1;             Number of edges in the triangulation.
    VecInt sega(nline);                 Allocate working space for two ends of each edge,
    VecInt segb(nline);                     edge length, and index on which we will sort.
    VecDoub segd(nline);                    Also the "mother" tree for equivalence classes.
    VecInt mo(npts);
    for (j=0; j<ntree; j++) {           Find all edges in the triangulation, store them
        if (thelist[j].stat == 0) continue;     and their lengths.
        tt = thelist[j];
        for (i=0,k=1; i<3; i++,k++) {
            if (k==3) k=0;
```

```
            if (tt.p[i] > tt.p[k]) continue;    Ensure we get each edge only once.
            if (tt.p[i] >= npts || tt.p[k] >= npts) continue;   No edges connect-
            sega[n] = tt.p[i];                                     ing to fictitious
            segb[n] = tt.p[k];                                     points.
            segd[n] = dist(pts[sega[n]],pts[segb[n]]);
            n++;
        }
    }
    Indexx idx(segd);                       Sort the edges by creating an index array.
    for (j=0; j<npts; j++) mo[j] = j;       Initialize equivalence relation tree.
    n = -1;
    for (i=0; i<nspan; i++) {               Add exactly nspan segments.
        for (;;) {                          Loop for the shortest valid segment n.
            jj = j = idx.el(sega,++n);
            kk = k = idx.el(segb,n);
            while (mo[jj] != jj) jj = mo[jj];     Track each end to its highest ances-
            while (mo[kk] != kk) kk = mo[kk];        tor.
            if (jj != kk) {                 The segment is valid only if it connects different
                minsega[i] = j;                highest ancestors.
                minsegb[i] = k;
                m = mo[jj] = kk;            Now, equate the highest ancestors, and retrace
                jj = j;                        our steps pointing all nodes encountered to
                while (mo[jj] != m) {          that highest node, necessary for speed of the
                    tmp = mo[jj];              algorithm.
                    mo[jj] = m;
                    jj = tmp;
                }
                kk = k;
                while (mo[kk] != m) {
                    tmp = mo[kk];
                    mo[kk] = m;
                    kk = tmp;
                }
                break;                      A segment has been successfully added.
            }
        }
    }
}
```

CITED REFERENCES AND FURTHER READING:

de Berg, M., van Kreveld, M., Overmars, M., and Schwarzkopf, O. 2000, *Computational Geometry: Algorithms and Applications*, 2nd revised ed. (Berlin: Springer), Chapters 7 and 11.

O'Rourke, J. 1998, *Computational Geometry in C*, 2nd ed. (Cambridge, UK: Cambridge University Press), Chapter 5.

21.8 Quadtrees and Octrees: Storing Geometrical Objects

Different from a KD tree is another kind of box tree, usually called a *quadtree* in two dimensions or a *octree* in three dimensions. Yes, we know that it ought to be spelled "octtree," not "octree," but the latter usage has become standard. We'll refer to quadtrees and octrees generically as "QO trees" and thus avoid linguistic controversy.

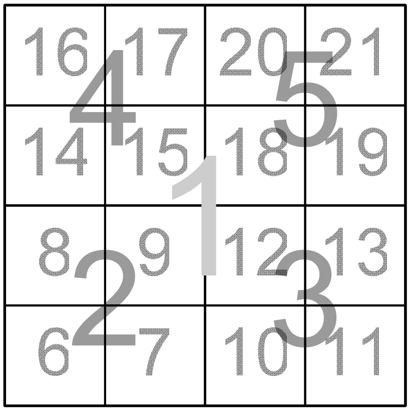

Figure 21.8.1. In a quadtree, the initial square 1 is first subdivided into squares 2, 3, 4, and 5. At the next level of subdivision, 2 is subdivided into 6, 7, 8, 9; 3 into 10, 11, 12, 13; and so forth.

QO trees start with a finite-sized box, usually square or cubical, rather than the near-infinite box used in the KD tree. A QO tree then subdivides each box not in one dimension at a time (as a KD tree) but rather in *all* dimensions. Thus, a square is subdivided into four daughter squares, a cube into eight daughter cubes — quite a brood. The coordinates of the subdivisions are taken to exactly bisect the mother box in each dimension, so all the boxes at one level of the tree are congruent, differing from the original box by a fixed power-of-two factor. Figure 21.8.1 illustrates this in the case of two dimensions.

QO trees thus provide a kind of addressing scheme for two- or three-dimensional space. Accordingly, they can be used to store and retrieve finite-sized geometrical objects that fit into the boxes of the tree at one or another level, and to test for intersections of such objects, for nearness relationships, and so on. The general idea (although there can be variations on this) is to store each object in the smallest box that completely contains it — or, in the case of a zero-sized object like a point, in the appropriate box at the deepest level of tree that we care to implement. Then, when doing a collision or nearness test, we traverse only those parts of the tree that are relevant, much as we did in the applications of KD trees.

Although we will illustrate only the most elementary of applications, QO trees are often at the heart of more complicated algorithms, for example [1-3],

- Hidden polygon removal in the visual plane (which projected polygons intersect a given pixel in the visual field?)
- Fast gravitational or Coulomb N-body calculations (store fictitious objects on various scales that sweep up the multipole moments of the collections of point masses that they contain) [4,5,6]
- Mesh generation (choose a local mesh scale to match the scale in the QO tree at which obstacles or boundaries are stored; the concept of a *balanced* QO tree is often used)
- Image compression (store slowly varying parts of the image as objects high in the tree, and prune unnecessary daughters).

A main weakness of QO trees follows just from their geometrical regularity. If a finite-sized object being stored in a QO tree falls on the boundary between two

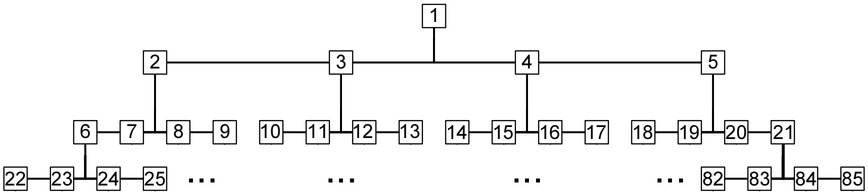

Figure 21.8.2. Quadtree shown in tree form. Because of its regularity, a quadtree's relationships can be described numerically. For example, the mother of box n is the integer part of $n/4$. The left daughter of box n is $4n - 2$.

boxes of about its size, then it can't be stored in either of them. Instead, it gets stored in the larger — sometimes very much larger — box that first completely contains it. If N "small" objects are stored, then the number that fall on boundaries of the highest-level boxes scales in two dimensions as $N^{1/2}$, or in three dimensions as $N^{2/3}$. These objects will thus end up stored in just a few boxes at the top of the tree, and they will participate in almost all operations that check for collisions or nearness. Thus, QO trees can usually effect a time savings that turns a naive algorithm scaling as N into one that scales as $N^{1/2}$ (in the two-dimensional case); but only rarely, or with specialized methods, can they get to that Nirvana of $\log N$ or constant scaling. Still, the square- (or, in three dimensions, cube-) root of a large number can be a large factor, and well worth saving in time. So QO trees are good to know about.

That same geometrical regularity of QO trees allows them to be implemented, at least optionally, as an efficient hash structure where most boxes in the tree, if they are empty, require no storage space. We will give such an implementation here, both for its intrinsic advantages and because it is fairly concise to code, using the `Hash` and `Mhash` classes from §7.6.

The key observations follow from Figure 21.8.2, which shows a QO tree laid out in tree form. The boxes are numbered as in Figure 21.8.1, starting with box 1 at the root of the tree. With this numbering scheme, there exist simple numerical relations between the mother and daughter boxes. This enables one to navigate the tree — up, down, and sideways — without the use of any stored pointers. In particular, if $k \geq 1$ is a box's number, the following relationships hold in D (for us, two or three) dimensions:

$$
\begin{aligned}
\text{mother } (k) &= \lfloor (k + 2^D - 2)/2^D \rfloor \\
\text{leftmost daughter } (k) &= 2^D k - 2^D + 2 \\
\text{rightmost daughter } (k) &= 2^D k + 1 \\
\text{total boxes through level p} &= [(2^D)^p - 1]/(2^D - 1)
\end{aligned}
\qquad (21.8.1)
$$

Note that the integer divide by 2^D implied by the $\lfloor\ \rfloor$ notation can be implemented simply as a right shift by D bits. You should check the formulas in (21.8.1) against Figure 21.8.2 to be sure that you understand how they work. The "levels" of the tree are numbered starting with $p = 1$ for (only) box 1. Notice that the mother of box 1 computes to 0, indicating that it has no mother; this is convenient for testing when to exit loops over ancestry.

Before we get to implementation details for the implementing class `Qotree`, we need to discuss the prerequisites for a class of geometrical objects to be stored

in the tree. Qotree will be templated by a type parameter elT representing those objects. To store an object myel of type elT, you must be sure to provide a method myel.isinbox() whose argument is a Box, and which returns 1 if myel is in the Box, or 0 otherwise. Similarly, to erase an object, you need to provide an == operator, to decide (by comparison) which is the object to be erased. Those two methods are all that Qotree needs for itself. However, many applications of Qotree (including some that we illustrate later in this section) need either or both of the methods myel.contains() and myel.collides(), the first returning whether myel contains a given point, the second returning whether myel collides with another element of type elT.

Here is a simple example of a class, representing a circle (when DIM is 2) or sphere (when DIM is 3), that has these methods and can thus be stored and processed with a Qotree:

sphcirc.h

```
template<Int DIM> struct Sphcirc {
Circle (DIM=2) or sphere (DIM=3) object, with methods suitable for use with Qotree.
    Point<DIM> center;
    Doub radius;
    Sphcirc() {}                                 Default constructor is needed to make arrays.
    Sphcirc(const Point<DIM> &mycenter, Doub myradius)   Construct by explicit center
        : center(mycenter), radius(myradius) {}             and radius.
    bool operator== (const Sphcirc &s) const {         Test if identical.
        return (radius == s.radius && center == s.center);
    }
    Int isinbox(const Box<DIM> &box) {          Is the circle/sphere inside a box?
        for (Int i=0; i<DIM; i++) {
            if ((center.x[i] - radius < box.lo.x[i]) ||
                (center.x[i] + radius > box.hi.x[i])) return 0;
        }
        return 1;
    }
    Int contains(const Point<DIM> &point) {    Is a given point inside the circle/sphere?
        if (dist(point,center) > radius) return 0;
        else return 1;
    }
    Int collides(const Sphcirc<DIM> &circ) {  Does it collide with another circle/sphere?
        if (dist(circ.center,center) > circ.radius+radius) return 0;
        else return 1;
    }
};
```

21.8.1 A Hashed QO Tree Implementation

We will implement the QO tree using two hash memories. First, there is an Mhash multimap memory (called elhash) whose keys are box numbers and whose stored elements are the geometrical objects that may be stored in the QO tree, with possibly multiple objects in a single box. Second, there is a single-valued Hash memory (called pophash) that associates an integer with every box that either (i) contains one or more elements (is "populated"), or (ii) is an ancestor to a box that is populated. In that integer, bit 0 (least significant bit) is used to indicate whether the box is populated, while bits $1 \dots 2^D$ (that is, $1 \dots 4$ or $1 \dots 8$) are used to indicate which (if any) daughters are themselves either populated or are an ancestor to a populated box. In other words, pophash, combined with the relationships in equation (21.8.1), substitutes for the entire structure of doubly linked pointers that might more conventionally implement the tree.

The maximum number of levels $p_{\max}$ that we can represent is limited only by the largest value that can be represented by the integer type that stores box numbers k. Using 32-bit signed integers, 16 levels are possible in two dimensions, since $(4^{16} - 1)/3 < 2^{31} - 1$

(cf. equation 21.8.1). In three dimensions, 11 levels can be represented, since $(8^{11} - 1)/7 < 2^{31} - 1$. Often there is no need for this much resolution ($\sim 10^9$ boxes), so we will provide for setting a smaller value of p_{max}, a good idea since the time to traverse one branch of the tree from root to leaf (a frequently occurring "atom" in other procedures) scales linearly with p_{max}.

qotree.h

```
template<class elT, Int DIM> struct Qotree {
Quadtree (DIM=2) or octree (DIM=3) object to store geometrical objects of type elT.
    static const Int PMAX = 32/DIM;          Roughly how many levels fit in 32 bits.
    static const Int QO = (1 << DIM);        I.e., 4 for quad-, 8 for oct-.
    static const Int QL = (QO - 2);          Offset constant to leftmost daughter.
    Int maxd;
    Doub blo[DIM];
    Doub bscale[DIM];
    Mhash<Int,elT,Hashfn1> elhash;           Contains stored elements hashed by box #.
    Hash<Int,Int,Hashfn1> pophash;           Contains node population info.
    Qotree(Int nh, Int nv, Int maxdep);      The constructor. See below.
    void setouterbox(Point<DIM> lo, Point<DIM> hi);  Set scale and position.
    Box<DIM> qobox(Int k);                   Return the box whose number is k.
    Int qowhichbox(elT tobj);                Return smallest box containing tobj.
    Int qostore(elT tobj);                   Store an elT object in the Qotree.
    Int qoerase(elT tobj);                   Erase an elT object in the Qotree.
    Int qoget(Int k, elT *list, Int nmax);      Retrieve all objects in box k.
    Int qodump(Int *k, elT *list, Int nmax);    Retrieve all objects.
    Int qocontainspt(Point<DIM> pt, elT *list, Int nmax);   See below.
    Int qocollides(elT qt, elT *list, Int nmax);            See below.
};

template<class elT, Int DIM>
Qotree<elT,DIM>::Qotree(Int nh, Int nv, Int maxdep) :
    elhash(nh, nv), maxd(maxdep), pophash(maxd*nh, maxd*nv) {
Constructor for a quad- (DIM=2) or oc- (DIM=3) tree that can store a max of nv elements of
type elT, using hash tables of length nh (typically ≈ nv). maxdep is the number of levels to be
represented.
    if (maxd > PMAX) throw("maxdep too large in Qotree");
    setouterbox(Point<DIM>(0.0,0.0,0.0),Point<DIM>(1.0,1.0,1.0));  Default scale.
}

template<class elT, Int DIM>
void Qotree<elT,DIM>::setouterbox(Point<DIM> lo, Point<DIM> hi) {
Sets the scale of Qotree to an outer box defined by points lo and hi. Must be called before
any elements are stored in the tree.
    for (Int j=0; j<DIM; j++) {
        blo[j] = lo.x[j];
        bscale[j] = hi.x[j] - lo.x[j];
    }
}
```

You will normally call `setouterbox()` immediately after invoking the `qotree` constructor to create a QO tree. Otherwise, you get the default box with a corner at the origin and unit size in each dimension.

Right away, we need two utility routines. The first takes a box's number (e.g., as in Figure 21.8.1) and returns the actual box (as a `Box<DIM>`). The second takes an object of the type to be stored in the tree (`elT`) and returns the number of the smallest box that contains it. It does this by starting at the top of the tree, trying each possible daughter, and moving deeper into the tree only when a containment is found.

qotree.h

```
template<class elT, Int DIM>
Box<DIM> Qotree<elT,DIM>::qobox(Int k) {
Returns the box indexed by k.
    Int j, kb;
    Point<DIM> plo, phi;
```

```
    Doub offset[DIM];
    Doub del = 1.0;
    for (j=0; j<DIM; j++) offset[j] = 0.0;
    while (k > 1) {                          Up through ancestors until get to root.
        kb = (k + QL) % QO;                  Which daughter is k? Add its offset.
        for (j=0; j<DIM; j++) { if (kb & (1 << j)) offset[j] += del; }
        k = (k + QL) >> DIM;                 Replace k by its mother,
        del *= 2.0;                          where offsets will be twice as big.
    }
    for (j=0; j<DIM; j++) {                  At the end, scale the offsets by the final del to
        plo.x[j] = blo[j] + bscale[j]*offset[j]/del;  make them metrically correct.
        phi.x[j] = blo[j] + bscale[j]*(offset[j]+1.0)/del;
    }
    return Box<DIM>(plo,phi);                Construct the box and return it.
}

template<class elT, Int DIM>
Int Qotree<elT,DIM>::qowhichbox(elT tobj) {
```
Return the box number of the smallest box that can contain an element `tobj`, without regard to whether `tobj` is already stored in the tree.
```
    Int p,k,kl,kr,ks=1;                      Answer is box 1 unless a smaller box found.
    for (p=2; p<=maxd; p++) {                Go down through the levels.
        kl = QO * ks - QL;                   Leftmost daughter.
        kr = kl + QO -1;                     Rightmost daughter.
        for (k=kl; k<=kr; k++) {             Do any daughters contain tobj?
            if (tobj.isinbox(qobox(k))) { ks = k; break; }
        }
        if (k > kr) break;                   No. Therefore, discontinue descent here.
    }
    return ks;
}
```

Now we are ready to store elements into the tree, or to erase elements previously stored. With `qowhichbox()`, above, and the methods that belong to the `Mhash`, it is trivial to do the actual store or erase. Trickier to code is to create or erase the trail of "breadcrumbs" in `pophash` that connect the box to its ancestors. When we erase, we must be sure not to cut off the trail to any remaining elements in the same box, or to elements in descendant boxes.

qotree.h
```
template<class elT, Int DIM>
Int Qotree<elT,DIM>::qostore(elT tobj){
```
Store the element `tobj` in the `Qotree`, and return the box number into which it was stored.
```
    Int k,ks,kks,km;
    ks = kks = qowhichbox(tobj);
    elhash.store(ks, tobj);                  Store the element in elhash
    pophash[ks] |= 1;                        and mark its box as populated.
    while (ks > 1){                          Now leave trail of breadcrumbs to the root mother.
        km = (ks + QL) >> DIM;               Mother of ks.
        k = ks - (QO*km - QL);               Which daughter of km is ks.
        ks = km;                             Now set the daughter bit in the mother.
        pophash[ks] |= (1 << (k+1));
    }
    return kks;
}

template<class elT, Int DIM>
Int Qotree<elT,DIM>::qoerase(elT tobj) {
```
Erase the element `tobj`, returning the box number into which it was stored or 0 if the element was not found in the `Qotree`. Note logic very similar to `qostore`.
```
    Int k,ks,kks,km;
    Int *ppop;
    ks = kks = qowhichbox(tobj);                 Find the box.
    if (elhash.erase(ks, tobj) == 0) return 0;      It ain't there!
    if (elhash.count(ks)) return kks;            Sisters still in same box, so we are done.
```

```
    ppop = &pophash[ks];                          Must now erase any unneeded breadcrumbs.
    *ppop &= ~((Uint)1);                          Unmark the pop bit.
    while (ks > 1) {                              Up through the ancestors...
        if (*ppop) break;                         Box is populated or has daughters, so done.
        pophash.erase(ks);                        Erase unneeded (zero) pophash entry.
        km = (ks + QL) >> DIM;                    Mother of ks.
        k = ks - (QO*km - QL);                    Which daughter of km is ks.
        ks = km;
        ppop = &pophash[ks];
        *ppop &= ~((Uint)(1 << (k+1)));           Unset the daughter bit in the mother.
    }
    return kks;
}
```

Finally, we need methods to retrieve elements previously stored, either those in a given box (by number), or else all the elements in the tree. In the former case, the `Mhash` does all the work. In the latter case, however, we must provide the machinery for a recursive search of the tree, since at any stage we may encounter a box with multiple populated daughters. Notice that the calling routine is responsible for supplying storage (as an array `list[]`) for the result and declaring the maximum number `nmax` of elements that it is prepared to accept.

qotree.h

```
template<class elT, Int DIM>
Int Qotree<elT,DIM>::qoget(Int k, elT *list, Int nmax) {
```

Retrieve all (or up to `nmax` if it is smaller) elements that are stored in box `k` of the `Qotree`. The elements are copied into `list[0..nlist-1]` and the value `nlist` ($\leq$ `nmax`) is returned.

```
    Int ks, pop, nlist;
    ks = k;
    nlist = 0;
    pophash.get(ks,pop);
    if ((pop & 1) && elhash.getinit(ks)) {
        while (nlist < nmax && elhash.getnext(list[nlist])) {nlist++;}
    }
    return nlist;
}
```

```
template<class elT, Int DIM>
Int Qotree<elT,DIM>::qodump(Int *klist, elT *list, Int nmax) {
```

Retrieve all (or up to `nmax` if it is smaller) elements that are stored anywhere in the `Qotree`, along with their corresponding box numbers. The elements are copied into `list[0..nlist-1]` and the value `nlist` ($\leq$ `nmax`) is returned. The box numbers are copied into `klist[0..nlist-1]`.

```
    Int nlist, ntask, ks, pop, k;
    Int tasklist[200];                            Stack of pending box numbers as we recur-
    nlist = 0;                                        sively traverse the tree.
    ntask = 1;
    tasklist[1] = 1;
    while (ntask) {                               As long as tasks remain...
        ks = tasklist[ntask--];
        if (pophash.get(ks,pop) == 0) continue;      Box empty and no daughters.
        if ((pop & 1) && elhash.getinit(ks)) {       The box is populated, so we output
            while (nlist < nmax && elhash.getnext(list[nlist])) {  its contents.
                klist[nlist] = ks;
                nlist++;
            }
        }
        if (nlist == nmax) break;                 No more room for output!
        k = QO*ks - QL;                           Leftmost daughter.
        while (pop >>= 1) {                       Loop over the daughter bits in pop.
            if (pop & 1) tasklist[++ntask] = k;      Daughter exists. Add to task list.
            k++;                                  Next daughter.
        }
    }
    return nlist;
}
```

The additional functions declared in `Qotree` pertain to applications, as we now discuss.

21.8.2 QO Tree Elementary Applications

Two important building blocks for applications of QO trees are, first, a routine that returns a list of all stored `elT` elements that intersect (i.e., contain) a specified point; and, second, a routine that returns a similar list of all stored `elT` elements that intersect (i.e., collide with) a specified `elT` element.

An element that intersects a point will evidently be stored in a box that is an ancestor to the box that the point is in, or else in the same box as the point. It takes just one pass down through the levels of the tree to find all such elements.

qotree.h

```
template<class elT, Int DIM>
Int Qotree<elT,DIM>::qocontainspt(Point<DIM>pt, elT *list, Int nmax) {
```

Retrieve all (or up to `nmax` if it is smaller) elements in `Qotree` that contain the point `pt`. The elements are copied into `list[0..nlist-1]` and the value `nlist` ($\leq$ `nmax`) is returned.

```
    Int j,k,ks,pop,nlist;
    Doub bblo[DIM], bbscale[DIM];
    for (j=0; j<DIM; j++) { bblo[j] = blo[j]; bbscale[j] = bscale[j]; }
    nlist = 0;
    ks = 1;                                 Start at the top of the tree.
    while (pophash.get(ks,pop)) {           Descend as long as something is there.
        if (pop & 1) {                      The box is populated, so we check its con-
            elhash.getinit(ks);                 tained elements,
            while (nlist < nmax && elhash.getnext(list[nlist])) {
                if (list[nlist].contains(pt)) {nlist++;} returning any that contain
            }                                                pt.
        }
        if ((pop >>= 1) == 0) break;        The box has no daughters, so we are done.
        for (k=0, j=0; j<DIM; j++) {        Compute k, the single daughter containing
            bbscale[j] *= 0.5;                  pt.
            if (pt.x[j] > bblo[j] + bbscale[j]) {
                k += (1 << j);
                bblo[j] += bbscale[j];
            }
        }
        if (((pop >> k) & 1) == 0) break;   No such daughter exists in the tree.
        ks = QO * ks - QL + k;              Daughter exists and is the next node to check.
    }
    return nlist;
}
```

When an element A intersects another element B, either A and B are in the same box, or else A is in an ancestor box to B, or else B is in an ancestor box to A. Equivalently, for a fixed A, we can find all intersecting B's by searching A's box, its ancestors, and its descendants. The latter search requires a task list stack, as we have seen before (e.g., in `qodump`).

qotree.h

```
template<class elT, Int DIM>
Int Qotree<elT,DIM>::qocollides(elT qt, elT *list, Int nmax) {
```

Retrieve all (or up to `nmax` if it is smaller) elements in `Qotree` that collide with an element `qt` (which needn't be in the tree itself). The elements are copied into `list[0..nlist-1]` and the value `nlist` ($\leq$ `nmax`) is returned.

```
    Int k,ks,kks,pop,nlist,ntask;
    Int tasklist[200];                  Stack of pending box numbers.
    nlist = 0;
    kks = ks = qowhichbox(qt);          kks saves the starting box.
    ntask = 0;
    while (ks > 0) {                    Put the starting box and all its ancestors on the
                                            task list.
```

```
        tasklist[++ntask] = ks;
        ks = (ks + QL) >> DIM;          Move to mother.
    }
    while (ntask) {
        ks = tasklist[ntask--];
        if (pophash.get(ks,pop) == 0) continue;   Box empty and no daughters.
        if (pop & 1) {                  The box is populated, so we check its contained
            elhash.getinit(ks);             elements,
            while (nlist < nmax && elhash.getnext(list[nlist])) {
                if (list[nlist].collides(qt)) {nlist++;}     returning any that col-
            }                                                    lide with qt.
        }
        if (ks >= kks) {                Recurse only for descendants, not ancestors!
            k = QO*ks - QL;             Leftmost daughter.
            while (pop >>= 1) {
                if (pop & 1)            Daughter exists. Add to task list.
                    tasklist[++ntask] = k;
                k++;                    Next daughter.
            }
        }
    }
    return nlist;
}
```

As an example of a simple application of a QO tree, let's replicate the functionality of `KDtree::locatenear` (§21.2) with a routine that finds all stored points within a specified radius r of a test point. Using the class `Sphcirc`, points are represented as circles/spheres of zero radius, the test point as a circle/sphere of radius r, and we use `qocollides` to find the collisions.

We implement this application as a structure, `Nearpoints`, whose constructor creates the QO tree out of a vector of points, and whose member function `locatenear` can then be called to find all stored points within any specified radius of any specified point.

```
template <int DIM> struct Nearpoints {                                  qotree.h
Object for constructing a QO tree containing a set of points, and for repeatedly querying which
stored points are within a specified radius of a specified new point.
    Int npts;
    Qotree<Sphcirc<DIM>,DIM> thetree;
    Sphcirc<DIM> *sphlist;
    Nearpoints(const vector< Point<DIM> > &pvec)
        : npts(pvec.size()), thetree(npts,npts,32/DIM) {
        Constructor. Creates the QO tree from a vector of points pvec.
        Int j,k;
        sphlist = new Sphcirc<DIM>[npts];
        Point<DIM> lo = pvec[0], hi = pvec[0];    Find bounding box for the points.
        for (j=1; j<npts; j++) for (k=0; k<DIM; k++) {
            if (pvec[j].x[k] < lo.x[k]) lo.x[k] = pvec[j].x[k];
            if (pvec[j].x[k] > hi.x[k]) hi.x[k] = pvec[j].x[k];
        }
        for (k=0; k<DIM; k++) {                   Expand it by 10% so that all points
            lo.x[k] -= 0.1*(hi.x[k]-lo.x[k]);         are well interior.
            hi.x[k] += 0.1*(hi.x[k]-lo.x[k]);
        }
        thetree.setouterbox(lo,hi);     Set the tree's outer box and store all the points.
        for (j=0; j<npts; j++) thetree.qostore(Sphcirc<DIM>(pvec[j],0.0));
    }
    ~Nearpoints() { delete [] sphlist; }
    Int locatenear(Point<DIM> pt, Doub r, Point<DIM> *list, Int nmax) {
```

```
    Once the tree is constructed, this function can be called repeatedly with varying points pt
    and radii r. It returns n, the number of stored points within radius r of pt (but no larger
    than nmax), and copies those points into list[0..n-1].
        Int j,n;
        n = thetree.qocollides(Sphcirc<DIM>(pt,r),sphlist,nmax);
        for (j=0; j<n; j++) list[j] = sphlist[j].center;
        return n;
    }
};
```

In practice, the routine above is rather slower than KDtree::locatenear for this application, because there is a lot of overhead involved in copying Point and Sphcirc elements around, and in computing Boxes as we drill down the tree. By contrast, KDtree is lean and mean, since it only stores points and, in our implementation, copies them internally to a fast store of coordinates.

Unlike the KD tree, however, the technique illustrated here can be generalized to much more complicated situations. For example, instead of being simple points, the stored objects could be broadcast reception areas for FM radio stations on a given frequency, and we might want to know where collisions occur with proposed new stations. The collides() function between two broadcast areas might involve a lengthy calculation taking into account their powers, the surrounding detailed topography, and so on. In such a case, the overhead of the QO tree might well be negligible as we seek to minimize the number of calls to collides().

As a second example of a simple application, consider a square Petri dish on which spores land, in random positions, one at a time. Each such spore quickly grows into a circular colony that just touches the nearest existing colony (or the edge of the dish), and then stops. (Don't ask us why. This is only an example.) What does the dish look like after N spores have landed?

Rather than give the code in detail, a simple description should suffice: The objects stored in the QO tree are circles. Looping over the number of spores, we pick a random location for each in turn. If the QO tree method qocontainspt() indicates that the location lies within an already-stored colony, go on to the next spore. Otherwise, start with a small trial radius and increase it (by doubling, e.g.) until qotreecollides() first indicates collisions. Now adjust the trial radius to be the minimum of distances to the colliding elements, add that colony to the tree, and go on to the next spore.

Figure 21.8.3 shows an example of the resulting configuration, after 1000 colonies have grown. (Another 3592 spores landed inside existing colonies and died immediately.)

CITED REFERENCES AND FURTHER READING:

de Berg, M., van Kreveld, M., Overmars, M., and Schwarzkopf, O. 2000, *Computational Geometry: Algorithms and Applications*, 2nd revised ed. (Berlin: Springer), Chapter 14.[1]

Samet, H. 1990, *The Design and Analysis of Spatial Data Structures* (Reading, MA: Addison-Wesley).[2]

Samet, H. 1990, *Applications of Spatial Data Structures: Computer Graphics, Image Processing, and GIS* (Reading, MA: Addison-Wesley).[3]

Pfalzner, S. and Gibbon, P. 1996, *Many-Body Tree Methods in Physics* (Cambridge, UK: Cambridge University Press).[4]

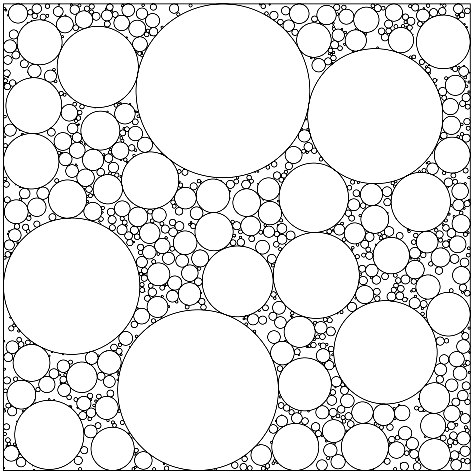

Figure 21.8.3. Spores land randomly on a square (!) Petri dish, and grow to colonies that barely touch the nearest pre-existing colony, or the edge of the dish. A QO tree can be used to keep track of collisions. Here, 1000 colonies have grown to their maximum size.

Greengard, L., and Wandzura, S., eds. 1998, "Fast Multipole Methods," special issue of *IEEE Computational Science and Engineering*, vol. 5, no. 3 (July–September), pp. 16–56.[5]

Gumerov, N.A., and Duraiswami, R. 2004, *Fast Multipole Methods for the Helmholtz Equation in Three Dimensions* (Amsterdam: Elsevier).[6]

CHAPTER 22

Less-Numerical Algorithms

22.0 Introduction

You can stop reading now. You are done with *Numerical Recipes*, as such. This final chapter is an idiosyncratic set of "*less*-numerical recipes" that, for one reason or another, we have decided to include between the covers of an otherwise *more*-numerically oriented book. Authors of computer science texts, we've noticed, like to throw in a token numerical subject (usually quite a dull one — quadrature, for example). We find that we are not free of the reverse tendency.

Our selection of material is not completely arbitrary. In §9.0 we promised to provide a simple plotting routine. Another promised topic, Gray codes, was already used in the construction of quasi-random sequences (§7.8) and here needs only some additional explication. Two other topics, on diagnosing a computer's floating-point parameters, and on arbitrary precision arithmetic, give additional insight into the machinery behind the casual assumption that computers are useful for doing things with real numbers (as opposed to integers or characters). The latter of these topics also shows a very different use for Chapter 12's fast Fourier transform.

The three other topics (checksums, Huffman, and arithmetic coding) involve different aspects of data coding, compression, and validation. The material here is intended to be somewhat less abstract, and somewhat more practical, than the discussion of coding in §16.2, where coding was used to illustrate statistical aspects of state estimation. If you handle a large amount of data (numerical data, even), then a passing familiarity with these subjects might at some point come in handy. In §13.6, for example, we already encountered a good use for Huffman coding.

But again, you don't have to read this chapter. (And you should learn about quadrature from Chapters 4 and 17, not from a computer science textbook!)

22.1 Plotting Simple Graphs

Yes, we all have our favorite plotting or graphics packages, and our favorite ways of generating plots from within C++ programs. But wait: Are your C++ pro-

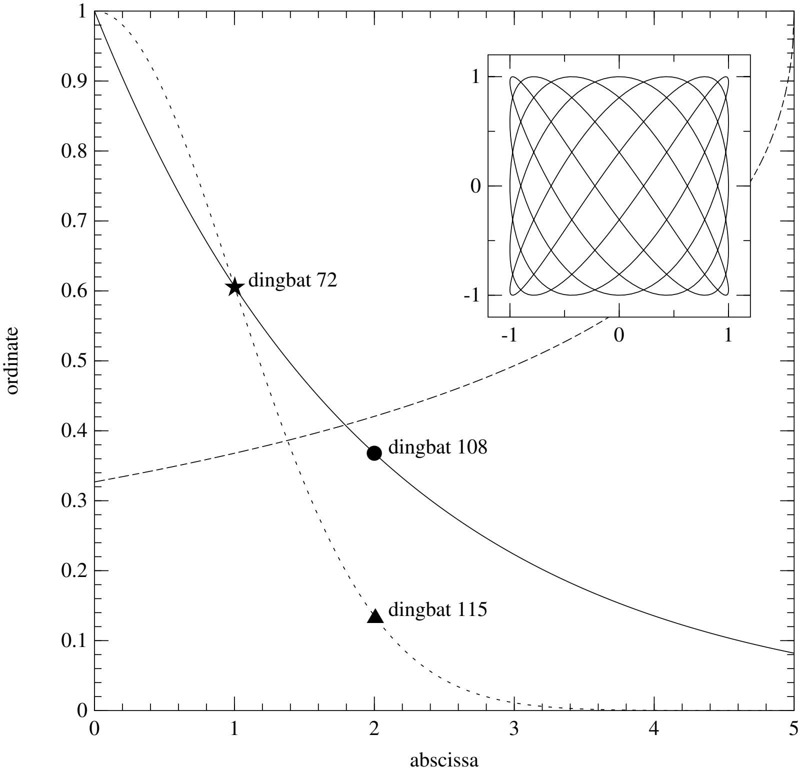

Figure 22.1.1. Simple plot generated using the objects `PSpage` and `PSplot`, which are wrappers for generating PostScript.

grams generating long text files of numbers, just so that you can read those numbers into a separate plotting or graphics package? If so, you might benefit from this section.

We find it useful to have on hand a couple of short C++ objects, implemented in simple source code, that generate simple plots. (By "simple," we mean "like most of the figures in this book.") We are then able to make plots from anywhere in our programs, whether as an aid in debugging or as final output. Equally important, we can make changes to the plotting source code at will, adding features or modifying the look of the plot.

One way of accomplishing these goals is by means of a C++ "wrapper" that does no more, nor less, than to write out a valid PostScript [1] file, which can be viewed or printed using a PostScript viewer such as the freely available Ghostscript/GSview [2]. In fact, the viewer can readily be invoked by a method within the wrapper object, so that the plot simply pops up in its own window on your screen.

An example will make this clearer. Figure 22.1.1 shows a sample plot that has a couple of x, y scaled boxes, some lines and points of varying types, and some text labels. Here is the code that generates the figure:

```
void psplot_example() {                                          psplotexample.h
Routine for creating Figure 22.1.1.
    VecDoub x1(500),x2(500),y1(500),y2(500),y3(500),y4(500);
    for (Int i=0;i<500;i++) {                        Generate some data.
```

```
        x1[i] = 5.*i/499.;
        y1[i] = exp(-0.5*x1[i]);
        y2[i] = exp(-0.5*SQR(x1[i]));
        y3[i] = exp(-0.5*sqrt(5.-x1[i]));
        x2[i] = cos(0.062957*i);
        y4[i] = sin(0.088141*i);
    }

    PSpage pg("d:\\nr3\\newchap20\\myplot.ps");     Instantiate a page.
    PSplot plot1(pg,100.,500.,100.,500.);          Instantiate a plot on the page. Po-
    plot1.setlimits(0.,5.,0.,1.);                      sition is specified in pt (72 pt =
    plot1.frame();                                     1 in, or 28 pt = 1 cm).
    plot1.autoscales();
    plot1.xlabel("abscissa");
    plot1.ylabel("ordinate");
    plot1.lineplot(x1,y1);
    plot1.setdash("2 4");
    plot1.lineplot(x1,y2);
    plot1.setdash("6 2 4 2");
    plot1.lineplot(x1,y3);
    plot1.setdash("");                             Unsets dash.
    plot1.pointsymbol(1.,exp(-0.5),72,16.);
    plot1.pointsymbol(2.,exp(-1.),108,12.);
    plot1.pointsymbol(2.,exp(-2.),115,12.);
    plot1.label("dingbat 72",1.1,exp(-0.5));
    plot1.label("dingbat 108",2.1,exp(-1.));
    plot1.label("dingbat 115",2.1,exp(-2.));

    PSplot plot2(pg,325.,475.,325.,475.);          Instantiate a second plot.
    plot2.clear();                                 Erase what's underneath it.
    plot2.setlimits(-1.2,1.2,-1.2,1.2);
    plot2.frame();
    plot2.scales(1.,0.5,1.,0.5);
    plot2.lineplot(x2,y4);

    pg.close();
    pg.display();                                  Pop up a window displaying the plot
}                                                      file.
```

The general idea is that a `PSpage` object (`pg` in the example above) represents a whole sheet of paper, or window on the screen. It can contain one or more `PSplot` objects. In the above example there are two: `plot1` and `plot2`. `PSplot` objects can be separate on the page, or overlapping. Each has its own x, y coordinate system, its own x- and y-axis labels, and so forth. With no more explanation than this, you should be able to find a program line above that corresponds to each feature in the figure. The last line makes the plot pop up on our screen.

Point symbols are referenced by their character number in the Zapf Dingbats font, which is built into PostScript. If you want to see all the possible symbols, a Web search for "LaTeX Postscript Dingbats" will turn up several charts. Or, just write a program to plot them all. (Hint: There are possibly useful symbols from 33 to 126, and from 161 to 254.)

A Webnote [3] gives the complete source code for the `PSpage` and `PSplot` objects, which is only about 150 lines long. In the course of writing this book, our personal version of the code expanded to about 450 lines. This is an order of magnitude or two less than the standard packages that are available in open source code, GNUPLOT, for example [4]. It is a question of trading off capability (theirs much greater) for ease of modifying the source code (you be the judge).

If you choose to go down this road, you'll soon want to learn more of PostScript as a language. A good reference is [5].

CITED REFERENCES AND FURTHER READING:

Adobe Systems, Inc. 1999, *PostScript Language Reference*, 3rd ed. (Reading, MA: Addison-Wesley).[1]

Ghostscript and GSview 2007+, at `http://www.cs.wisc.edu/~ghost/`.[2]

Numerical Recipes Software 2007, "Code for PSpage and PSplot," *Numerical Recipes Webnote No. 26*, at `http://www.nr.com/webnotes?26` [3]

GNUPLOT 2007+, at `http://www.gnuplot.info`.[4]

McGilton, H., and Campione, M. 1992, *PostScript by Example* (Reading, MA: Addison-Wesley).[5]

22.2 Diagnosing Machine Parameters

A convenient fiction is that a computer's floating-point arithmetic is "accurate enough." If you believe this fiction, then numerical analysis becomes a very clean subject. Roundoff error disappears, and many finite algorithms are exact. Only manageable truncation error (§1.1) stands between you and a perfect calculation. Sounds rather naive, doesn't it?

Yes, it is naive. Notwithstanding, we have adopted this fiction throughout most of this book. To do a good job of answering the question of how roundoff error propagates, or can be bounded, for every algorithm that we have discussed would be impractical. In fact, it would not be possible: Rigorous analysis of many practical algorithms has never been made, by us or anyone.

Almost all processors today share the same floating-point data representation, namely that specified in IEEE Standard 754-1985 [1], and therefore the same strengths and weaknesses as regards roundoff error. But this was not always so! The history of computing is full of machines with strange floating-point representations by modern standards. Many early computers had 36-bit words, typically partitioned as a sign bit, 8 bits of exponent, and 27 bits of mantissa. The influential IBM 7090/7094 series was of this type. The legendary CDC 6600 and 7600 machines, designed by Seymour Cray, had 60-bit words (sign, 11-bit exponent, 48-bit mantissa). A particularly odd design was the IBM STRETCH, whose 64 bits were allocated to an exponent flag bit, 10 exponent bits, the exponent sign, a 48-bit mantissa, its sign, and three flag bits. The exponent flag bit was used to signal overflow or underflow, while the other flag bits could be set by the user to indicate — anything! So let us all be grateful for IEEE 754.

Likewise, almost all numerical computing today is done in double precision, that is, in 64-bit words, what C++ defines as `double` and we denote as `Doub`. This, also, was not always so. It has happened (one might argue) because the availability of memory has increased even more rapidly than the appetite for it in numerical computation. Many programmers born before 1960 still feel a small frisson when they type `double` instead of `float`. Indeed, the vast majority of routines in this book will work just fine, for the vast majority of applications, with merely `float` precision. In most cases, the use of `double` simply serves to reinforce an erroneous belief in the above "convenient fiction."

Still, every once in a while, you will need to know what the limitations of your machine's floating-point arithmetic actually are — the more so when your treatment of floating-point roundoff error is going to be intuitive, experimental, or casual. This will certainly be true if you ever encounter a processor with nonstandard (that is, non-IEEE compliant) hardware. Such processors still do exist, though generally hidden away in embedded special-purpose devices.

If you are lucky, then calls to the methods in the C++ standard library class `numeric_limits` will tell you what you need to know. It is a good idea to familiarize yourself with that class, including some of its esoterica, like `round_style` and `has_denorm` [2].

A more experimental approach is to use methods that were developed to ferret out machine parameters in the bad old days before standards [3,4], especially parameters that were supposed to be transparent to the (ordinary) user. The object `Machar`, listed in full in a Webnote [5], gives an implementation of a number of of these methods. The quantities determined are

- `ibeta` is the radix in which numbers are represented, almost always 2, but historically sometimes 16, or even 10.
- `it` is the number of base-`ibeta` digits in the floating-point mantissa M.
- `machep` is the exponent of the smallest (most negative) power of `ibeta` that, added to 1.0, gives something different from 1.0.
- `eps` is the floating-point number $\mathtt{ibeta}^{\mathtt{machep}}$, loosely referred to as the "floating-point precision."
- `negep` is the exponent of the smallest power of `ibeta` that, subtracted from 1.0, gives something different from 1.0.
- `epsneg` is $\mathtt{ibeta}^{\mathtt{negep}}$, another way of defining floating-point precision. Not infrequently, `epsneg` is 0.5 times `eps`; occasionally `eps` and `epsneg` are equal.
- `iexp` is the number of bits in the exponent (including its sign or bias).
- `minexp` is the smallest (most negative) power of `ibeta` consistent with there being no leading zeros in the mantissa.
- `xmin` is the floating-point number $\mathtt{ibeta}^{\mathtt{minexp}}$, generally the smallest (in magnitude) useable floating value.
- `maxexp` is the smallest (positive) power of `ibeta` that causes overflow.
- `xmax` is $(1 - \mathtt{epsneg}) \times \mathtt{ibeta}^{\mathtt{maxexp}}$, generally the largest (in magnitude) useable floating value.
- `irnd` returns a code in the range $0 \ldots 5$, giving information on what kind of rounding is done in addition, and on how underflow is handled. See below.
- `ngrd` is the number of "guard digits" used when truncating the product of two mantissas to fit the representation.

The parameter `irnd` needs some additional explanation. In the IEEE standard, bit patterns correspond to exact, "representable" numbers. The specified method for rounding an addition is to add two representable numbers "exactly," and then round the sum to the closest representable number. If the sum is precisely halfway between two representable numbers, it should be rounded to the even one (low-order bit zero). The same behavior should hold for all the other arithmetic operations, that is, they should be done in a manner equivalent to infinite precision, and then rounded to the closest representable number.

If `irnd` returns 2 or 5, then your processor is compliant with this standard. If it

Sample Results Returned by Machar			
	IEEE-compliant processor		historical
precision	float	double	DEC-VAX
ibeta	2	2	2
it	24	53	24
machep	−23	−52	−24
eps	1.19×10^{-7}	2.22×10^{-16}	5.96×10^{-8}
negep	−24	−53	−24
epsneg	5.96×10^{-8}	1.11×10^{-16}	5.96×10^{-8}
iexp	8	11	8
minexp	−126	−1022	−128
xmin	1.18×10^{-38}	2.23×10^{-308}	2.94×10^{-39}
maxexp	128	1024	127
xmax	3.40×10^{38}	1.79×10^{308}	1.70×10^{38}
irnd	5	5	1
ngrd	0	0	0

returns 1 or 4, then it is doing some kind of rounding, but not the IEEE standard. If irnd returns 0 or 3, then it is truncating the result, not rounding it — not desirable.

The other issue addressed by irnd concerns underflow. If a floating value is less than xmin, many computers underflow its value to zero. Values irnd $= 0, 1,$ or 2 indicate this behavior. The IEEE standard specifies a more graceful kind of underflow: As a value becomes smaller than xmin, its exponent is frozen at the smallest allowed value while its mantissa is decreased, acquiring leading zeros and "gracefully" losing precision. This is indicated by irnd $= 3, 4,$ or 5.

Sometimes results can be compiler-dependent. For example, some compilers underflow intermediate results ungracefully, yielding irnd = 2 rather than 5.

Call the report method in Machar to see the comparison between its results and those returned by numeric_limits. Some values returned by Machar for IEEE compliant processors are given in the table above and compared with an important historical processor, the DEC-VAX. This processor, like its predecessor PDP-11, used a representation with a "phantom" leading 1 bit in the mantissa. You can see that this achieved a smaller eps for the same wordlength but could not underflow gracefully, since there were no denormalized numbers.

CITED REFERENCES AND FURTHER READING:

IEEE Standard for Binary Floating-Point Numbers, ANSI/IEEE Std 754–1985 (New York: IEEE, 1985).[1]

Josuttis, N.M. 1999, *The C++ Standard Library: A Tutorial and Reference* (Boston: Addison-Wesley), §4.3.[2]

Cody, W.J. 1988, "MACHAR: A Subroutine to Dynamically Determine Machine Parameters," *ACM Transactions on Mathematical Software*, vol. 14, pp. 303–311.[3]

Malcolm, M.A. 1972, "Algorithms to Reveal Properties of Floating-Point Arithmetic," *Communications of the ACM*, vol. 15, pp. 949–951.[4]

Numerical Recipes Software 2007, "Code for Machar," *Numerical Recipes Webnote No. 27*, at `http://www.nr.com/webnotes?27` [5]

Goldberg, D. 1991, "What Every Computer Scientist Should Know About Floating-Point Arithmetic," *ACM Computing Surveys*, vol. 23, pp. 5–48.

22.3 Gray Codes

A Gray code is a function $G(i)$ of the integers i that for each integer $N \geq 0$ is one-to-one for $0 \leq i \leq 2^N - 1$, and that has the following remarkable property: The binary representations of $G(i)$ and $G(i+1)$ differ in *exactly one bit*. An example of a Gray code (in fact, the most commonly used one) is the sequence 0000, 0001, 0011, 0010, 0110, 0111, 0101, 0100, 1100, 1101, 1111, 1110, 1010, 1011, 1001, and 1000, for $i = 0, \ldots, 15$. The algorithm for generating this code is simply to form the bitwise exclusive-or (XOR) of i with $i/2$ (integer part). Think about how the carries work when you add one to a number in binary, and you will be able to see why this works. You will also see that $G(i)$ and $G(i+1)$ differ in the bit position of the rightmost zero bit of i (prefixing a leading zero if necessary).

The spelling is "Gray," not "gray": The codes are named after one Frank Gray, who first patented the idea for use in shaft encoders. A shaft encoder is a wheel with concentric coded stripes, each of which is "read" by a fixed optical sensor or conducting brush. The idea is to generate a binary code describing the angle of the wheel. The obvious, but wrong, way to build a shaft encoder is to have one stripe (the innermost, say) present on half the wheel, but absent on the other half; the next stripe is present in quadrants 1 and 3; the next stripe is present in octants 1, 3, 5, and 7; and so on. The optical or electrical sensors together then read a direct binary code for the position of the wheel.

The reason this method is bad is that there is no way to guarantee that all the brushes will make or break contact *exactly* simultaneously as the wheel turns. Going from position 7 (0111) to 8 (1000), one might pass spuriously and transiently through 6 (0110), 14 (1110), and 10 (1010), as the different brushes make or break contact. Use of a Gray code on the encoding stripes guarantees that there is no transient state between 7 (0100 in the sequence above) and 8 (1100).

Of course we then need circuitry, or algorithmics, to translate from $G(i)$ to i. Figure 22.3.1(b) shows how this is done by a cascade of XOR gates. The idea is that each output bit should be the XOR of all more significant input bits. To do N bits of Gray code inversion requires $N-1$ steps (or gate delays) in the circuit. (Nevertheless, this is typically very fast in circuitry.) In a register with word-wide binary operations, we don't have to do N consecutive operations, but only $\ln_2 N$. The trick is to use the associativity of XOR and group the operations hierarchically. This involves sequential right-shifts by $1, 2, 4, 8, \ldots$ bits until the wordlength is exhausted. Here is a piece of code for doing both $G(i)$ and its inverse:

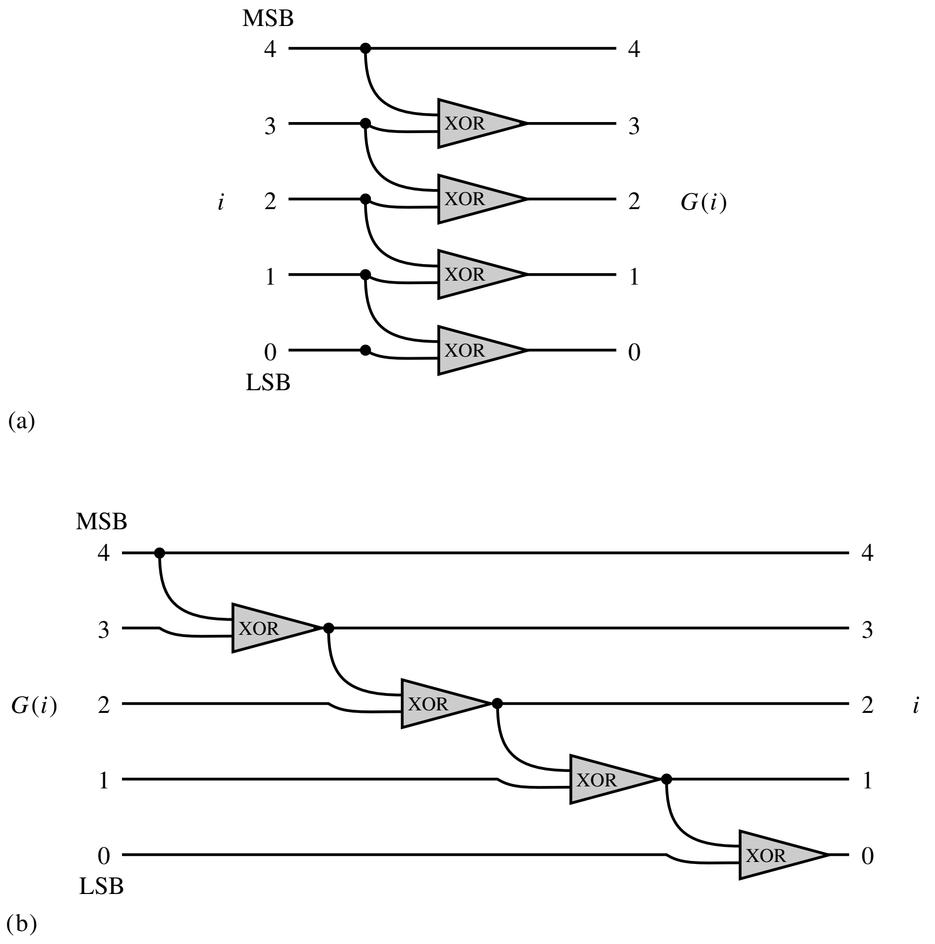

Figure 22.3.1. Single-bit operations for calculating the Gray code $G(i)$ from i (a), or the inverse (b). LSB and MSB indicate the least and most significant bits, respectively. XOR denotes exclusive-or.

`struct Gray {` igray.h

Methods for the Gray code and its inverse.

```
    Uint gray(const Uint n) {return n ^ (n >> 1);}
```
Return the Gray code of an integer n. This is the easy direction!

```
    Uint invgray(const Uint n) {
```
Return the inverse of the Gray code.

```
        Int ish=1;
        Uint ans=n,idiv;
        for (;;) {                         In hierarchical stages, starting with a one-bit right-shift,
            ans ^= (idiv=ans >> ish);      cause each bit to be XORed with all more sig-
            if (idiv <= 1 || ish == 16) return ans;   nificant bits.
            ish <<= 1;                     Double the amount of shift on the next cycle.
        }
    }
};
```

In numerical work, Gray codes can be useful when you need to do some task

that depends intimately on the bits of i, looping over many values of i. Then, if there are economies in repeating the task for values differing by only one bit, it makes sense to do things in Gray code order rather than consecutive order. We saw an example of this in §7.8, for the generation of quasi-random sequences.

CITED REFERENCES AND FURTHER READING:

Horowitz, P., and Hill, W. 1989, *The Art of Electronics*, 2nd ed. (New York: Cambridge University Press), §8.02.

Knuth, D.E. 2005, *Generating All Tuples and Permutations*, fascicle 2 of vol. 4 of *The Art of Computer Programming* (Upper Saddle River, NJ: Addison-Wesley), §7.2.1.1.

22.4 Cyclic Redundancy and Other Checksums

There are networks all around you: not just "the" Internet with its IP and TCP protocols, but also *embedded* networks that move bits around within a device or among closely coupled devices. Examples include the *SMBus* network that communicates power management information between smart batteries and the devices that they power, or the *Bluetooth* network that connects cell phones to nearby accessories. We wouldn't be overly surprised to find a network inside of our wristwatch or electric toothbrush!

Different networks have different protocols, but standard engineering practice is to package the raw information into packets with fixed or variable numbers of bits. Packet lengths are typically in the range from a few tens to a few thousand bits. Smaller would imply proportionally too much overhead per packet, while longer would make excessive demands on buffer sizes, collision avoidance, etc.

When a packet is sent from point A to point B, one wants to know that it has arrived without error. The simplest form of insurance is to add a "parity bit," chosen so as to make the total number of one-bits (versus zero-bits) either always even ("even parity") or always odd ("odd parity"). Any *single-bit* error in a packet will thereby be detected. When errors are sufficiently rare, or their consequence sufficiently minor, use of parity provides enough error detection. For example, the ASCII character set was originally designed for 7-bit characters, with an 8th parity bit.

Since the parity bit has two possible values (0 and 1), it has, on average, only a 50% chance of detecting an erroneous packet with *multiple* wrong bits. That is not nearly good enough for most applications. Most communications protocols [1] use a multibit generalization of the parity bit called a "cyclic redundancy check" or CRC. Often, the CRC is 16 bits (two bytes) long. Then the chance of a random set of errors going undetected is 1 in $2^{16} = 65536$.

Now enters mathematics. It is easy to find M-bit CRCs that have the property of detecting *all* errors that occur in M or fewer *consecutive* bits, for any length of message. (We prove this below.) Since noise in communication channels tends to be "bursty," with short sequences of adjacent bits getting corrupted, this consecutive-bit property is highly desirable. Furthermore, for packets with a fixed (or bounded) payload size of N bits, one can find CRCs that find all occurrences of D or fewer errors *anywhere* in the payload. Obviously, the game is to find the CRC that maximizes D. The value $D + 1$ is the *Hamming distance* of the CRC for that value of N using that checksum (cf. §16.2).

Useful 16-bit CRC Polynomials (after [3])			
j	Name	Polynomial	Best N (bits)
0		`0x755B` $= (x^3+x^2+1)^*(x^6+x^5+x^2+x+1)^*(x^7+x^3+1)^*$	242–2048+
1		`0xA7D3` $= (x^3+x^2+1)^*(x^6+x^5+x^2+x+1)^*(x^7+x^6+x^5+x^4+1)^*$	256–2048+
2	ANSI-16	`0x8005` $= (x+1)(x^{15}+x+1)^*$	242–2048+
3	CCITT-16	`0x1021` $= (x+1)(x^{15}+x^{14}+x^{13}+x^{12}+x^4+x^3+x^2+x+1)^*$	242–2048+
4		`0x5935` $= (x^{16}+x^{14}+x^{12}+x^{11}+x^8+x^5+x^4+x^2+1)$	136–241
5		`0x90D9` $= (x+1)(x^{15}+x^{11}+x^{10}+x^9+x^8+x^7+x^5+x^4+x^2+x+1)$	20–135
6	IEC-16	`0x5B93` $= (x+1)(x+1)(x^7+x^6+x^3+x+1)^*(x^7+x^6+x^5+x^4+x^3+x^2+1)^*$	20–112
7		`0x2D17` $= (x^2+x+1)^*(x^{14}+x^{13}+x^9+x^7+x^5+x^4+1)$	16–19
* denotes primitive factor			

The design of CRCs lies in the province of communications software experts and chip-level hardware designers — people with bits under their fingernails. A passing familiarity with some of the concepts involved can be useful, however, both because the mathematics involved has connections to other applications (for example, random number generation, cf. §7.1 and §7.5), and because you might actually want to add a couple of bytes of checksum to your own data records in some applications where you are handling, or moving, large amounts of data.

Sometimes CRCs can be used to compress data as they are recorded. If identical data records occur frequently, one can keep sorted in memory the CRCs of previously encountered records. A new record is archived in full if its CRC is different, otherwise only a pointer to a previous record need be archived. In this application one might use 8 bytes of CRC, to make the odds of mistakenly discarding a different data record tolerably small; or, if previous records can be randomly accessed, a full comparison can be made to decide whether records with identical CRCs are in fact identical.

Now let us briefly discuss the theory of CRCs. After that, we will give an implementation that generates 16-bit CRCs that are known to be particularly good, or else are enshrined as standard (and, it turns out, this is not the same thing!).

The mathematics underlying CRCs is "polynomials over the integers modulo 2." Any binary message can be thought of as a polynomial with coefficients 0 and 1. For example, the message "1100001101" is the polynomial $x^9 + x^8 + x^3 + x^2 + 1$. Since 0 and 1 are the only integers modulo 2, a power of x in the polynomial is either present (1) or absent (0).

An M-bit-long CRC is based on a polynomial of degree M, called the generator polynomial. Given the generator polynomial G (which can be written either in polynomial form or as a bit-string, e.g., 10001000000100001 for $x^{16} + x^{12} + x^5 + 1$), here is how you compute the CRC for a sequence of bits S: First, multiply S by x^M, that is, append M zero bits to it. Second, divide, by long division, G into Sx^M. Keep in mind that the subtractions in the long division are done modulo 2, so that there are never any "borrows": Modulo 2 subtraction is the same as logical exclusive-or (XOR). Third, ignore the quotient you get. Fourth, when you eventually get to a remainder, it is the CRC; call it C. C will be a polynomial of degree $M - 1$ or less, otherwise you would not have finished the long division. Therefore, in bit-string form, it has M bits, which may include leading zeros. (C might even be all zeros; see below.)

If you work through the above steps in an example, you will see that most of what you write down in the long-division tableau is superfluous. You are actually just left-shifting sequential bits of S, from the right, into an M-bit register. Every time a 1 bit gets shifted off the left end of this register, you zap the register by an XOR with the M low-order bits of G (that is, all the bits of G except its leading 1). When a 0 bit is shifted off the left end you don't zap the register. When the last bit that was originally part of S gets shifted off the left end of the register, what remains is the CRC.

You can immediately recognize how efficiently this procedure can be implemented in hardware. It requires only a shift register with a few hard-wired XOR taps into it. That is how CRCs are computed in communications devices, taking a tiny part of a single chip. In software, the implementation is not so elegant, since bit-shifting is not generally very efficient. One therefore typically finds (as in our implementation below) table-driven routines that pre-calculate the result of a bunch of shifts and XORs, say for each of 256 possible 8-bit inputs [2].

Every generator polynomial of degree M with a nonzero x^0 term yields a CRC that detects *all* possible combinations of errors in any *frame* of M consecutive bits. (A special case of this is that it detects any single-bit error in a message of arbitrary length N.) To see how this works, suppose two messages, S and T, differ only within a frame of M bits. Then their CRCs differ by an amount that is the remainder when G is divided into $(S - T)x^M \equiv R$. Now R has the form of leading zeros (which can be ignored), followed by some 1's in an M-bit frame, followed by trailing zeros (which are just multiplicative factors of x): $R = x^n F$, where F is a polynomial of degree at most $M - 1$ and $n > 0$. But since G has a nonzero x^0 term, it is not divisible by x. So G cannot divide R. Therefore S and T must have different CRCs.

What about two-bit errors, not necessarily in a frame of size M? That leads us to *primitive polynomials*: A polynomial over the integers modulo 2 may be irreducible, meaning that it can't be factored. A subset of the irreducible polynomials is the primitive polynomials. These generate maximum length sequences when used in shift registers, as described in §7.5. The polynomial $x^2 + 1$ is not irreducible: $x^2 + 1 = (x+1)(x+1)$, so it is also not primitive. The polynomial $x^4 + x^3 + x^2 + x + 1$ is irreducible, but it turns out not to be primitive. The polynomial $x^4 + x + 1$ is both irreducible and primitive.

Primitive polynomials are here interesting because they have a very high order. Don't confuse *order* with *degree*: The order e of a polynomial is the smallest integer e such that the polynomial divides (in the present mod 2 case) $x^e + 1$. Primitive

polynomials, it turns out, have the largest possible order e for their degree n, given by

$$e = 2^n - 1 \tag{22.4.1}$$

(In fact, this is why their shift registers have maximum length.) If two messages differ on exactly two bits, spaced k bits apart, then their difference is $x^k + 1$ times some trailing powers of x. If the generator G contains a primitive factor of order e, then G can't possibly divide this difference, as long as $k < e$.

Thus, a primitive factor of degree n guarantees two-bit error detection for spacings up to $2^n - 1$. For this reason, generators are often chosen to be primitive polynomials of degree M. Alternatively, the generator may be chosen be a primitive polynomial times $(1 + x)$, which turns out to detect parity errors for all message sizes N, while the range of two-bit detections is reduced only by a factor of 2.

A number of "standard" CRC polynomials were chosen by no other criteria, sometimes adding only the criterion that they should have only a small number of terms. (This was at one time important for hardware design.) For example, the CCITT (Comité Consultatif International Télégraphique et Téléphonique) has anointed $x^{16}+x^{12}+x^5+1$ as "CCITT-16"; it is the product of $x+1$ and a primitive polynomial. The polynomial ANSI-16 (see table on p. 1169) also has this character.

Similarly for some choices other than 16 bits: "CRC-12" is $(x+1)(x^{11}+x^2+1)$, the latter factor being primitive. The most common 32-bit CRC, "CRC-32," used in the ethernet standard (IEEE 802.3) and elsewhere, is $x^{32} + x^{26} + x^{23} + x^{22} + x^{16} + x^{12} + x^{11} + x^{10} + x^8 + x^7 + x^5 + x^4 + x^2 + x + 1$, which is primitive.

Now here is something relatively new in this ancient field [3]: For carefully chosen generators G, all two-bit errors in a packet with payload size N can be detected *even if* $e < N$. This is because the previous argument was sufficient, but not necessary: A cleverly chosen G can fail to divide $x^k - 1$ for other reasons than having a primitive factor of large order. This idea opens up the design space to search, essentially by brute force, for generators that have $D > 2$, that is, are capable of finding not just all two-bit errors, but all three-bit errors, all four-bit errors, etc., up to some bound that depends on N and M. Several of these "new" generators are shown in the table on p. 1169, which is based on [3] (which see for details), along with their recommended values of N. A generator that is good for large N is not necessarily good for small N, and vice versa, so you should stick to the recommended values. The hexadecimal values in the table give binary representations of the polynomials, with the convention that each must be prefaced by a leading 1 (the x^{16} term).

In most protocols, a transmitted block of data consists of some N data bits, directly followed by the M bits of their CRC (or the CRC XORed with a constant; see below). There are two equivalent ways of validating a block at the receiving end. Most obviously, the receiver can compute the CRC of the data bits, and compare it to the transmitted CRC bits. Less obviously, but more elegantly, the receiver can simply compute the CRC of the total block, with $N + M$ bits, and verify that a result of zero is obtained. Proof: The total block is the polynomial $Sx^M + C$ (data left-shifted to make room for the CRC bits). The definition of C is that $Sx^m = QG + C$, where Q is the discarded quotient. But then $Sx^M + C = QG + C + C = QG$ (remember modulo 2), which is a perfect multiple of G. It remains a multiple of G when it gets multiplied by an additional x^M on the receiving end, so it has a zero CRC, q.e.d.

A couple of small variations on the basic procedure need to be mentioned [1]: First, when the CRC is computed, the M-bit register need not be initialized to zero.

Initializing it to some other M-bit value (e.g., all 1's) in effect prefaces all blocks by a phantom message that would have given the initialization value as its remainder. It is advantageous to do this, since the CRC described thus far otherwise cannot detect the addition or removal of any number of initial zero bits. (Loss of an initial bit, or insertion of zero bits, are common "clocking errors.") Second, one can add (XOR) any M-bit constant K to the CRC before it is transmitted. This constant can either be XORed away at the receiving end, or else it just changes the expected CRC of the whole block by a known amount, namely the remainder of dividing G into Kx^M. The constant K is frequently "all bits," changing the CRC into its ones complement. This has the advantage of detecting another kind of error that the CRC would otherwise not find: deletion of an initial 1 bit in the message with spurious insertion of a 1 bit at the end of the block.

The following object `Icrc` implements the calculation of 16-bit CRCs for the generators listed in the table. The constructor sets which generator is to be used, and also whether the initial register should be all bits (the default) or zero. `Icrc` is loosely based on the function in [2]. Here is how to understand its operation: First look at the function `icrc1`. This is used only by the constructor, to initialize a table of length 256, incorporating one character into a 16-bit CRC register. The only trick used is that a character's bits are XORed into the most significant bits of the register, all eight together, instead of being fed into the least significant bit, one bit at a time, at the time of the register shift. This works because XOR is associative and commutative — we can feed in character bits *any* time before they will determine whether to zap with the generator polynomial.

Now look at the methods `crc` and `concat`. Go back to thinking about a character's bits being shifted into the CRC register from the least significant end. The key observation is that while 8 bits are being shifted into the register's low end, all the generator zapping is being determined by the bits already in the high end. Since XOR is commutative and associative, all we need is a table of the results of all this zapping, for each of 256 possible high-bit configurations. Then we can play catch-up and XOR an input character into the result of a lookup into this table. But this is exactly the table that was constructed by `icrc1`. References [2,4,5] give further details on table-driven CRC computations.

icrc.h
```
struct Icrc {
```
Object for computing 16-bit cyclic redundancy checksums.
```
    Uint jcrc,jfill,poly;
    static Uint icrctb[256];

    Icrc(const Int jpoly, const Bool fill=true) : jfill(fill ? 255 : 0) {
```
Constructor. Choose one of 8 generators (see table) by the value of `jpoly`. Initialize the CRC register to all bits if `fill` is true, otherwise to zero.
```
        Int j;
        Uint okpolys[8] = {0x755B,0xA7D3,0x8005,0x1021,0x5935,0x90D9,0x5B93,0x2D17};
```
Generator polynomials, see table.
```
        poly = okpolys[jpoly & 7];
        for (j=0;j<256;j++) {
            icrctb[j]=icrc1(j << 8,0);        Table of CRCs of all characters.
        }
        jcrc = (jfill | (jfill << 8));
    }
```

```
    Uint crc(const string &bufptr) {
    Initialize the CRC register, compute and return the 16-bit CRC for the string bufptr.
        jcrc = (jfill | (jfill << 8));
        return concat(bufptr);
    }

    Uint concat(const string &bufptr) {
    Without reinitializing the CRC register, compute and return the 16-bit CRC for the string
    bufptr. In effect, this appends bufptr to previous strings since the last call of crc and
    returns the overall CRC.
        Uint j,cword=jcrc,len=bufptr.size();
        for (j=0;j<len;j++) {          Loop over the characters in the string.
            cword=icrctb[Uchar(bufptr[j]) ^ hibyte(cword)] ^ (lobyte(cword) << 8);
        }
        return jcrc = cword;
    }

    Uint icrc1(const Uint jcrc, const Uchar onech) {
    Given a remainder up to now, return the new CRC after one character is added. Used by
    Icrc to initialize its table.
        Int i;
        Uint ans=(jcrc ^ onech << 8);
        for (i=0;i<8;i++) {              Here is where 8 one-bit shifts, and some XORs
            if (ans & 0x8000) ans = (ans <<= 1) ^ poly;      with the generator poly-
            else ans <<= 1;                                  nomial, are done.
            ans &= 0xffff;
        }
        return ans;
    }

    inline Uchar lobyte(const unsigned short x) {
        return (Uchar)(x & 0xff); }
    inline Uchar hibyte(const unsigned short x) {
        return (Uchar)((x >> 8) & 0xff); }
};
Uint Icrc::icrctb[256];
```

What if you need more than 16 bits of checksum? For a true 32-bit CRC, you will need to rewrite the routines given to work with a longer generating polynomial. For example, $x^{32} + x^7 + x^5 + x^3 + x^2 + x + 1$ is primitive modulo 2 and has nonleading, nonzero bits only in its least significant byte (which makes for some simplification). The idea of table lookup on only the most significant byte of the CRC register goes through unchanged.

Easier, if you don't care about the M-consecutive bit property of the checksum, is to just instantiate more than one copy of `Icrc`, each with a different generator (first argument in constructor). These provide statistically independent checks.

22.4.1 Other Kinds of Checksums

Quite different from CRCs are the various techniques used to append a decimal "check digit" to numbers that are handled by human beings (e.g., typed into a computer). Check digits need to be proof against the kinds of highly structured errors that humans tend to make, such as transposing consecutive digits. Wagner and Putter [6] give an interesting introduction to this subject, including specific algorithms.

Checksums now in widespread use vary from fair to poor. The 10-digit ISBN (International Standard Book Number) that you find on most books, including this

one, uses the check equation

$$10d_1 + 9d_2 + 8d_3 + \cdots + 2d_9 + d_{10} = 0 \pmod{11} \qquad (22.4.2)$$

where d_{10} is the right-hand check digit. The character "X" is used to represent a check digit value of 10. Another popular scheme is the so-called "IBM check," often used for account numbers (including, e.g., MasterCard). Here, the check equation is

$$2\#d_1 + d_2 + 2\#d_3 + d_4 + \cdots = 0 \pmod{10} \qquad (22.4.3)$$

where $2\#d$ means, "multiply d by two and add the resulting decimal digits." United States banks code checks with a nine-digit processing number whose check equation is

$$3a_1 + 7a_2 + a_3 + 3a_4 + 7a_5 + a_6 + 3a_7 + 7a_8 + a_9 = 0 \pmod{10} \qquad (22.4.4)$$

The familiar 12-digit Universal Product Code (UPC) is printed with both a decimal representation and a synonymous bar code. The digits are divided into a one-digit "category," a five-digit manufacturer, a five-digit product identification, and one-digit checksum. The check equation is

$$3a_1 + a_2 + 3a_3 + a_4 + 3a_5 + \cdots + 3a_{11} + a_{12} = 0 \pmod{10} \qquad (22.4.5)$$

The bar code put on many envelopes by the U.S. Postal Service is decoded by removing the single tall marker bars at each end and breaking the remaining bars into six or ten groups of five. In each group the five bars signify (from left to right) the values $7, 4, 2, 1, 0$. Exactly two of them will be tall. Their sum is the represented digit, except that zero is represented as $7 + 4$. The five- or nine-digit zip code is followed by a check digit, with the check equation

$$\sum d_i = 0 \pmod{10} \qquad (22.4.6)$$

None of these schemes is close to optimal. An elegant scheme due to Verhoeff is described in [6]. The underlying idea is to use the ten-element *dihedral group* D_5, which corresponds to the symmetries of a pentagon, instead of the cyclic group of the integers modulo 10. The check equation is

$$a_1 * f(a_2) * f^2(a_3) * \cdots * f^{n-1}(a_n) = 0 \qquad (22.4.7)$$

where $*$ is (noncommutative) multiplication in D_5, and f^i denotes the ith iteration of a certain fixed permutation. Verhoeff's method finds *all* single errors in a string, and *all* adjacent transpositions. It also finds about 95% of twin errors ($aa \rightarrow bb$), jump transpositions ($acb \rightarrow bca$), and jump twin errors ($aca \rightarrow bcb$). Here is an implementation:

decchk.h

```
Bool decchk(string str, char &ch) {
```

Decimal check digit computation or verification. Returns as `ch` a check digit for appending to `string[0..n-1]`, that is, for storing into `string[n]`. In this mode, ignore the returned boolean value. If `string[0..n-1]` already ends with a check digit (`string[n-1]`), returns the function value `true` if the check digit is valid, otherwise `false`. In this mode, ignore the returned value of `ch`. Note that `string` and `ch` contain ASCII characters corresponding to the digits 0-9, *not*

byte values in that range. Other ASCII characters are allowed in string, and are ignored in calculating the check digit.

```
    char c;
    Int j,k=0,m=0,n=str.length();
    static Int ip[10][8]={{0,1,5,8,9,4,2,7},{1,5,8,9,4,2,7,0},
        {2,7,0,1,5,8,9,4},{3,6,3,6,3,6,3,6},{4,2,7,0,1,5,8,9},
        {5,8,9,4,2,7,0,1},{6,3,6,3,6,3,6,3},{7,0,1,5,8,9,4,2},
        {8,9,4,2,7,0,1,5},{9,4,2,7,0,1,5,8}};
    static Int ij[10][10]={{0,1,2,3,4,5,6,7,8,9},{1,2,3,4,0,6,7,8,9,5},
        {2,3,4,0,1,7,8,9,5,6},{3,4,0,1,2,8,9,5,6,7},{4,0,1,2,3,9,5,6,7,8},
        {5,9,8,7,6,0,4,3,2,1},{6,5,9,8,7,1,0,4,3,2},{7,6,5,9,8,2,1,0,4,3},
        {8,7,6,5,9,3,2,1,0,4},{9,8,7,6,5,4,3,2,1,0}};
        Group multiplication and permutation tables.
    for (j=0;j<n;j++) {                     Look at successive characters.
        c=str[j];
        if (c >= 48 && c <= 57)             Ignore everything except digits.
            k=ij[k][ip[(c+2) % 10][7 & m++]];
    }
    for (j=0;j<10;j++)                      Find which appended digit will check properly.
        if (ij[k][ip[j][m & 7]] == 0) break;
    ch=char(j+48);                          Convert to ASCII.
    return k==0;
}
```

CITED REFERENCES AND FURTHER READING:

Saadawi, T.N., and Ammar, M.H. 1994, *Fundamentals of Telecommunication Networks* (New York: Wiley).[1]

LeVan, J. 1987, "A Fast CRC," *Byte*, vol. 12, pp. 339–341 (November).[2]

Koopman, P., and Chakravarty, T. 2004, "Cyclic Redundancy Code (CRC) Polynomial Selection for Embedded Networks," in *International Conference on Dependable Systems and Networks (DSN-2004)* (IEEE Computer Society).[3]

Sarwate, D.V. 1988, "Computation of Cyclic Redundancy Checks via Table Look-Up," *Communications of the ACM*, vol. 31, pp. 1008–1013.[4]

Griffiths, G., and Stones, G.C. 1987, "The Tea-Leaf Reader Algorithm: An Efficient Implementation of CRC-16 and CRC-32," *Communications of the ACM*, vol. 30, pp. 617–620.[5]

Wagner, N.R., and Putter, P.S. 1989, "Error Detecting Decimal Digits," *Communications of the ACM*, vol. 32, pp. 106–110.[6]

22.5 Huffman Coding and Compression of Data

A lossless data compression algorithm takes a string of symbols (typically ASCII characters or bytes) and translates it *reversibly* into another string, one that is *on the average* of shorter length. The words "on the average" are crucial; it is obvious that no reversible algorithm can make all strings shorter — there just aren't enough short strings to be in one-to-one correspondence with longer strings. Compression algorithms are possible only when, on the input side, some strings, or some input symbols, are more common than others. These can then be encoded in fewer bits than rarer input strings or symbols, giving a net average gain. We already quantified this idea, with the concept of *entropy*, in §14.7.

There exist many, quite different, compression techniques, corresponding to different ways of detecting and using departures from equiprobability in input strings.

In this section and the next we shall consider only *variable length codes* with *defined word* inputs. In these, the input is sliced into fixed units, for example ASCII characters, while the corresponding output comes in chunks of variable size. The simplest such method is Huffman coding [1], discussed in this section. Another example, *arithmetic compression*, is discussed in §22.6.

At the opposite extreme from defined-word, variable length codes are schemes that divide up the *input* into units of variable length (words or phrases of English text, for example) and then transmit these, often with a fixed length output code. The most widely used code of this general type is the Ziv-Lempel code [2]. References [3-5] give the flavor of some other compression techniques, with references to the large literature.

The idea behind Huffman coding is simply to use shorter bit patterns for more common characters. Suppose the input alphabet has N_{ch} characters, and that these occur in the input string with respective probabilities p_i, $i = 1, \dots, N_{\text{ch}}$, so that $\sum p_i = 1$. As we saw in §14.7, strings consisting of independently random sequences of these characters (a conservative, but not always realistic assumption) require, on the average, at least

$$H = -\sum p_i \log_2 p_i \tag{22.5.1}$$

bits per character, where H is the entropy of the probability distribution. Moreover, coding schemes exist that approach the bound arbitrarily closely. For the case of equiprobable characters, with all $p_i = 1/N_{\text{ch}}$, one easily sees that $H = \log_2 N_{\text{ch}}$, which is the case of no compression at all. Any other set of p_i's gives a smaller entropy, allowing some useful compression.

Notice that the bound of (22.5.1) would be achieved if we could encode character i with a code of length $L_i = -\log_2 p_i$ bits: Equation (22.5.1) would then be the average $\sum p_i L_i$. The trouble with such a scheme is that $-\log_2 p_i$ is not generally an integer. How can we encode the letter "Q" in 5.32 bits? Huffman coding makes a stab at this by, in effect, approximating all the probabilities p_i by integer powers of 1/2, so that all the L_i's are integral. If all the p_i's are in fact of this form, then a Huffman code does achieve the entropy bound H.

The construction of a Huffman code is best illustrated by example. Imagine a language, Vowellish, with the $N_{\text{ch}} = 5$ character alphabet A, E, I, O, and U, occurring with the respective probabilities 0.12, 0.42, 0.09, 0.30, and 0.07. Then the construction of a Huffman code for Vowellish is accomplished in the table on the next page.

Here is how it works, proceeding in sequence through N_{ch} stages, represented by the columns of the table. The first stage starts with N_{ch} nodes, one for each letter of the alphabet, containing their respective relative frequencies. At each stage, the two smallest probabilities are found, summed to make a new node, and then dropped from the list of active nodes. (A "block" denotes the stage where a node is dropped.) All active nodes (including the new composite) are then carried over to the next stage (column). In the table, the names assigned to new nodes (e.g., AUI) are inconsequential. In the example shown, it happens that (after stage 1) the two smallest nodes are always an original node and a composite one; this need not be true in general: The two smallest probabilities might be both original nodes, or both composites, or one of each. At the last stage, all nodes will have been collected into one grand composite of total probability 1.

Node	Stage:	1	2	3	4	5
1	A:	0.12	0.12 ■			
2	E:	0.42	0.42	0.42	0.42 ■	
3	I:	0.09 ■				
4	O:	0.30	0.30	0.30 ■		
5	U:	0.07 ■				
6		UI:	0.16 ■			
7			AUI:	0.28 ■		
8				AUIO:	0.58 ■	
9					EAUIO:	1.00

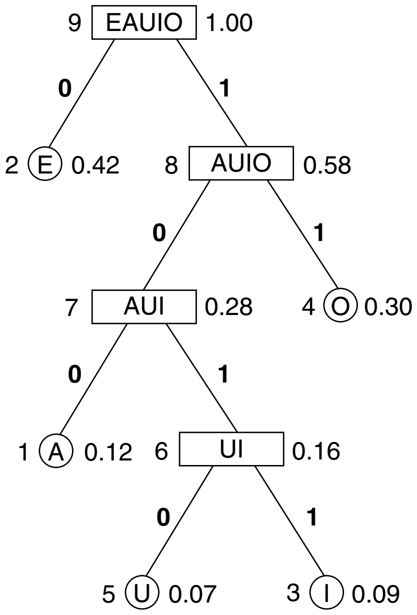

Figure 22.5.1. Huffman code for the fictitious language Vowellish, in tree form. A letter (A, E, I, O, or U) is encoded or decoded by traversing the tree from the top down; the code is the sequence of 0's and 1's on the branches. The value to the right of each node is its probability; to the left, its node number in the table.

Now, to see the code, you redraw the data in the table as a tree (Figure 22.5.1). As shown, each node of the tree corresponds to a node (row) in the table, indicated by the integer to its left and probability value to its right. Terminal nodes, so called, are shown as circles; these are single alphabetic characters. The branches of the tree are labeled 0 and 1. The code for a character is the sequence of zeros and ones that lead to it, from the top down. For example, E is simply 0, while U is 1010.

Any string of zeros and ones can now be decoded into an alphabetic sequence. Consider, for example, the string 1011111010. Starting at the top of the tree we

descend through 1011 to I, the first character. Since we have reached a terminal node, we reset to the top of the tree, next descending through 11 to O. Finally 1010 gives U. The string thus decodes to IOU.

These ideas are embodied in the following `Huffcode` object. The constructor lets you specify N_{ch}, and an integer frequency-of-occurrence table of length N_{ch} telling how often each character occurs in some large corpus of text. These integers are, of course, proportional to the p_i's. The reason for using integers is so that any two computers will produce exactly the same code from the same input data. This might not be true if we used floating-point values. The constructor utilizes a heap structure (see §8.3) for efficiency; for a detailed description, see Sedgewick [6].

Once you have created an instance of `Huffcode`, you code a message by calling `codeone` for each message character in turn. This writes bits into a byte array `code` that you supply as an argument. There is no message-dependent saved state, so you could interleave different messages if there were some reason to do so.

Decoding a Huffman-encoded message is slightly more complicated. The coding tree must be traversed from the top down, using up a variable number of bits. This is done by the method `decodeone`.

There is no such thing as an "end of message" (EOM) marker in Huffman codes — not unless you provide one. Successive calls to `decodeone` will happily decode bits into characters until your hardware traps an illegal memory read! That is because every path on the tree (cf. Figure 22.5.1) terminates in a valid character. In practice, one increases N_{ch} by 1, and gives the extra character a frequency of occurrence of 1 (versus large values for the other characters). The new character becomes the EOM marker. Similarly, one can add other extra characters for other "out-of-band" signaling. If these occur rarely, the overhead on the message is negligible.

huffcode.h

```
struct Huffcode {
```
Object for Huffman encoding and decoding.
```
    Int nch,nodemax,mq;
    Int ilong,nlong;
    VecInt ncod,left,right;
    VecUint icod;
    Uint setbit[32];

    Huffcode(const Int nnch, VecInt_I &nfreq)
    : nch(nnch), mq(2*nch-1), icod(mq), ncod(mq), left(mq), right(mq) {
```
Constructor. Given the frequency of occurrence table `nfreq[0..nnch-1]` for `nnch` characters, constructs the Huffman code. Also sets `ilong` and `nlong` as the character number that produced the longest code symbol, and the length of that symbol.
```
        Int ibit,j,node,k,n,nused;
        VecInt index(mq), nprob(mq), up(mq);
        for (j=0;j<32;j++) setbit[j] = 1 << j;
        for (nused=0,j=0;j<nch;j++) {
            nprob[j]=nfreq[j];
            icod[j]=ncod[j]=0;
            if (nfreq[j] != 0) index[nused++]=j;
        }
        for (j=nused-1;j>=0;j--)            Sort nprob into a heap structure in index.
            heep(index,nprob,nused,j);
        k=nch;
        while (nused > 1) {                 Combine heap nodes, remaking the heap at
            node=index[0];                      each stage.
            index[0]=index[(nused--)-1];
            heep(index,nprob,nused,0);
            nprob[k]=nprob[index[0]]+nprob[node];
```

```
        left[k]=node;                         Store left and right children of a node.
        right[k++]=index[0];
        up[index[0]] = -Int(k);               Indicate whether a node is a left or right child
        index[0]=k-1;                             of its parent.
        up[node]=k;
        heep(index,nprob,nused,0);
    }
    up[(nodemax=k)-1]=0;
    for (j=0;j<nch;j++) {                     Make the Huffman code from the tree.
        if (nprob[j] != 0) {
            for (n=0,ibit=0,node=up[j];node;node=up[node-1],ibit++) {
                if (node < 0) {
                    n |= setbit[ibit];
                    node = -node;
                }
            }
            icod[j]=n;
            ncod[j]=ibit;
        }
    }
    nlong=0;
    for (j=0;j<nch;j++) {
        if (ncod[j] > nlong) {
            nlong=ncod[j];
            ilong=j;
        }
    }
    if (nlong > numeric_limits<Uint>::digits)
        throw("Code too long in Huffcode.  See text.");
}
```

```
void codeone(const Int ich, char *code, Int &nb) {
```

Huffman encode the single character ich (in the range 0..nch-1), write the result to the byte array code starting at bit nb (whose smallest valid value is zero), and increment nb to the first unused bit. This routine is called repeatedly to encode consecutive characters in a message. The user is responsible for monitoring that the value of nb does not overrun the length of code.

```
    Int m,n,nc;
    if (ich >= nch) throw("bad ich (out of range) in Huffcode");
    if (ncod[ich]==0) throw("bad ich (zero prob) in Huffcode");
    for (n=ncod[ich]-1;n >= 0;n--,++nb) {        Loop over the bits in the stored
        nc=nb >> 3;                                  Huffman code for ich.
        m=nb & 7;
        if (m == 0) code[nc]=0;                  Set appropriate bits in code.
        if ((icod[ich] & setbit[n]) != 0) code[nc] |= setbit[m];
    }
}
```

```
Int decodeone(char *code, Int &nb) {
```

Starting at bit number nb in the byte array code, decode a single character (returned as ich in the range 0..nch-1) and increment nb appropriately. Repeated calls, starting with $nb = 0$, will return successive characters in a compressed message. The user is responsible for detecting EOM from the message content.

```
    Int nc;
    Int node=nodemax-1;
    for (;;) {                                Set node to the top of the decoding tree, and
        nc=nb >> 3;                               loop until a valid character is obtained.
        node=((code[nc] & setbit[7 & nb++]) != 0 ?
            right[node] : left[node]);
            Branch left or right in tree, depending on its value.
        if (node < nch) return node;          If we reach a terminal node, we have a com-
    }                                             plete character and can return.
}
```

```
    void heep(VecInt_IO &index, VecInt_IO &nprob, const Int n, const Int m) {
    Used by the constructor to maintain a heap structure in the array index[0..m-1].
        Int i=m,j,k;
        k=index[i];
        while (i < (n >> 1)) {
            if ((j = 2*i+1) < n-1
                && nprob[index[j]] > nprob[index[j+1]]) j++;
            if (nprob[k] <= nprob[index[j]]) break;
            index[i]=index[j];
            i=j;
        }
        index[i]=k;
    }
};
```

`Huffcode` requires that the longest code for a single character fits into your machine's integer wordlength (typically 32 bits), and will tell you if this is violated. If this happens, you'll need to increase the frequency-of-occurrence value for the rarest characters. This will affect your compression hardly at all.

It is a feature, not a bug, that `Huffcode` allows you to specify some characters as having zero frequency of occurrence, and then completely omits these from the code. This can be very useful when, for example, you want to compress a file consisting only of ASCII characters 0–9, `+`, `-`, and "`.`", as might occur in a file of numerical values. But don't then try to encode one of the omitted characters!

22.5.1 Run-Length Encoding

For the compression of highly correlated bit streams (for example the black or white values along a facsimile scan line), Huffman compression is often combined with *run-length encoding*: Instead of sending each bit, the input stream is converted to a series of integers indicating how many consecutive bits have the same value. These integers are then Huffman-compressed. The Group 3 CCITT facsimile standard functions in this manner, with a fixed, immutable, Huffman code, optimized for a set of eight standard documents [7].

CITED REFERENCES AND FURTHER READING:

Hamming, R.W. 1980, *Coding and Information Theory* (Englewood Cliffs, NJ: Prentice-Hall).

Huffman, D.A. 1952, "A Method for the Construction of Minimum-Redundancy Codes," *Proceedings of the Institute of Radio Engineers*, vol. 40, pp. 1098–1101.[1]

Ziv, J., and Lempel, A. 1978, "Compression of Individual Sequences via Variable-Rate Coding," *IEEE Transactions on Information Theory*, vol. IT-24, pp. 530–536.[2]

Sayood, K. 2005, *Introduction to Data Compression*, 3rd ed. (San Francisco: Morgan Kaufmann).[3]

Salomon, D. 2004, *Data Compression: The Complete Reference*, 3rd ed. (New York: Springer).[4]

Wayner, P. 1999, *Compression Algorithms for Real Programmers* (San Francisco: Morgan Kaufmann).[5]

Sedgewick, R. 1998, *Algorithms in C*, 3rd ed. (Reading, MA: Addison-Wesley), Chapter 22.[6]

Hunter, R., and Robinson, A.H. 1980, "International Digital Facsimile Coding Standard," *Proceedings of the IEEE*, vol. 68, pp. 854–867.[7]

22.6 Arithmetic Coding

We saw in the previous section, as well as §14.7, that a perfect coding scheme would use $L_i = -\log_2 p_i$ bits to encode character i (in the range $1 \le i \le N_{ch}$), if p_i is its probability of occurrence and characters occur independently randomly. Huffman coding gives a way of rounding the L_i's to close integer values and constructing a code with those lengths. *Arithmetic coding* [1], which we now discuss, actually does manage to encode characters using noninteger numbers of bits! It also provides a convenient way to output the result not as a stream of bits, but as a stream of symbols in any desired radix. This latter property is particularly useful if you want, e.g., to convert data from bytes (radix 256) to printable ASCII characters (radix 94), or to case-independent alphanumeric sequences containing only A-Z and 0-9 (radix 36).

In arithmetic coding, an input message of any length is represented as a real number R in the range $0 \le R < 1$. The longer the message, the more precision required of R. This is best illustrated by an example, so let us return to the fictitious language, Vowellish, of the previous section. Recall that Vowellish has a five-character alphabet (A, E, I, O, U), with occurrence probabilities 0.12, 0.42, 0.09, 0.30, and 0.07, respectively. Figure 22.6.1 shows how a message beginning "IOU" is encoded: The interval $[0, 1)$ is divided into segments corresponding to the five alphabetical characters; the length of a segment is the corresponding p_i. We see that the first message character, "I", narrows the range of R to $0.37 \le R < 0.46$. This interval is now subdivided into five subintervals, again with lengths proportional to the p_i's. The second message character, "O", narrows the range of R to $0.3763 \le R < 0.4033$. The "U" character further narrows the range to $0.37630 \le R < 0.37819$. *Any* value of R in this range can be sent as encoding "IOU". In particular, the binary fraction .011000001 is in this range, so "IOU" can be sent in 9 bits. (Huffman coding took 10 bits for this example; see §22.5.)

Of course there is the problem of knowing when to stop decoding. The fraction .011000001 represents not simply "IOU," but "IOU...," where the ellipses represent an infinite string of successor characters. We had a similar problem in Huffman coding, but there we would at least stop when we ran off the edge of the input buffer. Here, the real number .011000001 actually does represent an infinite message! Arithmetic coding therefore *always* must assume the existence of a special $N_{ch} + 1$st character, EOM (end of message), which occurs only once at the end of the input. Since EOM has a low probability of occurrence, it gets allocated only a very tiny piece of the number line.

In the above example, we gave R as a binary fraction. We could just as well have output it in any other radix, e.g., base 94 or base 36, whatever is convenient for the anticipated storage or communication channel.

You might wonder how one deals with the seemingly incredible precision required of R for a long message. The answer is that R is never actually represented all at once. At any give stage we have upper and lower bounds for R represented as a finite number of digits in the output radix. As digits of the upper and lower bounds become identical, we can left-shift them away and bring in new digits at the low-significance end. The object below has a parameter `NWK` for the number of working digits to keep around. This must be large enough to make the chance of an accidental degeneracy vanishingly small. (The object signals if a degeneracy ever occurs.)

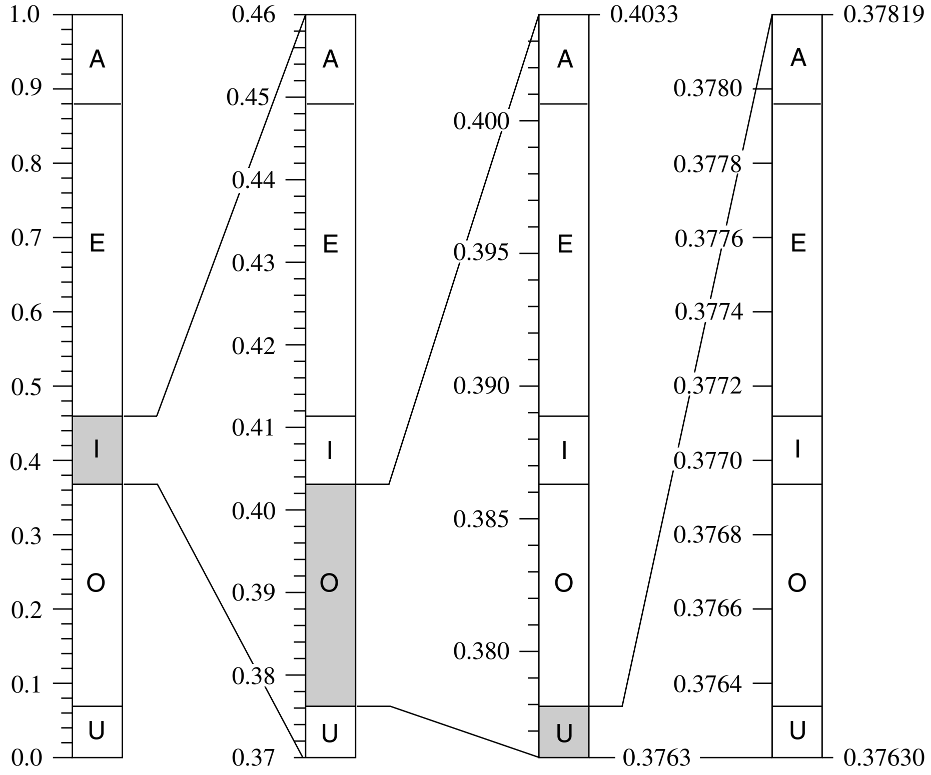

Figure 22.6.1. Arithmetic coding of the message "IOU..." in the fictitious language Vowellish. Successive characters give successively finer subdivisions of the initial interval between 0 and 1. The final value can be output as the digits of a fraction in any desired radix. Note how the subinterval allocated to a character is proportional to its probability of occurrence.

Since the process of discarding old digits and bringing in new ones is performed identically on encoding and decoding, everything stays synchronized.

In the `Arithcode` object below, the constructor has arguments to specify the number of characters and an integer frequency-of-occurrence table (as in `Huffcode`), plus an argument that allows you to specify an output radix for the code. Because there is some saved state between coding successive characters (the upper and lower bounds for R, for example), you must call `messageinit` prior to encoding or decoding the first character of a new message, and not interleave the encoding of different messages in a single instance of `Arithcode`. If you want to interleave messages, create more than one instance.

Successive calls to `codeone` for each input character encode the message. A final call with character `nch` (that is, one larger than your specified character set) adds the EOM marker and is mandatory. After this final call, `lcd` will be set to the number of bytes in the coded message (i.e., will point to the first unused location in `code`). The `decodeone` routine similarly returns successive characters of the decoded message in turn, with `nch` returned to indicate EOM.

Unlike the `Huffcode` object, `Arithcode` has no provision for omitting specified message characters from the code. Therefore, it also refuses to believe zero val-

ues in the table nfreq; a 0 is treated as if it were a 1. If you want to live dangerously, with a very slightly more efficient coding, you can change this in the constructor.

arithcode.h

```
struct Arithcode {
Object for arithmetic coding.
    Int nch,nrad,ncum;
    Uint jdif,nc,minint;
    VecUint ilob,iupb;
    VecInt ncumfq;
    static const Int NWK=20;            Number of working digits.

    Arithcode(VecInt_I &nfreq, const Int nnch, const Int nnrad)
    : nch(nnch), nrad(nnrad), ilob(NWK), iupb(NWK), ncumfq(nch+2) {
    Constructor. Given the frequency of occurrence table nfreq[0..nnch-1] for nnch charac-
    ters, constructs the Huffman code whose output has radix nnrad (which must be ≤ 256).
        Int j;
        if (nrad > 256) throw("output radix must be <= 256 in Arithcode");
        minint=numeric_limits<Uint>::max()/nrad;
        ncumfq[0]=0;
        for (j=1;j<=nch;j++) ncumfq[j]=ncumfq[j-1]+MAX(nfreq[j-1],1);
        ncum=ncumfq[nch+1]=ncumfq[nch]+1;
    }

    void messageinit() {
    Clear saved state for a new message (either encode or decode). This is mandatory before
    encoding or decoding the first character.
        Int j;
        jdif=nrad-1;
        for (j=NWK-1;j>=0;j--) {          Initialize enough digits of the upper and lower
            iupb[j]=nrad-1;                   bounds.
            ilob[j]=0;
            nc=j;
            if (jdif > minint) return;    Initialization complete.
            jdif=(jdif+1)*nrad-1;
        }
        throw("NWK too small in arcode.");
    }

    void codeone(const Int ich, char *code, Int &lcd) {
    Encode the single character ich in the range 0...nch-1 into the byte array code, starting
    at location code[lcd] and (if necessary) incrementing lcd so that, on return, it points to
    the first unused byte in code. A final call with ich=nch encodes "end of message." Byte
    values written into code will be in the range 0...nrad − 1.
        if (ich > nch) throw("bad ich in Arithcode");       Check for valid input char-
        advance(ich,code,lcd,1);                                acter.
    }

    Int decodeone(char *code, Int &lcd) {
    Decode and return a single message character, using code starting at location code[lcd],
    and (if necessary) increment lcd appropriately. Successive calls return successive message
    characters. The returned value nch indicates end of message (subsequent calls will return
    nonsense).
        Int ich;
        Uint j,ihi,ja,m;
        ja=(Uchar) code[lcd]-ilob[nc];
        for (j=nc+1;j<NWK;j++) {
            ja *= nrad;
            ja += Uchar(code[lcd+j-nc])-ilob[j];
        }
        ihi=nch+1;
        ich=0;
        while (ihi-ich > 1) {         If decoding, locate the character ich by bisection.
```

```
            m=(ich+ihi)>>1;
            if (ja >= multdiv(jdif,ncumfq[m],ncum)) ich=m;
            else ihi=m;
        }
        if (ich != nch) advance(ich,code,lcd,-1);
        return ich;
    }

    void advance(const Int ich, char *code, Int &lcd, const Int isign) {
```
Used internally. Operations common to encoding and decoding. Convert character ich to a new subrange [ilob,iupb).
```
        Uint j,k,jh,jl;
        jh=multdiv(jdif,ncumfq[ich+1],ncum);
        jl=multdiv(jdif,ncumfq[ich],ncum);
        jdif=jh-jl;
        arrsum(ilob,iupb,jh,NWK,nrad,nc);
        arrsum(ilob,ilob,jl,NWK,nrad,nc);
        for (j=nc;j<NWK;j++) {                 How many leading digits to output (if en-
            if (ich != nch && iupb[j] != ilob[j]) break;    coding) or skip over?
            if (isign > 0) code[lcd] = ilob[j];
            lcd++;
        }
        if (j+1 > NWK) return;                 Ran out of message. Did someone forget to
        nc=j;                                      encode a terminating ncd?
        for(j=0;jdif<minint;j++)               How many digits to shift?
            jdif *= nrad;
        if (j > nc) throw("NWK too small in arcode.");
        if (j != 0) {                          Shift them.
            for (k=nc;k<NWK;k++) {
                iupb[k-j]=iupb[k];
                ilob[k-j]=ilob[k];
            }
        }
        nc -= j;
        for (k=NWK-j;k<NWK;k++) iupb[k]=ilob[k]=0;
        return;                                Normal return.
    }

    inline Uint multdiv(const Uint j, const Uint k, const Uint m) {
```
Calculate (k*j)/m without overflow by use of double-length integers.
```
        return Uint((Ullong(j)*Ullong(k)/Ullong(m)));
    }

    void arrsum(VecUint_I &iin, VecUint_O &iout, Uint ja,
    const Int nwk, const Uint nrad, const Uint nc) {
```
Add the integer ja to the radix nrad multiple-precision integer iin[nc..nwk-1]. Return the result in iout[nc..nwk-1].
```
        Uint karry=0,j,jtmp;
        for (j=nwk-1;j>nc;j--) {
            jtmp=ja;
            ja /= nrad;
            iout[j]=iin[j]+(jtmp-ja*nrad)+karry;
            if (iout[j] >= nrad) {
                iout[j] -= nrad;
                karry=1;
            } else karry=0;
        }
        iout[nc]=iin[nc]+ja+karry;
    }
};
```

A few further notes: When an interval of size jdif is to be partitioned in the proportions of some n to some ntot, say, then we must compute (n*jdif)/ntot.

With ordinary integer arithmetic, the numerator is likely to overflow; and, unfortunately, an expression like `jdif/(ntot/n)` is not equivalent. We therefore need to use double-length integers, type `Ullong`, usually 64 bits, just for this operation.

The internally set variable `minint`, which is the minimum allowed number of discrete steps between the upper and lower bounds, determines when new low-significance digits are added. `minint` must be large enough to provide resolution of all the input characters. That is, we must have $p_i \times \texttt{minint} > 1$ for all i. A value of $100N_{\text{ch}}$, or $1.1/\min p_i$, whichever is larger, is generally adequate. However, for safety, the routine takes `minint` to be as large as possible, with the product `minint*nradd` just smaller than overflow. This results in some time inefficiency, and in a few unnecessary characters being output at the end of a message. You can decrease `minint` if you want to live closer to the edge.

If radix-changing, rather than compression, is your primary aim (for example to convert an arbitrary file into printable characters), then you are of course free to set all the components of `nfreq` equal, say, to 1.

While the output radix is limited to 256 (so that values fit into a byte), the input alphabet size $N_{\text{ch}} = \texttt{nch}$ can be less than, equal to, or greater than 256.

CITED REFERENCES AND FURTHER READING:

Sayood, K. 2005, *Introduction to Data Compression*, 3rd ed. (San Francisco: Morgan Kaufmann).

Salomon, D. 2004, *Data Compression: The Complete Reference*, 3rd ed. (New York: Springer).

Wayner, P. 1999, *Compression Algorithms for Real Programmers* (San Francisco: Morgan Kaufmann).

Witten, I.H., Neal, R.M., and Cleary, J.G. 1987, "Arithmetic Coding for Data Compression," *Communications of the ACM*, vol. 30, pp. 520–540.[1]

22.7 Arithmetic at Arbitrary Precision

Let's compute the number π to a couple of thousand decimal places. In doing so, we'll learn some things about multiple precision arithmetic on computers and meet quite an unusual application of the fast Fourier transform (FFT). We'll also develop a set of routines that you can use for other calculations at any desired level of arithmetic precision.

To start with, we need an analytic algorithm for π. Useful algorithms are quadratically convergent, i.e., they double the number of significant digits at each iteration. Quadratically convergent algorithms for π are based on the *AGM (arithmetic geometric mean)* method, which also finds application to the calculation of elliptic integrals (cf. §6.12) and in advanced implementations of the ADI method for elliptic partial differential equations (§20.5). Borwein and Borwein [1] treat this subject, which is beyond our scope here. One of their algorithms for π starts with the initializations

$$\begin{aligned} X_0 &= \sqrt{2} \\ \pi_0 &= 2 + \sqrt{2} \\ Y_0 &= \sqrt[4]{2} \end{aligned} \tag{22.7.1}$$

and then, for $i = 0, 1, \ldots,$ repeats the iteration

$$X_{i+1} = \frac{1}{2}\left(\sqrt{X_i} + \frac{1}{\sqrt{X_i}}\right)$$
$$\pi_{i+1} = \pi_i\left(\frac{X_{i+1}+1}{Y_i+1}\right) \tag{22.7.2}$$
$$Y_{i+1} = \frac{Y_i\sqrt{X_{i+1}} + \dfrac{1}{\sqrt{X_{i+1}}}}{Y_i+1}$$

The value π emerges as the limit π_∞.

Now to the question of how to do arithmetic to arbitrary precision: In a high-level language like C++, a natural choice is to work in radix (base) 256, so that character arrays can be directly interpreted as strings of digits. At the very end of our calculation, we will want to convert our answer to radix 10, but that is essentially a frill for the benefit of human ears, accustomed to the familiar chant, "three point one four one five nine...." For any less frivolous calculation, we would likely never leave base 256 (or the thence trivially reachable hexadecimal, octal, or binary bases).

We will adopt the convention of storing digit strings in the "human" ordering, that is, with the first stored digit in an array being most significant, the last stored digit being least significant. The opposite convention would, of course, also be possible. "Carries," where we need to partition a number larger than 255 into a low-order byte and a high-order carry, present a minor programming annoyance, solved, in the routines below, by the use of the functions `lobyte` and `hibyte`. It will be our usual convention to assume that the digit strings represent floating-point numbers with the radix point falling after the the first digit. When an operation results in a number that requires more digits in front of the decimal point, it is the responsibility of the user to shift the digits to the right and keep track of any excess factors of 256 that this implies.

It is easy at this point, following Knuth [2], to write a routines for the "fast" arithmetic operations: short addition (adding a single byte to a string), addition, subtraction, short multiplication (multiplying a string by a single byte), short division, ones-complement negation, and a couple of utility operations, copying and left-shifting strings. These are implemented in the following `MParith` object. The additional routines that are declared, but not defined, are discussed below.

mparith.h

```
struct MParith {
```
Multiple precision arithmetic operations done on character strings, interpreted as radix 256 numbers with the radix point after the first digit. Implementations for the simpler operations are listed here.

```
    void mpadd(VecUchar_O &w, VecUchar_I &u, VecUchar_I &v) {
```
Adds the unsigned radix 256 numbers u and v, yielding the unsigned result w. To achieve the full available accuracy, the array w must be longer, by one element, than the shorter of the two arrays u and v.

```
        Int j,n=u.size(),m=v.size(),p=w.size();
        Int n_min=MIN(n,m),p_min=MIN(n_min,p-1);
        Uint ireg=0;
        for (j=p_min-1;j>=0;j--) {
            ireg=u[j]+v[j]+hibyte(ireg);
            w[j+1]=lobyte(ireg);
        }
        w[0]=hibyte(ireg);
        if (p > p_min+1)
```

```
        for (j=p_min+1;j<p;j++) w[j]=0;
}
```

```
void mpsub(Int &is, VecUchar_O &w, VecUchar_I &u, VecUchar_I &v) {
```
Subtracts the unsigned radix 256 number v from u yielding the unsigned result w. If the result is negative (wraps around), is is returned as -1; otherwise it is returned as 0. To achieve the full available accuracy, the array w must be as long as the shorter of the two arrays u and v.
```
    Int j,n=u.size(),m=v.size(),p=w.size();
    Int n_min=MIN(n,m),p_min=MIN(n_min,p-1);
    Uint ireg=256;
    for (j=p_min-1;j>=0;j--) {
        ireg=255+u[j]-v[j]+hibyte(ireg);
        w[j]=lobyte(ireg);
    }
    is=hibyte(ireg)-1;
    if (p > p_min)
        for (j=p_min;j<p;j++) w[j]=0;
}
```

```
void mpsad(VecUchar_O &w, VecUchar_I &u, const Int iv) {
```
Short addition: The integer iv (in the range $0 \leq \text{iv} \leq 255$) is added to the least significant radix position of unsigned radix 256 number u, yielding result w. To ensure that the result does not require two digits before the radix point, one may first right-shift the operand u so that the first digit is 0, and keep track of multiples of 256 separately.
```
    Int j,n=u.size(),p=w.size();
    Uint ireg=256*iv;
    for (j=n-1;j>=0;j--) {
        ireg=u[j]+hibyte(ireg);
        if (j+1 < p) w[j+1]=lobyte(ireg);
    }
    w[0]=hibyte(ireg);
    for (j=n+1;j<p;j++) w[j]=0;
}
```

```
void mpsmu(VecUchar_O &w, VecUchar_I &u, const Int iv) {
```
Short multiplication: The unsigned radix 256 number u is multiplied by the integer iv (in the range $0 \leq \text{iv} \leq 255$), yielding result w. To ensure that the result does not require two digits before the radix point, one may first right-shift the operand u so that the first digit is 0, and keep track of multiples of 256 separately.
```
    Int j,n=u.size(),p=w.size();
    Uint ireg=0;
    for (j=n-1;j>=0;j--) {
        ireg=u[j]*iv+hibyte(ireg);
        if (j < p-1) w[j+1]=lobyte(ireg);
    }
    w[0]=hibyte(ireg);
    for (j=n+1;j<p;j++) w[j]=0;
}
```

```
void mpsdv(VecUchar_O &w, VecUchar_I &u, const Int iv, Int &ir) {
```
Short division: The unsigned radix 256 number u is divided by the integer iv (in the range $0 \leq \text{iv} \leq 255$), yielding a quotient w and a remainder ir (with $0 \leq \text{ir} \leq 255$). To achieve the full available accuracy, the array w must be as long as the array u.
```
    Int i,j,n=u.size(),p=w.size(),p_min=MIN(n,p);
    ir=0;
    for (j=0;j<p_min;j++) {
        i=256*ir+u[j];
        w[j]=Uchar(i/iv);
        ir=i % iv;
    }
    if (p > p_min)
        for (j=p_min;j<p;j++) w[j]=0;
}
```

```
    void mpneg(VecUchar_IO &u) {
```
Ones-complement negate the unsigned radix 256 number u.
```
        Int j,n=u.size();
        Uint ireg=256;
        for (j=n-1;j>=0;j--) {
            ireg=255-u[j]+hibyte(ireg);
            u[j]=lobyte(ireg);
        }
    }

    void mpmov(VecUchar_O &u, VecUchar_I &v) {
```
Move the unsigned radix 256 number v into u. To achieve full accuracy, the array v must be as long as the array u.
```
        Int j,n=u.size(),m=v.size(),n_min=MIN(n,m);
        for (j=0;j<n_min;j++) u[j]=v[j];
        if (n > n_min)
            for(j=n_min;j<n-1;j++) u[j]=0;
    }

    void mplsh(VecUchar_IO &u) {
```
Left-shift digits of unsigned radix 256 number u. The final element of the array is set to 0.
```
        Int j,n=u.size();
        for (j=0;j<n-1;j++) u[j]=u[j+1];
        u[n-1]=0;
    }

    Uchar lobyte(Uint x) {return (x & 0xff);}
    Uchar hibyte(Uint x) {return ((x >> 8) & 0xff);}
```
The following, more complicated, methods have discussion and implementation below.
```
    void mpmul(VecUchar_O &w, VecUchar_I &u, VecUchar_I &v);
    void mpinv(VecUchar_O &u, VecUchar_I &v);
    void mpdiv(VecUchar_O &q, VecUchar_O &r, VecUchar_I &u, VecUchar_I &v);
    void mpsqrt(VecUchar_O &w, VecUchar_O &u, VecUchar_I &v);
    void mp2dfr(VecUchar_IO &a, string &s);
    string mppi(const Int np);
};
```

Full multiplication of two strings of digits, if done by the traditional hand method, is not a fast operation: In multiplying two strings of length N, the multiplicand would be short-multiplied in turn by each byte of the multiplier, requiring $O(N^2)$ operations in all. We will see, however, that *all* the arithmetic operations on numbers of length N can in fact be done in $O(N \times \log N \times \log\log N)$ operations.

The trick is to recognize that multiplication is essentially a *convolution* (§13.1) of the digits of the multiplicand and multiplier, followed by some kind of carry operation. Consider, for example, two ways of writing the calculation 456×789:

$$
\begin{array}{r}
456 \\
\times\ 789 \\
\hline
4104 \\
3648 \\
3192 \\
\hline
359784
\end{array}
\qquad\qquad
\begin{array}{rrrrrr}
 & & & 4 & 5 & 6 \\
 & & \times & 7 & 8 & 9 \\
\hline
 & & & 36 & 45 & 54 \\
 & & 32 & 40 & 48 & \\
 & 28 & 35 & 42 & & \\
\hline
 & 28 & 67 & 118 & 93 & 54 \\
\hline
3 & 5 & 9 & 7 & 8 & 4
\end{array}
$$

The tableau on the left shows the conventional method of multiplication, in which three separate short multiplications of the full multiplicand (by 9, 8, and 7) are added

to obtain the final result. The tableau on the right shows a different method (sometimes taught for mental arithmetic), where the single-digit cross products are all computed (e.g. $8 \times 6 = 48$), then added in columns to obtain an incompletely carried result (here, the list 28, 67, 118, 93, 54). The final step is a single pass from right to left, recording the single least-significant digit and carrying the higher digit or digits into the total to the left (e.g. $93 + 5 = 98$, record the 8, carry 9).

You can see immediately that the column sums in the right-hand method are components of the convolution of the digit strings, for example $118 = 4 \times 9 + 5 \times 8 + 6 \times 7$. In §13.1, we learned how to compute the convolution of two vectors by the fast Fourier transform (FFT): Each vector is FFT'd, the two complex transforms are multiplied, and the result is inverse-FFT'd. Since the transforms are done with floating arithmetic, we need sufficient precision so that the exact integer value of each component of the result is discernible in the presence of roundoff error. We should therefore allow a (conservative) few times $\log_2(\log_2 N)$ bits for roundoff in the FFT. A number of length N bytes in radix 256 can generate convolution components as large as the order of $(256)^2 N$, thus requiring $16 + \log_2 N$ bits of precision for exact storage. If `it` is the number of bits in the floating mantissa (cf. §22.2), we obtain the condition

$$16 + \log_2 N + \text{few} \times \log_2 \log_2 N < \texttt{it} \qquad (22.7.3)$$

We see that single precision, say with `it` $= 24$, is inadequate for any interesting value of N, while double precision, say with `it` $= 53$, allows N to be greater than 10^6, corresponding to some millions of decimal digits. The use of `Doub` in the routines `realft` (§12.3) and `four1` (§12.2) is therefore a necessity, not merely a convenience, for this application.

```
void MParith::mpmul(VecUchar_O &w, VecUchar_I &u, VecUchar_I &v) {
```
mparith.h

Uses fast Fourier transform to multiply the unsigned radix 256 integers `u[0..n-1]` and `v[0..m-1]`, yielding a product `w[0..n+m-1]`.

```
    const Doub RX=256.0;
    Int j,nn=1,n=u.size(),m=v.size(),p=w.size(),n_max=MAX(m,n);
    Doub cy,t;
    while (nn < n_max) nn <<= 1;      Find the smallest usable power of 2 for the transform.
    nn <<= 1;
    VecDoub a(nn,0.0),b(nn,0.0);
    for (j=0;j<n;j++) a[j]=u[j];      Move U and V to double precision floating arrays.
    for (j=0;j<m;j++) b[j]=v[j];
    realft(a,1);                      Perform the convolution: First, the two Fourier trans-
    realft(b,1);                          forms.
    b[0] *= a[0];                     Then multiply the complex results (real and imagi-
    b[1] *= a[1];                         nary parts).
    for (j=2;j<nn;j+=2) {
        b[j]=(t=b[j])*a[j]-b[j+1]*a[j+1];
        b[j+1]=t*a[j+1]+b[j+1]*a[j];
    }
    realft(b,-1);                     Then do the inverse Fourier transform.
    cy=0.0;                           Make a final pass to do all the carries.
    for (j=nn-1;j>=0;j--) {
        t=b[j]/(nn >> 1)+cy+0.5;      The 0.5 allows for roundoff error.
        cy=Uint(t/RX);
        b[j]=t-cy*RX;
    }
    if (cy >= RX) throw("cannot happen in mpmul");
    for (j=0;j<p;j++) w[j]=0;
    w[0]=Uchar(cy);                   Copy answer to output.
    for (j=1;j<MIN(n+m,p);j++) w[j]=Uchar(b[j-1]);
}
```

With multiplication thus a "fast" operation, division is best performed by multiplying the dividend by the reciprocal of the divisor. The reciprocal of a value V is calculated by iteration of Newton's rule,

$$U_{i+1} = U_i(2 - VU_i) \tag{22.7.4}$$

which results in the quadratic convergence of U_∞ to $1/V$, as you can easily prove. (Many historical supercomputers, and some more recent RISC processors, actually use this iteration to perform divisions.) We can now see where the operations count $N \log N \log\log N$, mentioned above, originates: $N \log N$ is in the Fourier transform, with the iteration to converge Newton's rule giving an additional factor of $\log\log N$.

mparith.h

```
void MParith::mpinv(VecUchar_O &u, VecUchar_I &v) {
```
Character string v[0..m-1] is interpreted as a radix 256 number with the radix point after (nonzero) v[0]; u[0..n-1] is set to the most significant digits of its reciprocal, with the radix point after u[0].

```
    const Int MF=4;
    const Doub BI=1.0/256.0;
    Int i,j,n=u.size(),m=v.size(),mm=MIN(MF,m);
    Doub fu,fv=Doub(v[mm-1]);
    VecUchar s(n+m),r(2*n+m);
    for (j=mm-2;j>=0;j--) {            Use ordinary floating arithmetic to get an initial
        fv *= BI;                          approximation.
        fv += v[j];
    }
    fu=1.0/fv;
    for (j=0;j<n;j++) {
        i=Int(fu);
        u[j]=Uchar(i);
        fu=256.0*(fu-i);
    }
    for (;;) {                         Iterate Newton's rule to convergence.
        mpmul(s,u,v);                  Construct 2 - UV in S.
        mplsh(s);
        mpneg(s);
        s[0] += Uchar(2);              Multiply SU into U.
        mpmul(r,s,u);
        mplsh(r);
        mpmov(u,r);
        for (j=1;j<n-1;j++)            If fractional part of S is not zero, it has not
            if (s[j] != 0) break;          converged to 1.
        if (j==n-1) return;
    }
}
```

Division now follows as a simple corollary, with only the necessity of calculating the reciprocal to sufficient accuracy to get an exact quotient and remainder.

mparith.h

```
void MParith::mpdiv(VecUchar_O &q, VecUchar_O &r, VecUchar_I &u, VecUchar_I &v) {
```
Divides unsigned radix 256 integers u[0..n-1] by v[0..m-1] (with $m \le n$ required), yielding a quotient q[0..n-m] and a remainder r[0..m-1].

```
    const Int MACC=1;
    Int i,is,mm,n=u.size(),m=v.size(),p=r.size(),n_min=MIN(m,p);
    if (m > n) throw("Divisor longer than dividend in mpdiv");
    mm=m+MACC;
    VecUchar s(mm),rr(mm),ss(mm+1),qq(n-m+1),t(n);
    mpinv(s,v);                        Set S = 1/V.
    mpmul(rr,s,u);                     Set Q = SU.
    mpsad(ss,rr,1);
```

```
    mplsh(ss);
    mplsh(ss);
    mpmov(qq,ss);
    mpmov(q,qq);
    mpmul(t,qq,v);                          Multiply and subtract to get the remainder.
    mplsh(t);
    mpsub(is,t,u,t);
    if (is != 0) throw("MACC too small in mpdiv");
    for (i=0;i<n_min;i++) r[i]=t[i+n-m];
    if (p>m) for (i=m;i<p;i++) r[i]=0;
}
```

Square roots are calculated by a Newton's rule much like division. If

$$U_{i+1} = \frac{1}{2}U_i(3 - VU_i^2) \tag{22.7.5}$$

then U_∞ converges quadratically to $1/\sqrt{V}$. A final multiplication by V gives $\sqrt{V}$.

mparith.h

```
void MParith::mpsqrt(VecUchar_O &w, VecUchar_O &u, VecUchar_I &v) {
```

Character string v[0..m-1] is interpreted as a radix 256 number with the radix point after v[0]; w[0..n-1] is set to its square root (radix point after w[0]), and u[0..n-1] is set to the reciprocal thereof (radix point before u[0]). w and u need not be distinct, in which case they are set to the square root.

```
    const Int MF=3;
    const Doub BI=1.0/256.0;
    Int i,ir,j,n=u.size(),m=v.size(),mm=MIN(m,MF);
    VecUchar r(2*n),x(n+m),s(2*n+m),t(3*n+m);
    Doub fu,fv=Doub(v[mm-1]);
    for (j=mm-2;j>=0;j--) {                 Use ordinary floating arithmetic to get an initial ap-
        fv *= BI;                                proximation.
        fv += v[j];
    }
    fu=1.0/sqrt(fv);
    for (j=0;j<n;j++) {
        i=Int(fu);
        u[j]=Uchar(i);
        fu=256.0*(fu-i);
    }
    for (;;) {                              Iterate Newton's rule to convergence.
        mpmul(r,u,u);                       Construct S = (3 - VU^2)/2.
        mplsh(r);
        mpmul(s,r,v);
        mplsh(s);
        mpneg(s);
        s[0] += Uchar(3);
        mpsdv(s,s,2,ir);
        for (j=1;j<n-1;j++) {               If fractional part of S is not zero, it has not con-
            if (s[j] != 0) {                     verged to 1.
                mpmul(t,s,u);               Replace U by SU.
                mplsh(t);
                mpmov(u,t);
                break;
            }
        }
        if (j<n-1) continue;
        mpmul(x,u,v);                       Get square root from reciprocal and return.
        mplsh(x);
        mpmov(w,x);
        return;
    }
}
```

We already mentioned that radix conversion to decimal is a merely cosmetic operation that should normally be omitted. The simplest way to convert a fraction to decimal is to multiply it repeatedly by 10, picking off (and subtracting) the resulting integer part. This has an operations count of $O(N^2)$, however, since each liberated decimal digit takes an $O(N)$ operation. It *is* possible to do the radix conversion as a fast operation by a "divide-and-conquer" strategy, in which the fraction is (fast) multiplied by a large power of 10, enough to move about half the desired digits to the left of the radix point. The integer and fractional pieces are now processed independently, each further subdivided. If our goal were a few billion digits of π, instead of a few thousand, we would need to implement this scheme. For present purposes, the following lazy routine is adequate:

mparith.h

```
void MParith::mp2dfr(VecUchar_IO &a, string &s)
```
Converts a radix 256 fraction a[0..n-1] (radix point before a[0]) to a decimal fraction represented as an ASCII string s[0..m-1], where m is a returned value. The input array a[0..n-1] is destroyed. NOTE: For simplicity, this routine implements a slow ($\propto N^2$) algorithm. Fast ($\propto N \ln N$), more complicated, radix conversion algorithms do exist.
```
{
    const Uint IAZ=48;
    char buffer[4];
    Int j,m;

    Int n=a.size();
    m=Int(2.408*n);
    sprintf(buffer,"%d",a[0]);
    s=buffer;
    s += '.';
    mplsh(a);
    for (j=0;j<m;j++) {
        mpsmu(a,a,10);
        s += a[0]+IAZ;
        mplsh(a);
    }
}
```

Finally, then, we arrive at a routine implementing equations (22.7.1) and (22.7.2):

mparith.h

```
string MParith::mppi(const Int np) {
```
Demonstrate multiple precision routines by calculating and printing the first np bytes of π.
```
    const Uint IAOFF=48,MACC=2;
    Int ir,j,n=np+MACC;
    Uchar mm;
    string s;
    VecUchar x(n),y(n),sx(n),sxi(n),z(n),t(n),pi(n),ss(2*n),tt(2*n);
    t[0]=2;                                   Set T = 2.
    for (j=1;j<n;j++) t[j]=0;
    mpsqrt(x,x,t);                            Set X_0 = sqrt(2).
    mpadd(pi,t,x);                            Set pi_0 = 2 + sqrt(2).
    mplsh(pi);
    mpsqrt(sx,sxi,x);                         Set Y_0 = 2^(1/4).
    mpmov(y,sx);
    for (;;) {
        mpadd(z,sx,sxi);                      Set X_{i+1} = (X_i^{1/2} + X_i^{-1/2})/2.
        mplsh(z);
        mpsdv(x,z,2,ir);
        mpsqrt(sx,sxi,x);                     Form the temporary T = Y_i X_{i+1}^{1/2} + X_{i+1}^{-1/2}.
        mpmul(tt,y,sx);
        mplsh(tt);
```

```
        mpadd(tt,tt,sxi);
        mplsh(tt);
        x[0]++;                          Increment X_{i+1} and Y_i by 1.
        y[0]++;
        mpinv(ss,y);                     Set Y_{i+1} = T/(Y_i + 1).
        mpmul(y,tt,ss);
        mplsh(y);
        mpmul(tt,x,ss);                  Form temporary T = (X_{i+1} + 1)/(Y_i + 1).
        mplsh(tt);
        mpmul(ss,pi,tt);                 Set π_{i+1} = Tπ_i.
        mplsh(ss);
        mpmov(pi,ss);
        mm=tt[0]-1;                      If T = 1, then we have converged.
        for (j=1;j < n-1;j++)
            if (tt[j] != mm) break;
        if (j == n-1) {
            mp2dfr(pi,s);
```

Convert to decimal for printing. NOTE: The conversion routine, for this demonstration only, is a slow ($\propto N^2$) algorithm. Fast ($\propto N \ln N$), more complicated, radix conversion algorithms do exist.

```
            s.erase(Int(2.408*np),s.length());
            return s;
        }
    }
}
```

Figure 22.7.1 gives the result, computed with n = 1000. As an exercise, you might enjoy checking the first hundred digits of the figure against the first 12 terms of Ramanujan's celebrated identity [3]

$$\frac{1}{\pi} = \frac{\sqrt{8}}{9801} \sum_{n=0}^{\infty} \frac{(4n)!\,(1103 + 26390n)}{(n!\,396^n)^4} \tag{22.7.6}$$

using the above routines. You might also use the routines to verify that the number $2^{512} + 1$ is not a prime, but has factors 2,424,833 and 7,455,602,825,647,884,208,337,395,736,200,454,918,783,366,342,657 (which are in fact prime; the remaining prime factor being about 7.416×10^{98}) [4].

CITED REFERENCES AND FURTHER READING:

Borwein, J.M., and Borwein, P.B. 1987, *Pi and the AGM: A Study in Analytic Number Theory and Computational Complexity* (New York: Wiley).[1]

Knuth, D.E. 1997, *Seminumerical Algorithms*, 3rd ed., vol. 2 of *The Art of Computer Programming* (Reading, MA: Addison-Wesley), §4.3.[2]

Ramanujan, S. 1927, *Collected Papers of Srinivasa Ramanujan*, G.H. Hardy, P.V. Seshu Aiyar, and B.M. Wilson, eds. (Cambridge, UK: Cambridge University Press), pp. 23–39.[3]

Kolata, G. 1990, June 20, "Biggest Division a Giant Leap in Math," *The New York Times*.[4]

Kronsjö, L. 1987, *Algorithms: Their Complexity and Efficiency*, 2nd ed. (New York: Wiley).

3.14159265358979323846264338327950288419716939937510582097494459230781640628
620899862803482534211706798214808651328230664709384460955058223172535940812
848111745028410270193852110555964462294895493038196442881097566593344612847
564823378678316527120190914564856692346034861045432664821339360726024914127
372458700660631558817488152092096282925409171536436789259036001133053054882
046652138414695194151160943305727036575959195309218611738193261179310511854
807446237996274956735188575272489122793818301194912983367336244065664308602
139494639522473719070217986094370277053921717629317675238467481846766940513
200056812714526356082778577134275778960917363717872146844090122495343014654
958537105079227968925892354201995611212902196086403441815981362977477130996
051870721134999999837297804995105973173281609631859502445945534690830264252
230825334468503526193118817101000313783875288658753320838142061717766914730
359825349042875546873115956286388235378759375195778185778053217122680661300
192787661119590921642019893809525720106548586327886593615338182796823030195
203530185296899577362259941389124972177528347913151557485724245415069595082
953311686172785588907509838175463746493931925506040092770167113900984882401
285836160356370766010471018194295559619894676783744944825537977472684710404
753464620804668425906949129331367702898915210475216205696602405803815019351
125338243003558764024749647326391419927260426992279678235478163600934172164
121992458631503028618297455570674983850549458858692699569092721079750930295
532116534498720275596023648066549911988183479775356636980742654252786255181
841757467289097777279380008164706001614524919217321721477235014144197356854
816136115735255213347574184946843852332390739414333454776241686251898356948
556209921922218427255025425688767179049460165346680498862723279178608578438
382796797668145410095388378636095068006422512520511739298489608412848862694
560424196528502221066118630674427862203919494504712371378696095636437191728
746776465757396241389086583264599581339047802759009946576407895126946839835
259570982582262052248940772671947826848260147699090264013639443745530506820
349625245174939965143142980919065925093722169646151570985838741059788595977
297549893016175392846813826868386894277415599185592524595395943104997252468
084598727364469584865383673622262609912460805124388439045124413654976278079
771569143599770012961608944169486855584840635342207222582848864815845602850

Figure 22.7.1. The first 2398 decimal digits of π, computed by the routines in this section.

Index

Abstract Base Class (ABC) 24, 33, 34, 87, 114, 703, 874
Accelerated convergence of series 177, 211–218
Accuracy 8–12
- achievable in minimization 493, 497, 503
- achievable in root finding 448
- contrasted with fidelity 1037, 1046
- CPU different from memory 230
- vs. stability 907, 931, 932, 1035, 1050

Adams-Bashford-Moulton method 943
Adams' stopping criterion 467
Adaptive integration 901, 910–921, 928, 930, 935, 946, 995
- Monte Carlo 410–418
- PI stepsize control 915
- predictive stepsize control 939
- *see also* Adaptive quadrature

Adaptive quadrature 155, 167, 194–196
- and singularities 195
- termination criterion 194

Addition
- multiple precision 1186
- theorem, elliptic integrals 310

ADI (alternating direction implicit) method 1052, 1053, 1065, 1066, 1185
Adjoint operator 1071
Advanced topics (explanation) 6
Advective equation 1032
Affine scaling 543
Agglomerative clustering 873–882
AGM (arithmetic geometric mean) 1185
Airy function 254, 283, 289, 291
- routine for 290

Aitken's
- delta squared process 212, 214
- interpolation algorithm 118

Algorithms, less-numerical 1160–1193
Aliasing 606, 685
- *see also* Fourier transform

Alignment of strings by DP 559–562
All-poles or all-zeros models 681, 682
- *see also* Maximum entropy method (MEM); Periodogram

Alternating-direction implicit method (ADI) 1052, 1053, 1065, 1066, 1185
Alternating series 211, 216
Alternative extended Simpson's rule 160
AMD (approximate minimum degree) 544, 548
Amoeba 503
- *see also* Simplex, method of Nelder and Mead

Amplification factor 1033, 1035, 1038, 1045, 1046
Amplitude error 1036
Analog-to-digital converter 1018, 1166
Analyticity 246
Analyze/factorize/operate package 76, 1030
Anderson-Darling statistic 739
Andrew's sine 821
Angle
- between vectors 1120, 1121
- coordinates on n-sphere 1128
- exterior, of polygons 1122

Annealing, method of simulated 487, 488, 549–555
- assessment 554, 555
- for continuous variables 550, 552–554
- schedule 551, 552
- thermodynamic analogy 550
- traveling salesman problem 551, 552

ANSI-16 1171
ANSI/ISO C++ standard 5
Antonov-Saleev variant of Sobol' sequence 404–406, 408, 409
Apple Mac OS X 5
Approximate inverse of matrix 63
Approximation of functions 110
- by Chebyshev polynomials 234, 625
- by rational functions 247–251
- by wavelets 711, 712, 989
- Padé approximant 212, 245–247
- *see also* Fitting

Area
- polygon 1126
- sphere in n-dimensions 1128
- triangle 1111

Arithmetic
- arbitrary precision 1160, 1185–1193
- floating point 1163
- IEEE standard 1164, 1165
- rounding 1164, 1165
- 64 bit 341

Arithmetic coding 755, 1160, 1181–1185

Arithmetic-geometric mean (AGM) method 1185
Array
  assign function 27
  centered subarray of 115
  classes for 24–29
  resize function 27
  size function 27
  three-dimensional 36
  unit-offset 36
  zero-offset 36
Artificial viscosity 1037, 1042
Ascending transformation, elliptic integrals 310
ASCII character set 1168, 1175, 1181
assign 27
Associated Legendre polynomials 971
  recurrence relation for 294
  relation to Legendre polynomials 293
Association, measures of 721, 741, 758–761
Asymptotic series 210, 216
  exponential integral 216, 269
Attenuation factors 698
Autocorrelation
  in linear prediction 673–675
  use of FFT 648, 649
  Wiener-Khinchin theorem 602, 682
Autoregressive model (AR) *see* Maximum entropy method (MEM)
Average deviation of distribution 723
Averaging kernel, in Backus-Gilbert method 1014

B-spline 148
Backsubstitution 47, 49, 53, 56, 103
  complex equations 55
  direct for computing $\mathbf{A}^{-1} \cdot \mathbf{B}$ 53
  in band-diagonal matrix 60
  relaxation solution of boundary value problems 966
Backtracking 522
  in quasi-Newton methods 478–483
Backus-Gilbert method 1014–1016
Backward deflation 464, 465
Bader-Deuflhard method 940
Bahl-Cocke-Jelinek-Raviv algorithm
  forward-backward algorithm 867
Bairstow's method 466, 471
Balancing 592, 594
Band-diagonal matrix 56, 58–61
  backsubstitution 60
  LU decomposition 59
  multiply by vector 58
  storage 58
Band-pass filter 667, 670
  wavelets 701
Bandwidth limited function 605
Bank accounts, checksum for 1174
Bar codes, checksum for 1174
Barrier method 541
Bartels-Golub update 535
Bartlett window 657
Barycentric coordinates 1114, 1116
Barycentric rational interpolation 113, 127, 128
Base class 23
Base of representation 8, 1164
Basin of convergence 461, 463
Basis functions in general linear least squares 788
Baum-Welch re-estimation
  hidden Markov model 865–867
  relation to expectation-maximization 866
Bayes' theorem 774, 777, 825
Bayesian
  approach to inverse problems 1005, 1022
  contrasted with frequentist 774
  estimation of parameters by MCMC 774, 824–835
  lack of goodness-of-fit methods 779, 1010
  normalizing constant 779
  odds ratio 757, 779
  parameter estimation 777, 778
  prior 757, 775, 777, 1005
  views on straight line fitting 787
  vs. historic maximum entropy method 1022
Bayesian algorithms
  hidden Markov model 868
  Viterbi decoding 868
Bayesian networks 840, 841
  node parents 841
  nodes 840
  posterior probabilities 841
  prior probabilities 841
Bayesian re-estimation
  hidden Markov model 864–866
Belief networks 840
  forward-backward algorithm 867
Bellman-Dijkstra-Viterbi algorithm 556, 850, 853
Berlekamp-Massey decoding algorithm 852
Bernoulli number 164
Bessel functions 274–292
  asymptotic form 274, 279, 284
  complex 254
  continued fraction 283, 284, 287, 288
  fractional order 274, 283–292
  Miller's algorithm 221, 278
  modified 279–283
  modified, fractional order 287–289
  modified, normalization formula 282, 288
  modified, routines for 280
  normalization formula 221
  recurrence relation 219, 274, 275, 278, 281, 283–285
  reflection formulas 286
  reflection formulas, modified functions 289
  routines for 276, 286
  routines for modified functions 289
  series for 210, 274
  series for K_ν 288
  series for Y_ν 284, 285
  spherical 283, 291, 292
  turning point 283

Wronskian 283, 284, 287
Best-fit parameters 773, 781, 785, 822–824
see also Fitting
Beta function 256, 258, 259
incomplete *see* Incomplete beta function
Beta probability distribution 333, 334
deviates 371
gamma as limiting case 333
Betting 755–758, 760, 761
fair bet 755, 756, 758, 760, 761
proportional 758, 760
Bezier curve 148
BFGS algorithm *see* Broyden-Fletcher-Goldfarb-Shanno algorithm
Bias
of exponent 8
removal in linear prediction 145, 678, 679
Biconjugacy 88
Biconjugate gradient method
elliptic partial differential equations 1030
for sparse system 88, 716
preconditioning 89, 1030
Bicubic interpolation 136–138
Bicubic spline 135
Big-endian 9
Biharmonic equation 153
Bilinear interpolation 133, 134
Binary block code 851
Binomial coefficients 256, 258
recurrences for 258
Binomial probability function 258, 338, 339
deviates from 374–377
moments of 735
Poisson as limiting case 338
Binormal distribution 746, 813
Biorthogonality 88
Bisection 115, 460
compared to minimum bracketing 492
root finding 445, 447–449, 454, 492, 584
Bispectrum 604
Bit 8, 754–756, 760, 761
phantom 9
pop count 16
reversal in fast Fourier transform (FFT) 610, 638
Bit-parallel random comparison 374
Bit-twiddling hacks 16
Bitwise logical functions 1170
test if integer a power of 2 16, 611
trick for next power of 2 16, 361
Black-Scholes formula 329
BLAST (software) 562
BLAT (software) 562
Block-by-block method 994
Bluetooth 1168
Bode's rule 158
Boltzmann probability distribution 550
Boltzmann's constant 550
Bolyai-Gerwien theorem 1127
Bookie, information theory view of 758
`Bool` 25
Bootstrap method 809, 810
Bordering method for Toeplitz matrix 96
Borwein and Borwein method for π 1185
Boundary 196, 528, 955
Boundary conditions
for differential equations 900
for spheroidal harmonics 972, 973
in multigrid method 1072
initial value problems 900
partial differential equations 620, 1025, 1053–1058
two-point boundary value problems 900, 955–984
Boundary value problems 1026
see also Differential equations; Elliptic partial differential equations; Two-point boundary value problems
Bounds checking 35
in `vector` by `at` 35
Box 1099–1101
test if point inside 1100
tree of, as data structure 1101
Box-Muller algorithm for normal deviate 364
Bracketing
of function minimum 445, 490–496, 503
of roots 443, 445–447, 454, 455, 464, 465, 470, 492
Branch cut, for hypergeometric function 252–254
Break iteration 15
Brenner's FFT implementation 611, 628
Brent's method
minimization 489, 496–499, 785
minimization, using derivative 489, 499, 500
root finding 443, 449, 453–456, 459, 786
Broyden-Fletcher-Goldfarb-Shanno algorithm 490, 521–525
Broyden's method 474, 483–486
singular Jacobian 486
Bubble sort 420
Bugs, how to report 5
Bulirsch-Stoer
algorithm for rational function interpolation 125
for second order equations 929
method 252, 318, 900, 901, 909, 921–929, 942
method, dense output 927
method, implementation 927
method, stepsize control 924–926, 929
Burg's LP algorithm 677
Burn-in 826, 833–835
Butterfly 360, 361, 610
Byte 8

C (programming language) 1
`__FILE__` and `__LINE__` macros 30
idioms 16
syntax 12–17
C++

ANSI/ISO standard 5
C family syntax 12–17
const statement 31, 32
contiguous storage for vector 27
control structures 14, 15
error class 30
inline directive 29
NR not a textbook on 2
operator associativity 12
operator precedence 12
overloading 28
scope, temporary 20, 21
standard library 10, 24
templates 17, 22, 26, 33, 34, 419, 421
throw 30
try and catch 30
types 25
types used in NR 4
user-defined conversions 31
valarray class 25
vector class 24
virtual function 33
why used in NR 1
C# (programming language) 1, 12
Calendar algorithms 2, 3, 6, 7
Calibration 778
Cardinal functions 1089–1091
Cards, sorting a hand of 420, 422
Carlson's elliptic integrals 310–316
Carpe diem 830
catch 30
Cauchy principal value integrals 178
Cauchy probability distribution 322, 323
deviates from 367
see also Lorentzian probability distribution
Cauchy problem for partial differential equations 1024
Cavender-Felsenstein model 873
Cayley's representation of $\exp(-iHt)$ 1049
CCITT (Comité Consultatif International Télégraphique et Téléphonique) 1171, 1180
CCITT-16 1171
CDF *see* Cumulative Distribution Function
Center of mass 399, 400, 1113, 1127
Central limit theorem 777
Central tendency, measures of 721
Centroid *see* Center of mass
Change of variable
in integration 170–172, 995
in Monte Carlo integration 401
in probability distribution 362
Char 25
Character-based clustering methods 869
Characteristic polynomial
digital filter 670
eigensystems 563, 583, 665
linear prediction 676
matrix with a specified 469
of recurrence relation 221
of tridiagonal system 665
Characteristics of partial differential equations 1024–1026
Chebyshev acceleration in successive over-relaxation (SOR) 1064
Chebyshev approximation 95, 156, 232–239
Clenshaw-Curtis quadrature 241
Clenshaw's recurrence formula 236
coefficients for 234
contrasted with Padé approximation 245
derivative of approximated function 232, 240, 241
economization of series 243–245
even function 237
fast cosine transform and 625
for error function 264
gamma functions 285
integral of approximated function 240, 241
odd function 237
polynomial fits derived from 241, 243, 248
rational function 247–251
Remes exchange algorithm for filter 669
Chebyshev polynomials 183, 187, 233–239
basis functions for spectral methods 1085
continuous orthonormality 233
discrete orthonormality 233
explicit formulas for 233
formula for x^k in terms of 233
Check digit (decimal) 1173
Checksum 1160, 1168–1175
cyclic redundancy (CRC) 1168–1173
Chemical reaction networks 946–954
Chi-by-eye 774
Chi-square fitting *see* Fitting; Least-squares fitting
Chi-square probability function 330, 331, 732, 778, 779, 1003
as boundary of confidence region 812
deviates from 371
Chi-square test 731–734
and confidence limit estimation 812
chi-by-eye 774
chi-square-gamma test 735
degrees of freedom 732, 733
for binned data 731–734
for contingency table 742–745
for inverse problems 1003
for straight line fitting 781
for straight line fitting, errors in both coordinates 785
for two binned data sets 732
goodness-of-fit 780
how much $\Delta\chi^2$ is significant 816
least-squares fitting 778–780
and likelihood ratio test 735
modified Neyman 735
nonlinear models 799
small numbers of counts 734, 735
for two binned data sets 735
unequal size samples 733
Chirp signal 672
Cholesky decomposition 100–102, 525, 568

and covariance structure 378, 379
decorrelating random variables 379
multivariate Gaussian distribution 847, 848
operation count 100
pivoting 101
solution of normal equations 543, 790, 791
sparse decomposition 544, 548
Circle
inscribed or circumscribed 1112
largest empty 1147
random point on 1131
Circulant 700
Circumscribed circle (circumcircle) 1112
CLAPACK 567
Class 17–24
abstract base (ABC) 24, 33, 34, 87, 114, 703, 874
base class 23
derived 23
error class 30
inheritance 23, 24
is-a relationship 23
matrix 24–29
partial abstraction via 24
prerequisite relationship 23
`public` vs. `private` 17
pure virtual 34
suffix `_I`, `_O`, `_IO` 26, 32
templated 22, 33, 34
vector 24–29
see also Object
Class library 2
Classification 840–898
kernel methods 889, 892
support vector machine 883–898
Clenshaw-Curtis quadrature 156, 241, 624, 625
Clenshaw's recurrence formula 219, 222, 223
for Chebyshev polynomials 236
stability 223
Clock, program timing routine 355
Clocking errors 1172
CLP (linear programming package) 536
Clustering
agglomerative 873–882
hierarchical 868–882
k-means 848–850
neighbor-joining (NJ) 873, 878–882
cn function 316
Coarse-grid correction 1068
Coarse-to-fine operator 1068
Codes
binary block codes 851
codeword 851
correcting bit errors 855
error-correcting 851–855
Golay code 852
Hamming code 852
Hamming distance 851, 1168
hard-decision decoding 853
linear codes 851
minimal trellis 853
perfect code 852
Reed-Solomon 852, 855
soft-decision decoding 853
syndrome decoding 852
trellis 853, 856
turbo codes 855
Viterbi decoding 854
Coding
arithmetic 755, 1181–1185
checksums 1168–1175
compression 754, 756
decoding a Huffman-encoded message 1178
Huffman 713, 1175–1180
run-length 1180
variable length code 1176
Ziv-Lempel 1176
see also Arithmetic coding; Huffman coding
Coefficients
binomial 258
for Gaussian quadrature 179, 180
for Gaussian quadrature, nonclassical weight function 189–191, 995
for quadrature formulas 157–162, 995
Column operations on matrix 43, 45
Column totals 743, 759
Combinatorial minimization *see* Annealing
Comité Consultatif International Télégraphique et Téléphonique (CCITT) 1171, 1180
Communications protocol 1168
Comparison function for rejection method 366
Compiler
check on via constructors 36
tested 5
Complementary error function *see* Error function
Complete elliptic integral *see* Elliptic integrals
Complex arithmetic 225, 226
access vector as if complex 613, 620
avoidance of in path integration 253
`Complex` type 25
cubic equations 228, 229
linear equations 55
quadratic equations 227
Complex error function 302
Complex plane
fractal structure for Newton's rule 462
path integration for function evaluation 251–254, 318
poles in 124, 210, 252, 256, 670, 682, 922
Complex systems of linear equations 55
Compression of data 713, 715, 1160, 1175–1185
Computational geometry
floating point arithmetic in 1098
Computer graphics 1097
Computer vision 1097
Concordant pair for Kendall's tau 751
Condition number 69, 89, 791, 793
Conditional entropy 758–761
Confidence level 810, 811, 814–816
Confidence limits

and chi-square 811
bootstrap method 809, 810
by Monte Carlo simulation 807–810
confidence region, confidence interval 810, 811
from singular value decomposition (SVD) 816, 817
on estimated model parameters 807–817
Confluent hypergeometric function 254, 287
Conjugate directions 509, 511, 512, 516
Conjugate gradient method
and wavelets 716
biconjugate 88
compared to variable metric method 521
elliptic partial differential equations 1030
for minimization 489, 515–520, 1011, 1020
for sparse system 87–92, 716
minimum residual method 89
preconditioner 89, 90
Conservative differential equations 928, 930
`const`
correctness 26, 31, 32
protects container, not contents 31, 32
to protect data 32
Constellation in Viterbi decoding 855
Constrained linear inversion method 1006
Constrained linear optimization *see* Linear programming
Constrained optimization 487
Constraints
deterministic 1011–1013
linear 526, 530
Constructor 18, 27
Container, STL 421
Contingency coefficient C 743, 744
Contingency table 741–745, 753, 758, 759
statistics based on chi-square 742–745
statistics based on entropy 758–761
Continue statement 15
Continued fraction 206–209
and recurrence relation 222
Bessel functions 283, 284, 288
convergence criterion 208
equivalence transformation 208
evaluation 206–209
evaluation along with normalization condition 288
even and odd parts 208, 260, 267, 298, 301
exponential integral 267
Fresnel integral 298
incomplete beta function 270
incomplete gamma function 260
Lentz's method 207, 260
modified Lentz's method 208
Pincherle's theorem 222
ratio of Bessel functions 287
rational function approximation 207, 260
recurrence for evaluating 207, 208
sine and cosine integrals 301
Steed's method 207
tangent function 206
typography for 206
Continuous variable (statistics) 741
Control structures and scope 21
Convergence
accelerated, for series 177, 211–218
basin of 461, 463
criteria for 448, 493, 503, 598, 599, 802, 969
eigenvalues accelerated by shifting 585
exponential 174–178, 180, 238, 239, 1083–1096
golden ratio 449, 500
hyperlinear (series) 211
linear 448, 495
linear (series) 211
logarithmic (series) 211
Markov model 858
of algorithm for π 1185
of golden section search 494, 495
of Levenberg-Marquardt method 802
of QL method 584, 585
of Ridders' method 452
quadratic 64, 452, 459, 511, 512, 522, 1185
rate 448, 454, 457, 459
recurrence relation 222
series vs. continued fraction 206
spectral radius and 1061, 1066
Conversions, user-defined 31
Convex hull 1097, 1132, 1146
Convex sets, use in inverse problems 1011–1013
Convolution
and polynomial interpolation 129
denoted by asterisk 602
finite impulse response (FIR) 642, 643
multiple precision arithmetic 1188
multiplication as 1188
necessity for optimal filtering 645
of functions 602, 616, 617, 631
of large data sets 646, 647
overlap-add method 647
overlap-save method 646
relation to wavelet transform 700, 701
theorem 602, 641, 656
theorem, discrete 642, 643
treatment of end effects 643
use of FFT 641–647
wraparound problem 643
Cooley-Tukey FFT algorithm 616
Cornwell-Evans algorithm 1021
Corporate promotion ladder 427
Corrected distance transformation 873
Corrected two-pass algorithm 724
Correction, in multigrid method 1067
Correlation coefficient (linear) 745–748
Correlation function 602, 617
and Fourier transforms 602, 617
autocorrelation 602, 649, 673–675
theorem 602, 648
three-point 604
treatment of end effects 648

using FFT 648, 649
Wiener-Khinchin theorem 602, 682
Correlation, statistical 721, 741
among parameters in a fit 782, 793
Kendall's tau 749, 751–754
linear correlation coefficient 745–748, 783
linear related to least-squares fitting 745, 783
nonparametric or rank statistical 748–754
Spearman rank-order coefficient 749–751
sum squared difference of ranks 749
uncertainty coefficient 761
Coset leader 852
Cosine function, recurrence 219
Cosine integral 297, 300–302
continued fraction 301
routine for 301
series 301
Cosine transform *see* Fast Fourier transform (FFT); Fourier transform
Coulomb wave function 254, 283
Counts, small numbers of 734, 735
Courant condition 1034, 1036, 1038–1040, 1042
multidimensional 1051
Courant-Friedrichs-Lewy stability criterion *see* Courant condition
Covariance
a priori 824
from singular value decomposition (SVD) 817
in general linear least squares 790, 791, 794
in nonlinear models 802
in straight line fitting 782
matrix, and normal equations 790
matrix, Cholesky decomposition 101
matrix, is inverse of Hessian matrix 802
matrix, of errors 1003, 1015
matrix, when it is meaningful 812, 813
relation to chi-square 812–816
CR method *see* Cyclic reduction (CR)
Cramer's V 743, 744
Crank-Nicolson method 1045, 1049, 1051, 1052
Cray, Seymour 1163
CRC (cyclic redundancy check) 1168–1173
CRC-12 1171
Critical (Nyquist) sampling 605, 607, 653
Cross ⊗ (denotes matrix outer product) 78
Crosstabulation analysis 742
see also Contingency table
Crout's algorithm 49, 59
Cubic equations 227–229, 461
Cubic spline interpolation 120–124
see also Spline
Cumulative Distribution Function (cdf) 435
Curse of dimensionality 556, 891
Curvature matrix *see* Hessian matrix
Curve interpolation 147
Cycle, in multigrid method 1069
Cyclic Jacobi method 573
Cyclic reduction (CR) 224, 1054, 1057, 1058
Cyclic redundancy check (CRC) 1168–1173
Cyclic tridiagonal systems 79, 80

D.C. (direct current) 602
Danielson-Lanczos lemma 609, 610, 638
Data
continuous vs. binned 731
entropy 754–761, 1176
essay on 720
fitting 773–838
fraudulent 780
glitches in 777
iid (independent and identically distributed) 809
linearly separable 884
missing data points 150–154
modeling 773–838
smoothing 721, 766–772
statistical tests 720–772
unevenly or irregularly sampled 139–154, 685, 690, 771
use of CRCs in manipulating 1169
windowing 655–667
see also Statistical tests
Data compression 713, 715, 1160
arithmetic coding 1181–1185
cosine transform 625
Huffman coding 713, 1175–1181
linear predictive coding (LPC) 679–681
lossless 1175
Data Encryption Standard (DES) 358–361
Data type 8
DAUB4 700, 702, 706, 707, 711, 715
DAUB6 702
DAUB20 706
Daubechies wavelet coefficients 700–702, 704, 706–708, 715
Davidon-Fletcher-Powell algorithm 490, 521, 522
Dawson's integral 302, 304, 717
approximation for 303
routine for 303
DE rule 174
implementation 175
infinite range 176
Decoding
Berlekamp-Massey algorithm for Reed-Solomon code 852
directed graph 556, 850
hard-decision 853
hard-decision vs. soft-decision 855
maximum likelihood 854
Reed-Solomon codes 855
soft-decision decoding 853
syndrome decoding 852
Turbo codes 855
Viterbi algorithm 854
Viterbi, compared to hidden Markov model 867, 868
Decomposition *see* Cholesky decomposition; LU decomposition; QR decomposition; Singular value decomposition (SVD)

Deconvolution 645–647, 650
see also Convolution; Fast Fourier transform (FFT); Fourier transform
Decorrelating random variables 379
Defect, in multigrid method 1067
Deferred approach to the limit *see* Richardson's deferred approach to the limit
Deflation
of matrix 585
of polynomials 464–466, 471
Degeneracy
kernel 992
linear algebraic equations 73, 793
minimization principle 1002
Degrees of freedom 732, 733, 778, 779, 813–815
Delaunay triangulation 1097, 1131–1149
applications of 1141–1149
incremental constructions 1134
interpolation using 1141
largest minimum angle property 1134
minimum spanning tree 1147
not minimum weight 1134
Delone, B.N. *see* Delaunay Triangulation
Dense output, for differential equations 904, 915, 927
Dependencies, program 4
Dependency graph or matrix 949
Derivatives
approximation by sinc expansion 178
computation via Chebyshev approximation 232, 240, 241
computation via Savitzky-Golay filters 232, 769
matrix of first partial *see* Jacobian determinant
matrix of second partial *see* Hessian matrix
numerical computation 229–232, 480, 769, 936, 960, 978
of polynomial 202
use in optimization 499–502
Derived class 23
DES *see* Data Encryption Standard
Descending transformation, elliptic integrals 310
Descent direction 478, 484, 522
Descriptive statistics 720–772
see also Statistical tests
Design matrix 768, 788, 1002
Design of experiments 410
Detailed balance equation 825–827
Determinant 39, 54, 55
Devex 535
Deviates, random *see* Random deviates
DFP algorithm *see* Davidon-Fletcher-Powell algorithm
Diagonal dominance 57, 802, 987, 1060
Diagonal rational function 125
Diehard test, for random numbers 345
Difference equations, finite *see* Finite difference equations (FDEs)
Difference operator 212
Differential equations 899–954
accuracy vs. stability 907, 931
Adams-Bashforth-Moulton schemes 943
adaptive stepsize control 901, 910–921, 924–926, 929, 939, 941, 943, 944, 946
algebraically difficult sets 970
backward Euler's method 932
Bader-Deuflhard method for stiff 940
boundary conditions 900, 955, 962, 977
Bulirsch-Stoer method 252, 318, 900, 901, 909, 921–928, 942
Bulirsch-Stoer method for conservative equations 928, 930
comparison of methods 900, 901, 942, 946, 957
conservative 928, 930
dense output 904, 915, 927
discreteness effects 946–954
eigenvalue problem 958, 973, 977–981
embedded Runge-Kutta method 911, 936
equivalence of multistep and multivalue methods 945
Euler's method 900, 907, 931
forward Euler's method 931
free boundary problem 958, 983
global vs. local error 914
high-order implicit methods 934
implicit differencing 932, 933, 944
initial value problems 900
integrating to an unknown point 916
internal boundary conditions 983, 984
internal singular points 983, 984
interpolation on right-hand sides 115
Kaps-Rentrop method for stiff 934
local extrapolation 911
modified midpoint method 922, 923
multistep methods 900, 942–946
multivalue methods 942–946
Nordsieck method 944
order of method 907, 922
path integration for function evaluation 251–254, 318
predictor-corrector methods 900, 909, 934, 942–946
r.h.s. independent of x 932, 934
reduction to first-order sets 899, 956
relaxation method 957, 964–970
relaxation method, example of 971, 973–977
Rosenbrock methods for stiff 934–940
Runge-Kutta method 900, 907–921, 934, 942, 1096
Runge-Kutta method, high-order 907–910, 912
scaling stepsize to required accuracy 913, 914
second order 928, 930
semi-implicit differencing 934
semi-implicit Euler method 934, 940
semi-implicit extrapolation method 934, 935, 940, 941
semi-implicit midpoint rule 940
shooting method 956, 959–961

shooting method, example 971, 977–981
similarity to Volterra integral equations 993
singular points 921, 962, 983, 984
solving with sinc expansions 178
step doubling 910
stepsize control 901, 910–920, 924, 929, 938, 941, 944, 946
stepsize, danger of too small 920
stiff 901, 931–941
stiff methods compared 941
stochastic simulation 946–954
Stoermer's rule 928
see also Partial differential equations; Two-point boundary value problems
Differentiation matrix 1091
routine for 1092
Diffusion equation 1024, 1043–1049, 1059
Crank-Nicolson method 1045, 1049, 1051, 1052
forward time centered space (FTCS) 1044, 1046, 1059
implicit differencing 1045
multidimensional 1051, 1052
Digamma function 267
Digital filtering *see* Filter
Dihedral angle 1116
Dihedral group D_5 1174
Dimensionality, curse of 556, 891
Dimensions (units) 801
Diminishing increment sort 422
Dingbats, Zapf 1162
Dirac delta function 700, 987
Direct method *see* Periodogram
Direct methods for linear algebraic equations 40
Direct product *see* Outer product of matrices
Directed graph
Markov model 856
stages and states 556, 850
transition matrix 856
transition probability 856
trellis 856
Viterbi decoding 850
Direction numbers, Sobol's sequence 404
Direction of largest decrease 512
Direction set methods for minimization 489, 509–514
Dirichlet boundary conditions 1026, 1045, 1055, 1061, 1063
Discordant pair for Kendall's tau 751
Discrete convolution theorem 642, 643
Discrete Fourier transform (DFT) 605–608
approximation to continuous transform 607, 608
see also Fast Fourier transform (FFT)
Discrete optimization 536, 549
Discrete prolate spheroidal sequence (dpss) 662–667
Discretization error 173
Discriminant 227, 572
Dispersion 1036
DISPO *see* Savitzky-Golay filters
Dissipation, numerical 1035
Distance matrix 869
Distributions, statistical *see* Statistical distributions
Divergent series 210, 211, 216
Divide-and-conquer method 589
Division
complex 226
integer vs. floating 8
multiple precision 1190
of polynomials 204, 464, 471
dn function 316
DNA sequence 559–562, 869, 884
Do-while iteration 15
Dogleg step methods 486
Domain of integration 196
Dominant solution of recurrence relation 220
Dormand-Prince parameters 912, 920
Dot ·
denotes matrix multiplication 37
denotes row or column sums 759
`Doub` 25
Double exponential error distribution 820
Double root 443
Doubling rate 756
Downhill simplex method *see* Simplex, method of Nelder and Mead
DP *see* Dynamic programming
dpss (discrete prolate spheroidal sequence) 662–667
Dual problem 538, 886
Dual viewpoint, in multigrid method 1077
Duality gap 538
Duplication theorem, elliptic integrals 311
DWT (discrete wavelet transform) *see* Wavelet transform
Dynamic programming 555–562
Bellman-Dijkstra-Viterbi algorithm 556, 850
directed graph 556, 850

e-folding rate 756
Eardley's equivalence class method 440
Economization of power series 243–245
Eigensystems 563–599
and integral equations 987, 992
balancing matrix 592, 594
bounds on eigenvalues 64
calculation of few eigenvectors or eigenvalues 568, 598
canned routines 567
characteristic polynomial 563, 583
completeness 564, 565
defective 564, 591, 598, 599
deflation 585
degenerate eigenvalues 563, 565
divide-and-conquer method 589
eigenvalues 563
elimination method 567, 594
factorization method 567
fast Givens reduction 578

generalized eigenproblem 568, 569
Givens reduction 578–583
Givens transformation 587
Hermitian matrix 590
Hessenberg matrix 567, 585, 590–595, 598
Householder transformation 567, 578–584, 587, 590, 594
ill-conditioned eigenvalues 591, 592
implicit shifts 586–589
invariance under similarity transform 566
inverse iteration 568, 584, 589, 597–599
Jacobi transformation 567, 570–576, 578, 590, 599
left eigenvalues 565
list of tasks 568
Markov model transition matrix 858, 859
MRRR algorithm 589, 599
multiple eigenvalues 599
nonlinear 568, 569
nonsymmetric matrix 590–595
operation count of balancing 592
operation count of Givens reduction 578
operation count of Householder reduction 582
operation count of inverse iteration 598, 599
operation count of Jacobi method 573, 574
operation count of QL method 585, 588
operation count of QR method for Hessenberg matrices 596
operation count of reduction to Hessenberg form 594
orthogonality 564
polynomial roots and 469
QL method 584–586, 590
QR method 67, 567, 571, 584–586
QR method for Hessenberg matrices 596
real symmetric matrix 188, 576, 577, 582, 992
reduction to Hessenberg form 594, 595
relation to singular value decomposition (SVD) 569, 570
right eigenvalues 565
shifting eigenvalues 563, 585, 596
special matrices 568
termination criterion 598, 599
tridiagonal matrix 567, 576, 577, 583–589, 598
Eigenvalue and eigenvector, defined 563
Eigenvalue problem for differential equations 958, 973, 977–981
Eigenvalues and polynomial root finding 469
EISPACK 567
Electromagnetic potential 631
Elimination *see* Gaussian elimination
Ellipse in confidence limit estimation 811, 814, 815
Elliptic integrals 309–316, 1185
addition theorem 310
Carlson's forms and algorithms 310–316
Cauchy principal value 311
duplication theorem 311
Legendre 309, 314, 315
routines for 311–315
symmetric form 309, 310
Weierstrass 310
Elliptic partial differential equations 1024
alternating-direction implicit method (ADI) 1065, 1066, 1185
analyze/factorize/operate package 1030
biconjugate gradient method 1030
boundary conditions 1026
comparison of rapid methods 1058
conjugate gradient method 1030
cyclic reduction 1054, 1057, 1058
Fourier analysis and cyclic reduction (FACR) 1053–1058
Gauss-Seidel method 1060, 1061, 1068, 1078
Jacobi's method 1060, 1061, 1068
matrix methods 1028, 1030
multigrid method 1030, 1066–1083
rapid (Fourier) method 1029, 1054–1057
relaxation method 1028, 1059–1066
spectral methods 1096
successive over-relaxation (SOR) 1061–1066, 1070
EM algorithm *see* Expectation-maximization algorithm
Embedded networks 1168
Embedded Runge-Kutta method 911, 936
Encryption 358
Entropy 754–761, 1006, 1176
chain rule 759
conditional 758–761
of data 1017
relative 756
EOM (end of message) 1178, 1181
epsilon (ϵ) algorithm 212
Equality constraints 526, 528
Equations
cubic 227–229, 461
differential *see* Differential equations
normal (fitting) 768, 789–793, 1007
quadratic 10, 227–229
see also Differential equations; Partial differential equations; Root finding
Equilibrium, physical 825
Equivalence classes 419, 439–441
Equivalence transformation 208
Ergodic
Markov model 858
Ergodic property 825
Error 8–12
checksums for preventing 1172
clocking 1172
discretization 173
double exponential distribution 820
in multigrid method 1067
interpolation 113
local truncation 1077, 1078
Lorentzian distribution 820

nonnormal 779, 812, 818–824
relative truncation 1077
roundoff 10, 11, 229, 1163, 1164
series, advantage of an even 165, 923
systematic vs. statistical 778
trimming 173
truncation 11, 173, 229, 500, 910, 911, 1163
varieties of, in PDEs 1036–1038
see also Roundoff error
Error-correcting codes 851–855
Berlekamp-Massey decoding algorithm 852
binary block code 851
codeword 851
correcting bit errors 855
coset leader 852
Golay code 852
Hamming code 852
Hamming distance 851, 1168
hard-decision decoding 853
linear codes 851
minimal trellis 853
parity-check matrix 851
perfect code 852
Reed-Solomon 852, 855
soft-decision decoding 853
syndrome 852
syndrome decoding 852
trellis 853, 856
turbo codes 855
Viterbi decoding 854, 855
Error ellipse, how to draw 817, 847
Error function 259, 264–266, 718
approximation via sampling theorem 718, 719
Chebyshev approximation 264
complex 302
Fisher's z-transformation 747
inverse 264
relation to Dawson's integral 302
relation to Fresnel integrals 298
relation to incomplete gamma function 264
routine for 264
significance of correlation 746
sum squared difference of ranks 750
Error handling in programs 2, 30, 31, 35
Estimation of parameters *see* Fitting; Maximum likelihood estimate
Estimation of power spectrum 681–684
Euclid 1098
Euler equation (fluid flow) 1037
Euler-Maclaurin summation formula 164, 167
Euler's constant 267, 269, 300
Euler's method for differential equations 900, 907, 931
Euler's transformation 211, 212
Evaluation of functions *see* Function
Even and odd parts, of continued fraction 208, 260, 267
Even parity 1168
Exception handling in programs 2, 30, 31, 35
Expectation-maximization algorithm 842–844
expectation step (E-step) 843
for hidden Markov model 866
maximization step (M-step) 843
relation to Baum-Welch re-estimation 866
Explicit differencing 1032
Exponent in floating point format 8, 1164
Exponential convergence 174–178, 180, 238, 239, 1083–1096
Exponential integral 266–269
asymptotic expansion 269
continued fraction 267
recurrence relation 219
related to incomplete gamma function 267
relation to cosine integral 301
routine for Ei(x) 269
routine for $E_n(x)$ 268
series 267
Exponential probability distribution 326, 327, 686
deviate from 362
relation to Poisson process 369, 829
Extended midpoint rule 157, 161, 167
Extended precision, use in iterative improvement 62
Extended Simpson's rule 160, 994, 997
three-eighths rule 995
Extended trapezoidal rule 157, 159, 162, 167, 993
roundoff error 165
Extirpolation (so-called) 690, 691
Extrapolation 110–154
Bulirsch-Stoer method 922, 924
by linear prediction 673–681
differential equations 900
local 911
maximum entropy method as type of 683
polynomial 922, 924, 943
rational function 922
relation to interpolation 110
Romberg integration 166
see also Interpolation
Extremization *see* Minimization

F-distribution probability function 332, 333
deviates 371
F-test for differences of variances 728, 730
FACR *see* Fourier analysis and cyclic reduction (FACR)
Facsimile standard 1180
Factorial
evaluation of 210
relation to gamma function 256
representability 257
routine for 257
routine for log 258
False position 449, 452, 454
Family tree 440
FAS (full approximation storage algorithm) 1076–1083

Fast Fourier transform (FFT) 608–616, 640, 1160
alternative algorithms 615, 616
applications 640–719
approximation to continuous transform 608
bare routine for 611
bit reversal 610, 638
butterfly 360, 361, 610
Clenshaw-Curtis quadrature 241
convolution 616, 631, 641–647, 1189
convolution of large data sets 646, 647
Cooley-Tukey algorithm 616
correlation 648, 649
cosine transform 241, 624–627, 1056
cosine transform, second form 625, 1057
Danielson-Lanczos lemma 609, 610, 638
data sets not a power of 2 616
data smoothing 766, 767
data windowing 655–667
decimation-in-frequency algorithm 616
decimation-in-time algorithm 615
decomposition into blocks 614
differentiation matrix using 1092
discrete autocorrelation 649
discrete convolution theorem 642, 643
discrete correlation theorem 648
double frequency 690
endpoint corrections 694
external storage 637, 638
figures of merit for data windows 658
filtering 667–672
FIR filter 668, 669
for quadrature 156
for spherical harmonic transforms 296
four-step framework 615
Fourier integrals 692–699
Fourier integrals,infinite range 699
history 609
IIR filter 668–672
image processing 1010, 1012
integrals using 156
inverse of sine transform 623
large data sets 637, 638
leakage 655, 656
Lomb periodogram and 689
memory-local algorithm 638
multidimensional 627–630
multiple precision arithmetic 1185
multiple precision multiplication 1189
number-theoretic transforms 616
of real data in 2D and 3D 631–637
of real functions 617–627, 631–637
of single real function 618–620
of two real functions simultaneously 617, 618
operation count 609, 610
optimal (Wiener) filtering 649–652, 673, 674
order of storage in 611
parallel 614
partial differential equations 1029, 1054–1057
periodicity of 608
periodogram 653–656, 681, 683
power spectrum estimation 652–667
related algorithms 615, 616
Sande-Tukey algorithm 616
sine transform 620–623, 1055
Singleton's algorithm 637, 638
six-step framework 615
spectral methods 1086
treatment of end effects in convolution 643
treatment of end effects in correlation 648
Tukey's trick for frequency doubling 690
two real functions simultaneously 617
use in smoothing data 766, 767
virtual memory machine 638
Winograd algorithms 616
zoom transforms 615
see also Discrete Fourier transform (DFT); Fourier transform; Spectral density
Fast Legendre transform 295, 297
Fast multipole methods 140, 1150
FASTA (software) 562
Faure sequence 404
Fax (facsimile) Group 3 standard 1180
Feasible vector 526, 538
basis vector 528
Fermi-Dirac integral 178
FFT *see* Fast Fourier transform (FFT)
Field, in data record 428
Figure-of-merit function 773
`__FILE__` (ANSI C macro) 30
Fill-in, sparse linear equations 59, 76, 535, 544
Filon's method 698
Filter 667–672
acausal 668
bilinear transformation method 670, 672
by fast Fourier transform (FFT) 637, 649, 667–672
causal 668, 767, 770
characteristic polynomial 670
data smoothing 766
digital 667–672
DISPO 767
finite impulse response (FIR) 642, 643, 668, 669
homogeneous modes of 670
infinite impulse response (IIR) 668–672, 681
Kalman 824
linear 668–672
low-pass for smoothing 766
nonrecursive 668
optimal (Wiener) 645, 649–652, 673, 674, 767
quadrature mirror 701, 708
realizable 668, 670, 671
recursive 668–672, 681
Remes exchange algorithm 669
Savitzky-Golay 232, 766–772
stability of 670, 671
time domain 667–672
Fine-to-coarse operator 1068

Finite difference equations (FDEs) 964, 970, 981
accuracy of 1085
alternating-direction implicit method (ADI) 1052, 1053, 1065, 1066
art, not science 1035
Cayley's form for unitary operator 1049
Courant condition 1034, 1036, 1038, 1042
Courant condition (multidimensional) 1051
Crank-Nicolson method 1045, 1049, 1051, 1052
eigenmodes of 1033, 1034
explicit vs. implicit schemes 1033
forward Euler 1032
forward time centered space (FTCS) 1032, 1044, 1049, 1059
implicit scheme 1045
in relaxation methods 964
Lax method 1034–1036, 1042
Lax method (multidimensional) 1050, 1051
mesh drifting instability 1040
numerical derivatives 229
partial differential equations 1027
relation to spectral methods 1093
staggered leapfrog method 1038, 1039
two-step Lax-Wendroff method 1040
upwind differencing 1037, 1042
see also Partial differential equations
Finite element methods 132, 1030
Finite impulse response (FIR) 642, 643
FIR (finite impulse response) filter 668, 669
First-class objects 397
Fisher discriminant algorithm 892
Fisher's z-transformation 746
Fitting 773–838
basis functions 788
by Chebyshev approximation 234
by rational Chebyshev approximation 247–251
chi-square 778–780
confidence levels from singular value decomposition (SVD) 816, 817
confidence levels related to chi-square values 812–816
confidence limits on fitted parameters 807–817
covariance matrix not always meaningful 774, 812
degeneracy of parameters 797
exponential, an 797
freezing parameters in 791, 824
Gaussians, a sum of 805
general linear least squares 788–798
how much $\Delta\chi^2$ is significant 816
K–S test, caution regarding 740
Kalman filter 824
kriging 836–838
least squares 776–780
Legendre polynomials 797
Levenberg-Marquardt method 801–806, 1022
linear regression 780–785
Markov chain Monte Carlo 824–835
maximum likelihood estimation 777, 818
Monte Carlo simulation 740, 779, 807–810
multidimensional 798, 836–838
nonlinear models 799–806
nonlinear models, advanced methods 806
nonlinear problems that are linear 797
nonnormal errors 781, 812, 818–824
of sharp spectral features 682
polynomial 94, 129, 241, 243, 768, 788, 797
robust methods 818–824
standard (probable) errors on fitted parameters 781, 782, 786, 787, 790, 794, 795, 807–817
straight line 780–785, 822–824
straight line, errors in both coordinates 785–787
see also Error; Least-squares fitting; Maximum likelihood estimate; Robust estimation
Five-point difference star 1071
Fixed point format 8
Fletcher-Powell algorithm *see* Davidon-Fletcher-Powell algorithm
Fletcher-Reeves algorithm 489, 515–519
Floating point format 8–11, 1163–1165
care in numerical derivatives 229, 230
in computational geometry 1098
enabling exceptions 35, 575
history 1163
IEEE 9, 10, 34, 1164
little- vs. big-endian 9
NaN 34, 35
Flux-conservative initial value problems 1031–1043
FMG (full multigrid method) 1067, 1072–1076
`for` iteration 14
Formats of numbers 8–11, 1163–1165
Fortran 1
INTENT attribute 26
Forward-backward algorithm
as a sum-product algorithm 867
Bahl-Cocke-Jelinek-Raviv algorithm 867
belief propagation 867
compared to Viterbi decoding 867
hidden Markov model 861, 862, 864–867
renormalization 862
Forward deflation 464, 465
Forward difference operator 212
Forward Euler differencing 1032
Forward Time Centered Space *see* FTCS
Four-step framework, for FFT 615
Fourier analysis and cyclic reduction (FACR) 1054, 1058
Fourier and spectral applications 600, 640–719
Fourier integrals
attenuation factors 698
endpoint corrections 694
tail integration by parts 699
use of fast Fourier transform (FFT) 692–699

Fourier series as basis functions for spectral methods 1085
Fourier transform 110, 600–640
- aliasing 606, 685
- approximation of Dawson's integral 303
- autocorrelation 602
- basis functions compared 621
- contrasted with wavelet transform 699, 700, 711
- convolution 602, 616, 617, 631, 641–647, 1189
- correlation 602, 617, 648, 649
- cosine transform 241, 624–627, 1056
- cosine transform, second form 625, 1057
- critical sampling 605, 653, 655
- decomposition into blocks 614
- definition 600
- discrete Fourier transform (DFT) 233, 236, 605–608
- Gaussian function 717, 718
- image processing 1010, 1012
- infinite range 699
- inverse of discrete Fourier transform 608
- method for partial differential equations 1054–1057
- missing data 685
- missing data, fast algorithm 689–692
- Nyquist frequency 605, 607, 632, 653, 655, 685
- optimal (Wiener) filtering 649–652, 673, 674
- Parseval's theorem 602, 603, 608, 654
- power spectral density (PSD) 602, 603
- power spectrum estimation by FFT 652–667
- power spectrum estimation by maximum entropy method 681–684
- properties of 601
- sampling rate 605
- sampling theorem 605, 653, 655, 717–719
- scalings of 601
- significance of a peak in 686
- sine transform 620–623, 1055
- symmetries of 601
- uneven sampling, fast algorithm 689–692
- unevenly sampled data 685–692
- wavelets and 707, 708
- Wiener-Khinchin theorem 602, 674, 682
- *see also* Fast Fourier transform (FFT); Spectral density

Fractal region 462
Fractional step methods 1052
Fredholm alternative 987
Fredholm equations 986
- eigenvalue problems 987, 992
- error estimate in solution 991
- first kind 986
- Fredholm alternative 987
- homogeneous vs. inhomogeneous 987
- homogeneous, second kind 991
- ill-conditioned 987
- infinite range 995
- inverse problems 987, 1001–1006
- kernel 986
- nonlinear 988
- Nystrom method 989–992, 995
- product Nystrom method 995
- second kind 987–992
- subtraction of singularity 996
- symmetric kernel 992
- with singularities 995–1000
- with singularities, worked example 999, 1000
- *see also* Inverse problems

Frequency domain 600
Frequency spectrum *see* Fast Fourier transform (FFT)
Frequentist, contrasted with Bayesian 774
Fresnel integrals 297–300
- asymptotic form 298
- continued fraction 298
- routine for 299
- series 298

Friday the 13th 7
FSAL (first-same-as-last) 913
FTCS (forward time centered space) 1032, 1044, 1049
- stability of 1033, 1044, 1060

Full approximation storage (FAS) algorithm 1076–1083
Full conditional distribution 827
Full moon 7
Full multigrid method (FMG) 1067, 1072–1076
Full Newton methods, nonlinear least squares 806
Full pivoting 43
Full weighting 1071
Function
- Airy 254, 283, 289, 291
- approximation 110, 233–239
- associated Legendre polynomial 293, 971
- autocorrelation of 602
- bandwidth limited 605
- Bessel 219, 254, 274–292
- beta 258, 259
- branch cuts of 252–254
- chi-square probability 1003
- complex 251
- confluent hypergeometric 254, 287
- convolution of 602, 617
- correlation of 602, 617
- Coulomb wave 254, 283
- cumulative distribution (cdf) 320–339
- Dawson's integral 302, 304, 717
- digamma 267
- elliptic integrals 309–316, 1185
- error 264–266, 298, 302, 718, 746, 750
- error function 259
- evaluation 201–254
- evaluation by path integration 251–254, 318
- exponential integral 219, 266–269, 301
- factorial 256, 257
- Fermi-Dirac integral 178

Fresnel integral 297–300
functor 21–23, 444, 459, 905
gamma 256, 257
hypergeometric 252, 318–320
incomplete beta 270–273
incomplete gamma 259–263, 732, 779
inverse cumulative distribution 320–339
inverse hyperbolic 227, 310
inverse incomplete gamma 263
inverse of $x \log(x)$ 307–309, 335
inverse trigonometric 310
Jacobian elliptic 309, 316, 317
Kolmogorov-Smirnov probability 737, 763
Legendre polynomial 219, 293, 797
log factorial 258
logarithm 310
minimization 487–562
modified Bessel 279–283
modified Bessel, fractional order 287–289
object 21
path integration to evaluate 251–254
pathological 111, 445
probability 320–339
representations of 600
routine for plotting a 444
sine and cosine integrals 297, 300–302
sn, dn, cn 316, 317
spherical Bessel 283
spherical harmonics 292–297
spheroidal harmonic 971–981
statistical 320–339
templated 17, 22, 26
utility 17
virtual 33
Weber 254
Function object *see* Functor
Functional iteration, for implicit equations 943
Functor 21–23, 202, 204, 237, 240, 444, 459, 660, 905, 936, 940
FWHM (full width at half maximum) 659

g++ 5
Gambling 755–758, 760, 761
Gamma function 256, 257
and area of sphere 1129
complex 257
incomplete *see* Incomplete gamma function
Gamma probability distribution 331, 332
as limiting case of beta 333
deviates from 369
relation to Poisson process 829
sum rule for deviates 370
Gauss-Chebyshev integration 180, 183, 187, 625
Gauss-Hermite integration 183, 995
abscissas and weights 185
normalization 185
Gauss-Jacobi integration 183
abscissas and weights 186
Gauss-Jordan elimination 41–46, 75
operation count 47, 54
solution of normal equations 790
storage requirements 44
Gauss-Kronrod quadrature 192, 195
Gauss-Laguerre integration 183, 995
Gauss-Legendre integration 183, 193
see also Gaussian integration
Gauss-Lobatto quadrature 191, 192, 195, 241, 624, 1089
Gauss-Markov estimation 144
Gauss-Radau quadrature 191
Gauss-Seidel method (relaxation) 1060–1062, 1068
nonlinear 1078
Gauss transformation 310
Gaussian
Hardy's theorem on Fourier transforms 717
multivariate 378, 379, 842, 843, 847, 848, 1006, 1129, 1130
see also Gaussian (normal) distribution
Gaussian (normal) distribution 341, 776, 778, 1004
central limit theorem 777
Cholesky decomposition of 847, 848
deviates from 364, 365, 368, 686
kurtosis of 723, 724
multivariate 813, 842
semi-invariants of 725
sum of 12 uniform 377
tails compared to Poisson 778
two-dimensional (binormal) 746
variance of skewness of 723
see also Normal (Gaussian) distribution
Gaussian elimination 46–48, 65, 71
fill-in 59, 76, 535
in reduction to Hessenberg form 594
integral equations 993
operation count 47
relaxation solution of boundary value problems 966, 984
Gaussian integration 159, 179–193, 238, 296, 995, 997, 1086–1089
and orthogonal polynomials 181, 1087
calculation of abscissas and weights 182–188
discrete orthogonality relation 1087
error estimate in solution 991
exponential convergence of 180, 1089
extensions of 191–193, 1089
for integral equations 988, 990
from known recurrence relation 188, 189
Golub-Welsch algorithm for weights and abscissas 188
for incomplete beta function 271
for incomplete gamma function 260, 262
nonclassical weight function 189–191, 995
preassigned nodes 191
weight function $\log x$ 190, 191
weight functions 179–181, 995
Gaussian mixture model 842–848
Gaussian process regression 144, 836–838
Gear's method (stiff ODEs) 934, 941

Geiger counter 340
Gene sequencing
  alignment algorithms 559–562
  hidden Markov model 866
Generalized eigenvalue problems 568, 569
Generalized minimum residual method (GMRES) 89
Genetic algorithms 840
Geometric series 211, 214
Geophysics, use of Backus-Gilbert method 1016
Gerchberg-Saxton algorithm 1012
Ghostscript 1161
Gibbs sampler 827, 828
  recommended for discrete distributions 828
Gilbert and Sullivan 920
Gillespie method 947
Givens reduction 578–583, 587
  fast 578
  operation count 578
Glassman, A.J. 229
Global optimization 487, 488, 549–555, 774
  continuous variables 552–554
  difficulty of 803
Globally convergent
  minimization 521–525
  root finding 474, 477–486, 959, 960, 963
GLPK (linear programming package) 536
GMRES (generalized minimum residual method) 89
GNU C++ compiler 5
GNU Scientific Library 3
Gnuplot 1162
Godunov's method 1043
Golden mean (golden ratio) 11, 449, 494, 500
Golden section search 443, 489, 496
Goldman-Tucker theorem 539
Golub-Welsch algorithm, for Gaussian quadrature 188
Goodness-of-fit 773, 779, 782, 783, 787, 813
  no good Bayesian methods 779, 1010
Gram-Schmidt
  orthogonalization 105, 564, 565, 589, 598
  SVD as alternative to 74
Graphics, function plotting 444, 1160–1163
Gravitational potential 631
Gray code 405, 1160, 1166–1168
Greenbaum, A. 90
Gregorian calendar 6
Grid square 132
Gridding 150–154
Group, dihedral 1174
Guard digits 1164

Half weighting 1071
Halley's method 263, 264, 271, 335, 463
Halton's quasi-random sequence 404
Hamming code 852
Hamming distance 873
  error-correcting codes 851, 1168
Hamming window 658
Hamming's motto 443
Hann window 657
Hard-decision
  decoding 853
  error correction 855
Harmonic analysis *see* Fourier transform
Harwell-Boeing format 83
Hash
  collision strategy 387, 390
  examples 396
  function 352, 387–389
  key 387
  memory 392–397
  multimap memory 394–397
  table 386–392
  of whole array 358–361
Heap (data structure) 426, 434, 952, 1178
Heapsort 420, 426–428, 434
Helmholtz equation 1057
Hermite interpolation 916
Hermite polynomials 183, 185
Hermitian matrix 564, 590
Hertz (unit of frequency) 600
Hessenberg matrix 105, 567, 585, 590–595, 598
  QR algorithm 596
  see also Matrix
Hessian matrix 483, 510, 517, 521, 522, 799–801, 1011, 1020, 1021
  is inverse of covariance matrix 802
  second derivatives in 800, 801
Hidden Markov model 856–868
  backward estimate 861
  Baum-Welch re-estimation 865–867
  Bayesian nature of 868
  Bayesian posterior probability 860, 861, 864
  Bayesian re-estimation 864–866
  compared to Viterbi algorithm 867, 868
  convergence of Baum-Welch re-estimation 866
  expectation-maximization algorithm 866
  forward-backward algorithm 861, 862, 864–867
  forward estimate 860
  gene sequencing 866
  hidden state 859
  know intermediate states 864
  missing data 864
  observations 859
  re-estimation of symbol probability matrix 865
  re-estimation of transition probabilities 865
  renormalization 862
  speech recognition 866
  symbols 859
  trellis decoding 864
  variations 864
Hierarchical clustering 868–882
Hierarchically band-diagonal matrix 716
High-order not same as high-accuracy 112, 156, 238, 489, 500, 908, 911, 943

High-pass filter 667
Higher-order statistics 604
Hilbert matrix 94
Hilbert's Third Problem 1127
Histogram, variable-size bins 438
Historic maximum entropy method 1022
Hobson's choice 704
Homogeneous linear equations 69
Hook step methods 486
HOPDM (software) 548
Hotelling's method for matrix inverse 64, 716
Householder transformation 67, 567, 578–584, 586, 587, 590, 594
 in QR decomposition 103
 operation count 582
Huffman coding 680, 713, 1160, 1175–1181
Hull, convex 1097, 1132, 1146
Hyperbolic functions, explicit formulas for inverse 227
Hyperbolic partial differential equations 1024
 advective equation 1032
 flux-conservative initial value problems 1031–1043
Hypergeometric function 252, 318–320
 routine for 318, 319
Hypothesis, null 720

`_I` 26, 32, 36
IBM
 bad random number generator 344
 checksum 1174
 radix base for floating point arithmetic 592
ICF (intrinsic correlation function) model 1022
Identity (unit) matrix 39
Idioms 16
IEEE floating point format 9, 10, 34, 257
`if` structure 14
 warning about nesting 14
IIR (infinite impulse response) filter 668–672, 681
Ill-conditioned integral equations 987
Image processing 631, 1010
 as an inverse problem 1010
 cosine transform 625
 fast Fourier transform (FFT) 631, 637, 1010
 from modulus of Fourier transform 1012
 maximum entropy method (MEM) 1016–1022
 QO tree and 1150
 wavelet transform 713, 715
Implicit
 function theorem 442
 pivoting 44
 shifts in QL method 586–589
Implicit differencing 1033
 for diffusion equation 1045
 for stiff equations 932, 933, 944
Importance sampling, in Monte Carlo 411, 412, 414, 835, 836
Improper integrals 167–172
Impulse response function 641–643, 649, 668
IMSL 3, 40, 76, 466, 470, 568
IMT (Iri, Moriguti, Takasawa) rule 173
In-place selection 439
Include files 3, 4
Incomplete beta function 270–273
 for F-test 730
 for Student's t 729
 routine for 273
Incomplete gamma function 259–263
 deviates from 369
 for chi-square 732, 779
 inverse 263
Increment of linear congruential generator 343
Incremental quantile estimation 435
 changes with time 438
Indentation of blocks 14
Index table 419, 426, 428–431
Inequality constraints 526, 528, 538
Inference 840–898
Information
 mutual 758–761
 side 760, 761
 theory 754–761
Inheritance 23, 24
 examples of in NR 23
Initial value problems 900, 1024, 1026
 see also Differential equations;
Injection operator 1068
`inline` directive 29
Inscribed circle (incircle) 1112
Instability *see* Stability
Instantiation 18, 19
`Int`, `__int32`, `__int64` 25
Integer programming 536
Integral equations 986–1023
 adaptive stepsize control 995
 block-by-block method 994
 correspondence with linear algebraic equations 986
 degenerate kernel 992
 eigenvalue problems 987, 992
 error estimate in solution 991
 Fredholm 986, 989–992
 Fredholm alternative 987
 homogeneous, second kind 991
 ill-conditioned 987
 infinite range 995
 inverse problems 987, 1001–1006
 kernel 986
 nonlinear 988, 994
 Nystrom method 989–992, 995
 product Nystrom method 995
 solving with sinc expansions 178
 subtraction of singularity 996
 symmetric kernel 992
 unstable quadrature 994
 Volterra 988, 992–995
 wavelets 989
 with singularities 995–1000

with singularities, worked example 999, 1000
see also Inverse problems
Integral operator, wavelet approximation of 715, 989
Integration of functions 155–200
Chebyshev approximation 240, 241
cosine integrals 300
Fourier integrals 692–699
Fourier integrals, infinite range 699
Fresnel integrals 297
Gauss-Hermite 185
Gauss-Jacobi 186
Gauss-Laguerre 184
Gauss-Legendre 183
infinite ranges 176–178
integrals that are elliptic integrals 309
path integration 251–254
sine integrals 300
see also Quadrature
Integro-differential equations 989
INTENT attribute (Fortran) 26
Interior-point method 85, 536–549
see also Linear Programming
Intermediate value theorem 445
Interpolation 110–154
Aitken's algorithm 118
avoid in Fourier analysis 685
barycentric rational 113, 127, 128
bicubic 136–138
biharmonic 153
bilinear 133, 134
caution on high-order 112, 113
coefficients of polynomial 111, 129–131, 241, 243, 690
curve 139, 147
error estimates for 111
for computing Fourier integrals 694
for differential equation output 916
functions with poles 124
grid, on a 132–135
Hermite 916
inverse multiquadric 142
inverse quadratic 454, 496
irregular grid 139–149, 1097, 1141, 1142
kriging 144–147
Laplace/Poisson 150–154
minimum curvature 153
multidimensional 113, 132–135, 139–154
multigrid method, in 1070–1072
multiquadric 141
Neville's algorithm 118, 231, 924
normalized radial basis functions 140
Nystrom 990
open vs. closed curve 148
operation count for 111
operator 1068
order of 112
ordinary differential equations and 113
oscillations of polynomial 112, 129, 489, 500
parabolic, for minimum finding 496–499
polynomial 110, 118–120, 231, 924
pseudospectral method and 1087
radial basis functions 139–144
rational Chebyshev approximation 247–251
rational function 110, 113, 124–128, 245, 275, 922
reverse (extirpolation) 690, 691
scattered data 139–154
Shepard's method 140
spline 111, 120–124, 135
trigonometric 110
see also Fitting
Intersection
line and sphere 1121
line and triangle 1121
line segments 1118
lines 1117
QO tree used to find 1150
Intersections 1097
Interval variable (statistics) 741
Intrinsic correlation function (ICF) model 1022
Inverse function of $x \log(x)$ 307–309, 335
Inverse hyperbolic function 227, 310
Inverse iteration *see* Eigensystems
stable equilibrium of Markov model 859
Inverse multiquadric 142
Inverse problems 987, 1001–1006
and integral equations 987
Backus-Gilbert method 1014–1016
Bayesian approach 1005, 1022
central idea 1005
constrained linear inversion method 1006
data inversion 1014
deterministic constraints 1011–1013
Gerchberg-Saxton algorithm 1012
in geophysics 1016
incomplete Fourier coefficients 1018, 1020
linear regularization 1006–1013
maximum entropy method (MEM) 1016–1022
MEM demystified 1019, 1020
optimally localized average 1014–1016
Phillips-Twomey method 1006
principal solution 1004
regularization 1002–1006
regularizing operator 1004
stabilizing functional 1004
Tikhonov-Miller regularization 1007
trade-off curve 1002, 1016
two-dimensional regularization 1010, 1011
use of conjugate gradient minimization 1011, 1020
use of convex sets 1011–1013
use of Fourier transform 1010, 1012
Van Cittert's method 1011
Inverse quadratic interpolation 454, 496
Inverse response kernel, in Backus-Gilbert method 1014
Inverse trigonometric function 310
_IO 26, 32, 36

IQ (incremental quantile) agent 435
Irreducibility of Markov model 858
Irreducible polynomials modulo 2 382
Irregular grid, interpolation on 139–149, 1141, 1142
Is-a relationship 23
ISBN (International Standard Book Number)
  checksum 1173
Iterated integrals 196, 197
Iteration 14
  for linear algebraic equations 40
  functional 943
  in root finding 443
  required for two-point boundary value problems 955–957
  to improve solution of linear algebraic equations 61–65, 245
Iteration matrix 1060

Jacobi matrix, for Gaussian quadrature 188
Jacobi transformation (or rotation) 105, 567, 570–576, 578, 590, 599
  decorrelating random variables 380
Jacobi's method (relaxation) 1060, 1061, 1068
Jacobian determinant 364, 981
Jacobian elliptic functions 309, 316, 317
Jacobian matrix 475, 477, 480, 483, 540, 935, 936
  singular in Newton's rule 486
Java 1, 12
Jenkins-Traub method 470
Jordan curve theorem 1124
JPEG-2000 standard 712
Julian Day 3, 6
Jump transposition errors 1174

K-means clustering 848–850
K-S test *see* Kolmogorov-Smirnov test
Kalman filter 824
Kaps-Rentrop method 934
KD tree 1101–1110
  construction of 1102–1106
  number of boxes in 1102
Kelly's formula 758
Kendall's tau 749, 751–754
Kernel 986
  averaging, in Backus-Gilbert method 1014
  degenerate 992
  finite rank 992
  inverse response 1014
  separable 992
  singular 995
  symmetric 992
Kernel methods of classification 840, 889, 892
Keys used in sorting 428
KKT (Karush-Kuhn-Tucker) conditions 539, 542, 886, 889
Kolmogorov-Smirnov probability distribution 334–336
Kolmogorov-Smirnov test 731, 736–738, 819
  two-dimensional 762–766
  variants 738, 762
Kriging 139
  fitting by 836–838
  fitting not same as interpolation 838
  interpolation by 144–147
  is Gaussian process regression 837
  linear prediction and 674, 679
  nugget effect 838
Kuiper's statistic 739
Kullback-Leibler distance 756–758
  symmetrized 757
Kurtosis 723, 725

L-estimate 818
Lag 602, 648, 669
Lagged Fibonacci generator 354
Lagrange multiplier 758, 760, 1001
Lagrange's formula for polynomial interpolation 94, 118, 690, 691, 694, 1089, 1092
Laguerre polynomials 183
Laguerre's method 444, 466–469
  convergence 466
Lanczos lemma 609, 610
Lanczos method for gamma function 256
Landen transformation 310
LAPACK 40, 567
Laplace's equation 292, 1024
  see also Poisson equation
Laplace/Poisson interpolation 150–154
Las Vegas 744
Latin square or hypercube 409, 410
Latitude/longitude in n-dimensions 1128
Laurent series 681, 682
Lax method 1034–1036, 1042, 1050, 1051
  multidimensional 1050, 1051
Lax-Wendroff method 1040
LCG *see* Linear congruential random number generator
`ldexp` 207, 279, 283
LDL 544, 548
`Ldoub` 25
Leakage in power spectrum estimation 655, 656, 658, 662–665
Leakage width 658, 659
Leapfrog method 1038, 1039
Least-squares filters *see* Savitzky-Golay filters
Least-squares fitting 776–798
  degeneracies in 794, 795, 797
  Fourier components 686
  freezing parameters in 791, 824
  general linear case 788–798
  how much $\Delta\chi^2$ is significant 816
  Levenberg-Marquardt method 801–806, 1022
  Lomb periodogram 686
  maximum likelihood estimator 777
  method for smoothing data 768
  multidimensional 798
  nonlinear 486, 799–806, 1022
  nonlinear, advanced methods 806

normal equations 768, 789–793, 1007
normal equations often singular 793, 797
optimal (Wiener) filtering 650
QR method in 105, 791
rational Chebyshev approximation 249
relation to linear correlation 745, 783
Savitzky-Golay filter as 768
singular value decomposition (SVD) 39, 65–75, 249, 793
skewed by outliers 778
spectral analysis 686
standard (probable) errors on fitted parameters 794
weighted 777
see also Fitting
Left eigenvalues or eigenvectors 564, 565
Legendre elliptic integral *see* Elliptic integrals
Legendre polynomials 183, 293
basis functions for spectral methods 1086
fitting data to 797
recurrence relation 219
see also Associated Legendre polynomials; Spherical harmonics
Lehmer-Schur algorithm 470
Lemarie's wavelet 708
Lentz's method for continued fraction 207, 260
Lepage, P. 414
Leptokurtic distribution 723
Levenberg-Marquardt algorithm 486, 801–806, 1022
advanced implementation 806
Levin transformation 214
Levinson's method 96
Liapunov stability 933
Likelihood ratio 735, 757
Limbo 457
Limit cycle
Laguerre's method 466
Markov model 858
Line 1097, 1117–1121
closest approach of two 1121
closest approach to point 1121
distance of point to 1118
equation satisfied by 1117
in 3 dimensions 1121
intersection of two 1117
intersection with sphere 1121
intersection with triangle 1121
left-of relations 1118
segments 1118–1120
skew 1121
`__LINE__` (ANSI C macro) 30
Line minimization *see* Minimization, along a ray
Line search *see* Minimization, along a ray
Linear algebraic equations 37–109
and integral equations 986, 990
band-diagonal 58–61
biconjugate gradient method 88
Cholesky decomposition 100–102, 378, 379, 525, 543, 568, 791
complex 55
computing $\mathbf{A}^{-1} \cdot \mathbf{B}$ 53
conjugate gradient method 87–92, 716
cyclic tridiagonal 79, 80
direct methods 40, 76
Gauss-Jordan elimination 41–46
Gaussian elimination 46, 48
Hilbert matrix 94
Hotelling's method 64, 716
iterative improvement 61–65, 245, 548
iterative methods 40, 87–92
large sets of 38, 39
least-squares solution 65, 70, 73, 249, 793
LU decomposition 48–55, 245, 483, 484, 486, 534, 936, 990, 1008
nonsingular 38, 39
overdetermined 39, 249, 793, 1004
parallel solution 57
partitioned 81
QR decomposition 102–106, 483, 484, 486, 791
row vs. column elimination 45, 46
Schultz's method 64, 716
Sherman-Morrison formula 76–79, 94, 534
singular 38, 69, 73, 249, 793
singular value decomposition (SVD) 65–75, 249, 793, 1003
sparse 39, 58, 75–92, 534, 544, 548, 937, 1011
summary of tasks 39, 40
Toeplitz 93, 96–99, 245
Vandermonde 93–96, 130
wavelet solution 715, 716, 989
Woodbury formula 80, 81, 94
see also Eigensystems
Linear codes 851
Linear congruential random number generator 341, 343, 348
Linear constraints 526, 530
Linear convergence 448, 495
Linear correlation (statistics) 745–748
Linear dependency
constructing orthonormal basis 74, 105
in linear algebraic equations 38
of directions in N-dimensional space 511
Linear equations *see* Differential equations; Integral equations; Linear algebraic equations
Linear feedback shift register (LFSR) 346, 380–386
state vector 380
update rule 380
Linear inversion method, constrained 1006
Linear optimization 526
Linear prediction 673–681
characteristic polynomial 676
coefficients 673–681
compared with regularization 1008
contrasted to polynomial extrapolation 675, 677
is Gaussian process regression 837

kriging and 144
multidimensional 836–838
related to optimal filtering 673, 674
removal of bias in 145, 678, 679
stability 676
Linear predictive coding (LPC) 679–681
Linear programming 488, 526–549
affine scaling 543
artificial variables 530, 531
augmented equations 543, 548
auxiliary objective function 530
barrier method 541
basic variables 529, 531
boundary 528
bounded variables 535, 546
centering parameter 541
central path 540
complementarity condition 539
complementary slackness theorem 539
constraints 526, 530
cycling 534
degenerate basis 533
Devex 535
dual algorithm 535
dual feasible basis vector 538
dual interior-point method 542
dual problem 538, 539
duality gap 538
duality measure 541
efficiency 537, 541
ellipsoid method 537
equality constraints 526, 528
feasible basis vector 528, 529, 532
feasible vector 526, 538
free variables 538
fundamental theorem 528
Goldman-Tucker theorem 539
inequality constraints 526, 528, 538
infeasible method 537
interior-point method 85, 488, 536–549
KKT conditions 539, 542
logical variables 530, 538
long-step method 541
minimum ratio test 532
multiple pricing 535
nonbasic variables 529, 531
normal equations 85, 543, 548
objective function 526, 528, 530
optimal feasible vector 526, 528, 532
path-following method 541
phases one and two 530
predictor-corrector method 547
primal algorithm 535
primal-dual interior-point method 542
primal-dual solution 539
primal interior-point method 542
primal problem 538
reduced cost 531
scaling of variables 535, 546
short-step method 541
simplex method 488, 502, 526–536, 548
simplex vs. interior-point 548
slack variables 529, 531, 535, 538, 547
sparse linear algebra 534, 544, 548
stalling 534
standard form 529, 530, 538
steepest edge pricing 535
strict complementarity 539
strong duality theorem 539
structural variables 530
surplus variables 529
unbounded objective function 532, 538
vertex of simplex 528, 531
weak duality theorem 538
worked example 530–533
zero variables 530
Linear regression 780–787
see also Fitting
Linear regularization 1006–1013
Linearly separable data 884
LINPACK 40, 567
Little-endian 9, 34
`Llong` 25
Local extrapolation 911, 914
Local extremum 487, 551
Localization of roots *see* Bracketing
Log-sum-exp formula 844
Logarithmic function 310
barrier function 541
inverse of $x \log(x)$ 307–309, 335
Logistic probability distribution 324–326
deviates from 363
Lognormal probability distribution 328, 329, 827
Lomb periodogram method of spectral analysis 685–687
fast algorithm 689–692
`long long int` 25
Loops 14
Lorentzian distribution 322
Lorentzian probability distribution 820
Low-pass filter 667, 766
LP coefficients *see* Linear prediction
LPC (linear predictive coding) 679–681
lp_solve 535, 536
LU decomposition 48–55, 62, 65, 71, 75, 108, 475, 534, 790, 936
band-diagonal matrix 59
Bartels-Golub update 535
complex equations 55
Crout's algorithm 49, 59
fill-in, minimizing 535
for $\mathbf{A}^{-1} \cdot \mathbf{B}$ 53
for integral equations 990
for inverse iteration of eigenvectors 598
for inverse problems 1008
for matrix inverse 54
for nonlinear sets of equations 475, 486
for Padé approximant 245
for Toeplitz matrix 98
operation count 49, 54

pivoting 50, 535
repeated backsubstitution 54, 60
solution of linear algebraic equations 54
solution of normal equations 790
stable equilibrium of Markov model 859
threshold partial pivoting 535
Lucifer (encryption algorithm) 358
Lucy's Y^2 and Z^2 statistic 735
LUSOL 535

M-estimates 818
how to compute 821, 822
local 819–821
see also Maximum likelihood estimate
Machine accuracy 10, 1163
Machine learning 840
supervised 883
support vector machine 883–898
unsupervised 842, 868
Macintosh, *see* Apple Macintosh
Maehly's procedure 465, 472
Magic
in MEM image restoration 1019, 1020
in Padé approximation 246, 247
Mantissa in floating point format 8–10, 1164
Mantissa in floating-point format 1189
Maple (software) 3
Marginals 743, 759, 825
Markov chain 825
Markov chain Monte Carlo 551, 774, 824–836
acceptance probability 827, 832
best stepsize 832
burn-in 826, 833–835
candidate point 827
compared to Monte Carlo integration 825
convergence diagnostics 835
converges to sample, not population, values 834
correlated directions 831
correlation time 834
detailed balance equation 825, 827
ergodic average 834
ergodic behavior 825
fitting model parameters 825
full conditional distributions 827
Gibbs sampler 827, 828
and inverse problems 1006
lognormal steps 827
Metropolis-Hastings algorithm 826, 827
normalizing constant 825, 828, 835, 836
parallel computing 835
parameter uncertainties 833
proposal distribution 826–828, 835
proposal generator 830
rapid mixing 826, 831
variable dimension models 835
Markov model 856–868
aperiodic 858
as ensemble 857
convergence 858
corrected phylogenetic distance for 873
diagnosing 858, 859
directed graph 856
equilibrium distribution 857
ergodic 858
evolution in time 857
hidden 856–868
inverse iteration 859
irreducible 858
limit cycle 858
LU decomposition 859
multiple equilibria 859
population vector 857
transition matrix 856
transition probability 856
unstable equilibria 858, 859
Markowitz criterion 535
Marquardt method (least-squares fitting) 801–806, 1022
Mass, center of 399, 400
MasterCard checksum 1174
`MatDoub`, `MatInt`, etc. 26
Mathematica (software) 1, 3
Mathematical Center (Amsterdam) 454
Matlab 1, 3
Matrix 37, 38
approximation of 74, 75, 715
band-diagonal 56, 58–61, 76
band triangular 76
banded 40, 568
bidiagonal 67
block diagonal 76, 964, 966
block triangular 76
block tridiagonal 76
bordered 76
characteristic polynomial 563, 583
Cholesky decomposition 100–102, 378, 379, 525, 543, 568, 791
class for 24–29
column augmented 42, 43
complex 55
condition number 69, 89
curvature 800
cyclic banded 76
cyclic tridiagonal 79, 80
defective 564, 591, 598, 599
of derivatives *see* Hessian matrix; Jacobian determinant
design (fitting) 768, 788
determinant of 39, 54, 55
diagonalization 566
distance 869
elementary row and column operations 42, 43
finite differencing of partial differential equations 1027
Hermitian 564, 568, 590
Hermitian conjugate 564
Hessenberg 105, 567, 585, 590–596, 598
Hessian *see* Hessian matrix
hierarchically band-diagonal 716

Hilbert 94
identity 39
ill-conditioned 69, 71, 130, 131
indexed storage of 82–87
integral equations and 986, 990
inverse 39, 41, 47, 54, 76, 78, 81, 82, 106–108, 565
inverse by Hotelling's method 64, 716
inverse by Schultz's method 64, 716
inverse multiplied by a matrix 53
inverse, approximate 63
iteration for inverse 63–65, 716
Jacobi rotation 573
Jacobi transformation 567, 570–576, 578
Jacobian 935, 936
logical multiplication 949
lower triangular 48, 100, 988
Moore-Penrose inverse 70
multiplication denoted by dot 37
multiplication, optimizing order of 558, 559
norm 64
normal 564, 565
nullity 67, 68
nullspace 39, 67–70, 72, 563, 1002
orthogonal 103, 564, 579, 703, 1130
orthogonal transformation 566, 578, 584
orthonormal basis 74, 105
outer product denoted by ⊗ 78, 523
partitioning for determinant 82
partitioning for inverse 81, 82
positive-definite 40, 100, 543, 791
pseudoinverse 70, 73
QR decomposition 102–106, 483, 484, 486, 791
range 67, 68
rank 67
rank-nullity theorem 68
residual 63
responsibility 842
rotation 1097, 1130, 1131
row and column indices 38
row vs. column operations 45, 46
self-adjoint 564, 565
similarity transform 566, 567, 570, 592, 594
singular 69, 71, 73, 563
singular value decomposition 39, 65–75, 1003
sparse 39, 75–92, 534, 544, 548, 715, 937, 964, 966, 1011
special forms 40
splitting in relaxation method 1060
spread 1015
storage schemes in C++ 38
suffix `_I`, `_O`, `_IO` 26, 32, 36
symmetric 40, 100, 563, 565, 568, 571, 576–583, 992
Toeplitz 93, 96–99, 245
transpose of sparse 85
triangular 567
tridiagonal 40, 56–61, 75, 76, 78, 122, 188, 576–589, 598, 1045, 1057, 1058, 1066
tridiagonal with fringes 1028
unitary 564
updating 105, 106, 484
upper Hessenberg 594
upper triangular 48, 103
Vandermonde 93–96, 130
see also Eigensystems; `NRmatrix`
Matrix equations *see* Linear algebraic equations
Matterhorn 723
MAX utility function 17
Maximization *see* Minimization
Maximum entropy method (MEM) 681–684, 1006
algorithms for image restoration 1020
Bayesian 1022
Cornwell-Evans algorithm 1021
demystified 1019, 1020
for inverse problems 1016–1022
historic vs. Bayesian 1022
image restoration 1016–1022
intrinsic correlation function (ICF) model 1022
operation count 683
see also Linear prediction
Maximum likelihood
compared with probability 854
trellis decoding 854
Maximum likelihood estimate (M-estimates) 812, 818
chi-square test 812
defined 777
how to compute 821, 822
mean absolute deviation 820, 822
relation to least squares 777
Maxwell's equations 1032
MCMC *see* Markov chain Monte Carlo
Mean absolute deviation of distribution 723, 820
related to median 822
Mean value theorem 151
Mean(s)
of distribution 722, 723, 725
statistical differences between two 726–730
Measurement errors 773
Median 419
by selection 822
calculating 432
changes with time 438
incremental estimation 435
as L-estimate 818
of distribution 722, 725, 726
role in robust straight line fitting 822
Median-of-three, in Quicksort 423
MEM *see* Maximum entropy method (MEM)
Memory, using scope to manage 20
Merit function 773
for inverse problems 1004
for straight line fitting 781, 822

for straight line fitting, errors in both coordinates 785
in general linear least squares 788
nonlinear models 799
Mesh-drift instability 1040
Mesh generation 1150
Mesokurtic distribution 723
Message 754
Method of lines 1095
Method of regularization 1006
Metropolis algorithm 550, 552, 825
Metropolis-Hastings algorithm 551, 826, 827
Gibbs sampler as special case 827
Microsoft
integer types 26
NaN handling poor 35
Visual C++ 5
Windows 5
Midpoint method *see* Modified midpoint method; Semi-implicit midpoint rule
Mikado, or the Town of Titipu 920
Miller's algorithm 221, 278
Min-sum algorithm
dynamic programming 556
Viterbi decoding 867
MIN utility function 17
Minimal solution of recurrence relation 220, 221
Minimal trellis 853
Minimax
polynomial 235, 248
rational function 248, 249
Minimization 487–562
along a ray 88, 478, 489, 507–509, 511, 512, 519–521, 524, 540
annealing, method of simulated 487, 488, 549–555
bracketing of minimum 490–496, 503
Brent's method 489, 496–500, 785
Broyden-Fletcher-Goldfarb-Shanno algorithm 490, 521–525
by searching smaller subspaces 1021
chi-square 778–780, 799
choice of methods 488–490
combinatorial 549
conjugate gradient method 489, 515–520, 1011, 1020
convergence rate 495, 511
Davidon-Fletcher-Powell algorithm 490, 521, 522
degenerate 1002
direction set methods 489, 509–514
downhill simplex method 489, 502–507, 552, 821
finding best-fit parameters 773
Fletcher-Reeves algorithm 489, 515–519
functional 1001, 1002
global 487, 552–554, 774
globally convergent multidimensional 521–525
golden section search 492–496
in nonlinear model fitting 799
KKT conditions 539, 542
line methods 507–509
linear 526
multidimensional 502–525
of path length 555–562
Polak-Ribiere algorithm 489, 517
Powell's method 489, 502, 509–514
quasi-Newton methods 477, 489, 521–525
root finding and 476, 477
scaling of variables 523
steepest descent method 516, 1011
termination criterion 493, 503
use for sparse linear systems 87, 89
use in finding double roots 443
using derivatives 489, 499–502
variable metric methods 489, 521–525
see also Linear programming
Minimum curvature method 153
Minimum residual method, for sparse system 89
Minimum spanning tree 1147
MINPACK 806
Missing data 150–154, 685
in hidden Markov model 864
Mississippi River 552, 555
Mixture model, Gaussian 842–848
Mixture weight 843
Mode of distribution 722, 725, 726
Model-trust region 486, 806
Modeling of data *see* Fitting
Modes, homogeneous, of recursive filters 670
Modified Bessel functions *see* Bessel functions
Modified Lentz's method, for continued fractions 208
Modified midpoint method 922, 923
Modified moments 190
Modulation, trellis coded 855
Modulus of linear congruential generator 343
Moments
and quadrature formulas 996
filter that preserves 768
modified problem of 190
of distribution 721–726
problem of 94
semi-invariants 725
Monic polynomial 181
Monotonicity constraint, in upwind differencing 1042
Monte Carlo 197, 341, 397–418
adaptive 410–418
and Kolmogorov-Smirnov statistic 740, 762, 764
bootstrap method 809, 810
comparison of sampling methods 412–414
importance sampling 411, 412, 414, 835, 836
integration 156, 197, 397–403, 410–418
integration compared to MCMC 825
integration, recursive 416
integration, using Sobol' sequence 408, 409
integration, VEGAS algorithm 414–416

Markov chain 774, 824–836
partial differential equations 1030
quasi-random sequences in 403–410
quick and dirty 809, 810
recursive 410–418
significance of Lomb periodogram 686, 687
simulation of data 779, 807–810, 812
stratified sampling 412–414, 416
Moore-Penrose inverse 70
Mother functions 700
Mother Nature 807, 809
Moving average (MA) model 681
Moving window averaging 767
MRRR algorithm (Multiple Relatively Robust Representations) 589, 599
Muller's method 466, 473
Multidimensional
confidence levels of fitting 810, 812, 814, 816
data, use of binning 741
fitting 798, 836–838
Fourier transform 627–630
Fourier transform, real data 631–637
initial value problems 1049–1053
integrals 156, 196–199, 398, 410
interpolation 132–135, 139–154
Kolmogorov-Smirnov test 762–766
minimization 502–525
Monte Carlo integration 397–403, 410
normal (Gaussian) distribution 813
partial differential equations 1049–1053, 1083, 1095
root finding 442–486, 956, 959, 960, 963, 964
search using quasi-random sequence 404
secant method 474, 483
wavelet transform 712, 713
Multigrid method 1030, 1066–1083
avoid SOR 1070
boundary conditions 1072
choice of operators 1071
coarse-grid correction 1068
coarse-to-fine operator 1068
cycle 1069
dual viewpoint 1077
fine-to-coarse operator 1068
full approximation storage (FAS) algorithm 1076–1083
full multigrid method (FMG) 1067, 1072–1076
full weighting 1071
Gauss-Seidel relaxation 1069
half weighting 1071
importance of adjoint operator 1071
injection operator 1068
interpolation operator 1068
line relaxation 1070
local truncation error 1077, 1078
Newton's rule 1077, 1079
nonlinear equations 1077
nonlinear Gauss-Seidel relaxation 1078
odd-even ordering 1070, 1073
operation count 1067
prolongation operator 1068
recursive nature 1069
relative truncation error 1077
relaxation as smoothing operator 1069
restriction operator 1068
speeding up FMG algorithm 1076
stopping criterion 1078
straight injection 1071
symbol of operator 1070, 1071
use of Richardson extrapolation 1072
V-cycle 1069
W-cycle 1069
zebra relaxation 1070
Multiple precision arithmetic 1185–1193
Multiple roots 443, 464
Multiplication
complex 225
multiple precision 1188
Multiplicative linear congruential generator (MLCG) 341, 344, 348, 349
Multiplier of linear congruential generator 343
Multiply-with-carry (MWC) 347
Multipole methods, fast 140, 1150
Multiquadric 141
Multistep and multivalue methods (ODEs) 900, 942–946
see also Differential Equations; Predictor-corrector methods
Multitaper methods 662–665
Multivariate normal
deviates 378, 379
distribution 813, 847, 848
Murphy's Law 509
Mutual information 758–761

NAG 3, 40, 76, 568
Namespace, why no NR 36
NaN (not-a-number) 34, 35
how to set and test 34
`isnan` 35
quiet vs. signalling 35
Nat 755, 756, 760, 761
Natural cubic spline 122
Navier-Stokes equation 1035
Nearest neighbor 1097, 1101–1110, 1146
all points within specified radius 1109
Delaunay edges connect 1146
Needle, eye of (minimization) 503
Needleman-Wunsch algorithm 559
Negation, multiple precision 1186
Negentropy 1017–1019
Neighbor-joining (NJ) method 873, 878–882
Nelder-Mead minimization method 489, 502–507
Nested iteration 1072
Netlib 3
Networks 1168
Neumann boundary conditions 1026, 1045, 1056, 1057, 1063
Neural networks 840, 883

Neutrino 762
Neville's algorithm 118, 125, 166, 231
Newton-Cotes formulas 158, 179
  open 158, 159
Newton-Raphson method *see* Newton's rule
Newton's rule 182, 229, 443, 444, 456–462, 464, 466, 470, 584
  caution on use of numerical derivatives 459
  extended by Halley 463
  first published by Raphson 456
  for interior-point method 539
  for matrix inverse 64, 716
  for reciprocal of number 1190
  for square root of number 1191
  fractal domain of convergence 462
  globally convergent multidimensional 474, 477–486, 959, 960, 963
  in multidimensions 472–476, 959, 960, 963, 964
  in nonlinear multigrid 1077, 1079
  nonlinear Volterra equations 994
  safe 460
  scaling of variables 484
  singular Jacobian 486
  solving stiff ODEs 943, 944
  with backtracking 478–483
Next reaction method 952
Niederreiter sequence 404
NIST-STS, for random number tests 345
NL2SOL 806
Noise
  bursty 1168
  effect on maximum entropy method 683
  equivalent bandwidth 658
  fitting data that contains 770, 773
  model, for optimal filtering 651
Nominal variable (statistics) 741
Non-interfering directions *see* Conjugate directions
Nonexpansive projection operator 1012
Nonlinear eigenvalue problems 568, 569
Nonlinear equations
  finding roots of 442–486
  in MEM inverse problems 1018
  integral equations 988, 994
  multigrid method for elliptic PDEs 1077
Nonlinear instability 1037
Nonlinear programming 536
Nonnegativity constraints 526, 527
  barrier function 541
Nonparametric statistics 748–754
Nonpolynomial complete (NP-complete) 551
Nordsieck method 944
Norm, of matrix 64
Normal (Gaussian) distribution 320, 321, 341, 776, 778, 805, 1004
  central limit theorem 777
  deviates from 364, 365, 368, 377, 686
  kurtosis of 723, 724
  multivariate 378, 379, 813, 842, 843, 847, 848, 1006, 1129, 1130
  semi-invariants of 725
  sum of 12 uniform 377
  tails compared to Poisson 778
  two-dimensional (binormal) 746
  variance of skewness of 723
  see also Gaussian (normal) distribution
Normal equations (fitting) 40, 768, 789–793, 1002, 1007
  often are singular 793
Normal equations (interior-point method) 85, 543
Normalization
  normalizing constant 825, 828, 835, 836
  of Bessel functions 221
  of floating-point representation 9
  of functions 181, 973
  of modified Bessel functions 282
Normalized Radial Basis Functions 140
Not a Number *see* NaN
Notch filter 667, 671
NP-complete problem 551
`NR::`, why missing in 3rd ed. 36
`nr3.h` file 3, 4, 17, 28–30, 34–36
`NRmatrix` 26, 28, 29
  bounds checking 35
  instrumenting 36
  methods in 27
`NRvector` 26, 28, 29
  bounds checking 35
  instrumenting 36
  methods in 27
Nugget effect 838
  different from measurement error 838
Null hypothesis 720
Nullity 67, 68
Nullspace 39, 67–70, 72, 563, 1002
Number-theoretic transforms 616
`numeric_limits` 10, 34
Numerical derivatives 178, 229–232, 769
Numerical integration *see* Quadrature
Numerical Recipes
  bugs in 5
  compilers tested 5
  cookbook, not menu 3
  dependencies 4
  electronic versions 5
  how to use routines in 3
  is not a program library 2, 18
  is not a programming text 2
  machines tested 5
  obtaining source code 3
  types 25
  webnotes 4
Nyquist frequency 605, 607, 632, 653, 655, 685–687, 693
Nystrom method 989–992, 995
  product version 995

`_O` 26, 32, 36

Object 17–24
avoid copying large 36
constructor 18, 27
definition 18
destruction 20, 21
functor 21–23
grouping related functions 18
hides internal structure 17
inheritance 23, 24
instantiation 18, 19
multiple instances of 20
returning multiple values via 19
saving internal state 20
simple uses of 18–20
standardizing an interface 19
`struct` vs. `class` 17
see also Class
Object-oriented programming (OOP) 17–21, 23
Objective function 526, 528, 530
Oblateness parameter 971
Octave (software) 3
Octree *see* QO tree
Odd-even ordering
in Gauss-Seidel relaxation 1070, 1073
in successive over-relaxation (SOR) 1064
Odd parity 1168
Odds ratio 757
ODE *see* Differential equations
One-sided power spectral density 602
OOP *see* Object-oriented programming
Operation count
balancing 592
Baum-Welch re-estimation of hidden Markov model 865
Bessel function evaluation 278
bisection method 448
Cholesky decomposition 100
coefficients of interpolating polynomial 130
complex multiplication 108
cubic spline interpolation 122
evaluating polynomial 203
fast Fourier transform (FFT) 609, 610
Gauss-Jordan elimination 47, 54
Gaussian elimination 47
Givens reduction 578
Householder reduction 582
interpolation 111
inverse iteration 598, 599
iterative improvement 63
Jacobi transformation 573, 574
Kendall's tau 752
LU decomposition 49, 54
Markov model diagnosis 858
matrix inversion 108
matrix multiplication 107
maximum entropy method 683
multidimensional minimization 515
multigrid method 1067
multiplication 1188, 1190
polynomial evaluation 108, 203
QL method 585, 588
QR decomposition 103, 105
QR method for Hessenberg matrices 596
reduction to Hessenberg form 594
selection by partitioning 433
sorting 420, 422, 423
Toeplitz matrix 93
Vandermonde matrix 93
Operator
precedence, in C++ 12
splitting 1028, 1052, 1053, 1065
Optimal (Wiener) filtering 645, 649–652, 673, 674, 767
compared with regularization 1008
Optimal feasible vector 526, 528
Optimally Localized Average (OLA) 1014–1016
Optimization *see* Minimization
Options, financial 329
Ordinal variable (statistics) 741
Ordinary differential equations *see* Differential equations
Orthogonal *see* Orthonormal functions; Orthonormal polynomials
Orthogonal transformation 566, 578, 584, 699
Orthonormal basis, constructing 74, 105
Orthonormal functions 181, 292
Orthonormal polynomials
and Gaussian quadrature 181, 1087
Chebyshev 183, 187, 233
construct for arbitrary weight 189–191
Gaussian weights from recurrence 188, 189
Hermite 183
in Gauss-Hermite integration 185
Jacobi 183
Laguerre 183
Legendre 183
weight function log x 190, 191
Orthonormality 66, 68, 70, 181, 579
Out-of-band signaling 1178
Outer product of matrices (denoted by $\otimes$) 78, 523
Outgoing wave boundary conditions 1026
Outlier 723, 778, 779, 781, 818, 821
see also Robust estimation
Overcorrection 1061, 1062
Overflow 1164
in complex arithmetic 225, 226
Overlap-add and overlap-save methods 646, 647
Overrelaxation parameter 1062
choice of 1062–1064

p-value test 720
Packet-switched networks 1168
Padé approximant 125, 212, 245–247
Parabolic interpolation 496, 497
Parabolic partial differential equations 1024, 1043
Parallel axis theorem 413
Parallel programming
cyclic reduction 224

FFT 614
polynomial evaluation 205
recurrence relations 223, 224
recursive doubling 223
tridiagonal systems 57
Parameters in fitting function 776–780, 807–817
Parentheses, annoying 12
Parity bit 1168
Parity-check matrix 851
Parseval's theorem 602, 603, 654
discrete form 608
Parsimony, maximum 882
Partial abstraction 24
Partial differential equations 1024–1096
advective equation 1032
alternating-direction implicit method (ADI) 1052, 1053, 1065, 1066
amplification factor 1033, 1038
analyze/factorize/operate package 1030
artificial viscosity 1037, 1042
biconjugate gradient method 1030
boundary conditions 1025
boundary value problems 1025–1030, 1053–1058
Cauchy problem 1024
Cayley's form 1049
characteristics 1024–1026
Chebyshev acceleration 1064
classification of 1024–1030
comparison of rapid methods 1058
conjugate gradient method 1030
Courant condition 1034, 1036, 1038–1040, 1042
Courant condition (multidimensional) 1051
Crank-Nicolson method 1045, 1047, 1049, 1051, 1052
cyclic reduction (CR) method 1054, 1057, 1058
diffusion equation 1024, 1043–1049, 1051, 1052, 1059
Dirichlet boundary conditions 1026, 1045, 1055, 1061, 1063
elliptic, defined 1024
error, varieties of 1036–1038
explicit vs. implicit differencing 1033
FACR method 1058
finite difference method 1027
finite element methods 1030
flux-conservative initial value problems 1031–1043
forward Euler differencing 1032
forward time centered space (FTCS) 1032, 1044, 1049, 1059
Fourier analysis and cyclic reduction (FACR) 1053–1058
Gauss-Seidel method (relaxation) 1060, 1061, 1068, 1078
Godunov's method 1043
Helmholtz equation 1057
high-order methods, caution on 1050
hyperbolic 1024, 1031
implicit differencing 1045
inhomogeneous boundary conditions 1055
initial value problems 1024, 1026
initial value problems, recommendations on 1042
Jacobi's method (relaxation) 1060, 1061, 1068
Laplace's equation 1024
Lax method 1034–1036, 1042, 1050, 1051
Lax method (multidimensional) 1050, 1051
matrix methods 1028, 1030
mesh drift instability 1040
Monte Carlo methods 1030
multidimensional initial value problems 1049–1053
multigrid method 1029, 1066–1083
Neumann boundary conditions 1026, 1045, 1056, 1057, 1063
nonlinear diffusion equation 1047
nonlinear instability 1037
numerical dissipation or viscosity 1035
operator splitting 1028, 1052, 1053, 1065
outgoing wave boundary conditions 1026
parabolic 1024, 1043
periodic boundary conditions 1055, 1063
piecewise parabolic method (PPM) 1043
Poisson equation 1024, 1057
rapid (Fourier) methods 620, 1029, 1054
relaxation methods 1028, 1059–1066
Schrödinger equation 1048, 1049
second-order accuracy 1038–1042, 1045
shock 1037, 1042, 1043
sparse matrices from 76
spectral methods 239, 1030, 1083–1096
spectral radius 1061, 1066
stability vs. accuracy 1035
stability vs. efficiency 1027
staggered grids 625, 1057
staggered leapfrog method 1038, 1039
successive over-relaxation (SOR) 1061–1066, 1070
time splitting 1052, 1053, 1065
two-step Lax-Wendroff method 1040
upwind differencing 1037, 1042
variational methods 1030
varieties of error 1036–1038
von Neumann stability analysis 1033, 1034, 1036, 1039, 1045, 1046
wave equation 1024, 1031
see also Elliptic partial differential equations; Finite difference equations (FDEs)
Partial pivoting 43, 45, 535
Partition-exchange 423, 433
Partitioned matrix, inverse of 81, 82
Party tricks 106, 203
Parzen window 657
Pascal (language) 1
Path integration, for function evaluation 251–254, 318

Path length, minimization of 555–562
PAUP (software) 874
PBCG (preconditioned biconjugate gradient method) 89, 1030
PC methods *see* Predictor-corrector methods
PCx (software) 548
PDEs *see* Partial differential equations
PDF (probability density function) *see* Statistical distributions
Pearson's r 745
PECE method 944
Penalty function 541
Pentagon, symmetries of 1174
Percentile 320, 419, 435
Perfect code 852
Period of linear congruential generator 343
Periodic boundary conditions 1055, 1063
Periodogram 653–657, 681, 683
  Lomb's normalized 685–687, 689
  variance of 655, 656
Perron's theorems 221
Perturbation methods for matrix inversion 76–79
Peter Principle 427
Phantom bit 9
Phase error 1036
Phase-locked loop 824
Phi statistic 744
Phillips-Twomey method 1006
PHYLIP (software) 874
Phylogenetic tree *see* Tree, phylogenetic
π, computation of 1185
PI stepsize control 915
Piecewise parabolic method (PPM) 1043
Pigeonhole principle 387
Pincherle's theorem 222
Pivot element 43, 46, 47, 967
Pivoting 41, 43–45, 60, 76, 78, 101
  and QR decomposition 103, 105
  for tridiagonal systems 57
  full 43
  implicit 44, 51
  in LU decomposition 50
  in reduction to Hessenberg form 594
  in relaxation method 967
  Markowitz criterion 535
  partial 43, 45, 46, 50, 535
  threshold partial 535
Pixel 631, 714, 715, 1010, 1017
Planck's constant 1048
Plane rotation *see* Givens reduction; Jacobi transformation (or rotation)
Plane, defined by triangle 1115
Platykurtic distribution 723
Plotting of functions 444, 1160–1163
POCS (projection onto convex sets) 1012
Point 1099–1101
  closest approach of line to 1121
  distance between two 1099
  distance to line 1118
  projection into plane 1115
  random in triangle 1114
  random on sphere 1129, 1130
  test if inside box 1100
  test if inside polygon 1124
Poisson equation 631, 1024, 1057
Poisson probability function 336–338, 390
  as limiting case of binomial 338
  deviates from 372–374, 686
  moments of 725, 734
  semi-invariants of 725
  tails compared to Gaussian 778
Poisson process 362, 369, 829, 830
Polak-Ribiere algorithm 489, 517
Poles *see* Complex plane, poles in
Polishing of roots 459, 465, 471
Polygon 1097, 1122–1127
  area 1126
  Bolyai-Gerwien theorem 1127
  CCW vs. CW 1122
  centroid of 1127
  constructable by compass/straightedge 1127
  convex vs. concave 1122
  Jordan curve theorem 1124
  pentagon, symmetries of 1174
  removal of hidden 1150
  routine for classifying 1125
  simple vs. complex 1122, 1125
  sum of exterior angles 1122
  test if point inside 1124
  winding number 1122–1124
Polynomial interpolation 110, 118–120
  Aitken's algorithm 118
  coefficients for 129–131
  in Bulirsch-Stoer method 924
  in predictor-corrector method 943
  Lagrange's formula 94, 118, 1089, 1092
  multidimensional 132–135
  Neville's algorithm 118, 125, 166, 231, 924
  pathology in determining coefficients for 130
  Runge phenomenon 1090
  smoothing filters 768
  see also Interpolation
Polynomials 201–205
  algebraic manipulations 203
  approximation from Chebyshev coefficients 241, 243
  characteristic 469
  characteristic, for digital filters 670, 676
  characteristic, for eigenvalues of matrix 563, 583
  Chebyshev 187
  deflation 464–466, 471
  derivatives of 202
  division 95, 204, 464, 471
  evaluation of 201, 202
  evaluation of derivatives 202
  extrapolation in Bulirsch-Stoer method 922, 924
  extrapolation in Romberg integration 166
  fitting 94, 129, 241, 243, 768, 788, 797

generator for CRC 1170
ill-conditioned 463
irreducible modulo 2 382
matrix method for roots 469
minimax 235, 248
modulo 2 381, 1169
monic 181
multiplication 203, 204
operation count for 203
order, distinct from degree 1170
orthonormal 181, 1087
parallel evaluation 205
primitive modulo 2 382–386, 406
roots of 227–229, 463–473
shifting of 243
stopping criterion in root finding 467
Population count of bits 16
Population vector 857
Portable random number generator *see* Random number generator
Positive-definite matrix, testing for 101
Positivity constraints 526, 527
Postal Service (U.S.), barcode 1174
PostScript 1161
Powell's method 489, 502, 509–514
Power (in a signal) 602
Power of 2
next higher 16, 361
test if integer is a 16, 611
Power series 201–205, 209–218, 246
economization of 243, 244
Padé approximant of 245–247
Power spectral density *see* Fourier transform; Spectral density
Power spectrum 655
Bartlett window 657
data windowing 655–660
estimation by FFT 652–667
figures of merit for data windows 658
Hamming window 658
Hann window 657
leakage 655, 656, 658, 662–665
mean squared amplitude 653
multitaper methods 662–667
normalization conventions 652, 653
overlapping data segments 660–662
Parzen window 657
periodogram 653–657
power spectral density 652
PSD 652
Slepian tapers 662–667
square window 656
sum squared amplitude 653
time-integral squared amplitude 653
variance reduction in spectral estimation 656, 662
Welch window 658
Power spectrum estimation *see* Fourier transform; Spectral density
PPM (piecewise parabolic method) 1043
Precedence of operators, in C++ 12
Precision
floating point 1164
multiple 1185–1193
Preconditioned biconjugate gradient method (PBCG) 89
Preconditioning, in conjugate gradient methods 1030
Predictive stepsize control 939
Predictor-corrector methods 900, 909, 934, 942–946
Adams-Bashforth-Moulton schemes 943
adaptive order methods 946
compared to other methods 942, 946
fallacy of multiple correction 943
functional iteration vs. Newton's rule 944
multivalue compared with multistep 945, 946
Nordsieck method 944
starting and stopping 944
stepsize control 943, 944, 946
with fixed number of iterations 944
Prerequisite relationship 23
Primitive polynomials modulo 2 382–386, 406, 1170
Principal component analysis (PCA) 892
Principal directions 509, 512
Principal solution, of inverse problem 1004
Principal value integrals 178
Prior probability 757, 775, 841, 1005
smoothness 1006
Prize, $1000 offer revoked 342
Probability *see* Random number generator; Statistical tests; Statistical distributions
Process loss 658
Product Nystrom method 995
Products, reaction 947
Program(s)
as black boxes 67, 255, 443, 507
dependencies 4
NR not a program library 2
typography of 14
validation 5
Programming, NR not a textbook on 2
Projection onto convex sets (POCS) 1011–1013
generalizations 1013
Projection operator, nonexpansive 1012
Prolongation operator 1068
Proportional betting 758, 760
Proposal distribution 826–828, 835
Protocol, for communications 1168
PSD (power spectral density) *see* Fourier transform; Spectral density; Power spectrum
Pseudo-random numbers 340–386
Pseudoinverse 70
Pseudospectral method *see* Spectral methods
Puns, particularly bad 35, 202, 946, 958, 1098
Pure virtual class 34
Pyramidal algorithm 702, 703
Pythagorean theorem 1111
Pythagoreans 494

QO tree 1149–1158
applications of 1156–1158
intersecting objects 1150
use of hash in implementing 1151
QR decomposition 102–106, 483, 484, 486
and least squares 791
backsubstitution 103
operation count 103
pivoting 103
updating 105, 106, 483
use for orthonormal basis 74, 105
use for random rotation 1130
see also Eigensystems
Quadratic
convergence 64, 310, 452, 459, 511, 512, 522, 1185
equations 10, 227–229, 494, 572
interpolation 454, 466
programming 536, 884–886
Quadrature 155–200
adaptive 155, 167, 194–196, 241, 995
alternative extended Simpson's rule 160
and computer science 1160
arbitrary weight function 189–191, 995
Bode's rule 158
Cauchy principal values 178
change of variable in 170–172, 995
Chebyshev fitting 156, 240, 241
classical formulas for 156–162
Clenshaw-Curtis 156, 241, 624, 625
closed formulas 157–160
cubic splines 156
DE rule 174
error estimate in solution 991
extended formula of order $1/N^3$ 160
extended midpoint rule 161, 167
extended rules 159–162, 166, 993, 995, 997
extended Simpson's rule 160
extended trapezoidal rule 159, 162
for improper integrals 167–172, 995–1000
for integral equations 988, 993
Fourier integrals 692–699
Fourier integrals, infinite range 699
functors and 22
Gauss-Chebyshev 183, 187, 625
Gauss-Hermite 183, 995
Gauss-Jacobi 183
Gauss-Kronrod 192, 195
Gauss-Laguerre 183, 995
Gauss-Legendre 183, 193, 990, 996
Gauss-Lobatto 191, 192, 195, 241, 624
Gauss-Radau 191
Gaussian integration 159, 179–193, 238, 296, 988, 990, 995, 1086–1089
Gaussian integration, nonclassical weight function 189–191, 995
IMT rule 173
infinite ranges 176–178
Monte Carlo 156, 197, 397–403, 410
multidimensional 156, 196–199
Newton-Cotes formulas 158, 179
Newton-Cotes open formulas 158, 159
open formulas 157–162, 167
oscillatory function 217
related to differential equations 155
related to predictor-corrector methods 943
Romberg integration 156, 166, 169, 231, 923, 994
semi-open formulas 160–162
Simpson's rule 158, 165, 169, 698, 990, 994, 997
Simpson's three-eighths rule 158, 995, 997
singularity removal 170, 171, 173, 995
singularity removal, worked example 999, 1000
TANH rule 173
trapezoidal rule 158, 160, 162, 166, 173, 175, 178, 695, 698, 989, 993
using FFTs 156
variable transformation 172–178
weight function log x 190, 191
see also Integration of functions
Quadrature mirror filter 701, 708
Quadtree *see* QO tree
Quantile
changes with time 438
estimation 435
values 320, 419
Quantum mechanics, Uncertainty Principle 717
Quartet puzzling 882
Quartile value 419
Quasi-Newton methods for minimization 489, 521–525
Quasi-random sequence 403–410, 418, 1160, 1168
for Monte Carlo integration 408, 413, 418
Halton's 404
Sobol's 404–406
see also Random number generator
Quicksort 420, 422–426, 429, 433
Quotient-difference algorithm 206

R (programming language) 3
R-estimates 818
Racetrack betting 757, 760
Radial Basis Functions 139–144
Gaussian 142
inverse multiquadric 142
multiquadric 141
thin-plate spline 142
Wendland 142
Radioactive decay 362
Radix base for floating point arithmetic 592, 1164
Radix base for floating-point arithmetic 1186, 1192
Radix conversion 1181, 1185, 1192
Ramanujan's identity for π 1193
Random
angle variables 364
bits 380–386

byte 352
point in triangle 1114
point on circle 1131
point on sphere 1129, 1130
rotation matrix 1130, 1131
variables, decorrelating 379
walk 10
walk, multiplicative 329
Random deviates 340–386
angles 364
beta distribution 371
binomial 374–377
Cauchy distribution 367
chi-square distribution 371
exponential 362
F-distribution 371
faster 377
gamma distribution 369
Gaussian 341, 364, 365, 368, 377, 686, 1004
integer range 343
logistic 363
multivariate Gaussian 378, 379
normal 341, 364, 365, 368, 377, 686
Poisson 372–374, 686
quasi-random sequences 403–410, 1160, 1168
Rayleigh 365
squeeze 368
Student's-t distribution 371
sum of 12 uniform 377
trig functions 364
uniform 341–357
Random number generator
32-bit limited 355–357
Box-Muller algorithm 364
combined generators 342, 345–352
Data Encryption Standard 358–361
Diehard test 345
floating point 354
for everyday use 351
for hash function 387
for integer-valued probability distribution 372
hash function 352
highest quality 342, 351
inheritance 23
lagged Fibonnaci 354
linear congruential generator 341, 343, 348
linear feedback shift register (LSFR) 346, 380–386
MLCG 341, 344, 348, 349
multiply with carry method (MWC) 347
NIST-STS test 345
nonrandomness of low-order bits 344
planes, numbers lie on 344
primitive polynomials modulo 2 382
pseudo-DES 358
quasi-random sequences 403–410, 1160, 1168
Quicksort use of 423
random bits 380–386
random byte 352
ratio-of-uniforms method 367–371
recommended methods 345–352
rejection method 365–368
simulated annealing method 551, 552
spectral test 344
subtractive method 354
successor relation 350, 352
system-supplied 342
timings 355
transformation method 362–365
trick for trigonometric functions 364, 367
uniform 341–357
xorshift method 345
Random numbers *see* Monte Carlo; Random deviates
`RANDU`, infamous routine 344
Range 67, 68, 70
Rank (matrix) 67
kernel of finite 992
Rank (sorting) 419, 428–431
Rank (statistics) 748–754, 818
Kendall's tau 751–754
Spearman correlation coefficient 749–751
sum squared differences of 749
Rank-nullity theorem 68
Raphson, Joseph 456
Rate equations 947, 948
Ratio-of-uniforms method for random number generator 367–371
Ratio variable (statistics) 741
Rational Chebyshev approximation 247–251
Rational function 110, 201–205, 245, 248, 670
approximation for Bessel functions 275
approximation for continued fraction 207, 260
as power spectrum estimate 681
Chebyshev approximation 247–251
diagonal 125
evaluation of 204, 205
extrapolation in Bulirsch-Stoer method 922
interpolation and extrapolation using 110, 124–128, 245, 247–251, 922
minimax 248, 249
response of recursive filter 670
Rayleigh deviates 365
RBF *see* Radial Basis Functions
Reactions, chemical or nuclear 946–954
reaction products 947
Realizable (causal) 668, 670, 671
Rearranging *see* Sorting
Reciprocal, multiple precision 1190
Record, in data file 428
Recurrence relation 219–223
and continued fraction 222
associated Legendre polynomials 294
Bessel function 219, 274, 275, 278, 283–285
binomial coefficients 258
Bulirsch-Stoer 125

characteristic polynomial of tridiagonal matrix 583, 665
Clenshaw's recurrence formula 222, 223
continued fraction evaluation 207, 208
convergence 222
cosine function 219, 610
dominant solution 220
exponential integrals 219
gamma function 256
Golden Mean 11
hidden Markov model 861
Legendre polynomials 219
minimal vs. dominant solution 220
modified Bessel function 281
Neville's 118, 231
orthonormal polynomials 181
parallel evaluation 223, 224
Perron's theorems 221
Pincherle's theorem 222
polynomial interpolation 118, 119, 231
random number generator 343
rational function interpolation 125
sequence of trig functions 219
sine function 219, 610
spherical harmonics 294
stability of 12, 220, 222, 223, 275, 278, 282, 294
trig functions 687
weight of Gaussian quadrature 183

Recursive
doubling (parallel method) 223
Monte Carlo integration 410–418
multigrid method 1069
stratified sampling 416–418

Red-black *see* Odd-even ordering

Reduction of variance in Monte Carlo integration 402, 410

Reed-Solomon code 852, 855
Berlekamp-Massey algorithm 852
syndrome decoding 852

References (explanation) 6

Reflection formula for gamma function 256

Regula falsi (false position) 449

Regularity condition 983

Regularization
compared with optimal filtering 1008
constrained linear inversion method 1006
linear 1006–1013
nonlinear 1018
objective criterion 1009
of inverse problems 1002–1006
Phillips-Twomey method 1006
support vector machines 893
Tikhonov-Miller 1007
trade-off curve 1005
two-dimensional 1010, 1011
zeroth order 1002–1006
see also Inverse problems

Regularizing operator 1004

Rejection method for random number generator 365–368

Relative entropy 756

Relaxation method
automated allocation of mesh points 981–983
computation of spheroidal harmonics 971, 973–977
elliptic partial differential equations 1028, 1059–1066
example 971, 973–977
for algebraically difficult sets 970
for differential equations 957, 964–970
Gauss-Seidel method 1060, 1061, 1068, 1078
internal boundary conditions 983, 984
internal singular points 983, 984
Jacobi's method 1060, 1061, 1068
successive over-relaxation (SOR) 1061–1066, 1070
see also Multigrid method

Remes algorithms
exchange algorithm 669
for minimax rational function 249

Residual 63, 70, 88
in multigrid method 1067

`resize` 27

Resolution function, in Backus-Gilbert method 1014

Response function 641–643, 649

Responsibility matrix 842

Restriction operator 1068

Reward, $1000 offer revoked 342

Richardson's deferred approach to the limit 166, 169, 231, 900, 911, 921, 922, 994, 1072
see also Bulirsch-Stoer method

Richtmyer artificial viscosity 1042

Ridders' method
for numerical derivatives 231
root finding 443, 449, 452–454

Riemann shock problem 1043

Riemann zeta function 211

Right eigenvalues or eigenvectors 564, 565

Rights management 5

Rise/fall time 659

Robust estimation 723, 778, 818–824
Andrew's sine 821
average deviation 723
double exponential errors 820
Kalman filtering 824
Lorentzian errors 820
mean absolute deviation 723
nonparametric correlation 748–754
Tukey's biweight 821
use of a priori covariances 824
see also Statistical tests

Romberg integration 156, 166, 169, 231, 923, 994

Root finding 181, 182, 442–486
advanced implementations of Newton's rule 486
Bairstow's method 466, 471

bisection 445, 447–449, 454, 460, 492, 584, 822
bracketing of roots 443, 445–447, 454, 455, 464, 465, 470
Brent's method 443, 449, 453–456, 459, 786
Broyden's method 474, 483–486
compared with multidimensional minimization 476, 477
complex analytic functions 466
convergence criteria 448, 475
deflation of polynomials 464, 471
double root 443
eigenvalue methods 469, 470
false position 449, 452, 454
Halley's method 263, 264, 271, 335, 463
in complex plane 254
in one dimension 442
in relaxation method 964
in shooting method 956, 959
Jenkins-Traub method 470
Laguerre's method 444, 466–469
Lehmer-Schur algorithm 470
Maehly's procedure 465, 472
matrix method 469, 470
Muller's method 466, 473
multidimensional 442, 459
multiple roots 443
Newton's rule 182, 229, 443, 444, 456–462, 464, 466, 470–477, 539, 584, 944, 959, 964, 994, 1077, 1079, 1190, 1191
pathological cases 445, 457, 464, 474
polynomials 444, 463–473, 563
Ridders' method 443, 449, 452–454
root polishing 459, 465, 470–473
safe Newton's rule 460
secant method 449, 454, 466, 500
singular Jacobian in Newton's rule 486
stopping criterion for polynomials 467
use of minimum finding 443
using derivatives 456
without derivatives 456
zero suppression 473
see also Roots
Root polishing 459, 465, 470–473
Roots
Chebyshev polynomials 233
cubic equations 228
multiple 443, 466
nonlinear equations 442–486
polynomials 444, 464, 563
quadratic equations 227
reflection in unit circle 676
square, multiple precision 1191
see also Root finding
Rosenbrock method 934–940
compared with semi-implicit extrapolation 941
stepsize control 938
Rotation matrix 1097, 1130, 1131
Roundoff error 10, 11, 1163, 1164
bracketing a minimum 500
conjugate gradient method 1030
eigensystems 572, 573, 582, 584, 586, 591, 594
extended trapezoidal rule 165
general linear least squares 791, 795
graceful 1165
hardware aspects 1164
Householder reduction 581, 582
IEEE standard 1165
least-squares fitting 783, 791
Levenberg-Marquardt method 802
linear algebraic equations 38, 41, 43, 61, 72, 95
linear predictive coding (LPC) 680
magnification of 10, 11, 61
maximum entropy method (MEM) 683
multidimensional minimization 521, 525
multiple roots 464
numerical derivatives 229
recurrence relations 220
reduction to Hessenberg form 594
series 207, 210
straight line fitting 783
variance 724
Row
degeneracy 38
operations on matrix 42, 45
totals 743, 759
RSS algorithm 416, 417
RST properties (reflexive, symmetric, transitive) 440
Run-length encoding 1180
Runge-Kutta method 900, 901, 907–910, 935, 942, 1096
dense output 915
Dormand-Prince parameters 912, 920
embedded 911, 936
FSAL (first-same-as-last) 913
high-order 907–910, 912, 920
implementation 916–920
number of function evaluations 912
stepsize control 910–920
Runge phenomenon 1090
Rybicki, G.B. 96, 130, 183, 303, 634, 689, 717

Sampling
a distribution 825
importance 411, 412, 414
Latin square or hypercube 409, 410
Markov chain Monte Carlo 825
recursive stratified 416–418
stratified 412–414
uneven or irregular 685, 771
Sampling theorem 178, 239, 605, 653
for numerical approximation 717–719
Sande-Tukey FFT algorithm 616
Savitzky-Golay filters
for data smoothing 766–772
for numerical derivatives 232, 769

ScaLAPACK 40
Scallop loss 658
Schrödinger equation 1048, 1049
Schrage's algorithm 344
Schultz's method for matrix inverse 64, 716
Scilab (software) 3
Scope, temporary 20, 21
Searching
 an ordered table 114–118
 selection 431–439
 with correlated values 115
Secant method 443, 449, 454, 466, 500
 Broyden's method 483–486
 multidimensional (Broyden's) 474, 483–486
Second Euler-Maclaurin summation formula 167
Second order differential equations 928, 930
Seed of random number generator 343
Selection 419, 431–439
 by partition-exchange 433
 find m largest elements 434
 for median 822
 heap algorithm 434
 in place 432, 439
 incremental quantile estimation 435
 largest or smallest 434
 operation count 433, 439
 single-pass 432
 use to find median 726
Semi-implicit Euler method 934, 940
Semi-implicit extrapolation method 934, 935, 940, 941
 compared with Rosenbrock method 941
 stepsize control 941
Semi-implicit midpoint rule 940
Semi-invariants of a distribution 725
Sentinel, in Quicksort 424, 433
Separable kernel 992
Separation of variables 292
Sequence, alignment of by DP 559–562
Sequential quantile estimation 435
 changes with time 438
Series 209–218
 accelerating convergence of 177, 211–218
 alternating 211, 216
 asymptotic 210, 216
 Bessel function K_ν 288
 Bessel function Y_ν 284, 285
 Bessel functions 210, 274
 divergent 210, 211, 216
 economization 243–245
 ϵ algorithm 212
 Euler's transformation 211, 212
 exponential integral 267, 269
 Fresnel integral 298
 geometric 211, 214
 hypergeometric 252, 318
 hyperlinear convergence 211
 incomplete beta function 270
 incomplete gamma function 259
 Laurent 681, 682
 Levin transformation 214
 linear convergence 211
 logarithmic convergence 211
 relation to continued fractions 206
 Riemann zeta function 211
 roundoff error in 207
 sine and cosine integrals 301
 sine function 210
 Taylor 456, 510, 900, 911, 965, 969
 transformation of 211, 212
 van Wijngaarden's algorithm 217
Set bits, counting 16
Shaft encoder 1166
Shell algorithm (Shell's sort) 420–423
Shepard interpolation 140
Sherman-Morrison formula 76–79, 94, 483, 534
Shifting of eigenvalues 563, 585, 596
Shock wave 1037, 1042, 1043
Shooting method
 computation of spheroidal harmonics 979
 example 971, 977–981
 for differential equations 956, 959–961, 971, 977–981
 for difficult cases 962
 interior fitting point 962
Side information 760, 761
Sidelobe level 658
Sign bit in floating point format 8
SIGN utility function 17
Signal, bandwidth limited 605
Significance (statistical) 727
 of 2-d K-S test 763, 764
 one- vs. two-sided 747
 peak in Lomb periodogram 686, 687
 two-tailed 730
Similarity transform 566, 567, 570, 592, 594
Simplex
 defined 502
 method in linear programming 489, 502, 526–536, 548
 method of Nelder and Mead 489, 502–507, 552, 821
 use in simulated annealing 552
Simplex method *see* Linear Programming
Simpson's rule 156, 158, 160, 165, 169, 698, 990, 994
Simpson's three-eighths rule 158, 995, 997
Simulated annealing *see* Annealing, method of simulated
Simulation *see* Monte Carlo
Sinc expansion 178
Sine function
 evaluated from tan$(\theta/2)$ 219
 recurrence 219
 series 210
Sine integral 297, 300–302
 continued fraction 301
 routine for 301
 series 301
 see also Cosine integral

Sine transform *see* Fast Fourier transform (FFT); Fourier transform
Singleton's algorithm for FFT 637, 638
Singular value decomposition (SVD) 39, 65–75
  and least squares 65, 70, 73, 249, 791, 793
  and rational Chebyshev approximation 249
  approximation of matrices 74, 75
  basis for nullspace and range 68
  confidence levels from 816, 817
  covariance matrix 817
  fewer equations than unknowns 73
  for inverse problems 1003
  in minimization 512
  more equations than unknowns 73, 74
  of square matrix 69–73
  relation to eigendecomposition 569, 570
  use for ill-conditioned matrices 71, 73, 563
  use for orthonormal basis 74, 105
Singularities
  in integral equations 995–1000
  in integral equations, worked example 999, 1000
  in integrands 167, 173, 195, 995
  of hypergeometric function 252, 253, 318
  removal in numerical integration 170, 171, 173, 995
Singularity, subtraction of the 996
Six-step framework, for FFT 615
`size` 27
Skewness of distribution 723, 725
Slack variables 529, 538, 888
Slepian functions 662–667
SMBus 1168
Smith-Waterman algorithm 562
Smoothing
  data 129, 766–772
  in multigrid methods 1069
  operator in integral equations 987
Smoothness prior 1006
sn function 316
Sobol's quasi-random sequence 404–406
Soft-decision decoding 851–855
  error correction 855
  minimal trellis 853
  trellis 853, 856
  Viterbi algorithm 854
Software engineering 2
Sorting 419–441
  bubble sort cautioned against 420
  compared to selection 431
  eigenvectors 575
  Heapsort 420, 426–428, 434
  index table 419, 426, 428–431
  operation count 420, 422, 423
  Quicksort 420, 422–426, 429, 433
  rank table 419, 431
  ranking 428–431
  Shell's method 420–423
  straight insertion 420, 423, 575
Source code, obtaining NR 3
Sparse linear equations 39, 75–92, 534, 544, 548, 937
  band-diagonal 58
  Bartels-Golub update 535
  biconjugate gradient method 88, 716
  fill-in, minimizing 59, 76, 535, 544
  in inverse problems 1011
  indexed storage 82–87
  minimum residual method 89
  named patterns 76, 1028
  partial differential equations 1028
  relaxation method for boundary value problems 964
  wavelet transform 700, 716
  see also Matrix
Spearman rank-order coefficient 749–751, 819
Special functions *see* Function
Spectral analysis *see* Fourier transform; Periodogram
Spectral density
  one-sided PSD 602
  periodogram 681, 683
  power spectral density (PSD) 602, 603, 652
  power spectral density per unit time 603
  power spectrum estimation by MEM 681–684
  two-sided PSD 603
  see also Power spectrum
Spectral lines, how to smooth 767
Spectral methods 239, 1030, 1083–1096
  analytic example 1084
  and discontinuities 1083
  and Gaussian quadrature 1087–1089
  as finite difference methods 1093
  cardinal functions 1089–1091
  choice of basis functions 1085
  collocation method 1086
  contrasted with finite differencing 1083, 1085
  differentiation matrix 1091
  efficiency of 1083
  exponential convergence of 1085
  Galerkin method 1086
  grid point representation 1090
  interpolation of solution 1093
  method of lines 1095
  multidimensional equations 1095
  nonlinear equations 1094
  pseudospectral 1088
  tau method 1086
  variable coefficient equations 1094
  worked example 1094, 1095
Spectral radius 1061, 1066
Spectral test for random number generator 344
Spectrum *see* Fourier transform
Speech recognition by hidden Markov model 866
Sphere 1097, 1128–1130
  2- vs. 3-sphere 1128
  angular coordinates 1128
  find all points within a 1109
  intersection with line 1121
  random point on 1129, 1130

surface area in n-dimensions 1128
volume in n-dimensions 1129
Spherical Bessel functions 283
routine for 291
Spherical coordinates 1128
Spherical harmonics 292–297
basis functions for spectral methods 1085
fast transform 295, 297
orthogonality 292
routine for 294
stable recurrence for 294
table of 293
see also Associated Legendre polynomials
Spheroidal harmonics 971–981
boundary conditions 972, 973
normalization 973
routines for 975–977
Spline 111
cubic 120–124
gives tridiagonal system 122
interpolating 148
natural 122
operation count 122
two-dimensional (bicubic) 135
Spread matrix 1015
Square root
complex 226
multiple precision 1191
Square window 656
Squeeze, for computing random deviates 368
Stability 8–12
and stiff differential equations 932
Courant condition 1034, 1036, 1038, 1042, 1051
diffusion equation 1045, 1046
in quadrature solution of Volterra equation 994
mesh drift in PDEs 1040
nonlinear 1037
of Clenshaw's recurrence 223
of Gauss-Jordan elimination 41, 43
of implicit differencing 932, 1046
of Markov model 858, 859
of polynomial deflation 464, 465
of recurrence relations 220, 222, 223, 275, 278, 282, 294
partial differential equations 1026, 1033
von Neumann analysis for PDEs 1033, 1034, 1036, 1039, 1045, 1046
see also Accuracy
Stabilized Kolmogorov-Smirnov test 739
Stabilizing functional 1004
Stage, trellis 857
Staggered leapfrog method 1038, 1039
Standard (probable) errors 727, 781, 783, 786, 787, 790, 794, 807–817
Standard deviation
of a distribution 722, 723
of Fisher's z 747
of linear correlation coefficient 746
of sum squared difference of ranks 750
Standard Template Library (STL) containers 421
State change vector 947
Statistical distributions 320–339
beta 333, 334
binomial 338, 339
Cauchy 322, 323
chi-square 330, 331
density, change of variables in 362
exponential 326, 327
F-distribution 332, 333
full conditional 827
gamma 331, 332
Kolmogorov-Smirnov 334–336
logistic 324–326
lognormal 328, 329
Lorentzian 322
normal 320, 321
Poisson 336–338
Student's 323, 324
Weibull 327, 328
Statistical error 778
Statistical tests 720–772
Anderson-Darling 739
average deviation 723
bootstrap method 809, 810
chi-square 731–734, 742–745
contingency coefficient C 743, 744
contingency tables 741–745, 753, 758
correlation 721
Cramer's V 743, 744
difference of distributions 730–740
difference of means 727
difference of variances 728, 730
entropy measures of association 758–761
F-test 728, 730
Fisher's z-transformation 746
general paradigm 720
Kendall's tau 749, 751–754
Kolmogorov-Smirnov 731, 736–738, 762, 819
Kuiper's statistic 739
kurtosis 723, 725
L-estimates 818
linear correlation coefficient 745–748
Lucy's Y^2 and Z^2 735
M-estimates 818
mean 721–723, 725, 726
mean absolute deviation 723
measures of association 721, 741, 759
measures of central tendency 721–726
median 722, 725, 726, 818
mode 722, 725, 726
moments 721–726
nonparametric correlation 748–754
p-value test 720
Pearson's r 745
periodic signals 686, 687
phi statistic 744
R-estimates 818

rank correlation 748–754
robust 723, 749, 818–824
semi-invariants 725
shift vs. spread 739
significance 727
significance, one- vs. two-sided 730, 747
skewness 723, 725
small numbers of counts 734, 735
Spearman rank-order coefficient 749–751, 819
standard deviation 722, 723
strength vs. significance 727, 741
Student's t 727–730, 746
Student's t, for correlation 746
Student's t, paired samples 729
Student's t, Spearman rank-order coefficient 749
Student's t, unequal variances 728
sum squared difference of ranks 749
tail test 720
Tukey's trimean 818
two-dimensional 762–766
variance 721, 722, 724, 725, 729
Wilcoxon 818
see also Error; Robust estimation

Statistics, higher-order 604
Steed's method
Bessel functions 283, 287
continued fractions 207
Steepest descent method 516
in inverse problems 1011
Step
doubling 162, 174, 177, 910
tripling 168, 169
Stieltjes, procedure of 189
Stiff equations 901, 931–941
Kaps-Rentrop method 934
methods compared 941
predictor-corrector method 934
Rosenbrock method 934–940
scaling of variables 935
semi-implicit Euler method 940
semi-implicit extrapolation method 934, 935
semi-implicit midpoint rule 940
Stiff functions 111, 500
Stirling's approximation 256, 1017
STL *see* Standard Template Library
Stochastic simulation 946–954
when not to use 953
Stock market prices 329
Stoermer's rule 928
Stopping criterion
multigrid method 1078
polynomial root finding 467
Storage
band-diagonal matrix 58
sparse matrices 82–87
Straight injection 1071
Straight insertion 420, 423, 575
Straight-line fitting 780–785
errors in both coordinates 785–787
robust estimation 822–824
Strassen's fast matrix algorithms 107
Stratified sampling, Monte Carlo 412–414, 416
Strings, aligning by DP 559–562
`struct` *see* Class; Object
Student-t deviates 371
Student's probability distribution 323, 324
Cauchy as special case 323
normal as limiting case 323
Student's t-test
for correlation 746
for difference of means 727–730
for difference of means (paired samples) 729
for difference of means (unequal variances) 728
for difference of ranks 750
Spearman rank-order coefficient 749
Sturmian sequence 583
Sub-random sequences *see* Quasi-random sequence
Subtraction, multiple precision 1186
Subtractive method for random number generator 354
Successive over-relaxation (SOR) 1061–1066
bad in multigrid method 1070
Chebyshev acceleration 1064
choice of overrelaxation parameter 1062–1064
Successor relation, random generators 350
Sum-product algorithm 867
Sum squared difference of ranks 749
Sums *see* Series
Supernova 1987A 762
Support vector machine 883–898
dual formulation 886–889
kernel examples 891
kernel trick 889–892
linearly separable data 884
Mangasarian-Musicant variant 893–898
margin 884
regularization parameter 888, 893
SVMlight package 893
SVD *see* Singular value decomposition (SVD)
SVM *see* Support vector machine
SWAP utility function 17
Symbol, of operator 1070, 1071
Syndrome decoding
coset leader 852
error-correcting codes 852
Golay code 852
Hamming code 852
perfect code 852
Reed-Solomon code 852
Synthetic division 95, 202, 243, 464, 471
Systematic errors 778

Tableau (interpolation) 118, 125
Tail test 720
Tangent function, continued fraction 206

TANH rule 173
  infinite range 176
Taylor series 229, 456, 510, 900, 911, 944, 965, 969
Templates (C++) 17, 22, 26, 419, 421
Thermodynamics, and simulated annealing 550
Thin-plate spline 142
Three-dimensional array 36
Threshold partial pivoting 535
`throw` statement 30
Tides 677
Tikhonov-Miller regularization 1007
Time domain 600
Time reuse 952
Time splitting 1052, 1053, 1065
Timing, C routine for 355
TNT parsimony software 882
Toeplitz matrix 93, 96–99, 245
  LU decomposition 98
  new, fast algorithms 99
  nonsymmetric 96–98
Tongue twisters 431
Torus 400, 401, 408
Trade-off curve 1002, 1016
Transformation
  Gauss 310
  Landen 310
  method for random number generator 362–365
Transforms, number theoretic 616
Transition matrix
  directed graph 856
  eigenvalues and eigenvectors 858, 859
  Markov model 856
Transition probability
  directed graph 856
  Markov model 856
Transport error 1037
Transpose of sparse matrix 85
Trapezoidal rule 158, 160, 162, 166, 173, 175, 178, 695, 698, 989, 993
Traveling salesman problem 549, 551, 552
Tree
  data structure 1097
  KD *see* KD tree
  minimum spanning 1097, 1147
  of boxes as data structure 1101
  quadtree/octree *see* QO tree
Tree, phylogenetic 868–882
  additive 871
  agglomerative clustering 874–882
  branch length 870
  corrected distance transformation 873
  maximum likelihood 882
  maximum parsimony 882
  rooted vs. unrooted 871
  search over topologies 882
  software packages 874
  ultrametric 871
  UPGMA 877
  WPGMA 877
Trellis 853, 856
  directed graph 856
  maximum likelihood 854
  stage 857
Trellis coded modulation 855
Triangle 1097, 1111–1116
  angle between two 1116
  area of 1111
  centroid or barycenter 1113
  circumscribed circle (circumcircle) 1112
  in 3 dimensions 1114
  inscribed circle (incircle) 1112
  intersection with line 1121
  plane defined by 1115
  random point in 1114
Triangulation
  and interpolation 132
  applications of 1141–1149
  definition 1131
  Delaunay 1097, 1131–1149
  hashing and 1136
  incremental construction 1134
  interpolation using 1141
  largest minimum angle property 1134
  minimum weight 1134
  number of lines and triangles in 1132
Tridiagonal matrix 56–61, 188, 567, 598
  cyclic 79, 80
  eigenvalues 576, 577, 583–589, 665
  from cubic spline 122
  from operator splitting 1066
  in alternating-direction implicit method (ADI) 1066
  in cyclic reduction 1057, 1058
  parallel solution 57
  reduction of symmetric matrix to 576–583
  with fringes 1028
  see also Matrix
Trigonometric
  functions, tan$(\theta/2)$ as minimal 219
  functions, recurrence relation 219, 687
  interpolation 110
  solution of cubic equation 228
Trimming error 173
Truncation error 11, 173, 500, 910, 911, 1163
  exponentially decreasing 238
  in multigrid method 1077
  in numerical derivatives 229
`try` 30
Tukey's biweight 821
Tukey's trimean 818
Turbo codes 855
Twenty questions 755, 758, 761
Twin errors 1174
Two-dimensional *see* Multidimensional
Two-dimensional K–S test 762–766
Two-pass algorithm for variance 724
Two-point boundary value problems 900, 955–984

automated allocation of mesh points 981–983
boundary conditions 955, 962, 977
difficult cases 962
eigenvalue problem for differential equations 958, 973, 977–981
free boundary problem 958, 983
grid (mesh) points 957, 964, 981–983
internal boundary conditions 983, 984
internal singular points 983, 984
linear requires no iteration 961
multiple shooting 964
problems reducible to standard form 958
regularity condition 983
relaxation method 957, 964–970
relaxation method, example of 973–977
shooting method 956, 959–961, 971, 977–981
shooting method, example of 977–981
shooting to a fitting point 962
singular endpoints 962, 972, 978
see also Elliptic partial differential equations
Two-sided exponential error distribution 820
Two-sided power spectral density 603
Two-step Lax-Wendroff method 1040
Types used in NR 4, 25, 26

`Uchar` 25
`Uint` 25
`Ullong` 25
Ultrametric tree 871
Uncertainty coefficient 761
Uncertainty principle 717
Underflow, in IEEE arithmetic 9, 1165
Underrelaxation 1062
Uniform deviates *see* Random deviates, uniform
Unit-offset array 36
Unitary (function) 1048, 1049
Unitary (matrix) *see* Matrix
Universal Product Code (UPC) 1174
Unnormalized value 9
Unsupervised learning 842, 868
UPC checksum 1174
UPGMA 877
Upper Hessenberg matrix *see* Hessenberg matrix
Upwind differencing 1037, 1042
U.S. Postal Service barcode 1174
Utility functions 17

V-cycle 1069
`valarray` class 25
Validation of Numerical Recipes procedures 5
Valley, long or narrow 503, 509, 512, 516, 550, 552
Van Cittert's method 1011
Van Wijngaarden-Dekker-Brent method *see* Brent's method
Vandermonde matrix 93–96, 130
Variable length code 1176
Variable metric method 489, 521–525
compared to conjugate gradient method 521
Variable step-size integration 155, 167, 901, 924, 928–930, 938, 941, 943, 944, 946
Variance(s)
of distribution 721, 722, 725, 728–730
pooled 727
reduction of (in Monte Carlo) 402, 410
statistical differences between two 726–730
two-pass algorithm for computing 724
see also Covariance
Variational methods, partial differential equations 1030
Variogram 145, 837
various models for 837
`VecDoub`, `VecInt`, etc. 26
Vector
angle between two 1120, 1121
C++ `vector` class 24
class for 24–29
contiguous storage for 27
of matrices 36
suffix `_I`, `_O`, `_IO` 26, 32, 36
see also Array; `NRvector`
VEGAS algorithm for Monte Carlo 414–416
Verhoeff's algorithm for checksums 1174
Viète's formulas for cubic roots 228
Viscosity
artificial 1037, 1042
numerical 1035, 1042
Viterbi decoding 850–855
as a min-sum algorithm 867
Bayesian nature of 868
compared to forward-backward algorithm 867
compared to hidden Markov model 867, 868
constellation 855
directed graph 850
state defined 850
training 867
transition 850
with arbitrary transition probability 867
with parameter re-estimation 867
Volterra equations 988
adaptive stepsize control 995
analogy with ODEs 993
block-by-block method 994
first kind 988, 993
nonlinear 988, 994
second kind 988, 992–995
unstable quadrature 994
von Neumann-Richtmyer artificial viscosity 1042
von Neumann stability analysis for PDEs 1033, 1034, 1036, 1039, 1045, 1046
Voronoi diagram 1097, 1142–1146
and Delaunay triangulation 1143
avoiding obstacles 1147
Vowellish (coding example) 1176, 1181

W-cycle 1069
Wave equation 292, 1024, 1031
Wavelet transform 699–716
and Fourier domain 707, 708
appearance of wavelets 706, 707

approximation condition of order p 701
coefficient values 703, 704
contrasted with Fourier transform 699, 700, 711
Daubechies wavelet filter coefficients 700, 702, 704, 706–708, 715
detail information 701, 702
discrete wavelet transform (DWT) 702–706
DWT (discrete wavelet transform) 702–706
eliminating wraparound 703, 709
fast solution of linear equations 715, 716
filters 707, 708
for integral equations 989
image processing 713, 715
inheritance 23
inverse 703
JPEG-2000 712
Lemarie's wavelet 708
mother-function coefficient 703
mother functions 700
multidimensional 712, 713
nonsmoothness of wavelets 707
of linear operator 715
on the interval 709
pyramidal algorithm 702, 703
quadrature mirror filter 701
smooth information 701, 702
truncation 711, 712
wavelet filter coefficient 700, 703
wavelets 700, 706, 707
Wavelets *see* Wavelet transform
Weber function 254
Webnotes, Numerical Recipes 4
Weibull probability distribution 327, 328
Weighted Kolmogorov-Smirnov test 739
Weighted least-squares fitting *see* Least-squares fitting
Weighting, full vs. half in multigrid 1071
Weights for Gaussian quadrature 179, 180, 995
nonclassical weight function 189–191, 995
Welch window 658
`while` iteration 14
Wiener filtering 645, 649–652, 673, 674, 767
compared to regularization 1008
Wiener-Khinchin theorem 602, 674, 682
Wilcoxon test 818
Winding number 1122–1124
Window function 660
Bartlett 657
flat-topped 658, 659
Hamming 658
Hann 657
Parzen 657
Slepian 662
square 656
Welch 658
Winograd Fourier transform algorithms 616
Woodbury formula 80, 81, 94
Wordlength 8, 12
WPGMA 877
Wraparound
object for accessing vector 613
order for storing spectrum 611, 628, 632
problem in convolution 643
Wronskian, of Bessel functions 283, 284, 287

X-ray diffraction pattern, processing of 1012
Xorshift random number generator 345

Yale Sparse Matrix Package 76

Z-transform 670, 681
Z-transformation, Fisher's 746
Zapf Dingbats 1162
Zealots 1020
Zebra relaxation 1070
Zero contours 474
Zero-offset array 36
Zeroth-order regularization 1002–1006
Zip code, barcode for 1174
Ziv-Lempel compression 1176
ZooAnimal (OOP example) 23
Zoom transforms 615

CAMBRIDGE UNIVERSITY PRESS
and
NUMERICAL RECIPES SOFTWARE

Coupon for
Special Offer Number One

At a future time, this coupon will be exchangeable for a valuable benefit to owners of this Numerical Recipes, Third Edition book, ISBN 9780521866088. You must clip and mail this actual coupon, not a photocopy or fax. This coupon is valid only for Offer Number One, not for any other offer.

To find out about special offers, visit
http://www.nr.com/specialoffers.html

Special Offer Code ________________

Name ____

Address ____

____

Email ____

»»

CAMBRIDGE UNIVERSITY PRESS
and
NUMERICAL RECIPES SOFTWARE

Coupon for
Special Offer Number Two

At a future time, this coupon will be exchangeable for a valuable benefit to owners of this Numerical Recipes, Third Edition book, ISBN 9780521866088. You must clip and mail this actual coupon, not a photocopy or fax. This coupon is valid only for Offer Number Two, not for any other offer.

To find out about special offers, visit
http://www.nr.com/specialoffers.html

Special Offer Code ________________

Name ____

Address ____

____

Email ____

CAMBRIDGE UNIVERSITY PRESS
and
NUMERICAL RECIPES SOFTWARE

Coupon for
Special Offer Number Three

At a future time, this coupon will be exchangeable for a valuable benefit to owners of this Numerical Recipes, Third Edition book, ISBN 9780521866088. You must clip and mail this actual coupon, not a photocopy or fax. This coupon is valid only for Offer Number Three, not for any other offer.

To find out about special offers, visit
http://www.nr.com/specialoffers.html

Special Offer Code __________________

Name ____

Address ____

____

Email ____

»»

CAMBRIDGE UNIVERSITY PRESS
and
NUMERICAL RECIPES SOFTWARE

Coupon for
Special Offer Number Four

At a future time, this coupon will be exchangeable for a valuable benefit to owners of this Numerical Recipes, Third Edition book, ISBN 9780521866088. You must clip and mail this actual coupon, not a photocopy or fax. This coupon is valid only for Offer Number Four, not for any other offer.

To find out about special offers, visit
http://www.nr.com/specialoffers.html

Special Offer Code __________________

Name ____

Address ____

____

Email ____
