# Supplementary material for: Effective Local and Secondary Protein Structure Prediction by Combining a Neural Network-Based Approach with Extensive Feature Design and Selection without Reliance on Evolutionary Information
Source: Int J Mol Sci. 2023 Oct 27;24(21):15656. doi: 10.3390/ijms242115656 (PMC10648199; doi:10.3390/ijms242115656)
Supplement: Supplementary file 1 [file ijms-24-15656-s001.zip › Generation.Databases.And.Predictors.tar.gz!/foundation/BioPolymerMechanics/BioPolymerMechanics.plg]

```
# Build Log


### --------------------Configuration: BioPolymerMechanics - Win32 Debug--------------------


### Command Lines

Build : warning : failed to (or don't know how to) build 'D:\Agony\foundation\Common\Simple_test.cpp'

Creating temporary file "C:\TMP\RSPA18.tmp" with contents
[
/nologo /MLd /W3 /Gm /GX /ZI /Od /D "WIN32" /D "_DEBUG" /D "_CONSOLE" /D "_MBCS" /FR"Debug/" /Fp"Debug/BioPolymerMechanics.pch" /YX /Fo"Debug/" /Fd"Debug/" /FD /GZ /c 
"D:\Agony\foundation\BioPolymerMechanics\foundation\Aminoacid_name_translator.cpp"
"D:\Agony\foundation\BioPolymerMechanics\foundation\Atom.cpp"
"D:\Agony\foundation\BioPolymerMechanics\foundation\Atom_test.cpp"
"D:\Agony\foundation\BioPolymerMechanics\foundation\Core_attributes.cpp"
"D:\Agony\foundation\BioPolymerMechanics\foundation\Core_iterator.cpp"
"D:\Agony\foundation\BioPolymerMechanics\foundation\Core_iterator_test.cpp"
"D:\Agony\foundation\BioPolymerMechanics\foundation\Geometry_util\Geometry_util.cpp"
"D:\Agony\foundation\Common\get_extension_file_name.cpp"
"D:\Agony\foundation\Generality_Biopolymers\is_standard_one_letter_aminoacid_sequence.cpp"
"D:\Agony\foundation\Generality_Biopolymers\is_standard_three_letter_aminoacid.cpp"
"D:\Agony\foundation\BioPolymerMechanics\main.cpp"
"D:\Agony\foundation\BioPolymerMechanics\foundation\Model.cpp"
"D:\Agony\foundation\BioPolymerMechanics\foundation\Model_test.cpp"
"D:\Agony\foundation\Common\new_extensio_file_name.cpp"
"D:\Agony\foundation\Common\PutVaDouble.cpp"
"D:\Agony\foundation\Common\Simple_test.cpp"
]
Creating command line "cl.exe @C:\TMP\RSPA18.tmp" 
Creating temporary file "C:\TMP\RSPA19.tmp" with contents
[
kernel32.lib user32.lib gdi32.lib winspool.lib comdlg32.lib advapi32.lib shell32.lib ole32.lib oleaut32.lib uuid.lib odbc32.lib odbccp32.lib kernel32.lib user32.lib gdi32.lib winspool.lib comdlg32.lib advapi32.lib shell32.lib ole32.lib oleaut32.lib uuid.lib odbc32.lib odbccp32.lib /nologo /subsystem:console /incremental:yes /pdb:"Debug/BioPolymerMechanics.pdb" /debug /machine:I386 /out:"Debug/BioPolymerMechanics.exe" /pdbtype:sept 
.\Debug\Aminoacid_name_translator.obj
.\Debug\Atom.obj
.\Debug\Atom_test.obj
.\Debug\Core_attributes.obj
.\Debug\Core_iterator.obj
.\Debug\Core_iterator_test.obj
.\Debug\Geometry_util.obj
.\Debug\get_extension_file_name.obj
.\Debug\is_standard_one_letter_aminoacid_sequence.obj
.\Debug\is_standard_three_letter_aminoacid.obj
.\Debug\main.obj
.\Debug\Model.obj
.\Debug\Model_test.obj
.\Debug\new_extensio_file_name.obj
.\Debug\PutVaDouble.obj
.\Debug\Simple_test.obj
]
Creating command line "link.exe @C:\TMP\RSPA19.tmp"

### Output Window

Compiling...
Aminoacid_name_translator.cpp
d:\agony\foundation\biopolymermechanics\foundation\aminoacid_name_translator.cpp(5) : fatal error C1083: Cannot open include file: '..//..//Generality_Biopolymers//Generality_Biopolymers.h': No such file or directory
Atom.cpp
Atom_test.cpp
d:\agony\foundation\biopolymermechanics\foundation\atom_test.h(4) : fatal error C1083: Cannot open include file: '../../Common/Simple_test.h': No such file or directory
Core_attributes.cpp
Core_iterator.cpp
Core_iterator_test.cpp
d:\agony\foundation\biopolymermechanics\foundation\core_iterator_test.h(4) : fatal error C1083: Cannot open include file: '../../Common/Simple_test.h': No such file or directory
Geometry_util.cpp
get_extension_file_name.cpp
fatal error C1083: Cannot open source file: 'D:\Agony\foundation\Common\get_extension_file_name.cpp': No such file or directory
is_standard_one_letter_aminoacid_sequence.cpp
fatal error C1083: Cannot open source file: 'D:\Agony\foundation\Generality_Biopolymers\is_standard_one_letter_aminoacid_sequence.cpp': No such file or directory
is_standard_three_letter_aminoacid.cpp
fatal error C1083: Cannot open source file: 'D:\Agony\foundation\Generality_Biopolymers\is_standard_three_letter_aminoacid.cpp': No such file or directory
main.cpp
d:\agony\foundation\biopolymermechanics\foundation\atom_test.h(4) : fatal error C1083: Cannot open include file: '../../Common/Simple_test.h': No such file or directory
Model.cpp
d:\agony\foundation\biopolymermechanics\foundation\model.cpp(18) : fatal error C1083: Cannot open include file: '../../Common/CommonFunc.h': No such file or directory
Model_test.cpp
d:\agony\foundation\biopolymermechanics\foundation\model_test.h(4) : fatal error C1083: Cannot open include file: '../../Common/Simple_test.h': No such file or directory
new_extensio_file_name.cpp
fatal error C1083: Cannot open source file: 'D:\Agony\foundation\Common\new_extensio_file_name.cpp': No such file or directory
PutVaDouble.cpp
fatal error C1083: Cannot open source file: 'D:\Agony\foundation\Common\PutVaDouble.cpp': No such file or directory
Simple_test.cpp
fatal error C1083: Cannot open source file: 'D:\Agony\foundation\Common\Simple_test.cpp': No such file or directory
Error executing cl.exe.

### Results

BioPolymerMechanics.exe - 12 error(s), 1 warning(s)
```
