# Supplementary material for: Effective Local and Secondary Protein Structure Prediction by Combining a Neural Network-Based Approach with Extensive Feature Design and Selection without Reliance on Evolutionary Information
Source: Int J Mol Sci. 2023 Oct 27;24(21):15656. doi: 10.3390/ijms242115656 (PMC10648199; doi:10.3390/ijms242115656)
Supplement: Supplementary file 1 [file ijms-24-15656-s001.zip › Generation.Databases.And.Predictors.tar.gz!/foundation/Special_function/c6-4.pdf]

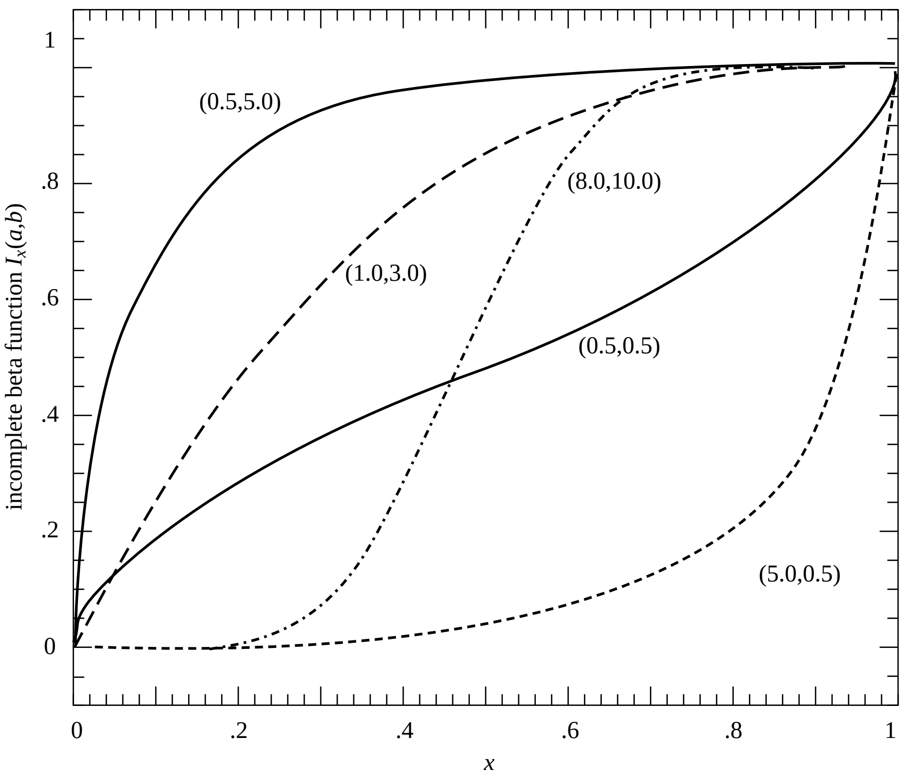

Figure 6.4.1. The incomplete beta function $I_x(a, b)$ for five different pairs of (a, b). Notice that the pairs $(0.5, 5.0)$ and $(5.0, 0.5)$ are symmetrically related as indicated in equation (6.4.3).

6.4 Incomplete Beta Function, Student's Distribution, F-Distribution, Cumulative Binomial Distribution

The incomplete beta function is defined by

$$I_x(a,b) \equiv \frac{B_x(a,b)}{B(a,b)} \equiv \frac{1}{B(a,b)} \int_0^x t^{a-1}(1-t)^{b-1}dt \qquad (a,b>0) \tag{6.4.1}$$

It has the limiting values

$$I_0(a,b) = 0 \qquad I_1(a,b) = 1 \tag{6.4.2}$$

and the symmetry relation

$$I_x(a,b) = 1 - I_{1-x}(b,a) \tag{6.4.3}$$

If a and b are both rather greater than one, then $I_x(a, b)$ rises from "near-zero" to "near-unity" quite sharply at about $x = a/(a+b)$. Figure 6.4.1 plots the function for several pairs (a, b).

Sample page from NUMERICAL RECIPES IN C: THE ART OF SCIENTIFIC COMPUTING (ISBN 0-521-43108-5)
Copyright (C) 1988-1992 by Cambridge University Press. Programs Copyright (C) 1988-1992 by Numerical Recipes Software.
Permission is granted for internet users to make one paper copy for their own personal use. Further reproduction, or any copying of machine-readable files (including this one) to any server computer, is strictly prohibited. To order Numerical Recipes books or CDROMs, visit website http://www.nr.com or call 1-800-872-7423 (North America only), or send email to directcustserv@cambridge.org (outside North America).

The incomplete beta function has a series expansion

$$I_x(a,b) = \frac{x^a(1-x)^b}{aB(a,b)}\left[1 + \sum_{n=0}^{\infty}\frac{B(a+1,n+1)}{B(a+b,n+1)}x^{n+1}\right], \tag{6.4.4}$$

but this does not prove to be very useful in its numerical evaluation. (Note, however, that the beta functions in the coefficients can be evaluated for each value of n with just the previous value and a few multiplies, using equations 6.1.9 and 6.1.3.)

The continued fraction representation proves to be much more useful,

$$I_x(a,b) = \frac{x^a(1-x)^b}{aB(a,b)}\left[\frac{1}{1+}\frac{d_1}{1+}\frac{d_2}{1+}\cdots\right] \tag{6.4.5}$$

where

$$\begin{aligned} d_{2m+1} &= -\frac{(a+m)(a+b+m)x}{(a+2m)(a+2m+1)} \\ d_{2m} &= \frac{m(b-m)x}{(a+2m-1)(a+2m)} \end{aligned} \tag{6.4.6}$$

This continued fraction converges rapidly for $x < (a+1)/(a+b+2)$, taking in the worst case $O(\sqrt{\max(a,b)})$ iterations. But for $x > (a+1)/(a+b+2)$ we can just use the symmetry relation (6.4.3) to obtain an equivalent computation where the continued fraction will also converge rapidly. Hence we have

```
#include <math.h>

float betai(float a, float b, float x)
Returns the incomplete beta function Ix(a,b).
{
    float betacf(float a, float b, float x);
    float gammln(float xx);
    void nrerror(char error_text[]);
    float bt;

    if (x < 0.0 || x > 1.0) nrerror("Bad x in routine betai");
    if (x == 0.0 || x == 1.0) bt=0.0;
    else                                    Factors in front of the continued fraction.
        bt=exp(gammln(a+b)-gammln(a)-gammln(b)+a*log(x)+b*log(1.0-x));
    if (x < (a+1.0)/(a+b+2.0))              Use continued fraction directly.
        return bt*betacf(a,b,x)/a;
    else                                    Use continued fraction after making the sym-
        return 1.0-bt*betacf(b,a,1.0-x)/b;     metry transformation.
}
```

which utilizes the continued fraction evaluation routine

```
#include <math.h>
#define MAXIT 100
#define EPS 3.0e-7
#define FPMIN 1.0e-30

float betacf(float a, float b, float x)
Used by betai: Evaluates continued fraction for incomplete beta function by modified Lentz's
method (§5.2).
{
    void nrerror(char error_text[]);
```

Sample page from NUMERICAL RECIPES IN C: THE ART OF SCIENTIFIC COMPUTING (ISBN 0-521-43108-5)
Copyright (C) 1988-1992 by Cambridge University Press. Programs Copyright (C) 1988-1992 by Numerical Recipes Software.
Permission is granted for internet users to make one paper copy for their own personal use. Further reproduction, or any copying of machine-readable files (including this one) to any server computer, is strictly prohibited. To order Numerical Recipes books or CDROMs, visit website
http://www.nr.com or call 1-800-872-7423 (North America only), or send email to directcustserv@cambridge.org (outside North America).

```
    int m,m2;
    float aa,c,d,del,h,qab,qam,qap;

    qab=a+b;                              These q's will be used in factors that occur
    qap=a+1.0;                                in the coefficients (6.4.6).
    qam=a-1.0;
    c=1.0;                                First step of Lentz's method.
    d=1.0-qab*x/qap;
    if (fabs(d) < FPMIN) d=FPMIN;
    d=1.0/d;
    h=d;
    for (m=1;m<=MAXIT;m++) {
        m2=2*m;
        aa=m*(b-m)*x/((qam+m2)*(a+m2));
        d=1.0+aa*d;                       One step (the even one) of the recurrence.
        if (fabs(d) < FPMIN) d=FPMIN;
        c=1.0+aa/c;
        if (fabs(c) < FPMIN) c=FPMIN;
        d=1.0/d;
        h *= d*c;
        aa = -(a+m)*(qab+m)*x/((a+m2)*(qap+m2));
        d=1.0+aa*d;                       Next step of the recurrence (the odd one).
        if (fabs(d) < FPMIN) d=FPMIN;
        c=1.0+aa/c;
        if (fabs(c) < FPMIN) c=FPMIN;
        d=1.0/d;
        del=d*c;
        h *= del;
        if (fabs(del-1.0) < EPS) break;   Are we done?
    }
    if (m > MAXIT) nrerror("a or b too big, or MAXIT too small in betacf");
    return h;
}
```

Student's Distribution Probability Function

Student's distribution, denoted $A(t|\nu)$, is useful in several statistical contexts, notably in the test of whether two observed distributions have the same mean. $A(t|\nu)$ is the probability, for ν degrees of freedom, that a certain statistic t (measuring the observed difference of means) would be smaller than the observed value if the means were in fact the same. (See Chapter 14 for further details.) Two means are significantly different if, e.g., $A(t|\nu) > 0.99$. In other words, $1 - A(t|\nu)$ is the significance level at which the hypothesis that the means are equal is disproved.

The mathematical definition of the function is

$$A(t|\nu) = \frac{1}{\nu^{1/2}B(\frac{1}{2}, \frac{\nu}{2})} \int_{-t}^{t} \left(1 + \frac{x^2}{\nu}\right)^{-\frac{\nu+1}{2}} dx \tag{6.4.7}$$

Limiting values are

$$A(0|\nu) = 0 \qquad A(\infty|\nu) = 1 \tag{6.4.8}$$

$A(t|\nu)$ is related to the incomplete beta function $I_x(a, b)$ by

$$A(t|\nu) = 1 - I_{\frac{\nu}{\nu+t^2}}\left(\frac{\nu}{2}, \frac{1}{2}\right) \tag{6.4.9}$$

Sample page from NUMERICAL RECIPES IN C: THE ART OF SCIENTIFIC COMPUTING (ISBN 0-521-43108-5)
Copyright (C) 1988-1992 by Cambridge University Press. Programs Copyright (C) 1988-1992 by Numerical Recipes Software.
Permission is granted for internet users to make one paper copy for their own personal use. Further reproduction, or any copying of machine-readable files (including this one) to any server computer, is strictly prohibited. To order Numerical Recipes books or CDROMs, visit website http://www.nr.com or call 1-800-872-7423 (North America only), or send email to directcustserv@cambridge.org (outside North America).

So, you can use (6.4.9) and the above routine `betai` to evaluate the function.

F-Distribution Probability Function

This function occurs in the statistical test of whether two observed samples have the same variance. A certain statistic F, essentially the ratio of the observed dispersion of the first sample to that of the second one, is calculated. (For further details, see Chapter 14.) The probability that F would be as *large* as it is if the first sample's underlying distribution actually has *smaller* variance than the second's is denoted $Q(F|\nu_1,\nu_2)$, where ν_1 and ν_2 are the number of degrees of freedom in the first and second samples, respectively. In other words, $Q(F|\nu_1,\nu_2)$ is the significance level at which the hypothesis "1 has smaller variance than 2" can be rejected. A small numerical value implies a very significant rejection, in turn implying high confidence in the hypothesis "1 has variance greater or equal to 2."

$Q(F|\nu_1,\nu_2)$ has the limiting values

$$Q(0|\nu_1,\nu_2)=1 \qquad Q(\infty|\nu_1,\nu_2)=0 \tag{6.4.10}$$

Its relation to the incomplete beta function $I_x(a,b)$ as evaluated by `betai` above is

$$Q(F|\nu_1,\nu_2)=I_{\frac{\nu_2}{\nu_2+\nu_1 F}}\left(\frac{\nu_2}{2},\frac{\nu_1}{2}\right) \tag{6.4.11}$$

Cumulative Binomial Probability Distribution

Suppose an event occurs with probability p per trial. Then the probability P of its occurring k *or more* times in n trials is termed a *cumulative binomial probability*, and is related to the incomplete beta function $I_x(a,b)$ as follows:

$$P\equiv\sum_{j=k}^{n}\binom{n}{j}p^j(1-p)^{n-j}=I_p(k,n-k+1) \tag{6.4.12}$$

For n larger than a dozen or so, `betai` is a much better way to evaluate the sum in (6.4.12) than would be the straightforward sum with concurrent computation of the binomial coefficients. (For n smaller than a dozen, either method is acceptable.)

CITED REFERENCES AND FURTHER READING:

Abramowitz, M., and Stegun, I.A. 1964, *Handbook of Mathematical Functions*, Applied Mathematics Series, Volume 55 (Washington: National Bureau of Standards; reprinted 1968 by Dover Publications, New York), Chapters 6 and 26.

Pearson, E., and Johnson, N. 1968, *Tables of the Incomplete Beta Function* (Cambridge: Cambridge University Press).

Sample page from NUMERICAL RECIPES IN C: THE ART OF SCIENTIFIC COMPUTING (ISBN 0-521-43108-5)
Copyright (C) 1988-1992 by Cambridge University Press. Programs Copyright (C) 1988-1992 by Numerical Recipes Software.
Permission is granted for internet users to make one paper copy for their own personal use. Further reproduction, or any copying of machine-readable files (including this one) to any server computer, is strictly prohibited. To order Numerical Recipes books or CDROMs, visit website http://www.nr.com or call 1-800-872-7423 (North America only), or send email to directcustserv@cambridge.org (outside North America).

6.5 Bessel Functions of Integer Order

This section and the next one present practical algorithms for computing various kinds of Bessel functions of integer order. In §6.7 we deal with fractional order. In fact, the more complicated routines for fractional order work fine for integer order too. For integer order, however, the routines in this section (and §6.6) are simpler and faster. Their only drawback is that they are limited by the precision of the underlying rational approximations. For full double precision, it is best to work with the routines for fractional order in §6.7.

For any real ν, the Bessel function $J_\nu(x)$ can be defined by the series representation

$$J_\nu(x) = \left(\frac{1}{2}x\right)^\nu \sum_{k=0}^{\infty} \frac{(-\frac{1}{4}x^2)^k}{k!\Gamma(\nu+k+1)} \tag{6.5.1}$$

The series converges for all x, but it is not computationally very useful for $x \gg 1$.

For ν *not* an integer the Bessel function $Y_\nu(x)$ is given by

$$Y_\nu(x) = \frac{J_\nu(x)\cos(\nu\pi) - J_{-\nu}(x)}{\sin(\nu\pi)} \tag{6.5.2}$$

The right-hand side goes to the correct limiting value $Y_n(x)$ as ν goes to some integer n, but this is also not computationally useful.

For arguments $x < \nu$, both Bessel functions look qualitatively like simple power laws, with the asymptotic forms for $0 < x \ll \nu$

$$\begin{aligned} J_\nu(x) &\sim \frac{1}{\Gamma(\nu+1)}\left(\frac{1}{2}x\right)^\nu \qquad \nu \geq 0 \\ Y_0(x) &\sim \frac{2}{\pi}\ln(x) \\ Y_\nu(x) &\sim -\frac{\Gamma(\nu)}{\pi}\left(\frac{1}{2}x\right)^{-\nu} \qquad \nu > 0 \end{aligned} \tag{6.5.3}$$

For $x > \nu$, both Bessel functions look qualitatively like sine or cosine waves whose amplitude decays as $x^{-1/2}$. The asymptotic forms for $x \gg \nu$ are

$$\begin{aligned} J_\nu(x) &\sim \sqrt{\frac{2}{\pi x}}\cos\left(x - \frac{1}{2}\nu\pi - \frac{1}{4}\pi\right) \\ Y_\nu(x) &\sim \sqrt{\frac{2}{\pi x}}\sin\left(x - \frac{1}{2}\nu\pi - \frac{1}{4}\pi\right) \end{aligned} \tag{6.5.4}$$

In the transition region where $x \sim \nu$, the typical amplitudes of the Bessel functions are on the order

$$\begin{aligned} J_\nu(\nu) &\sim \frac{2^{1/3}}{3^{2/3}\Gamma(\frac{2}{3})}\frac{1}{\nu^{1/3}} \sim \frac{0.4473}{\nu^{1/3}} \\ Y_\nu(\nu) &\sim -\frac{2^{1/3}}{3^{1/6}\Gamma(\frac{2}{3})}\frac{1}{\nu^{1/3}} \sim -\frac{0.7748}{\nu^{1/3}} \end{aligned} \tag{6.5.5}$$

Sample page from NUMERICAL RECIPES IN C: THE ART OF SCIENTIFIC COMPUTING (ISBN 0-521-43108-5)
Copyright (C) 1988-1992 by Cambridge University Press. Programs Copyright (C) 1988-1992 by Numerical Recipes Software.
Permission is granted for internet users to make one paper copy for their own personal use. Further reproduction, or any copying of machine-readable files (including this one) to any server computer, is strictly prohibited. To order Numerical Recipes books or CDROMs, visit website http://www.nr.com or call 1-800-872-7423 (North America only), or send email to directcustserv@cambridge.org (outside North America).
